# Using priorities between human and livestock bacterial antimicrobial resistance (AMR) to identify data gaps in livestock AMR surveillance

**DOI:** 10.1186/s12879-024-09847-3

**Published:** 2024-09-26

**Authors:** Narmada Venkateswaran, Lucien R. Swetschinski, Christina Fastl, Carlotta Di Bari, Nicola G. Criscuolo, Ranya Mulchandani, Cheng Zhao, Tomislav Meštrović, Kevin S. Ikuta, Sara Babo Martins, Lucy A. Coyne, João Sucena Afonso, Ben Huntington, Jonathan Rushton, Brecht Devleesschauwer, Benn Sartorius, Thomas P. Van Boeckel, David M. Pigott

**Affiliations:** 1 Global Burden of Animal Diseases Programme, https://animalhealthmetrics.org; 2 Institute for Health Metrics and Evaluation, Department of Health Metrics Sciences, University of Washington, Seattle, WA USA; 3 Department of Epidemiology and Public Health, Sciensano, Brussels, Belgium; 4 Health Geography and Policy Group, ETH Zürich, Zürich, Switzerland; 5 University Centre Varaždin, University North, Varaždin, Croatia; 6 David Geffen School of Medicine, University of California Los Angeles, Los Angeles, CA USA; 7 Division of Infectious Diseases, Veterans Affairs, Los Angeles, CA USA; 8 Institute of Infection, Veterinary and Ecological Sciences, University of Liverpool, Neston, UK; 9 Pengwern Animal Health Ltd, 259 Wallasey Village, Wallasey Wirral, Merseyside, UK; 10 Department of Translational Physiology, Infectiology and Public Health, Ghent University, Merelbeke, Belgium; 11 Faculty of Medicine, UQ Centre for Clinical Research (UQCCR), University of Queensland, Brisbane, Australia; 12 One Health Trust, Washington, D.C. USA; 13 National Office of Animal Health Ltd, 25 Town Square, Stevenage, SG1 1BP UK; 14 One Health Institute, University of Zürich, Zürich, Switzerland; 15 Spatial Epidemiology Lab, Université Libre de Bruxelles (ULB), Brussels, Belgium; 16 School of Public Health, The University of Sydney, Sydney, Australia

**Keywords:** Antimicrobial resistance, Data gaps, Surveillance, Livestock

## Abstract

**Background:**

Bacterial antimicrobial resistance (AMR) is a global threat to both humans and livestock. Despite this, there is limited global consensus on data-informed, priority areas for intervention in both sectors. We compare current livestock AMR data collection efforts with other variables pertinent to human and livestock AMR to identify critical data gaps and mutual priorities.

**Methods:**

We globally synthesized livestock AMR data from open-source surveillance reports and point prevalence surveys stratified for six pathogens (*Escherichia coli*, *Staphylococcus aureus*, non-typhoidal *Salmonella*, *Campylobacter* spp., *Enterococcus faecalis*, *Enterococcus faecium*) and eleven antimicrobial classes important in human and veterinary use, published between 2000 and 2020. We also included all livestock species represented in the data: cattle, chickens, pigs, sheep, turkeys, ducks, horses, buffaloes, and goats. We compared this data with intended priorities calculated from: disability-adjusted life years (DALYs), livestock antimicrobial usage (AMU), livestock biomass, and a global correlation exercise between livestock and human proportion of resistant isolates.

**Results:**

Resistance to fluoroquinolones and macrolides in *Staphylococcus aureus* were identified as priorities in many countries but, less than 10% of these reported livestock AMR data. Resistance data for *Escherichia coli* specific to cattle, chickens, and pigs, which we prioritized, were also well collected. AMR data collection on non-typhoidal *Salmonella* and other livestock species were often not prioritized. Of 232 categories prioritized by at least one country, data were only collected for 48% (*n* = 112).

**Conclusions:**

The lack of livestock AMR data globally for broad resistance in *Staphylococcus aureus* could underplay their zoonotic threat. Countries can bolster livestock AMR data collection, reporting, and intervention setting for *Staphylococcus aureus* as done for *Escherichia coli*. This framework can provide guidance on areas to strengthen AMR surveillance and decision-making for humans and livestock, and if done routinely, can adapt to resistance trends and priorities.

**Supplementary Information:**

The online version contains supplementary material available at 10.1186/s12879-024-09847-3.

## Background

In recent decades, bacterial antimicrobial resistance (AMR) has been identified as a global health threat; estimates indicate it caused 1.27 million human deaths worldwide in 2019 [1]. There are multiple mechanisms by which AMR emerges, such as the interaction between the human sector and the wider environment [2]. Livestock are often treated with the same antimicrobials as humans, and the use of antimicrobials for growth promotion in some countries and disease prevention instead of other hygiene practices creates more avenues for resistance to evolve [2, 3]. This leads to a complex interplay between antimicrobial usage (AMU) and resistance in humans, animals, and the environment [3].

Several exposure pathways have been proposed linking AMR in humans to the wider environment. These include active ingredients leaching into the environment, introduction into the food chain through animal source foods, or through direct transmission from infectious livestock [4–6]. Livestock AMR commonly arises from interactions with AMU; projections estimate global livestock AMU will increase by 8% between 2020 and 2030 given current trends of consumption [7]. Additionally, there is growing evidence showing significant associations between AMU and AMR in both food-producing animals and humans [8]. Due to global AMR and AMU data for livestock and humans being fragmented in availability and quality, there is limited understanding of this relationship in low- and middle- income countries (LMICs) [7, 9, 10]. Given the transmission dynamics of AMR between livestock and humans, when analyzing the trends in AMR in humans globally, it is important to understand the global data landscape of animal AMR, particularly when there is increasing uncertainty and concern about the degree to which the agricultural sector contributes to human AMR [3].

Although many human and animal health organizations have outlined AMR as a shared concern, and the need for coordinated monitoring and intervention efforts, there is limited understanding of the mutual antimicrobial class and pathogen combinations of concern on a global scale [11]. Recent research has assessed the global burden and geographic variation of AMR in humans and animals separately for well-represented antimicrobial class and pathogen combinations [1, 7, 12]. We aim to build upon these global analyses to highlight the global data gaps in livestock AMR against an evaluation of priorities among shared human and veterinary actors. Through this global analysis and identification of gaps, we present suggestions for strengthening current surveillance of AMR specific to pathogen, antimicrobial class, and livestock species.

## Methods

To identify antimicrobial classes of shared importance in humans and livestock, we utilized the main global antimicrobial prioritization frameworks pertaining to humans by World Health Organization (WHO) and to animals by World Organisation for Animal Health (WOAH) [13, 14]. We then evaluated the global coverage of livestock bacterial AMR data by pathogen-antimicrobial-class-livestock (referred to as a category) considering all pathogens available in this data. We created a composite indicator from globally available factors important in human and livestock bacterial AMR to determine category ranks: human disability adjusted life-years (DALYs) attributable to AMR, livestock AMU, livestock biomass, and a global correlation assessment between livestock and human AMR. We ordered categories within each country and designated the top 10% of values for our composite indicator in a country as priorities and compared the alignment of these to the availability of livestock AMR data on a national and global scale.

### Compilation of livestock AMR data sources

We used WHO and WOAH reports to identify antimicrobials of shared human and veterinary importance [13, 14]. The top two tiers were considered in both (WHO: “Highest priority of critical importance” and “High priority of critical importance”. WOAH: “Critically important” and “Highly important”). Only antimicrobial classes that fell into these tiers in both WHO and WOAH reports were included in subsequent analyses.

Livestock AMR data, specifically the proportion of resistant isolates, from 2000 to 2020 were extracted for each category of pathogen-antimicrobial-class-livestock. All pathogens which impacted both humans and livestock were considered. We used two different types of sources: routine surveillance systems and a published database of individual studies. Resistance data for high-income countries (HICs) were retrieved from various surveillance systems worldwide via open-access sites and reports; these included the European Food Safety Authority (EFSA), the National Antimicrobial Resistance Monitoring System (NARMS), the Japanese Veterinary Antimicrobial Resistance Monitoring System (NARMS). Resistance data for LMICs were sourced from resistancebank.org, a repository of point prevalence surveys collected from peer-review literature used as surrogates for systematic surveillance data in LMICs [15, 16]. For a detailed breakdown of countries covered, further information on the data sources, and additional methods, refer to Supplementary Material file 1 (SM1).

### Creation of composite indicator

**Fig. 1:**
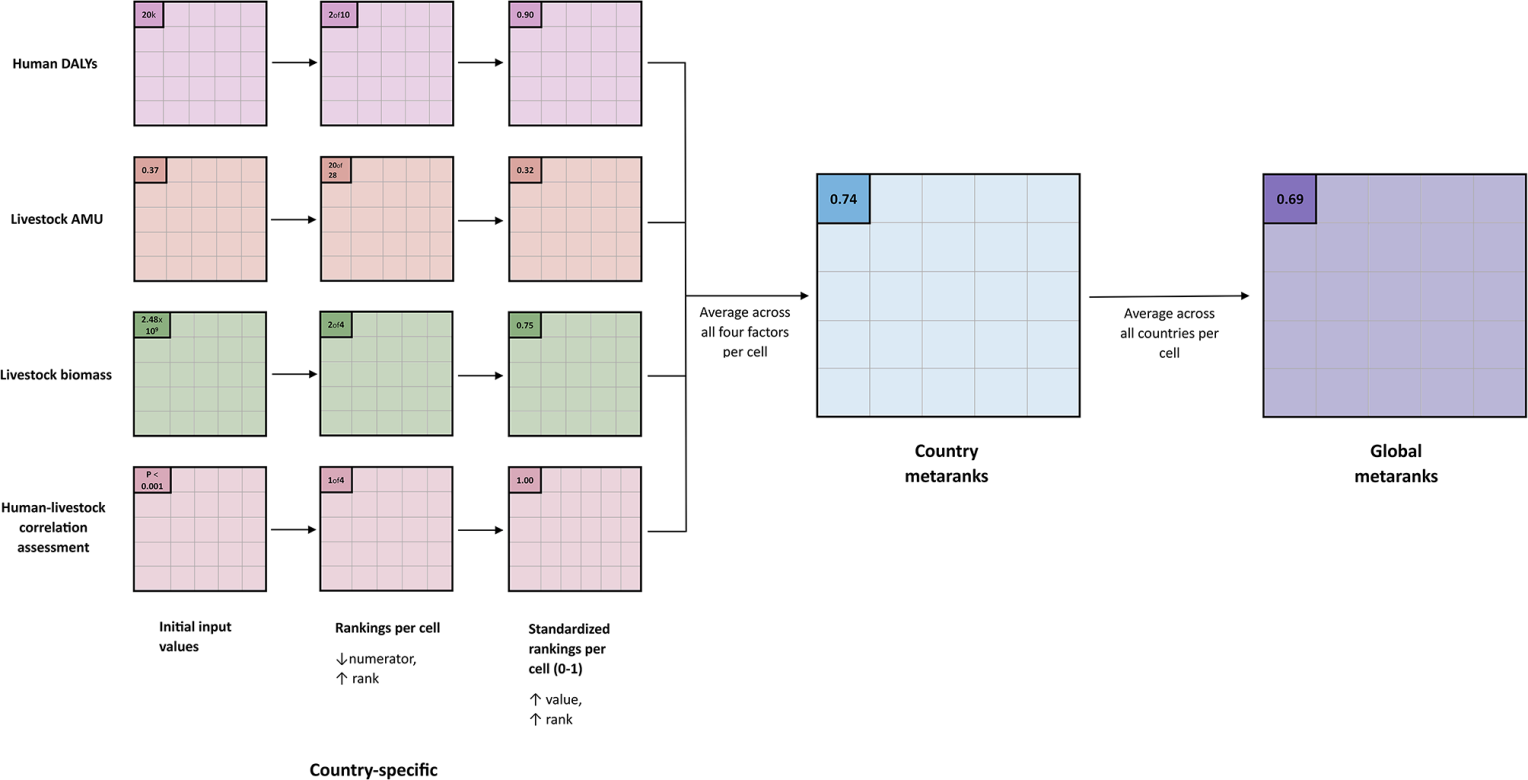
Diagram displaying the creation of the composite indicator (referenced here as metaranks) based on human disability adjusted life-years (DALYs) attributable to AMR, livestock AMU (mg/kg), livestock biomass (population correction units), and a global correlation assessment between livestock and human proportion of resistant isolates (represented as significance levels of Spearman correlation estimates) by antimicrobial class, pathogen, and livestock species (also represented here as a cell). Values displayed, from initial input values, to ranks, and final composite indicators, are taken from the example of fluoroquinolones, *E. coli* and pigs, with country-specific examples referencing the Philippines

For each specification of antimicrobial class, pathogen, and livestock species, we aimed to create a composite indicator that evaluated their relative importance (Fig. 1). We considered four factors: human DALYs attributable to AMR, livestock AMU (mg/kg), livestock biomass (population correction units), and a global correlation assessment between livestock and human proportion of resistant isolates. We chose these factors to represent the various transmission and impact points of AMR from livestock to humans in addition to having global coverage. Livestock AMU has been linked to the proliferation of livestock and human AMR, and biomass captures the size of the livestock population for which antimicrobials could be used and therefore propagate AMR [8, 17]. The correlation assessment aims to capture the pathway connecting livestock and human AMR, and human burden of disease due to AMR is captured through the inclusion of DALYs.

Human DALYs were derived from the Global Research on Antimicrobial Resistance (GRAM) project, where the class-pathogen-livestock combinations were ranked based upon the burden associated with their pathogen component [1]. Wherever a component was absent from the global dataset used, the component was assumed to have the lowest ranking - in the case of human DALY burden, no *Campylobacter*-related estimates were present, and as a result combinations including this element were assigned the lowest ranking. Livestock AMU, sourced from Mulchandani et al., was incorporated by ranking each class-pathogen-livestock combination based upon the aggregate country-specific usage of a specific antimicrobial class among each livestock species in that country [7]. Livestock biomass, also sourced from Mulchandani et al., was reported as a national tally for cattle, chickens, pigs, and sheep. Each class-pathogen-livestock category was ranked based upon this tally, with species with no estimated tally (namely turkeys, ducks, horses, buffaloes, and goats) assumed to be equal-tied for lowest rank. Finally, we incorporated evidence from a global correlation exercise evaluating the relatedness of the proportion of resistant isolates in livestock (sourced from the livestock AMR data considered) and humans (sourced from the GRAM project) [1]. Spearman correlation coefficients and *p*-values were calculated by each unique pathogen-antimicrobial-class-livestock category. Statistical significance levels were denoted as follows: *p* < 0.0001 (****), *p* < 0.001 (***), *p* < 0.01 (**), and *p* < 0.05 (*). Categories with positive correlations were ranked first by ascending order of significance (i.e. lower p-value), with negative correlations receiving the lowest rank. Ranks were determined at a global level, then all countries were assumed to receive the same rank for a particular category. For more details about ranking factors and assumptions, refer to SM1. Categories for which there were no values were assigned the lowest rank, and ranks were standardized using min-max standardization:

$$\:{X}_{stand}=\frac{X-{X}_{min}}{{{X}_{max}-X}_{min}}$$

X_stand_ is the standardized rank (between 0 and 1), X is the original rank, X_min_ is the minimum rank in the variable, and X_max_ is the maximum rank in the variable.

We aggregated the standardized ranks in each indicator to create a composite indicator per pathogen- antimicrobial class-livestock species for each country.

### Designation of ‘priorities’

The top 10% of ranked categories per country were designated as priorities and were compared to the livestock AMR data. Three classifications detailing this alignment were identified: prioritized categories without data, prioritized categories with data, and non-prioritized categories with data. This comparison was also propagated to a global level. Please refer to SM1 for more details on the creation of the composite indicator and priorities, and to SM2 for national level figures.

## Results

### Livestock AMR data gaps

**Fig. 2:**
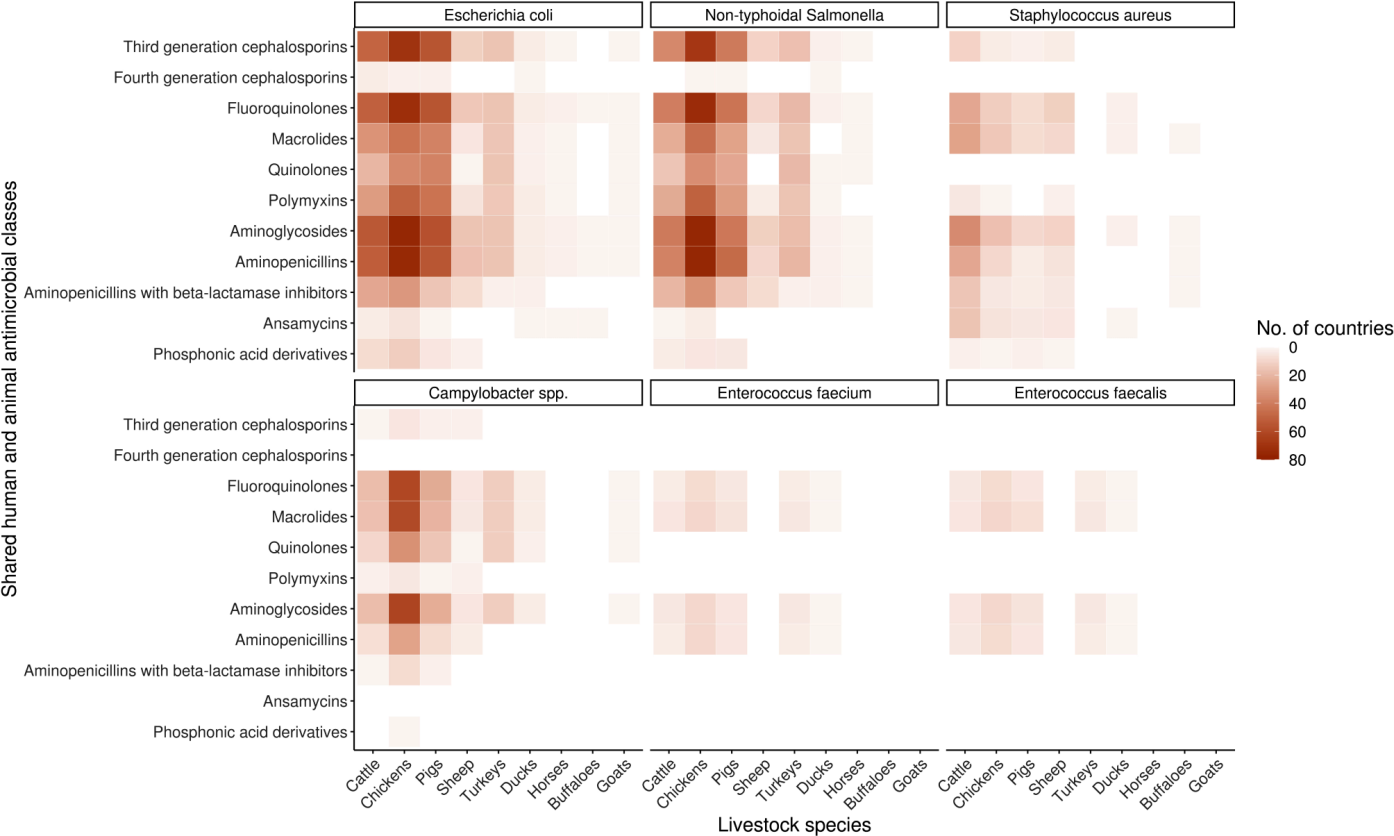
Livestock AMR data coverage for all countries extracted (*n* = 109) aggregated by livestock species, antimicrobial classes and pathogen species. Antimicrobials are ordered top to bottom by shared human and animal relevance. Dark brown shows the most number of countries that have AMR data for the specific antimicrobial class, pathogen, and livestock species. Lighter brown and peach indicate less number of countries with AMR data for the specific antimicrobial class, pathogen, and livestock species, and white shows there are no countries from data extracted that have AMR data for the specific antimicrobial class, pathogen, and livestock species

Eleven antimicrobials were identified of shared importance in both human and veterinary use: third generation cephalosporins, fourth generation cephalosporins, fluoroquinolones, macrolides, quinolones, polymyxins, aminoglycosides, aminopenicillins, aminopenicillins with beta-lactamase inhibitors, ansamycins, and phosphonic acid derivatives. The pathogens for which livestock AMR data was available for were: *Campylobacter* spp., *Escherichia coli*, *Enterococcus faecalis*, *Enterococcus faecium*, non-typhoidal *Salmonella*, and *Staphylococcus aureus*. With 109 countries (61% LMICs, 39% HICs) reporting livestock AMR data, the majority of data were specific to cattle, chickens, and pigs, with an emphasis on *E. coli* and non-typhoidal *Salmonella* resistance (Fig. 2).

Aminoglycoside-resistant *E. coli* and non-typhoidal *Salmonella*, as well as aminopenicillin-resistant non-typhoidal *Salmonella* in chickens were the most frequent categories (76 countries), followed by fluoroquinolone-resistant non-typhoidal *Salmonella* (*n* = 73), fluoroquinolone- (*n* = 72) and third-generation-cephalosporin- (*n* = 70) resistant *E. coli* in chickens. This was largely driven by high-income countries that primarily only tracked these three species and pathogens; for example, 41% of countries which reported for aminoglycoside-resistant *E. coli* in chickens were high-income (Fig. 2). Reporting on *Enterococcus* spp. and other livestock species was limited – for example, only ten countries reported data on macrolide-resistant *Enterococcus* spp. in chickens, and three countries reported data on fluoroquinolone-resistant *E. coli* in ducks. Most high-income countries did not report on resistant *S. aureus*, or only reported methicillin-resistant *S. aureus*. Reported data from LMICs were more broadly spread across categories, and some livestock species were more likely to be or were only reported through these countries (Fig. 2). Resistance data for buffaloes were only reported in China, and resistance to classes like ansamycins were reported by 22 LMICs. The scope of resistancebank.org did not include *Enterococcus* spp, hence no livestock AMR data from LMICs for this pathogen was available.

### Composite indicator patterns

When assessing human DALYs, *E. coli* resistance typically ranked higher than other pathogens; 118/194 countries ranked third-generation-cephalosporin-resistant-*E. coli* highest. There were no DALY estimates for resistant *Campylobacter* spp. With livestock AMU by country, aminopenicillin usage in sheep, and macrolide, aminopenicillin, and phosphonic acid derivatives’ usage in pigs were ranked higher compared to other categories. Usage of third and fourth generation cephalosporins were generally lower ranked than other categories. When considering livestock biomass, cattle were ranked the highest compared to chickens, pigs, and sheep in 110/194 countries. Looking at the global correlation assessment, the highest ranked categories were macrolide-resistant *S. aureus* in pigs; aminopenicillin-resistant *E. coli* in cattle and chickens; aminopenicillin-and-beta-lactamase-inhibitor-resistant *E. coli* in cattle; fluoroquinolone-resistant *E. coli* in cattle, chickens, and pigs; and third-generation-cephalosporin-resistant *E. coli* in cattle, chickens, and pigs. Categories using sheep AMR data like third-generation-cephalosporin-resistant *E. coli* in sheep were not significant and therefore ranked low.

**Fig. 3:**
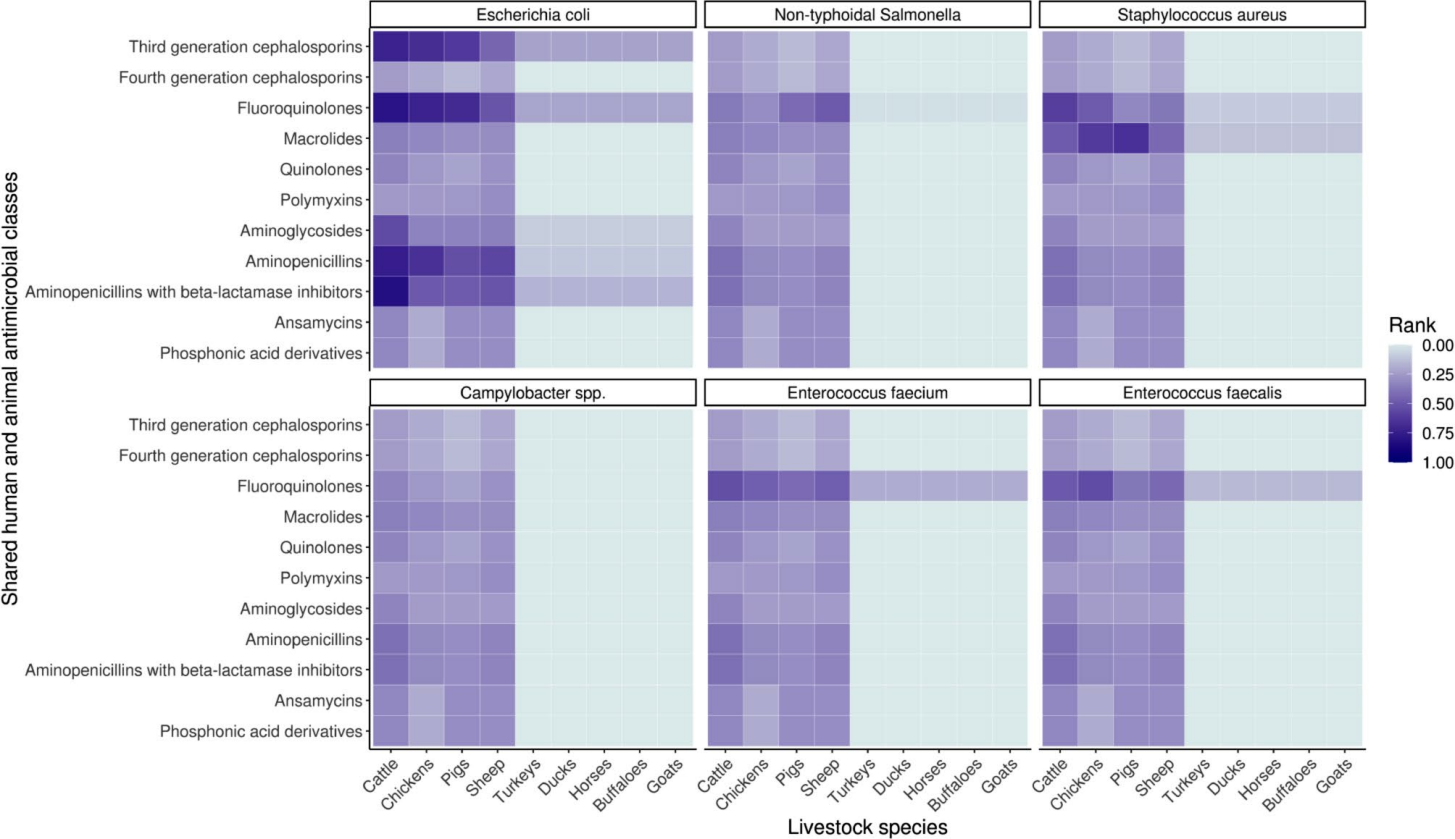
Global metaranks (*n* = 194) accounting for livestock antimicrobial usage (AMU), livestock biomass (population correction units), human DALYs attributable to AMR, and significance levels of correlations between human and livestock proportion of resistance. Global metaranks were calculated for a particular livestock species, antimicrobial classes and pathogen species combination. Antimicrobials are ordered top to bottom by shared human and animal relevance. Dark purple shows the highest metaranks calculated for the specific antimicrobial class, pathogen, and livestock species. Lighter purple and blue indicate a lower metarank for the specific antimicrobial class, pathogen, and livestock species

In aggregating these globally, fluoroquinolone-resistant *E. coli* in cattle was the highest ranked category, followed by aminopenicillin-and-beta-lactamase-inhibitor-resistant *E. coli* in cattle, and third-generation-cephalosporin-resistant *E. coli* in cattle (Fig. 3). Many patterns prevailed in the top ten ranked categories, where fluoroquinolone-, third-generation-cephalosporin-, and aminopenicillin-resistant *E. coli* in cattle, chickens, and pigs, as well as macrolide-resistant *S*. *aureus* in pigs were highest ranked in comparison to other categories in several countries. Resistant *Campylobacter* spp. and *Enterococcus spp.* in buffaloes, ducks, goats, horses, and turkeys across all considered antimicrobial classes were lower ranked compared to their other counterparts.

### Alignment of priorities and livestock AMR data gaps

In assessing our priorities generated from the top 10% of values from the composite indicator per country, we prioritized aminopenicillin-resistant *E. coli* in cattle, chickens, and sheep; aminopenicillin-and-beta-lactamase-inhibitor-resistant *E. coli* in cattle; fluoroquinolone-resistant *E. coli* in cattle, chickens, and pigs; third-generation-cephalosporin-resistant *E. coli* in cattle, chickens, and pigs; and macrolide-resistant *S. aureus* in pigs for all countries.

**Fig. 4:**
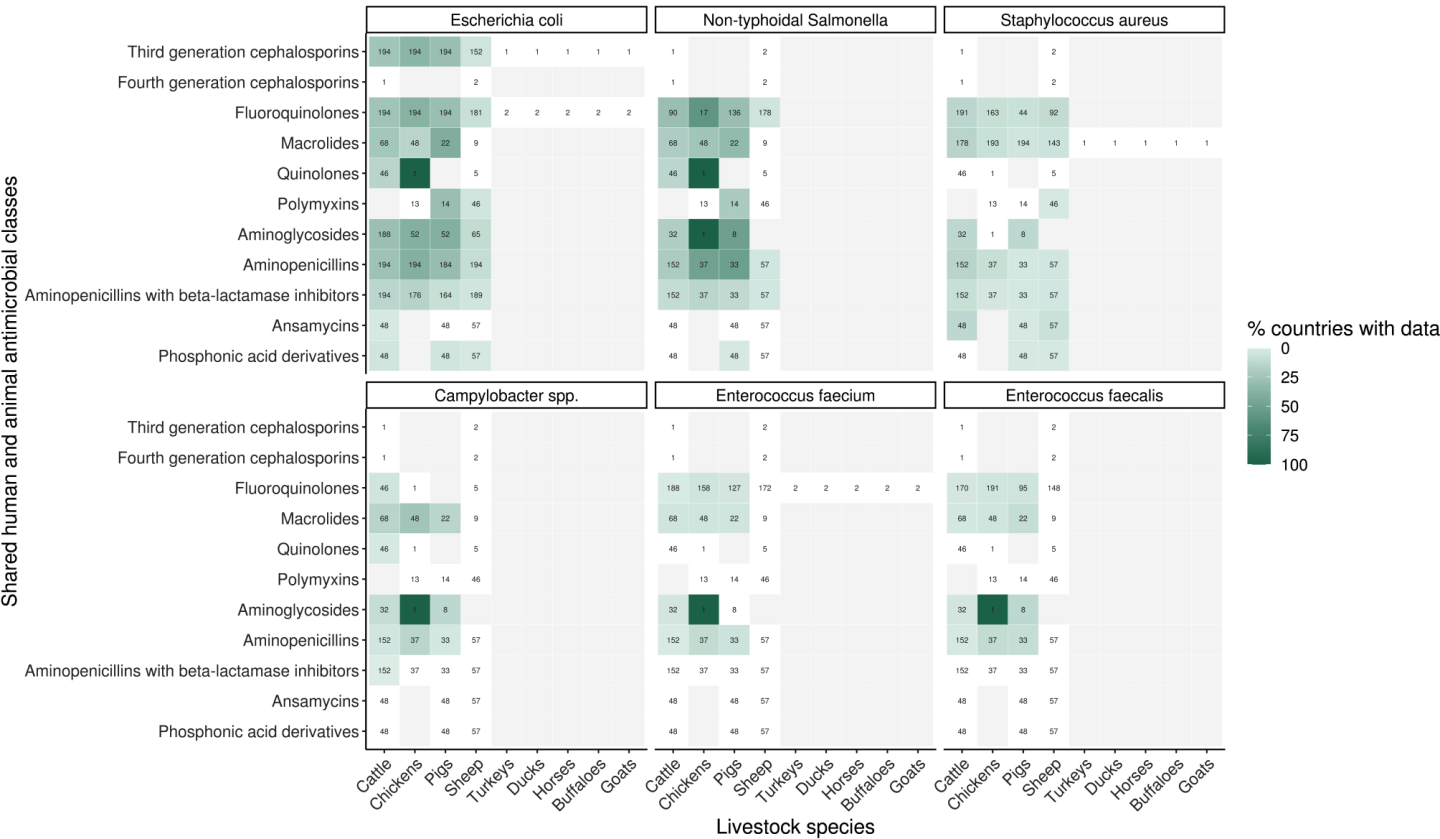
Global percentage of countries (*n* = 194) with data for a particular livestock species, antimicrobial classes and pathogen species combination if it was prioritized. Antimicrobials are ordered top to bottom by shared human and animal relevance. White and lighter colors relay a low percentage of countries with data, and darker colors indicate a higher percentage of countries with data. Cells for which no countries have prioritized the category are grey, and have no numbers stated. Numbers in each cell correspond to the number of countries that have prioritized that particular category

Of 232 categories prioritized in at least one country, data were only collected for 48% (*n* = 112) (Fig. 4). Macrolide-resistant *S. aureus* in chicken and pigs particularly had notable mismatches between prioritization and data collection; only 7% and 4% of countries collected data in these categories respectively despite being globally prioritized. Additionally, less than 15% of countries collected data for fluoroquinolone-resistant *S. aureus* in cattle (13%), chickens (8%), and pigs (7%). Fluoroquinolone-resistant non-typhoidal *Salmonella* in sheep was also similarly misaligned, where only 6% of the 178 countries that prioritized the category collected data for it. On the other hand, aminopenicillin- and fluoroquinolone-resistant *E. coli* data in chickens were reported the most by countries when prioritized at 39% and 37% respectively (at least 72/194 countries).

**Fig. 5:**
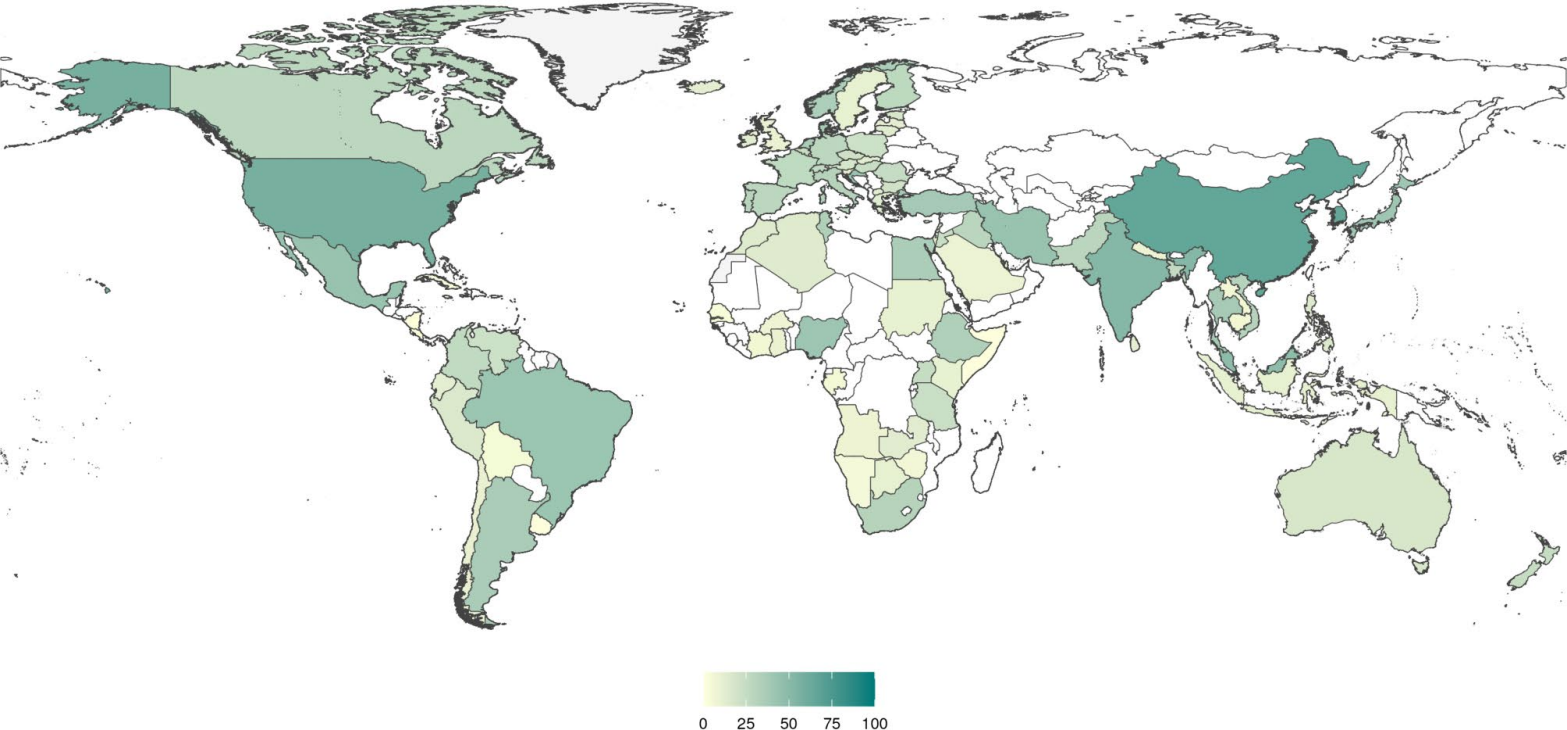
Map showing the percentage of priorities that have recorded livestock AMR data for a particular category of antimicrobial class, pathogen, and livestock species per country. Darker colors (more blue-grey) show a higher percentage of countries with livestock AMR data for prioritized categories, lighter colors (lighter blue and yellow) shows a lower percentage. White represents countries without livestock AMR data for prioritized categories, and locations without any prioritized categories are colored light grey

No country had livestock AMR data for all prioritized categories determined; China and the Republic of Korea had the highest percentage of prioritized categories with data at 66% (40/61) and 65% (39/60) respectively (Fig. 5). Approximately half or 95 of 194 countries (49%) did not have data for any of their indicated national priorities, whilst three quarters of countries had less than 20% of identified priorities with data.

## Discussion

Our study aimed to identify key gaps where strengthening AMR surveillance in livestock could provide potential benefits to both livestock and human AMR. There has been growing evidence of significant, bi-directional interactions between livestock and human AMR and AMU, therefore we wanted to account for these elements with specificity by pathogen-antimicrobial-class-livestock [8, 18]. We can then focus our attention onto data gaps within categories that we prioritized to engage data collection efforts in globally relevant areas in the human-livestock space, and better direct and utilize funds for global livestock AMR surveillance.

The most notable data gaps we found for prioritized categories were for *S. aureus*, where especially macrolide- and fluoroquinolone- resistant *S. aureus* in chickens and pigs had limited data in relation to their prioritization. Macrolides and fluoroquinolones have been identified as antimicrobials of high concern in both human and veterinary medicine [13, 14]. Macrolides are important in treating several common infections in pigs such as pneumonia, and the genetic mechanism encoding macrolide resistance in *S. aureus* has been identified as a global concern in due to the potential for horizontal gene transfer between bacterial species and genera [19]. Resistance to fluoroquinolones is also concerning because of the class’ use in treating a wide range of pathogens and infections in both human and veterinary medicine [20]. While currently most HICs only report data for methicillin-resistant *S. aureus* (MRSA) strains, collection efforts in these systems can be expanded to include *S. aureus* resistant to other antimicrobials in addition to methicillin. With *S. aureus* among the leading causes of human deaths due to AMR, expanding data monitoring to other antimicrobials can allow for better assessment of livestock resistance trends and potential impacts on human AMR [1]. Furthermore, we identified fluoroquinolone-resistant non-typhoidal *Salmonella* in sheep as an area for several countries to prioritize AMR data collection in. Fluoroquinolone-resistant *Salmonella* has been identified as a high priority pathogen by WHO, and could pose a zoonotic threat through human ingestion of contaminated animal products [21, 22]. Although limited countries have economically significant and sizeable sheep populations which has impacted the creation of livestock-based estimates, expanding monitoring efforts to include sheep samples in these countries could appropriately assess concern for this category [7].

Our prioritization framework provides a guide for countries to improve and target surveillance efforts to areas of most need. In the majority of countries that did not have livestock AMR data for prioritized categories, we found no reports or monitoring systems tracking livestock AMR. The majority of these were also LMICs, which often face additional technological, financial and resource limitations that impact the setup and operation of these monitoring systems [23]. Particularly with capacity limitations in clinical laboratories to perform microbiological cultures in LMICs and disconnect between clinical and laboratory data, this may result in missed detection of AMR-caused infections that contribute to accurate surveillance [24, 25]. With LMICs often having high burden due to AMR, expanding both clinical laboratory and surveillance infrastructure, increasing coordination between the two sectors, and accordingly enacting control strategies will be critical in assessing and tackling the threat of AMR in these countries [1, 25, 26]. Countries can model their efforts after existing task forces such as the Mapping AMR and AMU Partnership consortium, which has helped build capacity in 14 African countries, identifying critical gaps in infrastructure and providing actionable steps to improve capacity in tandem with major organizations [25]. Choosing pathogens, antimicrobial classes, and livestock species of greatest concern through prioritization can factor into the allocation of limited funds and resources in these countries.

For other countries where livestock AMR data is available for prioritized categories but missing data for others, existing systems can be broadened to track data that reflects all local concerns. No country had 100% alignment in livestock AMR data for prioritized categories. An area where countries are extensively monitoring livestock AMR that matches up with our global priorities is aminopenicillin- and fluoroquinolone-resistant *E. coli* monitoring in cattle, chickens, and pigs. This is supported by other reports where WHO has identified *E. coli* as a critical pathogen; significant links have been found between humans and livestock for fluoroquinolone- and aminopenicillin-resistant *E. coli*, and cattle, chickens, and pigs have been shown to contribute to over 90% of global animal biomass [7, 8, 21]. If similar efforts can be expanded to other areas of priority with limited data, we can better parse livestock resistance trends and corroborate areas of concern to implement behavioral interventions. Many countries have the capabilities and infrastructure to monitor AMR, as seen in examples where livestock AMR data was available for rarely prioritized categories such as fluoroquinolone-resistant non-typhoidal *Salmonella* in chickens; they can utilize these to strengthen categories with currently limited data.

In order to assign relevant priorities to and build dynamic surveillance systems, the framework outlined here should be conducted periodically to reflect changes in livestock usage and biomass patterns, as well as resulting human AMR burden. Other antimicrobial classes and pathogens may become a greater concern in the future and may therefore warrant identification as a new priority; performing this exercise iteratively can help adjust national and global priorities so that appropriate infrastructure can be expanded to include new surveillance categories.

### Limitations

The individual factors used in our study had limitations based on availability of data used to estimate them, which impacted prioritization. For example, DALYs attributed to resistant *Campylobacter* spp. were not available due to limited data and thus received the lowest rank, decreasing its likelihood for prioritization despite the pathogen being a shared concern in human and livestock sectors [1]. Facilitating data collection and reporting for these factors can better ensure priorities reflect current trends. Furthermore, with *Enterococcus* spp. data in LMICs not reflected in resistancebank.org, we were unable to assess if data gaps in categories pertaining to the pathogen were reflective of global monitoring efforts, or an artifact of the data sources we considered. With categories like fluoroquinolone-resistant *E. faecalis* in chickens being globally prioritized, including additional data sources detailing *Enterococcus* spp. resistance could better highlight true mismatches in livestock AMR data and our intended priorities.

When expanding beyond our analysis to consider the set of possible factors that are hypothesized to influence the transmission of resistant pathogens from livestock to humans, the constraint of global, quantitative data, becomes even more severe, and in some instances, data is incredibly limited. Not all farm-level contexts will be comparable, therefore further efforts to differentiate the risks represented by intensive farming systems versus extensive practices would be an important future dimension. Our current framework does not describe any possible differences between one herd of 1000 cows on one farm versus 10 herds of 100 cows in ten unconnected farms. Further epidemiological investigations are necessary to differentiate the relative impact of different transmission pathways too, whether food-borne, direct transmission, or even thinking about antimicrobial resistance as an environmental-scale problem where livestock usage of antibiotics contributes to broader resistance development, not just those mediated through livestock hosts. National-level policies may influence the relative importance of different sectors, or the ongoing relevance of priority sectors over time due to successful stewardship actions. Finding ways of tracking the existence of legislation, and more importantly, ongoing compliance with such legislation, will be an important feature of a future system more dynamically re-evaluating priority areas.

We also propagated assumptions for each indicator; all animals assumed to result in the same number of human DALYs for a particular antimicrobial class-pathogen combination, and correlations between livestock and human AMR were not detailed by transmission pathways for example. Contact rates between livestock and humans can vary with different production systems that can impact comparability between countries, and AMR transmission rates could differ between direct contact with livestock versus consumption of animal-sourced foods. Source attribution studies can help elucidate the differences between these modes of transmission, and annotated risks obtained from these varying by country and sector for example could replace the current correlation indicator to create more tailored priorities [27, 28]. Finally, where data or estimates were absent, we assumed that these categories had the lowest rank. While in many instances we believe it is likely that this assumption is correct, and that the absence of data is indicative of that drug being less widely used, or that livestock species being rarer than the others, there is the potential for specific combinations to currently be inappropriately characterized. While some categories require complete usage of this lowest rank, we do see overlapping ranges of country rankings in spite of their ranks being the end result of different amounts of assumptions, and therefore, we believe that the patterns we observe are meaningful and can support actions taken today, while also being sufficiently flexible to being revised should data become available, or estimates be revised. For more information related to this discussion, please reference SM1.

While it was important to incorporate a dimension that characterizes the specific risk that the animal sector has for a specific category among humans, observed data characterizing this is rare and certainly not globally complete for all our categories. We have relied on correlation as a proxy here. Should this element be removed however, the global results and global priority categories remain broadly consistent (categories that were in the top 10 remain in the top 25 for instance), and relative priorities among sectors remain intact. The main variation is seen when looking at the exhaustive list of priority categories within a specific country where some specific categories fall in the list, and others replace them - such variation however is to be anticipated within a composite indicator, and will naturally happen as and when further updates are incorporated.

Many organizations have outlined the need for a One Health approach to align human, animal, and environmental sectors together in defining national capacities and performance of systems related to AMR [29, 30]. We only focused on the human-livestock space, and thus did not include aquaculture, environmental samples, wild animals or pets. Considering these sectors in a similar indicator approach could give a more comprehensive perspective on how incomplete our current understanding of the risk of environmental transmission in propagating human AMR is. As we also needed to incorporate factors with global coverage in the creation of our composite indicator, we were unable to include other variables such as veterinary services and pathogen-specific characteristics that could further elucidate country-specific interventions for AMR. Additionally, in our consolidation of non-LMIC sources of livestock AMR data, we recognize that there may have been sources that were not captured in our exercise. For EU countries, most livestock AMR data were extracted from the European Food Safety Authority (EFSA) reporting system. Data were limited by requirements at time of submission by countries and may not entirely reflect what is collected at country level. Though we supplemented wherever possible with national reports for combinations of pathogen, antimicrobial class, and livestock species not reported in EFSA, further validation of livestock AMR data with national surveillance efforts in European countries can better identify data gaps. We also only used resistancebank.org for LMIC data sources as it is the most comprehensive database of livestock AMR data available for these countries, however, it is possible that additional resources for these countries may be reported elsewhere. A key part of utilizing this assessment is in consulting national data repositories to determine whether gaps identified in this exercise remain so. And though we were still able to identify meaningful global priorities, countries can repeat this methods framework as necessary to include both newer livestock AMR data and updated estimates for variables utilized in the prioritization framework and re-define national priorities.

## Conclusions

Our study provides a framework for countries to review and strengthen the collection of livestock AMR data aligned with local priorities relevant in the possible transmission of AMR from livestock to humans. When coupled with existing efforts in increasing capacity and coordination between clinical and monitoring infrastructure particularly in resource-limited settings, this can be a powerful tool for funding and resource allocation that prioritizes areas of most concern. With changing trends in resistance and constituent factors, regular evaluation of priorities can help countries dynamically adapt and address emerging threats as AMR continues to evolve.

## Electronic supplementary material

Below is the link to the electronic supplementary material.

Supplementary Material 1

Supplementary Material 2

## Data Availability

All livestock AMR data evaluated were obtained from open-access surveillance reports and data repositories that are listed and cited in full in SM1. The final dataset used in analyses can be obtained upon reasonable request from the corresponding author.

## Abbreviations

AMR: Antimicrobial resistance
AMU: Antimicrobial usage
LMICs: Low- and middle- income countries
WHO: World Health Organization
WOAH: World Organisation for Animal Health
DALYs: Disability adjusted life-years
HIC: High-income countries
SM1: Supplementary Material file 1
GRAM: Global Research on Antimicrobial Resistance
EFSA: European Food Safety Authority
