# Supplementary material for: Using priorities between human and livestock bacterial antimicrobial resistance (AMR) to identify data gaps in livestock AMR surveillance
Source: BMC Infect Dis. 2024 Sep 26;24:1027. doi: 10.1186/s12879-024-09847-3 (PMC11425882; doi:10.1186/s12879-024-09847-3)
Supplement: Supplementary file 1 — Supplementary Material 1 [file 12879_2024_9847_MOESM1_ESM.docx]

**Supplementary material 1**

This is the supplementary material file 1 to “Using Priorities between Human and Livestock Bacterial Antimicrobial Resistance (AMR) to Identify Data Gaps in Livestock AMR Surveillance”. Contained here are additional materials referenced from the manuscript, including methods and additional figures.

**Table of Contents**

[**Detailed methods** 1](#_Toc175172566)

[**Livestock data collection and classification** 1](#_Toc175172567)

[**Ranking and prioritization exercise** 2](#_Toc175172568)

[**Rank imputations and sensitivity analyses** 4](#_Toc175172569)

[**References** 6](#_Toc175172570)

[**Figures** 9](#_Toc175172571)

[**Classification of shared antimicrobial classes in human and veterinary medicine** 9](#_Toc175172572)

[**Data sources and classification** 10](#_Toc175172573)

[**Correlation assessment** 18](#_Toc175172574)

[**Global misalignments in livestock AMR data and created priorities** 20](#_Toc175172575)

[**Rank imputations and assumptions** 23](#_Toc175172576)

# **Detailed methods**

## **Livestock data collection and classification**

For livestock AMR data we consulted a variety of databases. Data collected from the year 2000 and later was included in our scope. Low-and-middle-income country (LMIC) AMR data, where scientific literature dominates, was sourced from a prior literature review exercise, housed in resistancebank.org (1–3). AMR data for the years 2000-2021 was collected for *Escherichia coli*, non-typhoidal *Salmonella*, *Staphylococcus aureus*, and *Campylobacter* spp. The year of reporting was chosen based on the sampling end date recorded in the database; if this information was not available then the start date was chosen, and if neither information was available then the date was imputed to be 3 years earlier than the recorded publication date. This imputation was selected based on the median difference between the start date and the publication date for resistancebank.org surveys with this information. For each survey, the livestock species surveyed, the pathogens identified, the drugs each pathogen was evaluated against and the proportion of those that were resistant are reported, in addition to a variety of pertinent metadata associated with sampling site, whether the animal was alive, or whether related product was tested. For non-LMIC countries, we went to national or regional surveillance programs to identify data. The livestock species included across all databases were cattle, chickens, pigs, turkeys, sheep, ducks, horses, buffaloes, and goats, with samples and isolates taken from both live animals, carcasses and food products, all of which were incorporated into the analysis. Only healthy animals (non-diseased) from indicator bacteria were included in our scope. Genetic strains conferring additional resistance (such as extended spectrum beta-lactamase (ESBL)-producing *E.coli*) were not differentiated from other types of resistant pathogens.

Data for European countries was primarily extracted from the European Food Safety Authority and European Centre for Disease Prevention and Control surveillance reports (4–6). AMR data for the years 2016-2020 was collected for *E.coli*, non-typhoidal *Salmonella*, *Enterococcus faecalis*, *Enterococcus faecium* and *Campylobacter* spp. for species cattle (general bovines and calves under 1 year old), chicken (broilers, breeders, and laying hens), turkeys (fattening), and pigs (fattening) for carcass, meat, and live samples. Additional AMR data from 2016-2020 was supplemented from national reports where available to obtain resistance values for combinations of pathogen, antimicrobial class, and livestock species not reported in EFSA. Data from the following countries’ national surveillance systems and reports were extracted based on this criteria: Denmark (DANMAP), Finland (FINRES-VET), Netherlands (MARAN), and Norway (NORM-VET) (7–25). Data for resistant *E. coli*, *E. faecalis*, *E. faecium*, *Campylobacter* spp., and non-typhoidal *Salmonella* for the United States of America (USA) was extracted from the National Antimicrobial Resistance Monitoring System (NARMS) database, an integrated surveillance system tracking enteric pathogens causing AMR (26). AMR phenotypic data for years 1997-2019 was extracted for retail, product and cecal samples. Data from 2014-2019 for Canada was extracted from the Canadian Integrated Program for Antimicrobial Resistance Surveillance (CIPARS) database, an integrated surveillance system (27–31). Data was collected for *E.coli*, *Campylobacter* spp., and non-typhoidal *Salmonella* AMR data was collected from retail meat, farm, abattoir and clinical surveillance for chicken, cattle, pig, turkey, and horse isolates. Data from 2014-2017 was extracted for Japan from the Japanese Veterinary Antimicrobial Resistance Monitoring System (JVARM) for pathogens *E.coli*, *E.faecalis*, *E.faecium*, *Campylobacter* spp., and non-typhoidal *Salmonella*, and livestock species cattle, chickens (broilers and layers), and pigs (32,33). Data from 2010-2020 was extracted for South Korea from the Korea Disease Control and Prevention Agency (KDCA) for pathogens *E. coli*, *E. faecalis*, *E. faecium*, *Campylobacter* spp., non-typhoidal *Salmonella* and *S. aureus* and livestock species cattle, chickens, pigs and ducks (34). Carcass and fecal samples were included from live animals and domestic food product data was included. Available data from 2010 was extracted from New Zealand from a survey compiling isolate data collected from fresh carcasses and food products for *E.coli*, *Campylobacter* spp., and non-typhoidal *Salmonella* from species cattle (very young calves), pigs, and chicken (35). Available data from 2004 was extracted from a pilot surveillance program in Australia looking at bacteria of animal origin (36). Data was extracted for the pathogens *E.coli*, *E.faecalis*, *E.faecium*, and *Campylobacter* spp.For a list of all the countries represented in the databases, see Table 1. Data distinguishing by production system was not considered in this analysis.

Antimicrobial class refers to a set of related antimicrobial agents often grouped by similarities in chemical structure, an example being fluoroquinolones. Antimicrobial compounds refer to the specific antimicrobial agent itself, an example being ciprofloxacin. Some antimicrobial compounds listed in databases are a combination of two antimicrobial compounds (e.g. cefepime and tazobactam); this notation has been preserved and the class notation includes the classes of both compounds (e.g. second generation cephalosporin with beta-lactamase inhibitors). Compounds used exclusively in veterinary medicine, and compound combinations that were only used in veterinary medicine were excluded (e.g. the combination lincomycin and spectinomycin is mainly authorized to treat pigs and/or poultry even though each compound alone is used in human medicine as well).

resistancebank.org notation categorizing antimicrobial class to compound was used for the other databases for the livestock categorization of antimicrobial class and compound, and analysis presented is on the antimicrobial class level. Fluoroquinolones were distinguished from quinolones as having a nitrogen atom in replacement of the eighth carbon atom backbone, as well as the addition of a fluorine atom (37). This distinct was applied to the analysis, starting from the prioritization exercise. Other compounds identified in other data sources not classified in resistancebank.org were defined according to similarities with other compounds listed in classes and verification online. Given differential classification, not all pathogen and antimicrobial combinations present in any single repository are necessarily present in each other database. Consequently, we have to match and aggregate different databases to match like-with-like. For example, one of the human estimates is specific to *S. aureus* and vancomycin (listed as the antimicrobial class in the GRAM project) (38). Vancomycin is defined as a glycopeptide according to the livestock categorization, and so livestock AMR data had to be manipulated to only consider vancomycin in the class of glycopeptides to be comparable to the human estimate. For more information regarding detailing database alignment of pathogens, and designated antimicrobial classes, refer to Table 2.

## **Ranking and prioritization exercise**

*Human DALYs attributable to AMR*

Following the first phase of the GRAM project’s efforts to model the burden of antimicrobial resistance in humans, we used 2019 estimates for the disability-adjusted life years (DALYs) attributable to AMR (38). These estimates were specific to country, antimicrobial class, and pathogen. The dataset was first cleaned and processed to only include the pathogens and antimicrobial classes identified as mutual concerns in human and animal health. We then ranked the combinations with the most DALYs per country. When applying these rankings to additional stratifications by livestock species, we assumed that every livestock species gets the same ranking within a particular antimicrobial class-pathogen combination and country specification. For example, the ranking for aminopenicillins, *E.coli* and cattle in Argentina would be the same as aminopenicillins, *E.coli* and chickens.

*Livestock antimicrobial usage (AMU)*

2020 national estimates for average consumption of antimicrobials per kilogram of animal produced (mg/PCU) were obtained for livestock species cattle, chickens, pigs, and sheep (39). These were specific to country, antimicrobial class, and livestock species. Chickens and pigs had additional stratifications in national estimates of AMU by intensive and extensive production; cattle and sheep did not. We ranked the highest AMU values per country. We first separated the dataset into extensive and intensive AMU data frames, and included cattle and sheep AMU values in both. We then ranked within each data frame, then averaged the rankings between the two dataframes for a particular country, livestock species, and antimicrobial class. Some antimicrobial classes estimates were generated encompassed two or more specific classes in our scope; we ranked with the existing classes and values, and assumed specific classes within had the same ranking. Classes for which this was done was third and fourth generation cephalosporins, penicillins (aminopenicillins, aminopenicillins with beta-lactamase inhibitors), quinolones (fluoroquinolones, other quinolones), other antibiotics (phosphonic acid derivatives, ansamycins). When applying these rankings to additional stratifications by pathogen, we assumed that every pathogen species gets the same ranking as a particular antimicrobial class and livestock species. For example, the ranking for aminopenicillins, *E.coli* and cattle in Argentina would be the same as aminopenicillins, *S.aureus* and cattle.

*Livestock biomass*

2020 estimates for population correction units (PCU), a biomass-correlationary measure, were obtained for cattle, chickens, pigs, and sheep (39). PCU is the total number of animals in a country, multiplied by the average weight of the animal at the time of treatment with antimicrobials. PCU estimates were specific to livestock species and country. Rankings were calculated of the livestock species with the highest PCU per country. When applying these rankings to additional stratifications by antimicrobial class and pathogen combinations, we assumed that every antimicrobial class and pathogen gets the same ranking within a particular livestock species and country specification. For example, the ranking for aminopenicillins, *E.coli* and cattle in Argentina would be the same as the ranking for fluoroquinolones, non-typhoidal *Salmonella* and cattle in Argentina.

*Significance of correlations between livestock and human AMR*

In order to compare livestock and human AMR, we used the proportion of resistance in both matched by year to generate scatterplots. For human AMR, we used estimates for the proportion of infections by a given pathogen that are resistant to a specific antimicrobial class. These estimates are available for the years 1990-2018, and are used for assessing the relationship between livestock and human metric data in a specific pathogen and antimicrobial combination (38). While the paper published only shows the most recent year results, estimates for the years 1990-2018 were calculated as part of their preparation from which we were able to source these estimates for. Human estimates for 2019 and 2020 were imputed to have the same estimates reported in 2018 for each antimicrobial class and pathogen combination and each country. Human AMR estimates due to resistant *Campylobacter* spp. were not generated, thus the pathogen was not considered in any future analysis. Similarly, human estimates for resistant pathogens to the One Health antimicrobial classes were not generated for classes fourth generation cephalosporins, polymyxins, ansamycins, and phosphonic acid derivatives, and so analyses were not included for these.

For livestock AMR, we calculated the average percentage of antimicrobial resistant isolates found in livestock animal sources by reported year, country and antibiotic class-pathogen combination. Initially there were 56342 data points for all livestock AMR data. An initial sensitivity analysis removed extreme livestock AMR metric values (classified as either 0% or 100%) with 5 or less isolates. 7592 data points were removed as a result of this sensitivity analysis. Additionally, we also implemented the assumption that resistance for one compound in a class naturally conferred resistance to others. In cases where the same sample was tested across two or more antimicrobials in the same class, only the resistance results of the one with the highest reported resistance was kept. Following this, 9910 data points were further removed, leaving the total number of data points for percentage of resistance in livestock at 38840. In joining with human AMR estimates, there were 9595 data points in total.

Livestock AMR data points were weighted with two variables, the total number of isolates present in each country specific to the reporting year, antimicrobial class, and pathogen combination, and PCU. The proportion of PCU for cattle, chickens, pigs, and sheep in relation to the total PCU per country in 2020 was used to weight the livestock AMR metric for livestock-specific points.^18^ Only data for cattle, chickens, pigs, and sheep were retained in the comparison after this weighting; this left 3669 data points in total.

Spearman correlations and p values were calculated for a particular antimicrobial class, pathogen, and livestock species. Statistical significance was denoted as follows: p<0.0001 (****), followed by p<0.001 (***), then p<0.01 (**), then p<0.05 (*). These categories of statistical significance were then ranked by the highest significance level and if they were positive. Negative correlations were given the lowest rank. Each country was assumed to have the same ranking for a particular antimicrobial class, pathogen, and livestock species. Refer to Supplementary Table 3 for these results.

*Creation of composite indicators*

For each category of antimicrobial class, pathogen, and livestock species per country, the standardized ranks from each individual factor were summed up to create country metaranks for each category. These country metaranks were summed up for each category to create global metaranks per country for each category.

*Priority ranking and monitoring data comparison*

The country metaranks for each category were first converted to binary form by taking priorities in the 90th percentile and above for each country. We then compared these to the binary AMR livestock monitoring data and created two comparison variables: prioritized categories with monitoring data and prioritized categories without monitoring data.

On the global scale, we created the same comparison variables by summing the number of countries that had prioritized categories with monitoring data, prioritized categories without monitoring data, and non-prioritized categories with monitoring data.

## **Rank imputations and sensitivity analyses**

When creating the metarank as mentioned above, for categories for which there were no estimates available for any of the factors, we imputed these to have the lowest rank. We calculated the sum of rank imputations for each country and category (same dimensions as our analysis in the main manuscript has been conducted in), where each country and category could have a maximum of 4 imputations that were used to generate a metarank.

We wanted to explore the impact of two decisions in our infrastructure:

1. the assignment of the lowest rank in situations where a factor had no data for the relevant category
2. the inclusion of the correlation factor in our composite indicator

Supplemental Figures 5 and 6 help describe this situation further. Supplemental Figure 5 indicates which categories had to invoke this assumption, and to what extent. Supplemental Figure 6 provides a boxplot showing the interquartile range, and range of countries with data within that assumption level. Unsurprisingly, we see strong structuring in metaranks as a function of the number of assumptions made. Combinations where all categories had to be assumed, such as the combination of *Enterococcus faecium* resistance to macrolides in goats, has a metarank of zero, definitionally so; there are no biomass estimates for goats, no AMU estimates for goats and macrolides, no human AMR DALYs associated with *Enterococcus faecium* and macrolides, and no livestock and human proportion of resistant isolates needed for the correlation assessment. As we lessen the number of assumptions made on imputation we see a progressive increase in mean global metarank, in a manner that is expected, and intended. Nevertheless, in spite of these strong structures leading to predictable differences in the mean, when we consider the interquartile range and the full range of the data, there is overlap between one category and the other, with some countries where a full set of data is incorporated having a metarank lower than that of even some countries where three inputs have been assumed, and have the “worst-case” assumption.

In evaluating the impact of the inclusion of the correlation factor in the composite indicator, we opted to compare and contrast two versions of the composite indicator, one with, and one without, the correlation factor included. We re-evaluated key results from the main manuscript (as seen in Supplemental Figures 7 and 8). With the original correlation dimension included, we noted that fluoroquinolone-resistant *E. coli* in cattle was the highest ranked category, followed by aminopenicillin-and-beta-lactamase-inhibitor-resistant *E. coli* in cattle, and third-generation-cephalosporin-resistant *E. coli* in cattle and with the correlation dimension removed, all three remain in the top ten global priority categories. Our further conclusions remain broadly true “Many patterns prevailed in the top ten ranked categories, where fluoroquinolone-, third-generation-cephalosporin-, and aminopenicillin-resistant *E. coli* in cattle, chickens, and pigs, as well as macrolide-resistant *S. aureus* in pigs were highest ranked in comparison to other categories in several countries.”; all these categories remain in the top 25 of ranked categories.

Where we do see differences is in specific countries, and changes in the numbers of countries in which that category was prioritized. For example, third-generation cephalosporins-resistant *E.coli* in cattle, chickens, and pigs was prioritized in all countries (n=194) when correlation was included, however with the removal of correlation, this dropped to 189, 181, and 153 countries respectively. Similarly with fluoroquinolone-resistant *S. aureus* in chickens, the removal of correlation resulted in a drop from 191 to 163 prioritizing the category. For other categories such as fluoroquinolones and *E. faecium*, the removal of correlation increased prioritization for cattle, chickens and pigs in 191, 177, and 146 countries from 188, 158, and 127 countries respectively.

Changes in ranks, and whether specific categories drop in or out of the exhaustive listing of all national priorities is inevitable as we alter the indicators included. It is reassuring that not all conclusions are driven by the inclusion of this feature, and that broad patterns that can be drawn from the analysis of relative importance of sectors at a global scale holds true. Importantly though we wanted to incorporate this dimension into the framework so that the intersectional risk of the livestock transmission pathway could be incorporated. While we still have to make assumptions that correlations act as proxy for the risk presented by animals to humans in specific categories, we hope that further investigations on this important topic can allow for more accurate rankings in this dimension.

# **References**

1. Van Boeckel TP, Pires J, Silvester R, et al. Global trends in antimicrobial resistance in animals in low- and middle-income countries. Science. 2019 Sep 20;365(6459).

2. resistancebank.org. resistancebank.org. Accessed 6 May 2022.

3. Criscuolo NG, Pires J, Zhao C, et al. resistancebank.org, an open-access repository for surveys of antimicrobial resistance in animals. Sci Data. 2021 Jul 22;8(1):189.

4. European Food Safety Authority, European Centre for Disease Prevention and Control. The European Union summary report on antimicrobial resistance in zoonotic and indicator bacteria from humans, animals and food in 2016. EFSA Journal. 2018 Feb 1;16(2).

5. European Food Safety Authority, European Centre for Disease Prevention and Control. The European Union Summary Report on Antimicrobial Resistance in zoonotic and indicator bacteria from humans, animals and food in 2017/2018. EFSA Journal. 2020 Mar 1;18(3).

6. European Food Safety Authority, European Centre for Disease Prevention and Control. The European Union Summary Report on Antimicrobial Resistance in zoonotic and indicator bacteria from humans, animals and food in 2019–2020. EFSA Journal. 2022 Mar 1;20(3).

7. National Food Institute, Technical University of Denmark, Statens Serum Institut. DANMAP 2016- Use of antimicrobial agents and occurrence of antimicrobial resistance in bacteria from food animals, food and humans in Denmark. 2017.

8. National Food Institute, Technical University of Denmark, Statens Serum Institut. DANMAP 2017- Use of antimicrobial agents and occurrence of antimicrobial resistance in bacteria from food animals, food and humans in Denmark. 2018.

9. National Food Institute, Technical University of Denmark, Statens Serum Institut. DANMAP 2018- Use of antimicrobial agents and occurrence of antimicrobial resistance in bacteria from food animals, food and humans in Denmark. 2019.

10. National Food Institute, Technical University of Denmark, Statens Serum Institut. DANMAP 2019- Use of antimicrobial agents and occurrence of antimicrobial resistance in bacteria from food animals, food and humans in Denmark. 2020.

11. National Food Institute, Technical University of Denmark, Statens Serum Institut. DANMAP 2020- Use of antimicrobial agents and occurrence of antimicrobial resistance in bacteria from food animals, food and humans in Denmark. 2021.

12. Finnish Food Authority, Finnish Medicines Agency Fimea, University of Helsinki. FINRES-Vet 2016-2017: Finnish Veterinary Antimicrobial Resistance Monitoring and Consumption of Antimicrobial Agents. 2018.

13. Finnish Food Authority, Finnish Medicines Agency Fimea, University of Helsinki. FINRES-Vet 2018: Finnish Veterinary Antimicrobial Resistance Monitoring and Consumption of Antimicrobial Agents. 2019.

14. Finnish Food Authority, Finnish Medicines Agency Fimea, University of Helsinki. FINRES-Vet 2019: Finnish Veterinary Antimicrobial Resistance Monitoring and Consumption of Antimicrobial Agents. 2020.

15. Finnish Food Authority, Finnish Medicines Agency Fimea, University of Helsinki. FINRES-Vet 2020: Finnish Veterinary Antimicrobial Resistance Monitoring and Consumption of Antimicrobial Agents. 2021.

16. National Institute for Public Health and the Environment. MARAN 2017: Monitoring of Antimicrobial Resistance and Antibiotic Usage in Animals in the Netherlands in 2016. 2017.

17. National Institute for Public Health and the Environment. MARAN 2018: Monitoring of Antimicrobial Resistance and Antibiotic Usage in Animals in the Netherlands in 2017. 2018.

18. National Institute of Public Health and the Environment. MARAN 2019: Monitoring of Antimicrobial Resistance and Antibiotic Usage in Animals in the Netherlands in 2018. 2019.

19. National Institute for Public Health and the Environment. MARAN 2020: Monitoring of Antimicrobial Resistance and Antibiotic Usage in Animals in the Netherlands in 2019. 2020.

20. National Institute of Public Health and the Environment. MARAN 2021: Monitoring of Antimicrobial Resistance and Antibiotic Usage in Animals in the Netherlands in 2020. 2021.

21. Norwegian Institute for Public Health, Norwegian Veterinary Institute, University Hospital of North Norway. NORM/NORM-VET 2016. Usage of Antimicrobial Agents and Occurrence of Antimicrobial Resistance in Norway. 2017.

22. Norwegian Institute for Public Health, Norwegian Veterinary Institute, University Hospital of North Norway. NORM/NORM-VET 2017. Usage of Antimicrobial Agents and Occurrence of Antimicrobial Resistance in Norway. 2018;1890–9965.

23. Norwegian Institute of Public Health, Norwegian Veterinary Institute, University Hospital of North Norway. NORM/NORM-VET 2018. Usage of Antimicrobial Agents and Occurrence of Antimicrobial Resistance in Norway. 2019;1890–9965.

24. Norwegian Institute of Public Health, Norwegian Veterinary Institute, University Hospital of North Norway. NORM/NORM-VET 2019. Usage of Antimicrobial Agents and Occurrence of Antimicrobial Resistance in Norway. 2020;1890–9965.

25. Norwegian Institute for Public Health, Norwegian Veterinary Institute, University Hospital of North Norway. NORM/NORM-VET 2020. Usage of Antimicrobial Agents and Occurrence of Antimicrobial Resistance in Norway. 2021;1890–9965.

26. Food and Drug Administration. NARMS Now: Integrated Data. <https://www.fda.gov/animal-veterinary/national-antimicrobial-resistance-monitoring-system/narms-now-integrated-data>. Accessed 10 Oct 2022.

27. Government of Canada. Canadian Integrated Program for Antimicrobial Resistance Surveillance (CIPARS) 2014: Annual report. Guelph; 2016.

28. Government of Canada. Canadian Integrated Program for Antimicrobial Resistance Surveillance (CIPARS) 2015: Annual report. Guelph; 2017.

29. Government of Canada. Canadian Integrated Program for Antimicrobial Resistance Surveillance (CIPARS) 2016: Annual report. 2018.

30. Government of Canada. Canadian Integrated Program for Antimicrobial Resistance Surveillance (CIPARS) 2017: Annual report. 2019.

31. Government of Canada. Canadian Integrated Program for Antimicrobial Resistance Surveillance (CIPARS) 2018: Annual report. 2020.

32. Government of Japan. Report on the Japanese Veterinary Antimicrobial Resistance Monitoring System 2014-2015. 2018.

33. Government of Japan. Report on the Japanese Veterinary Antimicrobial Resistance Monitoring System 2016-2017. 2020.

34. Korea Disease Control and Prevention Agency. One Health AMR Non-Human Surveillance System. <https://www.kdca.go.kr/nohas/en/statistics/selectIARStatisticsToSPECAFTab.do>. Accessed 19 Apr 2022.

35. Heffernan Helen, New Zealand. Ministry of Agriculture and Forestry. A baseline survey of antimicrobial resistance in bacteria from selected New Zealand foods, 2009-2010. Ministry of Agriculture and Forestry; 2011.

36. Government of Australia. Pilot Surveillance Program for Antimicrobial Resistance in Bacteria of Animal Origin. 2007.

37. Redgrave LS, Sutton SB, Webber MA, et al. Fluoroquinolone resistance: mechanisms, impact on bacteria, and role in evolutionary success. Trends Microbiol. 2014 Aug;22(8):438–45.

38. Antimicrobial Resistance Collaborators. Global burden of bacterial antimicrobial resistance in 2019: a systematic analysis. Lancet. 2022 Feb 12;399(10325):629–55.

39. Mulchandani R, Wang Y, Gilbert M, et al. Global trends in antimicrobial use in food-producing animals: 2020 to 2030. PLOS Glob Public Health. 2023 Feb 1;3(2):e0001305.

# **Figures**

## **Classification of shared antimicrobial classes in human and veterinary medicine**


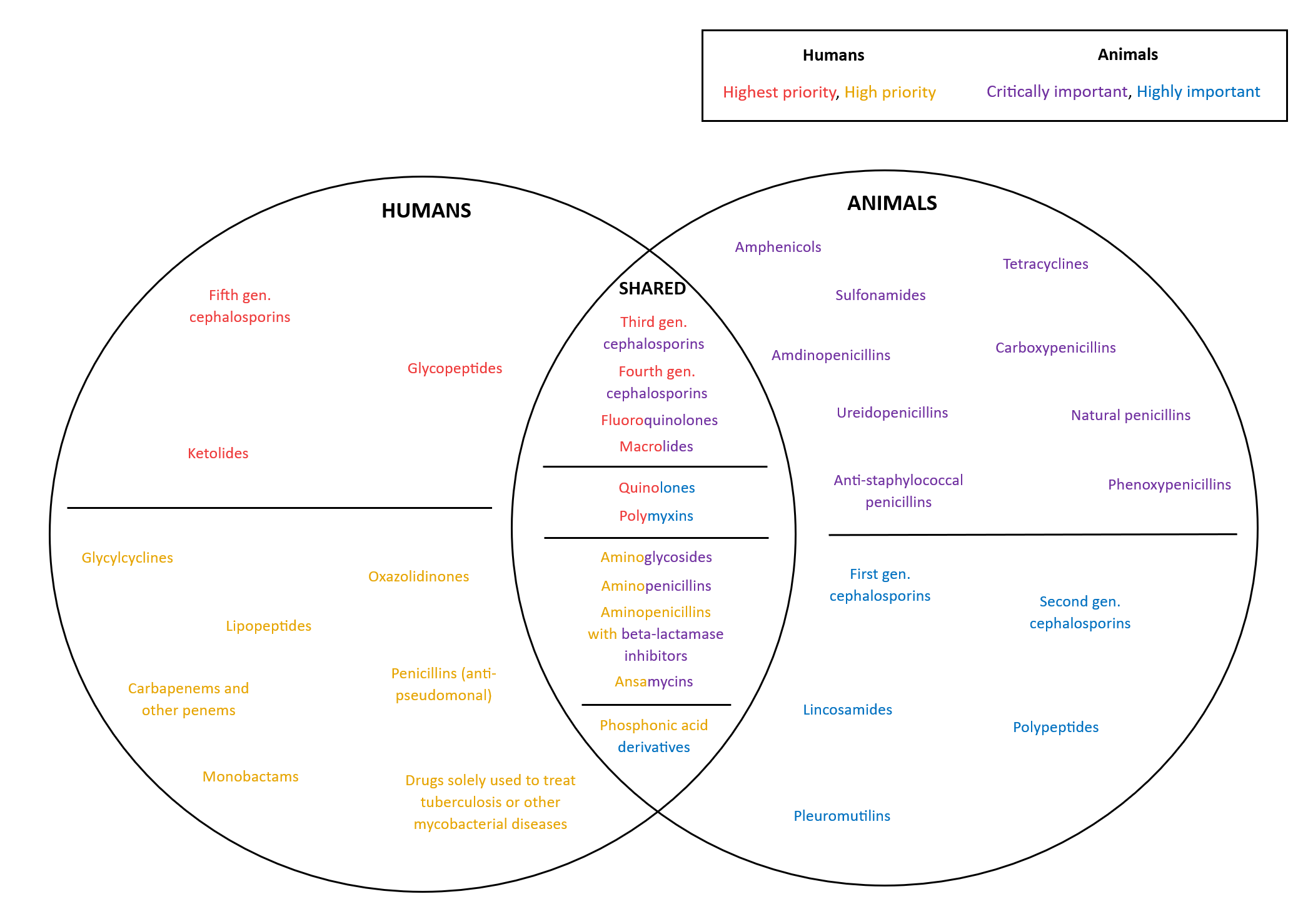


Supplemental Figure 1: Diagram of “highest priority” (red) and “high priority” (yellow) antimicrobial classes in humans (defined by the World Health Organization) with veterinary “critically important” (purple) and “highly important” (blue) antimicrobial classes (as identified by the World Organisation of Animal Health).

## **Data sources and classification**

| **Database included** | **Countries represented** | **Source** |
| --- | --- | --- |
| Resistance Bank | Algeria, Angola, Argentina, Bahrain, Bangladesh, Bhutan, Bolivia (Plurinational State of), Botswana, Brazil, Burkina Faso, Cambodia, Cameroon, Chad, Chile, Colombia, Costa Rica, Côte d'Ivoire, Cuba, Ecuador, Egypt, Ethiopia, Gabon, Gambia, Ghana, Grenada, India, Indonesia, Iran (Islamic Republic of), Iraq, Israel, Jamaica, Jordan, Kazakhstan, Kenya, Kuwait, Lao People's Democratic Republic, Lebanon, Malaysia, Mexico, Morocco, Myanmar, Namibia, Nepal, Nicaragua, Nigeria, Oman, Pakistan, Peru, Philippines, Qatar, Rwanda, Saint Lucia, Saudi Arabia, Senegal, Singapore, Somalia, South Africa, Sri Lanka, Thailand, Trinidad and Tobago, Tunisia, Türkiye, Uganda, United Republic of Tanzania, Uruguay, Venezuela (Bolivarian Republic of), Viet Nam, Zambia, Zimbabwe | https://resistancebank.org/ |
| European Food Safety Authority (EFSA) and European Centre for Disease Prevention and Control (ECDC)- EFSA-ECDC | Austria, Belgium, Bulgaria, Croatia, Cyprus, Czechia, Denmark, Estonia, Finland, France, Germany, Greece, Hungary, Iceland, Ireland, Italy, Latvia, Lithuania, Luxembourg, Malta, Netherlands, North Macedonia, Norway, Poland, Portugal, Romania, Slovakia, Slovenia, Spain, Sweden, Switzerland, United Kingdom | https://www.efsa.europa.eu/en/efsajournal/pub/6490 |
| Danish Integrated Antimicrobial Resistance Monitoring and Research Program (DANMAP) | Denmark | https://www.danmap.org/ |
| Finnish Veterinary Antimicrobial Resistance Monitoring and Consumption of Antimicrobial Agents (FINRES) | Finland | https://www.ruokavirasto.fi/en/animals/animal-medication/monitoring-of-antibiotic-resistance/finres-vet-reports/ |
| Monitoring of Antimicrobial Resistance and Antibiotic Usage in Animals in the Netherlands (MARAN) | Netherlands | https://swab.nl/en/nethmap-pvid369 |
| Norsk overvåkingssystem for antibiotikaresistens hos mikrober (NORM-VET) | Norway | https://www.vetinst.no/en/surveillance-programmes/norm-norm-vet-report |
| National Antimicrobial Resistance Monitoring System (NARMS) | United States of America | https://www.fda.gov/animal-veterinary/national-antimicrobial-resistance-monitoring-system/narms-now-integrated-data |
| Canadian Integrated Program for Antimicrobial Resistance Surveillance (CIPARS) | Canada | https://www.canada.ca/en/public-health/services/surveillance/canadian-integrated-program-antimicrobial-resistance-surveillance-cipars/cipars-reports.html |
| Japanese Veterinary Antimicrobial Resistance Monitoring System (JVARM) | Japan | https://www.maff.go.jp/nval/english/AMR/Monitoring/index.html |
| Korea Disease Control and Prevention Agency (KDCA) | South Korea | https://www.kdca.go.kr/nohas/en/statistics/selectIARStatisticsToSPECAFTab.do |
| New Zealand Food Safety Authority (NZFSA) | New Zealand | https://www.mpi.govt.nz/dmsdocument/21464-A-baseline-survey-of-antimicrobial-resistance-in-bacteria-from-selected-New-Zealand-foods-2009-2010 |
| Pilot surveillance program for antimicrobial resistance in bacteria of animal origin | Australia | https://www.agriculture.gov.au/agriculture-land/animal/health/amr/antimicrobial-resistance-bacteria-animal-origin#executive-summary |

Supplemental Table 1: Sources of livestock AMR data used in this analysis, with country and URL information

| **Class** | **Antimicrobial compound (abbreviation)** | **Antimicrobial compound (full name)** | **Source for classification** | **Type of compound** |
| --- | --- | --- | --- | --- |
| 1st Generation Cephalosporin | CFL | Cefalotin | Resistance Bank | Human and Veterinary use |
| 1st Generation Cephalosporin | CFZ | Cefazolin | Resistance Bank | Human and Veterinary use |
| 1st Generation Cephalosporin | CLX | Cefalexin | Resistance Bank | Human and Veterinary use |
| 1st Generation Cephalosporin | CFR | Cefadroxil | Resistance Bank | Human and Veterinary use |
| 1st Generation Cephalosporin | CPR | Cefradine | Resistance Bank | Human and Veterinary use |
| 1st Generation Cephalosporin | CLD | Cephaloridine | Resistance Bank | Human and Veterinary use |
| 1st Generation Cephalosporin | CLM | Cefalonium | Resistance Bank | Veterinary use only |
| 1st Generation Cephalosporin | CTZ | Ceftezole | Resistance Bank | Human and Veterinary use |
| 2nd Generation Cephalosporin | CXM | Cefuroxime | Resistance Bank | Human and Veterinary use |
| 2nd Generation Cephalosporin | CFC | Cefaclor | Resistance Bank | Human and Veterinary use |
| 2nd Generation Cephalosporin | CMD | Cefamandole | Resistance Bank | Human and Veterinary use |
| 3rd Generation Cephalosporin | CTX | Cefotaxime | Resistance Bank | Human and Veterinary use |
| 3rd Generation Cephalosporin | CRO | Ceftriaxone | Resistance Bank | Human and Veterinary use |
| 3rd Generation Cephalosporin | CAZ | Ceftazidime | Resistance Bank | Human and Veterinary use |
| 3rd Generation Cephalosporin | CFU | Ceftiofur | Resistance Bank | Veterinary use only |
| 3rd Generation Cephalosporin | CFP | Cefoperazone | Resistance Bank | Human and Veterinary use |
| 3rd Generation Cephalosporin | CFM | Cefixime | Resistance Bank | Human and Veterinary use |
| 3rd Generation Cephalosporin | CPD | Cefpodoxime | Resistance Bank | Human and Veterinary use |
| 3rd Generation Cephalosporin | CZM | Ceftizoxime | Resistance Bank | Human and Veterinary use |
| 3rd Generation Cephalosporin | MOX | Moxalactam | Resistance Bank | Human and Veterinary use |
| 3rd Generation Cephalosporin | CDR | Cefdinir | Resistance Bank | Human and Veterinary use |
| 4th Generation Cephalosporin | FEP | Cefepime | Google search | Human and Veterinary use |
| 4th Generation Cephalosporin | CFQ | Cefquinome | Resistance Bank | Veterinary use only |
| 4th Generation Cephalosporin | CPO | Cefpirome | Resistance Bank | Human and Veterinary use |
| 5th Generation Cephalosporin | CPT | Ceftaroline | Resistance Bank | Human and Veterinary use |
| Amidinopenicillins | MEC | Mecillinam | Resistance Bank | Human and Veterinary use |
| Aminocoumarins | NOV | Novobiocin | Resistance Bank | Veterinary use only |
| Aminocyclitols | SPT | Spectinomycin | Resistance Bank | Human and Veterinary use |
| Aminoglycosides | GEN | Gentamicin | Resistance Bank | Human and Veterinary use |
| Aminoglycosides | STR | Streptomycin | Resistance Bank | Human and Veterinary use |
| Aminoglycosides | AMK | Amikacin | Resistance Bank | Human and Veterinary use |
| Aminoglycosides | KAN | Kanamycin | Resistance Bank | Human and Veterinary use |
| Aminoglycosides | NEO | Neomycin | Resistance Bank | Human and Veterinary use |
| Aminoglycosides | TOB | Tobramycin | Resistance Bank | Human and Veterinary use |
| Aminoglycosides | APR | Apramycin | Resistance Bank | Veterinary use only |
| Aminoglycosides | NET | Netilmycin | Resistance Bank | Human and Veterinary use |
| Aminoglycosides | ISP | Isepamicin | Resistance Bank | Human and Veterinary use |
| Aminopenicillins | AMP | Ampicillin | Resistance Bank | Human and Veterinary use |
| Aminopenicillins | AMX | Amoxicillin | Resistance Bank | Human and Veterinary use |
| Aminopenicillins | PIP | Piperacillin | Resistance Bank | Human and Veterinary use |
| Aminopenicillins with Beta-Lactamase Inhibitors | AMC | Amoxicillin-Clavulanic acid | Resistance Bank | Human and Veterinary use |
| Aminopenicillins with Beta-Lactamase Inhibitors | PIT | Piperacillin-Tazobactam | Resistance Bank | Human and Veterinary use |
| Aminopenicillins with Beta-Lactamase Inhibitors | SAM | Ampicillin-Sulbactam | Resistance Bank | Human and Veterinary use |
| Aminopenicillins with Beta-Lactamase Inhibitors | TIM | Ticarcillin-Clavulanic acid | Resistance Bank | Human and Veterinary use |
| Amphenicols | CHL | Chloramphenicol | Resistance Bank | Human and Veterinary use |
| Amphenicols | FFC | Florfenicol | Resistance Bank | Veterinary use only |
| Amphenicols | TFC | Thiamphenicol | Resistance Bank | Human and Veterinary use |
| Ansamycins | RIF | Rifampicin | Resistance Bank | Human and Veterinary use |
| Carbapenems | IPM | Imipenem | Resistance Bank | Human and Veterinary use |
| Carbapenems | MEM | Meropenem | Resistance Bank | Human and Veterinary use |
| Carbapenems | ERT | Ertapenem | Resistance Bank | Human and Veterinary use |
| Carbapenems | DOR | Doripenem | Resistance Bank | Human and Veterinary use |
| Cephamycin | FOX | Cefoxitin | Resistance Bank | Human and Veterinary use |
| Cephamycin | CTT | Cefotetan | Resistance Bank | Human and Veterinary use |
| Cyclic Polypeptides | BAC | Bacitracin | Resistance Bank | Human and Veterinary use |
| Cytotoxic Chemotherapy |  | Bleomycin | Google search (NARMS data) | Human and Veterinary use |
| Glycopeptides | VAN | Vancomycin | Resistance Bank | Human and Veterinary use |
| Glycopeptides | TEC | Teicoplanin | Resistance Bank | Human and Veterinary use |
| Glycylcyclines | TIG | Tigecycline | Resistance Bank | Human and Veterinary use |
| Lincosamides | CLI | Clindamycin | Resistance Bank | Human and Veterinary use |
| Lincosamides | LIN | Lincomycin | Resistance Bank | Human and Veterinary use |
| Lincosamides | PIR | Pirlimycin | Resistance Bank | Veterinary use only |
| Lipopeptides | DAP | Daptomycin | Resistance Bank | Human and Veterinary use |
| Macrolides | ERY | Erythromycin | Resistance Bank | Human and Veterinary use |
| Macrolides | AZM | Azithromycin | Resistance Bank | Human and Veterinary use |
| Macrolides | TYL | Tylosin | Resistance Bank | Veterinary use only |
| Macrolides | TIL | Tilmicosin | Resistance Bank | Veterinary use only |
| Macrolides | CLR | Clarithromycin | Resistance Bank | Human and Veterinary use |
| Macrolides | TEL | Telithromycin | Resistance Bank | Human and Veterinary use |
| Macrolides | ROX | Roxithromycin | Resistance Bank | Human and Veterinary use |
| Macrolides | ASP | Acetylspiramycin | Resistance Bank | Human and Veterinary use |
| Macrolides | MID | Midecamycin | Resistance Bank | Human and Veterinary use |
| Macrolides | SPI | Spiramycin | Resistance Bank | Human and Veterinary use |
| Macrolides | OLE | Oleandomycin | Resistance Bank | Human and Veterinary use |
| Monobactams | ATM | Aztreonam | Resistance Bank | Human and Veterinary use |
| Nitrofurans | NIT | Nitrofurantoin | Resistance Bank | Human and Veterinary use |
| Nitrofurans | FRZ | Furazolidone | Resistance Bank | Veterinary use only |
| Nitrofurans | FUR | Furazidin | Resistance Bank | Human and Veterinary use |
| Nitroimidazoles | MTD | Metronidazole | Resistance Bank | Human and Veterinary use |
| Oxazolidinones | LIZ | Linezolid | Resistance Bank | Human and Veterinary use |
| Penicillins | CAR | Carbenicillin | Resistance Bank | Human and Veterinary use |
| Penicillins | TIC | Ticarcillin | Resistance Bank | Human and Veterinary use |
| Penicillins | AZL | Azlocillin | Resistance Bank | Human and Veterinary use |
| Penicillins (Anti-Staphylococcal) | OXA | Oxacillin | Resistance Bank | Human and Veterinary use |
| Penicillins (Anti-Staphylococcal) | MET | Methicillin | Resistance Bank | Human and Veterinary use |
| Penicillins (Anti-Staphylococcal) | CLO | Cloxacillin | Resistance Bank | Human and Veterinary use |
| Penicillins (Anti-Staphylococcal) | DIC | Dicloxacillin | Resistance Bank | Veterinary use only |
| Penicillins (Anti-Staphylococcal) | FLU | Flucloxacillin | Resistance Bank | Human and Veterinary use |
| Penicillins (Anti-Staphylococcal) | NAF | Nafcillin | Resistance Bank | Human and Veterinary use |
| Penicillins (Narrow Spectrum) | PEN | Penicillin | Resistance Bank | Human and Veterinary use |
| Phosphonic Acid | FOF | Fosfomycin | Resistance Bank | Human and Veterinary use |
| Pleuromutilins | TIA | Tiamulin | Resistance Bank | Veterinary use only |
| Pleuromutilins | VAL | Valnemulin | Resistance Bank | Veterinary use only |
| Polymyxins | CST | Colistin | Resistance Bank | Human and Veterinary use |
| Polymyxins | PMB | Polymixin B | Resistance Bank | Human and Veterinary use |
| Pseudomonic Acids | MUP | Mupirocin | Resistance Bank | Human and Veterinary use |
| Fluoroquinolones | CIP | Ciprofloxacin | Resistance Bank | Human and Veterinary use |
| Quinolones | NAL | Nalidixic acid | Resistance Bank | Human and Veterinary use |
| Fluoroquinolones | ENR | Enrofloxacin | Resistance Bank | Veterinary use only |
| Fluoroquinolones | NOR | Norfloxacin | Resistance Bank | Human and Veterinary use |
| Fluoroquinolones | OFX | Ofloxacin | Resistance Bank | Human and Veterinary use |
| Fluoroquinolones | LVX | Levofloxacin | Resistance Bank | Human and Veterinary use |
| Fluoroquinolones | PEF | Pefloxacin | Resistance Bank | Human and Veterinary use |
| Fluoroquinolones | GAT | Gatifloxacin | Resistance Bank | Human and Veterinary use |
| Fluoroquinolones | LOM | Lomefloxacin | Resistance Bank | Human and Veterinary use |
| Quinolones | FLQ | Flumequine | Resistance Bank | Human and Veterinary use |
| Fluoroquinolones | DAN | Danofloxacin | Resistance Bank | Veterinary use only |
| Fluoroquinolones | MXF | Moxifloxacin | Resistance Bank | Human and Veterinary use |
| Fluoroquinolones | ENO | Enoxacin | Resistance Bank | Human and Veterinary use |
| Fluoroquinolones | SAR | Sarafloxacin | Resistance Bank | Human and Veterinary use |
| Fluoroquinolones | PIM | Pipemidic acid | Resistance Bank | Human and Veterinary use |
| Fluoroquinolones | MRB | Marbofloxacin | Resistance Bank | Veterinary use only |
| Fluoroquinolones | DIF | Difloxacin | Resistance Bank | Veterinary use only |
| Quinolones | OXO | Oxolinic acid | Resistance Bank | Human and Veterinary use |
| Fluoroquinolones | GEM | Gemifloxacin | Resistance Bank | Human and Veterinary use |
| Steroid Antibacterials | FUS | Fusidic acid/Fusidate | Resistance Bank | Human and Veterinary use |
| Streptogramins | Q-D | Quinupristin-Dalfopristin | Resistance Bank | Human and Veterinary use |
| Streptogramins | PRI | Pristinamycin | Resistance Bank | Human and Veterinary use |
| Streptogramins | VRG | Virginiamycin | Resistance Bank | Human and Veterinary use |
| Sulfonamides, Trimethoprim and Combinations | SXT | Sulfamethoxazole-Trimethoprim | Resistance Bank | Human and Veterinary use |
| Sulfonamides, Trimethoprim and Combinations | TMP | Trimethoprim | Resistance Bank | Human and Veterinary use |
| Sulfonamides, Trimethoprim and Combinations | SMZ | Sulfamethoxazole | Resistance Bank | Human and Veterinary use |
| Sulfonamides, Trimethoprim and Combinations | SOX | Sulfafurazole/Sulfisoxazole | Resistance Bank | Human and Veterinary use |
| Sulfonamides, Trimethoprim and Combinations |  | Sulfamethoxazole-Sulfisoxazole | NARMS data inclusion | Human and Veterinary use |
| Sulfonamides, Trimethoprim and Combinations | SSS | Sulphonamides | Resistance Bank | Human and Veterinary use |
| Sulfonamides, Trimethoprim and Combinations | SUD | Sulfadiazine | Resistance Bank | Human and Veterinary use |
| Sulfonamides, Trimethoprim and Combinations | SMN | Sulfamonomethoxine | Resistance Bank | Veterinary use only |
| Sulfonamides, Trimethoprim and Combinations | SUT | Sulfonamides-Trimethoprim | Resistance Bank | Human and Veterinary use |
| Sulfonamides, Trimethoprim and Combinations | SPZ | Sulfaphenazole | Resistance Bank | Human and Veterinary use |
| Sulfonamides, Trimethoprim and Combinations | SPX | Sulfamethizole | Resistance Bank | Human and Veterinary use |
| Sulfonamides, Trimethoprim and Combinations | TDZ | Trimethoprim + Sulfadiazine | Resistance Bank | Veterinary use only |
| Sulfonamides, Trimethoprim and Combinations | SFX | Sulfadimethoxine | Resistance Bank | Human and Veterinary use |
| Tetracyclines | TET | Tetracycline | Resistance Bank | Human and Veterinary use |
| Tetracyclines | DOX | Doxycycline | Resistance Bank | Human and Veterinary use |
| Tetracyclines | OXT | Oxytetracycline | Resistance Bank | Human and Veterinary use |
| Tetracyclines | MIN | Minocycline | Resistance Bank | Human and Veterinary use |
| Tetracyclines | CTE | Chlortetracycline | Resistance Bank | Human and Veterinary use |
| Tetracyclines | DEM | Demeclocycline | Resistance Bank | Human and Veterinary use |
| Quinoxaline | OLA | Olaquindox | This study (google search) | Veterinary use only |
| Quinoxaline | MEQ | Mequindox | This study (google search) | Veterinary use only |
| Quinoxaline | MEZ | Mezlocillin | This study (google search) | Human and Veterinary use |
| 3rd Generation Cephalosporin with Beta-Lactamase Inhibitors | CAC | Ceftazidime-Clavulanic acid | This study (based on Resistance Bank) | Human and Veterinary use |
| 3rd Generation Cephalosporin with Beta-Lactamase Inhibitors | CRS | Ceftriaxone + Sulbactam | This study (based on Resistance Bank) | Human and Veterinary use |
| 3rd Generation Cephalosporin with Beta-Lactamase Inhibitors | CTC | CTX-Clavulanic acid | This study (based on Resistance Bank) | Human and Veterinary use |
| 3rd Generation Cephalosporin with Beta-Lactamase Inhibitors | SFP | Sulbactam + Cefoperazone | This study (based on Resistance Bank) | Human and Veterinary use |
| Quinoxaline | CRB | Carbadox | This study (google search) | Veterinary use only |
| Lincosamides with Aminocyclitols | LIS | Lincomycin + Spectinomycin | This study (based on Resistance Bank) | Veterinary use only |
| Beta-Lactamase Inhibitors | CLA | Clavulanic acid | This study (based on Resistance Bank) | Human and Veterinary use |
| 3rd Generation Cephalosporin with Beta-Lactamase Inhibitors | CRT | Ceftriaxone + Tazobactam | This study (based on Resistance Bank) | Human and Veterinary use |
| Aminopenicillins with Penicillins (Anti-Staphylococcal) | ACL | Amoxicillin/Cloxacillin | This study (based on Resistance Bank) | Human and Veterinary use |
| 2nd Generation Cephalosporin with Beta-Lactamase Inhibitors | FET | Cefepime + Tazobactam | This study (based on Resistance Bank) | Human and Veterinary use |
| Penicillins (Narrow Spectrum) with Aminoglycosides | PES | Penicillin & Streptomycin | This study (based on Resistance Bank) | Human and Veterinary use |
| Sulfonamides, Trimethoprim and Combinations |  | Sulfisoxazole | This study (based on Resistance Bank) | Human and Veterinary use |
| Terpine glycosides |  | Flavomycin | This study (google search) | Veterinary use only |
| Orthosomycin |  | Avilamycin | This study (google search) | Veterinary use only |
| Aminopenicillins with Penicillins |  | Ampicillin-Penicillin | This study | Human and Veterinary use |
| Aminopenicillins |  | Dihydrostreptomycin | This study (google search) | Veterinary use only |
| Ionophore |  | Salinomycin | This study (google search) | Human and Veterinary use |

Supplemental Table 2: Scope of compounds with antimicrobial class classifications and source definitions identified in this study.

##

##

## **Correlation assessment**

| **Category** | **Spearman rho coefficient** | ***p*-value** | **Significance level** |
| --- | --- | --- | --- |
| **Aminoglycosides & *Escherichia coli*- Cattle** | **0.153665** | **0.027823** | ***** |
| **Aminoglycosides & *Escherichia coli*- Chickens** | **-0.01943** | **0.751126** |  |
| **Aminoglycosides & *Escherichia coli*- Pigs** | **0.010565** | **0.889322** |  |
| **Aminoglycosides & *Escherichia coli*- Sheep** | **-0.05317** | **0.754635** |  |
| **Aminopenicillins & *Escherichia coli*- Cattle** | **0.53254** | **1.33E-15** | ******** |
| **Aminopenicillins & *Escherichia coli*- Chickens** | **0.324723** | **9.51E-08** | ******** |
| **Aminopenicillins & *Escherichia coli*- Pigs** | **0.18769** | **0.014541** | ***** |
| **Aminopenicillins & *Escherichia coli*- Sheep** | **0.375564** | **0.02861** | ***** |
| **Aminopenicillins with beta-lactamase inhibitors & *Escherichia coli*- Cattle** | **0.461161** | **3.29E-06** | ******** |
| **Aminopenicillins with beta-lactamase inhibitors & *Escherichia co*li- Chickens** | **0.07599** | **0.434415** |  |
| **Aminopenicillins with beta-lactamase inhibitors & *Escherichia coli-* Pigs** | **0.073054** | **0.563044** |  |
| **Aminopenicillins with beta-lactamase inhibitors & *Escherichia coli*- Sheep** | **-0.17186** | **0.524498** |  |
| **Fluoroquinolones & *Enterococcus faecalis-* Cattle** | **0.23258** | **0.224701** |  |
| **Fluoroquinolones & *Enterococcus faecalis*- Chickens** | **0.403709** | **0.016172** | ***** |
| **Fluoroquinolones & *Enterococcus faecalis*- Pigs** | **0.137207** | **0.469672** |  |
| **Fluoroquinolones & *Enterococcus faecium*- Cattle** | **0.131179** | **0.505813** |  |
| **Fluoroquinolones & *Enterococcus faecium*- Chickens** | **-0.03821** | **0.830102** |  |
| **Fluoroquinolones & *Enterococcus faecium*- Pigs** | **0.127911** | **0.508453** |  |
| **Fluoroquinolones & *Escherichia coli*- Cattle** | **0.326217** | **2.88E-05** | ******** |
| **Fluoroquinolones & *Escherichia coli*- Chickens** | **0.303053** | **4.05E-06** | ******** |
| **Fluoroquinolones & *Escherichia coli*- Pigs** | **0.499485** | **2.62E-09** | ******** |
| **Fluoroquinolones & *Escherichia coli*- Sheep** | **0.139493** | **0.454209** |  |
| **Fluoroquinolones & Non-typhoidal *Salmonella*- Cattle** | **0.150511** | **0.121757** |  |
| **Fluoroquinolones & Non-typhoidal *Salmonella*- Chickens** | **-0.03749** | **0.559977** |  |
| **Fluoroquinolones & Non-typhoidal *Salmonella*- Pigs** | **0.234455** | **0.005137** | ****** |
| **Fluoroquinolones & Non-typhoidal *Salmonella*- Sheep** | **0.663382** | **0.007015** | ****** |
| **Fluoroquinolones & *Staphylococcus aureus*- Cattle** | **0.272095** | **0.009894** | ****** |
| **Fluoroquinolones & *Staphylococcus aureus*- Chickens** | **0.400168** | **0.017222** | ***** |
| **Fluoroquinolones & *Staphylococcus aureus*- Pigs** | **0.009686** | **0.958755** |  |
| **Fluoroquinolones & *Staphylococcus aureus*- Sheep** | **0.358528** | **0.172683** |  |
| **Macrolides & *Staphylococcus aureus-* Cattle** | **-0.03292** | **0.74636** |  |
| **Macrolides & *Staphylococcus aureus-* Chickens** | **0.517192** | **0.001046** | ****** |
| **Macrolides & *Staphylococcus aureus*- Pigs** | **0.742131** | **1.76E-06** | ******** |
| **Macrolides & *Staphylococcus aureus-* Sheep** | **0.044781** | **0.869199** |  |
| **Third generation cephalosporins & *Escherichia coli*- Cattle** | **0.447823** | **4.59E-08** | ******** |
| **Third generation cephalosporins & *Escherichia coli*- Chickens** | **0.345873** | **9.55E-07** | ******** |
| **Third generation cephalosporins & *Escherichia col*i- Pigs** | **0.496746** | **2.19E-08** | ******** |
| **Third generation cephalosporins & *Escherichia coli*- Sheep** | **0.384356** | **0.08538** |  |

Supplemental Table 3: Reported Spearman correlation values for combinations matched by both human (proportion of infections that are drug resistant in humans) and livestock AMR metrics (percent of isolates that are antimicrobial resistant in livestock) values. Other livestock species (namely turkeys, duck, horses, buffaloes, goats) were not included due to a lack of or limited livestock AMR data, and some combinations such as Fluoroquinolones and *Enterococcus* spp. only had sufficient data points for cattle, chickens, and pigs. Statistical significance is denoted as follows: p<0.0001 (****), followed by p<0.001 (***), then p<0.01 (**), then p<0.05 (*).

##

##

## **Global misalignments in livestock AMR data and created priorities**


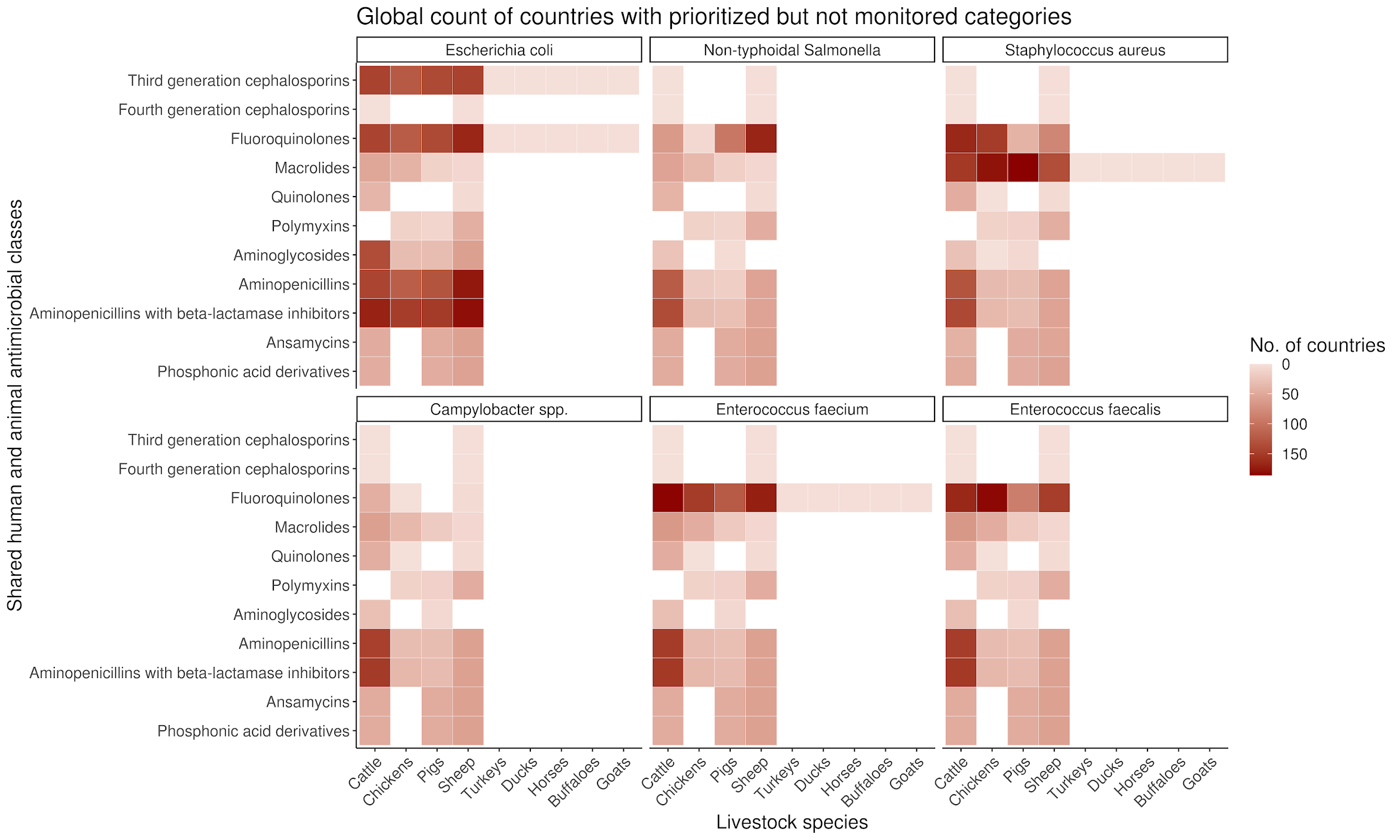


Supplemental Figure 2: Global aggregation of prioritized categories in countries (top 10% of values in composite indicator per country) without livestock AMR data for a particular combination of antimicrobial class, pathogen, and livestock species. Darker red show categories with higher number of countries that satisfy criteria, lighter red show categories with lower consensus of countries, and white shows no countries that satisfy criteria.


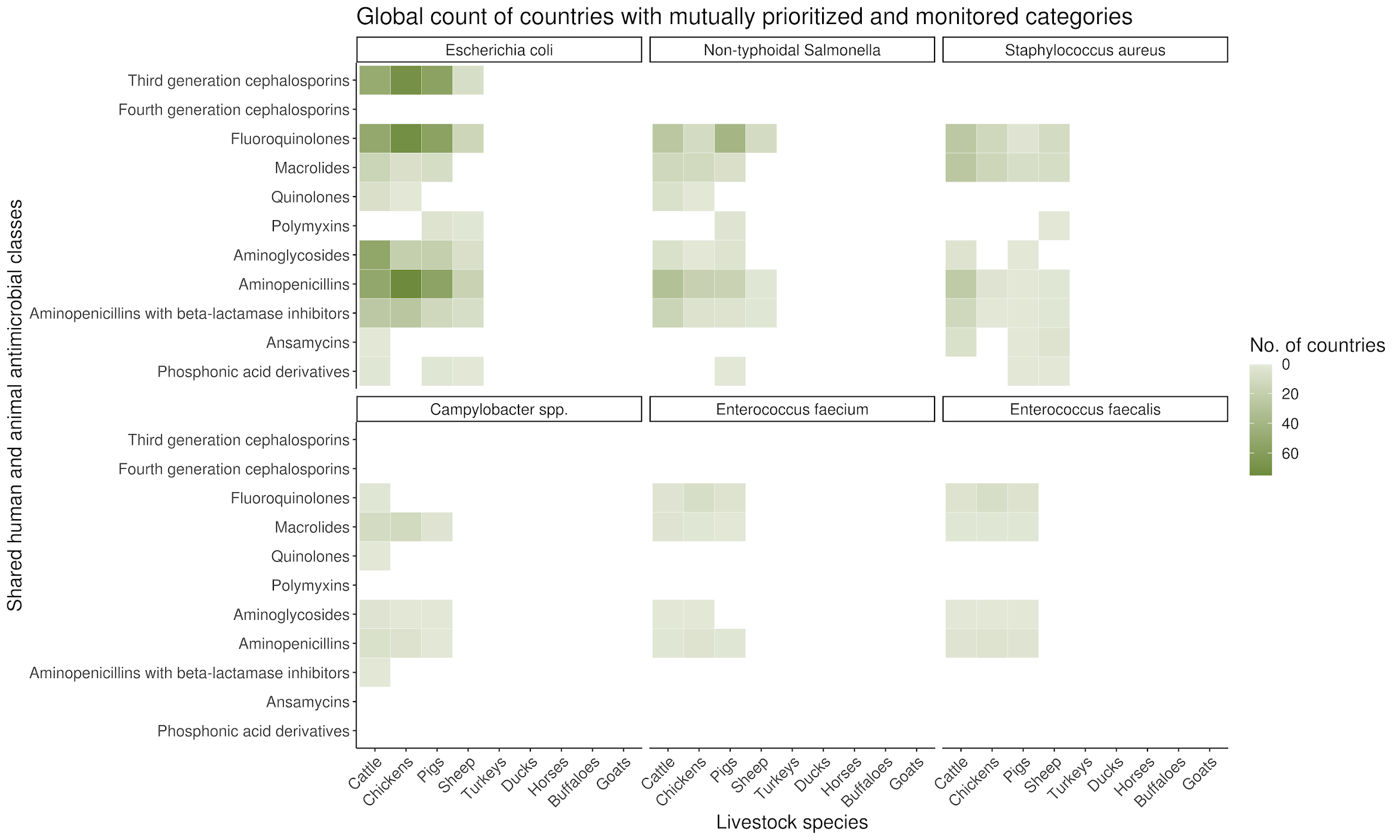


Supplemental Figure 3: Global aggregation of prioritized categories in countries (top 10% of values in composite indicator per country) with livestock AMR data for a particular combination of antimicrobial class, pathogen, and livestock species. Darker green show categories with higher number of countries that satisfy criteria, lighter green show categories with lower consensus of countries, and white shows no countries that satisfy criteria.


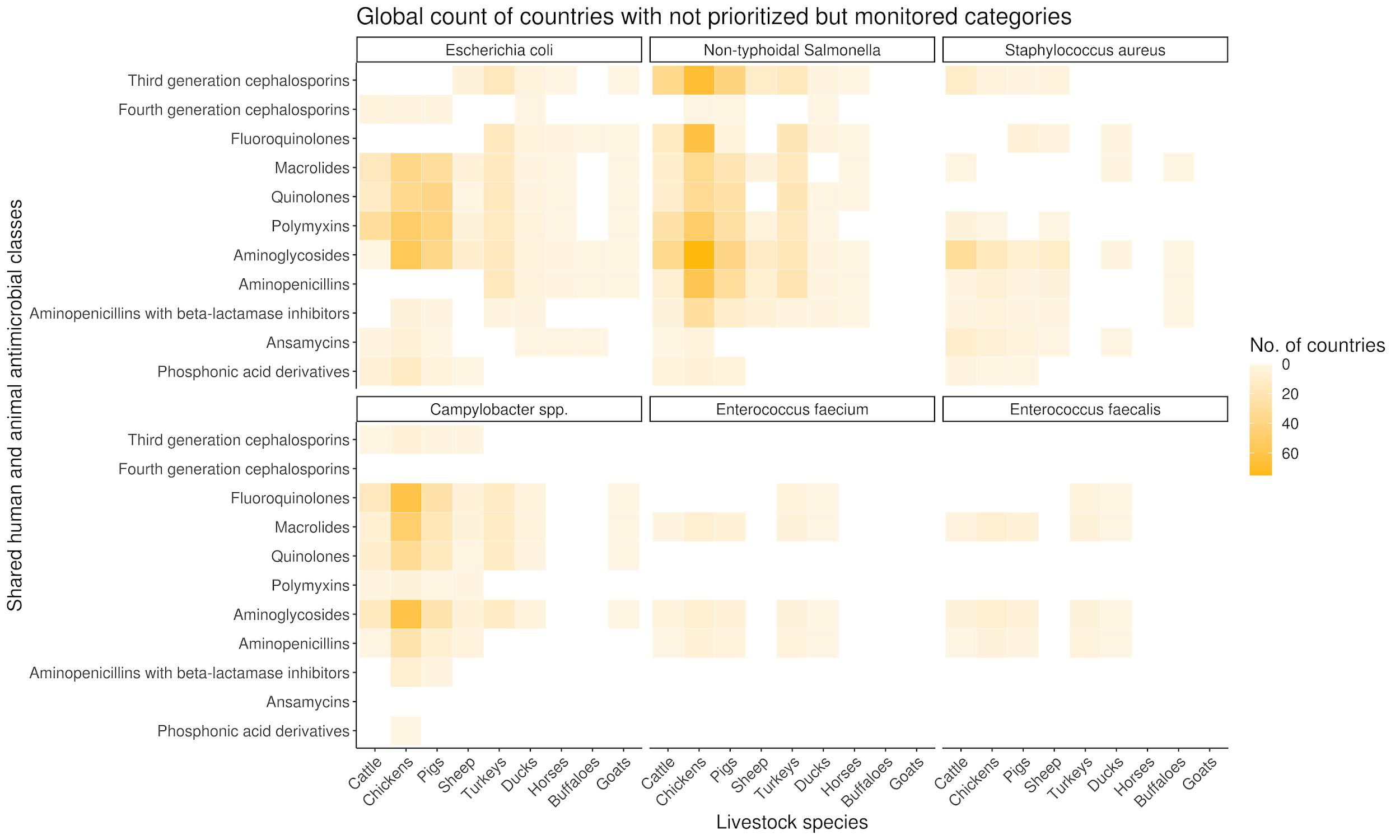


Supplemental Figure 4: Global aggregation of non-prioritized categories in countries (priority being defined as the top 10% of values in composite indicator per country) with livestock AMR data for a particular combination of antimicrobial class, pathogen, and livestock species. Darker yellow show categories with higher number of countries that satisfy criteria, lighter yellow show categories with lower consensus of countries, and white shows no countries that satisfy criteria.

## **Rank imputations and assumptions**


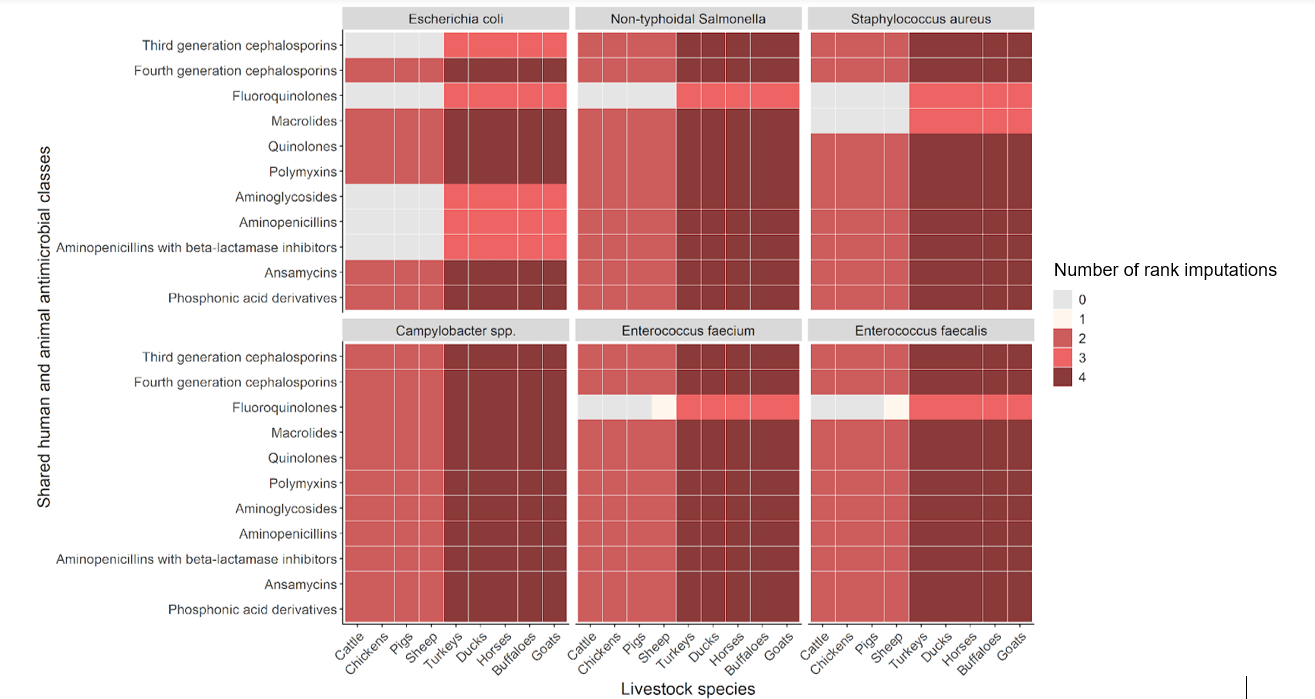


Supplemental Figure 5: Heatmap showing the most common number of rank imputations used to calculate global metaranks for a particular category of antimicrobial class, pathogen, and livestock species. Rank imputation was defined as the lowest rank assigned to a particular variable in the ranking exercise due to a lack of estimates, where each category could have a maximum of 4 rank imputations. The mode number of rank imputations from all countries considered (n=194) is plotted, where darker red shows a higher number of rank imputations, and lighter red shows a lower number of rank imputations with grey indicating no imputations were used (i.e. estimates were available for the specified category). The maximum number of assumptions that could be used were 4, one corresponding to each variable.


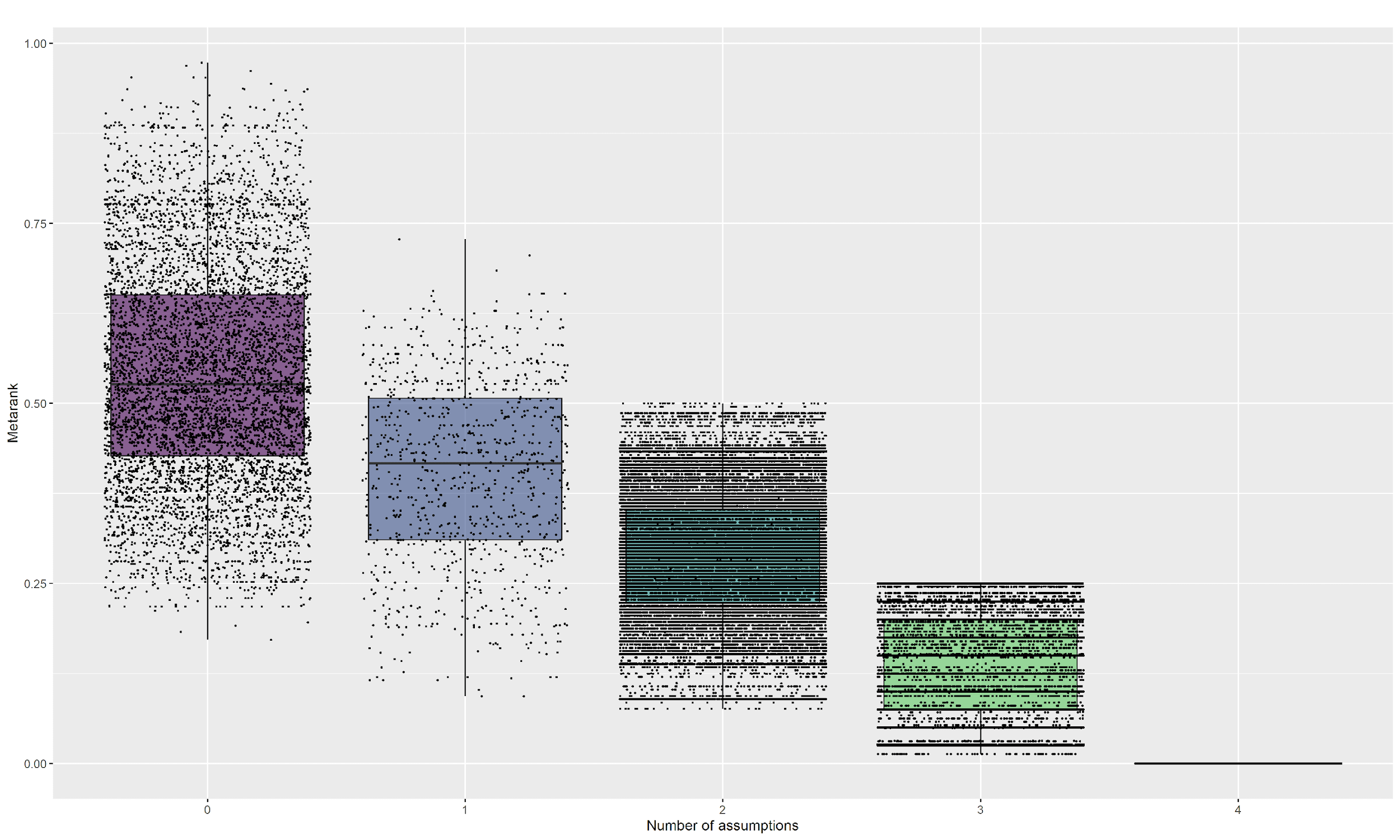


Supplemental Figure 6: Boxplot showing the most common number of rank imputations used to calculate global metaranks for a particular category of antimicrobial class, pathogen, and livestock species, with each scatter point representing a country-specific category. Rank imputation was defined as the lowest rank assigned to a particular variable in the ranking exercise due to a lack of estimates, where each category could have a maximum of 4 rank imputations. The maximum number of assumptions that could be used were 4, one corresponding to each variable.

**
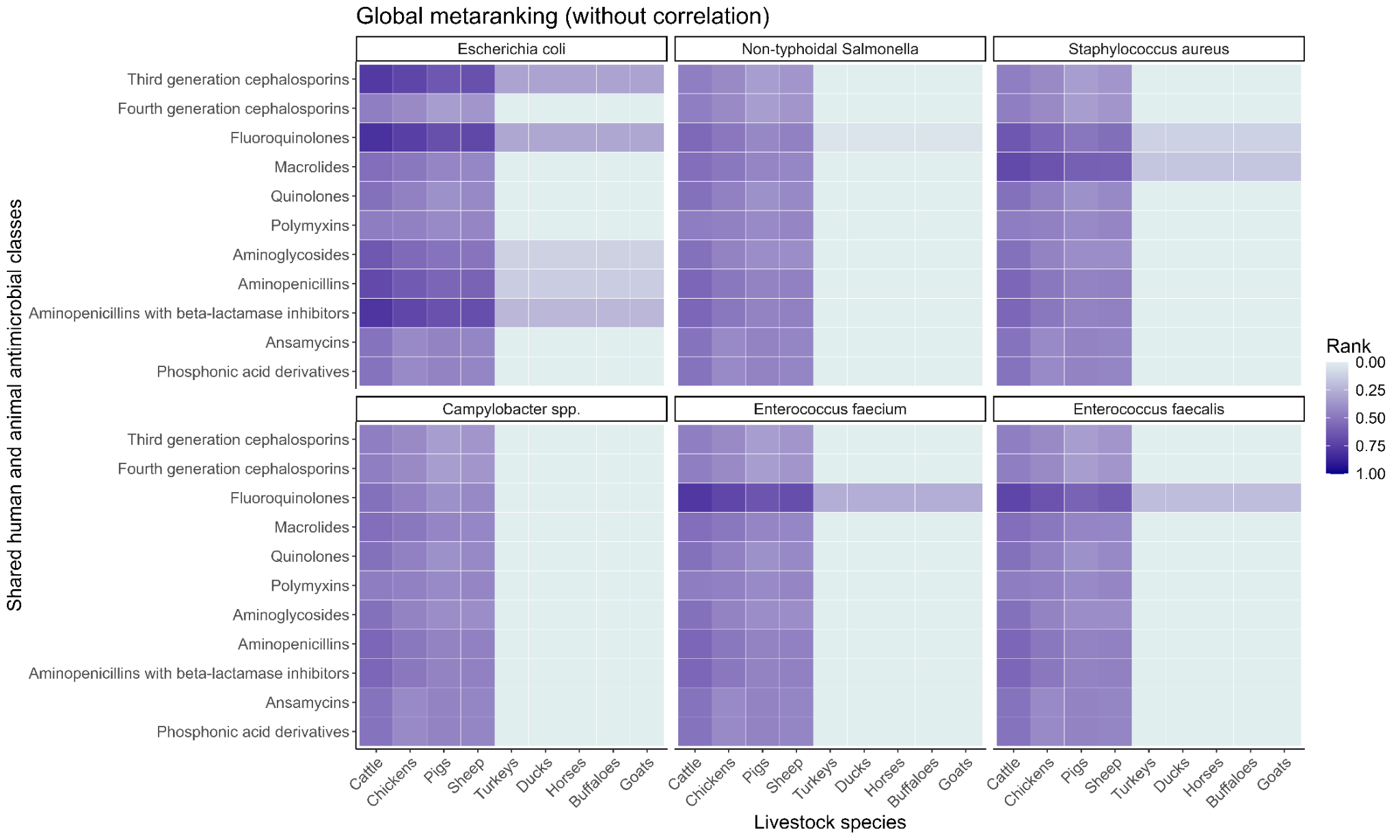
**

Supplemental Figure 7: Global metaranks (n=194) accounting for livestock antimicrobial usage (AMU), livestock biomass (population correction units), and human DALYs attributable to AMR. Global metaranks were calculated for a particular livestock species, antimicrobial classes and pathogen species combination. Antimicrobials are ordered top to bottom by shared human and animal relevance. Dark purple shows the highest metaranks calculated for the specific antimicrobial class, pathogen, and livestock species. Lighter purple and blue indicate a lower metarank for the specific antimicrobial class, pathogen, and livestock species.

**
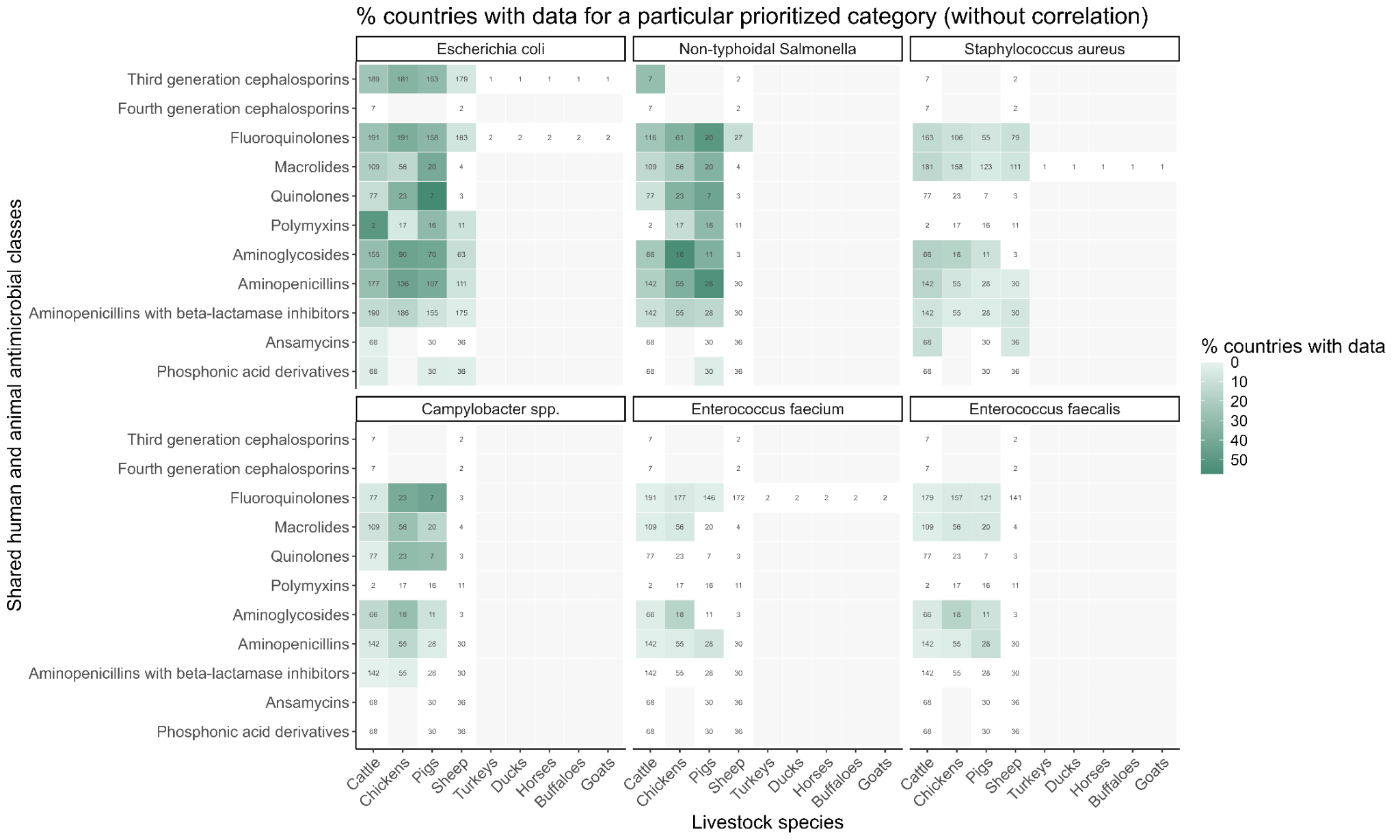
**

Supplemental Figure 8: Global percentage of countries (n=194) with data for a particular livestock species, antimicrobial classes and pathogen species combination if it was prioritized (where priorities here have been determined without the correlation assessment). Antimicrobials are ordered top to bottom by shared human and animal relevance. White and lighter colors relay a low percentage of countries with data, and darker colors indicate a higher percentage of countries with data. Cells for which no countries have prioritized the category are grey. Numbers in each cell correspond to the number of countries that have prioritized that particular category.
