# Supplementary material for: Using priorities between human and livestock bacterial antimicrobial resistance (AMR) to identify data gaps in livestock AMR surveillance
Source: BMC Infect Dis. 2024 Sep 26;24:1027. doi: 10.1186/s12879-024-09847-3 (PMC11425882; doi:10.1186/s12879-024-09847-3)
Supplement: Supplementary file 2 — Supplementary Material 2 [file 12879_2024_9847_MOESM2_ESM.pdf]

Supplementary material 2

This is supplement 2 to the manuscript "Using Priorities between Human and Livestock Bacterial Antimicrobial Resistance (AMR) to Identify Data Gaps in Livestock AMR Surveillance". This contains all national figures generated from ranking individual factors important in human and animal antimicrobial resistance (AMR) burden, creating the composite indicator integrating these factors, designating priorities from the top 10% of the composite indicator, and the gaps between these priorities and collected livestock AMR data. Each figure displayed is specific to a country, apart from the global correlation exercise where every country receives the same rankings each other.

The document is structured by country, ordered alphabetically, with one plot per page. 194 countries are shown, which have been chosen and defined as such by the Global Burden of Disease study. The order of the plots is as follows: ranking of human AMR DALYs; ranking of livestock antimicrobial usage (AMU); ranking of livestock biomass; ranking of significance levels between livestock and human proportions of resistance (global correlation exercise); composite indicator averaging ranks from human AMR DALYs, livestock AMU, livestock biomass, and global correlation exercise; livestock AMR data; and misalignment between intended priorities and livestock AMR data. Specificity of these plots is by pathogen-antimicrobial-class-livestock-species (referred to as a category), and considers the same 594 categories as depicted in the manuscript figures 2-5.

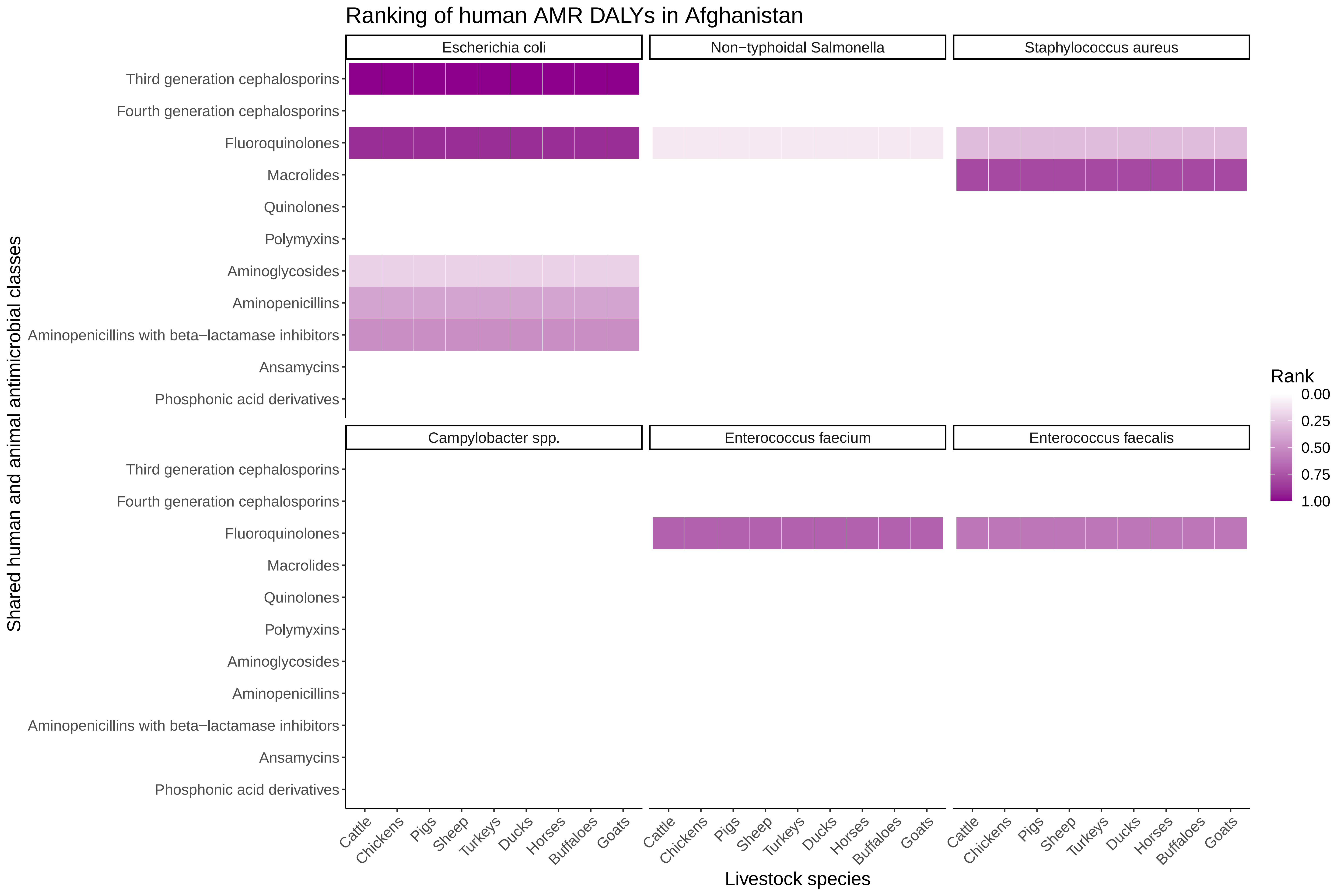

Ranking of human AMR DALYs in Afghanistan
Escherichia coli
Non−typhoidal Salmonella
Staphylococcus aureus
Campylobacter spp.
Enterococcus faecium
Enterococcus faecalis
Third generation cephalosporins
Fourth generation cephalosporins
Fluoroquinolones
Macrolides
Quinolones
Polymyxins
Aminoglycosides
Aminopenicillins
Aminopenicillins with beta−lactamase inhibitors
Ansamycins
Phosphonic acid derivatives
Shared human and animal antimicrobial classes
Cattle
Chickens
Pigs
Sheep
Turkeys
Ducks
Horses
Buffaloes
Goats
Livestock species
Rank
0.00
0.25
0.50
0.75
1.00

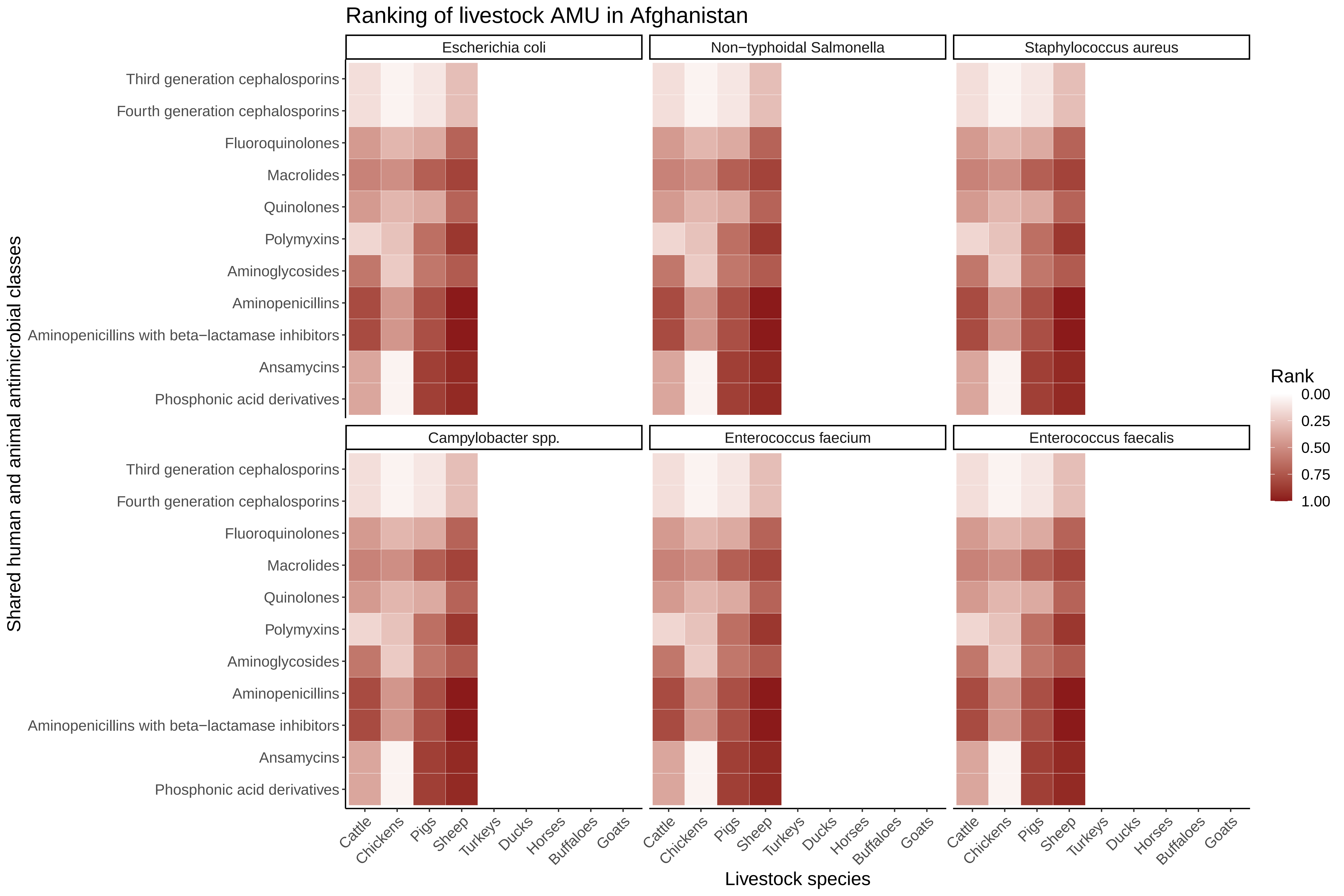

Ranking of livestock AMU in Afghanistan
Escherichia coli
Non−typhoidal Salmonella
Staphylococcus aureus
Campylobacter spp.
Enterococcus faecium
Enterococcus faecalis
Shared human and animal antimicrobial classes
Third generation cephalosporins
Fourth generation cephalosporins
Fluoroquinolones
Macrolides
Quinolones
Polymyxins
Aminoglycosides
Aminopenicillins
Aminopenicillins with beta−lactamase inhibitors
Ansamycins
Phosphonic acid derivatives
Livestock species
Cattle
Chickens
Pigs
Sheep
Turkeys
Ducks
Horses
Buffaloes
Goats
Rank
0.00
0.25
0.50
0.75
1.00

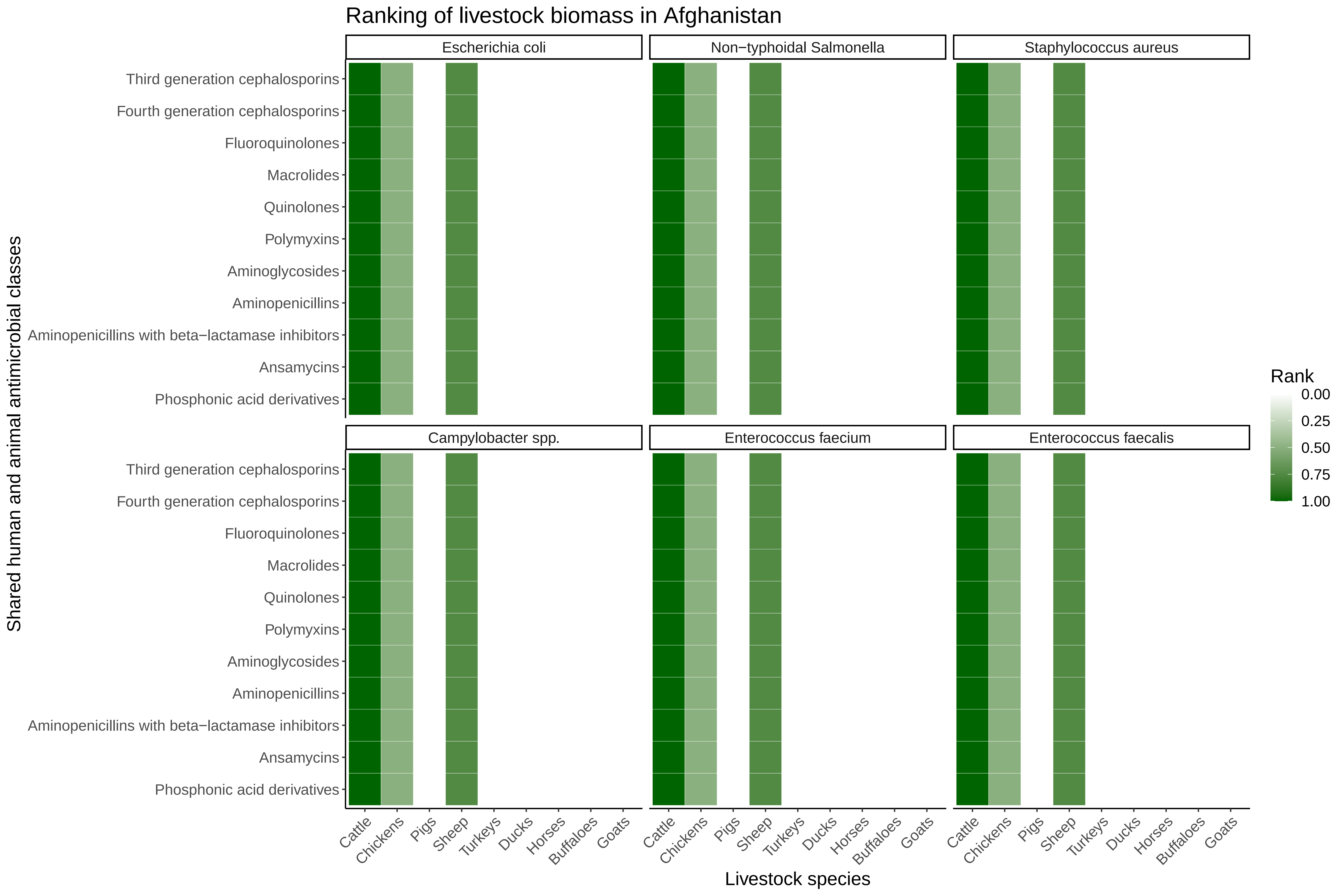
Ranking of livestock biomass in Afghanistan
Escherichia coli
Non−typhoidal Salmonella
Staphylococcus aureus
Campylobacter spp.
Enterococcus faecium
Enterococcus faecalis
Third generation cephalosporins
Fourth generation cephalosporins
Fluoroquinolones
Macrolides
Quinolones
Polymyxins
Aminoglycosides
Aminopenicillins
Aminopenicillins with beta−lactamase inhibitors
Ansamycins
Phosphonic acid derivatives
Shared human and animal antimicrobial classes
Cattle
Chickens
Pigs
Sheep
Turkeys
Ducks
Horses
Buffaloes
Goats
Livestock species
Rank
0.00
0.25
0.50
0.75
1.00

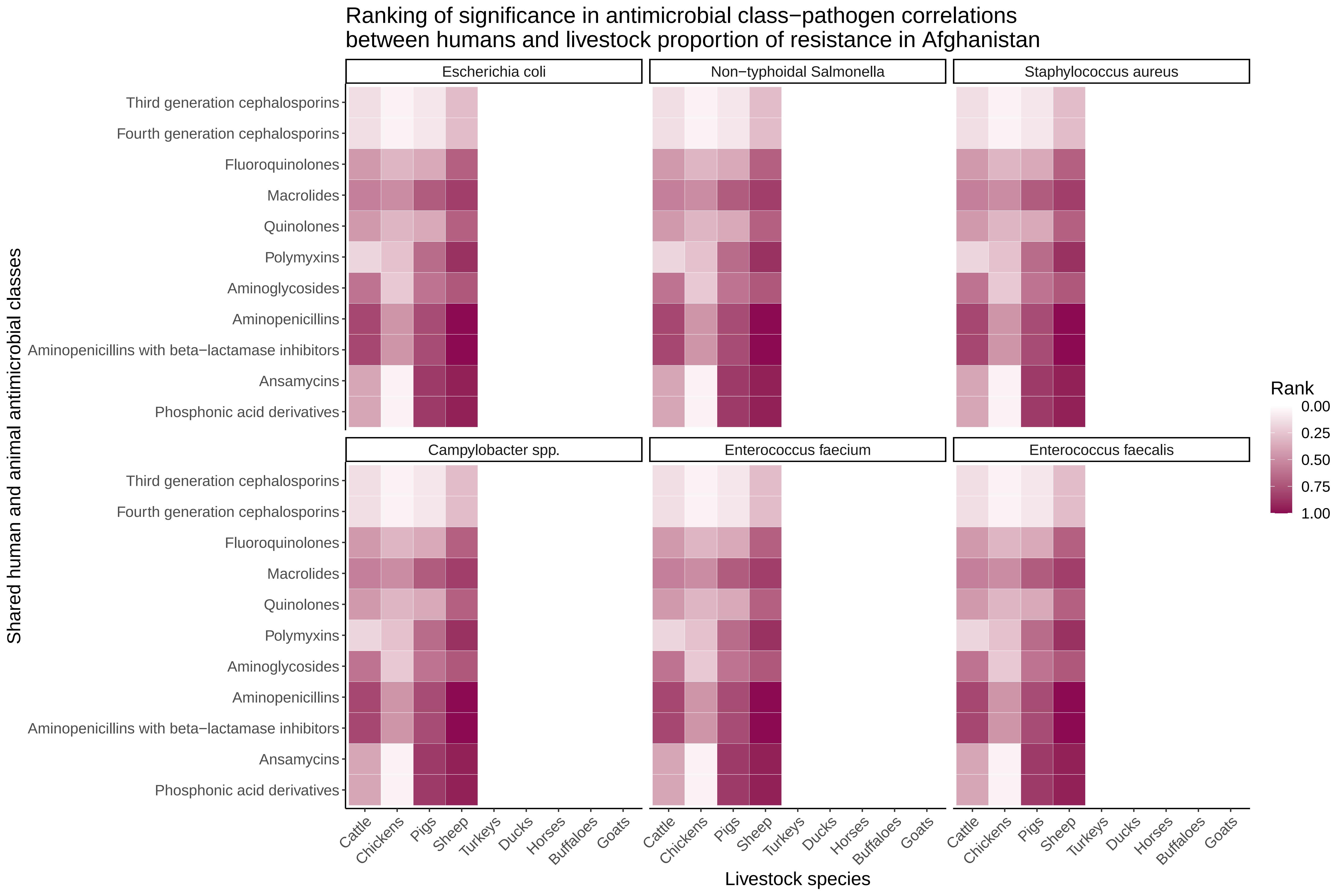
Ranking of significance in antimicrobial class−pathogen correlations
between humans and livestock proportion of resistance in Afghanistan
Escherichia coli
Non−typhoidal Salmonella
Staphylococcus aureus
Campylobacter spp.
Enterococcus faecium
Enterococcus faecalis
Third generation cephalosporins
Fourth generation cephalosporins
Fluoroquinolones
Macrolides
Quinolones
Polymyxins
Aminoglycosides
Aminopenicillins
Aminopenicillins with beta−lactamase inhibitors
Ansamycins
Phosphonic acid derivatives
Shared human and animal antimicrobial classes
Cattle
Chickens
Pigs
Sheep
Turkeys
Ducks
Horses
Buffaloes
Goats
Livestock species
Rank
0.00
0.25
0.50
0.75
1.00

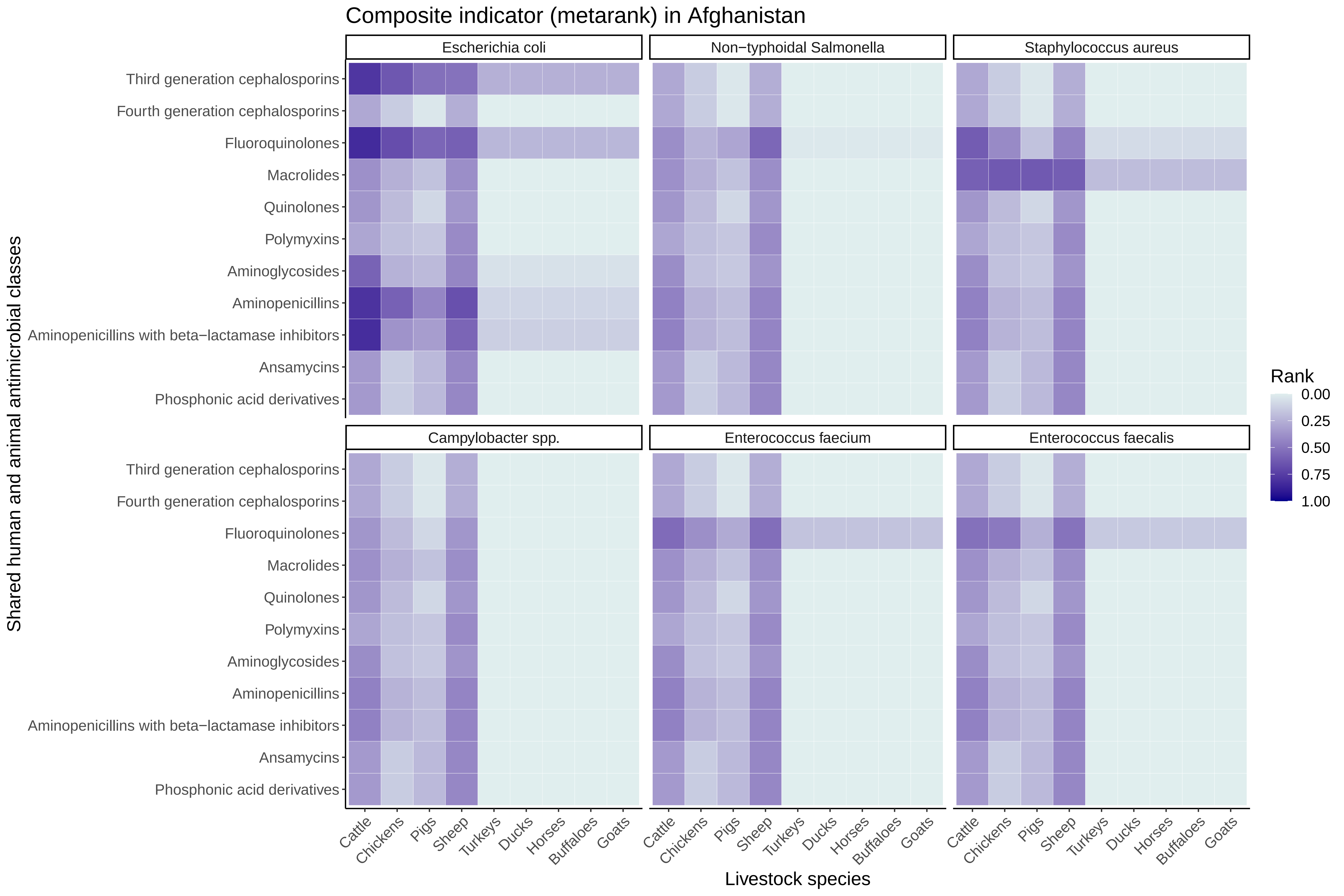

Composite indicator (metarank) in Afghanistan
Escherichia coli
Non−typhoidal Salmonella
Staphylococcus aureus
Campylobacter spp.
Enterococcus faecium
Enterococcus faecalis
Shared human and animal antimicrobial classes
Third generation cephalosporins
Fourth generation cephalosporins
Fluoroquinolones
Macrolides
Quinolones
Polymyxins
Aminoglycosides
Aminopenicillins
Aminopenicillins with beta−lactamase inhibitors
Ansamycins
Phosphonic acid derivatives
Cattle
Chickens
Pigs
Sheep
Turkeys
Ducks
Horses
Buffaloes
Goats
Livestock species
Rank
0.00
0.25
0.50
0.75
1.00

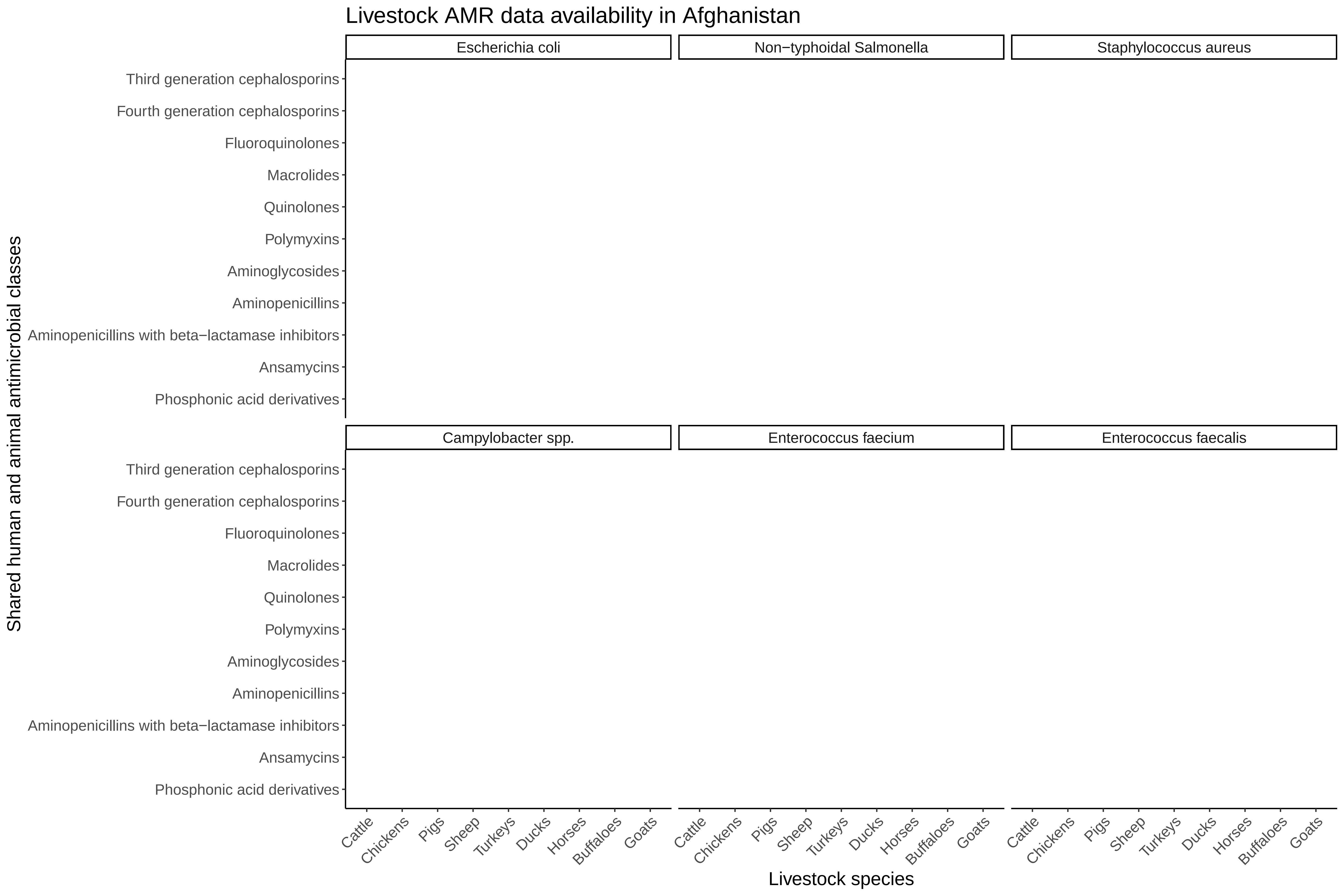
Livestock AMR data availability in Afghanistan
Escherichia coli
Non−typhoidal Salmonella
Staphylococcus aureus
Campylobacter spp.
Enterococcus faecium
Enterococcus faecalis
Third generation cephalosporins
Fourth generation cephalosporins
Fluoroquinolones
Macrolides
Quinolones
Polymyxins
Aminoglycosides
Aminopenicillins
Aminopenicillins with beta−lactamase inhibitors
Ansamycins
Phosphonic acid derivatives
Shared human and animal antimicrobial classes
Cattle
Chickens
Pigs
Sheep
Turkeys
Ducks
Horses
Buffaloes
Goats
Livestock species

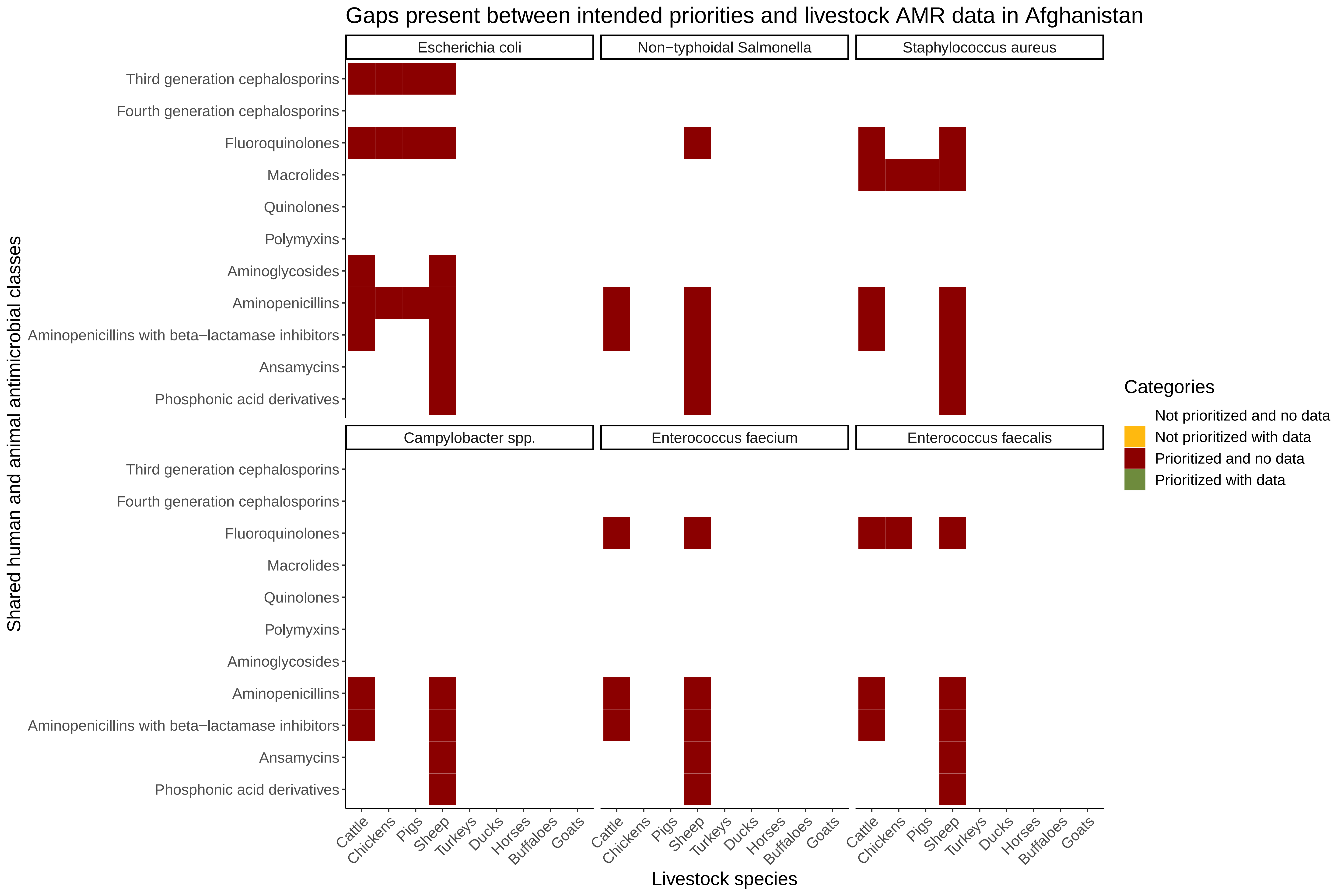

Gaps present between intended priorities and livestock AMR data in Afghanistan
Escherichia coli
Non−typhoidal Salmonella
Staphylococcus aureus
Campylobacter spp.
Enterococcus faecium
Enterococcus faecalis
Third generation cephalosporins
Fourth generation cephalosporins
Fluoroquinolones
Macrolides
Quinolones
Polymyxins
Aminoglycosides
Aminopenicillins
Aminopenicillins with beta−lactamase inhibitors
Ansamycins
Phosphonic acid derivatives
Shared human and animal antimicrobial classes
Cattle
Chickens
Pigs
Sheep
Turkeys
Ducks
Horses
Buffaloes
Goats
Livestock species
Categories
Not prioritized and no data
Not prioritized with data
Prioritized and no data
Prioritized with data

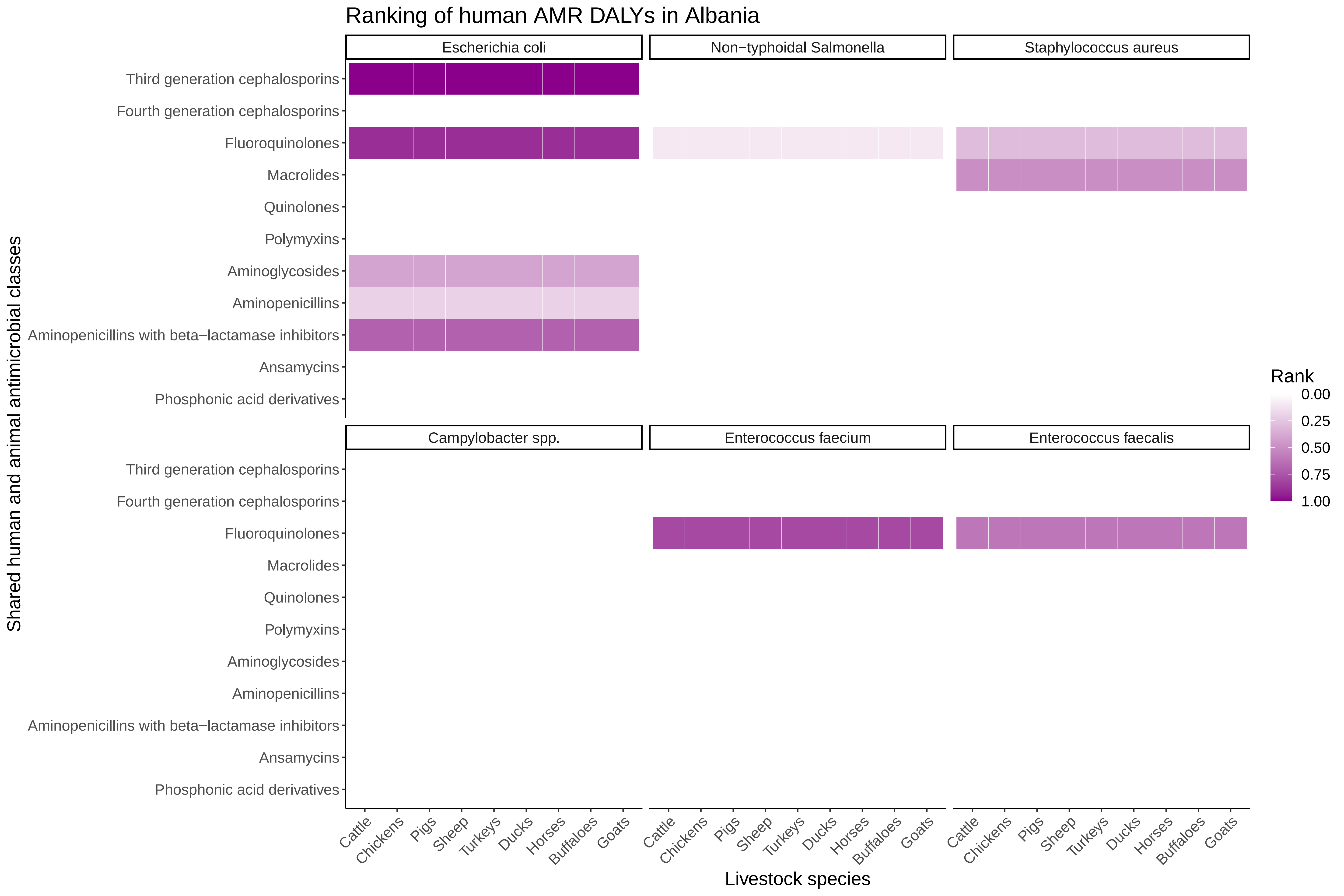

Ranking of human AMR DALYs in Albania
Escherichia coli
Non−typhoidal Salmonella
Staphylococcus aureus
Campylobacter spp.
Enterococcus faecium
Enterococcus faecalis
Third generation cephalosporins
Fourth generation cephalosporins
Fluoroquinolones
Macrolides
Quinolones
Polymyxins
Aminoglycosides
Aminopenicillins
Aminopenicillins with beta−lactamase inhibitors
Ansamycins
Phosphonic acid derivatives
Shared human and animal antimicrobial classes
Cattle
Chickens
Pigs
Sheep
Turkeys
Ducks
Horses
Buffaloes
Goats
Livestock species
Rank
0.00
0.25
0.50
0.75
1.00

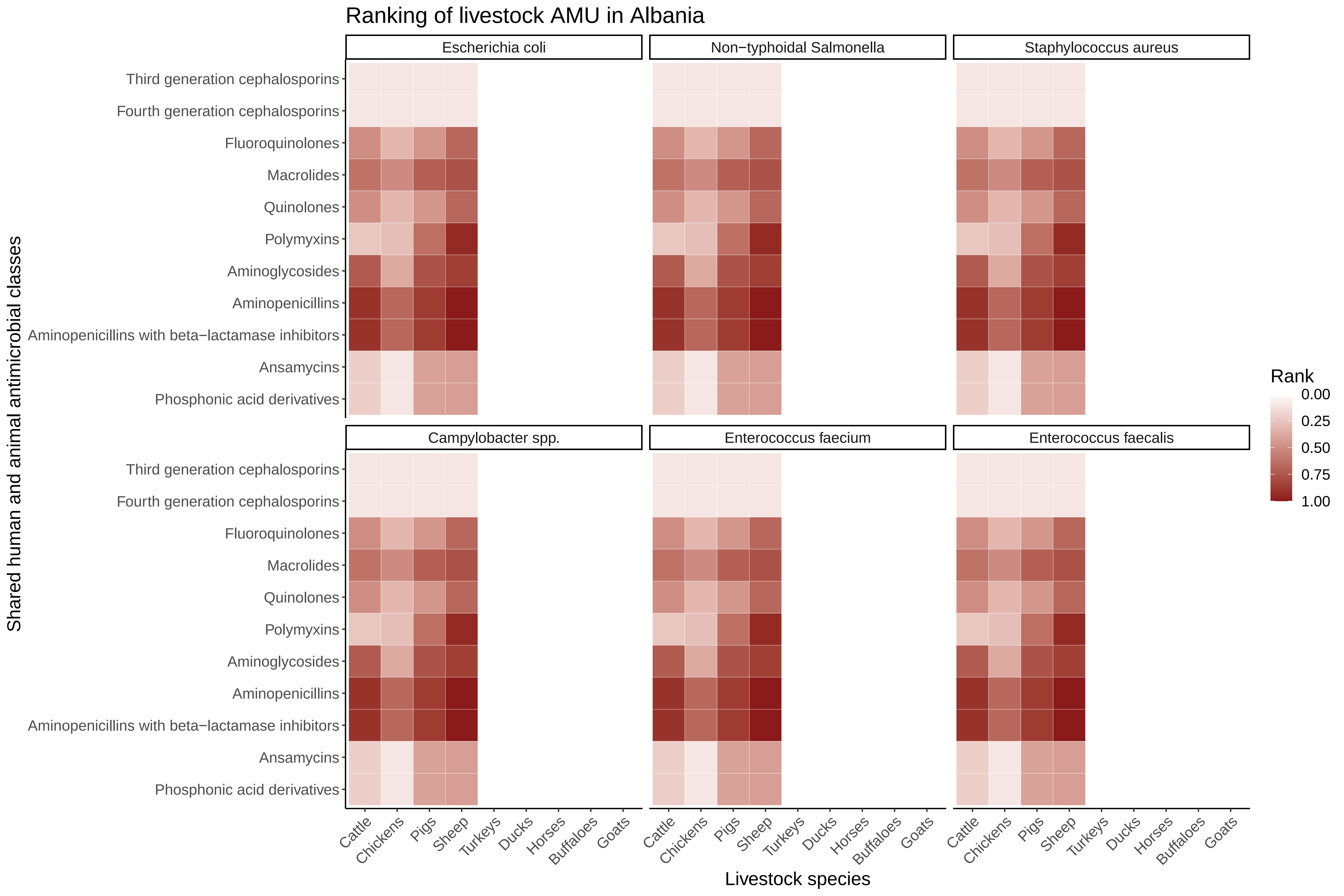
Ranking of livestock AMU in Albania
Escherichia coli
Non−typhoidal Salmonella
Staphylococcus aureus
Campylobacter spp.
Enterococcus faecium
Enterococcus faecalis
Third generation cephalosporins
Fourth generation cephalosporins
Fluoroquinolones
Macrolides
Quinolones
Polymyxins
Aminoglycosides
Aminopenicillins
Aminopenicillins with beta−lactamase inhibitors
Ansamycins
Phosphonic acid derivatives
Shared human and animal antimicrobial classes
Cattle
Chickens
Pigs
Sheep
Turkeys
Ducks
Horses
Buffaloes
Goats
Livestock species
Rank
0.00
0.25
0.50
0.75
1.00

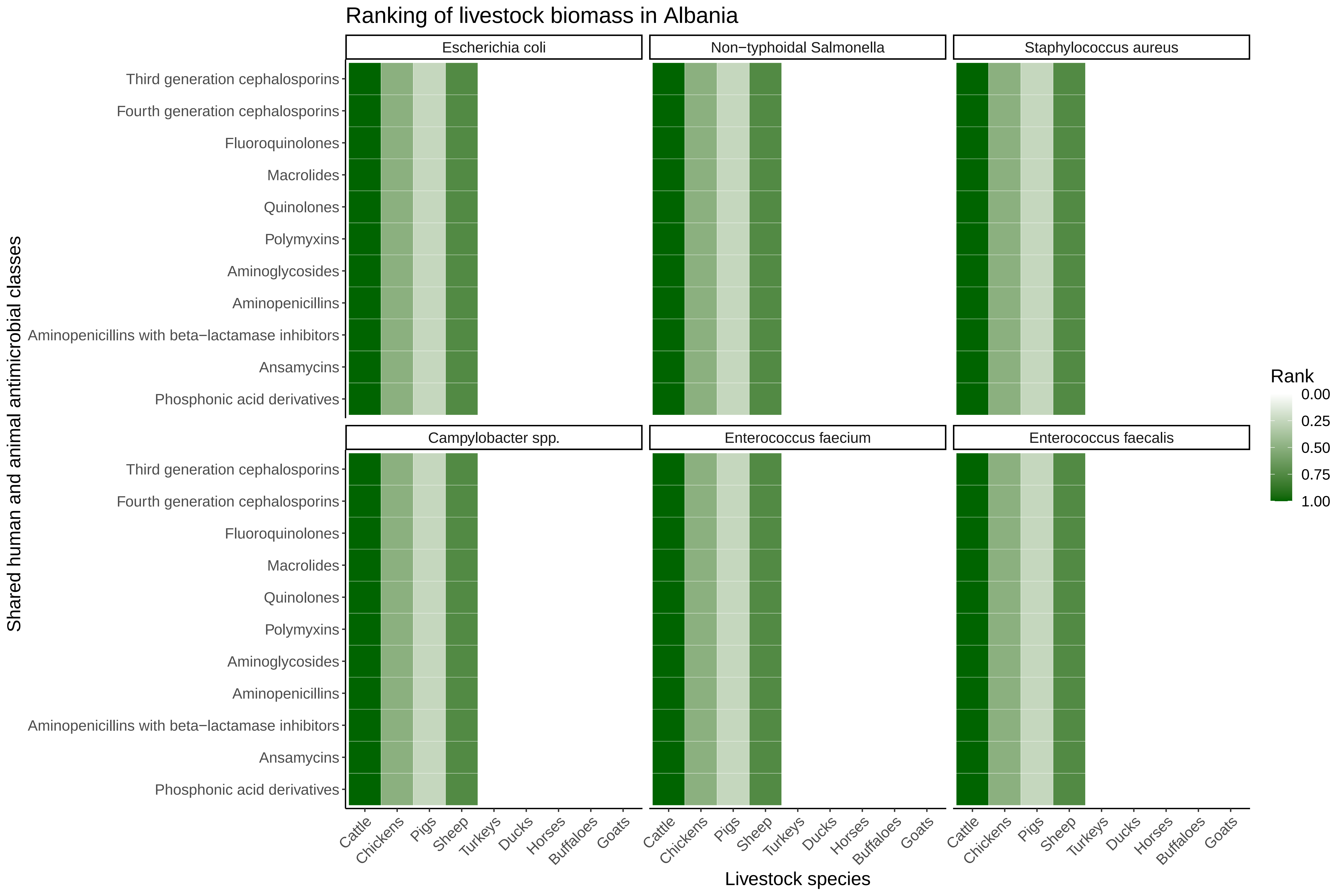
Ranking of livestock biomass in Albania
Escherichia coli
Non−typhoidal Salmonella
Staphylococcus aureus
Campylobacter spp.
Enterococcus faecium
Enterococcus faecalis
Shared human and animal antimicrobial classes
Third generation cephalosporins
Fourth generation cephalosporins
Fluoroquinolones
Macrolides
Quinolones
Polymyxins
Aminoglycosides
Aminopenicillins
Aminopenicillins with beta−lactamase inhibitors
Ansamycins
Phosphonic acid derivatives
Cattle
Chickens
Pigs
Sheep
Turkeys
Ducks
Horses
Buffaloes
Goats
Livestock species
Rank
0.00
0.25
0.50
0.75
1.00

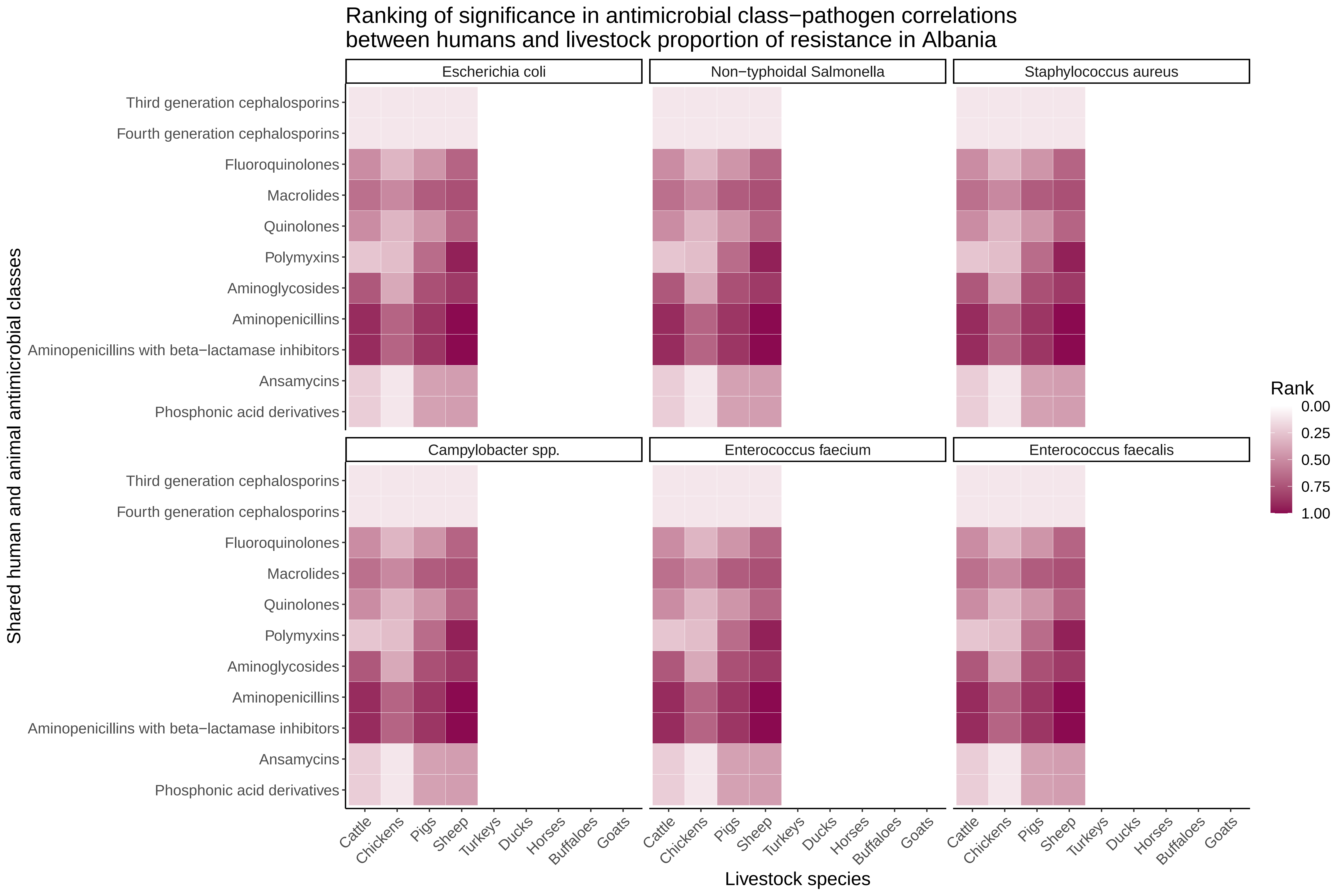

Ranking of significance in antimicrobial class−pathogen correlations
between humans and livestock proportion of resistance in Albania
Escherichia coli
Non−typhoidal Salmonella
Staphylococcus aureus
Campylobacter spp.
Enterococcus faecium
Enterococcus faecalis
Third generation cephalosporins
Fourth generation cephalosporins
Fluoroquinolones
Macrolides
Quinolones
Polymyxins
Aminoglycosides
Aminopenicillins
Aminopenicillins with beta−lactamase inhibitors
Ansamycins
Phosphonic acid derivatives
Shared human and animal antimicrobial classes
Cattle
Chickens
Pigs
Sheep
Turkeys
Ducks
Horses
Buffaloes
Goats
Livestock species
Rank
0.00
0.25
0.50
0.75
1.00

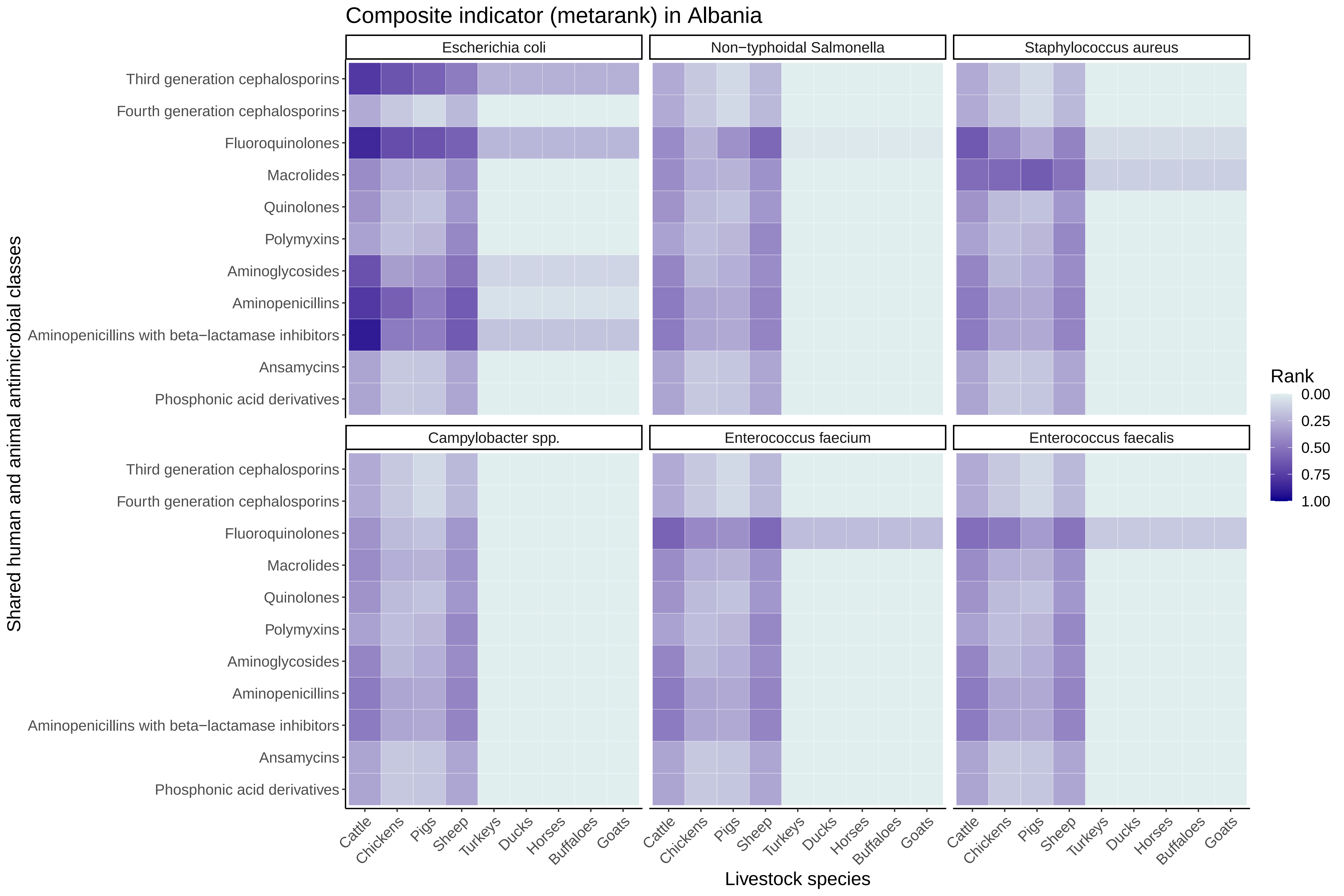

Composite indicator (metarank) in Albania
Escherichia coli
Non−typhoidal Salmonella
Staphylococcus aureus
Campylobacter spp.
Enterococcus faecium
Enterococcus faecalis
Third generation cephalosporins
Fourth generation cephalosporins
Fluoroquinolones
Macrolides
Quinolones
Polymyxins
Aminoglycosides
Aminopenicillins
Aminopenicillins with beta−lactamase inhibitors
Ansamycins
Phosphonic acid derivatives
Shared human and animal antimicrobial classes
Cattle
Chickens
Pigs
Sheep
Turkeys
Ducks
Horses
Buffaloes
Goats
Livestock species
Rank
0.00
0.25
0.50
0.75
1.00

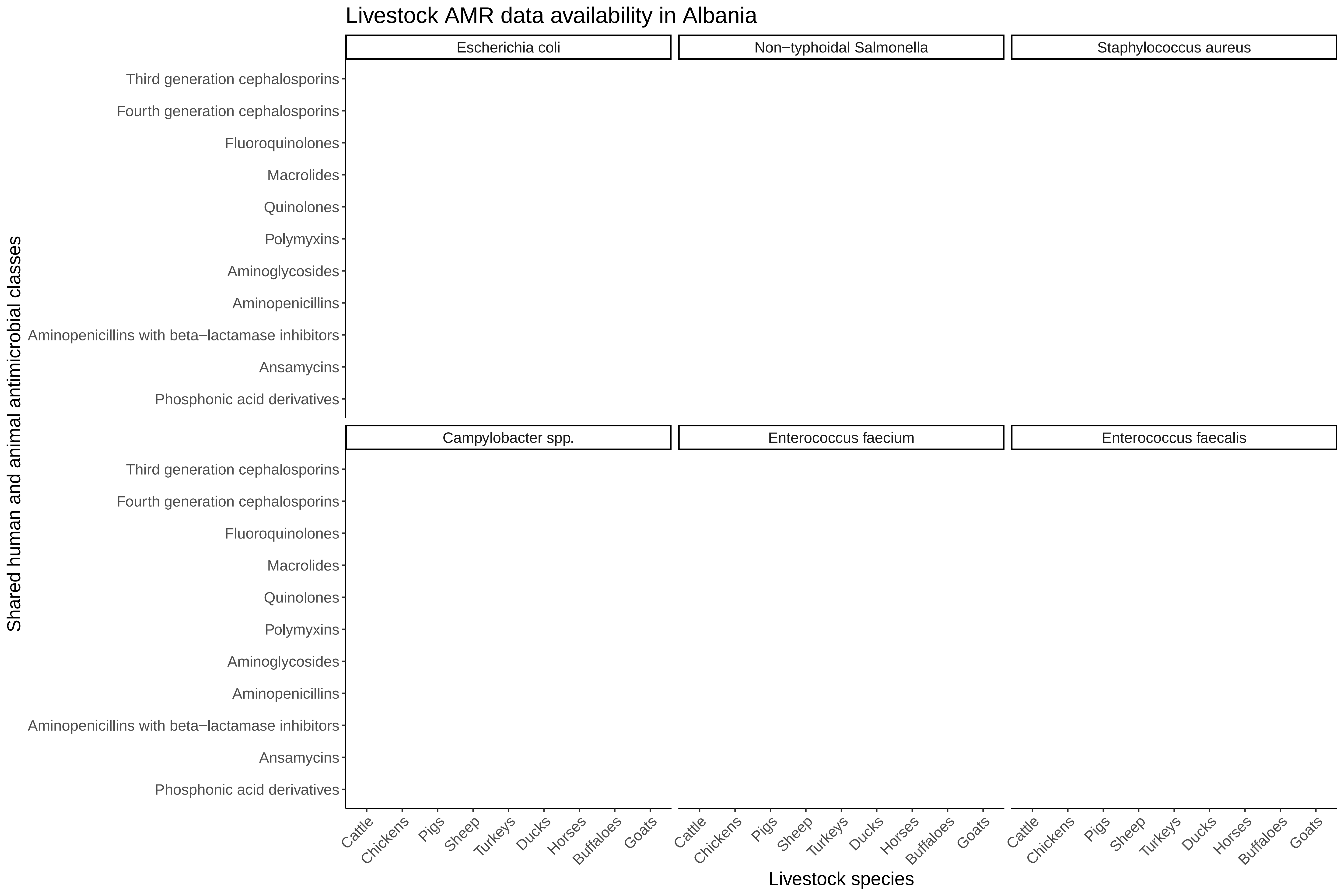
Livestock AMR data availability in Albania
Escherichia coli
Non−typhoidal Salmonella
Staphylococcus aureus
Campylobacter spp.
Enterococcus faecium
Enterococcus faecalis
Third generation cephalosporins
Fourth generation cephalosporins
Fluoroquinolones
Macrolides
Quinolones
Polymyxins
Aminoglycosides
Aminopenicillins
Aminopenicillins with beta−lactamase inhibitors
Ansamycins
Phosphonic acid derivatives
Shared human and animal antimicrobial classes
Cattle
Chickens
Pigs
Sheep
Turkeys
Ducks
Horses
Buffaloes
Goats
Livestock species

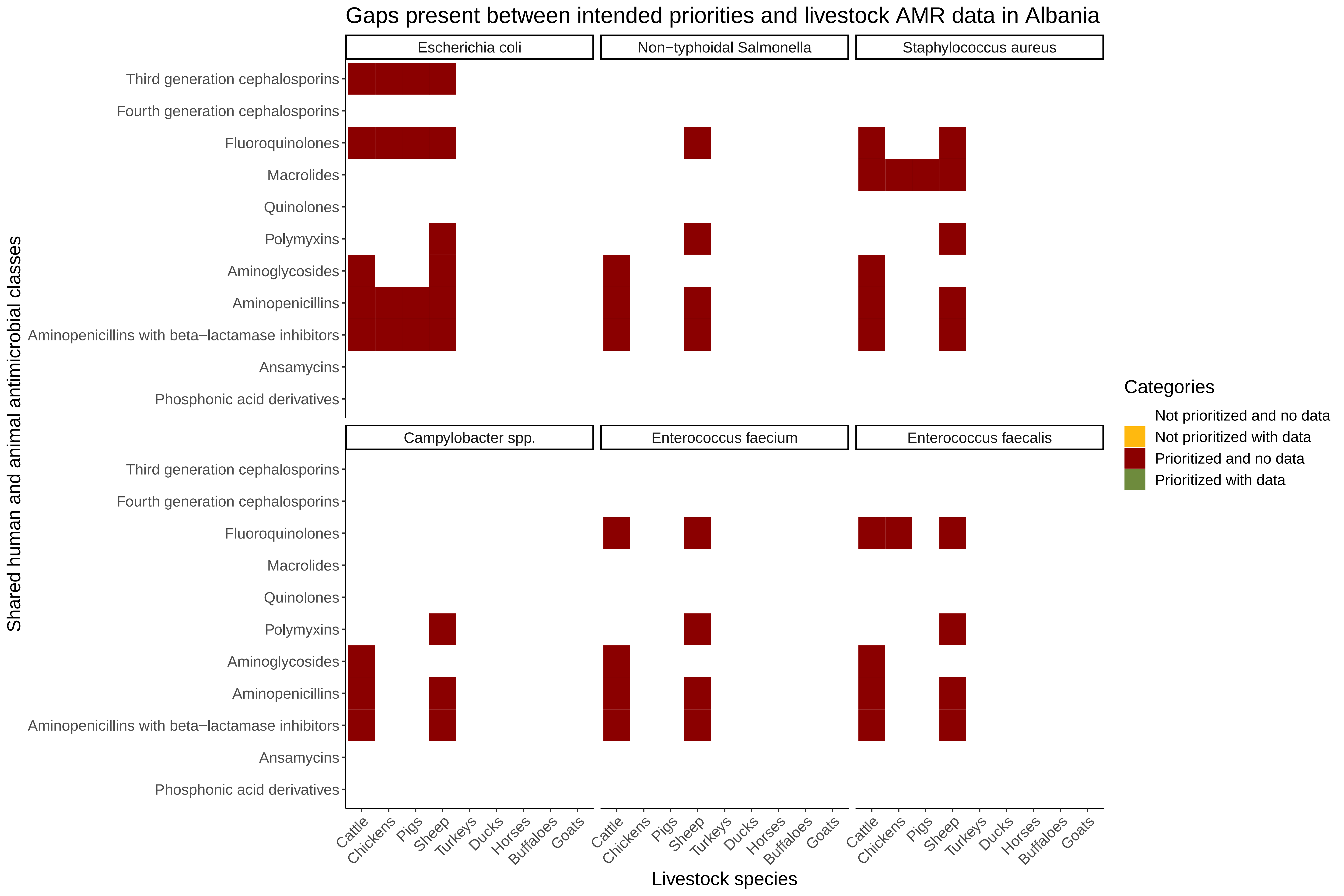

Gaps present between intended priorities and livestock AMR data in Albania
Escherichia coli
Non−typhoidal Salmonella
Staphylococcus aureus
Campylobacter spp.
Enterococcus faecium
Enterococcus faecalis
Third generation cephalosporins
Fourth generation cephalosporins
Fluoroquinolones
Macrolides
Quinolones
Polymyxins
Aminoglycosides
Aminopenicillins
Aminopenicillins with beta−lactamase inhibitors
Ansamycins
Phosphonic acid derivatives
Shared human and animal antimicrobial classes
Cattle
Chickens
Pigs
Sheep
Turkeys
Ducks
Horses
Buffaloes
Goats
Livestock species
Categories
Not prioritized and no data
Not prioritized with data
Prioritized and no data
Prioritized with data

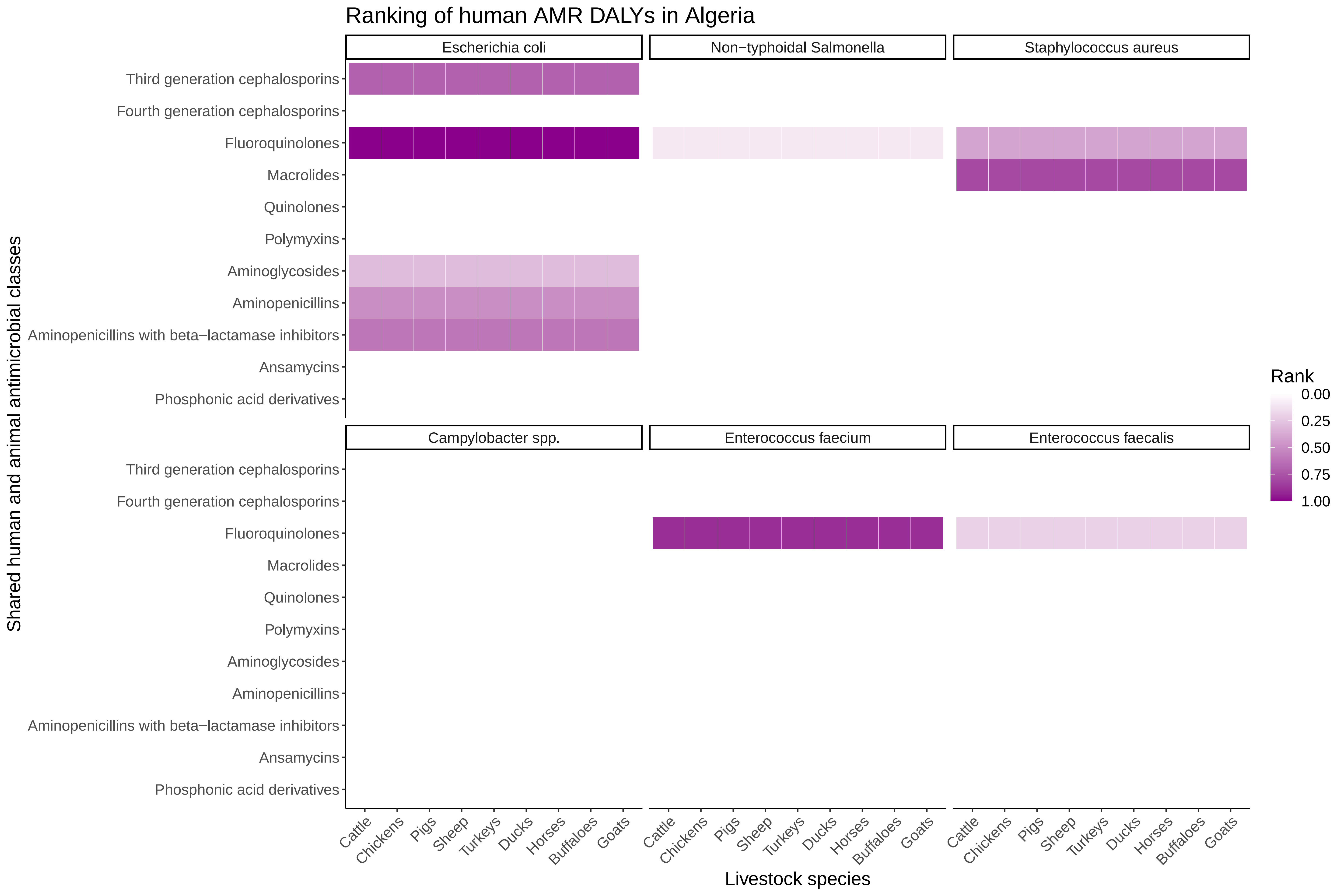

Ranking of human AMR DALYs in Algeria
Escherichia coli
Non−typhoidal Salmonella
Staphylococcus aureus
Campylobacter spp.
Enterococcus faecium
Enterococcus faecalis
Third generation cephalosporins
Fourth generation cephalosporins
Fluoroquinolones
Macrolides
Quinolones
Polymyxins
Aminoglycosides
Aminopenicillins
Aminopenicillins with beta−lactamase inhibitors
Ansamycins
Phosphonic acid derivatives
Shared human and animal antimicrobial classes
Cattle
Chickens
Pigs
Sheep
Turkeys
Ducks
Horses
Buffaloes
Goats
Livestock species
Rank
0.00
0.25
0.50
0.75
1.00

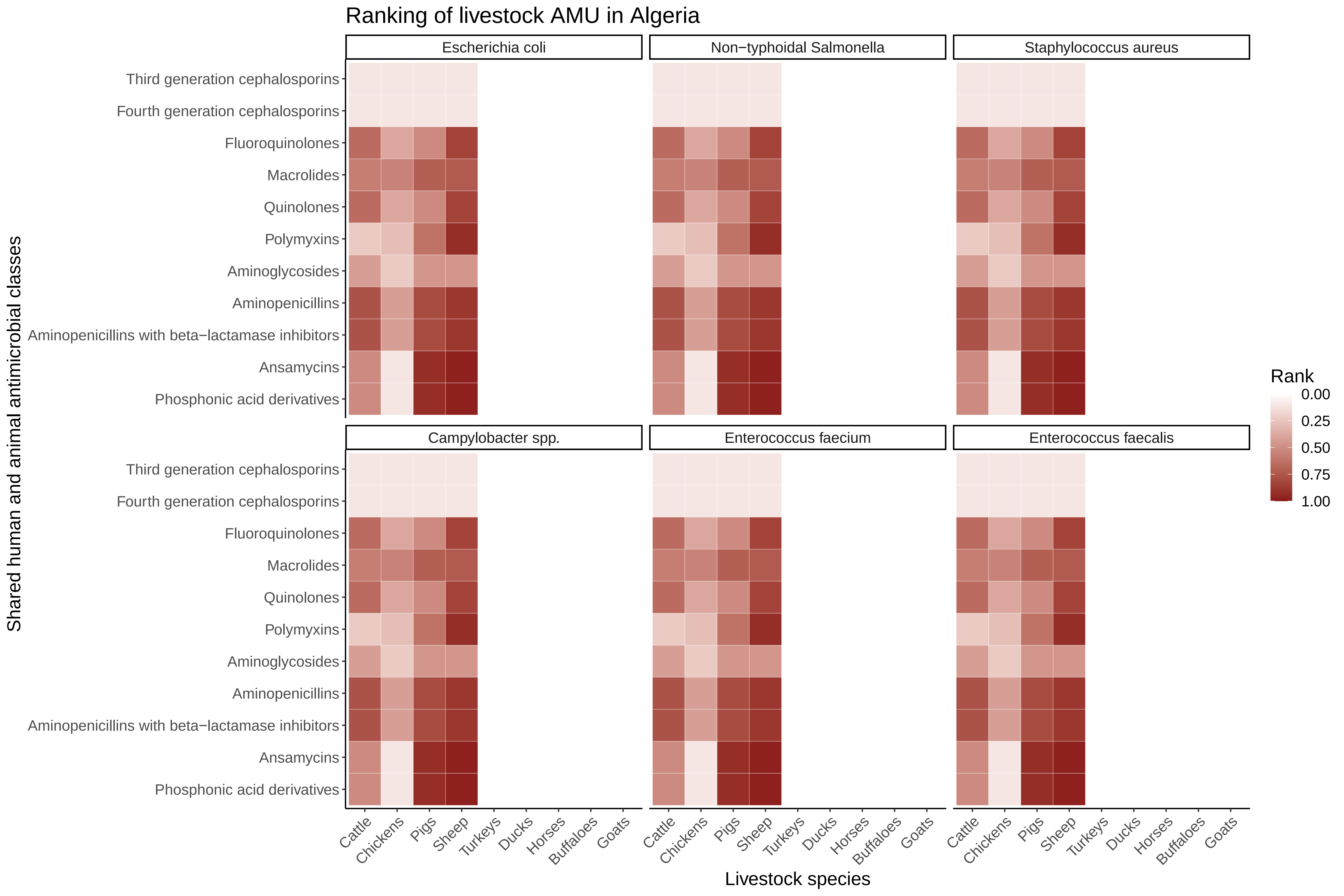
Ranking of livestock AMU in Algeria
Escherichia coli
Non−typhoidal Salmonella
Staphylococcus aureus
Campylobacter spp.
Enterococcus faecium
Enterococcus faecalis
Shared human and animal antimicrobial classes
Third generation cephalosporins
Fourth generation cephalosporins
Fluoroquinolones
Macrolides
Quinolones
Polymyxins
Aminoglycosides
Aminopenicillins
Aminopenicillins with beta−lactamase inhibitors
Ansamycins
Phosphonic acid derivatives
Cattle
Chickens
Pigs
Sheep
Turkeys
Ducks
Horses
Buffaloes
Goats
Livestock species
Rank
0.00
0.25
0.50
0.75
1.00

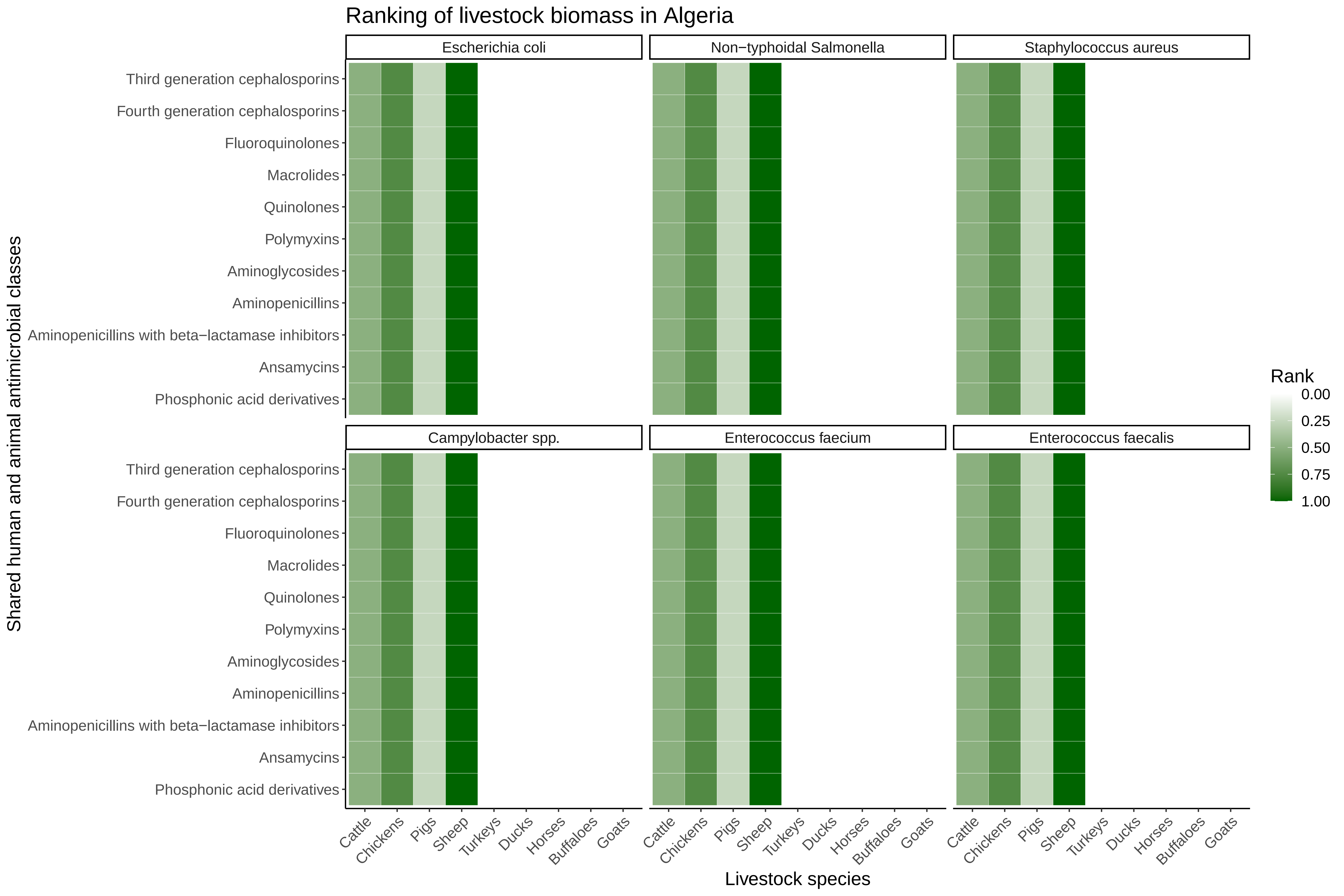

Ranking of livestock biomass in Algeria
Escherichia coli
Non−typhoidal Salmonella
Staphylococcus aureus
Campylobacter spp.
Enterococcus faecium
Enterococcus faecalis
Third generation cephalosporins
Fourth generation cephalosporins
Fluoroquinolones
Macrolides
Quinolones
Polymyxins
Aminoglycosides
Aminopenicillins
Aminopenicillins with beta−lactamase inhibitors
Ansamycins
Phosphonic acid derivatives
Shared human and animal antimicrobial classes
Cattle
Chickens
Pigs
Sheep
Turkeys
Ducks
Horses
Buffaloes
Goats
Livestock species
Rank
0.00
0.25
0.50
0.75
1.00

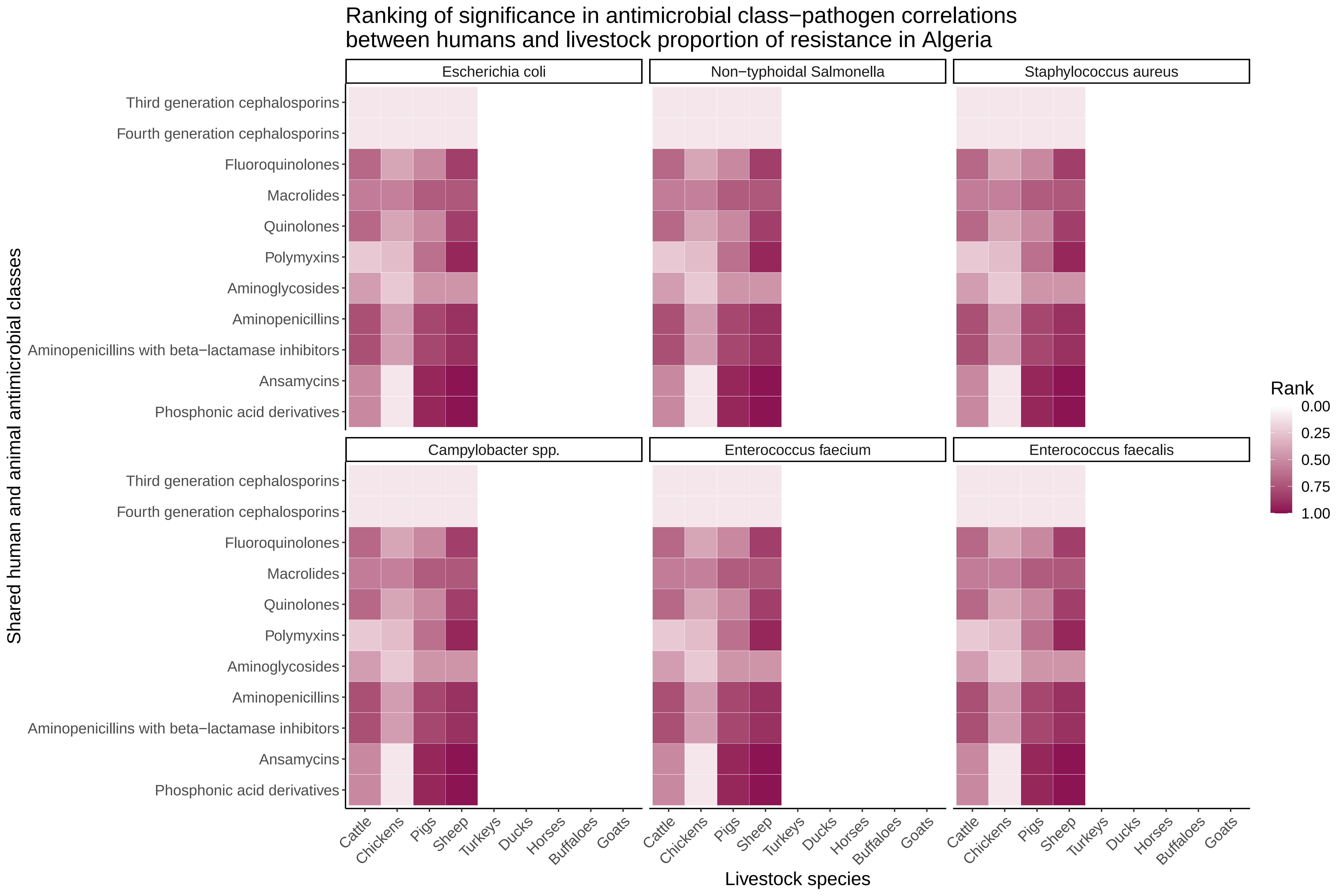

Ranking of significance in antimicrobial class−pathogen correlations
between humans and livestock proportion of resistance in Algeria
Escherichia coli
Non−typhoidal Salmonella
Staphylococcus aureus
Campylobacter spp.
Enterococcus faecium
Enterococcus faecalis
Shared human and animal antimicrobial classes
Third generation cephalosporins
Fourth generation cephalosporins
Fluoroquinolones
Macrolides
Quinolones
Polymyxins
Aminoglycosides
Aminopenicillins
Aminopenicillins with beta−lactamase inhibitors
Ansamycins
Phosphonic acid derivatives
Livestock species
Cattle
Chickens
Pigs
Sheep
Turkeys
Ducks
Horses
Buffaloes
Goats
Rank
0.00
0.25
0.50
0.75
1.00

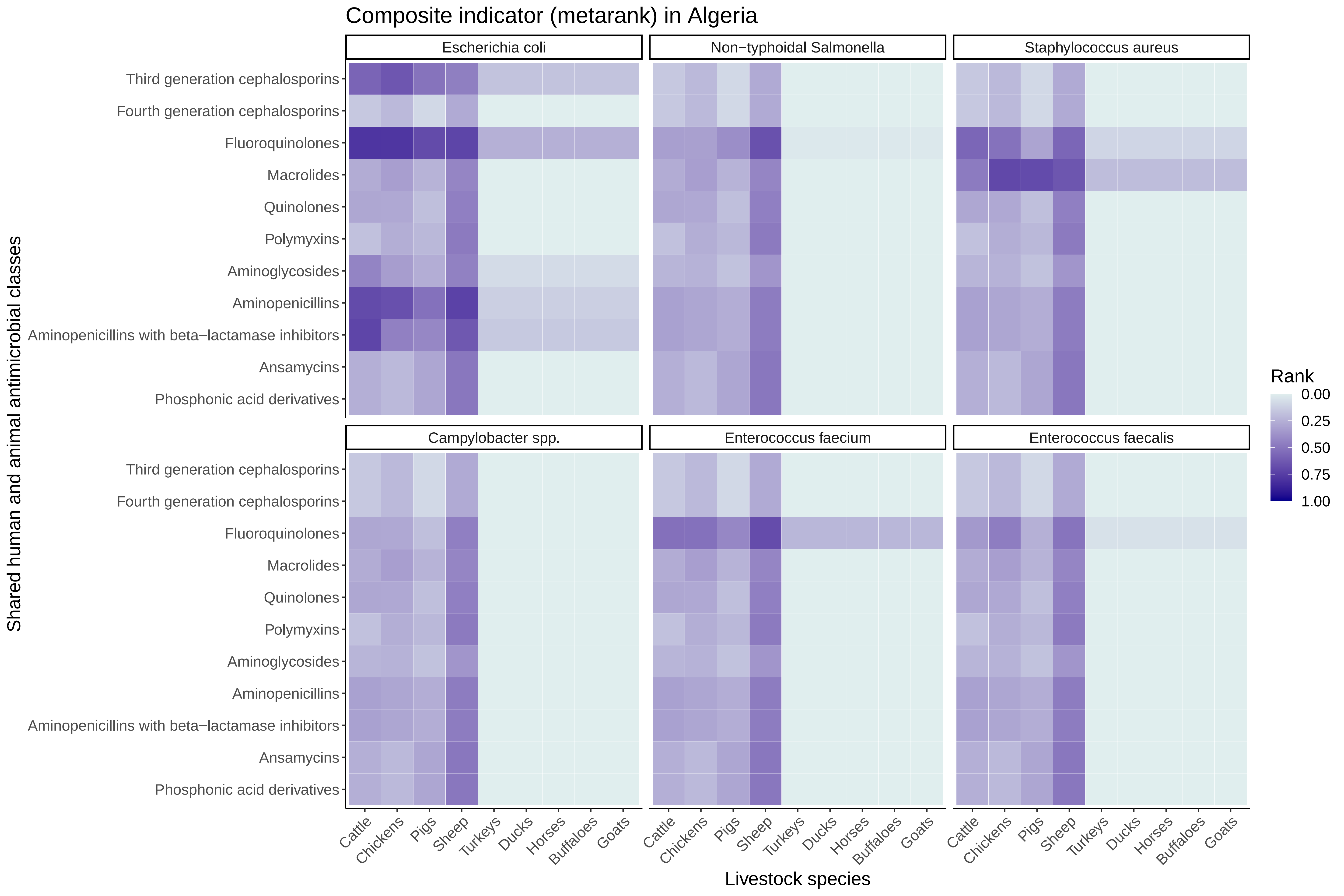
Composite indicator (metarank) in Algeria
Escherichia coli
Non−typhoidal Salmonella
Staphylococcus aureus
Campylobacter spp.
Enterococcus faecium
Enterococcus faecalis
Shared human and animal antimicrobial classes
Third generation cephalosporins
Fourth generation cephalosporins
Fluoroquinolones
Macrolides
Quinolones
Polymyxins
Aminoglycosides
Aminopenicillins
Aminopenicillins with beta−lactamase inhibitors
Ansamycins
Phosphonic acid derivatives
Cattle
Chickens
Pigs
Sheep
Turkeys
Ducks
Horses
Buffaloes
Goats
Livestock species
Rank
0.00
0.25
0.50
0.75
1.00

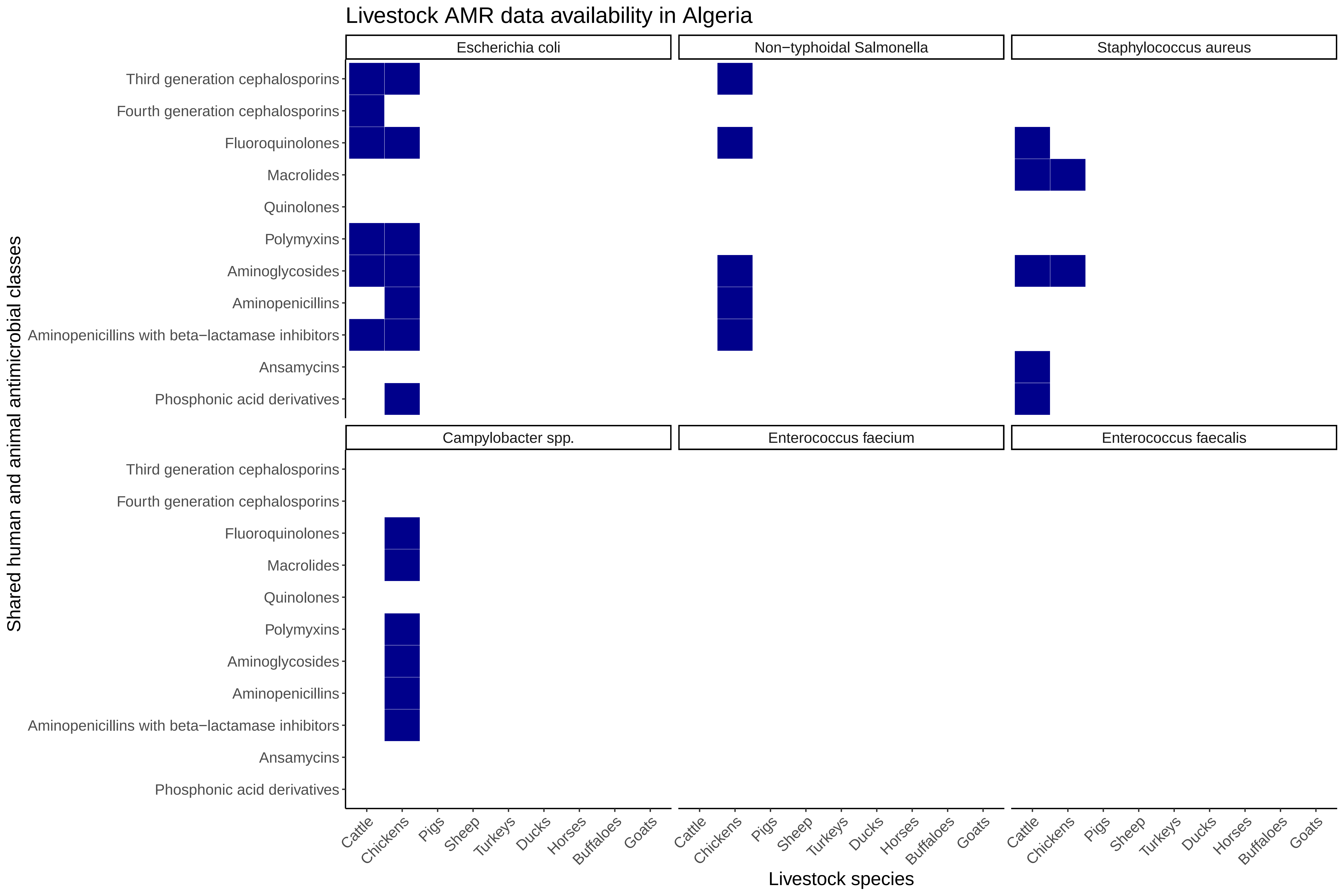

Livestock AMR data availability in Algeria
Escherichia coli
Non−typhoidal Salmonella
Staphylococcus aureus
Campylobacter spp.
Enterococcus faecium
Enterococcus faecalis
Shared human and animal antimicrobial classes
Third generation cephalosporins
Fourth generation cephalosporins
Fluoroquinolones
Macrolides
Quinolones
Polymyxins
Aminoglycosides
Aminopenicillins
Aminopenicillins with beta−lactamase inhibitors
Ansamycins
Phosphonic acid derivatives
Cattle
Chickens
Pigs
Sheep
Turkeys
Ducks
Horses
Buffaloes
Goats
Livestock species

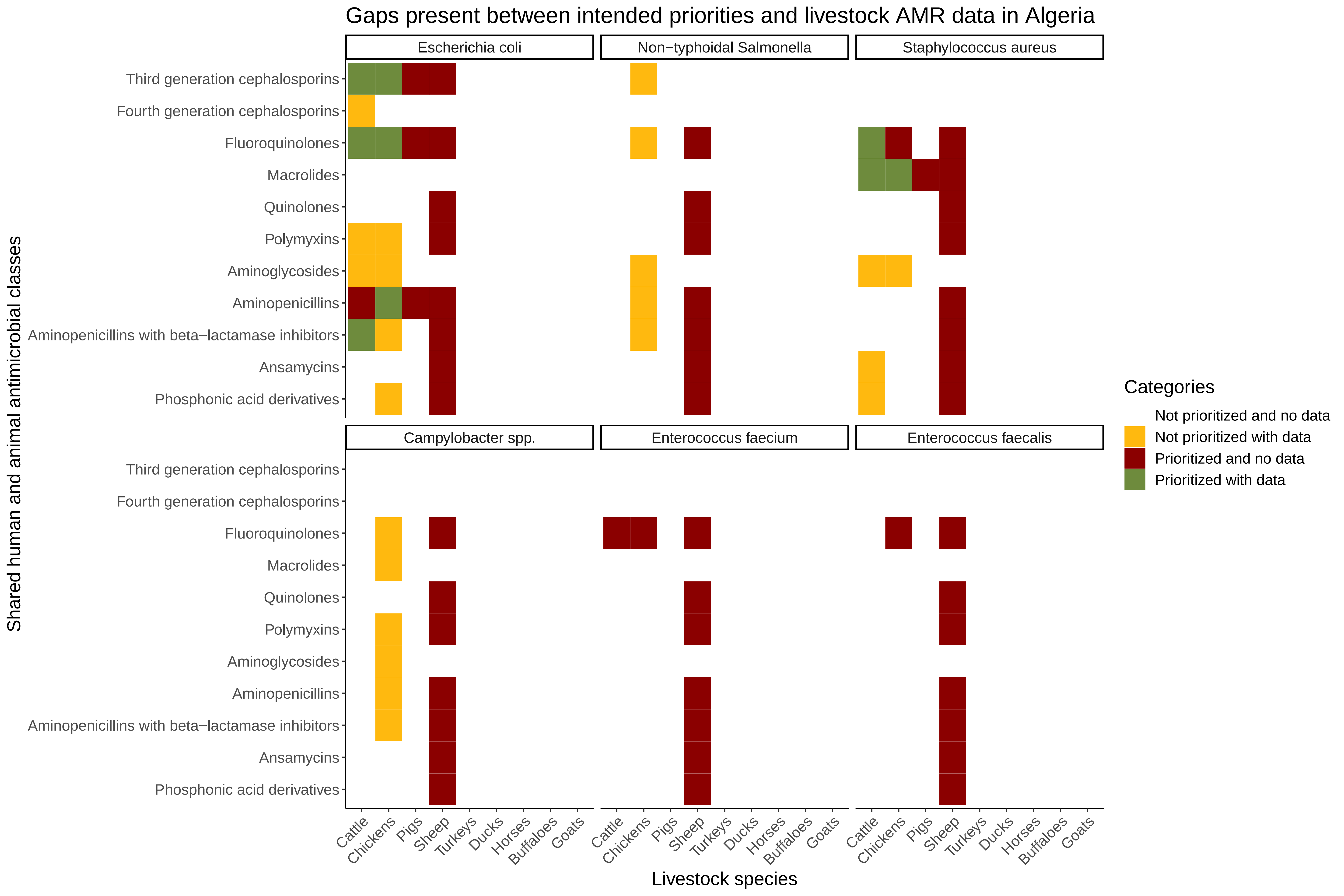

Gaps present between intended priorities and livestock AMR data in Algeria
Escherichia coli
Non−typhoidal Salmonella
Staphylococcus aureus
Campylobacter spp.
Enterococcus faecium
Enterococcus faecalis
Third generation cephalosporins
Fourth generation cephalosporins
Fluoroquinolones
Macrolides
Quinolones
Polymyxins
Aminoglycosides
Aminopenicillins
Aminopenicillins with beta−lactamase inhibitors
Ansamycins
Phosphonic acid derivatives
Shared human and animal antimicrobial classes
Cattle
Chickens
Pigs
Sheep
Turkeys
Ducks
Horses
Buffaloes
Goats
Livestock species
Categories
Not prioritized and no data
Not prioritized with data
Prioritized and no data
Prioritized with data

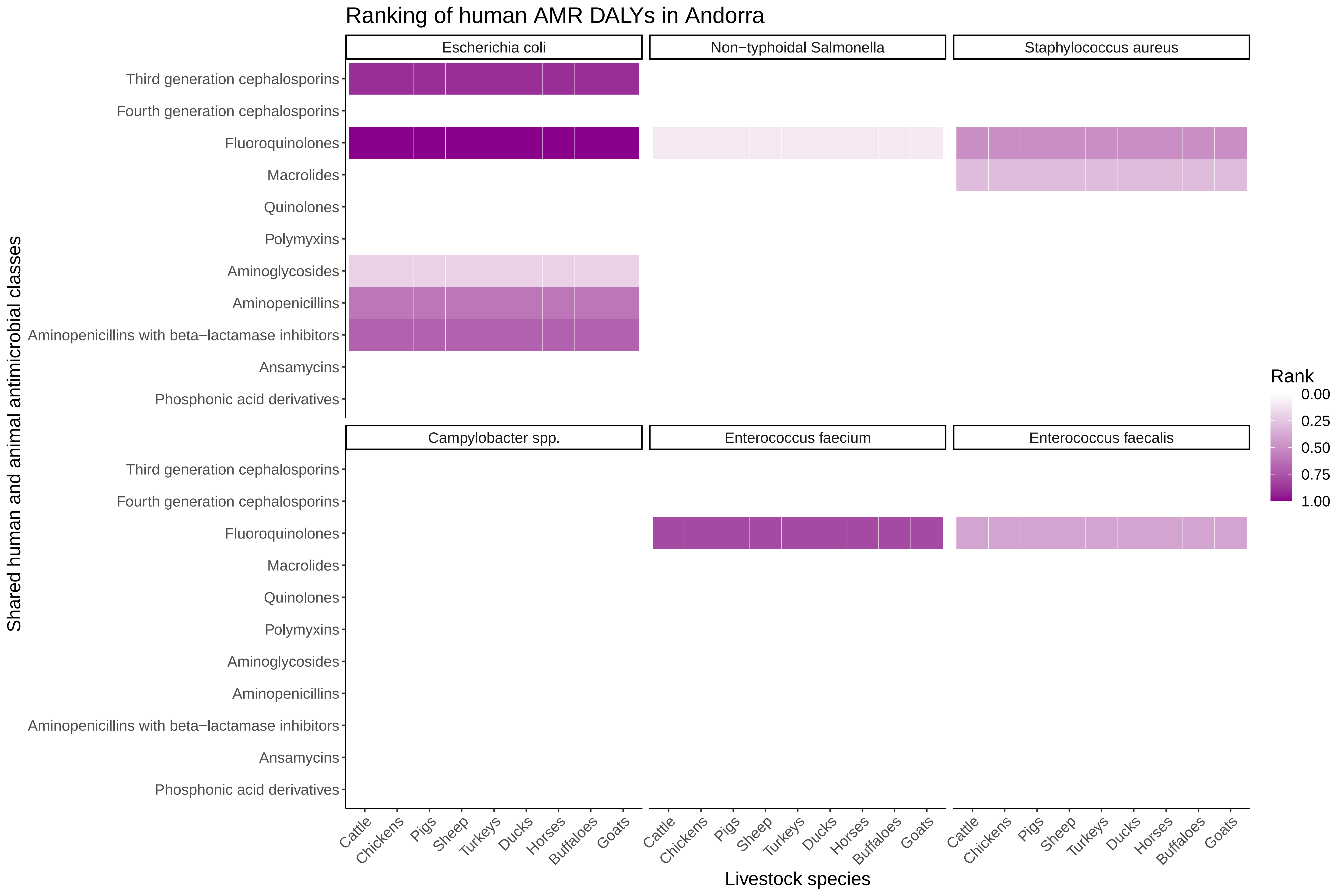

Ranking of human AMR DALYs in Andorra
Escherichia coli
Non−typhoidal Salmonella
Staphylococcus aureus
Campylobacter spp.
Enterococcus faecium
Enterococcus faecalis
Shared human and animal antimicrobial classes
Third generation cephalosporins
Fourth generation cephalosporins
Fluoroquinolones
Macrolides
Quinolones
Polymyxins
Aminoglycosides
Aminopenicillins
Aminopenicillins with beta−lactamase inhibitors
Ansamycins
Phosphonic acid derivatives
Cattle
Chickens
Pigs
Sheep
Turkeys
Ducks
Horses
Buffaloes
Goats
Livestock species
Rank
0.00
0.25
0.50
0.75
1.00

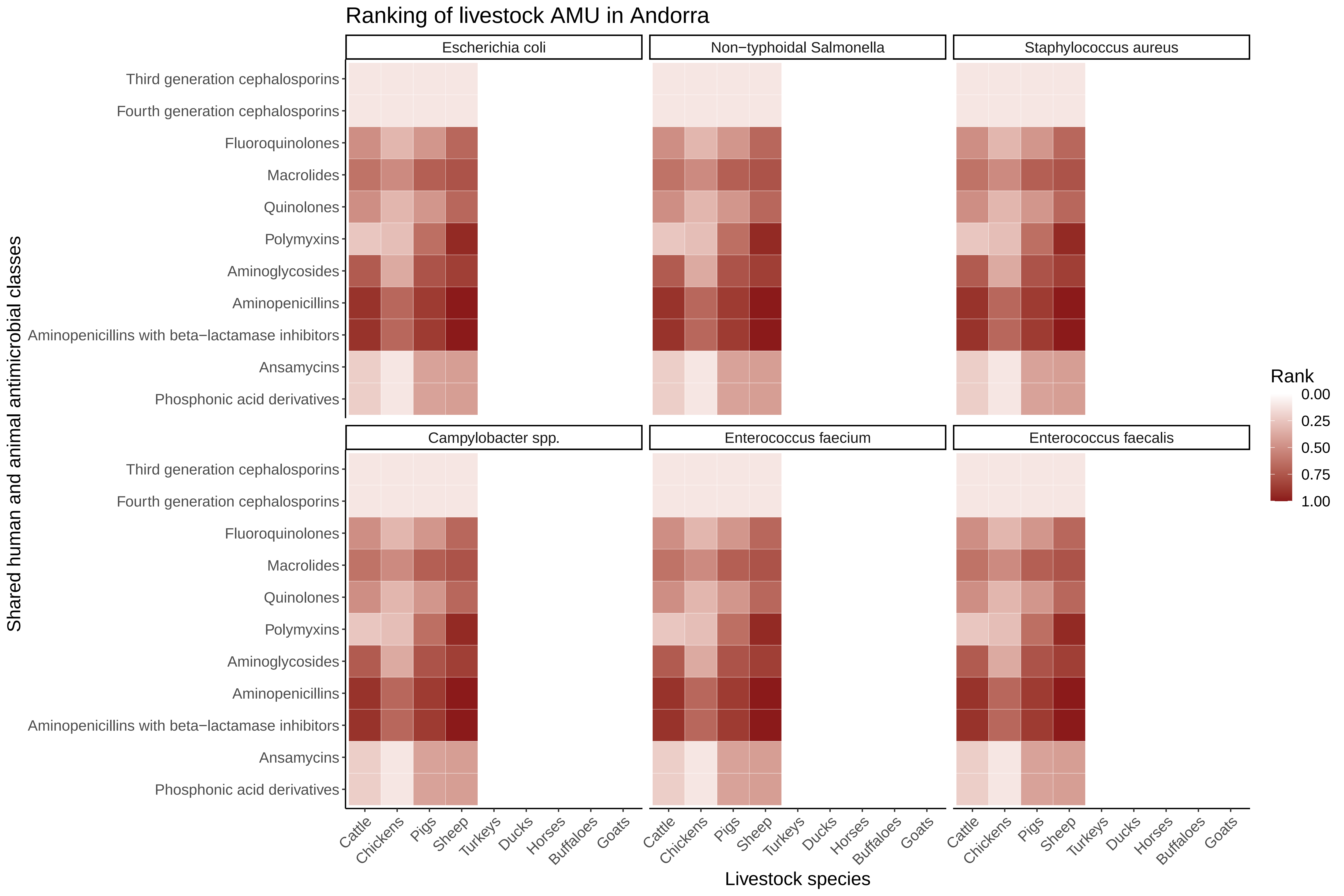
Ranking of livestock AMU in Andorra
Escherichia coli
Non−typhoidal Salmonella
Staphylococcus aureus
Campylobacter spp.
Enterococcus faecium
Enterococcus faecalis
Third generation cephalosporins
Fourth generation cephalosporins
Fluoroquinolones
Macrolides
Quinolones
Polymyxins
Aminoglycosides
Aminopenicillins
Aminopenicillins with beta−lactamase inhibitors
Ansamycins
Phosphonic acid derivatives
Shared human and animal antimicrobial classes
Cattle
Chickens
Pigs
Sheep
Turkeys
Ducks
Horses
Buffaloes
Goats
Livestock species
Rank
0.00
0.25
0.50
0.75
1.00

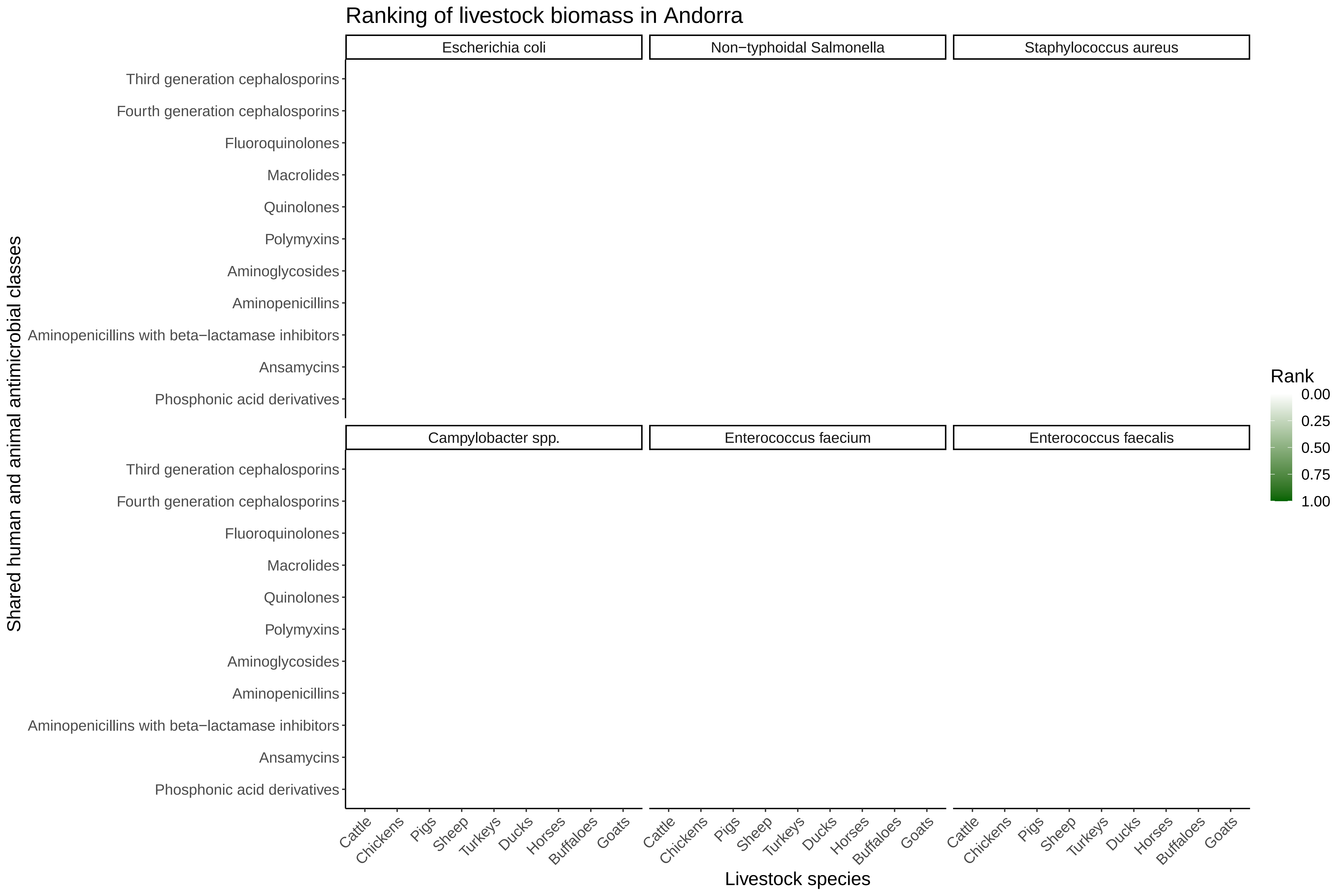
Ranking of livestock biomass in Andorra
Escherichia coli
Non−typhoidal Salmonella
Staphylococcus aureus
Campylobacter spp.
Enterococcus faecium
Enterococcus faecalis
Shared human and animal antimicrobial classes
Third generation cephalosporins
Fourth generation cephalosporins
Fluoroquinolones
Macrolides
Quinolones
Polymyxins
Aminoglycosides
Aminopenicillins
Aminopenicillins with beta−lactamase inhibitors
Ansamycins
Phosphonic acid derivatives
Cattle
Chickens
Pigs
Sheep
Turkeys
Ducks
Horses
Buffaloes
Goats
Livestock species
Rank
0.00
0.25
0.50
0.75
1.00

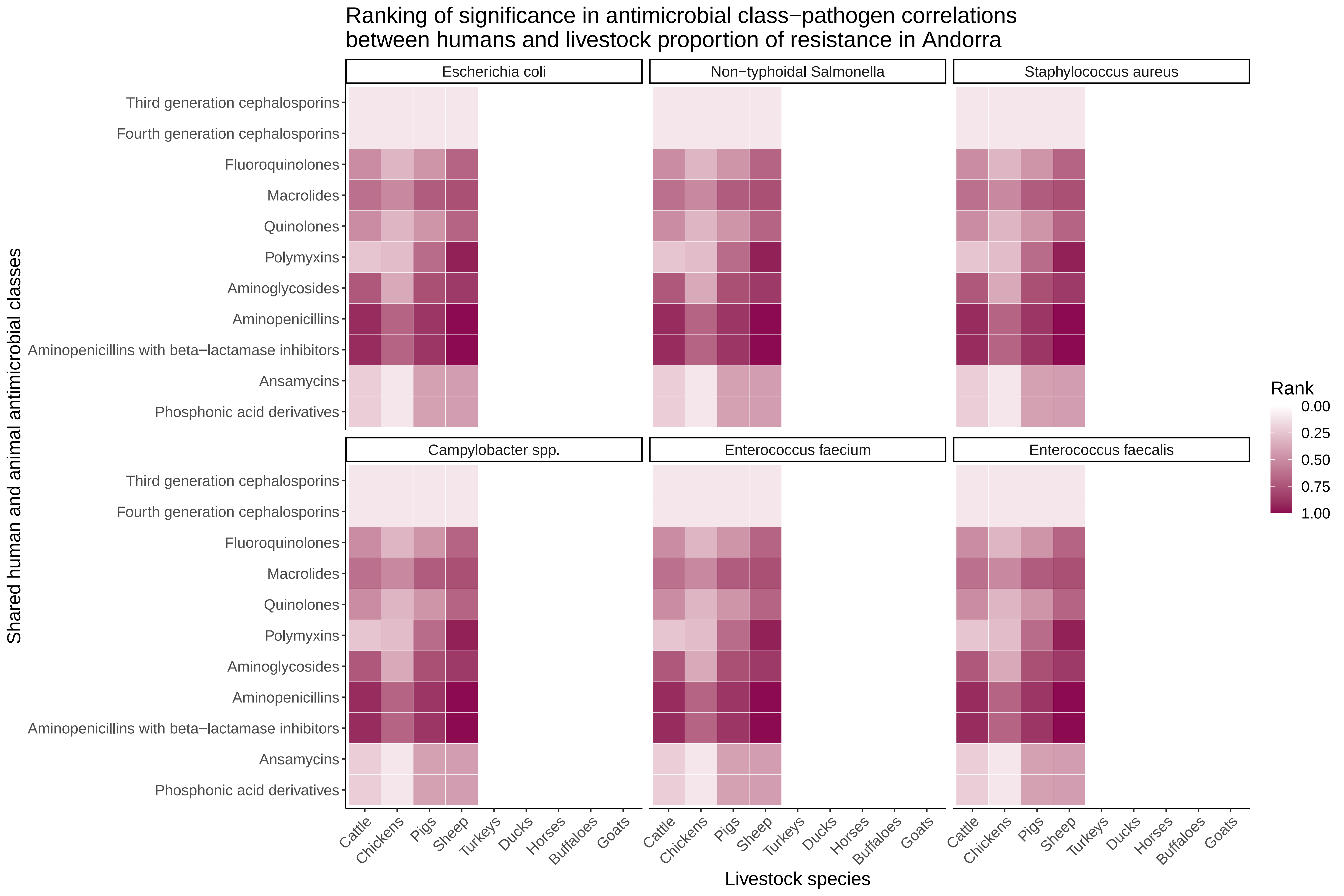
Ranking of significance in antimicrobial class−pathogen correlations
between humans and livestock proportion of resistance in Andorra
Escherichia coli
Non−typhoidal Salmonella
Staphylococcus aureus
Campylobacter spp.
Enterococcus faecium
Enterococcus faecalis
Third generation cephalosporins
Fourth generation cephalosporins
Fluoroquinolones
Macrolides
Quinolones
Polymyxins
Aminoglycosides
Aminopenicillins
Aminopenicillins with beta−lactamase inhibitors
Ansamycins
Phosphonic acid derivatives
Shared human and animal antimicrobial classes
Cattle
Chickens
Pigs
Sheep
Turkeys
Ducks
Horses
Buffaloes
Goats
Livestock species
Rank
0.00
0.25
0.50
0.75
1.00

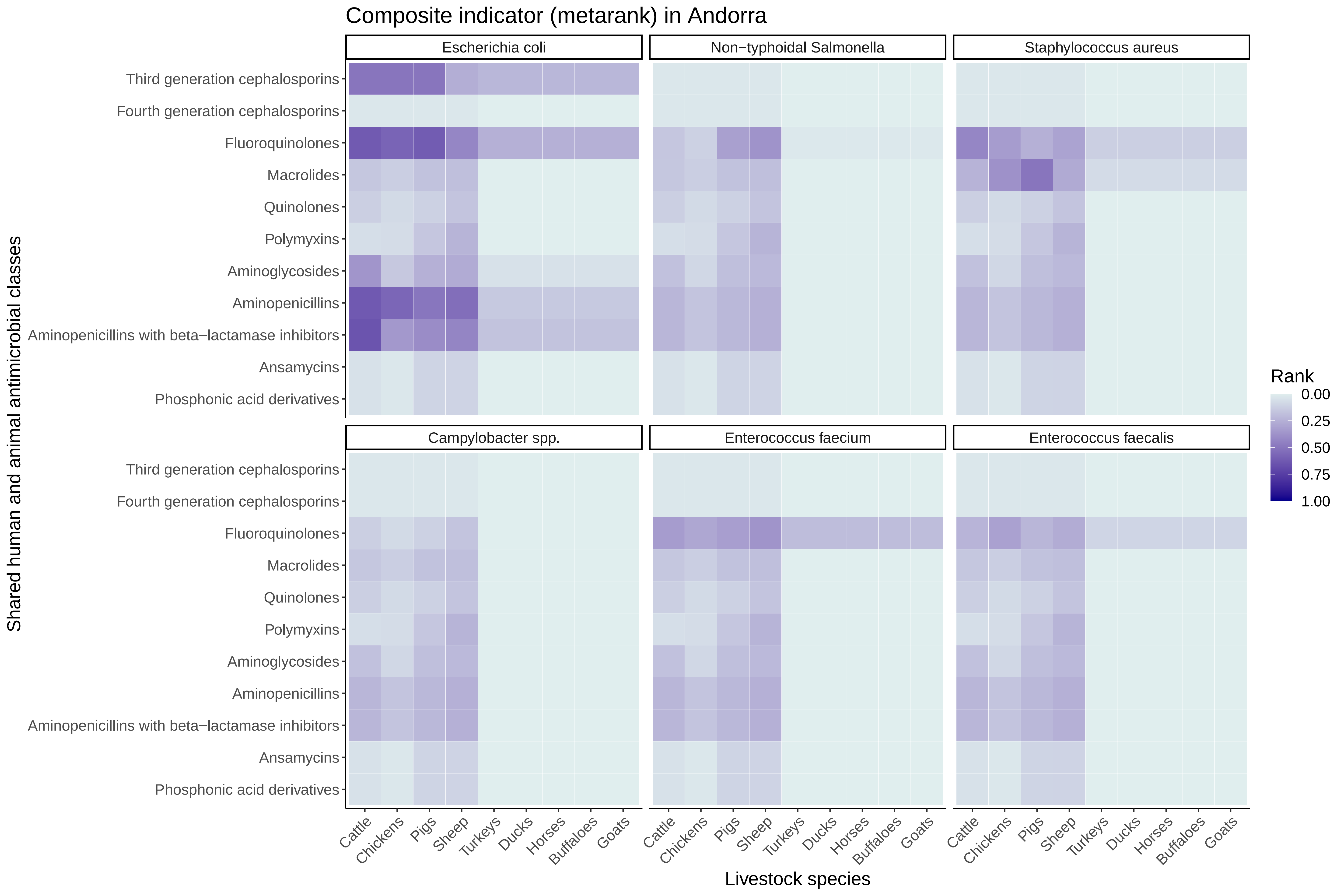

Composite indicator (metarank) in Andorra
Escherichia coli
Non−typhoidal Salmonella
Staphylococcus aureus
Campylobacter spp.
Enterococcus faecium
Enterococcus faecalis
Third generation cephalosporins
Fourth generation cephalosporins
Fluoroquinolones
Macrolides
Quinolones
Polymyxins
Aminoglycosides
Aminopenicillins
Aminopenicillins with beta−lactamase inhibitors
Ansamycins
Phosphonic acid derivatives
Shared human and animal antimicrobial classes
Cattle
Chickens
Pigs
Sheep
Turkeys
Ducks
Horses
Buffaloes
Goats
Livestock species
Rank
0.00
0.25
0.50
0.75
1.00

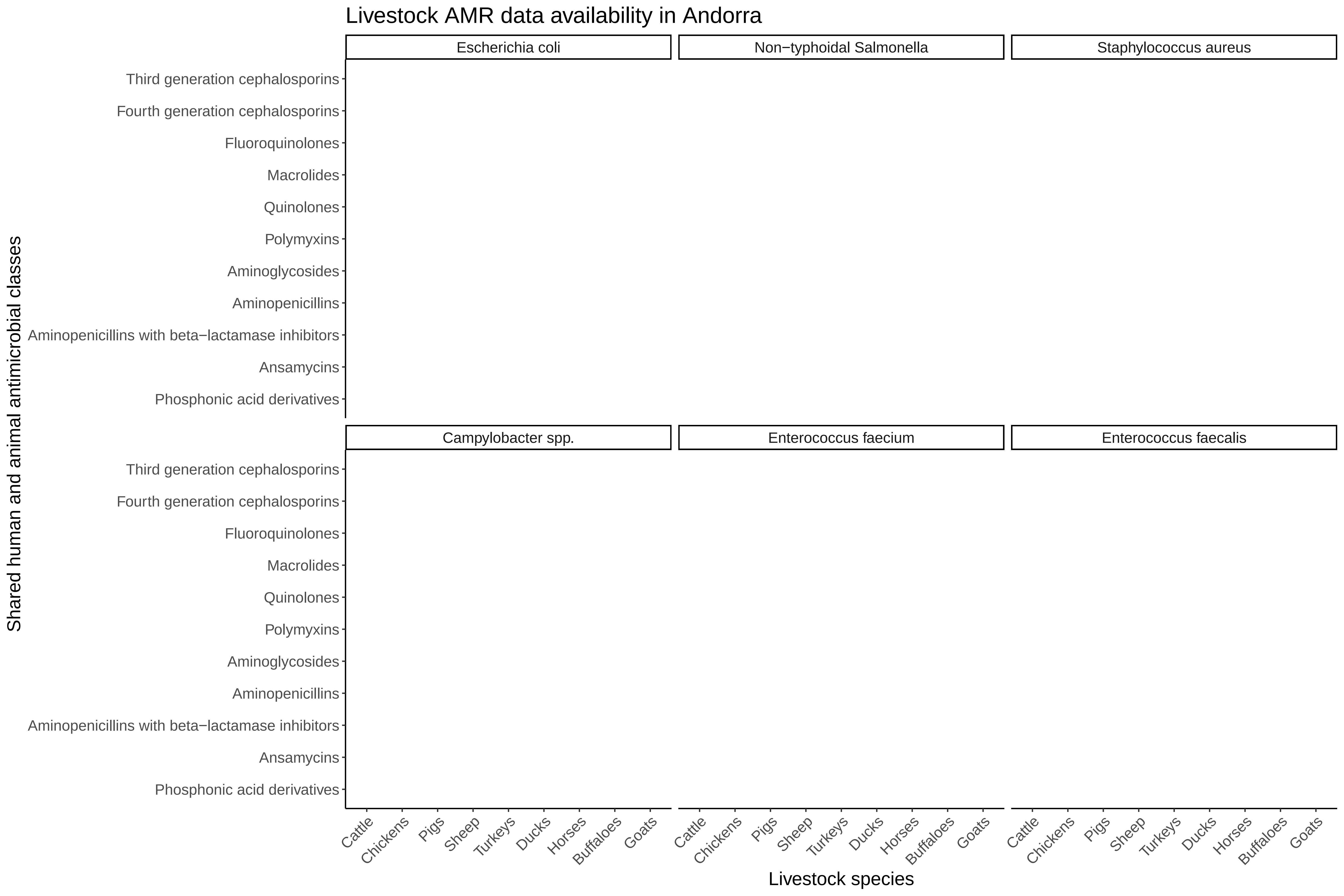

Livestock AMR data availability in Andorra
Escherichia coli
Non−typhoidal Salmonella
Staphylococcus aureus
Campylobacter spp.
Enterococcus faecium
Enterococcus faecalis
Third generation cephalosporins
Fourth generation cephalosporins
Fluoroquinolones
Macrolides
Quinolones
Polymyxins
Aminoglycosides
Aminopenicillins
Aminopenicillins with beta−lactamase inhibitors
Ansamycins
Phosphonic acid derivatives
Shared human and animal antimicrobial classes
Cattle
Chickens
Pigs
Sheep
Turkeys
Ducks
Horses
Buffaloes
Goats
Livestock species

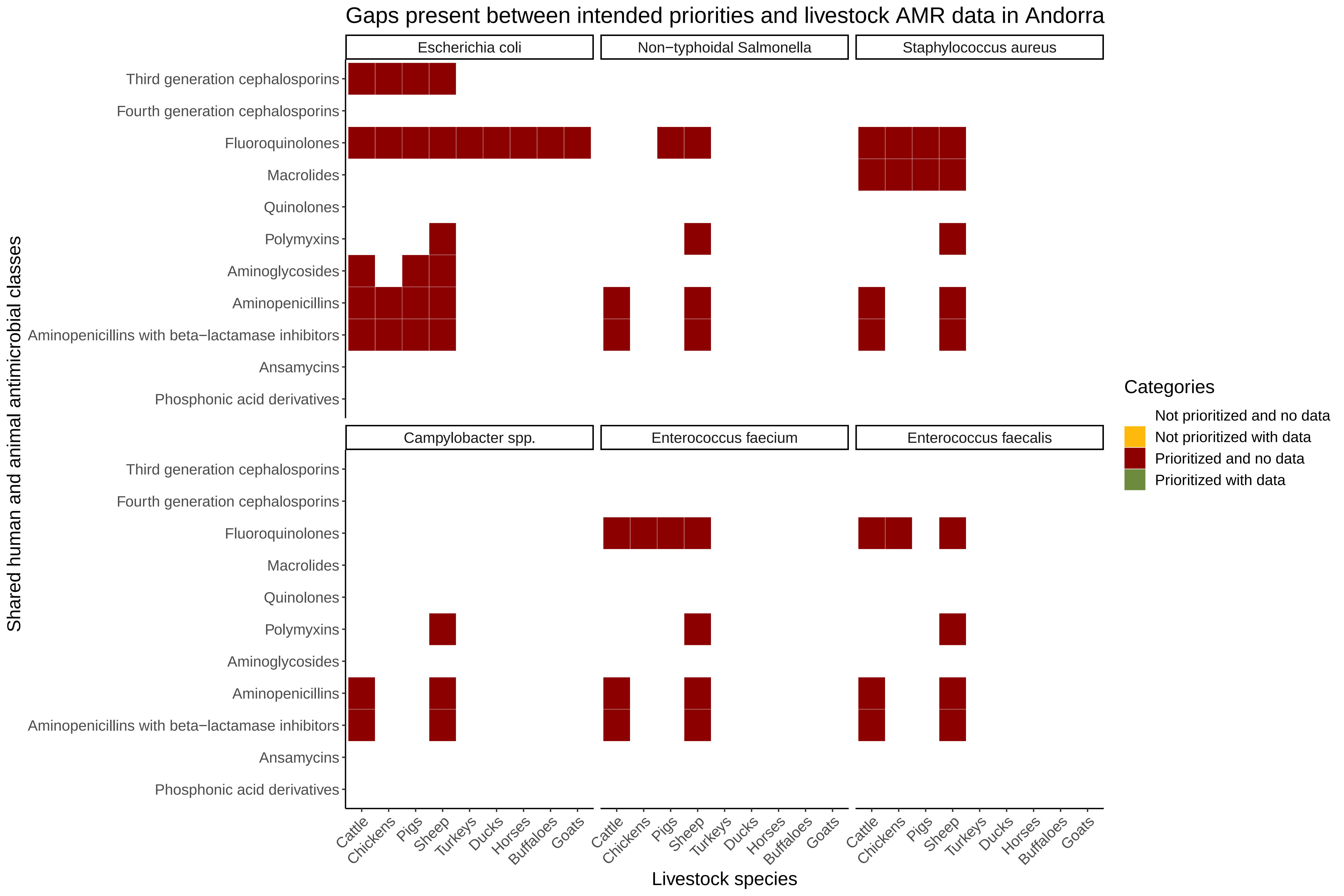

Gaps present between intended priorities and livestock AMR data in Andorra
Escherichia coli
Non−typhoidal Salmonella
Staphylococcus aureus
Campylobacter spp.
Enterococcus faecium
Enterococcus faecalis
Third generation cephalosporins
Fourth generation cephalosporins
Fluoroquinolones
Macrolides
Quinolones
Polymyxins
Aminoglycosides
Aminopenicillins
Aminopenicillins with beta−lactamase inhibitors
Ansamycins
Phosphonic acid derivatives
Cattle
Chickens
Pigs
Sheep
Turkeys
Ducks
Horses
Buffaloes
Goats
Shared human and animal antimicrobial classes
Livestock species
Categories
Not prioritized and no data
Not prioritized with data
Prioritized and no data
Prioritized with data

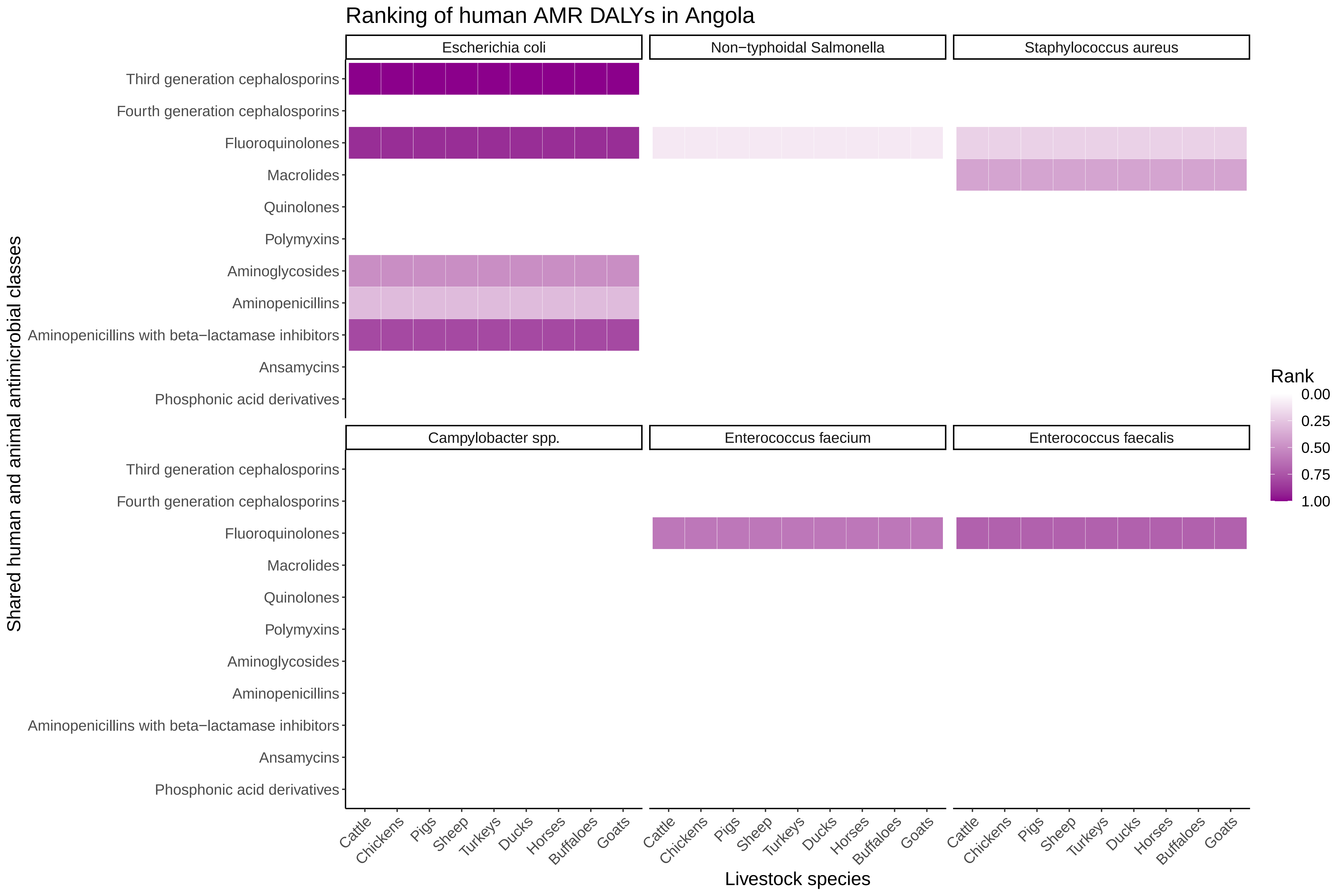
Ranking of human AMR DALYs in Angola
Escherichia coli
Non−typhoidal Salmonella
Staphylococcus aureus
Campylobacter spp.
Enterococcus faecium
Enterococcus faecalis
Third generation cephalosporins
Fourth generation cephalosporins
Fluoroquinolones
Macrolides
Quinolones
Polymyxins
Aminoglycosides
Aminopenicillins
Aminopenicillins with beta−lactamase inhibitors
Ansamycins
Phosphonic acid derivatives
Shared human and animal antimicrobial classes
Cattle
Chickens
Pigs
Sheep
Turkeys
Ducks
Horses
Buffaloes
Goats
Livestock species
Rank
0.00
0.25
0.50
0.75
1.00

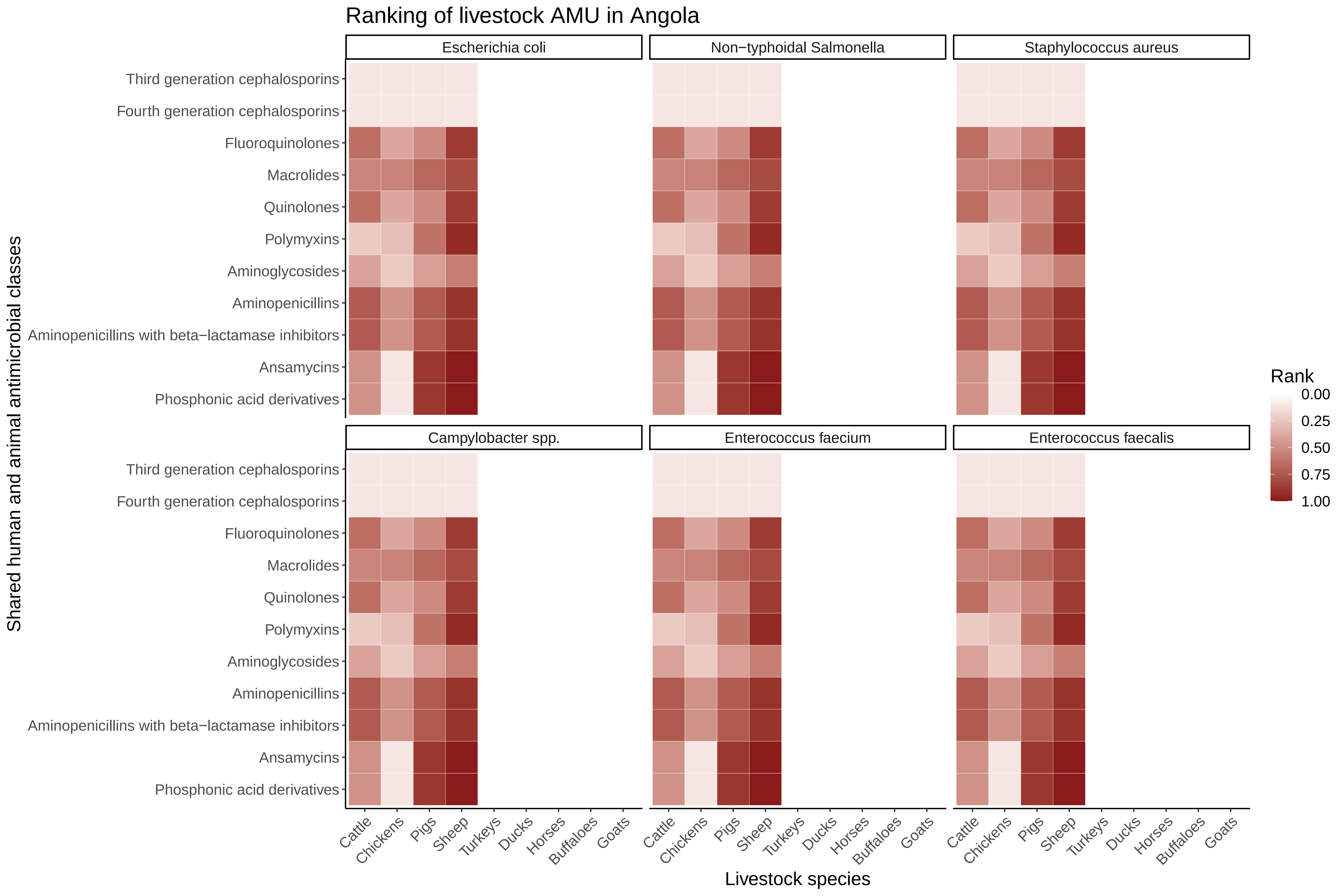

Ranking of livestock AMU in Angola
Escherichia coli
Non−typhoidal Salmonella
Staphylococcus aureus
Campylobacter spp.
Enterococcus faecium
Enterococcus faecalis
Shared human and animal antimicrobial classes
Third generation cephalosporins
Fourth generation cephalosporins
Fluoroquinolones
Macrolides
Quinolones
Polymyxins
Aminoglycosides
Aminopenicillins
Aminopenicillins with beta−lactamase inhibitors
Ansamycins
Phosphonic acid derivatives
Cattle
Chickens
Pigs
Sheep
Turkeys
Ducks
Horses
Buffaloes
Goats
Livestock species
Rank
0.00
0.25
0.50
0.75
1.00

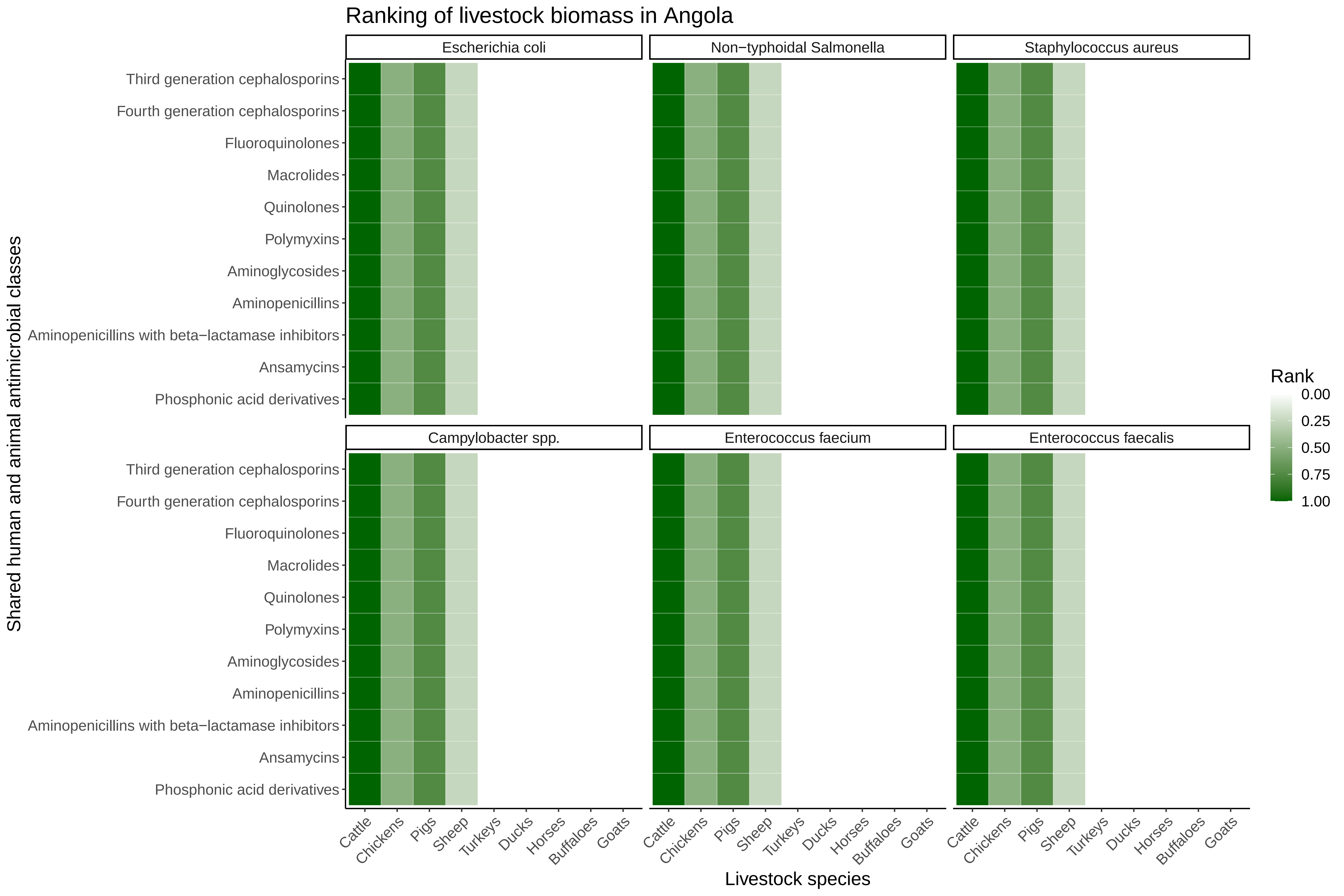

Ranking of livestock biomass in Angola
Escherichia coli
Non−typhoidal Salmonella
Staphylococcus aureus
Campylobacter spp.
Enterococcus faecium
Enterococcus faecalis
Third generation cephalosporins
Fourth generation cephalosporins
Fluoroquinolones
Macrolides
Quinolones
Polymyxins
Aminoglycosides
Aminopenicillins
Aminopenicillins with beta−lactamase inhibitors
Ansamycins
Phosphonic acid derivatives
Shared human and animal antimicrobial classes
Cattle
Chickens
Pigs
Sheep
Turkeys
Ducks
Horses
Buffaloes
Goats
Livestock species
Rank
0.00
0.25
0.50
0.75
1.00

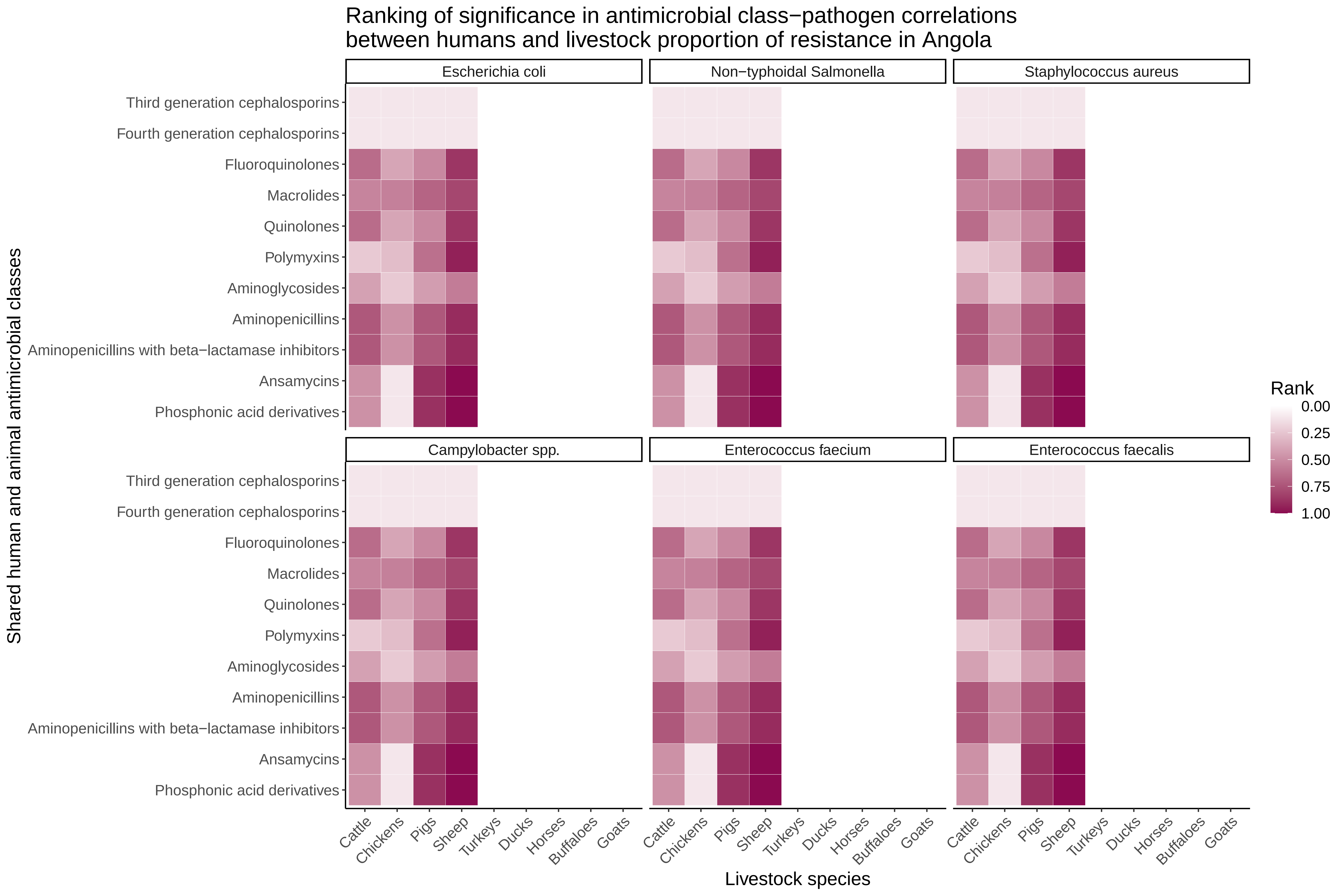

Ranking of significance in antimicrobial class−pathogen correlations
between humans and livestock proportion of resistance in Angola
Escherichia coli
Non−typhoidal Salmonella
Staphylococcus aureus
Campylobacter spp.
Enterococcus faecium
Enterococcus faecalis
Third generation cephalosporins
Fourth generation cephalosporins
Fluoroquinolones
Macrolides
Quinolones
Polymyxins
Aminoglycosides
Aminopenicillins
Aminopenicillins with beta−lactamase inhibitors
Ansamycins
Phosphonic acid derivatives
Shared human and animal antimicrobial classes
Cattle
Chickens
Pigs
Sheep
Turkeys
Ducks
Horses
Buffaloes
Goats
Livestock species
Rank
0.00
0.25
0.50
0.75
1.00

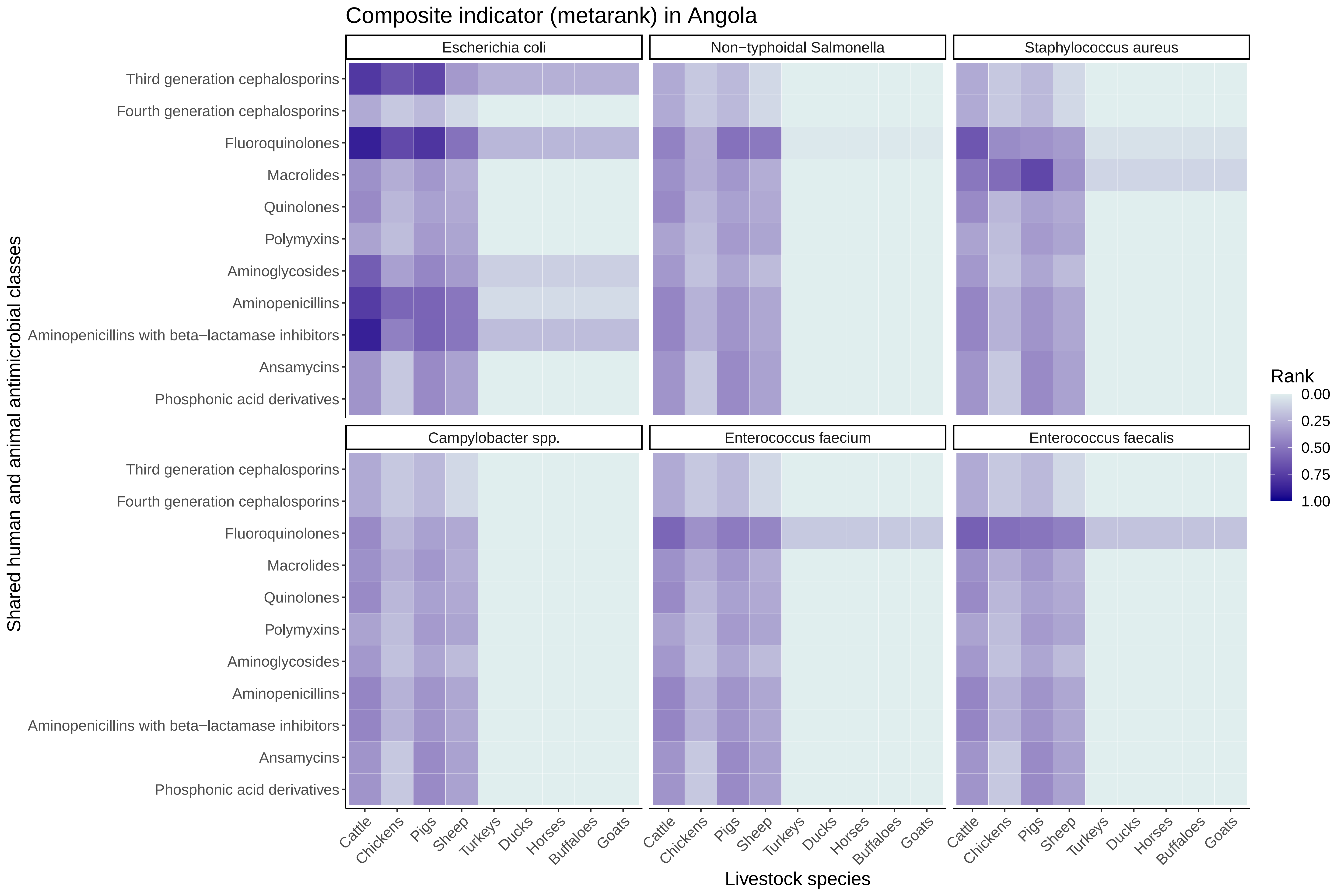
Composite indicator (metarank) in Angola
Escherichia coli
Non−typhoidal Salmonella
Staphylococcus aureus
Campylobacter spp.
Enterococcus faecium
Enterococcus faecalis
Third generation cephalosporins
Fourth generation cephalosporins
Fluoroquinolones
Macrolides
Quinolones
Polymyxins
Aminoglycosides
Aminopenicillins
Aminopenicillins with beta−lactamase inhibitors
Ansamycins
Phosphonic acid derivatives
Shared human and animal antimicrobial classes
Cattle
Chickens
Pigs
Sheep
Turkeys
Ducks
Horses
Buffaloes
Goats
Livestock species
Rank
0.00
0.25
0.50
0.75
1.00

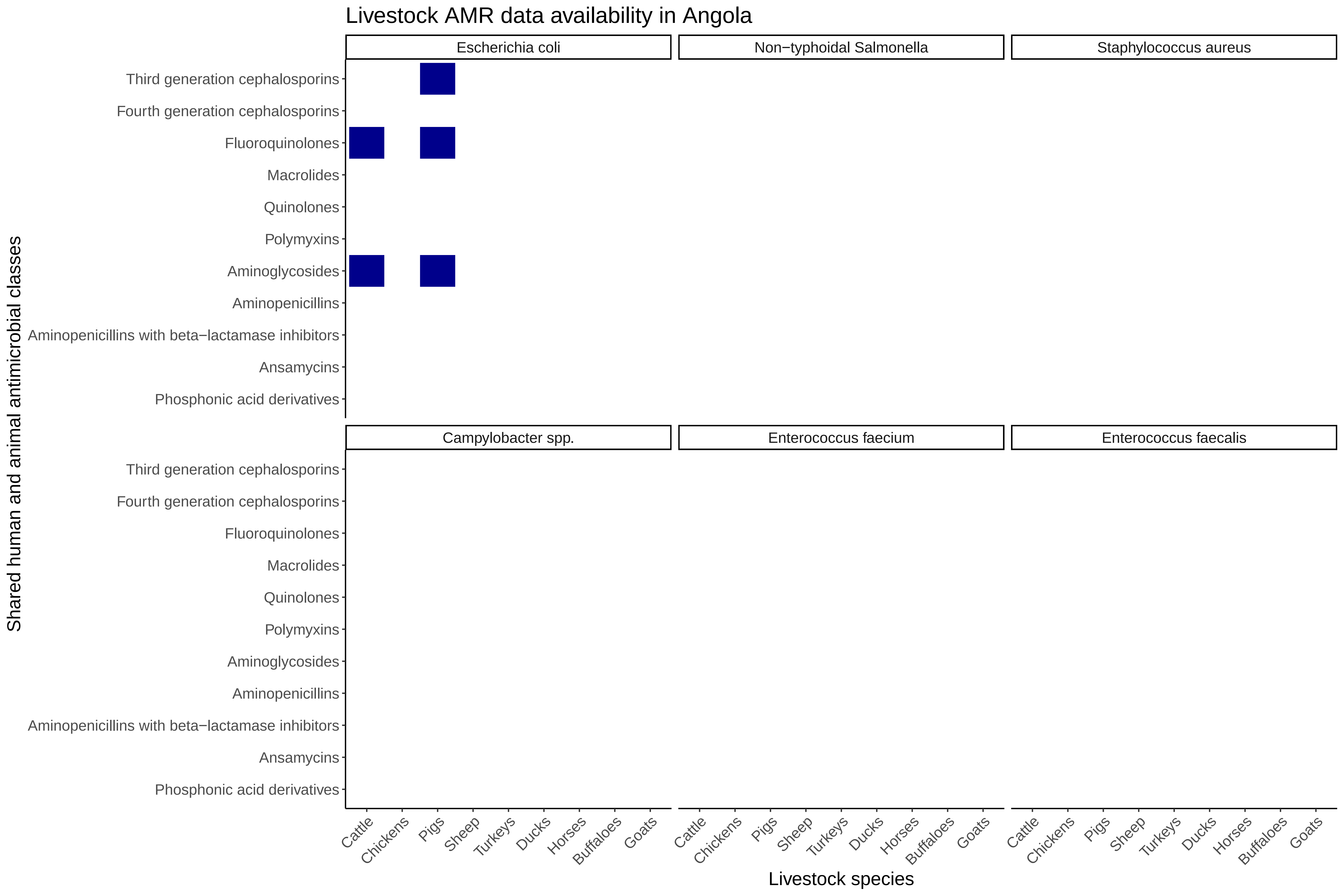

Livestock AMR data availability in Angola
Escherichia coli
Non−typhoidal Salmonella
Staphylococcus aureus
Campylobacter spp.
Enterococcus faecium
Enterococcus faecalis
Shared human and animal antimicrobial classes
Third generation cephalosporins
Fourth generation cephalosporins
Fluoroquinolones
Macrolides
Quinolones
Polymyxins
Aminoglycosides
Aminopenicillins
Aminopenicillins with beta−lactamase inhibitors
Ansamycins
Phosphonic acid derivatives
Cattle
Chickens
Pigs
Sheep
Turkeys
Ducks
Horses
Buffaloes
Goats
Livestock species

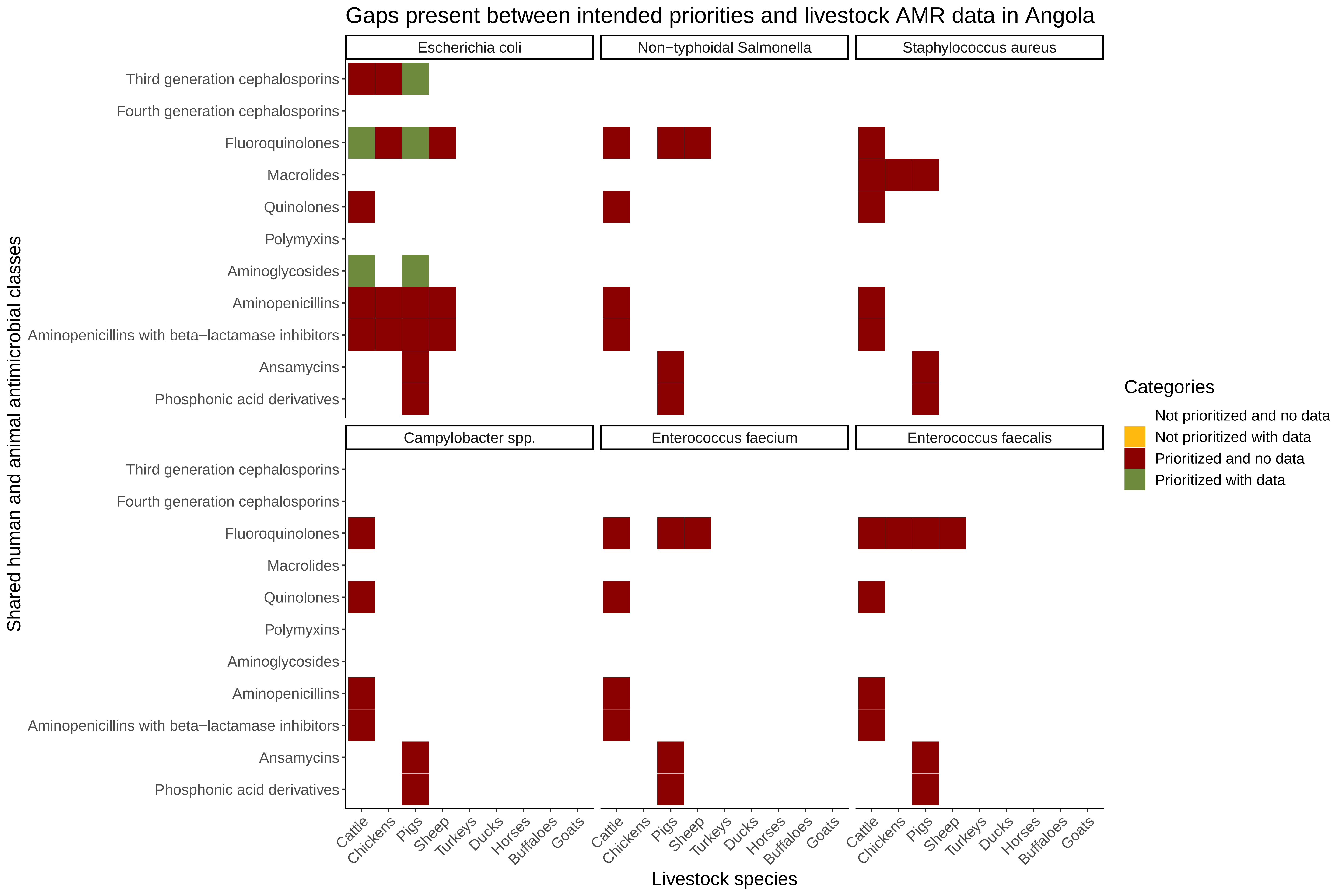
Gaps present between intended priorities and livestock AMR data in Angola
Escherichia coli
Non−typhoidal Salmonella
Staphylococcus aureus
Campylobacter spp.
Enterococcus faecium
Enterococcus faecalis
Third generation cephalosporins
Fourth generation cephalosporins
Fluoroquinolones
Macrolides
Quinolones
Polymyxins
Aminoglycosides
Aminopenicillins
Aminopenicillins with beta−lactamase inhibitors
Ansamycins
Phosphonic acid derivatives
Shared human and animal antimicrobial classes
Cattle
Chickens
Pigs
Sheep
Turkeys
Ducks
Horses
Buffaloes
Goats
Livestock species
Categories
Not prioritized and no data
Not prioritized with data
Prioritized and no data
Prioritized with data

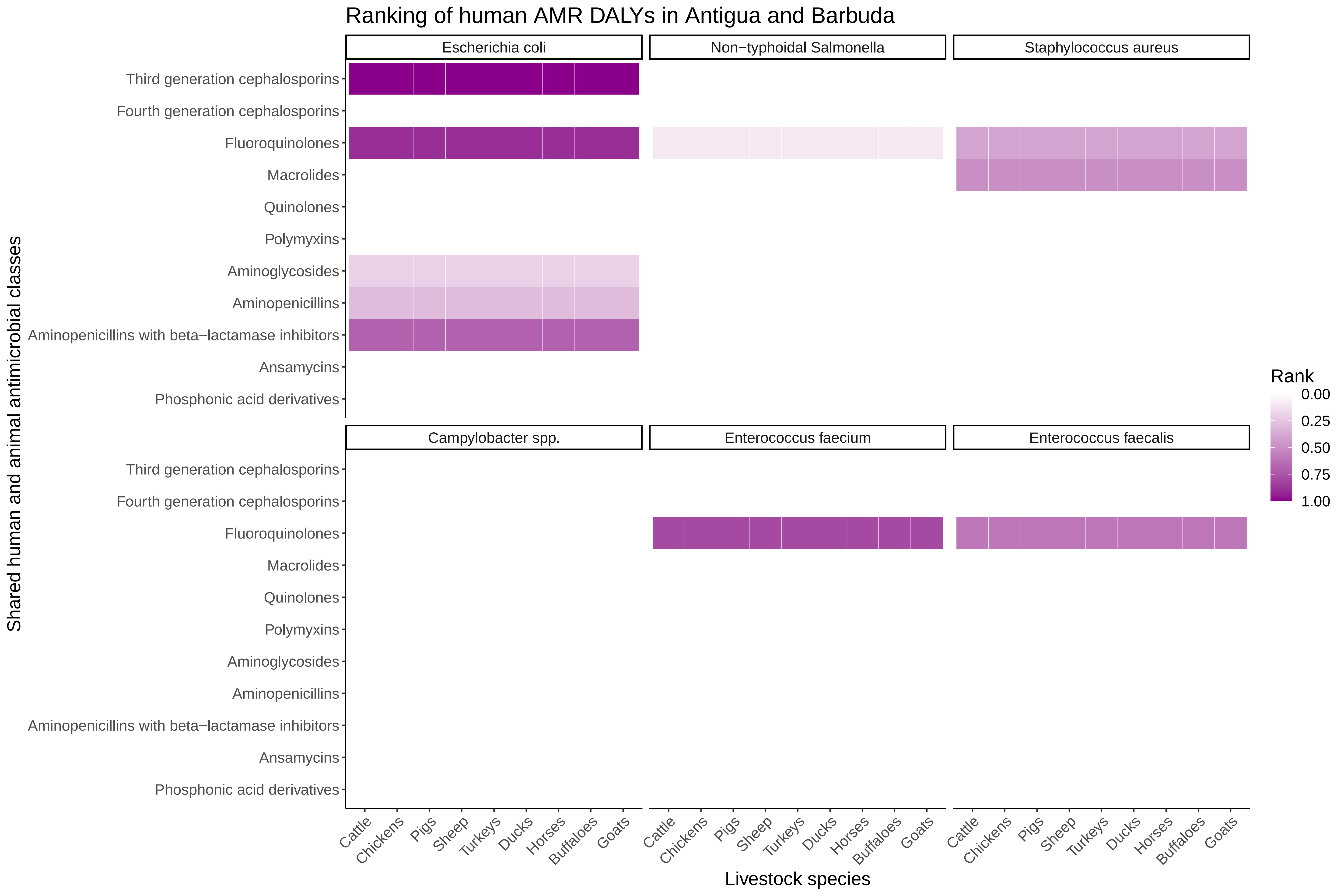

Ranking of human AMR DALYs in Antigua and Barbuda
Escherichia coli
Non−typhoidal Salmonella
Staphylococcus aureus
Campylobacter spp.
Enterococcus faecium
Enterococcus faecalis
Shared human and animal antimicrobial classes
Third generation cephalosporins
Fourth generation cephalosporins
Fluoroquinolones
Macrolides
Quinolones
Polymyxins
Aminoglycosides
Aminopenicillins
Aminopenicillins with beta−lactamase inhibitors
Ansamycins
Phosphonic acid derivatives
Livestock species
Cattle
Chickens
Pigs
Sheep
Turkeys
Ducks
Horses
Buffaloes
Goats
Rank
0.00
0.25
0.50
0.75
1.00

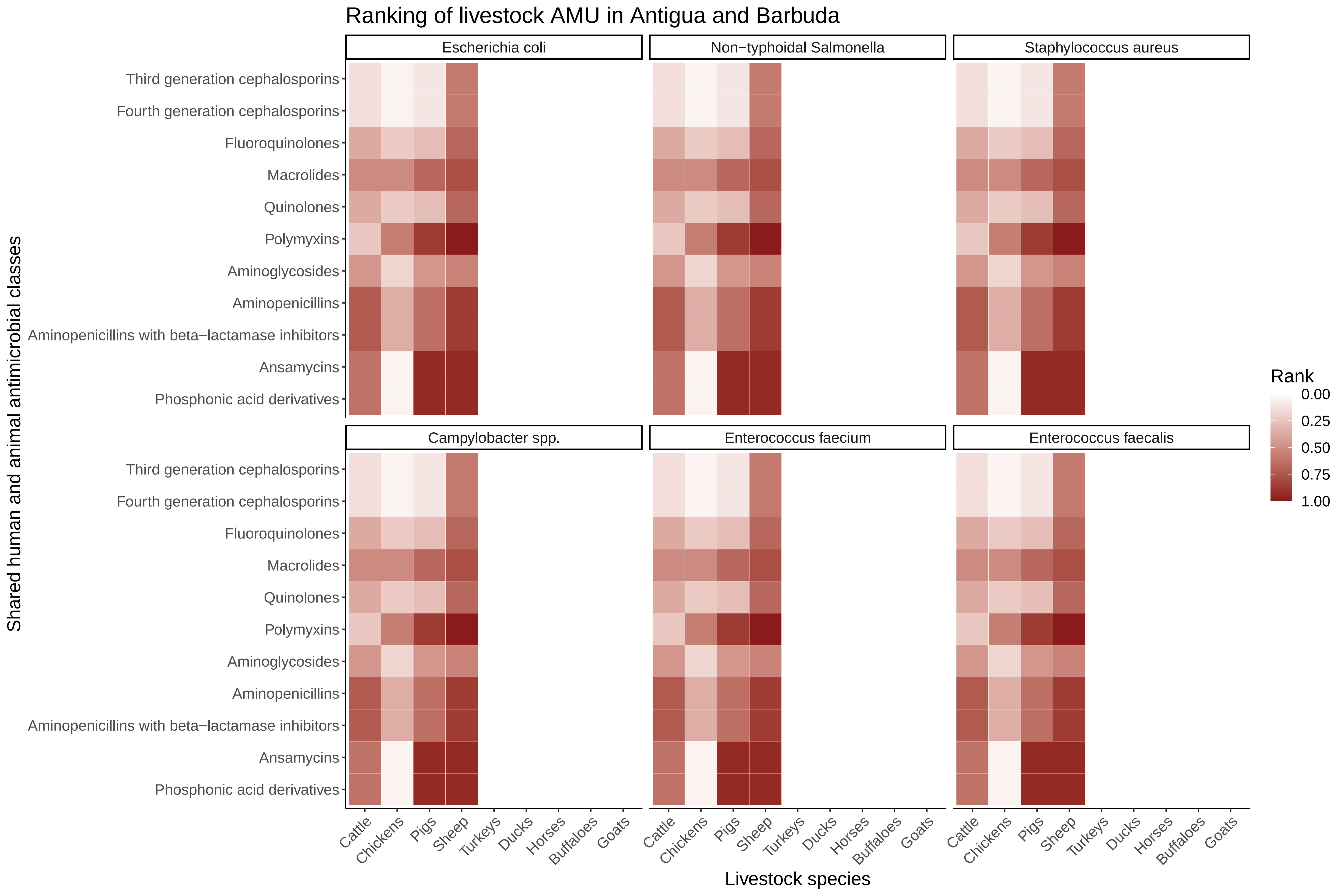
Ranking of livestock AMU in Antigua and Barbuda
Escherichia coli
Non−typhoidal Salmonella
Staphylococcus aureus
Campylobacter spp.
Enterococcus faecium
Enterococcus faecalis
Shared human and animal antimicrobial classes
Third generation cephalosporins
Fourth generation cephalosporins
Fluoroquinolones
Macrolides
Quinolones
Polymyxins
Aminoglycosides
Aminopenicillins
Aminopenicillins with beta−lactamase inhibitors
Ansamycins
Phosphonic acid derivatives
Livestock species
Cattle
Chickens
Pigs
Sheep
Turkeys
Ducks
Horses
Buffaloes
Goats
Rank
0.00
0.25
0.50
0.75
1.00

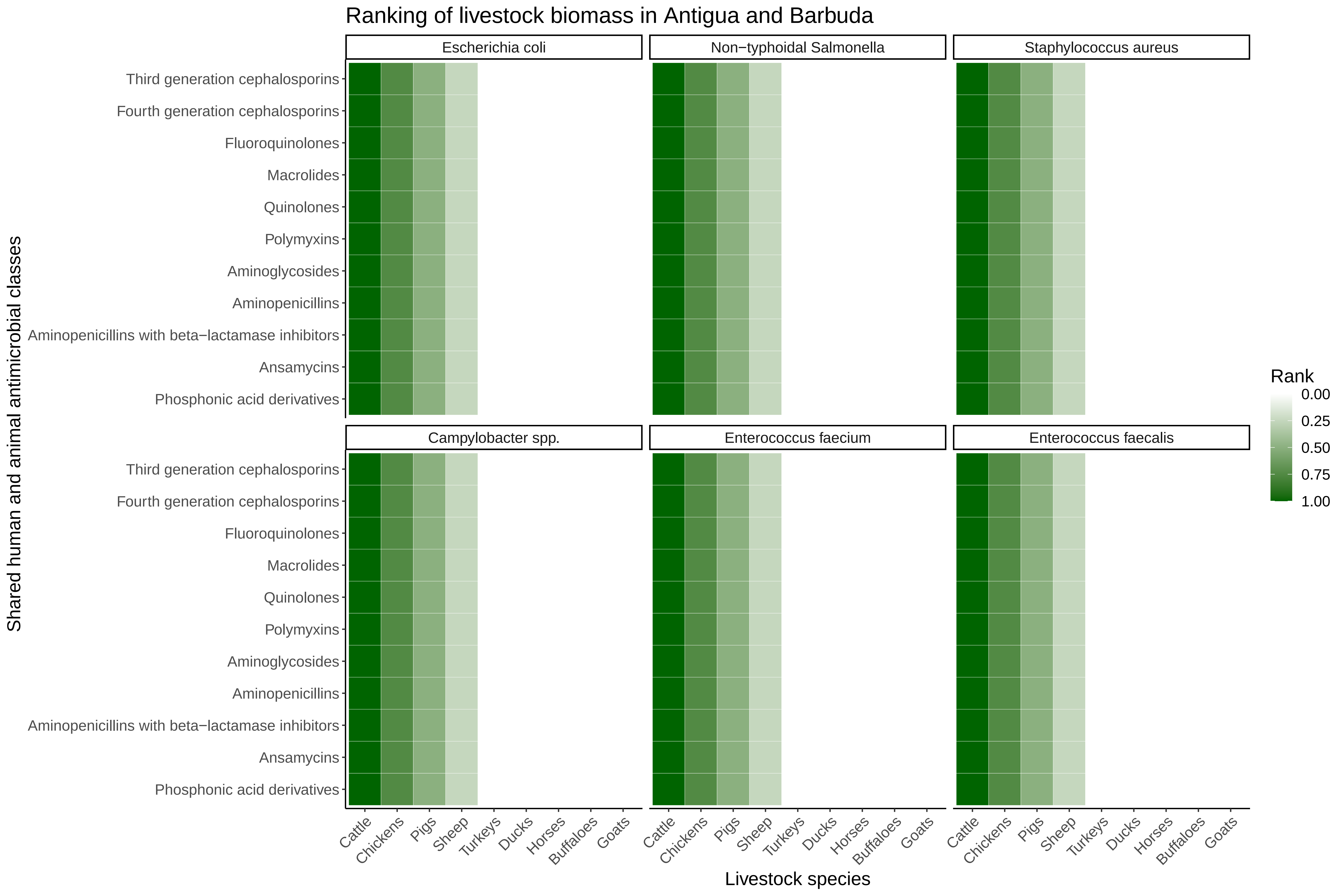
Ranking of livestock biomass in Antigua and Barbuda
Escherichia coli
Non−typhoidal Salmonella
Staphylococcus aureus
Campylobacter spp.
Enterococcus faecium
Enterococcus faecalis
Third generation cephalosporins
Fourth generation cephalosporins
Fluoroquinolones
Macrolides
Quinolones
Polymyxins
Aminoglycosides
Aminopenicillins
Aminopenicillins with beta−lactamase inhibitors
Ansamycins
Phosphonic acid derivatives
Shared human and animal antimicrobial classes
Cattle
Chickens
Pigs
Sheep
Turkeys
Ducks
Horses
Buffaloes
Goats
Livestock species
Rank
0.00
0.25
0.50
0.75
1.00

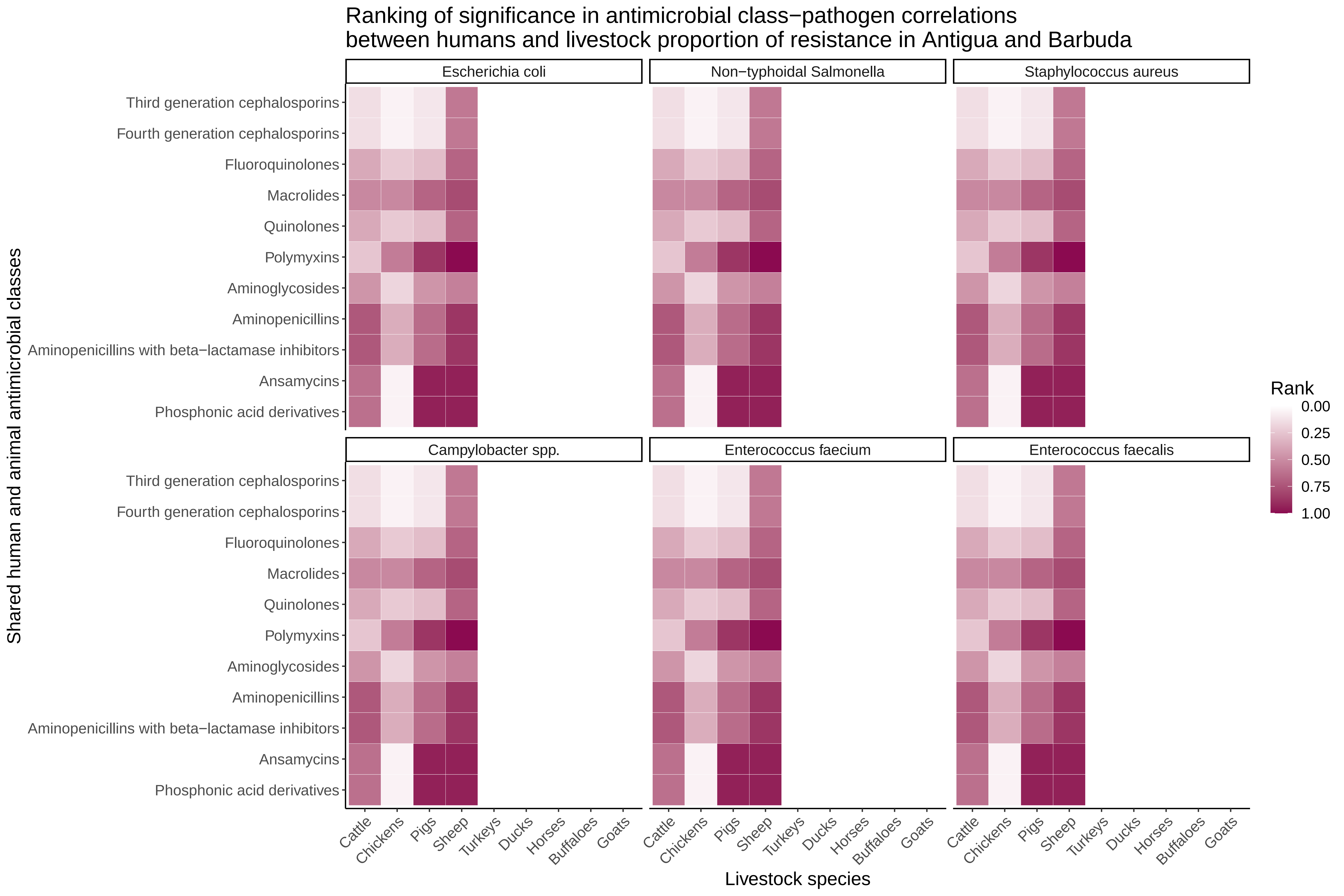

Ranking of significance in antimicrobial class−pathogen correlations
between humans and livestock proportion of resistance in Antigua and Barbuda
Escherichia coli
Non−typhoidal Salmonella
Staphylococcus aureus
Campylobacter spp.
Enterococcus faecium
Enterococcus faecalis
Third generation cephalosporins
Fourth generation cephalosporins
Fluoroquinolones
Macrolides
Quinolones
Polymyxins
Aminoglycosides
Aminopenicillins
Aminopenicillins with beta−lactamase inhibitors
Ansamycins
Phosphonic acid derivatives
Shared human and animal antimicrobial classes
Cattle
Chickens
Pigs
Sheep
Turkeys
Ducks
Horses
Buffaloes
Goats
Livestock species
Rank
0.00
0.25
0.50
0.75
1.00

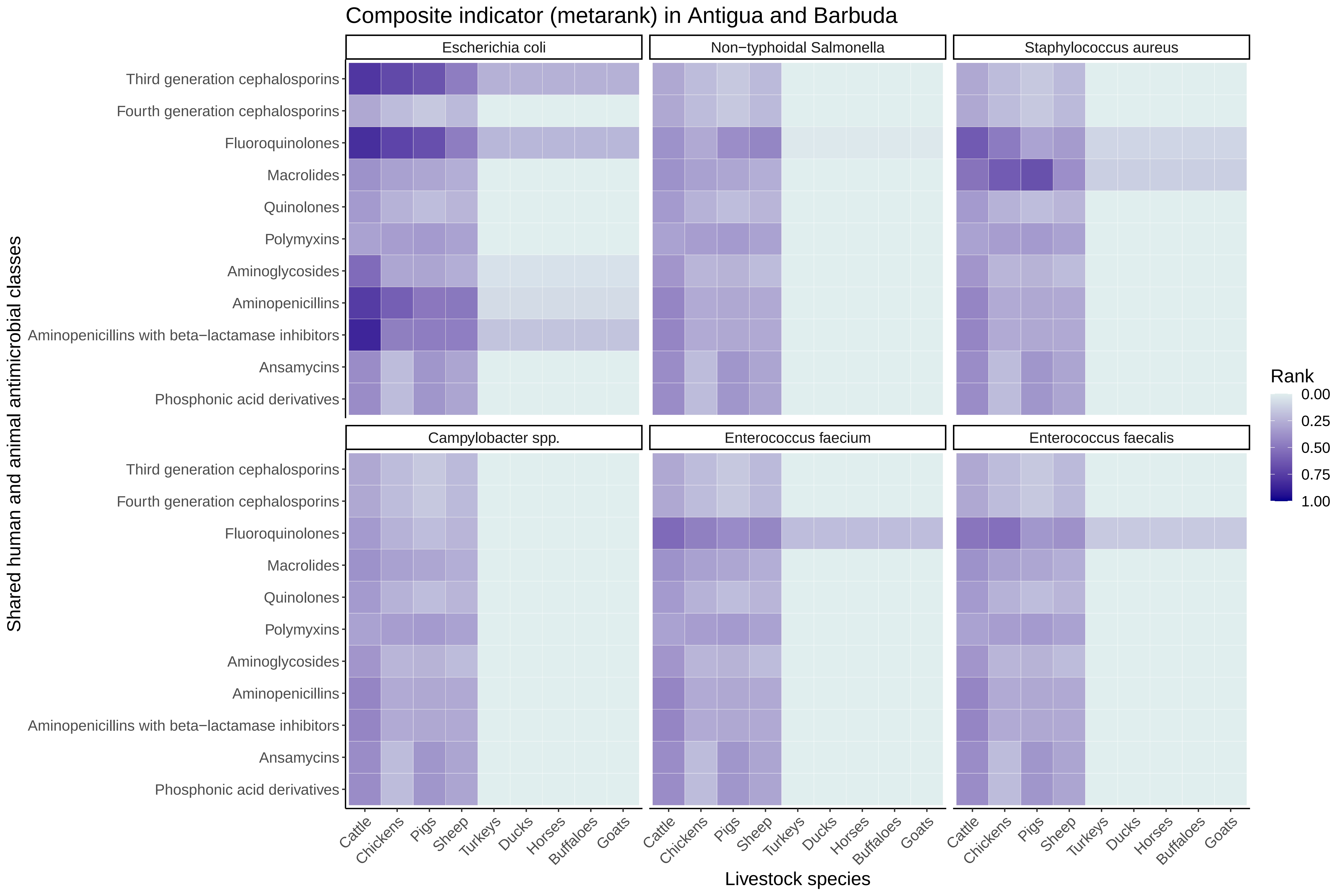

Composite indicator (metarank) in Antigua and Barbuda
Escherichia coli
Non−typhoidal Salmonella
Staphylococcus aureus
Campylobacter spp.
Enterococcus faecium
Enterococcus faecalis
Third generation cephalosporins
Fourth generation cephalosporins
Fluoroquinolones
Macrolides
Quinolones
Polymyxins
Aminoglycosides
Aminopenicillins
Aminopenicillins with beta−lactamase inhibitors
Ansamycins
Phosphonic acid derivatives
Shared human and animal antimicrobial classes
Cattle
Chickens
Pigs
Sheep
Turkeys
Ducks
Horses
Buffaloes
Goats
Livestock species
Rank
0.00
0.25
0.50
0.75
1.00

Livestock AMR data availability in Antigua and Barbuda

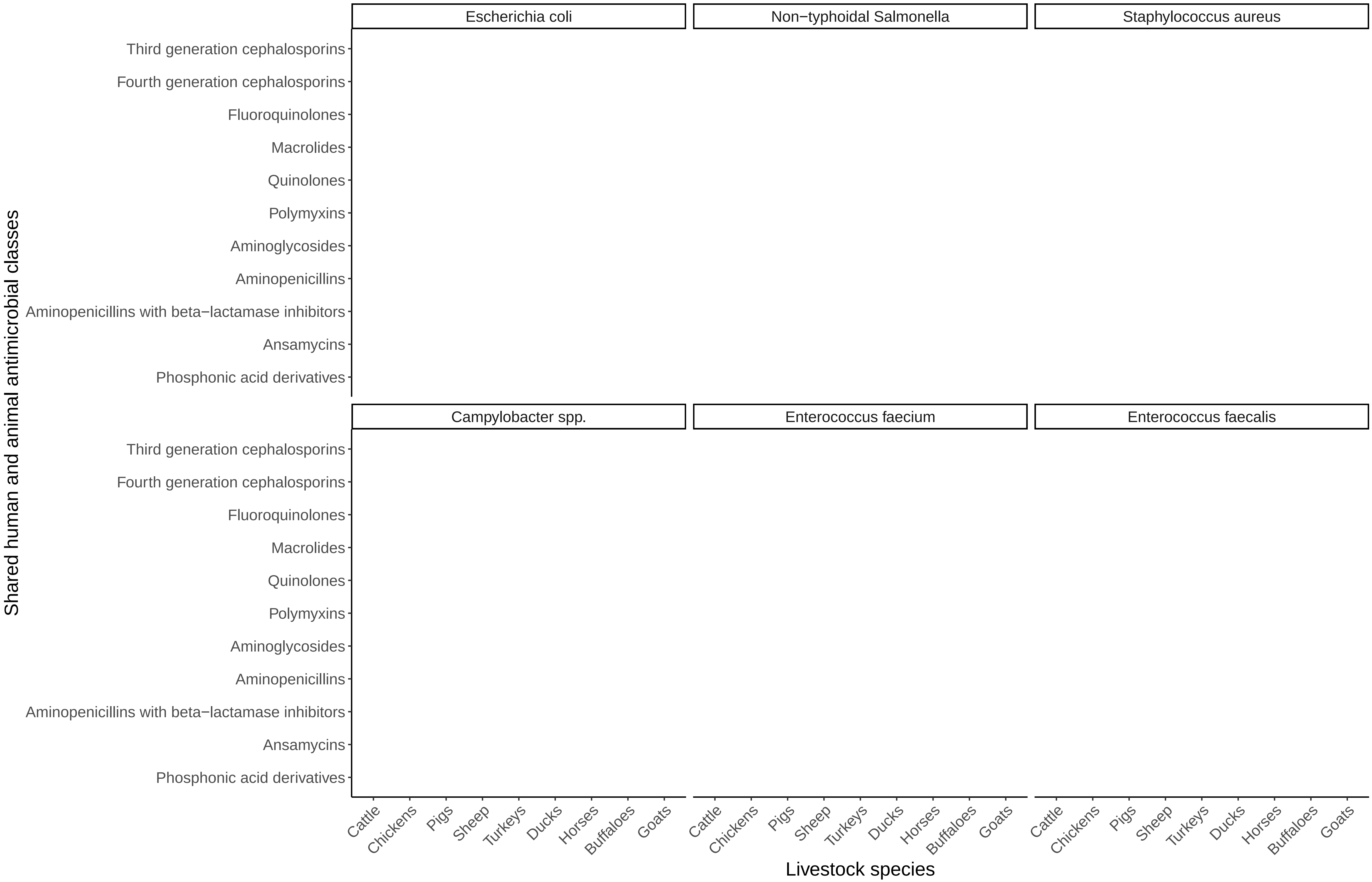

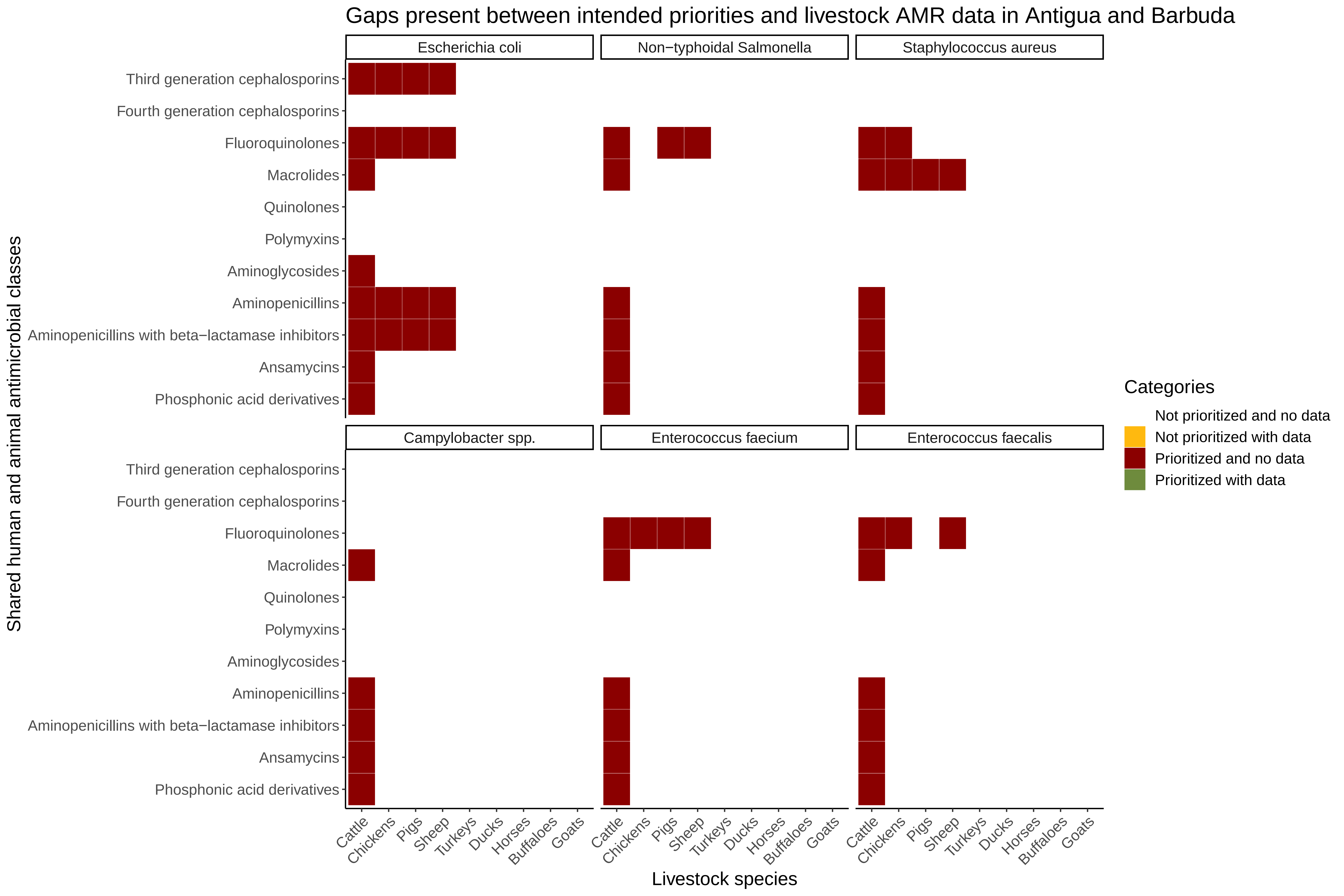

Gaps present between intended priorities and livestock AMR data in Antigua and Barbuda
Escherichia coli
Non−typhoidal Salmonella
Staphylococcus aureus
Campylobacter spp.
Enterococcus faecium
Enterococcus faecalis
Shared human and animal antimicrobial classes
Third generation cephalosporins
Fourth generation cephalosporins
Fluoroquinolones
Macrolides
Quinolones
Polymyxins
Aminoglycosides
Aminopenicillins
Aminopenicillins with beta−lactamase inhibitors
Ansamycins
Phosphonic acid derivatives
Livestock species
Cattle
Chickens
Pigs
Sheep
Turkeys
Ducks
Horses
Buffaloes
Goats
Categories
Not prioritized and no data
Not prioritized with data
Prioritized and no data
Prioritized with data

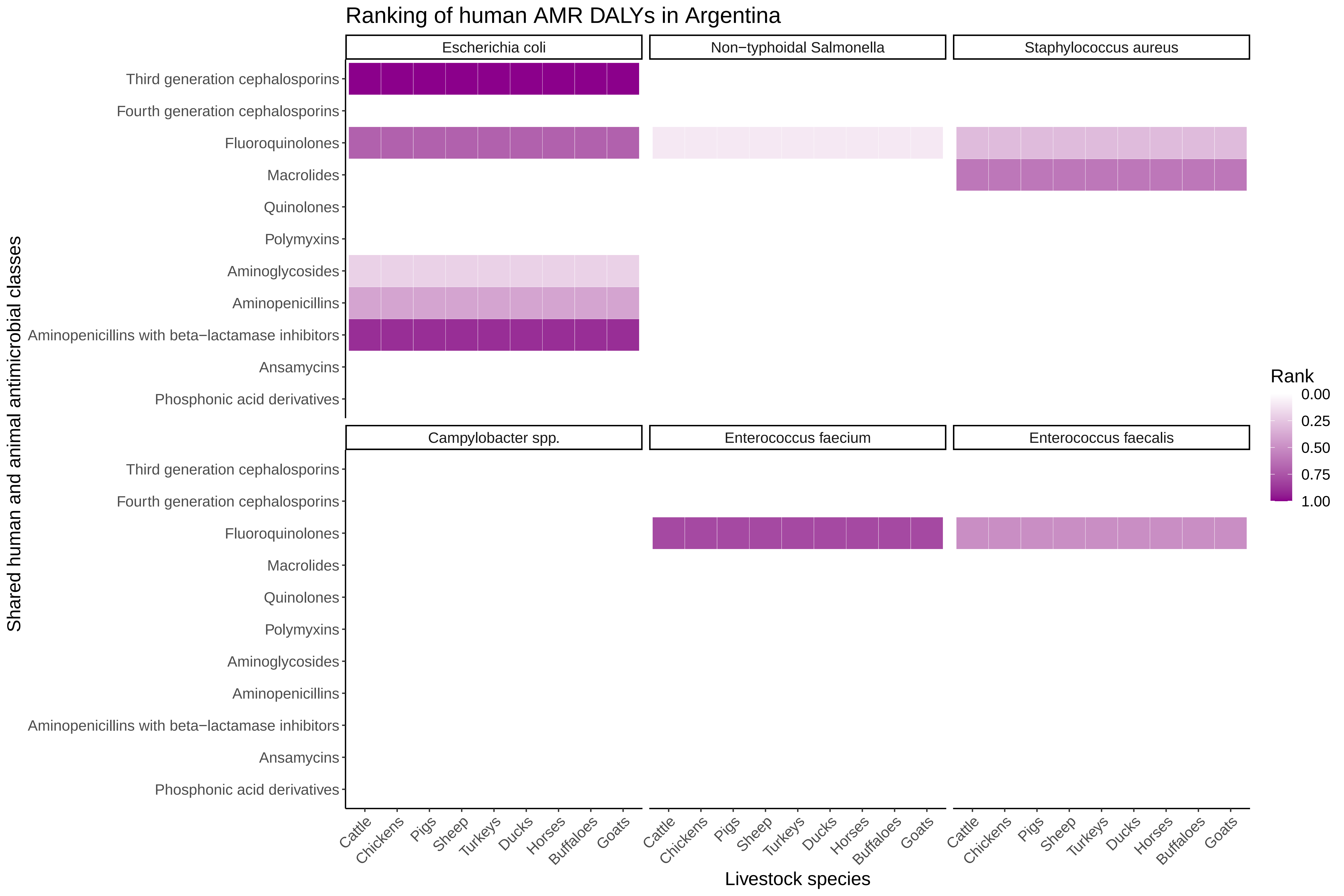

Ranking of human AMR DALYs in Argentina
Escherichia coli
Non−typhoidal Salmonella
Staphylococcus aureus
Campylobacter spp.
Enterococcus faecium
Enterococcus faecalis
Third generation cephalosporins
Fourth generation cephalosporins
Fluoroquinolones
Macrolides
Quinolones
Polymyxins
Aminoglycosides
Aminopenicillins
Aminopenicillins with beta−lactamase inhibitors
Ansamycins
Phosphonic acid derivatives
Shared human and animal antimicrobial classes
Cattle
Chickens
Pigs
Sheep
Turkeys
Ducks
Horses
Buffaloes
Goats
Livestock species
Rank
0.00
0.25
0.50
0.75
1.00

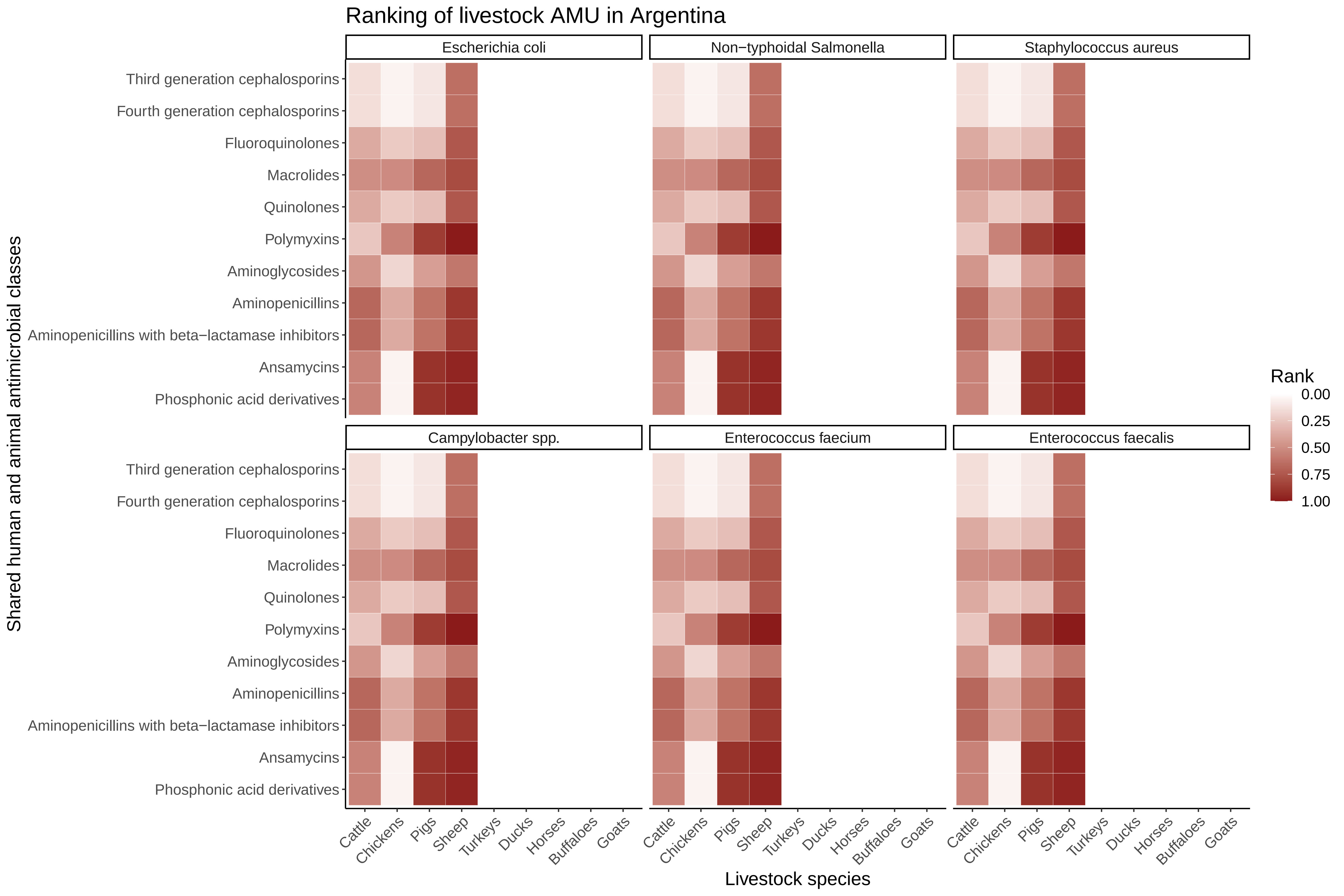

Ranking of livestock AMU in Argentina
Escherichia coli
Non−typhoidal Salmonella
Staphylococcus aureus
Campylobacter spp.
Enterococcus faecium
Enterococcus faecalis
Shared human and animal antimicrobial classes
Third generation cephalosporins
Fourth generation cephalosporins
Fluoroquinolones
Macrolides
Quinolones
Polymyxins
Aminoglycosides
Aminopenicillins
Aminopenicillins with beta−lactamase inhibitors
Ansamycins
Phosphonic acid derivatives
Cattle
Chickens
Pigs
Sheep
Turkeys
Ducks
Horses
Buffaloes
Goats
Livestock species
Rank
0.00
0.25
0.50
0.75
1.00

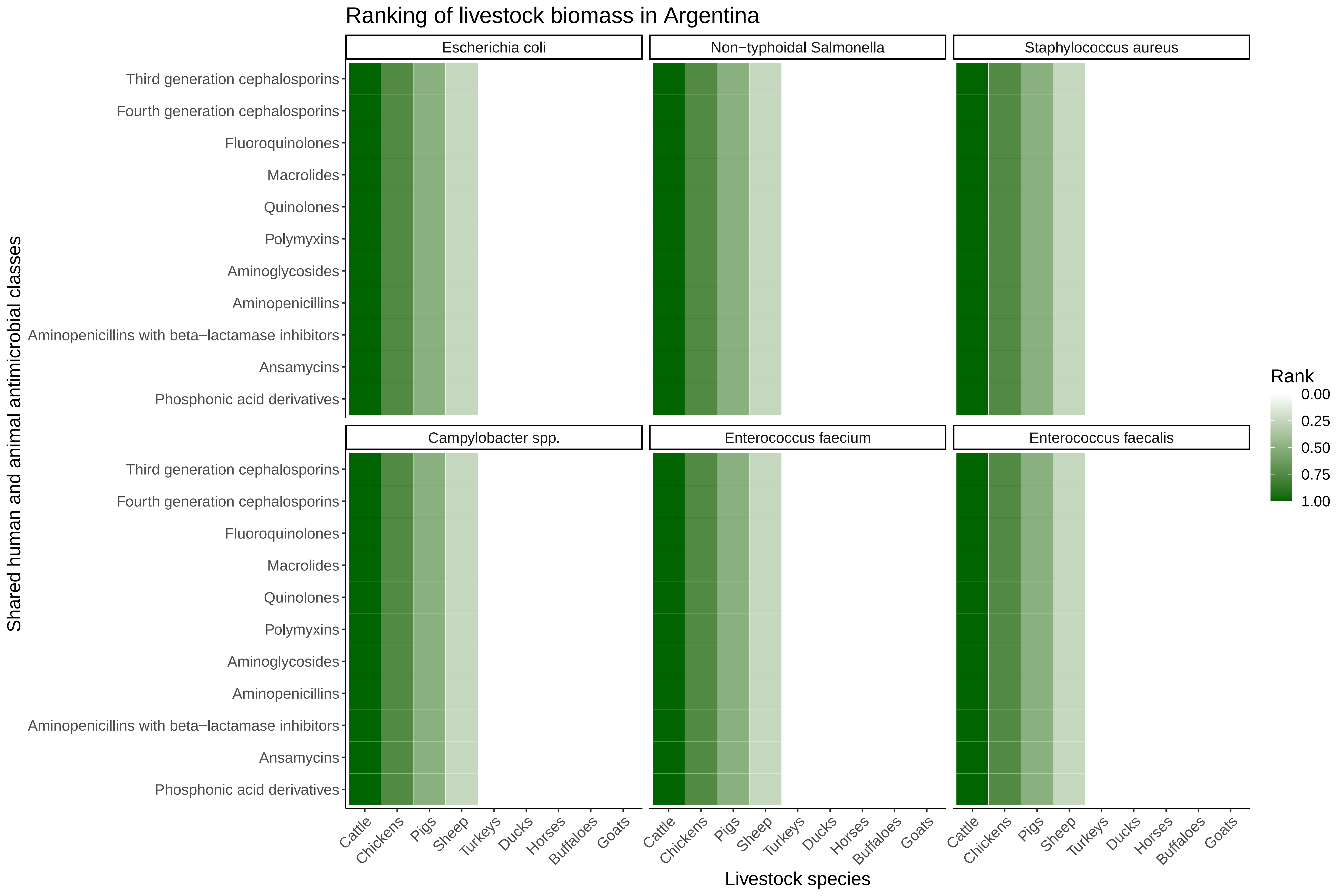
Ranking of livestock biomass in Argentina
Escherichia coli
Non−typhoidal Salmonella
Staphylococcus aureus
Campylobacter spp.
Enterococcus faecium
Enterococcus faecalis
Third generation cephalosporins
Fourth generation cephalosporins
Fluoroquinolones
Macrolides
Quinolones
Polymyxins
Aminoglycosides
Aminopenicillins
Aminopenicillins with beta−lactamase inhibitors
Ansamycins
Phosphonic acid derivatives
Shared human and animal antimicrobial classes
Cattle
Chickens
Pigs
Sheep
Turkeys
Ducks
Horses
Buffaloes
Goats
Livestock species
Rank
0.00
0.25
0.50
0.75
1.00

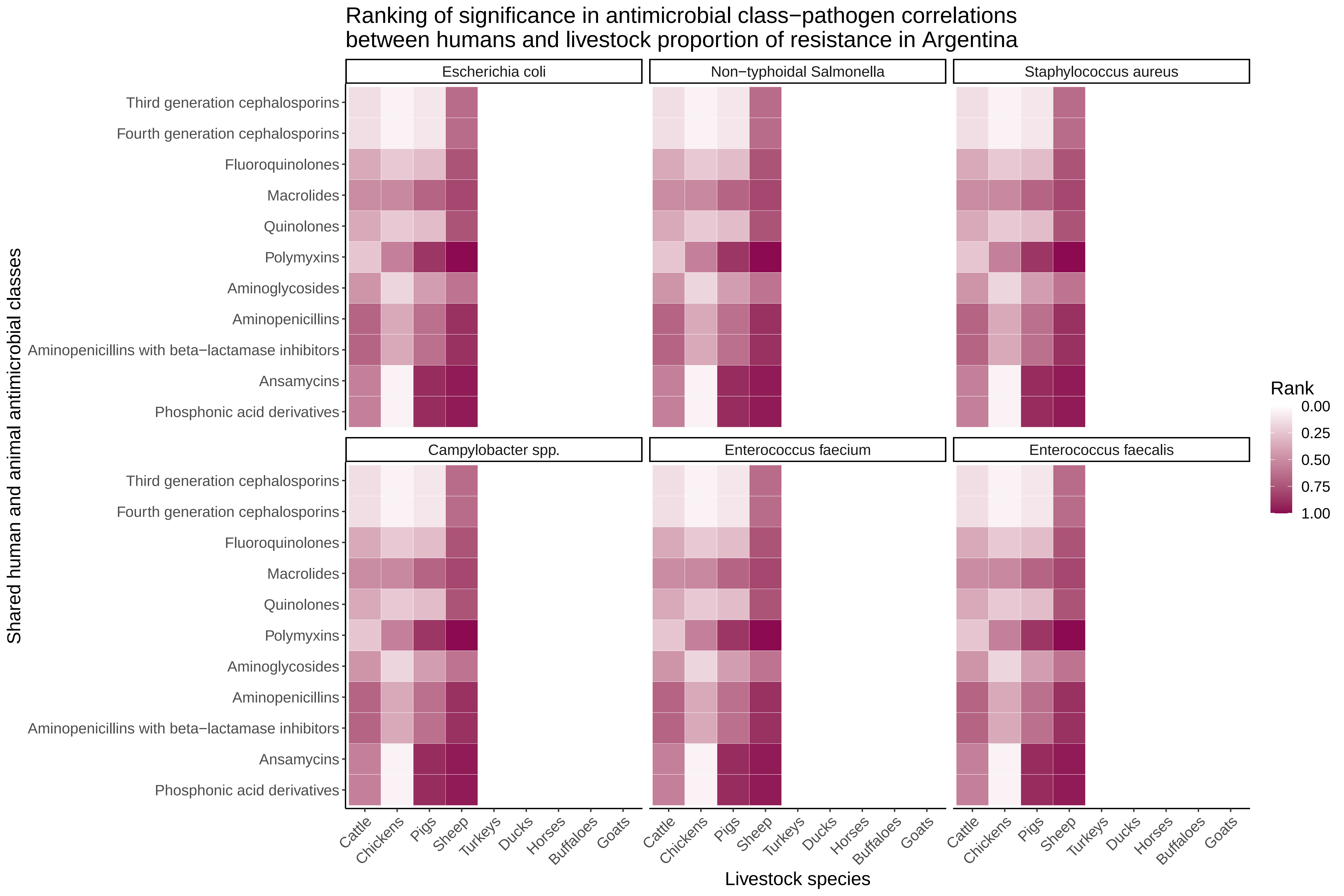
Ranking of significance in antimicrobial class−pathogen correlations
between humans and livestock proportion of resistance in Argentina
Escherichia coli
Non−typhoidal Salmonella
Staphylococcus aureus
Campylobacter spp.
Enterococcus faecium
Enterococcus faecalis
Shared human and animal antimicrobial classes
Third generation cephalosporins
Fourth generation cephalosporins
Fluoroquinolones
Macrolides
Quinolones
Polymyxins
Aminoglycosides
Aminopenicillins
Aminopenicillins with beta−lactamase inhibitors
Ansamycins
Phosphonic acid derivatives
Livestock species
Cattle
Chickens
Pigs
Sheep
Turkeys
Ducks
Horses
Buffaloes
Goats
Rank
0.00
0.25
0.50
0.75
1.00

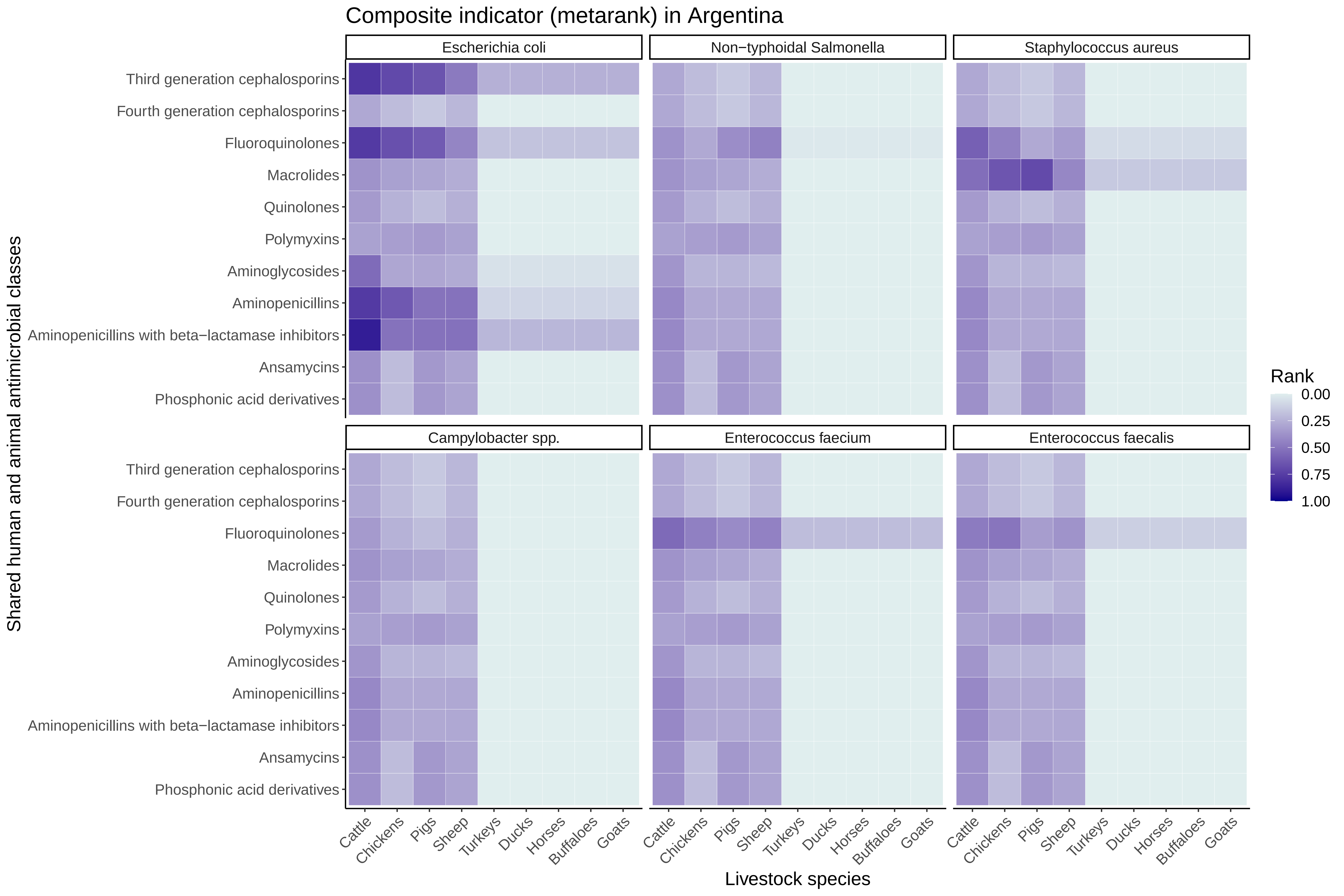
Composite indicator (metarank) in Argentina
Escherichia coli
Non−typhoidal Salmonella
Staphylococcus aureus
Campylobacter spp.
Enterococcus faecium
Enterococcus faecalis
Shared human and animal antimicrobial classes
Third generation cephalosporins
Fourth generation cephalosporins
Fluoroquinolones
Macrolides
Quinolones
Polymyxins
Aminoglycosides
Aminopenicillins
Aminopenicillins with beta−lactamase inhibitors
Ansamycins
Phosphonic acid derivatives
Cattle
Chickens
Pigs
Sheep
Turkeys
Ducks
Horses
Buffaloes
Goats
Livestock species
Rank
0.00
0.25
0.50
0.75
1.00

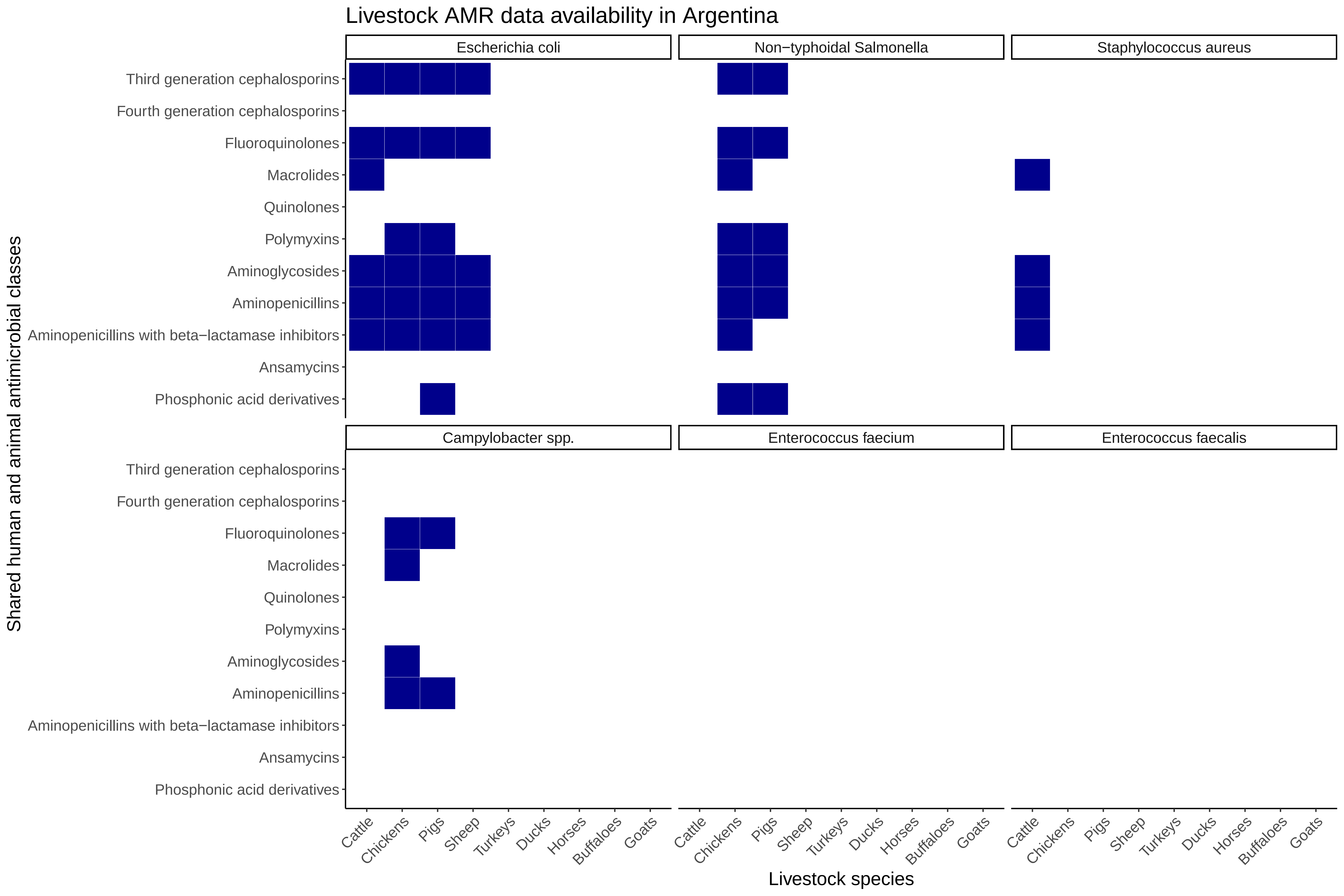

Livestock AMR data availability in Argentina
Escherichia coli
Non−typhoidal Salmonella
Staphylococcus aureus
Campylobacter spp.
Enterococcus faecium
Enterococcus faecalis
Third generation cephalosporins
Fourth generation cephalosporins
Fluoroquinolones
Macrolides
Quinolones
Polymyxins
Aminoglycosides
Aminopenicillins
Aminopenicillins with beta−lactamase inhibitors
Ansamycins
Phosphonic acid derivatives
Shared human and animal antimicrobial classes
Cattle
Chickens
Pigs
Sheep
Turkeys
Ducks
Horses
Buffaloes
Goats
Livestock species

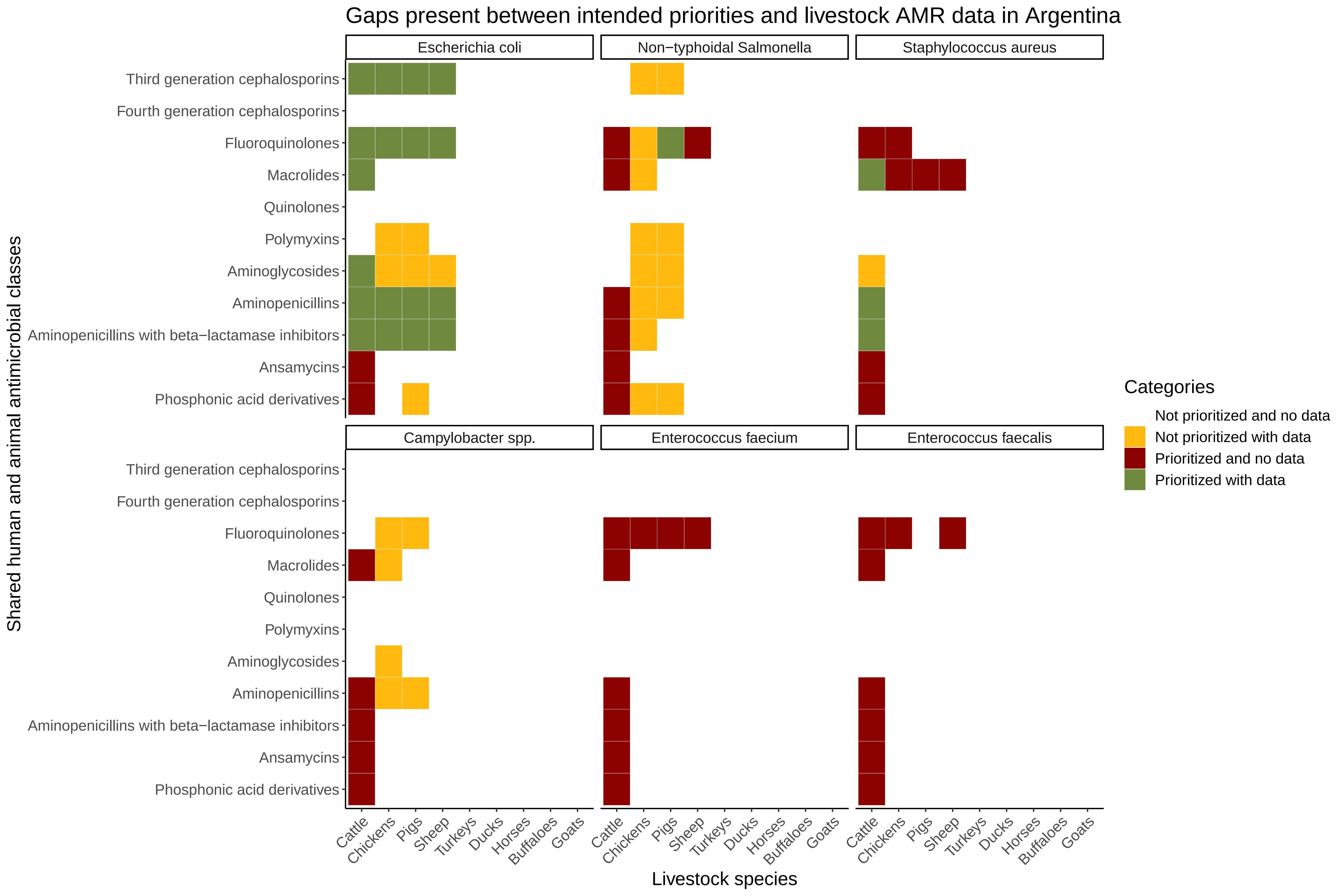

Gaps present between intended priorities and livestock AMR data in Argentina
Escherichia coli
Non−typhoidal Salmonella
Staphylococcus aureus
Campylobacter spp.
Enterococcus faecium
Enterococcus faecalis
Third generation cephalosporins
Fourth generation cephalosporins
Fluoroquinolones
Macrolides
Quinolones
Polymyxins
Aminoglycosides
Aminopenicillins
Aminopenicillins with beta−lactamase inhibitors
Ansamycins
Phosphonic acid derivatives
Shared human and animal antimicrobial classes
Cattle
Chickens
Pigs
Sheep
Turkeys
Ducks
Horses
Buffaloes
Goats
Livestock species
Categories
Not prioritized and no data
Not prioritized with data
Prioritized and no data
Prioritized with data

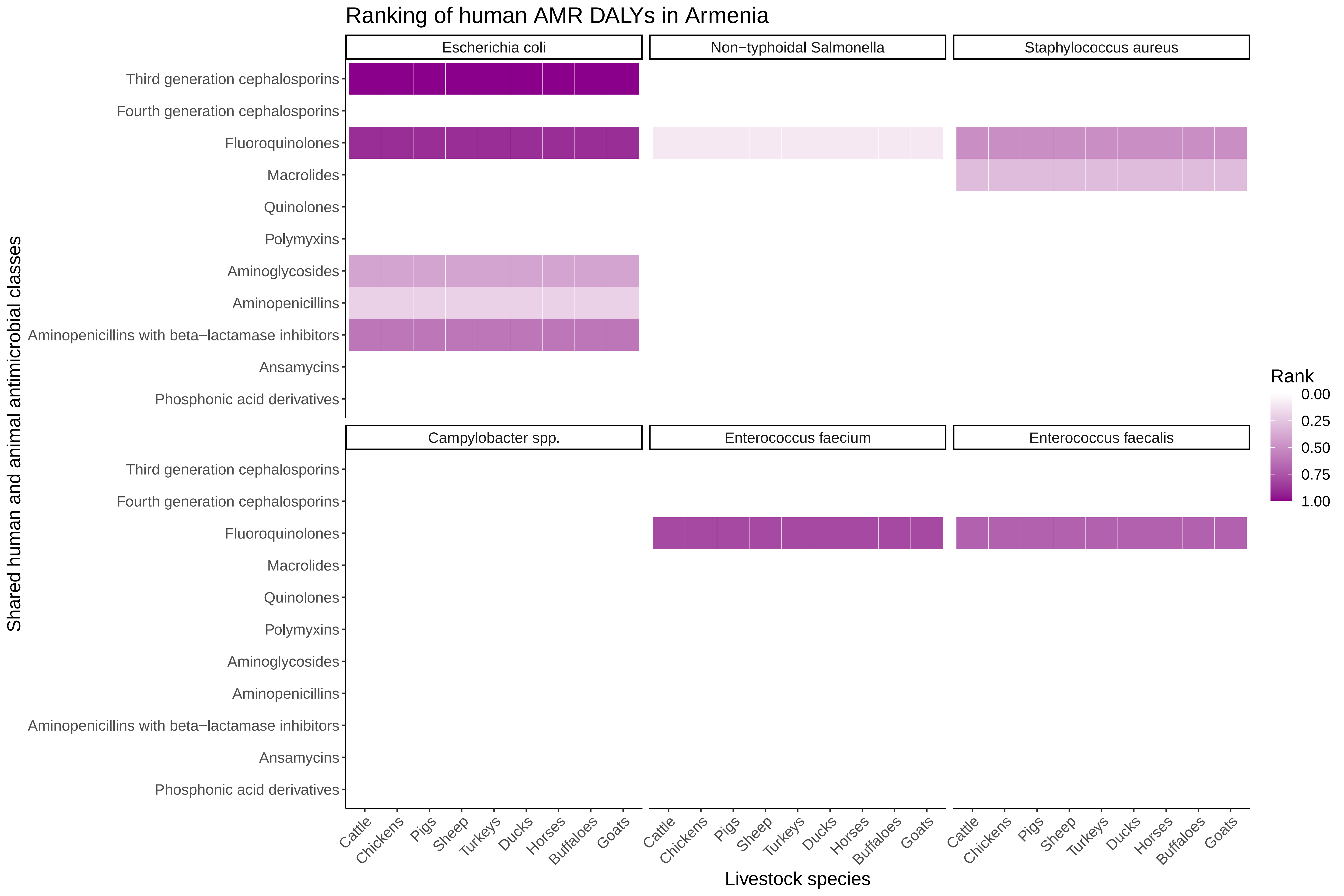

Ranking of human AMR DALYs in Armenia
Escherichia coli
Non−typhoidal Salmonella
Staphylococcus aureus
Campylobacter spp.
Enterococcus faecium
Enterococcus faecalis
Third generation cephalosporins
Fourth generation cephalosporins
Fluoroquinolones
Macrolides
Quinolones
Polymyxins
Aminoglycosides
Aminopenicillins
Aminopenicillins with beta−lactamase inhibitors
Ansamycins
Phosphonic acid derivatives
Shared human and animal antimicrobial classes
Cattle
Chickens
Pigs
Sheep
Turkeys
Ducks
Horses
Buffaloes
Goats
Livestock species
Rank
0.00
0.25
0.50
0.75
1.00

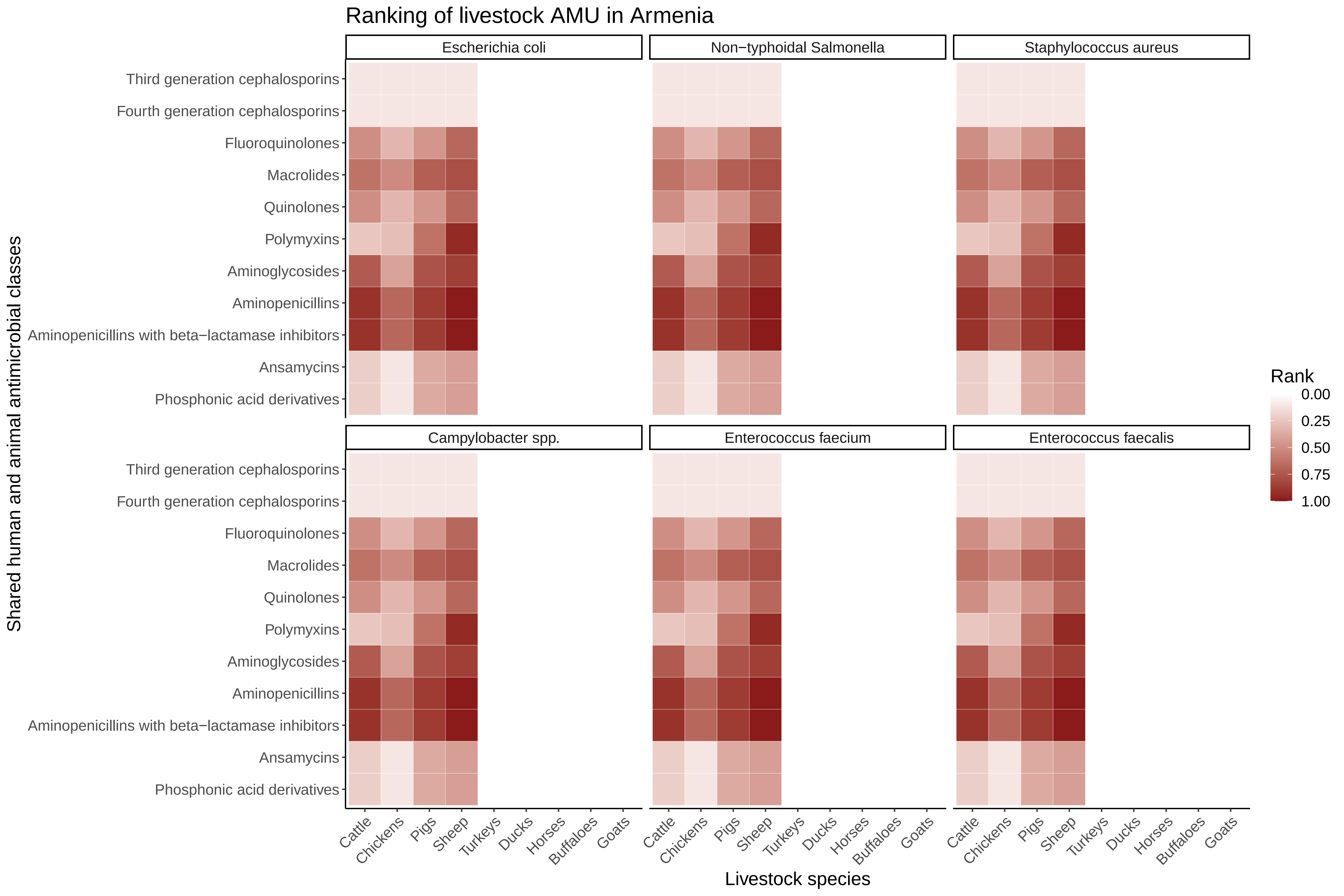

Ranking of livestock AMU in Armenia
Escherichia coli
Non−typhoidal Salmonella
Staphylococcus aureus
Campylobacter spp.
Enterococcus faecium
Enterococcus faecalis
Third generation cephalosporins
Fourth generation cephalosporins
Fluoroquinolones
Macrolides
Quinolones
Polymyxins
Aminoglycosides
Aminopenicillins
Aminopenicillins with beta−lactamase inhibitors
Ansamycins
Phosphonic acid derivatives
Shared human and animal antimicrobial classes
Cattle
Chickens
Pigs
Sheep
Turkeys
Ducks
Horses
Buffaloes
Goats
Livestock species
Rank
0.00
0.25
0.50
0.75
1.00

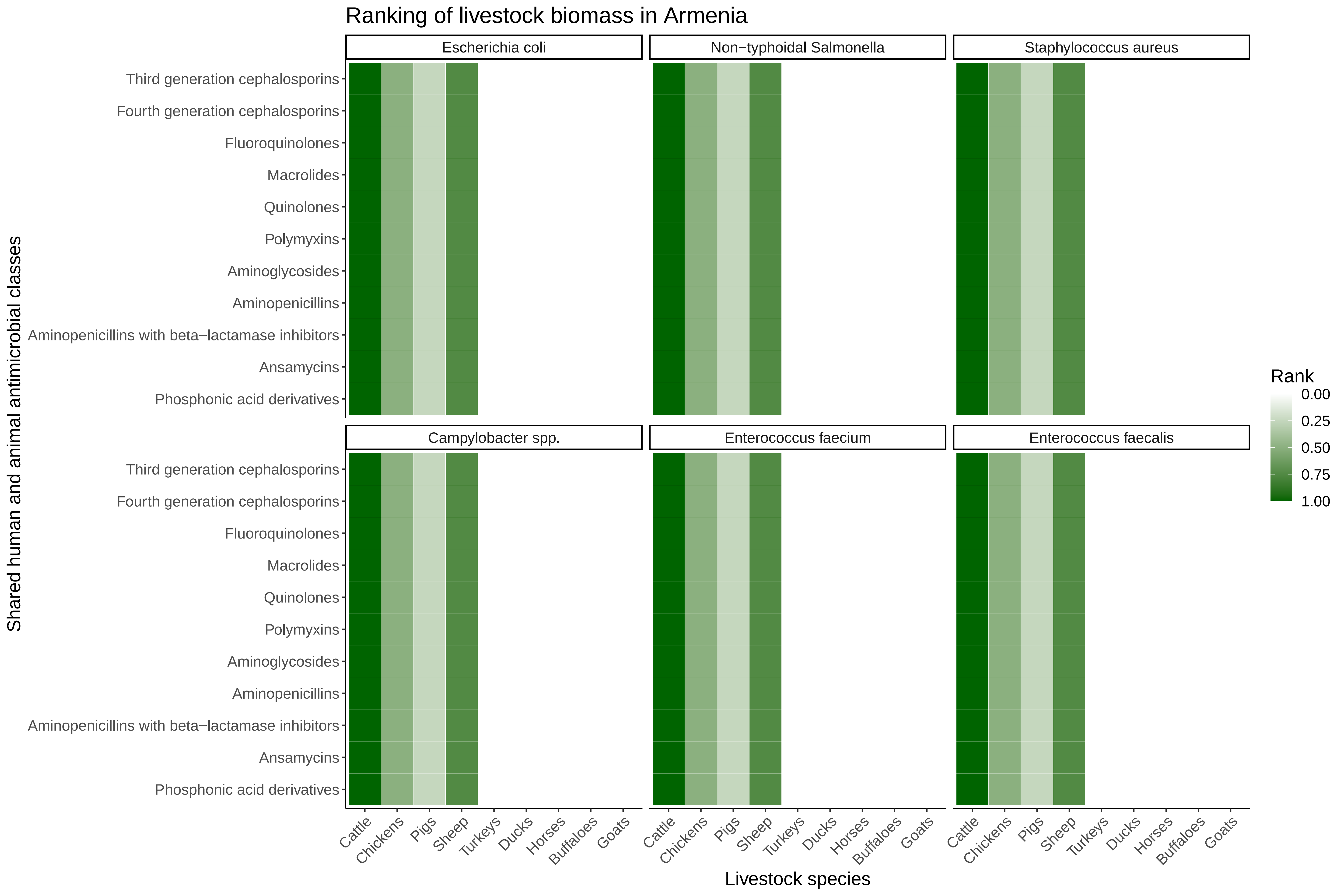

Ranking of livestock biomass in Armenia
Escherichia coli
Non−typhoidal Salmonella
Staphylococcus aureus
Campylobacter spp.
Enterococcus faecium
Enterococcus faecalis
Third generation cephalosporins
Fourth generation cephalosporins
Fluoroquinolones
Macrolides
Quinolones
Polymyxins
Aminoglycosides
Aminopenicillins
Aminopenicillins with beta−lactamase inhibitors
Ansamycins
Phosphonic acid derivatives
Shared human and animal antimicrobial classes
Cattle
Chickens
Pigs
Sheep
Turkeys
Ducks
Horses
Buffaloes
Goats
Livestock species
Rank
0.00
0.25
0.50
0.75
1.00

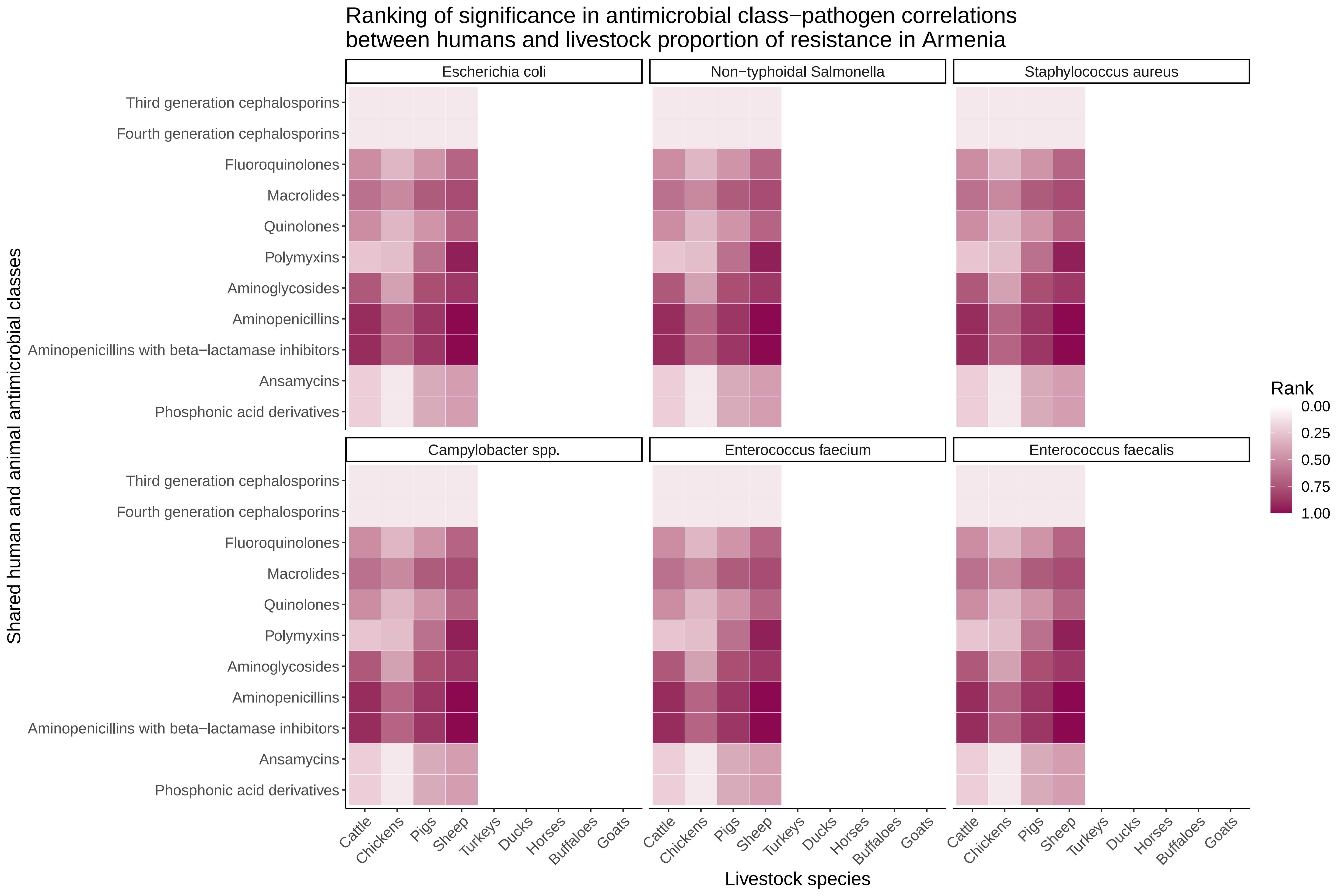
Ranking of significance in antimicrobial class−pathogen correlations
between humans and livestock proportion of resistance in Armenia
Escherichia coli
Non−typhoidal Salmonella
Staphylococcus aureus
Campylobacter spp.
Enterococcus faecium
Enterococcus faecalis
Third generation cephalosporins
Fourth generation cephalosporins
Fluoroquinolones
Macrolides
Quinolones
Polymyxins
Aminoglycosides
Aminopenicillins
Aminopenicillins with beta−lactamase inhibitors
Ansamycins
Phosphonic acid derivatives
Shared human and animal antimicrobial classes
Cattle
Chickens
Pigs
Sheep
Turkeys
Ducks
Horses
Buffaloes
Goats
Livestock species
Rank
0.00
0.25
0.50
0.75
1.00

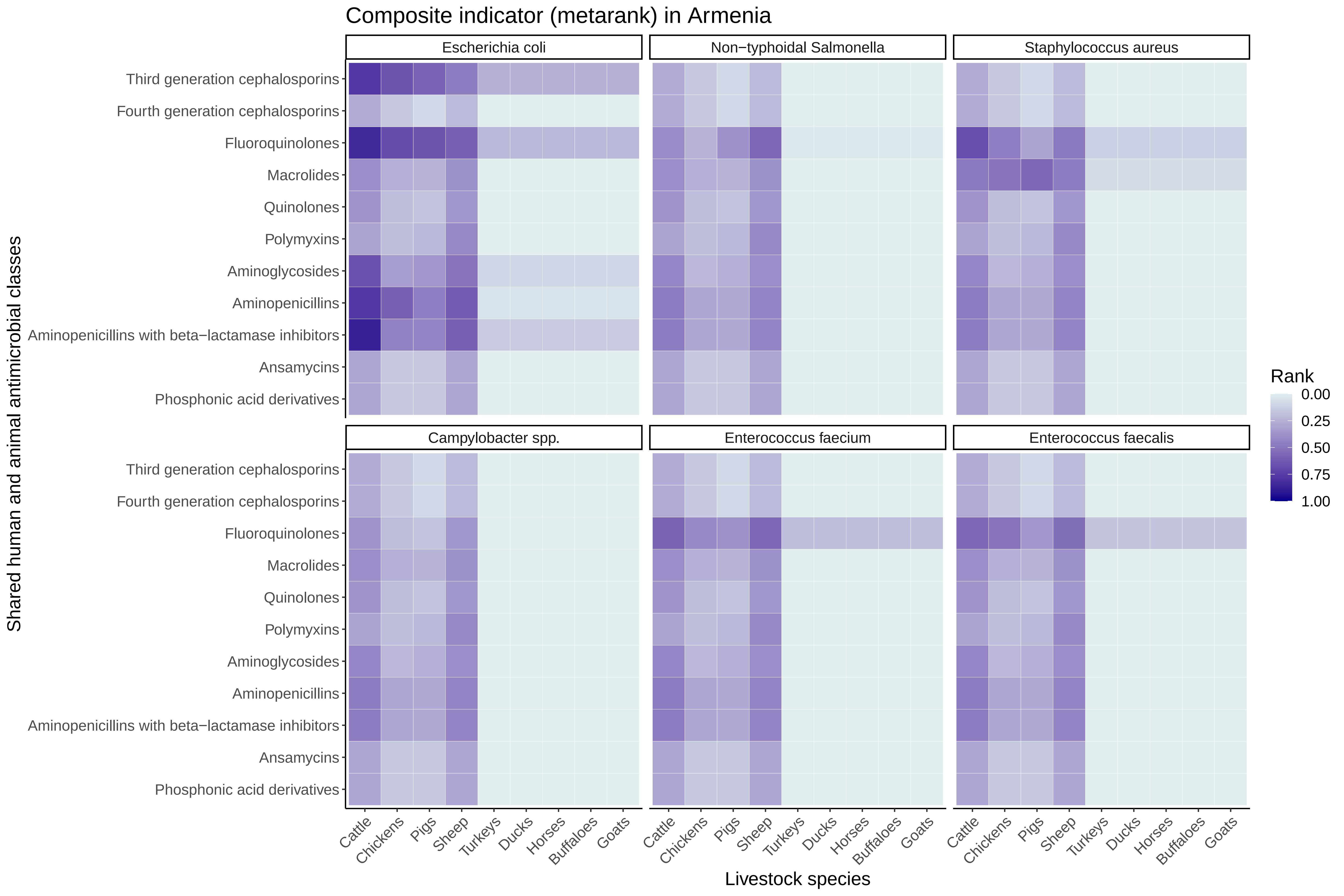
Composite indicator (metarank) in Armenia
Escherichia coli
Non−typhoidal Salmonella
Staphylococcus aureus
Campylobacter spp.
Enterococcus faecium
Enterococcus faecalis
Third generation cephalosporins
Fourth generation cephalosporins
Fluoroquinolones
Macrolides
Quinolones
Polymyxins
Aminoglycosides
Aminopenicillins
Aminopenicillins with beta−lactamase inhibitors
Ansamycins
Phosphonic acid derivatives
Shared human and animal antimicrobial classes
Cattle
Chickens
Pigs
Sheep
Turkeys
Ducks
Horses
Buffaloes
Goats
Livestock species
Rank
0.00
0.25
0.50
0.75
1.00

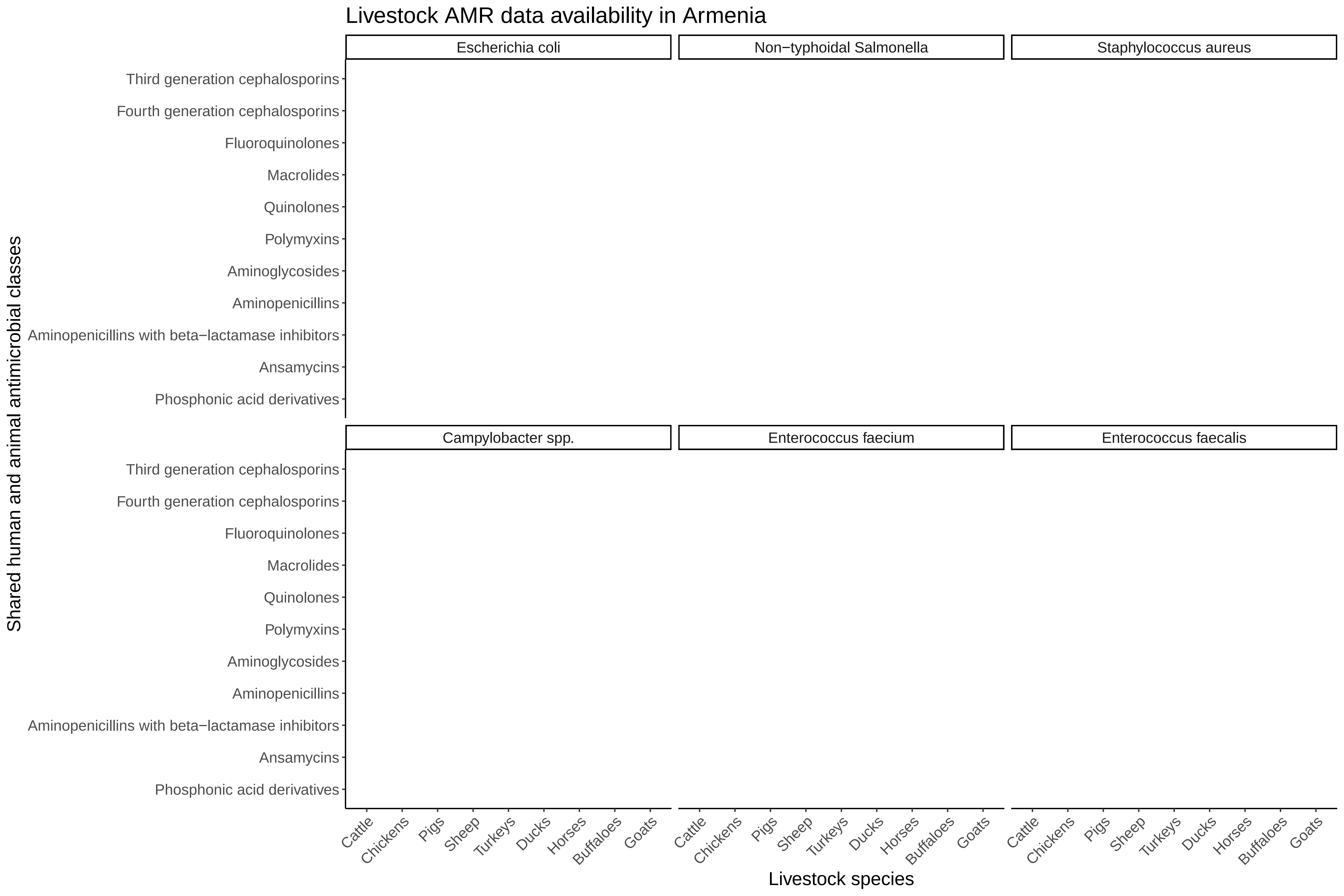
Livestock AMR data availability in Armenia
Escherichia coli
Non−typhoidal Salmonella
Staphylococcus aureus
Campylobacter spp.
Enterococcus faecium
Enterococcus faecalis
Shared human and animal antimicrobial classes
Third generation cephalosporins
Fourth generation cephalosporins
Fluoroquinolones
Macrolides
Quinolones
Polymyxins
Aminoglycosides
Aminopenicillins
Aminopenicillins with beta−lactamase inhibitors
Ansamycins
Phosphonic acid derivatives
Cattle
Chickens
Pigs
Sheep
Turkeys
Ducks
Horses
Buffaloes
Goats
Livestock species

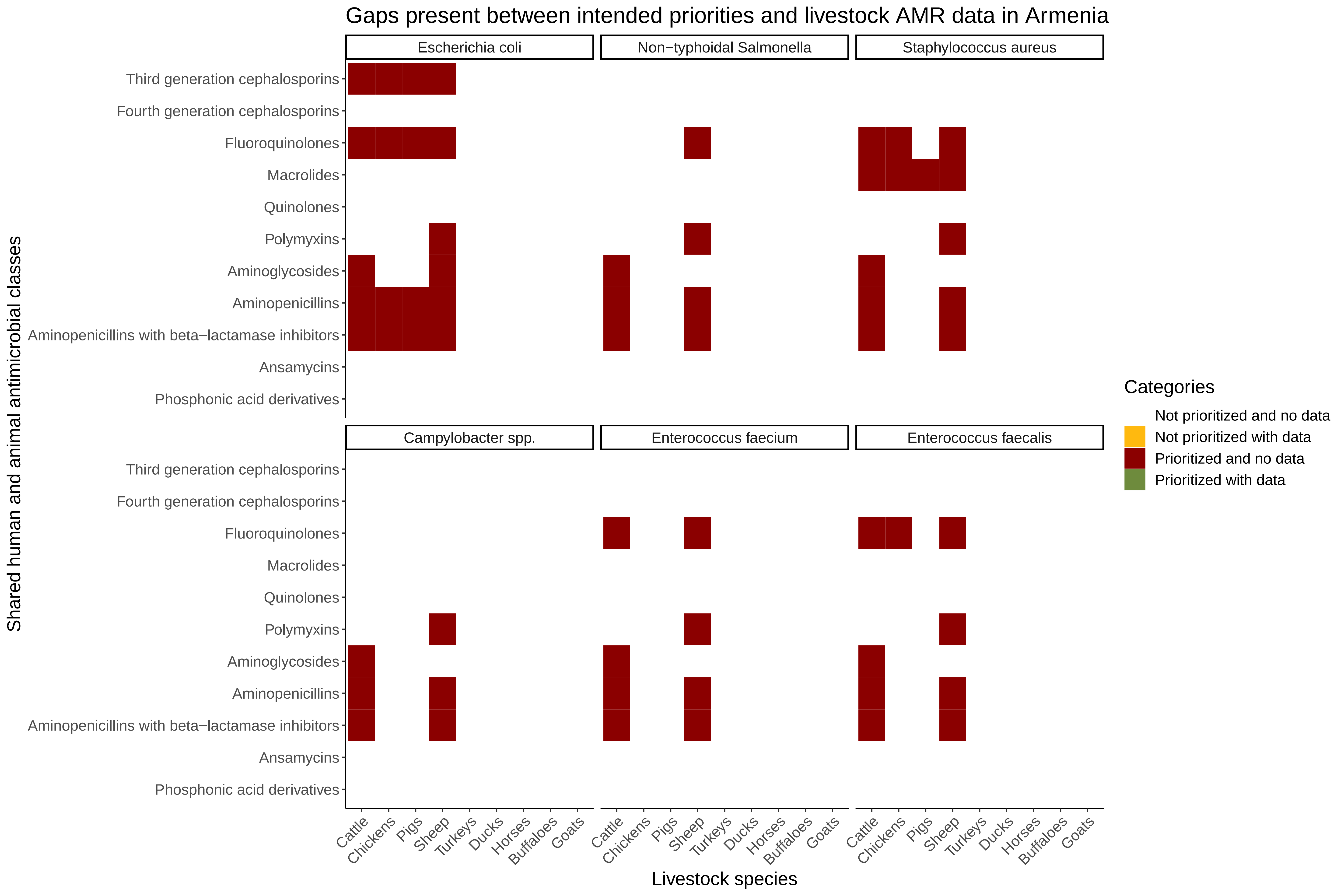

Gaps present between intended priorities and livestock AMR data in Armenia
Escherichia coli
Non−typhoidal Salmonella
Staphylococcus aureus
Campylobacter spp.
Enterococcus faecium
Enterococcus faecalis
Third generation cephalosporins
Fourth generation cephalosporins
Fluoroquinolones
Macrolides
Quinolones
Polymyxins
Aminoglycosides
Aminopenicillins
Aminopenicillins with beta−lactamase inhibitors
Ansamycins
Phosphonic acid derivatives
Shared human and animal antimicrobial classes
Cattle
Chickens
Pigs
Sheep
Turkeys
Ducks
Horses
Buffaloes
Goats
Livestock species
Categories
Not prioritized and no data
Not prioritized with data
Prioritized and no data
Prioritized with data

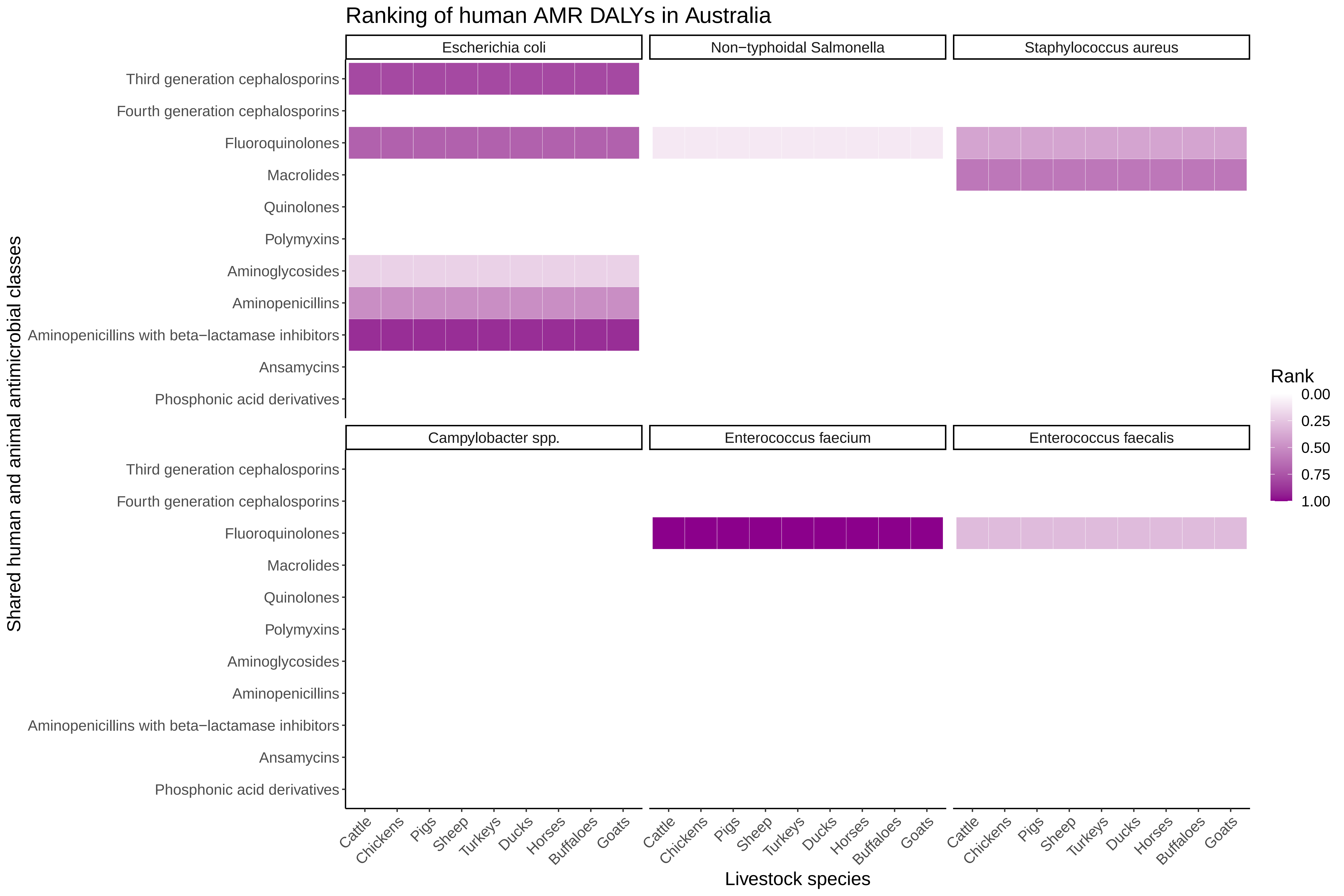
Ranking of human AMR DALYs in Australia
Escherichia coli
Non−typhoidal Salmonella
Staphylococcus aureus
Campylobacter spp.
Enterococcus faecium
Enterococcus faecalis
Third generation cephalosporins
Fourth generation cephalosporins
Fluoroquinolones
Macrolides
Quinolones
Polymyxins
Aminoglycosides
Aminopenicillins
Aminopenicillins with beta−lactamase inhibitors
Ansamycins
Phosphonic acid derivatives
Shared human and animal antimicrobial classes
Cattle
Chickens
Pigs
Sheep
Turkeys
Ducks
Horses
Buffaloes
Goats
Livestock species
Rank
0.00
0.25
0.50
0.75
1.00

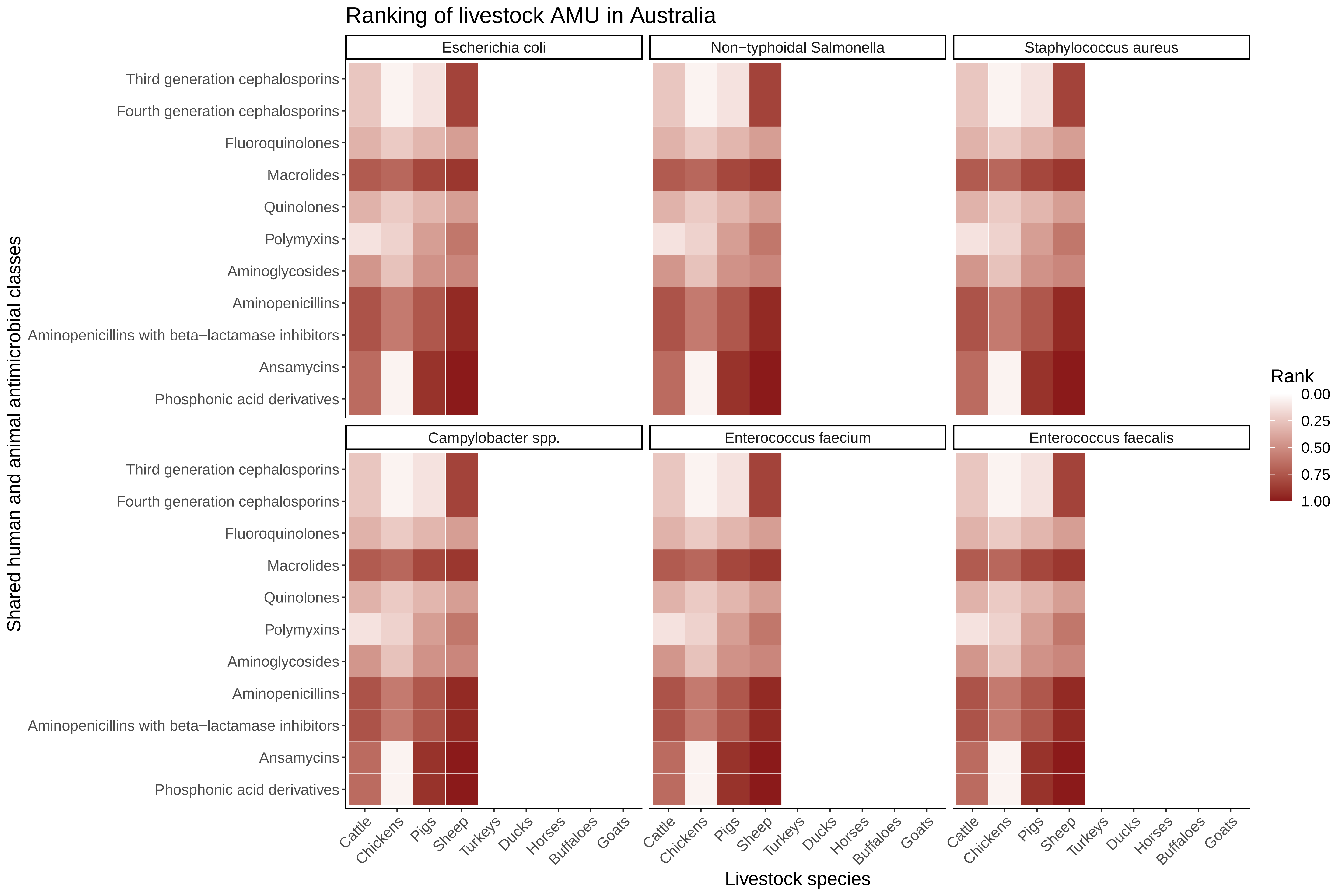

Ranking of livestock AMU in Australia
Escherichia coli
Non−typhoidal Salmonella
Staphylococcus aureus
Campylobacter spp.
Enterococcus faecium
Enterococcus faecalis
Third generation cephalosporins
Fourth generation cephalosporins
Fluoroquinolones
Macrolides
Quinolones
Polymyxins
Aminoglycosides
Aminopenicillins
Aminopenicillins with beta−lactamase inhibitors
Ansamycins
Phosphonic acid derivatives
Shared human and animal antimicrobial classes
Cattle
Chickens
Pigs
Sheep
Turkeys
Ducks
Horses
Buffaloes
Goats
Livestock species
Rank
0.00
0.25
0.50
0.75
1.00

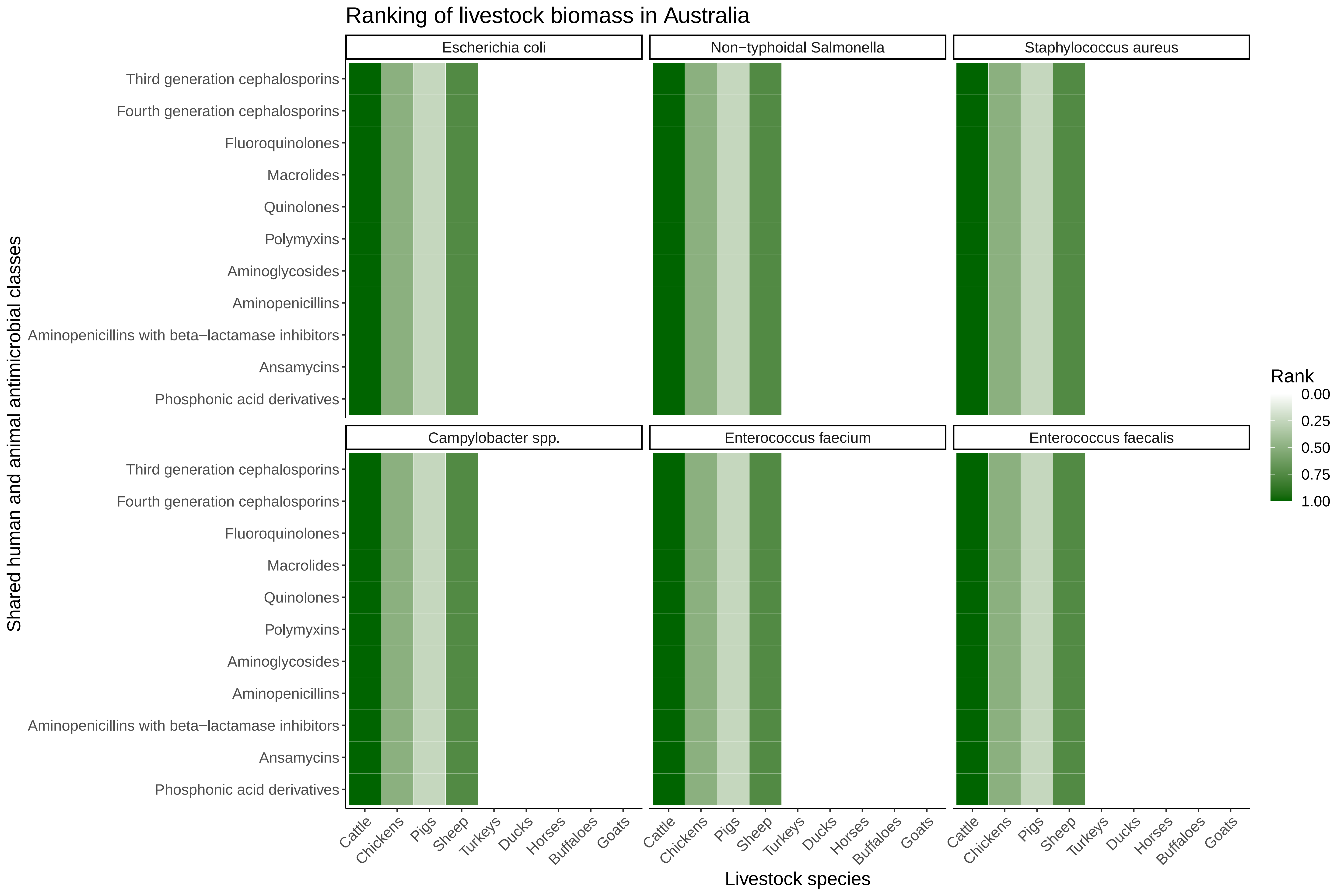

Ranking of livestock biomass in Australia
Escherichia coli
Non−typhoidal Salmonella
Staphylococcus aureus
Campylobacter spp.
Enterococcus faecium
Enterococcus faecalis
Shared human and animal antimicrobial classes
Third generation cephalosporins
Fourth generation cephalosporins
Fluoroquinolones
Macrolides
Quinolones
Polymyxins
Aminoglycosides
Aminopenicillins
Aminopenicillins with beta−lactamase inhibitors
Ansamycins
Phosphonic acid derivatives
Cattle
Chickens
Pigs
Sheep
Turkeys
Ducks
Horses
Buffaloes
Goats
Livestock species
Rank
0.00
0.25
0.50
0.75
1.00

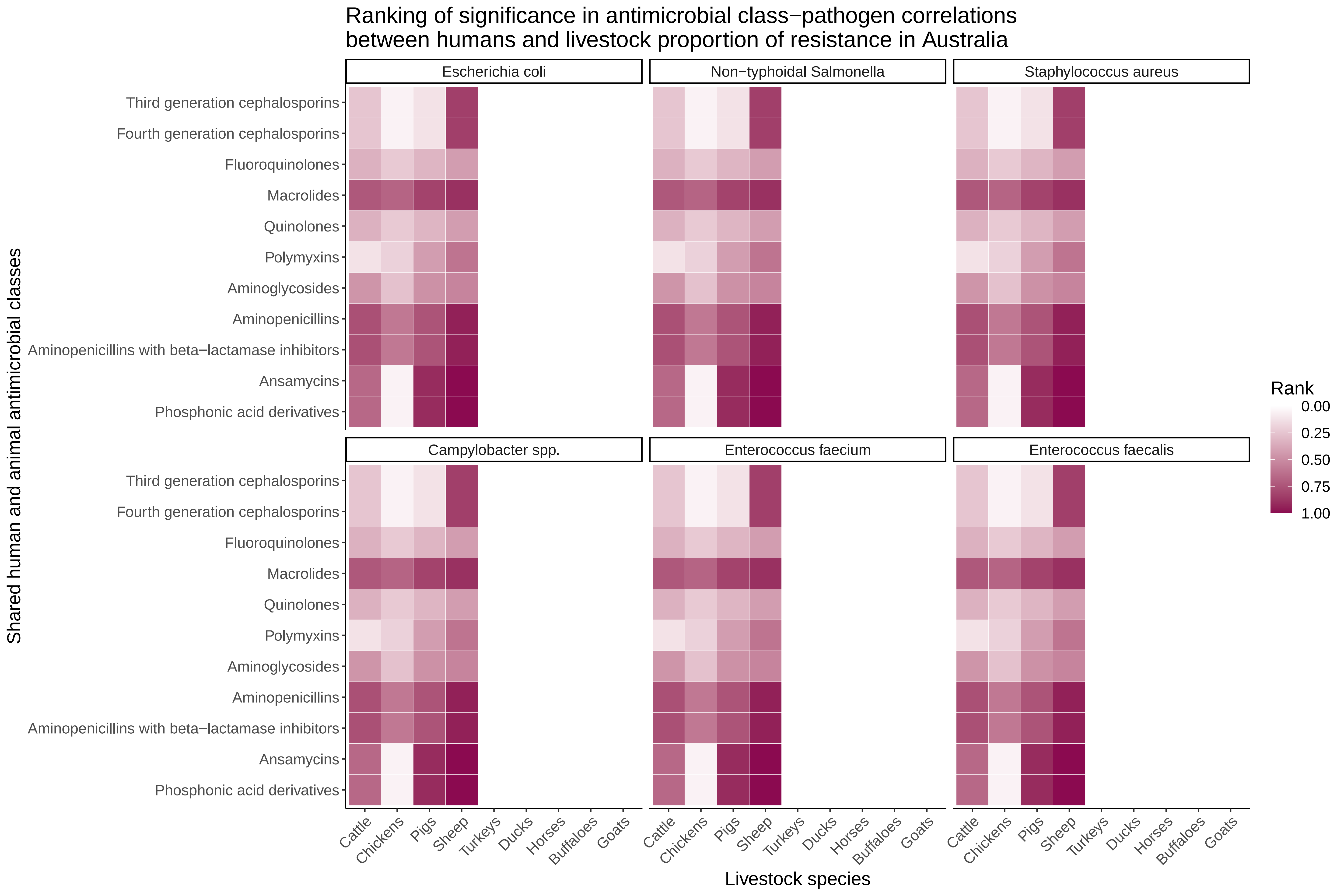

Ranking of significance in antimicrobial class−pathogen correlations
between humans and livestock proportion of resistance in Australia
Escherichia coli
Non−typhoidal Salmonella
Staphylococcus aureus
Campylobacter spp.
Enterococcus faecium
Enterococcus faecalis
Third generation cephalosporins
Fourth generation cephalosporins
Fluoroquinolones
Macrolides
Quinolones
Polymyxins
Aminoglycosides
Aminopenicillins
Aminopenicillins with beta−lactamase inhibitors
Ansamycins
Phosphonic acid derivatives
Shared human and animal antimicrobial classes
Cattle
Chickens
Pigs
Sheep
Turkeys
Ducks
Horses
Buffaloes
Goats
Livestock species
Rank
0.00
0.25
0.50
0.75
1.00

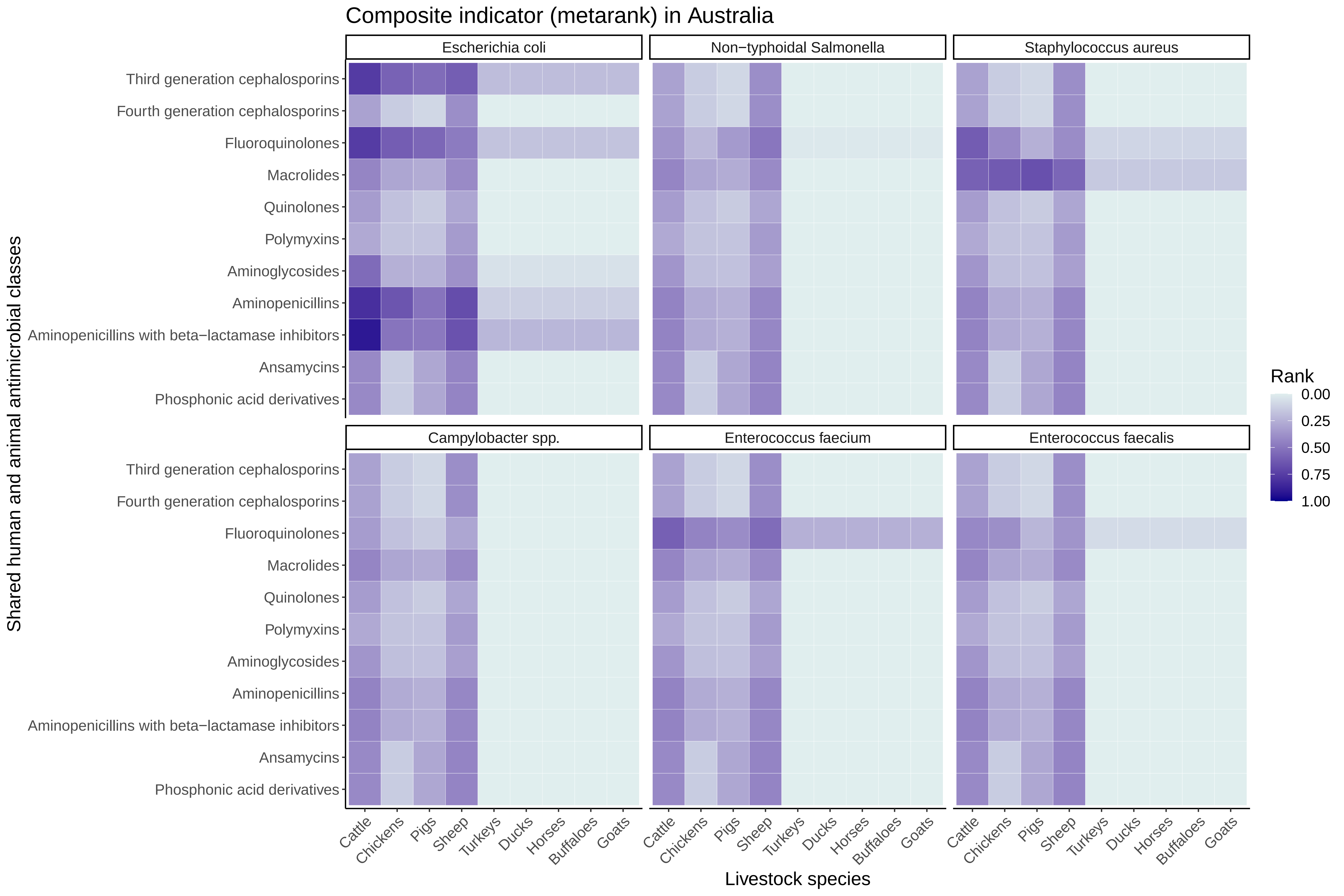

Composite indicator (metarank) in Australia
Escherichia coli
Non−typhoidal Salmonella
Staphylococcus aureus
Campylobacter spp.
Enterococcus faecium
Enterococcus faecalis
Third generation cephalosporins
Fourth generation cephalosporins
Fluoroquinolones
Macrolides
Quinolones
Polymyxins
Aminoglycosides
Aminopenicillins
Aminopenicillins with beta−lactamase inhibitors
Ansamycins
Phosphonic acid derivatives
Shared human and animal antimicrobial classes
Cattle
Chickens
Pigs
Sheep
Turkeys
Ducks
Horses
Buffaloes
Goats
Livestock species
Rank
0.00
0.25
0.50
0.75
1.00

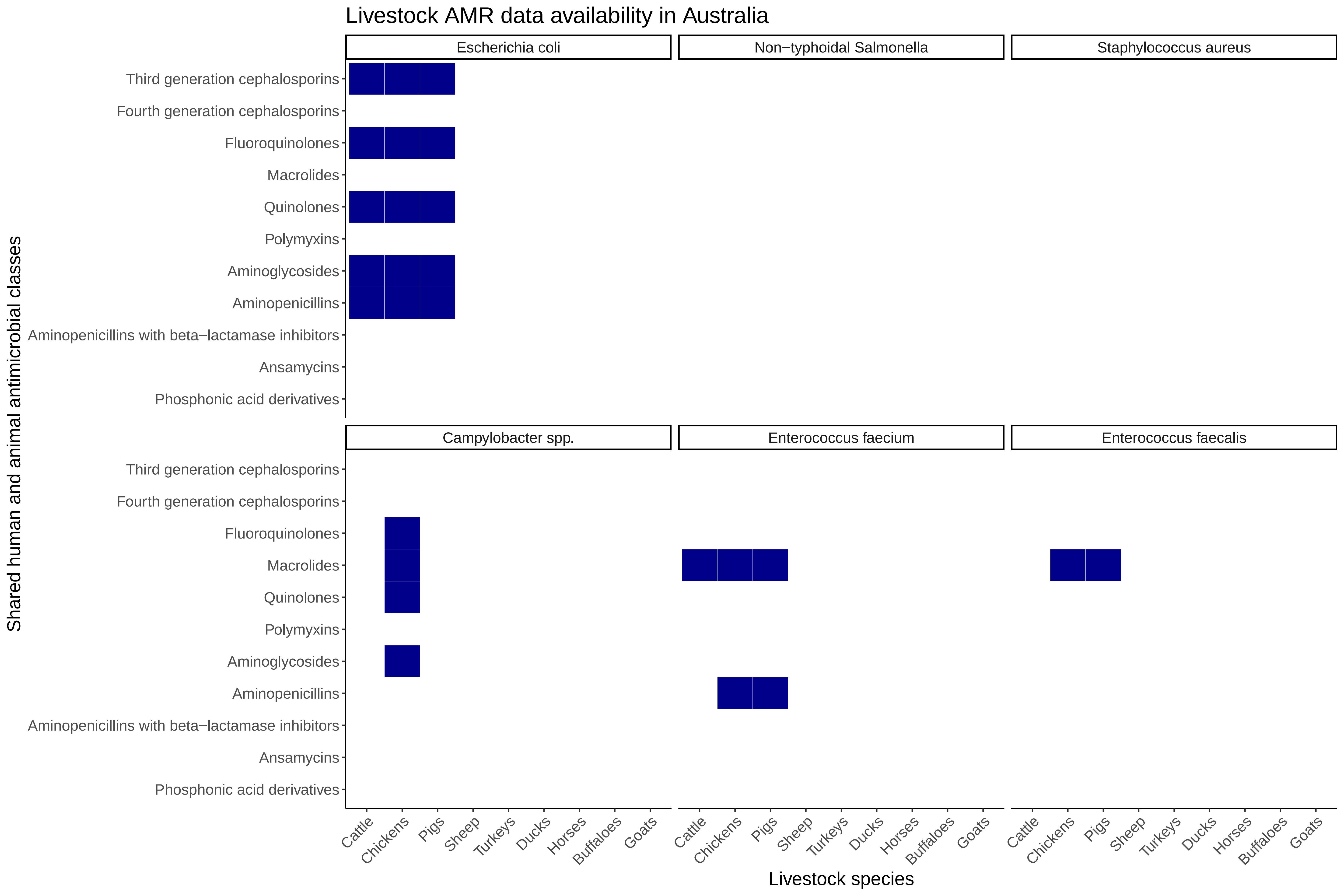

Livestock AMR data availability in Australia
Escherichia coli
Non−typhoidal Salmonella
Staphylococcus aureus
Campylobacter spp.
Enterococcus faecium
Enterococcus faecalis
Shared human and animal antimicrobial classes
Third generation cephalosporins
Fourth generation cephalosporins
Fluoroquinolones
Macrolides
Quinolones
Polymyxins
Aminoglycosides
Aminopenicillins
Aminopenicillins with beta−lactamase inhibitors
Ansamycins
Phosphonic acid derivatives
Cattle
Chickens
Pigs
Sheep
Turkeys
Ducks
Horses
Buffaloes
Goats
Livestock species

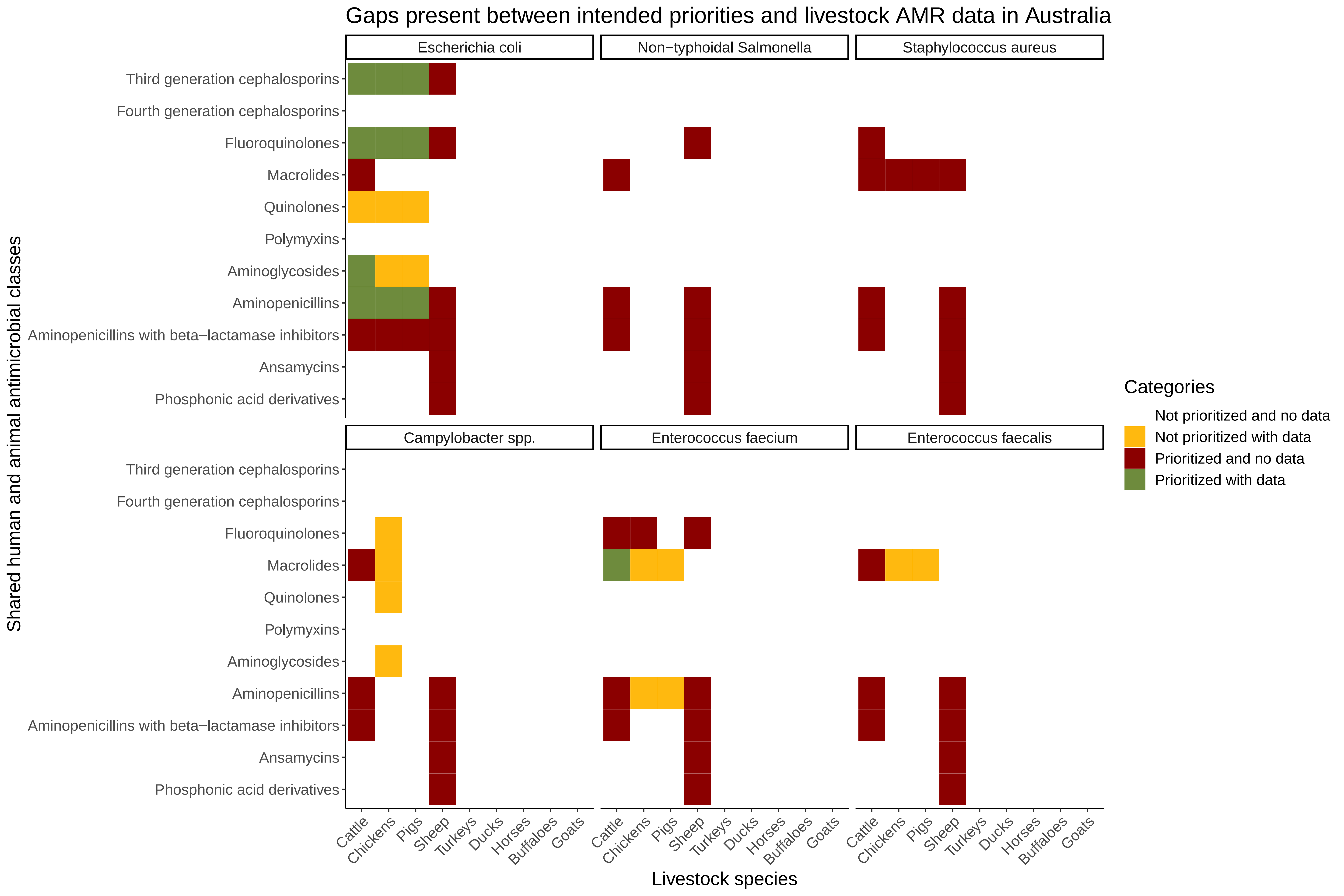
Gaps present between intended priorities and livestock AMR data in Australia
Escherichia coli
Non−typhoidal Salmonella
Staphylococcus aureus
Campylobacter spp.
Enterococcus faecium
Enterococcus faecalis
Third generation cephalosporins
Fourth generation cephalosporins
Fluoroquinolones
Macrolides
Quinolones
Polymyxins
Aminoglycosides
Aminopenicillins
Aminopenicillins with beta−lactamase inhibitors
Ansamycins
Phosphonic acid derivatives
Shared human and animal antimicrobial classes
Cattle
Chickens
Pigs
Sheep
Turkeys
Ducks
Horses
Buffaloes
Goats
Livestock species
Categories
Not prioritized and no data
Not prioritized with data
Prioritized and no data
Prioritized with data

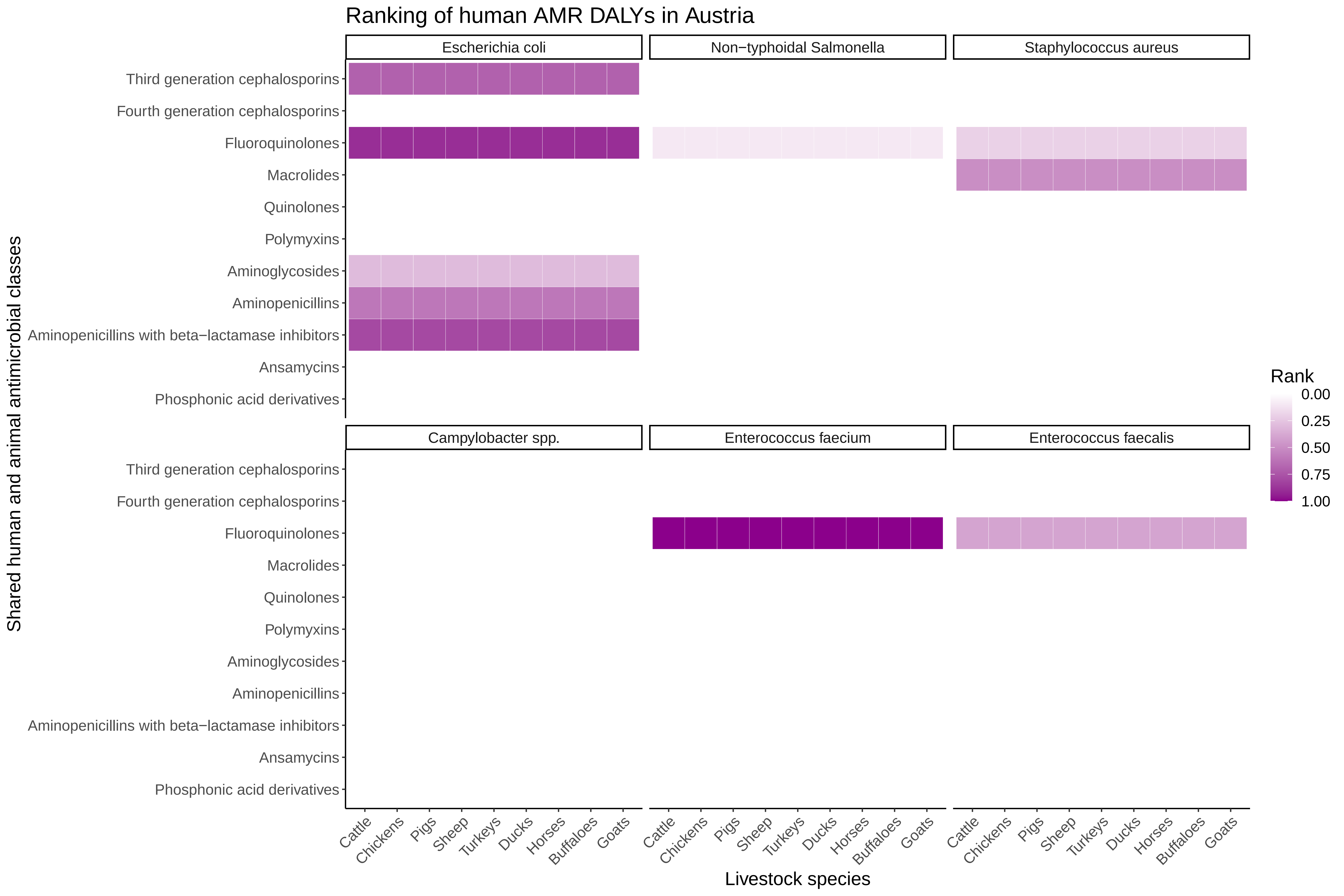

Ranking of human AMR DALYs in Austria
Escherichia coli
Non−typhoidal Salmonella
Staphylococcus aureus
Campylobacter spp.
Enterococcus faecium
Enterococcus faecalis
Third generation cephalosporins
Fourth generation cephalosporins
Fluoroquinolones
Macrolides
Quinolones
Polymyxins
Aminoglycosides
Aminopenicillins
Aminopenicillins with beta−lactamase inhibitors
Ansamycins
Phosphonic acid derivatives
Shared human and animal antimicrobial classes
Cattle
Chickens
Pigs
Sheep
Turkeys
Ducks
Horses
Buffaloes
Goats
Livestock species
Rank
0.00
0.25
0.50
0.75
1.00

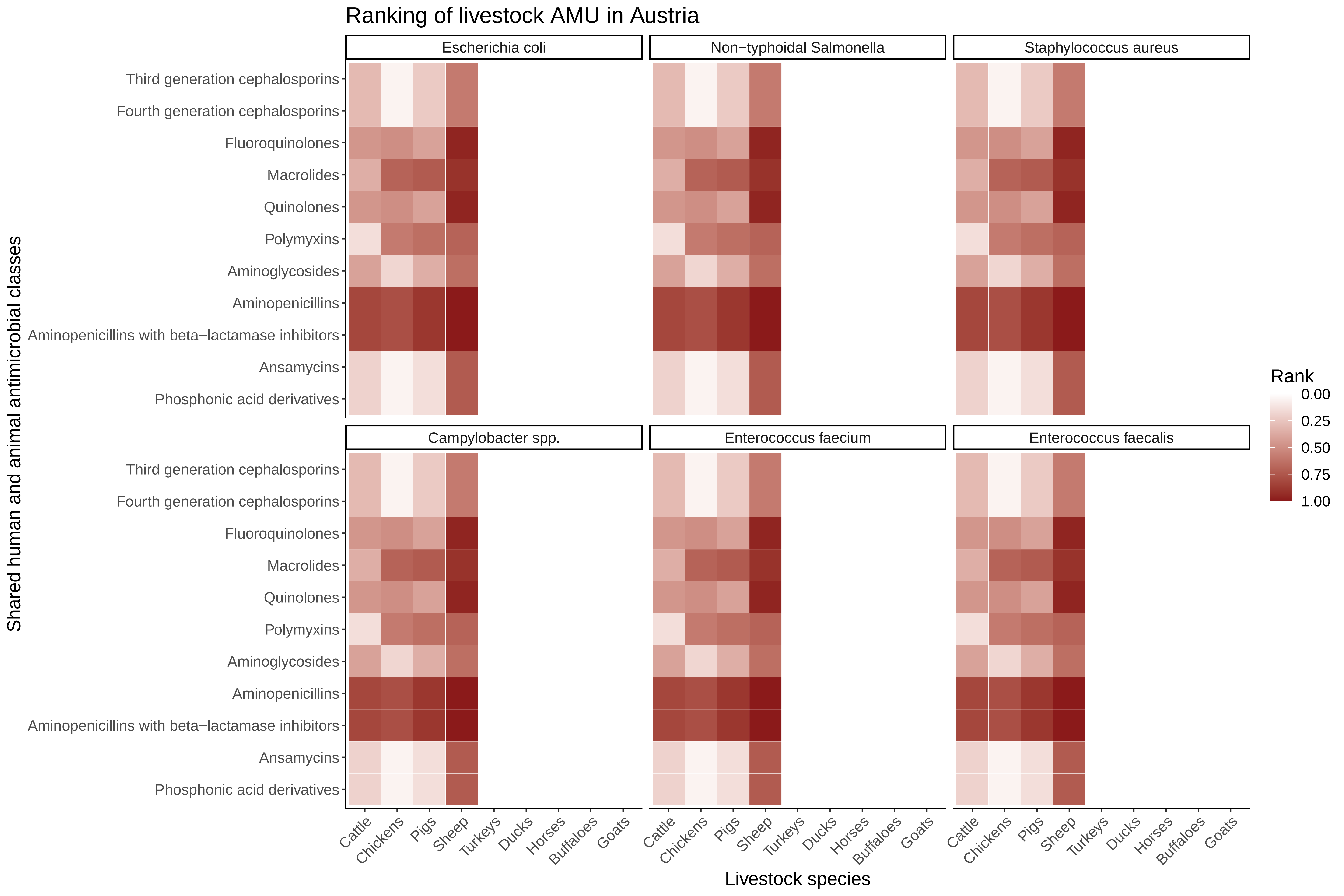

Ranking of livestock AMU in Austria
Escherichia coli
Non−typhoidal Salmonella
Staphylococcus aureus
Campylobacter spp.
Enterococcus faecium
Enterococcus faecalis
Third generation cephalosporins
Fourth generation cephalosporins
Fluoroquinolones
Macrolides
Quinolones
Polymyxins
Aminoglycosides
Aminopenicillins
Aminopenicillins with beta−lactamase inhibitors
Ansamycins
Phosphonic acid derivatives
Shared human and animal antimicrobial classes
Cattle
Chickens
Pigs
Sheep
Turkeys
Ducks
Horses
Buffaloes
Goats
Livestock species
Rank
0.00
0.25
0.50
0.75
1.00

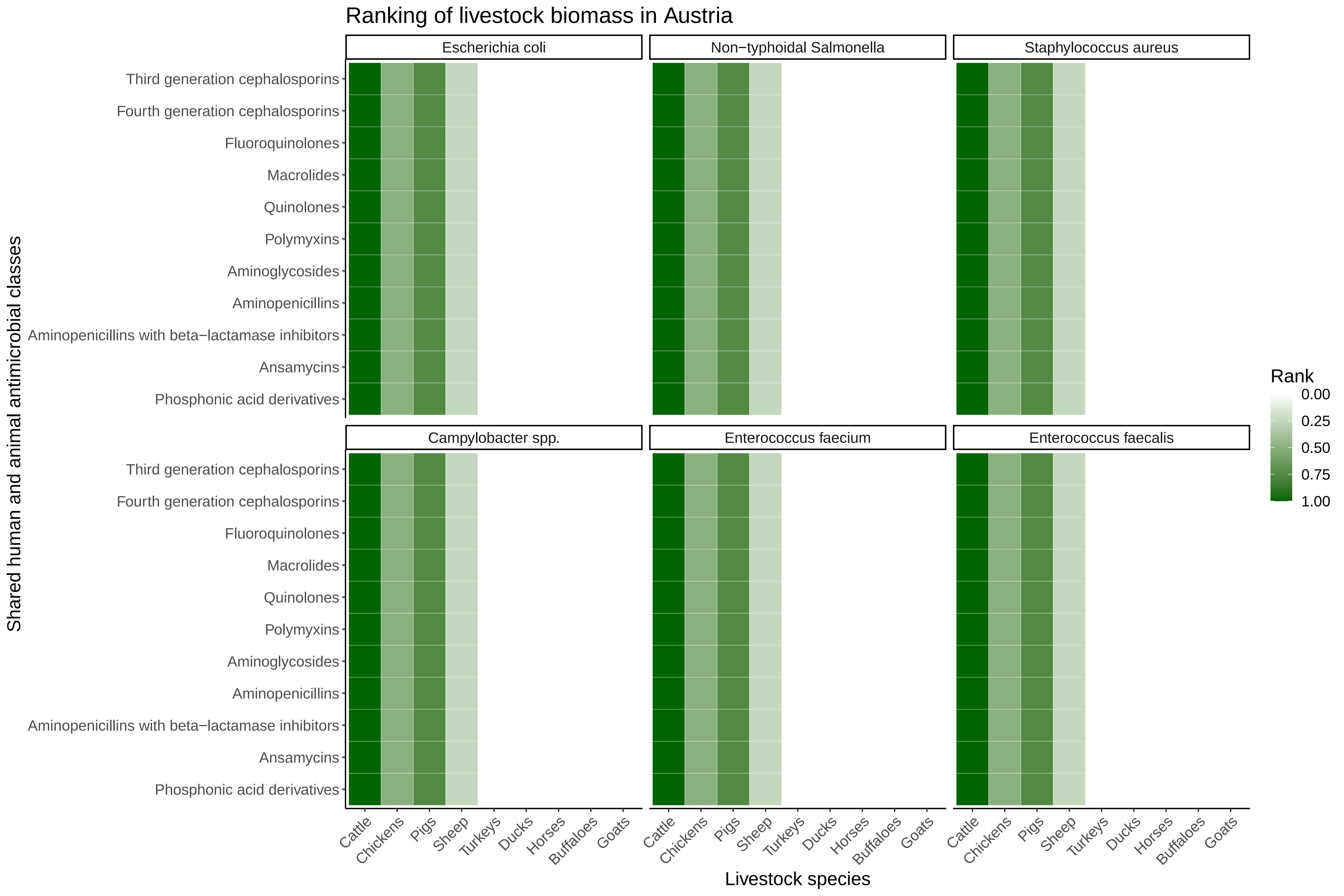

Ranking of livestock biomass in Austria
Escherichia coli
Non−typhoidal Salmonella
Staphylococcus aureus
Campylobacter spp.
Enterococcus faecium
Enterococcus faecalis
Third generation cephalosporins
Fourth generation cephalosporins
Fluoroquinolones
Macrolides
Quinolones
Polymyxins
Aminoglycosides
Aminopenicillins
Aminopenicillins with beta−lactamase inhibitors
Ansamycins
Phosphonic acid derivatives
Shared human and animal antimicrobial classes
Cattle
Chickens
Pigs
Sheep
Turkeys
Ducks
Horses
Buffaloes
Goats
Livestock species
Rank
0.00
0.25
0.50
0.75
1.00

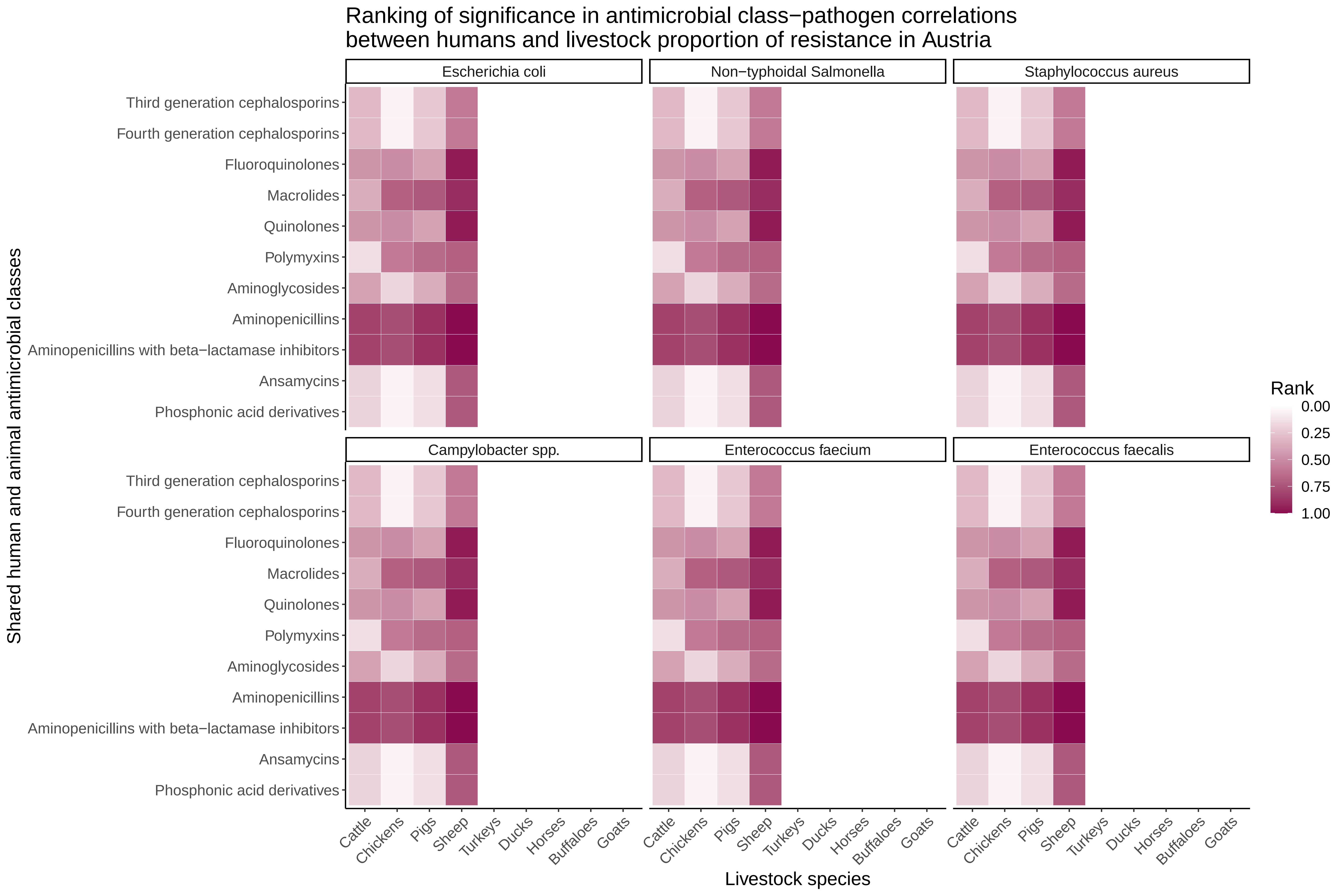

Ranking of significance in antimicrobial class−pathogen correlations
between humans and livestock proportion of resistance in Austria
Escherichia coli
Non−typhoidal Salmonella
Staphylococcus aureus
Campylobacter spp.
Enterococcus faecium
Enterococcus faecalis
Third generation cephalosporins
Fourth generation cephalosporins
Fluoroquinolones
Macrolides
Quinolones
Polymyxins
Aminoglycosides
Aminopenicillins
Aminopenicillins with beta−lactamase inhibitors
Ansamycins
Phosphonic acid derivatives
Shared human and animal antimicrobial classes
Cattle
Chickens
Pigs
Sheep
Turkeys
Ducks
Horses
Buffaloes
Goats
Livestock species
Rank
0.00
0.25
0.50
0.75
1.00

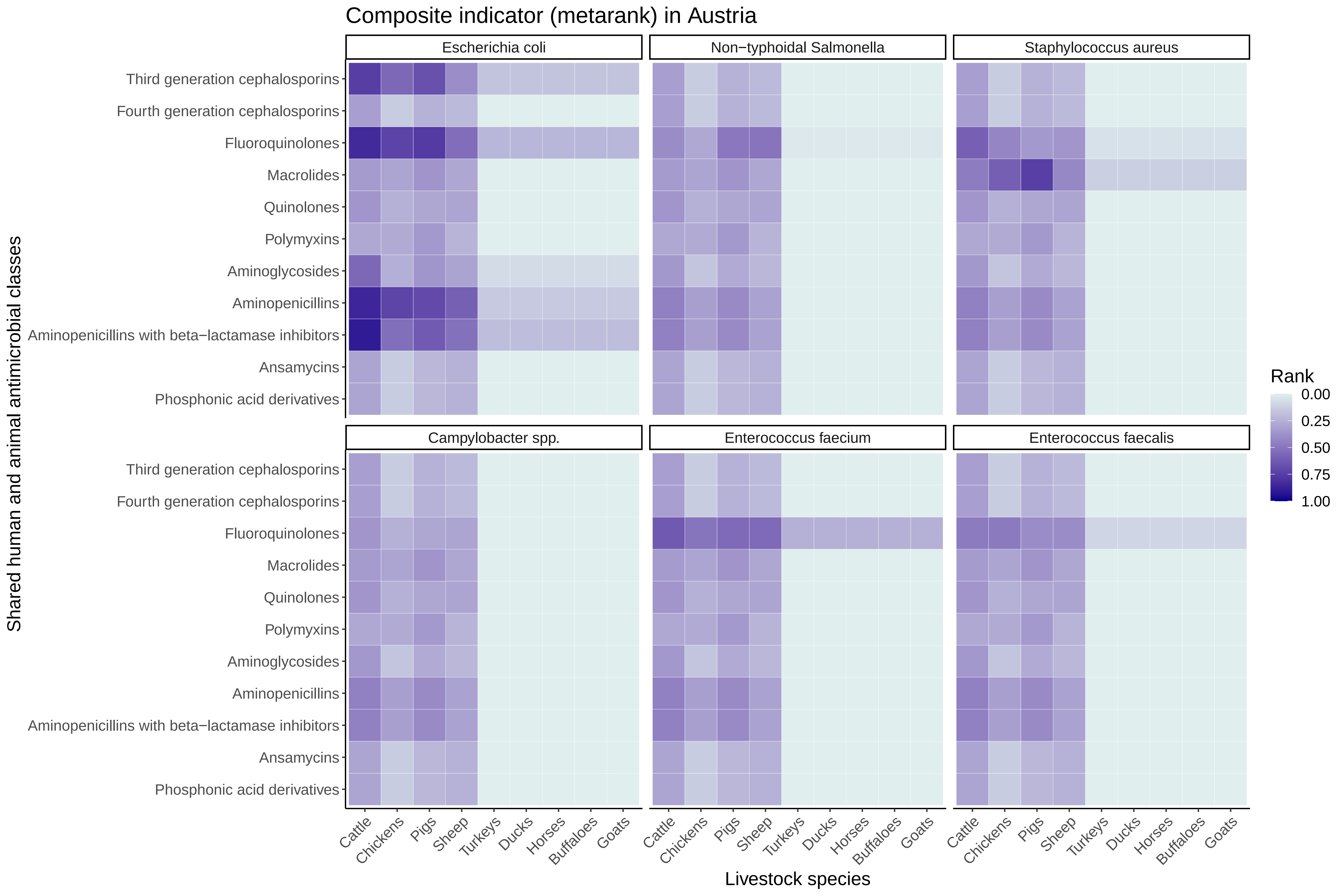

Composite indicator (metarank) in Austria
Escherichia coli
Non−typhoidal Salmonella
Staphylococcus aureus
Campylobacter spp.
Enterococcus faecium
Enterococcus faecalis
Shared human and animal antimicrobial classes
Third generation cephalosporins
Fourth generation cephalosporins
Fluoroquinolones
Macrolides
Quinolones
Polymyxins
Aminoglycosides
Aminopenicillins
Aminopenicillins with beta−lactamase inhibitors
Ansamycins
Phosphonic acid derivatives
Cattle
Chickens
Pigs
Sheep
Turkeys
Ducks
Horses
Buffaloes
Goats
Livestock species
Rank
0.00
0.25
0.50
0.75
1.00

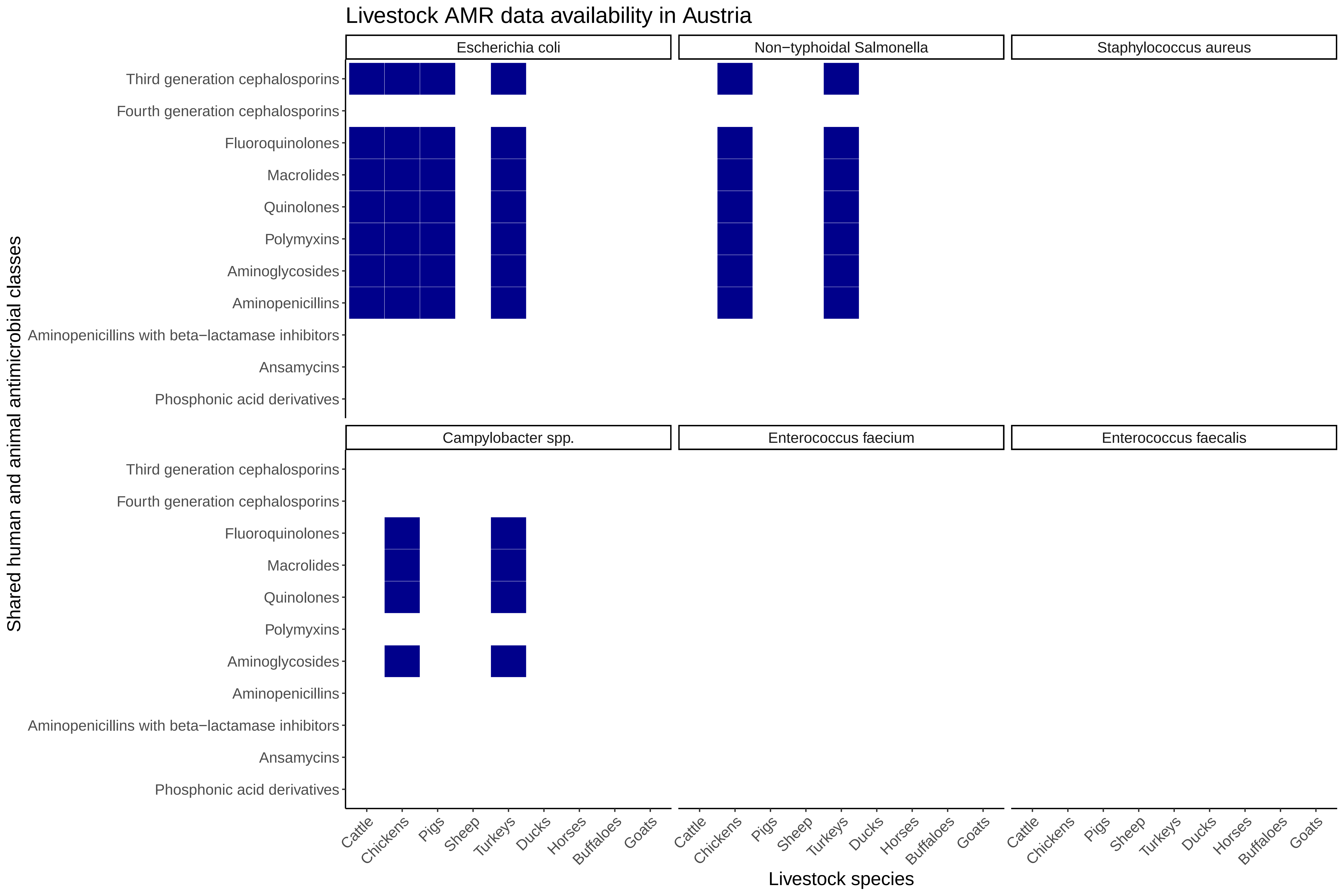

Livestock AMR data availability in Austria
Escherichia coli
Non−typhoidal Salmonella
Staphylococcus aureus
Campylobacter spp.
Enterococcus faecium
Enterococcus faecalis
Shared human and animal antimicrobial classes
Third generation cephalosporins
Fourth generation cephalosporins
Fluoroquinolones
Macrolides
Quinolones
Polymyxins
Aminoglycosides
Aminopenicillins
Aminopenicillins with beta−lactamase inhibitors
Ansamycins
Phosphonic acid derivatives
Cattle
Chickens
Pigs
Sheep
Turkeys
Ducks
Horses
Buffaloes
Goats
Livestock species

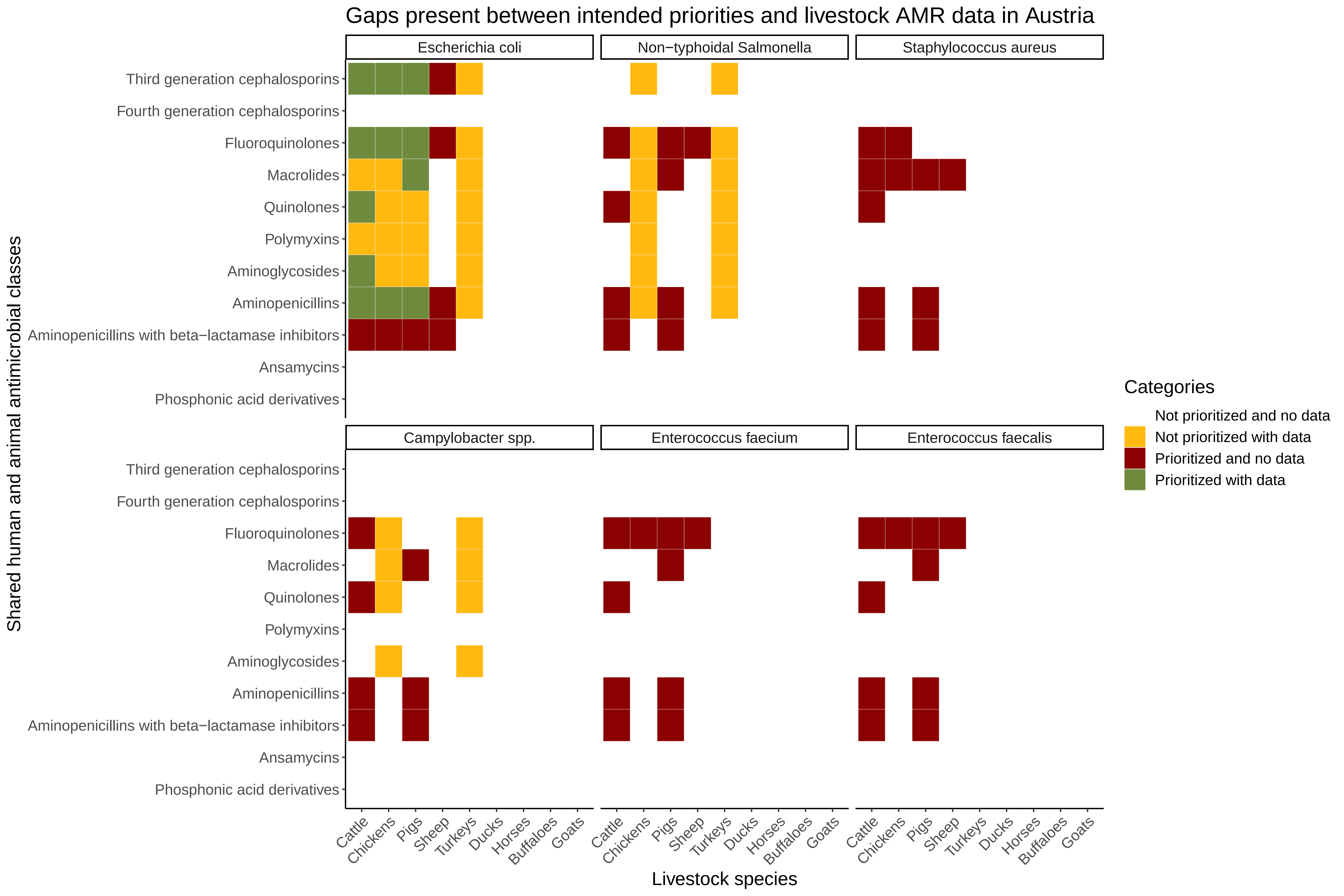

Gaps present between intended priorities and livestock AMR data in Austria
Escherichia coli
Non−typhoidal Salmonella
Staphylococcus aureus
Campylobacter spp.
Enterococcus faecium
Enterococcus faecalis
Third generation cephalosporins
Fourth generation cephalosporins
Fluoroquinolones
Macrolides
Quinolones
Polymyxins
Aminoglycosides
Aminopenicillins
Aminopenicillins with beta−lactamase inhibitors
Ansamycins
Phosphonic acid derivatives
Shared human and animal antimicrobial classes
Cattle
Chickens
Pigs
Sheep
Turkeys
Ducks
Horses
Buffaloes
Goats
Livestock species
Categories
Not prioritized and no data
Not prioritized with data
Prioritized and no data
Prioritized with data

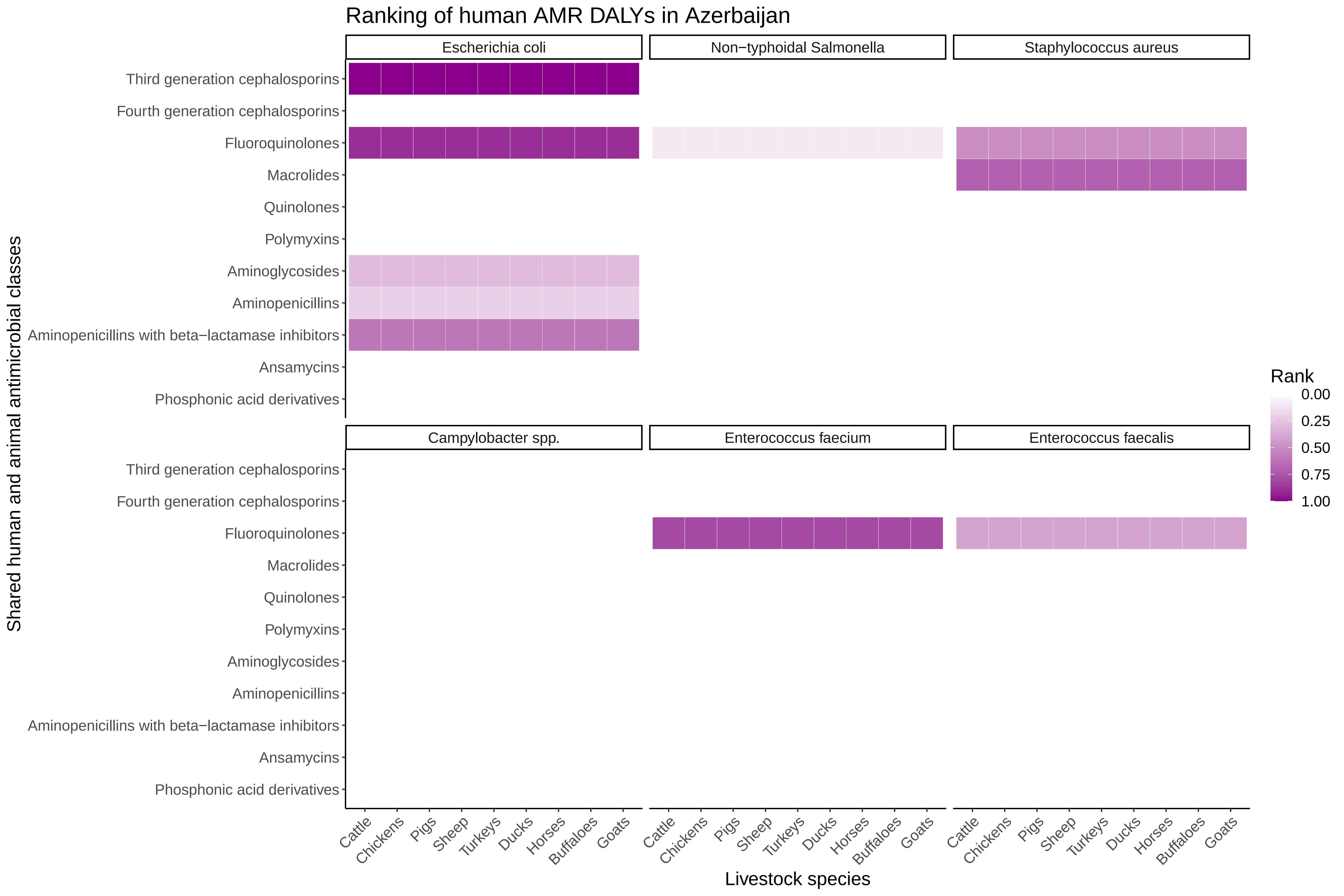

Ranking of human AMR DALYs in Azerbaijan
Escherichia coli
Non−typhoidal Salmonella
Staphylococcus aureus
Campylobacter spp.
Enterococcus faecium
Enterococcus faecalis
Third generation cephalosporins
Fourth generation cephalosporins
Fluoroquinolones
Macrolides
Quinolones
Polymyxins
Aminoglycosides
Aminopenicillins
Aminopenicillins with beta−lactamase inhibitors
Ansamycins
Phosphonic acid derivatives
Shared human and animal antimicrobial classes
Cattle
Chickens
Pigs
Sheep
Turkeys
Ducks
Horses
Buffaloes
Goats
Livestock species
Rank
0.00
0.25
0.50
0.75
1.00

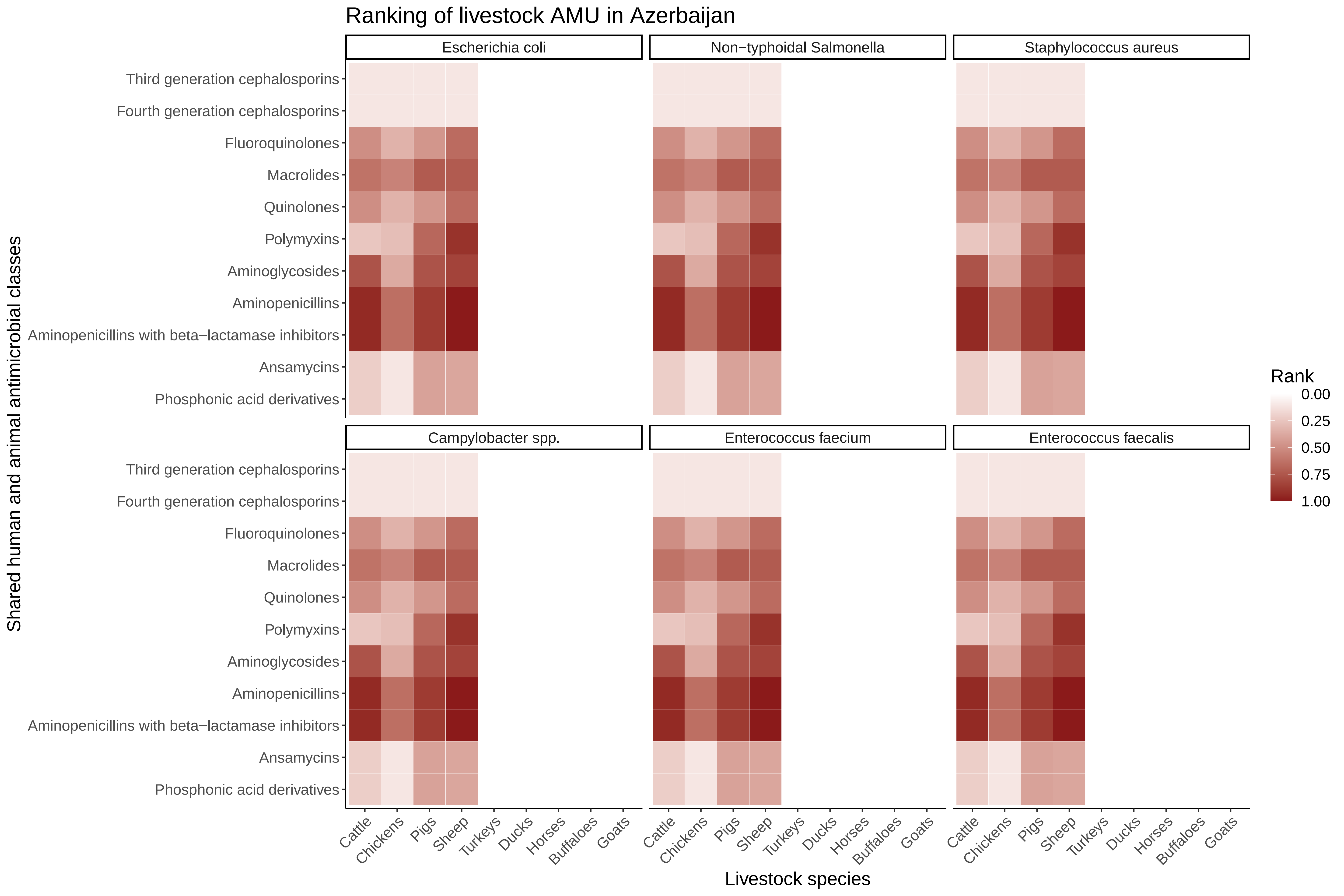
Ranking of livestock AMU in Azerbaijan
Escherichia coli
Non−typhoidal Salmonella
Staphylococcus aureus
Campylobacter spp.
Enterococcus faecium
Enterococcus faecalis
Shared human and animal antimicrobial classes
Third generation cephalosporins
Fourth generation cephalosporins
Fluoroquinolones
Macrolides
Quinolones
Polymyxins
Aminoglycosides
Aminopenicillins
Aminopenicillins with beta−lactamase inhibitors
Ansamycins
Phosphonic acid derivatives
Cattle
Chickens
Pigs
Sheep
Turkeys
Ducks
Horses
Buffaloes
Goats
Livestock species
Rank
0.00
0.25
0.50
0.75
1.00

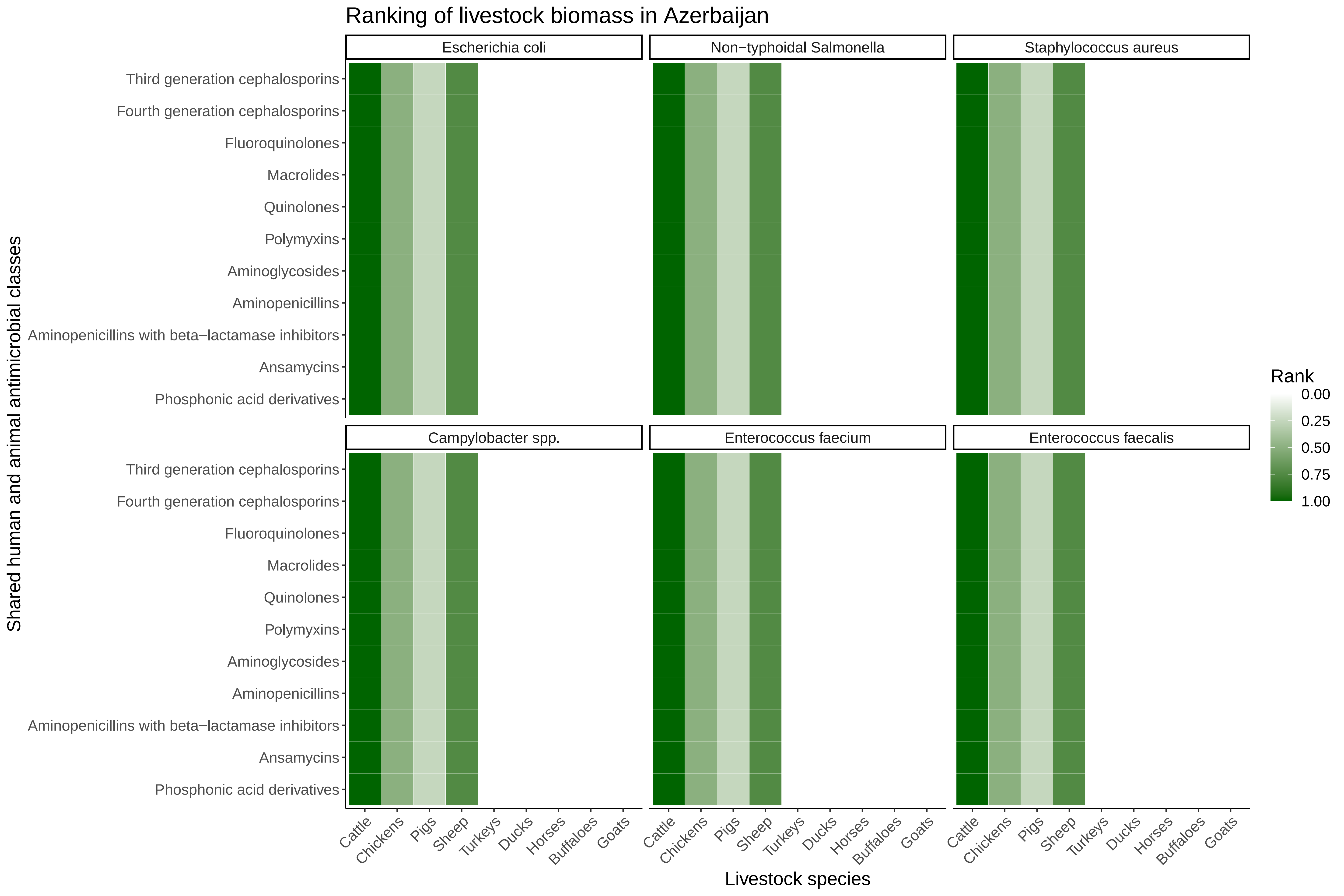

Ranking of livestock biomass in Azerbaijan
Escherichia coli
Non−typhoidal Salmonella
Staphylococcus aureus
Campylobacter spp.
Enterococcus faecium
Enterococcus faecalis
Third generation cephalosporins
Fourth generation cephalosporins
Fluoroquinolones
Macrolides
Quinolones
Polymyxins
Aminoglycosides
Aminopenicillins
Aminopenicillins with beta−lactamase inhibitors
Ansamycins
Phosphonic acid derivatives
Shared human and animal antimicrobial classes
Cattle
Chickens
Pigs
Sheep
Turkeys
Ducks
Horses
Buffaloes
Goats
Livestock species
Rank
0.00
0.25
0.50
0.75
1.00

Ranking of significance in antimicrobial class−pathogen correlations between humans and livestock proportion of resistance in Azerbaijan

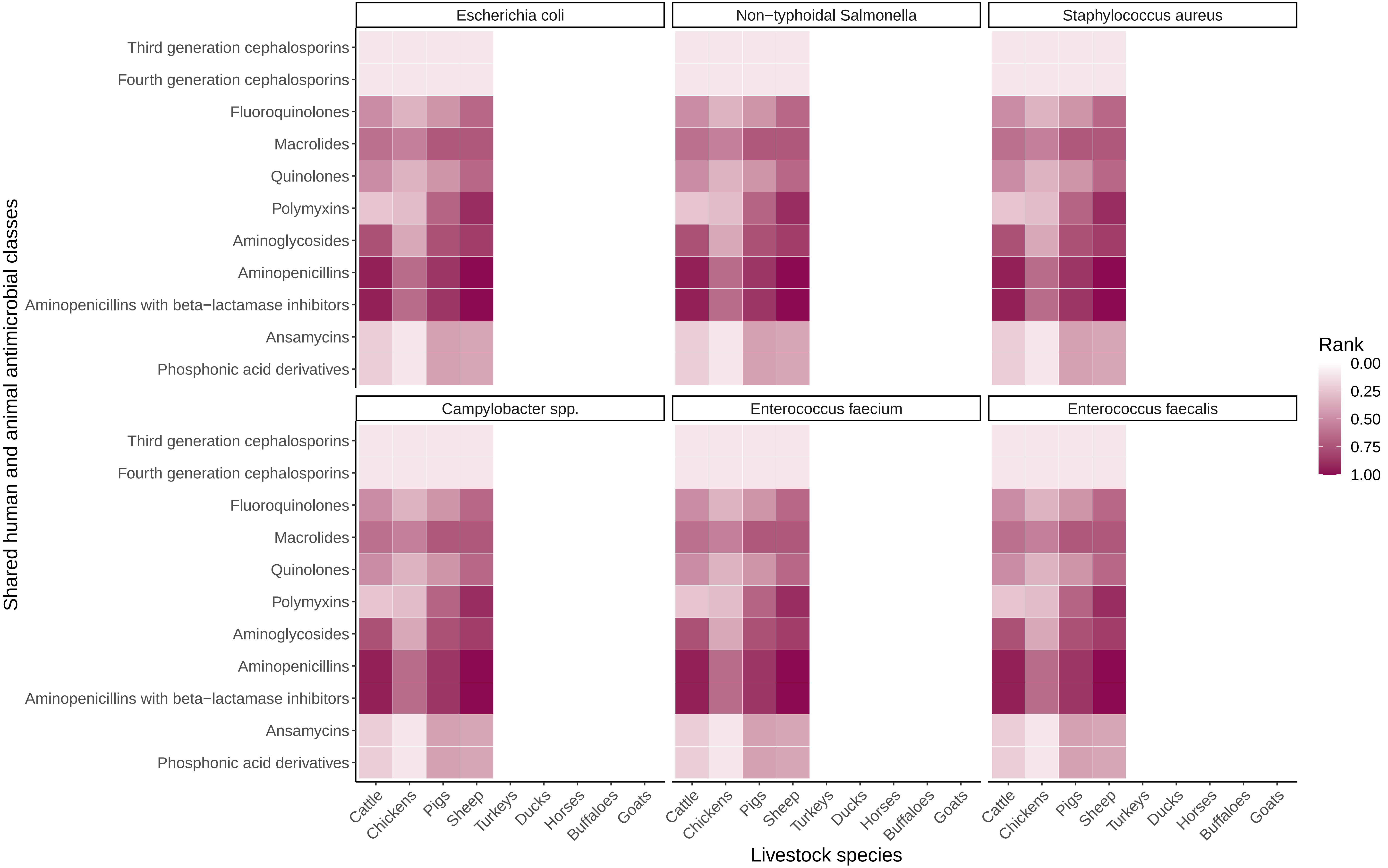

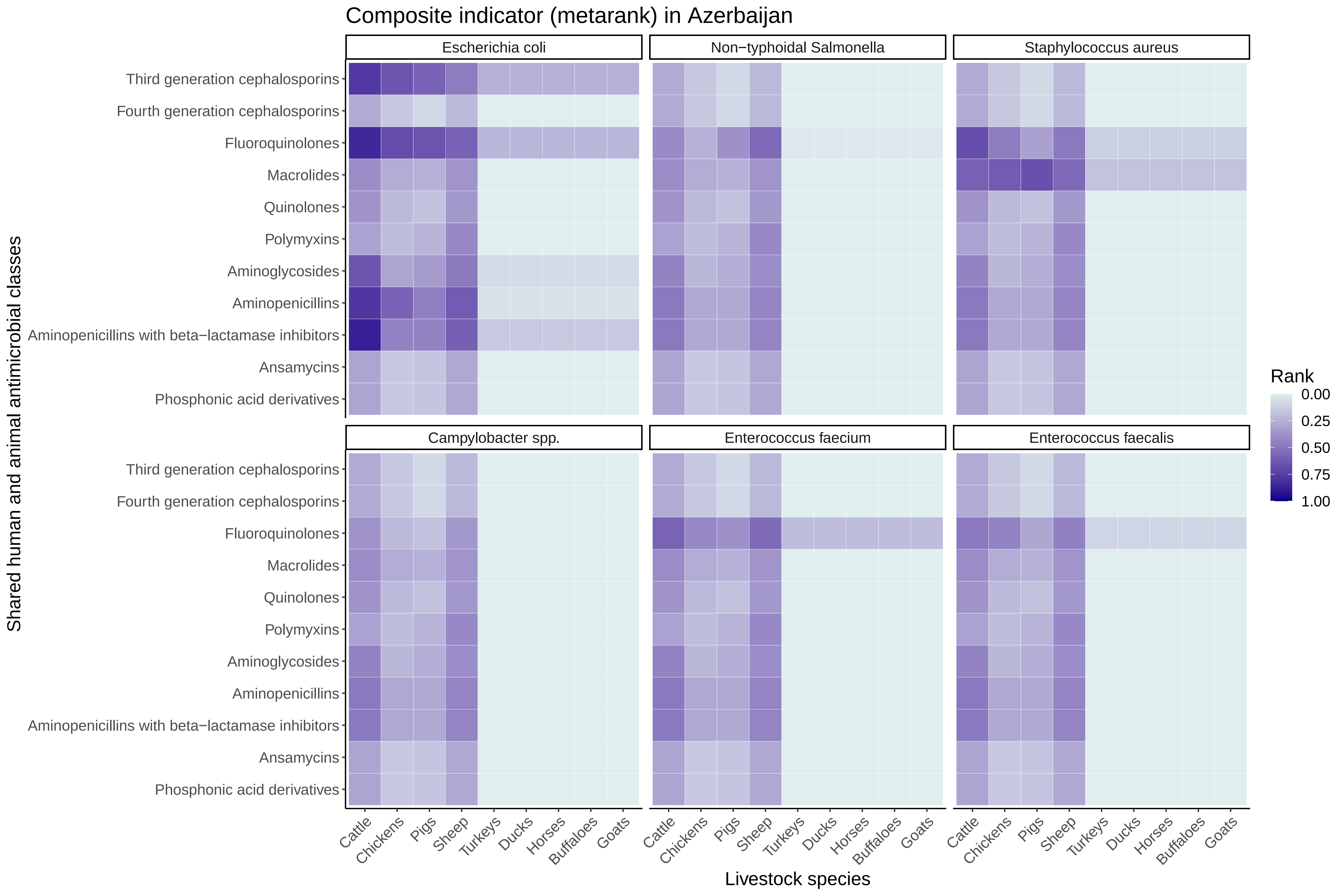
Composite indicator (metarank) in Azerbaijan
Escherichia coli
Non−typhoidal Salmonella
Staphylococcus aureus
Campylobacter spp.
Enterococcus faecium
Enterococcus faecalis
Shared human and animal antimicrobial classes
Third generation cephalosporins
Fourth generation cephalosporins
Fluoroquinolones
Macrolides
Quinolones
Polymyxins
Aminoglycosides
Aminopenicillins
Aminopenicillins with beta−lactamase inhibitors
Ansamycins
Phosphonic acid derivatives
Cattle
Chickens
Pigs
Sheep
Turkeys
Ducks
Horses
Buffaloes
Goats
Livestock species
Rank
0.00
0.25
0.50
0.75
1.00

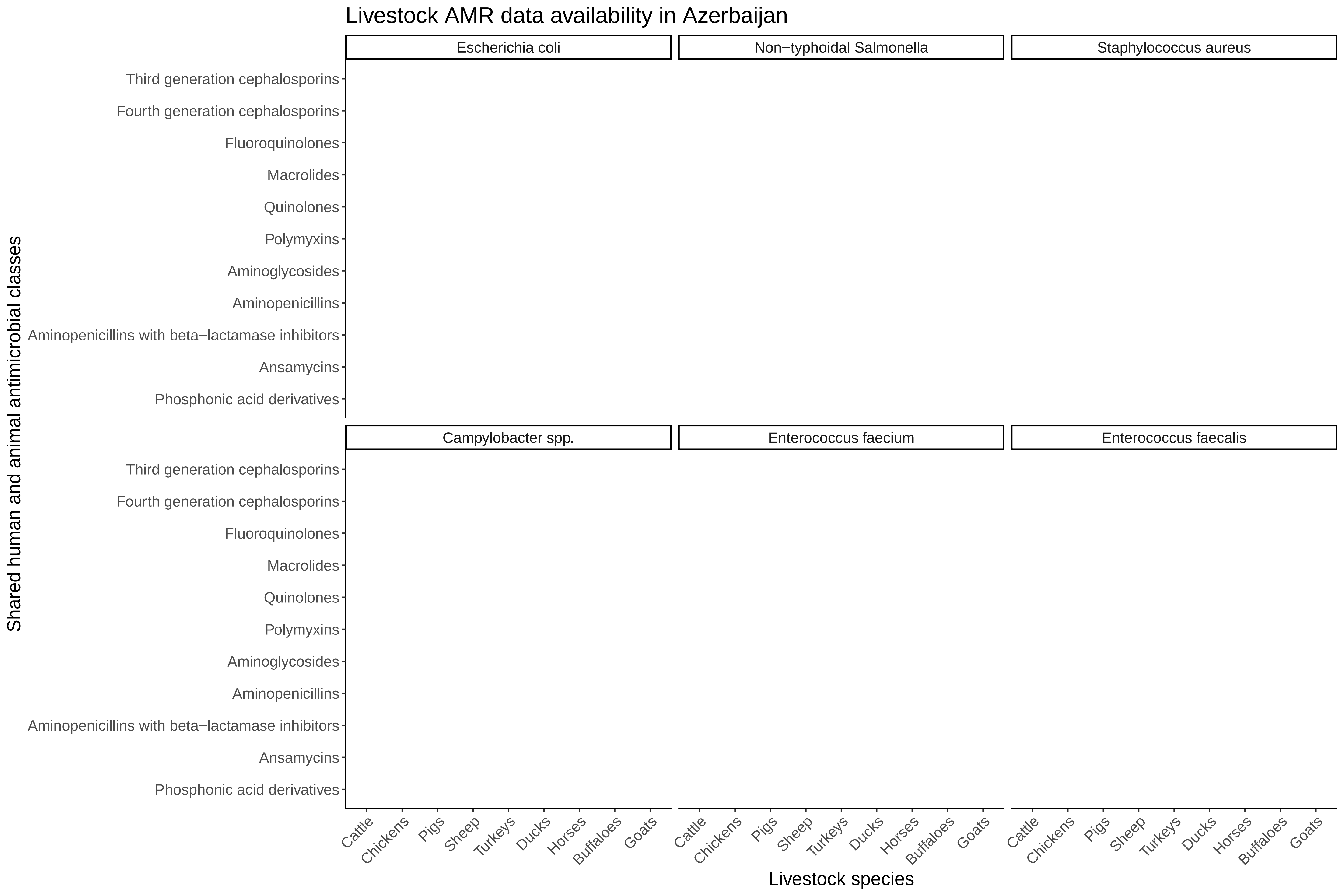
Livestock AMR data availability in Azerbaijan
Escherichia coli
Non−typhoidal Salmonella
Staphylococcus aureus
Campylobacter spp.
Enterococcus faecium
Enterococcus faecalis
Shared human and animal antimicrobial classes
Third generation cephalosporins
Fourth generation cephalosporins
Fluoroquinolones
Macrolides
Quinolones
Polymyxins
Aminoglycosides
Aminopenicillins
Aminopenicillins with beta−lactamase inhibitors
Ansamycins
Phosphonic acid derivatives
Cattle
Chickens
Pigs
Sheep
Turkeys
Ducks
Horses
Buffaloes
Goats
Livestock species

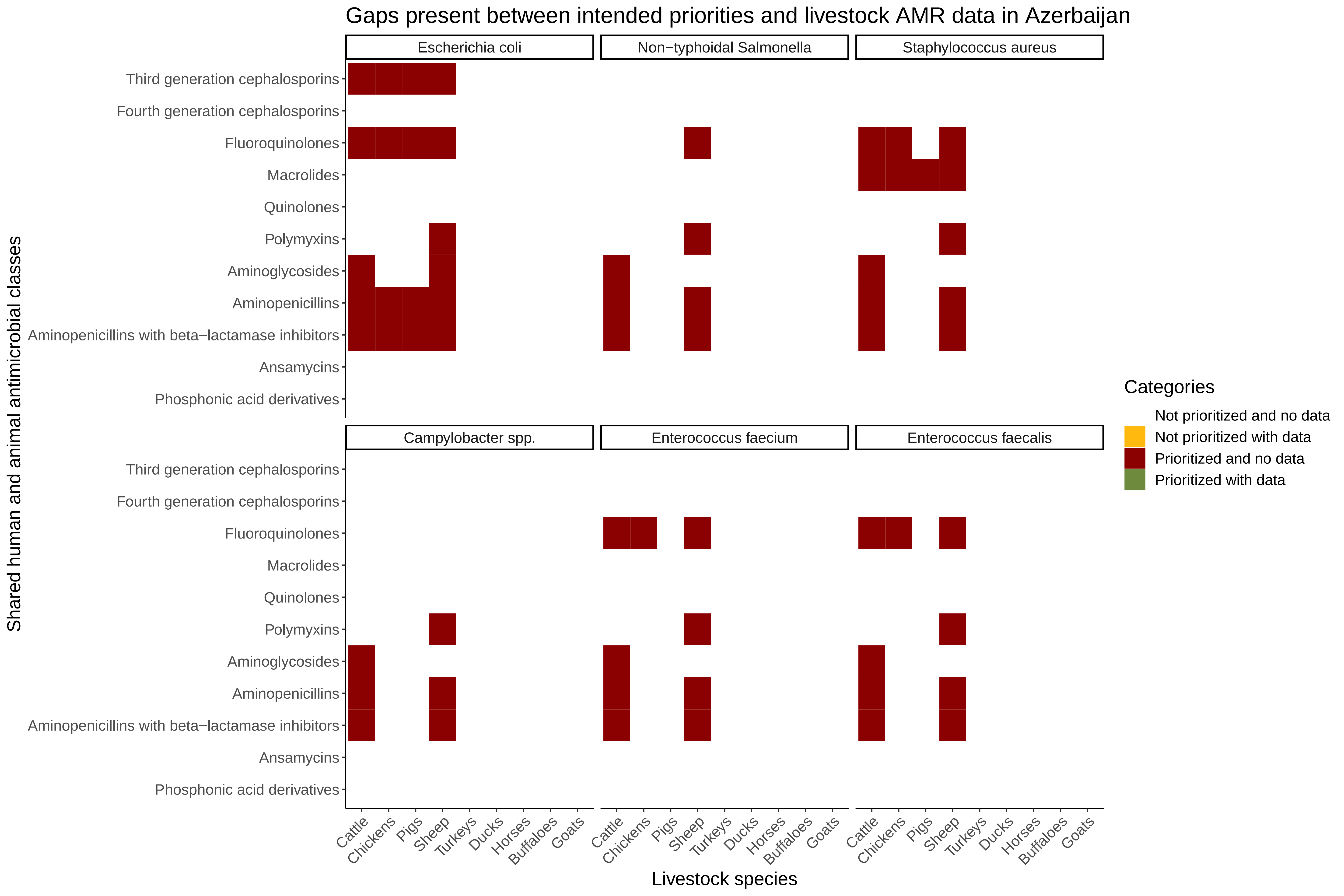

Gaps present between intended priorities and livestock AMR data in Azerbaijan
Escherichia coli
Non−typhoidal Salmonella
Staphylococcus aureus
Campylobacter spp.
Enterococcus faecium
Enterococcus faecalis
Third generation cephalosporins
Fourth generation cephalosporins
Fluoroquinolones
Macrolides
Quinolones
Polymyxins
Aminoglycosides
Aminopenicillins
Aminopenicillins with beta−lactamase inhibitors
Ansamycins
Phosphonic acid derivatives
Shared human and animal antimicrobial classes
Cattle
Chickens
Pigs
Sheep
Turkeys
Ducks
Horses
Buffaloes
Goats
Livestock species
Categories
Not prioritized and no data
Not prioritized with data
Prioritized and no data
Prioritized with data

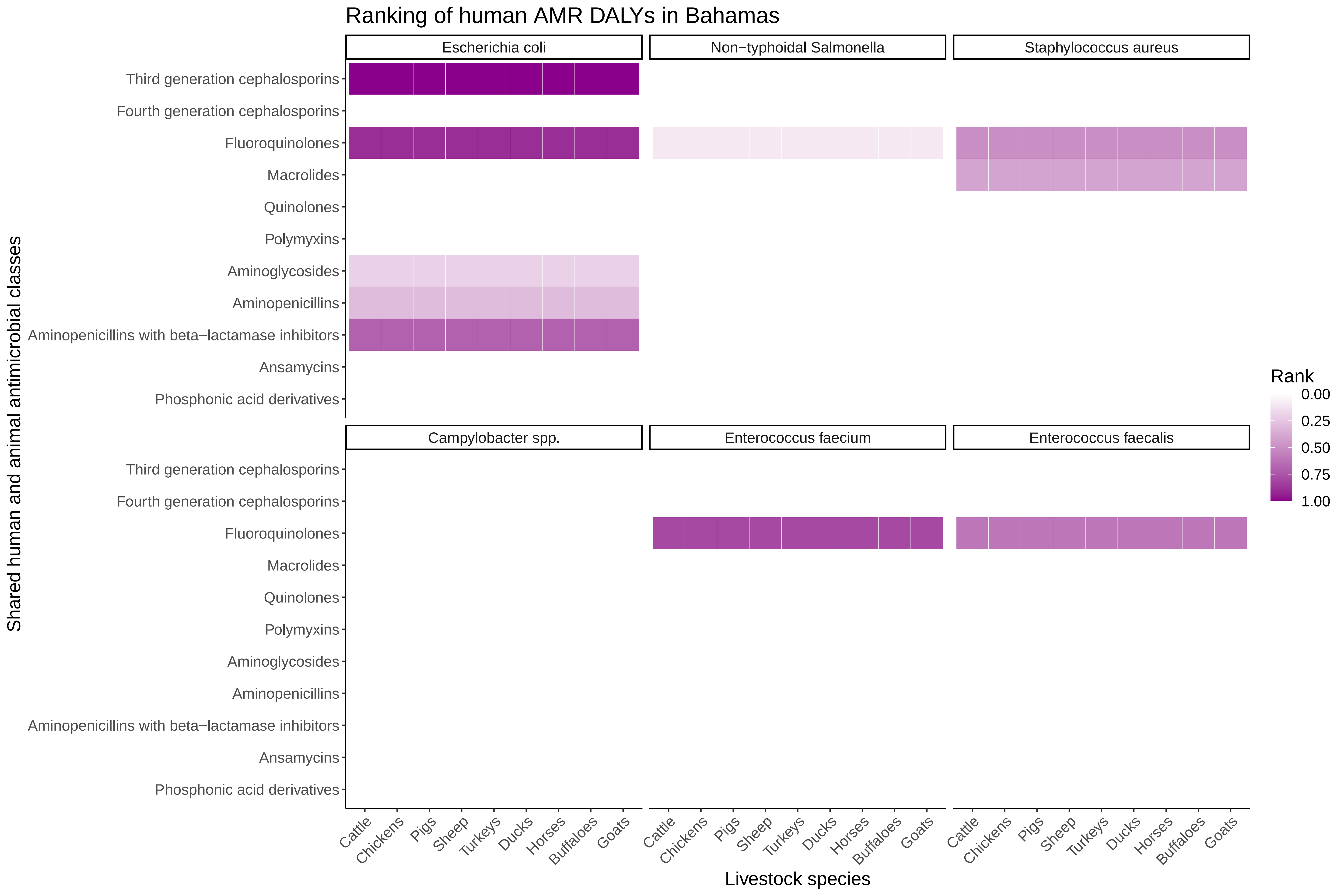

Ranking of human AMR DALYs in Bahamas
Escherichia coli
Non−typhoidal Salmonella
Staphylococcus aureus
Campylobacter spp.
Enterococcus faecium
Enterococcus faecalis
Third generation cephalosporins
Fourth generation cephalosporins
Fluoroquinolones
Macrolides
Quinolones
Polymyxins
Aminoglycosides
Aminopenicillins
Aminopenicillins with beta−lactamase inhibitors
Ansamycins
Phosphonic acid derivatives
Shared human and animal antimicrobial classes
Cattle
Chickens
Pigs
Sheep
Turkeys
Ducks
Horses
Buffaloes
Goats
Livestock species
Rank
0.00
0.25
0.50
0.75
1.00

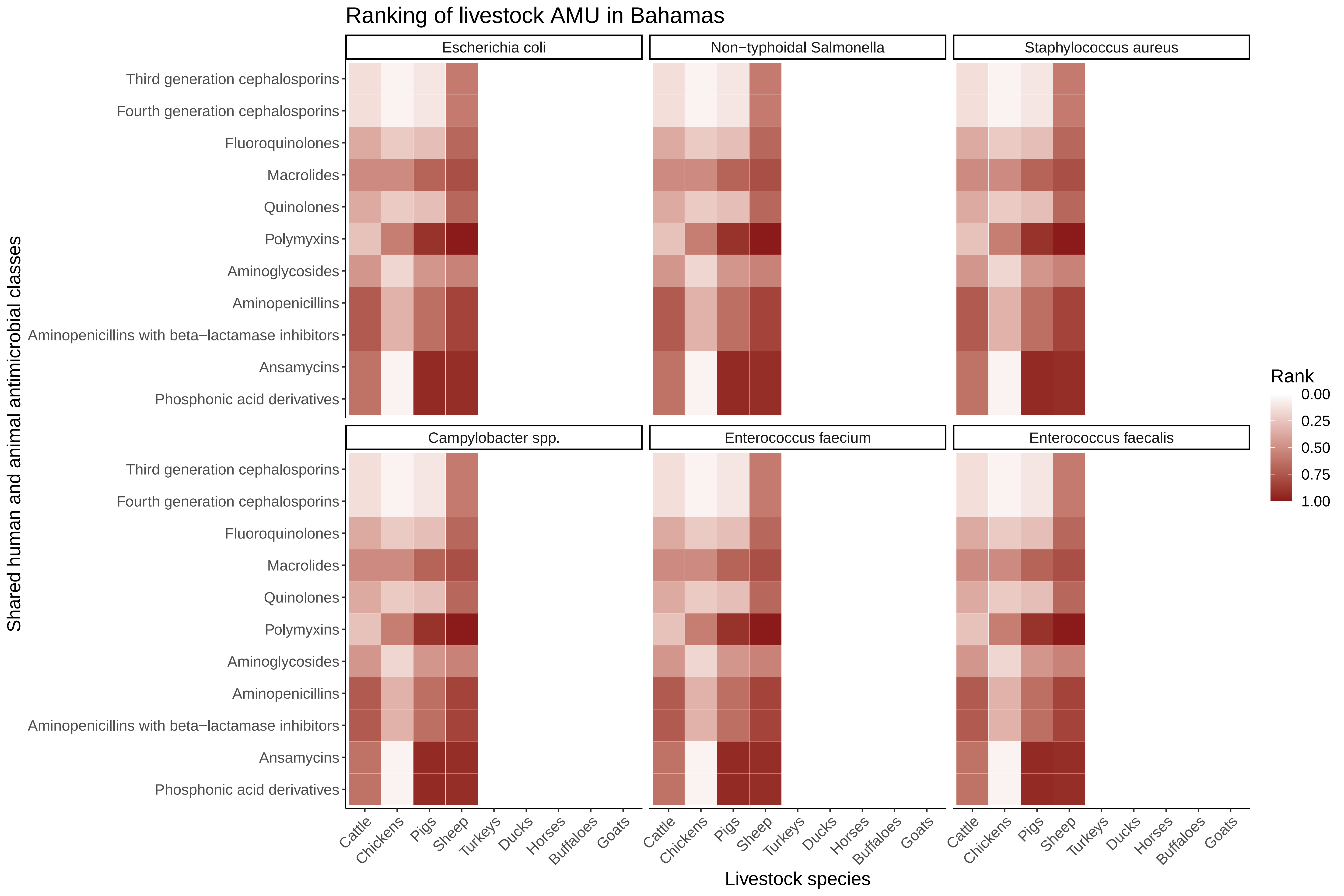

Ranking of livestock AMU in Bahamas
Escherichia coli
Non−typhoidal Salmonella
Staphylococcus aureus
Campylobacter spp.
Enterococcus faecium
Enterococcus faecalis
Third generation cephalosporins
Fourth generation cephalosporins
Fluoroquinolones
Macrolides
Quinolones
Polymyxins
Aminoglycosides
Aminopenicillins
Aminopenicillins with beta−lactamase inhibitors
Ansamycins
Phosphonic acid derivatives
Shared human and animal antimicrobial classes
Cattle
Chickens
Pigs
Sheep
Turkeys
Ducks
Horses
Buffaloes
Goats
Livestock species
Rank
0.00
0.25
0.50
0.75
1.00

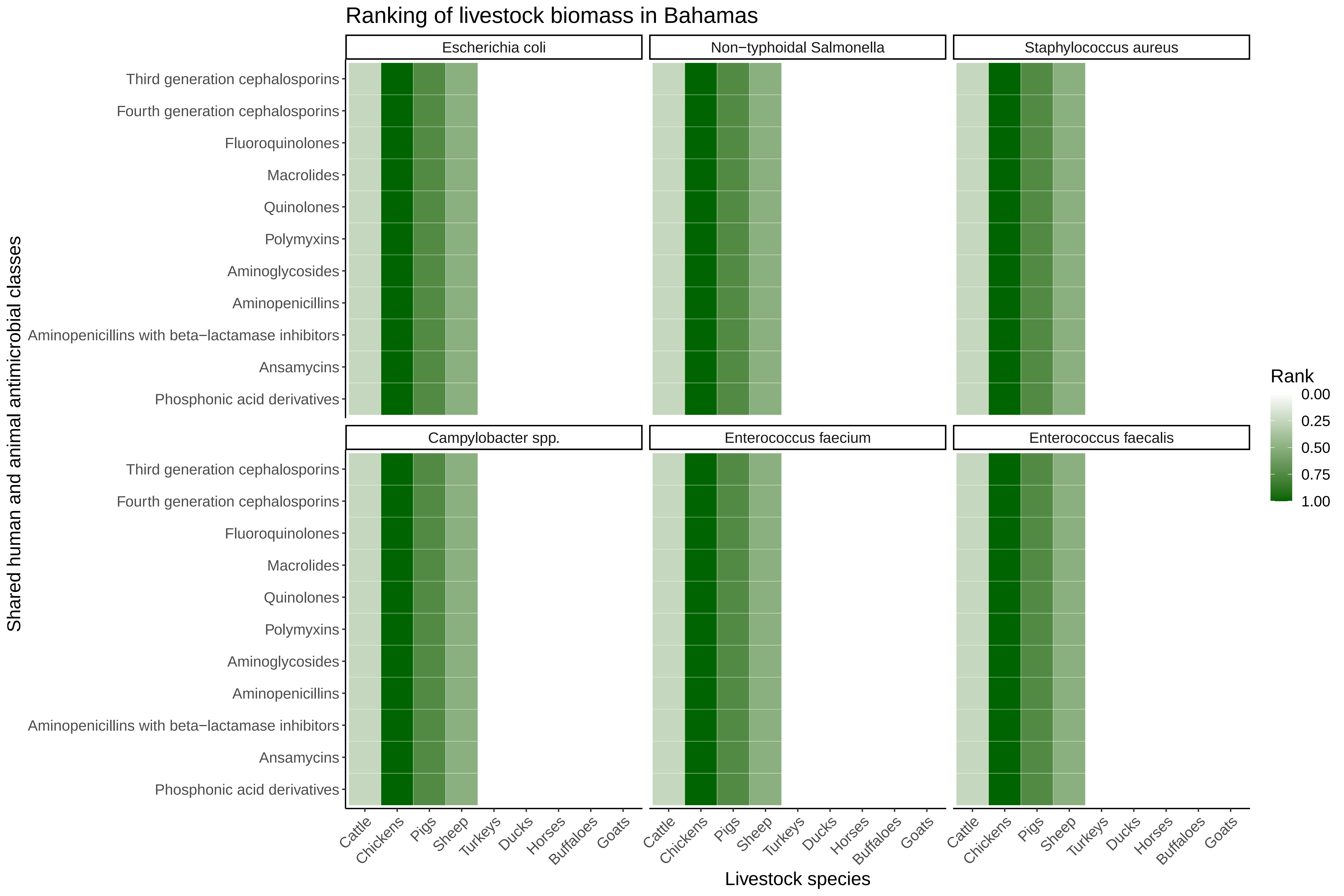

Ranking of livestock biomass in Bahamas
Escherichia coli
Non−typhoidal Salmonella
Staphylococcus aureus
Campylobacter spp.
Enterococcus faecium
Enterococcus faecalis
Third generation cephalosporins
Fourth generation cephalosporins
Fluoroquinolones
Macrolides
Quinolones
Polymyxins
Aminoglycosides
Aminopenicillins
Aminopenicillins with beta−lactamase inhibitors
Ansamycins
Phosphonic acid derivatives
Shared human and animal antimicrobial classes
Cattle
Chickens
Pigs
Sheep
Turkeys
Ducks
Horses
Buffaloes
Goats
Livestock species
Rank
0.00
0.25
0.50
0.75
1.00

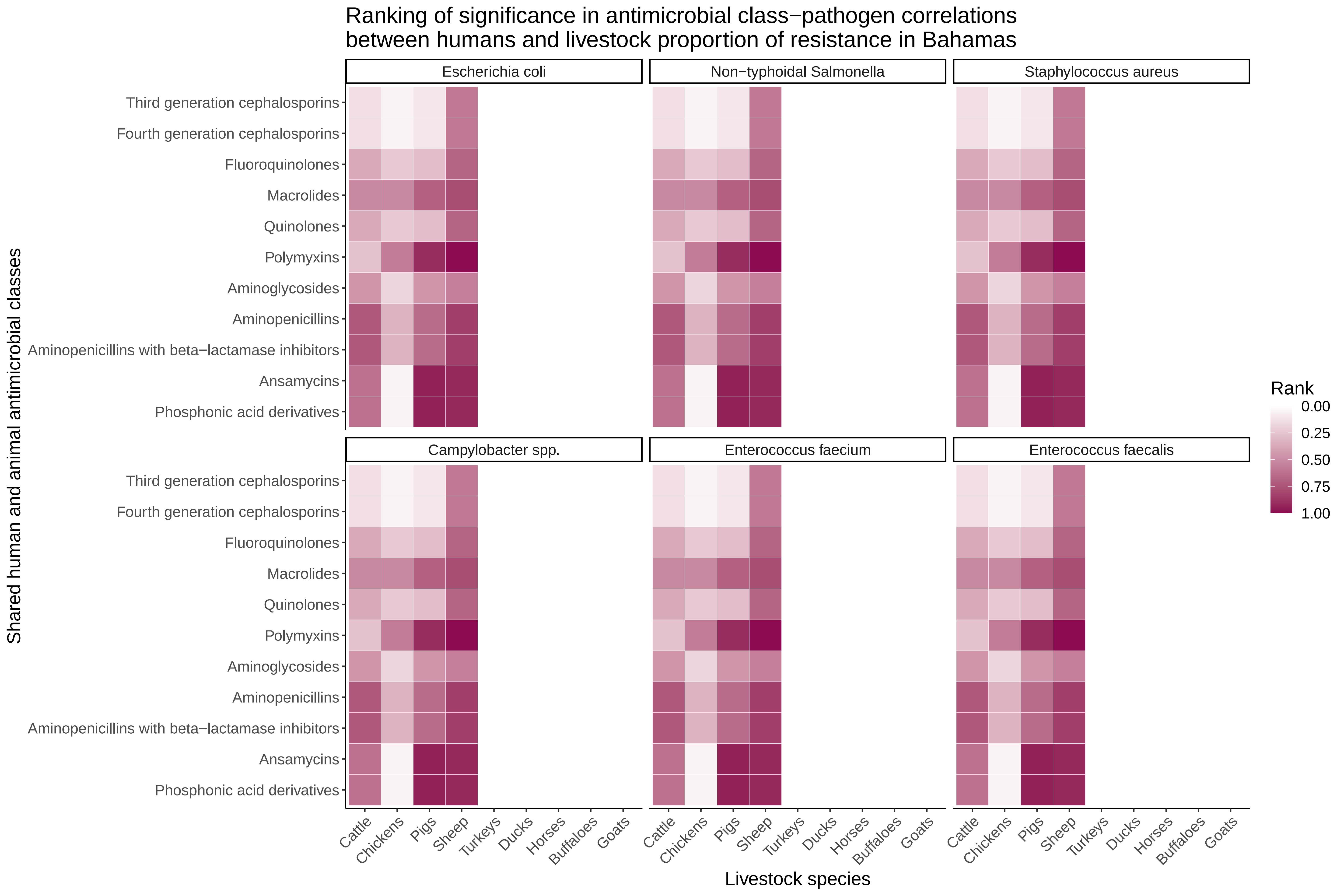
Ranking of significance in antimicrobial class−pathogen correlations
between humans and livestock proportion of resistance in Bahamas
Escherichia coli
Non−typhoidal Salmonella
Staphylococcus aureus
Campylobacter spp.
Enterococcus faecium
Enterococcus faecalis
Third generation cephalosporins
Fourth generation cephalosporins
Fluoroquinolones
Macrolides
Quinolones
Polymyxins
Aminoglycosides
Aminopenicillins
Aminopenicillins with beta−lactamase inhibitors
Ansamycins
Phosphonic acid derivatives
Shared human and animal antimicrobial classes
Cattle
Chickens
Pigs
Sheep
Turkeys
Ducks
Horses
Buffaloes
Goats
Livestock species
Rank
0.00
0.25
0.50
0.75
1.00

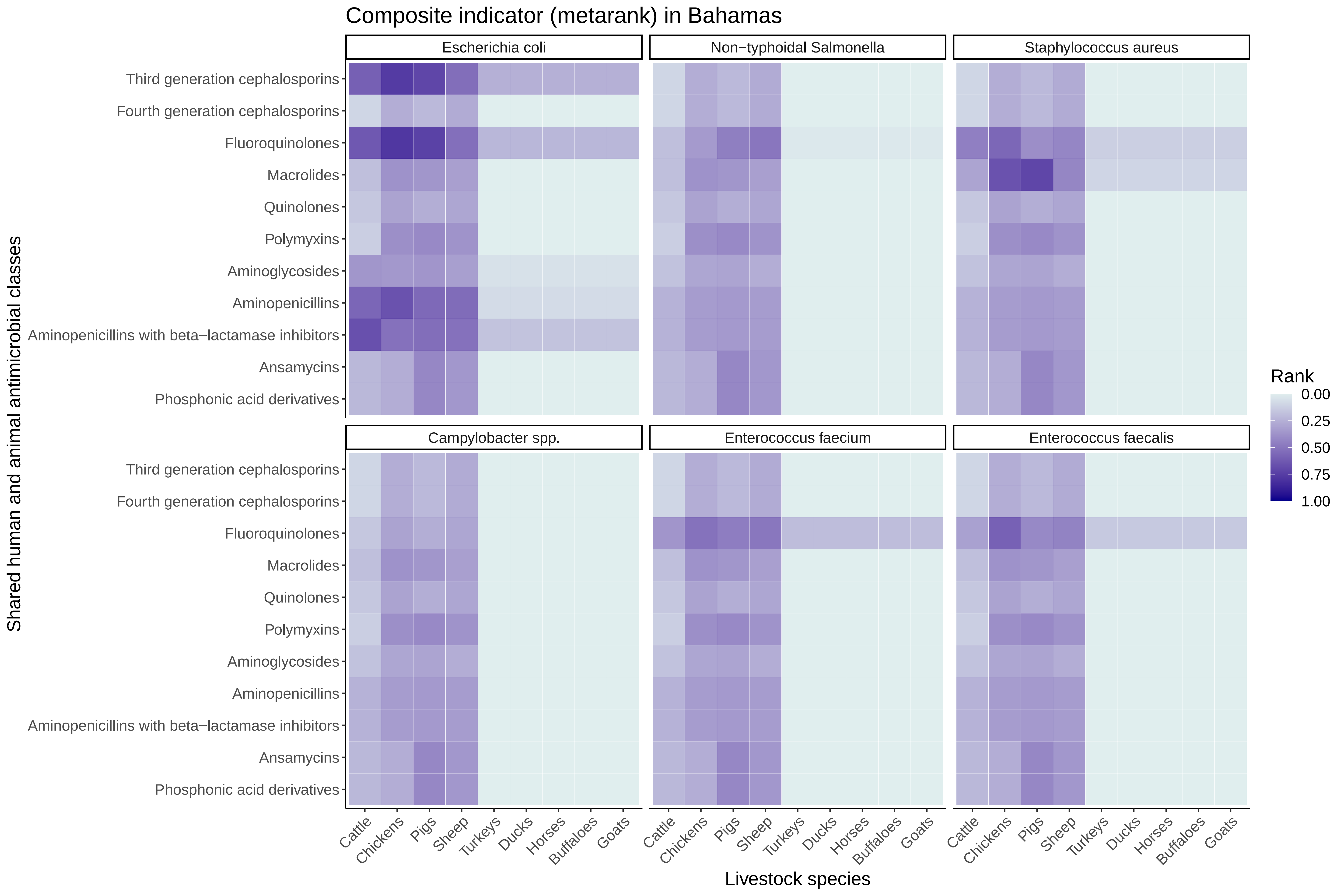
Composite indicator (metarank) in Bahamas
Escherichia coli
Non−typhoidal Salmonella
Staphylococcus aureus
Campylobacter spp.
Enterococcus faecium
Enterococcus faecalis
Shared human and animal antimicrobial classes
Third generation cephalosporins
Fourth generation cephalosporins
Fluoroquinolones
Macrolides
Quinolones
Polymyxins
Aminoglycosides
Aminopenicillins
Aminopenicillins with beta−lactamase inhibitors
Ansamycins
Phosphonic acid derivatives
Livestock species
Cattle
Chickens
Pigs
Sheep
Turkeys
Ducks
Horses
Buffaloes
Goats
Rank
0.00
0.25
0.50
0.75
1.00

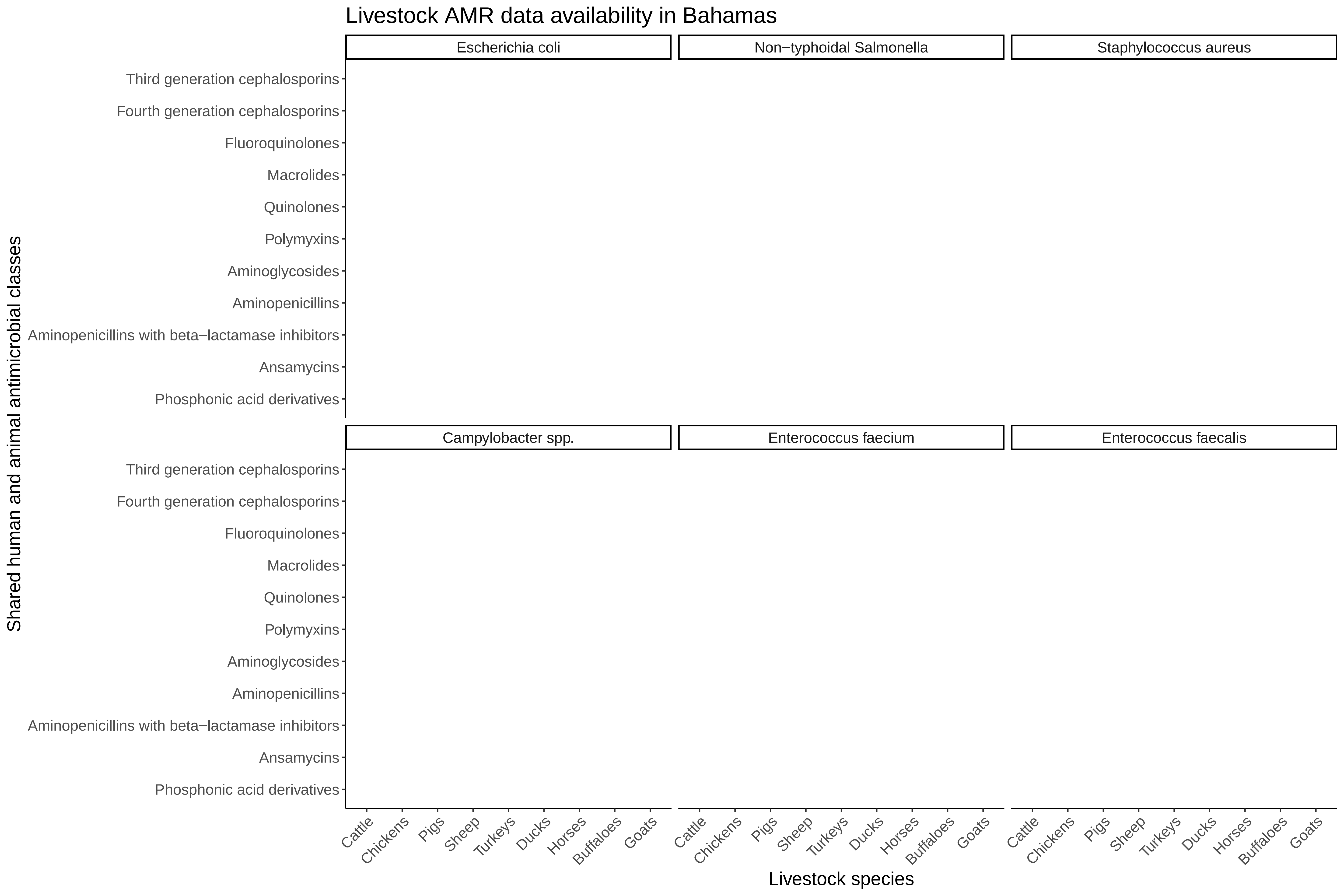

Livestock AMR data availability in Bahamas
Escherichia coli
Non−typhoidal Salmonella
Staphylococcus aureus
Campylobacter spp.
Enterococcus faecium
Enterococcus faecalis
Third generation cephalosporins
Fourth generation cephalosporins
Fluoroquinolones
Macrolides
Quinolones
Polymyxins
Aminoglycosides
Aminopenicillins
Aminopenicillins with beta−lactamase inhibitors
Ansamycins
Phosphonic acid derivatives
Shared human and animal antimicrobial classes
Cattle
Chickens
Pigs
Sheep
Turkeys
Ducks
Horses
Buffaloes
Goats
Livestock species

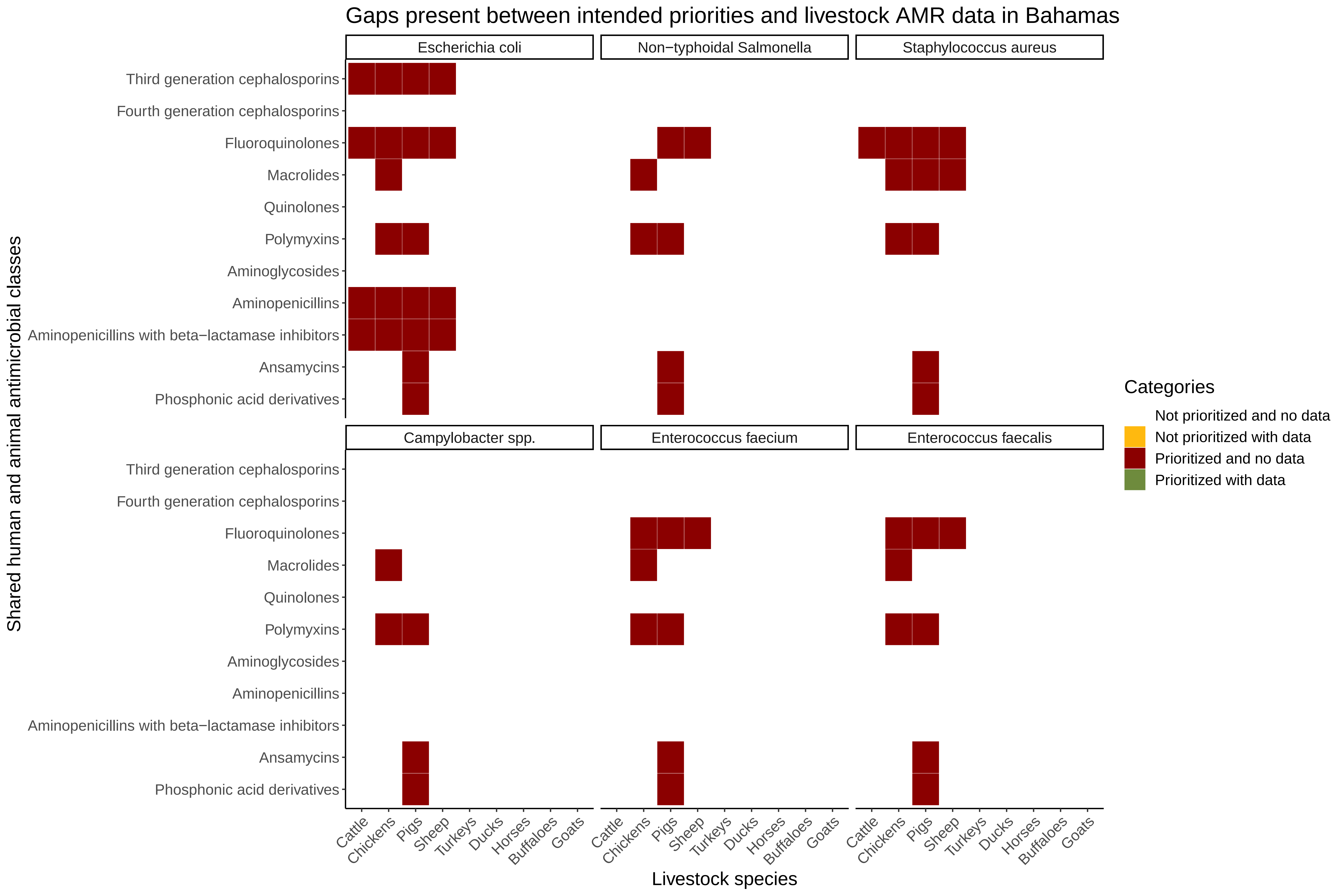

Gaps present between intended priorities and livestock AMR data in Bahamas
Escherichia coli
Non−typhoidal Salmonella
Staphylococcus aureus
Campylobacter spp.
Enterococcus faecium
Enterococcus faecalis
Third generation cephalosporins
Fourth generation cephalosporins
Fluoroquinolones
Macrolides
Quinolones
Polymyxins
Aminoglycosides
Aminopenicillins
Aminopenicillins with beta−lactamase inhibitors
Ansamycins
Phosphonic acid derivatives
Shared human and animal antimicrobial classes
Cattle
Chickens
Pigs
Sheep
Turkeys
Ducks
Horses
Buffaloes
Goats
Livestock species
Categories
Not prioritized and no data
Not prioritized with data
Prioritized and no data
Prioritized with data

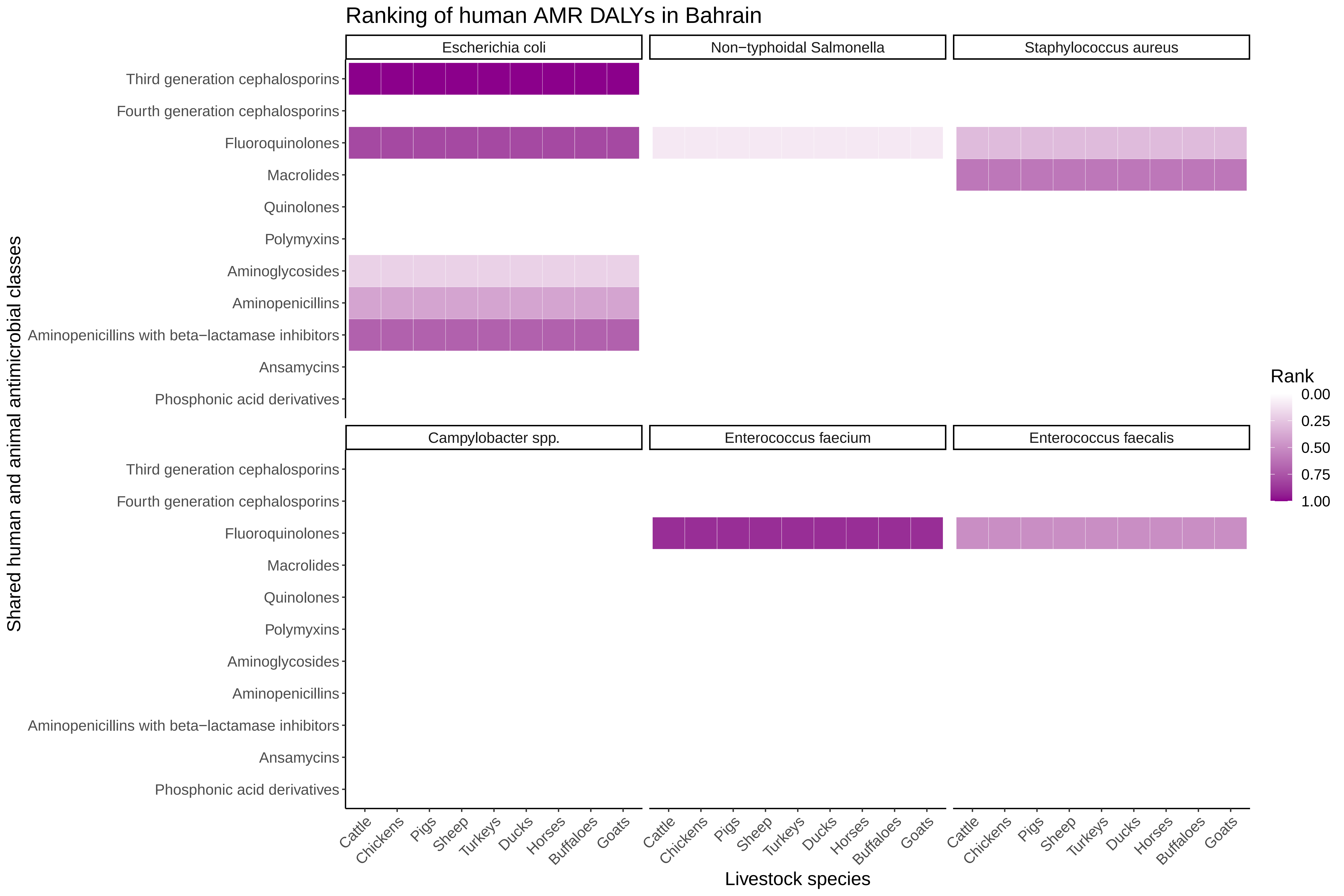

Ranking of human AMR DALYs in Bahrain
Escherichia coli
Non−typhoidal Salmonella
Staphylococcus aureus
Campylobacter spp.
Enterococcus faecium
Enterococcus faecalis
Third generation cephalosporins
Fourth generation cephalosporins
Fluoroquinolones
Macrolides
Quinolones
Polymyxins
Aminoglycosides
Aminopenicillins
Aminopenicillins with beta−lactamase inhibitors
Ansamycins
Phosphonic acid derivatives
Shared human and animal antimicrobial classes
Cattle
Chickens
Pigs
Sheep
Turkeys
Ducks
Horses
Buffaloes
Goats
Livestock species
Rank
0.00
0.25
0.50
0.75
1.00

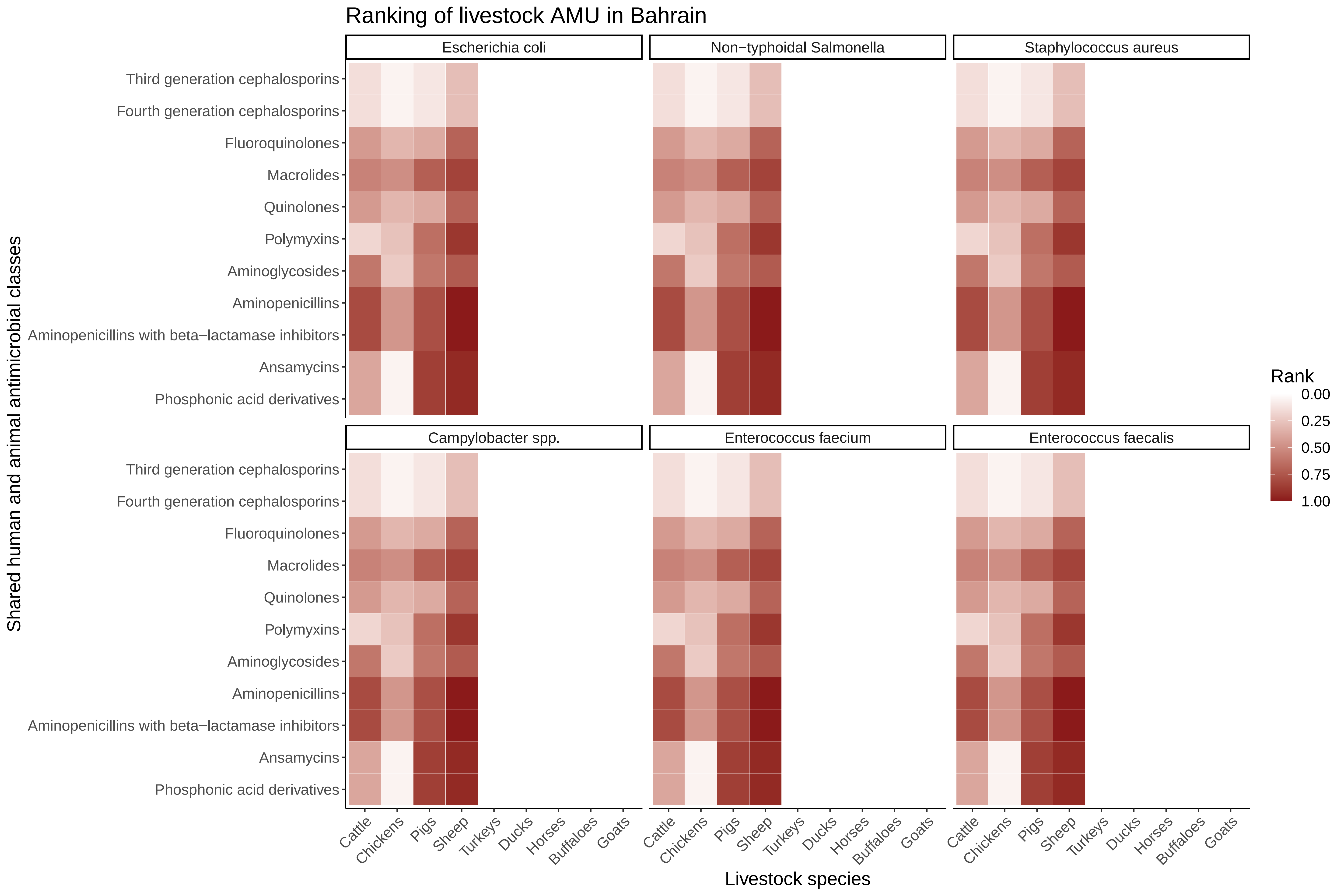

Ranking of livestock AMU in Bahrain
Escherichia coli
Non−typhoidal Salmonella
Staphylococcus aureus
Campylobacter spp.
Enterococcus faecium
Enterococcus faecalis
Third generation cephalosporins
Fourth generation cephalosporins
Fluoroquinolones
Macrolides
Quinolones
Polymyxins
Aminoglycosides
Aminopenicillins
Aminopenicillins with beta−lactamase inhibitors
Ansamycins
Phosphonic acid derivatives
Shared human and animal antimicrobial classes
Cattle
Chickens
Pigs
Sheep
Turkeys
Ducks
Horses
Buffaloes
Goats
Livestock species
Rank
0.00
0.25
0.50
0.75
1.00

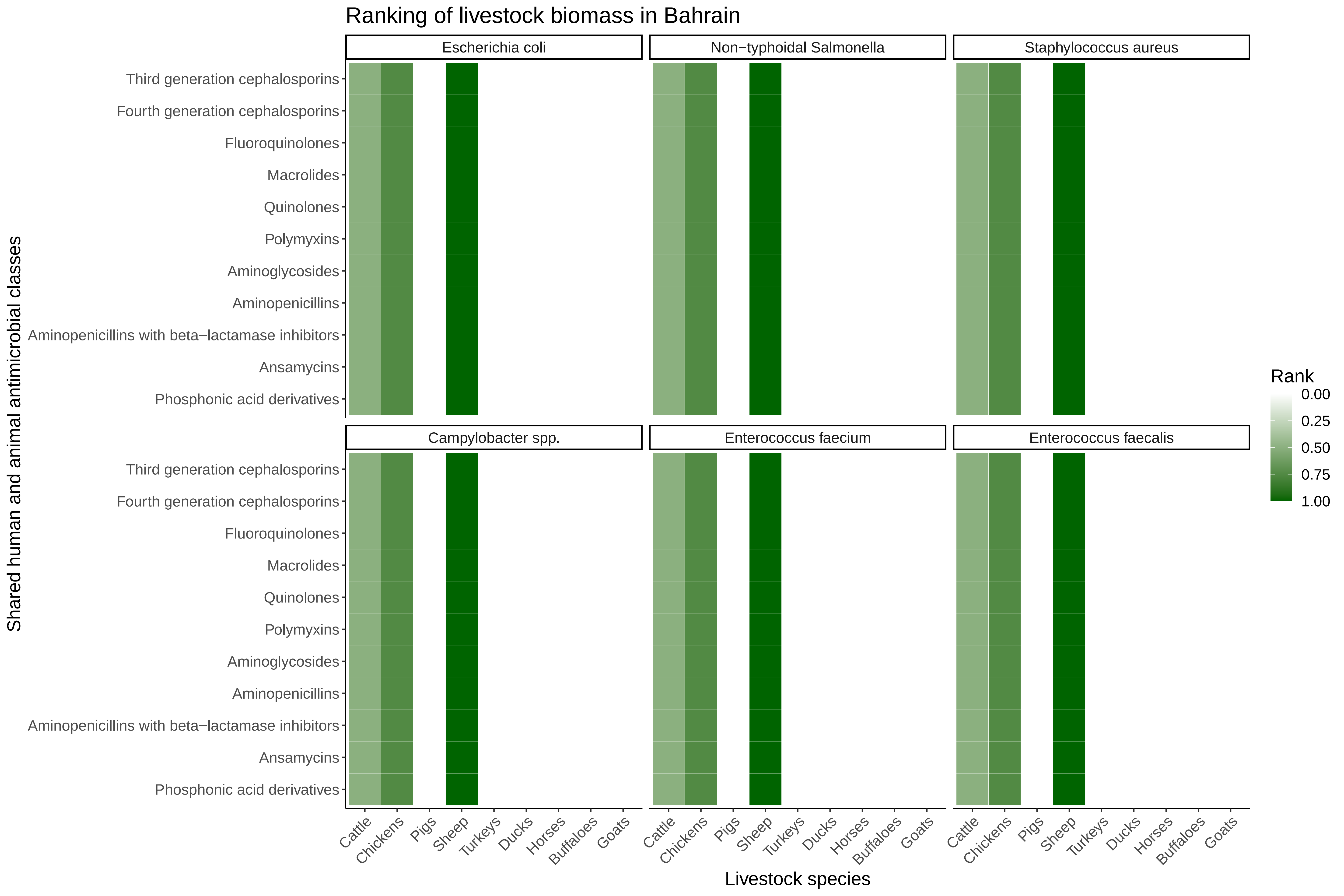
Ranking of livestock biomass in Bahrain
Escherichia coli
Non−typhoidal Salmonella
Staphylococcus aureus
Campylobacter spp.
Enterococcus faecium
Enterococcus faecalis
Third generation cephalosporins
Fourth generation cephalosporins
Fluoroquinolones
Macrolides
Quinolones
Polymyxins
Aminoglycosides
Aminopenicillins
Aminopenicillins with beta−lactamase inhibitors
Ansamycins
Phosphonic acid derivatives
Shared human and animal antimicrobial classes
Cattle
Chickens
Pigs
Sheep
Turkeys
Ducks
Horses
Buffaloes
Goats
Livestock species
Rank
0.00
0.25
0.50
0.75
1.00

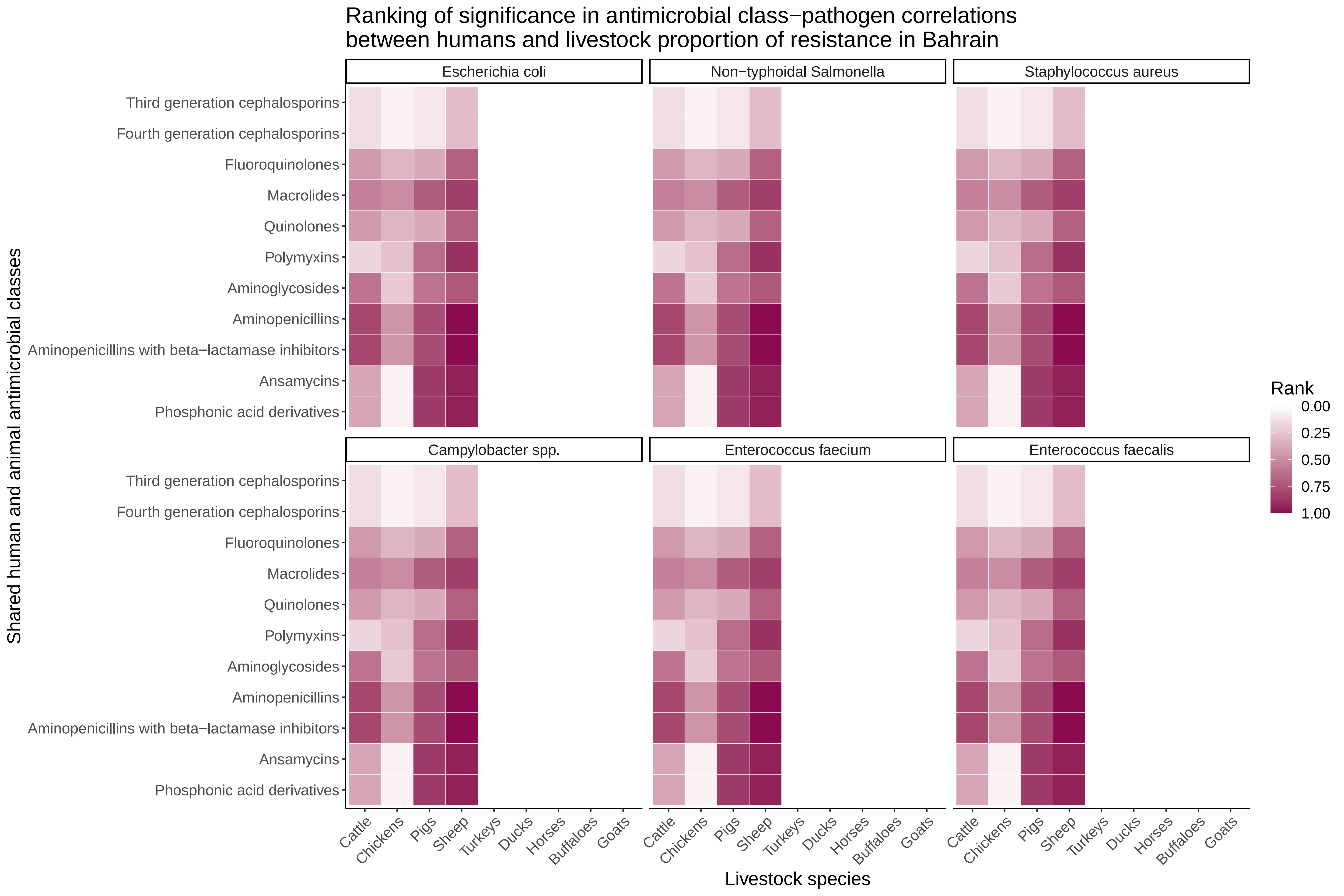
Ranking of significance in antimicrobial class−pathogen correlations
between humans and livestock proportion of resistance in Bahrain
Escherichia coli
Non−typhoidal Salmonella
Staphylococcus aureus
Campylobacter spp.
Enterococcus faecium
Enterococcus faecalis
Third generation cephalosporins
Fourth generation cephalosporins
Fluoroquinolones
Macrolides
Quinolones
Polymyxins
Aminoglycosides
Aminopenicillins
Aminopenicillins with beta−lactamase inhibitors
Ansamycins
Phosphonic acid derivatives
Shared human and animal antimicrobial classes
Cattle
Chickens
Pigs
Sheep
Turkeys
Ducks
Horses
Buffaloes
Goats
Livestock species
Rank
0.00
0.25
0.50
0.75
1.00

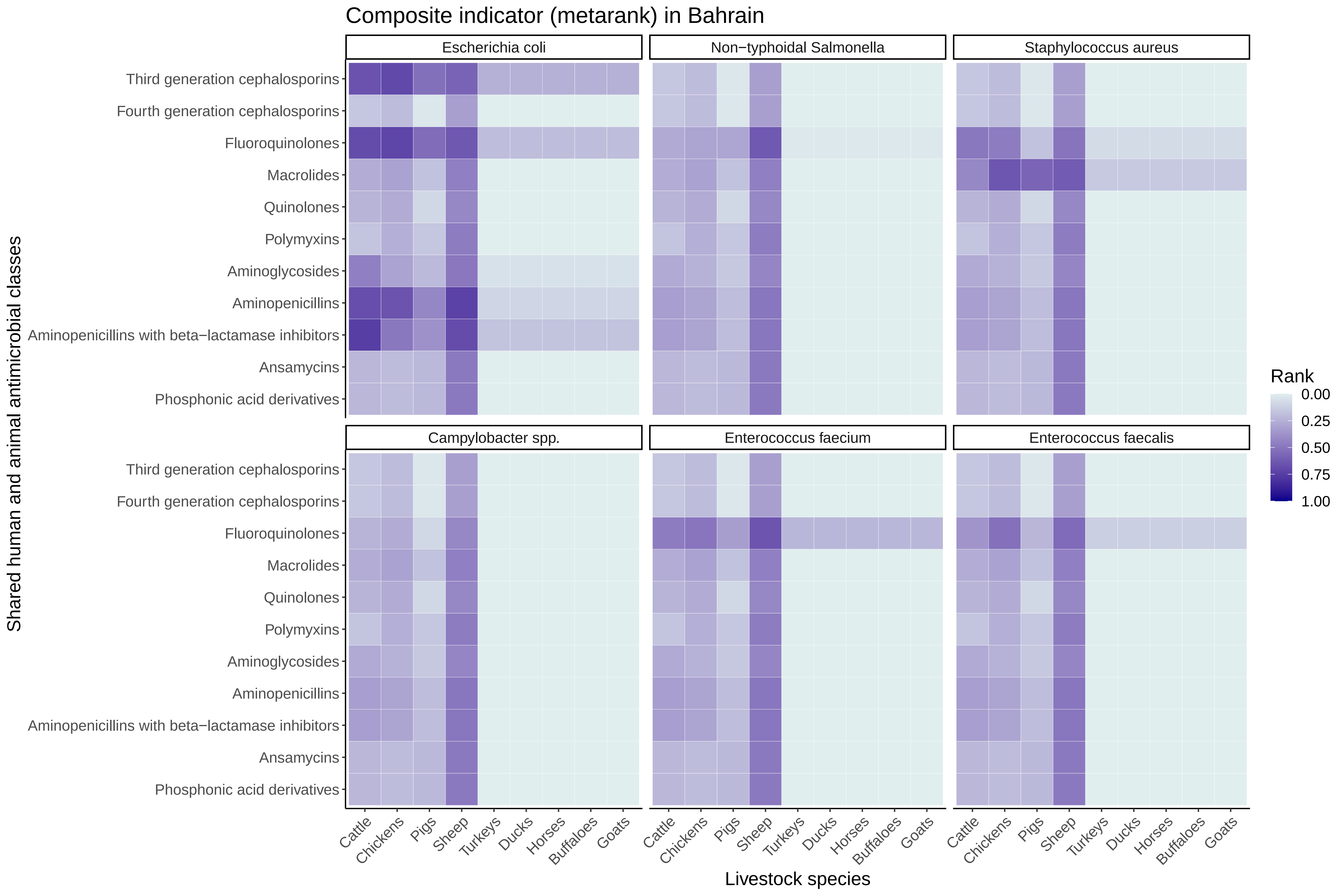
Composite indicator (metarank) in Bahrain
Escherichia coli
Non−typhoidal Salmonella
Staphylococcus aureus
Campylobacter spp.
Enterococcus faecium
Enterococcus faecalis
Shared human and animal antimicrobial classes
Third generation cephalosporins
Fourth generation cephalosporins
Fluoroquinolones
Macrolides
Quinolones
Polymyxins
Aminoglycosides
Aminopenicillins
Aminopenicillins with beta−lactamase inhibitors
Ansamycins
Phosphonic acid derivatives
Cattle
Chickens
Pigs
Sheep
Turkeys
Ducks
Horses
Buffaloes
Goats
Livestock species
Rank
0.00
0.25
0.50
0.75
1.00

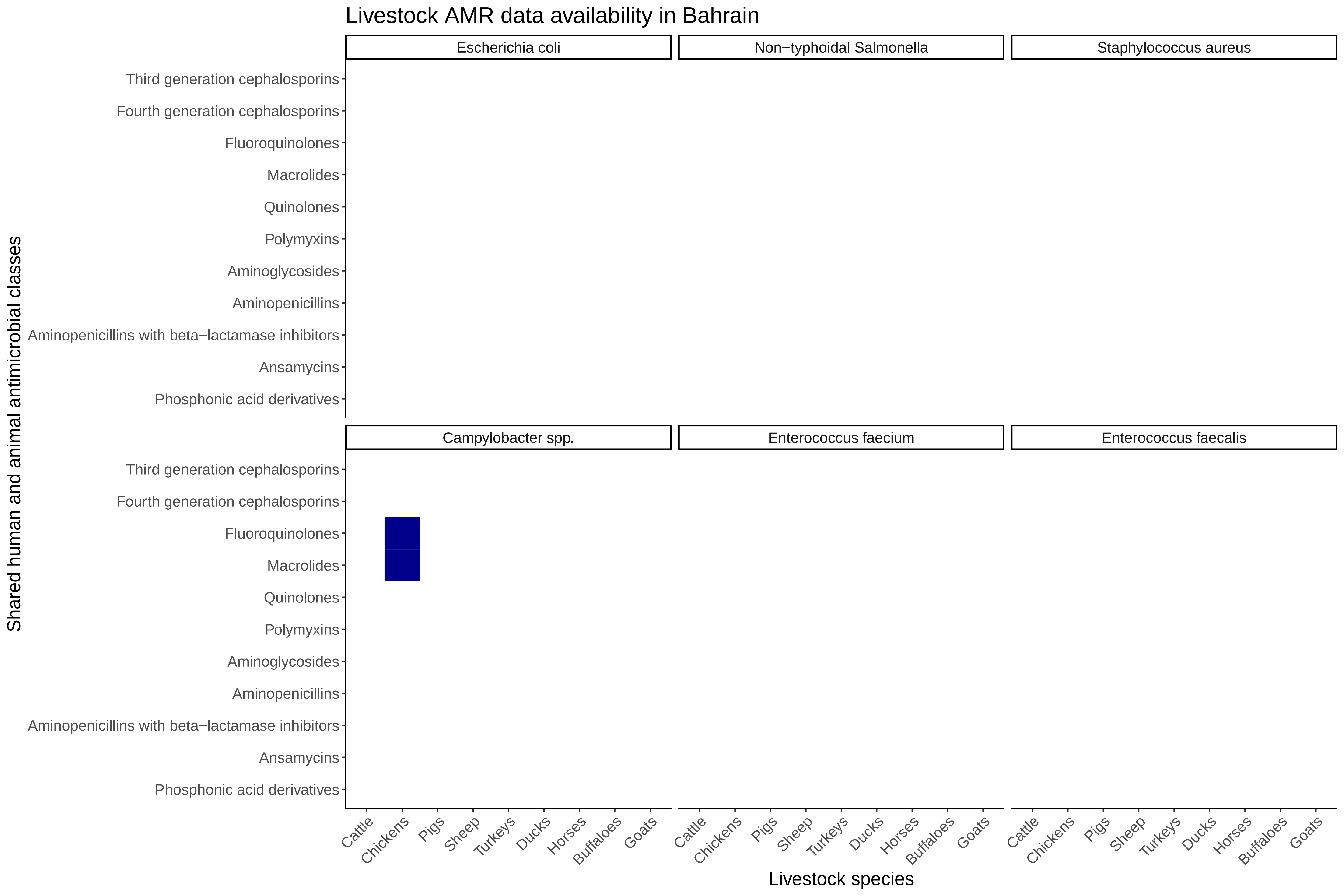

Livestock AMR data availability in Bahrain
Escherichia coli
Non−typhoidal Salmonella
Staphylococcus aureus
Campylobacter spp.
Enterococcus faecium
Enterococcus faecalis
Third generation cephalosporins
Fourth generation cephalosporins
Fluoroquinolones
Macrolides
Quinolones
Polymyxins
Aminoglycosides
Aminopenicillins
Aminopenicillins with beta−lactamase inhibitors
Ansamycins
Phosphonic acid derivatives
Shared human and animal antimicrobial classes
Cattle
Chickens
Pigs
Sheep
Turkeys
Ducks
Horses
Buffaloes
Goats
Livestock species

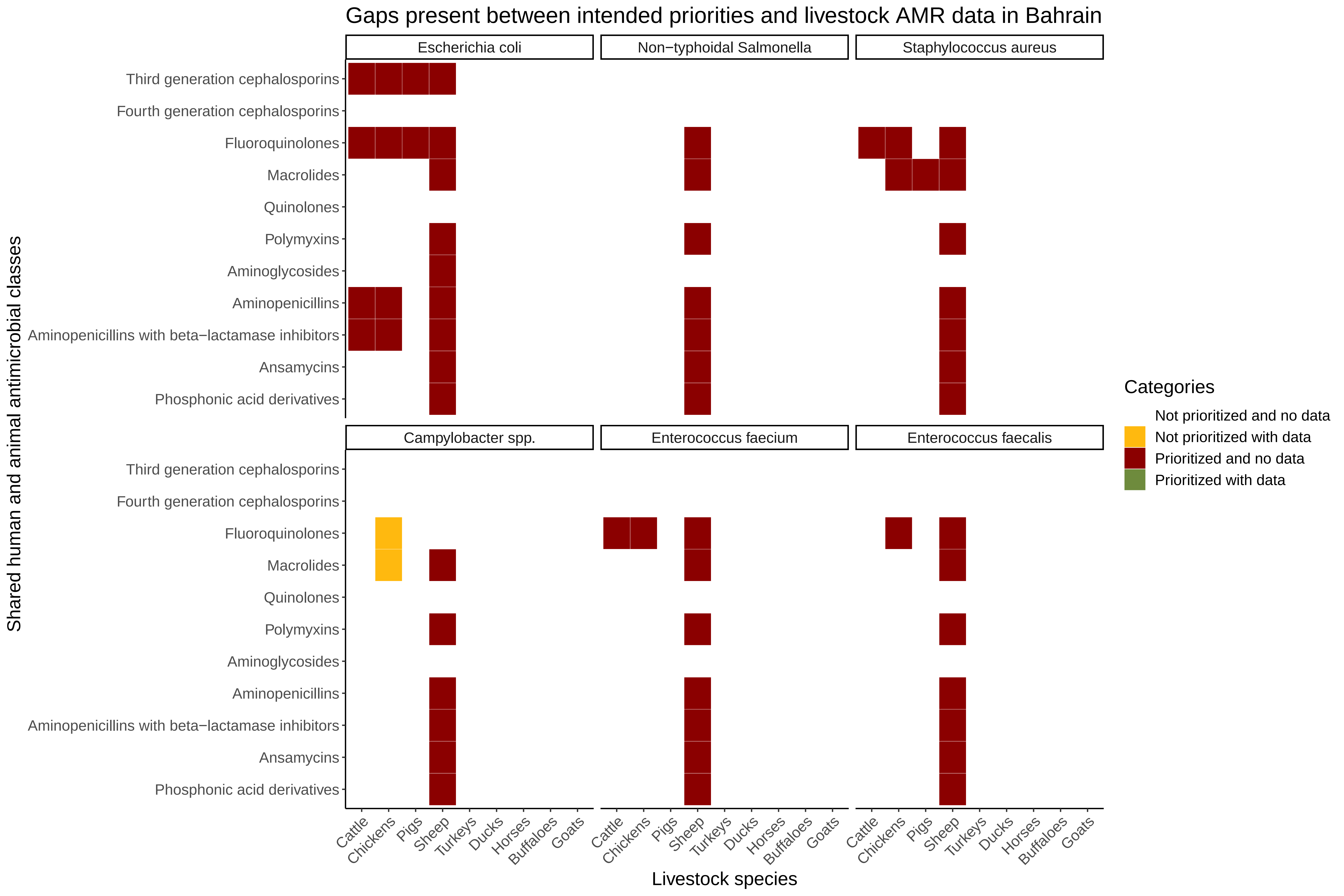

Gaps present between intended priorities and livestock AMR data in Bahrain
Escherichia coli
Non−typhoidal Salmonella
Staphylococcus aureus
Campylobacter spp.
Enterococcus faecium
Enterococcus faecalis
Third generation cephalosporins
Fourth generation cephalosporins
Fluoroquinolones
Macrolides
Quinolones
Polymyxins
Aminoglycosides
Aminopenicillins
Aminopenicillins with beta−lactamase inhibitors
Ansamycins
Phosphonic acid derivatives
Shared human and animal antimicrobial classes
Cattle
Chickens
Pigs
Sheep
Turkeys
Ducks
Horses
Buffaloes
Goats
Livestock species
Categories
Not prioritized and no data
Not prioritized with data
Prioritized and no data
Prioritized with data

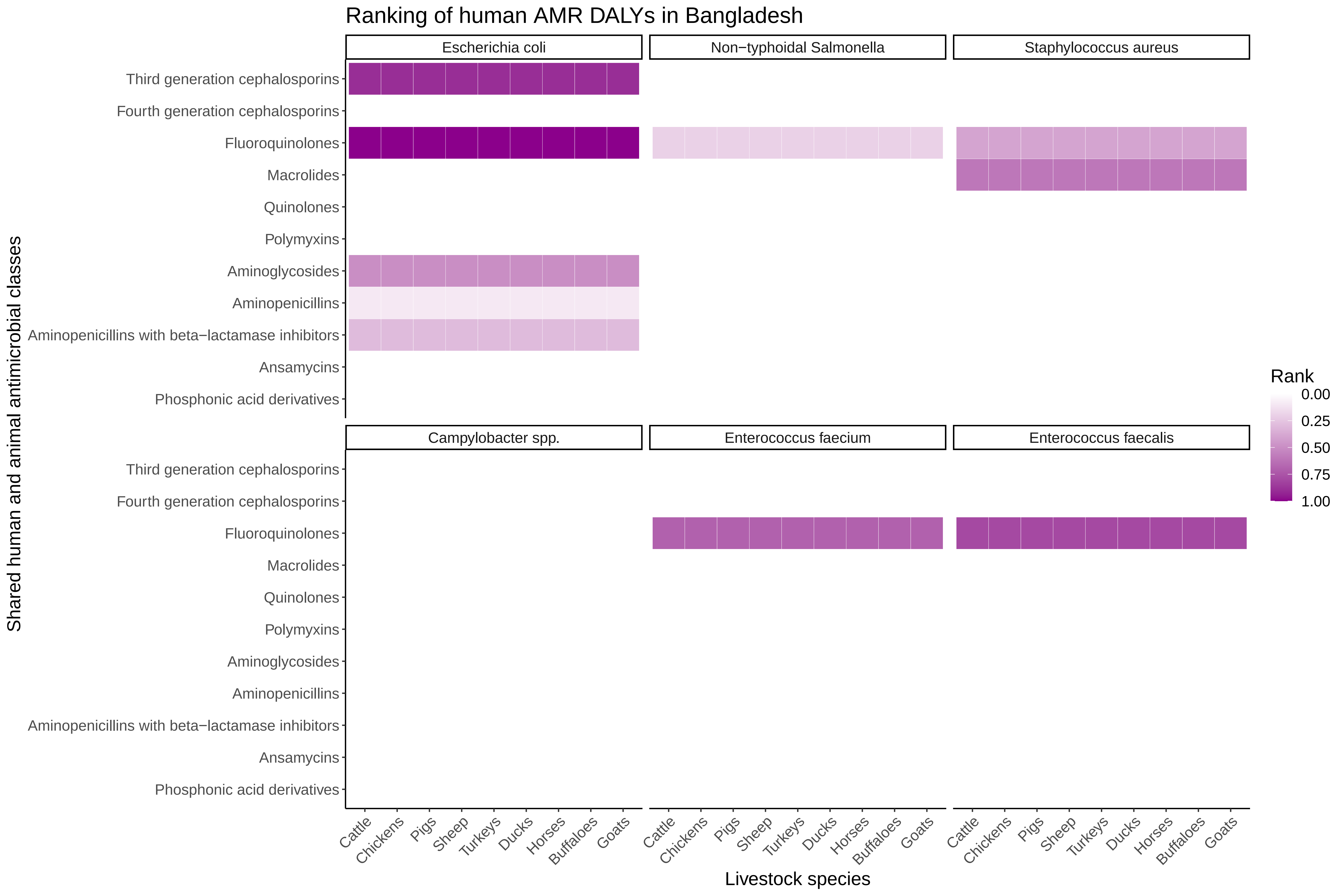

Ranking of human AMR DALYs in Bangladesh
Escherichia coli
Non−typhoidal Salmonella
Staphylococcus aureus
Campylobacter spp.
Enterococcus faecium
Enterococcus faecalis
Shared human and animal antimicrobial classes
Third generation cephalosporins
Fourth generation cephalosporins
Fluoroquinolones
Macrolides
Quinolones
Polymyxins
Aminoglycosides
Aminopenicillins
Aminopenicillins with beta−lactamase inhibitors
Ansamycins
Phosphonic acid derivatives
Cattle
Chickens
Pigs
Sheep
Turkeys
Ducks
Horses
Buffaloes
Goats
Livestock species
Rank
0.00
0.25
0.50
0.75
1.00

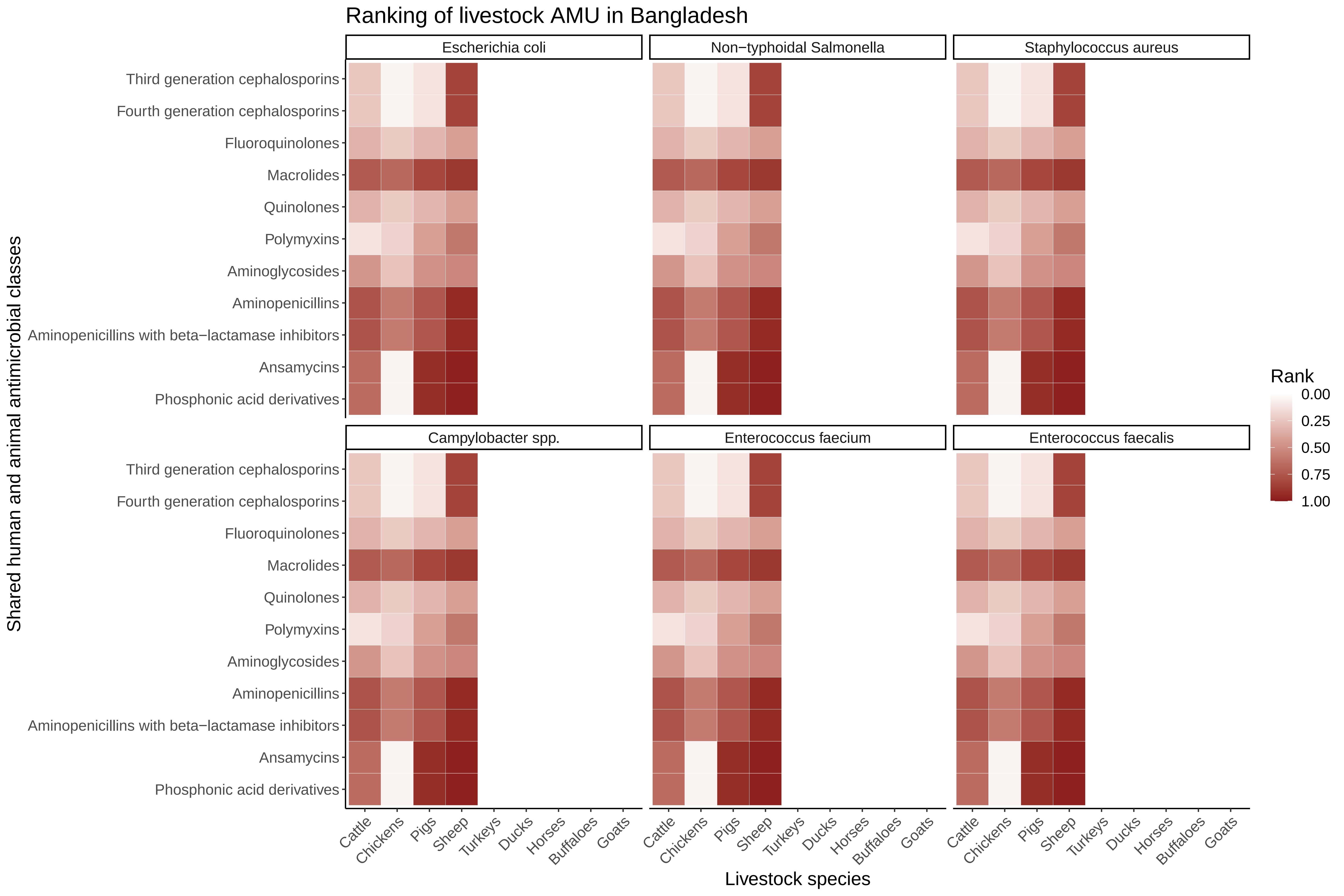

Ranking of livestock AMU in Bangladesh
Escherichia coli
Non−typhoidal Salmonella
Staphylococcus aureus
Campylobacter spp.
Enterococcus faecium
Enterococcus faecalis
Third generation cephalosporins
Fourth generation cephalosporins
Fluoroquinolones
Macrolides
Quinolones
Polymyxins
Aminoglycosides
Aminopenicillins
Aminopenicillins with beta−lactamase inhibitors
Ansamycins
Phosphonic acid derivatives
Shared human and animal antimicrobial classes
Cattle
Chickens
Pigs
Sheep
Turkeys
Ducks
Horses
Buffaloes
Goats
Livestock species
Rank
0.00
0.25
0.50
0.75
1.00

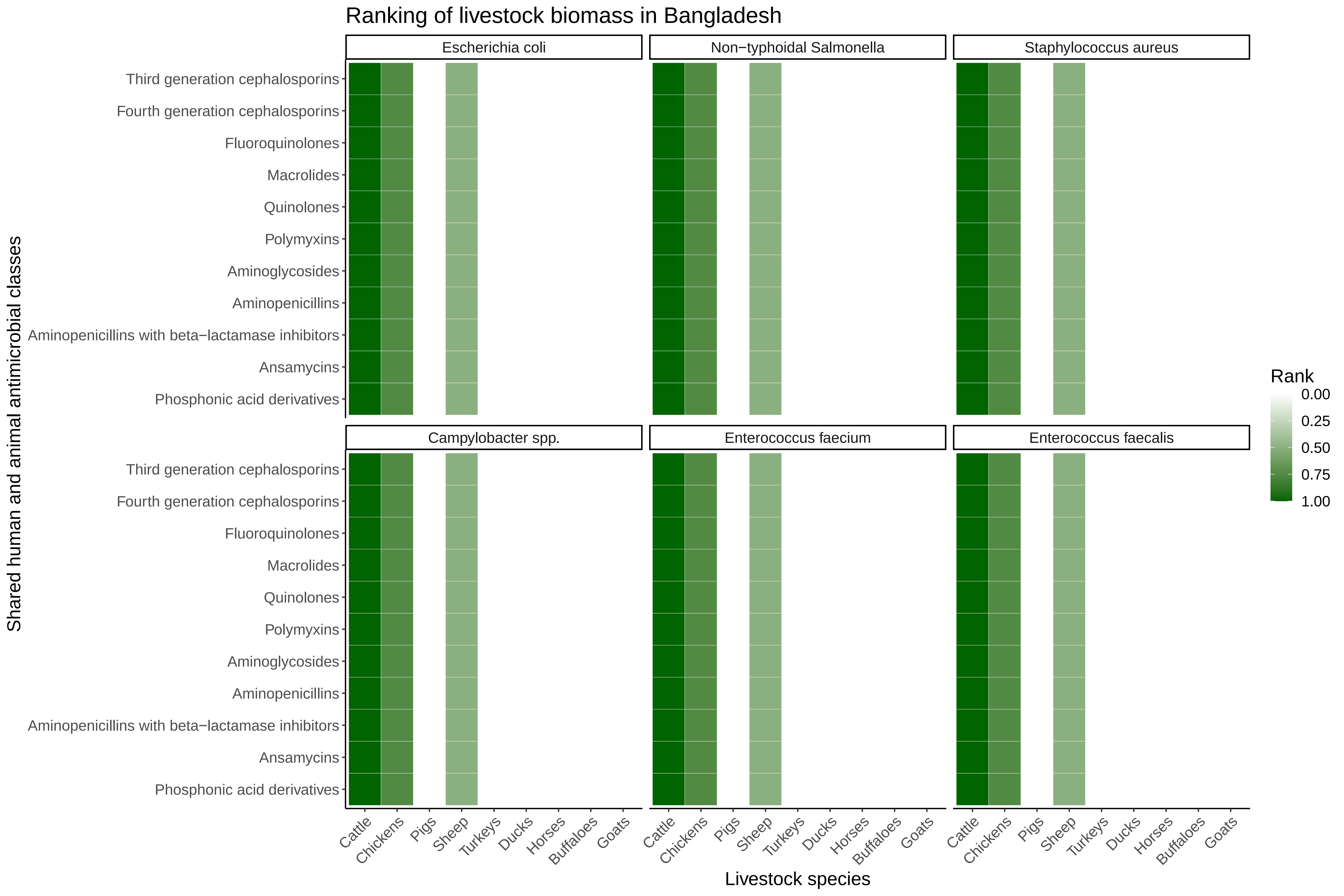

Ranking of livestock biomass in Bangladesh
Escherichia coli
Non−typhoidal Salmonella
Staphylococcus aureus
Campylobacter spp.
Enterococcus faecium
Enterococcus faecalis
Third generation cephalosporins
Fourth generation cephalosporins
Fluoroquinolones
Macrolides
Quinolones
Polymyxins
Aminoglycosides
Aminopenicillins
Aminopenicillins with beta−lactamase inhibitors
Ansamycins
Phosphonic acid derivatives
Shared human and animal antimicrobial classes
Cattle
Chickens
Pigs
Sheep
Turkeys
Ducks
Horses
Buffaloes
Goats
Livestock species
Rank
0.00
0.25
0.50
0.75
1.00

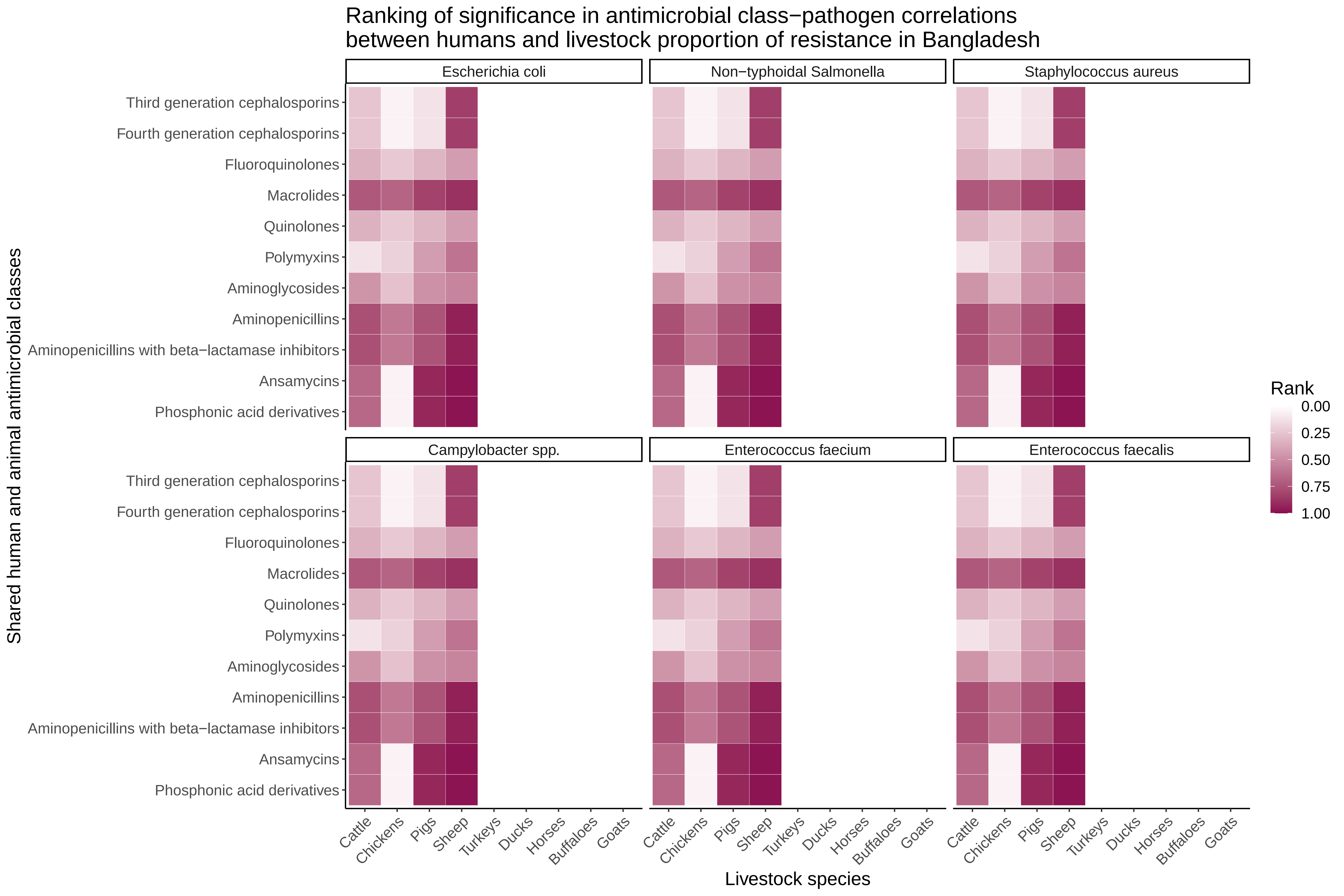
Ranking of significance in antimicrobial class−pathogen correlations
between humans and livestock proportion of resistance in Bangladesh
Escherichia coli
Non−typhoidal Salmonella
Staphylococcus aureus
Campylobacter spp.
Enterococcus faecium
Enterococcus faecalis
Third generation cephalosporins
Fourth generation cephalosporins
Fluoroquinolones
Macrolides
Quinolones
Polymyxins
Aminoglycosides
Aminopenicillins
Aminopenicillins with beta−lactamase inhibitors
Ansamycins
Phosphonic acid derivatives
Shared human and animal antimicrobial classes
Cattle
Chickens
Pigs
Sheep
Turkeys
Ducks
Horses
Buffaloes
Goats
Livestock species
Rank
0.00
0.25
0.50
0.75
1.00

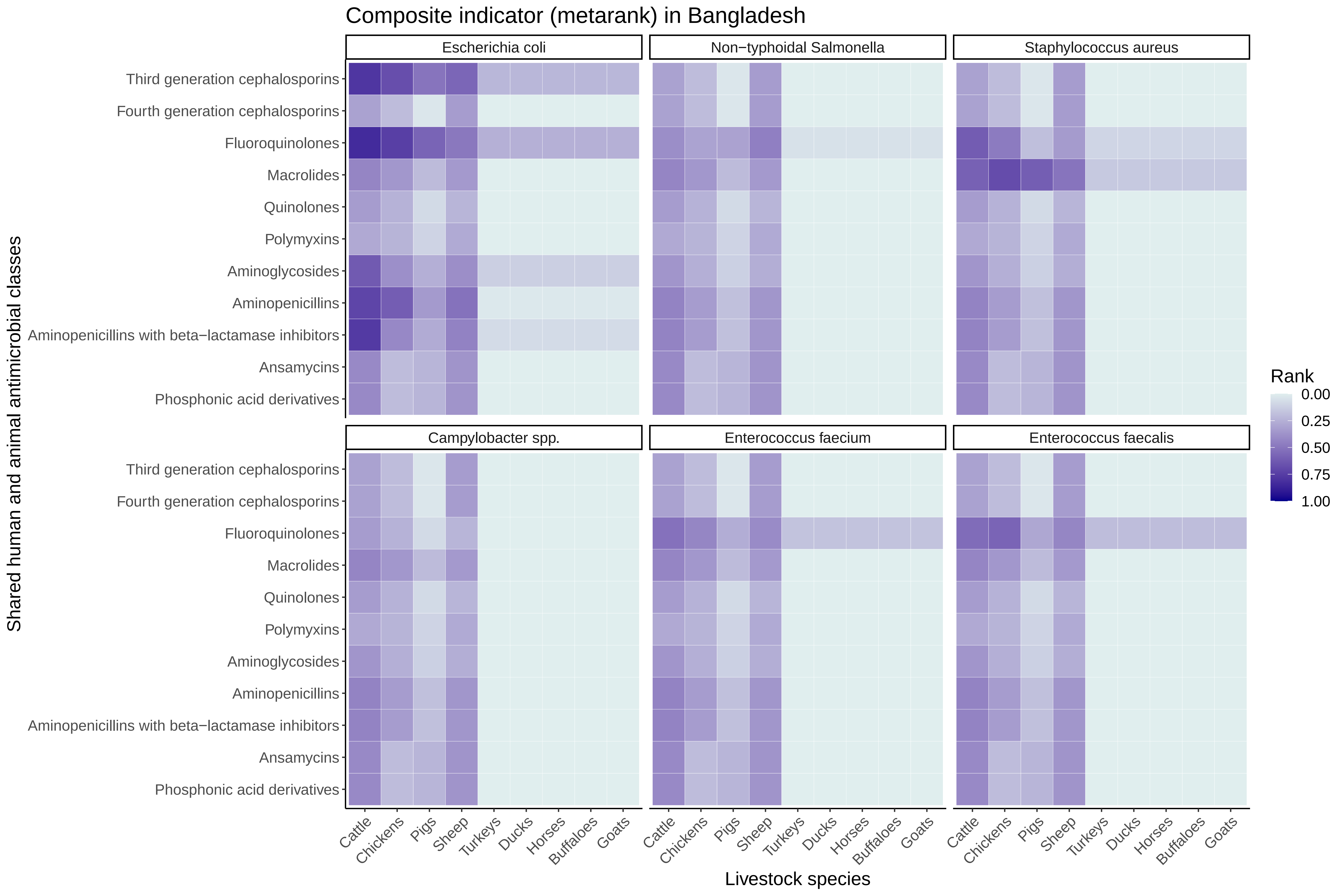

Composite indicator (metarank) in Bangladesh
Escherichia coli
Non−typhoidal Salmonella
Staphylococcus aureus
Campylobacter spp.
Enterococcus faecium
Enterococcus faecalis
Third generation cephalosporins
Fourth generation cephalosporins
Fluoroquinolones
Macrolides
Quinolones
Polymyxins
Aminoglycosides
Aminopenicillins
Aminopenicillins with beta−lactamase inhibitors
Ansamycins
Phosphonic acid derivatives
Shared human and animal antimicrobial classes
Cattle
Chickens
Pigs
Sheep
Turkeys
Ducks
Horses
Buffaloes
Goats
Livestock species
Rank
0.00
0.25
0.50
0.75
1.00

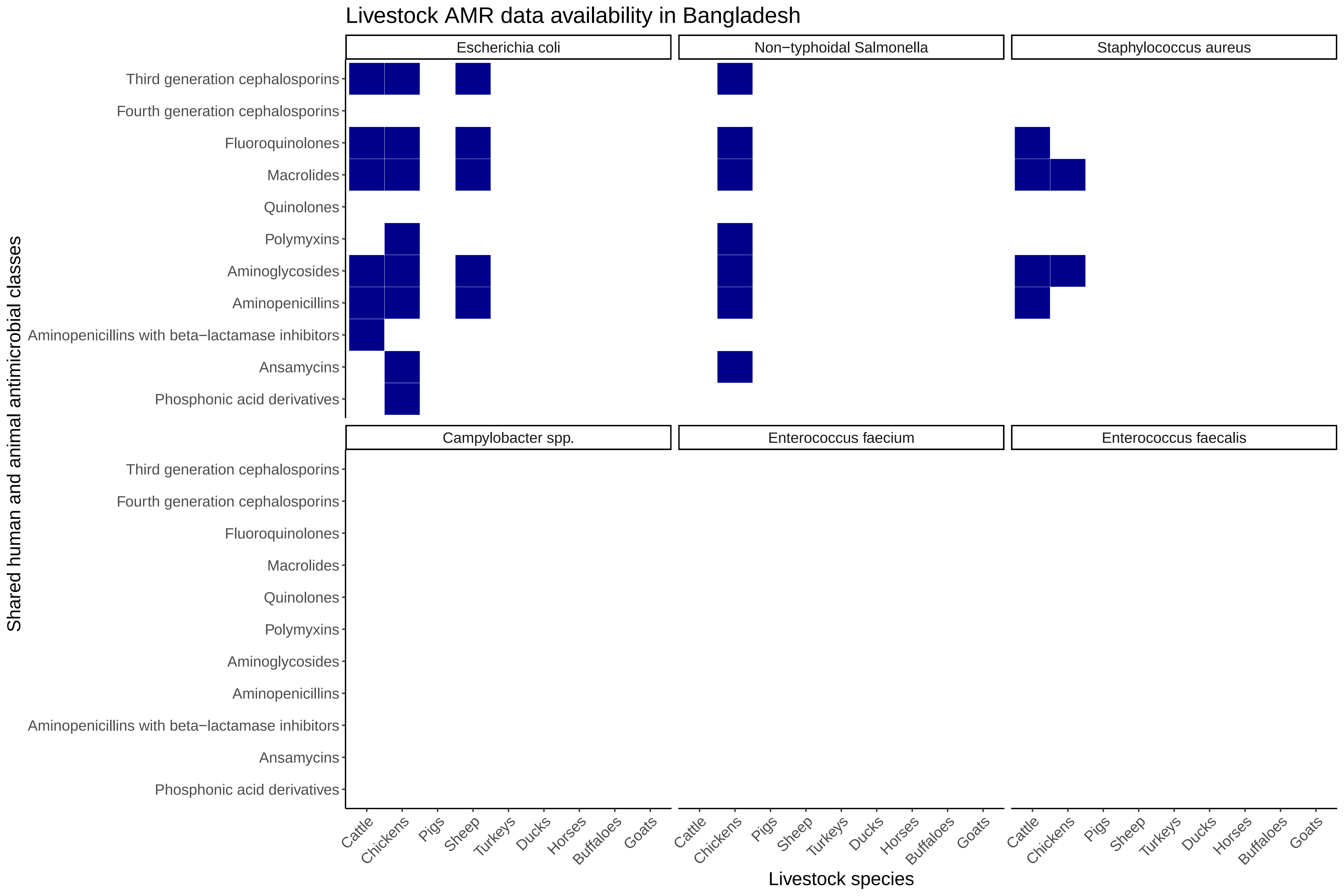

Livestock AMR data availability in Bangladesh
Escherichia coli
Non−typhoidal Salmonella
Staphylococcus aureus
Campylobacter spp.
Enterococcus faecium
Enterococcus faecalis
Shared human and animal antimicrobial classes
Third generation cephalosporins
Fourth generation cephalosporins
Fluoroquinolones
Macrolides
Quinolones
Polymyxins
Aminoglycosides
Aminopenicillins
Aminopenicillins with beta−lactamase inhibitors
Ansamycins
Phosphonic acid derivatives
Cattle
Chickens
Pigs
Sheep
Turkeys
Ducks
Horses
Buffaloes
Goats
Livestock species

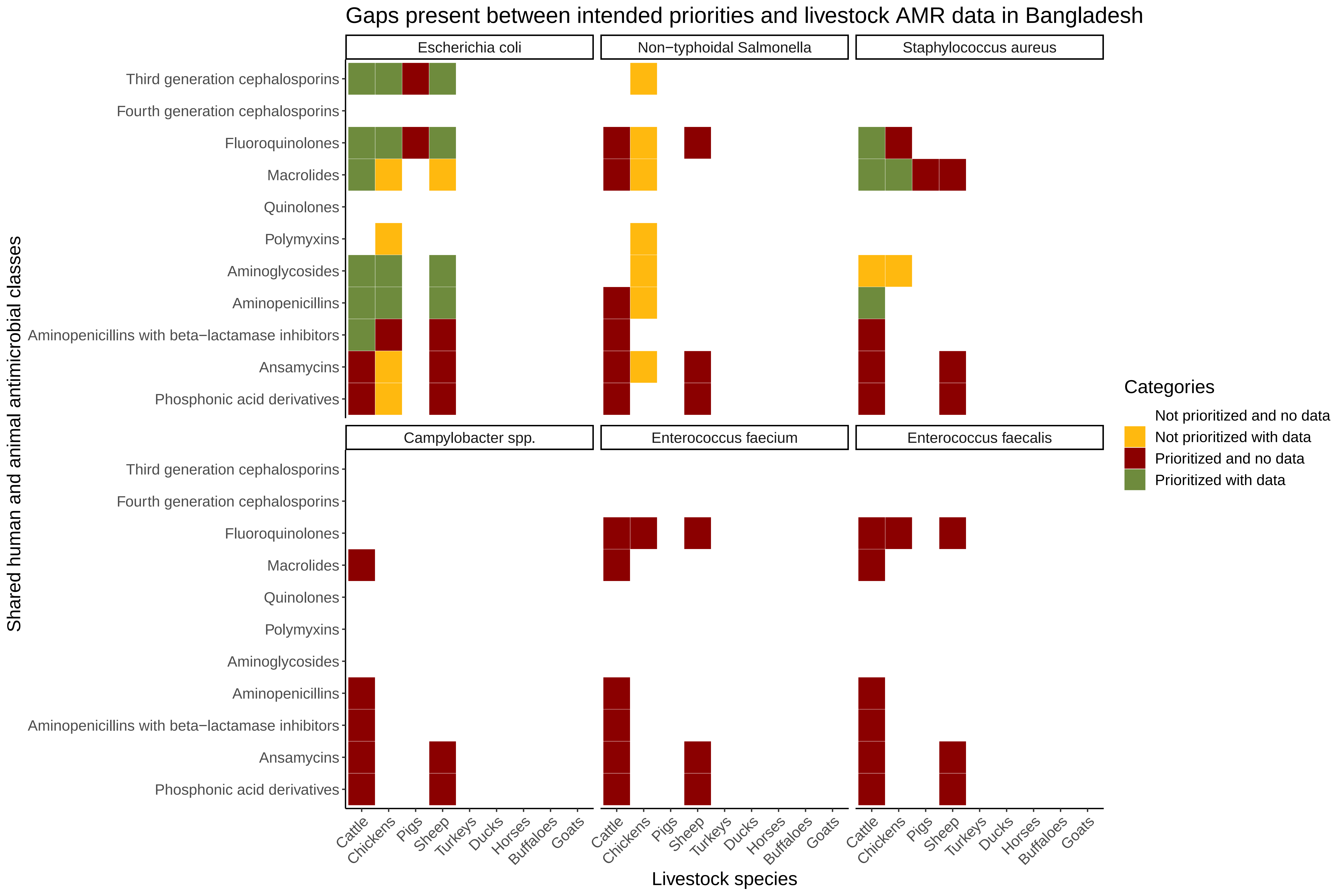

Gaps present between intended priorities and livestock AMR data in Bangladesh
Escherichia coli
Non−typhoidal Salmonella
Staphylococcus aureus
Campylobacter spp.
Enterococcus faecium
Enterococcus faecalis
Third generation cephalosporins
Fourth generation cephalosporins
Fluoroquinolones
Macrolides
Quinolones
Polymyxins
Aminoglycosides
Aminopenicillins
Aminopenicillins with beta−lactamase inhibitors
Ansamycins
Phosphonic acid derivatives
Shared human and animal antimicrobial classes
Cattle
Chickens
Pigs
Sheep
Turkeys
Ducks
Horses
Buffaloes
Goats
Livestock species
Categories
Not prioritized and no data
Not prioritized with data
Prioritized and no data
Prioritized with data

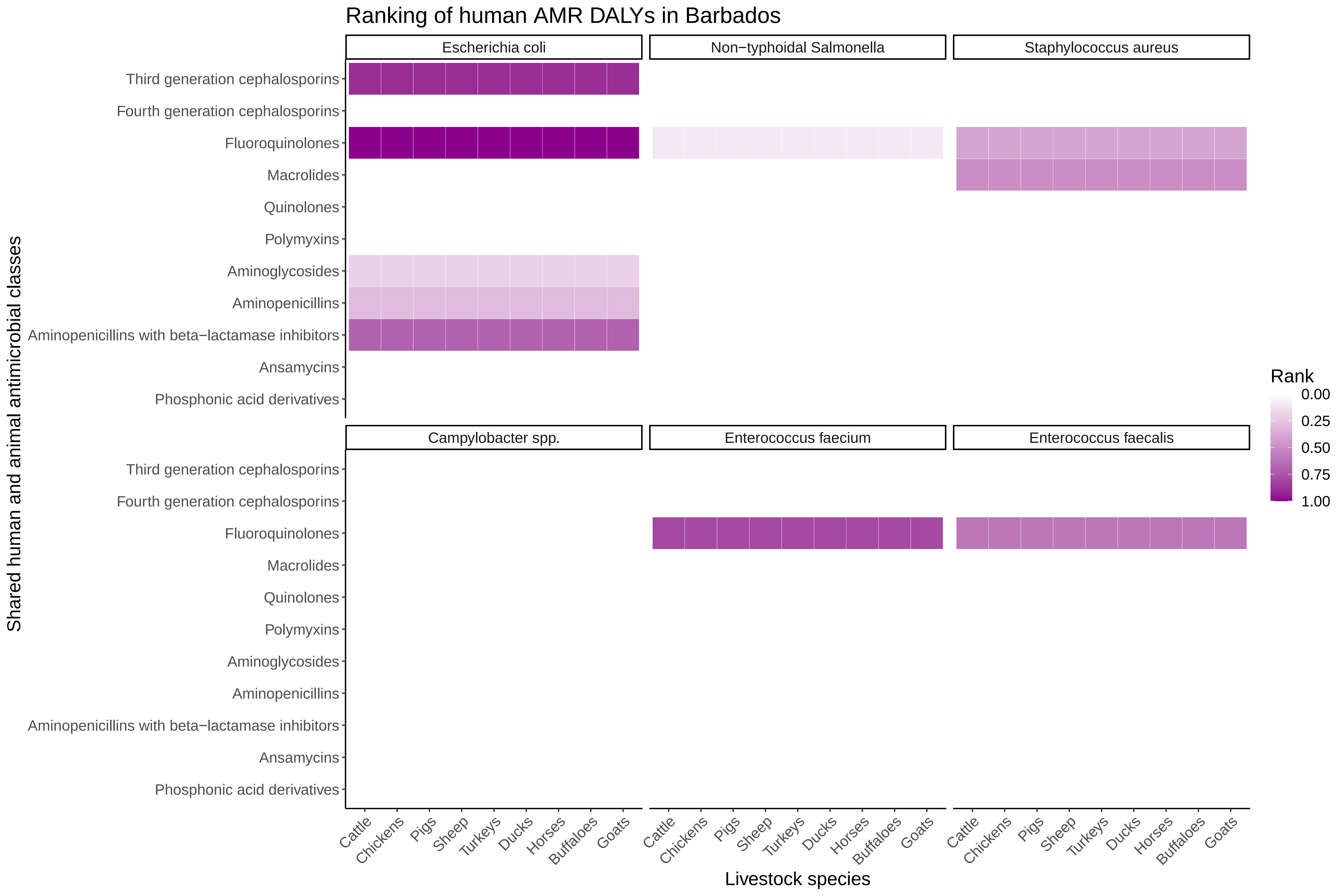
Ranking of human AMR DALYs in Barbados
Escherichia coli
Non−typhoidal Salmonella
Staphylococcus aureus
Campylobacter spp.
Enterococcus faecium
Enterococcus faecalis
Shared human and animal antimicrobial classes
Third generation cephalosporins
Fourth generation cephalosporins
Fluoroquinolones
Macrolides
Quinolones
Polymyxins
Aminoglycosides
Aminopenicillins
Aminopenicillins with beta−lactamase inhibitors
Ansamycins
Phosphonic acid derivatives
Cattle
Chickens
Pigs
Sheep
Turkeys
Ducks
Horses
Buffaloes
Goats
Livestock species
Rank
0.00
0.25
0.50
0.75
1.00

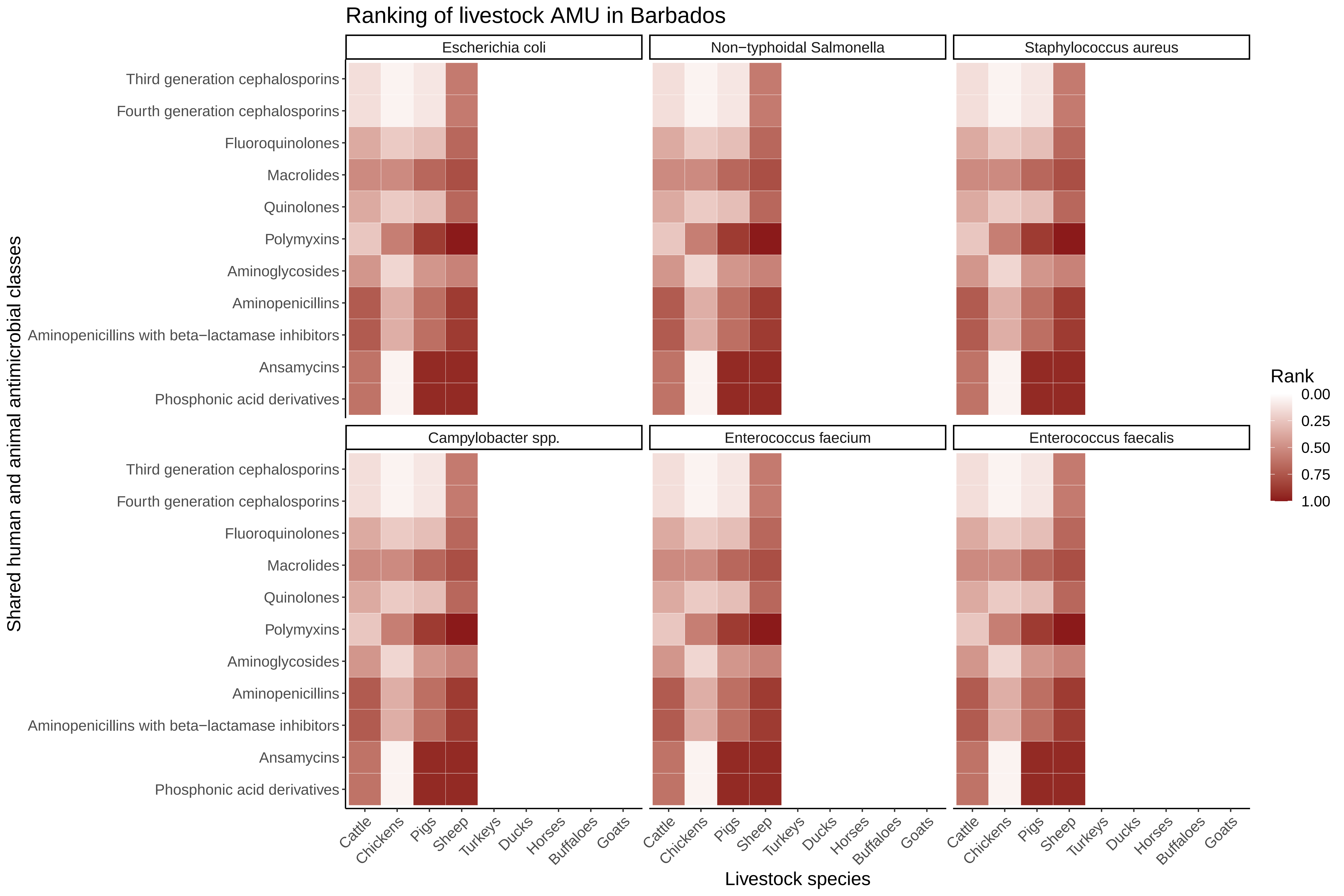

Ranking of livestock AMU in Barbados
Escherichia coli
Non−typhoidal Salmonella
Staphylococcus aureus
Campylobacter spp.
Enterococcus faecium
Enterococcus faecalis
Shared human and animal antimicrobial classes
Third generation cephalosporins
Fourth generation cephalosporins
Fluoroquinolones
Macrolides
Quinolones
Polymyxins
Aminoglycosides
Aminopenicillins
Aminopenicillins with beta−lactamase inhibitors
Ansamycins
Phosphonic acid derivatives
Cattle
Chickens
Pigs
Sheep
Turkeys
Ducks
Horses
Buffaloes
Goats
Livestock species
Rank
0.00
0.25
0.50
0.75
1.00

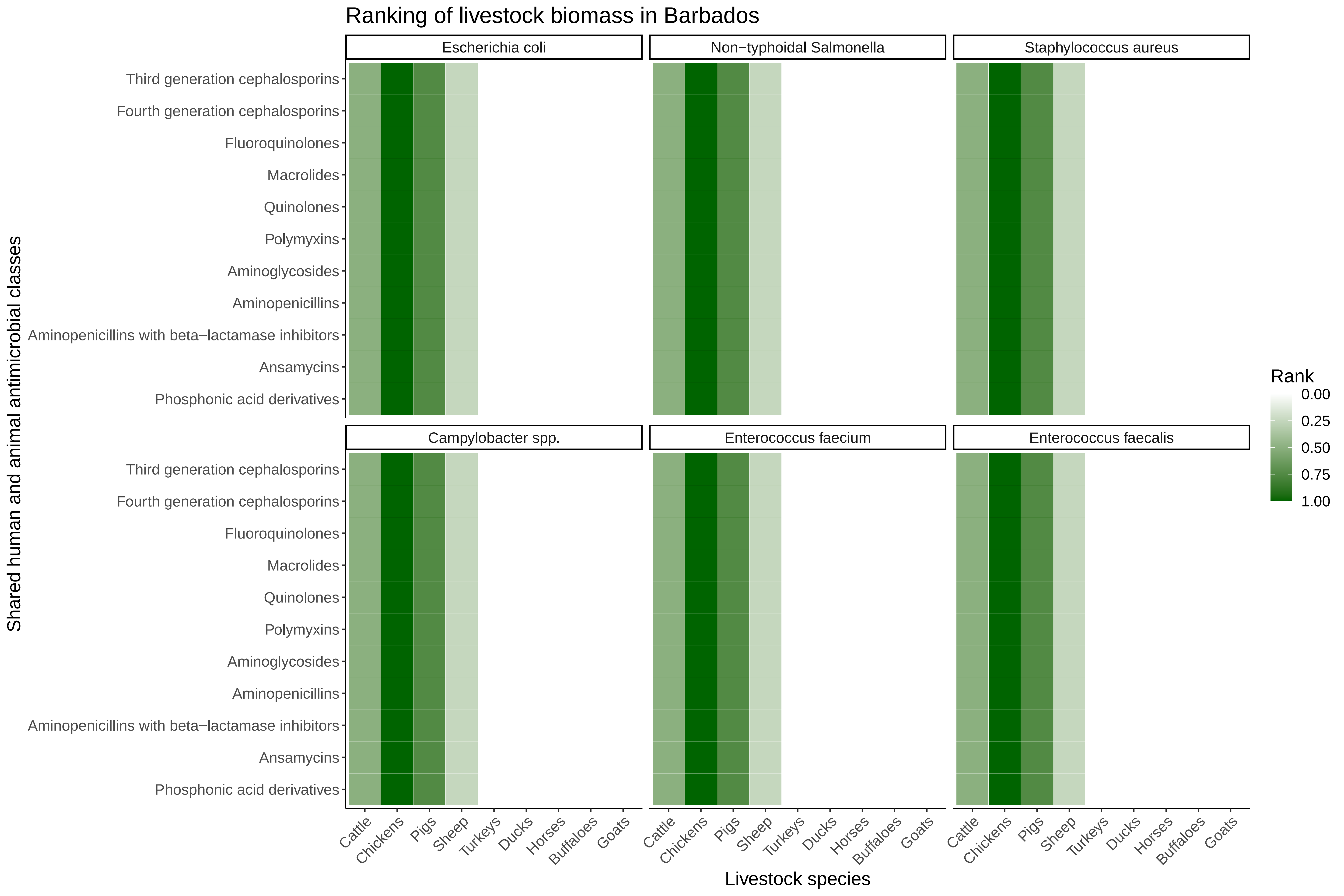
Ranking of livestock biomass in Barbados
Escherichia coli
Non−typhoidal Salmonella
Staphylococcus aureus
Campylobacter spp.
Enterococcus faecium
Enterococcus faecalis
Shared human and animal antimicrobial classes
Third generation cephalosporins
Fourth generation cephalosporins
Fluoroquinolones
Macrolides
Quinolones
Polymyxins
Aminoglycosides
Aminopenicillins
Aminopenicillins with beta−lactamase inhibitors
Ansamycins
Phosphonic acid derivatives
Livestock species
Cattle
Chickens
Pigs
Sheep
Turkeys
Ducks
Horses
Buffaloes
Goats
Rank
0.00
0.25
0.50
0.75
1.00

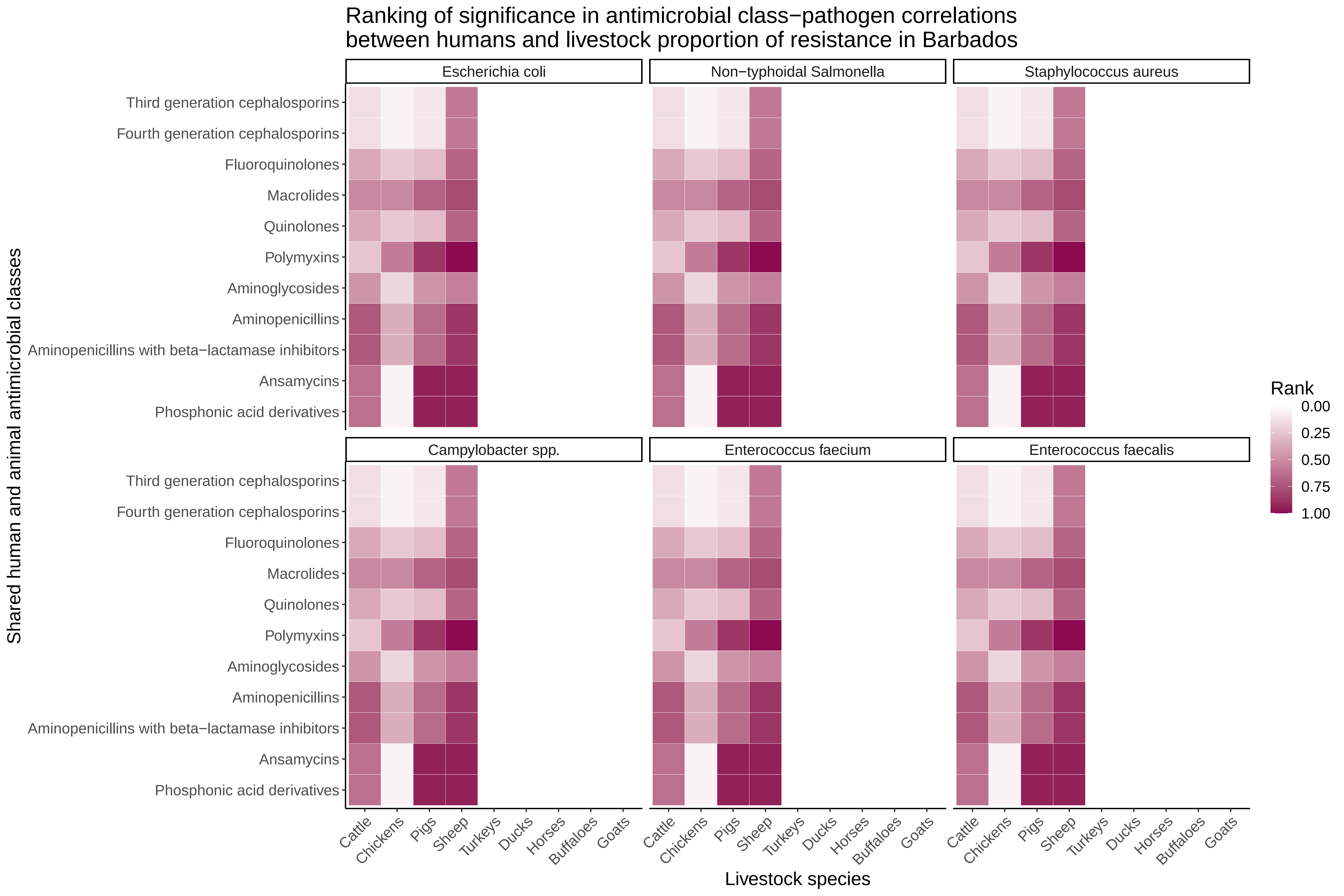

Ranking of significance in antimicrobial class−pathogen correlations
between humans and livestock proportion of resistance in Barbados
Escherichia coli
Non−typhoidal Salmonella
Staphylococcus aureus
Campylobacter spp.
Enterococcus faecium
Enterococcus faecalis
Third generation cephalosporins
Fourth generation cephalosporins
Fluoroquinolones
Macrolides
Quinolones
Polymyxins
Aminoglycosides
Aminopenicillins
Aminopenicillins with beta−lactamase inhibitors
Ansamycins
Phosphonic acid derivatives
Shared human and animal antimicrobial classes
Cattle
Chickens
Pigs
Sheep
Turkeys
Ducks
Horses
Buffaloes
Goats
Livestock species
Rank
0.00
0.25
0.50
0.75
1.00

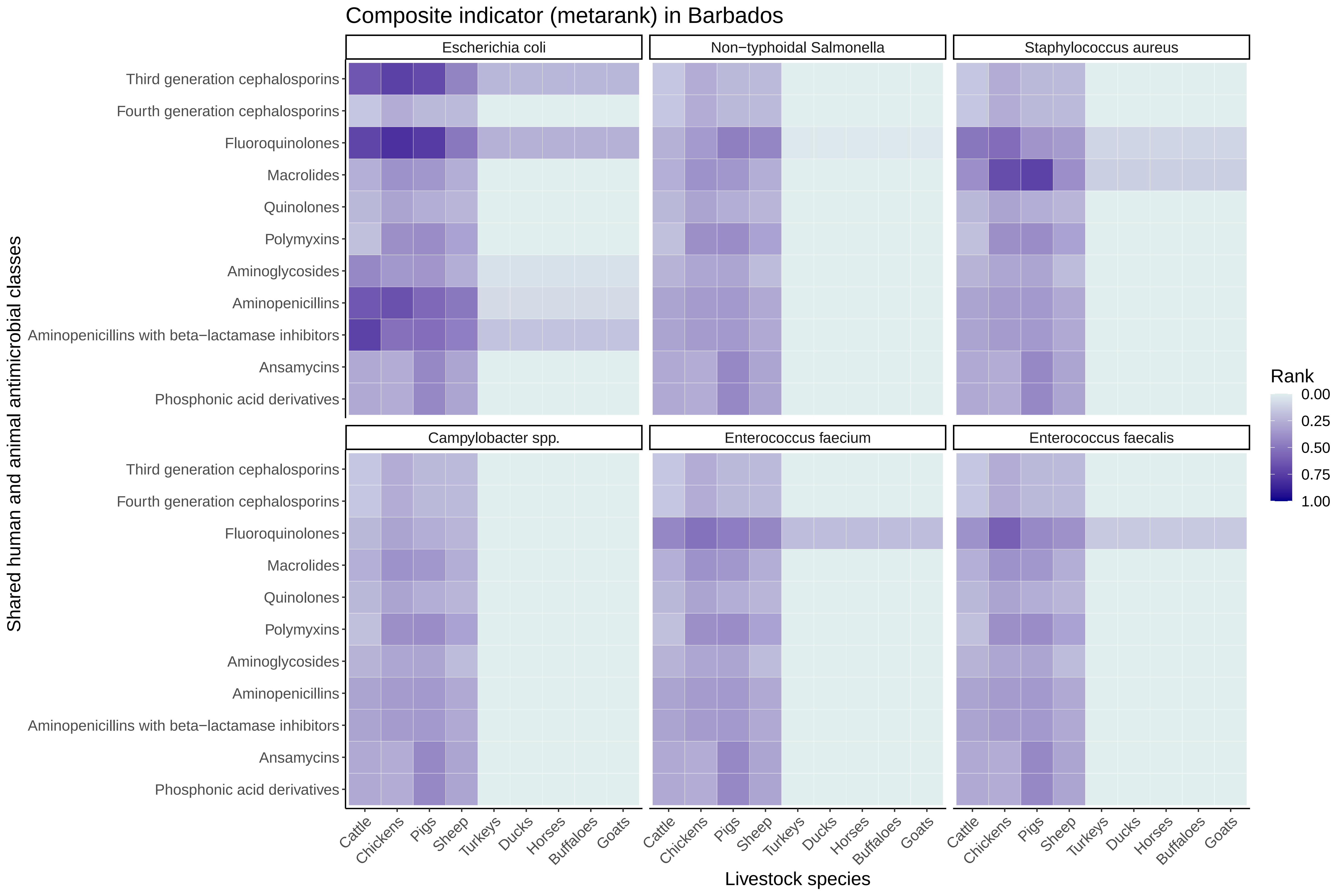

Composite indicator (metarank) in Barbados
Escherichia coli
Non−typhoidal Salmonella
Staphylococcus aureus
Campylobacter spp.
Enterococcus faecium
Enterococcus faecalis
Shared human and animal antimicrobial classes
Third generation cephalosporins
Fourth generation cephalosporins
Fluoroquinolones
Macrolides
Quinolones
Polymyxins
Aminoglycosides
Aminopenicillins
Aminopenicillins with beta−lactamase inhibitors
Ansamycins
Phosphonic acid derivatives
Cattle
Chickens
Pigs
Sheep
Turkeys
Ducks
Horses
Buffaloes
Goats
Livestock species
Rank
0.00
0.25
0.50
0.75
1.00

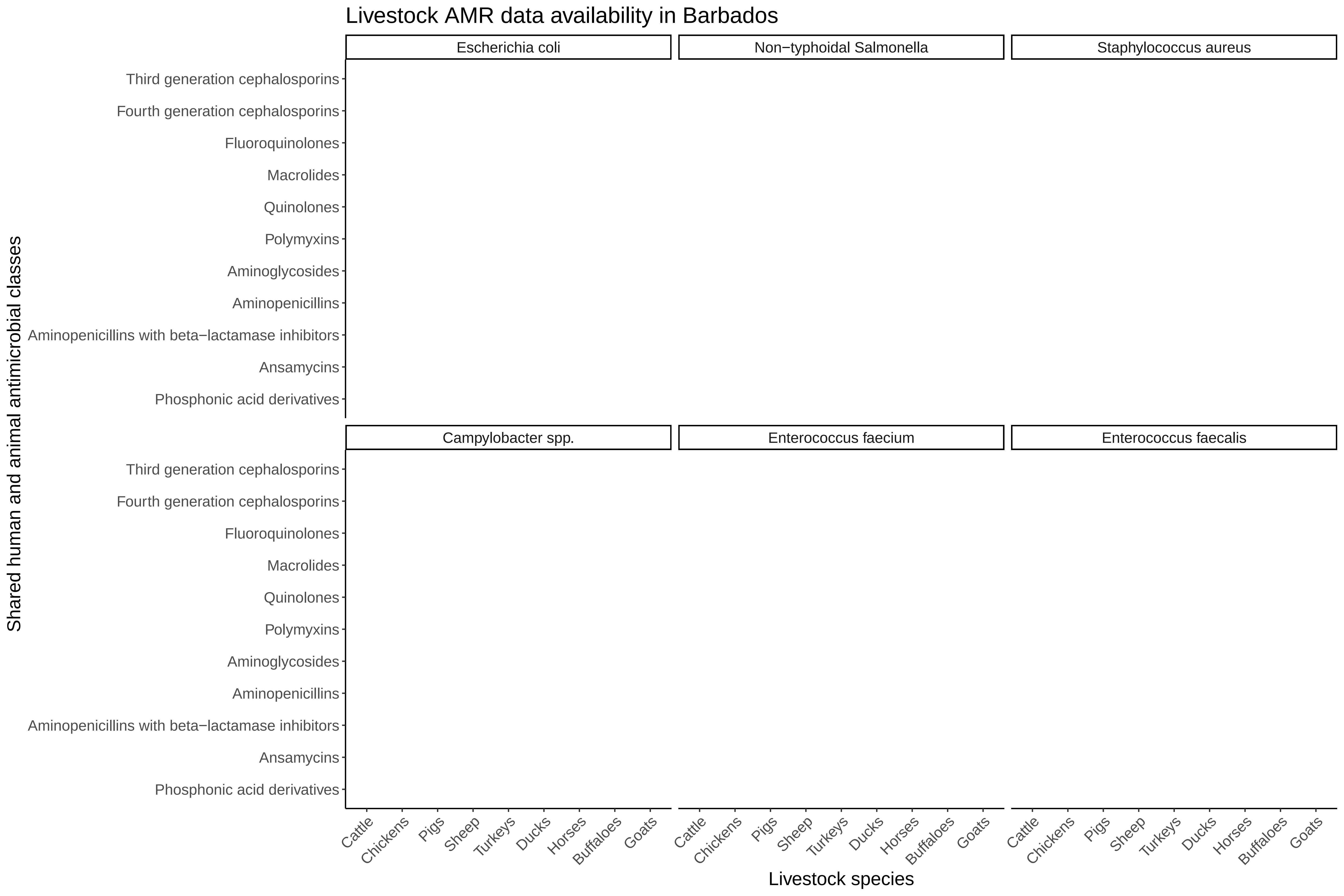
Livestock AMR data availability in Barbados
Escherichia coli
Non−typhoidal Salmonella
Staphylococcus aureus
Campylobacter spp.
Enterococcus faecium
Enterococcus faecalis
Third generation cephalosporins
Fourth generation cephalosporins
Fluoroquinolones
Macrolides
Quinolones
Polymyxins
Aminoglycosides
Aminopenicillins
Aminopenicillins with beta−lactamase inhibitors
Ansamycins
Phosphonic acid derivatives
Shared human and animal antimicrobial classes
Cattle
Chickens
Pigs
Sheep
Turkeys
Ducks
Horses
Buffaloes
Goats
Livestock species

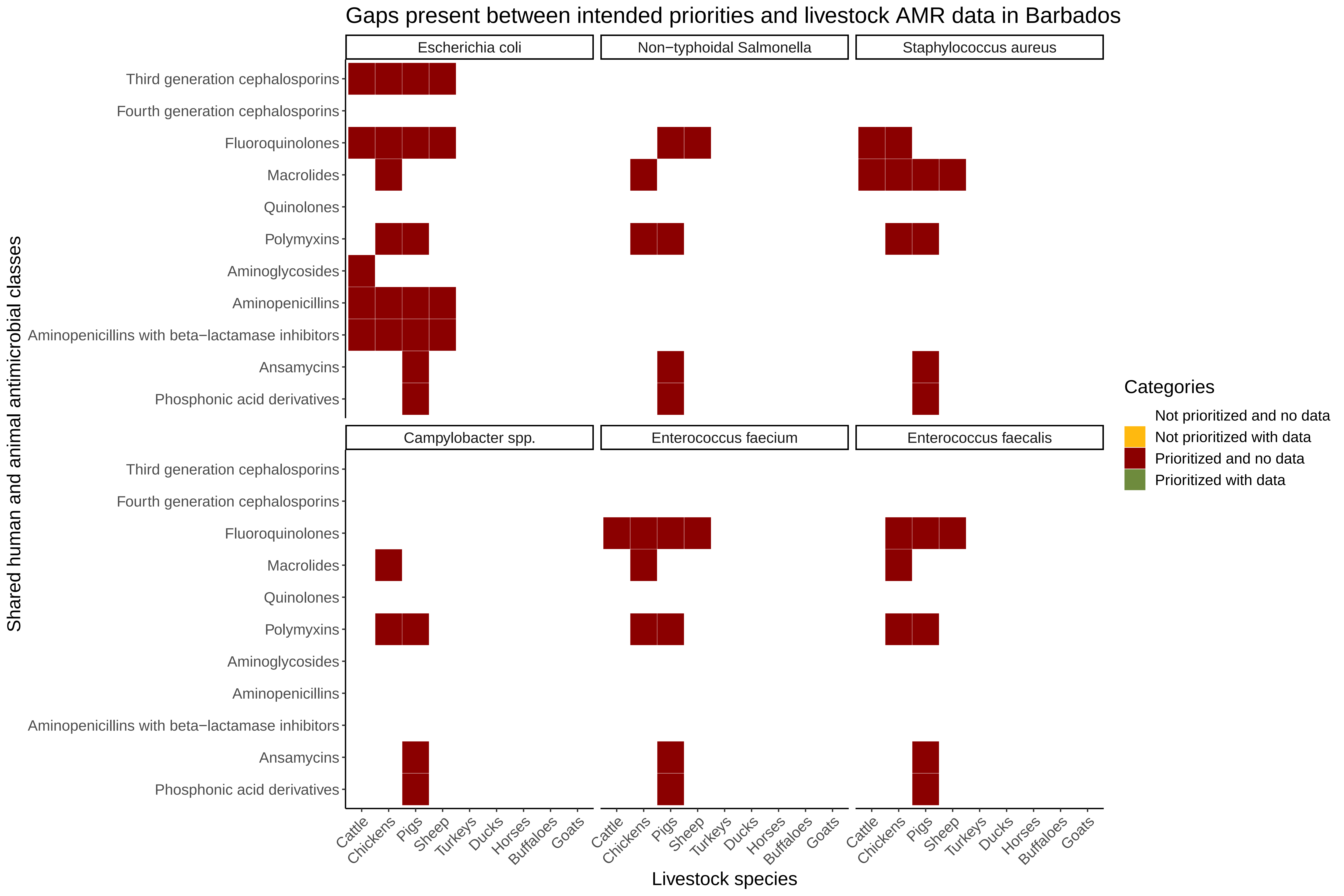

Gaps present between intended priorities and livestock AMR data in Barbados
Escherichia coli
Non−typhoidal Salmonella
Staphylococcus aureus
Campylobacter spp.
Enterococcus faecium
Enterococcus faecalis
Third generation cephalosporins
Fourth generation cephalosporins
Fluoroquinolones
Macrolides
Quinolones
Polymyxins
Aminoglycosides
Aminopenicillins
Aminopenicillins with beta−lactamase inhibitors
Ansamycins
Phosphonic acid derivatives
Shared human and animal antimicrobial classes
Cattle
Chickens
Pigs
Sheep
Turkeys
Ducks
Horses
Buffaloes
Goats
Livestock species
Categories
Not prioritized and no data
Not prioritized with data
Prioritized and no data
Prioritized with data

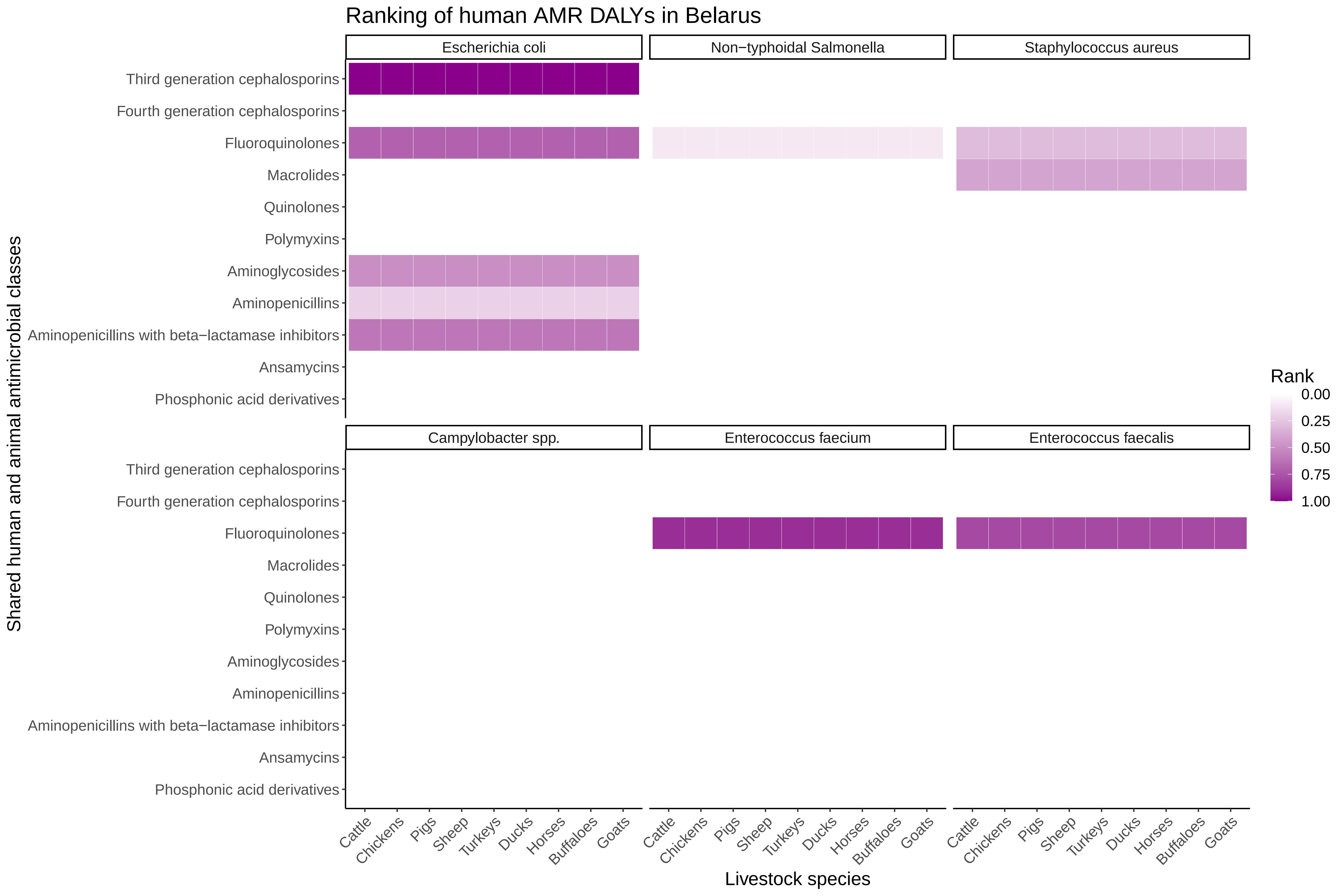

Ranking of human AMR DALYs in Belarus
Escherichia coli
Non−typhoidal Salmonella
Staphylococcus aureus
Campylobacter spp.
Enterococcus faecium
Enterococcus faecalis
Third generation cephalosporins
Fourth generation cephalosporins
Fluoroquinolones
Macrolides
Quinolones
Polymyxins
Aminoglycosides
Aminopenicillins
Aminopenicillins with beta−lactamase inhibitors
Ansamycins
Phosphonic acid derivatives
Shared human and animal antimicrobial classes
Cattle
Chickens
Pigs
Sheep
Turkeys
Ducks
Horses
Buffaloes
Goats
Livestock species
Rank
0.00
0.25
0.50
0.75
1.00

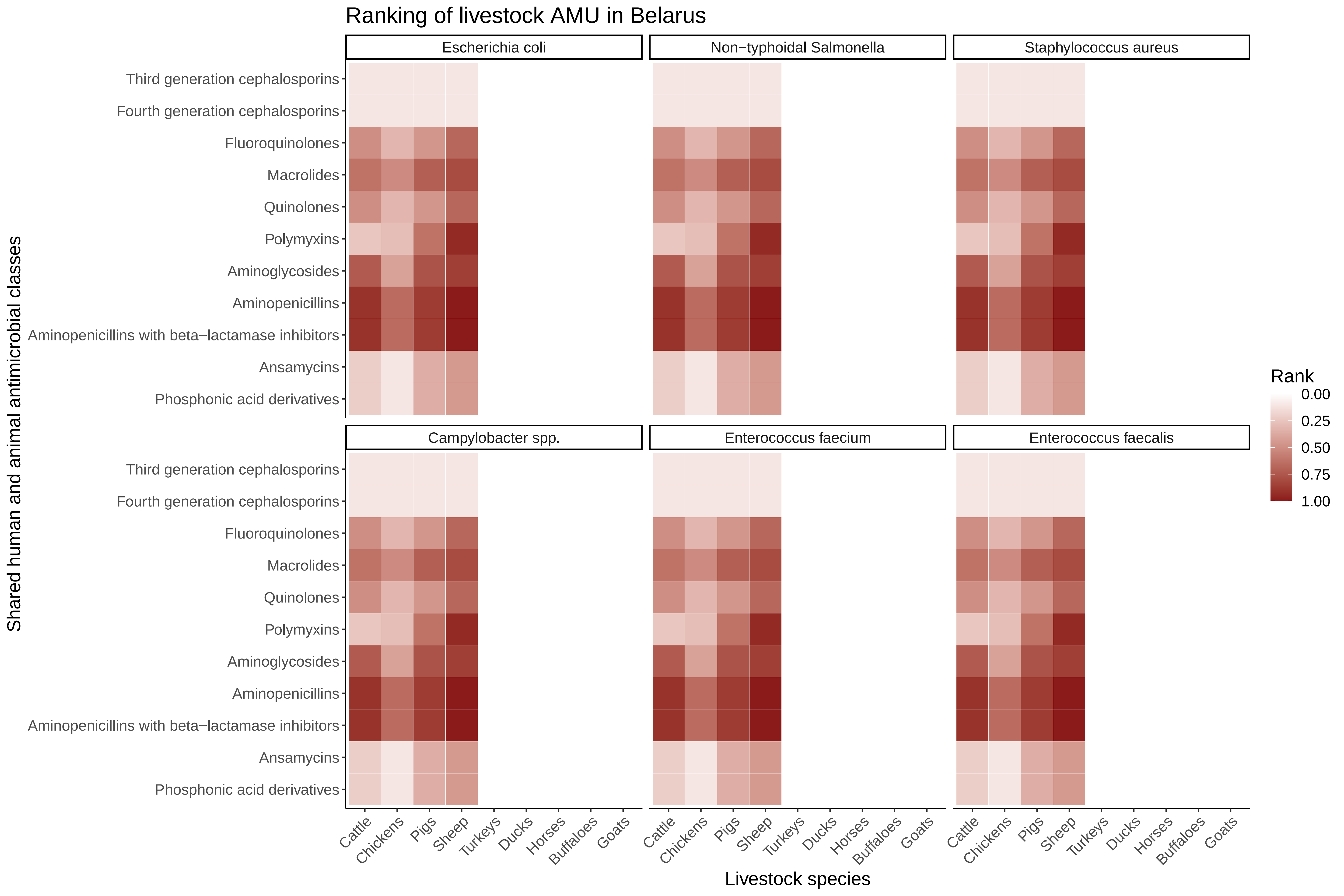
Ranking of livestock AMU in Belarus
Escherichia coli
Non−typhoidal Salmonella
Staphylococcus aureus
Campylobacter spp.
Enterococcus faecium
Enterococcus faecalis
Shared human and animal antimicrobial classes
Third generation cephalosporins
Fourth generation cephalosporins
Fluoroquinolones
Macrolides
Quinolones
Polymyxins
Aminoglycosides
Aminopenicillins
Aminopenicillins with beta−lactamase inhibitors
Ansamycins
Phosphonic acid derivatives
Livestock species
Cattle
Chickens
Pigs
Sheep
Turkeys
Ducks
Horses
Buffaloes
Goats
Rank
0.00
0.25
0.50
0.75
1.00

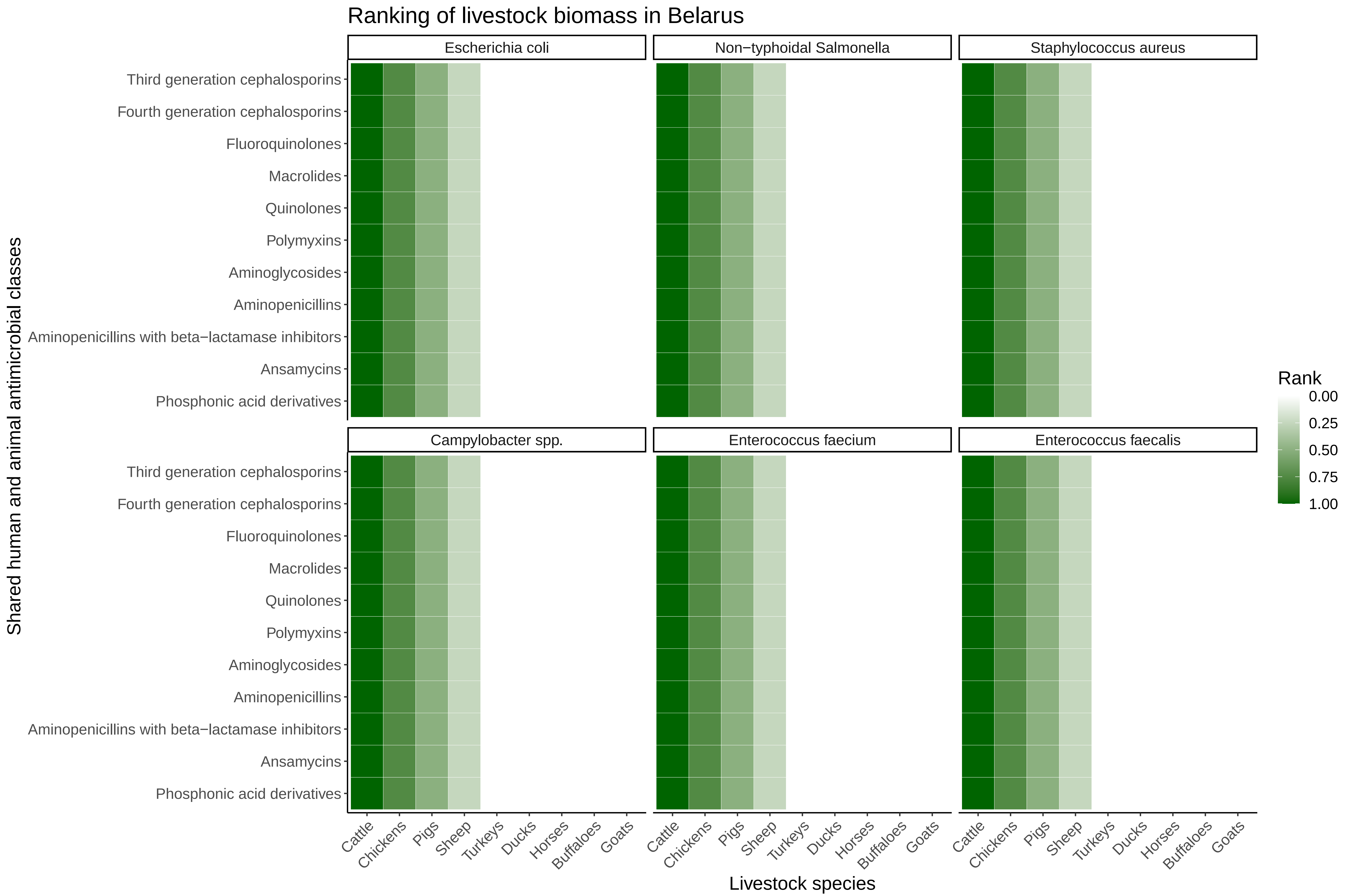

Ranking of livestock biomass in Belarus
Escherichia coli
Non−typhoidal Salmonella
Staphylococcus aureus
Campylobacter spp.
Enterococcus faecium
Enterococcus faecalis
Third generation cephalosporins
Fourth generation cephalosporins
Fluoroquinolones
Macrolides
Quinolones
Polymyxins
Aminoglycosides
Aminopenicillins
Aminopenicillins with beta−lactamase inhibitors
Ansamycins
Phosphonic acid derivatives
Shared human and animal antimicrobial classes
Cattle
Chickens
Pigs
Sheep
Turkeys
Ducks
Horses
Buffaloes
Goats
Livestock species
Rank
0.00
0.25
0.50
0.75
1.00

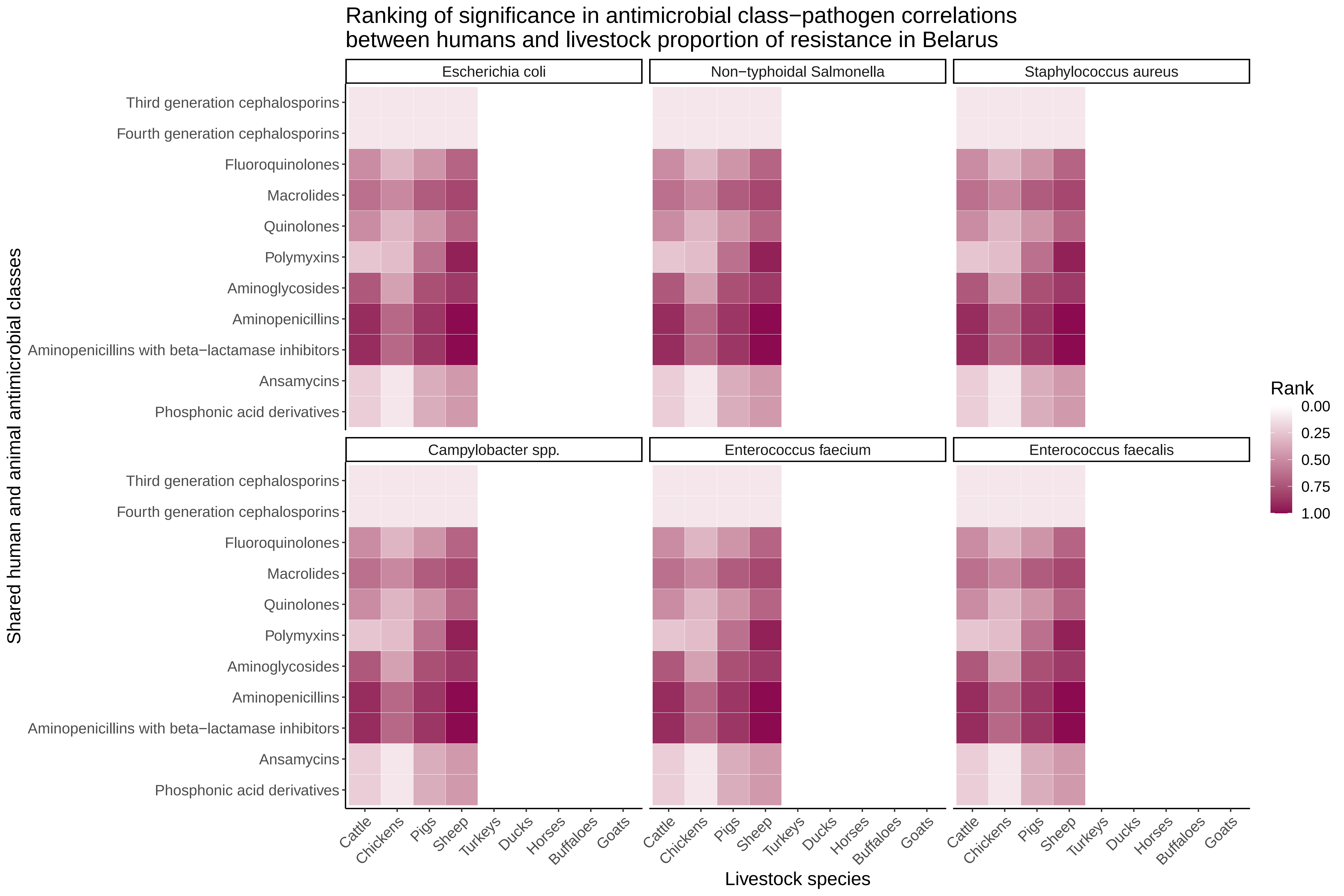

Ranking of significance in antimicrobial class−pathogen correlations
between humans and livestock proportion of resistance in Belarus
Escherichia coli
Non−typhoidal Salmonella
Staphylococcus aureus
Campylobacter spp.
Enterococcus faecium
Enterococcus faecalis
Third generation cephalosporins
Fourth generation cephalosporins
Fluoroquinolones
Macrolides
Quinolones
Polymyxins
Aminoglycosides
Aminopenicillins
Aminopenicillins with beta−lactamase inhibitors
Ansamycins
Phosphonic acid derivatives
Shared human and animal antimicrobial classes
Cattle
Chickens
Pigs
Sheep
Turkeys
Ducks
Horses
Buffaloes
Goats
Livestock species
Rank
0.00
0.25
0.50
0.75
1.00

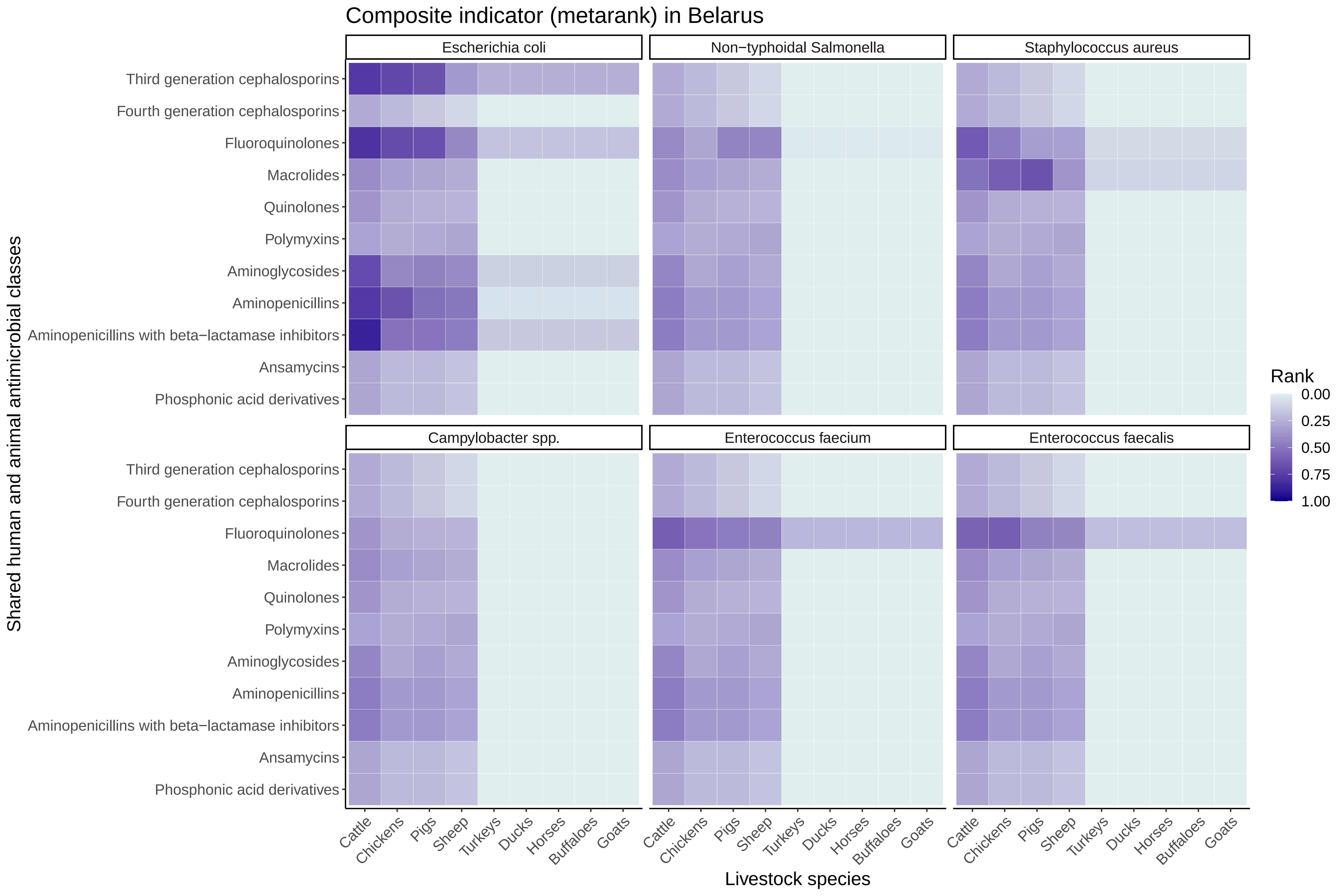

Composite indicator (metarank) in Belarus
Escherichia coli
Non−typhoidal Salmonella
Staphylococcus aureus
Campylobacter spp.
Enterococcus faecium
Enterococcus faecalis
Shared human and animal antimicrobial classes
Third generation cephalosporins
Fourth generation cephalosporins
Fluoroquinolones
Macrolides
Quinolones
Polymyxins
Aminoglycosides
Aminopenicillins
Aminopenicillins with beta−lactamase inhibitors
Ansamycins
Phosphonic acid derivatives
Cattle
Chickens
Pigs
Sheep
Turkeys
Ducks
Horses
Buffaloes
Goats
Livestock species
Rank
0.00
0.25
0.50
0.75
1.00

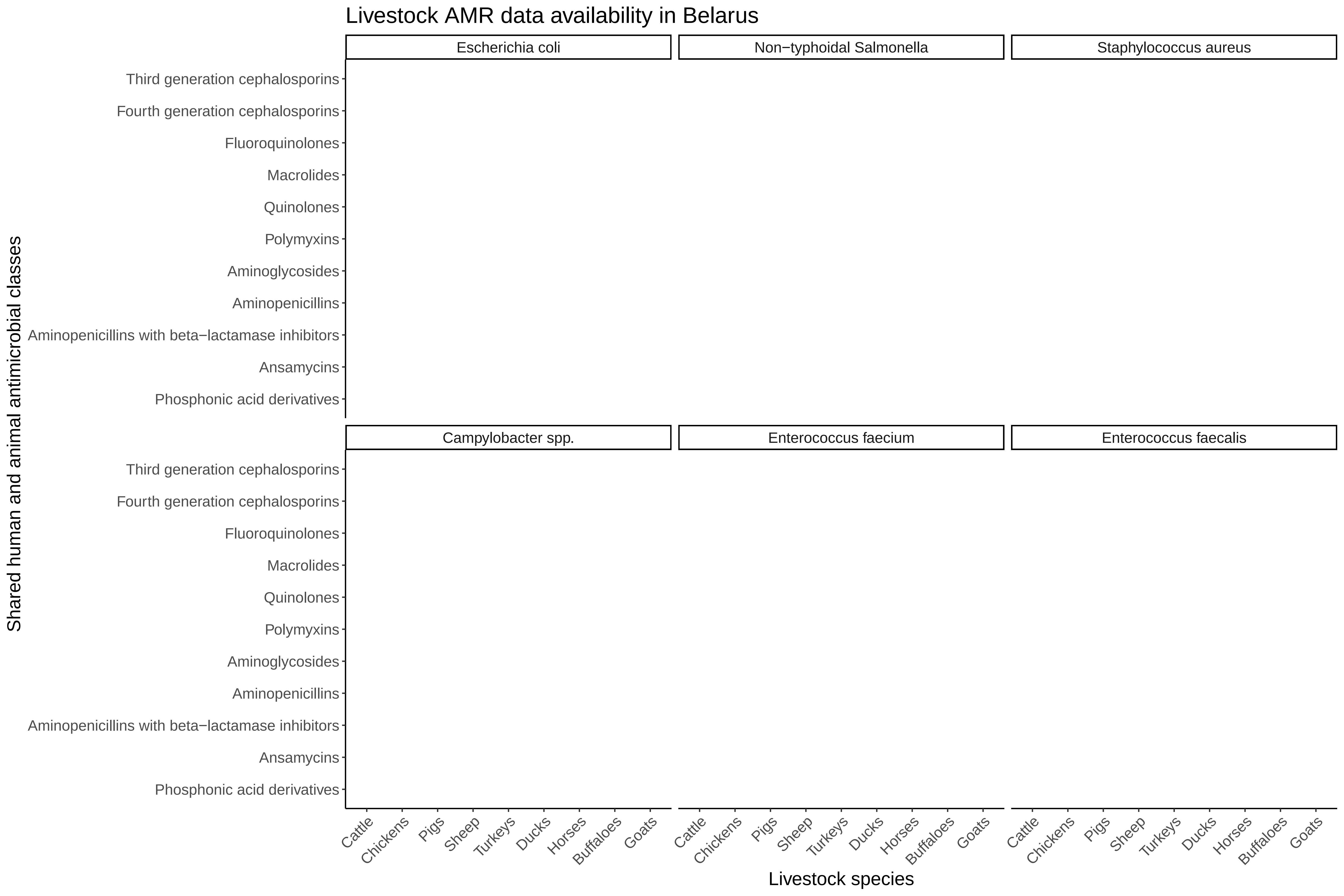
Livestock AMR data availability in Belarus
Escherichia coli
Non−typhoidal Salmonella
Staphylococcus aureus
Campylobacter spp.
Enterococcus faecium
Enterococcus faecalis
Shared human and animal antimicrobial classes
Third generation cephalosporins
Fourth generation cephalosporins
Fluoroquinolones
Macrolides
Quinolones
Polymyxins
Aminoglycosides
Aminopenicillins
Aminopenicillins with beta−lactamase inhibitors
Ansamycins
Phosphonic acid derivatives
Cattle
Chickens
Pigs
Sheep
Turkeys
Ducks
Horses
Buffaloes
Goats
Livestock species

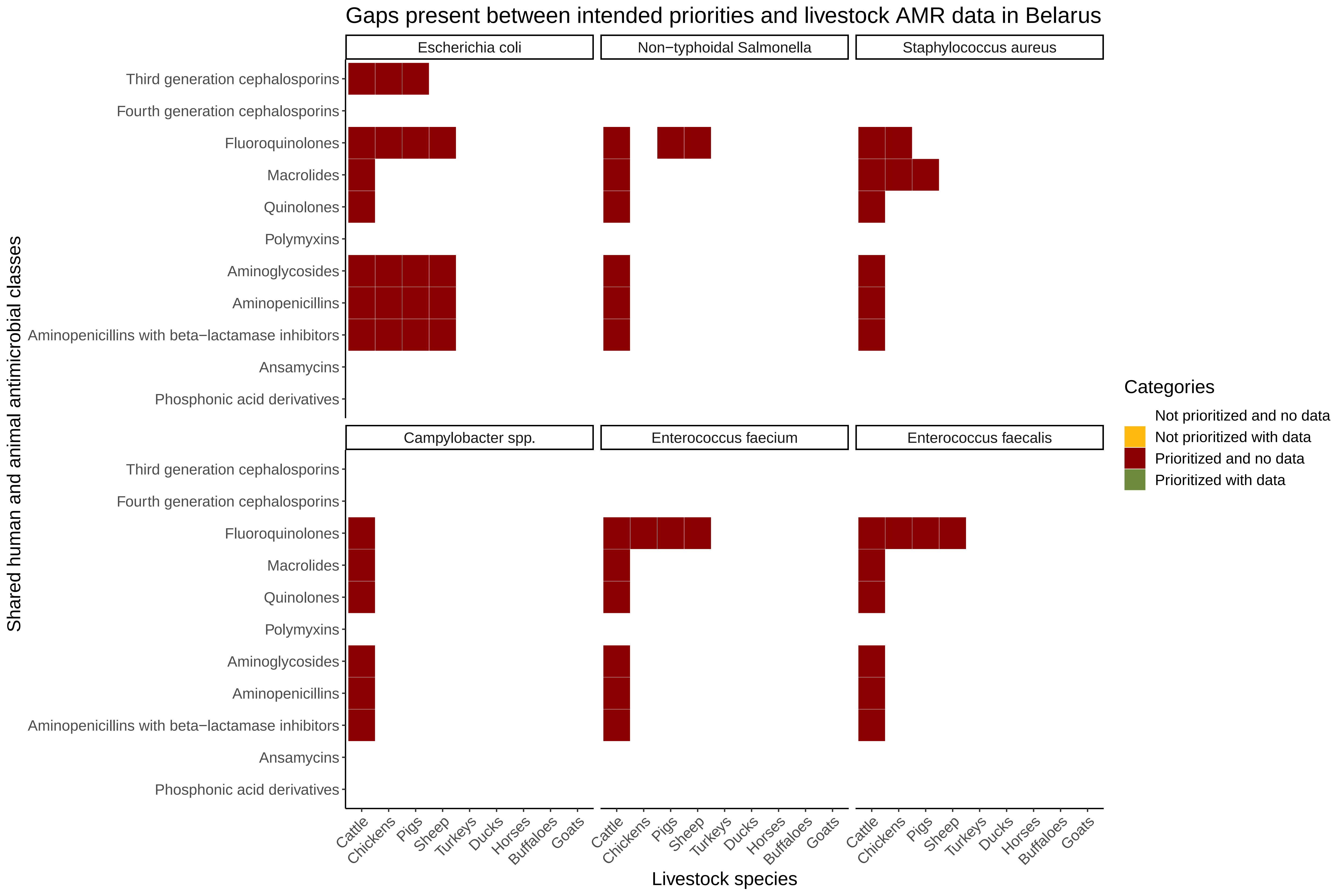
Gaps present between intended priorities and livestock AMR data in Belarus
Escherichia coli
Non−typhoidal Salmonella
Staphylococcus aureus
Campylobacter spp.
Enterococcus faecium
Enterococcus faecalis
Third generation cephalosporins
Fourth generation cephalosporins
Fluoroquinolones
Macrolides
Quinolones
Polymyxins
Aminoglycosides
Aminopenicillins
Aminopenicillins with beta−lactamase inhibitors
Ansamycins
Phosphonic acid derivatives
Shared human and animal antimicrobial classes
Cattle
Chickens
Pigs
Sheep
Turkeys
Ducks
Horses
Buffaloes
Goats
Livestock species
Categories
Not prioritized and no data
Not prioritized with data
Prioritized and no data
Prioritized with data

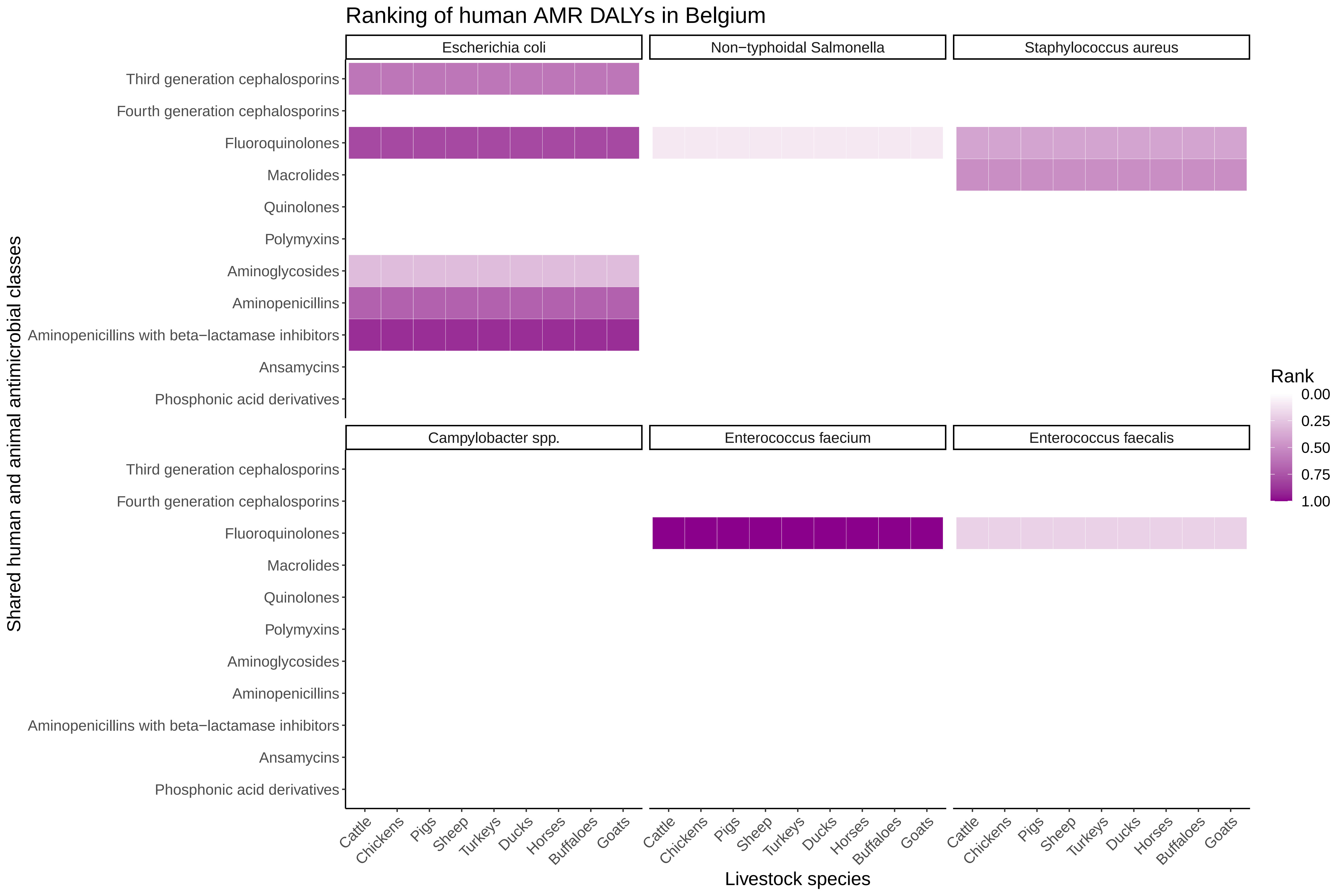

Ranking of human AMR DALYs in Belgium
Escherichia coli
Non−typhoidal Salmonella
Staphylococcus aureus
Campylobacter spp.
Enterococcus faecium
Enterococcus faecalis
Third generation cephalosporins
Fourth generation cephalosporins
Fluoroquinolones
Macrolides
Quinolones
Polymyxins
Aminoglycosides
Aminopenicillins
Aminopenicillins with beta−lactamase inhibitors
Ansamycins
Phosphonic acid derivatives
Shared human and animal antimicrobial classes
Cattle
Chickens
Pigs
Sheep
Turkeys
Ducks
Horses
Buffaloes
Goats
Livestock species
Rank
0.00
0.25
0.50
0.75
1.00

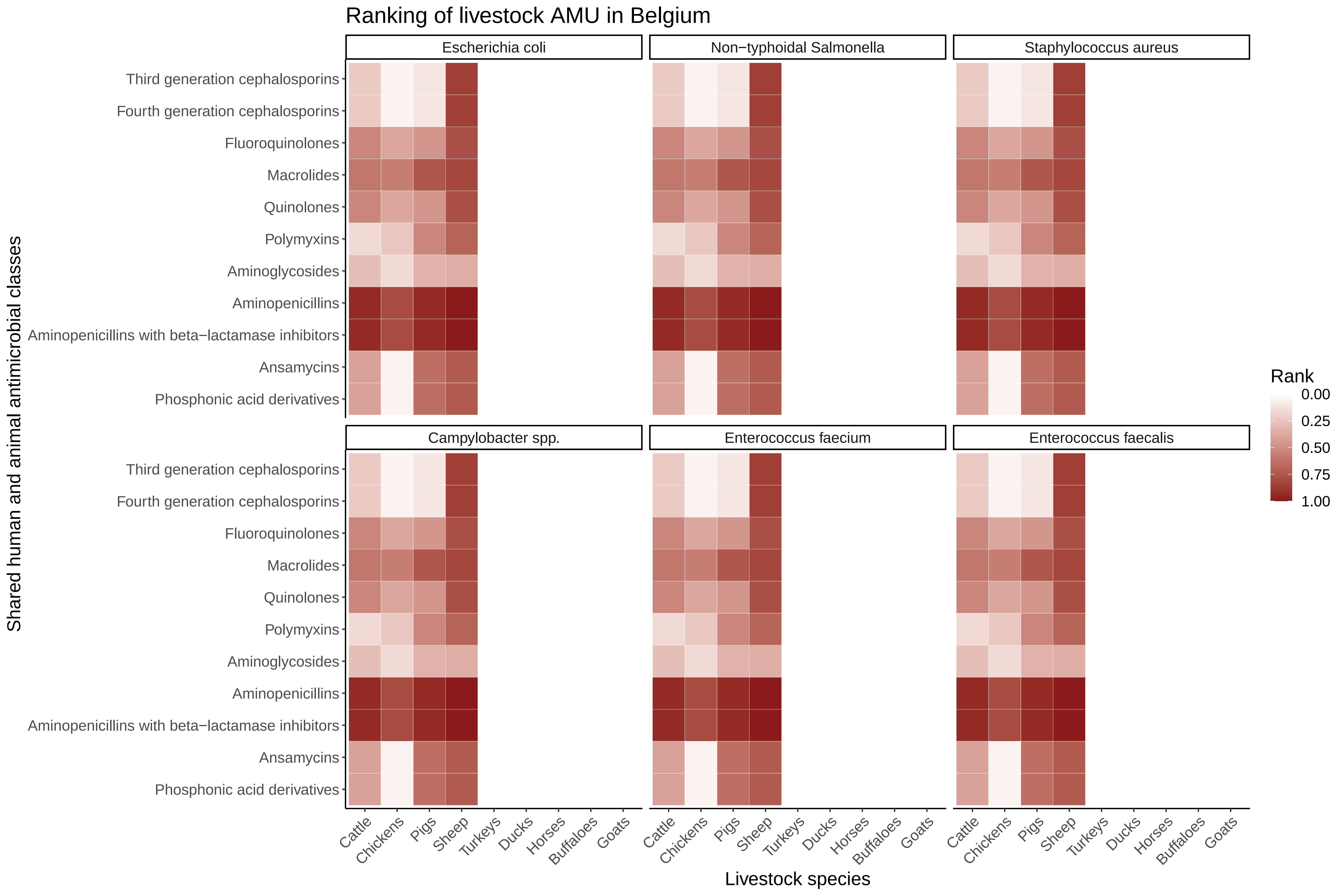
Ranking of livestock AMU in Belgium
Escherichia coli
Non−typhoidal Salmonella
Staphylococcus aureus
Campylobacter spp.
Enterococcus faecium
Enterococcus faecalis
Third generation cephalosporins
Fourth generation cephalosporins
Fluoroquinolones
Macrolides
Quinolones
Polymyxins
Aminoglycosides
Aminopenicillins
Aminopenicillins with beta−lactamase inhibitors
Ansamycins
Phosphonic acid derivatives
Shared human and animal antimicrobial classes
Cattle
Chickens
Pigs
Sheep
Turkeys
Ducks
Horses
Buffaloes
Goats
Livestock species
Rank
0.00
0.25
0.50
0.75
1.00

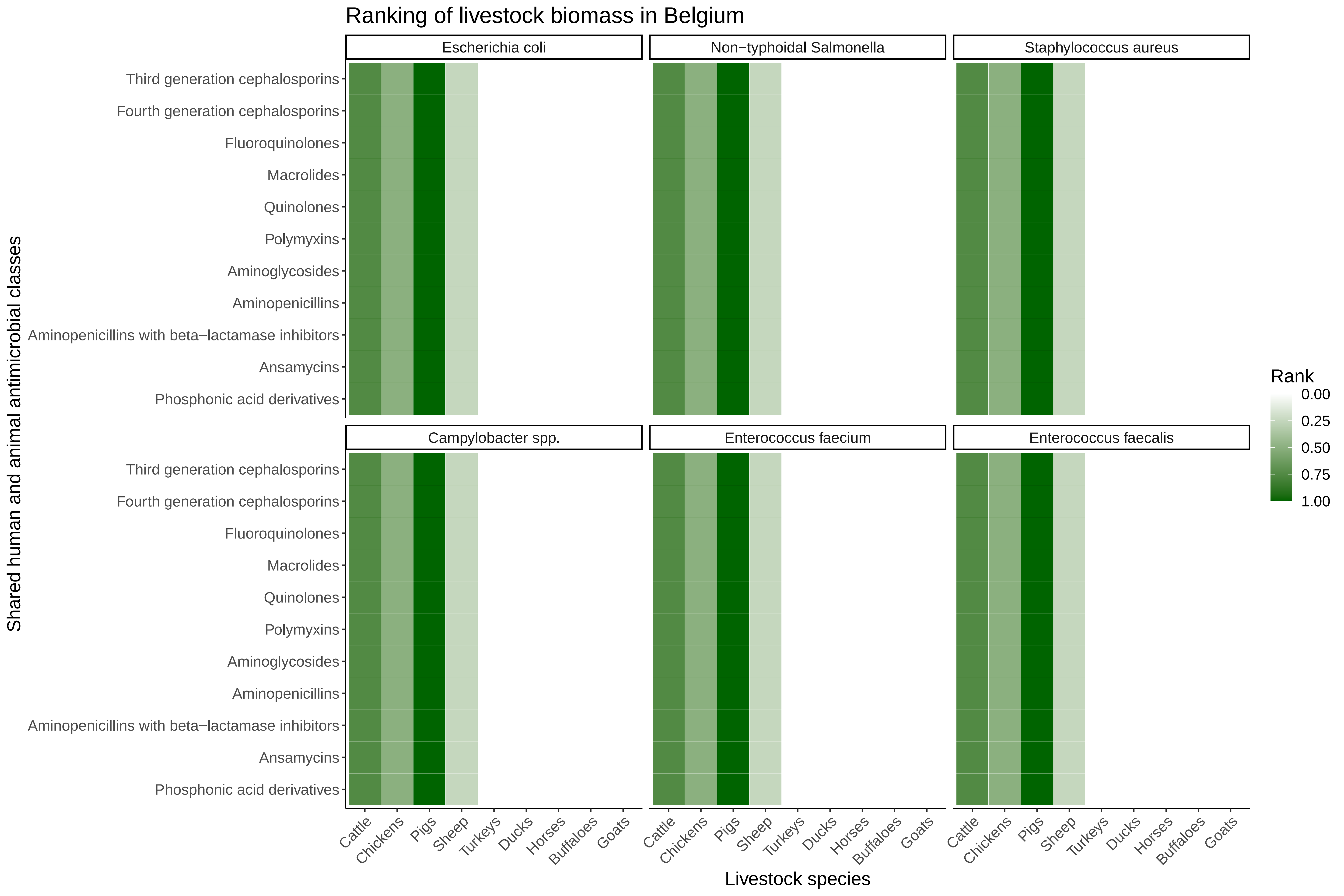

Ranking of livestock biomass in Belgium
Escherichia coli
Non−typhoidal Salmonella
Staphylococcus aureus
Campylobacter spp.
Enterococcus faecium
Enterococcus faecalis
Shared human and animal antimicrobial classes
Third generation cephalosporins
Fourth generation cephalosporins
Fluoroquinolones
Macrolides
Quinolones
Polymyxins
Aminoglycosides
Aminopenicillins
Aminopenicillins with beta−lactamase inhibitors
Ansamycins
Phosphonic acid derivatives
Cattle
Chickens
Pigs
Sheep
Turkeys
Ducks
Horses
Buffaloes
Goats
Livestock species
Rank
0.00
0.25
0.50
0.75
1.00

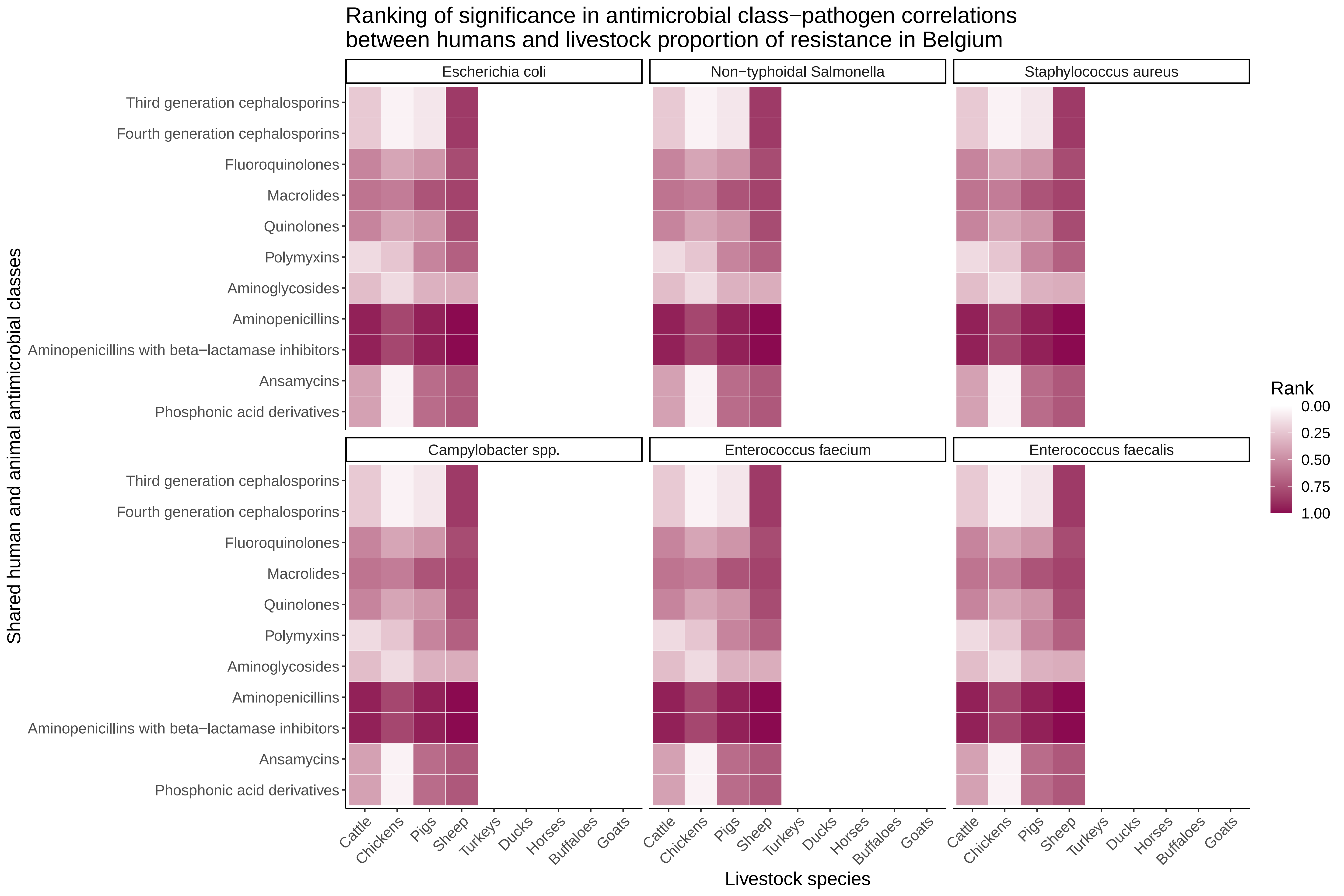

Ranking of significance in antimicrobial class−pathogen correlations
between humans and livestock proportion of resistance in Belgium
Escherichia coli
Non−typhoidal Salmonella
Staphylococcus aureus
Campylobacter spp.
Enterococcus faecium
Enterococcus faecalis
Third generation cephalosporins
Fourth generation cephalosporins
Fluoroquinolones
Macrolides
Quinolones
Polymyxins
Aminoglycosides
Aminopenicillins
Aminopenicillins with beta−lactamase inhibitors
Ansamycins
Phosphonic acid derivatives
Shared human and animal antimicrobial classes
Cattle
Chickens
Pigs
Sheep
Turkeys
Ducks
Horses
Buffaloes
Goats
Livestock species
Rank
0.00
0.25
0.50
0.75
1.00

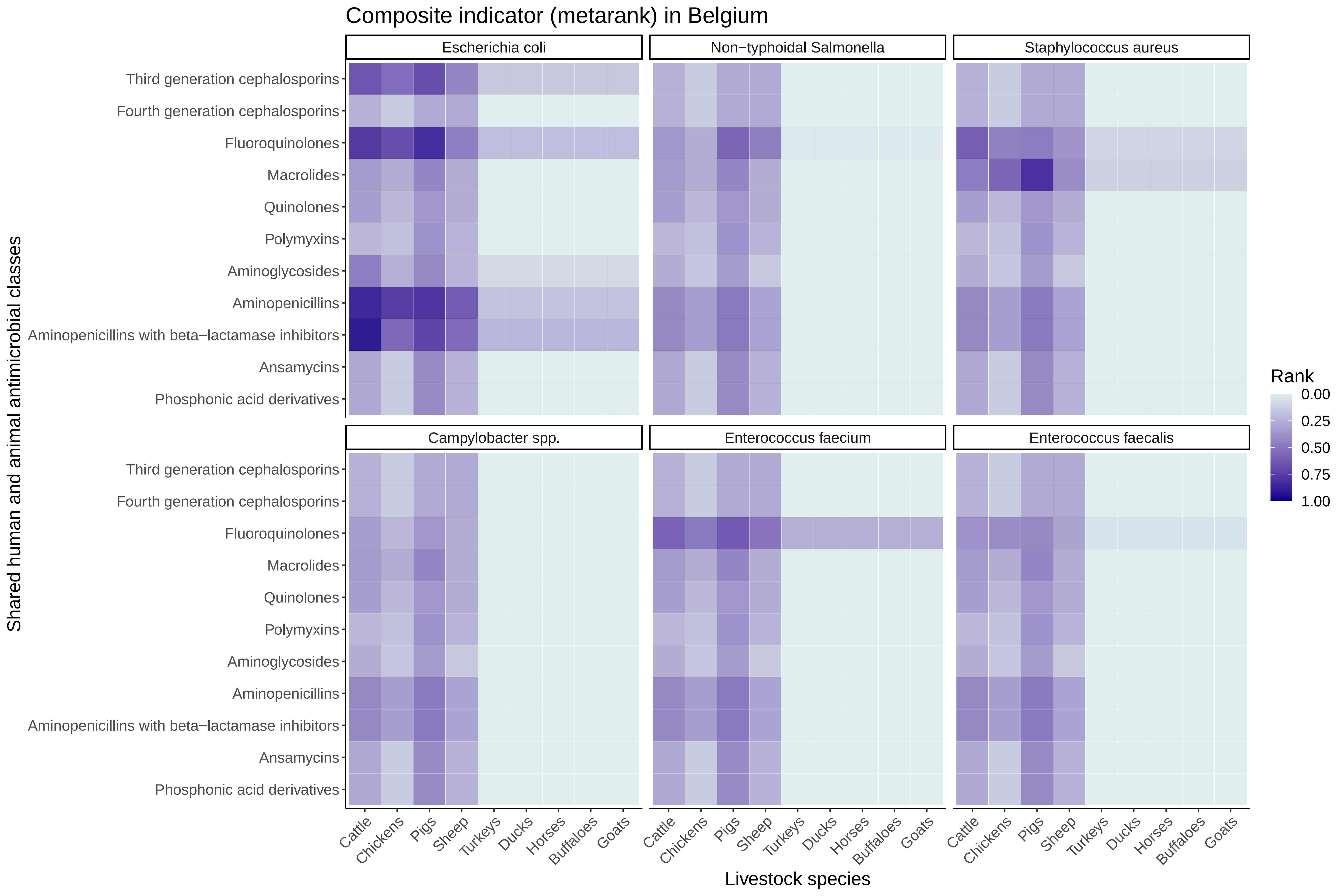

Composite indicator (metarank) in Belgium
Escherichia coli
Non−typhoidal Salmonella
Staphylococcus aureus
Campylobacter spp.
Enterococcus faecium
Enterococcus faecalis
Shared human and animal antimicrobial classes
Third generation cephalosporins
Fourth generation cephalosporins
Fluoroquinolones
Macrolides
Quinolones
Polymyxins
Aminoglycosides
Aminopenicillins
Aminopenicillins with beta−lactamase inhibitors
Ansamycins
Phosphonic acid derivatives
Cattle
Chickens
Pigs
Sheep
Turkeys
Ducks
Horses
Buffaloes
Goats
Livestock species
Rank
0.00
0.25
0.50
0.75
1.00

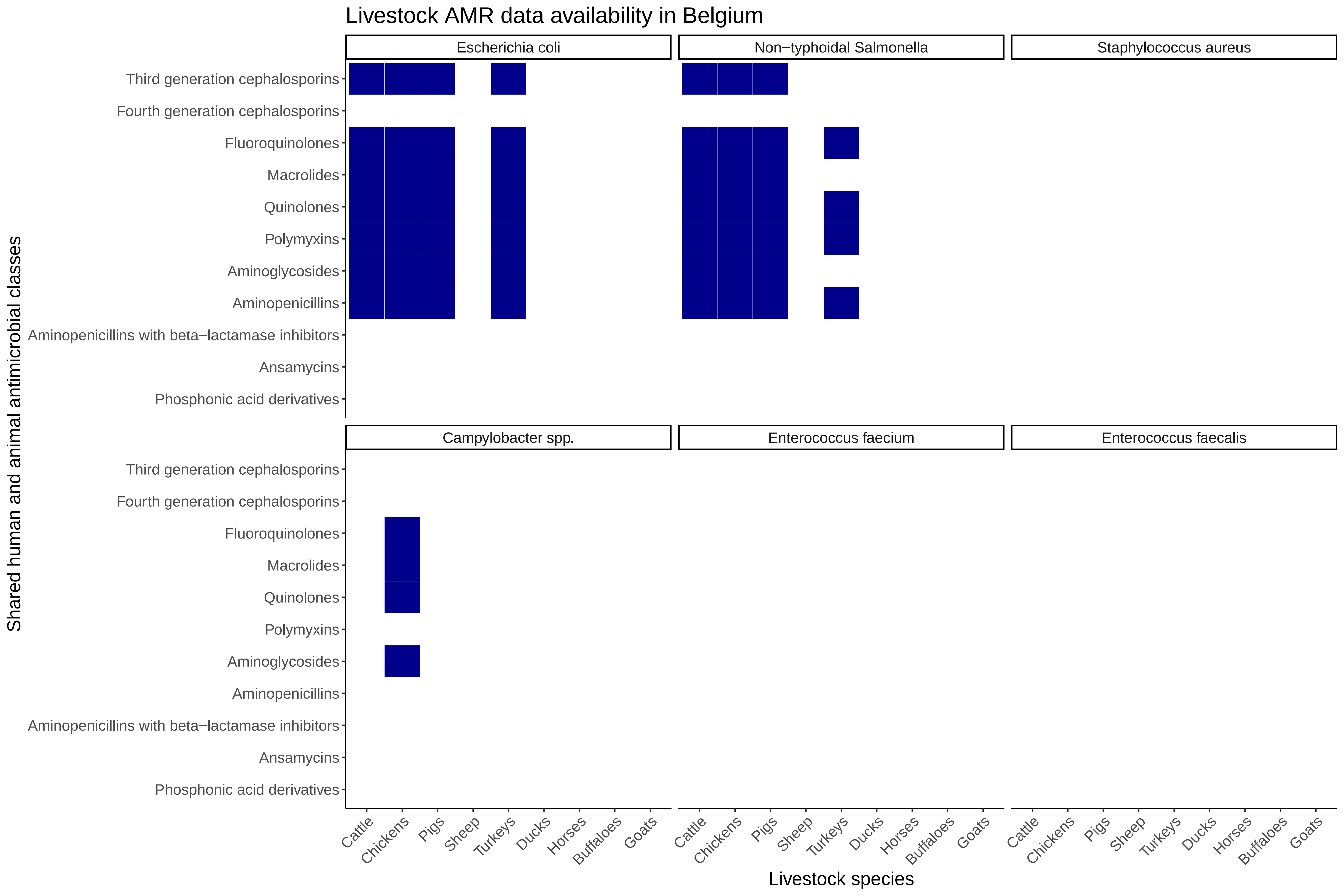
Livestock AMR data availability in Belgium
Escherichia coli
Non−typhoidal Salmonella
Staphylococcus aureus
Campylobacter spp.
Enterococcus faecium
Enterococcus faecalis
Shared human and animal antimicrobial classes
Third generation cephalosporins
Fourth generation cephalosporins
Fluoroquinolones
Macrolides
Quinolones
Polymyxins
Aminoglycosides
Aminopenicillins
Aminopenicillins with beta−lactamase inhibitors
Ansamycins
Phosphonic acid derivatives
Cattle
Chickens
Pigs
Sheep
Turkeys
Ducks
Horses
Buffaloes
Goats
Livestock species

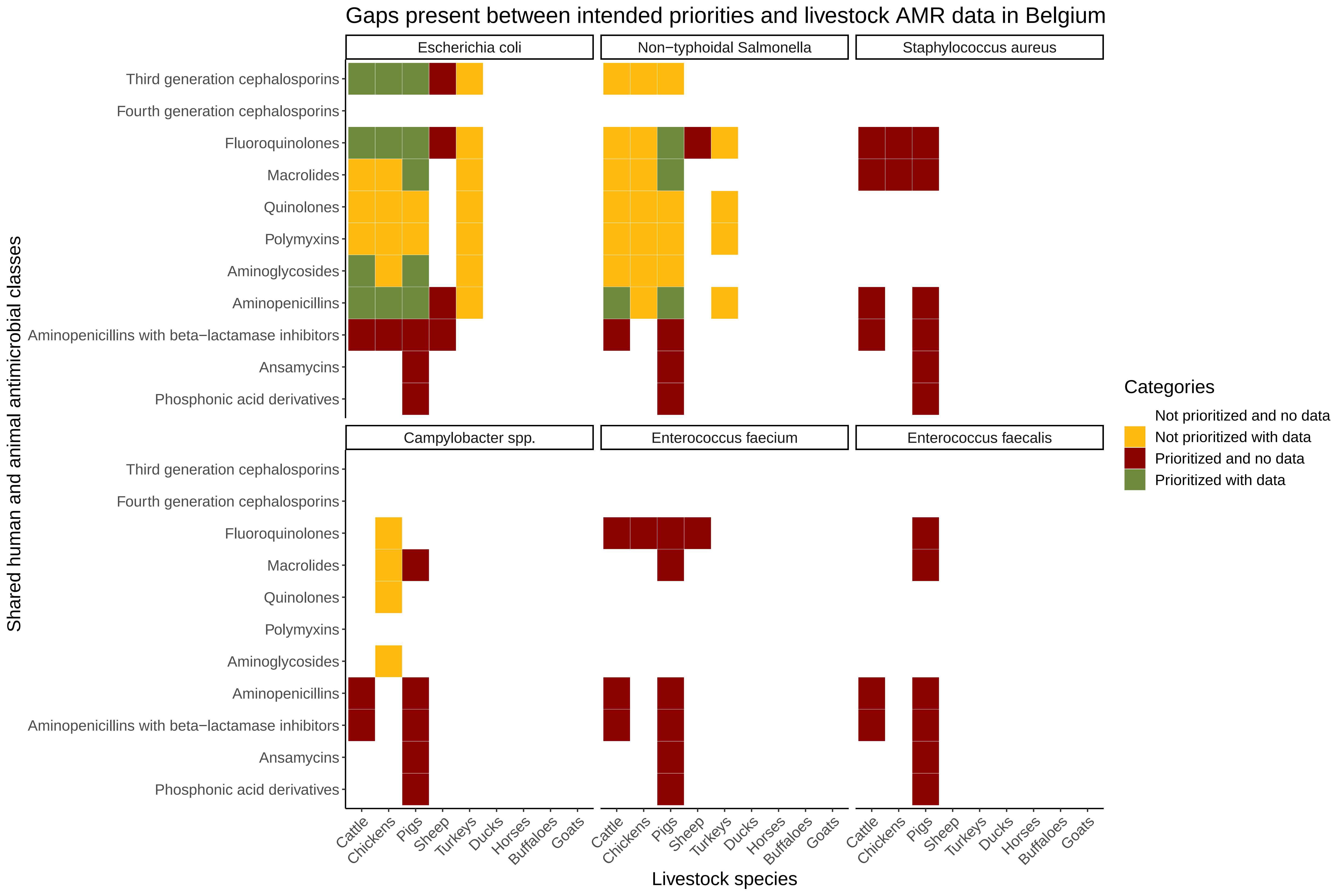

Gaps present between intended priorities and livestock AMR data in Belgium
Escherichia coli
Non−typhoidal Salmonella
Staphylococcus aureus
Campylobacter spp.
Enterococcus faecium
Enterococcus faecalis
Third generation cephalosporins
Fourth generation cephalosporins
Fluoroquinolones
Macrolides
Quinolones
Polymyxins
Aminoglycosides
Aminopenicillins
Aminopenicillins with beta−lactamase inhibitors
Ansamycins
Phosphonic acid derivatives
Shared human and animal antimicrobial classes
Cattle
Chickens
Pigs
Sheep
Turkeys
Ducks
Horses
Buffaloes
Goats
Livestock species
Categories
Not prioritized and no data
Not prioritized with data
Prioritized and no data
Prioritized with data

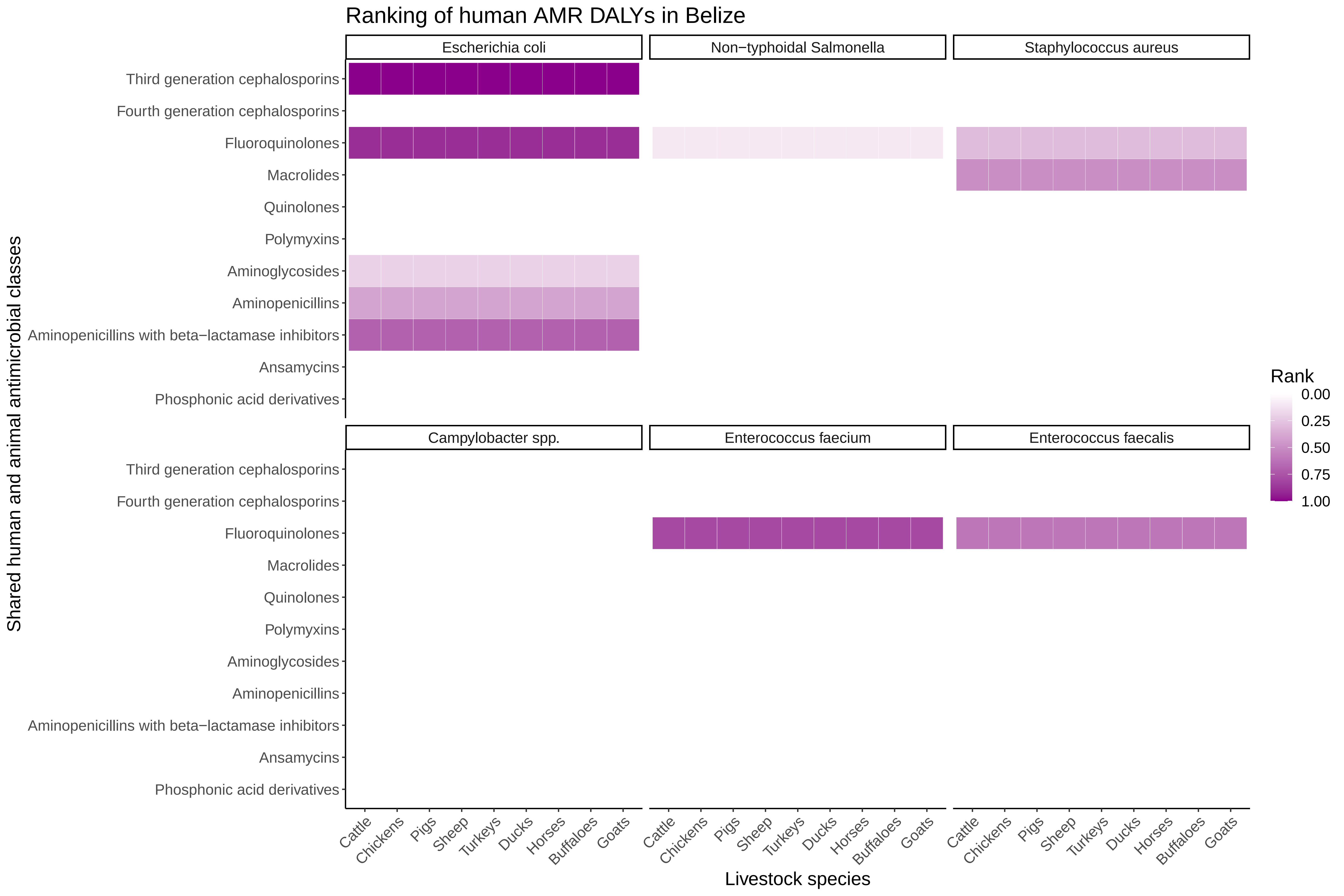

Ranking of human AMR DALYs in Belize
Escherichia coli
Non−typhoidal Salmonella
Staphylococcus aureus
Campylobacter spp.
Enterococcus faecium
Enterococcus faecalis
Third generation cephalosporins
Fourth generation cephalosporins
Fluoroquinolones
Macrolides
Quinolones
Polymyxins
Aminoglycosides
Aminopenicillins
Aminopenicillins with beta−lactamase inhibitors
Ansamycins
Phosphonic acid derivatives
Shared human and animal antimicrobial classes
Cattle
Chickens
Pigs
Sheep
Turkeys
Ducks
Horses
Buffaloes
Goats
Livestock species
Rank
0.00
0.25
0.50
0.75
1.00

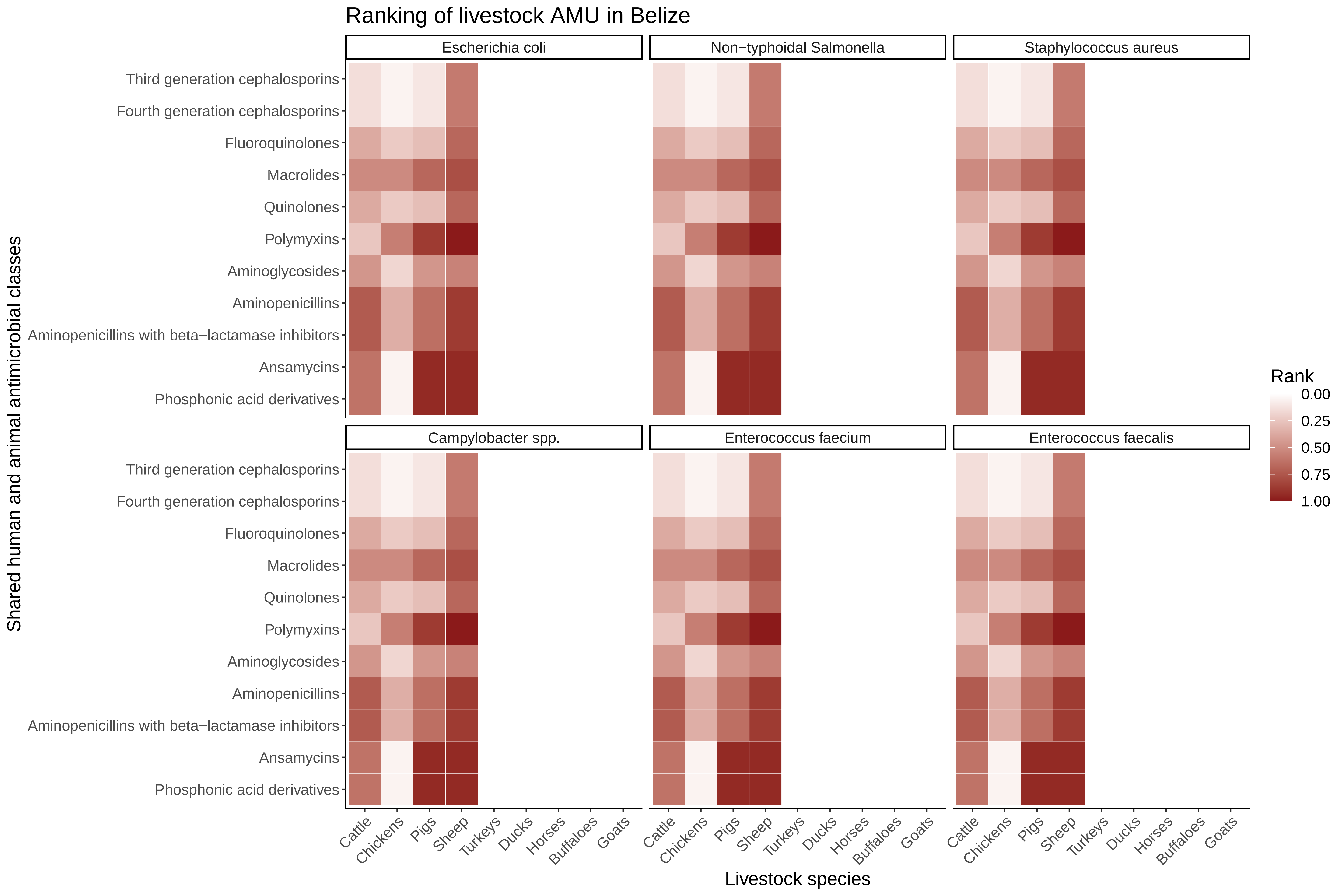

Ranking of livestock AMU in Belize
Escherichia coli
Non−typhoidal Salmonella
Staphylococcus aureus
Campylobacter spp.
Enterococcus faecium
Enterococcus faecalis
Shared human and animal antimicrobial classes
Third generation cephalosporins
Fourth generation cephalosporins
Fluoroquinolones
Macrolides
Quinolones
Polymyxins
Aminoglycosides
Aminopenicillins
Aminopenicillins with beta−lactamase inhibitors
Ansamycins
Phosphonic acid derivatives
Livestock species
Cattle
Chickens
Pigs
Sheep
Turkeys
Ducks
Horses
Buffaloes
Goats
Rank
0.00
0.25
0.50
0.75
1.00

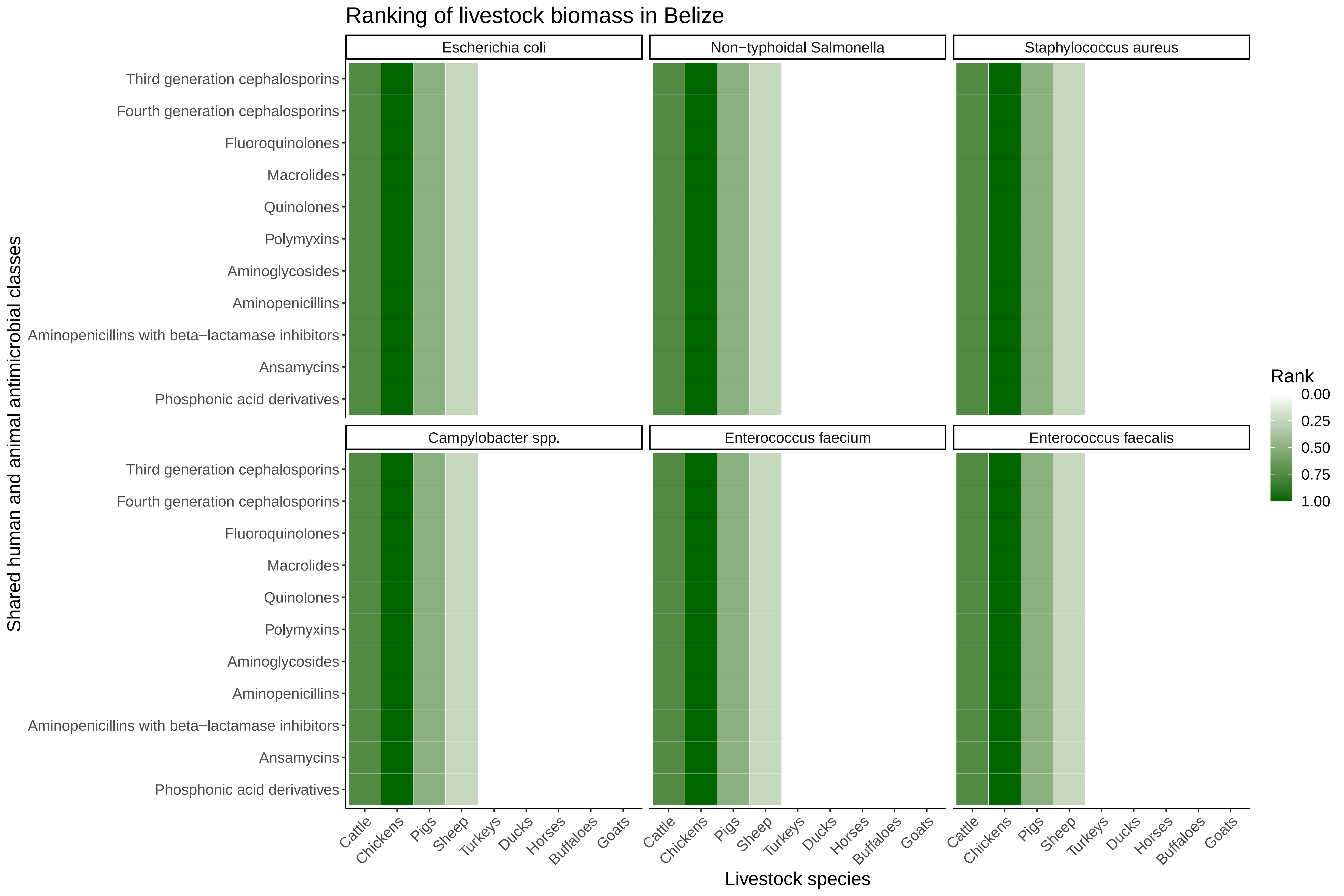
Ranking of livestock biomass in Belize
Escherichia coli
Non−typhoidal Salmonella
Staphylococcus aureus
Campylobacter spp.
Enterococcus faecium
Enterococcus faecalis
Third generation cephalosporins
Fourth generation cephalosporins
Fluoroquinolones
Macrolides
Quinolones
Polymyxins
Aminoglycosides
Aminopenicillins
Aminopenicillins with beta−lactamase inhibitors
Ansamycins
Phosphonic acid derivatives
Shared human and animal antimicrobial classes
Cattle
Chickens
Pigs
Sheep
Turkeys
Ducks
Horses
Buffaloes
Goats
Livestock species
Rank
0.00
0.25
0.50
0.75
1.00

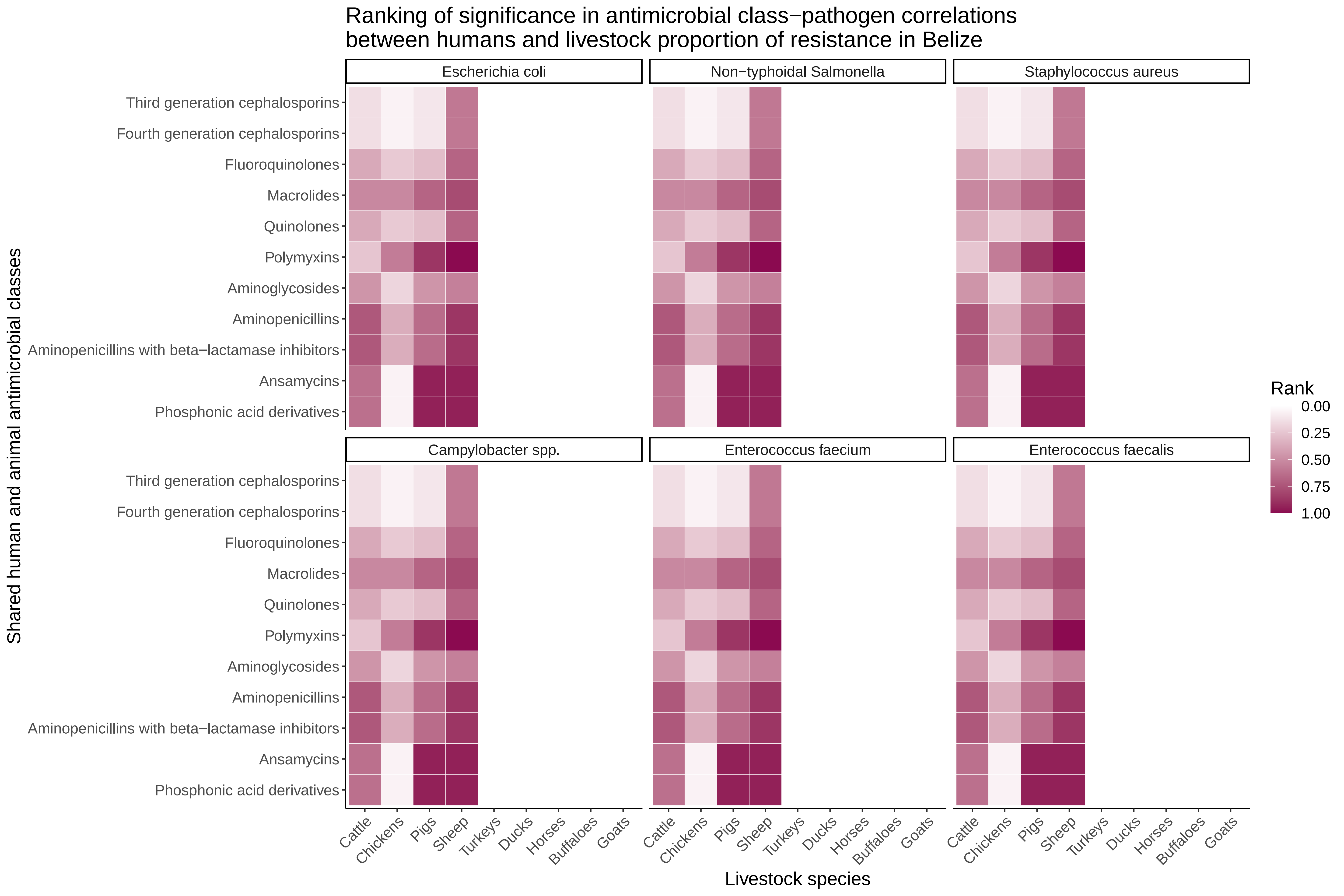
Ranking of significance in antimicrobial class−pathogen correlations
between humans and livestock proportion of resistance in Belize
Escherichia coli
Non−typhoidal Salmonella
Staphylococcus aureus
Campylobacter spp.
Enterococcus faecium
Enterococcus faecalis
Third generation cephalosporins
Fourth generation cephalosporins
Fluoroquinolones
Macrolides
Quinolones
Polymyxins
Aminoglycosides
Aminopenicillins
Aminopenicillins with beta−lactamase inhibitors
Ansamycins
Phosphonic acid derivatives
Shared human and animal antimicrobial classes
Cattle
Chickens
Pigs
Sheep
Turkeys
Ducks
Horses
Buffaloes
Goats
Livestock species
Rank
0.00
0.25
0.50
0.75
1.00

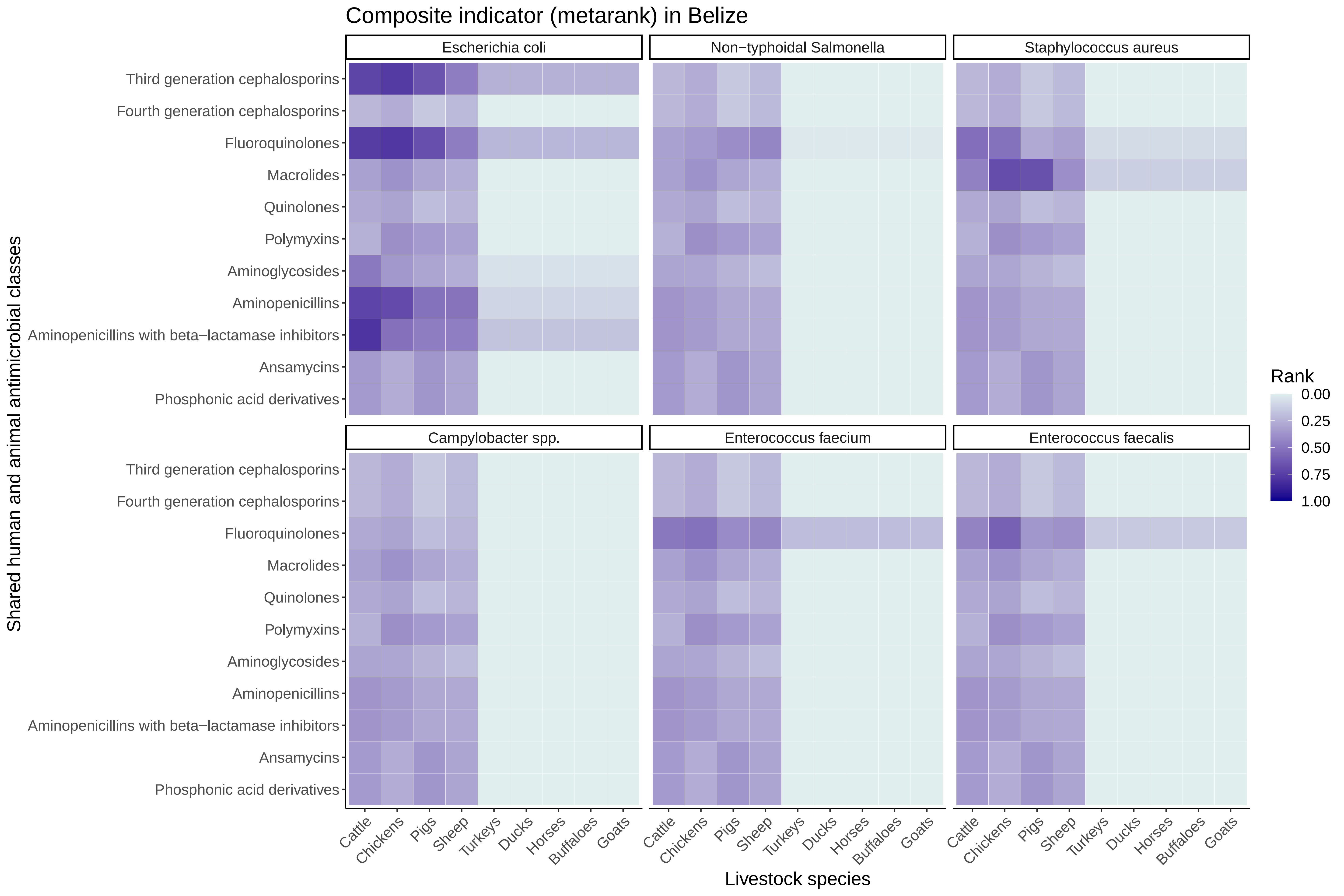

Composite indicator (metarank) in Belize
Escherichia coli
Non−typhoidal Salmonella
Staphylococcus aureus
Campylobacter spp.
Enterococcus faecium
Enterococcus faecalis
Shared human and animal antimicrobial classes
Third generation cephalosporins
Fourth generation cephalosporins
Fluoroquinolones
Macrolides
Quinolones
Polymyxins
Aminoglycosides
Aminopenicillins
Aminopenicillins with beta−lactamase inhibitors
Ansamycins
Phosphonic acid derivatives
Cattle
Chickens
Pigs
Sheep
Turkeys
Ducks
Horses
Buffaloes
Goats
Livestock species
Rank
0.00
0.25
0.50
0.75
1.00

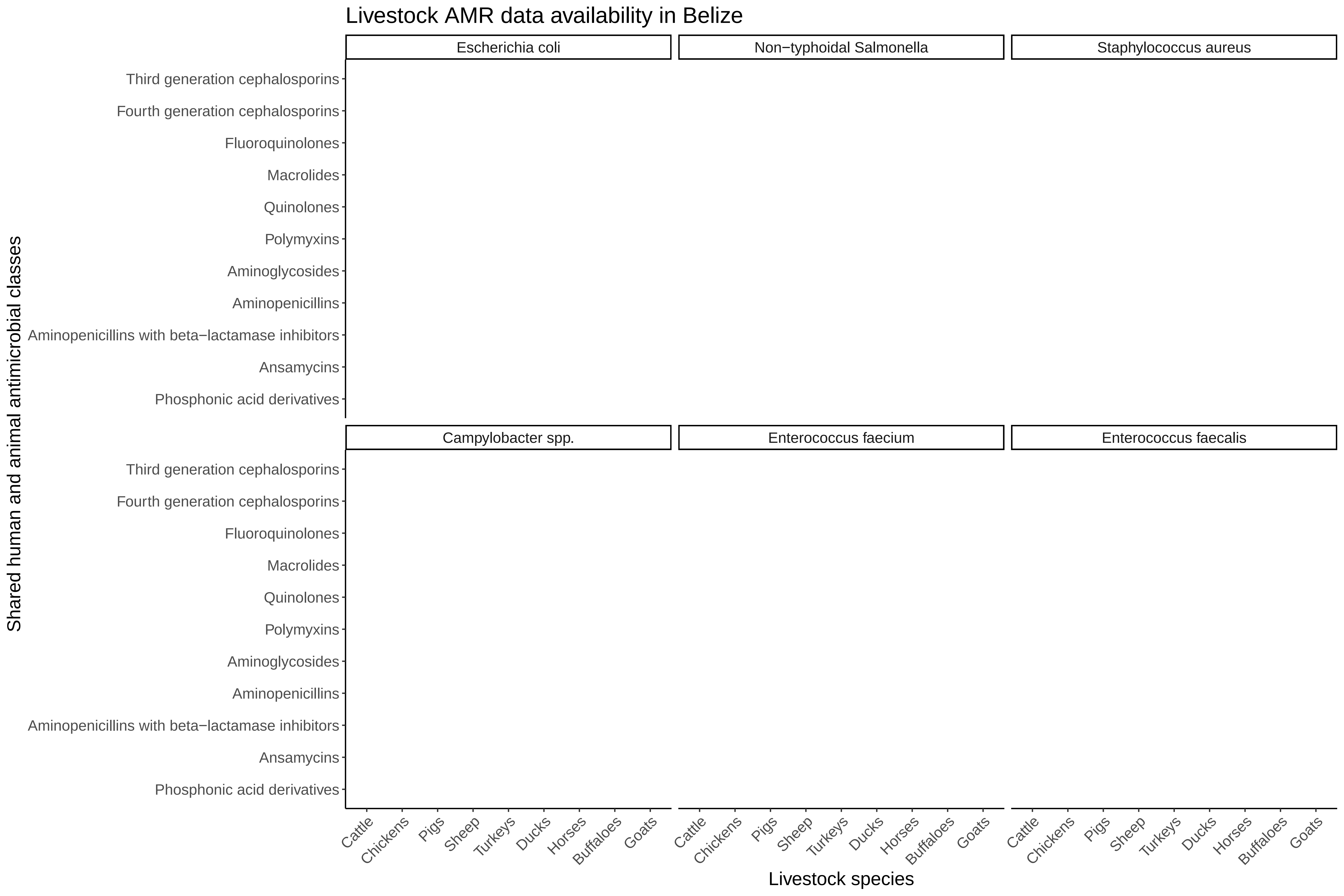
Livestock AMR data availability in Belize
Escherichia coli
Non−typhoidal Salmonella
Staphylococcus aureus
Campylobacter spp.
Enterococcus faecium
Enterococcus faecalis
Shared human and animal antimicrobial classes
Third generation cephalosporins
Fourth generation cephalosporins
Fluoroquinolones
Macrolides
Quinolones
Polymyxins
Aminoglycosides
Aminopenicillins
Aminopenicillins with beta−lactamase inhibitors
Ansamycins
Phosphonic acid derivatives
Cattle
Chickens
Pigs
Sheep
Turkeys
Ducks
Horses
Buffaloes
Goats
Livestock species

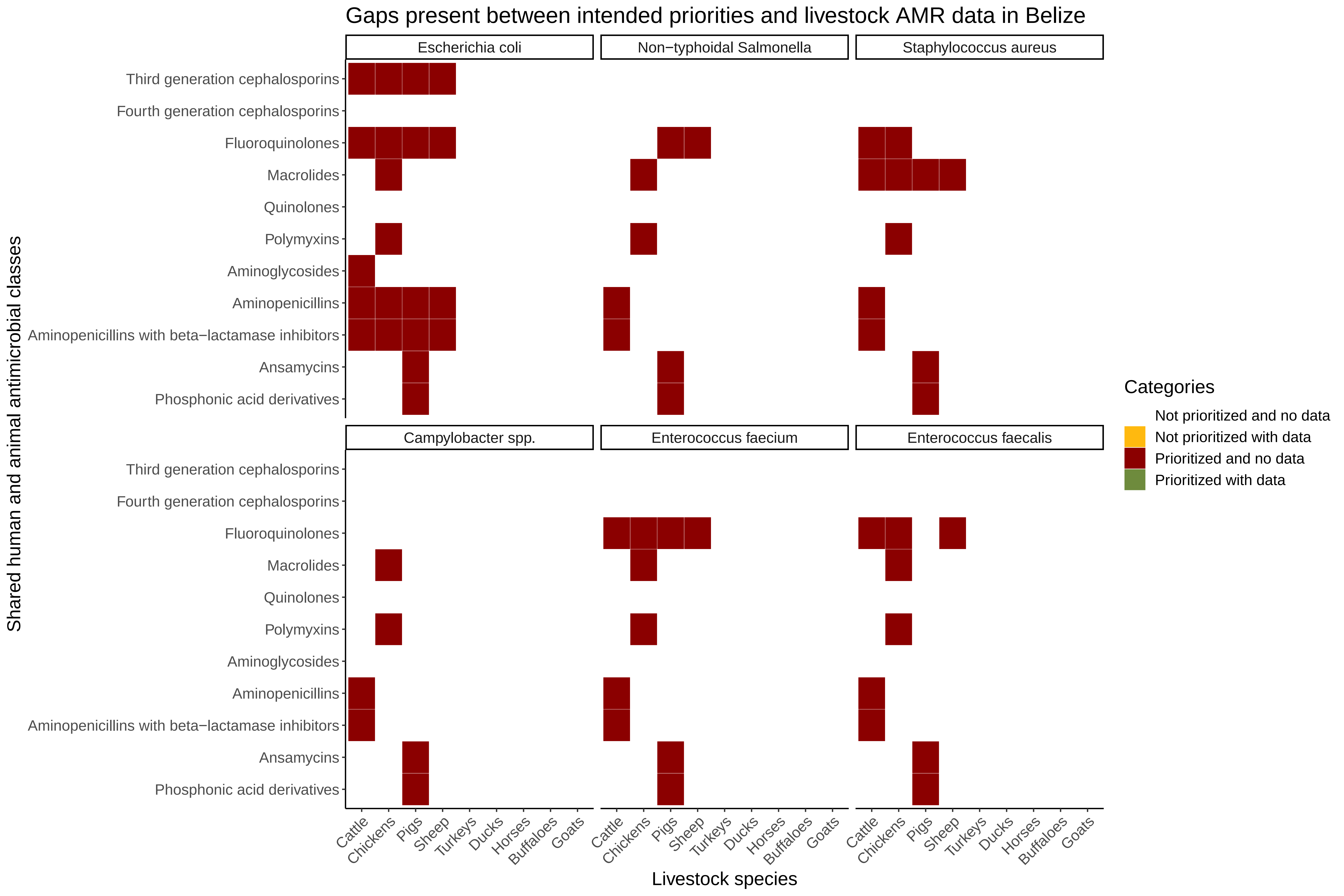

Gaps present between intended priorities and livestock AMR data in Belize
Escherichia coli
Non−typhoidal Salmonella
Staphylococcus aureus
Campylobacter spp.
Enterococcus faecium
Enterococcus faecalis
Third generation cephalosporins
Fourth generation cephalosporins
Fluoroquinolones
Macrolides
Quinolones
Polymyxins
Aminoglycosides
Aminopenicillins
Aminopenicillins with beta−lactamase inhibitors
Ansamycins
Phosphonic acid derivatives
Shared human and animal antimicrobial classes
Cattle
Chickens
Pigs
Sheep
Turkeys
Ducks
Horses
Buffaloes
Goats
Livestock species
Categories
Not prioritized and no data
Not prioritized with data
Prioritized and no data
Prioritized with data

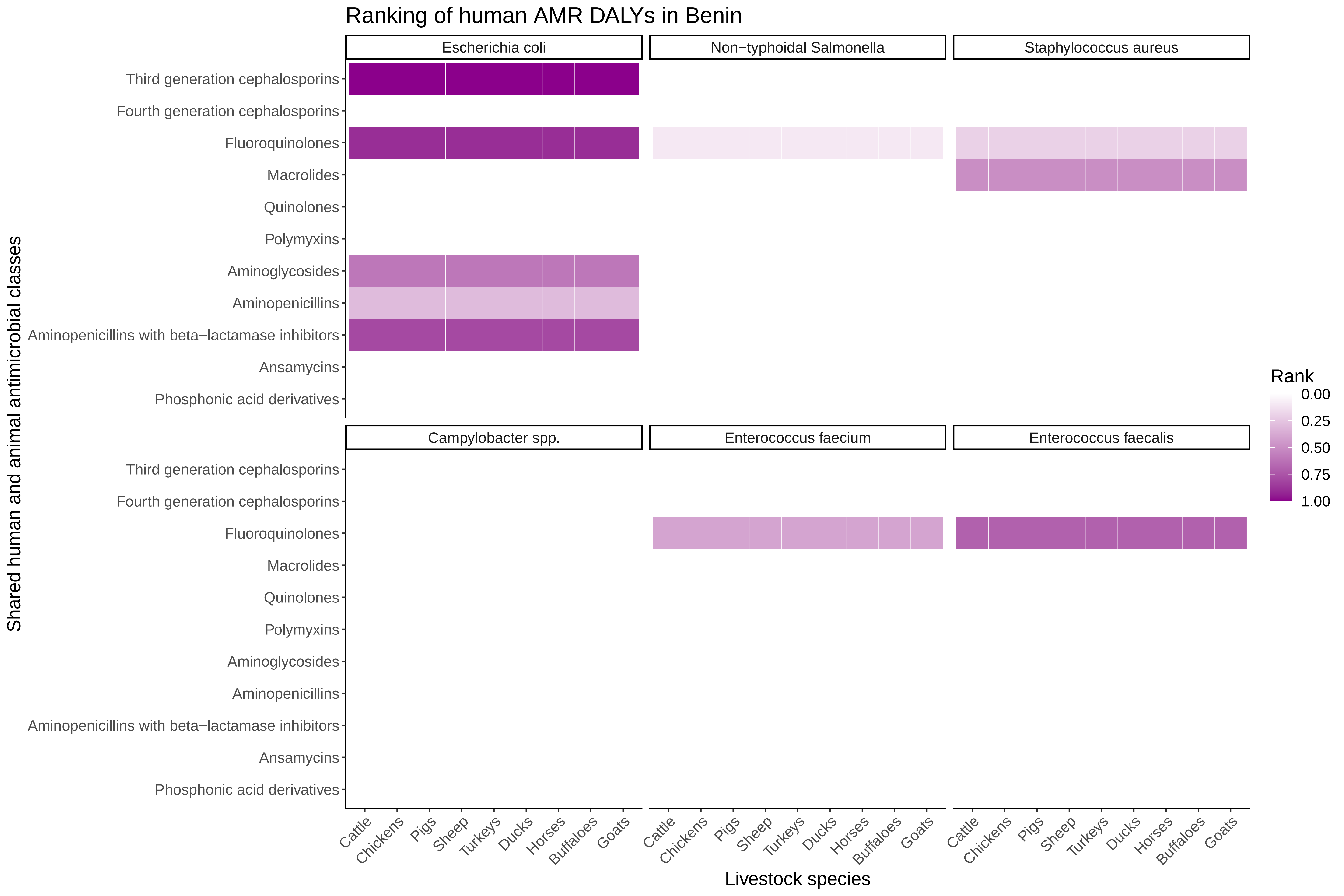
Ranking of human AMR DALYs in Benin
Escherichia coli
Non−typhoidal Salmonella
Staphylococcus aureus
Campylobacter spp.
Enterococcus faecium
Enterococcus faecalis
Shared human and animal antimicrobial classes
Third generation cephalosporins
Fourth generation cephalosporins
Fluoroquinolones
Macrolides
Quinolones
Polymyxins
Aminoglycosides
Aminopenicillins
Aminopenicillins with beta−lactamase inhibitors
Ansamycins
Phosphonic acid derivatives
Cattle
Chickens
Pigs
Sheep
Turkeys
Ducks
Horses
Buffaloes
Goats
Livestock species
Rank
0.00
0.25
0.50
0.75
1.00

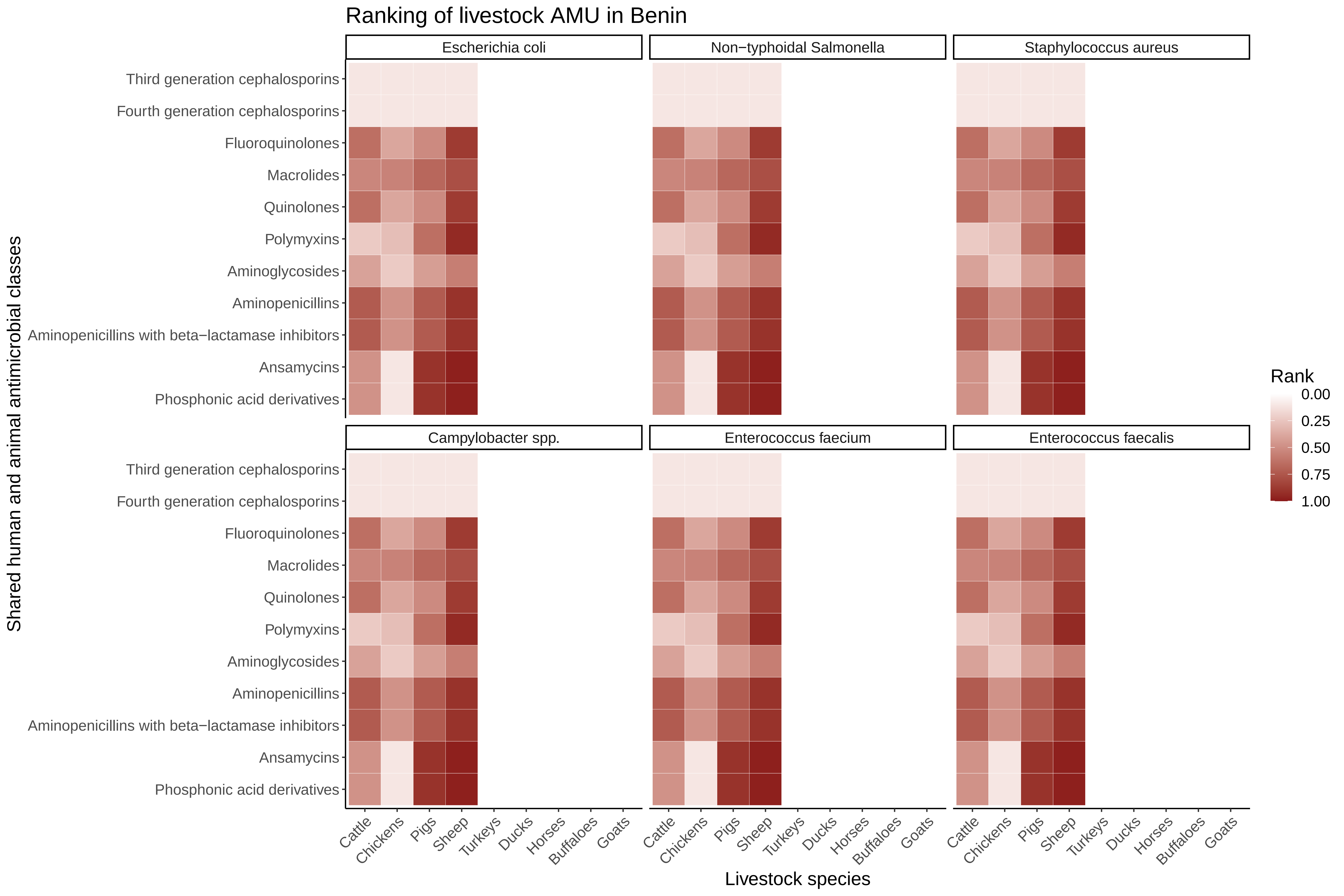

Ranking of livestock AMU in Benin
Escherichia coli
Non−typhoidal Salmonella
Staphylococcus aureus
Campylobacter spp.
Enterococcus faecium
Enterococcus faecalis
Third generation cephalosporins
Fourth generation cephalosporins
Fluoroquinolones
Macrolides
Quinolones
Polymyxins
Aminoglycosides
Aminopenicillins
Aminopenicillins with beta−lactamase inhibitors
Ansamycins
Phosphonic acid derivatives
Shared human and animal antimicrobial classes
Cattle
Chickens
Pigs
Sheep
Turkeys
Ducks
Horses
Buffaloes
Goats
Livestock species
Rank
0.00
0.25
0.50
0.75
1.00

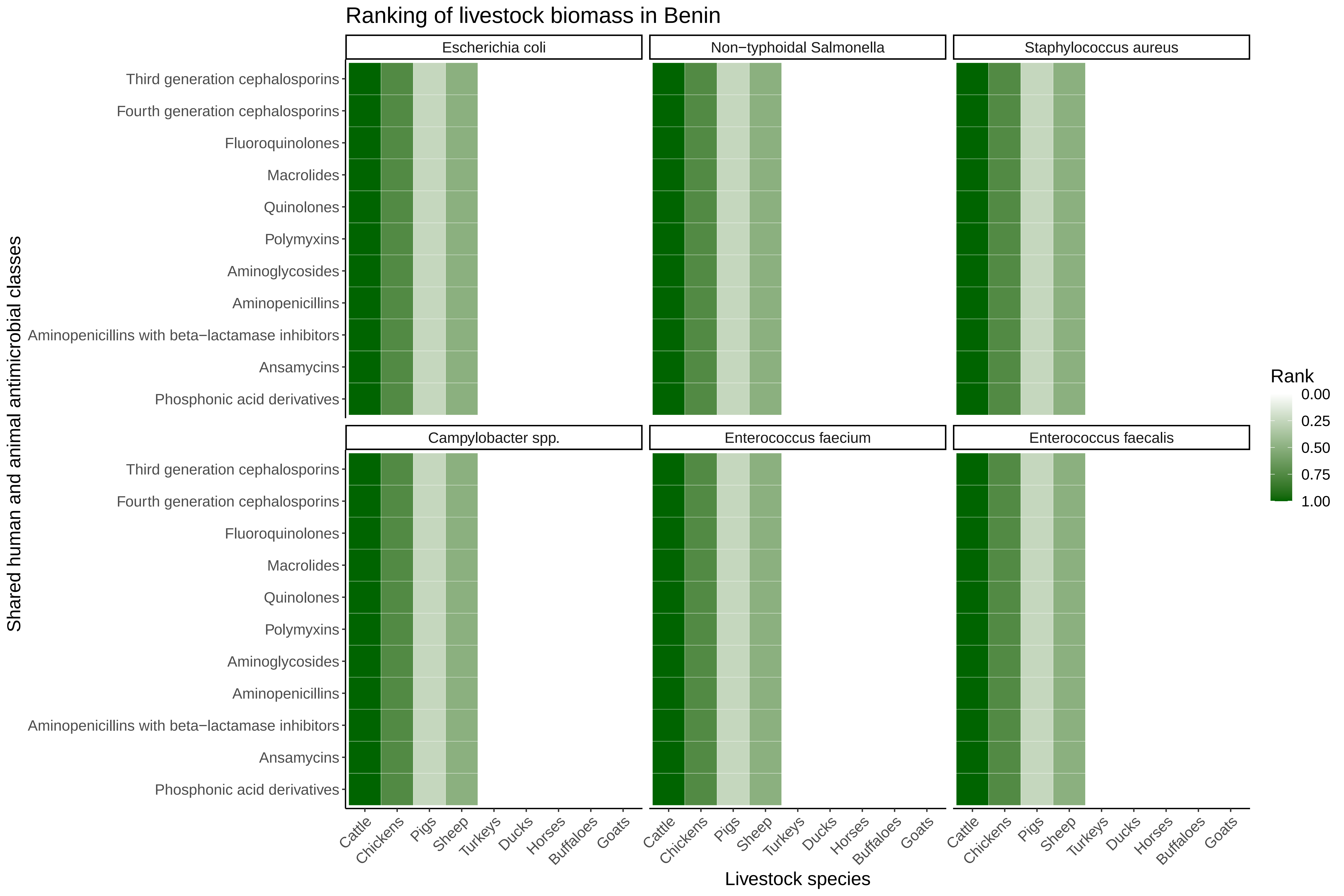

Ranking of livestock biomass in Benin
Escherichia coli
Non−typhoidal Salmonella
Staphylococcus aureus
Campylobacter spp.
Enterococcus faecium
Enterococcus faecalis
Third generation cephalosporins
Fourth generation cephalosporins
Fluoroquinolones
Macrolides
Quinolones
Polymyxins
Aminoglycosides
Aminopenicillins
Aminopenicillins with beta−lactamase inhibitors
Ansamycins
Phosphonic acid derivatives
Shared human and animal antimicrobial classes
Cattle
Chickens
Pigs
Sheep
Turkeys
Ducks
Horses
Buffaloes
Goats
Livestock species
Rank
0.00
0.25
0.50
0.75
1.00

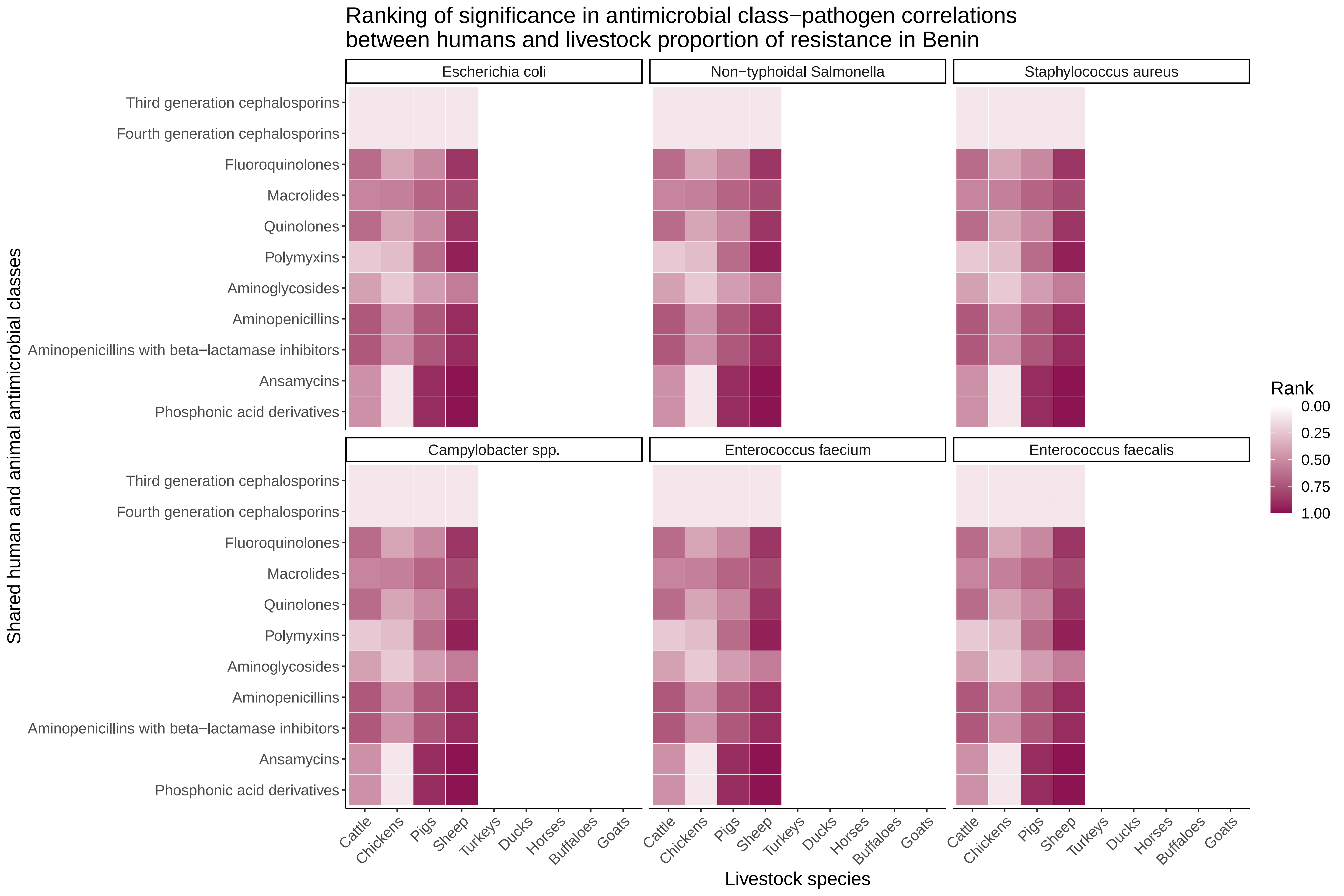
Ranking of significance in antimicrobial class−pathogen correlations
between humans and livestock proportion of resistance in Benin
Escherichia coli
Non−typhoidal Salmonella
Staphylococcus aureus
Campylobacter spp.
Enterococcus faecium
Enterococcus faecalis
Third generation cephalosporins
Fourth generation cephalosporins
Fluoroquinolones
Macrolides
Quinolones
Polymyxins
Aminoglycosides
Aminopenicillins
Aminopenicillins with beta−lactamase inhibitors
Ansamycins
Phosphonic acid derivatives
Shared human and animal antimicrobial classes
Cattle
Chickens
Pigs
Sheep
Turkeys
Ducks
Horses
Buffaloes
Goats
Livestock species
Rank
0.00
0.25
0.50
0.75
1.00

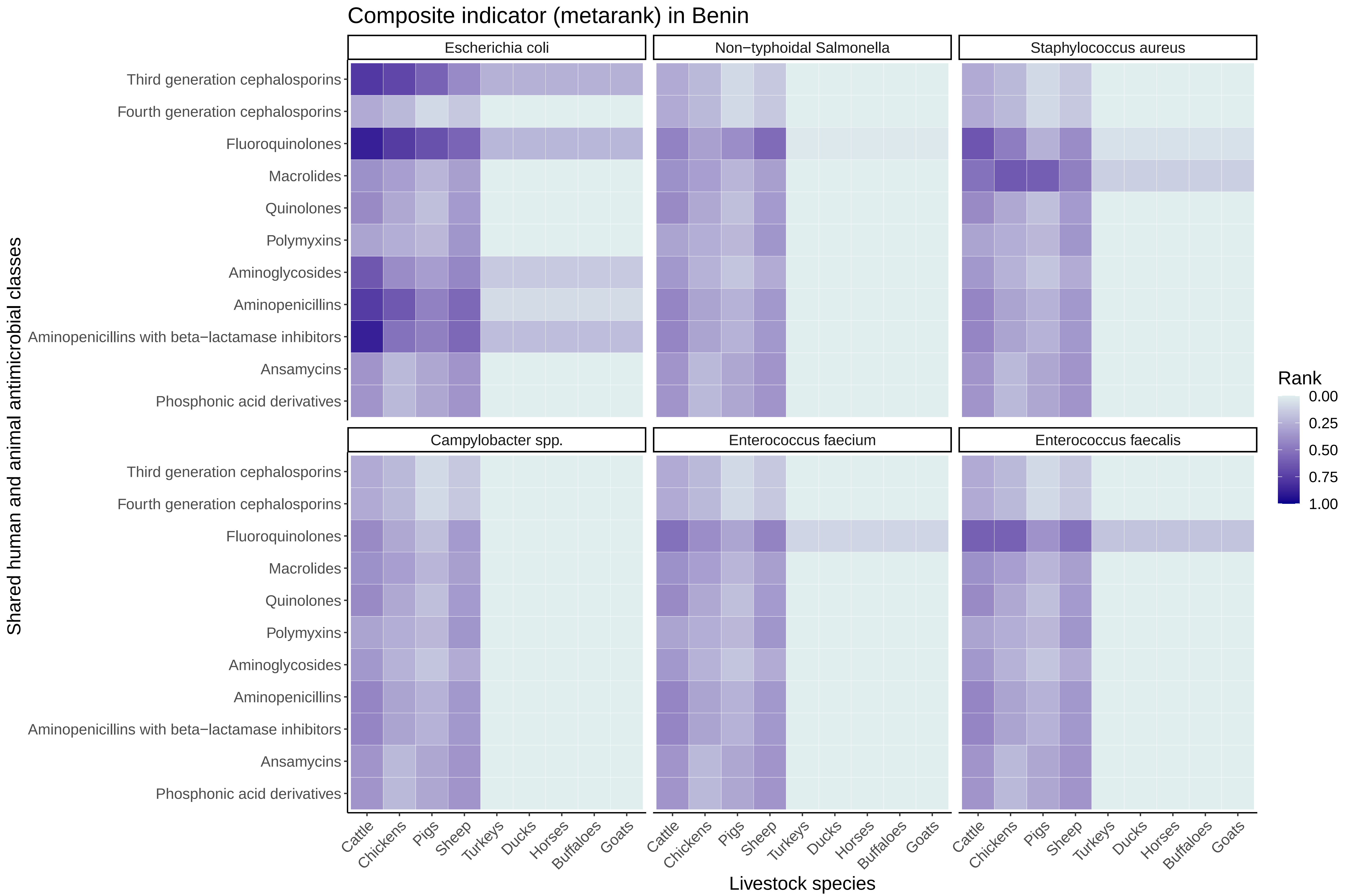
Composite indicator (metarank) in Benin
Escherichia coli
Non−typhoidal Salmonella
Staphylococcus aureus
Campylobacter spp.
Enterococcus faecium
Enterococcus faecalis
Shared human and animal antimicrobial classes
Third generation cephalosporins
Fourth generation cephalosporins
Fluoroquinolones
Macrolides
Quinolones
Polymyxins
Aminoglycosides
Aminopenicillins
Aminopenicillins with beta−lactamase inhibitors
Ansamycins
Phosphonic acid derivatives
Livestock species
Cattle
Chickens
Pigs
Sheep
Turkeys
Ducks
Horses
Buffaloes
Goats
Rank
0.00
0.25
0.50
0.75
1.00

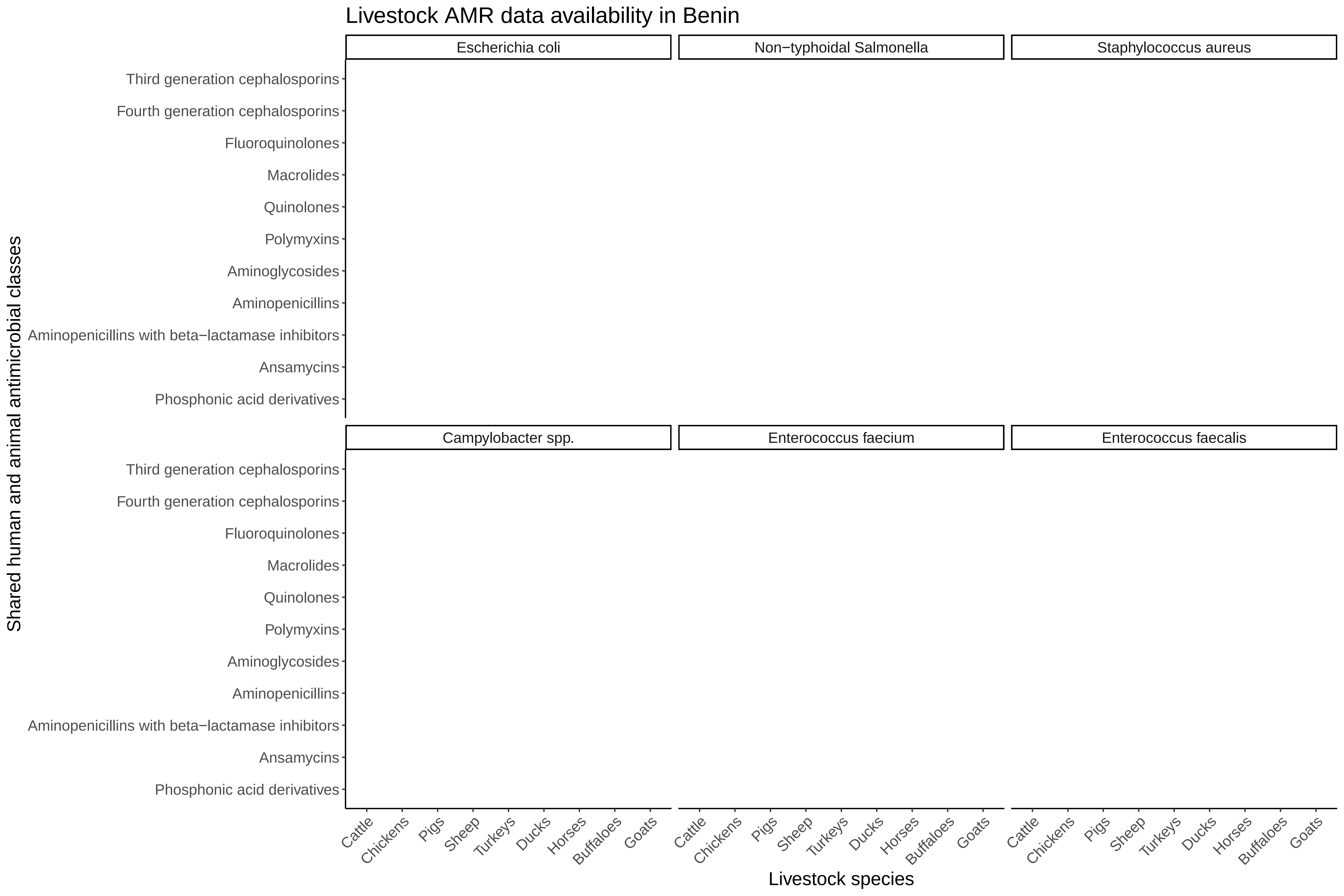
Livestock AMR data availability in Benin
Escherichia coli
Non−typhoidal Salmonella
Staphylococcus aureus
Campylobacter spp.
Enterococcus faecium
Enterococcus faecalis
Shared human and animal antimicrobial classes
Third generation cephalosporins
Fourth generation cephalosporins
Fluoroquinolones
Macrolides
Quinolones
Polymyxins
Aminoglycosides
Aminopenicillins
Aminopenicillins with beta−lactamase inhibitors
Ansamycins
Phosphonic acid derivatives
Cattle
Chickens
Pigs
Sheep
Turkeys
Ducks
Horses
Buffaloes
Goats
Livestock species

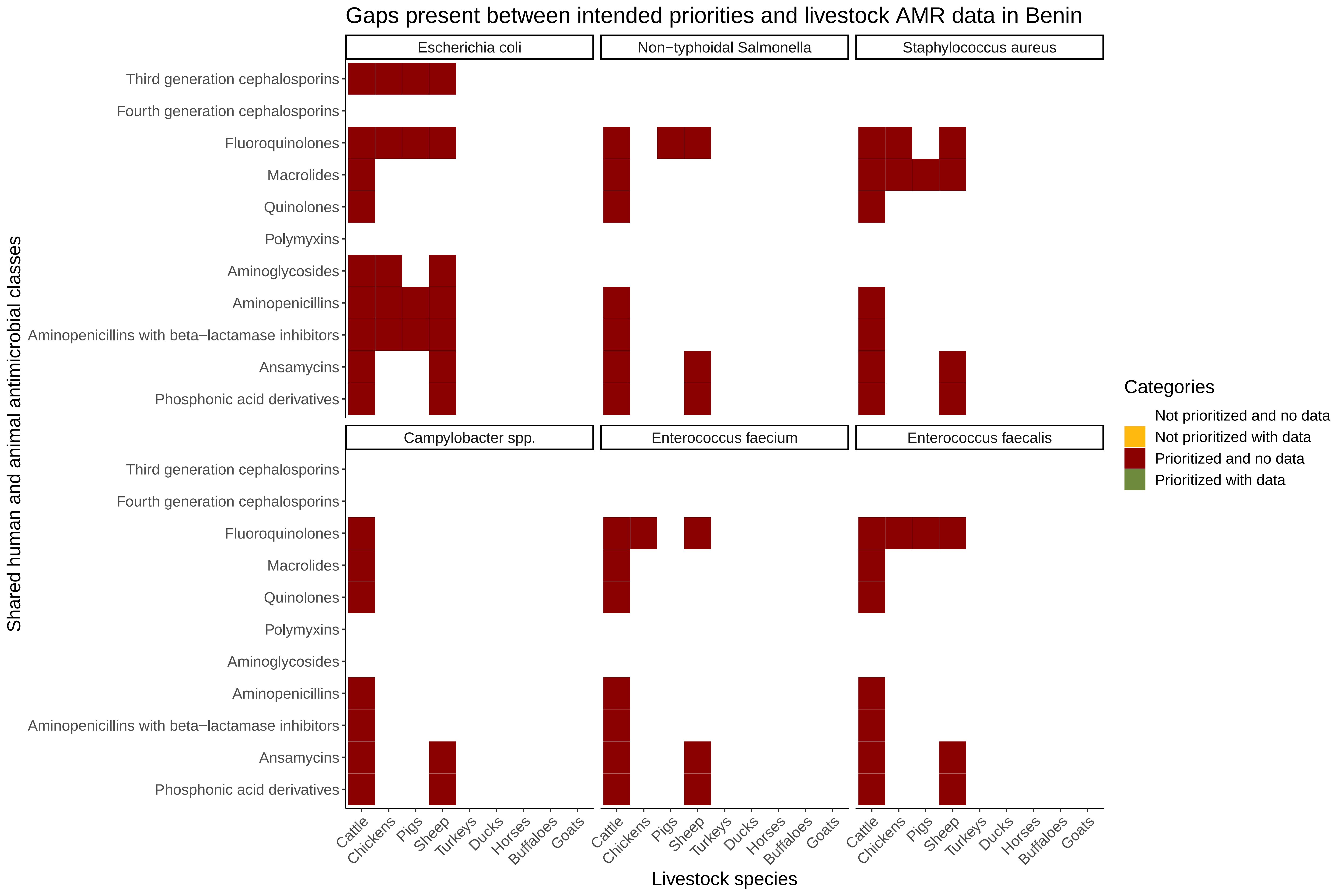
Gaps present between intended priorities and livestock AMR data in Benin
Escherichia coli
Non−typhoidal Salmonella
Staphylococcus aureus
Campylobacter spp.
Enterococcus faecium
Enterococcus faecalis
Shared human and animal antimicrobial classes
Third generation cephalosporins
Fourth generation cephalosporins
Fluoroquinolones
Macrolides
Quinolones
Polymyxins
Aminoglycosides
Aminopenicillins
Aminopenicillins with beta−lactamase inhibitors
Ansamycins
Phosphonic acid derivatives
Livestock species
Cattle
Chickens
Pigs
Sheep
Turkeys
Ducks
Horses
Buffaloes
Goats
Categories
Not prioritized and no data
Not prioritized with data
Prioritized and no data
Prioritized with data

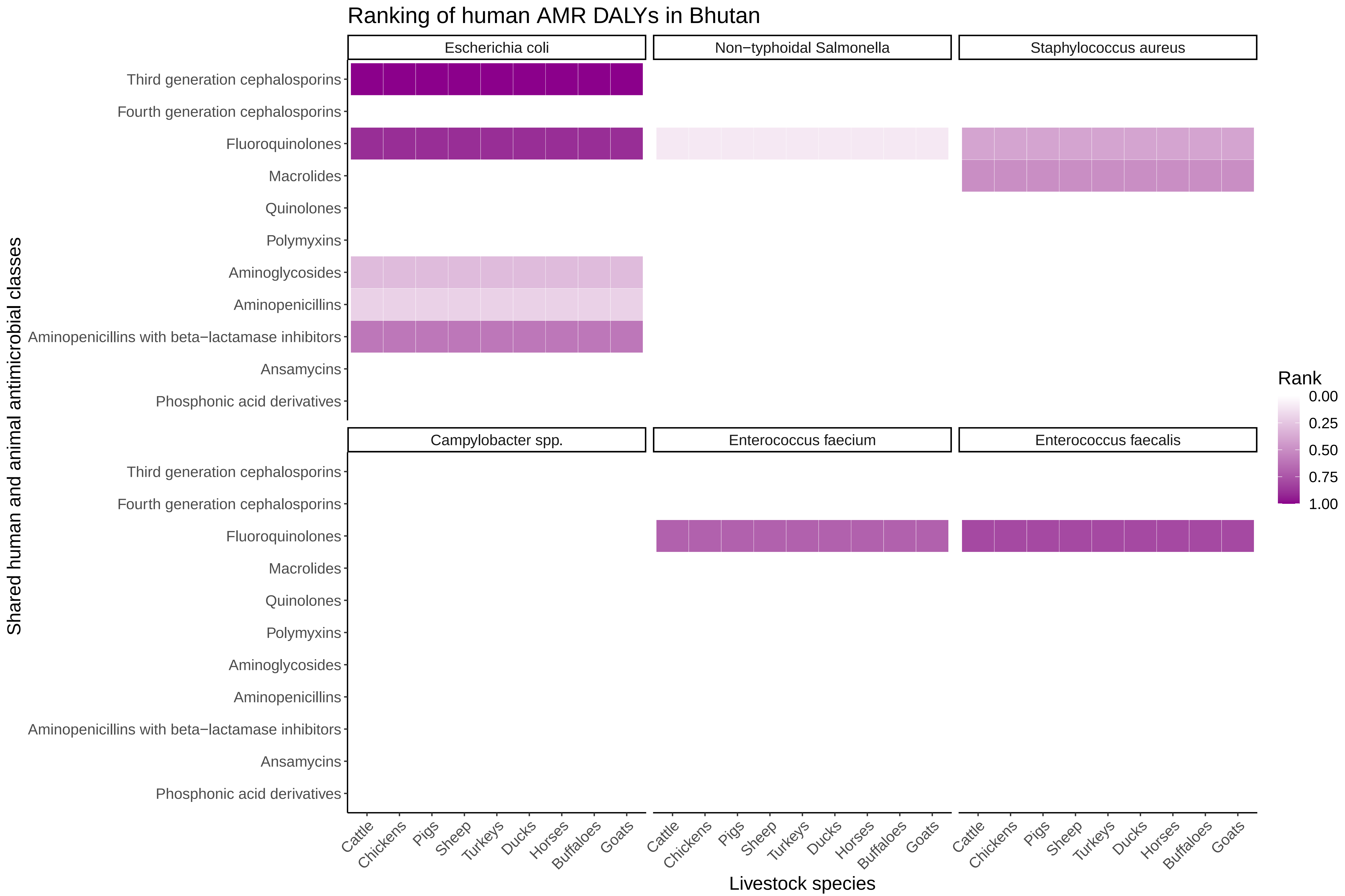

Ranking of human AMR DALYs in Bhutan
Escherichia coli
Non−typhoidal Salmonella
Staphylococcus aureus
Campylobacter spp.
Enterococcus faecium
Enterococcus faecalis
Third generation cephalosporins
Fourth generation cephalosporins
Fluoroquinolones
Macrolides
Quinolones
Polymyxins
Aminoglycosides
Aminopenicillins
Aminopenicillins with beta−lactamase inhibitors
Ansamycins
Phosphonic acid derivatives
Shared human and animal antimicrobial classes
Cattle
Chickens
Pigs
Sheep
Turkeys
Ducks
Horses
Buffaloes
Goats
Livestock species
Rank
0.00
0.25
0.50
0.75
1.00

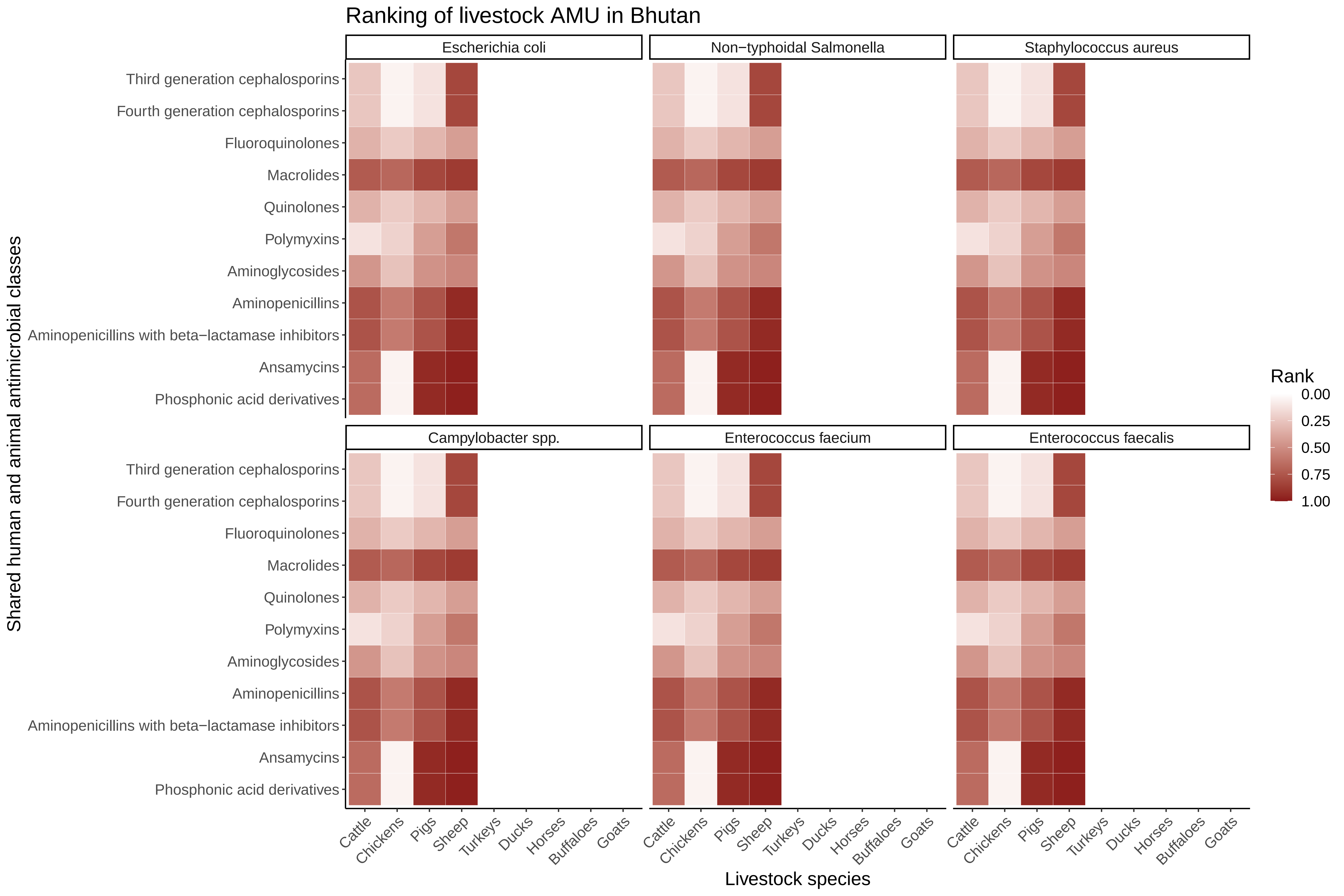

Ranking of livestock AMU in Bhutan
Escherichia coli
Non−typhoidal Salmonella
Staphylococcus aureus
Campylobacter spp.
Enterococcus faecium
Enterococcus faecalis
Third generation cephalosporins
Fourth generation cephalosporins
Fluoroquinolones
Macrolides
Quinolones
Polymyxins
Aminoglycosides
Aminopenicillins
Aminopenicillins with beta−lactamase inhibitors
Ansamycins
Phosphonic acid derivatives
Shared human and animal antimicrobial classes
Cattle
Chickens
Pigs
Sheep
Turkeys
Ducks
Horses
Buffaloes
Goats
Livestock species
Rank
0.00
0.25
0.50
0.75
1.00

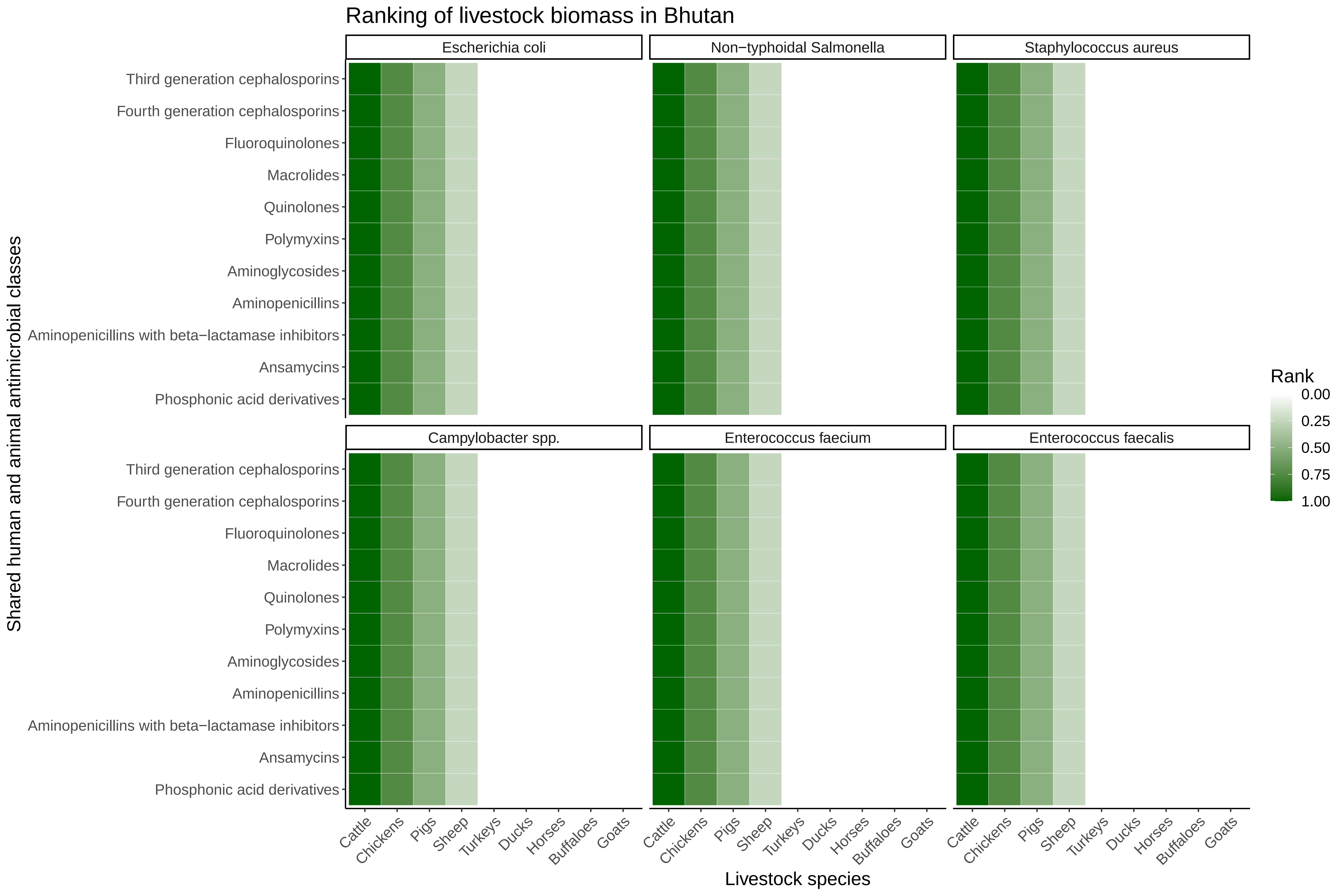

Ranking of livestock biomass in Bhutan
Escherichia coli
Non−typhoidal Salmonella
Staphylococcus aureus
Campylobacter spp.
Enterococcus faecium
Enterococcus faecalis
Third generation cephalosporins
Fourth generation cephalosporins
Fluoroquinolones
Macrolides
Quinolones
Polymyxins
Aminoglycosides
Aminopenicillins
Aminopenicillins with beta−lactamase inhibitors
Ansamycins
Phosphonic acid derivatives
Shared human and animal antimicrobial classes
Cattle
Chickens
Pigs
Sheep
Turkeys
Ducks
Horses
Buffaloes
Goats
Livestock species
Rank
0.00
0.25
0.50
0.75
1.00

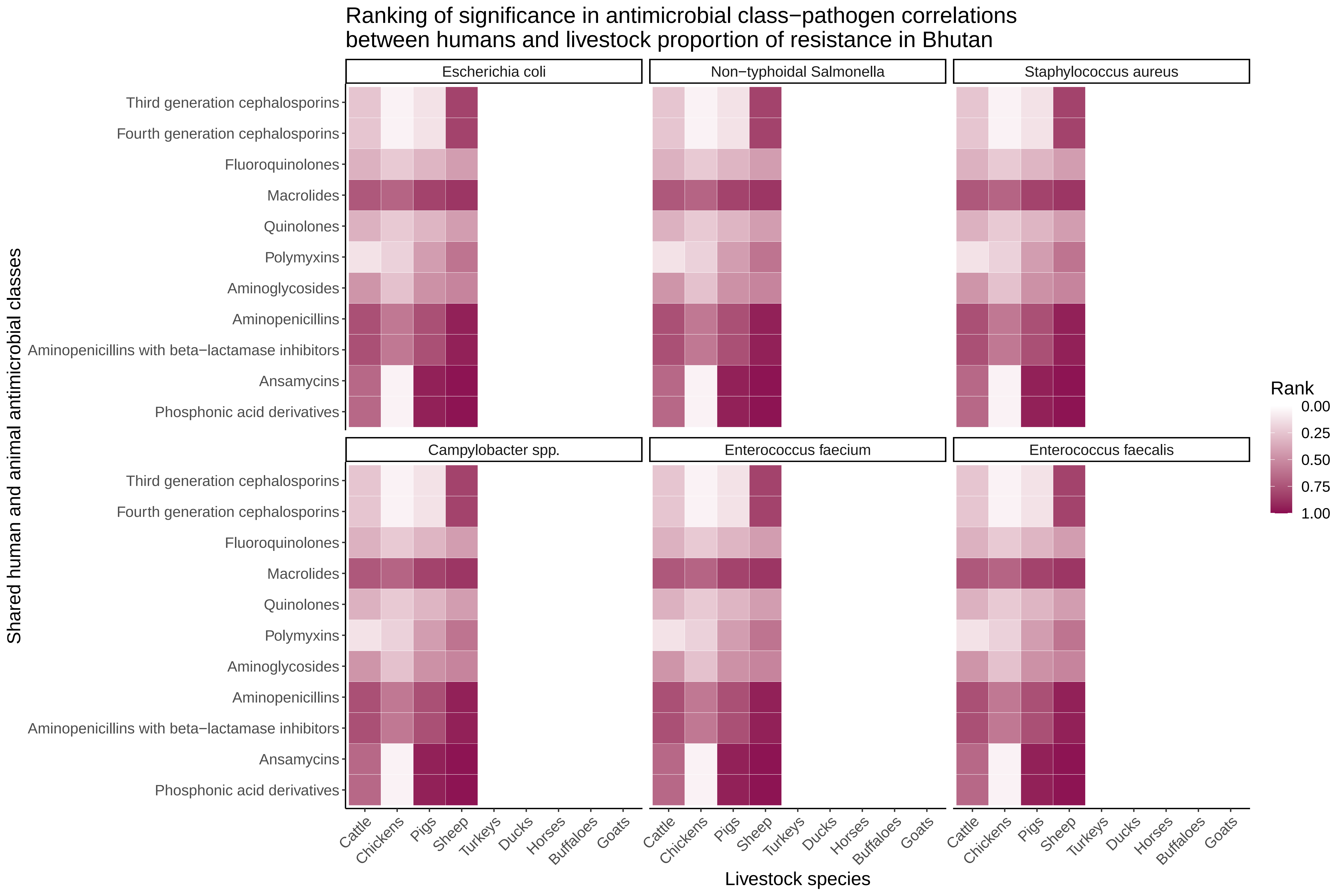

Ranking of significance in antimicrobial class−pathogen correlations
between humans and livestock proportion of resistance in Bhutan
Escherichia coli
Non−typhoidal Salmonella
Staphylococcus aureus
Campylobacter spp.
Enterococcus faecium
Enterococcus faecalis
Third generation cephalosporins
Fourth generation cephalosporins
Fluoroquinolones
Macrolides
Quinolones
Polymyxins
Aminoglycosides
Aminopenicillins
Aminopenicillins with beta−lactamase inhibitors
Ansamycins
Phosphonic acid derivatives
Shared human and animal antimicrobial classes
Cattle
Chickens
Pigs
Sheep
Turkeys
Ducks
Horses
Buffaloes
Goats
Livestock species
Rank
0.00
0.25
0.50
0.75
1.00

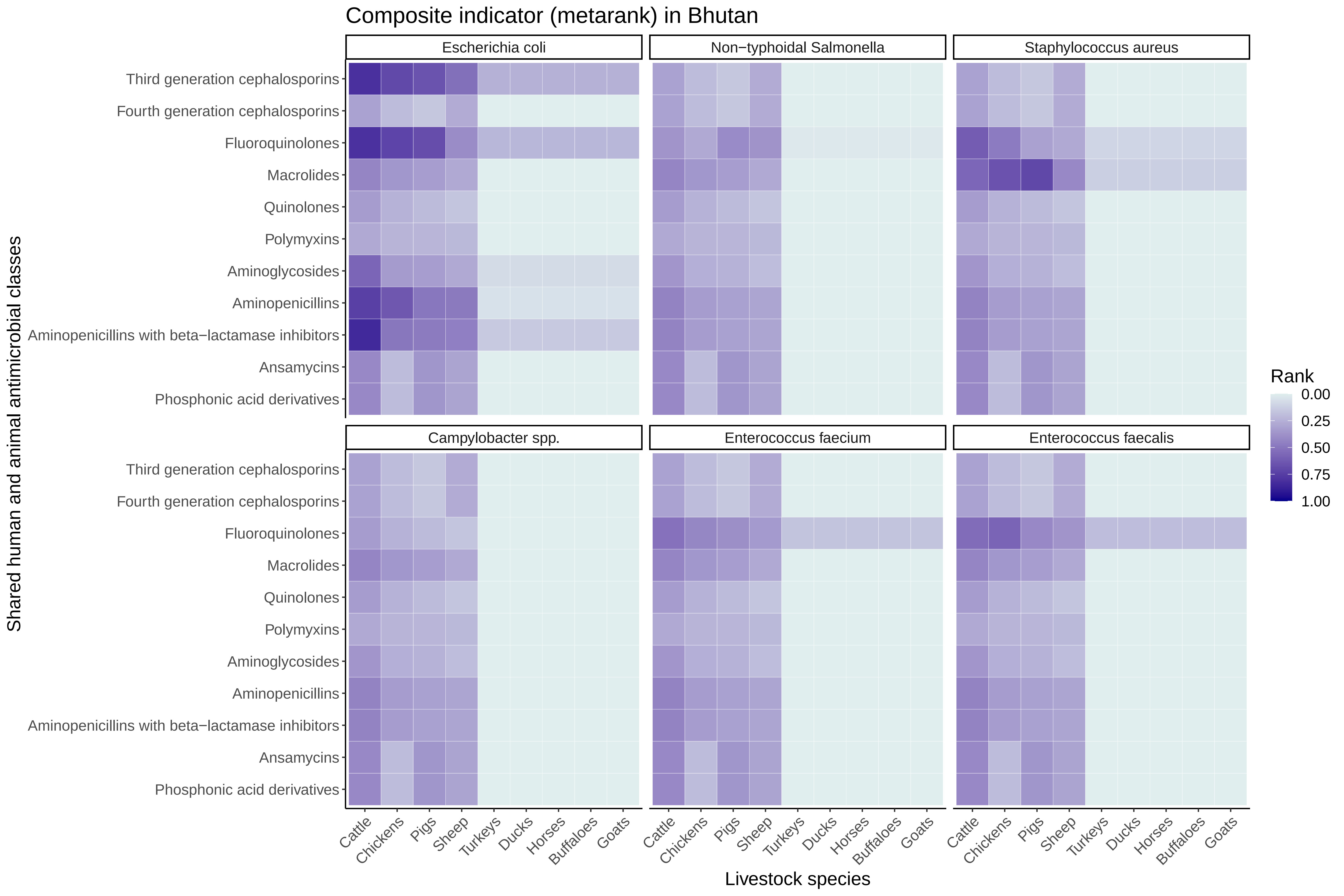

Composite indicator (metarank) in Bhutan
Escherichia coli
Non−typhoidal Salmonella
Staphylococcus aureus
Campylobacter spp.
Enterococcus faecium
Enterococcus faecalis
Third generation cephalosporins
Fourth generation cephalosporins
Fluoroquinolones
Macrolides
Quinolones
Polymyxins
Aminoglycosides
Aminopenicillins
Aminopenicillins with beta−lactamase inhibitors
Ansamycins
Phosphonic acid derivatives
Shared human and animal antimicrobial classes
Cattle
Chickens
Pigs
Sheep
Turkeys
Ducks
Horses
Buffaloes
Goats
Livestock species
Rank
0.00
0.25
0.50
0.75
1.00

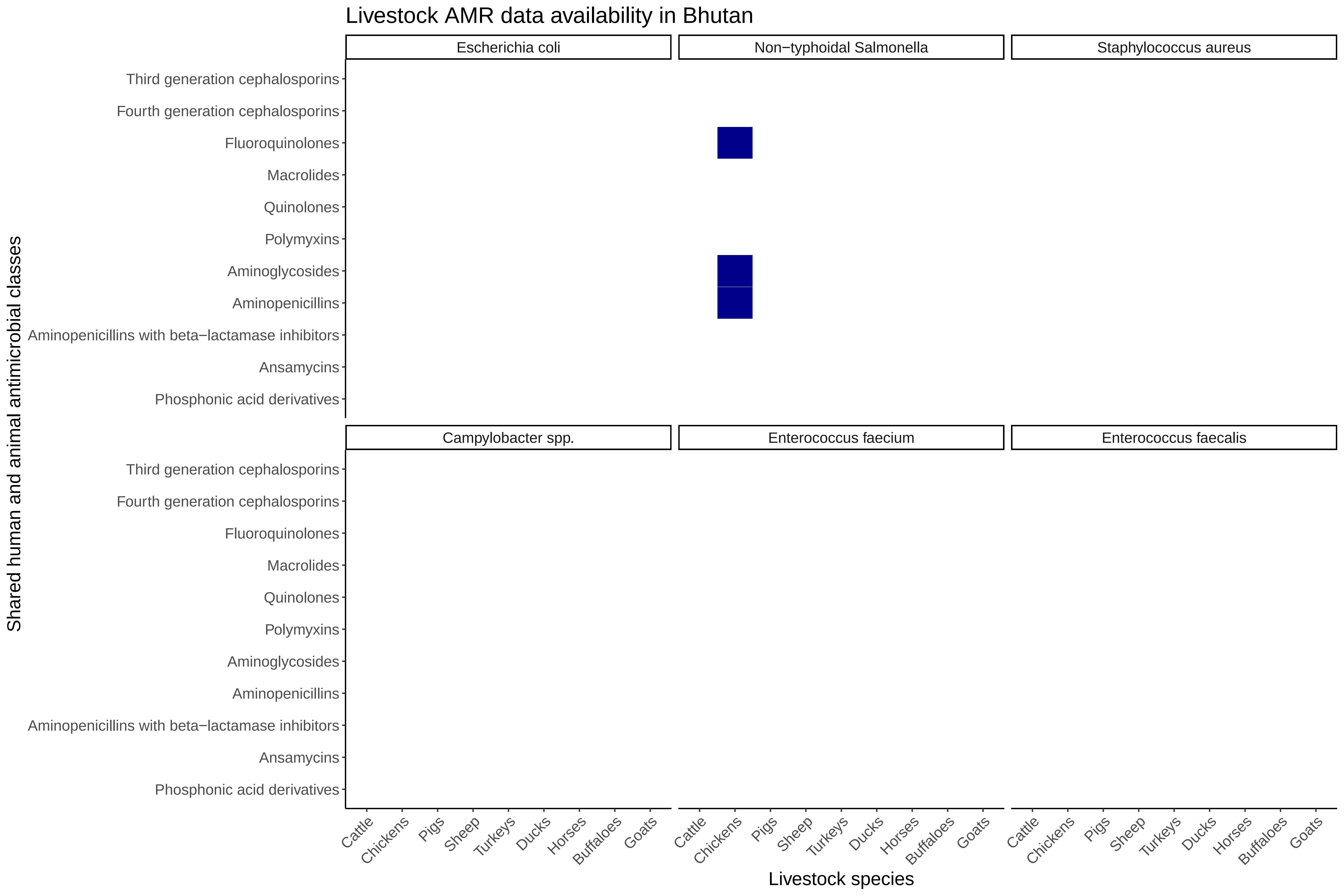
Livestock AMR data availability in Bhutan
Escherichia coli
Non−typhoidal Salmonella
Staphylococcus aureus
Campylobacter spp.
Enterococcus faecium
Enterococcus faecalis
Third generation cephalosporins
Fourth generation cephalosporins
Fluoroquinolones
Macrolides
Quinolones
Polymyxins
Aminoglycosides
Aminopenicillins
Aminopenicillins with beta−lactamase inhibitors
Ansamycins
Phosphonic acid derivatives
Shared human and animal antimicrobial classes
Cattle
Chickens
Pigs
Sheep
Turkeys
Ducks
Horses
Buffaloes
Goats
Livestock species

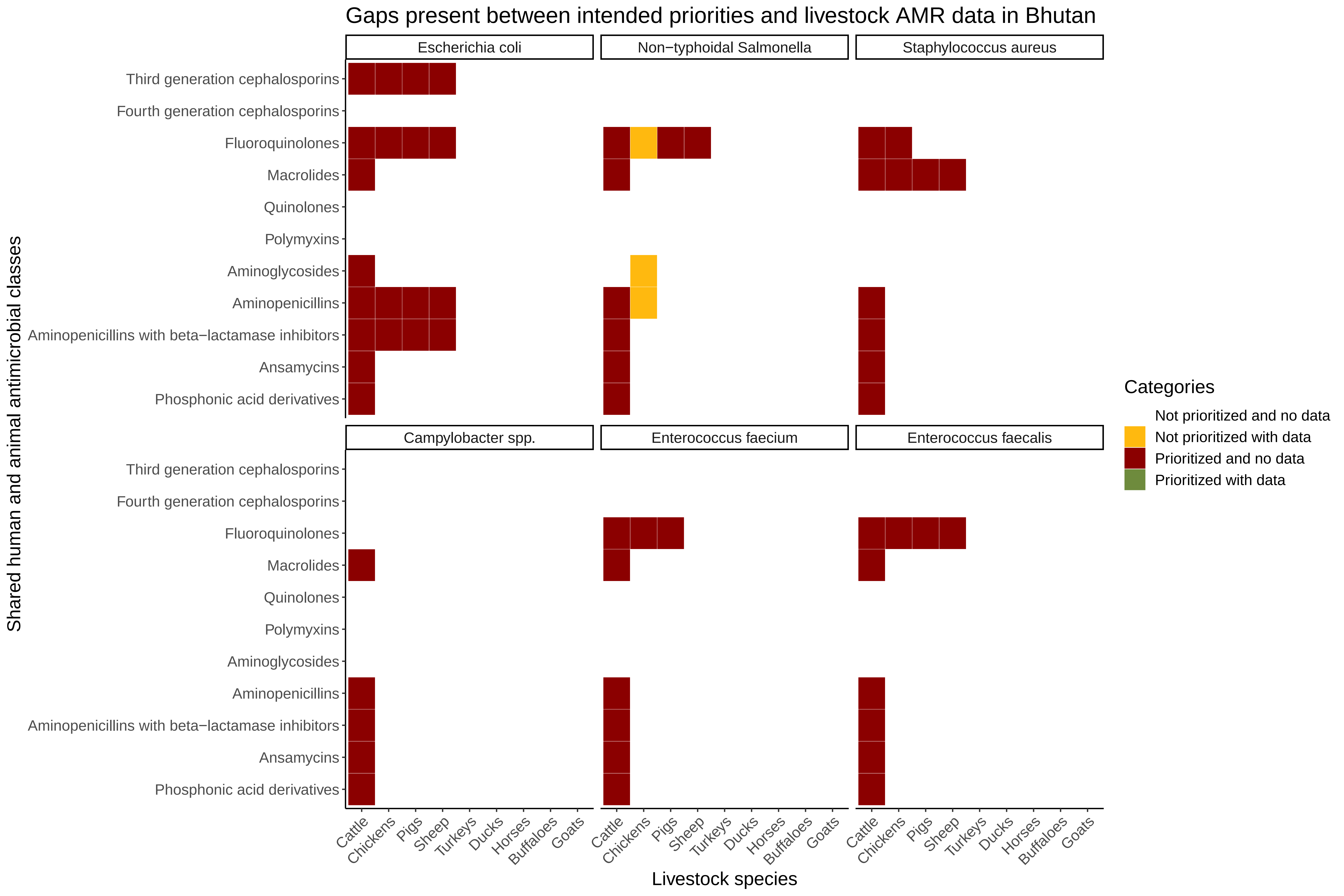
Gaps present between intended priorities and livestock AMR data in Bhutan
Escherichia coli
Non−typhoidal Salmonella
Staphylococcus aureus
Campylobacter spp.
Enterococcus faecium
Enterococcus faecalis
Third generation cephalosporins
Fourth generation cephalosporins
Fluoroquinolones
Macrolides
Quinolones
Polymyxins
Aminoglycosides
Aminopenicillins
Aminopenicillins with beta−lactamase inhibitors
Ansamycins
Phosphonic acid derivatives
Shared human and animal antimicrobial classes
Cattle
Chickens
Pigs
Sheep
Turkeys
Ducks
Horses
Buffaloes
Goats
Livestock species
Categories
Not prioritized and no data
Not prioritized with data
Prioritized and no data
Prioritized with data

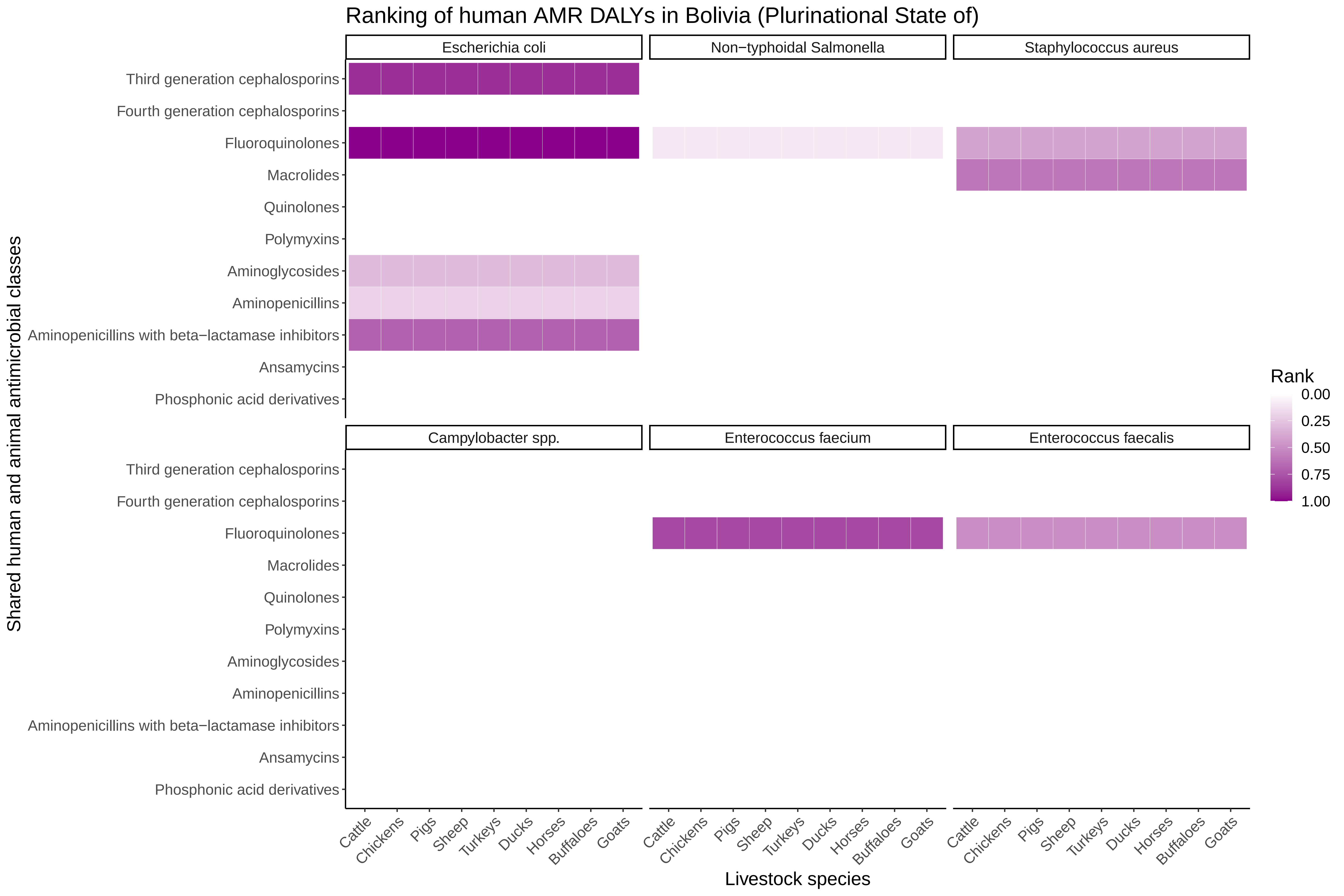

Ranking of human AMR DALYs in Bolivia (Plurinational State of)
Escherichia coli
Non−typhoidal Salmonella
Staphylococcus aureus
Campylobacter spp.
Enterococcus faecium
Enterococcus faecalis
Shared human and animal antimicrobial classes
Third generation cephalosporins
Fourth generation cephalosporins
Fluoroquinolones
Macrolides
Quinolones
Polymyxins
Aminoglycosides
Aminopenicillins
Aminopenicillins with beta−lactamase inhibitors
Ansamycins
Phosphonic acid derivatives
Livestock species
Cattle
Chickens
Pigs
Sheep
Turkeys
Ducks
Horses
Buffaloes
Goats
Rank
0.00
0.25
0.50
0.75
1.00

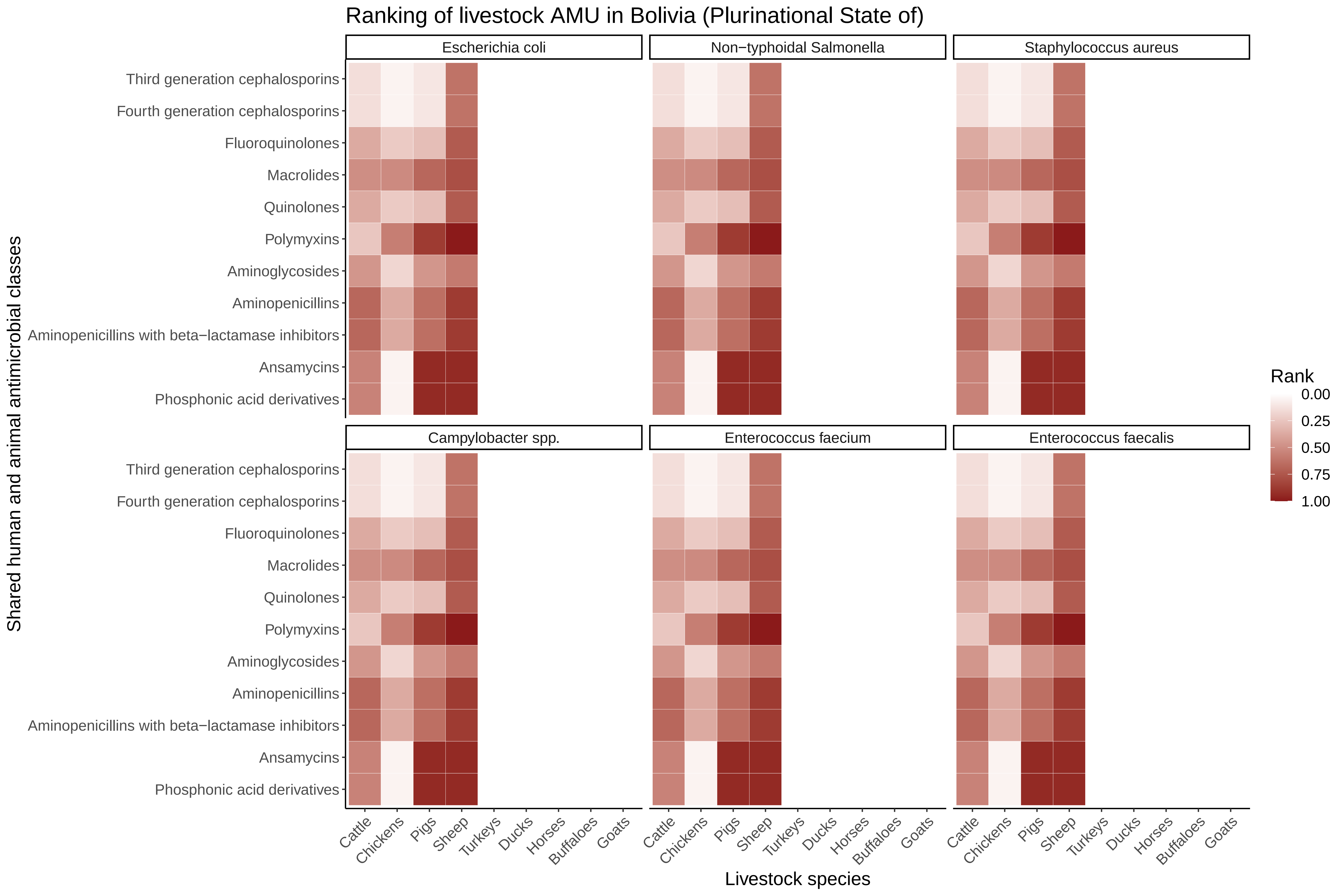

Ranking of livestock AMU in Bolivia (Plurinational State of)
Escherichia coli
Non−typhoidal Salmonella
Staphylococcus aureus
Campylobacter spp.
Enterococcus faecium
Enterococcus faecalis
Shared human and animal antimicrobial classes
Third generation cephalosporins
Fourth generation cephalosporins
Fluoroquinolones
Macrolides
Quinolones
Polymyxins
Aminoglycosides
Aminopenicillins
Aminopenicillins with beta−lactamase inhibitors
Ansamycins
Phosphonic acid derivatives
Rank
0.00
0.25
0.50
0.75
1.00
Cattle
Chickens
Pigs
Sheep
Turkeys
Ducks
Horses
Buffaloes
Goats
Livestock species

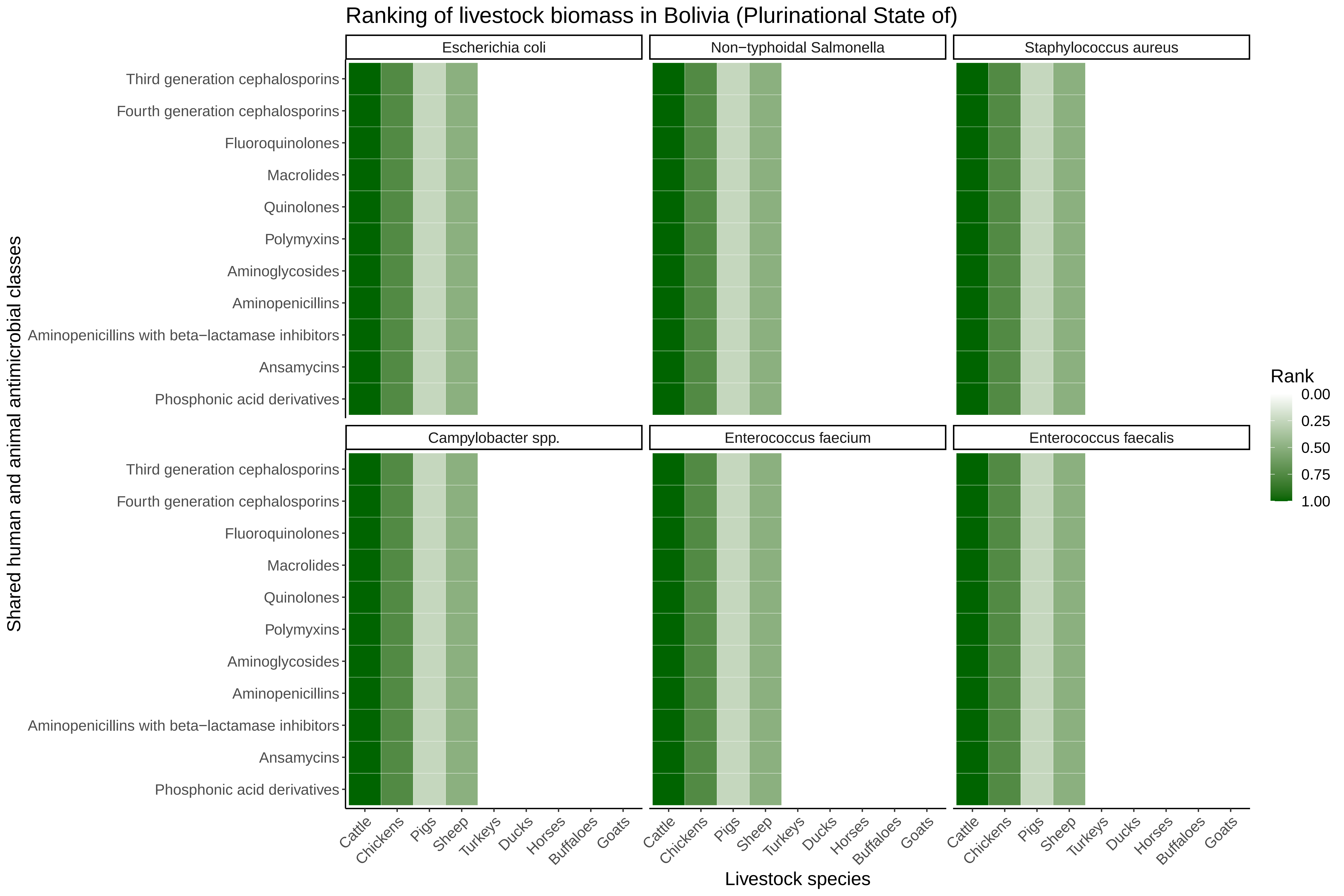

Ranking of livestock biomass in Bolivia (Plurinational State of)
Escherichia coli
Non−typhoidal Salmonella
Staphylococcus aureus
Campylobacter spp.
Enterococcus faecium
Enterococcus faecalis
Third generation cephalosporins
Fourth generation cephalosporins
Fluoroquinolones
Macrolides
Quinolones
Polymyxins
Aminoglycosides
Aminopenicillins
Aminopenicillins with beta−lactamase inhibitors
Ansamycins
Phosphonic acid derivatives
Shared human and animal antimicrobial classes
Cattle
Chickens
Pigs
Sheep
Turkeys
Ducks
Horses
Buffaloes
Goats
Livestock species
Rank
0.00
0.25
0.50
0.75
1.00

Ranking of significance in antimicrobial class−pathogen correlations between humans and livestock proportion of resistance in Bolivia (Plurinational State of)

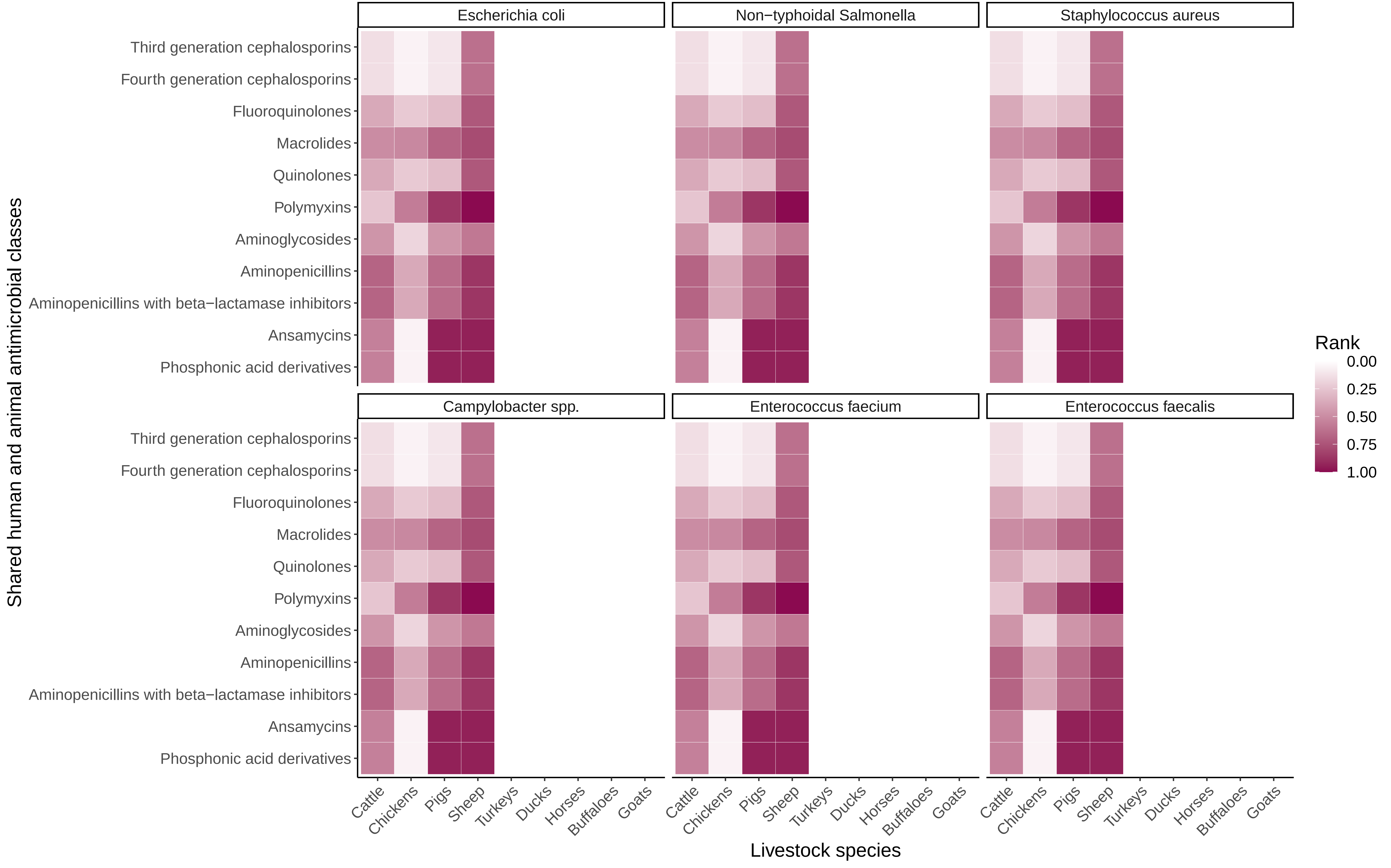

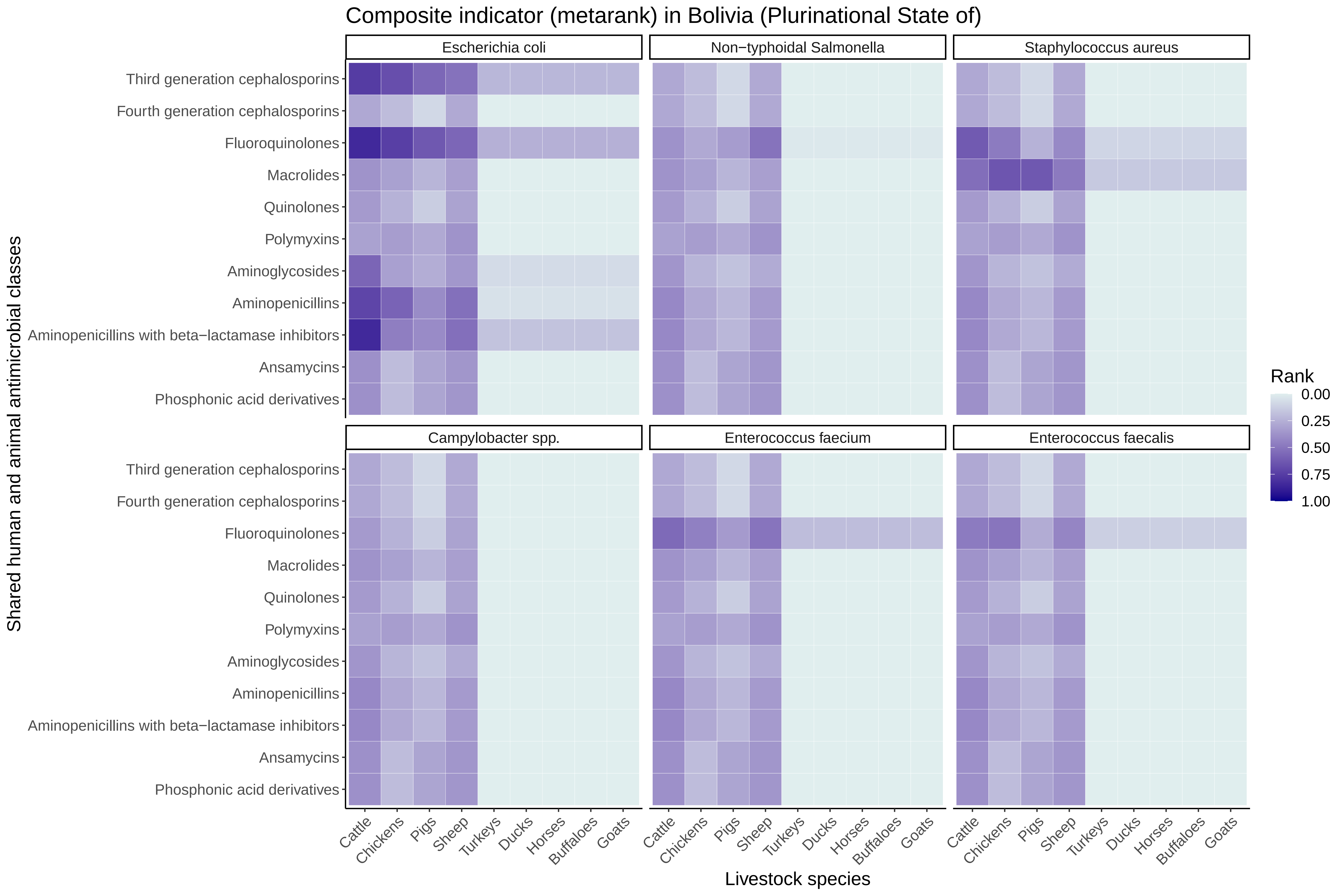
Composite indicator (metarank) in Bolivia (Plurinational State of)
Escherichia coli
Non−typhoidal Salmonella
Staphylococcus aureus
Campylobacter spp.
Enterococcus faecium
Enterococcus faecalis
Shared human and animal antimicrobial classes
Third generation cephalosporins
Fourth generation cephalosporins
Fluoroquinolones
Macrolides
Quinolones
Polymyxins
Aminoglycosides
Aminopenicillins
Aminopenicillins with beta−lactamase inhibitors
Ansamycins
Phosphonic acid derivatives
Livestock species
Cattle
Chickens
Pigs
Sheep
Turkeys
Ducks
Horses
Buffaloes
Goats
Rank
0.00
0.25
0.50
0.75
1.00

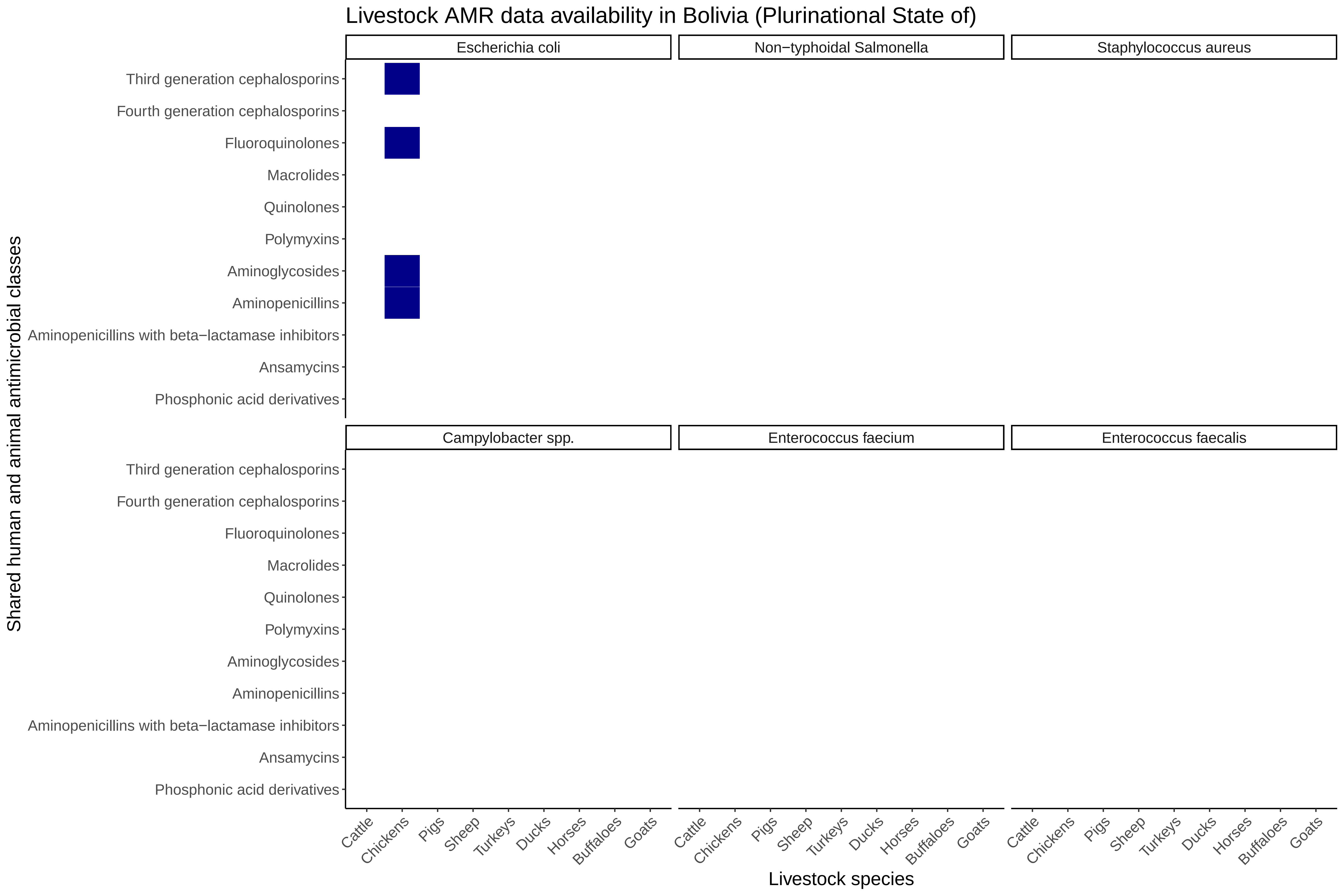
Livestock AMR data availability in Bolivia (Plurinational State of)
Escherichia coli
Non−typhoidal Salmonella
Staphylococcus aureus
Campylobacter spp.
Enterococcus faecium
Enterococcus faecalis
Shared human and animal antimicrobial classes
Third generation cephalosporins
Fourth generation cephalosporins
Fluoroquinolones
Macrolides
Quinolones
Polymyxins
Aminoglycosides
Aminopenicillins
Aminopenicillins with beta−lactamase inhibitors
Ansamycins
Phosphonic acid derivatives
Cattle
Chickens
Pigs
Sheep
Turkeys
Ducks
Horses
Buffaloes
Goats
Livestock species

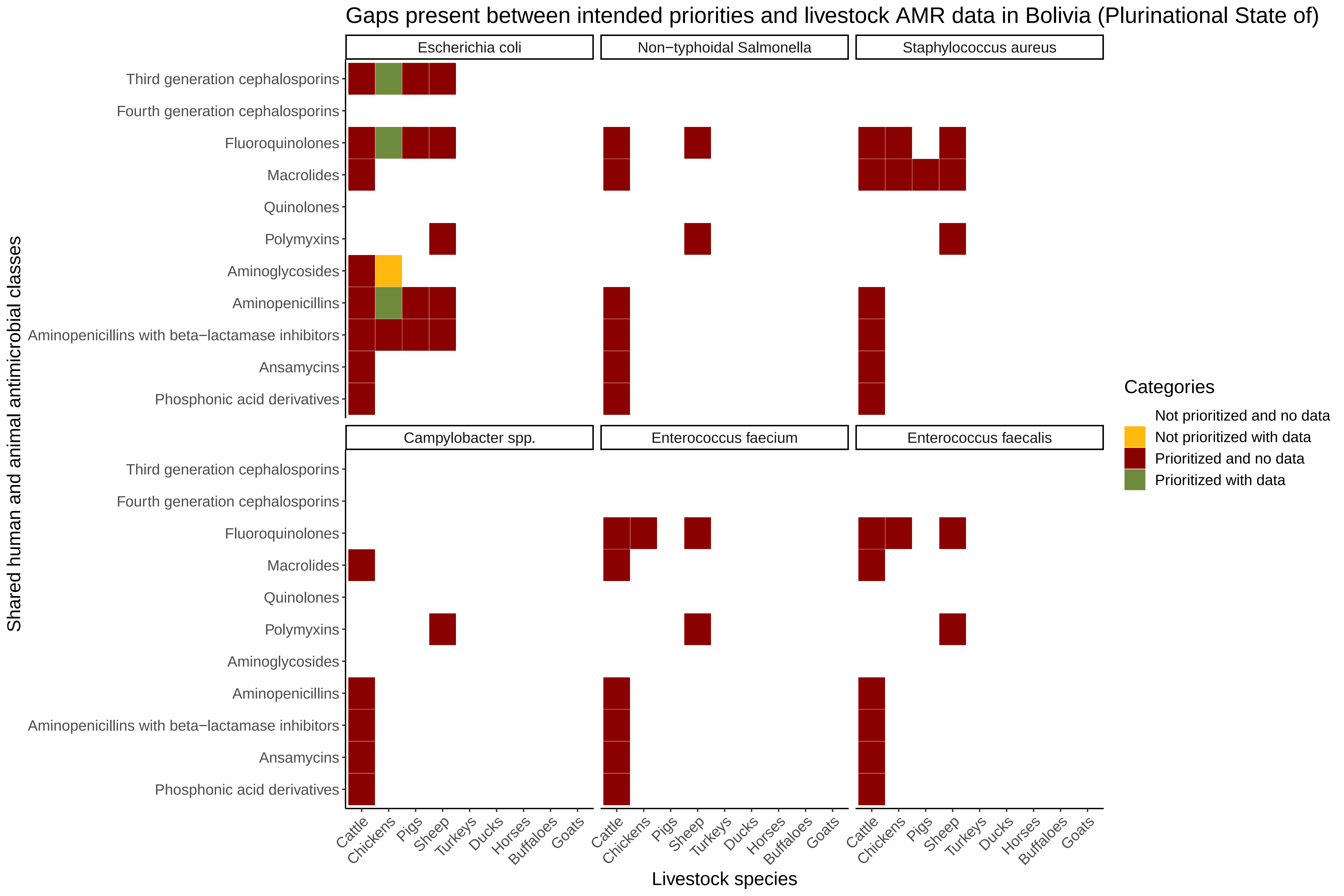

Gaps present between intended priorities and livestock AMR data in Bolivia (Plurinational State of)
Escherichia coli
Non−typhoidal Salmonella
Staphylococcus aureus
Campylobacter spp.
Enterococcus faecium
Enterococcus faecalis
Shared human and animal antimicrobial classes
Third generation cephalosporins
Fourth generation cephalosporins
Fluoroquinolones
Macrolides
Quinolones
Polymyxins
Aminoglycosides
Aminopenicillins
Aminopenicillins with beta−lactamase inhibitors
Ansamycins
Phosphonic acid derivatives
Livestock species
Cattle
Chickens
Pigs
Sheep
Turkeys
Ducks
Horses
Buffaloes
Goats
Categories
Not prioritized and no data
Not prioritized with data
Prioritized and no data
Prioritized with data

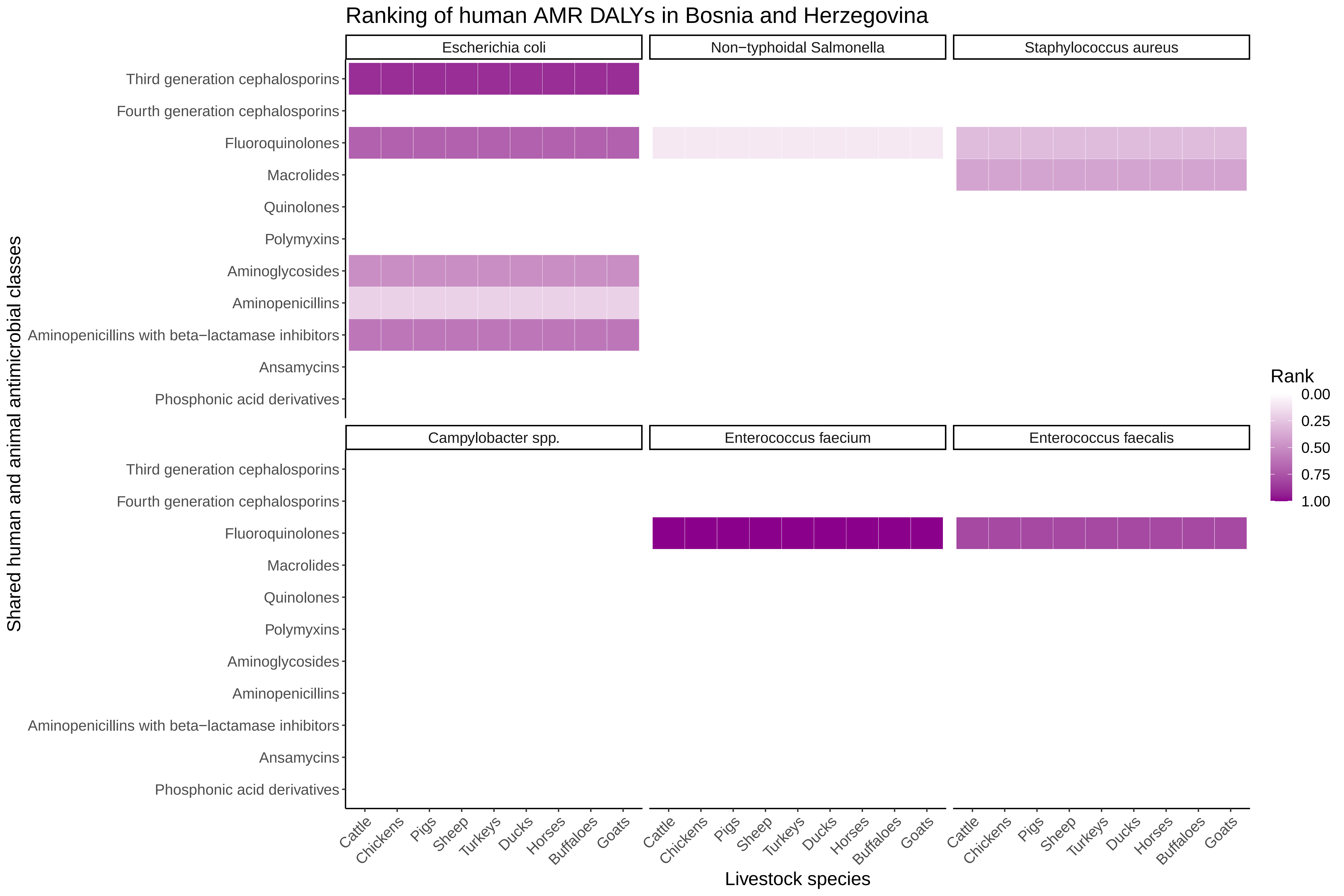

Ranking of human AMR DALYs in Bosnia and Herzegovina
Escherichia coli
Non−typhoidal Salmonella
Staphylococcus aureus
Campylobacter spp.
Enterococcus faecium
Enterococcus faecalis
Third generation cephalosporins
Fourth generation cephalosporins
Fluoroquinolones
Macrolides
Quinolones
Polymyxins
Aminoglycosides
Aminopenicillins
Aminopenicillins with beta−lactamase inhibitors
Ansamycins
Phosphonic acid derivatives
Shared human and animal antimicrobial classes
Cattle
Chickens
Pigs
Sheep
Turkeys
Ducks
Horses
Buffaloes
Goats
Livestock species
Rank
0.00
0.25
0.50
0.75
1.00

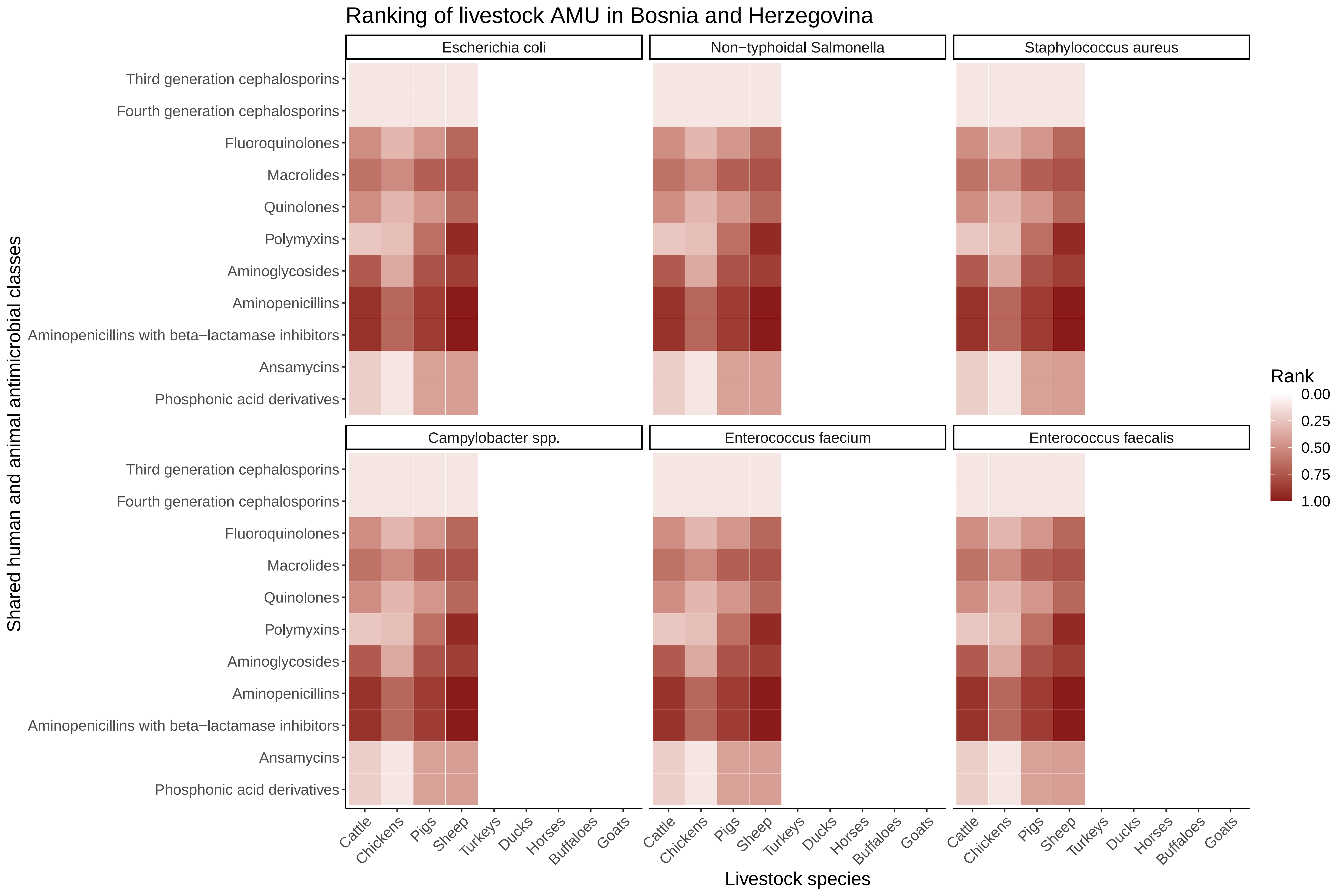

Ranking of livestock AMU in Bosnia and Herzegovina
Escherichia coli
Non−typhoidal Salmonella
Staphylococcus aureus
Campylobacter spp.
Enterococcus faecium
Enterococcus faecalis
Shared human and animal antimicrobial classes
Third generation cephalosporins
Fourth generation cephalosporins
Fluoroquinolones
Macrolides
Quinolones
Polymyxins
Aminoglycosides
Aminopenicillins
Aminopenicillins with beta−lactamase inhibitors
Ansamycins
Phosphonic acid derivatives
Rank
0.00
0.25
0.50
0.75
1.00
Cattle
Chickens
Pigs
Sheep
Turkeys
Ducks
Horses
Buffaloes
Goats
Livestock species

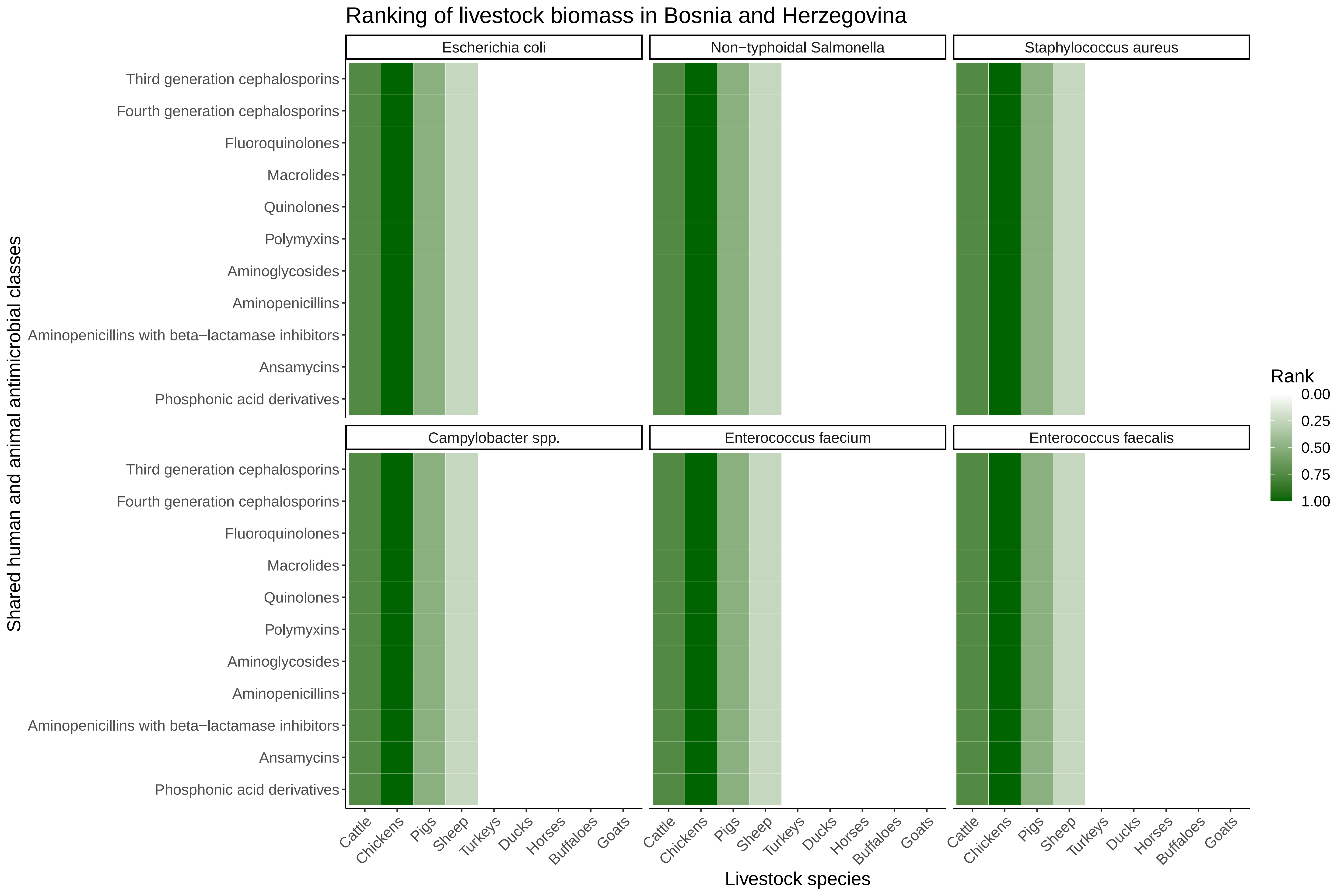
Ranking of livestock biomass in Bosnia and Herzegovina
Escherichia coli
Non−typhoidal Salmonella
Staphylococcus aureus
Campylobacter spp.
Enterococcus faecium
Enterococcus faecalis
Third generation cephalosporins
Fourth generation cephalosporins
Fluoroquinolones
Macrolides
Quinolones
Polymyxins
Aminoglycosides
Aminopenicillins
Aminopenicillins with beta−lactamase inhibitors
Ansamycins
Phosphonic acid derivatives
Shared human and animal antimicrobial classes
Cattle
Chickens
Pigs
Sheep
Turkeys
Ducks
Horses
Buffaloes
Goats
Livestock species
Rank
0.00
0.25
0.50
0.75
1.00

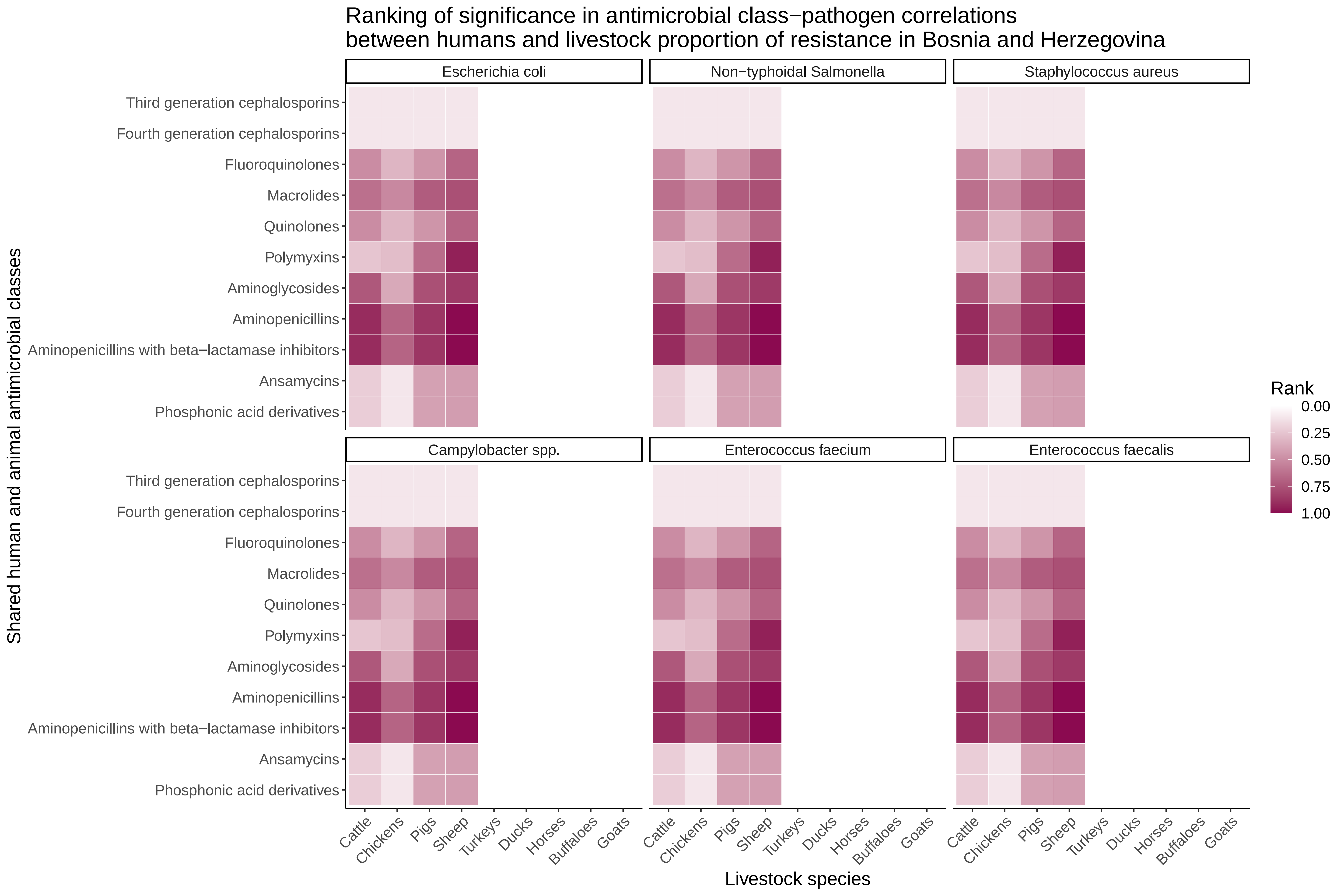

Ranking of significance in antimicrobial class−pathogen correlations
between humans and livestock proportion of resistance in Bosnia and Herzegovina
Escherichia coli
Non−typhoidal Salmonella
Staphylococcus aureus
Campylobacter spp.
Enterococcus faecium
Enterococcus faecalis
Shared human and animal antimicrobial classes
Third generation cephalosporins
Fourth generation cephalosporins
Fluoroquinolones
Macrolides
Quinolones
Polymyxins
Aminoglycosides
Aminopenicillins
Aminopenicillins with beta−lactamase inhibitors
Ansamycins
Phosphonic acid derivatives
Livestock species
Cattle
Chickens
Pigs
Sheep
Turkeys
Ducks
Horses
Buffaloes
Goats
Rank
0.00
0.25
0.50
0.75
1.00

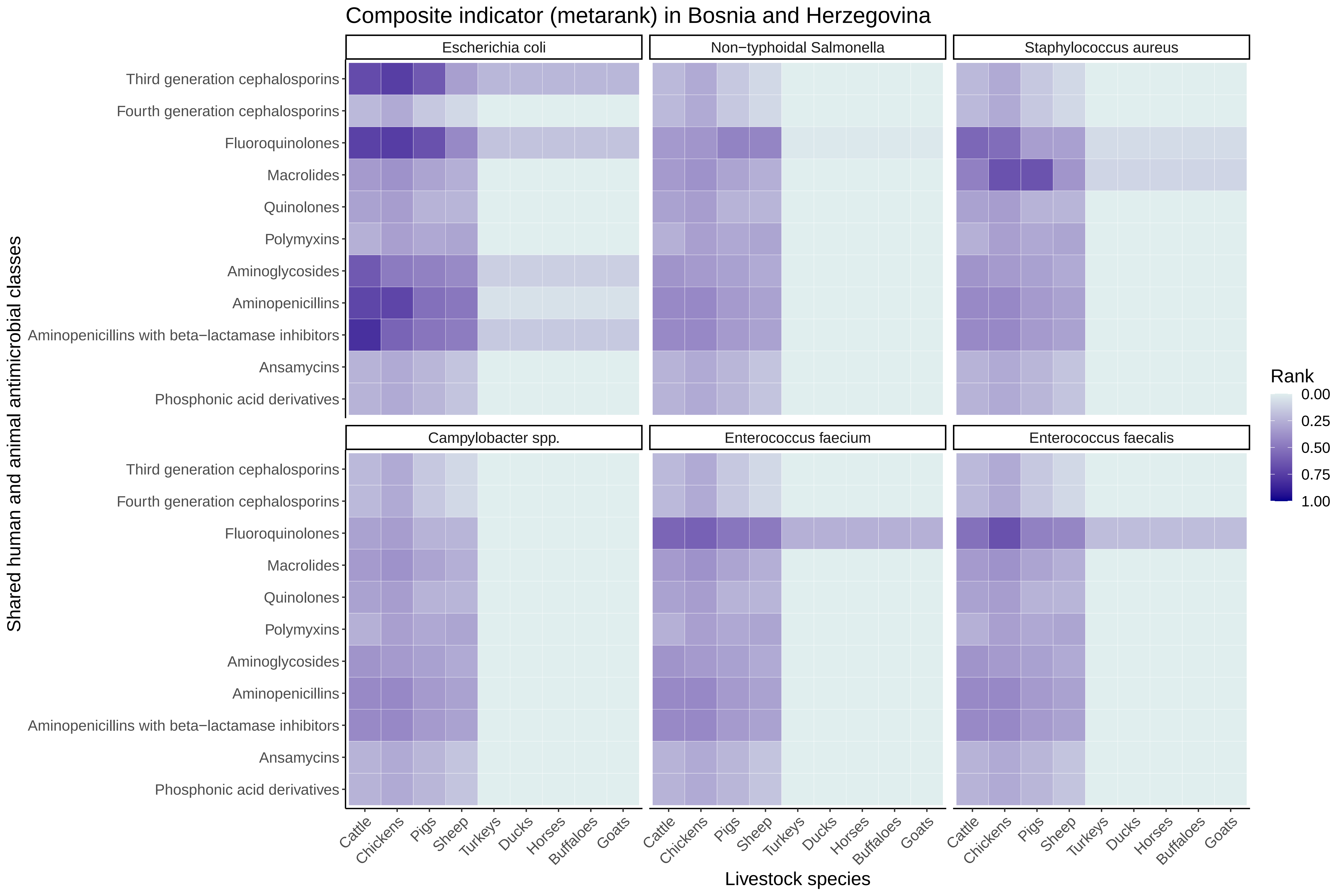

Composite indicator (metarank) in Bosnia and Herzegovina
Escherichia coli
Non−typhoidal Salmonella
Staphylococcus aureus
Campylobacter spp.
Enterococcus faecium
Enterococcus faecalis
Shared human and animal antimicrobial classes
Third generation cephalosporins
Fourth generation cephalosporins
Fluoroquinolones
Macrolides
Quinolones
Polymyxins
Aminoglycosides
Aminopenicillins
Aminopenicillins with beta−lactamase inhibitors
Ansamycins
Phosphonic acid derivatives
Cattle
Chickens
Pigs
Sheep
Turkeys
Ducks
Horses
Buffaloes
Goats
Livestock species
Rank
0.00
0.25
0.50
0.75
1.00

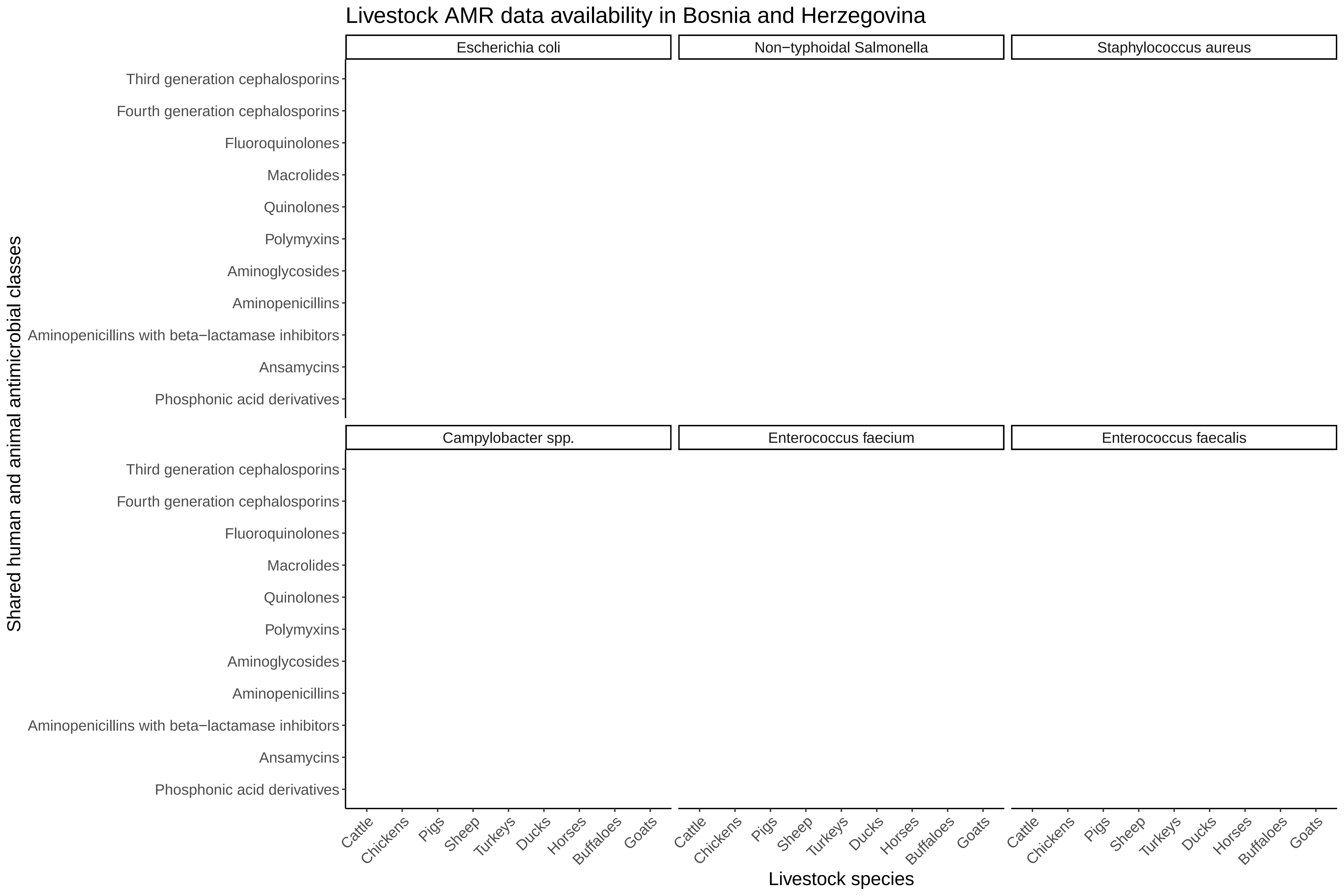
Livestock AMR data availability in Bosnia and Herzegovina
Escherichia coli
Non−typhoidal Salmonella
Staphylococcus aureus
Campylobacter spp.
Enterococcus faecium
Enterococcus faecalis
Third generation cephalosporins
Fourth generation cephalosporins
Fluoroquinolones
Macrolides
Quinolones
Polymyxins
Aminoglycosides
Aminopenicillins
Aminopenicillins with beta−lactamase inhibitors
Ansamycins
Phosphonic acid derivatives
Shared human and animal antimicrobial classes
Cattle
Chickens
Pigs
Sheep
Turkeys
Ducks
Horses
Buffaloes
Goats
Livestock species

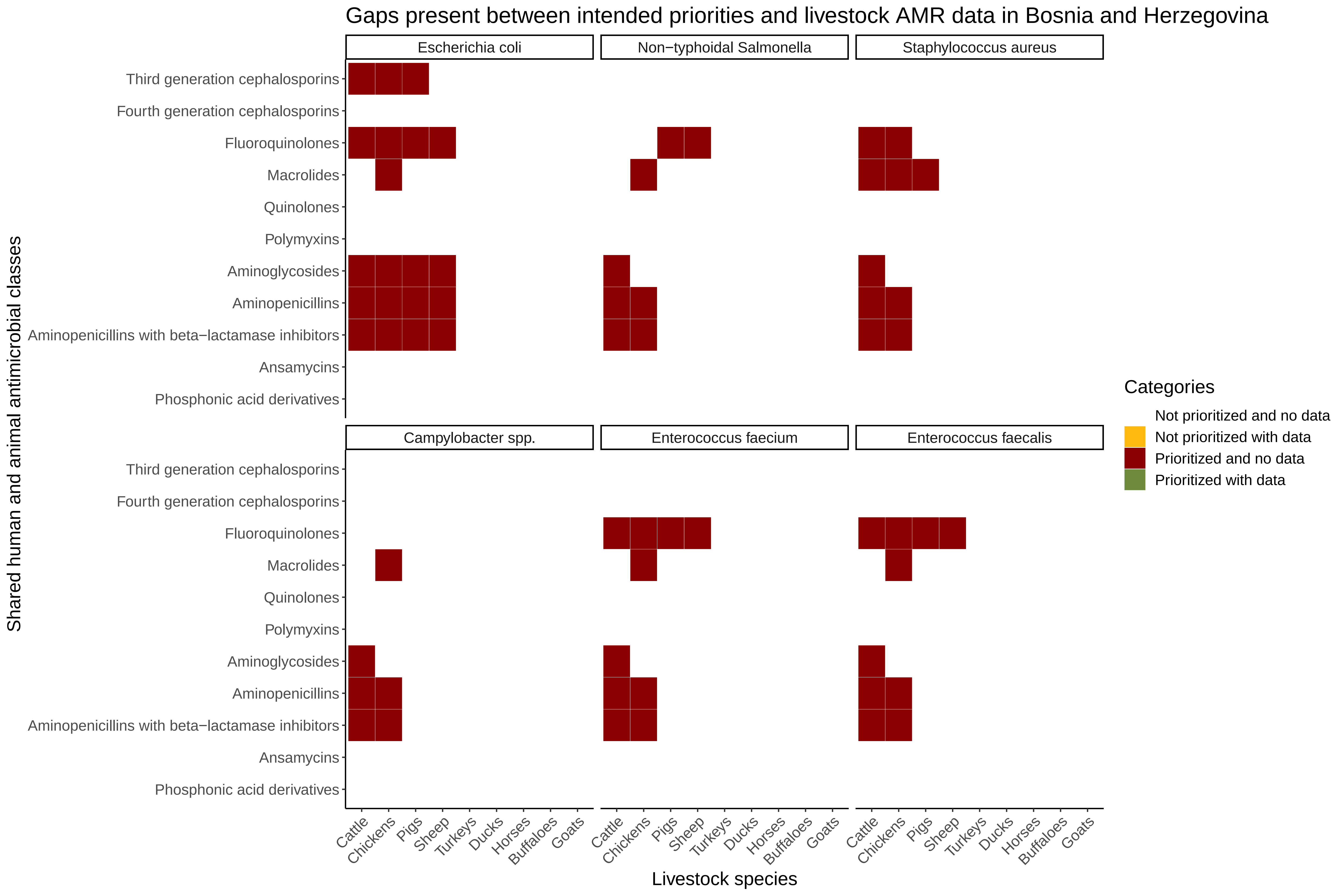
Gaps present between intended priorities and livestock AMR data in Bosnia and Herzegovina
Escherichia coli
Non−typhoidal Salmonella
Staphylococcus aureus
Campylobacter spp.
Enterococcus faecium
Enterococcus faecalis
Shared human and animal antimicrobial classes
Third generation cephalosporins
Fourth generation cephalosporins
Fluoroquinolones
Macrolides
Quinolones
Polymyxins
Aminoglycosides
Aminopenicillins
Aminopenicillins with beta−lactamase inhibitors
Ansamycins
Phosphonic acid derivatives
Cattle
Chickens
Pigs
Sheep
Turkeys
Ducks
Horses
Buffaloes
Goats
Livestock species
Categories
Not prioritized and no data
Not prioritized with data
Prioritized and no data
Prioritized with data

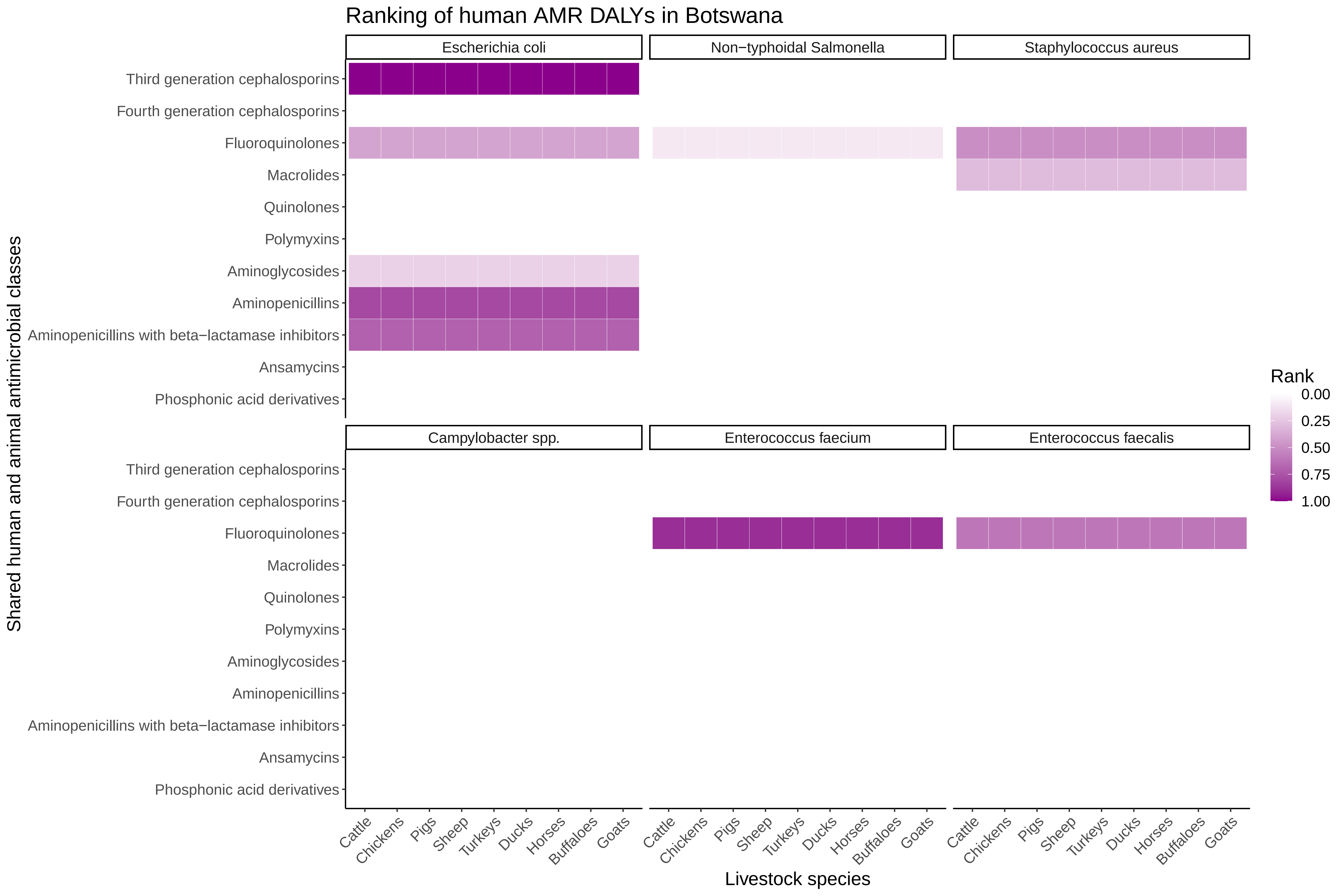

Ranking of human AMR DALYs in Botswana
Escherichia coli
Non−typhoidal Salmonella
Staphylococcus aureus
Campylobacter spp.
Enterococcus faecium
Enterococcus faecalis
Shared human and animal antimicrobial classes
Third generation cephalosporins
Fourth generation cephalosporins
Fluoroquinolones
Macrolides
Quinolones
Polymyxins
Aminoglycosides
Aminopenicillins
Aminopenicillins with beta−lactamase inhibitors
Ansamycins
Phosphonic acid derivatives
Livestock species
Cattle
Chickens
Pigs
Sheep
Turkeys
Ducks
Horses
Buffaloes
Goats
Rank
0.00
0.25
0.50
0.75
1.00

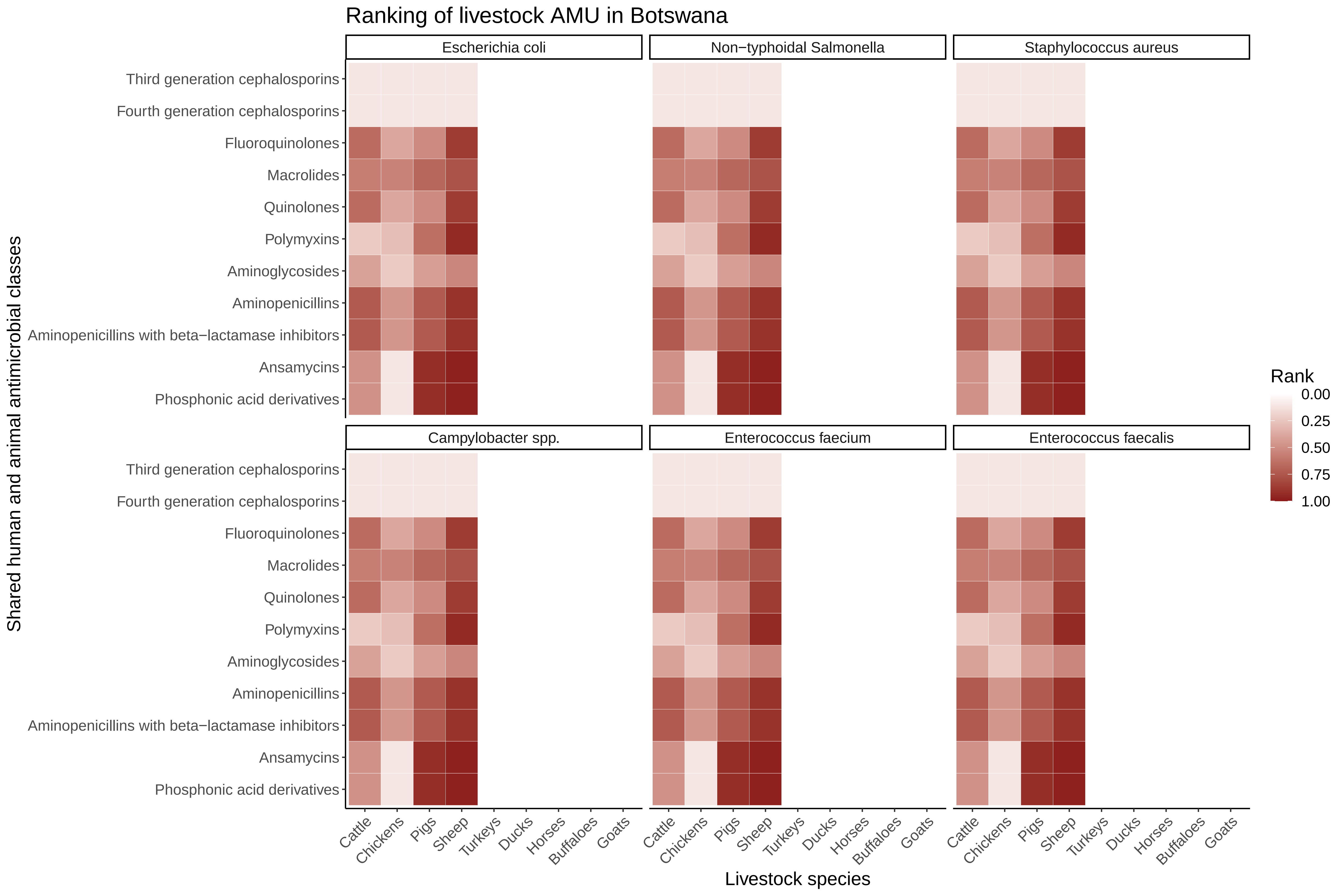
Ranking of livestock AMU in Botswana
Escherichia coli
Non−typhoidal Salmonella
Staphylococcus aureus
Campylobacter spp.
Enterococcus faecium
Enterococcus faecalis
Third generation cephalosporins
Fourth generation cephalosporins
Fluoroquinolones
Macrolides
Quinolones
Polymyxins
Aminoglycosides
Aminopenicillins
Aminopenicillins with beta−lactamase inhibitors
Ansamycins
Phosphonic acid derivatives
Shared human and animal antimicrobial classes
Cattle
Chickens
Pigs
Sheep
Turkeys
Ducks
Horses
Buffaloes
Goats
Livestock species
Rank
0.00
0.25
0.50
0.75
1.00

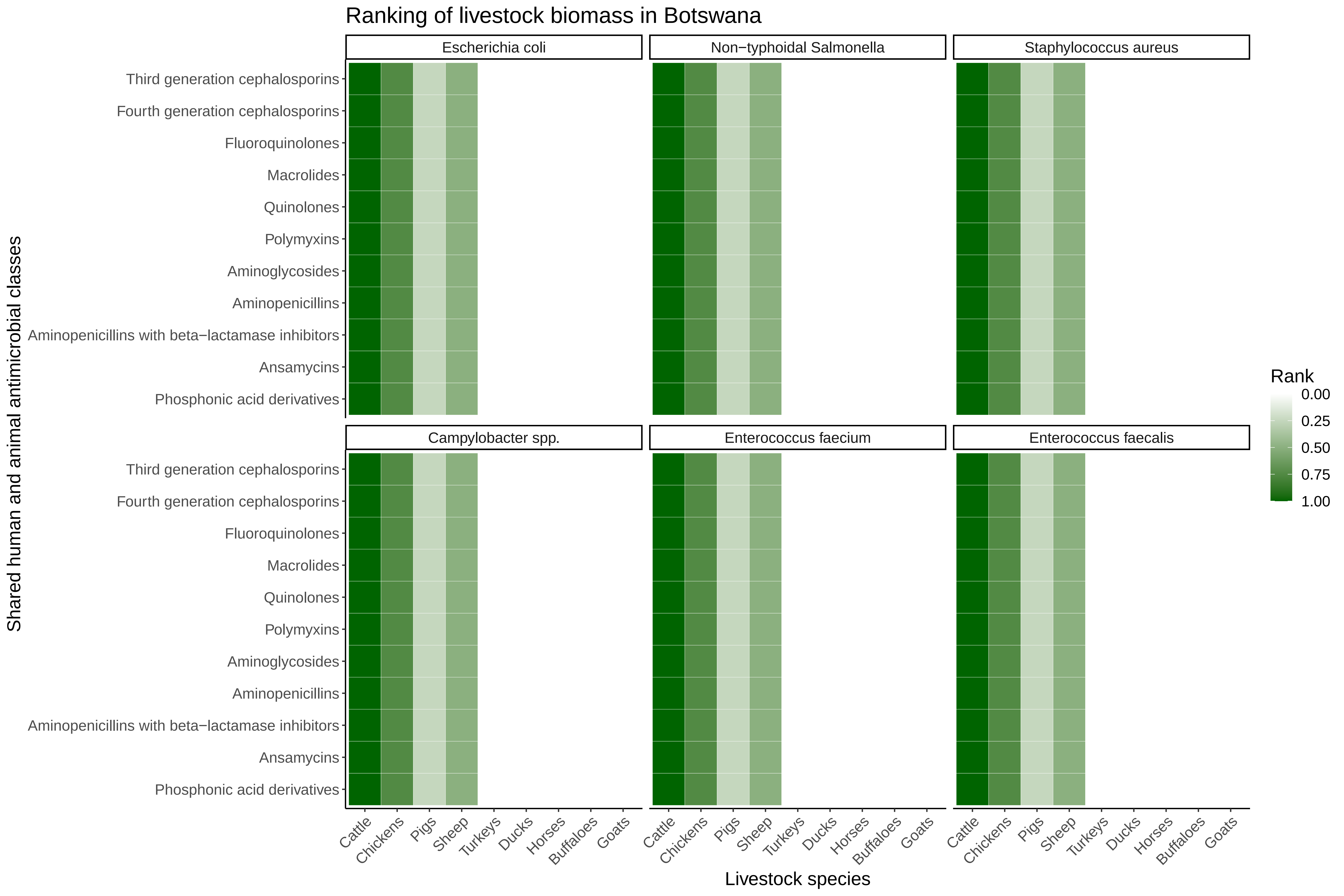

Ranking of livestock biomass in Botswana
Escherichia coli
Non−typhoidal Salmonella
Staphylococcus aureus
Campylobacter spp.
Enterococcus faecium
Enterococcus faecalis
Third generation cephalosporins
Fourth generation cephalosporins
Fluoroquinolones
Macrolides
Quinolones
Polymyxins
Aminoglycosides
Aminopenicillins
Aminopenicillins with beta−lactamase inhibitors
Ansamycins
Phosphonic acid derivatives
Shared human and animal antimicrobial classes
Cattle
Chickens
Pigs
Sheep
Turkeys
Ducks
Horses
Buffaloes
Goats
Livestock species
Rank
0.00
0.25
0.50
0.75
1.00

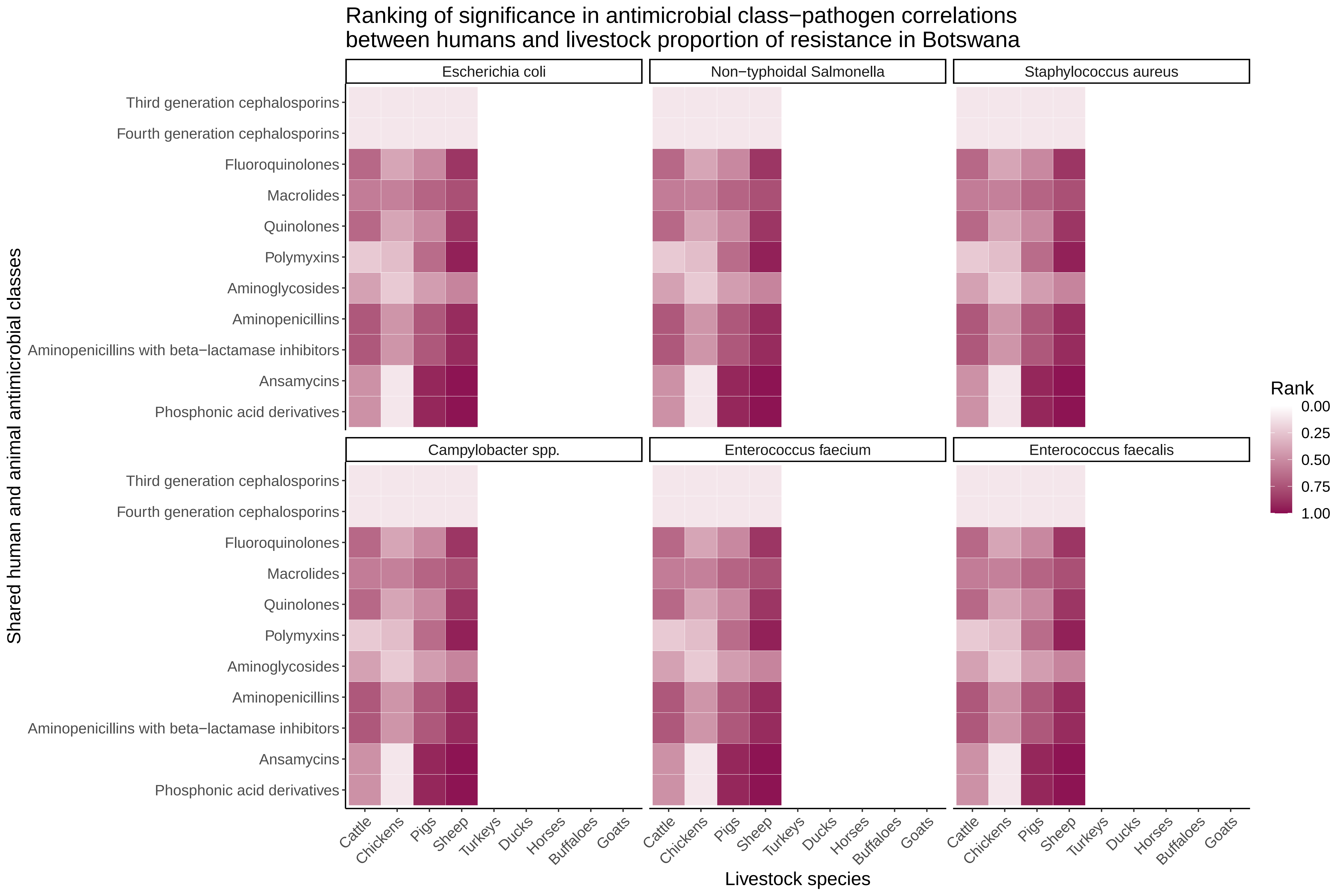

Ranking of significance in antimicrobial class−pathogen correlations
between humans and livestock proportion of resistance in Botswana
Escherichia coli
Non−typhoidal Salmonella
Staphylococcus aureus
Campylobacter spp.
Enterococcus faecium
Enterococcus faecalis
Third generation cephalosporins
Fourth generation cephalosporins
Fluoroquinolones
Macrolides
Quinolones
Polymyxins
Aminoglycosides
Aminopenicillins
Aminopenicillins with beta−lactamase inhibitors
Ansamycins
Phosphonic acid derivatives
Shared human and animal antimicrobial classes
Cattle
Chickens
Pigs
Sheep
Turkeys
Ducks
Horses
Buffaloes
Goats
Livestock species
Rank
0.00
0.25
0.50
0.75
1.00

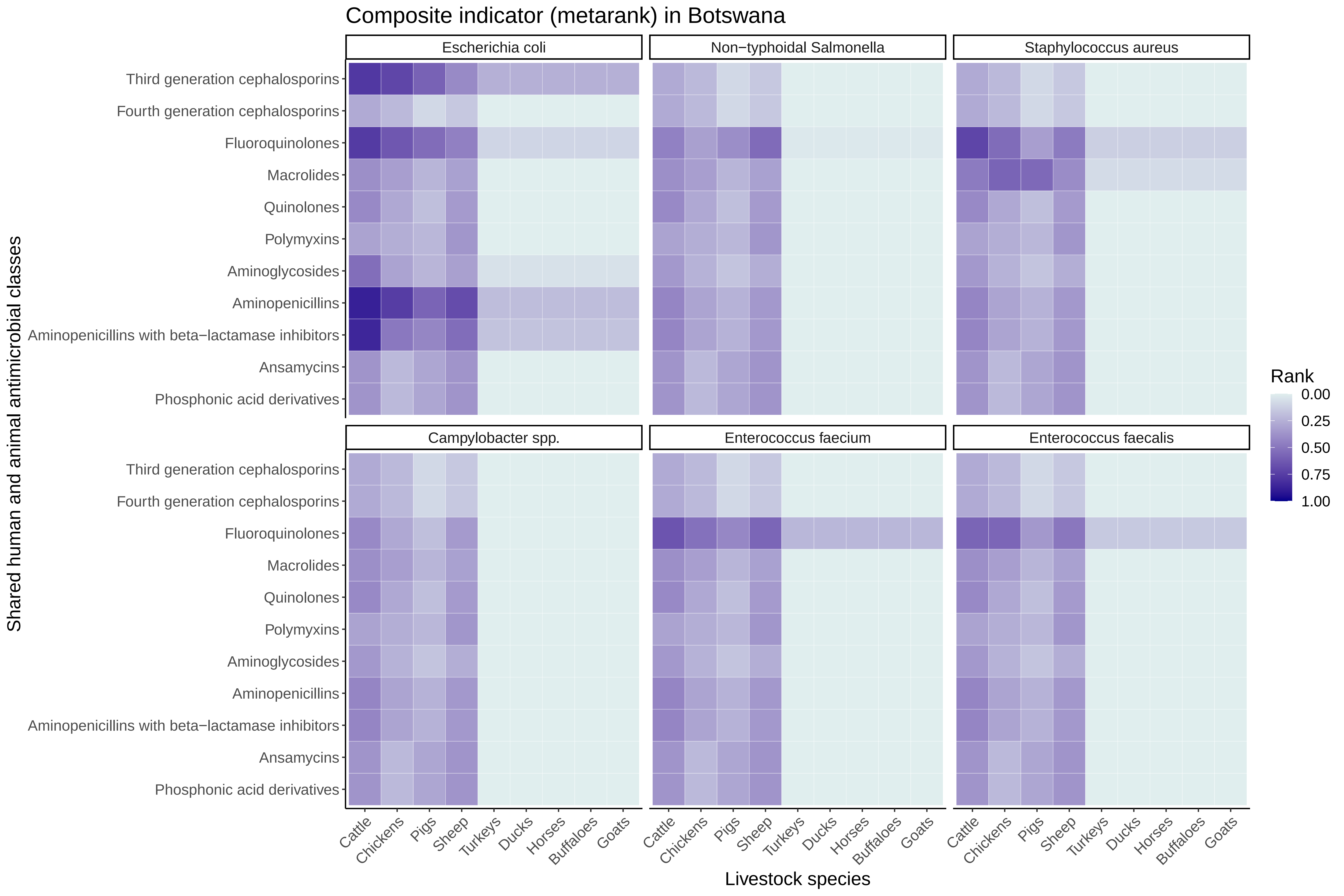
Composite indicator (metarank) in Botswana
Escherichia coli
Non−typhoidal Salmonella
Staphylococcus aureus
Campylobacter spp.
Enterococcus faecium
Enterococcus faecalis
Shared human and animal antimicrobial classes
Third generation cephalosporins
Fourth generation cephalosporins
Fluoroquinolones
Macrolides
Quinolones
Polymyxins
Aminoglycosides
Aminopenicillins
Aminopenicillins with beta−lactamase inhibitors
Ansamycins
Phosphonic acid derivatives
Cattle
Chickens
Pigs
Sheep
Turkeys
Ducks
Horses
Buffaloes
Goats
Livestock species
Rank
0.00
0.25
0.50
0.75
1.00

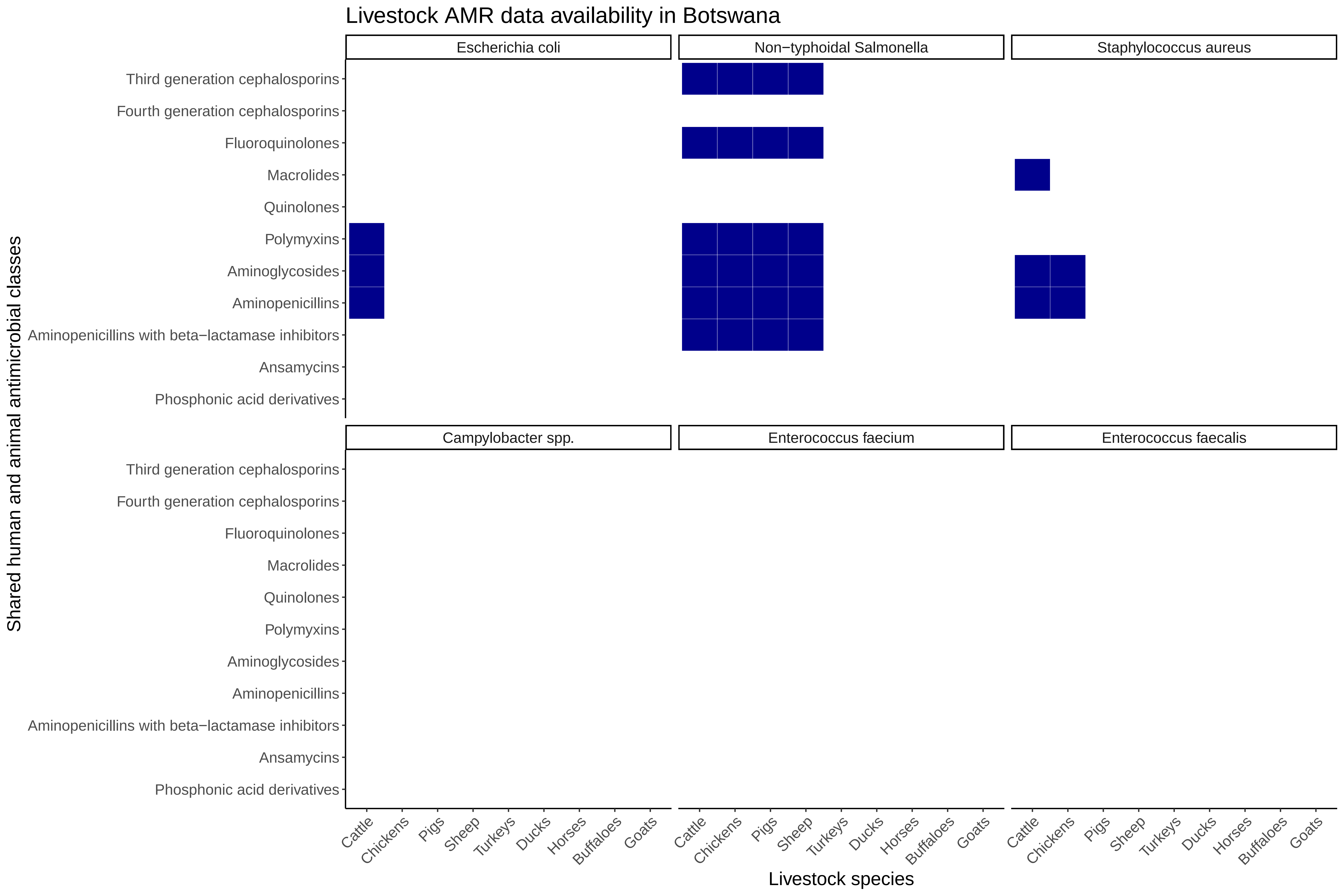

Livestock AMR data availability in Botswana
Escherichia coli
Non−typhoidal Salmonella
Staphylococcus aureus
Campylobacter spp.
Enterococcus faecium
Enterococcus faecalis
Third generation cephalosporins
Fourth generation cephalosporins
Fluoroquinolones
Macrolides
Quinolones
Polymyxins
Aminoglycosides
Aminopenicillins
Aminopenicillins with beta−lactamase inhibitors
Ansamycins
Phosphonic acid derivatives
Shared human and animal antimicrobial classes
Cattle
Chickens
Pigs
Sheep
Turkeys
Ducks
Horses
Buffaloes
Goats
Livestock species

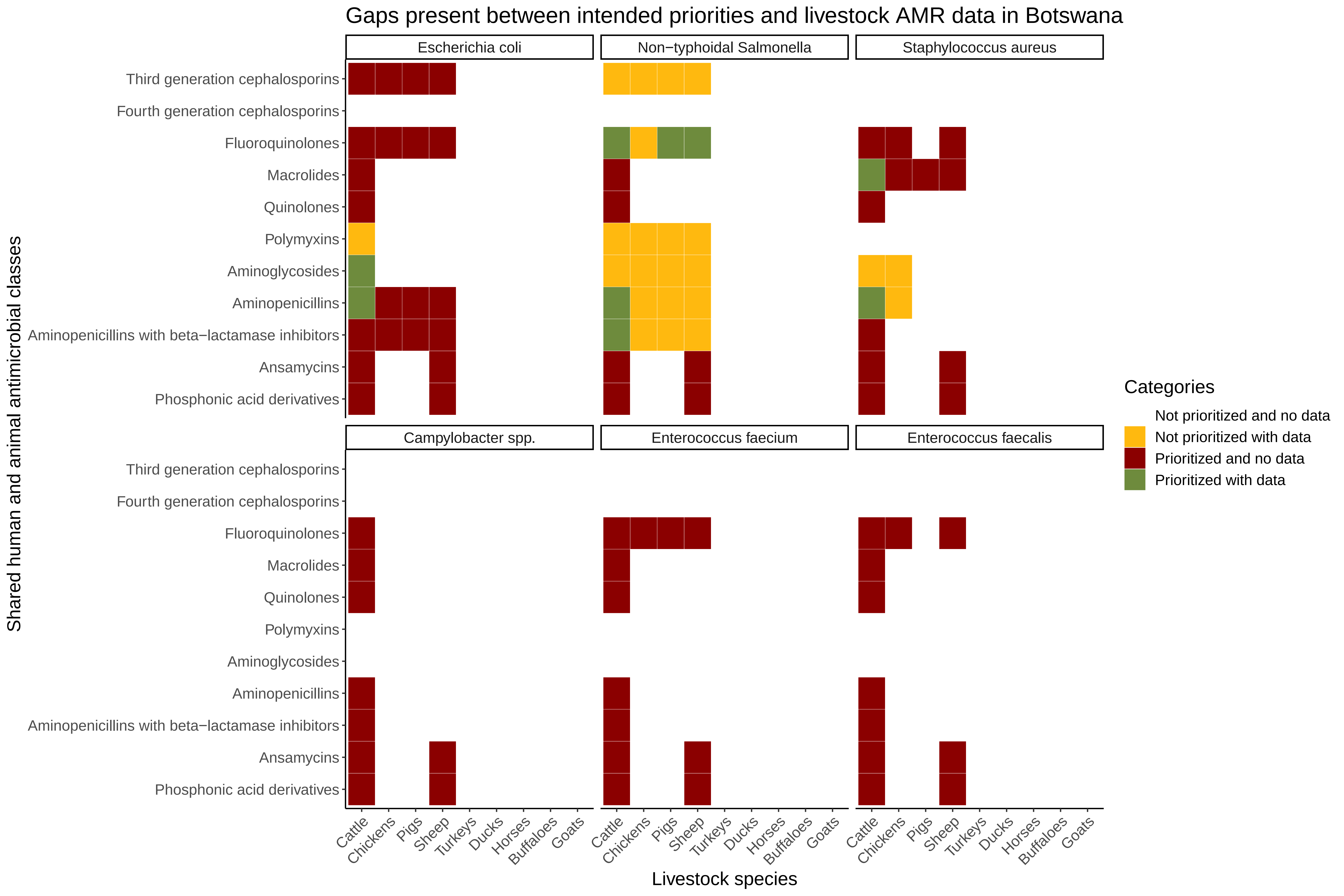

Gaps present between intended priorities and livestock AMR data in Botswana
Escherichia coli
Non−typhoidal Salmonella
Staphylococcus aureus
Campylobacter spp.
Enterococcus faecium
Enterococcus faecalis
Third generation cephalosporins
Fourth generation cephalosporins
Fluoroquinolones
Macrolides
Quinolones
Polymyxins
Aminoglycosides
Aminopenicillins
Aminopenicillins with beta−lactamase inhibitors
Ansamycins
Phosphonic acid derivatives
Shared human and animal antimicrobial classes
Cattle
Chickens
Pigs
Sheep
Turkeys
Ducks
Horses
Buffaloes
Goats
Livestock species
Categories
Not prioritized and no data
Not prioritized with data
Prioritized and no data
Prioritized with data

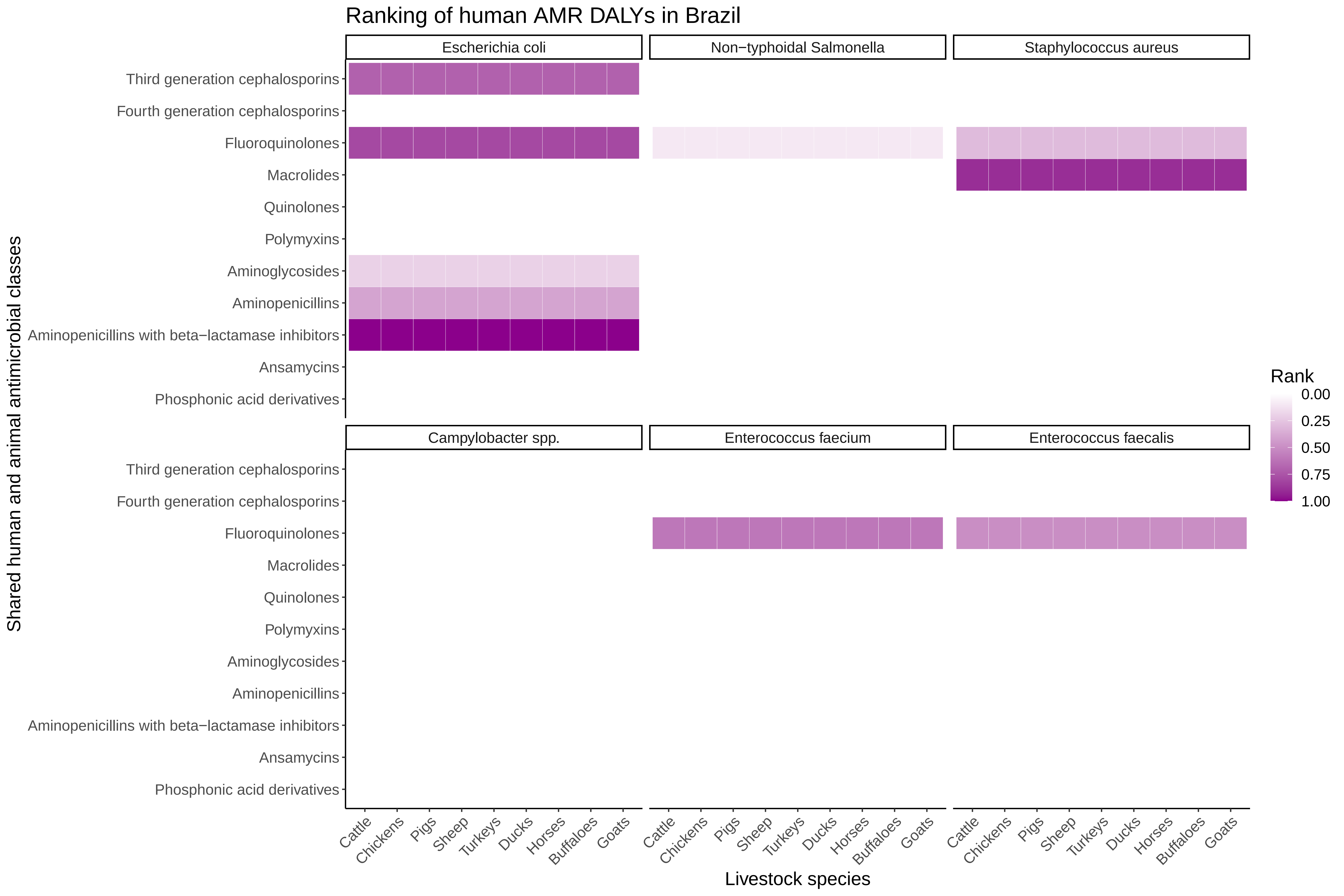
Ranking of human AMR DALYs in Brazil
Escherichia coli
Non−typhoidal Salmonella
Staphylococcus aureus
Campylobacter spp.
Enterococcus faecium
Enterococcus faecalis
Shared human and animal antimicrobial classes
Third generation cephalosporins
Fourth generation cephalosporins
Fluoroquinolones
Macrolides
Quinolones
Polymyxins
Aminoglycosides
Aminopenicillins
Aminopenicillins with beta−lactamase inhibitors
Ansamycins
Phosphonic acid derivatives
Cattle
Chickens
Pigs
Sheep
Turkeys
Ducks
Horses
Buffaloes
Goats
Livestock species
Rank
0.00
0.25
0.50
0.75
1.00

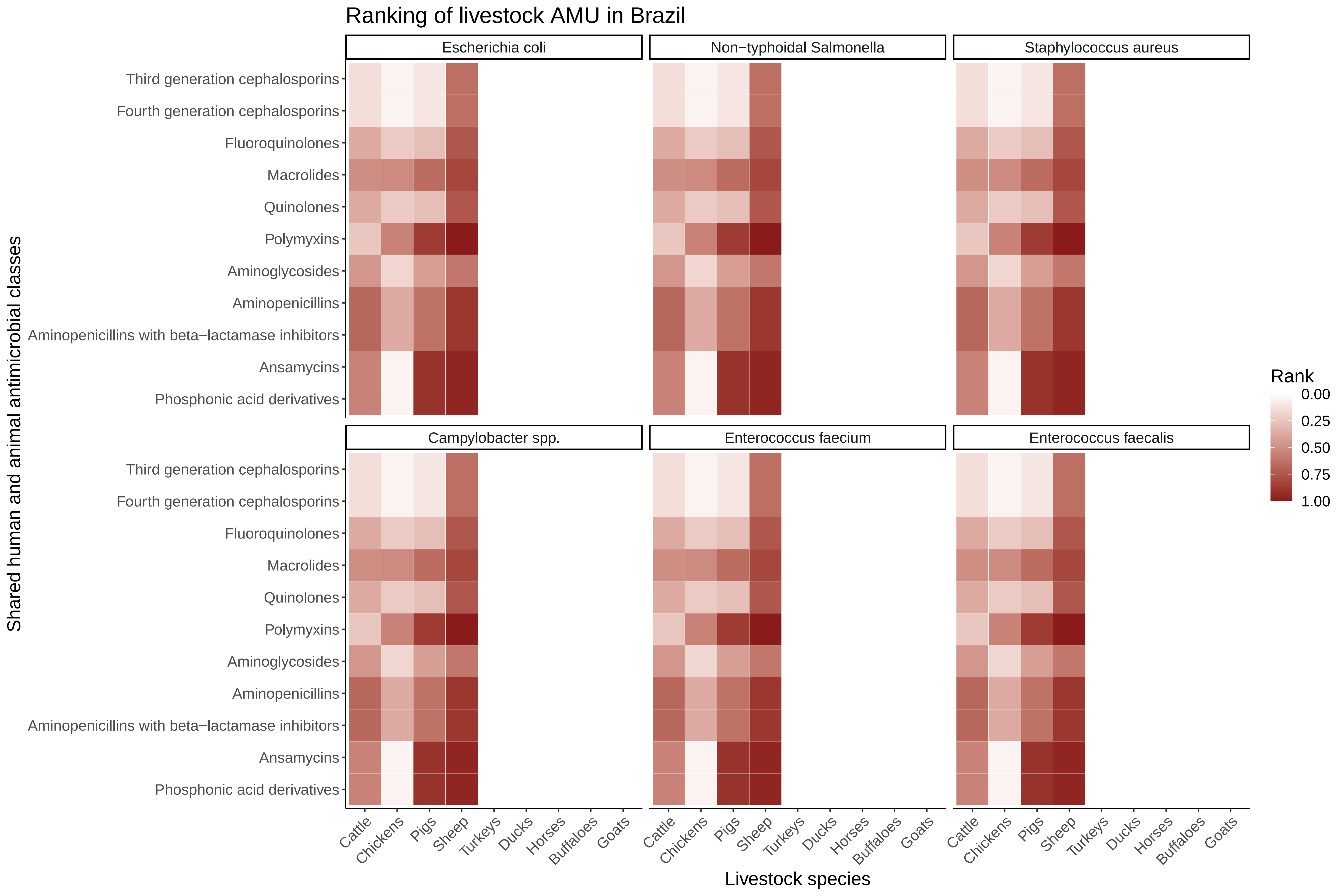
Ranking of livestock AMU in Brazil
Escherichia coli
Non−typhoidal Salmonella
Staphylococcus aureus
Campylobacter spp.
Enterococcus faecium
Enterococcus faecalis
Shared human and animal antimicrobial classes
Third generation cephalosporins
Fourth generation cephalosporins
Fluoroquinolones
Macrolides
Quinolones
Polymyxins
Aminoglycosides
Aminopenicillins
Aminopenicillins with beta−lactamase inhibitors
Ansamycins
Phosphonic acid derivatives
Cattle
Chickens
Pigs
Sheep
Turkeys
Ducks
Horses
Buffaloes
Goats
Livestock species
Rank
0.00
0.25
0.50
0.75
1.00

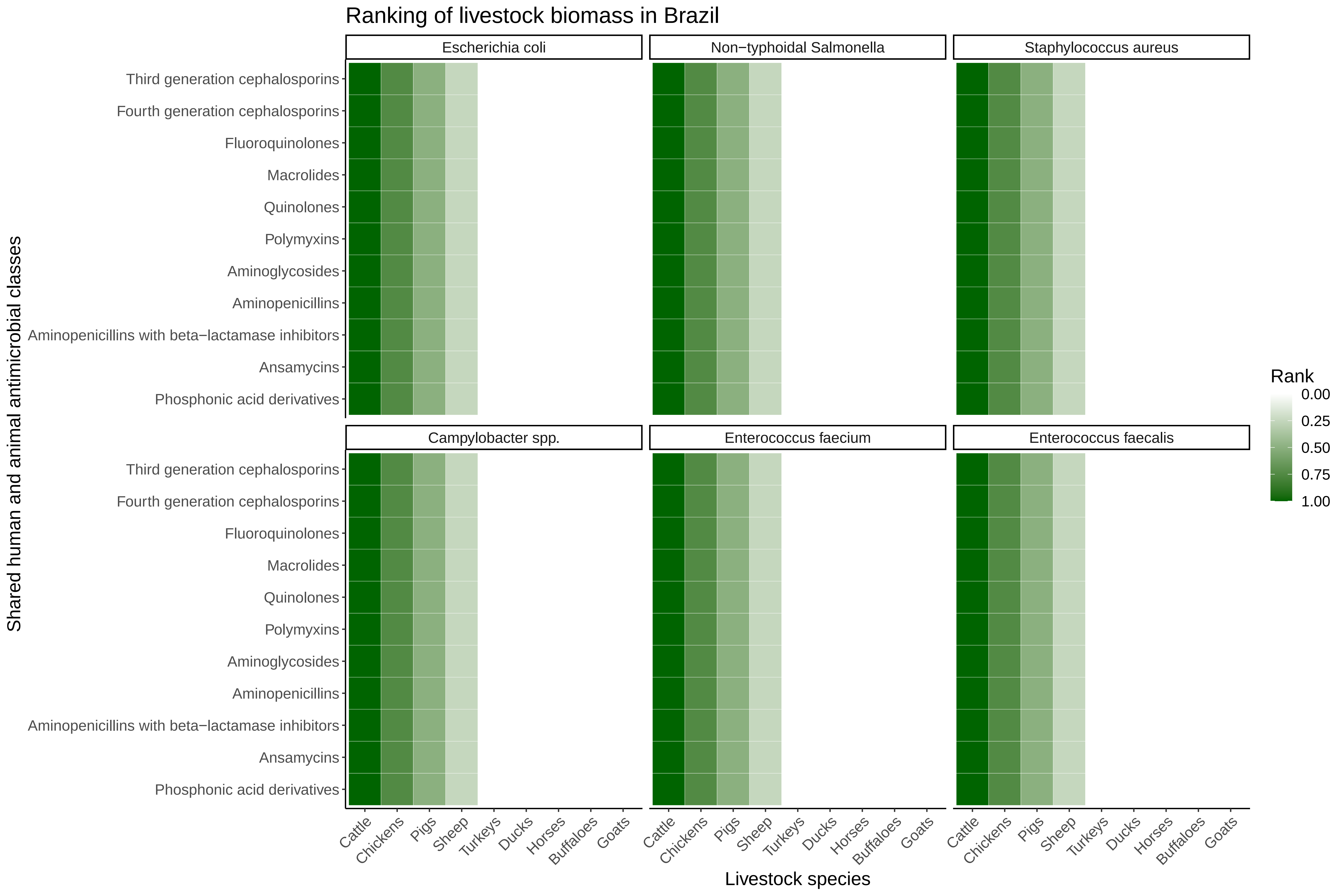

Ranking of livestock biomass in Brazil
Escherichia coli
Non−typhoidal Salmonella
Staphylococcus aureus
Campylobacter spp.
Enterococcus faecium
Enterococcus faecalis
Third generation cephalosporins
Fourth generation cephalosporins
Fluoroquinolones
Macrolides
Quinolones
Polymyxins
Aminoglycosides
Aminopenicillins
Aminopenicillins with beta−lactamase inhibitors
Ansamycins
Phosphonic acid derivatives
Shared human and animal antimicrobial classes
Cattle
Chickens
Pigs
Sheep
Turkeys
Ducks
Horses
Buffaloes
Goats
Livestock species
Rank
0.00
0.25
0.50
0.75
1.00

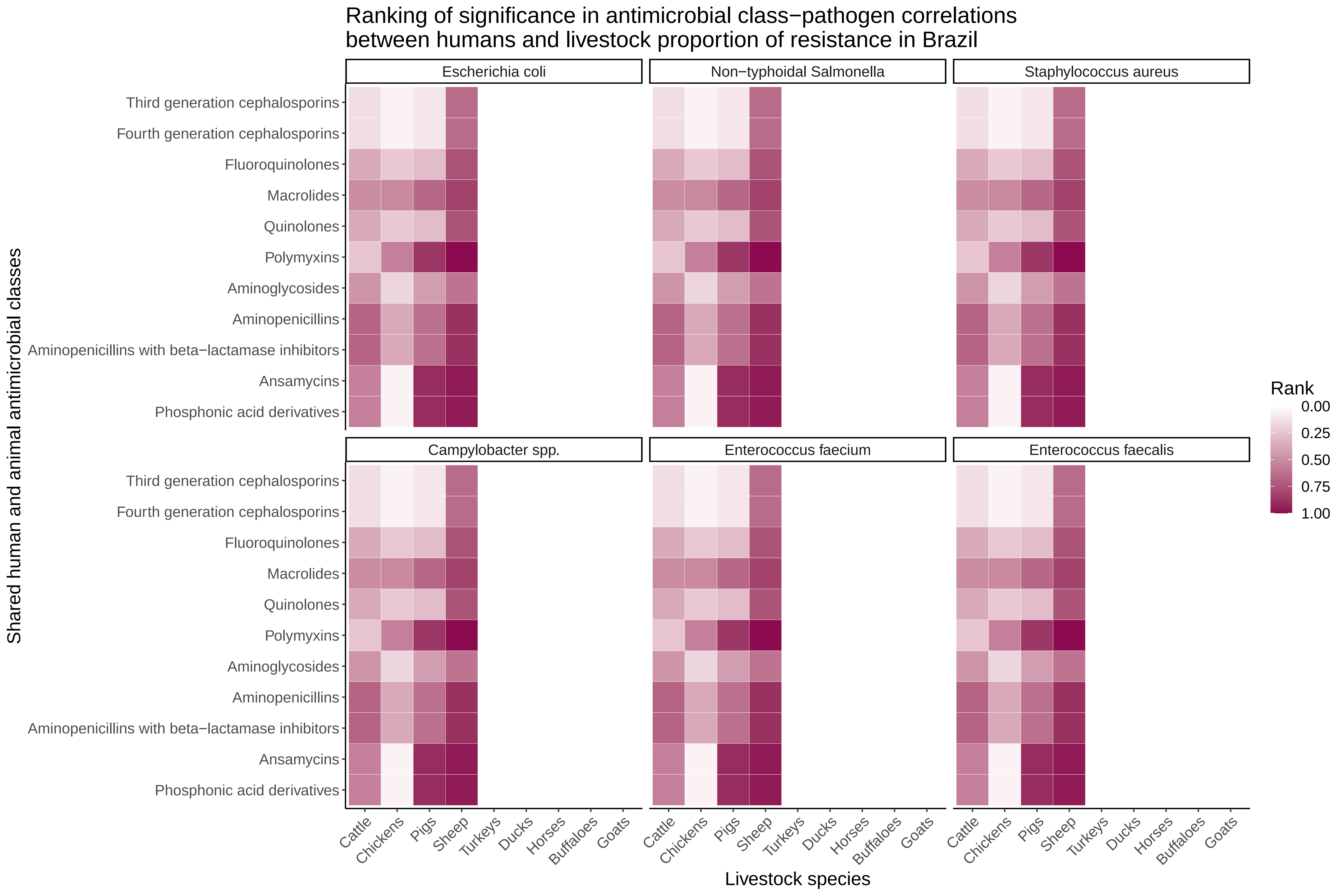

Ranking of significance in antimicrobial class−pathogen correlations
between humans and livestock proportion of resistance in Brazil
Escherichia coli
Non−typhoidal Salmonella
Staphylococcus aureus
Campylobacter spp.
Enterococcus faecium
Enterococcus faecalis
Third generation cephalosporins
Fourth generation cephalosporins
Fluoroquinolones
Macrolides
Quinolones
Polymyxins
Aminoglycosides
Aminopenicillins
Aminopenicillins with beta−lactamase inhibitors
Ansamycins
Phosphonic acid derivatives
Shared human and animal antimicrobial classes
Cattle
Chickens
Pigs
Sheep
Turkeys
Ducks
Horses
Buffaloes
Goats
Livestock species
Rank
0.00
0.25
0.50
0.75
1.00

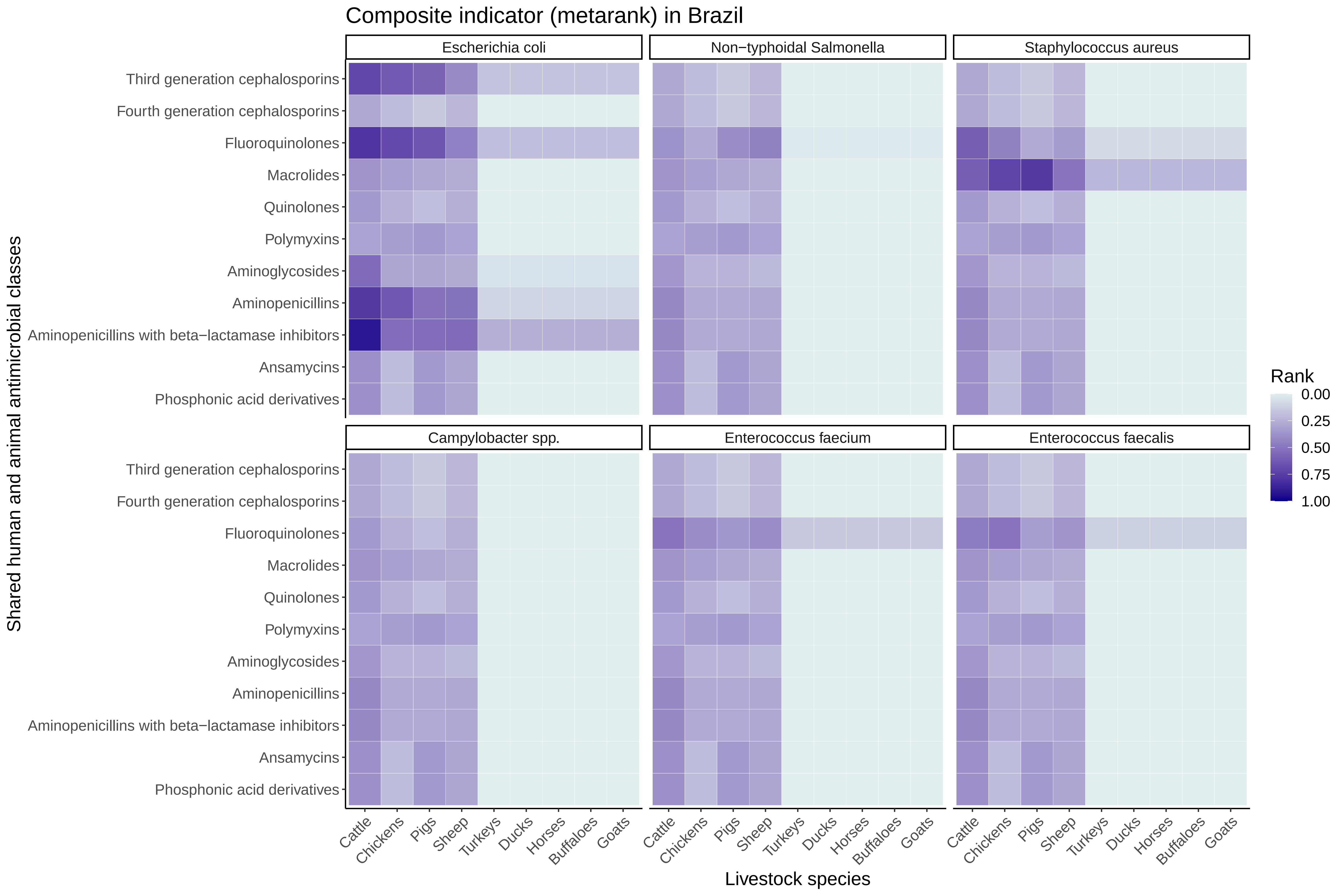
Composite indicator (metarank) in Brazil
Escherichia coli
Non−typhoidal Salmonella
Staphylococcus aureus
Campylobacter spp.
Enterococcus faecium
Enterococcus faecalis
Third generation cephalosporins
Fourth generation cephalosporins
Fluoroquinolones
Macrolides
Quinolones
Polymyxins
Aminoglycosides
Aminopenicillins
Aminopenicillins with beta−lactamase inhibitors
Ansamycins
Phosphonic acid derivatives
Cattle
Chickens
Pigs
Sheep
Turkeys
Ducks
Horses
Buffaloes
Goats
Shared human and animal antimicrobial classes
Livestock species
Rank
0.00
0.25
0.50
0.75
1.00

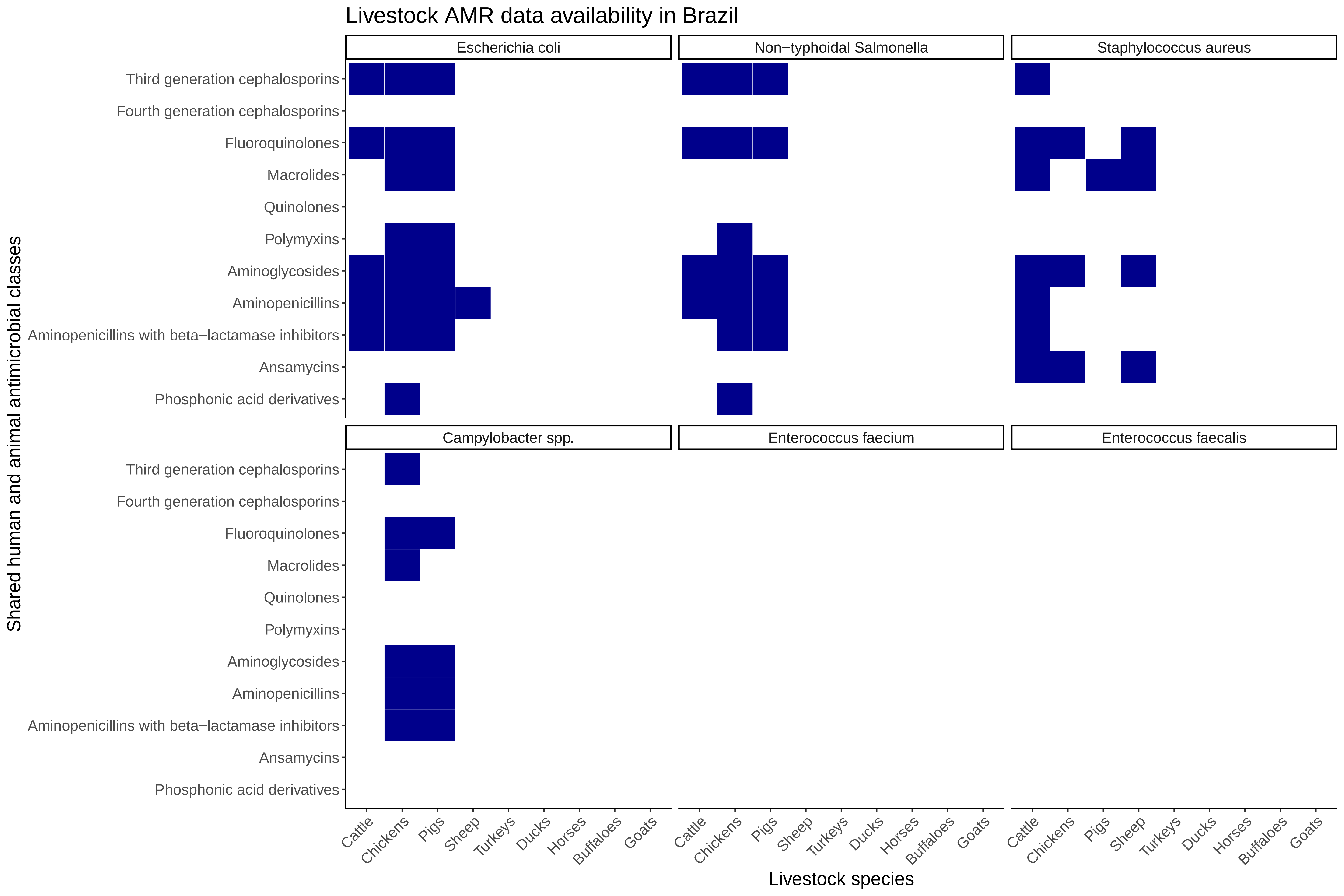

Livestock AMR data availability in Brazil
Escherichia coli
Non−typhoidal Salmonella
Staphylococcus aureus
Campylobacter spp.
Enterococcus faecium
Enterococcus faecalis
Shared human and animal antimicrobial classes
Third generation cephalosporins
Fourth generation cephalosporins
Fluoroquinolones
Macrolides
Quinolones
Polymyxins
Aminoglycosides
Aminopenicillins
Aminopenicillins with beta−lactamase inhibitors
Ansamycins
Phosphonic acid derivatives
Cattle
Chickens
Pigs
Sheep
Turkeys
Ducks
Horses
Buffaloes
Goats
Livestock species

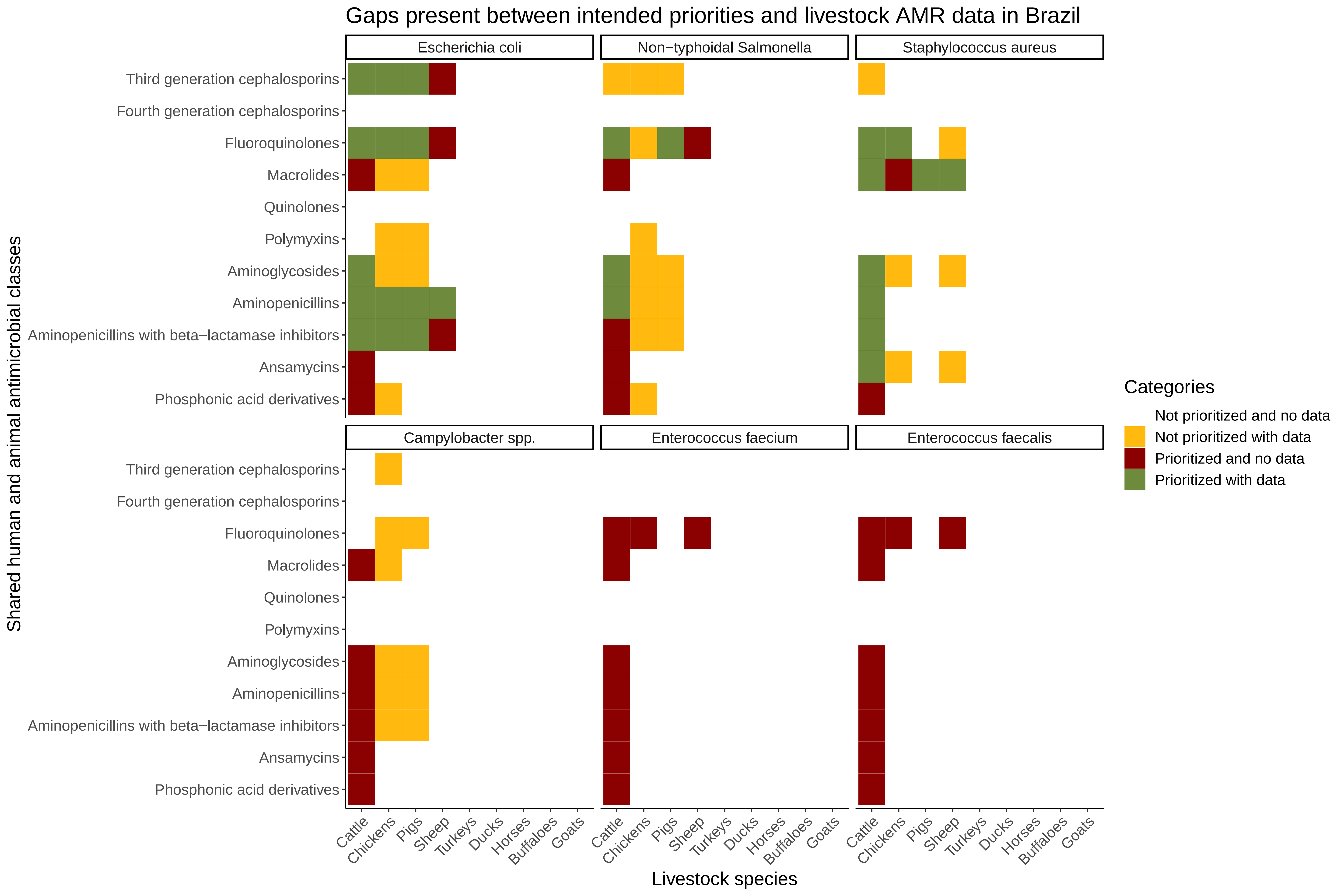

Gaps present between intended priorities and livestock AMR data in Brazil
Escherichia coli
Non−typhoidal Salmonella
Staphylococcus aureus
Campylobacter spp.
Enterococcus faecium
Enterococcus faecalis
Third generation cephalosporins
Fourth generation cephalosporins
Fluoroquinolones
Macrolides
Quinolones
Polymyxins
Aminoglycosides
Aminopenicillins
Aminopenicillins with beta−lactamase inhibitors
Ansamycins
Phosphonic acid derivatives
Shared human and animal antimicrobial classes
Cattle
Chickens
Pigs
Sheep
Turkeys
Ducks
Horses
Buffaloes
Goats
Livestock species
Categories
Not prioritized and no data
Not prioritized with data
Prioritized and no data
Prioritized with data

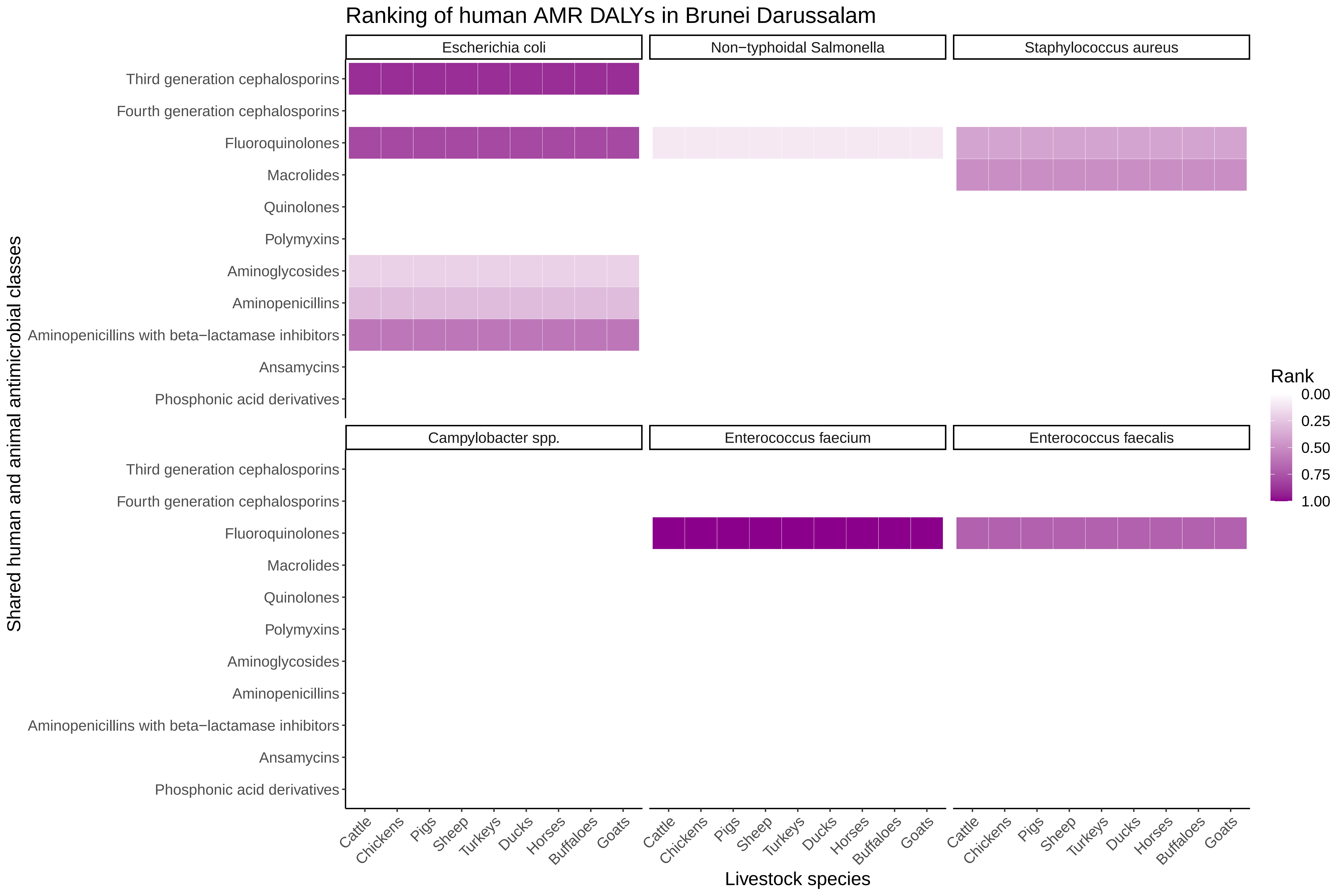
Ranking of human AMR DALYs in Brunei Darussalam
Escherichia coli
Non−typhoidal Salmonella
Staphylococcus aureus
Campylobacter spp.
Enterococcus faecium
Enterococcus faecalis
Third generation cephalosporins
Fourth generation cephalosporins
Fluoroquinolones
Macrolides
Quinolones
Polymyxins
Aminoglycosides
Aminopenicillins
Aminopenicillins with beta−lactamase inhibitors
Ansamycins
Phosphonic acid derivatives
Shared human and animal antimicrobial classes
Cattle
Chickens
Pigs
Sheep
Turkeys
Ducks
Horses
Buffaloes
Goats
Livestock species
Rank
0.00
0.25
0.50
0.75
1.00

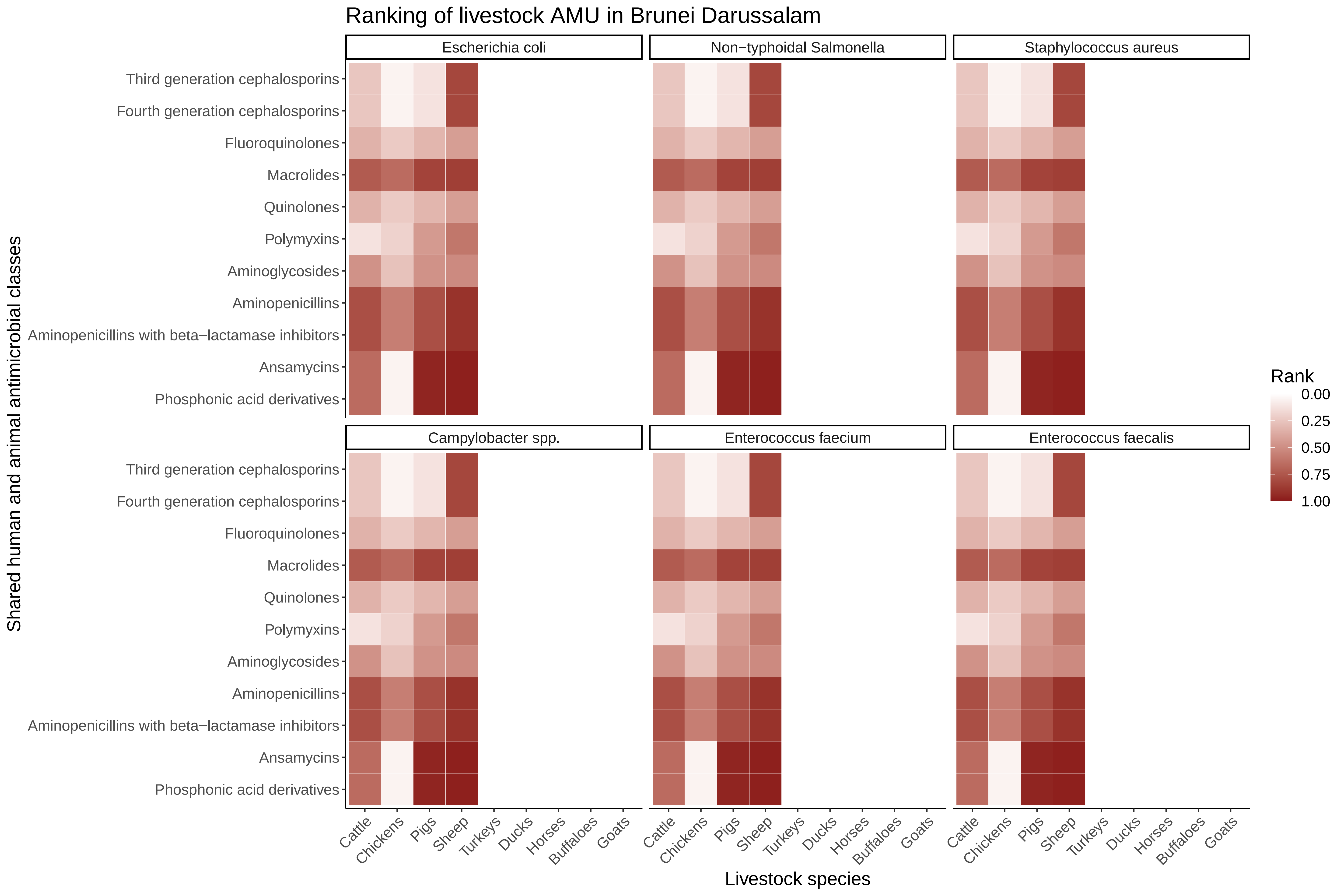
Ranking of livestock AMU in Brunei Darussalam
Escherichia coli
Non−typhoidal Salmonella
Staphylococcus aureus
Campylobacter spp.
Enterococcus faecium
Enterococcus faecalis
Shared human and animal antimicrobial classes
Third generation cephalosporins
Fourth generation cephalosporins
Fluoroquinolones
Macrolides
Quinolones
Polymyxins
Aminoglycosides
Aminopenicillins
Aminopenicillins with beta−lactamase inhibitors
Ansamycins
Phosphonic acid derivatives
Cattle
Chickens
Pigs
Sheep
Turkeys
Ducks
Horses
Buffaloes
Goats
Livestock species
Rank
0.00
0.25
0.50
0.75
1.00

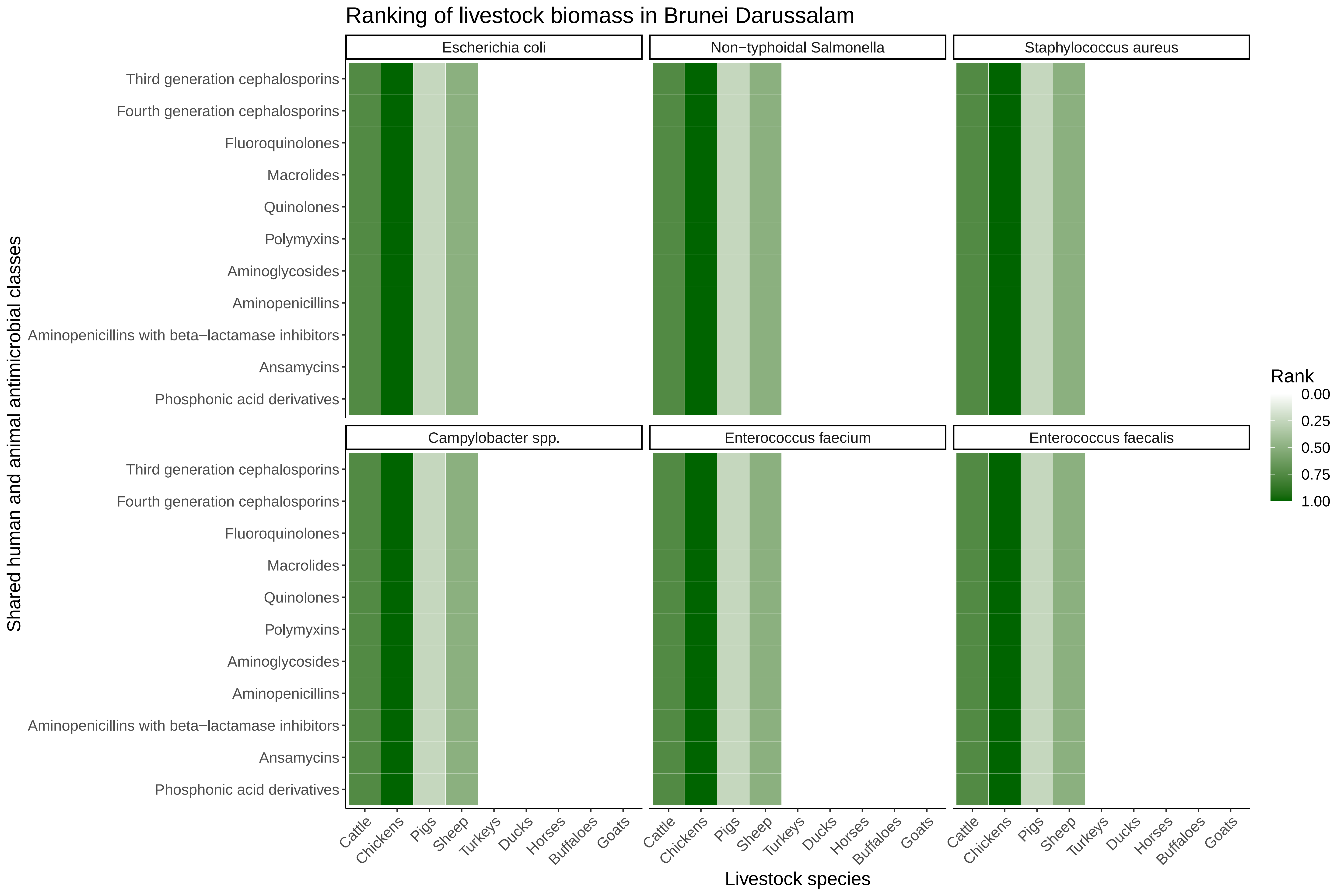
Ranking of livestock biomass in Brunei Darussalam
Escherichia coli
Non−typhoidal Salmonella
Staphylococcus aureus
Campylobacter spp.
Enterococcus faecium
Enterococcus faecalis
Third generation cephalosporins
Fourth generation cephalosporins
Fluoroquinolones
Macrolides
Quinolones
Polymyxins
Aminoglycosides
Aminopenicillins
Aminopenicillins with beta−lactamase inhibitors
Ansamycins
Phosphonic acid derivatives
Shared human and animal antimicrobial classes
Cattle
Chickens
Pigs
Sheep
Turkeys
Ducks
Horses
Buffaloes
Goats
Livestock species
Rank
0.00
0.25
0.50
0.75
1.00

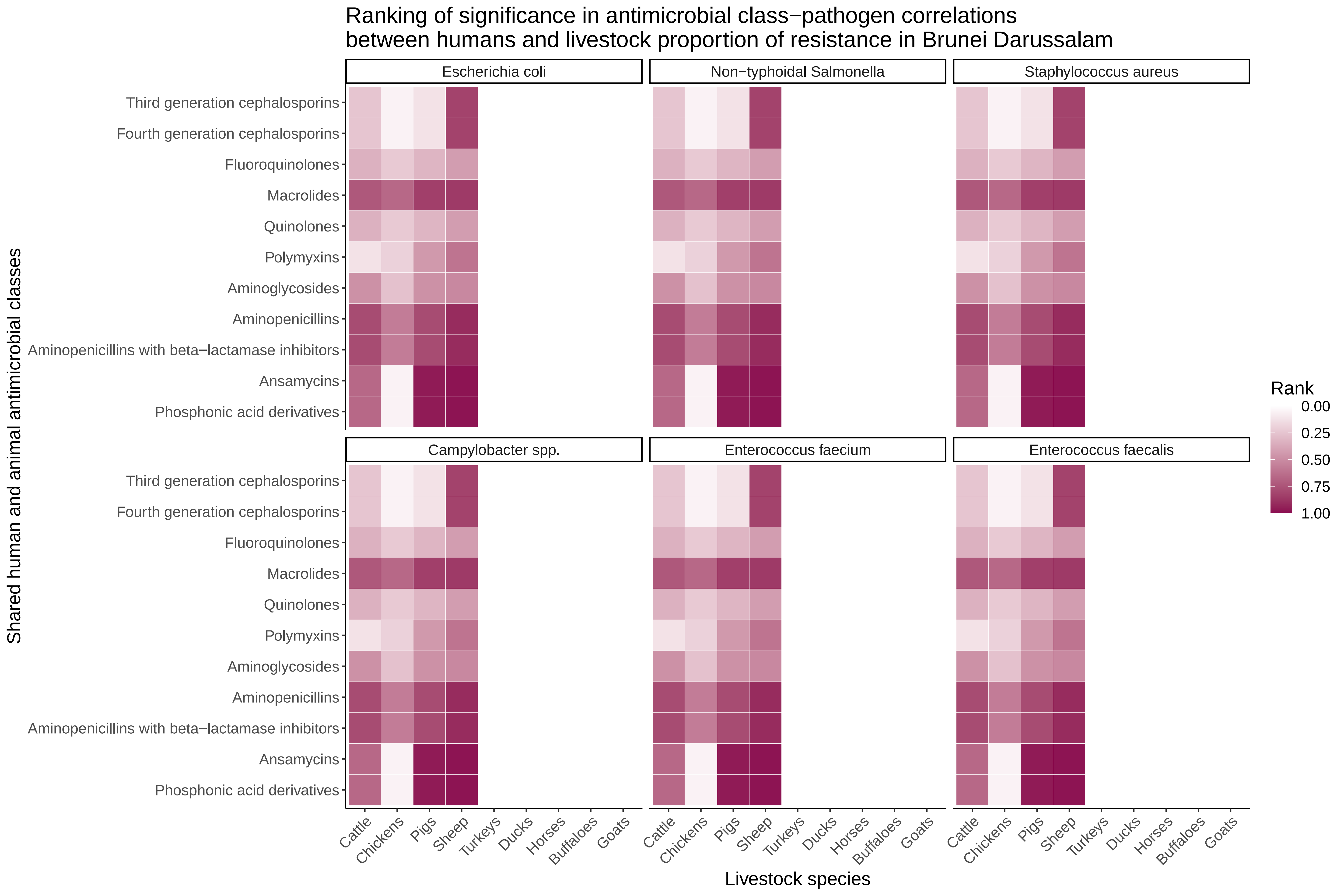

Ranking of significance in antimicrobial class−pathogen correlations
between humans and livestock proportion of resistance in Brunei Darussalam
Escherichia coli
Non−typhoidal Salmonella
Staphylococcus aureus
Campylobacter spp.
Enterococcus faecium
Enterococcus faecalis
Shared human and animal antimicrobial classes
Third generation cephalosporins
Fourth generation cephalosporins
Fluoroquinolones
Macrolides
Quinolones
Polymyxins
Aminoglycosides
Aminopenicillins
Aminopenicillins with beta−lactamase inhibitors
Ansamycins
Phosphonic acid derivatives
Livestock species
Cattle
Chickens
Pigs
Sheep
Turkeys
Ducks
Horses
Buffaloes
Goats
Rank
0.00
0.25
0.50
0.75
1.00

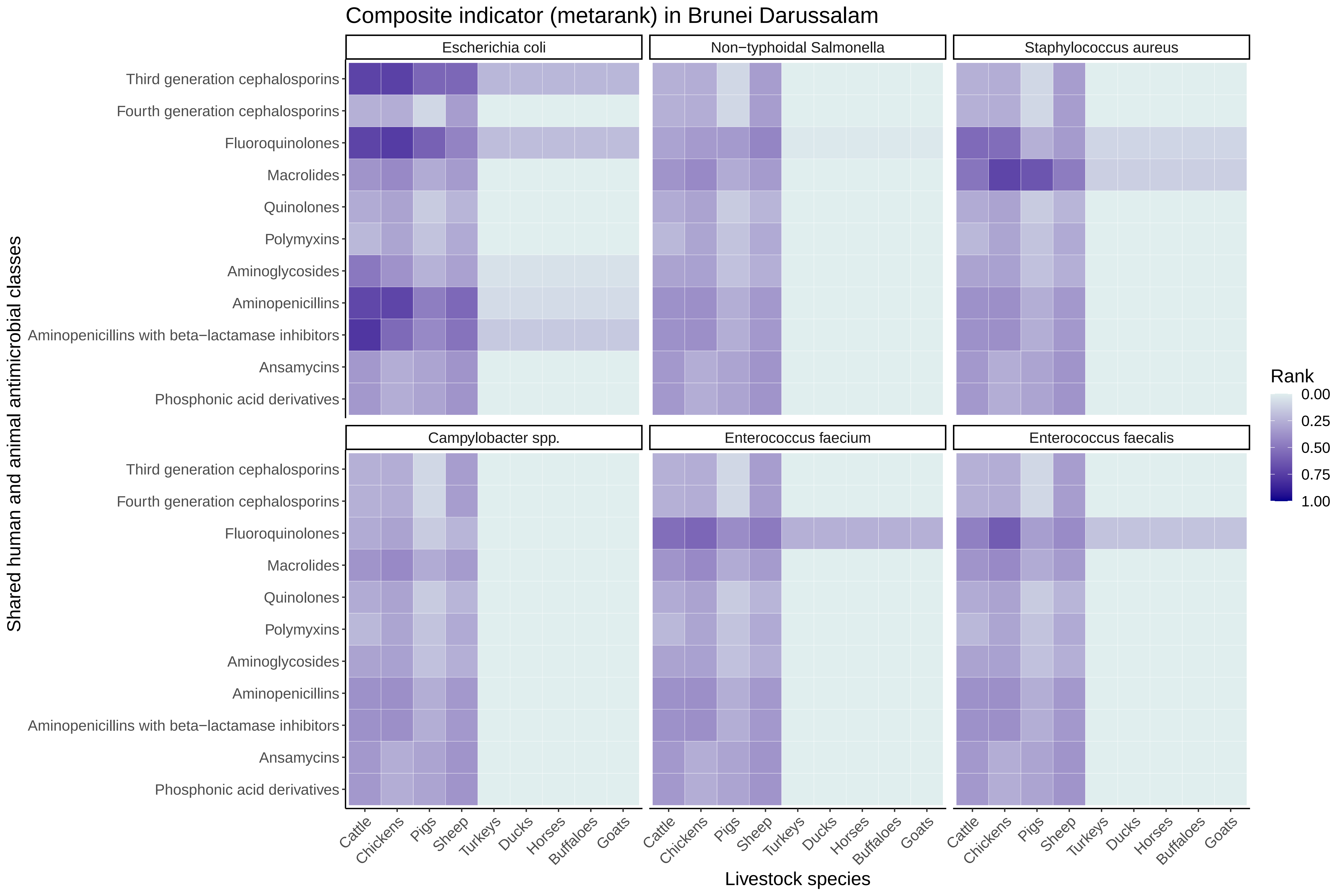

Composite indicator (metarank) in Brunei Darussalam
Escherichia coli
Non−typhoidal Salmonella
Staphylococcus aureus
Campylobacter spp.
Enterococcus faecium
Enterococcus faecalis
Shared human and animal antimicrobial classes
Third generation cephalosporins
Fourth generation cephalosporins
Fluoroquinolones
Macrolides
Quinolones
Polymyxins
Aminoglycosides
Aminopenicillins
Aminopenicillins with beta−lactamase inhibitors
Ansamycins
Phosphonic acid derivatives
Cattle
Chickens
Pigs
Sheep
Turkeys
Ducks
Horses
Buffaloes
Goats
Livestock species
Rank
0.00
0.25
0.50
0.75
1.00

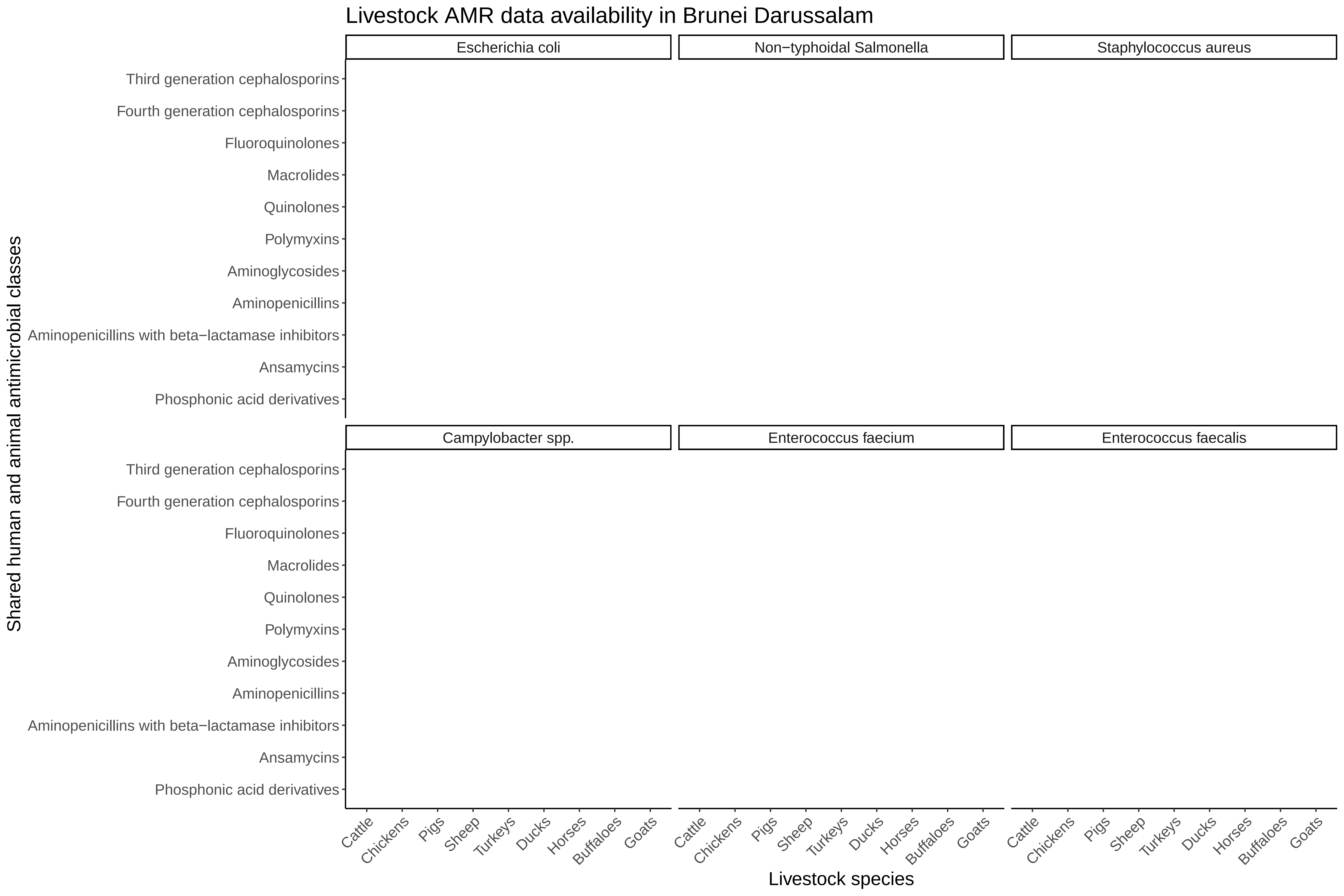
Livestock AMR data availability in Brunei Darussalam
Escherichia coli
Non−typhoidal Salmonella
Staphylococcus aureus
Campylobacter spp.
Enterococcus faecium
Enterococcus faecalis
Shared human and animal antimicrobial classes
Third generation cephalosporins
Fourth generation cephalosporins
Fluoroquinolones
Macrolides
Quinolones
Polymyxins
Aminoglycosides
Aminopenicillins
Aminopenicillins with beta−lactamase inhibitors
Ansamycins
Phosphonic acid derivatives
Cattle
Chickens
Pigs
Sheep
Turkeys
Ducks
Horses
Buffaloes
Goats
Livestock species

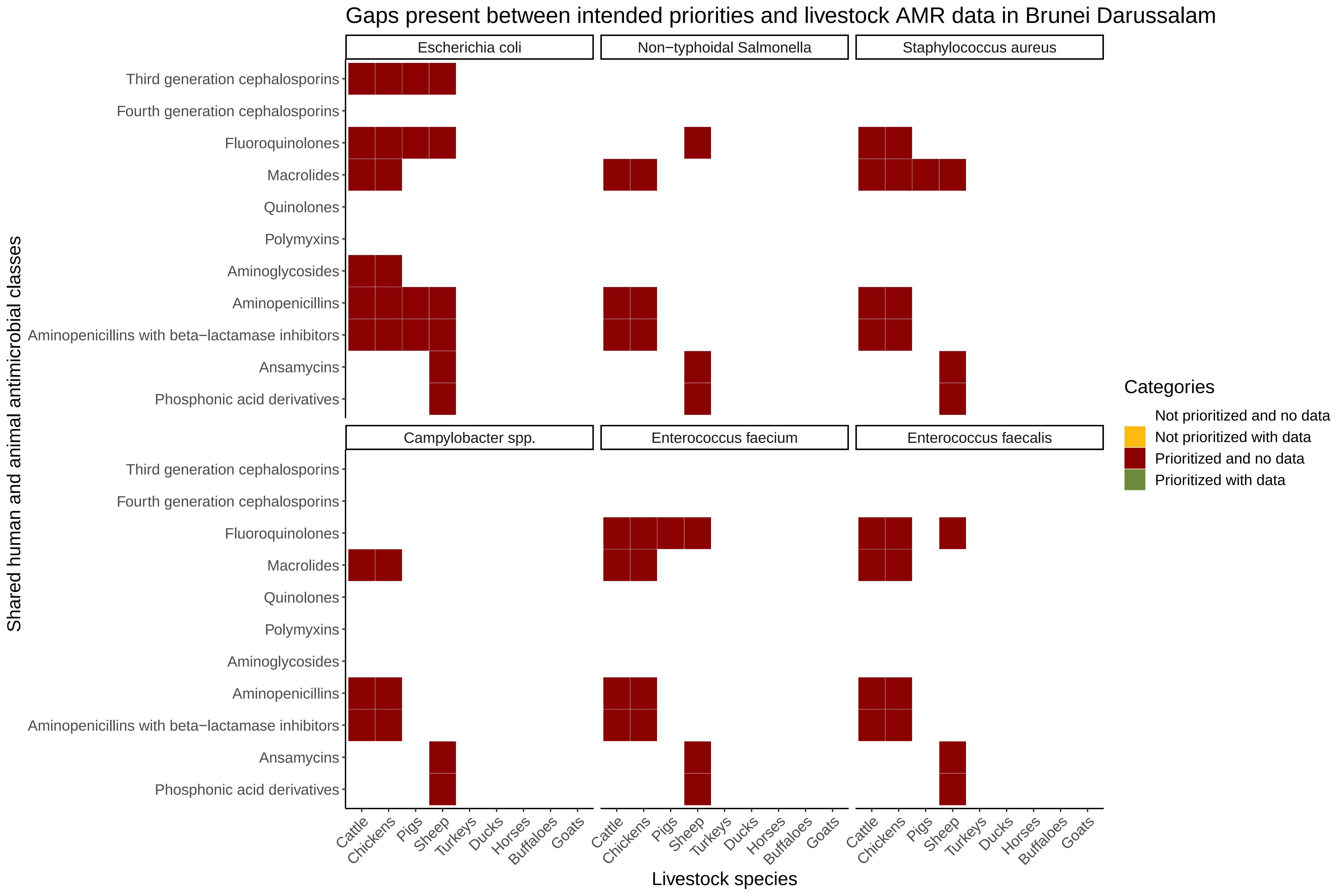
Gaps present between intended priorities and livestock AMR data in Brunei Darussalam
Escherichia coli
Non−typhoidal Salmonella
Staphylococcus aureus
Campylobacter spp.
Enterococcus faecium
Enterococcus faecalis
Third generation cephalosporins
Fourth generation cephalosporins
Fluoroquinolones
Macrolides
Quinolones
Polymyxins
Aminoglycosides
Aminopenicillins
Aminopenicillins with beta−lactamase inhibitors
Ansamycins
Phosphonic acid derivatives
Shared human and animal antimicrobial classes
Cattle
Chickens
Pigs
Sheep
Turkeys
Ducks
Horses
Buffaloes
Goats
Livestock species
Categories
Not prioritized and no data
Not prioritized with data
Prioritized and no data
Prioritized with data

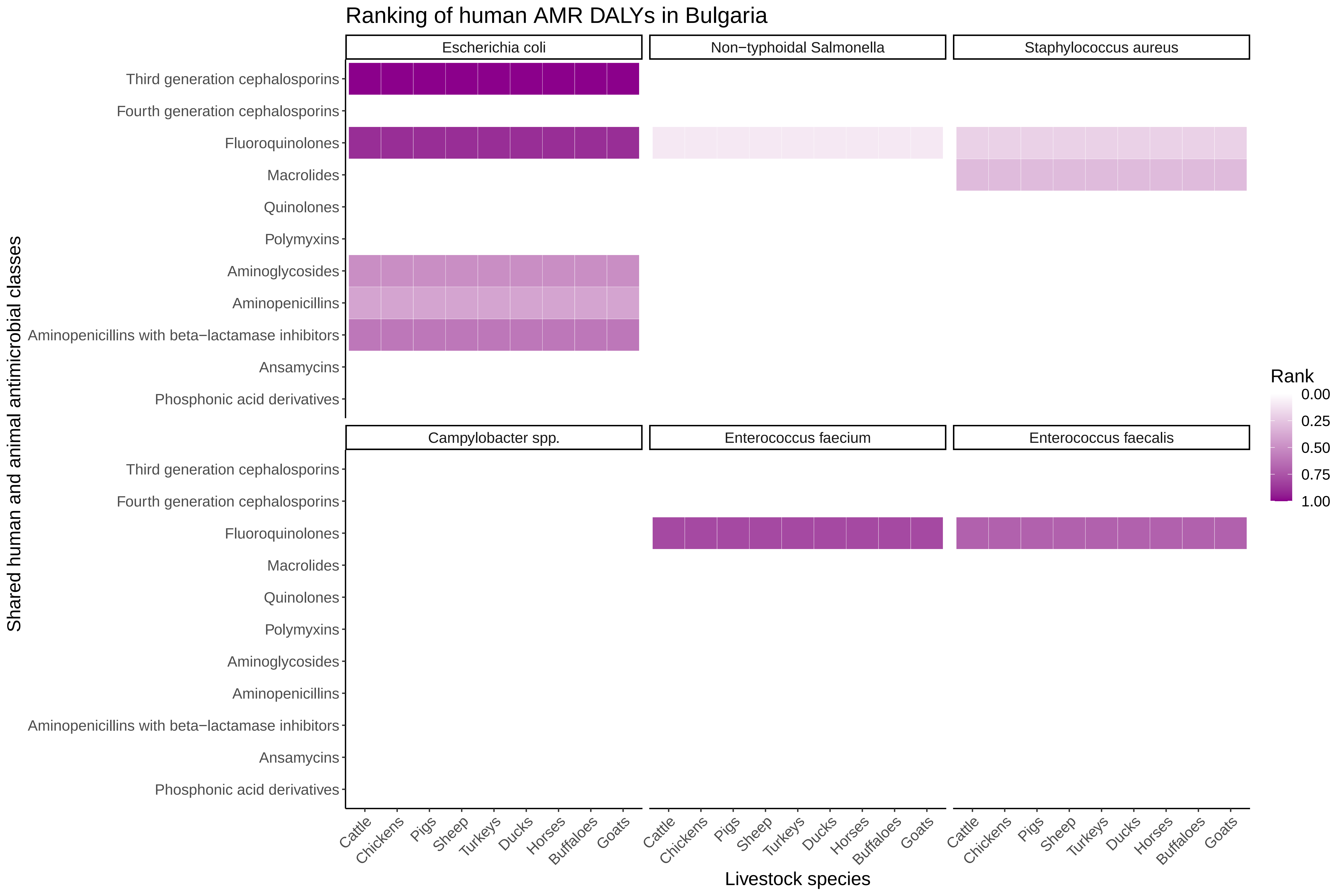

Ranking of human AMR DALYs in Bulgaria
Escherichia coli
Non−typhoidal Salmonella
Staphylococcus aureus
Campylobacter spp.
Enterococcus faecium
Enterococcus faecalis
Third generation cephalosporins
Fourth generation cephalosporins
Fluoroquinolones
Macrolides
Quinolones
Polymyxins
Aminoglycosides
Aminopenicillins
Aminopenicillins with beta−lactamase inhibitors
Ansamycins
Phosphonic acid derivatives
Shared human and animal antimicrobial classes
Cattle
Chickens
Pigs
Sheep
Turkeys
Ducks
Horses
Buffaloes
Goats
Livestock species
Rank
0.00
0.25
0.50
0.75
1.00

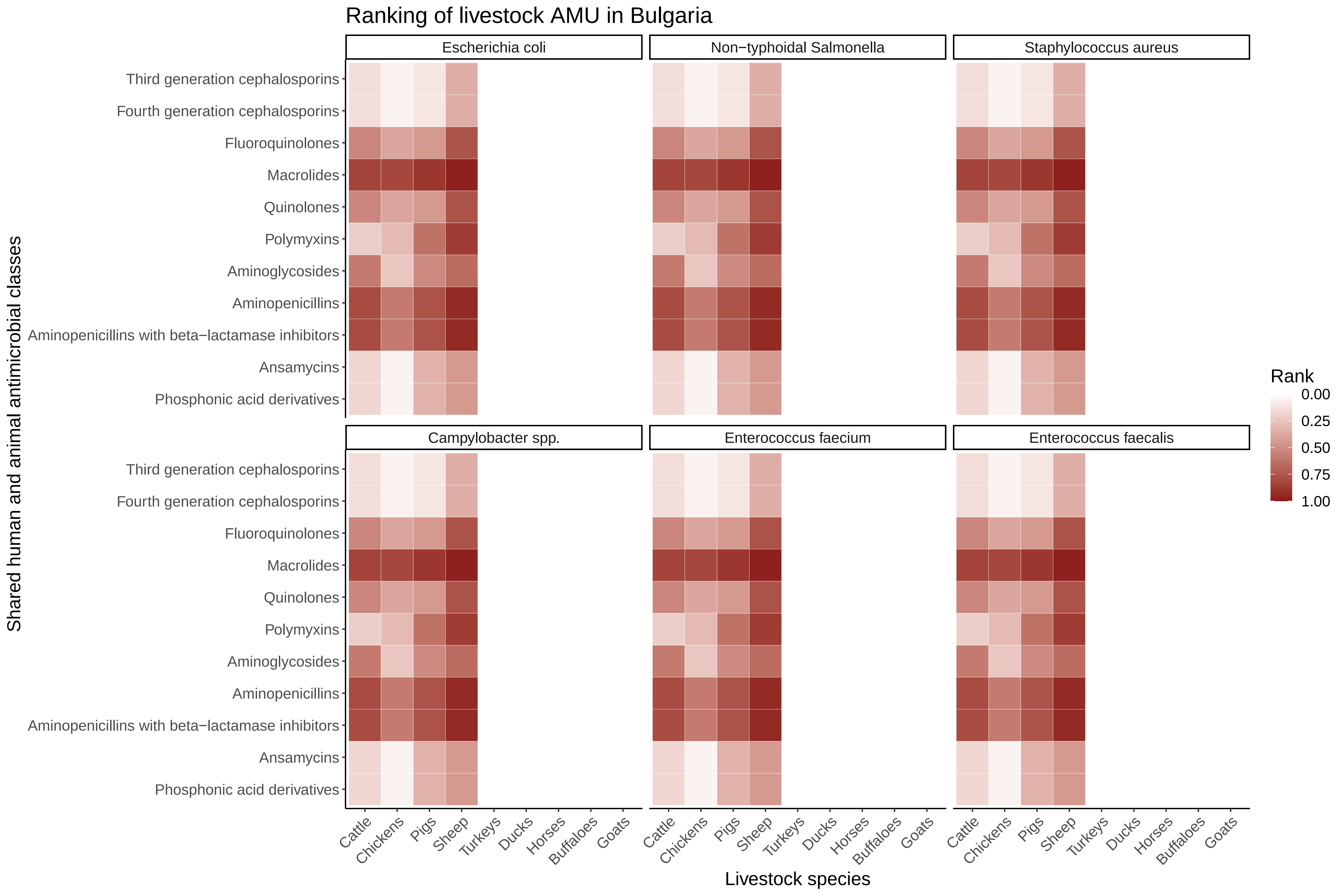

Ranking of livestock AMU in Bulgaria
Escherichia coli
Non−typhoidal Salmonella
Staphylococcus aureus
Campylobacter spp.
Enterococcus faecium
Enterococcus faecalis
Shared human and animal antimicrobial classes
Third generation cephalosporins
Fourth generation cephalosporins
Fluoroquinolones
Macrolides
Quinolones
Polymyxins
Aminoglycosides
Aminopenicillins
Aminopenicillins with beta−lactamase inhibitors
Ansamycins
Phosphonic acid derivatives
Livestock species
Cattle
Chickens
Pigs
Sheep
Turkeys
Ducks
Horses
Buffaloes
Goats
Rank
0.00
0.25
0.50
0.75
1.00

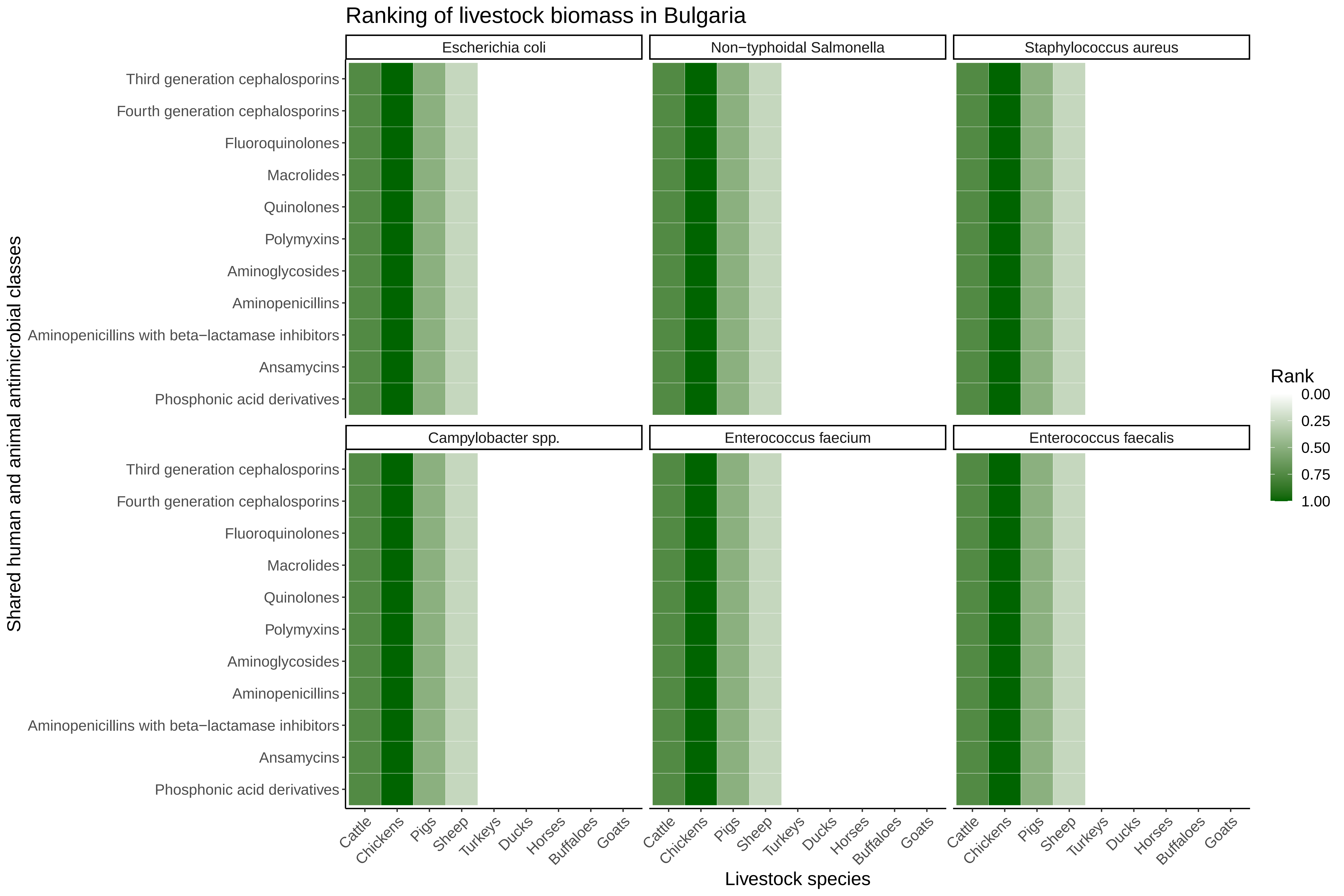

Ranking of livestock biomass in Bulgaria
Escherichia coli
Non−typhoidal Salmonella
Staphylococcus aureus
Campylobacter spp.
Enterococcus faecium
Enterococcus faecalis
Third generation cephalosporins
Fourth generation cephalosporins
Fluoroquinolones
Macrolides
Quinolones
Polymyxins
Aminoglycosides
Aminopenicillins
Aminopenicillins with beta−lactamase inhibitors
Ansamycins
Phosphonic acid derivatives
Shared human and animal antimicrobial classes
Cattle
Chickens
Pigs
Sheep
Turkeys
Ducks
Horses
Buffaloes
Goats
Livestock species
Rank
0.00
0.25
0.50
0.75
1.00

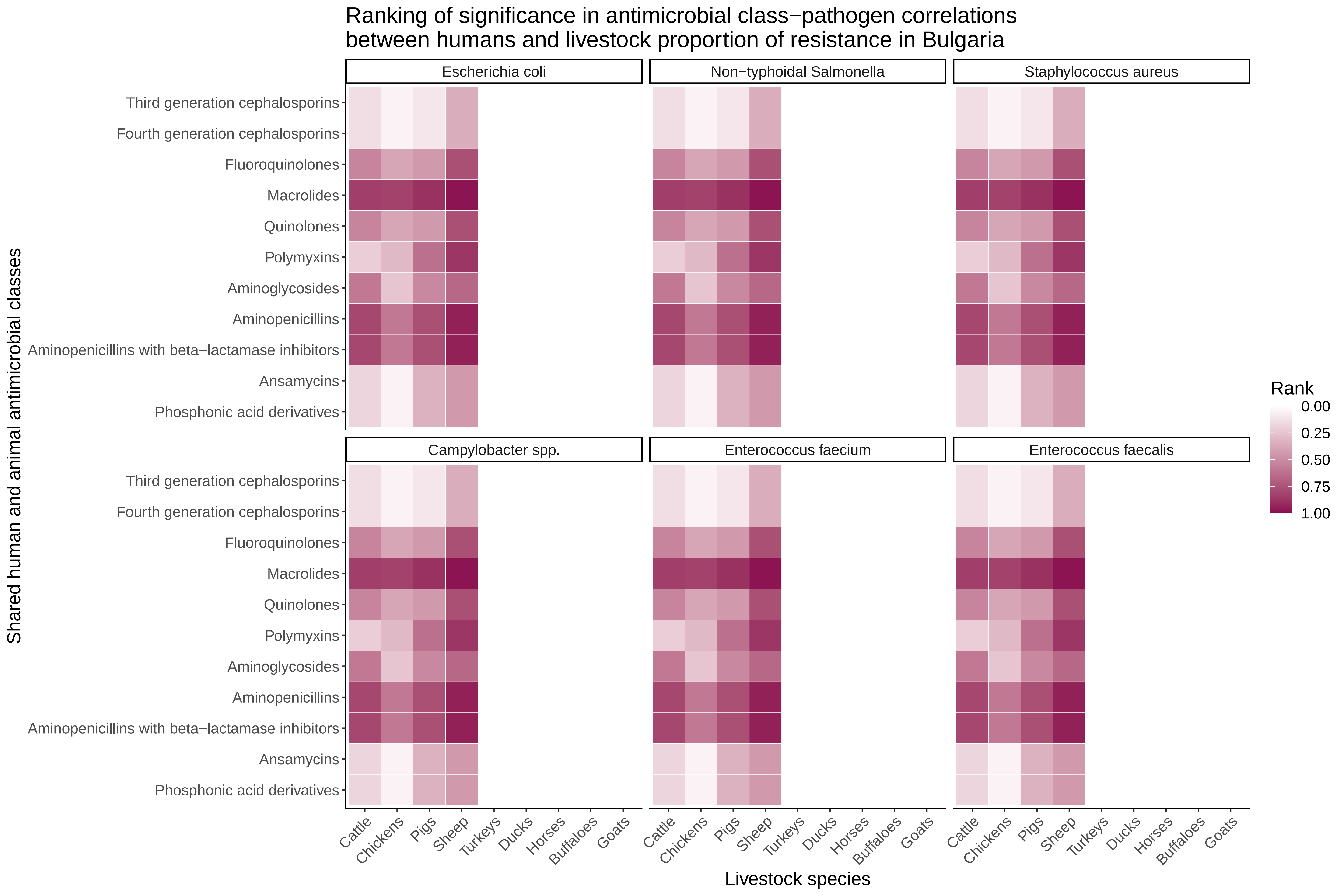
Ranking of significance in antimicrobial class−pathogen correlations
between humans and livestock proportion of resistance in Bulgaria
Escherichia coli
Non−typhoidal Salmonella
Staphylococcus aureus
Campylobacter spp.
Enterococcus faecium
Enterococcus faecalis
Third generation cephalosporins
Fourth generation cephalosporins
Fluoroquinolones
Macrolides
Quinolones
Polymyxins
Aminoglycosides
Aminopenicillins
Aminopenicillins with beta−lactamase inhibitors
Ansamycins
Phosphonic acid derivatives
Shared human and animal antimicrobial classes
Cattle
Chickens
Pigs
Sheep
Turkeys
Ducks
Horses
Buffaloes
Goats
Livestock species
Rank
0.00
0.25
0.50
0.75
1.00

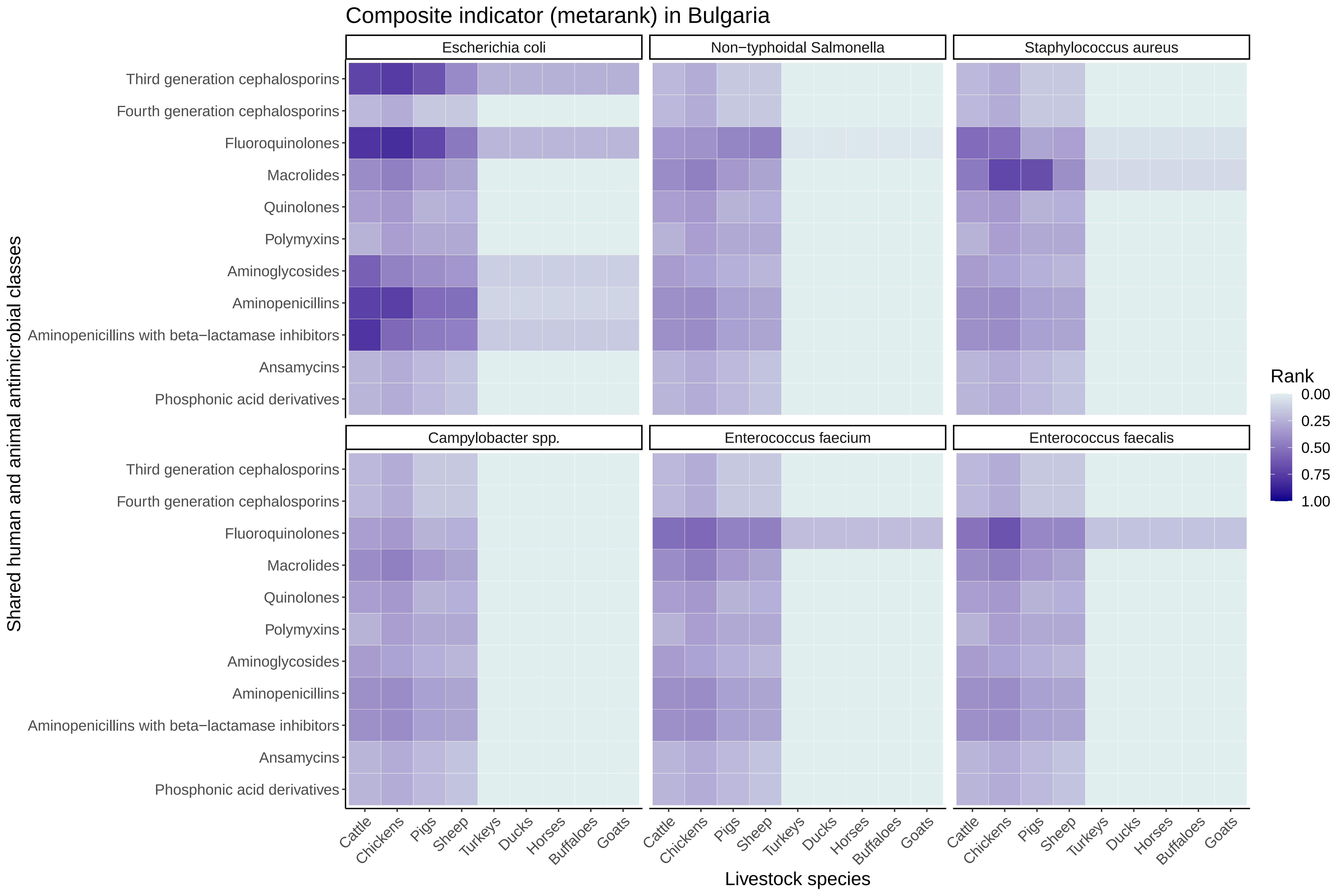

Composite indicator (metarank) in Bulgaria
Escherichia coli
Non−typhoidal Salmonella
Staphylococcus aureus
Campylobacter spp.
Enterococcus faecium
Enterococcus faecalis
Third generation cephalosporins
Fourth generation cephalosporins
Fluoroquinolones
Macrolides
Quinolones
Polymyxins
Aminoglycosides
Aminopenicillins
Aminopenicillins with beta−lactamase inhibitors
Ansamycins
Phosphonic acid derivatives
Shared human and animal antimicrobial classes
Cattle
Chickens
Pigs
Sheep
Turkeys
Ducks
Horses
Buffaloes
Goats
Livestock species
Rank
0.00
0.25
0.50
0.75
1.00

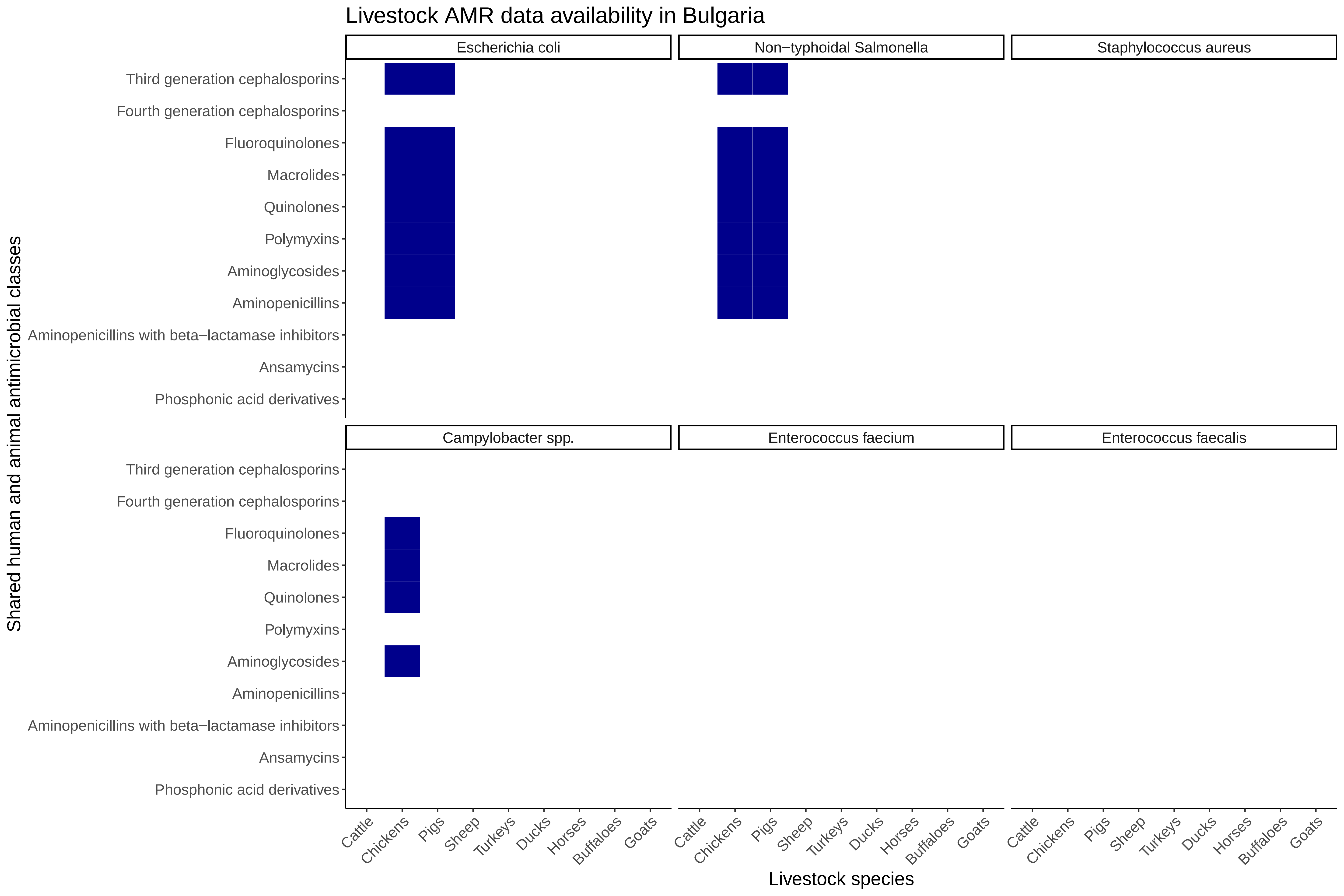

Livestock AMR data availability in Bulgaria
Escherichia coli
Non−typhoidal Salmonella
Staphylococcus aureus
Campylobacter spp.
Enterococcus faecium
Enterococcus faecalis
Third generation cephalosporins
Fourth generation cephalosporins
Fluoroquinolones
Macrolides
Quinolones
Polymyxins
Aminoglycosides
Aminopenicillins
Aminopenicillins with beta−lactamase inhibitors
Ansamycins
Phosphonic acid derivatives
Shared human and animal antimicrobial classes
Cattle
Chickens
Pigs
Sheep
Turkeys
Ducks
Horses
Buffaloes
Goats
Livestock species

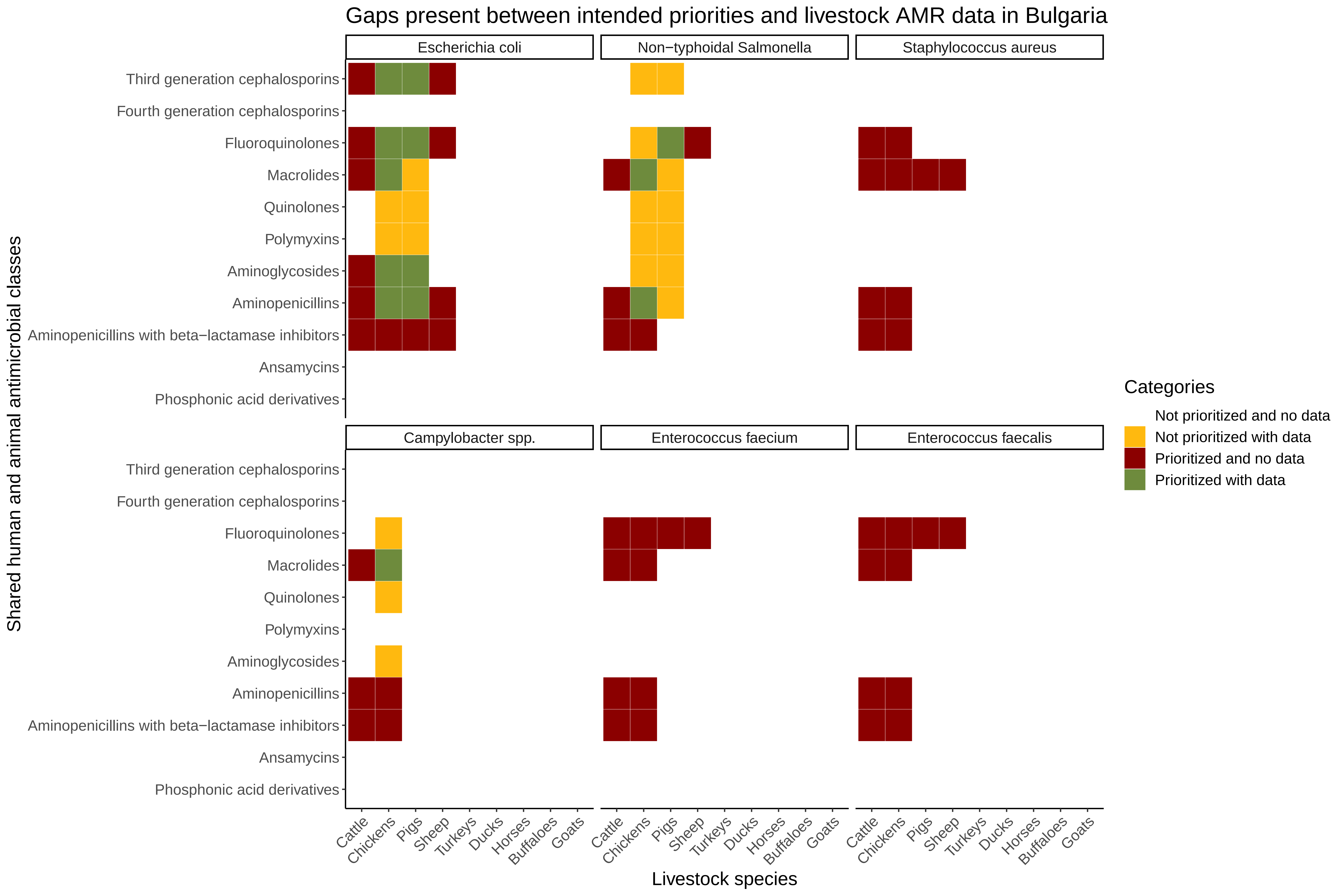
Gaps present between intended priorities and livestock AMR data in Bulgaria
Escherichia coli
Non−typhoidal Salmonella
Staphylococcus aureus
Campylobacter spp.
Enterococcus faecium
Enterococcus faecalis
Third generation cephalosporins
Fourth generation cephalosporins
Fluoroquinolones
Macrolides
Quinolones
Polymyxins
Aminoglycosides
Aminopenicillins
Aminopenicillins with beta−lactamase inhibitors
Ansamycins
Phosphonic acid derivatives
Shared human and animal antimicrobial classes
Cattle
Chickens
Pigs
Sheep
Turkeys
Ducks
Horses
Buffaloes
Goats
Livestock species
Categories
Not prioritized and no data
Not prioritized with data
Prioritized and no data
Prioritized with data

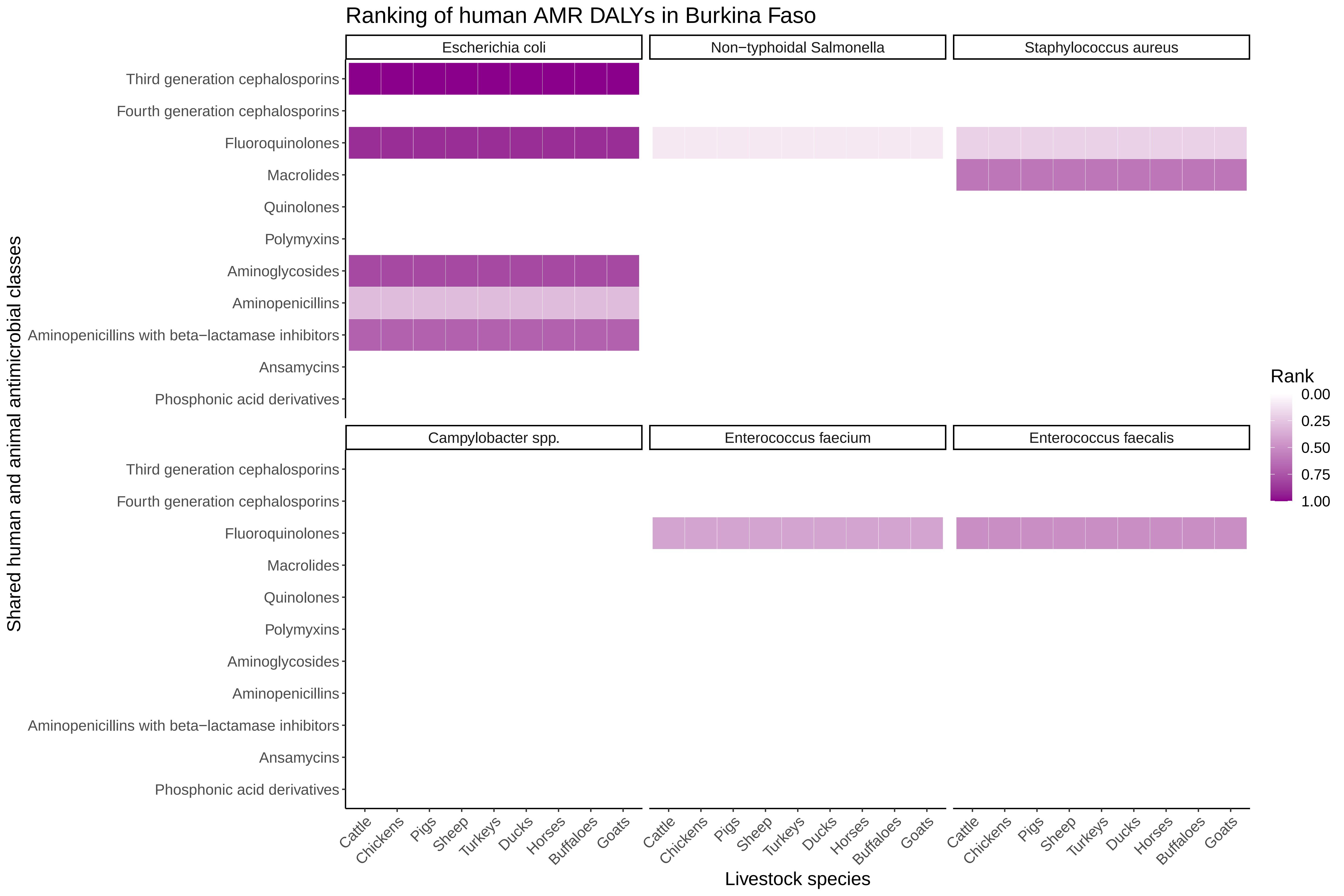

Ranking of human AMR DALYs in Burkina Faso
Escherichia coli
Non−typhoidal Salmonella
Staphylococcus aureus
Campylobacter spp.
Enterococcus faecium
Enterococcus faecalis
Third generation cephalosporins
Fourth generation cephalosporins
Fluoroquinolones
Macrolides
Quinolones
Polymyxins
Aminoglycosides
Aminopenicillins
Aminopenicillins with beta−lactamase inhibitors
Ansamycins
Phosphonic acid derivatives
Shared human and animal antimicrobial classes
Cattle
Chickens
Pigs
Sheep
Turkeys
Ducks
Horses
Buffaloes
Goats
Livestock species
Rank
0.00
0.25
0.50
0.75
1.00

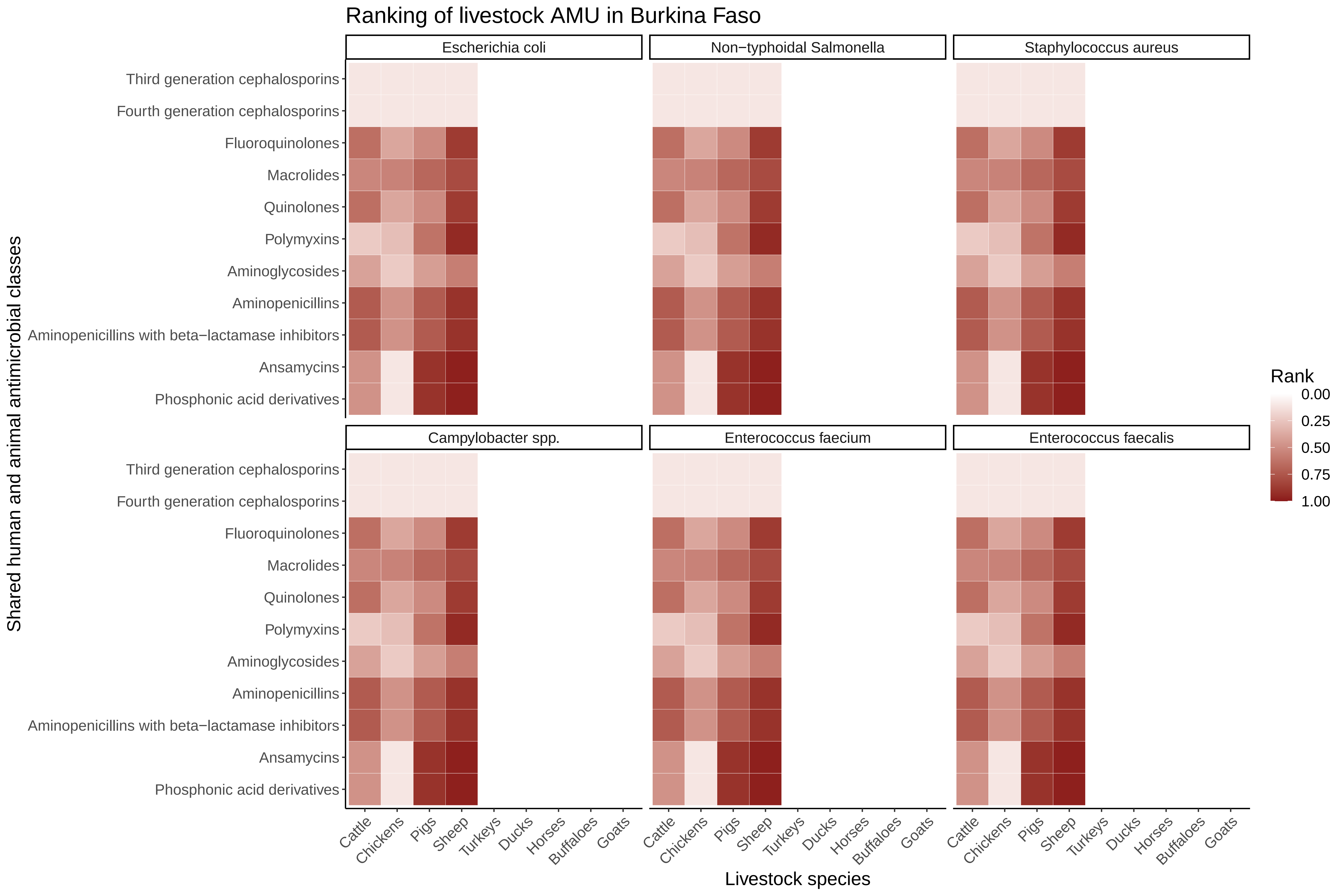

Ranking of livestock AMU in Burkina Faso
Escherichia coli
Non−typhoidal Salmonella
Staphylococcus aureus
Campylobacter spp.
Enterococcus faecium
Enterococcus faecalis
Third generation cephalosporins
Fourth generation cephalosporins
Fluoroquinolones
Macrolides
Quinolones
Polymyxins
Aminoglycosides
Aminopenicillins
Aminopenicillins with beta−lactamase inhibitors
Ansamycins
Phosphonic acid derivatives
Shared human and animal antimicrobial classes
Cattle
Chickens
Pigs
Sheep
Turkeys
Ducks
Horses
Buffaloes
Goats
Livestock species
Rank
0.00
0.25
0.50
0.75
1.00

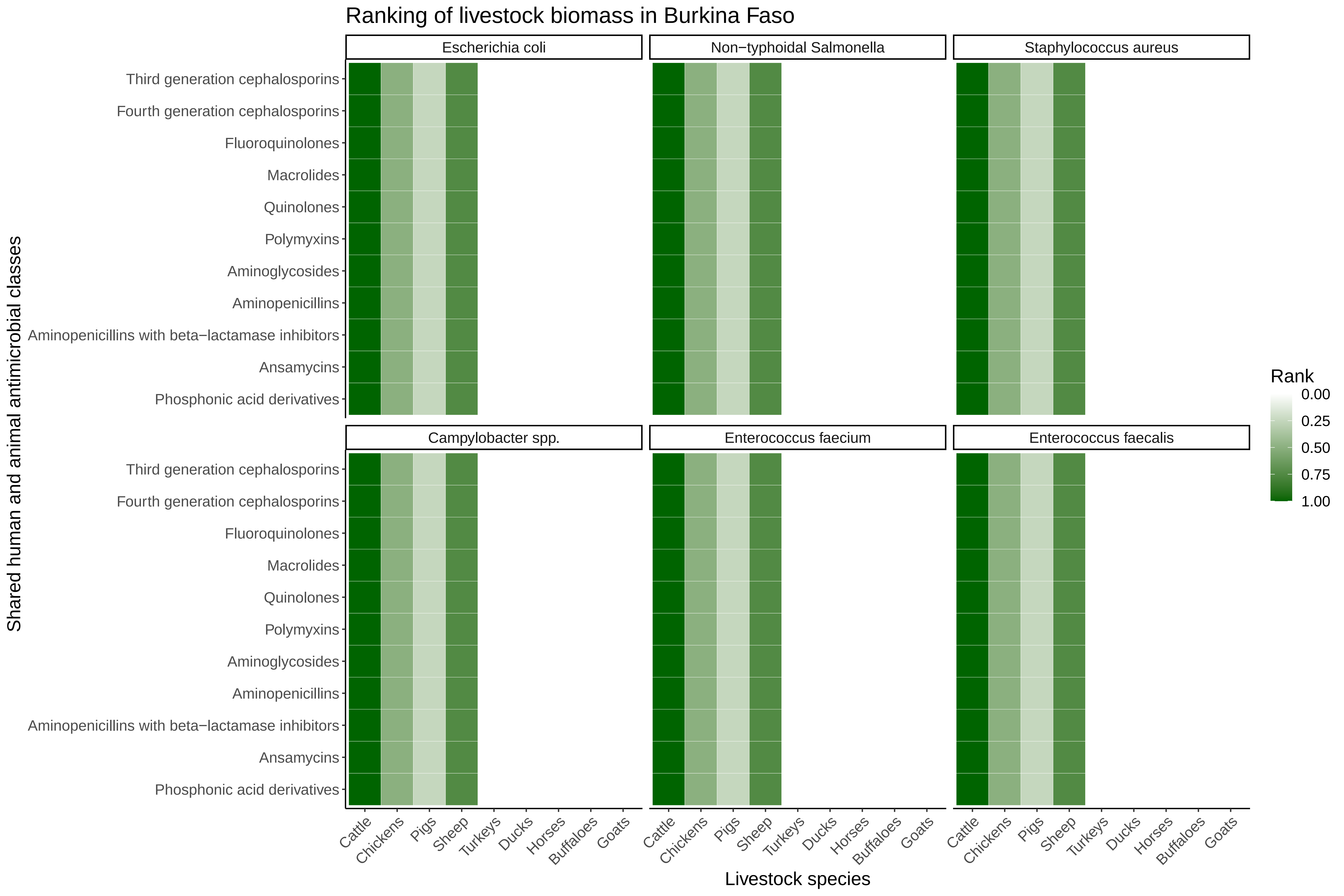

Ranking of livestock biomass in Burkina Faso
Escherichia coli
Non−typhoidal Salmonella
Staphylococcus aureus
Campylobacter spp.
Enterococcus faecium
Enterococcus faecalis
Shared human and animal antimicrobial classes
Third generation cephalosporins
Fourth generation cephalosporins
Fluoroquinolones
Macrolides
Quinolones
Polymyxins
Aminoglycosides
Aminopenicillins
Aminopenicillins with beta−lactamase inhibitors
Ansamycins
Phosphonic acid derivatives
Livestock species
Cattle
Chickens
Pigs
Sheep
Turkeys
Ducks
Horses
Buffaloes
Goats
Rank
0.00
0.25
0.50
0.75
1.00

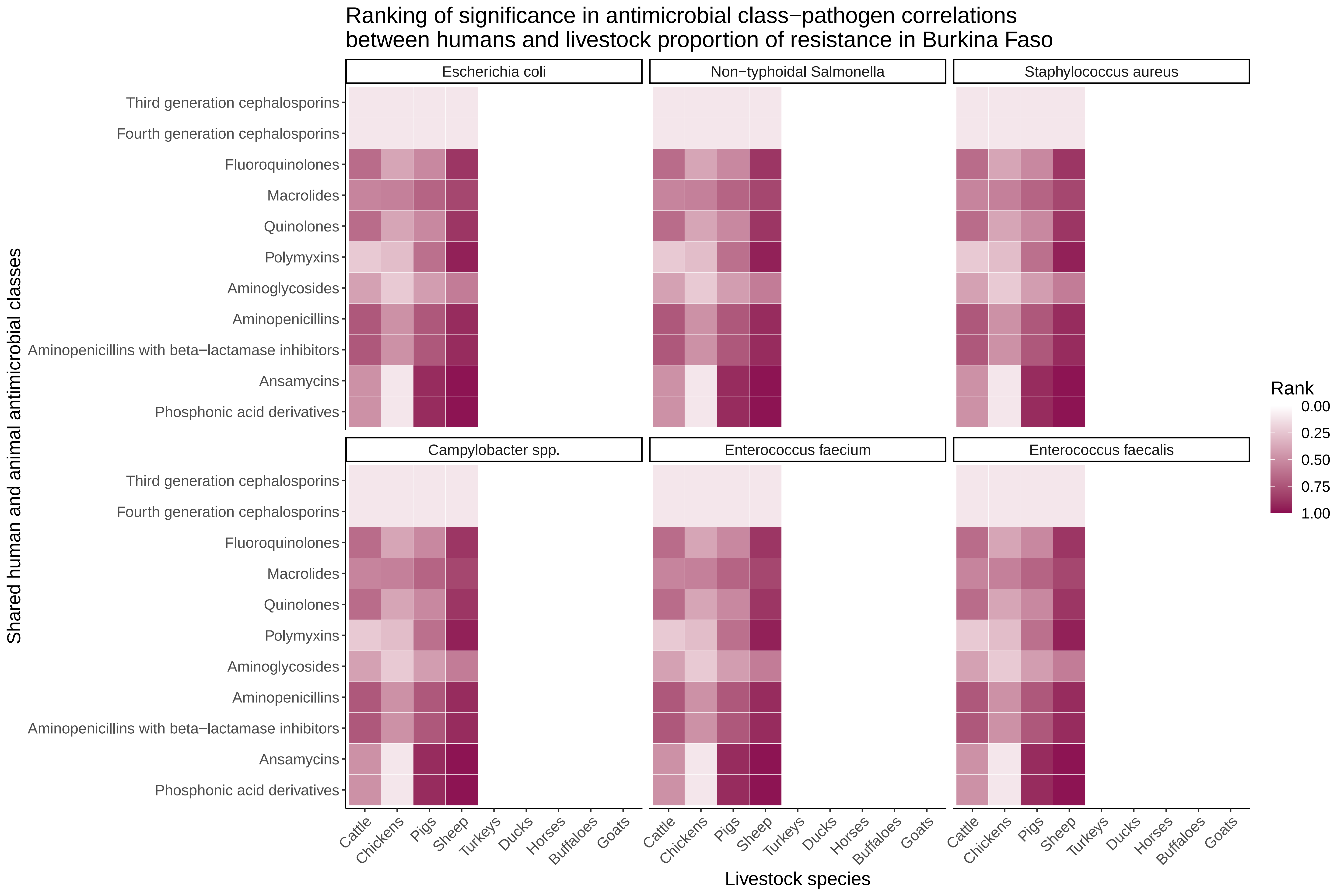

Ranking of significance in antimicrobial class−pathogen correlations between humans and livestock proportion of resistance in Burkina Faso
Escherichia coli
Non−typhoidal Salmonella
Staphylococcus aureus
Campylobacter spp.
Enterococcus faecium
Enterococcus faecalis
Shared human and animal antimicrobial classes
Third generation cephalosporins
Fourth generation cephalosporins
Fluoroquinolones
Macrolides
Quinolones
Polymyxins
Aminoglycosides
Aminopenicillins
Aminopenicillins with beta−lactamase inhibitors
Ansamycins
Phosphonic acid derivatives
Livestock species
Cattle
Chickens
Pigs
Sheep
Turkeys
Ducks
Horses
Buffaloes
Goats
Rank
0.00
0.25
0.50
0.75
1.00

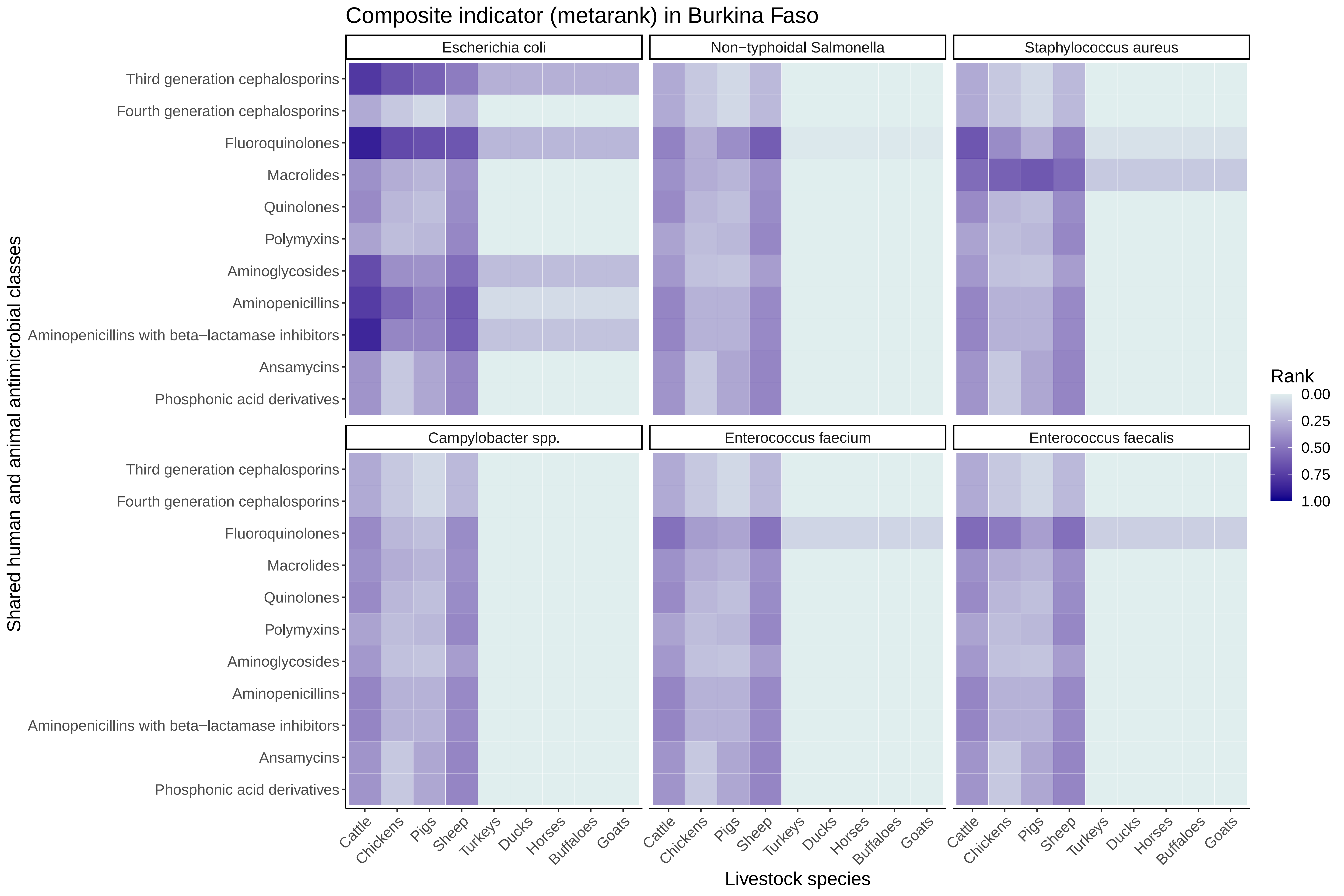

Composite indicator (metarank) in Burkina Faso
Escherichia coli
Non−typhoidal Salmonella
Staphylococcus aureus
Campylobacter spp.
Enterococcus faecium
Enterococcus faecalis
Shared human and animal antimicrobial classes
Third generation cephalosporins
Fourth generation cephalosporins
Fluoroquinolones
Macrolides
Quinolones
Polymyxins
Aminoglycosides
Aminopenicillins
Aminopenicillins with beta−lactamase inhibitors
Ansamycins
Phosphonic acid derivatives
Cattle
Chickens
Pigs
Sheep
Turkeys
Ducks
Horses
Buffaloes
Goats
Livestock species
Rank
0.00
0.25
0.50
0.75
1.00

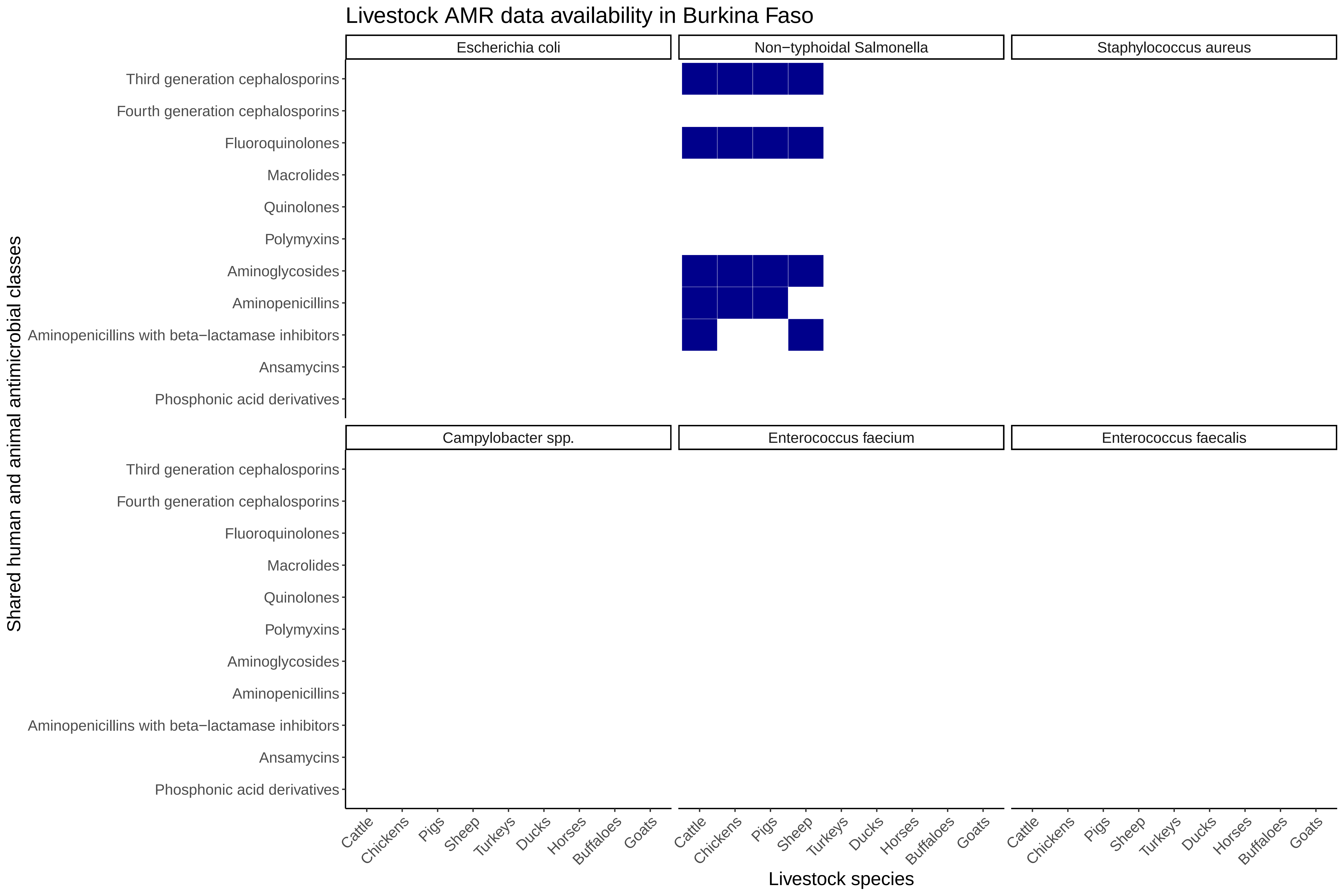

Livestock AMR data availability in Burkina Faso
Escherichia coli
Non−typhoidal Salmonella
Staphylococcus aureus
Campylobacter spp.
Enterococcus faecium
Enterococcus faecalis
Shared human and animal antimicrobial classes
Third generation cephalosporins
Fourth generation cephalosporins
Fluoroquinolones
Macrolides
Quinolones
Polymyxins
Aminoglycosides
Aminopenicillins
Aminopenicillins with beta−lactamase inhibitors
Ansamycins
Phosphonic acid derivatives
Livestock species
Cattle
Chickens
Pigs
Sheep
Turkeys
Ducks
Horses
Buffaloes
Goats

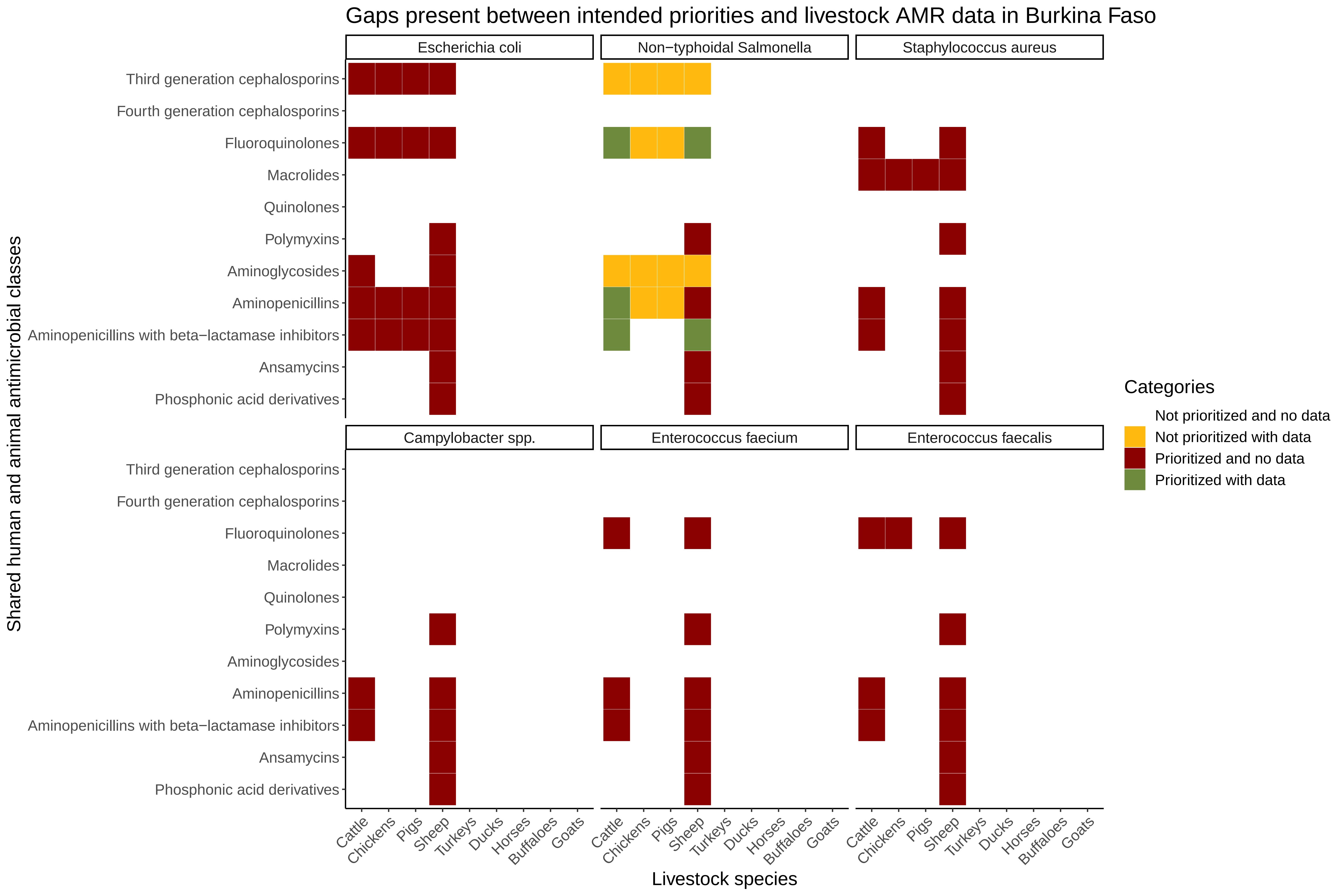

Gaps present between intended priorities and livestock AMR data in Burkina Faso
Escherichia coli
Non−typhoidal Salmonella
Staphylococcus aureus
Campylobacter spp.
Enterococcus faecium
Enterococcus faecalis
Shared human and animal antimicrobial classes
Third generation cephalosporins
Fourth generation cephalosporins
Fluoroquinolones
Macrolides
Quinolones
Polymyxins
Aminoglycosides
Aminopenicillins
Aminopenicillins with beta−lactamase inhibitors
Ansamycins
Phosphonic acid derivatives
Cattle
Chickens
Pigs
Sheep
Turkeys
Ducks
Horses
Buffaloes
Goats
Livestock species
Categories
Not prioritized and no data
Not prioritized with data
Prioritized and no data
Prioritized with data

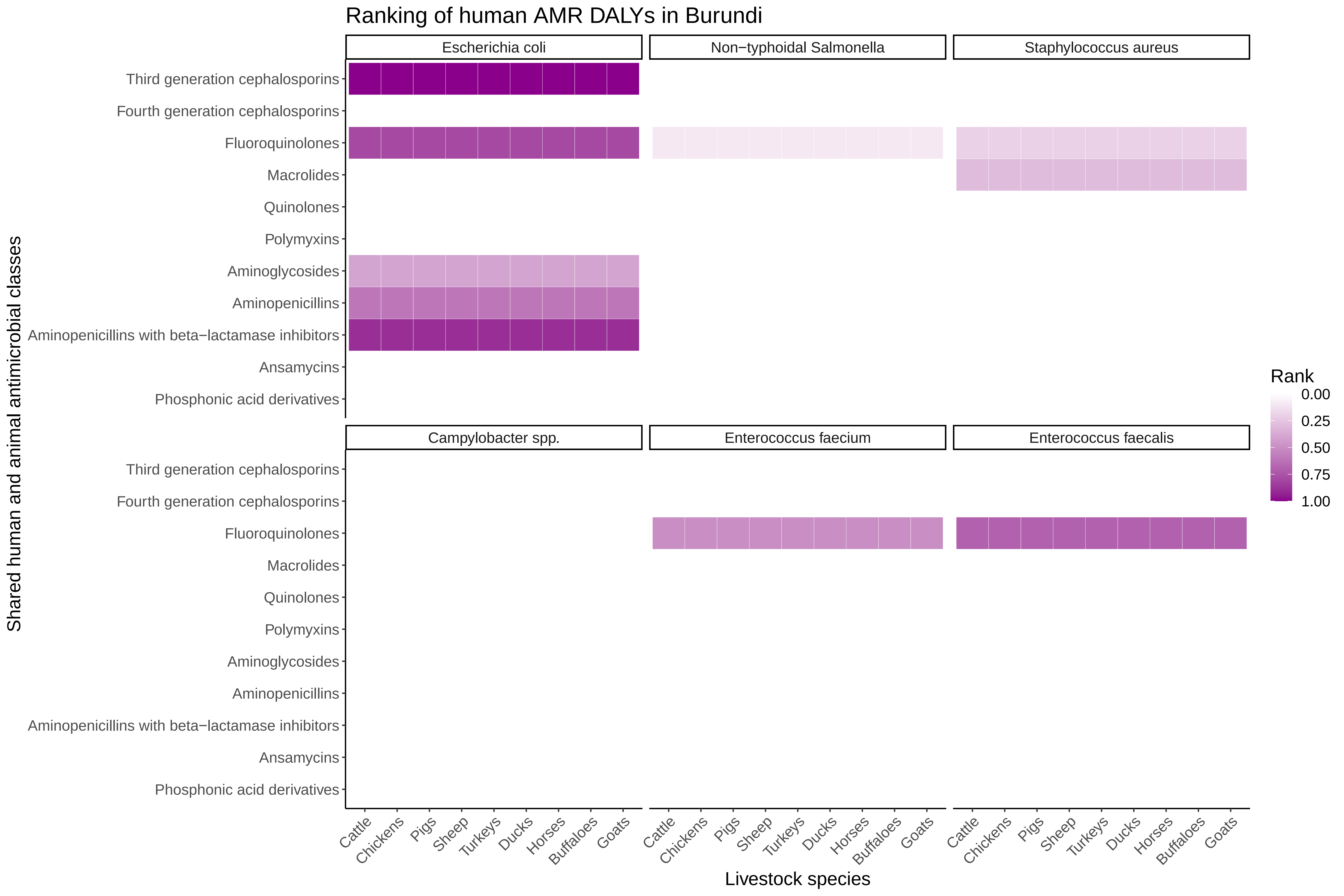
Ranking of human AMR DALYs in Burundi
Escherichia coli
Non−typhoidal Salmonella
Staphylococcus aureus
Campylobacter spp.
Enterococcus faecium
Enterococcus faecalis
Third generation cephalosporins
Fourth generation cephalosporins
Fluoroquinolones
Macrolides
Quinolones
Polymyxins
Aminoglycosides
Aminopenicillins
Aminopenicillins with beta−lactamase inhibitors
Ansamycins
Phosphonic acid derivatives
Shared human and animal antimicrobial classes
Cattle
Chickens
Pigs
Sheep
Turkeys
Ducks
Horses
Buffaloes
Goats
Livestock species
Rank
0.00
0.25
0.50
0.75
1.00

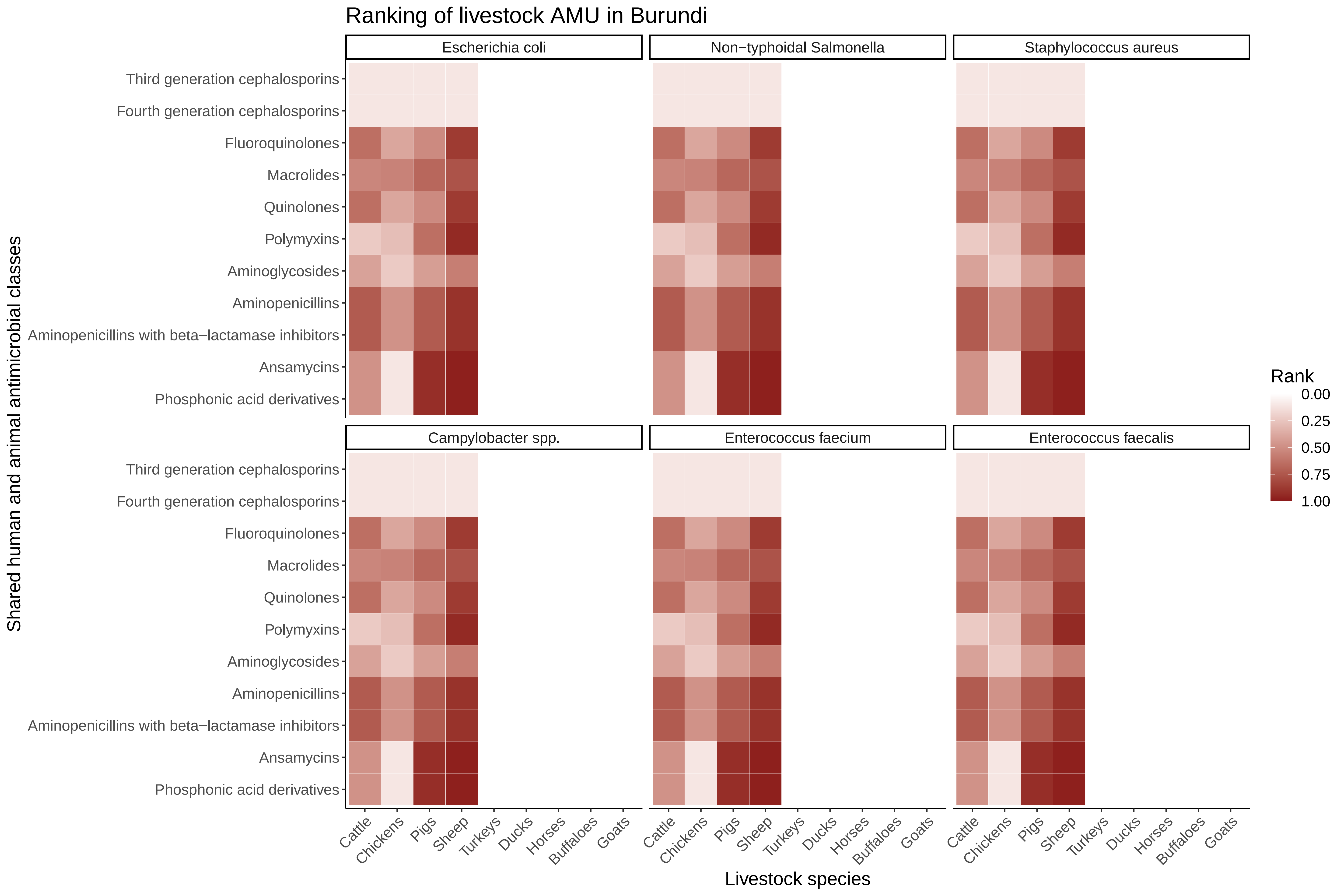
Ranking of livestock AMU in Burundi
Escherichia coli
Non−typhoidal Salmonella
Staphylococcus aureus
Campylobacter spp.
Enterococcus faecium
Enterococcus faecalis
Third generation cephalosporins
Fourth generation cephalosporins
Fluoroquinolones
Macrolides
Quinolones
Polymyxins
Aminoglycosides
Aminopenicillins
Aminopenicillins with beta−lactamase inhibitors
Ansamycins
Phosphonic acid derivatives
Shared human and animal antimicrobial classes
Cattle
Chickens
Pigs
Sheep
Turkeys
Ducks
Horses
Buffaloes
Goats
Livestock species
Rank
0.00
0.25
0.50
0.75
1.00

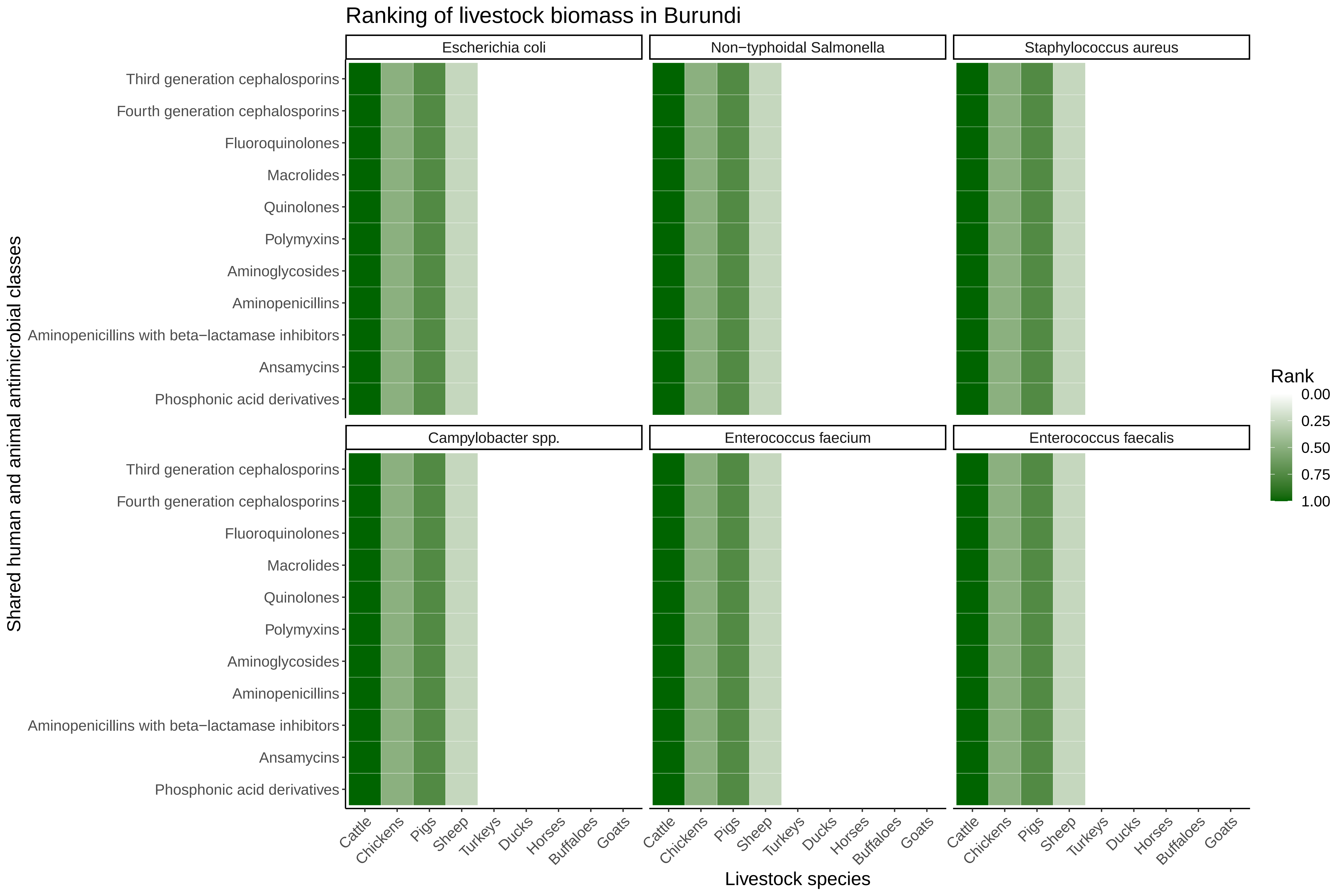
Ranking of livestock biomass in Burundi
Escherichia coli
Non−typhoidal Salmonella
Staphylococcus aureus
Campylobacter spp.
Enterococcus faecium
Enterococcus faecalis
Third generation cephalosporins
Fourth generation cephalosporins
Fluoroquinolones
Macrolides
Quinolones
Polymyxins
Aminoglycosides
Aminopenicillins
Aminopenicillins with beta−lactamase inhibitors
Ansamycins
Phosphonic acid derivatives
Shared human and animal antimicrobial classes
Cattle
Chickens
Pigs
Sheep
Turkeys
Ducks
Horses
Buffaloes
Goats
Livestock species
Rank
0.00
0.25
0.50
0.75
1.00

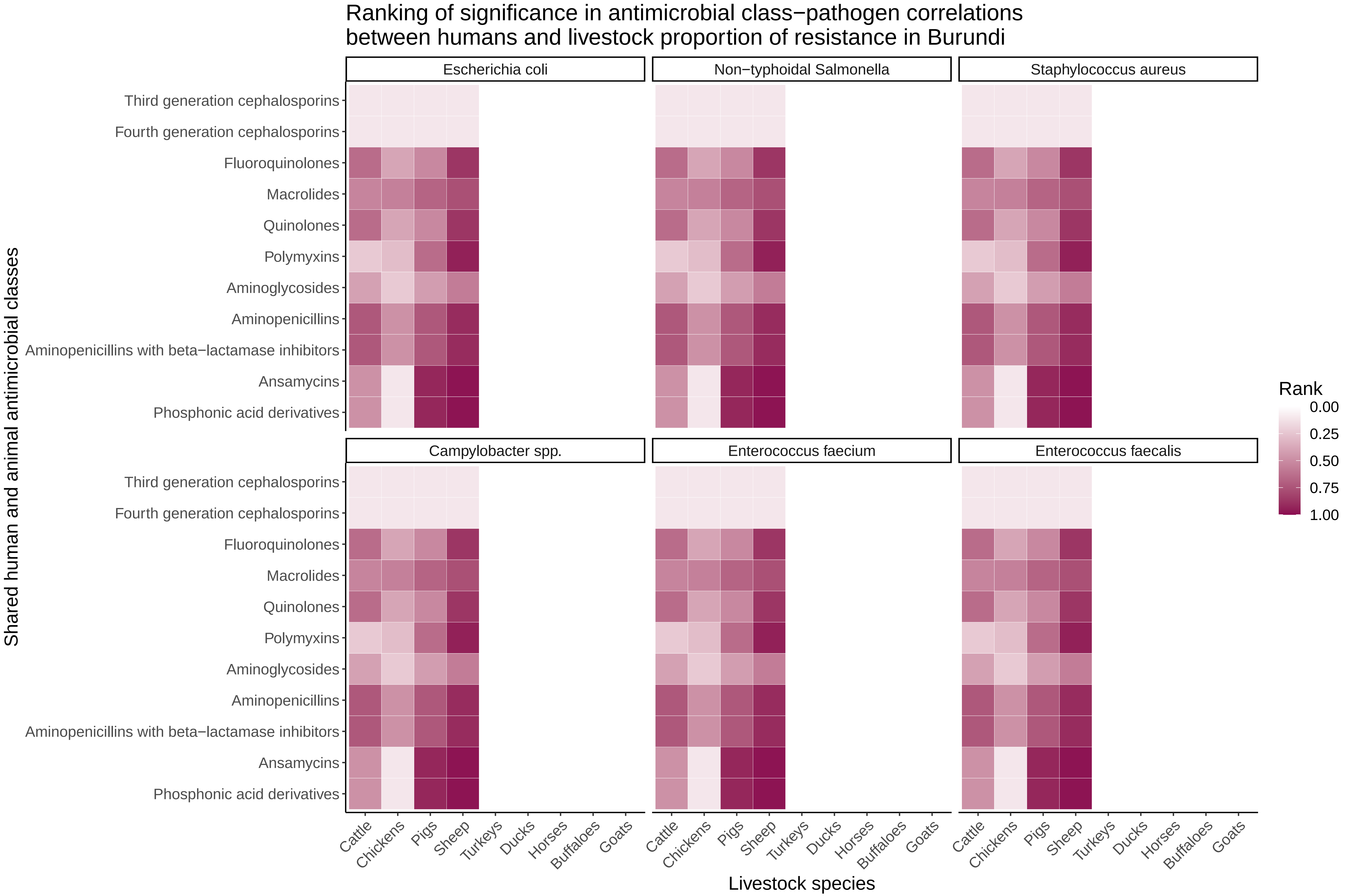
Ranking of significance in antimicrobial class−pathogen correlations
between humans and livestock proportion of resistance in Burundi
Escherichia coli
Non−typhoidal Salmonella
Staphylococcus aureus
Campylobacter spp.
Enterococcus faecium
Enterococcus faecalis
Shared human and animal antimicrobial classes
Third generation cephalosporins
Fourth generation cephalosporins
Fluoroquinolones
Macrolides
Quinolones
Polymyxins
Aminoglycosides
Aminopenicillins
Aminopenicillins with beta−lactamase inhibitors
Ansamycins
Phosphonic acid derivatives
Cattle
Chickens
Pigs
Sheep
Turkeys
Ducks
Horses
Buffaloes
Goats
Livestock species
Rank
0.00
0.25
0.50
0.75
1.00

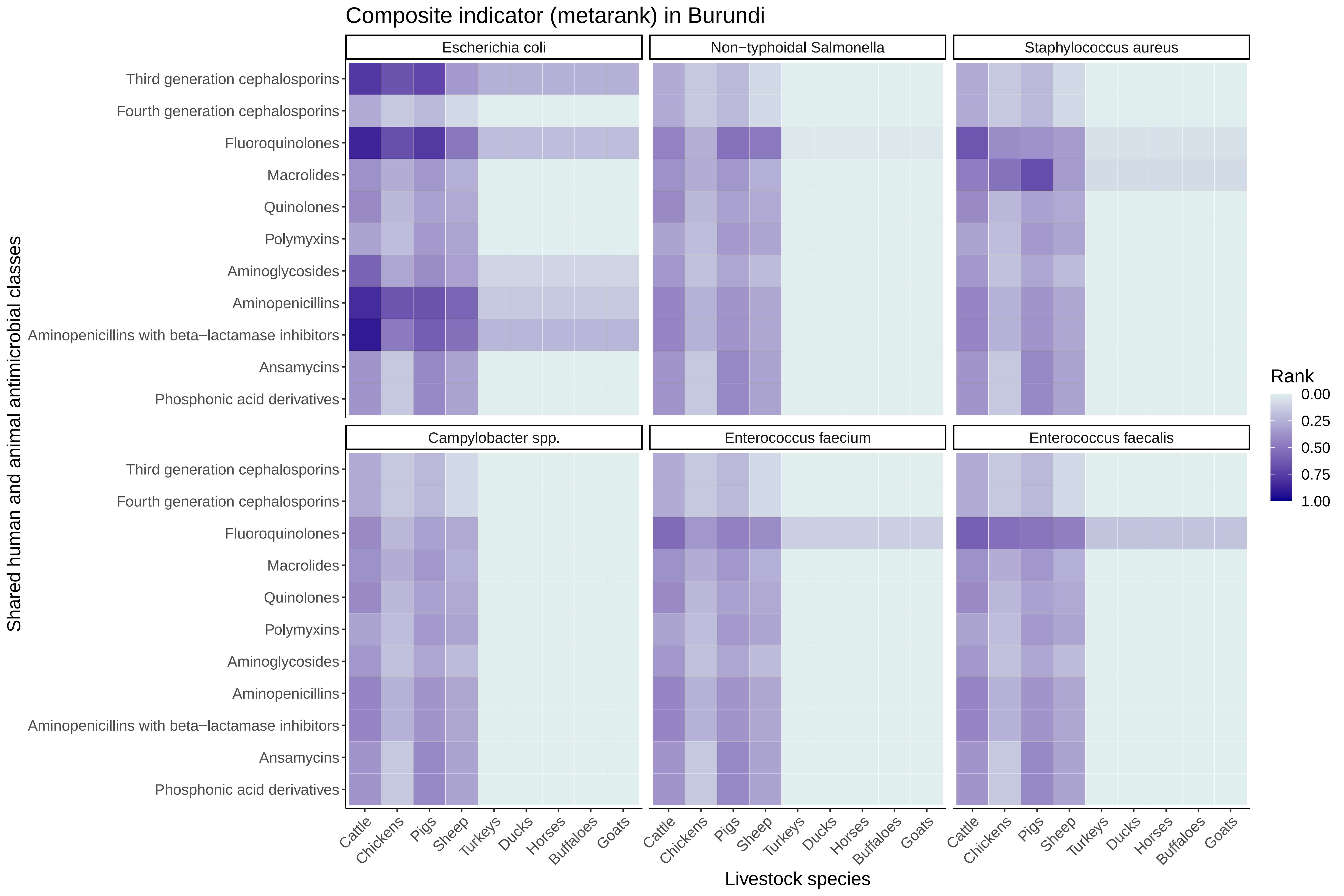

Composite indicator (metarank) in Burundi
Escherichia coli
Non−typhoidal Salmonella
Staphylococcus aureus
Campylobacter spp.
Enterococcus faecium
Enterococcus faecalis
Shared human and animal antimicrobial classes
Third generation cephalosporins
Fourth generation cephalosporins
Fluoroquinolones
Macrolides
Quinolones
Polymyxins
Aminoglycosides
Aminopenicillins
Aminopenicillins with beta−lactamase inhibitors
Ansamycins
Phosphonic acid derivatives
Livestock species
Cattle
Chickens
Pigs
Sheep
Turkeys
Ducks
Horses
Buffaloes
Goats
Rank
0.00
0.25
0.50
0.75
1.00

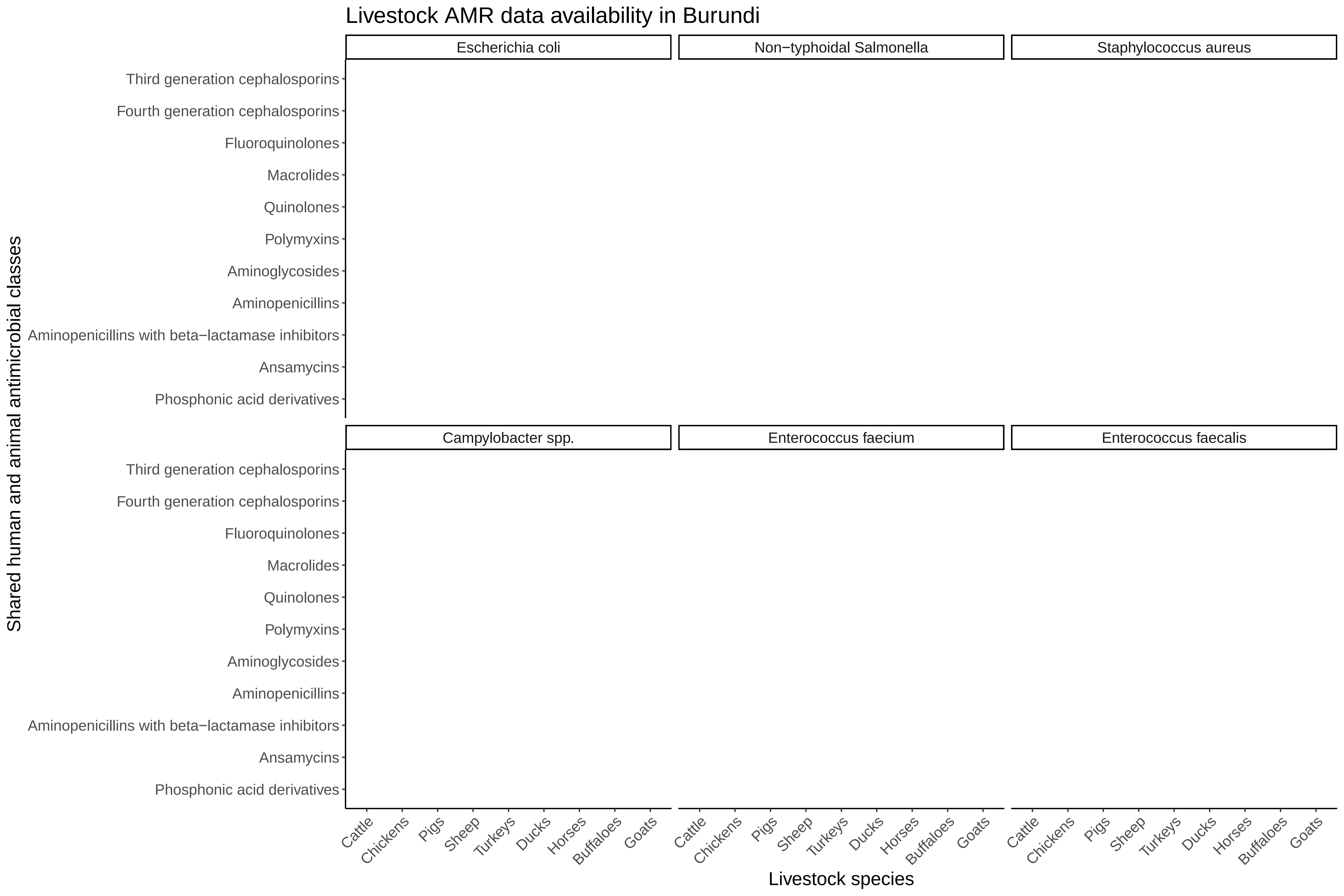

Livestock AMR data availability in Burundi
Escherichia coli
Non−typhoidal Salmonella
Staphylococcus aureus
Campylobacter spp.
Enterococcus faecium
Enterococcus faecalis
Third generation cephalosporins
Fourth generation cephalosporins
Fluoroquinolones
Macrolides
Quinolones
Polymyxins
Aminoglycosides
Aminopenicillins
Aminopenicillins with beta−lactamase inhibitors
Ansamycins
Phosphonic acid derivatives
Shared human and animal antimicrobial classes
Cattle
Chickens
Pigs
Sheep
Turkeys
Ducks
Horses
Buffaloes
Goats
Livestock species

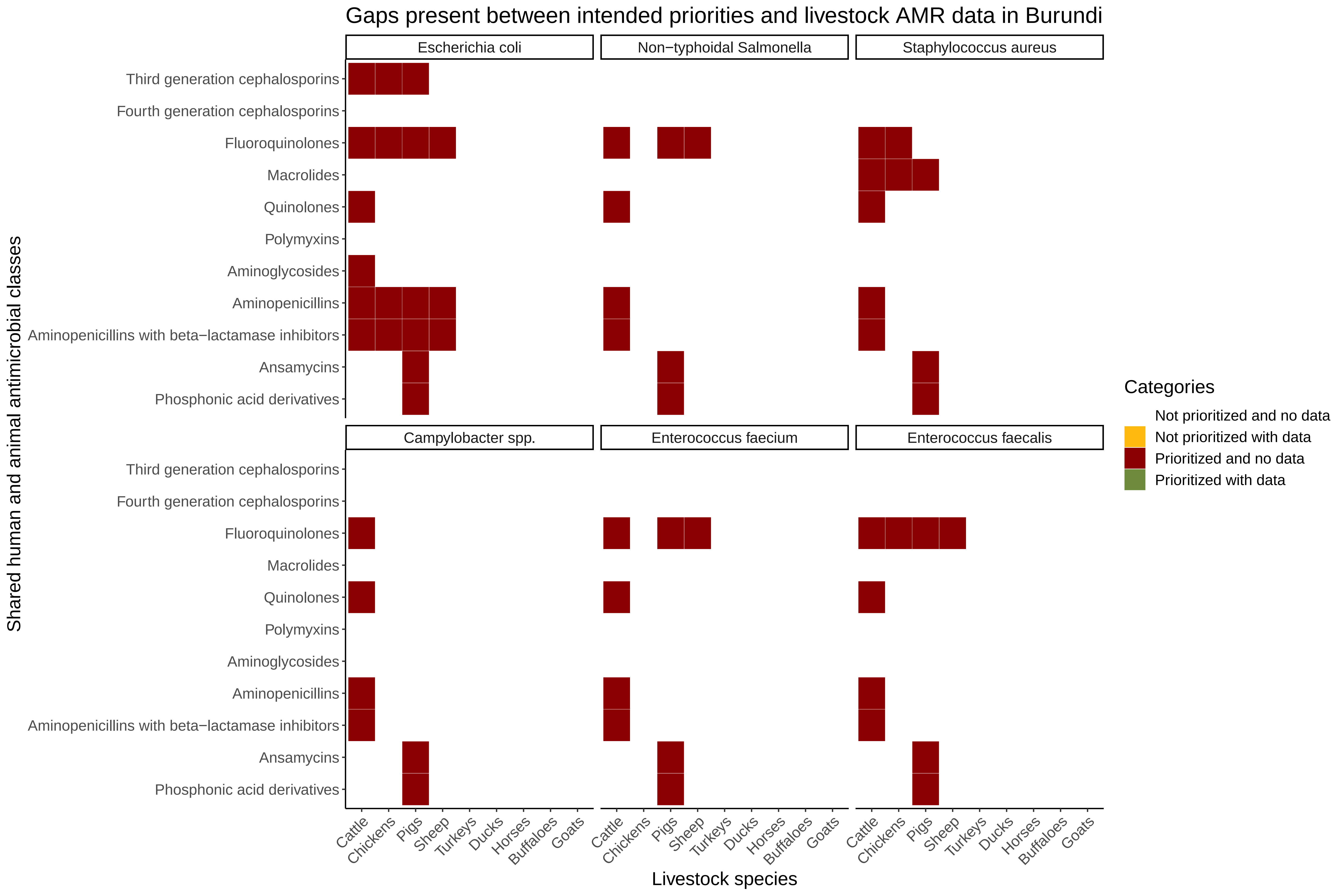

Gaps present between intended priorities and livestock AMR data in Burundi
Escherichia coli
Non−typhoidal Salmonella
Staphylococcus aureus
Campylobacter spp.
Enterococcus faecium
Enterococcus faecalis
Shared human and animal antimicrobial classes
Third generation cephalosporins
Fourth generation cephalosporins
Fluoroquinolones
Macrolides
Quinolones
Polymyxins
Aminoglycosides
Aminopenicillins
Aminopenicillins with beta−lactamase inhibitors
Ansamycins
Phosphonic acid derivatives
Cattle
Chickens
Pigs
Sheep
Turkeys
Ducks
Horses
Buffaloes
Goats
Livestock species
Categories
Not prioritized and no data
Not prioritized with data
Prioritized and no data
Prioritized with data

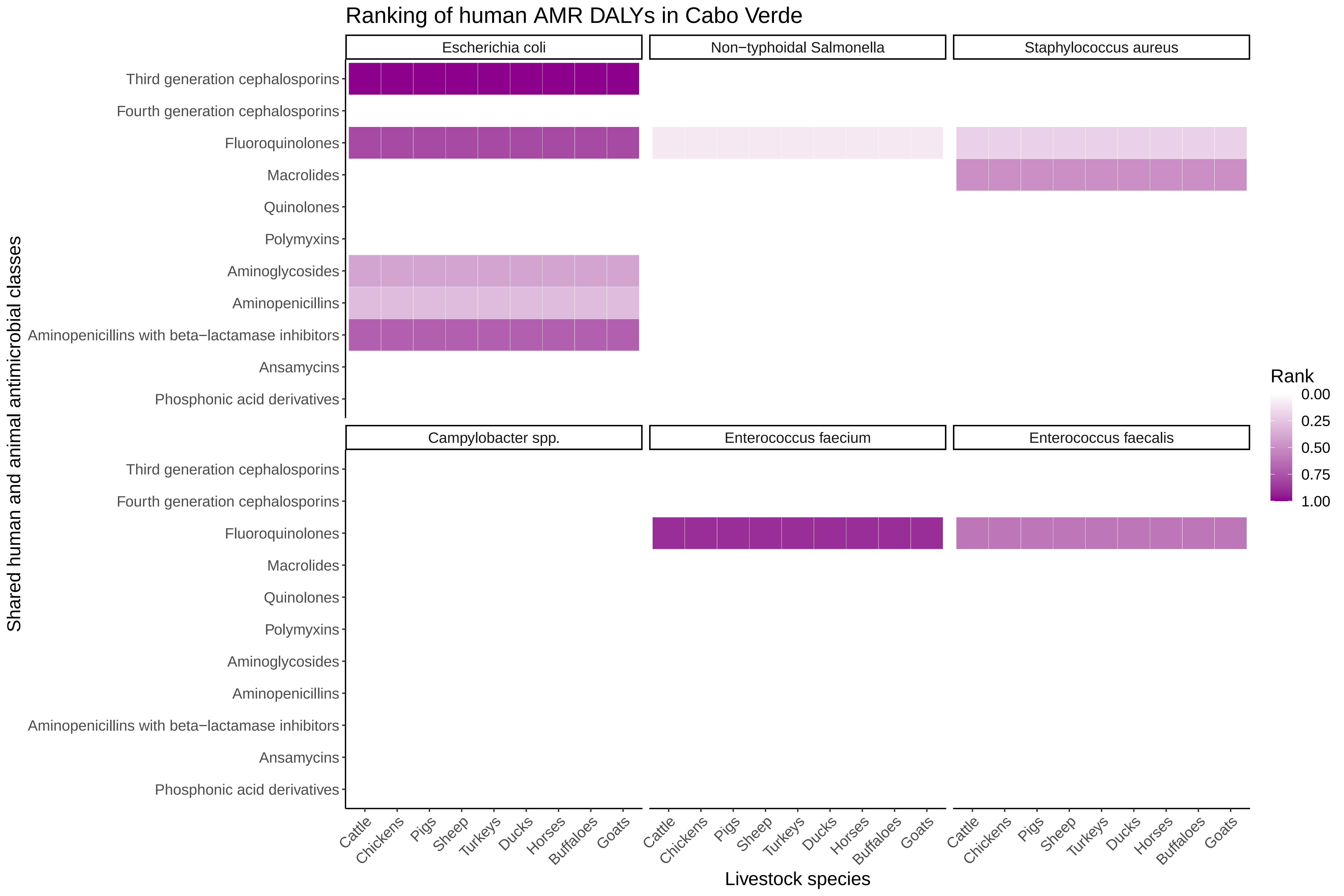

Ranking of human AMR DALYs in Cabo Verde
Escherichia coli
Non−typhoidal Salmonella
Staphylococcus aureus
Campylobacter spp.
Enterococcus faecium
Enterococcus faecalis
Shared human and animal antimicrobial classes
Third generation cephalosporins
Fourth generation cephalosporins
Fluoroquinolones
Macrolides
Quinolones
Polymyxins
Aminoglycosides
Aminopenicillins
Aminopenicillins with beta−lactamase inhibitors
Ansamycins
Phosphonic acid derivatives
Cattle
Chickens
Pigs
Sheep
Turkeys
Ducks
Horses
Buffaloes
Goats
Livestock species
Rank
0.00
0.25
0.50
0.75
1.00

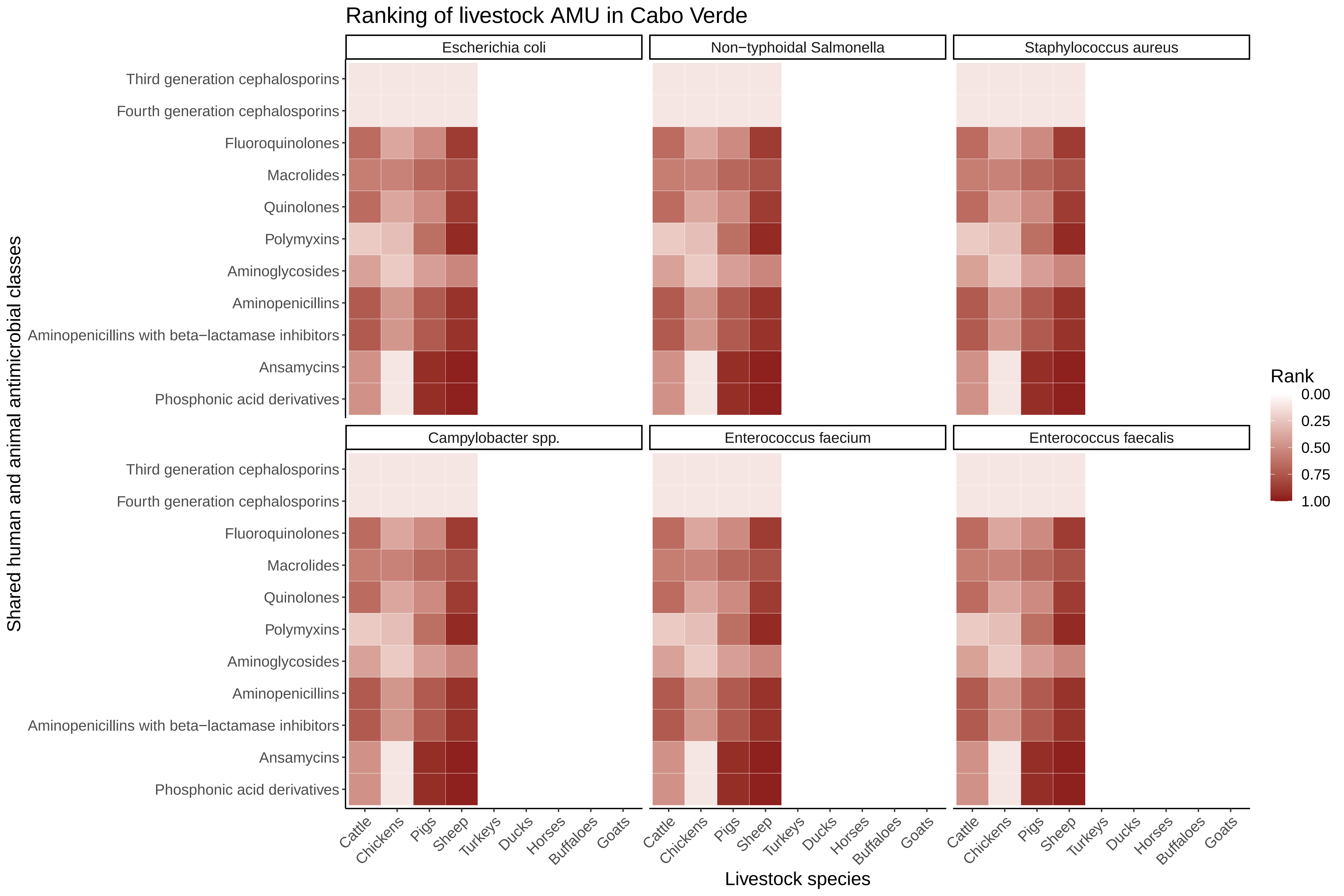

Ranking of livestock AMU in Cabo Verde
Escherichia coli
Non−typhoidal Salmonella
Staphylococcus aureus
Campylobacter spp.
Enterococcus faecium
Enterococcus faecalis
Shared human and animal antimicrobial classes
Third generation cephalosporins
Fourth generation cephalosporins
Fluoroquinolones
Macrolides
Quinolones
Polymyxins
Aminoglycosides
Aminopenicillins
Aminopenicillins with beta−lactamase inhibitors
Ansamycins
Phosphonic acid derivatives
Cattle
Chickens
Pigs
Sheep
Turkeys
Ducks
Horses
Buffaloes
Goats
Livestock species
Rank
0.00
0.25
0.50
0.75
1.00

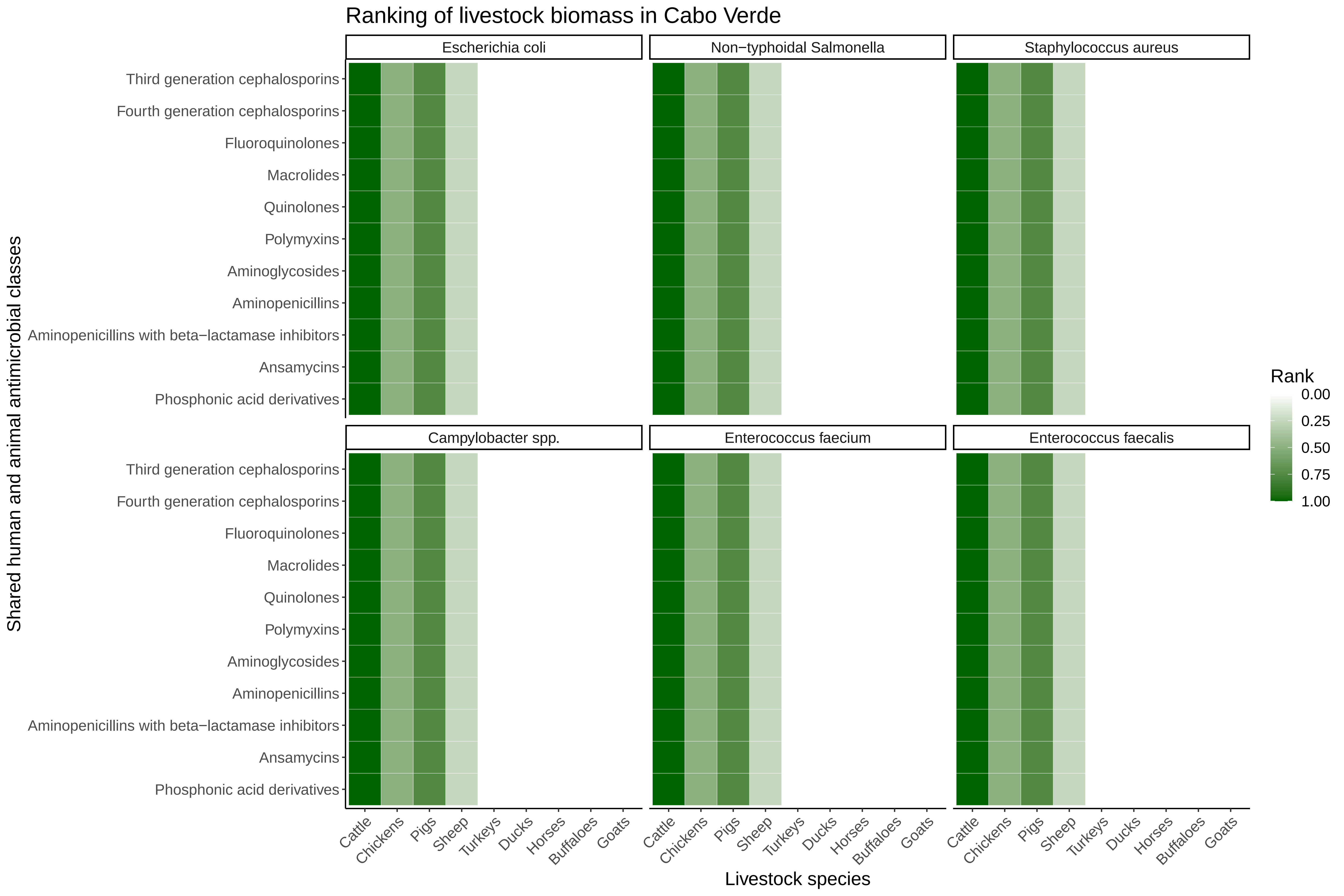

Ranking of livestock biomass in Cabo Verde
Escherichia coli
Non−typhoidal Salmonella
Staphylococcus aureus
Campylobacter spp.
Enterococcus faecium
Enterococcus faecalis
Third generation cephalosporins
Fourth generation cephalosporins
Fluoroquinolones
Macrolides
Quinolones
Polymyxins
Aminoglycosides
Aminopenicillins
Aminopenicillins with beta−lactamase inhibitors
Ansamycins
Phosphonic acid derivatives
Shared human and animal antimicrobial classes
Cattle
Chickens
Pigs
Sheep
Turkeys
Ducks
Horses
Buffaloes
Goats
Livestock species
Rank
0.00
0.25
0.50
0.75
1.00

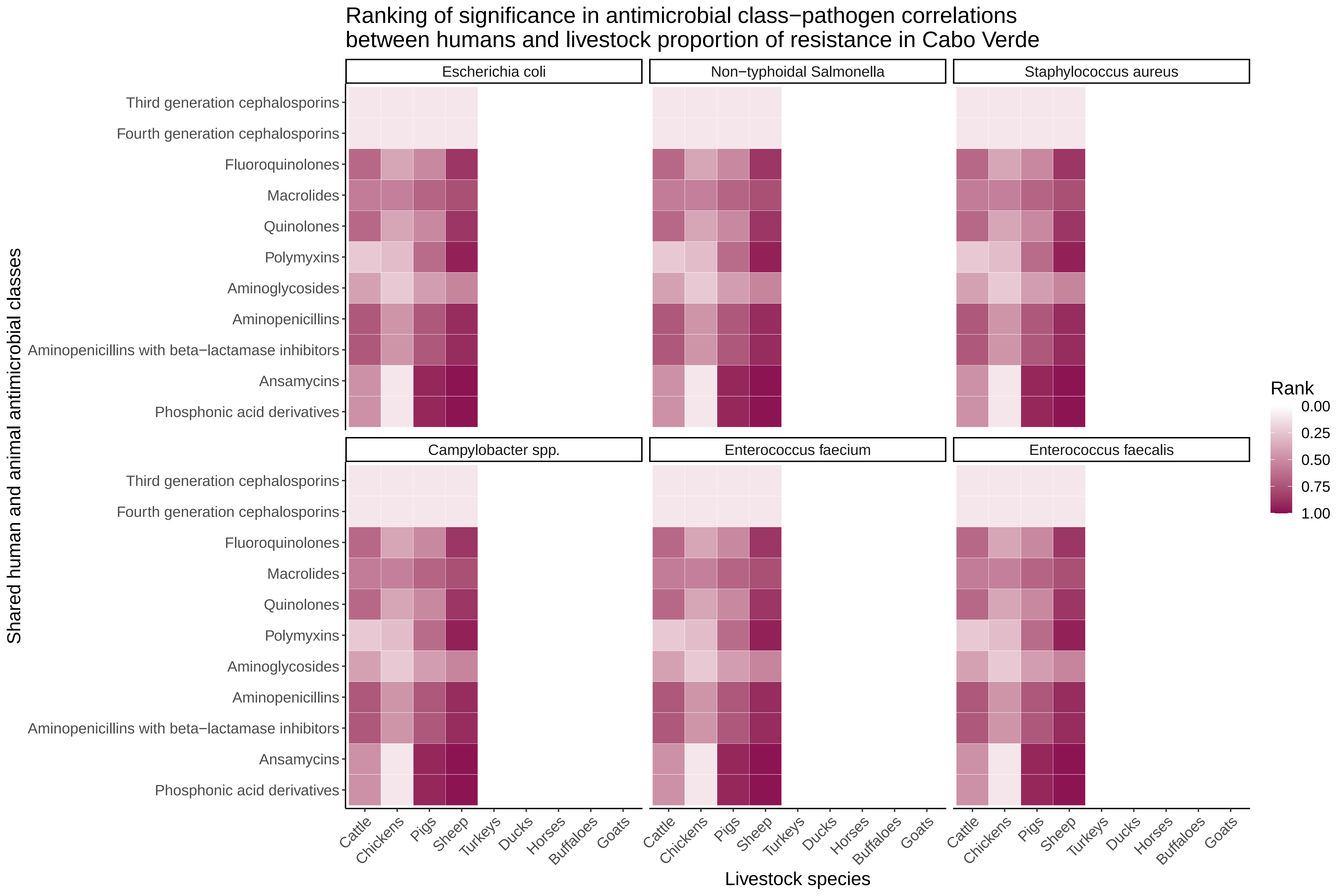

Ranking of significance in antimicrobial class−pathogen correlations
between humans and livestock proportion of resistance in Cabo Verde
Escherichia coli
Non−typhoidal Salmonella
Staphylococcus aureus
Campylobacter spp.
Enterococcus faecium
Enterococcus faecalis
Third generation cephalosporins
Fourth generation cephalosporins
Fluoroquinolones
Macrolides
Quinolones
Polymyxins
Aminoglycosides
Aminopenicillins
Aminopenicillins with beta−lactamase inhibitors
Ansamycins
Phosphonic acid derivatives
Shared human and animal antimicrobial classes
Cattle
Chickens
Pigs
Sheep
Turkeys
Ducks
Horses
Buffaloes
Goats
Livestock species
Rank
0.00
0.25
0.50
0.75
1.00

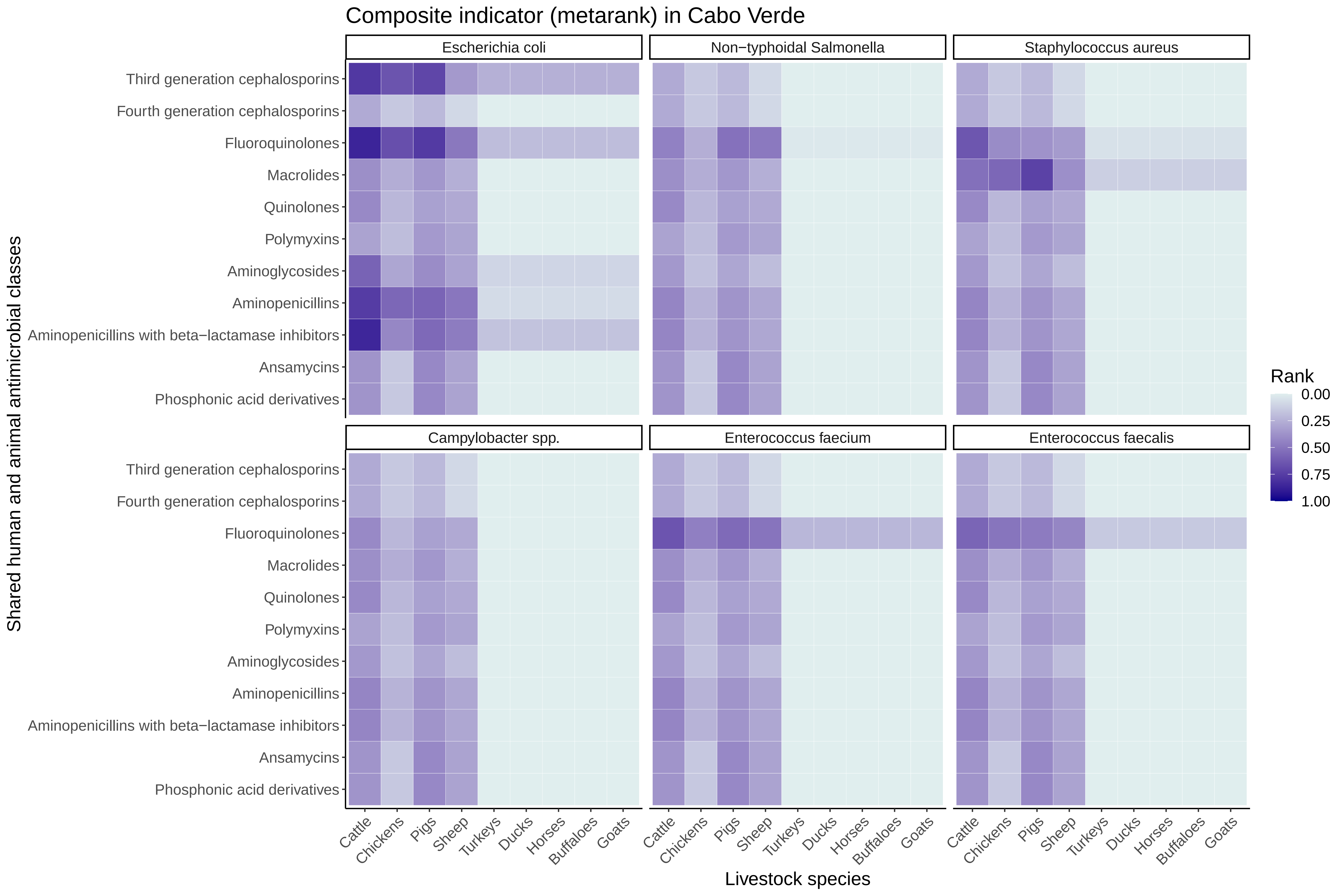

Composite indicator (metarank) in Cabo Verde
Escherichia coli
Non−typhoidal Salmonella
Staphylococcus aureus
Campylobacter spp.
Enterococcus faecium
Enterococcus faecalis
Shared human and animal antimicrobial classes
Third generation cephalosporins
Fourth generation cephalosporins
Fluoroquinolones
Macrolides
Quinolones
Polymyxins
Aminoglycosides
Aminopenicillins
Aminopenicillins with beta−lactamase inhibitors
Ansamycins
Phosphonic acid derivatives
Cattle
Chickens
Pigs
Sheep
Turkeys
Ducks
Horses
Buffaloes
Goats
Livestock species
Rank
0.00
0.25
0.50
0.75
1.00

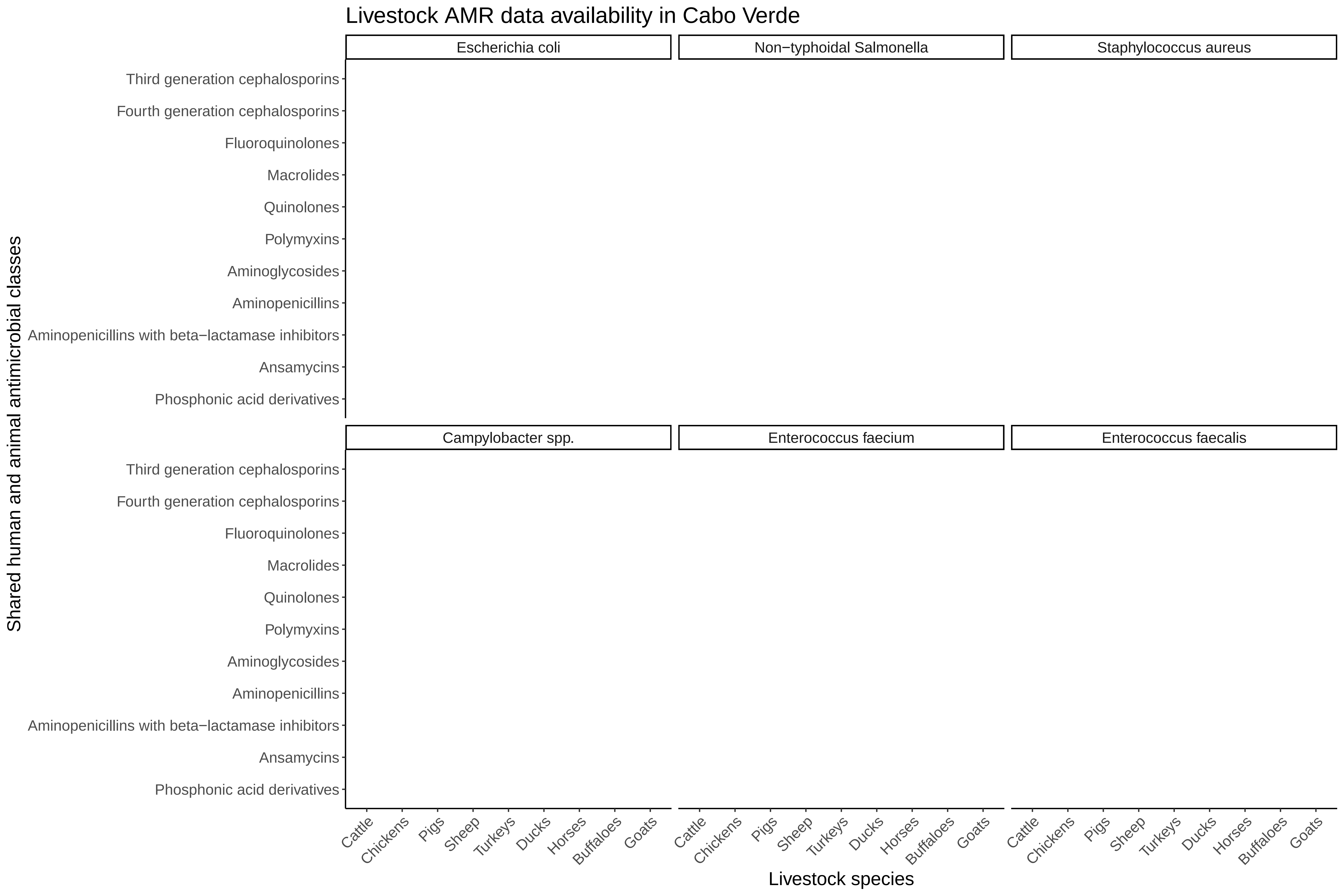
Livestock AMR data availability in Cabo Verde
Escherichia coli
Non−typhoidal Salmonella
Staphylococcus aureus
Campylobacter spp.
Enterococcus faecium
Enterococcus faecalis
Shared human and animal antimicrobial classes
Third generation cephalosporins
Fourth generation cephalosporins
Fluoroquinolones
Macrolides
Quinolones
Polymyxins
Aminoglycosides
Aminopenicillins
Aminopenicillins with beta−lactamase inhibitors
Ansamycins
Phosphonic acid derivatives
Cattle
Chickens
Pigs
Sheep
Turkeys
Ducks
Horses
Buffaloes
Goats
Livestock species

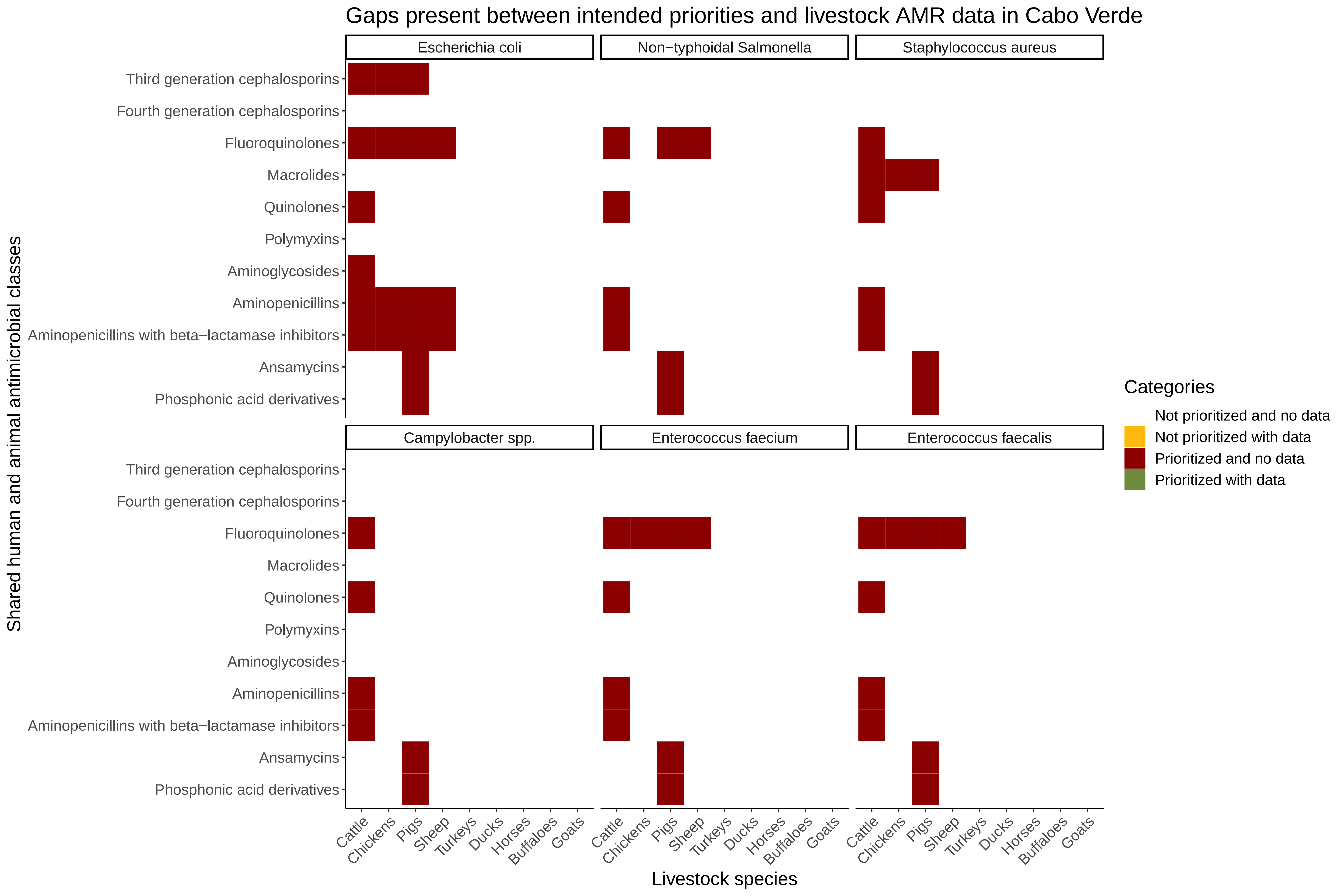

Gaps present between intended priorities and livestock AMR data in Cabo Verde
Escherichia coli
Non−typhoidal Salmonella
Staphylococcus aureus
Campylobacter spp.
Enterococcus faecium
Enterococcus faecalis
Third generation cephalosporins
Fourth generation cephalosporins
Fluoroquinolones
Macrolides
Quinolones
Polymyxins
Aminoglycosides
Aminopenicillins
Aminopenicillins with beta−lactamase inhibitors
Ansamycins
Phosphonic acid derivatives
Shared human and animal antimicrobial classes
Cattle
Chickens
Pigs
Sheep
Turkeys
Ducks
Horses
Buffaloes
Goats
Livestock species
Categories
Not prioritized and no data
Not prioritized with data
Prioritized and no data
Prioritized with data

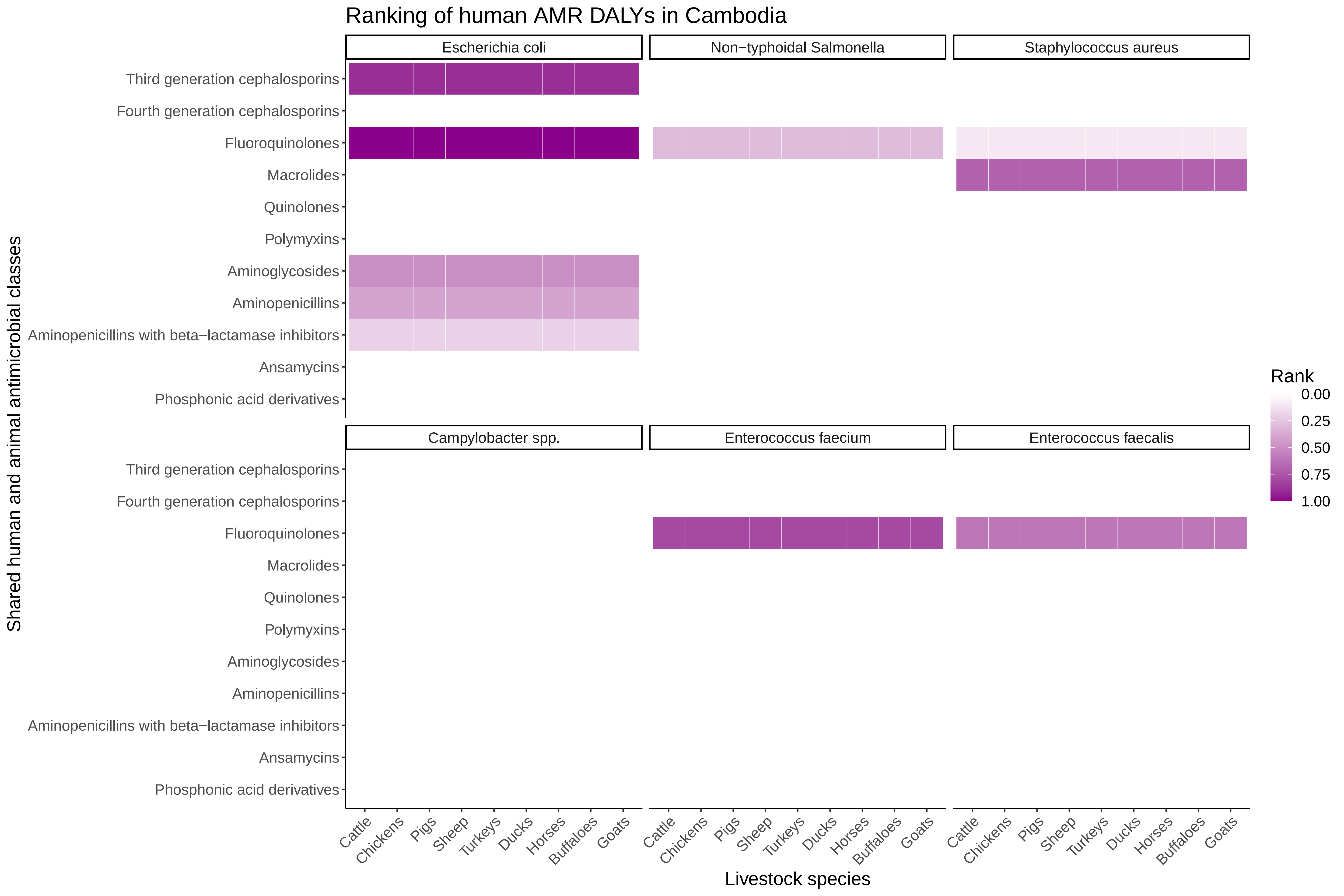
Ranking of human AMR DALYs in Cambodia
Escherichia coli
Non−typhoidal Salmonella
Staphylococcus aureus
Campylobacter spp.
Enterococcus faecium
Enterococcus faecalis
Third generation cephalosporins
Fourth generation cephalosporins
Fluoroquinolones
Macrolides
Quinolones
Polymyxins
Aminoglycosides
Aminopenicillins
Aminopenicillins with beta−lactamase inhibitors
Ansamycins
Phosphonic acid derivatives
Shared human and animal antimicrobial classes
Cattle
Chickens
Pigs
Sheep
Turkeys
Ducks
Horses
Buffaloes
Goats
Livestock species
Rank
0.00
0.25
0.50
0.75
1.00

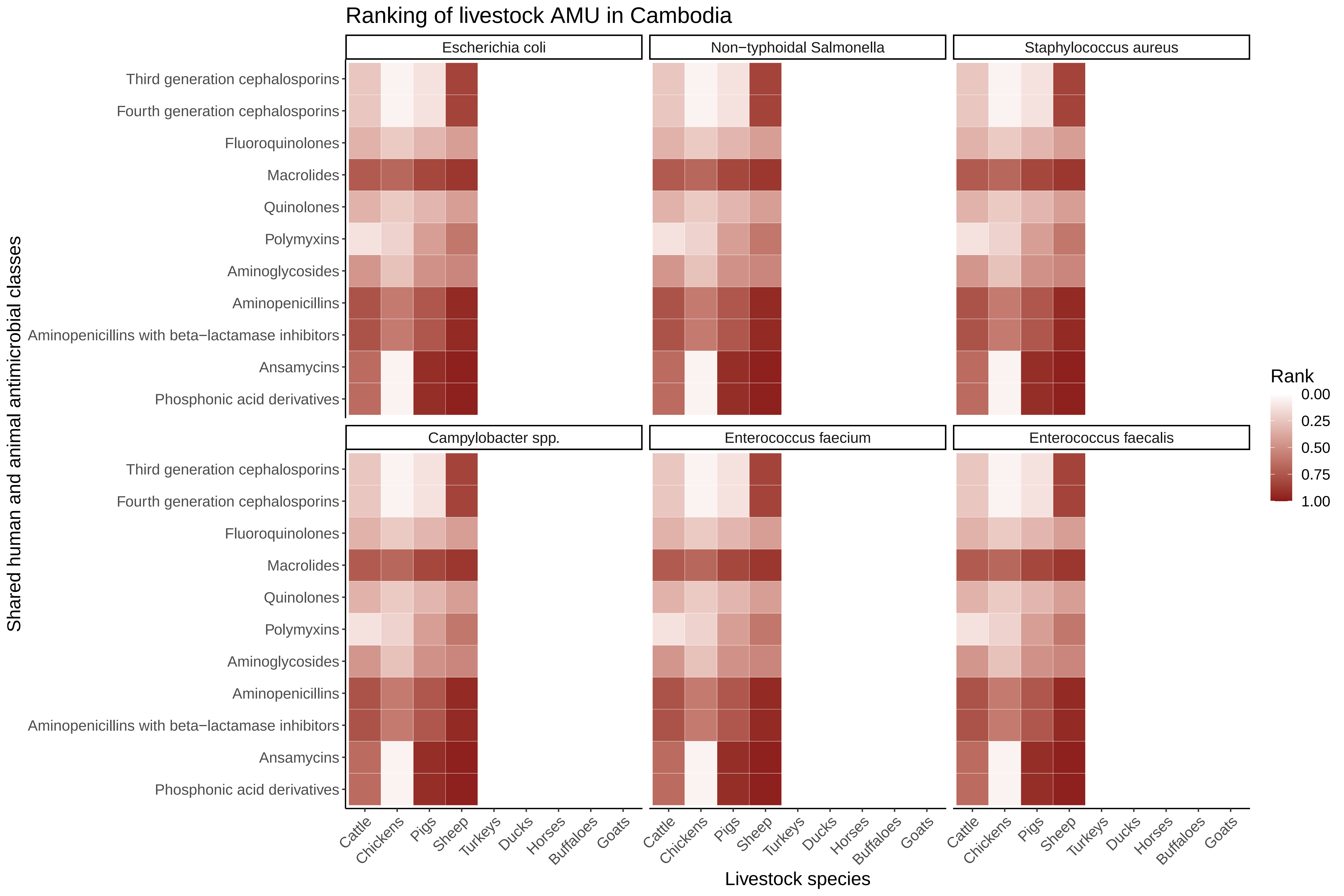

Ranking of livestock AMU in Cambodia
Escherichia coli
Non−typhoidal Salmonella
Staphylococcus aureus
Campylobacter spp.
Enterococcus faecium
Enterococcus faecalis
Shared human and animal antimicrobial classes
Third generation cephalosporins
Fourth generation cephalosporins
Fluoroquinolones
Macrolides
Quinolones
Polymyxins
Aminoglycosides
Aminopenicillins
Aminopenicillins with beta−lactamase inhibitors
Ansamycins
Phosphonic acid derivatives
Livestock species
Cattle
Chickens
Pigs
Sheep
Turkeys
Ducks
Horses
Buffaloes
Goats
Rank
0.00
0.25
0.50
0.75
1.00

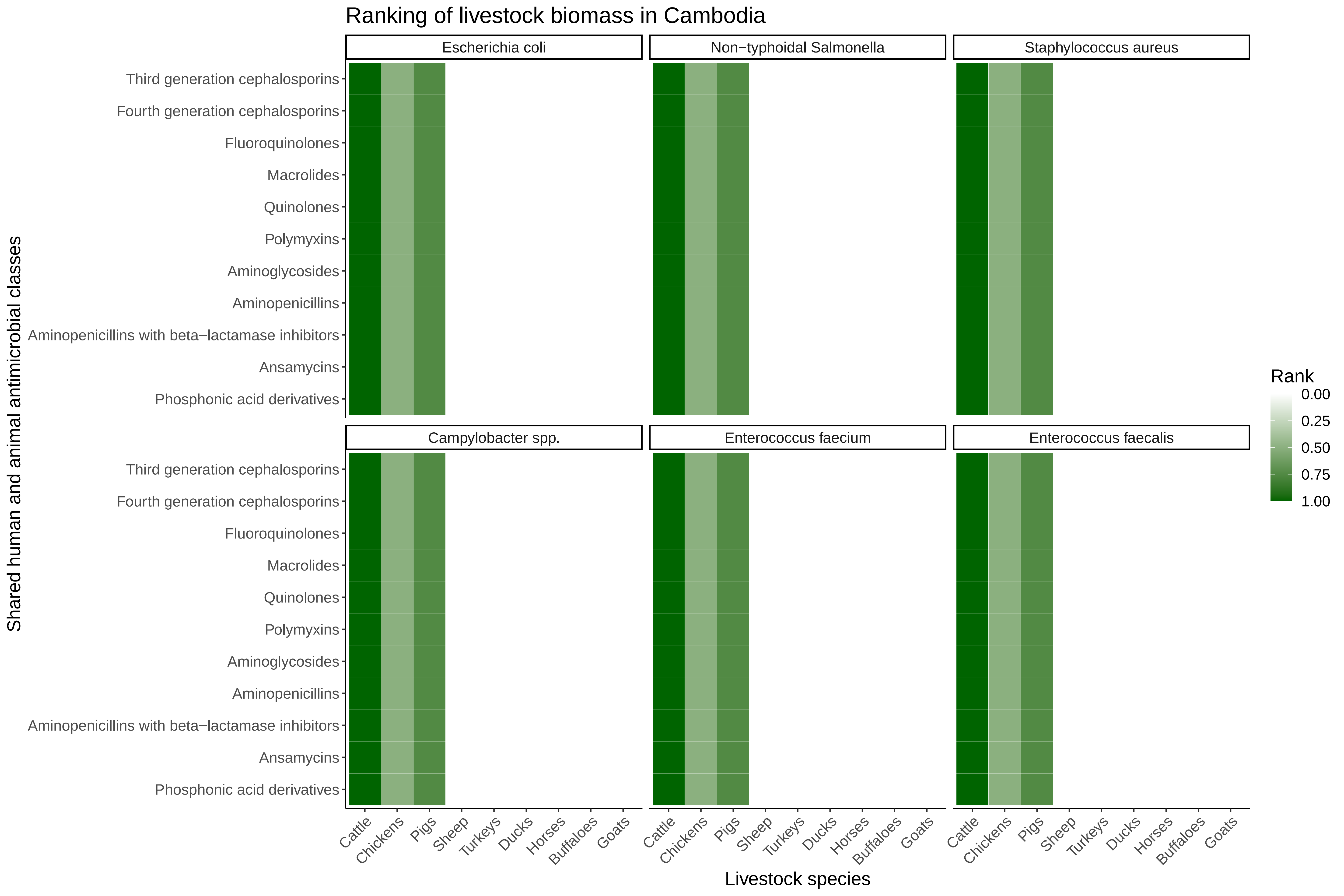
Ranking of livestock biomass in Cambodia
Escherichia coli
Non−typhoidal Salmonella
Staphylococcus aureus
Campylobacter spp.
Enterococcus faecium
Enterococcus faecalis
Third generation cephalosporins
Fourth generation cephalosporins
Fluoroquinolones
Macrolides
Quinolones
Polymyxins
Aminoglycosides
Aminopenicillins
Aminopenicillins with beta−lactamase inhibitors
Ansamycins
Phosphonic acid derivatives
Shared human and animal antimicrobial classes
Cattle
Chickens
Pigs
Sheep
Turkeys
Ducks
Horses
Buffaloes
Goats
Livestock species
Rank
0.00
0.25
0.50
0.75
1.00

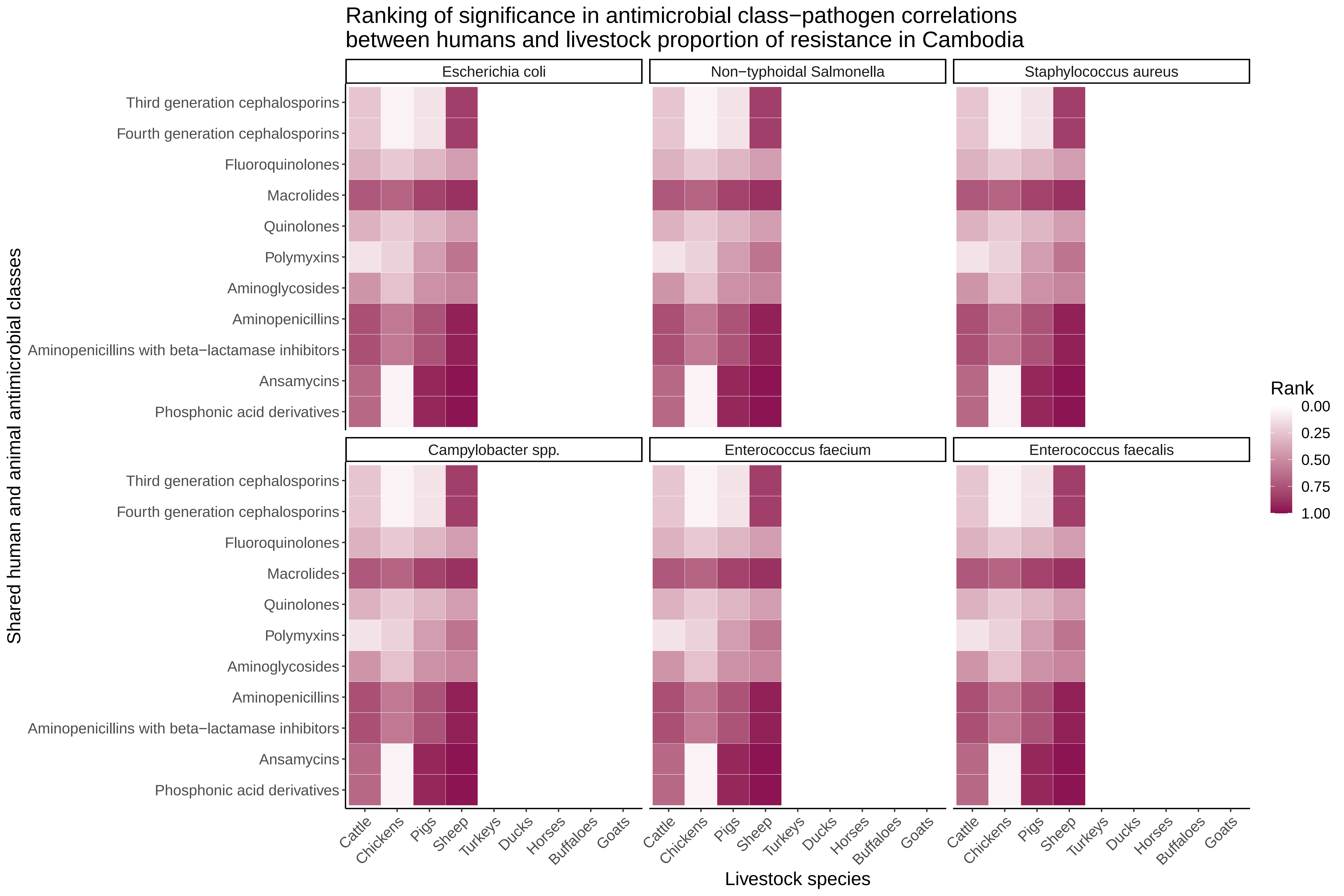

Ranking of significance in antimicrobial class−pathogen correlations
between humans and livestock proportion of resistance in Cambodia
Escherichia coli
Non−typhoidal Salmonella
Staphylococcus aureus
Campylobacter spp.
Enterococcus faecium
Enterococcus faecalis
Third generation cephalosporins
Fourth generation cephalosporins
Fluoroquinolones
Macrolides
Quinolones
Polymyxins
Aminoglycosides
Aminopenicillins
Aminopenicillins with beta−lactamase inhibitors
Ansamycins
Phosphonic acid derivatives
Shared human and animal antimicrobial classes
Cattle
Chickens
Pigs
Sheep
Turkeys
Ducks
Horses
Buffaloes
Goats
Livestock species
Rank
0.00
0.25
0.50
0.75
1.00

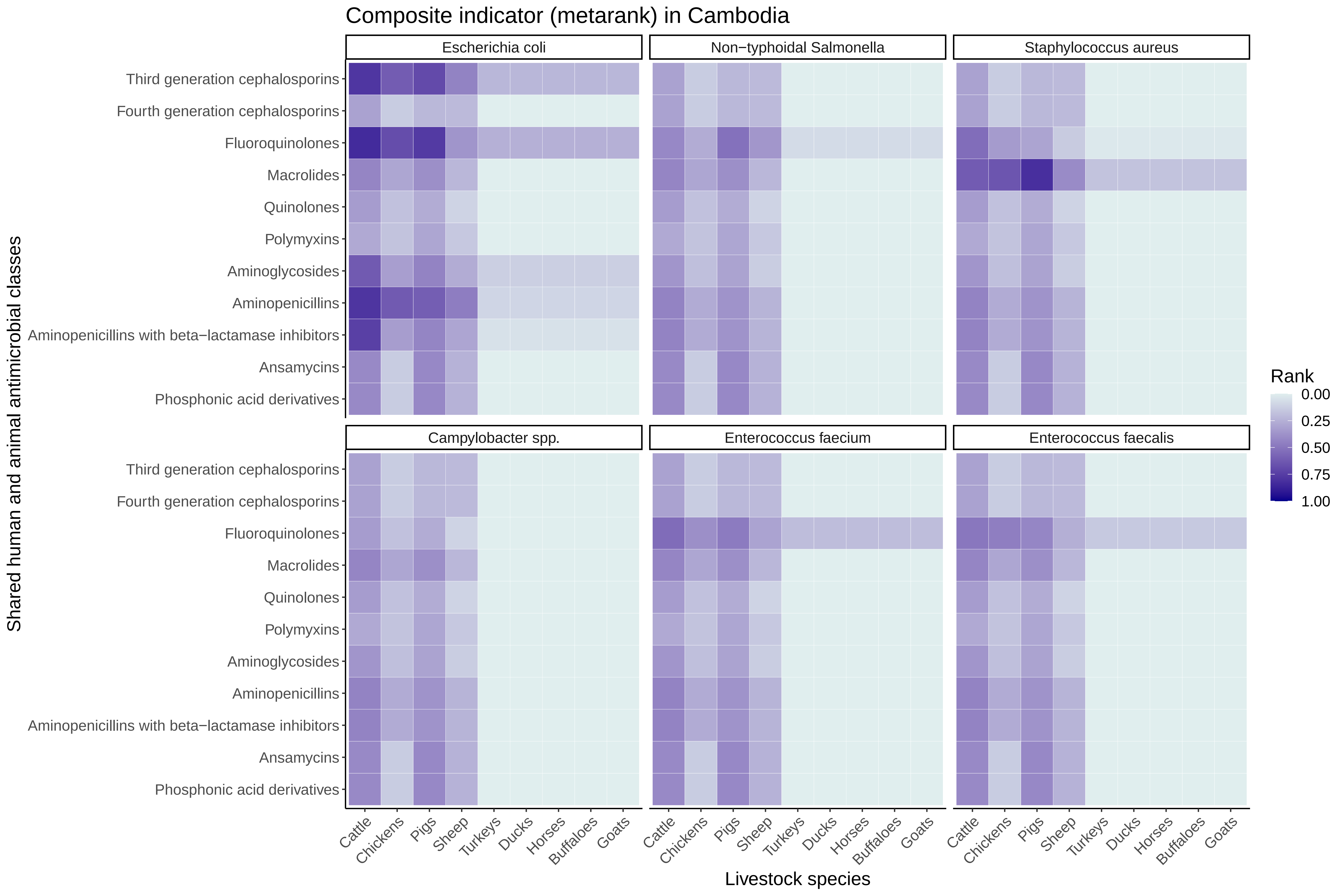

Composite indicator (metarank) in Cambodia
Escherichia coli
Non−typhoidal Salmonella
Staphylococcus aureus
Campylobacter spp.
Enterococcus faecium
Enterococcus faecalis
Third generation cephalosporins
Fourth generation cephalosporins
Fluoroquinolones
Macrolides
Quinolones
Polymyxins
Aminoglycosides
Aminopenicillins
Aminopenicillins with beta−lactamase inhibitors
Ansamycins
Phosphonic acid derivatives
Shared human and animal antimicrobial classes
Cattle
Chickens
Pigs
Sheep
Turkeys
Ducks
Horses
Buffaloes
Goats
Livestock species
Rank
0.00
0.25
0.50
0.75
1.00

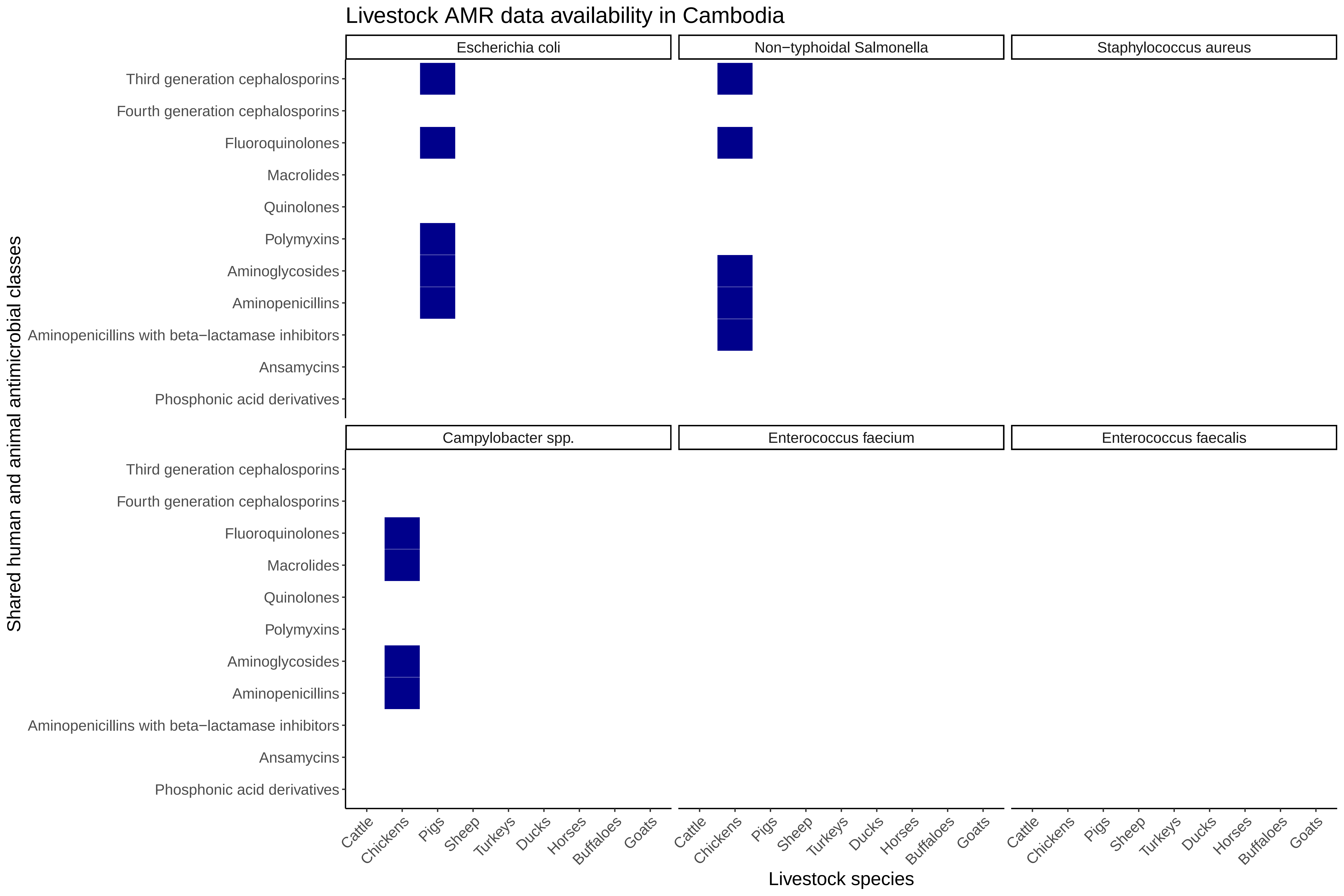

Livestock AMR data availability in Cambodia
Escherichia coli
Non−typhoidal Salmonella
Staphylococcus aureus
Campylobacter spp.
Enterococcus faecium
Enterococcus faecalis
Shared human and animal antimicrobial classes
Third generation cephalosporins
Fourth generation cephalosporins
Fluoroquinolones
Macrolides
Quinolones
Polymyxins
Aminoglycosides
Aminopenicillins
Aminopenicillins with beta−lactamase inhibitors
Ansamycins
Phosphonic acid derivatives
Cattle
Chickens
Pigs
Sheep
Turkeys
Ducks
Horses
Buffaloes
Goats
Livestock species

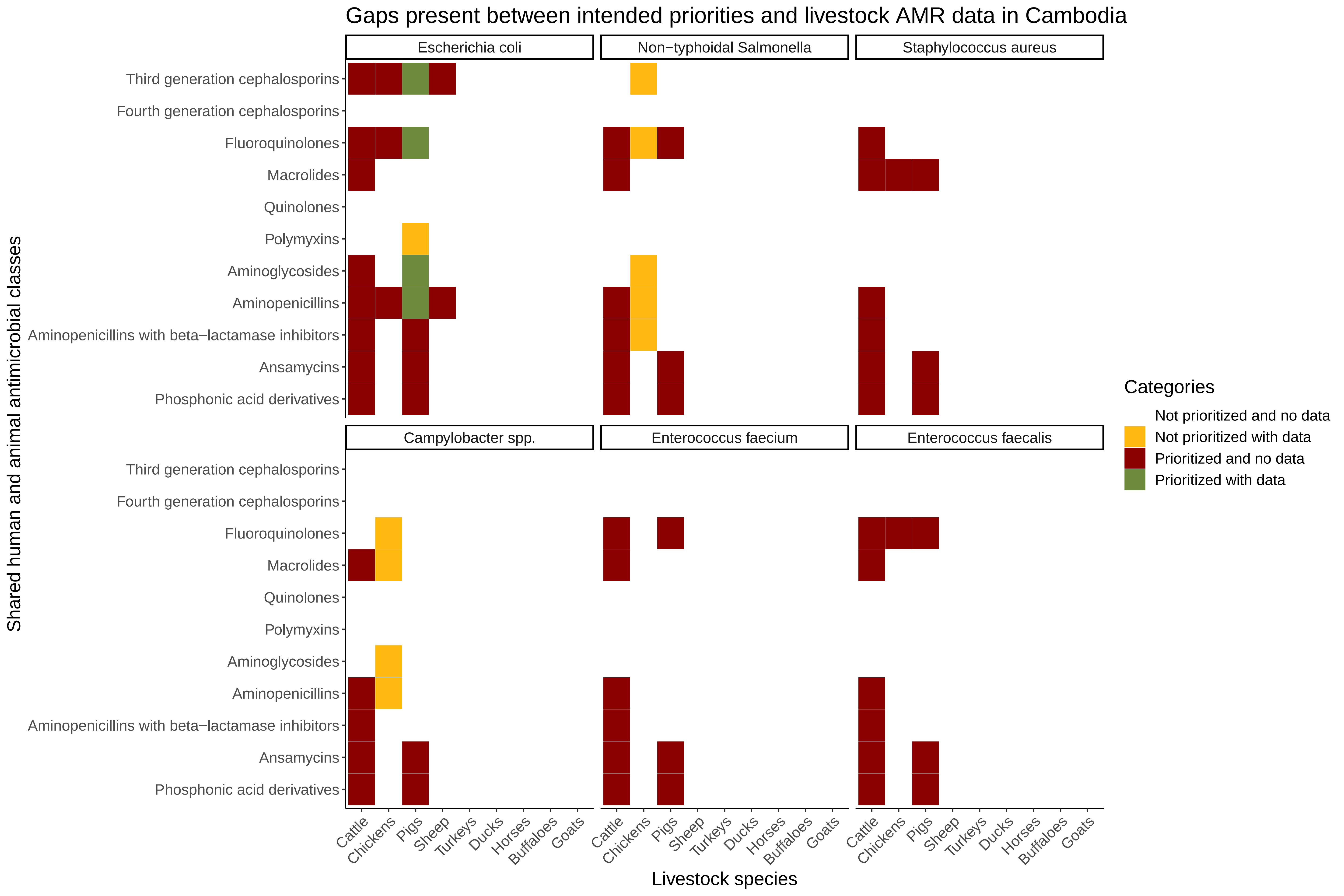

Gaps present between intended priorities and livestock AMR data in Cambodia
Escherichia coli
Non−typhoidal Salmonella
Staphylococcus aureus
Campylobacter spp.
Enterococcus faecium
Enterococcus faecalis
Third generation cephalosporins
Fourth generation cephalosporins
Fluoroquinolones
Macrolides
Quinolones
Polymyxins
Aminoglycosides
Aminopenicillins
Aminopenicillins with beta−lactamase inhibitors
Ansamycins
Phosphonic acid derivatives
Shared human and animal antimicrobial classes
Cattle
Chickens
Pigs
Sheep
Turkeys
Ducks
Horses
Buffaloes
Goats
Livestock species
Categories
Not prioritized and no data
Not prioritized with data
Prioritized and no data
Prioritized with data

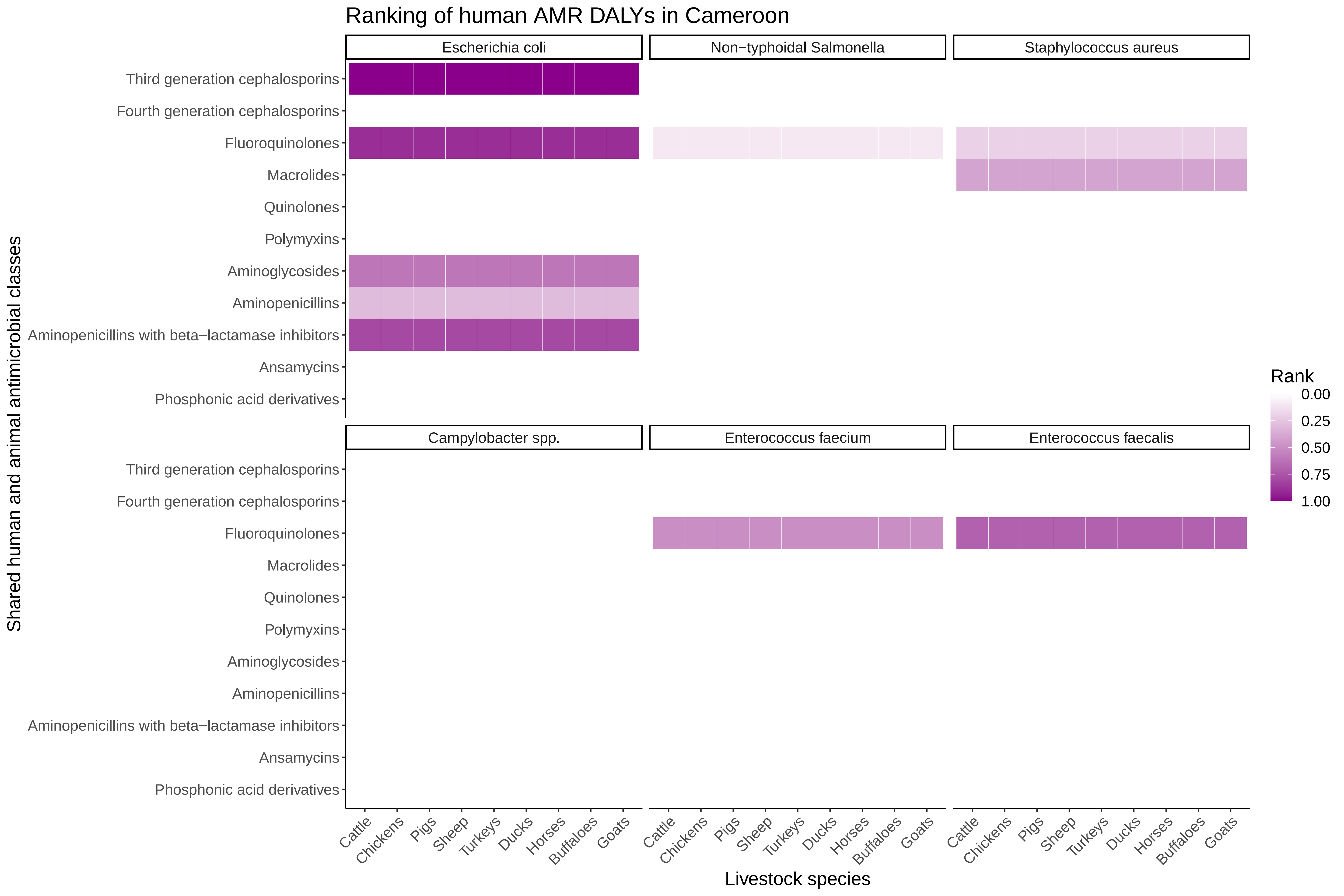
Ranking of human AMR DALYs in Cameroon
Escherichia coli
Non−typhoidal Salmonella
Staphylococcus aureus
Campylobacter spp.
Enterococcus faecium
Enterococcus faecalis
Shared human and animal antimicrobial classes
Third generation cephalosporins
Fourth generation cephalosporins
Fluoroquinolones
Macrolides
Quinolones
Polymyxins
Aminoglycosides
Aminopenicillins
Aminopenicillins with beta−lactamase inhibitors
Ansamycins
Phosphonic acid derivatives
Cattle
Chickens
Pigs
Sheep
Turkeys
Ducks
Horses
Buffaloes
Goats
Livestock species
Rank
0.00
0.25
0.50
0.75
1.00

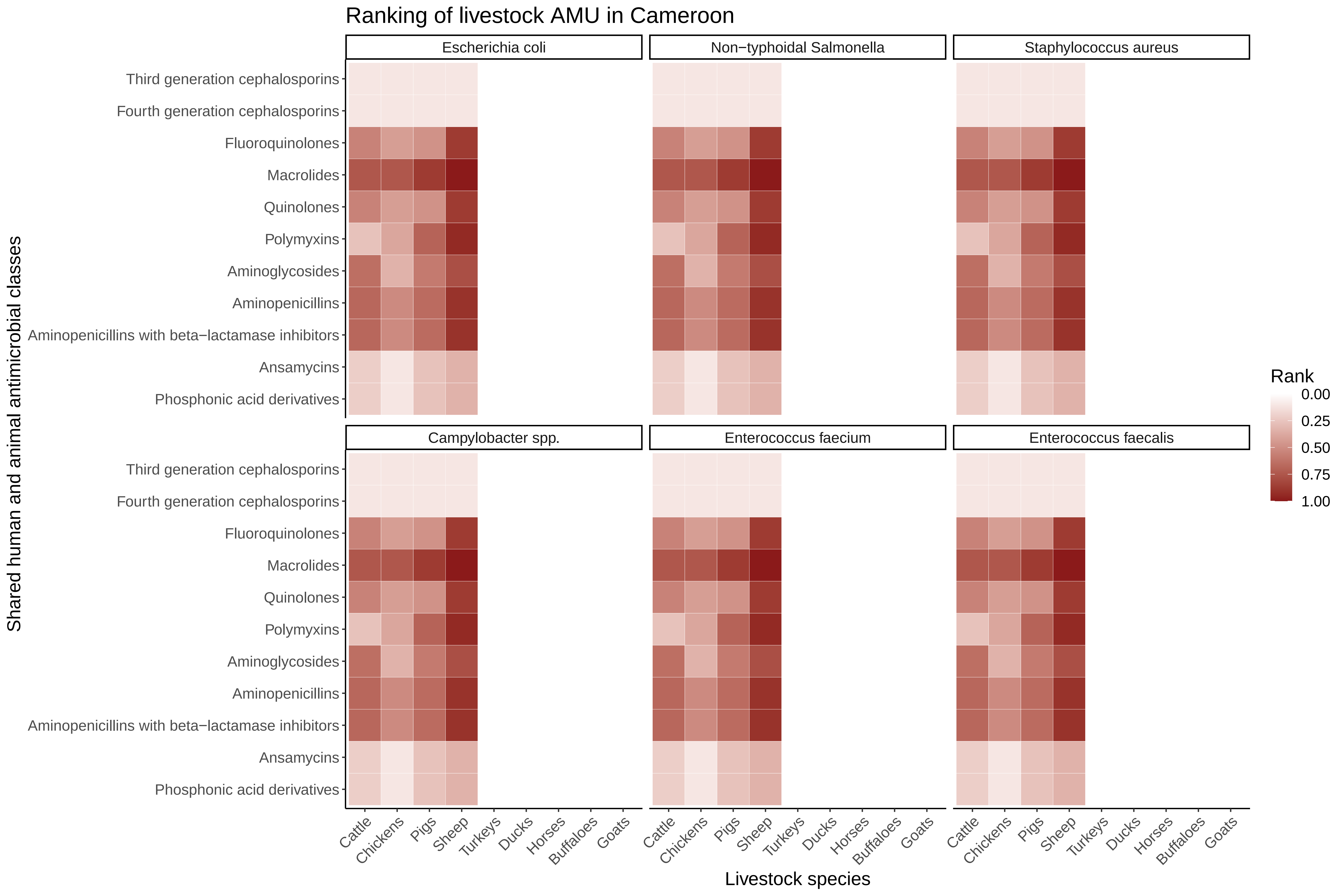

Ranking of livestock AMU in Cameroon
Escherichia coli
Non−typhoidal Salmonella
Staphylococcus aureus
Campylobacter spp.
Enterococcus faecium
Enterococcus faecalis
Third generation cephalosporins
Fourth generation cephalosporins
Fluoroquinolones
Macrolides
Quinolones
Polymyxins
Aminoglycosides
Aminopenicillins
Aminopenicillins with beta−lactamase inhibitors
Ansamycins
Phosphonic acid derivatives
Shared human and animal antimicrobial classes
Cattle
Chickens
Pigs
Sheep
Turkeys
Ducks
Horses
Buffaloes
Goats
Livestock species
Rank
0.00
0.25
0.50
0.75
1.00

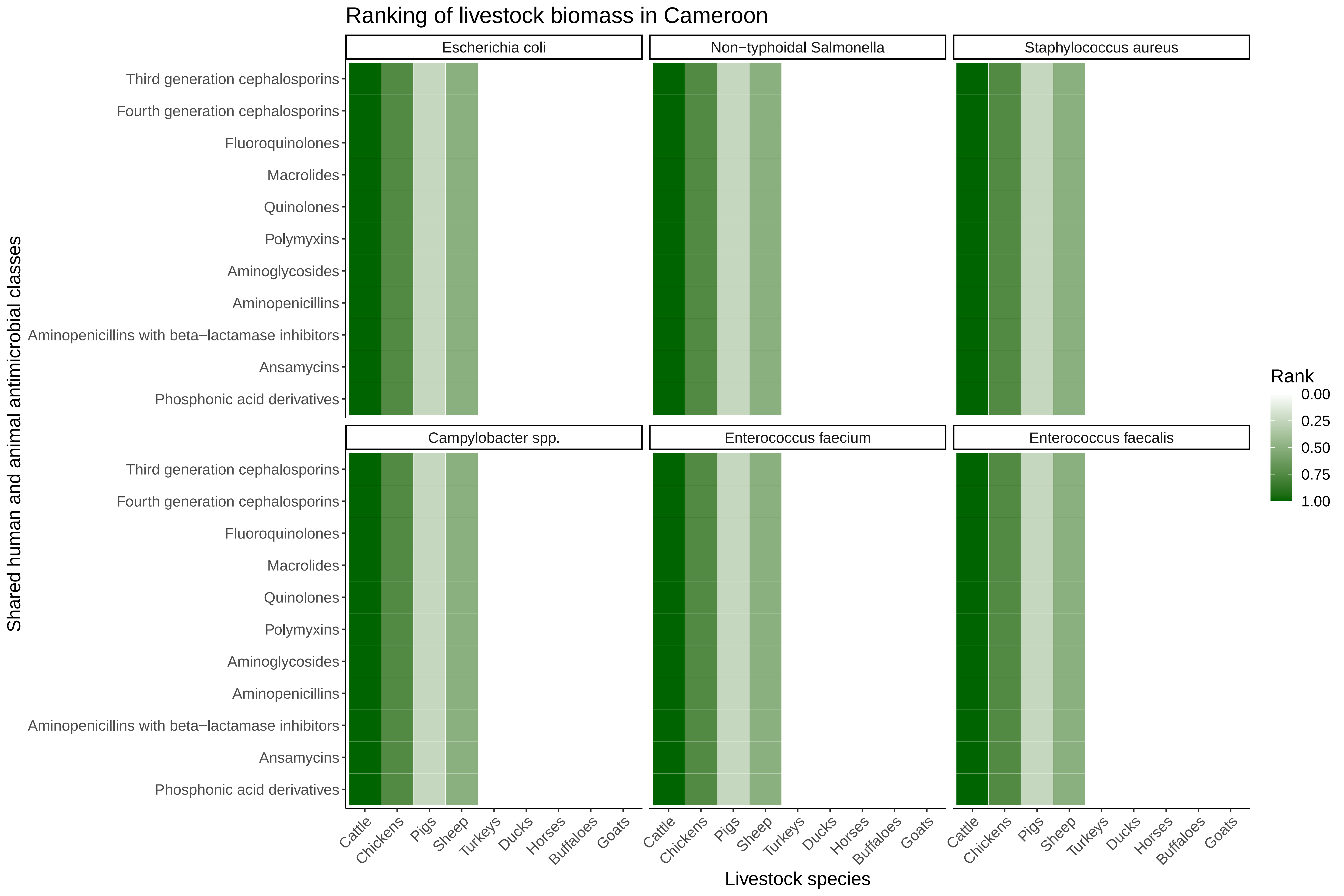

Ranking of livestock biomass in Cameroon
Escherichia coli
Non−typhoidal Salmonella
Staphylococcus aureus
Campylobacter spp.
Enterococcus faecium
Enterococcus faecalis
Shared human and animal antimicrobial classes
Third generation cephalosporins
Fourth generation cephalosporins
Fluoroquinolones
Macrolides
Quinolones
Polymyxins
Aminoglycosides
Aminopenicillins
Aminopenicillins with beta−lactamase inhibitors
Ansamycins
Phosphonic acid derivatives
Livestock species
Cattle
Chickens
Pigs
Sheep
Turkeys
Ducks
Horses
Buffaloes
Goats
Rank
0.00
0.25
0.50
0.75
1.00

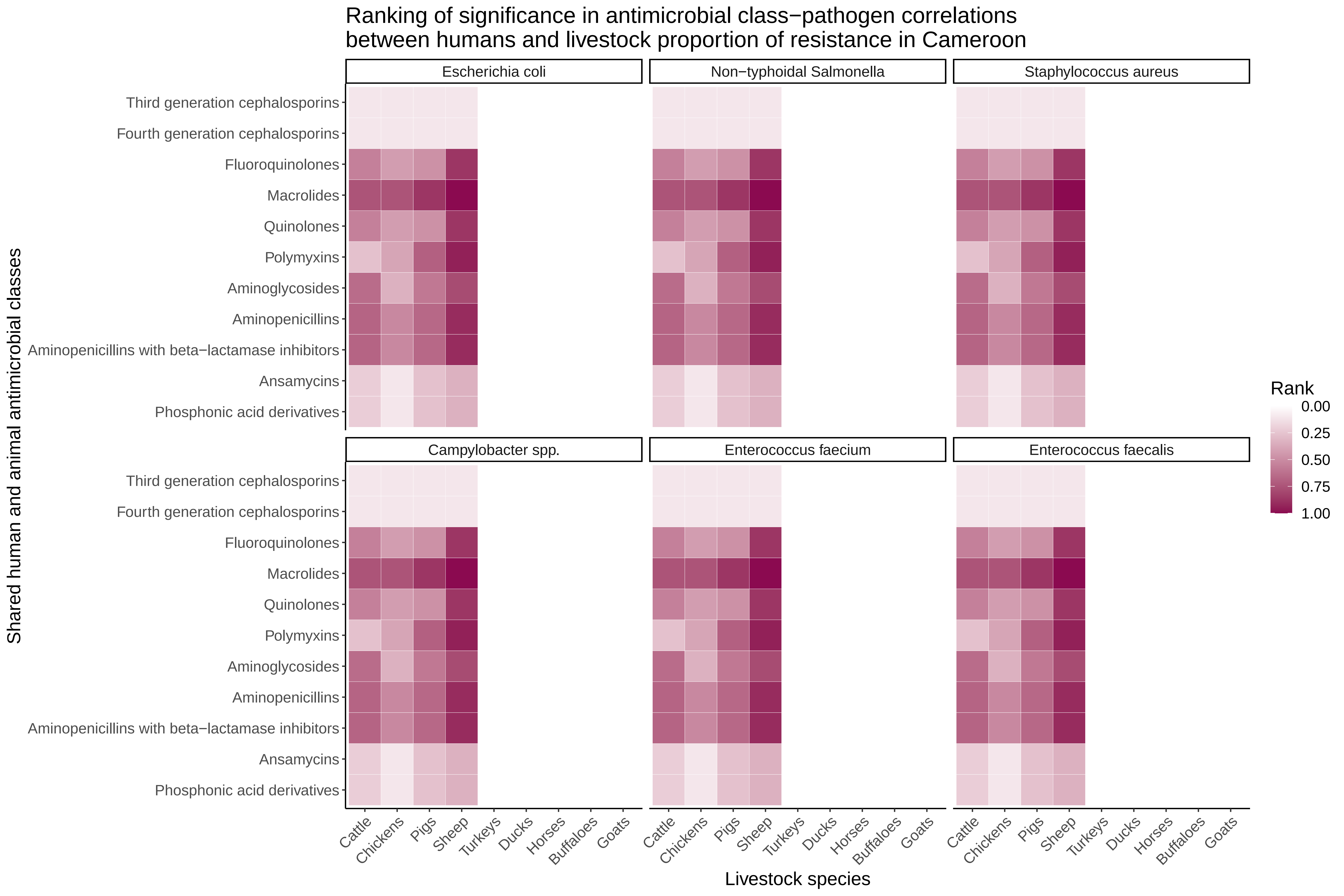

Ranking of significance in antimicrobial class−pathogen correlations
between humans and livestock proportion of resistance in Cameroon
Escherichia coli
Non−typhoidal Salmonella
Staphylococcus aureus
Campylobacter spp.
Enterococcus faecium
Enterococcus faecalis
Shared human and animal antimicrobial classes
Third generation cephalosporins
Fourth generation cephalosporins
Fluoroquinolones
Macrolides
Quinolones
Polymyxins
Aminoglycosides
Aminopenicillins
Aminopenicillins with beta−lactamase inhibitors
Ansamycins
Phosphonic acid derivatives
Cattle
Chickens
Pigs
Sheep
Turkeys
Ducks
Horses
Buffaloes
Goats
Livestock species
Rank
0.00
0.25
0.50
0.75
1.00

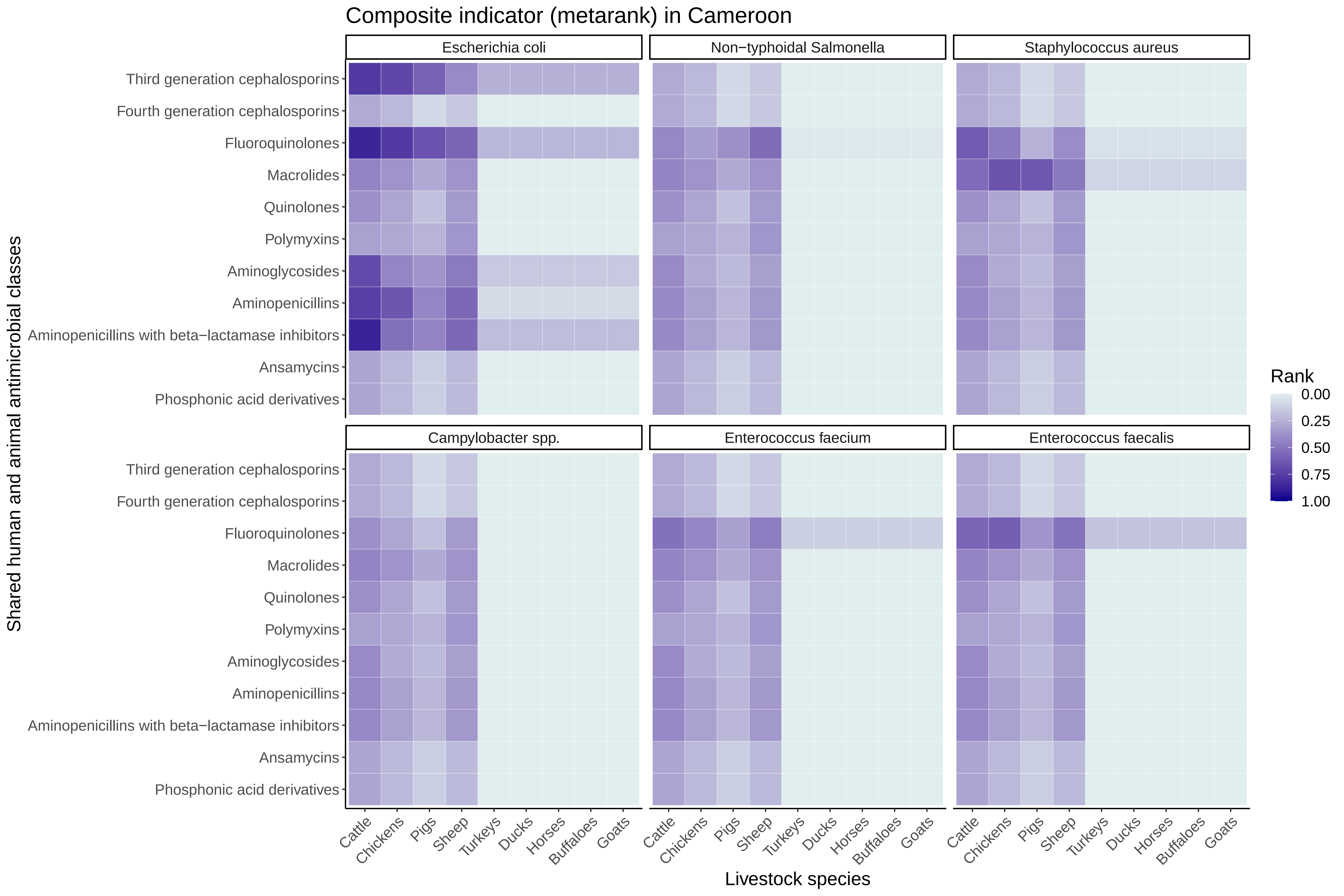

Composite indicator (metarank) in Cameroon
Escherichia coli
Non−typhoidal Salmonella
Staphylococcus aureus
Campylobacter spp.
Enterococcus faecium
Enterococcus faecalis
Third generation cephalosporins
Fourth generation cephalosporins
Fluoroquinolones
Macrolides
Quinolones
Polymyxins
Aminoglycosides
Aminopenicillins
Aminopenicillins with beta−lactamase inhibitors
Ansamycins
Phosphonic acid derivatives
Shared human and animal antimicrobial classes
Cattle
Chickens
Pigs
Sheep
Turkeys
Ducks
Horses
Buffaloes
Goats
Livestock species
Rank
0.00
0.25
0.50
0.75
1.00

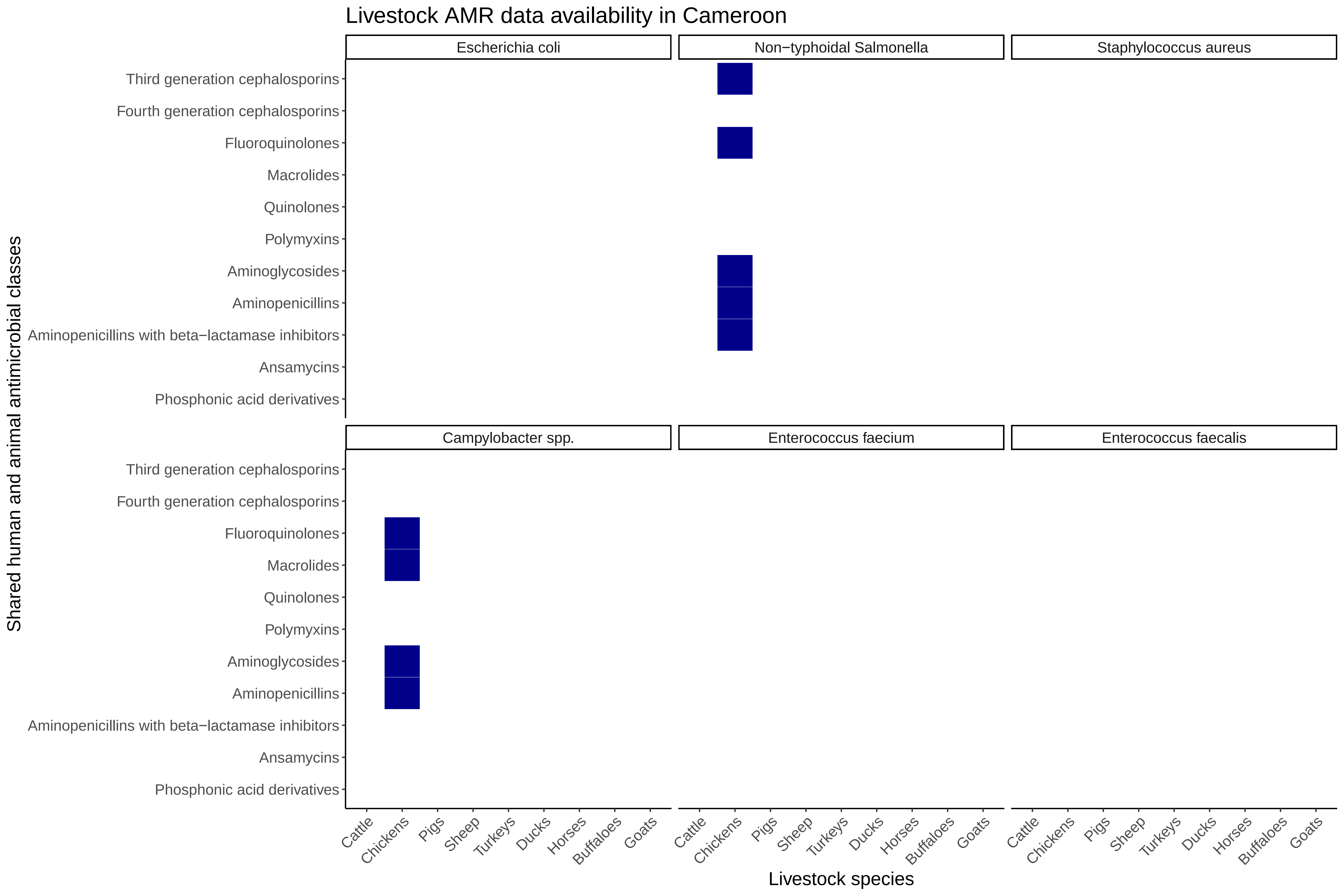

Livestock AMR data availability in Cameroon
Escherichia coli
Non−typhoidal Salmonella
Staphylococcus aureus
Campylobacter spp.
Enterococcus faecium
Enterococcus faecalis
Third generation cephalosporins
Fourth generation cephalosporins
Fluoroquinolones
Macrolides
Quinolones
Polymyxins
Aminoglycosides
Aminopenicillins
Aminopenicillins with beta−lactamase inhibitors
Ansamycins
Phosphonic acid derivatives
Shared human and animal antimicrobial classes
Cattle
Chickens
Pigs
Sheep
Turkeys
Ducks
Horses
Buffaloes
Goats
Livestock species

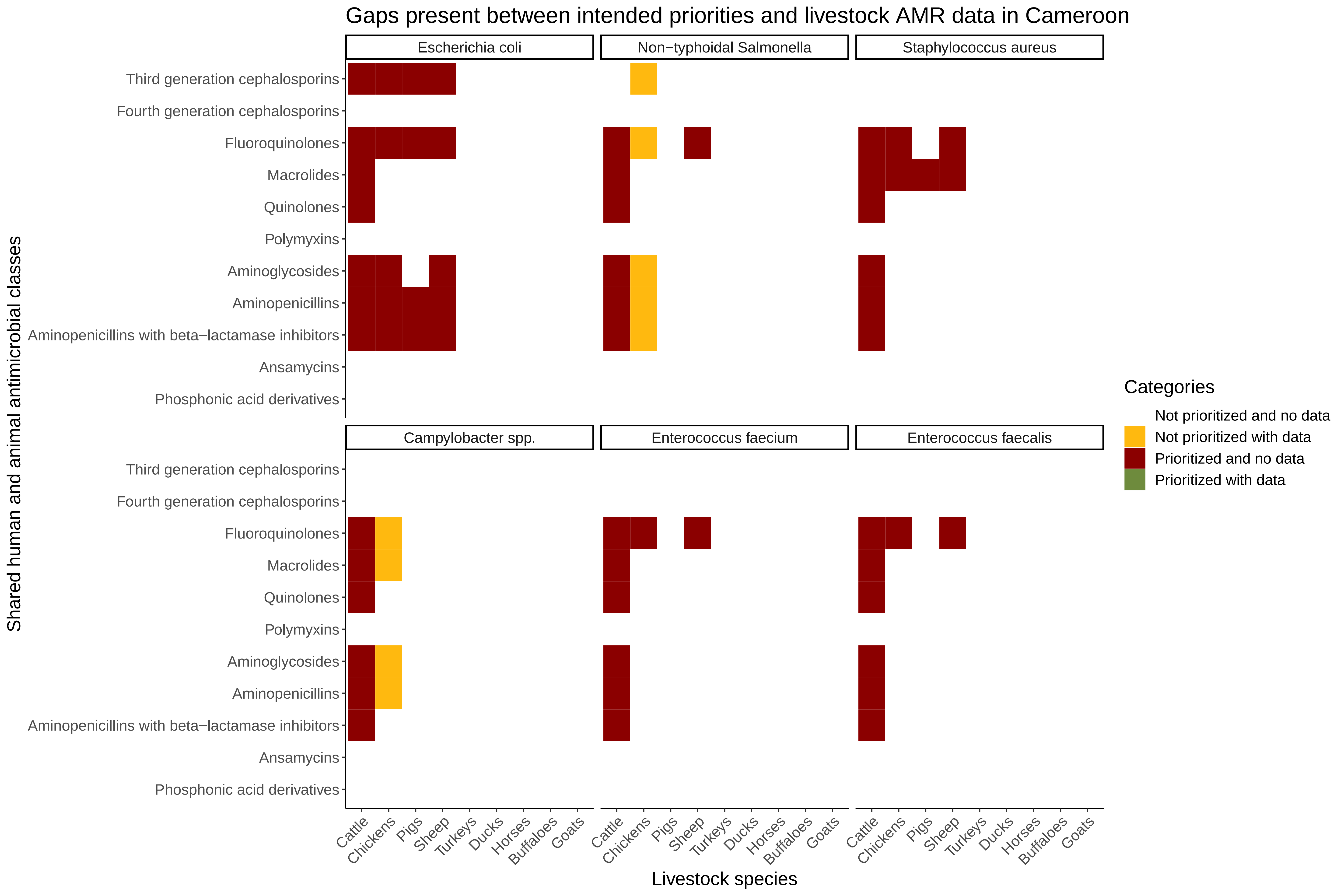
Gaps present between intended priorities and livestock AMR data in Cameroon
Escherichia coli
Non−typhoidal Salmonella
Staphylococcus aureus
Campylobacter spp.
Enterococcus faecium
Enterococcus faecalis
Shared human and animal antimicrobial classes
Third generation cephalosporins
Fourth generation cephalosporins
Fluoroquinolones
Macrolides
Quinolones
Polymyxins
Aminoglycosides
Aminopenicillins
Aminopenicillins with beta−lactamase inhibitors
Ansamycins
Phosphonic acid derivatives
Livestock species
Cattle
Chickens
Pigs
Sheep
Turkeys
Ducks
Horses
Buffaloes
Goats
Categories
Not prioritized and no data
Not prioritized with data
Prioritized and no data
Prioritized with data

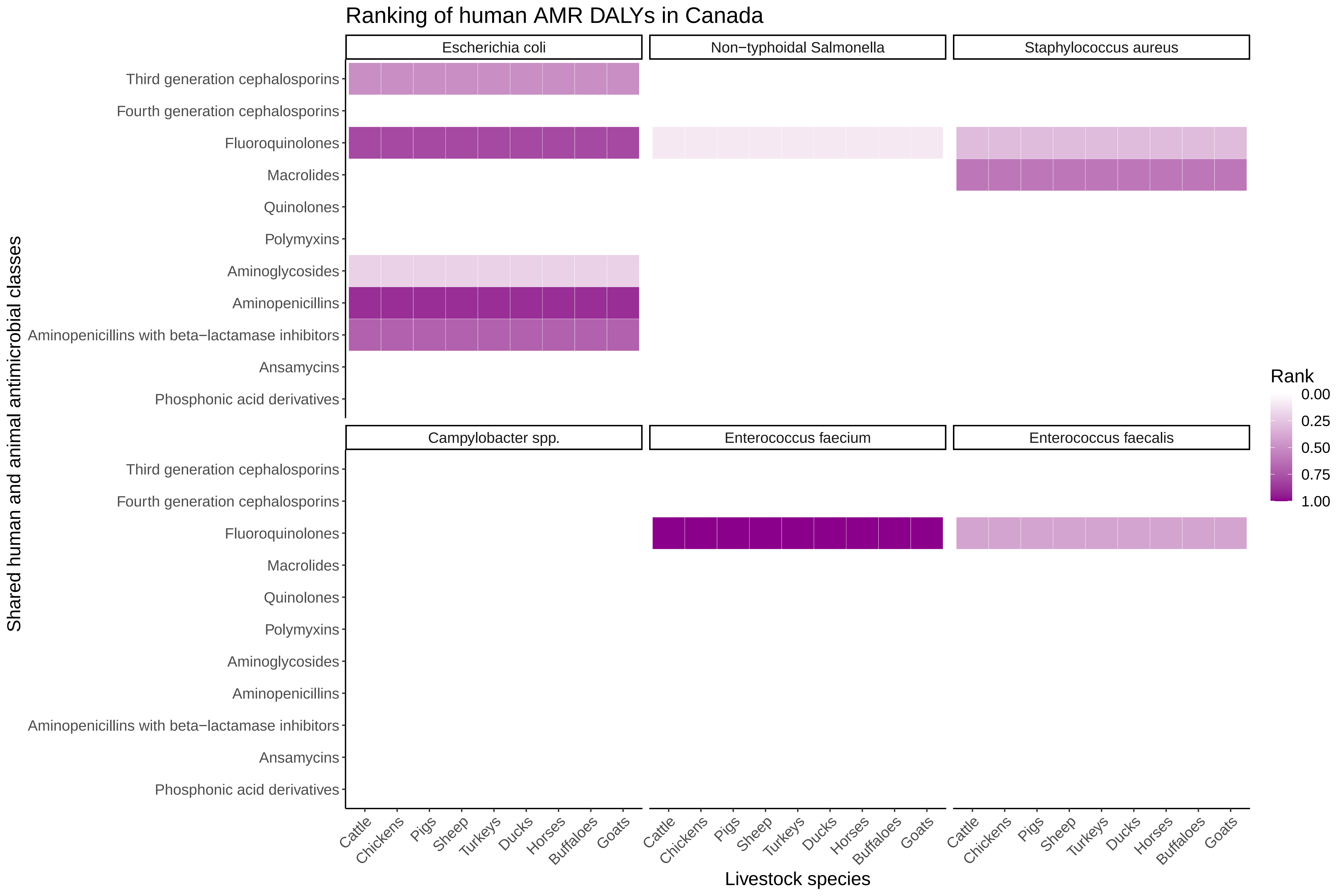

Ranking of human AMR DALYs in Canada
Escherichia coli
Non−typhoidal Salmonella
Staphylococcus aureus
Campylobacter spp.
Enterococcus faecium
Enterococcus faecalis
Third generation cephalosporins
Fourth generation cephalosporins
Fluoroquinolones
Macrolides
Quinolones
Polymyxins
Aminoglycosides
Aminopenicillins
Aminopenicillins with beta−lactamase inhibitors
Ansamycins
Phosphonic acid derivatives
Shared human and animal antimicrobial classes
Cattle
Chickens
Pigs
Sheep
Turkeys
Ducks
Horses
Buffaloes
Goats
Livestock species
Rank
0.00
0.25
0.50
0.75
1.00

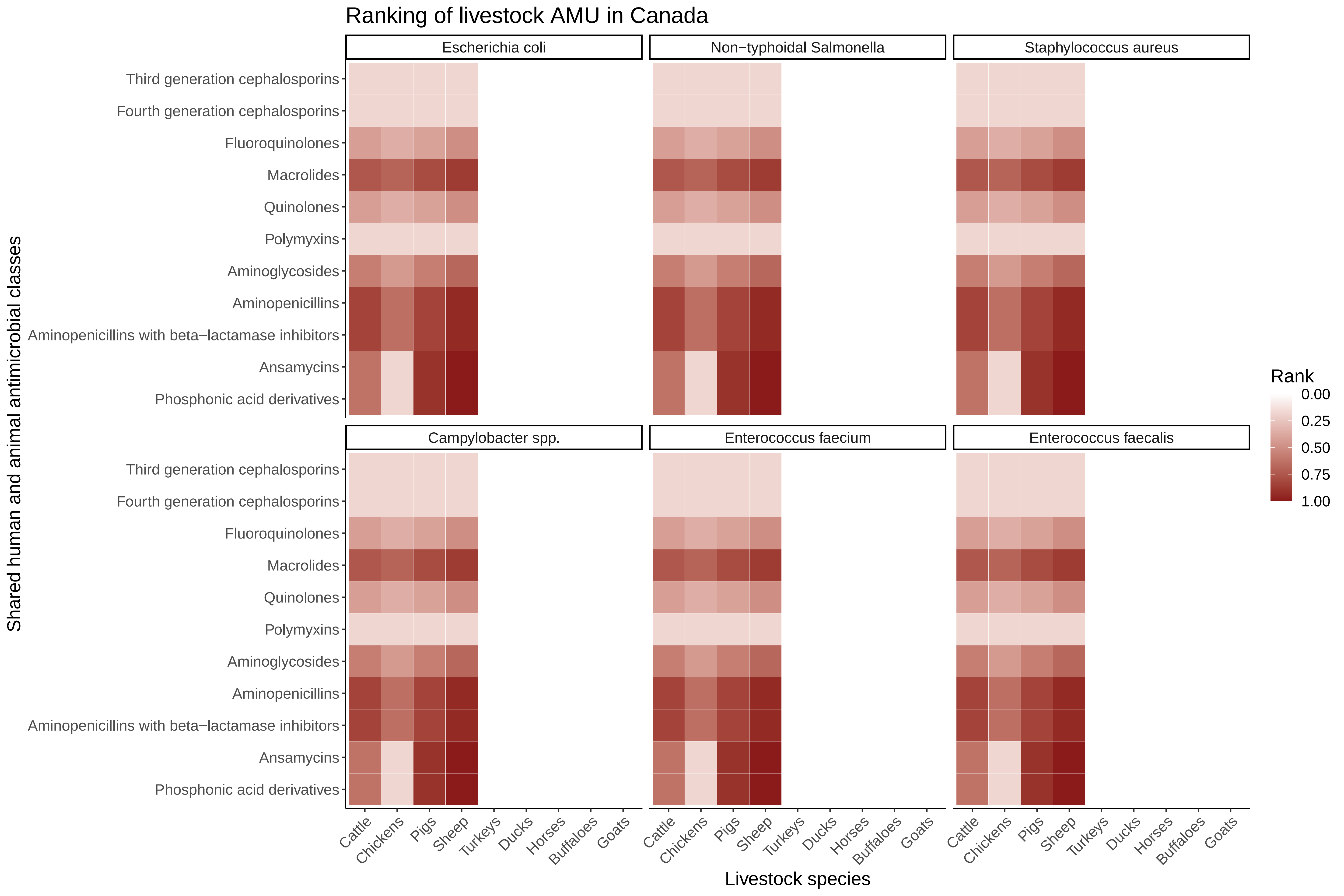
Ranking of livestock AMU in Canada
Escherichia coli
Non−typhoidal Salmonella
Staphylococcus aureus
Campylobacter spp.
Enterococcus faecium
Enterococcus faecalis
Shared human and animal antimicrobial classes
Third generation cephalosporins
Fourth generation cephalosporins
Fluoroquinolones
Macrolides
Quinolones
Polymyxins
Aminoglycosides
Aminopenicillins
Aminopenicillins with beta−lactamase inhibitors
Ansamycins
Phosphonic acid derivatives
Cattle
Chickens
Pigs
Sheep
Turkeys
Ducks
Horses
Buffaloes
Goats
Livestock species
Rank
0.00
0.25
0.50
0.75
1.00

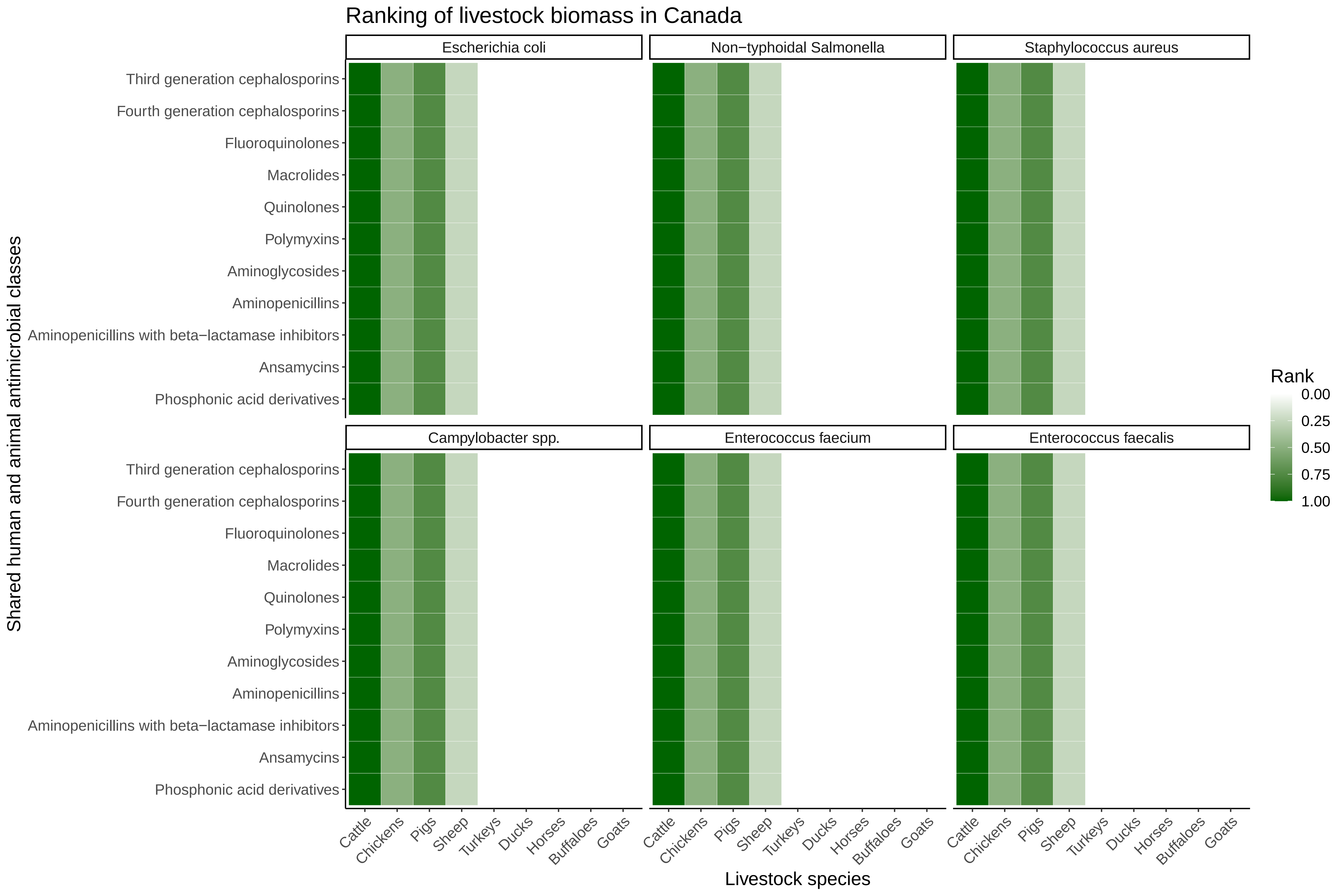

Ranking of livestock biomass in Canada
Escherichia coli
Non−typhoidal Salmonella
Staphylococcus aureus
Campylobacter spp.
Enterococcus faecium
Enterococcus faecalis
Third generation cephalosporins
Fourth generation cephalosporins
Fluoroquinolones
Macrolides
Quinolones
Polymyxins
Aminoglycosides
Aminopenicillins
Aminopenicillins with beta−lactamase inhibitors
Ansamycins
Phosphonic acid derivatives
Shared human and animal antimicrobial classes
Cattle
Chickens
Pigs
Sheep
Turkeys
Ducks
Horses
Buffaloes
Goats
Livestock species
Rank
0.00
0.25
0.50
0.75
1.00

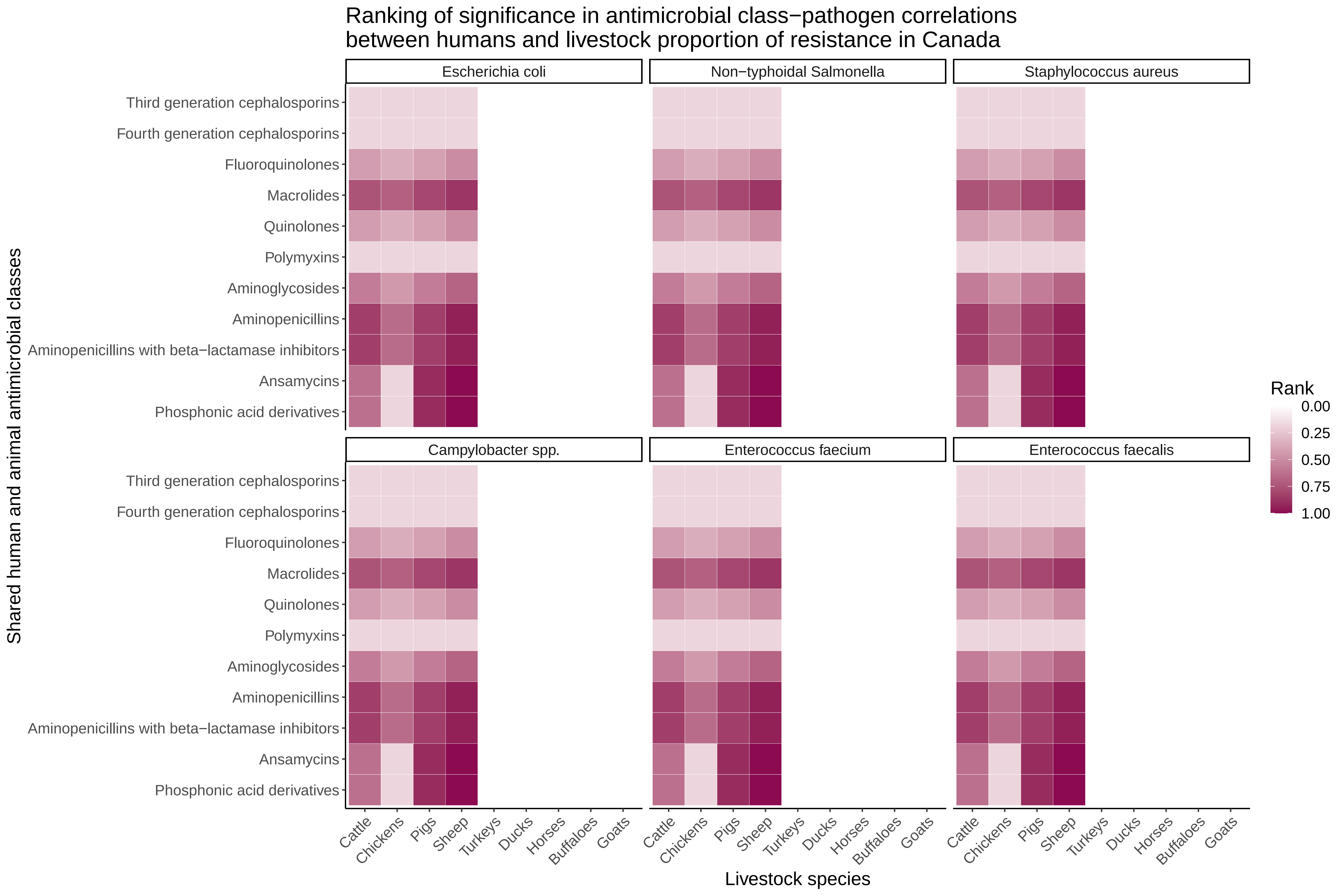

Ranking of significance in antimicrobial class−pathogen correlations between humans and livestock proportion of resistance in Canada
Escherichia coli
Non−typhoidal Salmonella
Staphylococcus aureus
Campylobacter spp.
Enterococcus faecium
Enterococcus faecalis
Shared human and animal antimicrobial classes
Third generation cephalosporins
Fourth generation cephalosporins
Fluoroquinolones
Macrolides
Quinolones
Polymyxins
Aminoglycosides
Aminopenicillins
Aminopenicillins with beta−lactamase inhibitors
Ansamycins
Phosphonic acid derivatives
Livestock species
Cattle
Chickens
Pigs
Sheep
Turkeys
Ducks
Horses
Buffaloes
Goats
Rank
0.00
0.25
0.50
0.75
1.00

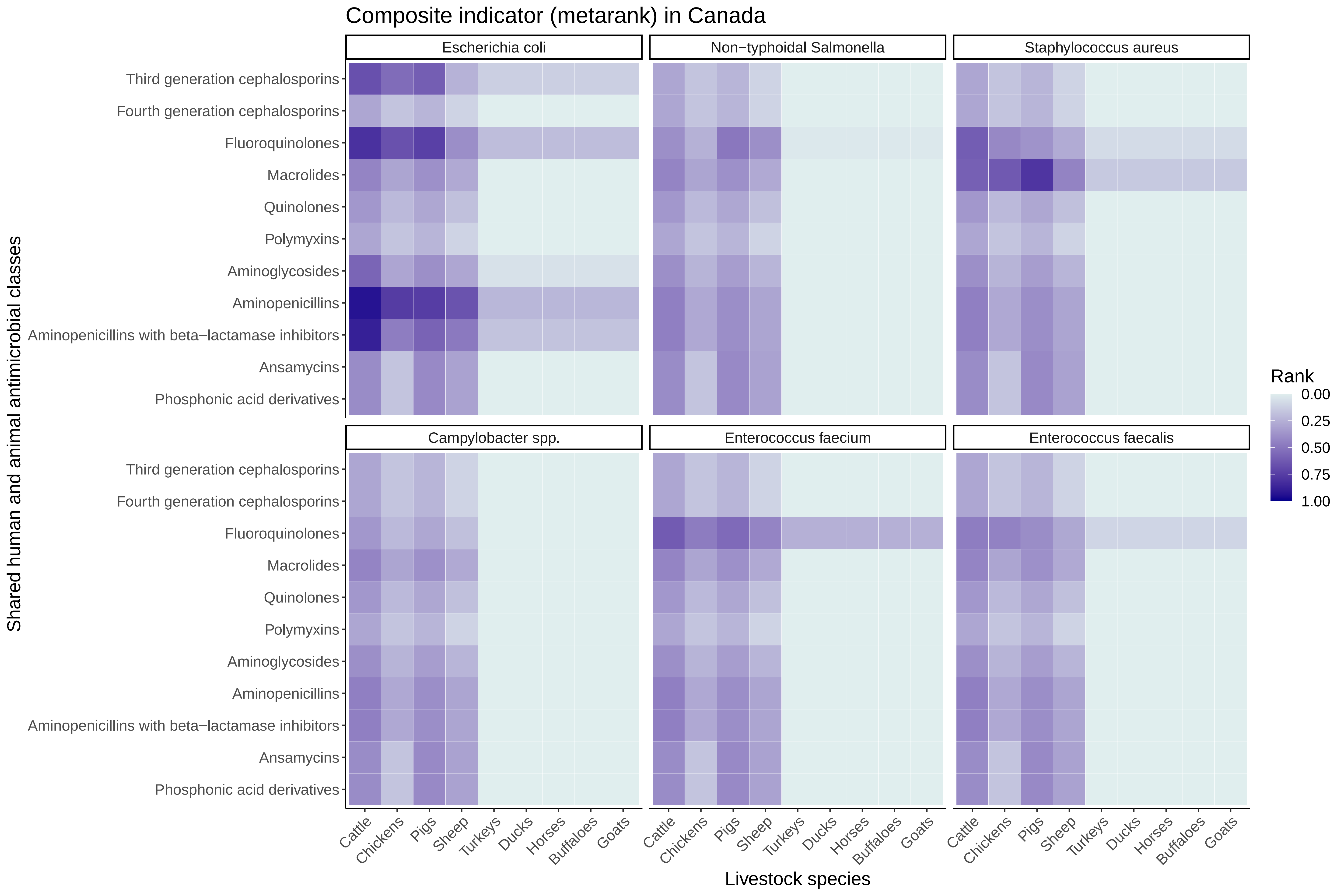

Composite indicator (metarank) in Canada
Escherichia coli
Non−typhoidal Salmonella
Staphylococcus aureus
Campylobacter spp.
Enterococcus faecium
Enterococcus faecalis
Shared human and animal antimicrobial classes
Third generation cephalosporins
Fourth generation cephalosporins
Fluoroquinolones
Macrolides
Quinolones
Polymyxins
Aminoglycosides
Aminopenicillins
Aminopenicillins with beta−lactamase inhibitors
Ansamycins
Phosphonic acid derivatives
Cattle
Chickens
Pigs
Sheep
Turkeys
Ducks
Horses
Buffaloes
Goats
Livestock species
Rank
0.00
0.25
0.50
0.75
1.00

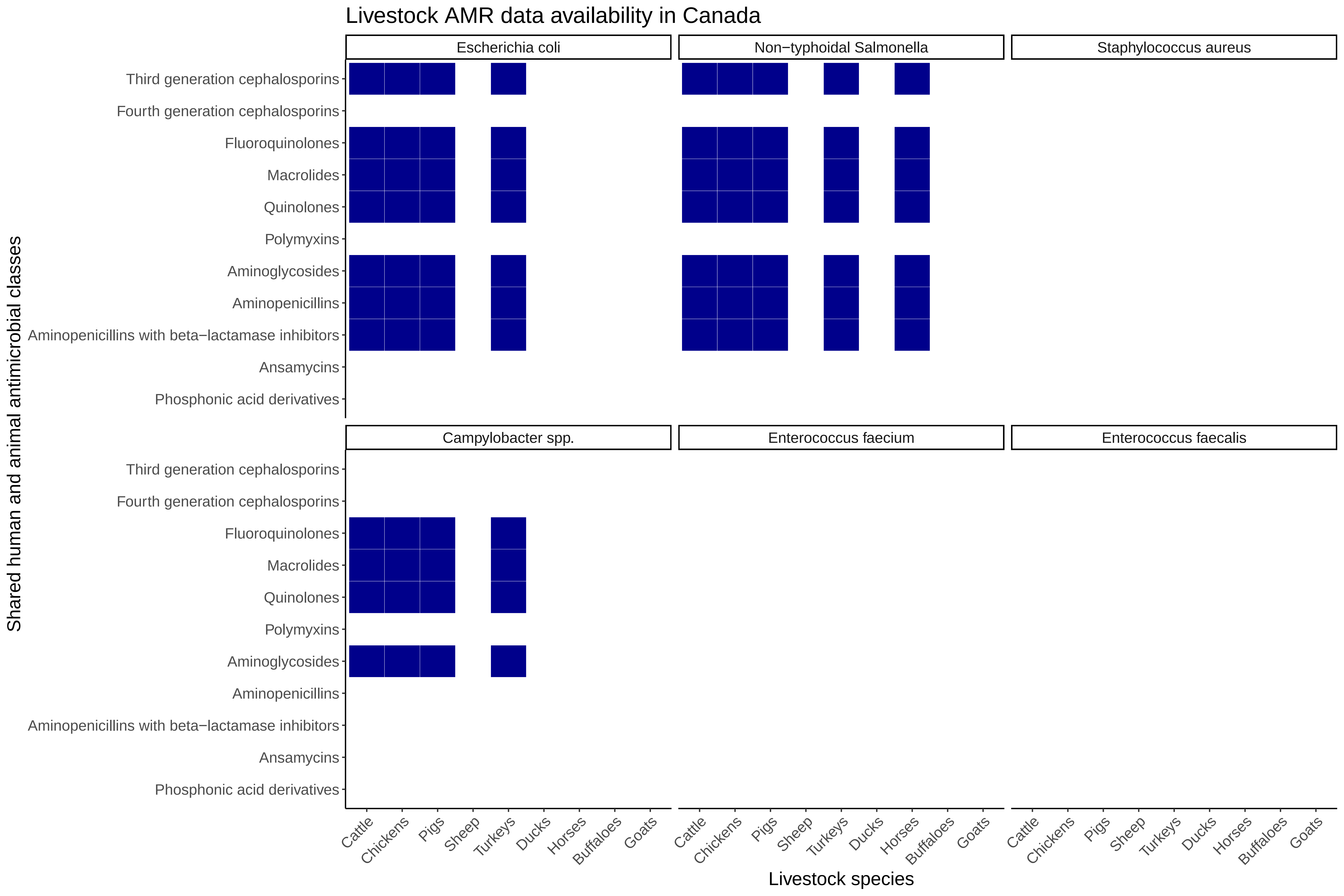

Livestock AMR data availability in Canada
Escherichia coli
Non−typhoidal Salmonella
Staphylococcus aureus
Campylobacter spp.
Enterococcus faecium
Enterococcus faecalis
Shared human and animal antimicrobial classes
Third generation cephalosporins
Fourth generation cephalosporins
Fluoroquinolones
Macrolides
Quinolones
Polymyxins
Aminoglycosides
Aminopenicillins
Aminopenicillins with beta−lactamase inhibitors
Ansamycins
Phosphonic acid derivatives
Cattle
Chickens
Pigs
Sheep
Turkeys
Ducks
Horses
Buffaloes
Goats
Livestock species

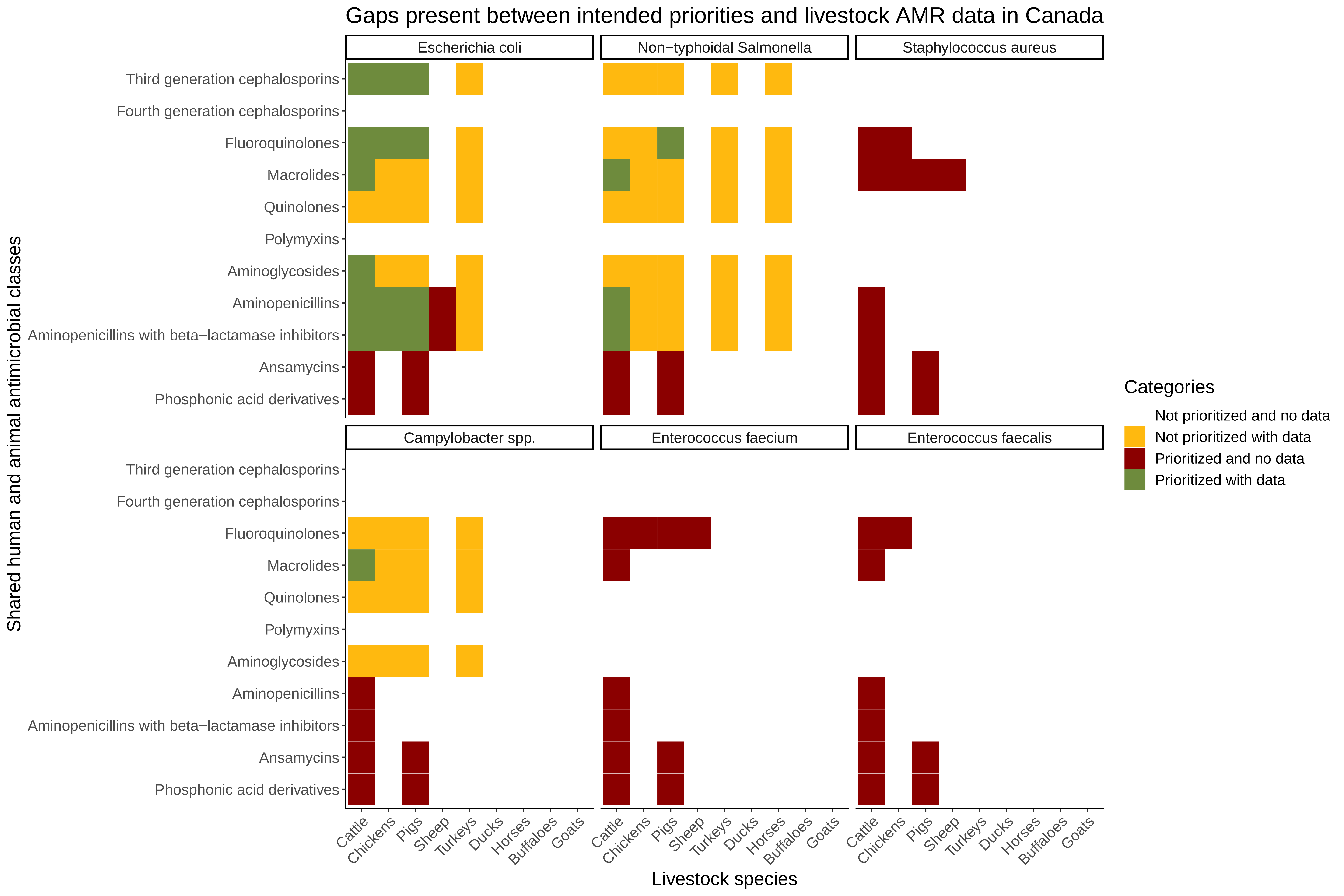

Gaps present between intended priorities and livestock AMR data in Canada
Escherichia coli
Non−typhoidal Salmonella
Staphylococcus aureus
Campylobacter spp.
Enterococcus faecium
Enterococcus faecalis
Third generation cephalosporins
Fourth generation cephalosporins
Fluoroquinolones
Macrolides
Quinolones
Polymyxins
Aminoglycosides
Aminopenicillins
Aminopenicillins with beta−lactamase inhibitors
Ansamycins
Phosphonic acid derivatives
Shared human and animal antimicrobial classes
Cattle
Chickens
Pigs
Sheep
Turkeys
Ducks
Horses
Buffaloes
Goats
Livestock species
Categories
Not prioritized and no data
Not prioritized with data
Prioritized and no data
Prioritized with data

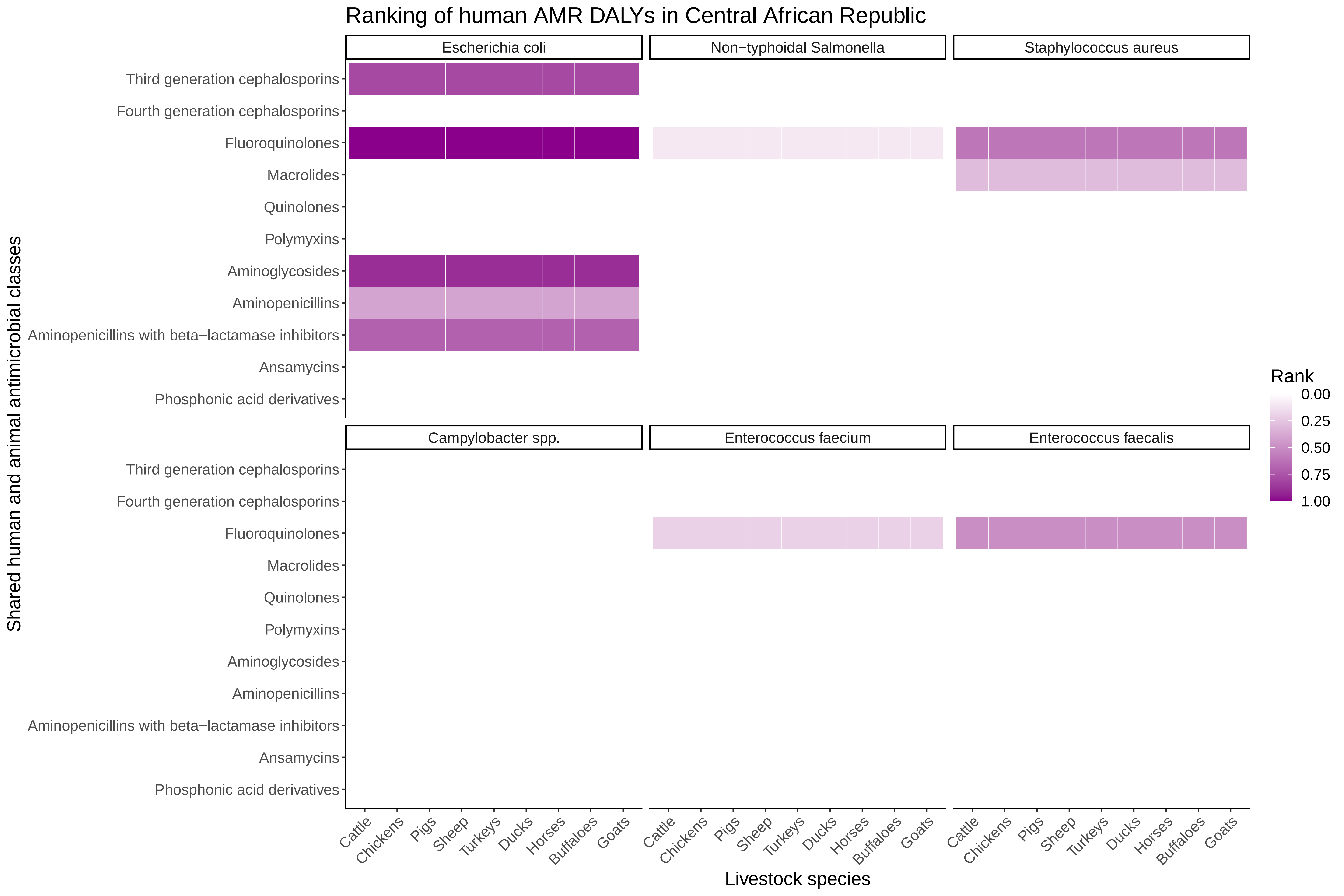
Ranking of human AMR DALYs in Central African Republic
Escherichia coli
Non−typhoidal Salmonella
Staphylococcus aureus
Campylobacter spp.
Enterococcus faecium
Enterococcus faecalis
Third generation cephalosporins
Fourth generation cephalosporins
Fluoroquinolones
Macrolides
Quinolones
Polymyxins
Aminoglycosides
Aminopenicillins
Aminopenicillins with beta−lactamase inhibitors
Ansamycins
Phosphonic acid derivatives
Shared human and animal antimicrobial classes
Cattle
Chickens
Pigs
Sheep
Turkeys
Ducks
Horses
Buffaloes
Goats
Livestock species
Rank
0.00
0.25
0.50
0.75
1.00

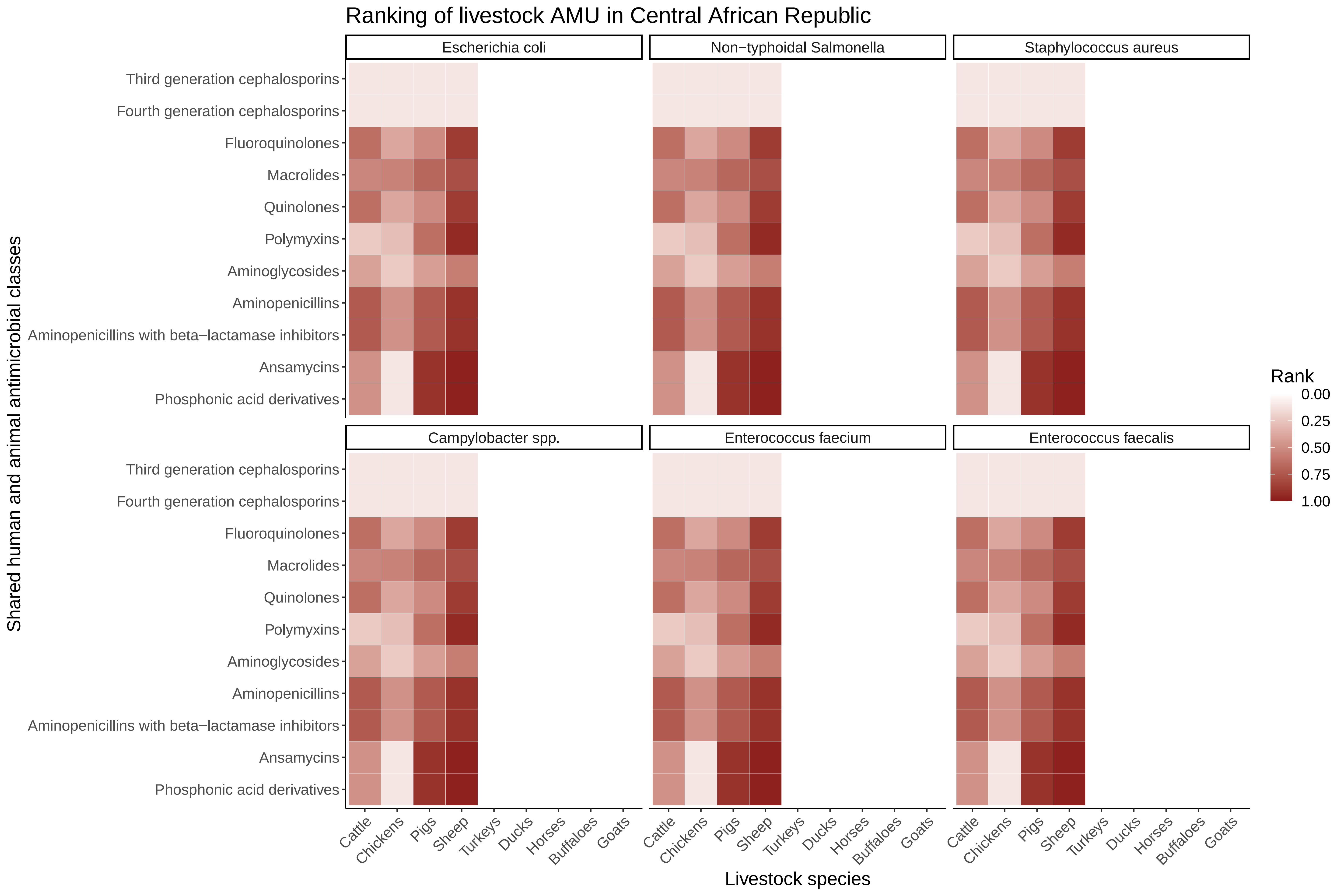

Ranking of livestock AMU in Central African Republic
Escherichia coli
Non−typhoidal Salmonella
Staphylococcus aureus
Campylobacter spp.
Enterococcus faecium
Enterococcus faecalis
Third generation cephalosporins
Fourth generation cephalosporins
Fluoroquinolones
Macrolides
Quinolones
Polymyxins
Aminoglycosides
Aminopenicillins
Aminopenicillins with beta−lactamase inhibitors
Ansamycins
Phosphonic acid derivatives
Shared human and animal antimicrobial classes
Cattle
Chickens
Pigs
Sheep
Turkeys
Ducks
Horses
Buffaloes
Goats
Livestock species
Rank
0.00
0.25
0.50
0.75
1.00

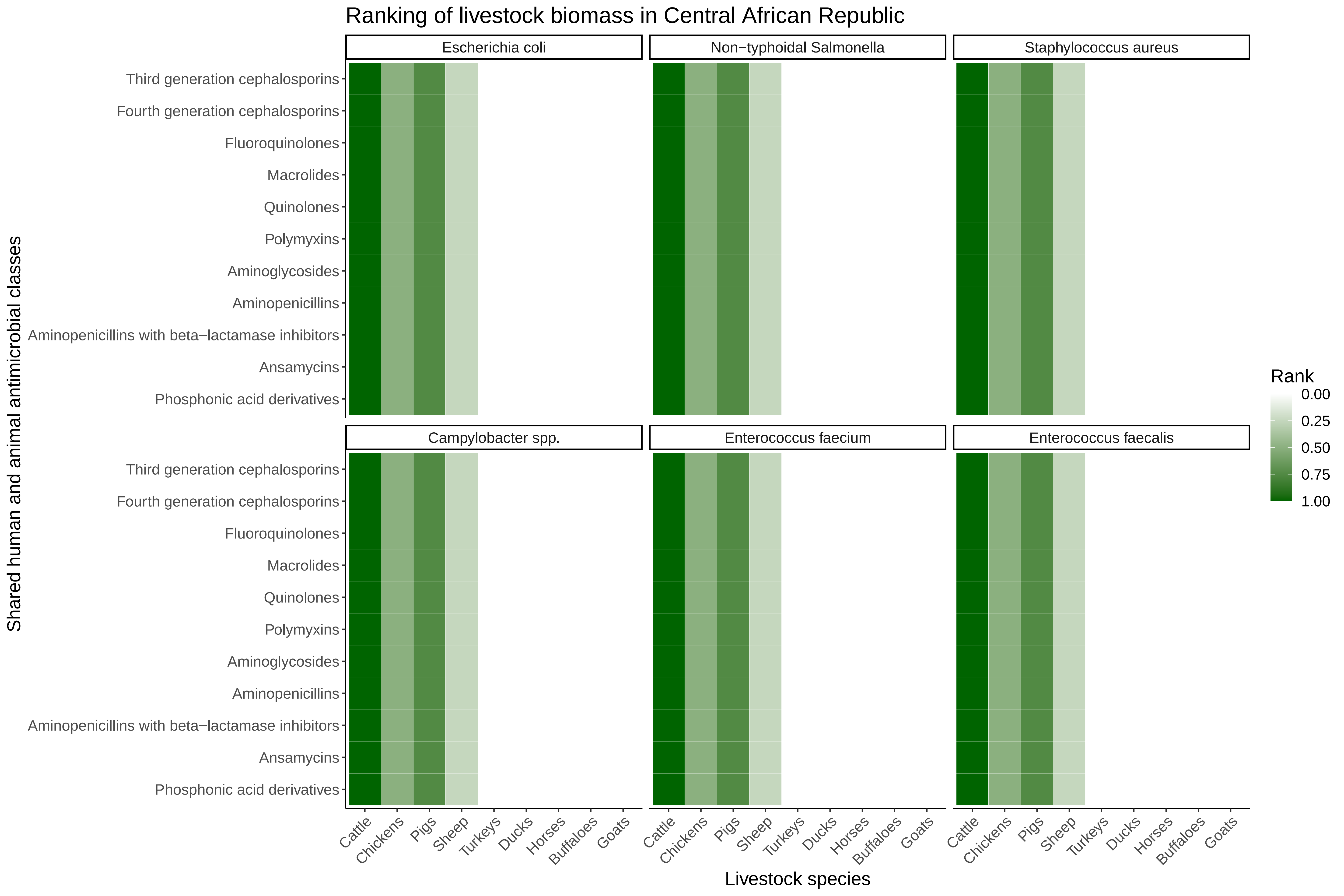

Ranking of livestock biomass in Central African Republic
Escherichia coli
Non−typhoidal Salmonella
Staphylococcus aureus
Campylobacter spp.
Enterococcus faecium
Enterococcus faecalis
Third generation cephalosporins
Fourth generation cephalosporins
Fluoroquinolones
Macrolides
Quinolones
Polymyxins
Aminoglycosides
Aminopenicillins
Aminopenicillins with beta−lactamase inhibitors
Ansamycins
Phosphonic acid derivatives
Shared human and animal antimicrobial classes
Cattle
Chickens
Pigs
Sheep
Turkeys
Ducks
Horses
Buffaloes
Goats
Livestock species
Rank
0.00
0.25
0.50
0.75
1.00

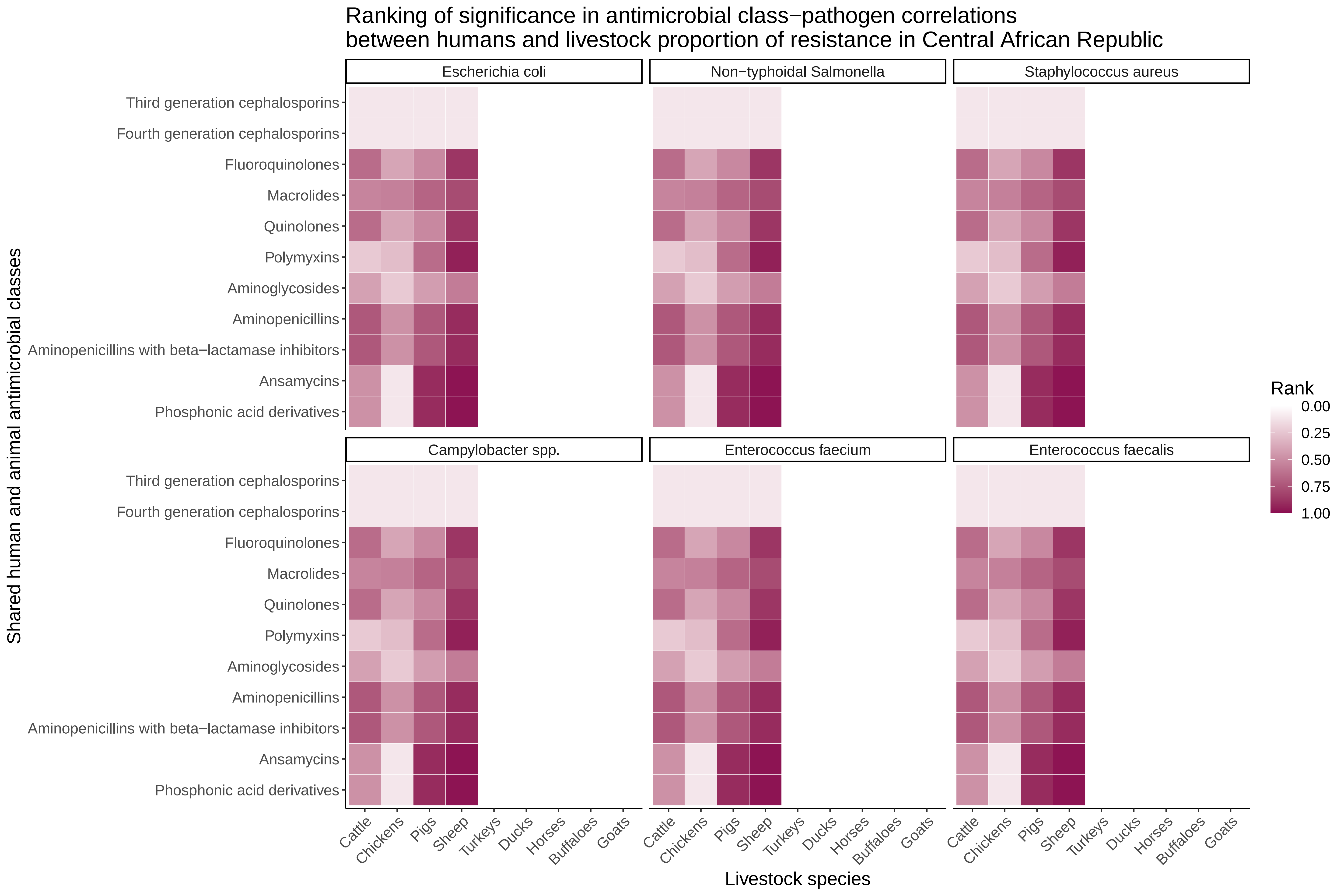
Ranking of significance in antimicrobial class−pathogen correlations
between humans and livestock proportion of resistance in Central African Republic
Escherichia coli
Non−typhoidal Salmonella
Staphylococcus aureus
Campylobacter spp.
Enterococcus faecium
Enterococcus faecalis
Third generation cephalosporins
Fourth generation cephalosporins
Fluoroquinolones
Macrolides
Quinolones
Polymyxins
Aminoglycosides
Aminopenicillins
Aminopenicillins with beta−lactamase inhibitors
Ansamycins
Phosphonic acid derivatives
Shared human and animal antimicrobial classes
Cattle
Chickens
Pigs
Sheep
Turkeys
Ducks
Horses
Buffaloes
Goats
Livestock species
Rank
0.00
0.25
0.50
0.75
1.00

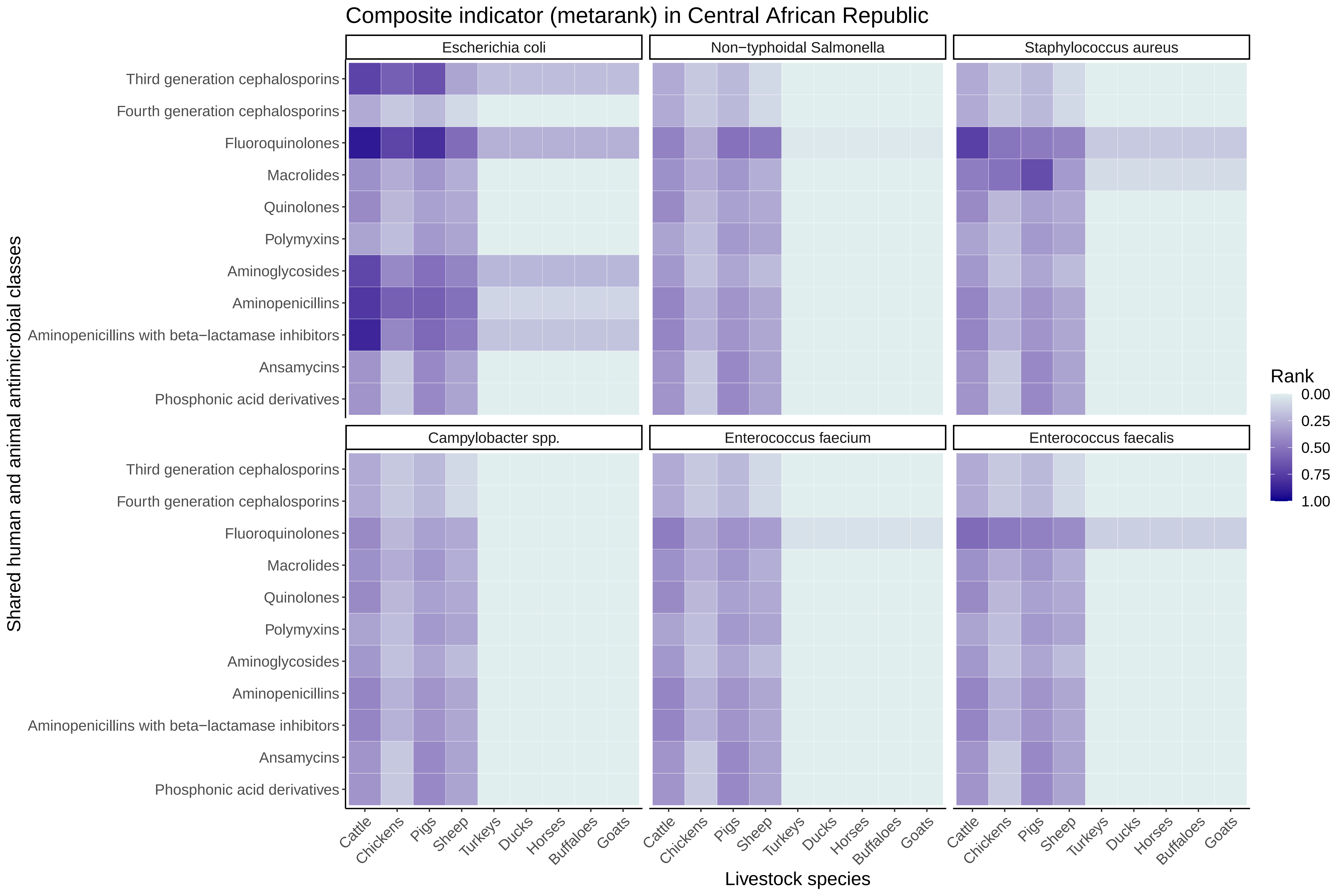

Composite indicator (metarank) in Central African Republic
Escherichia coli
Non−typhoidal Salmonella
Staphylococcus aureus
Campylobacter spp.
Enterococcus faecium
Enterococcus faecalis
Shared human and animal antimicrobial classes
Third generation cephalosporins
Fourth generation cephalosporins
Fluoroquinolones
Macrolides
Quinolones
Polymyxins
Aminoglycosides
Aminopenicillins
Aminopenicillins with beta−lactamase inhibitors
Ansamycins
Phosphonic acid derivatives
Cattle
Chickens
Pigs
Sheep
Turkeys
Ducks
Horses
Buffaloes
Goats
Livestock species
Rank
0.00
0.25
0.50
0.75
1.00

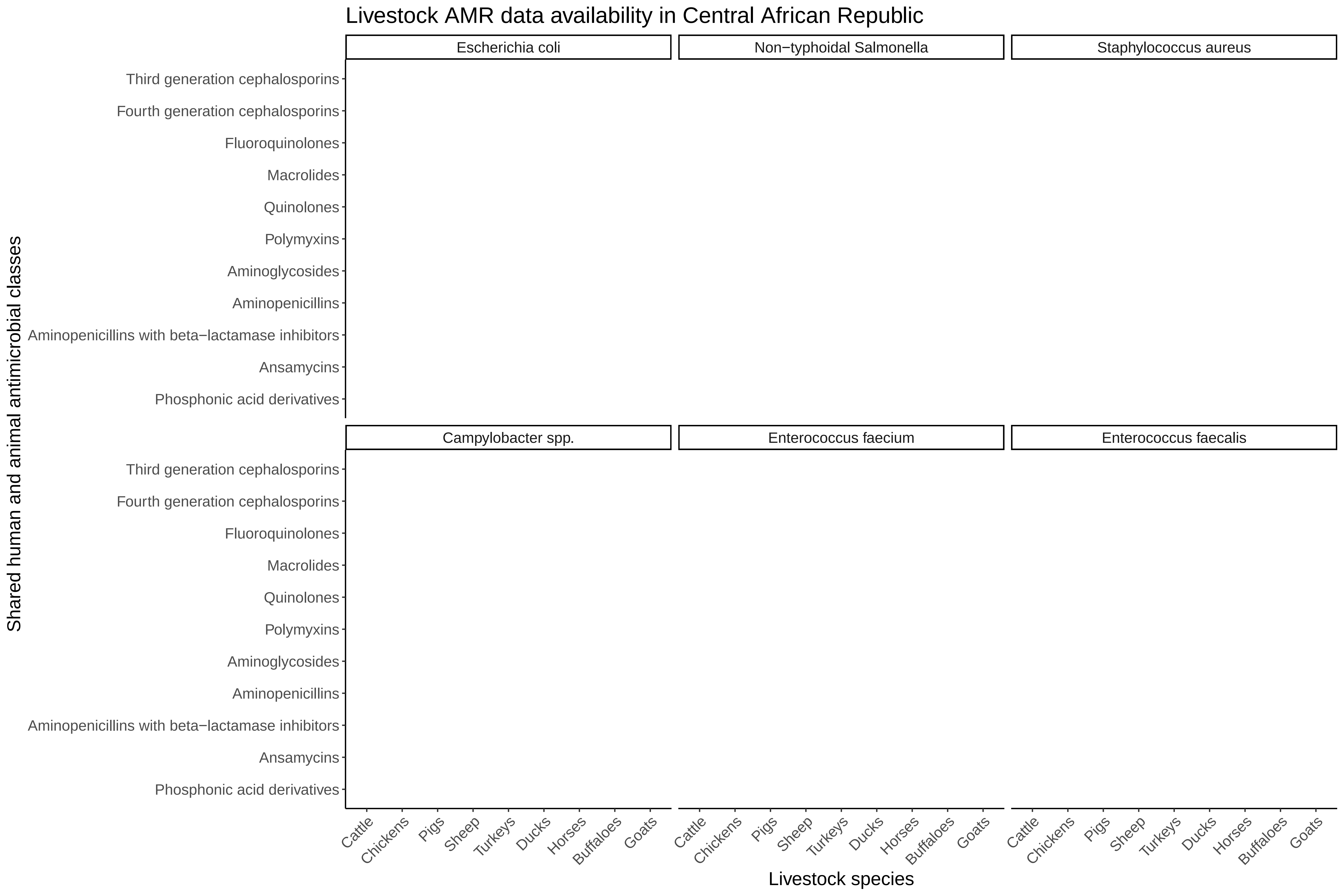
Livestock AMR data availability in Central African Republic
Escherichia coli
Non−typhoidal Salmonella
Staphylococcus aureus
Campylobacter spp.
Enterococcus faecium
Enterococcus faecalis
Shared human and animal antimicrobial classes
Third generation cephalosporins
Fourth generation cephalosporins
Fluoroquinolones
Macrolides
Quinolones
Polymyxins
Aminoglycosides
Aminopenicillins
Aminopenicillins with beta−lactamase inhibitors
Ansamycins
Phosphonic acid derivatives
Cattle
Chickens
Pigs
Sheep
Turkeys
Ducks
Horses
Buffaloes
Goats
Livestock species

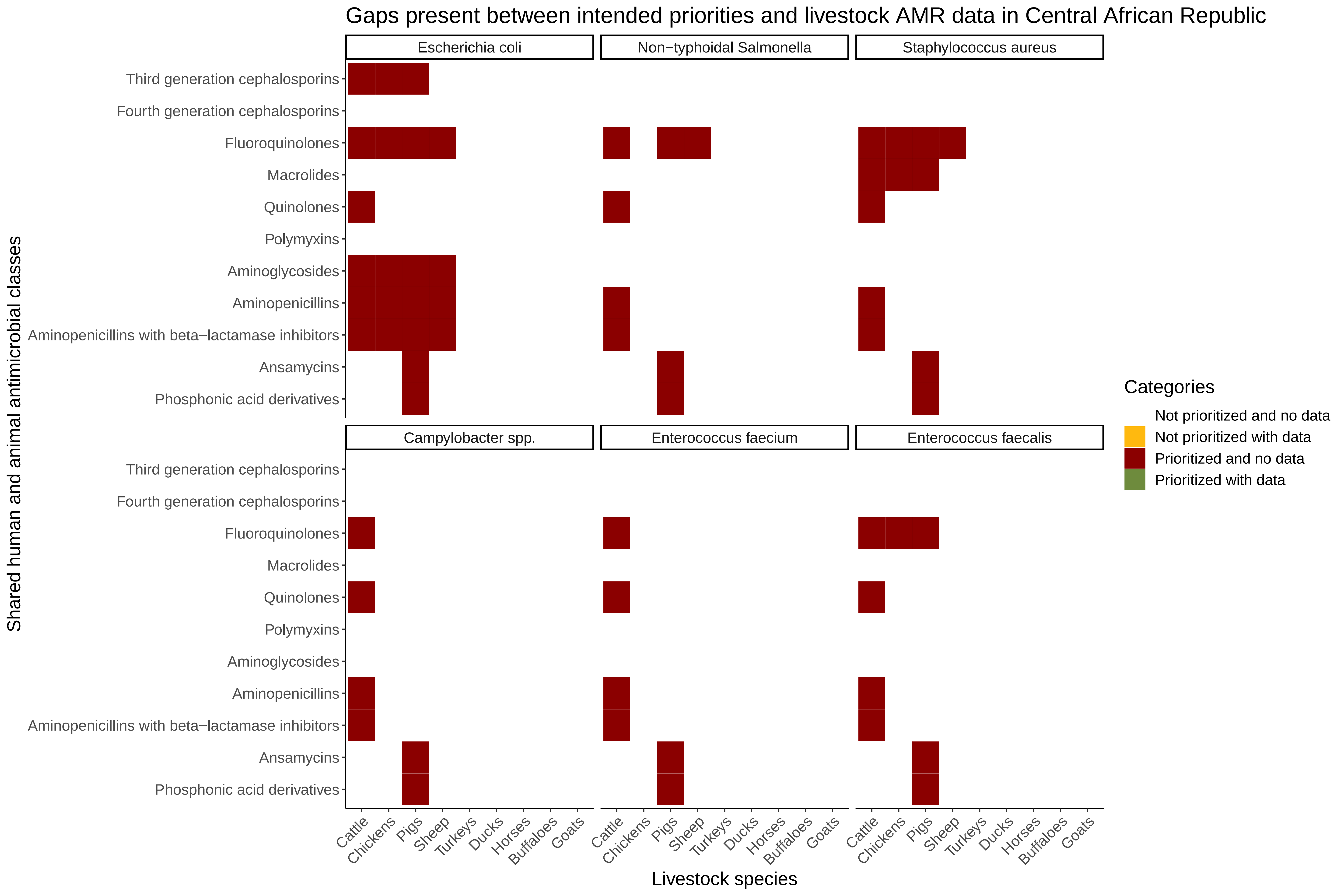

Gaps present between intended priorities and livestock AMR data in Central African Republic
Escherichia coli
Non−typhoidal Salmonella
Staphylococcus aureus
Campylobacter spp.
Enterococcus faecium
Enterococcus faecalis
Shared human and animal antimicrobial classes
Third generation cephalosporins
Fourth generation cephalosporins
Fluoroquinolones
Macrolides
Quinolones
Polymyxins
Aminoglycosides
Aminopenicillins
Aminopenicillins with beta−lactamase inhibitors
Ansamycins
Phosphonic acid derivatives
Cattle
Chickens
Pigs
Sheep
Turkeys
Ducks
Horses
Buffaloes
Goats
Livestock species
Categories
Not prioritized and no data
Not prioritized with data
Prioritized and no data
Prioritized with data

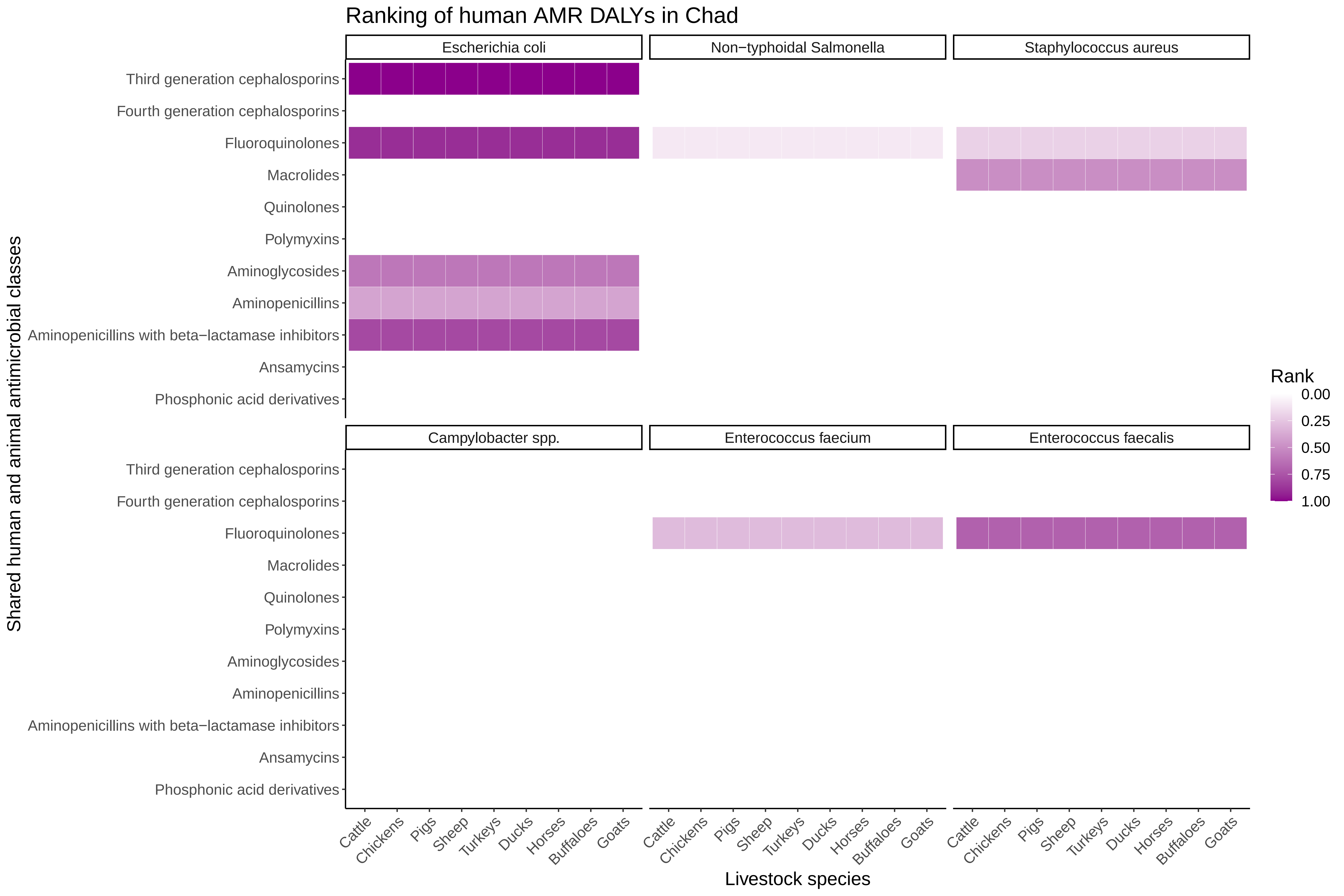
Ranking of human AMR DALYs in Chad
Escherichia coli
Non−typhoidal Salmonella
Staphylococcus aureus
Campylobacter spp.
Enterococcus faecium
Enterococcus faecalis
Third generation cephalosporins
Fourth generation cephalosporins
Fluoroquinolones
Macrolides
Quinolones
Polymyxins
Aminoglycosides
Aminopenicillins
Aminopenicillins with beta−lactamase inhibitors
Ansamycins
Phosphonic acid derivatives
Shared human and animal antimicrobial classes
Cattle
Chickens
Pigs
Sheep
Turkeys
Ducks
Horses
Buffaloes
Goats
Livestock species
Rank
0.00
0.25
0.50
0.75
1.00

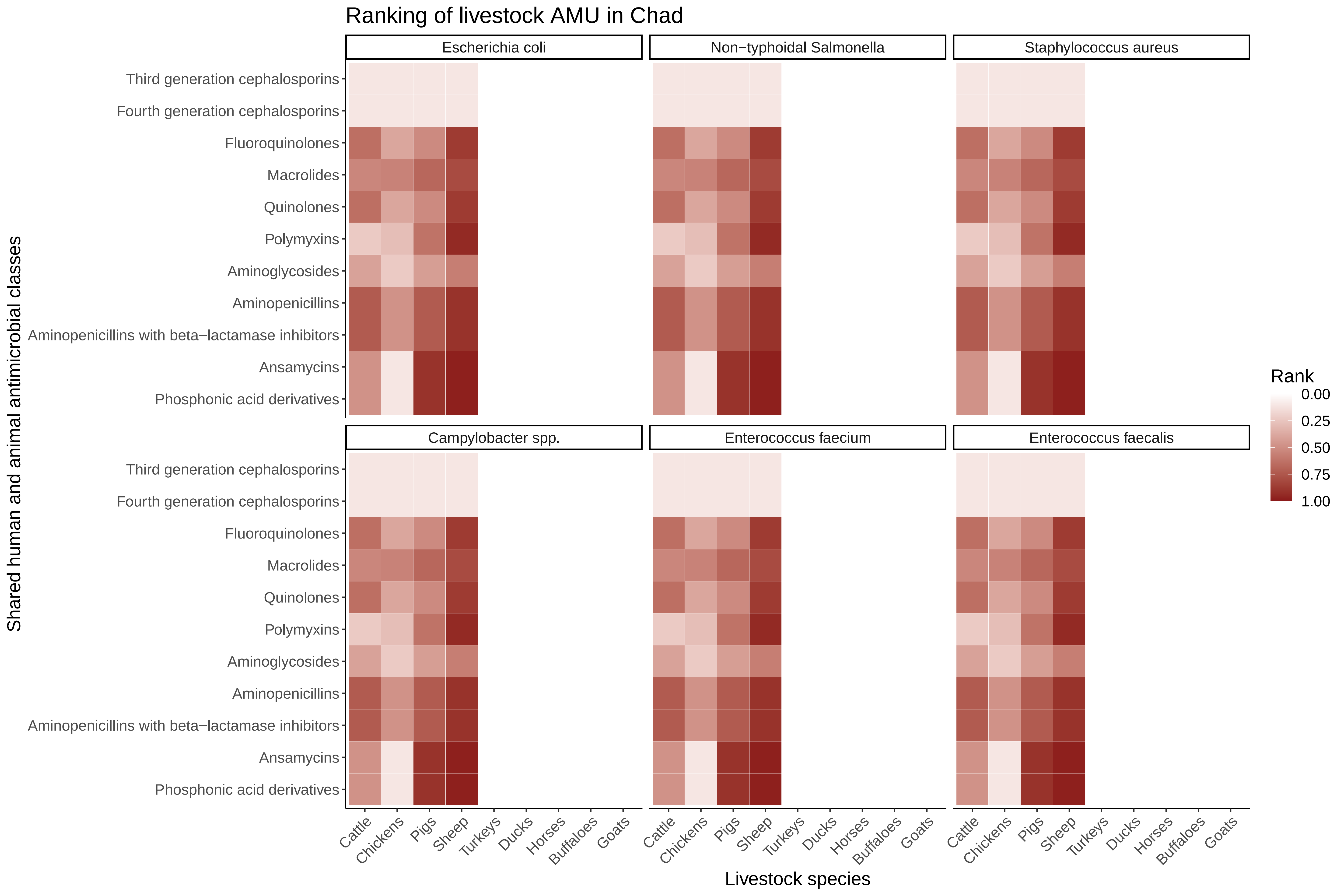

Ranking of livestock AMU in Chad
Escherichia coli
Non−typhoidal Salmonella
Staphylococcus aureus
Campylobacter spp.
Enterococcus faecium
Enterococcus faecalis
Third generation cephalosporins
Fourth generation cephalosporins
Fluoroquinolones
Macrolides
Quinolones
Polymyxins
Aminoglycosides
Aminopenicillins
Aminopenicillins with beta−lactamase inhibitors
Ansamycins
Phosphonic acid derivatives
Shared human and animal antimicrobial classes
Cattle
Chickens
Pigs
Sheep
Turkeys
Ducks
Horses
Buffaloes
Goats
Livestock species
Rank
0.00
0.25
0.50
0.75
1.00

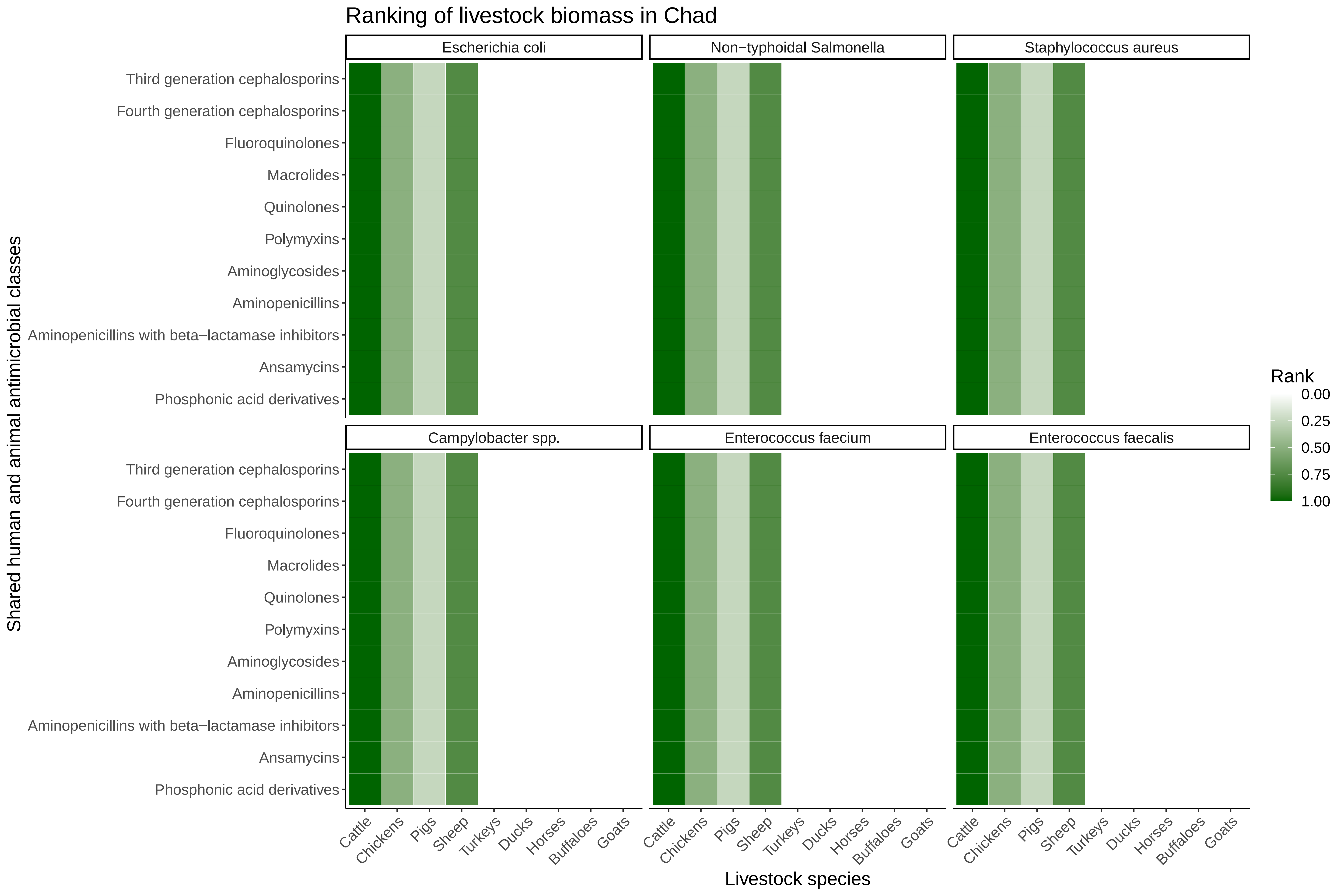
Ranking of livestock biomass in Chad
Escherichia coli
Non−typhoidal Salmonella
Staphylococcus aureus
Campylobacter spp.
Enterococcus faecium
Enterococcus faecalis
Third generation cephalosporins
Fourth generation cephalosporins
Fluoroquinolones
Macrolides
Quinolones
Polymyxins
Aminoglycosides
Aminopenicillins
Aminopenicillins with beta−lactamase inhibitors
Ansamycins
Phosphonic acid derivatives
Shared human and animal antimicrobial classes
Cattle
Chickens
Pigs
Sheep
Turkeys
Ducks
Horses
Buffaloes
Goats
Livestock species
Rank
0.00
0.25
0.50
0.75
1.00

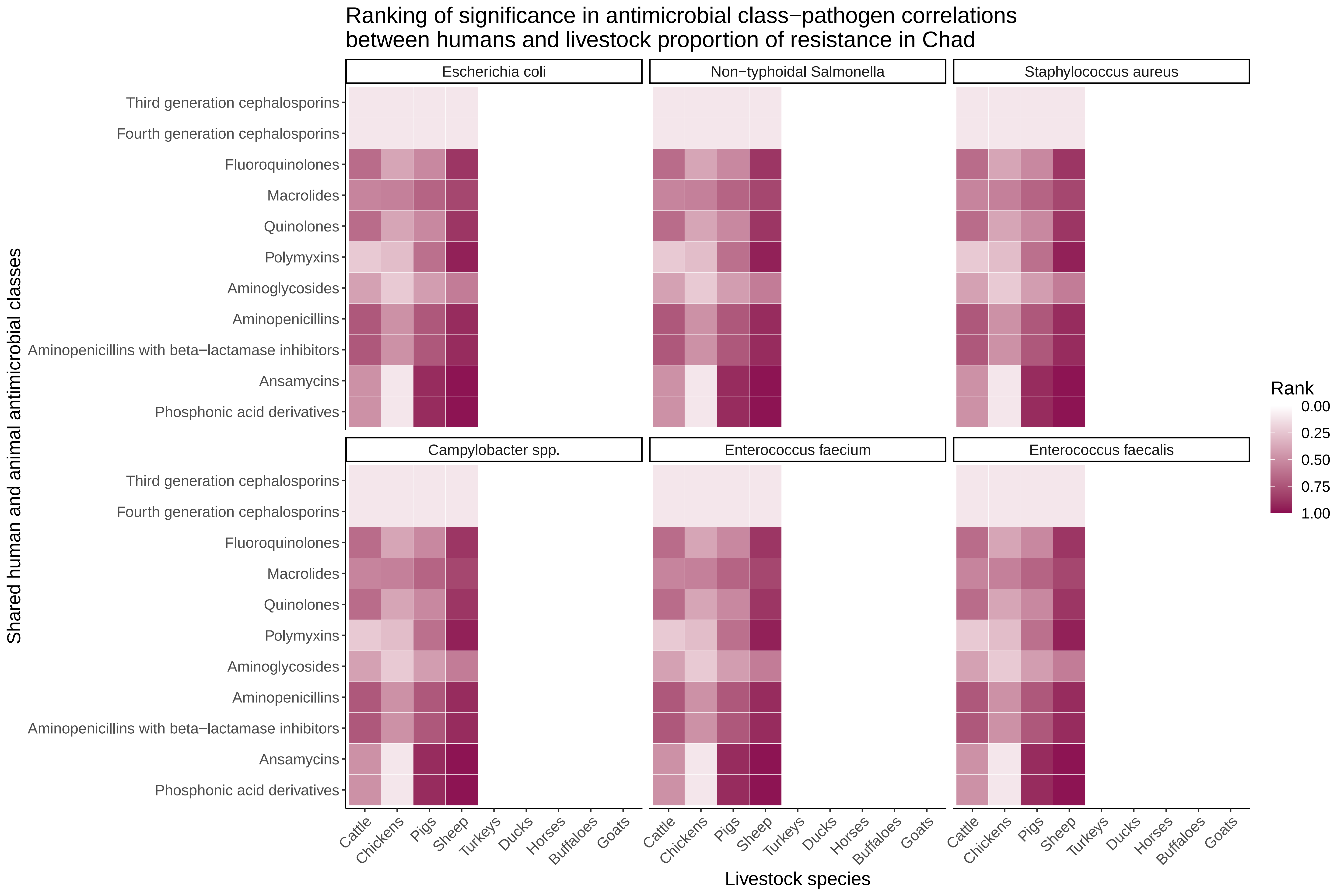
Ranking of significance in antimicrobial class−pathogen correlations
between humans and livestock proportion of resistance in Chad
Escherichia coli
Non−typhoidal Salmonella
Staphylococcus aureus
Campylobacter spp.
Enterococcus faecium
Enterococcus faecalis
Third generation cephalosporins
Fourth generation cephalosporins
Fluoroquinolones
Macrolides
Quinolones
Polymyxins
Aminoglycosides
Aminopenicillins
Aminopenicillins with beta−lactamase inhibitors
Ansamycins
Phosphonic acid derivatives
Shared human and animal antimicrobial classes
Cattle
Chickens
Pigs
Sheep
Turkeys
Ducks
Horses
Buffaloes
Goats
Livestock species
Rank
0.00
0.25
0.50
0.75
1.00

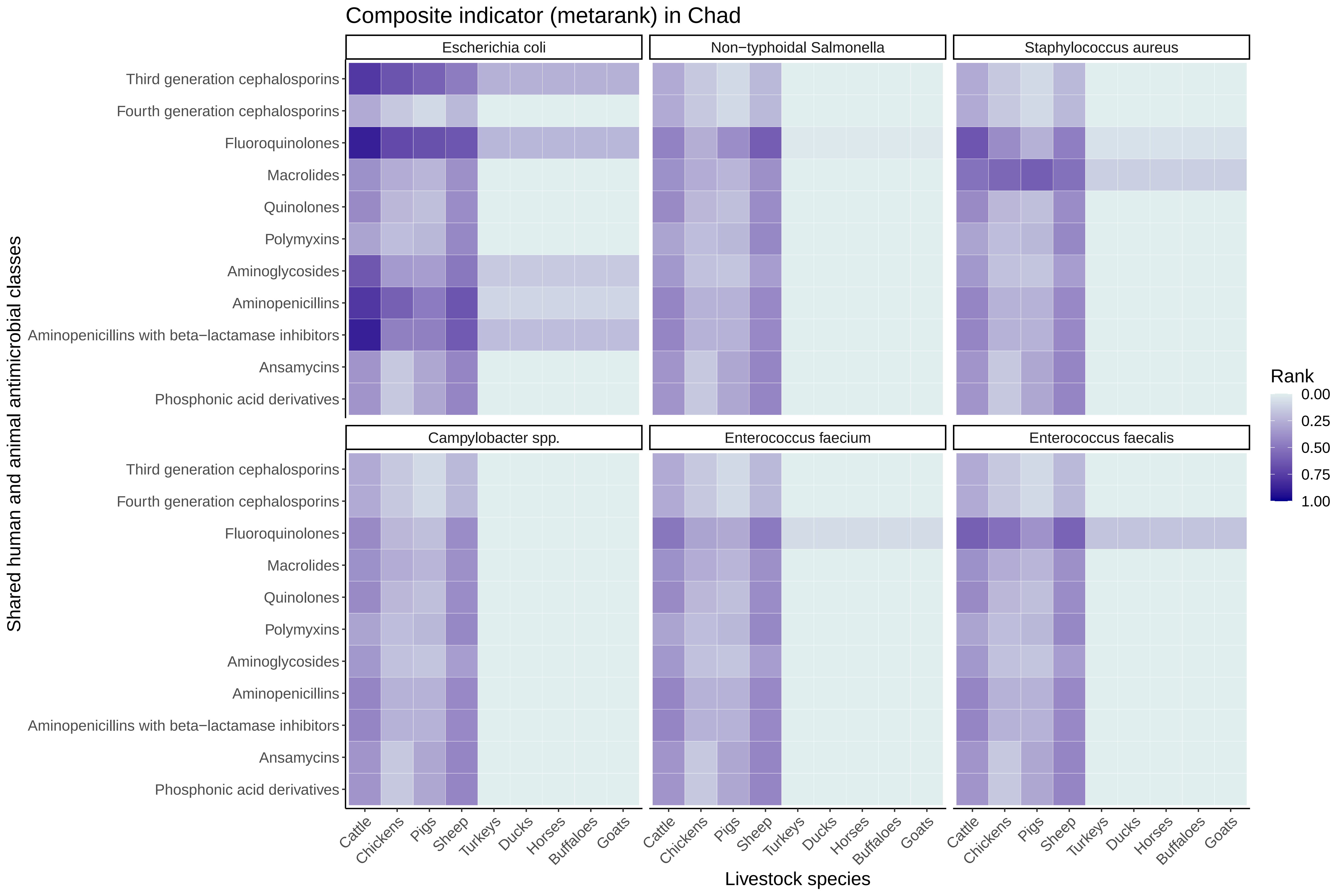

Composite indicator (metarank) in Chad
Escherichia coli
Non−typhoidal Salmonella
Staphylococcus aureus
Campylobacter spp.
Enterococcus faecium
Enterococcus faecalis
Shared human and animal antimicrobial classes
Third generation cephalosporins
Fourth generation cephalosporins
Fluoroquinolones
Macrolides
Quinolones
Polymyxins
Aminoglycosides
Aminopenicillins
Aminopenicillins with beta−lactamase inhibitors
Ansamycins
Phosphonic acid derivatives
Cattle
Chickens
Pigs
Sheep
Turkeys
Ducks
Horses
Buffaloes
Goats
Livestock species
Rank
0.00
0.25
0.50
0.75
1.00

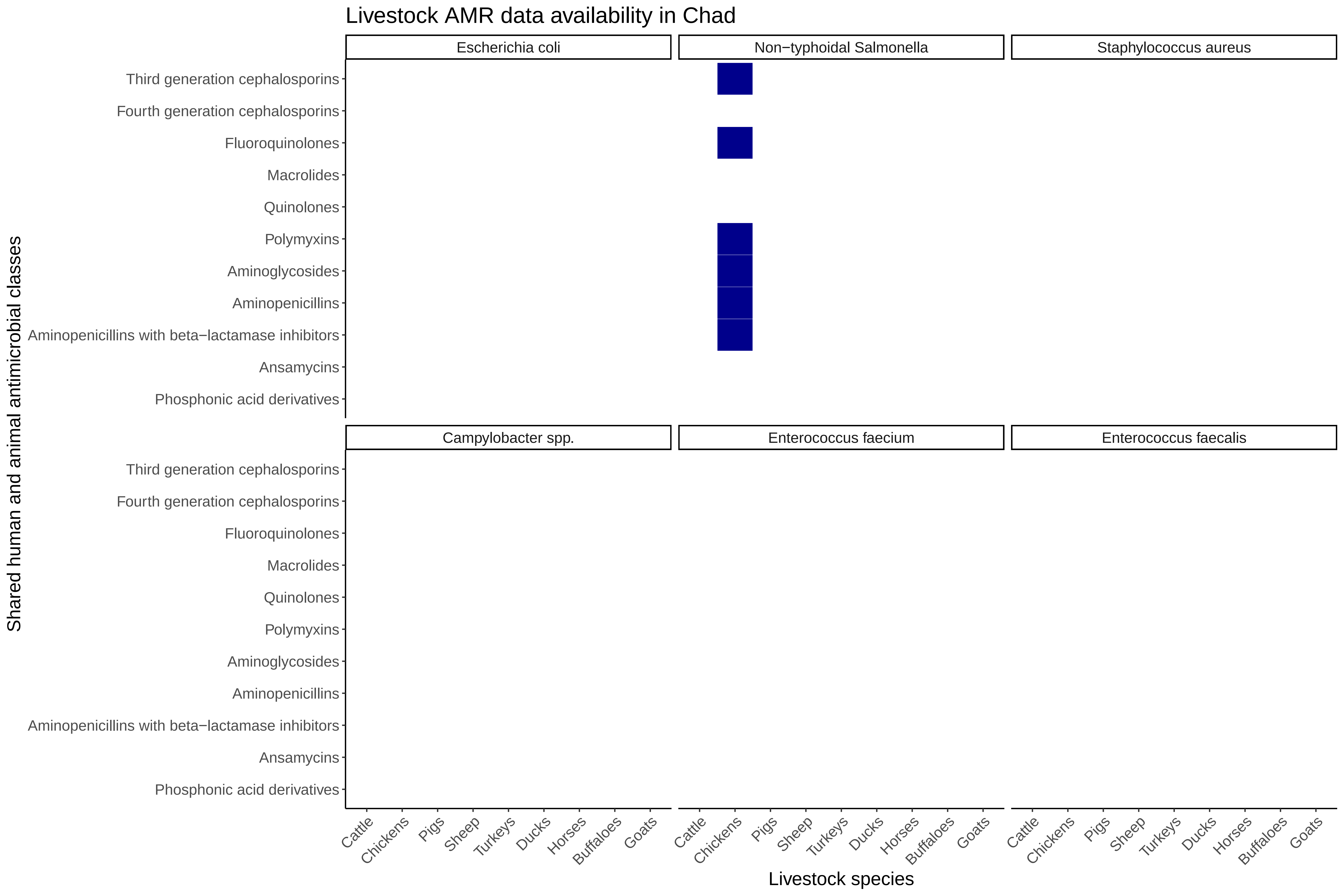
Livestock AMR data availability in Chad
Escherichia coli
Non−typhoidal Salmonella
Staphylococcus aureus
Campylobacter spp.
Enterococcus faecium
Enterococcus faecalis
Shared human and animal antimicrobial classes
Third generation cephalosporins
Fourth generation cephalosporins
Fluoroquinolones
Macrolides
Quinolones
Polymyxins
Aminoglycosides
Aminopenicillins
Aminopenicillins with beta−lactamase inhibitors
Ansamycins
Phosphonic acid derivatives
Cattle
Chickens
Pigs
Sheep
Turkeys
Ducks
Horses
Buffaloes
Goats
Livestock species

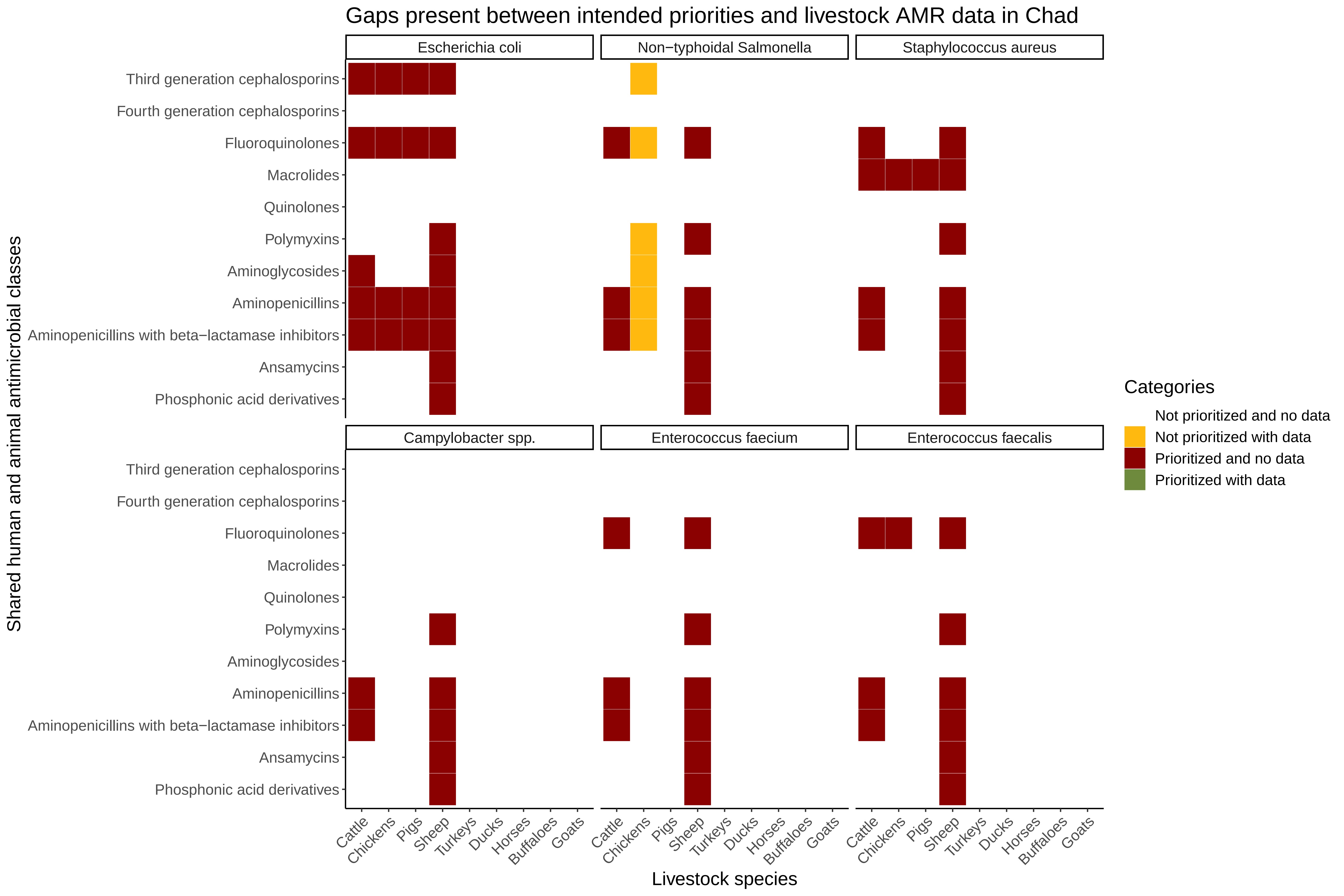

Gaps present between intended priorities and livestock AMR data in Chad
Escherichia coli
Non−typhoidal Salmonella
Staphylococcus aureus
Campylobacter spp.
Enterococcus faecium
Enterococcus faecalis
Third generation cephalosporins
Fourth generation cephalosporins
Fluoroquinolones
Macrolides
Quinolones
Polymyxins
Aminoglycosides
Aminopenicillins
Aminopenicillins with beta−lactamase inhibitors
Ansamycins
Phosphonic acid derivatives
Shared human and animal antimicrobial classes
Cattle
Chickens
Pigs
Sheep
Turkeys
Ducks
Horses
Buffaloes
Goats
Livestock species
Categories
Not prioritized and no data
Not prioritized with data
Prioritized and no data
Prioritized with data

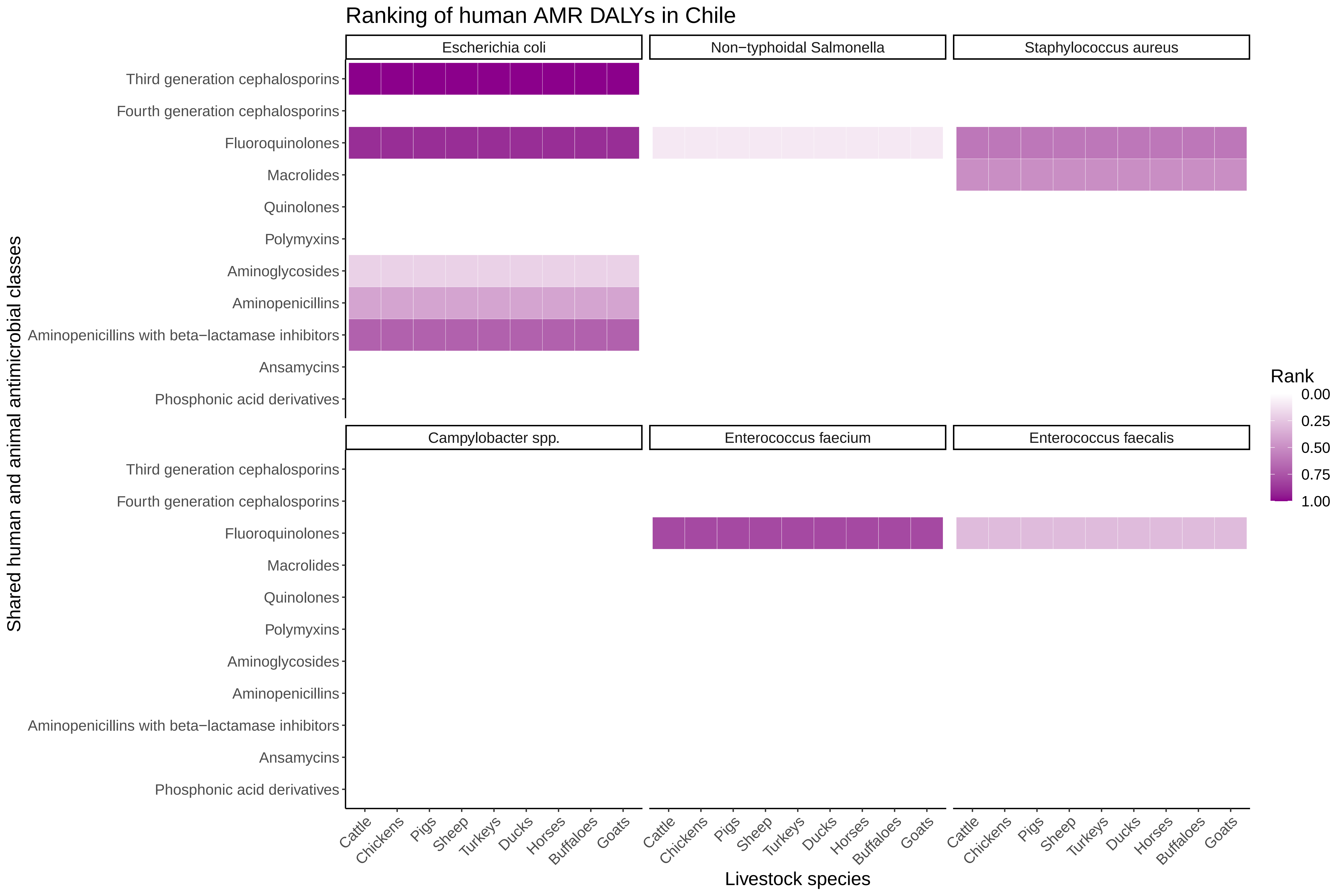

Ranking of human AMR DALYs in Chile
Escherichia coli
Non−typhoidal Salmonella
Staphylococcus aureus
Campylobacter spp.
Enterococcus faecium
Enterococcus faecalis
Third generation cephalosporins
Fourth generation cephalosporins
Fluoroquinolones
Macrolides
Quinolones
Polymyxins
Aminoglycosides
Aminopenicillins
Aminopenicillins with beta−lactamase inhibitors
Ansamycins
Phosphonic acid derivatives
Shared human and animal antimicrobial classes
Cattle
Chickens
Pigs
Sheep
Turkeys
Ducks
Horses
Buffaloes
Goats
Livestock species
Rank
0.00
0.25
0.50
0.75
1.00

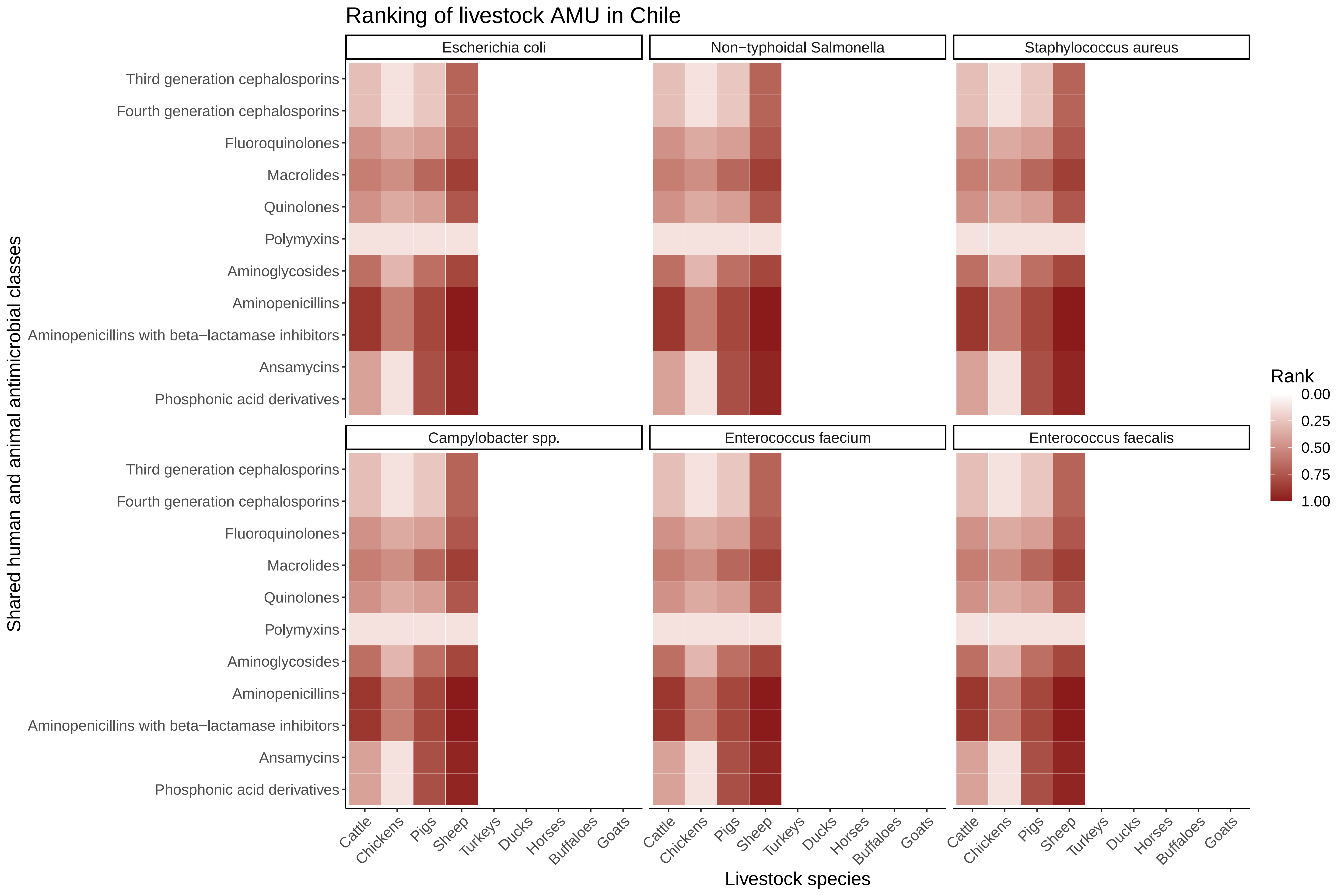
Ranking of livestock AMU in Chile
Escherichia coli
Non−typhoidal Salmonella
Staphylococcus aureus
Campylobacter spp.
Enterococcus faecium
Enterococcus faecalis
Shared human and animal antimicrobial classes
Third generation cephalosporins
Fourth generation cephalosporins
Fluoroquinolones
Macrolides
Quinolones
Polymyxins
Aminoglycosides
Aminopenicillins
Aminopenicillins with beta−lactamase inhibitors
Ansamycins
Phosphonic acid derivatives
Cattle
Chickens
Pigs
Sheep
Turkeys
Ducks
Horses
Buffaloes
Goats
Livestock species
Rank
0.00
0.25
0.50
0.75
1.00

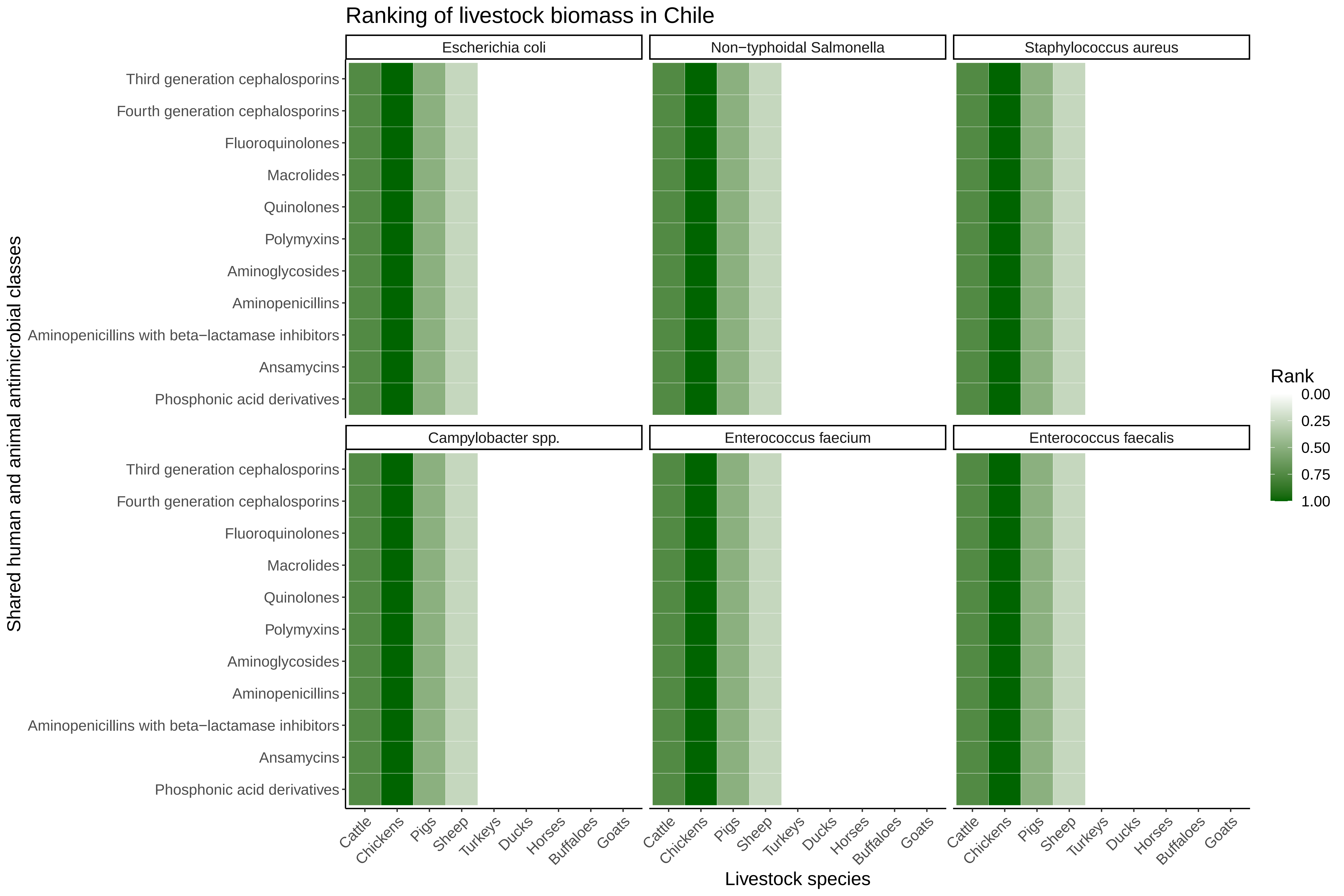

Ranking of livestock biomass in Chile
Escherichia coli
Non−typhoidal Salmonella
Staphylococcus aureus
Campylobacter spp.
Enterococcus faecium
Enterococcus faecalis
Shared human and animal antimicrobial classes
Third generation cephalosporins
Fourth generation cephalosporins
Fluoroquinolones
Macrolides
Quinolones
Polymyxins
Aminoglycosides
Aminopenicillins
Aminopenicillins with beta−lactamase inhibitors
Ansamycins
Phosphonic acid derivatives
Livestock species
Cattle
Chickens
Pigs
Sheep
Turkeys
Ducks
Horses
Buffaloes
Goats
Rank
0.00
0.25
0.50
0.75
1.00

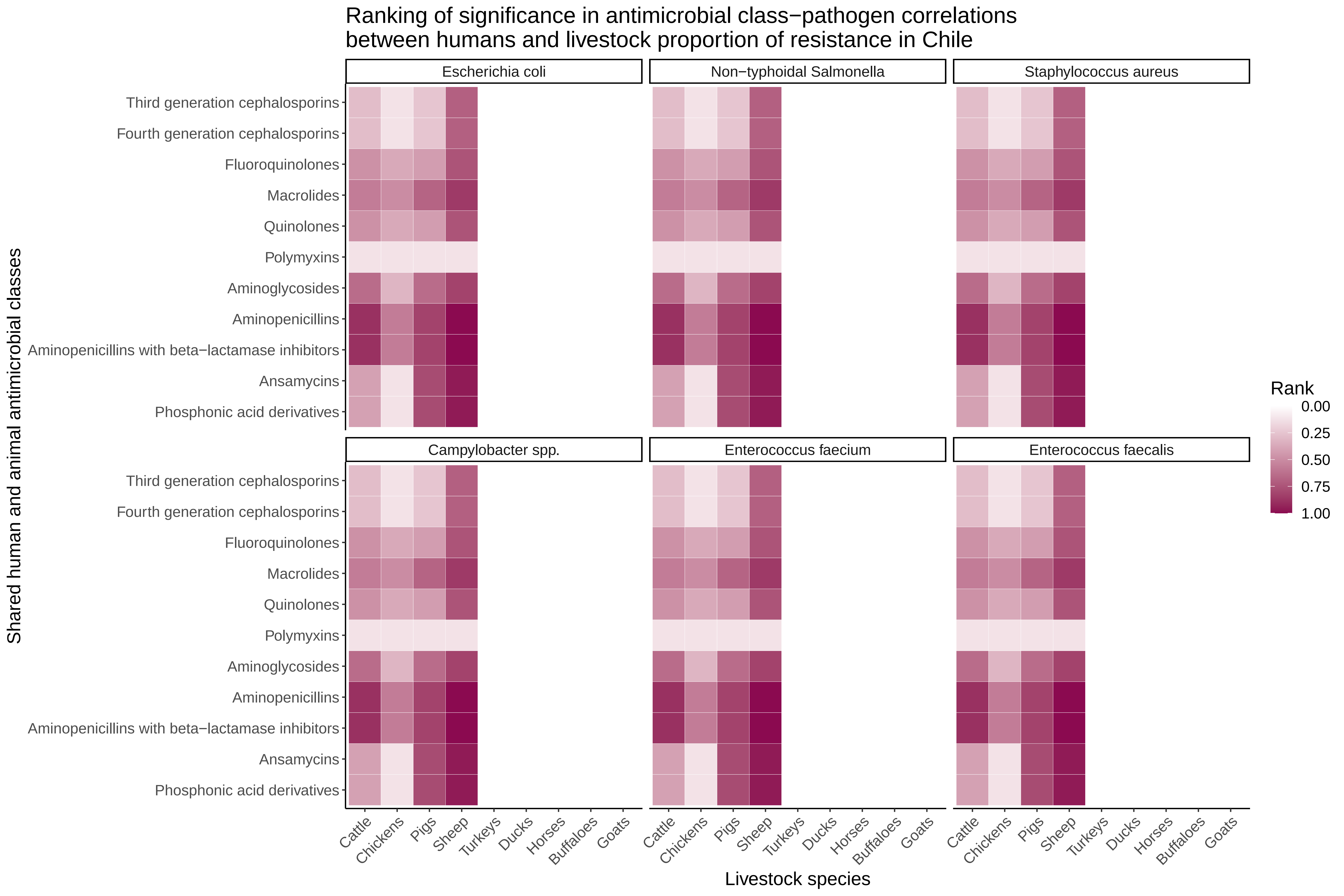

Ranking of significance in antimicrobial class−pathogen correlations
between humans and livestock proportion of resistance in Chile
Escherichia coli
Non−typhoidal Salmonella
Staphylococcus aureus
Campylobacter spp.
Enterococcus faecium
Enterococcus faecalis
Shared human and animal antimicrobial classes
Third generation cephalosporins
Fourth generation cephalosporins
Fluoroquinolones
Macrolides
Quinolones
Polymyxins
Aminoglycosides
Aminopenicillins
Aminopenicillins with beta−lactamase inhibitors
Ansamycins
Phosphonic acid derivatives
Livestock species
Cattle
Chickens
Pigs
Sheep
Turkeys
Ducks
Horses
Buffaloes
Goats
Rank
0.00
0.25
0.50
0.75
1.00

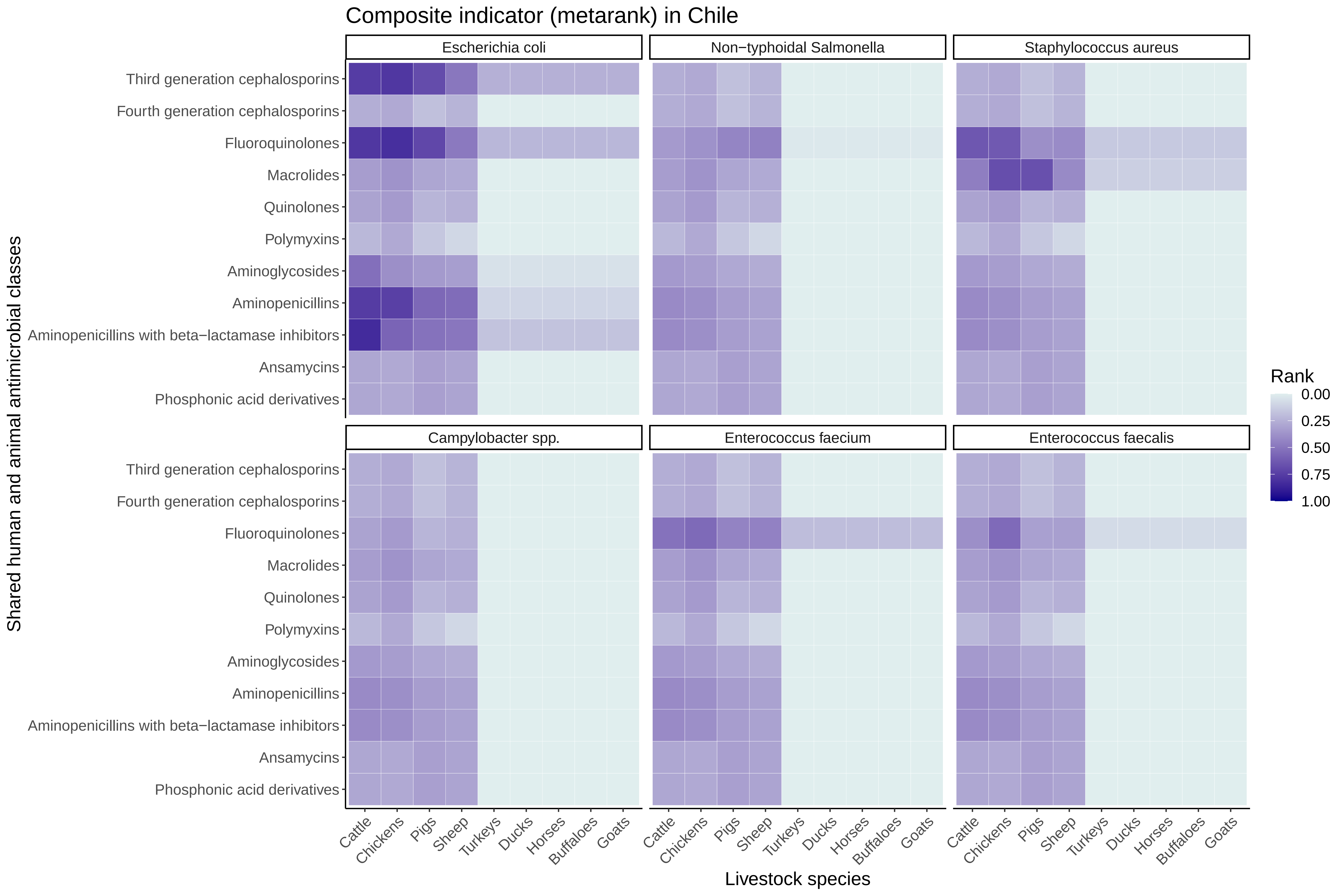
Composite indicator (metarank) in Chile
Escherichia coli
Non−typhoidal Salmonella
Staphylococcus aureus
Campylobacter spp.
Enterococcus faecium
Enterococcus faecalis
Third generation cephalosporins
Fourth generation cephalosporins
Fluoroquinolones
Macrolides
Quinolones
Polymyxins
Aminoglycosides
Aminopenicillins
Aminopenicillins with beta−lactamase inhibitors
Ansamycins
Phosphonic acid derivatives
Shared human and animal antimicrobial classes
Cattle
Chickens
Pigs
Sheep
Turkeys
Ducks
Horses
Buffaloes
Goats
Livestock species
Rank
0.00
0.25
0.50
0.75
1.00

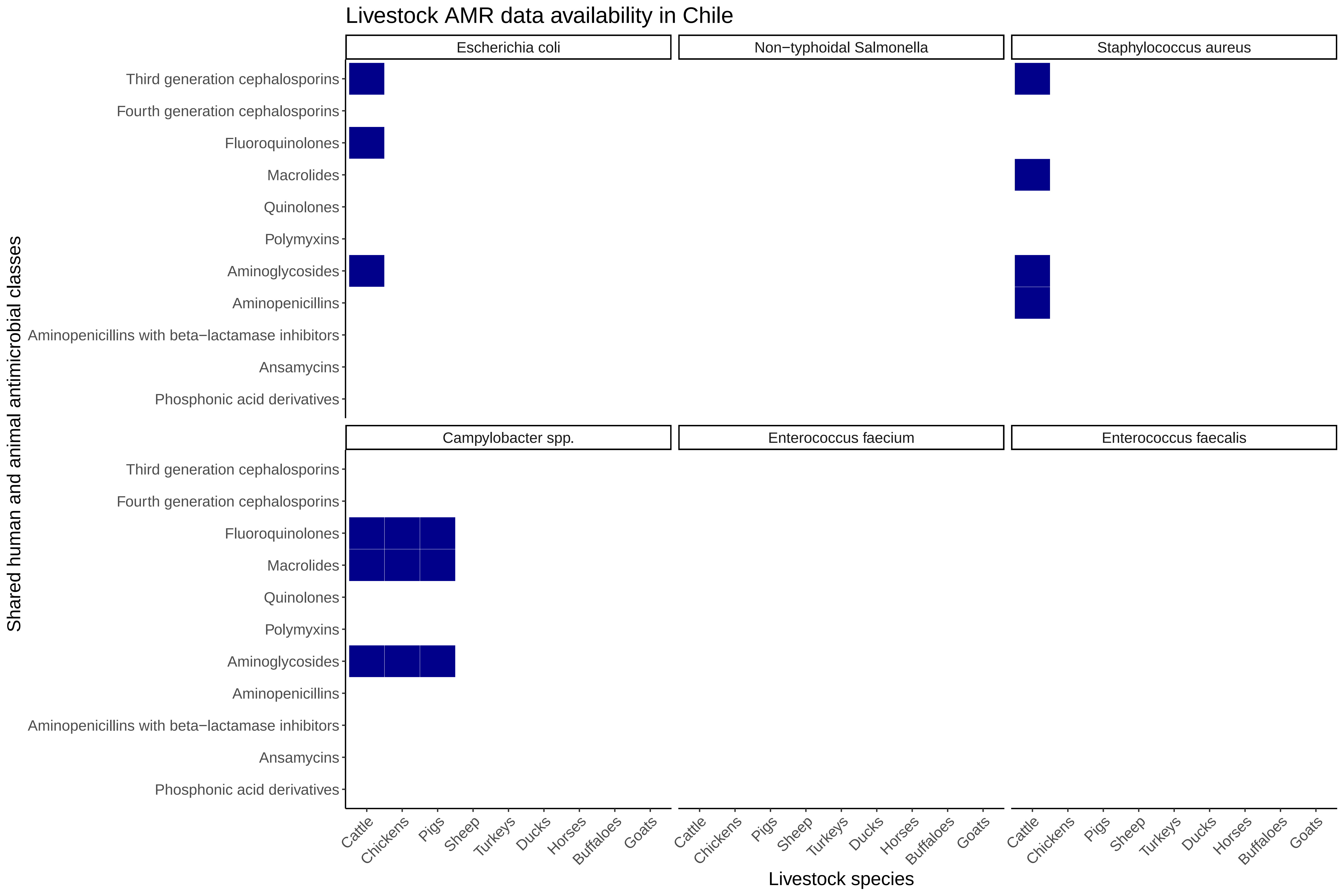

Livestock AMR data availability in Chile
Escherichia coli
Non−typhoidal Salmonella
Staphylococcus aureus
Campylobacter spp.
Enterococcus faecium
Enterococcus faecalis
Shared human and animal antimicrobial classes
Third generation cephalosporins
Fourth generation cephalosporins
Fluoroquinolones
Macrolides
Quinolones
Polymyxins
Aminoglycosides
Aminopenicillins
Aminopenicillins with beta−lactamase inhibitors
Ansamycins
Phosphonic acid derivatives
Cattle
Chickens
Pigs
Sheep
Turkeys
Ducks
Horses
Buffaloes
Goats
Livestock species

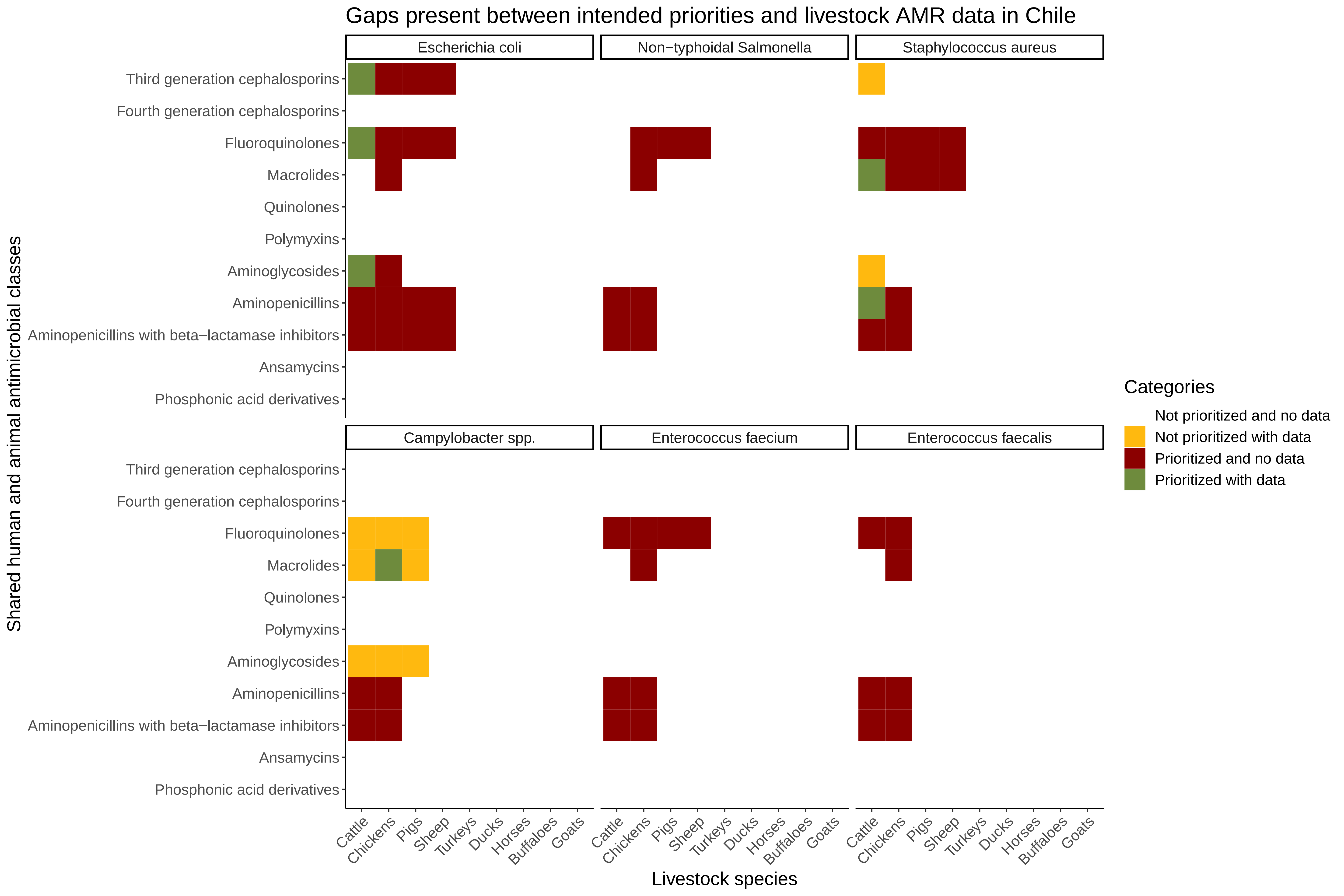

Gaps present between intended priorities and livestock AMR data in Chile
Escherichia coli
Non−typhoidal Salmonella
Staphylococcus aureus
Campylobacter spp.
Enterococcus faecium
Enterococcus faecalis
Third generation cephalosporins
Fourth generation cephalosporins
Fluoroquinolones
Macrolides
Quinolones
Polymyxins
Aminoglycosides
Aminopenicillins
Aminopenicillins with beta−lactamase inhibitors
Ansamycins
Phosphonic acid derivatives
Shared human and animal antimicrobial classes
Cattle
Chickens
Pigs
Sheep
Turkeys
Ducks
Horses
Buffaloes
Goats
Livestock species
Categories
Not prioritized and no data
Not prioritized with data
Prioritized and no data
Prioritized with data

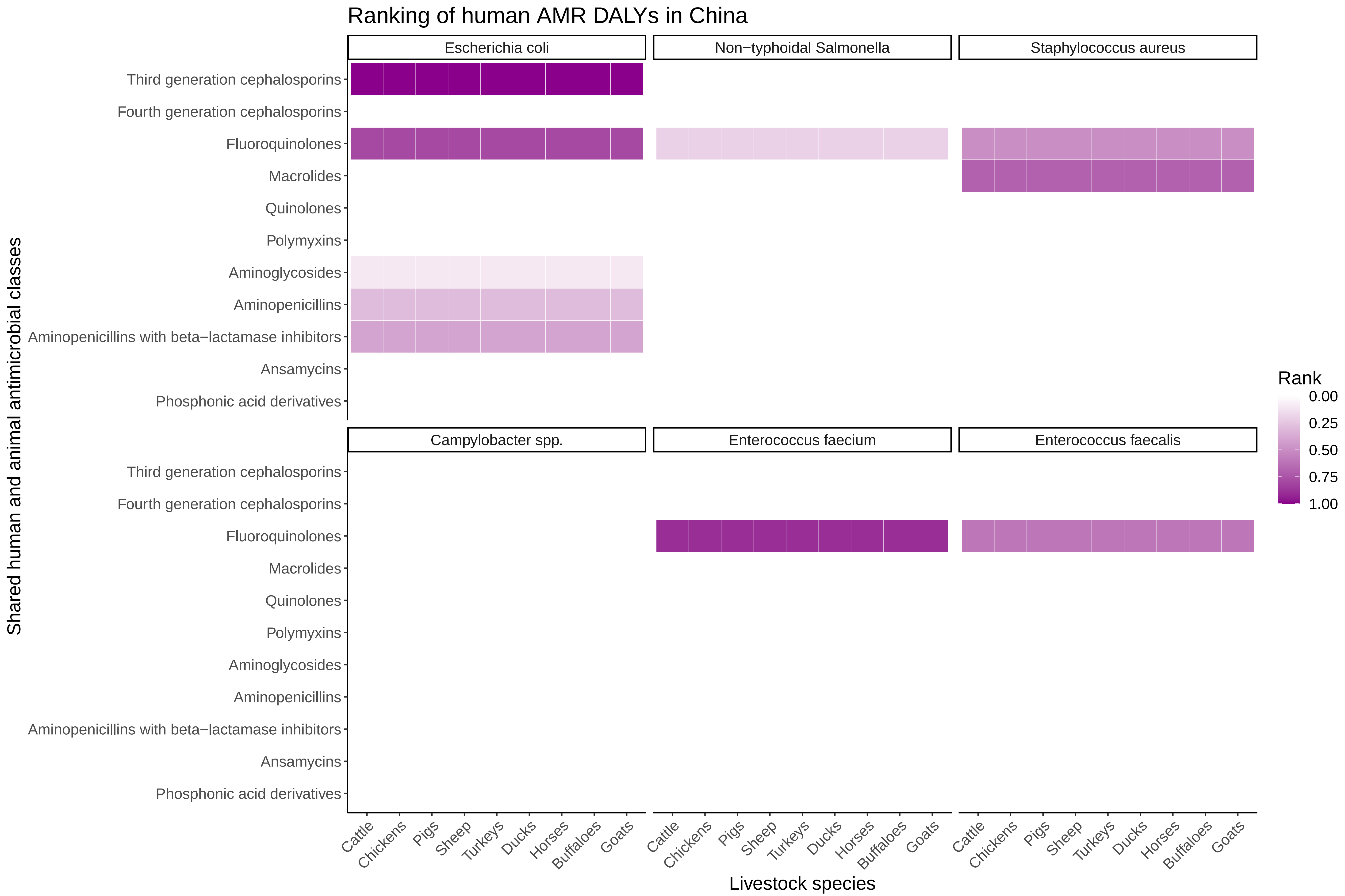

Ranking of human AMR DALYs in China
Escherichia coli
Non−typhoidal Salmonella
Staphylococcus aureus
Campylobacter spp.
Enterococcus faecium
Enterococcus faecalis
Shared human and animal antimicrobial classes
Third generation cephalosporins
Fourth generation cephalosporins
Fluoroquinolones
Macrolides
Quinolones
Polymyxins
Aminoglycosides
Aminopenicillins
Aminopenicillins with beta−lactamase inhibitors
Ansamycins
Phosphonic acid derivatives
Livestock species
Cattle
Chickens
Pigs
Sheep
Turkeys
Ducks
Horses
Buffaloes
Goats
Rank
0.00
0.25
0.50
0.75
1.00

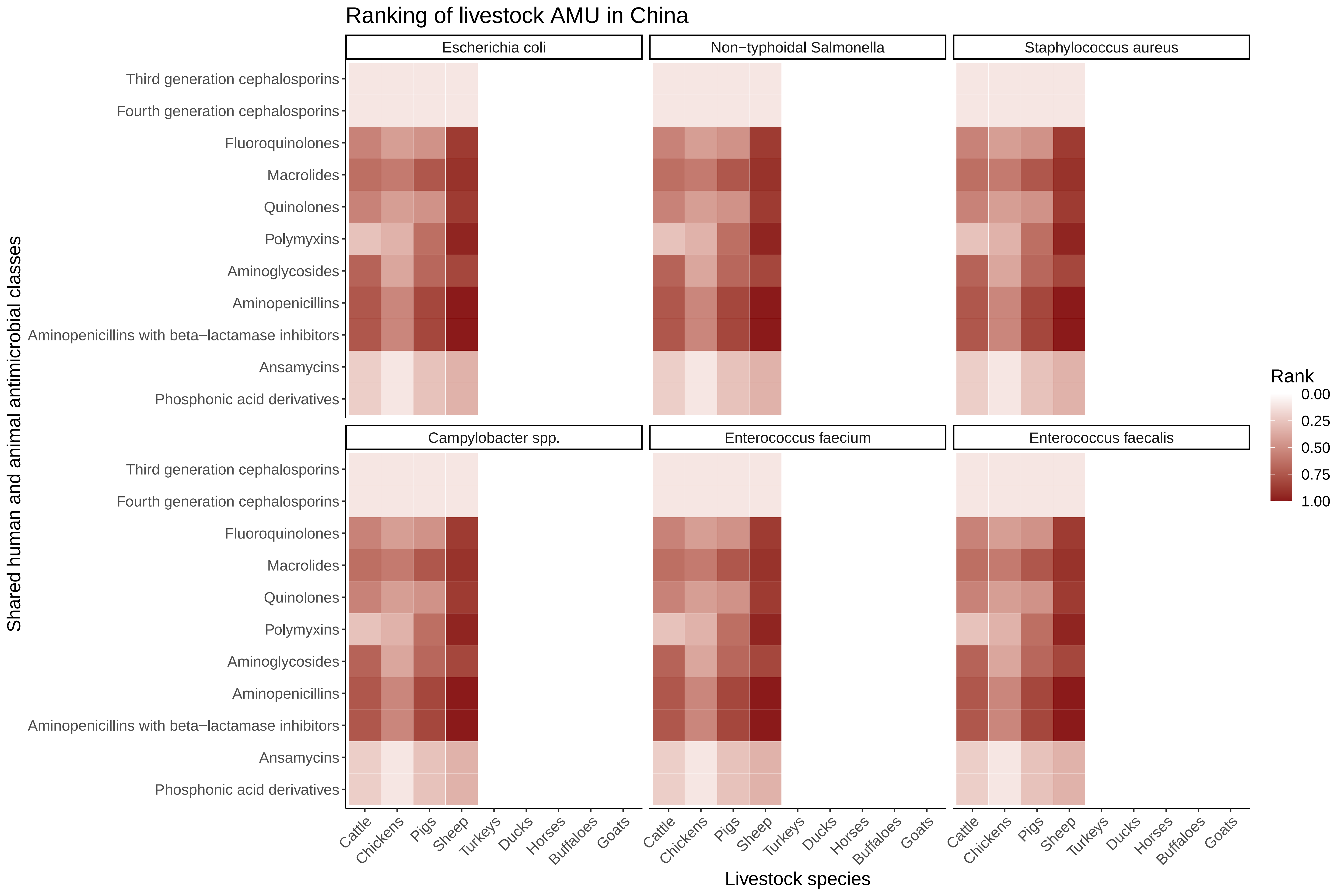
Ranking of livestock AMU in China
Escherichia coli
Non−typhoidal Salmonella
Staphylococcus aureus
Campylobacter spp.
Enterococcus faecium
Enterococcus faecalis
Shared human and animal antimicrobial classes
Third generation cephalosporins
Fourth generation cephalosporins
Fluoroquinolones
Macrolides
Quinolones
Polymyxins
Aminoglycosides
Aminopenicillins
Aminopenicillins with beta−lactamase inhibitors
Ansamycins
Phosphonic acid derivatives
Cattle
Chickens
Pigs
Sheep
Turkeys
Ducks
Horses
Buffaloes
Goats
Livestock species
Rank
0.00
0.25
0.50
0.75
1.00

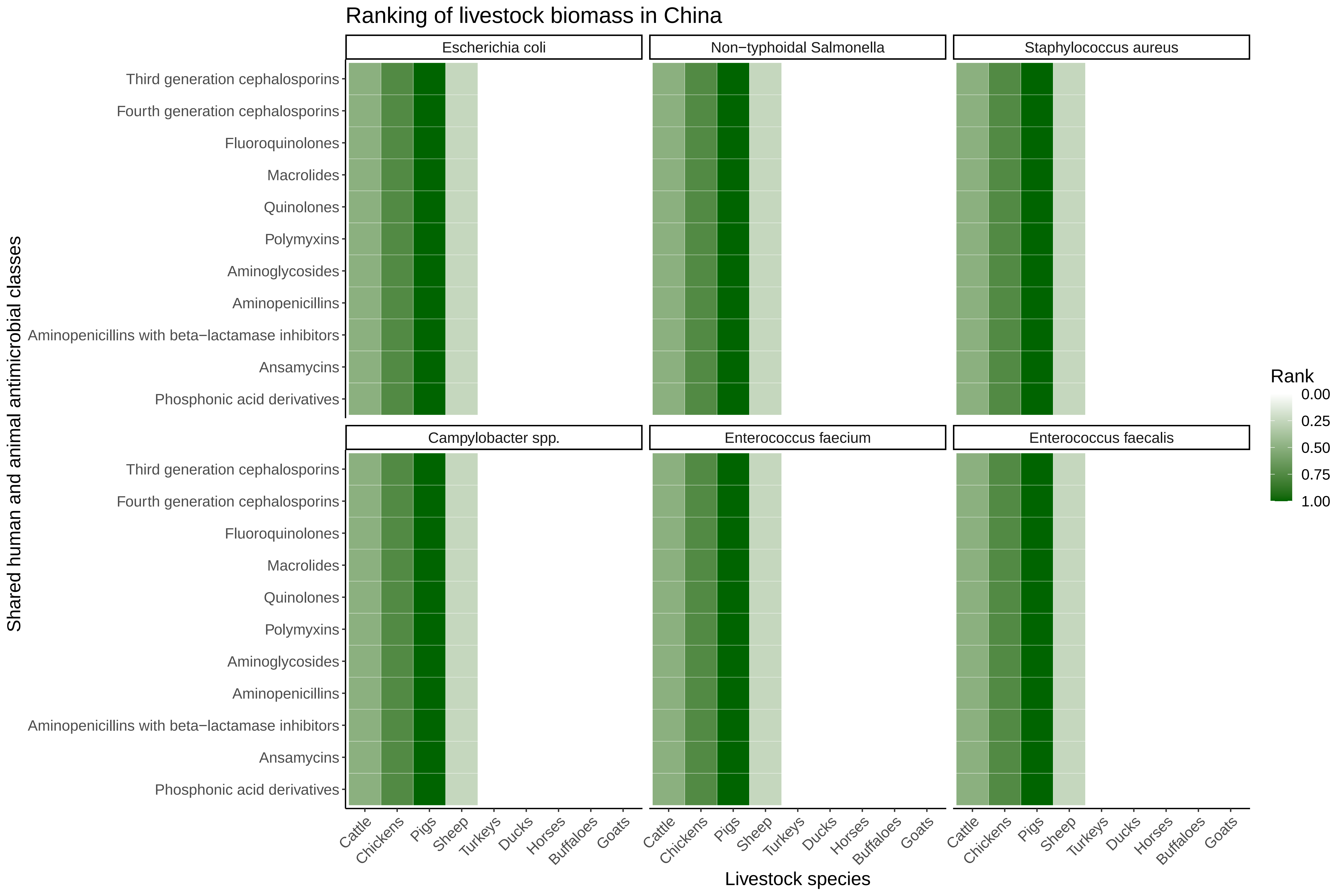

Ranking of livestock biomass in China
Escherichia coli
Non−typhoidal Salmonella
Staphylococcus aureus
Campylobacter spp.
Enterococcus faecium
Enterococcus faecalis
Third generation cephalosporins
Fourth generation cephalosporins
Fluoroquinolones
Macrolides
Quinolones
Polymyxins
Aminoglycosides
Aminopenicillins
Aminopenicillins with beta−lactamase inhibitors
Ansamycins
Phosphonic acid derivatives
Shared human and animal antimicrobial classes
Cattle
Chickens
Pigs
Sheep
Turkeys
Ducks
Horses
Buffaloes
Goats
Livestock species
Rank
0.00
0.25
0.50
0.75
1.00

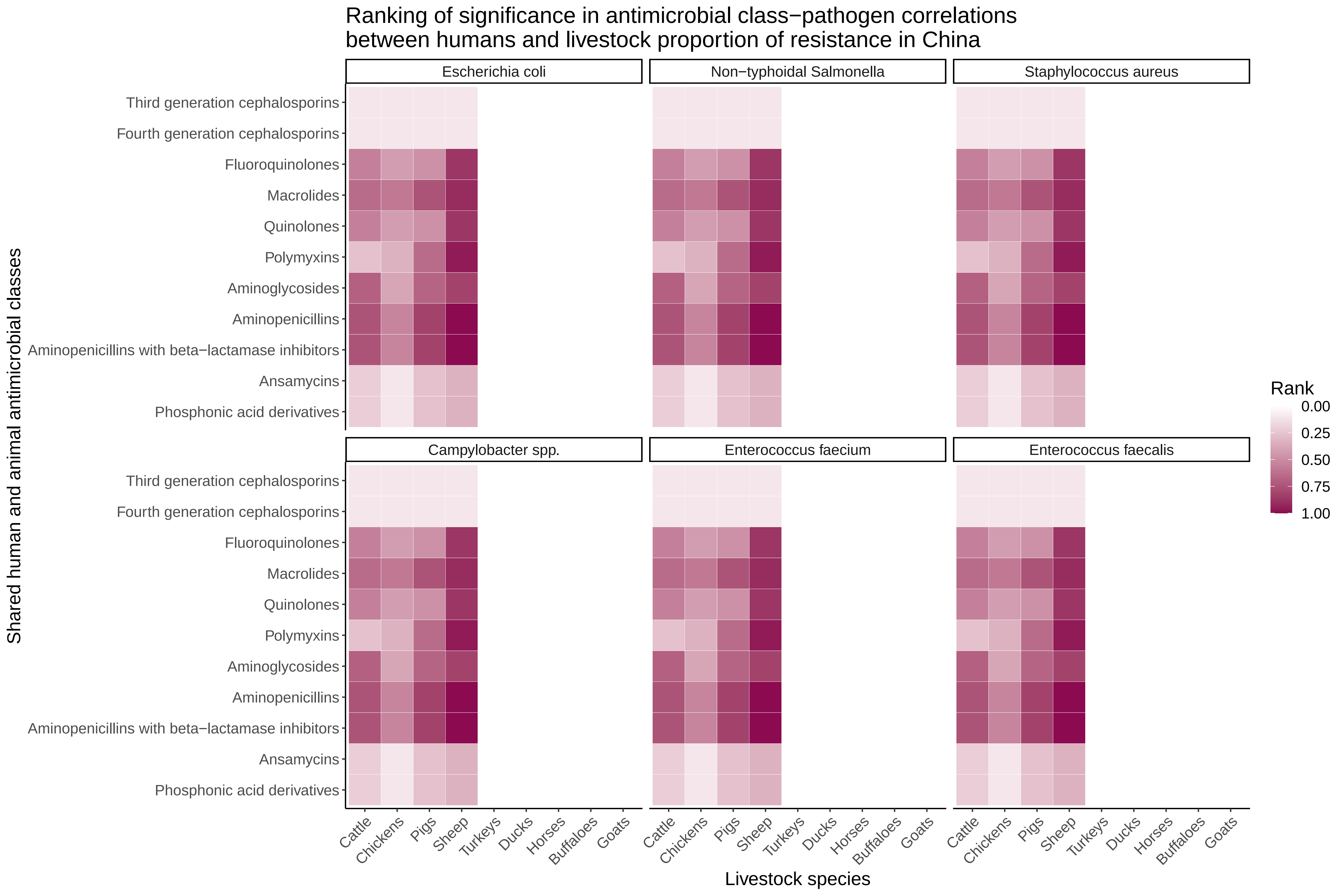
Ranking of significance in antimicrobial class−pathogen correlations
between humans and livestock proportion of resistance in China
Escherichia coli
Non−typhoidal Salmonella
Staphylococcus aureus
Campylobacter spp.
Enterococcus faecium
Enterococcus faecalis
Third generation cephalosporins
Fourth generation cephalosporins
Fluoroquinolones
Macrolides
Quinolones
Polymyxins
Aminoglycosides
Aminopenicillins
Aminopenicillins with beta−lactamase inhibitors
Ansamycins
Phosphonic acid derivatives
Shared human and animal antimicrobial classes
Cattle
Chickens
Pigs
Sheep
Turkeys
Ducks
Horses
Buffaloes
Goats
Livestock species
Rank
0.00
0.25
0.50
0.75
1.00

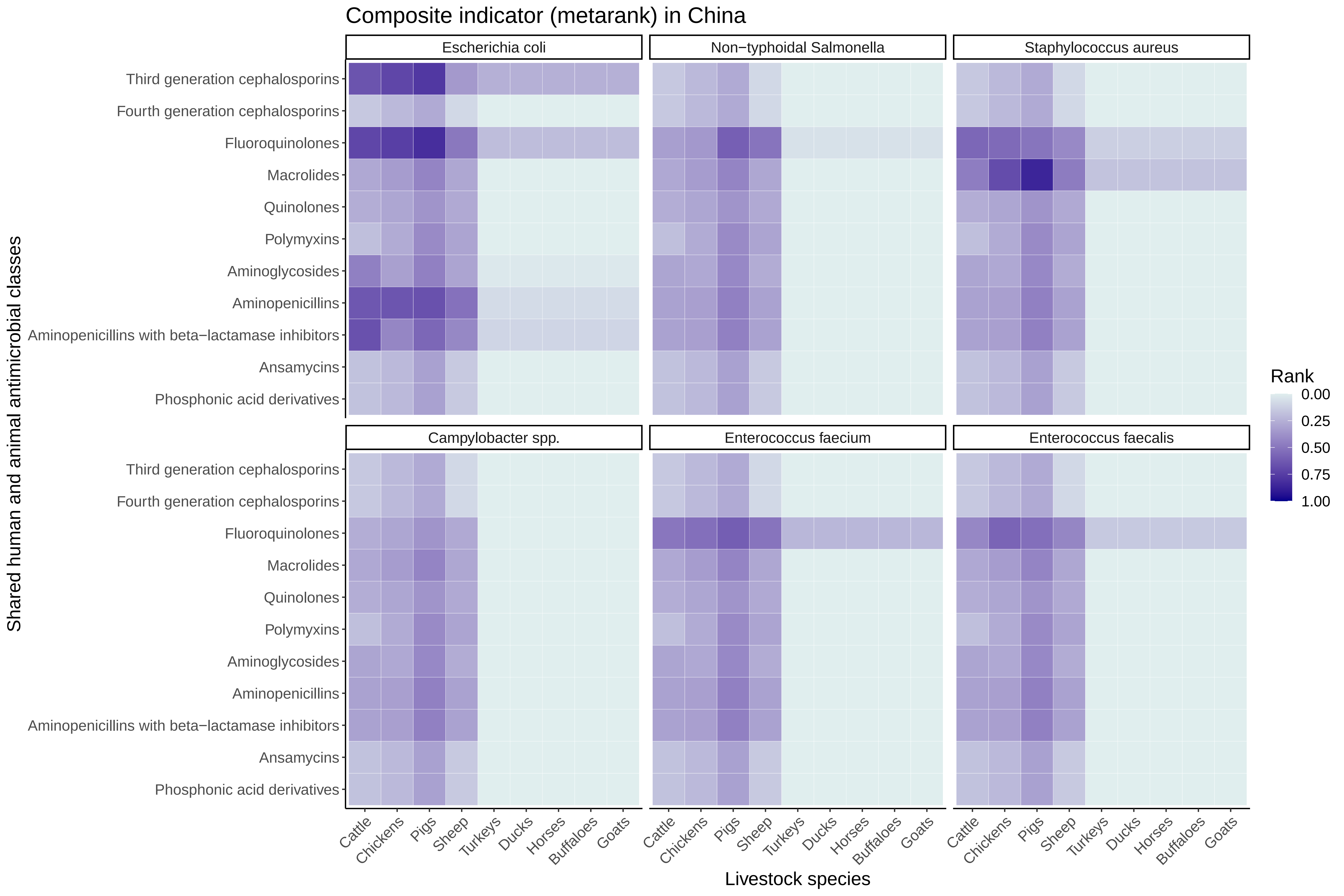

Composite indicator (metarank) in China
Escherichia coli
Non−typhoidal Salmonella
Staphylococcus aureus
Campylobacter spp.
Enterococcus faecium
Enterococcus faecalis
Shared human and animal antimicrobial classes
Third generation cephalosporins
Fourth generation cephalosporins
Fluoroquinolones
Macrolides
Quinolones
Polymyxins
Aminoglycosides
Aminopenicillins
Aminopenicillins with beta−lactamase inhibitors
Ansamycins
Phosphonic acid derivatives
Cattle
Chickens
Pigs
Sheep
Turkeys
Ducks
Horses
Buffaloes
Goats
Livestock species
Rank
0.00
0.25
0.50
0.75
1.00

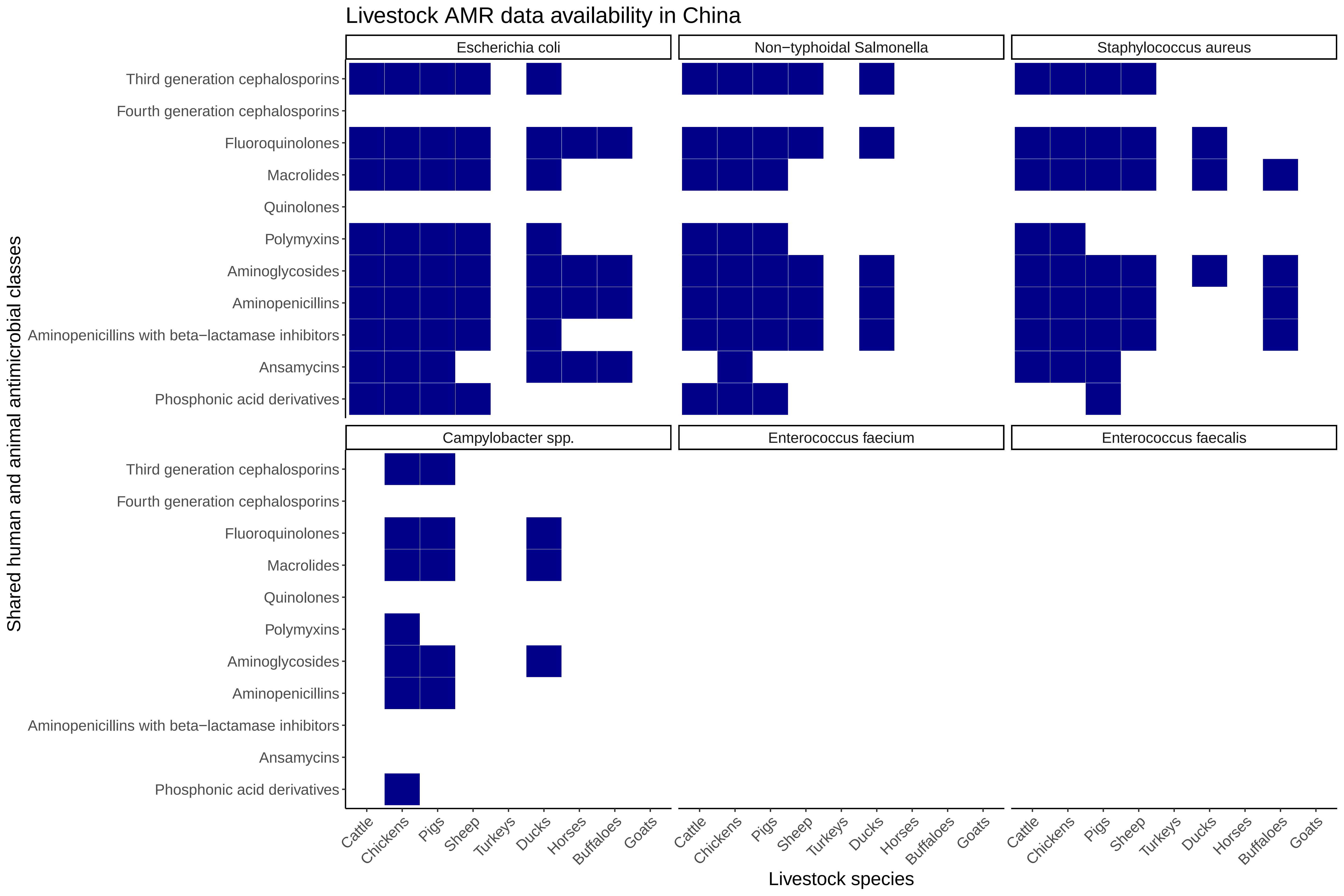

Livestock AMR data availability in China
Escherichia coli
Non−typhoidal Salmonella
Staphylococcus aureus
Campylobacter spp.
Enterococcus faecium
Enterococcus faecalis
Shared human and animal antimicrobial classes
Third generation cephalosporins
Fourth generation cephalosporins
Fluoroquinolones
Macrolides
Quinolones
Polymyxins
Aminoglycosides
Aminopenicillins
Aminopenicillins with beta−lactamase inhibitors
Ansamycins
Phosphonic acid derivatives
Cattle
Chickens
Pigs
Sheep
Turkeys
Ducks
Horses
Buffaloes
Goats
Livestock species

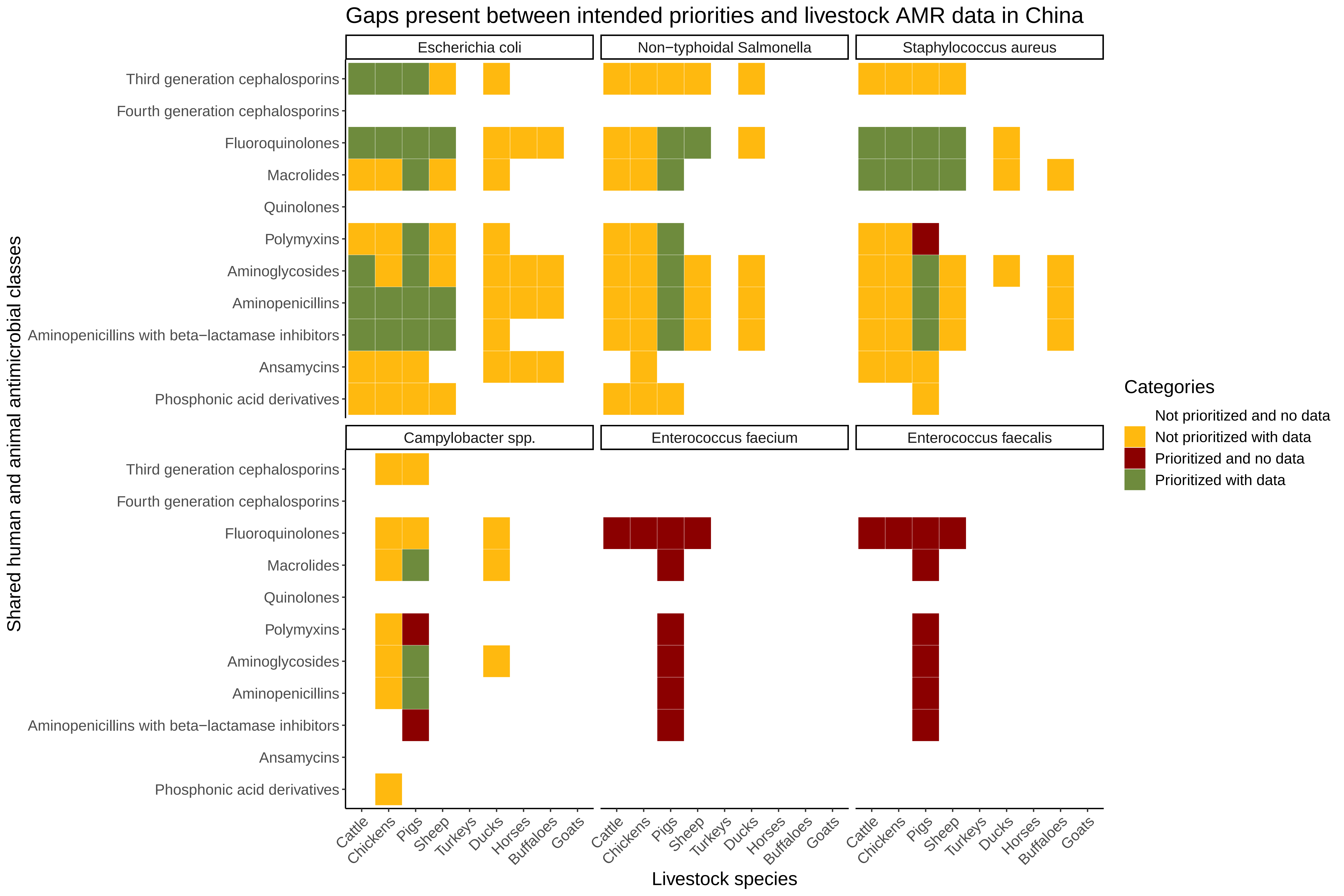
Gaps present between intended priorities and livestock AMR data in China
Escherichia coli
Non−typhoidal Salmonella
Staphylococcus aureus
Campylobacter spp.
Enterococcus faecium
Enterococcus faecalis
Shared human and animal antimicrobial classes
Third generation cephalosporins
Fourth generation cephalosporins
Fluoroquinolones
Macrolides
Quinolones
Polymyxins
Aminoglycosides
Aminopenicillins
Aminopenicillins with beta−lactamase inhibitors
Ansamycins
Phosphonic acid derivatives
Livestock species
Cattle
Chickens
Pigs
Sheep
Turkeys
Ducks
Horses
Buffaloes
Goats
Categories
Not prioritized and no data
Not prioritized with data
Prioritized and no data
Prioritized with data

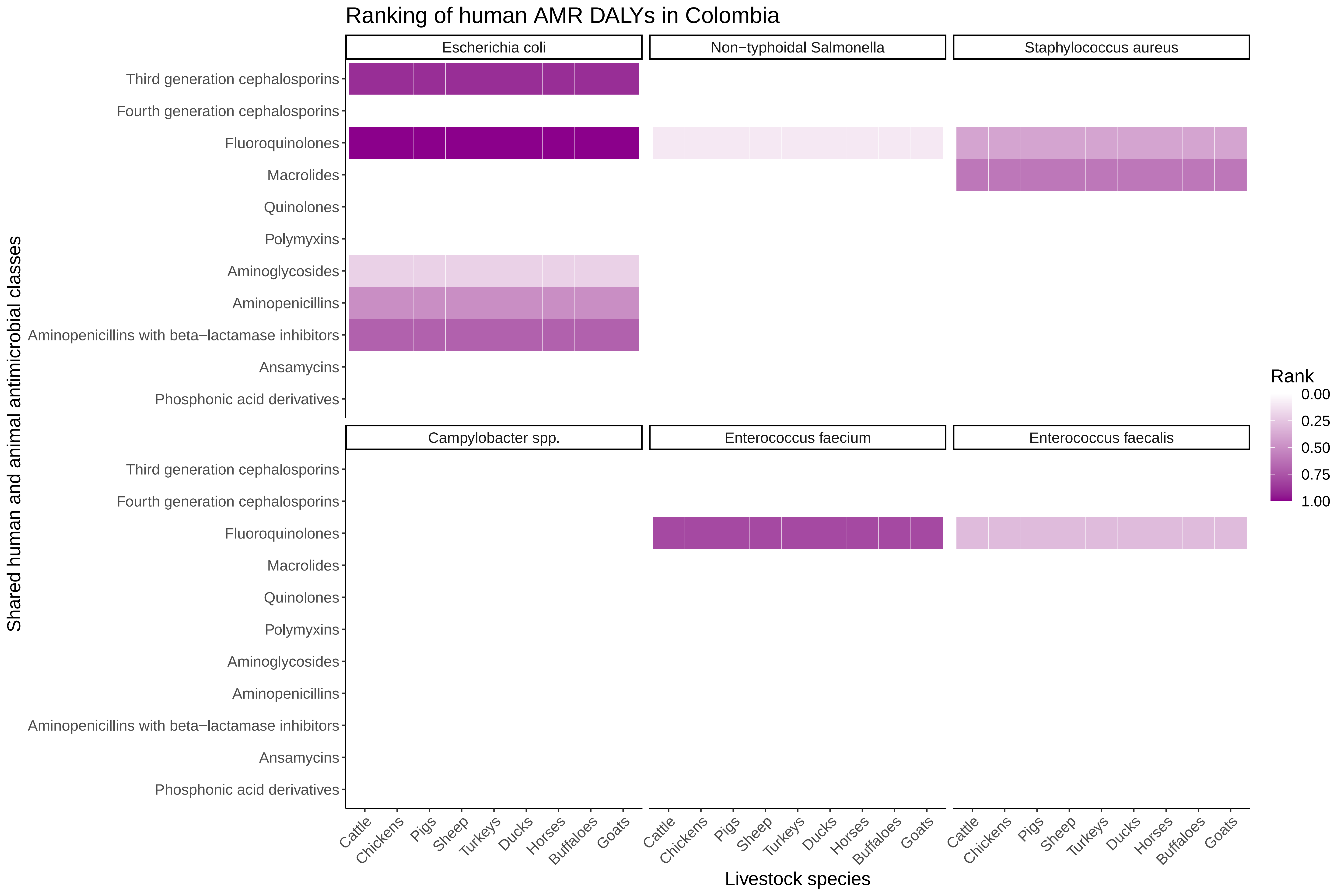
Ranking of human AMR DALYs in Colombia
Escherichia coli
Non−typhoidal Salmonella
Staphylococcus aureus
Campylobacter spp.
Enterococcus faecium
Enterococcus faecalis
Third generation cephalosporins
Fourth generation cephalosporins
Fluoroquinolones
Macrolides
Quinolones
Polymyxins
Aminoglycosides
Aminopenicillins
Aminopenicillins with beta−lactamase inhibitors
Ansamycins
Phosphonic acid derivatives
Shared human and animal antimicrobial classes
Cattle
Chickens
Pigs
Sheep
Turkeys
Ducks
Horses
Buffaloes
Goats
Livestock species
Rank
0.00
0.25
0.50
0.75
1.00

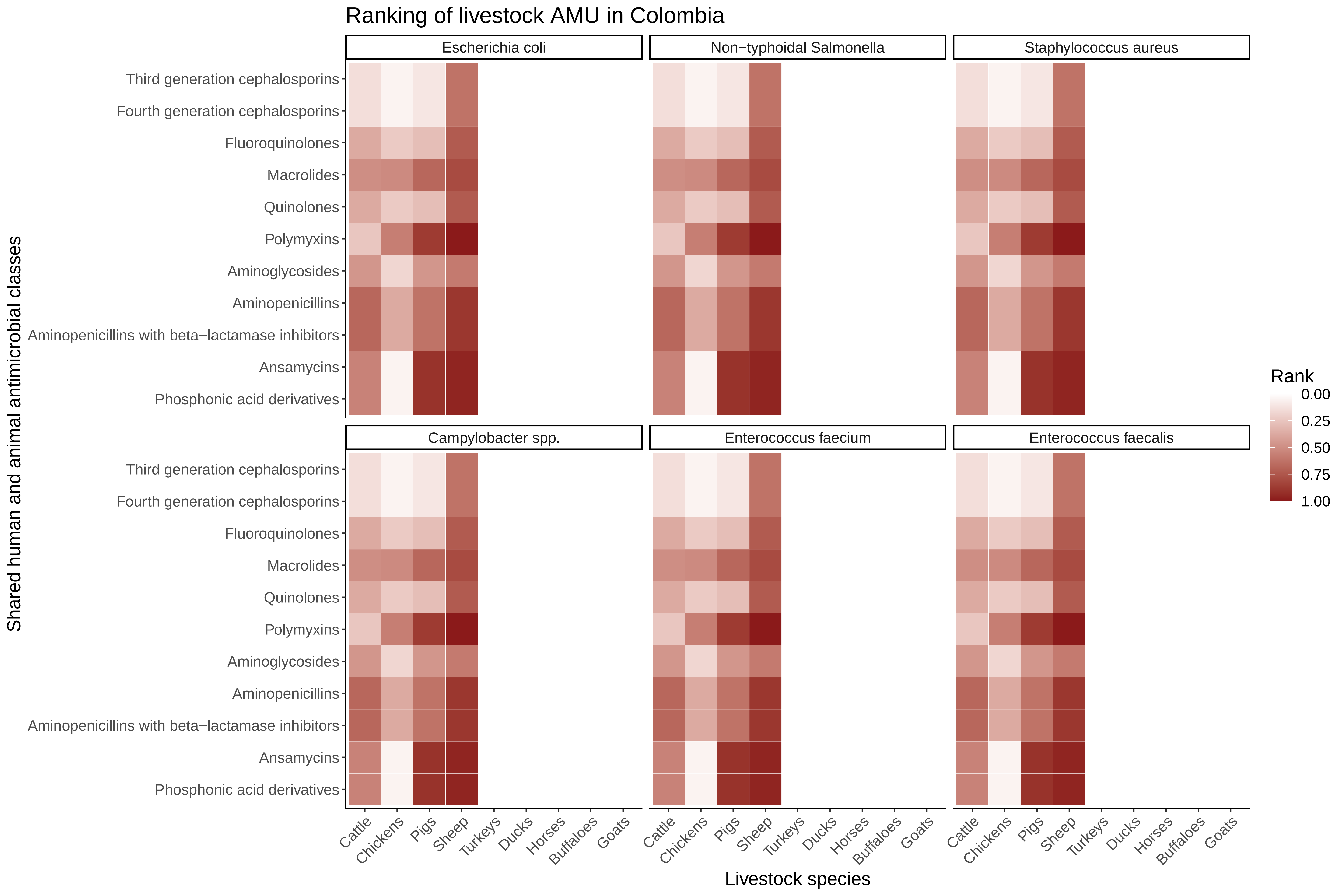

Ranking of livestock AMU in Colombia
Escherichia coli
Non−typhoidal Salmonella
Staphylococcus aureus
Campylobacter spp.
Enterococcus faecium
Enterococcus faecalis
Shared human and animal antimicrobial classes
Third generation cephalosporins
Fourth generation cephalosporins
Fluoroquinolones
Macrolides
Quinolones
Polymyxins
Aminoglycosides
Aminopenicillins
Aminopenicillins with beta−lactamase inhibitors
Ansamycins
Phosphonic acid derivatives
Livestock species
Cattle
Chickens
Pigs
Sheep
Turkeys
Ducks
Horses
Buffaloes
Goats
Rank
0.00
0.25
0.50
0.75
1.00

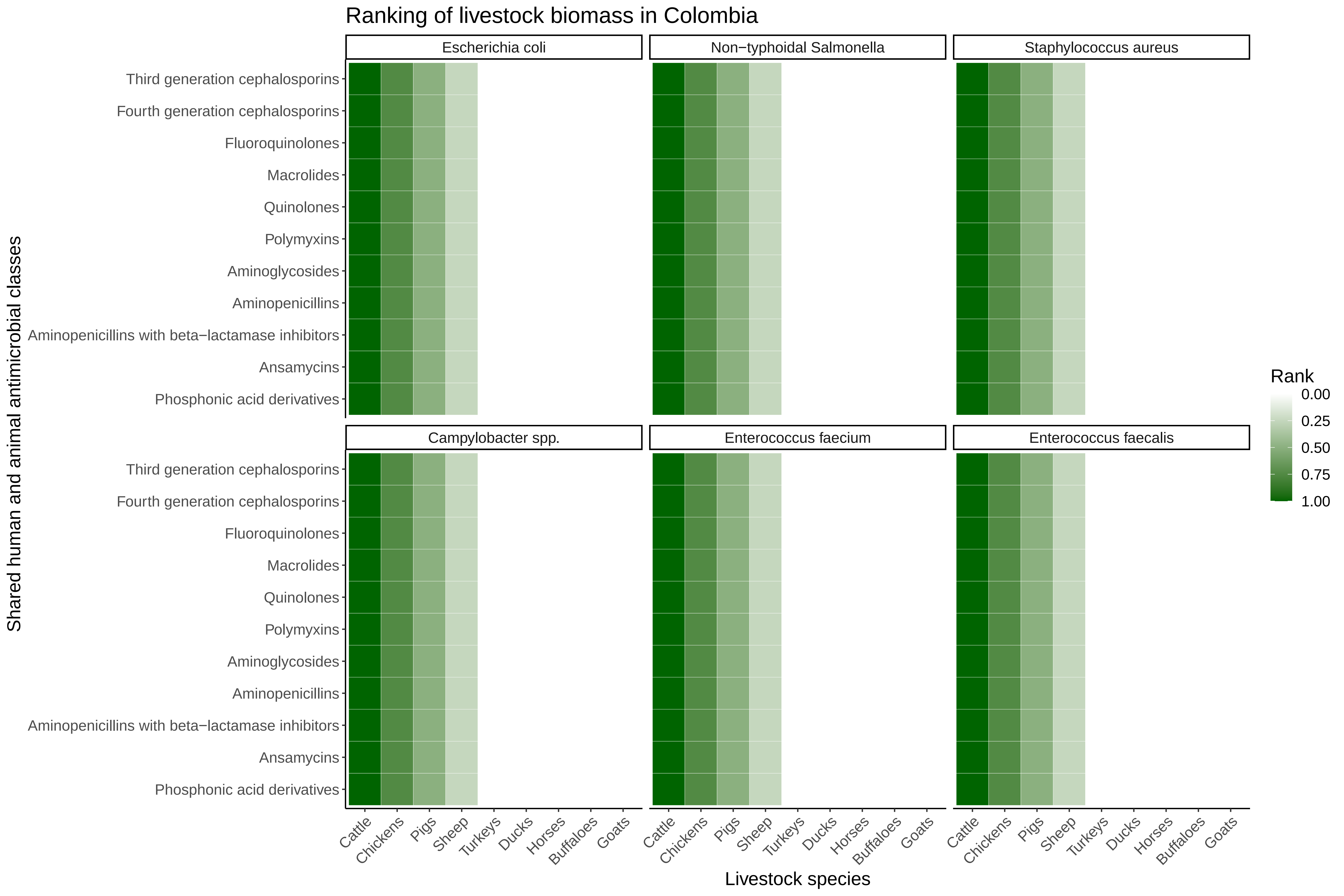
Ranking of livestock biomass in Colombia
Escherichia coli
Non−typhoidal Salmonella
Staphylococcus aureus
Campylobacter spp.
Enterococcus faecium
Enterococcus faecalis
Third generation cephalosporins
Fourth generation cephalosporins
Fluoroquinolones
Macrolides
Quinolones
Polymyxins
Aminoglycosides
Aminopenicillins
Aminopenicillins with beta−lactamase inhibitors
Ansamycins
Phosphonic acid derivatives
Shared human and animal antimicrobial classes
Cattle
Chickens
Pigs
Sheep
Turkeys
Ducks
Horses
Buffaloes
Goats
Livestock species
Rank
0.00
0.25
0.50
0.75
1.00

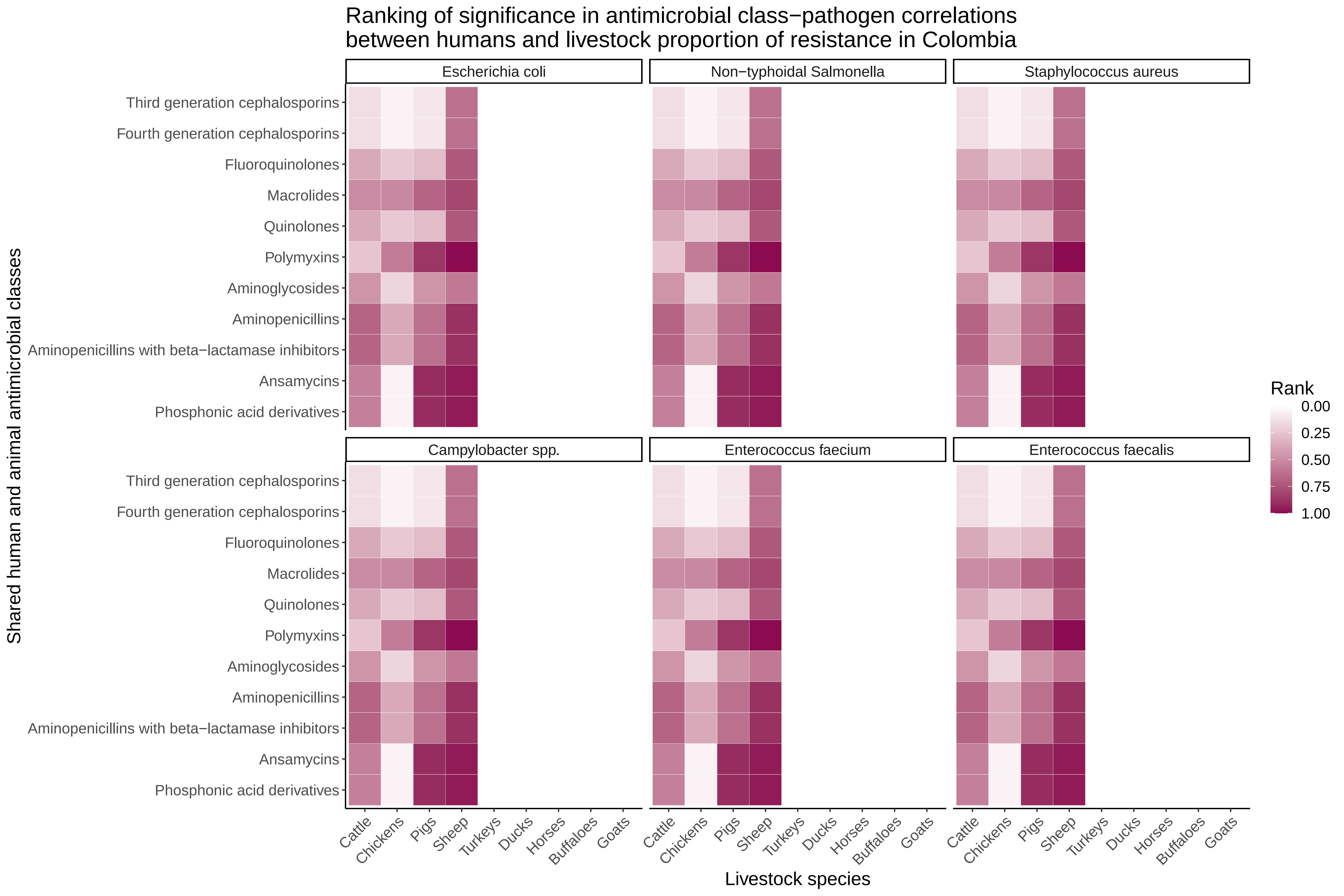

Ranking of significance in antimicrobial class−pathogen correlations
between humans and livestock proportion of resistance in Colombia
Escherichia coli
Non−typhoidal Salmonella
Staphylococcus aureus
Campylobacter spp.
Enterococcus faecium
Enterococcus faecalis
Third generation cephalosporins
Fourth generation cephalosporins
Fluoroquinolones
Macrolides
Quinolones
Polymyxins
Aminoglycosides
Aminopenicillins
Aminopenicillins with beta−lactamase inhibitors
Ansamycins
Phosphonic acid derivatives
Shared human and animal antimicrobial classes
Cattle
Chickens
Pigs
Sheep
Turkeys
Ducks
Horses
Buffaloes
Goats
Livestock species
Rank
0.00
0.25
0.50
0.75
1.00

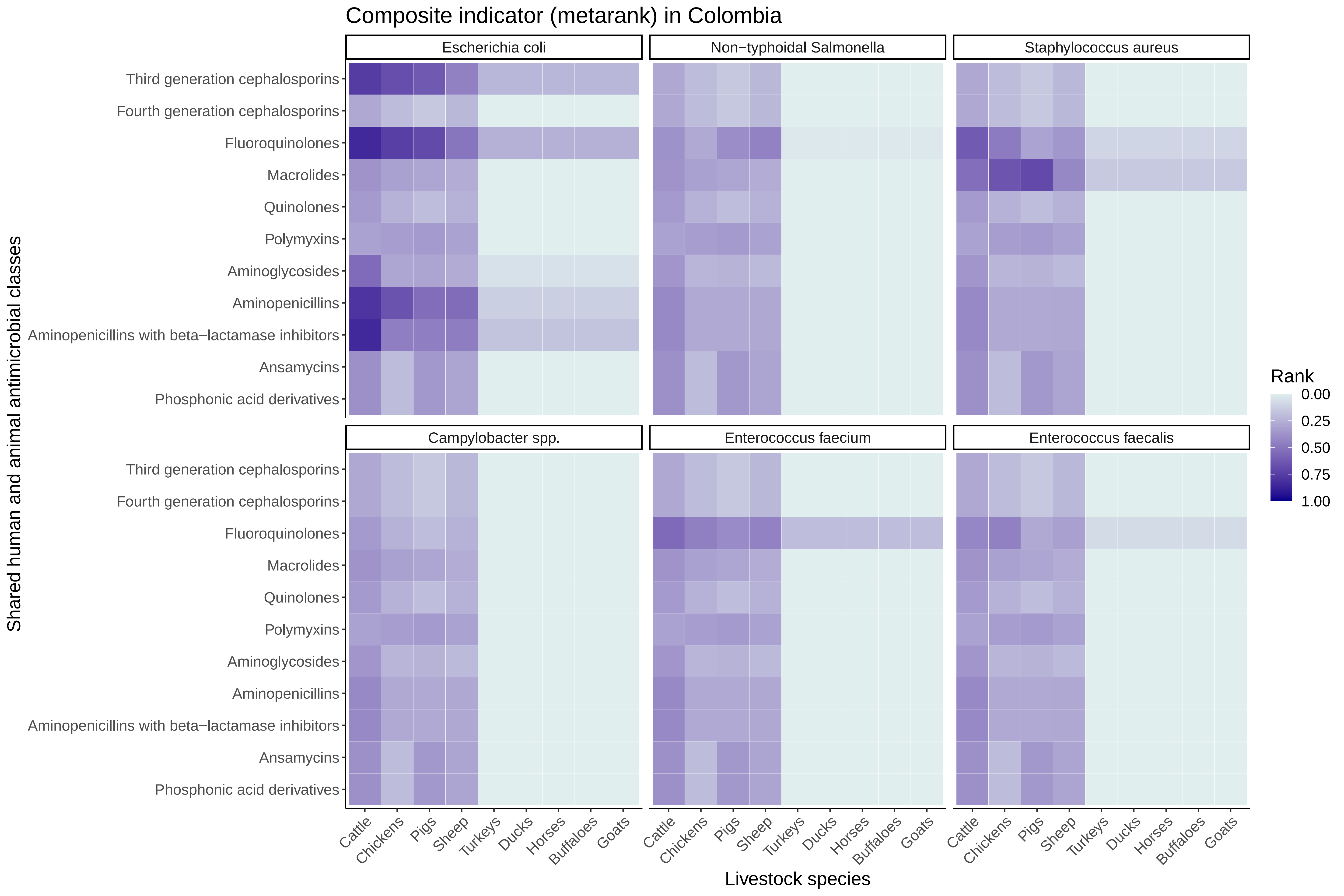
Composite indicator (metarank) in Colombia
Escherichia coli
Non−typhoidal Salmonella
Staphylococcus aureus
Campylobacter spp.
Enterococcus faecium
Enterococcus faecalis
Third generation cephalosporins
Fourth generation cephalosporins
Fluoroquinolones
Macrolides
Quinolones
Polymyxins
Aminoglycosides
Aminopenicillins
Aminopenicillins with beta−lactamase inhibitors
Ansamycins
Phosphonic acid derivatives
Shared human and animal antimicrobial classes
Cattle
Chickens
Pigs
Sheep
Turkeys
Ducks
Horses
Buffaloes
Goats
Livestock species
Rank
0.00
0.25
0.50
0.75
1.00

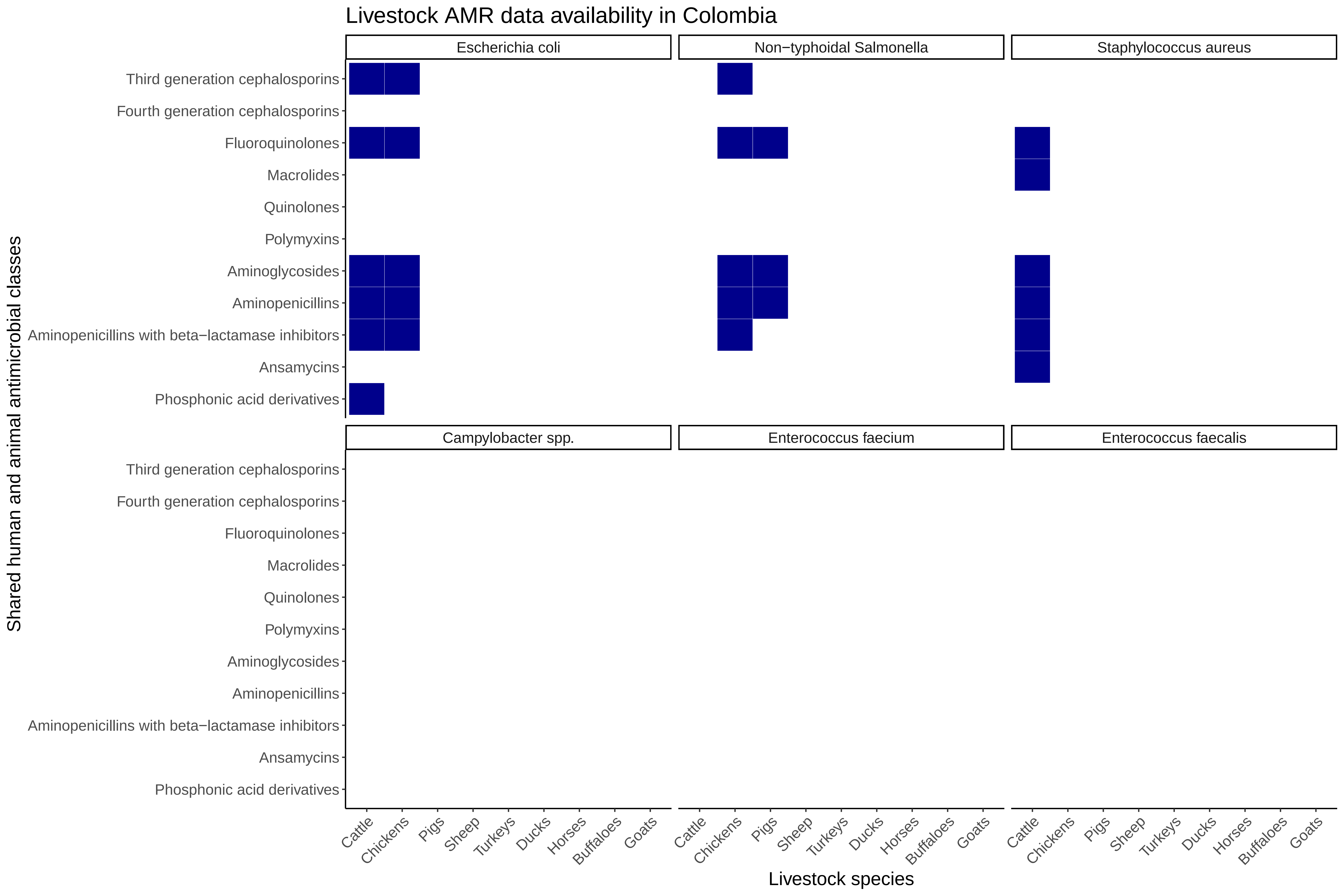

Livestock AMR data availability in Colombia
Escherichia coli
Non−typhoidal Salmonella
Staphylococcus aureus
Campylobacter spp.
Enterococcus faecium
Enterococcus faecalis
Third generation cephalosporins
Fourth generation cephalosporins
Fluoroquinolones
Macrolides
Quinolones
Polymyxins
Aminoglycosides
Aminopenicillins
Aminopenicillins with beta−lactamase inhibitors
Ansamycins
Phosphonic acid derivatives
Shared human and animal antimicrobial classes
Cattle
Chickens
Pigs
Sheep
Turkeys
Ducks
Horses
Buffaloes
Goats
Livestock species

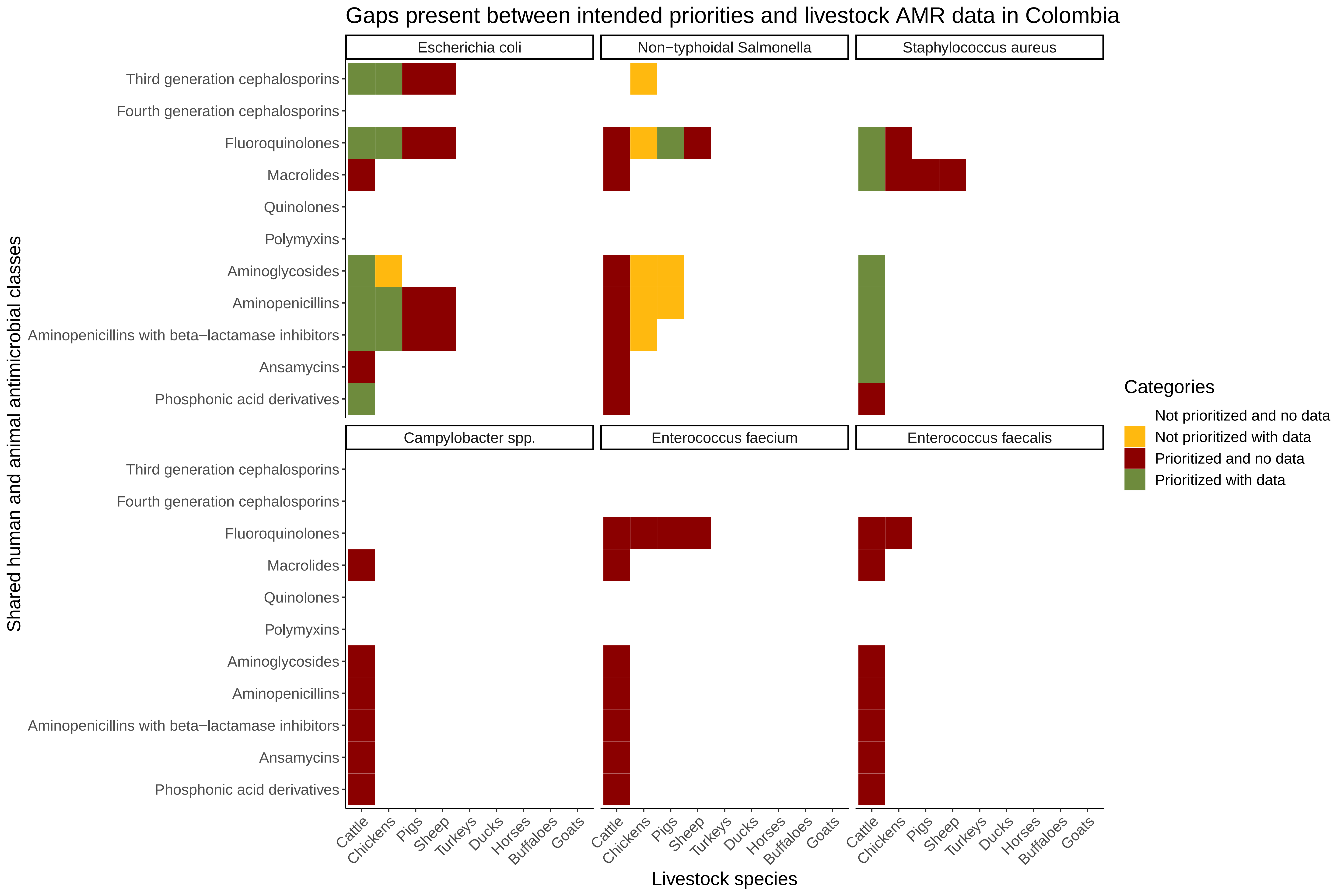

Gaps present between intended priorities and livestock AMR data in Colombia
Escherichia coli
Non−typhoidal Salmonella
Staphylococcus aureus
Campylobacter spp.
Enterococcus faecium
Enterococcus faecalis
Shared human and animal antimicrobial classes
Third generation cephalosporins
Fourth generation cephalosporins
Fluoroquinolones
Macrolides
Quinolones
Polymyxins
Aminoglycosides
Aminopenicillins
Aminopenicillins with beta−lactamase inhibitors
Ansamycins
Phosphonic acid derivatives
Cattle
Chickens
Pigs
Sheep
Turkeys
Ducks
Horses
Buffaloes
Goats
Livestock species
Categories
Not prioritized and no data
Not prioritized with data
Prioritized and no data
Prioritized with data

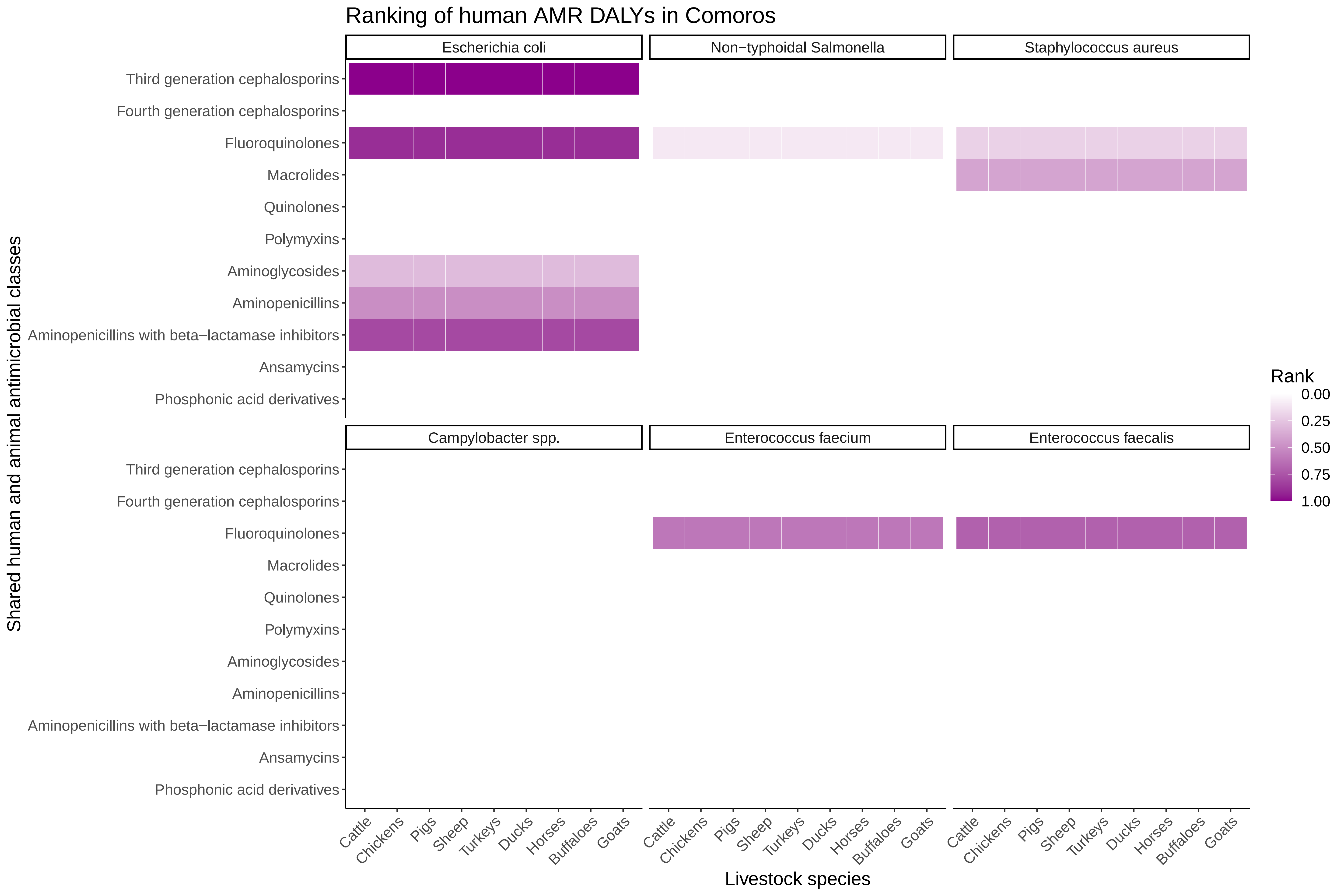

Ranking of human AMR DALYs in Comoros
Escherichia coli
Non−typhoidal Salmonella
Staphylococcus aureus
Campylobacter spp.
Enterococcus faecium
Enterococcus faecalis
Third generation cephalosporins
Fourth generation cephalosporins
Fluoroquinolones
Macrolides
Quinolones
Polymyxins
Aminoglycosides
Aminopenicillins
Aminopenicillins with beta−lactamase inhibitors
Ansamycins
Phosphonic acid derivatives
Shared human and animal antimicrobial classes
Cattle
Chickens
Pigs
Sheep
Turkeys
Ducks
Horses
Buffaloes
Goats
Livestock species
Rank
0.00
0.25
0.50
0.75
1.00

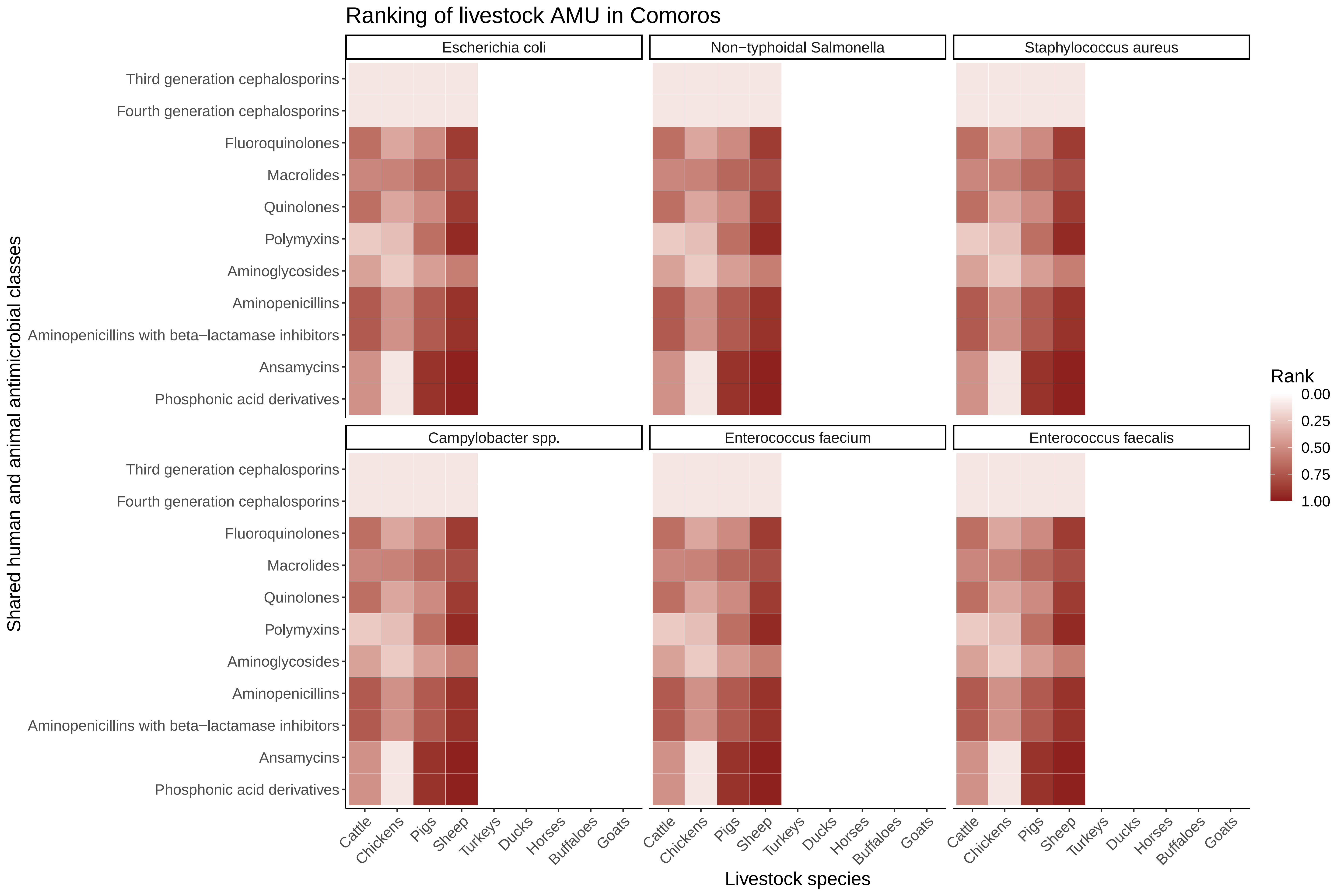

Ranking of livestock AMU in Comoros
Escherichia coli
Non−typhoidal Salmonella
Staphylococcus aureus
Campylobacter spp.
Enterococcus faecium
Enterococcus faecalis
Shared human and animal antimicrobial classes
Third generation cephalosporins
Fourth generation cephalosporins
Fluoroquinolones
Macrolides
Quinolones
Polymyxins
Aminoglycosides
Aminopenicillins
Aminopenicillins with beta−lactamase inhibitors
Ansamycins
Phosphonic acid derivatives
Cattle
Chickens
Pigs
Sheep
Turkeys
Ducks
Horses
Buffaloes
Goats
Livestock species
Rank
0.00
0.25
0.50
0.75
1.00

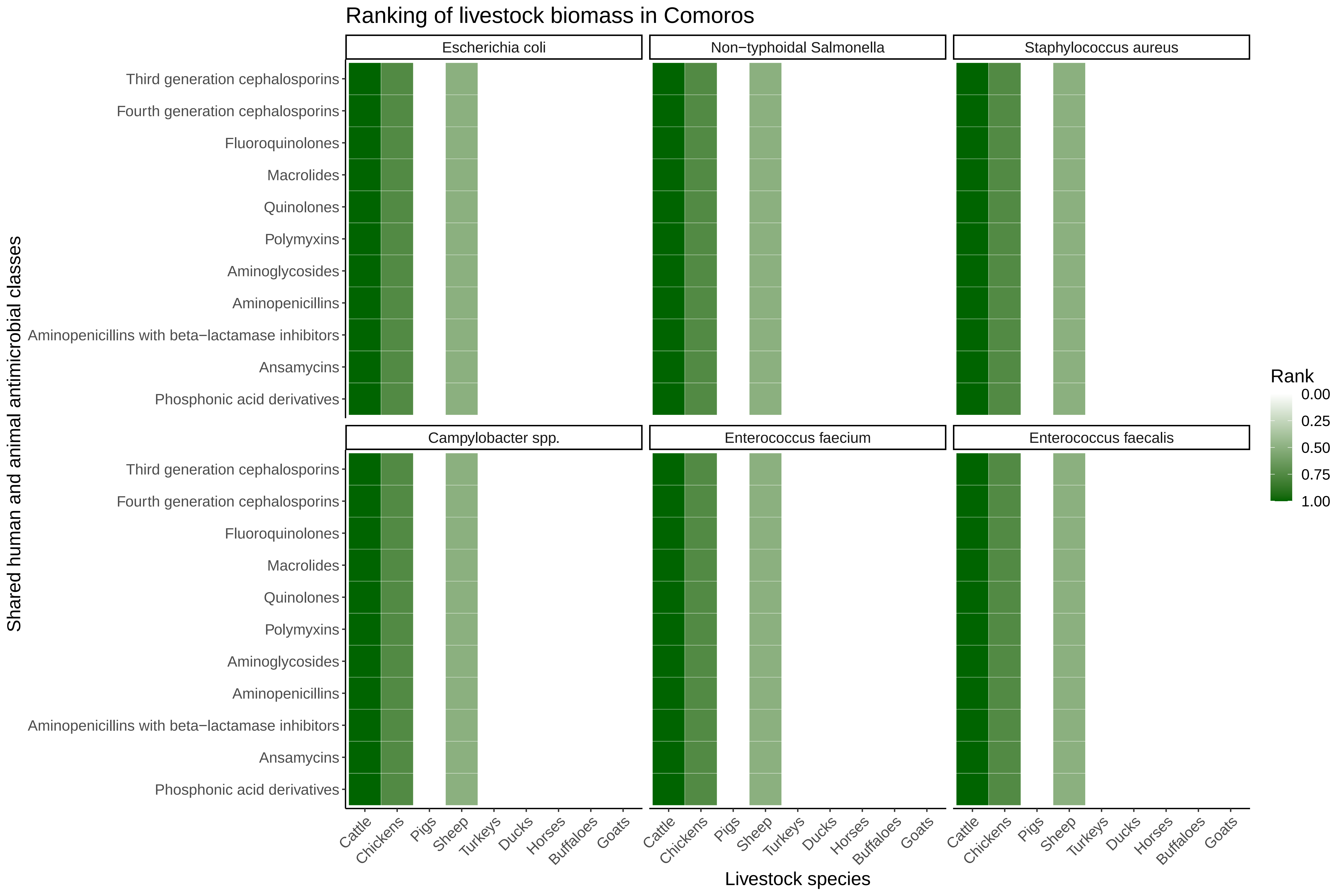

Ranking of livestock biomass in Comoros
Escherichia coli
Non−typhoidal Salmonella
Staphylococcus aureus
Campylobacter spp.
Enterococcus faecium
Enterococcus faecalis
Third generation cephalosporins
Fourth generation cephalosporins
Fluoroquinolones
Macrolides
Quinolones
Polymyxins
Aminoglycosides
Aminopenicillins
Aminopenicillins with beta−lactamase inhibitors
Ansamycins
Phosphonic acid derivatives
Shared human and animal antimicrobial classes
Cattle
Chickens
Pigs
Sheep
Turkeys
Ducks
Horses
Buffaloes
Goats
Livestock species
Rank
0.00
0.25
0.50
0.75
1.00

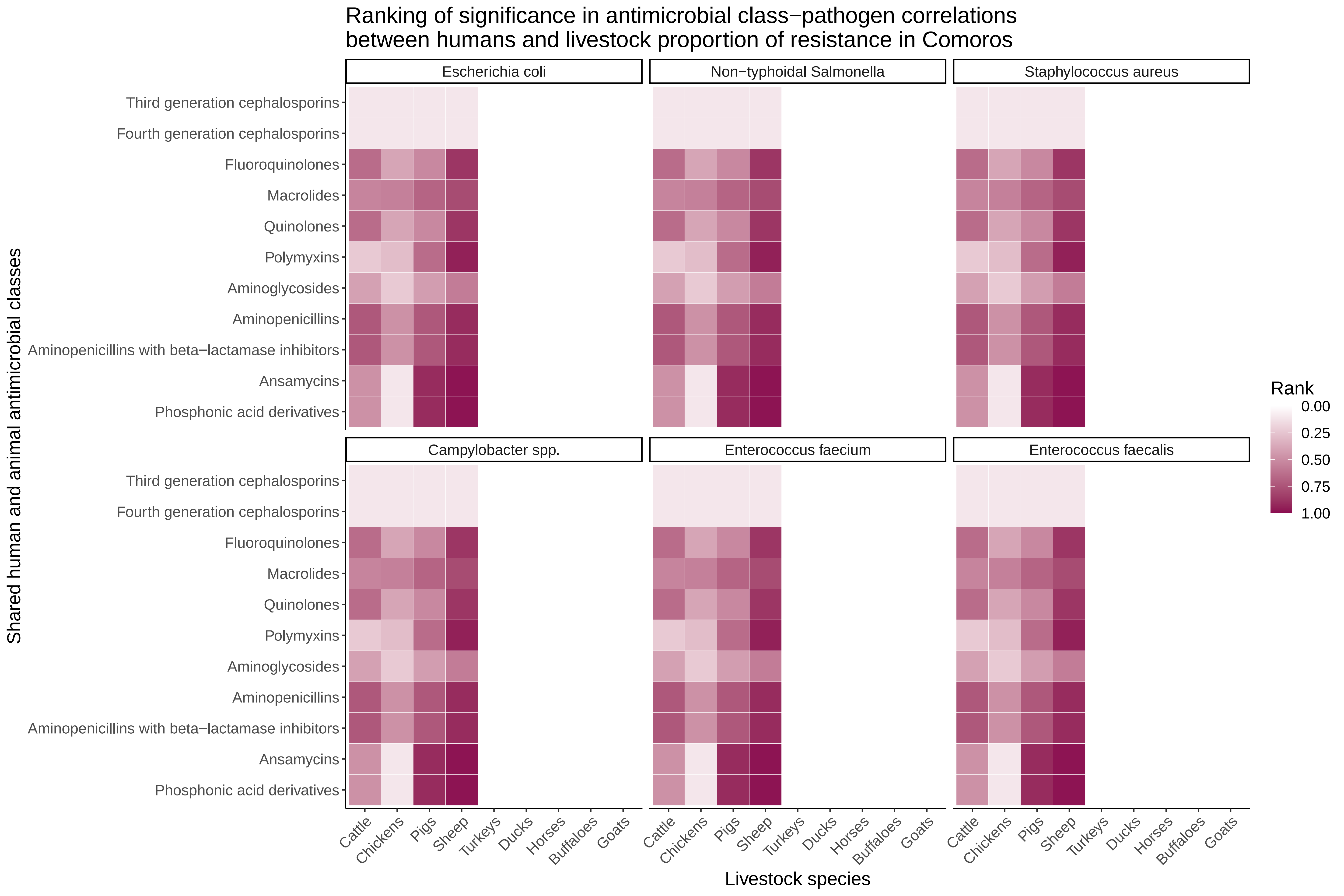
Ranking of significance in antimicrobial class−pathogen correlations
between humans and livestock proportion of resistance in Comoros
Escherichia coli
Non−typhoidal Salmonella
Staphylococcus aureus
Campylobacter spp.
Enterococcus faecium
Enterococcus faecalis
Shared human and animal antimicrobial classes
Third generation cephalosporins
Fourth generation cephalosporins
Fluoroquinolones
Macrolides
Quinolones
Polymyxins
Aminoglycosides
Aminopenicillins
Aminopenicillins with beta−lactamase inhibitors
Ansamycins
Phosphonic acid derivatives
Livestock species
Cattle
Chickens
Pigs
Sheep
Turkeys
Ducks
Horses
Buffaloes
Goats
Rank
0.00
0.25
0.50
0.75
1.00

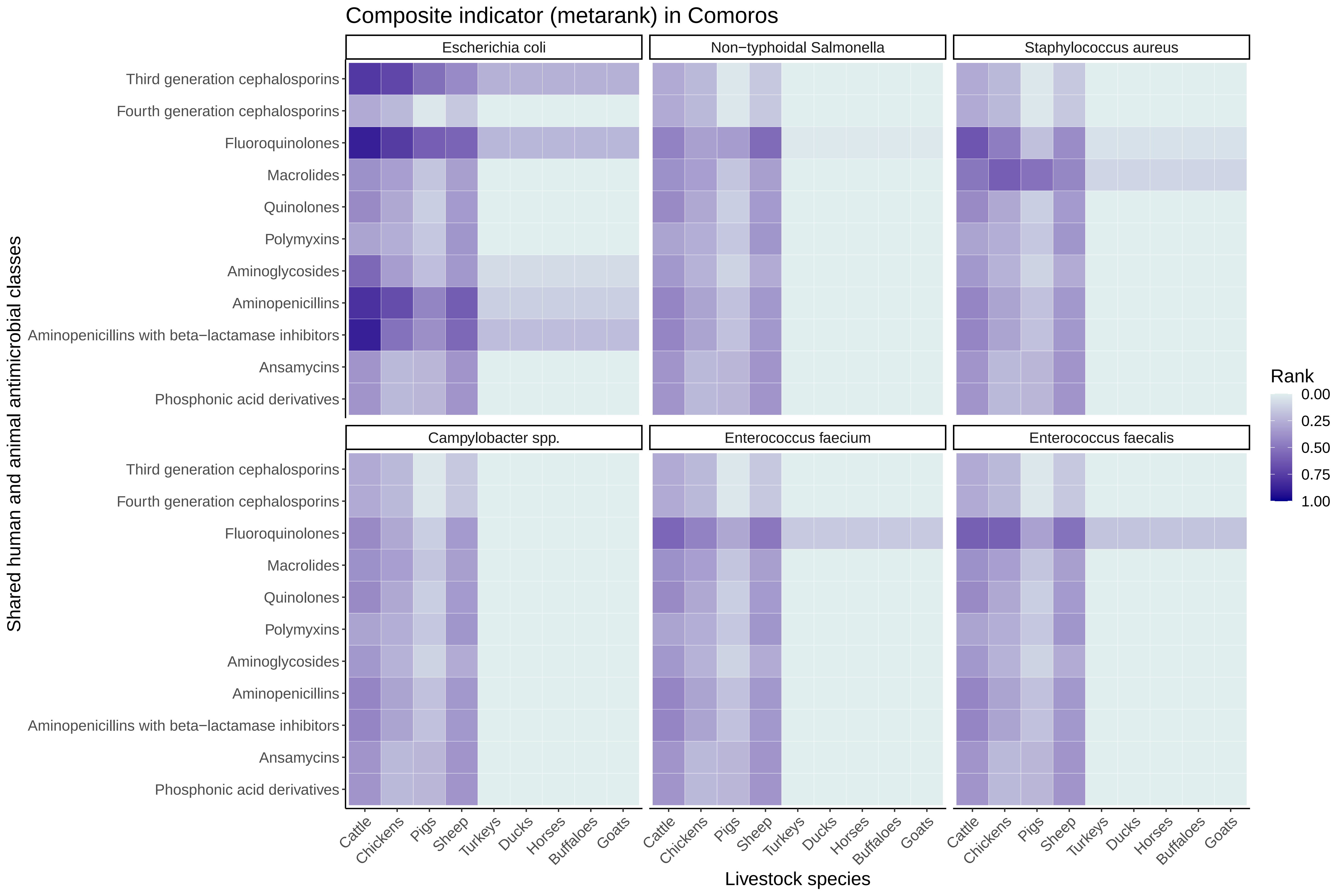
Composite indicator (metarank) in Comoros
Escherichia coli
Non−typhoidal Salmonella
Staphylococcus aureus
Campylobacter spp.
Enterococcus faecium
Enterococcus faecalis
Shared human and animal antimicrobial classes
Third generation cephalosporins
Fourth generation cephalosporins
Fluoroquinolones
Macrolides
Quinolones
Polymyxins
Aminoglycosides
Aminopenicillins
Aminopenicillins with beta−lactamase inhibitors
Ansamycins
Phosphonic acid derivatives
Cattle
Chickens
Pigs
Sheep
Turkeys
Ducks
Horses
Buffaloes
Goats
Livestock species
Rank
0.00
0.25
0.50
0.75
1.00

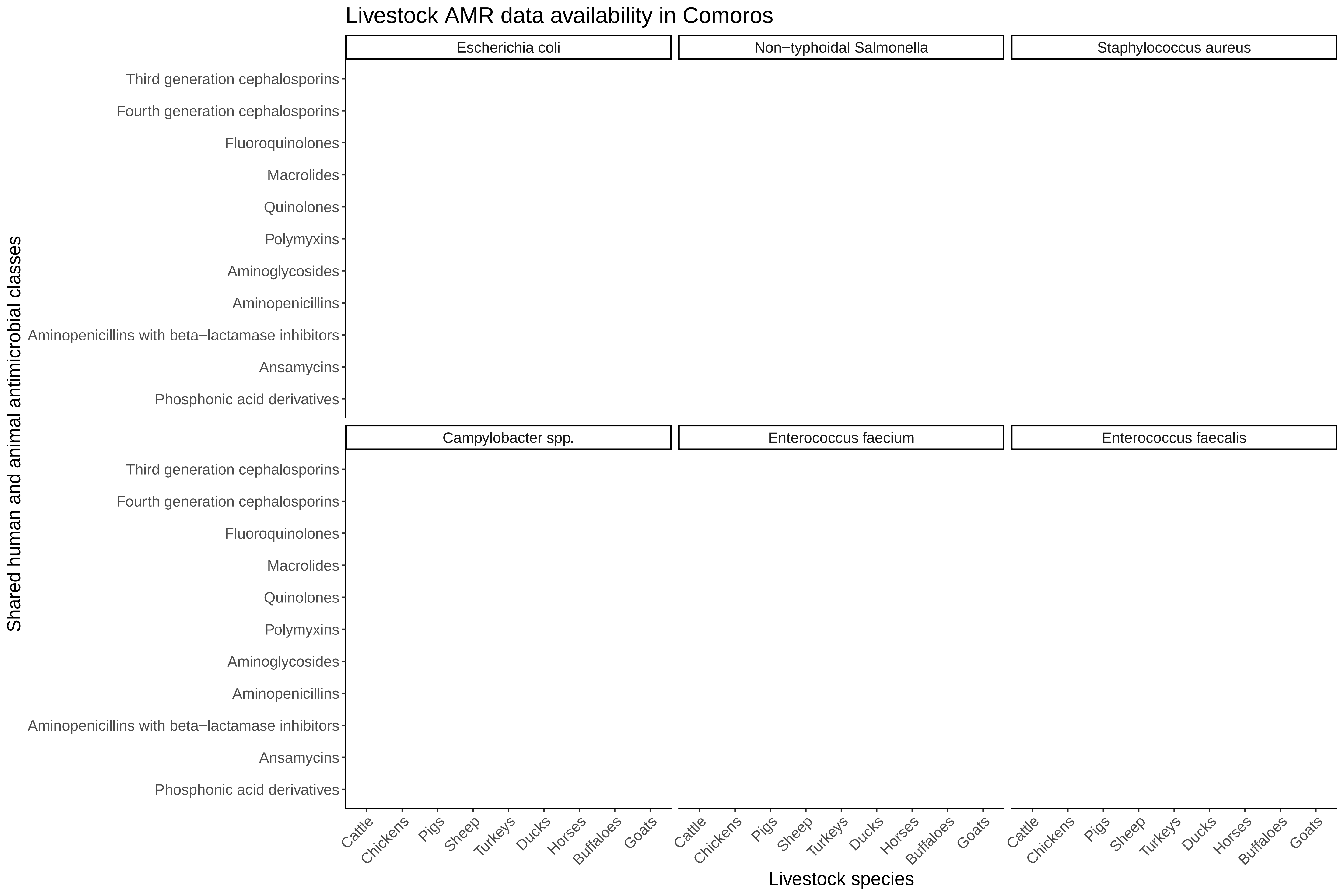
Livestock AMR data availability in Comoros
Escherichia coli
Non−typhoidal Salmonella
Staphylococcus aureus
Campylobacter spp.
Enterococcus faecium
Enterococcus faecalis
Shared human and animal antimicrobial classes
Third generation cephalosporins
Fourth generation cephalosporins
Fluoroquinolones
Macrolides
Quinolones
Polymyxins
Aminoglycosides
Aminopenicillins
Aminopenicillins with beta−lactamase inhibitors
Ansamycins
Phosphonic acid derivatives
Cattle
Chickens
Pigs
Sheep
Turkeys
Ducks
Horses
Buffaloes
Goats
Livestock species

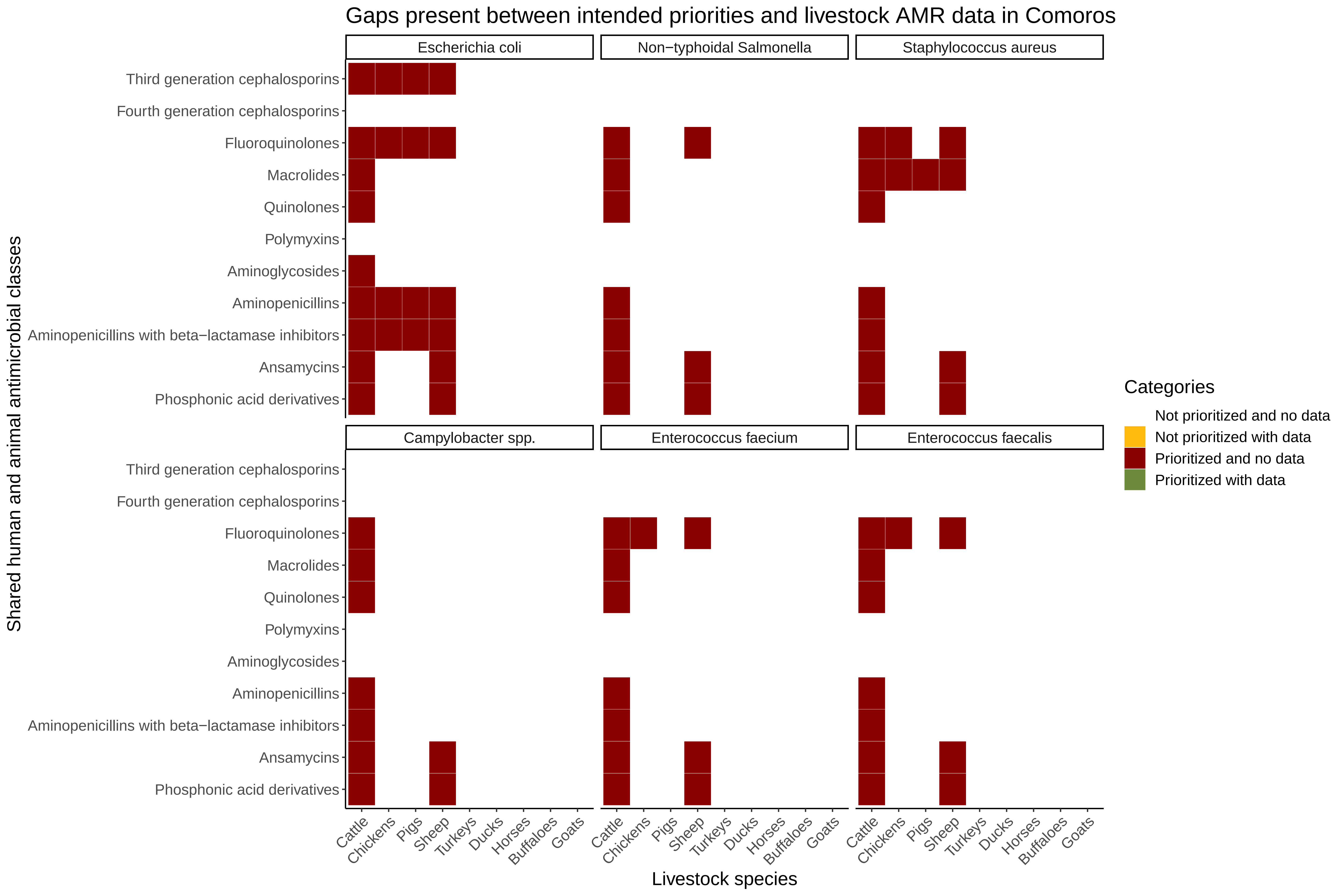

Gaps present between intended priorities and livestock AMR data in Comoros
Escherichia coli
Non−typhoidal Salmonella
Staphylococcus aureus
Campylobacter spp.
Enterococcus faecium
Enterococcus faecalis
Third generation cephalosporins
Fourth generation cephalosporins
Fluoroquinolones
Macrolides
Quinolones
Polymyxins
Aminoglycosides
Aminopenicillins
Aminopenicillins with beta−lactamase inhibitors
Ansamycins
Phosphonic acid derivatives
Shared human and animal antimicrobial classes
Cattle
Chickens
Pigs
Sheep
Turkeys
Ducks
Horses
Buffaloes
Goats
Livestock species
Categories
Not prioritized and no data
Not prioritized with data
Prioritized and no data
Prioritized with data

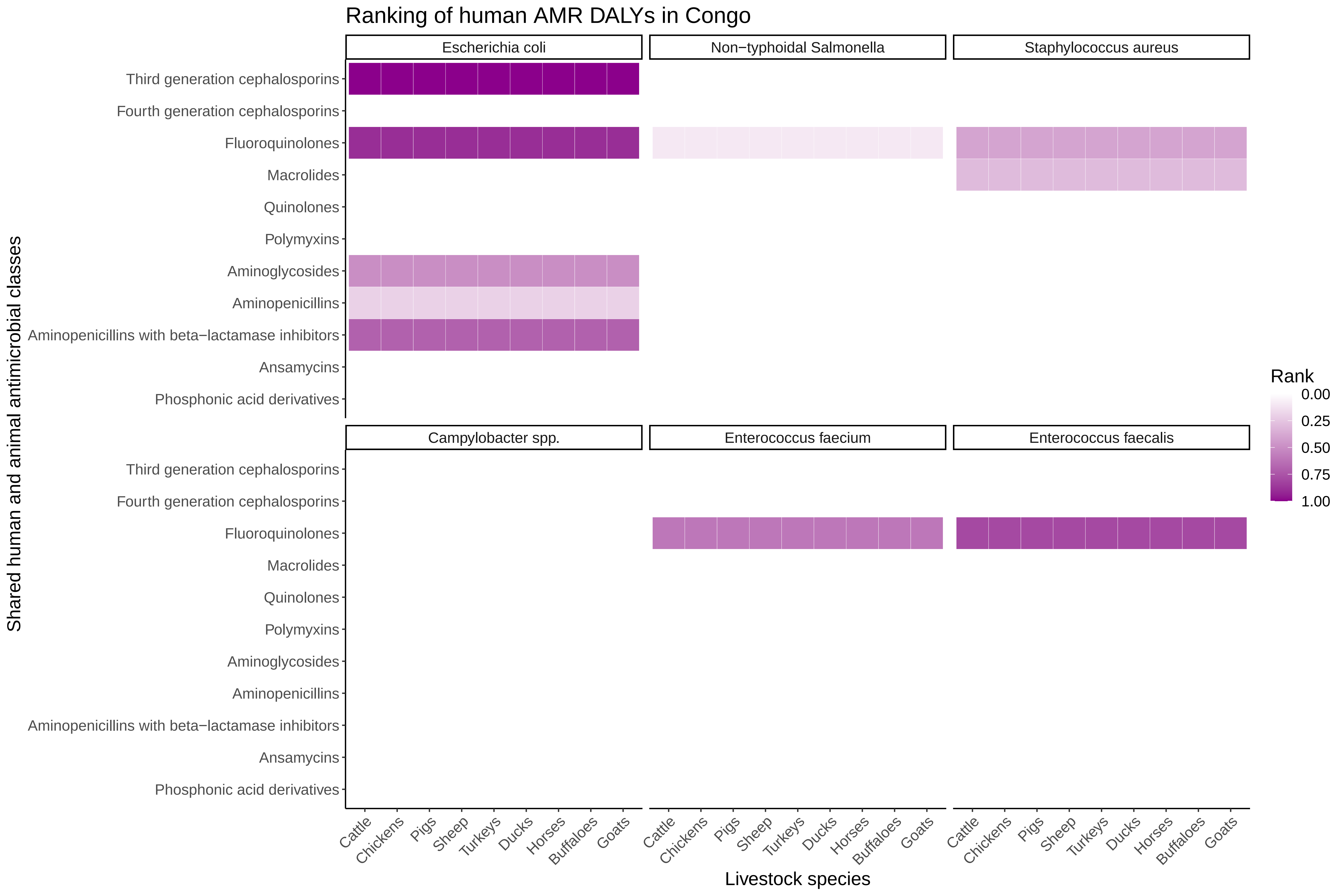

Ranking of human AMR DALYs in Congo
Escherichia coli
Non−typhoidal Salmonella
Staphylococcus aureus
Campylobacter spp.
Enterococcus faecium
Enterococcus faecalis
Third generation cephalosporins
Fourth generation cephalosporins
Fluoroquinolones
Macrolides
Quinolones
Polymyxins
Aminoglycosides
Aminopenicillins
Aminopenicillins with beta−lactamase inhibitors
Ansamycins
Phosphonic acid derivatives
Shared human and animal antimicrobial classes
Cattle
Chickens
Pigs
Sheep
Turkeys
Ducks
Horses
Buffaloes
Goats
Livestock species
Rank
0.00
0.25
0.50
0.75
1.00

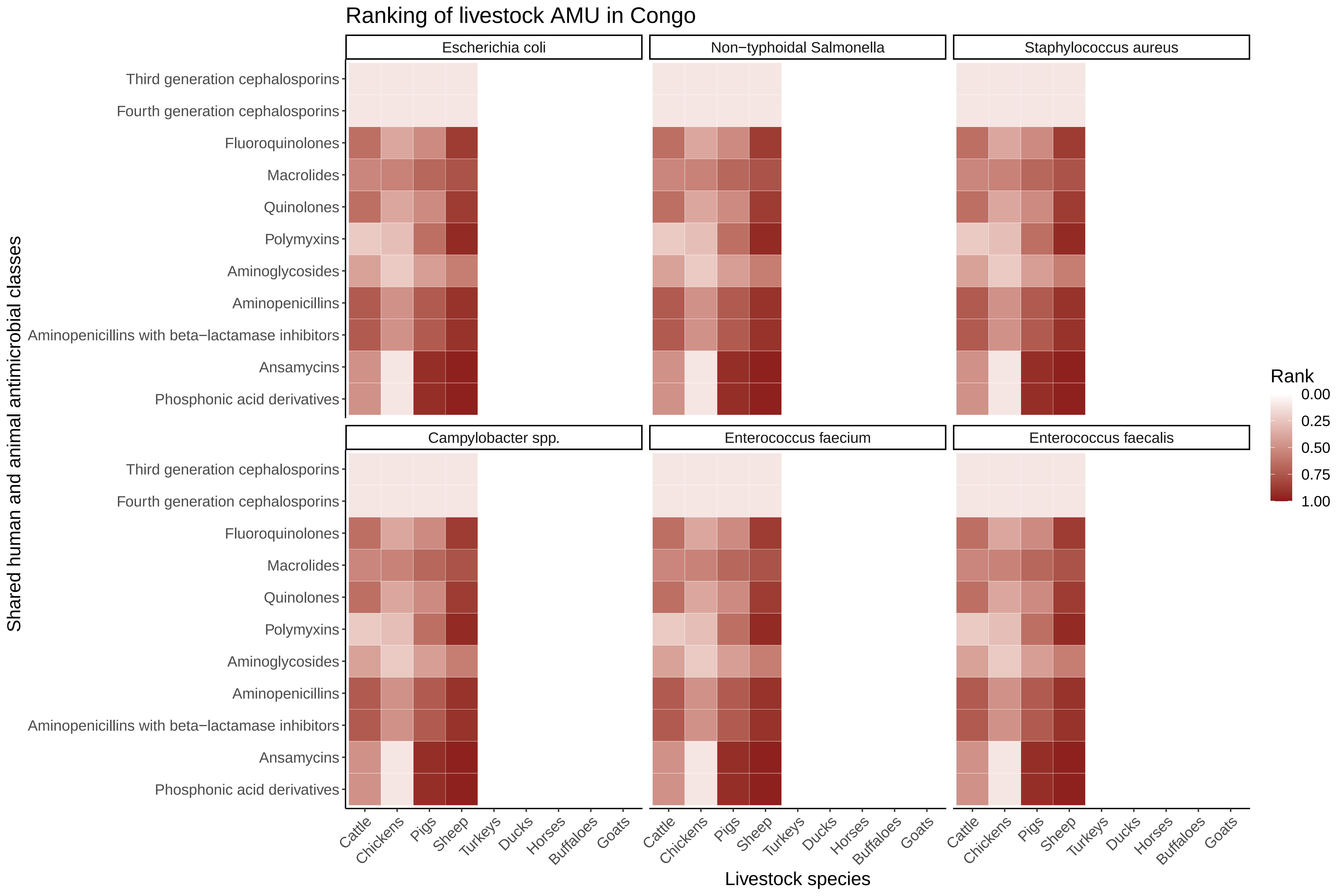

Ranking of livestock AMU in Congo
Escherichia coli
Non−typhoidal Salmonella
Staphylococcus aureus
Campylobacter spp.
Enterococcus faecium
Enterococcus faecalis
Third generation cephalosporins
Fourth generation cephalosporins
Fluoroquinolones
Macrolides
Quinolones
Polymyxins
Aminoglycosides
Aminopenicillins
Aminopenicillins with beta−lactamase inhibitors
Ansamycins
Phosphonic acid derivatives
Shared human and animal antimicrobial classes
Cattle
Chickens
Pigs
Sheep
Turkeys
Ducks
Horses
Buffaloes
Goats
Livestock species
Rank
0.00
0.25
0.50
0.75
1.00

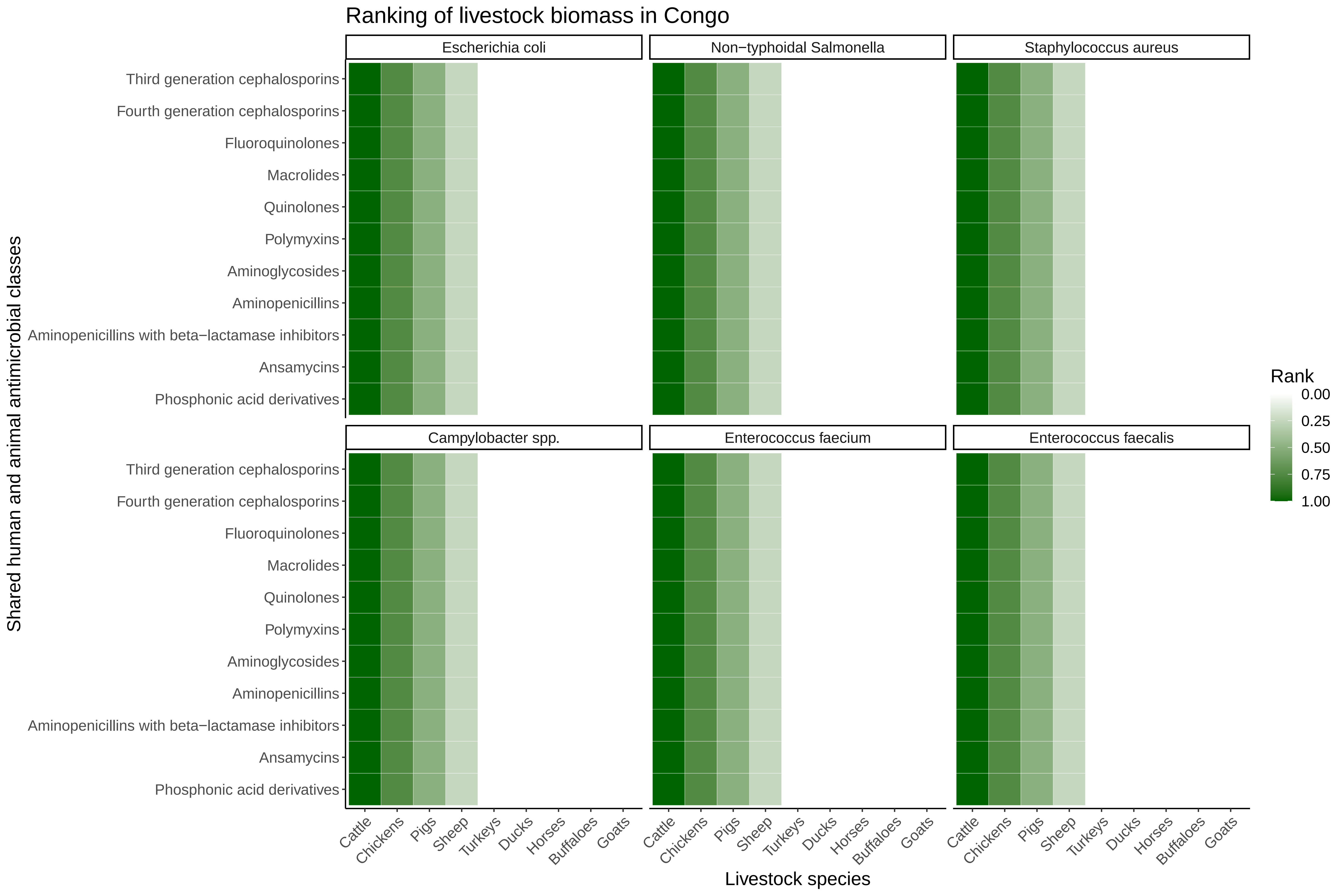
Ranking of livestock biomass in Congo
Escherichia coli
Non−typhoidal Salmonella
Staphylococcus aureus
Campylobacter spp.
Enterococcus faecium
Enterococcus faecalis
Third generation cephalosporins
Fourth generation cephalosporins
Fluoroquinolones
Macrolides
Quinolones
Polymyxins
Aminoglycosides
Aminopenicillins
Aminopenicillins with beta−lactamase inhibitors
Ansamycins
Phosphonic acid derivatives
Shared human and animal antimicrobial classes
Cattle
Chickens
Pigs
Sheep
Turkeys
Ducks
Horses
Buffaloes
Goats
Livestock species
Rank
0.00
0.25
0.50
0.75
1.00

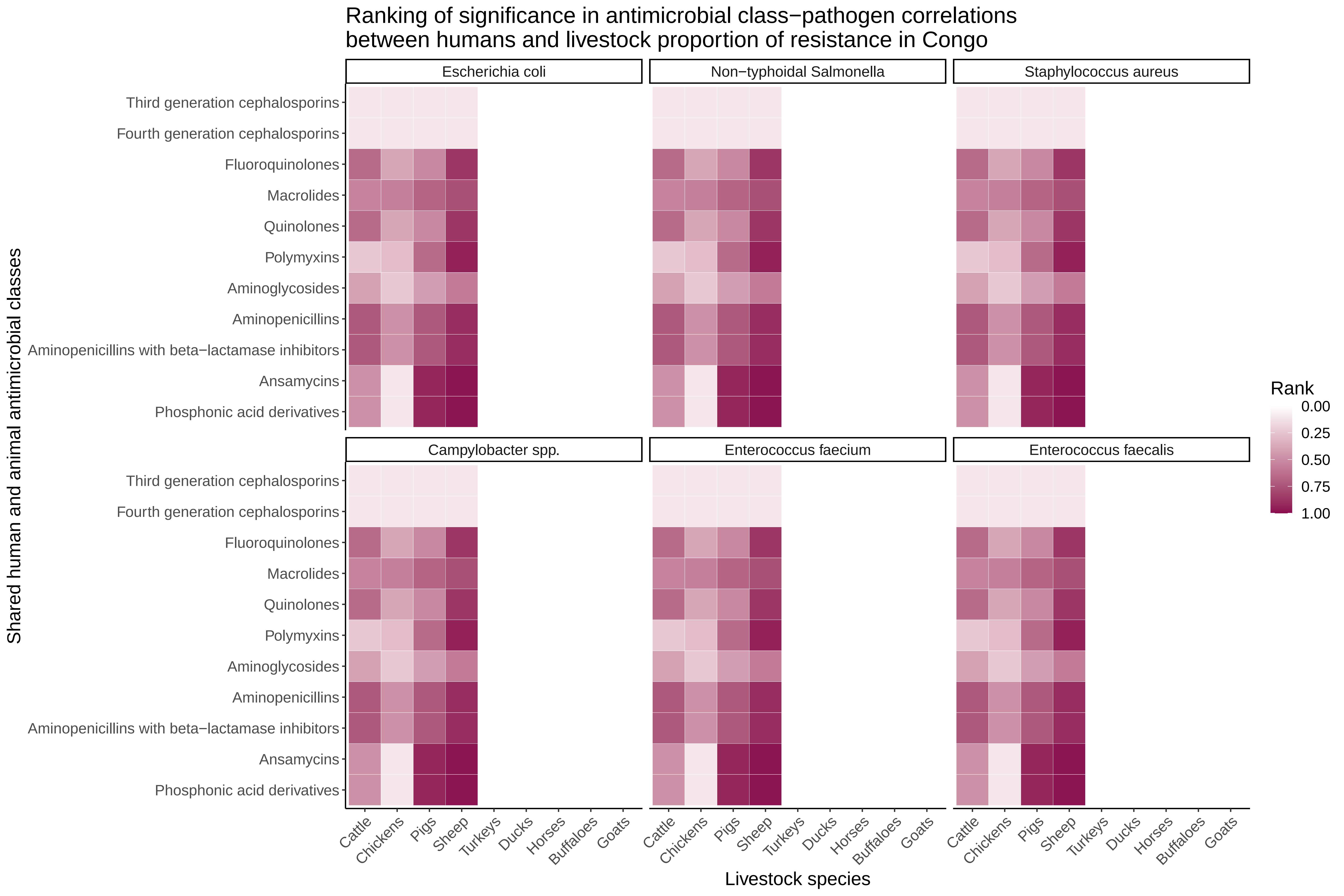
Ranking of significance in antimicrobial class−pathogen correlations
between humans and livestock proportion of resistance in Congo
Escherichia coli
Non−typhoidal Salmonella
Staphylococcus aureus
Campylobacter spp.
Enterococcus faecium
Enterococcus faecalis
Third generation cephalosporins
Fourth generation cephalosporins
Fluoroquinolones
Macrolides
Quinolones
Polymyxins
Aminoglycosides
Aminopenicillins
Aminopenicillins with beta−lactamase inhibitors
Ansamycins
Phosphonic acid derivatives
Shared human and animal antimicrobial classes
Cattle
Chickens
Pigs
Sheep
Turkeys
Ducks
Horses
Buffaloes
Goats
Livestock species
Rank
0.00
0.25
0.50
0.75
1.00

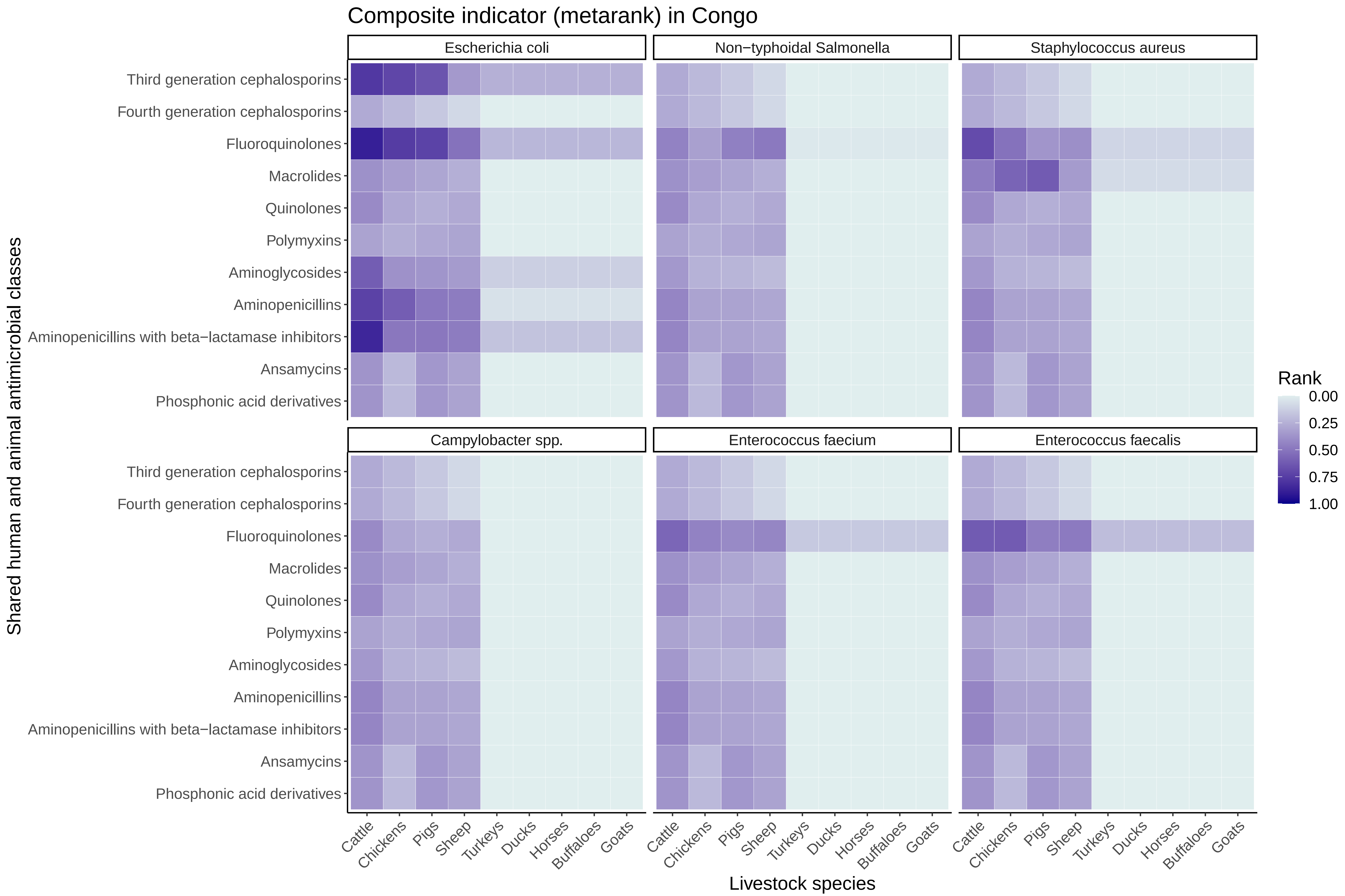

Composite indicator (metarank) in Congo
Escherichia coli
Non−typhoidal Salmonella
Staphylococcus aureus
Campylobacter spp.
Enterococcus faecium
Enterococcus faecalis
Shared human and animal antimicrobial classes
Third generation cephalosporins
Fourth generation cephalosporins
Fluoroquinolones
Macrolides
Quinolones
Polymyxins
Aminoglycosides
Aminopenicillins
Aminopenicillins with beta−lactamase inhibitors
Ansamycins
Phosphonic acid derivatives
Livestock species
Cattle
Chickens
Pigs
Sheep
Turkeys
Ducks
Horses
Buffaloes
Goats
Rank
0.00
0.25
0.50
0.75
1.00

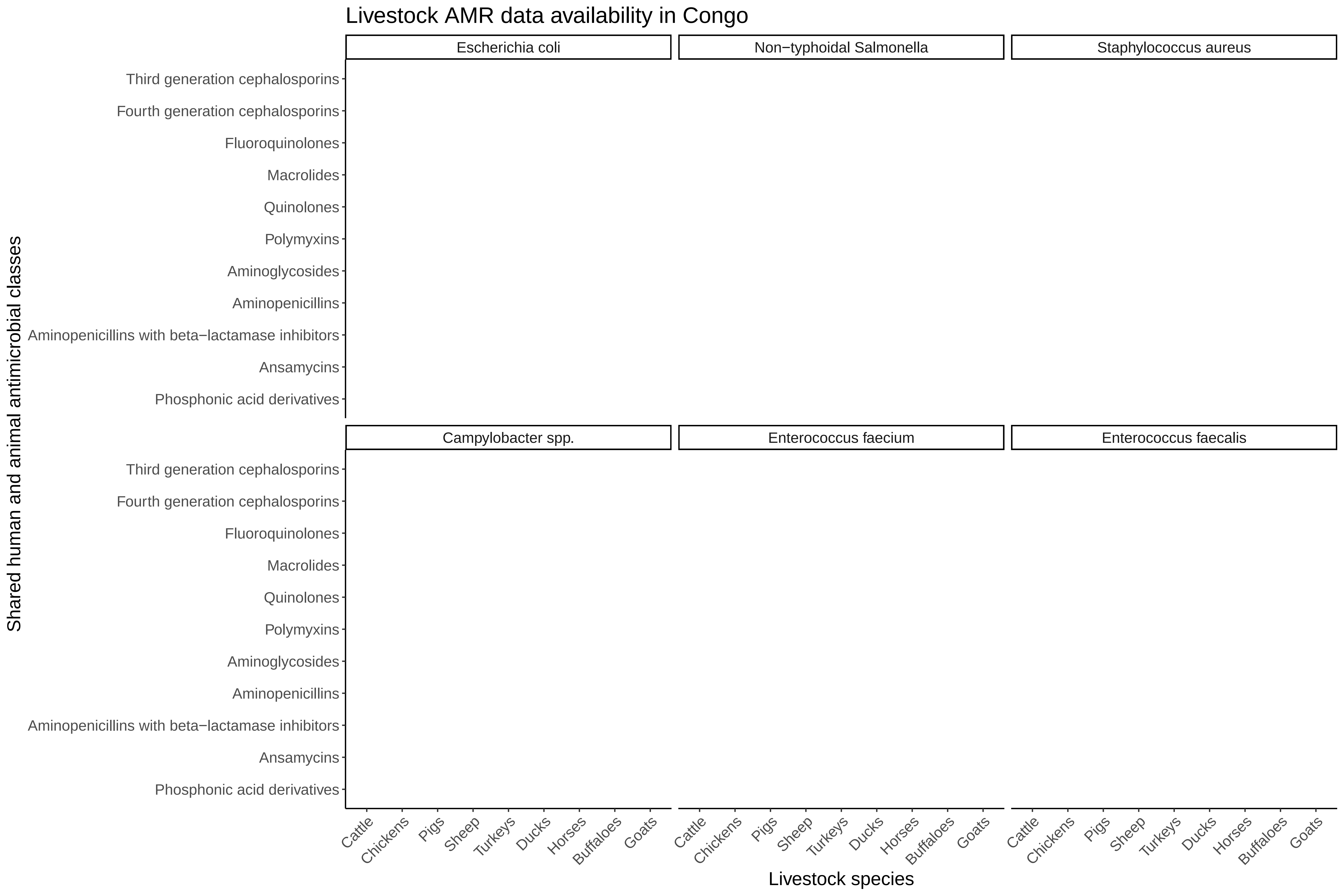
Livestock AMR data availability in Congo
Escherichia coli
Non−typhoidal Salmonella
Staphylococcus aureus
Campylobacter spp.
Enterococcus faecium
Enterococcus faecalis
Shared human and animal antimicrobial classes
Third generation cephalosporins
Fourth generation cephalosporins
Fluoroquinolones
Macrolides
Quinolones
Polymyxins
Aminoglycosides
Aminopenicillins
Aminopenicillins with beta−lactamase inhibitors
Ansamycins
Phosphonic acid derivatives
Cattle
Chickens
Pigs
Sheep
Turkeys
Ducks
Horses
Buffaloes
Goats
Livestock species

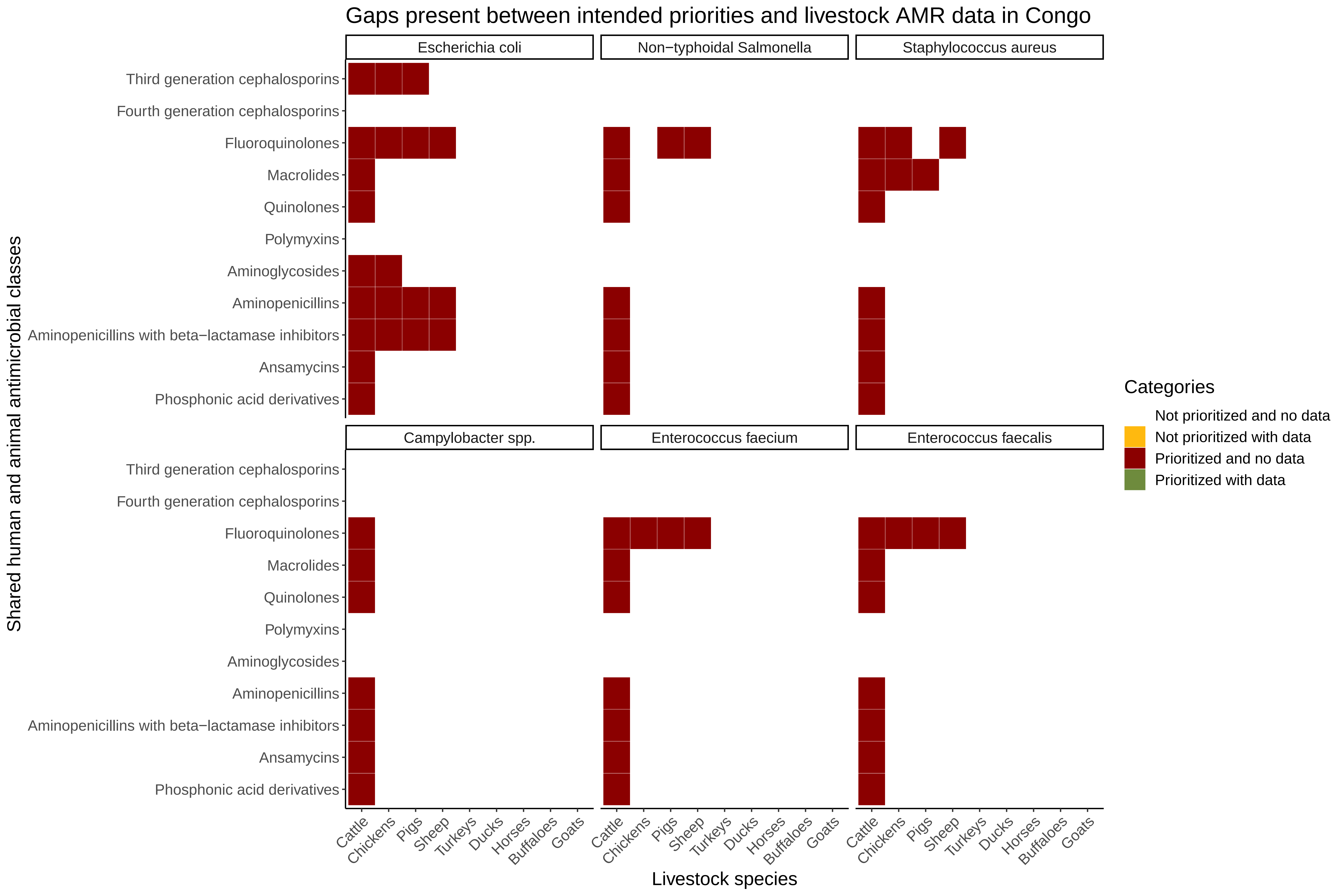
Gaps present between intended priorities and livestock AMR data in Congo
Escherichia coli
Non−typhoidal Salmonella
Staphylococcus aureus
Campylobacter spp.
Enterococcus faecium
Enterococcus faecalis
Shared human and animal antimicrobial classes
Third generation cephalosporins
Fourth generation cephalosporins
Fluoroquinolones
Macrolides
Quinolones
Polymyxins
Aminoglycosides
Aminopenicillins
Aminopenicillins with beta−lactamase inhibitors
Ansamycins
Phosphonic acid derivatives
Cattle
Chickens
Pigs
Sheep
Turkeys
Ducks
Horses
Buffaloes
Goats
Livestock species
Categories
Not prioritized and no data
Not prioritized with data
Prioritized and no data
Prioritized with data

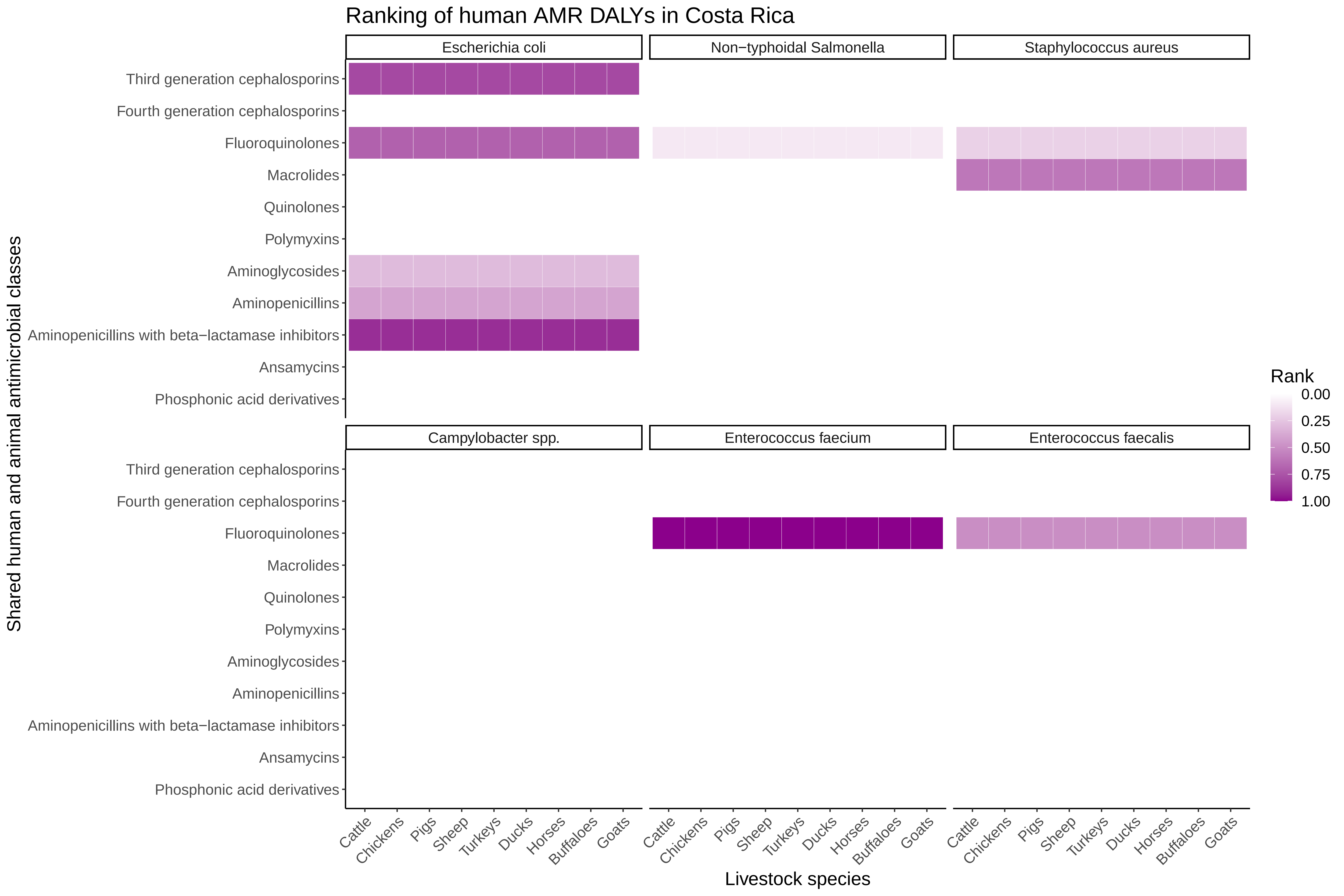

Ranking of human AMR DALYs in Costa Rica
Escherichia coli
Non−typhoidal Salmonella
Staphylococcus aureus
Campylobacter spp.
Enterococcus faecium
Enterococcus faecalis
Third generation cephalosporins
Fourth generation cephalosporins
Fluoroquinolones
Macrolides
Quinolones
Polymyxins
Aminoglycosides
Aminopenicillins
Aminopenicillins with beta−lactamase inhibitors
Ansamycins
Phosphonic acid derivatives
Shared human and animal antimicrobial classes
Cattle
Chickens
Pigs
Sheep
Turkeys
Ducks
Horses
Buffaloes
Goats
Livestock species
Rank
0.00
0.25
0.50
0.75
1.00

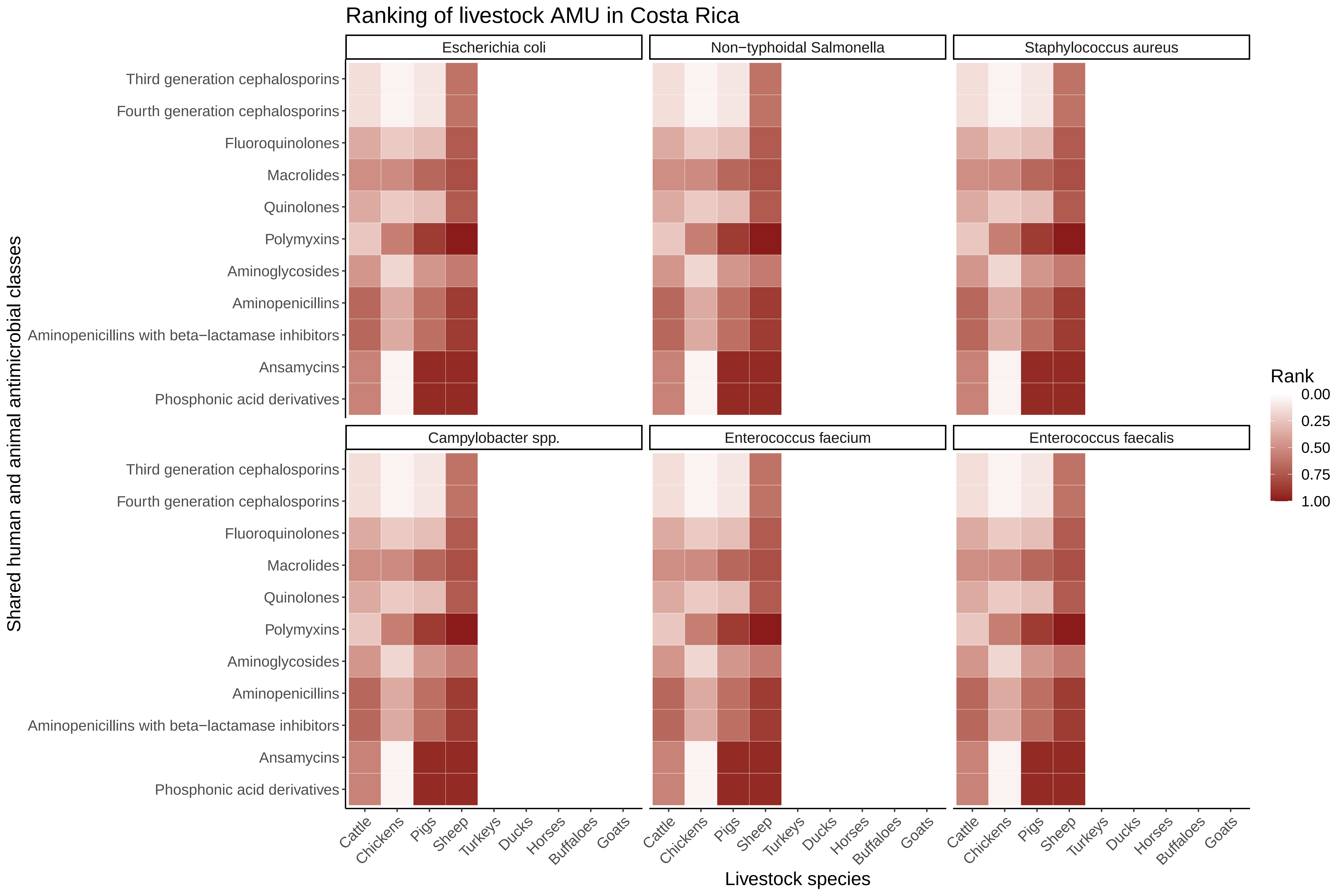

Ranking of livestock AMU in Costa Rica
Escherichia coli
Non−typhoidal Salmonella
Staphylococcus aureus
Campylobacter spp.
Enterococcus faecium
Enterococcus faecalis
Third generation cephalosporins
Fourth generation cephalosporins
Fluoroquinolones
Macrolides
Quinolones
Polymyxins
Aminoglycosides
Aminopenicillins
Aminopenicillins with beta−lactamase inhibitors
Ansamycins
Phosphonic acid derivatives
Shared human and animal antimicrobial classes
Cattle
Chickens
Pigs
Sheep
Turkeys
Ducks
Horses
Buffaloes
Goats
Livestock species
Rank
0.00
0.25
0.50
0.75
1.00

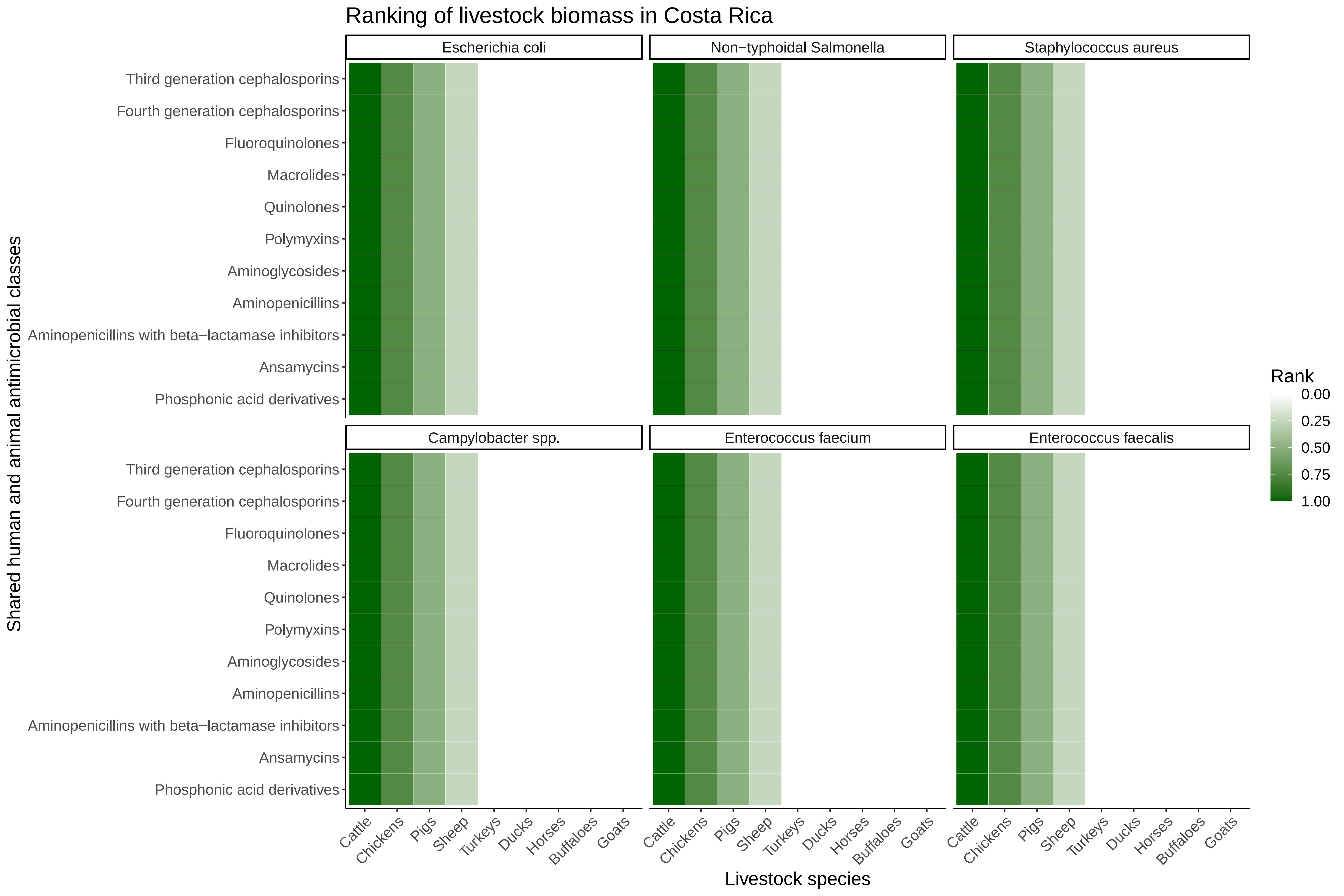
Ranking of livestock biomass in Costa Rica
Escherichia coli
Non−typhoidal Salmonella
Staphylococcus aureus
Campylobacter spp.
Enterococcus faecium
Enterococcus faecalis
Shared human and animal antimicrobial classes
Third generation cephalosporins
Fourth generation cephalosporins
Fluoroquinolones
Macrolides
Quinolones
Polymyxins
Aminoglycosides
Aminopenicillins
Aminopenicillins with beta−lactamase inhibitors
Ansamycins
Phosphonic acid derivatives
Livestock species
Cattle
Chickens
Pigs
Sheep
Turkeys
Ducks
Horses
Buffaloes
Goats
Rank
0.00
0.25
0.50
0.75
1.00

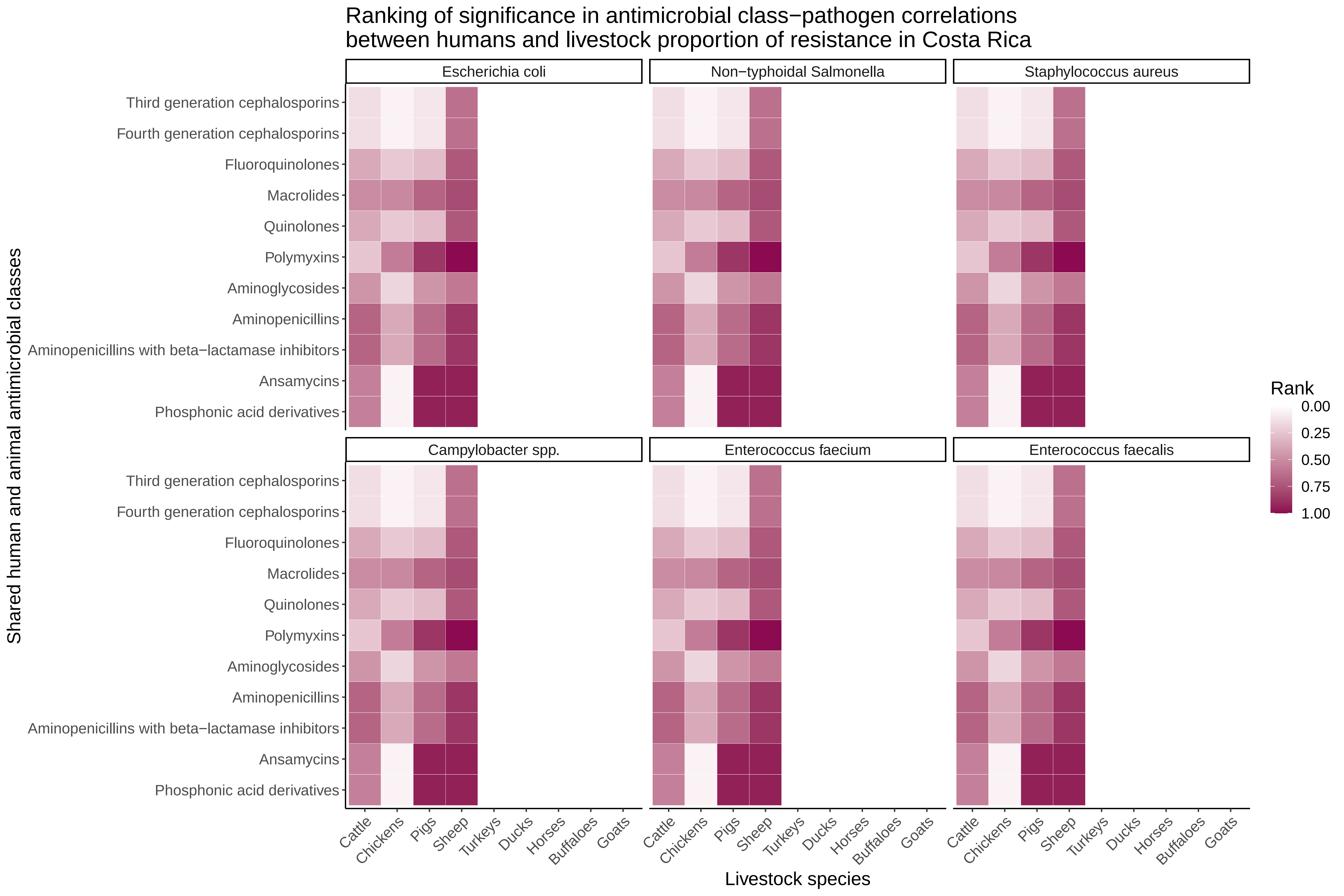

Ranking of significance in antimicrobial class−pathogen correlations
between humans and livestock proportion of resistance in Costa Rica
Escherichia coli
Non−typhoidal Salmonella
Staphylococcus aureus
Campylobacter spp.
Enterococcus faecium
Enterococcus faecalis
Third generation cephalosporins
Fourth generation cephalosporins
Fluoroquinolones
Macrolides
Quinolones
Polymyxins
Aminoglycosides
Aminopenicillins
Aminopenicillins with beta−lactamase inhibitors
Ansamycins
Phosphonic acid derivatives
Shared human and animal antimicrobial classes
Cattle
Chickens
Pigs
Sheep
Turkeys
Ducks
Horses
Buffaloes
Goats
Livestock species
Rank
0.00
0.25
0.50
0.75
1.00

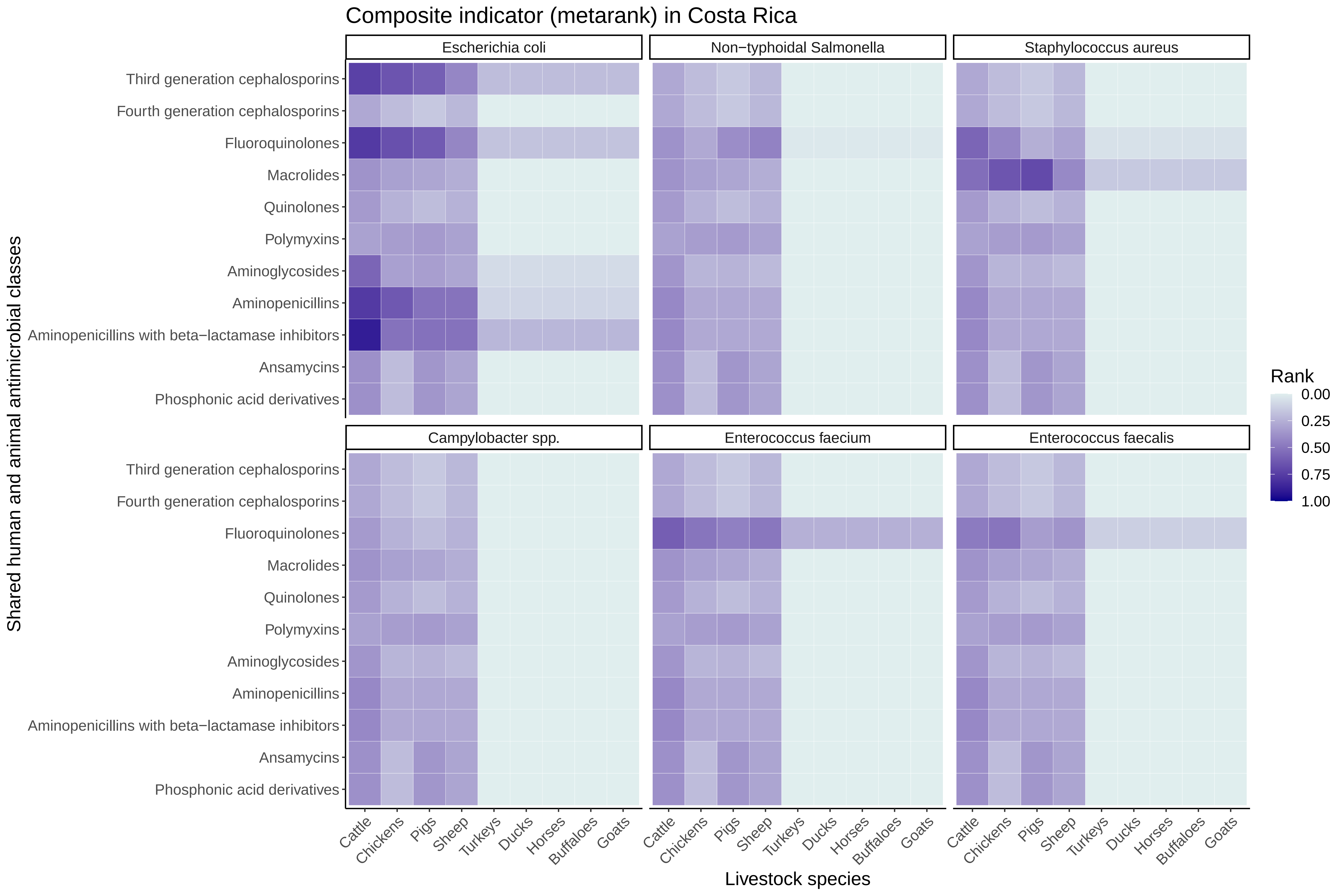
Composite indicator (metarank) in Costa Rica
Escherichia coli
Non−typhoidal Salmonella
Staphylococcus aureus
Campylobacter spp.
Enterococcus faecium
Enterococcus faecalis
Shared human and animal antimicrobial classes
Third generation cephalosporins
Fourth generation cephalosporins
Fluoroquinolones
Macrolides
Quinolones
Polymyxins
Aminoglycosides
Aminopenicillins
Aminopenicillins with beta−lactamase inhibitors
Ansamycins
Phosphonic acid derivatives
Cattle
Chickens
Pigs
Sheep
Turkeys
Ducks
Horses
Buffaloes
Goats
Livestock species
Rank
0.00
0.25
0.50
0.75
1.00

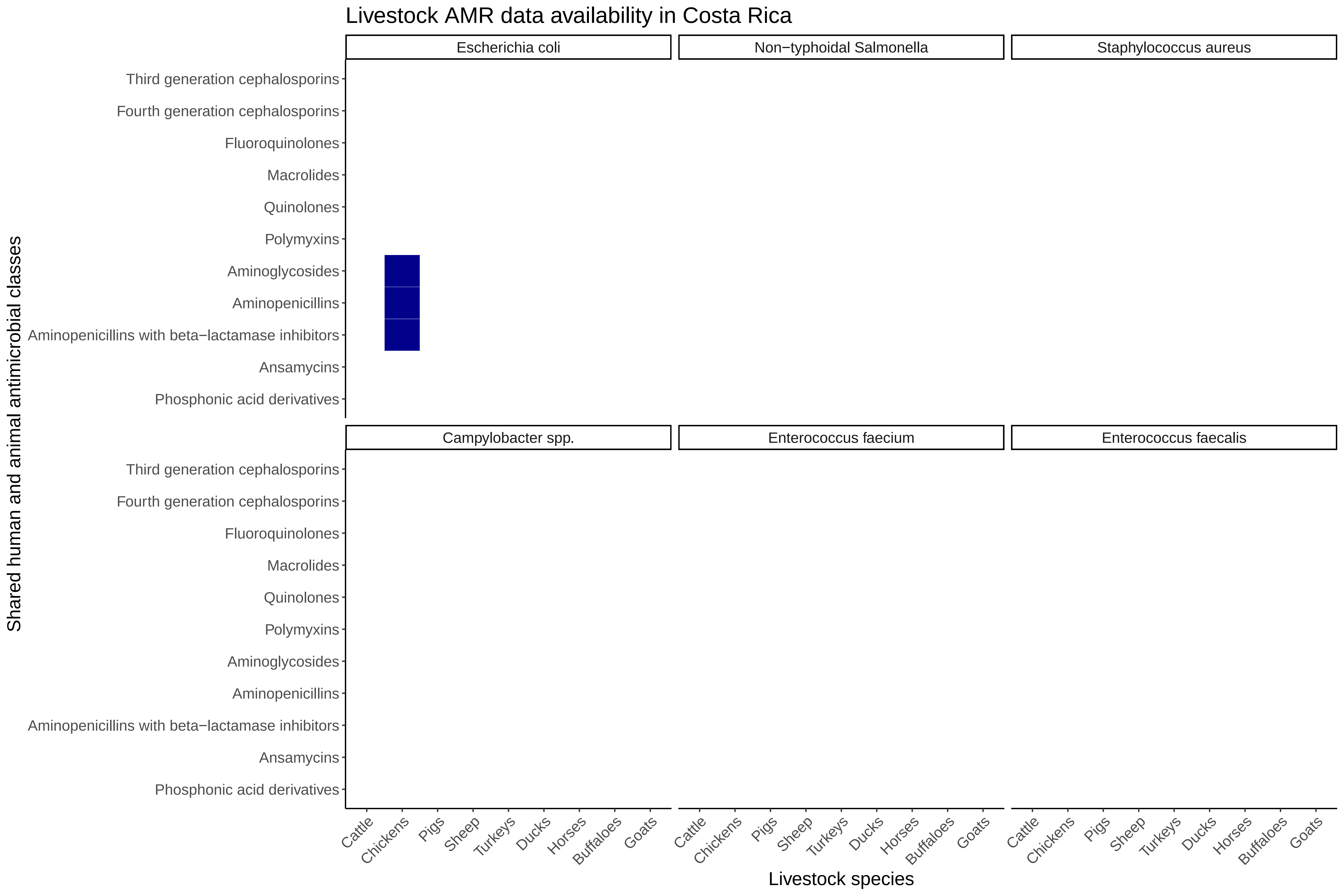

Livestock AMR data availability in Costa Rica
Escherichia coli
Non−typhoidal Salmonella
Staphylococcus aureus
Campylobacter spp.
Enterococcus faecium
Enterococcus faecalis
Shared human and animal antimicrobial classes
Third generation cephalosporins
Fourth generation cephalosporins
Fluoroquinolones
Macrolides
Quinolones
Polymyxins
Aminoglycosides
Aminopenicillins
Aminopenicillins with beta−lactamase inhibitors
Ansamycins
Phosphonic acid derivatives
Cattle
Chickens
Pigs
Sheep
Turkeys
Ducks
Horses
Buffaloes
Goats
Livestock species

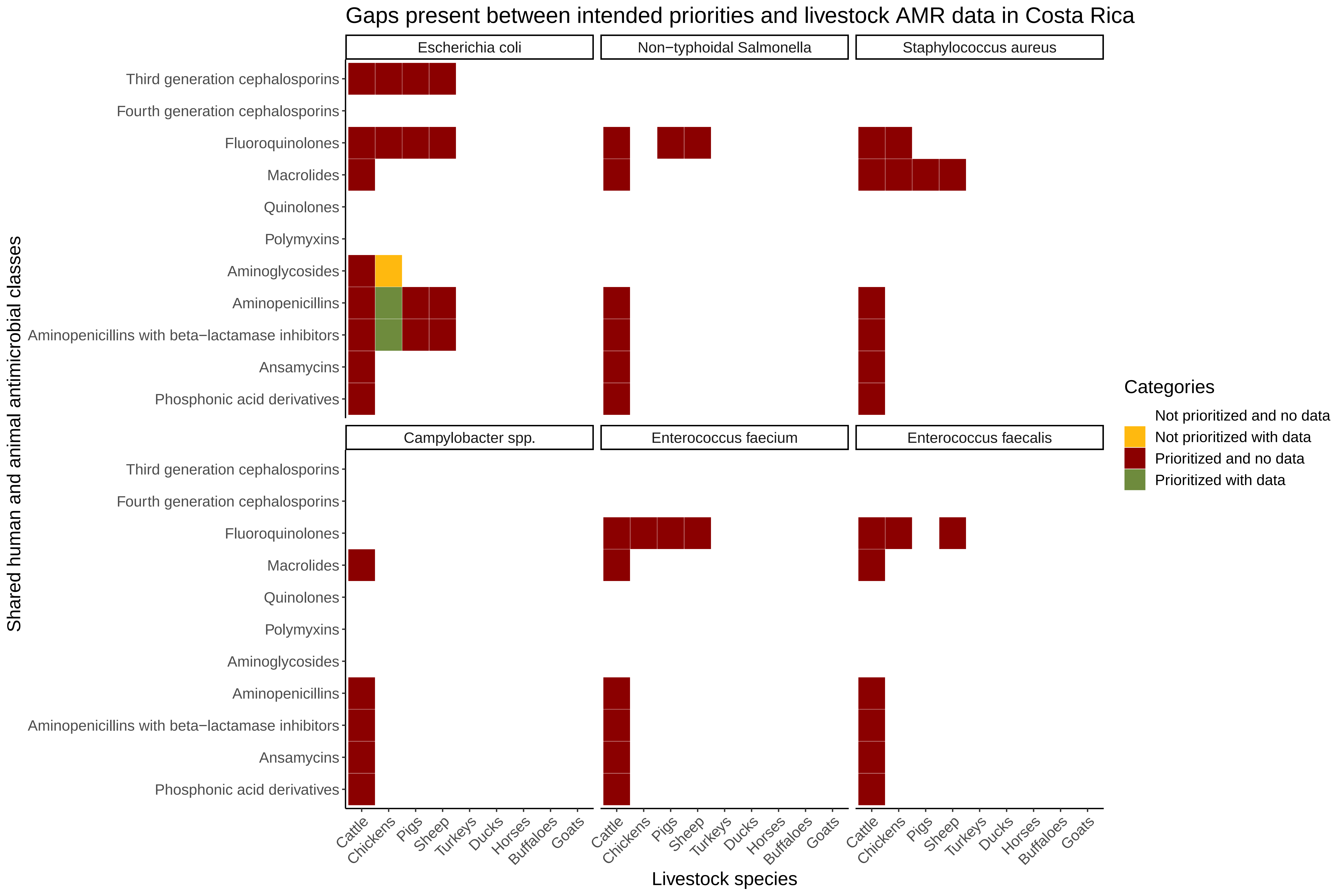

Gaps present between intended priorities and livestock AMR data in Costa Rica
Escherichia coli
Non−typhoidal Salmonella
Staphylococcus aureus
Campylobacter spp.
Enterococcus faecium
Enterococcus faecalis
Third generation cephalosporins
Fourth generation cephalosporins
Fluoroquinolones
Macrolides
Quinolones
Polymyxins
Aminoglycosides
Aminopenicillins
Aminopenicillins with beta−lactamase inhibitors
Ansamycins
Phosphonic acid derivatives
Shared human and animal antimicrobial classes
Cattle
Chickens
Pigs
Sheep
Turkeys
Ducks
Horses
Buffaloes
Goats
Livestock species
Categories
Not prioritized and no data
Not prioritized with data
Prioritized and no data
Prioritized with data

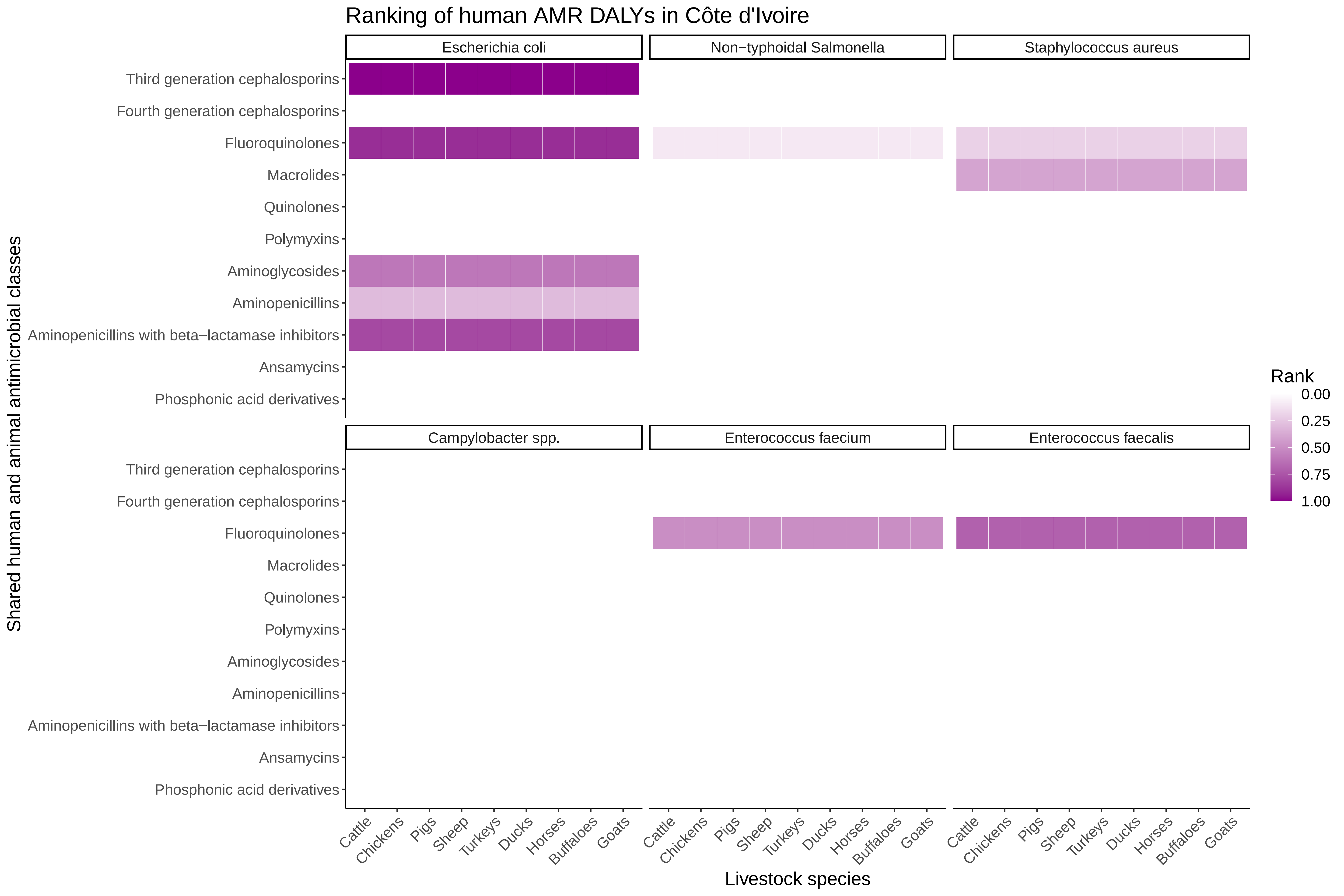
Ranking of human AMR DALYs in Côte d'Ivoire
Escherichia coli
Non−typhoidal Salmonella
Staphylococcus aureus
Campylobacter spp.
Enterococcus faecium
Enterococcus faecalis
Third generation cephalosporins
Fourth generation cephalosporins
Fluoroquinolones
Macrolides
Quinolones
Polymyxins
Aminoglycosides
Aminopenicillins
Aminopenicillins with beta−lactamase inhibitors
Ansamycins
Phosphonic acid derivatives
Shared human and animal antimicrobial classes
Cattle
Chickens
Pigs
Sheep
Turkeys
Ducks
Horses
Buffaloes
Goats
Livestock species
Rank
0.00
0.25
0.50
0.75
1.00

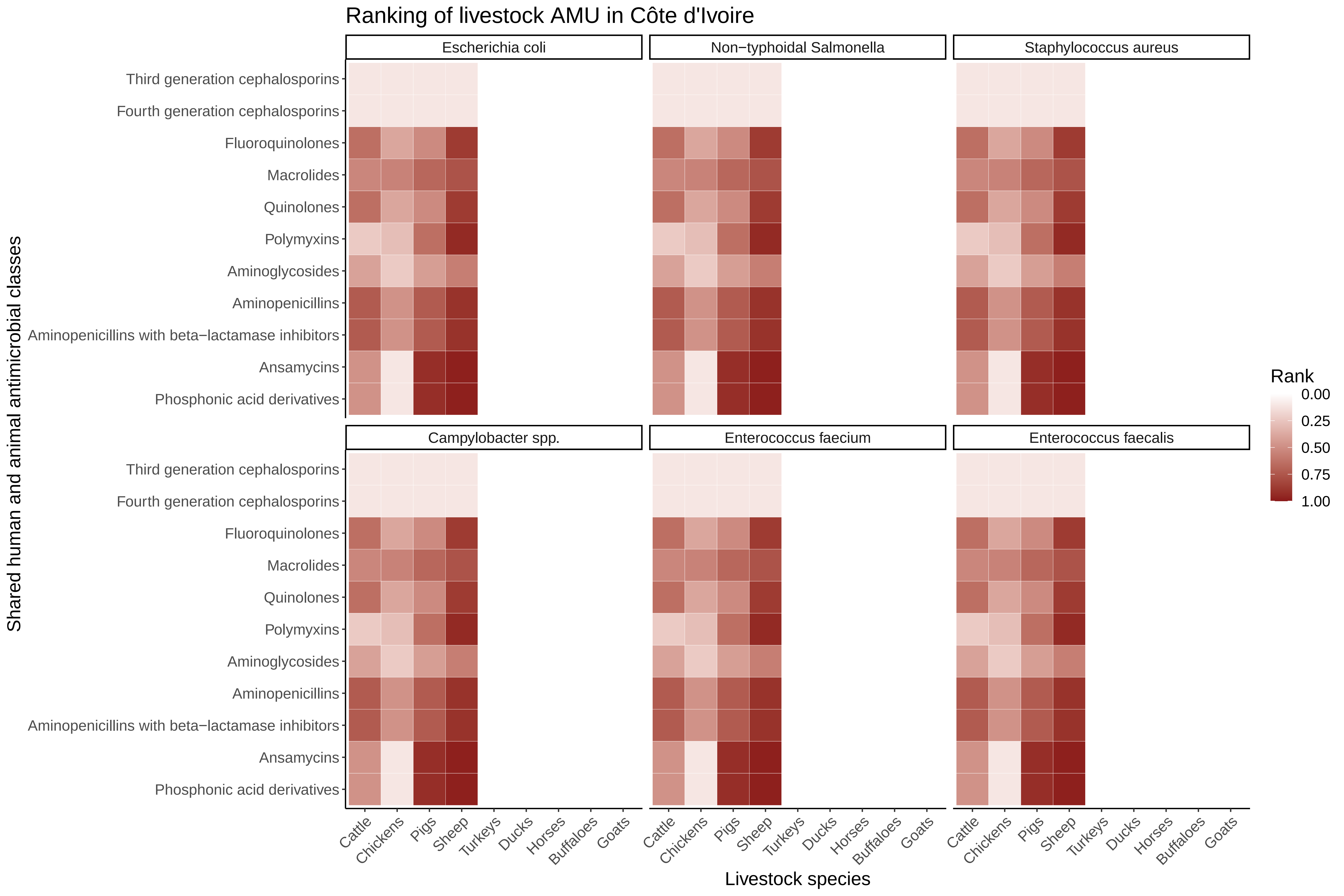
Ranking of livestock AMU in Côte d'Ivoire
Escherichia coli
Non−typhoidal Salmonella
Staphylococcus aureus
Campylobacter spp.
Enterococcus faecium
Enterococcus faecalis
Third generation cephalosporins
Fourth generation cephalosporins
Fluoroquinolones
Macrolides
Quinolones
Polymyxins
Aminoglycosides
Aminopenicillins
Aminopenicillins with beta−lactamase inhibitors
Ansamycins
Phosphonic acid derivatives
Shared human and animal antimicrobial classes
Cattle
Chickens
Pigs
Sheep
Turkeys
Ducks
Horses
Buffaloes
Goats
Livestock species
Rank
0.00
0.25
0.50
0.75
1.00

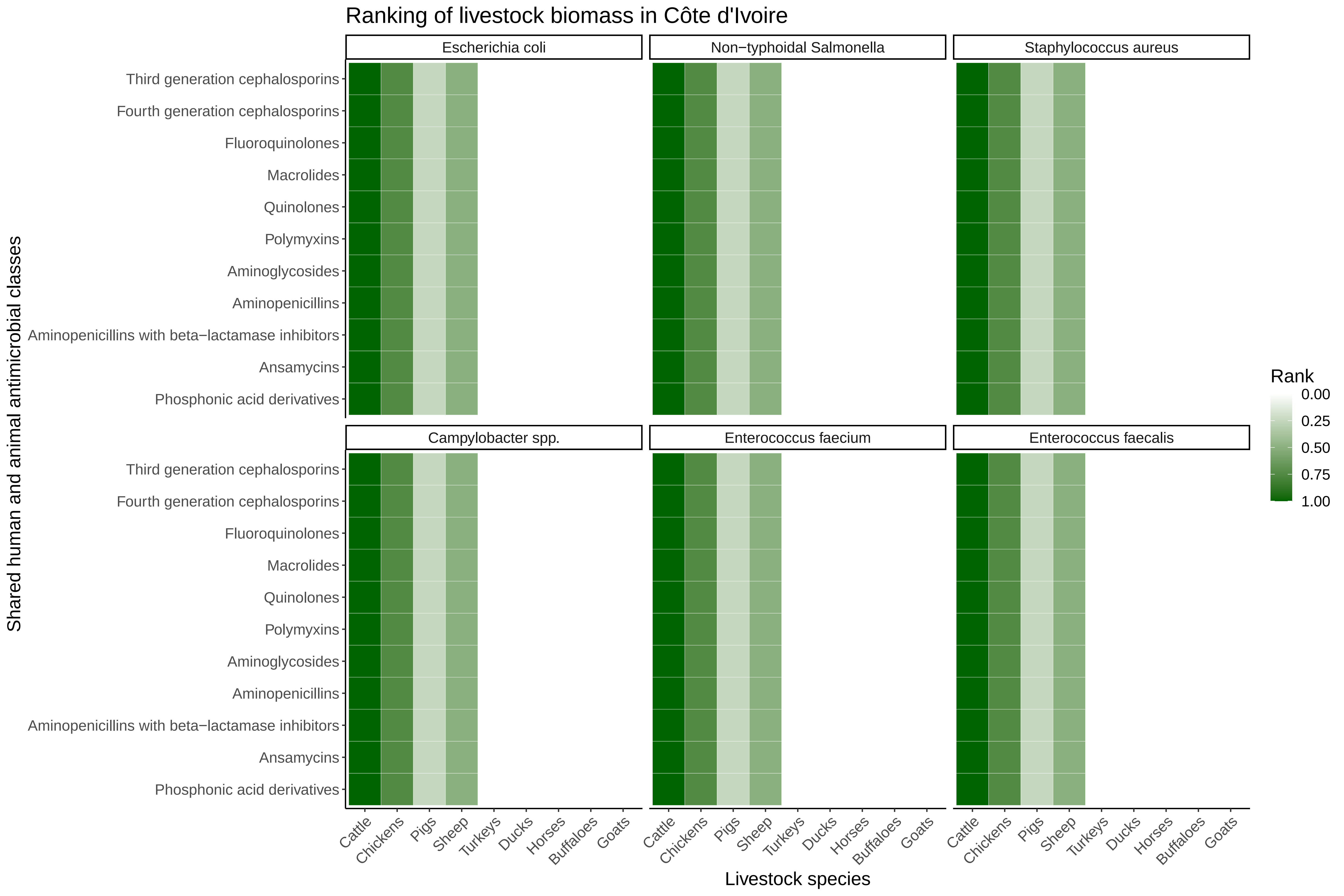

Ranking of livestock biomass in Côte d'Ivoire
Escherichia coli
Non−typhoidal Salmonella
Staphylococcus aureus
Campylobacter spp.
Enterococcus faecium
Enterococcus faecalis
Shared human and animal antimicrobial classes
Third generation cephalosporins
Fourth generation cephalosporins
Fluoroquinolones
Macrolides
Quinolones
Polymyxins
Aminoglycosides
Aminopenicillins
Aminopenicillins with beta−lactamase inhibitors
Ansamycins
Phosphonic acid derivatives
Livestock species
Cattle
Chickens
Pigs
Sheep
Turkeys
Ducks
Horses
Buffaloes
Goats
Rank
0.00
0.25
0.50
0.75
1.00

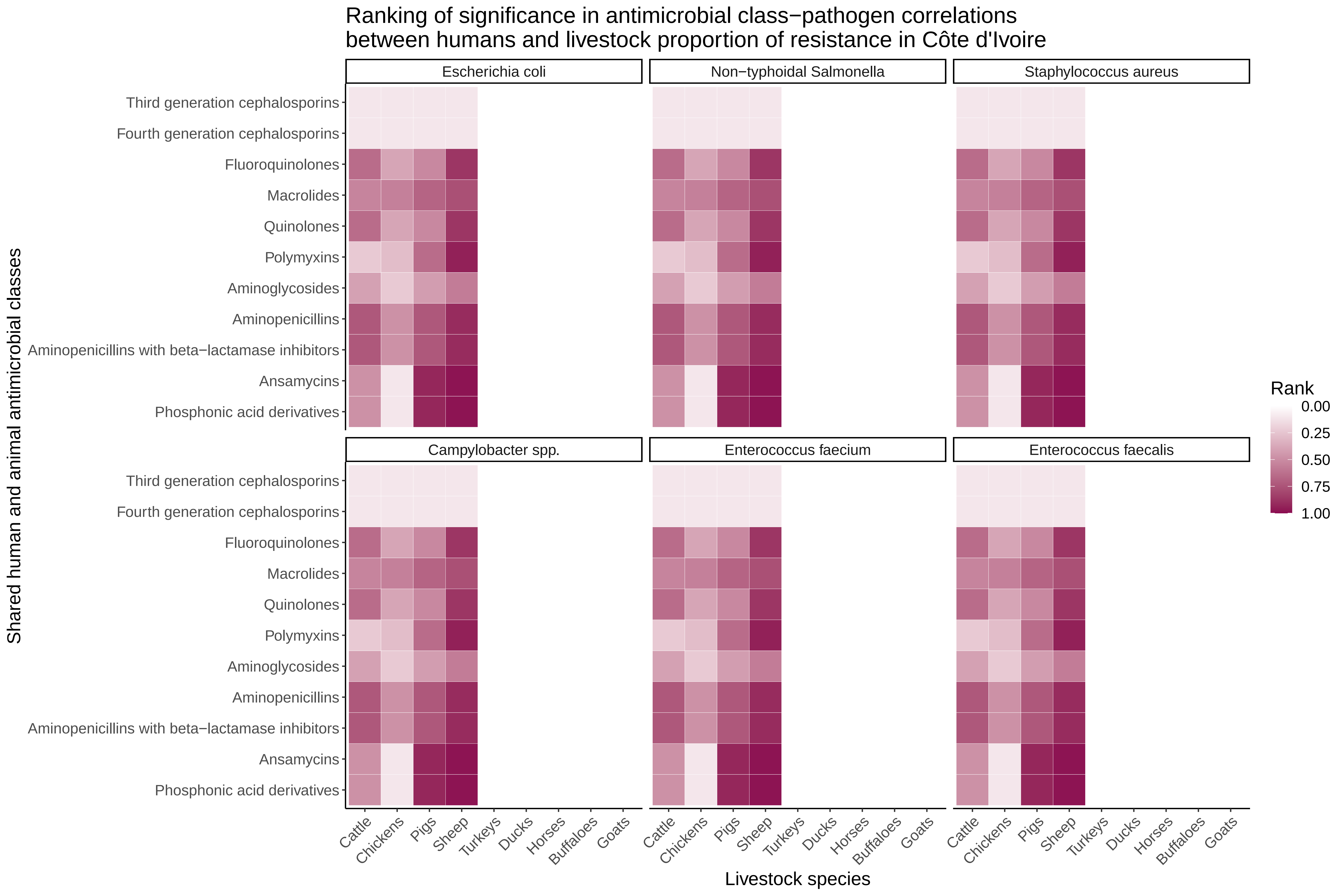
Ranking of significance in antimicrobial class−pathogen correlations
between humans and livestock proportion of resistance in Côte d'Ivoire
Escherichia coli
Non−typhoidal Salmonella
Staphylococcus aureus
Campylobacter spp.
Enterococcus faecium
Enterococcus faecalis
Shared human and animal antimicrobial classes
Third generation cephalosporins
Fourth generation cephalosporins
Fluoroquinolones
Macrolides
Quinolones
Polymyxins
Aminoglycosides
Aminopenicillins
Aminopenicillins with beta−lactamase inhibitors
Ansamycins
Phosphonic acid derivatives
Livestock species
Cattle
Chickens
Pigs
Sheep
Turkeys
Ducks
Horses
Buffaloes
Goats
Rank
0.00
0.25
0.50
0.75
1.00

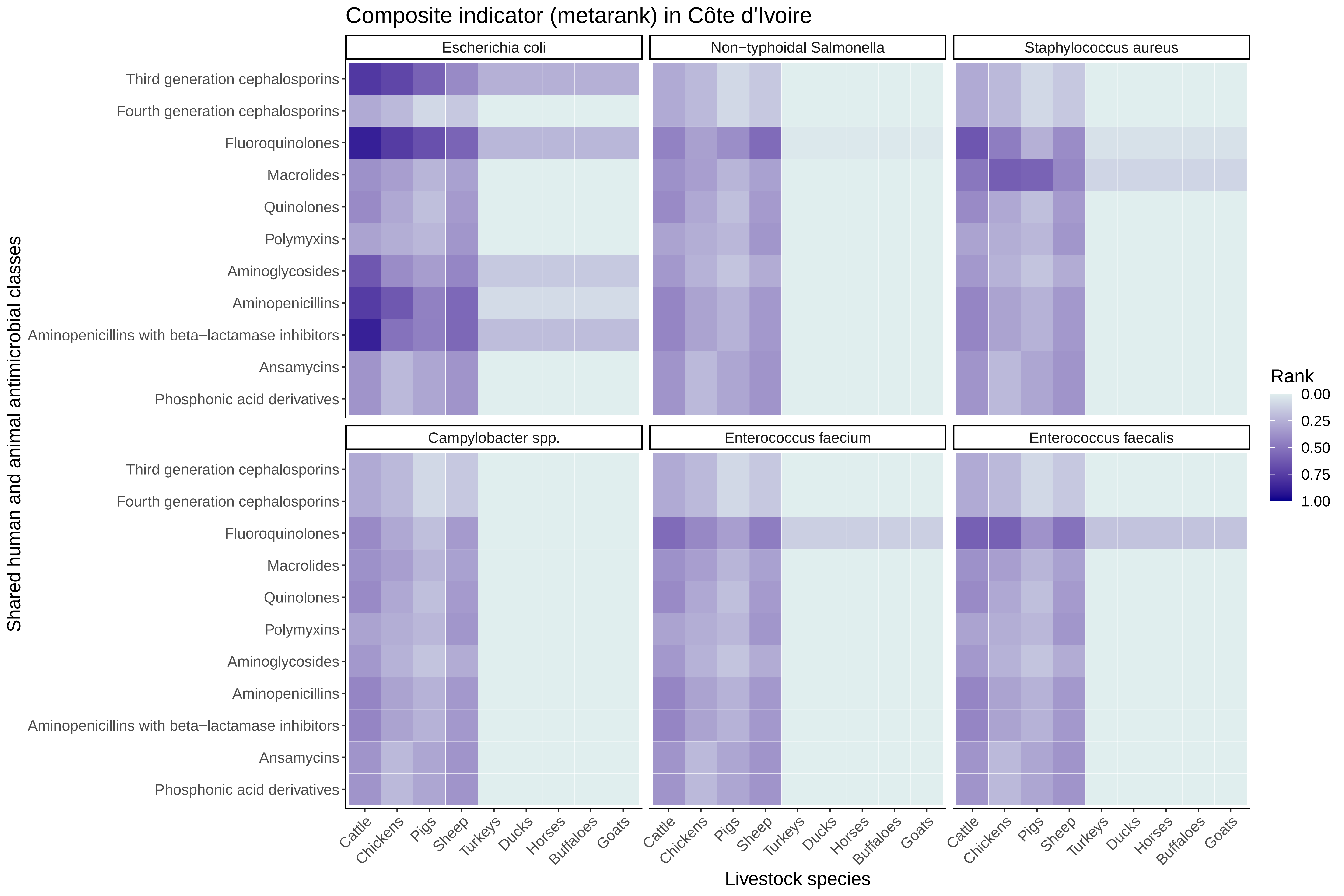
Composite indicator (metarank) in Côte d'Ivoire
Escherichia coli
Non−typhoidal Salmonella
Staphylococcus aureus
Campylobacter spp.
Enterococcus faecium
Enterococcus faecalis
Third generation cephalosporins
Fourth generation cephalosporins
Fluoroquinolones
Macrolides
Quinolones
Polymyxins
Aminoglycosides
Aminopenicillins
Aminopenicillins with beta−lactamase inhibitors
Ansamycins
Phosphonic acid derivatives
Shared human and animal antimicrobial classes
Cattle
Chickens
Pigs
Sheep
Turkeys
Ducks
Horses
Buffaloes
Goats
Livestock species
Rank
0.00
0.25
0.50
0.75
1.00

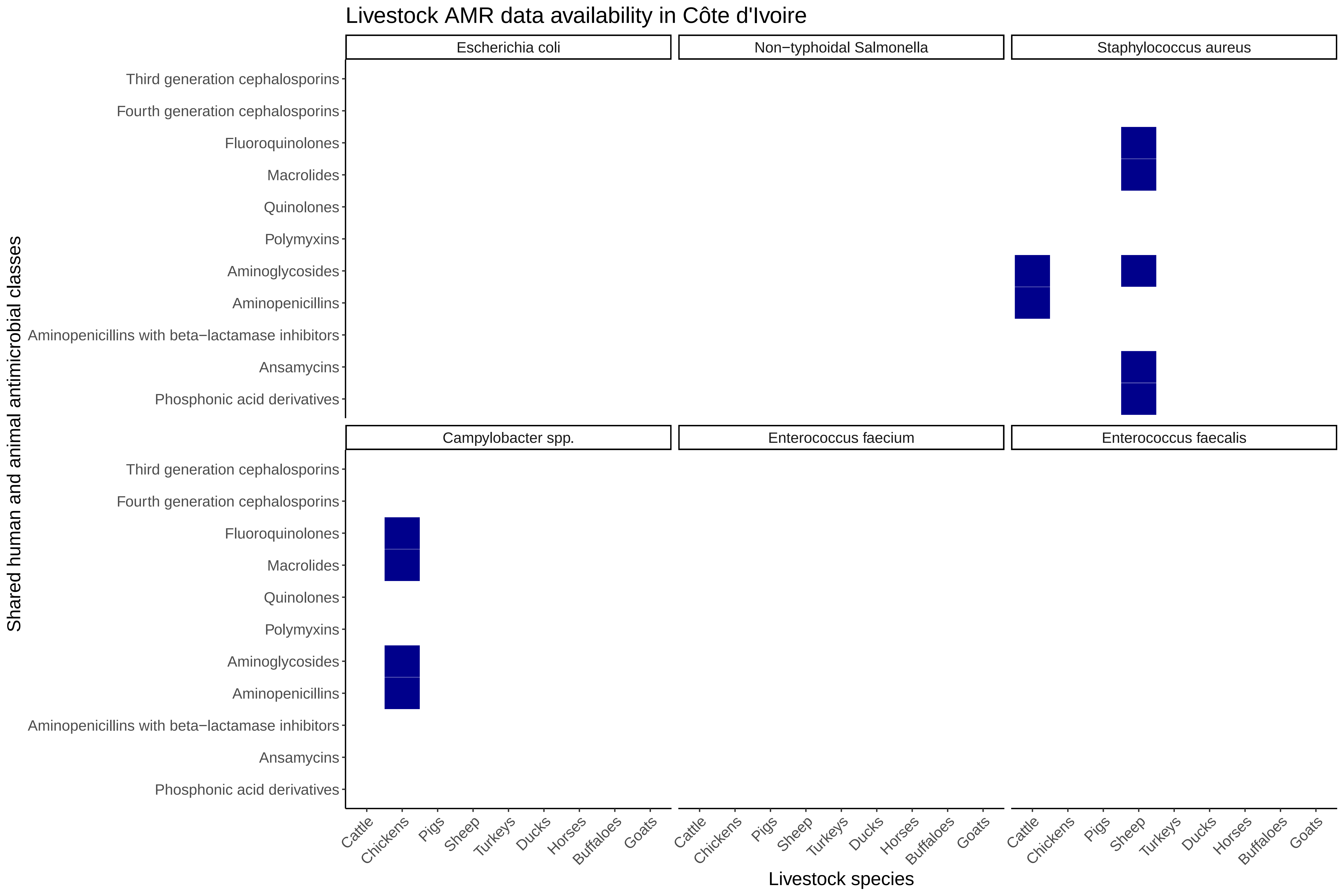
Livestock AMR data availability in Côte d'Ivoire
Escherichia coli
Non−typhoidal Salmonella
Staphylococcus aureus
Campylobacter spp.
Enterococcus faecium
Enterococcus faecalis
Shared human and animal antimicrobial classes
Third generation cephalosporins
Fourth generation cephalosporins
Fluoroquinolones
Macrolides
Quinolones
Polymyxins
Aminoglycosides
Aminopenicillins
Aminopenicillins with beta−lactamase inhibitors
Ansamycins
Phosphonic acid derivatives
Cattle
Chickens
Pigs
Sheep
Turkeys
Ducks
Horses
Buffaloes
Goats
Livestock species

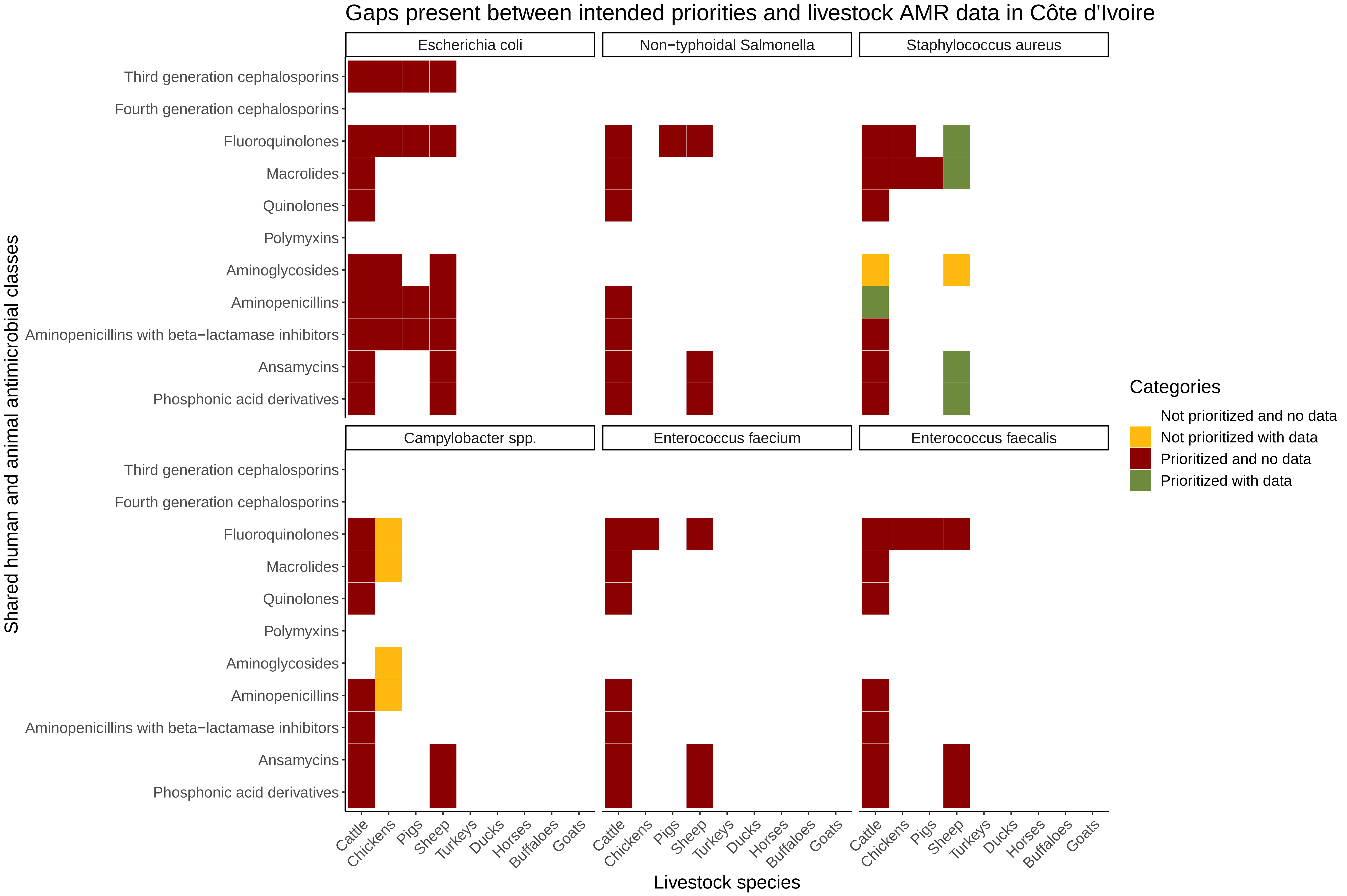

Gaps present between intended priorities and livestock AMR data in Côte d'Ivoire
Escherichia coli
Non−typhoidal Salmonella
Staphylococcus aureus
Campylobacter spp.
Enterococcus faecium
Enterococcus faecalis
Third generation cephalosporins
Fourth generation cephalosporins
Fluoroquinolones
Macrolides
Quinolones
Polymyxins
Aminoglycosides
Aminopenicillins
Aminopenicillins with beta−lactamase inhibitors
Ansamycins
Phosphonic acid derivatives
Shared human and animal antimicrobial classes
Cattle
Chickens
Pigs
Sheep
Turkeys
Ducks
Horses
Buffaloes
Goats
Livestock species
Categories
Not prioritized and no data
Not prioritized with data
Prioritized and no data
Prioritized with data

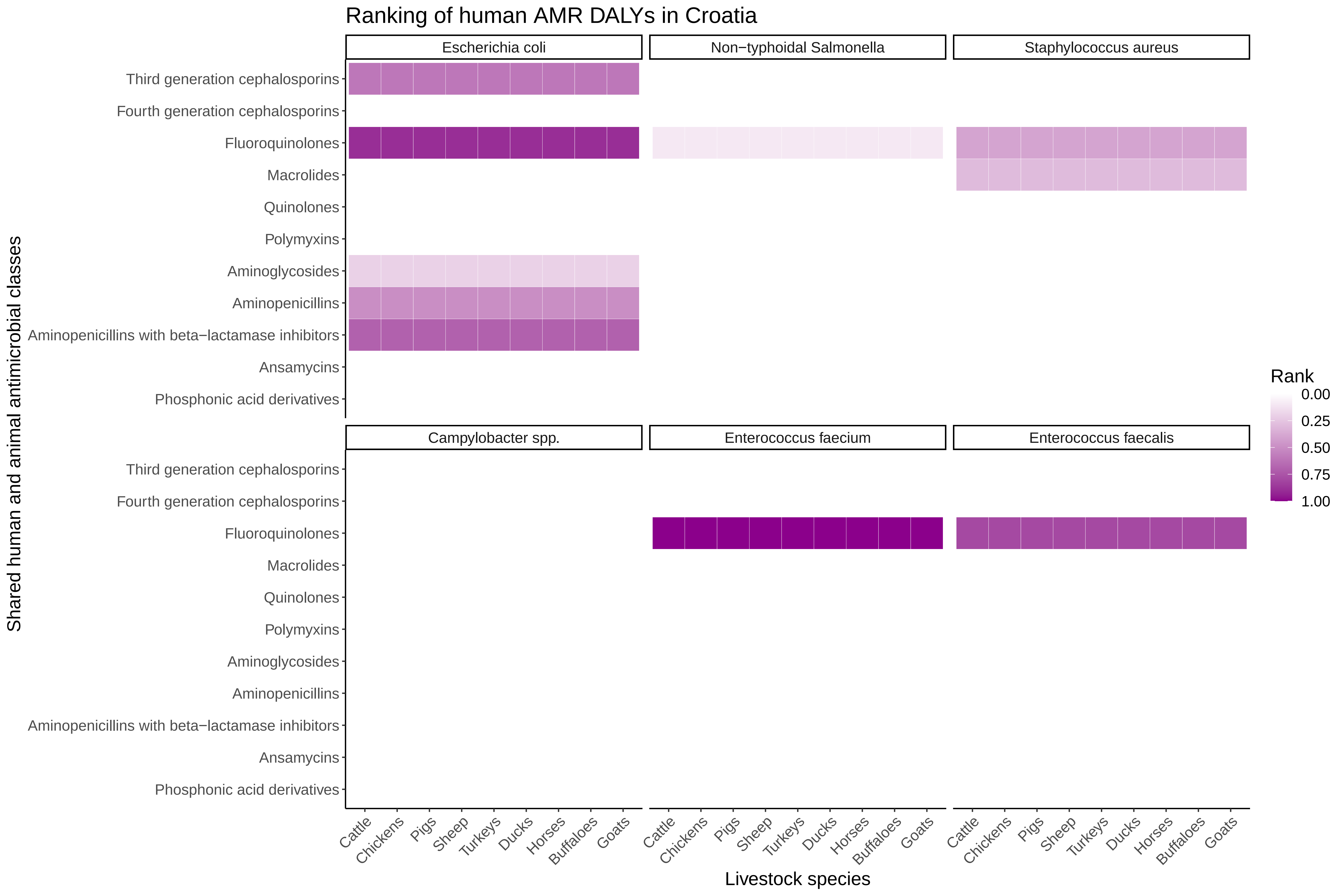

Ranking of human AMR DALYs in Croatia
Escherichia coli
Non−typhoidal Salmonella
Staphylococcus aureus
Campylobacter spp.
Enterococcus faecium
Enterococcus faecalis
Third generation cephalosporins
Fourth generation cephalosporins
Fluoroquinolones
Macrolides
Quinolones
Polymyxins
Aminoglycosides
Aminopenicillins
Aminopenicillins with beta−lactamase inhibitors
Ansamycins
Phosphonic acid derivatives
Shared human and animal antimicrobial classes
Cattle
Chickens
Pigs
Sheep
Turkeys
Ducks
Horses
Buffaloes
Goats
Livestock species
Rank
0.00
0.25
0.50
0.75
1.00

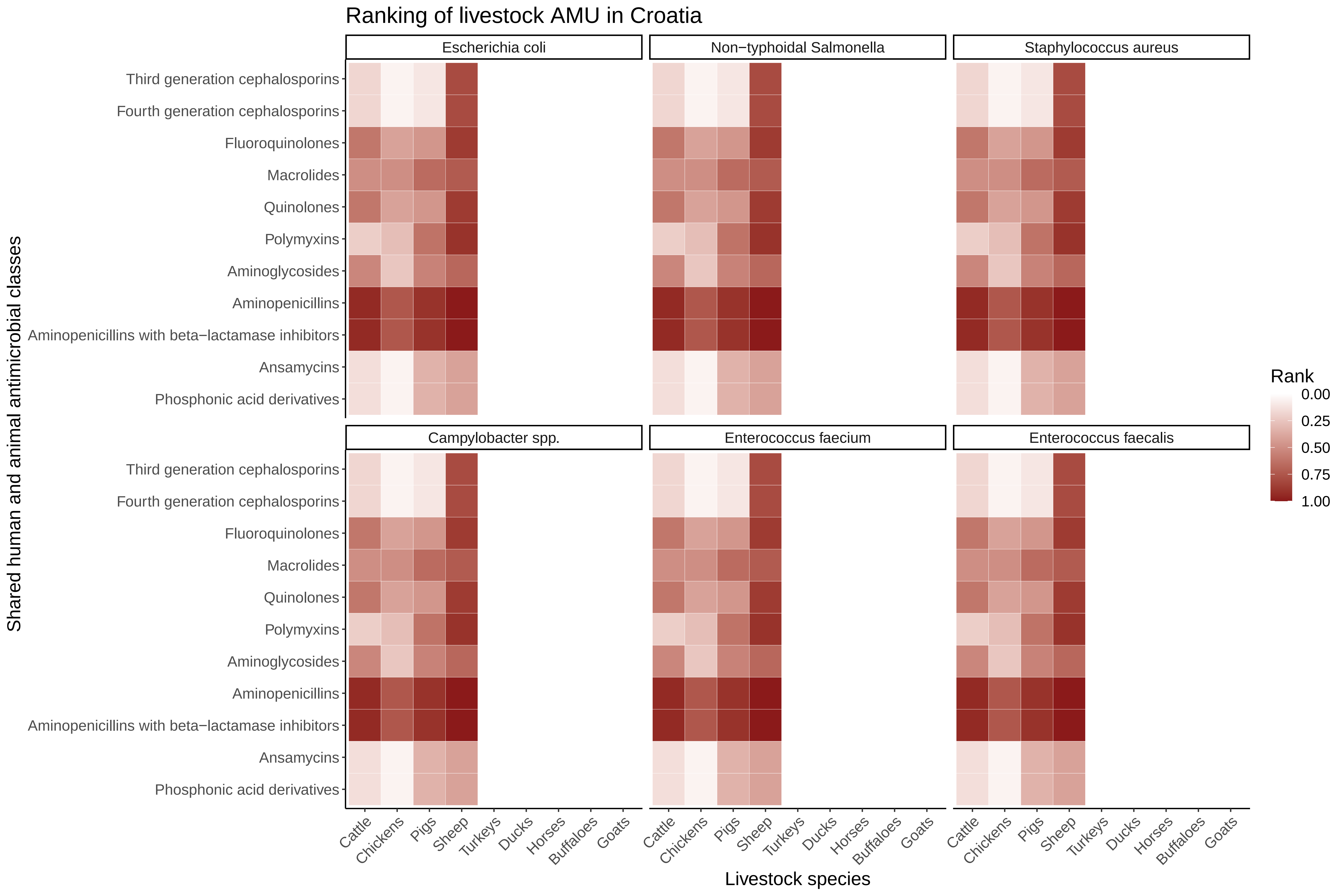

Ranking of livestock AMU in Croatia
Escherichia coli
Non−typhoidal Salmonella
Staphylococcus aureus
Campylobacter spp.
Enterococcus faecium
Enterococcus faecalis
Shared human and animal antimicrobial classes
Third generation cephalosporins
Fourth generation cephalosporins
Fluoroquinolones
Macrolides
Quinolones
Polymyxins
Aminoglycosides
Aminopenicillins
Aminopenicillins with beta−lactamase inhibitors
Ansamycins
Phosphonic acid derivatives
Cattle
Chickens
Pigs
Sheep
Turkeys
Ducks
Horses
Buffaloes
Goats
Livestock species
Rank
0.00
0.25
0.50
0.75
1.00

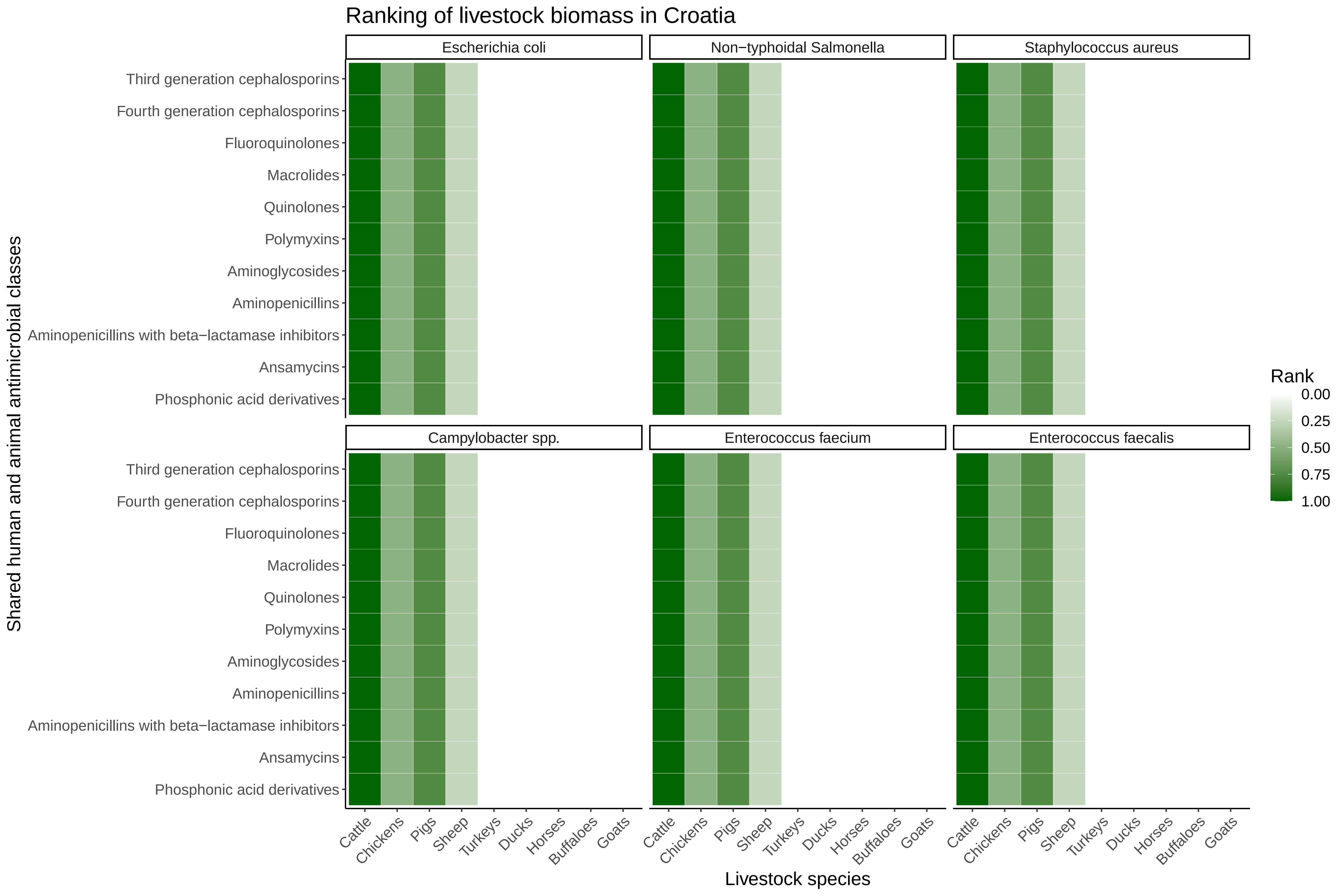
Ranking of livestock biomass in Croatia
Escherichia coli
Non−typhoidal Salmonella
Staphylococcus aureus
Campylobacter spp.
Enterococcus faecium
Enterococcus faecalis
Third generation cephalosporins
Fourth generation cephalosporins
Fluoroquinolones
Macrolides
Quinolones
Polymyxins
Aminoglycosides
Aminopenicillins
Aminopenicillins with beta−lactamase inhibitors
Ansamycins
Phosphonic acid derivatives
Shared human and animal antimicrobial classes
Cattle
Chickens
Pigs
Sheep
Turkeys
Ducks
Horses
Buffaloes
Goats
Livestock species
Rank
0.00
0.25
0.50
0.75
1.00

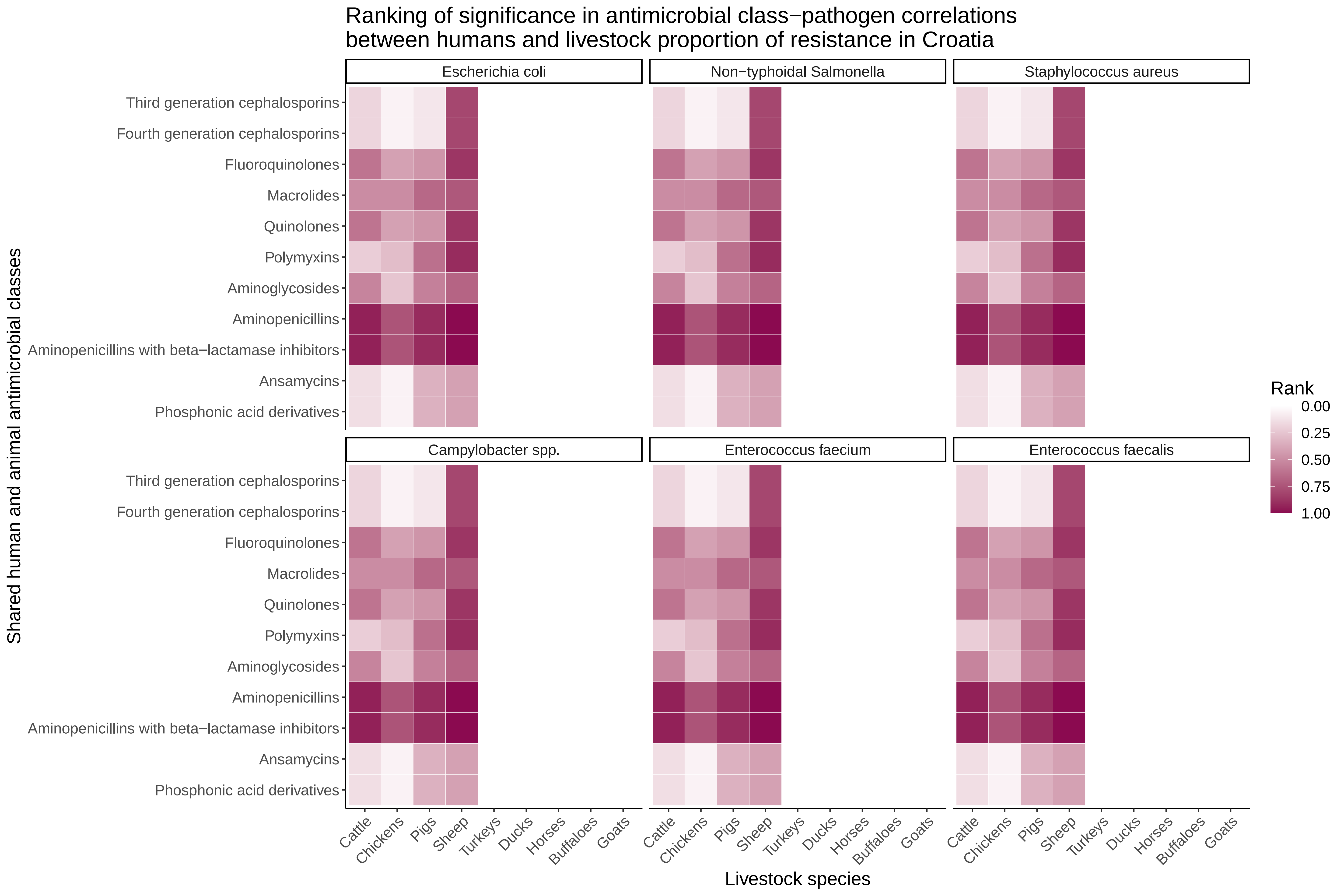
Ranking of significance in antimicrobial class−pathogen correlations
between humans and livestock proportion of resistance in Croatia
Escherichia coli
Non−typhoidal Salmonella
Staphylococcus aureus
Campylobacter spp.
Enterococcus faecium
Enterococcus faecalis
Third generation cephalosporins
Fourth generation cephalosporins
Fluoroquinolones
Macrolides
Quinolones
Polymyxins
Aminoglycosides
Aminopenicillins
Aminopenicillins with beta−lactamase inhibitors
Ansamycins
Phosphonic acid derivatives
Shared human and animal antimicrobial classes
Cattle
Chickens
Pigs
Sheep
Turkeys
Ducks
Horses
Buffaloes
Goats
Livestock species
Rank
0.00
0.25
0.50
0.75
1.00

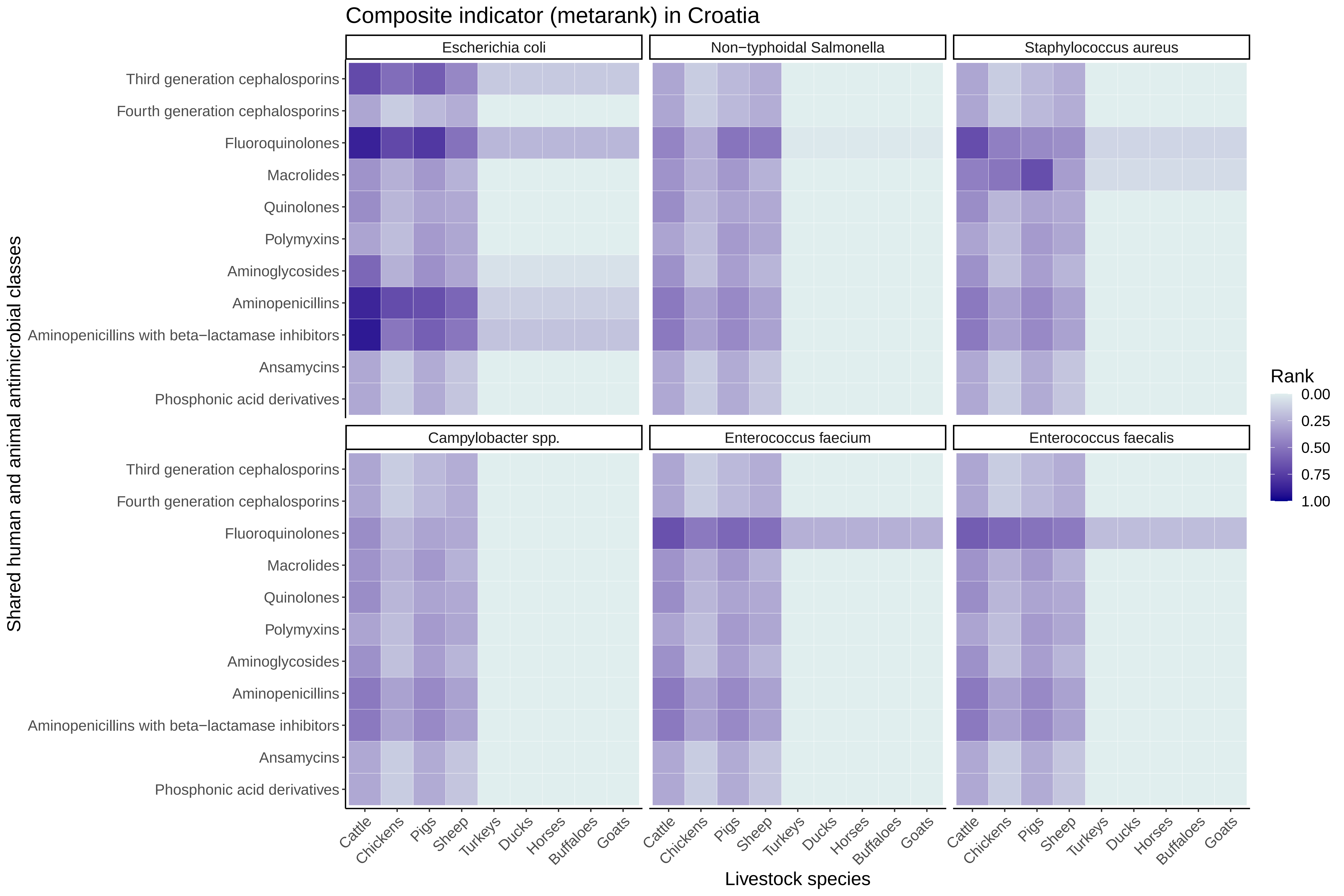

Composite indicator (metarank) in Croatia
Escherichia coli
Non−typhoidal Salmonella
Staphylococcus aureus
Campylobacter spp.
Enterococcus faecium
Enterococcus faecalis
Shared human and animal antimicrobial classes
Third generation cephalosporins
Fourth generation cephalosporins
Fluoroquinolones
Macrolides
Quinolones
Polymyxins
Aminoglycosides
Aminopenicillins
Aminopenicillins with beta−lactamase inhibitors
Ansamycins
Phosphonic acid derivatives
Cattle
Chickens
Pigs
Sheep
Turkeys
Ducks
Horses
Buffaloes
Goats
Livestock species
Rank
0.00
0.25
0.50
0.75
1.00

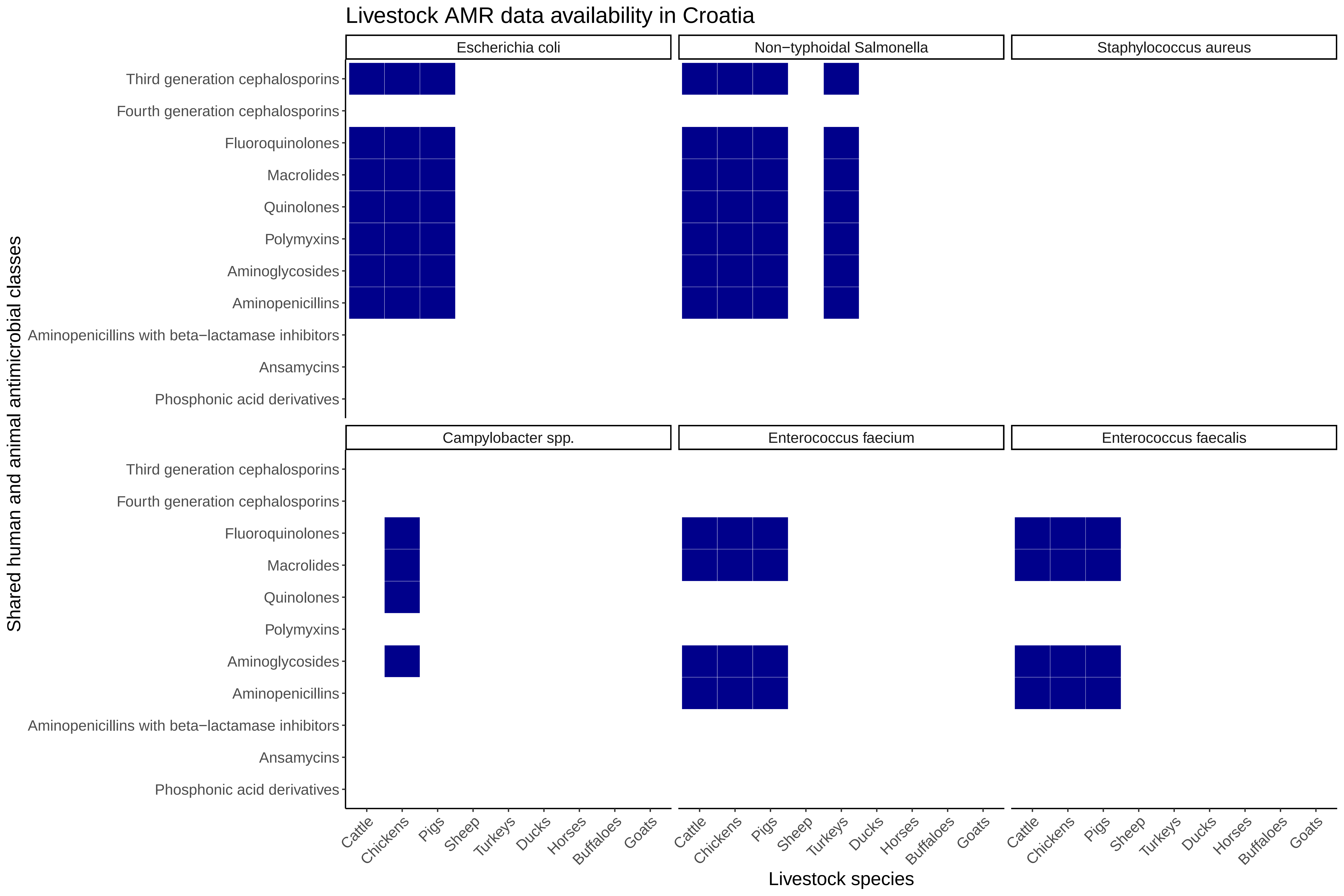

Livestock AMR data availability in Croatia
Escherichia coli
Non−typhoidal Salmonella
Staphylococcus aureus
Campylobacter spp.
Enterococcus faecium
Enterococcus faecalis
Shared human and animal antimicrobial classes
Third generation cephalosporins
Fourth generation cephalosporins
Fluoroquinolones
Macrolides
Quinolones
Polymyxins
Aminoglycosides
Aminopenicillins
Aminopenicillins with beta−lactamase inhibitors
Ansamycins
Phosphonic acid derivatives
Cattle
Chickens
Pigs
Sheep
Turkeys
Ducks
Horses
Buffaloes
Goats
Livestock species

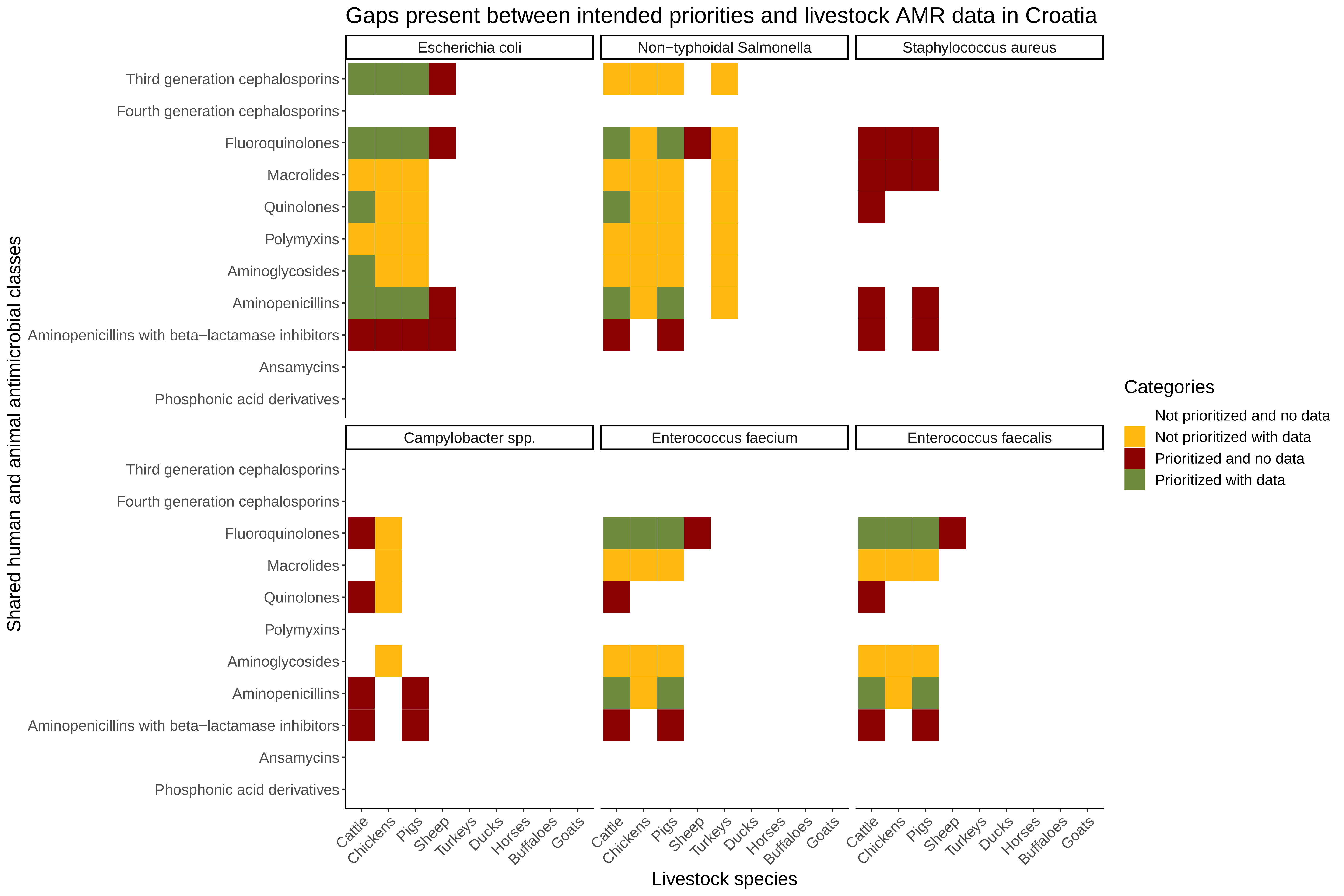
Gaps present between intended priorities and livestock AMR data in Croatia
Escherichia coli
Non−typhoidal Salmonella
Staphylococcus aureus
Campylobacter spp.
Enterococcus faecium
Enterococcus faecalis
Shared human and animal antimicrobial classes
Third generation cephalosporins
Fourth generation cephalosporins
Fluoroquinolones
Macrolides
Quinolones
Polymyxins
Aminoglycosides
Aminopenicillins
Aminopenicillins with beta−lactamase inhibitors
Ansamycins
Phosphonic acid derivatives
Cattle
Chickens
Pigs
Sheep
Turkeys
Ducks
Horses
Buffaloes
Goats
Livestock species
Categories
Not prioritized and no data
Not prioritized with data
Prioritized and no data
Prioritized with data

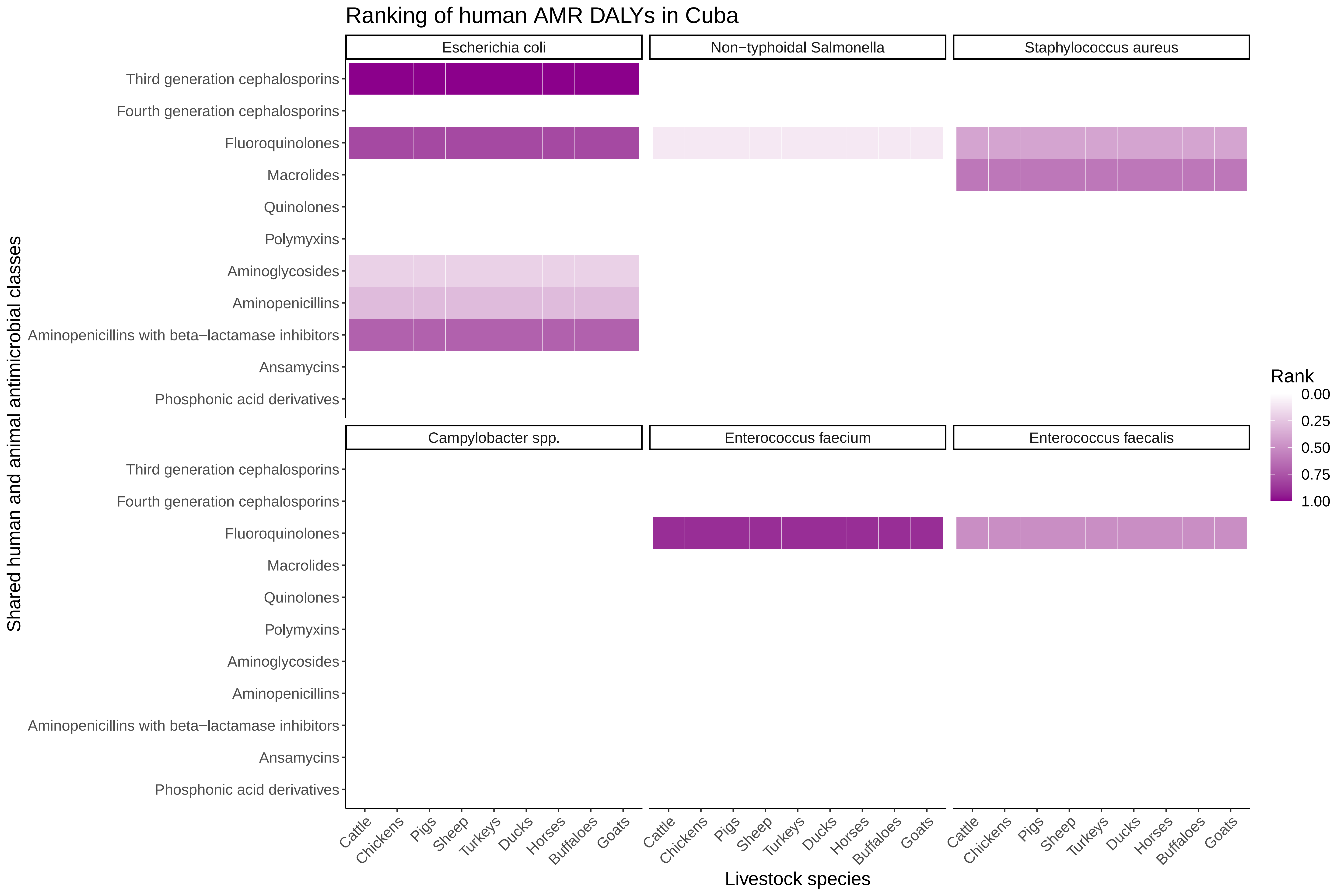

Ranking of human AMR DALYs in Cuba
Escherichia coli
Non−typhoidal Salmonella
Staphylococcus aureus
Campylobacter spp.
Enterococcus faecium
Enterococcus faecalis
Shared human and animal antimicrobial classes
Third generation cephalosporins
Fourth generation cephalosporins
Fluoroquinolones
Macrolides
Quinolones
Polymyxins
Aminoglycosides
Aminopenicillins
Aminopenicillins with beta−lactamase inhibitors
Ansamycins
Phosphonic acid derivatives
Livestock species
Cattle
Chickens
Pigs
Sheep
Turkeys
Ducks
Horses
Buffaloes
Goats
Rank
0.00
0.25
0.50
0.75
1.00

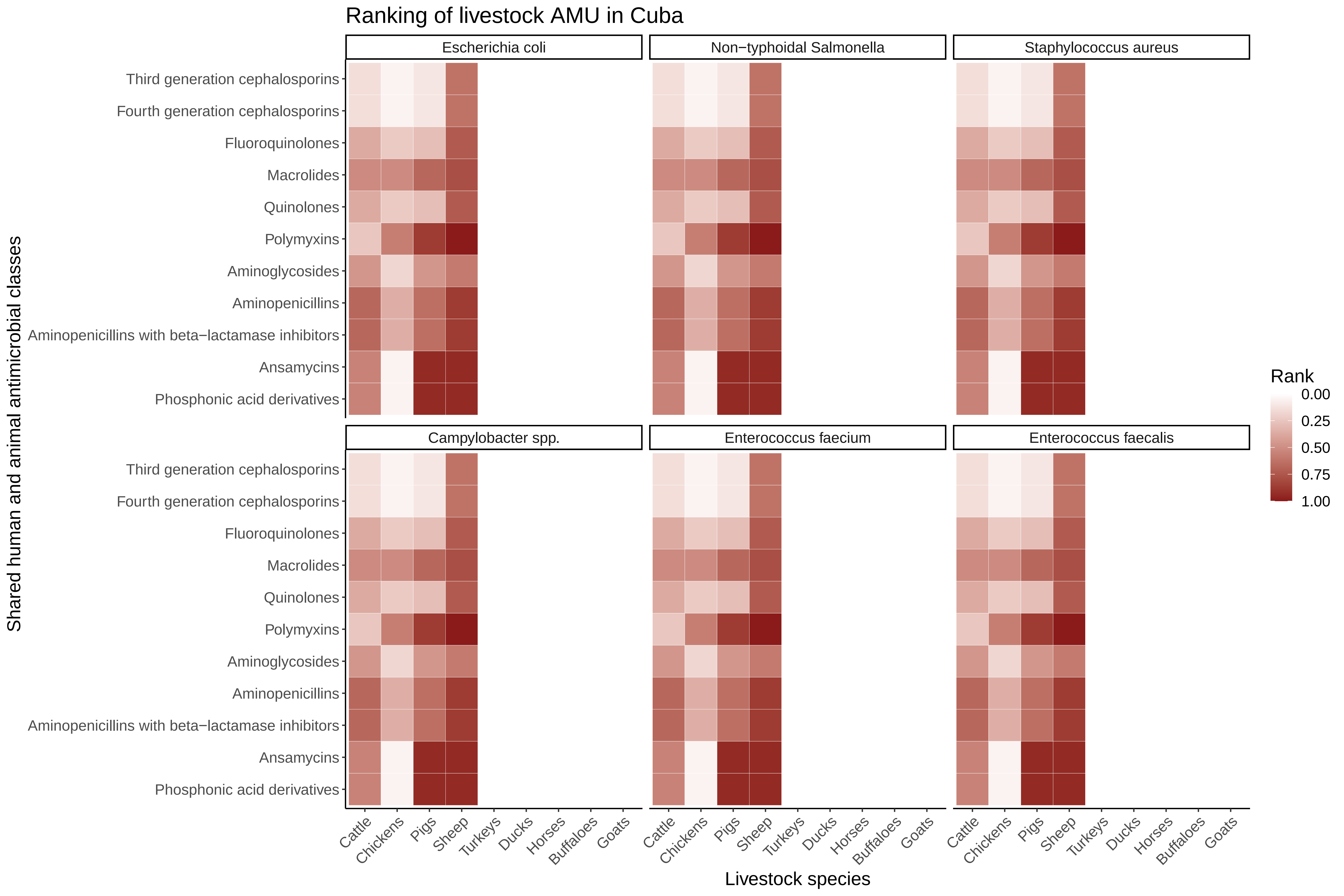
Ranking of livestock AMU in Cuba
Escherichia coli
Non−typhoidal Salmonella
Staphylococcus aureus
Campylobacter spp.
Enterococcus faecium
Enterococcus faecalis
Third generation cephalosporins
Fourth generation cephalosporins
Fluoroquinolones
Macrolides
Quinolones
Polymyxins
Aminoglycosides
Aminopenicillins
Aminopenicillins with beta−lactamase inhibitors
Ansamycins
Phosphonic acid derivatives
Shared human and animal antimicrobial classes
Cattle
Chickens
Pigs
Sheep
Turkeys
Ducks
Horses
Buffaloes
Goats
Livestock species
Rank
0.00
0.25
0.50
0.75
1.00

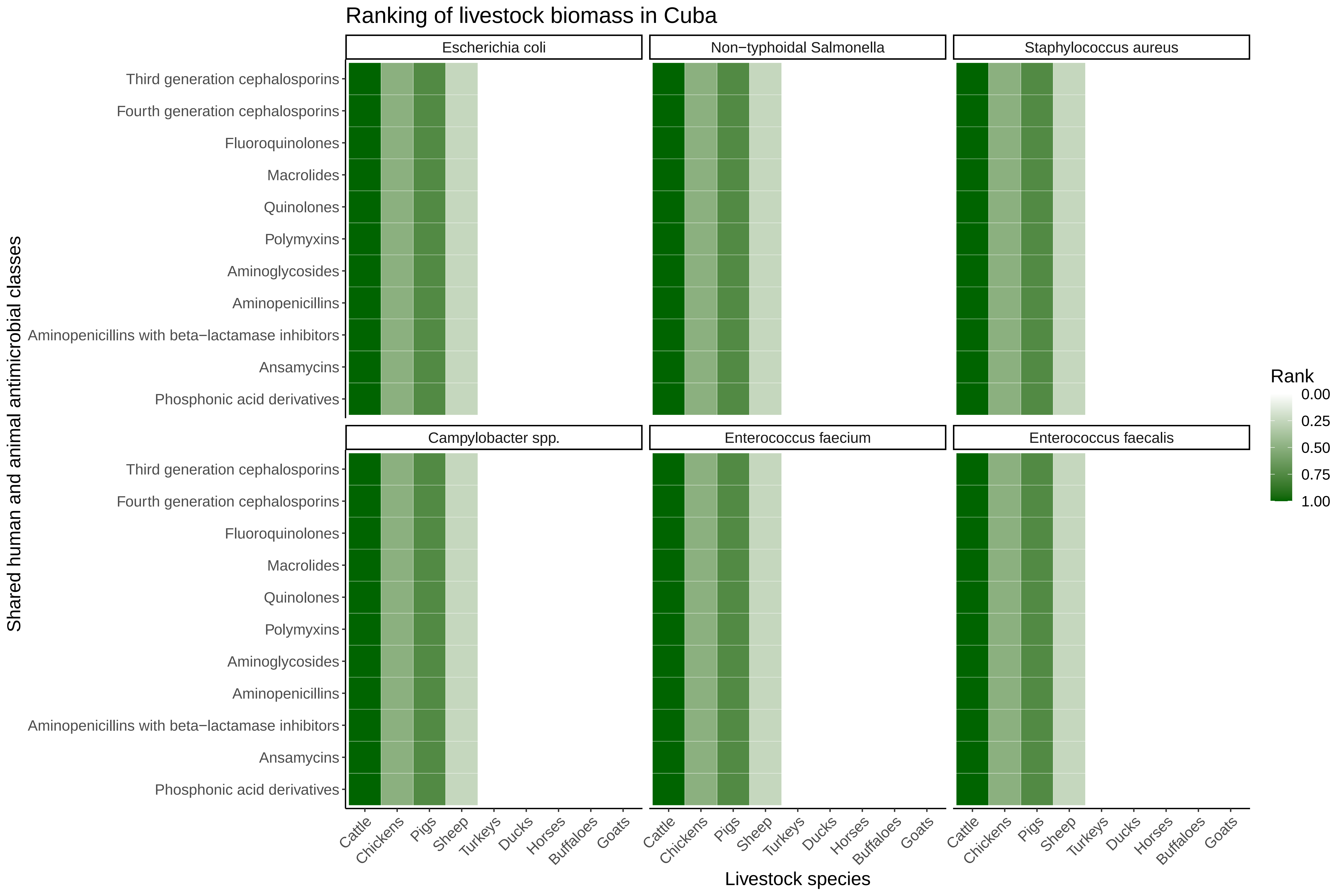

Ranking of livestock biomass in Cuba
Escherichia coli
Non−typhoidal Salmonella
Staphylococcus aureus
Campylobacter spp.
Enterococcus faecium
Enterococcus faecalis
Third generation cephalosporins
Fourth generation cephalosporins
Fluoroquinolones
Macrolides
Quinolones
Polymyxins
Aminoglycosides
Aminopenicillins
Aminopenicillins with beta−lactamase inhibitors
Ansamycins
Phosphonic acid derivatives
Shared human and animal antimicrobial classes
Cattle
Chickens
Pigs
Sheep
Turkeys
Ducks
Horses
Buffaloes
Goats
Livestock species
Rank
0.00
0.25
0.50
0.75
1.00

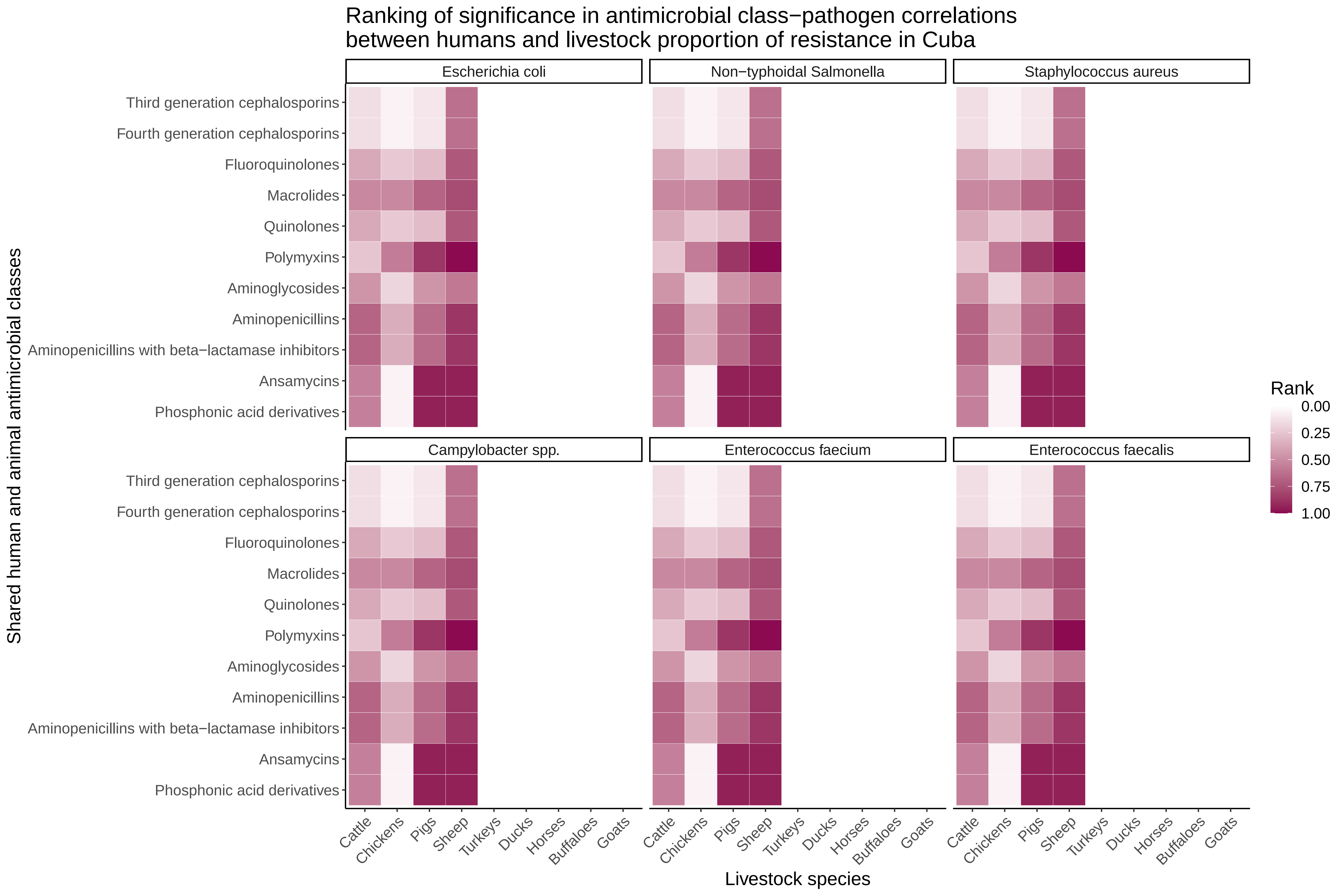

Ranking of significance in antimicrobial class−pathogen correlations
between humans and livestock proportion of resistance in Cuba
Escherichia coli
Non−typhoidal Salmonella
Staphylococcus aureus
Campylobacter spp.
Enterococcus faecium
Enterococcus faecalis
Shared human and animal antimicrobial classes
Third generation cephalosporins
Fourth generation cephalosporins
Fluoroquinolones
Macrolides
Quinolones
Polymyxins
Aminoglycosides
Aminopenicillins
Aminopenicillins with beta−lactamase inhibitors
Ansamycins
Phosphonic acid derivatives
Rank
0.00
0.25
0.50
0.75
1.00
Cattle
Chickens
Pigs
Sheep
Turkeys
Ducks
Horses
Buffaloes
Goats
Livestock species

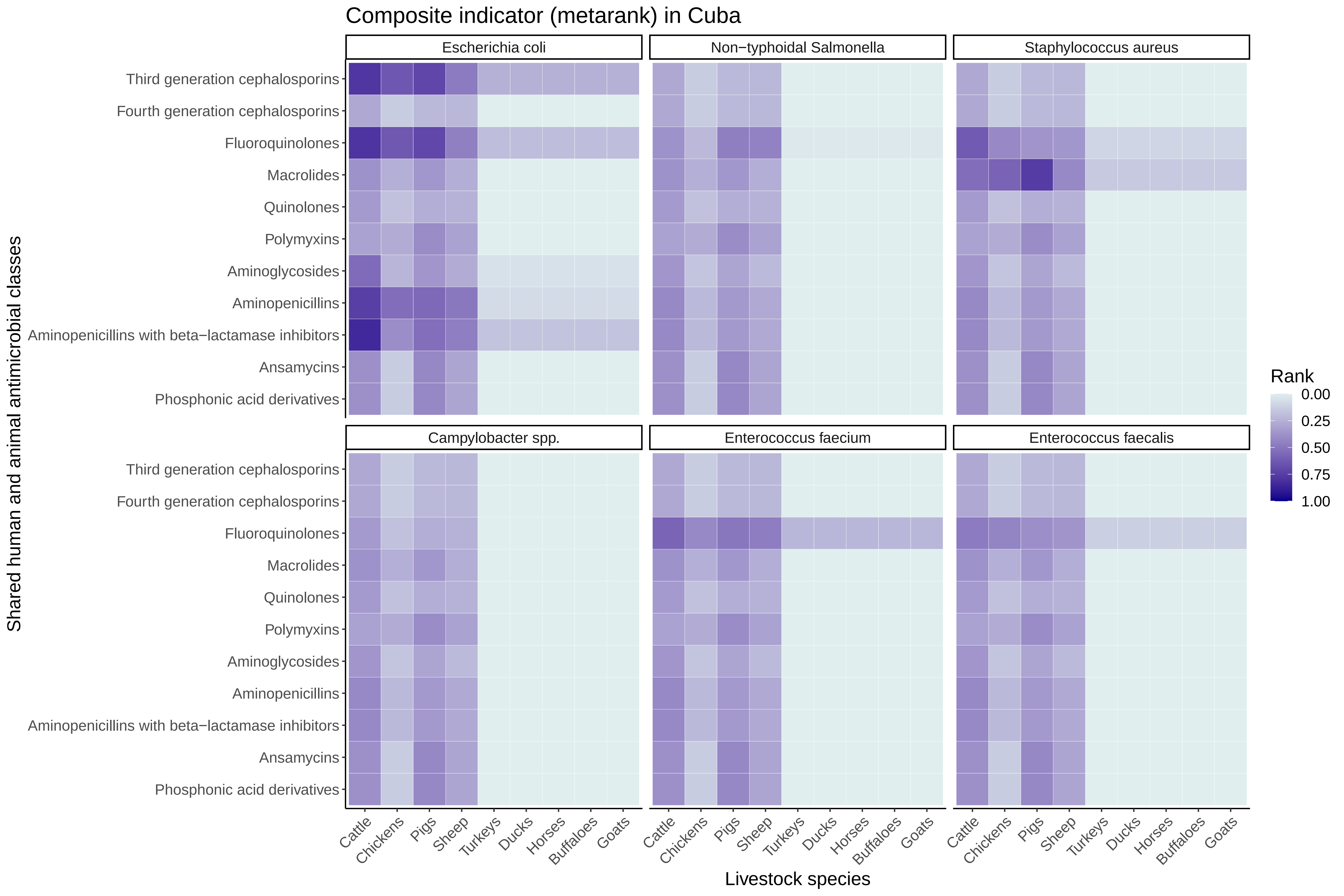

Composite indicator (metarank) in Cuba
Escherichia coli
Non−typhoidal Salmonella
Staphylococcus aureus
Campylobacter spp.
Enterococcus faecium
Enterococcus faecalis
Third generation cephalosporins
Fourth generation cephalosporins
Fluoroquinolones
Macrolides
Quinolones
Polymyxins
Aminoglycosides
Aminopenicillins
Aminopenicillins with beta−lactamase inhibitors
Ansamycins
Phosphonic acid derivatives
Shared human and animal antimicrobial classes
Cattle
Chickens
Pigs
Sheep
Turkeys
Ducks
Horses
Buffaloes
Goats
Livestock species
Rank
0.00
0.25
0.50
0.75
1.00

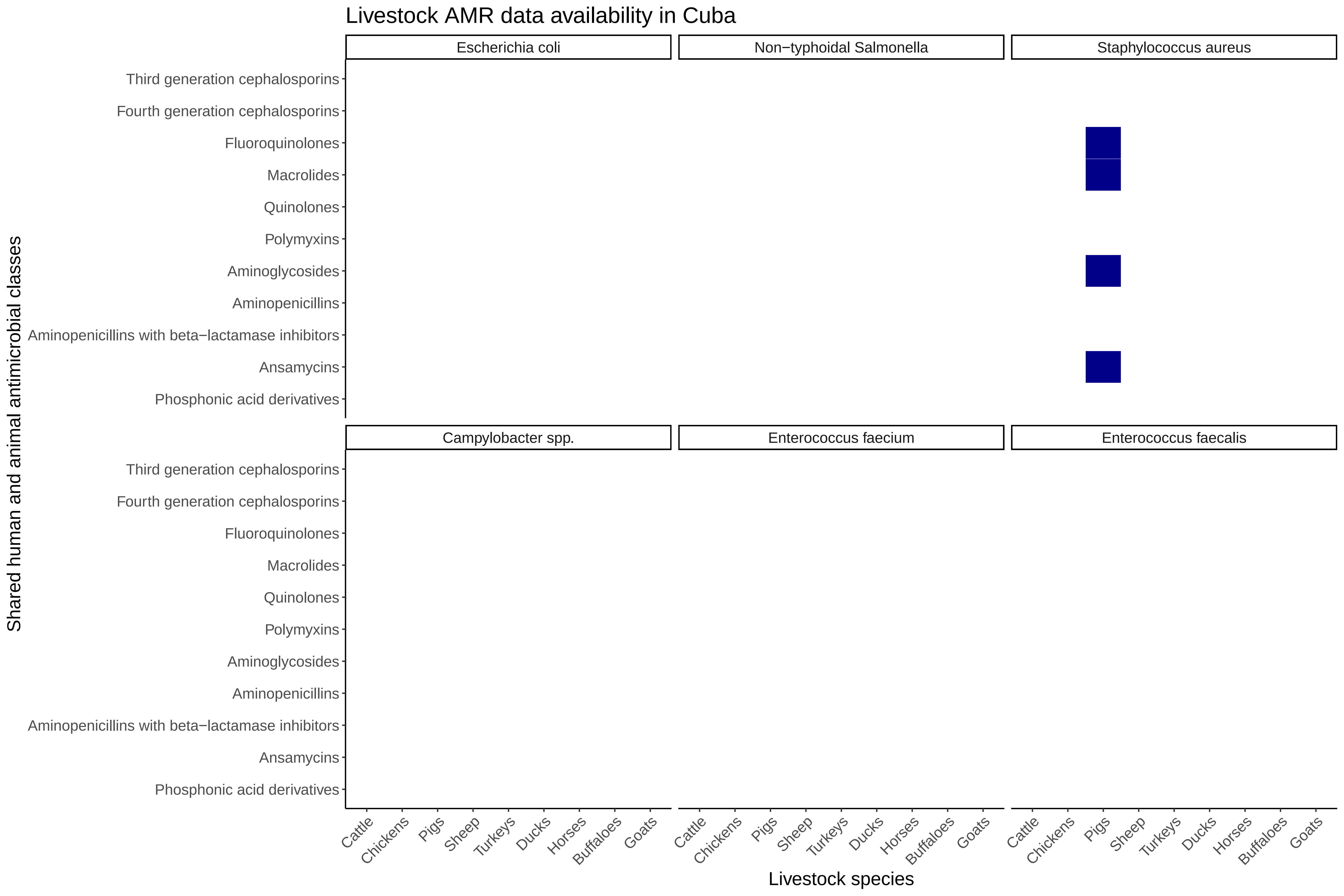
Livestock AMR data availability in Cuba
Escherichia coli
Non−typhoidal Salmonella
Staphylococcus aureus
Campylobacter spp.
Enterococcus faecium
Enterococcus faecalis
Shared human and animal antimicrobial classes
Third generation cephalosporins
Fourth generation cephalosporins
Fluoroquinolones
Macrolides
Quinolones
Polymyxins
Aminoglycosides
Aminopenicillins
Aminopenicillins with beta−lactamase inhibitors
Ansamycins
Phosphonic acid derivatives
Cattle
Chickens
Pigs
Sheep
Turkeys
Ducks
Horses
Buffaloes
Goats
Livestock species

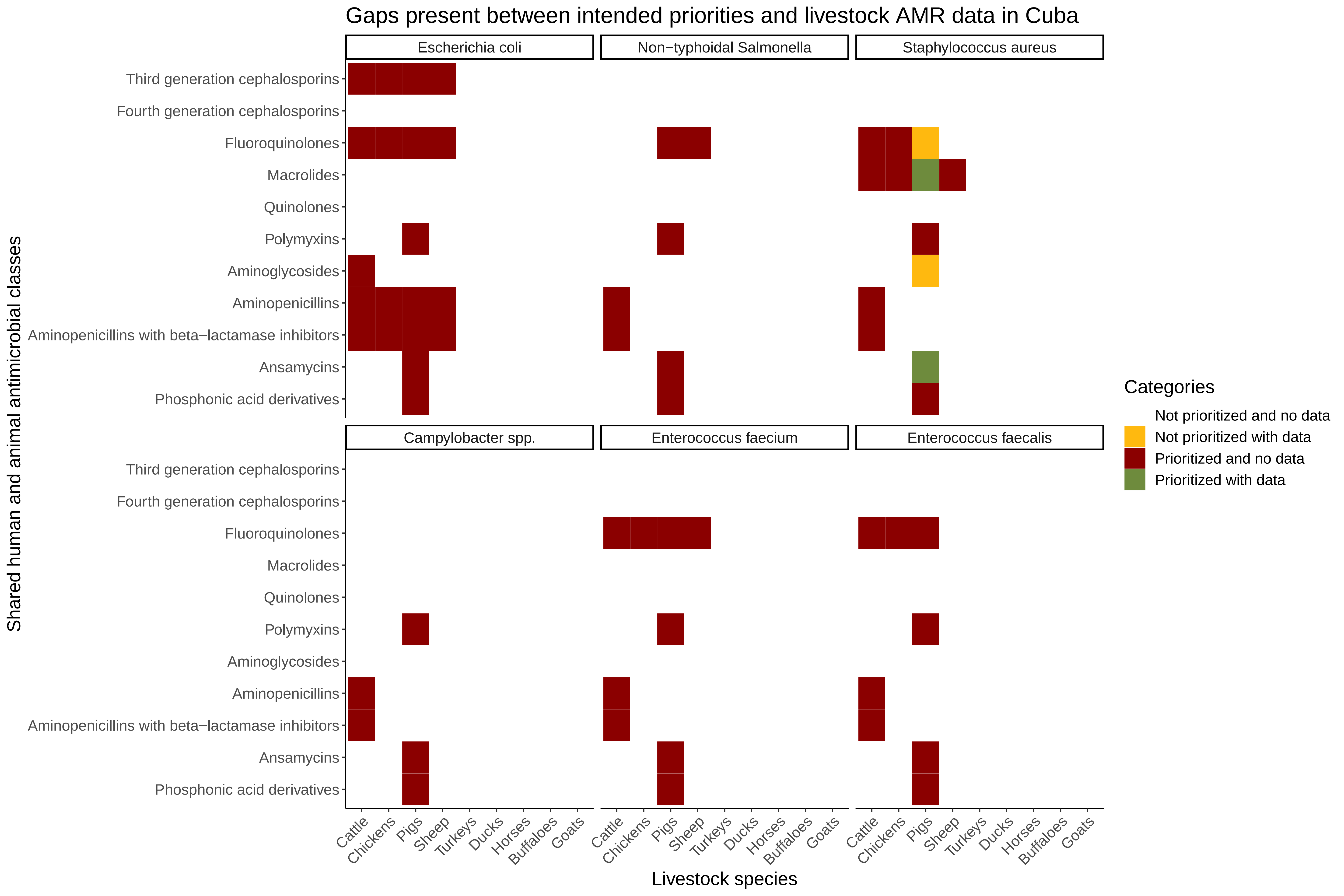

Gaps present between intended priorities and livestock AMR data in Cuba
Escherichia coli
Non−typhoidal Salmonella
Staphylococcus aureus
Campylobacter spp.
Enterococcus faecium
Enterococcus faecalis
Third generation cephalosporins
Fourth generation cephalosporins
Fluoroquinolones
Macrolides
Quinolones
Polymyxins
Aminoglycosides
Aminopenicillins
Aminopenicillins with beta−lactamase inhibitors
Ansamycins
Phosphonic acid derivatives
Shared human and animal antimicrobial classes
Cattle
Chickens
Pigs
Sheep
Turkeys
Ducks
Horses
Buffaloes
Goats
Livestock species
Categories
Not prioritized and no data
Not prioritized with data
Prioritized and no data
Prioritized with data

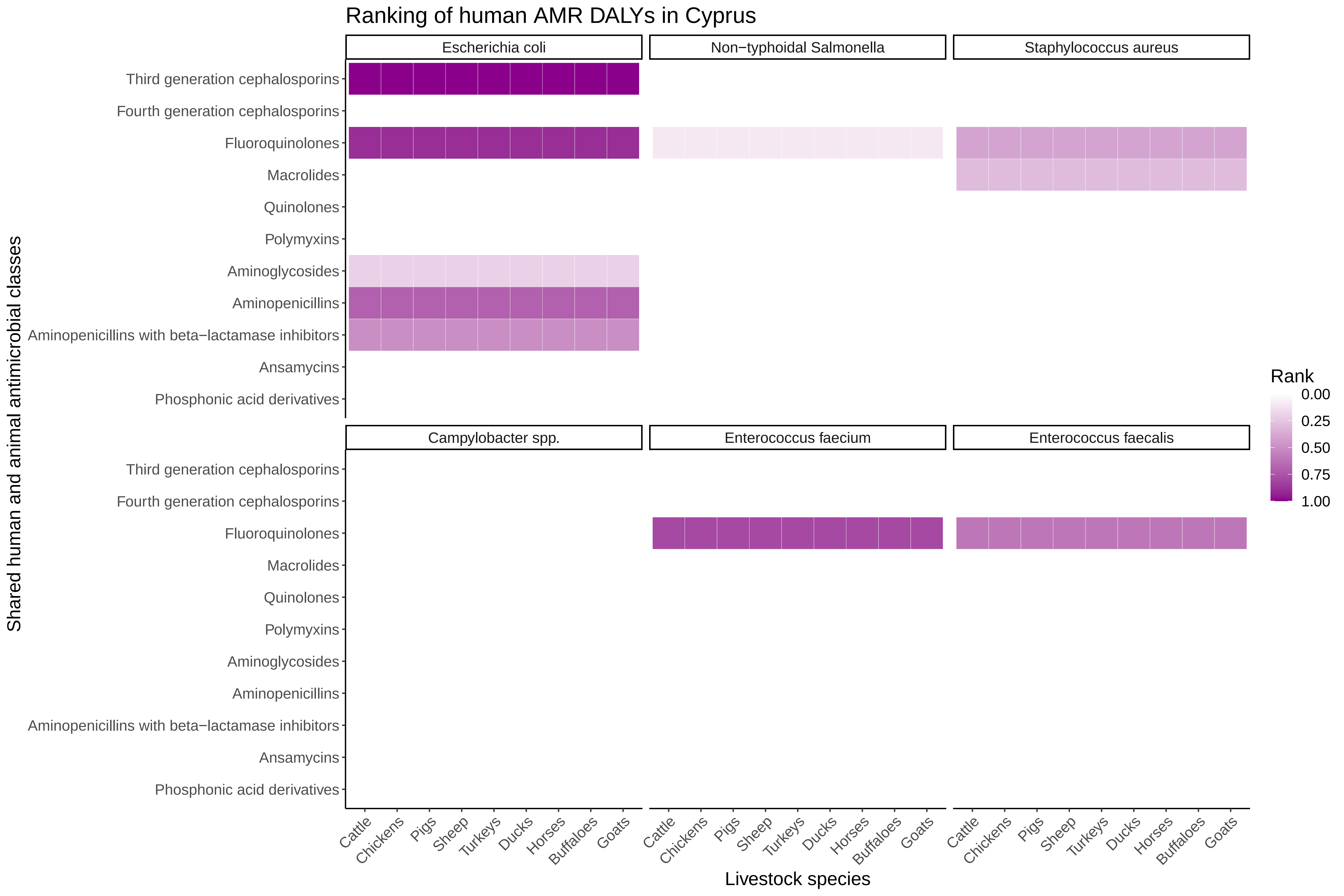
Ranking of human AMR DALYs in Cyprus
Escherichia coli
Non−typhoidal Salmonella
Staphylococcus aureus
Campylobacter spp.
Enterococcus faecium
Enterococcus faecalis
Third generation cephalosporins
Fourth generation cephalosporins
Fluoroquinolones
Macrolides
Quinolones
Polymyxins
Aminoglycosides
Aminopenicillins
Aminopenicillins with beta−lactamase inhibitors
Ansamycins
Phosphonic acid derivatives
Shared human and animal antimicrobial classes
Cattle
Chickens
Pigs
Sheep
Turkeys
Ducks
Horses
Buffaloes
Goats
Livestock species
Rank
0.00
0.25
0.50
0.75
1.00

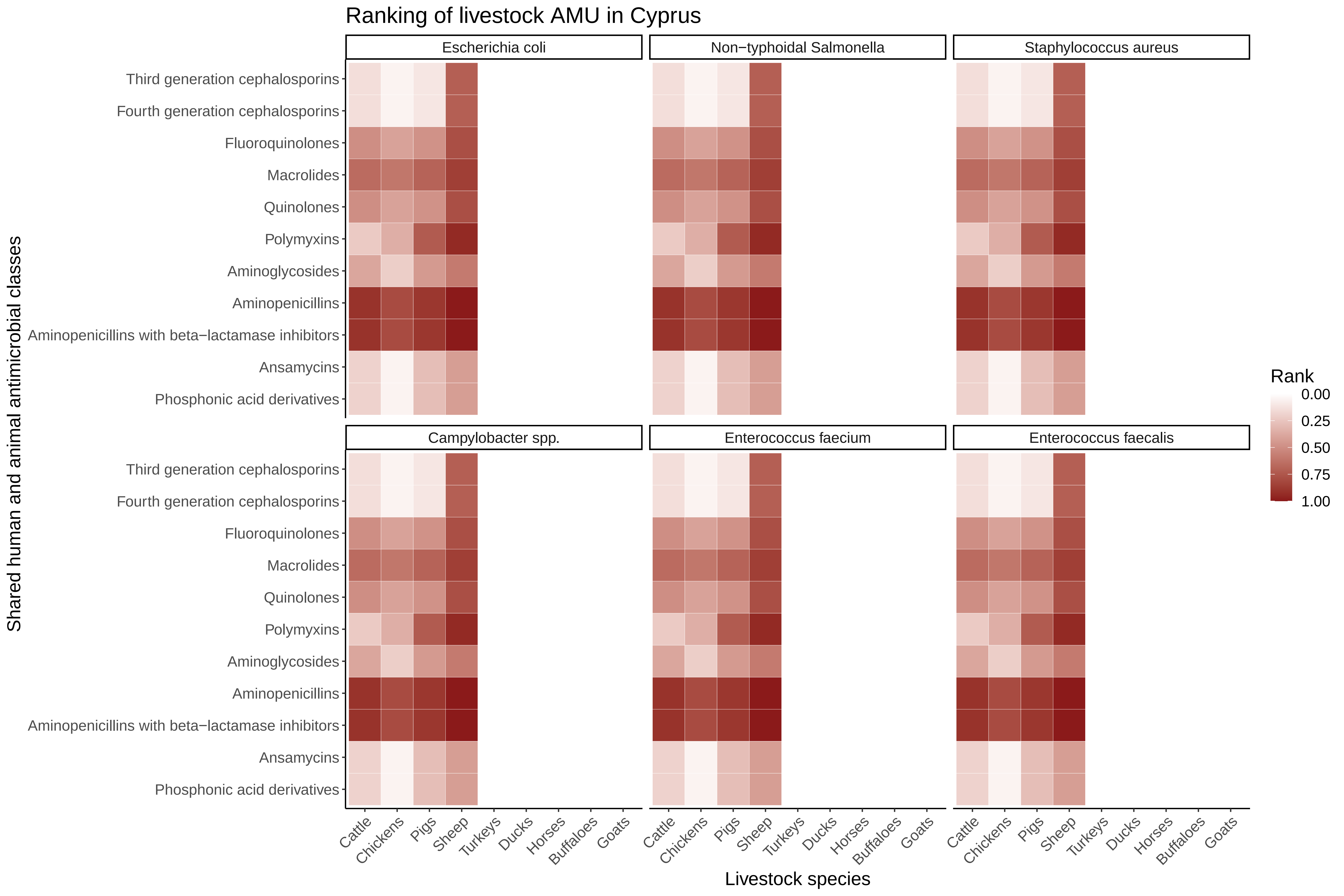
Ranking of livestock AMU in Cyprus
Escherichia coli
Non−typhoidal Salmonella
Staphylococcus aureus
Campylobacter spp.
Enterococcus faecium
Enterococcus faecalis
Shared human and animal antimicrobial classes
Third generation cephalosporins
Fourth generation cephalosporins
Fluoroquinolones
Macrolides
Quinolones
Polymyxins
Aminoglycosides
Aminopenicillins
Aminopenicillins with beta−lactamase inhibitors
Ansamycins
Phosphonic acid derivatives
Cattle
Chickens
Pigs
Sheep
Turkeys
Ducks
Horses
Buffaloes
Goats
Livestock species
Rank
0.00
0.25
0.50
0.75
1.00

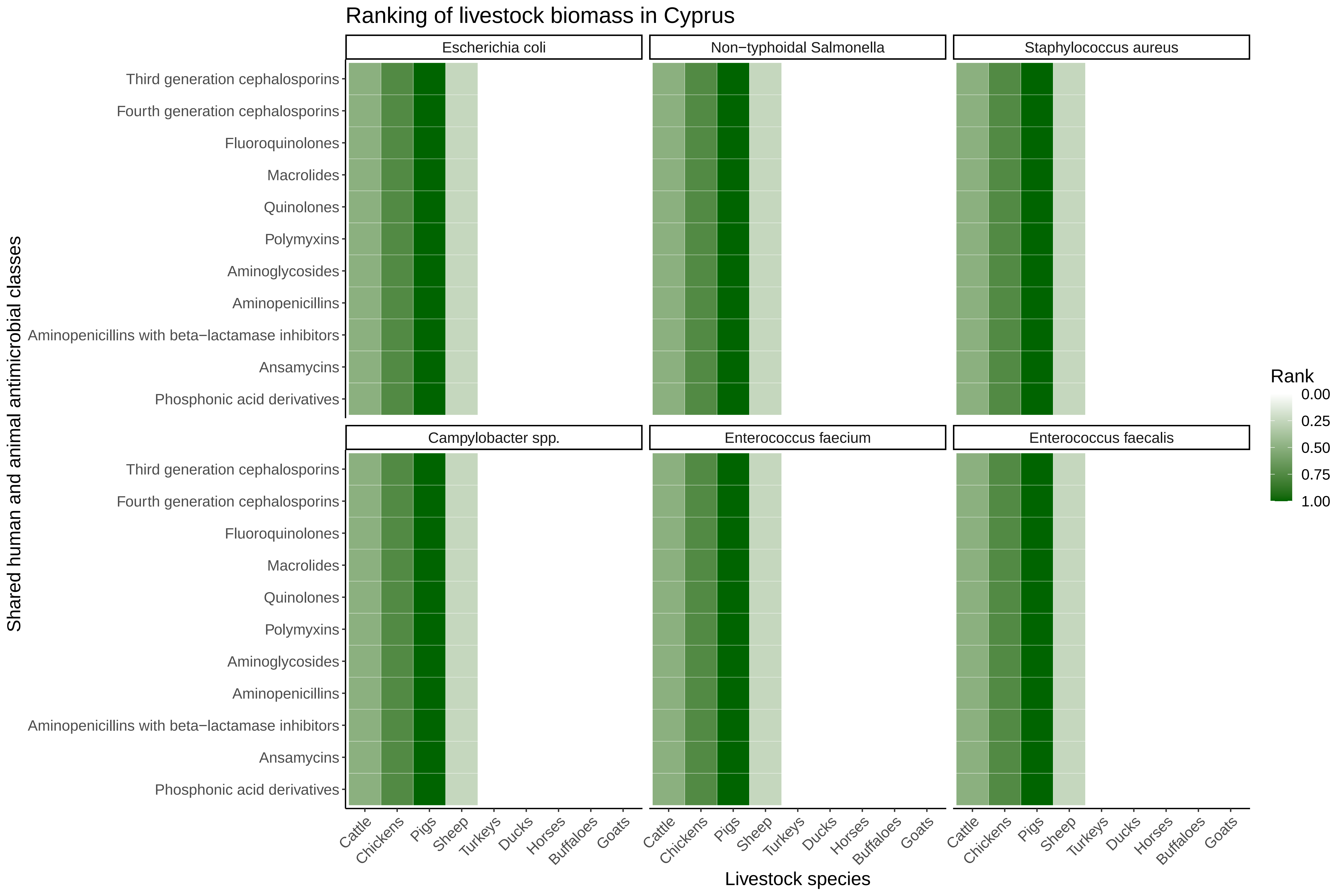

Ranking of livestock biomass in Cyprus
Escherichia coli
Non−typhoidal Salmonella
Staphylococcus aureus
Campylobacter spp.
Enterococcus faecium
Enterococcus faecalis
Third generation cephalosporins
Fourth generation cephalosporins
Fluoroquinolones
Macrolides
Quinolones
Polymyxins
Aminoglycosides
Aminopenicillins
Aminopenicillins with beta−lactamase inhibitors
Ansamycins
Phosphonic acid derivatives
Shared human and animal antimicrobial classes
Cattle
Chickens
Pigs
Sheep
Turkeys
Ducks
Horses
Buffaloes
Goats
Livestock species
Rank
0.00
0.25
0.50
0.75
1.00

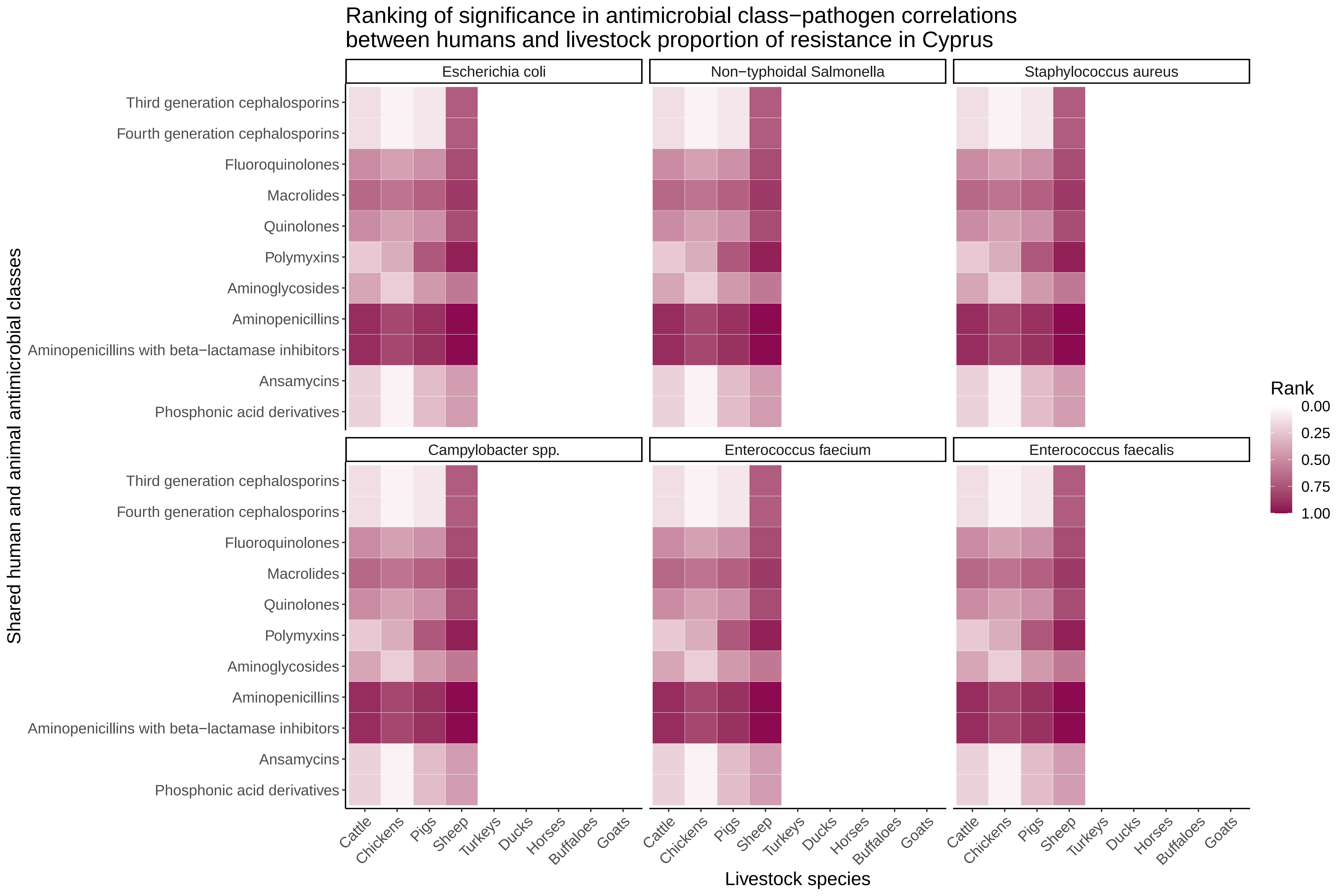
Ranking of significance in antimicrobial class−pathogen correlations
between humans and livestock proportion of resistance in Cyprus
Escherichia coli
Non−typhoidal Salmonella
Staphylococcus aureus
Campylobacter spp.
Enterococcus faecium
Enterococcus faecalis
Third generation cephalosporins
Fourth generation cephalosporins
Fluoroquinolones
Macrolides
Quinolones
Polymyxins
Aminoglycosides
Aminopenicillins
Aminopenicillins with beta−lactamase inhibitors
Ansamycins
Phosphonic acid derivatives
Shared human and animal antimicrobial classes
Cattle
Chickens
Pigs
Sheep
Turkeys
Ducks
Horses
Buffaloes
Goats
Livestock species
Rank
0.00
0.25
0.50
0.75
1.00

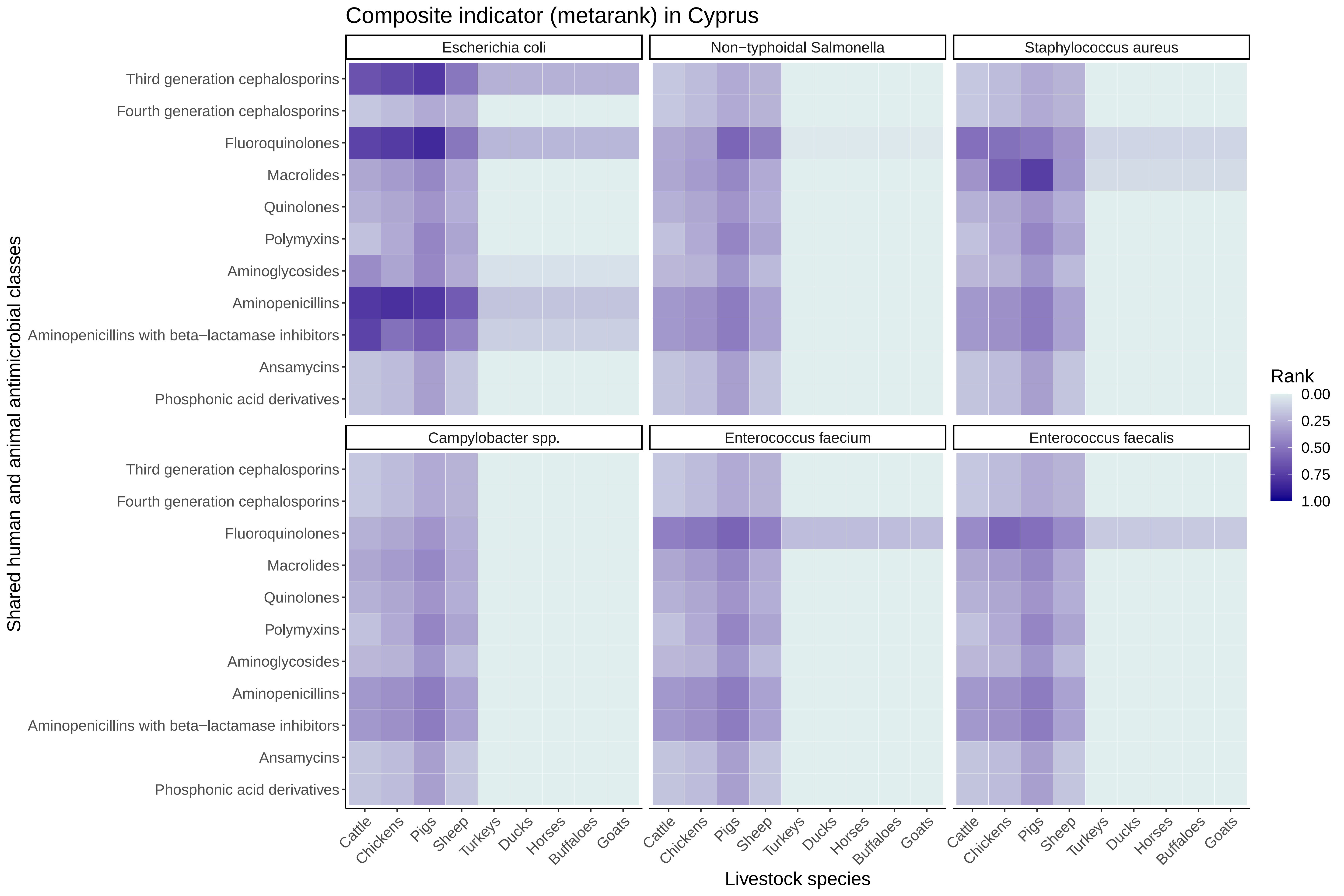

Composite indicator (metarank) in Cyprus
Escherichia coli
Non−typhoidal Salmonella
Staphylococcus aureus
Campylobacter spp.
Enterococcus faecium
Enterococcus faecalis
Shared human and animal antimicrobial classes
Third generation cephalosporins
Fourth generation cephalosporins
Fluoroquinolones
Macrolides
Quinolones
Polymyxins
Aminoglycosides
Aminopenicillins
Aminopenicillins with beta−lactamase inhibitors
Ansamycins
Phosphonic acid derivatives
Cattle
Chickens
Pigs
Sheep
Turkeys
Ducks
Horses
Buffaloes
Goats
Livestock species
Rank
0.00
0.25
0.50
0.75
1.00

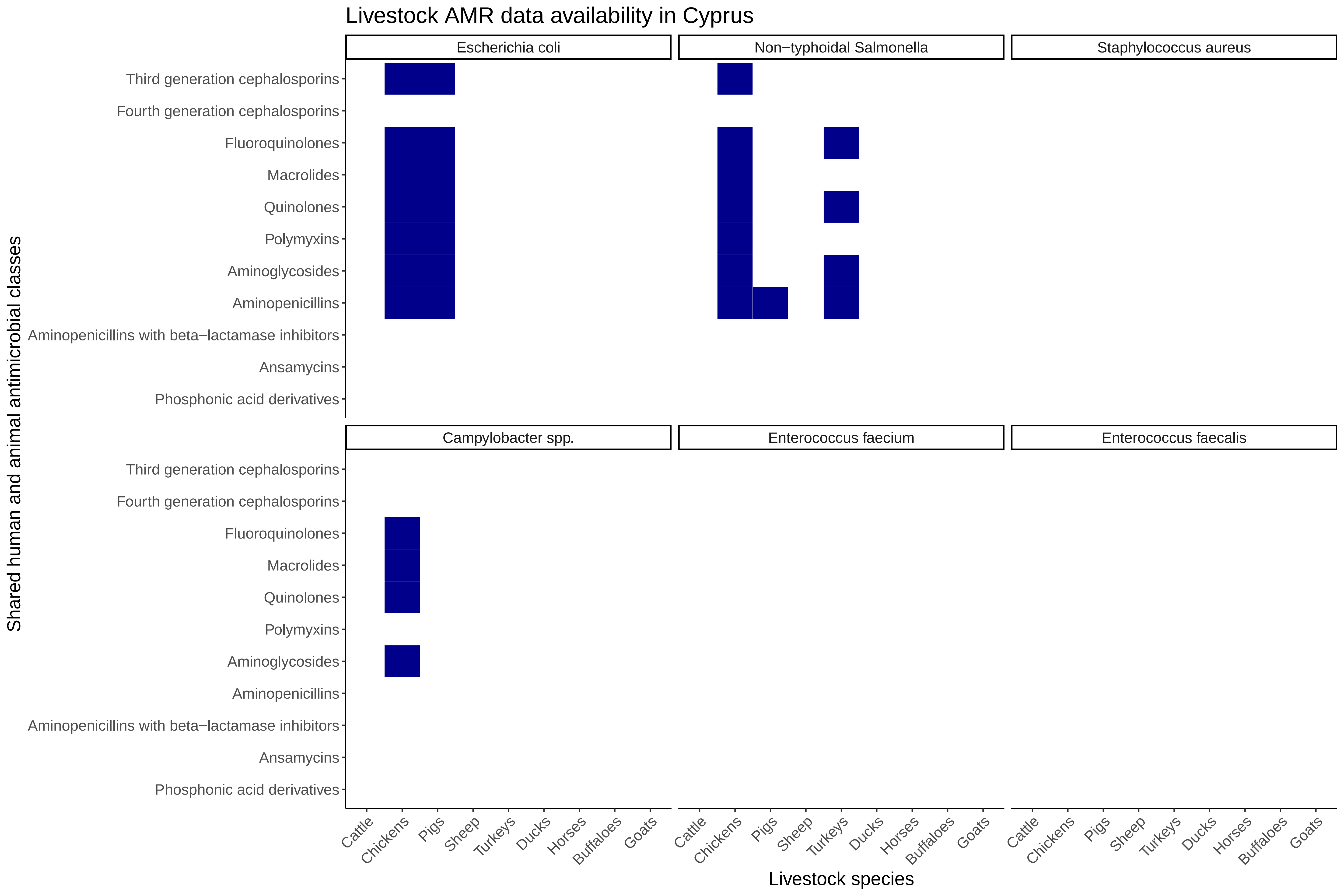
Livestock AMR data availability in Cyprus
Escherichia coli
Non−typhoidal Salmonella
Staphylococcus aureus
Campylobacter spp.
Enterococcus faecium
Enterococcus faecalis
Shared human and animal antimicrobial classes
Third generation cephalosporins
Fourth generation cephalosporins
Fluoroquinolones
Macrolides
Quinolones
Polymyxins
Aminoglycosides
Aminopenicillins
Aminopenicillins with beta−lactamase inhibitors
Ansamycins
Phosphonic acid derivatives
Cattle
Chickens
Pigs
Sheep
Turkeys
Ducks
Horses
Buffaloes
Goats
Livestock species

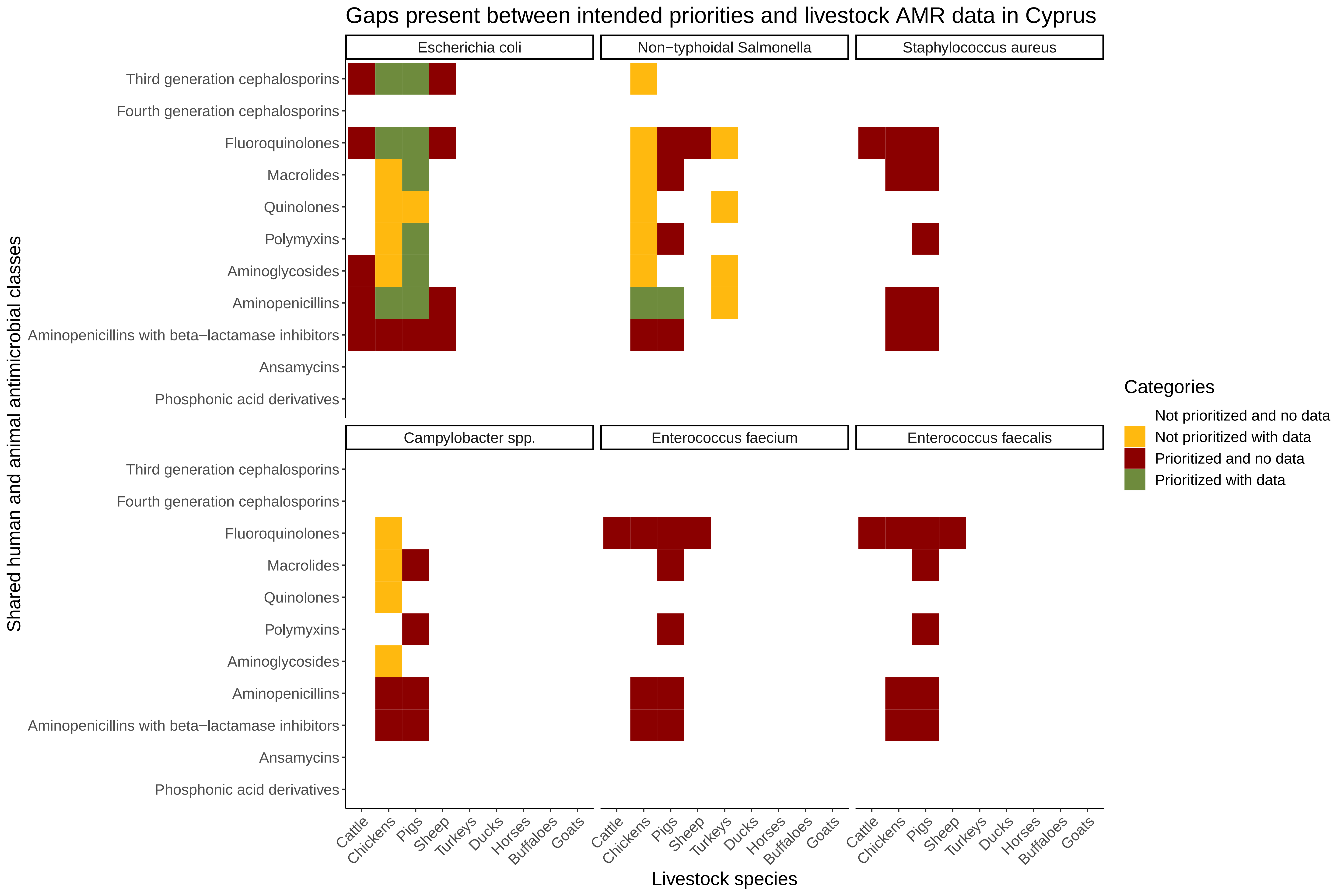

Gaps present between intended priorities and livestock AMR data in Cyprus
Escherichia coli
Non−typhoidal Salmonella
Staphylococcus aureus
Campylobacter spp.
Enterococcus faecium
Enterococcus faecalis
Shared human and animal antimicrobial classes
Third generation cephalosporins
Fourth generation cephalosporins
Fluoroquinolones
Macrolides
Quinolones
Polymyxins
Aminoglycosides
Aminopenicillins
Aminopenicillins with beta−lactamase inhibitors
Ansamycins
Phosphonic acid derivatives
Livestock species
Cattle
Chickens
Pigs
Sheep
Turkeys
Ducks
Horses
Buffaloes
Goats
Categories
Not prioritized and no data
Not prioritized with data
Prioritized and no data
Prioritized with data

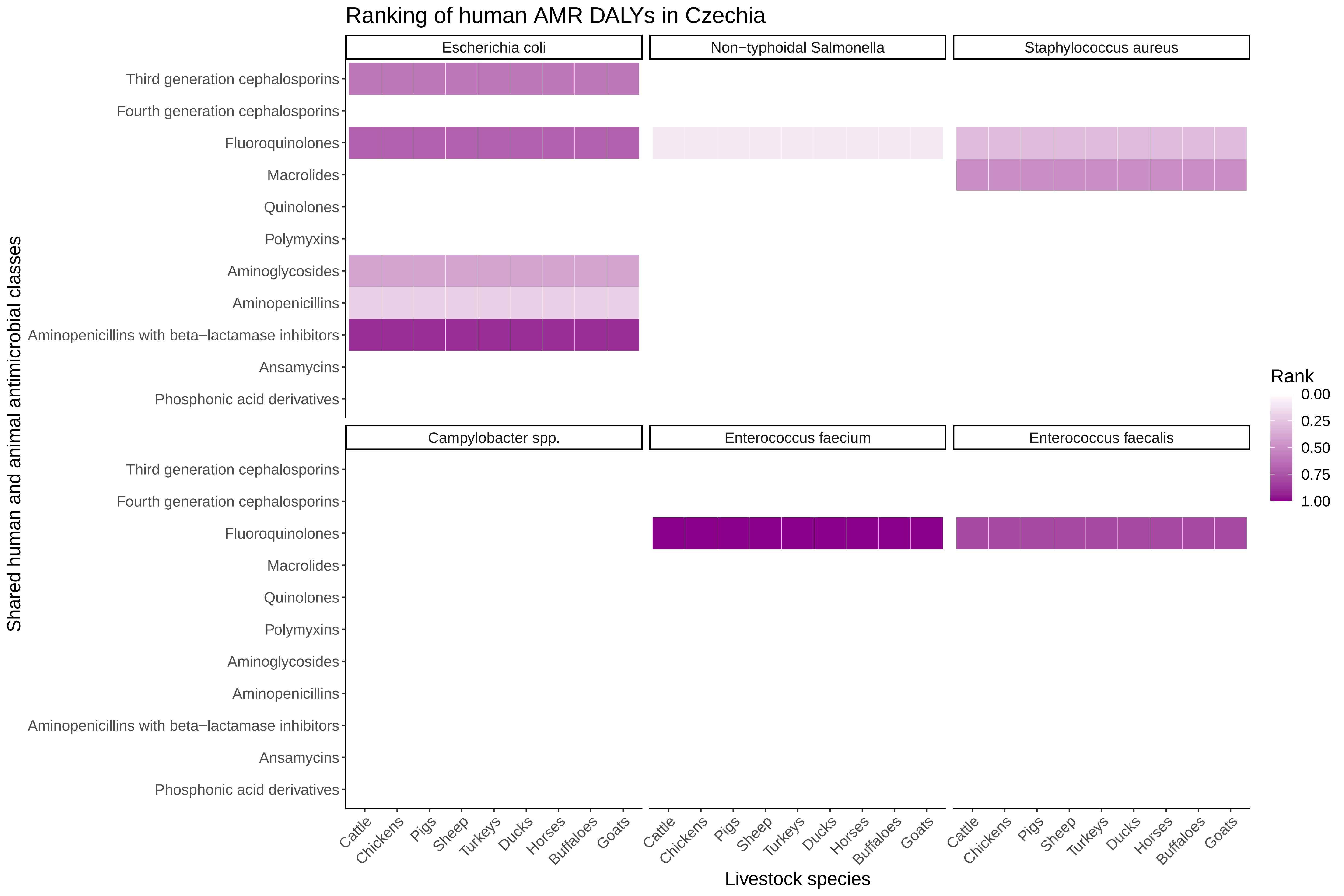

Ranking of human AMR DALYs in Czechia
Escherichia coli
Non−typhoidal Salmonella
Staphylococcus aureus
Campylobacter spp.
Enterococcus faecium
Enterococcus faecalis
Third generation cephalosporins
Fourth generation cephalosporins
Fluoroquinolones
Macrolides
Quinolones
Polymyxins
Aminoglycosides
Aminopenicillins
Aminopenicillins with beta−lactamase inhibitors
Ansamycins
Phosphonic acid derivatives
Shared human and animal antimicrobial classes
Cattle
Chickens
Pigs
Sheep
Turkeys
Ducks
Horses
Buffaloes
Goats
Livestock species
Rank
0.00
0.25
0.50
0.75
1.00

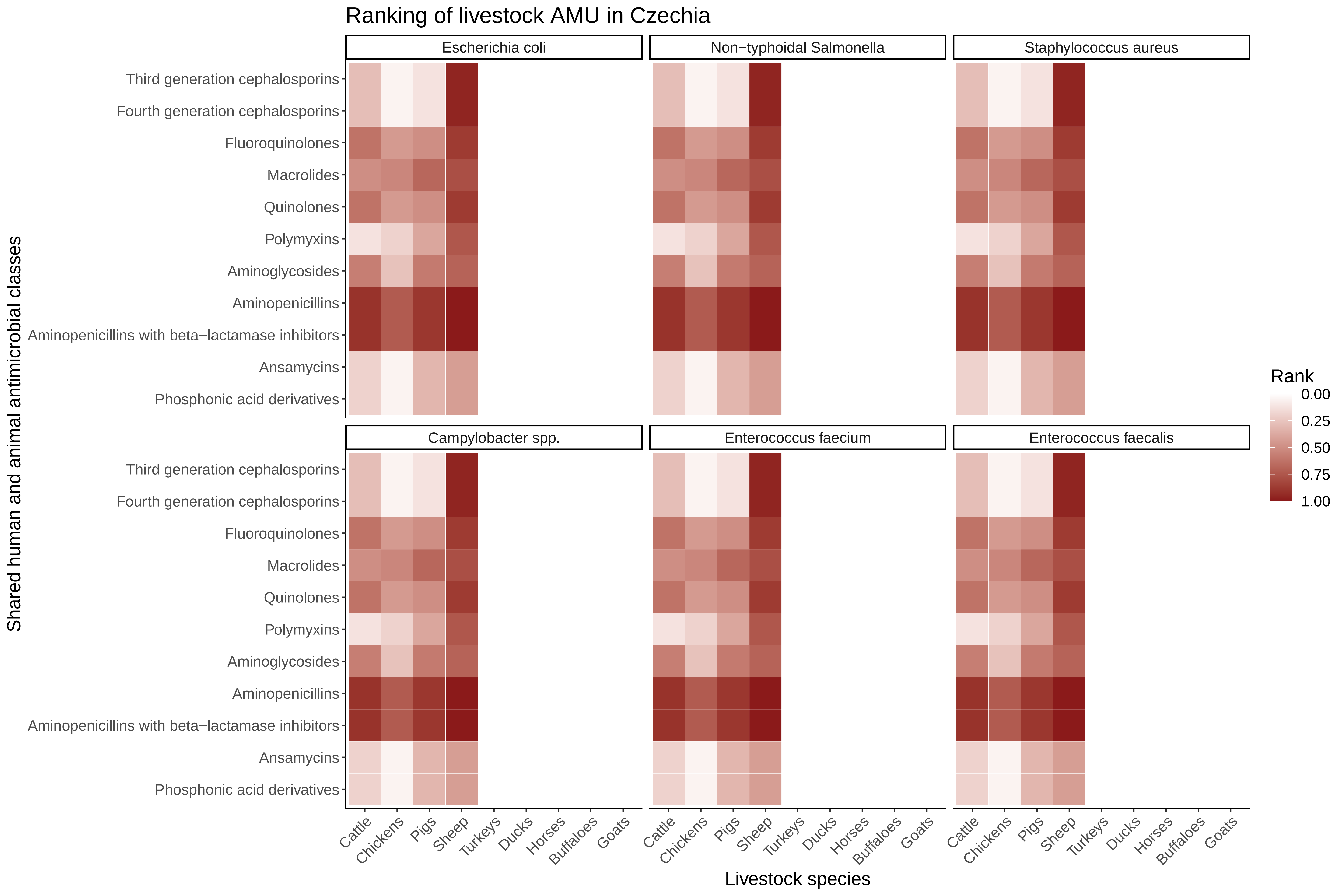

Ranking of livestock AMU in Czechia
Escherichia coli
Non−typhoidal Salmonella
Staphylococcus aureus
Campylobacter spp.
Enterococcus faecium
Enterococcus faecalis
Shared human and animal antimicrobial classes
Third generation cephalosporins
Fourth generation cephalosporins
Fluoroquinolones
Macrolides
Quinolones
Polymyxins
Aminoglycosides
Aminopenicillins
Aminopenicillins with beta−lactamase inhibitors
Ansamycins
Phosphonic acid derivatives
Cattle
Chickens
Pigs
Sheep
Turkeys
Ducks
Horses
Buffaloes
Goats
Livestock species
Rank
0.00
0.25
0.50
0.75
1.00

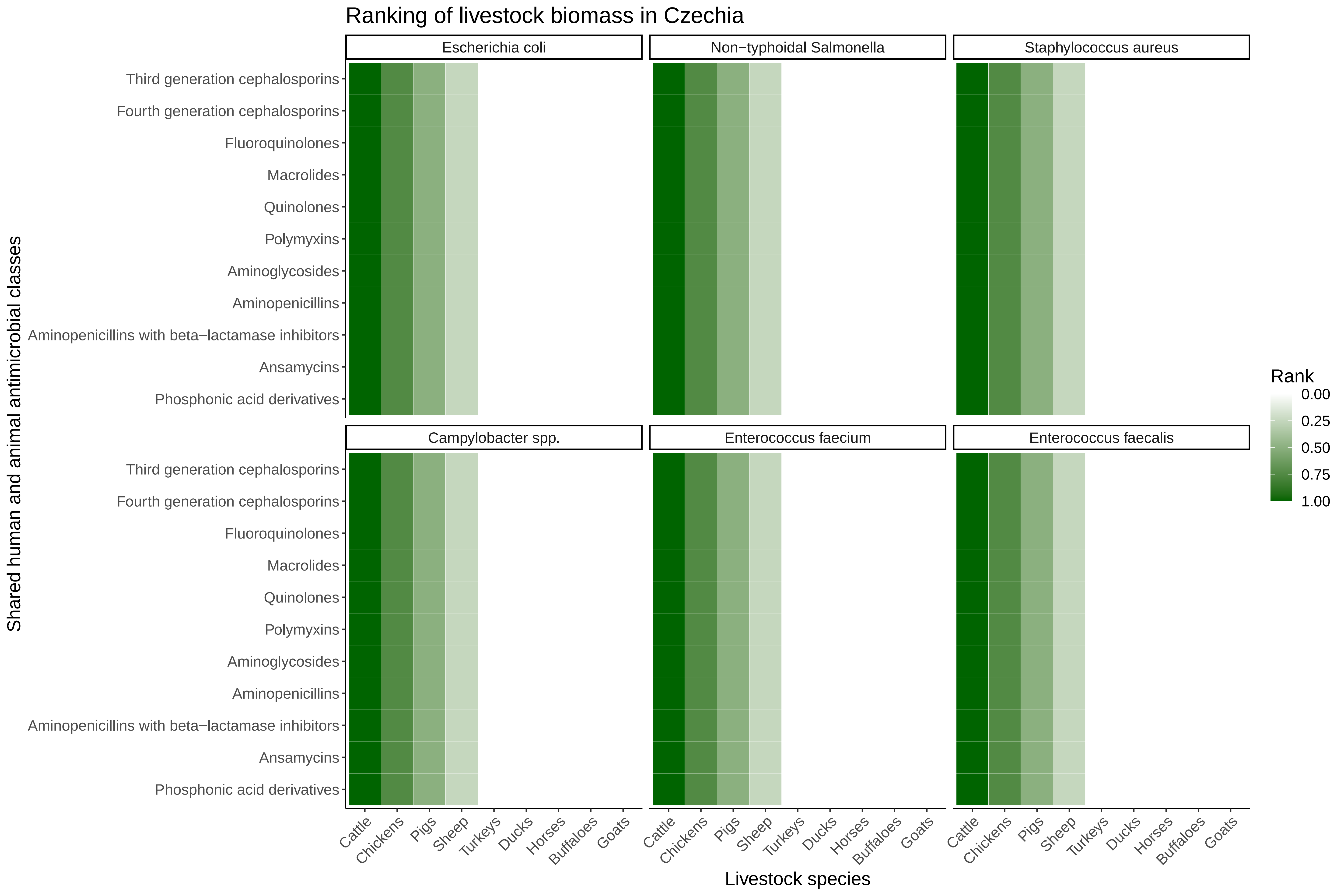

Ranking of livestock biomass in Czechia
Escherichia coli
Non−typhoidal Salmonella
Staphylococcus aureus
Campylobacter spp.
Enterococcus faecium
Enterococcus faecalis
Shared human and animal antimicrobial classes
Third generation cephalosporins
Fourth generation cephalosporins
Fluoroquinolones
Macrolides
Quinolones
Polymyxins
Aminoglycosides
Aminopenicillins
Aminopenicillins with beta−lactamase inhibitors
Ansamycins
Phosphonic acid derivatives
Livestock species
Cattle
Chickens
Pigs
Sheep
Turkeys
Ducks
Horses
Buffaloes
Goats
Rank
0.00
0.25
0.50
0.75
1.00

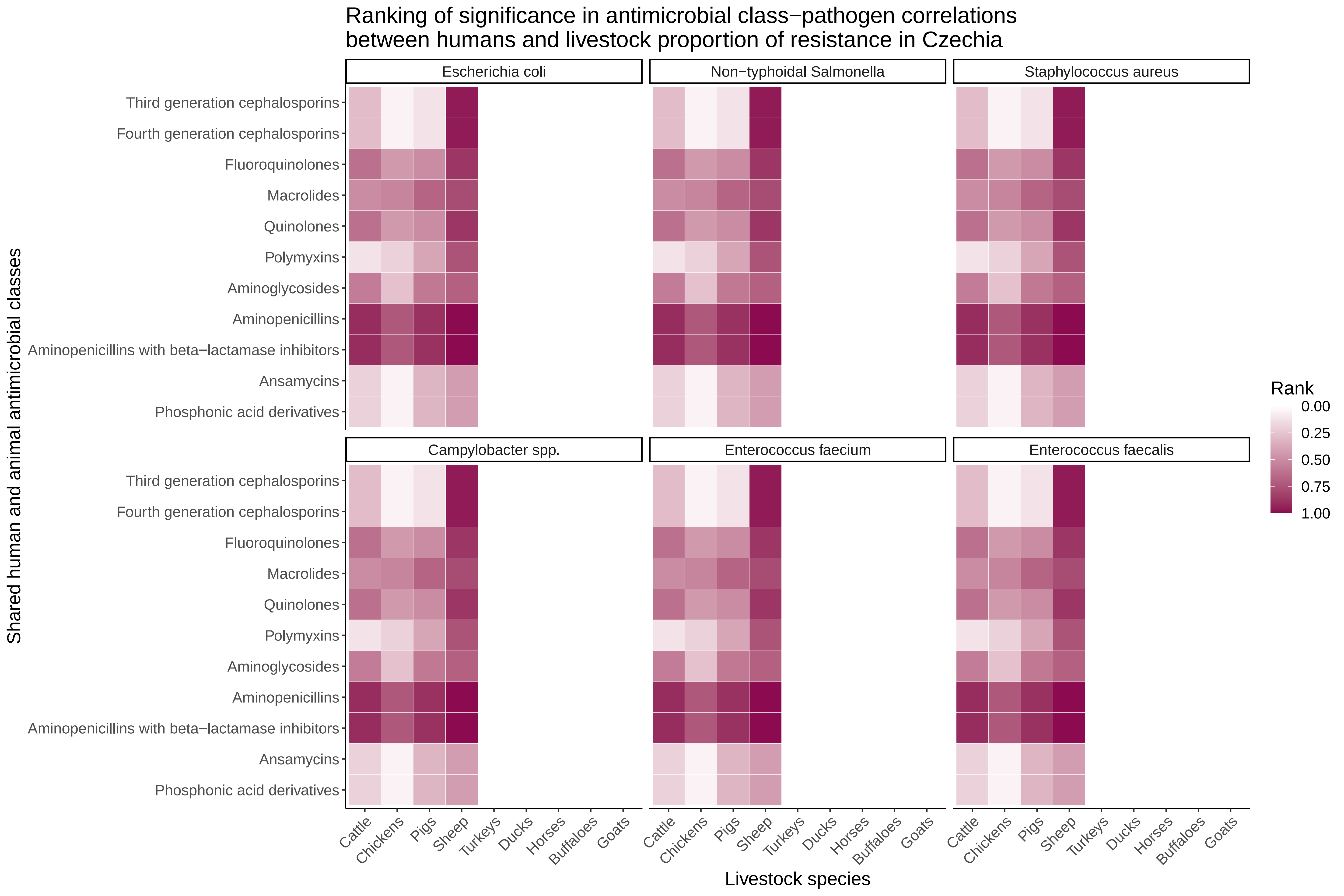

Ranking of significance in antimicrobial class−pathogen correlations
between humans and livestock proportion of resistance in Czechia
Escherichia coli
Non−typhoidal Salmonella
Staphylococcus aureus
Campylobacter spp.
Enterococcus faecium
Enterococcus faecalis
Third generation cephalosporins
Fourth generation cephalosporins
Fluoroquinolones
Macrolides
Quinolones
Polymyxins
Aminoglycosides
Aminopenicillins
Aminopenicillins with beta−lactamase inhibitors
Ansamycins
Phosphonic acid derivatives
Shared human and animal antimicrobial classes
Cattle
Chickens
Pigs
Sheep
Turkeys
Ducks
Horses
Buffaloes
Goats
Livestock species
Rank
0.00
0.25
0.50
0.75
1.00

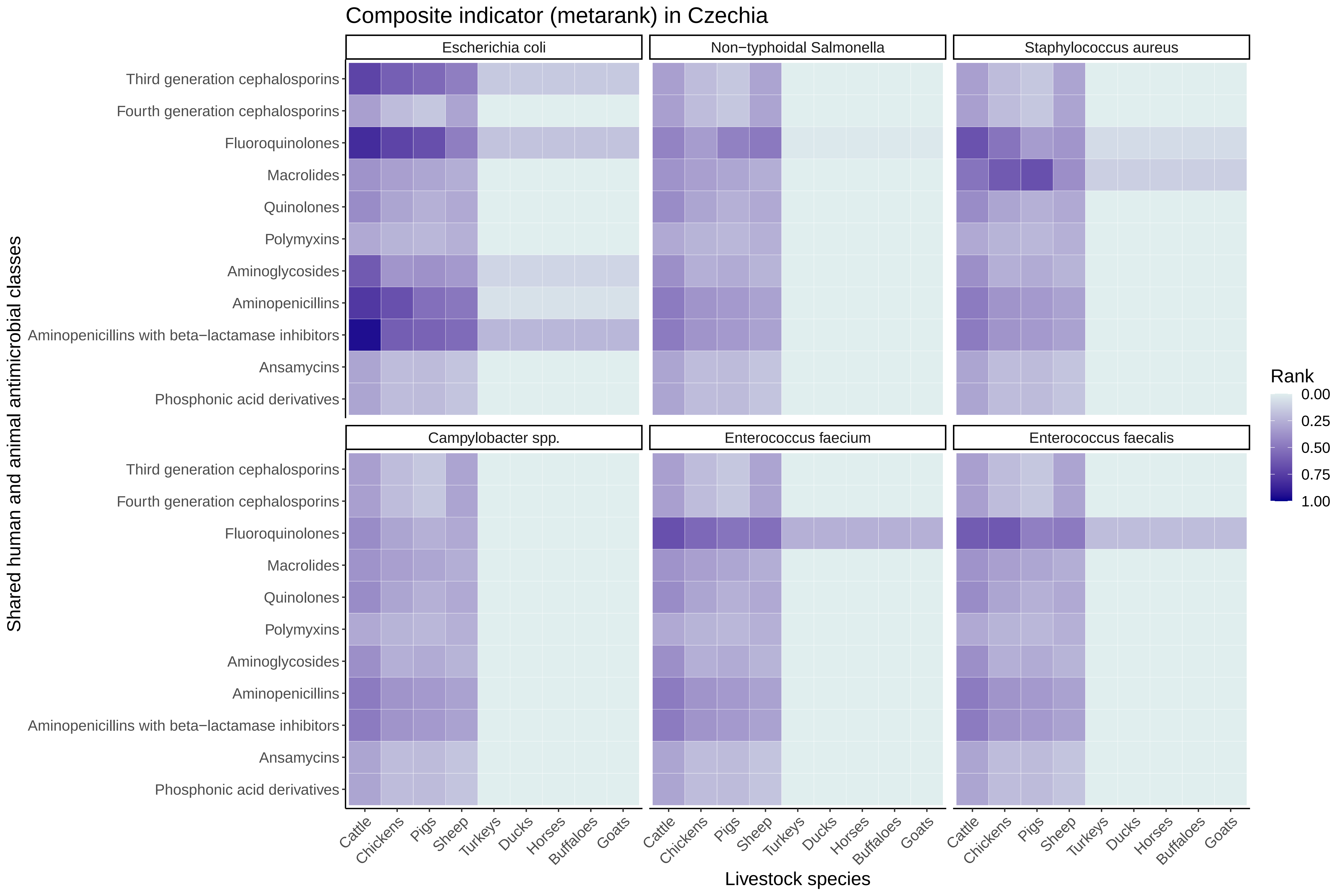

Composite indicator (metarank) in Czechia
Escherichia coli
Non−typhoidal Salmonella
Staphylococcus aureus
Campylobacter spp.
Enterococcus faecium
Enterococcus faecalis
Shared human and animal antimicrobial classes
Third generation cephalosporins
Fourth generation cephalosporins
Fluoroquinolones
Macrolides
Quinolones
Polymyxins
Aminoglycosides
Aminopenicillins
Aminopenicillins with beta−lactamase inhibitors
Ansamycins
Phosphonic acid derivatives
Livestock species
Cattle
Chickens
Pigs
Sheep
Turkeys
Ducks
Horses
Buffaloes
Goats
Rank
0.00
0.25
0.50
0.75
1.00

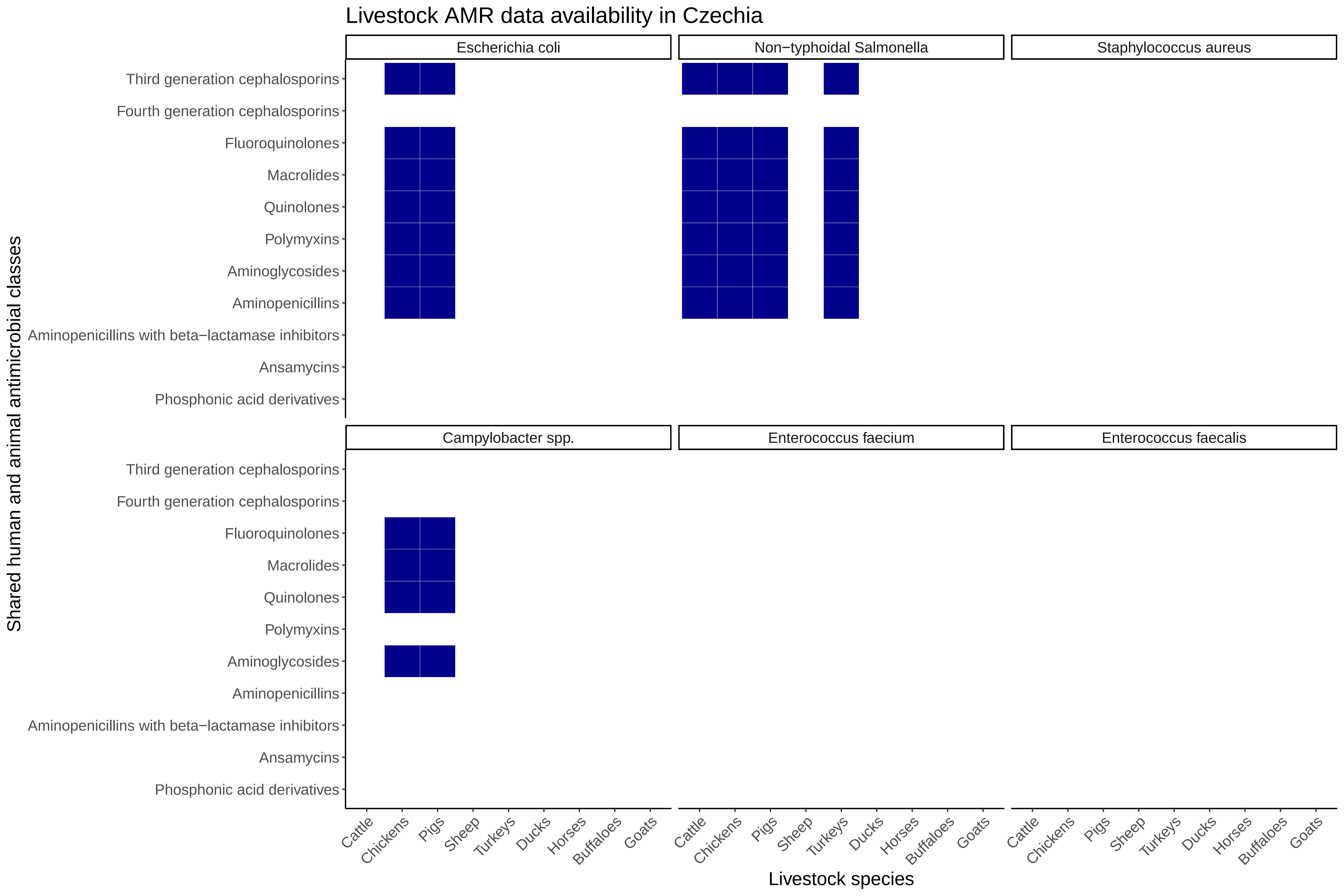
Livestock AMR data availability in Czechia
Escherichia coli
Non−typhoidal Salmonella
Staphylococcus aureus
Campylobacter spp.
Enterococcus faecium
Enterococcus faecalis
Third generation cephalosporins
Fourth generation cephalosporins
Fluoroquinolones
Macrolides
Quinolones
Polymyxins
Aminoglycosides
Aminopenicillins
Aminopenicillins with beta−lactamase inhibitors
Ansamycins
Phosphonic acid derivatives
Shared human and animal antimicrobial classes
Cattle
Chickens
Pigs
Sheep
Turkeys
Ducks
Horses
Buffaloes
Goats
Livestock species

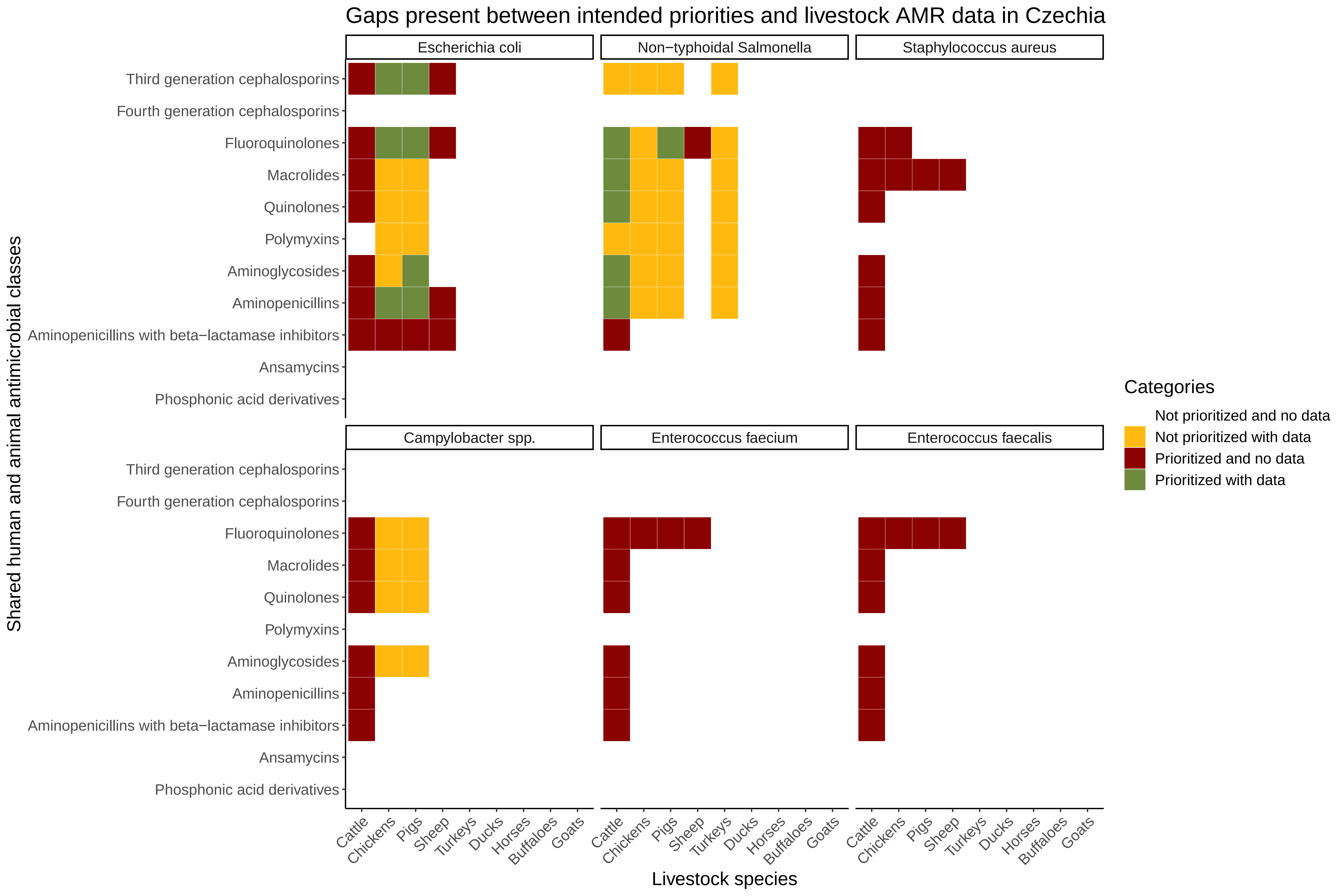

Gaps present between intended priorities and livestock AMR data in Czechia
Escherichia coli
Non−typhoidal Salmonella
Staphylococcus aureus
Campylobacter spp.
Enterococcus faecium
Enterococcus faecalis
Third generation cephalosporins
Fourth generation cephalosporins
Fluoroquinolones
Macrolides
Quinolones
Polymyxins
Aminoglycosides
Aminopenicillins
Aminopenicillins with beta−lactamase inhibitors
Ansamycins
Phosphonic acid derivatives
Shared human and animal antimicrobial classes
Cattle
Chickens
Pigs
Sheep
Turkeys
Ducks
Horses
Buffaloes
Goats
Livestock species
Categories
Not prioritized and no data
Not prioritized with data
Prioritized and no data
Prioritized with data

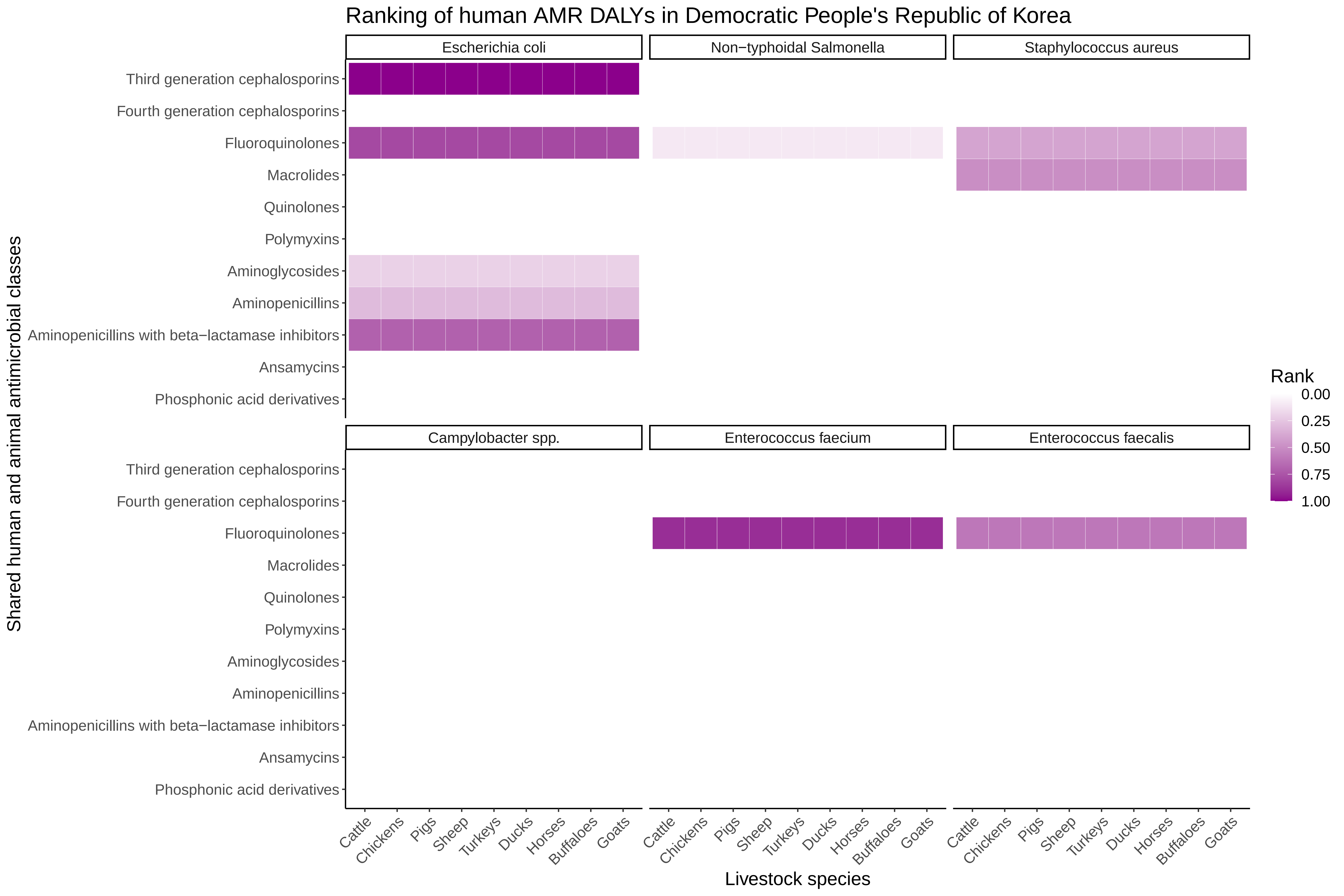
Ranking of human AMR DALYs in Democratic People's Republic of Korea
Escherichia coli
Non−typhoidal Salmonella
Staphylococcus aureus
Campylobacter spp.
Enterococcus faecium
Enterococcus faecalis
Shared human and animal antimicrobial classes
Third generation cephalosporins
Fourth generation cephalosporins
Fluoroquinolones
Macrolides
Quinolones
Polymyxins
Aminoglycosides
Aminopenicillins
Aminopenicillins with beta−lactamase inhibitors
Ansamycins
Phosphonic acid derivatives
Cattle
Chickens
Pigs
Sheep
Turkeys
Ducks
Horses
Buffaloes
Goats
Livestock species
Rank
0.00
0.25
0.50
0.75
1.00

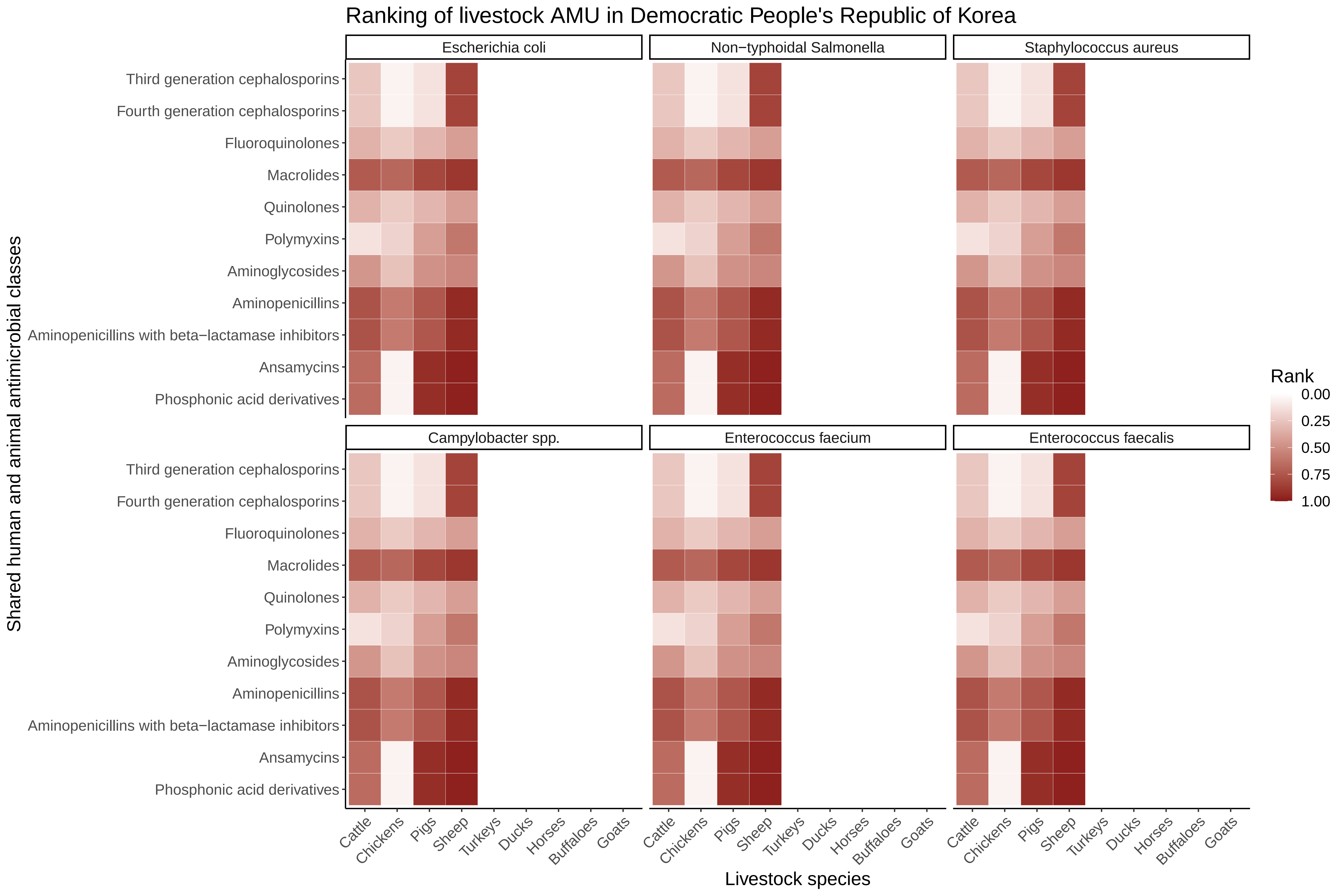

Ranking of livestock AMU in Democratic People's Republic of Korea
Escherichia coli
Non−typhoidal Salmonella
Staphylococcus aureus
Campylobacter spp.
Enterococcus faecium
Enterococcus faecalis
Third generation cephalosporins
Fourth generation cephalosporins
Fluoroquinolones
Macrolides
Quinolones
Polymyxins
Aminoglycosides
Aminopenicillins
Aminopenicillins with beta−lactamase inhibitors
Ansamycins
Phosphonic acid derivatives
Shared human and animal antimicrobial classes
Cattle
Chickens
Pigs
Sheep
Turkeys
Ducks
Horses
Buffaloes
Goats
Livestock species
Rank
0.00
0.25
0.50
0.75
1.00

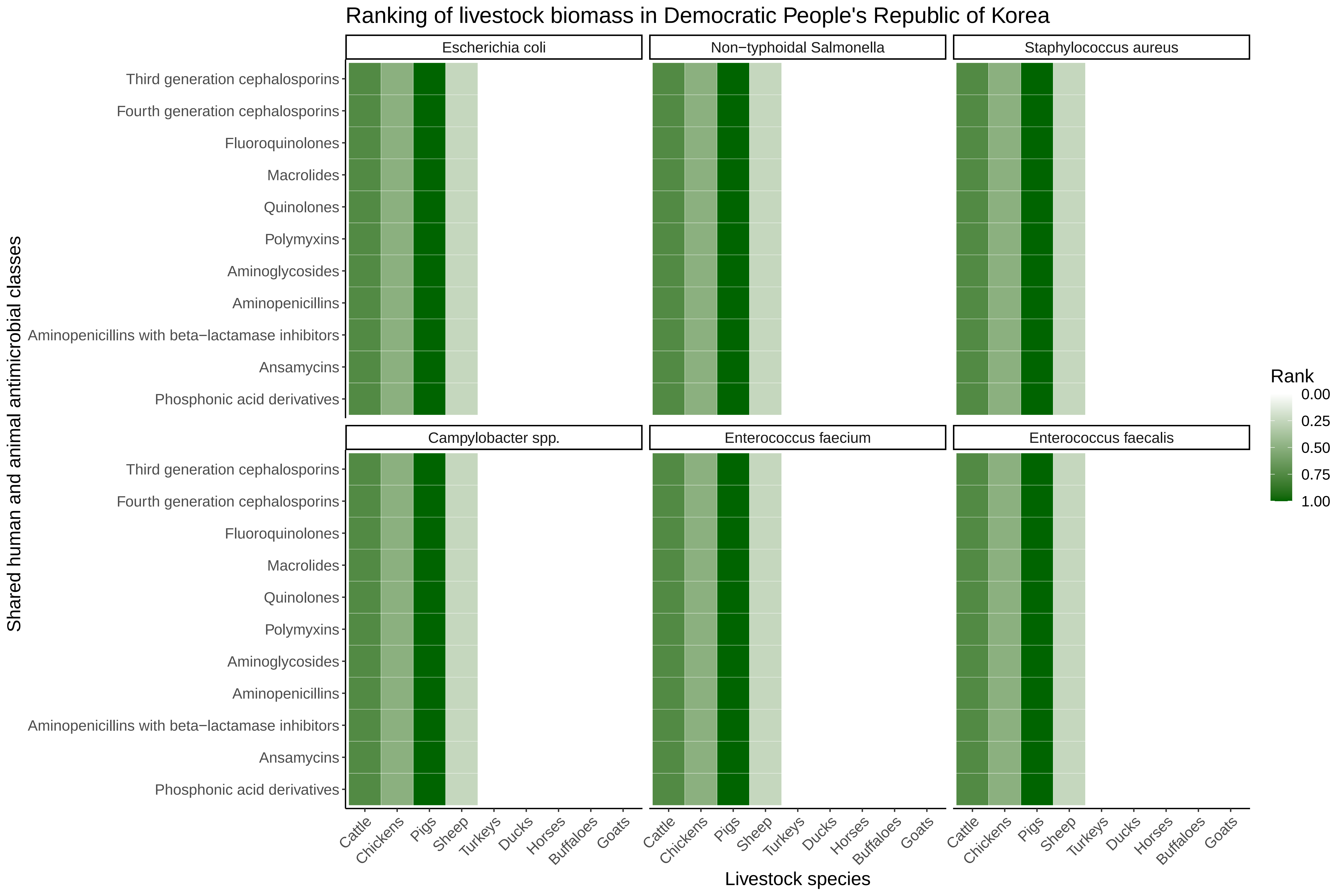

Ranking of livestock biomass in Democratic People's Republic of Korea
Escherichia coli
Non−typhoidal Salmonella
Staphylococcus aureus
Campylobacter spp.
Enterococcus faecium
Enterococcus faecalis
Third generation cephalosporins
Fourth generation cephalosporins
Fluoroquinolones
Macrolides
Quinolones
Polymyxins
Aminoglycosides
Aminopenicillins
Aminopenicillins with beta−lactamase inhibitors
Ansamycins
Phosphonic acid derivatives
Shared human and animal antimicrobial classes
Cattle
Chickens
Pigs
Sheep
Turkeys
Ducks
Horses
Buffaloes
Goats
Livestock species
Rank
0.00
0.25
0.50
0.75
1.00

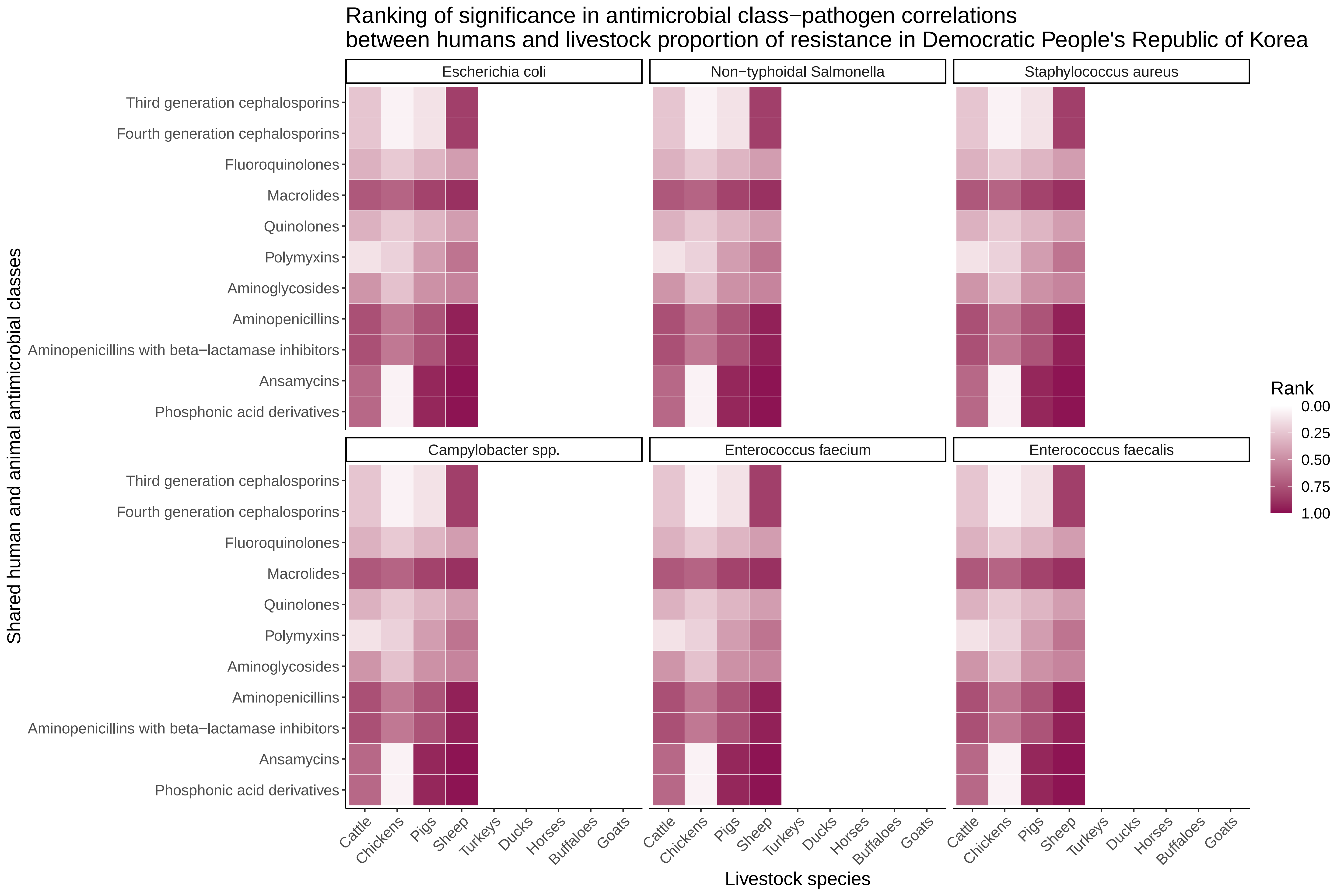
Ranking of significance in antimicrobial class−pathogen correlations
between humans and livestock proportion of resistance in Democratic People's Republic of Korea
Escherichia coli
Non−typhoidal Salmonella
Staphylococcus aureus
Campylobacter spp.
Enterococcus faecium
Enterococcus faecalis
Third generation cephalosporins
Fourth generation cephalosporins
Fluoroquinolones
Macrolides
Quinolones
Polymyxins
Aminoglycosides
Aminopenicillins
Aminopenicillins with beta−lactamase inhibitors
Ansamycins
Phosphonic acid derivatives
Shared human and animal antimicrobial classes
Cattle
Chickens
Pigs
Sheep
Turkeys
Ducks
Horses
Buffaloes
Goats
Livestock species
Rank
0.00
0.25
0.50
0.75
1.00

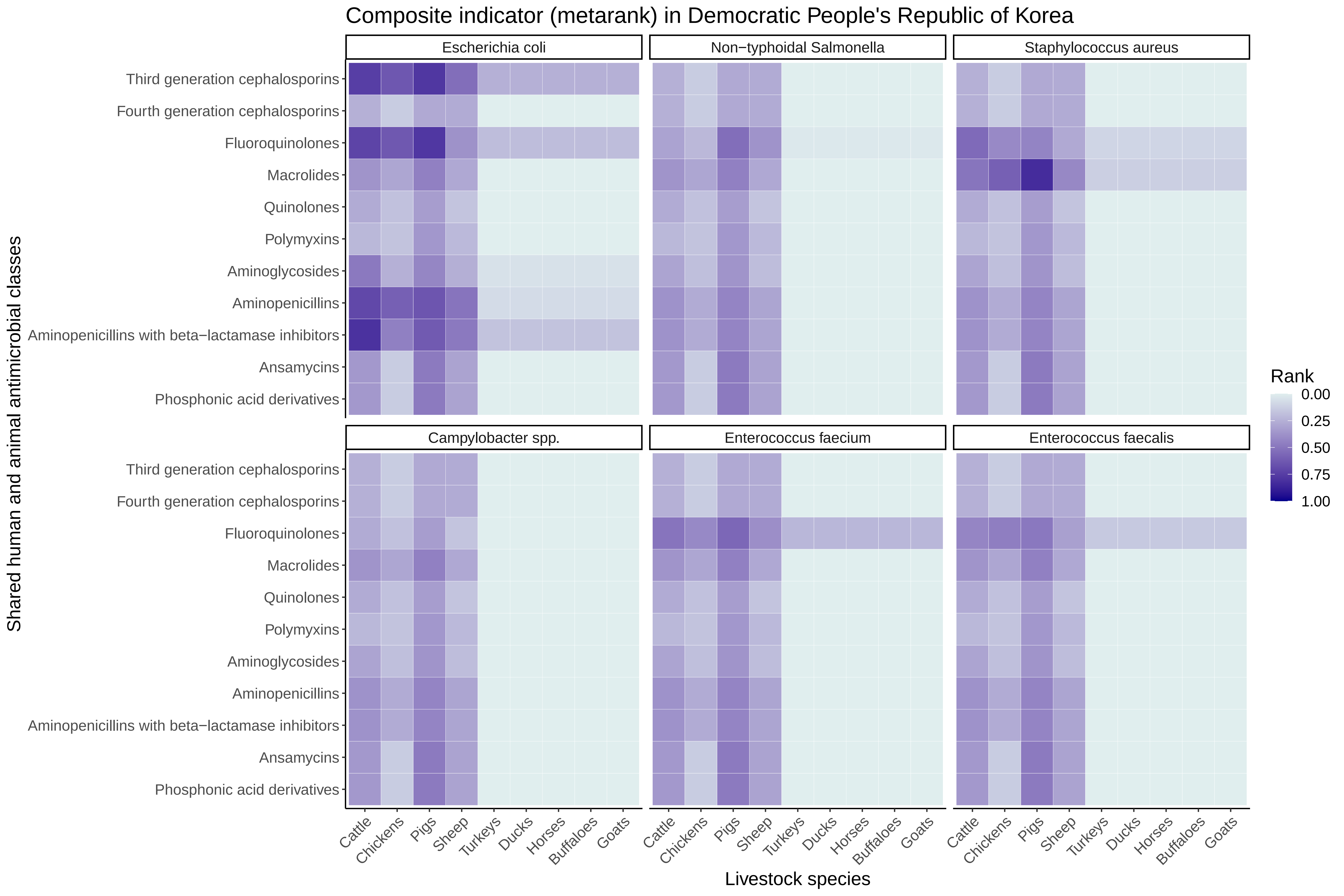
Composite indicator (metarank) in Democratic People's Republic of Korea
Escherichia coli
Non−typhoidal Salmonella
Staphylococcus aureus
Campylobacter spp.
Enterococcus faecium
Enterococcus faecalis
Shared human and animal antimicrobial classes
Third generation cephalosporins
Fourth generation cephalosporins
Fluoroquinolones
Macrolides
Quinolones
Polymyxins
Aminoglycosides
Aminopenicillins
Aminopenicillins with beta−lactamase inhibitors
Ansamycins
Phosphonic acid derivatives
Livestock species
Cattle
Chickens
Pigs
Sheep
Turkeys
Ducks
Horses
Buffaloes
Goats
Rank
0.00
0.25
0.50
0.75
1.00

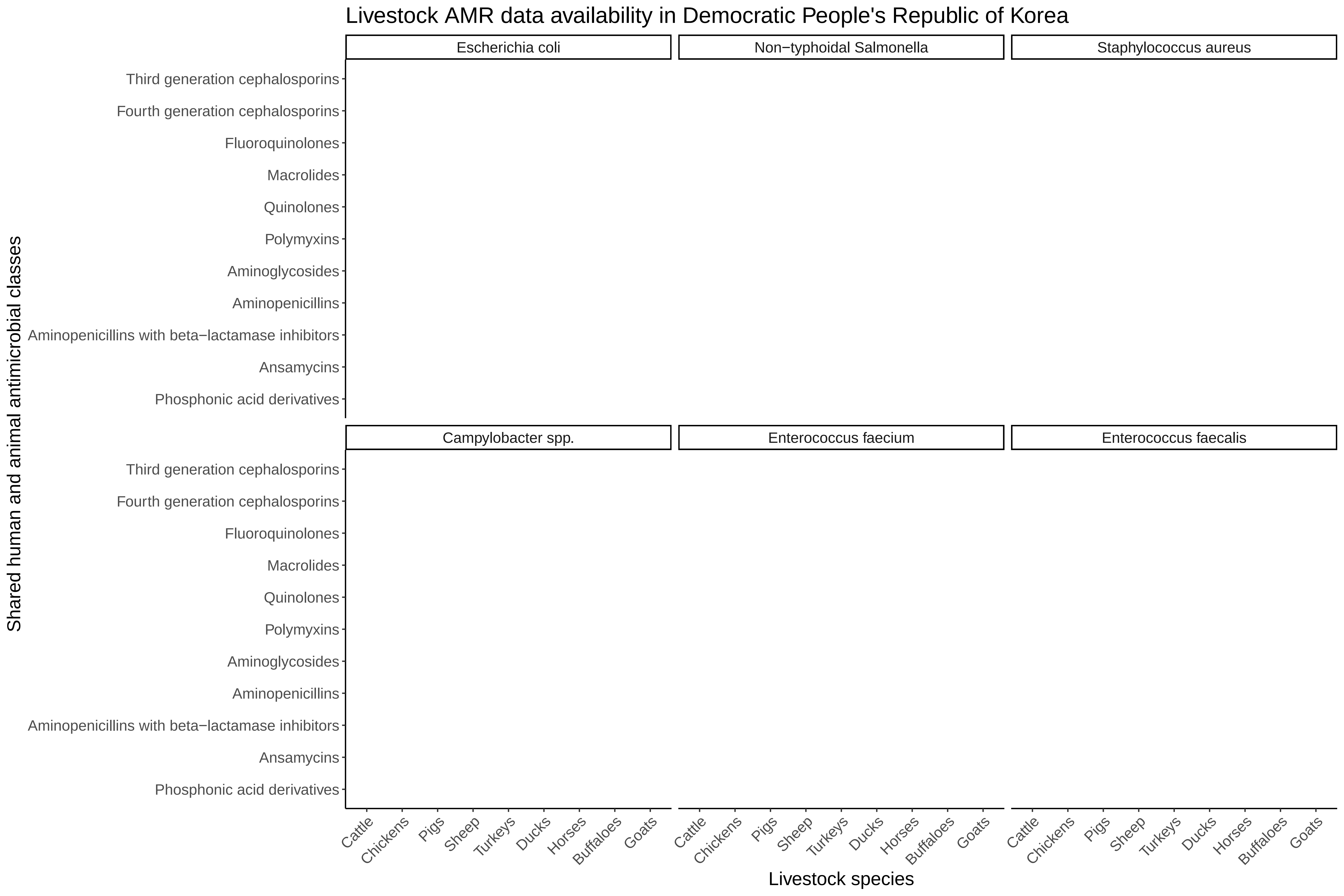
Livestock AMR data availability in Democratic People's Republic of Korea
Escherichia coli
Non−typhoidal Salmonella
Staphylococcus aureus
Campylobacter spp.
Enterococcus faecium
Enterococcus faecalis
Shared human and animal antimicrobial classes
Third generation cephalosporins
Fourth generation cephalosporins
Fluoroquinolones
Macrolides
Quinolones
Polymyxins
Aminoglycosides
Aminopenicillins
Aminopenicillins with beta−lactamase inhibitors
Ansamycins
Phosphonic acid derivatives
Cattle
Chickens
Pigs
Sheep
Turkeys
Ducks
Horses
Buffaloes
Goats
Livestock species

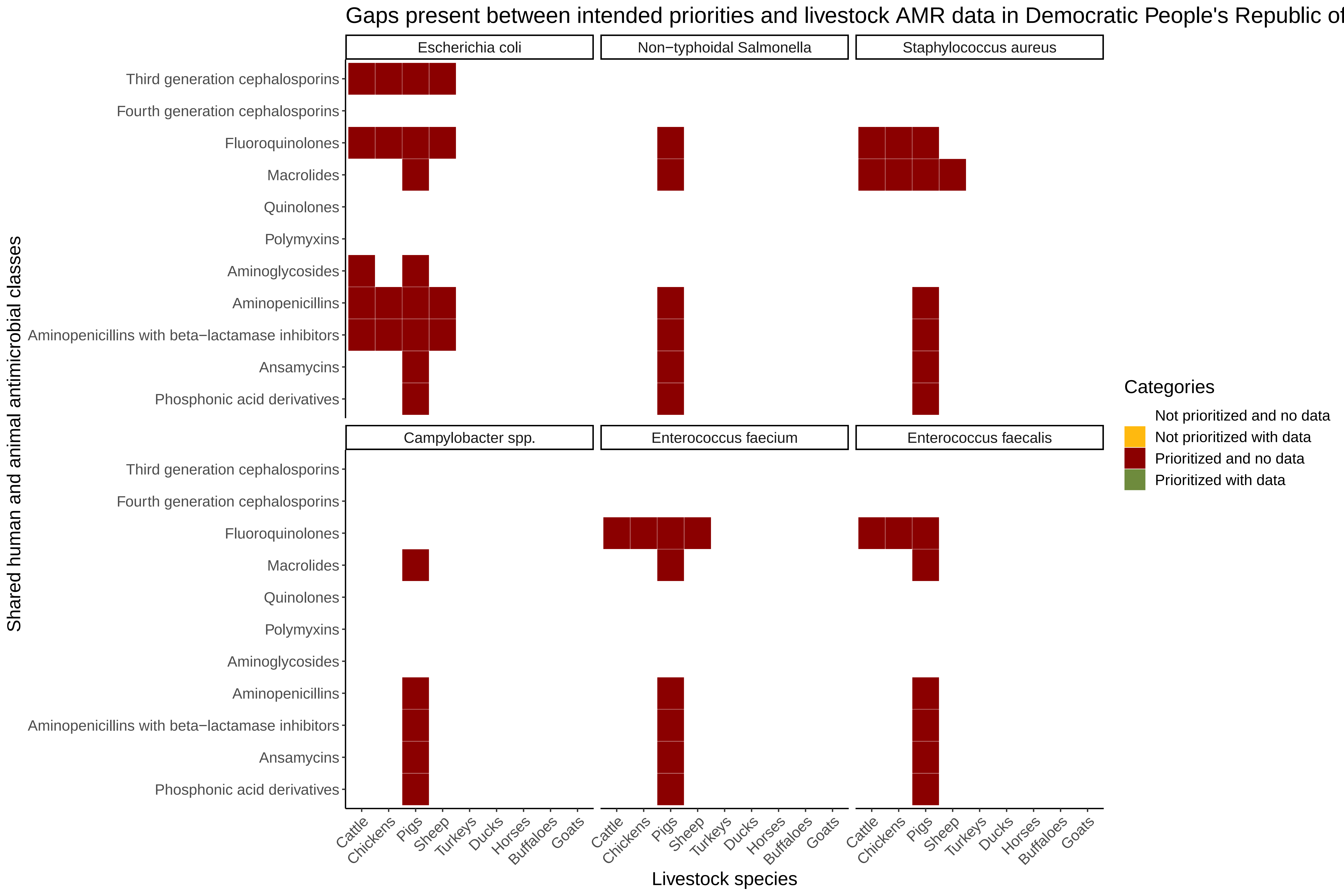
Gaps present between intended priorities and livestock AMR data in Democratic People's Republic of
Escherichia coli
Non−typhoidal Salmonella
Staphylococcus aureus
Campylobacter spp.
Enterococcus faecium
Enterococcus faecalis
Third generation cephalosporins
Fourth generation cephalosporins
Fluoroquinolones
Macrolides
Quinolones
Polymyxins
Aminoglycosides
Aminopenicillins
Aminopenicillins with beta−lactamase inhibitors
Ansamycins
Phosphonic acid derivatives
Shared human and animal antimicrobial classes
Cattle
Chickens
Pigs
Sheep
Turkeys
Ducks
Horses
Buffaloes
Goats
Livestock species
Categories
Not prioritized and no data
Not prioritized with data
Prioritized and no data
Prioritized with data

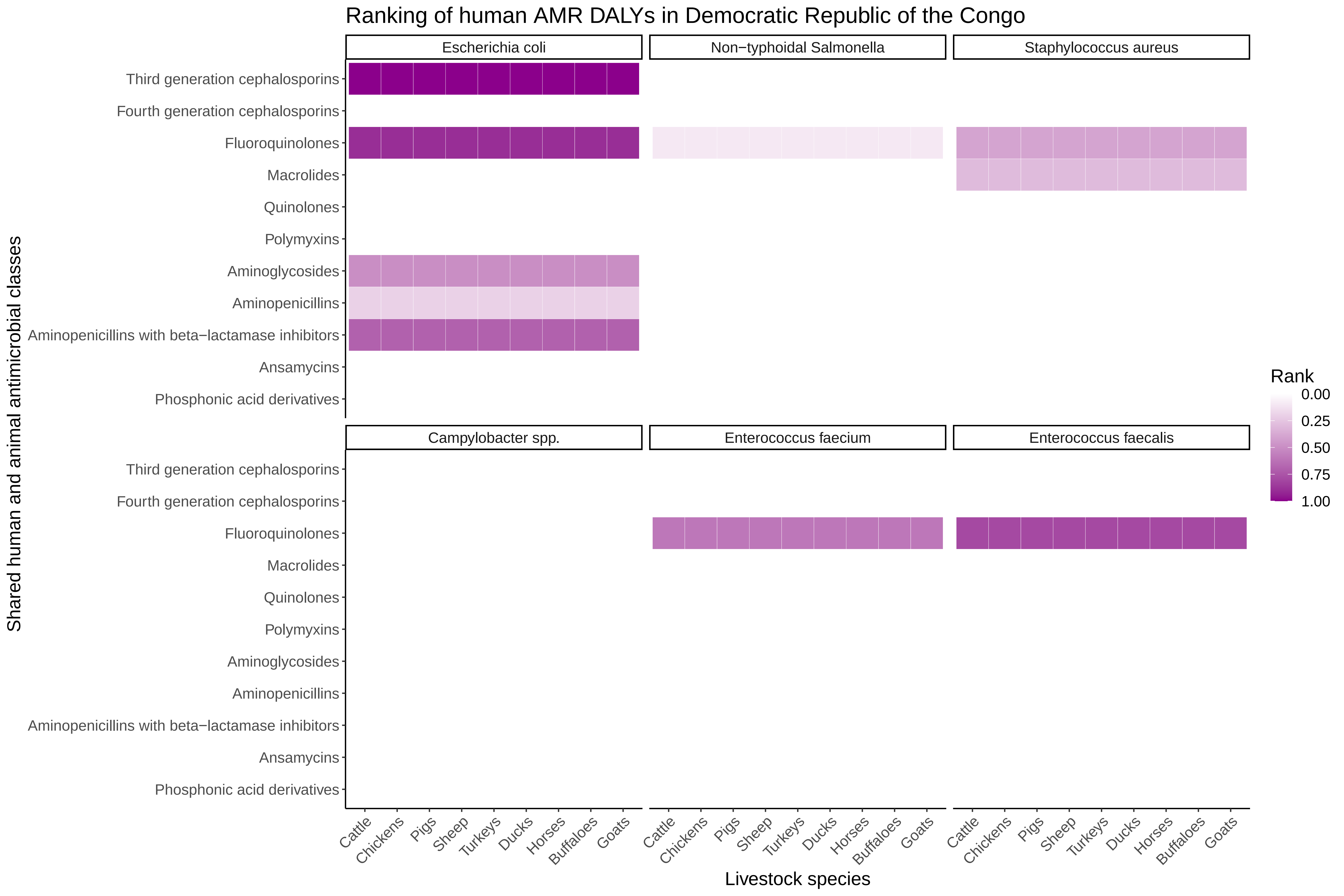

Ranking of human AMR DALYs in Democratic Republic of the Congo
Escherichia coli
Non−typhoidal Salmonella
Staphylococcus aureus
Campylobacter spp.
Enterococcus faecium
Enterococcus faecalis
Shared human and animal antimicrobial classes
Third generation cephalosporins
Fourth generation cephalosporins
Fluoroquinolones
Macrolides
Quinolones
Polymyxins
Aminoglycosides
Aminopenicillins
Aminopenicillins with beta−lactamase inhibitors
Ansamycins
Phosphonic acid derivatives
Cattle
Chickens
Pigs
Sheep
Turkeys
Ducks
Horses
Buffaloes
Goats
Livestock species
Rank
0.00
0.25
0.50
0.75
1.00

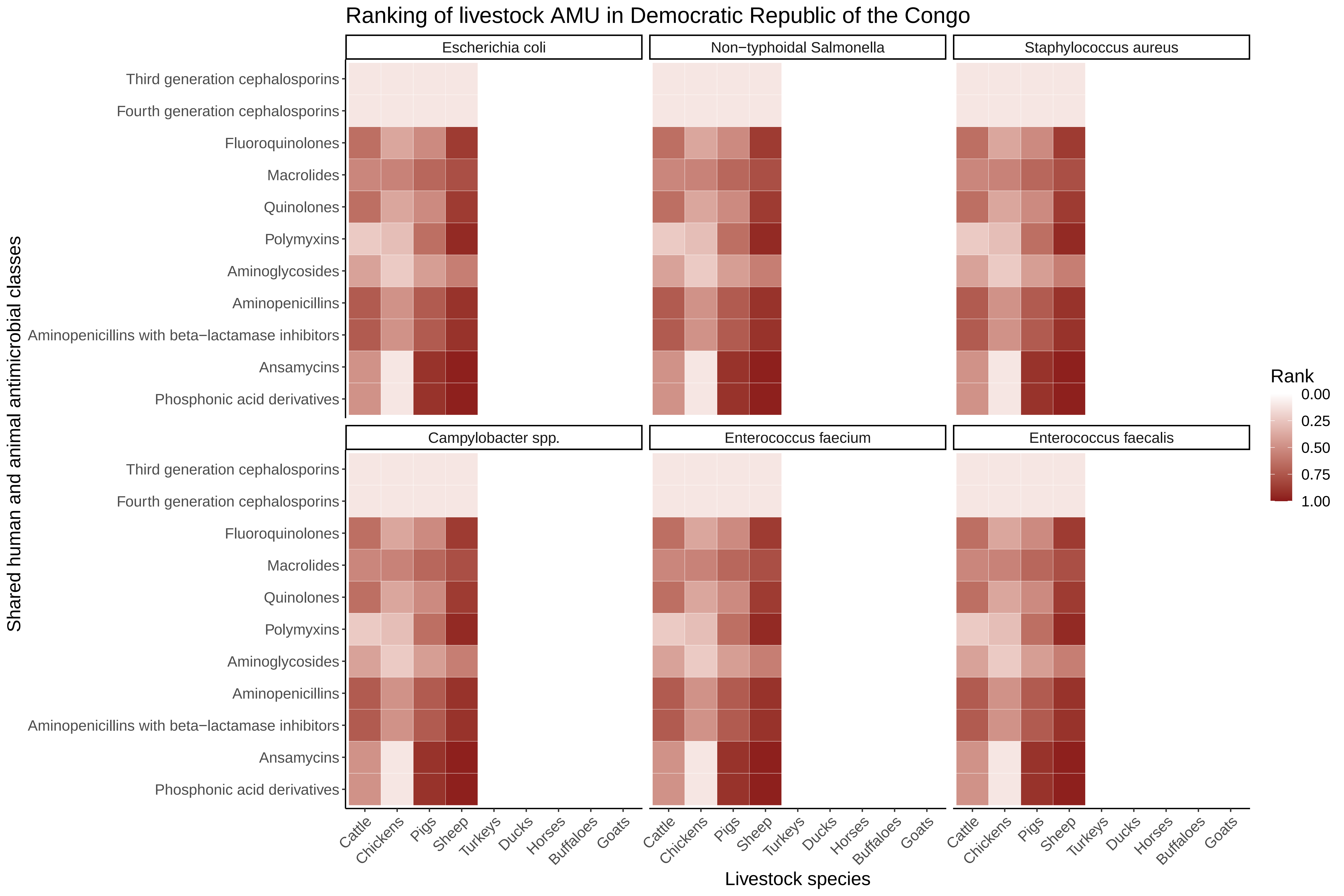

Ranking of livestock AMU in Democratic Republic of the Congo
Escherichia coli
Non−typhoidal Salmonella
Staphylococcus aureus
Campylobacter spp.
Enterococcus faecium
Enterococcus faecalis
Shared human and animal antimicrobial classes
Third generation cephalosporins
Fourth generation cephalosporins
Fluoroquinolones
Macrolides
Quinolones
Polymyxins
Aminoglycosides
Aminopenicillins
Aminopenicillins with beta−lactamase inhibitors
Ansamycins
Phosphonic acid derivatives
Livestock species
Cattle
Chickens
Pigs
Sheep
Turkeys
Ducks
Horses
Buffaloes
Goats
Rank
0.00
0.25
0.50
0.75
1.00

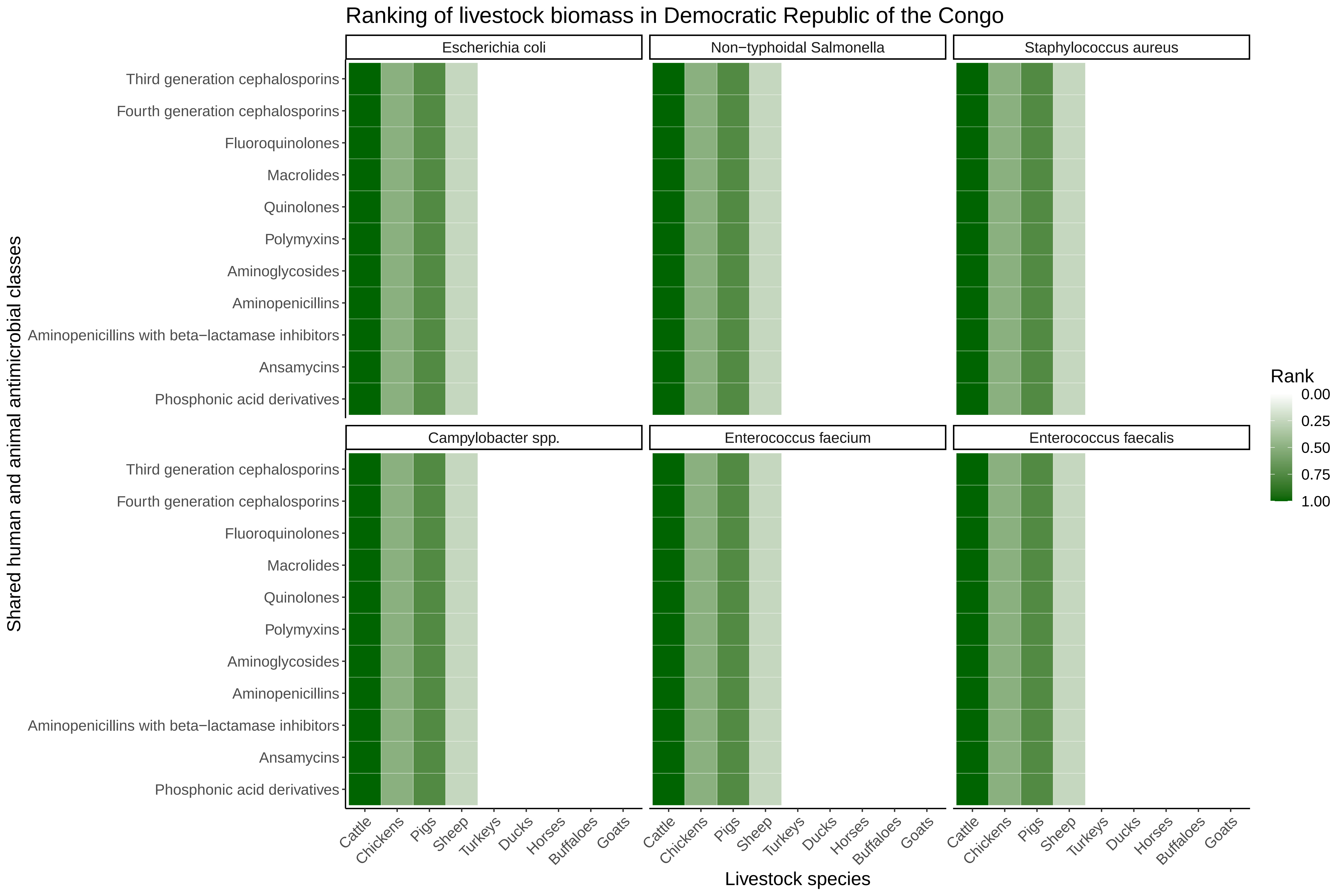
Ranking of livestock biomass in Democratic Republic of the Congo
Escherichia coli
Non−typhoidal Salmonella
Staphylococcus aureus
Campylobacter spp.
Enterococcus faecium
Enterococcus faecalis
Shared human and animal antimicrobial classes
Third generation cephalosporins
Fourth generation cephalosporins
Fluoroquinolones
Macrolides
Quinolones
Polymyxins
Aminoglycosides
Aminopenicillins
Aminopenicillins with beta−lactamase inhibitors
Ansamycins
Phosphonic acid derivatives
Cattle
Chickens
Pigs
Sheep
Turkeys
Ducks
Horses
Buffaloes
Goats
Livestock species
Rank
0.00
0.25
0.50
0.75
1.00

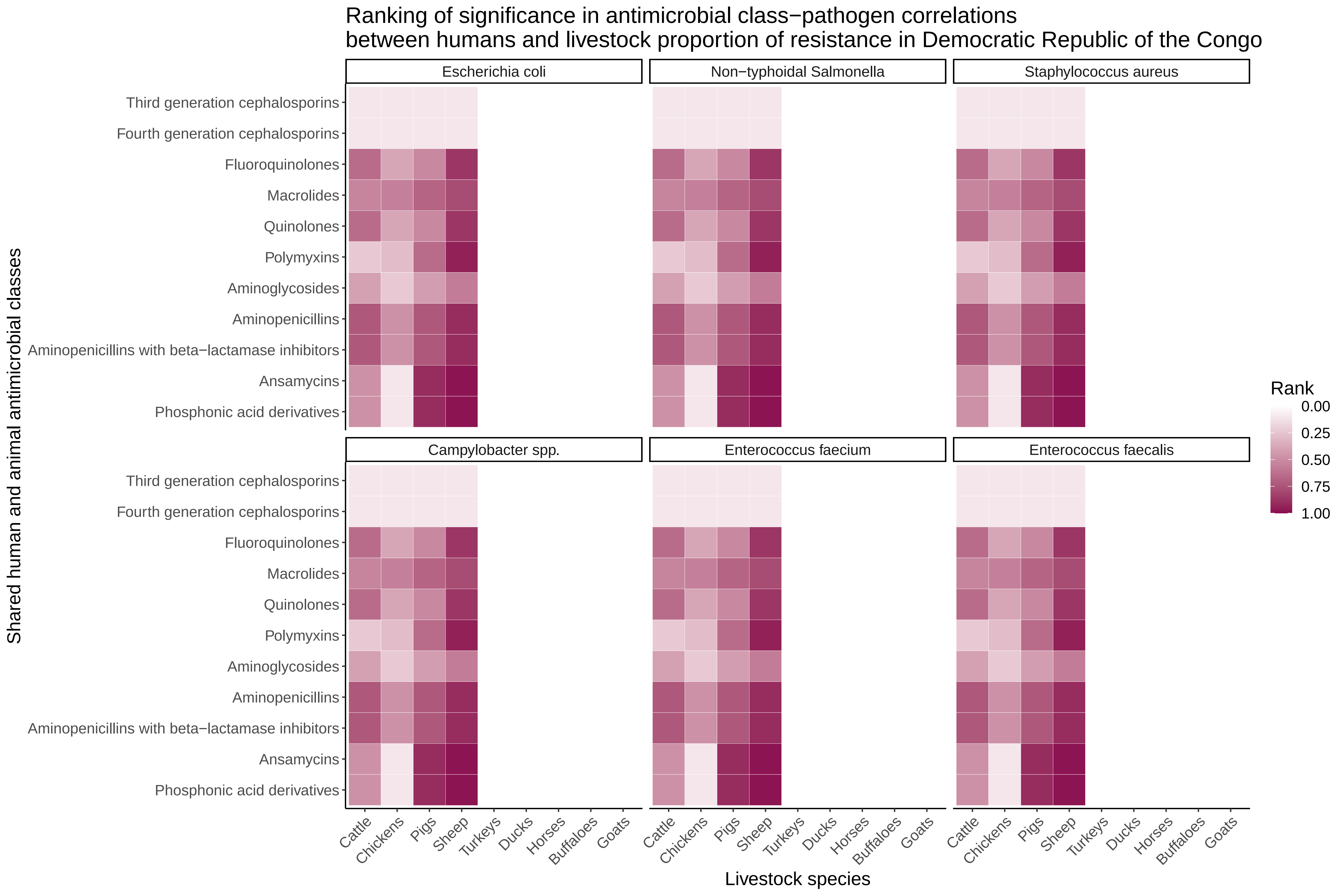
Ranking of significance in antimicrobial class−pathogen correlations
between humans and livestock proportion of resistance in Democratic Republic of the Congo
Escherichia coli
Non−typhoidal Salmonella
Staphylococcus aureus
Campylobacter spp.
Enterococcus faecium
Enterococcus faecalis
Third generation cephalosporins
Fourth generation cephalosporins
Fluoroquinolones
Macrolides
Quinolones
Polymyxins
Aminoglycosides
Aminopenicillins
Aminopenicillins with beta−lactamase inhibitors
Ansamycins
Phosphonic acid derivatives
Shared human and animal antimicrobial classes
Cattle
Chickens
Pigs
Sheep
Turkeys
Ducks
Horses
Buffaloes
Goats
Livestock species
Rank
0.00
0.25
0.50
0.75
1.00

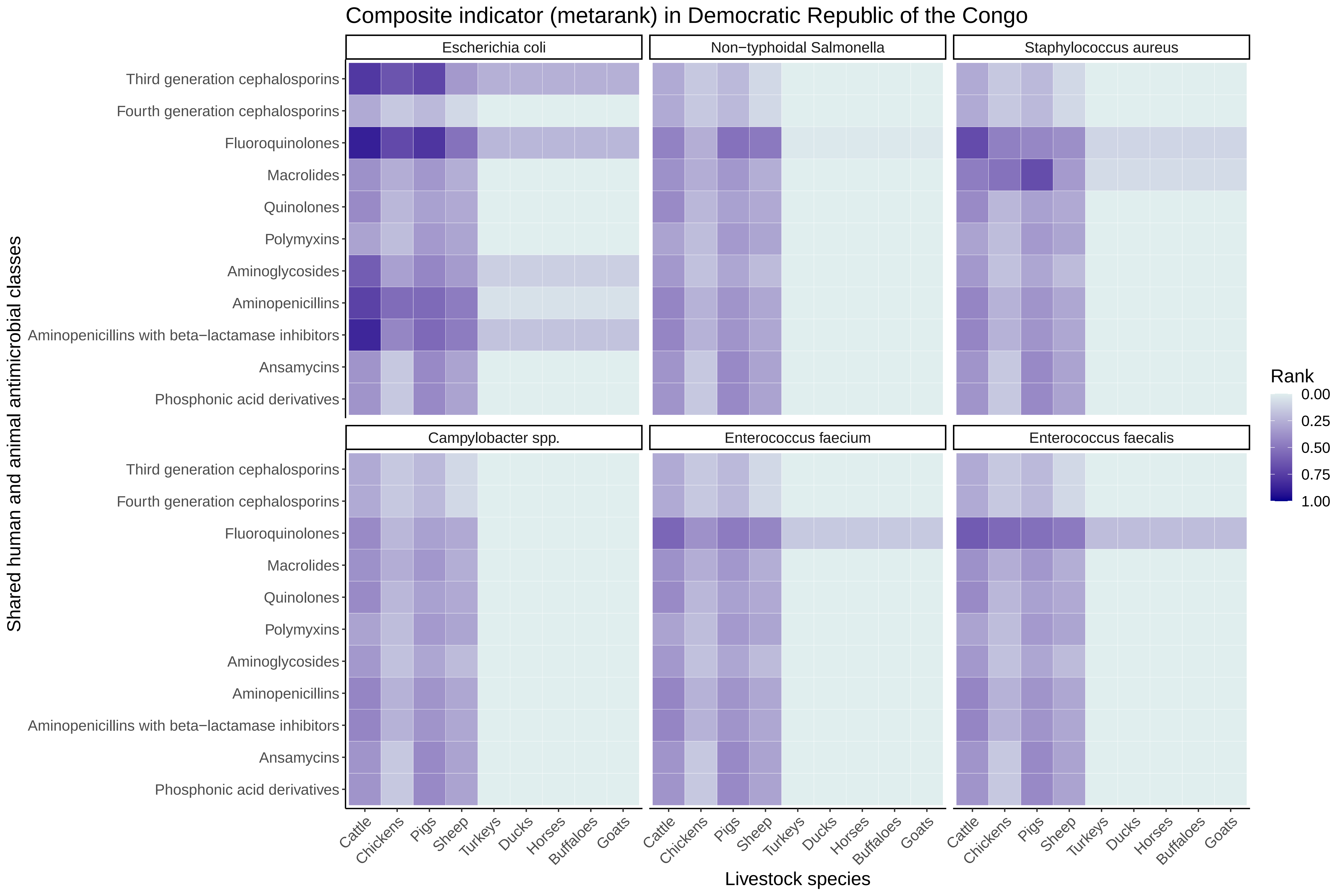

Composite indicator (metarank) in Democratic Republic of the Congo
Escherichia coli
Non−typhoidal Salmonella
Staphylococcus aureus
Campylobacter spp.
Enterococcus faecium
Enterococcus faecalis
Shared human and animal antimicrobial classes
Third generation cephalosporins
Fourth generation cephalosporins
Fluoroquinolones
Macrolides
Quinolones
Polymyxins
Aminoglycosides
Aminopenicillins
Aminopenicillins with beta−lactamase inhibitors
Ansamycins
Phosphonic acid derivatives
Livestock species
Cattle
Chickens
Pigs
Sheep
Turkeys
Ducks
Horses
Buffaloes
Goats
Rank
0.00
0.25
0.50
0.75
1.00

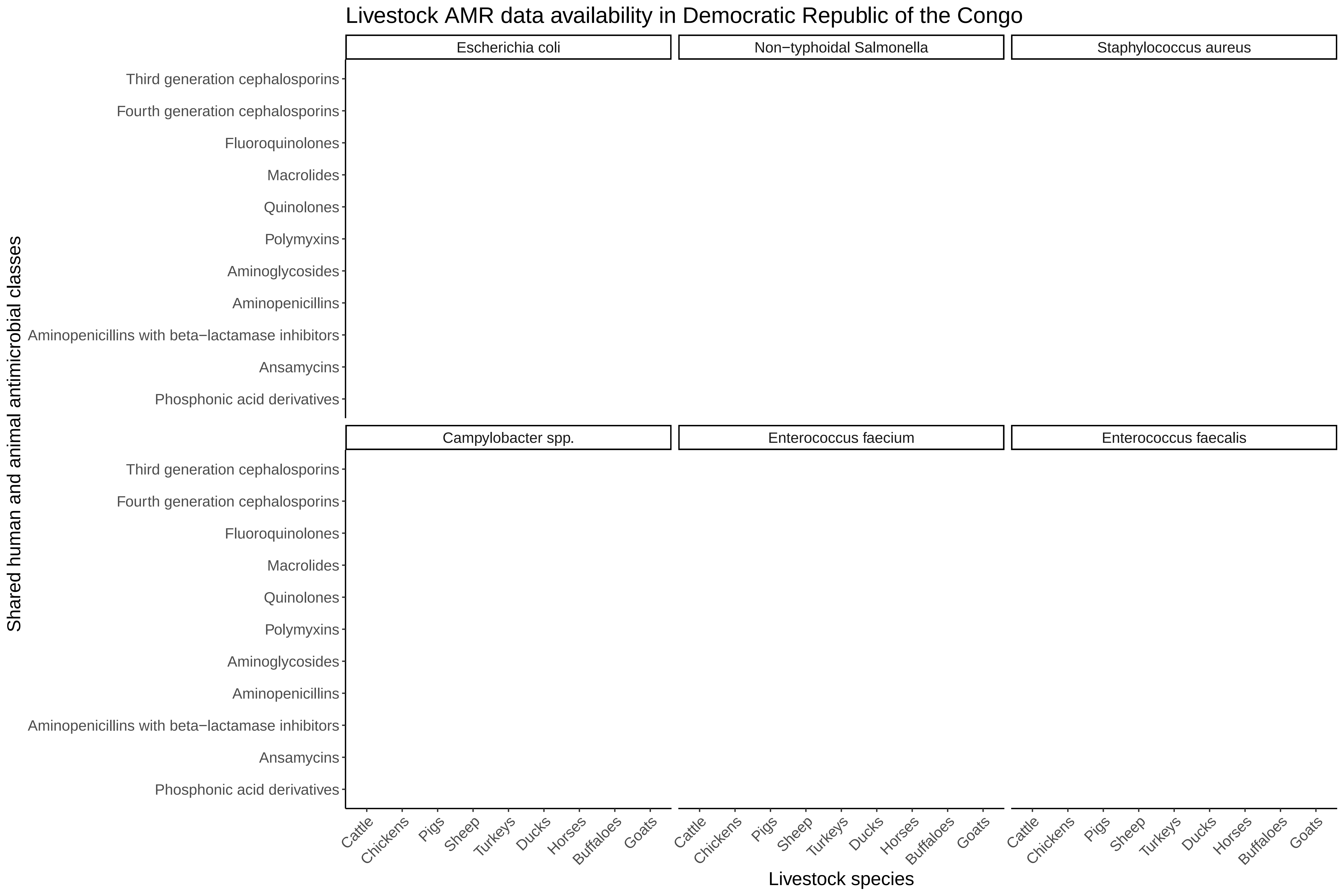
Livestock AMR data availability in Democratic Republic of the Congo
Escherichia coli
Non−typhoidal Salmonella
Staphylococcus aureus
Campylobacter spp.
Enterococcus faecium
Enterococcus faecalis
Shared human and animal antimicrobial classes
Third generation cephalosporins
Fourth generation cephalosporins
Fluoroquinolones
Macrolides
Quinolones
Polymyxins
Aminoglycosides
Aminopenicillins
Aminopenicillins with beta−lactamase inhibitors
Ansamycins
Phosphonic acid derivatives
Cattle
Chickens
Pigs
Sheep
Turkeys
Ducks
Horses
Buffaloes
Goats
Livestock species

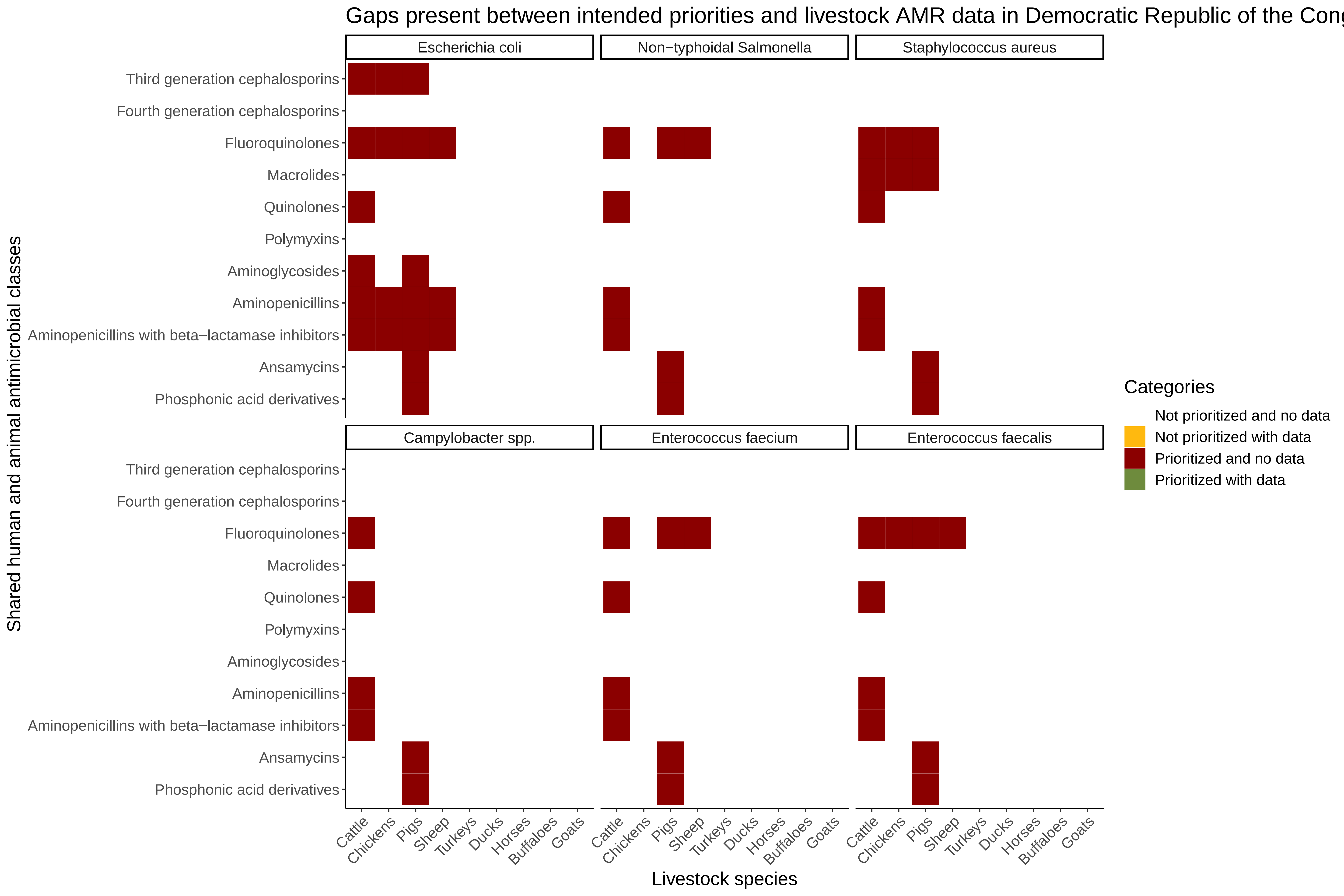

Gaps present between intended priorities and livestock AMR data in Democratic Republic of the Cong
Escherichia coli
Non−typhoidal Salmonella
Staphylococcus aureus
Campylobacter spp.
Enterococcus faecium
Enterococcus faecalis
Third generation cephalosporins
Fourth generation cephalosporins
Fluoroquinolones
Macrolides
Quinolones
Polymyxins
Aminoglycosides
Aminopenicillins
Aminopenicillins with beta−lactamase inhibitors
Ansamycins
Phosphonic acid derivatives
Shared human and animal antimicrobial classes
Cattle
Chickens
Pigs
Sheep
Turkeys
Ducks
Horses
Buffaloes
Goats
Livestock species
Categories
Not prioritized and no data
Not prioritized with data
Prioritized and no data
Prioritized with data

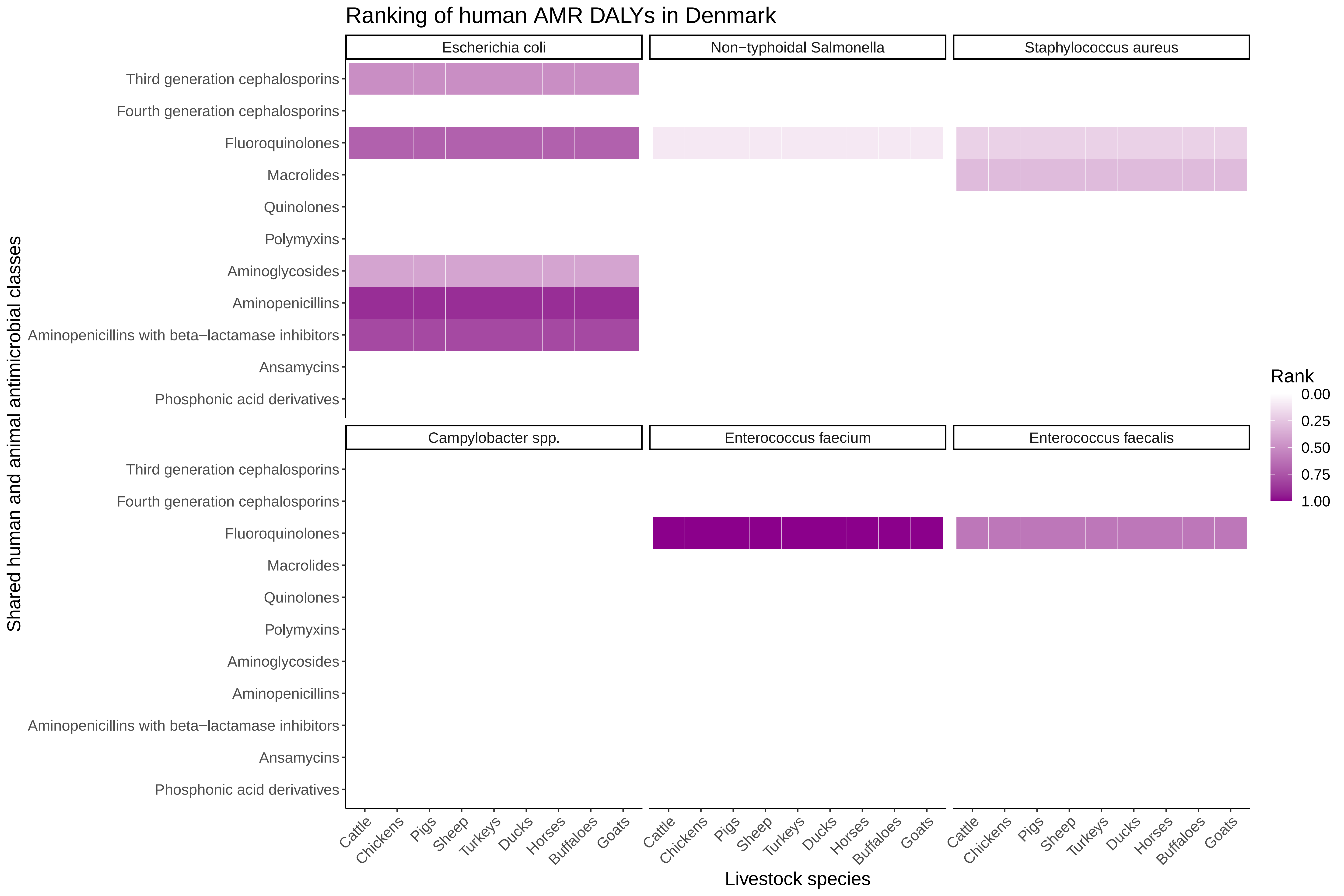
Ranking of human AMR DALYs in Denmark
Escherichia coli
Non−typhoidal Salmonella
Staphylococcus aureus
Campylobacter spp.
Enterococcus faecium
Enterococcus faecalis
Third generation cephalosporins
Fourth generation cephalosporins
Fluoroquinolones
Macrolides
Quinolones
Polymyxins
Aminoglycosides
Aminopenicillins
Aminopenicillins with beta−lactamase inhibitors
Ansamycins
Phosphonic acid derivatives
Shared human and animal antimicrobial classes
Cattle
Chickens
Pigs
Sheep
Turkeys
Ducks
Horses
Buffaloes
Goats
Livestock species
Rank
0.00
0.25
0.50
0.75
1.00

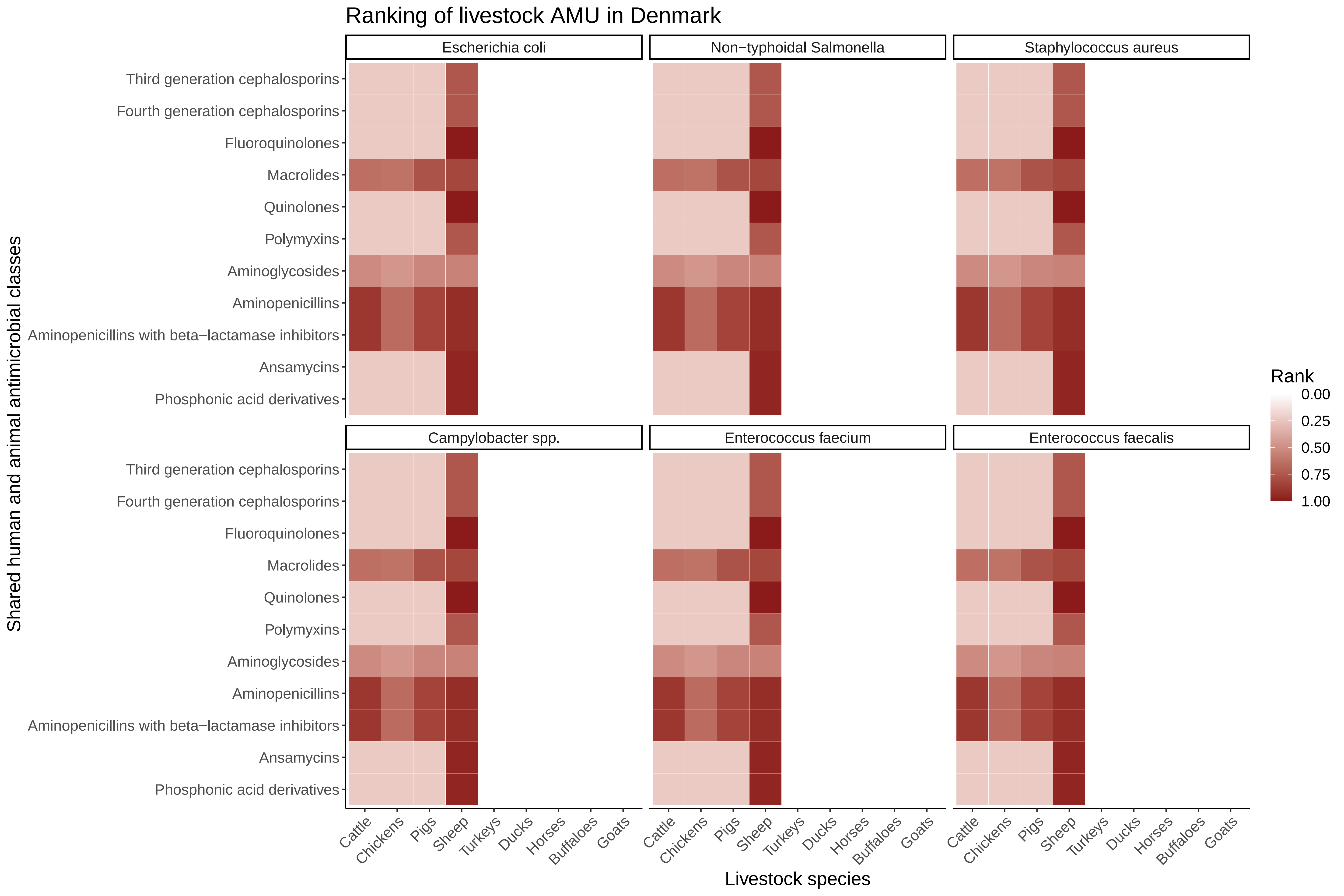

Ranking of livestock AMU in Denmark
Escherichia coli
Non−typhoidal Salmonella
Staphylococcus aureus
Campylobacter spp.
Enterococcus faecium
Enterococcus faecalis
Third generation cephalosporins
Fourth generation cephalosporins
Fluoroquinolones
Macrolides
Quinolones
Polymyxins
Aminoglycosides
Aminopenicillins
Aminopenicillins with beta−lactamase inhibitors
Ansamycins
Phosphonic acid derivatives
Shared human and animal antimicrobial classes
Cattle
Chickens
Pigs
Sheep
Turkeys
Ducks
Horses
Buffaloes
Goats
Livestock species
Rank
0.00
0.25
0.50
0.75
1.00

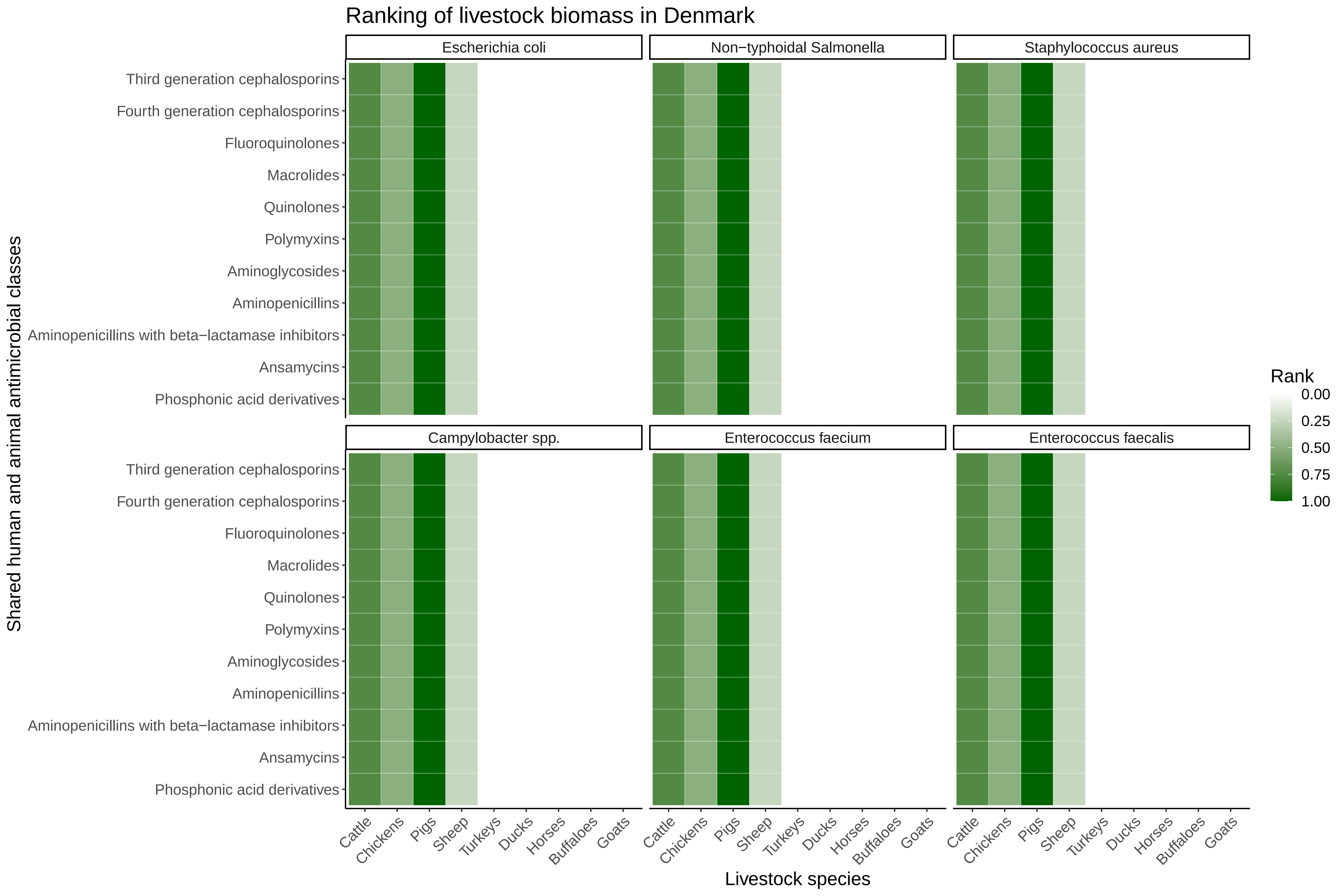
Ranking of livestock biomass in Denmark
Escherichia coli
Non−typhoidal Salmonella
Staphylococcus aureus
Campylobacter spp.
Enterococcus faecium
Enterococcus faecalis
Third generation cephalosporins
Fourth generation cephalosporins
Fluoroquinolones
Macrolides
Quinolones
Polymyxins
Aminoglycosides
Aminopenicillins
Aminopenicillins with beta−lactamase inhibitors
Ansamycins
Phosphonic acid derivatives
Shared human and animal antimicrobial classes
Cattle
Chickens
Pigs
Sheep
Turkeys
Ducks
Horses
Buffaloes
Goats
Livestock species
Rank
0.00
0.25
0.50
0.75
1.00

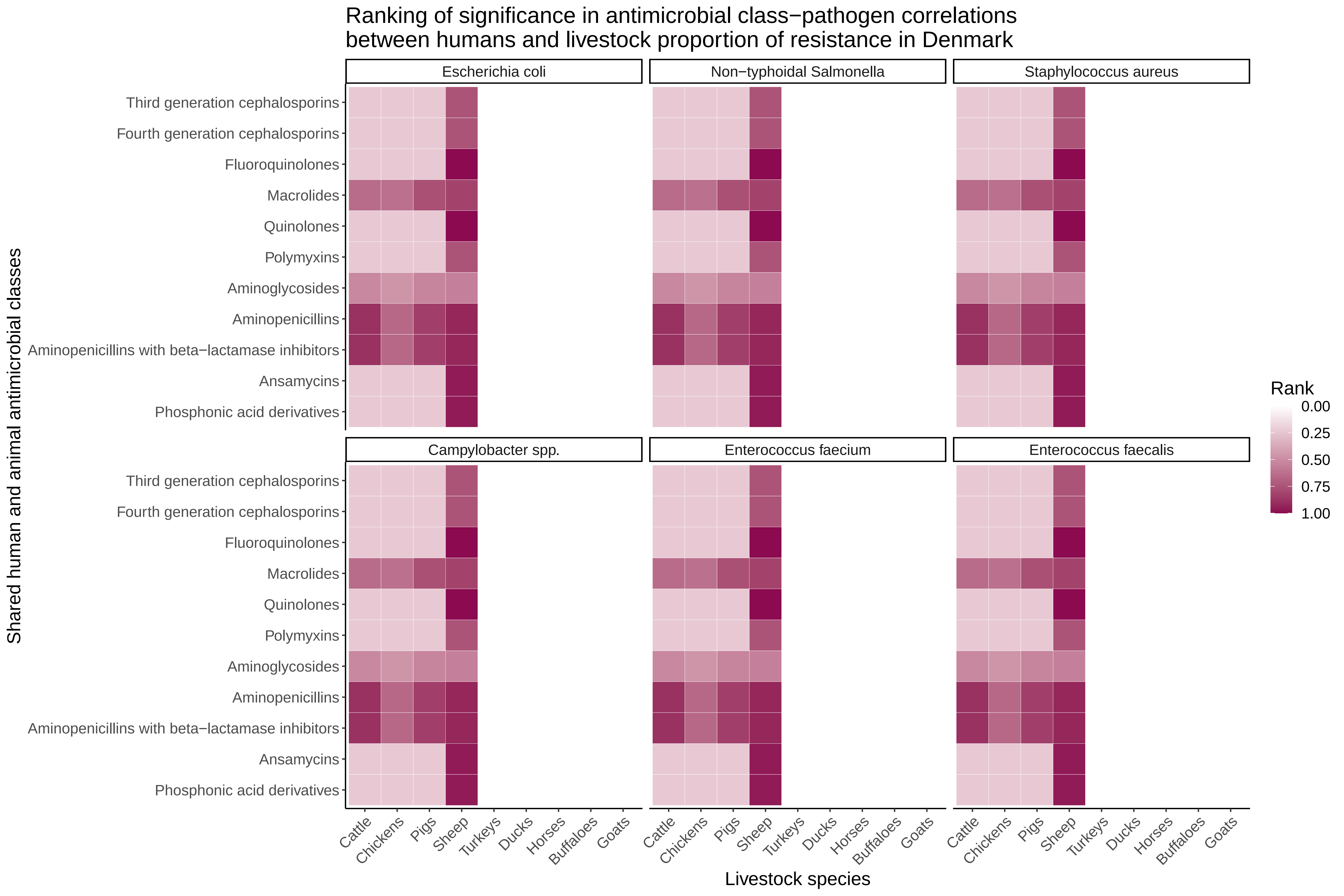

Ranking of significance in antimicrobial class−pathogen correlations
between humans and livestock proportion of resistance in Denmark
Escherichia coli
Non−typhoidal Salmonella
Staphylococcus aureus
Campylobacter spp.
Enterococcus faecium
Enterococcus faecalis
Third generation cephalosporins
Fourth generation cephalosporins
Fluoroquinolones
Macrolides
Quinolones
Polymyxins
Aminoglycosides
Aminopenicillins
Aminopenicillins with beta−lactamase inhibitors
Ansamycins
Phosphonic acid derivatives
Shared human and animal antimicrobial classes
Cattle
Chickens
Pigs
Sheep
Turkeys
Ducks
Horses
Buffaloes
Goats
Livestock species
Rank
0.00
0.25
0.50
0.75
1.00

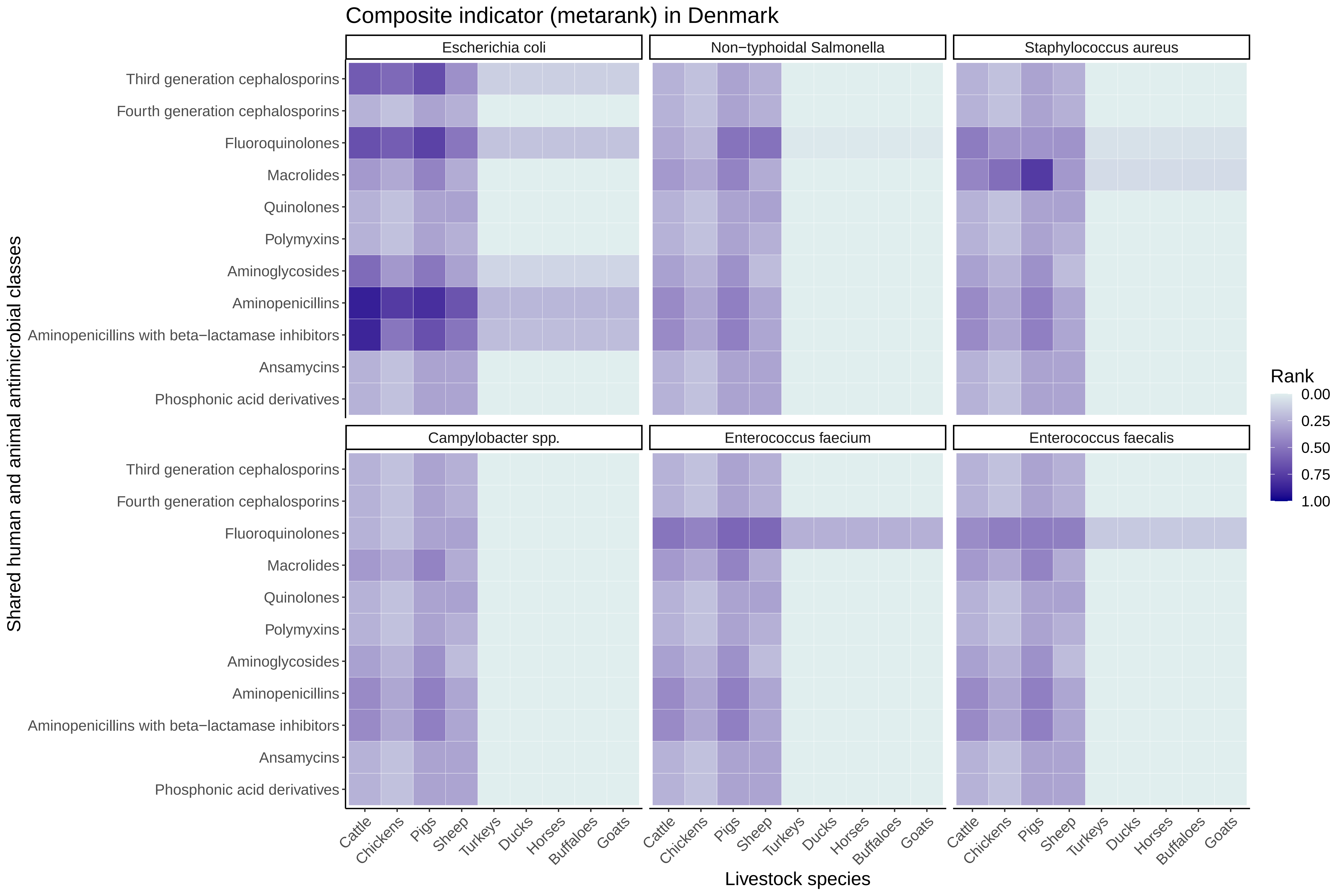
Composite indicator (metarank) in Denmark
Escherichia coli
Non−typhoidal Salmonella
Staphylococcus aureus
Campylobacter spp.
Enterococcus faecium
Enterococcus faecalis
Shared human and animal antimicrobial classes
Third generation cephalosporins
Fourth generation cephalosporins
Fluoroquinolones
Macrolides
Quinolones
Polymyxins
Aminoglycosides
Aminopenicillins
Aminopenicillins with beta−lactamase inhibitors
Ansamycins
Phosphonic acid derivatives
Livestock species
Cattle
Chickens
Pigs
Sheep
Turkeys
Ducks
Horses
Buffaloes
Goats
Rank
0.00
0.25
0.50
0.75
1.00

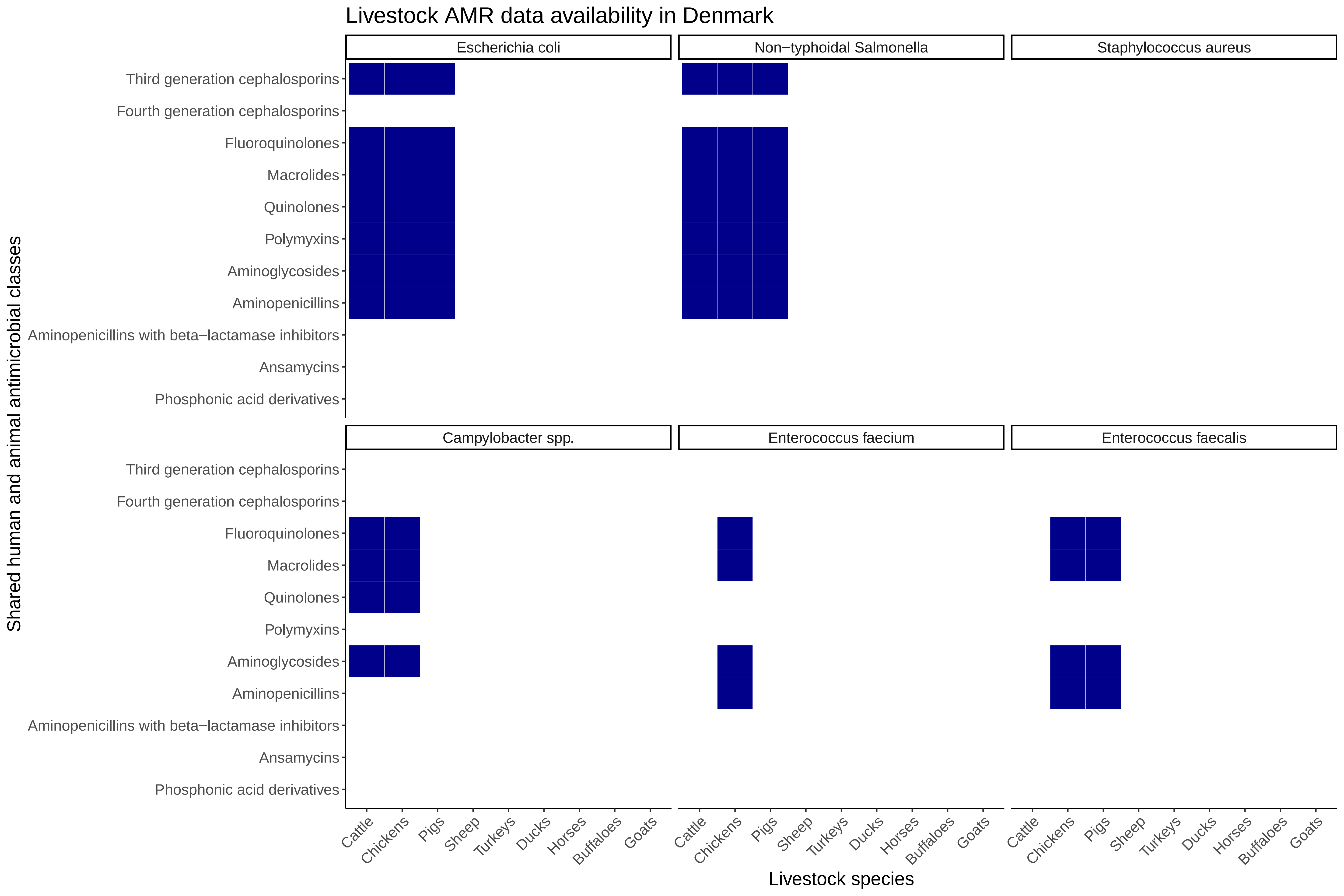
Livestock AMR data availability in Denmark
Escherichia coli
Non−typhoidal Salmonella
Staphylococcus aureus
Campylobacter spp.
Enterococcus faecium
Enterococcus faecalis
Shared human and animal antimicrobial classes
Third generation cephalosporins
Fourth generation cephalosporins
Fluoroquinolones
Macrolides
Quinolones
Polymyxins
Aminoglycosides
Aminopenicillins
Aminopenicillins with beta−lactamase inhibitors
Ansamycins
Phosphonic acid derivatives
Cattle
Chickens
Pigs
Sheep
Turkeys
Ducks
Horses
Buffaloes
Goats
Livestock species

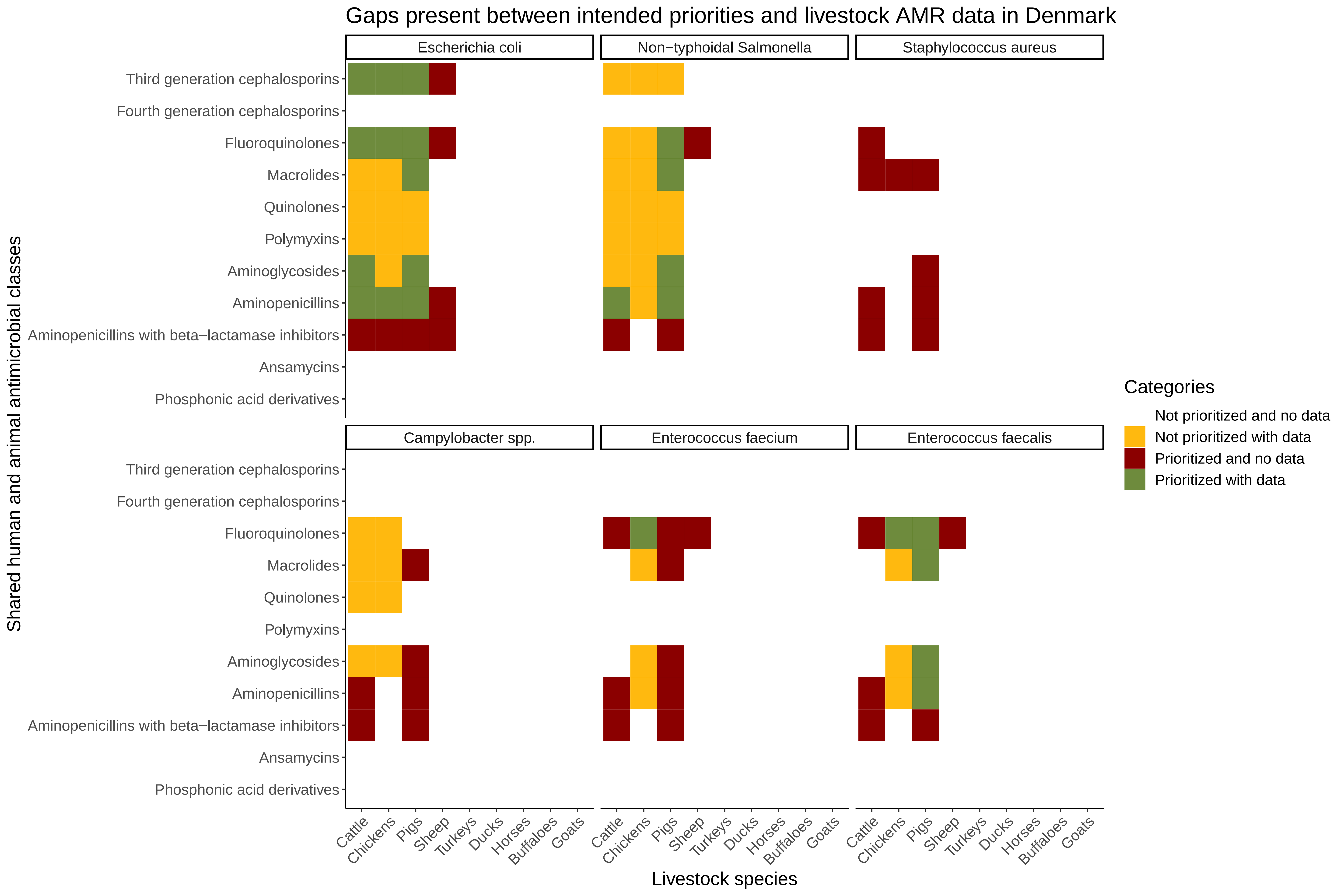

Gaps present between intended priorities and livestock AMR data in Denmark
Escherichia coli
Non−typhoidal Salmonella
Staphylococcus aureus
Campylobacter spp.
Enterococcus faecium
Enterococcus faecalis
Third generation cephalosporins
Fourth generation cephalosporins
Fluoroquinolones
Macrolides
Quinolones
Polymyxins
Aminoglycosides
Aminopenicillins
Aminopenicillins with beta−lactamase inhibitors
Ansamycins
Phosphonic acid derivatives
Shared human and animal antimicrobial classes
Cattle
Chickens
Pigs
Sheep
Turkeys
Ducks
Horses
Buffaloes
Goats
Livestock species
Categories
Not prioritized and no data
Not prioritized with data
Prioritized and no data
Prioritized with data

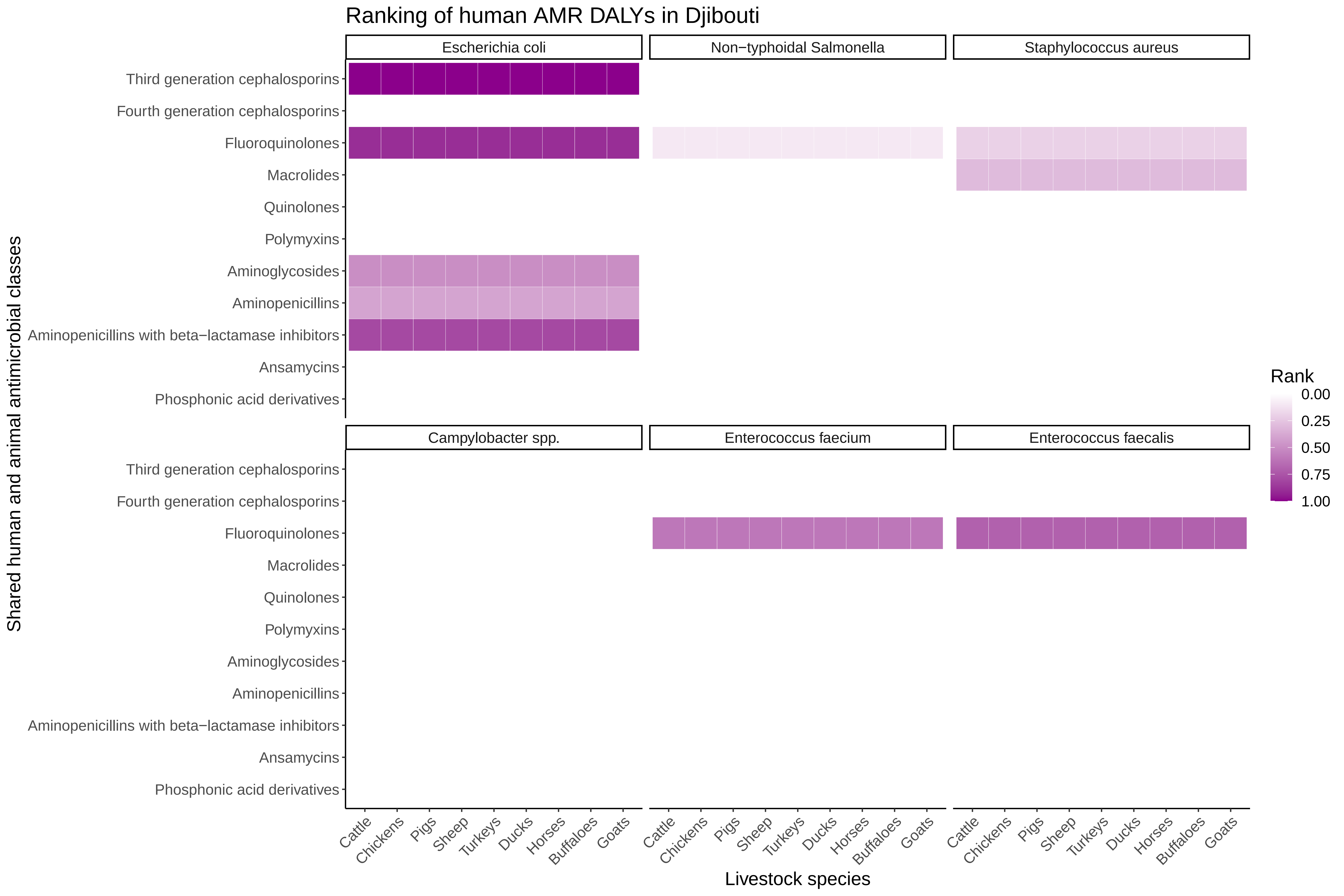

Ranking of human AMR DALYs in Djibouti
Escherichia coli
Non−typhoidal Salmonella
Staphylococcus aureus
Campylobacter spp.
Enterococcus faecium
Enterococcus faecalis
Third generation cephalosporins
Fourth generation cephalosporins
Fluoroquinolones
Macrolides
Quinolones
Polymyxins
Aminoglycosides
Aminopenicillins
Aminopenicillins with beta−lactamase inhibitors
Ansamycins
Phosphonic acid derivatives
Shared human and animal antimicrobial classes
Cattle
Chickens
Pigs
Sheep
Turkeys
Ducks
Horses
Buffaloes
Goats
Livestock species
Rank
0.00
0.25
0.50
0.75
1.00

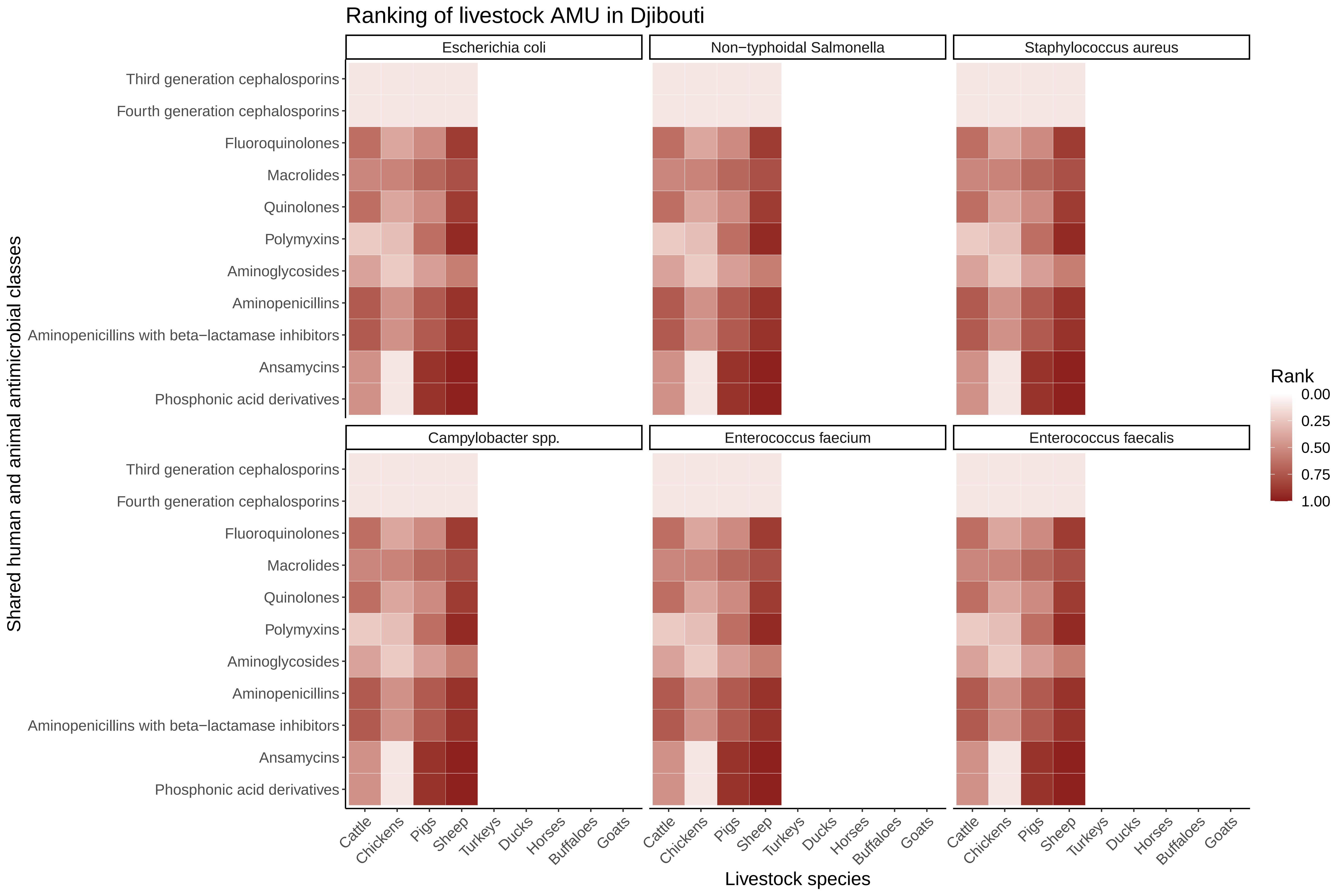

Ranking of livestock AMU in Djibouti
Escherichia coli
Non−typhoidal Salmonella
Staphylococcus aureus
Campylobacter spp.
Enterococcus faecium
Enterococcus faecalis
Shared human and animal antimicrobial classes
Third generation cephalosporins
Fourth generation cephalosporins
Fluoroquinolones
Macrolides
Quinolones
Polymyxins
Aminoglycosides
Aminopenicillins
Aminopenicillins with beta−lactamase inhibitors
Ansamycins
Phosphonic acid derivatives
Cattle
Chickens
Pigs
Sheep
Turkeys
Ducks
Horses
Buffaloes
Goats
Livestock species
Rank
0.00
0.25
0.50
0.75
1.00

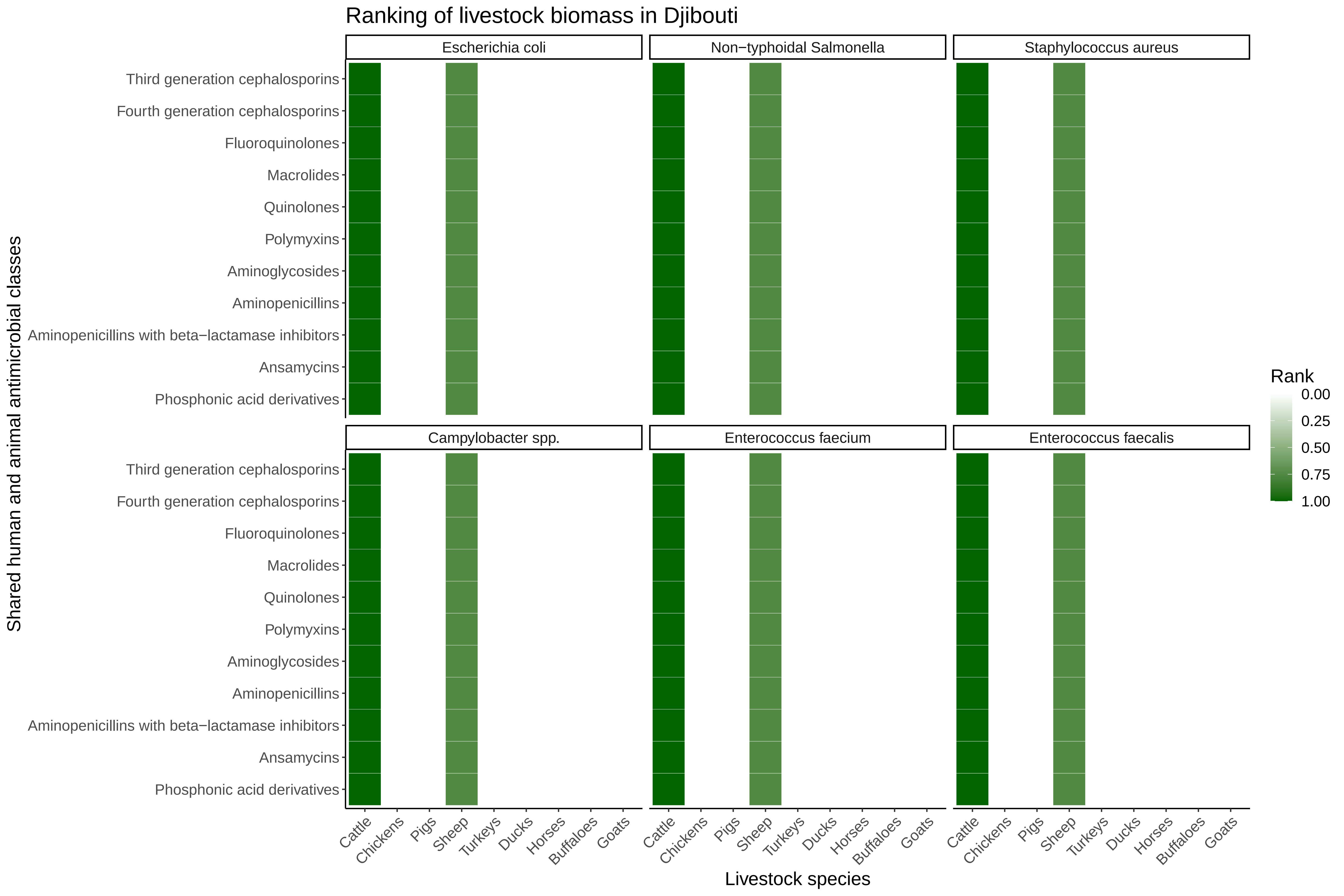

Ranking of livestock biomass in Djibouti
Escherichia coli
Non−typhoidal Salmonella
Staphylococcus aureus
Campylobacter spp.
Enterococcus faecium
Enterococcus faecalis
Third generation cephalosporins
Fourth generation cephalosporins
Fluoroquinolones
Macrolides
Quinolones
Polymyxins
Aminoglycosides
Aminopenicillins
Aminopenicillins with beta−lactamase inhibitors
Ansamycins
Phosphonic acid derivatives
Shared human and animal antimicrobial classes
Cattle
Chickens
Pigs
Sheep
Turkeys
Ducks
Horses
Buffaloes
Goats
Livestock species
Rank
0.00
0.25
0.50
0.75
1.00

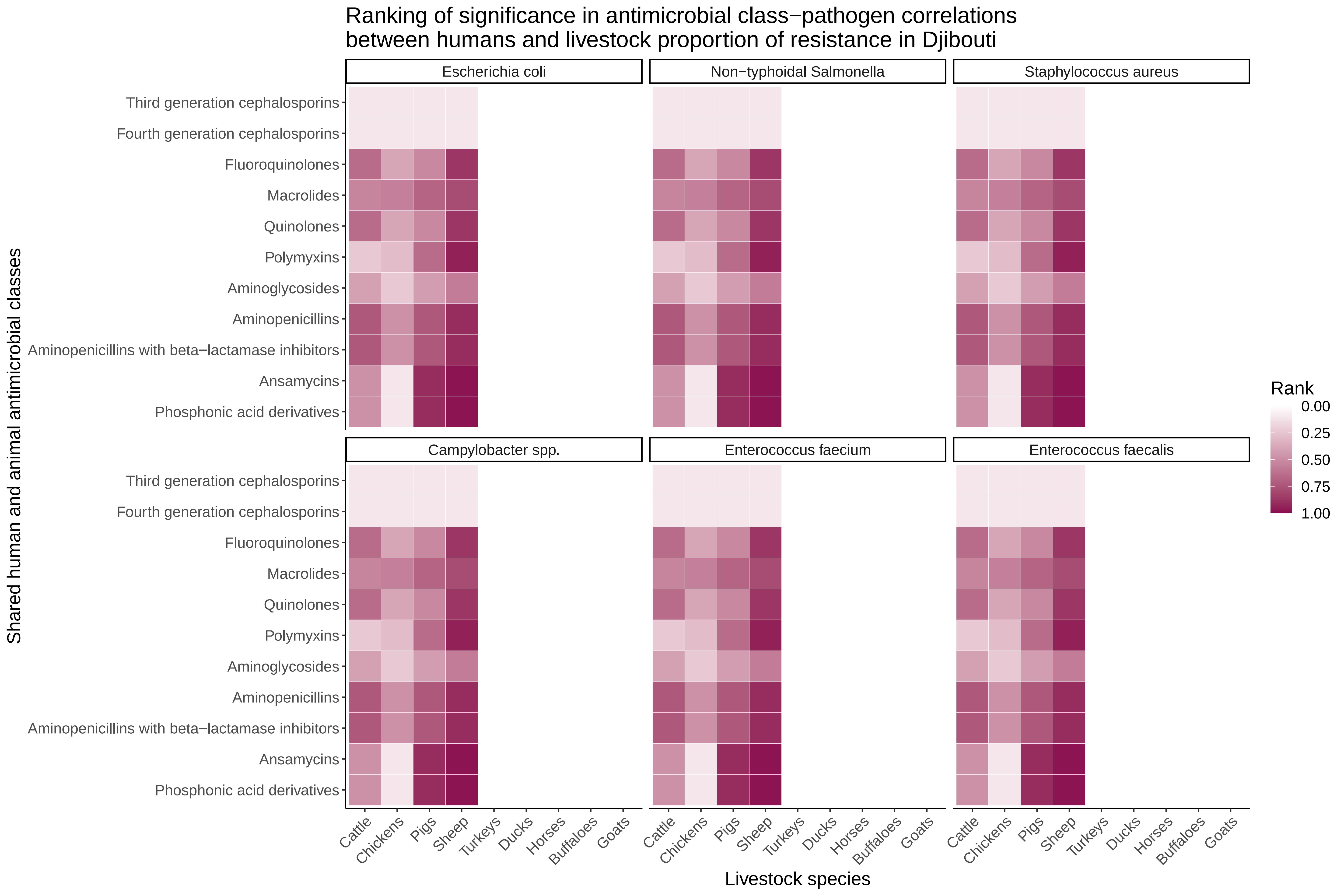
Ranking of significance in antimicrobial class−pathogen correlations
between humans and livestock proportion of resistance in Djibouti
Escherichia coli
Non−typhoidal Salmonella
Staphylococcus aureus
Campylobacter spp.
Enterococcus faecium
Enterococcus faecalis
Shared human and animal antimicrobial classes
Third generation cephalosporins
Fourth generation cephalosporins
Fluoroquinolones
Macrolides
Quinolones
Polymyxins
Aminoglycosides
Aminopenicillins
Aminopenicillins with beta−lactamase inhibitors
Ansamycins
Phosphonic acid derivatives
Livestock species
Cattle
Chickens
Pigs
Sheep
Turkeys
Ducks
Horses
Buffaloes
Goats
Rank
0.00
0.25
0.50
0.75
1.00

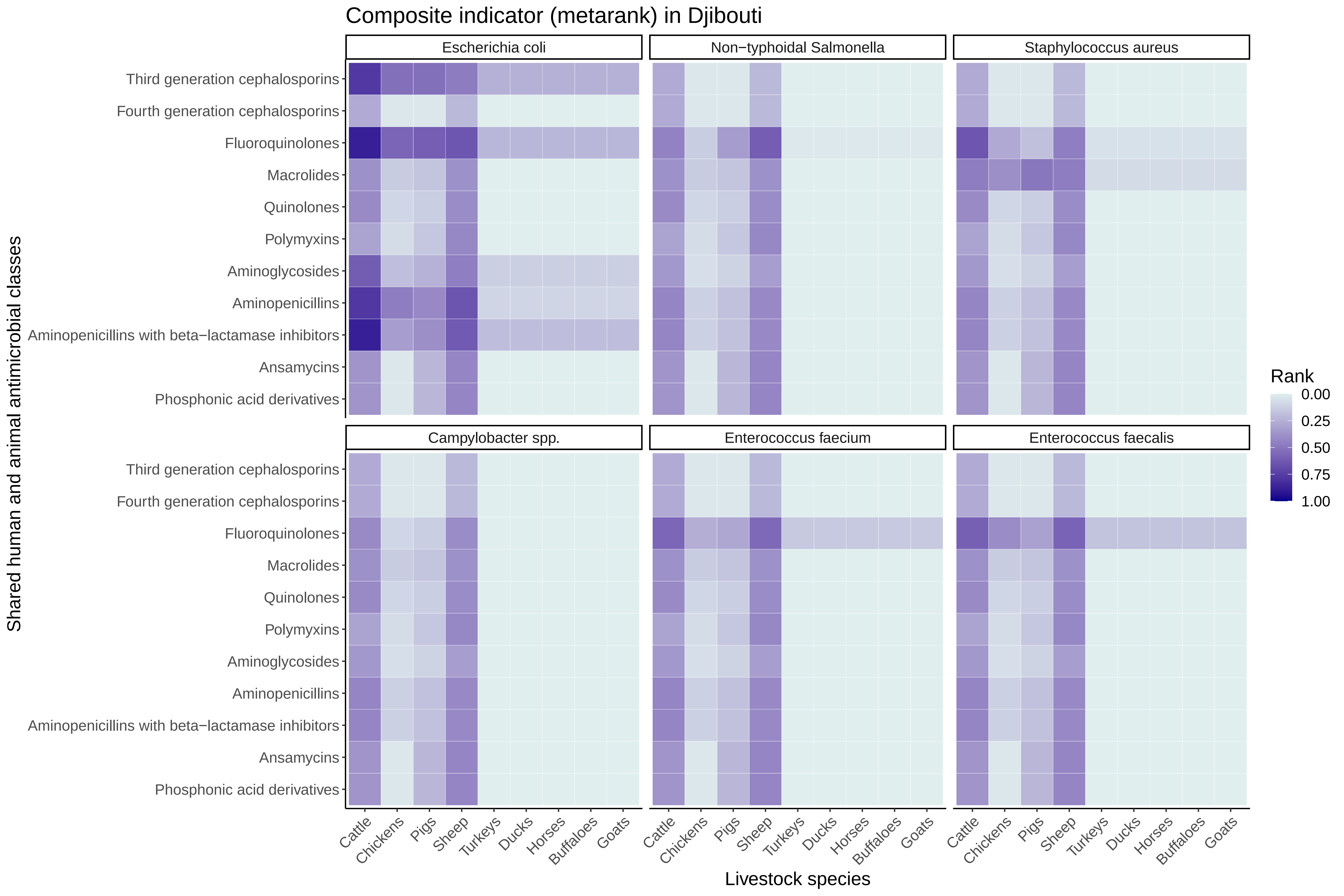

Composite indicator (metarank) in Djibouti
Escherichia coli
Non−typhoidal Salmonella
Staphylococcus aureus
Campylobacter spp.
Enterococcus faecium
Enterococcus faecalis
Third generation cephalosporins
Fourth generation cephalosporins
Fluoroquinolones
Macrolides
Quinolones
Polymyxins
Aminoglycosides
Aminopenicillins
Aminopenicillins with beta−lactamase inhibitors
Ansamycins
Phosphonic acid derivatives
Shared human and animal antimicrobial classes
Cattle
Chickens
Pigs
Sheep
Turkeys
Ducks
Horses
Buffaloes
Goats
Livestock species
Rank
0.00
0.25
0.50
0.75
1.00

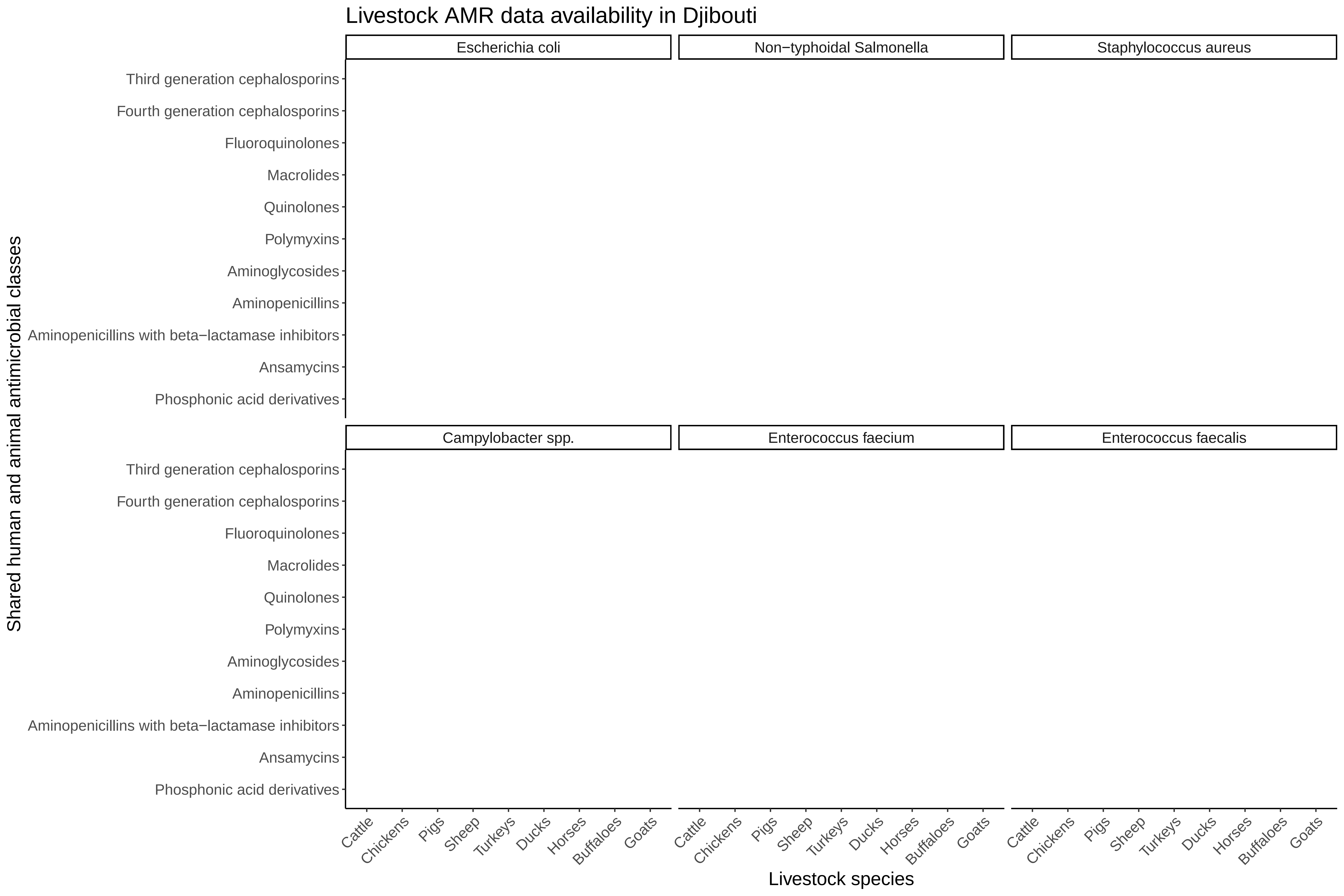
Livestock AMR data availability in Djibouti
Escherichia coli
Non−typhoidal Salmonella
Staphylococcus aureus
Campylobacter spp.
Enterococcus faecium
Enterococcus faecalis
Third generation cephalosporins
Fourth generation cephalosporins
Fluoroquinolones
Macrolides
Quinolones
Polymyxins
Aminoglycosides
Aminopenicillins
Aminopenicillins with beta−lactamase inhibitors
Ansamycins
Phosphonic acid derivatives
Shared human and animal antimicrobial classes
Cattle
Chickens
Pigs
Sheep
Turkeys
Ducks
Horses
Buffaloes
Goats
Livestock species

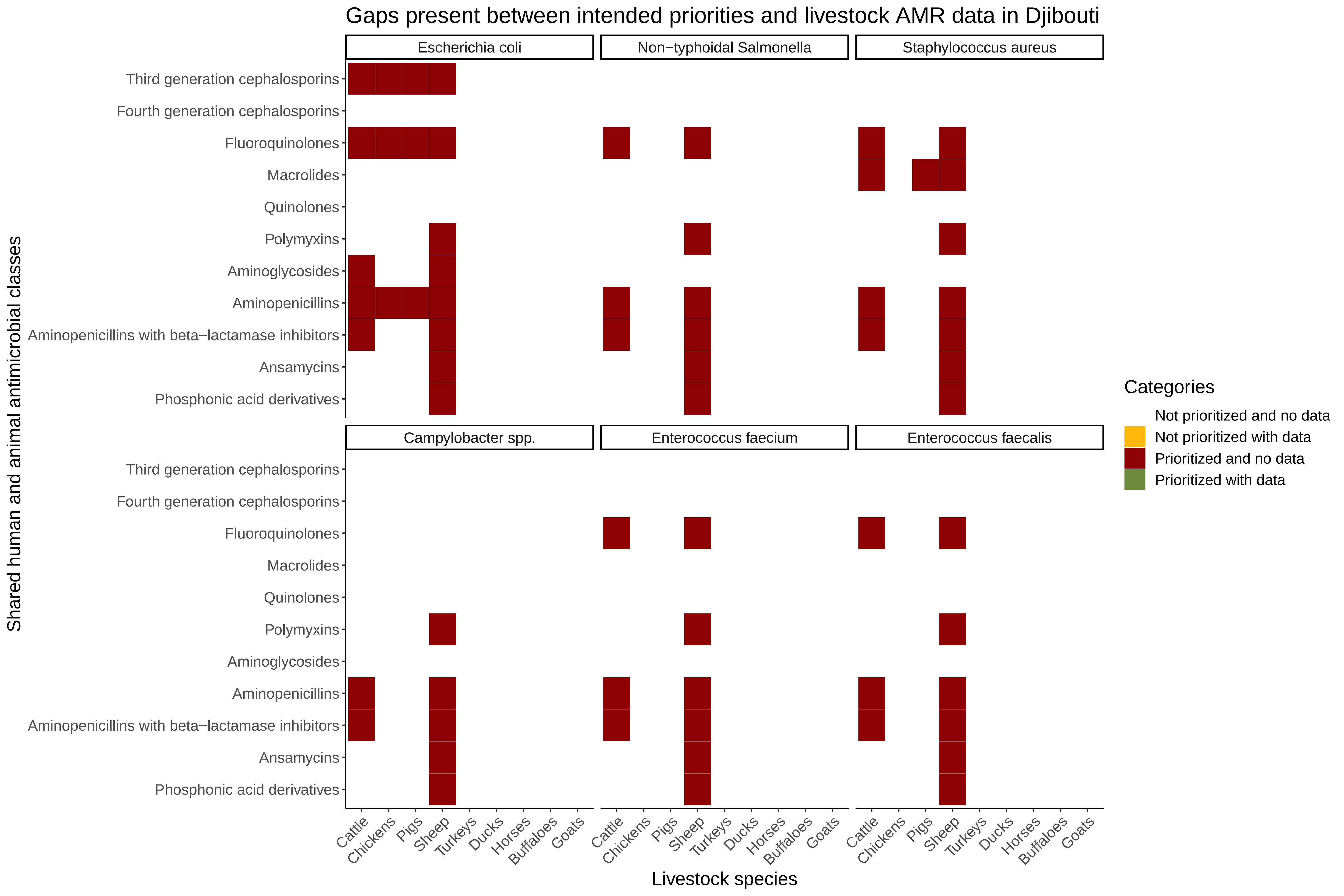

Gaps present between intended priorities and livestock AMR data in Djibouti
Escherichia coli
Non−typhoidal Salmonella
Staphylococcus aureus
Campylobacter spp.
Enterococcus faecium
Enterococcus faecalis
Shared human and animal antimicrobial classes
Third generation cephalosporins
Fourth generation cephalosporins
Fluoroquinolones
Macrolides
Quinolones
Polymyxins
Aminoglycosides
Aminopenicillins
Aminopenicillins with beta−lactamase inhibitors
Ansamycins
Phosphonic acid derivatives
Livestock species
Cattle
Chickens
Pigs
Sheep
Turkeys
Ducks
Horses
Buffaloes
Goats
Categories
Not prioritized and no data
Not prioritized with data
Prioritized and no data
Prioritized with data

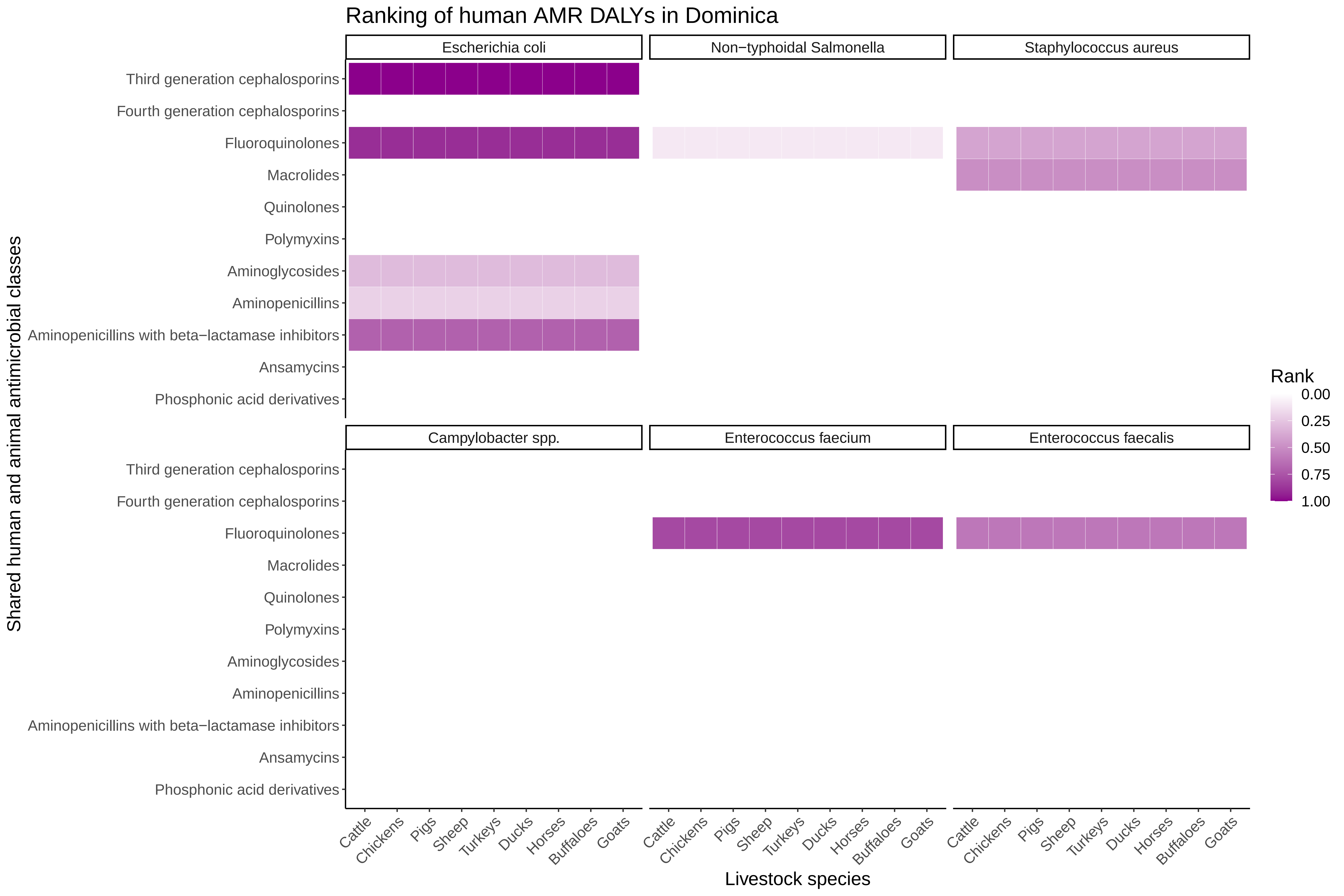

Ranking of human AMR DALYs in Dominica
Escherichia coli
Non−typhoidal Salmonella
Staphylococcus aureus
Campylobacter spp.
Enterococcus faecium
Enterococcus faecalis
Third generation cephalosporins
Fourth generation cephalosporins
Fluoroquinolones
Macrolides
Quinolones
Polymyxins
Aminoglycosides
Aminopenicillins
Aminopenicillins with beta−lactamase inhibitors
Ansamycins
Phosphonic acid derivatives
Shared human and animal antimicrobial classes
Cattle
Chickens
Pigs
Sheep
Turkeys
Ducks
Horses
Buffaloes
Goats
Livestock species
Rank
0.00
0.25
0.50
0.75
1.00

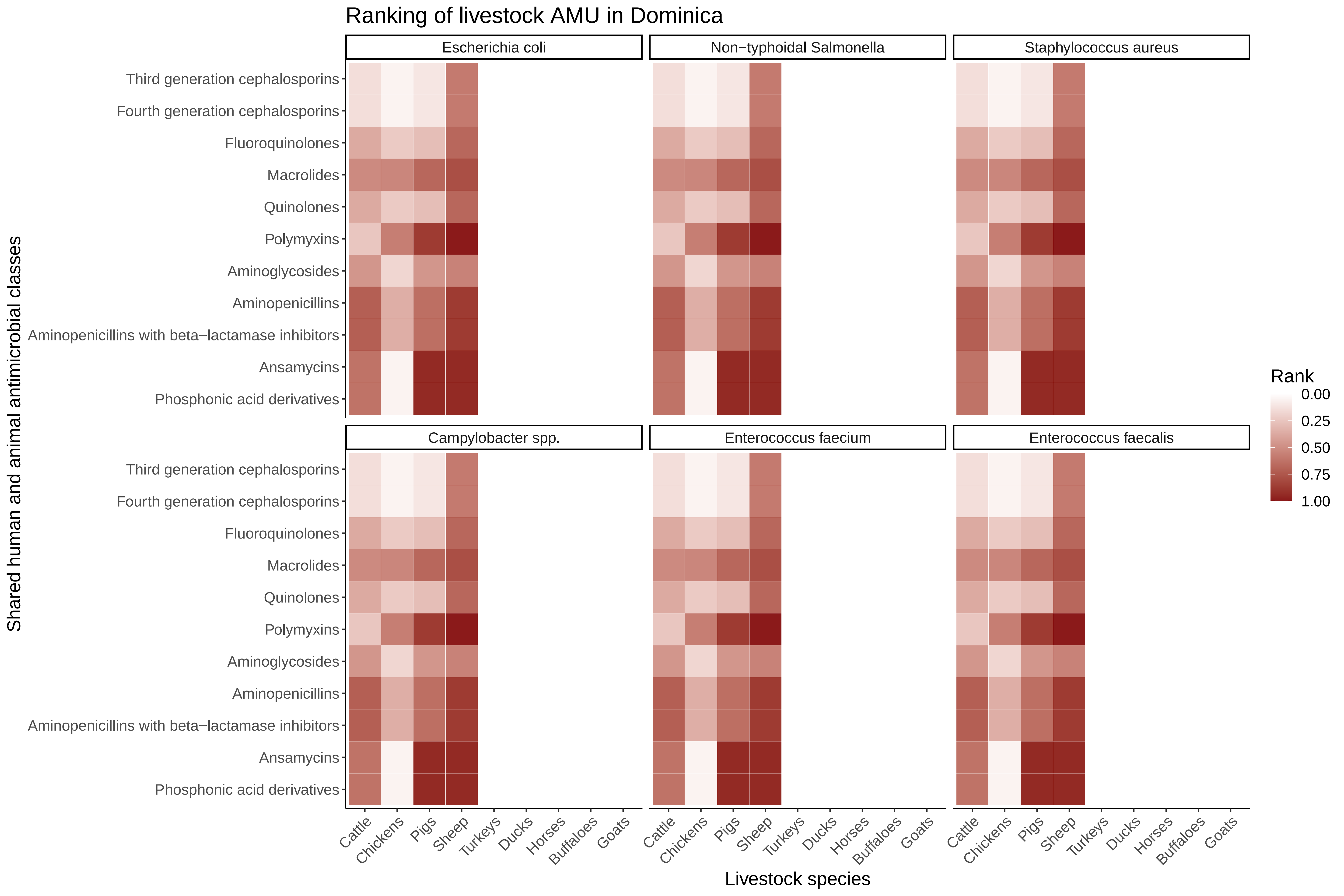
Ranking of livestock AMU in Dominica
Escherichia coli
Non−typhoidal Salmonella
Staphylococcus aureus
Campylobacter spp.
Enterococcus faecium
Enterococcus faecalis
Shared human and animal antimicrobial classes
Third generation cephalosporins
Fourth generation cephalosporins
Fluoroquinolones
Macrolides
Quinolones
Polymyxins
Aminoglycosides
Aminopenicillins
Aminopenicillins with beta−lactamase inhibitors
Ansamycins
Phosphonic acid derivatives
Livestock species
Cattle
Chickens
Pigs
Sheep
Turkeys
Ducks
Horses
Buffaloes
Goats
Rank
0.00
0.25
0.50
0.75
1.00

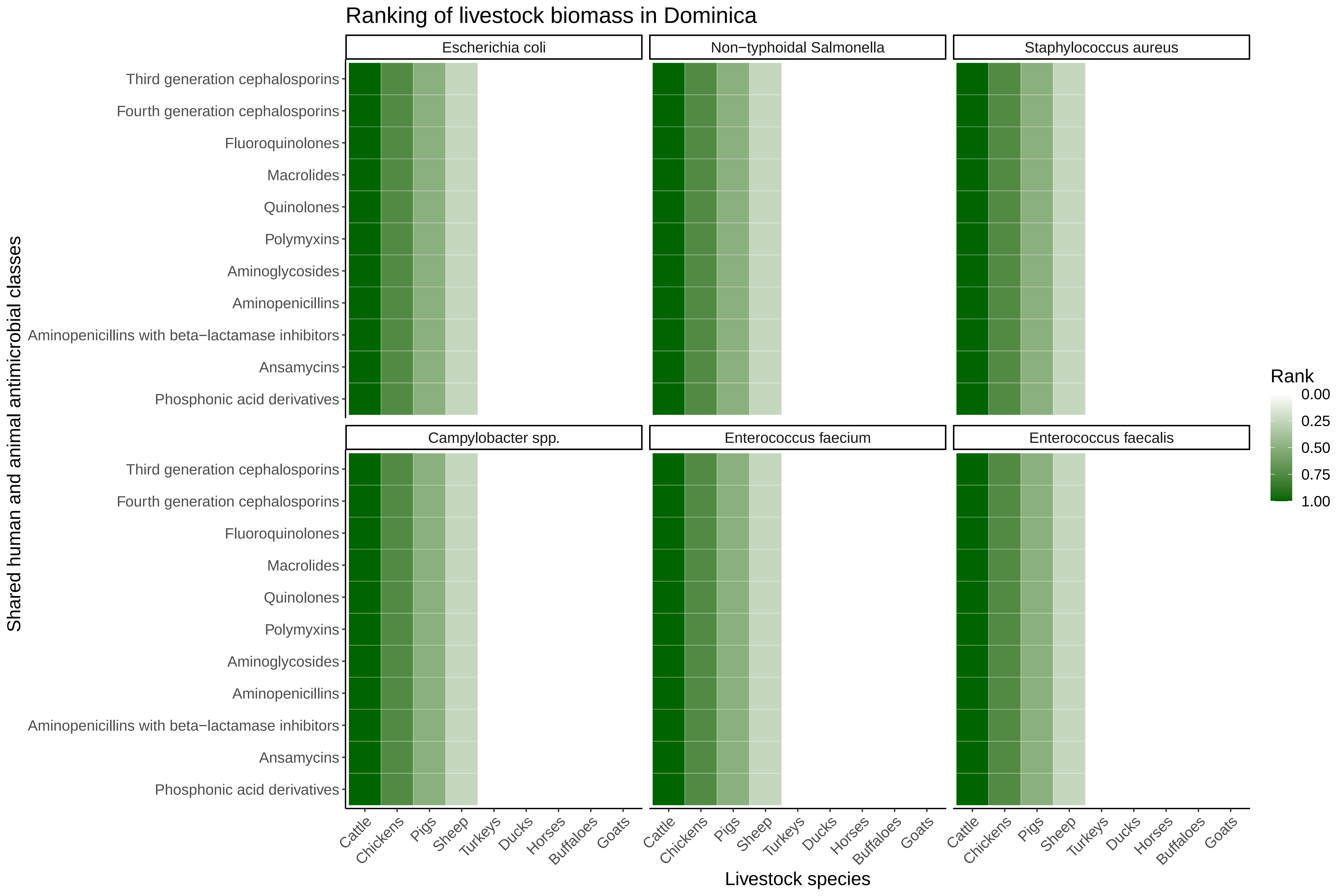

Ranking of livestock biomass in Dominica
Escherichia coli
Non−typhoidal Salmonella
Staphylococcus aureus
Campylobacter spp.
Enterococcus faecium
Enterococcus faecalis
Shared human and animal antimicrobial classes
Third generation cephalosporins
Fourth generation cephalosporins
Fluoroquinolones
Macrolides
Quinolones
Polymyxins
Aminoglycosides
Aminopenicillins
Aminopenicillins with beta−lactamase inhibitors
Ansamycins
Phosphonic acid derivatives
Livestock species
Cattle
Chickens
Pigs
Sheep
Turkeys
Ducks
Horses
Buffaloes
Goats
Rank
0.00
0.25
0.50
0.75
1.00

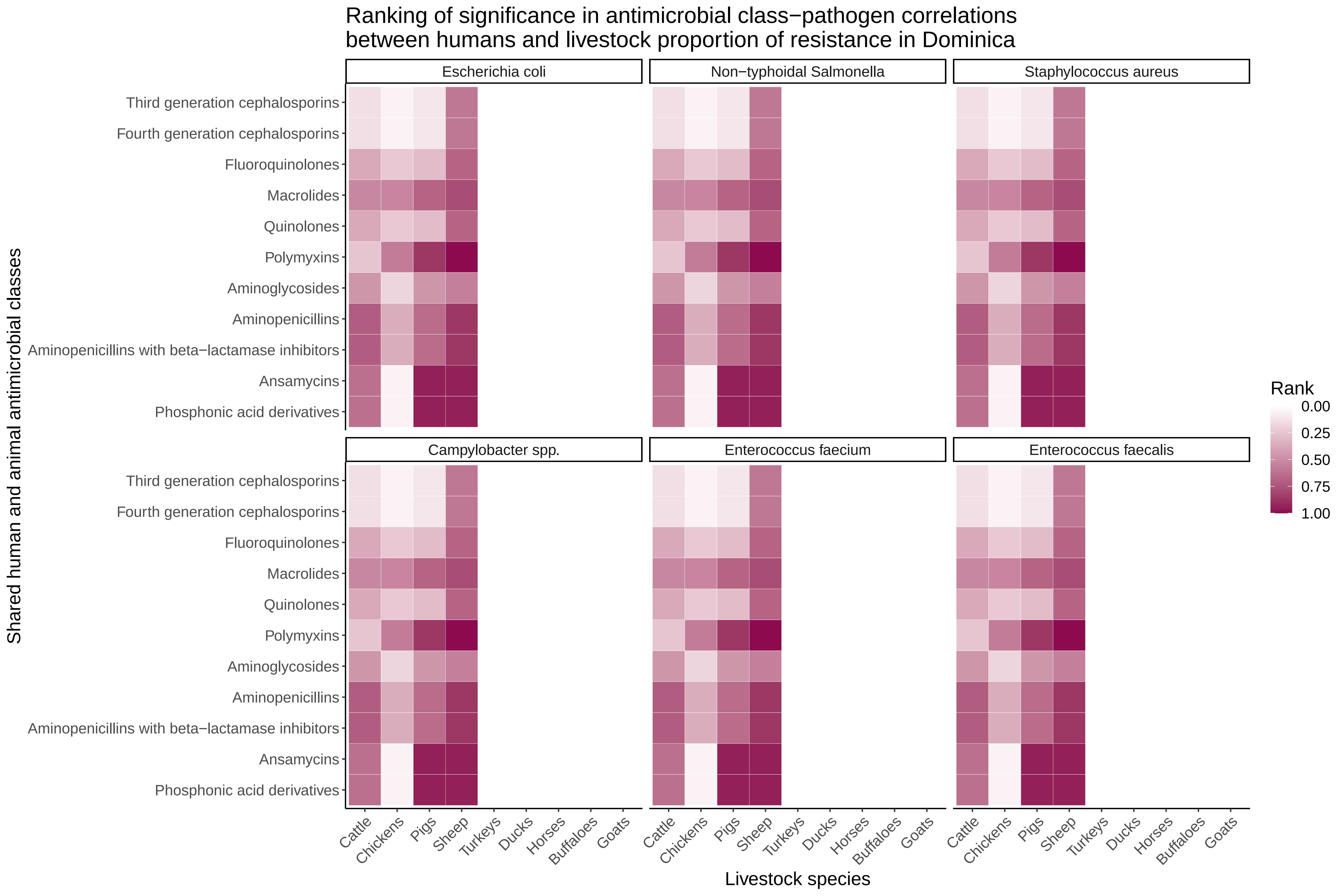

Ranking of significance in antimicrobial class−pathogen correlations
between humans and livestock proportion of resistance in Dominica
Escherichia coli
Non−typhoidal Salmonella
Staphylococcus aureus
Campylobacter spp.
Enterococcus faecium
Enterococcus faecalis
Third generation cephalosporins
Fourth generation cephalosporins
Fluoroquinolones
Macrolides
Quinolones
Polymyxins
Aminoglycosides
Aminopenicillins
Aminopenicillins with beta−lactamase inhibitors
Ansamycins
Phosphonic acid derivatives
Shared human and animal antimicrobial classes
Cattle
Chickens
Pigs
Sheep
Turkeys
Ducks
Horses
Buffaloes
Goats
Livestock species
Rank
0.00
0.25
0.50
0.75
1.00

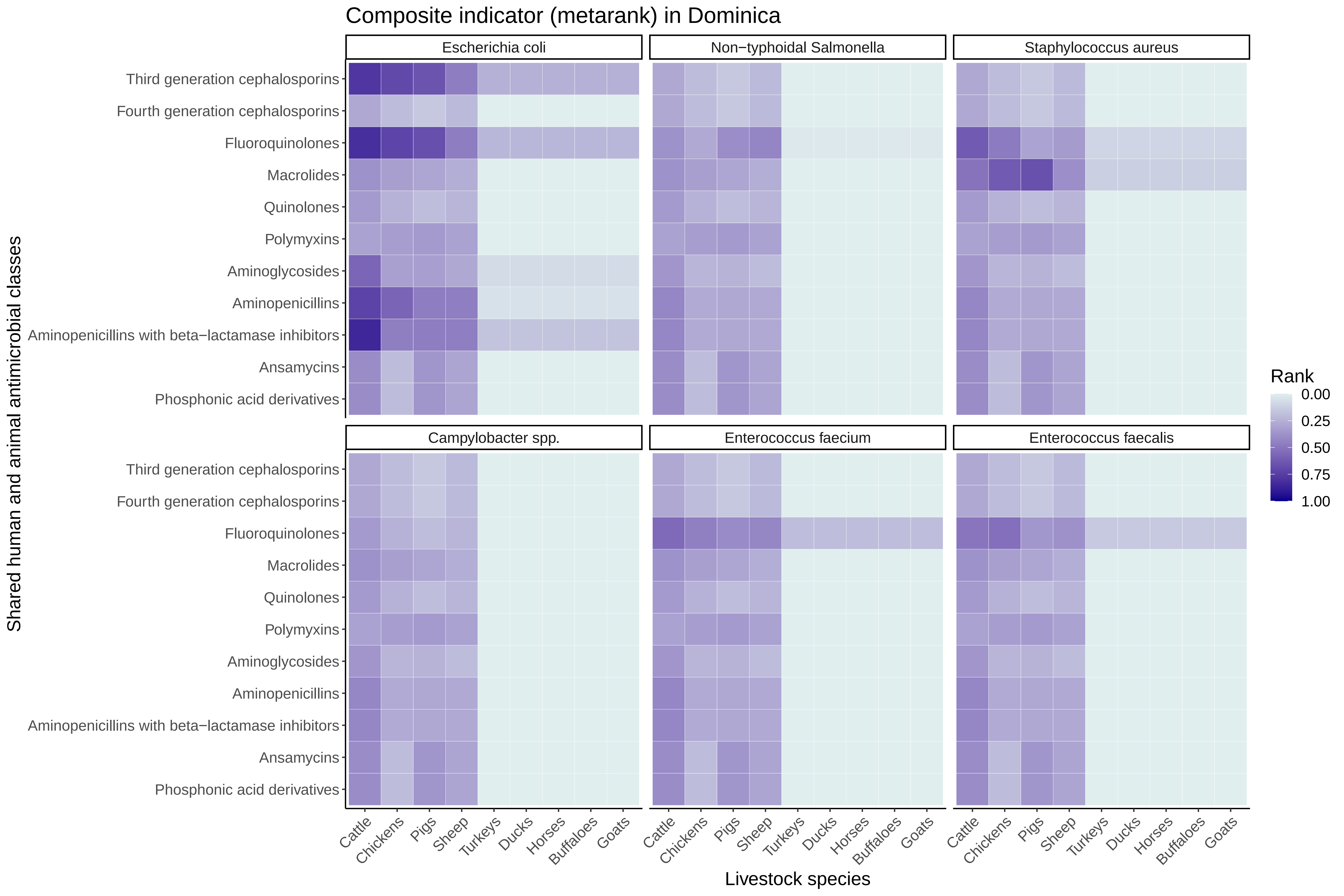
Composite indicator (metarank) in Dominica
Escherichia coli
Non−typhoidal Salmonella
Staphylococcus aureus
Campylobacter spp.
Enterococcus faecium
Enterococcus faecalis
Shared human and animal antimicrobial classes
Third generation cephalosporins
Fourth generation cephalosporins
Fluoroquinolones
Macrolides
Quinolones
Polymyxins
Aminoglycosides
Aminopenicillins
Aminopenicillins with beta−lactamase inhibitors
Ansamycins
Phosphonic acid derivatives
Livestock species
Cattle
Chickens
Pigs
Sheep
Turkeys
Ducks
Horses
Buffaloes
Goats
Rank
0.00
0.25
0.50
0.75
1.00

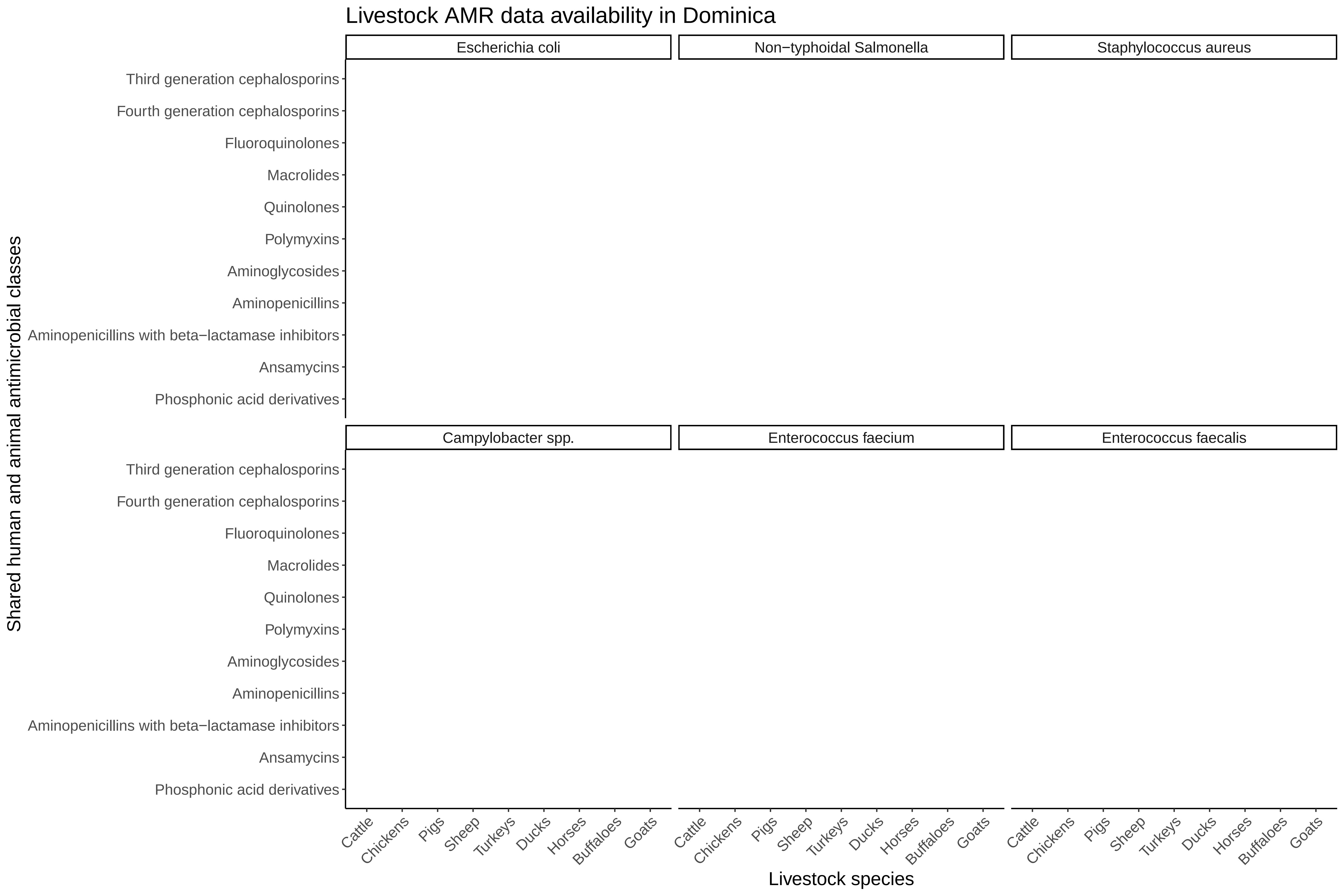

Livestock AMR data availability in Dominica
Escherichia coli
Non−typhoidal Salmonella
Staphylococcus aureus
Campylobacter spp.
Enterococcus faecium
Enterococcus faecalis
Shared human and animal antimicrobial classes
Third generation cephalosporins
Fourth generation cephalosporins
Fluoroquinolones
Macrolides
Quinolones
Polymyxins
Aminoglycosides
Aminopenicillins
Aminopenicillins with beta−lactamase inhibitors
Ansamycins
Phosphonic acid derivatives
Cattle
Chickens
Pigs
Sheep
Turkeys
Ducks
Horses
Buffaloes
Goats
Livestock species

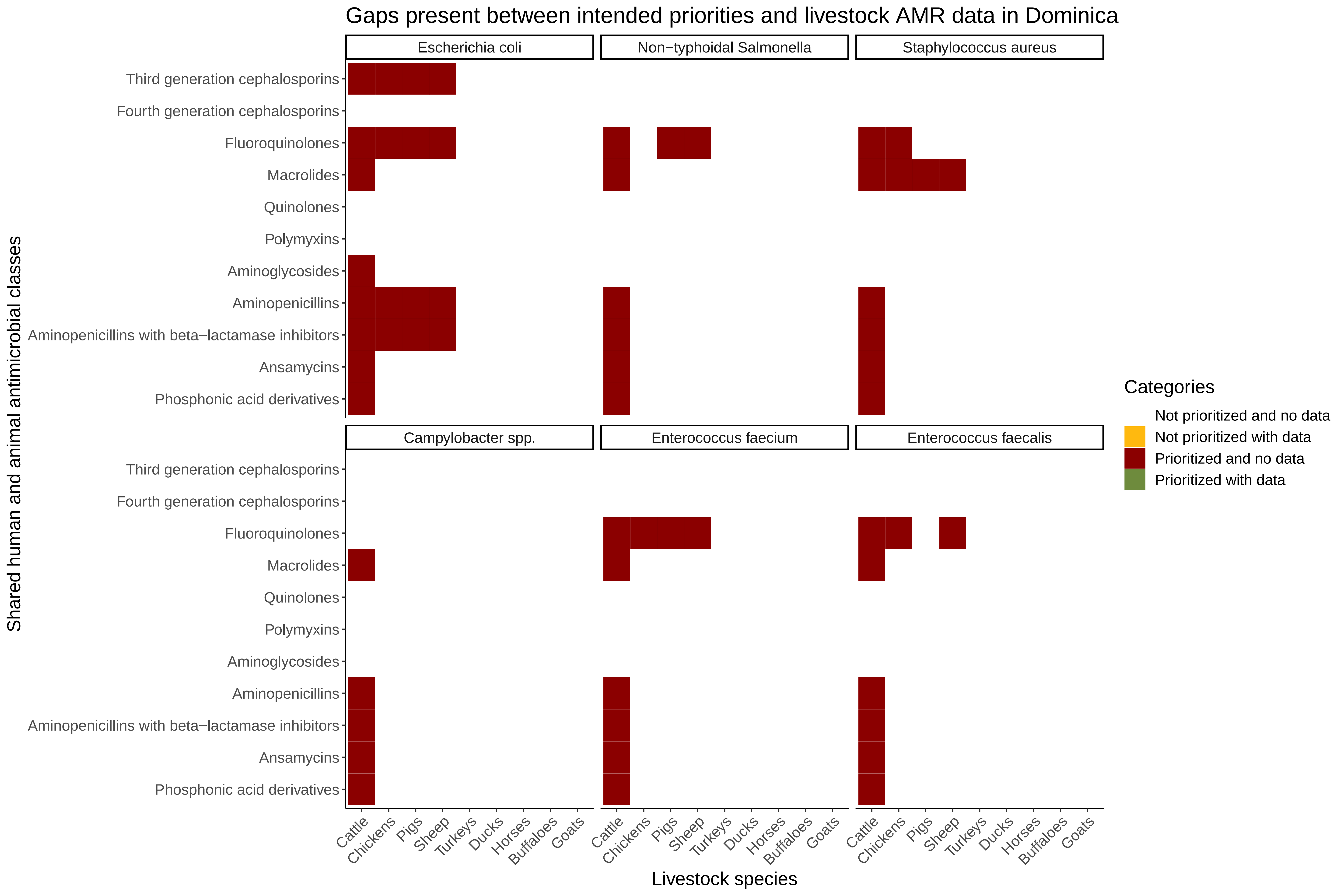

Gaps present between intended priorities and livestock AMR data in Dominica
Escherichia coli
Non−typhoidal Salmonella
Staphylococcus aureus
Campylobacter spp.
Enterococcus faecium
Enterococcus faecalis
Third generation cephalosporins
Fourth generation cephalosporins
Fluoroquinolones
Macrolides
Quinolones
Polymyxins
Aminoglycosides
Aminopenicillins
Aminopenicillins with beta−lactamase inhibitors
Ansamycins
Phosphonic acid derivatives
Shared human and animal antimicrobial classes
Cattle
Chickens
Pigs
Sheep
Turkeys
Ducks
Horses
Buffaloes
Goats
Livestock species
Categories
Not prioritized and no data
Not prioritized with data
Prioritized and no data
Prioritized with data

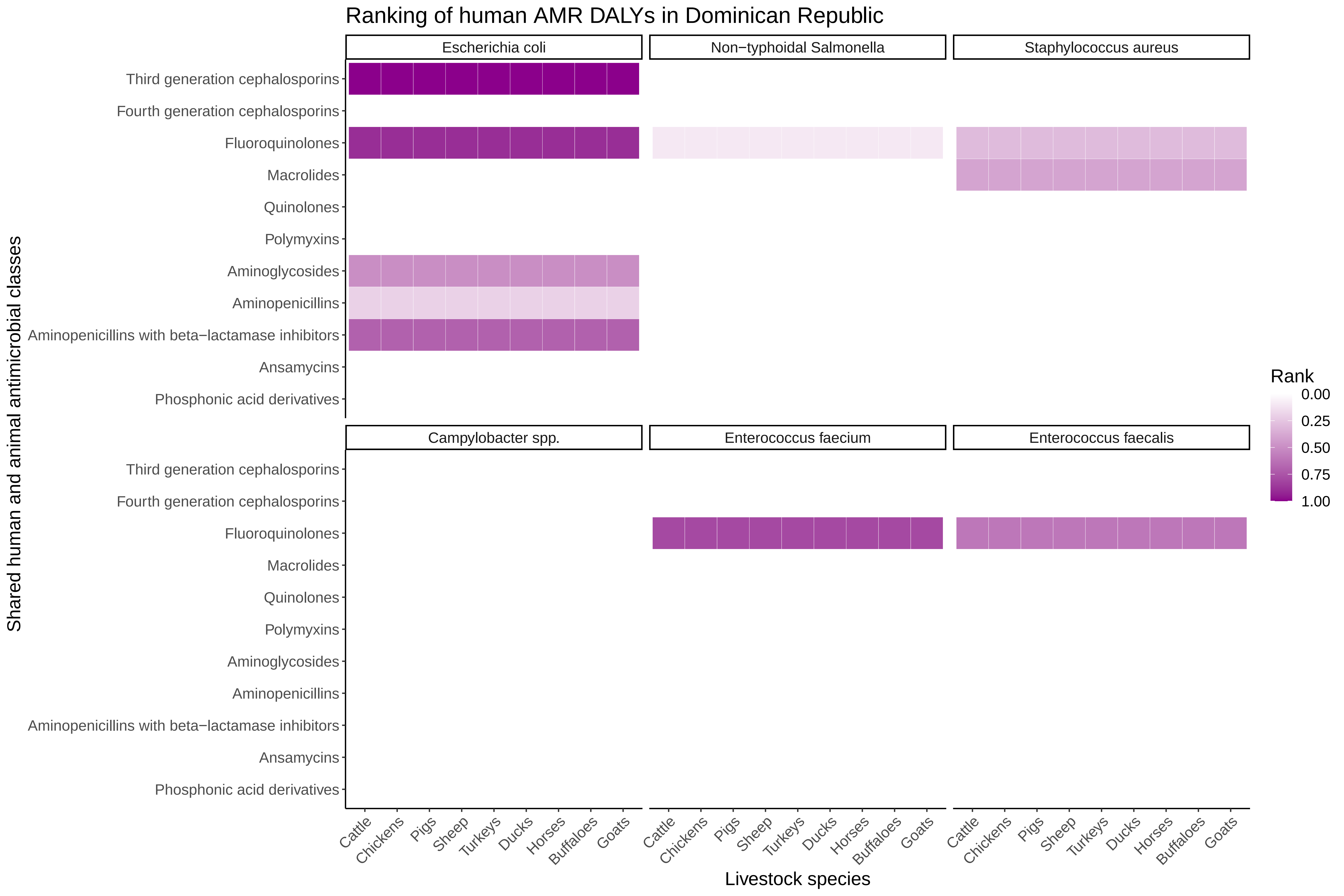

Ranking of human AMR DALYs in Dominican Republic
Escherichia coli
Non−typhoidal Salmonella
Staphylococcus aureus
Campylobacter spp.
Enterococcus faecium
Enterococcus faecalis
Third generation cephalosporins
Fourth generation cephalosporins
Fluoroquinolones
Macrolides
Quinolones
Polymyxins
Aminoglycosides
Aminopenicillins
Aminopenicillins with beta−lactamase inhibitors
Ansamycins
Phosphonic acid derivatives
Shared human and animal antimicrobial classes
Cattle
Chickens
Pigs
Sheep
Turkeys
Ducks
Horses
Buffaloes
Goats
Livestock species
Rank
0.00
0.25
0.50
0.75
1.00

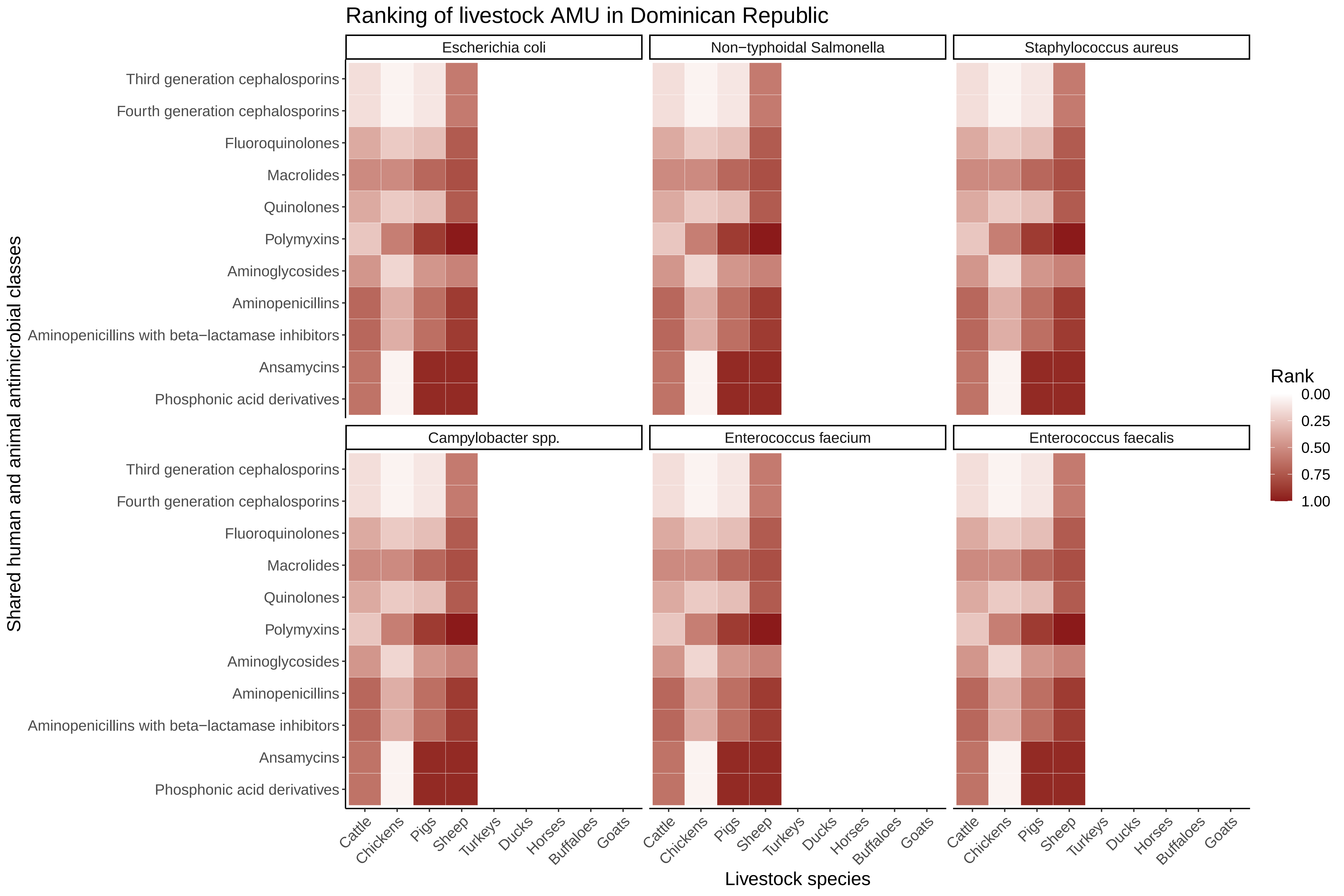

Ranking of livestock AMU in Dominican Republic
Escherichia coli
Non−typhoidal Salmonella
Staphylococcus aureus
Campylobacter spp.
Enterococcus faecium
Enterococcus faecalis
Third generation cephalosporins
Fourth generation cephalosporins
Fluoroquinolones
Macrolides
Quinolones
Polymyxins
Aminoglycosides
Aminopenicillins
Aminopenicillins with beta−lactamase inhibitors
Ansamycins
Phosphonic acid derivatives
Shared human and animal antimicrobial classes
Cattle
Chickens
Pigs
Sheep
Turkeys
Ducks
Horses
Buffaloes
Goats
Livestock species
Rank
0.00
0.25
0.50
0.75
1.00

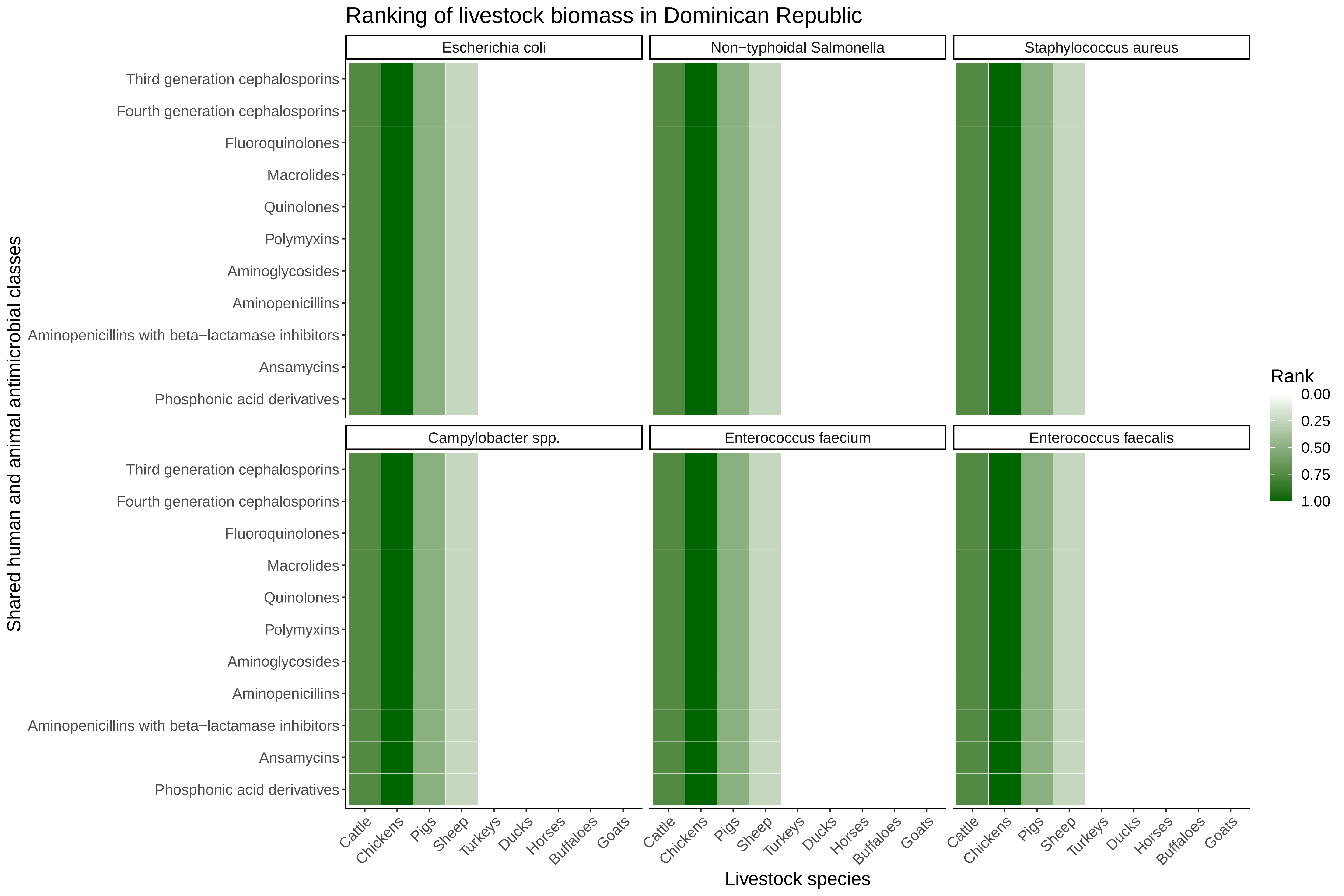
Ranking of livestock biomass in Dominican Republic
Escherichia coli
Non−typhoidal Salmonella
Staphylococcus aureus
Campylobacter spp.
Enterococcus faecium
Enterococcus faecalis
Shared human and animal antimicrobial classes
Third generation cephalosporins
Fourth generation cephalosporins
Fluoroquinolones
Macrolides
Quinolones
Polymyxins
Aminoglycosides
Aminopenicillins
Aminopenicillins with beta−lactamase inhibitors
Ansamycins
Phosphonic acid derivatives
Cattle
Chickens
Pigs
Sheep
Turkeys
Ducks
Horses
Buffaloes
Goats
Livestock species
Rank
0.00
0.25
0.50
0.75
1.00

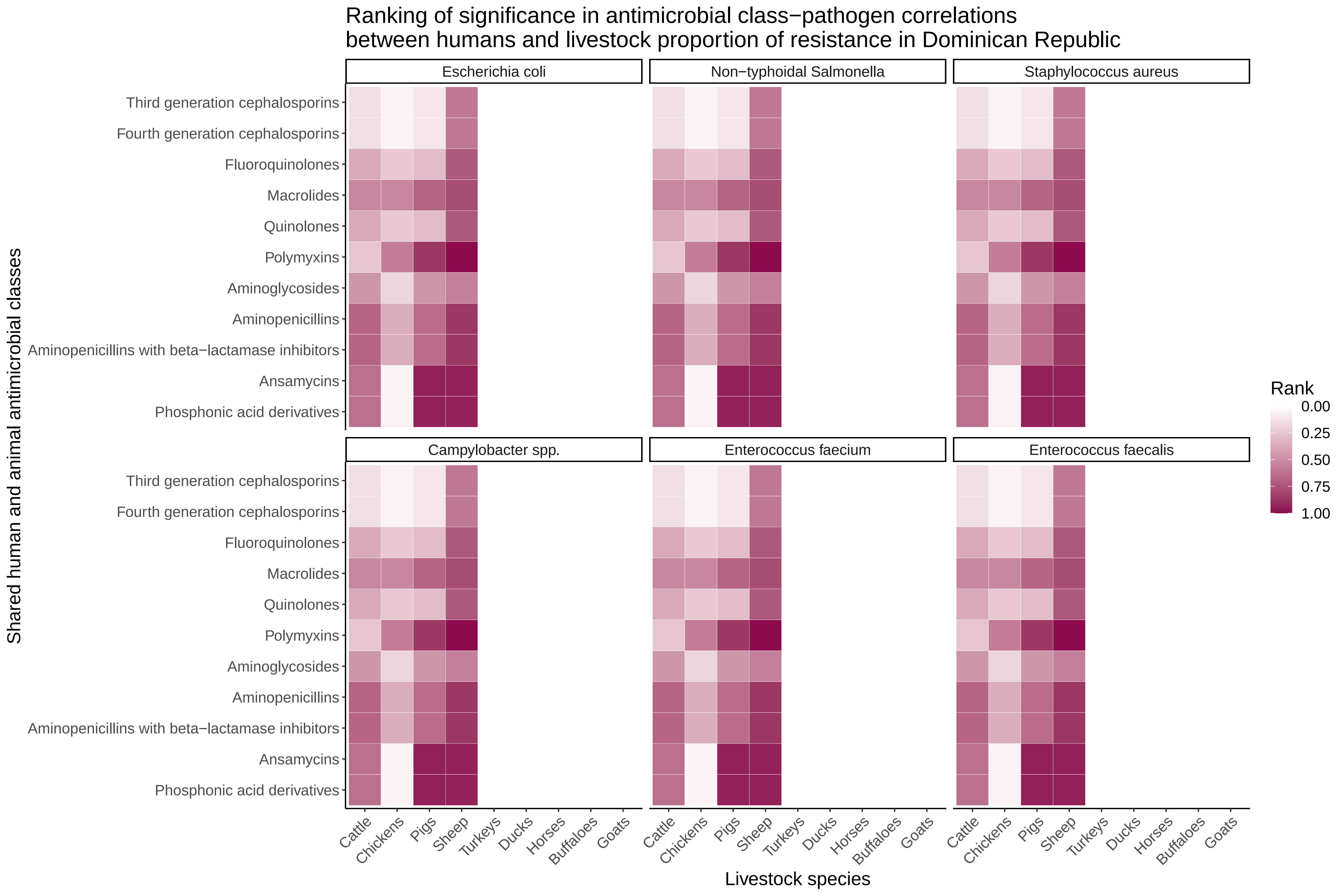

Ranking of significance in antimicrobial class−pathogen correlations
between humans and livestock proportion of resistance in Dominican Republic
Escherichia coli
Non−typhoidal Salmonella
Staphylococcus aureus
Campylobacter spp.
Enterococcus faecium
Enterococcus faecalis
Third generation cephalosporins
Fourth generation cephalosporins
Fluoroquinolones
Macrolides
Quinolones
Polymyxins
Aminoglycosides
Aminopenicillins
Aminopenicillins with beta−lactamase inhibitors
Ansamycins
Phosphonic acid derivatives
Shared human and animal antimicrobial classes
Cattle
Chickens
Pigs
Sheep
Turkeys
Ducks
Horses
Buffaloes
Goats
Livestock species
Rank
0.00
0.25
0.50
0.75
1.00

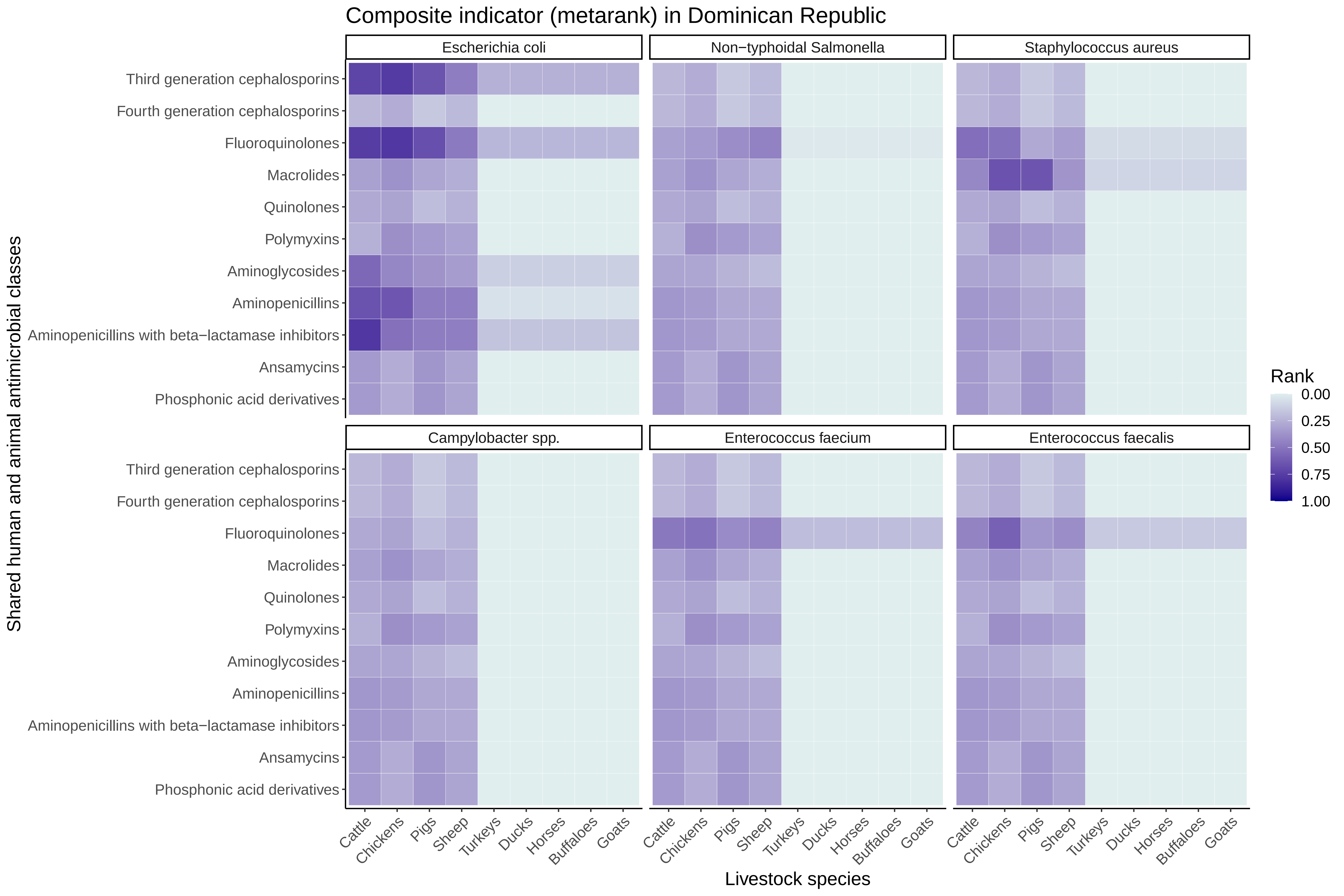

Composite indicator (metarank) in Dominican Republic
Escherichia coli
Non−typhoidal Salmonella
Staphylococcus aureus
Campylobacter spp.
Enterococcus faecium
Enterococcus faecalis
Third generation cephalosporins
Fourth generation cephalosporins
Fluoroquinolones
Macrolides
Quinolones
Polymyxins
Aminoglycosides
Aminopenicillins
Aminopenicillins with beta−lactamase inhibitors
Ansamycins
Phosphonic acid derivatives
Shared human and animal antimicrobial classes
Cattle
Chickens
Pigs
Sheep
Turkeys
Ducks
Horses
Buffaloes
Goats
Livestock species
Rank
0.00
0.25
0.50
0.75
1.00

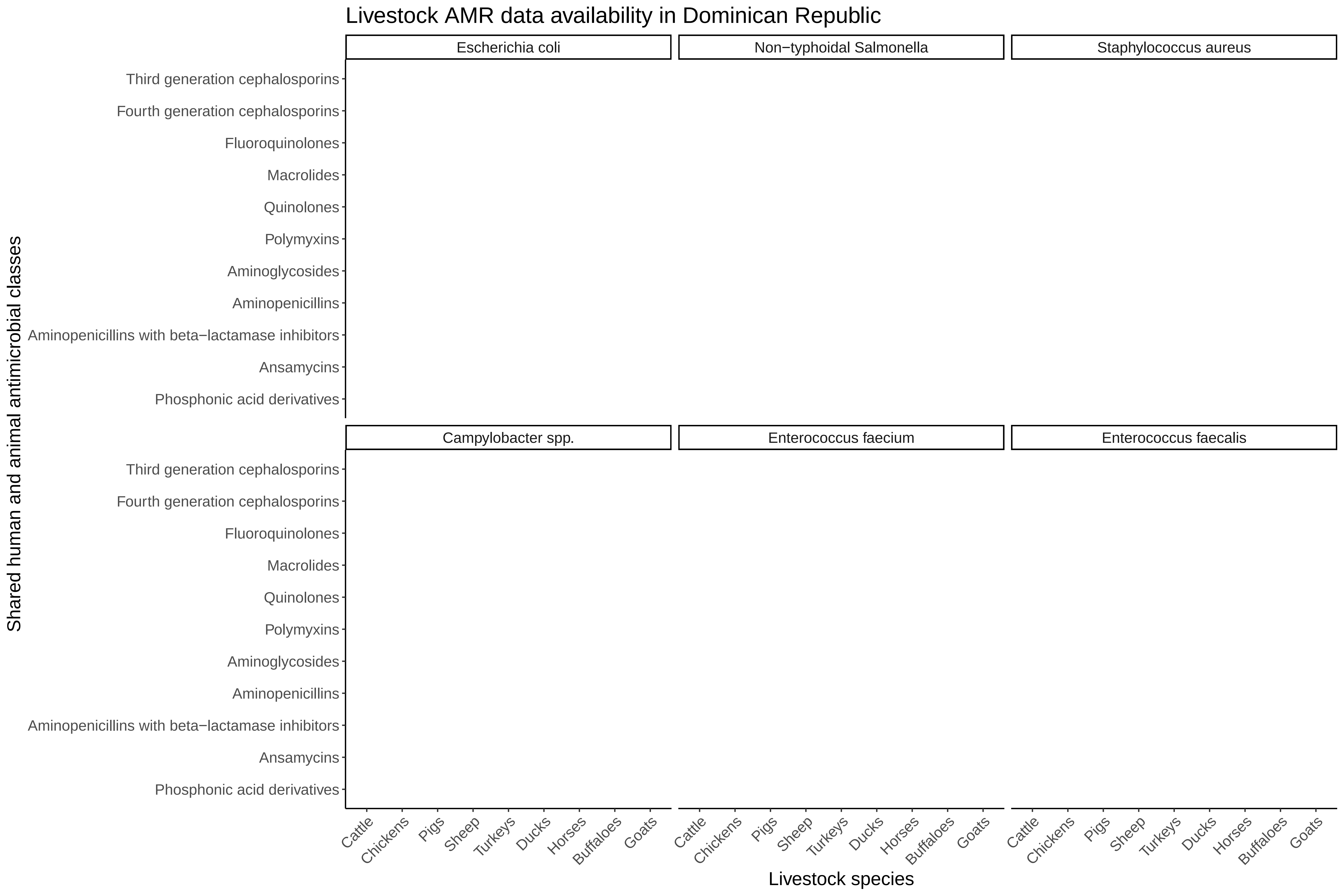
Livestock AMR data availability in Dominican Republic
Escherichia coli
Non−typhoidal Salmonella
Staphylococcus aureus
Campylobacter spp.
Enterococcus faecium
Enterococcus faecalis
Third generation cephalosporins
Fourth generation cephalosporins
Fluoroquinolones
Macrolides
Quinolones
Polymyxins
Aminoglycosides
Aminopenicillins
Aminopenicillins with beta−lactamase inhibitors
Ansamycins
Phosphonic acid derivatives
Shared human and animal antimicrobial classes
Cattle
Chickens
Pigs
Sheep
Turkeys
Ducks
Horses
Buffaloes
Goats
Livestock species

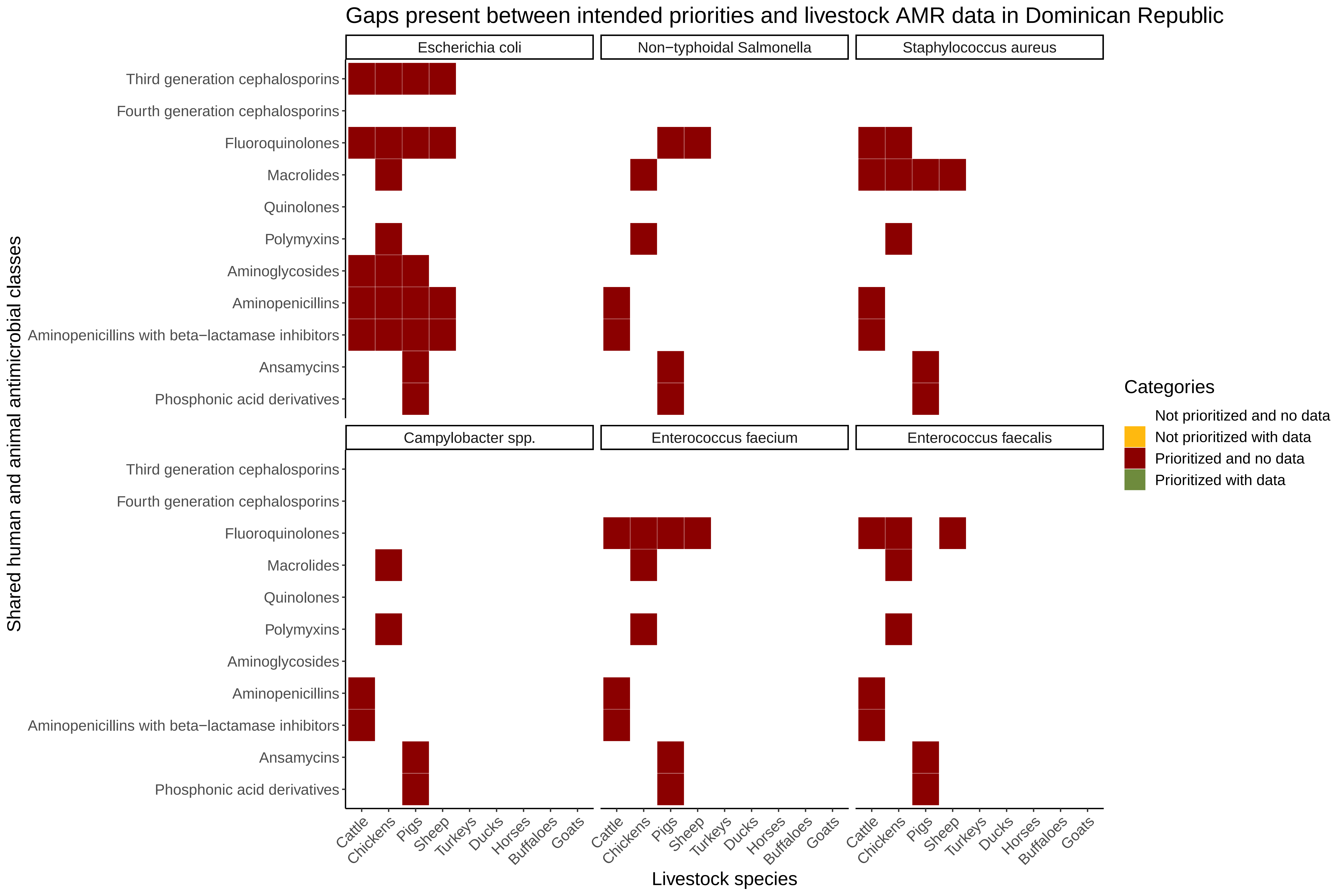

Gaps present between intended priorities and livestock AMR data in Dominican Republic
Escherichia coli
Non−typhoidal Salmonella
Staphylococcus aureus
Campylobacter spp.
Enterococcus faecium
Enterococcus faecalis
Third generation cephalosporins
Fourth generation cephalosporins
Fluoroquinolones
Macrolides
Quinolones
Polymyxins
Aminoglycosides
Aminopenicillins
Aminopenicillins with beta−lactamase inhibitors
Ansamycins
Phosphonic acid derivatives
Shared human and animal antimicrobial classes
Cattle
Chickens
Pigs
Sheep
Turkeys
Ducks
Horses
Buffaloes
Goats
Livestock species
Categories
Not prioritized and no data
Not prioritized with data
Prioritized and no data
Prioritized with data

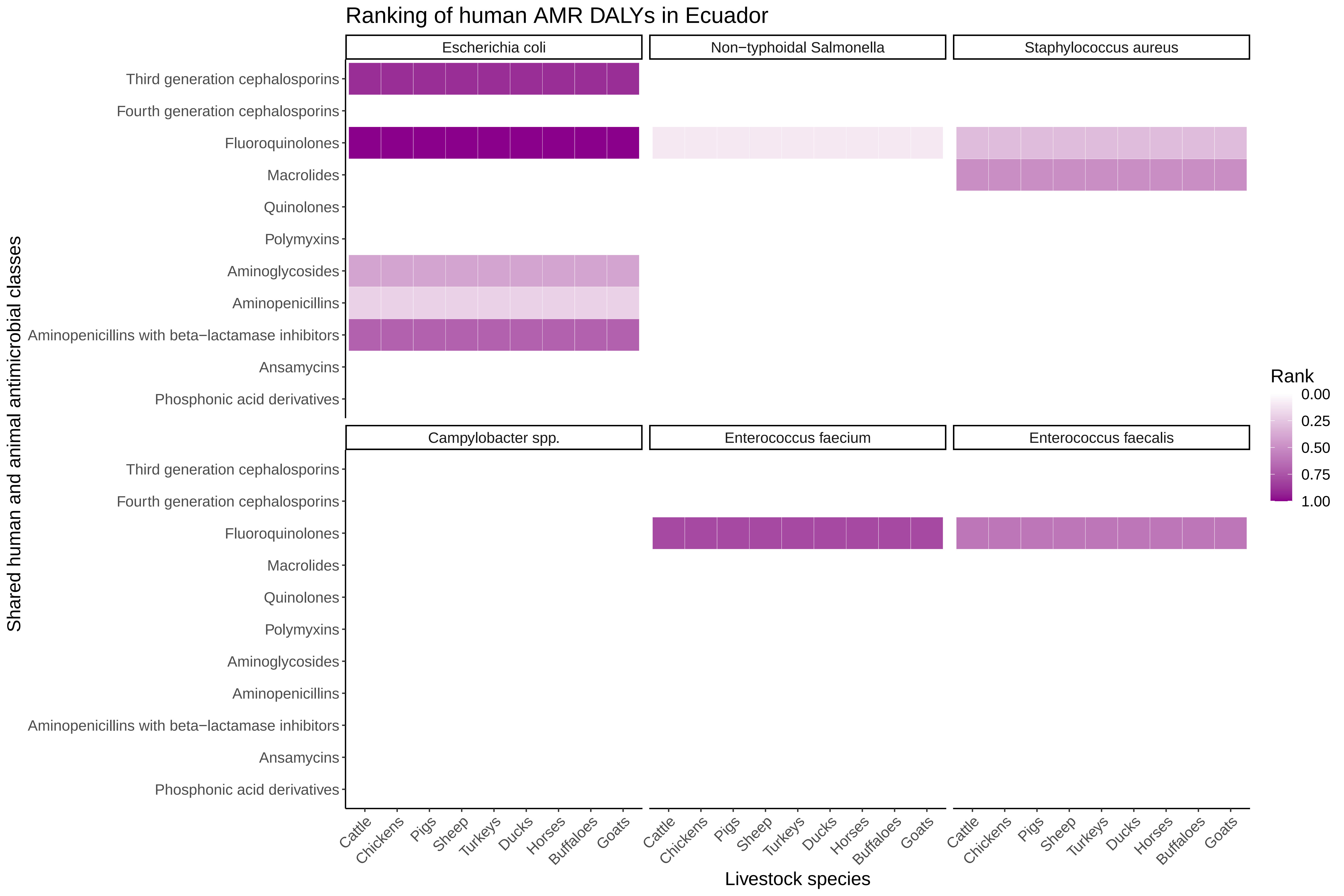

Ranking of human AMR DALYs in Ecuador
Escherichia coli
Non−typhoidal Salmonella
Staphylococcus aureus
Campylobacter spp.
Enterococcus faecium
Enterococcus faecalis
Third generation cephalosporins
Fourth generation cephalosporins
Fluoroquinolones
Macrolides
Quinolones
Polymyxins
Aminoglycosides
Aminopenicillins
Aminopenicillins with beta−lactamase inhibitors
Ansamycins
Phosphonic acid derivatives
Shared human and animal antimicrobial classes
Cattle
Chickens
Pigs
Sheep
Turkeys
Ducks
Horses
Buffaloes
Goats
Livestock species
Rank
0.00
0.25
0.50
0.75
1.00

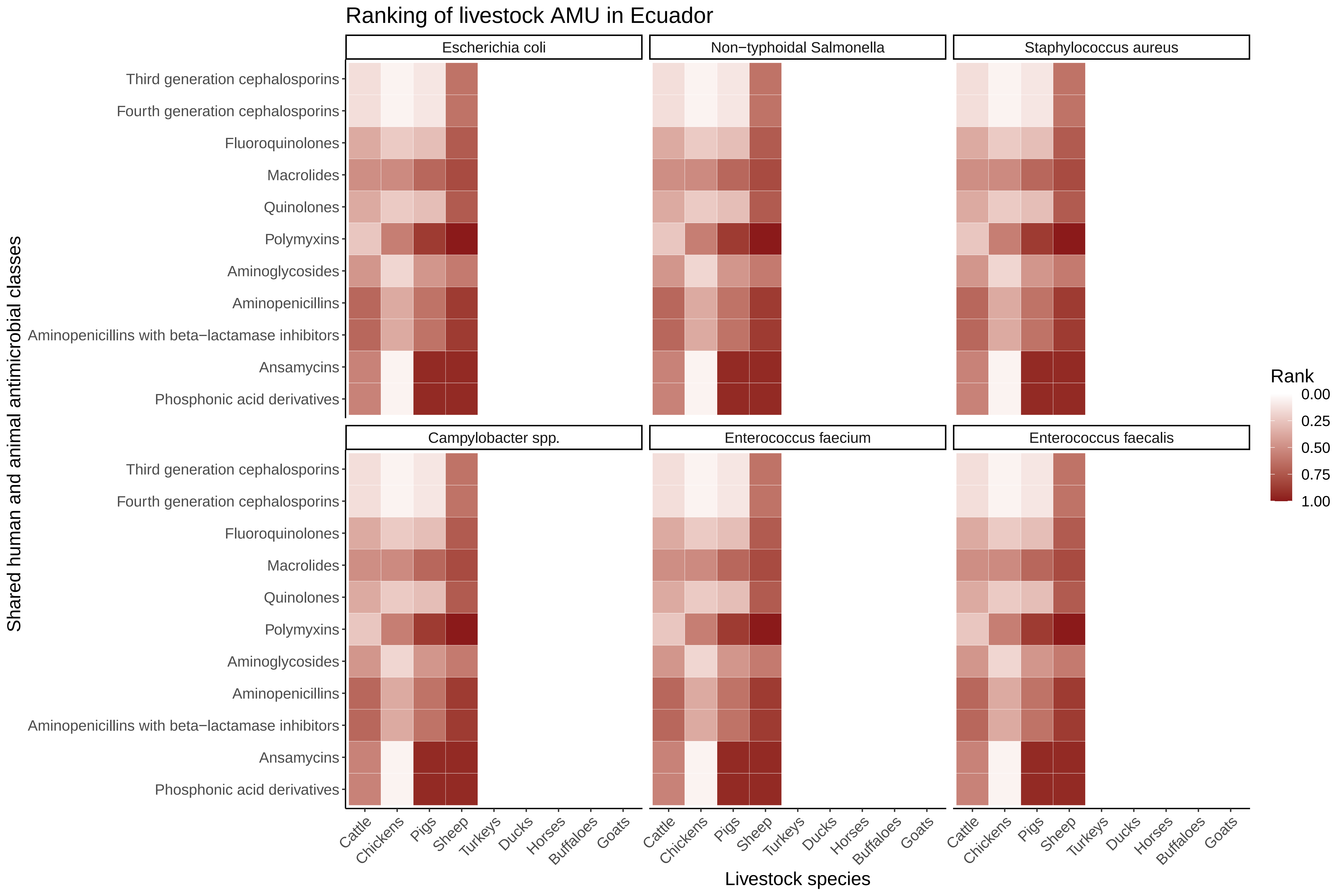

Ranking of livestock AMU in Ecuador
Escherichia coli
Non−typhoidal Salmonella
Staphylococcus aureus
Campylobacter spp.
Enterococcus faecium
Enterococcus faecalis
Shared human and animal antimicrobial classes
Third generation cephalosporins
Fourth generation cephalosporins
Fluoroquinolones
Macrolides
Quinolones
Polymyxins
Aminoglycosides
Aminopenicillins
Aminopenicillins with beta−lactamase inhibitors
Ansamycins
Phosphonic acid derivatives
Cattle
Chickens
Pigs
Sheep
Turkeys
Ducks
Horses
Buffaloes
Goats
Livestock species
Rank
0.00
0.25
0.50
0.75
1.00

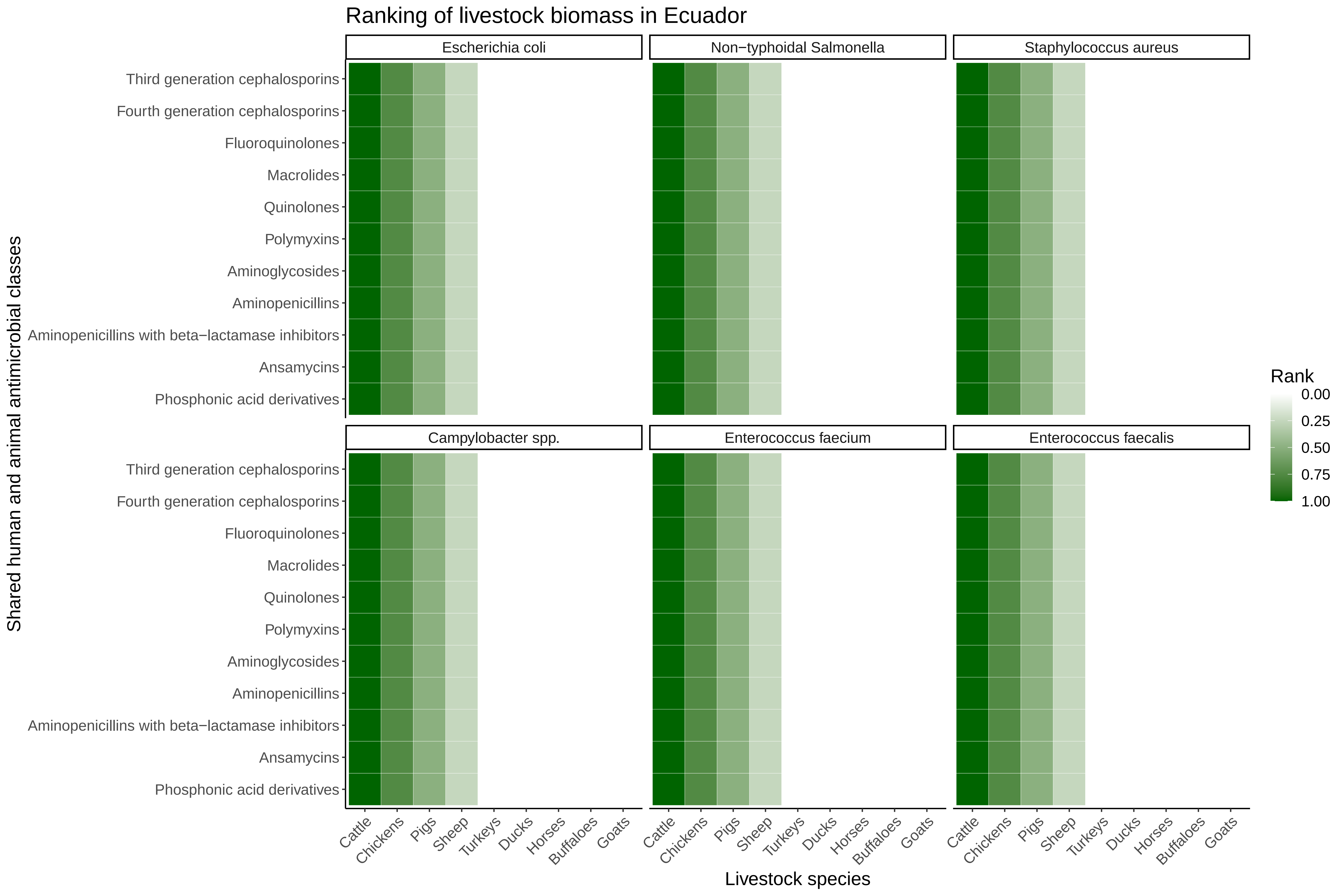

Ranking of livestock biomass in Ecuador
Escherichia coli
Non−typhoidal Salmonella
Staphylococcus aureus
Campylobacter spp.
Enterococcus faecium
Enterococcus faecalis
Third generation cephalosporins
Fourth generation cephalosporins
Fluoroquinolones
Macrolides
Quinolones
Polymyxins
Aminoglycosides
Aminopenicillins
Aminopenicillins with beta−lactamase inhibitors
Ansamycins
Phosphonic acid derivatives
Shared human and animal antimicrobial classes
Cattle
Chickens
Pigs
Sheep
Turkeys
Ducks
Horses
Buffaloes
Goats
Livestock species
Rank
0.00
0.25
0.50
0.75
1.00

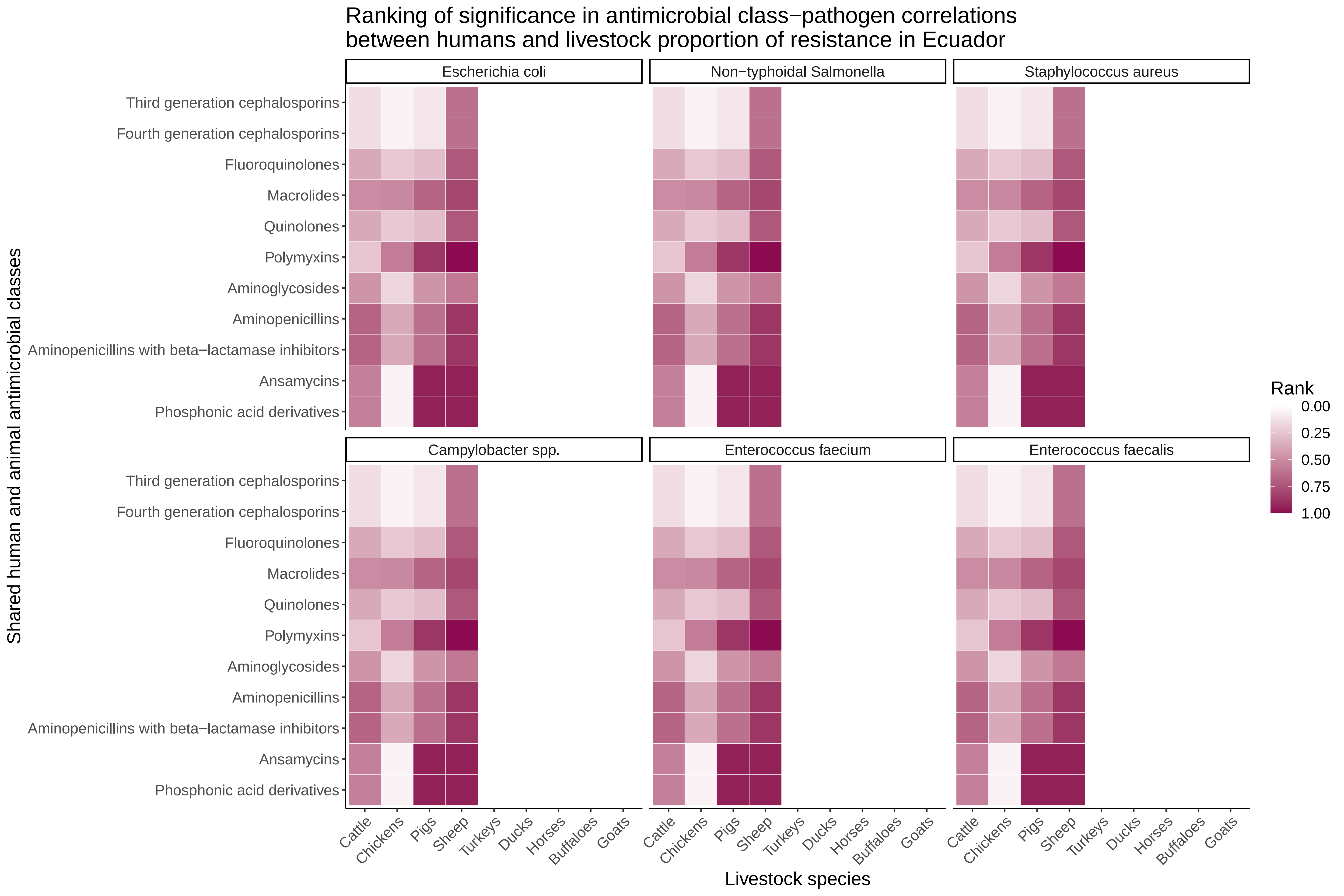

Ranking of significance in antimicrobial class−pathogen correlations
between humans and livestock proportion of resistance in Ecuador
Escherichia coli
Non−typhoidal Salmonella
Staphylococcus aureus
Campylobacter spp.
Enterococcus faecium
Enterococcus faecalis
Third generation cephalosporins
Fourth generation cephalosporins
Fluoroquinolones
Macrolides
Quinolones
Polymyxins
Aminoglycosides
Aminopenicillins
Aminopenicillins with beta−lactamase inhibitors
Ansamycins
Phosphonic acid derivatives
Shared human and animal antimicrobial classes
Cattle
Chickens
Pigs
Sheep
Turkeys
Ducks
Horses
Buffaloes
Goats
Livestock species
Rank
0.00
0.25
0.50
0.75
1.00

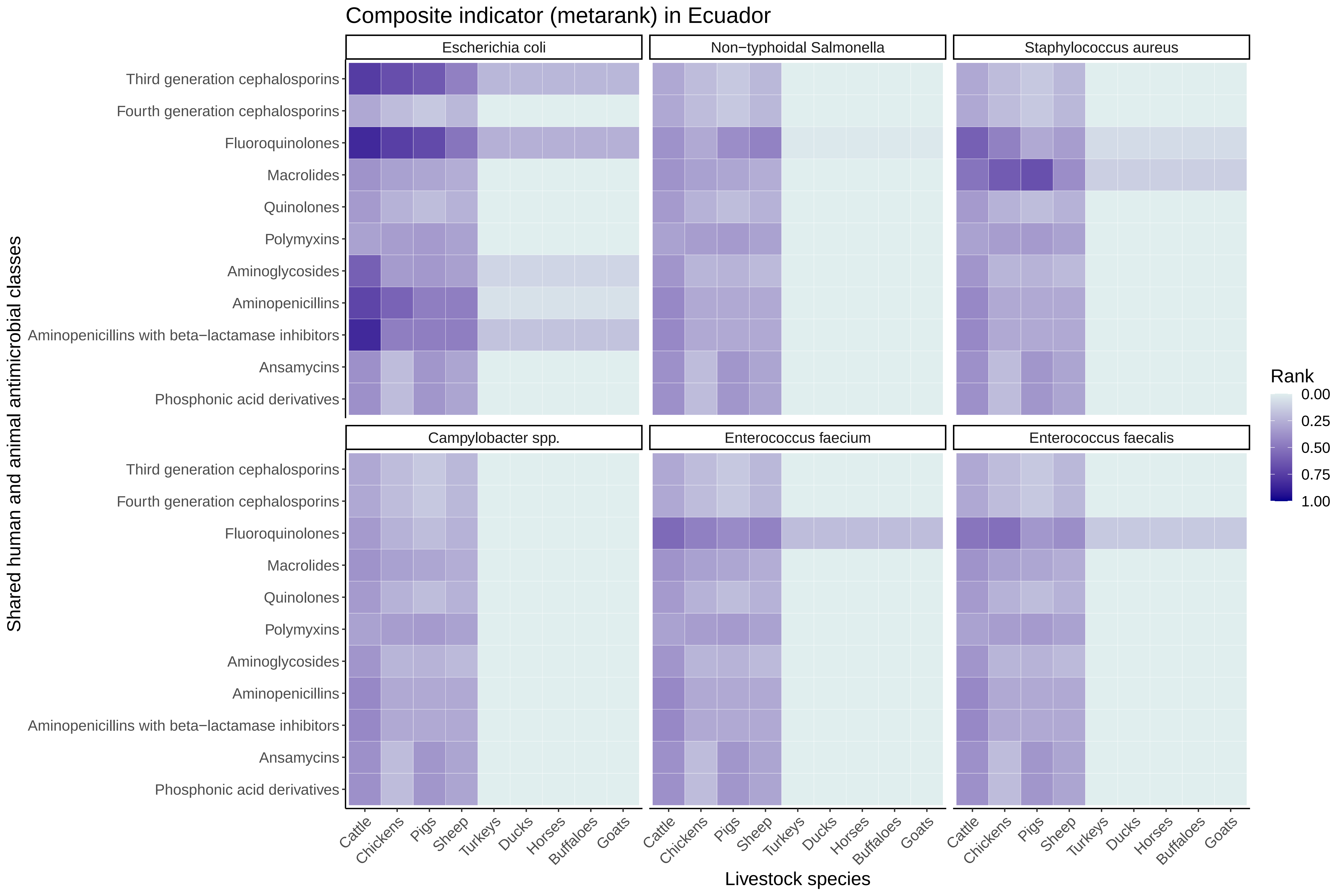
Composite indicator (metarank) in Ecuador
Escherichia coli
Non−typhoidal Salmonella
Staphylococcus aureus
Campylobacter spp.
Enterococcus faecium
Enterococcus faecalis
Shared human and animal antimicrobial classes
Third generation cephalosporins
Fourth generation cephalosporins
Fluoroquinolones
Macrolides
Quinolones
Polymyxins
Aminoglycosides
Aminopenicillins
Aminopenicillins with beta−lactamase inhibitors
Ansamycins
Phosphonic acid derivatives
Cattle
Chickens
Pigs
Sheep
Turkeys
Ducks
Horses
Buffaloes
Goats
Livestock species
Rank
0.00
0.25
0.50
0.75
1.00

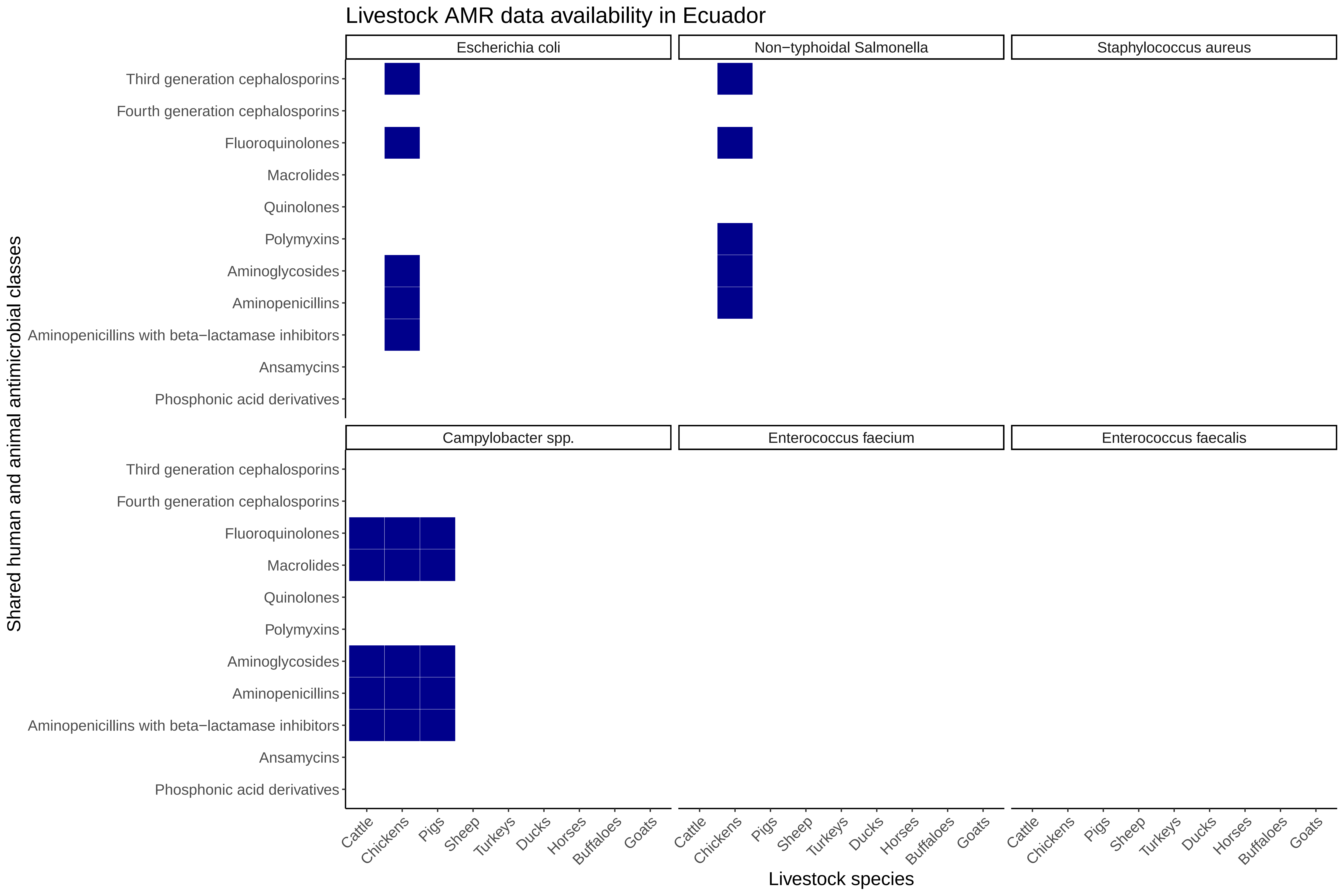
Livestock AMR data availability in Ecuador
Escherichia coli
Non−typhoidal Salmonella
Staphylococcus aureus
Campylobacter spp.
Enterococcus faecium
Enterococcus faecalis
Shared human and animal antimicrobial classes
Third generation cephalosporins
Fourth generation cephalosporins
Fluoroquinolones
Macrolides
Quinolones
Polymyxins
Aminoglycosides
Aminopenicillins
Aminopenicillins with beta−lactamase inhibitors
Ansamycins
Phosphonic acid derivatives
Cattle
Chickens
Pigs
Sheep
Turkeys
Ducks
Horses
Buffaloes
Goats
Livestock species

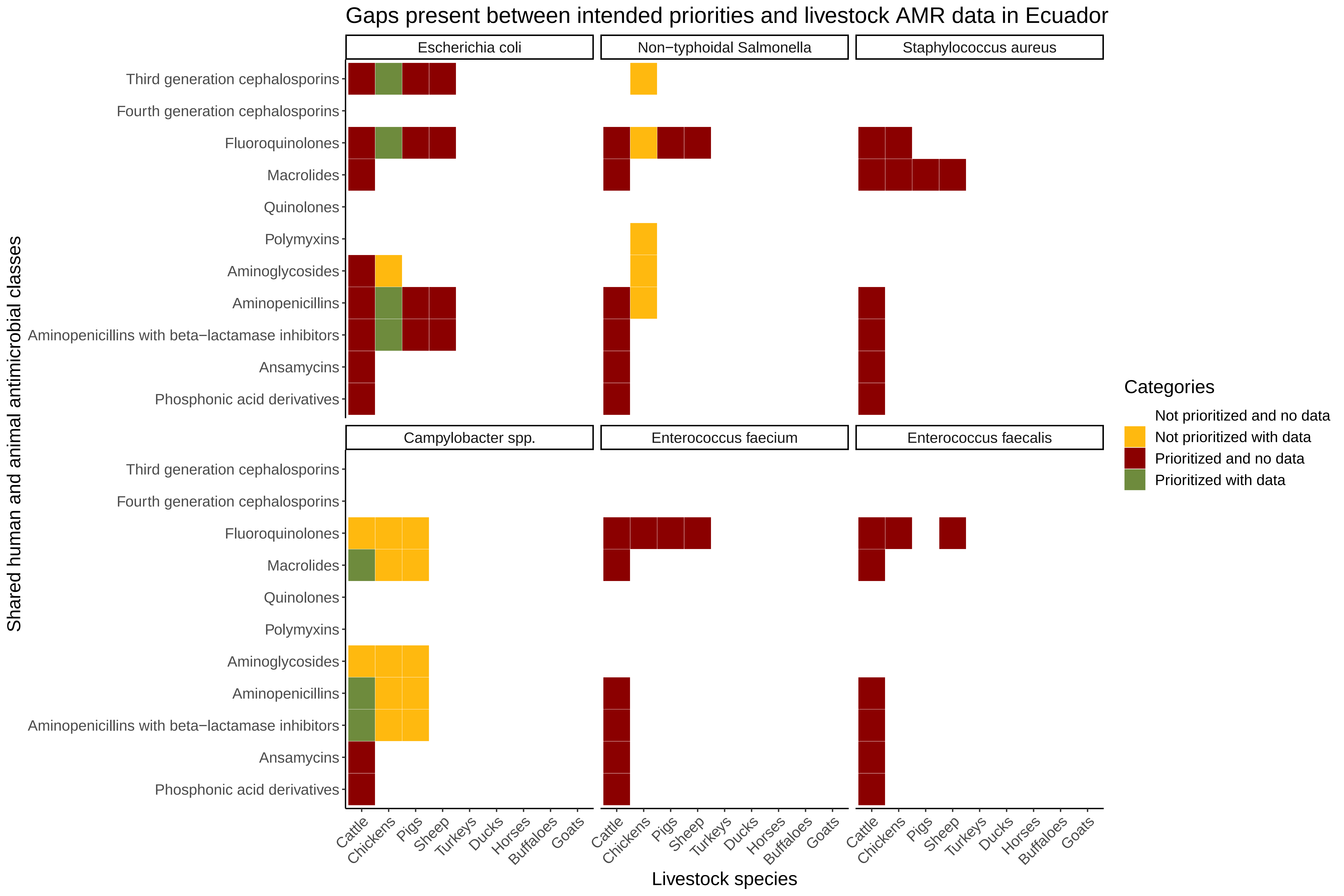
Gaps present between intended priorities and livestock AMR data in Ecuador
Escherichia coli
Non−typhoidal Salmonella
Staphylococcus aureus
Campylobacter spp.
Enterococcus faecium
Enterococcus faecalis
Third generation cephalosporins
Fourth generation cephalosporins
Fluoroquinolones
Macrolides
Quinolones
Polymyxins
Aminoglycosides
Aminopenicillins
Aminopenicillins with beta−lactamase inhibitors
Ansamycins
Phosphonic acid derivatives
Shared human and animal antimicrobial classes
Cattle
Chickens
Pigs
Sheep
Turkeys
Ducks
Horses
Buffaloes
Goats
Livestock species
Categories
Not prioritized and no data
Not prioritized with data
Prioritized and no data
Prioritized with data

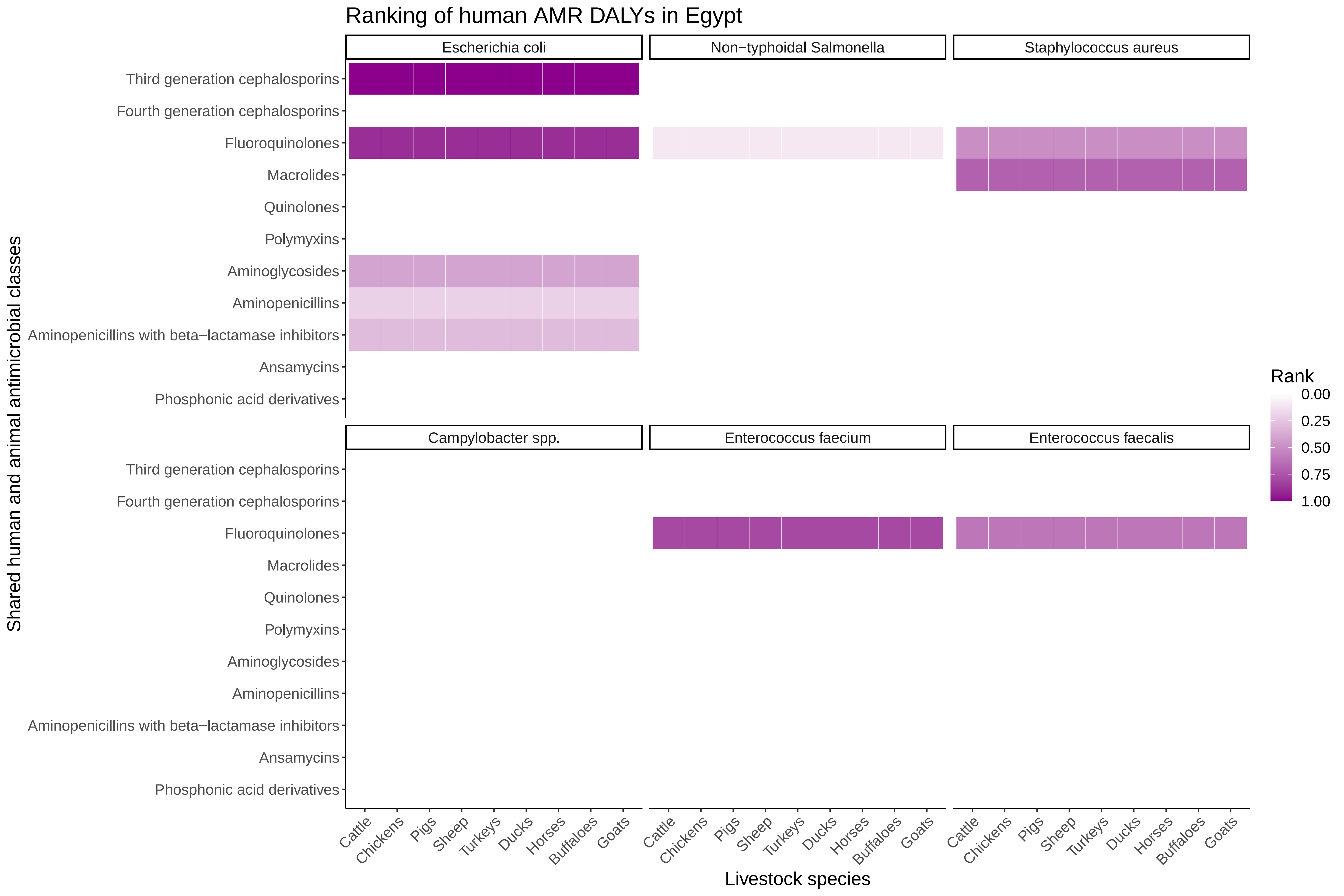

Ranking of human AMR DALYs in Egypt
Escherichia coli
Non−typhoidal Salmonella
Staphylococcus aureus
Campylobacter spp.
Enterococcus faecium
Enterococcus faecalis
Third generation cephalosporins
Fourth generation cephalosporins
Fluoroquinolones
Macrolides
Quinolones
Polymyxins
Aminoglycosides
Aminopenicillins
Aminopenicillins with beta−lactamase inhibitors
Ansamycins
Phosphonic acid derivatives
Shared human and animal antimicrobial classes
Cattle
Chickens
Pigs
Sheep
Turkeys
Ducks
Horses
Buffaloes
Goats
Livestock species
Rank
0.00
0.25
0.50
0.75
1.00

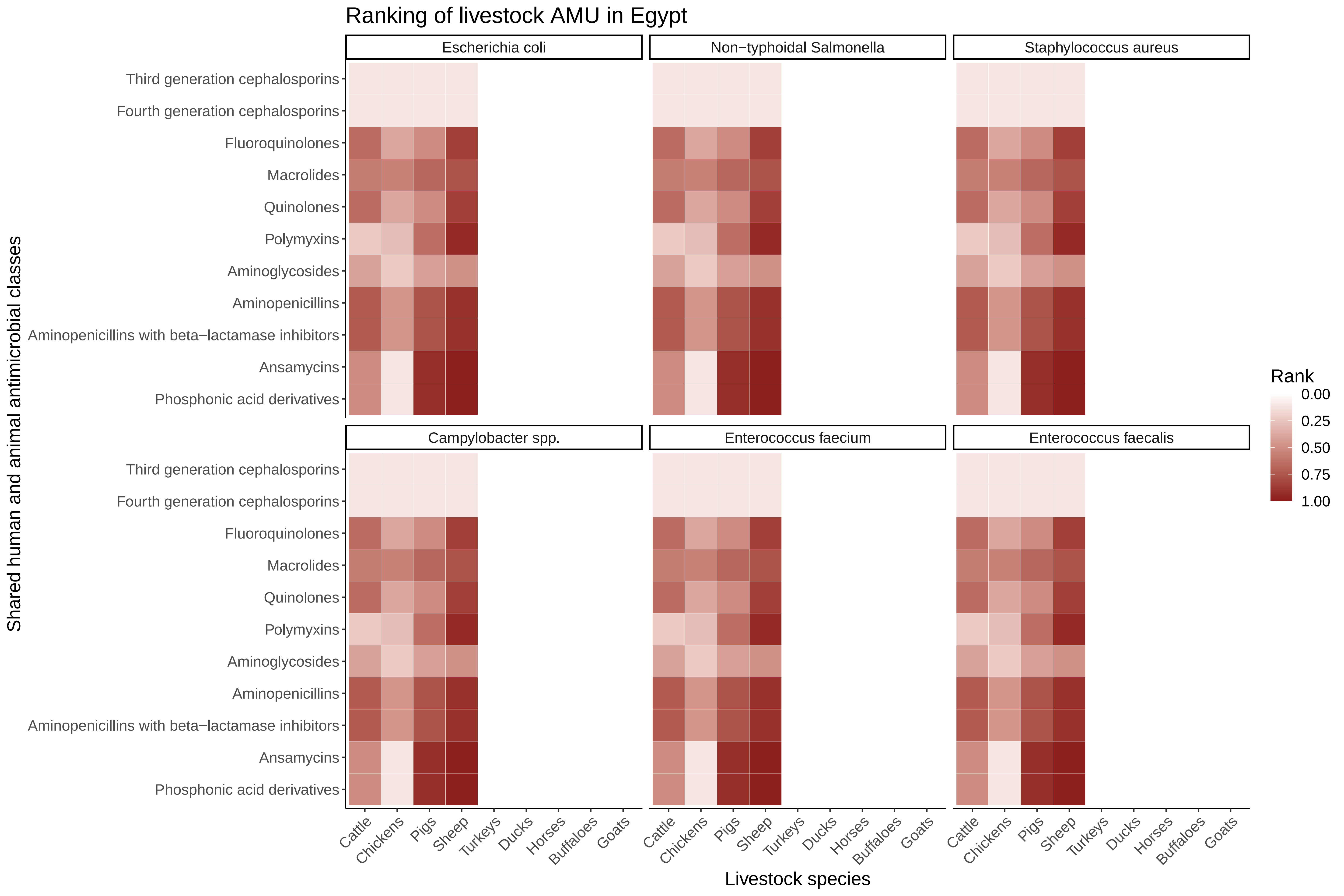

Ranking of livestock AMU in Egypt
Escherichia coli
Non−typhoidal Salmonella
Staphylococcus aureus
Campylobacter spp.
Enterococcus faecium
Enterococcus faecalis
Third generation cephalosporins
Fourth generation cephalosporins
Fluoroquinolones
Macrolides
Quinolones
Polymyxins
Aminoglycosides
Aminopenicillins
Aminopenicillins with beta−lactamase inhibitors
Ansamycins
Phosphonic acid derivatives
Shared human and animal antimicrobial classes
Cattle
Chickens
Pigs
Sheep
Turkeys
Ducks
Horses
Buffaloes
Goats
Livestock species
Rank
0.00
0.25
0.50
0.75
1.00

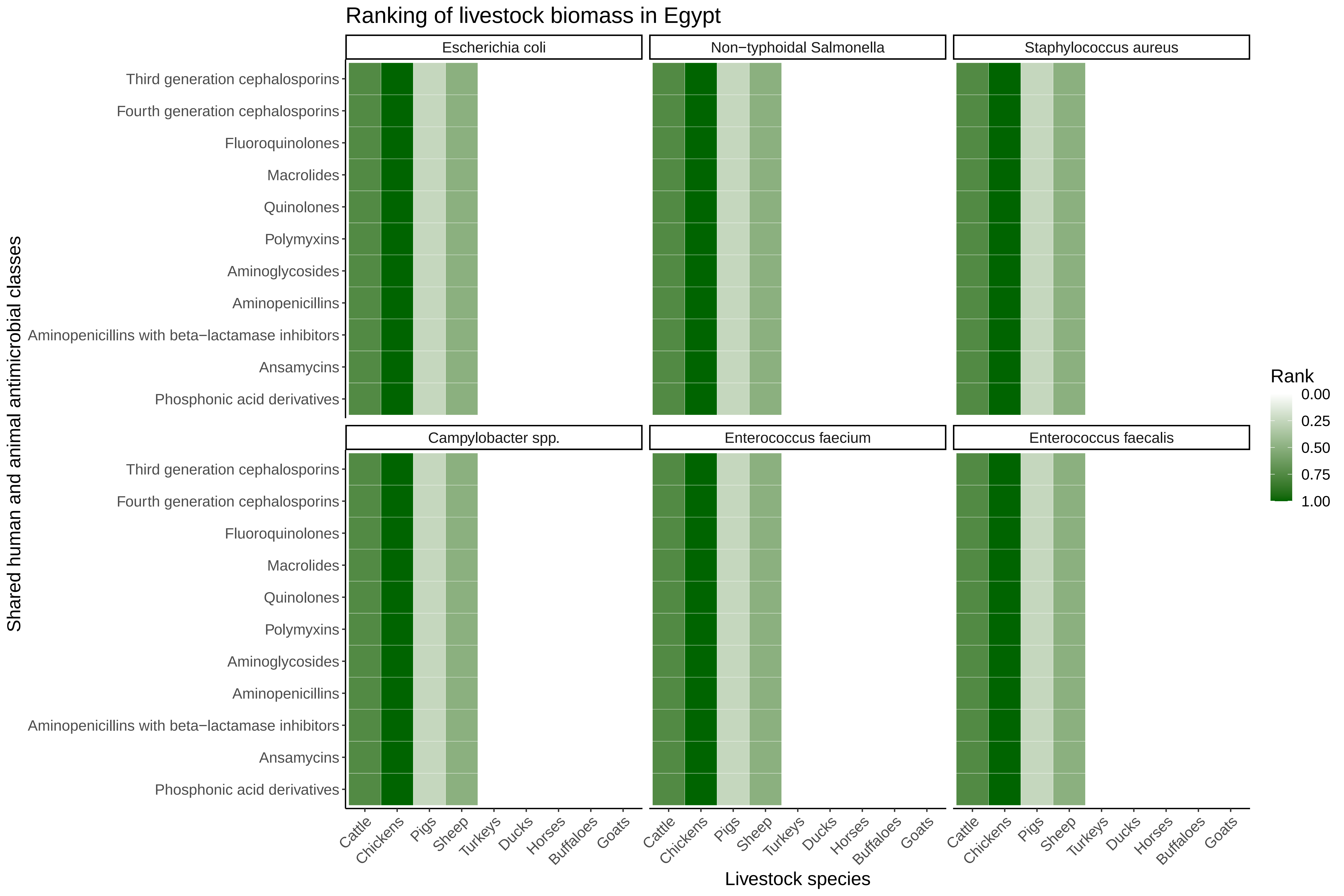

Ranking of livestock biomass in Egypt
Escherichia coli
Non−typhoidal Salmonella
Staphylococcus aureus
Campylobacter spp.
Enterococcus faecium
Enterococcus faecalis
Shared human and animal antimicrobial classes
Third generation cephalosporins
Fourth generation cephalosporins
Fluoroquinolones
Macrolides
Quinolones
Polymyxins
Aminoglycosides
Aminopenicillins
Aminopenicillins with beta−lactamase inhibitors
Ansamycins
Phosphonic acid derivatives
Livestock species
Cattle
Chickens
Pigs
Sheep
Turkeys
Ducks
Horses
Buffaloes
Goats
Rank
0.00
0.25
0.50
0.75
1.00

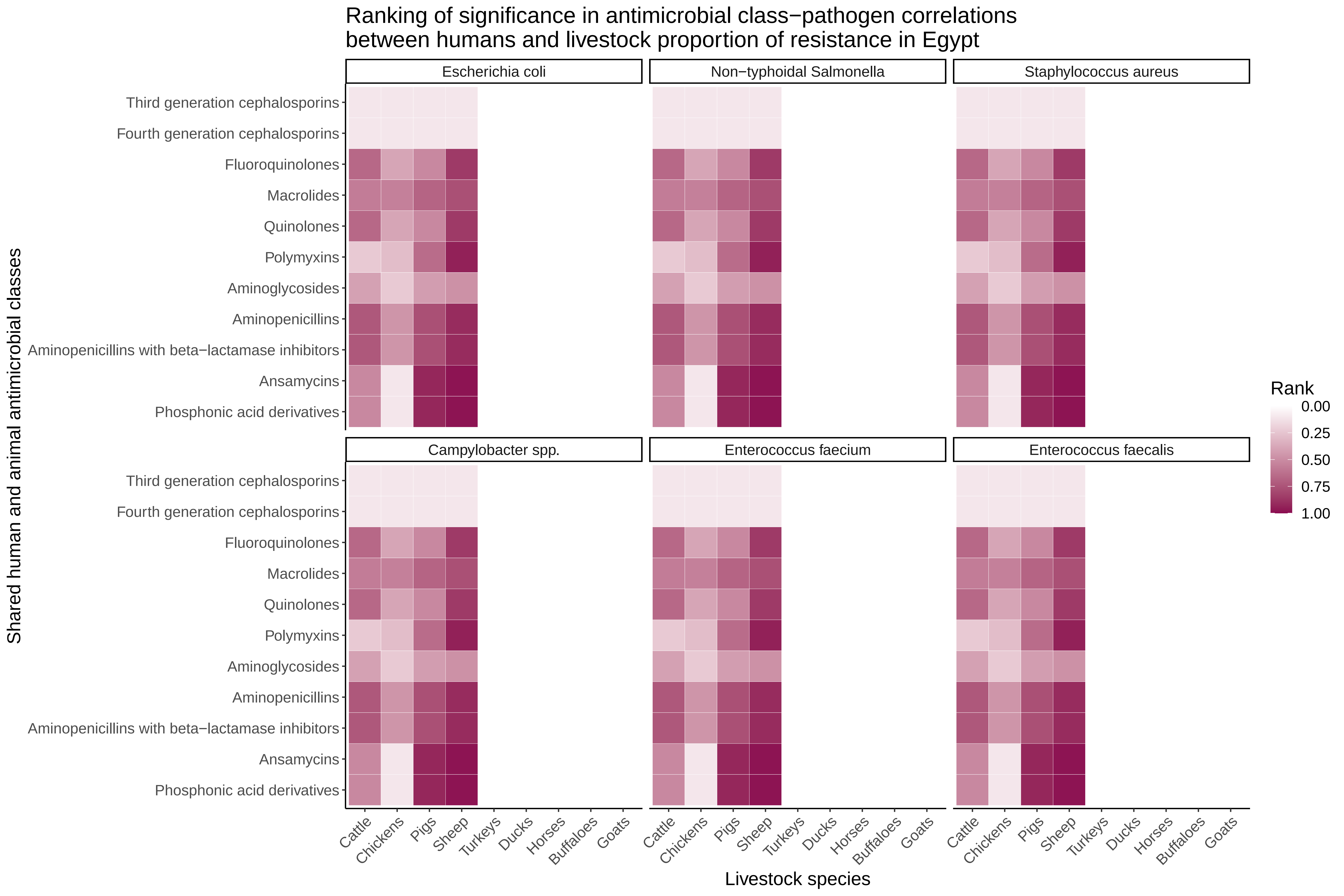

Ranking of significance in antimicrobial class−pathogen correlations
between humans and livestock proportion of resistance in Egypt
Escherichia coli
Non−typhoidal Salmonella
Staphylococcus aureus
Campylobacter spp.
Enterococcus faecium
Enterococcus faecalis
Third generation cephalosporins
Fourth generation cephalosporins
Fluoroquinolones
Macrolides
Quinolones
Polymyxins
Aminoglycosides
Aminopenicillins
Aminopenicillins with beta−lactamase inhibitors
Ansamycins
Phosphonic acid derivatives
Shared human and animal antimicrobial classes
Cattle
Chickens
Pigs
Sheep
Turkeys
Ducks
Horses
Buffaloes
Goats
Livestock species
Rank
0.00
0.25
0.50
0.75
1.00

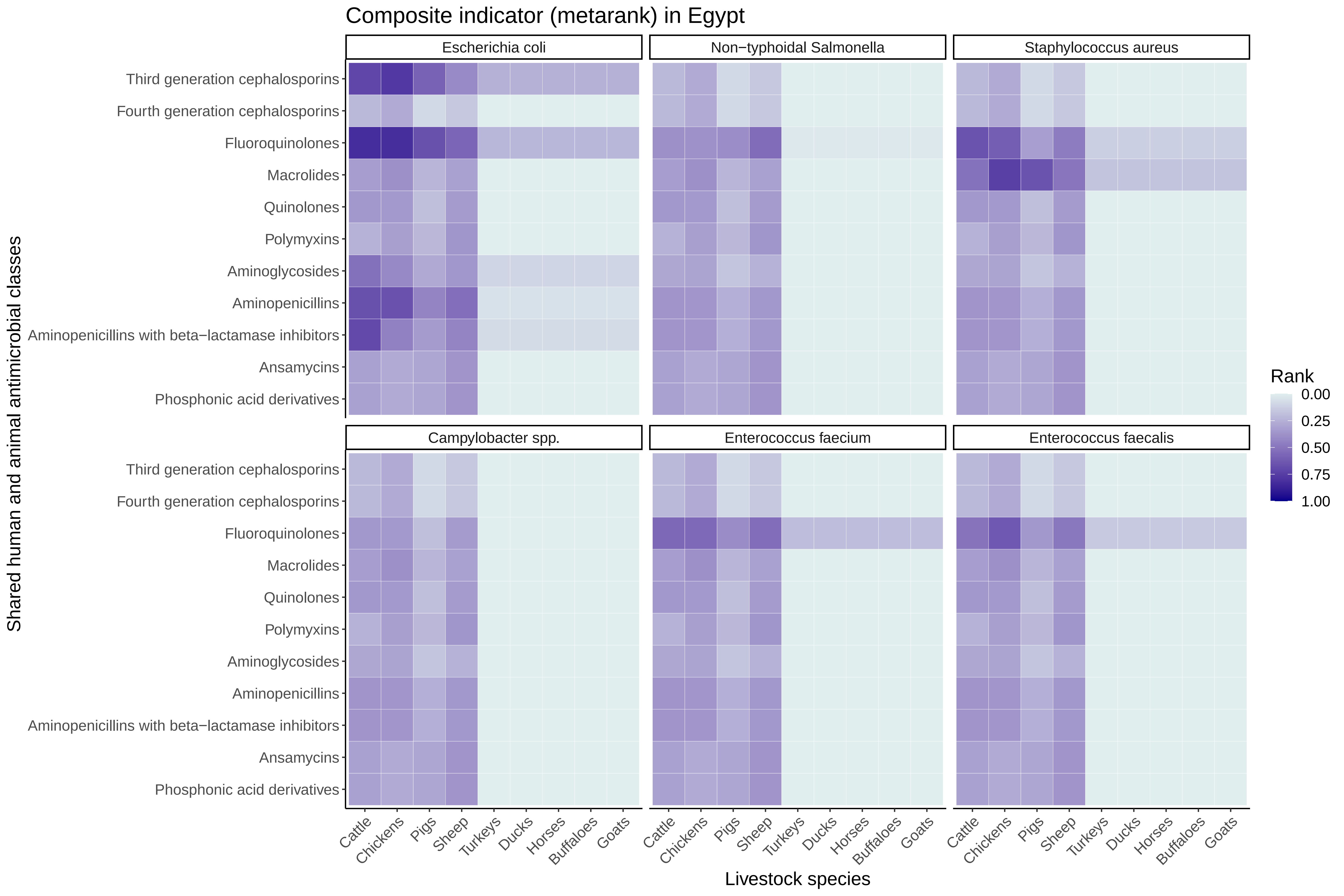

Composite indicator (metarank) in Egypt
Escherichia coli
Non−typhoidal Salmonella
Staphylococcus aureus
Campylobacter spp.
Enterococcus faecium
Enterococcus faecalis
Shared human and animal antimicrobial classes
Third generation cephalosporins
Fourth generation cephalosporins
Fluoroquinolones
Macrolides
Quinolones
Polymyxins
Aminoglycosides
Aminopenicillins
Aminopenicillins with beta−lactamase inhibitors
Ansamycins
Phosphonic acid derivatives
Cattle
Chickens
Pigs
Sheep
Turkeys
Ducks
Horses
Buffaloes
Goats
Livestock species
Rank
0.00
0.25
0.50
0.75
1.00

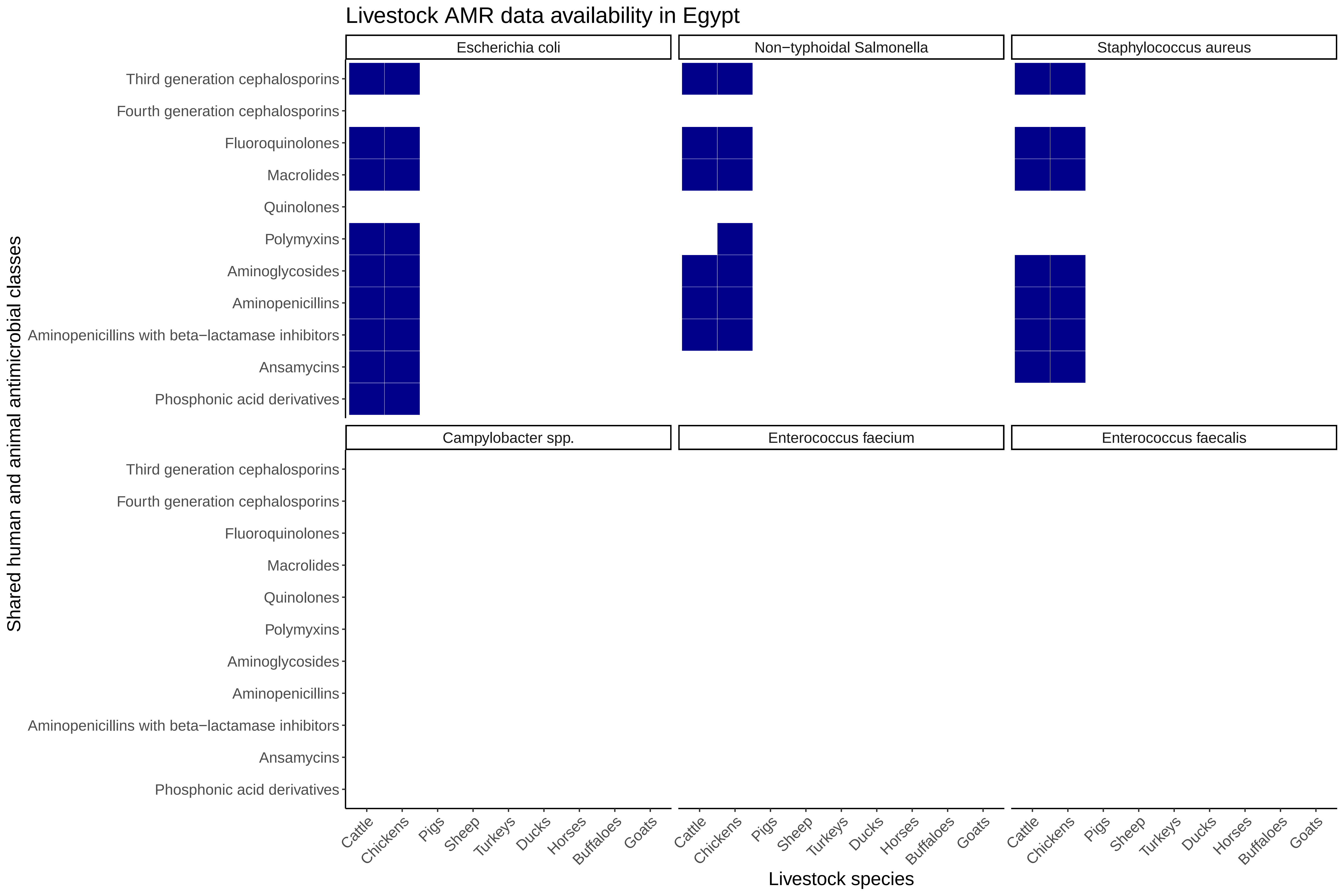

Livestock AMR data availability in Egypt
Escherichia coli
Non−typhoidal Salmonella
Staphylococcus aureus
Campylobacter spp.
Enterococcus faecium
Enterococcus faecalis
Third generation cephalosporins
Fourth generation cephalosporins
Fluoroquinolones
Macrolides
Quinolones
Polymyxins
Aminoglycosides
Aminopenicillins
Aminopenicillins with beta−lactamase inhibitors
Ansamycins
Phosphonic acid derivatives
Shared human and animal antimicrobial classes
Cattle
Chickens
Pigs
Sheep
Turkeys
Ducks
Horses
Buffaloes
Goats
Livestock species

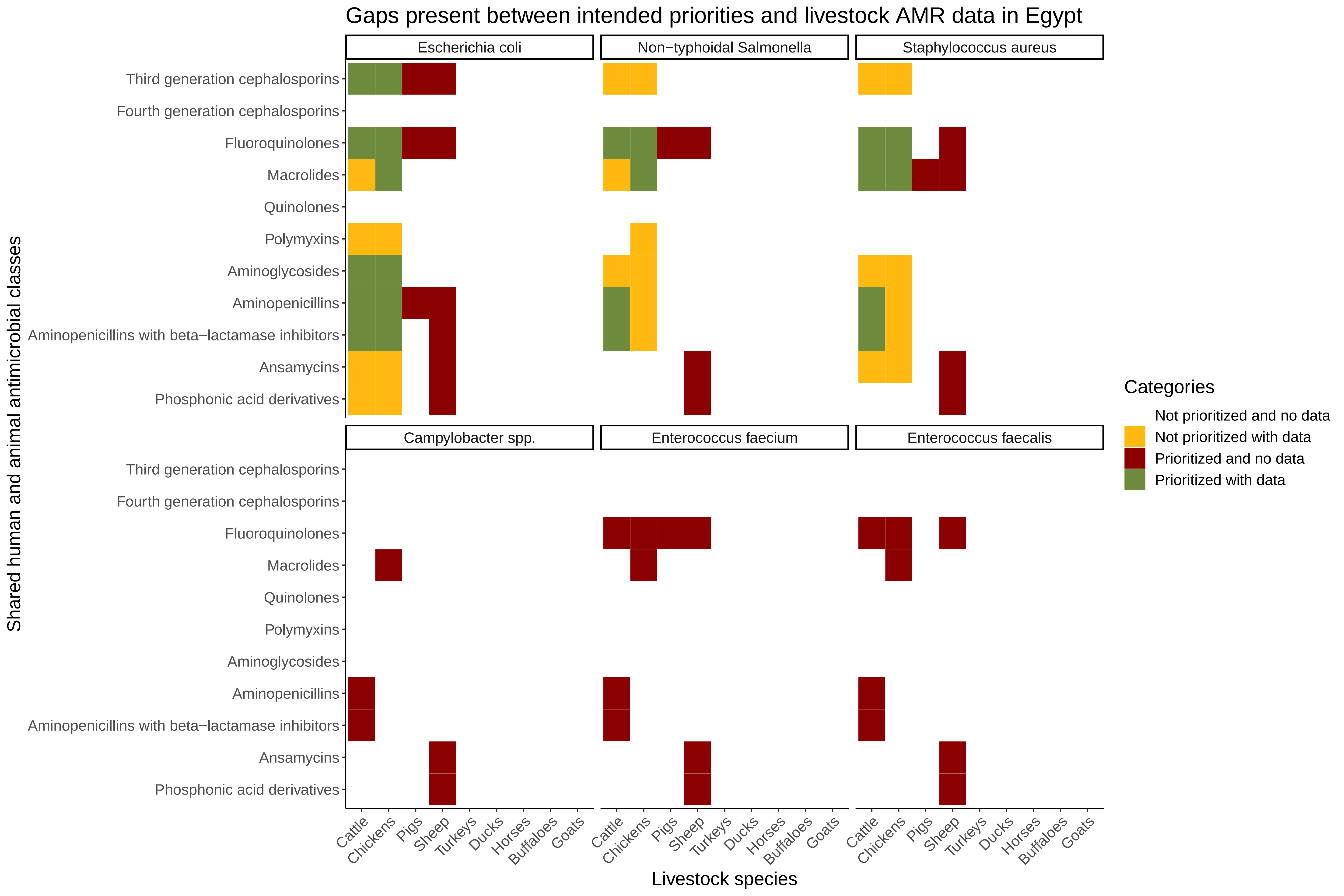

Gaps present between intended priorities and livestock AMR data in Egypt
Escherichia coli
Non−typhoidal Salmonella
Staphylococcus aureus
Campylobacter spp.
Enterococcus faecium
Enterococcus faecalis
Third generation cephalosporins
Fourth generation cephalosporins
Fluoroquinolones
Macrolides
Quinolones
Polymyxins
Aminoglycosides
Aminopenicillins
Aminopenicillins with beta−lactamase inhibitors
Ansamycins
Phosphonic acid derivatives
Shared human and animal antimicrobial classes
Cattle
Chickens
Pigs
Sheep
Turkeys
Ducks
Horses
Buffaloes
Goats
Livestock species
Categories
Not prioritized and no data
Not prioritized with data
Prioritized and no data
Prioritized with data

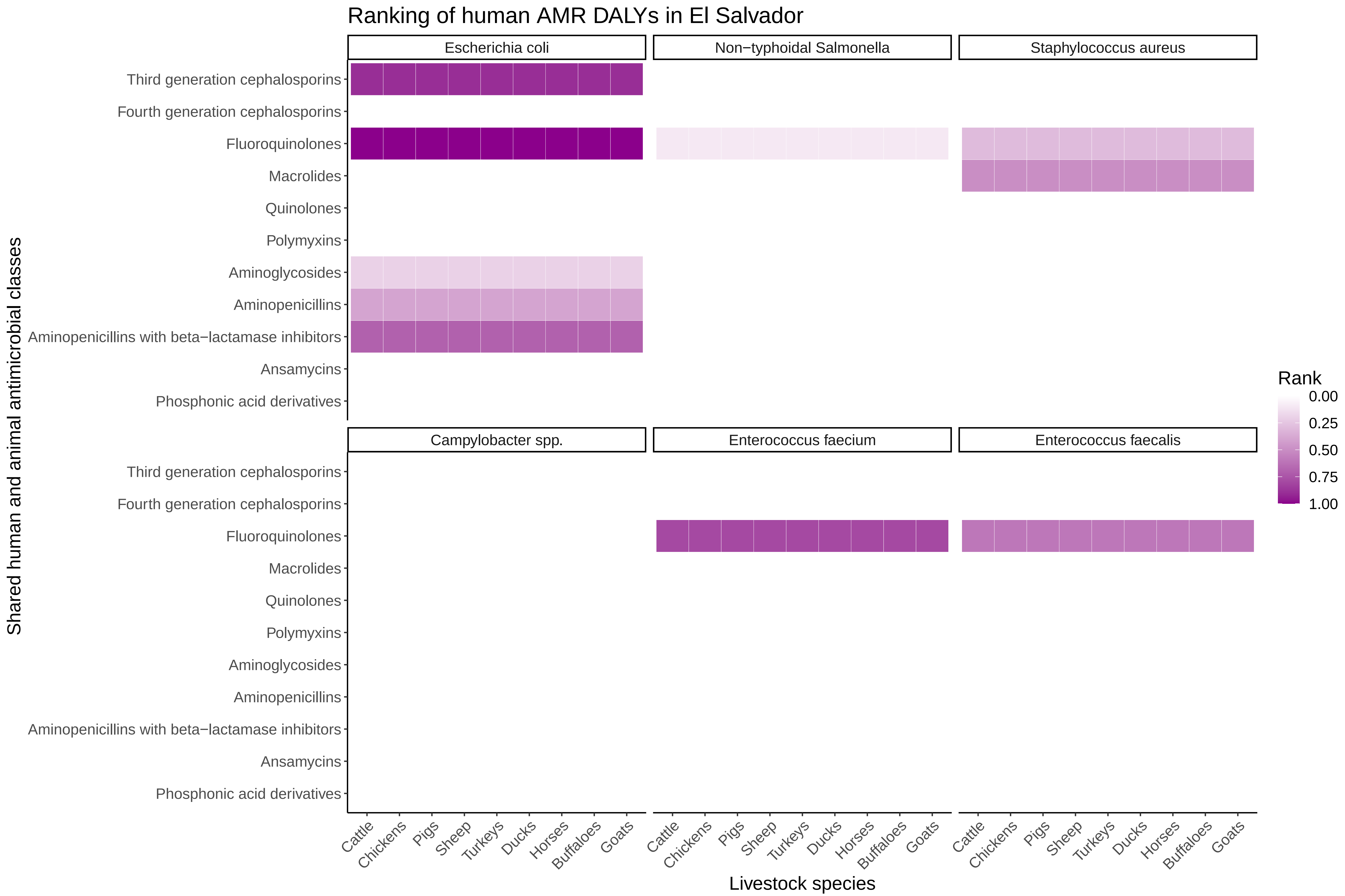

Ranking of human AMR DALYs in El Salvador
Escherichia coli
Non−typhoidal Salmonella
Staphylococcus aureus
Campylobacter spp.
Enterococcus faecium
Enterococcus faecalis
Third generation cephalosporins
Fourth generation cephalosporins
Fluoroquinolones
Macrolides
Quinolones
Polymyxins
Aminoglycosides
Aminopenicillins
Aminopenicillins with beta−lactamase inhibitors
Ansamycins
Phosphonic acid derivatives
Shared human and animal antimicrobial classes
Cattle
Chickens
Pigs
Sheep
Turkeys
Ducks
Horses
Buffaloes
Goats
Livestock species
Rank
0.00
0.25
0.50
0.75
1.00

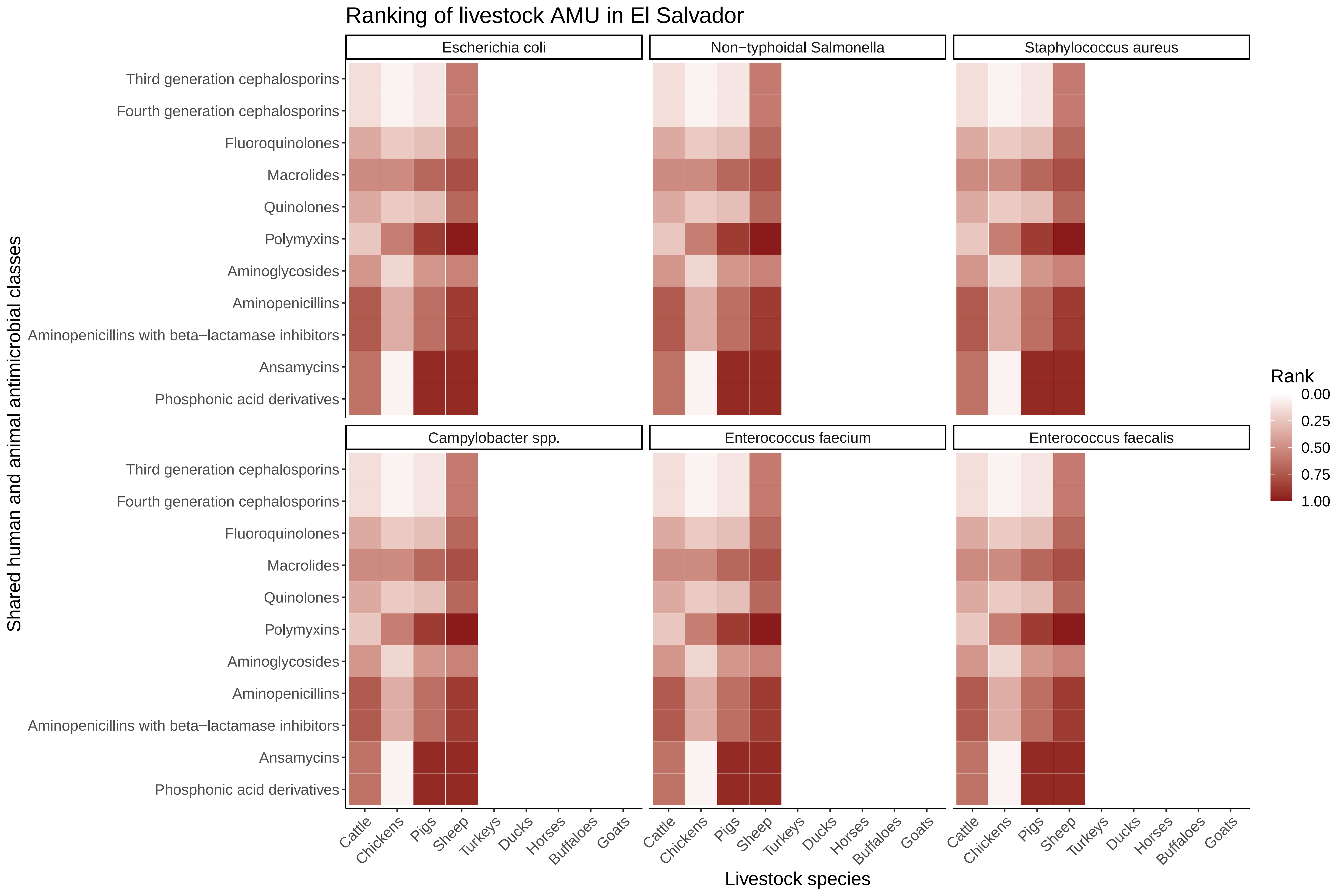

Ranking of livestock AMU in El Salvador
Escherichia coli
Non−typhoidal Salmonella
Staphylococcus aureus
Campylobacter spp.
Enterococcus faecium
Enterococcus faecalis
Shared human and animal antimicrobial classes
Third generation cephalosporins
Fourth generation cephalosporins
Fluoroquinolones
Macrolides
Quinolones
Polymyxins
Aminoglycosides
Aminopenicillins
Aminopenicillins with beta−lactamase inhibitors
Ansamycins
Phosphonic acid derivatives
Livestock species
Cattle
Chickens
Pigs
Sheep
Turkeys
Ducks
Horses
Buffaloes
Goats
Rank
0.00
0.25
0.50
0.75
1.00

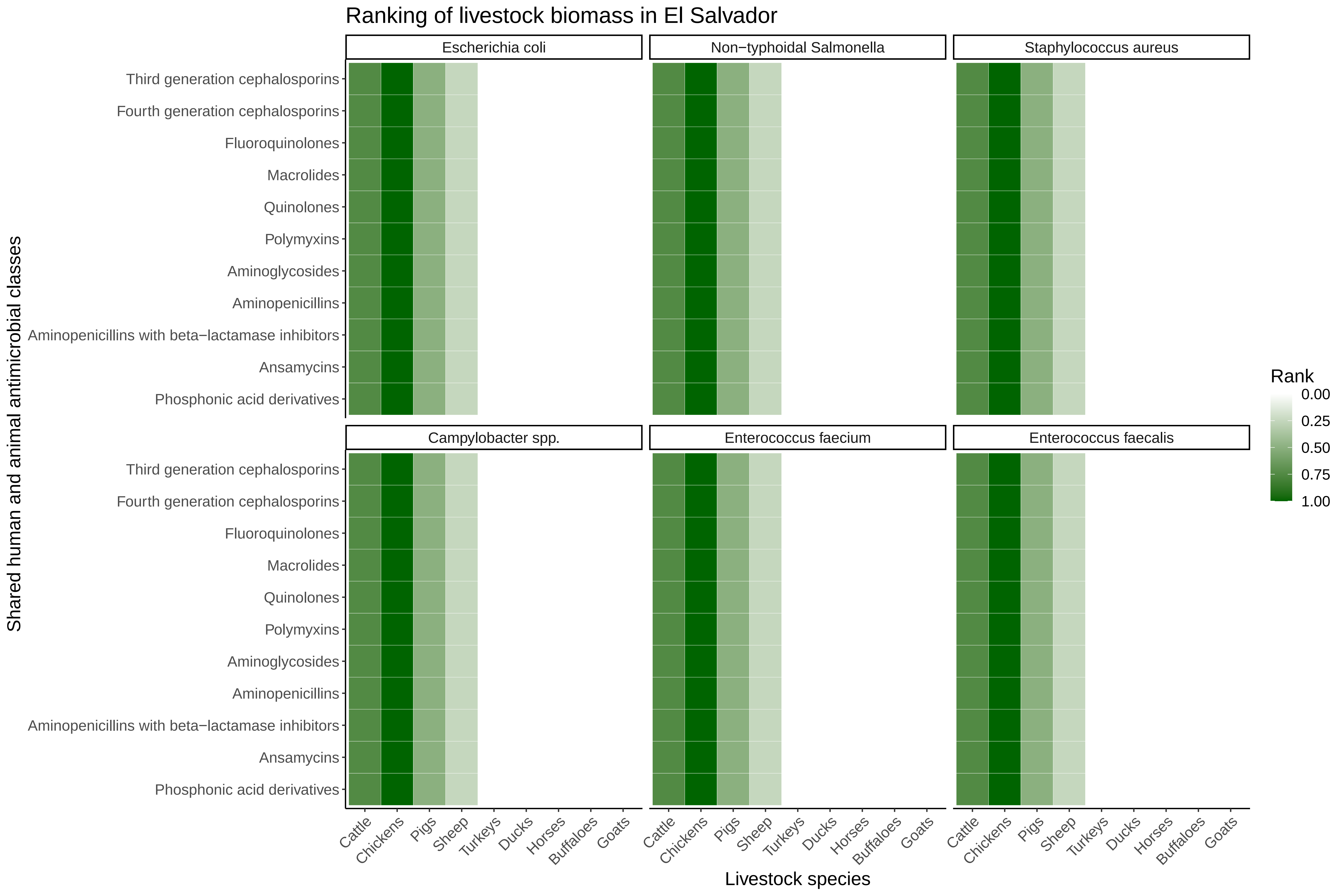

Ranking of livestock biomass in El Salvador
Escherichia coli
Non−typhoidal Salmonella
Staphylococcus aureus
Campylobacter spp.
Enterococcus faecium
Enterococcus faecalis
Third generation cephalosporins
Fourth generation cephalosporins
Fluoroquinolones
Macrolides
Quinolones
Polymyxins
Aminoglycosides
Aminopenicillins
Aminopenicillins with beta−lactamase inhibitors
Ansamycins
Phosphonic acid derivatives
Shared human and animal antimicrobial classes
Cattle
Chickens
Pigs
Sheep
Turkeys
Ducks
Horses
Buffaloes
Goats
Livestock species
Rank
0.00
0.25
0.50
0.75
1.00

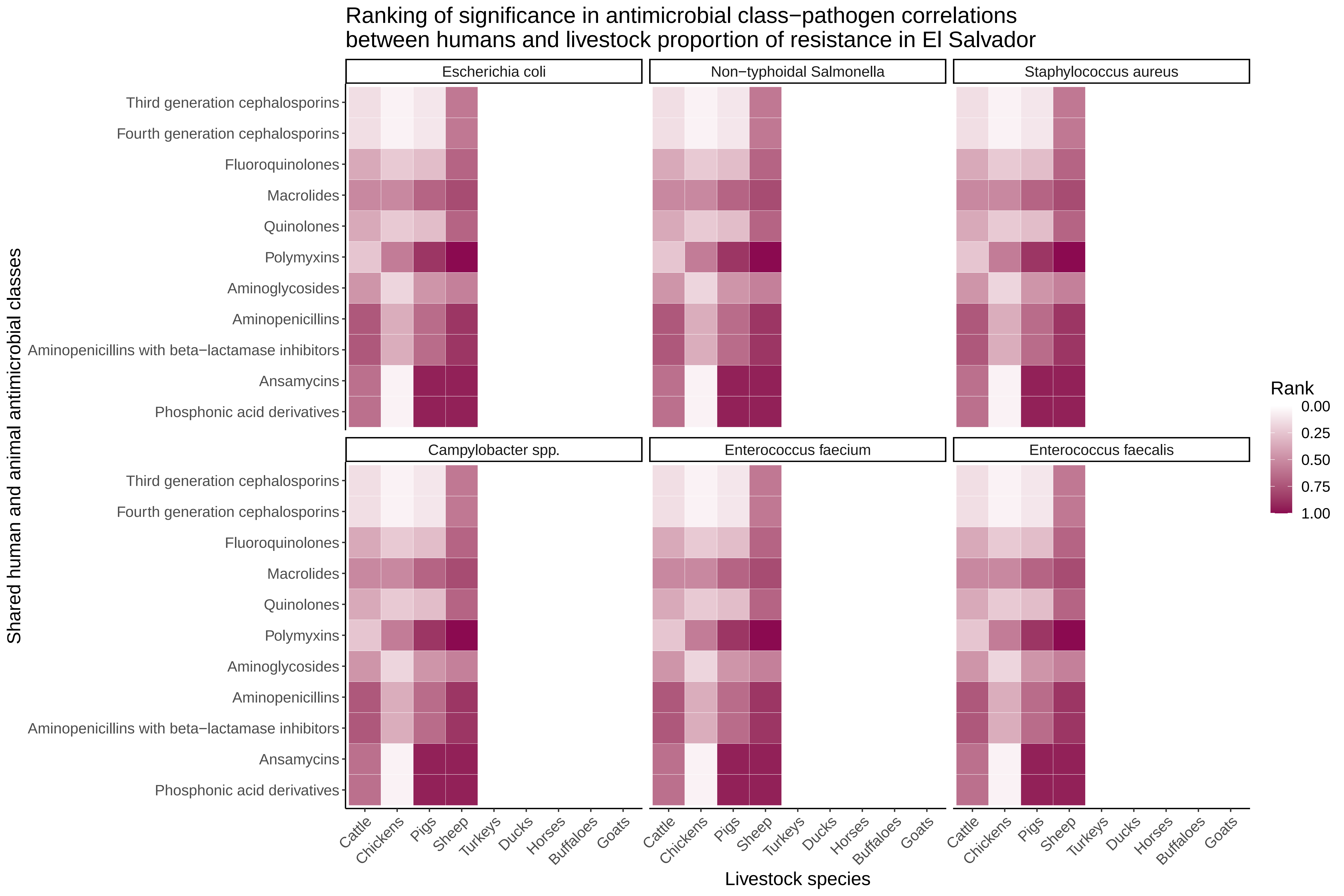

Ranking of significance in antimicrobial class−pathogen correlations
between humans and livestock proportion of resistance in El Salvador
Escherichia coli
Non−typhoidal Salmonella
Staphylococcus aureus
Campylobacter spp.
Enterococcus faecium
Enterococcus faecalis
Third generation cephalosporins
Fourth generation cephalosporins
Fluoroquinolones
Macrolides
Quinolones
Polymyxins
Aminoglycosides
Aminopenicillins
Aminopenicillins with beta−lactamase inhibitors
Ansamycins
Phosphonic acid derivatives
Shared human and animal antimicrobial classes
Cattle
Chickens
Pigs
Sheep
Turkeys
Ducks
Horses
Buffaloes
Goats
Livestock species
Rank
0.00
0.25
0.50
0.75
1.00

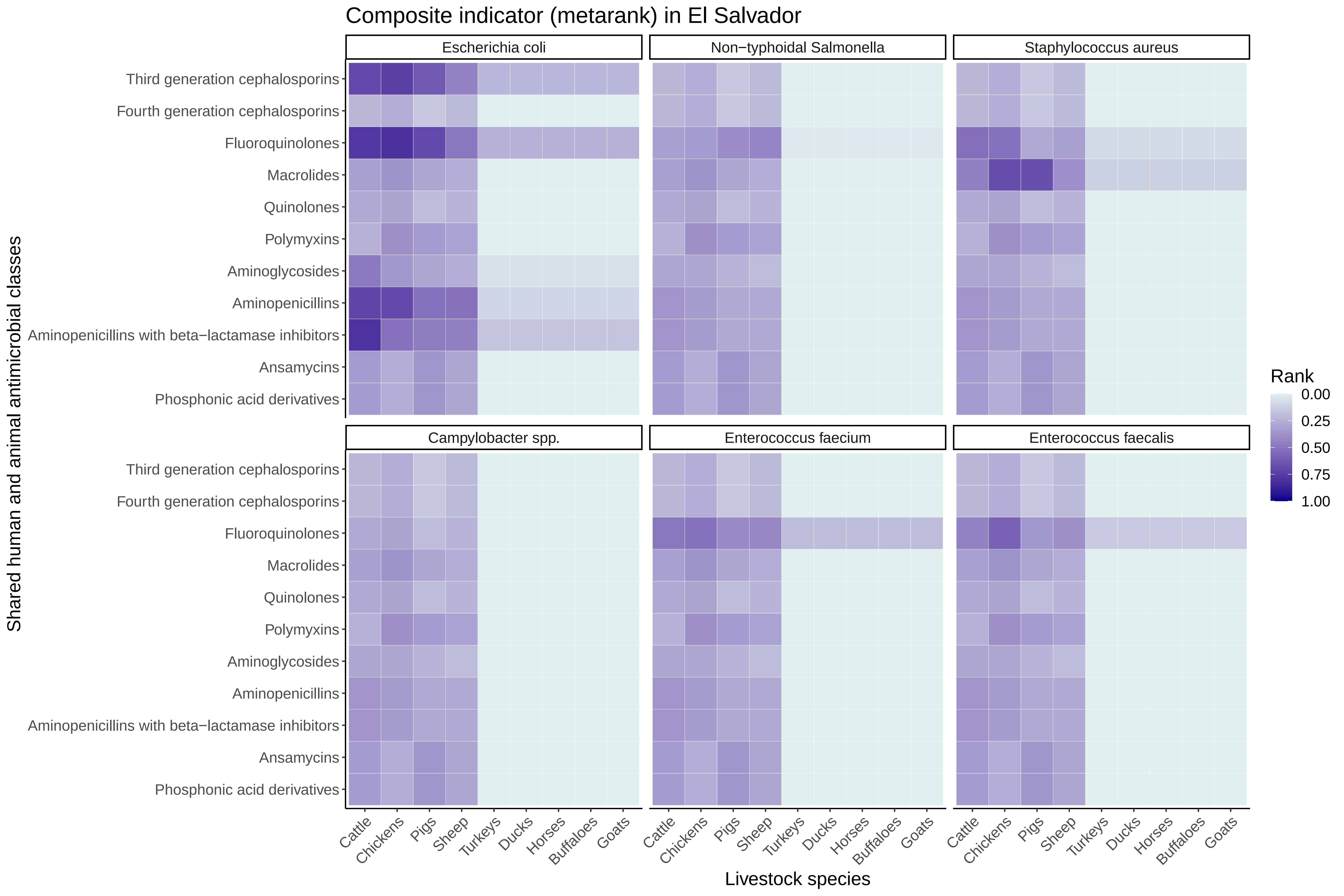

Composite indicator (metarank) in El Salvador
Escherichia coli
Non−typhoidal Salmonella
Staphylococcus aureus
Campylobacter spp.
Enterococcus faecium
Enterococcus faecalis
Shared human and animal antimicrobial classes
Third generation cephalosporins
Fourth generation cephalosporins
Fluoroquinolones
Macrolides
Quinolones
Polymyxins
Aminoglycosides
Aminopenicillins
Aminopenicillins with beta−lactamase inhibitors
Ansamycins
Phosphonic acid derivatives
Cattle
Chickens
Pigs
Sheep
Turkeys
Ducks
Horses
Buffaloes
Goats
Livestock species
Rank
0.00
0.25
0.50
0.75
1.00

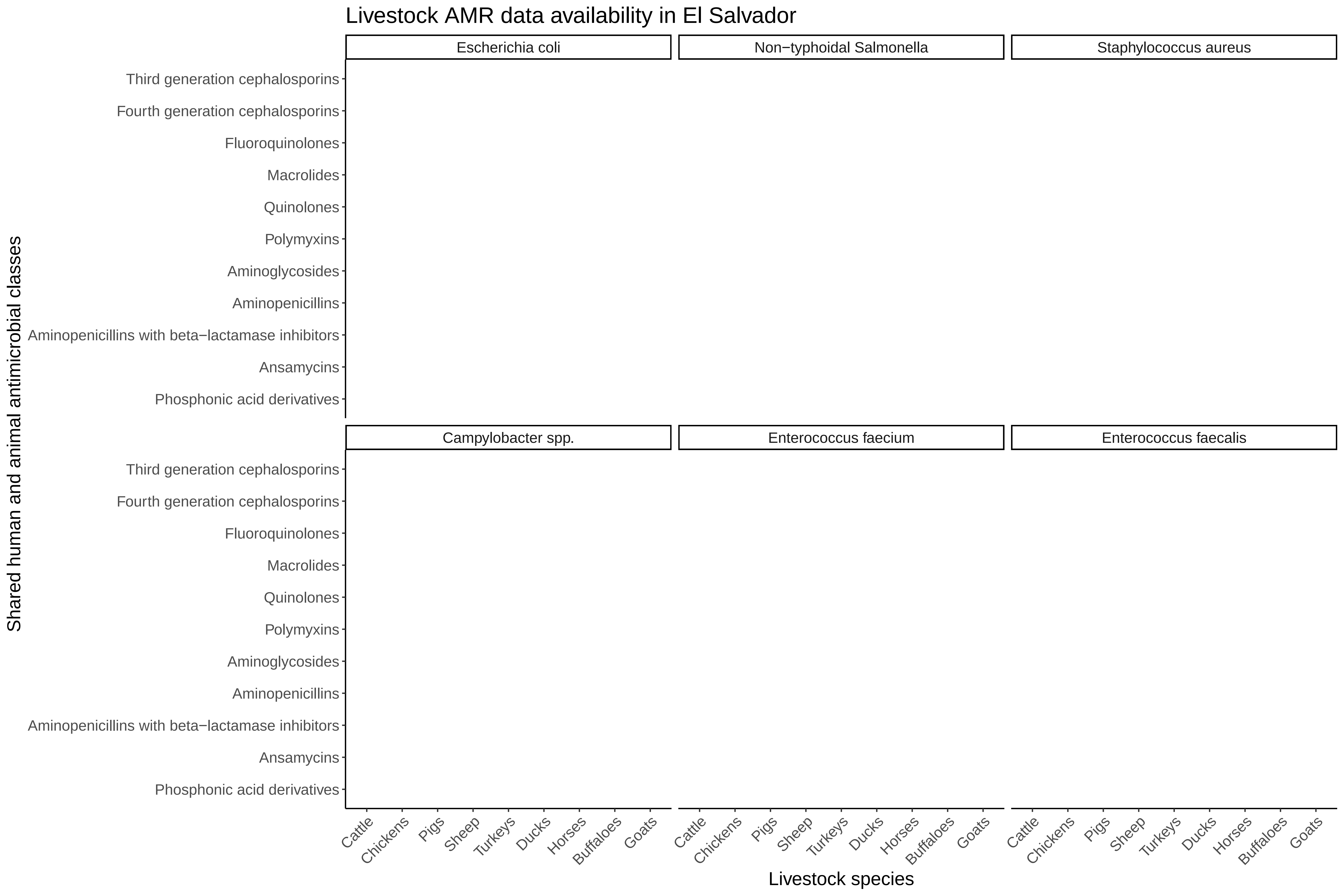
Livestock AMR data availability in El Salvador
Escherichia coli
Non−typhoidal Salmonella
Staphylococcus aureus
Campylobacter spp.
Enterococcus faecium
Enterococcus faecalis
Shared human and animal antimicrobial classes
Third generation cephalosporins
Fourth generation cephalosporins
Fluoroquinolones
Macrolides
Quinolones
Polymyxins
Aminoglycosides
Aminopenicillins
Aminopenicillins with beta−lactamase inhibitors
Ansamycins
Phosphonic acid derivatives
Cattle
Chickens
Pigs
Sheep
Turkeys
Ducks
Horses
Buffaloes
Goats
Livestock species

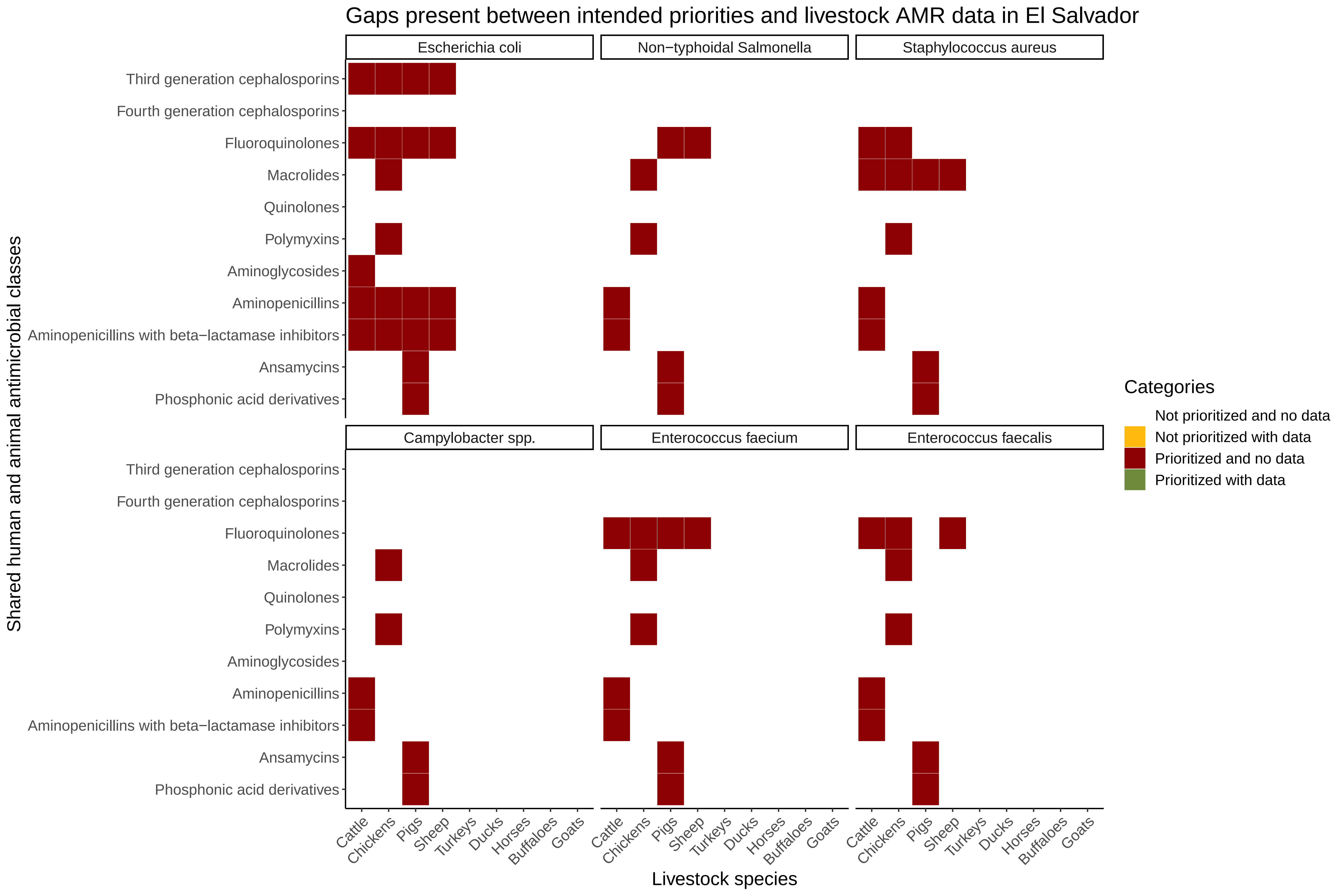

Gaps present between intended priorities and livestock AMR data in El Salvador
Escherichia coli
Non−typhoidal Salmonella
Staphylococcus aureus
Campylobacter spp.
Enterococcus faecium
Enterococcus faecalis
Shared human and animal antimicrobial classes
Third generation cephalosporins
Fourth generation cephalosporins
Fluoroquinolones
Macrolides
Quinolones
Polymyxins
Aminoglycosides
Aminopenicillins
Aminopenicillins with beta−lactamase inhibitors
Ansamycins
Phosphonic acid derivatives
Livestock species
Cattle
Chickens
Pigs
Sheep
Turkeys
Ducks
Horses
Buffaloes
Goats
Categories
Not prioritized and no data
Not prioritized with data
Prioritized and no data
Prioritized with data

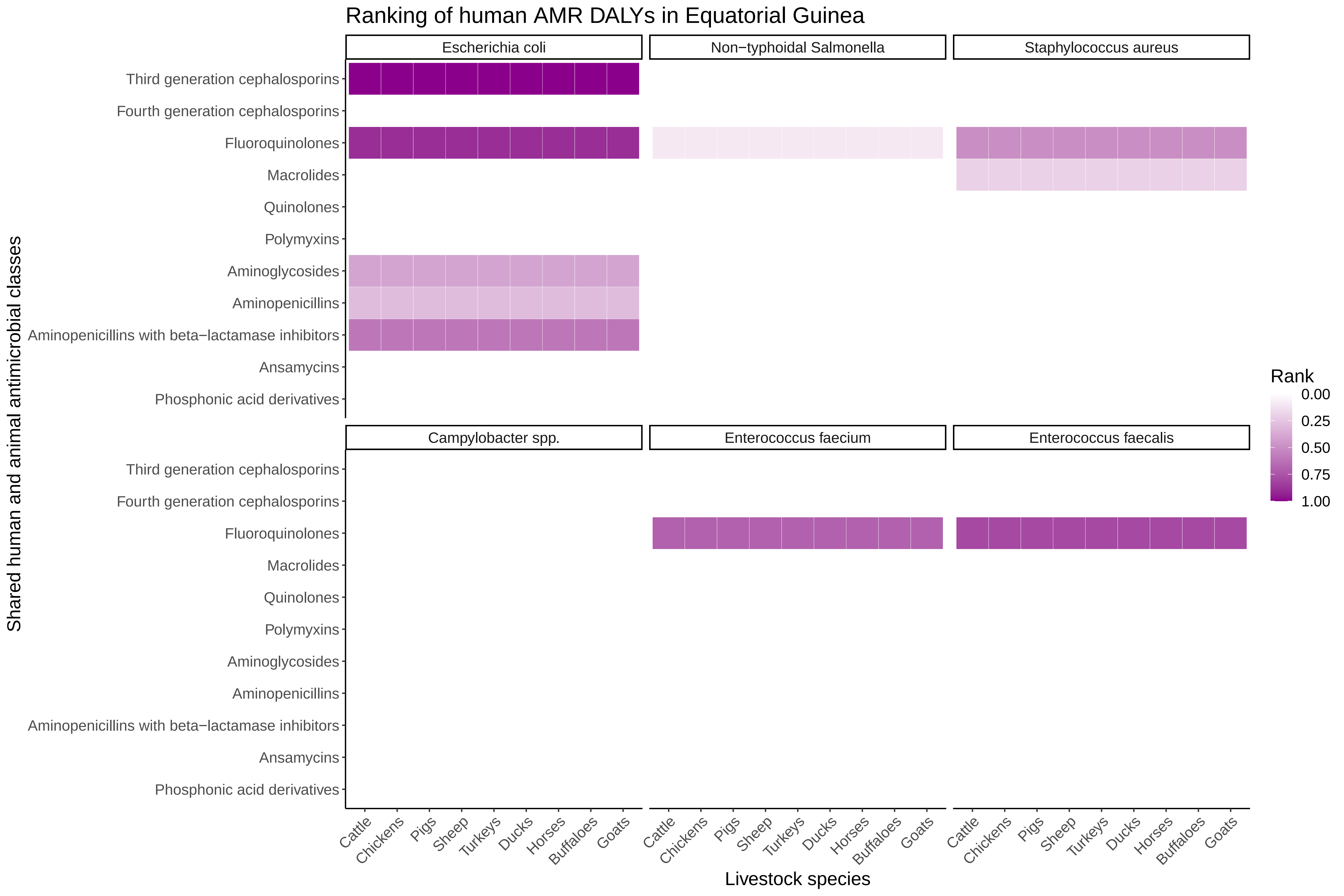

Ranking of human AMR DALYs in Equatorial Guinea
Escherichia coli
Non−typhoidal Salmonella
Staphylococcus aureus
Campylobacter spp.
Enterococcus faecium
Enterococcus faecalis
Third generation cephalosporins
Fourth generation cephalosporins
Fluoroquinolones
Macrolides
Quinolones
Polymyxins
Aminoglycosides
Aminopenicillins
Aminopenicillins with beta−lactamase inhibitors
Ansamycins
Phosphonic acid derivatives
Shared human and animal antimicrobial classes
Cattle
Chickens
Pigs
Sheep
Turkeys
Ducks
Horses
Buffaloes
Goats
Livestock species
Rank
0.00
0.25
0.50
0.75
1.00

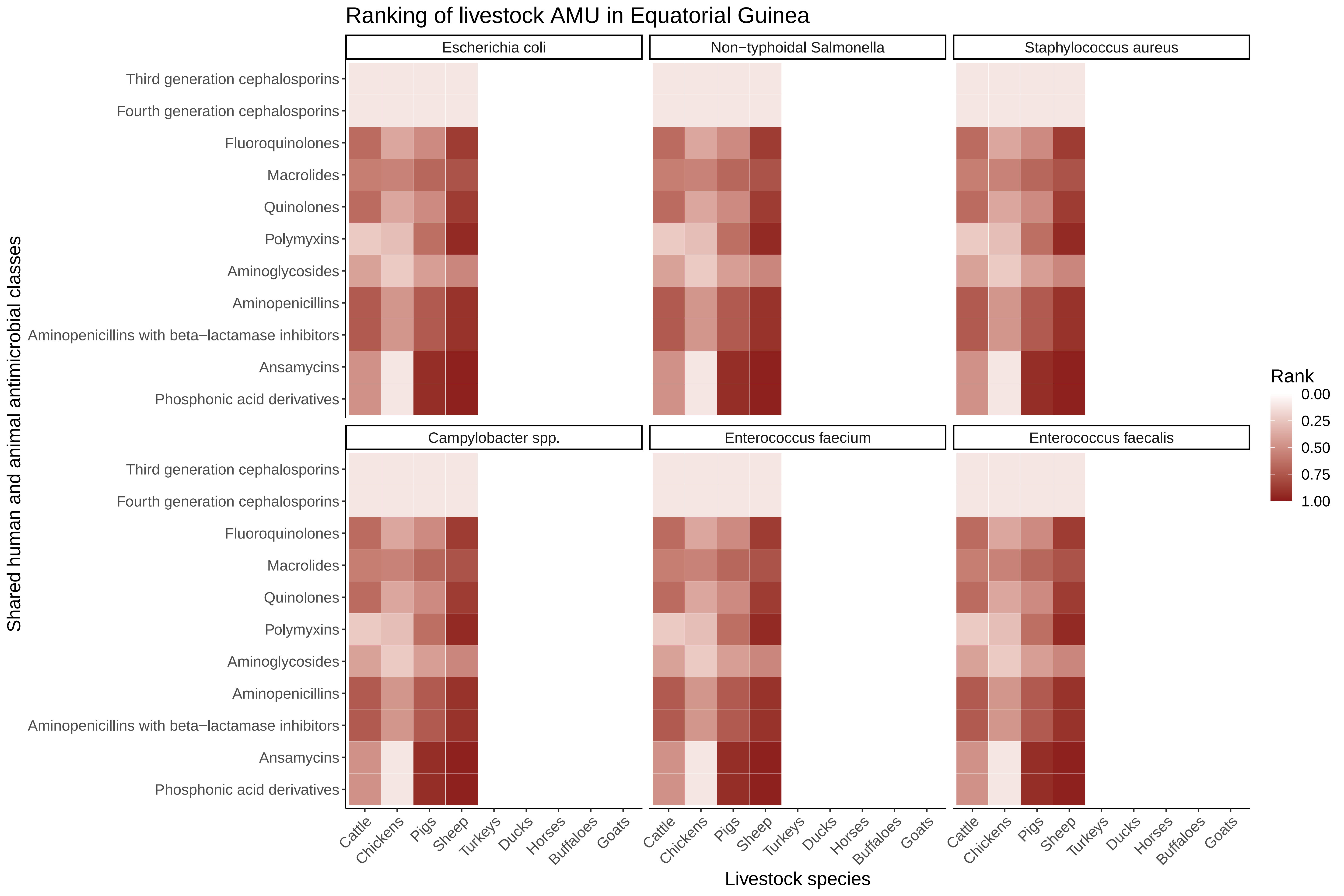

Ranking of livestock AMU in Equatorial Guinea
Escherichia coli
Non−typhoidal Salmonella
Staphylococcus aureus
Campylobacter spp.
Enterococcus faecium
Enterococcus faecalis
Third generation cephalosporins
Fourth generation cephalosporins
Fluoroquinolones
Macrolides
Quinolones
Polymyxins
Aminoglycosides
Aminopenicillins
Aminopenicillins with beta−lactamase inhibitors
Ansamycins
Phosphonic acid derivatives
Shared human and animal antimicrobial classes
Cattle
Chickens
Pigs
Sheep
Turkeys
Ducks
Horses
Buffaloes
Goats
Livestock species
Rank
0.00
0.25
0.50
0.75
1.00

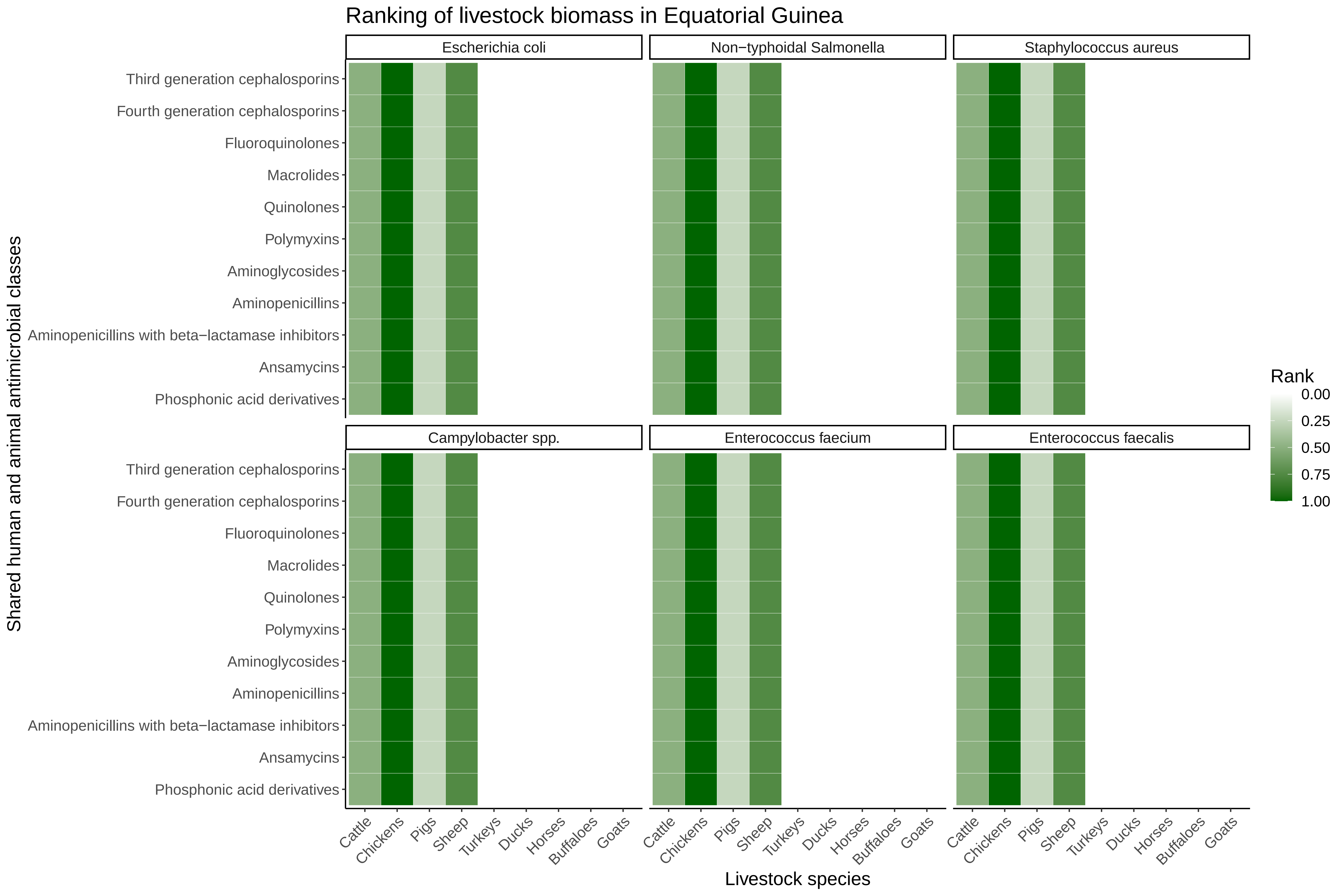

Ranking of livestock biomass in Equatorial Guinea
Escherichia coli
Non−typhoidal Salmonella
Staphylococcus aureus
Campylobacter spp.
Enterococcus faecium
Enterococcus faecalis
Third generation cephalosporins
Fourth generation cephalosporins
Fluoroquinolones
Macrolides
Quinolones
Polymyxins
Aminoglycosides
Aminopenicillins
Aminopenicillins with beta−lactamase inhibitors
Ansamycins
Phosphonic acid derivatives
Shared human and animal antimicrobial classes
Cattle
Chickens
Pigs
Sheep
Turkeys
Ducks
Horses
Buffaloes
Goats
Livestock species
Rank
0.00
0.25
0.50
0.75
1.00

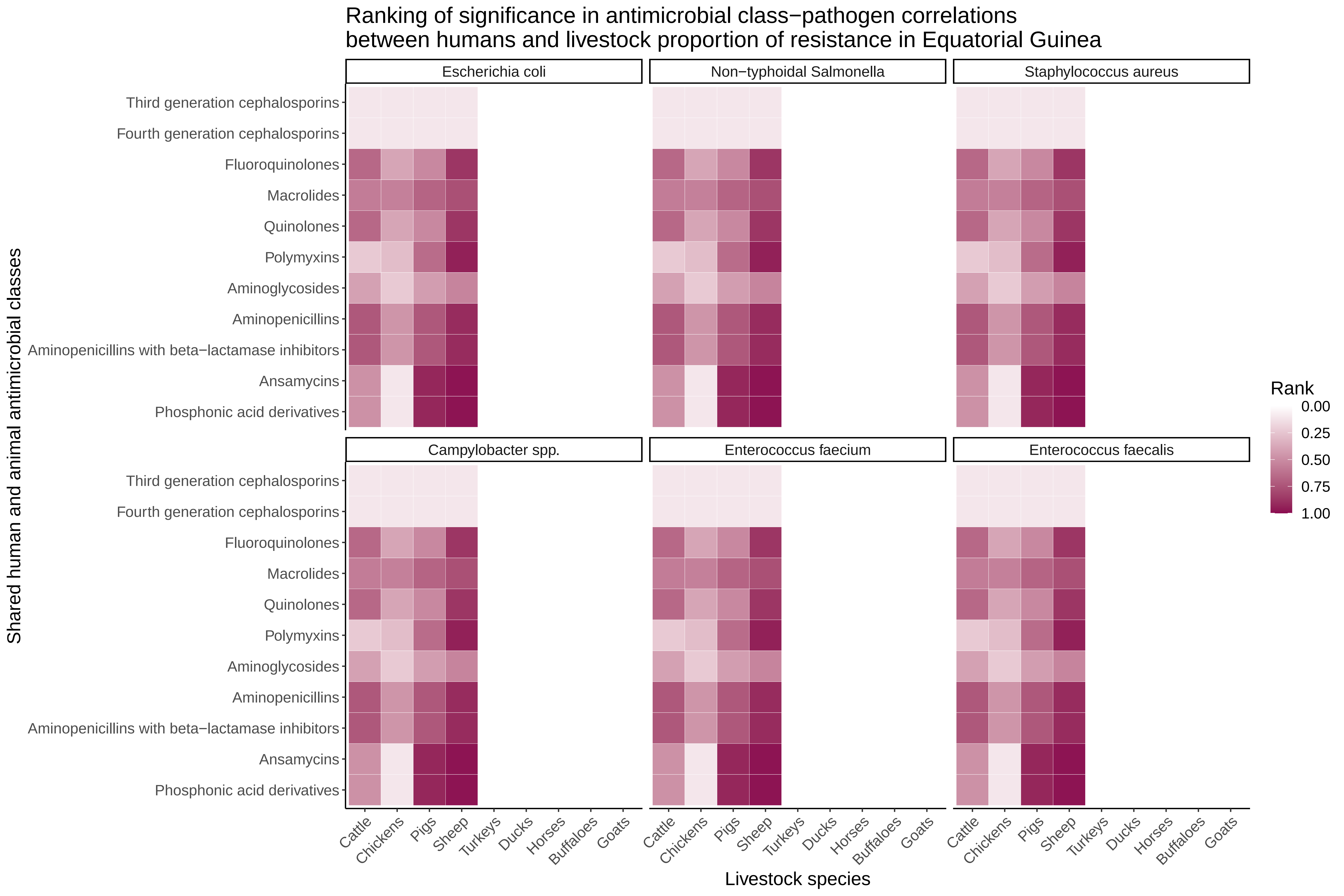
Ranking of significance in antimicrobial class−pathogen correlations
between humans and livestock proportion of resistance in Equatorial Guinea
Escherichia coli
Non−typhoidal Salmonella
Staphylococcus aureus
Campylobacter spp.
Enterococcus faecium
Enterococcus faecalis
Third generation cephalosporins
Fourth generation cephalosporins
Fluoroquinolones
Macrolides
Quinolones
Polymyxins
Aminoglycosides
Aminopenicillins
Aminopenicillins with beta−lactamase inhibitors
Ansamycins
Phosphonic acid derivatives
Shared human and animal antimicrobial classes
Cattle
Chickens
Pigs
Sheep
Turkeys
Ducks
Horses
Buffaloes
Goats
Livestock species
Rank
0.00
0.25
0.50
0.75
1.00

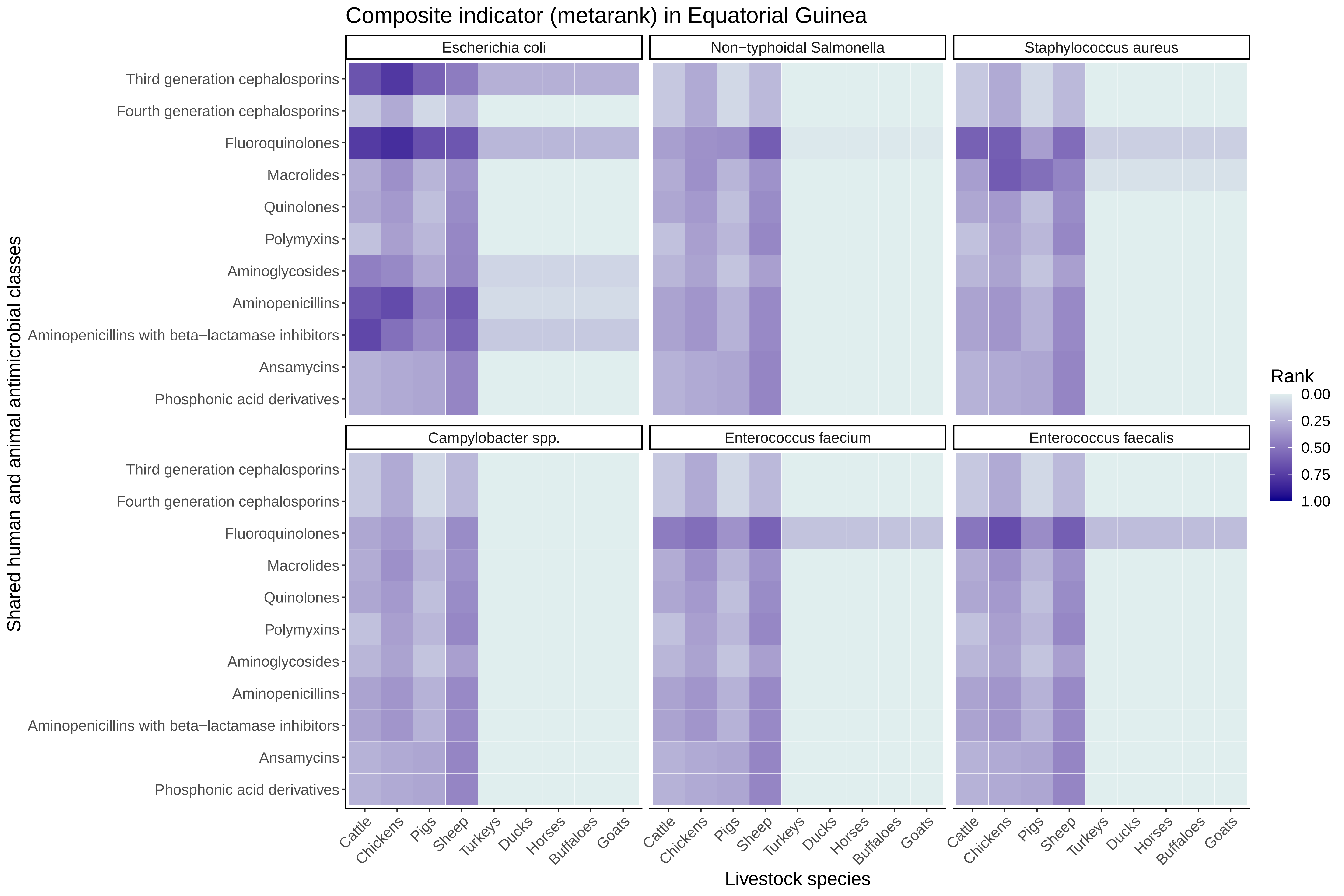

Composite indicator (metarank) in Equatorial Guinea
Escherichia coli
Non−typhoidal Salmonella
Staphylococcus aureus
Campylobacter spp.
Enterococcus faecium
Enterococcus faecalis
Shared human and animal antimicrobial classes
Third generation cephalosporins
Fourth generation cephalosporins
Fluoroquinolones
Macrolides
Quinolones
Polymyxins
Aminoglycosides
Aminopenicillins
Aminopenicillins with beta−lactamase inhibitors
Ansamycins
Phosphonic acid derivatives
Cattle
Chickens
Pigs
Sheep
Turkeys
Ducks
Horses
Buffaloes
Goats
Livestock species
Rank
0.00
0.25
0.50
0.75
1.00

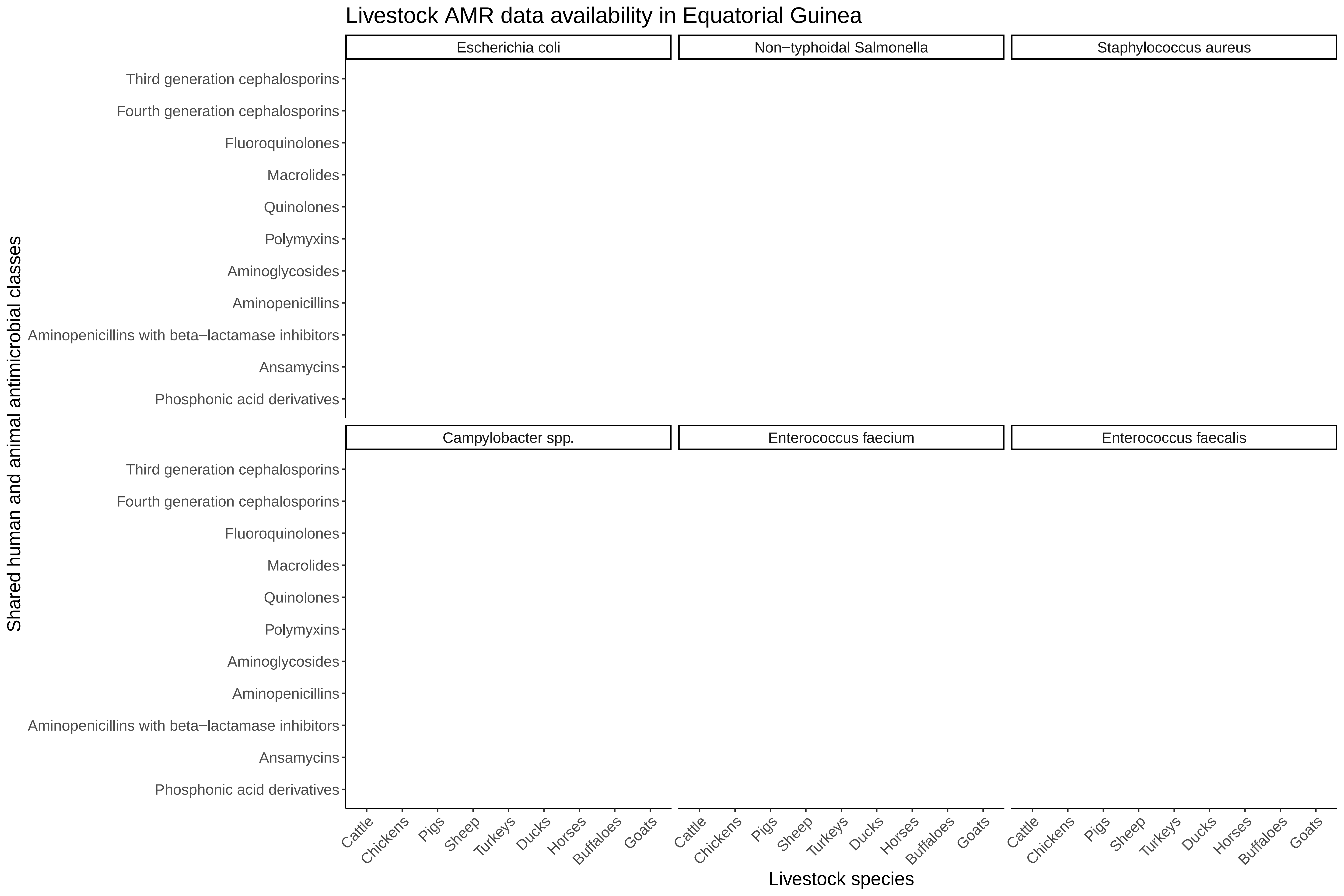

Livestock AMR data availability in Equatorial Guinea
Escherichia coli
Non−typhoidal Salmonella
Staphylococcus aureus
Campylobacter spp.
Enterococcus faecium
Enterococcus faecalis
Shared human and animal antimicrobial classes
Third generation cephalosporins
Fourth generation cephalosporins
Fluoroquinolones
Macrolides
Quinolones
Polymyxins
Aminoglycosides
Aminopenicillins
Aminopenicillins with beta−lactamase inhibitors
Ansamycins
Phosphonic acid derivatives
Cattle
Chickens
Pigs
Sheep
Turkeys
Ducks
Horses
Buffaloes
Goats
Livestock species

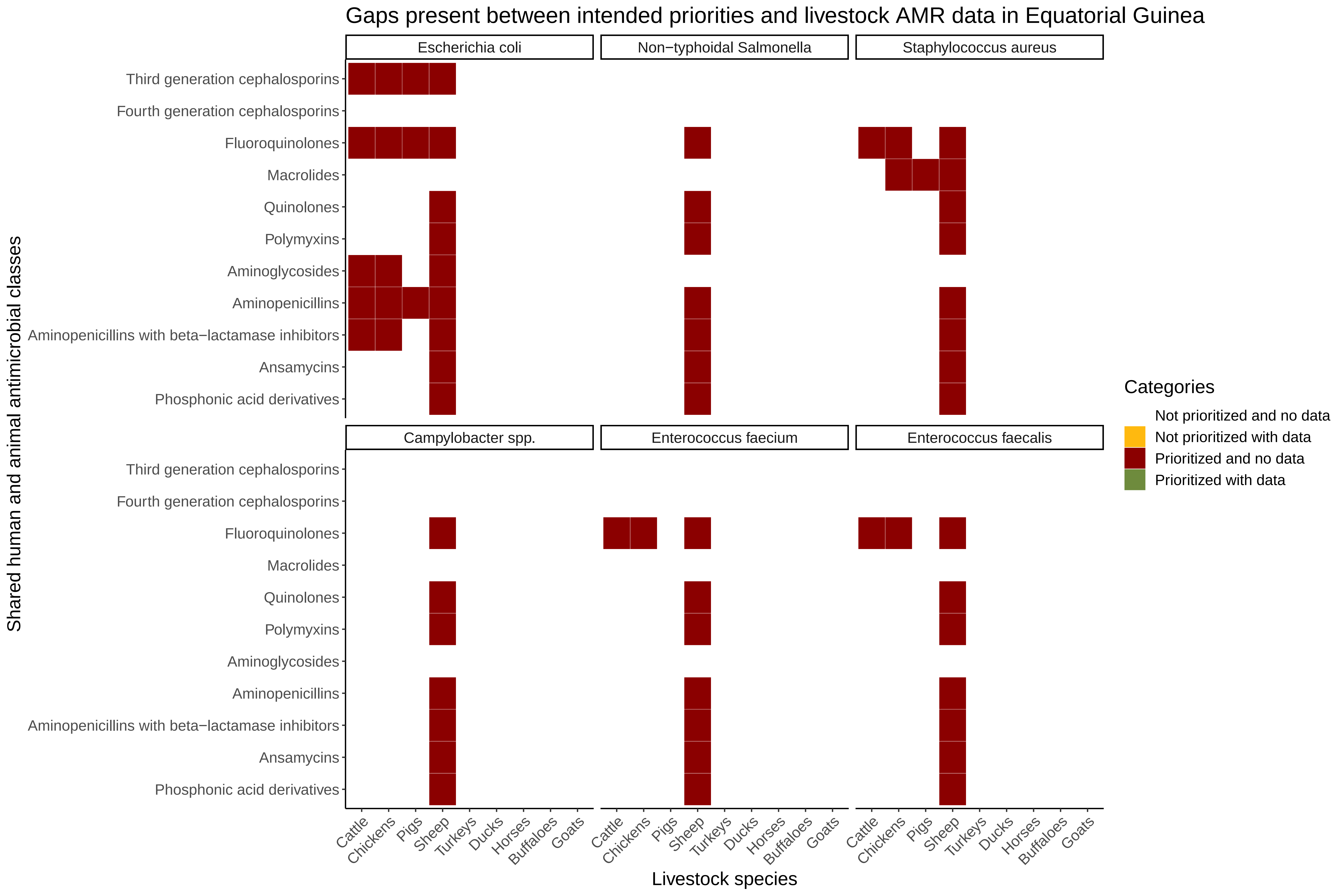

Gaps present between intended priorities and livestock AMR data in Equatorial Guinea
Escherichia coli
Non−typhoidal Salmonella
Staphylococcus aureus
Campylobacter spp.
Enterococcus faecium
Enterococcus faecalis
Third generation cephalosporins
Fourth generation cephalosporins
Fluoroquinolones
Macrolides
Quinolones
Polymyxins
Aminoglycosides
Aminopenicillins
Aminopenicillins with beta−lactamase inhibitors
Ansamycins
Phosphonic acid derivatives
Shared human and animal antimicrobial classes
Cattle
Chickens
Pigs
Sheep
Turkeys
Ducks
Horses
Buffaloes
Goats
Livestock species
Categories
Not prioritized and no data
Not prioritized with data
Prioritized and no data
Prioritized with data

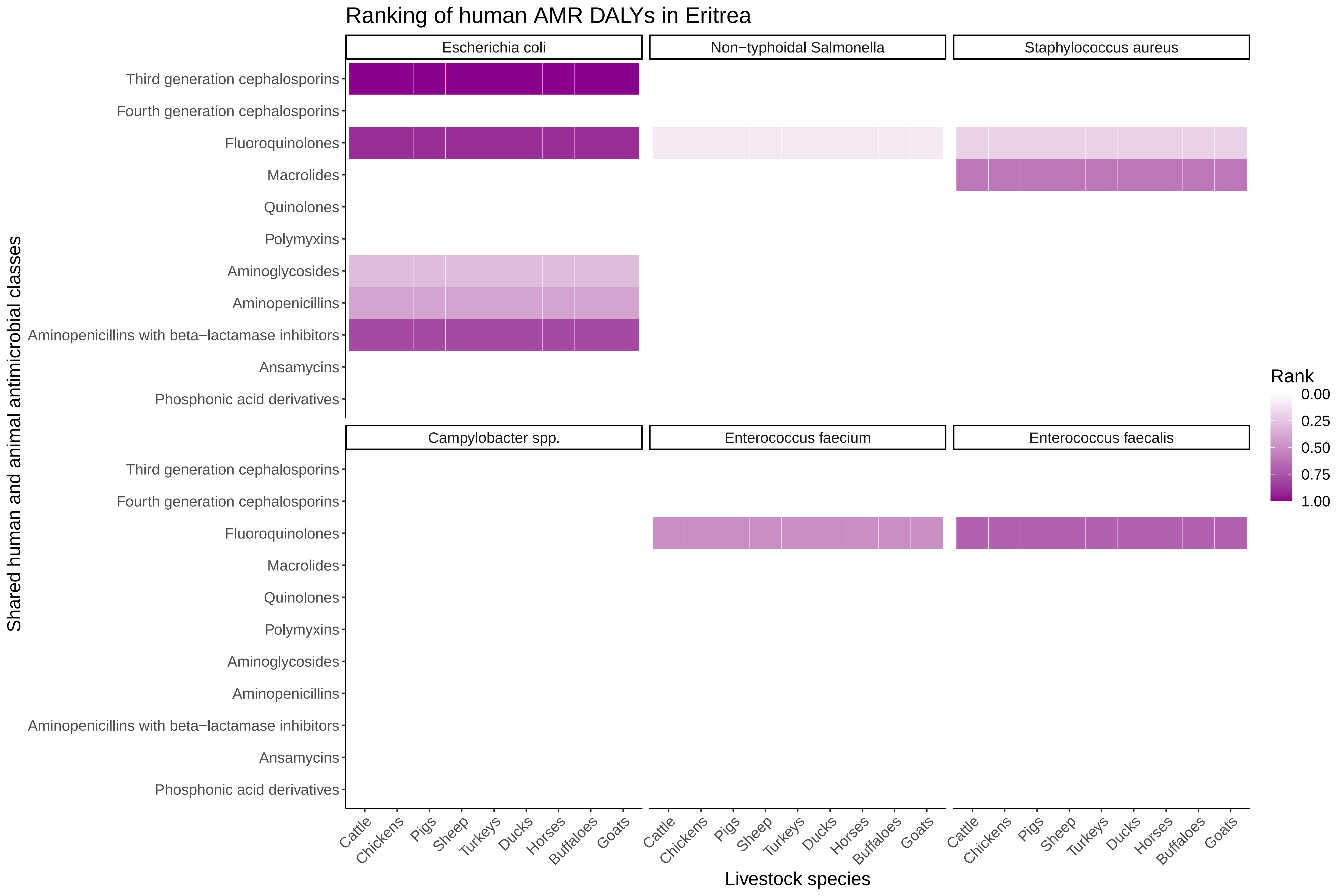

Ranking of human AMR DALYs in Eritrea
Escherichia coli
Non−typhoidal Salmonella
Staphylococcus aureus
Campylobacter spp.
Enterococcus faecium
Enterococcus faecalis
Third generation cephalosporins
Fourth generation cephalosporins
Fluoroquinolones
Macrolides
Quinolones
Polymyxins
Aminoglycosides
Aminopenicillins
Aminopenicillins with beta−lactamase inhibitors
Ansamycins
Phosphonic acid derivatives
Shared human and animal antimicrobial classes
Cattle
Chickens
Pigs
Sheep
Turkeys
Ducks
Horses
Buffaloes
Goats
Livestock species
Rank
0.00
0.25
0.50
0.75
1.00

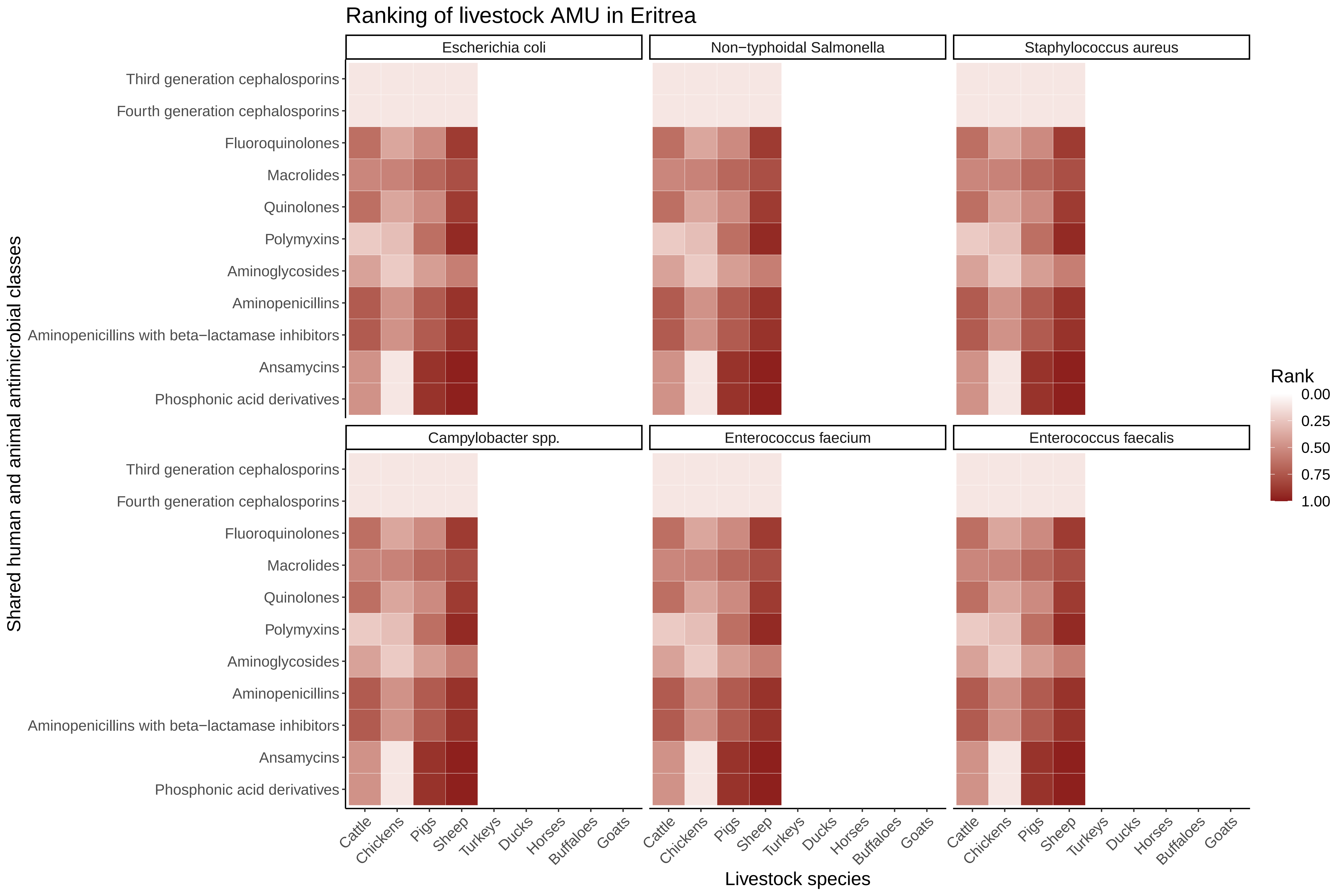
Ranking of livestock AMU in Eritrea
Escherichia coli
Non−typhoidal Salmonella
Staphylococcus aureus
Campylobacter spp.
Enterococcus faecium
Enterococcus faecalis
Third generation cephalosporins
Fourth generation cephalosporins
Fluoroquinolones
Macrolides
Quinolones
Polymyxins
Aminoglycosides
Aminopenicillins
Aminopenicillins with beta−lactamase inhibitors
Ansamycins
Phosphonic acid derivatives
Shared human and animal antimicrobial classes
Cattle
Chickens
Pigs
Sheep
Turkeys
Ducks
Horses
Buffaloes
Goats
Livestock species
Rank
0.00
0.25
0.50
0.75
1.00

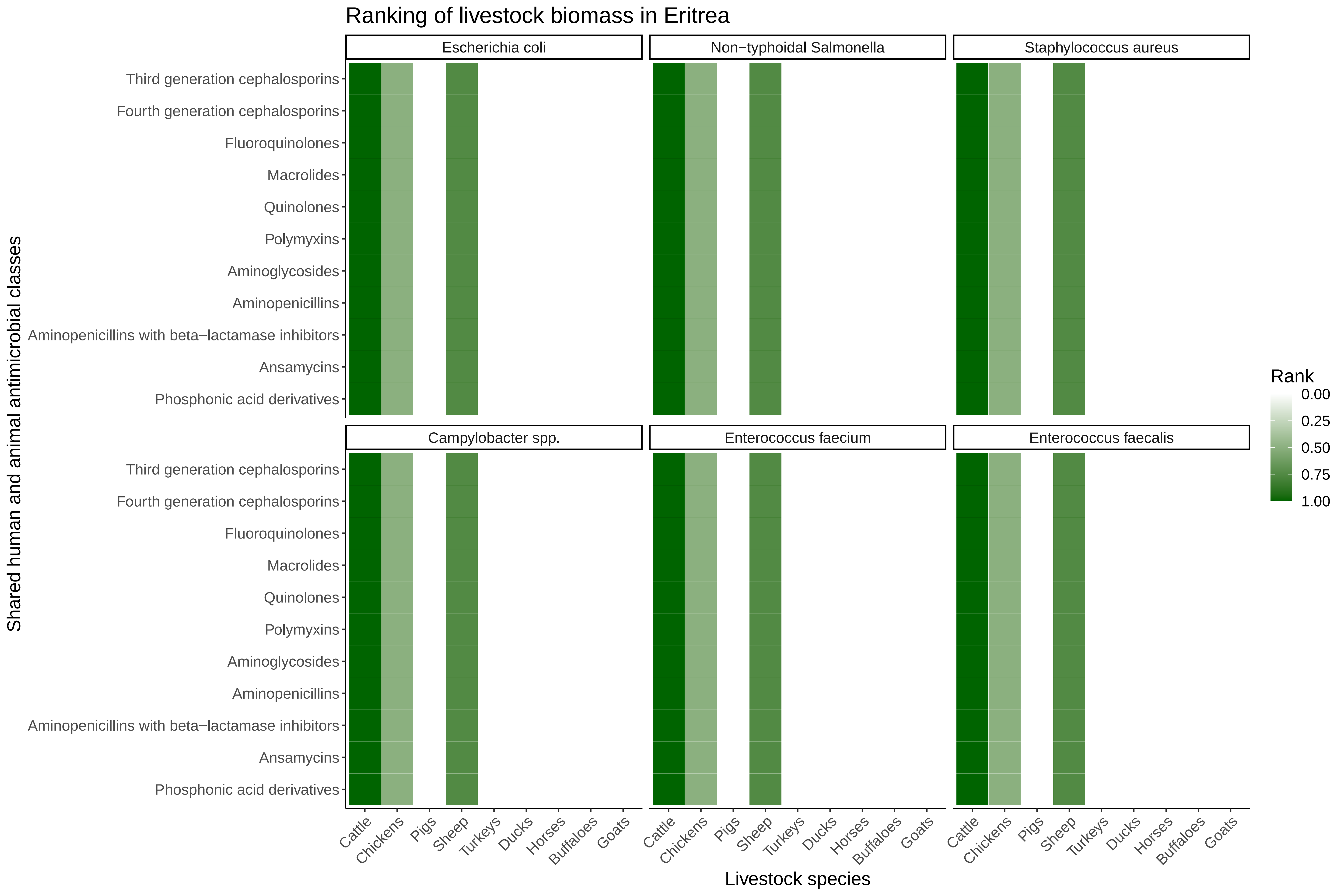

Ranking of livestock biomass in Eritrea
Escherichia coli
Non−typhoidal Salmonella
Staphylococcus aureus
Campylobacter spp.
Enterococcus faecium
Enterococcus faecalis
Shared human and animal antimicrobial classes
Third generation cephalosporins
Fourth generation cephalosporins
Fluoroquinolones
Macrolides
Quinolones
Polymyxins
Aminoglycosides
Aminopenicillins
Aminopenicillins with beta−lactamase inhibitors
Ansamycins
Phosphonic acid derivatives
Cattle
Chickens
Pigs
Sheep
Turkeys
Ducks
Horses
Buffaloes
Goats
Livestock species
Rank
0.00
0.25
0.50
0.75
1.00

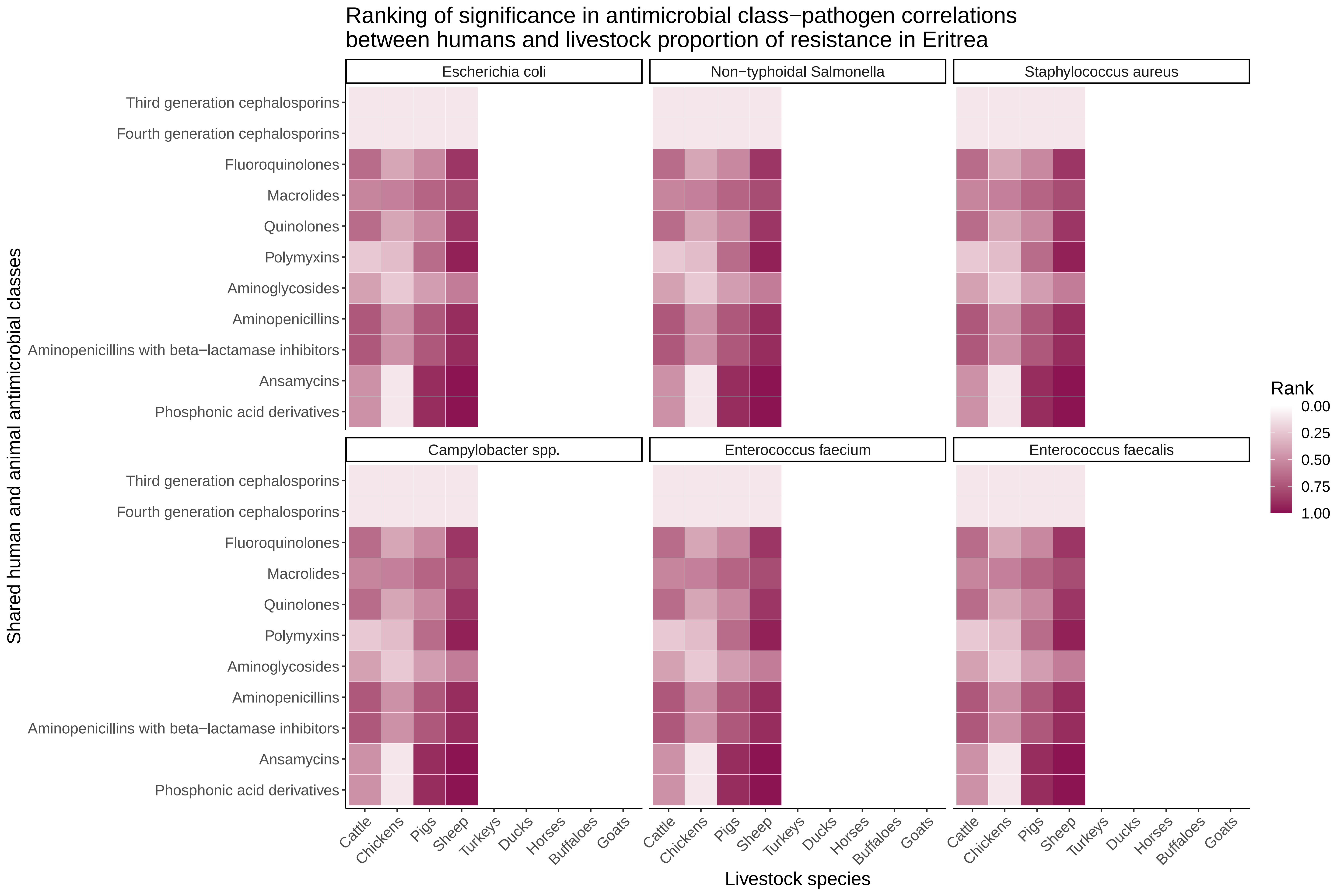

Ranking of significance in antimicrobial class−pathogen correlations
between humans and livestock proportion of resistance in Eritrea
Escherichia coli
Non−typhoidal Salmonella
Staphylococcus aureus
Campylobacter spp.
Enterococcus faecium
Enterococcus faecalis
Shared human and animal antimicrobial classes
Third generation cephalosporins
Fourth generation cephalosporins
Fluoroquinolones
Macrolides
Quinolones
Polymyxins
Aminoglycosides
Aminopenicillins
Aminopenicillins with beta−lactamase inhibitors
Ansamycins
Phosphonic acid derivatives
Livestock species
Cattle
Chickens
Pigs
Sheep
Turkeys
Ducks
Horses
Buffaloes
Goats
Rank
0.00
0.25
0.50
0.75
1.00

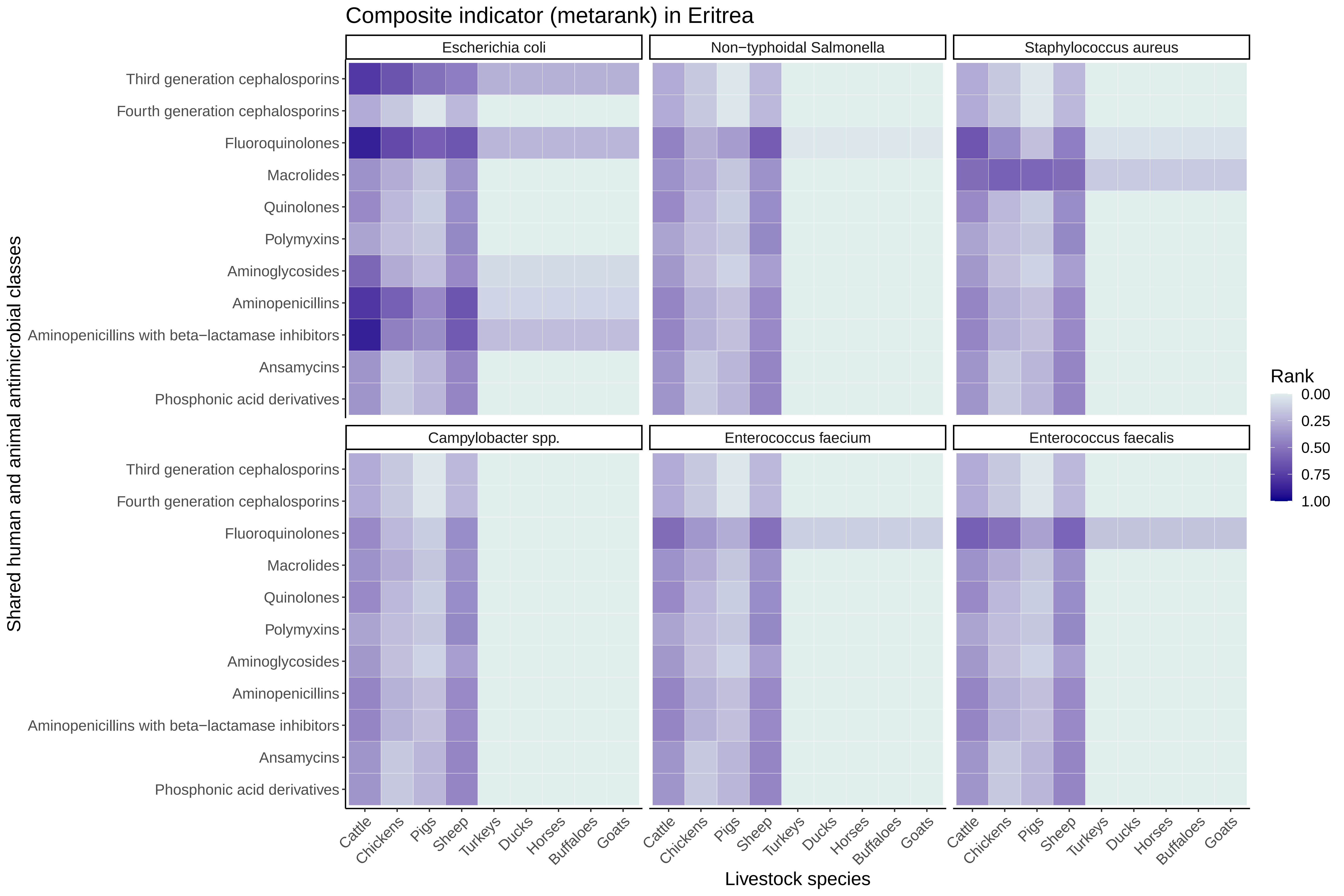

Composite indicator (metarank) in Eritrea
Escherichia coli
Non−typhoidal Salmonella
Staphylococcus aureus
Campylobacter spp.
Enterococcus faecium
Enterococcus faecalis
Shared human and animal antimicrobial classes
Third generation cephalosporins
Fourth generation cephalosporins
Fluoroquinolones
Macrolides
Quinolones
Polymyxins
Aminoglycosides
Aminopenicillins
Aminopenicillins with beta−lactamase inhibitors
Ansamycins
Phosphonic acid derivatives
Livestock species
Cattle
Chickens
Pigs
Sheep
Turkeys
Ducks
Horses
Buffaloes
Goats
Rank
0.00
0.25
0.50
0.75
1.00

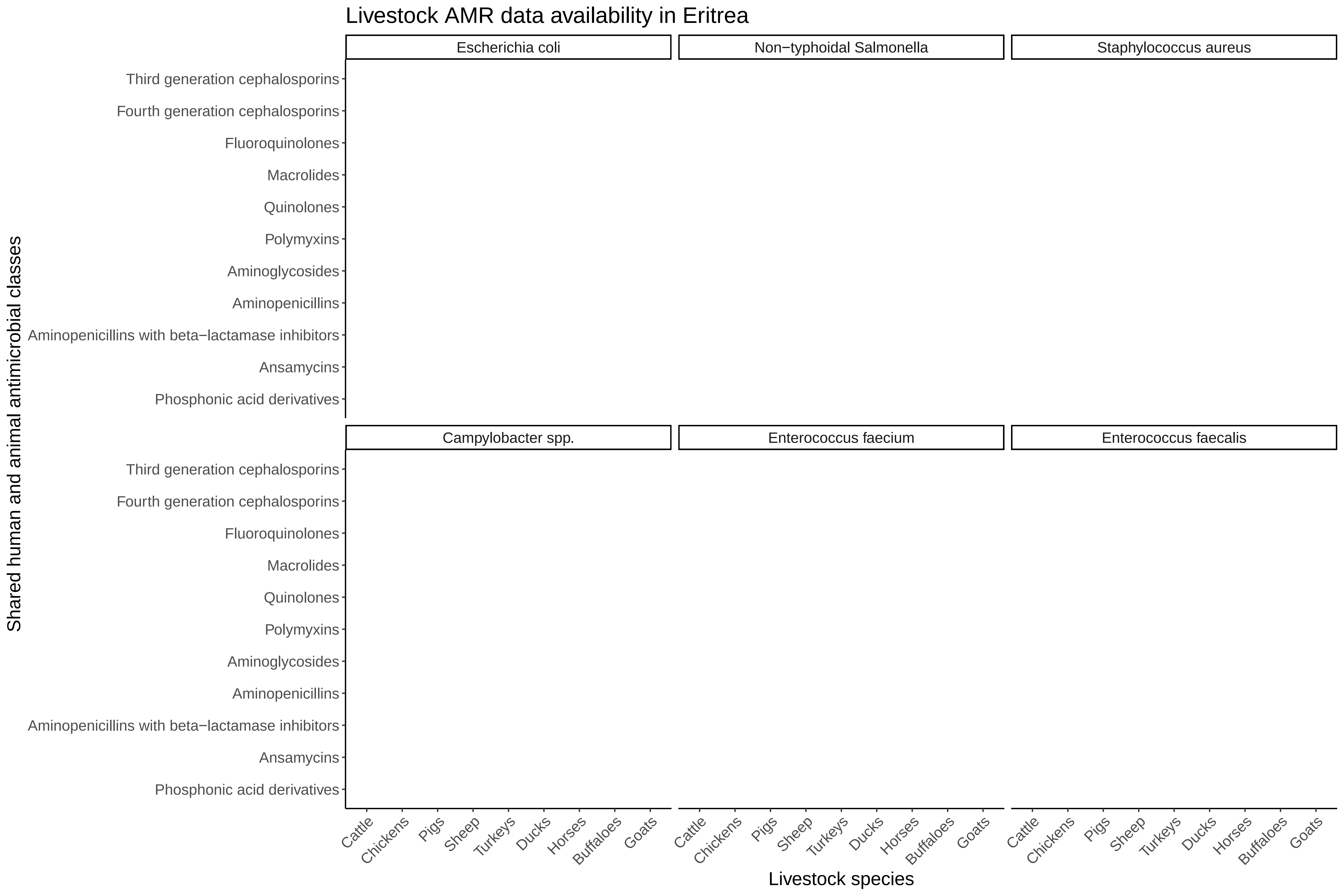

Livestock AMR data availability in Eritrea
Escherichia coli
Non−typhoidal Salmonella
Staphylococcus aureus
Campylobacter spp.
Enterococcus faecium
Enterococcus faecalis
Third generation cephalosporins
Fourth generation cephalosporins
Fluoroquinolones
Macrolides
Quinolones
Polymyxins
Aminoglycosides
Aminopenicillins
Aminopenicillins with beta−lactamase inhibitors
Ansamycins
Phosphonic acid derivatives
Shared human and animal antimicrobial classes
Cattle
Chickens
Pigs
Sheep
Turkeys
Ducks
Horses
Buffaloes
Goats
Livestock species

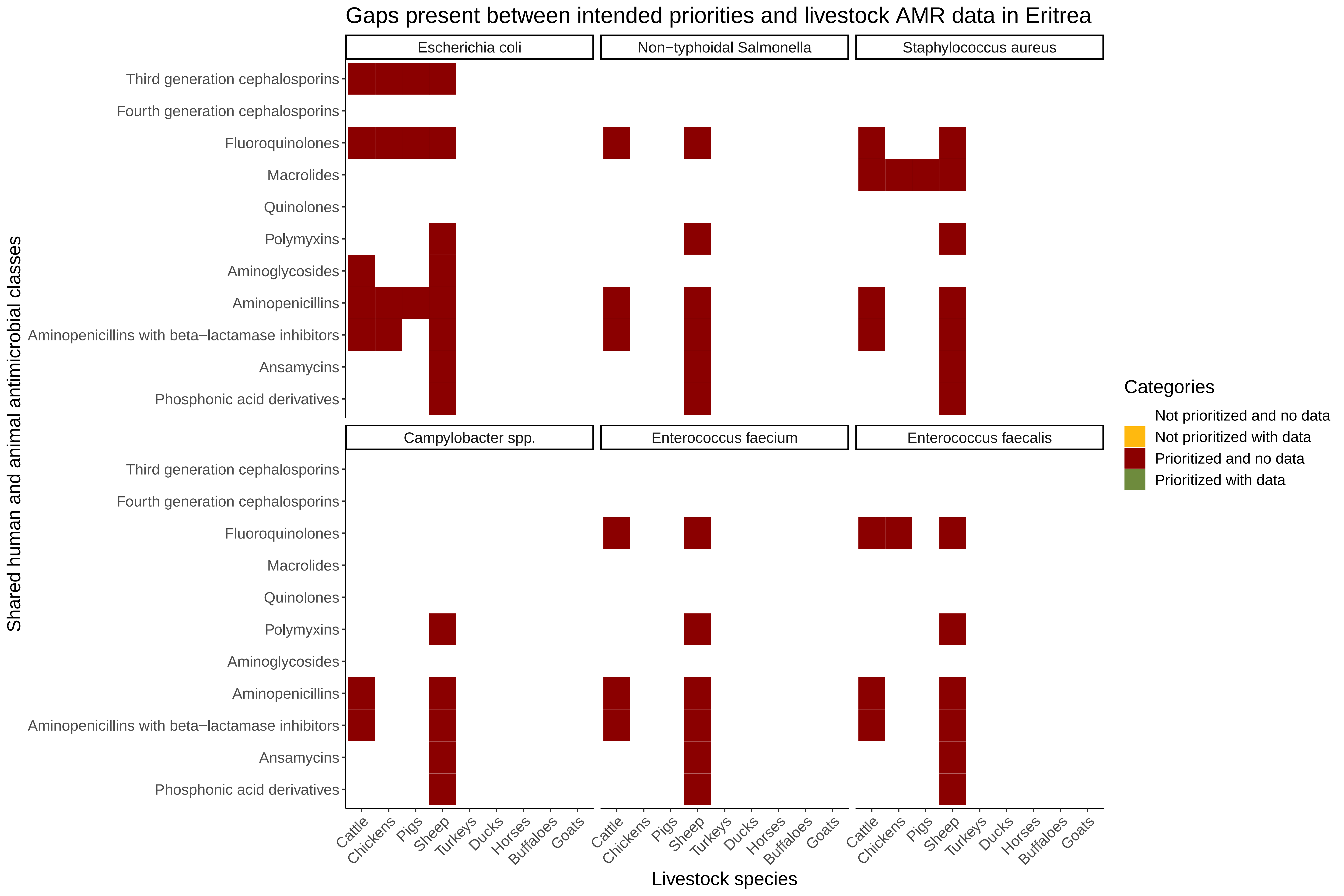
Gaps present between intended priorities and livestock AMR data in Eritrea
Escherichia coli
Non−typhoidal Salmonella
Staphylococcus aureus
Campylobacter spp.
Enterococcus faecium
Enterococcus faecalis
Third generation cephalosporins
Fourth generation cephalosporins
Fluoroquinolones
Macrolides
Quinolones
Polymyxins
Aminoglycosides
Aminopenicillins
Aminopenicillins with beta−lactamase inhibitors
Ansamycins
Phosphonic acid derivatives
Shared human and animal antimicrobial classes
Cattle
Chickens
Pigs
Sheep
Turkeys
Ducks
Horses
Buffaloes
Goats
Livestock species
Categories
Not prioritized and no data
Not prioritized with data
Prioritized and no data
Prioritized with data

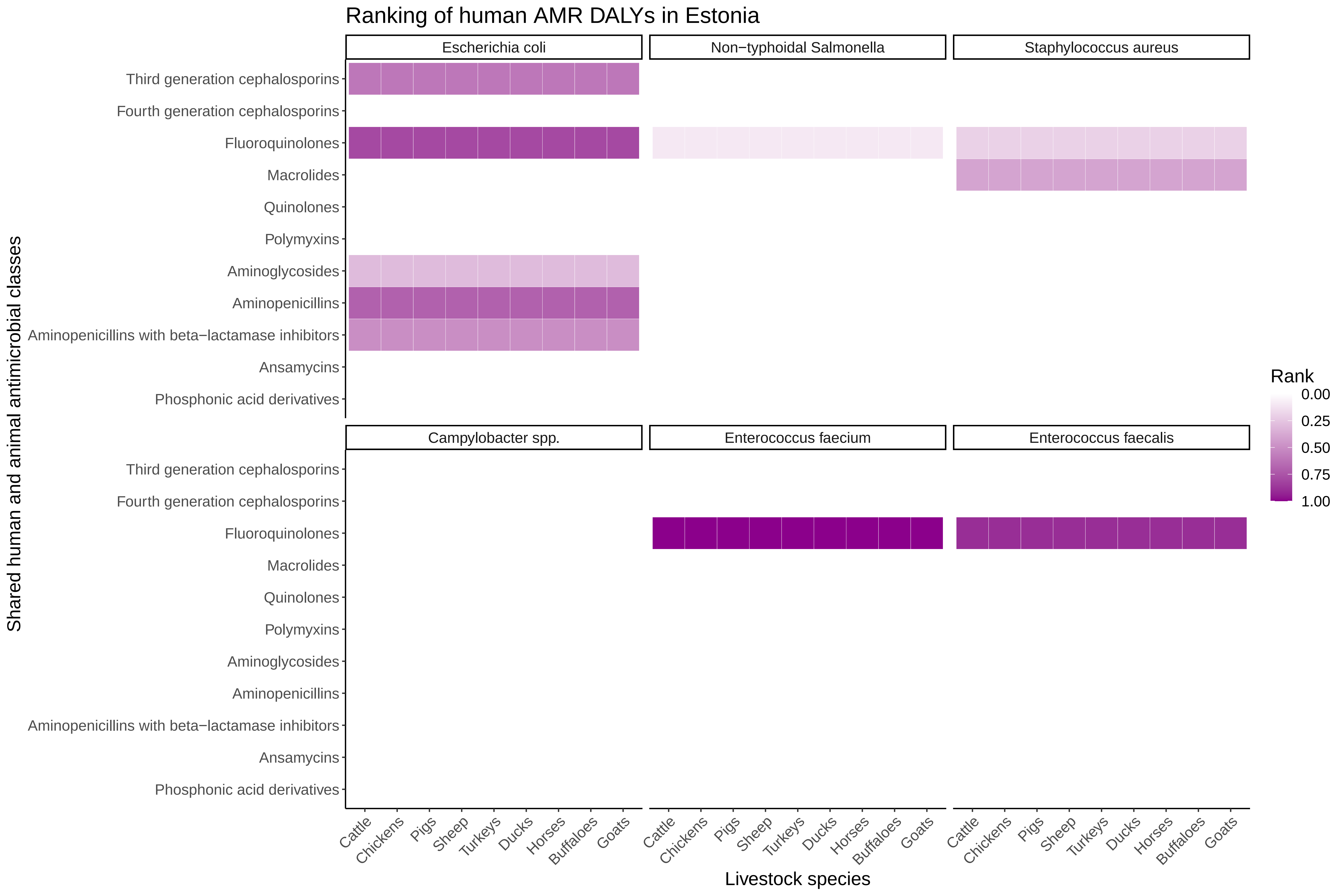
Ranking of human AMR DALYs in Estonia
Escherichia coli
Non−typhoidal Salmonella
Staphylococcus aureus
Campylobacter spp.
Enterococcus faecium
Enterococcus faecalis
Shared human and animal antimicrobial classes
Third generation cephalosporins
Fourth generation cephalosporins
Fluoroquinolones
Macrolides
Quinolones
Polymyxins
Aminoglycosides
Aminopenicillins
Aminopenicillins with beta−lactamase inhibitors
Ansamycins
Phosphonic acid derivatives
Cattle
Chickens
Pigs
Sheep
Turkeys
Ducks
Horses
Buffaloes
Goats
Livestock species
Rank
0.00
0.25
0.50
0.75
1.00

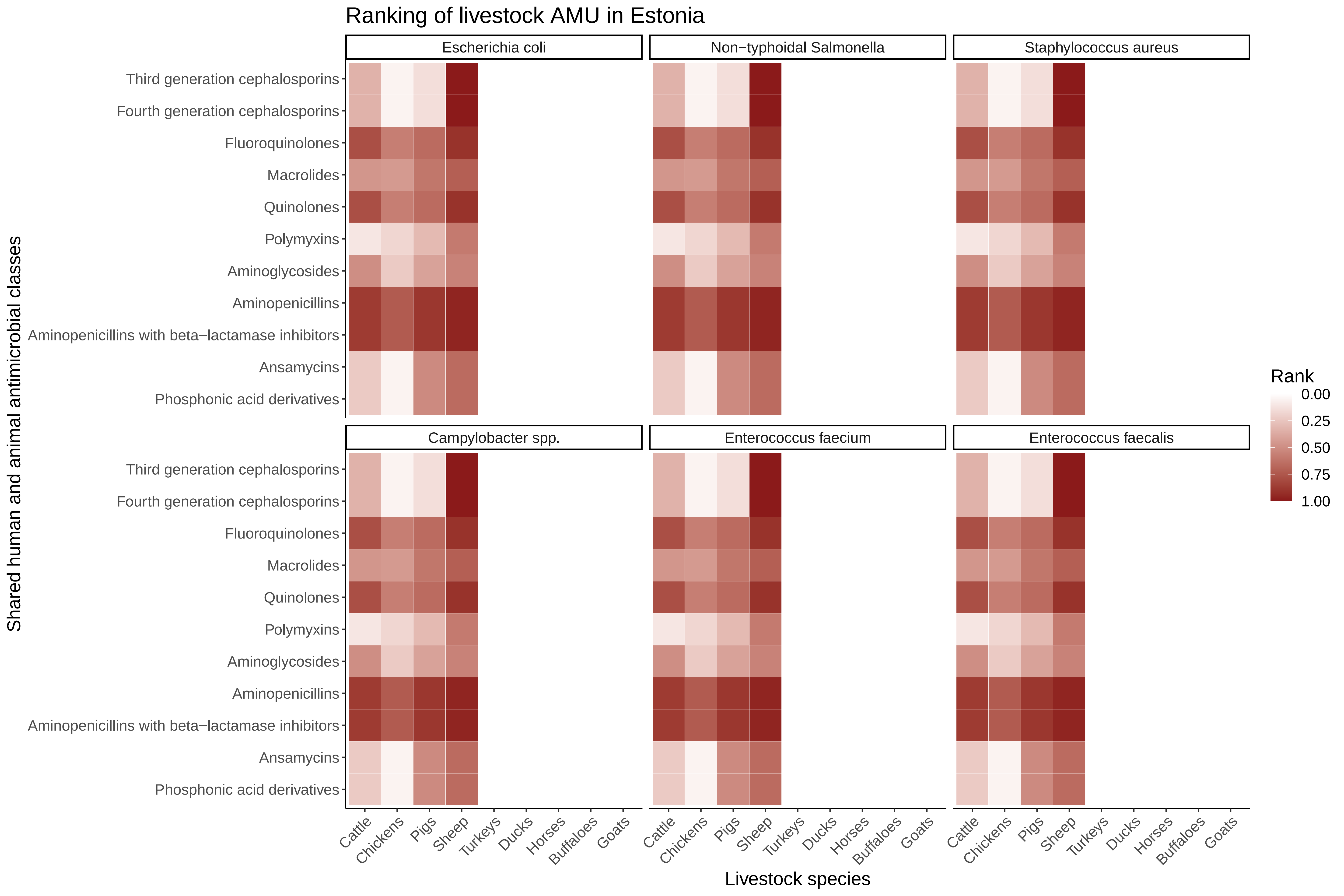
Ranking of livestock AMU in Estonia
Escherichia coli
Non−typhoidal Salmonella
Staphylococcus aureus
Campylobacter spp.
Enterococcus faecium
Enterococcus faecalis
Shared human and animal antimicrobial classes
Third generation cephalosporins
Fourth generation cephalosporins
Fluoroquinolones
Macrolides
Quinolones
Polymyxins
Aminoglycosides
Aminopenicillins
Aminopenicillins with beta−lactamase inhibitors
Ansamycins
Phosphonic acid derivatives
Livestock species
Cattle
Chickens
Pigs
Sheep
Turkeys
Ducks
Horses
Buffaloes
Goats
Rank
0.00
0.25
0.50
0.75
1.00

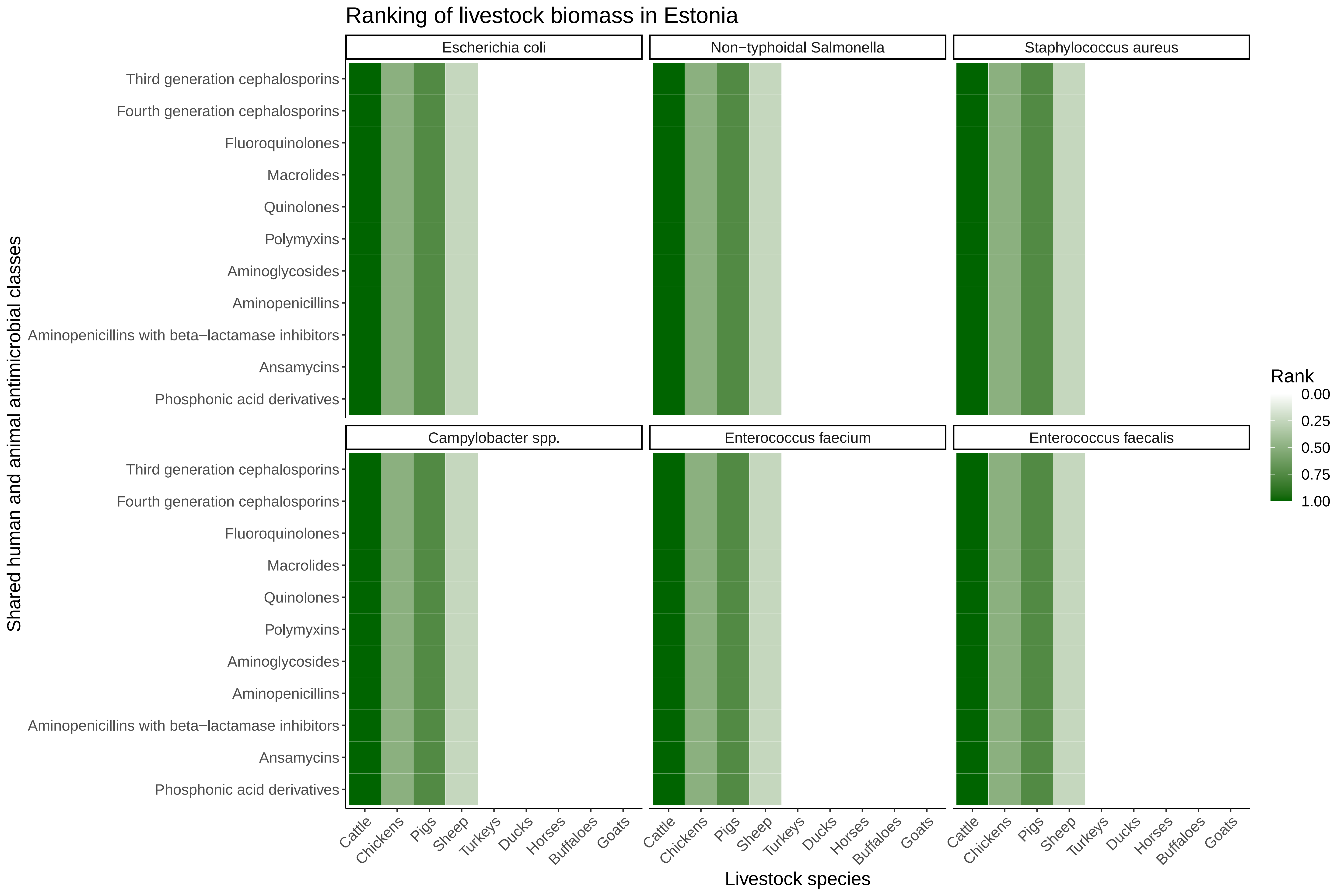
Ranking of livestock biomass in Estonia
Escherichia coli
Non−typhoidal Salmonella
Staphylococcus aureus
Campylobacter spp.
Enterococcus faecium
Enterococcus faecalis
Third generation cephalosporins
Fourth generation cephalosporins
Fluoroquinolones
Macrolides
Quinolones
Polymyxins
Aminoglycosides
Aminopenicillins
Aminopenicillins with beta−lactamase inhibitors
Ansamycins
Phosphonic acid derivatives
Shared human and animal antimicrobial classes
Cattle
Chickens
Pigs
Sheep
Turkeys
Ducks
Horses
Buffaloes
Goats
Livestock species
Rank
0.00
0.25
0.50
0.75
1.00

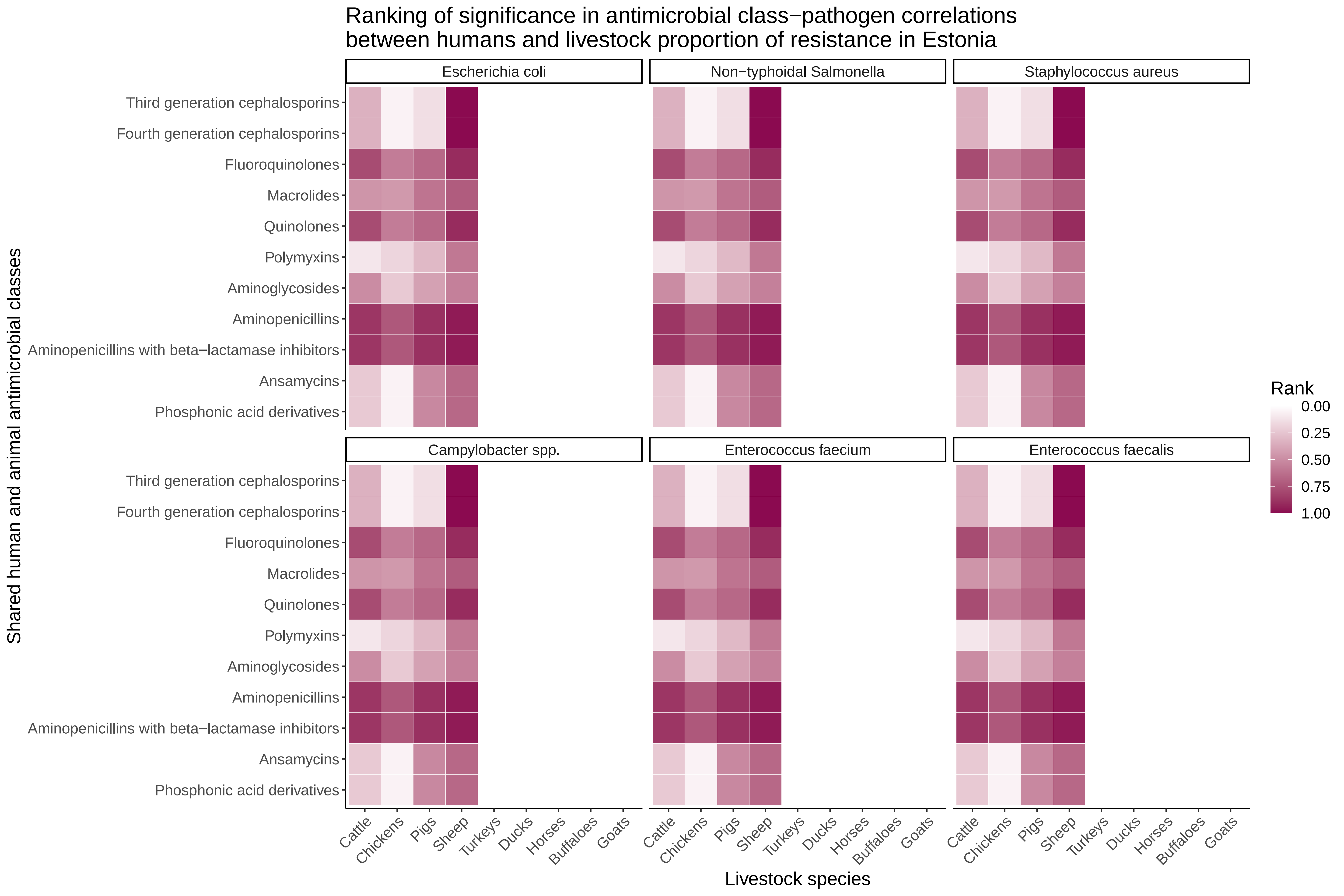

Ranking of significance in antimicrobial class−pathogen correlations
between humans and livestock proportion of resistance in Estonia
Escherichia coli
Non−typhoidal Salmonella
Staphylococcus aureus
Campylobacter spp.
Enterococcus faecium
Enterococcus faecalis
Third generation cephalosporins
Fourth generation cephalosporins
Fluoroquinolones
Macrolides
Quinolones
Polymyxins
Aminoglycosides
Aminopenicillins
Aminopenicillins with beta−lactamase inhibitors
Ansamycins
Phosphonic acid derivatives
Shared human and animal antimicrobial classes
Cattle
Chickens
Pigs
Sheep
Turkeys
Ducks
Horses
Buffaloes
Goats
Livestock species
Rank
0.00
0.25
0.50
0.75
1.00

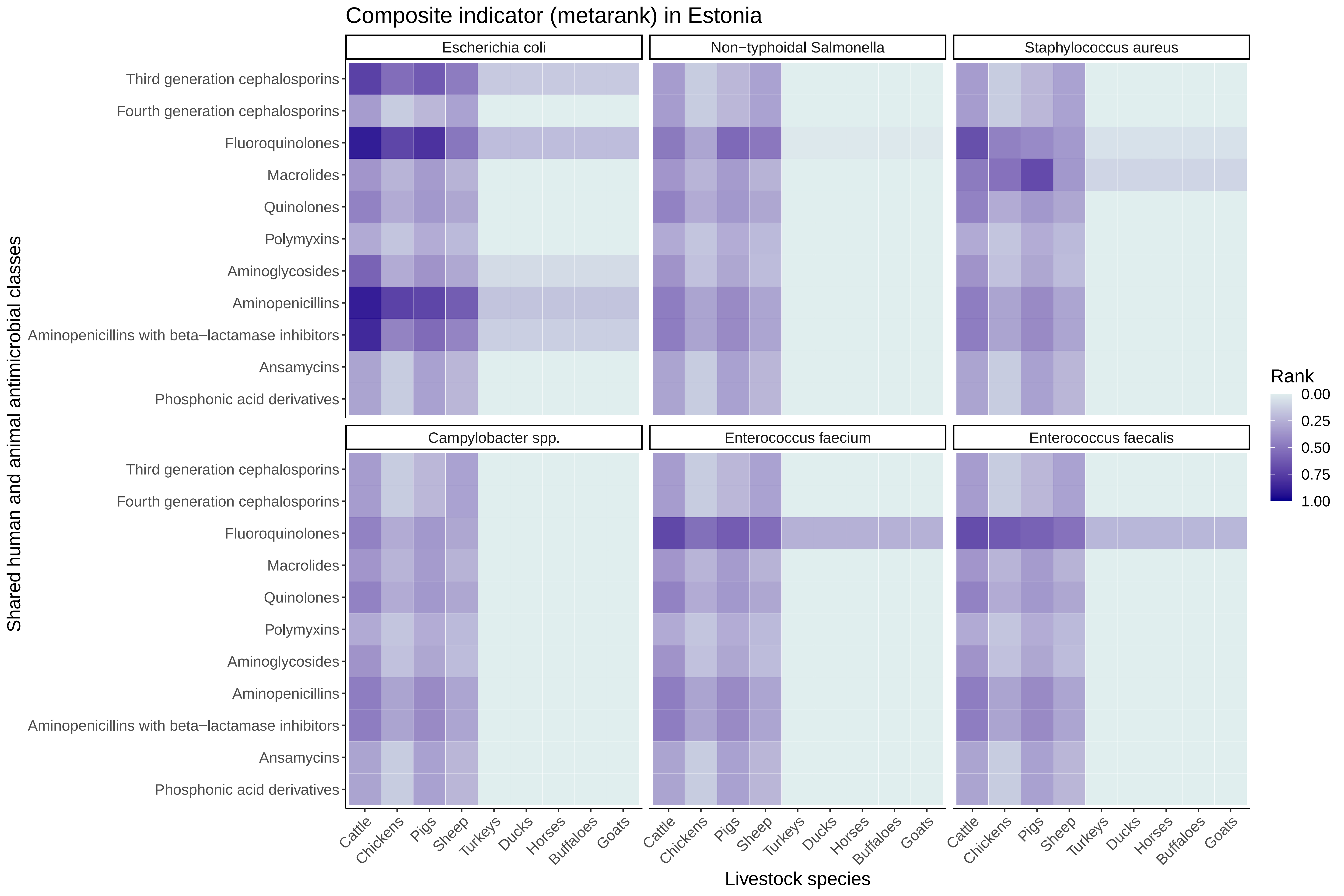
Composite indicator (metarank) in Estonia
Escherichia coli
Non−typhoidal Salmonella
Staphylococcus aureus
Campylobacter spp.
Enterococcus faecium
Enterococcus faecalis
Third generation cephalosporins
Fourth generation cephalosporins
Fluoroquinolones
Macrolides
Quinolones
Polymyxins
Aminoglycosides
Aminopenicillins
Aminopenicillins with beta−lactamase inhibitors
Ansamycins
Phosphonic acid derivatives
Shared human and animal antimicrobial classes
Cattle
Chickens
Pigs
Sheep
Turkeys
Ducks
Horses
Buffaloes
Goats
Livestock species
Rank
0.00
0.25
0.50
0.75
1.00

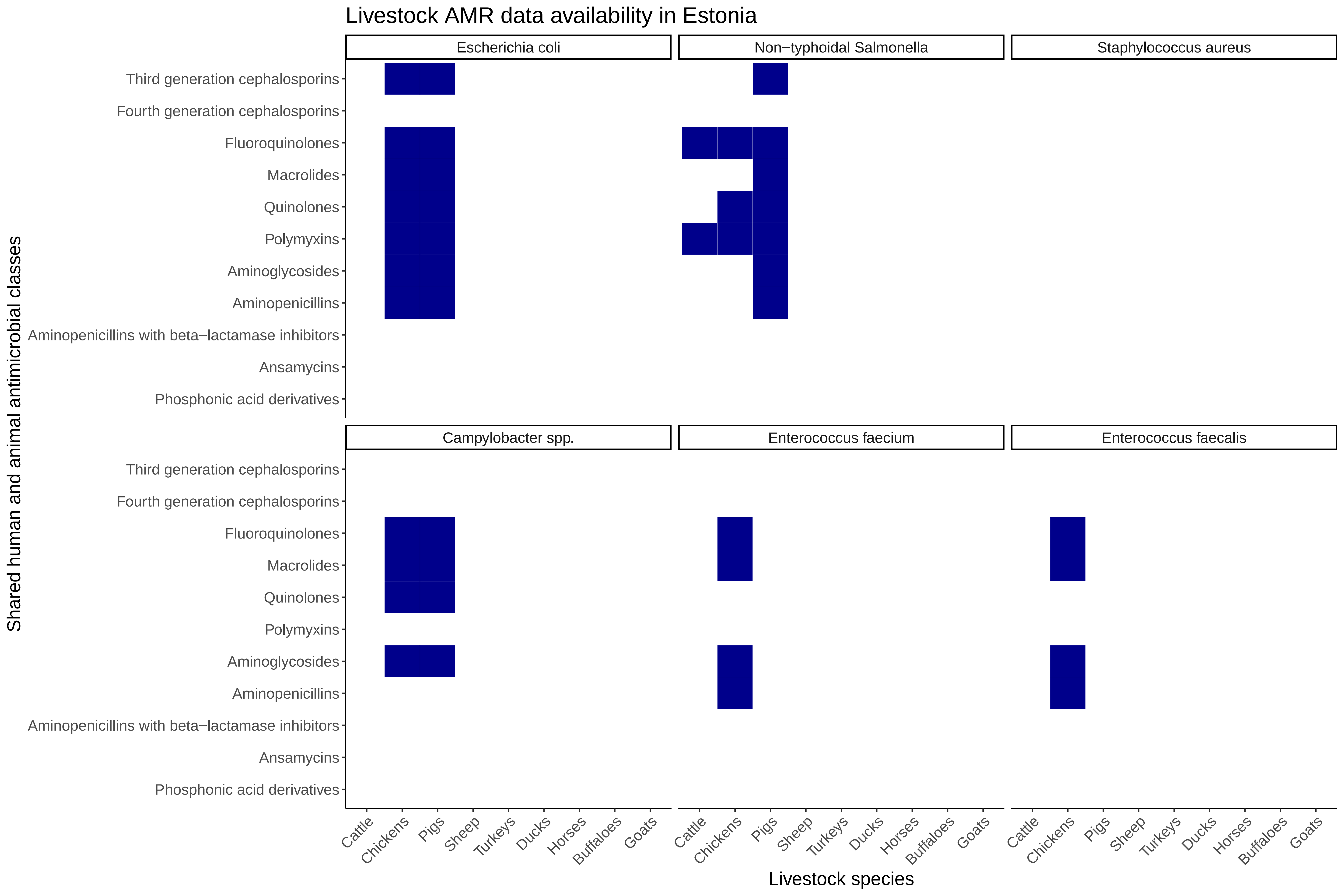

Livestock AMR data availability in Estonia
Escherichia coli
Non−typhoidal Salmonella
Staphylococcus aureus
Campylobacter spp.
Enterococcus faecium
Enterococcus faecalis
Third generation cephalosporins
Fourth generation cephalosporins
Fluoroquinolones
Macrolides
Quinolones
Polymyxins
Aminoglycosides
Aminopenicillins
Aminopenicillins with beta−lactamase inhibitors
Ansamycins
Phosphonic acid derivatives
Shared human and animal antimicrobial classes
Cattle
Chickens
Pigs
Sheep
Turkeys
Ducks
Horses
Buffaloes
Goats
Livestock species

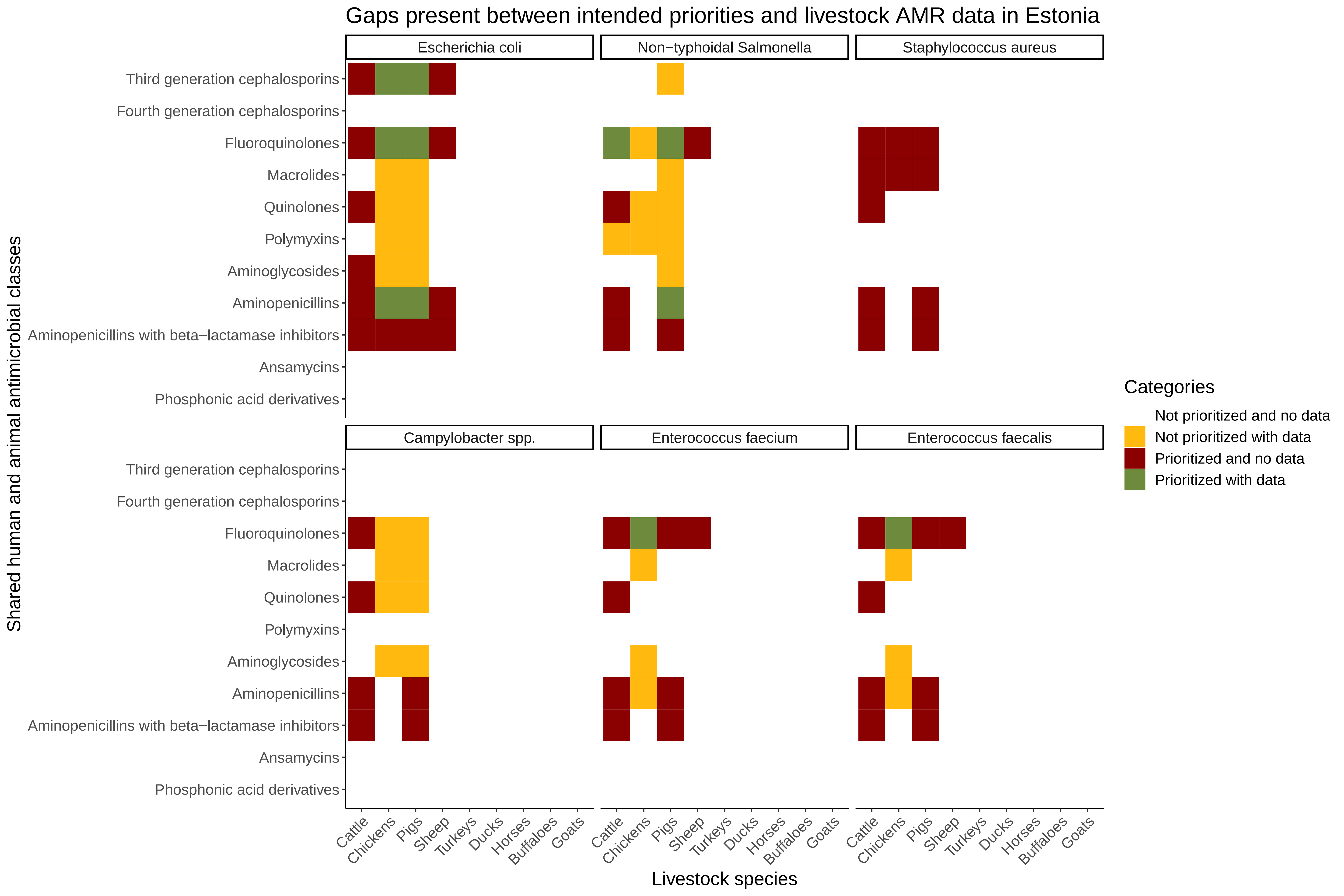
Gaps present between intended priorities and livestock AMR data in Estonia
Escherichia coli
Non−typhoidal Salmonella
Staphylococcus aureus
Campylobacter spp.
Enterococcus faecium
Enterococcus faecalis
Third generation cephalosporins
Fourth generation cephalosporins
Fluoroquinolones
Macrolides
Quinolones
Polymyxins
Aminoglycosides
Aminopenicillins
Aminopenicillins with beta−lactamase inhibitors
Ansamycins
Phosphonic acid derivatives
Shared human and animal antimicrobial classes
Cattle
Chickens
Pigs
Sheep
Turkeys
Ducks
Horses
Buffaloes
Goats
Livestock species
Categories
Not prioritized and no data
Not prioritized with data
Prioritized and no data
Prioritized with data

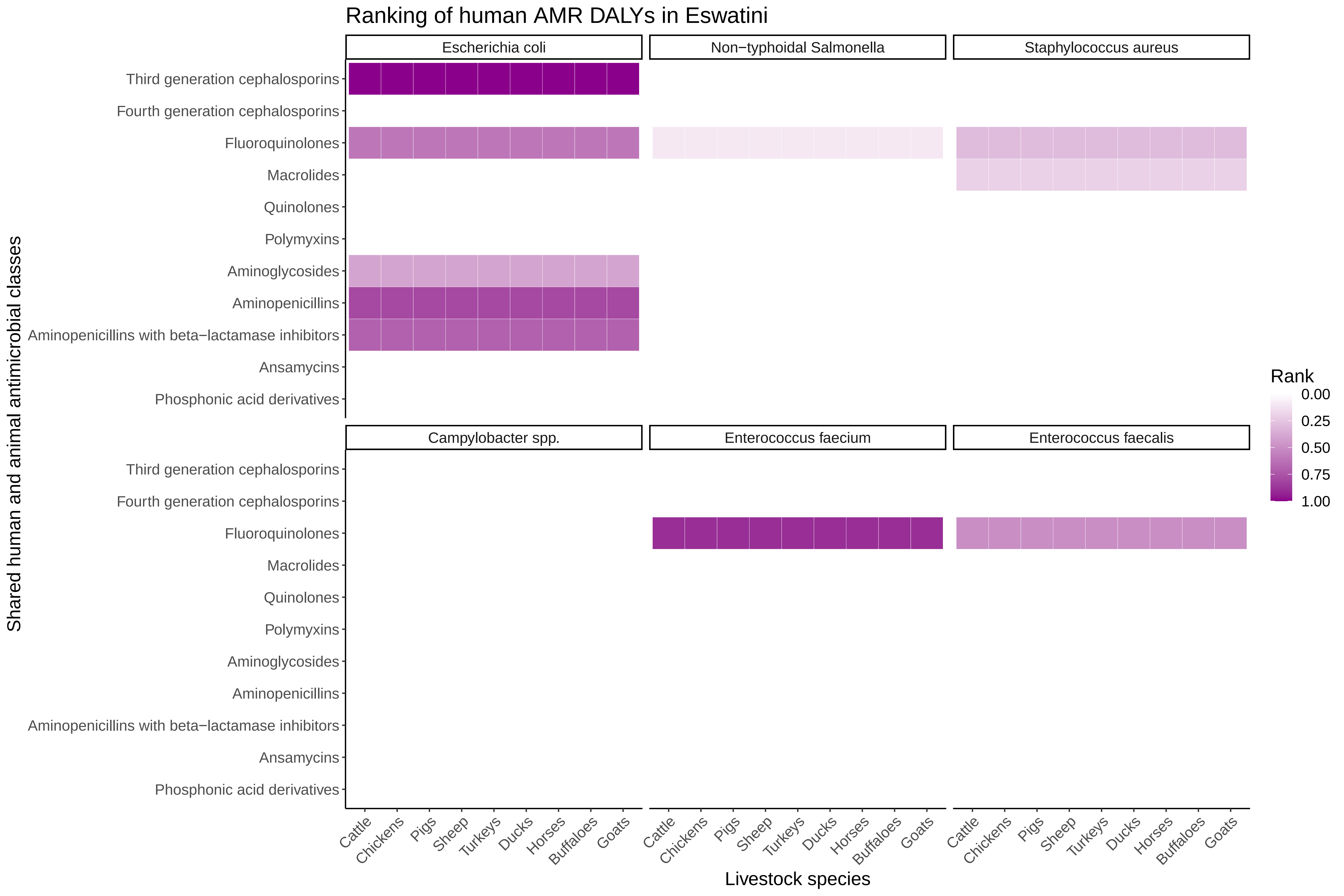

Ranking of human AMR DALYs in Eswatini
Escherichia coli
Non−typhoidal Salmonella
Staphylococcus aureus
Campylobacter spp.
Enterococcus faecium
Enterococcus faecalis
Third generation cephalosporins
Fourth generation cephalosporins
Fluoroquinolones
Macrolides
Quinolones
Polymyxins
Aminoglycosides
Aminopenicillins
Aminopenicillins with beta−lactamase inhibitors
Ansamycins
Phosphonic acid derivatives
Shared human and animal antimicrobial classes
Cattle
Chickens
Pigs
Sheep
Turkeys
Ducks
Horses
Buffaloes
Goats
Livestock species
Rank
0.00
0.25
0.50
0.75
1.00

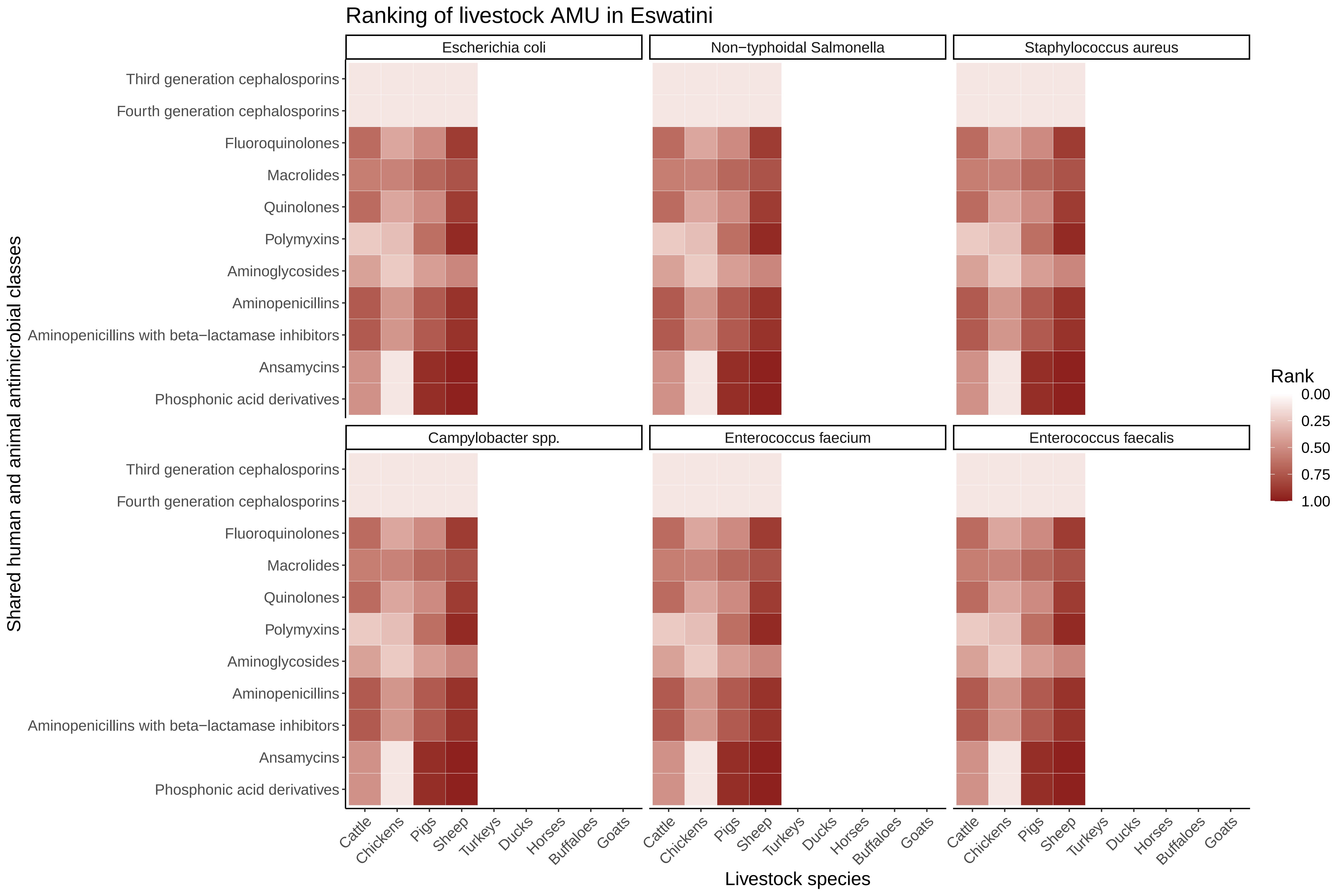

Ranking of livestock AMU in Eswatini
Escherichia coli
Non−typhoidal Salmonella
Staphylococcus aureus
Campylobacter spp.
Enterococcus faecium
Enterococcus faecalis
Shared human and animal antimicrobial classes
Third generation cephalosporins
Fourth generation cephalosporins
Fluoroquinolones
Macrolides
Quinolones
Polymyxins
Aminoglycosides
Aminopenicillins
Aminopenicillins with beta−lactamase inhibitors
Ansamycins
Phosphonic acid derivatives
Livestock species
Cattle
Chickens
Pigs
Sheep
Turkeys
Ducks
Horses
Buffaloes
Goats
Rank
0.00
0.25
0.50
0.75
1.00

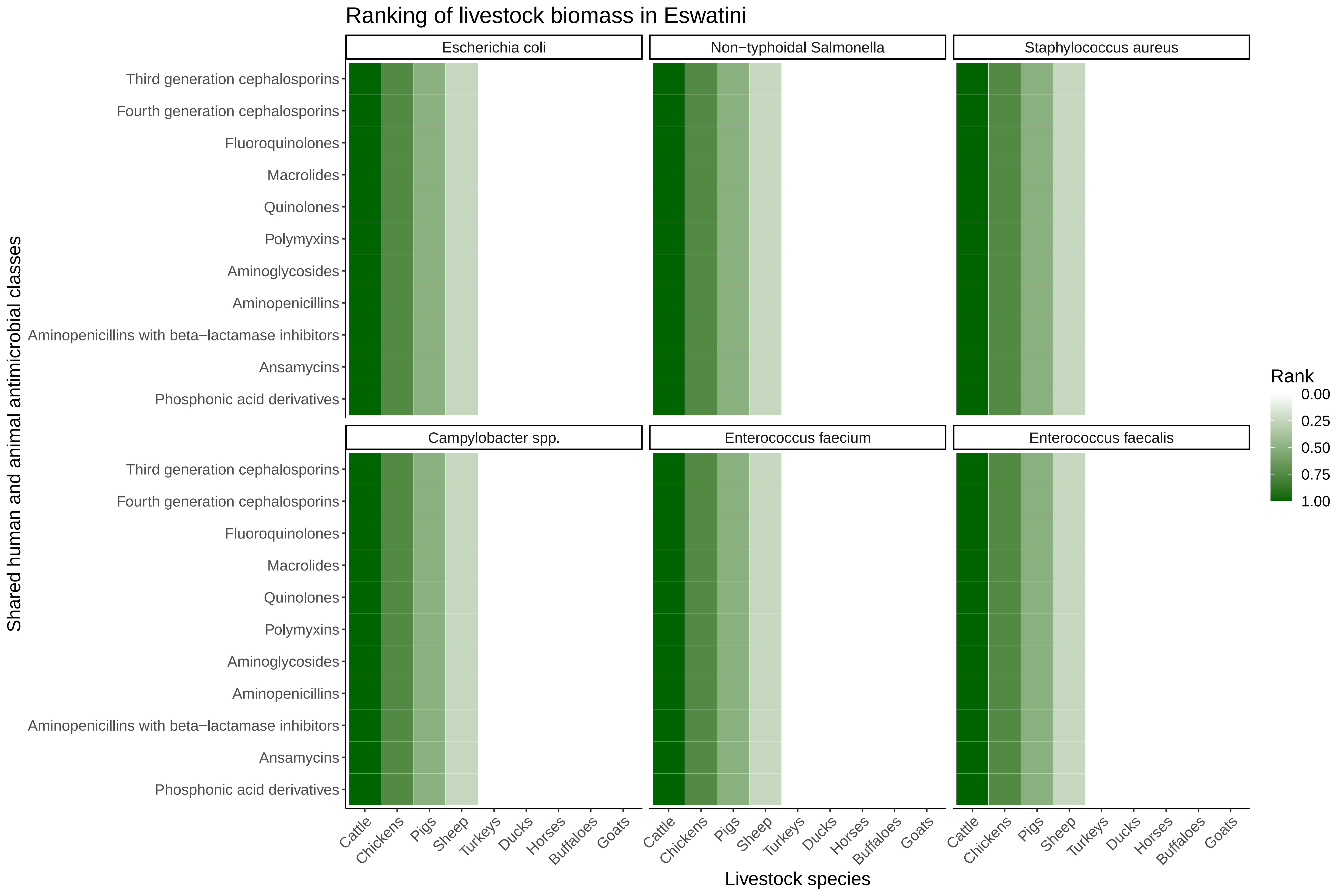

Ranking of livestock biomass in Eswatini
Escherichia coli
Non−typhoidal Salmonella
Staphylococcus aureus
Campylobacter spp.
Enterococcus faecium
Enterococcus faecalis
Shared human and animal antimicrobial classes
Third generation cephalosporins
Fourth generation cephalosporins
Fluoroquinolones
Macrolides
Quinolones
Polymyxins
Aminoglycosides
Aminopenicillins
Aminopenicillins with beta−lactamase inhibitors
Ansamycins
Phosphonic acid derivatives
Cattle
Chickens
Pigs
Sheep
Turkeys
Ducks
Horses
Buffaloes
Goats
Livestock species
Rank
0.00
0.25
0.50
0.75
1.00

Ranking of significance in antimicrobial class−pathogen correlations between humans and livestock proportion of resistance in Eswatini

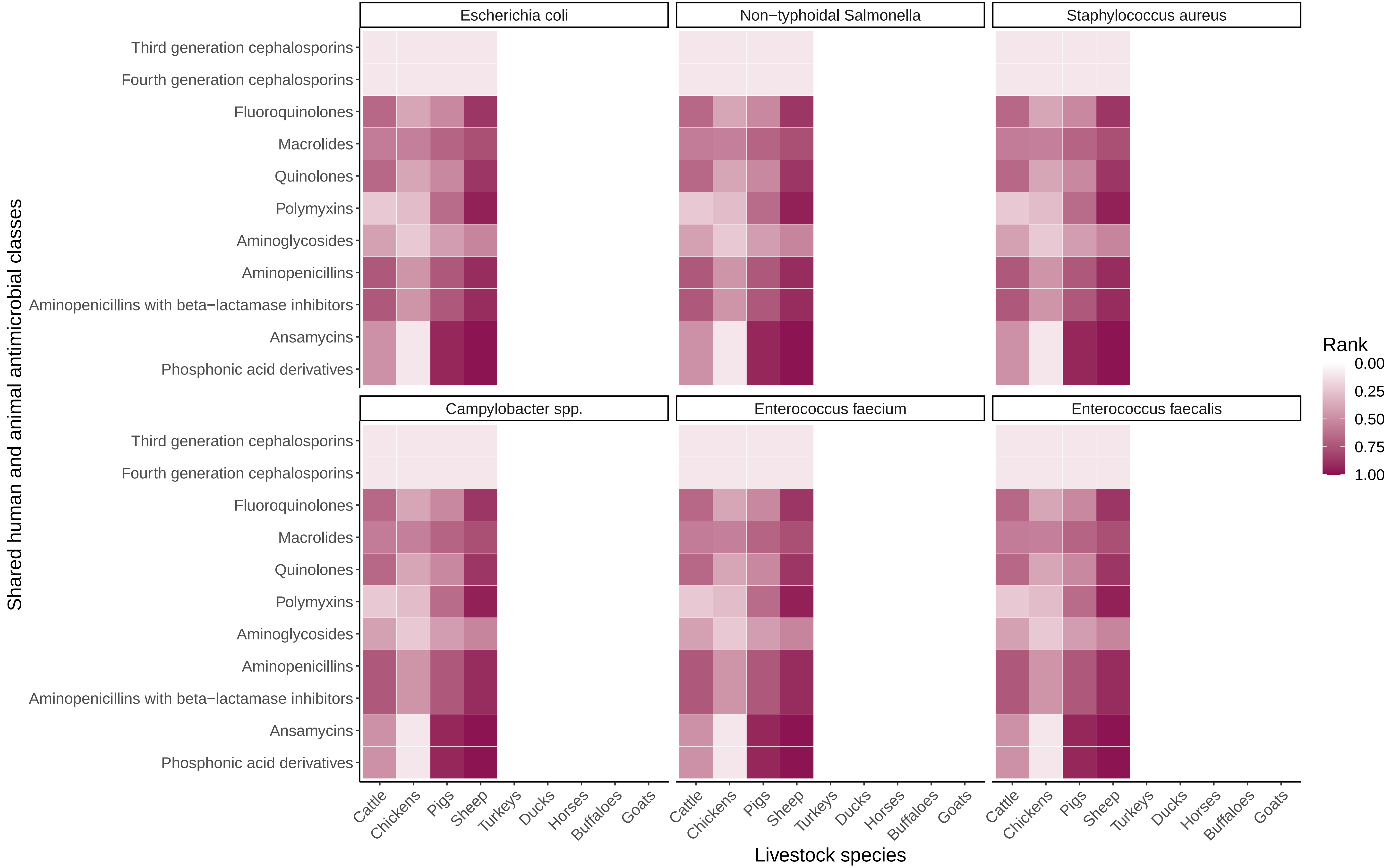

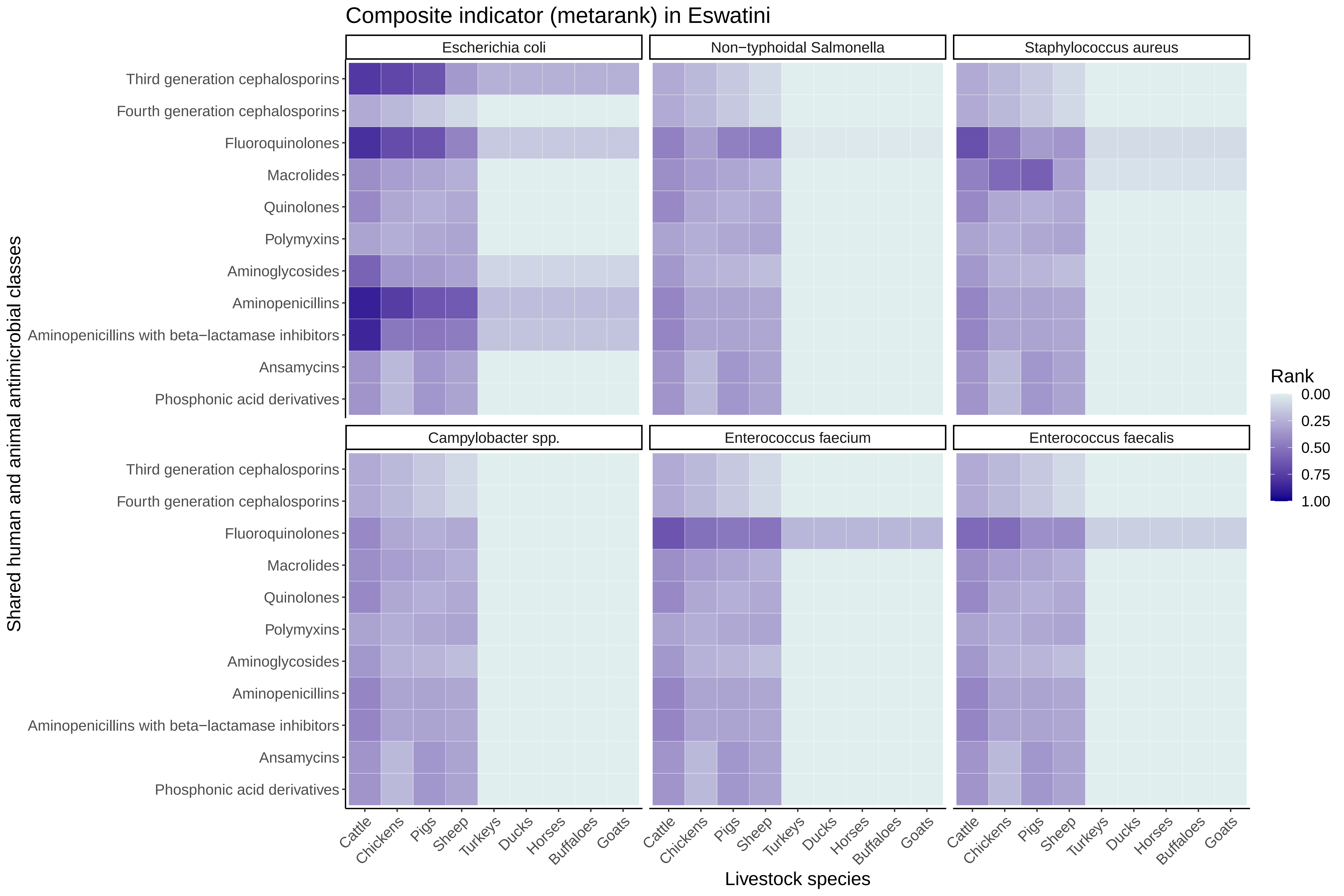

Composite indicator (metarank) in Eswatini
Escherichia coli
Non−typhoidal Salmonella
Staphylococcus aureus
Campylobacter spp.
Enterococcus faecium
Enterococcus faecalis
Shared human and animal antimicrobial classes
Third generation cephalosporins
Fourth generation cephalosporins
Fluoroquinolones
Macrolides
Quinolones
Polymyxins
Aminoglycosides
Aminopenicillins
Aminopenicillins with beta−lactamase inhibitors
Ansamycins
Phosphonic acid derivatives
Cattle
Chickens
Pigs
Sheep
Turkeys
Ducks
Horses
Buffaloes
Goats
Livestock species
Rank
0.00
0.25
0.50
0.75
1.00

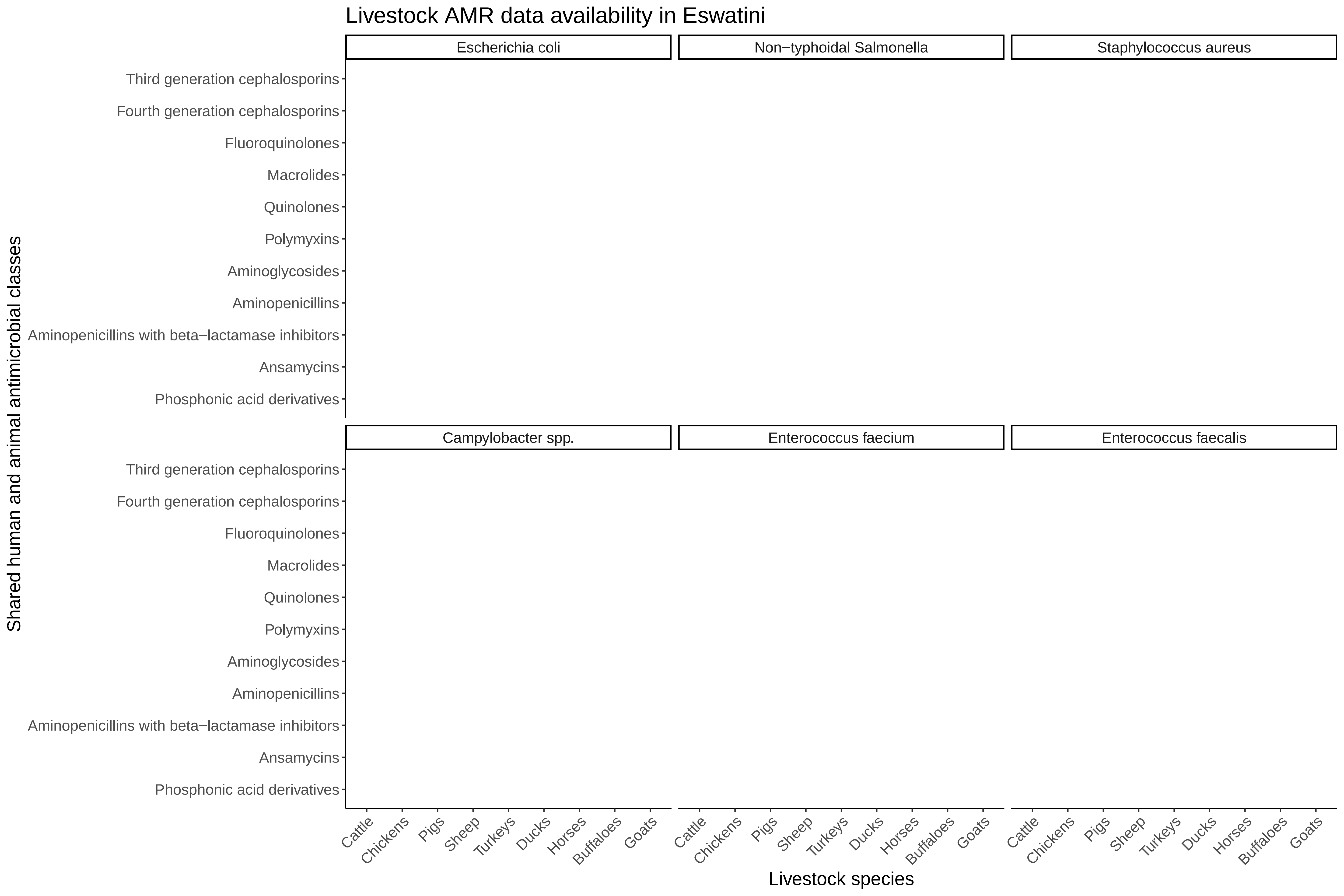
Livestock AMR data availability in Eswatini
Escherichia coli
Non−typhoidal Salmonella
Staphylococcus aureus
Campylobacter spp.
Enterococcus faecium
Enterococcus faecalis
Third generation cephalosporins
Fourth generation cephalosporins
Fluoroquinolones
Macrolides
Quinolones
Polymyxins
Aminoglycosides
Aminopenicillins
Aminopenicillins with beta−lactamase inhibitors
Ansamycins
Phosphonic acid derivatives
Shared human and animal antimicrobial classes
Cattle
Chickens
Pigs
Sheep
Turkeys
Ducks
Horses
Buffaloes
Goats
Livestock species

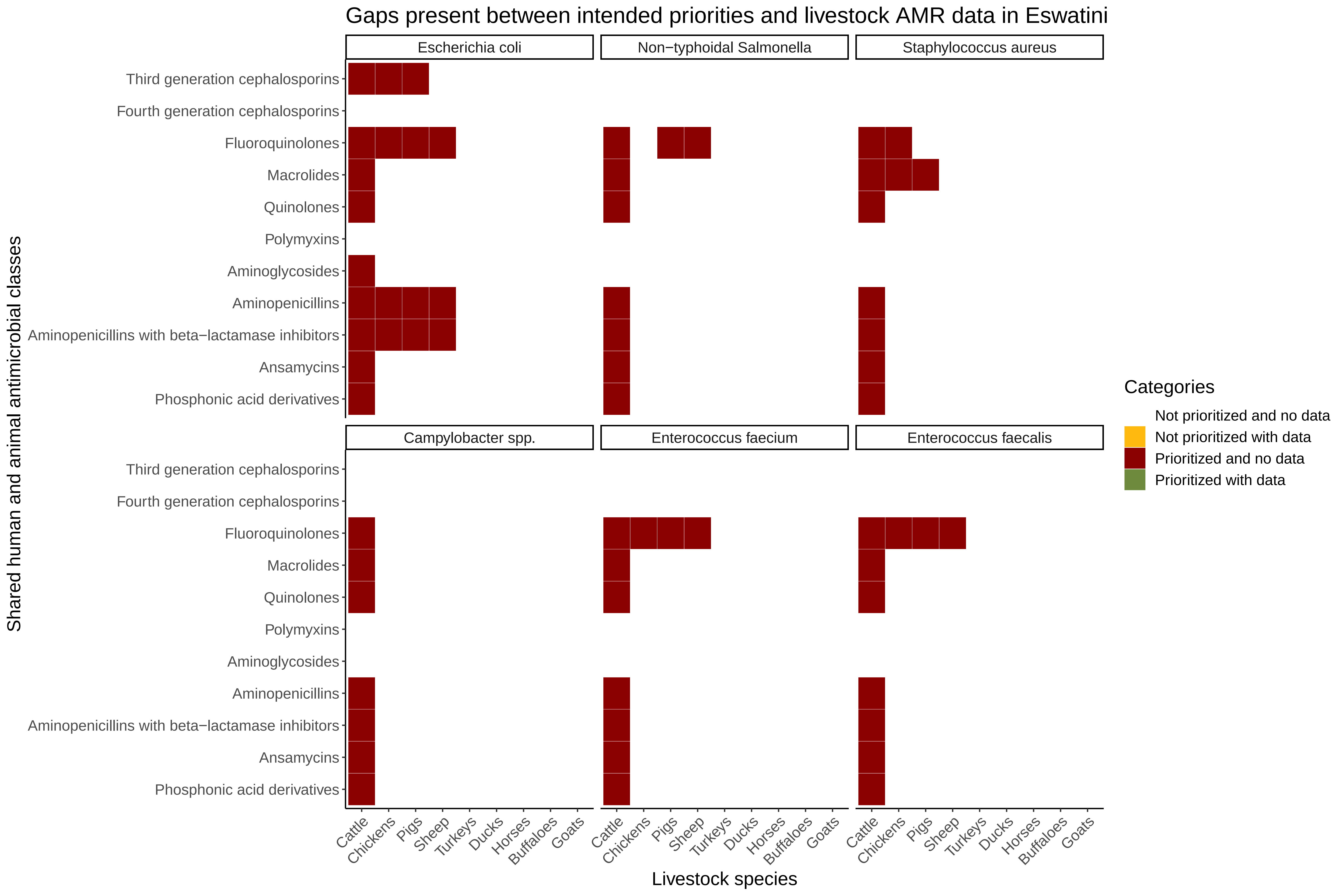

Gaps present between intended priorities and livestock AMR data in Eswatini
Escherichia coli
Non−typhoidal Salmonella
Staphylococcus aureus
Campylobacter spp.
Enterococcus faecium
Enterococcus faecalis
Third generation cephalosporins
Fourth generation cephalosporins
Fluoroquinolones
Macrolides
Quinolones
Polymyxins
Aminoglycosides
Aminopenicillins
Aminopenicillins with beta−lactamase inhibitors
Ansamycins
Phosphonic acid derivatives
Shared human and animal antimicrobial classes
Cattle
Chickens
Pigs
Sheep
Turkeys
Ducks
Horses
Buffaloes
Goats
Livestock species
Categories
Not prioritized and no data
Not prioritized with data
Prioritized and no data
Prioritized with data

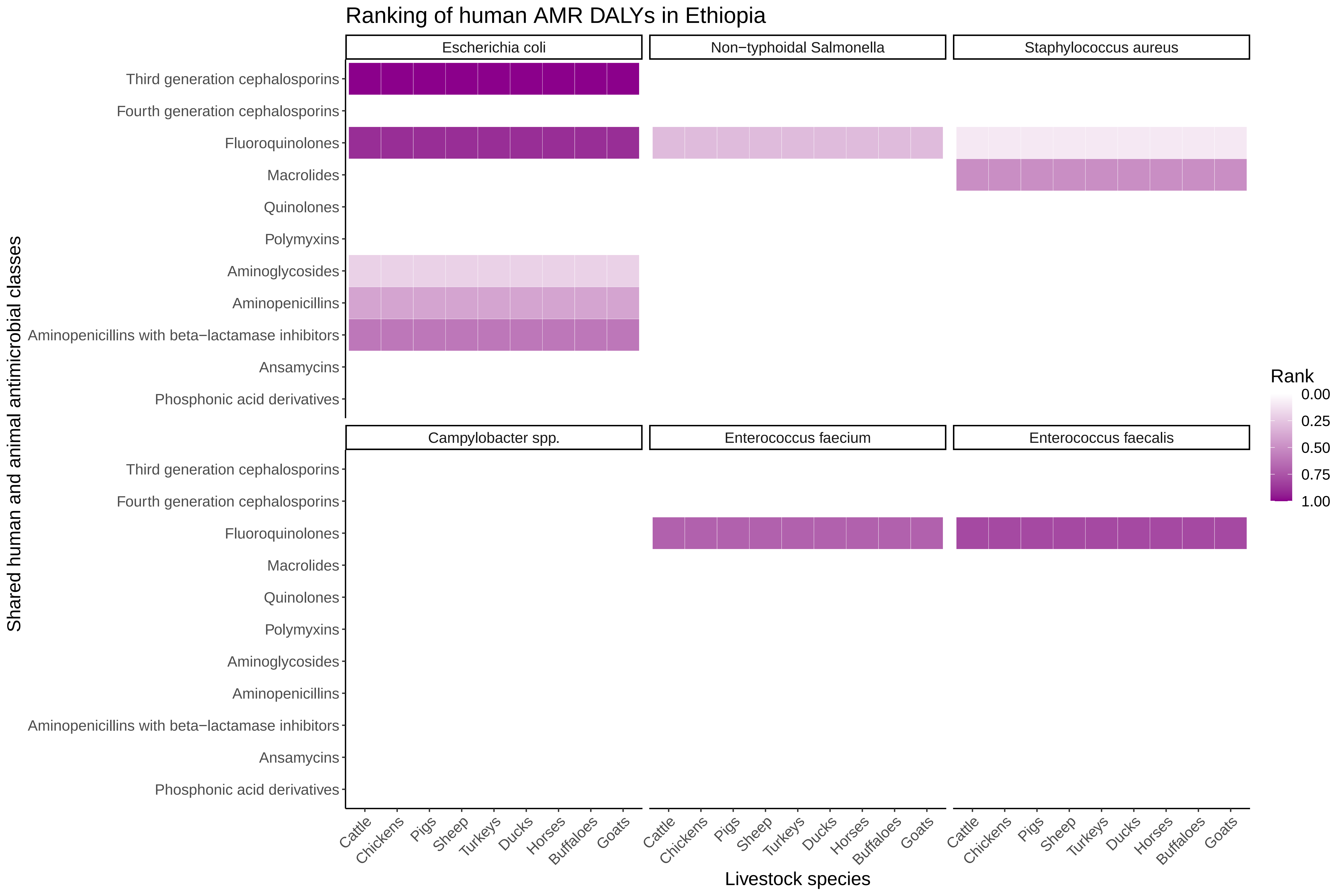
Ranking of human AMR DALYs in Ethiopia
Escherichia coli
Non−typhoidal Salmonella
Staphylococcus aureus
Campylobacter spp.
Enterococcus faecium
Enterococcus faecalis
Third generation cephalosporins
Fourth generation cephalosporins
Fluoroquinolones
Macrolides
Quinolones
Polymyxins
Aminoglycosides
Aminopenicillins
Aminopenicillins with beta−lactamase inhibitors
Ansamycins
Phosphonic acid derivatives
Shared human and animal antimicrobial classes
Cattle
Chickens
Pigs
Sheep
Turkeys
Ducks
Horses
Buffaloes
Goats
Livestock species
Rank
0.00
0.25
0.50
0.75
1.00

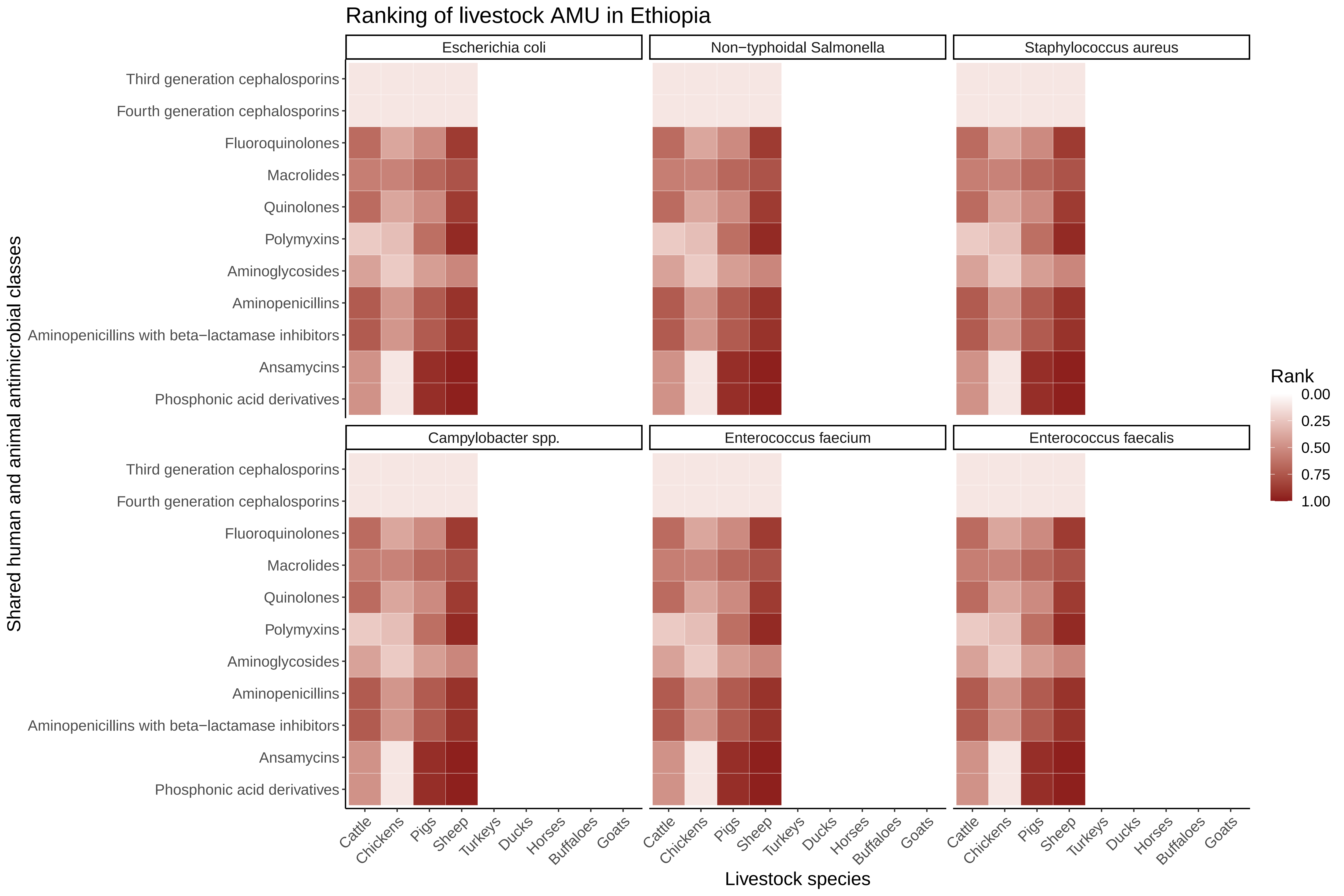
Ranking of livestock AMU in Ethiopia
Escherichia coli
Non−typhoidal Salmonella
Staphylococcus aureus
Campylobacter spp.
Enterococcus faecium
Enterococcus faecalis
Third generation cephalosporins
Fourth generation cephalosporins
Fluoroquinolones
Macrolides
Quinolones
Polymyxins
Aminoglycosides
Aminopenicillins
Aminopenicillins with beta−lactamase inhibitors
Ansamycins
Phosphonic acid derivatives
Shared human and animal antimicrobial classes
Cattle
Chickens
Pigs
Sheep
Turkeys
Ducks
Horses
Buffaloes
Goats
Livestock species
Rank
0.00
0.25
0.50
0.75
1.00

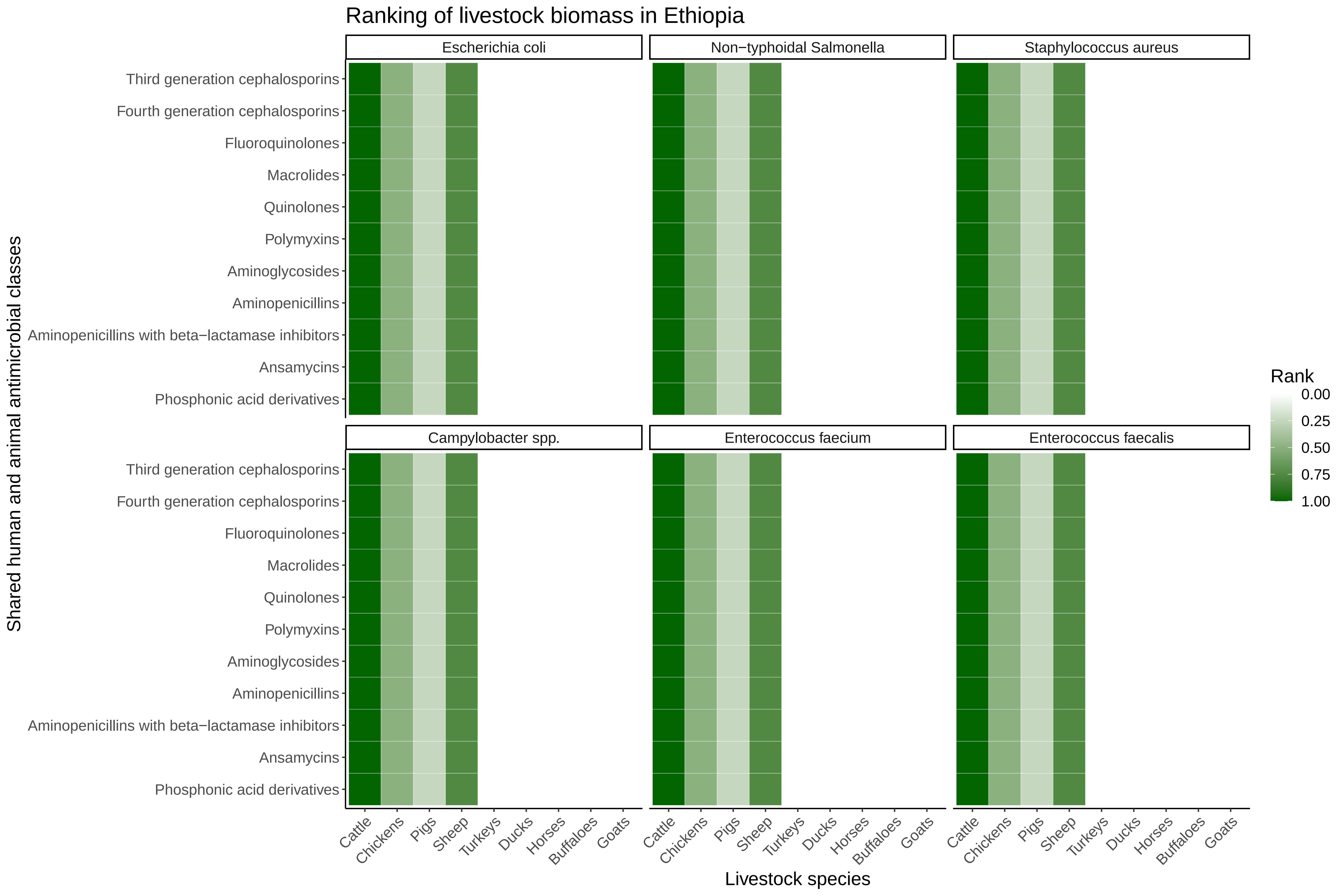
Ranking of livestock biomass in Ethiopia
Escherichia coli
Non−typhoidal Salmonella
Staphylococcus aureus
Campylobacter spp.
Enterococcus faecium
Enterococcus faecalis
Third generation cephalosporins
Fourth generation cephalosporins
Fluoroquinolones
Macrolides
Quinolones
Polymyxins
Aminoglycosides
Aminopenicillins
Aminopenicillins with beta−lactamase inhibitors
Ansamycins
Phosphonic acid derivatives
Shared human and animal antimicrobial classes
Cattle
Chickens
Pigs
Sheep
Turkeys
Ducks
Horses
Buffaloes
Goats
Livestock species
Rank
0.00
0.25
0.50
0.75
1.00

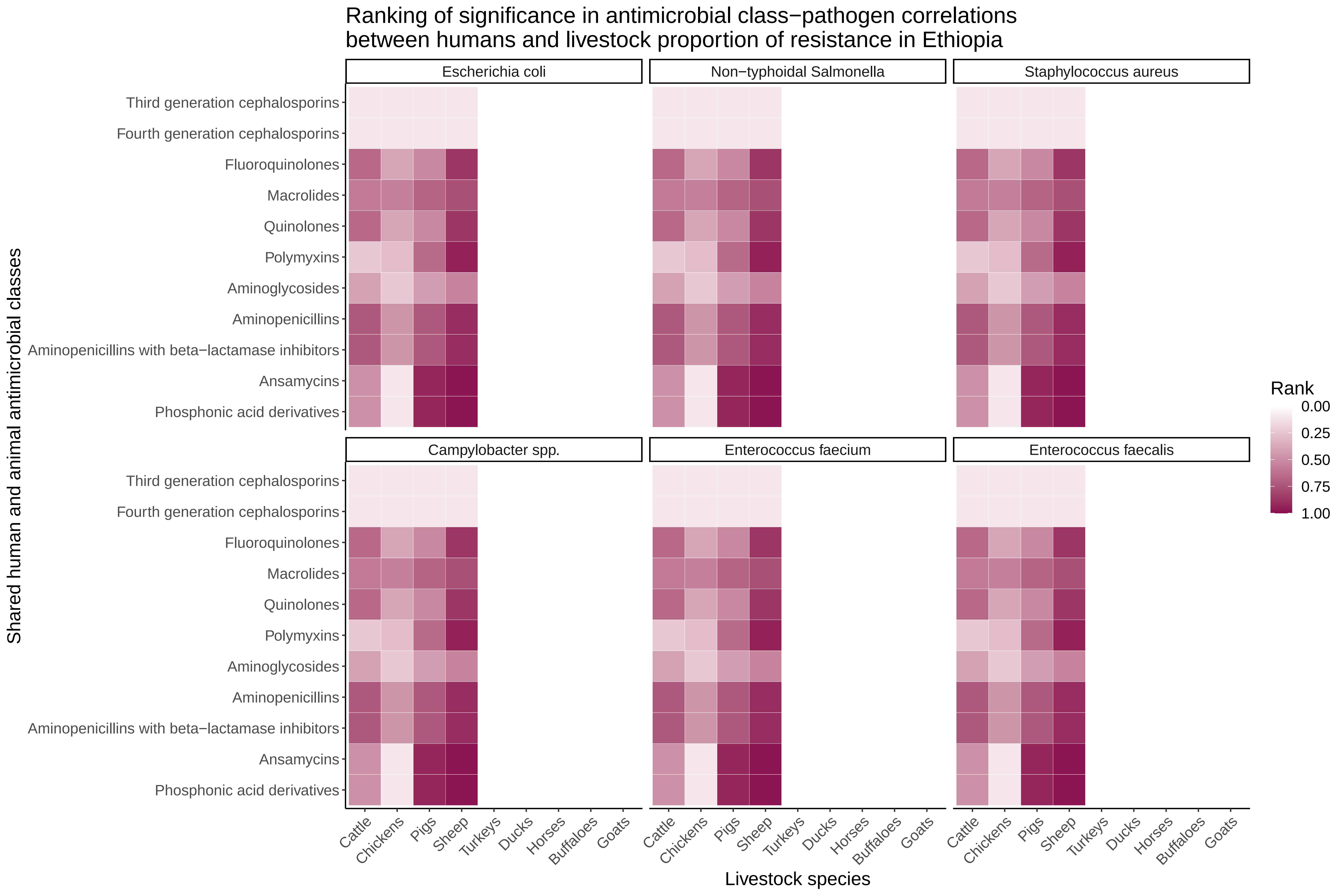

Ranking of significance in antimicrobial class−pathogen correlations between humans and livestock proportion of resistance in Ethiopia
Escherichia coli
Non−typhoidal Salmonella
Staphylococcus aureus
Campylobacter spp.
Enterococcus faecium
Enterococcus faecalis
Third generation cephalosporins
Fourth generation cephalosporins
Fluoroquinolones
Macrolides
Quinolones
Polymyxins
Aminoglycosides
Aminopenicillins
Aminopenicillins with beta−lactamase inhibitors
Ansamycins
Phosphonic acid derivatives
Shared human and animal antimicrobial classes
Cattle
Chickens
Pigs
Sheep
Turkeys
Ducks
Horses
Buffaloes
Goats
Livestock species
Rank
0.00
0.25
0.50
0.75
1.00

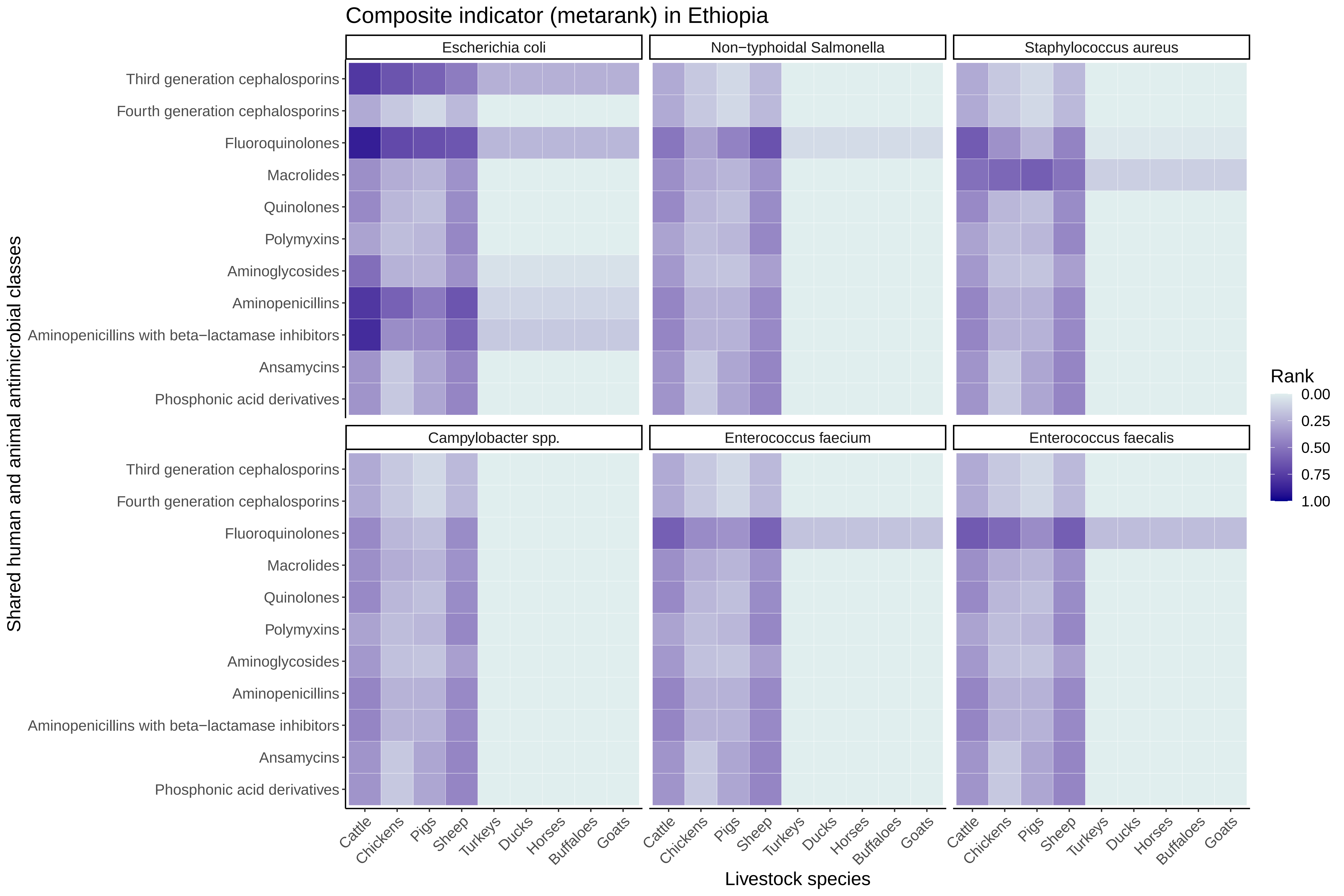

Composite indicator (metarank) in Ethiopia
Escherichia coli
Non−typhoidal Salmonella
Staphylococcus aureus
Campylobacter spp.
Enterococcus faecium
Enterococcus faecalis
Third generation cephalosporins
Fourth generation cephalosporins
Fluoroquinolones
Macrolides
Quinolones
Polymyxins
Aminoglycosides
Aminopenicillins
Aminopenicillins with beta−lactamase inhibitors
Ansamycins
Phosphonic acid derivatives
Shared human and animal antimicrobial classes
Cattle
Chickens
Pigs
Sheep
Turkeys
Ducks
Horses
Buffaloes
Goats
Livestock species
Rank
0.00
0.25
0.50
0.75
1.00

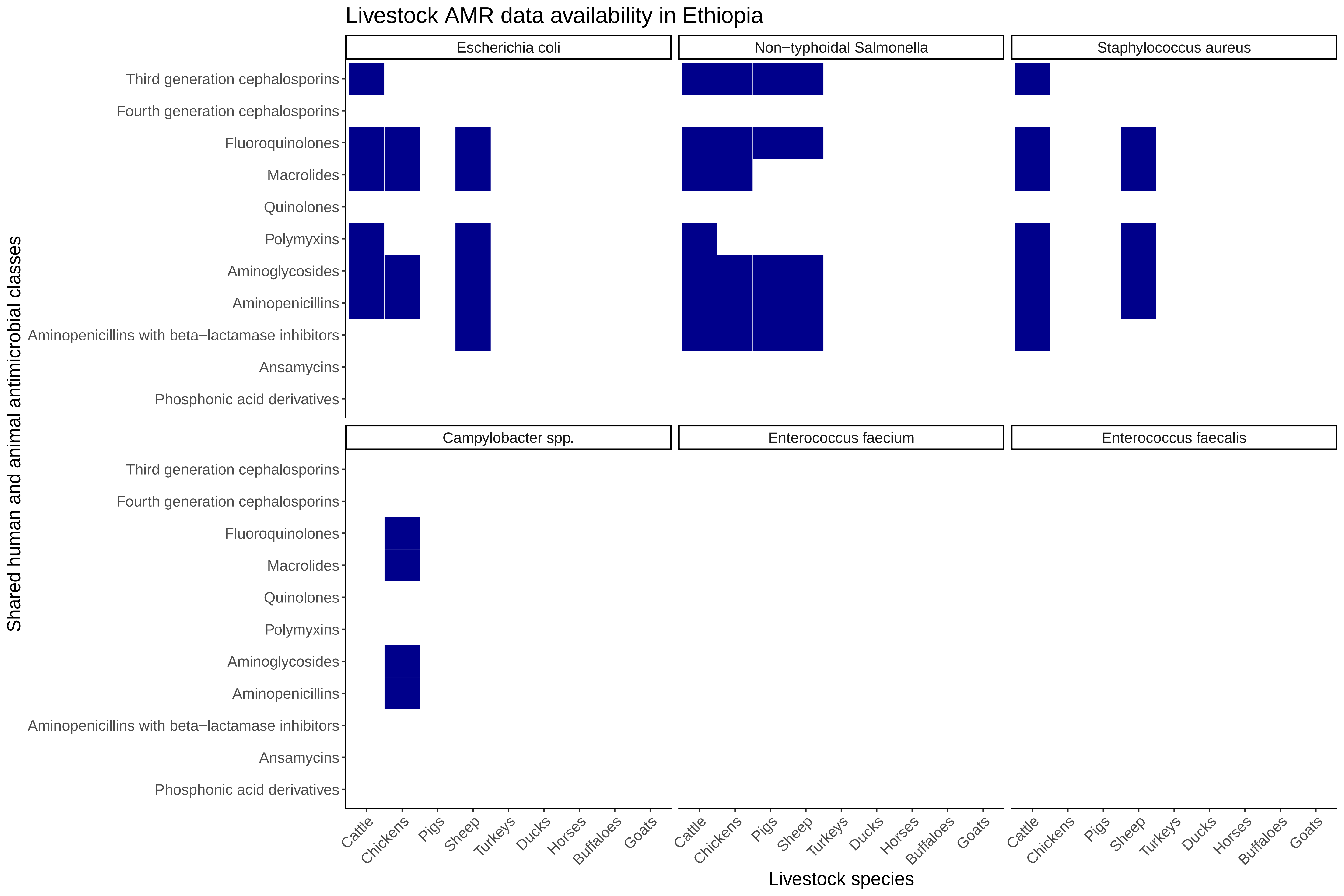

Livestock AMR data availability in Ethiopia
Escherichia coli
Non−typhoidal Salmonella
Staphylococcus aureus
Campylobacter spp.
Enterococcus faecium
Enterococcus faecalis
Third generation cephalosporins
Fourth generation cephalosporins
Fluoroquinolones
Macrolides
Quinolones
Polymyxins
Aminoglycosides
Aminopenicillins
Aminopenicillins with beta−lactamase inhibitors
Ansamycins
Phosphonic acid derivatives
Shared human and animal antimicrobial classes
Cattle
Chickens
Pigs
Sheep
Turkeys
Ducks
Horses
Buffaloes
Goats
Livestock species

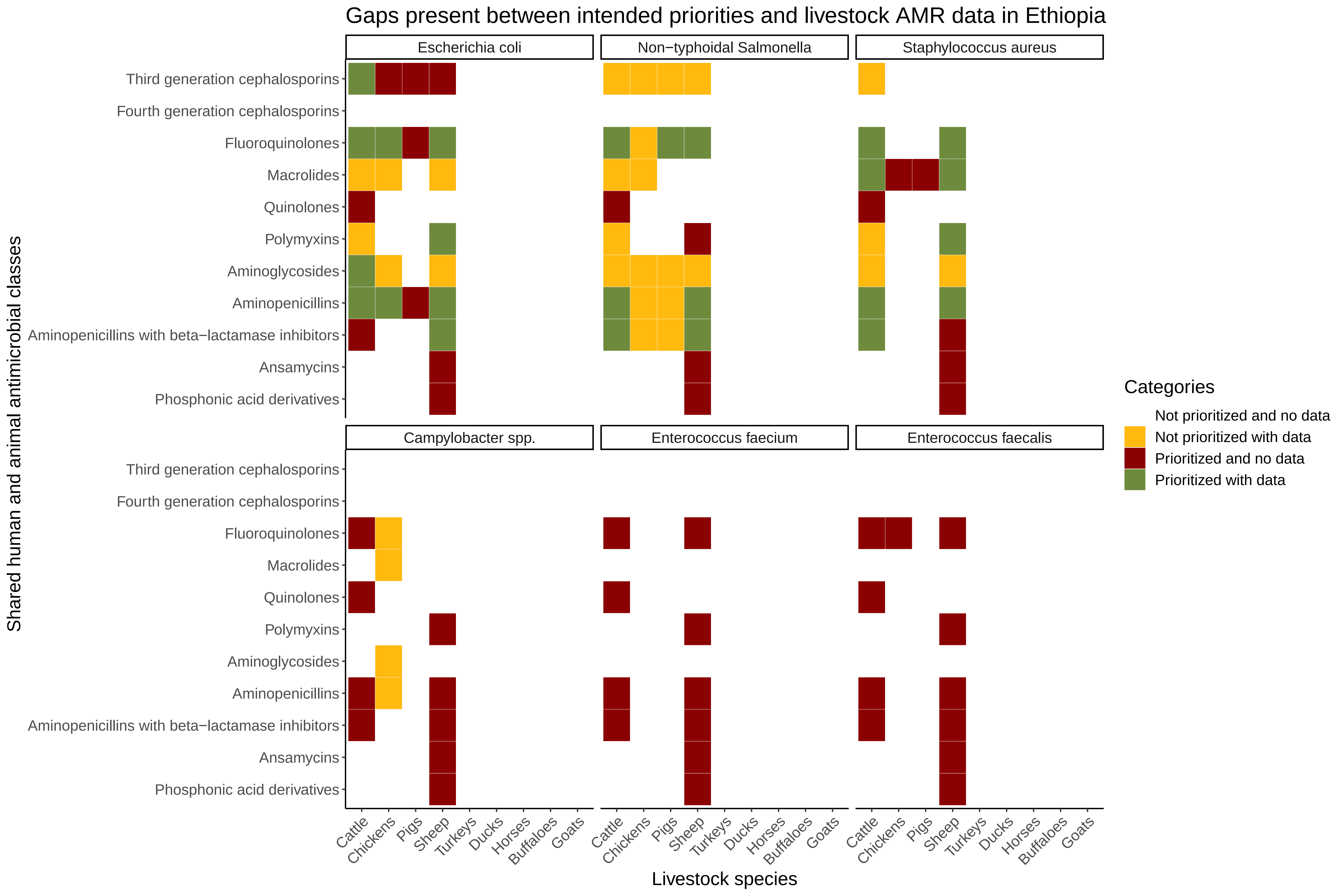

Gaps present between intended priorities and livestock AMR data in Ethiopia
Escherichia coli
Non−typhoidal Salmonella
Staphylococcus aureus
Campylobacter spp.
Enterococcus faecium
Enterococcus faecalis
Third generation cephalosporins
Fourth generation cephalosporins
Fluoroquinolones
Macrolides
Quinolones
Polymyxins
Aminoglycosides
Aminopenicillins
Aminopenicillins with beta−lactamase inhibitors
Ansamycins
Phosphonic acid derivatives
Cattle
Chickens
Pigs
Sheep
Turkeys
Ducks
Horses
Buffaloes
Goats
Livestock species
Shared human and animal antimicrobial classes
Categories
Not prioritized and no data
Not prioritized with data
Prioritized and no data
Prioritized with data

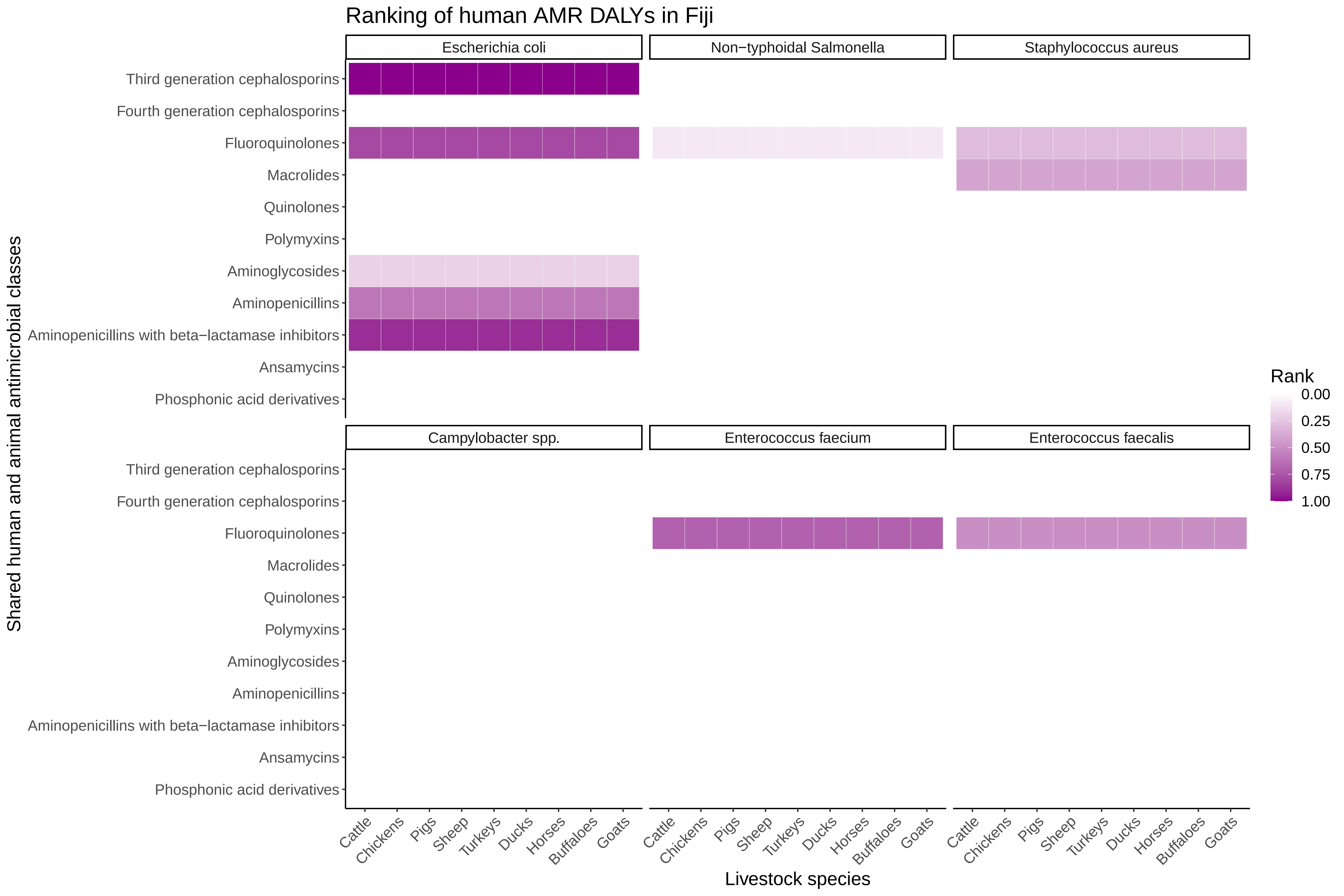
Ranking of human AMR DALYs in Fiji
Escherichia coli
Non−typhoidal Salmonella
Staphylococcus aureus
Campylobacter spp.
Enterococcus faecium
Enterococcus faecalis
Third generation cephalosporins
Fourth generation cephalosporins
Fluoroquinolones
Macrolides
Quinolones
Polymyxins
Aminoglycosides
Aminopenicillins
Aminopenicillins with beta−lactamase inhibitors
Ansamycins
Phosphonic acid derivatives
Shared human and animal antimicrobial classes
Cattle
Chickens
Pigs
Sheep
Turkeys
Ducks
Horses
Buffaloes
Goats
Livestock species
Rank
0.00
0.25
0.50
0.75
1.00

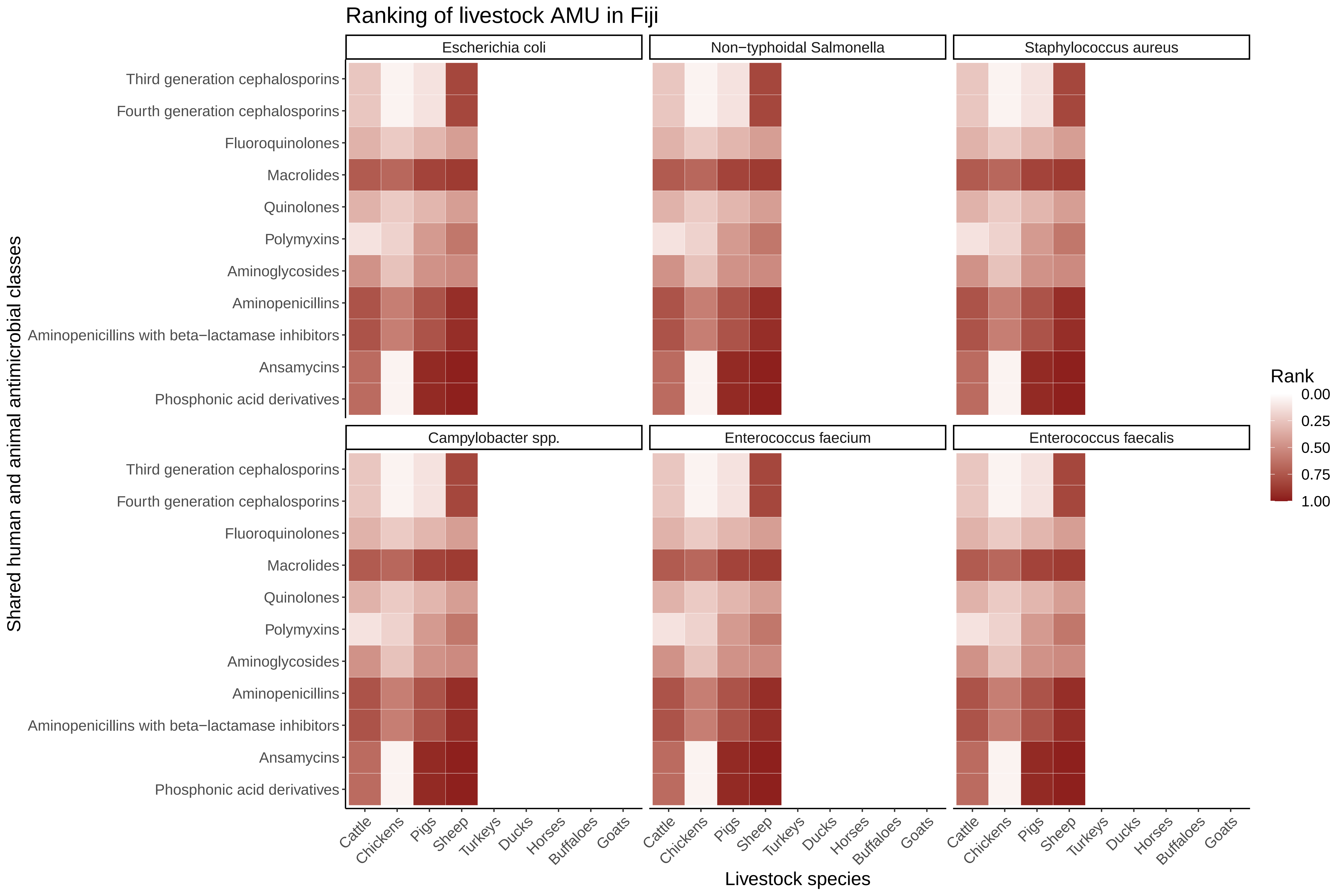
Ranking of livestock AMU in Fiji
Escherichia coli
Non−typhoidal Salmonella
Staphylococcus aureus
Campylobacter spp.
Enterococcus faecium
Enterococcus faecalis
Third generation cephalosporins
Fourth generation cephalosporins
Fluoroquinolones
Macrolides
Quinolones
Polymyxins
Aminoglycosides
Aminopenicillins
Aminopenicillins with beta−lactamase inhibitors
Ansamycins
Phosphonic acid derivatives
Shared human and animal antimicrobial classes
Cattle
Chickens
Pigs
Sheep
Turkeys
Ducks
Horses
Buffaloes
Goats
Livestock species
Rank
0.00
0.25
0.50
0.75
1.00

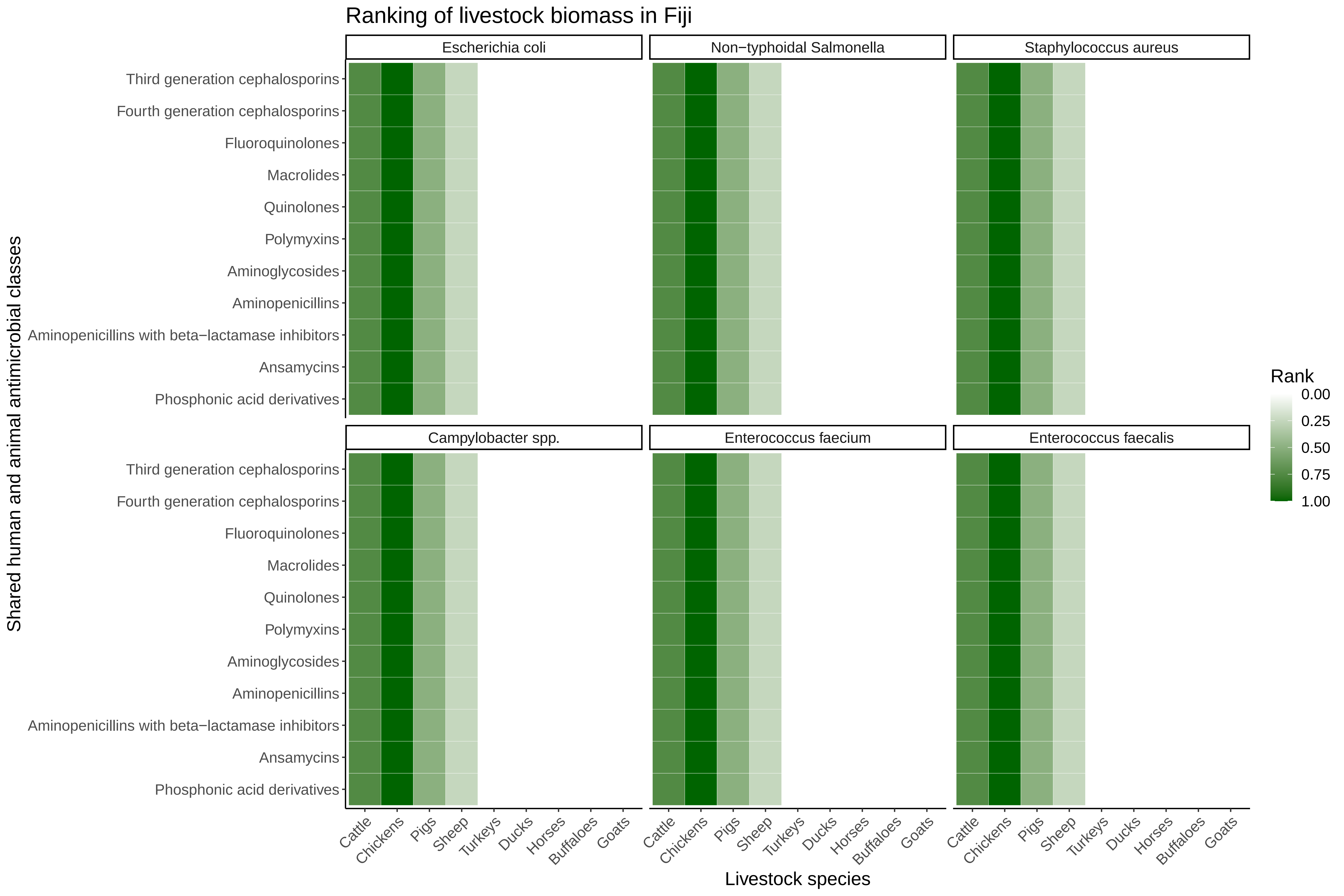
Ranking of livestock biomass in Fiji
Escherichia coli
Non−typhoidal Salmonella
Staphylococcus aureus
Campylobacter spp.
Enterococcus faecium
Enterococcus faecalis
Third generation cephalosporins
Fourth generation cephalosporins
Fluoroquinolones
Macrolides
Quinolones
Polymyxins
Aminoglycosides
Aminopenicillins
Aminopenicillins with beta−lactamase inhibitors
Ansamycins
Phosphonic acid derivatives
Shared human and animal antimicrobial classes
Cattle
Chickens
Pigs
Sheep
Turkeys
Ducks
Horses
Buffaloes
Goats
Livestock species
Rank
0.00
0.25
0.50
0.75
1.00

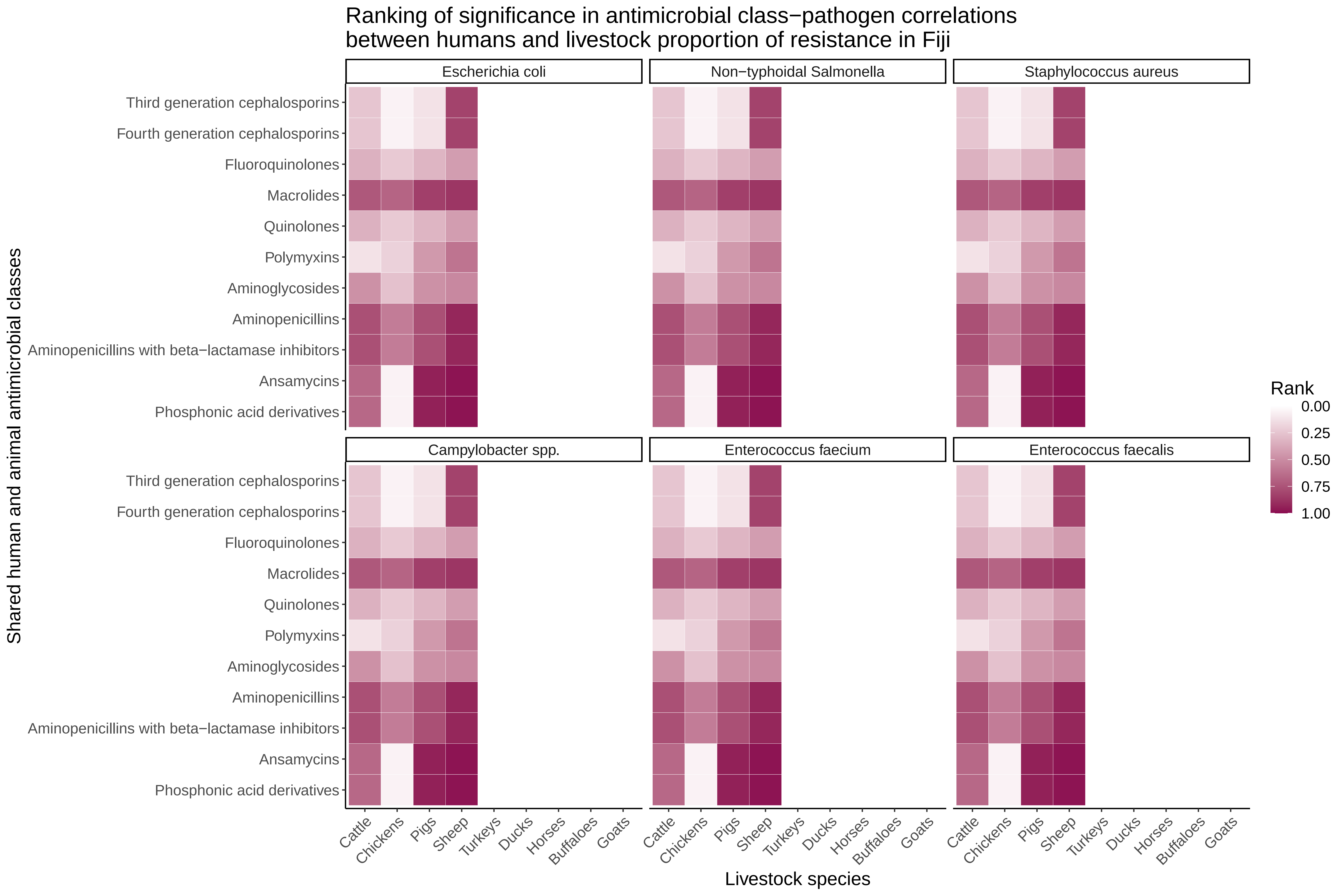

Ranking of significance in antimicrobial class−pathogen correlations
between humans and livestock proportion of resistance in Fiji
Escherichia coli
Non−typhoidal Salmonella
Staphylococcus aureus
Campylobacter spp.
Enterococcus faecium
Enterococcus faecalis
Third generation cephalosporins
Fourth generation cephalosporins
Fluoroquinolones
Macrolides
Quinolones
Polymyxins
Aminoglycosides
Aminopenicillins
Aminopenicillins with beta−lactamase inhibitors
Ansamycins
Phosphonic acid derivatives
Shared human and animal antimicrobial classes
Cattle
Chickens
Pigs
Sheep
Turkeys
Ducks
Horses
Buffaloes
Goats
Livestock species
Rank
0.00
0.25
0.50
0.75
1.00

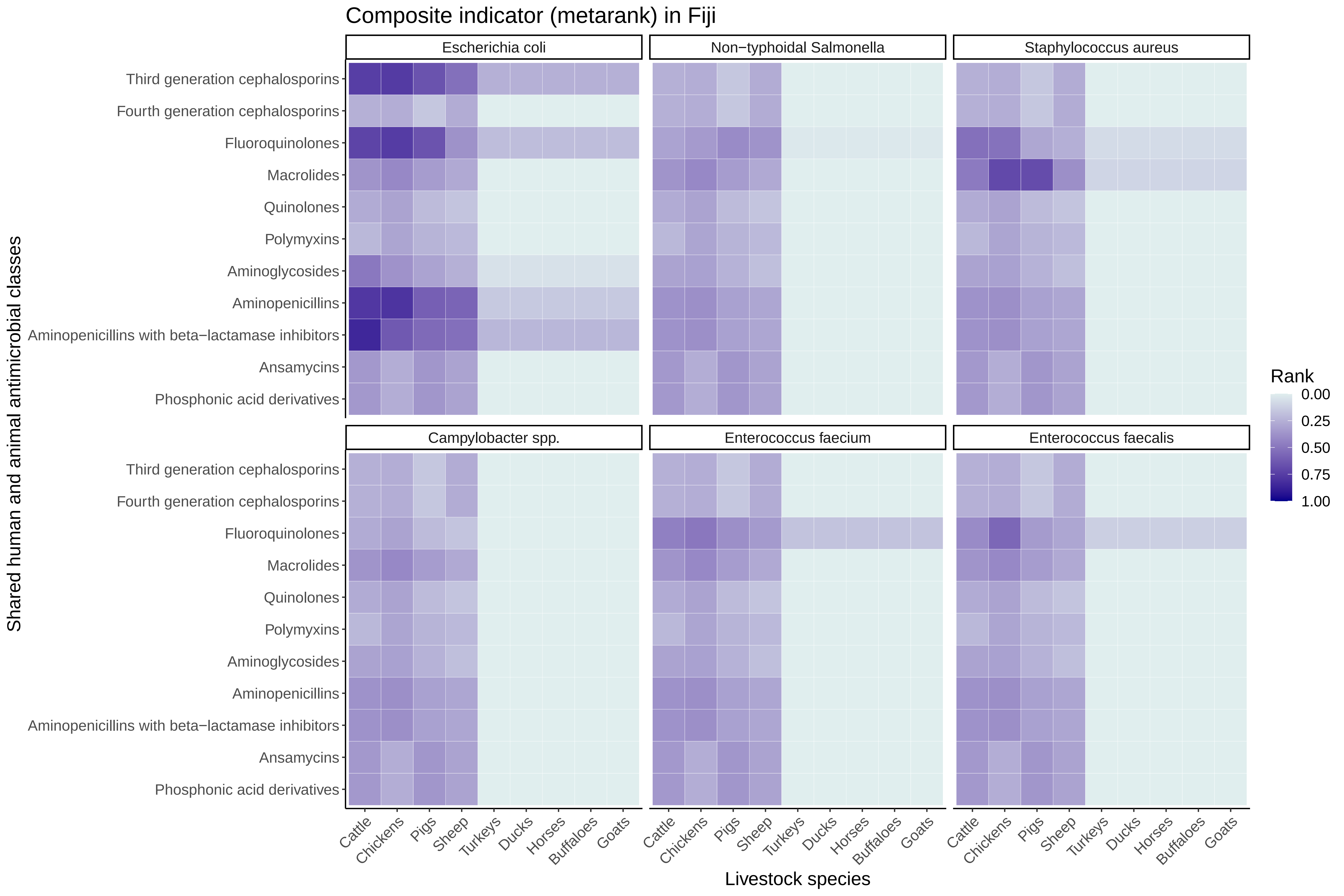

Composite indicator (metarank) in Fiji
Escherichia coli
Non−typhoidal Salmonella
Staphylococcus aureus
Campylobacter spp.
Enterococcus faecium
Enterococcus faecalis
Shared human and animal antimicrobial classes
Third generation cephalosporins
Fourth generation cephalosporins
Fluoroquinolones
Macrolides
Quinolones
Polymyxins
Aminoglycosides
Aminopenicillins
Aminopenicillins with beta−lactamase inhibitors
Ansamycins
Phosphonic acid derivatives
Livestock species
Cattle
Chickens
Pigs
Sheep
Turkeys
Ducks
Horses
Buffaloes
Goats
Rank
0.00
0.25
0.50
0.75
1.00

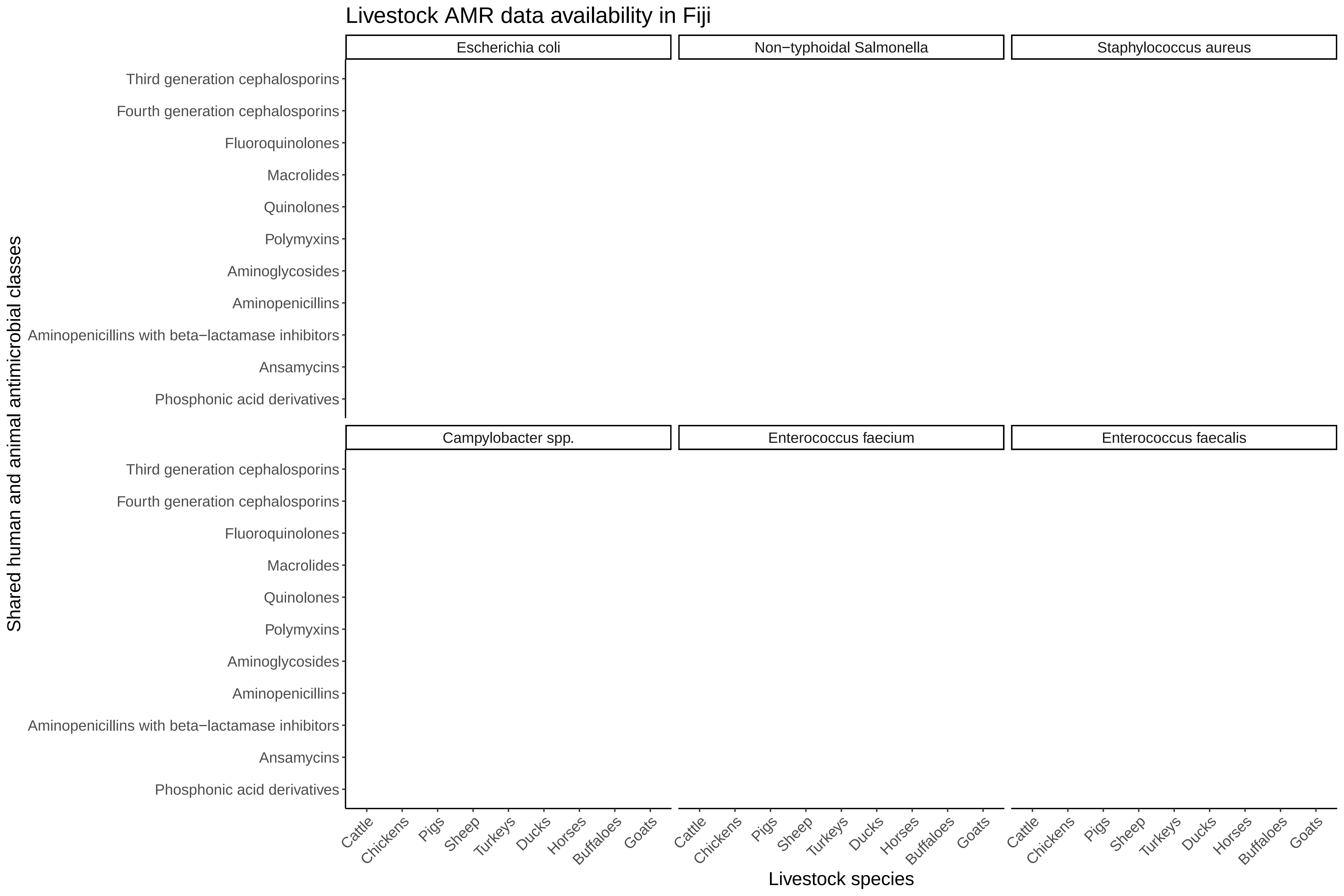
Livestock AMR data availability in Fiji
Escherichia coli
Non−typhoidal Salmonella
Staphylococcus aureus
Campylobacter spp.
Enterococcus faecium
Enterococcus faecalis
Shared human and animal antimicrobial classes
Third generation cephalosporins
Fourth generation cephalosporins
Fluoroquinolones
Macrolides
Quinolones
Polymyxins
Aminoglycosides
Aminopenicillins
Aminopenicillins with beta−lactamase inhibitors
Ansamycins
Phosphonic acid derivatives
Cattle
Chickens
Pigs
Sheep
Turkeys
Ducks
Horses
Buffaloes
Goats
Livestock species

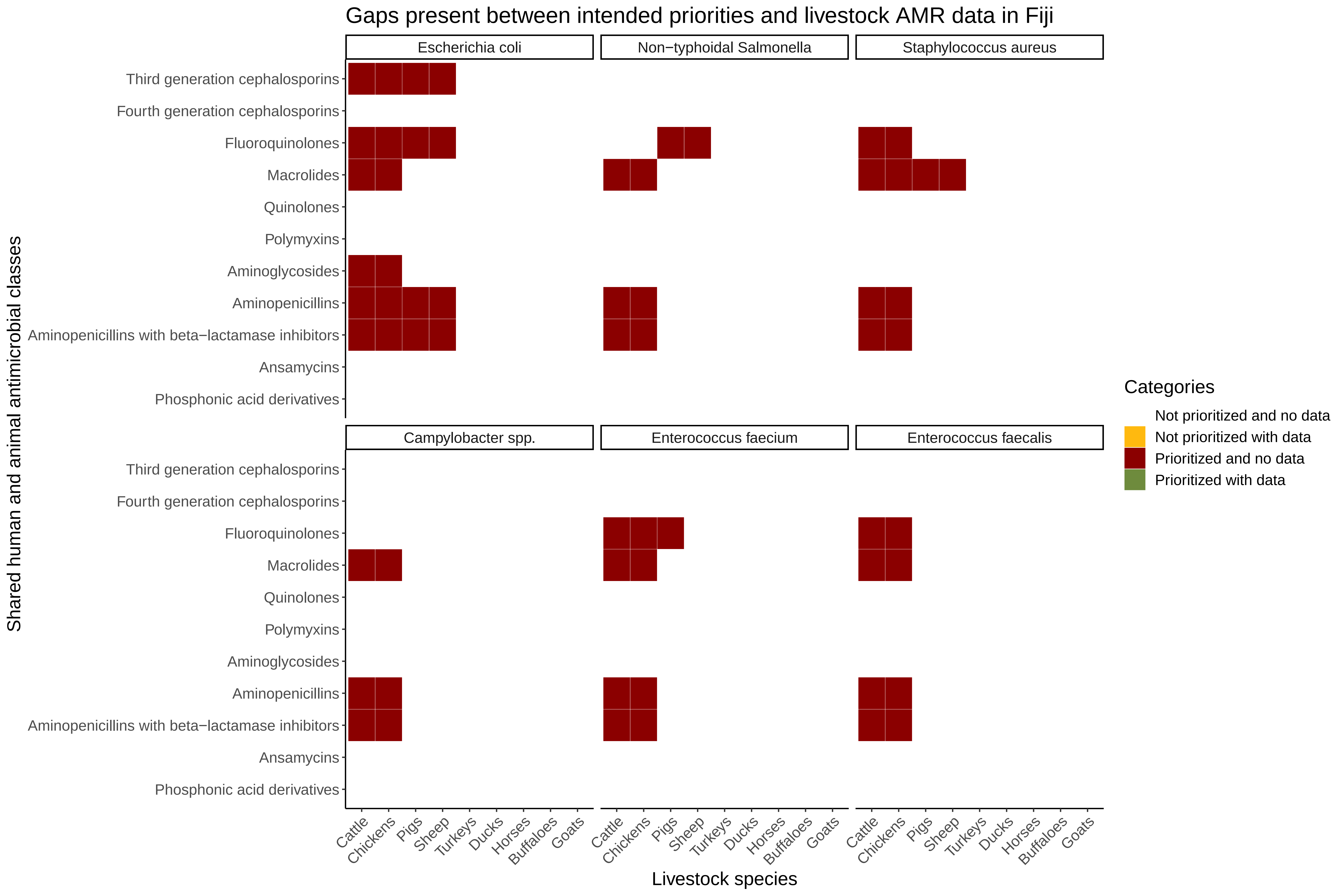

Gaps present between intended priorities and livestock AMR data in Fiji
Escherichia coli
Non−typhoidal Salmonella
Staphylococcus aureus
Campylobacter spp.
Enterococcus faecium
Enterococcus faecalis
Shared human and animal antimicrobial classes
Third generation cephalosporins
Fourth generation cephalosporins
Fluoroquinolones
Macrolides
Quinolones
Polymyxins
Aminoglycosides
Aminopenicillins
Aminopenicillins with beta−lactamase inhibitors
Ansamycins
Phosphonic acid derivatives
Livestock species
Cattle
Chickens
Pigs
Sheep
Turkeys
Ducks
Horses
Buffaloes
Goats
Categories
Not prioritized and no data
Not prioritized with data
Prioritized and no data
Prioritized with data

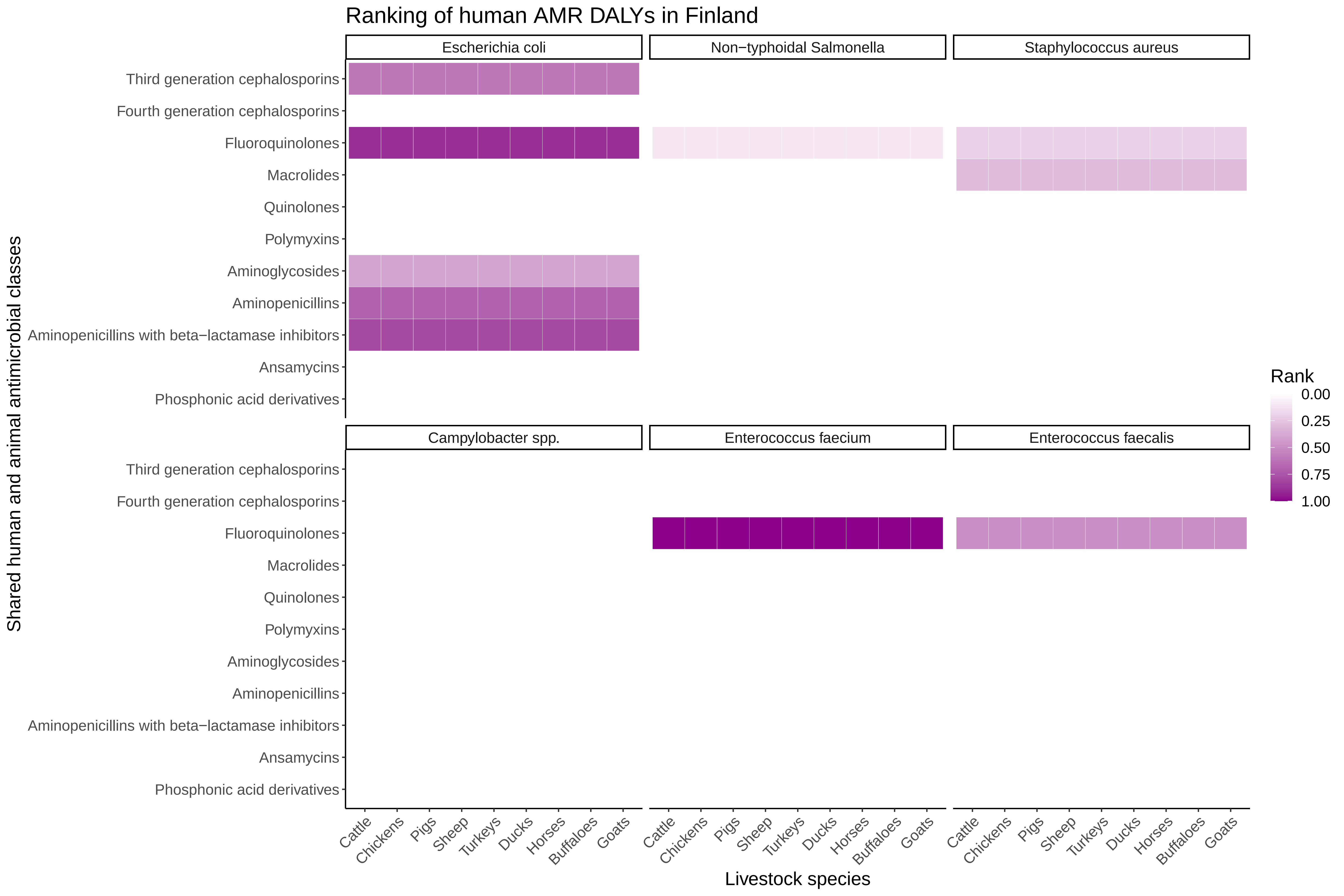
Ranking of human AMR DALYs in Finland
Escherichia coli
Non−typhoidal Salmonella
Staphylococcus aureus
Campylobacter spp.
Enterococcus faecium
Enterococcus faecalis
Shared human and animal antimicrobial classes
Third generation cephalosporins
Fourth generation cephalosporins
Fluoroquinolones
Macrolides
Quinolones
Polymyxins
Aminoglycosides
Aminopenicillins
Aminopenicillins with beta−lactamase inhibitors
Ansamycins
Phosphonic acid derivatives
Livestock species
Cattle
Chickens
Pigs
Sheep
Turkeys
Ducks
Horses
Buffaloes
Goats
Rank
0.00
0.25
0.50
0.75
1.00

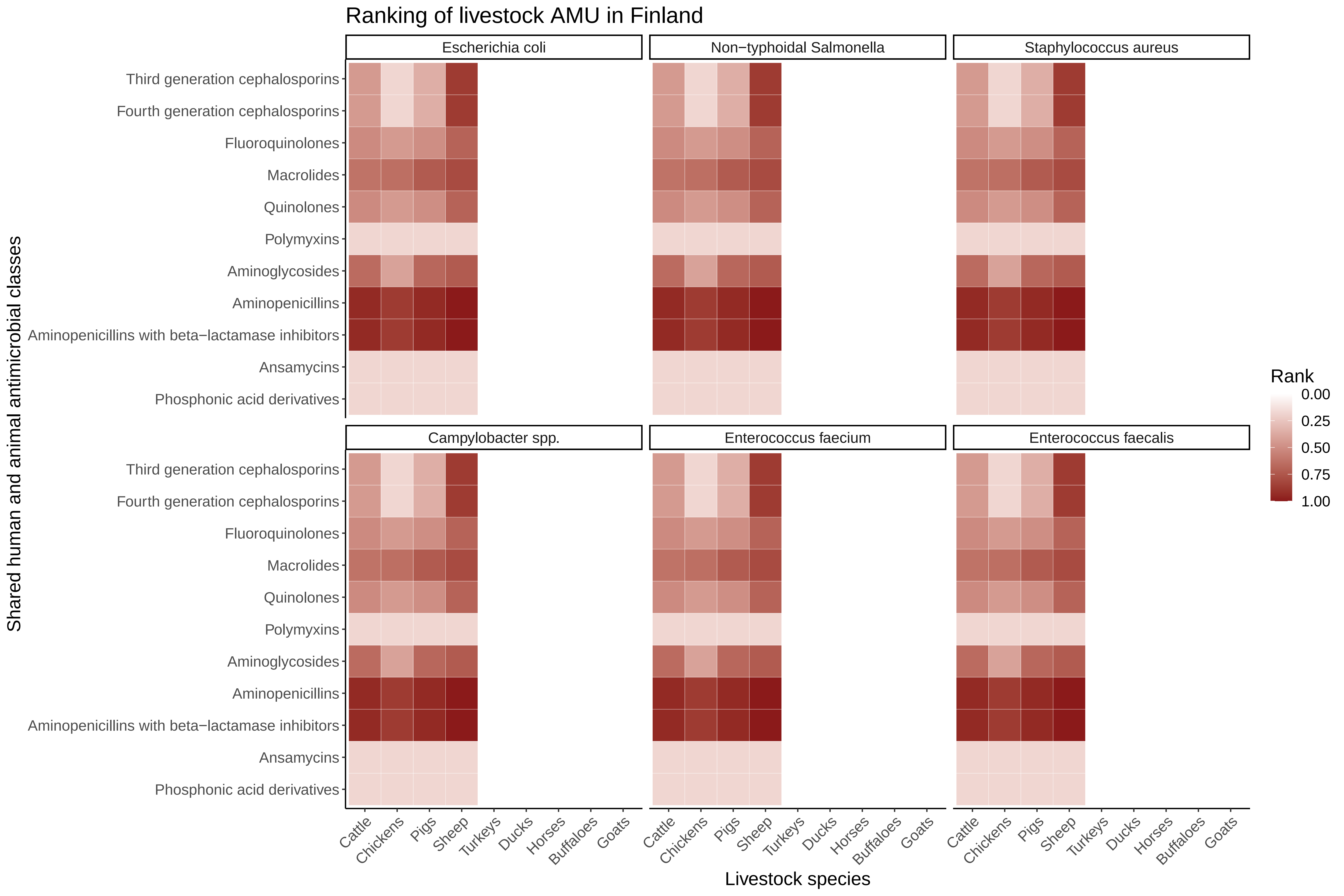

Ranking of livestock AMU in Finland
Escherichia coli
Non−typhoidal Salmonella
Staphylococcus aureus
Campylobacter spp.
Enterococcus faecium
Enterococcus faecalis
Third generation cephalosporins
Fourth generation cephalosporins
Fluoroquinolones
Macrolides
Quinolones
Polymyxins
Aminoglycosides
Aminopenicillins
Aminopenicillins with beta−lactamase inhibitors
Ansamycins
Phosphonic acid derivatives
Shared human and animal antimicrobial classes
Cattle
Chickens
Pigs
Sheep
Turkeys
Ducks
Horses
Buffaloes
Goats
Livestock species
Rank
0.00
0.25
0.50
0.75
1.00

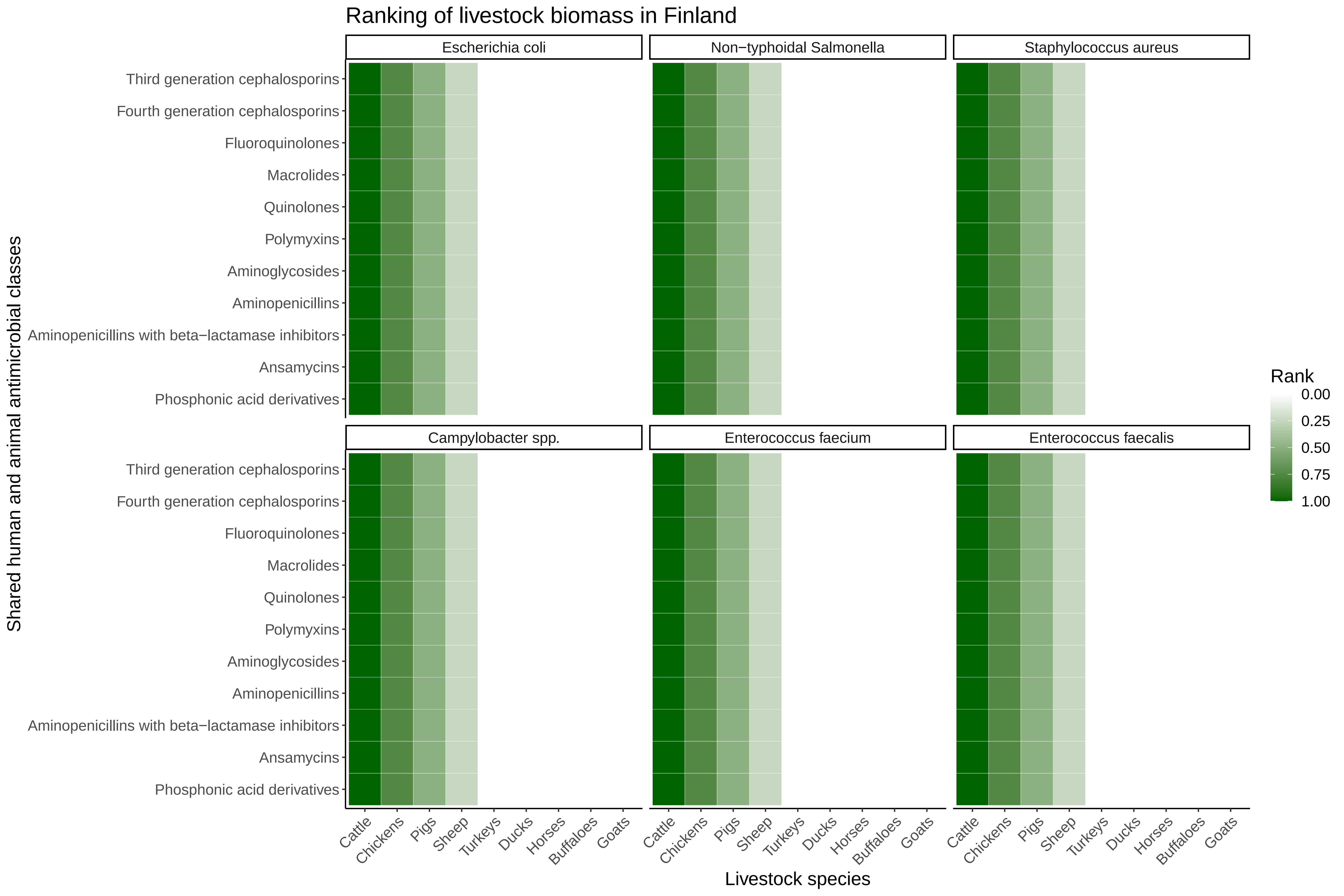

Ranking of livestock biomass in Finland
Escherichia coli
Non−typhoidal Salmonella
Staphylococcus aureus
Campylobacter spp.
Enterococcus faecium
Enterococcus faecalis
Shared human and animal antimicrobial classes
Third generation cephalosporins
Fourth generation cephalosporins
Fluoroquinolones
Macrolides
Quinolones
Polymyxins
Aminoglycosides
Aminopenicillins
Aminopenicillins with beta−lactamase inhibitors
Ansamycins
Phosphonic acid derivatives
Cattle
Chickens
Pigs
Sheep
Turkeys
Ducks
Horses
Buffaloes
Goats
Livestock species
Rank
0.00
0.25
0.50
0.75
1.00

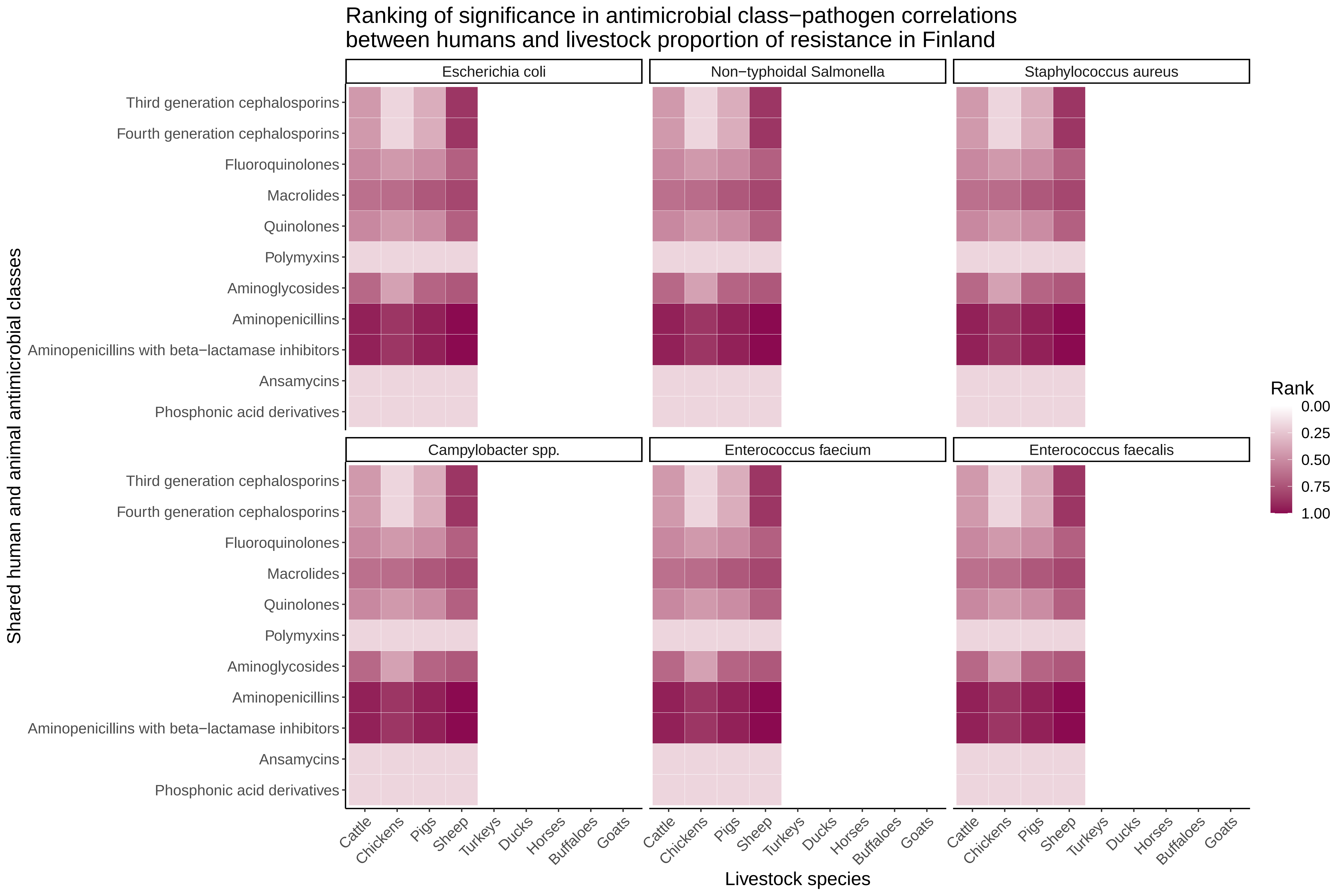

Ranking of significance in antimicrobial class−pathogen correlations between humans and livestock proportion of resistance in Finland
Escherichia coli
Non−typhoidal Salmonella
Staphylococcus aureus
Campylobacter spp.
Enterococcus faecium
Enterococcus faecalis
Third generation cephalosporins
Fourth generation cephalosporins
Fluoroquinolones
Macrolides
Quinolones
Polymyxins
Aminoglycosides
Aminopenicillins
Aminopenicillins with beta−lactamase inhibitors
Ansamycins
Phosphonic acid derivatives
Shared human and animal antimicrobial classes
Cattle
Chickens
Pigs
Sheep
Turkeys
Ducks
Horses
Buffaloes
Goats
Livestock species
Rank
0.00
0.25
0.50
0.75
1.00

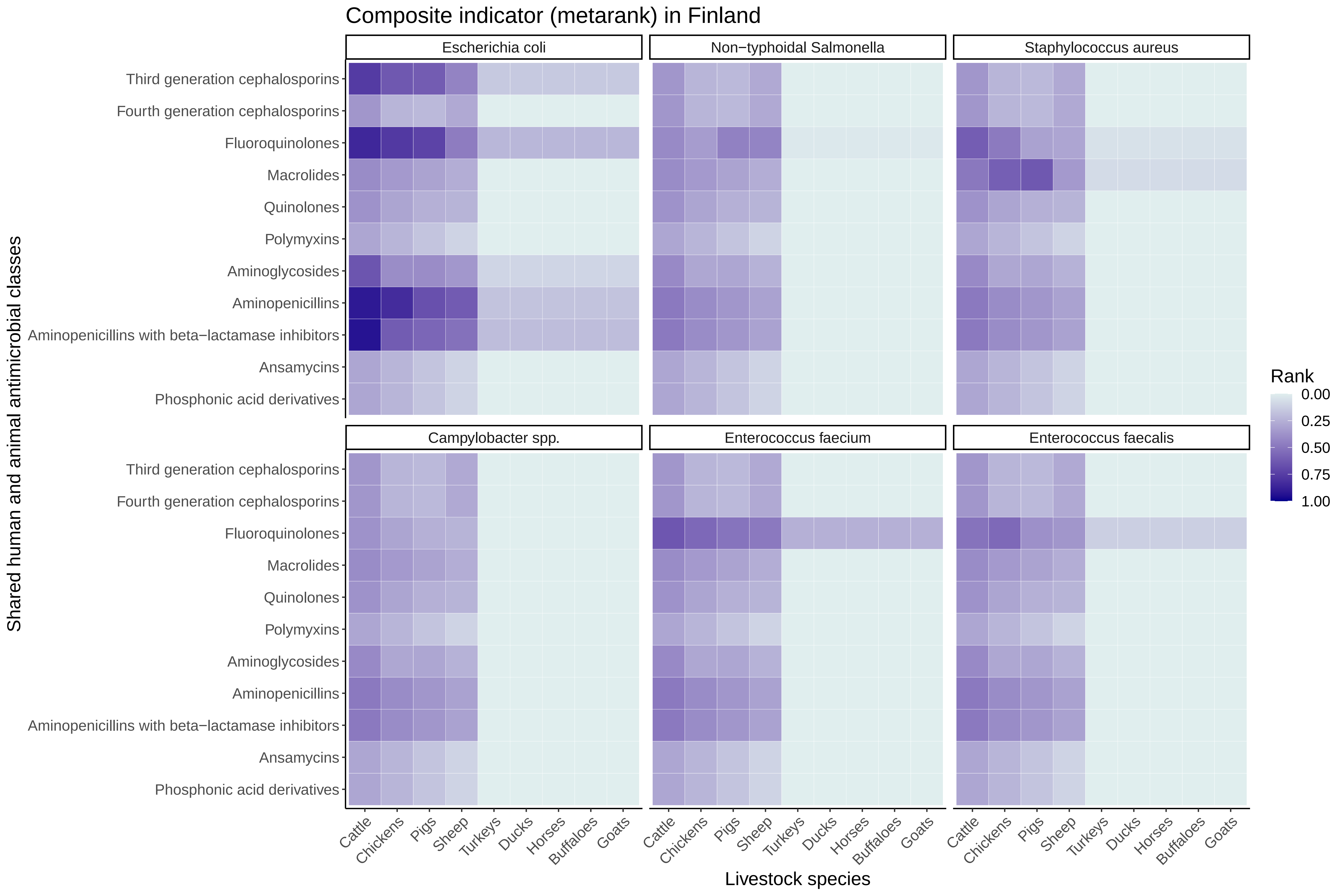

Composite indicator (metarank) in Finland
Escherichia coli
Non−typhoidal Salmonella
Staphylococcus aureus
Campylobacter spp.
Enterococcus faecium
Enterococcus faecalis
Shared human and animal antimicrobial classes
Third generation cephalosporins
Fourth generation cephalosporins
Fluoroquinolones
Macrolides
Quinolones
Polymyxins
Aminoglycosides
Aminopenicillins
Aminopenicillins with beta−lactamase inhibitors
Ansamycins
Phosphonic acid derivatives
Cattle
Chickens
Pigs
Sheep
Turkeys
Ducks
Horses
Buffaloes
Goats
Livestock species
Rank
0.00
0.25
0.50
0.75
1.00

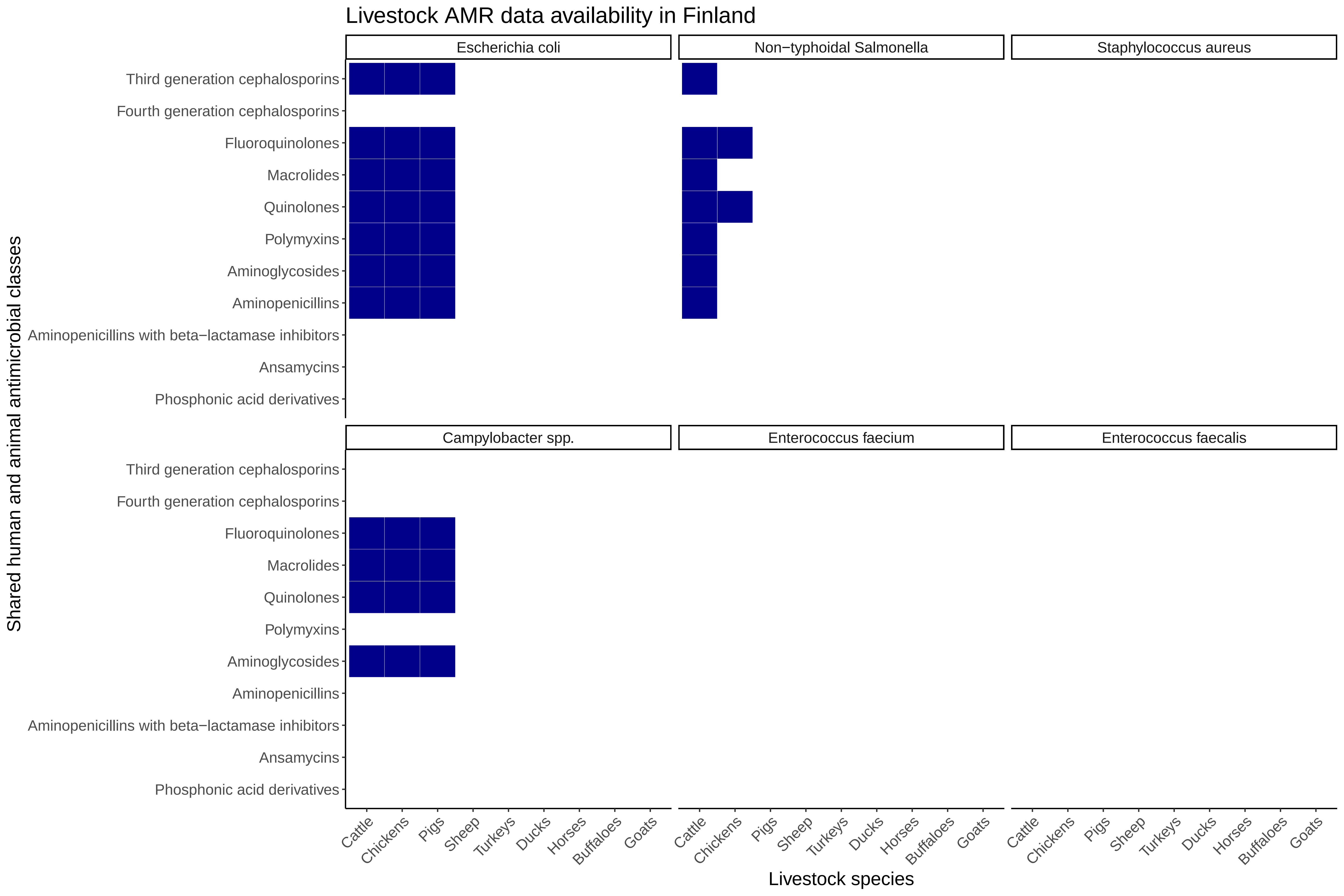

Livestock AMR data availability in Finland
Escherichia coli
Non−typhoidal Salmonella
Staphylococcus aureus
Campylobacter spp.
Enterococcus faecium
Enterococcus faecalis
Shared human and animal antimicrobial classes
Third generation cephalosporins
Fourth generation cephalosporins
Fluoroquinolones
Macrolides
Quinolones
Polymyxins
Aminoglycosides
Aminopenicillins
Aminopenicillins with beta−lactamase inhibitors
Ansamycins
Phosphonic acid derivatives
Cattle
Chickens
Pigs
Sheep
Turkeys
Ducks
Horses
Buffaloes
Goats
Livestock species

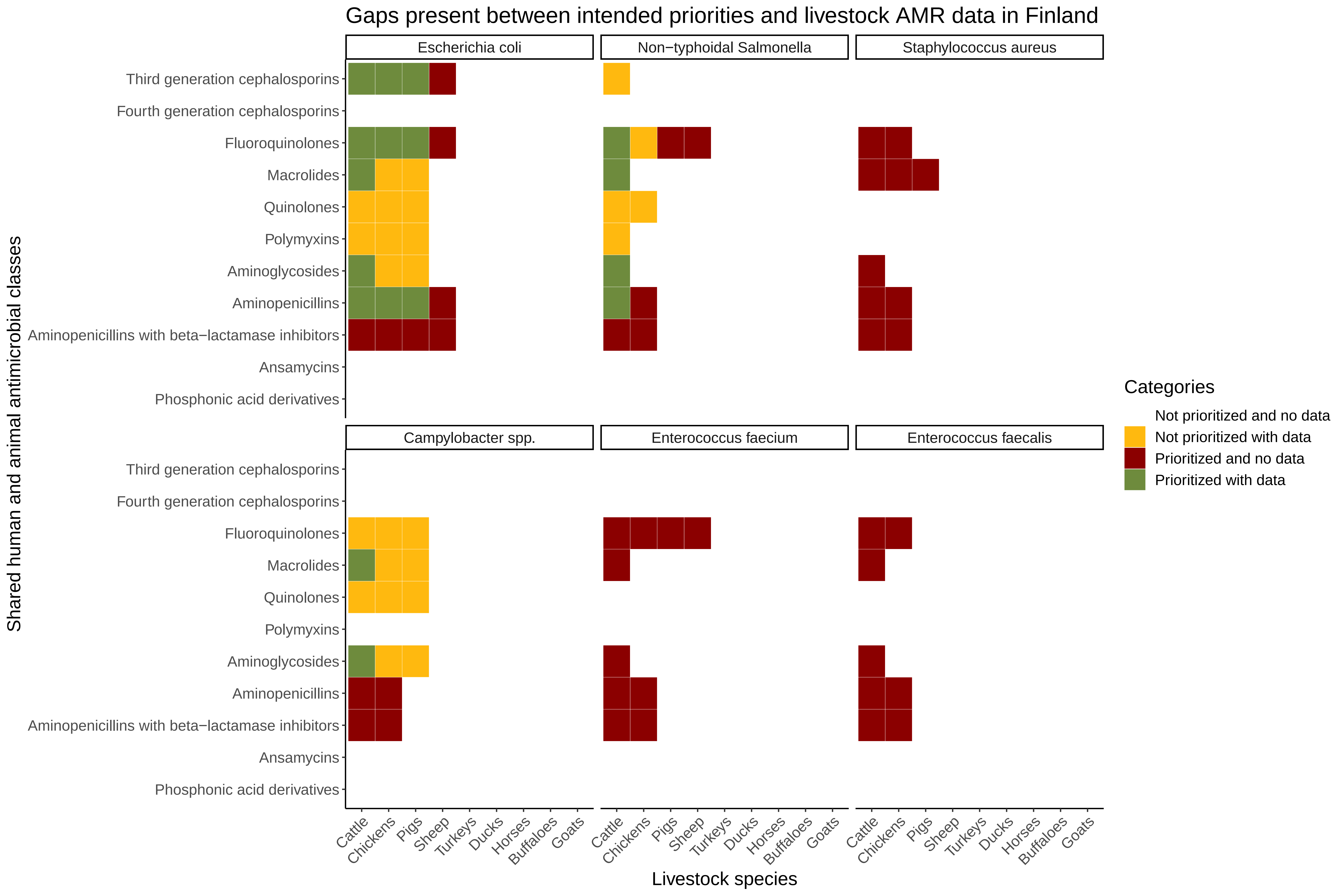
Gaps present between intended priorities and livestock AMR data in Finland
Escherichia coli
Non−typhoidal Salmonella
Staphylococcus aureus
Campylobacter spp.
Enterococcus faecium
Enterococcus faecalis
Third generation cephalosporins
Fourth generation cephalosporins
Fluoroquinolones
Macrolides
Quinolones
Polymyxins
Aminoglycosides
Aminopenicillins
Aminopenicillins with beta−lactamase inhibitors
Ansamycins
Phosphonic acid derivatives
Shared human and animal antimicrobial classes
Cattle
Chickens
Pigs
Sheep
Turkeys
Ducks
Horses
Buffaloes
Goats
Livestock species
Categories
Not prioritized and no data
Not prioritized with data
Prioritized and no data
Prioritized with data

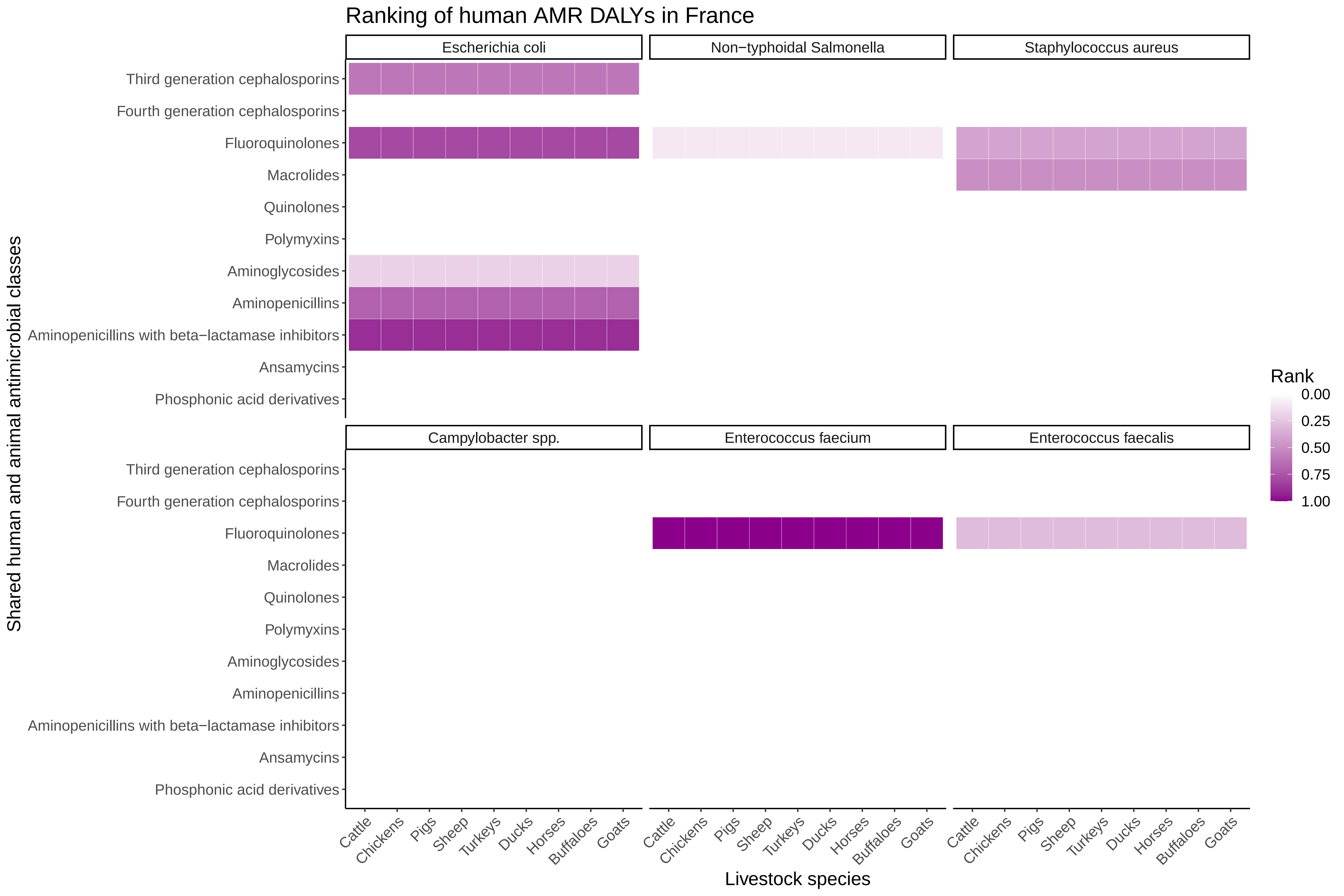

Ranking of human AMR DALYs in France
Escherichia coli
Non−typhoidal Salmonella
Staphylococcus aureus
Campylobacter spp.
Enterococcus faecium
Enterococcus faecalis
Third generation cephalosporins
Fourth generation cephalosporins
Fluoroquinolones
Macrolides
Quinolones
Polymyxins
Aminoglycosides
Aminopenicillins
Aminopenicillins with beta−lactamase inhibitors
Ansamycins
Phosphonic acid derivatives
Shared human and animal antimicrobial classes
Cattle
Chickens
Pigs
Sheep
Turkeys
Ducks
Horses
Buffaloes
Goats
Livestock species
Rank
0.00
0.25
0.50
0.75
1.00

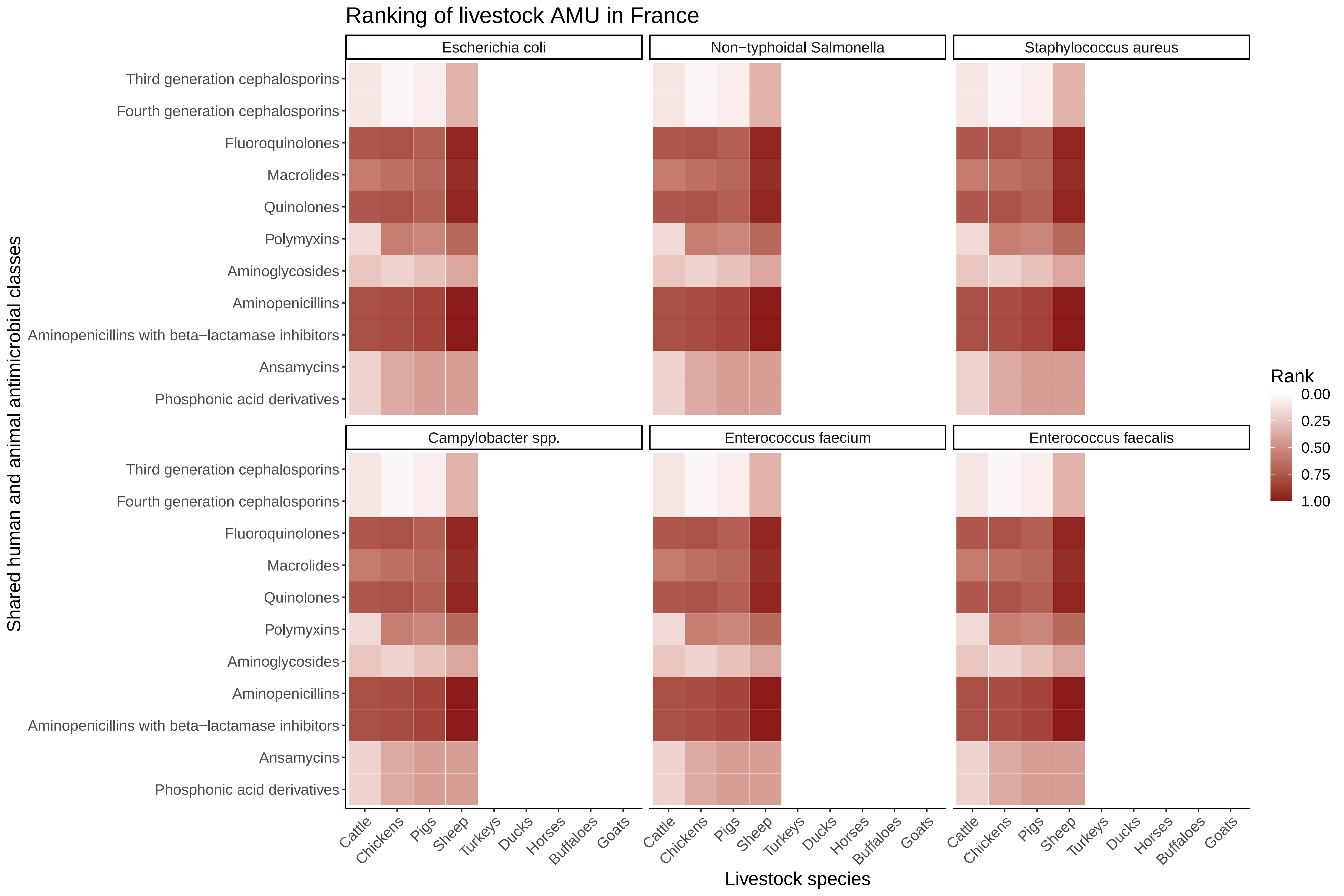

Ranking of livestock AMU in France
Escherichia coli
Non−typhoidal Salmonella
Staphylococcus aureus
Campylobacter spp.
Enterococcus faecium
Enterococcus faecalis
Shared human and animal antimicrobial classes
Third generation cephalosporins
Fourth generation cephalosporins
Fluoroquinolones
Macrolides
Quinolones
Polymyxins
Aminoglycosides
Aminopenicillins
Aminopenicillins with beta−lactamase inhibitors
Ansamycins
Phosphonic acid derivatives
Cattle
Chickens
Pigs
Sheep
Turkeys
Ducks
Horses
Buffaloes
Goats
Livestock species
Rank
0.00
0.25
0.50
0.75
1.00

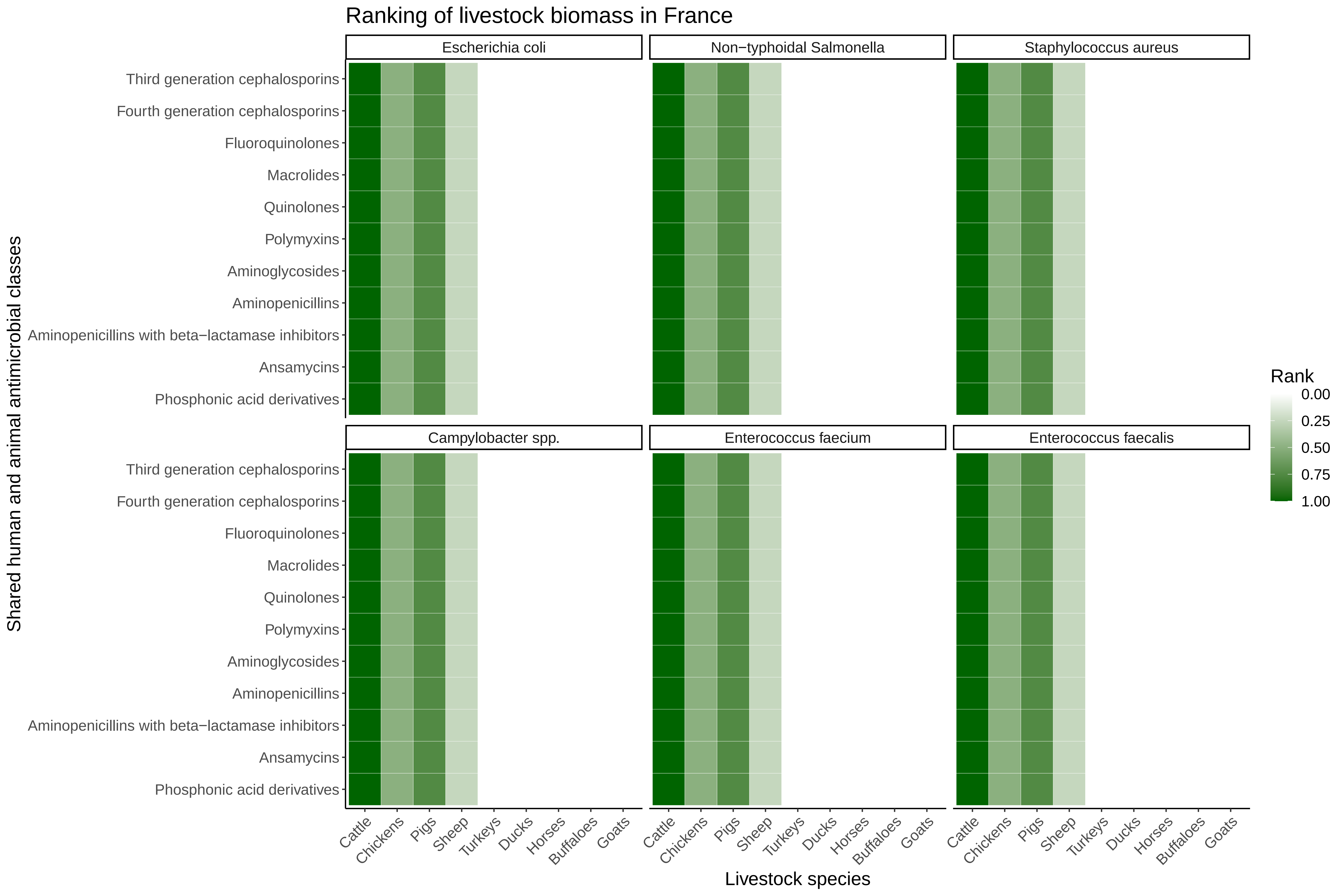

Ranking of livestock biomass in France
Escherichia coli
Non−typhoidal Salmonella
Staphylococcus aureus
Campylobacter spp.
Enterococcus faecium
Enterococcus faecalis
Shared human and animal antimicrobial classes
Third generation cephalosporins
Fourth generation cephalosporins
Fluoroquinolones
Macrolides
Quinolones
Polymyxins
Aminoglycosides
Aminopenicillins
Aminopenicillins with beta−lactamase inhibitors
Ansamycins
Phosphonic acid derivatives
Livestock species
Cattle
Chickens
Pigs
Sheep
Turkeys
Ducks
Horses
Buffaloes
Goats
Rank
0.00
0.25
0.50
0.75
1.00

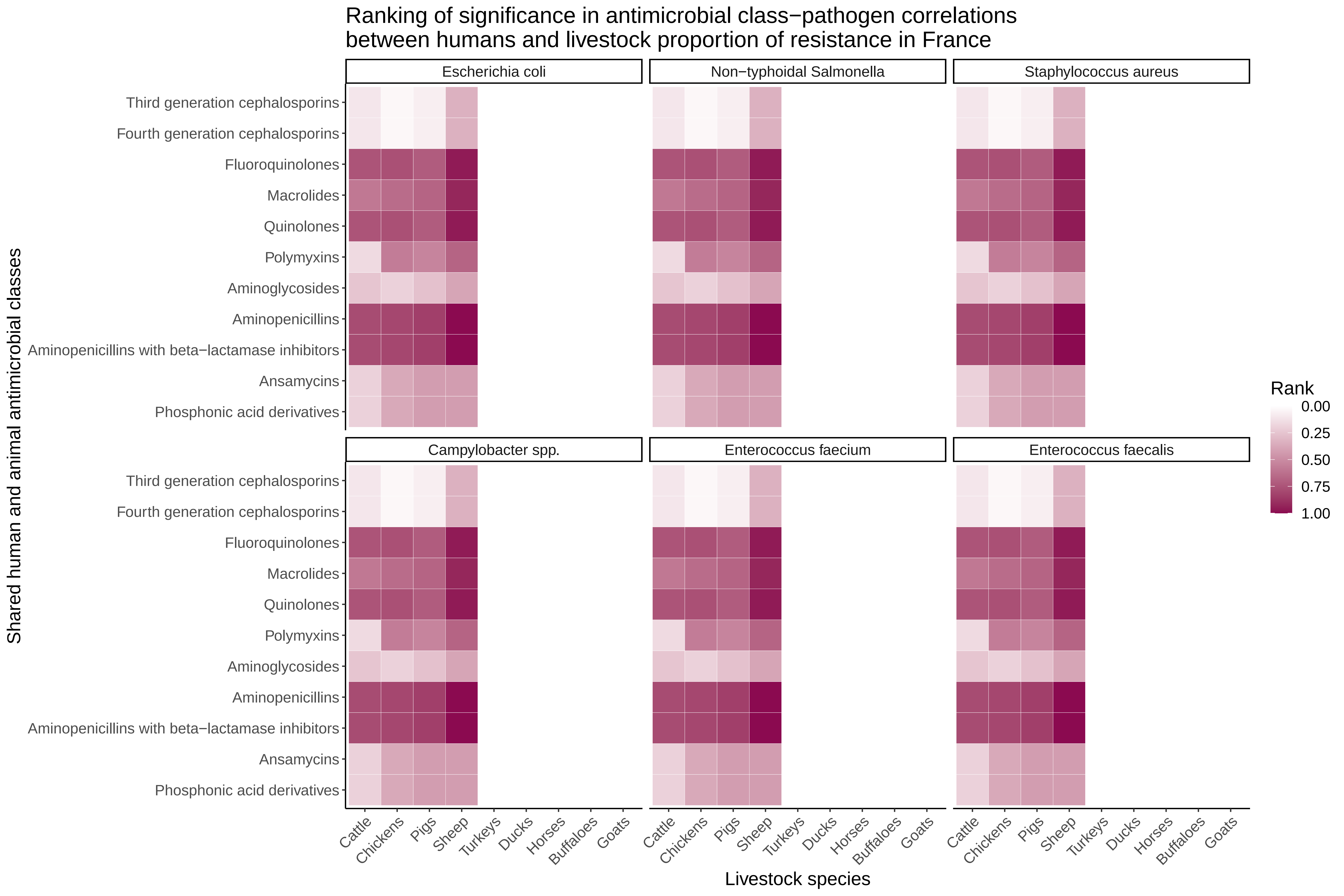

Ranking of significance in antimicrobial class−pathogen correlations
between humans and livestock proportion of resistance in France
Escherichia coli
Non−typhoidal Salmonella
Staphylococcus aureus
Campylobacter spp.
Enterococcus faecium
Enterococcus faecalis
Third generation cephalosporins
Fourth generation cephalosporins
Fluoroquinolones
Macrolides
Quinolones
Polymyxins
Aminoglycosides
Aminopenicillins
Aminopenicillins with beta−lactamase inhibitors
Ansamycins
Phosphonic acid derivatives
Shared human and animal antimicrobial classes
Cattle
Chickens
Pigs
Sheep
Turkeys
Ducks
Horses
Buffaloes
Goats
Livestock species
Rank
0.00
0.25
0.50
0.75
1.00

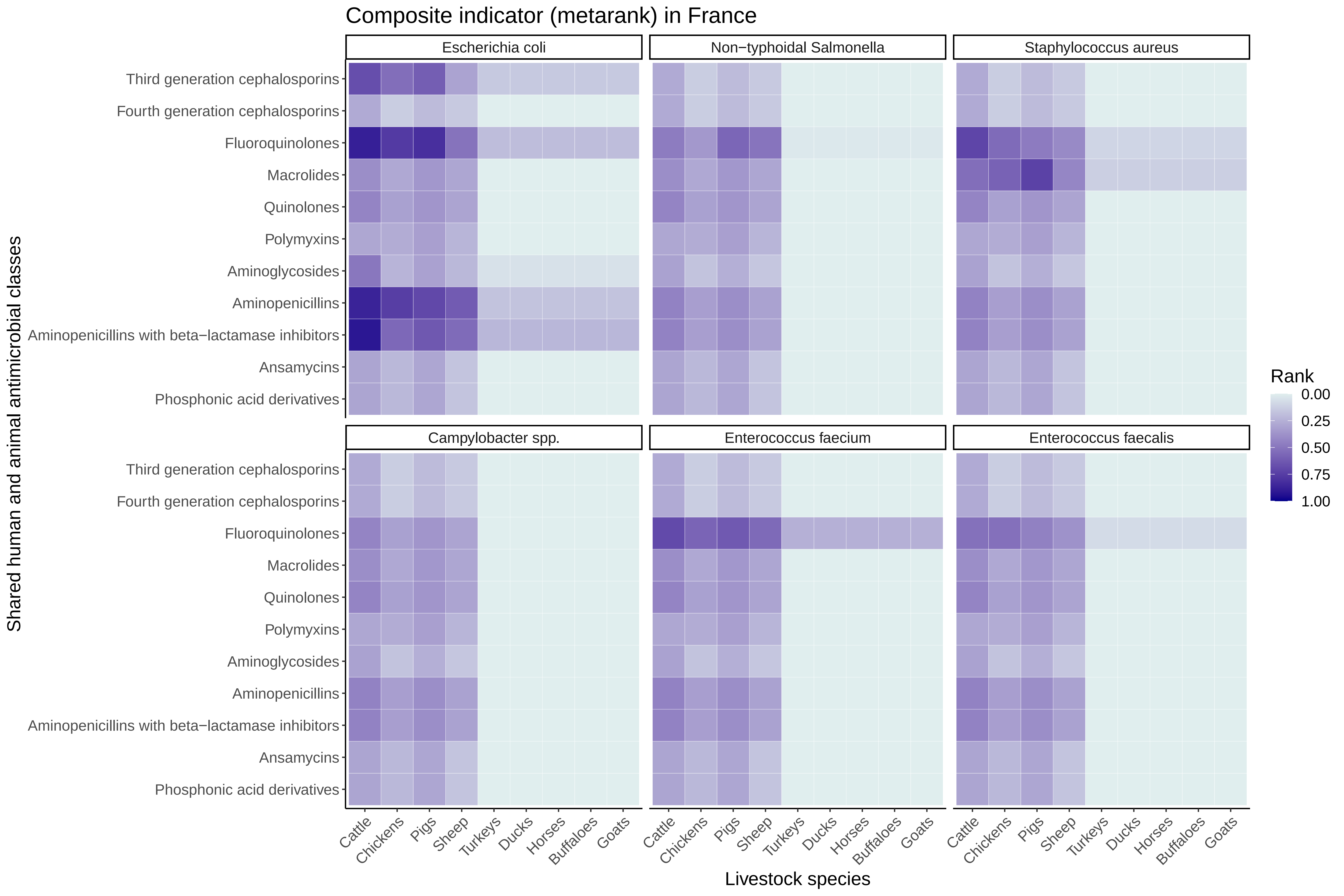

Composite indicator (metarank) in France
Escherichia coli
Non−typhoidal Salmonella
Staphylococcus aureus
Campylobacter spp.
Enterococcus faecium
Enterococcus faecalis
Shared human and animal antimicrobial classes
Third generation cephalosporins
Fourth generation cephalosporins
Fluoroquinolones
Macrolides
Quinolones
Polymyxins
Aminoglycosides
Aminopenicillins
Aminopenicillins with beta−lactamase inhibitors
Ansamycins
Phosphonic acid derivatives
Livestock species
Cattle
Chickens
Pigs
Sheep
Turkeys
Ducks
Horses
Buffaloes
Goats
Rank
0.00
0.25
0.50
0.75
1.00

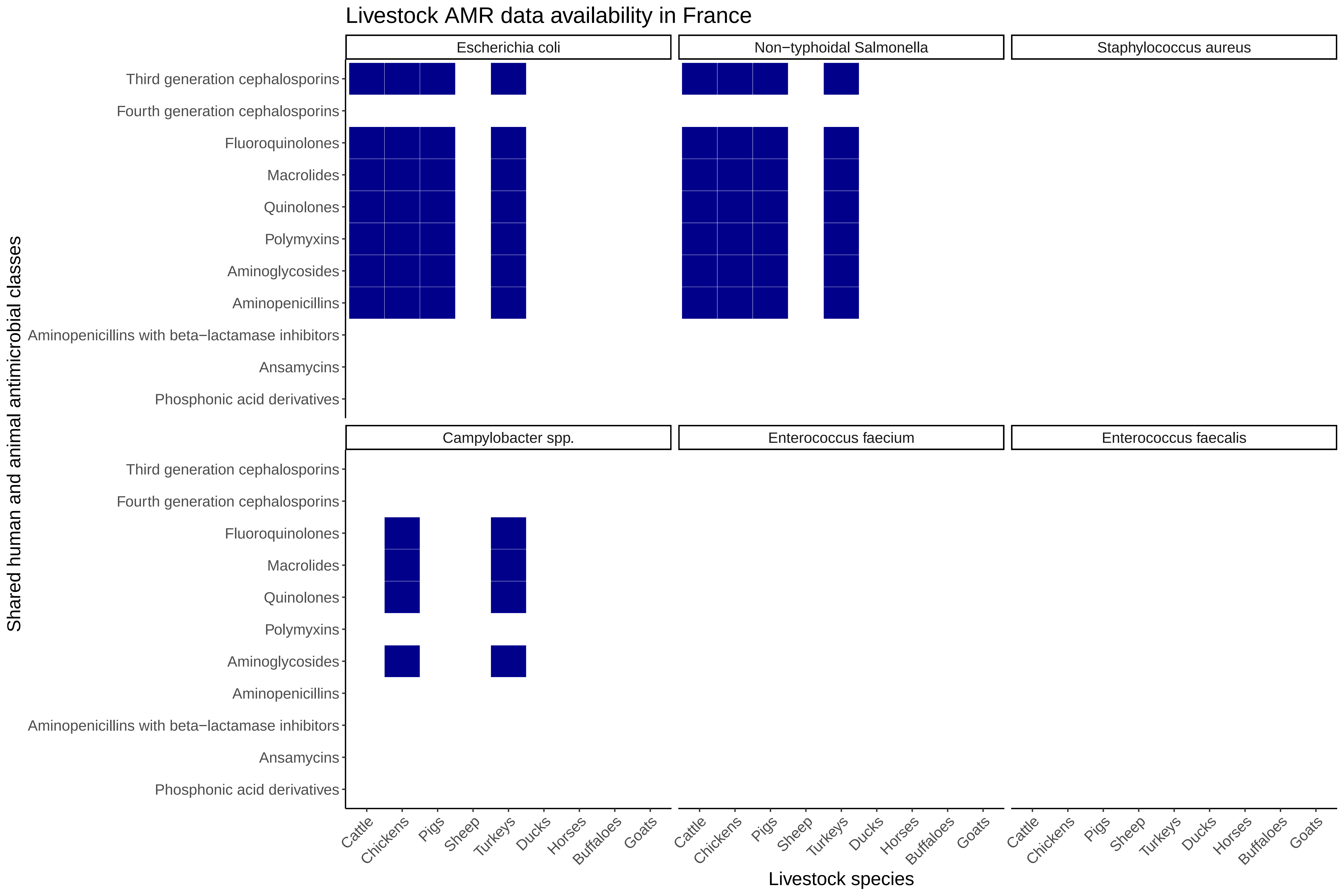

Livestock AMR data availability in France
Escherichia coli
Non−typhoidal Salmonella
Staphylococcus aureus
Campylobacter spp.
Enterococcus faecium
Enterococcus faecalis
Third generation cephalosporins
Fourth generation cephalosporins
Fluoroquinolones
Macrolides
Quinolones
Polymyxins
Aminoglycosides
Aminopenicillins
Aminopenicillins with beta−lactamase inhibitors
Ansamycins
Phosphonic acid derivatives
Shared human and animal antimicrobial classes
Cattle
Chickens
Pigs
Sheep
Turkeys
Ducks
Horses
Buffaloes
Goats
Livestock species

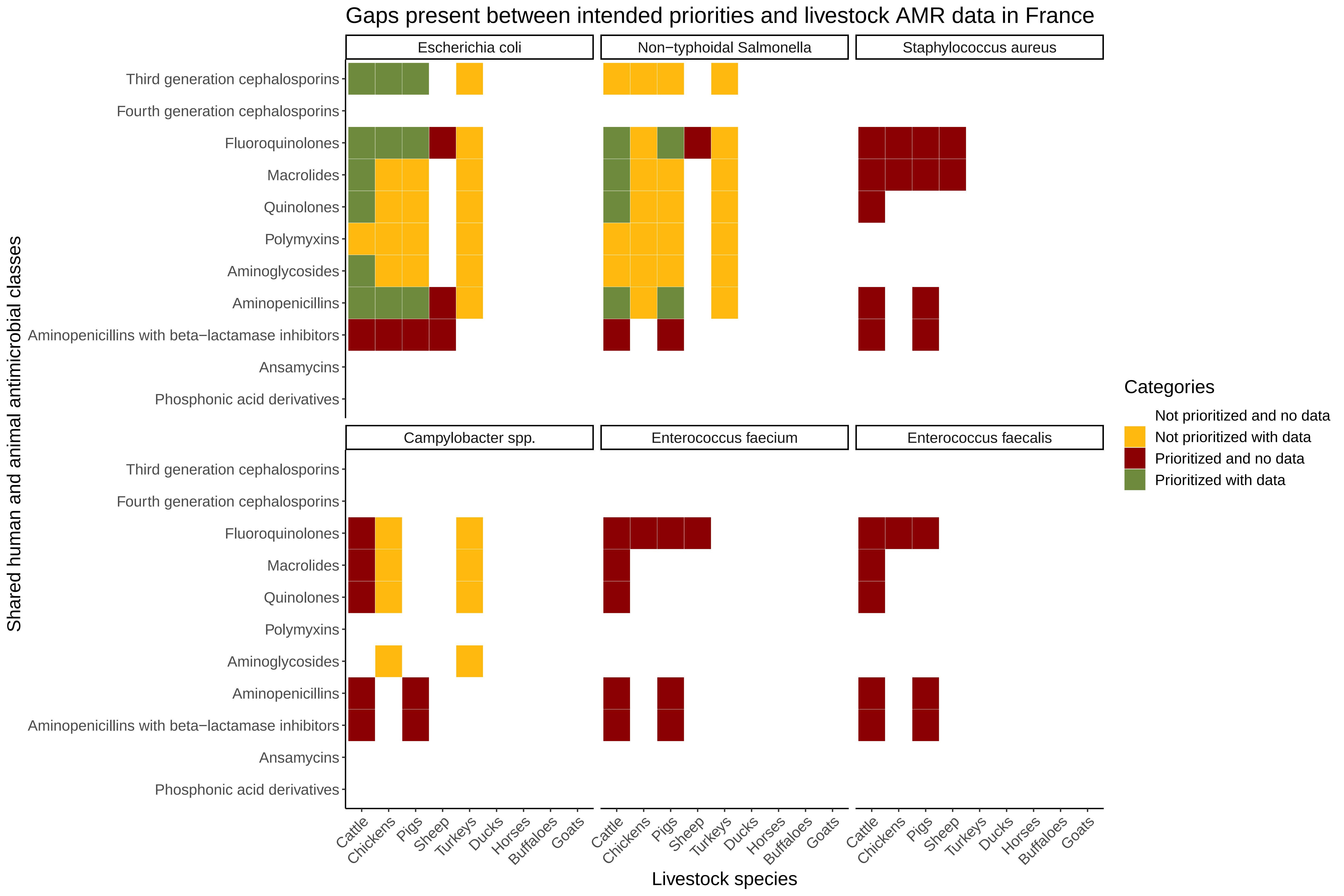

Gaps present between intended priorities and livestock AMR data in France
Escherichia coli
Non−typhoidal Salmonella
Staphylococcus aureus
Campylobacter spp.
Enterococcus faecium
Enterococcus faecalis
Third generation cephalosporins
Fourth generation cephalosporins
Fluoroquinolones
Macrolides
Quinolones
Polymyxins
Aminoglycosides
Aminopenicillins
Aminopenicillins with beta−lactamase inhibitors
Ansamycins
Phosphonic acid derivatives
Shared human and animal antimicrobial classes
Cattle
Chickens
Pigs
Sheep
Turkeys
Ducks
Horses
Buffaloes
Goats
Livestock species
Categories
Not prioritized and no data
Not prioritized with data
Prioritized and no data
Prioritized with data

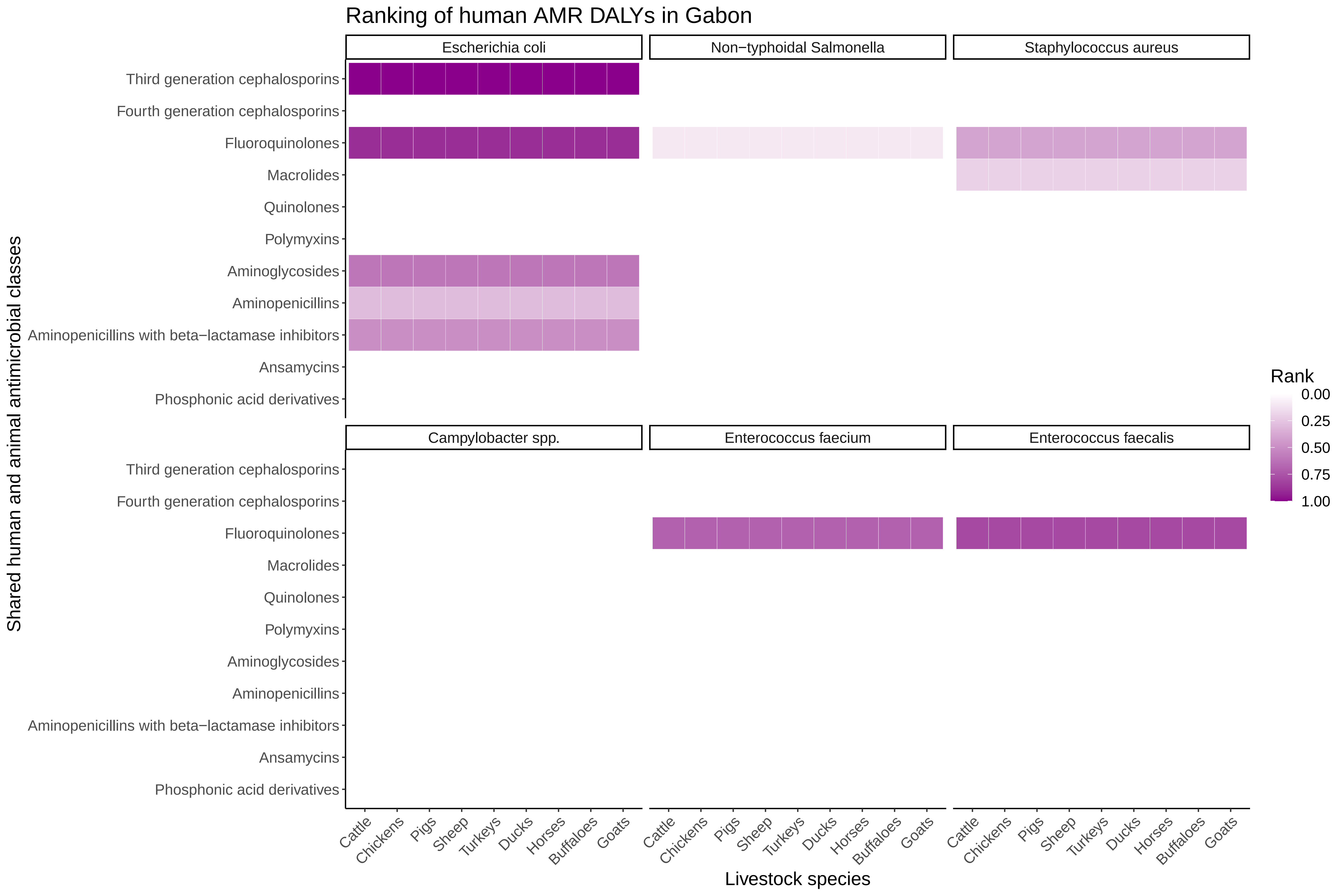
Ranking of human AMR DALYs in Gabon
Escherichia coli
Non−typhoidal Salmonella
Staphylococcus aureus
Campylobacter spp.
Enterococcus faecium
Enterococcus faecalis
Third generation cephalosporins
Fourth generation cephalosporins
Fluoroquinolones
Macrolides
Quinolones
Polymyxins
Aminoglycosides
Aminopenicillins
Aminopenicillins with beta−lactamase inhibitors
Ansamycins
Phosphonic acid derivatives
Shared human and animal antimicrobial classes
Cattle
Chickens
Pigs
Sheep
Turkeys
Ducks
Horses
Buffaloes
Goats
Livestock species
Rank
0.00
0.25
0.50
0.75
1.00

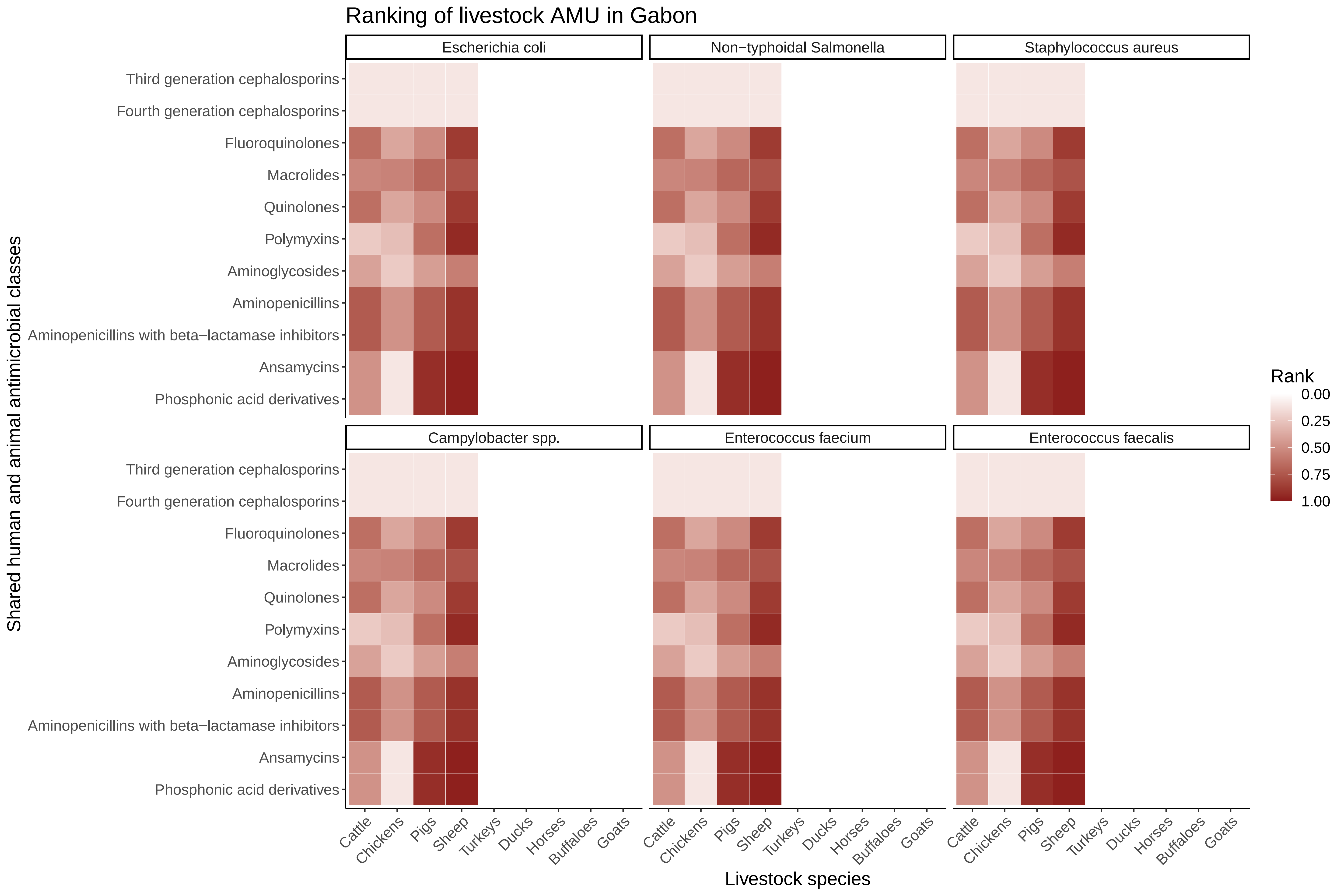

Ranking of livestock AMU in Gabon
Escherichia coli
Non−typhoidal Salmonella
Staphylococcus aureus
Campylobacter spp.
Enterococcus faecium
Enterococcus faecalis
Third generation cephalosporins
Fourth generation cephalosporins
Fluoroquinolones
Macrolides
Quinolones
Polymyxins
Aminoglycosides
Aminopenicillins
Aminopenicillins with beta−lactamase inhibitors
Ansamycins
Phosphonic acid derivatives
Shared human and animal antimicrobial classes
Cattle
Chickens
Pigs
Sheep
Turkeys
Ducks
Horses
Buffaloes
Goats
Livestock species
Rank
0.00
0.25
0.50
0.75
1.00

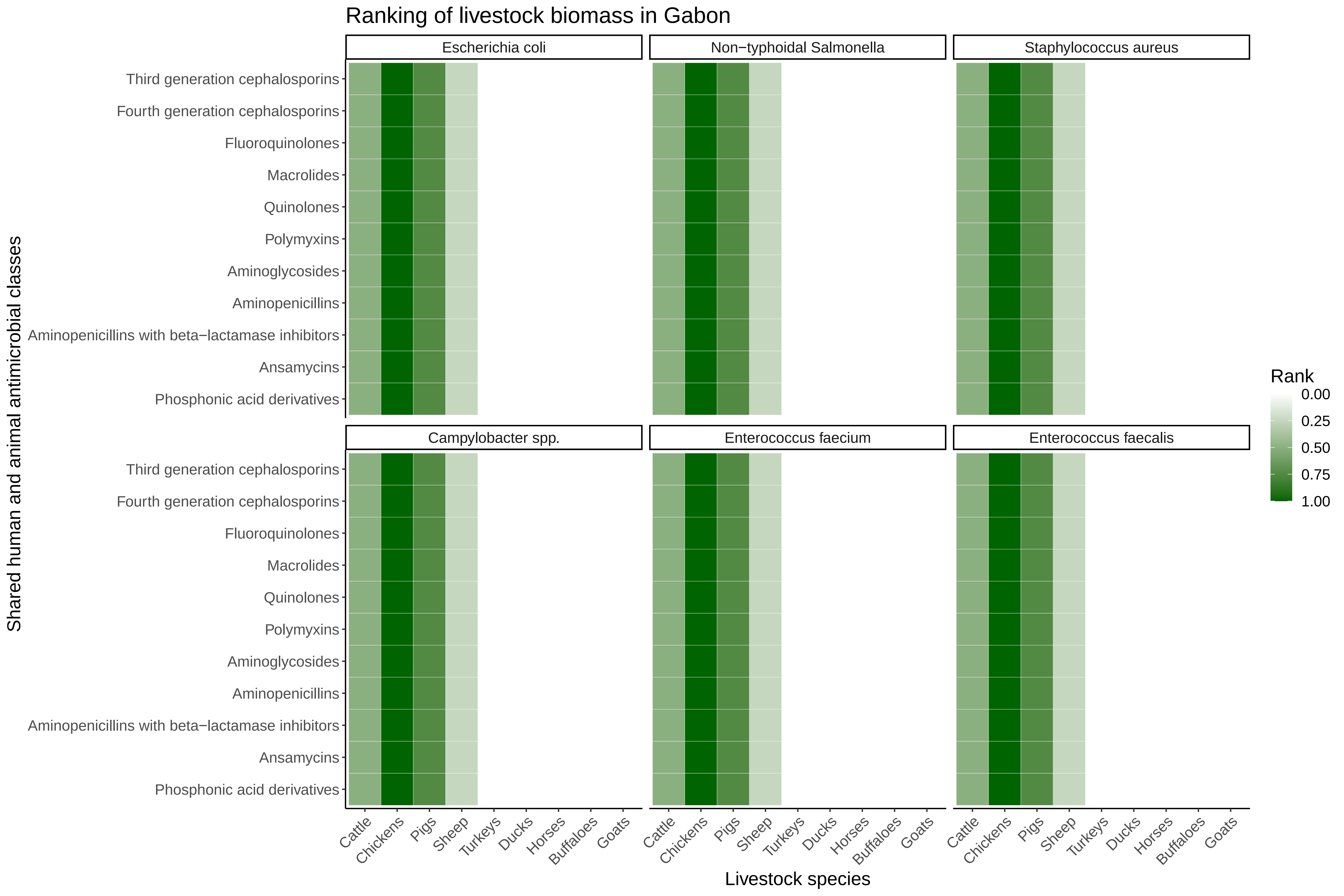

Ranking of livestock biomass in Gabon
Escherichia coli
Non−typhoidal Salmonella
Staphylococcus aureus
Campylobacter spp.
Enterococcus faecium
Enterococcus faecalis
Shared human and animal antimicrobial classes
Third generation cephalosporins
Fourth generation cephalosporins
Fluoroquinolones
Macrolides
Quinolones
Polymyxins
Aminoglycosides
Aminopenicillins
Aminopenicillins with beta−lactamase inhibitors
Ansamycins
Phosphonic acid derivatives
Livestock species
Cattle
Chickens
Pigs
Sheep
Turkeys
Ducks
Horses
Buffaloes
Goats
Rank
0.00
0.25
0.50
0.75
1.00

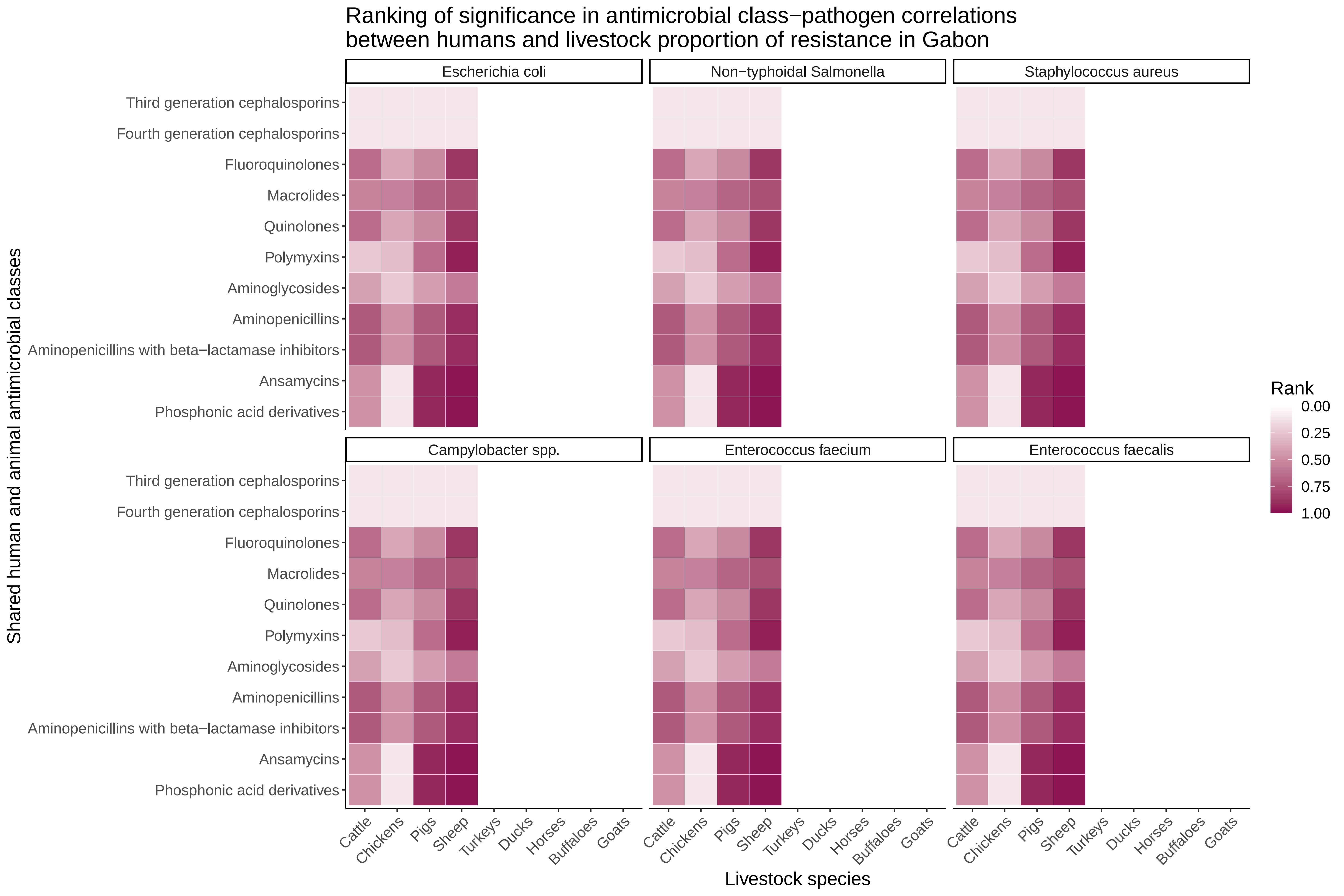

Ranking of significance in antimicrobial class−pathogen correlations
between humans and livestock proportion of resistance in Gabon
Escherichia coli
Non−typhoidal Salmonella
Staphylococcus aureus
Campylobacter spp.
Enterococcus faecium
Enterococcus faecalis
Shared human and animal antimicrobial classes
Third generation cephalosporins
Fourth generation cephalosporins
Fluoroquinolones
Macrolides
Quinolones
Polymyxins
Aminoglycosides
Aminopenicillins
Aminopenicillins with beta−lactamase inhibitors
Ansamycins
Phosphonic acid derivatives
Livestock species
Cattle
Chickens
Pigs
Sheep
Turkeys
Ducks
Horses
Buffaloes
Goats
Rank
0.00
0.25
0.50
0.75
1.00

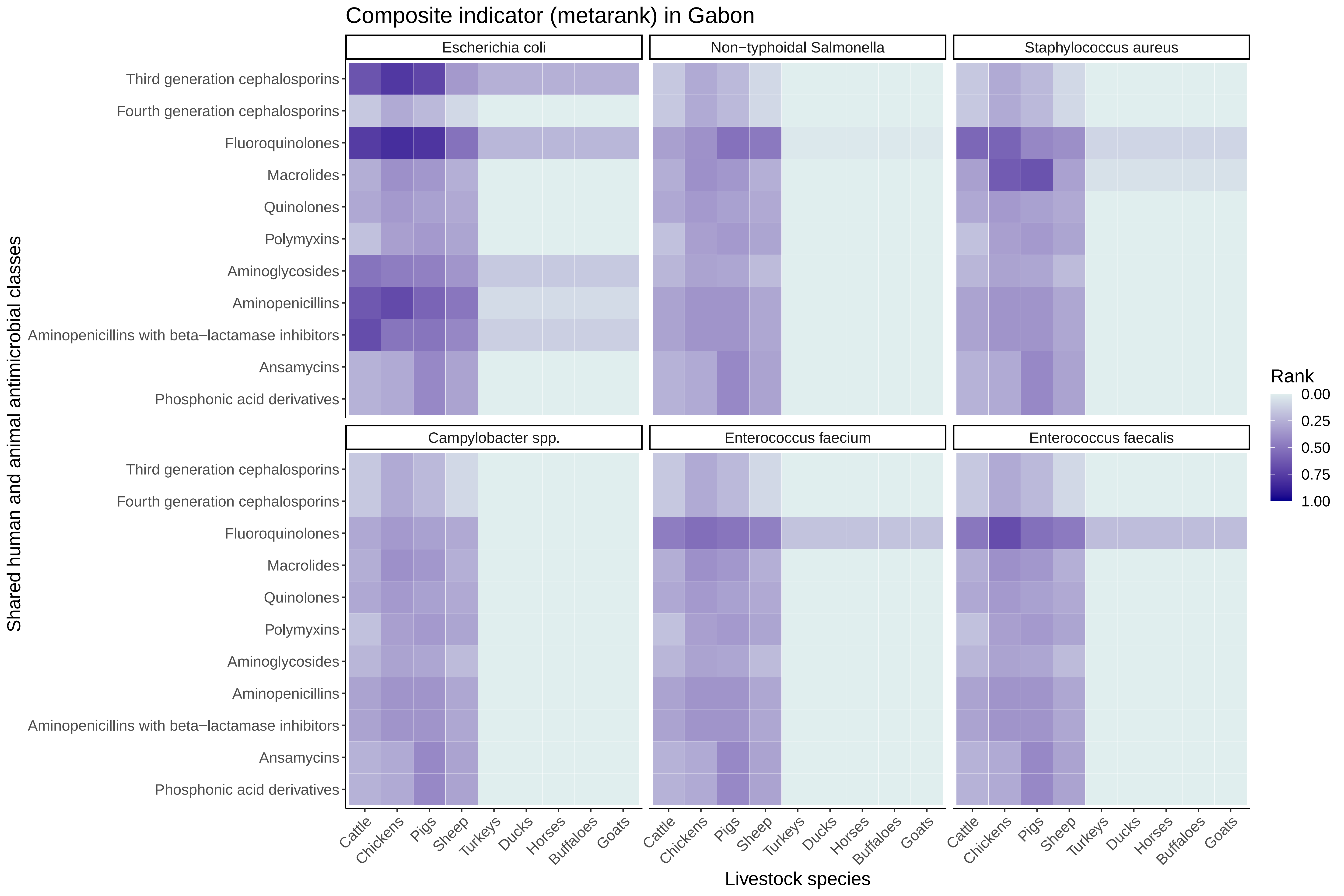

Composite indicator (metarank) in Gabon
Escherichia coli
Non−typhoidal Salmonella
Staphylococcus aureus
Campylobacter spp.
Enterococcus faecium
Enterococcus faecalis
Third generation cephalosporins
Fourth generation cephalosporins
Fluoroquinolones
Macrolides
Quinolones
Polymyxins
Aminoglycosides
Aminopenicillins
Aminopenicillins with beta−lactamase inhibitors
Ansamycins
Phosphonic acid derivatives
Shared human and animal antimicrobial classes
Cattle
Chickens
Pigs
Sheep
Turkeys
Ducks
Horses
Buffaloes
Goats
Livestock species
Rank
0.00
0.25
0.50
0.75
1.00

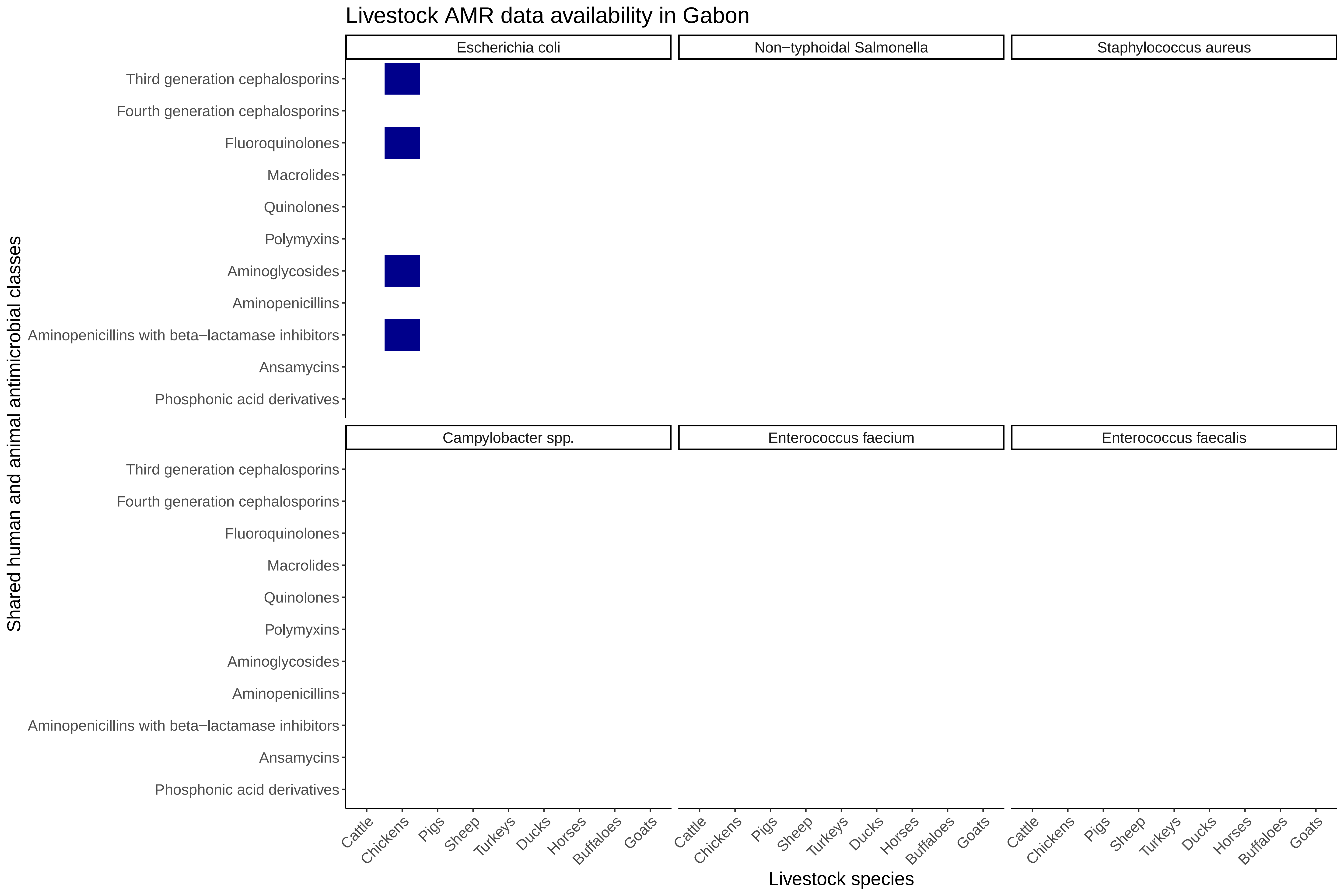
Livestock AMR data availability in Gabon
Escherichia coli
Non−typhoidal Salmonella
Staphylococcus aureus
Campylobacter spp.
Enterococcus faecium
Enterococcus faecalis
Shared human and animal antimicrobial classes
Third generation cephalosporins
Fourth generation cephalosporins
Fluoroquinolones
Macrolides
Quinolones
Polymyxins
Aminoglycosides
Aminopenicillins
Aminopenicillins with beta−lactamase inhibitors
Ansamycins
Phosphonic acid derivatives
Cattle
Chickens
Pigs
Sheep
Turkeys
Ducks
Horses
Buffaloes
Goats
Livestock species

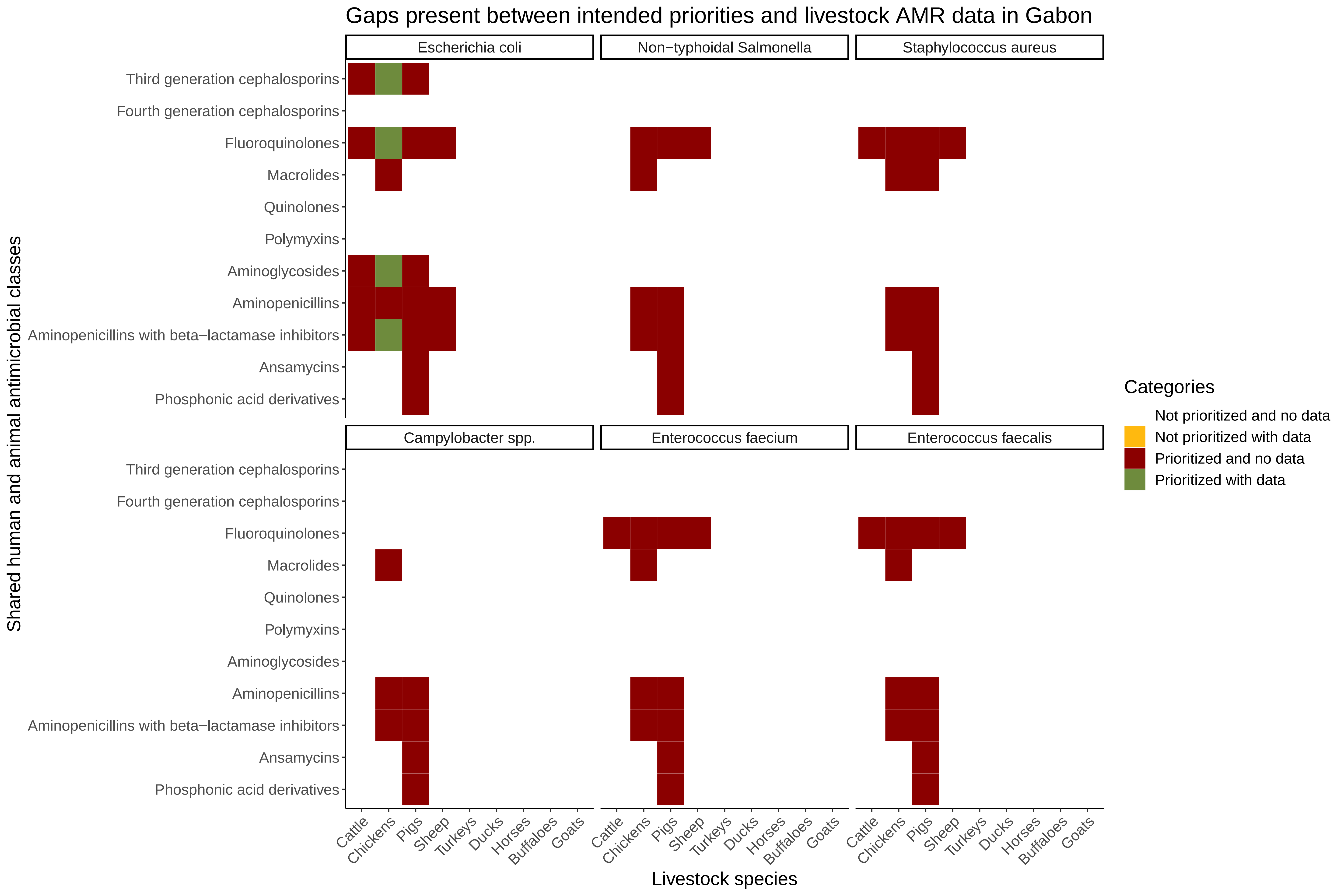

Gaps present between intended priorities and livestock AMR data in Gabon
Escherichia coli
Non−typhoidal Salmonella
Staphylococcus aureus
Campylobacter spp.
Enterococcus faecium
Enterococcus faecalis
Third generation cephalosporins
Fourth generation cephalosporins
Fluoroquinolones
Macrolides
Quinolones
Polymyxins
Aminoglycosides
Aminopenicillins
Aminopenicillins with beta−lactamase inhibitors
Ansamycins
Phosphonic acid derivatives
Shared human and animal antimicrobial classes
Cattle
Chickens
Pigs
Sheep
Turkeys
Ducks
Horses
Buffaloes
Goats
Livestock species
Categories
Not prioritized and no data
Not prioritized with data
Prioritized and no data
Prioritized with data

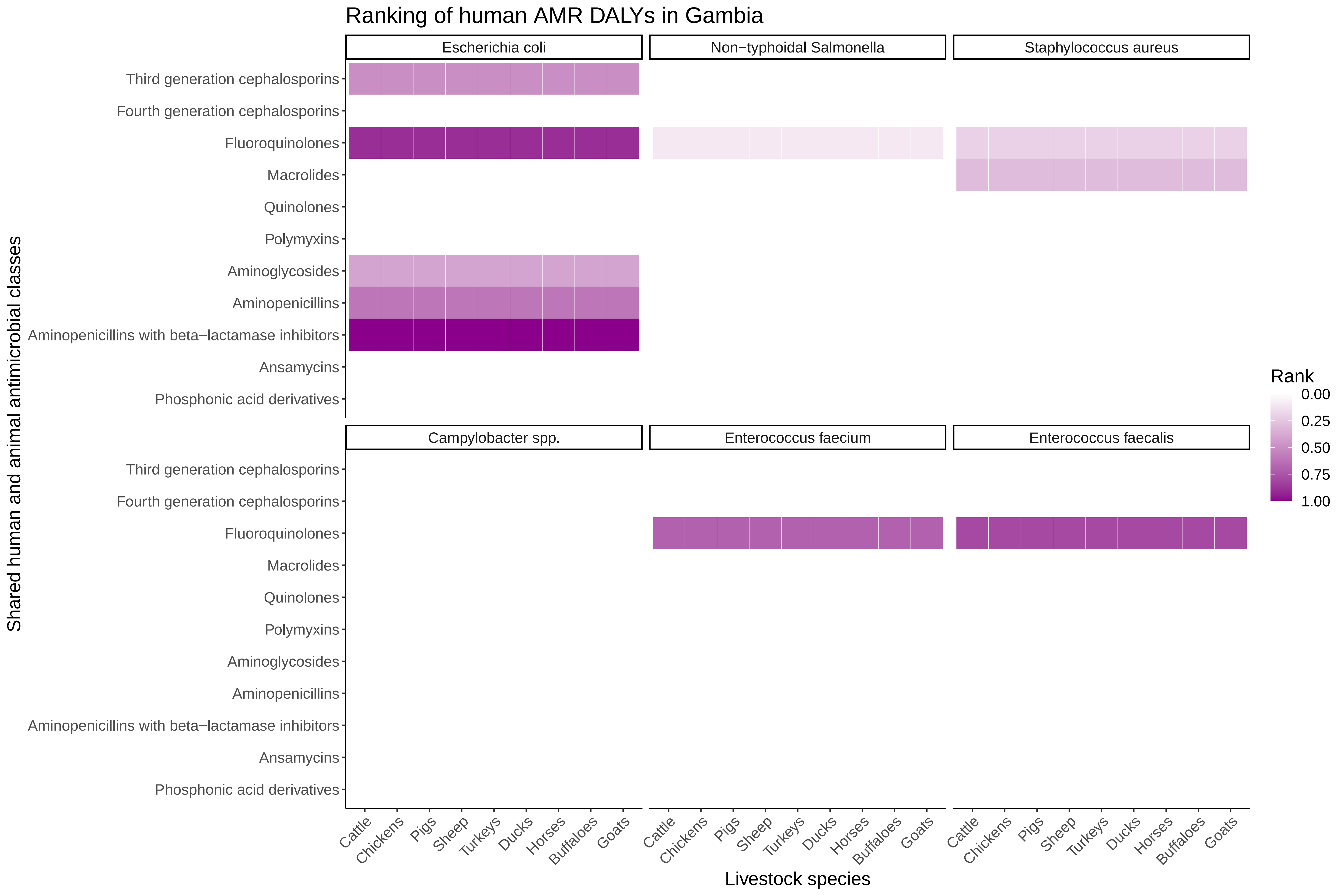

Ranking of human AMR DALYs in Gambia
Escherichia coli
Non−typhoidal Salmonella
Staphylococcus aureus
Campylobacter spp.
Enterococcus faecium
Enterococcus faecalis
Third generation cephalosporins
Fourth generation cephalosporins
Fluoroquinolones
Macrolides
Quinolones
Polymyxins
Aminoglycosides
Aminopenicillins
Aminopenicillins with beta−lactamase inhibitors
Ansamycins
Phosphonic acid derivatives
Shared human and animal antimicrobial classes
Cattle
Chickens
Pigs
Sheep
Turkeys
Ducks
Horses
Buffaloes
Goats
Livestock species
Rank
0.00
0.25
0.50
0.75
1.00

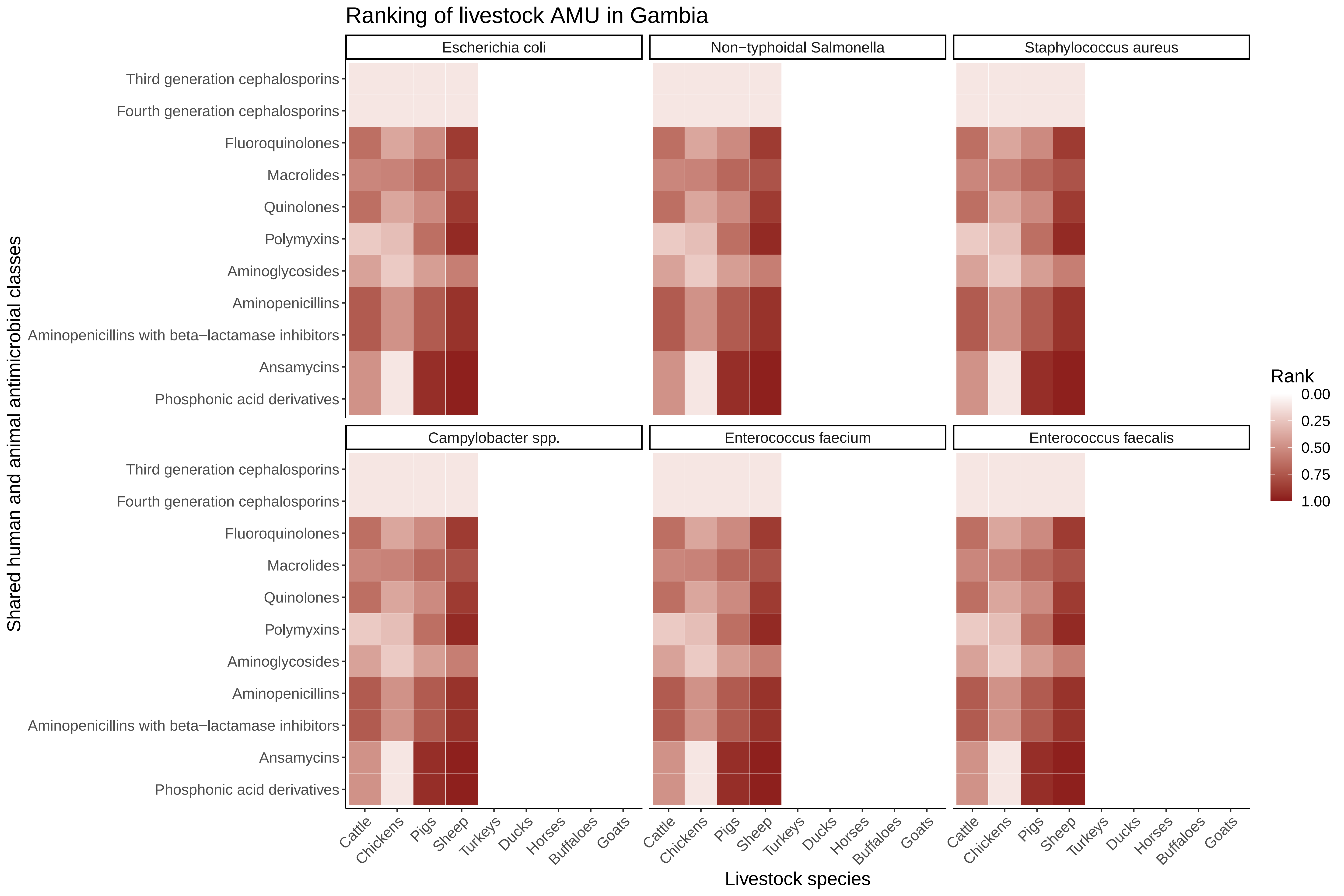

Ranking of livestock AMU in Gambia
Escherichia coli
Non−typhoidal Salmonella
Staphylococcus aureus
Campylobacter spp.
Enterococcus faecium
Enterococcus faecalis
Third generation cephalosporins
Fourth generation cephalosporins
Fluoroquinolones
Macrolides
Quinolones
Polymyxins
Aminoglycosides
Aminopenicillins
Aminopenicillins with beta−lactamase inhibitors
Ansamycins
Phosphonic acid derivatives
Shared human and animal antimicrobial classes
Cattle
Chickens
Pigs
Sheep
Turkeys
Ducks
Horses
Buffaloes
Goats
Livestock species
Rank
0.00
0.25
0.50
0.75
1.00

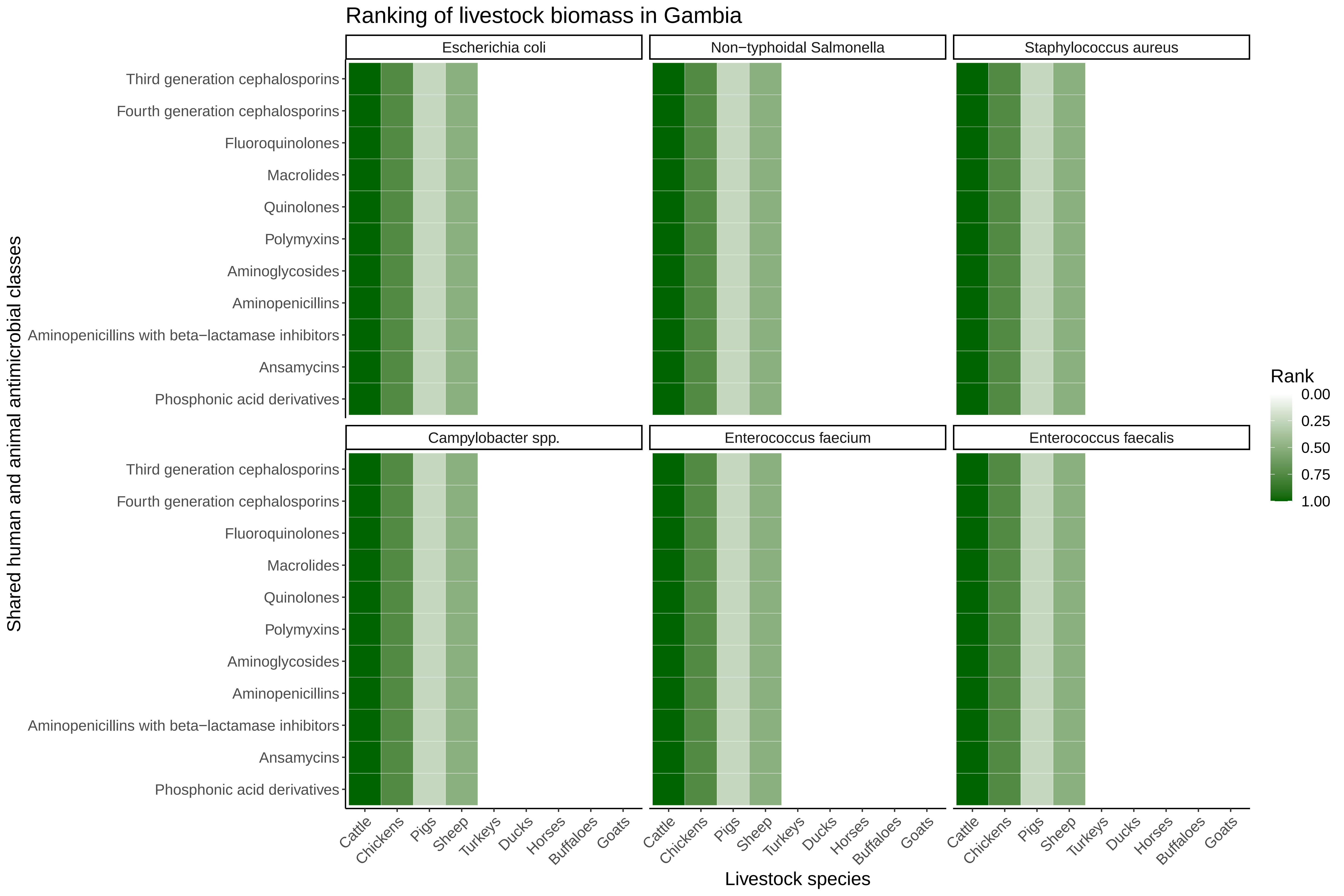

Ranking of livestock biomass in Gambia
Escherichia coli
Non−typhoidal Salmonella
Staphylococcus aureus
Campylobacter spp.
Enterococcus faecium
Enterococcus faecalis
Shared human and animal antimicrobial classes
Third generation cephalosporins
Fourth generation cephalosporins
Fluoroquinolones
Macrolides
Quinolones
Polymyxins
Aminoglycosides
Aminopenicillins
Aminopenicillins with beta−lactamase inhibitors
Ansamycins
Phosphonic acid derivatives
Livestock species
Cattle
Chickens
Pigs
Sheep
Turkeys
Ducks
Horses
Buffaloes
Goats
Rank
0.00
0.25
0.50
0.75
1.00

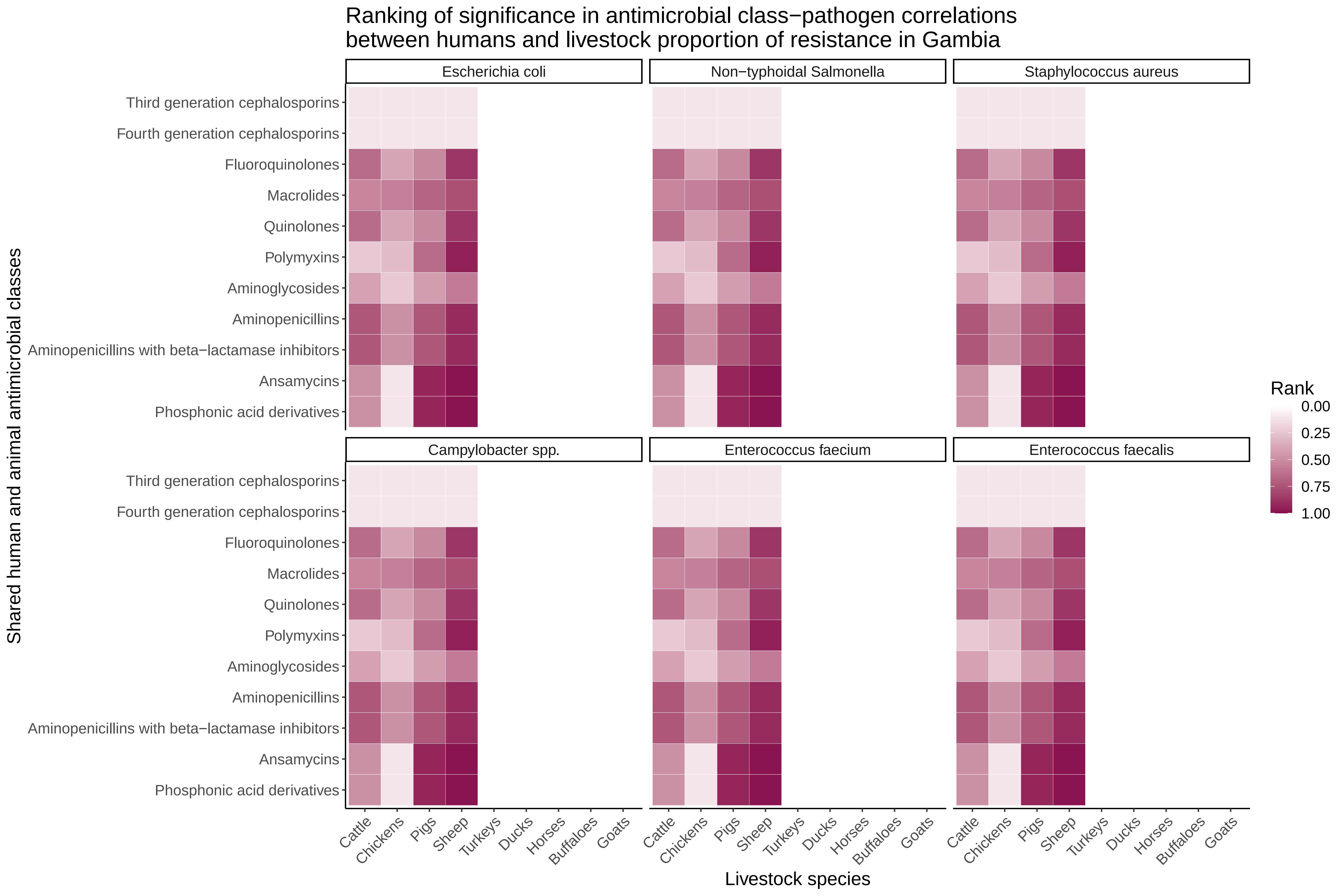
Ranking of significance in antimicrobial class−pathogen correlations
between humans and livestock proportion of resistance in Gambia
Escherichia coli
Non−typhoidal Salmonella
Staphylococcus aureus
Campylobacter spp.
Enterococcus faecium
Enterococcus faecalis
Third generation cephalosporins
Fourth generation cephalosporins
Fluoroquinolones
Macrolides
Quinolones
Polymyxins
Aminoglycosides
Aminopenicillins
Aminopenicillins with beta−lactamase inhibitors
Ansamycins
Phosphonic acid derivatives
Shared human and animal antimicrobial classes
Cattle
Chickens
Pigs
Sheep
Turkeys
Ducks
Horses
Buffaloes
Goats
Livestock species
Rank
0.00
0.25
0.50
0.75
1.00

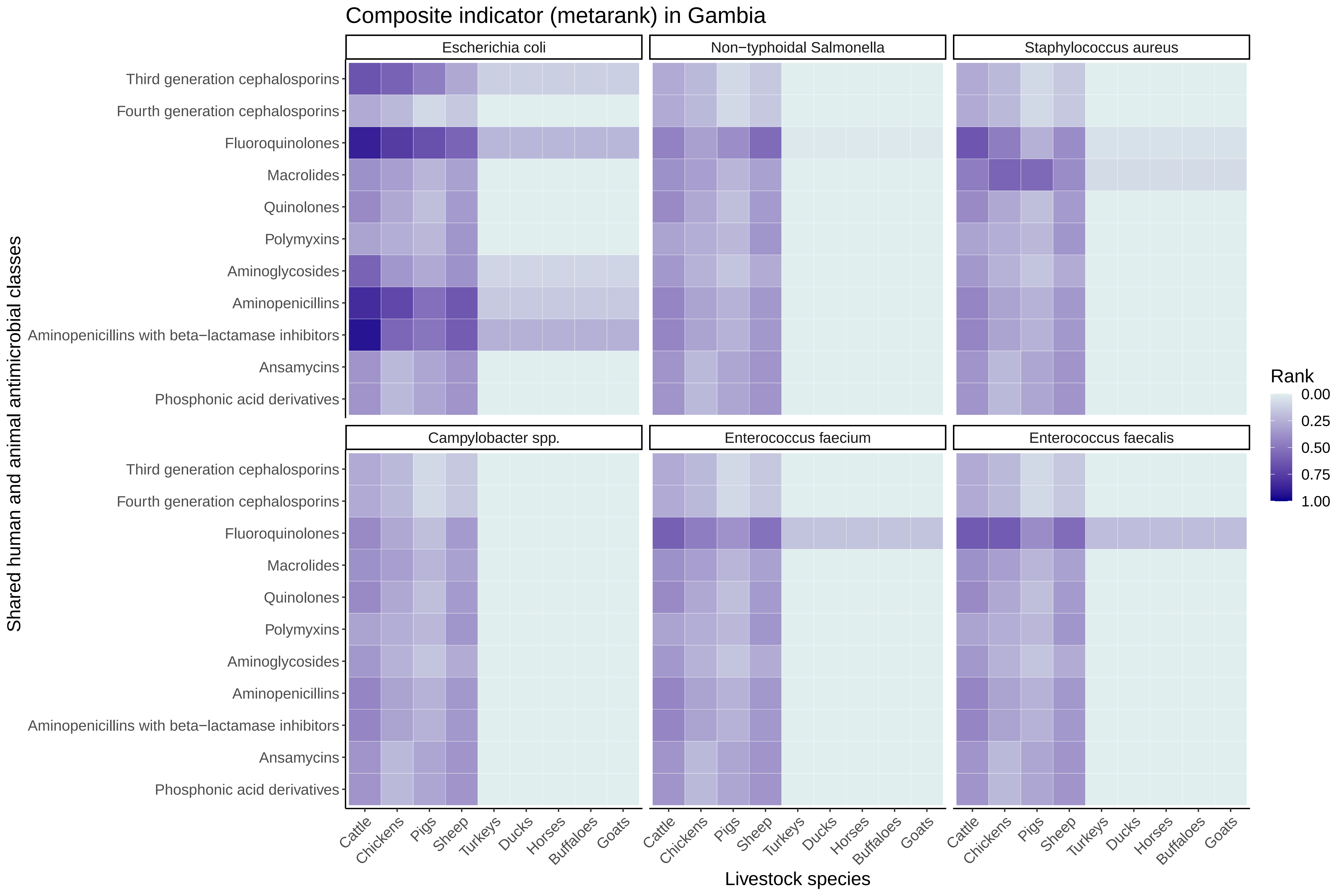
Composite indicator (metarank) in Gambia
Escherichia coli
Non−typhoidal Salmonella
Staphylococcus aureus
Campylobacter spp.
Enterococcus faecium
Enterococcus faecalis
Third generation cephalosporins
Fourth generation cephalosporins
Fluoroquinolones
Macrolides
Quinolones
Polymyxins
Aminoglycosides
Aminopenicillins
Aminopenicillins with beta−lactamase inhibitors
Ansamycins
Phosphonic acid derivatives
Shared human and animal antimicrobial classes
Cattle
Chickens
Pigs
Sheep
Turkeys
Ducks
Horses
Buffaloes
Goats
Livestock species
Rank
0.00
0.25
0.50
0.75
1.00

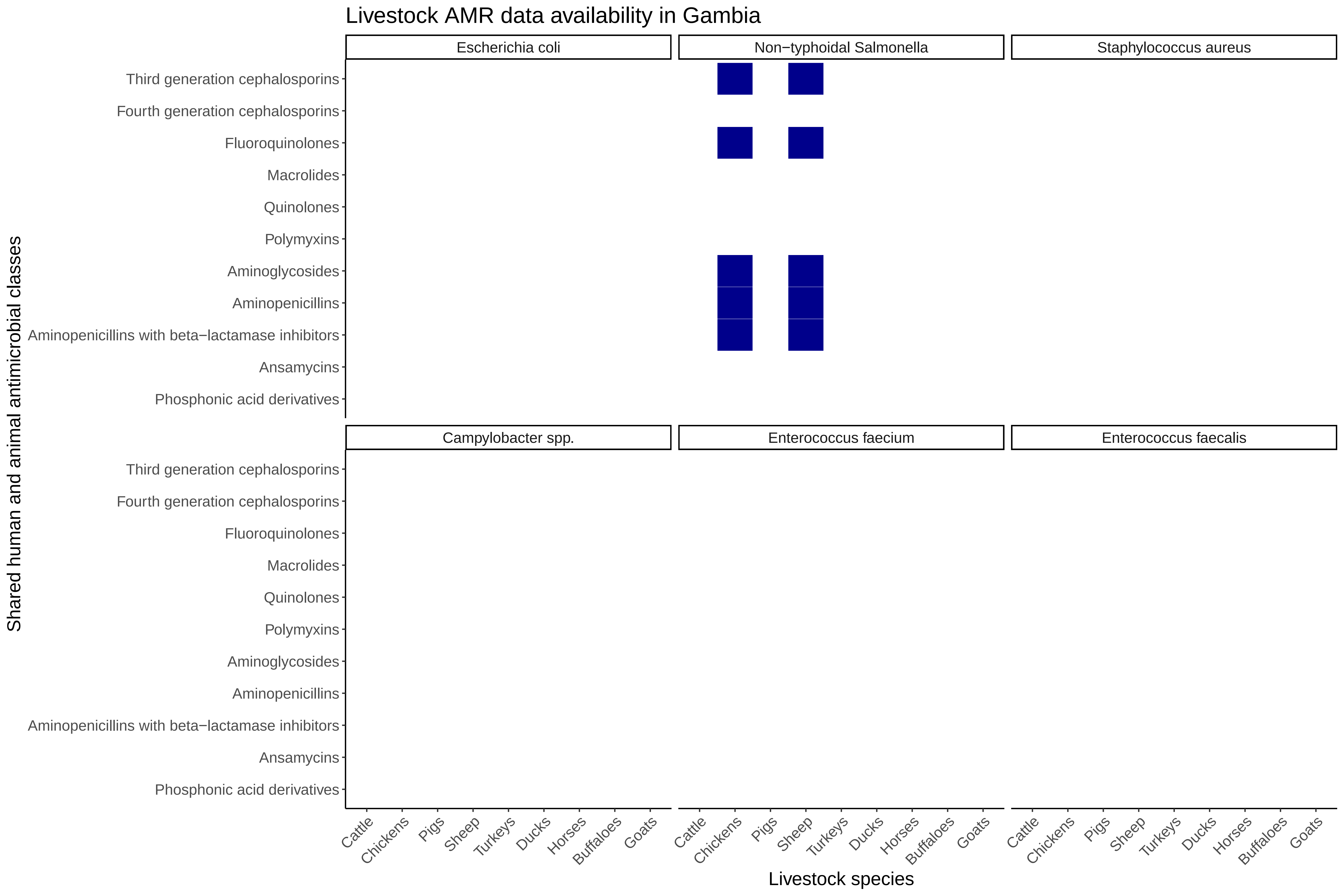

Livestock AMR data availability in Gambia
Escherichia coli
Non−typhoidal Salmonella
Staphylococcus aureus
Campylobacter spp.
Enterococcus faecium
Enterococcus faecalis
Shared human and animal antimicrobial classes
Third generation cephalosporins
Fourth generation cephalosporins
Fluoroquinolones
Macrolides
Quinolones
Polymyxins
Aminoglycosides
Aminopenicillins
Aminopenicillins with beta−lactamase inhibitors
Ansamycins
Phosphonic acid derivatives
Cattle
Chickens
Pigs
Sheep
Turkeys
Ducks
Horses
Buffaloes
Goats
Livestock species

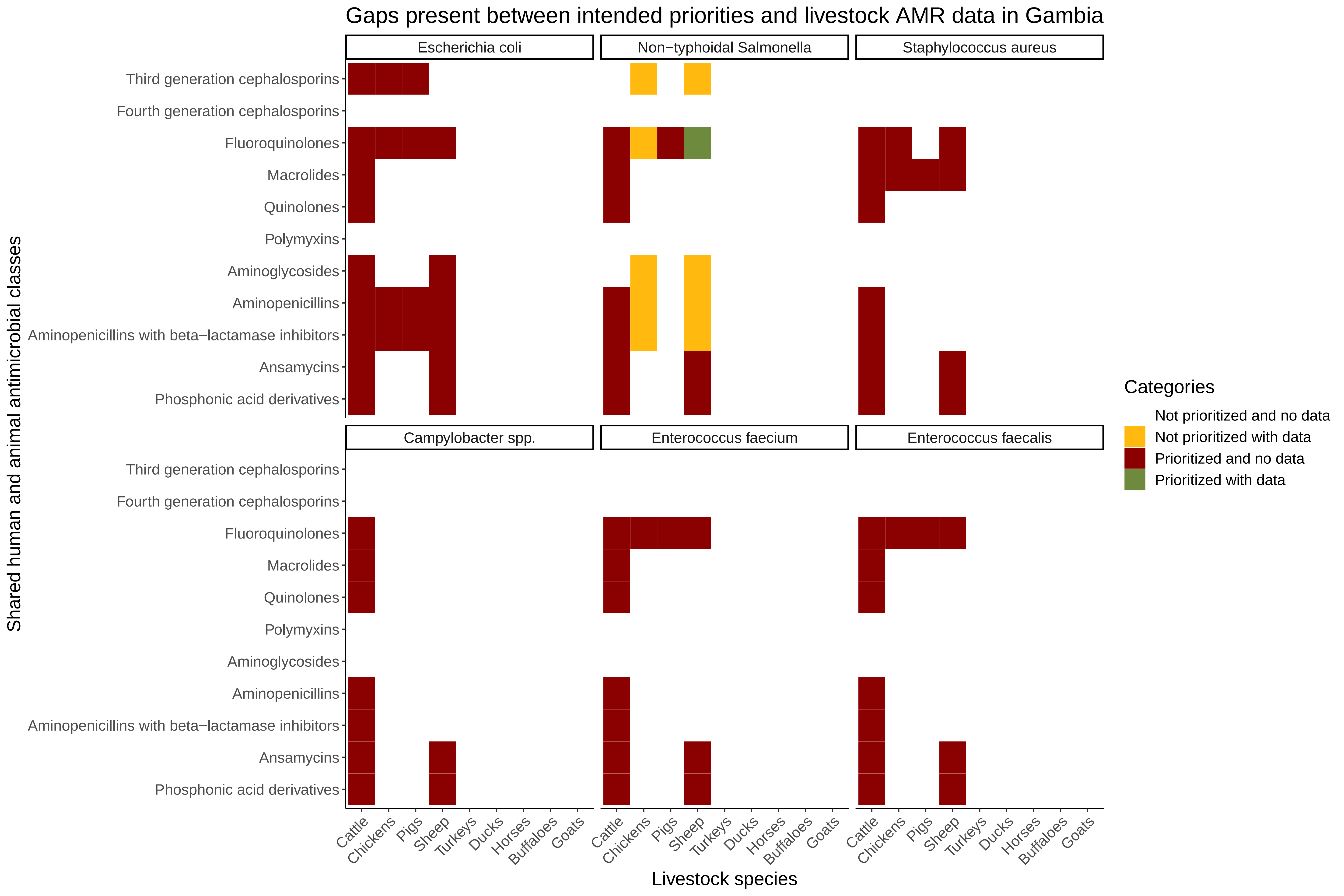
Gaps present between intended priorities and livestock AMR data in Gambia
Escherichia coli
Non−typhoidal Salmonella
Staphylococcus aureus
Campylobacter spp.
Enterococcus faecium
Enterococcus faecalis
Shared human and animal antimicrobial classes
Third generation cephalosporins
Fourth generation cephalosporins
Fluoroquinolones
Macrolides
Quinolones
Polymyxins
Aminoglycosides
Aminopenicillins
Aminopenicillins with beta−lactamase inhibitors
Ansamycins
Phosphonic acid derivatives
Cattle
Chickens
Pigs
Sheep
Turkeys
Ducks
Horses
Buffaloes
Goats
Livestock species
Categories
Not prioritized and no data
Not prioritized with data
Prioritized and no data
Prioritized with data

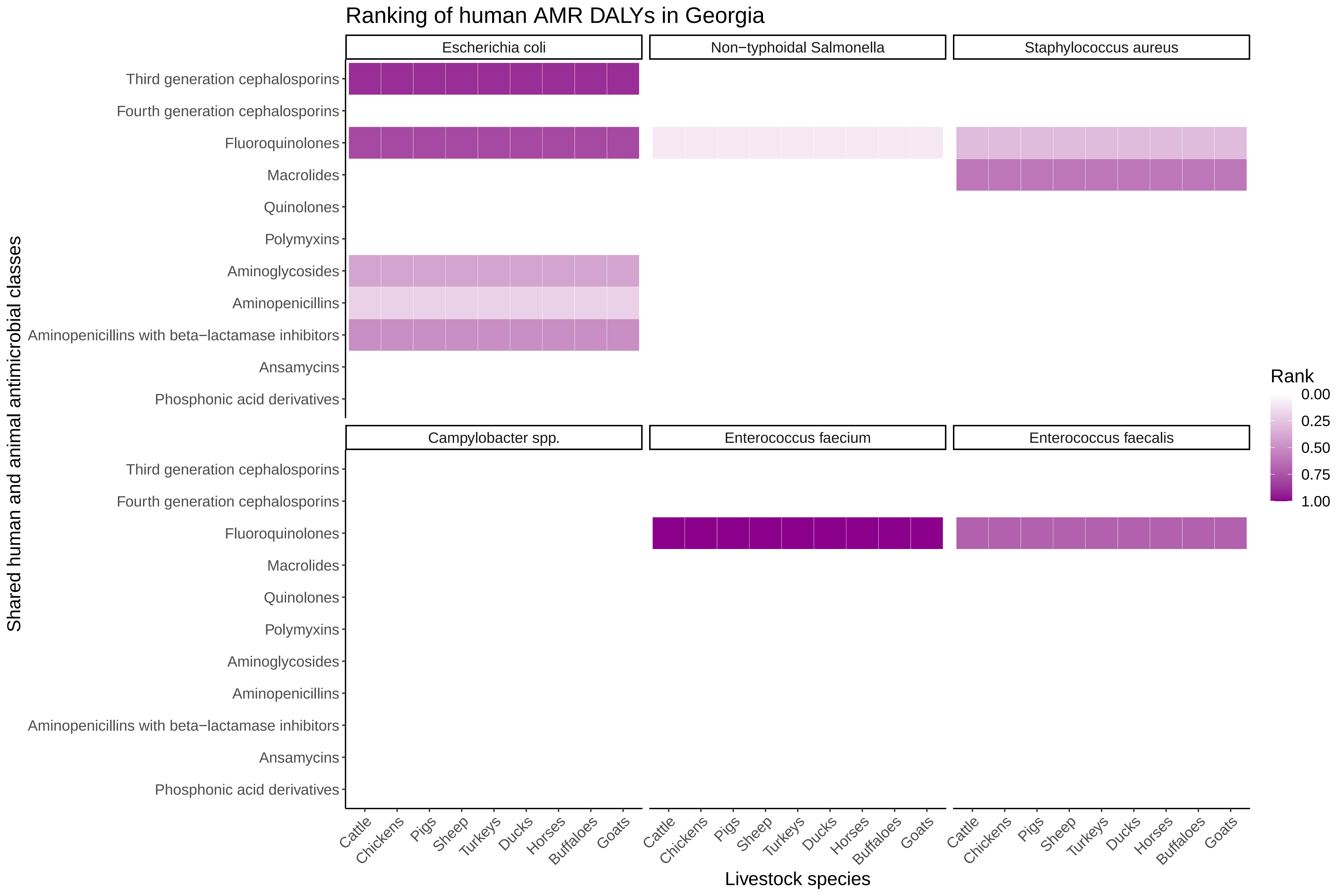
Ranking of human AMR DALYs in Georgia
Escherichia coli
Non−typhoidal Salmonella
Staphylococcus aureus
Campylobacter spp.
Enterococcus faecium
Enterococcus faecalis
Third generation cephalosporins
Fourth generation cephalosporins
Fluoroquinolones
Macrolides
Quinolones
Polymyxins
Aminoglycosides
Aminopenicillins
Aminopenicillins with beta−lactamase inhibitors
Ansamycins
Phosphonic acid derivatives
Shared human and animal antimicrobial classes
Cattle
Chickens
Pigs
Sheep
Turkeys
Ducks
Horses
Buffaloes
Goats
Livestock species
Rank
0.00
0.25
0.50
0.75
1.00

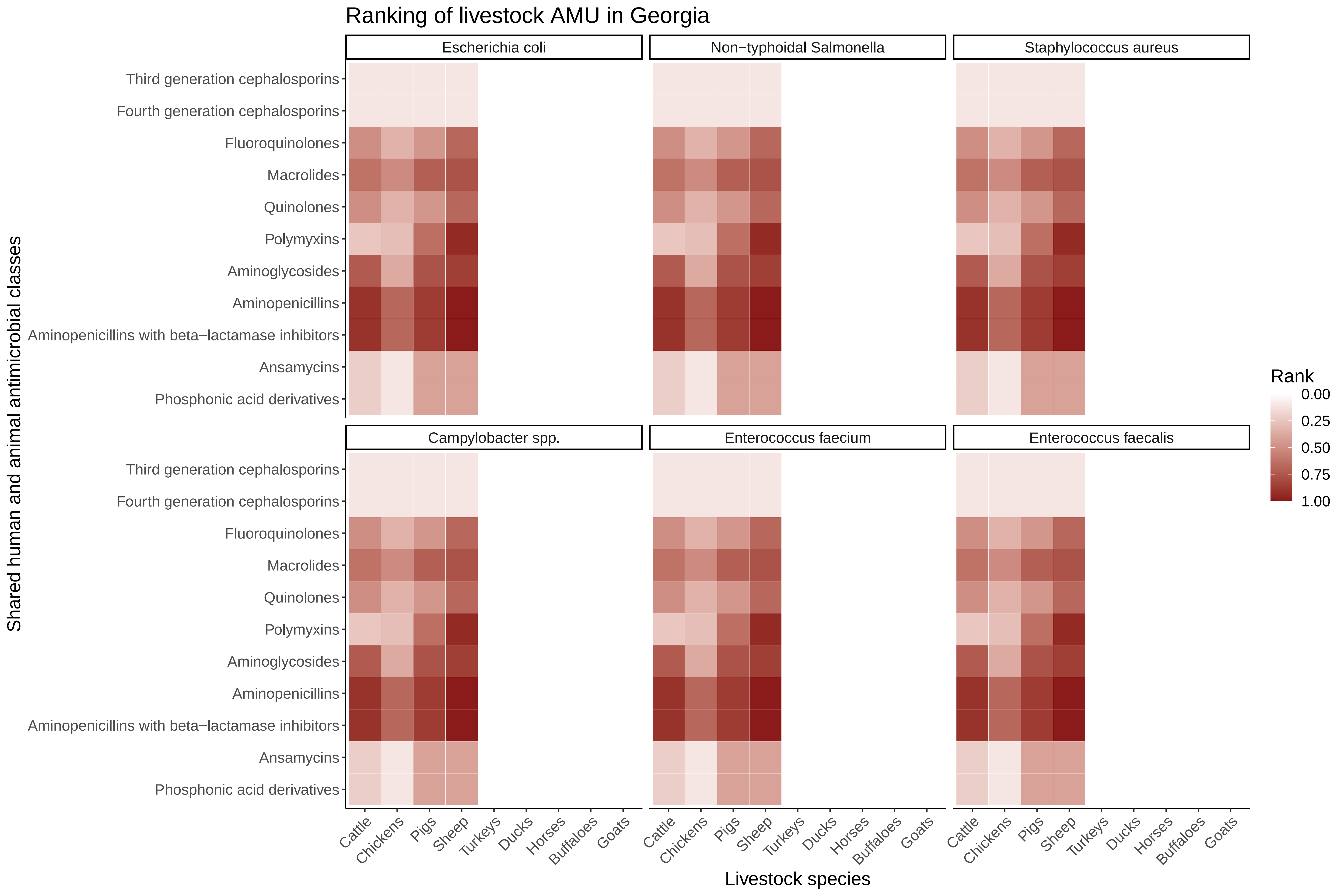

Ranking of livestock AMU in Georgia
Escherichia coli
Non−typhoidal Salmonella
Staphylococcus aureus
Campylobacter spp.
Enterococcus faecium
Enterococcus faecalis
Third generation cephalosporins
Fourth generation cephalosporins
Fluoroquinolones
Macrolides
Quinolones
Polymyxins
Aminoglycosides
Aminopenicillins
Aminopenicillins with beta−lactamase inhibitors
Ansamycins
Phosphonic acid derivatives
Shared human and animal antimicrobial classes
Cattle
Chickens
Pigs
Sheep
Turkeys
Ducks
Horses
Buffaloes
Goats
Livestock species
Rank
0.00
0.25
0.50
0.75
1.00

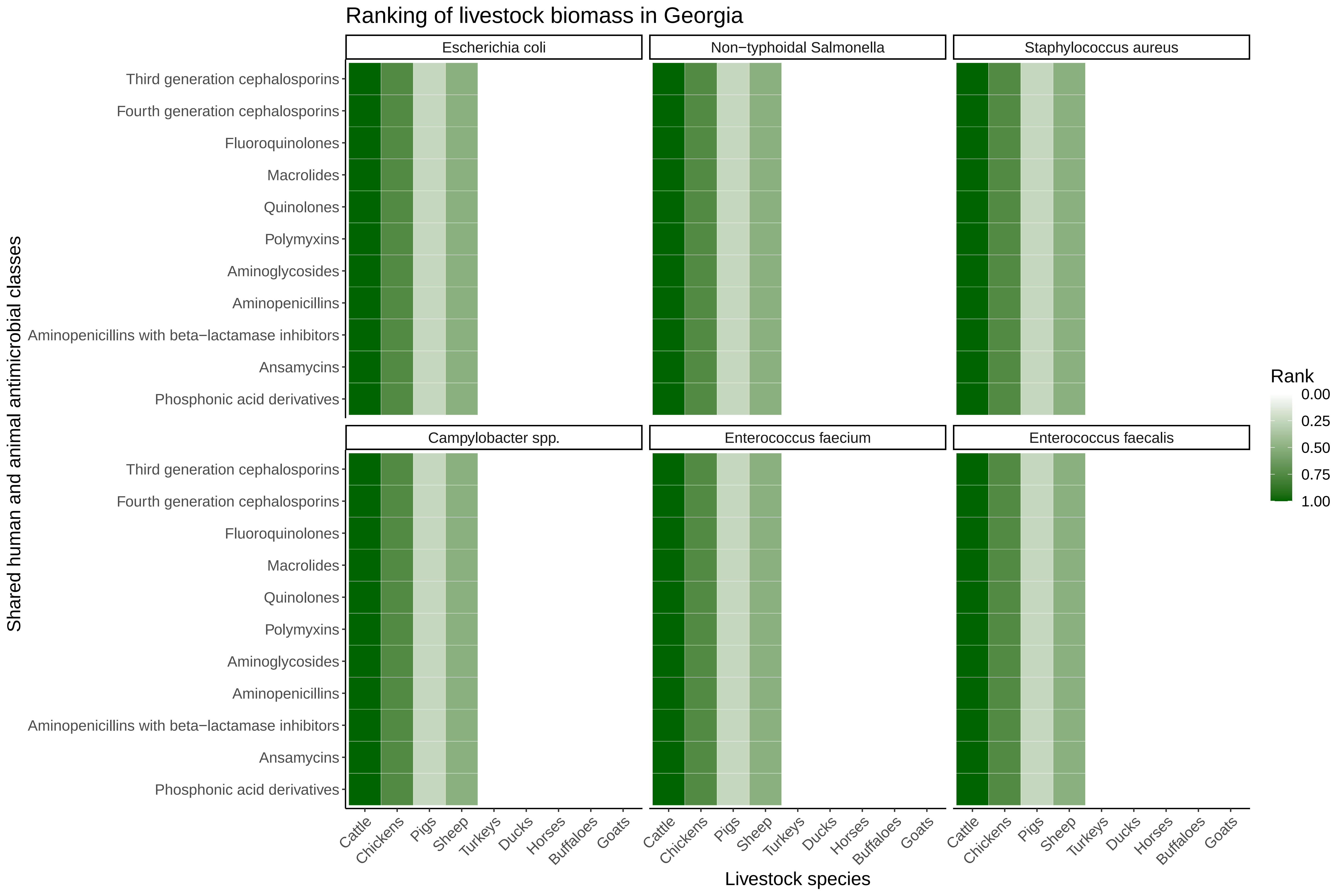
Ranking of livestock biomass in Georgia
Escherichia coli
Non−typhoidal Salmonella
Staphylococcus aureus
Campylobacter spp.
Enterococcus faecium
Enterococcus faecalis
Third generation cephalosporins
Fourth generation cephalosporins
Fluoroquinolones
Macrolides
Quinolones
Polymyxins
Aminoglycosides
Aminopenicillins
Aminopenicillins with beta−lactamase inhibitors
Ansamycins
Phosphonic acid derivatives
Shared human and animal antimicrobial classes
Cattle
Chickens
Pigs
Sheep
Turkeys
Ducks
Horses
Buffaloes
Goats
Livestock species
Rank
0.00
0.25
0.50
0.75
1.00

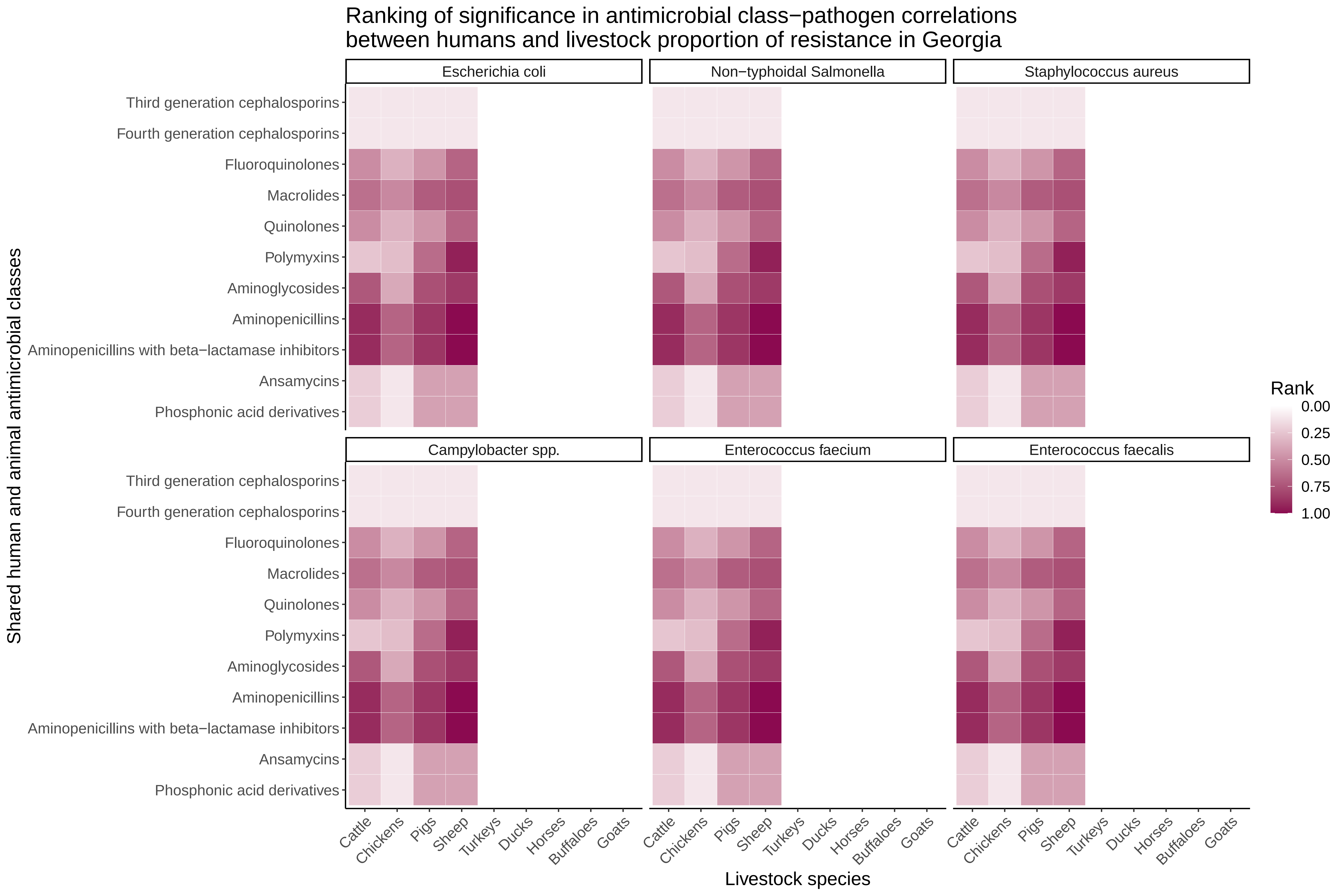

Ranking of significance in antimicrobial class−pathogen correlations between humans and livestock proportion of resistance in Georgia
Escherichia coli
Non−typhoidal Salmonella
Staphylococcus aureus
Campylobacter spp.
Enterococcus faecium
Enterococcus faecalis
Third generation cephalosporins
Fourth generation cephalosporins
Fluoroquinolones
Macrolides
Quinolones
Polymyxins
Aminoglycosides
Aminopenicillins
Aminopenicillins with beta−lactamase inhibitors
Ansamycins
Phosphonic acid derivatives
Shared human and animal antimicrobial classes
Cattle
Chickens
Pigs
Sheep
Turkeys
Ducks
Horses
Buffaloes
Goats
Livestock species
Rank
0.00
0.25
0.50
0.75
1.00

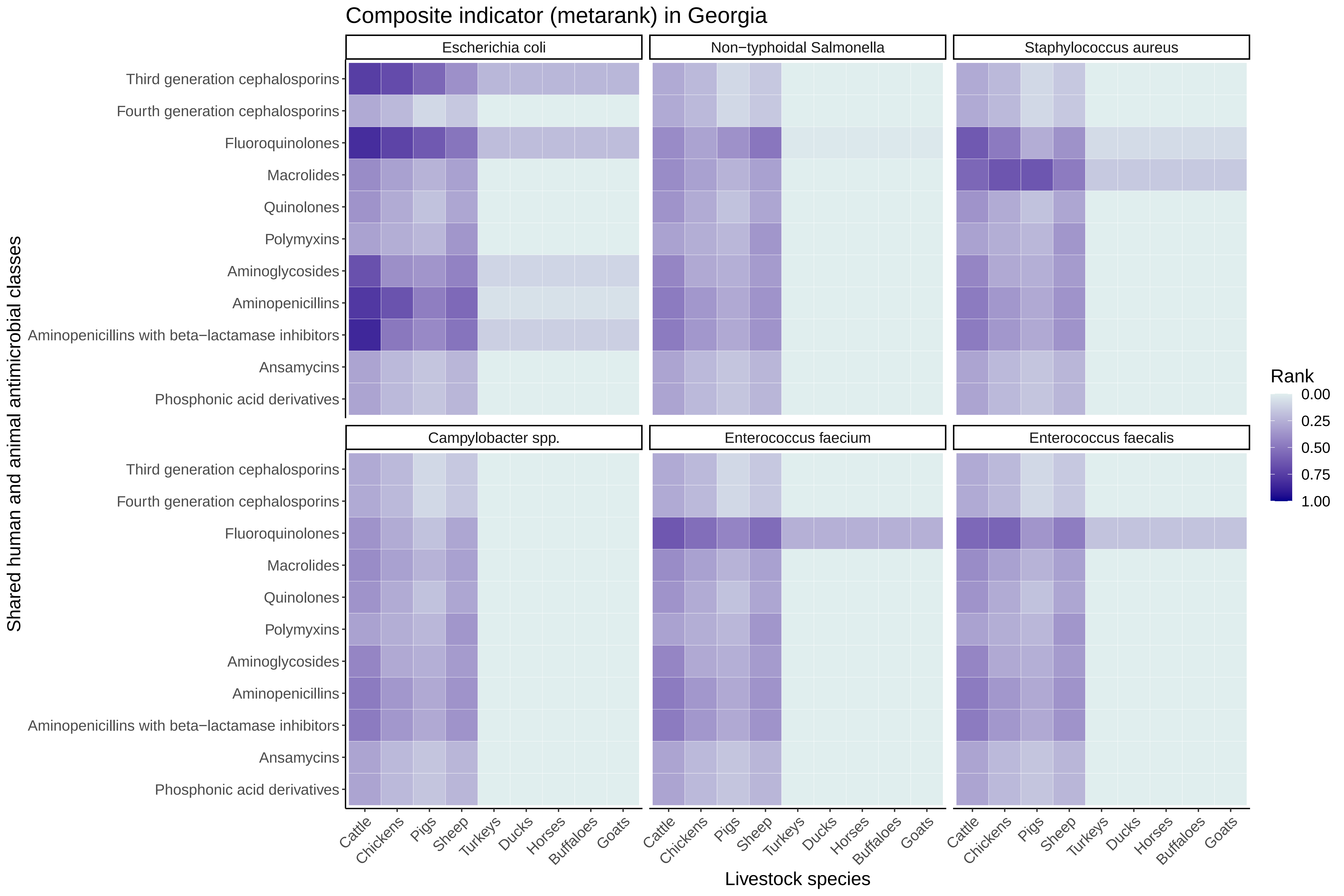

Composite indicator (metarank) in Georgia
Escherichia coli
Non−typhoidal Salmonella
Staphylococcus aureus
Campylobacter spp.
Enterococcus faecium
Enterococcus faecalis
Shared human and animal antimicrobial classes
Third generation cephalosporins
Fourth generation cephalosporins
Fluoroquinolones
Macrolides
Quinolones
Polymyxins
Aminoglycosides
Aminopenicillins
Aminopenicillins with beta−lactamase inhibitors
Ansamycins
Phosphonic acid derivatives
Livestock species
Cattle
Chickens
Pigs
Sheep
Turkeys
Ducks
Horses
Buffaloes
Goats
Rank
0.00
0.25
0.50
0.75
1.00

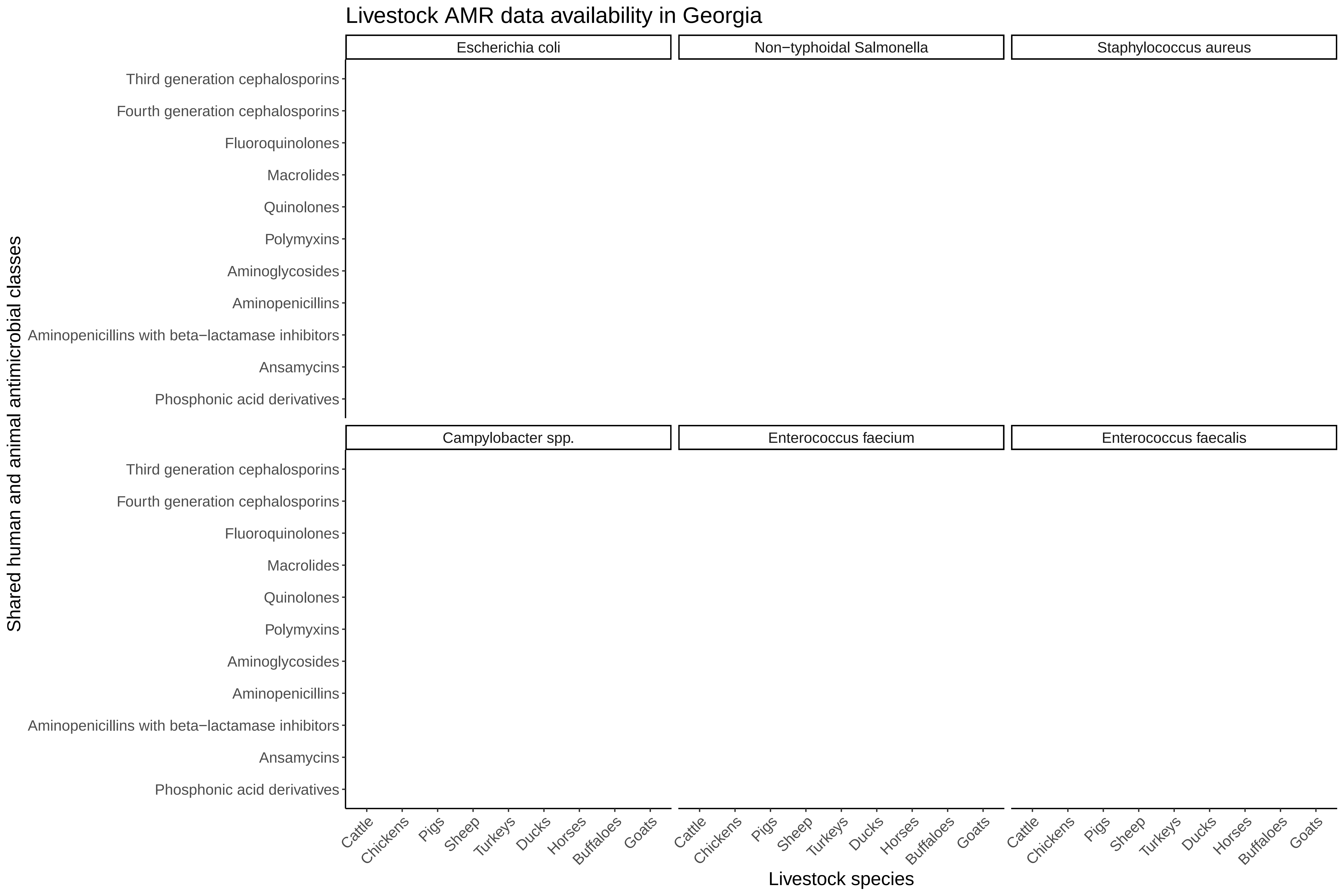
Livestock AMR data availability in Georgia
Escherichia coli
Non−typhoidal Salmonella
Staphylococcus aureus
Campylobacter spp.
Enterococcus faecium
Enterococcus faecalis
Shared human and animal antimicrobial classes
Third generation cephalosporins
Fourth generation cephalosporins
Fluoroquinolones
Macrolides
Quinolones
Polymyxins
Aminoglycosides
Aminopenicillins
Aminopenicillins with beta−lactamase inhibitors
Ansamycins
Phosphonic acid derivatives
Cattle
Chickens
Pigs
Sheep
Turkeys
Ducks
Horses
Buffaloes
Goats
Livestock species

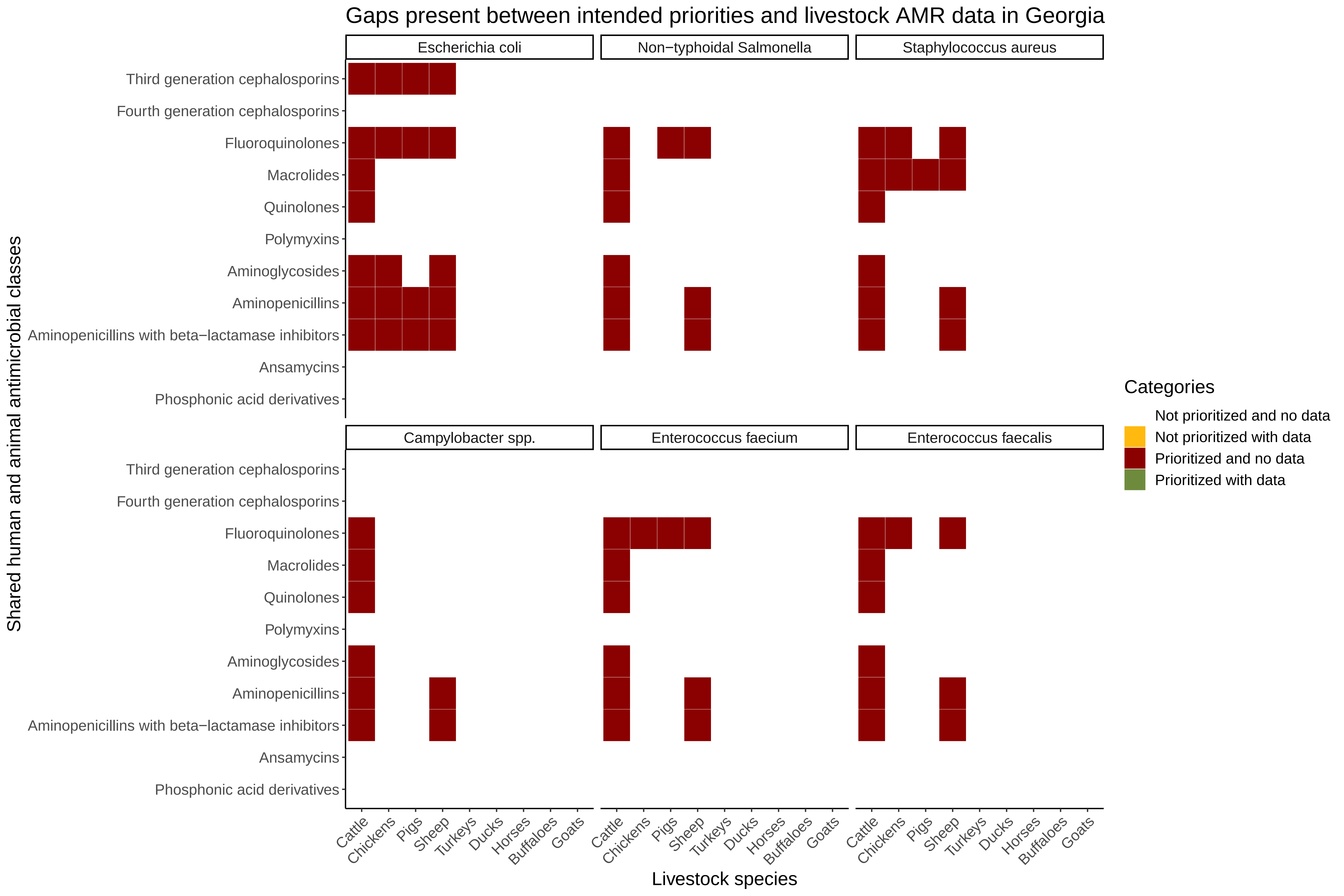

Gaps present between intended priorities and livestock AMR data in Georgia
Escherichia coli
Non−typhoidal Salmonella
Staphylococcus aureus
Campylobacter spp.
Enterococcus faecium
Enterococcus faecalis
Third generation cephalosporins
Fourth generation cephalosporins
Fluoroquinolones
Macrolides
Quinolones
Polymyxins
Aminoglycosides
Aminopenicillins
Aminopenicillins with beta−lactamase inhibitors
Ansamycins
Phosphonic acid derivatives
Shared human and animal antimicrobial classes
Cattle
Chickens
Pigs
Sheep
Turkeys
Ducks
Horses
Buffaloes
Goats
Livestock species
Categories
Not prioritized and no data
Not prioritized with data
Prioritized and no data
Prioritized with data

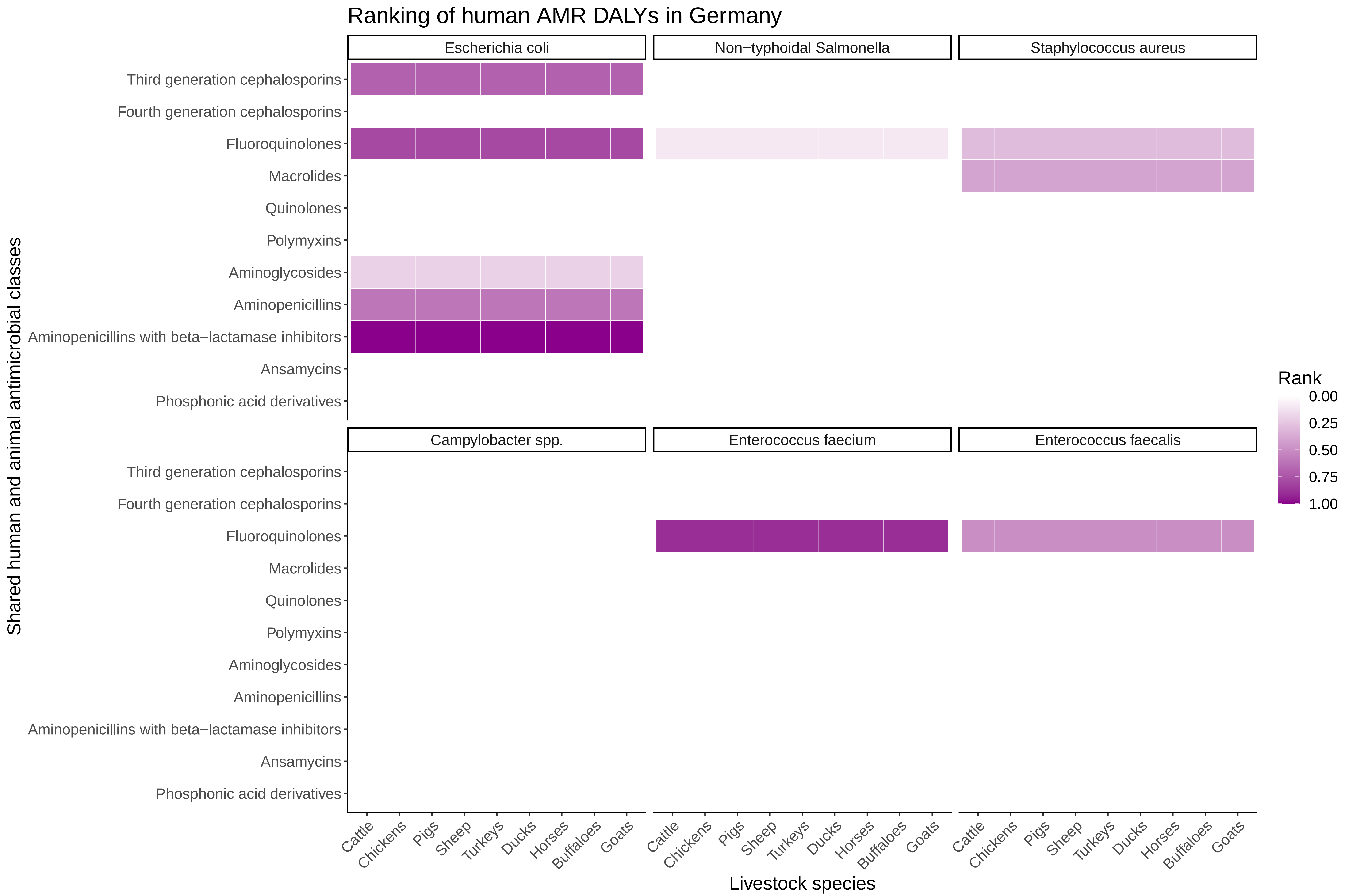
Ranking of human AMR DALYs in Germany
Escherichia coli
Non−typhoidal Salmonella
Staphylococcus aureus
Campylobacter spp.
Enterococcus faecium
Enterococcus faecalis
Shared human and animal antimicrobial classes
Third generation cephalosporins
Fourth generation cephalosporins
Fluoroquinolones
Macrolides
Quinolones
Polymyxins
Aminoglycosides
Aminopenicillins
Aminopenicillins with beta−lactamase inhibitors
Ansamycins
Phosphonic acid derivatives
Cattle
Chickens
Pigs
Sheep
Turkeys
Ducks
Horses
Buffaloes
Goats
Livestock species
Rank
0.00
0.25
0.50
0.75
1.00

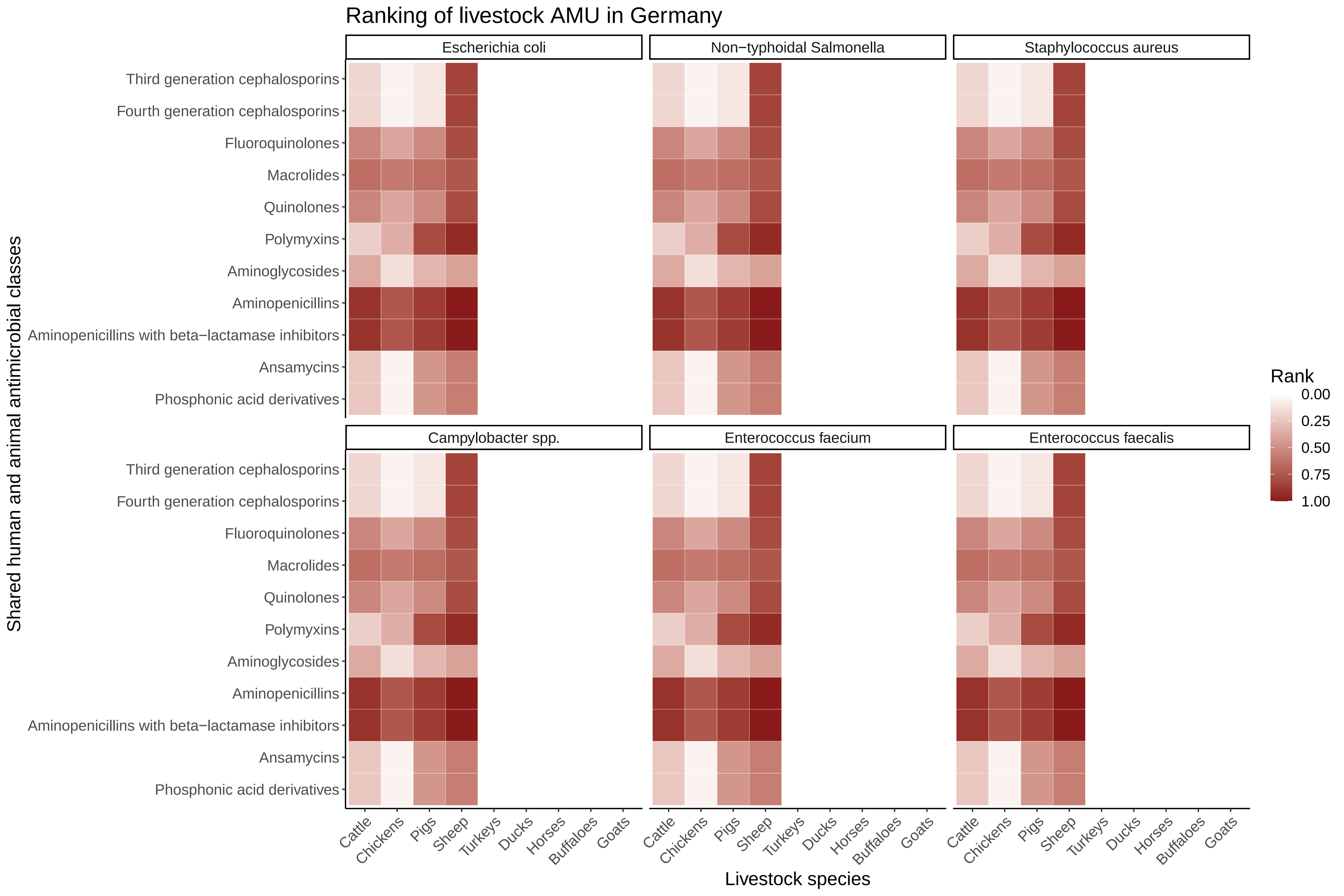

Ranking of livestock AMU in Germany
Escherichia coli
Non−typhoidal Salmonella
Staphylococcus aureus
Campylobacter spp.
Enterococcus faecium
Enterococcus faecalis
Shared human and animal antimicrobial classes
Third generation cephalosporins
Fourth generation cephalosporins
Fluoroquinolones
Macrolides
Quinolones
Polymyxins
Aminoglycosides
Aminopenicillins
Aminopenicillins with beta−lactamase inhibitors
Ansamycins
Phosphonic acid derivatives
Livestock species
Cattle
Chickens
Pigs
Sheep
Turkeys
Ducks
Horses
Buffaloes
Goats
Rank
0.00
0.25
0.50
0.75
1.00

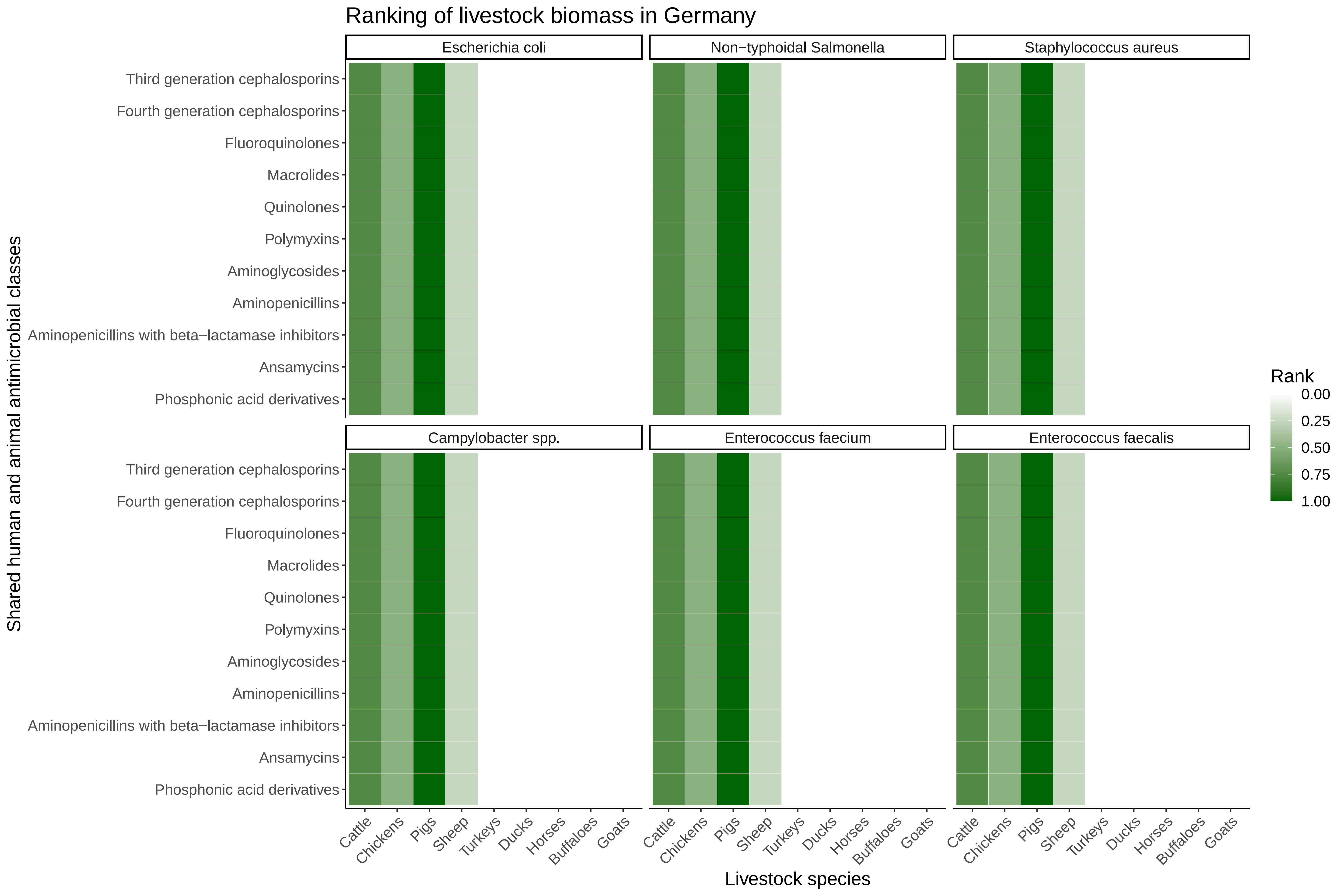

Ranking of livestock biomass in Germany
Escherichia coli
Non−typhoidal Salmonella
Staphylococcus aureus
Campylobacter spp.
Enterococcus faecium
Enterococcus faecalis
Third generation cephalosporins
Fourth generation cephalosporins
Fluoroquinolones
Macrolides
Quinolones
Polymyxins
Aminoglycosides
Aminopenicillins
Aminopenicillins with beta−lactamase inhibitors
Ansamycins
Phosphonic acid derivatives
Shared human and animal antimicrobial classes
Cattle
Chickens
Pigs
Sheep
Turkeys
Ducks
Horses
Buffaloes
Goats
Livestock species
Rank
0.00
0.25
0.50
0.75
1.00

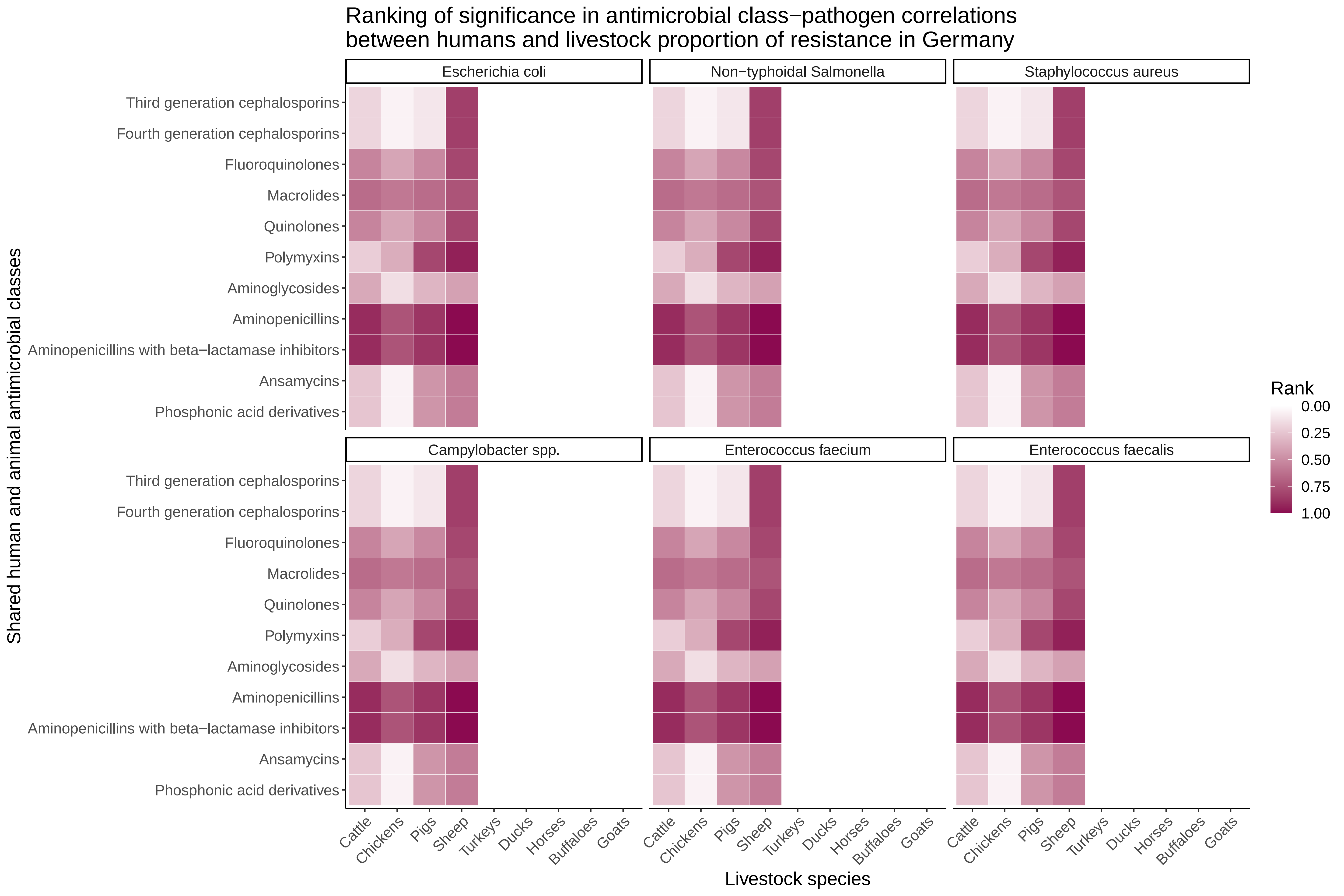

Ranking of significance in antimicrobial class−pathogen correlations
between humans and livestock proportion of resistance in Germany
Escherichia coli
Non−typhoidal Salmonella
Staphylococcus aureus
Campylobacter spp.
Enterococcus faecium
Enterococcus faecalis
Shared human and animal antimicrobial classes
Third generation cephalosporins
Fourth generation cephalosporins
Fluoroquinolones
Macrolides
Quinolones
Polymyxins
Aminoglycosides
Aminopenicillins
Aminopenicillins with beta−lactamase inhibitors
Ansamycins
Phosphonic acid derivatives
Livestock species
Cattle
Chickens
Pigs
Sheep
Turkeys
Ducks
Horses
Buffaloes
Goats
Rank
0.00
0.25
0.50
0.75
1.00

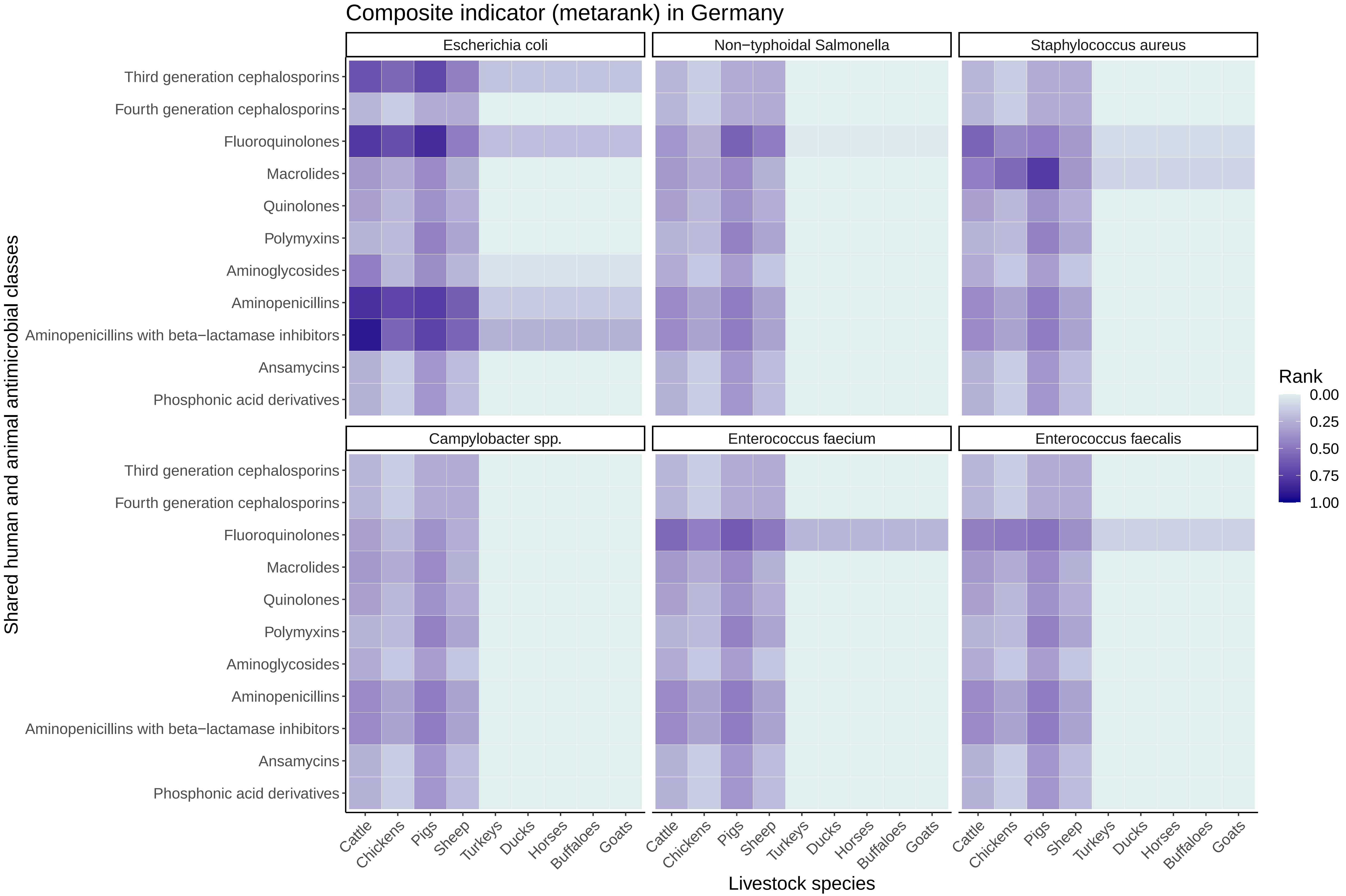

Composite indicator (metarank) in Germany
Escherichia coli
Non−typhoidal Salmonella
Staphylococcus aureus
Campylobacter spp.
Enterococcus faecium
Enterococcus faecalis
Shared human and animal antimicrobial classes
Third generation cephalosporins
Fourth generation cephalosporins
Fluoroquinolones
Macrolides
Quinolones
Polymyxins
Aminoglycosides
Aminopenicillins
Aminopenicillins with beta−lactamase inhibitors
Ansamycins
Phosphonic acid derivatives
Cattle
Chickens
Pigs
Sheep
Turkeys
Ducks
Horses
Buffaloes
Goats
Livestock species
Rank
0.00
0.25
0.50
0.75
1.00

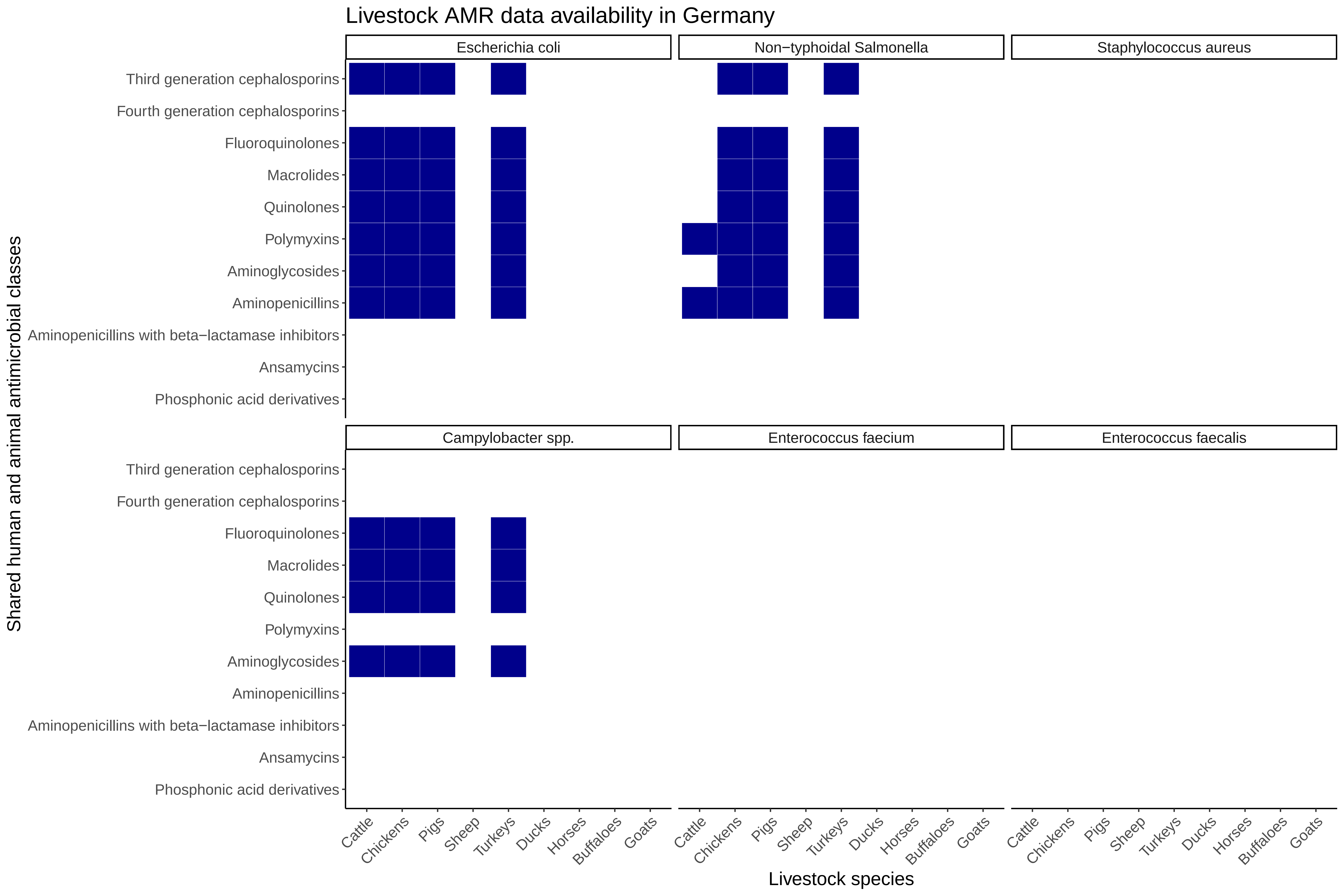

Livestock AMR data availability in Germany
Escherichia coli
Non−typhoidal Salmonella
Staphylococcus aureus
Campylobacter spp.
Enterococcus faecium
Enterococcus faecalis
Shared human and animal antimicrobial classes
Third generation cephalosporins
Fourth generation cephalosporins
Fluoroquinolones
Macrolides
Quinolones
Polymyxins
Aminoglycosides
Aminopenicillins
Aminopenicillins with beta−lactamase inhibitors
Ansamycins
Phosphonic acid derivatives
Cattle
Chickens
Pigs
Sheep
Turkeys
Ducks
Horses
Buffaloes
Goats
Livestock species

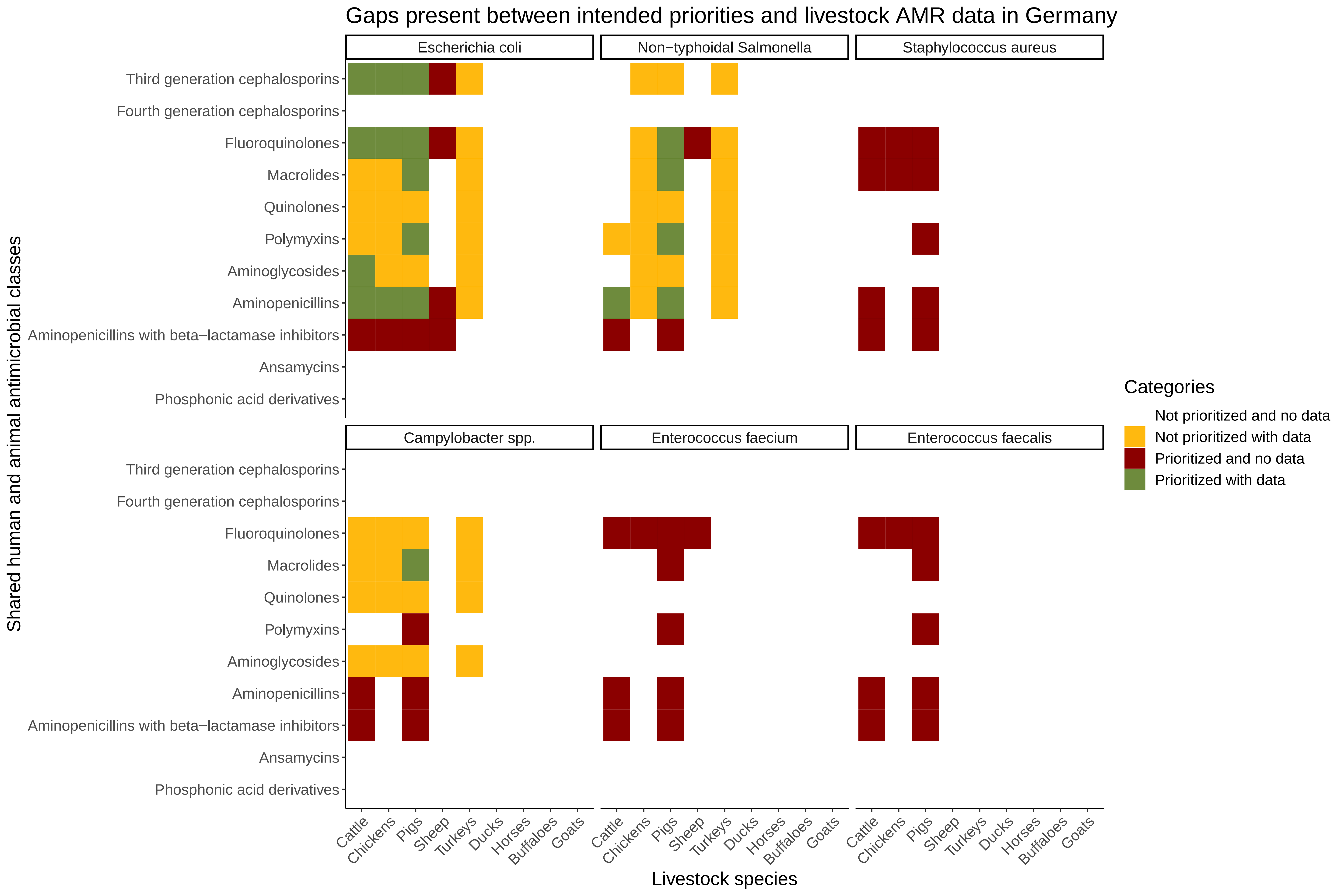
Gaps present between intended priorities and livestock AMR data in Germany
Escherichia coli
Non−typhoidal Salmonella
Staphylococcus aureus
Campylobacter spp.
Enterococcus faecium
Enterococcus faecalis
Third generation cephalosporins
Fourth generation cephalosporins
Fluoroquinolones
Macrolides
Quinolones
Polymyxins
Aminoglycosides
Aminopenicillins
Aminopenicillins with beta−lactamase inhibitors
Ansamycins
Phosphonic acid derivatives
Shared human and animal antimicrobial classes
Cattle
Chickens
Pigs
Sheep
Turkeys
Ducks
Horses
Buffaloes
Goats
Livestock species
Categories
Not prioritized and no data
Not prioritized with data
Prioritized and no data
Prioritized with data

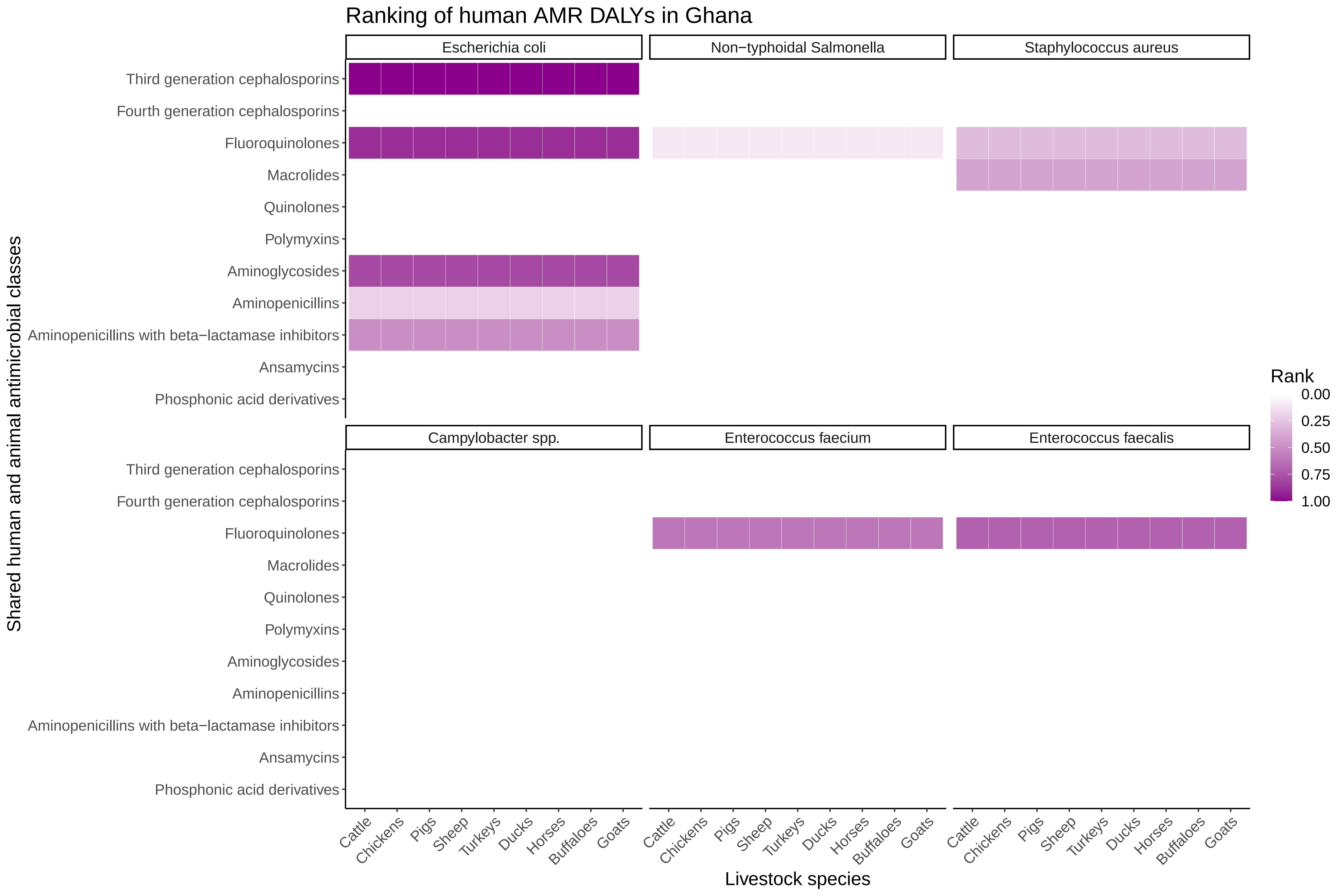

Ranking of human AMR DALYs in Ghana
Escherichia coli
Non−typhoidal Salmonella
Staphylococcus aureus
Campylobacter spp.
Enterococcus faecium
Enterococcus faecalis
Third generation cephalosporins
Fourth generation cephalosporins
Fluoroquinolones
Macrolides
Quinolones
Polymyxins
Aminoglycosides
Aminopenicillins
Aminopenicillins with beta−lactamase inhibitors
Ansamycins
Phosphonic acid derivatives
Shared human and animal antimicrobial classes
Cattle
Chickens
Pigs
Sheep
Turkeys
Ducks
Horses
Buffaloes
Goats
Livestock species
Rank
0.00
0.25
0.50
0.75
1.00

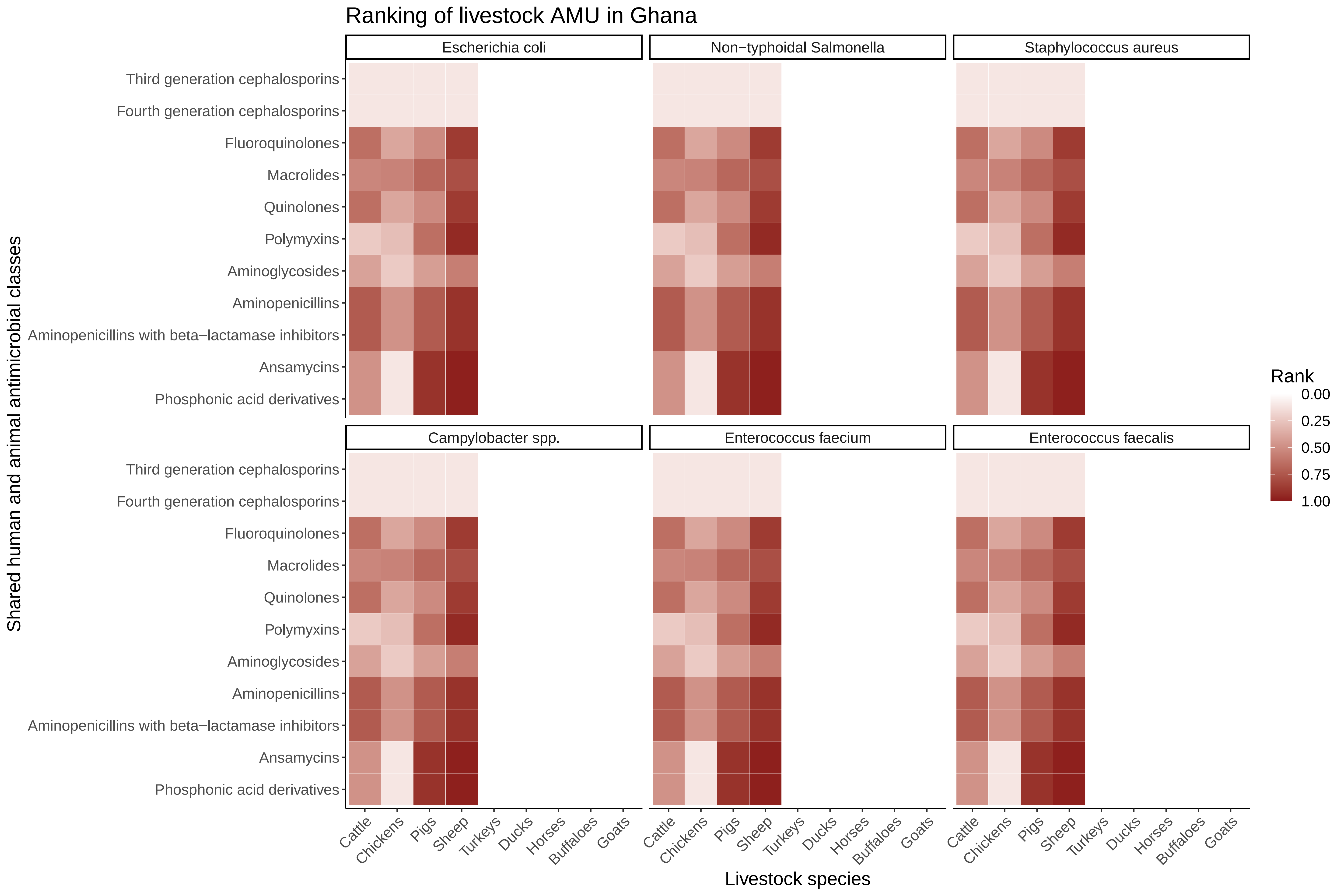

Ranking of livestock AMU in Ghana
Escherichia coli
Non−typhoidal Salmonella
Staphylococcus aureus
Campylobacter spp.
Enterococcus faecium
Enterococcus faecalis
Shared human and animal antimicrobial classes
Third generation cephalosporins
Fourth generation cephalosporins
Fluoroquinolones
Macrolides
Quinolones
Polymyxins
Aminoglycosides
Aminopenicillins
Aminopenicillins with beta−lactamase inhibitors
Ansamycins
Phosphonic acid derivatives
Cattle
Chickens
Pigs
Sheep
Turkeys
Ducks
Horses
Buffaloes
Goats
Livestock species
Rank
0.00
0.25
0.50
0.75
1.00

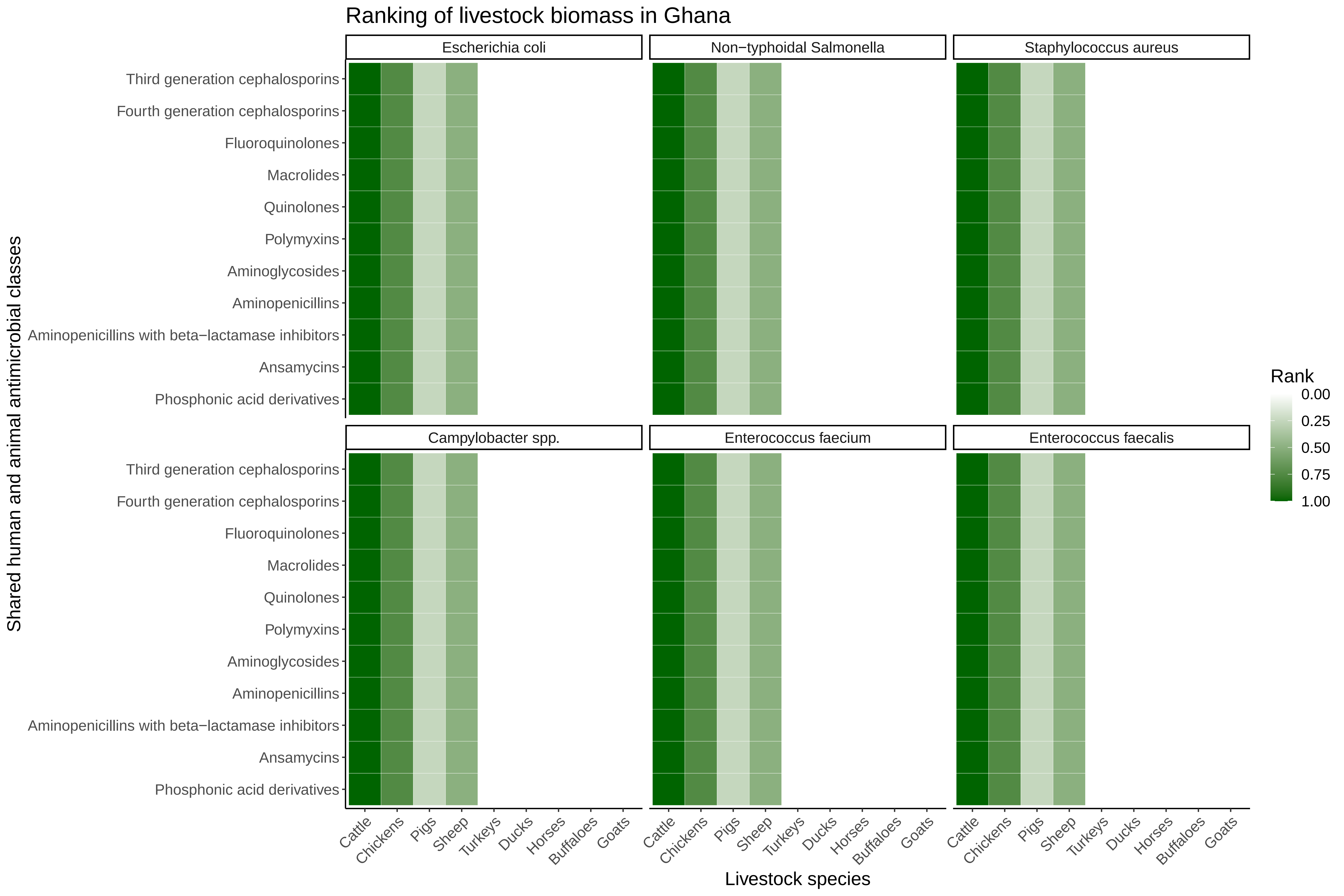

Ranking of livestock biomass in Ghana
Escherichia coli
Non−typhoidal Salmonella
Staphylococcus aureus
Campylobacter spp.
Enterococcus faecium
Enterococcus faecalis
Third generation cephalosporins
Fourth generation cephalosporins
Fluoroquinolones
Macrolides
Quinolones
Polymyxins
Aminoglycosides
Aminopenicillins
Aminopenicillins with beta−lactamase inhibitors
Ansamycins
Phosphonic acid derivatives
Shared human and animal antimicrobial classes
Cattle
Chickens
Pigs
Sheep
Turkeys
Ducks
Horses
Buffaloes
Goats
Livestock species
Rank
0.00
0.25
0.50
0.75
1.00

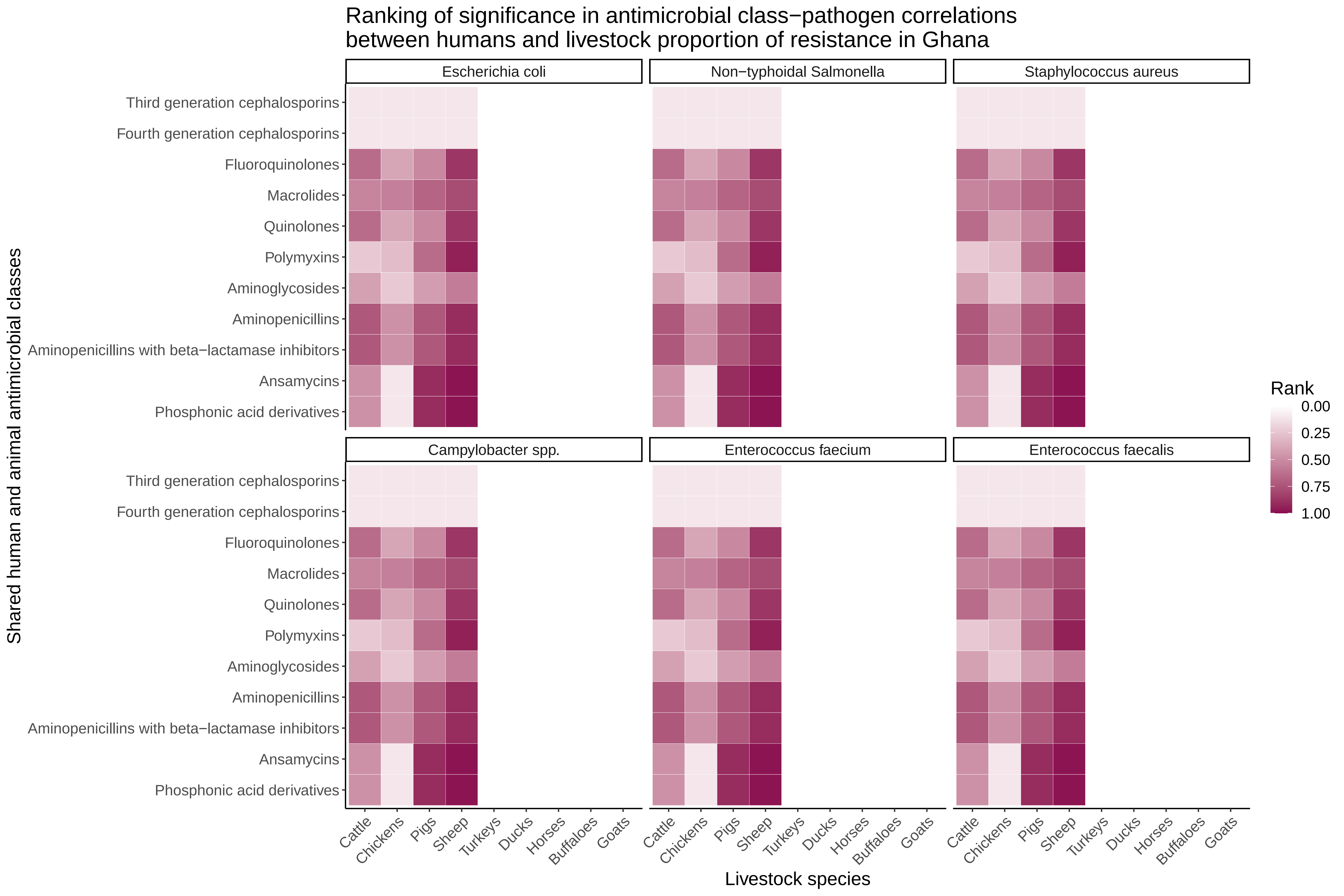
Ranking of significance in antimicrobial class−pathogen correlations
between humans and livestock proportion of resistance in Ghana
Escherichia coli
Non−typhoidal Salmonella
Staphylococcus aureus
Campylobacter spp.
Enterococcus faecium
Enterococcus faecalis
Shared human and animal antimicrobial classes
Third generation cephalosporins
Fourth generation cephalosporins
Fluoroquinolones
Macrolides
Quinolones
Polymyxins
Aminoglycosides
Aminopenicillins
Aminopenicillins with beta−lactamase inhibitors
Ansamycins
Phosphonic acid derivatives
Livestock species
Cattle
Chickens
Pigs
Sheep
Turkeys
Ducks
Horses
Buffaloes
Goats
Rank
0.00
0.25
0.50
0.75
1.00

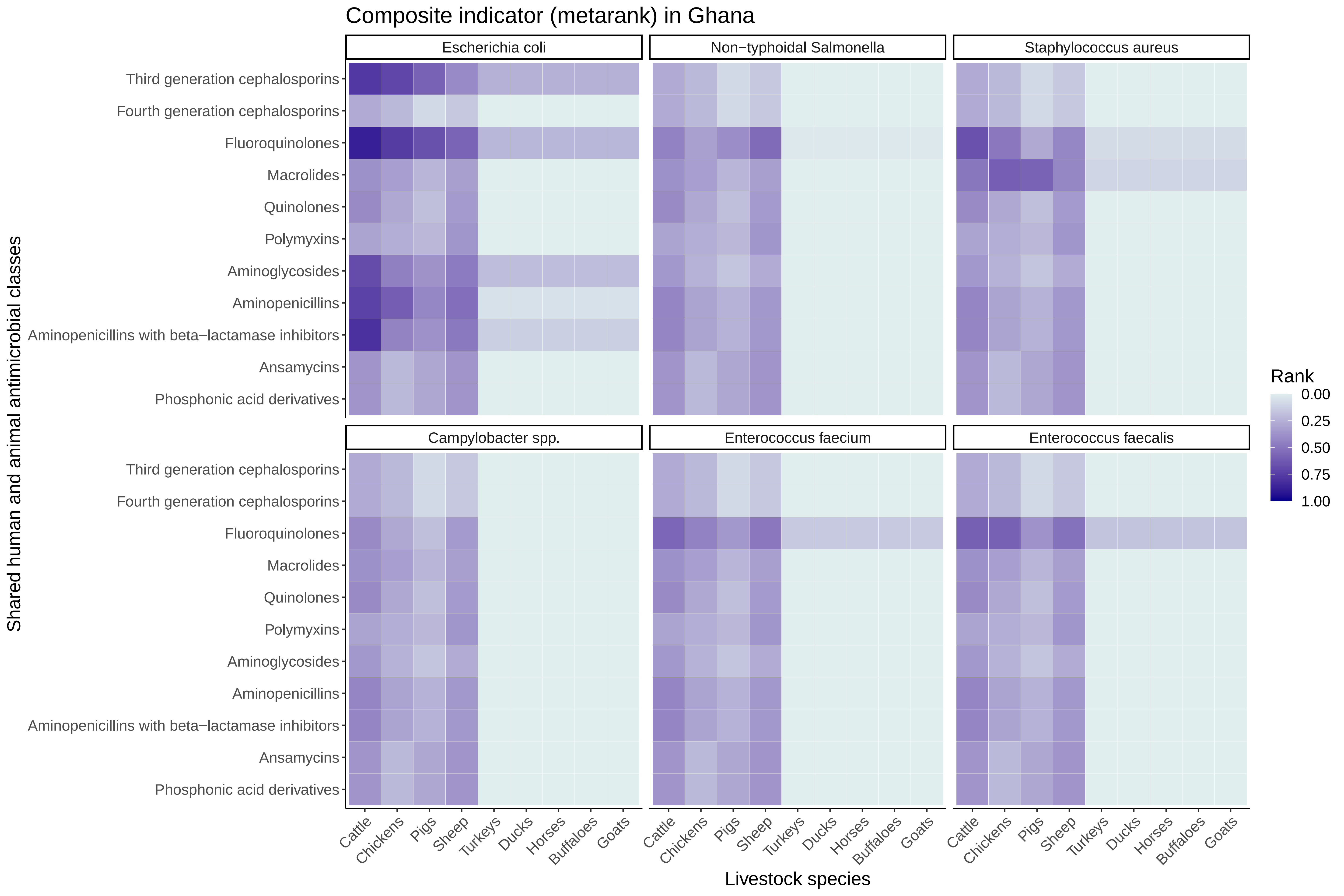
Composite indicator (metarank) in Ghana
Escherichia coli
Non−typhoidal Salmonella
Staphylococcus aureus
Campylobacter spp.
Enterococcus faecium
Enterococcus faecalis
Third generation cephalosporins
Fourth generation cephalosporins
Fluoroquinolones
Macrolides
Quinolones
Polymyxins
Aminoglycosides
Aminopenicillins
Aminopenicillins with beta−lactamase inhibitors
Ansamycins
Phosphonic acid derivatives
Shared human and animal antimicrobial classes
Cattle
Chickens
Pigs
Sheep
Turkeys
Ducks
Horses
Buffaloes
Goats
Livestock species
Rank
0.00
0.25
0.50
0.75
1.00

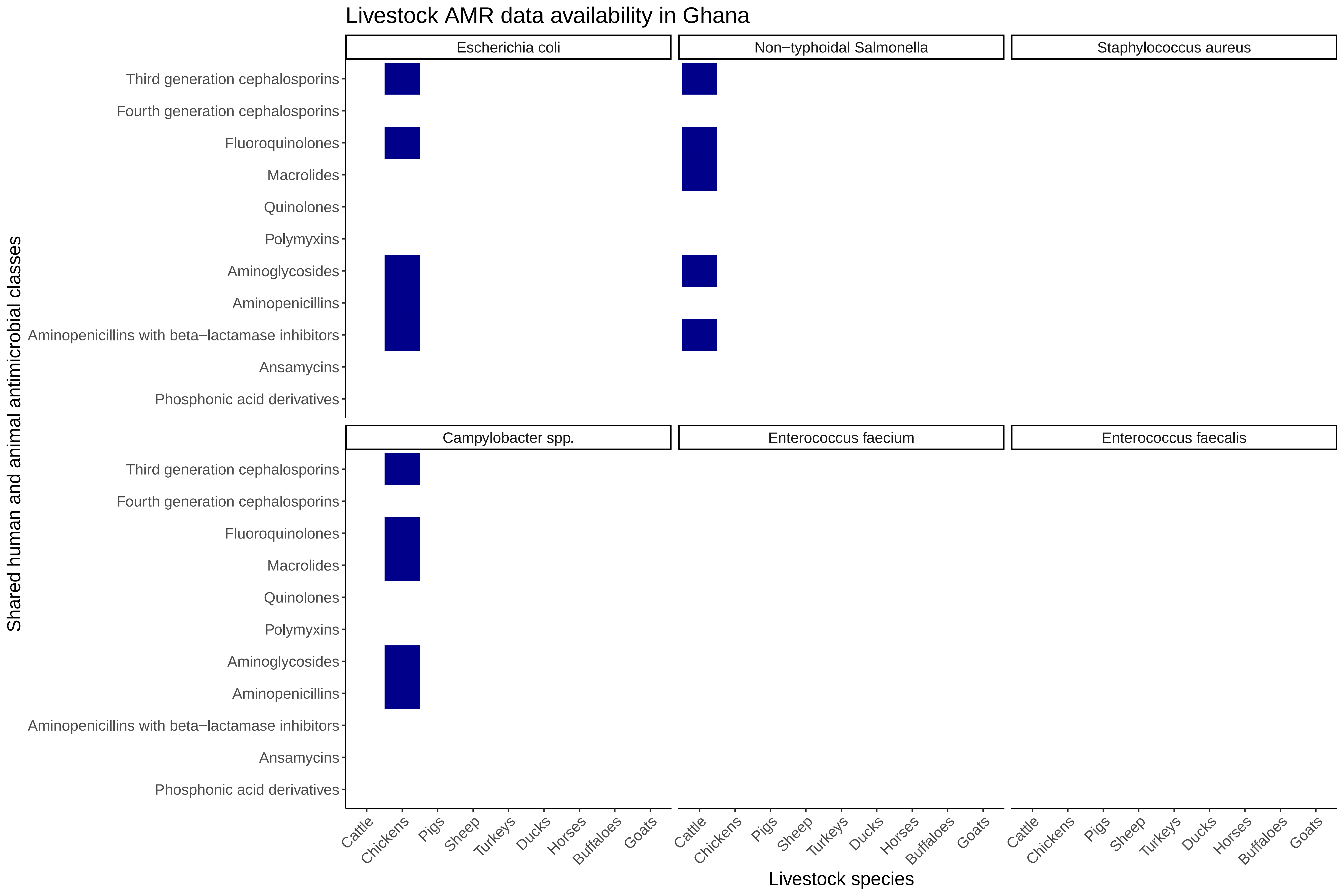
Livestock AMR data availability in Ghana
Escherichia coli
Non−typhoidal Salmonella
Staphylococcus aureus
Campylobacter spp.
Enterococcus faecium
Enterococcus faecalis
Third generation cephalosporins
Fourth generation cephalosporins
Fluoroquinolones
Macrolides
Quinolones
Polymyxins
Aminoglycosides
Aminopenicillins
Aminopenicillins with beta−lactamase inhibitors
Ansamycins
Phosphonic acid derivatives
Shared human and animal antimicrobial classes
Cattle
Chickens
Pigs
Sheep
Turkeys
Ducks
Horses
Buffaloes
Goats
Livestock species

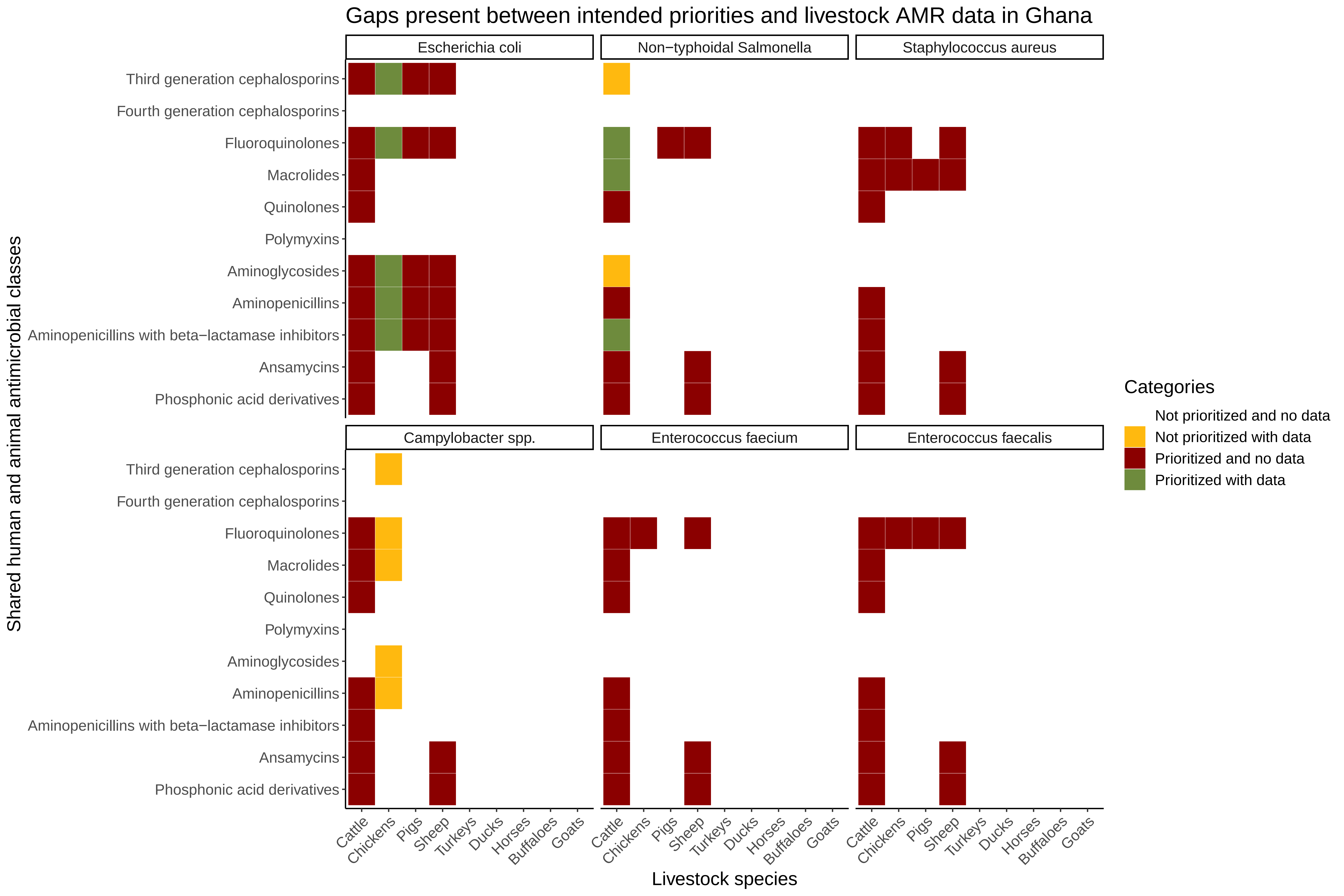
Gaps present between intended priorities and livestock AMR data in Ghana
Escherichia coli
Non−typhoidal Salmonella
Staphylococcus aureus
Campylobacter spp.
Enterococcus faecium
Enterococcus faecalis
Shared human and animal antimicrobial classes
Third generation cephalosporins
Fourth generation cephalosporins
Fluoroquinolones
Macrolides
Quinolones
Polymyxins
Aminoglycosides
Aminopenicillins
Aminopenicillins with beta−lactamase inhibitors
Ansamycins
Phosphonic acid derivatives
Cattle
Chickens
Pigs
Sheep
Turkeys
Ducks
Horses
Buffaloes
Goats
Livestock species
Categories
Not prioritized and no data
Not prioritized with data
Prioritized and no data
Prioritized with data

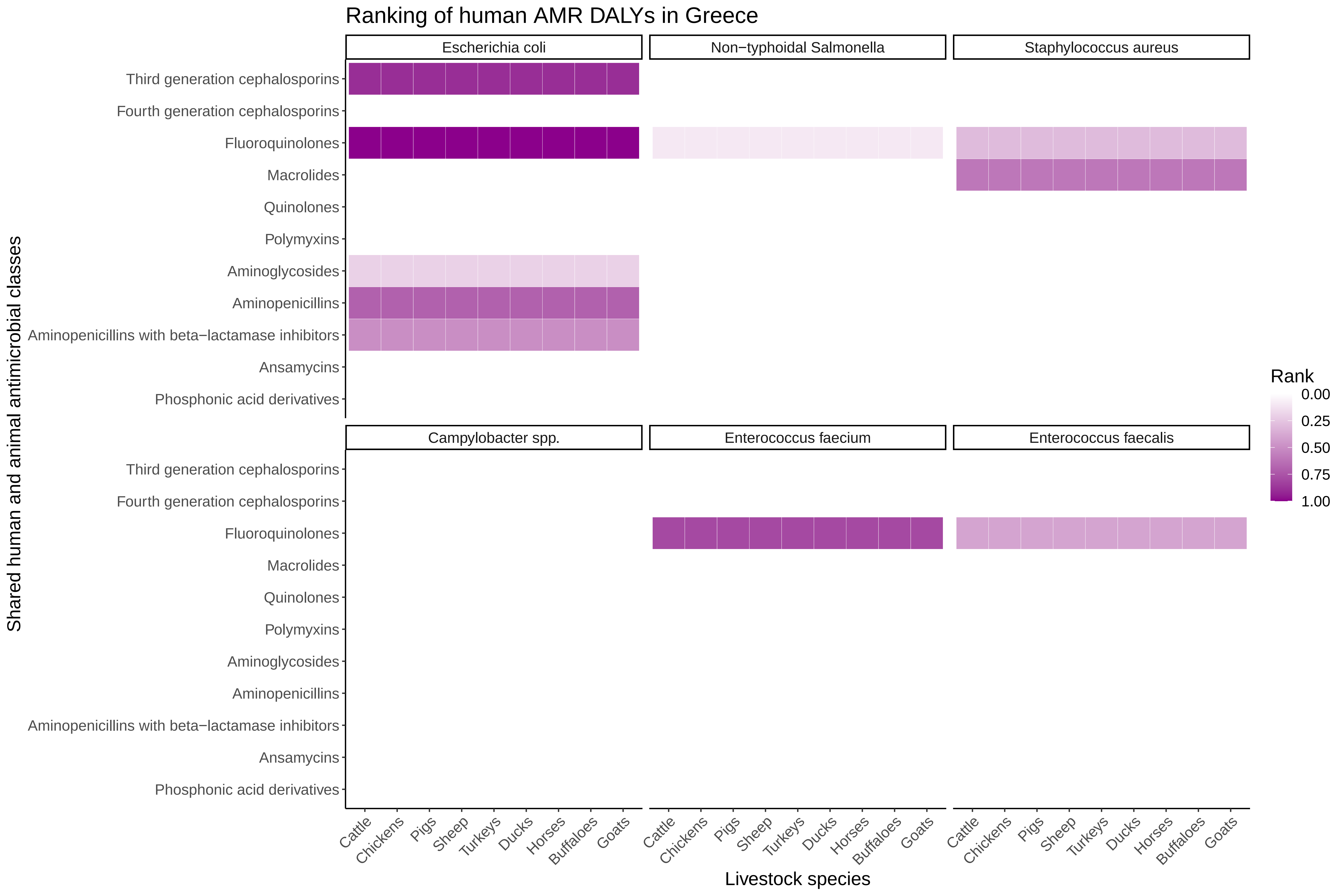

Ranking of human AMR DALYs in Greece
Escherichia coli
Non−typhoidal Salmonella
Staphylococcus aureus
Campylobacter spp.
Enterococcus faecium
Enterococcus faecalis
Third generation cephalosporins
Fourth generation cephalosporins
Fluoroquinolones
Macrolides
Quinolones
Polymyxins
Aminoglycosides
Aminopenicillins
Aminopenicillins with beta−lactamase inhibitors
Ansamycins
Phosphonic acid derivatives
Shared human and animal antimicrobial classes
Cattle
Chickens
Pigs
Sheep
Turkeys
Ducks
Horses
Buffaloes
Goats
Livestock species
Rank
0.00
0.25
0.50
0.75
1.00

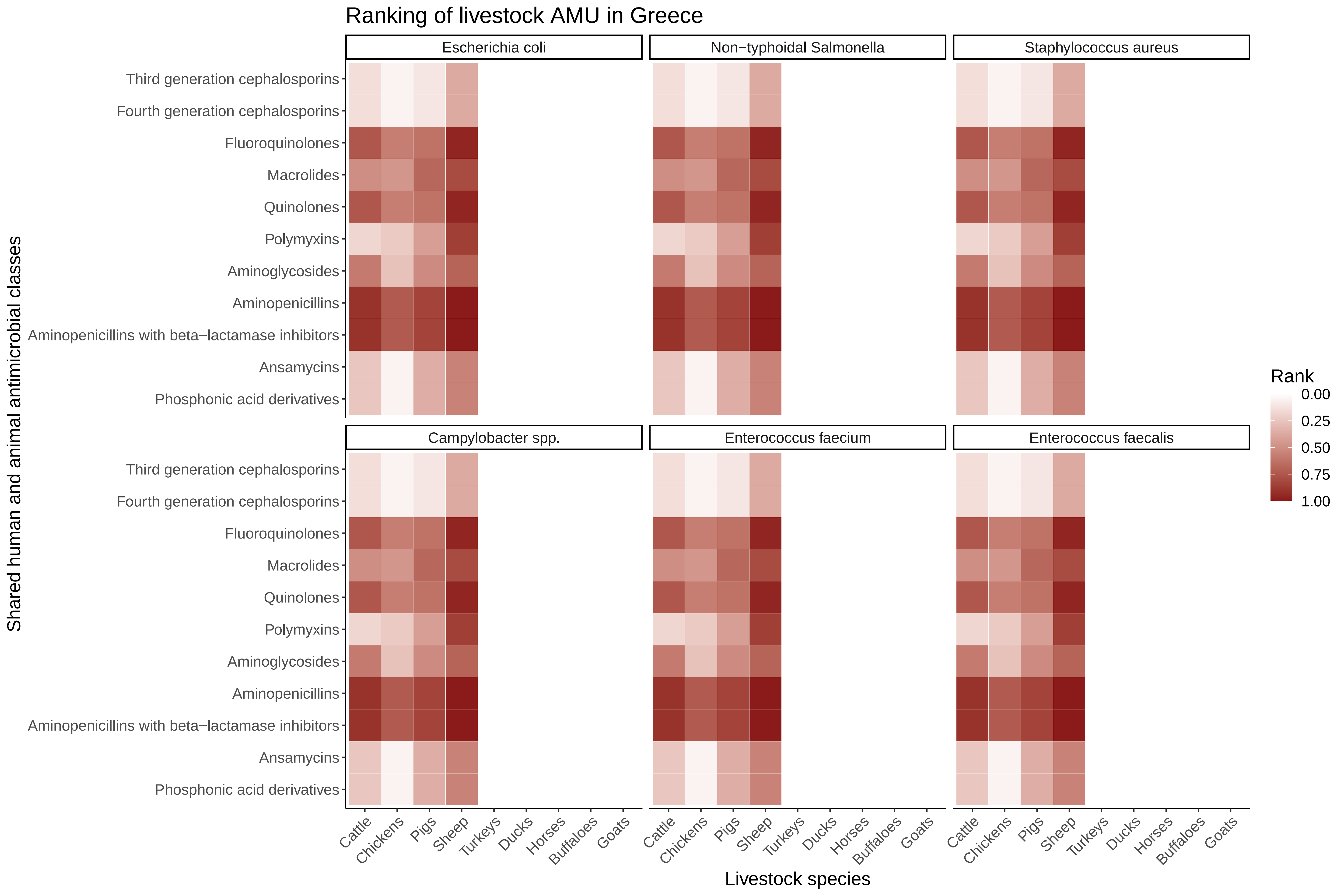
Ranking of livestock AMU in Greece
Escherichia coli
Non−typhoidal Salmonella
Staphylococcus aureus
Campylobacter spp.
Enterococcus faecium
Enterococcus faecalis
Third generation cephalosporins
Fourth generation cephalosporins
Fluoroquinolones
Macrolides
Quinolones
Polymyxins
Aminoglycosides
Aminopenicillins
Aminopenicillins with beta−lactamase inhibitors
Ansamycins
Phosphonic acid derivatives
Shared human and animal antimicrobial classes
Cattle
Chickens
Pigs
Sheep
Turkeys
Ducks
Horses
Buffaloes
Goats
Livestock species
Rank
0.00
0.25
0.50
0.75
1.00

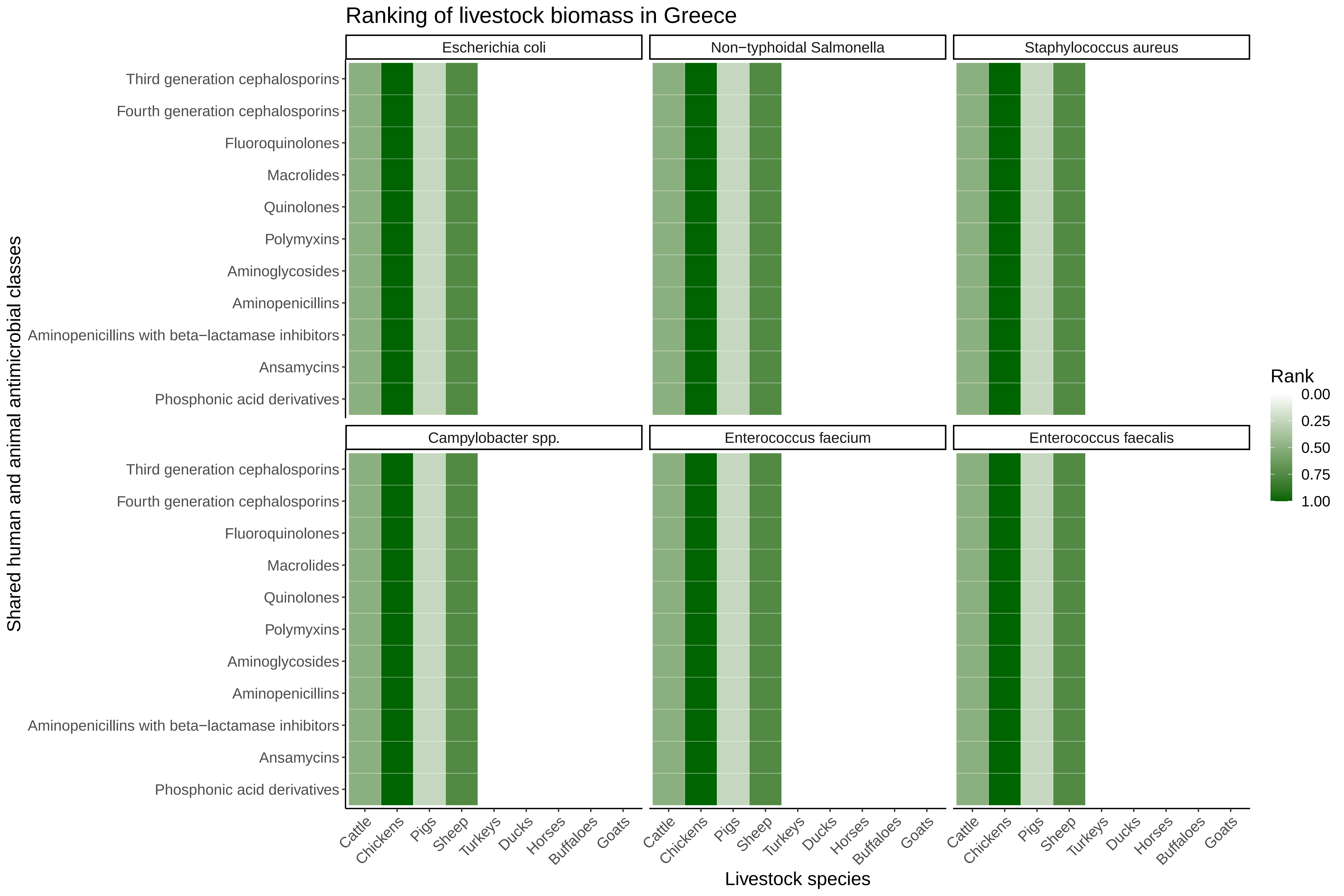

Ranking of livestock biomass in Greece
Escherichia coli
Non−typhoidal Salmonella
Staphylococcus aureus
Campylobacter spp.
Enterococcus faecium
Enterococcus faecalis
Third generation cephalosporins
Fourth generation cephalosporins
Fluoroquinolones
Macrolides
Quinolones
Polymyxins
Aminoglycosides
Aminopenicillins
Aminopenicillins with beta−lactamase inhibitors
Ansamycins
Phosphonic acid derivatives
Shared human and animal antimicrobial classes
Cattle
Chickens
Pigs
Sheep
Turkeys
Ducks
Horses
Buffaloes
Goats
Livestock species
Rank
0.00
0.25
0.50
0.75
1.00

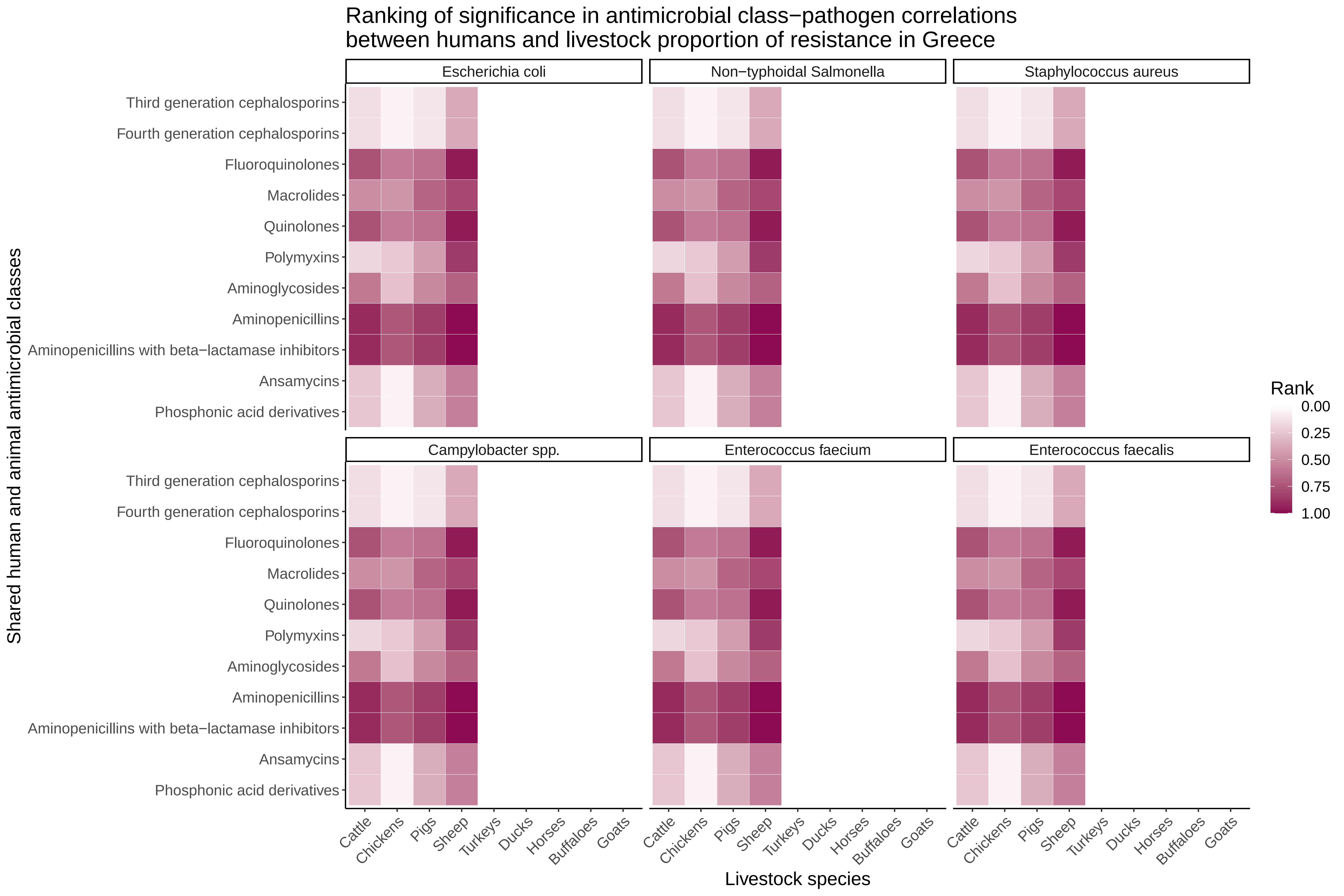

Ranking of significance in antimicrobial class−pathogen correlations
between humans and livestock proportion of resistance in Greece
Escherichia coli
Non−typhoidal Salmonella
Staphylococcus aureus
Campylobacter spp.
Enterococcus faecium
Enterococcus faecalis
Third generation cephalosporins
Fourth generation cephalosporins
Fluoroquinolones
Macrolides
Quinolones
Polymyxins
Aminoglycosides
Aminopenicillins
Aminopenicillins with beta−lactamase inhibitors
Ansamycins
Phosphonic acid derivatives
Shared human and animal antimicrobial classes
Cattle
Chickens
Pigs
Sheep
Turkeys
Ducks
Horses
Buffaloes
Goats
Livestock species
Rank
0.00
0.25
0.50
0.75
1.00

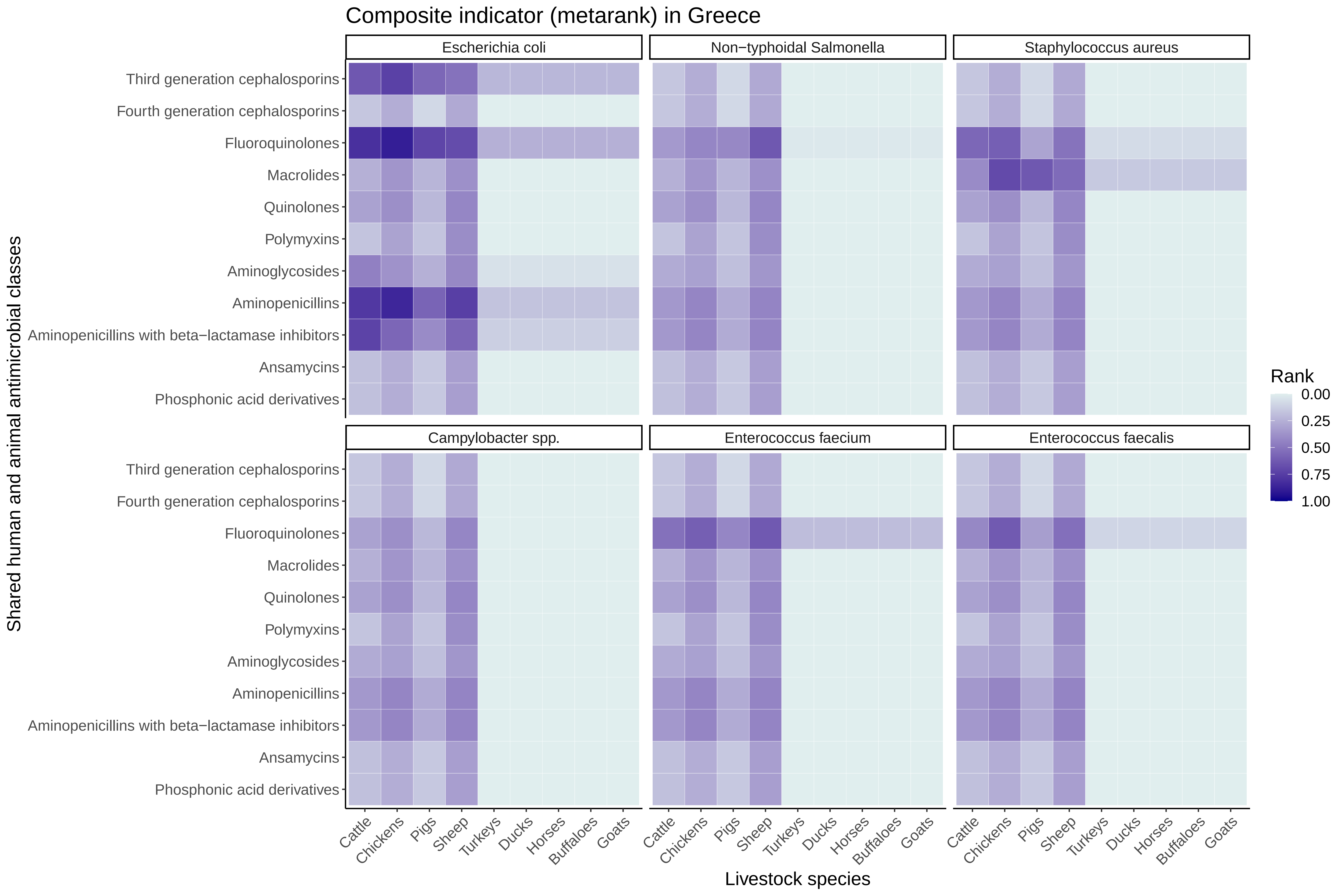

Composite indicator (metarank) in Greece
Escherichia coli
Non−typhoidal Salmonella
Staphylococcus aureus
Campylobacter spp.
Enterococcus faecium
Enterococcus faecalis
Shared human and animal antimicrobial classes
Third generation cephalosporins
Fourth generation cephalosporins
Fluoroquinolones
Macrolides
Quinolones
Polymyxins
Aminoglycosides
Aminopenicillins
Aminopenicillins with beta−lactamase inhibitors
Ansamycins
Phosphonic acid derivatives
Cattle
Chickens
Pigs
Sheep
Turkeys
Ducks
Horses
Buffaloes
Goats
Livestock species
Rank
0.00
0.25
0.50
0.75
1.00

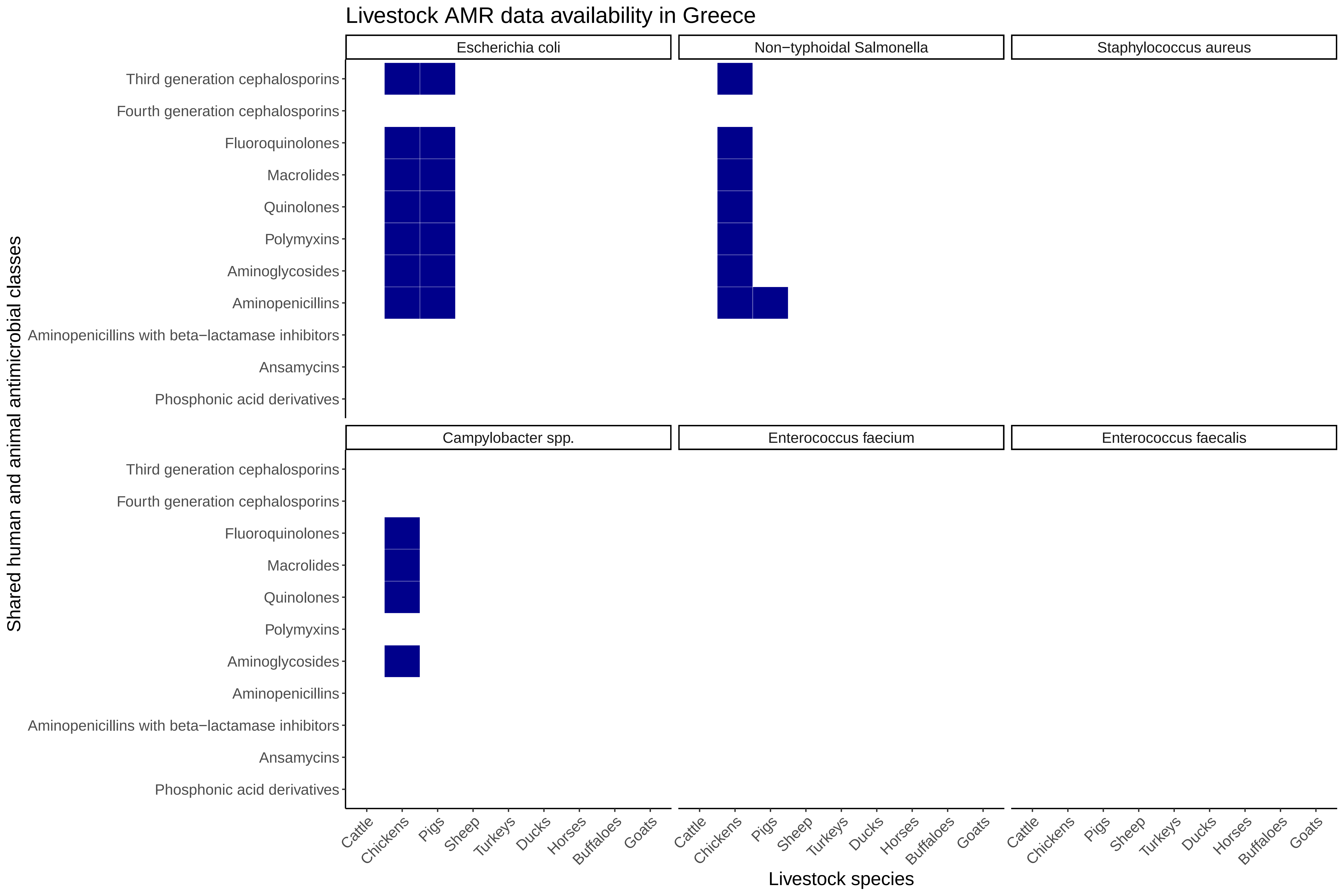

Livestock AMR data availability in Greece
Escherichia coli
Non−typhoidal Salmonella
Staphylococcus aureus
Campylobacter spp.
Enterococcus faecium
Enterococcus faecalis
Shared human and animal antimicrobial classes
Third generation cephalosporins
Fourth generation cephalosporins
Fluoroquinolones
Macrolides
Quinolones
Polymyxins
Aminoglycosides
Aminopenicillins
Aminopenicillins with beta−lactamase inhibitors
Ansamycins
Phosphonic acid derivatives
Cattle
Chickens
Pigs
Sheep
Turkeys
Ducks
Horses
Buffaloes
Goats
Livestock species

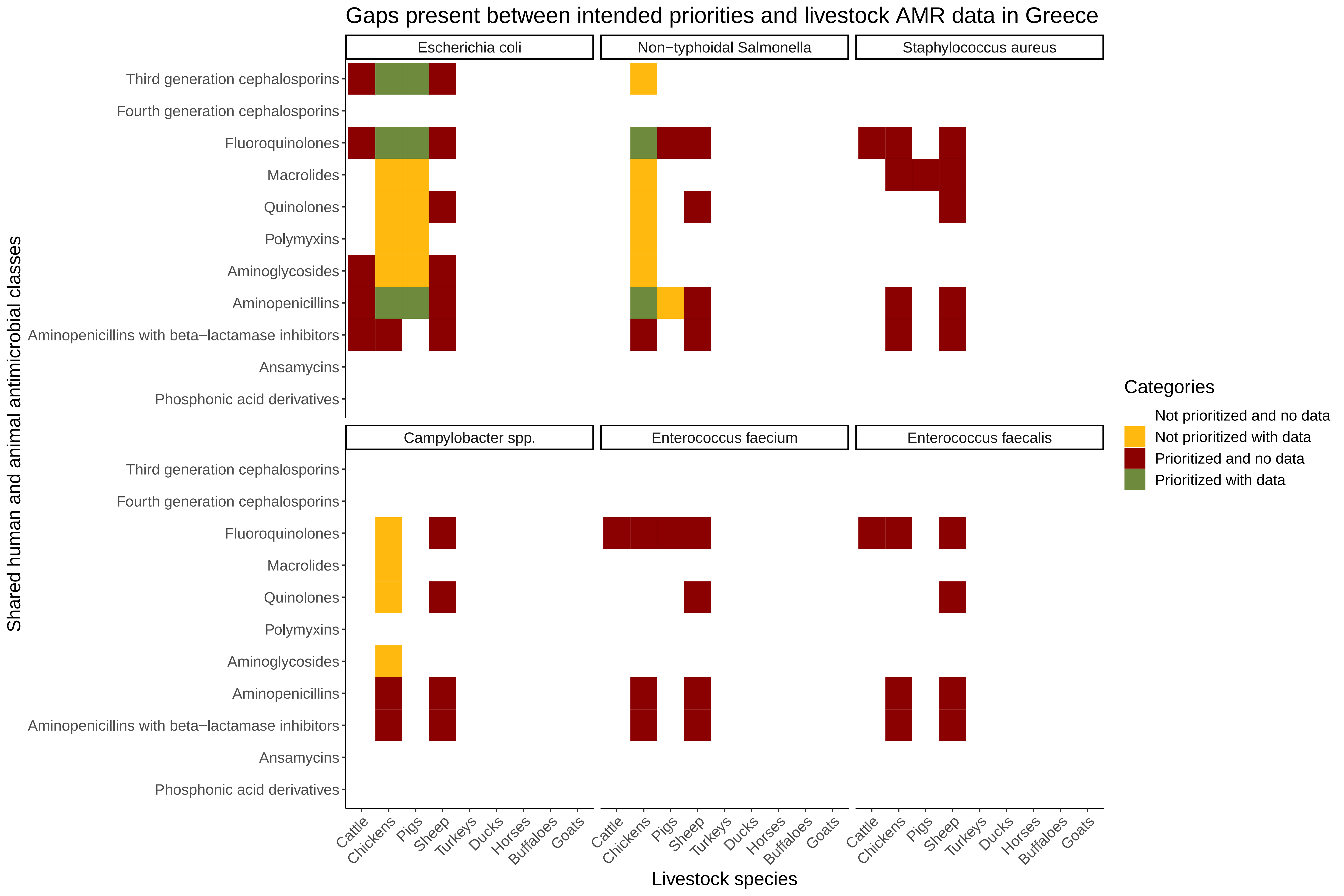

Gaps present between intended priorities and livestock AMR data in Greece
Escherichia coli
Non−typhoidal Salmonella
Staphylococcus aureus
Campylobacter spp.
Enterococcus faecium
Enterococcus faecalis
Third generation cephalosporins
Fourth generation cephalosporins
Fluoroquinolones
Macrolides
Quinolones
Polymyxins
Aminoglycosides
Aminopenicillins
Aminopenicillins with beta−lactamase inhibitors
Ansamycins
Phosphonic acid derivatives
Shared human and animal antimicrobial classes
Cattle
Chickens
Pigs
Sheep
Turkeys
Ducks
Horses
Buffaloes
Goats
Livestock species
Categories
Not prioritized and no data
Not prioritized with data
Prioritized and no data
Prioritized with data

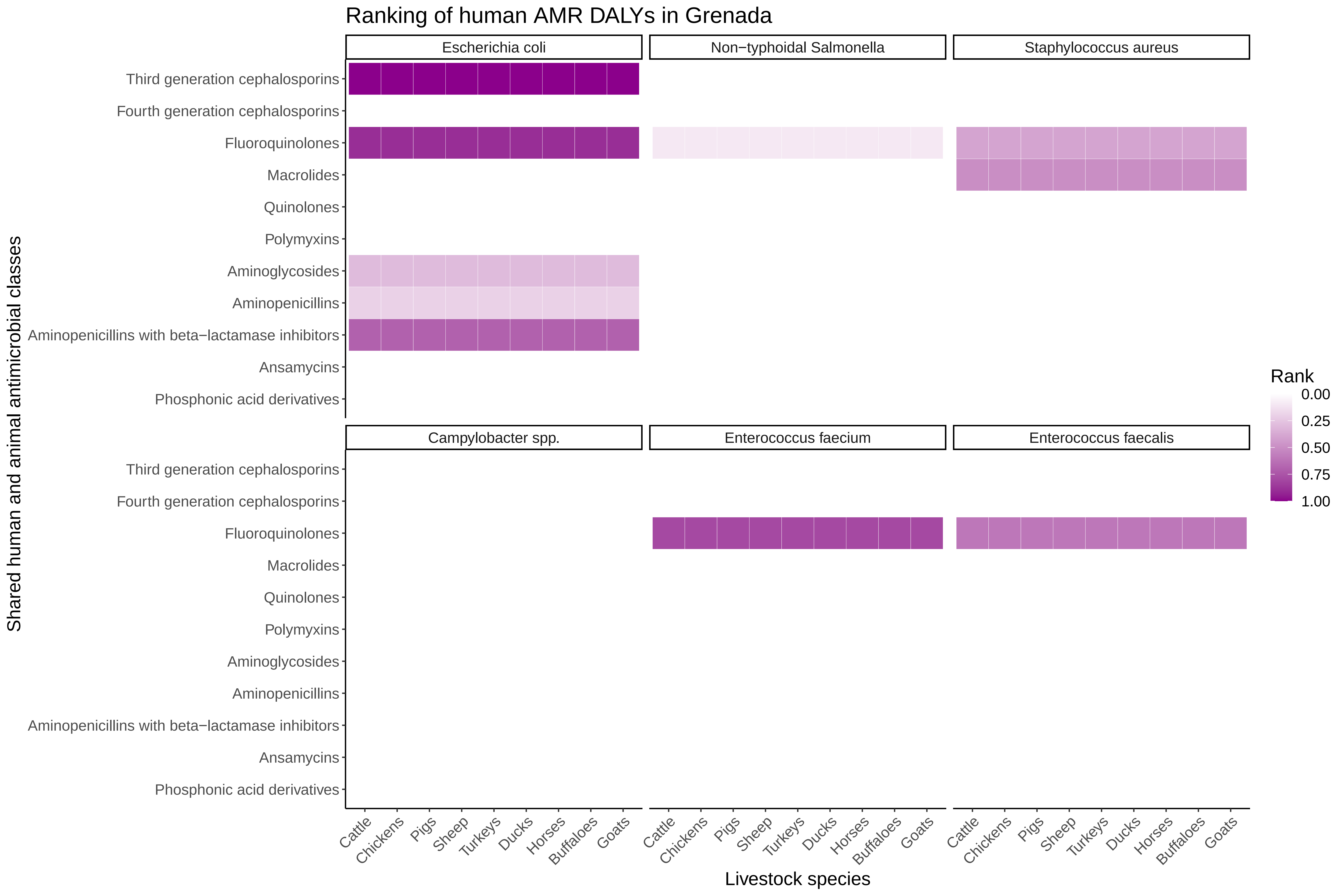
Ranking of human AMR DALYs in Grenada
Escherichia coli
Non−typhoidal Salmonella
Staphylococcus aureus
Campylobacter spp.
Enterococcus faecium
Enterococcus faecalis
Third generation cephalosporins
Fourth generation cephalosporins
Fluoroquinolones
Macrolides
Quinolones
Polymyxins
Aminoglycosides
Aminopenicillins
Aminopenicillins with beta−lactamase inhibitors
Ansamycins
Phosphonic acid derivatives
Shared human and animal antimicrobial classes
Cattle
Chickens
Pigs
Sheep
Turkeys
Ducks
Horses
Buffaloes
Goats
Livestock species
Rank
0.00
0.25
0.50
0.75
1.00

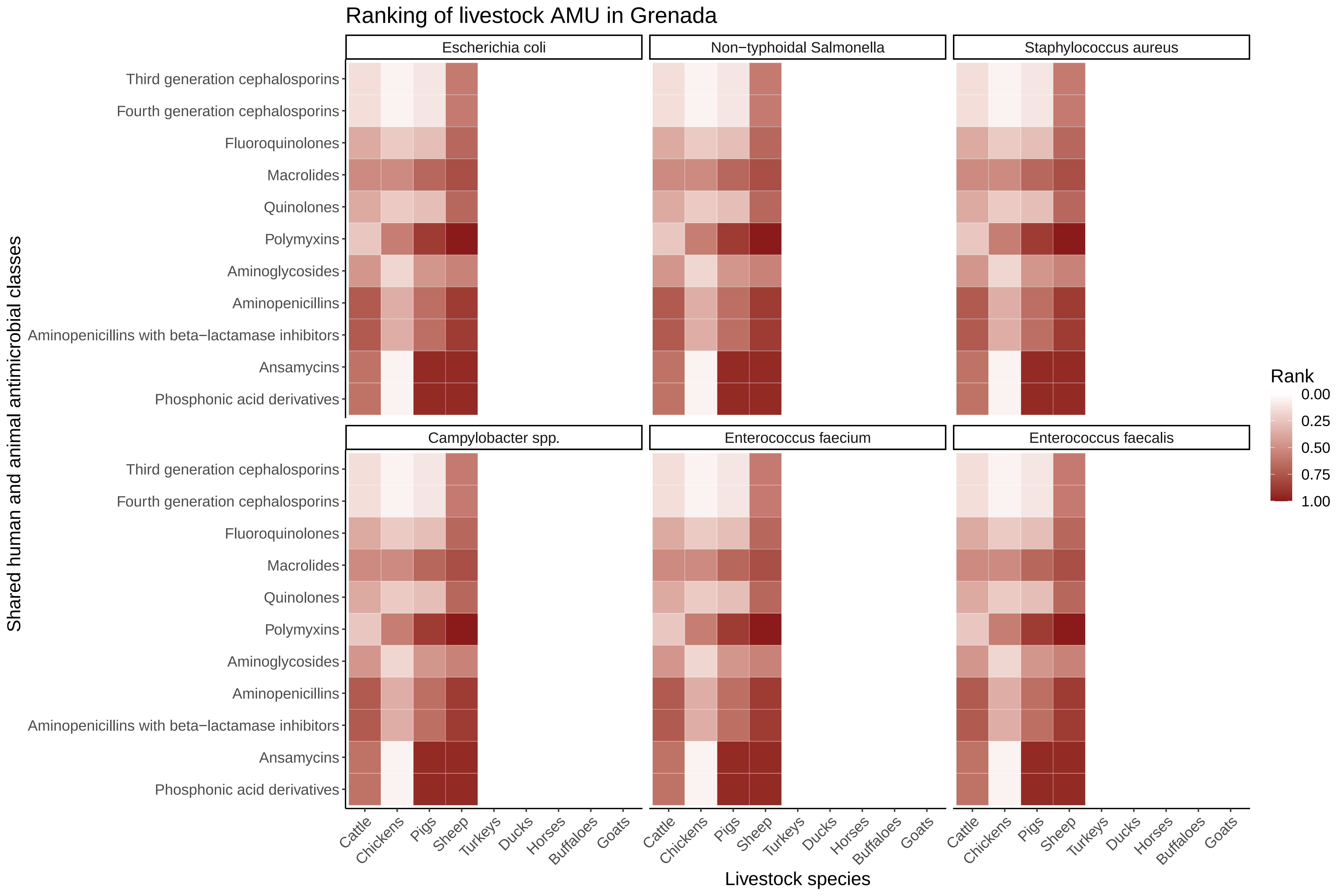
Ranking of livestock AMU in Grenada
Escherichia coli
Non−typhoidal Salmonella
Staphylococcus aureus
Campylobacter spp.
Enterococcus faecium
Enterococcus faecalis
Third generation cephalosporins
Fourth generation cephalosporins
Fluoroquinolones
Macrolides
Quinolones
Polymyxins
Aminoglycosides
Aminopenicillins
Aminopenicillins with beta−lactamase inhibitors
Ansamycins
Phosphonic acid derivatives
Shared human and animal antimicrobial classes
Cattle
Chickens
Pigs
Sheep
Turkeys
Ducks
Horses
Buffaloes
Goats
Livestock species
Rank
0.00
0.25
0.50
0.75
1.00

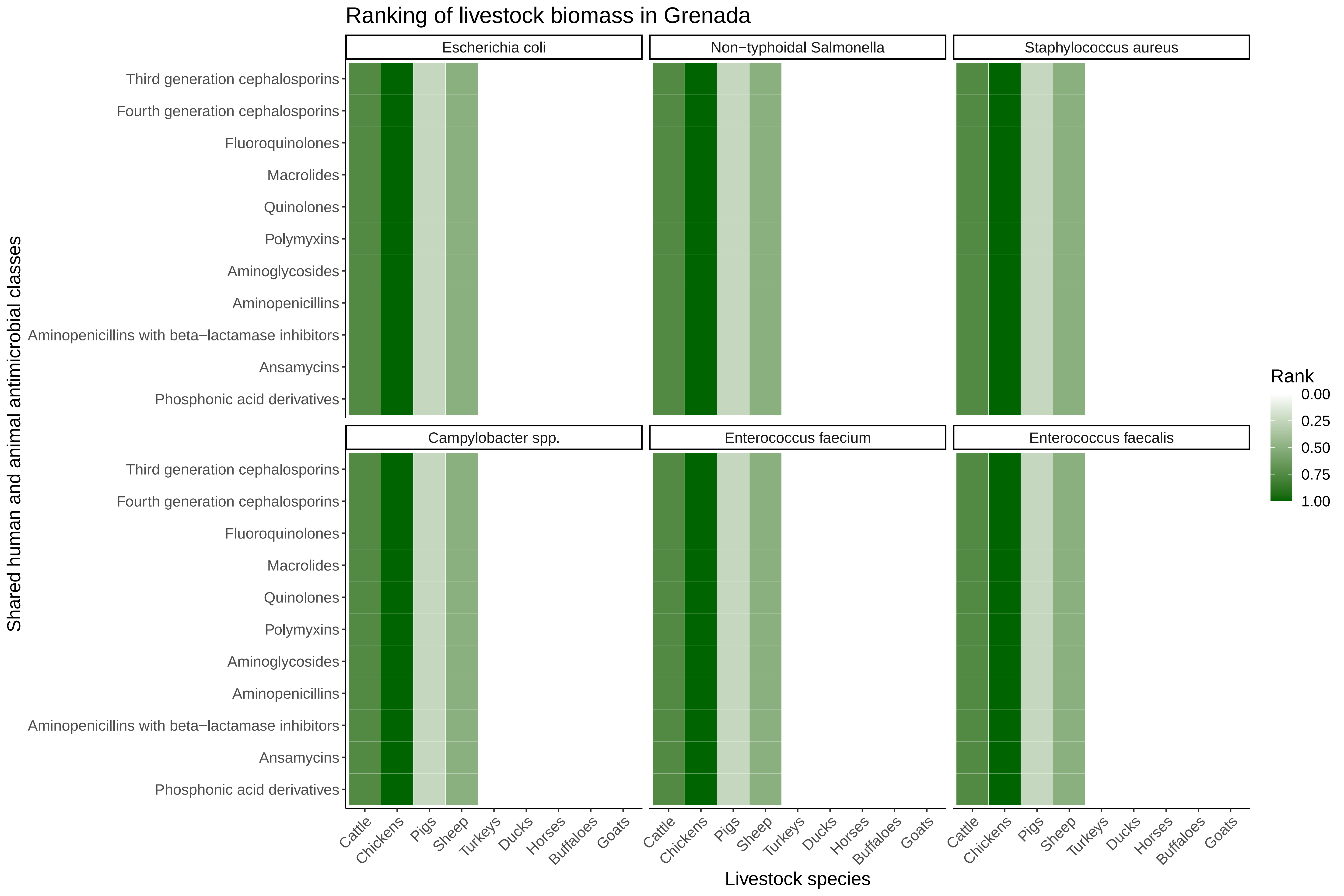
Ranking of livestock biomass in Grenada
Escherichia coli
Non−typhoidal Salmonella
Staphylococcus aureus
Campylobacter spp.
Enterococcus faecium
Enterococcus faecalis
Third generation cephalosporins
Fourth generation cephalosporins
Fluoroquinolones
Macrolides
Quinolones
Polymyxins
Aminoglycosides
Aminopenicillins
Aminopenicillins with beta−lactamase inhibitors
Ansamycins
Phosphonic acid derivatives
Shared human and animal antimicrobial classes
Cattle
Chickens
Pigs
Sheep
Turkeys
Ducks
Horses
Buffaloes
Goats
Livestock species
Rank
0.00
0.25
0.50
0.75
1.00

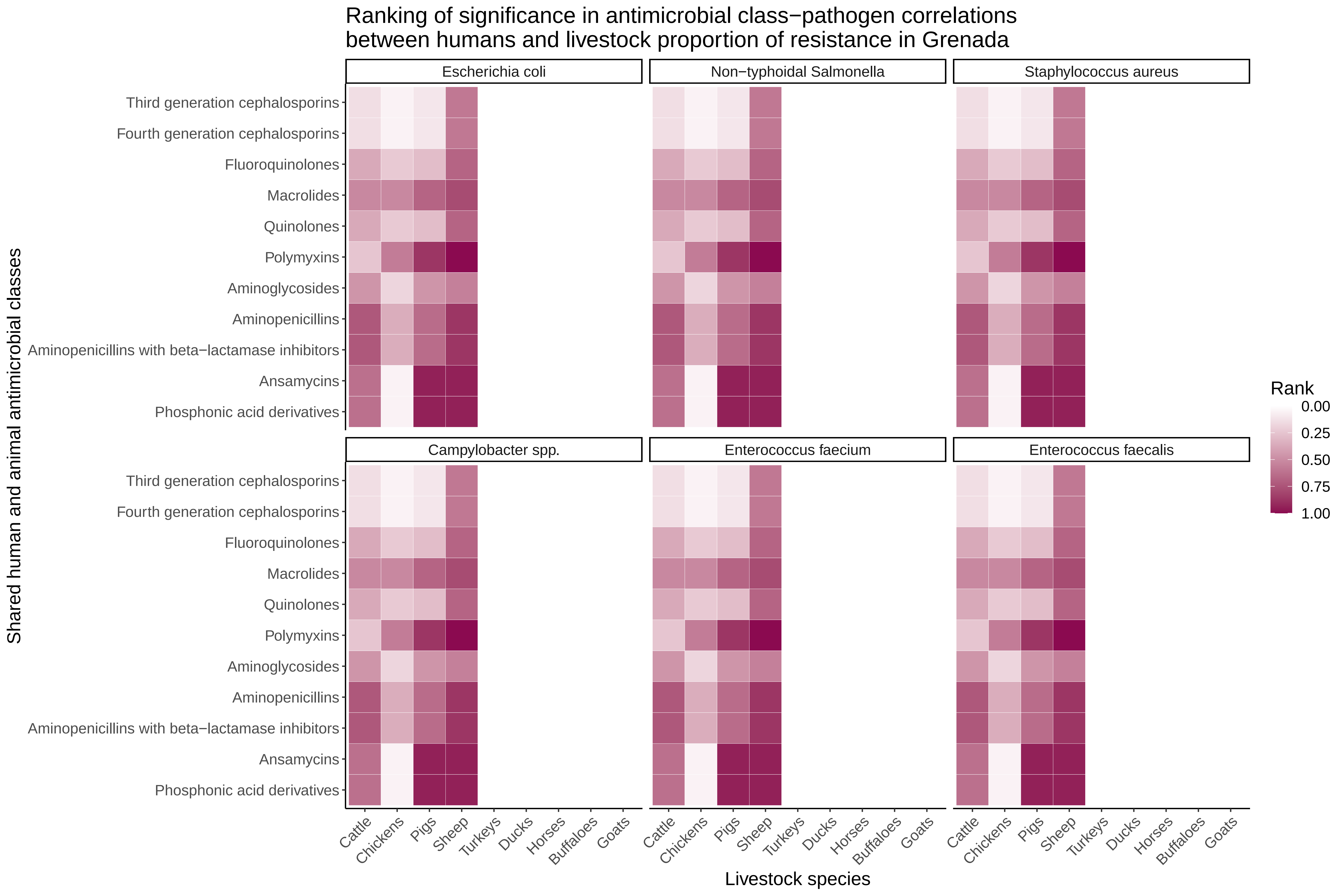

Ranking of significance in antimicrobial class−pathogen correlations
between humans and livestock proportion of resistance in Grenada
Escherichia coli
Non−typhoidal Salmonella
Staphylococcus aureus
Campylobacter spp.
Enterococcus faecium
Enterococcus faecalis
Third generation cephalosporins
Fourth generation cephalosporins
Fluoroquinolones
Macrolides
Quinolones
Polymyxins
Aminoglycosides
Aminopenicillins
Aminopenicillins with beta−lactamase inhibitors
Ansamycins
Phosphonic acid derivatives
Shared human and animal antimicrobial classes
Cattle
Chickens
Pigs
Sheep
Turkeys
Ducks
Horses
Buffaloes
Goats
Livestock species
Rank
0.00
0.25
0.50
0.75
1.00

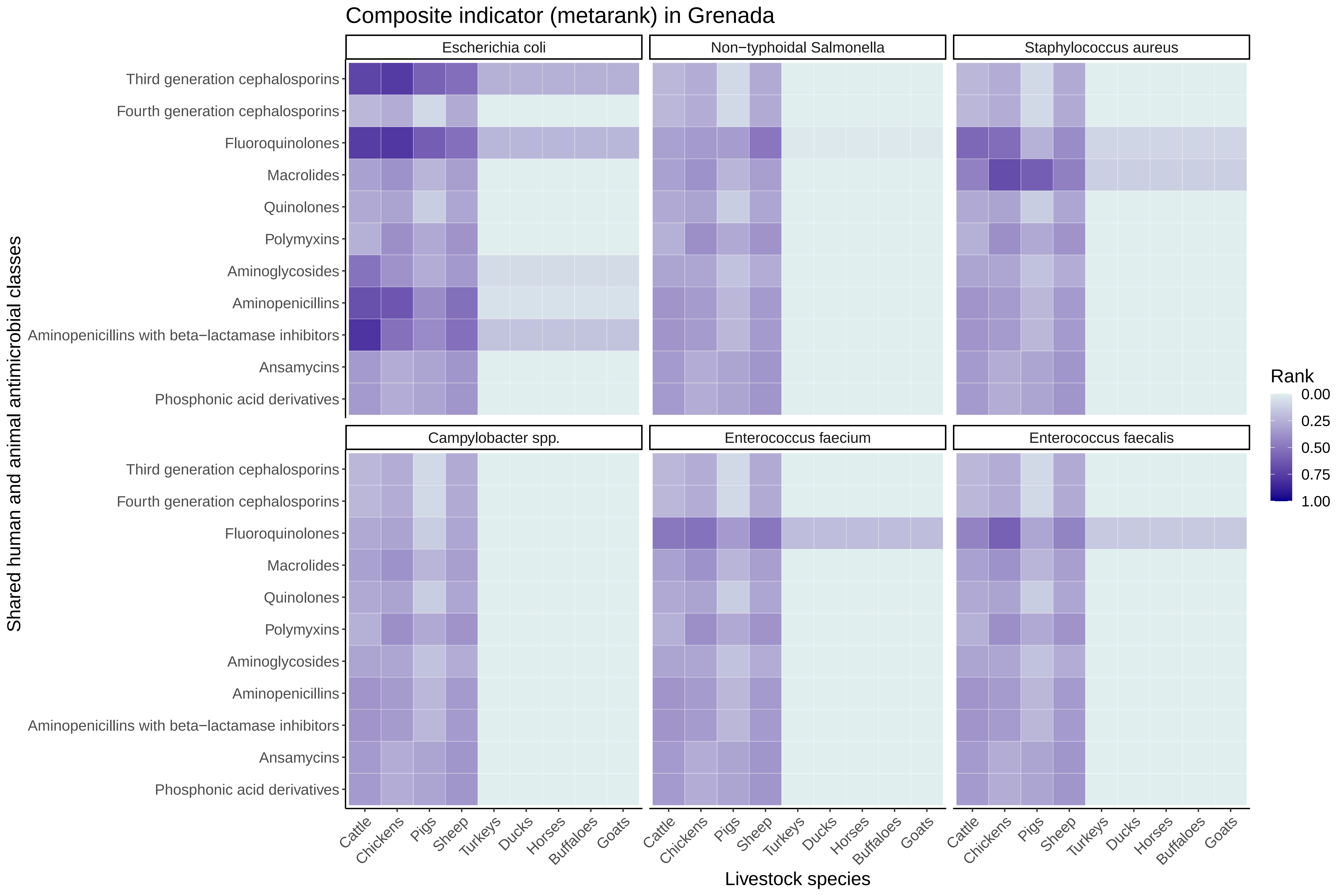

Composite indicator (metarank) in Grenada
Escherichia coli
Non−typhoidal Salmonella
Staphylococcus aureus
Campylobacter spp.
Enterococcus faecium
Enterococcus faecalis
Third generation cephalosporins
Fourth generation cephalosporins
Fluoroquinolones
Macrolides
Quinolones
Polymyxins
Aminoglycosides
Aminopenicillins
Aminopenicillins with beta−lactamase inhibitors
Ansamycins
Phosphonic acid derivatives
Shared human and animal antimicrobial classes
Cattle
Chickens
Pigs
Sheep
Turkeys
Ducks
Horses
Buffaloes
Goats
Livestock species
Rank
0.00
0.25
0.50
0.75
1.00

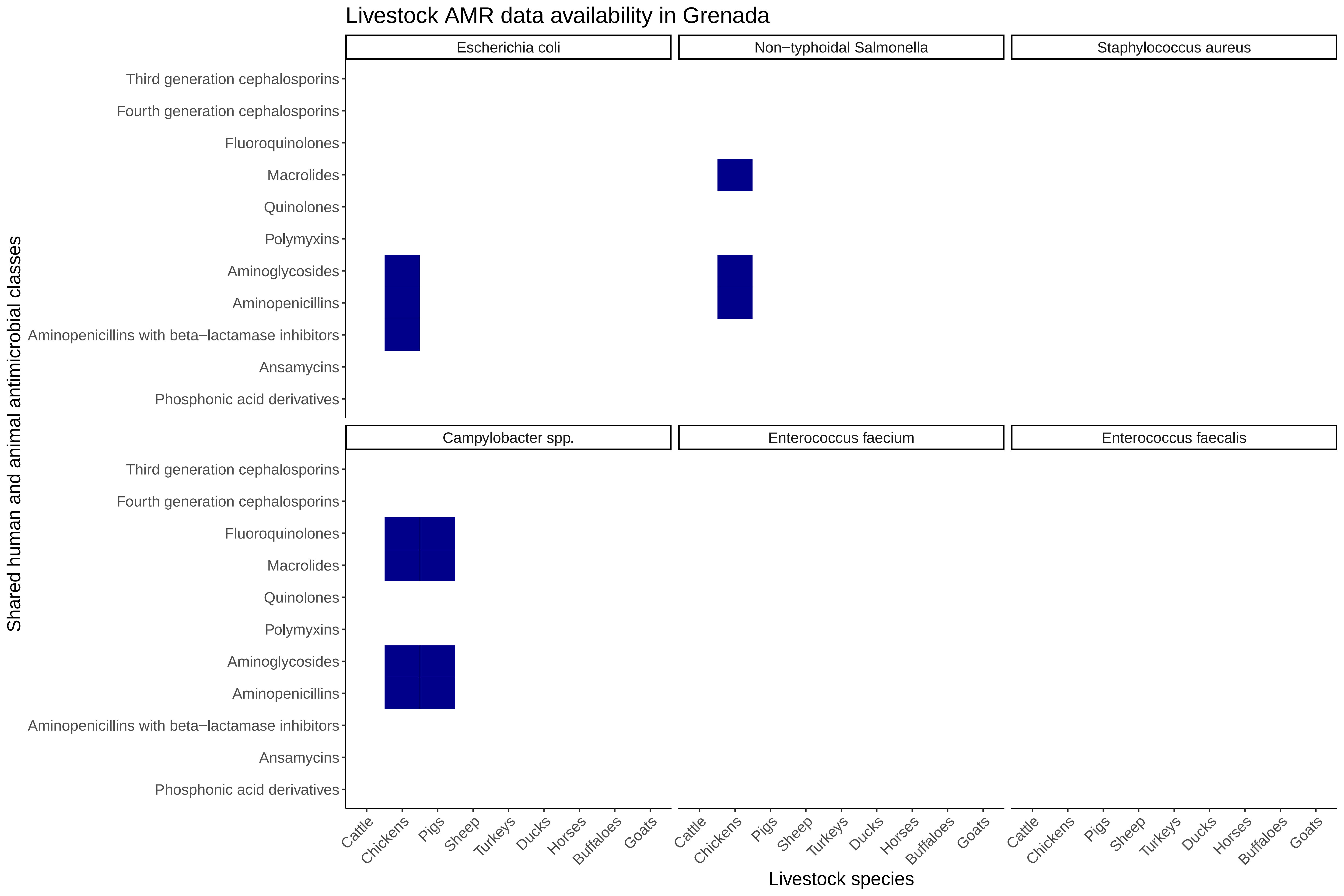
Livestock AMR data availability in Grenada
Escherichia coli
Non−typhoidal Salmonella
Staphylococcus aureus
Campylobacter spp.
Enterococcus faecium
Enterococcus faecalis
Shared human and animal antimicrobial classes
Third generation cephalosporins
Fourth generation cephalosporins
Fluoroquinolones
Macrolides
Quinolones
Polymyxins
Aminoglycosides
Aminopenicillins
Aminopenicillins with beta−lactamase inhibitors
Ansamycins
Phosphonic acid derivatives
Cattle
Chickens
Pigs
Sheep
Turkeys
Ducks
Horses
Buffaloes
Goats
Livestock species

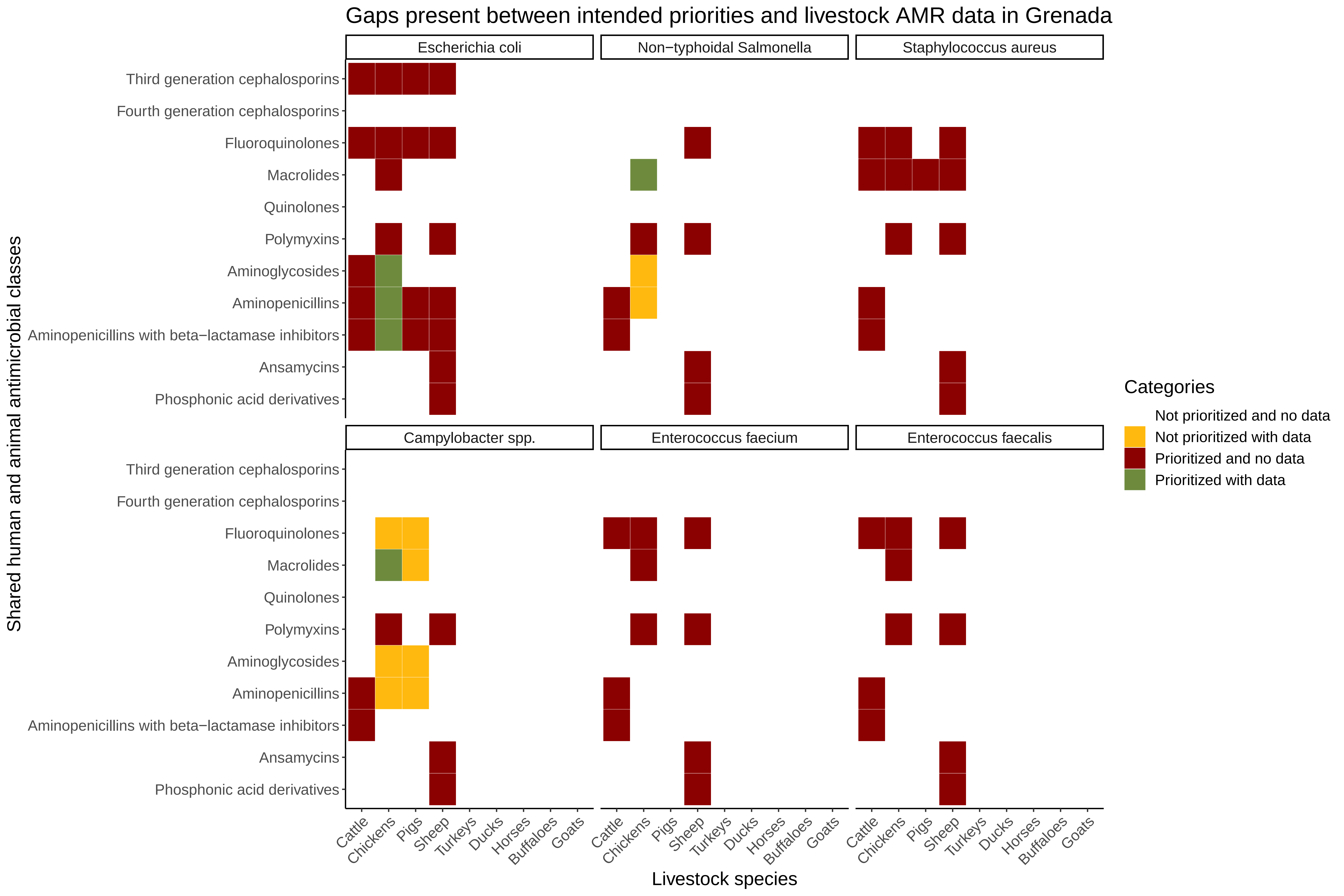

Gaps present between intended priorities and livestock AMR data in Grenada
Escherichia coli
Non−typhoidal Salmonella
Staphylococcus aureus
Campylobacter spp.
Enterococcus faecium
Enterococcus faecalis
Third generation cephalosporins
Fourth generation cephalosporins
Fluoroquinolones
Macrolides
Quinolones
Polymyxins
Aminoglycosides
Aminopenicillins
Aminopenicillins with beta−lactamase inhibitors
Ansamycins
Phosphonic acid derivatives
Shared human and animal antimicrobial classes
Cattle
Chickens
Pigs
Sheep
Turkeys
Ducks
Horses
Buffaloes
Goats
Livestock species
Categories
Not prioritized and no data
Not prioritized with data
Prioritized and no data
Prioritized with data

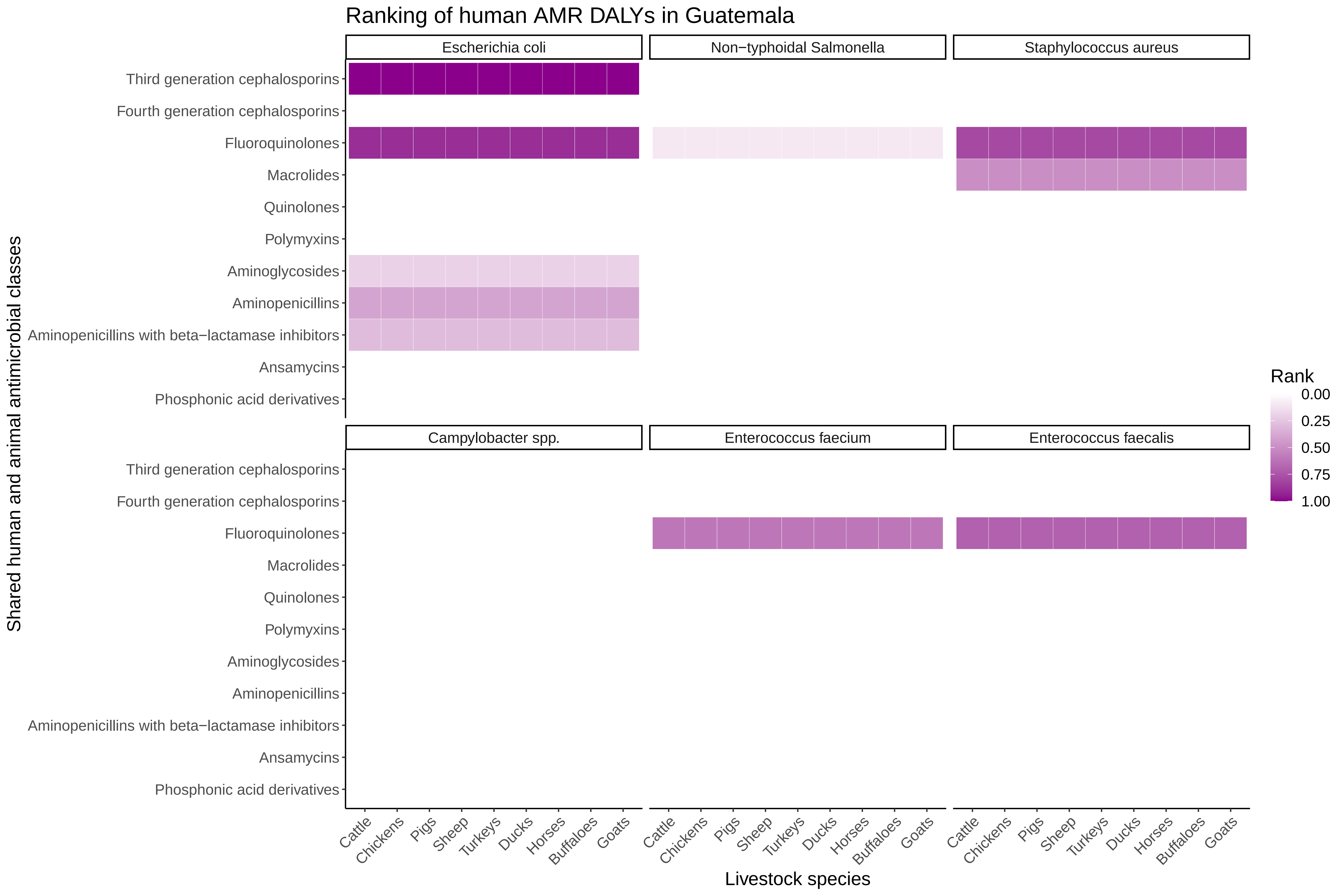

Ranking of human AMR DALYs in Guatemala
Escherichia coli
Non−typhoidal Salmonella
Staphylococcus aureus
Campylobacter spp.
Enterococcus faecium
Enterococcus faecalis
Shared human and animal antimicrobial classes
Third generation cephalosporins
Fourth generation cephalosporins
Fluoroquinolones
Macrolides
Quinolones
Polymyxins
Aminoglycosides
Aminopenicillins
Aminopenicillins with beta−lactamase inhibitors
Ansamycins
Phosphonic acid derivatives
Cattle
Chickens
Pigs
Sheep
Turkeys
Ducks
Horses
Buffaloes
Goats
Livestock species
Rank
0.00
0.25
0.50
0.75
1.00

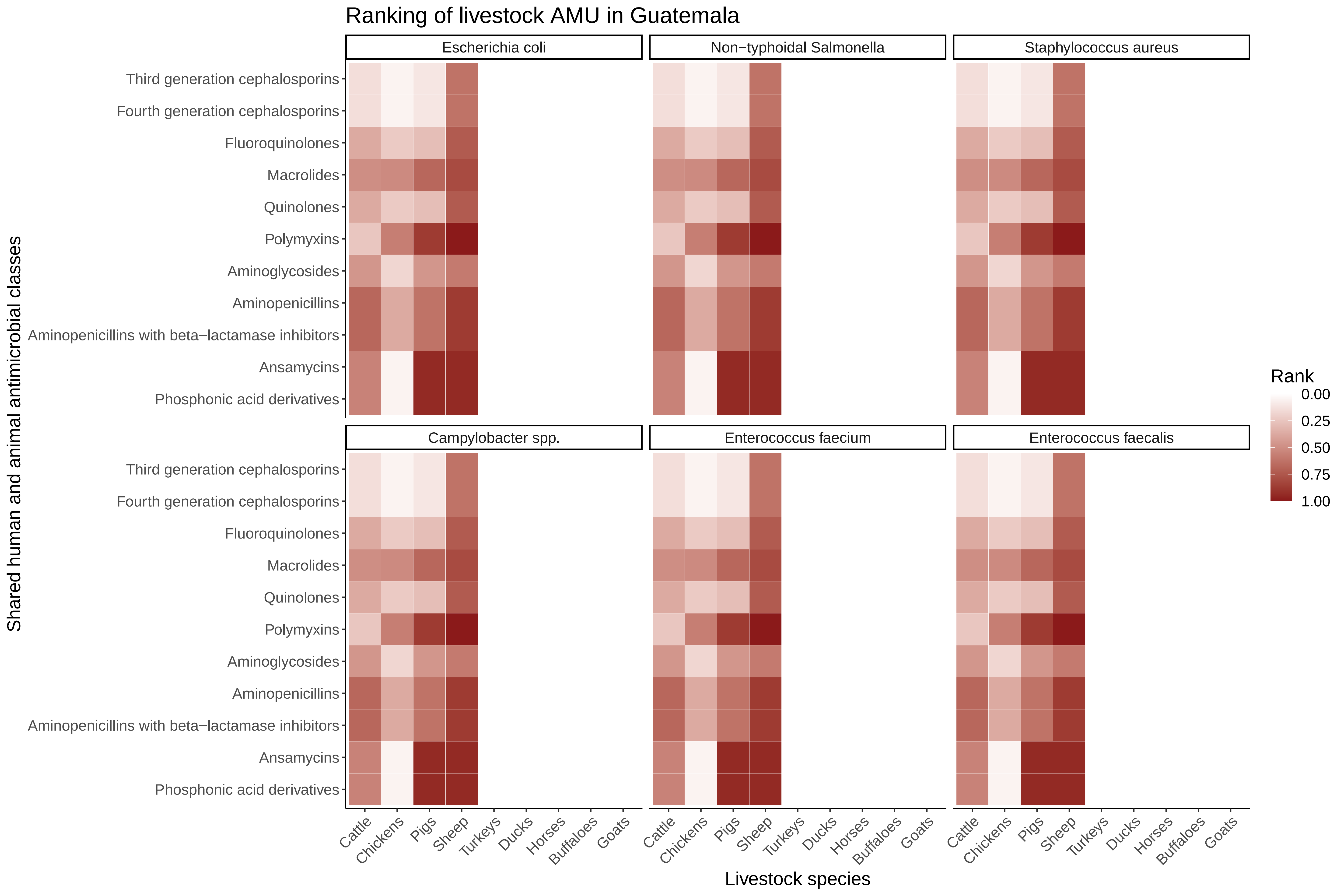

Ranking of livestock AMU in Guatemala
Escherichia coli
Non−typhoidal Salmonella
Staphylococcus aureus
Campylobacter spp.
Enterococcus faecium
Enterococcus faecalis
Third generation cephalosporins
Fourth generation cephalosporins
Fluoroquinolones
Macrolides
Quinolones
Polymyxins
Aminoglycosides
Aminopenicillins
Aminopenicillins with beta−lactamase inhibitors
Ansamycins
Phosphonic acid derivatives
Shared human and animal antimicrobial classes
Cattle
Chickens
Pigs
Sheep
Turkeys
Ducks
Horses
Buffaloes
Goats
Livestock species
Rank
0.00
0.25
0.50
0.75
1.00

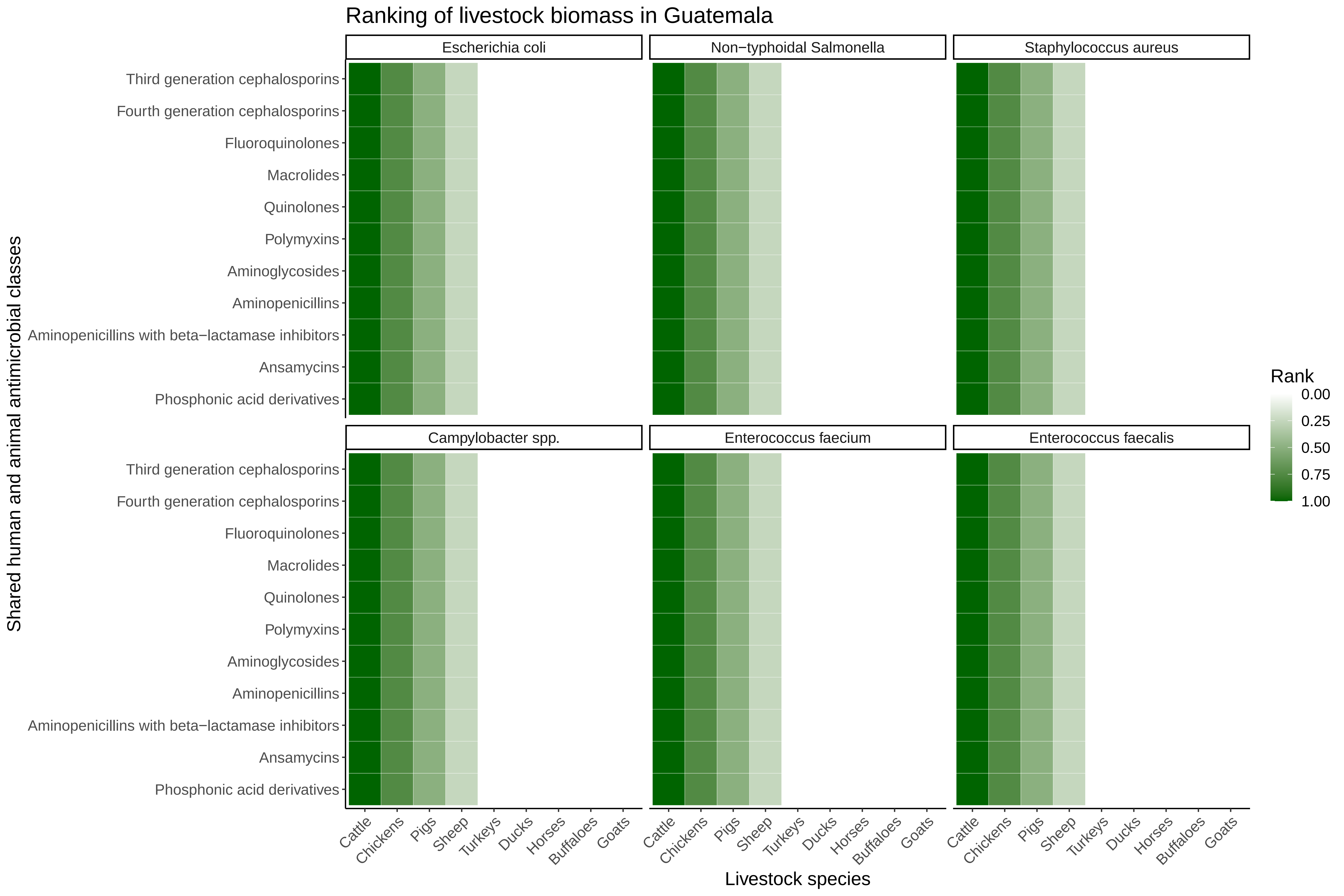
Ranking of livestock biomass in Guatemala
Escherichia coli
Non−typhoidal Salmonella
Staphylococcus aureus
Campylobacter spp.
Enterococcus faecium
Enterococcus faecalis
Third generation cephalosporins
Fourth generation cephalosporins
Fluoroquinolones
Macrolides
Quinolones
Polymyxins
Aminoglycosides
Aminopenicillins
Aminopenicillins with beta−lactamase inhibitors
Ansamycins
Phosphonic acid derivatives
Shared human and animal antimicrobial classes
Cattle
Chickens
Pigs
Sheep
Turkeys
Ducks
Horses
Buffaloes
Goats
Livestock species
Rank
0.00
0.25
0.50
0.75
1.00

Ranking of significance in antimicrobial class−pathogen correlations between humans and livestock proportion of resistance in Guatemala

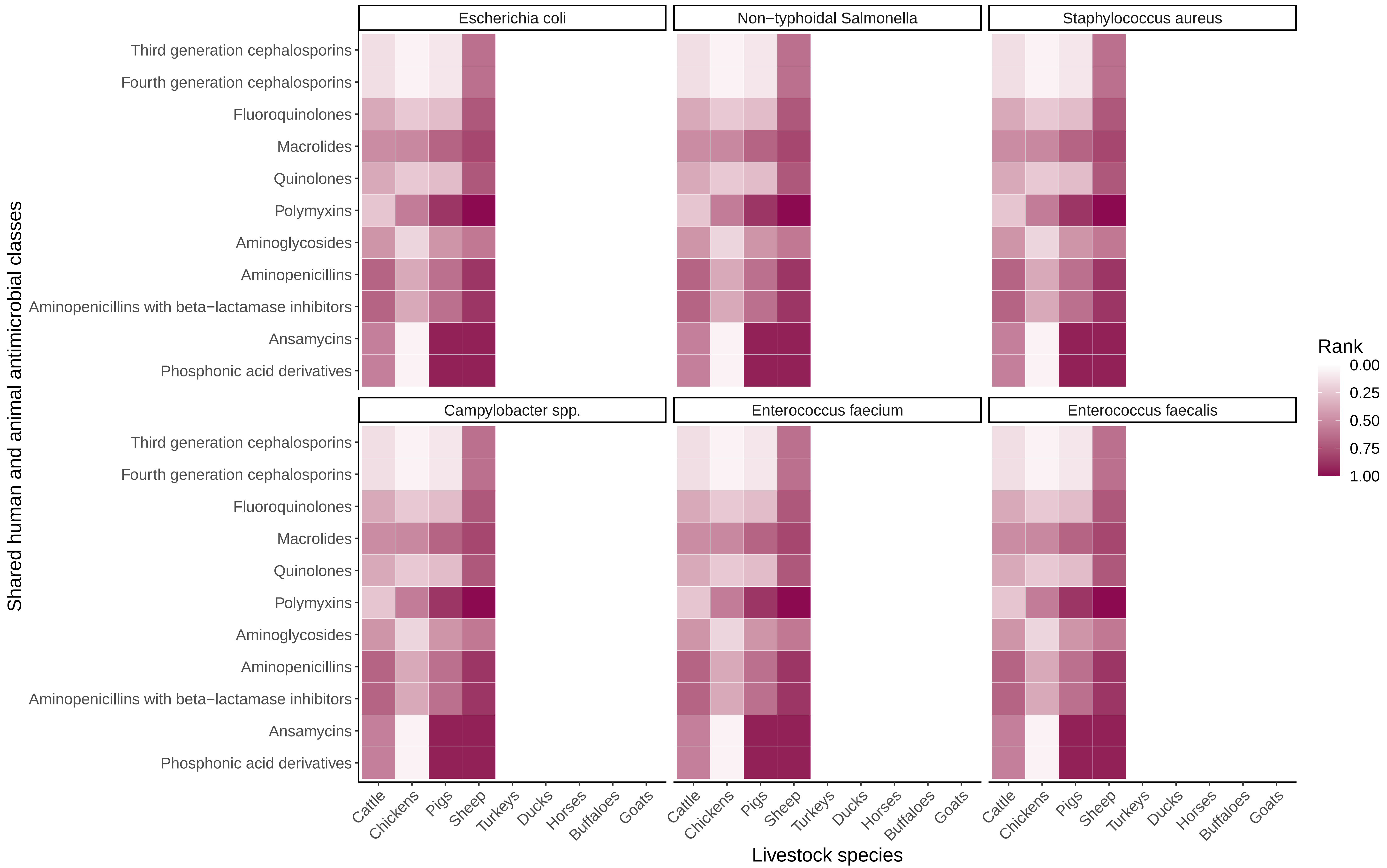

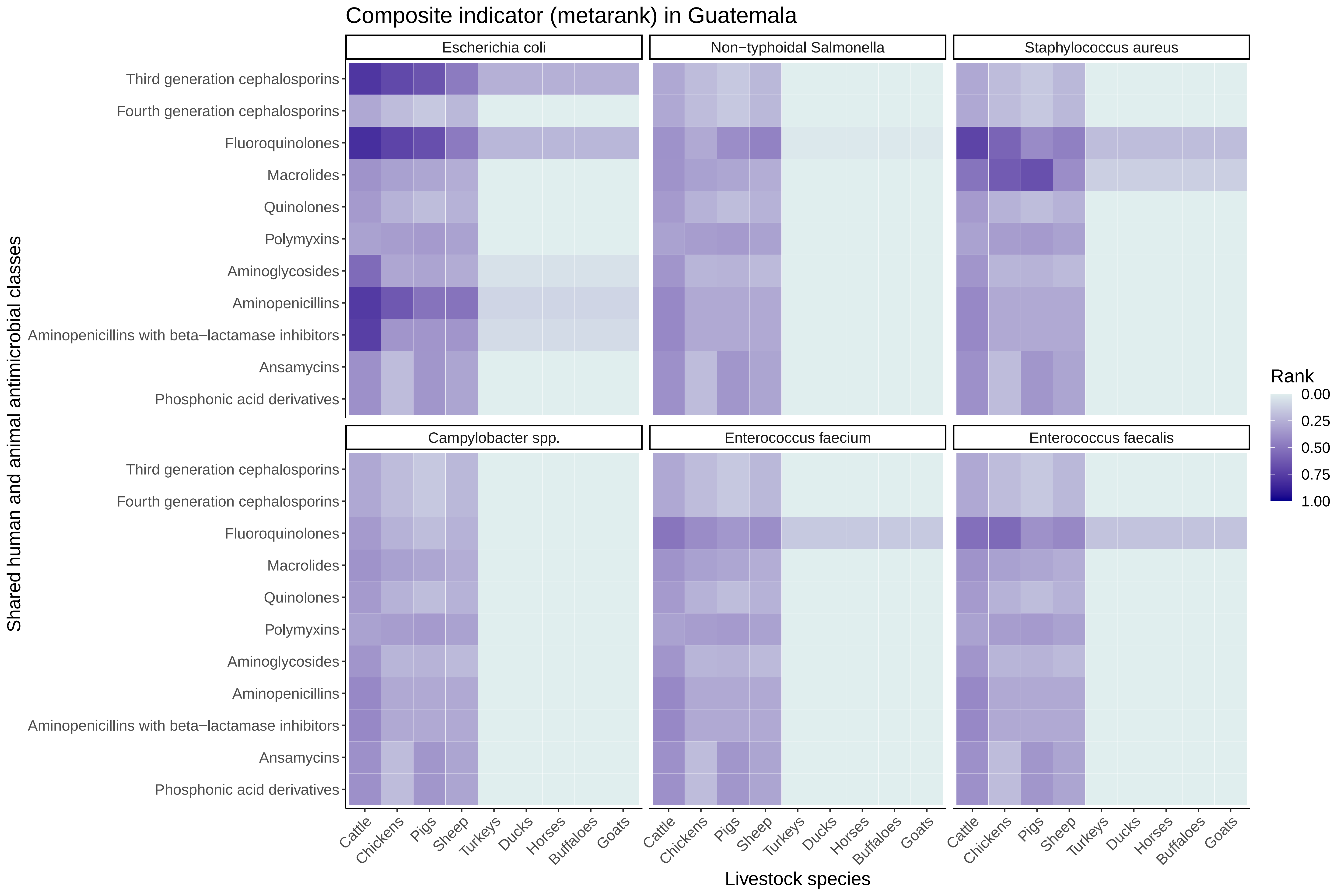
Composite indicator (metarank) in Guatemala
Escherichia coli
Non−typhoidal Salmonella
Staphylococcus aureus
Campylobacter spp.
Enterococcus faecium
Enterococcus faecalis
Shared human and animal antimicrobial classes
Third generation cephalosporins
Fourth generation cephalosporins
Fluoroquinolones
Macrolides
Quinolones
Polymyxins
Aminoglycosides
Aminopenicillins
Aminopenicillins with beta−lactamase inhibitors
Ansamycins
Phosphonic acid derivatives
Cattle
Chickens
Pigs
Sheep
Turkeys
Ducks
Horses
Buffaloes
Goats
Livestock species
Rank
0.00
0.25
0.50
0.75
1.00

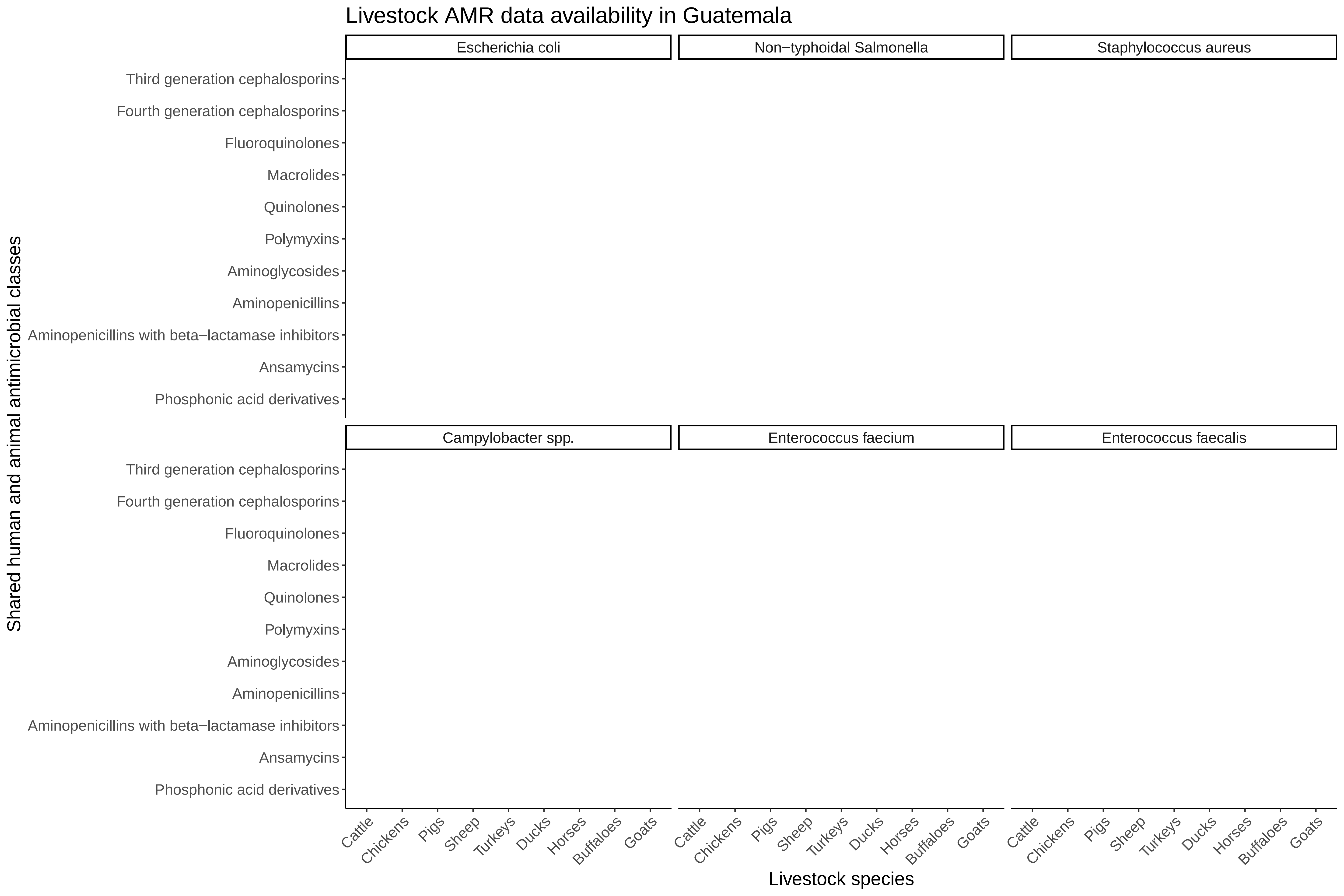
Livestock AMR data availability in Guatemala
Escherichia coli
Non−typhoidal Salmonella
Staphylococcus aureus
Campylobacter spp.
Enterococcus faecium
Enterococcus faecalis
Third generation cephalosporins
Fourth generation cephalosporins
Fluoroquinolones
Macrolides
Quinolones
Polymyxins
Aminoglycosides
Aminopenicillins
Aminopenicillins with beta−lactamase inhibitors
Ansamycins
Phosphonic acid derivatives
Shared human and animal antimicrobial classes
Cattle
Chickens
Pigs
Sheep
Turkeys
Ducks
Horses
Buffaloes
Goats
Livestock species

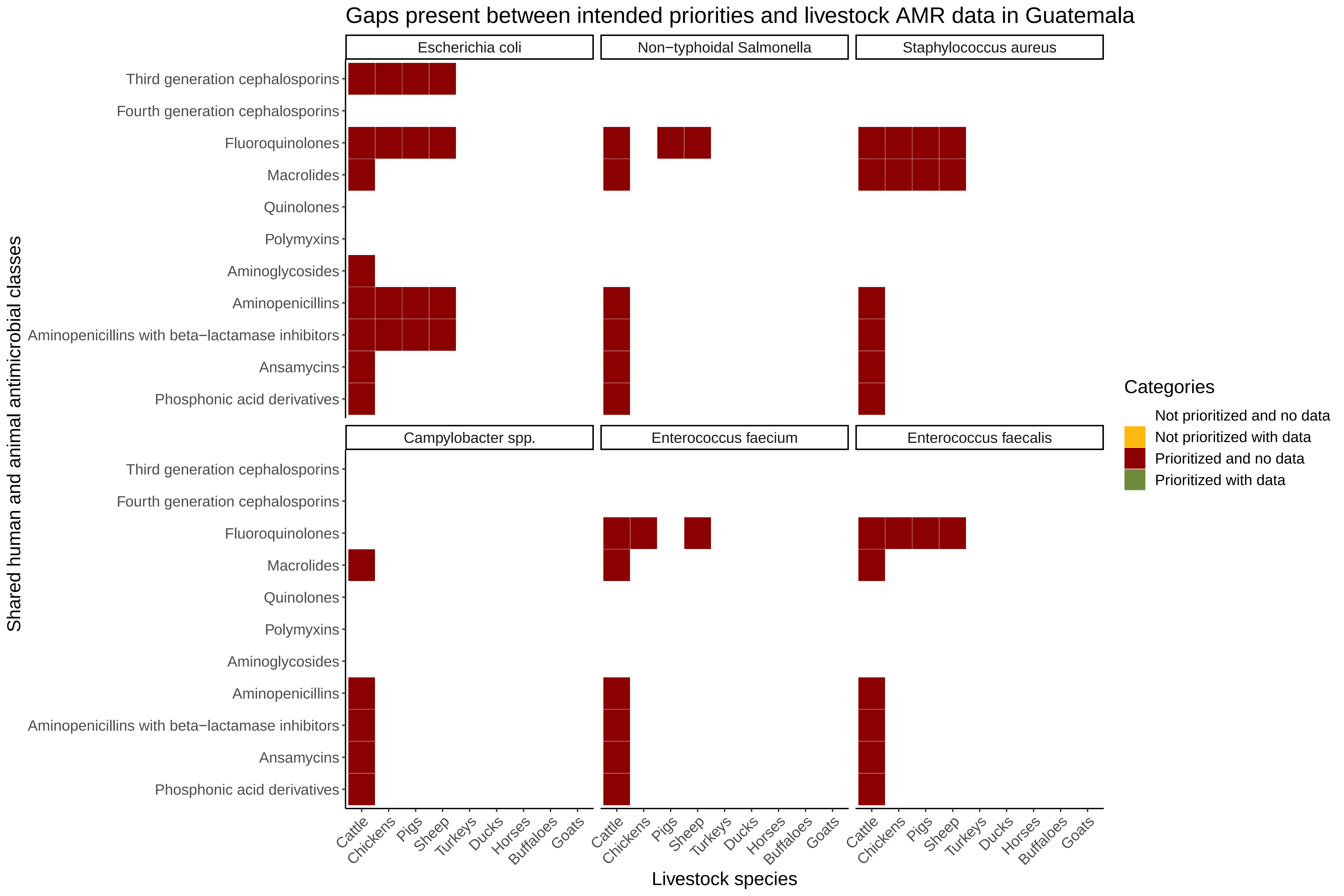
Gaps present between intended priorities and livestock AMR data in Guatemala
Escherichia coli
Non−typhoidal Salmonella
Staphylococcus aureus
Campylobacter spp.
Enterococcus faecium
Enterococcus faecalis
Third generation cephalosporins
Fourth generation cephalosporins
Fluoroquinolones
Macrolides
Quinolones
Polymyxins
Aminoglycosides
Aminopenicillins
Aminopenicillins with beta−lactamase inhibitors
Ansamycins
Phosphonic acid derivatives
Shared human and animal antimicrobial classes
Cattle
Chickens
Pigs
Sheep
Turkeys
Ducks
Horses
Buffaloes
Goats
Livestock species
Categories
Not prioritized and no data
Not prioritized with data
Prioritized and no data
Prioritized with data

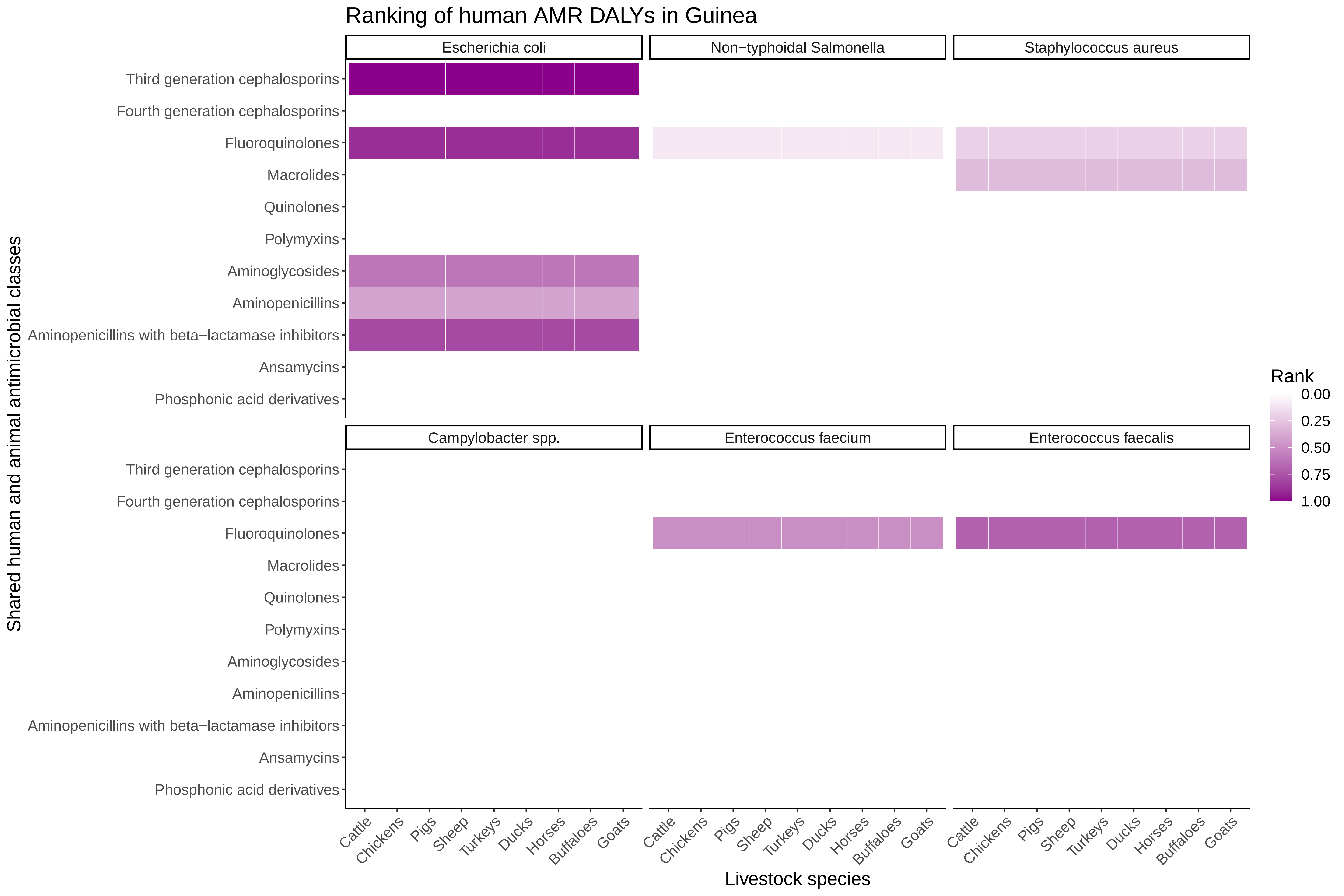

Ranking of human AMR DALYs in Guinea
Escherichia coli
Non−typhoidal Salmonella
Staphylococcus aureus
Campylobacter spp.
Enterococcus faecium
Enterococcus faecalis
Third generation cephalosporins
Fourth generation cephalosporins
Fluoroquinolones
Macrolides
Quinolones
Polymyxins
Aminoglycosides
Aminopenicillins
Aminopenicillins with beta−lactamase inhibitors
Ansamycins
Phosphonic acid derivatives
Shared human and animal antimicrobial classes
Cattle
Chickens
Pigs
Sheep
Turkeys
Ducks
Horses
Buffaloes
Goats
Livestock species
Rank
0.00
0.25
0.50
0.75
1.00

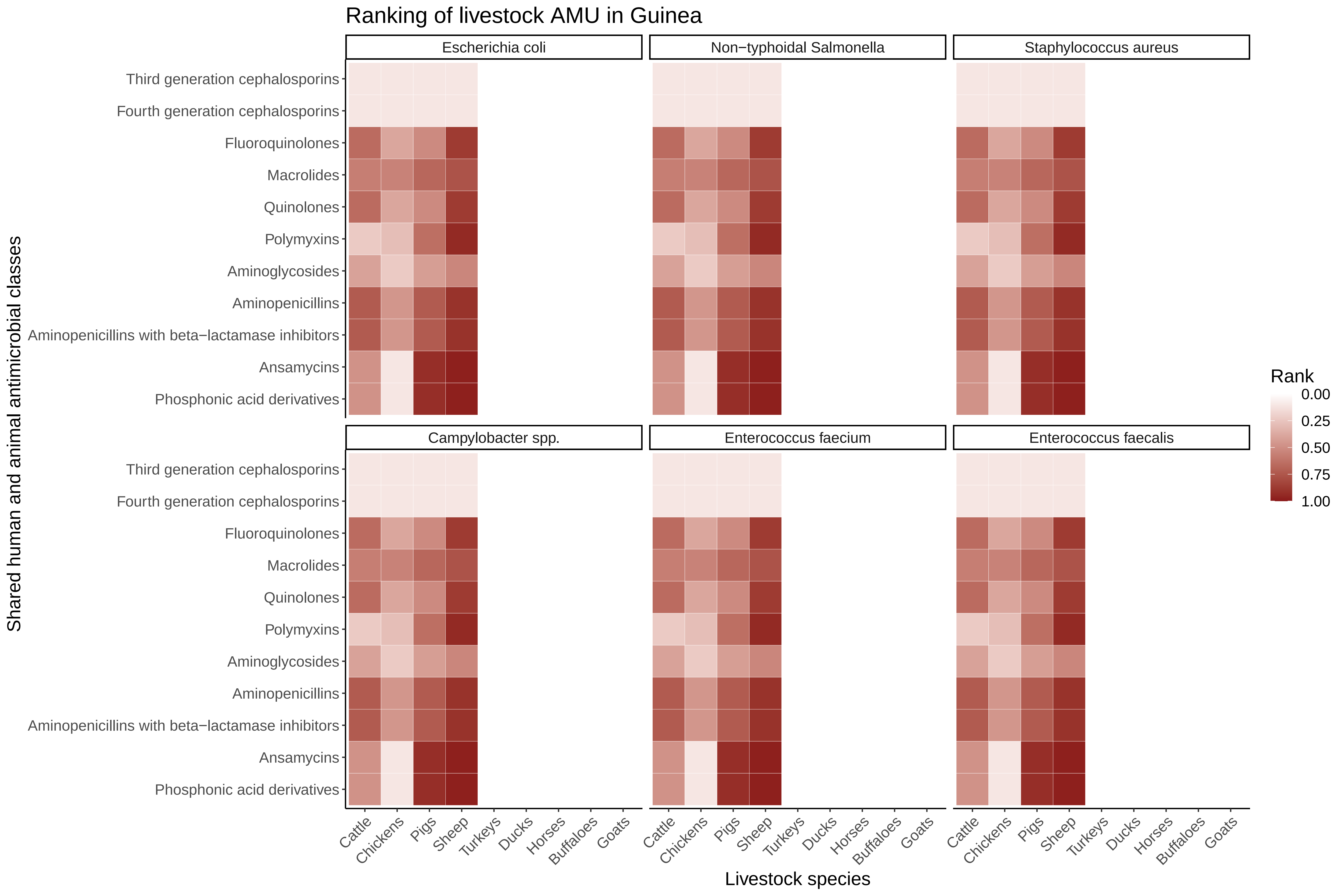
Ranking of livestock AMU in Guinea
Escherichia coli
Non−typhoidal Salmonella
Staphylococcus aureus
Campylobacter spp.
Enterococcus faecium
Enterococcus faecalis
Third generation cephalosporins
Fourth generation cephalosporins
Fluoroquinolones
Macrolides
Quinolones
Polymyxins
Aminoglycosides
Aminopenicillins
Aminopenicillins with beta−lactamase inhibitors
Ansamycins
Phosphonic acid derivatives
Shared human and animal antimicrobial classes
Cattle
Chickens
Pigs
Sheep
Turkeys
Ducks
Horses
Buffaloes
Goats
Livestock species
Rank
0.00
0.25
0.50
0.75
1.00

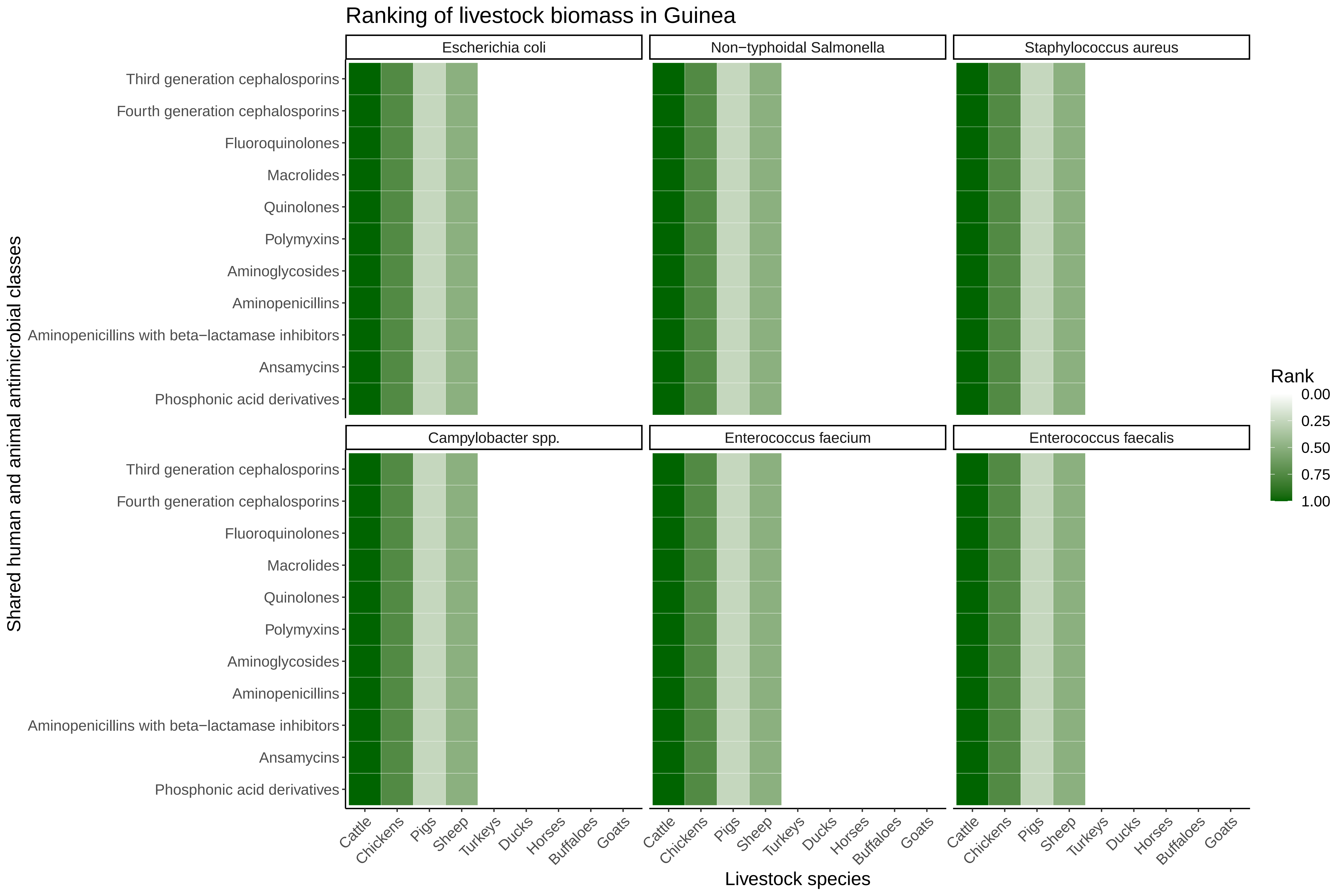

Ranking of livestock biomass in Guinea
Escherichia coli
Non−typhoidal Salmonella
Staphylococcus aureus
Campylobacter spp.
Enterococcus faecium
Enterococcus faecalis
Third generation cephalosporins
Fourth generation cephalosporins
Fluoroquinolones
Macrolides
Quinolones
Polymyxins
Aminoglycosides
Aminopenicillins
Aminopenicillins with beta−lactamase inhibitors
Ansamycins
Phosphonic acid derivatives
Shared human and animal antimicrobial classes
Cattle
Chickens
Pigs
Sheep
Turkeys
Ducks
Horses
Buffaloes
Goats
Livestock species
Rank
0.00
0.25
0.50
0.75
1.00

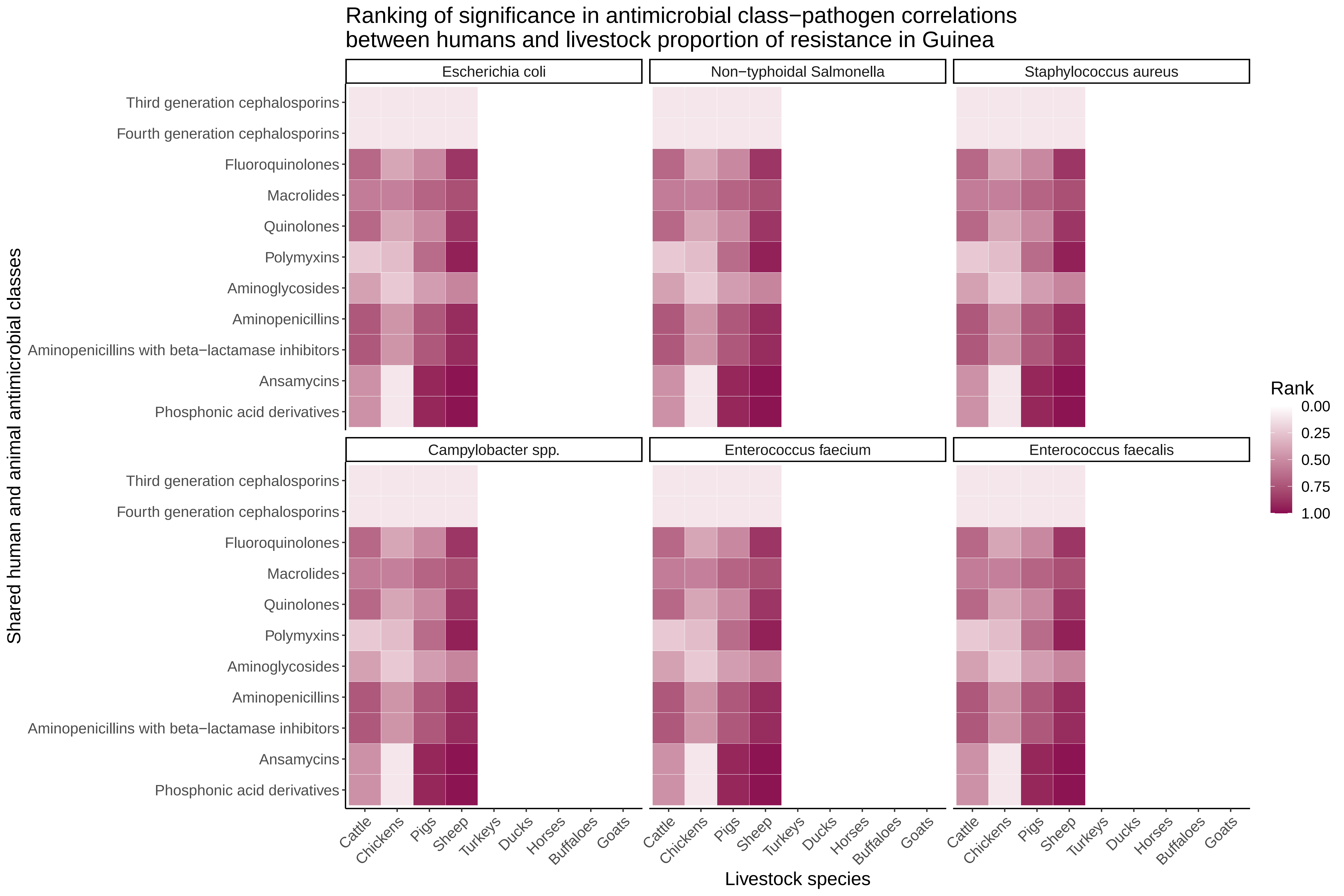
Ranking of significance in antimicrobial class−pathogen correlations
between humans and livestock proportion of resistance in Guinea
Escherichia coli
Non−typhoidal Salmonella
Staphylococcus aureus
Campylobacter spp.
Enterococcus faecium
Enterococcus faecalis
Third generation cephalosporins
Fourth generation cephalosporins
Fluoroquinolones
Macrolides
Quinolones
Polymyxins
Aminoglycosides
Aminopenicillins
Aminopenicillins with beta−lactamase inhibitors
Ansamycins
Phosphonic acid derivatives
Shared human and animal antimicrobial classes
Cattle
Chickens
Pigs
Sheep
Turkeys
Ducks
Horses
Buffaloes
Goats
Livestock species
Rank
0.00
0.25
0.50
0.75
1.00

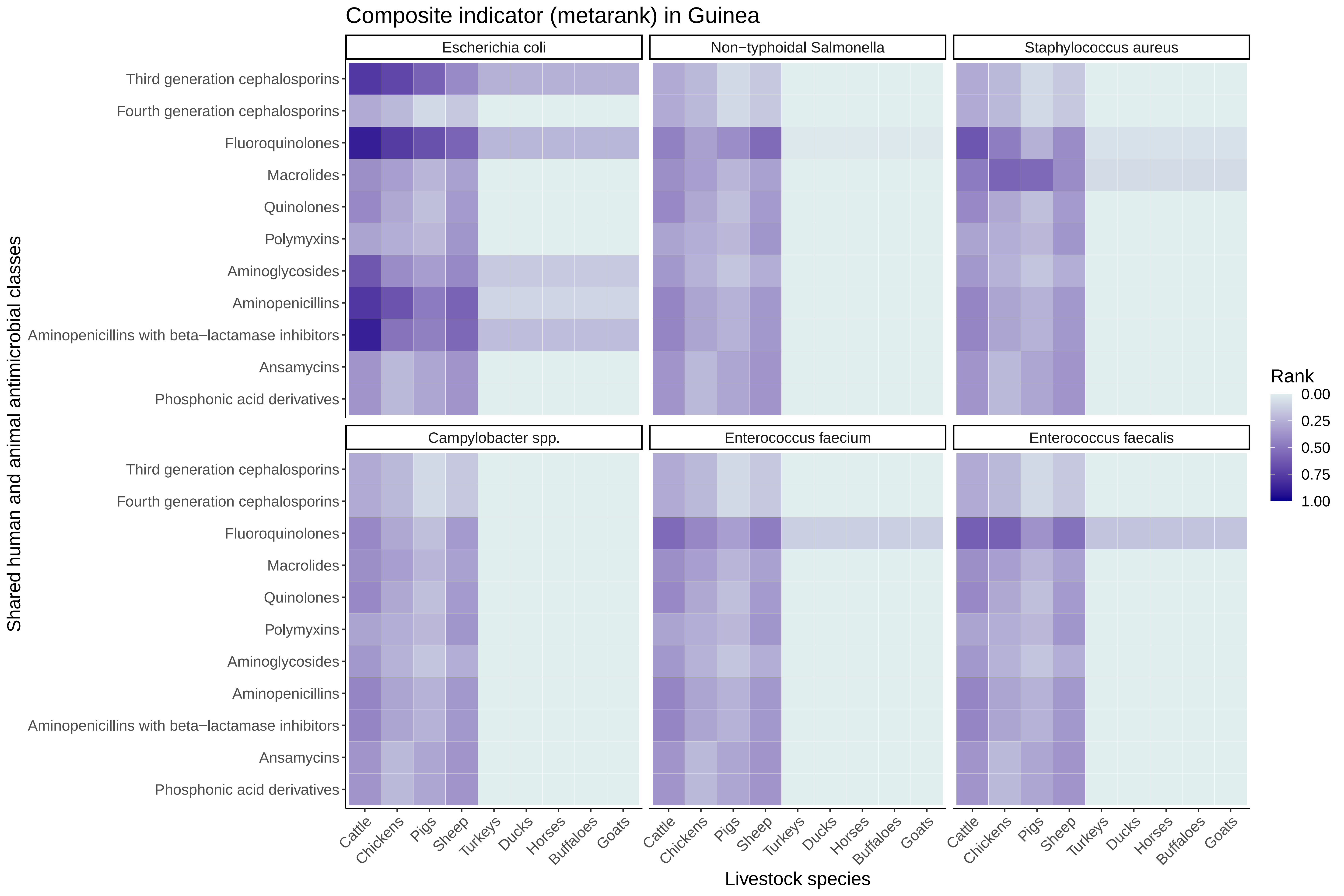

Composite indicator (metarank) in Guinea
Escherichia coli
Non−typhoidal Salmonella
Staphylococcus aureus
Campylobacter spp.
Enterococcus faecium
Enterococcus faecalis
Shared human and animal antimicrobial classes
Third generation cephalosporins
Fourth generation cephalosporins
Fluoroquinolones
Macrolides
Quinolones
Polymyxins
Aminoglycosides
Aminopenicillins
Aminopenicillins with beta−lactamase inhibitors
Ansamycins
Phosphonic acid derivatives
Cattle
Chickens
Pigs
Sheep
Turkeys
Ducks
Horses
Buffaloes
Goats
Livestock species
Rank
0.00
0.25
0.50
0.75
1.00

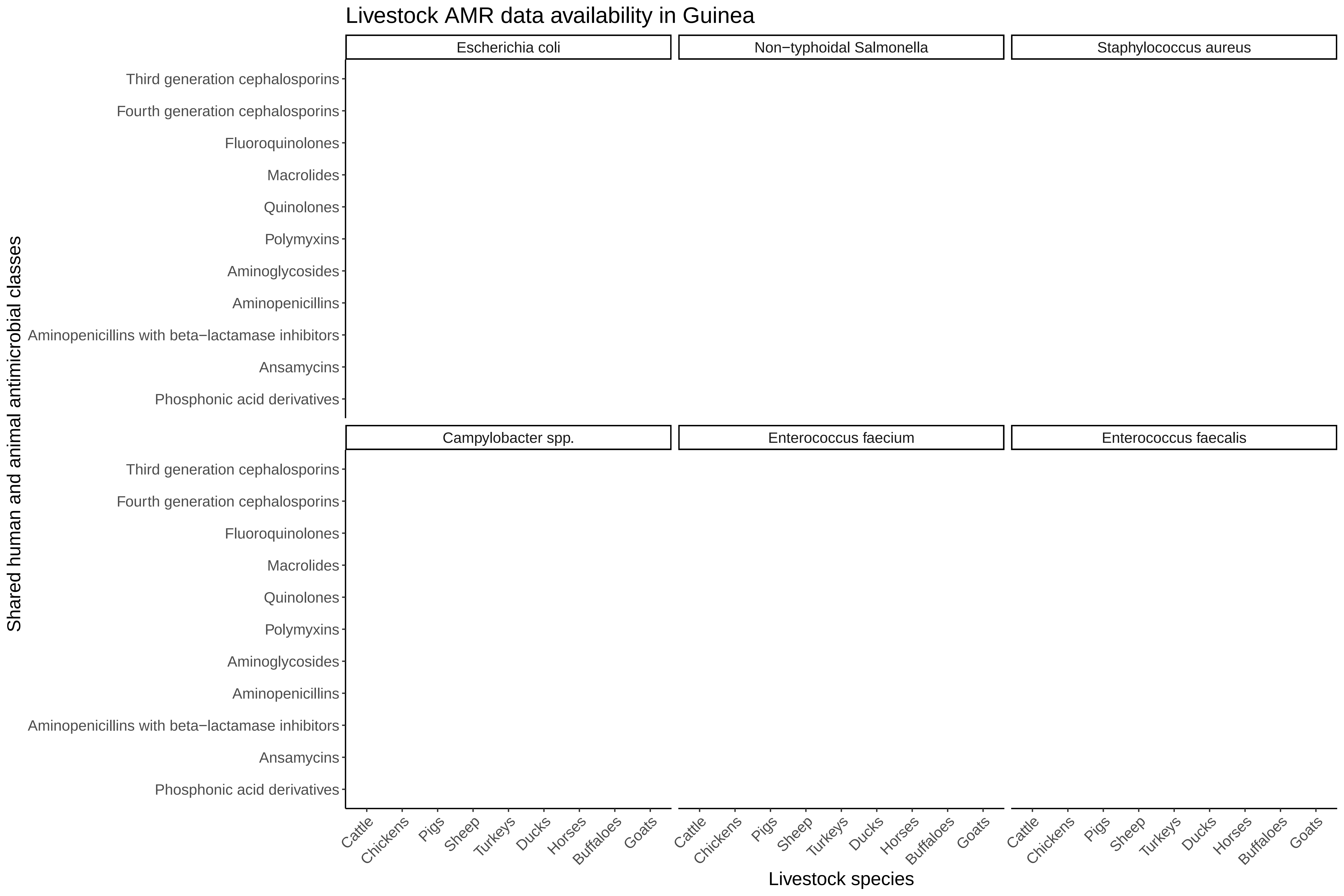
Livestock AMR data availability in Guinea
Escherichia coli
Non−typhoidal Salmonella
Staphylococcus aureus
Campylobacter spp.
Enterococcus faecium
Enterococcus faecalis
Third generation cephalosporins
Fourth generation cephalosporins
Fluoroquinolones
Macrolides
Quinolones
Polymyxins
Aminoglycosides
Aminopenicillins
Aminopenicillins with beta−lactamase inhibitors
Ansamycins
Phosphonic acid derivatives
Shared human and animal antimicrobial classes
Cattle
Chickens
Pigs
Sheep
Turkeys
Ducks
Horses
Buffaloes
Goats
Livestock species

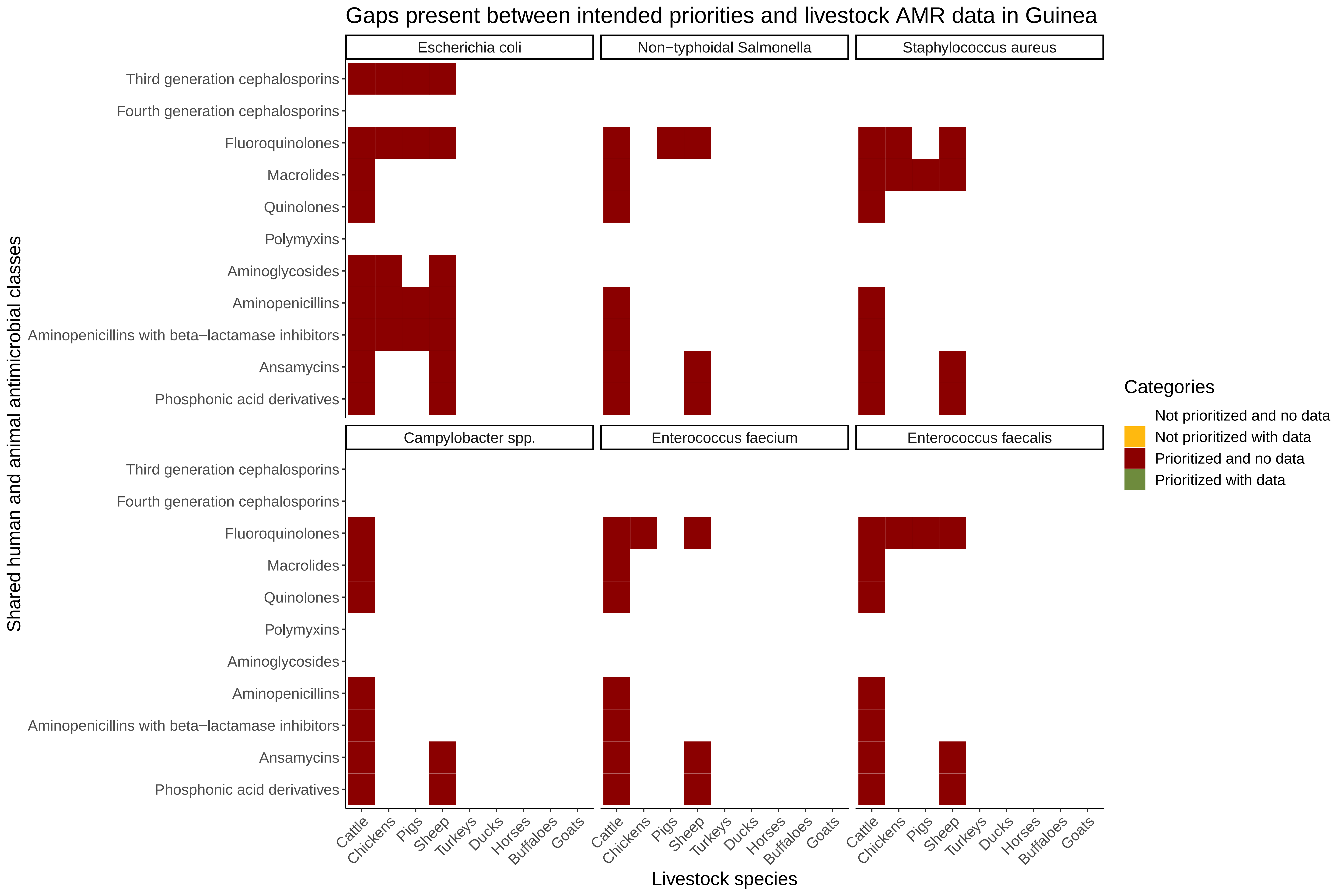

Gaps present between intended priorities and livestock AMR data in Guinea
Escherichia coli
Non−typhoidal Salmonella
Staphylococcus aureus
Campylobacter spp.
Enterococcus faecium
Enterococcus faecalis
Third generation cephalosporins
Fourth generation cephalosporins
Fluoroquinolones
Macrolides
Quinolones
Polymyxins
Aminoglycosides
Aminopenicillins
Aminopenicillins with beta−lactamase inhibitors
Ansamycins
Phosphonic acid derivatives
Shared human and animal antimicrobial classes
Cattle
Chickens
Pigs
Sheep
Turkeys
Ducks
Horses
Buffaloes
Goats
Livestock species
Categories
Not prioritized and no data
Not prioritized with data
Prioritized and no data
Prioritized with data

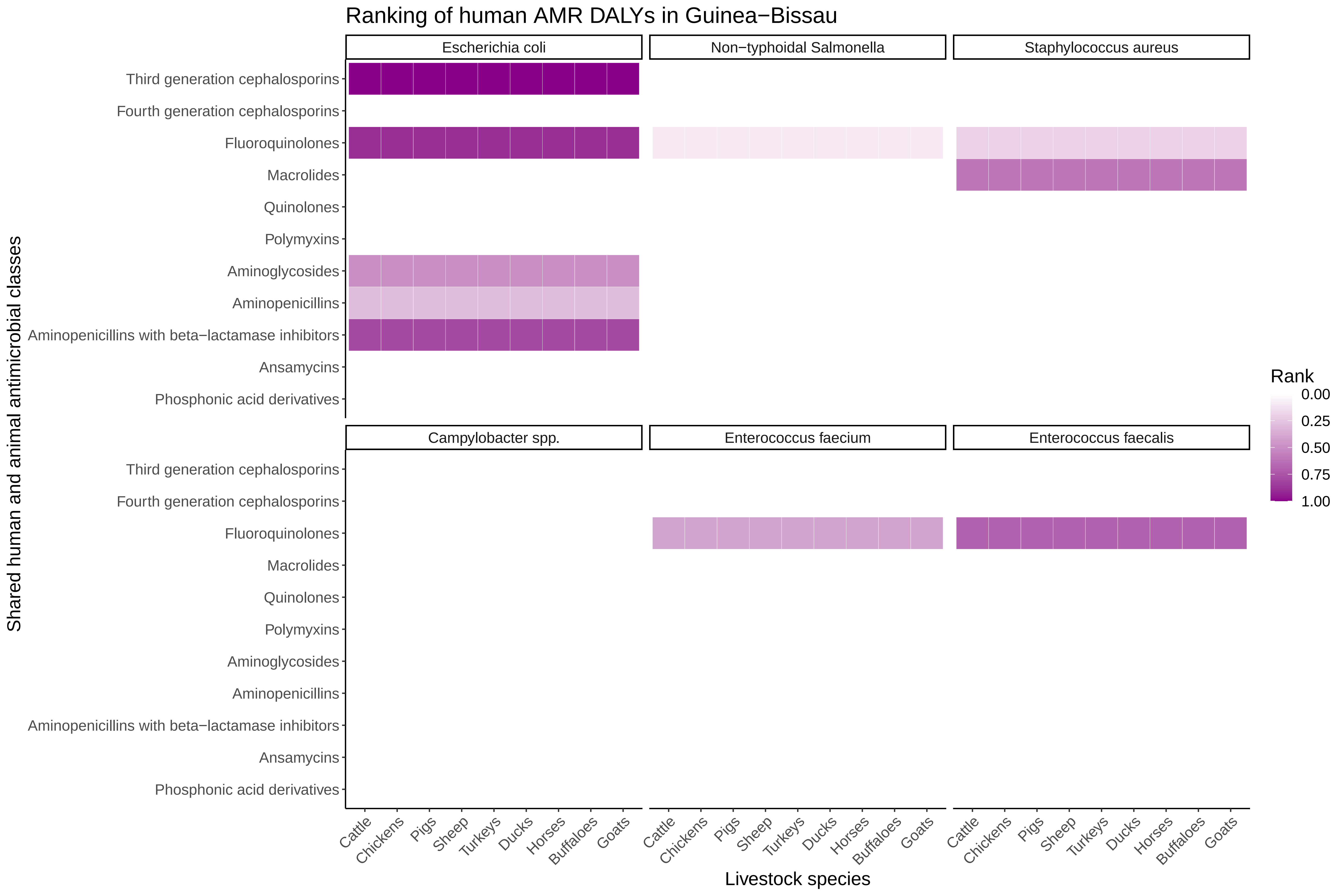

Ranking of human AMR DALYs in Guinea−Bissau
Escherichia coli
Non−typhoidal Salmonella
Staphylococcus aureus
Campylobacter spp.
Enterococcus faecium
Enterococcus faecalis
Third generation cephalosporins
Fourth generation cephalosporins
Fluoroquinolones
Macrolides
Quinolones
Polymyxins
Aminoglycosides
Aminopenicillins
Aminopenicillins with beta−lactamase inhibitors
Ansamycins
Phosphonic acid derivatives
Shared human and animal antimicrobial classes
Cattle
Chickens
Pigs
Sheep
Turkeys
Ducks
Horses
Buffaloes
Goats
Livestock species
Rank
0.00
0.25
0.50
0.75
1.00

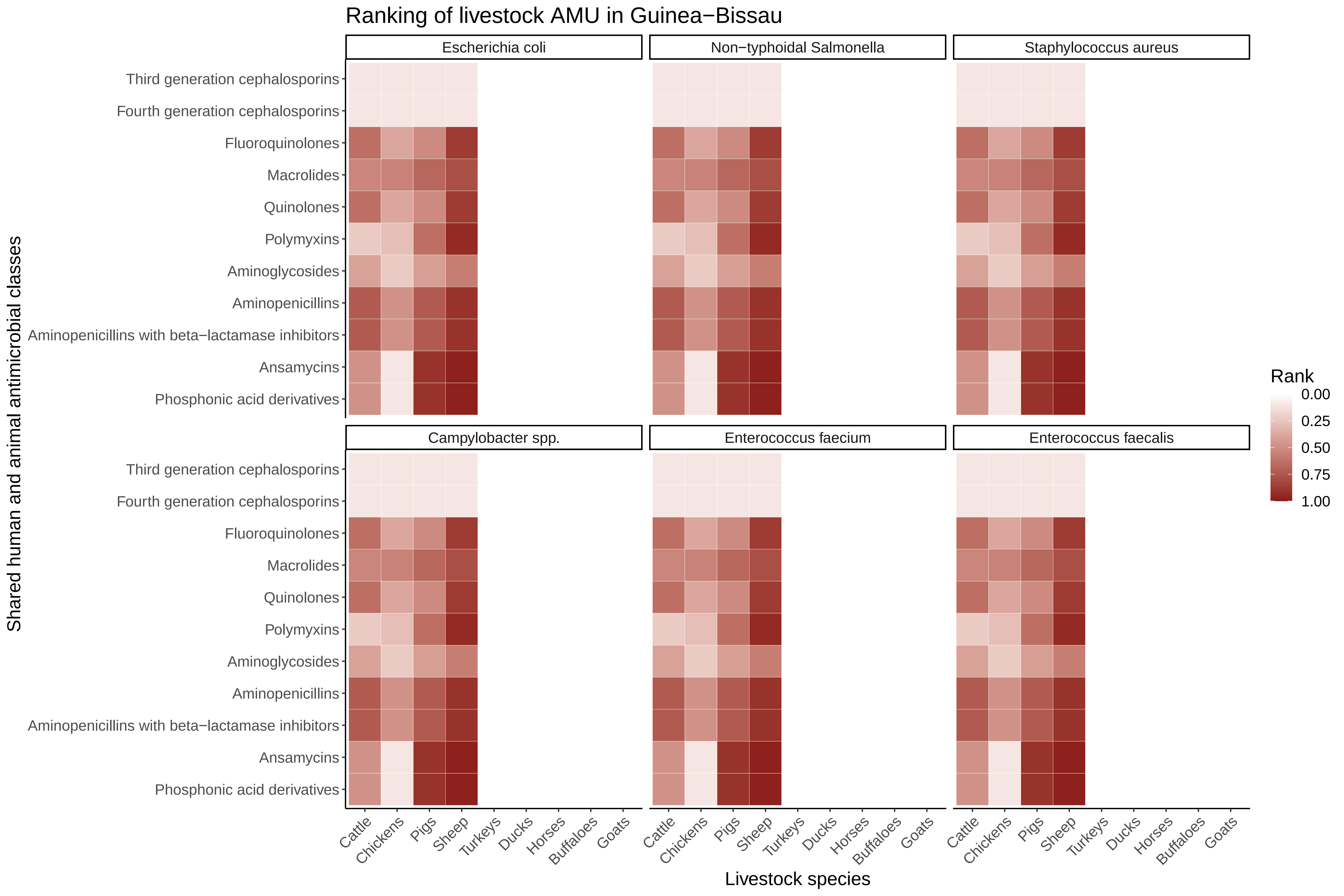
Ranking of livestock AMU in Guinea−Bissau
Escherichia coli
Non−typhoidal Salmonella
Staphylococcus aureus
Campylobacter spp.
Enterococcus faecium
Enterococcus faecalis
Shared human and animal antimicrobial classes
Third generation cephalosporins
Fourth generation cephalosporins
Fluoroquinolones
Macrolides
Quinolones
Polymyxins
Aminoglycosides
Aminopenicillins
Aminopenicillins with beta−lactamase inhibitors
Ansamycins
Phosphonic acid derivatives
Livestock species
Cattle
Chickens
Pigs
Sheep
Turkeys
Ducks
Horses
Buffaloes
Goats
Rank
0.00
0.25
0.50
0.75
1.00

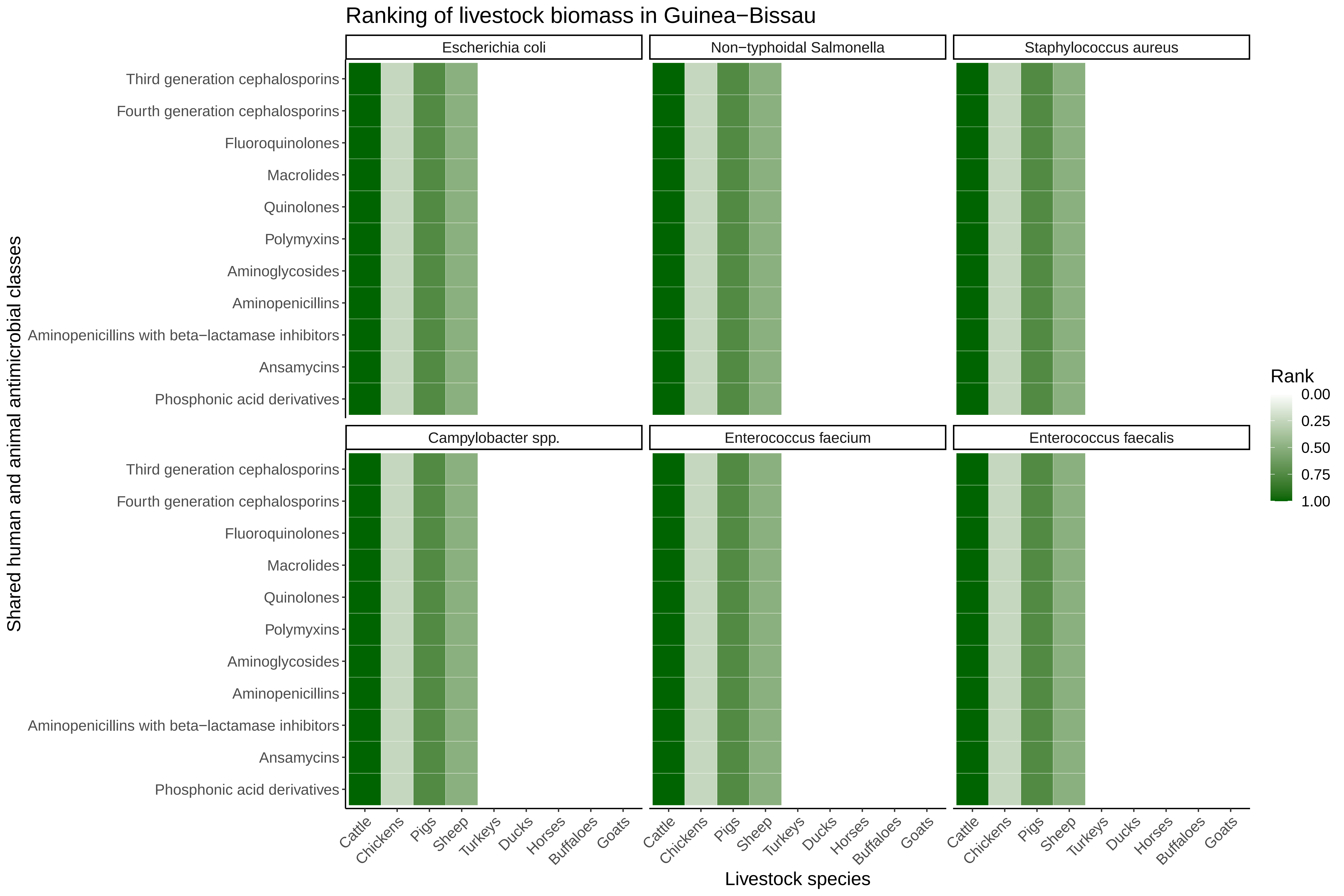
Ranking of livestock biomass in Guinea−Bissau
Escherichia coli
Non−typhoidal Salmonella
Staphylococcus aureus
Campylobacter spp.
Enterococcus faecium
Enterococcus faecalis
Third generation cephalosporins
Fourth generation cephalosporins
Fluoroquinolones
Macrolides
Quinolones
Polymyxins
Aminoglycosides
Aminopenicillins
Aminopenicillins with beta−lactamase inhibitors
Ansamycins
Phosphonic acid derivatives
Shared human and animal antimicrobial classes
Cattle
Chickens
Pigs
Sheep
Turkeys
Ducks
Horses
Buffaloes
Goats
Livestock species
Rank
0.00
0.25
0.50
0.75
1.00

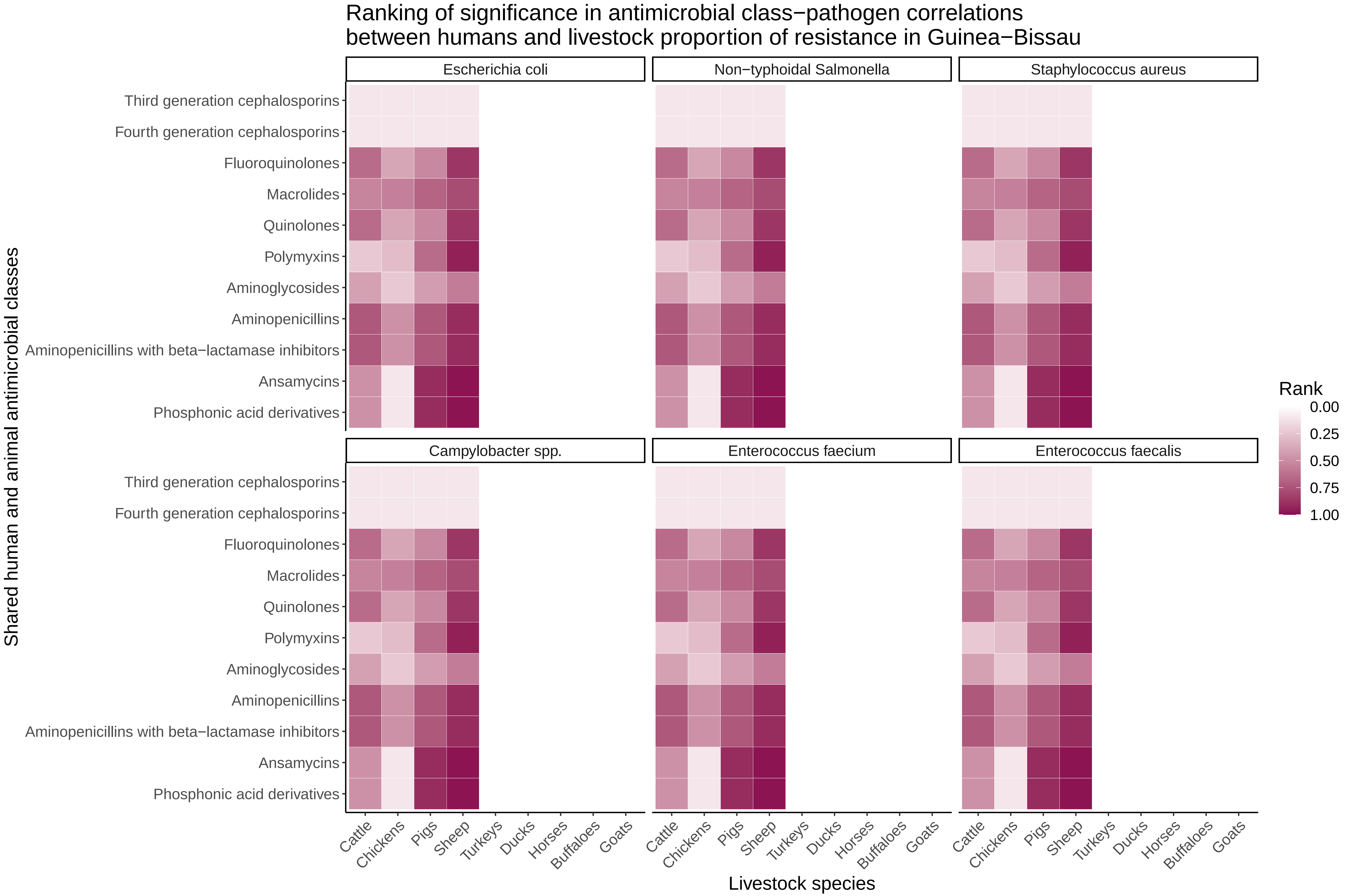

Ranking of significance in antimicrobial class−pathogen correlations
between humans and livestock proportion of resistance in Guinea−Bissau
Escherichia coli
Non−typhoidal Salmonella
Staphylococcus aureus
Campylobacter spp.
Enterococcus faecium
Enterococcus faecalis
Third generation cephalosporins
Fourth generation cephalosporins
Fluoroquinolones
Macrolides
Quinolones
Polymyxins
Aminoglycosides
Aminopenicillins
Aminopenicillins with beta−lactamase inhibitors
Ansamycins
Phosphonic acid derivatives
Shared human and animal antimicrobial classes
Cattle
Chickens
Pigs
Sheep
Turkeys
Ducks
Horses
Buffaloes
Goats
Livestock species
Rank
0.00
0.25
0.50
0.75
1.00

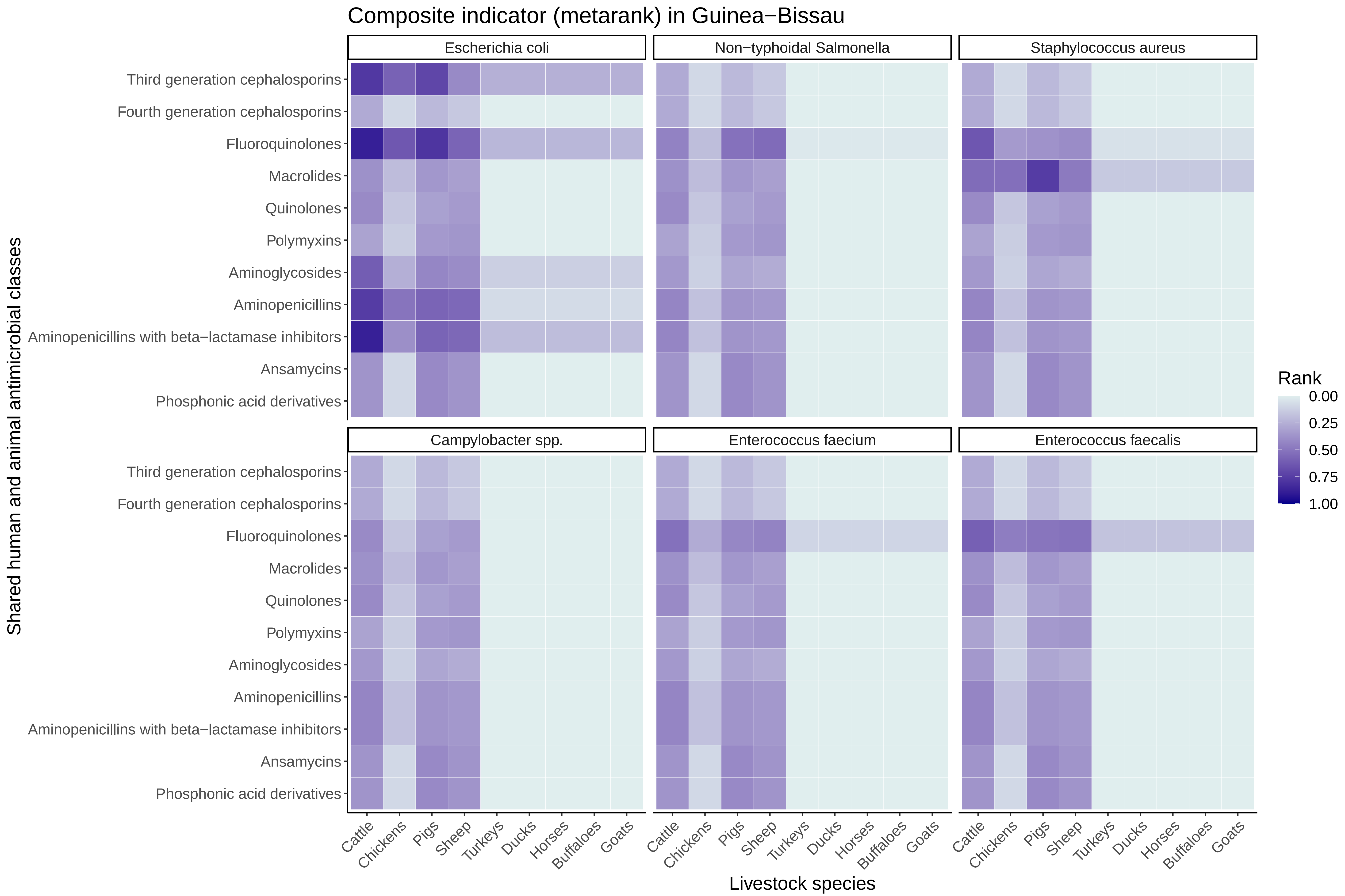

Composite indicator (metarank) in Guinea−Bissau
Escherichia coli
Non−typhoidal Salmonella
Staphylococcus aureus
Campylobacter spp.
Enterococcus faecium
Enterococcus faecalis
Shared human and animal antimicrobial classes
Third generation cephalosporins
Fourth generation cephalosporins
Fluoroquinolones
Macrolides
Quinolones
Polymyxins
Aminoglycosides
Aminopenicillins
Aminopenicillins with beta−lactamase inhibitors
Ansamycins
Phosphonic acid derivatives
Livestock species
Cattle
Chickens
Pigs
Sheep
Turkeys
Ducks
Horses
Buffaloes
Goats
Rank
0.00
0.25
0.50
0.75
1.00

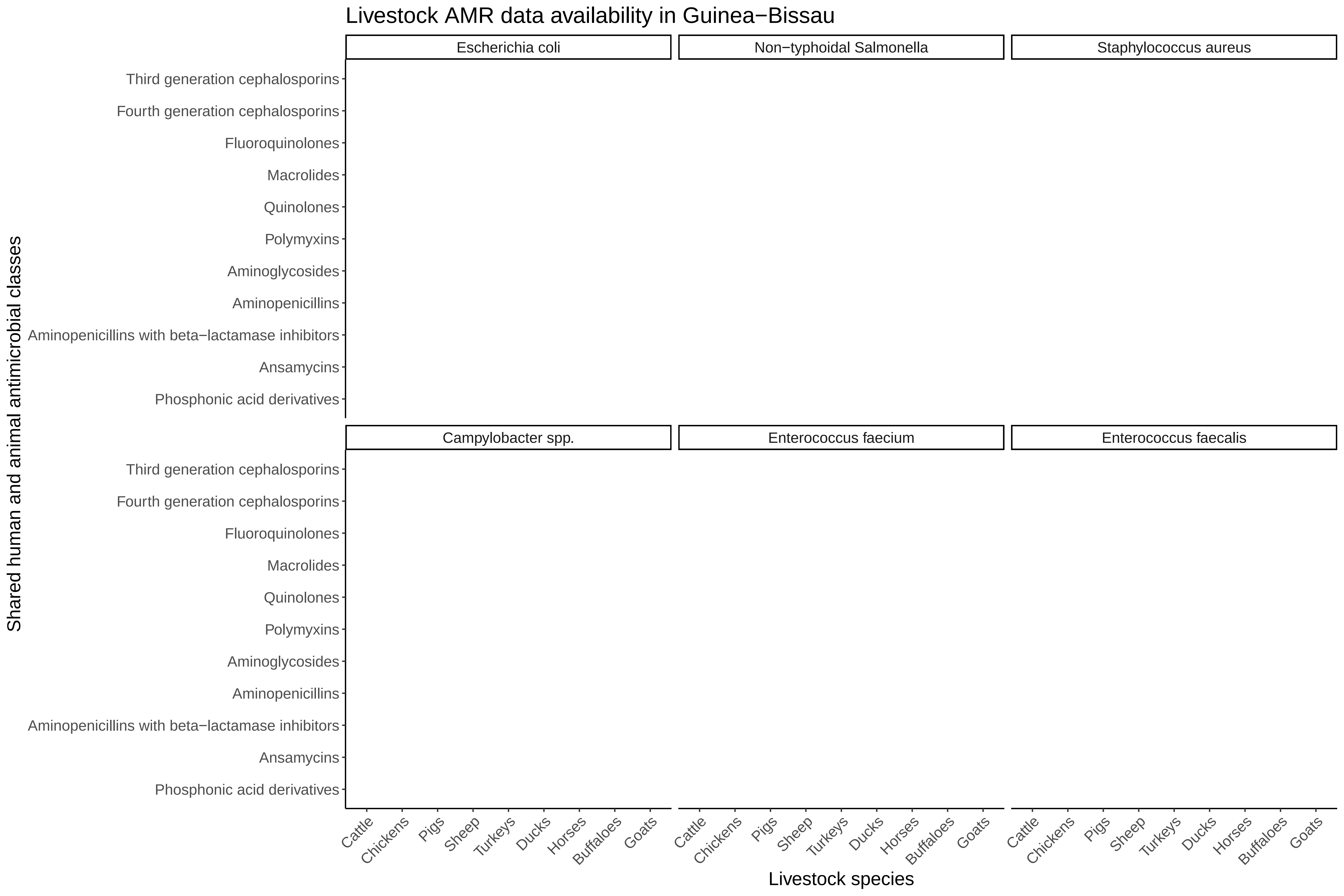

Livestock AMR data availability in Guinea−Bissau
Escherichia coli
Non−typhoidal Salmonella
Staphylococcus aureus
Campylobacter spp.
Enterococcus faecium
Enterococcus faecalis
Shared human and animal antimicrobial classes
Third generation cephalosporins
Fourth generation cephalosporins
Fluoroquinolones
Macrolides
Quinolones
Polymyxins
Aminoglycosides
Aminopenicillins
Aminopenicillins with beta−lactamase inhibitors
Ansamycins
Phosphonic acid derivatives
Cattle
Chickens
Pigs
Sheep
Turkeys
Ducks
Horses
Buffaloes
Goats
Livestock species

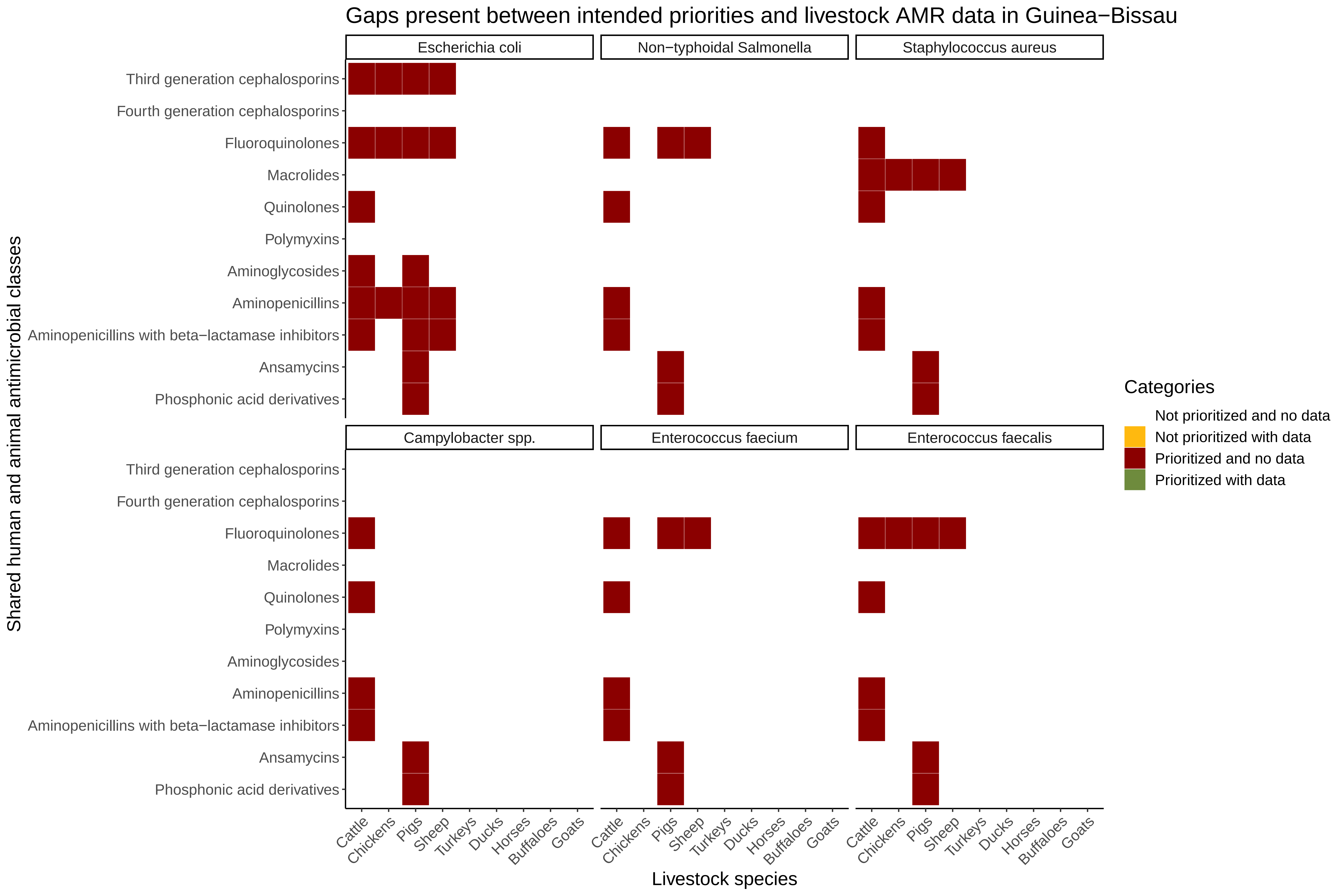

Gaps present between intended priorities and livestock AMR data in Guinea−Bissau
Escherichia coli
Non−typhoidal Salmonella
Staphylococcus aureus
Campylobacter spp.
Enterococcus faecium
Enterococcus faecalis
Shared human and animal antimicrobial classes
Third generation cephalosporins
Fourth generation cephalosporins
Fluoroquinolones
Macrolides
Quinolones
Polymyxins
Aminoglycosides
Aminopenicillins
Aminopenicillins with beta−lactamase inhibitors
Ansamycins
Phosphonic acid derivatives
Cattle
Chickens
Pigs
Sheep
Turkeys
Ducks
Horses
Buffaloes
Goats
Livestock species
Categories
Not prioritized and no data
Not prioritized with data
Prioritized and no data
Prioritized with data

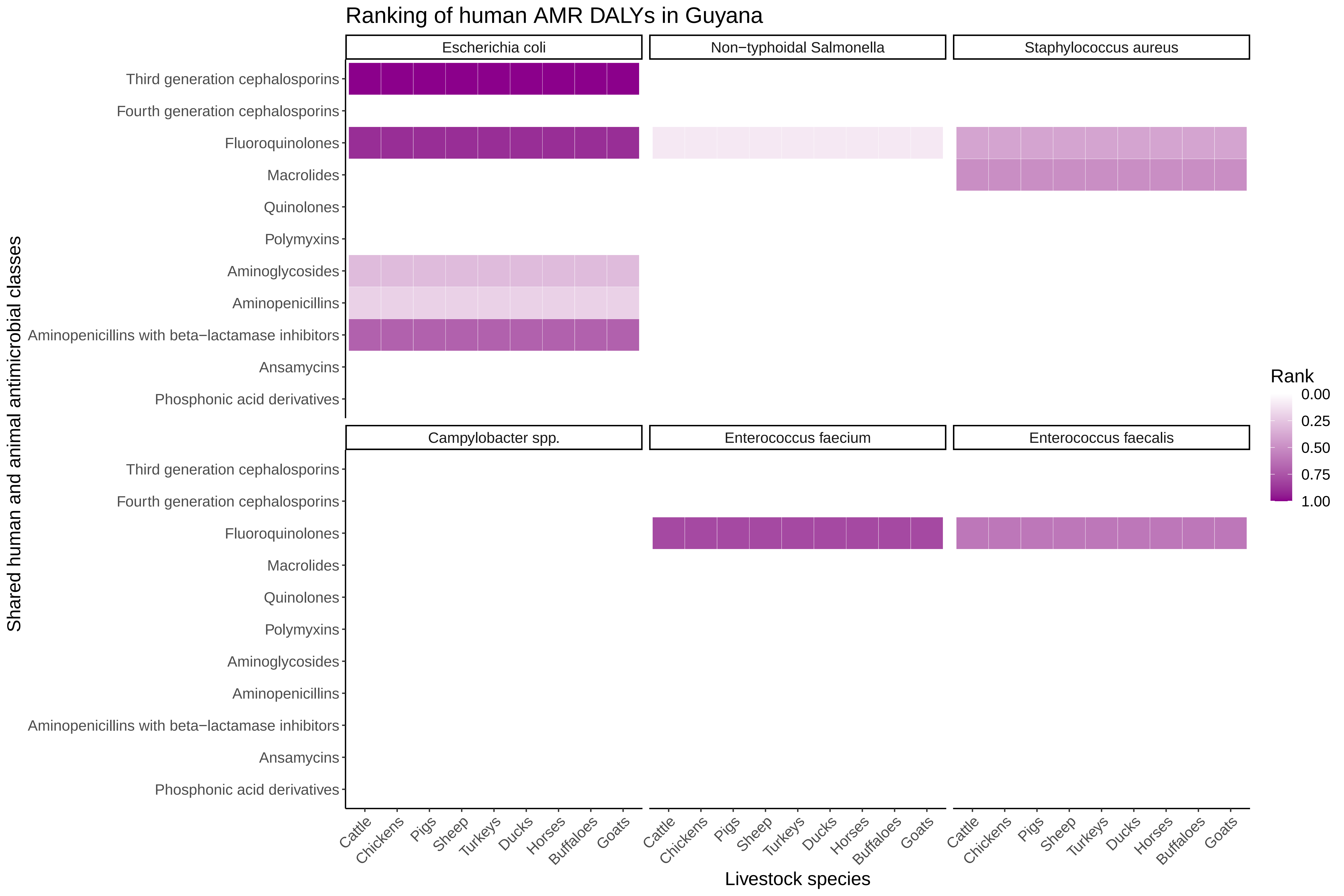

Ranking of human AMR DALYs in Guyana
Escherichia coli
Non−typhoidal Salmonella
Staphylococcus aureus
Campylobacter spp.
Enterococcus faecium
Enterococcus faecalis
Shared human and animal antimicrobial classes
Third generation cephalosporins
Fourth generation cephalosporins
Fluoroquinolones
Macrolides
Quinolones
Polymyxins
Aminoglycosides
Aminopenicillins
Aminopenicillins with beta−lactamase inhibitors
Ansamycins
Phosphonic acid derivatives
Cattle
Chickens
Pigs
Sheep
Turkeys
Ducks
Horses
Buffaloes
Goats
Livestock species
Rank
0.00
0.25
0.50
0.75
1.00

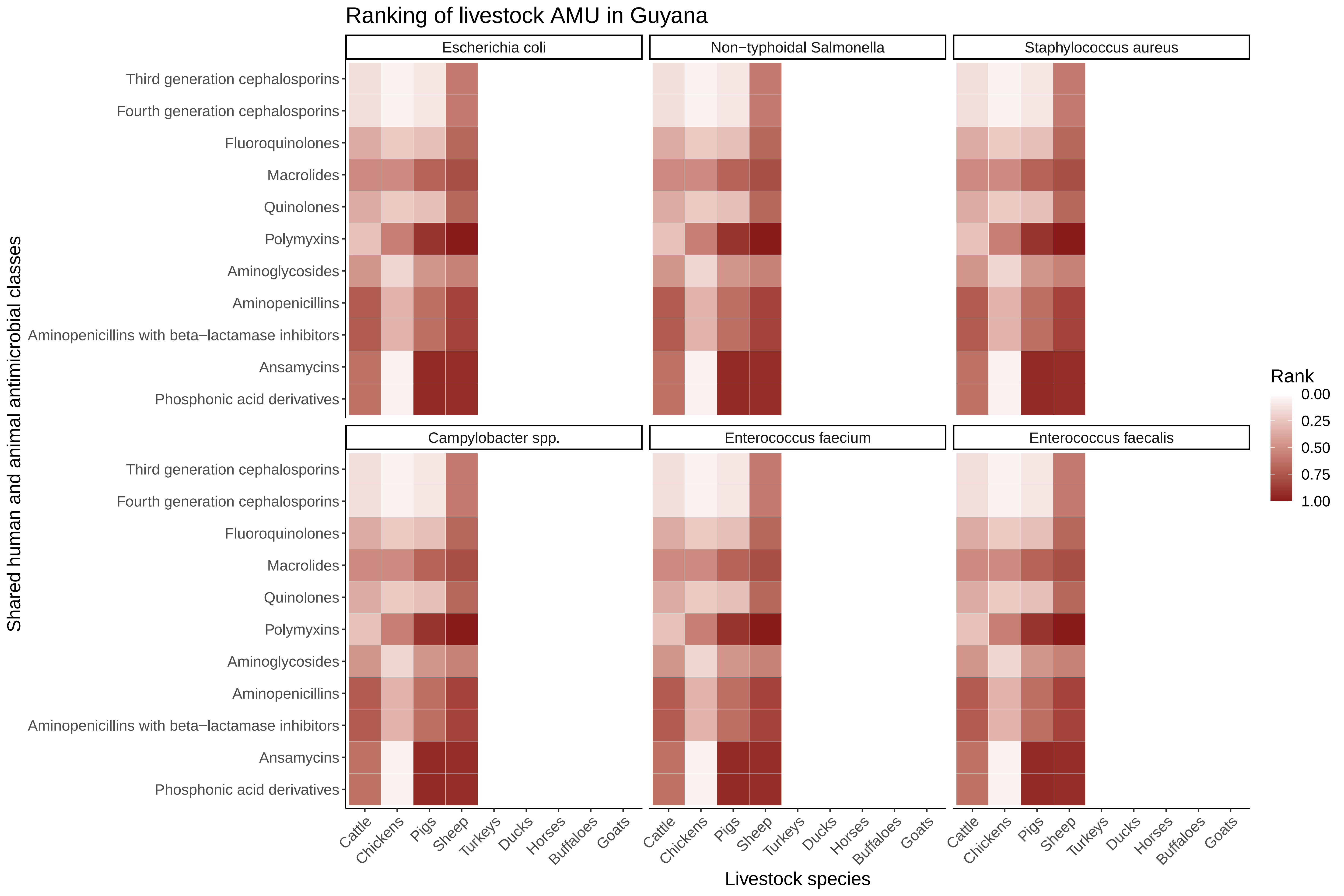

Ranking of livestock AMU in Guyana
Escherichia coli
Non−typhoidal Salmonella
Staphylococcus aureus
Campylobacter spp.
Enterococcus faecium
Enterococcus faecalis
Shared human and animal antimicrobial classes
Third generation cephalosporins
Fourth generation cephalosporins
Fluoroquinolones
Macrolides
Quinolones
Polymyxins
Aminoglycosides
Aminopenicillins
Aminopenicillins with beta−lactamase inhibitors
Ansamycins
Phosphonic acid derivatives
Livestock species
Cattle
Chickens
Pigs
Sheep
Turkeys
Ducks
Horses
Buffaloes
Goats
Rank
0.00
0.25
0.50
0.75
1.00

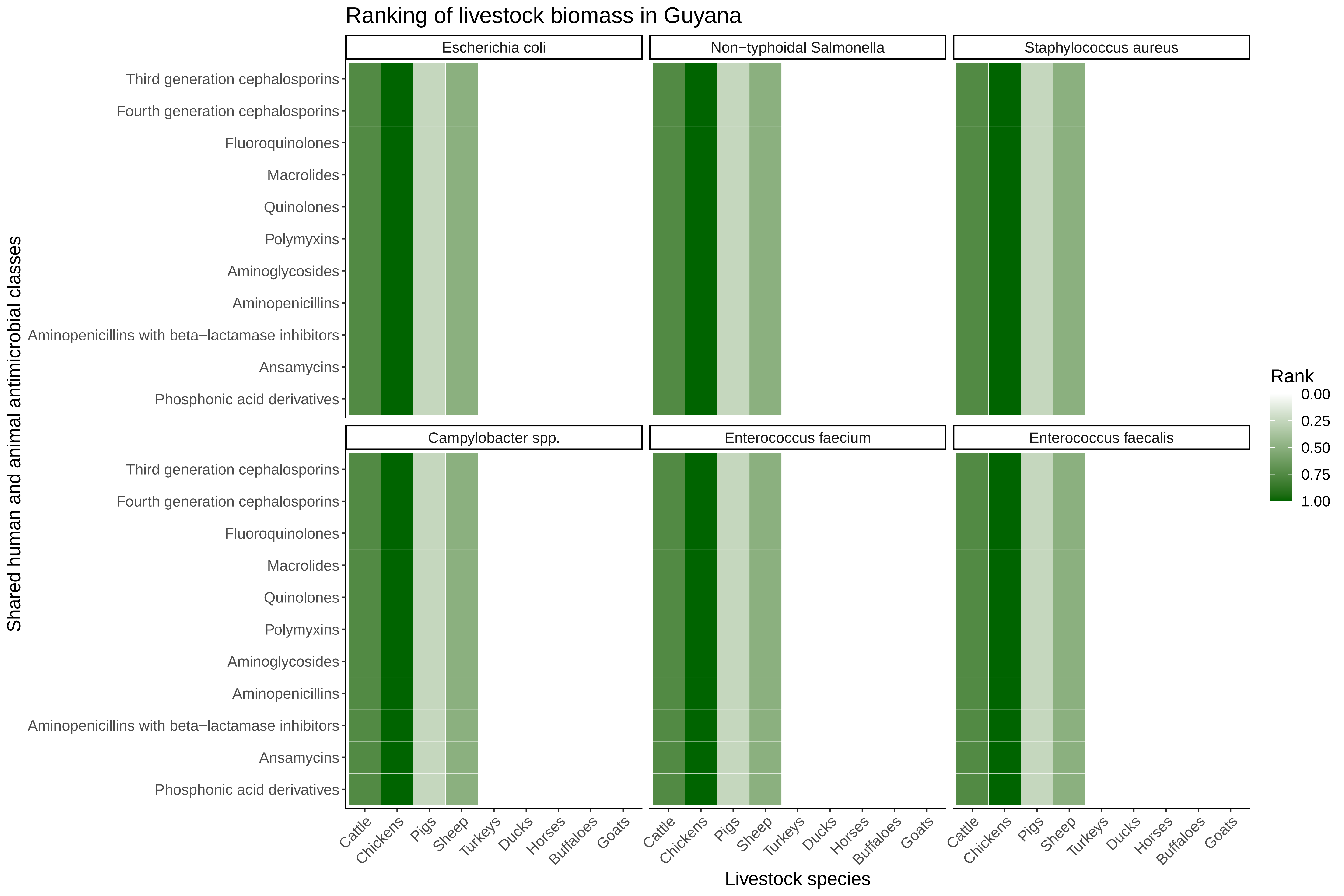

Ranking of livestock biomass in Guyana
Escherichia coli
Non−typhoidal Salmonella
Staphylococcus aureus
Campylobacter spp.
Enterococcus faecium
Enterococcus faecalis
Shared human and animal antimicrobial classes
Third generation cephalosporins
Fourth generation cephalosporins
Fluoroquinolones
Macrolides
Quinolones
Polymyxins
Aminoglycosides
Aminopenicillins
Aminopenicillins with beta−lactamase inhibitors
Ansamycins
Phosphonic acid derivatives
Livestock species
Cattle
Chickens
Pigs
Sheep
Turkeys
Ducks
Horses
Buffaloes
Goats
Rank
0.00
0.25
0.50
0.75
1.00

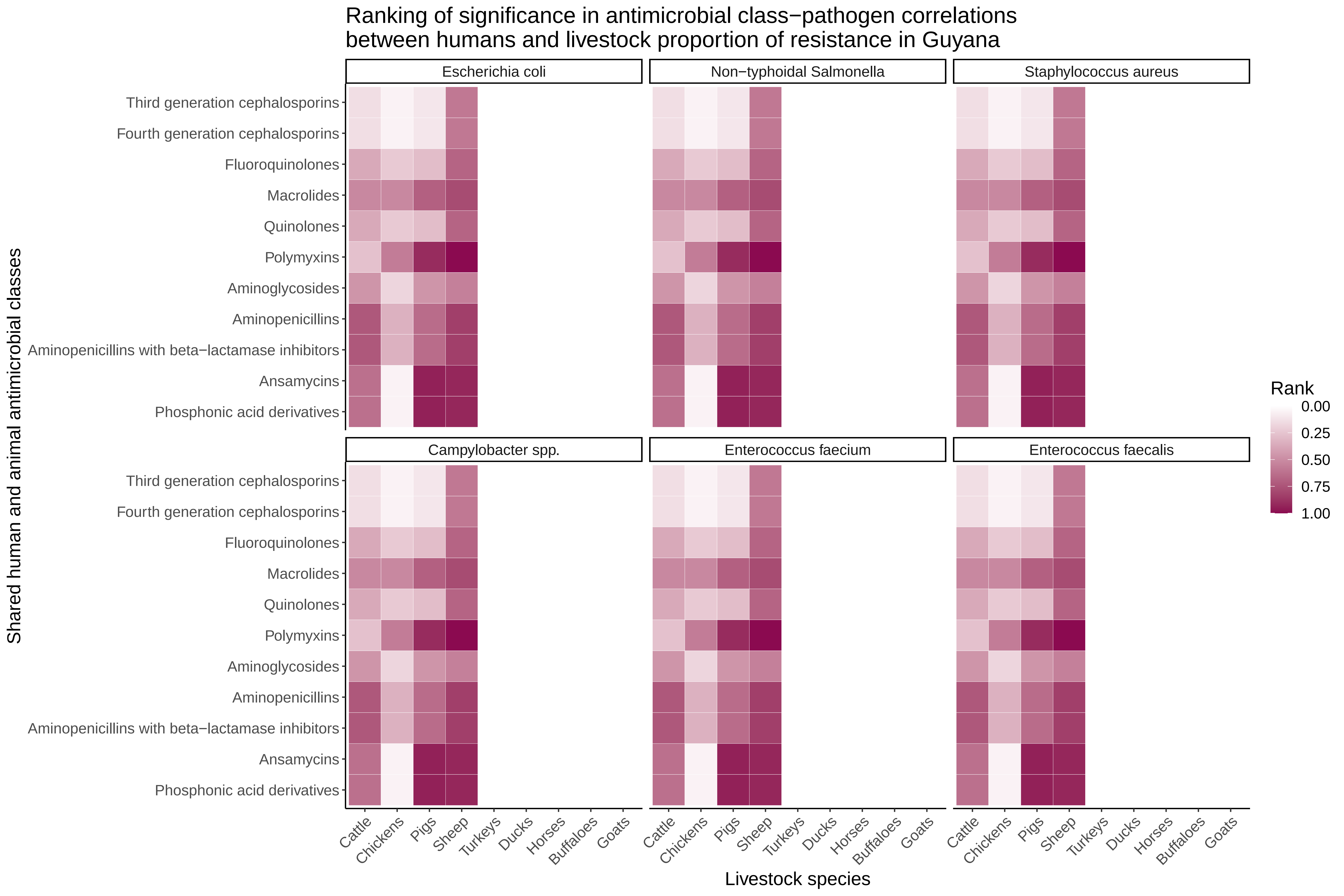

Ranking of significance in antimicrobial class−pathogen correlations
between humans and livestock proportion of resistance in Guyana
Escherichia coli
Non−typhoidal Salmonella
Staphylococcus aureus
Campylobacter spp.
Enterococcus faecium
Enterococcus faecalis
Third generation cephalosporins
Fourth generation cephalosporins
Fluoroquinolones
Macrolides
Quinolones
Polymyxins
Aminoglycosides
Aminopenicillins
Aminopenicillins with beta−lactamase inhibitors
Ansamycins
Phosphonic acid derivatives
Shared human and animal antimicrobial classes
Cattle
Chickens
Pigs
Sheep
Turkeys
Ducks
Horses
Buffaloes
Goats
Livestock species
Rank
0.00
0.25
0.50
0.75
1.00

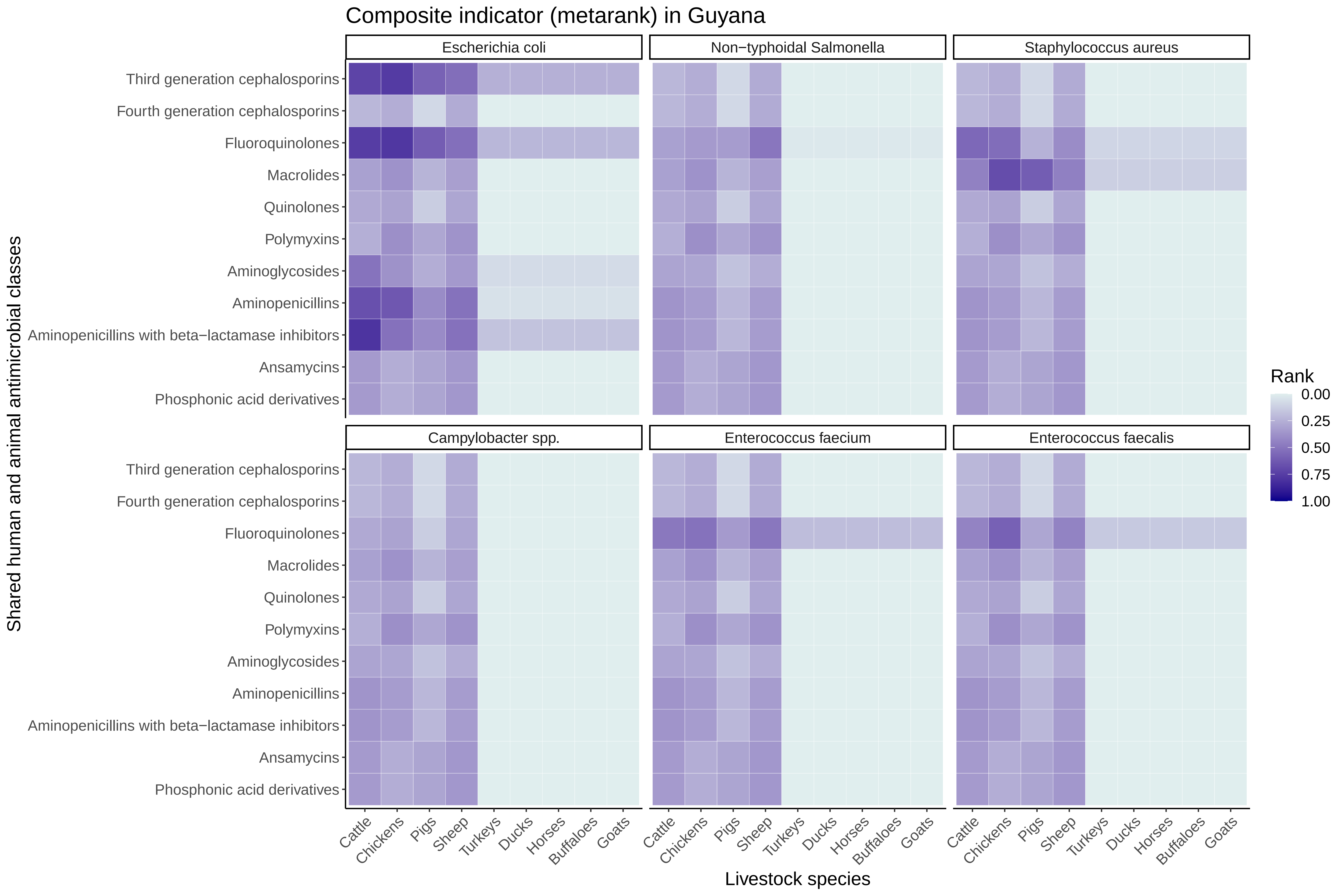

Composite indicator (metarank) in Guyana
Escherichia coli
Non−typhoidal Salmonella
Staphylococcus aureus
Campylobacter spp.
Enterococcus faecium
Enterococcus faecalis
Shared human and animal antimicrobial classes
Third generation cephalosporins
Fourth generation cephalosporins
Fluoroquinolones
Macrolides
Quinolones
Polymyxins
Aminoglycosides
Aminopenicillins
Aminopenicillins with beta−lactamase inhibitors
Ansamycins
Phosphonic acid derivatives
Cattle
Chickens
Pigs
Sheep
Turkeys
Ducks
Horses
Buffaloes
Goats
Livestock species
Rank
0.00
0.25
0.50
0.75
1.00

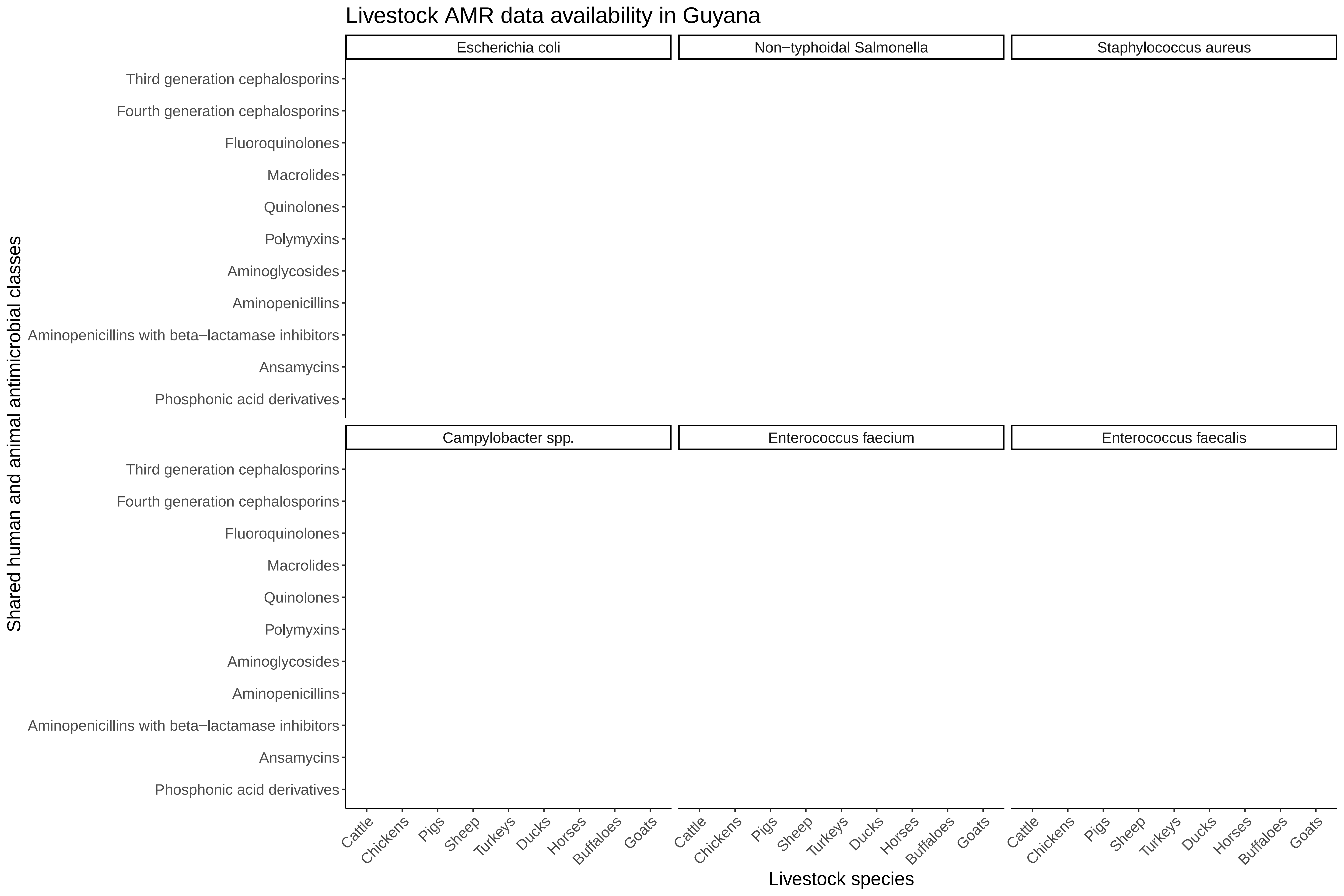

Livestock AMR data availability in Guyana
Escherichia coli
Non−typhoidal Salmonella
Staphylococcus aureus
Campylobacter spp.
Enterococcus faecium
Enterococcus faecalis
Shared human and animal antimicrobial classes
Third generation cephalosporins
Fourth generation cephalosporins
Fluoroquinolones
Macrolides
Quinolones
Polymyxins
Aminoglycosides
Aminopenicillins
Aminopenicillins with beta−lactamase inhibitors
Ansamycins
Phosphonic acid derivatives
Cattle
Chickens
Pigs
Sheep
Turkeys
Ducks
Horses
Buffaloes
Goats
Livestock species

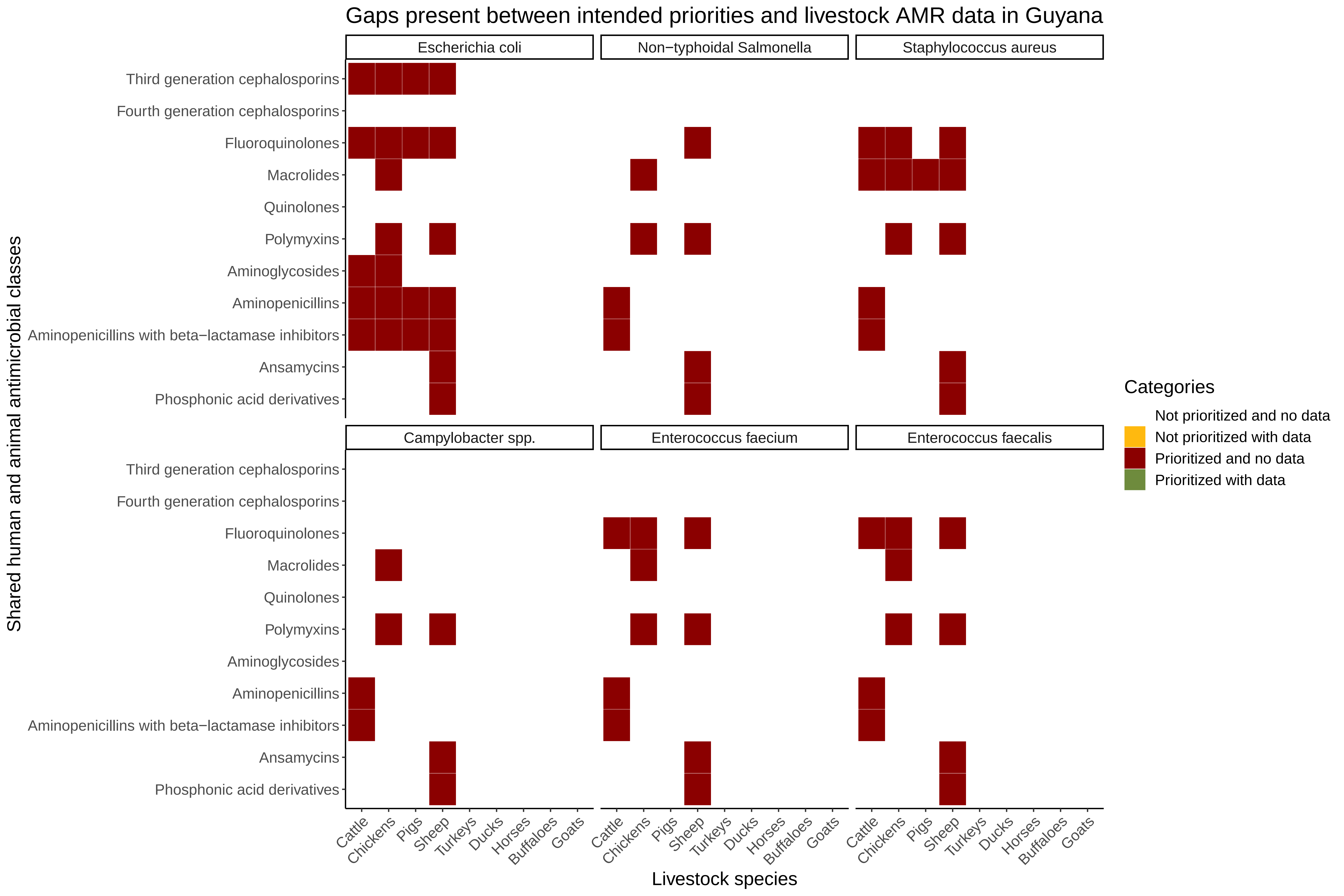

Gaps present between intended priorities and livestock AMR data in Guyana
Escherichia coli
Non−typhoidal Salmonella
Staphylococcus aureus
Campylobacter spp.
Enterococcus faecium
Enterococcus faecalis
Third generation cephalosporins
Fourth generation cephalosporins
Fluoroquinolones
Macrolides
Quinolones
Polymyxins
Aminoglycosides
Aminopenicillins
Aminopenicillins with beta−lactamase inhibitors
Ansamycins
Phosphonic acid derivatives
Shared human and animal antimicrobial classes
Cattle
Chickens
Pigs
Sheep
Turkeys
Ducks
Horses
Buffaloes
Goats
Livestock species
Categories
Not prioritized and no data
Not prioritized with data
Prioritized and no data
Prioritized with data

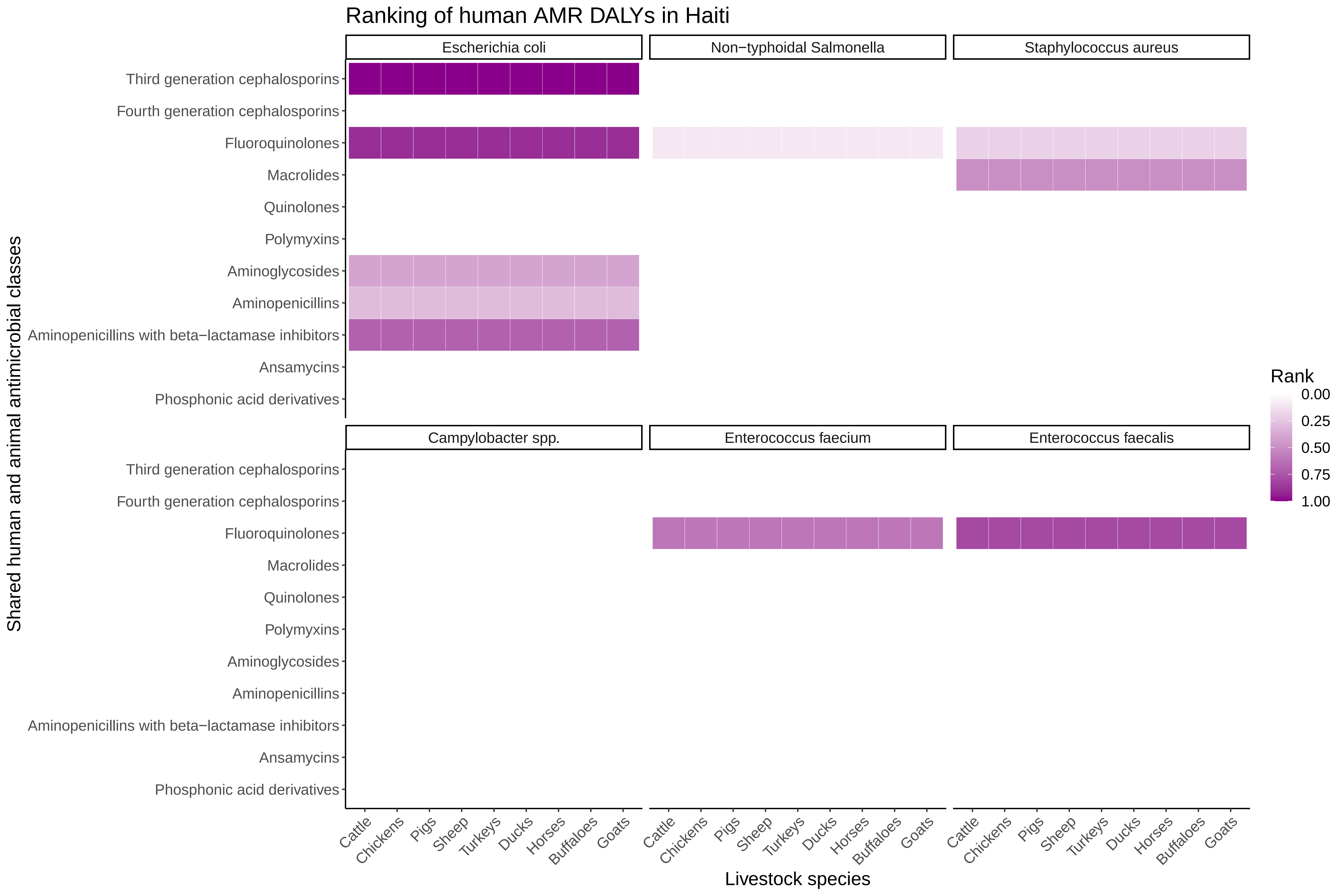
Ranking of human AMR DALYs in Haiti
Escherichia coli
Non−typhoidal Salmonella
Staphylococcus aureus
Campylobacter spp.
Enterococcus faecium
Enterococcus faecalis
Shared human and animal antimicrobial classes
Third generation cephalosporins
Fourth generation cephalosporins
Fluoroquinolones
Macrolides
Quinolones
Polymyxins
Aminoglycosides
Aminopenicillins
Aminopenicillins with beta−lactamase inhibitors
Ansamycins
Phosphonic acid derivatives
Livestock species
Cattle
Chickens
Pigs
Sheep
Turkeys
Ducks
Horses
Buffaloes
Goats
Rank
0.00
0.25
0.50
0.75
1.00

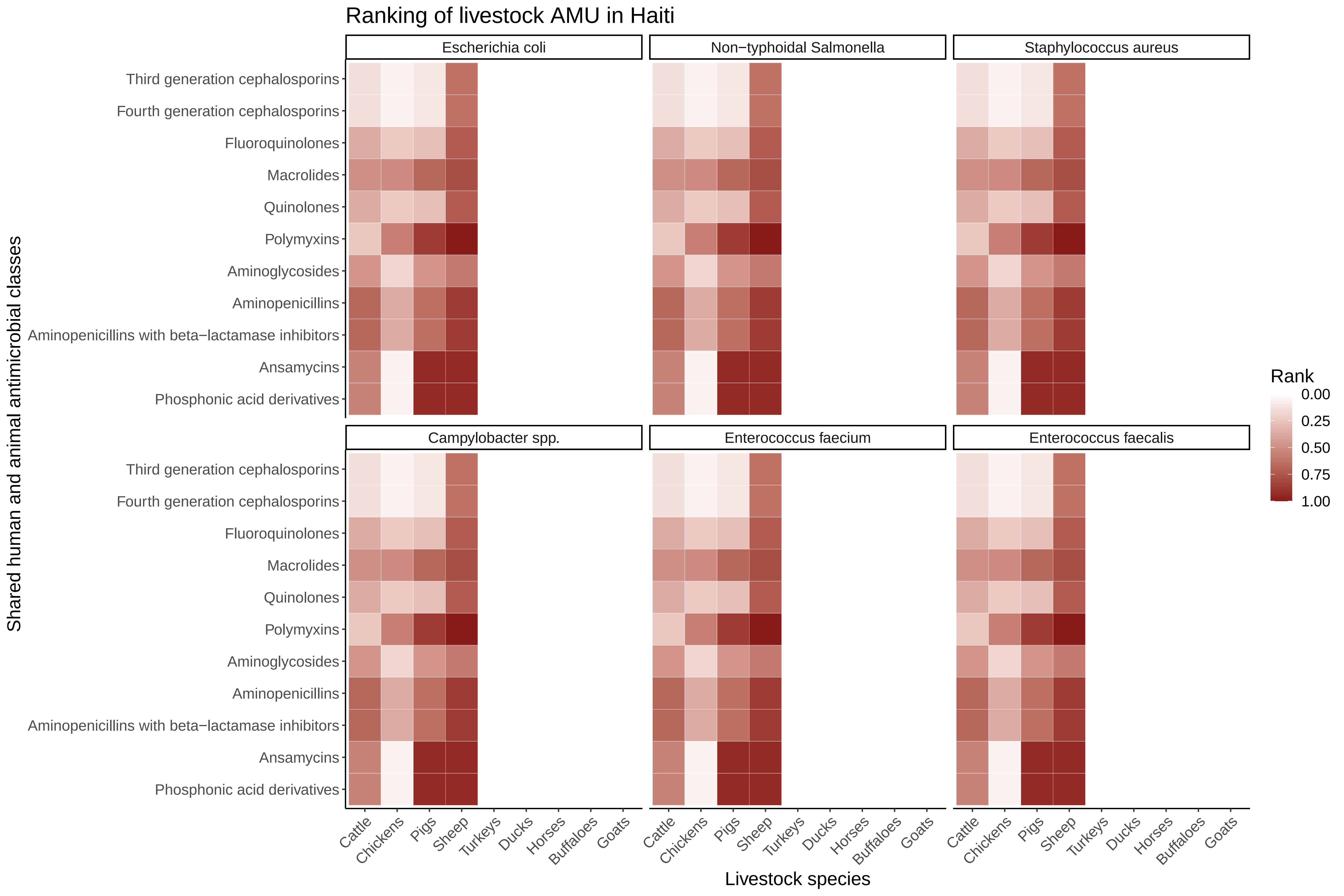

Ranking of livestock AMU in Haiti
Escherichia coli
Non−typhoidal Salmonella
Staphylococcus aureus
Campylobacter spp.
Enterococcus faecium
Enterococcus faecalis
Shared human and animal antimicrobial classes
Third generation cephalosporins
Fourth generation cephalosporins
Fluoroquinolones
Macrolides
Quinolones
Polymyxins
Aminoglycosides
Aminopenicillins
Aminopenicillins with beta−lactamase inhibitors
Ansamycins
Phosphonic acid derivatives
Cattle
Chickens
Pigs
Sheep
Turkeys
Ducks
Horses
Buffaloes
Goats
Livestock species
Rank
0.00
0.25
0.50
0.75
1.00

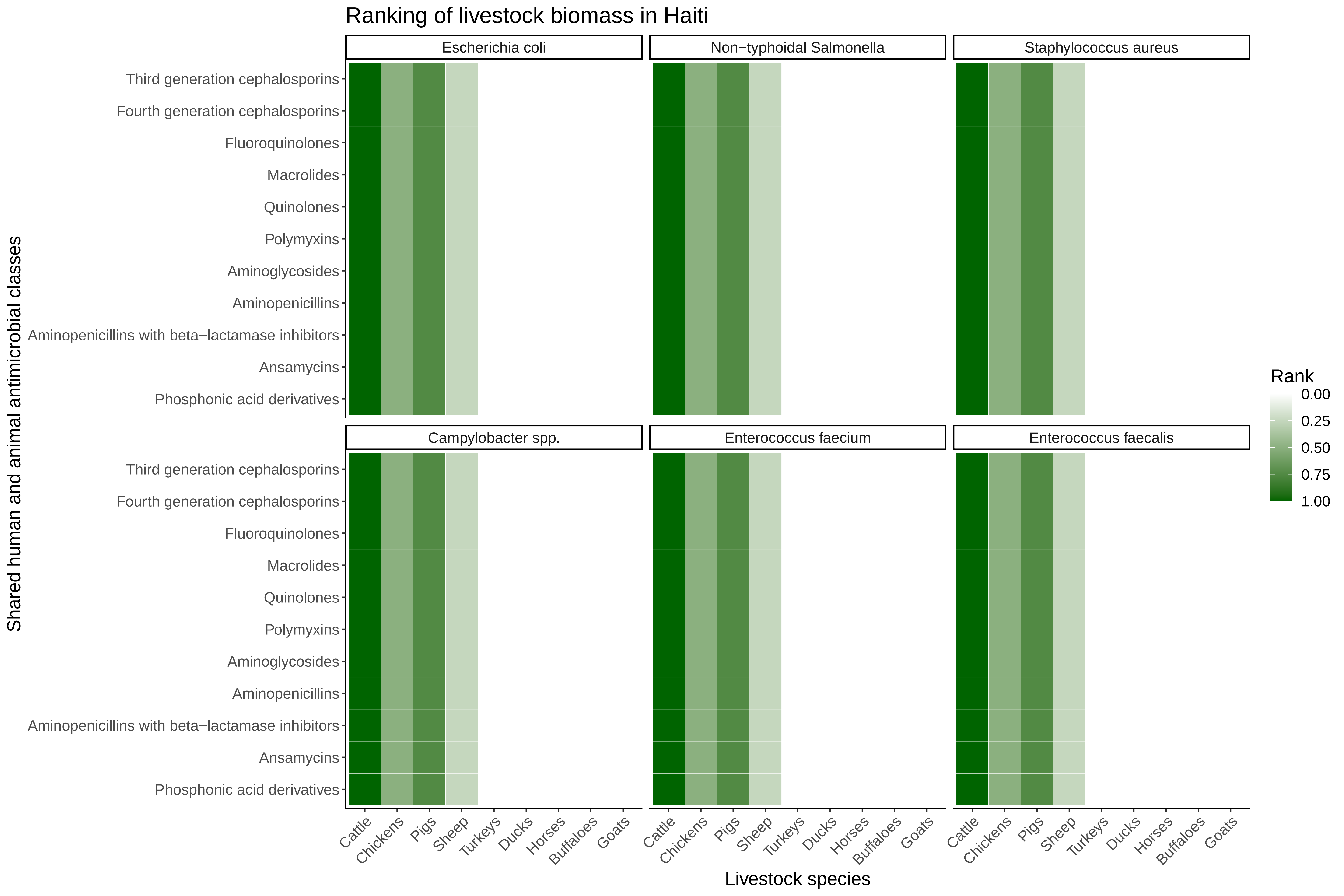

Ranking of livestock biomass in Haiti
Escherichia coli
Non−typhoidal Salmonella
Staphylococcus aureus
Campylobacter spp.
Enterococcus faecium
Enterococcus faecalis
Third generation cephalosporins
Fourth generation cephalosporins
Fluoroquinolones
Macrolides
Quinolones
Polymyxins
Aminoglycosides
Aminopenicillins
Aminopenicillins with beta−lactamase inhibitors
Ansamycins
Phosphonic acid derivatives
Shared human and animal antimicrobial classes
Cattle
Chickens
Pigs
Sheep
Turkeys
Ducks
Horses
Buffaloes
Goats
Livestock species
Rank
0.00
0.25
0.50
0.75
1.00

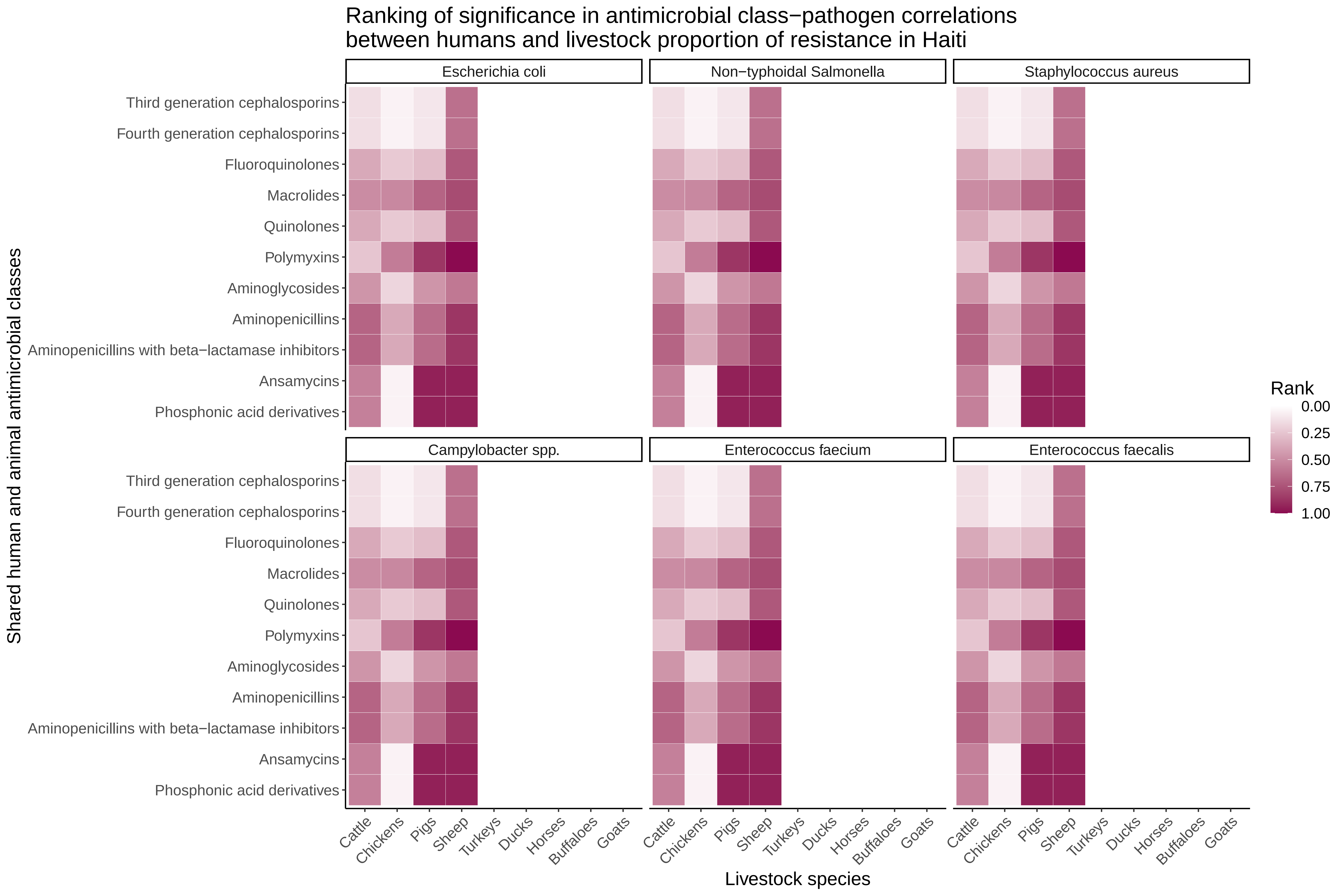
Ranking of significance in antimicrobial class−pathogen correlations
between humans and livestock proportion of resistance in Haiti
Escherichia coli
Non−typhoidal Salmonella
Staphylococcus aureus
Campylobacter spp.
Enterococcus faecium
Enterococcus faecalis
Third generation cephalosporins
Fourth generation cephalosporins
Fluoroquinolones
Macrolides
Quinolones
Polymyxins
Aminoglycosides
Aminopenicillins
Aminopenicillins with beta−lactamase inhibitors
Ansamycins
Phosphonic acid derivatives
Shared human and animal antimicrobial classes
Cattle
Chickens
Pigs
Sheep
Turkeys
Ducks
Horses
Buffaloes
Goats
Livestock species
Rank
0.00
0.25
0.50
0.75
1.00

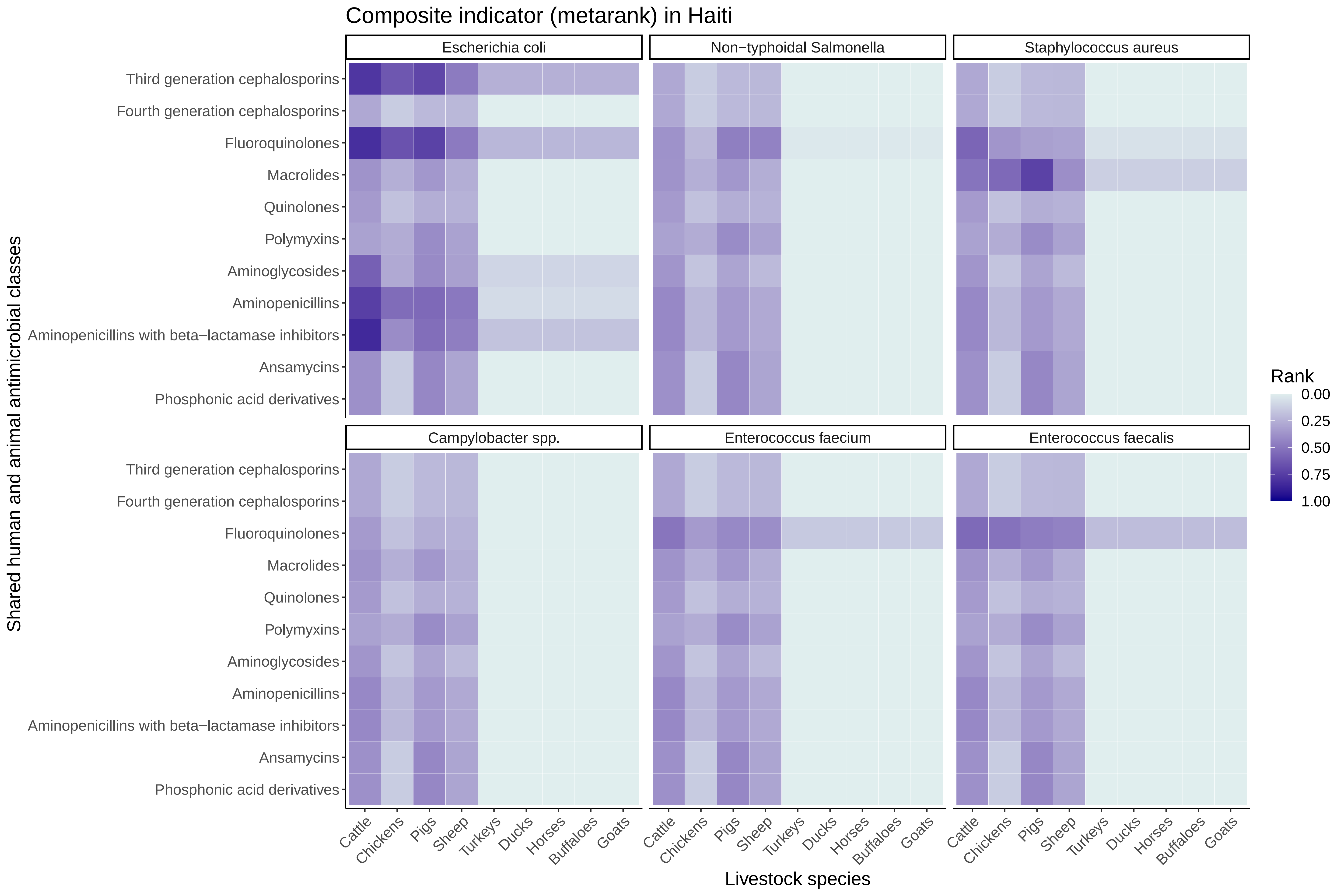

Composite indicator (metarank) in Haiti
Escherichia coli
Non−typhoidal Salmonella
Staphylococcus aureus
Campylobacter spp.
Enterococcus faecium
Enterococcus faecalis
Shared human and animal antimicrobial classes
Third generation cephalosporins
Fourth generation cephalosporins
Fluoroquinolones
Macrolides
Quinolones
Polymyxins
Aminoglycosides
Aminopenicillins
Aminopenicillins with beta−lactamase inhibitors
Ansamycins
Phosphonic acid derivatives
Cattle
Chickens
Pigs
Sheep
Turkeys
Ducks
Horses
Buffaloes
Goats
Livestock species
Rank
0.00
0.25
0.50
0.75
1.00

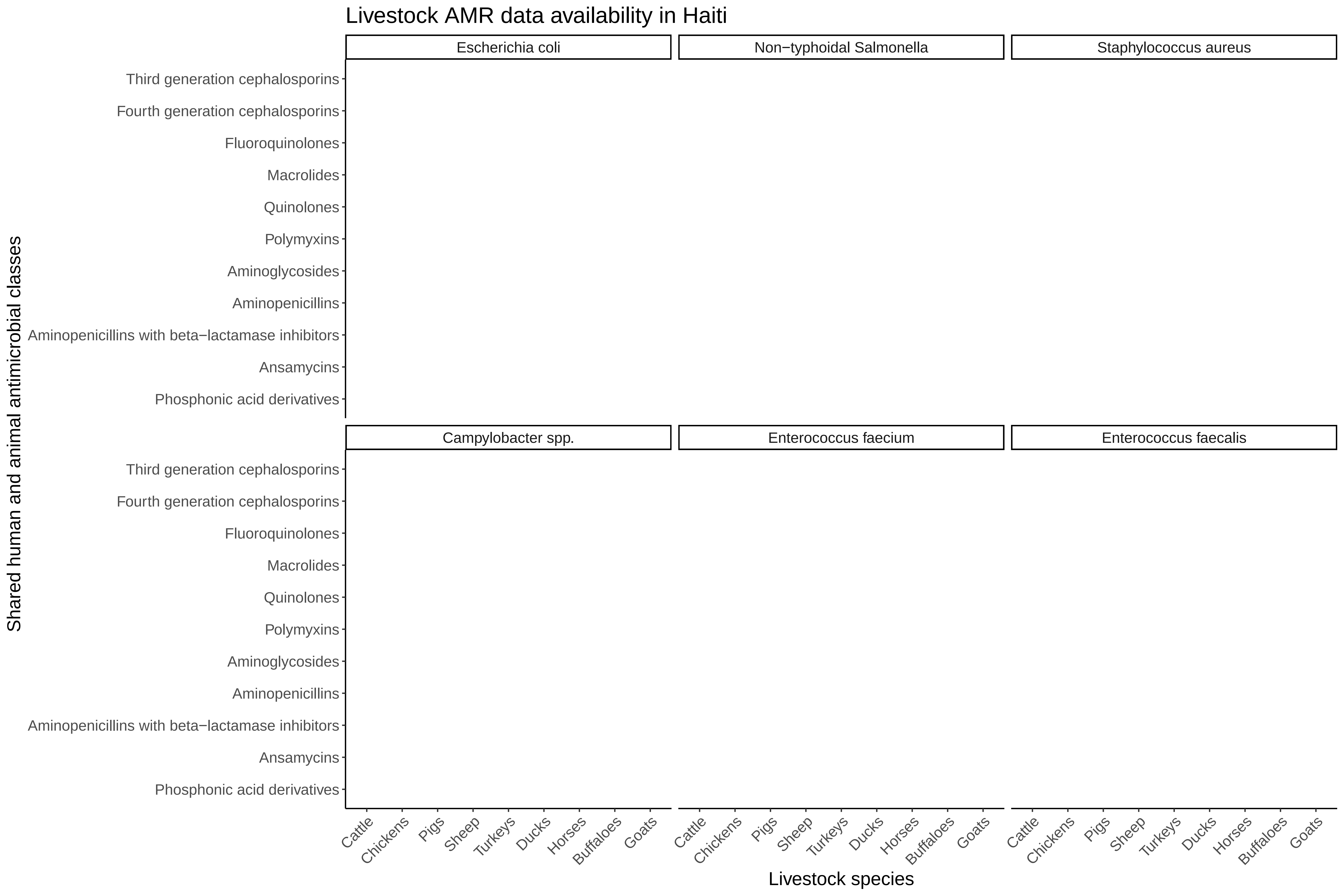
Livestock AMR data availability in Haiti
Escherichia coli
Non−typhoidal Salmonella
Staphylococcus aureus
Campylobacter spp.
Enterococcus faecium
Enterococcus faecalis
Shared human and animal antimicrobial classes
Third generation cephalosporins
Fourth generation cephalosporins
Fluoroquinolones
Macrolides
Quinolones
Polymyxins
Aminoglycosides
Aminopenicillins
Aminopenicillins with beta−lactamase inhibitors
Ansamycins
Phosphonic acid derivatives
Cattle
Chickens
Pigs
Sheep
Turkeys
Ducks
Horses
Buffaloes
Goats
Livestock species

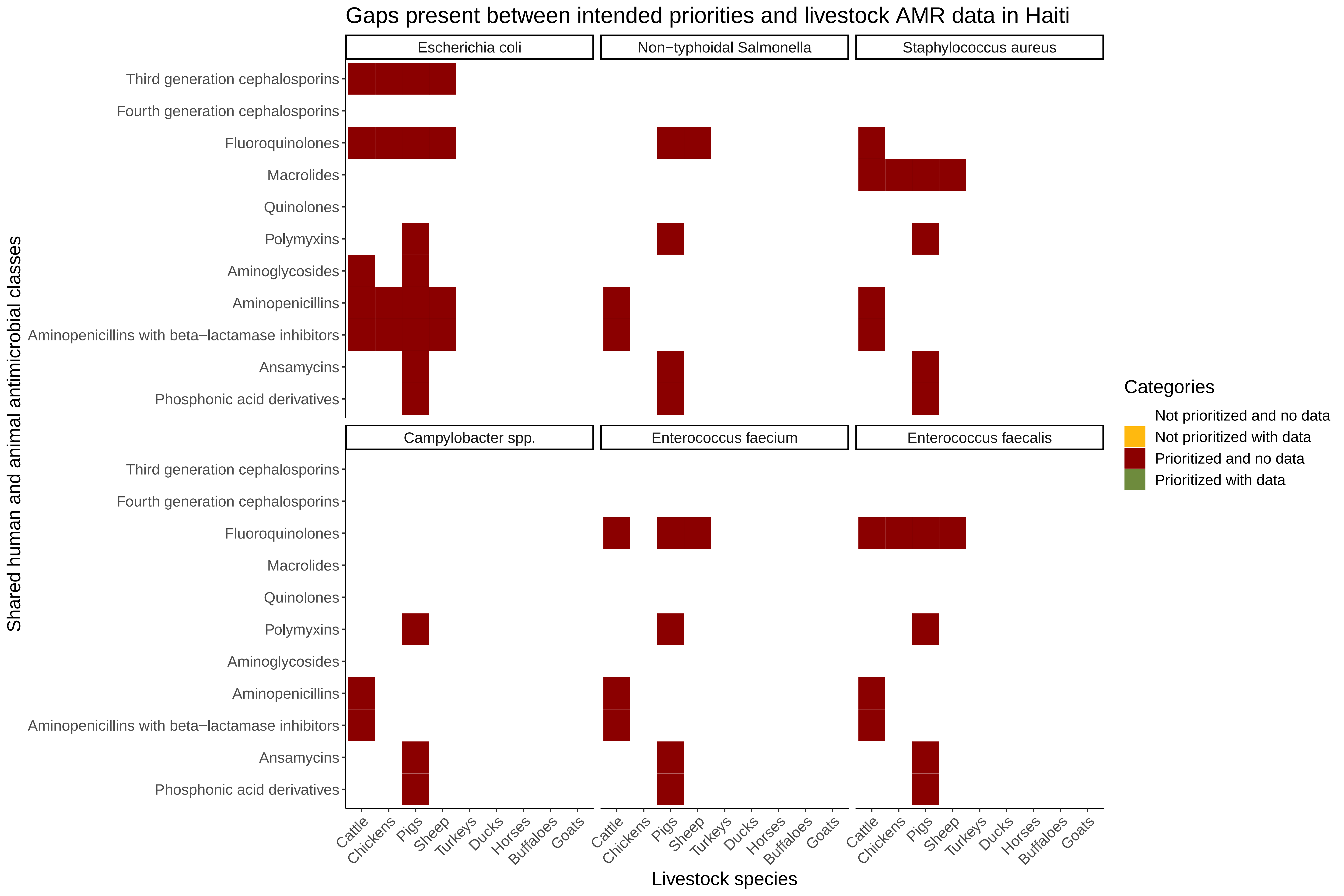
Gaps present between intended priorities and livestock AMR data in Haiti
Escherichia coli
Non−typhoidal Salmonella
Staphylococcus aureus
Campylobacter spp.
Enterococcus faecium
Enterococcus faecalis
Shared human and animal antimicrobial classes
Third generation cephalosporins
Fourth generation cephalosporins
Fluoroquinolones
Macrolides
Quinolones
Polymyxins
Aminoglycosides
Aminopenicillins
Aminopenicillins with beta−lactamase inhibitors
Ansamycins
Phosphonic acid derivatives
Cattle
Chickens
Pigs
Sheep
Turkeys
Ducks
Horses
Buffaloes
Goats
Livestock species
Categories
Not prioritized and no data
Not prioritized with data
Prioritized and no data
Prioritized with data

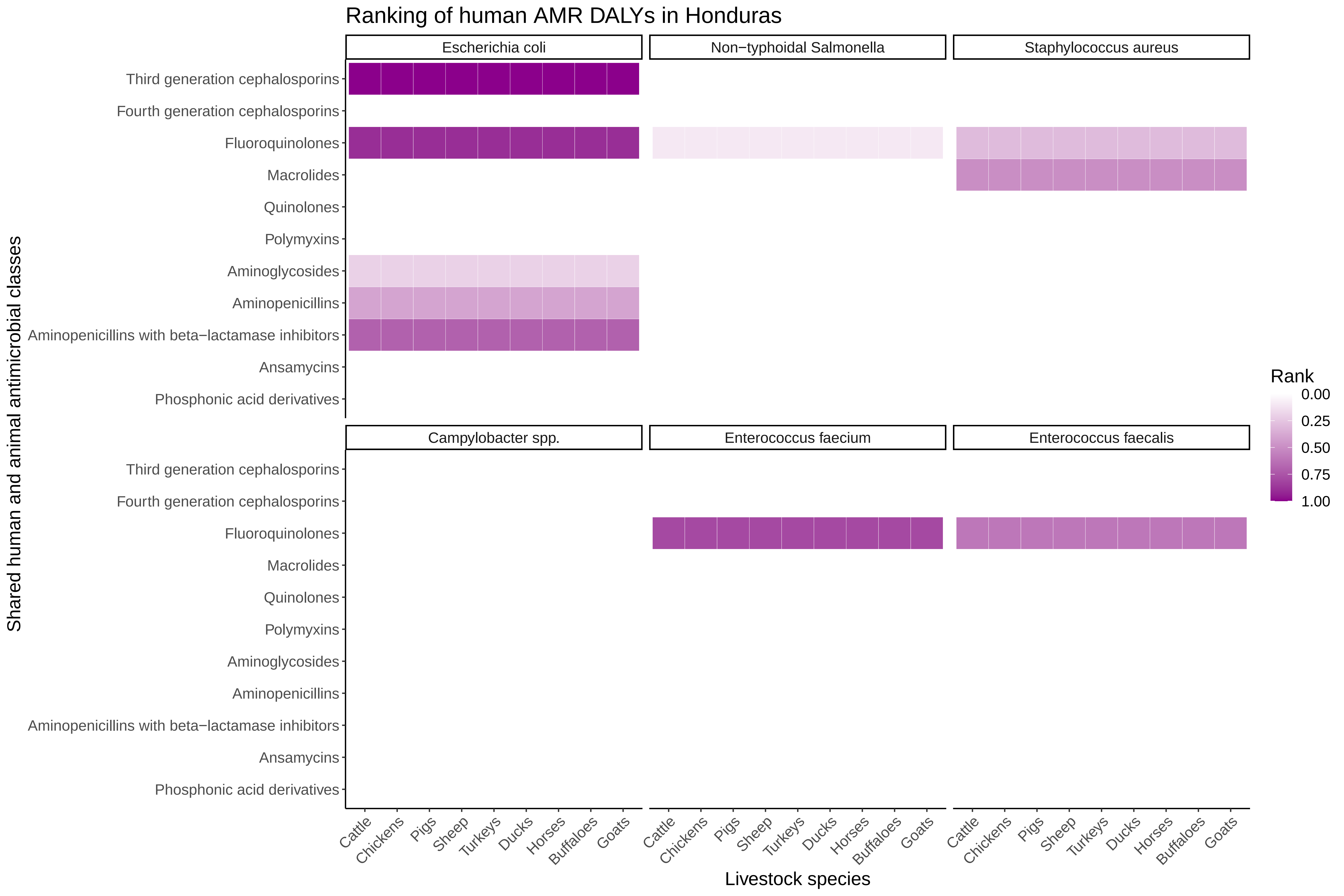

Ranking of human AMR DALYs in Honduras
Escherichia coli
Non−typhoidal Salmonella
Staphylococcus aureus
Campylobacter spp.
Enterococcus faecium
Enterococcus faecalis
Third generation cephalosporins
Fourth generation cephalosporins
Fluoroquinolones
Macrolides
Quinolones
Polymyxins
Aminoglycosides
Aminopenicillins
Aminopenicillins with beta−lactamase inhibitors
Ansamycins
Phosphonic acid derivatives
Shared human and animal antimicrobial classes
Cattle
Chickens
Pigs
Sheep
Turkeys
Ducks
Horses
Buffaloes
Goats
Livestock species
Rank
0.00
0.25
0.50
0.75
1.00

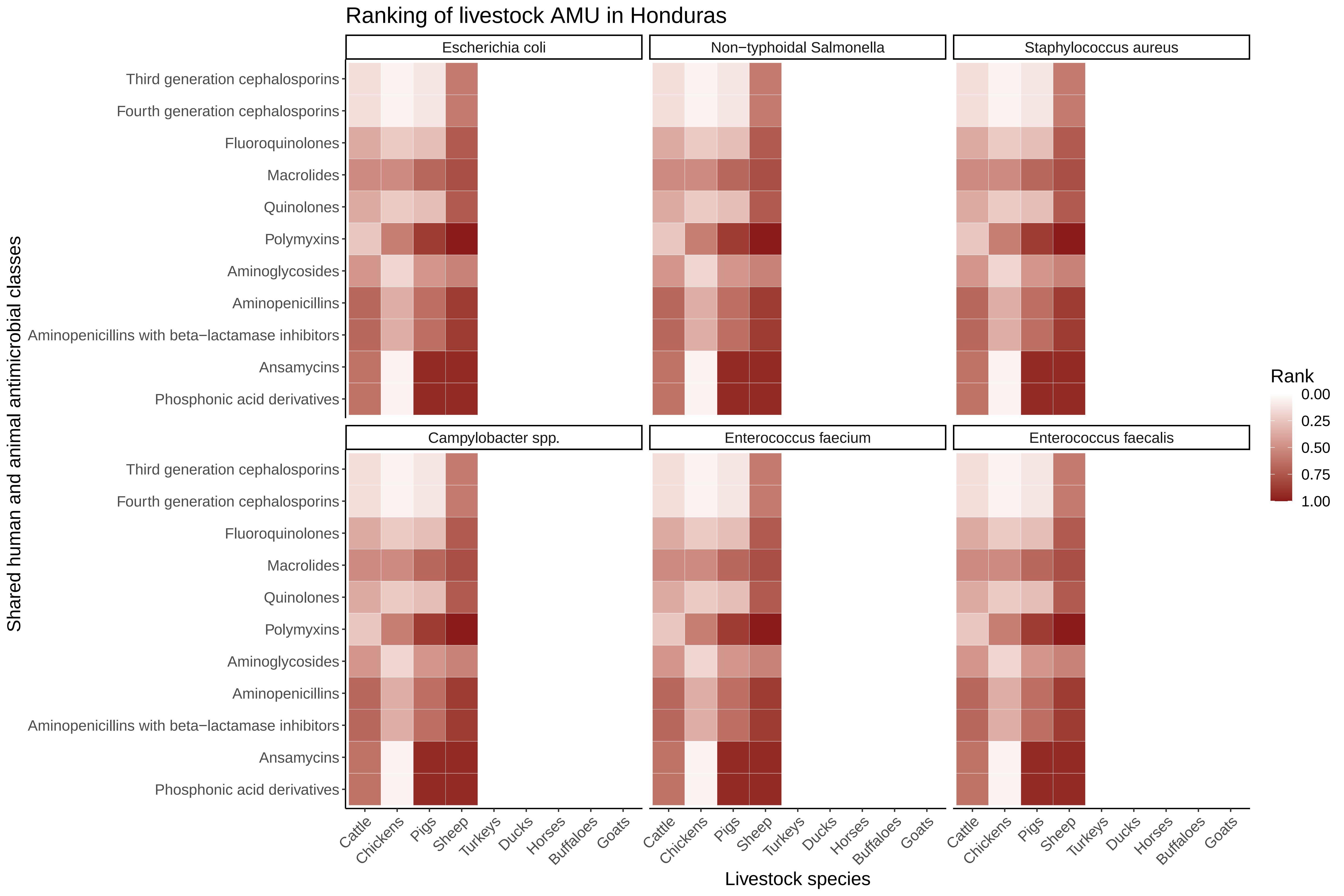
Ranking of livestock AMU in Honduras
Escherichia coli
Non−typhoidal Salmonella
Staphylococcus aureus
Campylobacter spp.
Enterococcus faecium
Enterococcus faecalis
Third generation cephalosporins
Fourth generation cephalosporins
Fluoroquinolones
Macrolides
Quinolones
Polymyxins
Aminoglycosides
Aminopenicillins
Aminopenicillins with beta−lactamase inhibitors
Ansamycins
Phosphonic acid derivatives
Shared human and animal antimicrobial classes
Cattle
Chickens
Pigs
Sheep
Turkeys
Ducks
Horses
Buffaloes
Goats
Livestock species
Rank
0.00
0.25
0.50
0.75
1.00

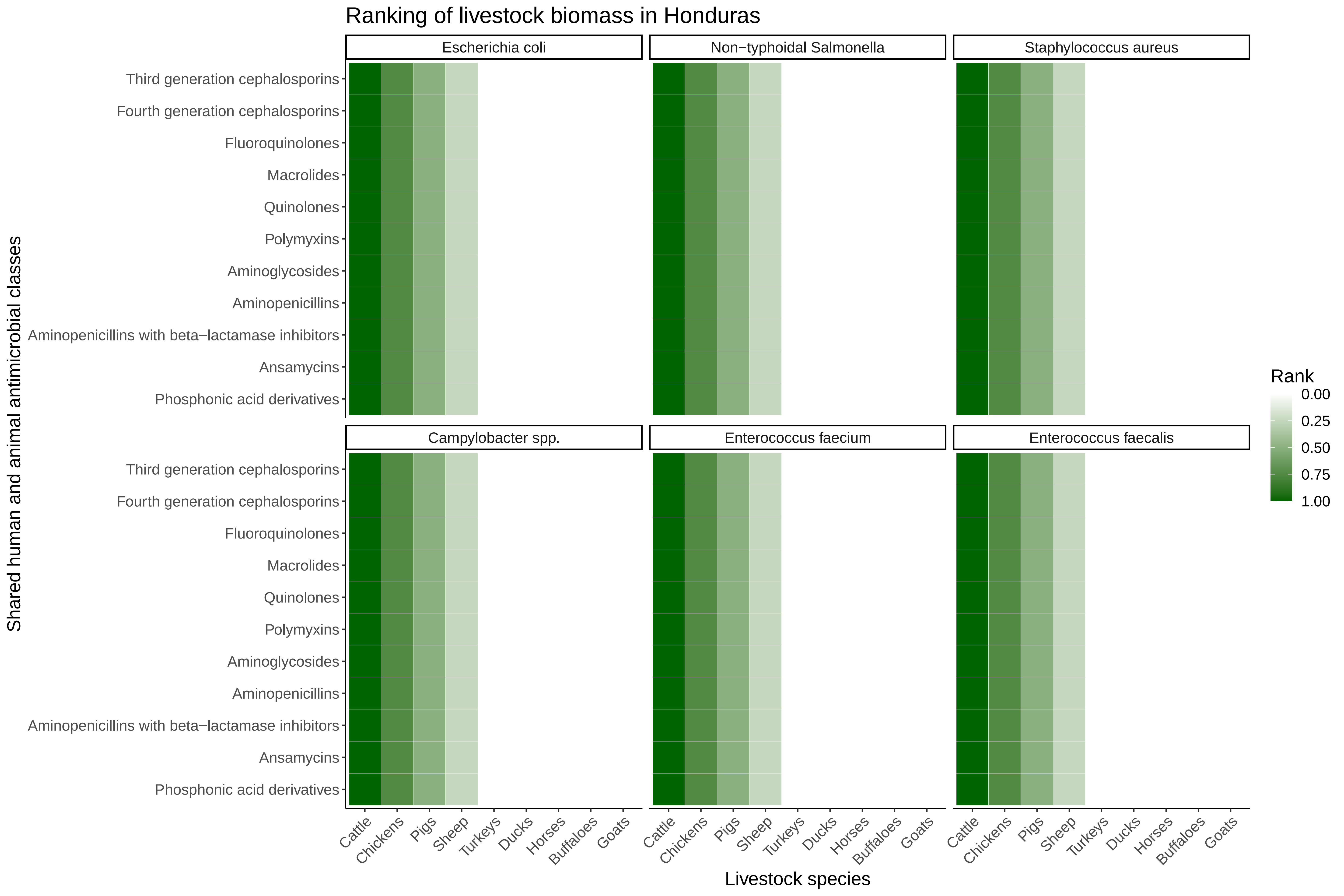

Ranking of livestock biomass in Honduras
Escherichia coli
Non−typhoidal Salmonella
Staphylococcus aureus
Campylobacter spp.
Enterococcus faecium
Enterococcus faecalis
Shared human and animal antimicrobial classes
Third generation cephalosporins
Fourth generation cephalosporins
Fluoroquinolones
Macrolides
Quinolones
Polymyxins
Aminoglycosides
Aminopenicillins
Aminopenicillins with beta−lactamase inhibitors
Ansamycins
Phosphonic acid derivatives
Cattle
Chickens
Pigs
Sheep
Turkeys
Ducks
Horses
Buffaloes
Goats
Livestock species
Rank
0.00
0.25
0.50
0.75
1.00

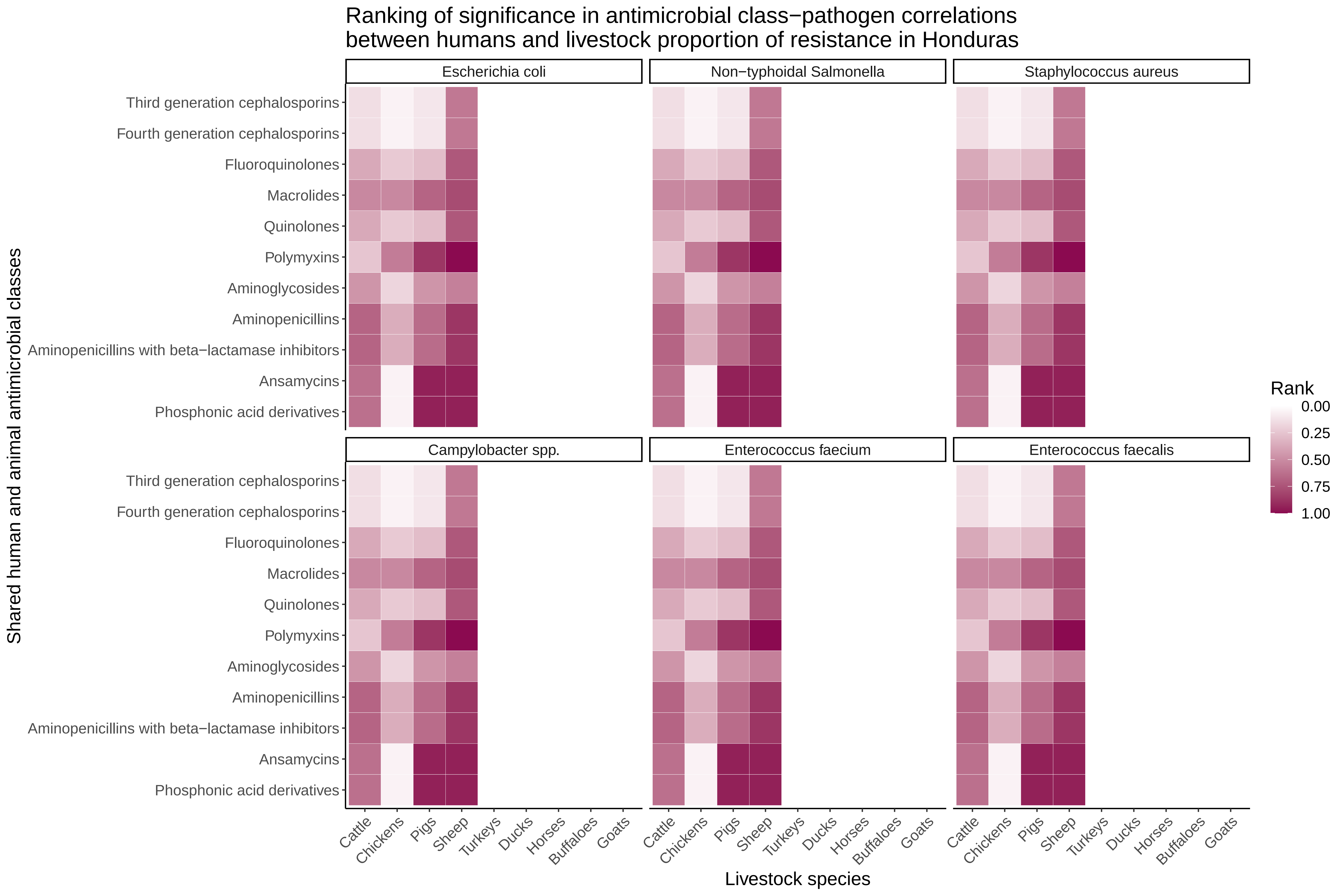

Ranking of significance in antimicrobial class−pathogen correlations
between humans and livestock proportion of resistance in Honduras
Escherichia coli
Non−typhoidal Salmonella
Staphylococcus aureus
Campylobacter spp.
Enterococcus faecium
Enterococcus faecalis
Shared human and animal antimicrobial classes
Third generation cephalosporins
Fourth generation cephalosporins
Fluoroquinolones
Macrolides
Quinolones
Polymyxins
Aminoglycosides
Aminopenicillins
Aminopenicillins with beta−lactamase inhibitors
Ansamycins
Phosphonic acid derivatives
Livestock species
Cattle
Chickens
Pigs
Sheep
Turkeys
Ducks
Horses
Buffaloes
Goats
Rank
0.00
0.25
0.50
0.75
1.00

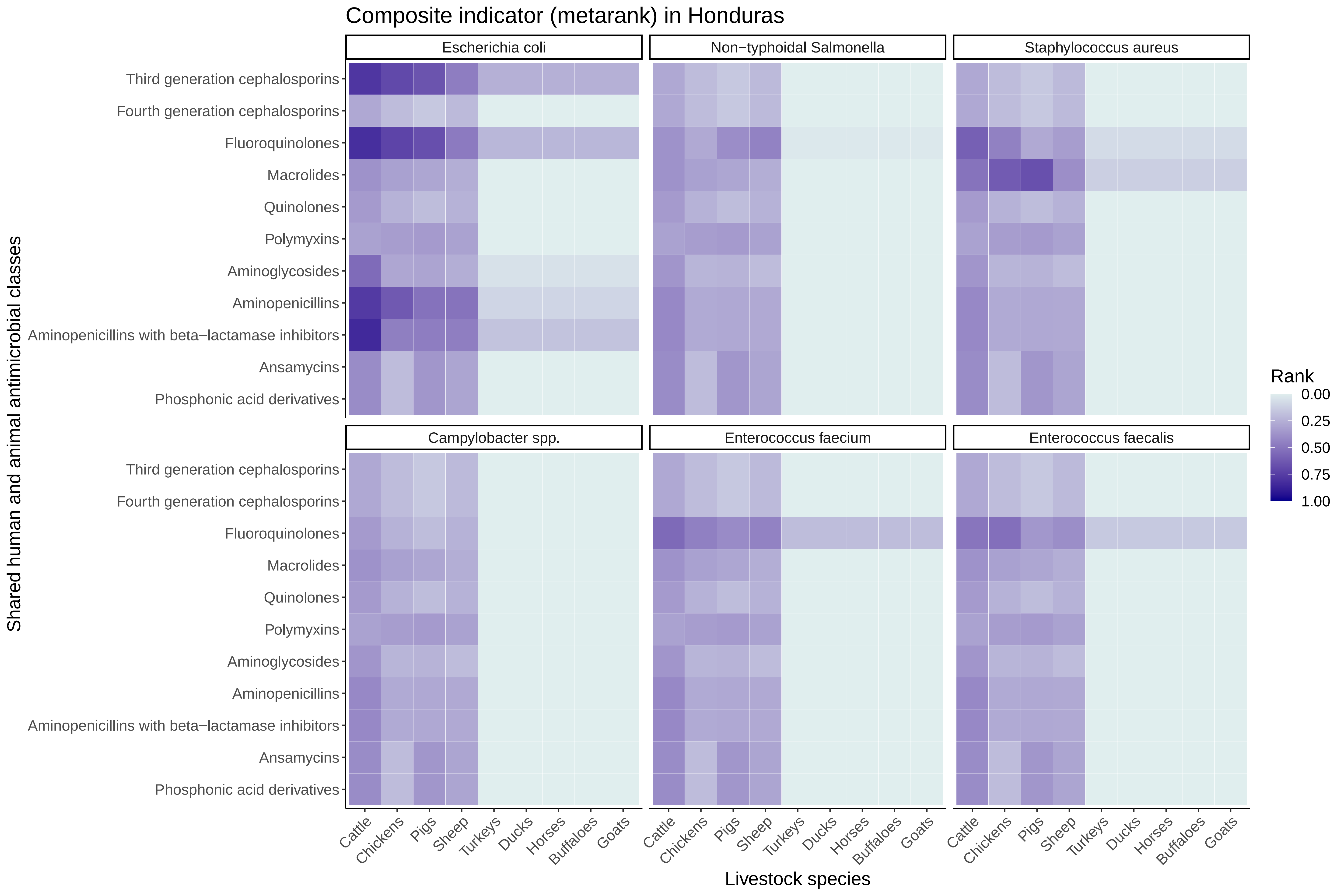

Composite indicator (metarank) in Honduras
Escherichia coli
Non−typhoidal Salmonella
Staphylococcus aureus
Campylobacter spp.
Enterococcus faecium
Enterococcus faecalis
Third generation cephalosporins
Fourth generation cephalosporins
Fluoroquinolones
Macrolides
Quinolones
Polymyxins
Aminoglycosides
Aminopenicillins
Aminopenicillins with beta−lactamase inhibitors
Ansamycins
Phosphonic acid derivatives
Shared human and animal antimicrobial classes
Cattle
Chickens
Pigs
Sheep
Turkeys
Ducks
Horses
Buffaloes
Goats
Livestock species
Rank
0.00
0.25
0.50
0.75
1.00

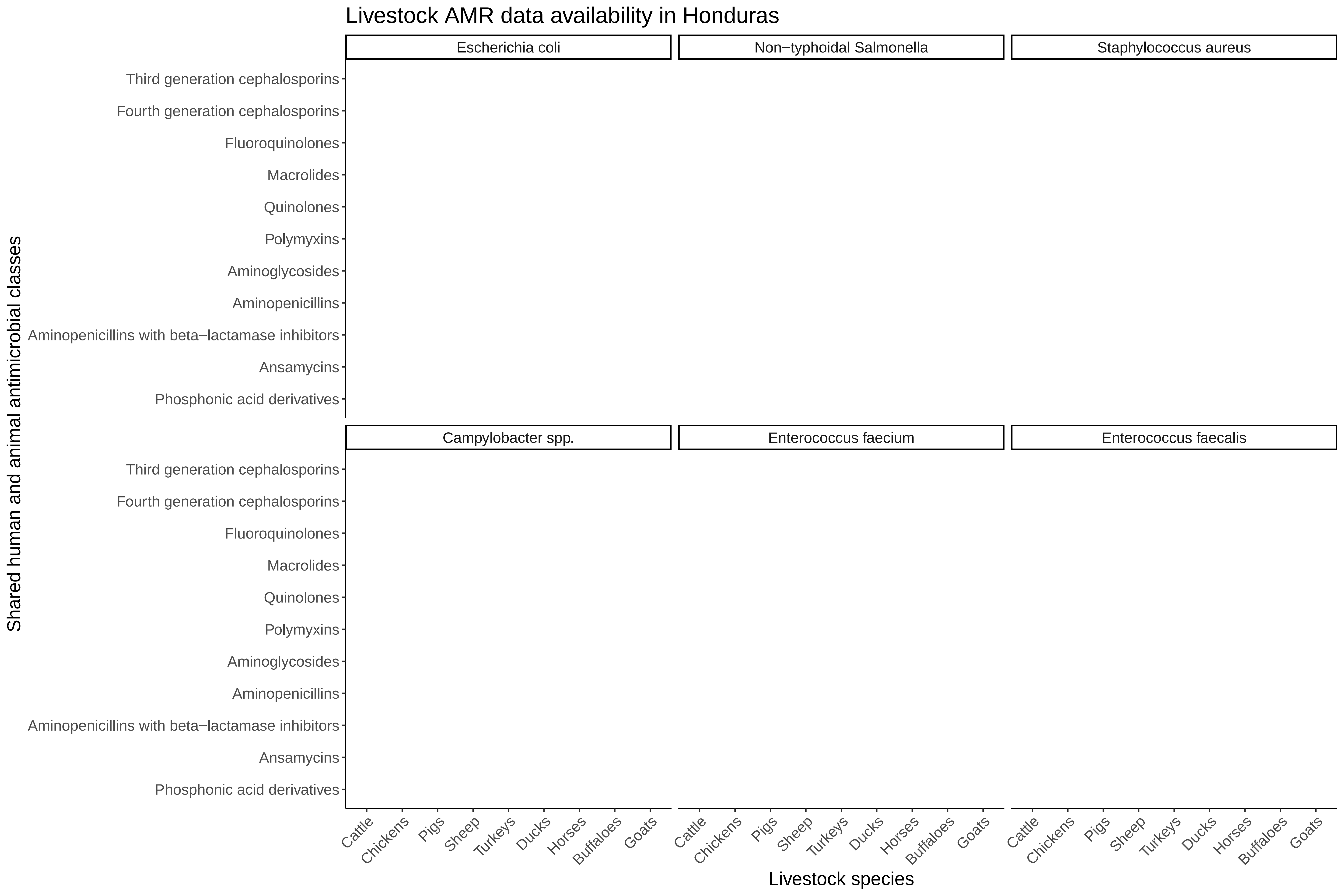
Livestock AMR data availability in Honduras
Escherichia coli
Non−typhoidal Salmonella
Staphylococcus aureus
Campylobacter spp.
Enterococcus faecium
Enterococcus faecalis
Shared human and animal antimicrobial classes
Third generation cephalosporins
Fourth generation cephalosporins
Fluoroquinolones
Macrolides
Quinolones
Polymyxins
Aminoglycosides
Aminopenicillins
Aminopenicillins with beta−lactamase inhibitors
Ansamycins
Phosphonic acid derivatives
Cattle
Chickens
Pigs
Sheep
Turkeys
Ducks
Horses
Buffaloes
Goats
Livestock species

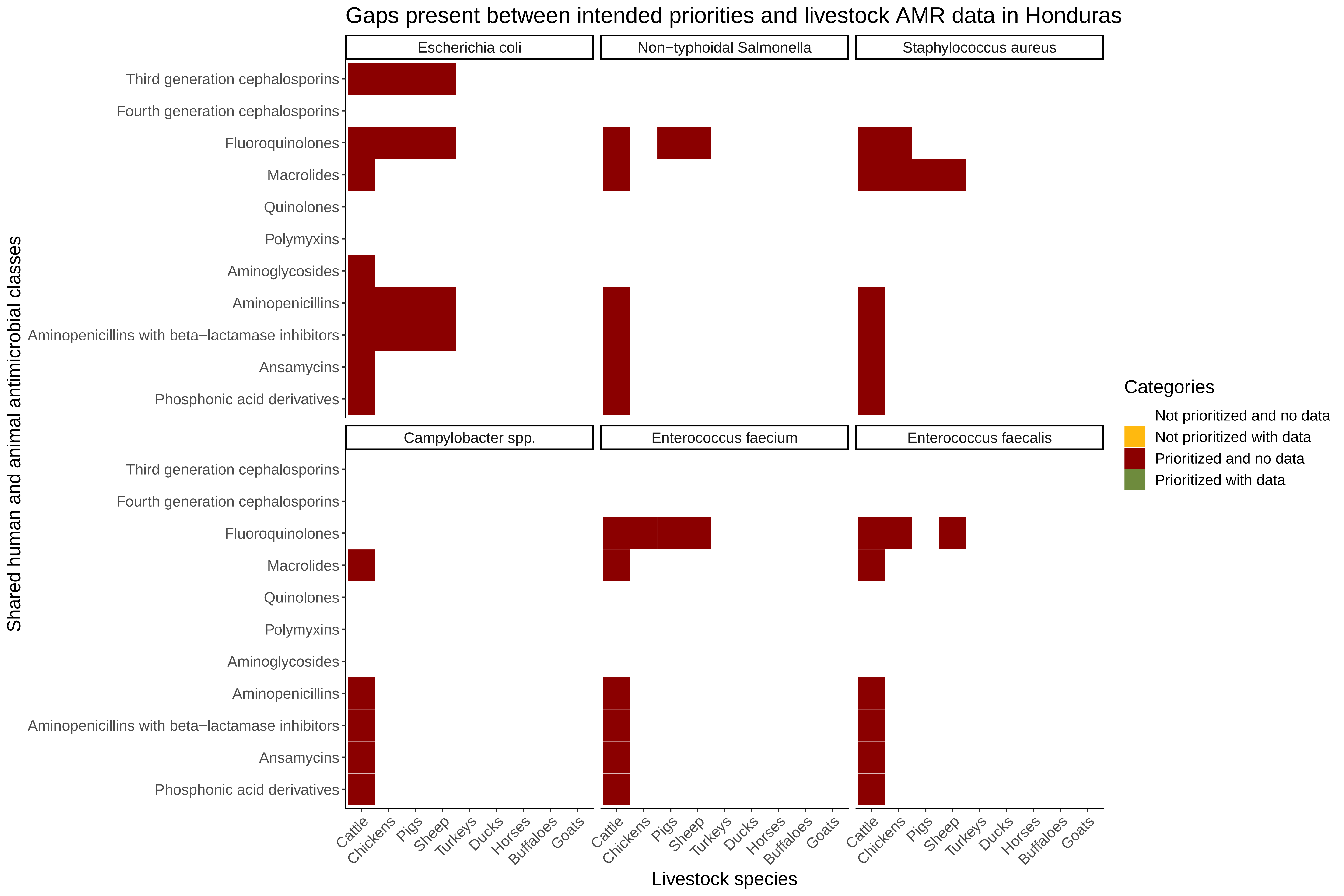

Gaps present between intended priorities and livestock AMR data in Honduras
Escherichia coli
Non−typhoidal Salmonella
Staphylococcus aureus
Campylobacter spp.
Enterococcus faecium
Enterococcus faecalis
Third generation cephalosporins
Fourth generation cephalosporins
Fluoroquinolones
Macrolides
Quinolones
Polymyxins
Aminoglycosides
Aminopenicillins
Aminopenicillins with beta−lactamase inhibitors
Ansamycins
Phosphonic acid derivatives
Shared human and animal antimicrobial classes
Cattle
Chickens
Pigs
Sheep
Turkeys
Ducks
Horses
Buffaloes
Goats
Livestock species
Categories
Not prioritized and no data
Not prioritized with data
Prioritized and no data
Prioritized with data

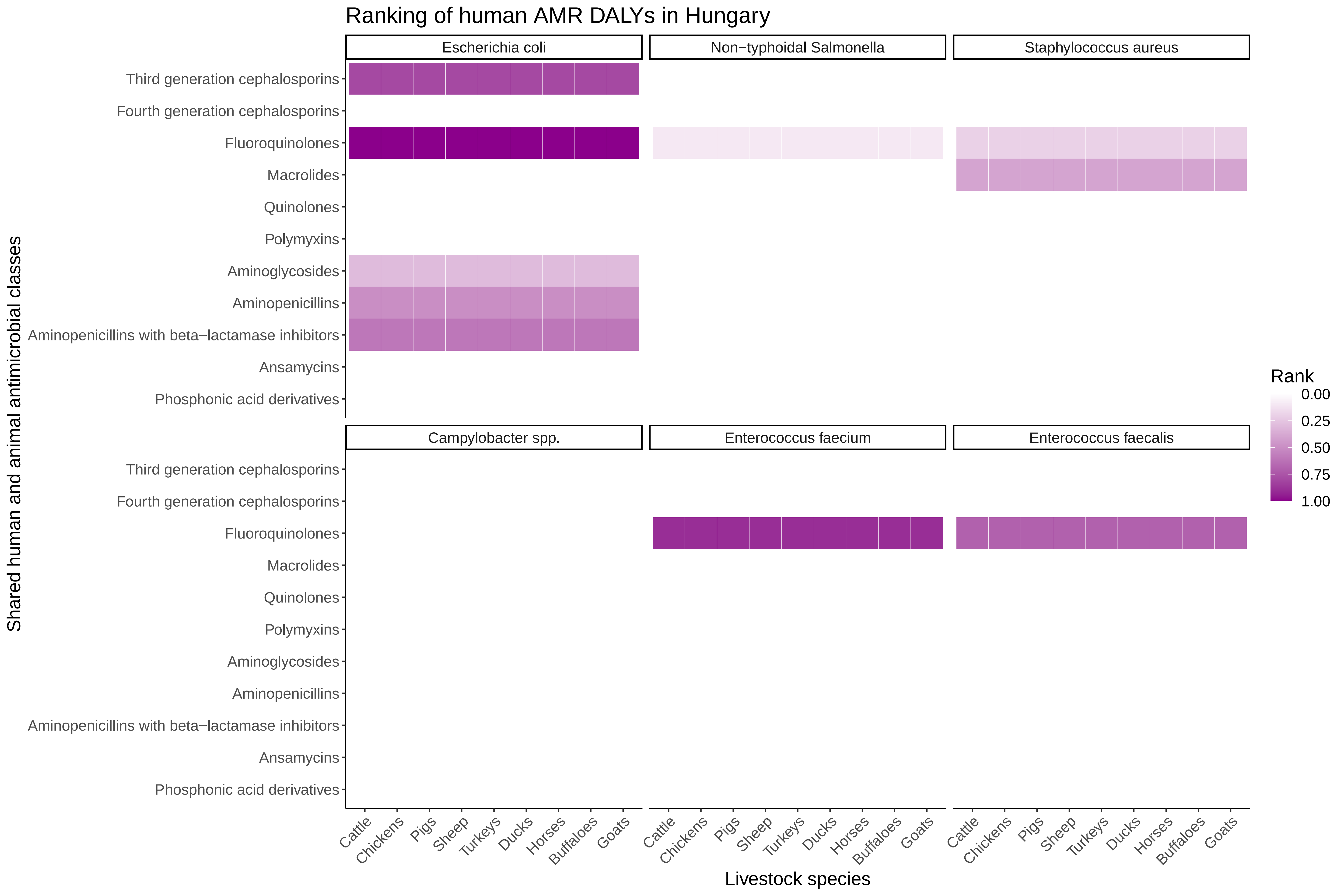
Ranking of human AMR DALYs in Hungary
Escherichia coli
Non−typhoidal Salmonella
Staphylococcus aureus
Campylobacter spp.
Enterococcus faecium
Enterococcus faecalis
Third generation cephalosporins
Fourth generation cephalosporins
Fluoroquinolones
Macrolides
Quinolones
Polymyxins
Aminoglycosides
Aminopenicillins
Aminopenicillins with beta−lactamase inhibitors
Ansamycins
Phosphonic acid derivatives
Shared human and animal antimicrobial classes
Cattle
Chickens
Pigs
Sheep
Turkeys
Ducks
Horses
Buffaloes
Goats
Livestock species
Rank
0.00
0.25
0.50
0.75
1.00

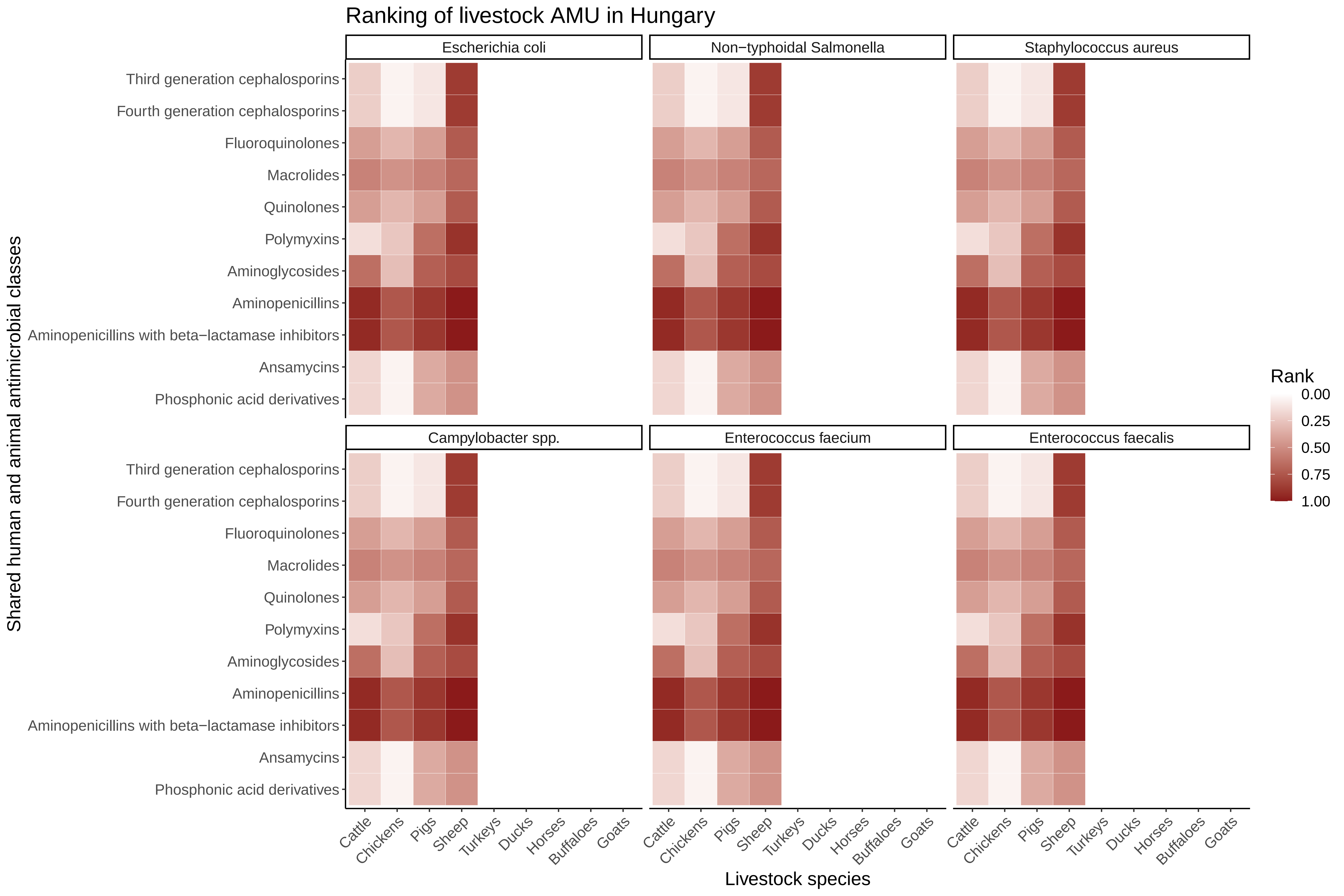

Ranking of livestock AMU in Hungary
Escherichia coli
Non−typhoidal Salmonella
Staphylococcus aureus
Campylobacter spp.
Enterococcus faecium
Enterococcus faecalis
Shared human and animal antimicrobial classes
Third generation cephalosporins
Fourth generation cephalosporins
Fluoroquinolones
Macrolides
Quinolones
Polymyxins
Aminoglycosides
Aminopenicillins
Aminopenicillins with beta−lactamase inhibitors
Ansamycins
Phosphonic acid derivatives
Cattle
Chickens
Pigs
Sheep
Turkeys
Ducks
Horses
Buffaloes
Goats
Livestock species
Rank
0.00
0.25
0.50
0.75
1.00

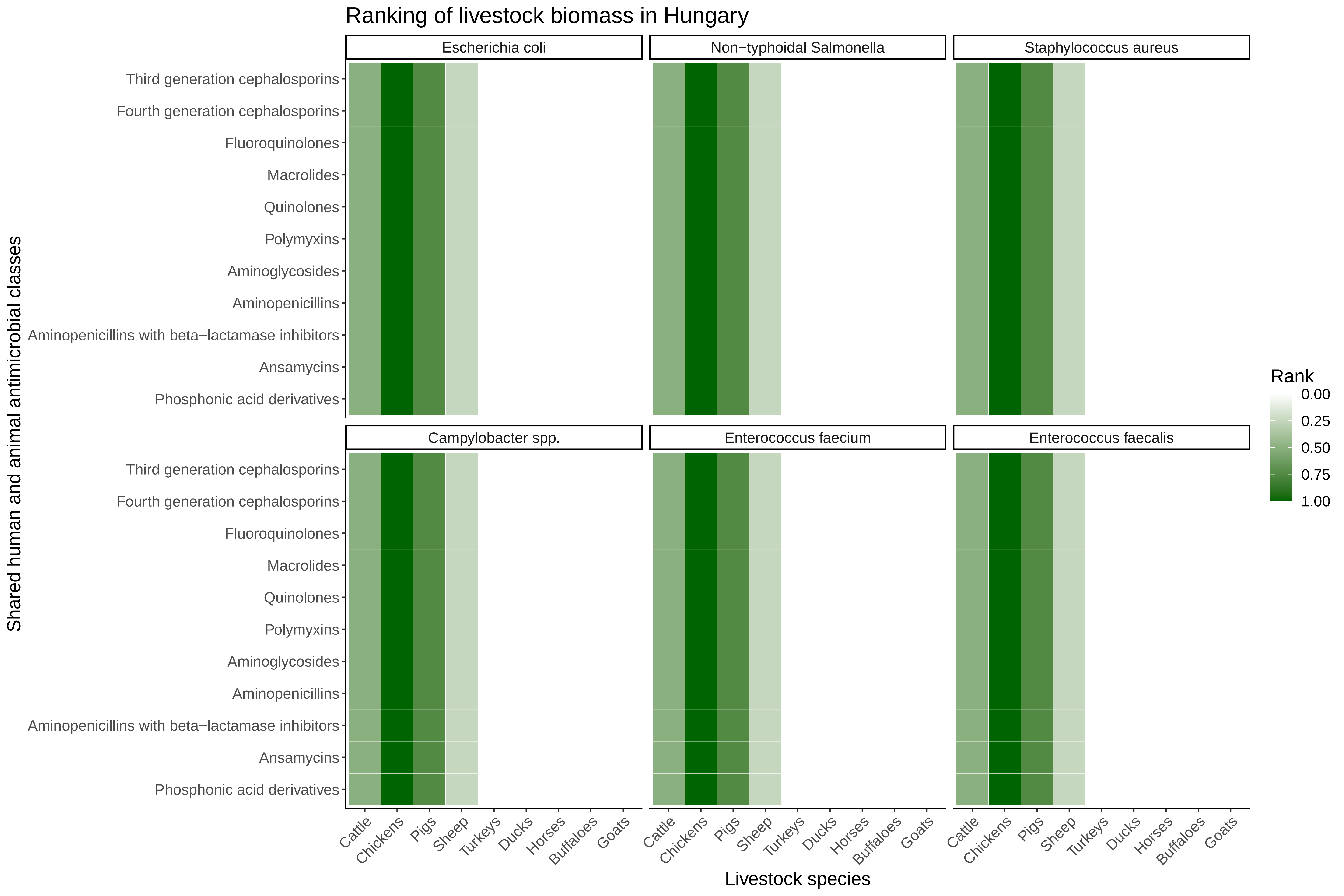

Ranking of livestock biomass in Hungary
Escherichia coli
Non−typhoidal Salmonella
Staphylococcus aureus
Campylobacter spp.
Enterococcus faecium
Enterococcus faecalis
Shared human and animal antimicrobial classes
Third generation cephalosporins
Fourth generation cephalosporins
Fluoroquinolones
Macrolides
Quinolones
Polymyxins
Aminoglycosides
Aminopenicillins
Aminopenicillins with beta−lactamase inhibitors
Ansamycins
Phosphonic acid derivatives
Cattle
Chickens
Pigs
Sheep
Turkeys
Ducks
Horses
Buffaloes
Goats
Livestock species
Rank
0.00
0.25
0.50
0.75
1.00

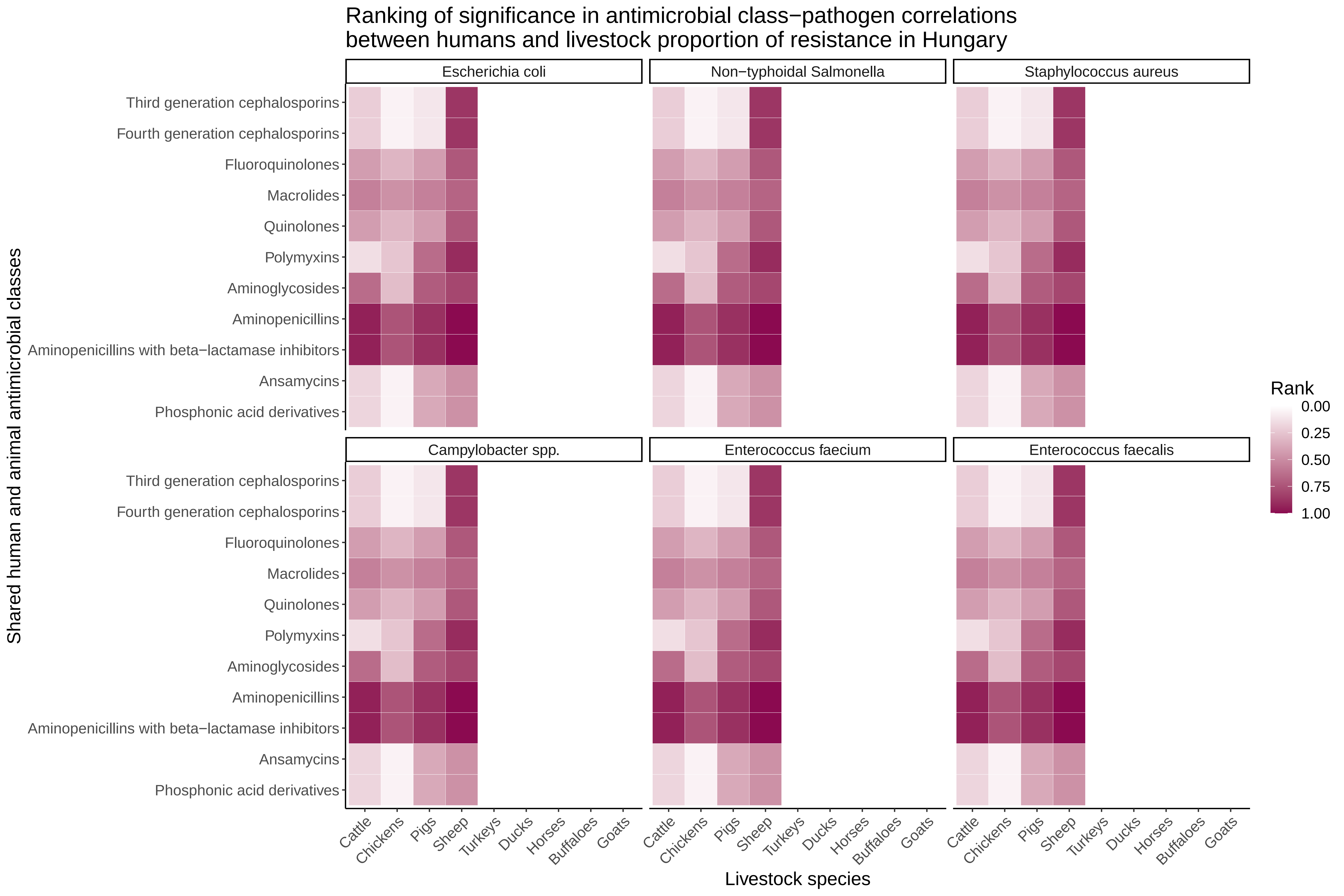

Ranking of significance in antimicrobial class−pathogen correlations
between humans and livestock proportion of resistance in Hungary
Escherichia coli
Non−typhoidal Salmonella
Staphylococcus aureus
Campylobacter spp.
Enterococcus faecium
Enterococcus faecalis
Third generation cephalosporins
Fourth generation cephalosporins
Fluoroquinolones
Macrolides
Quinolones
Polymyxins
Aminoglycosides
Aminopenicillins
Aminopenicillins with beta−lactamase inhibitors
Ansamycins
Phosphonic acid derivatives
Shared human and animal antimicrobial classes
Cattle
Chickens
Pigs
Sheep
Turkeys
Ducks
Horses
Buffaloes
Goats
Livestock species
Rank
0.00
0.25
0.50
0.75
1.00

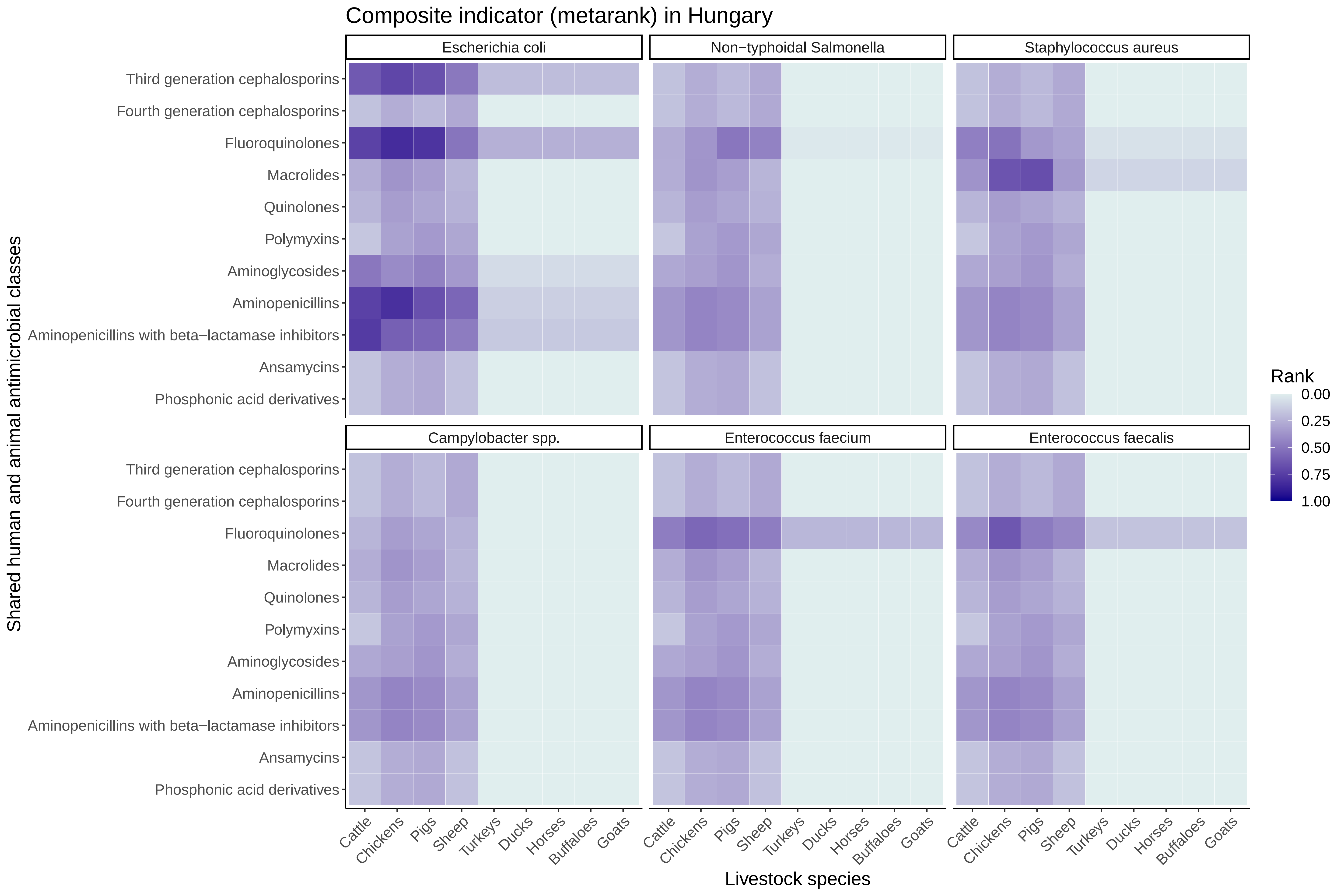

Composite indicator (metarank) in Hungary
Escherichia coli
Non−typhoidal Salmonella
Staphylococcus aureus
Campylobacter spp.
Enterococcus faecium
Enterococcus faecalis
Third generation cephalosporins
Fourth generation cephalosporins
Fluoroquinolones
Macrolides
Quinolones
Polymyxins
Aminoglycosides
Aminopenicillins
Aminopenicillins with beta−lactamase inhibitors
Ansamycins
Phosphonic acid derivatives
Shared human and animal antimicrobial classes
Cattle
Chickens
Pigs
Sheep
Turkeys
Ducks
Horses
Buffaloes
Goats
Livestock species
Rank
0.00
0.25
0.50
0.75
1.00

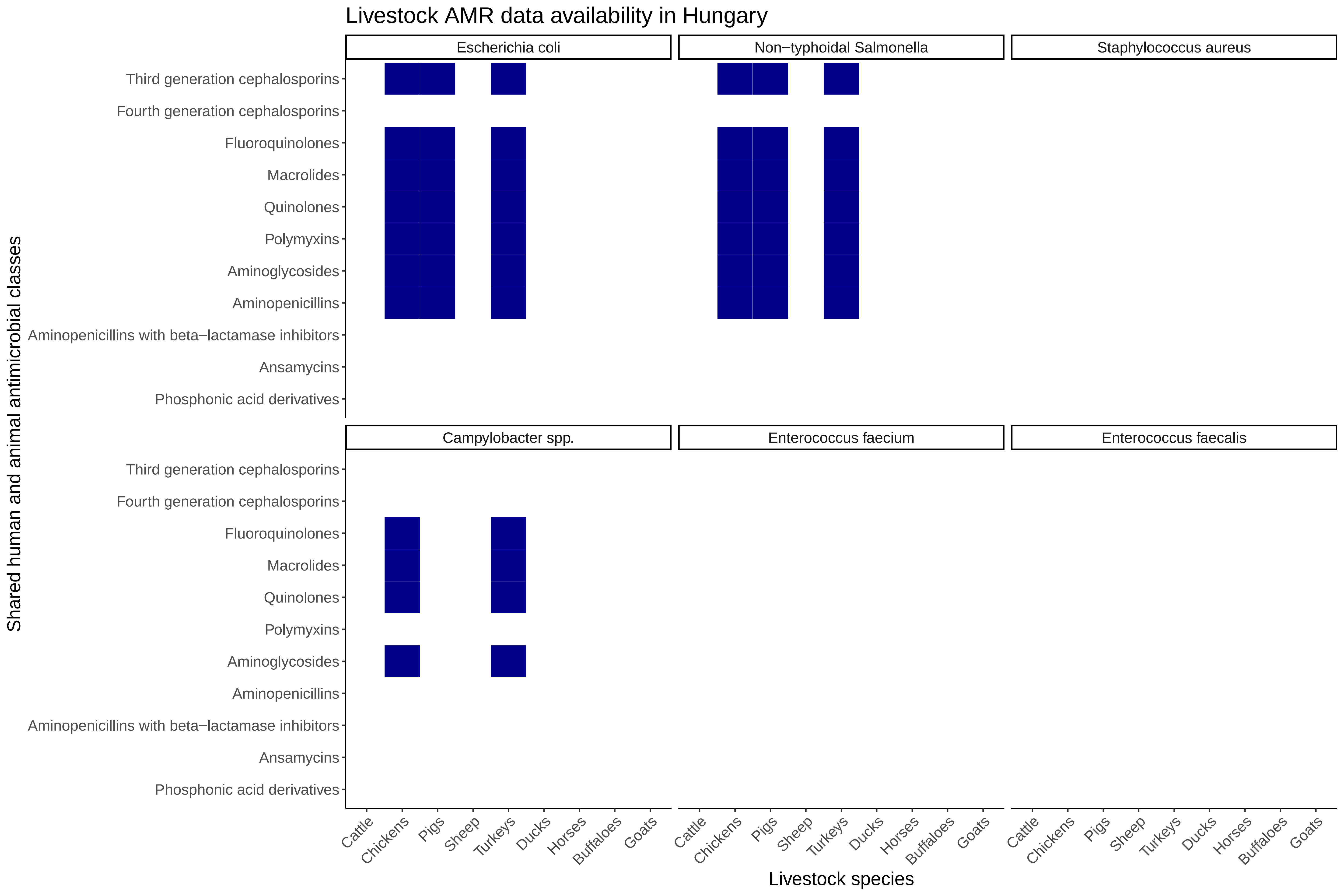

Livestock AMR data availability in Hungary
Escherichia coli
Non−typhoidal Salmonella
Staphylococcus aureus
Campylobacter spp.
Enterococcus faecium
Enterococcus faecalis
Shared human and animal antimicrobial classes
Third generation cephalosporins
Fourth generation cephalosporins
Fluoroquinolones
Macrolides
Quinolones
Polymyxins
Aminoglycosides
Aminopenicillins
Aminopenicillins with beta−lactamase inhibitors
Ansamycins
Phosphonic acid derivatives
Cattle
Chickens
Pigs
Sheep
Turkeys
Ducks
Horses
Buffaloes
Goats
Livestock species

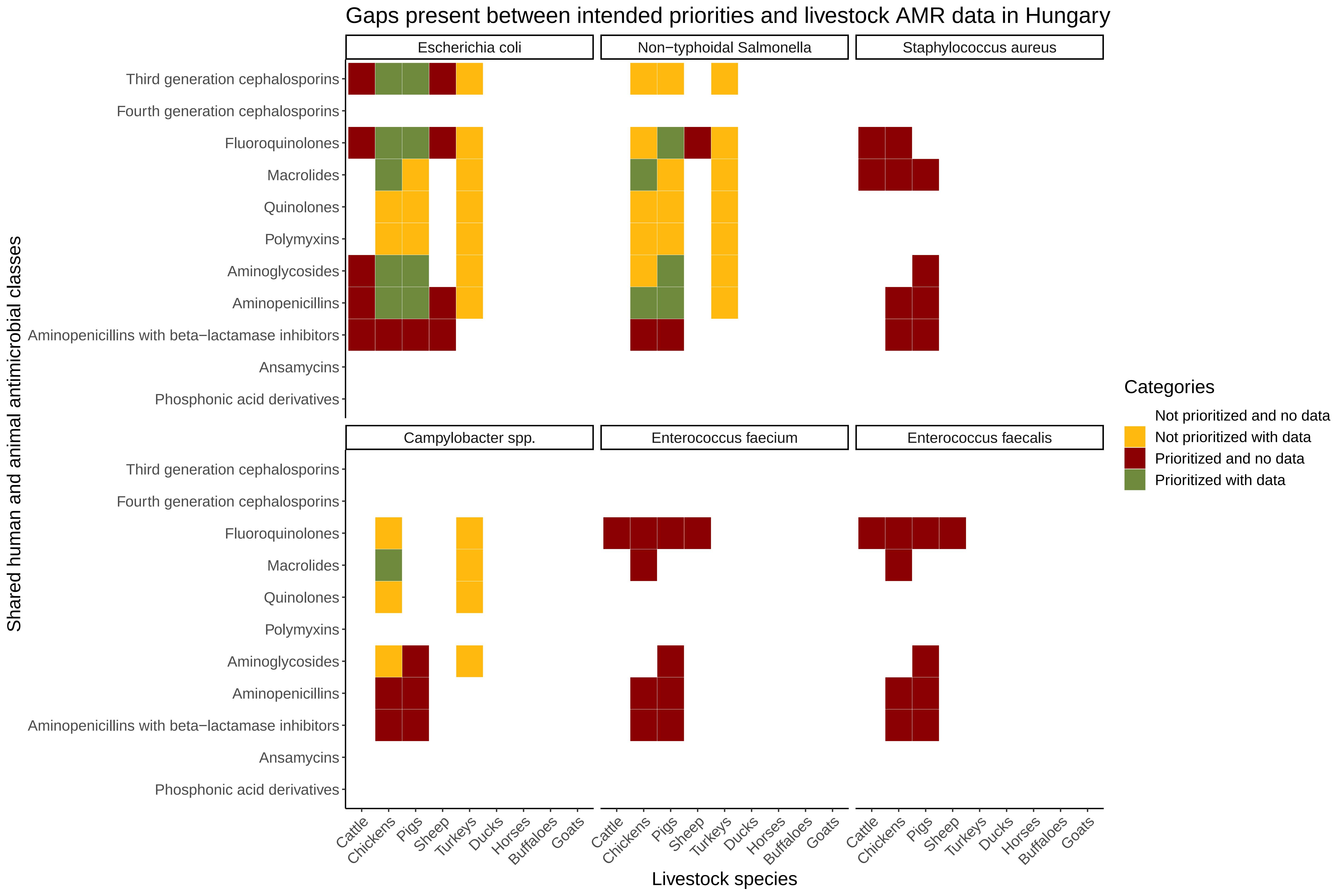

Gaps present between intended priorities and livestock AMR data in Hungary
Escherichia coli
Non−typhoidal Salmonella
Staphylococcus aureus
Campylobacter spp.
Enterococcus faecium
Enterococcus faecalis
Third generation cephalosporins
Fourth generation cephalosporins
Fluoroquinolones
Macrolides
Quinolones
Polymyxins
Aminoglycosides
Aminopenicillins
Aminopenicillins with beta−lactamase inhibitors
Ansamycins
Phosphonic acid derivatives
Shared human and animal antimicrobial classes
Cattle
Chickens
Pigs
Sheep
Turkeys
Ducks
Horses
Buffaloes
Goats
Livestock species
Categories
Not prioritized and no data
Not prioritized with data
Prioritized and no data
Prioritized with data

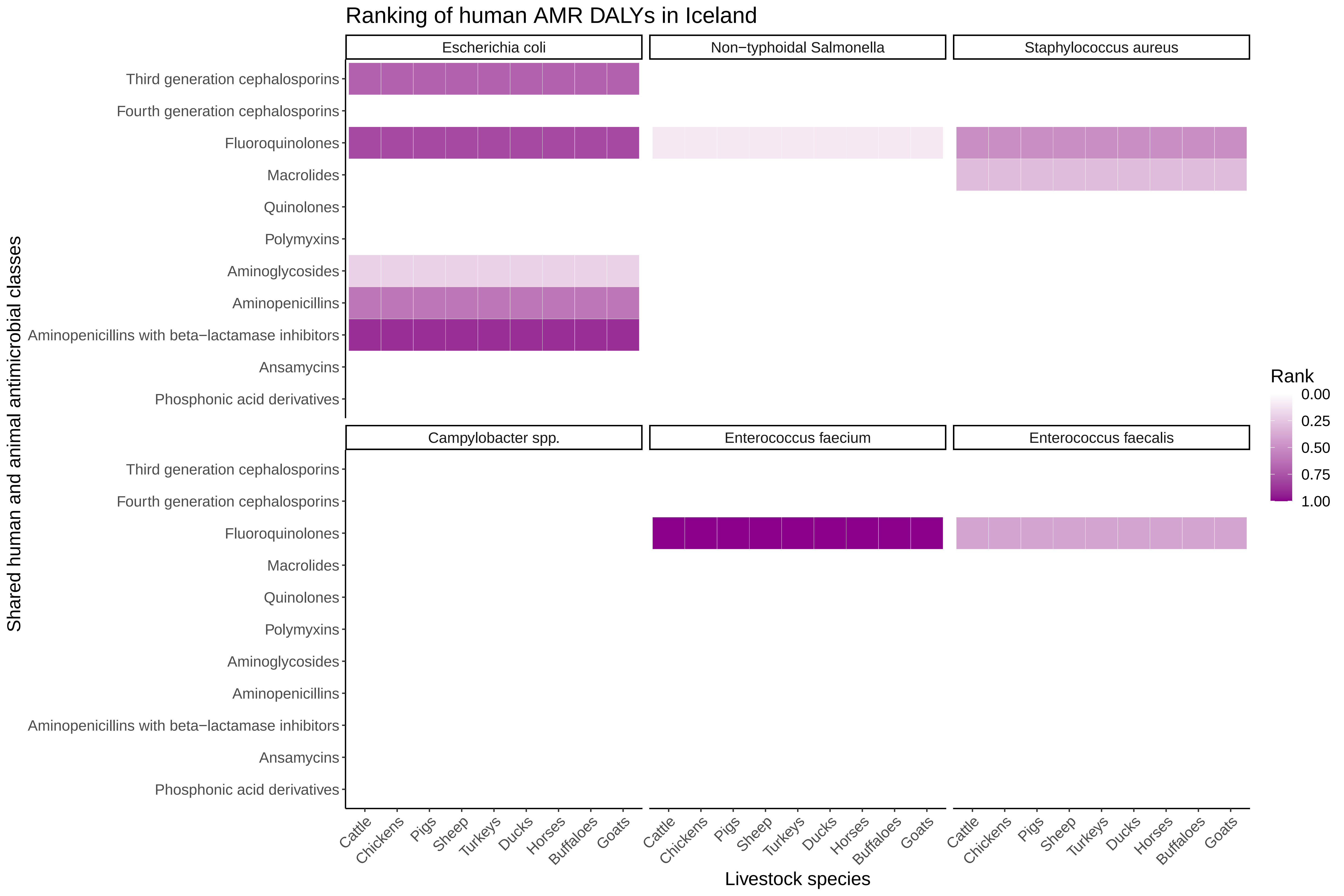
Ranking of human AMR DALYs in Iceland
Escherichia coli
Non−typhoidal Salmonella
Staphylococcus aureus
Campylobacter spp.
Enterococcus faecium
Enterococcus faecalis
Third generation cephalosporins
Fourth generation cephalosporins
Fluoroquinolones
Macrolides
Quinolones
Polymyxins
Aminoglycosides
Aminopenicillins
Aminopenicillins with beta−lactamase inhibitors
Ansamycins
Phosphonic acid derivatives
Shared human and animal antimicrobial classes
Cattle
Chickens
Pigs
Sheep
Turkeys
Ducks
Horses
Buffaloes
Goats
Livestock species
Rank
0.00
0.25
0.50
0.75
1.00

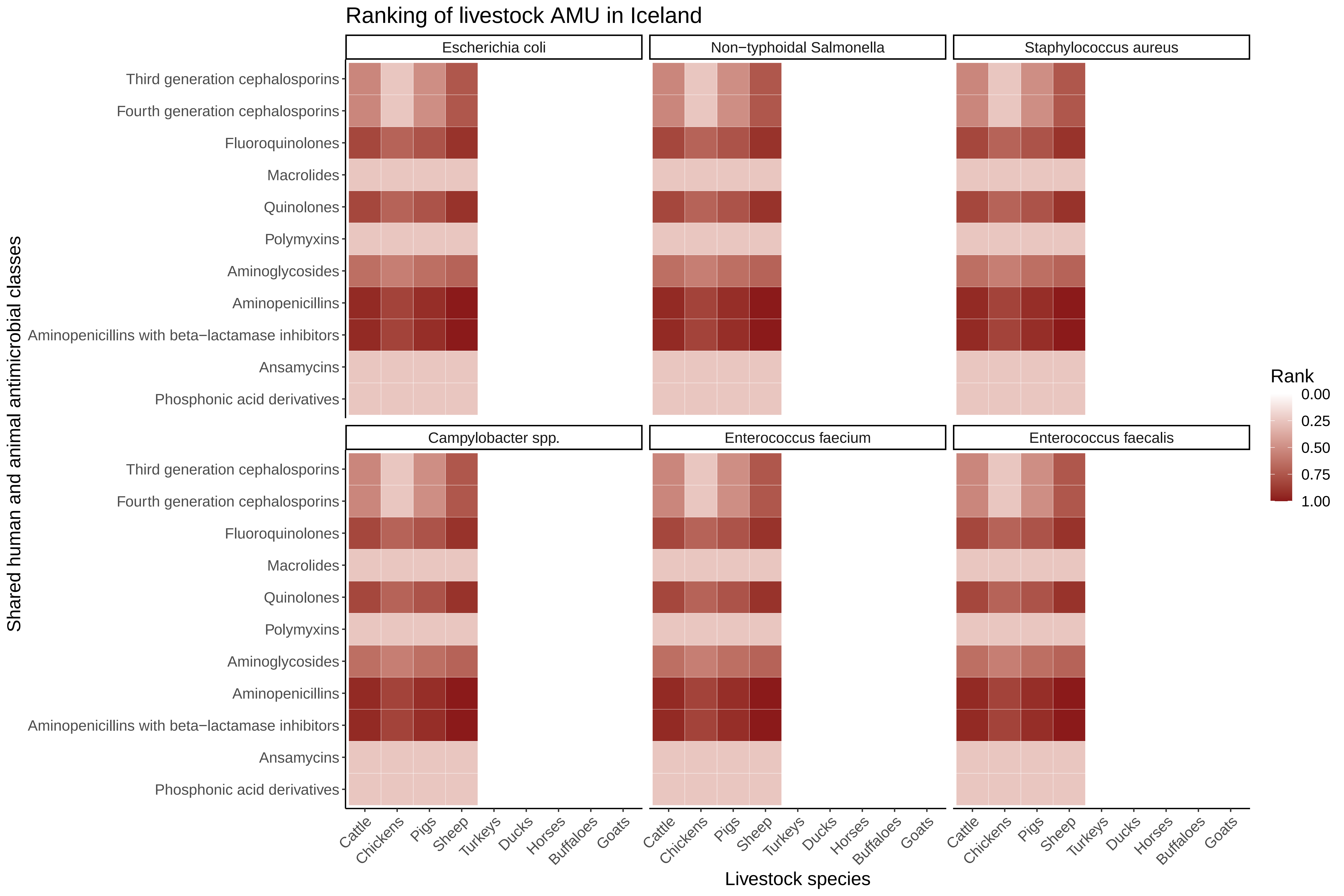
Ranking of livestock AMU in Iceland
Escherichia coli
Non−typhoidal Salmonella
Staphylococcus aureus
Campylobacter spp.
Enterococcus faecium
Enterococcus faecalis
Third generation cephalosporins
Fourth generation cephalosporins
Fluoroquinolones
Macrolides
Quinolones
Polymyxins
Aminoglycosides
Aminopenicillins
Aminopenicillins with beta−lactamase inhibitors
Ansamycins
Phosphonic acid derivatives
Shared human and animal antimicrobial classes
Cattle
Chickens
Pigs
Sheep
Turkeys
Ducks
Horses
Buffaloes
Goats
Livestock species
Rank
0.00
0.25
0.50
0.75
1.00

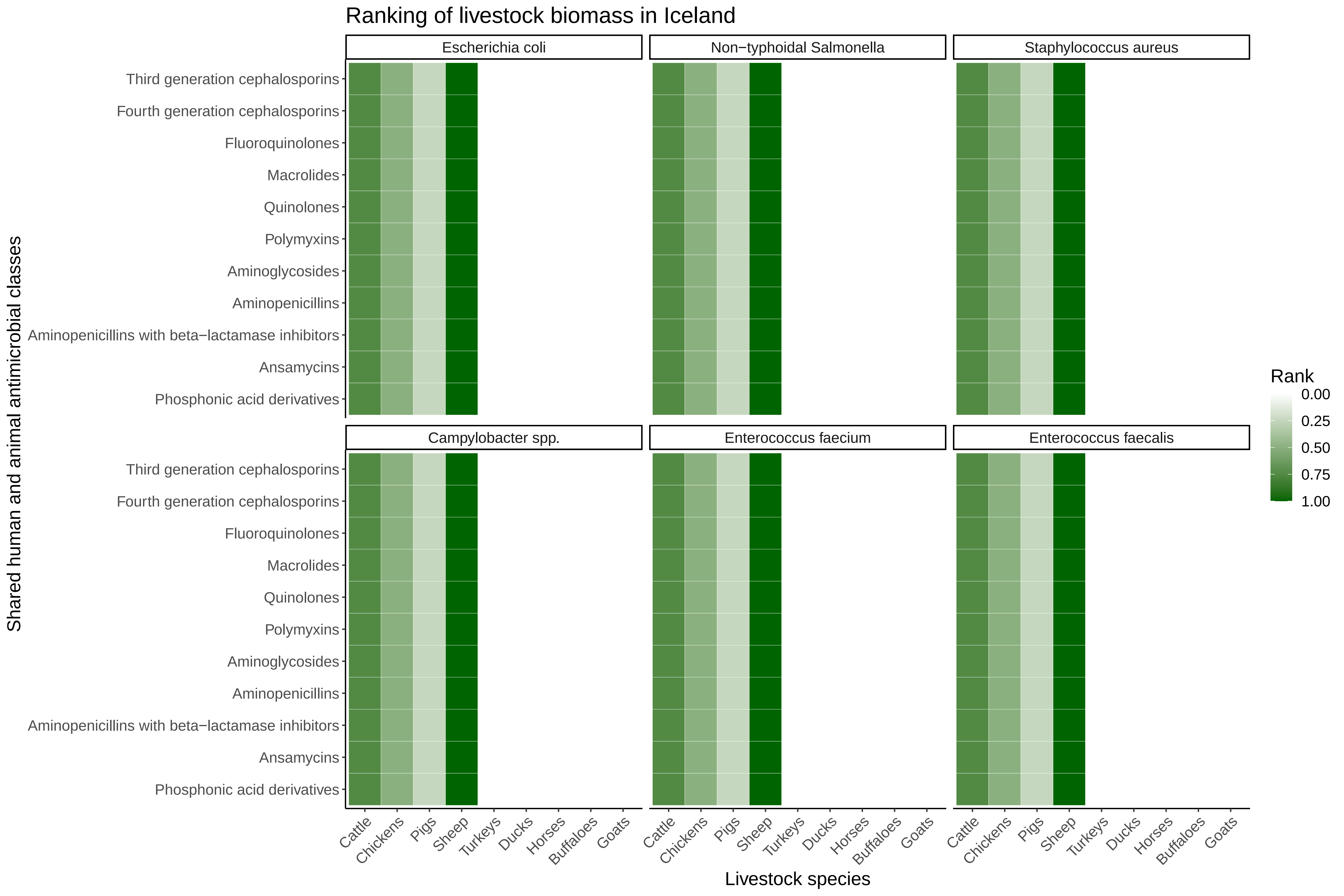

Ranking of livestock biomass in Iceland
Escherichia coli
Non−typhoidal Salmonella
Staphylococcus aureus
Campylobacter spp.
Enterococcus faecium
Enterococcus faecalis
Shared human and animal antimicrobial classes
Third generation cephalosporins
Fourth generation cephalosporins
Fluoroquinolones
Macrolides
Quinolones
Polymyxins
Aminoglycosides
Aminopenicillins
Aminopenicillins with beta−lactamase inhibitors
Ansamycins
Phosphonic acid derivatives
Cattle
Chickens
Pigs
Sheep
Turkeys
Ducks
Horses
Buffaloes
Goats
Livestock species
Rank
0.00
0.25
0.50
0.75
1.00

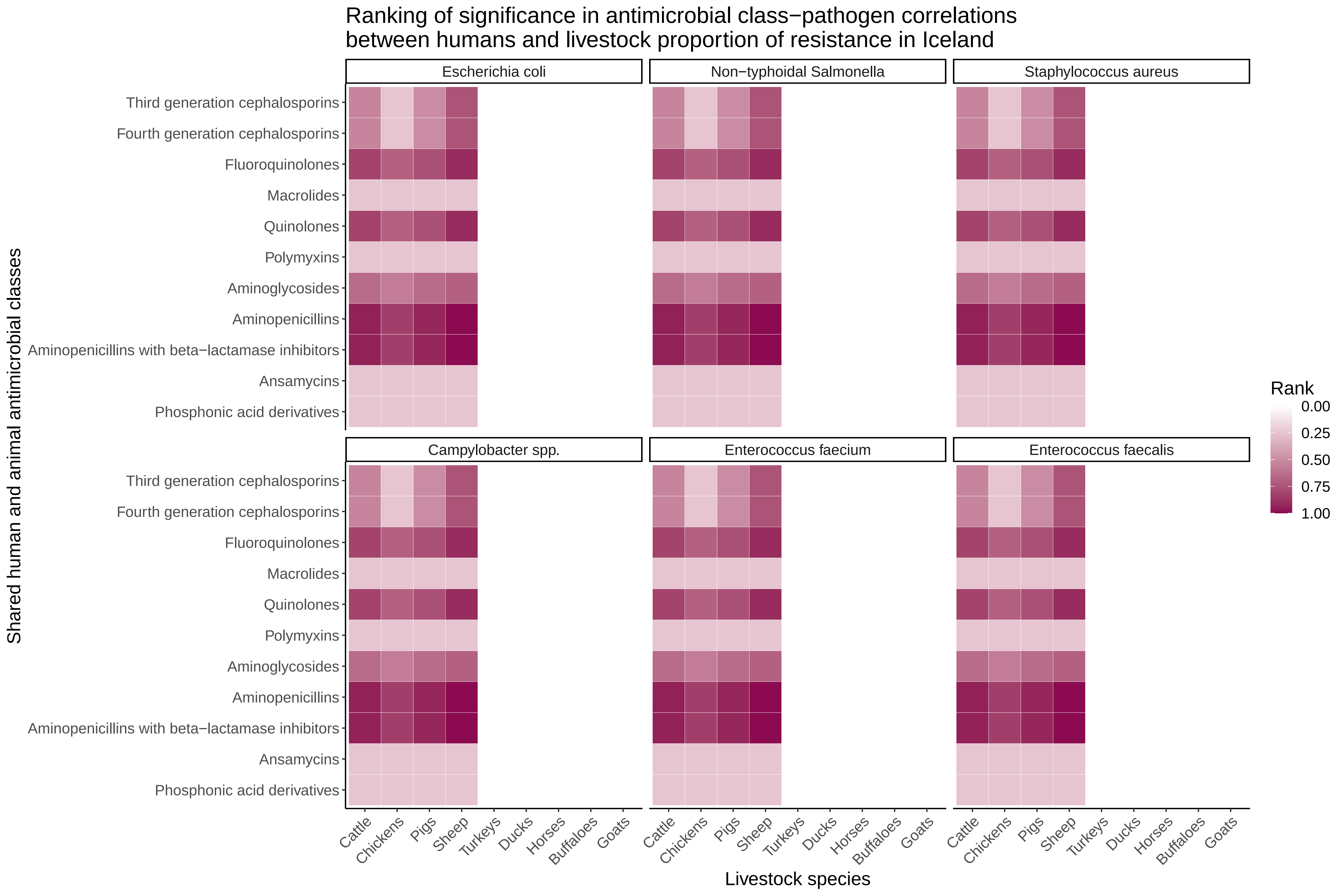

Ranking of significance in antimicrobial class−pathogen correlations between humans and livestock proportion of resistance in Iceland
Escherichia coli
Non−typhoidal Salmonella
Staphylococcus aureus
Campylobacter spp.
Enterococcus faecium
Enterococcus faecalis
Third generation cephalosporins
Fourth generation cephalosporins
Fluoroquinolones
Macrolides
Quinolones
Polymyxins
Aminoglycosides
Aminopenicillins
Aminopenicillins with beta−lactamase inhibitors
Ansamycins
Phosphonic acid derivatives
Shared human and animal antimicrobial classes
Cattle
Chickens
Pigs
Sheep
Turkeys
Ducks
Horses
Buffaloes
Goats
Livestock species
Rank
0.00
0.25
0.50
0.75
1.00

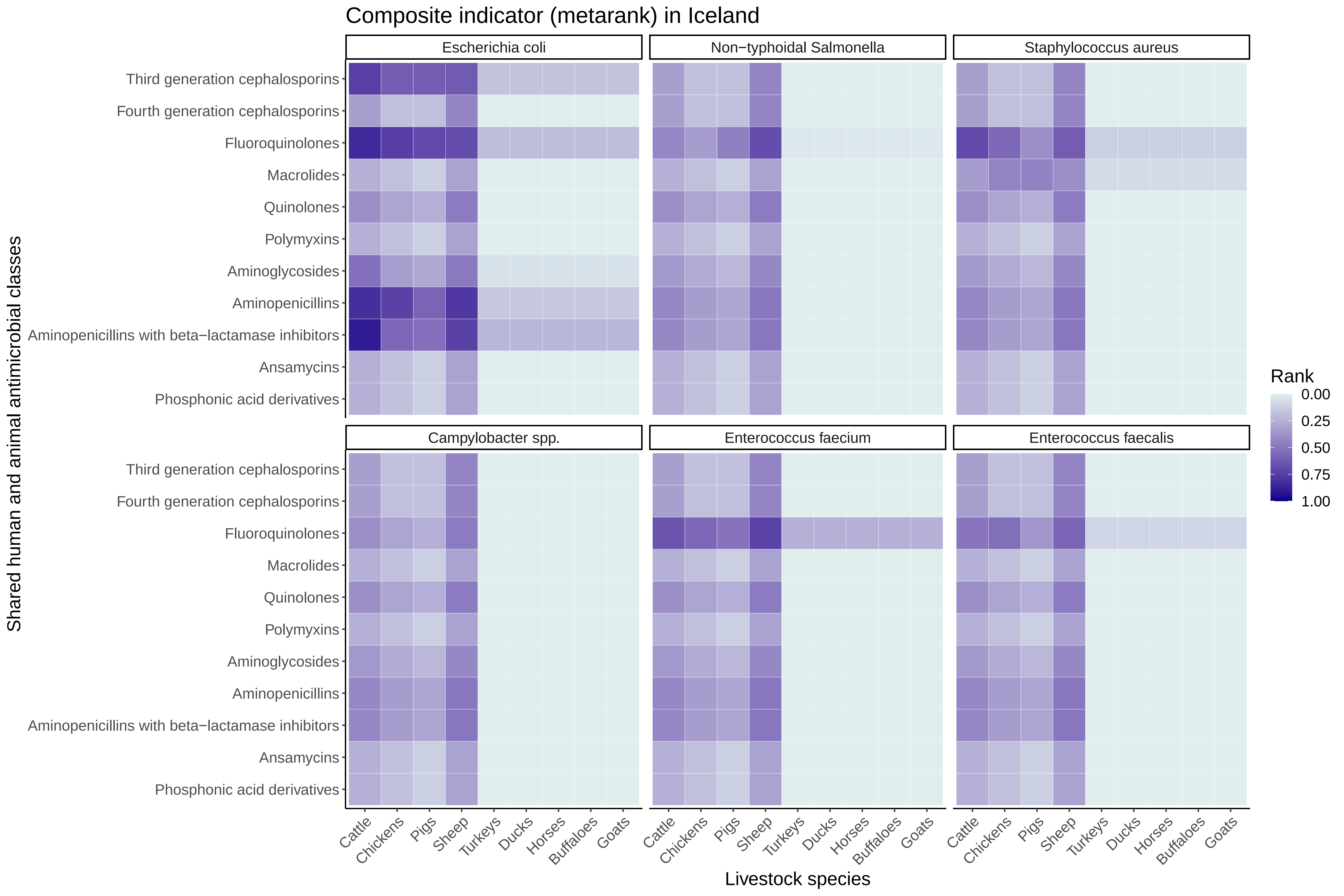

Composite indicator (metarank) in Iceland
Escherichia coli
Non−typhoidal Salmonella
Staphylococcus aureus
Campylobacter spp.
Enterococcus faecium
Enterococcus faecalis
Shared human and animal antimicrobial classes
Third generation cephalosporins
Fourth generation cephalosporins
Fluoroquinolones
Macrolides
Quinolones
Polymyxins
Aminoglycosides
Aminopenicillins
Aminopenicillins with beta−lactamase inhibitors
Ansamycins
Phosphonic acid derivatives
Cattle
Chickens
Pigs
Sheep
Turkeys
Ducks
Horses
Buffaloes
Goats
Livestock species
Rank
0.00
0.25
0.50
0.75
1.00

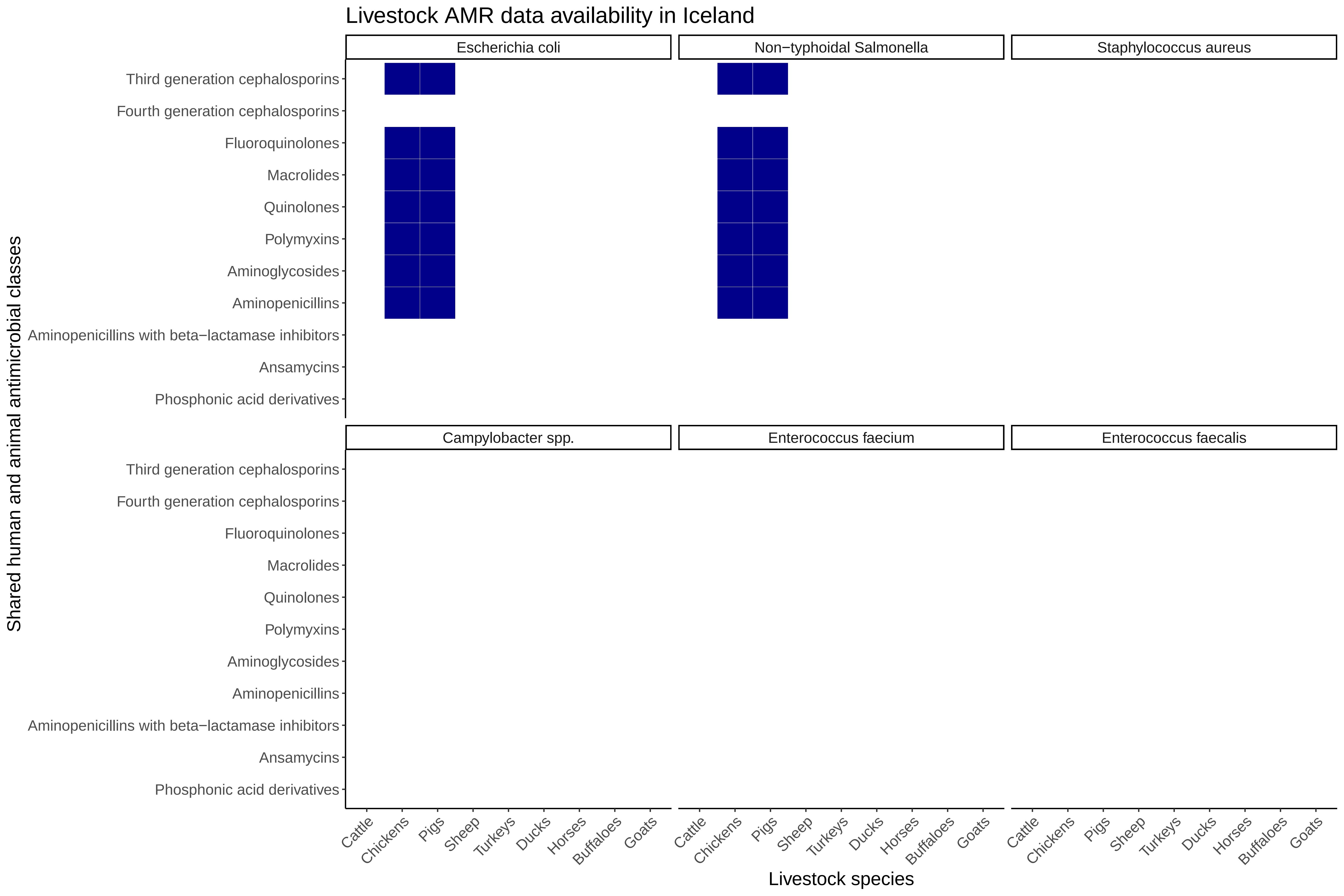

Livestock AMR data availability in Iceland
Escherichia coli
Non−typhoidal Salmonella
Staphylococcus aureus
Campylobacter spp.
Enterococcus faecium
Enterococcus faecalis
Shared human and animal antimicrobial classes
Third generation cephalosporins
Fourth generation cephalosporins
Fluoroquinolones
Macrolides
Quinolones
Polymyxins
Aminoglycosides
Aminopenicillins
Aminopenicillins with beta−lactamase inhibitors
Ansamycins
Phosphonic acid derivatives
Cattle
Chickens
Pigs
Sheep
Turkeys
Ducks
Horses
Buffaloes
Goats
Livestock species

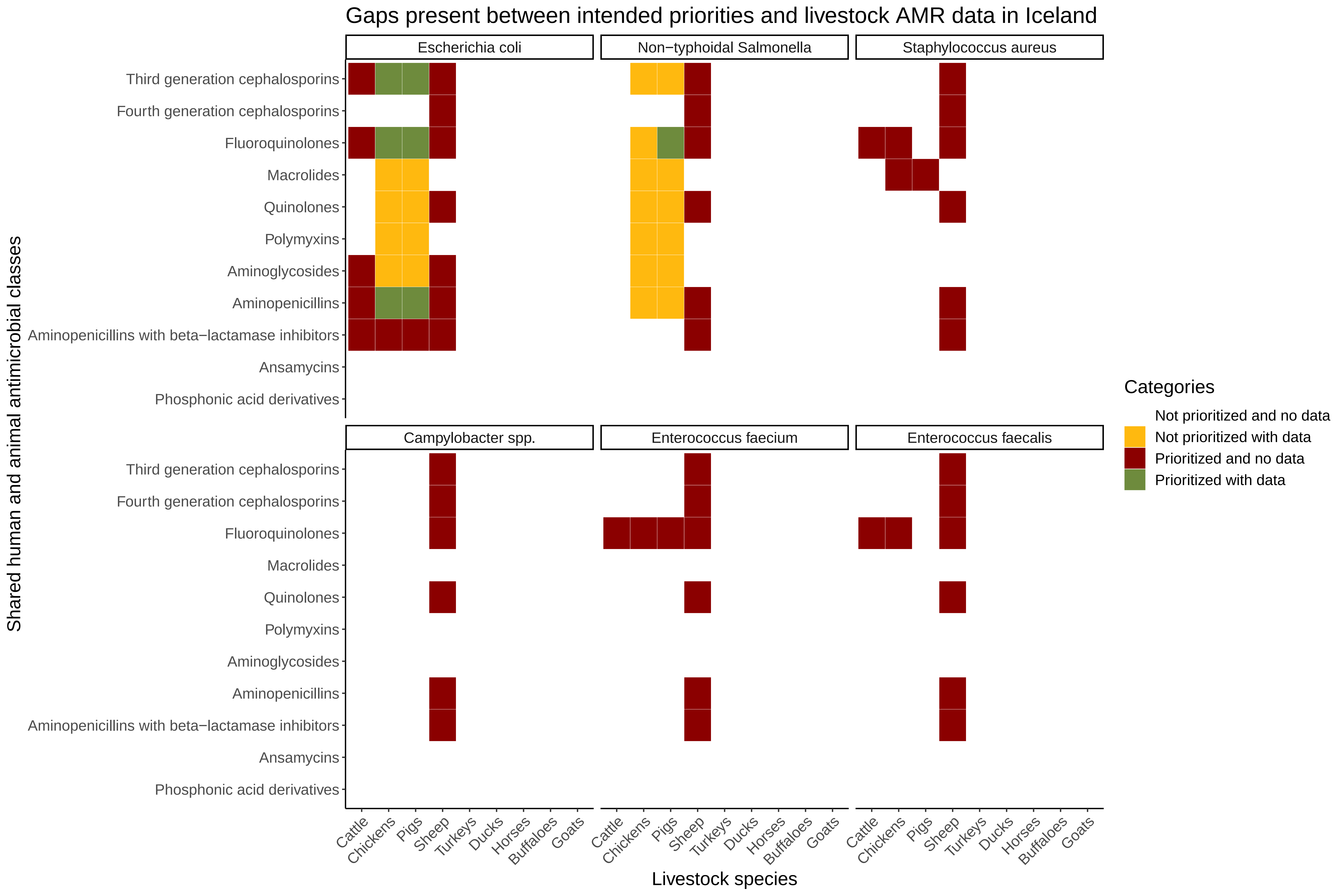
Gaps present between intended priorities and livestock AMR data in Iceland
Escherichia coli
Non−typhoidal Salmonella
Staphylococcus aureus
Campylobacter spp.
Enterococcus faecium
Enterococcus faecalis
Third generation cephalosporins
Fourth generation cephalosporins
Fluoroquinolones
Macrolides
Quinolones
Polymyxins
Aminoglycosides
Aminopenicillins
Aminopenicillins with beta−lactamase inhibitors
Ansamycins
Phosphonic acid derivatives
Shared human and animal antimicrobial classes
Cattle
Chickens
Pigs
Sheep
Turkeys
Ducks
Horses
Buffaloes
Goats
Livestock species
Categories
Not prioritized and no data
Not prioritized with data
Prioritized and no data
Prioritized with data

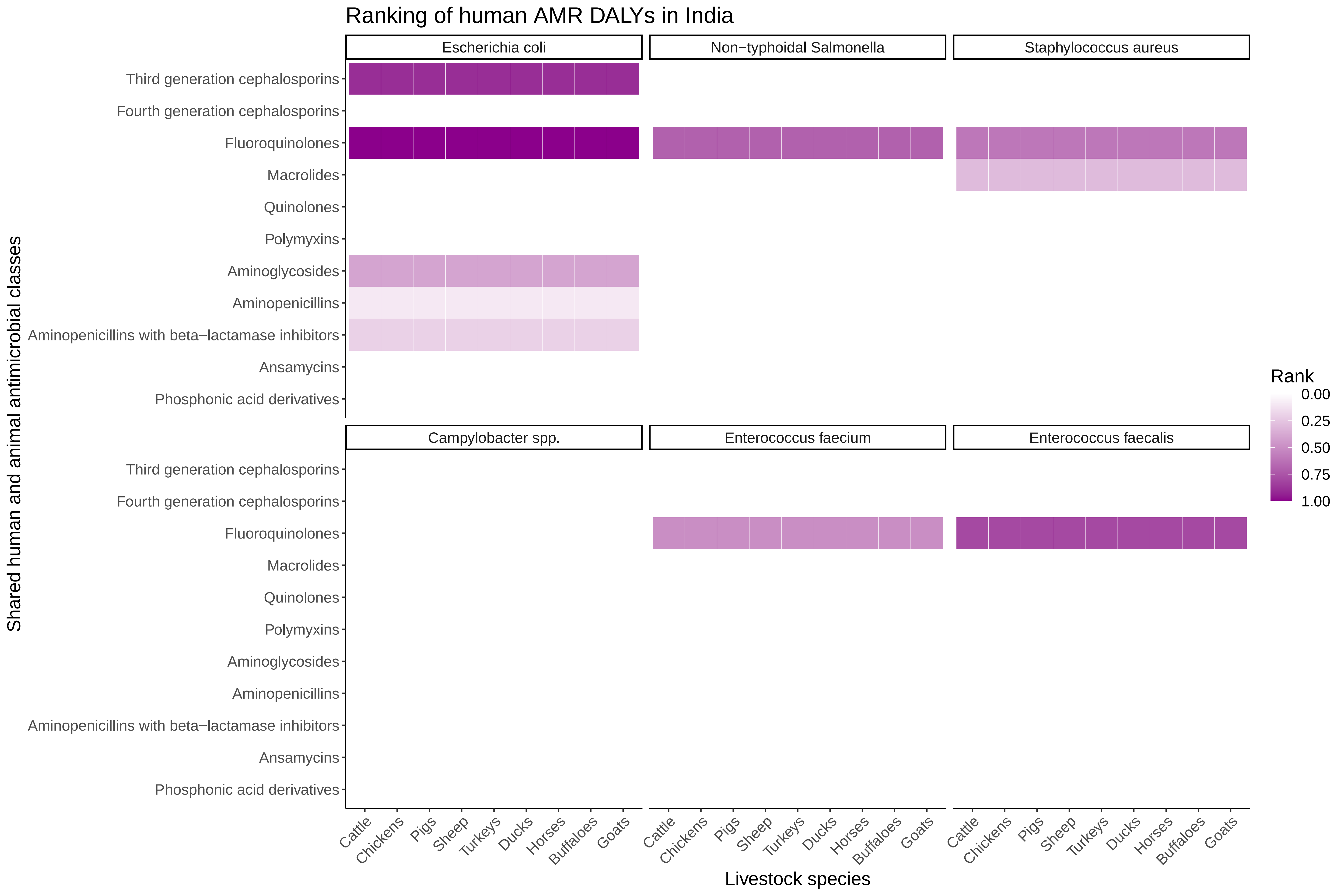

Ranking of human AMR DALYs in India
Escherichia coli
Non−typhoidal Salmonella
Staphylococcus aureus
Campylobacter spp.
Enterococcus faecium
Enterococcus faecalis
Shared human and animal antimicrobial classes
Third generation cephalosporins
Fourth generation cephalosporins
Fluoroquinolones
Macrolides
Quinolones
Polymyxins
Aminoglycosides
Aminopenicillins
Aminopenicillins with beta−lactamase inhibitors
Ansamycins
Phosphonic acid derivatives
Rank
0.00
0.25
0.50
0.75
1.00
Cattle
Chickens
Pigs
Sheep
Turkeys
Ducks
Horses
Buffaloes
Goats
Livestock species

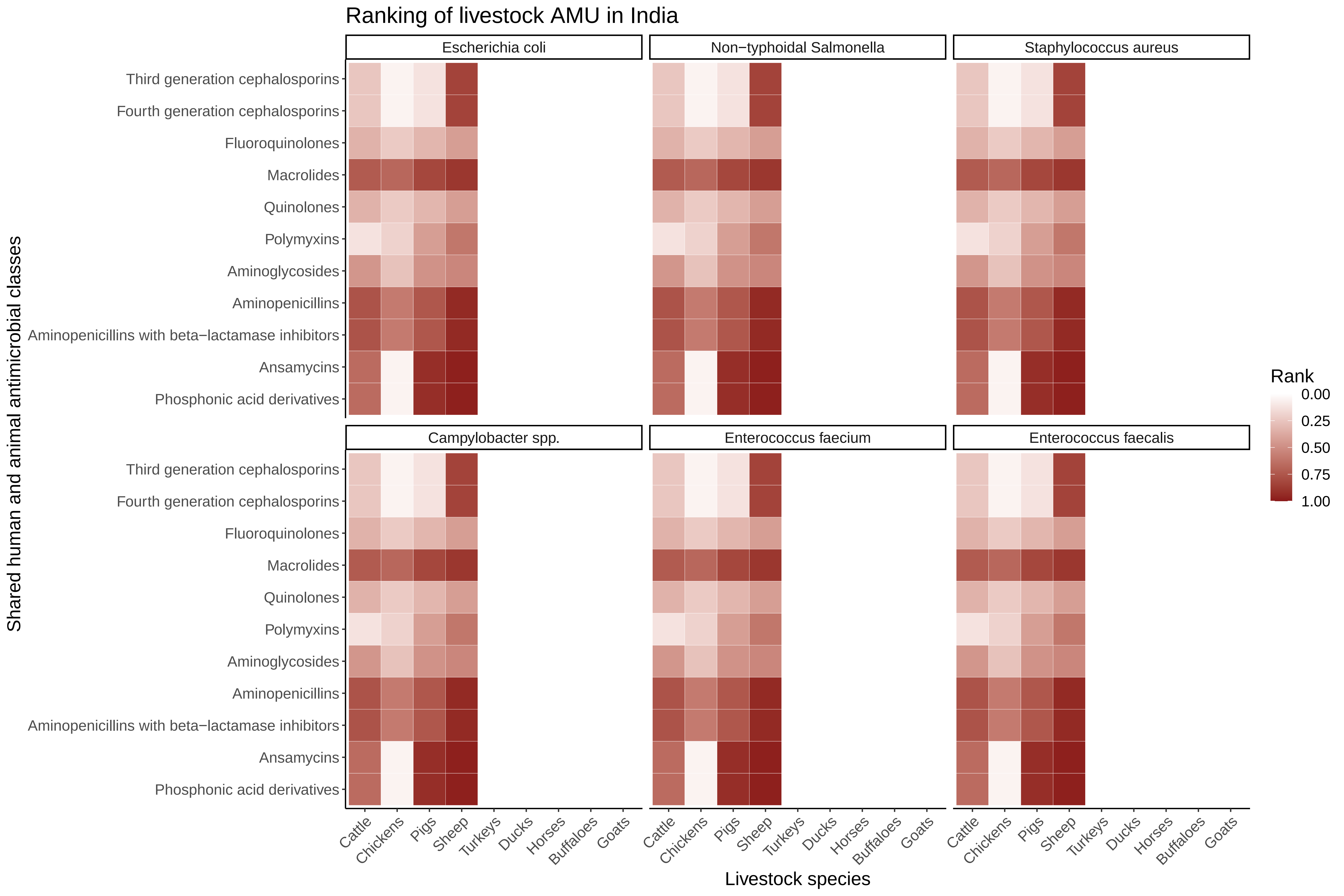

Ranking of livestock AMU in India
Escherichia coli
Non−typhoidal Salmonella
Staphylococcus aureus
Campylobacter spp.
Enterococcus faecium
Enterococcus faecalis
Third generation cephalosporins
Fourth generation cephalosporins
Fluoroquinolones
Macrolides
Quinolones
Polymyxins
Aminoglycosides
Aminopenicillins
Aminopenicillins with beta−lactamase inhibitors
Ansamycins
Phosphonic acid derivatives
Shared human and animal antimicrobial classes
Cattle
Chickens
Pigs
Sheep
Turkeys
Ducks
Horses
Buffaloes
Goats
Livestock species
Rank
0.00
0.25
0.50
0.75
1.00

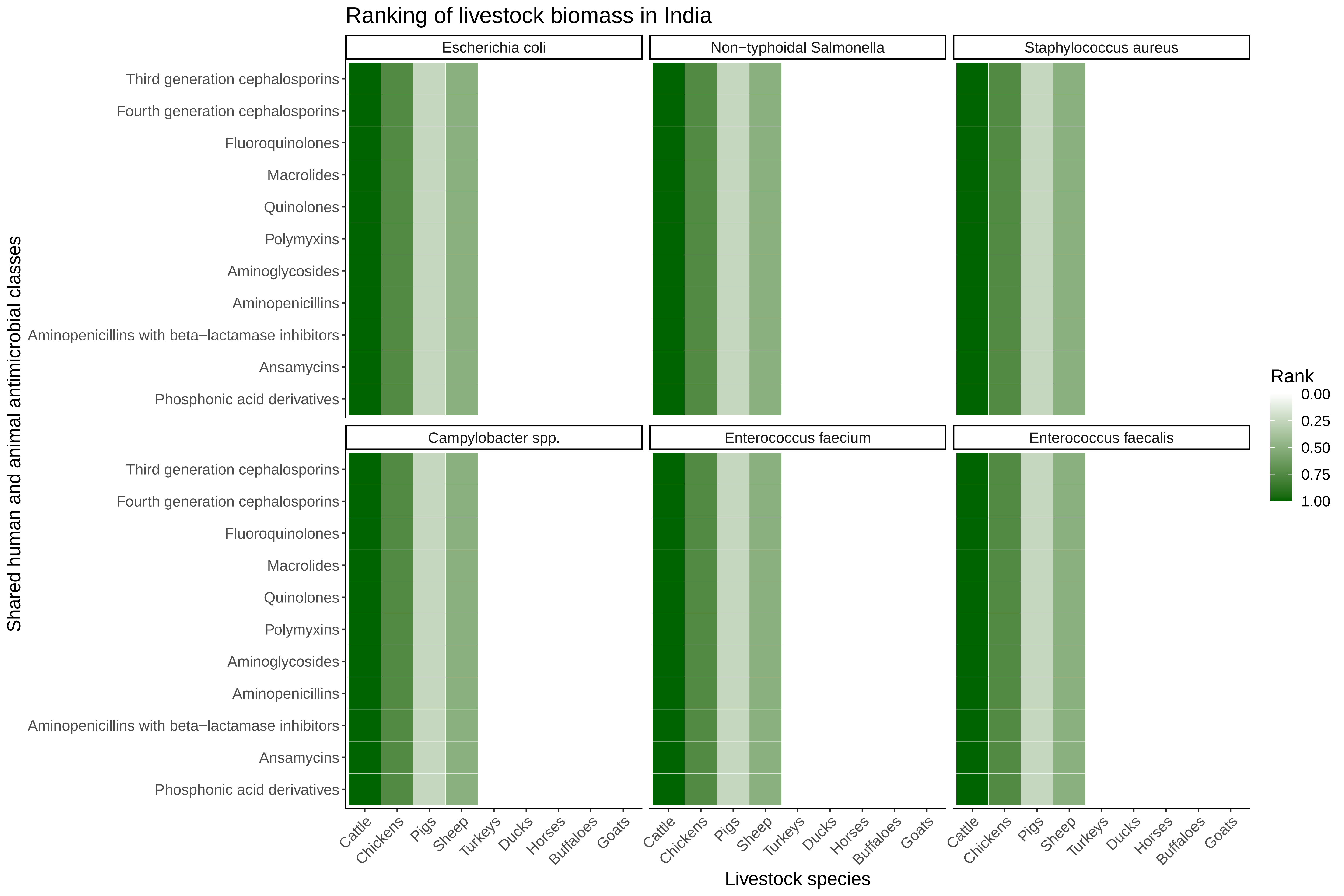

Ranking of livestock biomass in India
Escherichia coli
Non−typhoidal Salmonella
Staphylococcus aureus
Campylobacter spp.
Enterococcus faecium
Enterococcus faecalis
Third generation cephalosporins
Fourth generation cephalosporins
Fluoroquinolones
Macrolides
Quinolones
Polymyxins
Aminoglycosides
Aminopenicillins
Aminopenicillins with beta−lactamase inhibitors
Ansamycins
Phosphonic acid derivatives
Shared human and animal antimicrobial classes
Cattle
Chickens
Pigs
Sheep
Turkeys
Ducks
Horses
Buffaloes
Goats
Livestock species
Rank
0.00
0.25
0.50
0.75
1.00

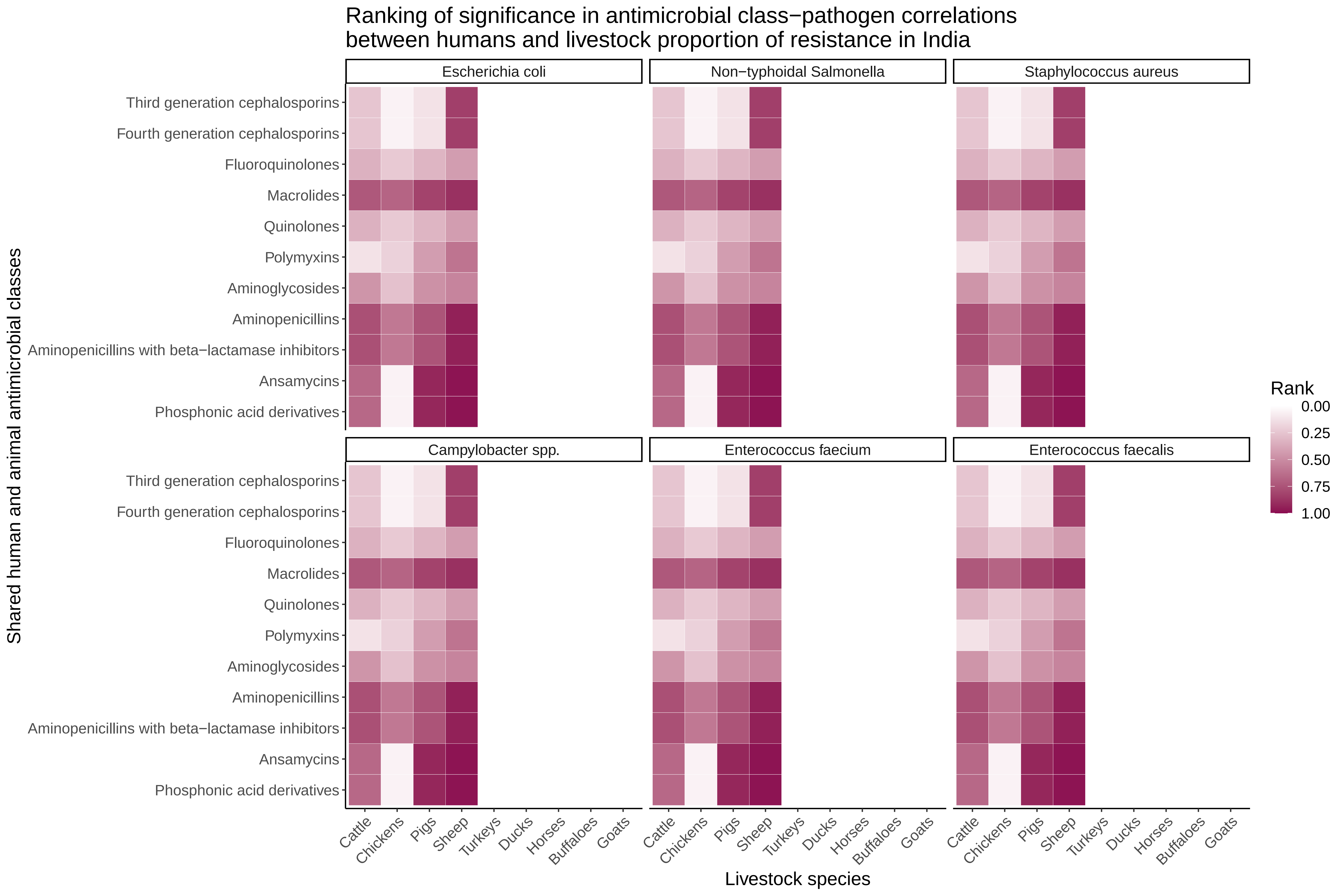

Ranking of significance in antimicrobial class−pathogen correlations
between humans and livestock proportion of resistance in India
Escherichia coli
Non−typhoidal Salmonella
Staphylococcus aureus
Campylobacter spp.
Enterococcus faecium
Enterococcus faecalis
Third generation cephalosporins
Fourth generation cephalosporins
Fluoroquinolones
Macrolides
Quinolones
Polymyxins
Aminoglycosides
Aminopenicillins
Aminopenicillins with beta−lactamase inhibitors
Ansamycins
Phosphonic acid derivatives
Shared human and animal antimicrobial classes
Cattle
Chickens
Pigs
Sheep
Turkeys
Ducks
Horses
Buffaloes
Goats
Livestock species
Rank
0.00
0.25
0.50
0.75
1.00

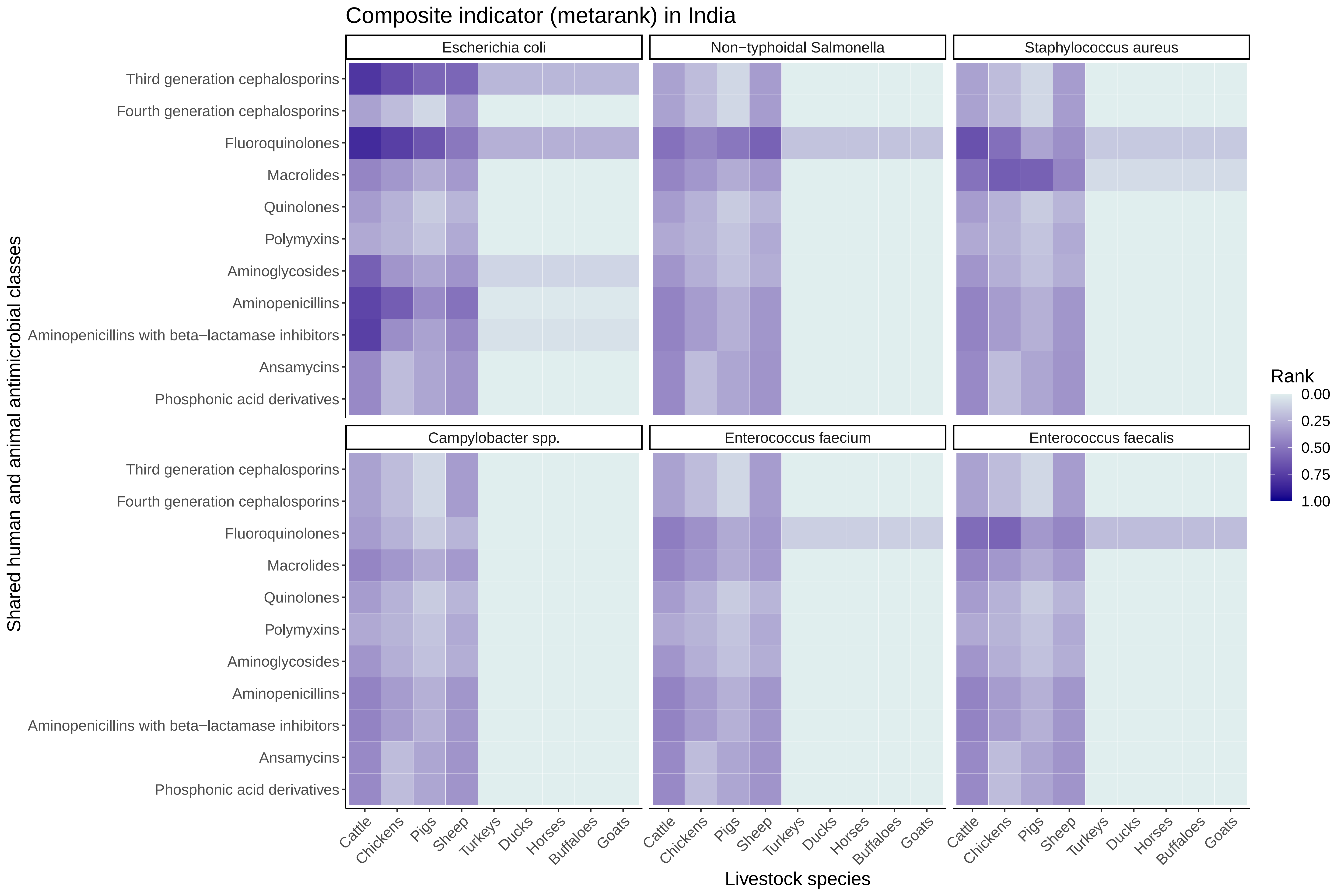

Composite indicator (metarank) in India
Escherichia coli
Non−typhoidal Salmonella
Staphylococcus aureus
Campylobacter spp.
Enterococcus faecium
Enterococcus faecalis
Shared human and animal antimicrobial classes
Third generation cephalosporins
Fourth generation cephalosporins
Fluoroquinolones
Macrolides
Quinolones
Polymyxins
Aminoglycosides
Aminopenicillins
Aminopenicillins with beta−lactamase inhibitors
Ansamycins
Phosphonic acid derivatives
Livestock species
Cattle
Chickens
Pigs
Sheep
Turkeys
Ducks
Horses
Buffaloes
Goats
Rank
0.00
0.25
0.50
0.75
1.00

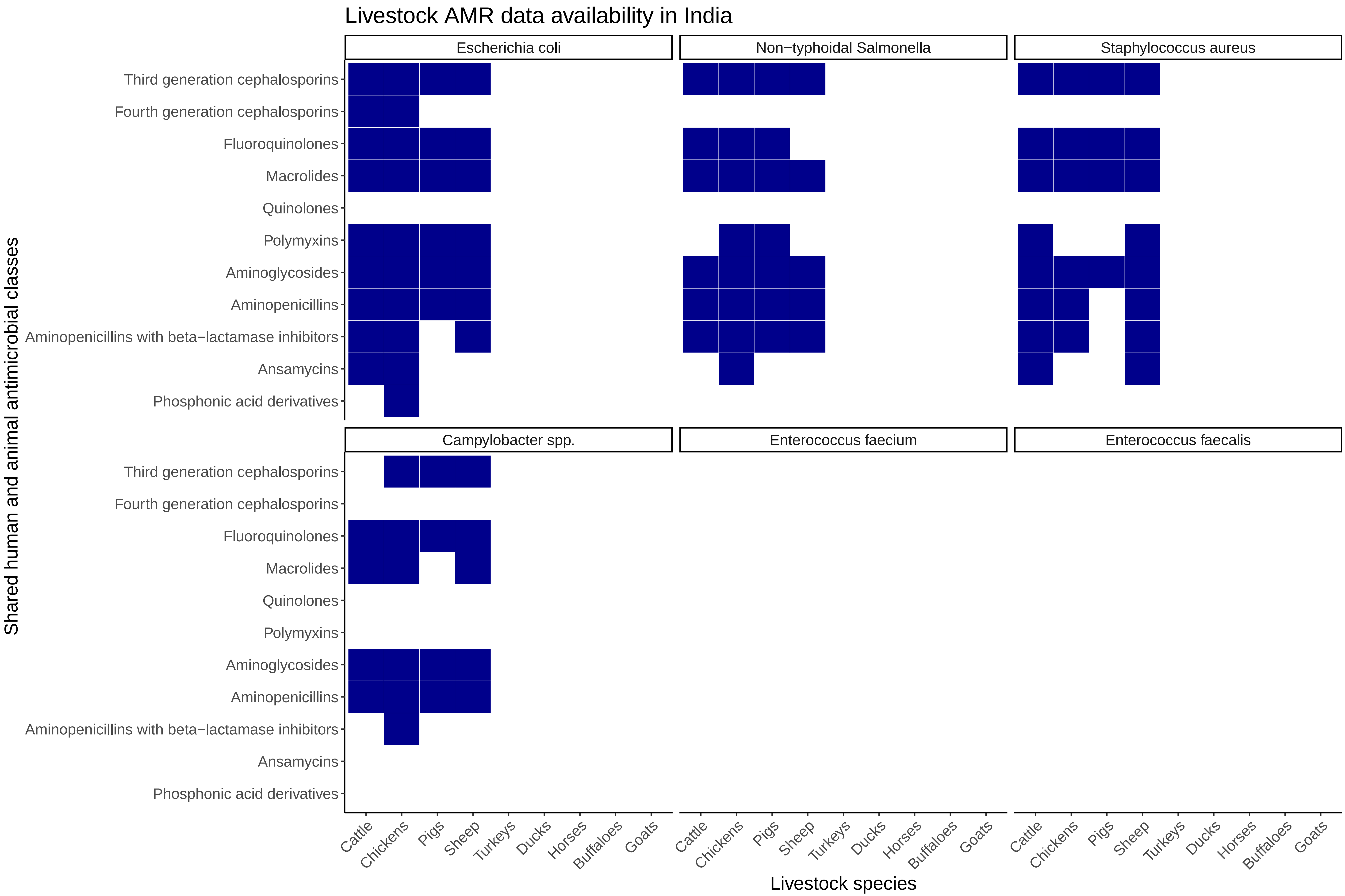

Livestock AMR data availability in India
Escherichia coli
Non−typhoidal Salmonella
Staphylococcus aureus
Campylobacter spp.
Enterococcus faecium
Enterococcus faecalis
Shared human and animal antimicrobial classes
Third generation cephalosporins
Fourth generation cephalosporins
Fluoroquinolones
Macrolides
Quinolones
Polymyxins
Aminoglycosides
Aminopenicillins
Aminopenicillins with beta−lactamase inhibitors
Ansamycins
Phosphonic acid derivatives
Cattle
Chickens
Pigs
Sheep
Turkeys
Ducks
Horses
Buffaloes
Goats
Livestock species

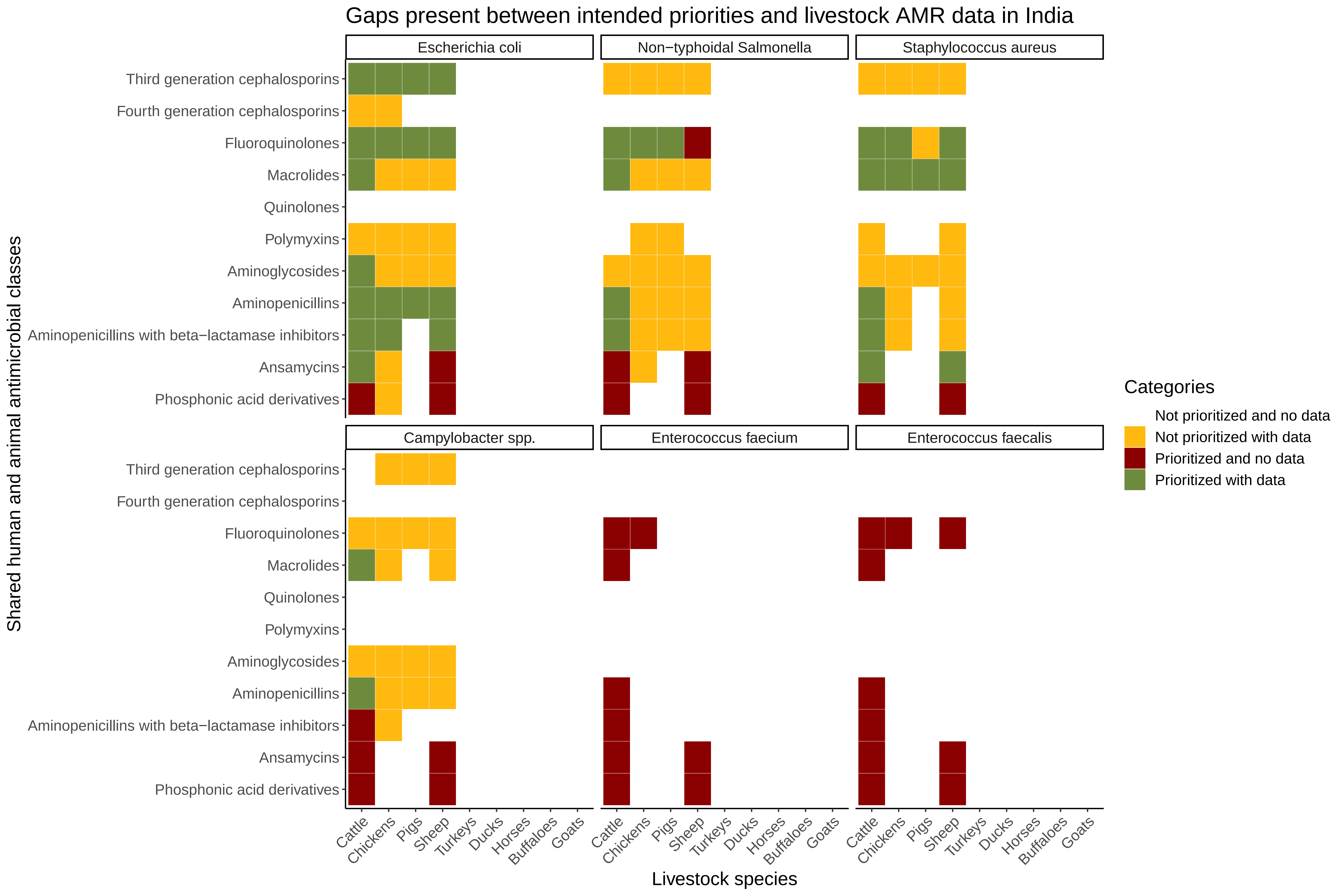

Gaps present between intended priorities and livestock AMR data in India
Escherichia coli
Non−typhoidal Salmonella
Staphylococcus aureus
Campylobacter spp.
Enterococcus faecium
Enterococcus faecalis
Shared human and animal antimicrobial classes
Third generation cephalosporins
Fourth generation cephalosporins
Fluoroquinolones
Macrolides
Quinolones
Polymyxins
Aminoglycosides
Aminopenicillins
Aminopenicillins with beta−lactamase inhibitors
Ansamycins
Phosphonic acid derivatives
Livestock species
Cattle
Chickens
Pigs
Sheep
Turkeys
Ducks
Horses
Buffaloes
Goats
Categories
Not prioritized and no data
Not prioritized with data
Prioritized and no data
Prioritized with data

Ranking of human AMR DALYs in Indonesia

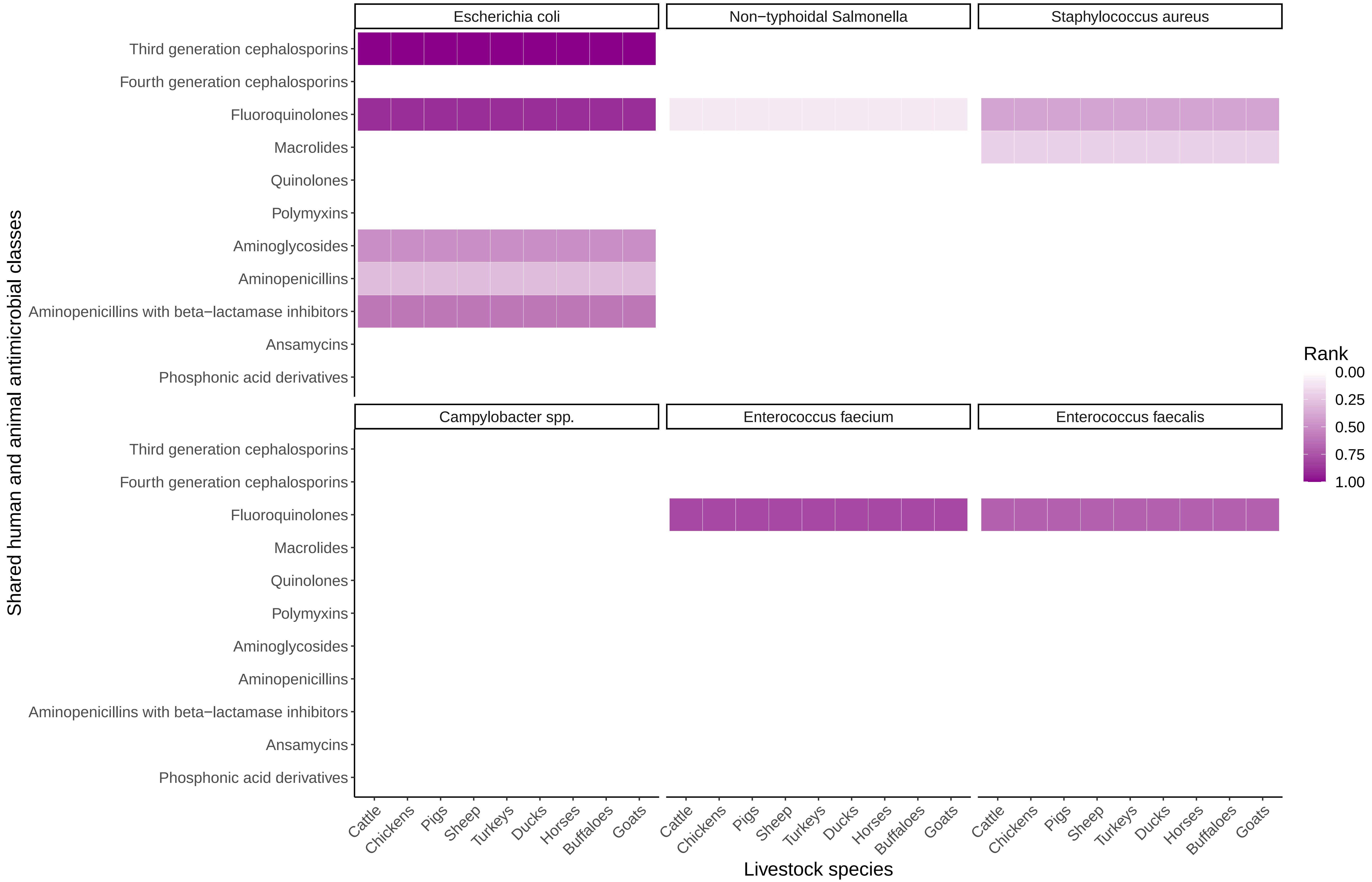

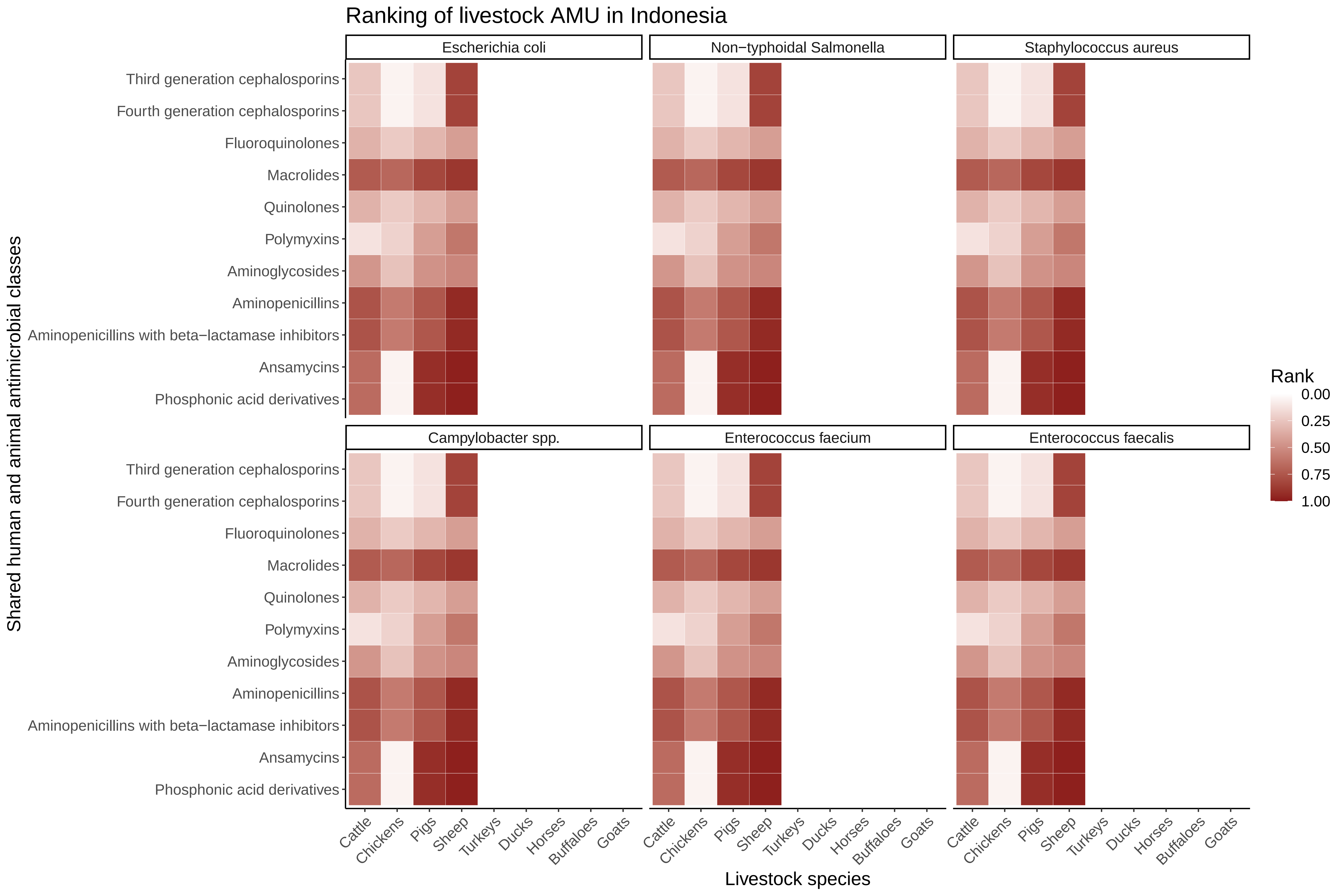

Ranking of livestock AMU in Indonesia
Escherichia coli
Non−typhoidal Salmonella
Staphylococcus aureus
Campylobacter spp.
Enterococcus faecium
Enterococcus faecalis
Third generation cephalosporins
Fourth generation cephalosporins
Fluoroquinolones
Macrolides
Quinolones
Polymyxins
Aminoglycosides
Aminopenicillins
Aminopenicillins with beta−lactamase inhibitors
Ansamycins
Phosphonic acid derivatives
Shared human and animal antimicrobial classes
Cattle
Chickens
Pigs
Sheep
Turkeys
Ducks
Horses
Buffaloes
Goats
Livestock species
Rank
0.00
0.25
0.50
0.75
1.00

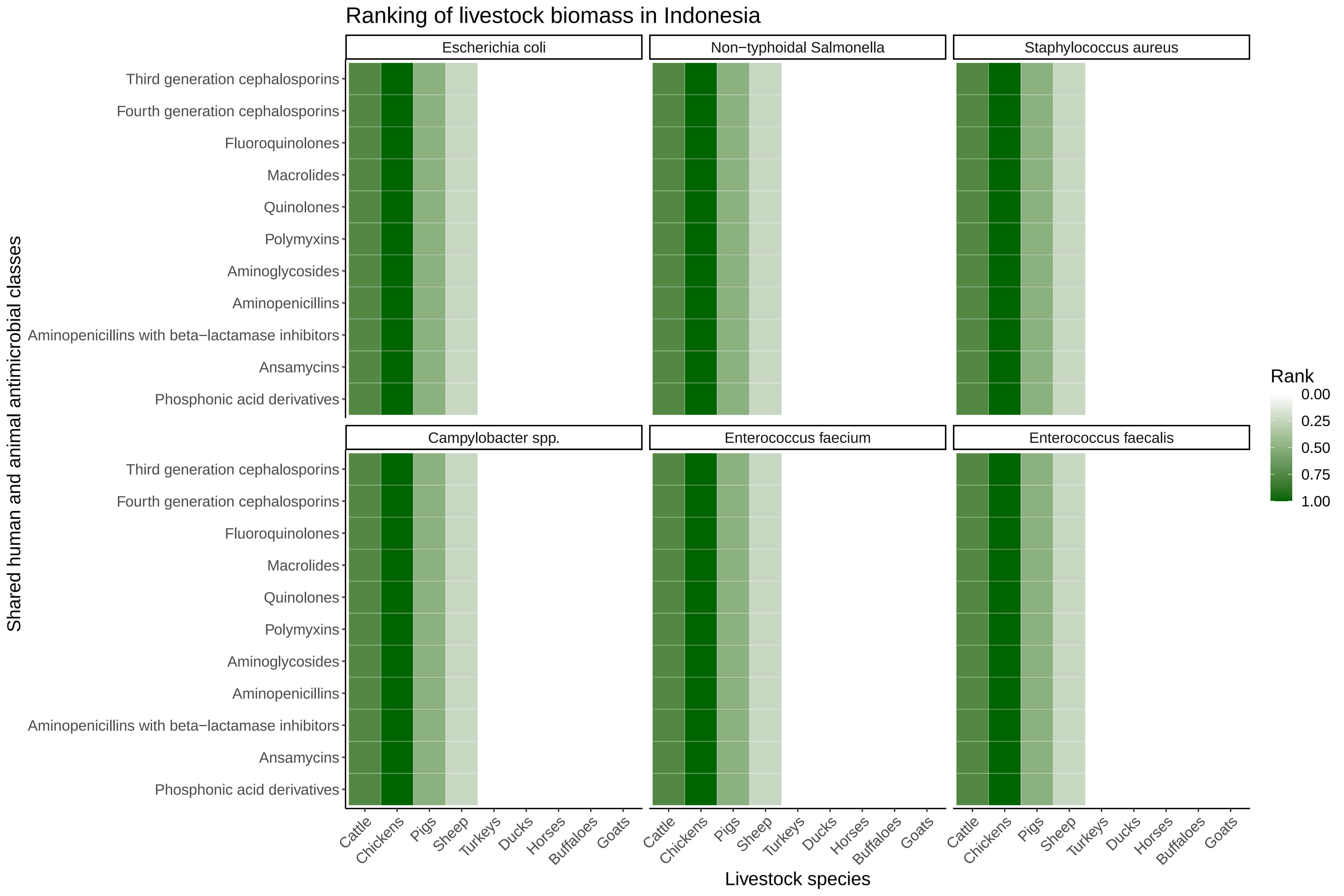

Ranking of livestock biomass in Indonesia
Escherichia coli
Non−typhoidal Salmonella
Staphylococcus aureus
Campylobacter spp.
Enterococcus faecium
Enterococcus faecalis
Shared human and animal antimicrobial classes
Third generation cephalosporins
Fourth generation cephalosporins
Fluoroquinolones
Macrolides
Quinolones
Polymyxins
Aminoglycosides
Aminopenicillins
Aminopenicillins with beta−lactamase inhibitors
Ansamycins
Phosphonic acid derivatives
Cattle
Chickens
Pigs
Sheep
Turkeys
Ducks
Horses
Buffaloes
Goats
Livestock species
Rank
0.00
0.25
0.50
0.75
1.00

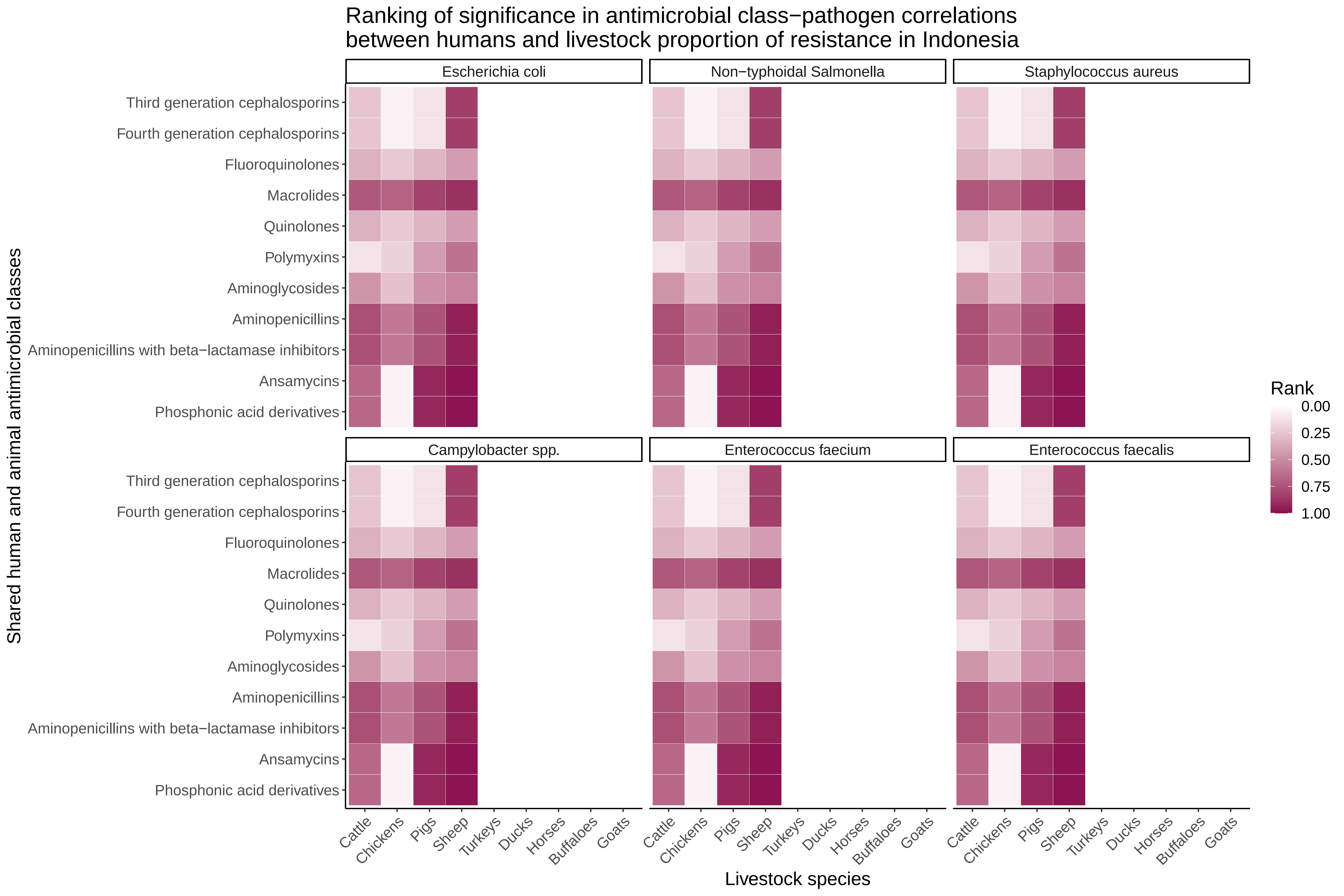

Ranking of significance in antimicrobial class−pathogen correlations
between humans and livestock proportion of resistance in Indonesia
Escherichia coli
Non−typhoidal Salmonella
Staphylococcus aureus
Campylobacter spp.
Enterococcus faecium
Enterococcus faecalis
Third generation cephalosporins
Fourth generation cephalosporins
Fluoroquinolones
Macrolides
Quinolones
Polymyxins
Aminoglycosides
Aminopenicillins
Aminopenicillins with beta−lactamase inhibitors
Ansamycins
Phosphonic acid derivatives
Shared human and animal antimicrobial classes
Cattle
Chickens
Pigs
Sheep
Turkeys
Ducks
Horses
Buffaloes
Goats
Livestock species
Rank
0.00
0.25
0.50
0.75
1.00

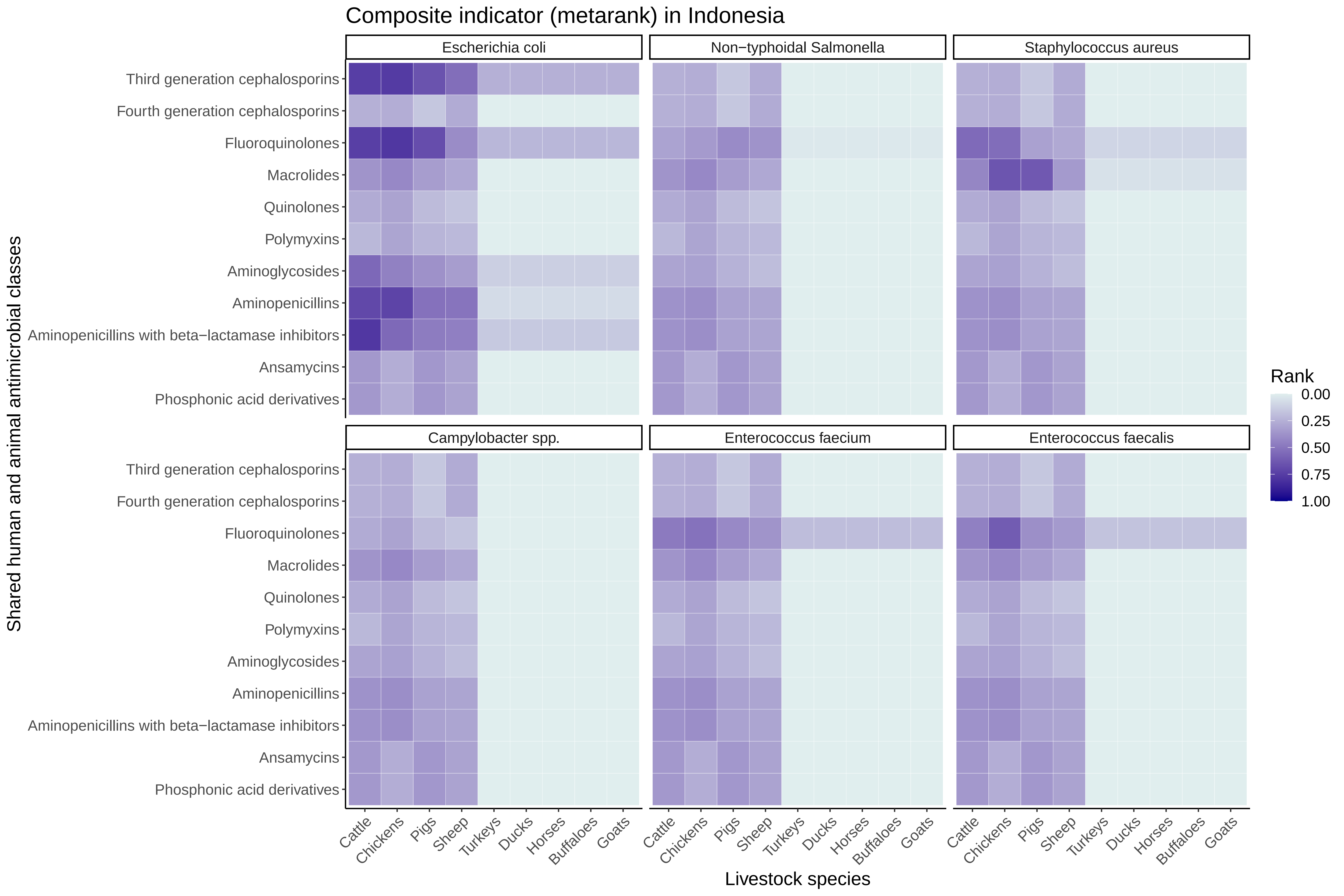

Composite indicator (metarank) in Indonesia
Escherichia coli
Non−typhoidal Salmonella
Staphylococcus aureus
Campylobacter spp.
Enterococcus faecium
Enterococcus faecalis
Shared human and animal antimicrobial classes
Third generation cephalosporins
Fourth generation cephalosporins
Fluoroquinolones
Macrolides
Quinolones
Polymyxins
Aminoglycosides
Aminopenicillins
Aminopenicillins with beta−lactamase inhibitors
Ansamycins
Phosphonic acid derivatives
Cattle
Chickens
Pigs
Sheep
Turkeys
Ducks
Horses
Buffaloes
Goats
Livestock species
Rank
0.00
0.25
0.50
0.75
1.00

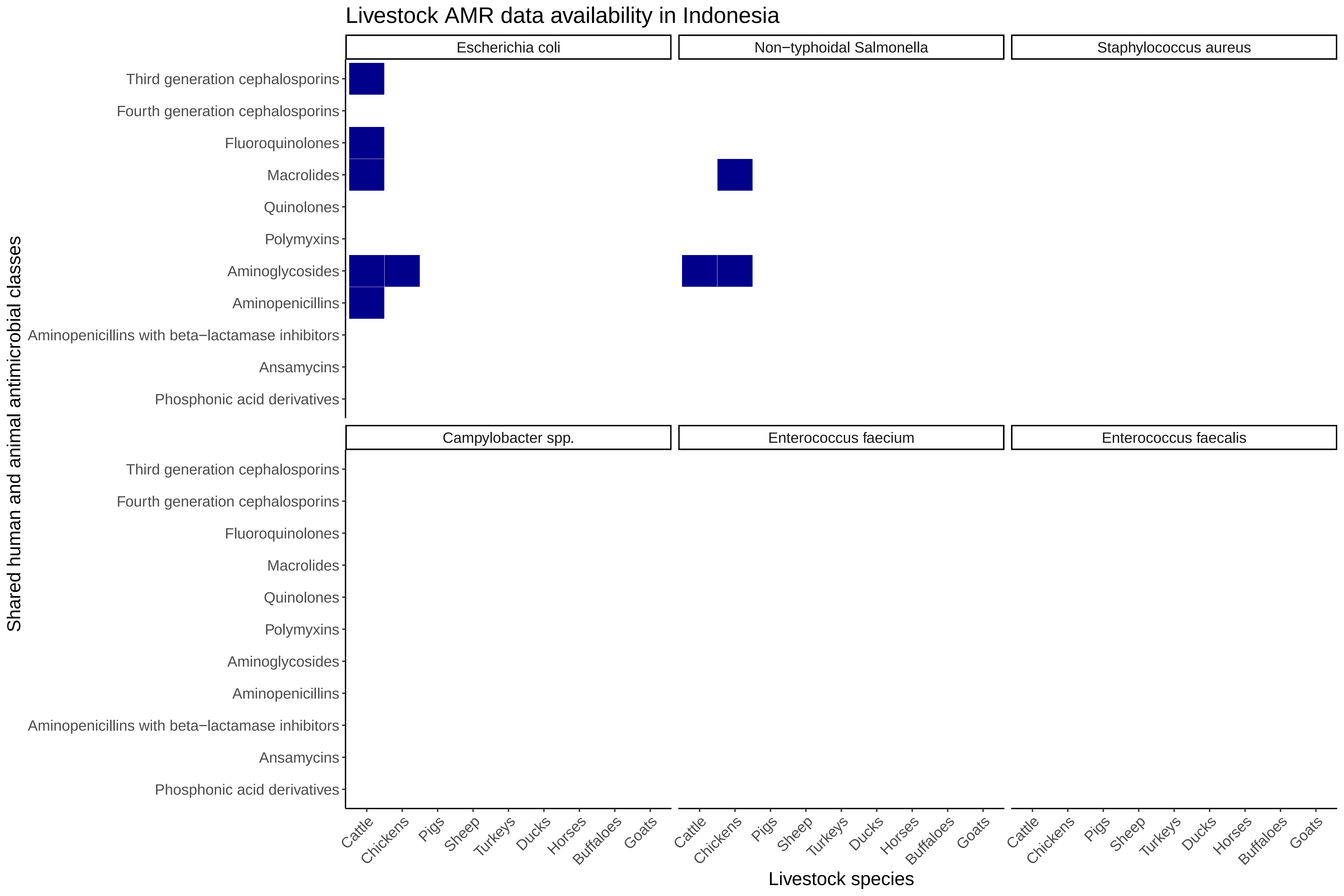

Livestock AMR data availability in Indonesia
Escherichia coli
Non−typhoidal Salmonella
Staphylococcus aureus
Campylobacter spp.
Enterococcus faecium
Enterococcus faecalis
Shared human and animal antimicrobial classes
Third generation cephalosporins
Fourth generation cephalosporins
Fluoroquinolones
Macrolides
Quinolones
Polymyxins
Aminoglycosides
Aminopenicillins
Aminopenicillins with beta−lactamase inhibitors
Ansamycins
Phosphonic acid derivatives
Cattle
Chickens
Pigs
Sheep
Turkeys
Ducks
Horses
Buffaloes
Goats
Livestock species

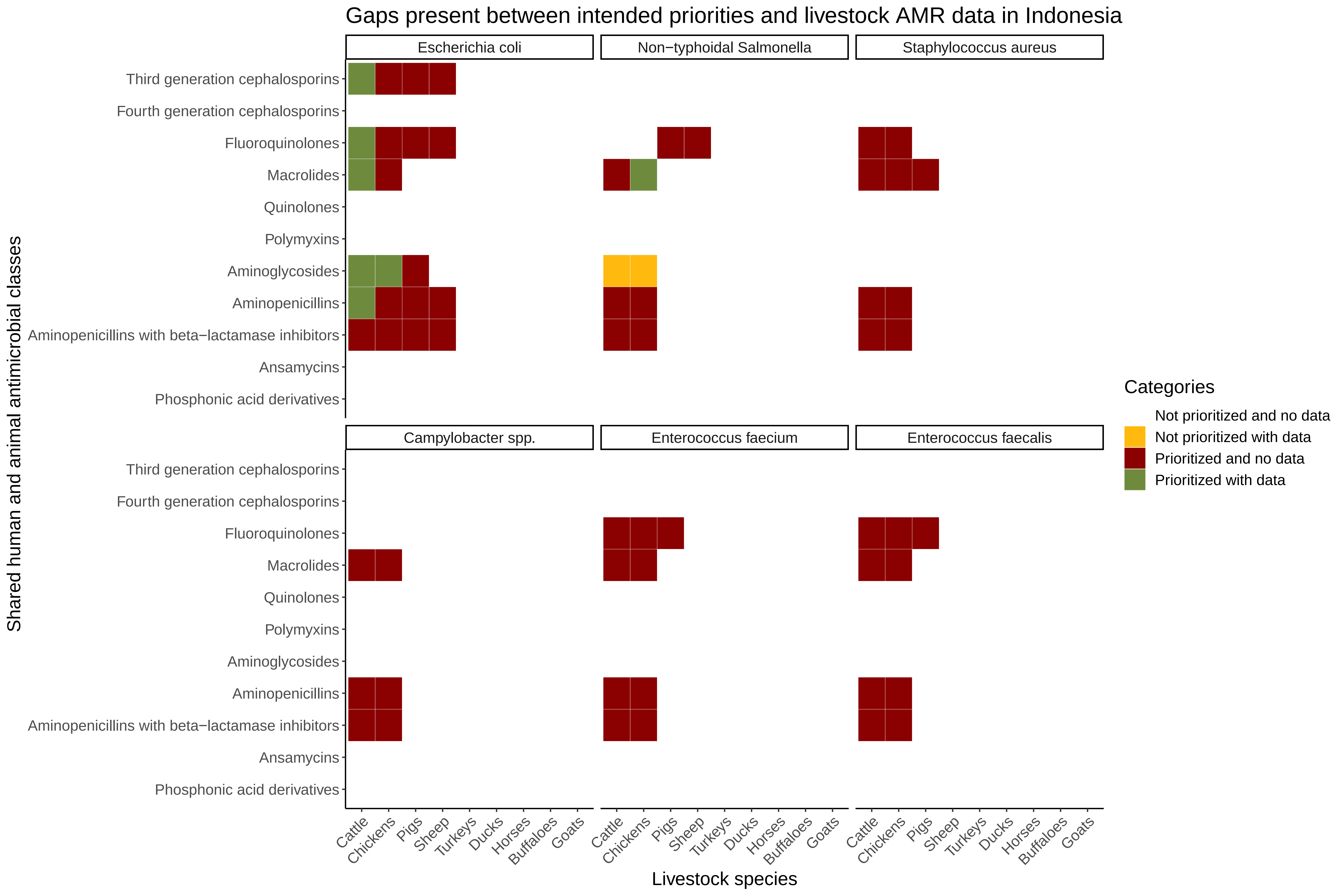

Gaps present between intended priorities and livestock AMR data in Indonesia
Escherichia coli
Non−typhoidal Salmonella
Staphylococcus aureus
Campylobacter spp.
Enterococcus faecium
Enterococcus faecalis
Shared human and animal antimicrobial classes
Third generation cephalosporins
Fourth generation cephalosporins
Fluoroquinolones
Macrolides
Quinolones
Polymyxins
Aminoglycosides
Aminopenicillins
Aminopenicillins with beta−lactamase inhibitors
Ansamycins
Phosphonic acid derivatives
Cattle
Chickens
Pigs
Sheep
Turkeys
Ducks
Horses
Buffaloes
Goats
Livestock species
Categories
Not prioritized and no data
Not prioritized with data
Prioritized and no data
Prioritized with data

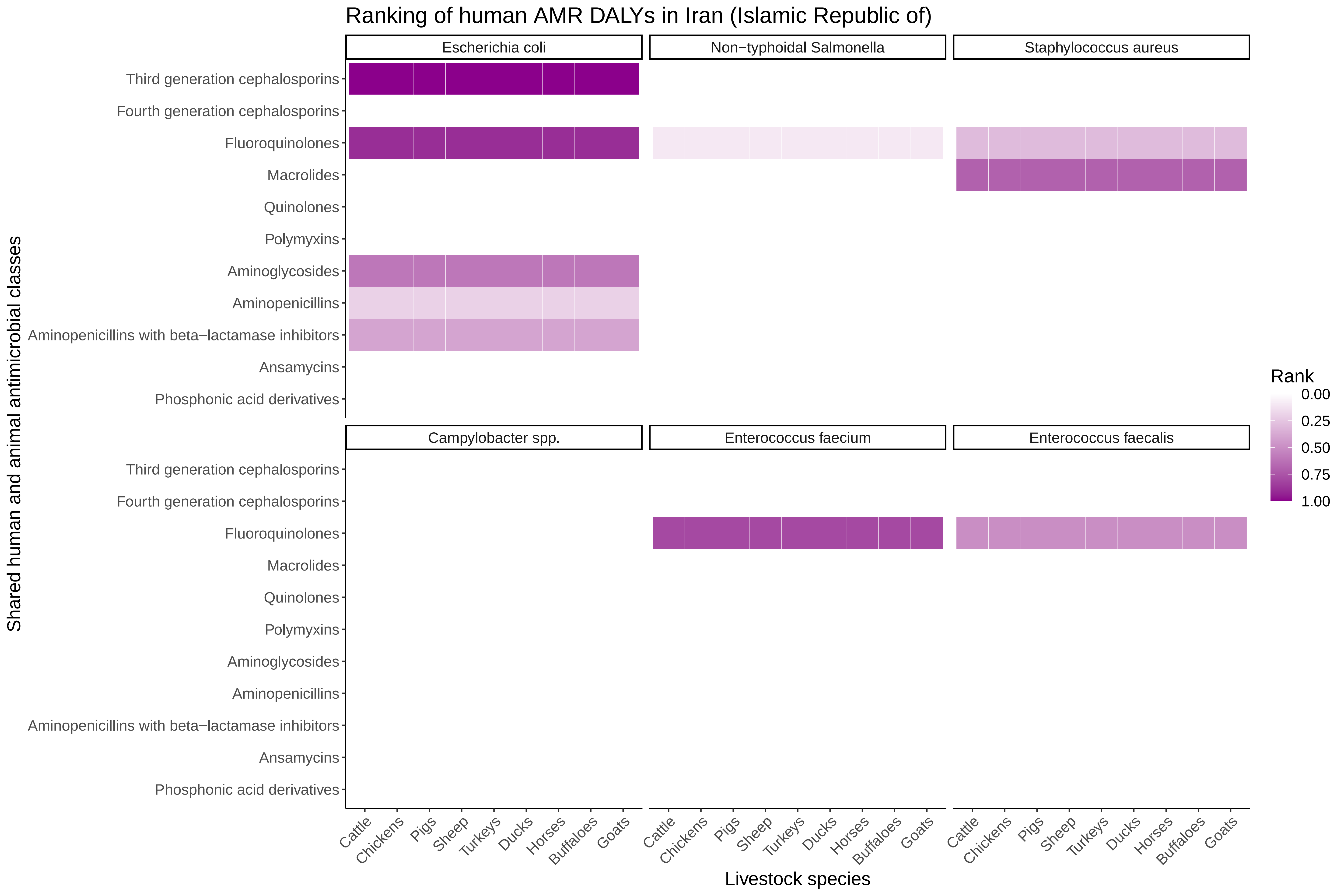
Ranking of human AMR DALYs in Iran (Islamic Republic of)
Escherichia coli
Non−typhoidal Salmonella
Staphylococcus aureus
Campylobacter spp.
Enterococcus faecium
Enterococcus faecalis
Third generation cephalosporins
Fourth generation cephalosporins
Fluoroquinolones
Macrolides
Quinolones
Polymyxins
Aminoglycosides
Aminopenicillins
Aminopenicillins with beta−lactamase inhibitors
Ansamycins
Phosphonic acid derivatives
Shared human and animal antimicrobial classes
Rank
0.00
0.25
0.50
0.75
1.00
Cattle
Chickens
Pigs
Sheep
Turkeys
Ducks
Horses
Buffaloes
Goats
Livestock species

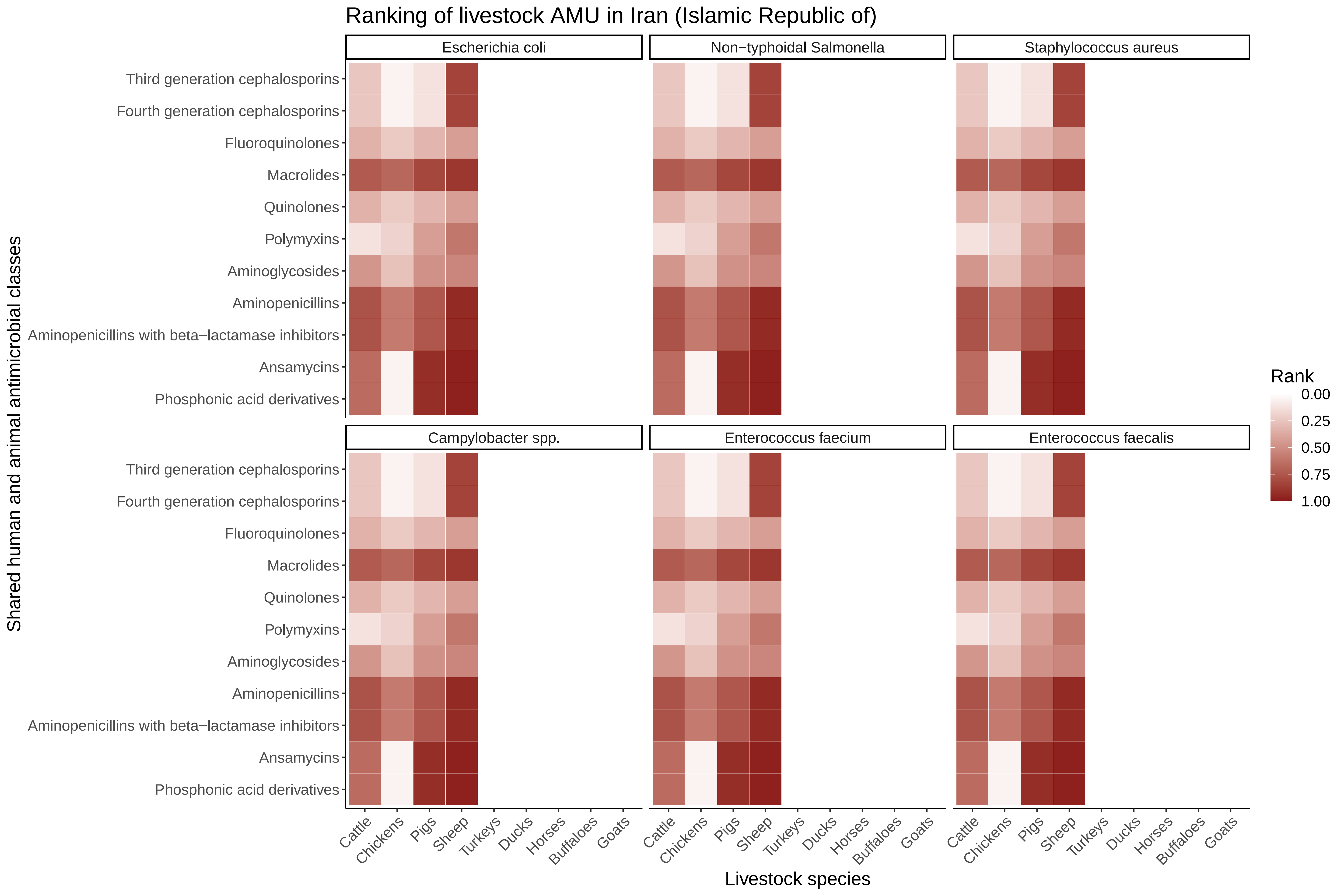

Ranking of livestock AMU in Iran (Islamic Republic of)
Escherichia coli
Non−typhoidal Salmonella
Staphylococcus aureus
Campylobacter spp.
Enterococcus faecium
Enterococcus faecalis
Shared human and animal antimicrobial classes
Third generation cephalosporins
Fourth generation cephalosporins
Fluoroquinolones
Macrolides
Quinolones
Polymyxins
Aminoglycosides
Aminopenicillins
Aminopenicillins with beta−lactamase inhibitors
Ansamycins
Phosphonic acid derivatives
Livestock species
Cattle
Chickens
Pigs
Sheep
Turkeys
Ducks
Horses
Buffaloes
Goats
Rank
0.00
0.25
0.50
0.75
1.00

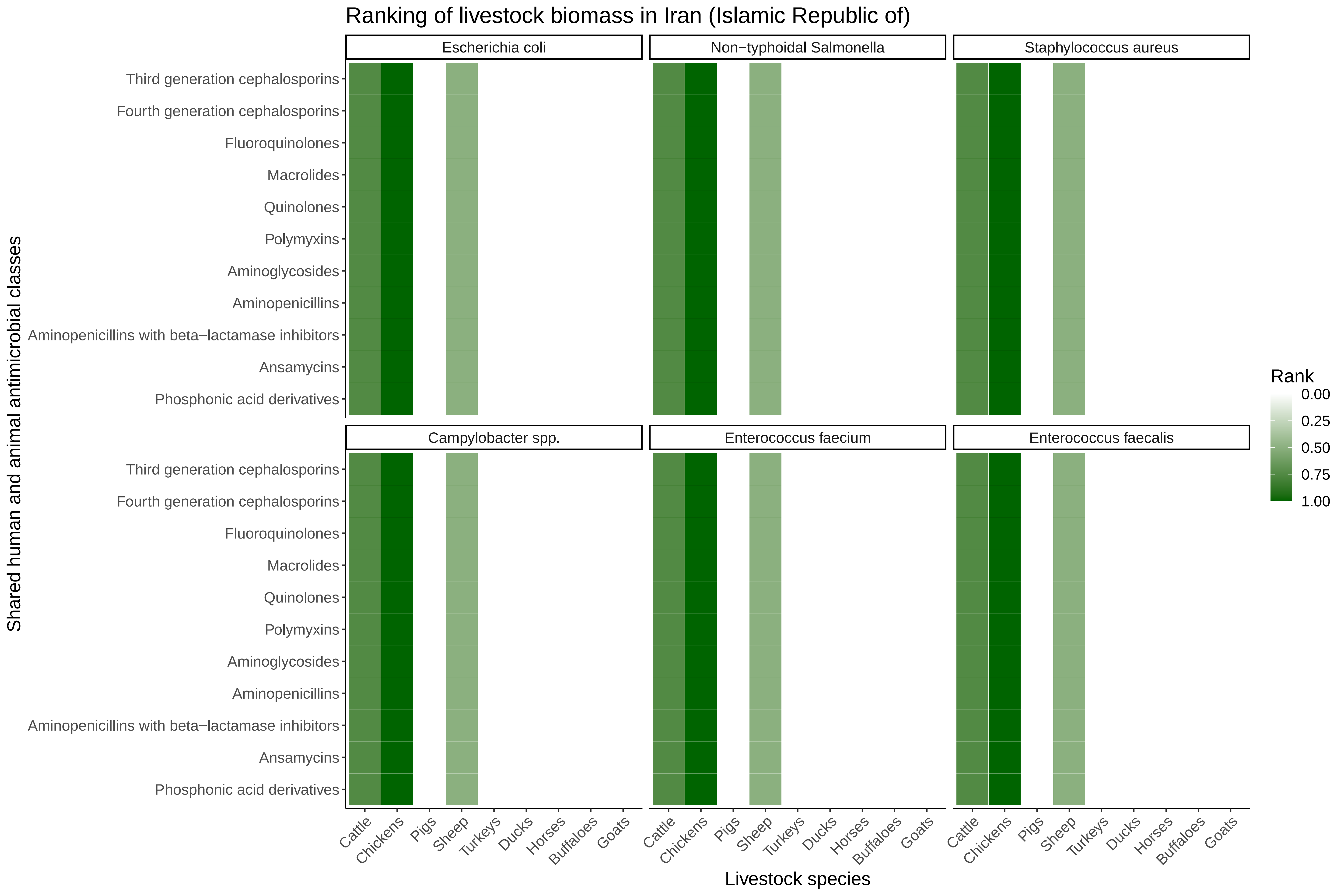

Ranking of livestock biomass in Iran (Islamic Republic of)
Escherichia coli
Non−typhoidal Salmonella
Staphylococcus aureus
Campylobacter spp.
Enterococcus faecium
Enterococcus faecalis
Shared human and animal antimicrobial classes
Third generation cephalosporins
Fourth generation cephalosporins
Fluoroquinolones
Macrolides
Quinolones
Polymyxins
Aminoglycosides
Aminopenicillins
Aminopenicillins with beta−lactamase inhibitors
Ansamycins
Phosphonic acid derivatives
Livestock species
Cattle
Chickens
Pigs
Sheep
Turkeys
Ducks
Horses
Buffaloes
Goats
Rank
0.00
0.25
0.50
0.75
1.00

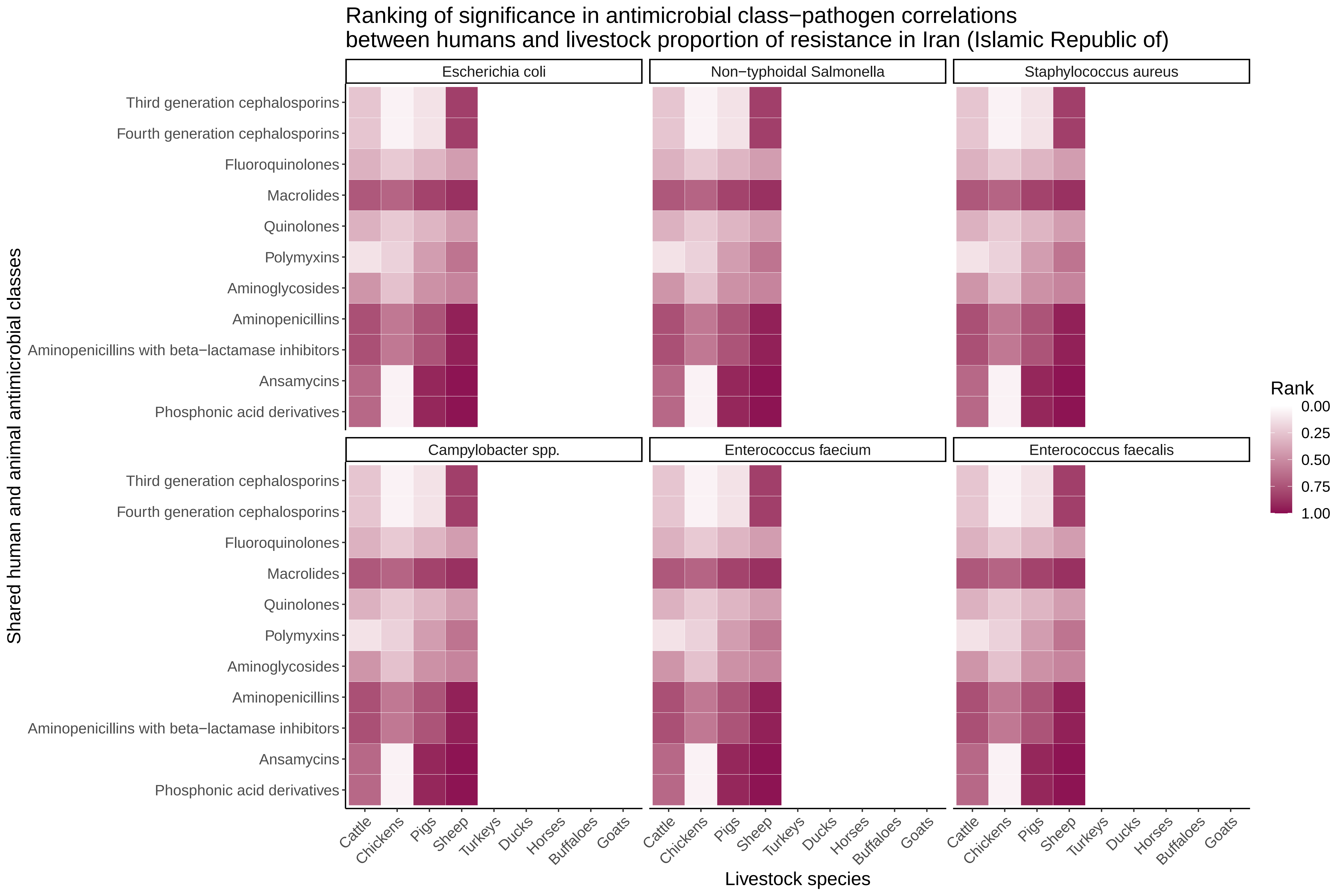

Ranking of significance in antimicrobial class−pathogen correlations
between humans and livestock proportion of resistance in Iran (Islamic Republic of)
Escherichia coli
Non−typhoidal Salmonella
Staphylococcus aureus
Campylobacter spp.
Enterococcus faecium
Enterococcus faecalis
Third generation cephalosporins
Fourth generation cephalosporins
Fluoroquinolones
Macrolides
Quinolones
Polymyxins
Aminoglycosides
Aminopenicillins
Aminopenicillins with beta−lactamase inhibitors
Ansamycins
Phosphonic acid derivatives
Shared human and animal antimicrobial classes
Cattle
Chickens
Pigs
Sheep
Turkeys
Ducks
Horses
Buffaloes
Goats
Livestock species
Rank
0.00
0.25
0.50
0.75
1.00

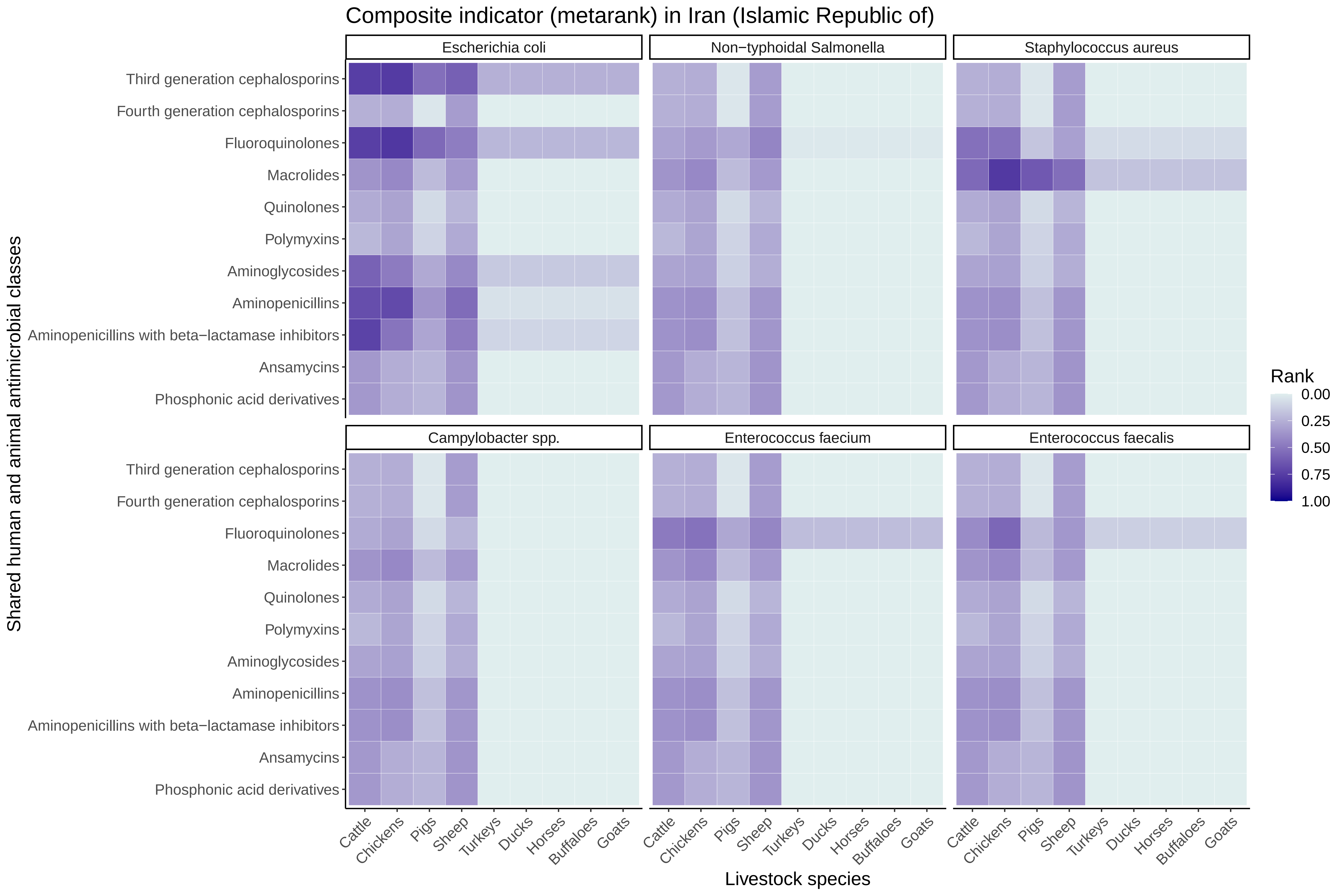

Composite indicator (metarank) in Iran (Islamic Republic of)
Escherichia coli
Non−typhoidal Salmonella
Staphylococcus aureus
Campylobacter spp.
Enterococcus faecium
Enterococcus faecalis
Shared human and animal antimicrobial classes
Third generation cephalosporins
Fourth generation cephalosporins
Fluoroquinolones
Macrolides
Quinolones
Polymyxins
Aminoglycosides
Aminopenicillins
Aminopenicillins with beta−lactamase inhibitors
Ansamycins
Phosphonic acid derivatives
Cattle
Chickens
Pigs
Sheep
Turkeys
Ducks
Horses
Buffaloes
Goats
Livestock species
Rank
0.00
0.25
0.50
0.75
1.00

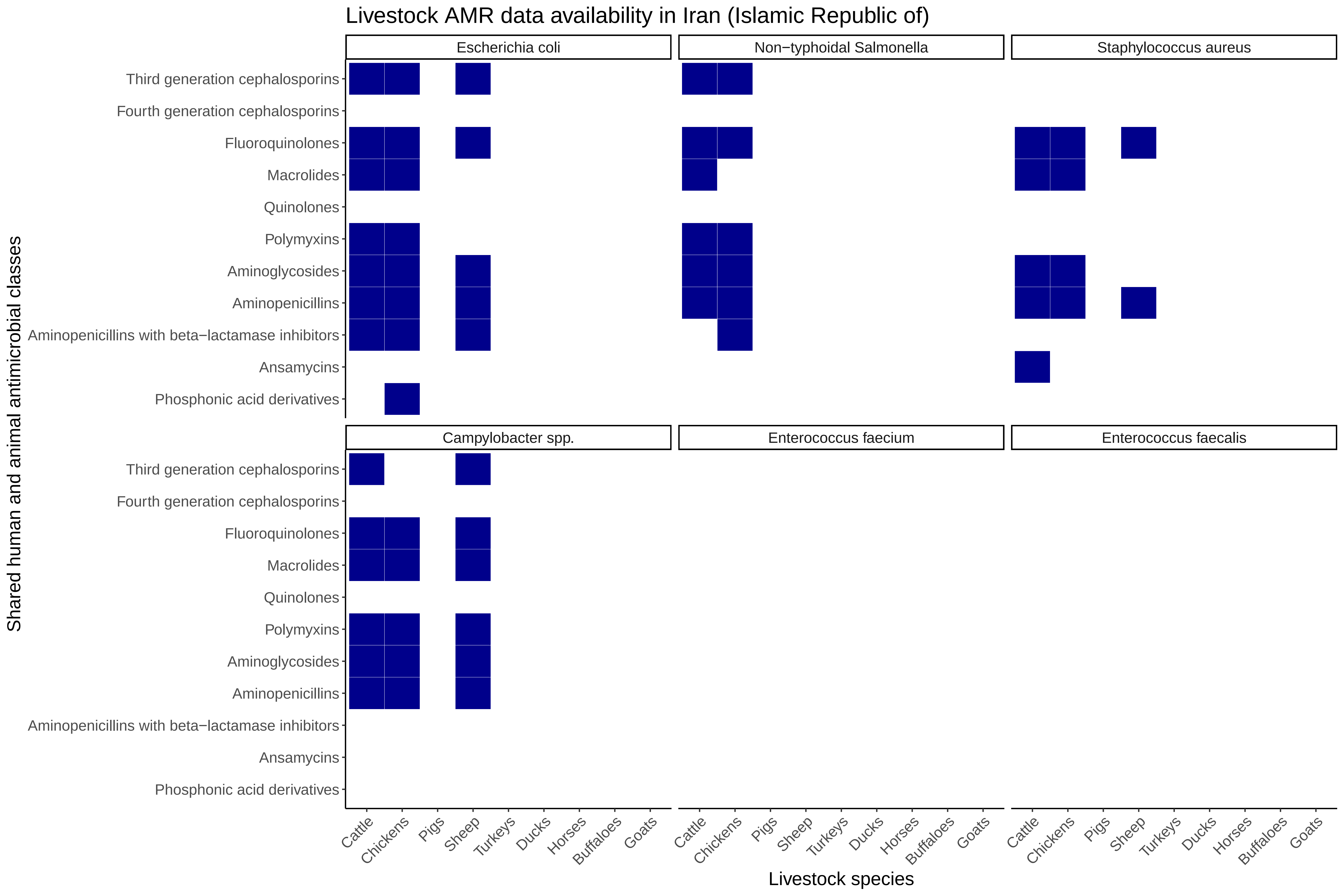

Livestock AMR data availability in Iran (Islamic Republic of)
Escherichia coli
Non−typhoidal Salmonella
Staphylococcus aureus
Campylobacter spp.
Enterococcus faecium
Enterococcus faecalis
Shared human and animal antimicrobial classes
Third generation cephalosporins
Fourth generation cephalosporins
Fluoroquinolones
Macrolides
Quinolones
Polymyxins
Aminoglycosides
Aminopenicillins
Aminopenicillins with beta−lactamase inhibitors
Ansamycins
Phosphonic acid derivatives
Livestock species
Cattle
Chickens
Pigs
Sheep
Turkeys
Ducks
Horses
Buffaloes
Goats

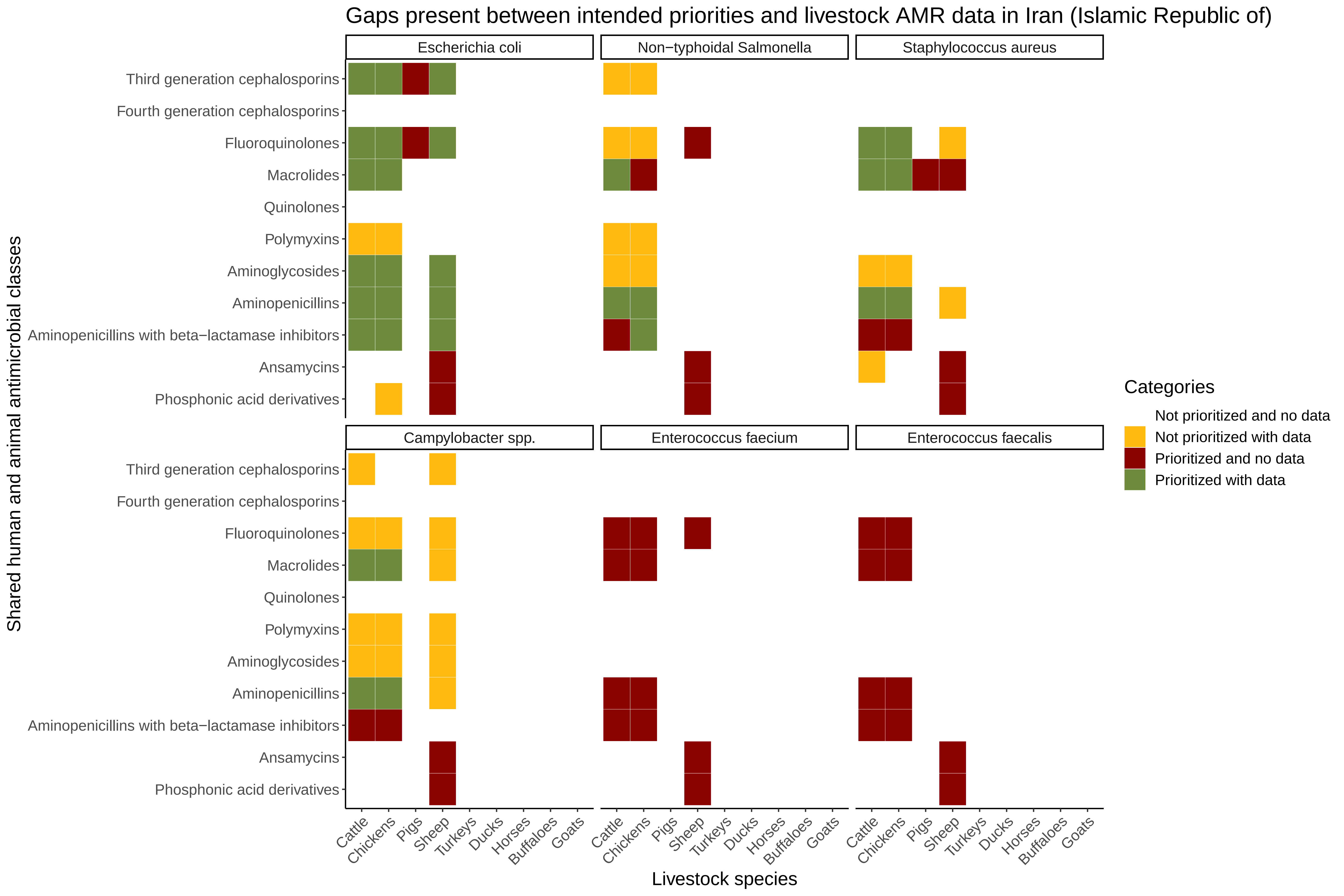

Gaps present between intended priorities and livestock AMR data in Iran (Islamic Republic of)
Escherichia coli
Non−typhoidal Salmonella
Staphylococcus aureus
Campylobacter spp.
Enterococcus faecium
Enterococcus faecalis
Shared human and animal antimicrobial classes
Third generation cephalosporins
Fourth generation cephalosporins
Fluoroquinolones
Macrolides
Quinolones
Polymyxins
Aminoglycosides
Aminopenicillins
Aminopenicillins with beta−lactamase inhibitors
Ansamycins
Phosphonic acid derivatives
Livestock species
Cattle
Chickens
Pigs
Sheep
Turkeys
Ducks
Horses
Buffaloes
Goats
Categories
Not prioritized and no data
Not prioritized with data
Prioritized and no data
Prioritized with data

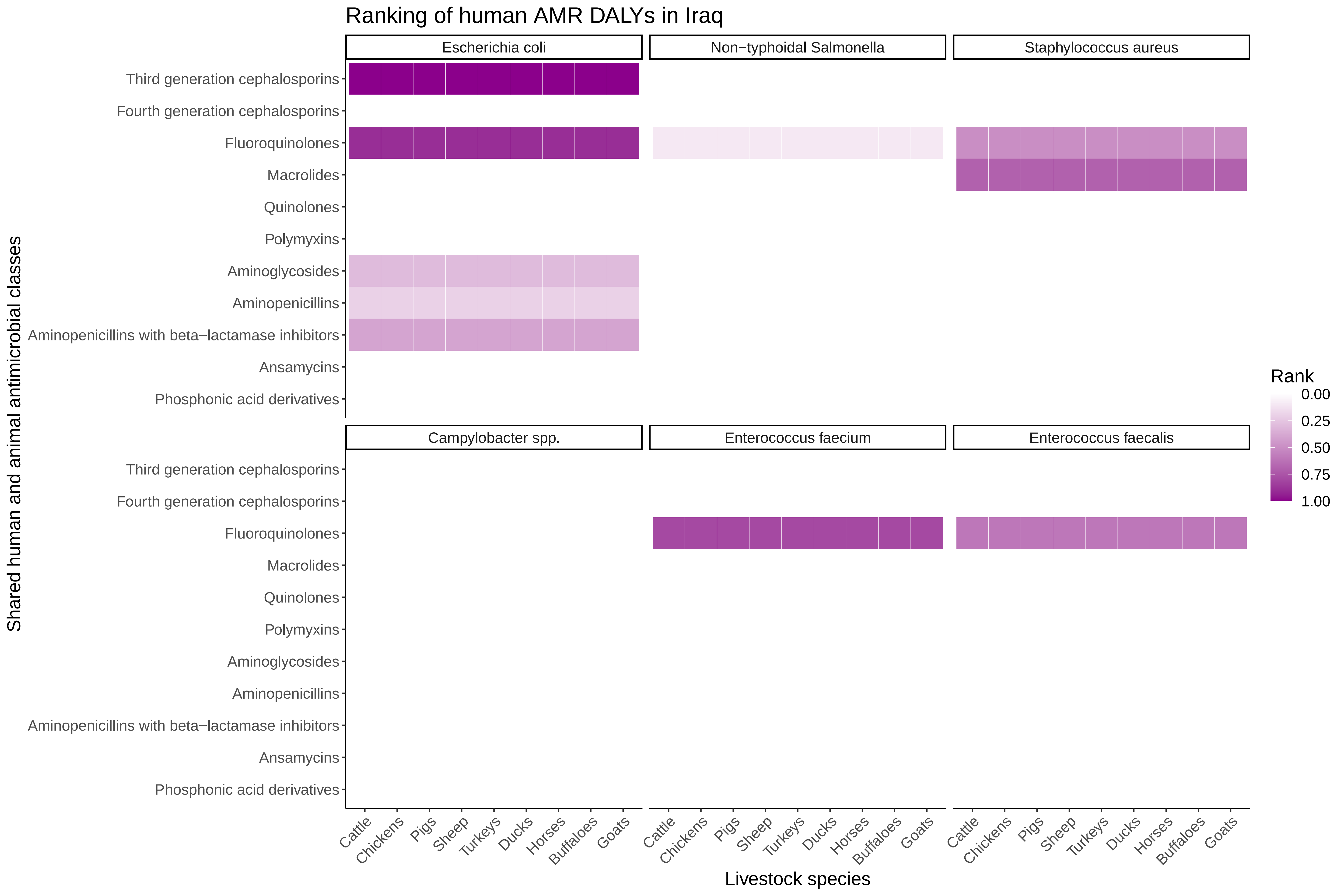

Ranking of human AMR DALYs in Iraq
Escherichia coli
Non−typhoidal Salmonella
Staphylococcus aureus
Campylobacter spp.
Enterococcus faecium
Enterococcus faecalis
Third generation cephalosporins
Fourth generation cephalosporins
Fluoroquinolones
Macrolides
Quinolones
Polymyxins
Aminoglycosides
Aminopenicillins
Aminopenicillins with beta−lactamase inhibitors
Ansamycins
Phosphonic acid derivatives
Shared human and animal antimicrobial classes
Cattle
Chickens
Pigs
Sheep
Turkeys
Ducks
Horses
Buffaloes
Goats
Livestock species
Rank
0.00
0.25
0.50
0.75
1.00

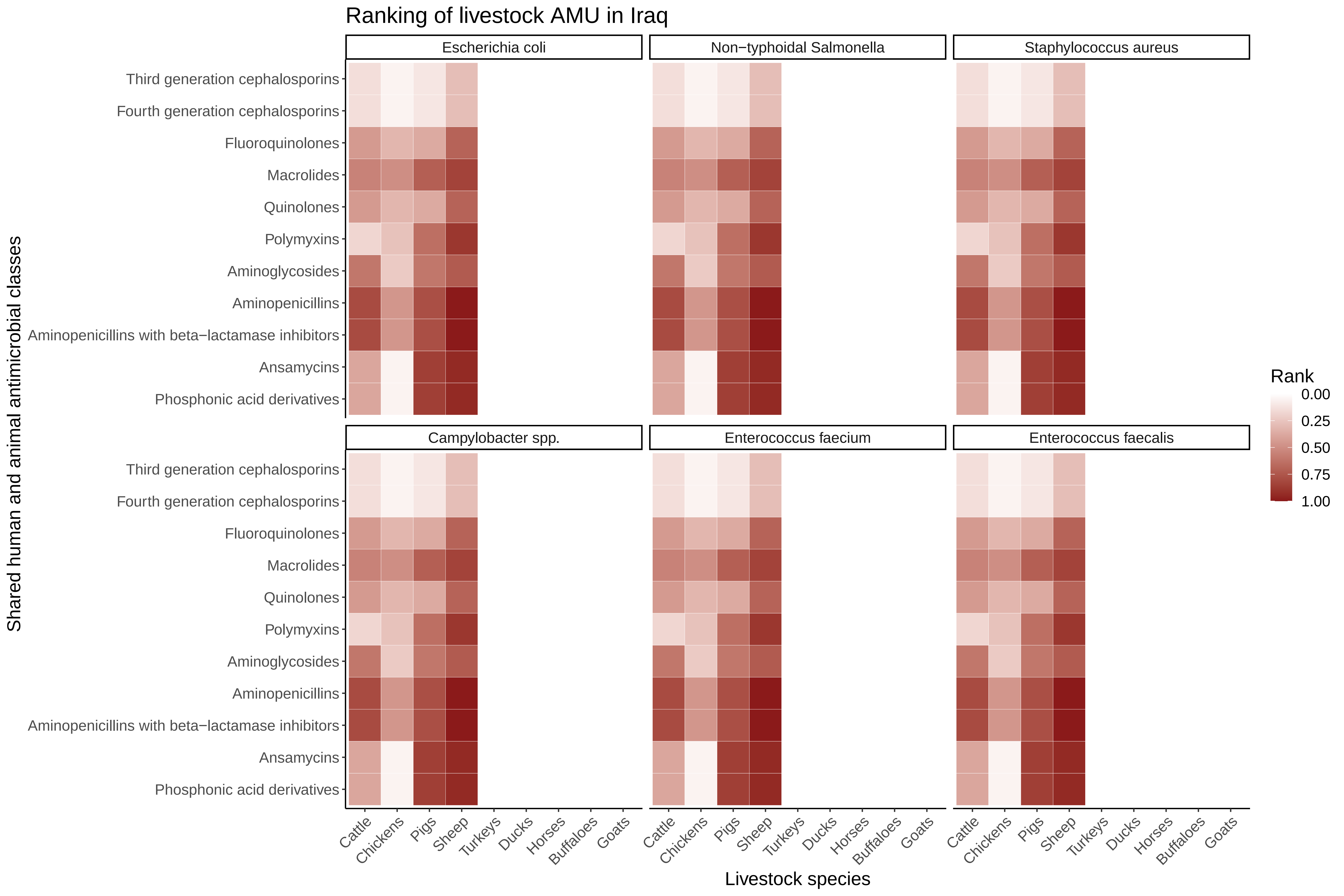

Ranking of livestock AMU in Iraq
Escherichia coli
Non−typhoidal Salmonella
Staphylococcus aureus
Campylobacter spp.
Enterococcus faecium
Enterococcus faecalis
Shared human and animal antimicrobial classes
Third generation cephalosporins
Fourth generation cephalosporins
Fluoroquinolones
Macrolides
Quinolones
Polymyxins
Aminoglycosides
Aminopenicillins
Aminopenicillins with beta−lactamase inhibitors
Ansamycins
Phosphonic acid derivatives
Cattle
Chickens
Pigs
Sheep
Turkeys
Ducks
Horses
Buffaloes
Goats
Livestock species
Rank
0.00
0.25
0.50
0.75
1.00

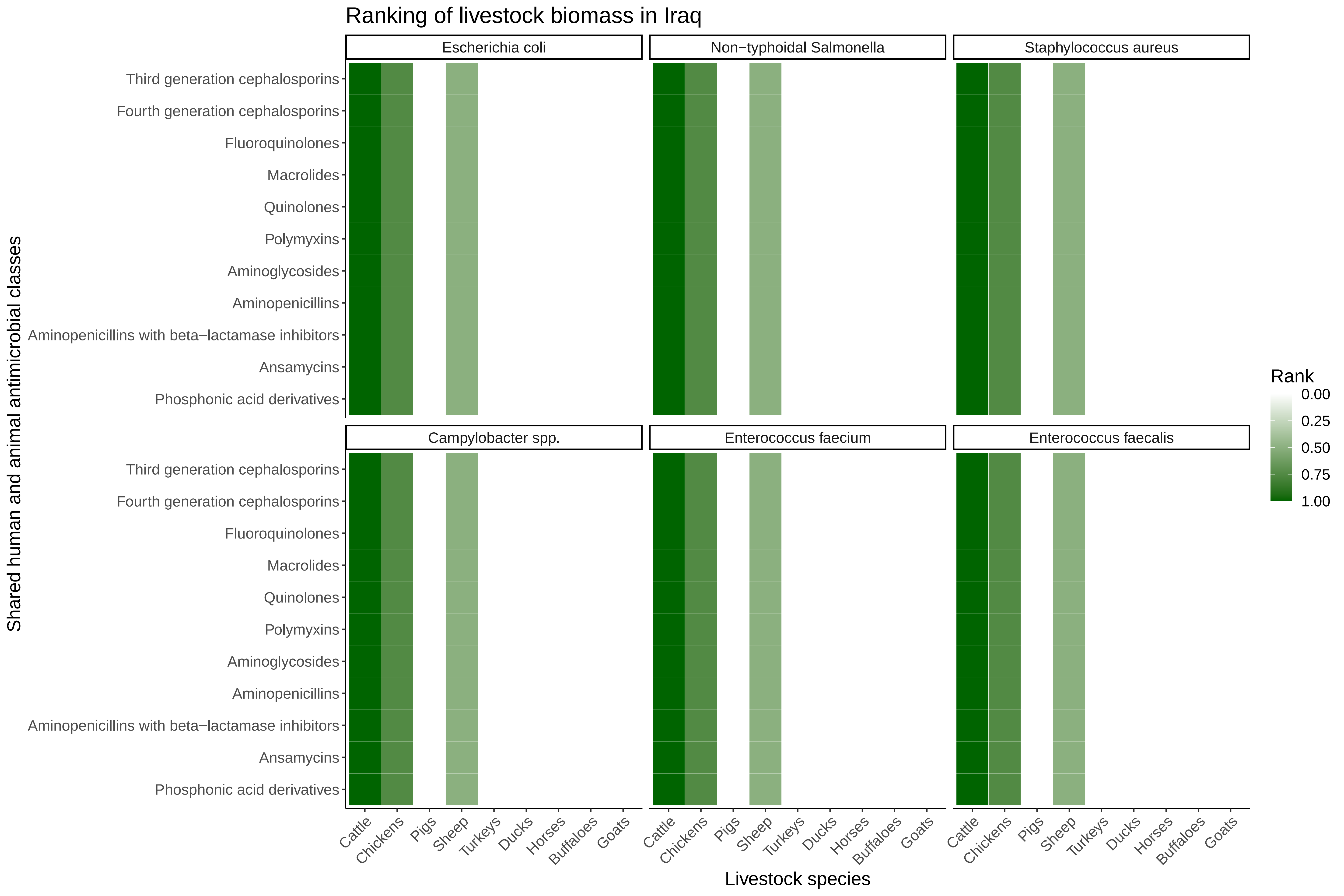

Ranking of livestock biomass in Iraq
Escherichia coli
Non−typhoidal Salmonella
Staphylococcus aureus
Campylobacter spp.
Enterococcus faecium
Enterococcus faecalis
Third generation cephalosporins
Fourth generation cephalosporins
Fluoroquinolones
Macrolides
Quinolones
Polymyxins
Aminoglycosides
Aminopenicillins
Aminopenicillins with beta−lactamase inhibitors
Ansamycins
Phosphonic acid derivatives
Shared human and animal antimicrobial classes
Cattle
Chickens
Pigs
Sheep
Turkeys
Ducks
Horses
Buffaloes
Goats
Livestock species
Rank
0.00
0.25
0.50
0.75
1.00

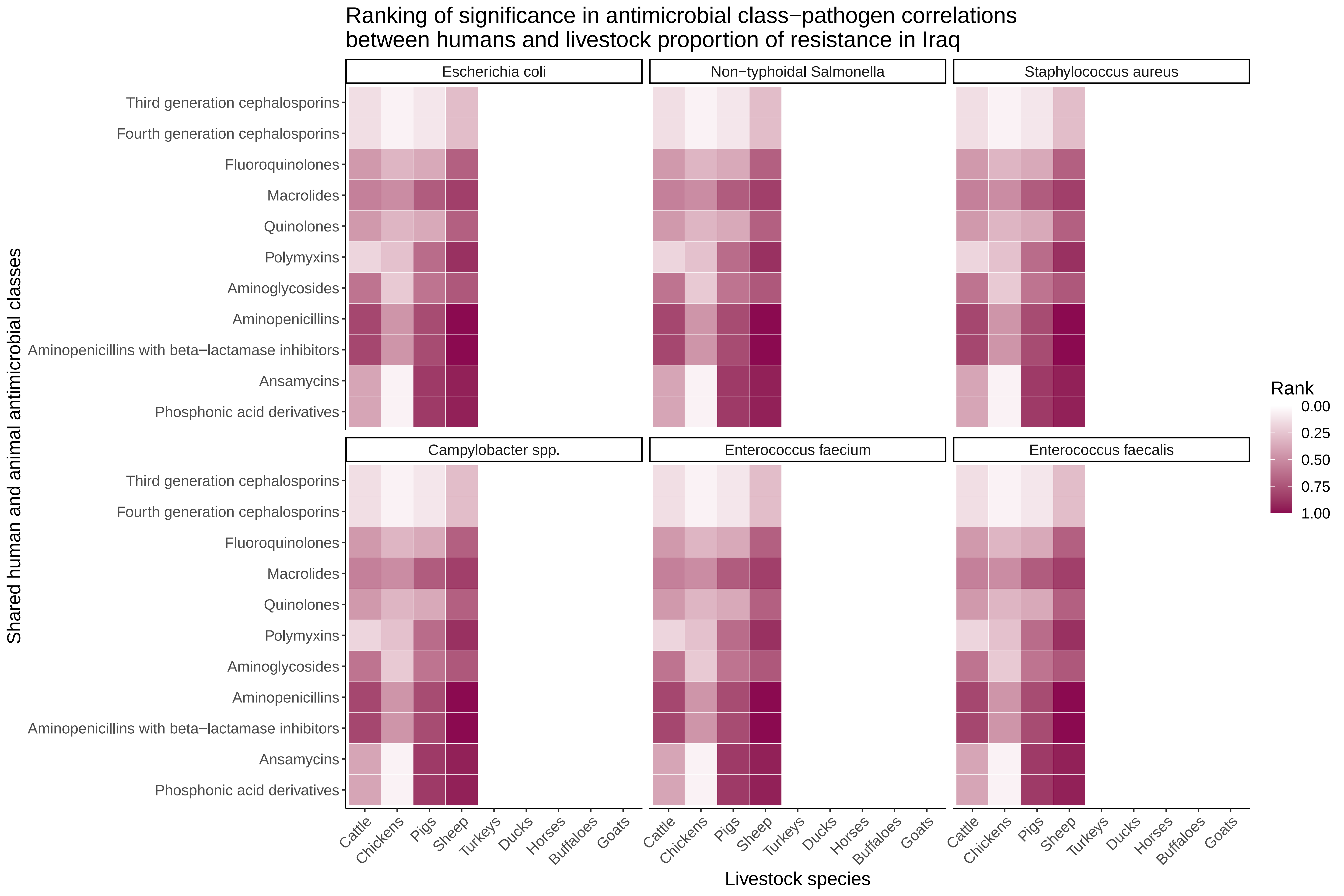

Ranking of significance in antimicrobial class−pathogen correlations
between humans and livestock proportion of resistance in Iraq
Escherichia coli
Non−typhoidal Salmonella
Staphylococcus aureus
Campylobacter spp.
Enterococcus faecium
Enterococcus faecalis
Shared human and animal antimicrobial classes
Third generation cephalosporins
Fourth generation cephalosporins
Fluoroquinolones
Macrolides
Quinolones
Polymyxins
Aminoglycosides
Aminopenicillins
Aminopenicillins with beta−lactamase inhibitors
Ansamycins
Phosphonic acid derivatives
Livestock species
Cattle
Chickens
Pigs
Sheep
Turkeys
Ducks
Horses
Buffaloes
Goats
Rank
0.00
0.25
0.50
0.75
1.00

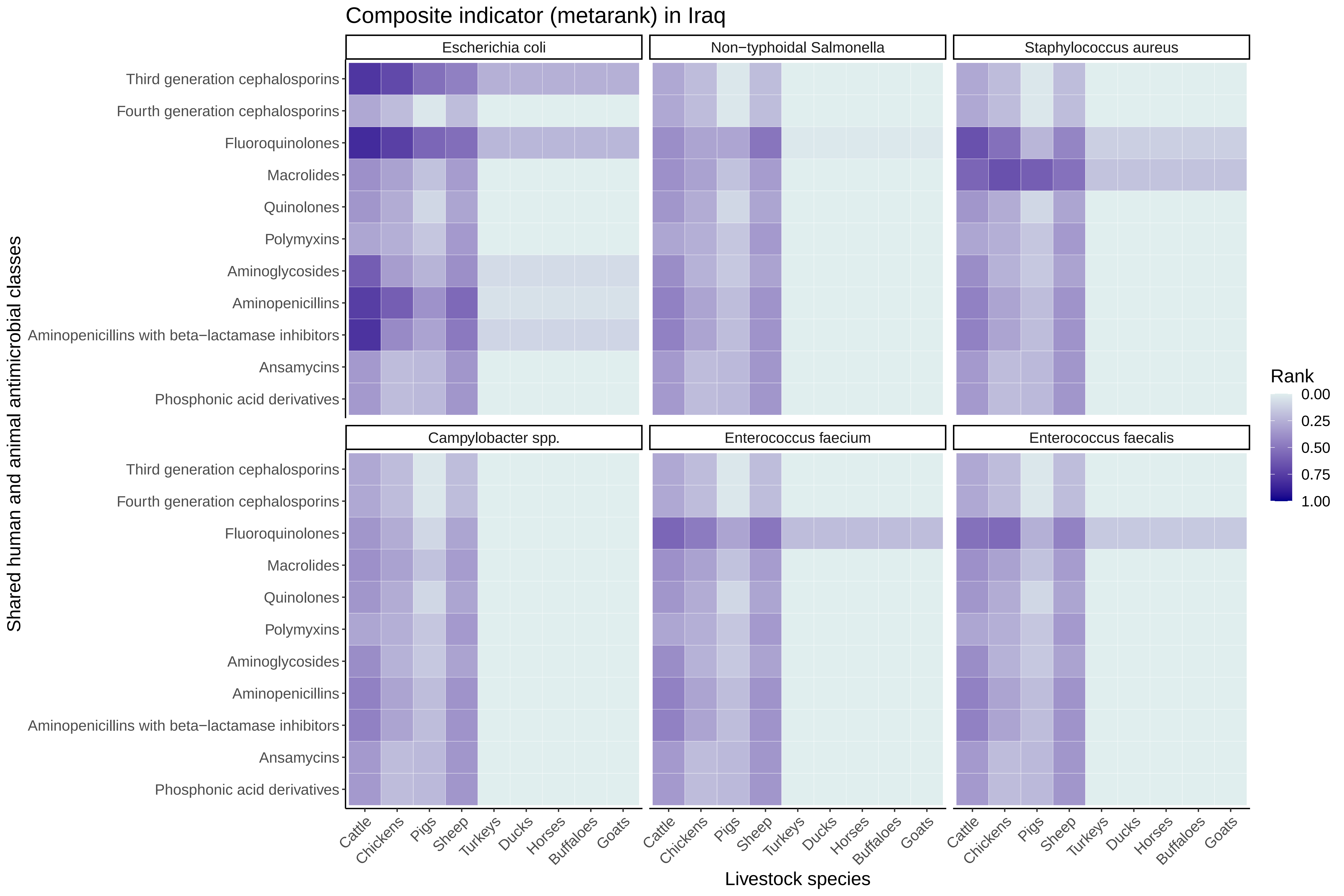

Composite indicator (metarank) in Iraq
Escherichia coli
Non−typhoidal Salmonella
Staphylococcus aureus
Campylobacter spp.
Enterococcus faecium
Enterococcus faecalis
Shared human and animal antimicrobial classes
Third generation cephalosporins
Fourth generation cephalosporins
Fluoroquinolones
Macrolides
Quinolones
Polymyxins
Aminoglycosides
Aminopenicillins
Aminopenicillins with beta−lactamase inhibitors
Ansamycins
Phosphonic acid derivatives
Cattle
Chickens
Pigs
Sheep
Turkeys
Ducks
Horses
Buffaloes
Goats
Livestock species
Rank
0.00
0.25
0.50
0.75
1.00

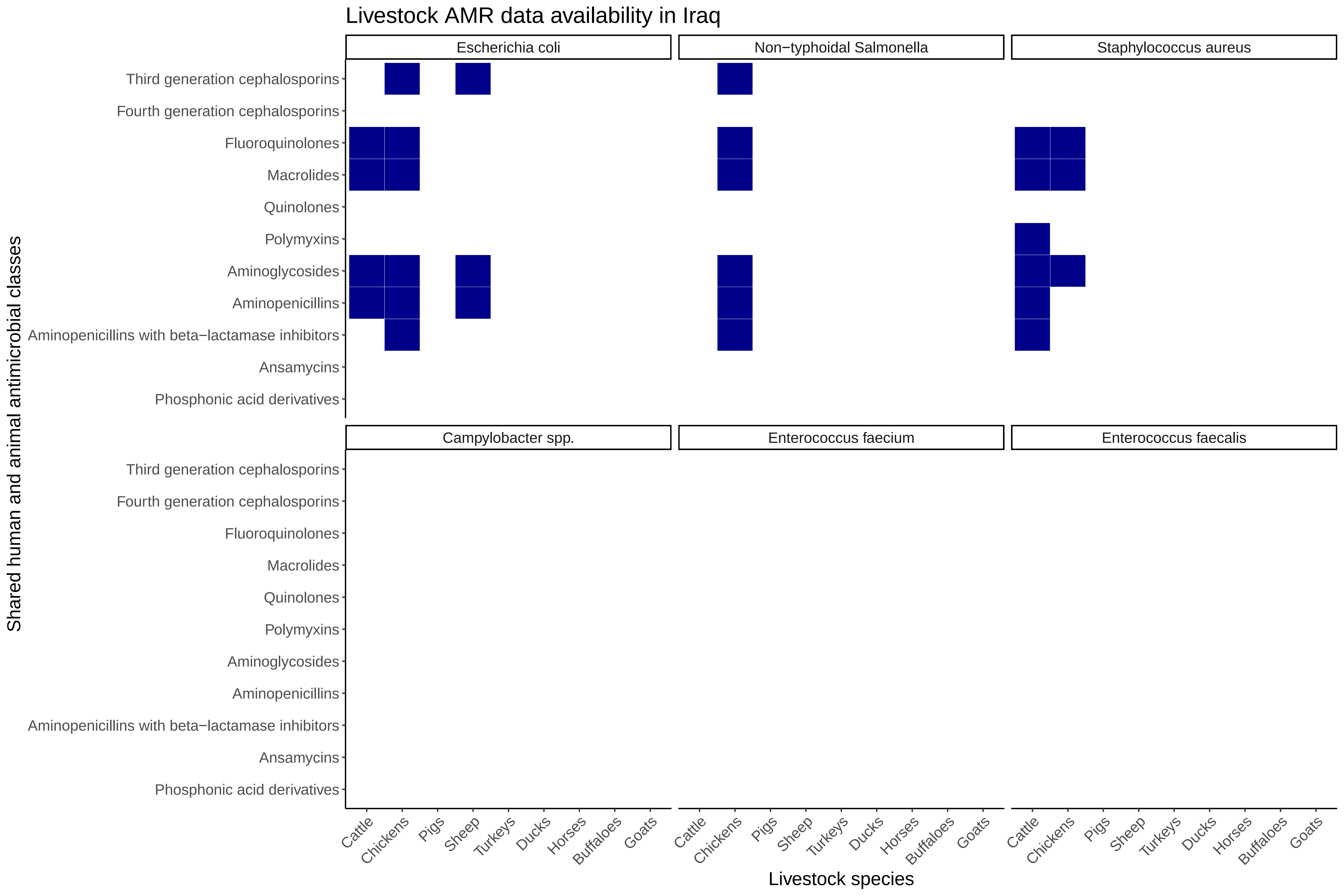

Livestock AMR data availability in Iraq
Escherichia coli
Non−typhoidal Salmonella
Staphylococcus aureus
Campylobacter spp.
Enterococcus faecium
Enterococcus faecalis
Shared human and animal antimicrobial classes
Third generation cephalosporins
Fourth generation cephalosporins
Fluoroquinolones
Macrolides
Quinolones
Polymyxins
Aminoglycosides
Aminopenicillins
Aminopenicillins with beta−lactamase inhibitors
Ansamycins
Phosphonic acid derivatives
Cattle
Chickens
Pigs
Sheep
Turkeys
Ducks
Horses
Buffaloes
Goats
Livestock species

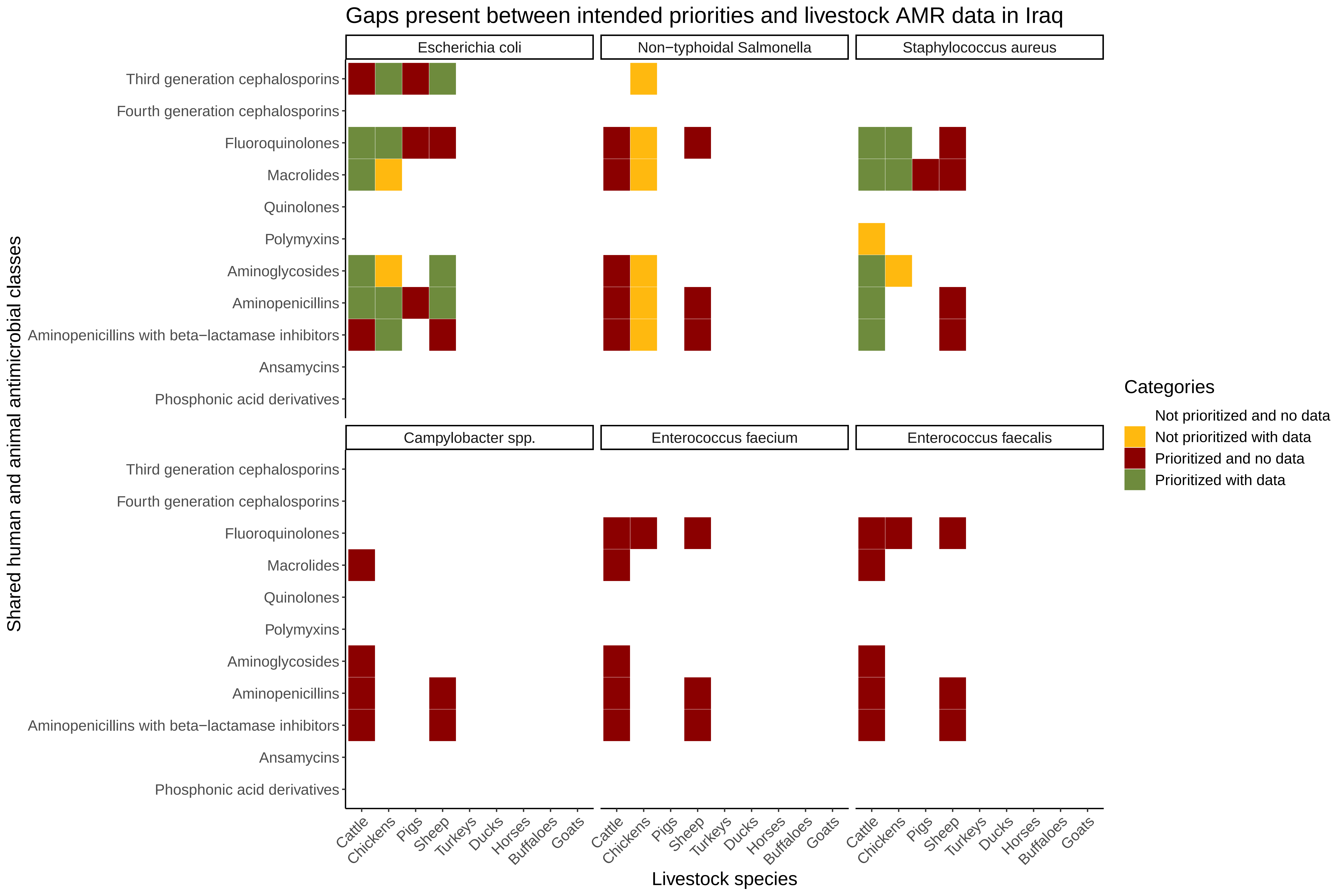

Gaps present between intended priorities and livestock AMR data in Iraq
Escherichia coli
Non−typhoidal Salmonella
Staphylococcus aureus
Campylobacter spp.
Enterococcus faecium
Enterococcus faecalis
Third generation cephalosporins
Fourth generation cephalosporins
Fluoroquinolones
Macrolides
Quinolones
Polymyxins
Aminoglycosides
Aminopenicillins
Aminopenicillins with beta−lactamase inhibitors
Ansamycins
Phosphonic acid derivatives
Cattle
Chickens
Pigs
Sheep
Turkeys
Ducks
Horses
Buffaloes
Goats
Shared human and animal antimicrobial classes
Livestock species
Categories
Not prioritized and no data
Not prioritized with data
Prioritized and no data
Prioritized with data

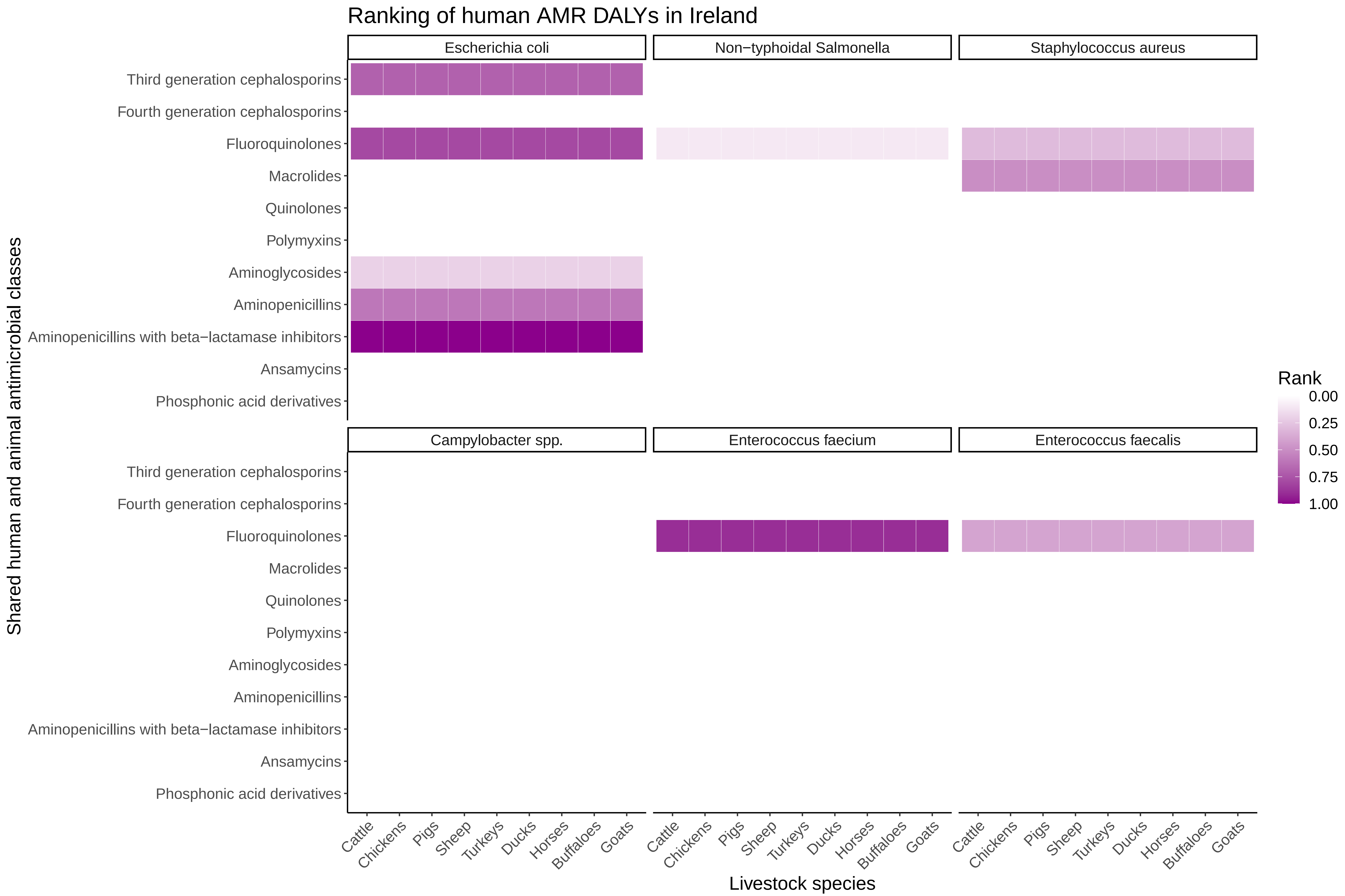
Ranking of human AMR DALYs in Ireland
Escherichia coli
Non−typhoidal Salmonella
Staphylococcus aureus
Campylobacter spp.
Enterococcus faecium
Enterococcus faecalis
Shared human and animal antimicrobial classes
Third generation cephalosporins
Fourth generation cephalosporins
Fluoroquinolones
Macrolides
Quinolones
Polymyxins
Aminoglycosides
Aminopenicillins
Aminopenicillins with beta−lactamase inhibitors
Ansamycins
Phosphonic acid derivatives
Cattle
Chickens
Pigs
Sheep
Turkeys
Ducks
Horses
Buffaloes
Goats
Livestock species
Rank
0.00
0.25
0.50
0.75
1.00

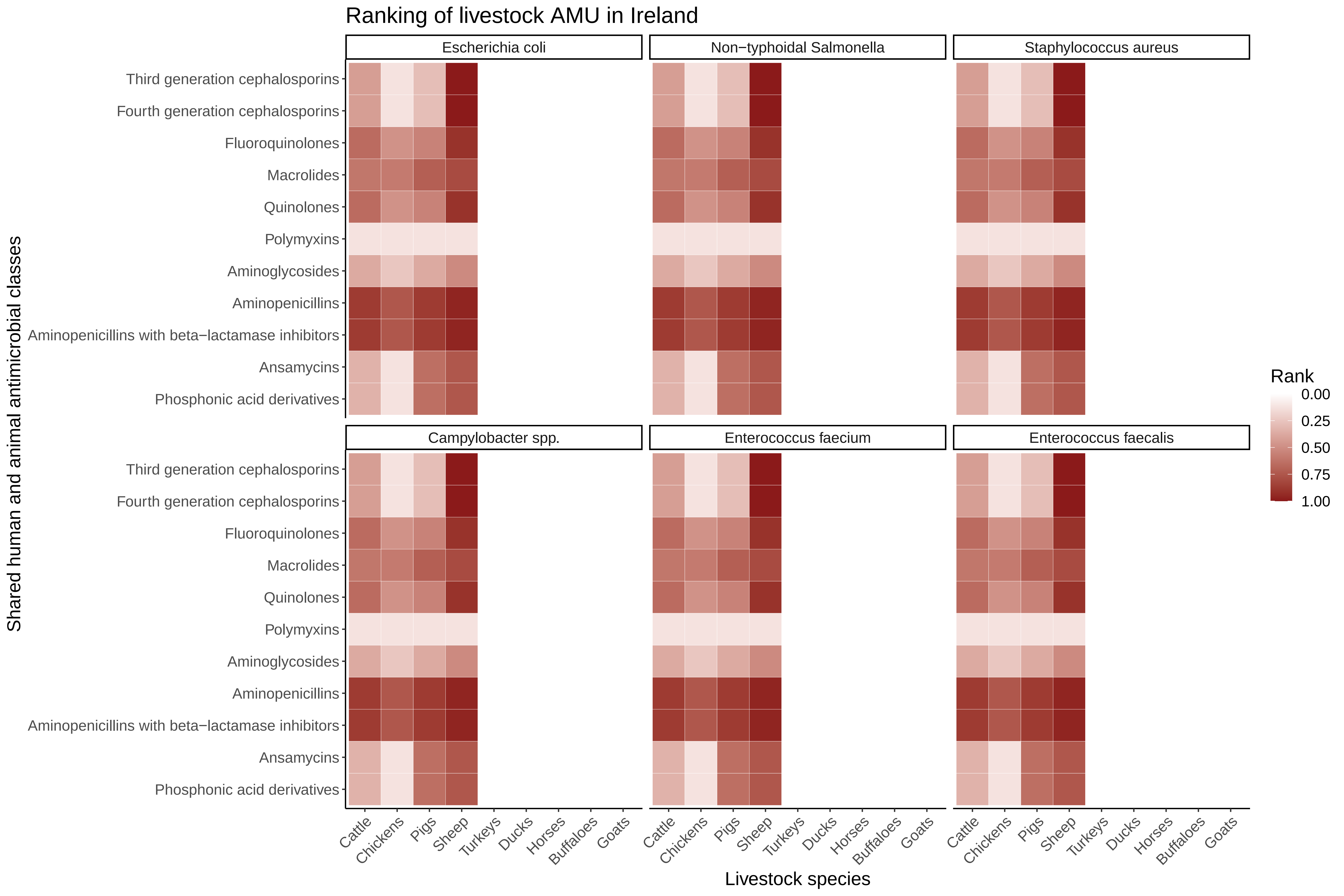

Ranking of livestock AMU in Ireland
Escherichia coli
Non−typhoidal Salmonella
Staphylococcus aureus
Campylobacter spp.
Enterococcus faecium
Enterococcus faecalis
Shared human and animal antimicrobial classes
Third generation cephalosporins
Fourth generation cephalosporins
Fluoroquinolones
Macrolides
Quinolones
Polymyxins
Aminoglycosides
Aminopenicillins
Aminopenicillins with beta−lactamase inhibitors
Ansamycins
Phosphonic acid derivatives
Livestock species
Cattle
Chickens
Pigs
Sheep
Turkeys
Ducks
Horses
Buffaloes
Goats
Rank
0.00
0.25
0.50
0.75
1.00

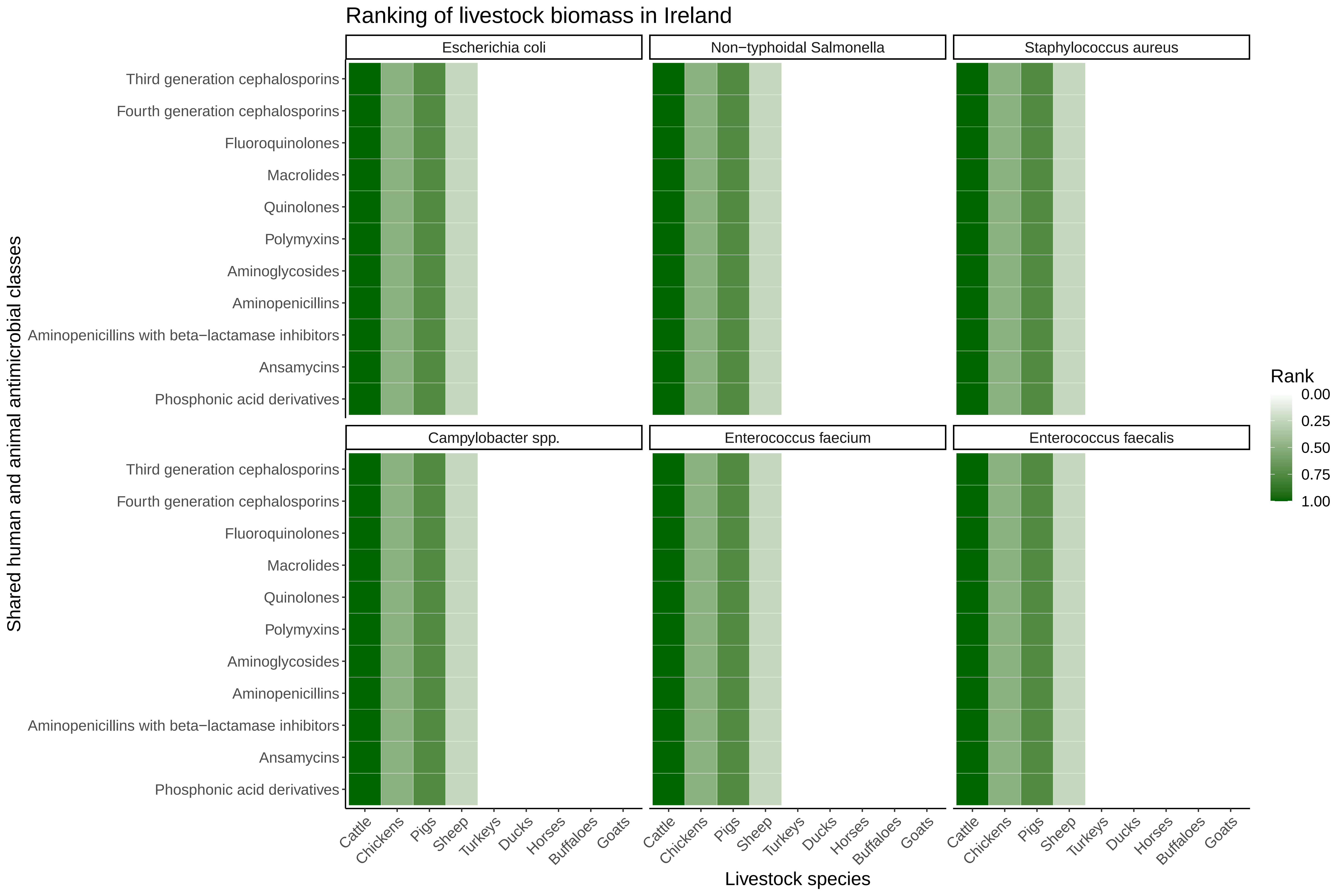

Ranking of livestock biomass in Ireland
Escherichia coli
Non−typhoidal Salmonella
Staphylococcus aureus
Campylobacter spp.
Enterococcus faecium
Enterococcus faecalis
Third generation cephalosporins
Fourth generation cephalosporins
Fluoroquinolones
Macrolides
Quinolones
Polymyxins
Aminoglycosides
Aminopenicillins
Aminopenicillins with beta−lactamase inhibitors
Ansamycins
Phosphonic acid derivatives
Shared human and animal antimicrobial classes
Cattle
Chickens
Pigs
Sheep
Turkeys
Ducks
Horses
Buffaloes
Goats
Livestock species
Rank
0.00
0.25
0.50
0.75
1.00

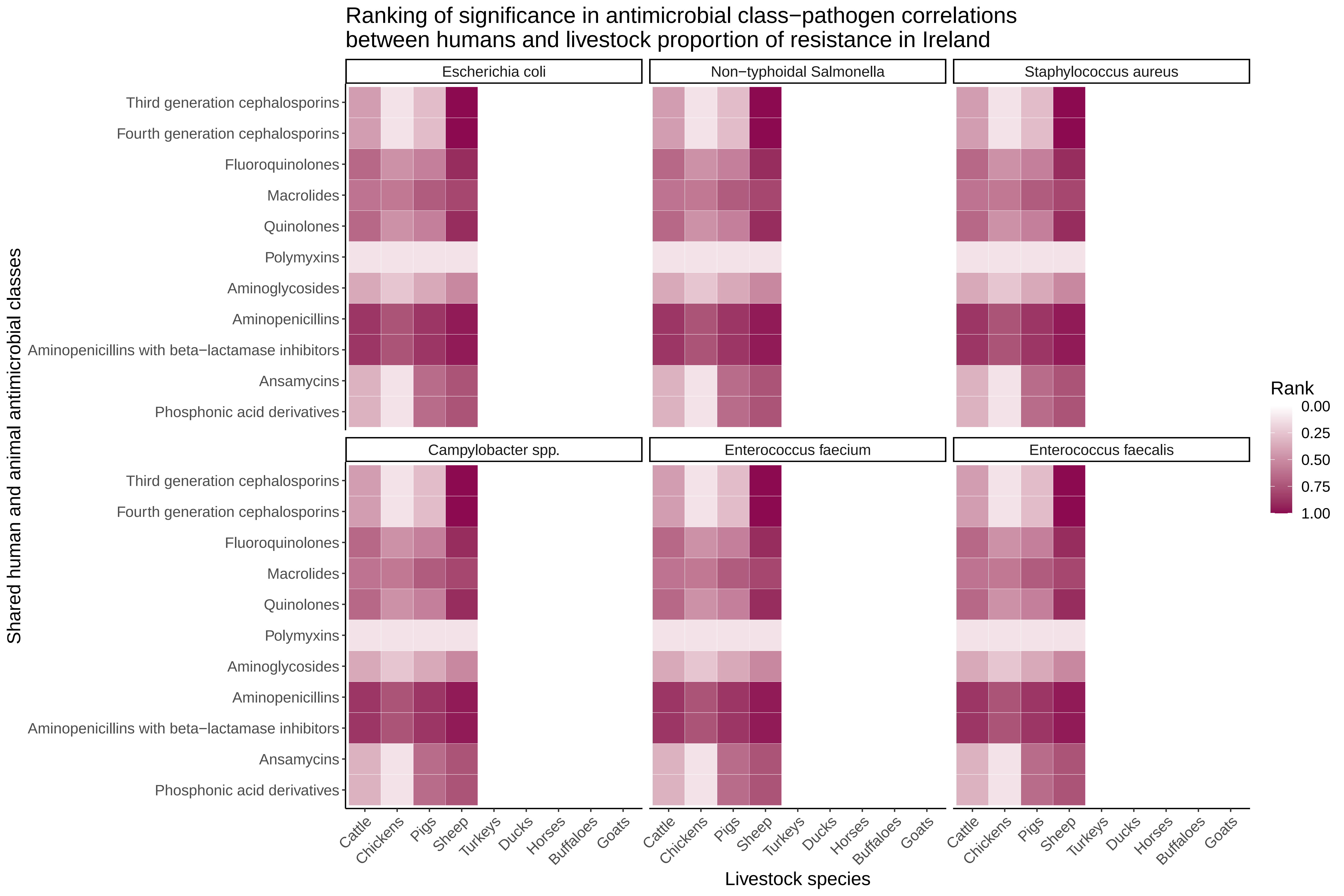
Ranking of significance in antimicrobial class−pathogen correlations
between humans and livestock proportion of resistance in Ireland
Escherichia coli
Non−typhoidal Salmonella
Staphylococcus aureus
Campylobacter spp.
Enterococcus faecium
Enterococcus faecalis
Third generation cephalosporins
Fourth generation cephalosporins
Fluoroquinolones
Macrolides
Quinolones
Polymyxins
Aminoglycosides
Aminopenicillins
Aminopenicillins with beta−lactamase inhibitors
Ansamycins
Phosphonic acid derivatives
Shared human and animal antimicrobial classes
Cattle
Chickens
Pigs
Sheep
Turkeys
Ducks
Horses
Buffaloes
Goats
Livestock species
Rank
0.00
0.25
0.50
0.75
1.00

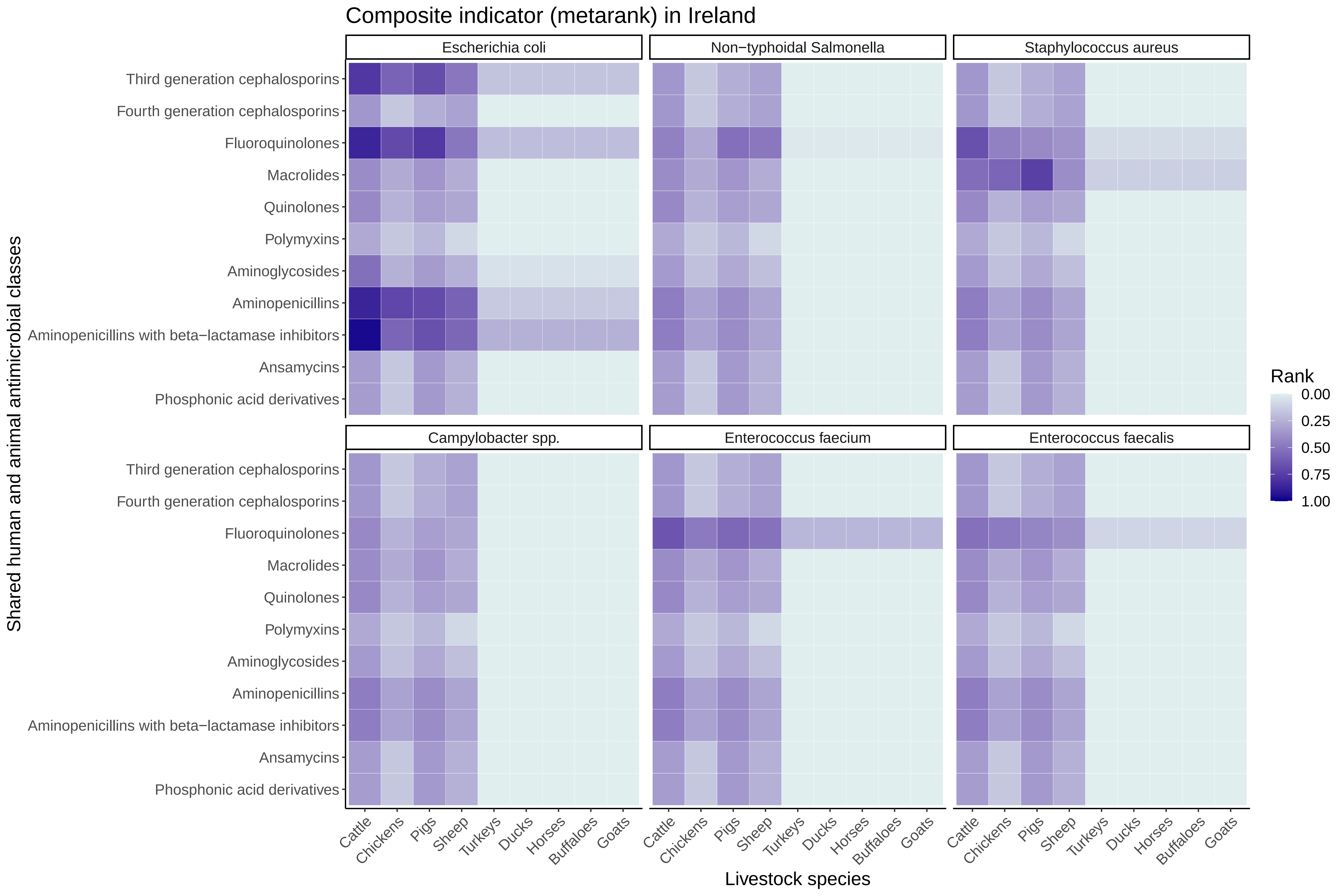

Composite indicator (metarank) in Ireland
Escherichia coli
Non−typhoidal Salmonella
Staphylococcus aureus
Campylobacter spp.
Enterococcus faecium
Enterococcus faecalis
Shared human and animal antimicrobial classes
Third generation cephalosporins
Fourth generation cephalosporins
Fluoroquinolones
Macrolides
Quinolones
Polymyxins
Aminoglycosides
Aminopenicillins
Aminopenicillins with beta−lactamase inhibitors
Ansamycins
Phosphonic acid derivatives
Cattle
Chickens
Pigs
Sheep
Turkeys
Ducks
Horses
Buffaloes
Goats
Livestock species
Rank
0.00
0.25
0.50
0.75
1.00

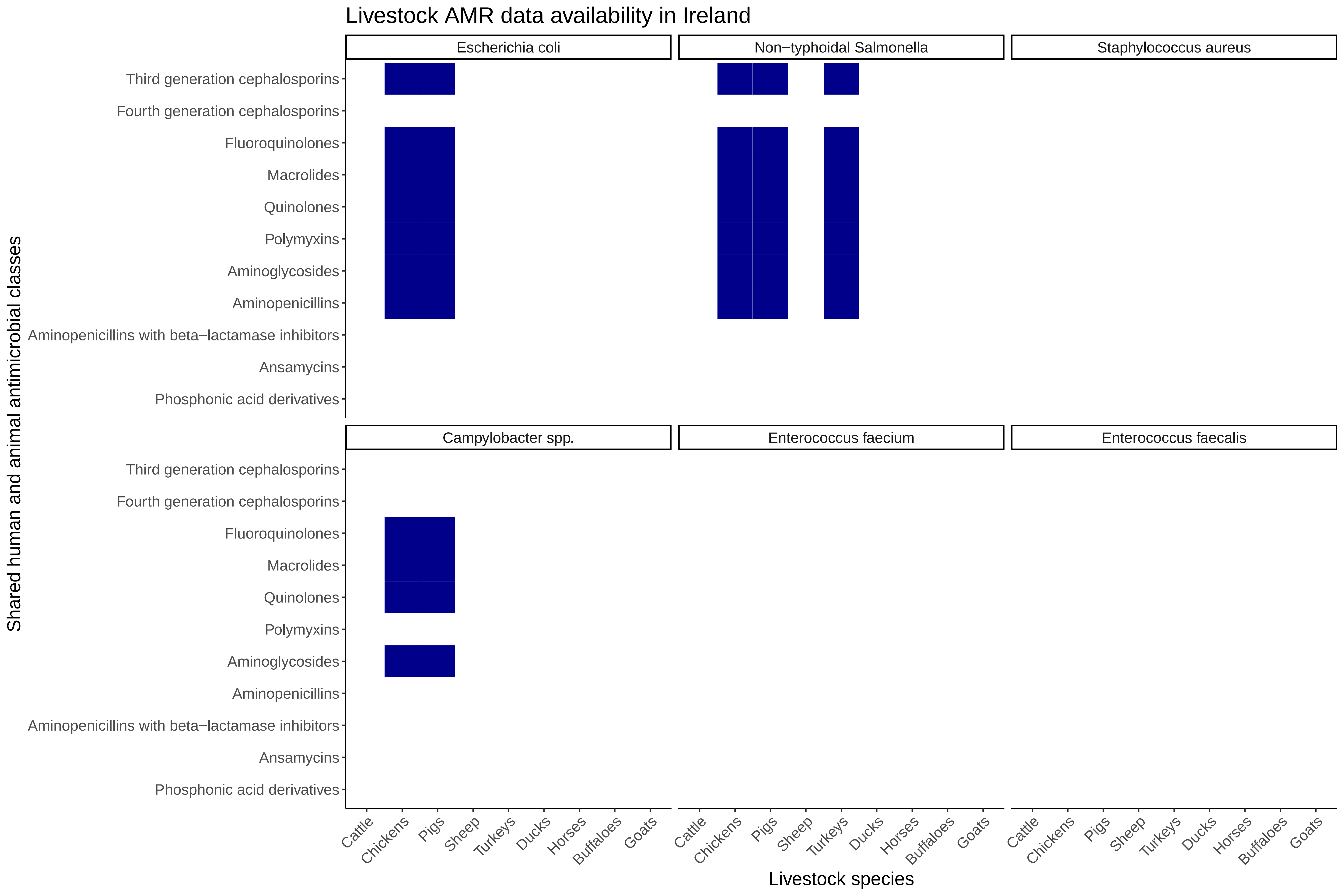

Livestock AMR data availability in Ireland
Escherichia coli
Non−typhoidal Salmonella
Staphylococcus aureus
Campylobacter spp.
Enterococcus faecium
Enterococcus faecalis
Shared human and animal antimicrobial classes
Third generation cephalosporins
Fourth generation cephalosporins
Fluoroquinolones
Macrolides
Quinolones
Polymyxins
Aminoglycosides
Aminopenicillins
Aminopenicillins with beta−lactamase inhibitors
Ansamycins
Phosphonic acid derivatives
Cattle
Chickens
Pigs
Sheep
Turkeys
Ducks
Horses
Buffaloes
Goats
Livestock species

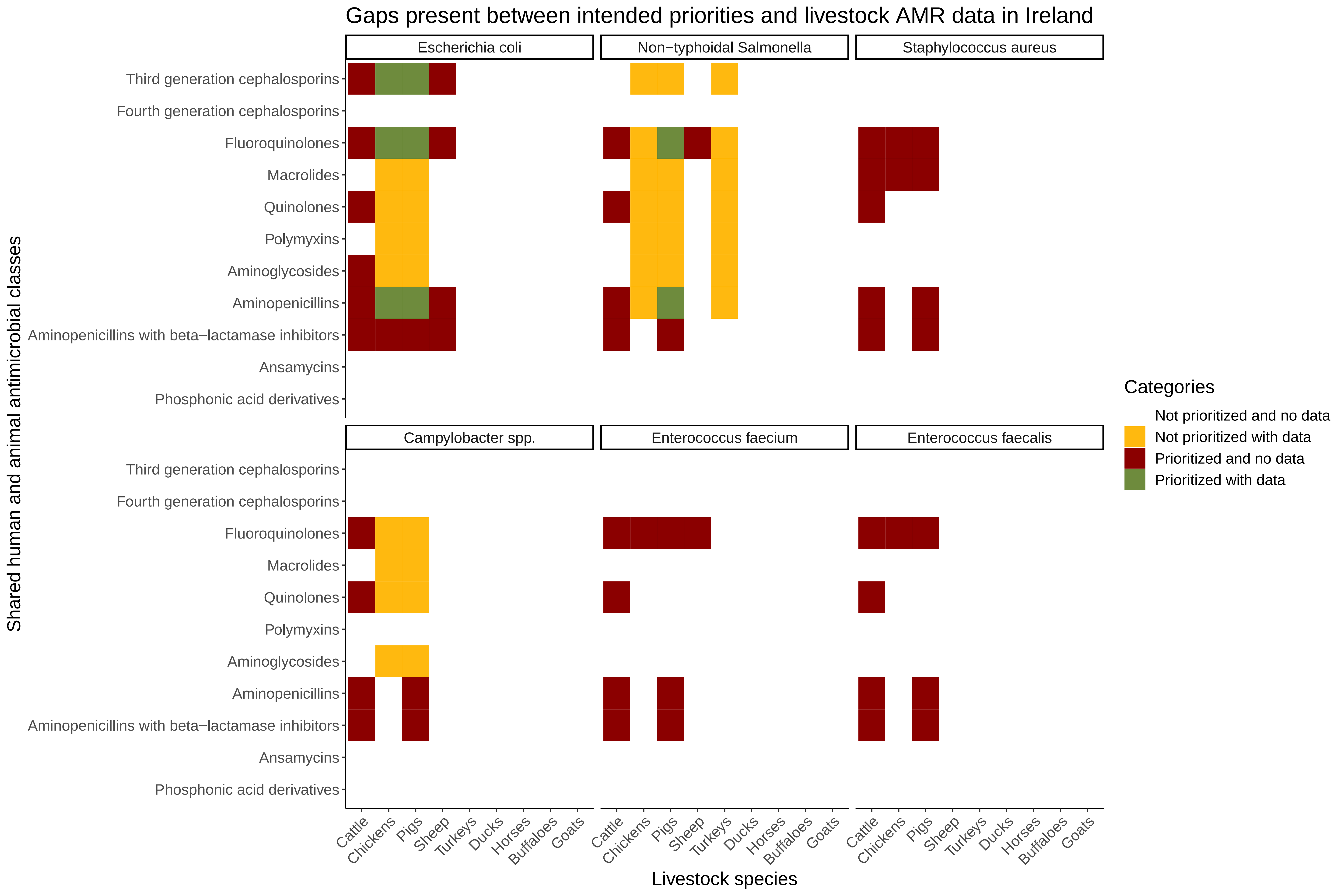

Gaps present between intended priorities and livestock AMR data in Ireland
Escherichia coli
Non−typhoidal Salmonella
Staphylococcus aureus
Campylobacter spp.
Enterococcus faecium
Enterococcus faecalis
Third generation cephalosporins
Fourth generation cephalosporins
Fluoroquinolones
Macrolides
Quinolones
Polymyxins
Aminoglycosides
Aminopenicillins
Aminopenicillins with beta−lactamase inhibitors
Ansamycins
Phosphonic acid derivatives
Shared human and animal antimicrobial classes
Cattle
Chickens
Pigs
Sheep
Turkeys
Ducks
Horses
Buffaloes
Goats
Livestock species
Categories
Not prioritized and no data
Not prioritized with data
Prioritized and no data
Prioritized with data

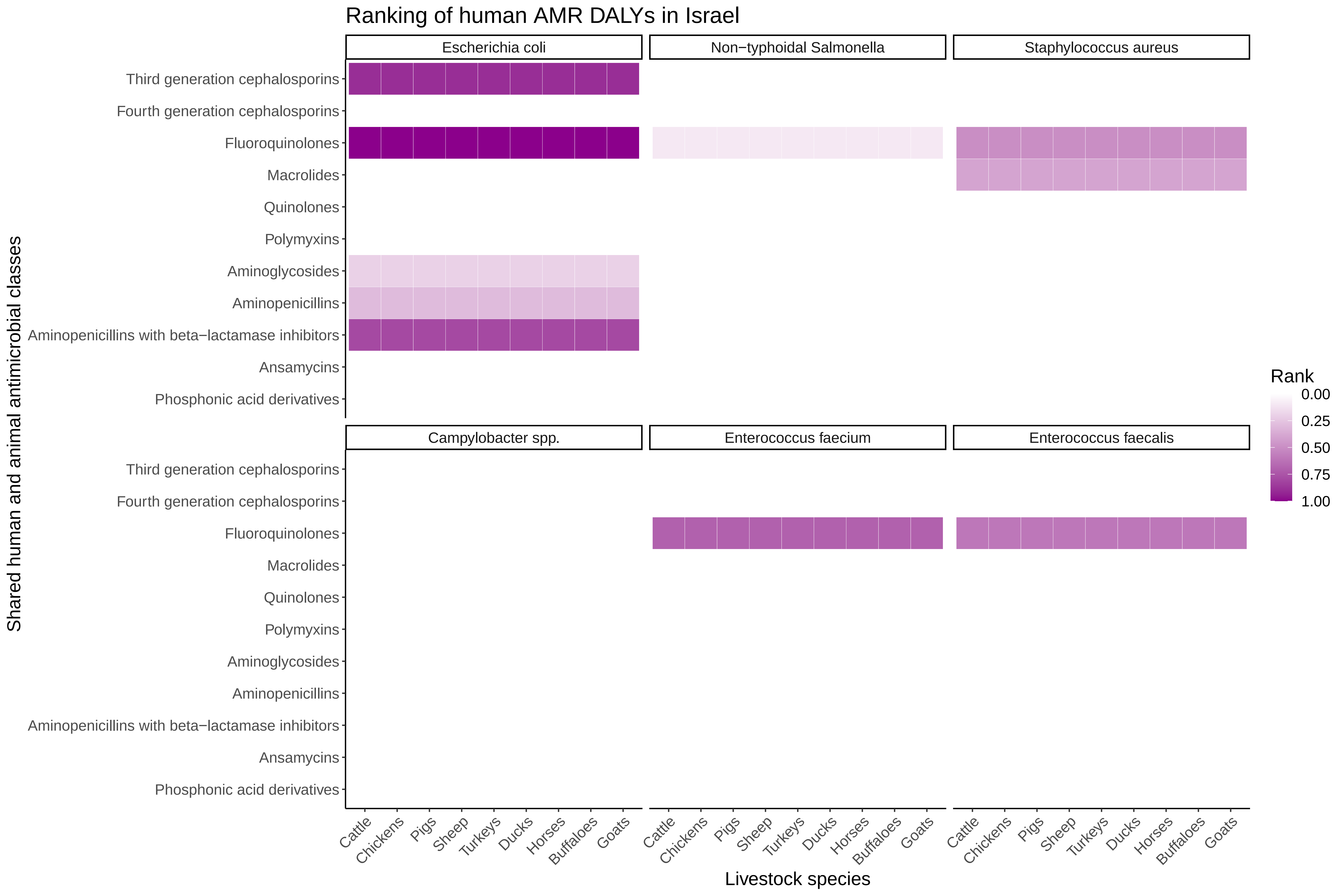

Ranking of human AMR DALYs in Israel
Escherichia coli
Non−typhoidal Salmonella
Staphylococcus aureus
Campylobacter spp.
Enterococcus faecium
Enterococcus faecalis
Third generation cephalosporins
Fourth generation cephalosporins
Fluoroquinolones
Macrolides
Quinolones
Polymyxins
Aminoglycosides
Aminopenicillins
Aminopenicillins with beta−lactamase inhibitors
Ansamycins
Phosphonic acid derivatives
Shared human and animal antimicrobial classes
Cattle
Chickens
Pigs
Sheep
Turkeys
Ducks
Horses
Buffaloes
Goats
Livestock species
Rank
0.00
0.25
0.50
0.75
1.00

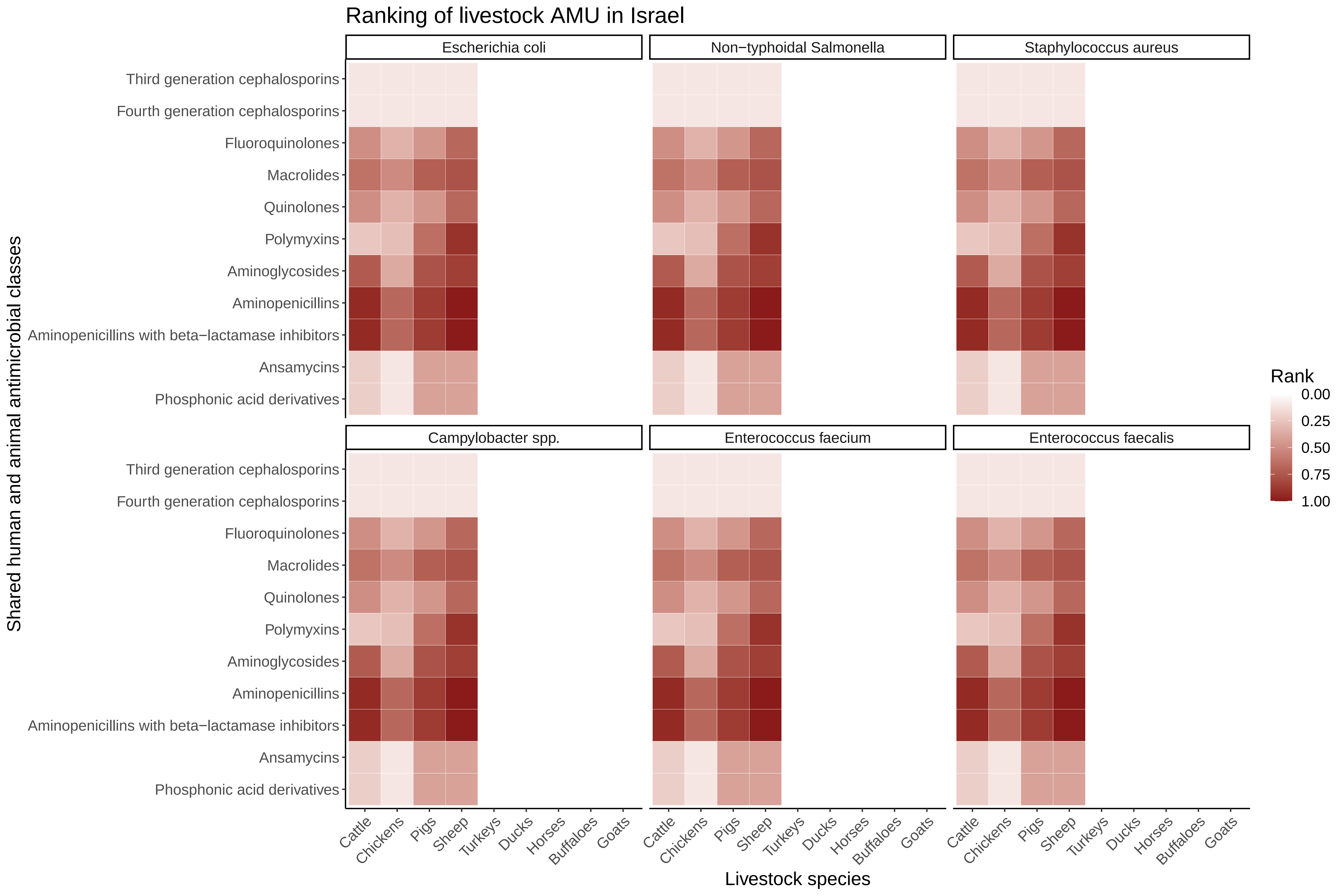

Ranking of livestock AMU in Israel
Escherichia coli
Non−typhoidal Salmonella
Staphylococcus aureus
Campylobacter spp.
Enterococcus faecium
Enterococcus faecalis
Third generation cephalosporins
Fourth generation cephalosporins
Fluoroquinolones
Macrolides
Quinolones
Polymyxins
Aminoglycosides
Aminopenicillins
Aminopenicillins with beta−lactamase inhibitors
Ansamycins
Phosphonic acid derivatives
Shared human and animal antimicrobial classes
Cattle
Chickens
Pigs
Sheep
Turkeys
Ducks
Horses
Buffaloes
Goats
Livestock species
Rank
0.00
0.25
0.50
0.75
1.00

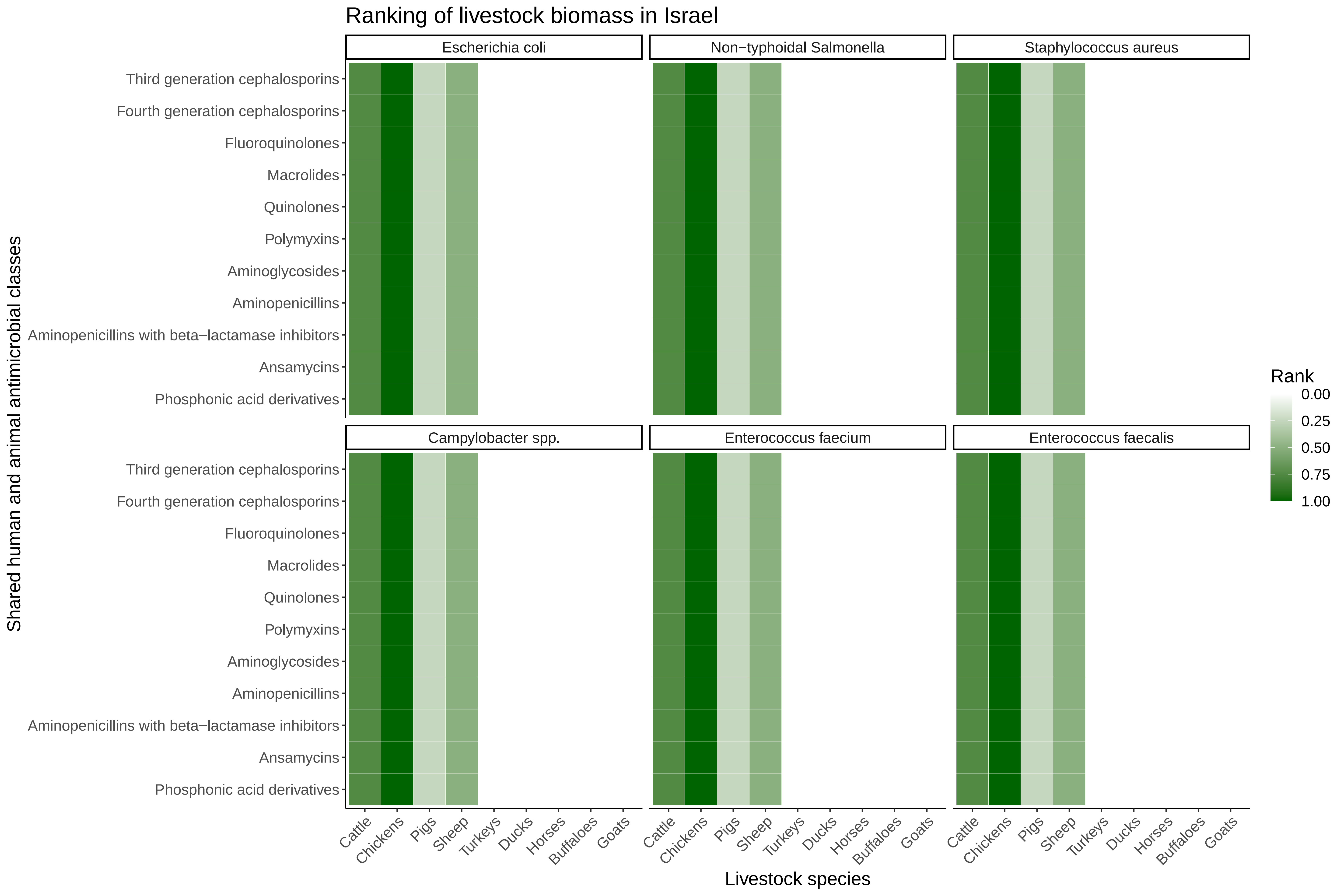
Ranking of livestock biomass in Israel
Escherichia coli
Non−typhoidal Salmonella
Staphylococcus aureus
Campylobacter spp.
Enterococcus faecium
Enterococcus faecalis
Third generation cephalosporins
Fourth generation cephalosporins
Fluoroquinolones
Macrolides
Quinolones
Polymyxins
Aminoglycosides
Aminopenicillins
Aminopenicillins with beta−lactamase inhibitors
Ansamycins
Phosphonic acid derivatives
Shared human and animal antimicrobial classes
Cattle
Chickens
Pigs
Sheep
Turkeys
Ducks
Horses
Buffaloes
Goats
Livestock species
Rank
0.00
0.25
0.50
0.75
1.00

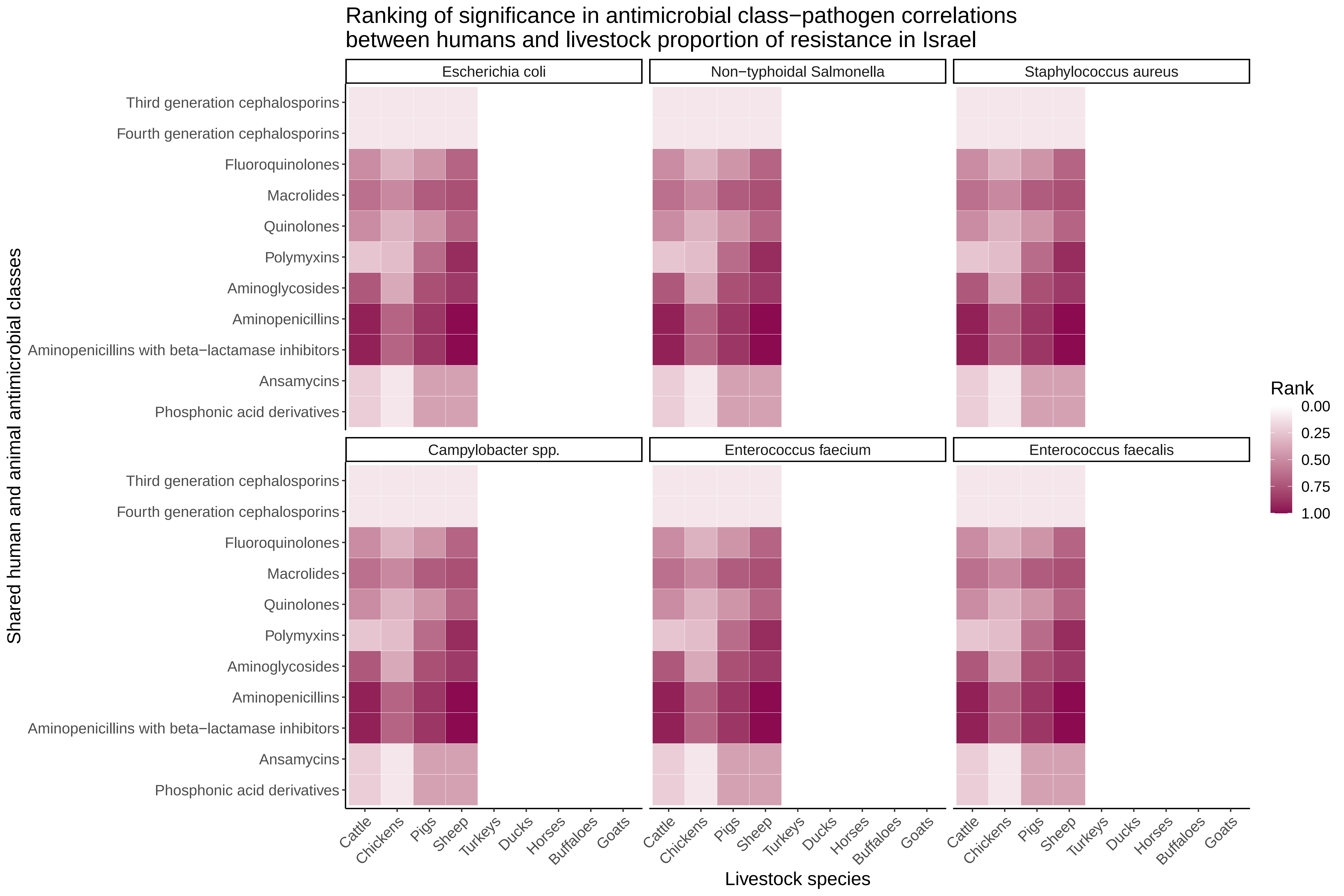

Ranking of significance in antimicrobial class−pathogen correlations
between humans and livestock proportion of resistance in Israel
Escherichia coli
Non−typhoidal Salmonella
Staphylococcus aureus
Campylobacter spp.
Enterococcus faecium
Enterococcus faecalis
Third generation cephalosporins
Fourth generation cephalosporins
Fluoroquinolones
Macrolides
Quinolones
Polymyxins
Aminoglycosides
Aminopenicillins
Aminopenicillins with beta−lactamase inhibitors
Ansamycins
Phosphonic acid derivatives
Shared human and animal antimicrobial classes
Cattle
Chickens
Pigs
Sheep
Turkeys
Ducks
Horses
Buffaloes
Goats
Livestock species
Rank
0.00
0.25
0.50
0.75
1.00

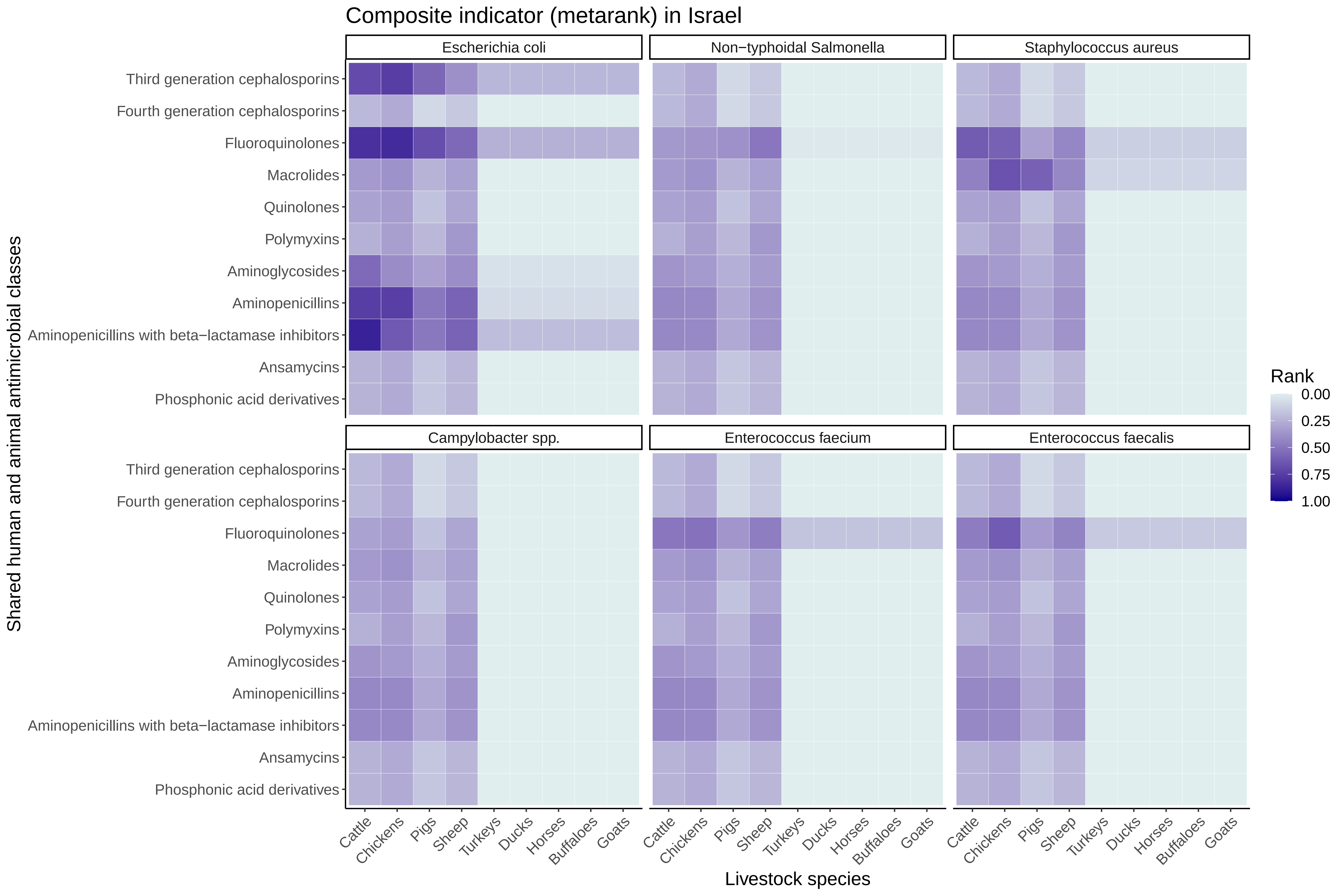
Composite indicator (metarank) in Israel
Escherichia coli
Non−typhoidal Salmonella
Staphylococcus aureus
Campylobacter spp.
Enterococcus faecium
Enterococcus faecalis
Shared human and animal antimicrobial classes
Third generation cephalosporins
Fourth generation cephalosporins
Fluoroquinolones
Macrolides
Quinolones
Polymyxins
Aminoglycosides
Aminopenicillins
Aminopenicillins with beta−lactamase inhibitors
Ansamycins
Phosphonic acid derivatives
Cattle
Chickens
Pigs
Sheep
Turkeys
Ducks
Horses
Buffaloes
Goats
Livestock species
Rank
0.00
0.25
0.50
0.75
1.00

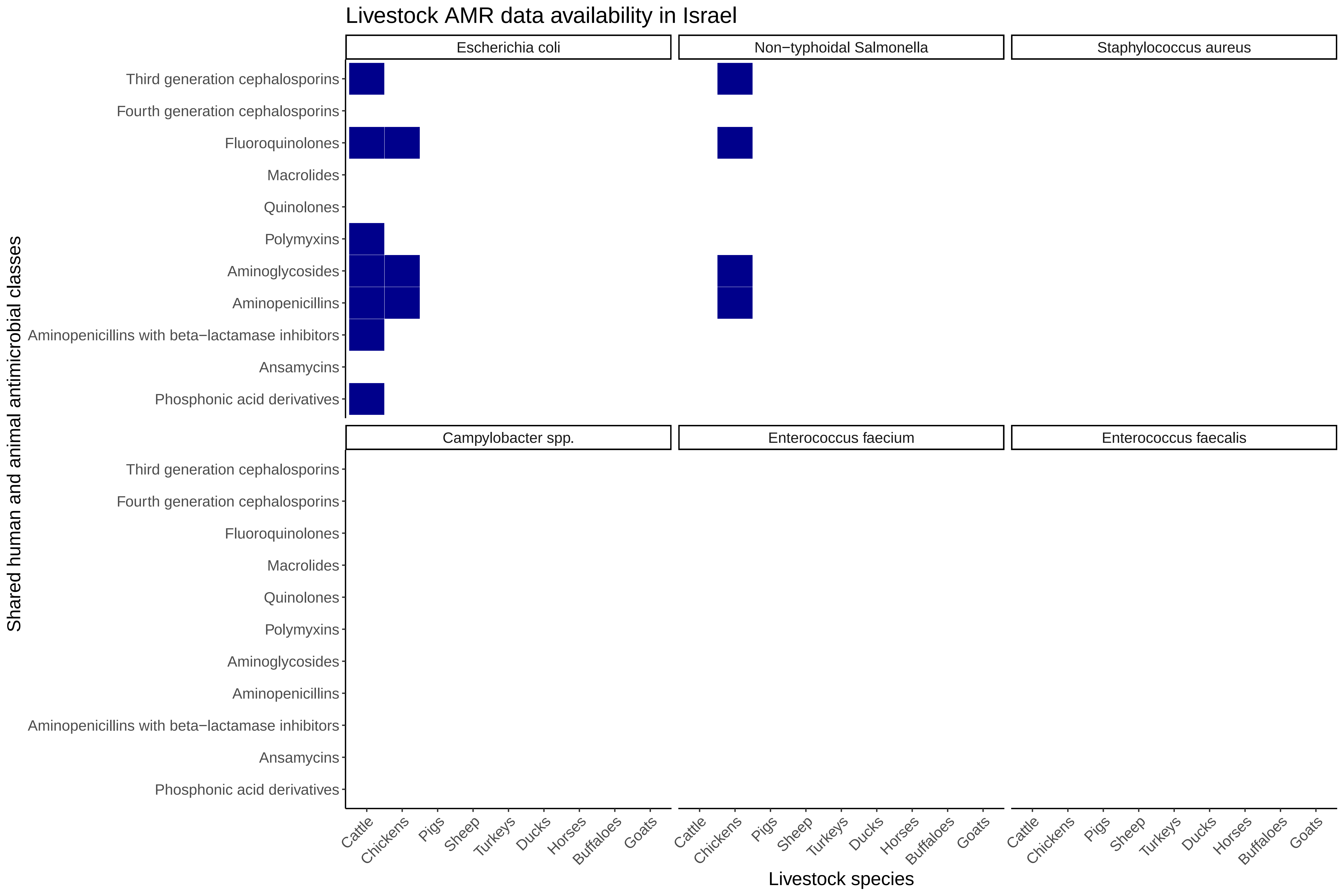

Livestock AMR data availability in Israel
Escherichia coli
Non−typhoidal Salmonella
Staphylococcus aureus
Campylobacter spp.
Enterococcus faecium
Enterococcus faecalis
Shared human and animal antimicrobial classes
Third generation cephalosporins
Fourth generation cephalosporins
Fluoroquinolones
Macrolides
Quinolones
Polymyxins
Aminoglycosides
Aminopenicillins
Aminopenicillins with beta−lactamase inhibitors
Ansamycins
Phosphonic acid derivatives
Cattle
Chickens
Pigs
Sheep
Turkeys
Ducks
Horses
Buffaloes
Goats
Livestock species

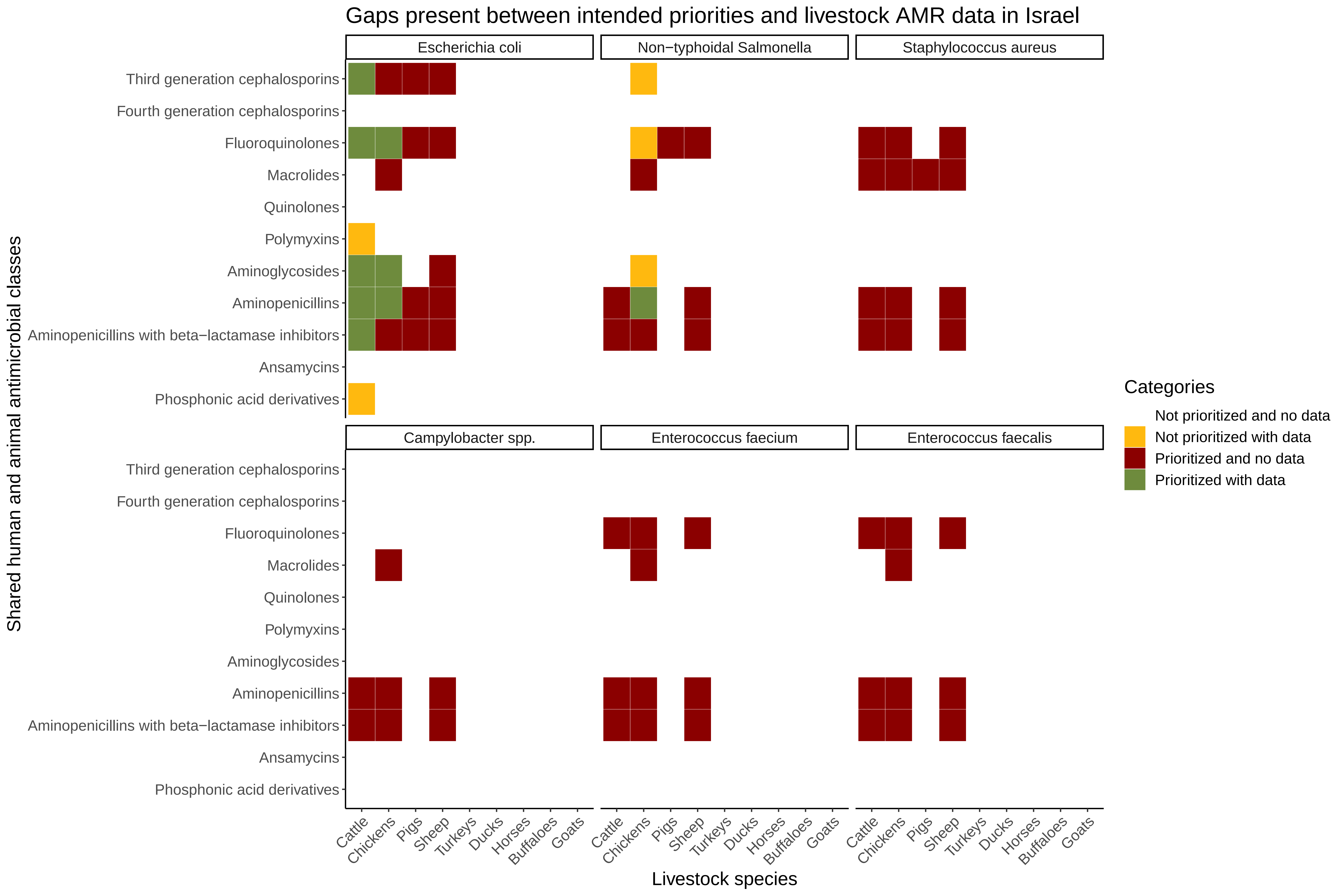

Gaps present between intended priorities and livestock AMR data in Israel
Escherichia coli
Non−typhoidal Salmonella
Staphylococcus aureus
Campylobacter spp.
Enterococcus faecium
Enterococcus faecalis
Shared human and animal antimicrobial classes
Third generation cephalosporins
Fourth generation cephalosporins
Fluoroquinolones
Macrolides
Quinolones
Polymyxins
Aminoglycosides
Aminopenicillins
Aminopenicillins with beta−lactamase inhibitors
Ansamycins
Phosphonic acid derivatives
Livestock species
Cattle
Chickens
Pigs
Sheep
Turkeys
Ducks
Horses
Buffaloes
Goats
Categories
Not prioritized and no data
Not prioritized with data
Prioritized and no data
Prioritized with data

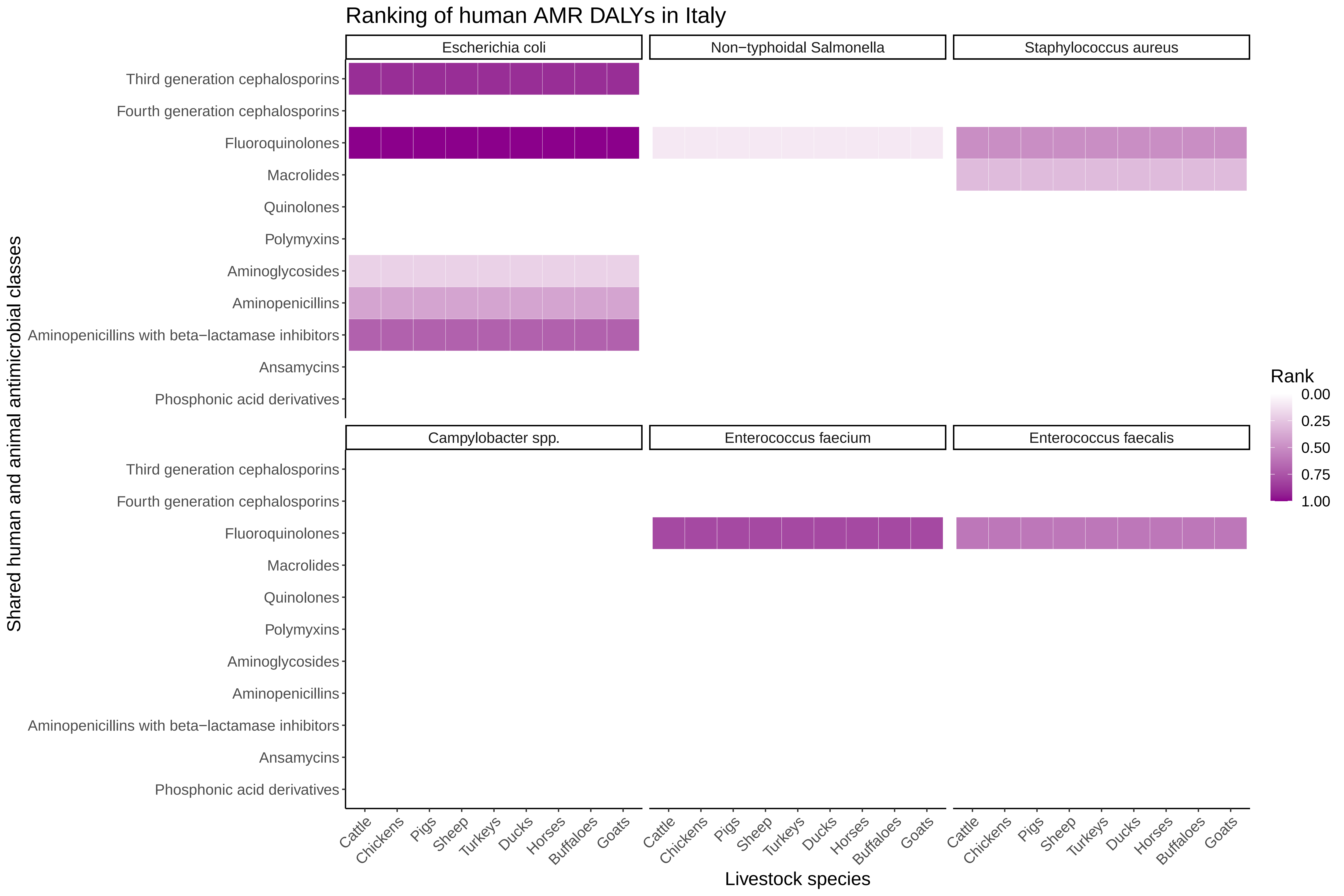
Ranking of human AMR DALYs in Italy
Escherichia coli
Non−typhoidal Salmonella
Staphylococcus aureus
Campylobacter spp.
Enterococcus faecium
Enterococcus faecalis
Shared human and animal antimicrobial classes
Third generation cephalosporins
Fourth generation cephalosporins
Fluoroquinolones
Macrolides
Quinolones
Polymyxins
Aminoglycosides
Aminopenicillins
Aminopenicillins with beta−lactamase inhibitors
Ansamycins
Phosphonic acid derivatives
Cattle
Chickens
Pigs
Sheep
Turkeys
Ducks
Horses
Buffaloes
Goats
Livestock species
Rank
0.00
0.25
0.50
0.75
1.00

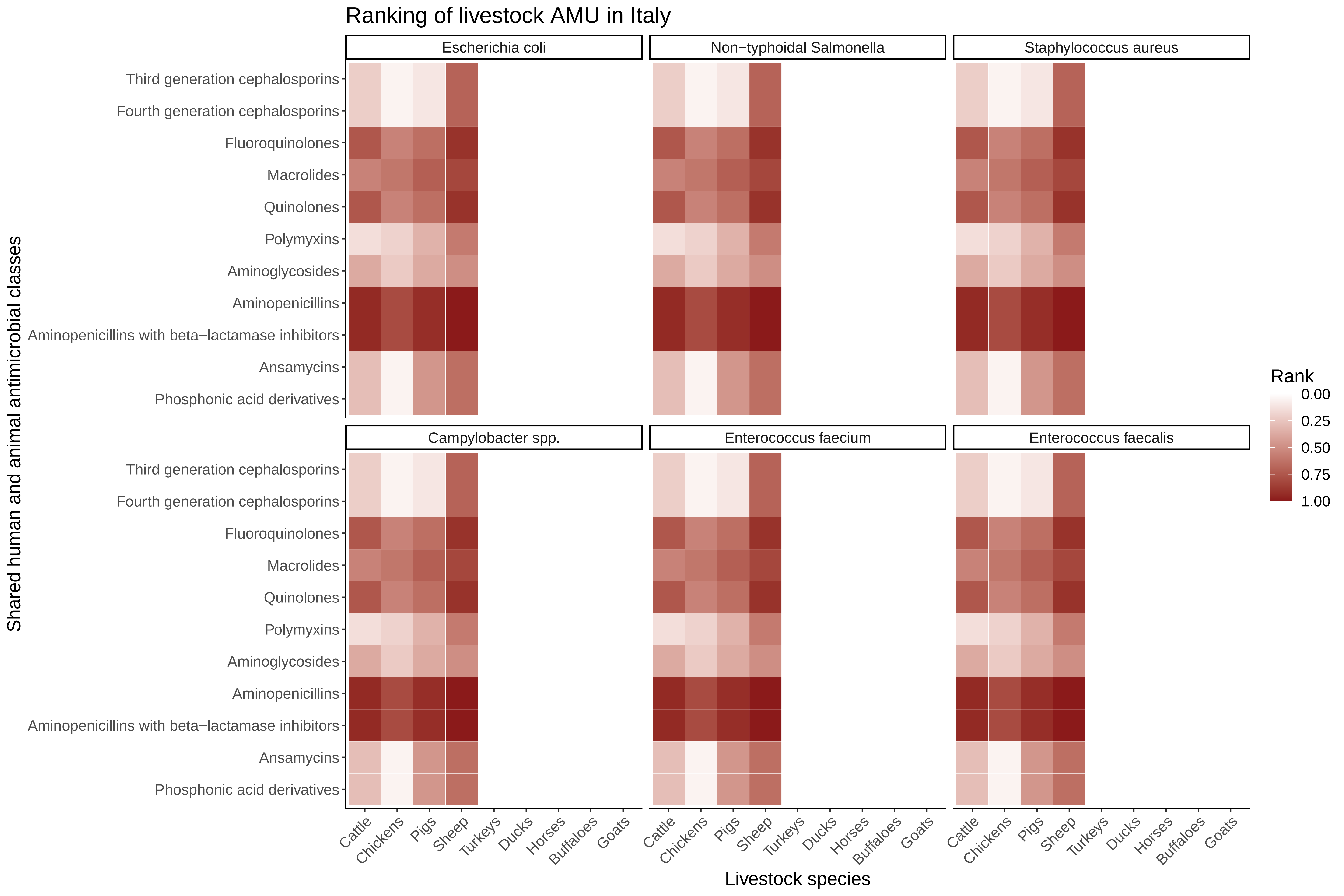
Ranking of livestock AMU in Italy
Escherichia coli
Non−typhoidal Salmonella
Staphylococcus aureus
Campylobacter spp.
Enterococcus faecium
Enterococcus faecalis
Third generation cephalosporins
Fourth generation cephalosporins
Fluoroquinolones
Macrolides
Quinolones
Polymyxins
Aminoglycosides
Aminopenicillins
Aminopenicillins with beta−lactamase inhibitors
Ansamycins
Phosphonic acid derivatives
Shared human and animal antimicrobial classes
Cattle
Chickens
Pigs
Sheep
Turkeys
Ducks
Horses
Buffaloes
Goats
Livestock species
Rank
0.00
0.25
0.50
0.75
1.00

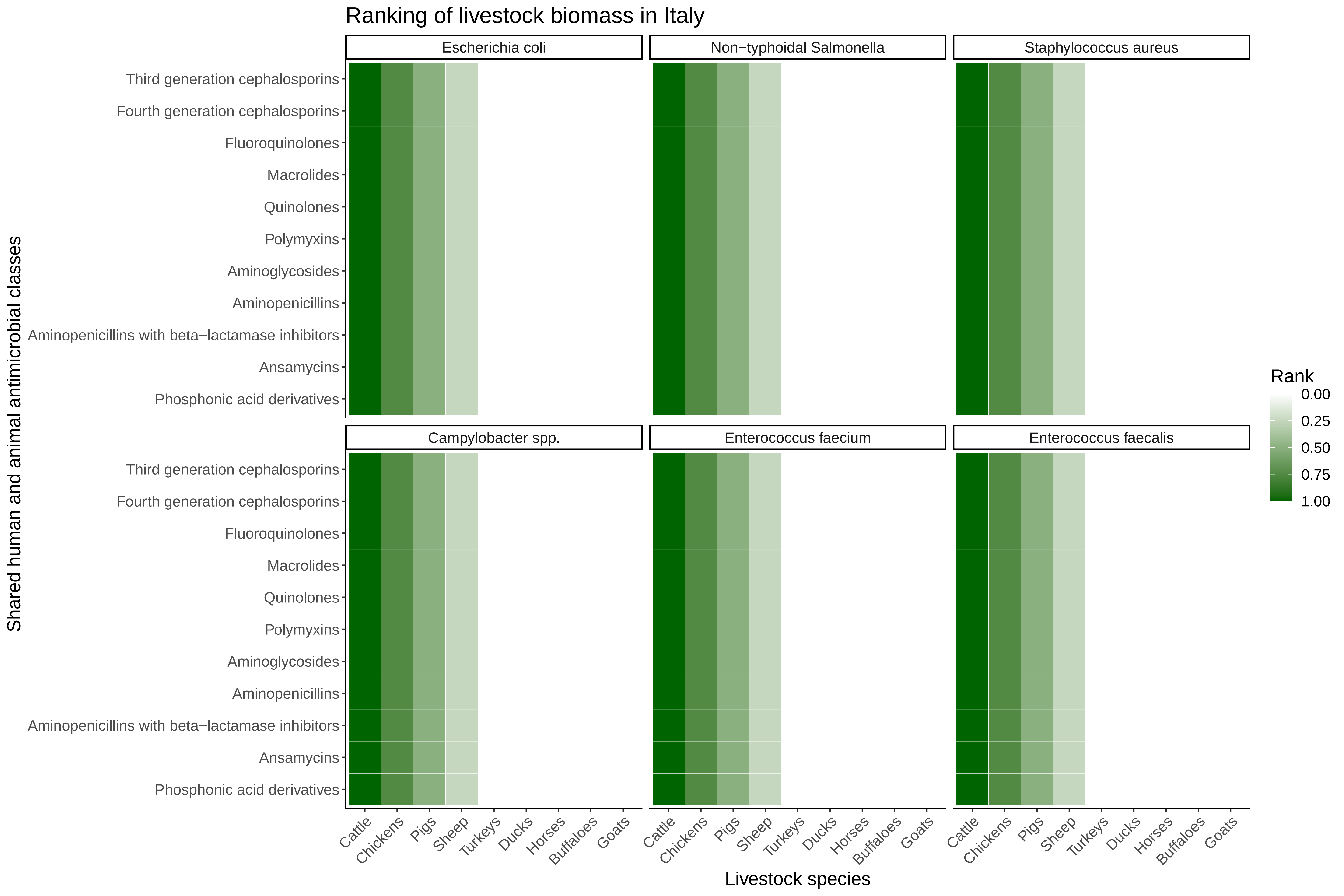
Ranking of livestock biomass in Italy
Escherichia coli
Non−typhoidal Salmonella
Staphylococcus aureus
Campylobacter spp.
Enterococcus faecium
Enterococcus faecalis
Third generation cephalosporins
Fourth generation cephalosporins
Fluoroquinolones
Macrolides
Quinolones
Polymyxins
Aminoglycosides
Aminopenicillins
Aminopenicillins with beta−lactamase inhibitors
Ansamycins
Phosphonic acid derivatives
Shared human and animal antimicrobial classes
Cattle
Chickens
Pigs
Sheep
Turkeys
Ducks
Horses
Buffaloes
Goats
Livestock species
Rank
0.00
0.25
0.50
0.75
1.00

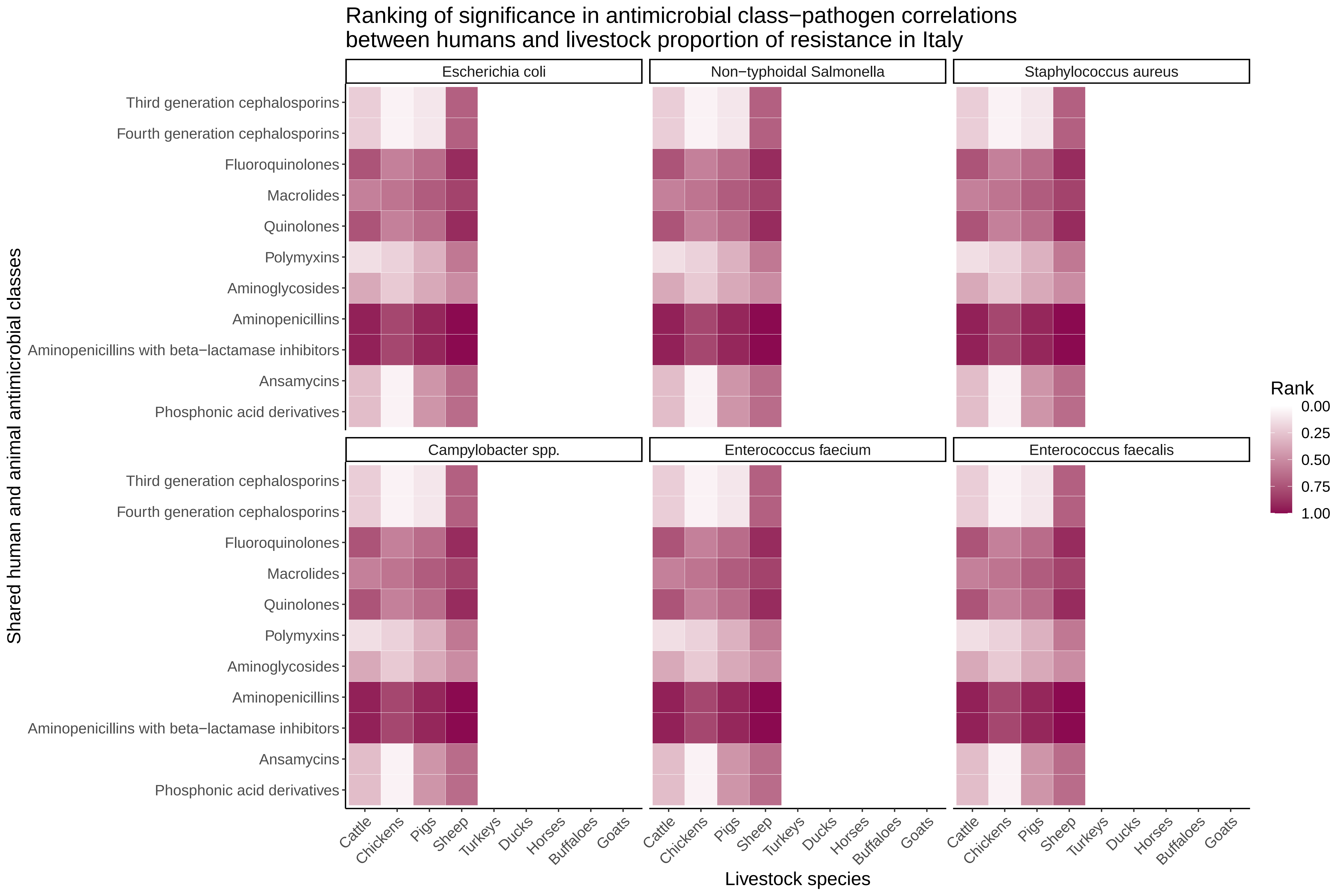
Ranking of significance in antimicrobial class−pathogen correlations
between humans and livestock proportion of resistance in Italy
Escherichia coli
Non−typhoidal Salmonella
Staphylococcus aureus
Campylobacter spp.
Enterococcus faecium
Enterococcus faecalis
Third generation cephalosporins
Fourth generation cephalosporins
Fluoroquinolones
Macrolides
Quinolones
Polymyxins
Aminoglycosides
Aminopenicillins
Aminopenicillins with beta−lactamase inhibitors
Ansamycins
Phosphonic acid derivatives
Shared human and animal antimicrobial classes
Cattle
Chickens
Pigs
Sheep
Turkeys
Ducks
Horses
Buffaloes
Goats
Livestock species
Rank
0.00
0.25
0.50
0.75
1.00

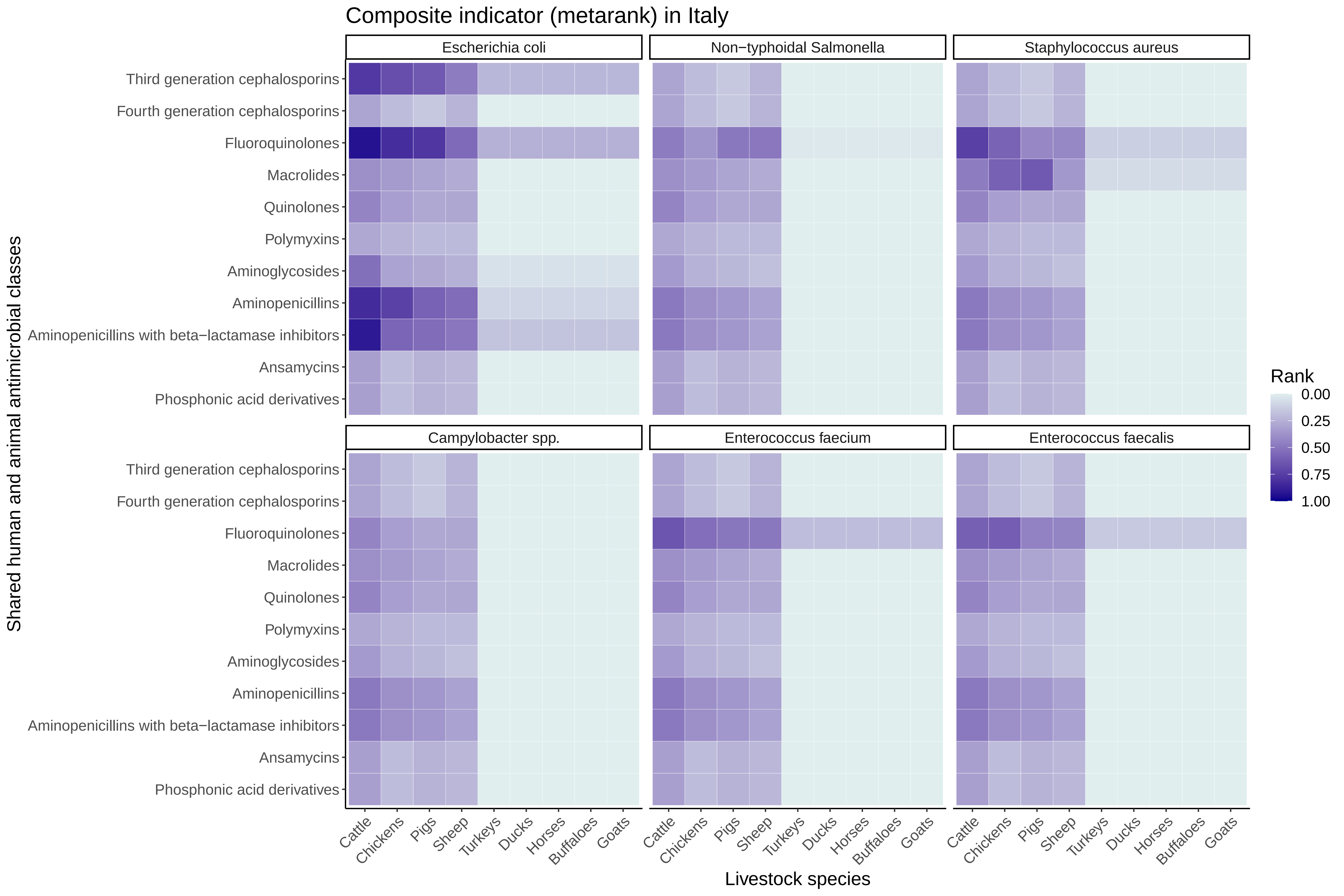
Composite indicator (metarank) in Italy
Escherichia coli
Non−typhoidal Salmonella
Staphylococcus aureus
Campylobacter spp.
Enterococcus faecium
Enterococcus faecalis
Third generation cephalosporins
Fourth generation cephalosporins
Fluoroquinolones
Macrolides
Quinolones
Polymyxins
Aminoglycosides
Aminopenicillins
Aminopenicillins with beta−lactamase inhibitors
Ansamycins
Phosphonic acid derivatives
Shared human and animal antimicrobial classes
Cattle
Chickens
Pigs
Sheep
Turkeys
Ducks
Horses
Buffaloes
Goats
Livestock species
Rank
0.00
0.25
0.50
0.75
1.00

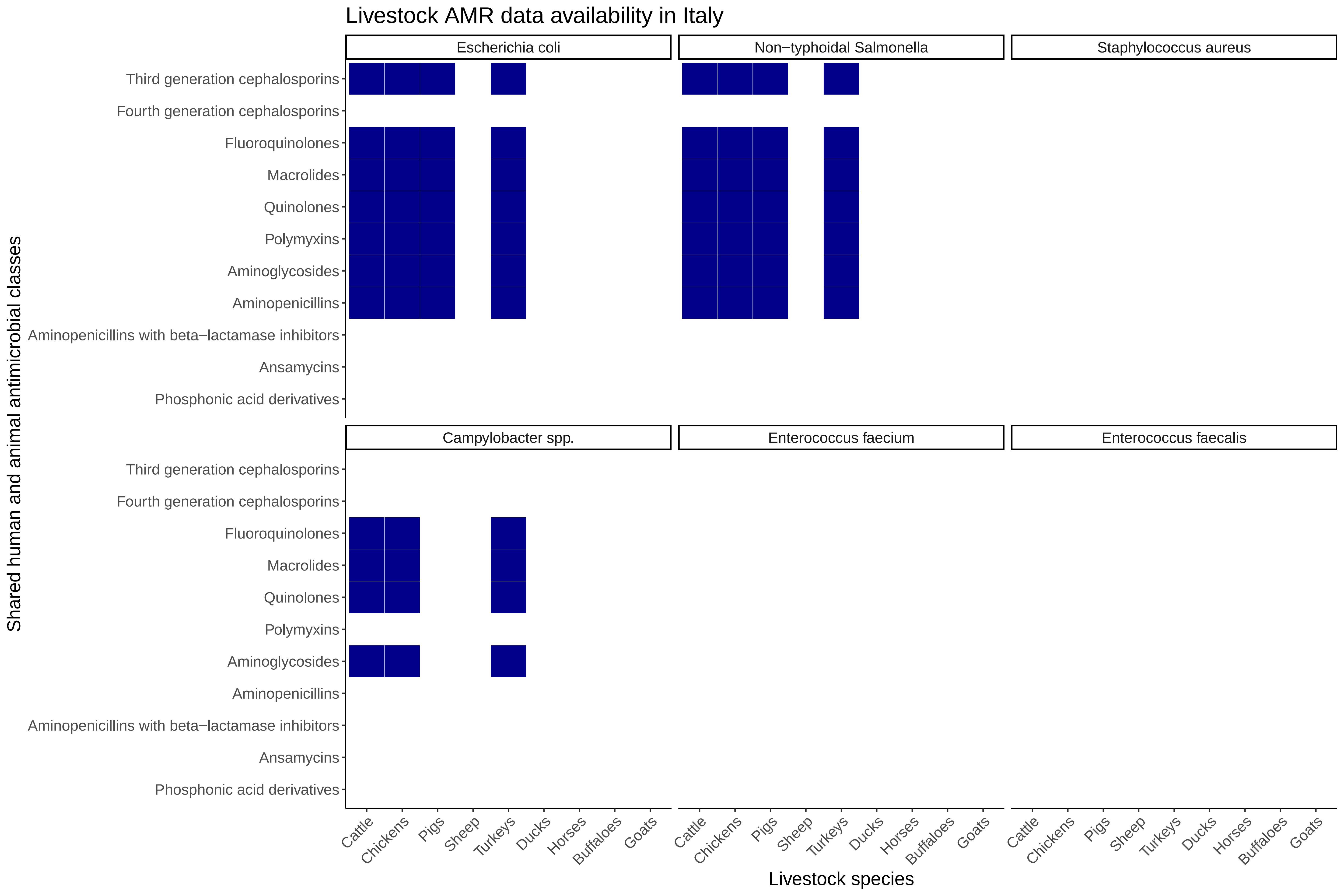
Livestock AMR data availability in Italy
Escherichia coli
Non−typhoidal Salmonella
Staphylococcus aureus
Campylobacter spp.
Enterococcus faecium
Enterococcus faecalis
Shared human and animal antimicrobial classes
Third generation cephalosporins
Fourth generation cephalosporins
Fluoroquinolones
Macrolides
Quinolones
Polymyxins
Aminoglycosides
Aminopenicillins
Aminopenicillins with beta−lactamase inhibitors
Ansamycins
Phosphonic acid derivatives
Cattle
Chickens
Pigs
Sheep
Turkeys
Ducks
Horses
Buffaloes
Goats
Livestock species

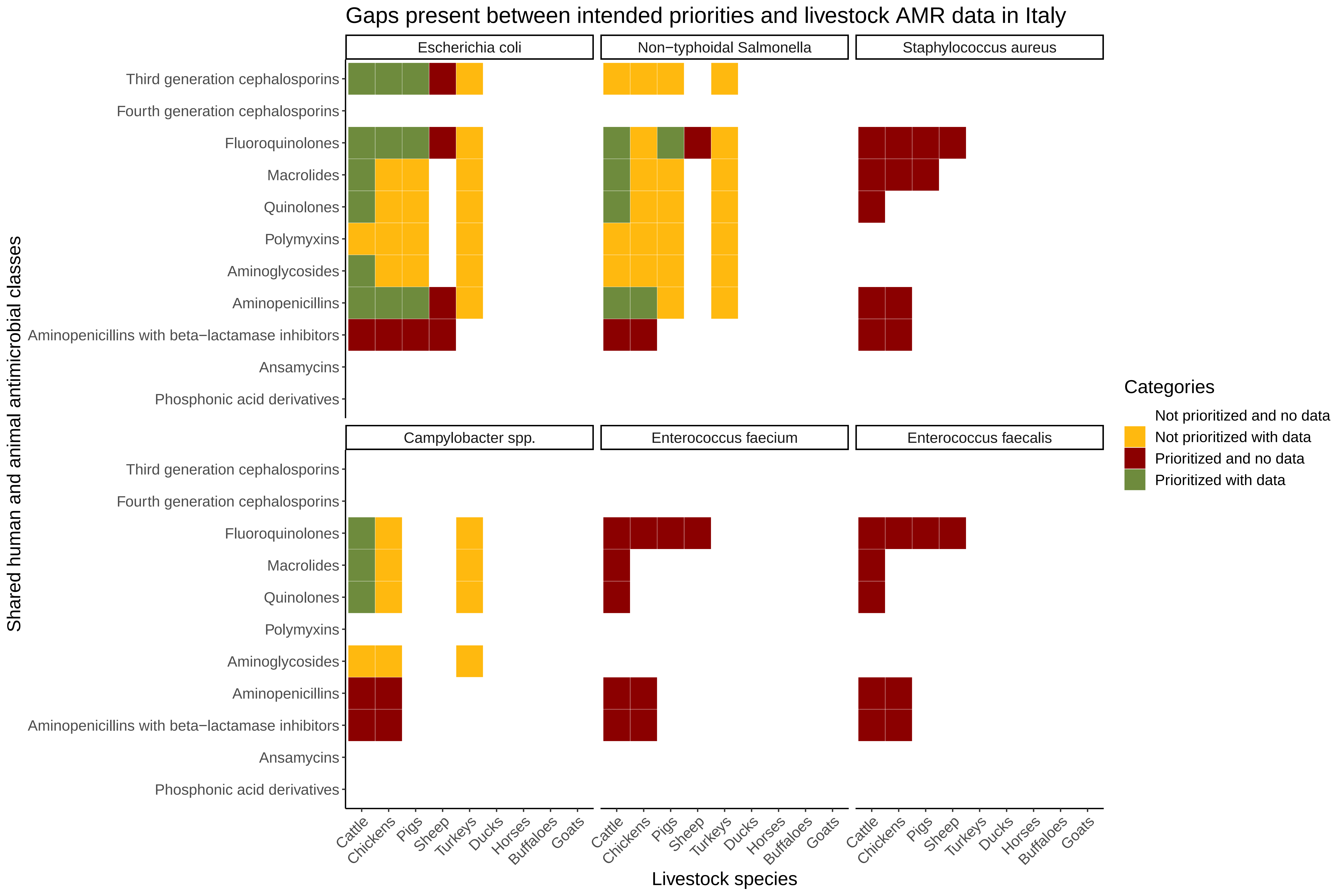

Gaps present between intended priorities and livestock AMR data in Italy
Escherichia coli
Non−typhoidal Salmonella
Staphylococcus aureus
Campylobacter spp.
Enterococcus faecium
Enterococcus faecalis
Shared human and animal antimicrobial classes
Third generation cephalosporins
Fourth generation cephalosporins
Fluoroquinolones
Macrolides
Quinolones
Polymyxins
Aminoglycosides
Aminopenicillins
Aminopenicillins with beta−lactamase inhibitors
Ansamycins
Phosphonic acid derivatives
Livestock species
Cattle
Chickens
Pigs
Sheep
Turkeys
Ducks
Horses
Buffaloes
Goats
Categories
Not prioritized and no data
Not prioritized with data
Prioritized and no data
Prioritized with data

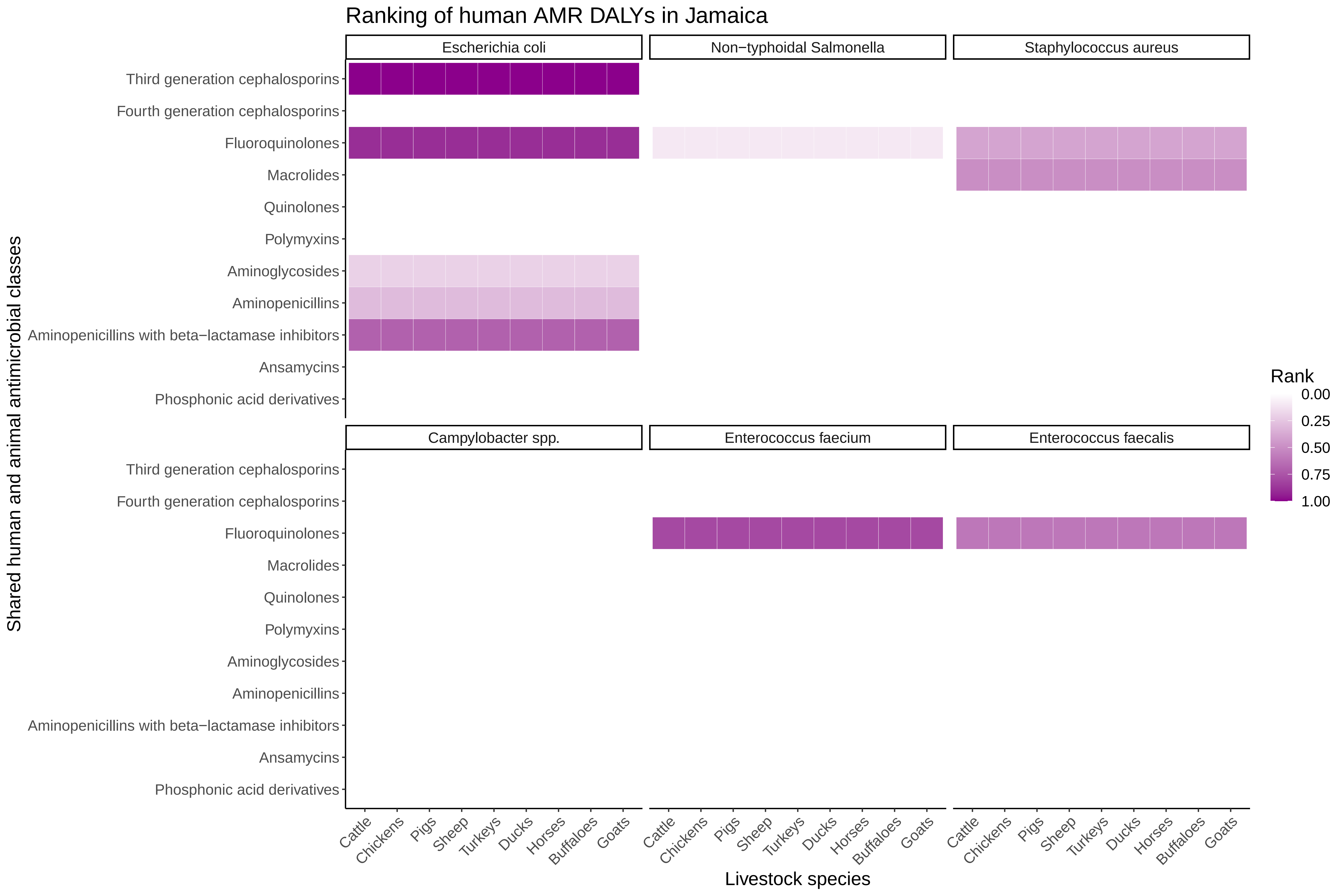

Ranking of human AMR DALYs in Jamaica
Escherichia coli
Non−typhoidal Salmonella
Staphylococcus aureus
Campylobacter spp.
Enterococcus faecium
Enterococcus faecalis
Third generation cephalosporins
Fourth generation cephalosporins
Fluoroquinolones
Macrolides
Quinolones
Polymyxins
Aminoglycosides
Aminopenicillins
Aminopenicillins with beta−lactamase inhibitors
Ansamycins
Phosphonic acid derivatives
Shared human and animal antimicrobial classes
Cattle
Chickens
Pigs
Sheep
Turkeys
Ducks
Horses
Buffaloes
Goats
Livestock species
Rank
0.00
0.25
0.50
0.75
1.00

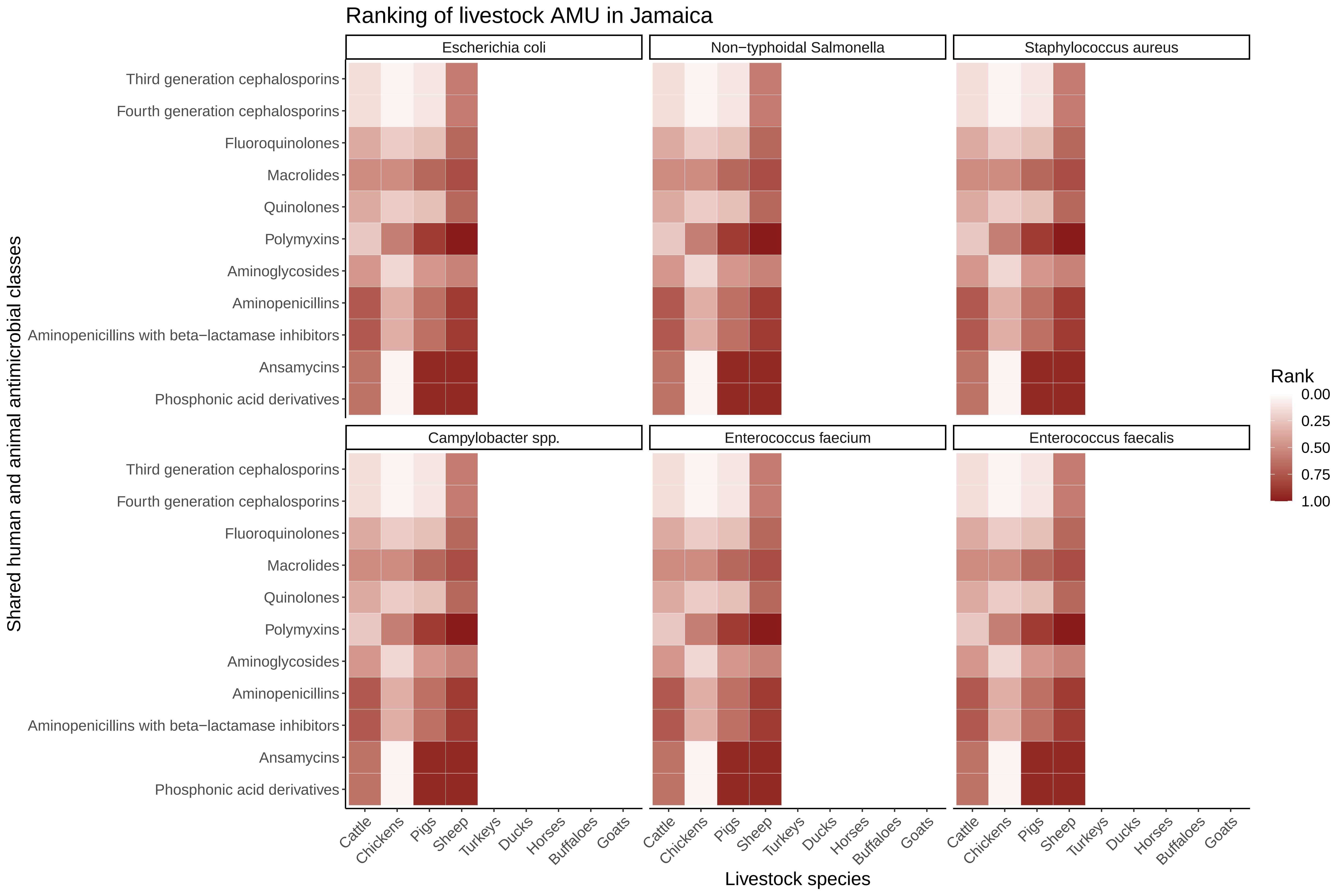

Ranking of livestock AMU in Jamaica
Escherichia coli
Non−typhoidal Salmonella
Staphylococcus aureus
Campylobacter spp.
Enterococcus faecium
Enterococcus faecalis
Third generation cephalosporins
Fourth generation cephalosporins
Fluoroquinolones
Macrolides
Quinolones
Polymyxins
Aminoglycosides
Aminopenicillins
Aminopenicillins with beta−lactamase inhibitors
Ansamycins
Phosphonic acid derivatives
Shared human and animal antimicrobial classes
Cattle
Chickens
Pigs
Sheep
Turkeys
Ducks
Horses
Buffaloes
Goats
Livestock species
Rank
0.00
0.25
0.50
0.75
1.00

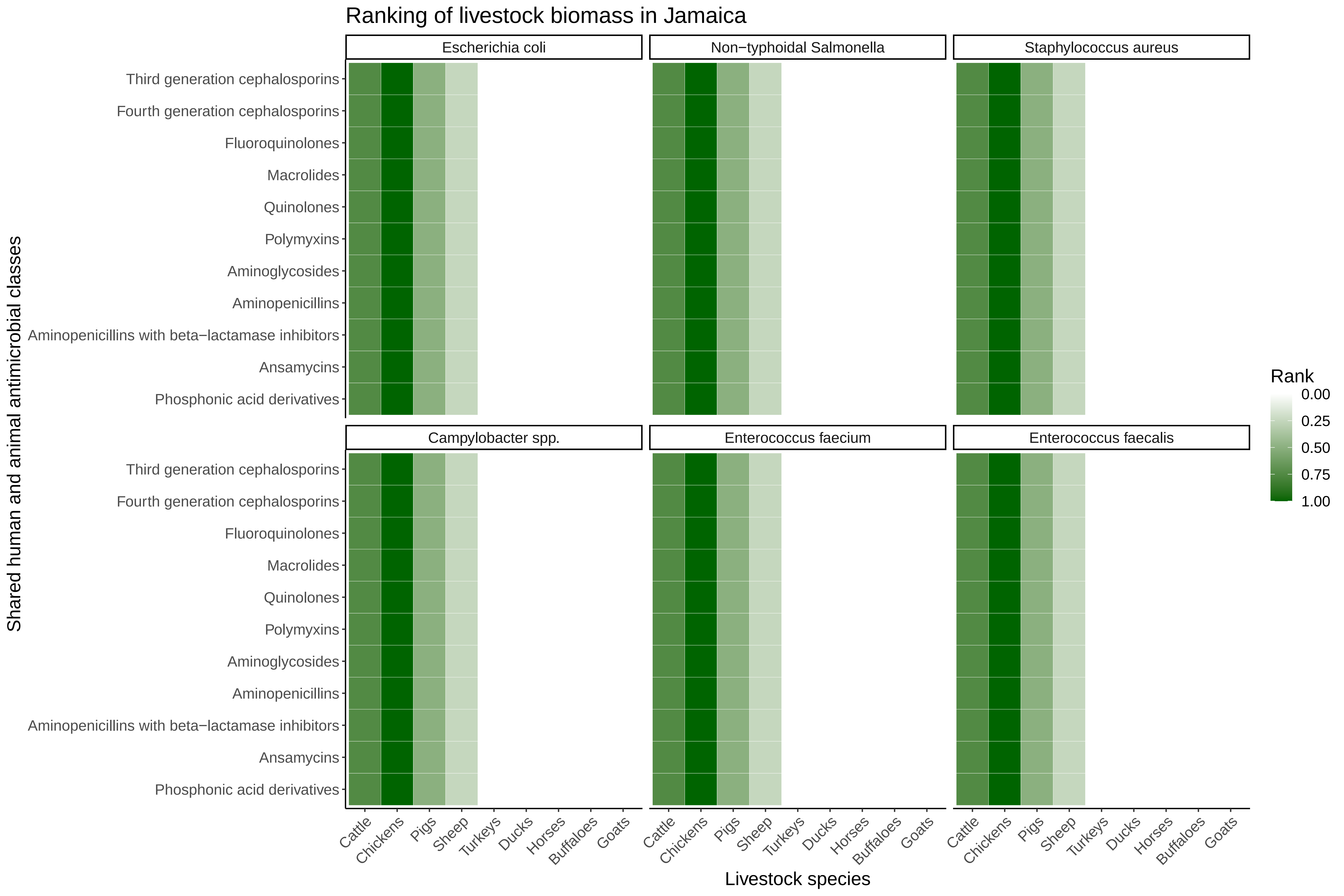
Ranking of livestock biomass in Jamaica
Escherichia coli
Non−typhoidal Salmonella
Staphylococcus aureus
Campylobacter spp.
Enterococcus faecium
Enterococcus faecalis
Shared human and animal antimicrobial classes
Third generation cephalosporins
Fourth generation cephalosporins
Fluoroquinolones
Macrolides
Quinolones
Polymyxins
Aminoglycosides
Aminopenicillins
Aminopenicillins with beta−lactamase inhibitors
Ansamycins
Phosphonic acid derivatives
Cattle
Chickens
Pigs
Sheep
Turkeys
Ducks
Horses
Buffaloes
Goats
Livestock species
Rank
0.00
0.25
0.50
0.75
1.00

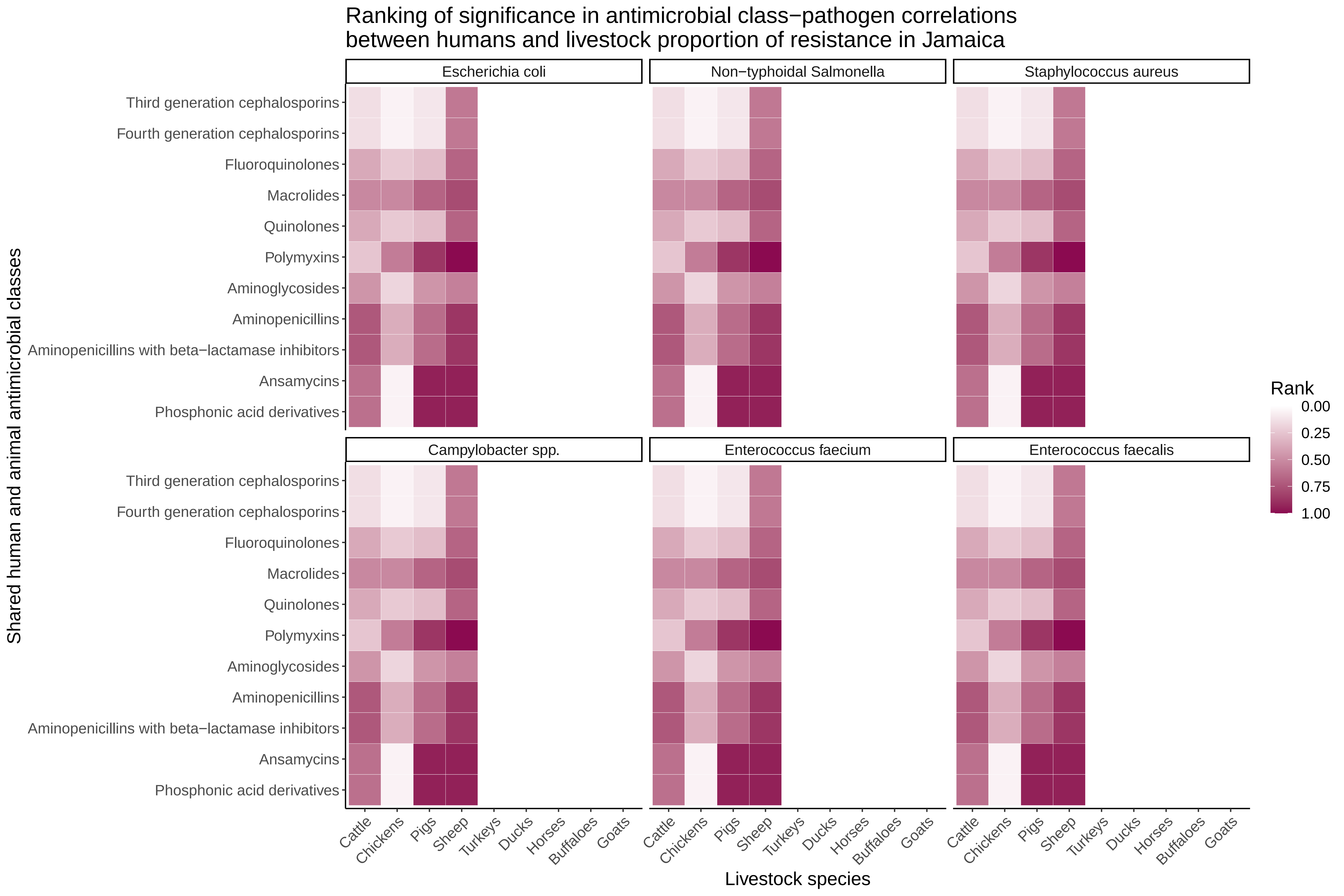

Ranking of significance in antimicrobial class−pathogen correlations
between humans and livestock proportion of resistance in Jamaica
Escherichia coli
Non−typhoidal Salmonella
Staphylococcus aureus
Campylobacter spp.
Enterococcus faecium
Enterococcus faecalis
Third generation cephalosporins
Fourth generation cephalosporins
Fluoroquinolones
Macrolides
Quinolones
Polymyxins
Aminoglycosides
Aminopenicillins
Aminopenicillins with beta−lactamase inhibitors
Ansamycins
Phosphonic acid derivatives
Shared human and animal antimicrobial classes
Cattle
Chickens
Pigs
Sheep
Turkeys
Ducks
Horses
Buffaloes
Goats
Livestock species
Rank
0.00
0.25
0.50
0.75
1.00

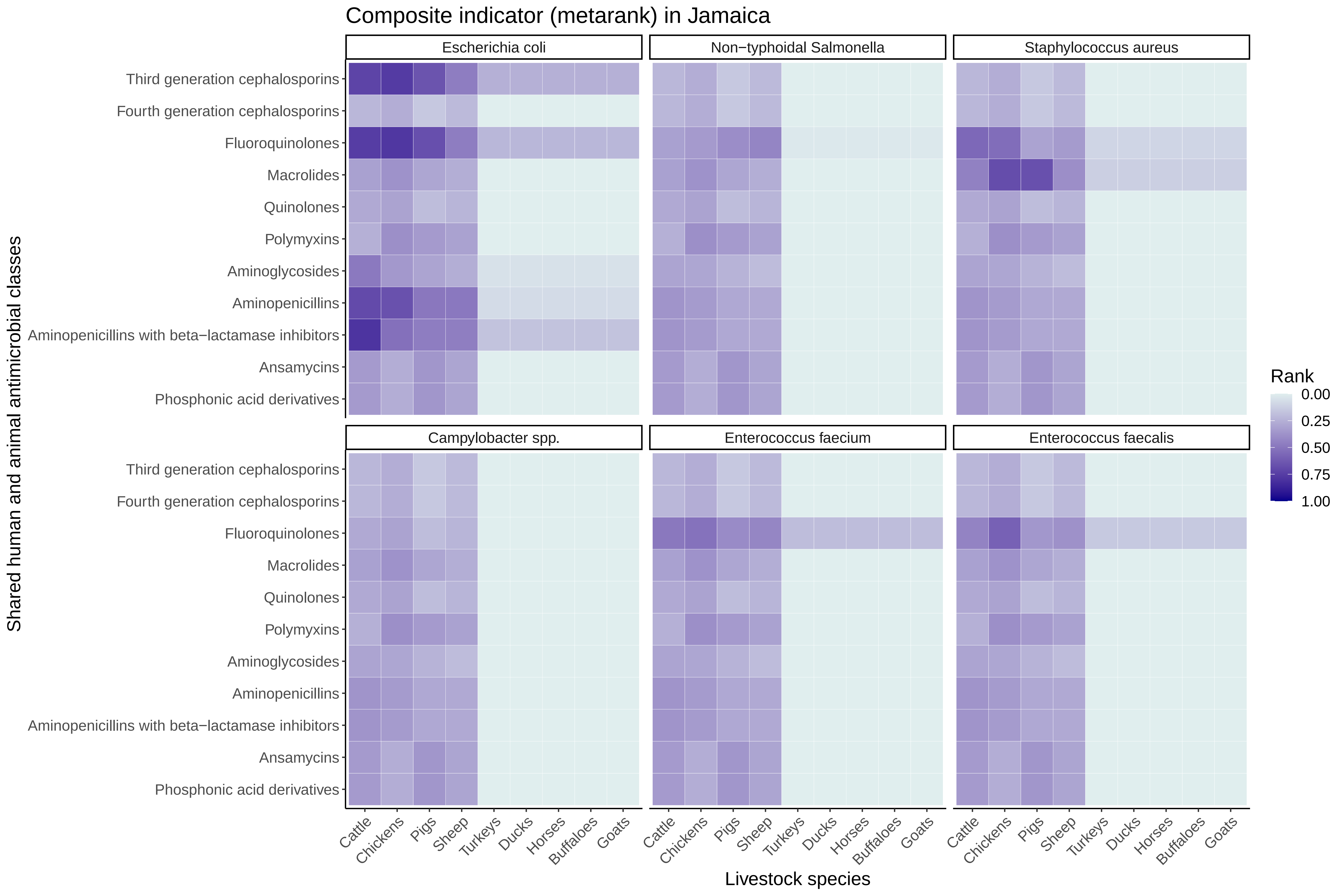
Composite indicator (metarank) in Jamaica
Escherichia coli
Non−typhoidal Salmonella
Staphylococcus aureus
Campylobacter spp.
Enterococcus faecium
Enterococcus faecalis
Shared human and animal antimicrobial classes
Third generation cephalosporins
Fourth generation cephalosporins
Fluoroquinolones
Macrolides
Quinolones
Polymyxins
Aminoglycosides
Aminopenicillins
Aminopenicillins with beta−lactamase inhibitors
Ansamycins
Phosphonic acid derivatives
Livestock species
Cattle
Chickens
Pigs
Sheep
Turkeys
Ducks
Horses
Buffaloes
Goats
Rank
0.00
0.25
0.50
0.75
1.00

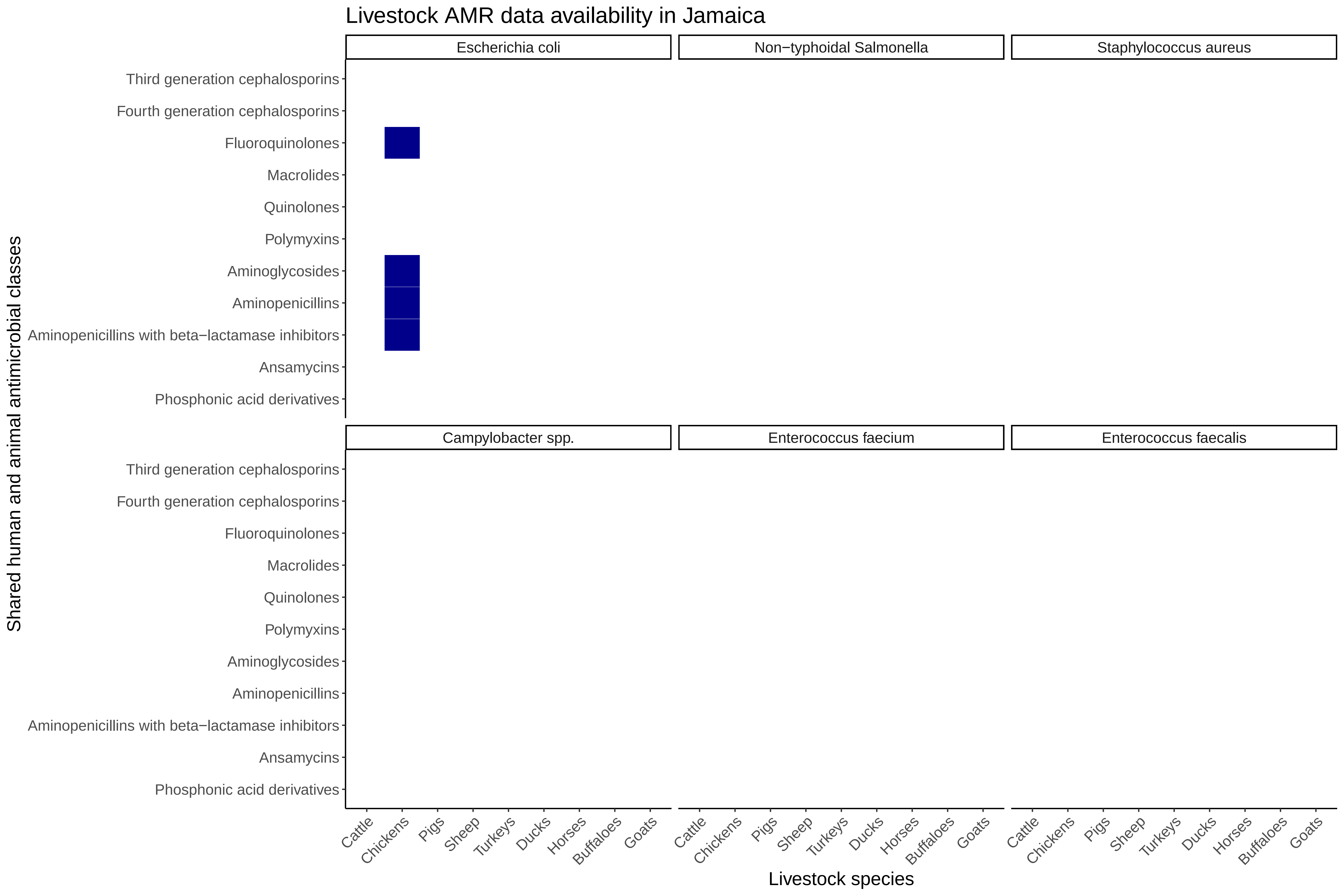
Livestock AMR data availability in Jamaica
Escherichia coli
Non−typhoidal Salmonella
Staphylococcus aureus
Campylobacter spp.
Enterococcus faecium
Enterococcus faecalis
Shared human and animal antimicrobial classes
Third generation cephalosporins
Fourth generation cephalosporins
Fluoroquinolones
Macrolides
Quinolones
Polymyxins
Aminoglycosides
Aminopenicillins
Aminopenicillins with beta−lactamase inhibitors
Ansamycins
Phosphonic acid derivatives
Cattle
Chickens
Pigs
Sheep
Turkeys
Ducks
Horses
Buffaloes
Goats
Livestock species

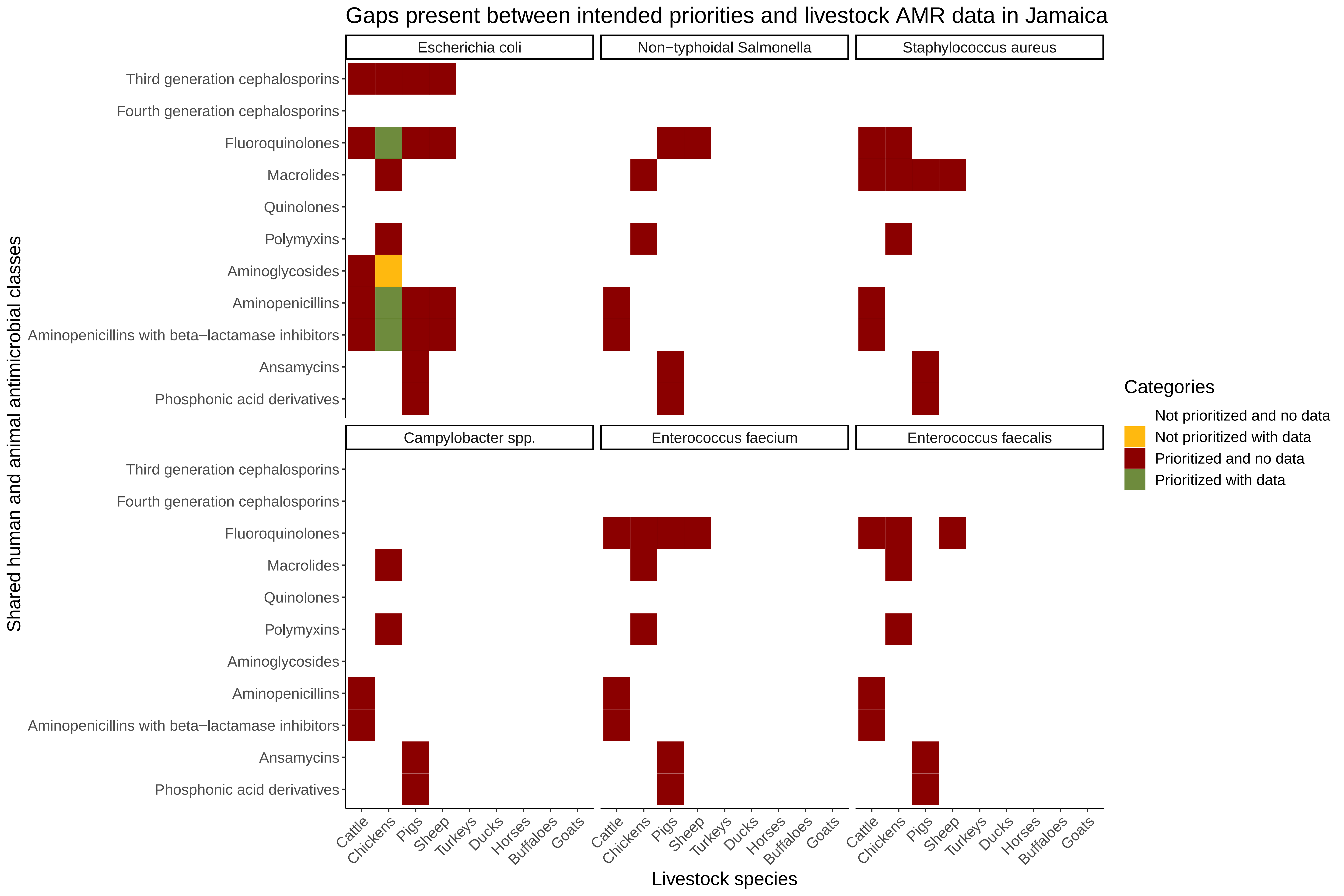

Gaps present between intended priorities and livestock AMR data in Jamaica
Escherichia coli
Non−typhoidal Salmonella
Staphylococcus aureus
Campylobacter spp.
Enterococcus faecium
Enterococcus faecalis
Third generation cephalosporins
Fourth generation cephalosporins
Fluoroquinolones
Macrolides
Quinolones
Polymyxins
Aminoglycosides
Aminopenicillins
Aminopenicillins with beta−lactamase inhibitors
Ansamycins
Phosphonic acid derivatives
Shared human and animal antimicrobial classes
Cattle
Chickens
Pigs
Sheep
Turkeys
Ducks
Horses
Buffaloes
Goats
Livestock species
Categories
Not prioritized and no data
Not prioritized with data
Prioritized and no data
Prioritized with data

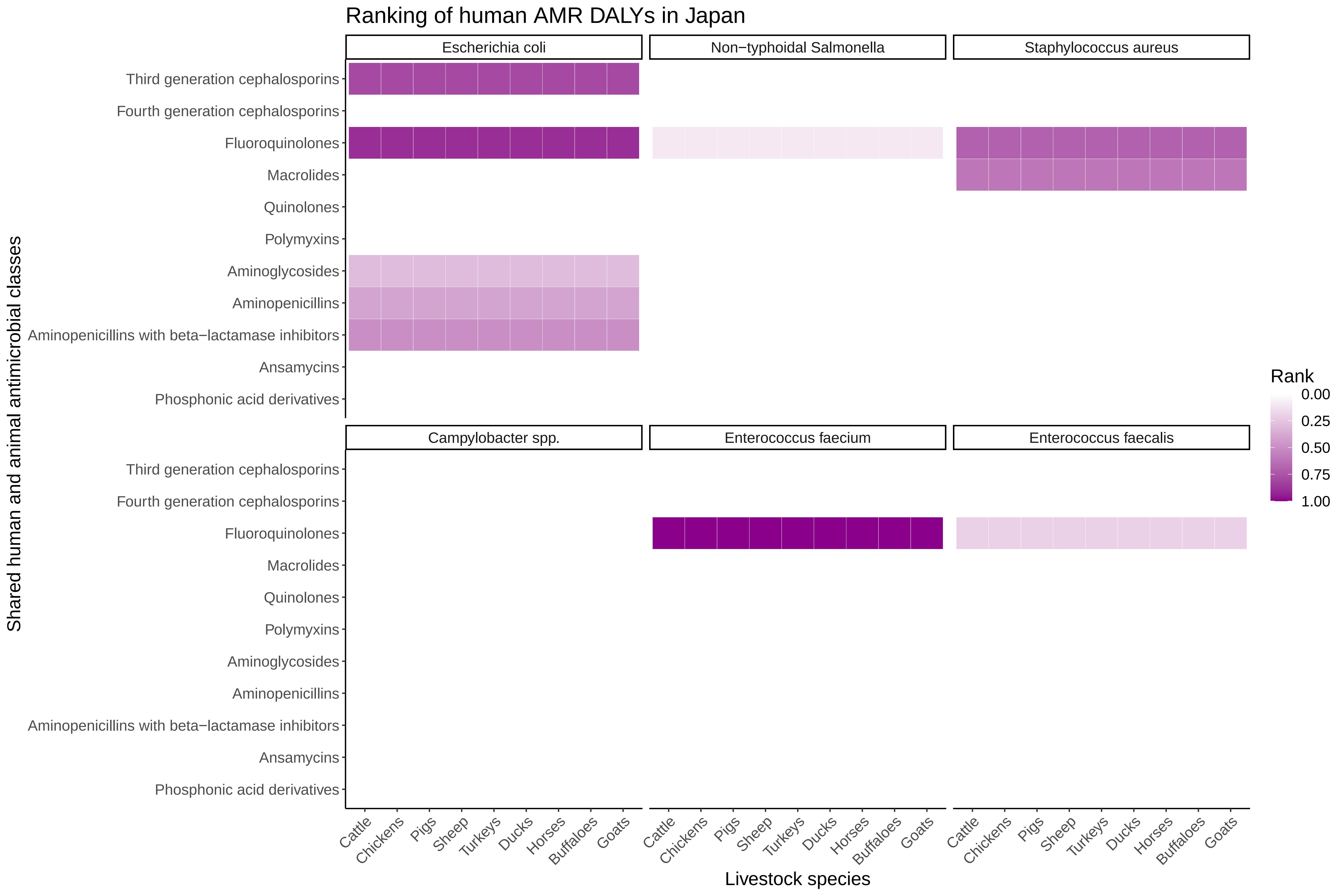

Ranking of human AMR DALYs in Japan
Escherichia coli
Non−typhoidal Salmonella
Staphylococcus aureus
Campylobacter spp.
Enterococcus faecium
Enterococcus faecalis
Third generation cephalosporins
Fourth generation cephalosporins
Fluoroquinolones
Macrolides
Quinolones
Polymyxins
Aminoglycosides
Aminopenicillins
Aminopenicillins with beta−lactamase inhibitors
Ansamycins
Phosphonic acid derivatives
Shared human and animal antimicrobial classes
Cattle
Chickens
Pigs
Sheep
Turkeys
Ducks
Horses
Buffaloes
Goats
Livestock species
Rank
0.00
0.25
0.50
0.75
1.00

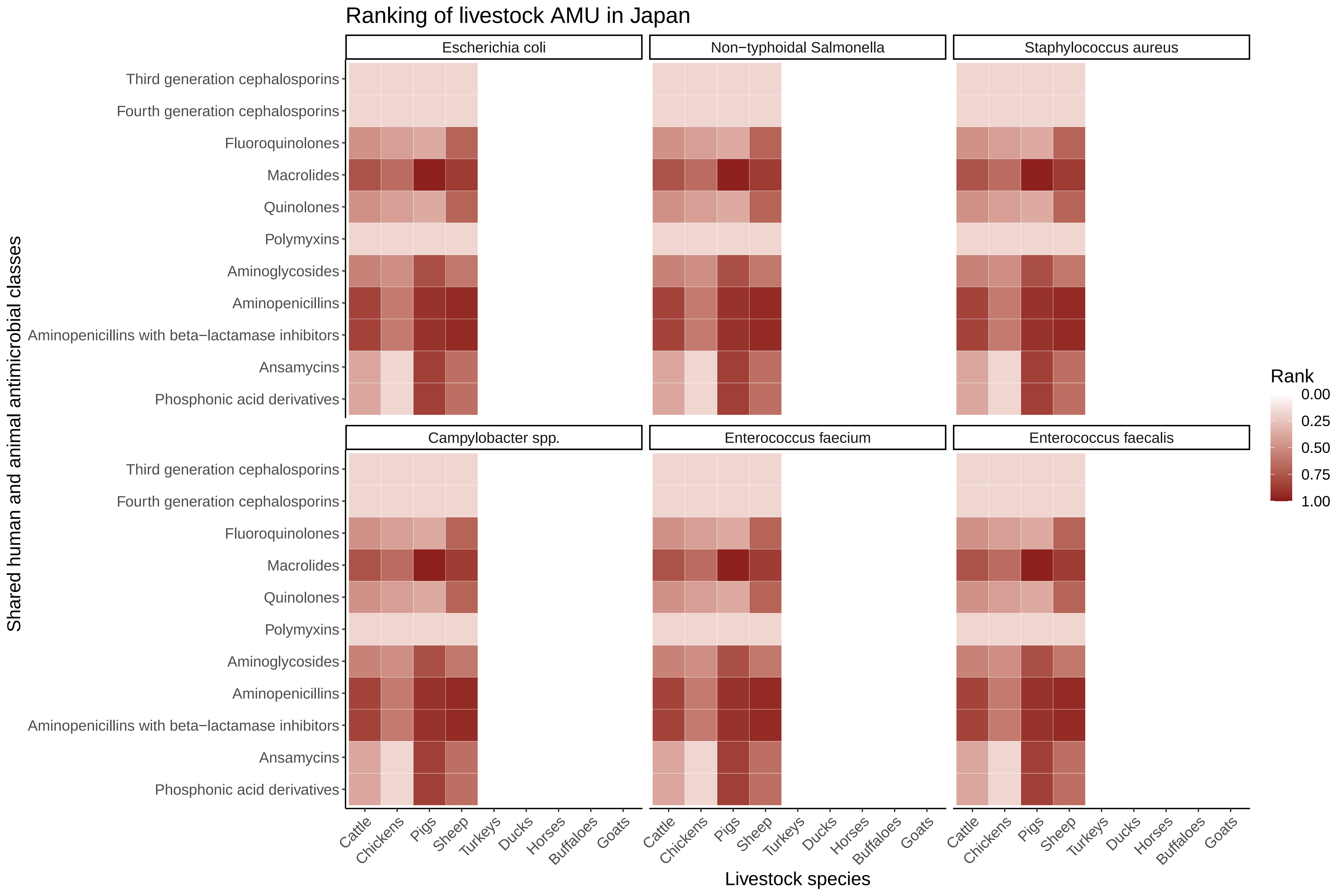
Ranking of livestock AMU in Japan
Escherichia coli
Non−typhoidal Salmonella
Staphylococcus aureus
Campylobacter spp.
Enterococcus faecium
Enterococcus faecalis
Third generation cephalosporins
Fourth generation cephalosporins
Fluoroquinolones
Macrolides
Quinolones
Polymyxins
Aminoglycosides
Aminopenicillins
Aminopenicillins with beta−lactamase inhibitors
Ansamycins
Phosphonic acid derivatives
Shared human and animal antimicrobial classes
Cattle
Chickens
Pigs
Sheep
Turkeys
Ducks
Horses
Buffaloes
Goats
Livestock species
Rank
0.00
0.25
0.50
0.75
1.00

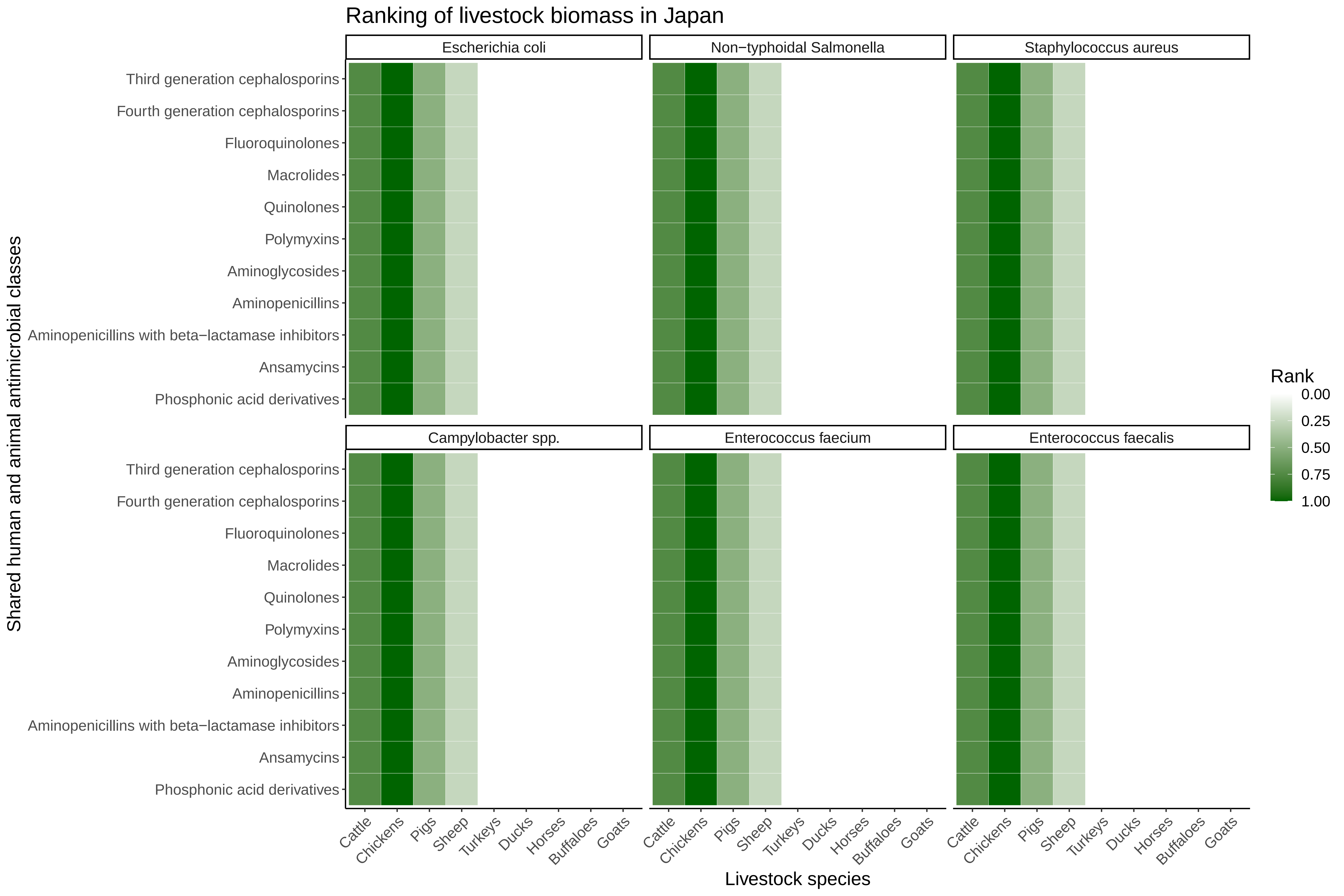
Ranking of livestock biomass in Japan
Escherichia coli
Non−typhoidal Salmonella
Staphylococcus aureus
Campylobacter spp.
Enterococcus faecium
Enterococcus faecalis
Third generation cephalosporins
Fourth generation cephalosporins
Fluoroquinolones
Macrolides
Quinolones
Polymyxins
Aminoglycosides
Aminopenicillins
Aminopenicillins with beta−lactamase inhibitors
Ansamycins
Phosphonic acid derivatives
Shared human and animal antimicrobial classes
Cattle
Chickens
Pigs
Sheep
Turkeys
Ducks
Horses
Buffaloes
Goats
Livestock species
Rank
0.00
0.25
0.50
0.75
1.00

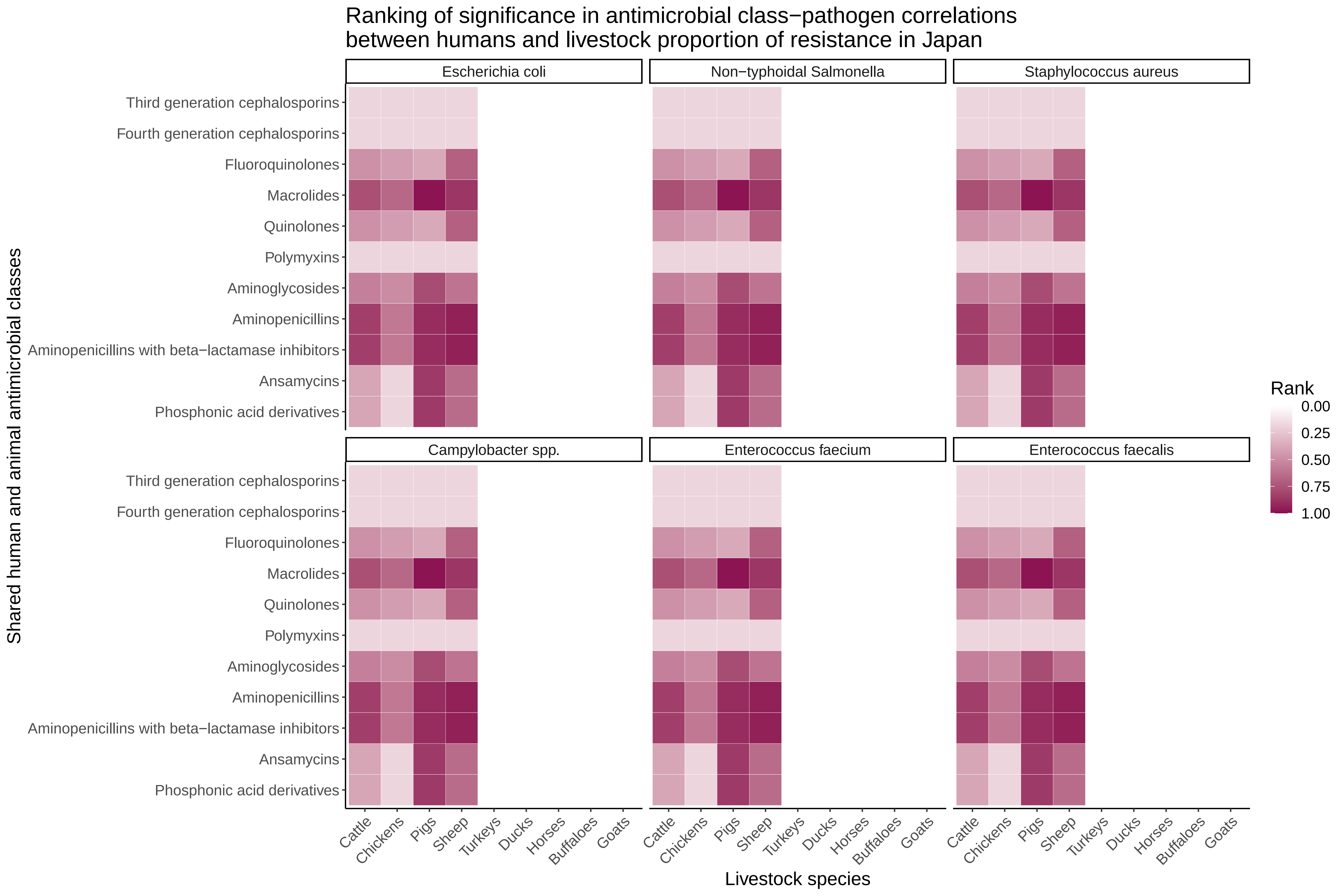
Ranking of significance in antimicrobial class−pathogen correlations
between humans and livestock proportion of resistance in Japan
Escherichia coli
Non−typhoidal Salmonella
Staphylococcus aureus
Campylobacter spp.
Enterococcus faecium
Enterococcus faecalis
Third generation cephalosporins
Fourth generation cephalosporins
Fluoroquinolones
Macrolides
Quinolones
Polymyxins
Aminoglycosides
Aminopenicillins
Aminopenicillins with beta−lactamase inhibitors
Ansamycins
Phosphonic acid derivatives
Shared human and animal antimicrobial classes
Cattle
Chickens
Pigs
Sheep
Turkeys
Ducks
Horses
Buffaloes
Goats
Livestock species
Rank
0.00
0.25
0.50
0.75
1.00

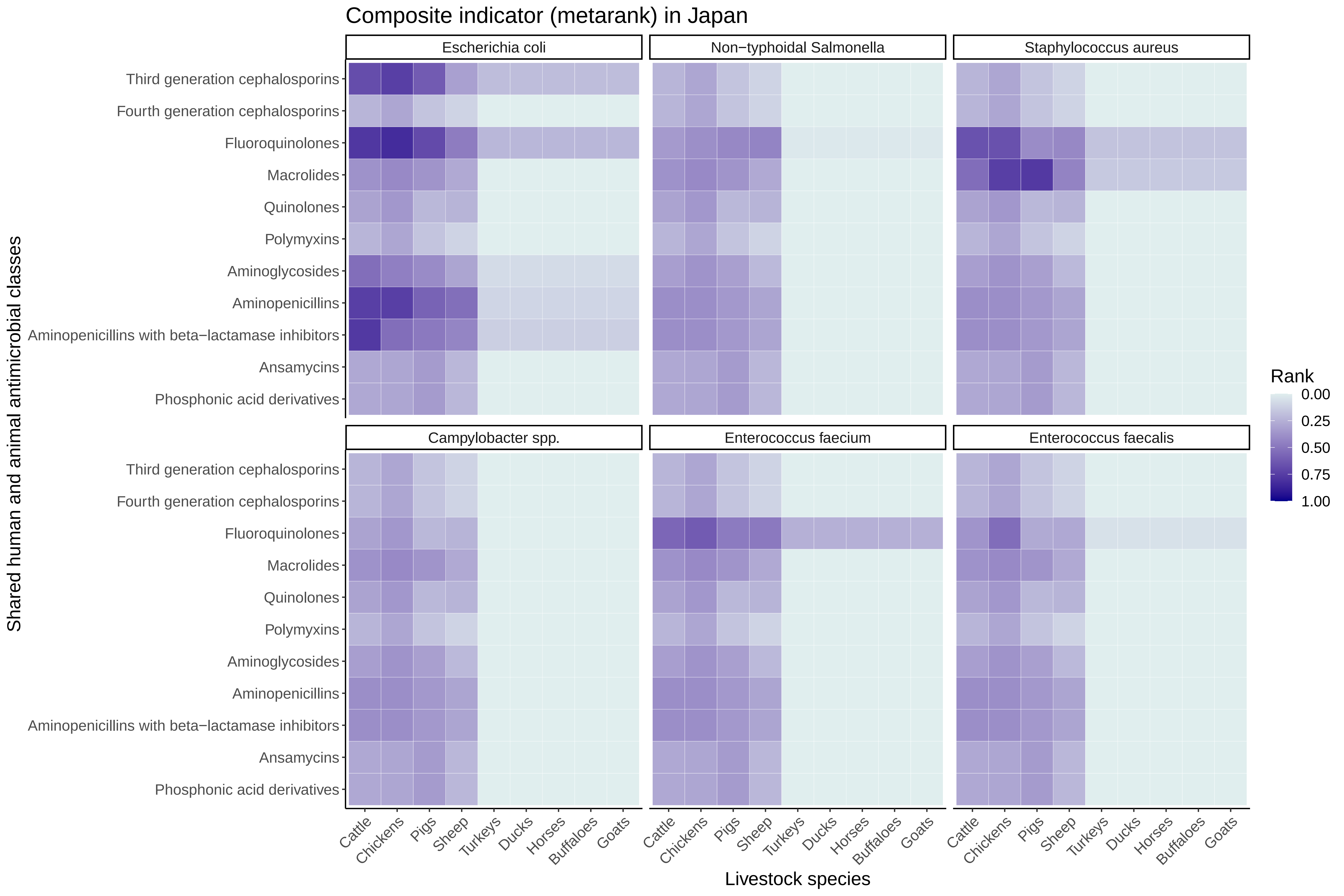

Composite indicator (metarank) in Japan
Escherichia coli
Non−typhoidal Salmonella
Staphylococcus aureus
Campylobacter spp.
Enterococcus faecium
Enterococcus faecalis
Shared human and animal antimicrobial classes
Third generation cephalosporins
Fourth generation cephalosporins
Fluoroquinolones
Macrolides
Quinolones
Polymyxins
Aminoglycosides
Aminopenicillins
Aminopenicillins with beta−lactamase inhibitors
Ansamycins
Phosphonic acid derivatives
Cattle
Chickens
Pigs
Sheep
Turkeys
Ducks
Horses
Buffaloes
Goats
Livestock species
Rank
0.00
0.25
0.50
0.75
1.00

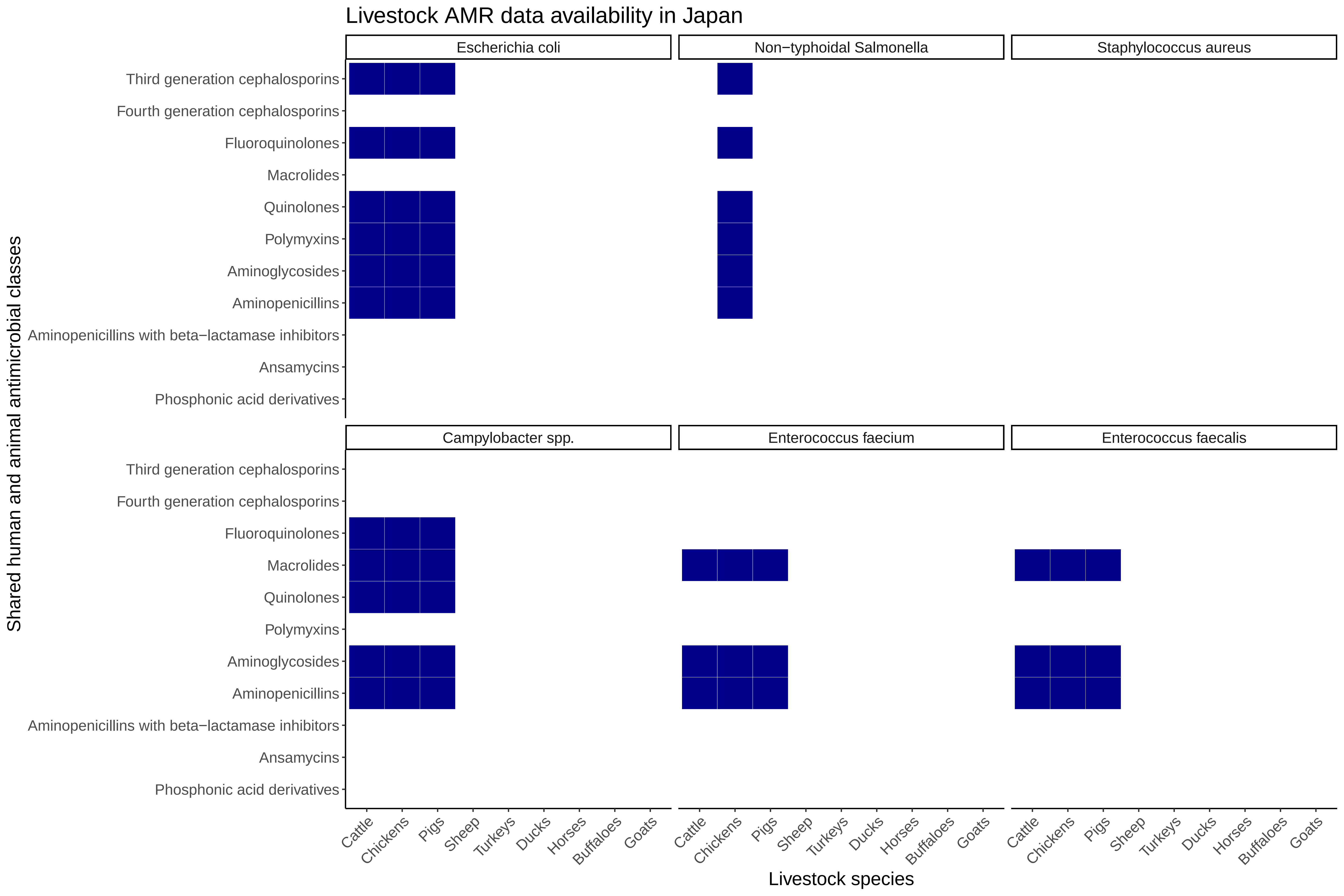
Livestock AMR data availability in Japan
Escherichia coli
Non−typhoidal Salmonella
Staphylococcus aureus
Campylobacter spp.
Enterococcus faecium
Enterococcus faecalis
Shared human and animal antimicrobial classes
Third generation cephalosporins
Fourth generation cephalosporins
Fluoroquinolones
Macrolides
Quinolones
Polymyxins
Aminoglycosides
Aminopenicillins
Aminopenicillins with beta−lactamase inhibitors
Ansamycins
Phosphonic acid derivatives
Cattle
Chickens
Pigs
Sheep
Turkeys
Ducks
Horses
Buffaloes
Goats
Livestock species

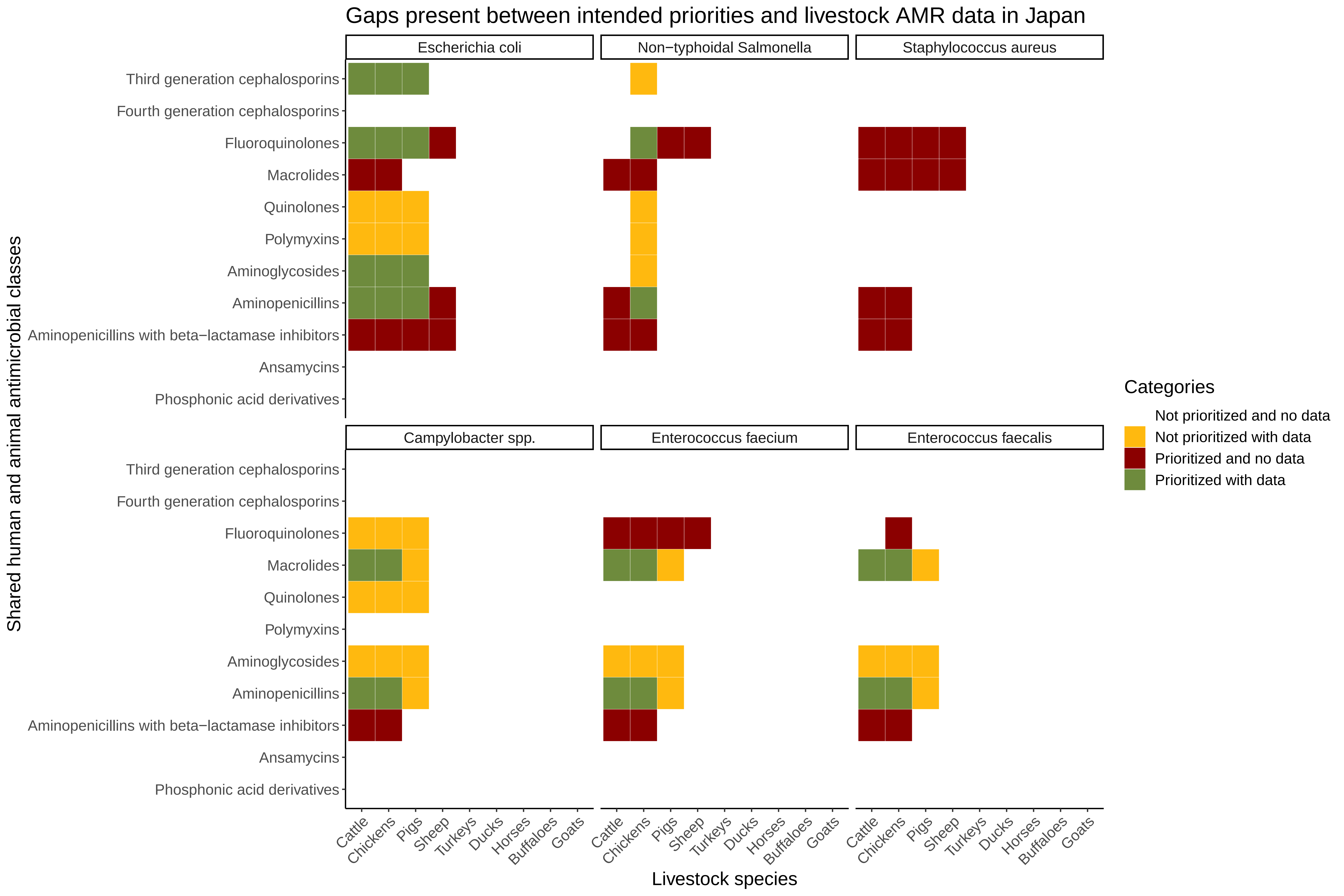

Gaps present between intended priorities and livestock AMR data in Japan
Escherichia coli
Non−typhoidal Salmonella
Staphylococcus aureus
Campylobacter spp.
Enterococcus faecium
Enterococcus faecalis
Shared human and animal antimicrobial classes
Third generation cephalosporins
Fourth generation cephalosporins
Fluoroquinolones
Macrolides
Quinolones
Polymyxins
Aminoglycosides
Aminopenicillins
Aminopenicillins with beta−lactamase inhibitors
Ansamycins
Phosphonic acid derivatives
Cattle
Chickens
Pigs
Sheep
Turkeys
Ducks
Horses
Buffaloes
Goats
Livestock species
Categories
Not prioritized and no data
Not prioritized with data
Prioritized and no data
Prioritized with data

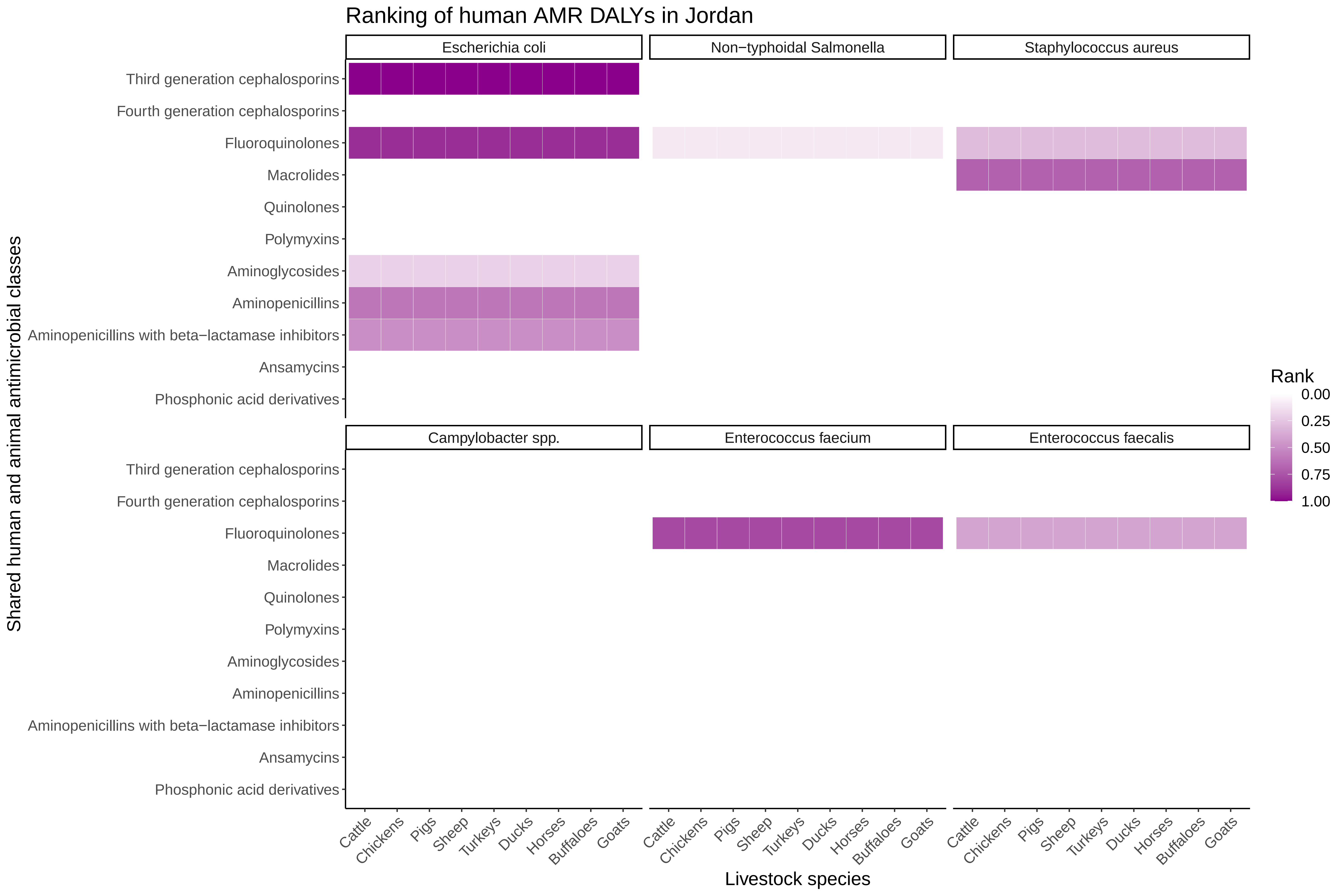

Ranking of human AMR DALYs in Jordan
Escherichia coli
Non−typhoidal Salmonella
Staphylococcus aureus
Campylobacter spp.
Enterococcus faecium
Enterococcus faecalis
Third generation cephalosporins
Fourth generation cephalosporins
Fluoroquinolones
Macrolides
Quinolones
Polymyxins
Aminoglycosides
Aminopenicillins
Aminopenicillins with beta−lactamase inhibitors
Ansamycins
Phosphonic acid derivatives
Shared human and animal antimicrobial classes
Cattle
Chickens
Pigs
Sheep
Turkeys
Ducks
Horses
Buffaloes
Goats
Livestock species
Rank
0.00
0.25
0.50
0.75
1.00

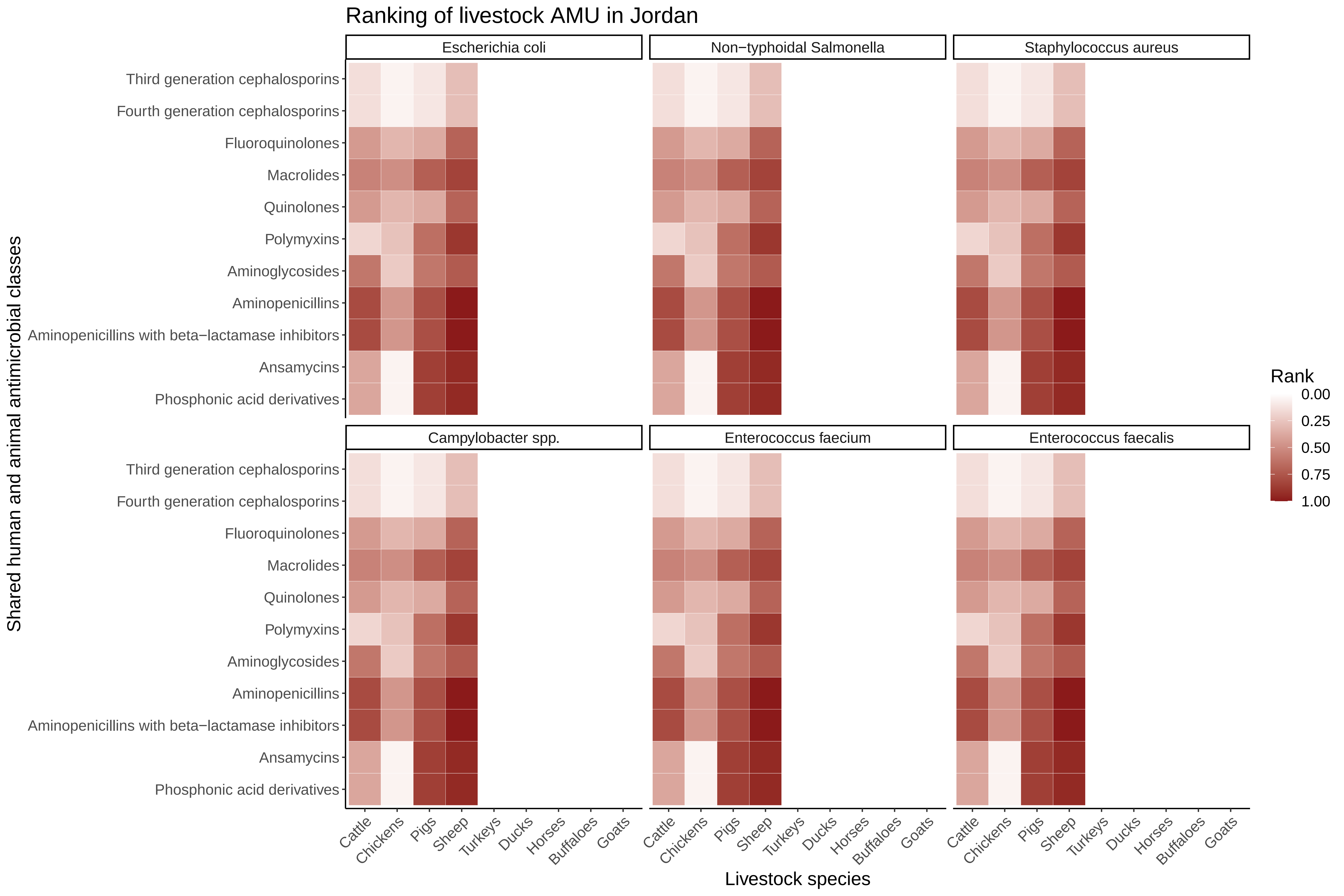
Ranking of livestock AMU in Jordan
Escherichia coli
Non−typhoidal Salmonella
Staphylococcus aureus
Campylobacter spp.
Enterococcus faecium
Enterococcus faecalis
Shared human and animal antimicrobial classes
Third generation cephalosporins
Fourth generation cephalosporins
Fluoroquinolones
Macrolides
Quinolones
Polymyxins
Aminoglycosides
Aminopenicillins
Aminopenicillins with beta−lactamase inhibitors
Ansamycins
Phosphonic acid derivatives
Livestock species
Cattle
Chickens
Pigs
Sheep
Turkeys
Ducks
Horses
Buffaloes
Goats
Rank
0.00
0.25
0.50
0.75
1.00

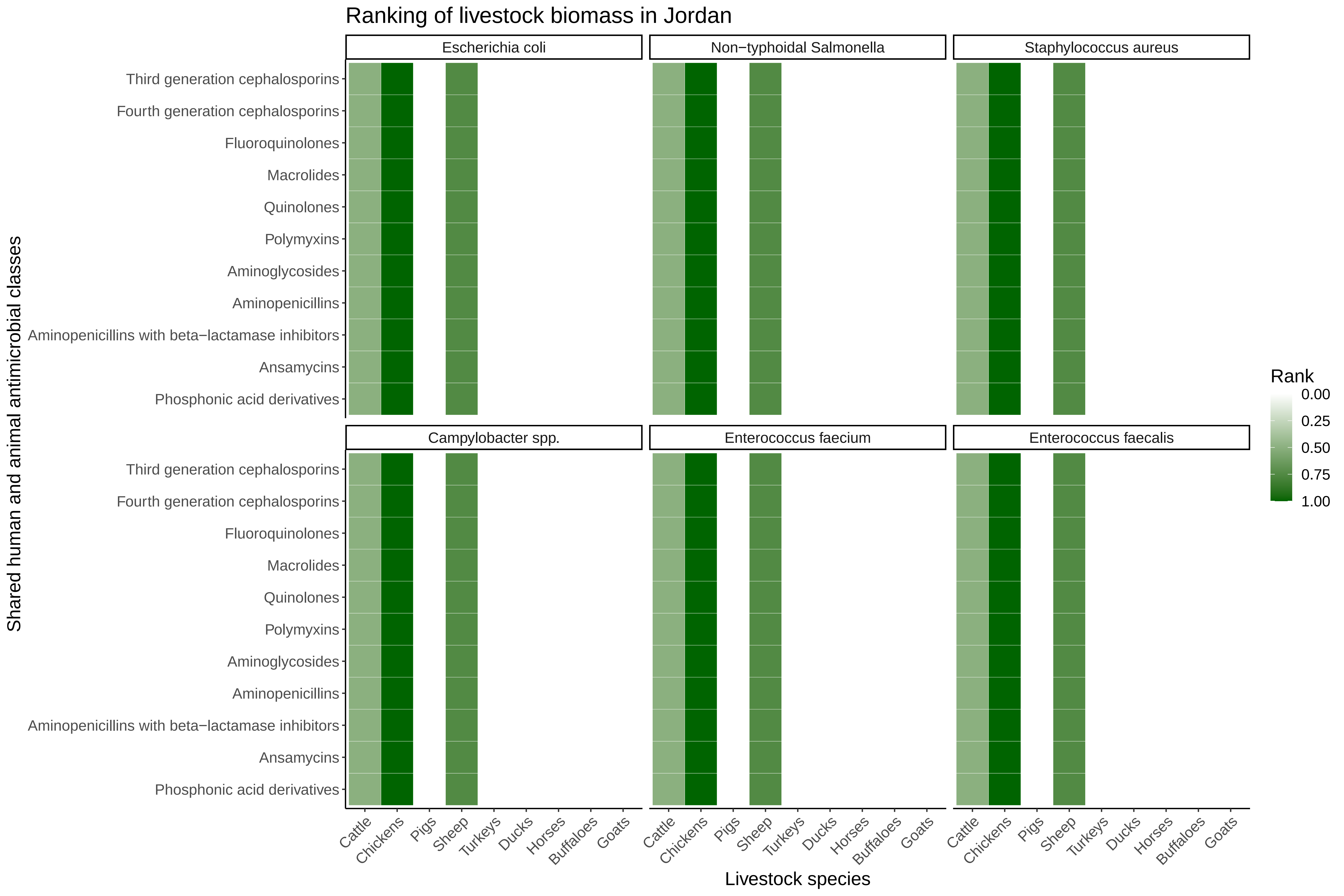

Ranking of livestock biomass in Jordan
Escherichia coli
Non−typhoidal Salmonella
Staphylococcus aureus
Campylobacter spp.
Enterococcus faecium
Enterococcus faecalis
Third generation cephalosporins
Fourth generation cephalosporins
Fluoroquinolones
Macrolides
Quinolones
Polymyxins
Aminoglycosides
Aminopenicillins
Aminopenicillins with beta−lactamase inhibitors
Ansamycins
Phosphonic acid derivatives
Shared human and animal antimicrobial classes
Cattle
Chickens
Pigs
Sheep
Turkeys
Ducks
Horses
Buffaloes
Goats
Livestock species
Rank
0.00
0.25
0.50
0.75
1.00

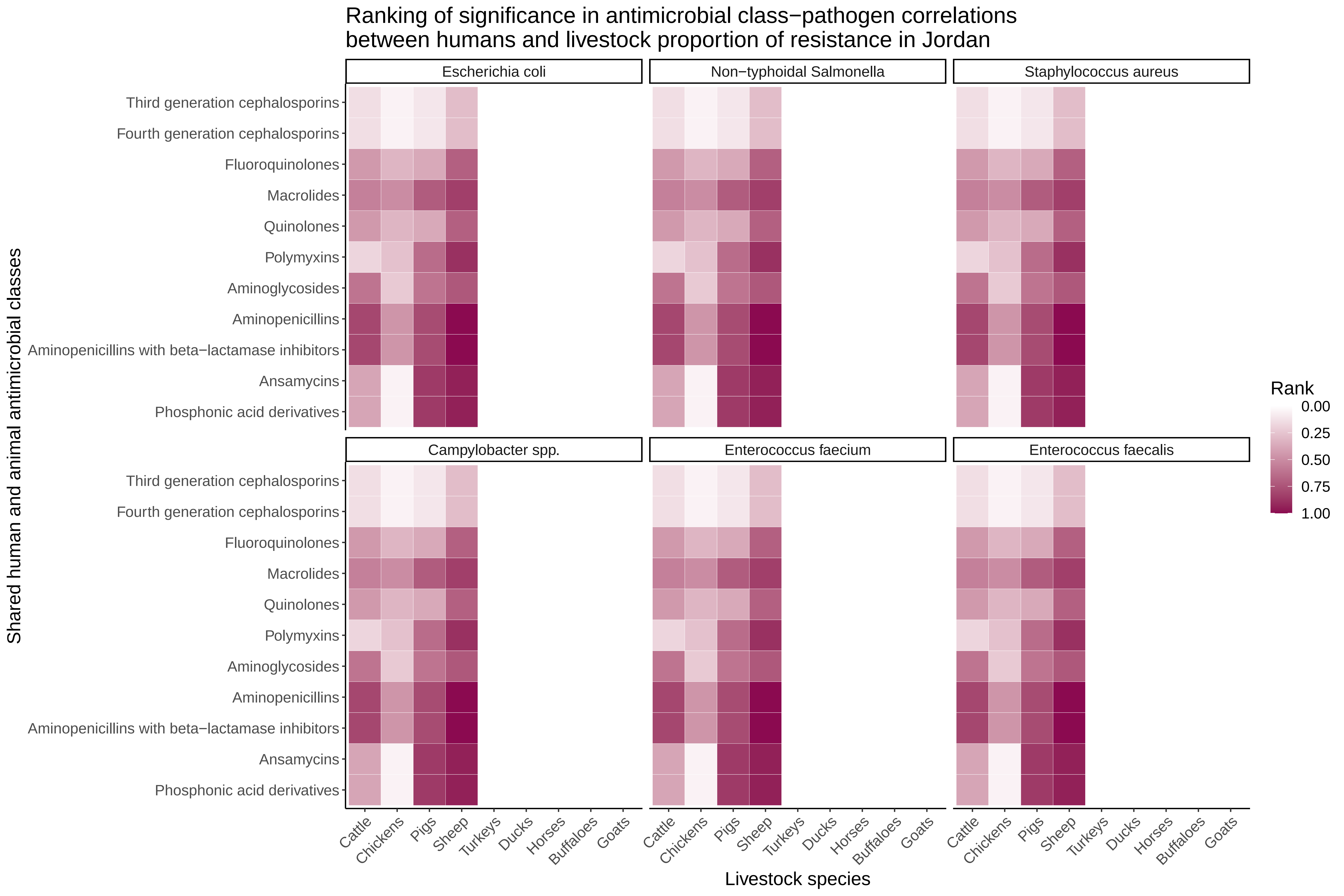

Ranking of significance in antimicrobial class−pathogen correlations between humans and livestock proportion of resistance in Jordan
Escherichia coli
Non−typhoidal Salmonella
Staphylococcus aureus
Campylobacter spp.
Enterococcus faecium
Enterococcus faecalis
Third generation cephalosporins
Fourth generation cephalosporins
Fluoroquinolones
Macrolides
Quinolones
Polymyxins
Aminoglycosides
Aminopenicillins
Aminopenicillins with beta−lactamase inhibitors
Ansamycins
Phosphonic acid derivatives
Shared human and animal antimicrobial classes
Cattle
Chickens
Pigs
Sheep
Turkeys
Ducks
Horses
Buffaloes
Goats
Livestock species
Rank
0.00
0.25
0.50
0.75
1.00

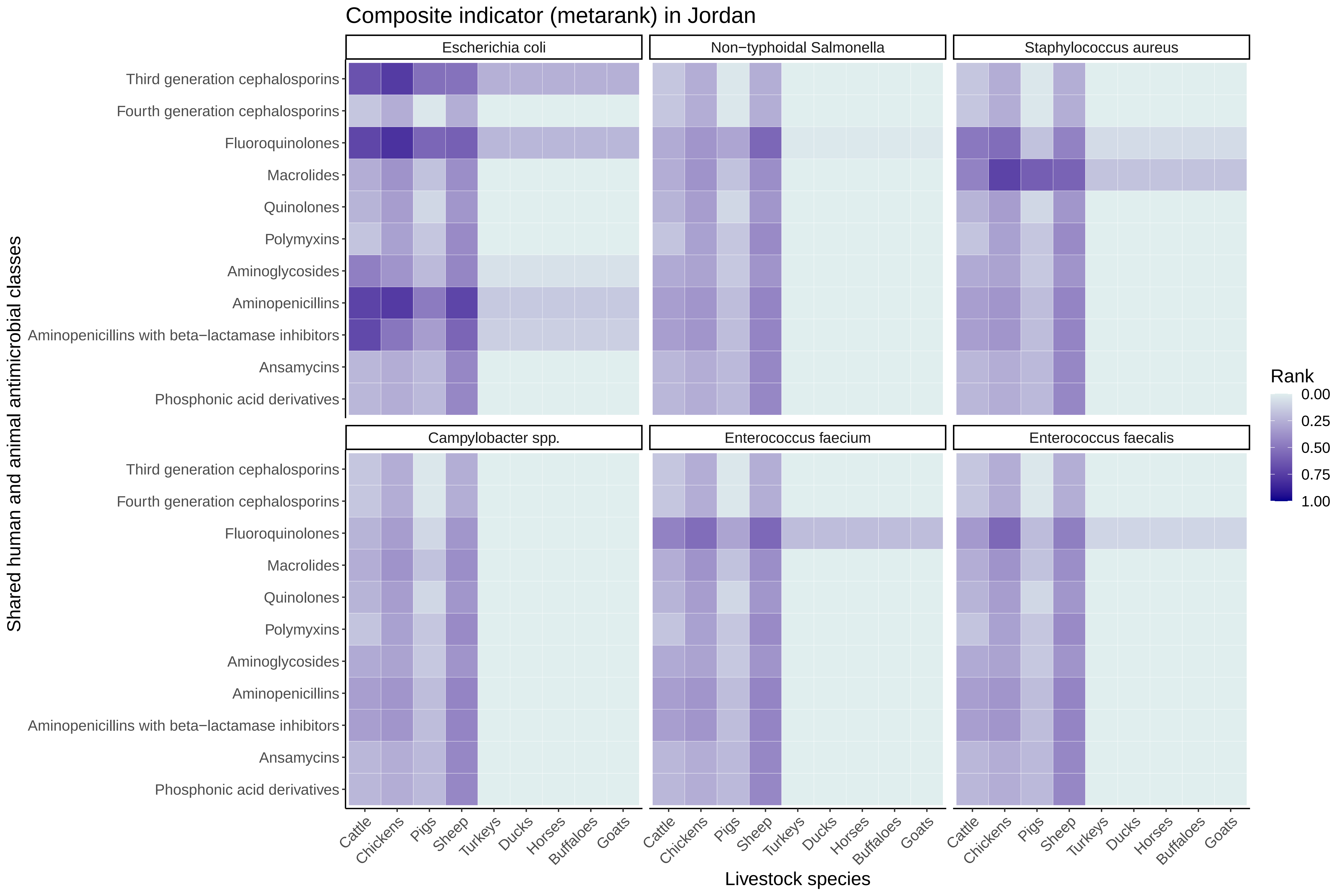

Composite indicator (metarank) in Jordan
Escherichia coli
Non−typhoidal Salmonella
Staphylococcus aureus
Campylobacter spp.
Enterococcus faecium
Enterococcus faecalis
Shared human and animal antimicrobial classes
Third generation cephalosporins
Fourth generation cephalosporins
Fluoroquinolones
Macrolides
Quinolones
Polymyxins
Aminoglycosides
Aminopenicillins
Aminopenicillins with beta−lactamase inhibitors
Ansamycins
Phosphonic acid derivatives
Cattle
Chickens
Pigs
Sheep
Turkeys
Ducks
Horses
Buffaloes
Goats
Livestock species
Rank
0.00
0.25
0.50
0.75
1.00

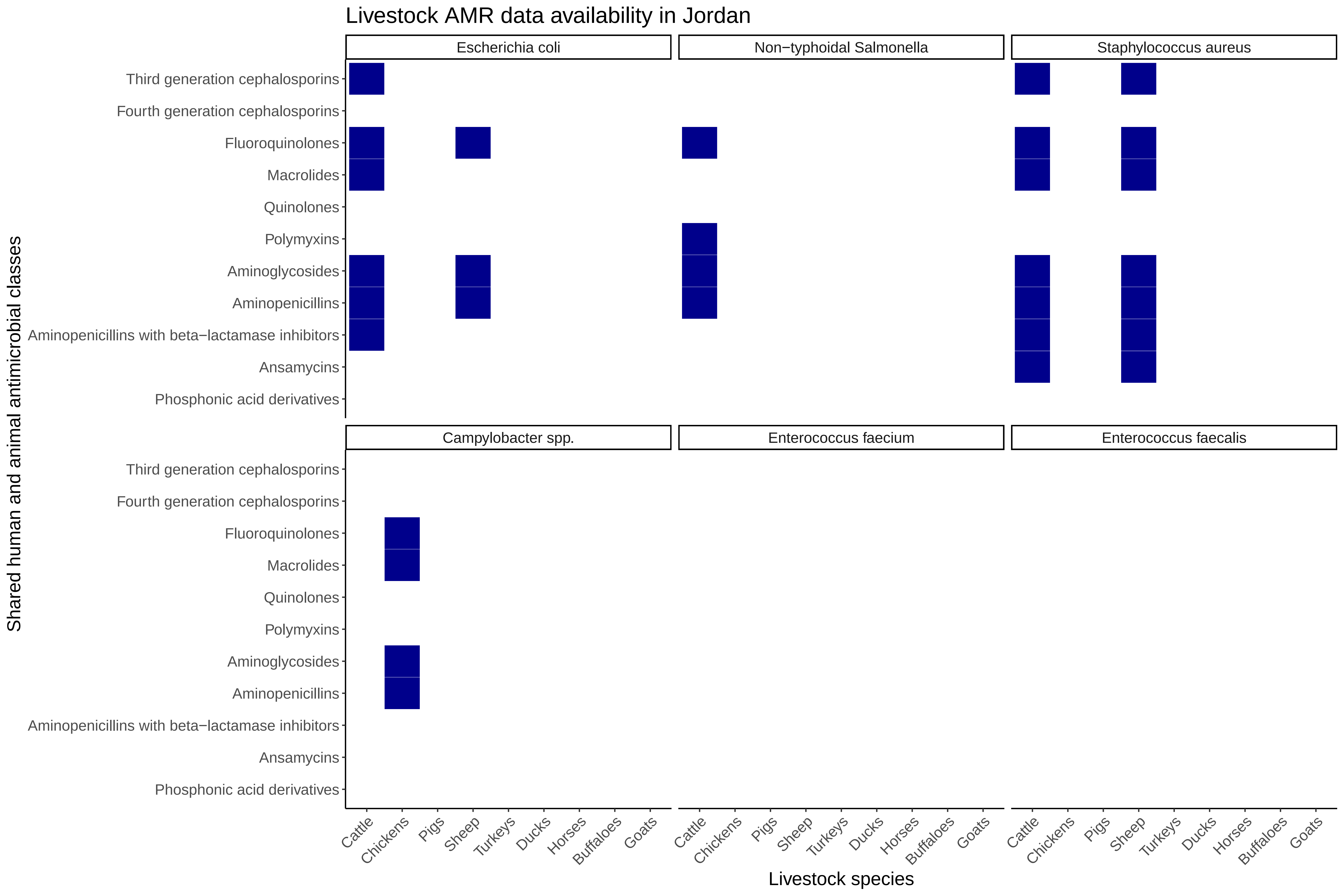
Livestock AMR data availability in Jordan
Escherichia coli
Non−typhoidal Salmonella
Staphylococcus aureus
Campylobacter spp.
Enterococcus faecium
Enterococcus faecalis
Shared human and animal antimicrobial classes
Third generation cephalosporins
Fourth generation cephalosporins
Fluoroquinolones
Macrolides
Quinolones
Polymyxins
Aminoglycosides
Aminopenicillins
Aminopenicillins with beta−lactamase inhibitors
Ansamycins
Phosphonic acid derivatives
Cattle
Chickens
Pigs
Sheep
Turkeys
Ducks
Horses
Buffaloes
Goats
Livestock species

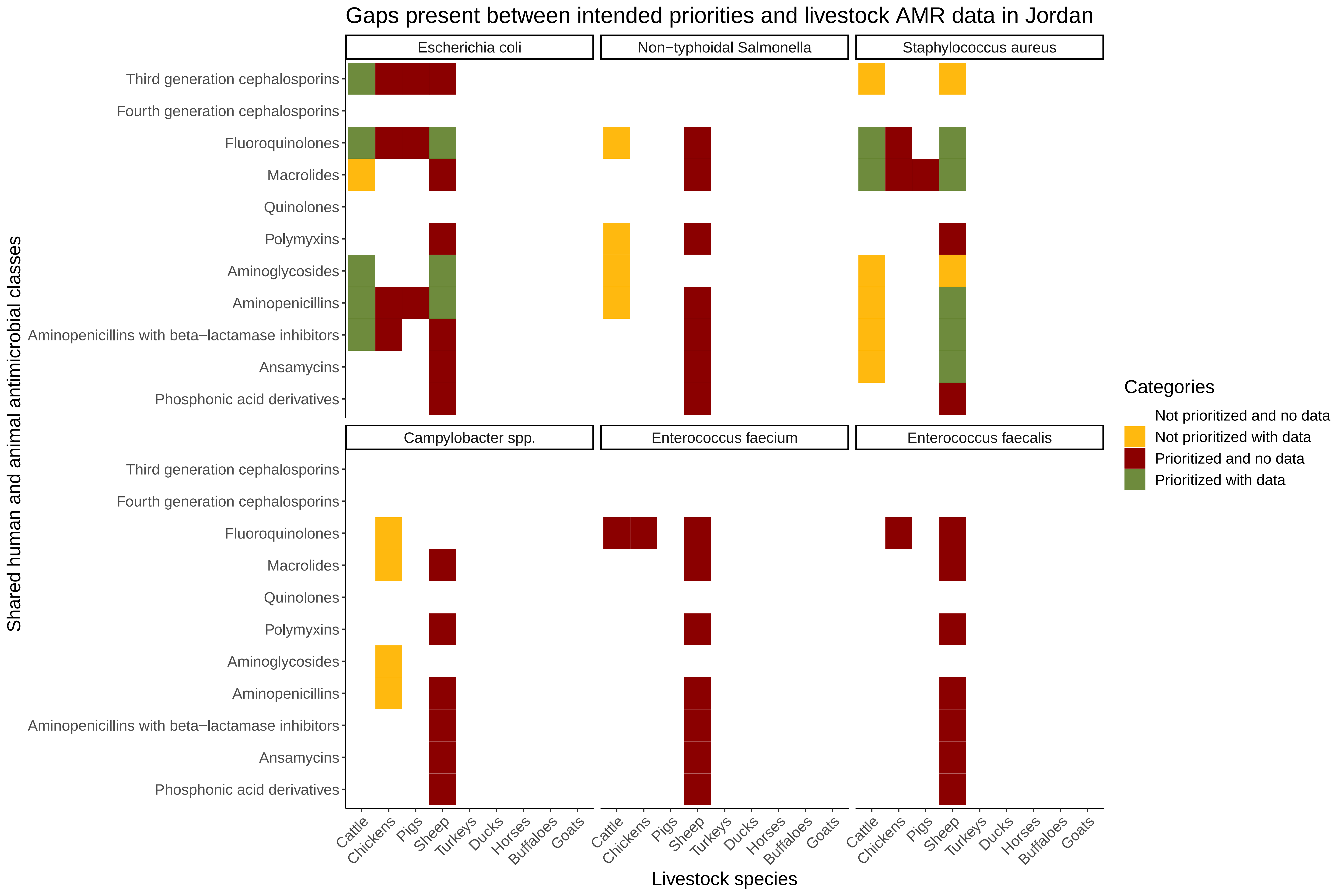

Gaps present between intended priorities and livestock AMR data in Jordan
Escherichia coli
Non−typhoidal Salmonella
Staphylococcus aureus
Campylobacter spp.
Enterococcus faecium
Enterococcus faecalis
Third generation cephalosporins
Fourth generation cephalosporins
Fluoroquinolones
Macrolides
Quinolones
Polymyxins
Aminoglycosides
Aminopenicillins
Aminopenicillins with beta−lactamase inhibitors
Ansamycins
Phosphonic acid derivatives
Shared human and animal antimicrobial classes
Cattle
Chickens
Pigs
Sheep
Turkeys
Ducks
Horses
Buffaloes
Goats
Livestock species
Categories
Not prioritized and no data
Not prioritized with data
Prioritized and no data
Prioritized with data

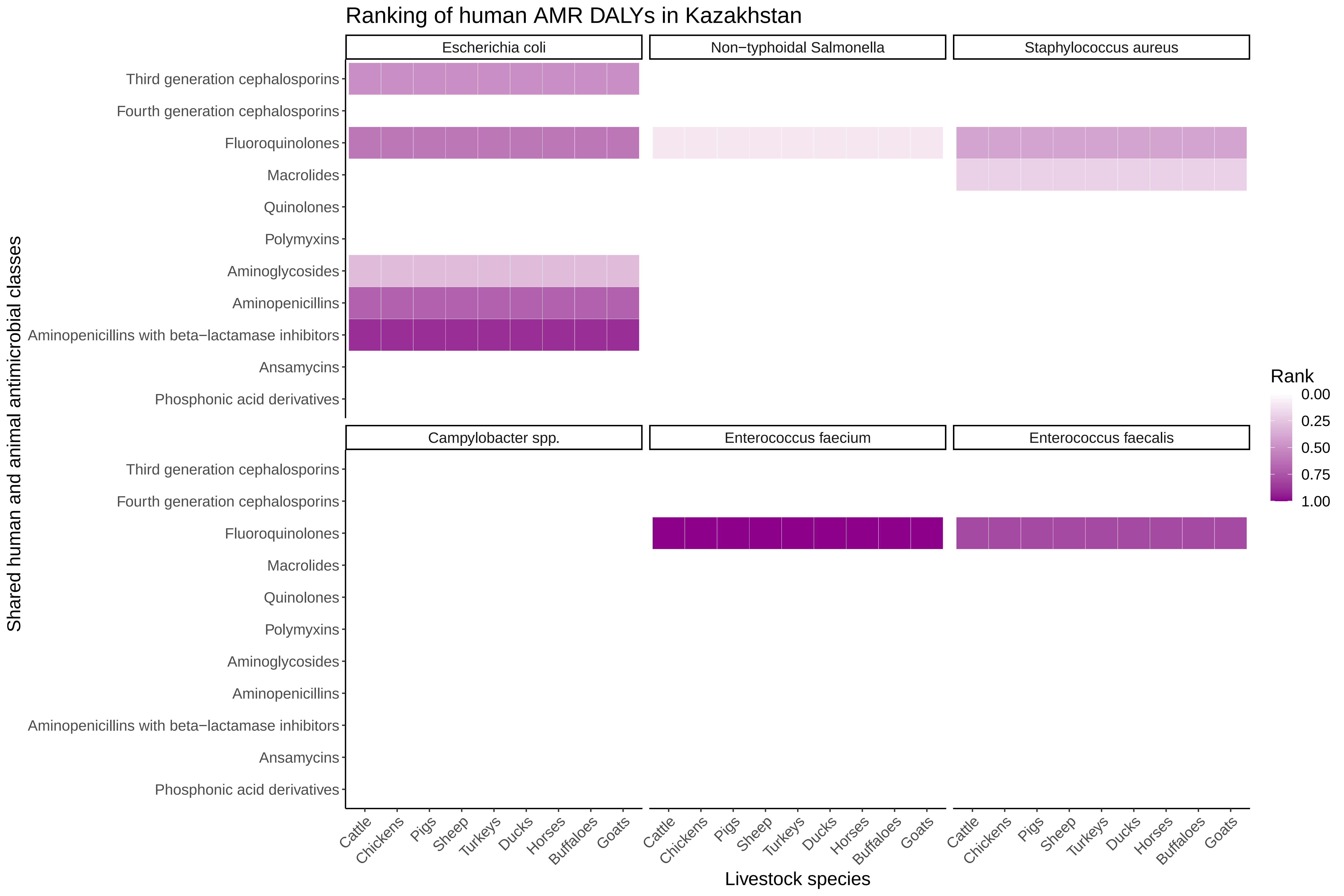
Ranking of human AMR DALYs in Kazakhstan
Escherichia coli
Non−typhoidal Salmonella
Staphylococcus aureus
Campylobacter spp.
Enterococcus faecium
Enterococcus faecalis
Shared human and animal antimicrobial classes
Third generation cephalosporins
Fourth generation cephalosporins
Fluoroquinolones
Macrolides
Quinolones
Polymyxins
Aminoglycosides
Aminopenicillins
Aminopenicillins with beta−lactamase inhibitors
Ansamycins
Phosphonic acid derivatives
Livestock species
Cattle
Chickens
Pigs
Sheep
Turkeys
Ducks
Horses
Buffaloes
Goats
Rank
0.00
0.25
0.50
0.75
1.00

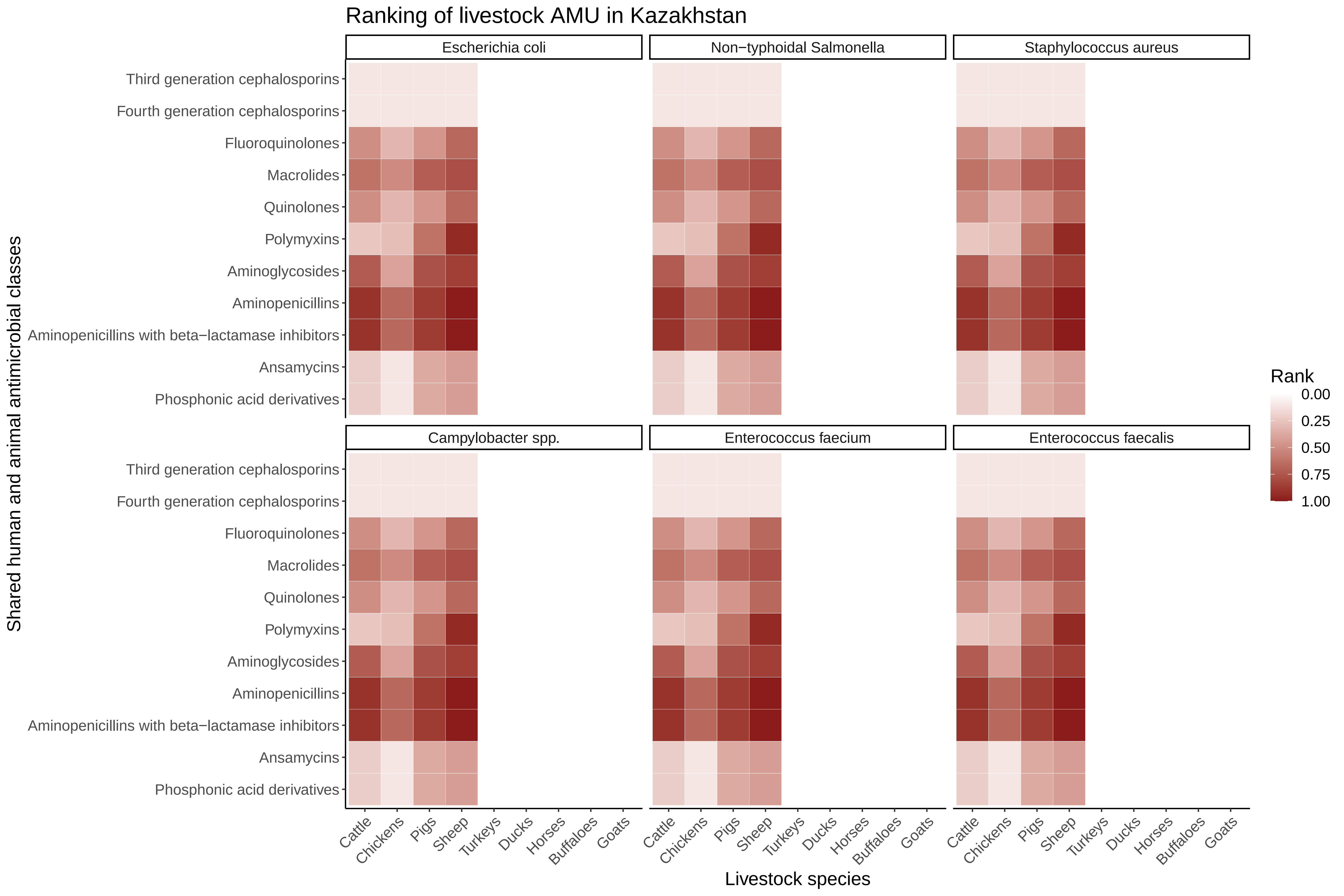

Ranking of livestock AMU in Kazakhstan
Escherichia coli
Non−typhoidal Salmonella
Staphylococcus aureus
Campylobacter spp.
Enterococcus faecium
Enterococcus faecalis
Third generation cephalosporins
Fourth generation cephalosporins
Fluoroquinolones
Macrolides
Quinolones
Polymyxins
Aminoglycosides
Aminopenicillins
Aminopenicillins with beta−lactamase inhibitors
Ansamycins
Phosphonic acid derivatives
Shared human and animal antimicrobial classes
Cattle
Chickens
Pigs
Sheep
Turkeys
Ducks
Horses
Buffaloes
Goats
Livestock species
Rank
0.00
0.25
0.50
0.75
1.00

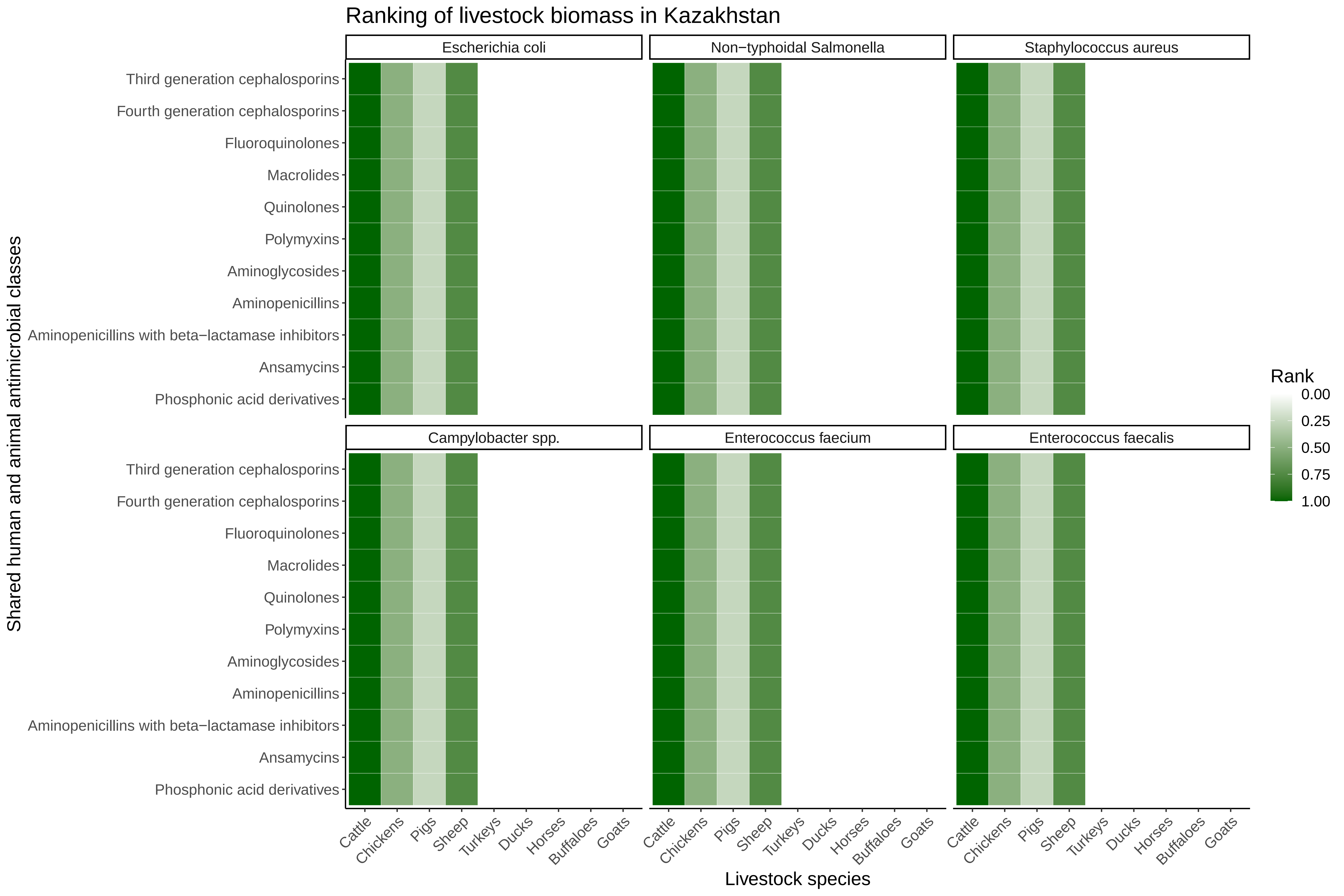

Ranking of livestock biomass in Kazakhstan
Escherichia coli
Non−typhoidal Salmonella
Staphylococcus aureus
Campylobacter spp.
Enterococcus faecium
Enterococcus faecalis
Third generation cephalosporins
Fourth generation cephalosporins
Fluoroquinolones
Macrolides
Quinolones
Polymyxins
Aminoglycosides
Aminopenicillins
Aminopenicillins with beta−lactamase inhibitors
Ansamycins
Phosphonic acid derivatives
Shared human and animal antimicrobial classes
Cattle
Chickens
Pigs
Sheep
Turkeys
Ducks
Horses
Buffaloes
Goats
Livestock species
Rank
0.00
0.25
0.50
0.75
1.00

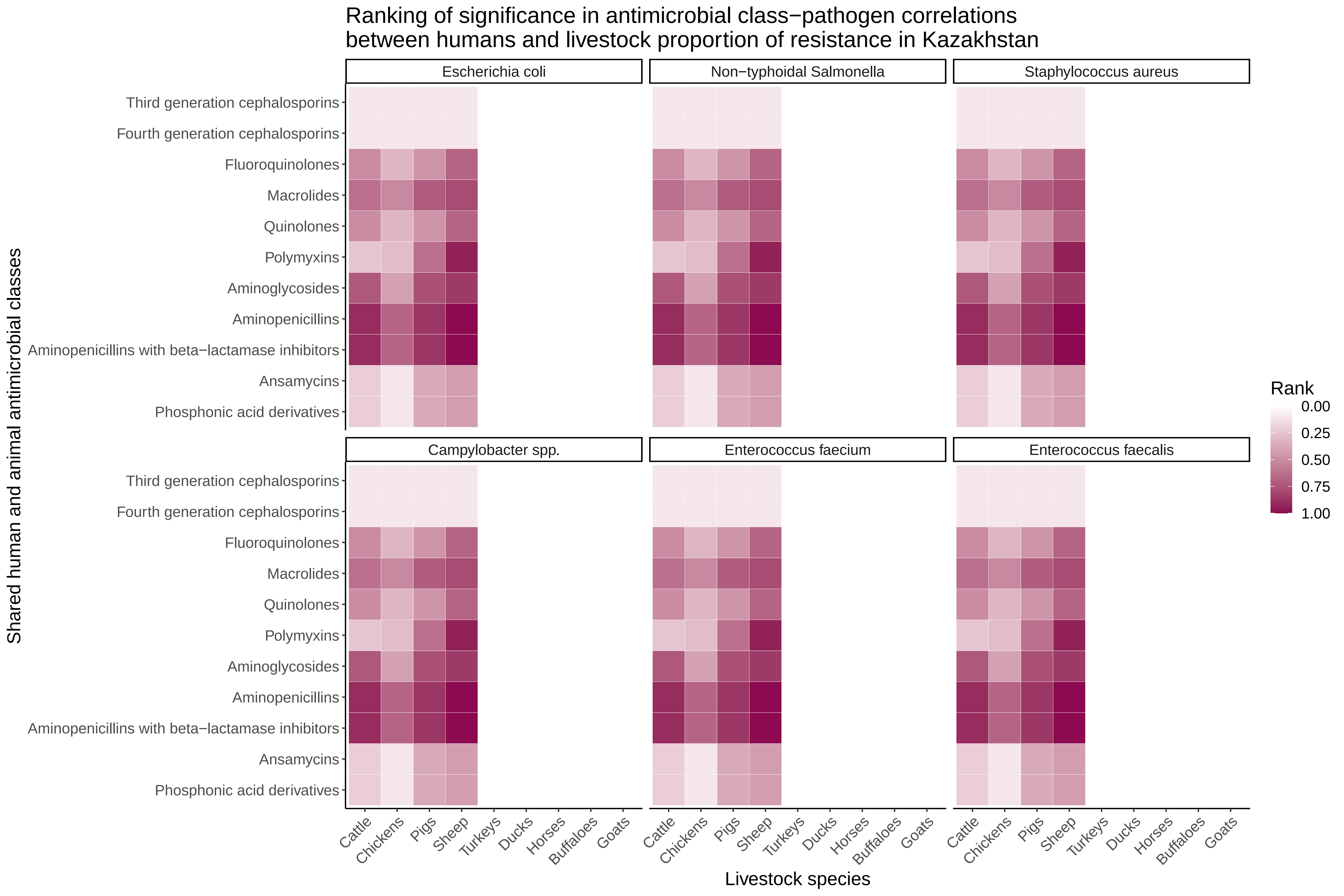

Ranking of significance in antimicrobial class−pathogen correlations
between humans and livestock proportion of resistance in Kazakhstan
Escherichia coli
Non−typhoidal Salmonella
Staphylococcus aureus
Campylobacter spp.
Enterococcus faecium
Enterococcus faecalis
Shared human and animal antimicrobial classes
Third generation cephalosporins
Fourth generation cephalosporins
Fluoroquinolones
Macrolides
Quinolones
Polymyxins
Aminoglycosides
Aminopenicillins
Aminopenicillins with beta−lactamase inhibitors
Ansamycins
Phosphonic acid derivatives
Cattle
Chickens
Pigs
Sheep
Turkeys
Ducks
Horses
Buffaloes
Goats
Livestock species
Rank
0.00
0.25
0.50
0.75
1.00

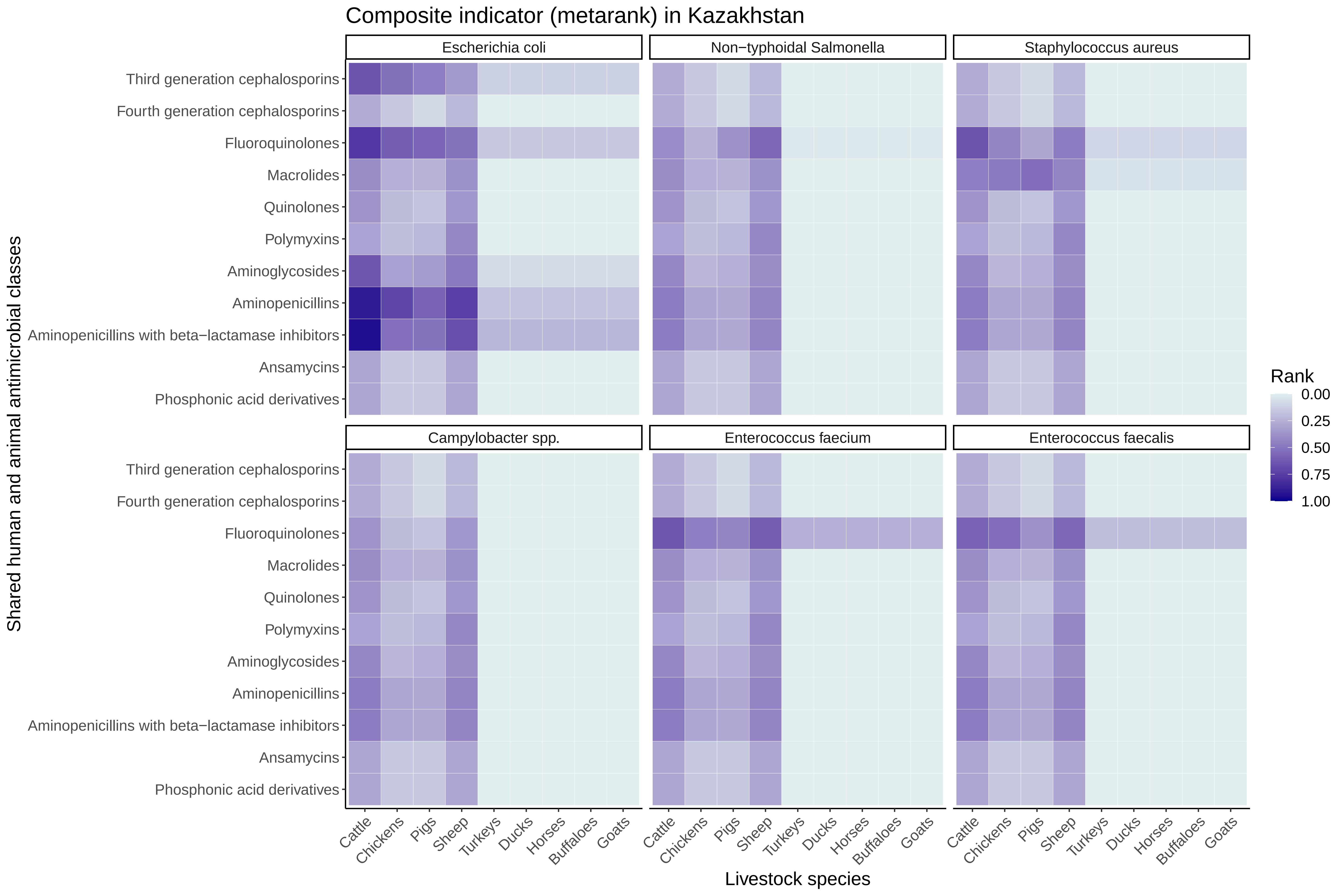

Composite indicator (metarank) in Kazakhstan
Escherichia coli
Non−typhoidal Salmonella
Staphylococcus aureus
Campylobacter spp.
Enterococcus faecium
Enterococcus faecalis
Third generation cephalosporins
Fourth generation cephalosporins
Fluoroquinolones
Macrolides
Quinolones
Polymyxins
Aminoglycosides
Aminopenicillins
Aminopenicillins with beta−lactamase inhibitors
Ansamycins
Phosphonic acid derivatives
Shared human and animal antimicrobial classes
Cattle
Chickens
Pigs
Sheep
Turkeys
Ducks
Horses
Buffaloes
Goats
Livestock species
Rank
0.00
0.25
0.50
0.75
1.00

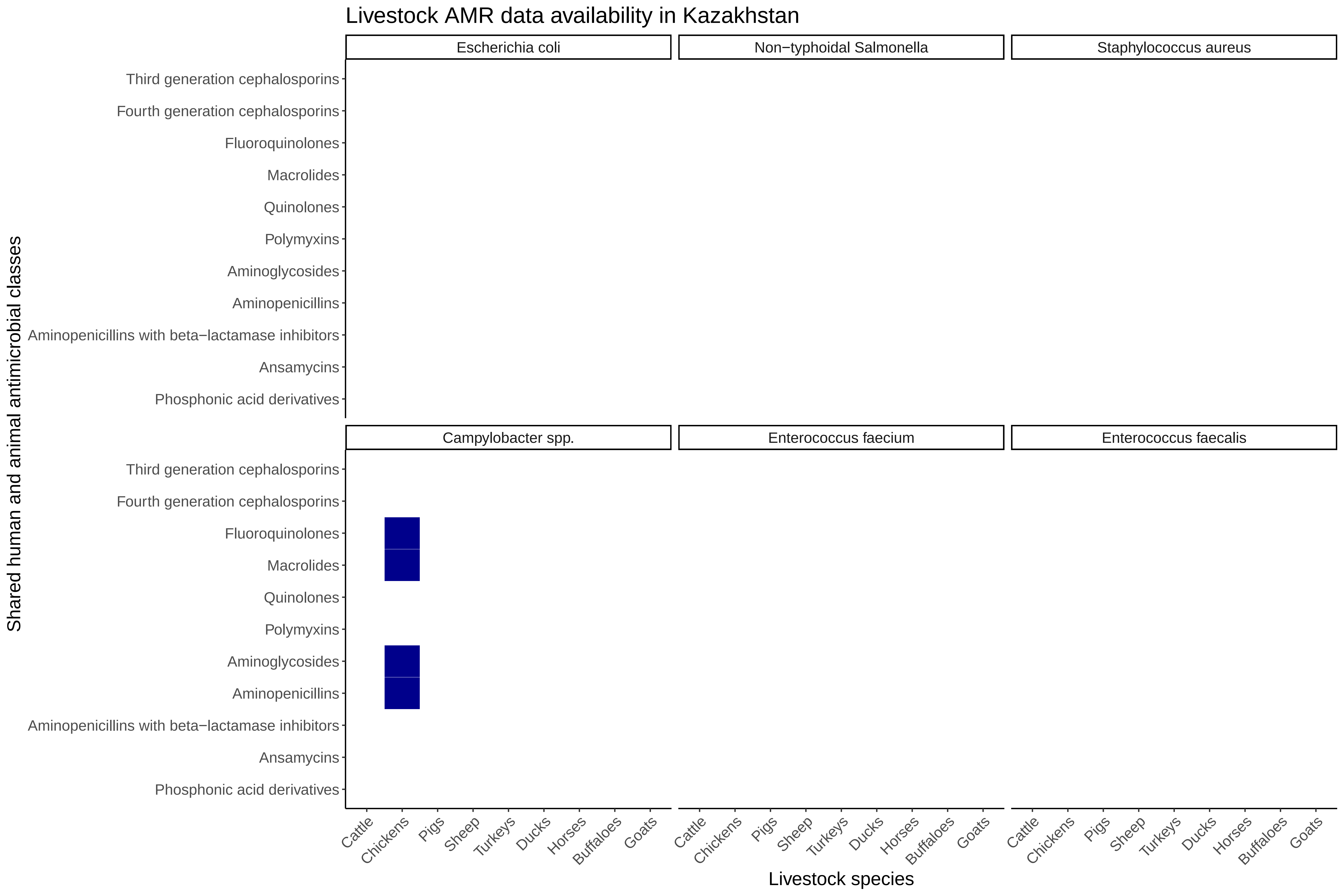

Livestock AMR data availability in Kazakhstan
Escherichia coli
Non−typhoidal Salmonella
Staphylococcus aureus
Campylobacter spp.
Enterococcus faecium
Enterococcus faecalis
Shared human and animal antimicrobial classes
Third generation cephalosporins
Fourth generation cephalosporins
Fluoroquinolones
Macrolides
Quinolones
Polymyxins
Aminoglycosides
Aminopenicillins
Aminopenicillins with beta−lactamase inhibitors
Ansamycins
Phosphonic acid derivatives
Cattle
Chickens
Pigs
Sheep
Turkeys
Ducks
Horses
Buffaloes
Goats
Livestock species

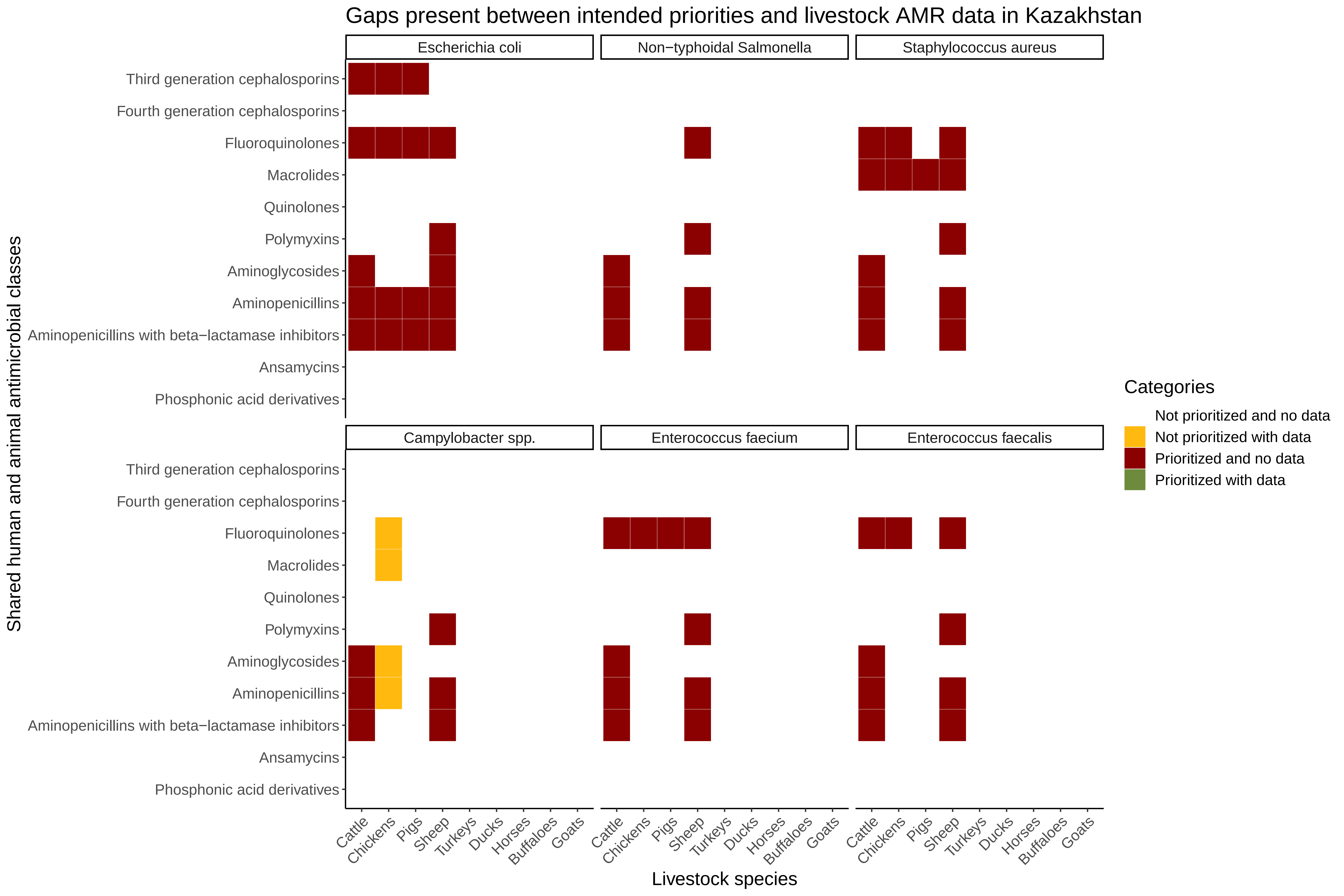

Gaps present between intended priorities and livestock AMR data in Kazakhstan
Escherichia coli
Non−typhoidal Salmonella
Staphylococcus aureus
Campylobacter spp.
Enterococcus faecium
Enterococcus faecalis
Third generation cephalosporins
Fourth generation cephalosporins
Fluoroquinolones
Macrolides
Quinolones
Polymyxins
Aminoglycosides
Aminopenicillins
Aminopenicillins with beta−lactamase inhibitors
Ansamycins
Phosphonic acid derivatives
Shared human and animal antimicrobial classes
Cattle
Chickens
Pigs
Sheep
Turkeys
Ducks
Horses
Buffaloes
Goats
Livestock species
Categories
Not prioritized and no data
Not prioritized with data
Prioritized and no data
Prioritized with data

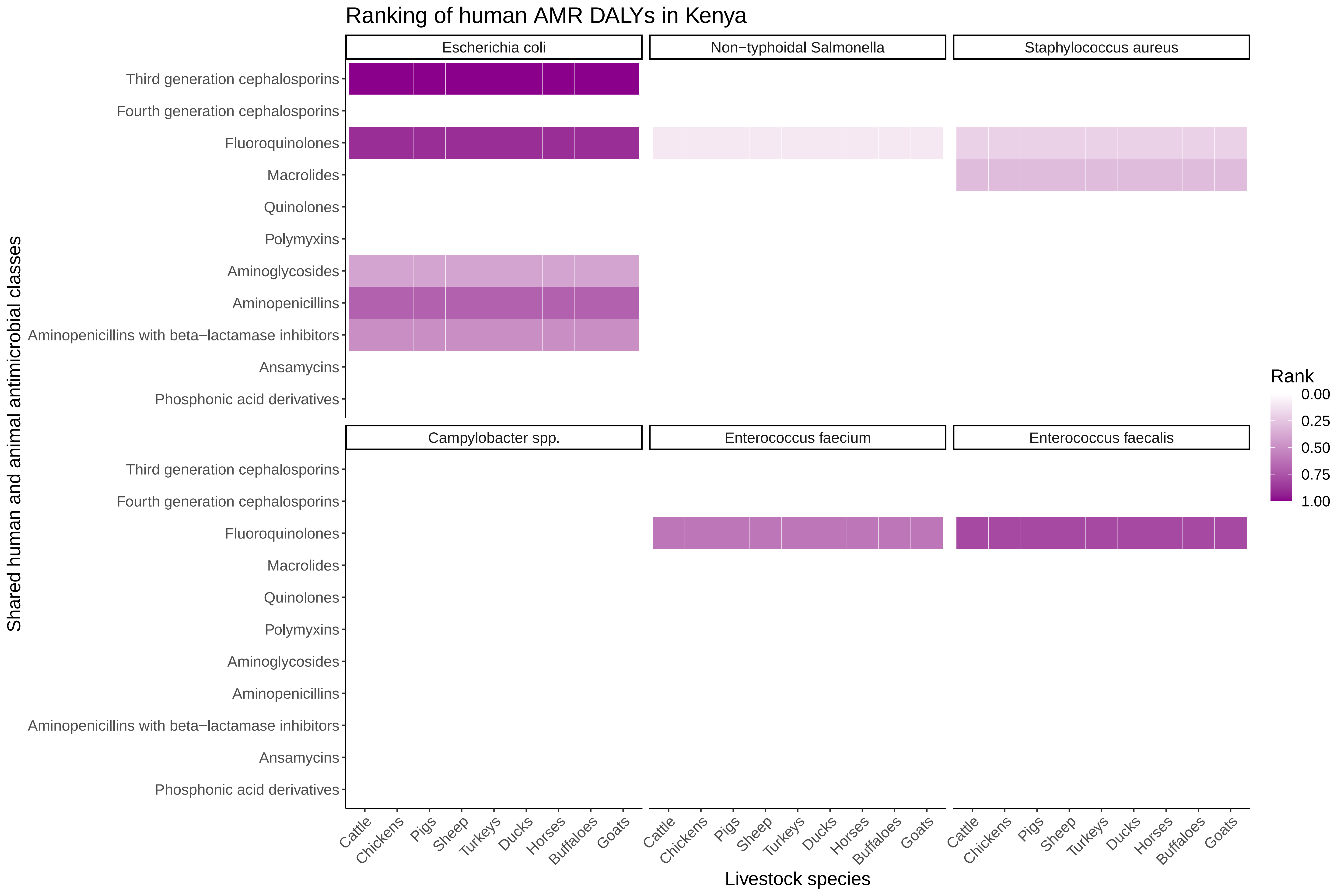

Ranking of human AMR DALYs in Kenya
Escherichia coli
Non−typhoidal Salmonella
Staphylococcus aureus
Campylobacter spp.
Enterococcus faecium
Enterococcus faecalis
Third generation cephalosporins
Fourth generation cephalosporins
Fluoroquinolones
Macrolides
Quinolones
Polymyxins
Aminoglycosides
Aminopenicillins
Aminopenicillins with beta−lactamase inhibitors
Ansamycins
Phosphonic acid derivatives
Shared human and animal antimicrobial classes
Cattle
Chickens
Pigs
Sheep
Turkeys
Ducks
Horses
Buffaloes
Goats
Livestock species
Rank
0.00
0.25
0.50
0.75
1.00

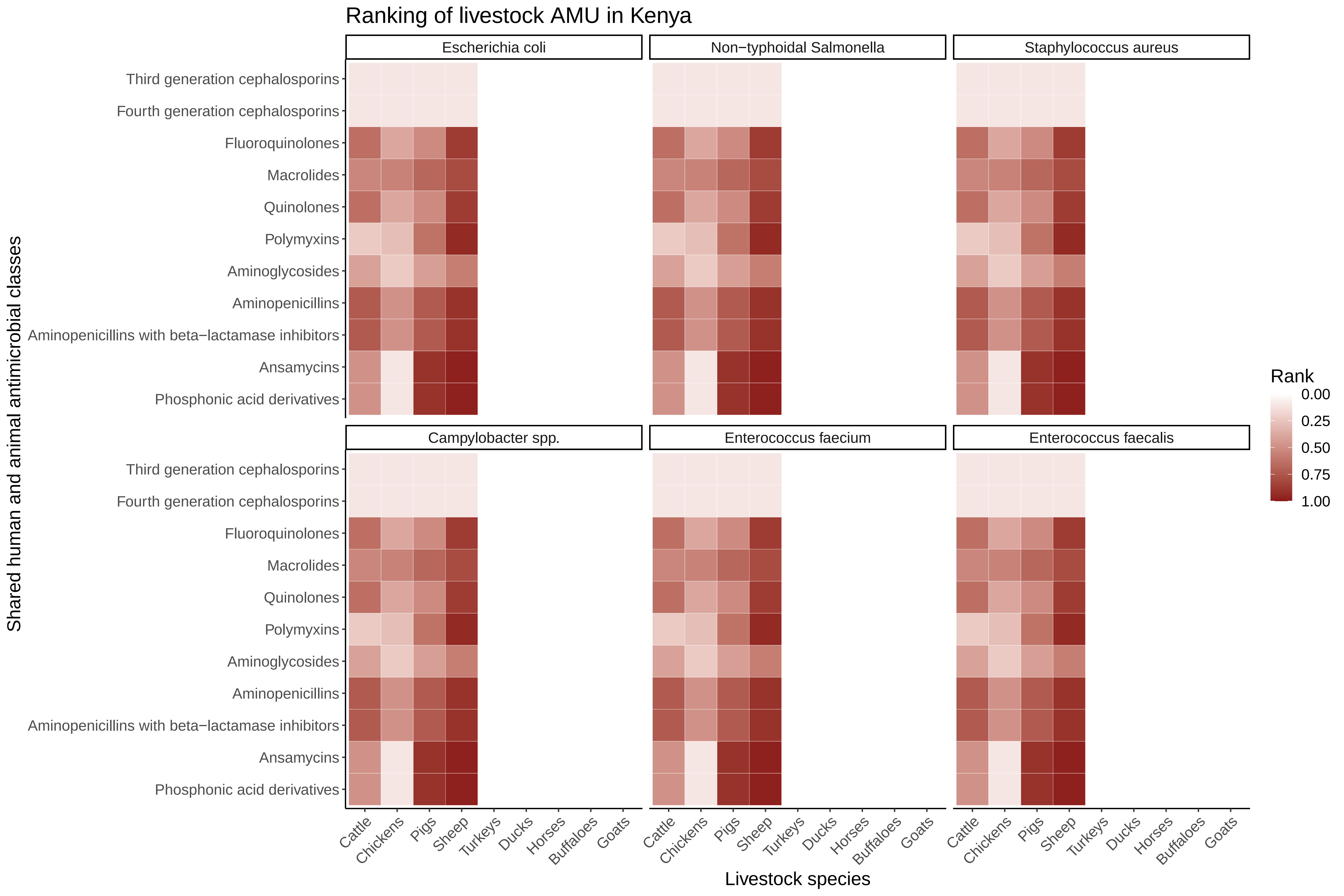

Ranking of livestock AMU in Kenya
Escherichia coli
Non−typhoidal Salmonella
Staphylococcus aureus
Campylobacter spp.
Enterococcus faecium
Enterococcus faecalis
Third generation cephalosporins
Fourth generation cephalosporins
Fluoroquinolones
Macrolides
Quinolones
Polymyxins
Aminoglycosides
Aminopenicillins
Aminopenicillins with beta−lactamase inhibitors
Ansamycins
Phosphonic acid derivatives
Shared human and animal antimicrobial classes
Cattle
Chickens
Pigs
Sheep
Turkeys
Ducks
Horses
Buffaloes
Goats
Livestock species
Rank
0.00
0.25
0.50
0.75
1.00

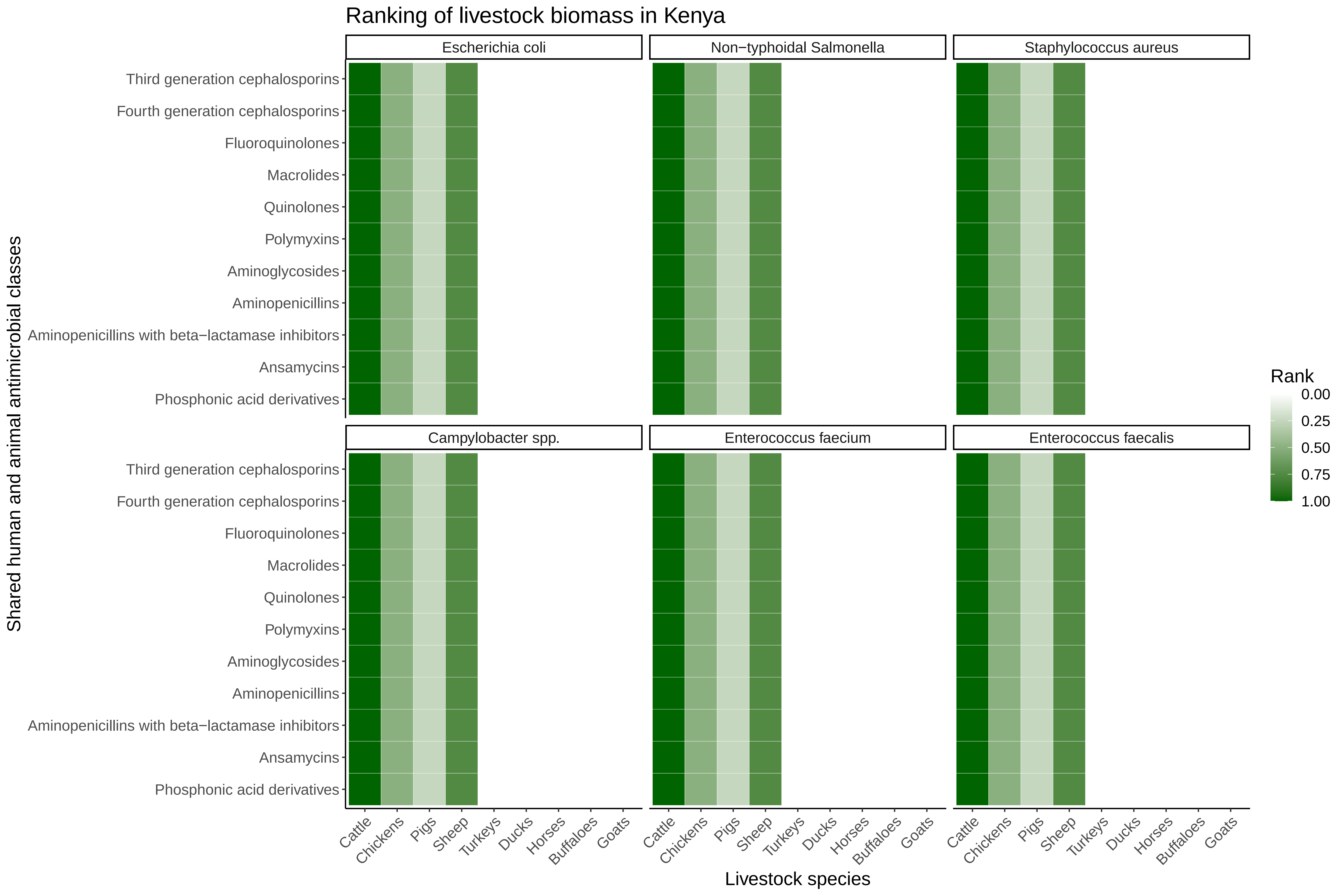

Ranking of livestock biomass in Kenya
Escherichia coli
Non−typhoidal Salmonella
Staphylococcus aureus
Campylobacter spp.
Enterococcus faecium
Enterococcus faecalis
Shared human and animal antimicrobial classes
Third generation cephalosporins
Fourth generation cephalosporins
Fluoroquinolones
Macrolides
Quinolones
Polymyxins
Aminoglycosides
Aminopenicillins
Aminopenicillins with beta−lactamase inhibitors
Ansamycins
Phosphonic acid derivatives
Livestock species
Cattle
Chickens
Pigs
Sheep
Turkeys
Ducks
Horses
Buffaloes
Goats
Rank
0.00
0.25
0.50
0.75
1.00

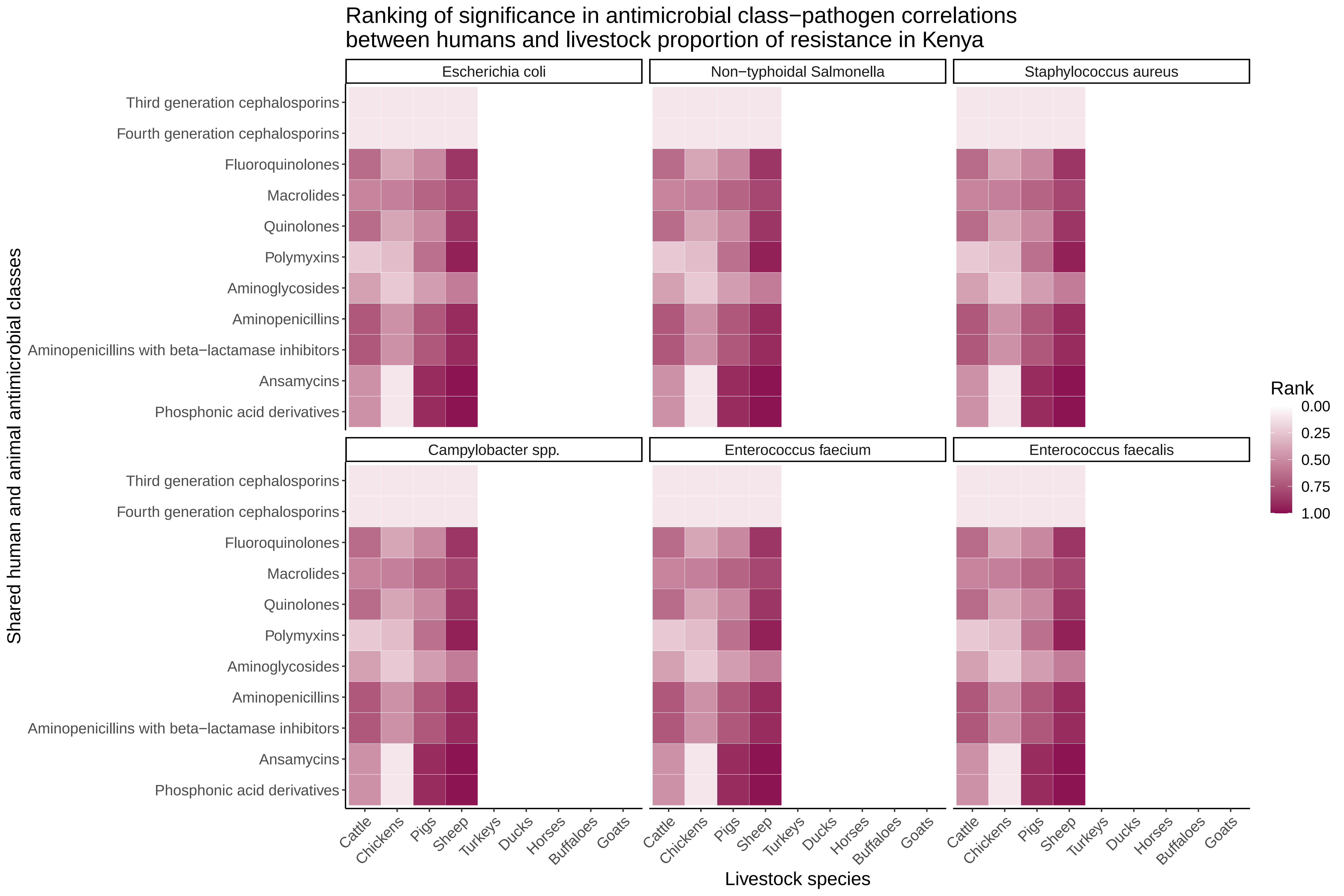

Ranking of significance in antimicrobial class−pathogen correlations
between humans and livestock proportion of resistance in Kenya
Escherichia coli
Non−typhoidal Salmonella
Staphylococcus aureus
Campylobacter spp.
Enterococcus faecium
Enterococcus faecalis
Third generation cephalosporins
Fourth generation cephalosporins
Fluoroquinolones
Macrolides
Quinolones
Polymyxins
Aminoglycosides
Aminopenicillins
Aminopenicillins with beta−lactamase inhibitors
Ansamycins
Phosphonic acid derivatives
Shared human and animal antimicrobial classes
Cattle
Chickens
Pigs
Sheep
Turkeys
Ducks
Horses
Buffaloes
Goats
Livestock species
Rank
0.00
0.25
0.50
0.75
1.00

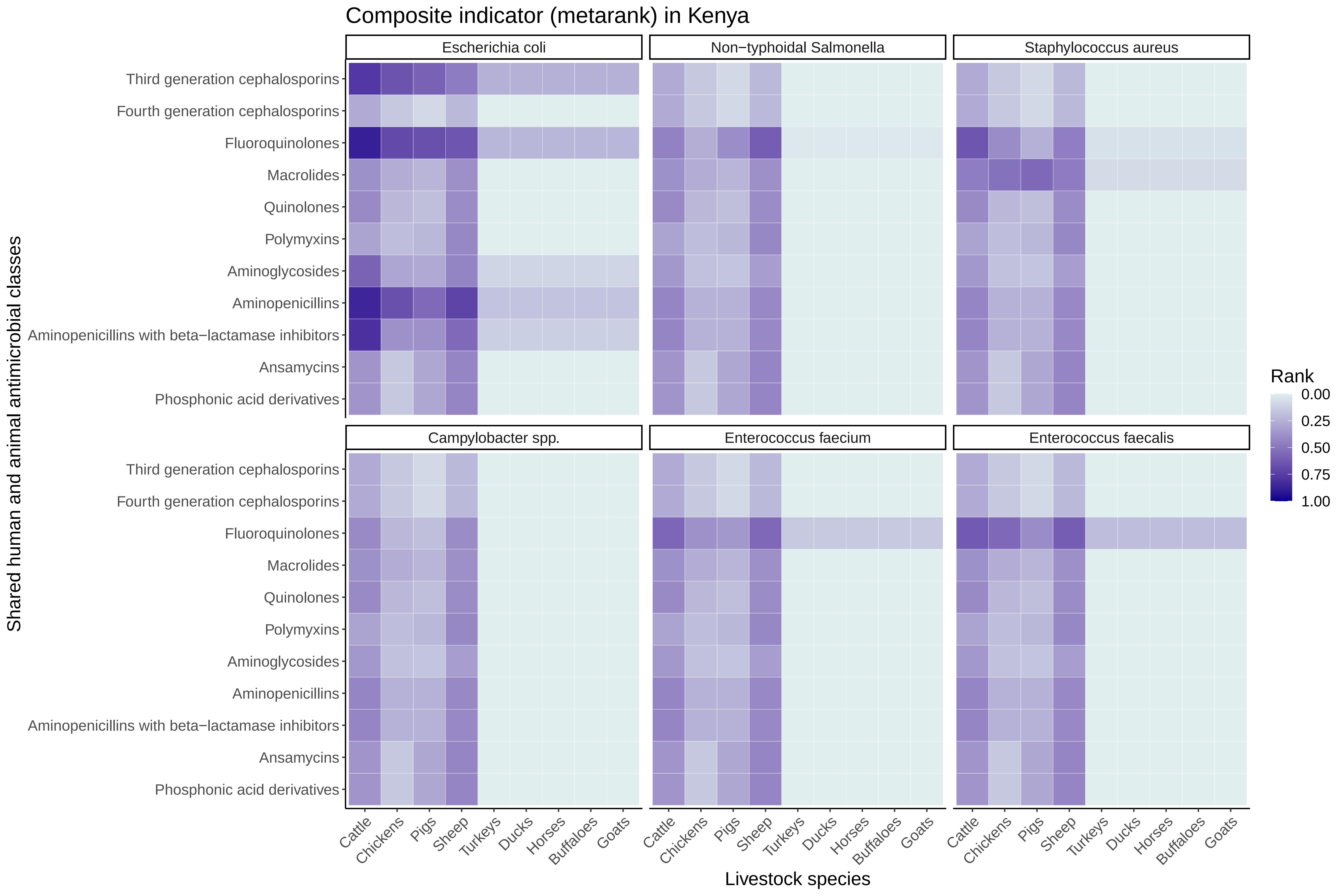
Composite indicator (metarank) in Kenya
Escherichia coli
Non−typhoidal Salmonella
Staphylococcus aureus
Campylobacter spp.
Enterococcus faecium
Enterococcus faecalis
Third generation cephalosporins
Fourth generation cephalosporins
Fluoroquinolones
Macrolides
Quinolones
Polymyxins
Aminoglycosides
Aminopenicillins
Aminopenicillins with beta−lactamase inhibitors
Ansamycins
Phosphonic acid derivatives
Cattle
Chickens
Pigs
Sheep
Turkeys
Ducks
Horses
Buffaloes
Goats
Livestock species
Shared human and animal antimicrobial classes
Rank
0.00
0.25
0.50
0.75
1.00

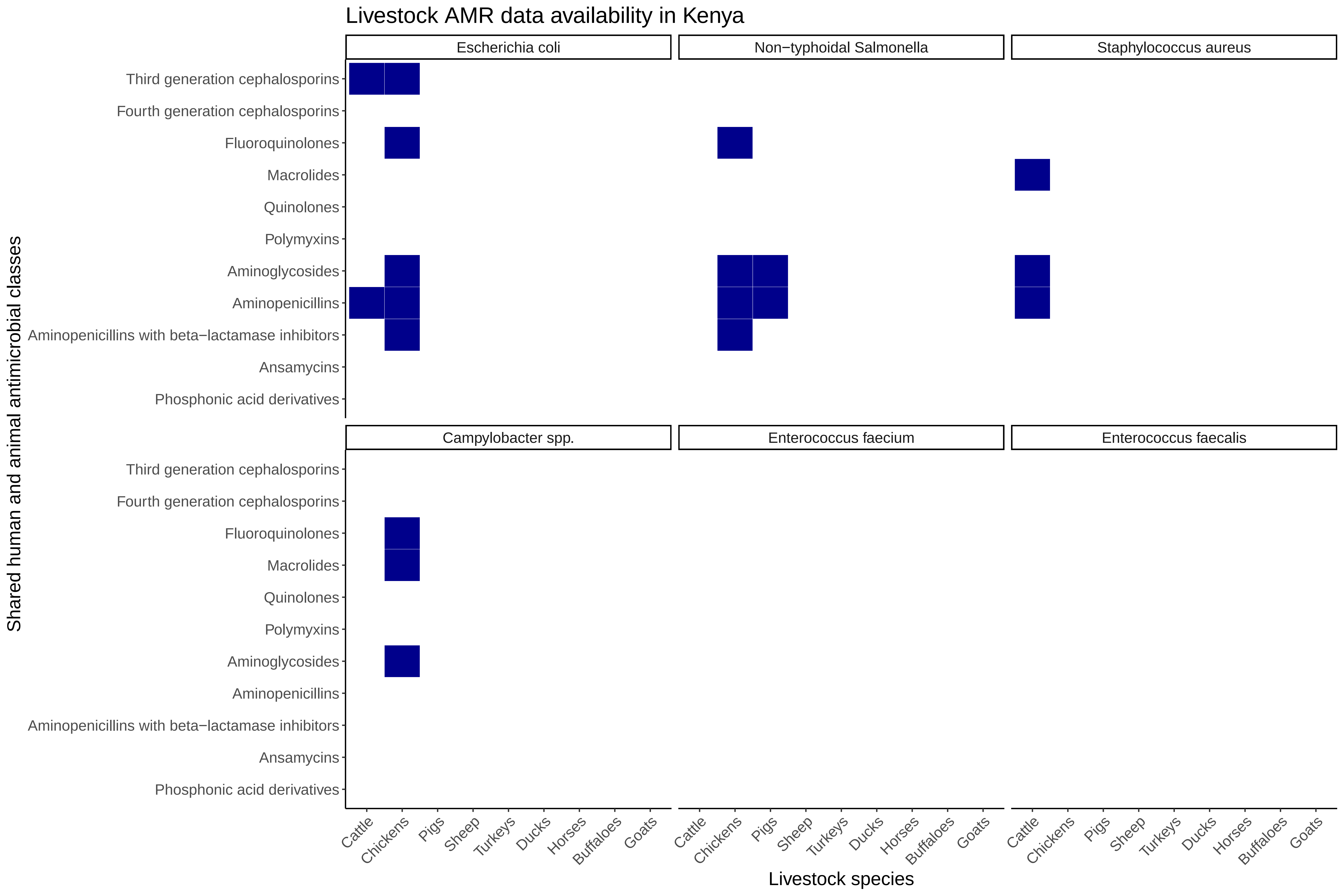

Livestock AMR data availability in Kenya
Escherichia coli
Non−typhoidal Salmonella
Staphylococcus aureus
Campylobacter spp.
Enterococcus faecium
Enterococcus faecalis
Shared human and animal antimicrobial classes
Third generation cephalosporins
Fourth generation cephalosporins
Fluoroquinolones
Macrolides
Quinolones
Polymyxins
Aminoglycosides
Aminopenicillins
Aminopenicillins with beta−lactamase inhibitors
Ansamycins
Phosphonic acid derivatives
Cattle
Chickens
Pigs
Sheep
Turkeys
Ducks
Horses
Buffaloes
Goats
Livestock species

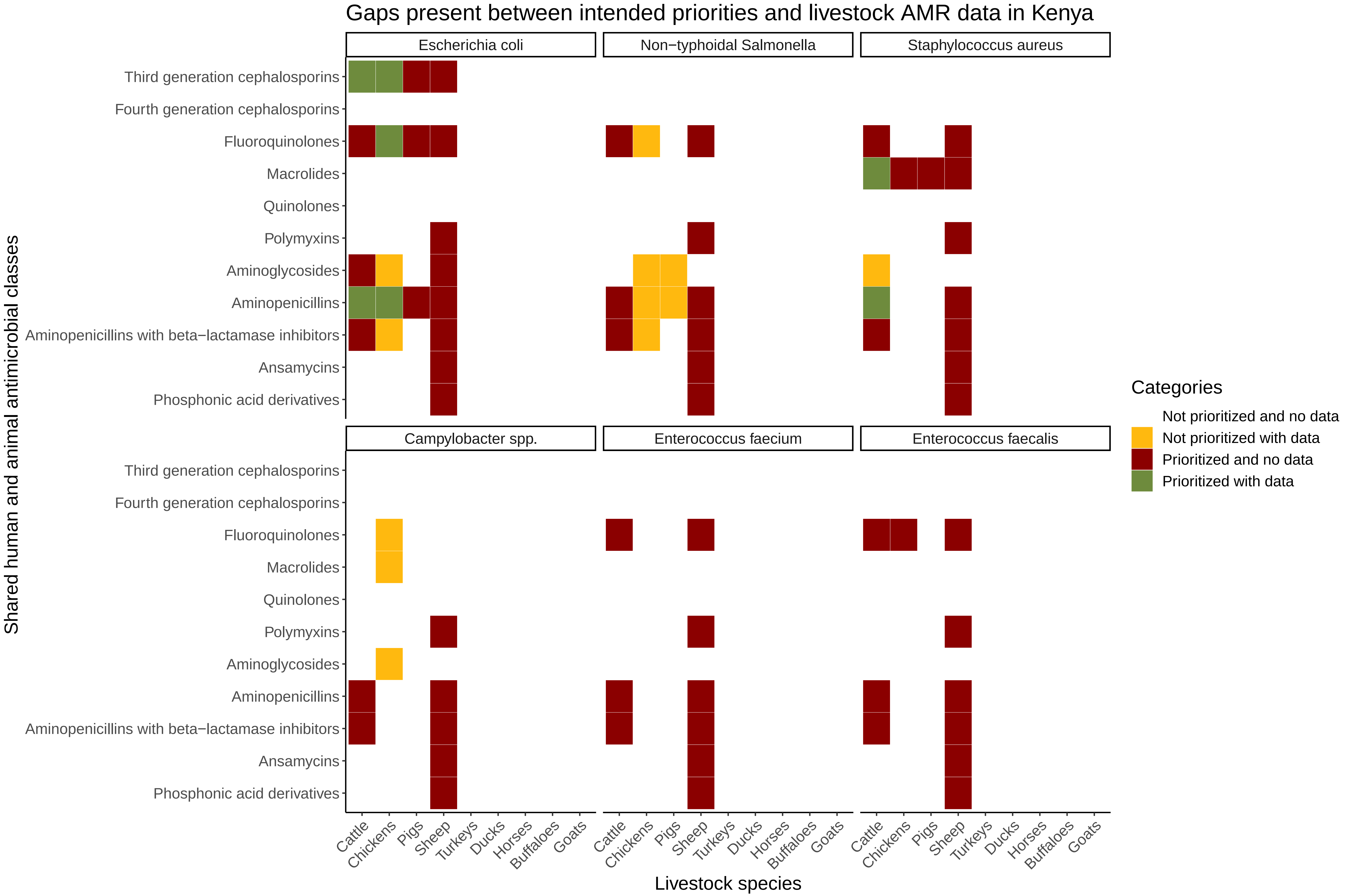

Gaps present between intended priorities and livestock AMR data in Kenya
Escherichia coli
Non−typhoidal Salmonella
Staphylococcus aureus
Campylobacter spp.
Enterococcus faecium
Enterococcus faecalis
Third generation cephalosporins
Fourth generation cephalosporins
Fluoroquinolones
Macrolides
Quinolones
Polymyxins
Aminoglycosides
Aminopenicillins
Aminopenicillins with beta−lactamase inhibitors
Ansamycins
Phosphonic acid derivatives
Shared human and animal antimicrobial classes
Cattle
Chickens
Pigs
Sheep
Turkeys
Ducks
Horses
Buffaloes
Goats
Livestock species
Categories
Not prioritized and no data
Not prioritized with data
Prioritized and no data
Prioritized with data

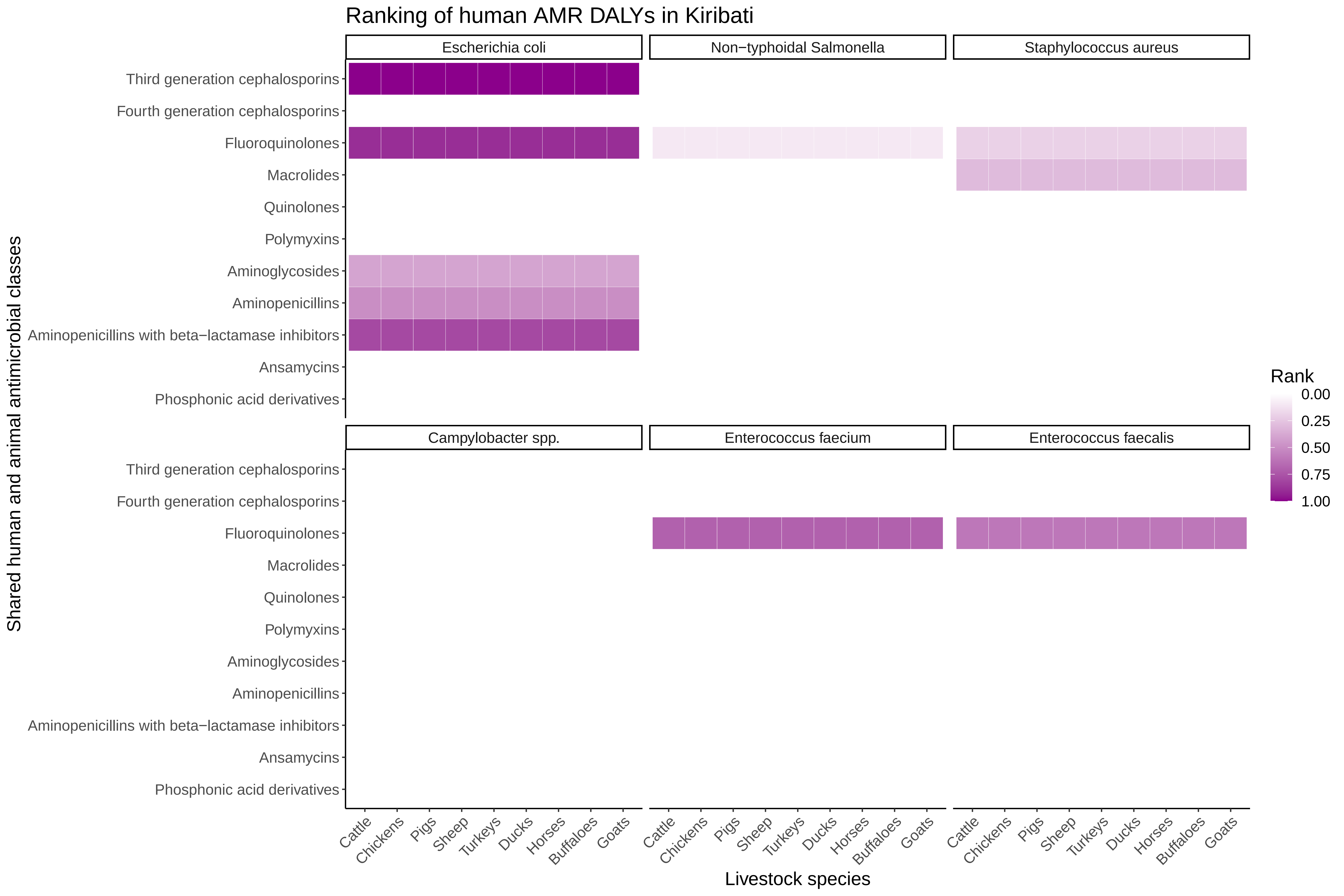
Ranking of human AMR DALYs in Kiribati
Escherichia coli
Non−typhoidal Salmonella
Staphylococcus aureus
Campylobacter spp.
Enterococcus faecium
Enterococcus faecalis
Shared human and animal antimicrobial classes
Third generation cephalosporins
Fourth generation cephalosporins
Fluoroquinolones
Macrolides
Quinolones
Polymyxins
Aminoglycosides
Aminopenicillins
Aminopenicillins with beta−lactamase inhibitors
Ansamycins
Phosphonic acid derivatives
Cattle
Chickens
Pigs
Sheep
Turkeys
Ducks
Horses
Buffaloes
Goats
Livestock species
Rank
0.00
0.25
0.50
0.75
1.00

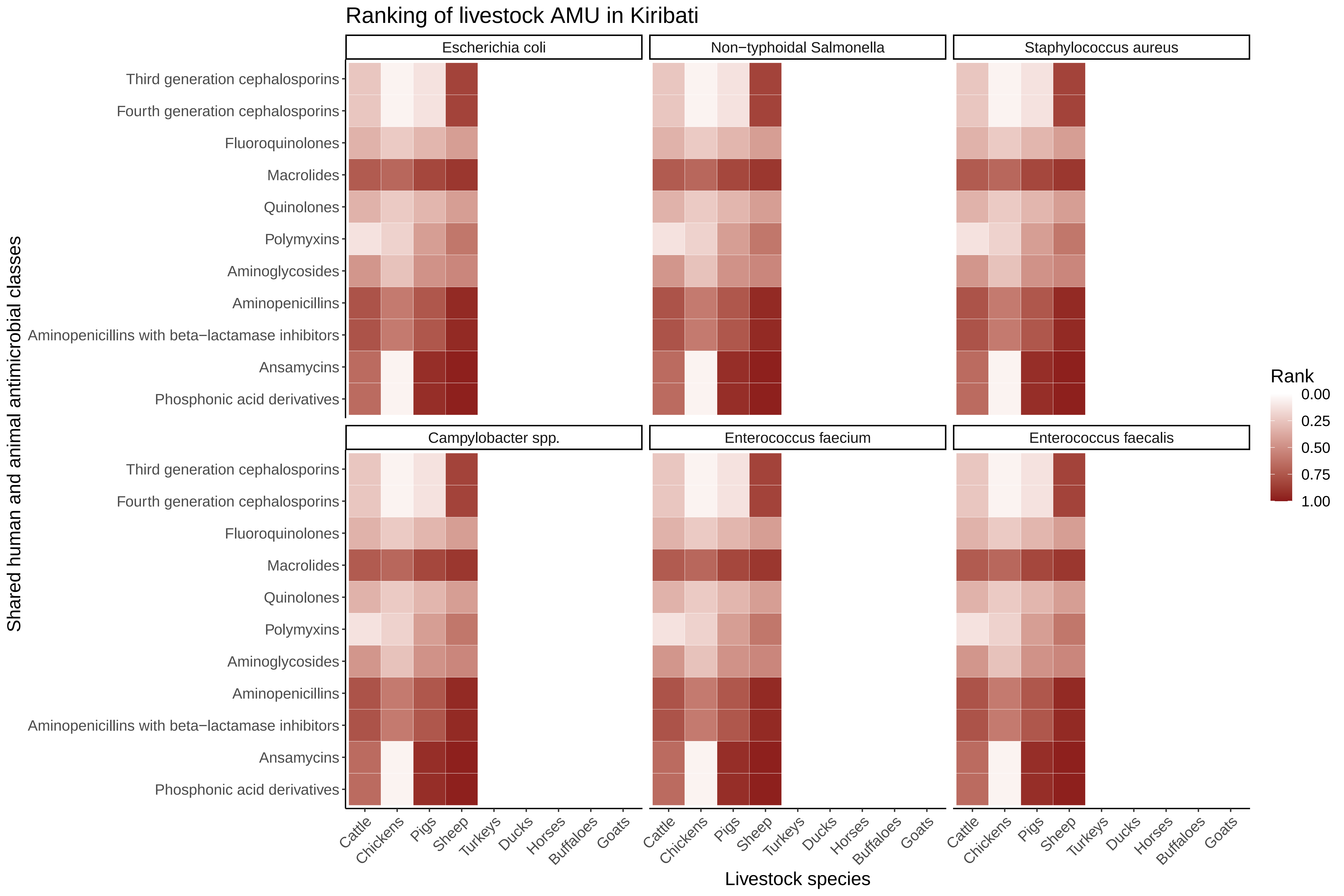

Ranking of livestock AMU in Kiribati
Escherichia coli
Non−typhoidal Salmonella
Staphylococcus aureus
Campylobacter spp.
Enterococcus faecium
Enterococcus faecalis
Third generation cephalosporins
Fourth generation cephalosporins
Fluoroquinolones
Macrolides
Quinolones
Polymyxins
Aminoglycosides
Aminopenicillins
Aminopenicillins with beta−lactamase inhibitors
Ansamycins
Phosphonic acid derivatives
Shared human and animal antimicrobial classes
Cattle
Chickens
Pigs
Sheep
Turkeys
Ducks
Horses
Buffaloes
Goats
Livestock species
Rank
0.00
0.25
0.50
0.75
1.00

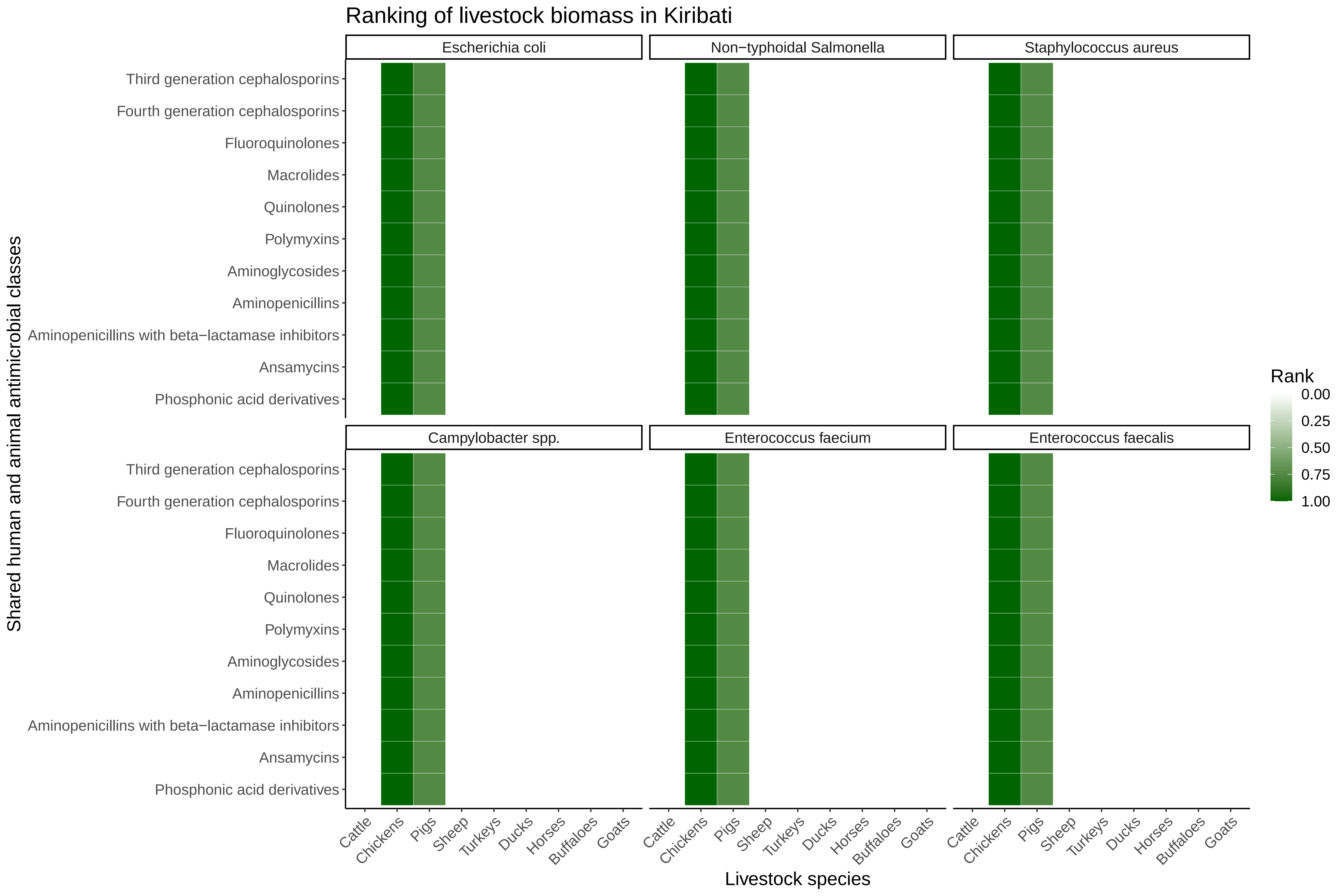
Ranking of livestock biomass in Kiribati
Escherichia coli
Non−typhoidal Salmonella
Staphylococcus aureus
Campylobacter spp.
Enterococcus faecium
Enterococcus faecalis
Third generation cephalosporins
Fourth generation cephalosporins
Fluoroquinolones
Macrolides
Quinolones
Polymyxins
Aminoglycosides
Aminopenicillins
Aminopenicillins with beta−lactamase inhibitors
Ansamycins
Phosphonic acid derivatives
Shared human and animal antimicrobial classes
Cattle
Chickens
Pigs
Sheep
Turkeys
Ducks
Horses
Buffaloes
Goats
Livestock species
Rank
0.00
0.25
0.50
0.75
1.00

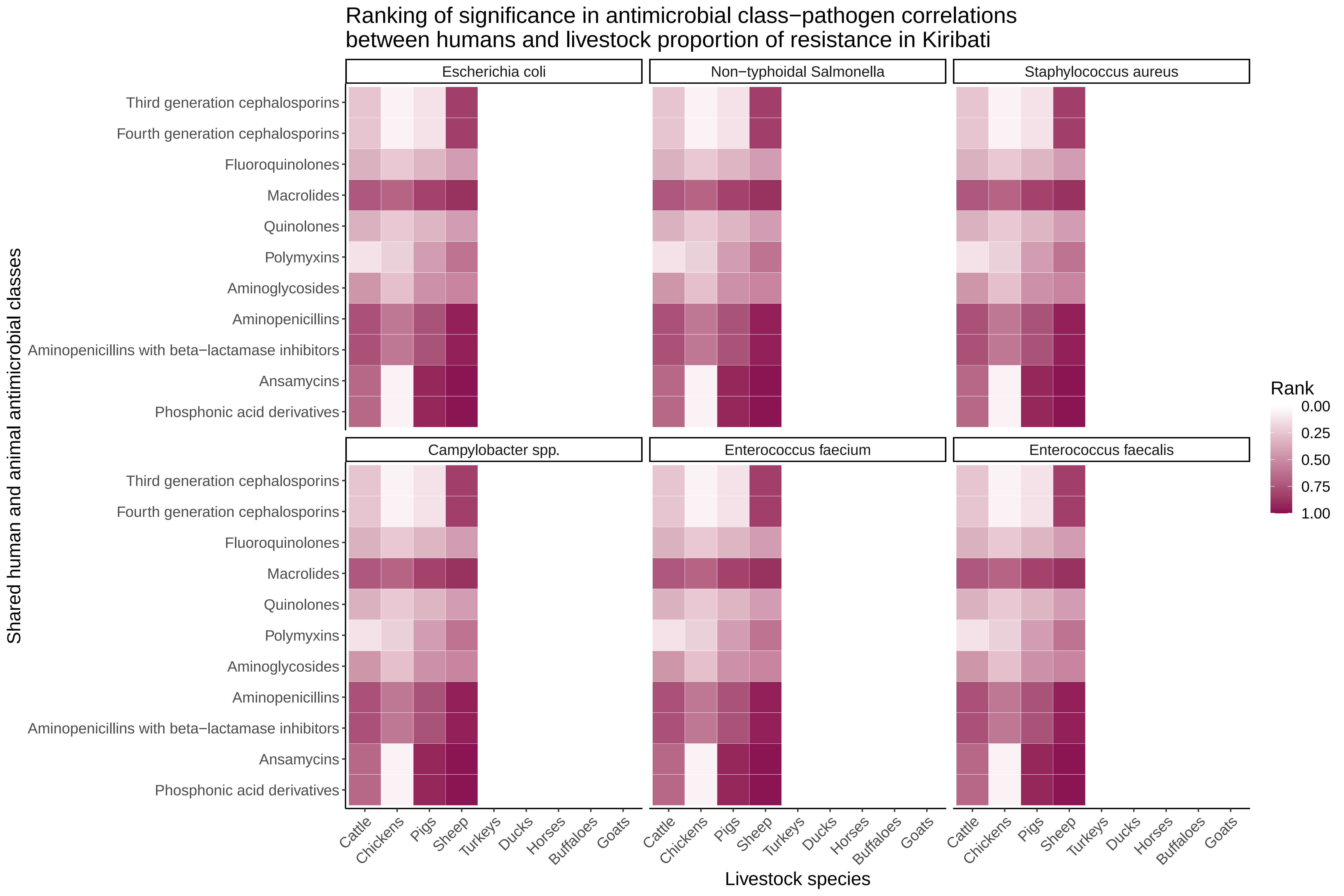

Ranking of significance in antimicrobial class−pathogen correlations
between humans and livestock proportion of resistance in Kiribati
Escherichia coli
Non−typhoidal Salmonella
Staphylococcus aureus
Campylobacter spp.
Enterococcus faecium
Enterococcus faecalis
Third generation cephalosporins
Fourth generation cephalosporins
Fluoroquinolones
Macrolides
Quinolones
Polymyxins
Aminoglycosides
Aminopenicillins
Aminopenicillins with beta−lactamase inhibitors
Ansamycins
Phosphonic acid derivatives
Shared human and animal antimicrobial classes
Cattle
Chickens
Pigs
Sheep
Turkeys
Ducks
Horses
Buffaloes
Goats
Livestock species
Rank
0.00
0.25
0.50
0.75
1.00

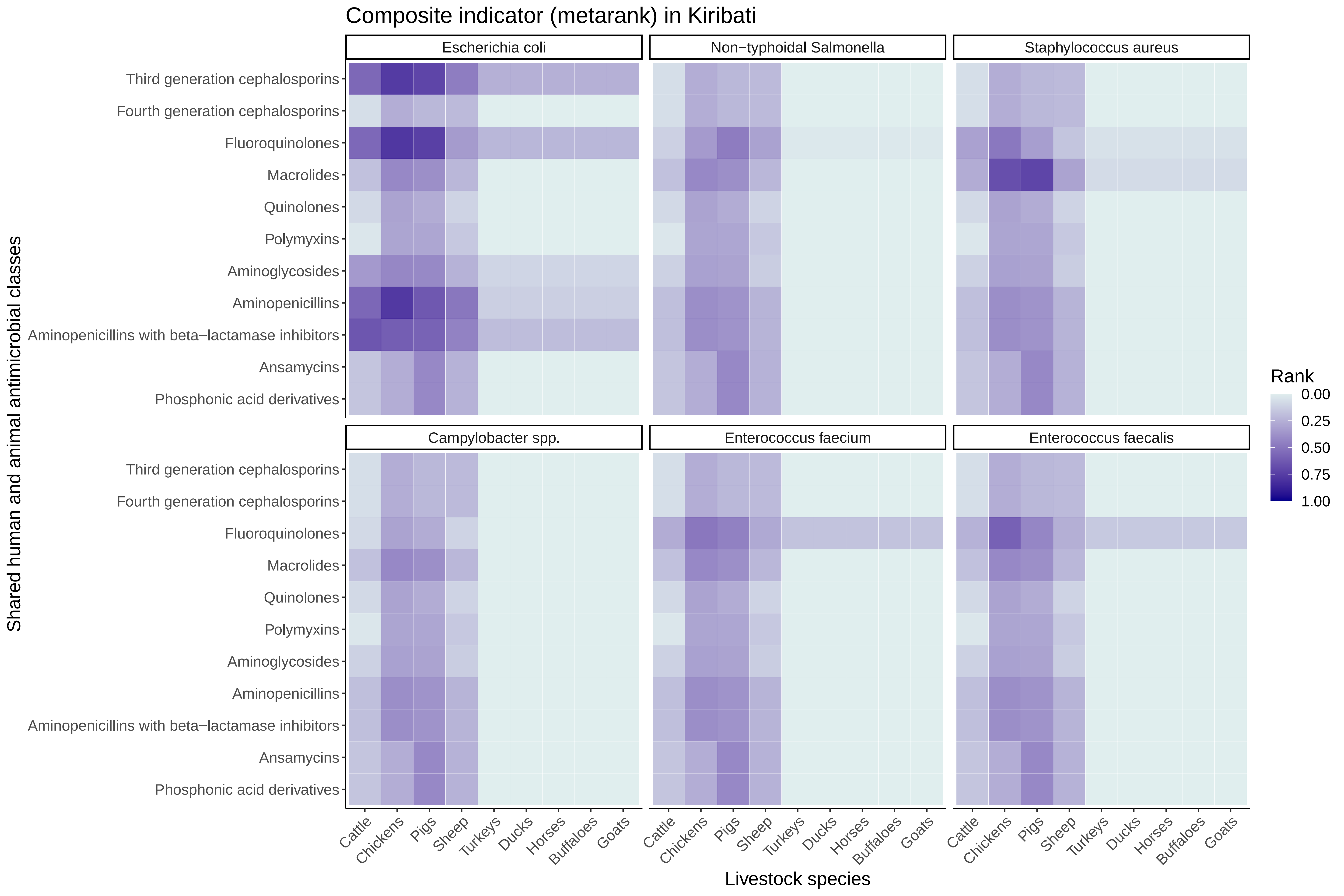

Composite indicator (metarank) in Kiribati
Escherichia coli
Non−typhoidal Salmonella
Staphylococcus aureus
Campylobacter spp.
Enterococcus faecium
Enterococcus faecalis
Shared human and animal antimicrobial classes
Third generation cephalosporins
Fourth generation cephalosporins
Fluoroquinolones
Macrolides
Quinolones
Polymyxins
Aminoglycosides
Aminopenicillins
Aminopenicillins with beta−lactamase inhibitors
Ansamycins
Phosphonic acid derivatives
Cattle
Chickens
Pigs
Sheep
Turkeys
Ducks
Horses
Buffaloes
Goats
Livestock species
Rank
0.00
0.25
0.50
0.75
1.00

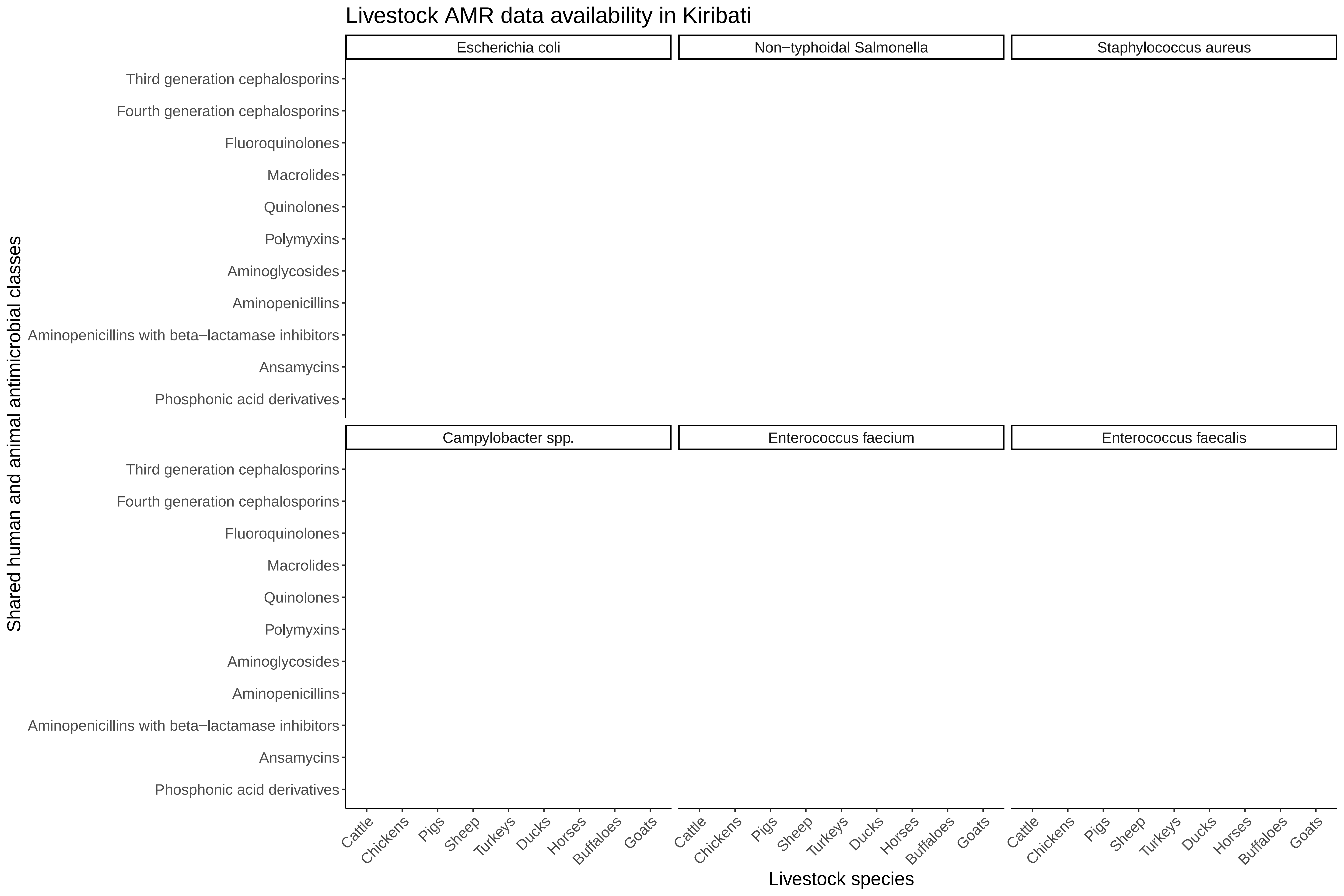
Livestock AMR data availability in Kiribati
Escherichia coli
Non−typhoidal Salmonella
Staphylococcus aureus
Campylobacter spp.
Enterococcus faecium
Enterococcus faecalis
Shared human and animal antimicrobial classes
Third generation cephalosporins
Fourth generation cephalosporins
Fluoroquinolones
Macrolides
Quinolones
Polymyxins
Aminoglycosides
Aminopenicillins
Aminopenicillins with beta−lactamase inhibitors
Ansamycins
Phosphonic acid derivatives
Cattle
Chickens
Pigs
Sheep
Turkeys
Ducks
Horses
Buffaloes
Goats
Livestock species

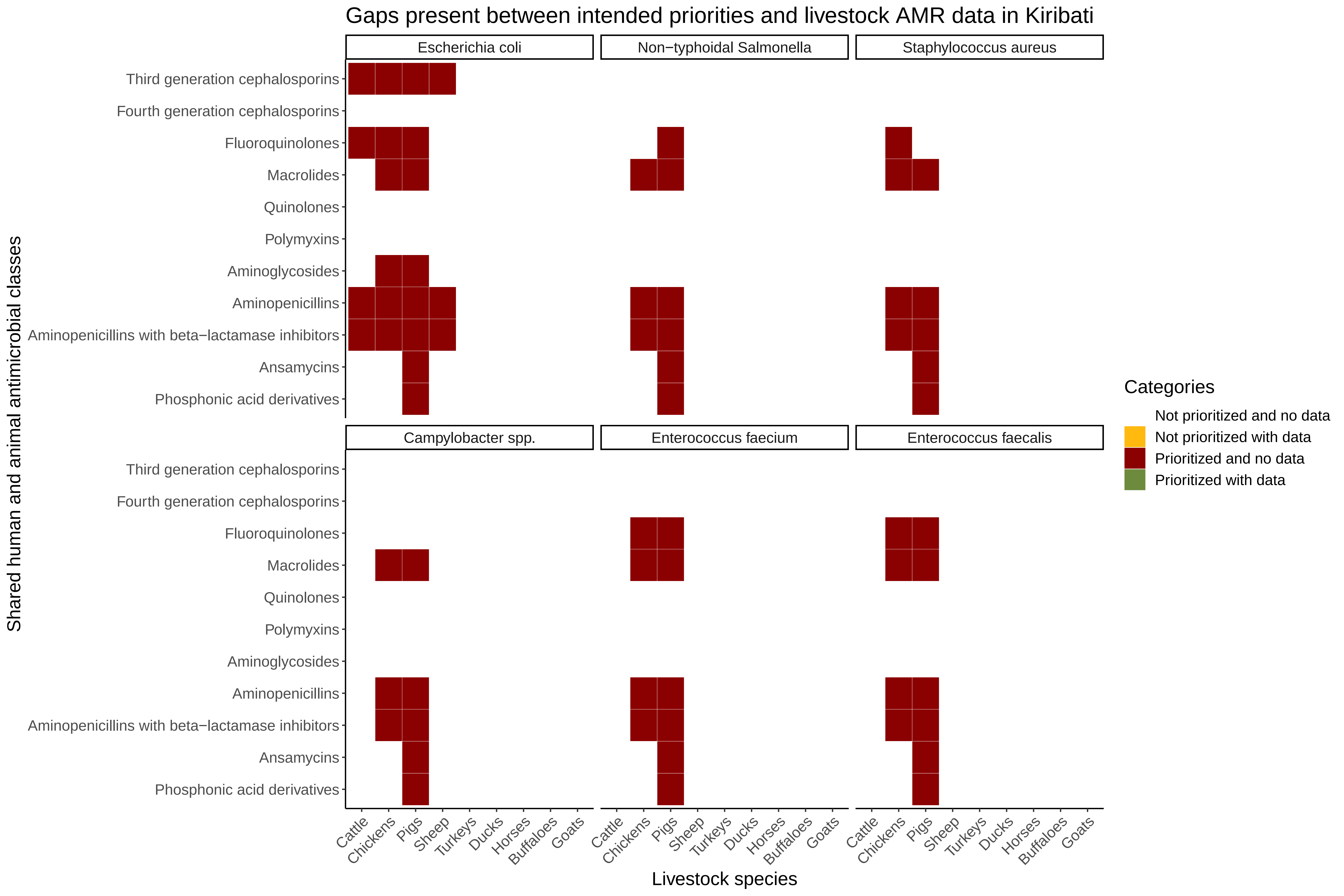

Gaps present between intended priorities and livestock AMR data in Kiribati
Escherichia coli
Non−typhoidal Salmonella
Staphylococcus aureus
Campylobacter spp.
Enterococcus faecium
Enterococcus faecalis
Third generation cephalosporins
Fourth generation cephalosporins
Fluoroquinolones
Macrolides
Quinolones
Polymyxins
Aminoglycosides
Aminopenicillins
Aminopenicillins with beta−lactamase inhibitors
Ansamycins
Phosphonic acid derivatives
Cattle
Chickens
Pigs
Sheep
Turkeys
Ducks
Horses
Buffaloes
Goats
Livestock species
Shared human and animal antimicrobial classes
Categories
Not prioritized and no data
Not prioritized with data
Prioritized and no data
Prioritized with data

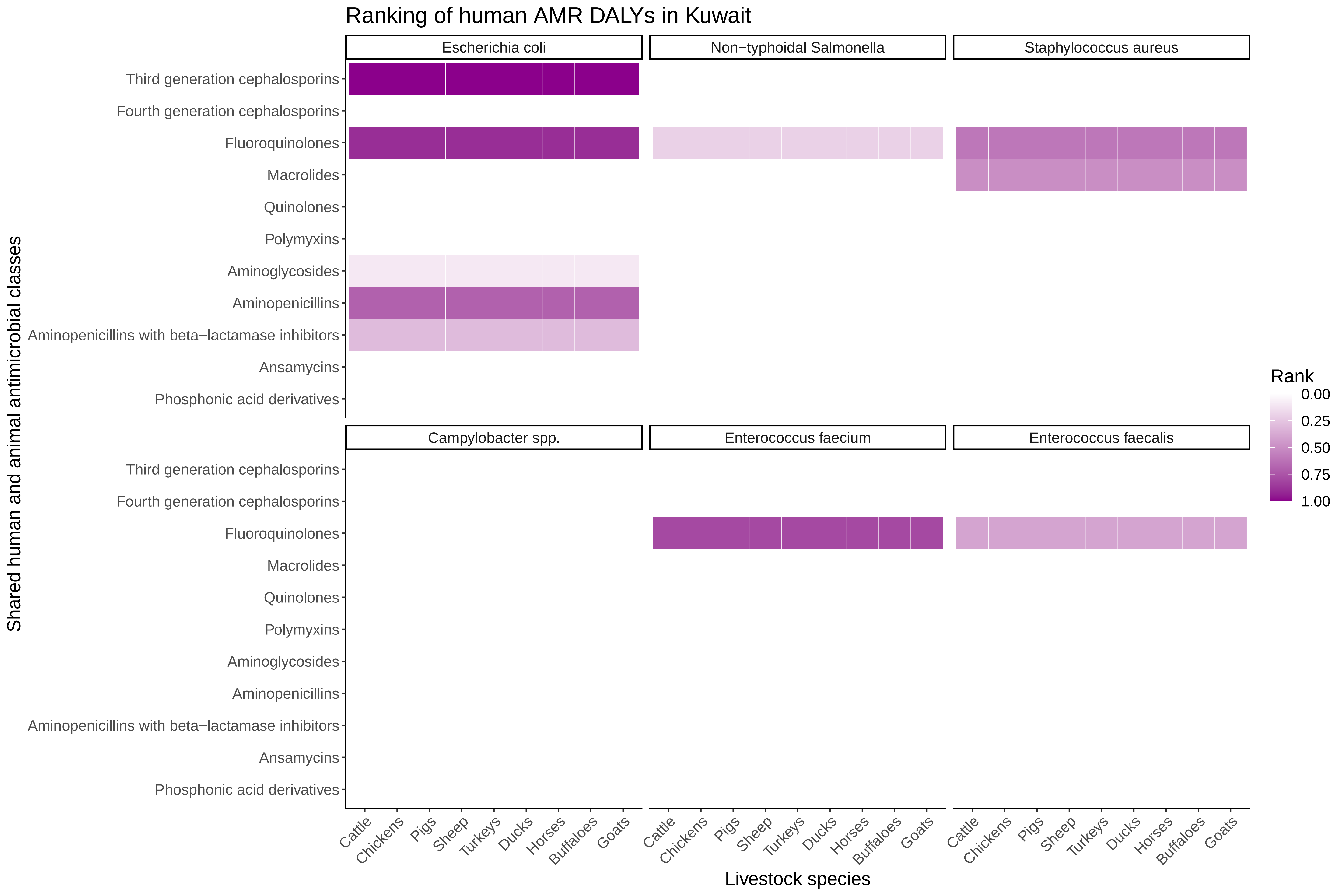
Ranking of human AMR DALYs in Kuwait
Escherichia coli
Non−typhoidal Salmonella
Staphylococcus aureus
Campylobacter spp.
Enterococcus faecium
Enterococcus faecalis
Third generation cephalosporins
Fourth generation cephalosporins
Fluoroquinolones
Macrolides
Quinolones
Polymyxins
Aminoglycosides
Aminopenicillins
Aminopenicillins with beta−lactamase inhibitors
Ansamycins
Phosphonic acid derivatives
Shared human and animal antimicrobial classes
Cattle
Chickens
Pigs
Sheep
Turkeys
Ducks
Horses
Buffaloes
Goats
Livestock species
Rank
0.00
0.25
0.50
0.75
1.00

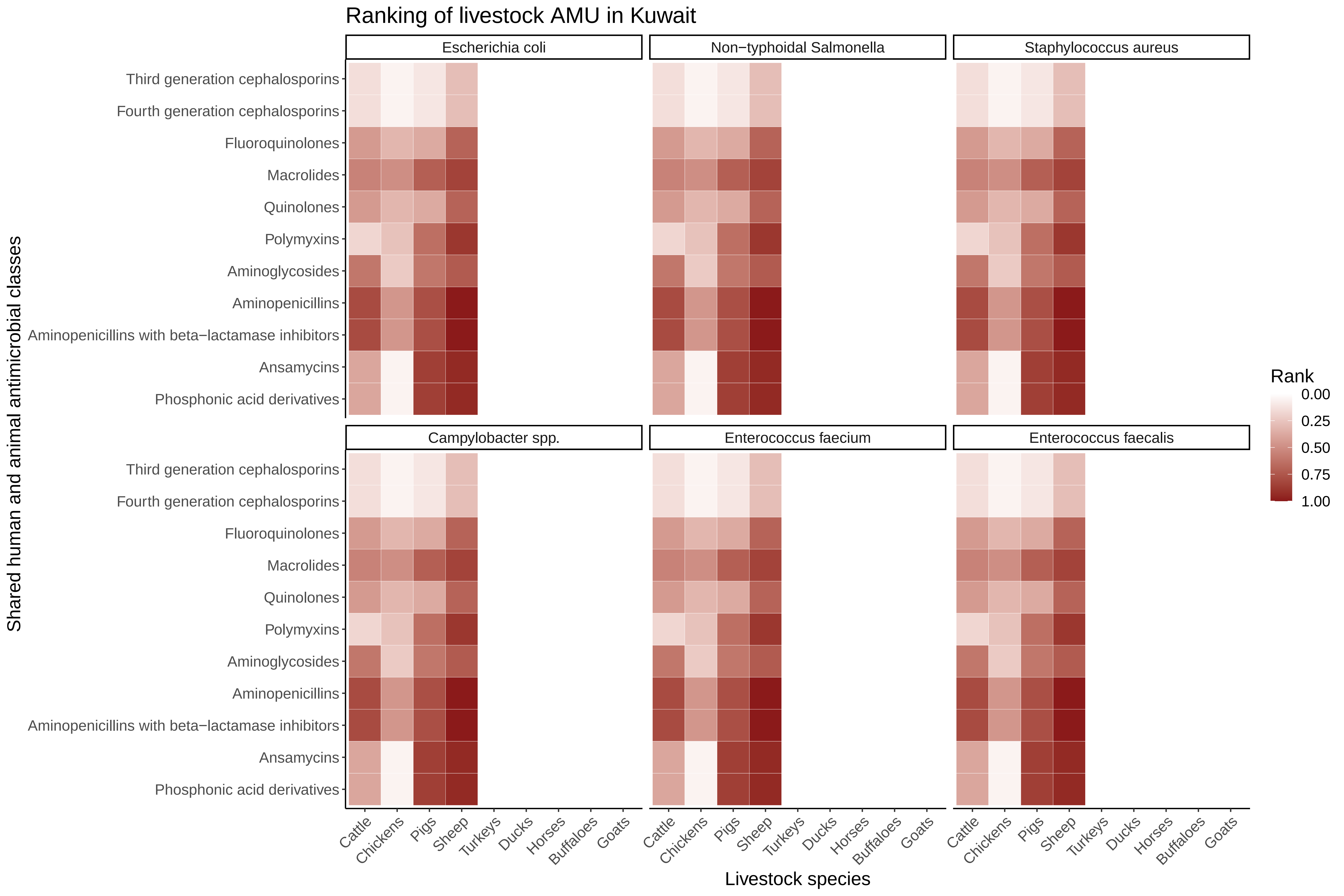

Ranking of livestock AMU in Kuwait
Escherichia coli
Non−typhoidal Salmonella
Staphylococcus aureus
Campylobacter spp.
Enterococcus faecium
Enterococcus faecalis
Shared human and animal antimicrobial classes
Third generation cephalosporins
Fourth generation cephalosporins
Fluoroquinolones
Macrolides
Quinolones
Polymyxins
Aminoglycosides
Aminopenicillins
Aminopenicillins with beta−lactamase inhibitors
Ansamycins
Phosphonic acid derivatives
Cattle
Chickens
Pigs
Sheep
Turkeys
Ducks
Horses
Buffaloes
Goats
Livestock species
Rank
0.00
0.25
0.50
0.75
1.00

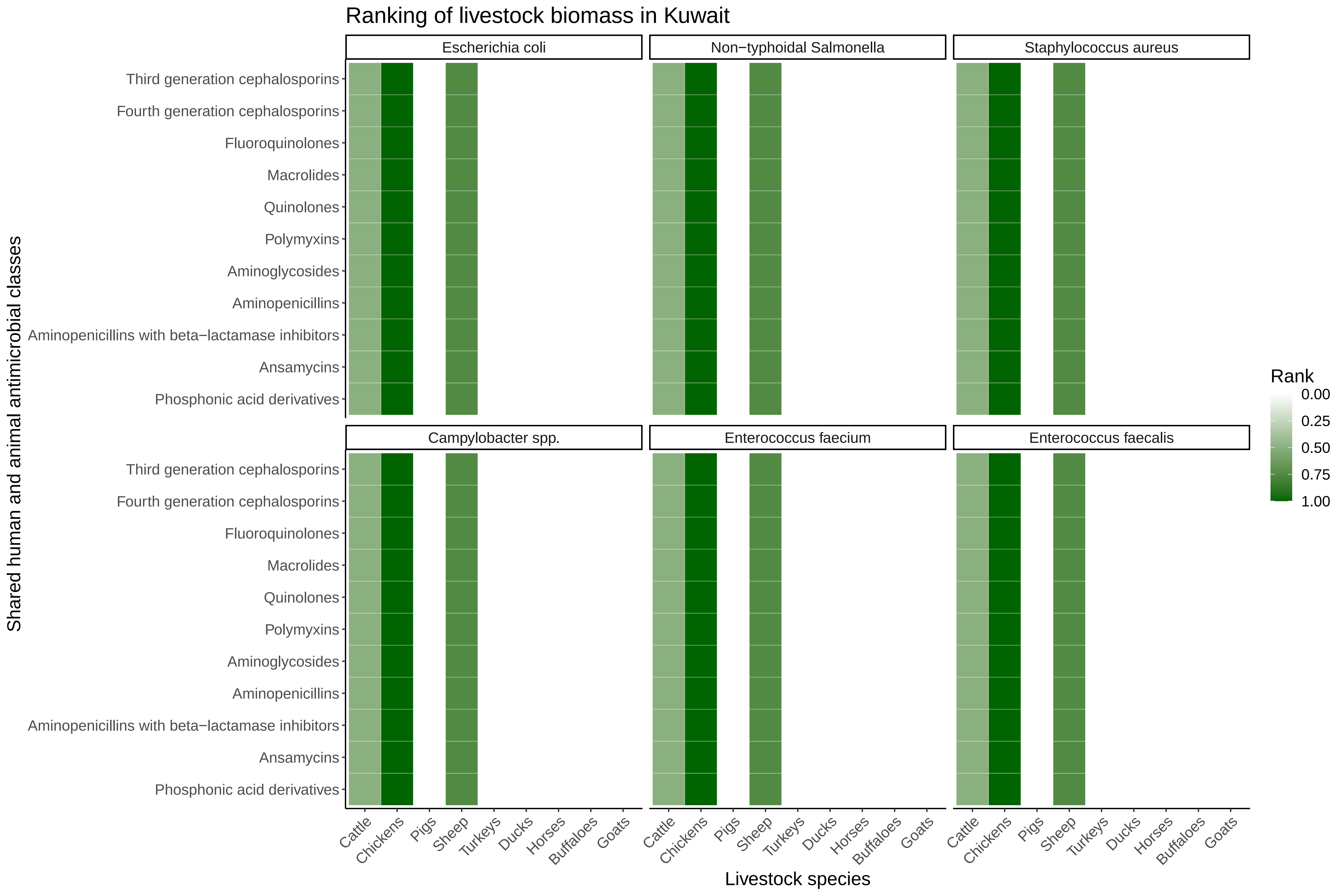

Ranking of livestock biomass in Kuwait
Escherichia coli
Non−typhoidal Salmonella
Staphylococcus aureus
Campylobacter spp.
Enterococcus faecium
Enterococcus faecalis
Third generation cephalosporins
Fourth generation cephalosporins
Fluoroquinolones
Macrolides
Quinolones
Polymyxins
Aminoglycosides
Aminopenicillins
Aminopenicillins with beta−lactamase inhibitors
Ansamycins
Phosphonic acid derivatives
Shared human and animal antimicrobial classes
Cattle
Chickens
Pigs
Sheep
Turkeys
Ducks
Horses
Buffaloes
Goats
Livestock species
Rank
0.00
0.25
0.50
0.75
1.00

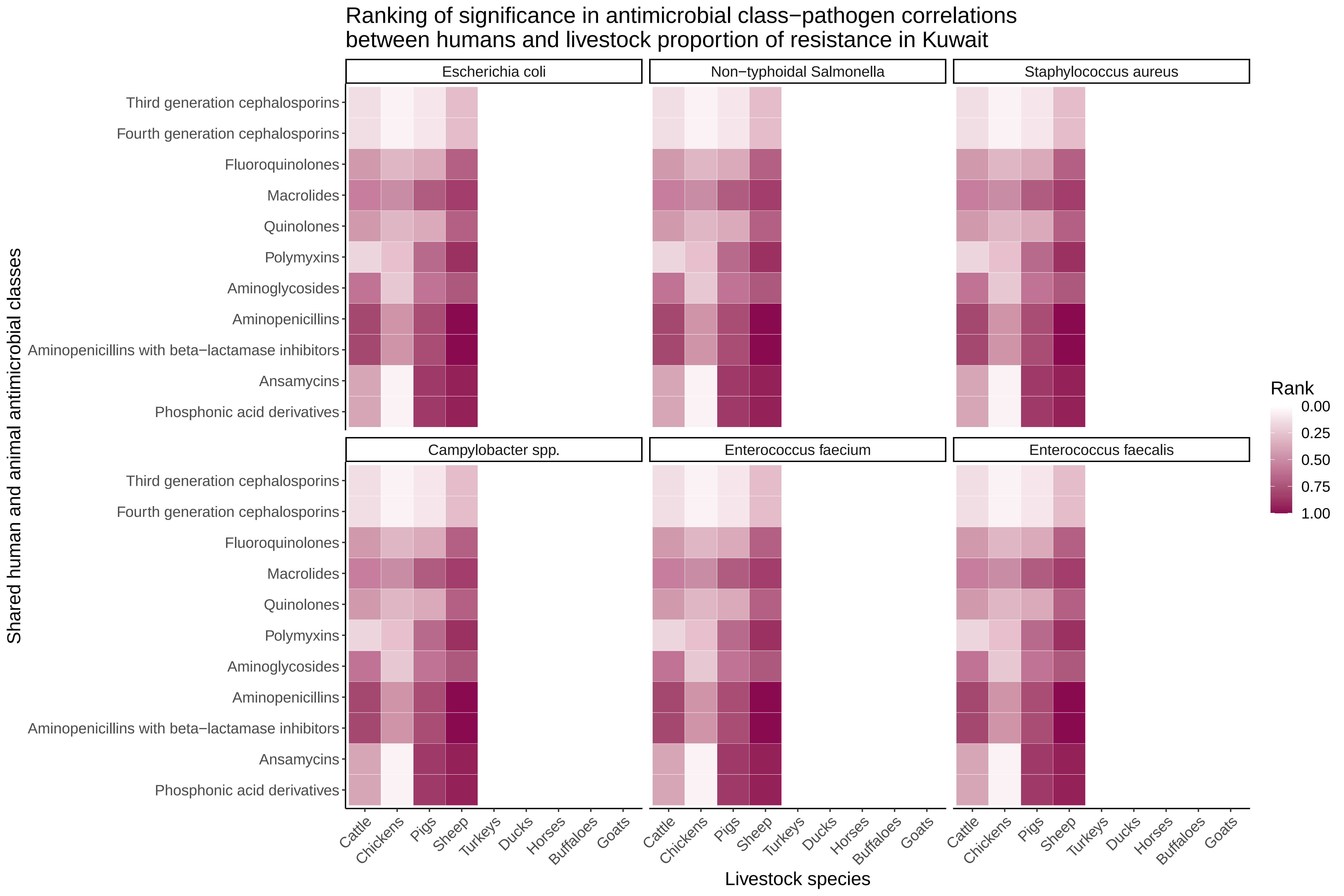

Ranking of significance in antimicrobial class−pathogen correlations
between humans and livestock proportion of resistance in Kuwait
Escherichia coli
Non−typhoidal Salmonella
Staphylococcus aureus
Campylobacter spp.
Enterococcus faecium
Enterococcus faecalis
Third generation cephalosporins
Fourth generation cephalosporins
Fluoroquinolones
Macrolides
Quinolones
Polymyxins
Aminoglycosides
Aminopenicillins
Aminopenicillins with beta−lactamase inhibitors
Ansamycins
Phosphonic acid derivatives
Shared human and animal antimicrobial classes
Cattle
Chickens
Pigs
Sheep
Turkeys
Ducks
Horses
Buffaloes
Goats
Livestock species
Rank
0.00
0.25
0.50
0.75
1.00

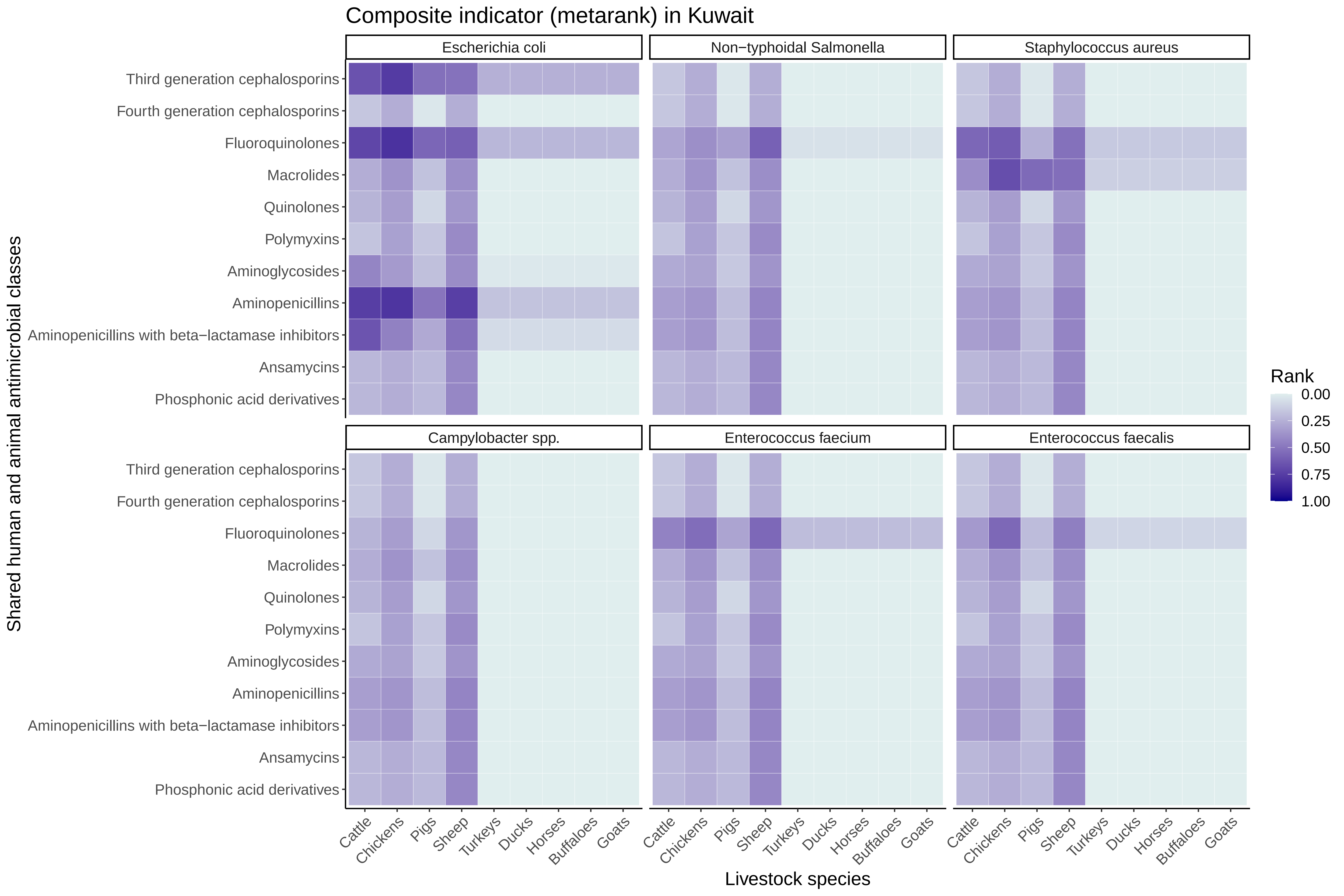

Composite indicator (metarank) in Kuwait
Escherichia coli
Non−typhoidal Salmonella
Staphylococcus aureus
Campylobacter spp.
Enterococcus faecium
Enterococcus faecalis
Third generation cephalosporins
Fourth generation cephalosporins
Fluoroquinolones
Macrolides
Quinolones
Polymyxins
Aminoglycosides
Aminopenicillins
Aminopenicillins with beta−lactamase inhibitors
Ansamycins
Phosphonic acid derivatives
Shared human and animal antimicrobial classes
Cattle
Chickens
Pigs
Sheep
Turkeys
Ducks
Horses
Buffaloes
Goats
Livestock species
Rank
0.00
0.25
0.50
0.75
1.00

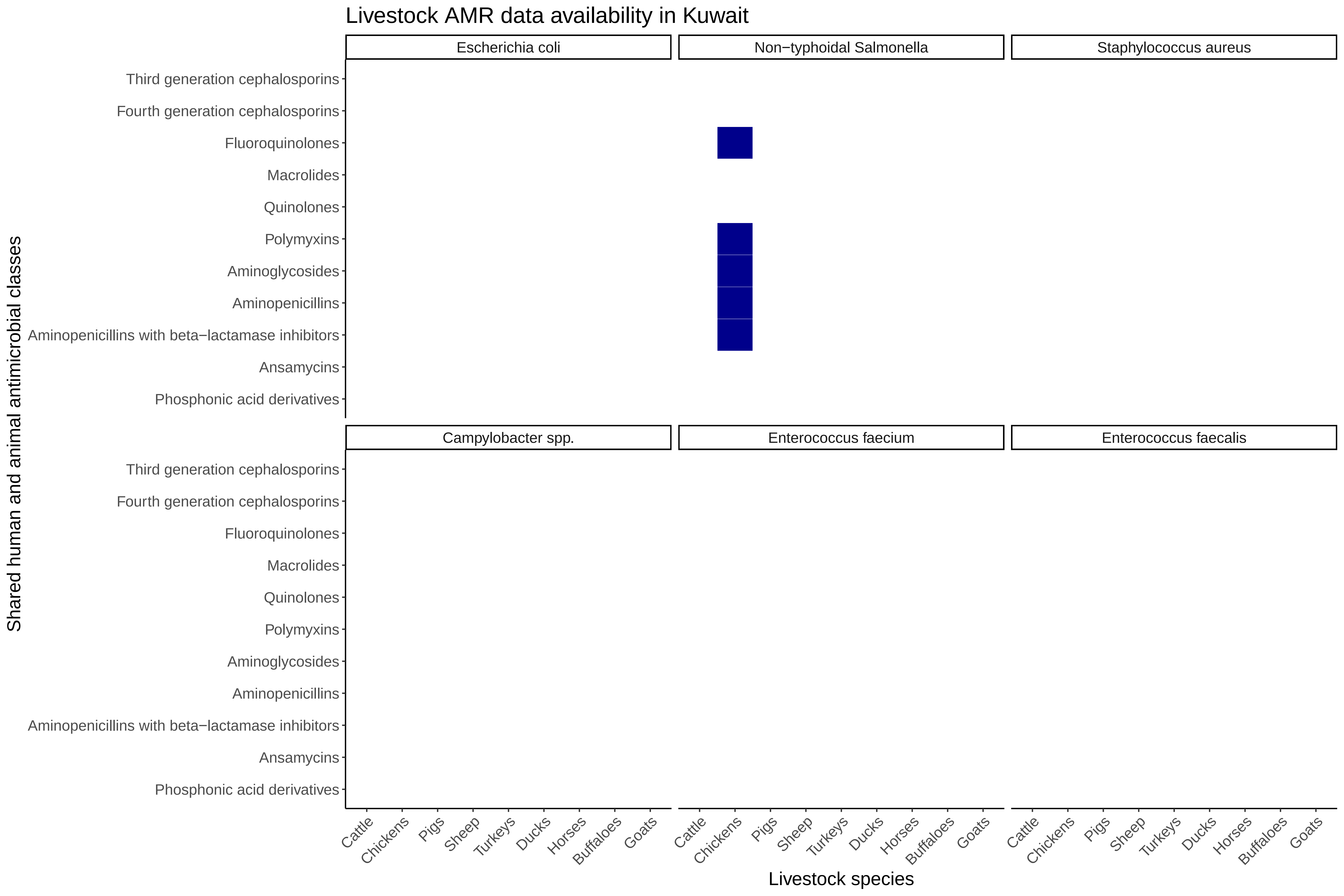
Livestock AMR data availability in Kuwait
Escherichia coli
Non−typhoidal Salmonella
Staphylococcus aureus
Campylobacter spp.
Enterococcus faecium
Enterococcus faecalis
Shared human and animal antimicrobial classes
Third generation cephalosporins
Fourth generation cephalosporins
Fluoroquinolones
Macrolides
Quinolones
Polymyxins
Aminoglycosides
Aminopenicillins
Aminopenicillins with beta−lactamase inhibitors
Ansamycins
Phosphonic acid derivatives
Cattle
Chickens
Pigs
Sheep
Turkeys
Ducks
Horses
Buffaloes
Goats
Livestock species

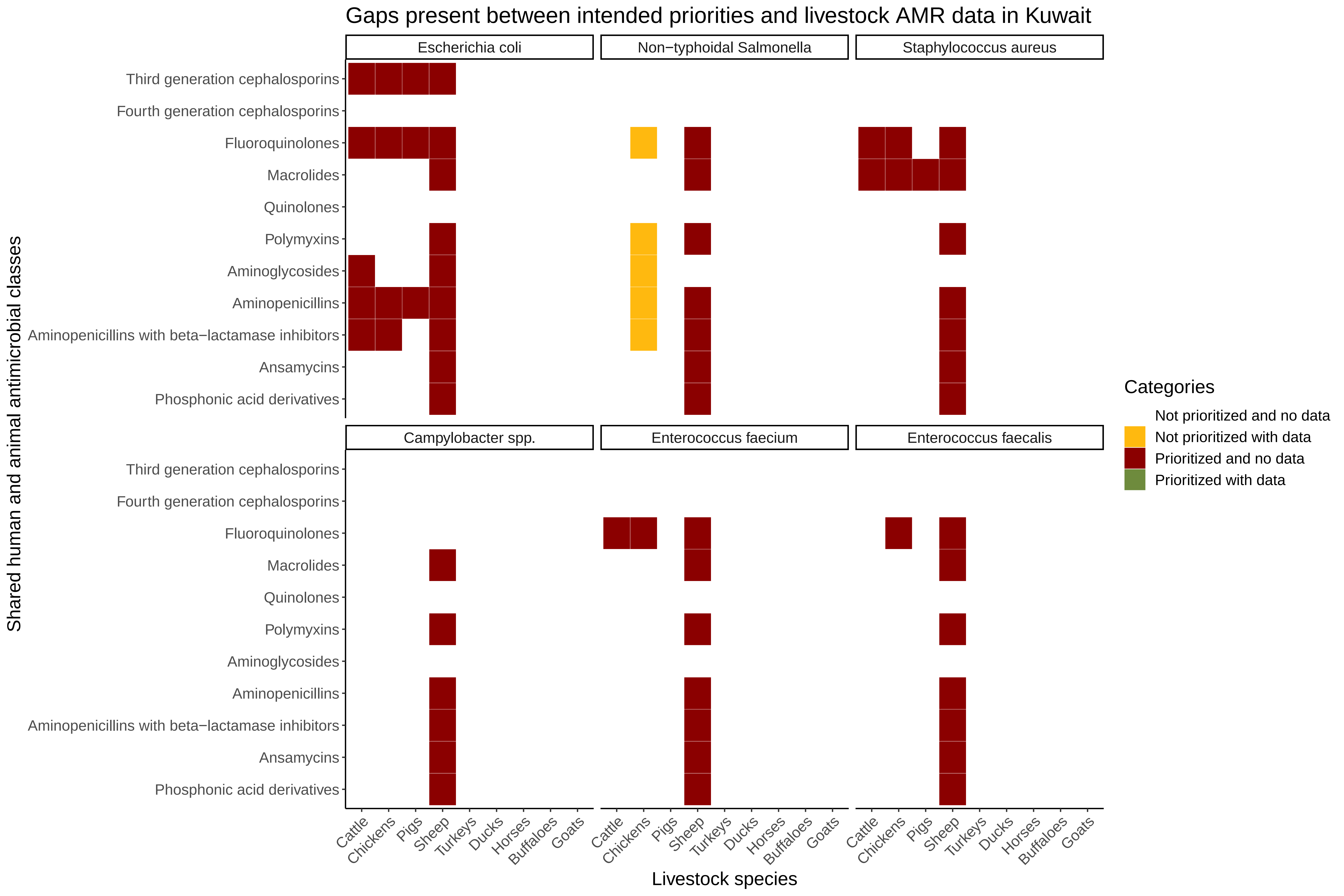

Gaps present between intended priorities and livestock AMR data in Kuwait
Escherichia coli
Non−typhoidal Salmonella
Staphylococcus aureus
Campylobacter spp.
Enterococcus faecium
Enterococcus faecalis
Shared human and animal antimicrobial classes
Third generation cephalosporins
Fourth generation cephalosporins
Fluoroquinolones
Macrolides
Quinolones
Polymyxins
Aminoglycosides
Aminopenicillins
Aminopenicillins with beta−lactamase inhibitors
Ansamycins
Phosphonic acid derivatives
Cattle
Chickens
Pigs
Sheep
Turkeys
Ducks
Horses
Buffaloes
Goats
Livestock species
Categories
Not prioritized and no data
Not prioritized with data
Prioritized and no data
Prioritized with data

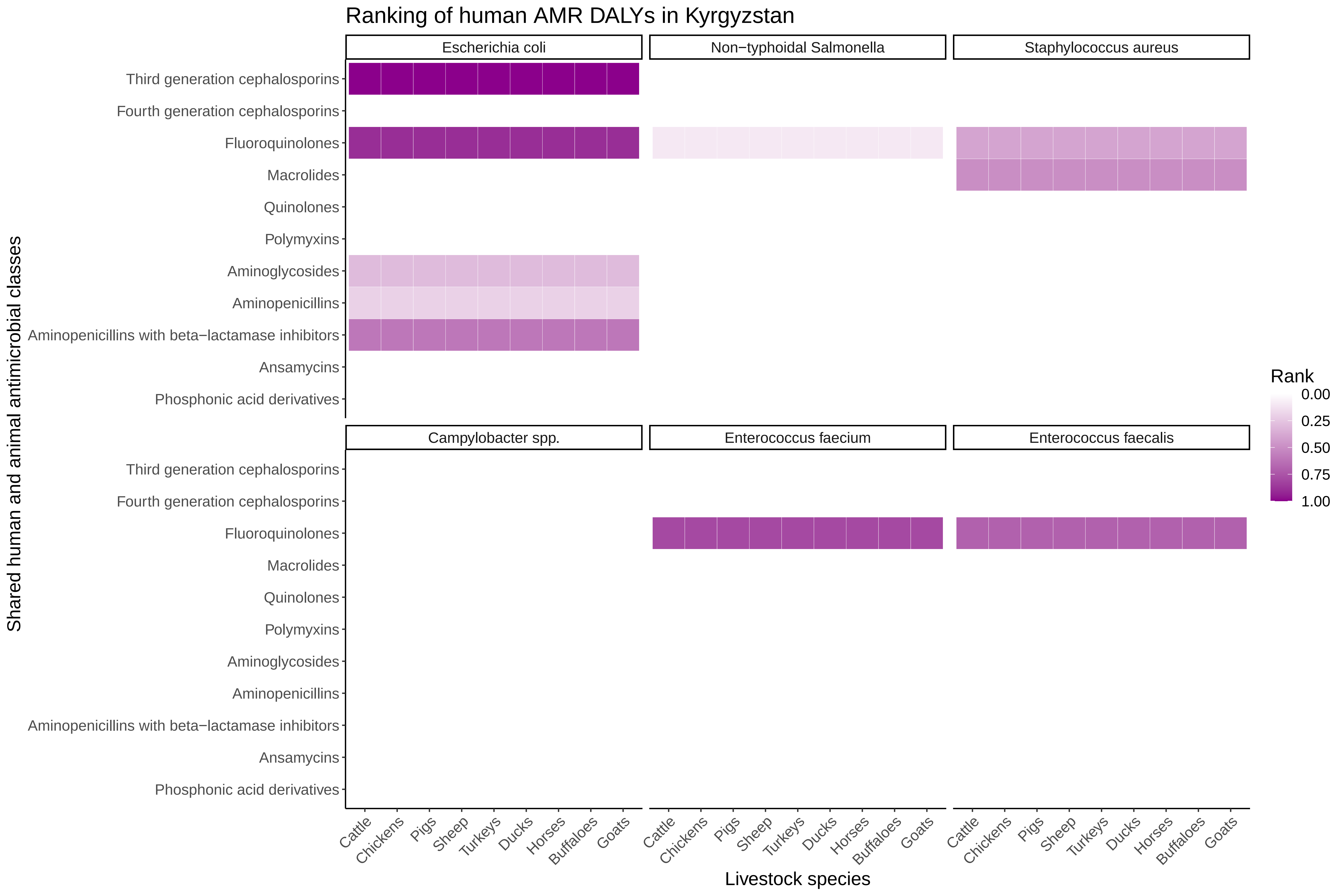
Ranking of human AMR DALYs in Kyrgyzstan
Escherichia coli
Non−typhoidal Salmonella
Staphylococcus aureus
Campylobacter spp.
Enterococcus faecium
Enterococcus faecalis
Shared human and animal antimicrobial classes
Third generation cephalosporins
Fourth generation cephalosporins
Fluoroquinolones
Macrolides
Quinolones
Polymyxins
Aminoglycosides
Aminopenicillins
Aminopenicillins with beta−lactamase inhibitors
Ansamycins
Phosphonic acid derivatives
Livestock species
Cattle
Chickens
Pigs
Sheep
Turkeys
Ducks
Horses
Buffaloes
Goats
Rank
0.00
0.25
0.50
0.75
1.00

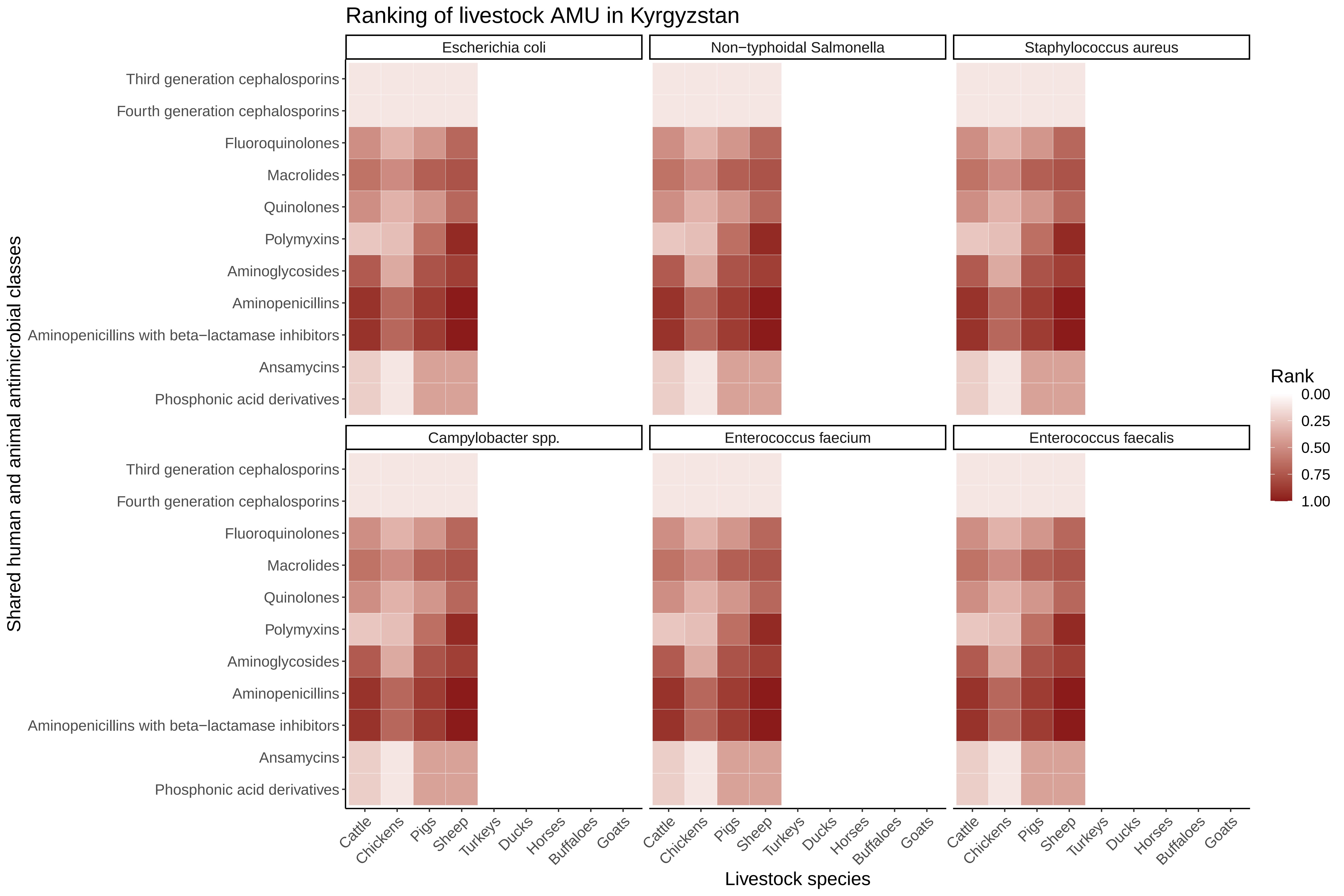
Ranking of livestock AMU in Kyrgyzstan
Escherichia coli
Non−typhoidal Salmonella
Staphylococcus aureus
Campylobacter spp.
Enterococcus faecium
Enterococcus faecalis
Shared human and animal antimicrobial classes
Third generation cephalosporins
Fourth generation cephalosporins
Fluoroquinolones
Macrolides
Quinolones
Polymyxins
Aminoglycosides
Aminopenicillins
Aminopenicillins with beta−lactamase inhibitors
Ansamycins
Phosphonic acid derivatives
Cattle
Chickens
Pigs
Sheep
Turkeys
Ducks
Horses
Buffaloes
Goats
Livestock species
Rank
0.00
0.25
0.50
0.75
1.00

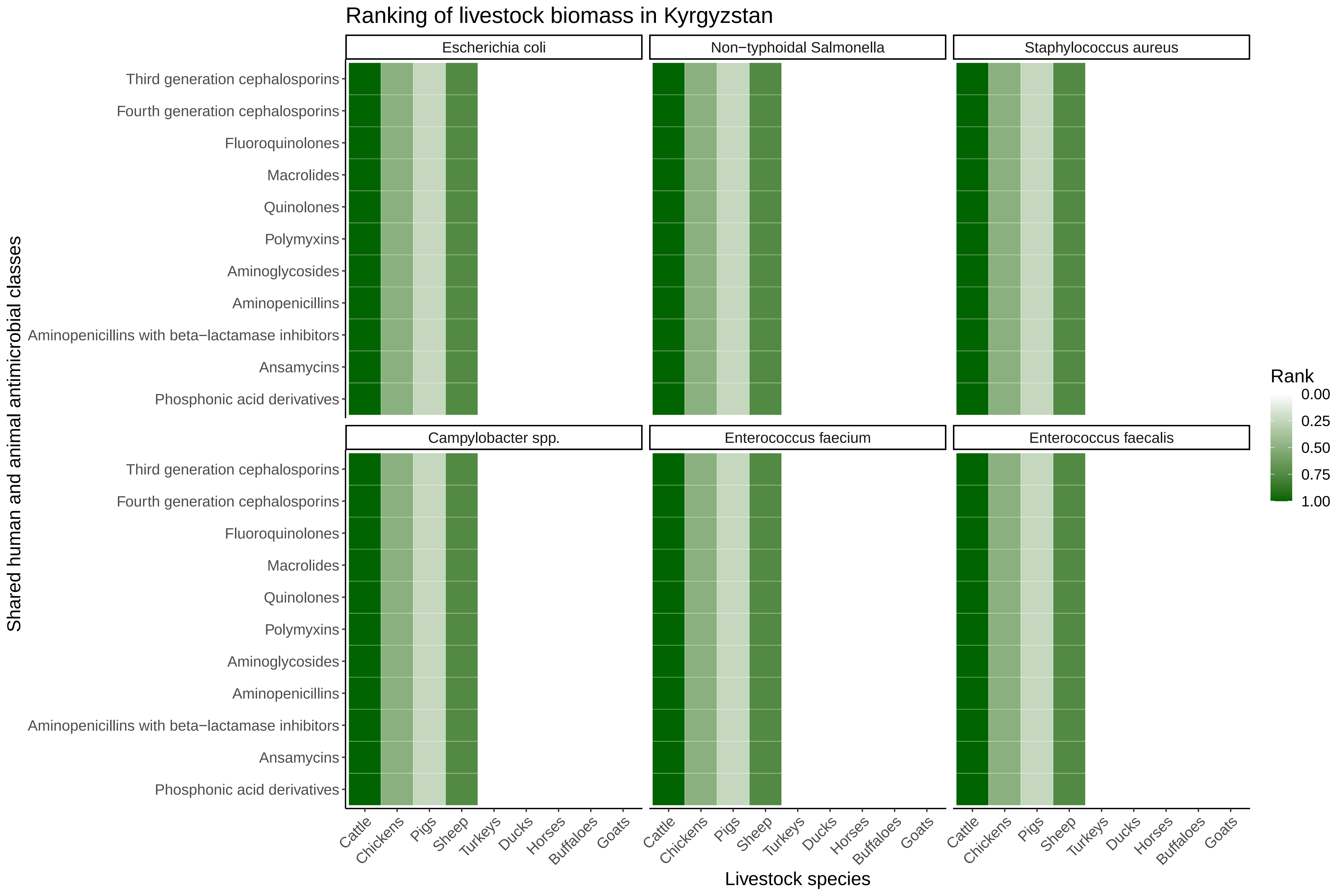

Ranking of livestock biomass in Kyrgyzstan
Escherichia coli
Non−typhoidal Salmonella
Staphylococcus aureus
Campylobacter spp.
Enterococcus faecium
Enterococcus faecalis
Third generation cephalosporins
Fourth generation cephalosporins
Fluoroquinolones
Macrolides
Quinolones
Polymyxins
Aminoglycosides
Aminopenicillins
Aminopenicillins with beta−lactamase inhibitors
Ansamycins
Phosphonic acid derivatives
Shared human and animal antimicrobial classes
Cattle
Chickens
Pigs
Sheep
Turkeys
Ducks
Horses
Buffaloes
Goats
Livestock species
Rank
0.00
0.25
0.50
0.75
1.00

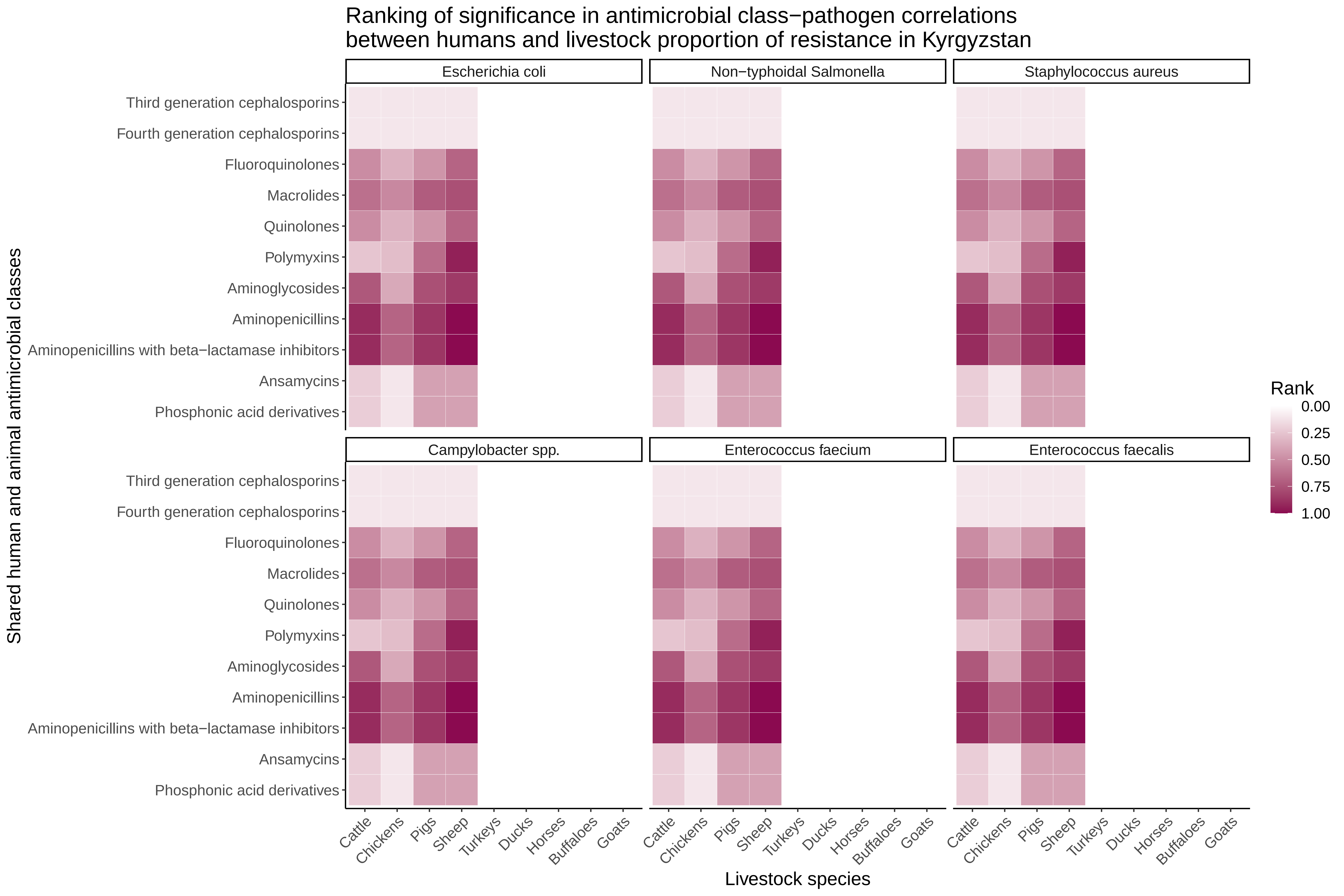

Ranking of significance in antimicrobial class−pathogen correlations
between humans and livestock proportion of resistance in Kyrgyzstan
Escherichia coli
Non−typhoidal Salmonella
Staphylococcus aureus
Campylobacter spp.
Enterococcus faecium
Enterococcus faecalis
Third generation cephalosporins
Fourth generation cephalosporins
Fluoroquinolones
Macrolides
Quinolones
Polymyxins
Aminoglycosides
Aminopenicillins
Aminopenicillins with beta−lactamase inhibitors
Ansamycins
Phosphonic acid derivatives
Shared human and animal antimicrobial classes
Cattle
Chickens
Pigs
Sheep
Turkeys
Ducks
Horses
Buffaloes
Goats
Livestock species
Rank
0.00
0.25
0.50
0.75
1.00

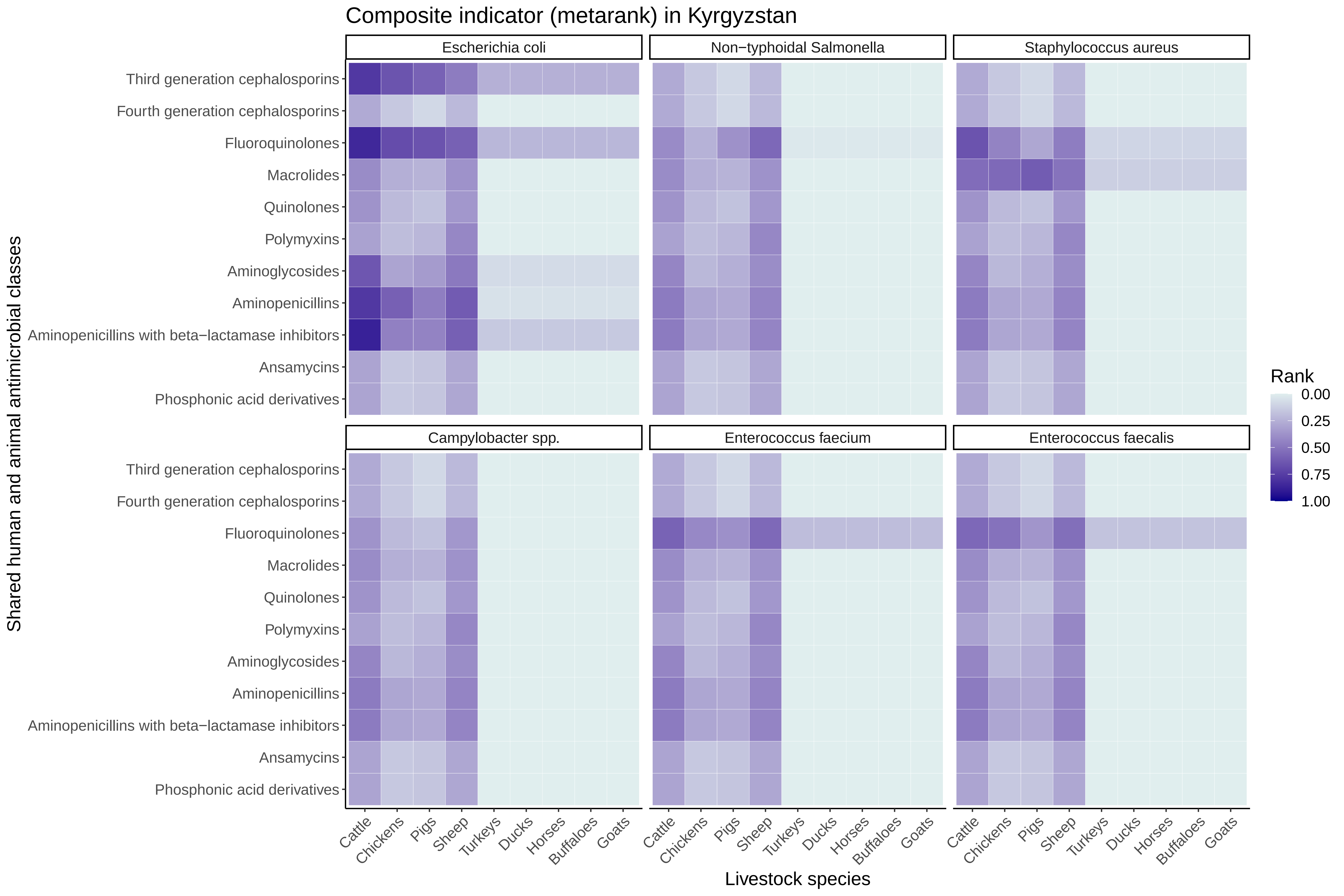
Composite indicator (metarank) in Kyrgyzstan
Escherichia coli
Non−typhoidal Salmonella
Staphylococcus aureus
Campylobacter spp.
Enterococcus faecium
Enterococcus faecalis
Shared human and animal antimicrobial classes
Third generation cephalosporins
Fourth generation cephalosporins
Fluoroquinolones
Macrolides
Quinolones
Polymyxins
Aminoglycosides
Aminopenicillins
Aminopenicillins with beta−lactamase inhibitors
Ansamycins
Phosphonic acid derivatives
Cattle
Chickens
Pigs
Sheep
Turkeys
Ducks
Horses
Buffaloes
Goats
Livestock species
Rank
0.00
0.25
0.50
0.75
1.00

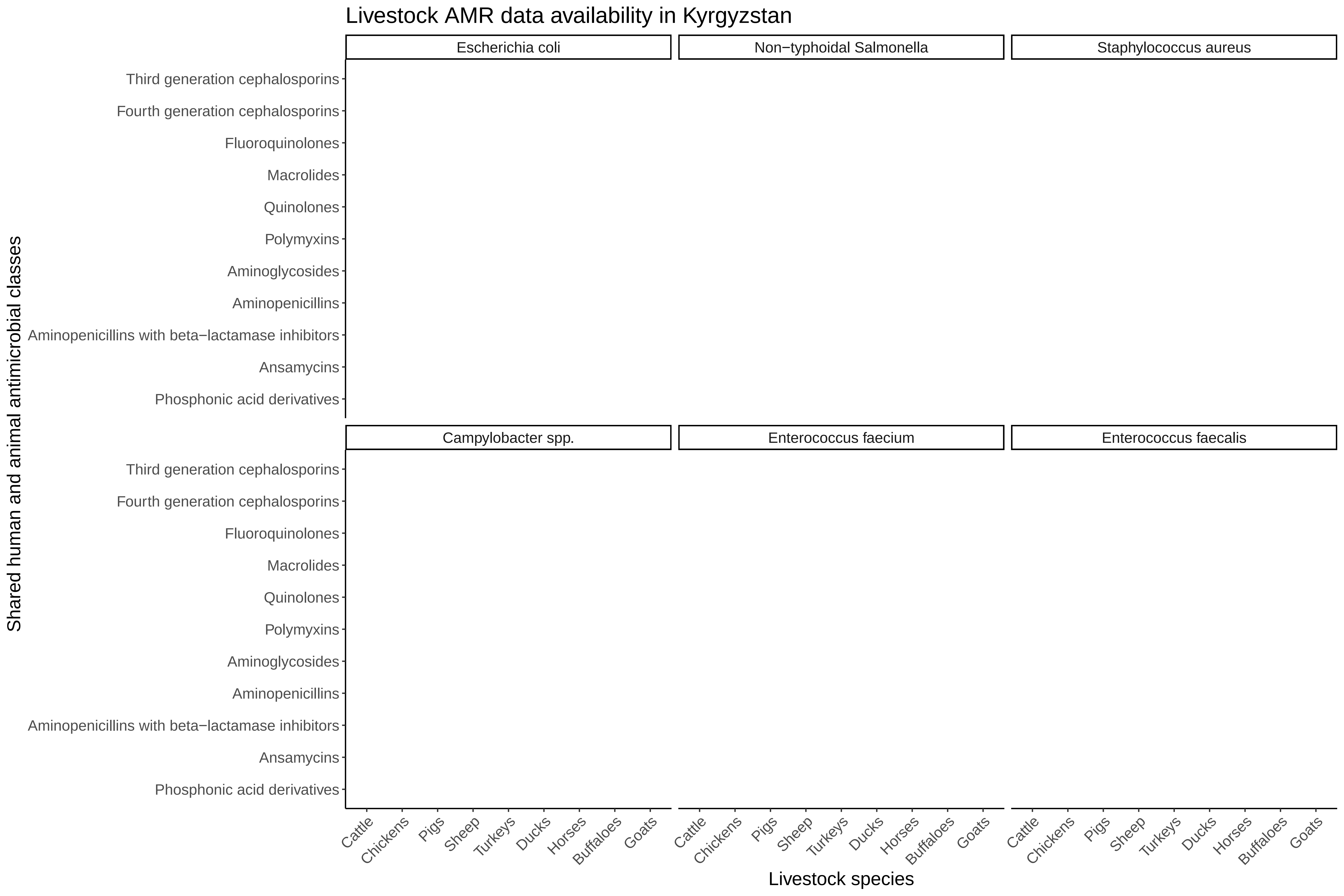

Livestock AMR data availability in Kyrgyzstan
Escherichia coli
Non−typhoidal Salmonella
Staphylococcus aureus
Campylobacter spp.
Enterococcus faecium
Enterococcus faecalis
Third generation cephalosporins
Fourth generation cephalosporins
Fluoroquinolones
Macrolides
Quinolones
Polymyxins
Aminoglycosides
Aminopenicillins
Aminopenicillins with beta−lactamase inhibitors
Ansamycins
Phosphonic acid derivatives
Shared human and animal antimicrobial classes
Cattle
Chickens
Pigs
Sheep
Turkeys
Ducks
Horses
Buffaloes
Goats
Livestock species

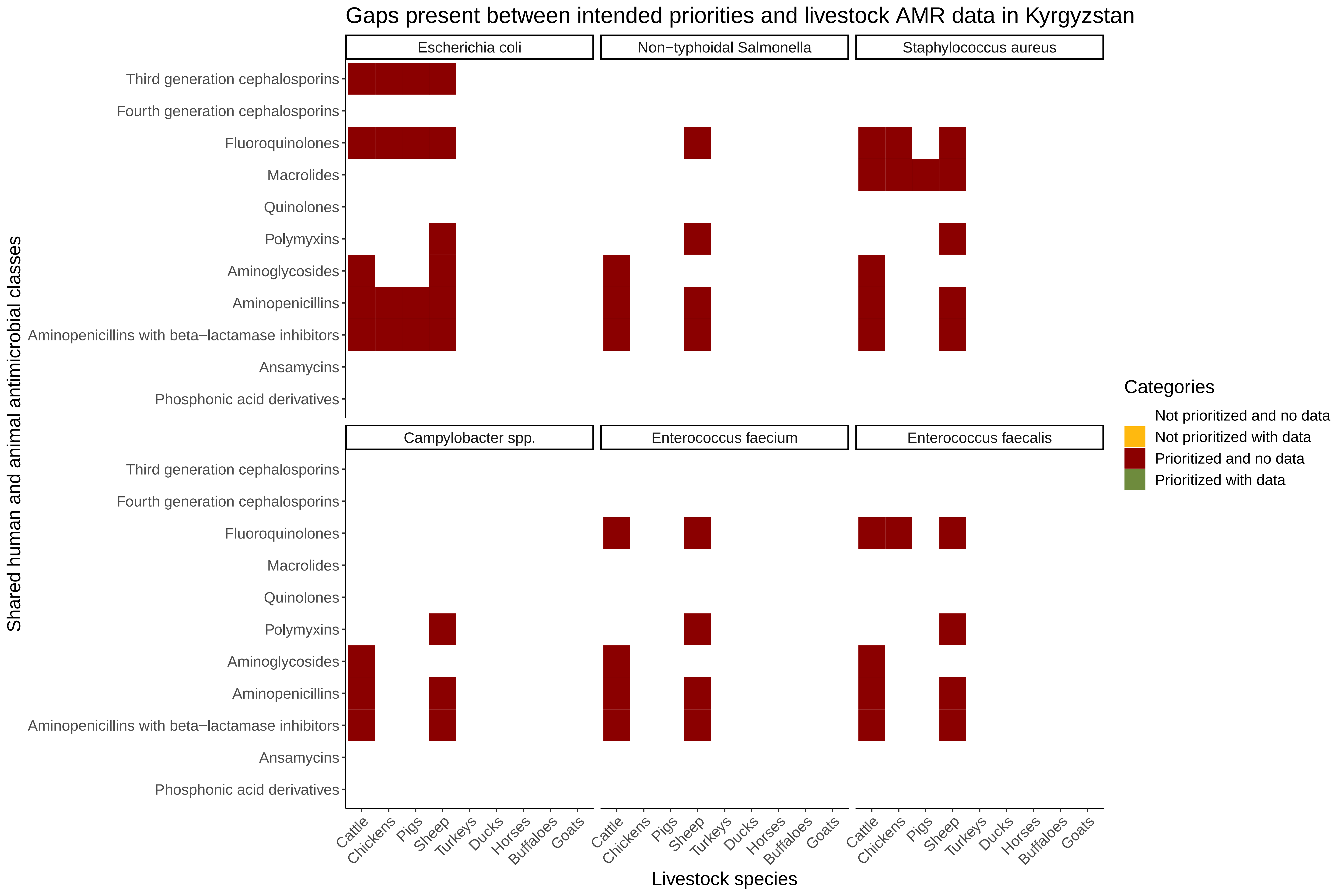

Gaps present between intended priorities and livestock AMR data in Kyrgyzstan
Escherichia coli
Non−typhoidal Salmonella
Staphylococcus aureus
Campylobacter spp.
Enterococcus faecium
Enterococcus faecalis
Shared human and animal antimicrobial classes
Third generation cephalosporins
Fourth generation cephalosporins
Fluoroquinolones
Macrolides
Quinolones
Polymyxins
Aminoglycosides
Aminopenicillins
Aminopenicillins with beta−lactamase inhibitors
Ansamycins
Phosphonic acid derivatives
Cattle
Chickens
Pigs
Sheep
Turkeys
Ducks
Horses
Buffaloes
Goats
Livestock species
Categories
Not prioritized and no data
Not prioritized with data
Prioritized and no data
Prioritized with data

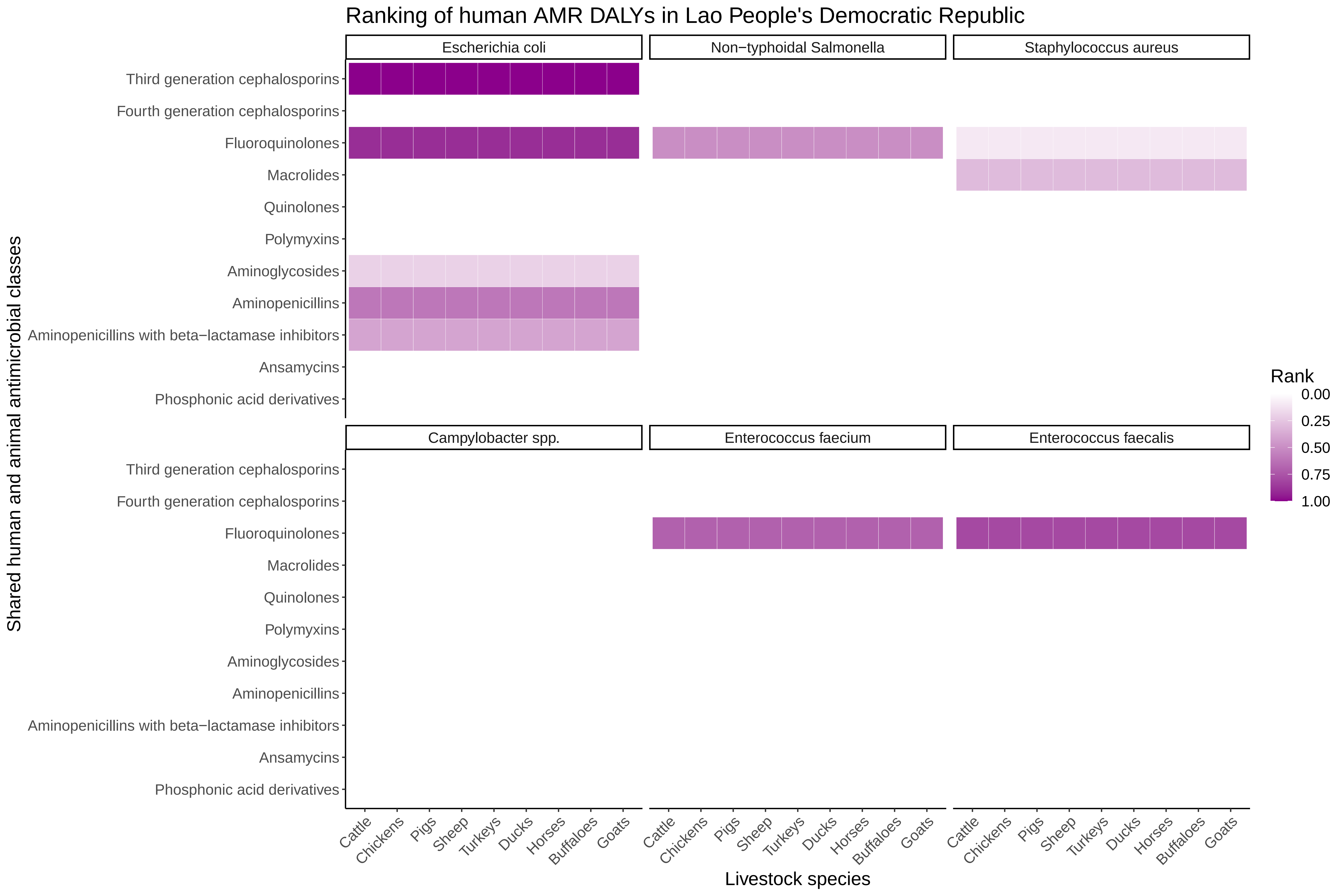
Ranking of human AMR DALYs in Lao People's Democratic Republic
Escherichia coli
Non−typhoidal Salmonella
Staphylococcus aureus
Campylobacter spp.
Enterococcus faecium
Enterococcus faecalis
Third generation cephalosporins
Fourth generation cephalosporins
Fluoroquinolones
Macrolides
Quinolones
Polymyxins
Aminoglycosides
Aminopenicillins
Aminopenicillins with beta−lactamase inhibitors
Ansamycins
Phosphonic acid derivatives
Shared human and animal antimicrobial classes
Cattle
Chickens
Pigs
Sheep
Turkeys
Ducks
Horses
Buffaloes
Goats
Livestock species
Rank
0.00
0.25
0.50
0.75
1.00

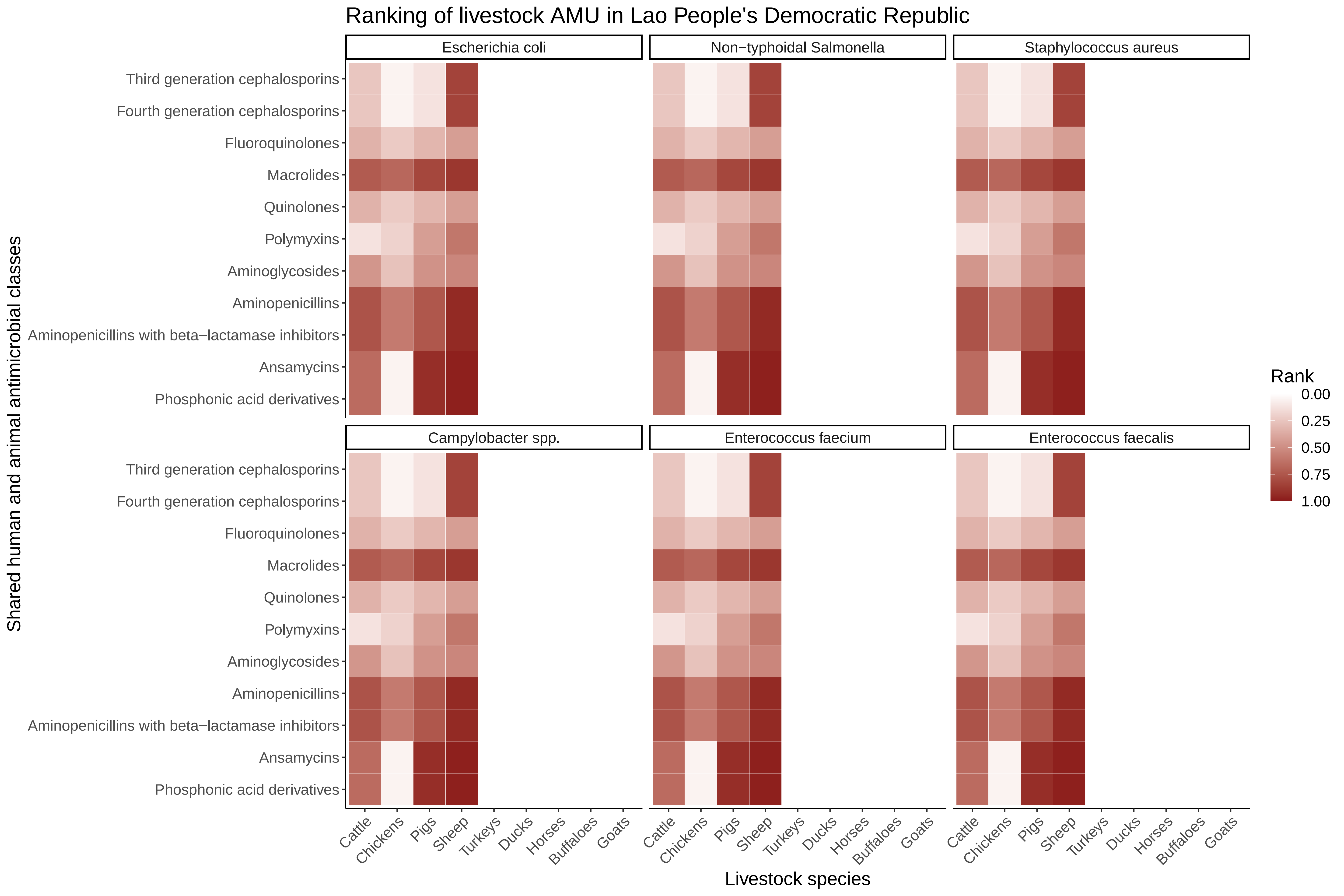

Ranking of livestock AMU in Lao People's Democratic Republic
Escherichia coli
Non−typhoidal Salmonella
Staphylococcus aureus
Campylobacter spp.
Enterococcus faecium
Enterococcus faecalis
Third generation cephalosporins
Fourth generation cephalosporins
Fluoroquinolones
Macrolides
Quinolones
Polymyxins
Aminoglycosides
Aminopenicillins
Aminopenicillins with beta−lactamase inhibitors
Ansamycins
Phosphonic acid derivatives
Shared human and animal antimicrobial classes
Cattle
Chickens
Pigs
Sheep
Turkeys
Ducks
Horses
Buffaloes
Goats
Livestock species
Rank
0.00
0.25
0.50
0.75
1.00

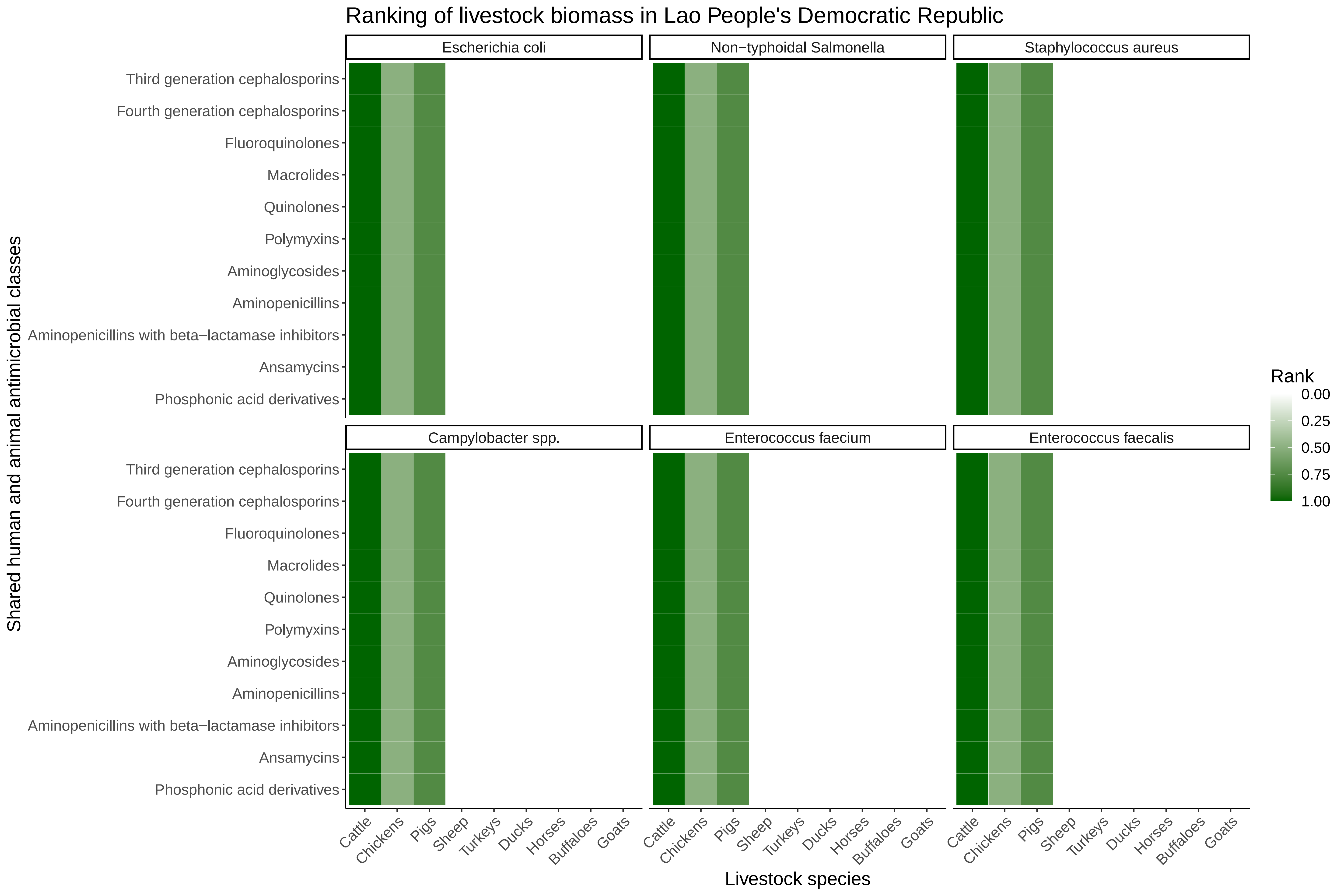
Ranking of livestock biomass in Lao People's Democratic Republic
Escherichia coli
Non−typhoidal Salmonella
Staphylococcus aureus
Campylobacter spp.
Enterococcus faecium
Enterococcus faecalis
Third generation cephalosporins
Fourth generation cephalosporins
Fluoroquinolones
Macrolides
Quinolones
Polymyxins
Aminoglycosides
Aminopenicillins
Aminopenicillins with beta−lactamase inhibitors
Ansamycins
Phosphonic acid derivatives
Shared human and animal antimicrobial classes
Cattle
Chickens
Pigs
Sheep
Turkeys
Ducks
Horses
Buffaloes
Goats
Livestock species
Rank
0.00
0.25
0.50
0.75
1.00

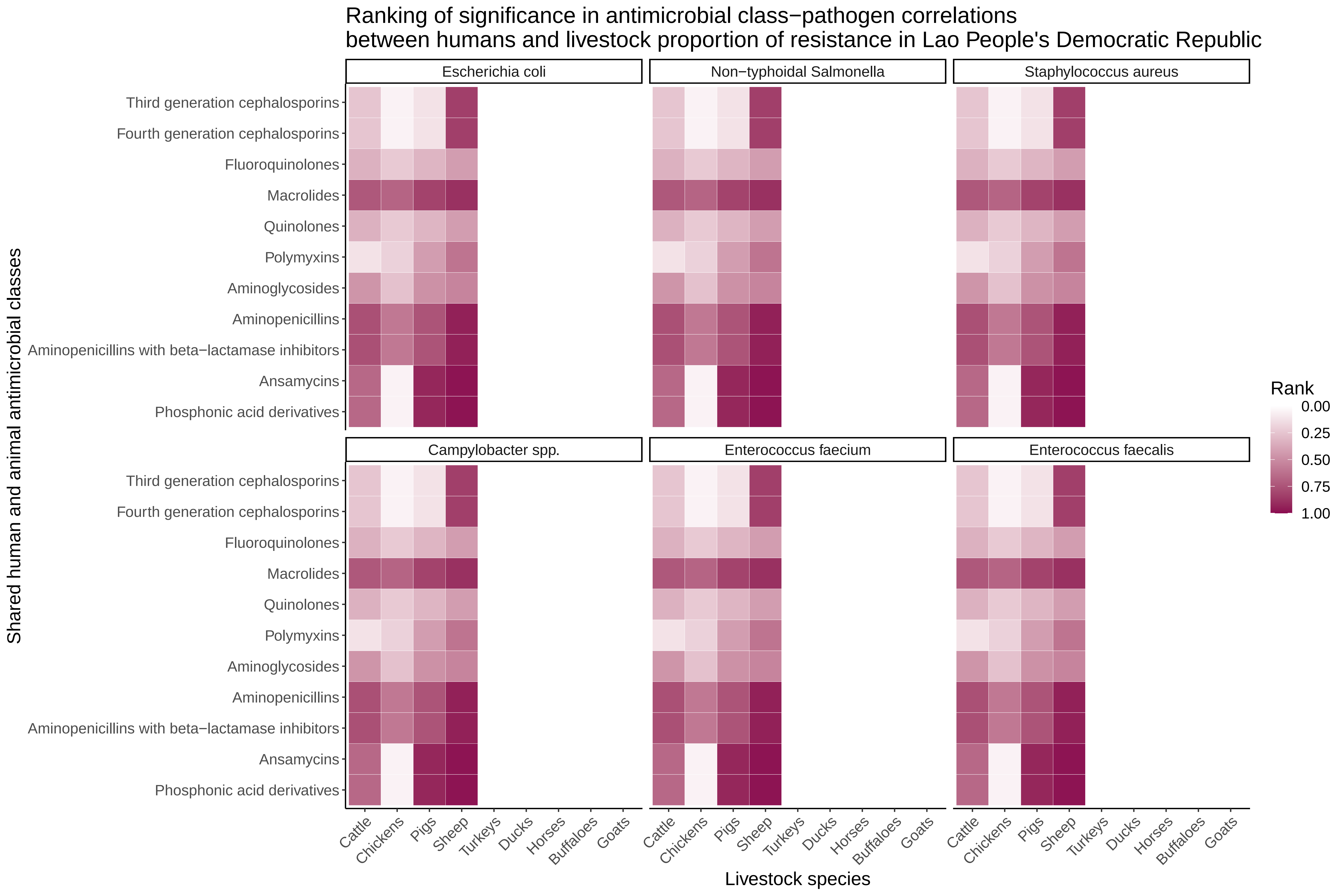

Ranking of significance in antimicrobial class−pathogen correlations
between humans and livestock proportion of resistance in Lao People's Democratic Republic
Escherichia coli
Non−typhoidal Salmonella
Staphylococcus aureus
Campylobacter spp.
Enterococcus faecium
Enterococcus faecalis
Shared human and animal antimicrobial classes
Third generation cephalosporins
Fourth generation cephalosporins
Fluoroquinolones
Macrolides
Quinolones
Polymyxins
Aminoglycosides
Aminopenicillins
Aminopenicillins with beta−lactamase inhibitors
Ansamycins
Phosphonic acid derivatives
Livestock species
Cattle
Chickens
Pigs
Sheep
Turkeys
Ducks
Horses
Buffaloes
Goats
Rank
0.00
0.25
0.50
0.75
1.00

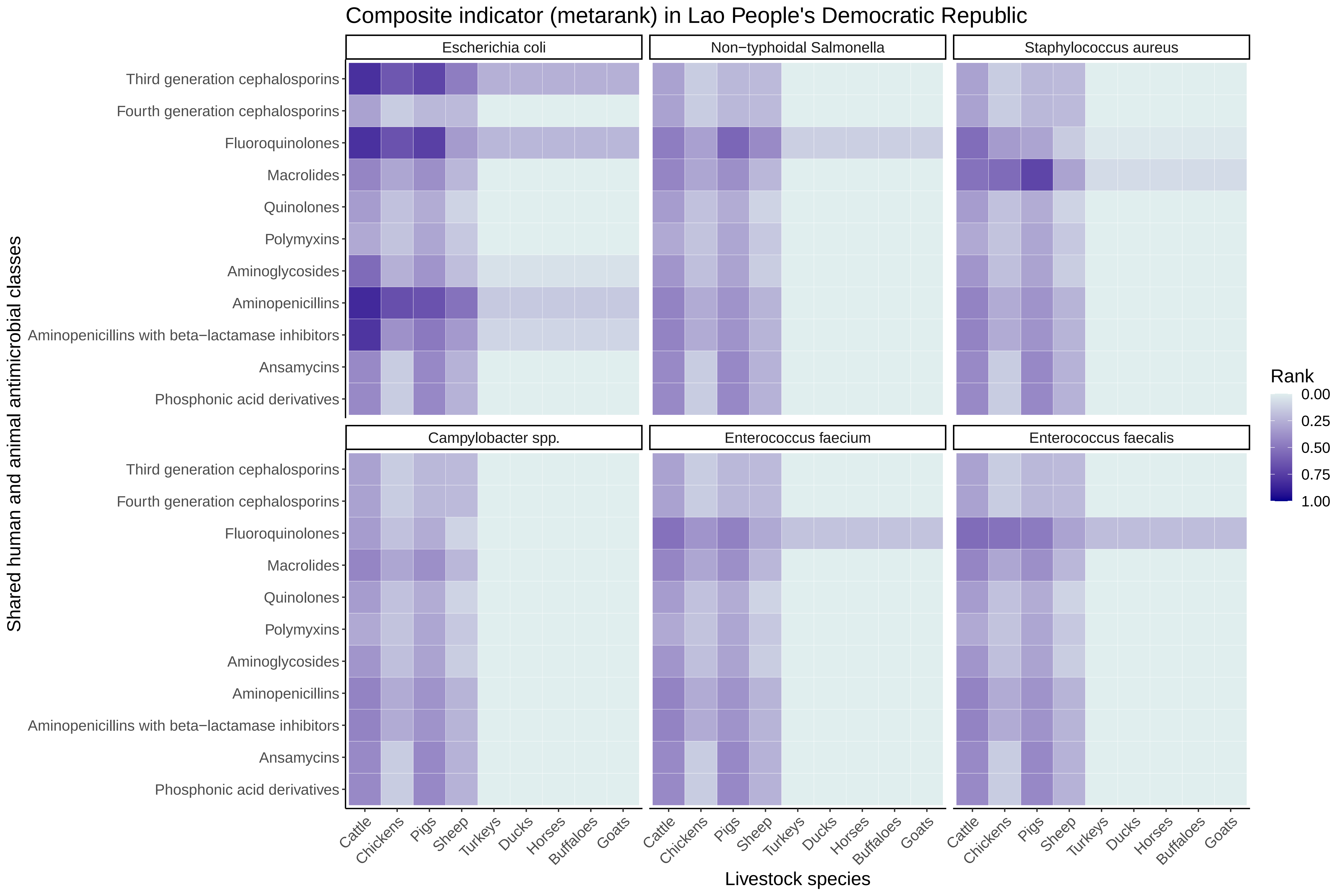

Composite indicator (metarank) in Lao People's Democratic Republic
Escherichia coli
Non−typhoidal Salmonella
Staphylococcus aureus
Campylobacter spp.
Enterococcus faecium
Enterococcus faecalis
Third generation cephalosporins
Fourth generation cephalosporins
Fluoroquinolones
Macrolides
Quinolones
Polymyxins
Aminoglycosides
Aminopenicillins
Aminopenicillins with beta−lactamase inhibitors
Ansamycins
Phosphonic acid derivatives
Shared human and animal antimicrobial classes
Cattle
Chickens
Pigs
Sheep
Turkeys
Ducks
Horses
Buffaloes
Goats
Livestock species
Rank
0.00
0.25
0.50
0.75
1.00

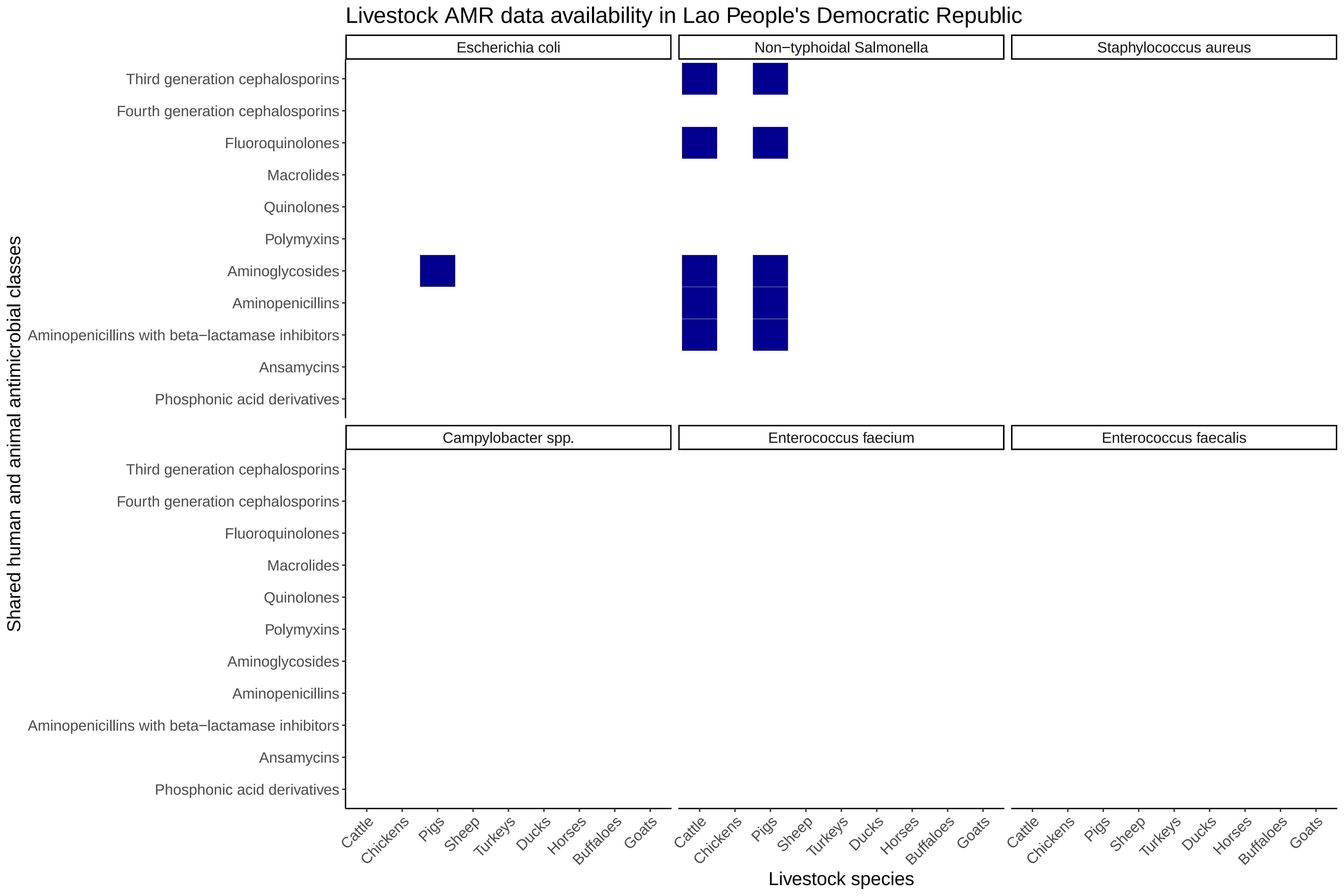

Livestock AMR data availability in Lao People's Democratic Republic
Escherichia coli
Non−typhoidal Salmonella
Staphylococcus aureus
Campylobacter spp.
Enterococcus faecium
Enterococcus faecalis
Shared human and animal antimicrobial classes
Third generation cephalosporins
Fourth generation cephalosporins
Fluoroquinolones
Macrolides
Quinolones
Polymyxins
Aminoglycosides
Aminopenicillins
Aminopenicillins with beta−lactamase inhibitors
Ansamycins
Phosphonic acid derivatives
Cattle
Chickens
Pigs
Sheep
Turkeys
Ducks
Horses
Buffaloes
Goats
Livestock species

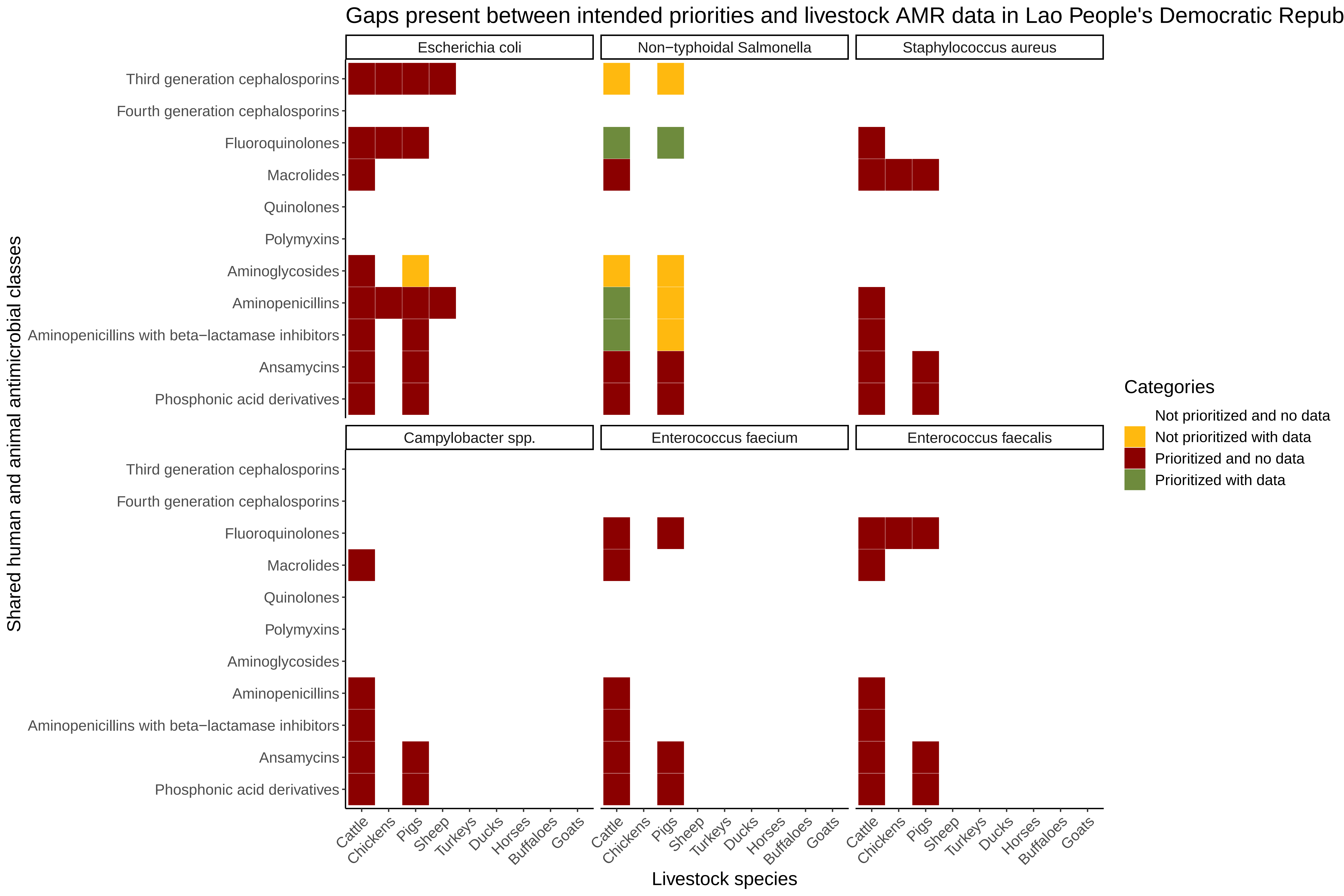

Gaps present between intended priorities and livestock AMR data in Lao People's Democratic Repub
Escherichia coli
Non−typhoidal Salmonella
Staphylococcus aureus
Campylobacter spp.
Enterococcus faecium
Enterococcus faecalis
Third generation cephalosporins
Fourth generation cephalosporins
Fluoroquinolones
Macrolides
Quinolones
Polymyxins
Aminoglycosides
Aminopenicillins
Aminopenicillins with beta−lactamase inhibitors
Ansamycins
Phosphonic acid derivatives
Cattle
Chickens
Pigs
Sheep
Turkeys
Ducks
Horses
Buffaloes
Goats
Livestock species
Shared human and animal antimicrobial classes
Categories
Not prioritized and no data
Not prioritized with data
Prioritized and no data
Prioritized with data

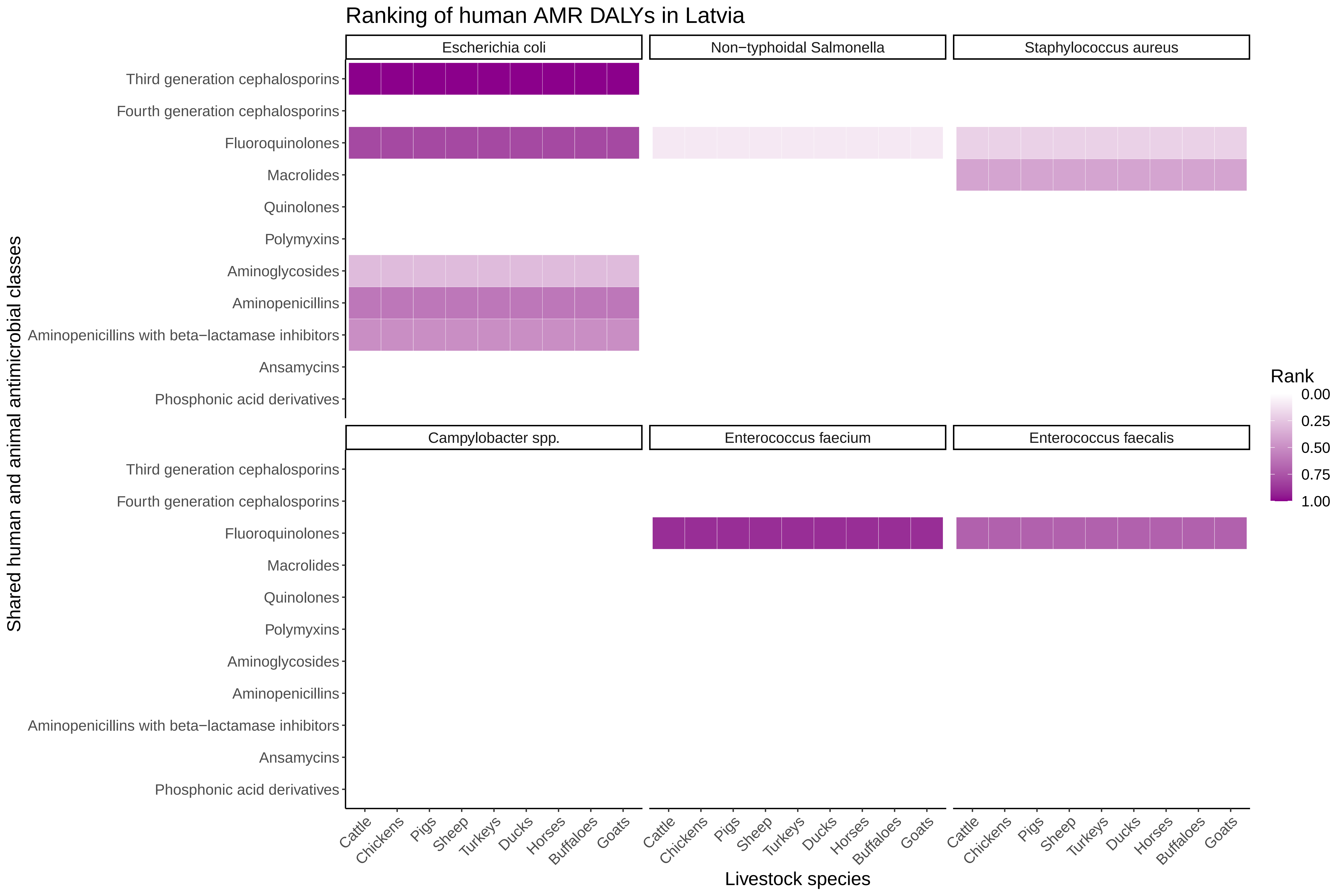

Ranking of human AMR DALYs in Latvia
Escherichia coli
Non−typhoidal Salmonella
Staphylococcus aureus
Campylobacter spp.
Enterococcus faecium
Enterococcus faecalis
Third generation cephalosporins
Fourth generation cephalosporins
Fluoroquinolones
Macrolides
Quinolones
Polymyxins
Aminoglycosides
Aminopenicillins
Aminopenicillins with beta−lactamase inhibitors
Ansamycins
Phosphonic acid derivatives
Shared human and animal antimicrobial classes
Cattle
Chickens
Pigs
Sheep
Turkeys
Ducks
Horses
Buffaloes
Goats
Livestock species
Rank
0.00
0.25
0.50
0.75
1.00

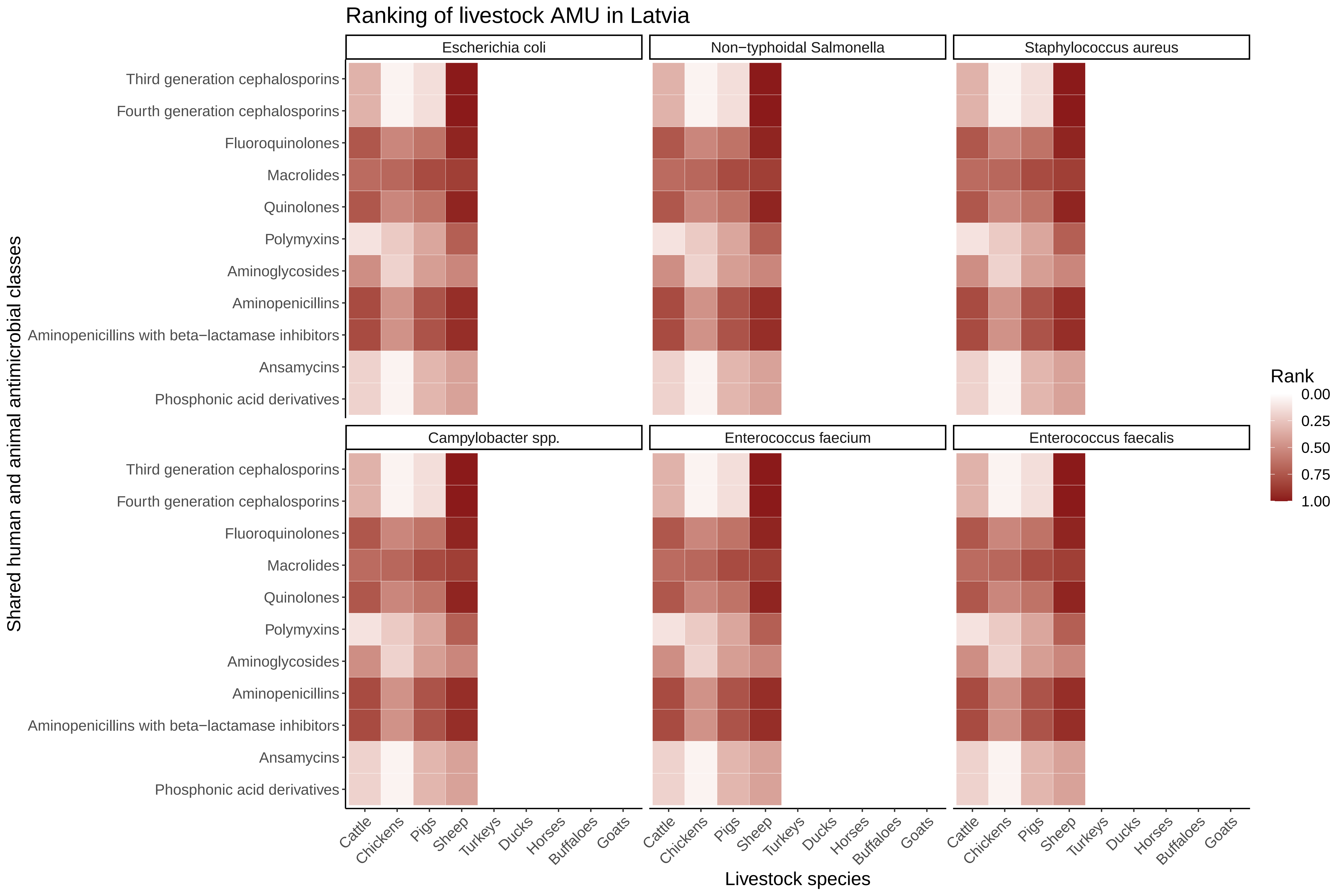
Ranking of livestock AMU in Latvia
Escherichia coli
Non−typhoidal Salmonella
Staphylococcus aureus
Campylobacter spp.
Enterococcus faecium
Enterococcus faecalis
Third generation cephalosporins
Fourth generation cephalosporins
Fluoroquinolones
Macrolides
Quinolones
Polymyxins
Aminoglycosides
Aminopenicillins
Aminopenicillins with beta−lactamase inhibitors
Ansamycins
Phosphonic acid derivatives
Cattle
Chickens
Pigs
Sheep
Turkeys
Ducks
Horses
Buffaloes
Goats
Livestock species
Shared human and animal antimicrobial classes
Rank
0.00
0.25
0.50
0.75
1.00

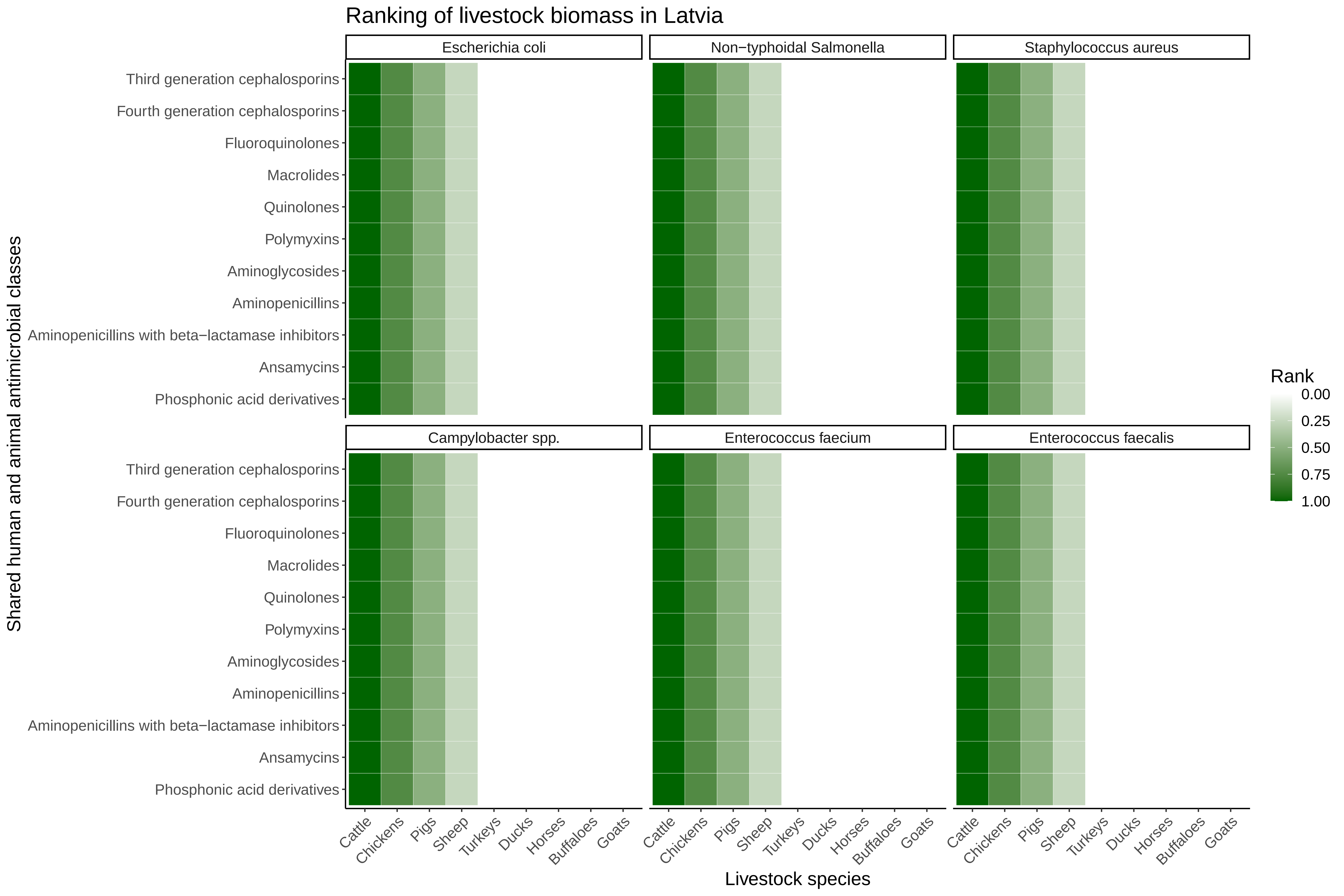

Ranking of livestock biomass in Latvia
Escherichia coli
Non−typhoidal Salmonella
Staphylococcus aureus
Campylobacter spp.
Enterococcus faecium
Enterococcus faecalis
Shared human and animal antimicrobial classes
Third generation cephalosporins
Fourth generation cephalosporins
Fluoroquinolones
Macrolides
Quinolones
Polymyxins
Aminoglycosides
Aminopenicillins
Aminopenicillins with beta−lactamase inhibitors
Ansamycins
Phosphonic acid derivatives
Cattle
Chickens
Pigs
Sheep
Turkeys
Ducks
Horses
Buffaloes
Goats
Livestock species
Rank
0.00
0.25
0.50
0.75
1.00

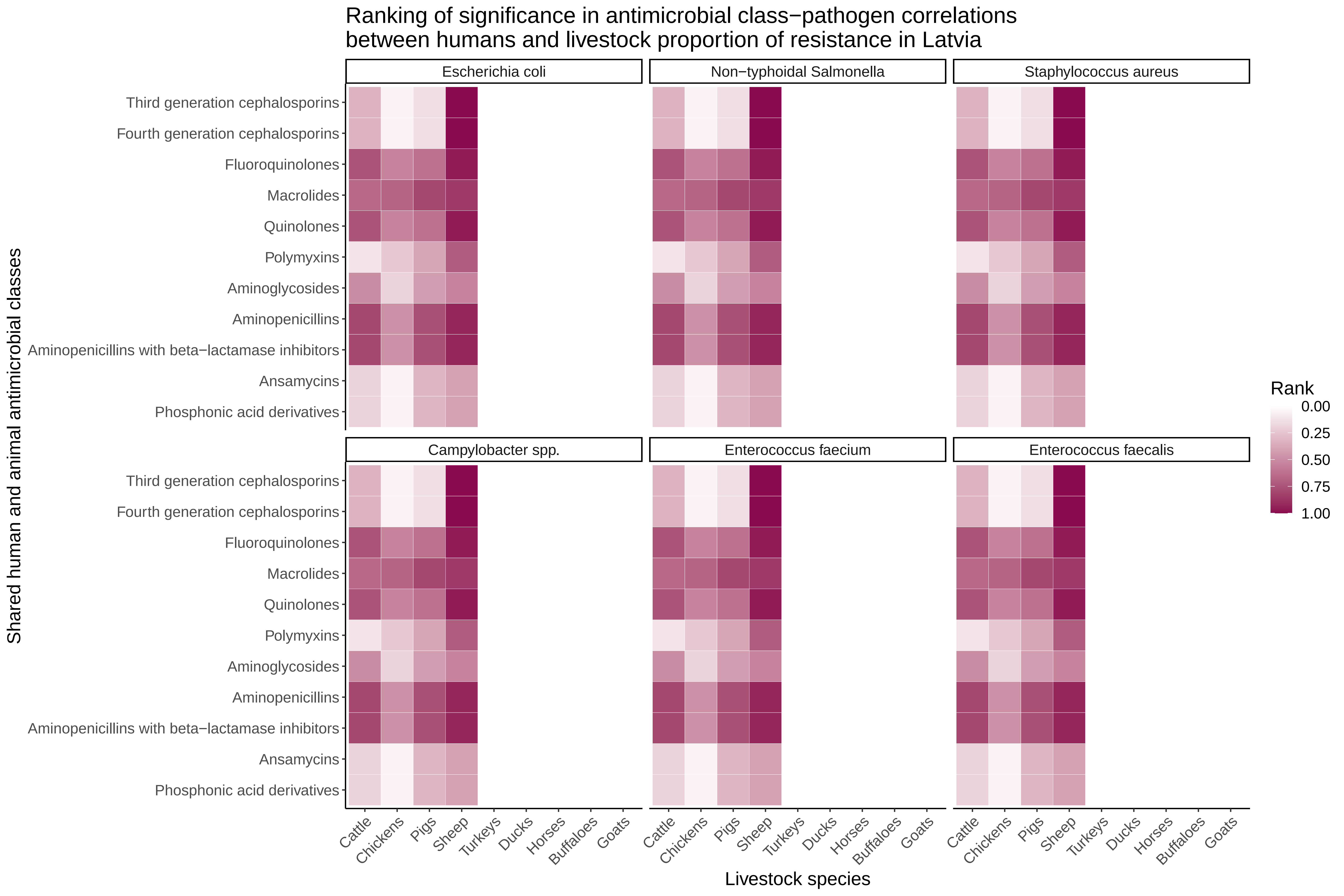
Ranking of significance in antimicrobial class−pathogen correlations
between humans and livestock proportion of resistance in Latvia
Escherichia coli
Non−typhoidal Salmonella
Staphylococcus aureus
Campylobacter spp.
Enterococcus faecium
Enterococcus faecalis
Third generation cephalosporins
Fourth generation cephalosporins
Fluoroquinolones
Macrolides
Quinolones
Polymyxins
Aminoglycosides
Aminopenicillins
Aminopenicillins with beta−lactamase inhibitors
Ansamycins
Phosphonic acid derivatives
Shared human and animal antimicrobial classes
Cattle
Chickens
Pigs
Sheep
Turkeys
Ducks
Horses
Buffaloes
Goats
Livestock species
Rank
0.00
0.25
0.50
0.75
1.00

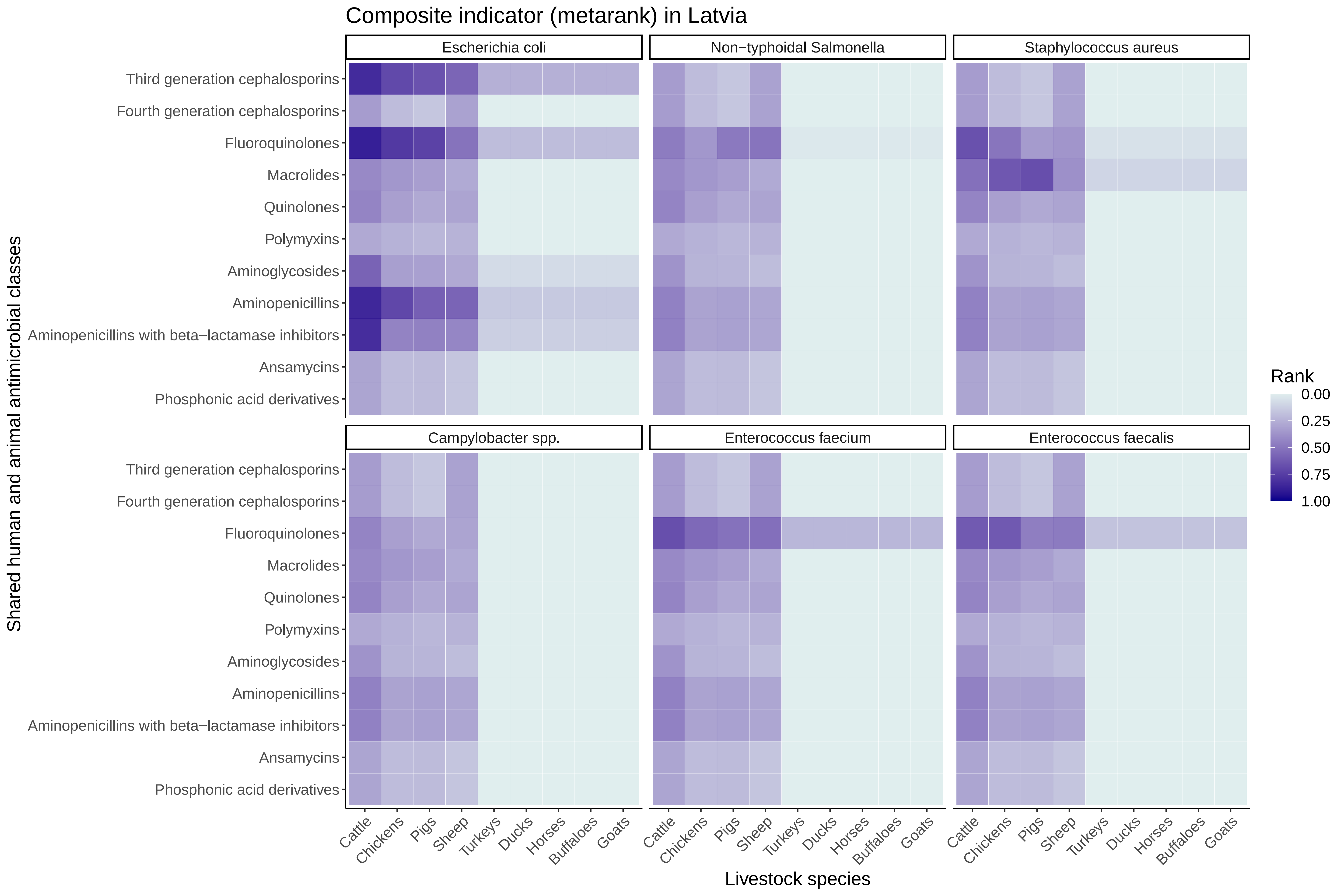

Composite indicator (metarank) in Latvia
Escherichia coli
Non−typhoidal Salmonella
Staphylococcus aureus
Campylobacter spp.
Enterococcus faecium
Enterococcus faecalis
Third generation cephalosporins
Fourth generation cephalosporins
Fluoroquinolones
Macrolides
Quinolones
Polymyxins
Aminoglycosides
Aminopenicillins
Aminopenicillins with beta−lactamase inhibitors
Ansamycins
Phosphonic acid derivatives
Shared human and animal antimicrobial classes
Cattle
Chickens
Pigs
Sheep
Turkeys
Ducks
Horses
Buffaloes
Goats
Livestock species
Rank
0.00
0.25
0.50
0.75
1.00

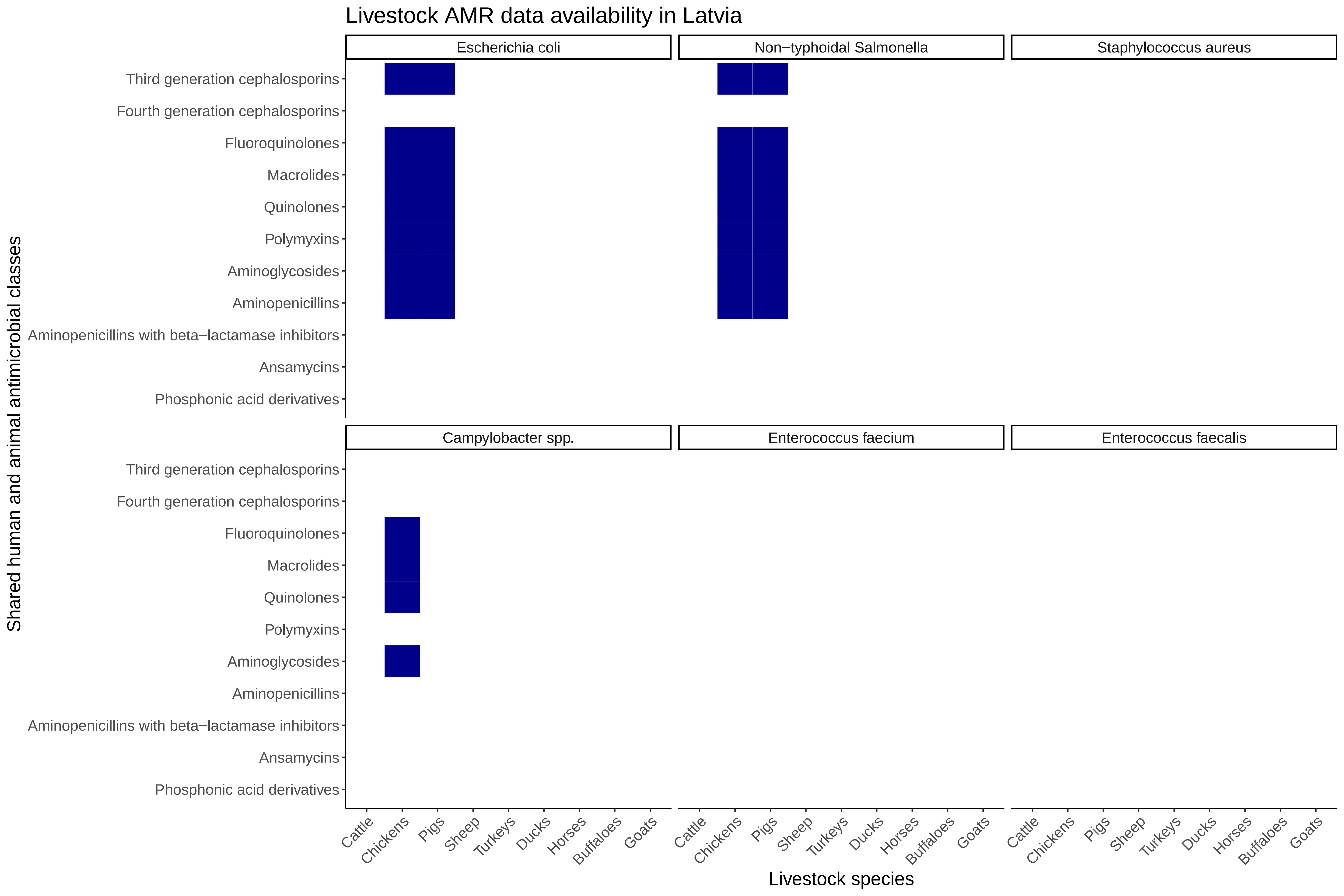
Livestock AMR data availability in Latvia
Escherichia coli
Non−typhoidal Salmonella
Staphylococcus aureus
Campylobacter spp.
Enterococcus faecium
Enterococcus faecalis
Shared human and animal antimicrobial classes
Third generation cephalosporins
Fourth generation cephalosporins
Fluoroquinolones
Macrolides
Quinolones
Polymyxins
Aminoglycosides
Aminopenicillins
Aminopenicillins with beta−lactamase inhibitors
Ansamycins
Phosphonic acid derivatives
Cattle
Chickens
Pigs
Sheep
Turkeys
Ducks
Horses
Buffaloes
Goats
Livestock species

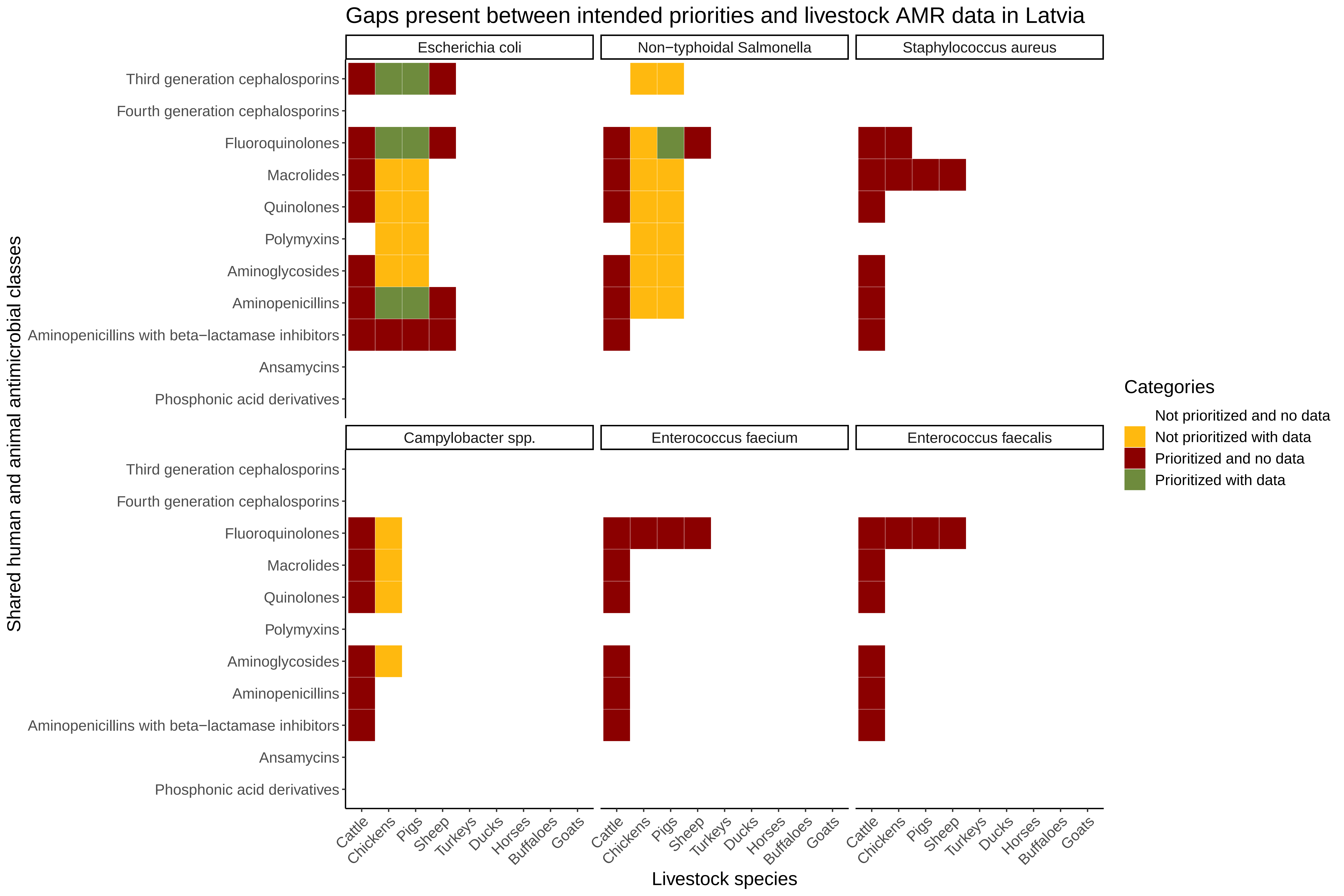

Gaps present between intended priorities and livestock AMR data in Latvia
Escherichia coli
Non−typhoidal Salmonella
Staphylococcus aureus
Campylobacter spp.
Enterococcus faecium
Enterococcus faecalis
Third generation cephalosporins
Fourth generation cephalosporins
Fluoroquinolones
Macrolides
Quinolones
Polymyxins
Aminoglycosides
Aminopenicillins
Aminopenicillins with beta−lactamase inhibitors
Ansamycins
Phosphonic acid derivatives
Shared human and animal antimicrobial classes
Cattle
Chickens
Pigs
Sheep
Turkeys
Ducks
Horses
Buffaloes
Goats
Livestock species
Categories
Not prioritized and no data
Not prioritized with data
Prioritized and no data
Prioritized with data

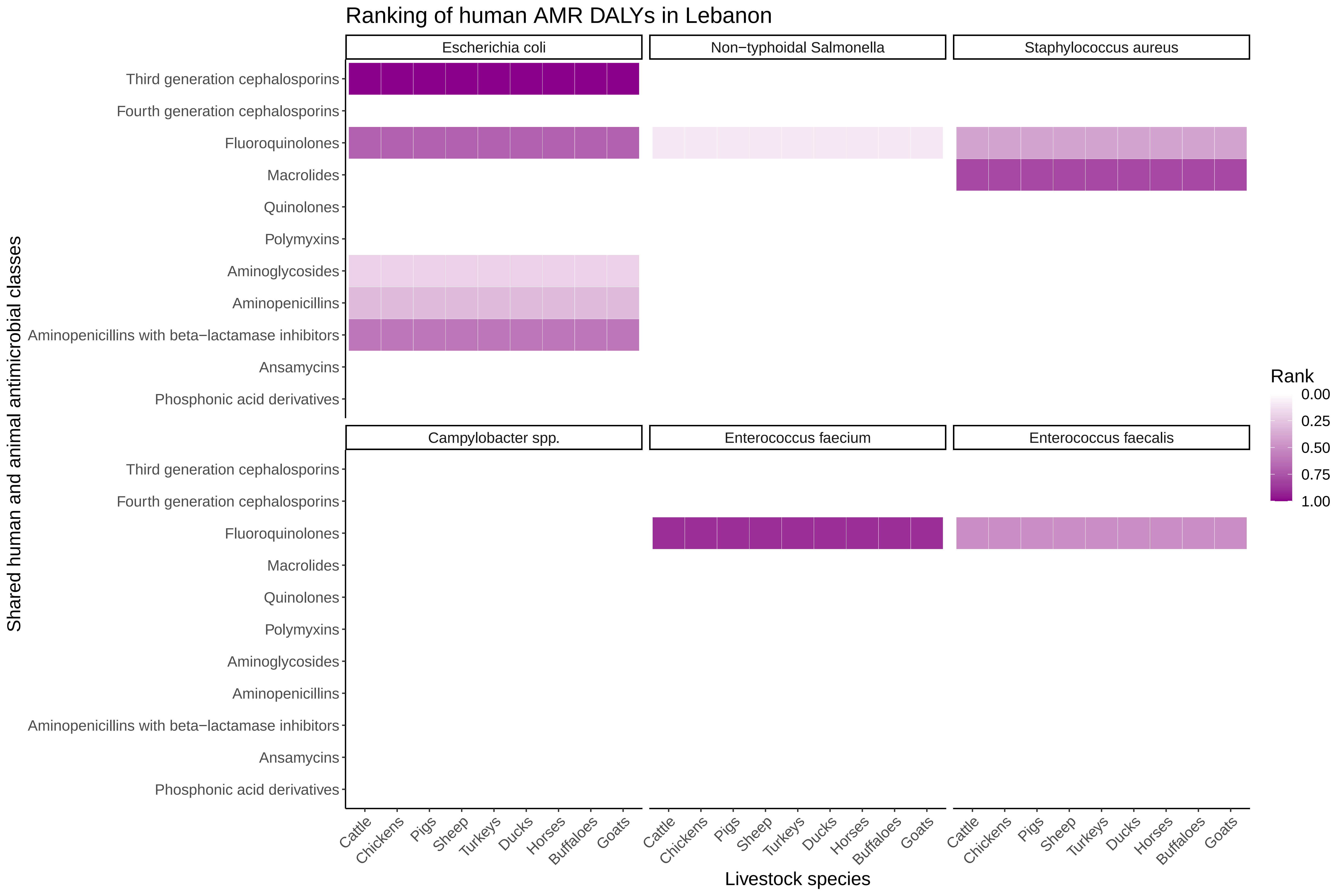

Ranking of human AMR DALYs in Lebanon
Escherichia coli
Non−typhoidal Salmonella
Staphylococcus aureus
Campylobacter spp.
Enterococcus faecium
Enterococcus faecalis
Third generation cephalosporins
Fourth generation cephalosporins
Fluoroquinolones
Macrolides
Quinolones
Polymyxins
Aminoglycosides
Aminopenicillins
Aminopenicillins with beta−lactamase inhibitors
Ansamycins
Phosphonic acid derivatives
Shared human and animal antimicrobial classes
Cattle
Chickens
Pigs
Sheep
Turkeys
Ducks
Horses
Buffaloes
Goats
Livestock species
Rank
0.00
0.25
0.50
0.75
1.00

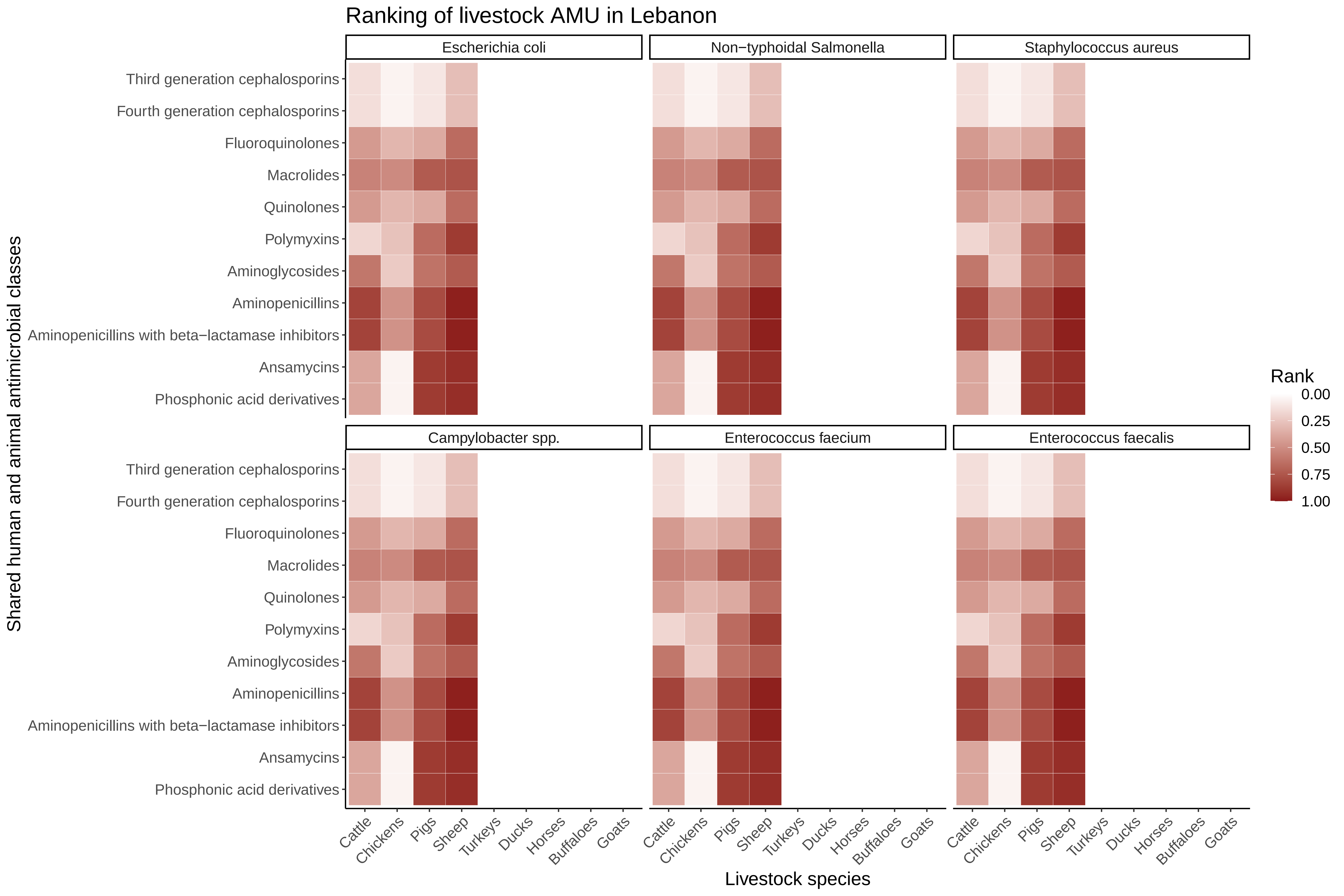

Ranking of livestock AMU in Lebanon
Escherichia coli
Non−typhoidal Salmonella
Staphylococcus aureus
Campylobacter spp.
Enterococcus faecium
Enterococcus faecalis
Third generation cephalosporins
Fourth generation cephalosporins
Fluoroquinolones
Macrolides
Quinolones
Polymyxins
Aminoglycosides
Aminopenicillins
Aminopenicillins with beta−lactamase inhibitors
Ansamycins
Phosphonic acid derivatives
Shared human and animal antimicrobial classes
Cattle
Chickens
Pigs
Sheep
Turkeys
Ducks
Horses
Buffaloes
Goats
Livestock species
Rank
0.00
0.25
0.50
0.75
1.00

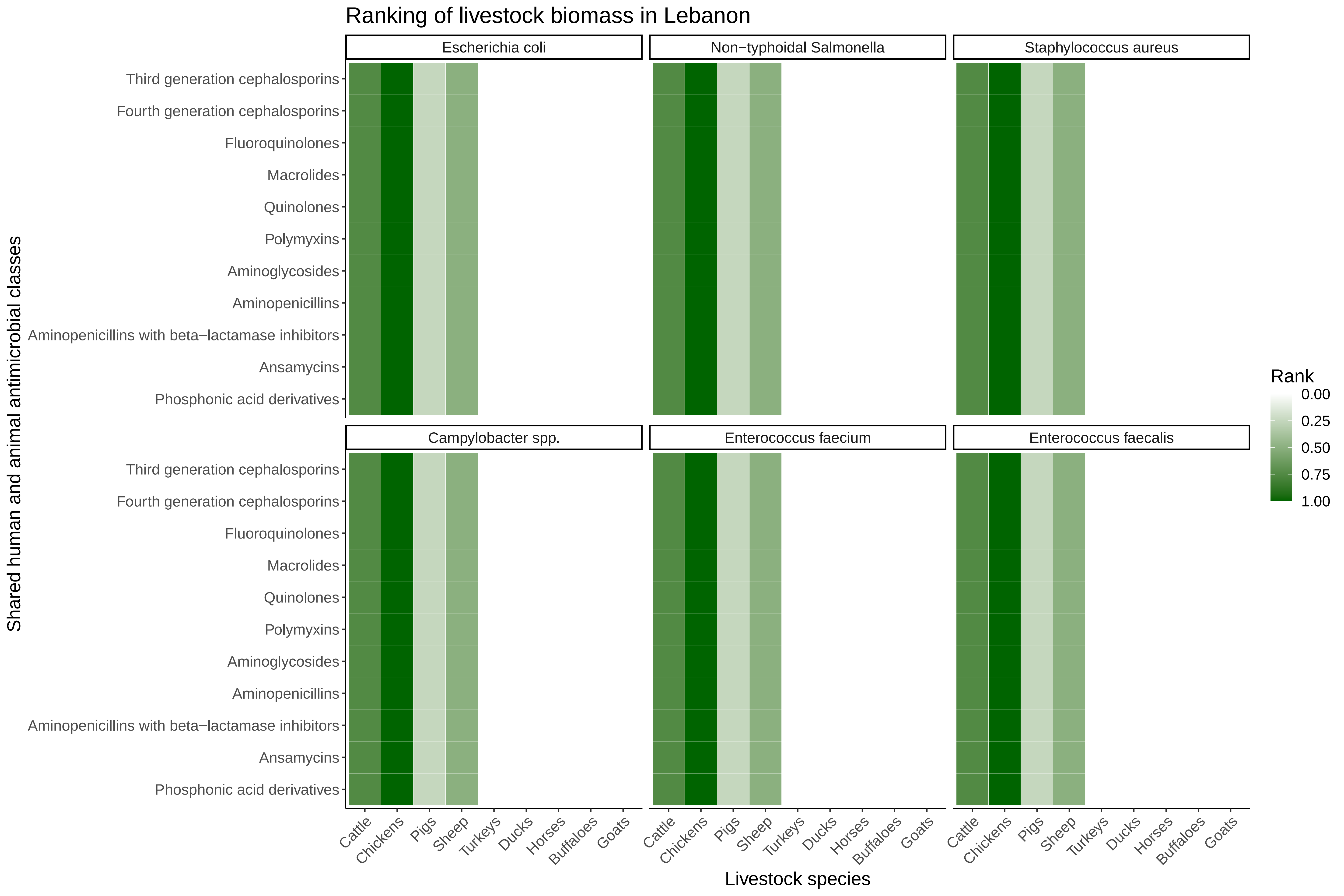

Ranking of livestock biomass in Lebanon
Escherichia coli
Non−typhoidal Salmonella
Staphylococcus aureus
Campylobacter spp.
Enterococcus faecium
Enterococcus faecalis
Third generation cephalosporins
Fourth generation cephalosporins
Fluoroquinolones
Macrolides
Quinolones
Polymyxins
Aminoglycosides
Aminopenicillins
Aminopenicillins with beta−lactamase inhibitors
Ansamycins
Phosphonic acid derivatives
Shared human and animal antimicrobial classes
Cattle
Chickens
Pigs
Sheep
Turkeys
Ducks
Horses
Buffaloes
Goats
Livestock species
Rank
0.00
0.25
0.50
0.75
1.00

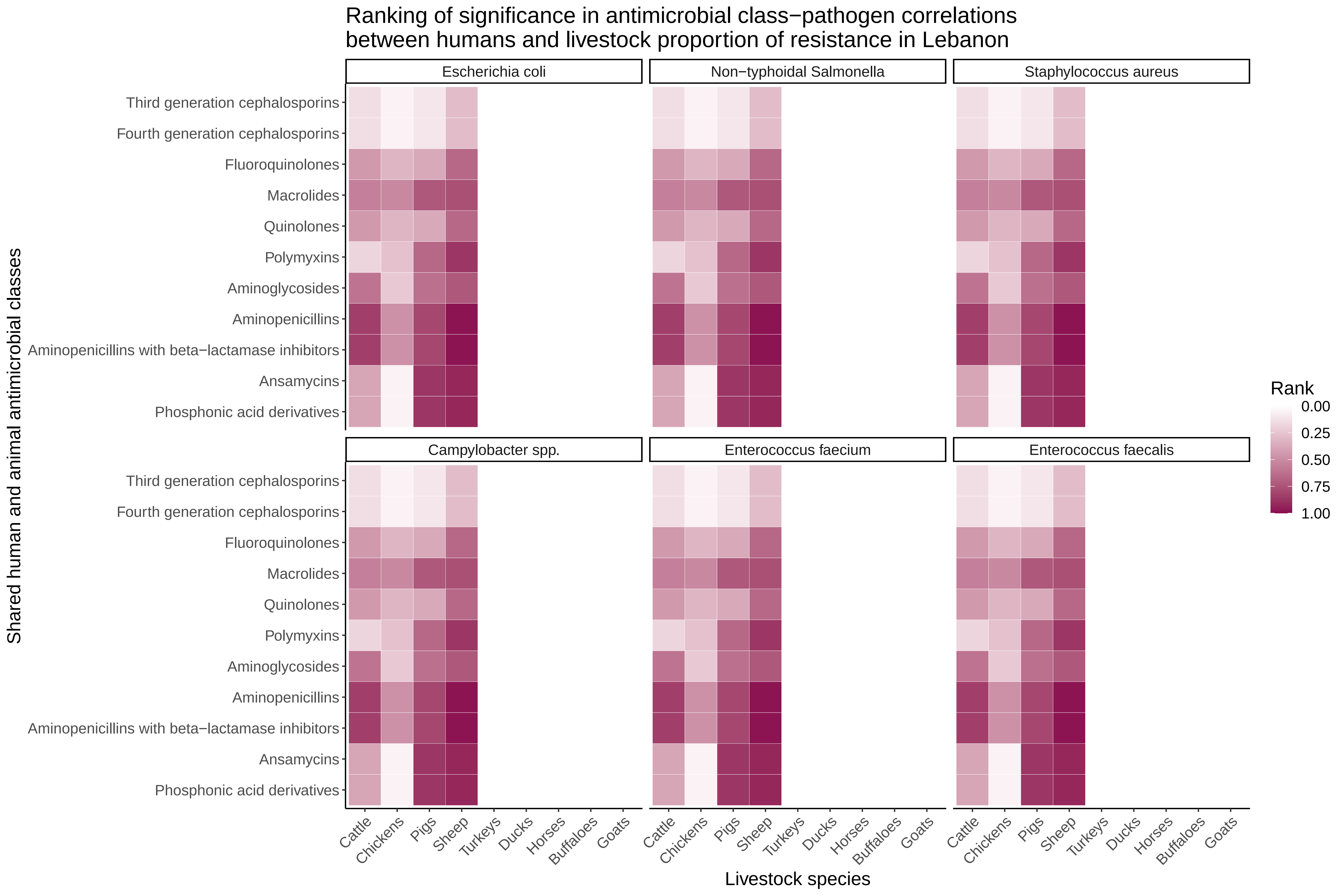
Ranking of significance in antimicrobial class−pathogen correlations
between humans and livestock proportion of resistance in Lebanon
Escherichia coli
Non−typhoidal Salmonella
Staphylococcus aureus
Campylobacter spp.
Enterococcus faecium
Enterococcus faecalis
Shared human and animal antimicrobial classes
Third generation cephalosporins
Fourth generation cephalosporins
Fluoroquinolones
Macrolides
Quinolones
Polymyxins
Aminoglycosides
Aminopenicillins
Aminopenicillins with beta−lactamase inhibitors
Ansamycins
Phosphonic acid derivatives
Livestock species
Cattle
Chickens
Pigs
Sheep
Turkeys
Ducks
Horses
Buffaloes
Goats
Rank
0.00
0.25
0.50
0.75
1.00

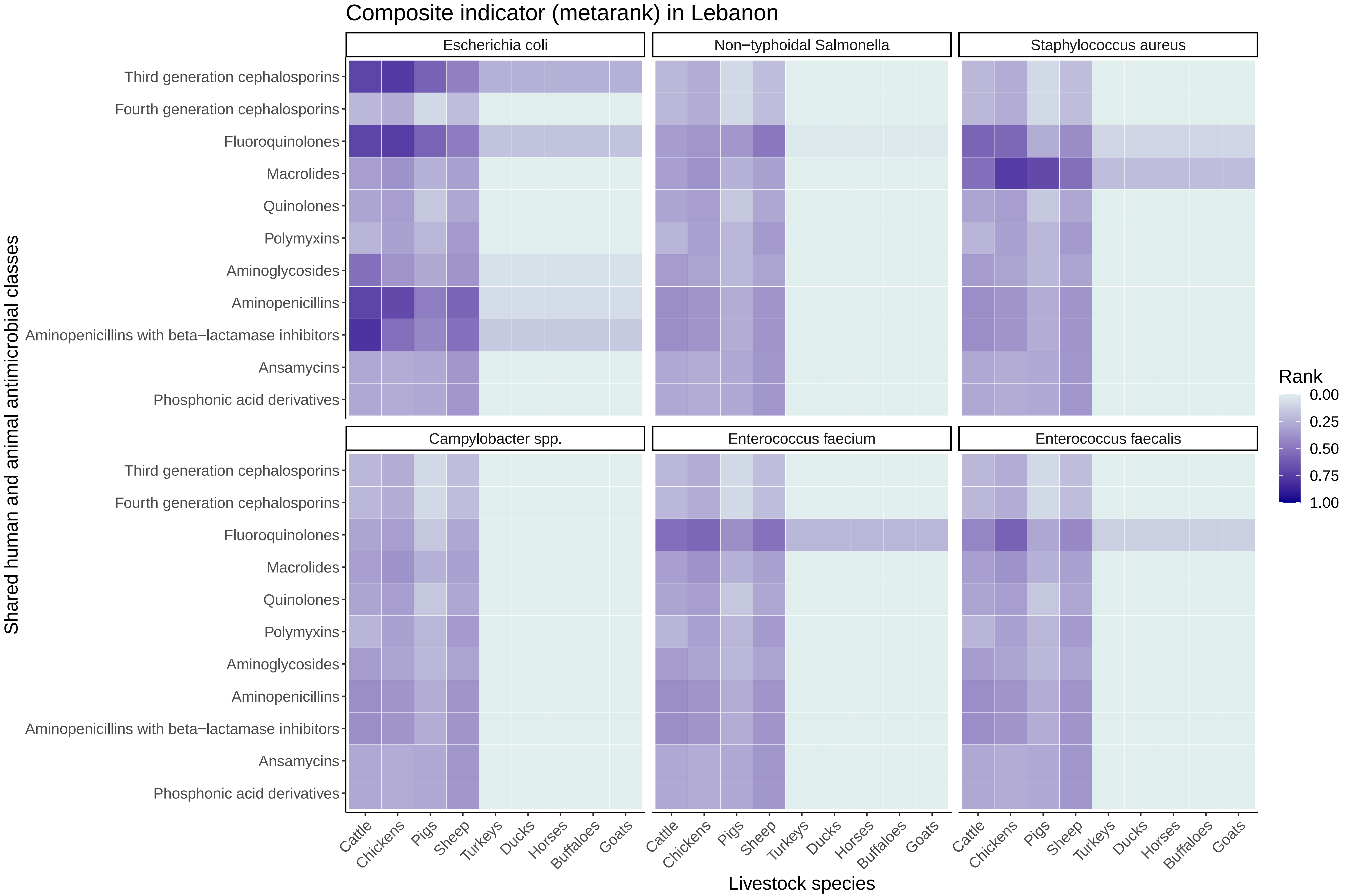

Composite indicator (metarank) in Lebanon
Escherichia coli
Non−typhoidal Salmonella
Staphylococcus aureus
Campylobacter spp.
Enterococcus faecium
Enterococcus faecalis
Shared human and animal antimicrobial classes
Third generation cephalosporins
Fourth generation cephalosporins
Fluoroquinolones
Macrolides
Quinolones
Polymyxins
Aminoglycosides
Aminopenicillins
Aminopenicillins with beta−lactamase inhibitors
Ansamycins
Phosphonic acid derivatives
Cattle
Chickens
Pigs
Sheep
Turkeys
Ducks
Horses
Buffaloes
Goats
Livestock species
Rank
0.00
0.25
0.50
0.75
1.00

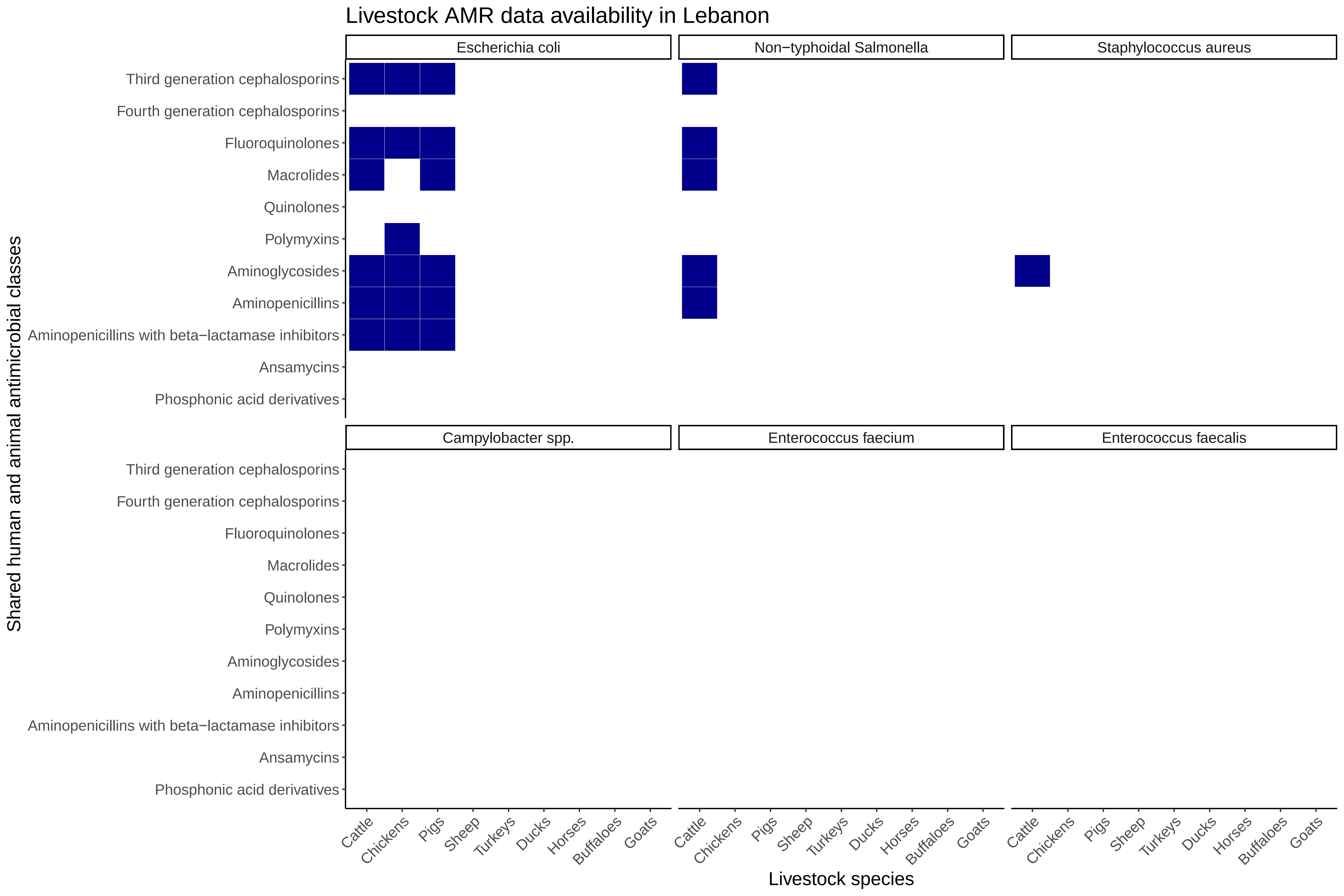

Livestock AMR data availability in Lebanon
Escherichia coli
Non−typhoidal Salmonella
Staphylococcus aureus
Campylobacter spp.
Enterococcus faecium
Enterococcus faecalis
Shared human and animal antimicrobial classes
Third generation cephalosporins
Fourth generation cephalosporins
Fluoroquinolones
Macrolides
Quinolones
Polymyxins
Aminoglycosides
Aminopenicillins
Aminopenicillins with beta−lactamase inhibitors
Ansamycins
Phosphonic acid derivatives
Cattle
Chickens
Pigs
Sheep
Turkeys
Ducks
Horses
Buffaloes
Goats
Livestock species

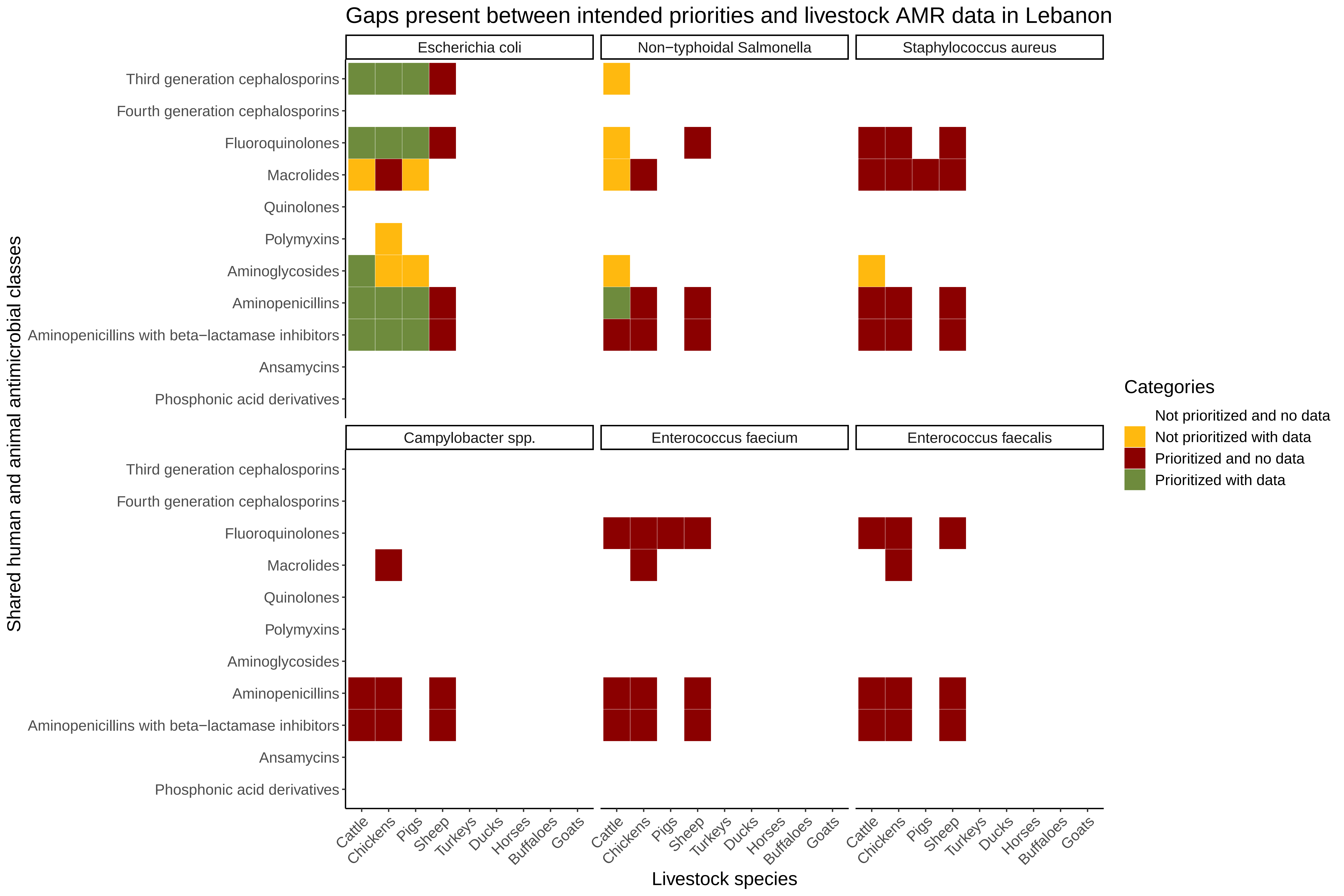

Gaps present between intended priorities and livestock AMR data in Lebanon
Escherichia coli
Non−typhoidal Salmonella
Staphylococcus aureus
Campylobacter spp.
Enterococcus faecium
Enterococcus faecalis
Third generation cephalosporins
Fourth generation cephalosporins
Fluoroquinolones
Macrolides
Quinolones
Polymyxins
Aminoglycosides
Aminopenicillins
Aminopenicillins with beta−lactamase inhibitors
Ansamycins
Phosphonic acid derivatives
Shared human and animal antimicrobial classes
Cattle
Chickens
Pigs
Sheep
Turkeys
Ducks
Horses
Buffaloes
Goats
Livestock species
Categories
Not prioritized and no data
Not prioritized with data
Prioritized and no data
Prioritized with data

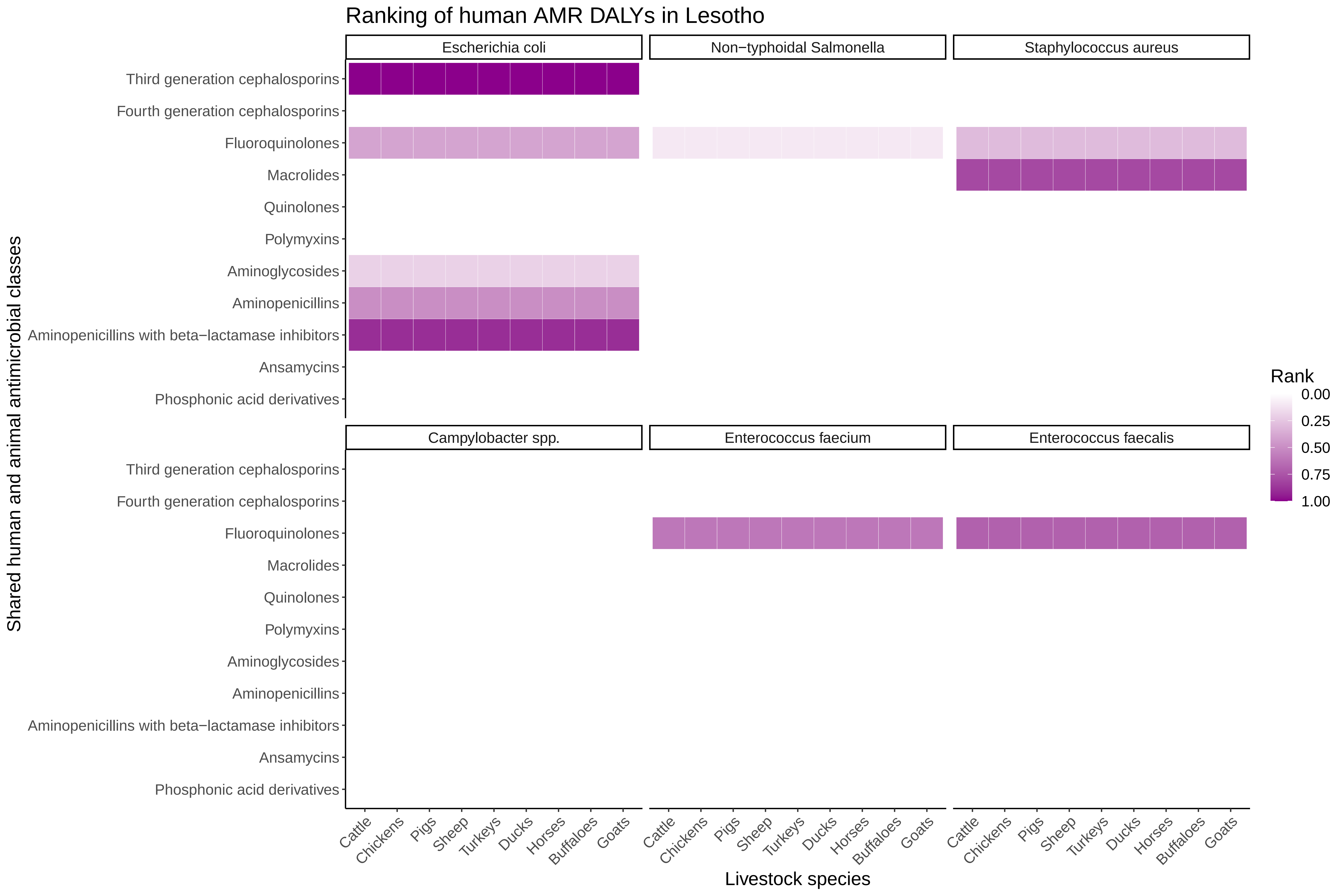

Ranking of human AMR DALYs in Lesotho
Escherichia coli
Non−typhoidal Salmonella
Staphylococcus aureus
Campylobacter spp.
Enterococcus faecium
Enterococcus faecalis
Third generation cephalosporins
Fourth generation cephalosporins
Fluoroquinolones
Macrolides
Quinolones
Polymyxins
Aminoglycosides
Aminopenicillins
Aminopenicillins with beta−lactamase inhibitors
Ansamycins
Phosphonic acid derivatives
Shared human and animal antimicrobial classes
Cattle
Chickens
Pigs
Sheep
Turkeys
Ducks
Horses
Buffaloes
Goats
Livestock species
Rank
0.00
0.25
0.50
0.75
1.00

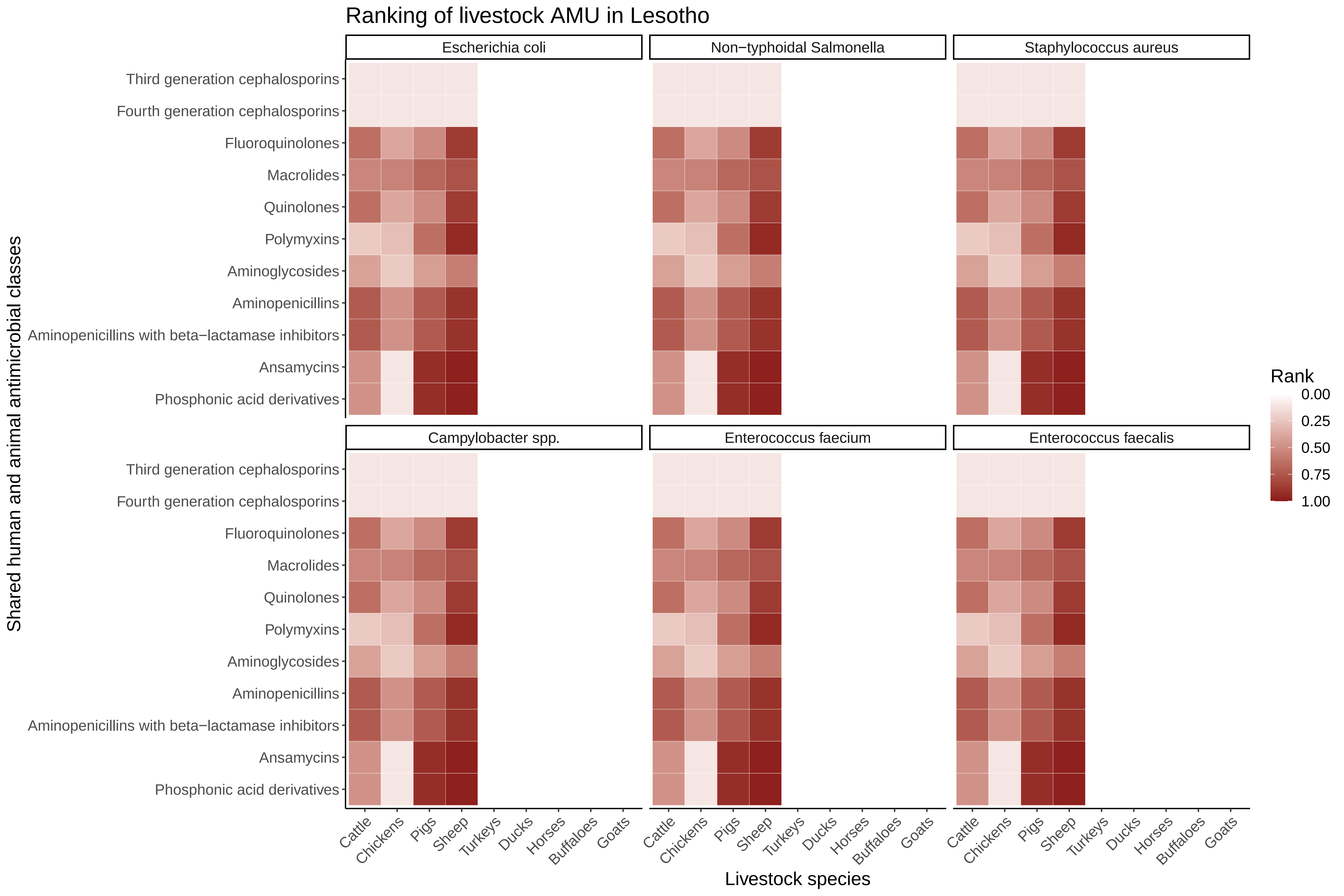

Ranking of livestock AMU in Lesotho
Escherichia coli
Non−typhoidal Salmonella
Staphylococcus aureus
Campylobacter spp.
Enterococcus faecium
Enterococcus faecalis
Shared human and animal antimicrobial classes
Third generation cephalosporins
Fourth generation cephalosporins
Fluoroquinolones
Macrolides
Quinolones
Polymyxins
Aminoglycosides
Aminopenicillins
Aminopenicillins with beta−lactamase inhibitors
Ansamycins
Phosphonic acid derivatives
Livestock species
Cattle
Chickens
Pigs
Sheep
Turkeys
Ducks
Horses
Buffaloes
Goats
Rank
0.00
0.25
0.50
0.75
1.00

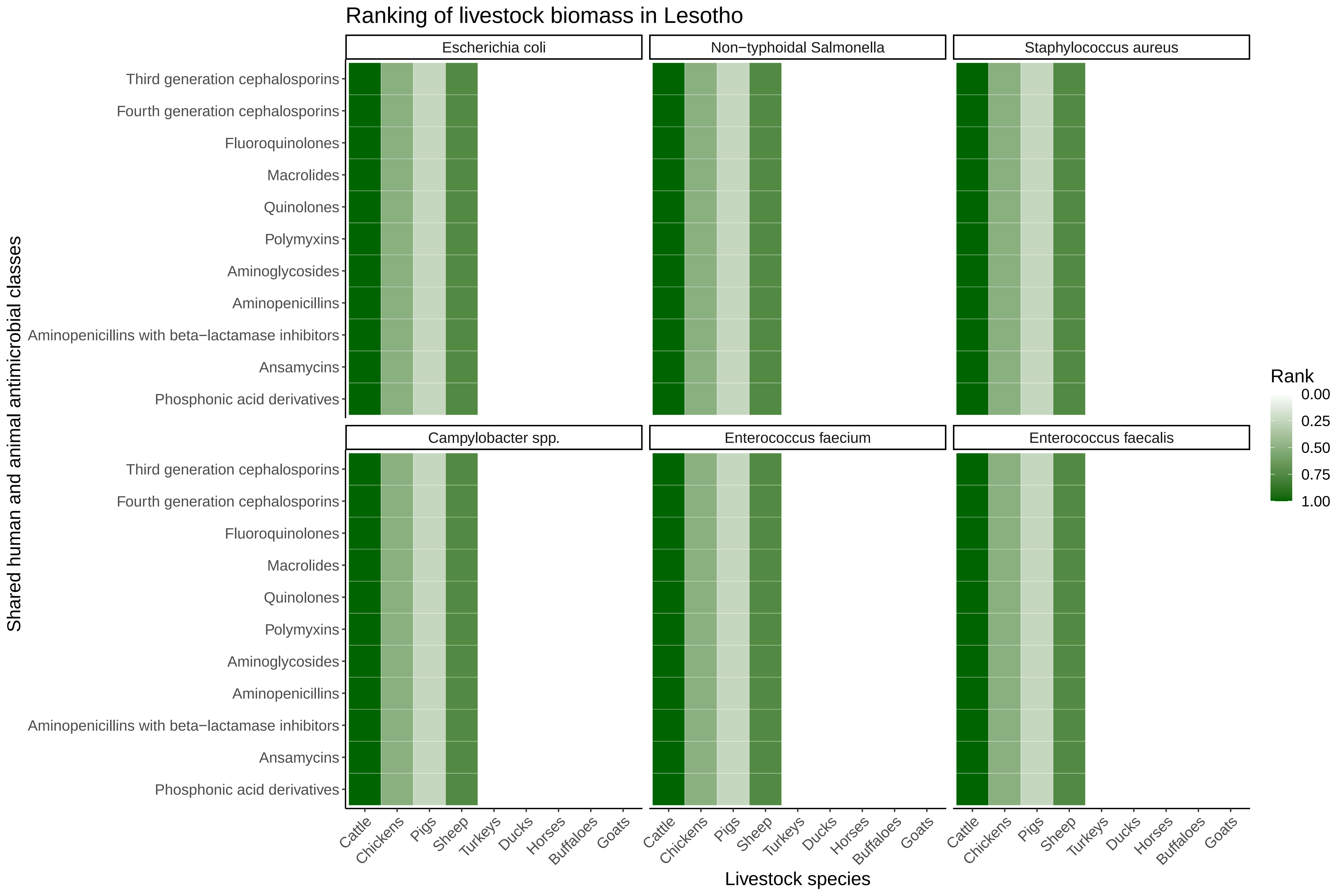

Ranking of livestock biomass in Lesotho
Escherichia coli
Non−typhoidal Salmonella
Staphylococcus aureus
Campylobacter spp.
Enterococcus faecium
Enterococcus faecalis
Shared human and animal antimicrobial classes
Third generation cephalosporins
Fourth generation cephalosporins
Fluoroquinolones
Macrolides
Quinolones
Polymyxins
Aminoglycosides
Aminopenicillins
Aminopenicillins with beta−lactamase inhibitors
Ansamycins
Phosphonic acid derivatives
Livestock species
Cattle
Chickens
Pigs
Sheep
Turkeys
Ducks
Horses
Buffaloes
Goats
Rank
0.00
0.25
0.50
0.75
1.00

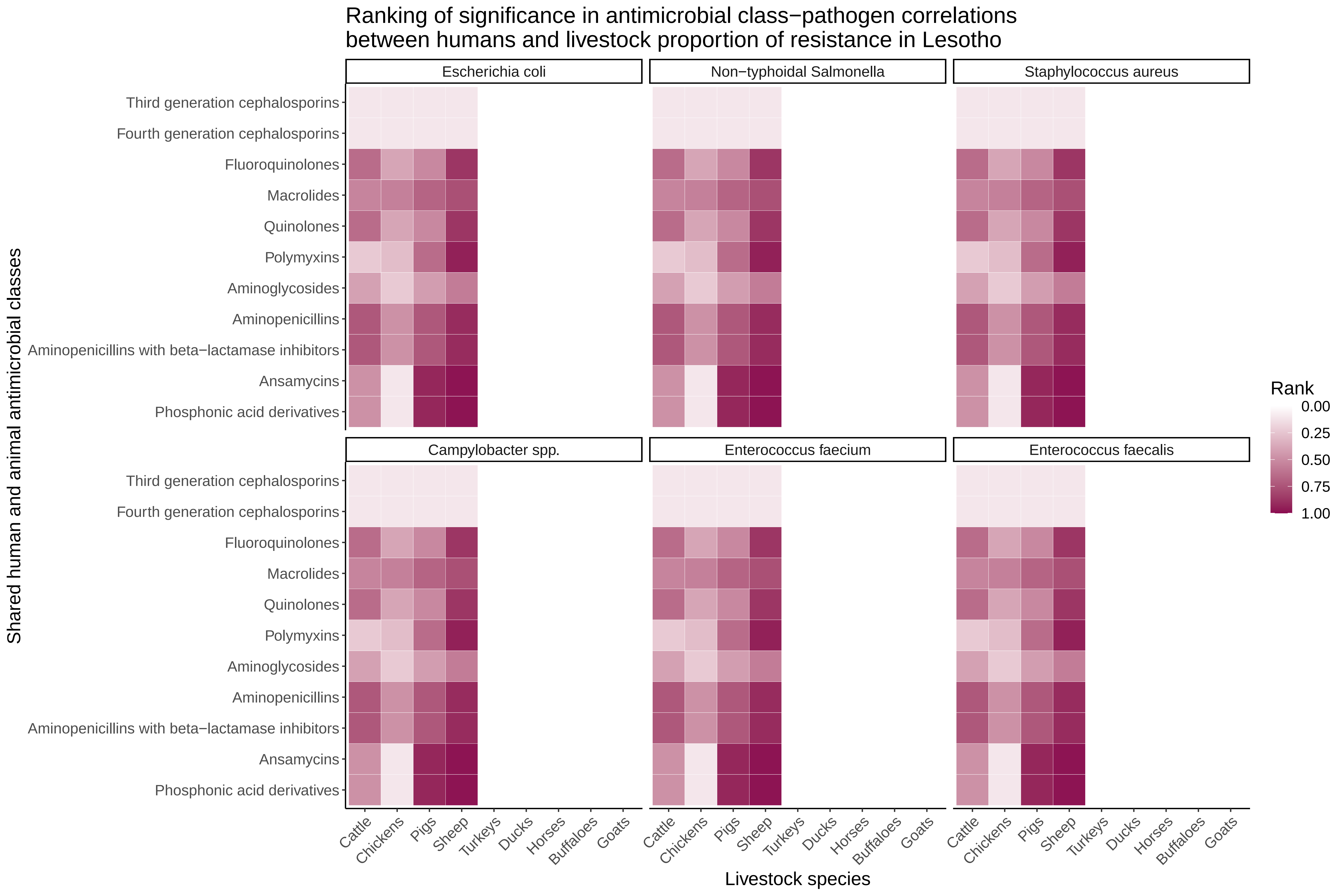

Ranking of significance in antimicrobial class−pathogen correlations between humans and livestock proportion of resistance in Lesotho
Escherichia coli
Non−typhoidal Salmonella
Staphylococcus aureus
Campylobacter spp.
Enterococcus faecium
Enterococcus faecalis
Shared human and animal antimicrobial classes
Third generation cephalosporins
Fourth generation cephalosporins
Fluoroquinolones
Macrolides
Quinolones
Polymyxins
Aminoglycosides
Aminopenicillins
Aminopenicillins with beta−lactamase inhibitors
Ansamycins
Phosphonic acid derivatives
Livestock species
Cattle
Chickens
Pigs
Sheep
Turkeys
Ducks
Horses
Buffaloes
Goats
Rank
0.00
0.25
0.50
0.75
1.00

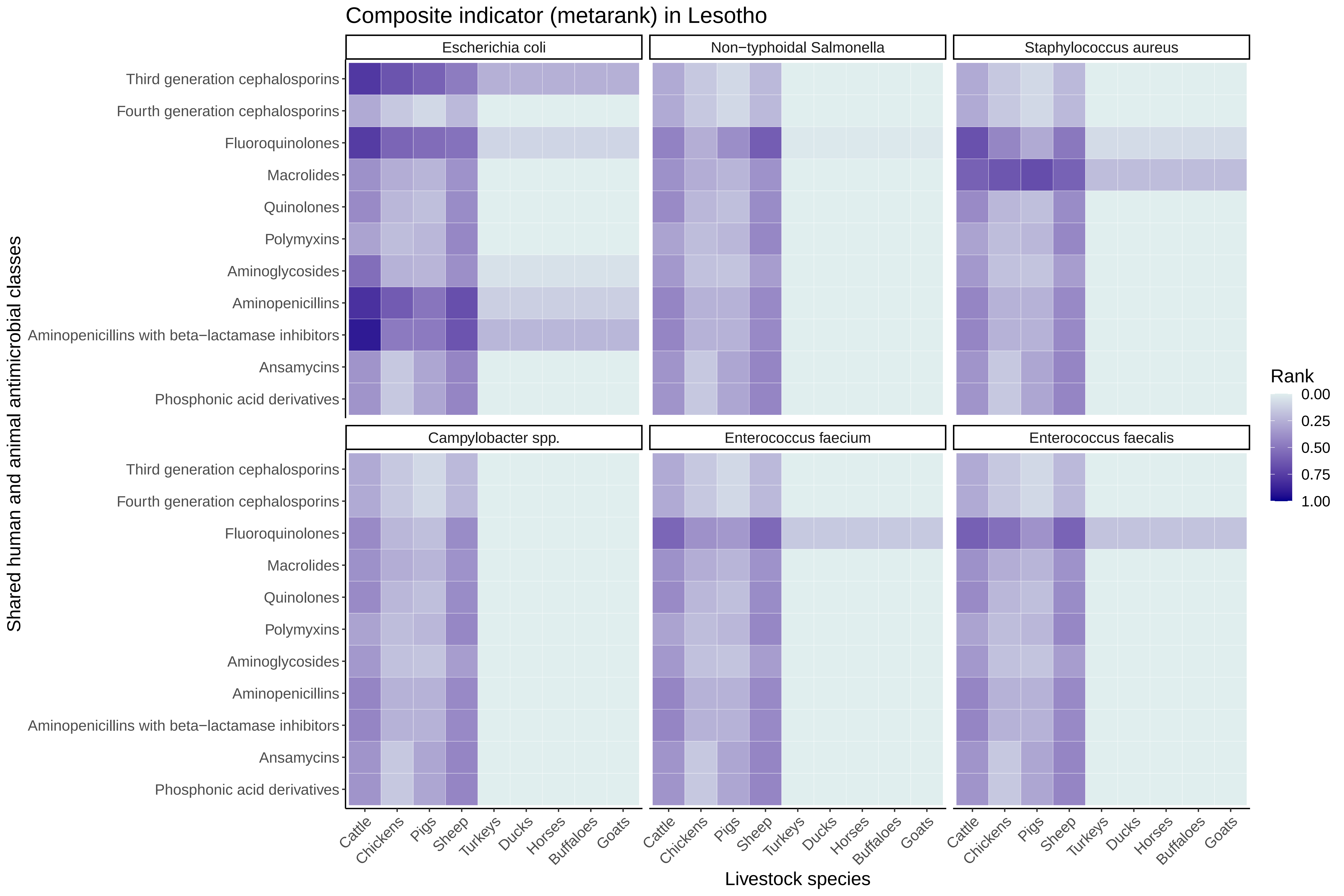

Composite indicator (metarank) in Lesotho
Escherichia coli
Non−typhoidal Salmonella
Staphylococcus aureus
Campylobacter spp.
Enterococcus faecium
Enterococcus faecalis
Shared human and animal antimicrobial classes
Third generation cephalosporins
Fourth generation cephalosporins
Fluoroquinolones
Macrolides
Quinolones
Polymyxins
Aminoglycosides
Aminopenicillins
Aminopenicillins with beta−lactamase inhibitors
Ansamycins
Phosphonic acid derivatives
Cattle
Chickens
Pigs
Sheep
Turkeys
Ducks
Horses
Buffaloes
Goats
Livestock species
Rank
0.00
0.25
0.50
0.75
1.00

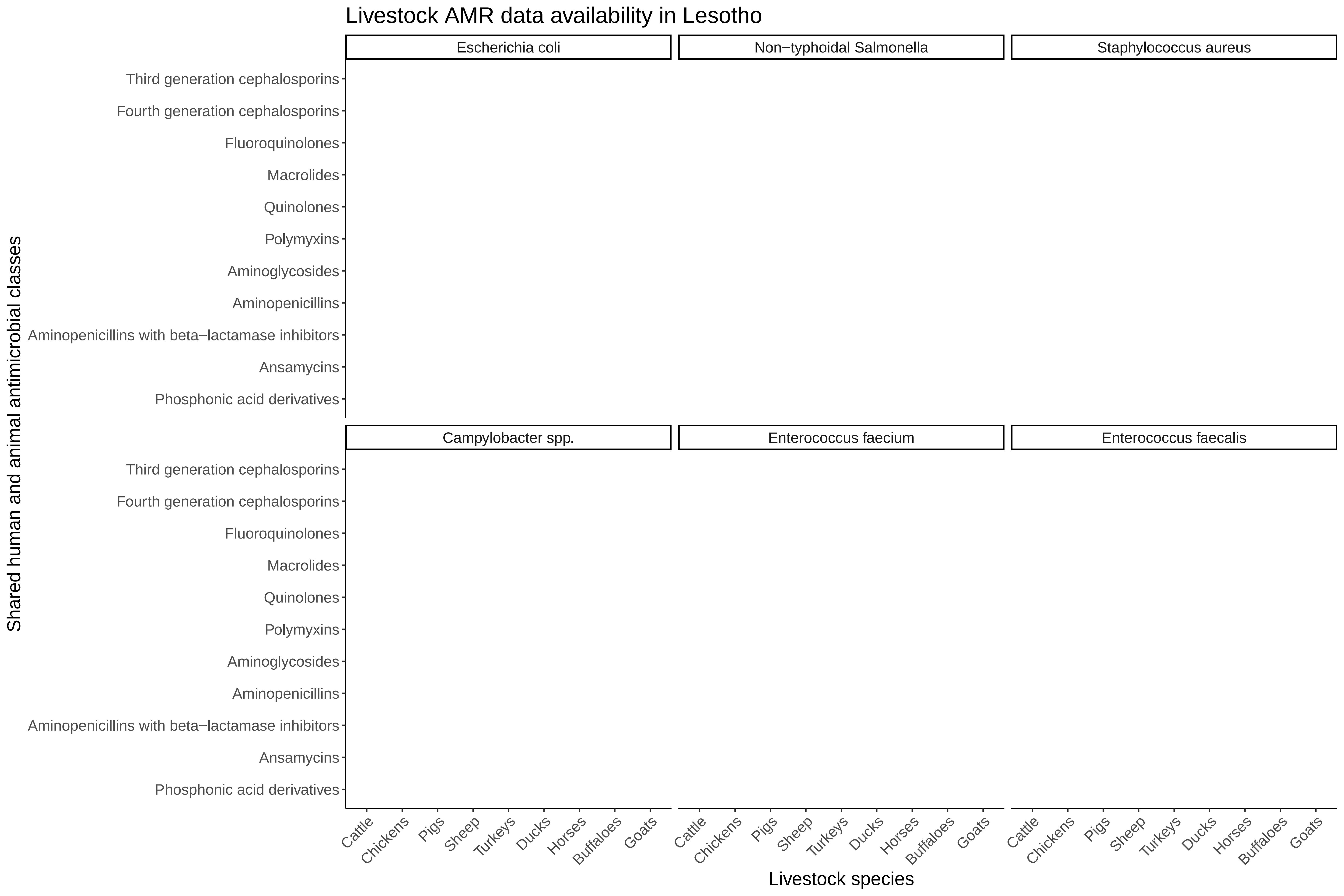
Livestock AMR data availability in Lesotho
Escherichia coli
Non−typhoidal Salmonella
Staphylococcus aureus
Campylobacter spp.
Enterococcus faecium
Enterococcus faecalis
Third generation cephalosporins
Fourth generation cephalosporins
Fluoroquinolones
Macrolides
Quinolones
Polymyxins
Aminoglycosides
Aminopenicillins
Aminopenicillins with beta−lactamase inhibitors
Ansamycins
Phosphonic acid derivatives
Cattle
Chickens
Pigs
Sheep
Turkeys
Ducks
Horses
Buffaloes
Goats
Shared human and animal antimicrobial classes
Livestock species

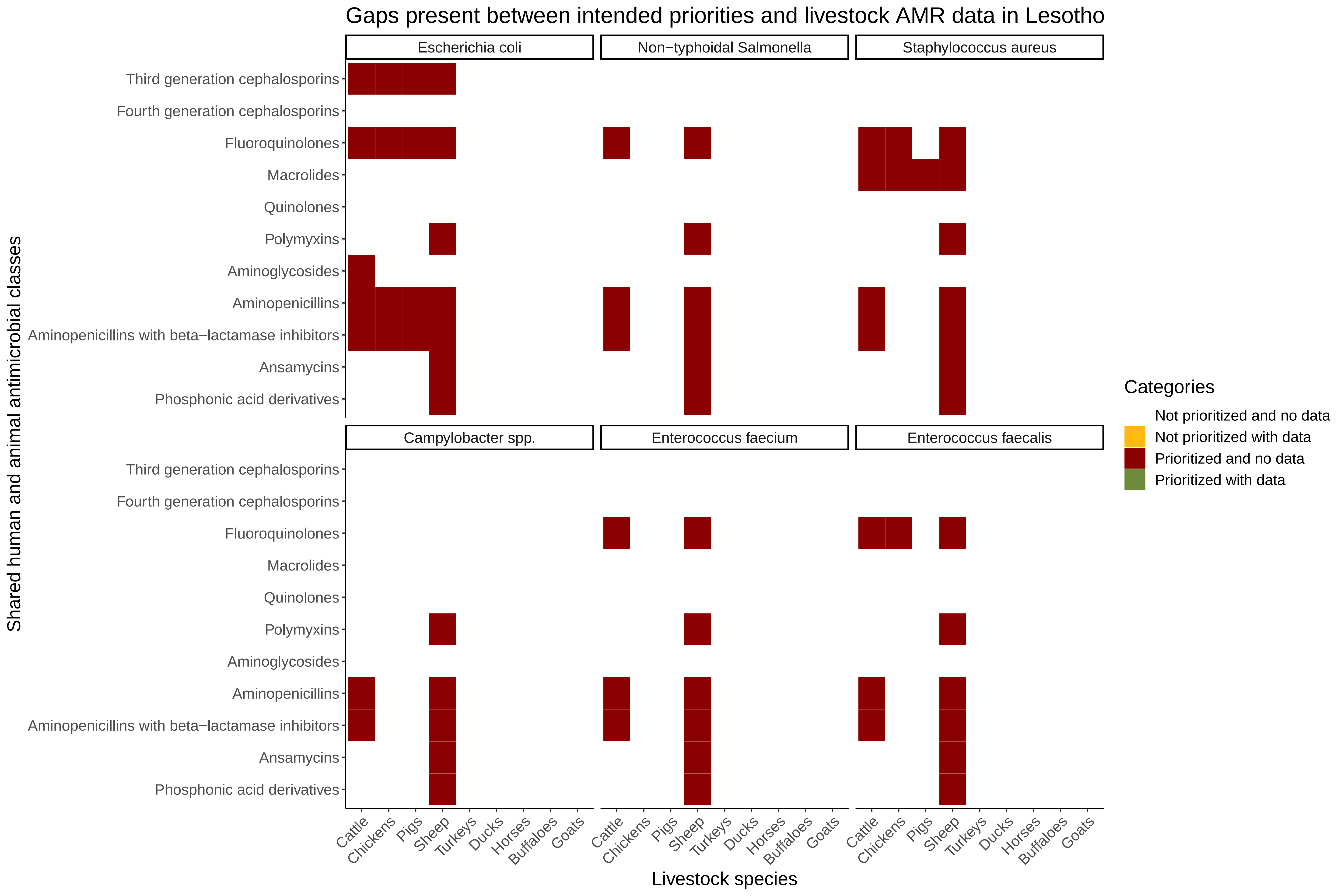

Gaps present between intended priorities and livestock AMR data in Lesotho
Escherichia coli
Non−typhoidal Salmonella
Staphylococcus aureus
Campylobacter spp.
Enterococcus faecium
Enterococcus faecalis
Third generation cephalosporins
Fourth generation cephalosporins
Fluoroquinolones
Macrolides
Quinolones
Polymyxins
Aminoglycosides
Aminopenicillins
Aminopenicillins with beta−lactamase inhibitors
Ansamycins
Phosphonic acid derivatives
Shared human and animal antimicrobial classes
Cattle
Chickens
Pigs
Sheep
Turkeys
Ducks
Horses
Buffaloes
Goats
Livestock species
Categories
Not prioritized and no data
Not prioritized with data
Prioritized and no data
Prioritized with data

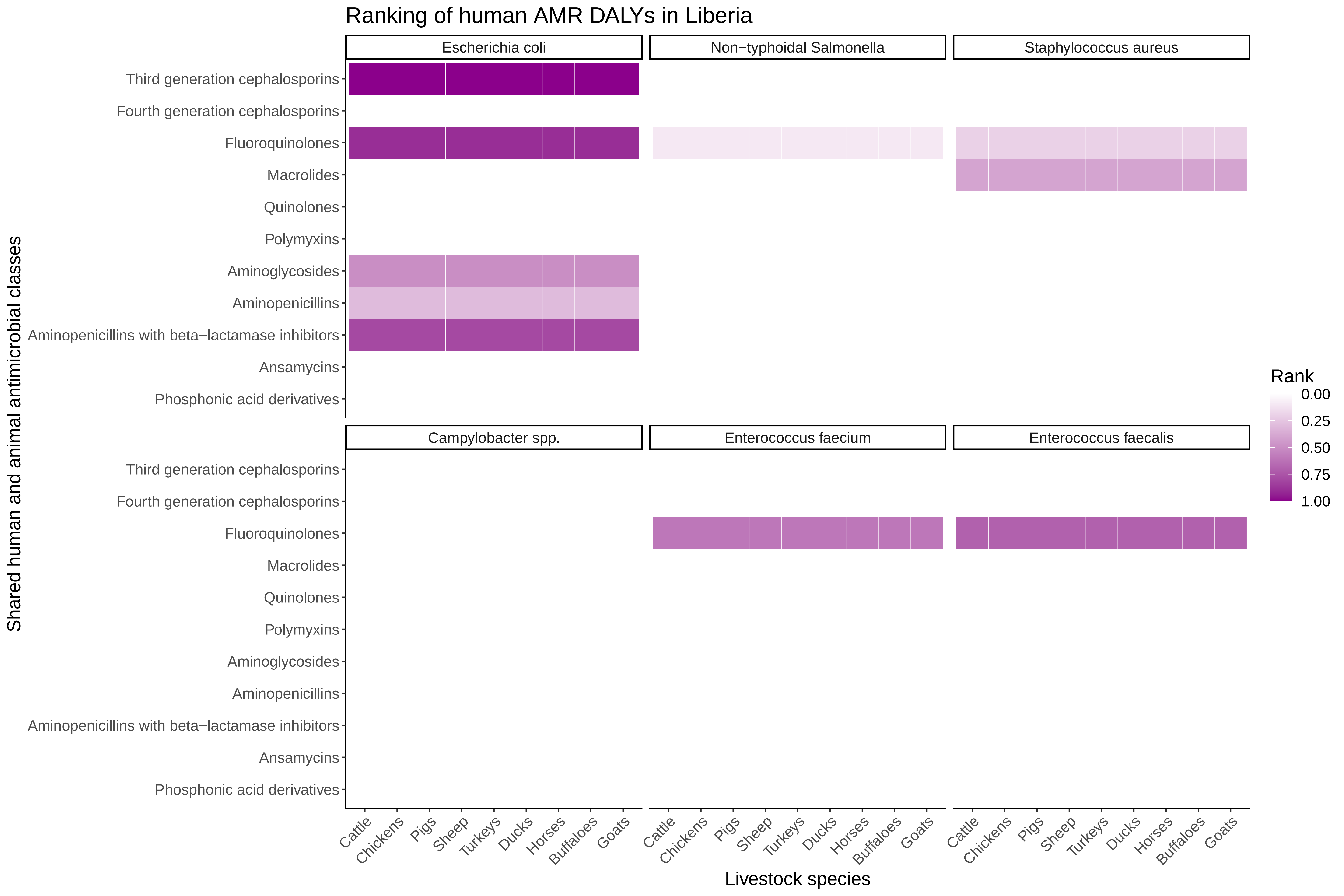
Ranking of human AMR DALYs in Liberia
Escherichia coli
Non−typhoidal Salmonella
Staphylococcus aureus
Campylobacter spp.
Enterococcus faecium
Enterococcus faecalis
Third generation cephalosporins
Fourth generation cephalosporins
Fluoroquinolones
Macrolides
Quinolones
Polymyxins
Aminoglycosides
Aminopenicillins
Aminopenicillins with beta−lactamase inhibitors
Ansamycins
Phosphonic acid derivatives
Shared human and animal antimicrobial classes
Cattle
Chickens
Pigs
Sheep
Turkeys
Ducks
Horses
Buffaloes
Goats
Livestock species
Rank
0.00
0.25
0.50
0.75
1.00

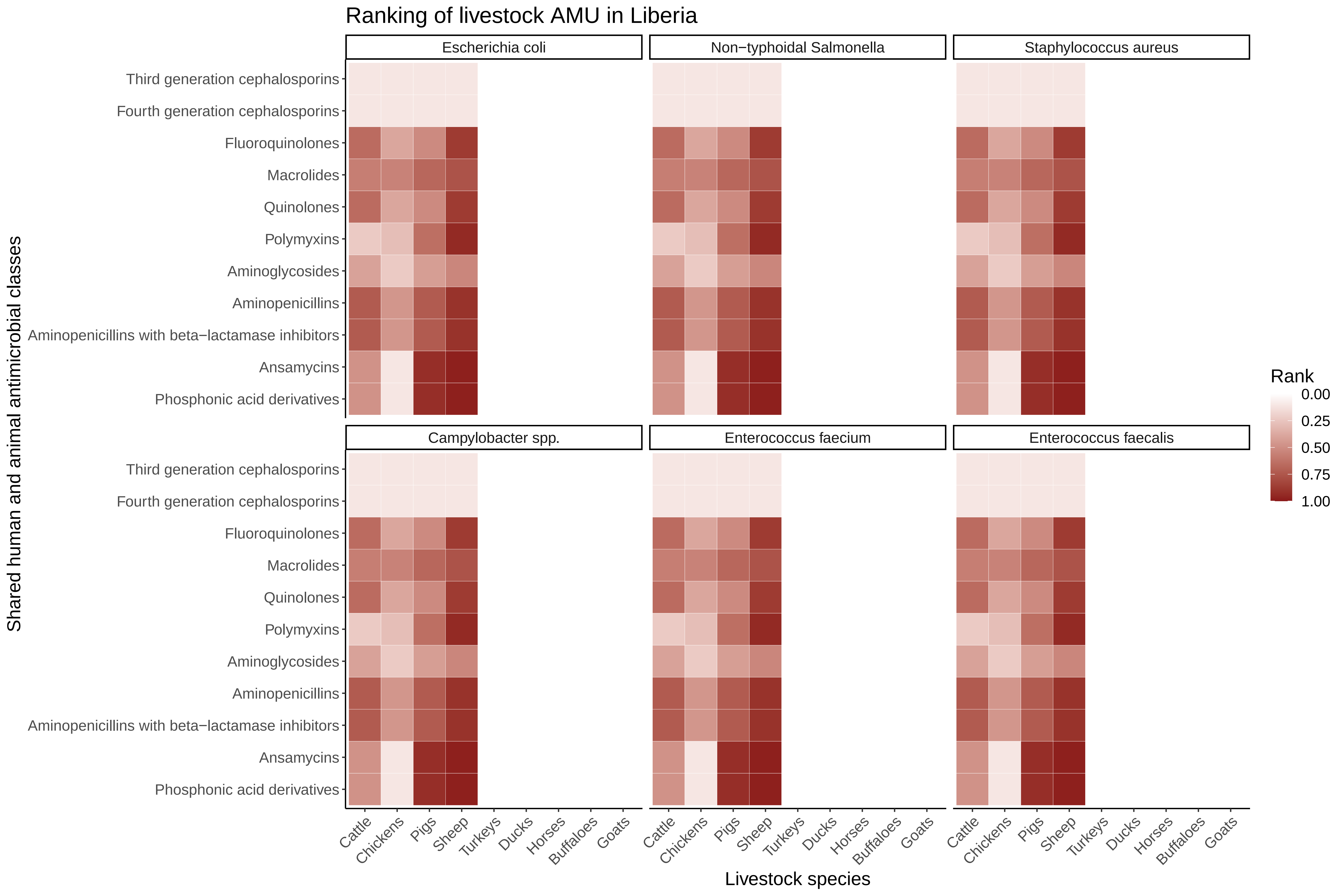

Ranking of livestock AMU in Liberia
Escherichia coli
Non−typhoidal Salmonella
Staphylococcus aureus
Campylobacter spp.
Enterococcus faecium
Enterococcus faecalis
Shared human and animal antimicrobial classes
Third generation cephalosporins
Fourth generation cephalosporins
Fluoroquinolones
Macrolides
Quinolones
Polymyxins
Aminoglycosides
Aminopenicillins
Aminopenicillins with beta−lactamase inhibitors
Ansamycins
Phosphonic acid derivatives
Cattle
Chickens
Pigs
Sheep
Turkeys
Ducks
Horses
Buffaloes
Goats
Livestock species
Rank
0.00
0.25
0.50
0.75
1.00

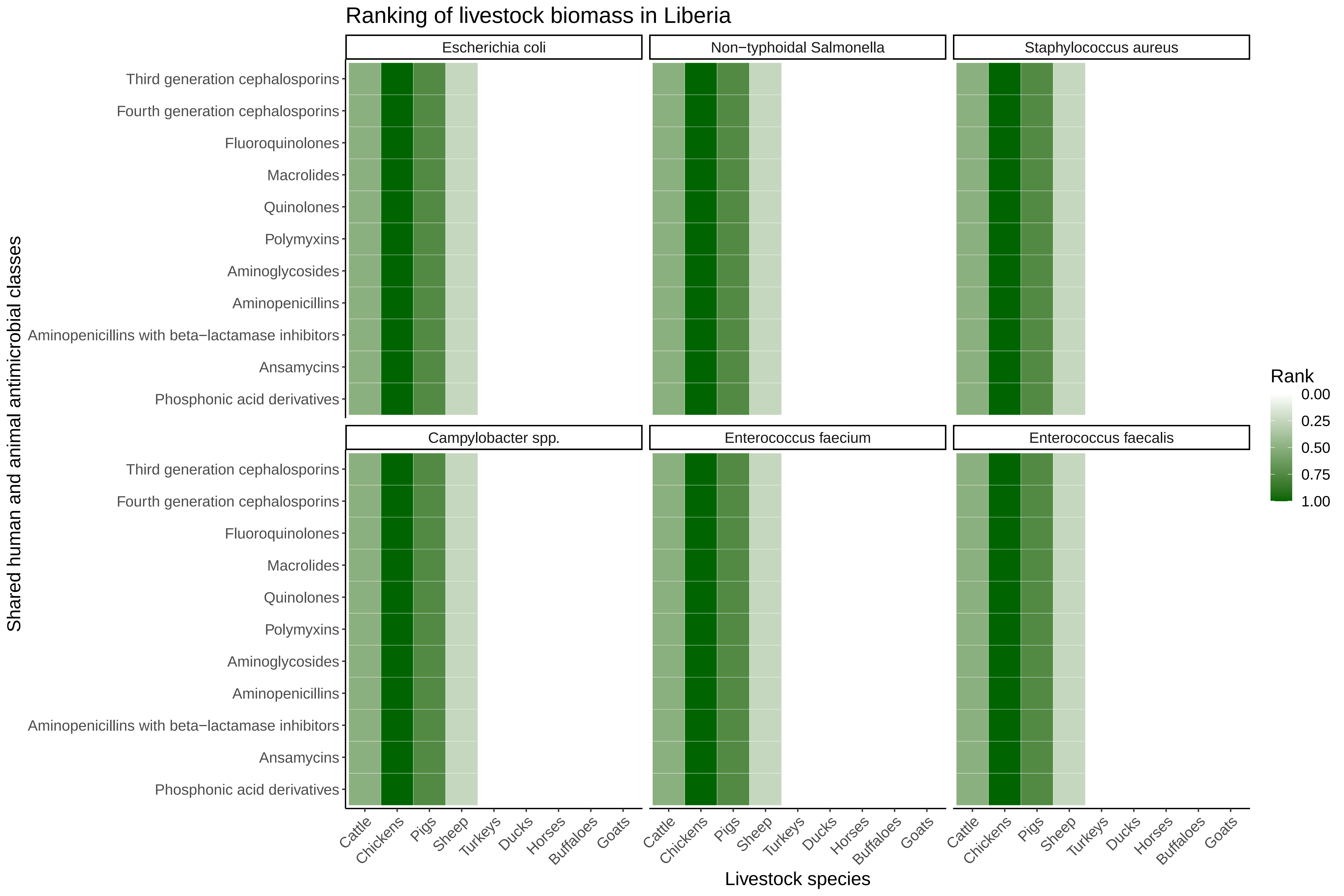
Ranking of livestock biomass in Liberia
Escherichia coli
Non−typhoidal Salmonella
Staphylococcus aureus
Campylobacter spp.
Enterococcus faecium
Enterococcus faecalis
Shared human and animal antimicrobial classes
Third generation cephalosporins
Fourth generation cephalosporins
Fluoroquinolones
Macrolides
Quinolones
Polymyxins
Aminoglycosides
Aminopenicillins
Aminopenicillins with beta−lactamase inhibitors
Ansamycins
Phosphonic acid derivatives
Cattle
Chickens
Pigs
Sheep
Turkeys
Ducks
Horses
Buffaloes
Goats
Livestock species
Rank
0.00
0.25
0.50
0.75
1.00

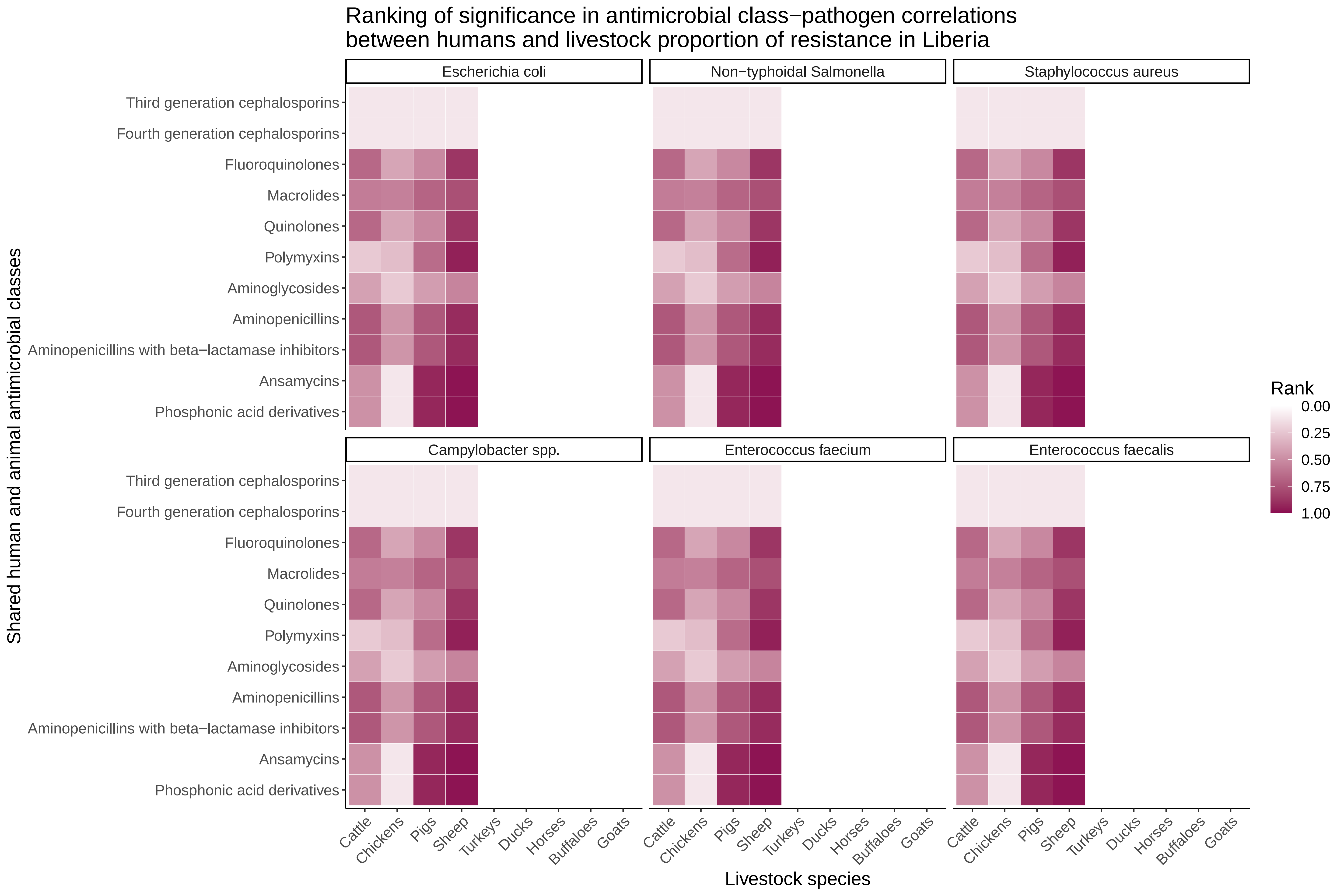

Ranking of significance in antimicrobial class−pathogen correlations
between humans and livestock proportion of resistance in Liberia
Escherichia coli
Non−typhoidal Salmonella
Staphylococcus aureus
Campylobacter spp.
Enterococcus faecium
Enterococcus faecalis
Third generation cephalosporins
Fourth generation cephalosporins
Fluoroquinolones
Macrolides
Quinolones
Polymyxins
Aminoglycosides
Aminopenicillins
Aminopenicillins with beta−lactamase inhibitors
Ansamycins
Phosphonic acid derivatives
Shared human and animal antimicrobial classes
Cattle
Chickens
Pigs
Sheep
Turkeys
Ducks
Horses
Buffaloes
Goats
Livestock species
Rank
0.00
0.25
0.50
0.75
1.00

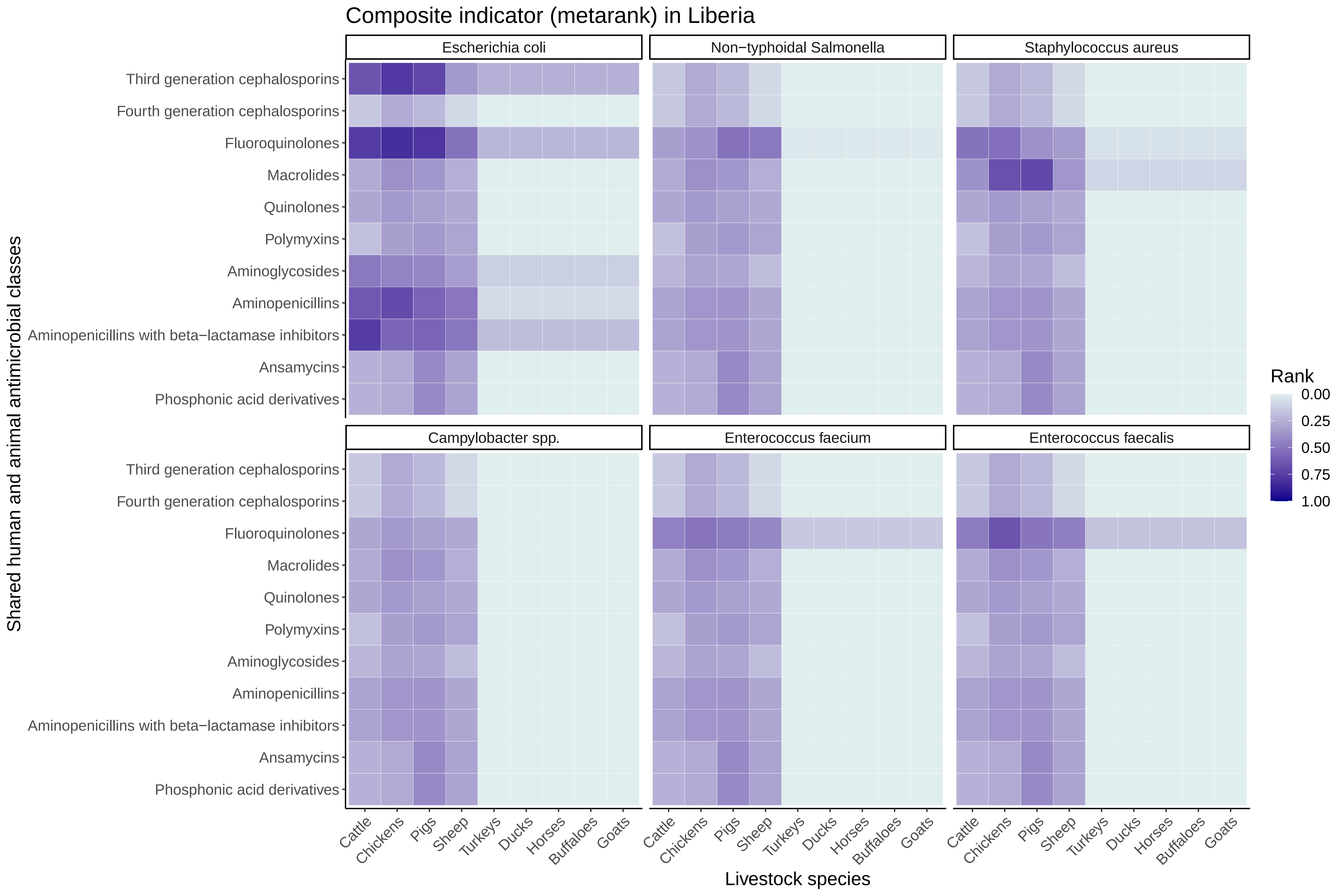

Composite indicator (metarank) in Liberia
Escherichia coli
Non−typhoidal Salmonella
Staphylococcus aureus
Campylobacter spp.
Enterococcus faecium
Enterococcus faecalis
Shared human and animal antimicrobial classes
Third generation cephalosporins
Fourth generation cephalosporins
Fluoroquinolones
Macrolides
Quinolones
Polymyxins
Aminoglycosides
Aminopenicillins
Aminopenicillins with beta−lactamase inhibitors
Ansamycins
Phosphonic acid derivatives
Cattle
Chickens
Pigs
Sheep
Turkeys
Ducks
Horses
Buffaloes
Goats
Livestock species
Rank
0.00
0.25
0.50
0.75
1.00

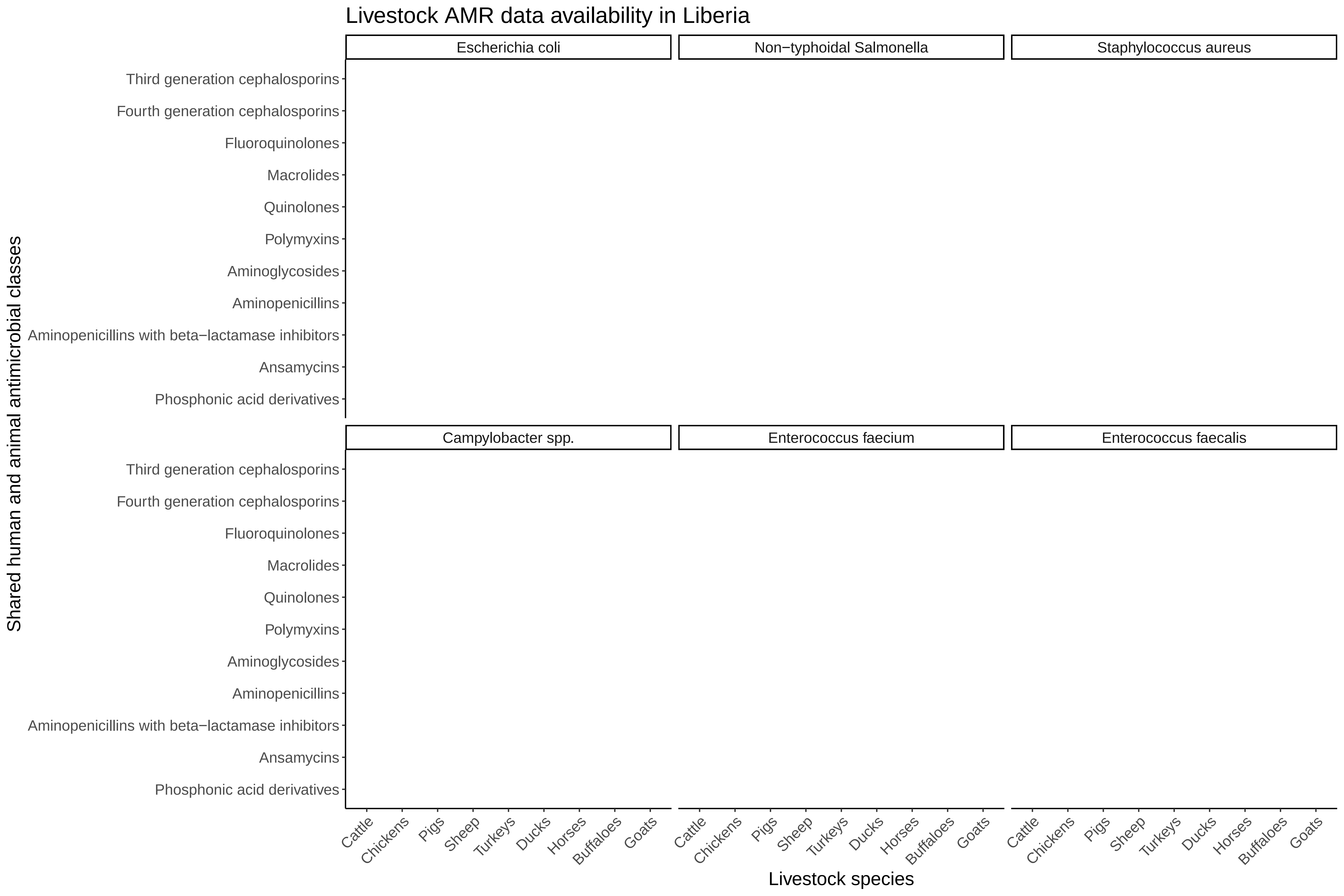

Livestock AMR data availability in Liberia
Escherichia coli
Non−typhoidal Salmonella
Staphylococcus aureus
Campylobacter spp.
Enterococcus faecium
Enterococcus faecalis
Third generation cephalosporins
Fourth generation cephalosporins
Fluoroquinolones
Macrolides
Quinolones
Polymyxins
Aminoglycosides
Aminopenicillins
Aminopenicillins with beta−lactamase inhibitors
Ansamycins
Phosphonic acid derivatives
Shared human and animal antimicrobial classes
Cattle
Chickens
Pigs
Sheep
Turkeys
Ducks
Horses
Buffaloes
Goats
Livestock species

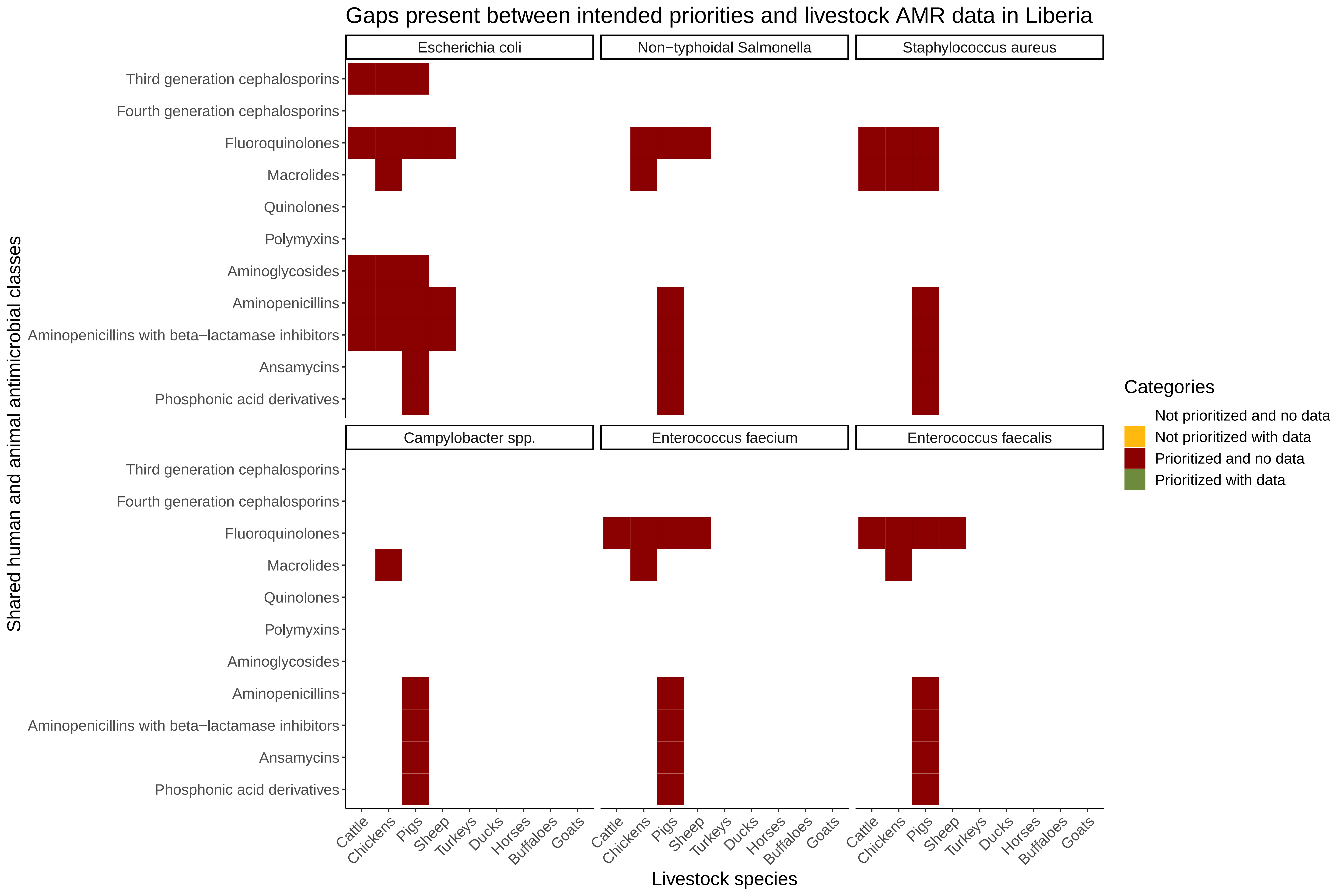

Gaps present between intended priorities and livestock AMR data in Liberia
Escherichia coli
Non−typhoidal Salmonella
Staphylococcus aureus
Campylobacter spp.
Enterococcus faecium
Enterococcus faecalis
Third generation cephalosporins
Fourth generation cephalosporins
Fluoroquinolones
Macrolides
Quinolones
Polymyxins
Aminoglycosides
Aminopenicillins
Aminopenicillins with beta−lactamase inhibitors
Ansamycins
Phosphonic acid derivatives
Shared human and animal antimicrobial classes
Cattle
Chickens
Pigs
Sheep
Turkeys
Ducks
Horses
Buffaloes
Goats
Livestock species
Categories
Not prioritized and no data
Not prioritized with data
Prioritized and no data
Prioritized with data

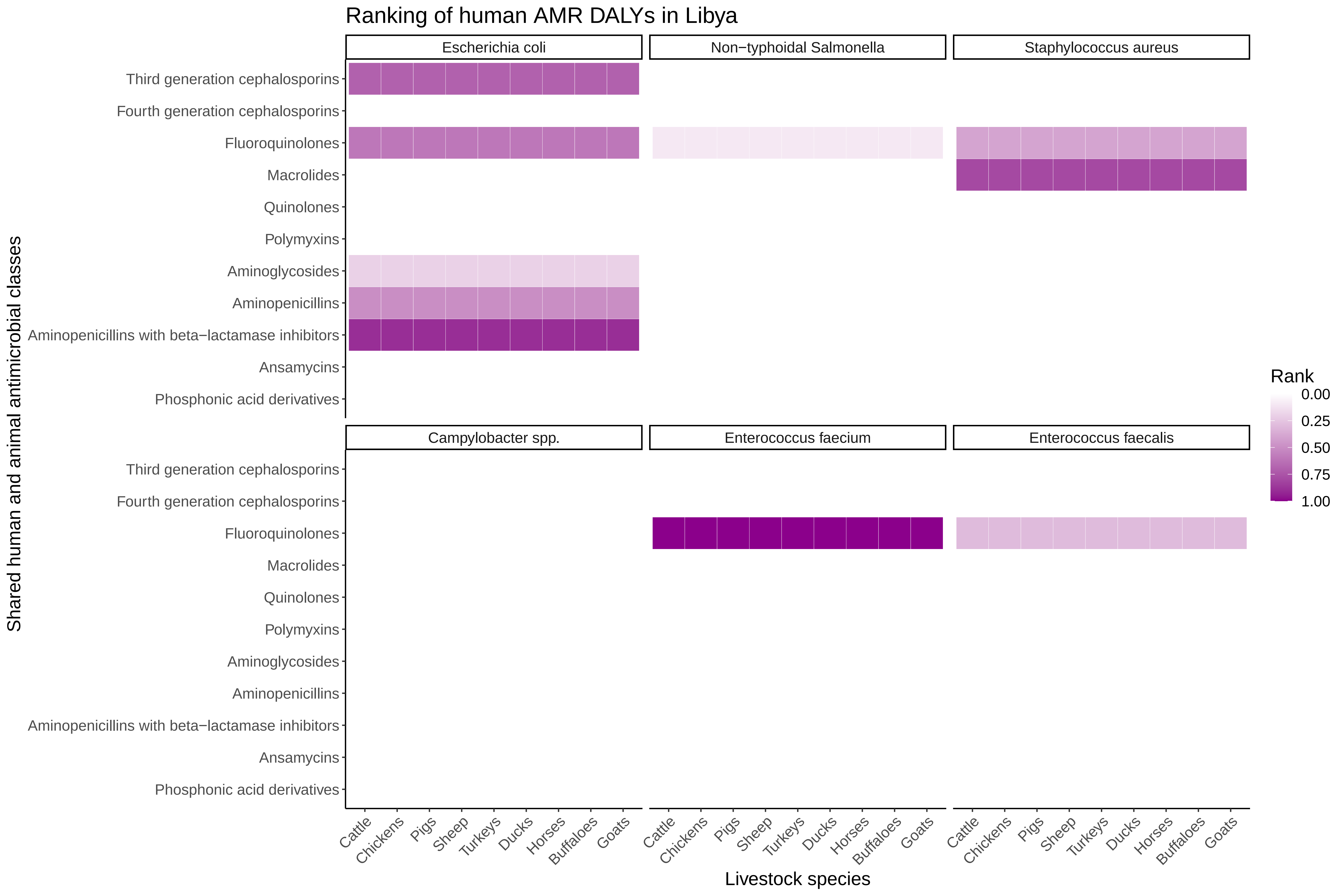

Ranking of human AMR DALYs in Libya
Escherichia coli
Non−typhoidal Salmonella
Staphylococcus aureus
Campylobacter spp.
Enterococcus faecium
Enterococcus faecalis
Third generation cephalosporins
Fourth generation cephalosporins
Fluoroquinolones
Macrolides
Quinolones
Polymyxins
Aminoglycosides
Aminopenicillins
Aminopenicillins with beta−lactamase inhibitors
Ansamycins
Phosphonic acid derivatives
Shared human and animal antimicrobial classes
Cattle
Chickens
Pigs
Sheep
Turkeys
Ducks
Horses
Buffaloes
Goats
Livestock species
Rank
0.00
0.25
0.50
0.75
1.00

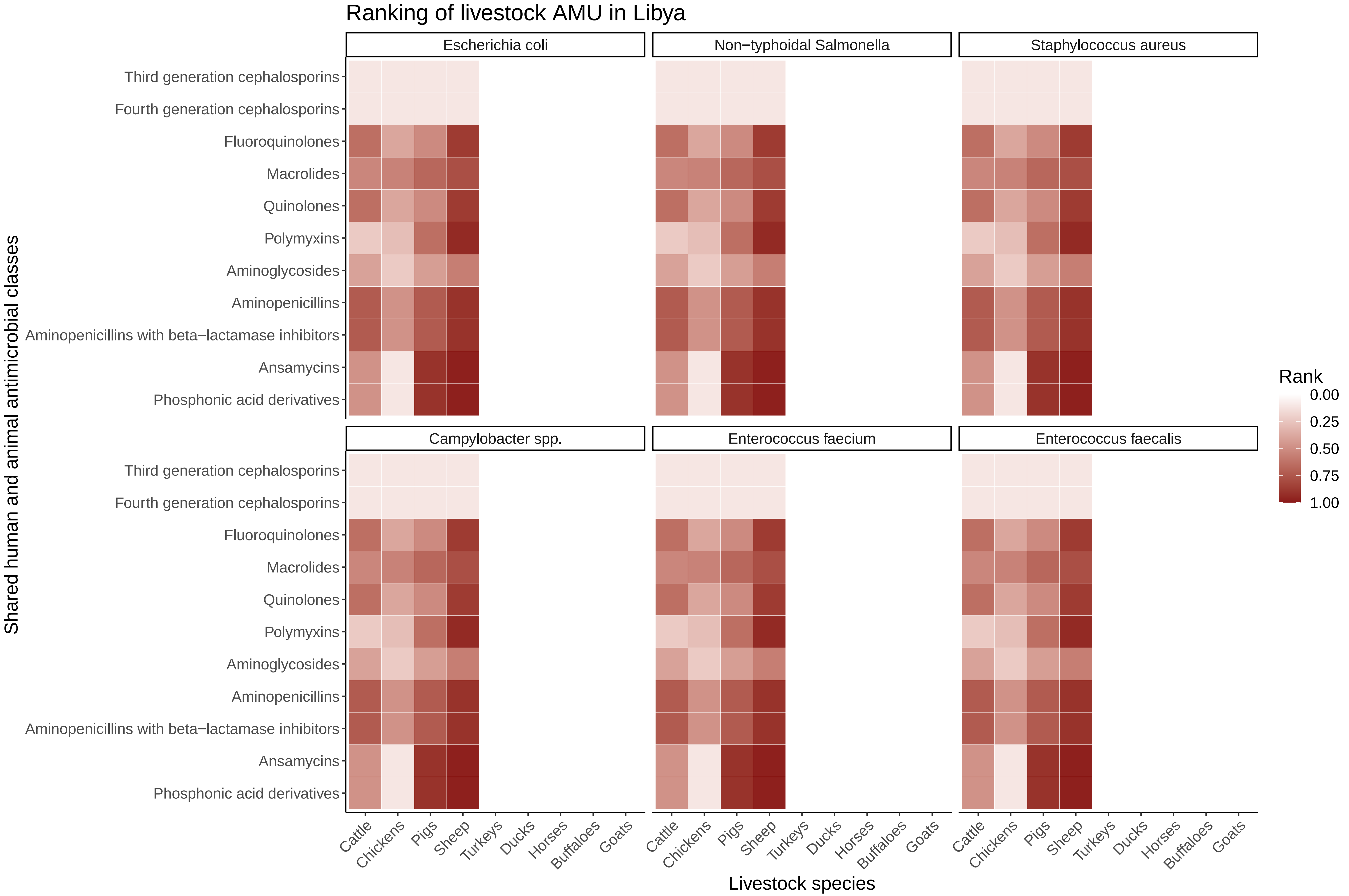

Ranking of livestock AMU in Libya
Escherichia coli
Non−typhoidal Salmonella
Staphylococcus aureus
Campylobacter spp.
Enterococcus faecium
Enterococcus faecalis
Third generation cephalosporins
Fourth generation cephalosporins
Fluoroquinolones
Macrolides
Quinolones
Polymyxins
Aminoglycosides
Aminopenicillins
Aminopenicillins with beta−lactamase inhibitors
Ansamycins
Phosphonic acid derivatives
Shared human and animal antimicrobial classes
Cattle
Chickens
Pigs
Sheep
Turkeys
Ducks
Horses
Buffaloes
Goats
Livestock species
Rank
0.00
0.25
0.50
0.75
1.00

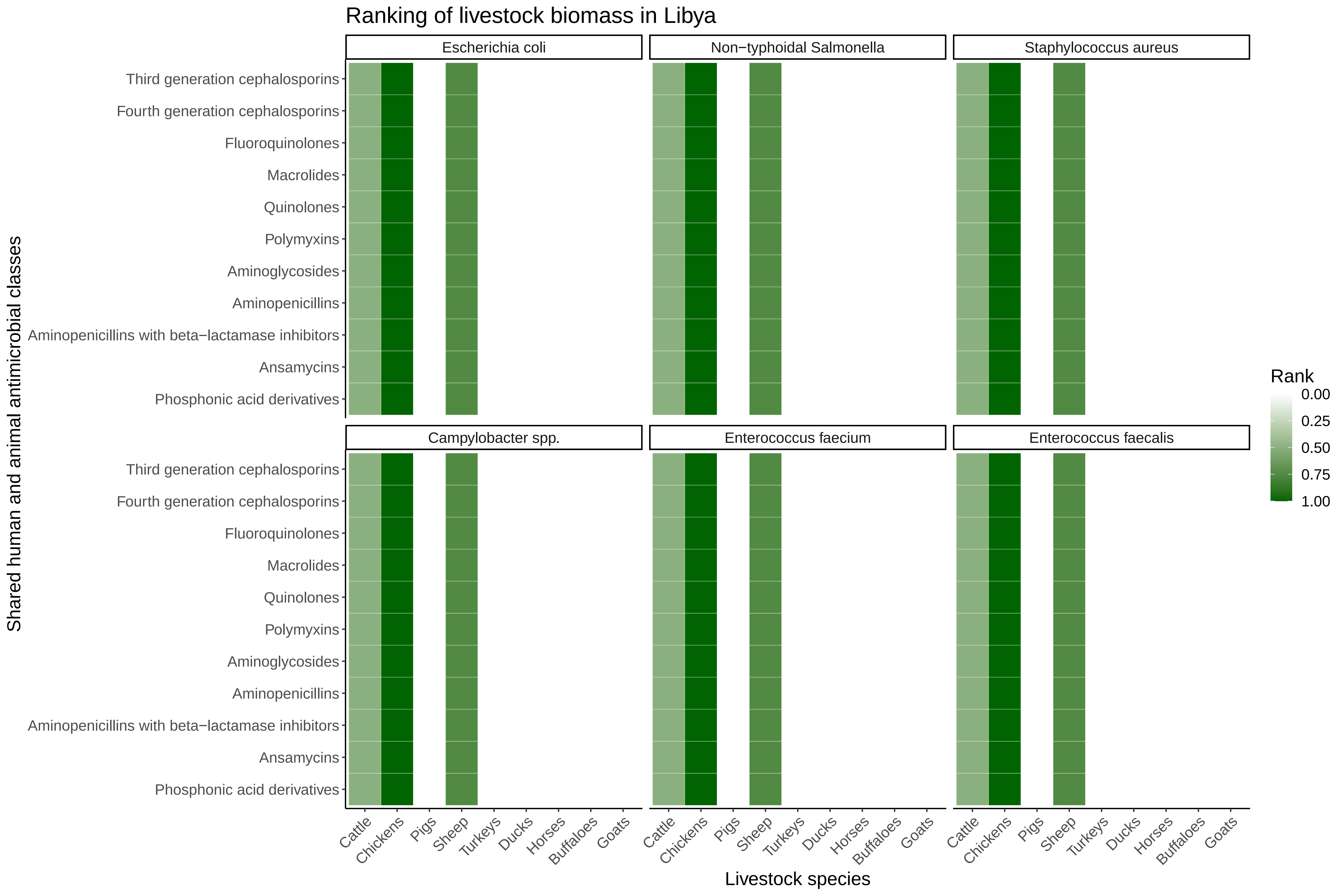

Ranking of livestock biomass in Libya
Escherichia coli
Non−typhoidal Salmonella
Staphylococcus aureus
Campylobacter spp.
Enterococcus faecium
Enterococcus faecalis
Third generation cephalosporins
Fourth generation cephalosporins
Fluoroquinolones
Macrolides
Quinolones
Polymyxins
Aminoglycosides
Aminopenicillins
Aminopenicillins with beta−lactamase inhibitors
Ansamycins
Phosphonic acid derivatives
Shared human and animal antimicrobial classes
Cattle
Chickens
Pigs
Sheep
Turkeys
Ducks
Horses
Buffaloes
Goats
Livestock species
Rank
0.00
0.25
0.50
0.75
1.00

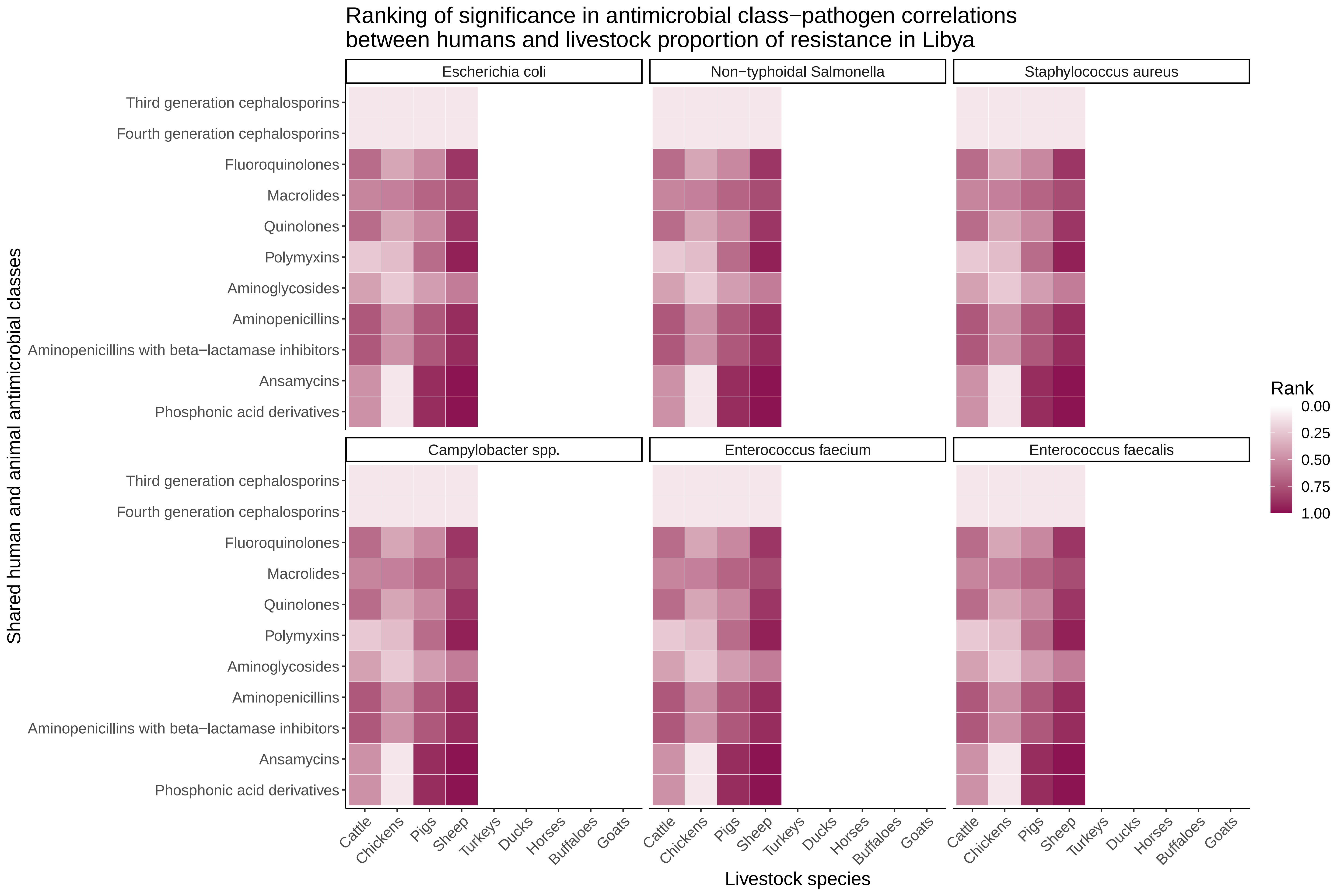

Ranking of significance in antimicrobial class−pathogen correlations between humans and livestock proportion of resistance in Libya
Escherichia coli
Non−typhoidal Salmonella
Staphylococcus aureus
Campylobacter spp.
Enterococcus faecium
Enterococcus faecalis
Third generation cephalosporins
Fourth generation cephalosporins
Fluoroquinolones
Macrolides
Quinolones
Polymyxins
Aminoglycosides
Aminopenicillins
Aminopenicillins with beta−lactamase inhibitors
Ansamycins
Phosphonic acid derivatives
Shared human and animal antimicrobial classes
Cattle
Chickens
Pigs
Sheep
Turkeys
Ducks
Horses
Buffaloes
Goats
Livestock species
Rank
0.00
0.25
0.50
0.75
1.00

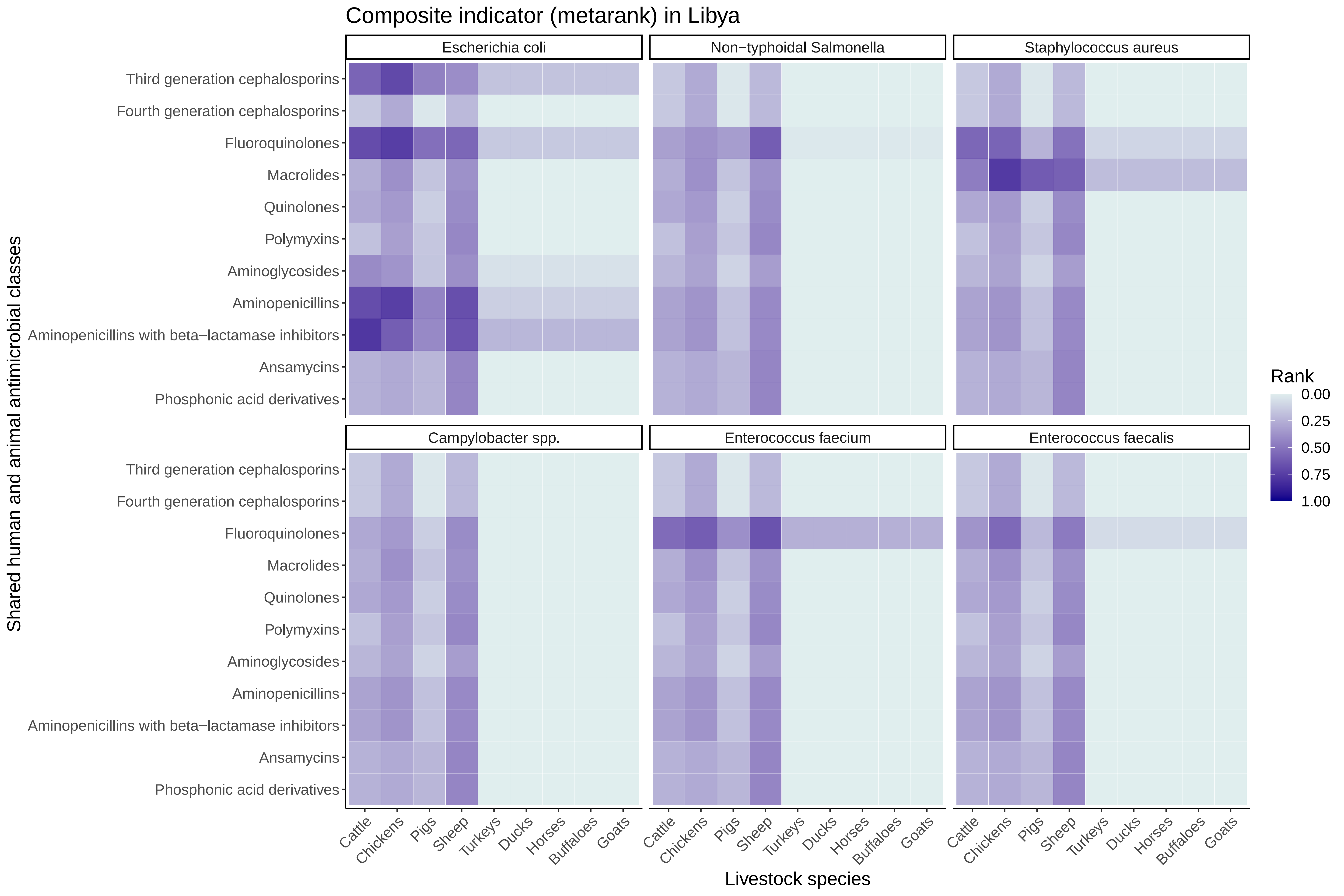

Composite indicator (metarank) in Libya
Escherichia coli
Non−typhoidal Salmonella
Staphylococcus aureus
Campylobacter spp.
Enterococcus faecium
Enterococcus faecalis
Shared human and animal antimicrobial classes
Third generation cephalosporins
Fourth generation cephalosporins
Fluoroquinolones
Macrolides
Quinolones
Polymyxins
Aminoglycosides
Aminopenicillins
Aminopenicillins with beta−lactamase inhibitors
Ansamycins
Phosphonic acid derivatives
Livestock species
Cattle
Chickens
Pigs
Sheep
Turkeys
Ducks
Horses
Buffaloes
Goats
Rank
0.00
0.25
0.50
0.75
1.00

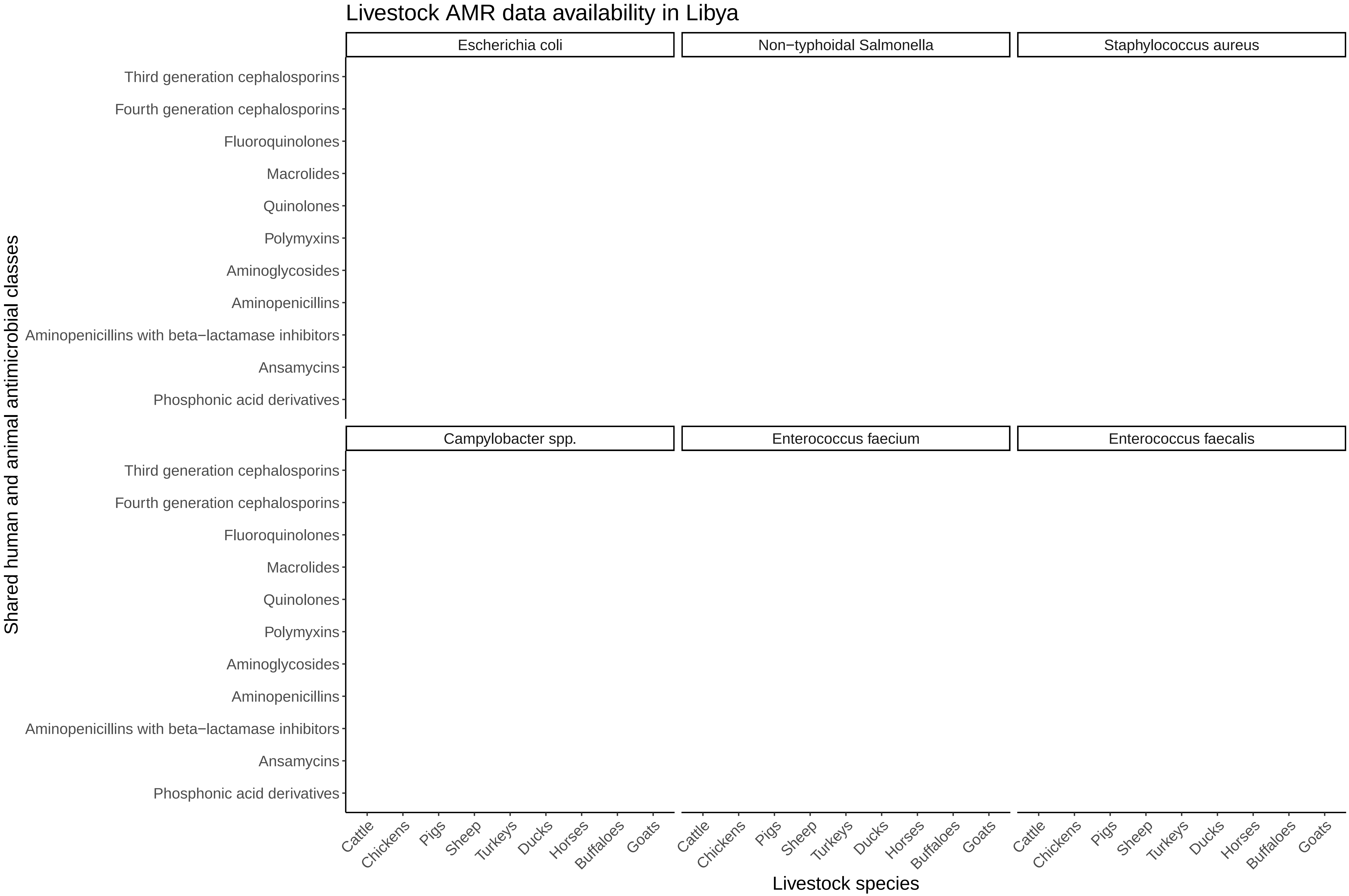
Livestock AMR data availability in Libya
Escherichia coli
Non−typhoidal Salmonella
Staphylococcus aureus
Campylobacter spp.
Enterococcus faecium
Enterococcus faecalis
Shared human and animal antimicrobial classes
Third generation cephalosporins
Fourth generation cephalosporins
Fluoroquinolones
Macrolides
Quinolones
Polymyxins
Aminoglycosides
Aminopenicillins
Aminopenicillins with beta−lactamase inhibitors
Ansamycins
Phosphonic acid derivatives
Cattle
Chickens
Pigs
Sheep
Turkeys
Ducks
Horses
Buffaloes
Goats
Livestock species

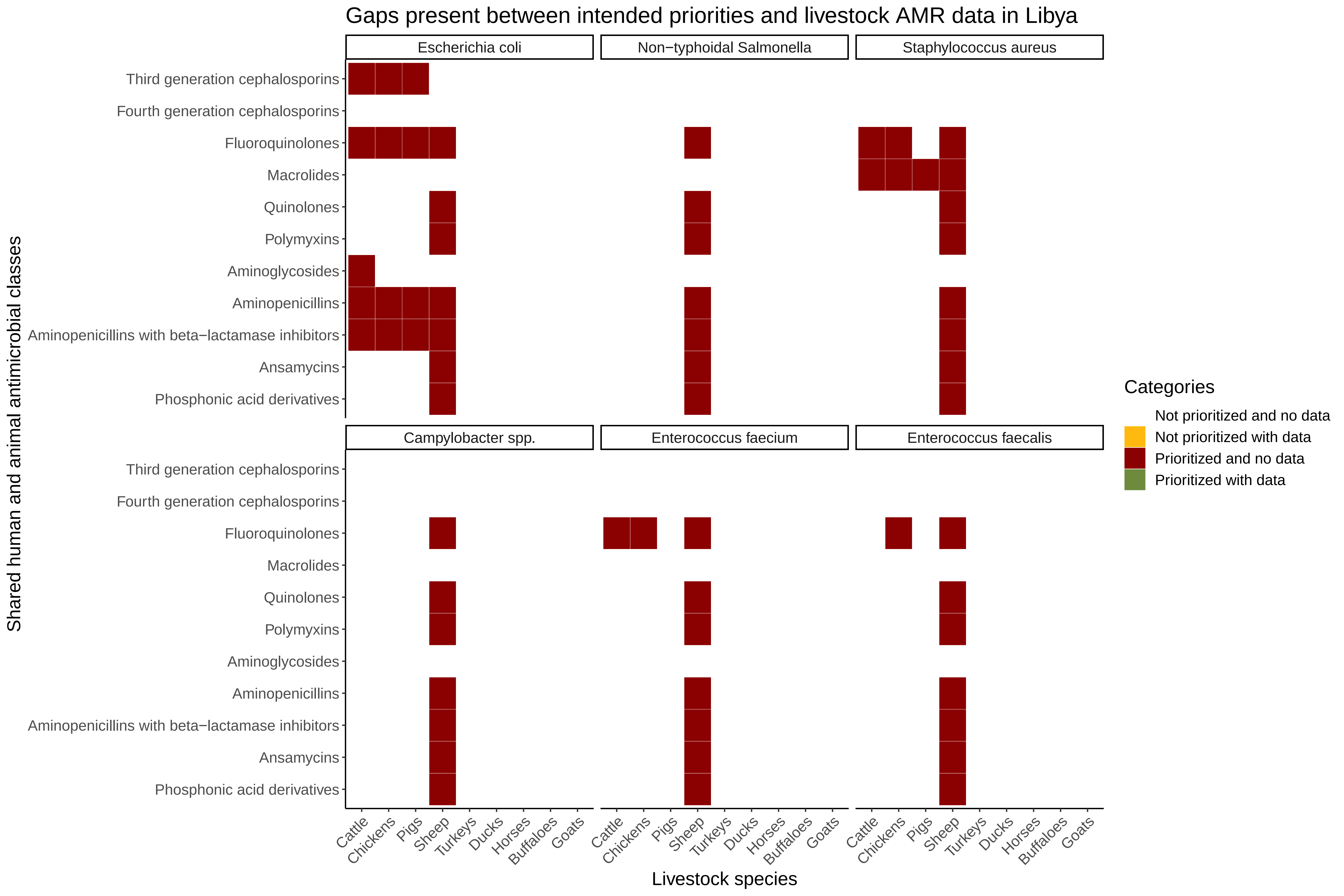

Gaps present between intended priorities and livestock AMR data in Libya
Escherichia coli
Non−typhoidal Salmonella
Staphylococcus aureus
Campylobacter spp.
Enterococcus faecium
Enterococcus faecalis
Shared human and animal antimicrobial classes
Third generation cephalosporins
Fourth generation cephalosporins
Fluoroquinolones
Macrolides
Quinolones
Polymyxins
Aminoglycosides
Aminopenicillins
Aminopenicillins with beta−lactamase inhibitors
Ansamycins
Phosphonic acid derivatives
Cattle
Chickens
Pigs
Sheep
Turkeys
Ducks
Horses
Buffaloes
Goats
Livestock species
Categories
Not prioritized and no data
Not prioritized with data
Prioritized and no data
Prioritized with data

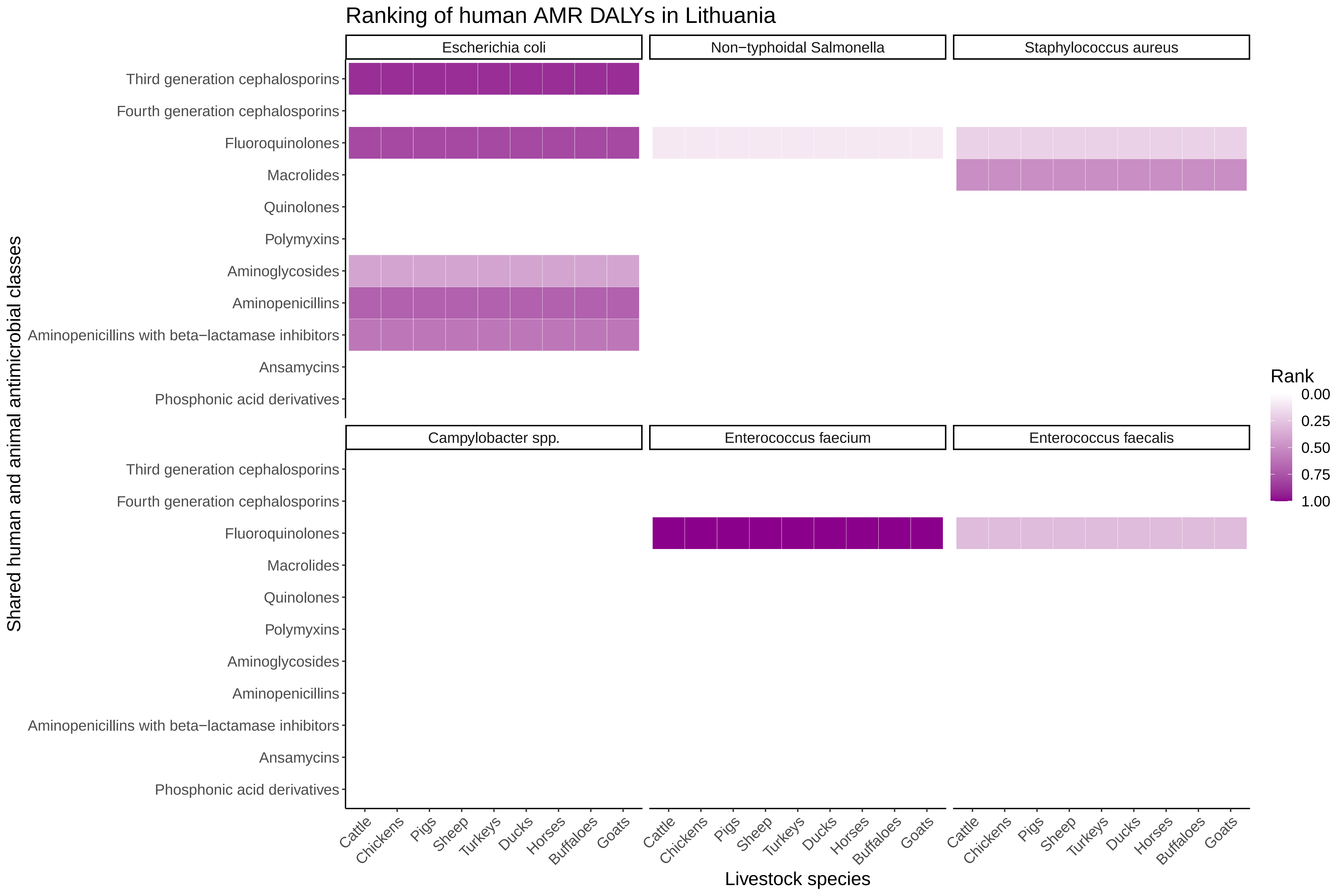

Ranking of human AMR DALYs in Lithuania
Escherichia coli
Non−typhoidal Salmonella
Staphylococcus aureus
Campylobacter spp.
Enterococcus faecium
Enterococcus faecalis
Third generation cephalosporins
Fourth generation cephalosporins
Fluoroquinolones
Macrolides
Quinolones
Polymyxins
Aminoglycosides
Aminopenicillins
Aminopenicillins with beta−lactamase inhibitors
Ansamycins
Phosphonic acid derivatives
Shared human and animal antimicrobial classes
Cattle
Chickens
Pigs
Sheep
Turkeys
Ducks
Horses
Buffaloes
Goats
Livestock species
Rank
0.00
0.25
0.50
0.75
1.00

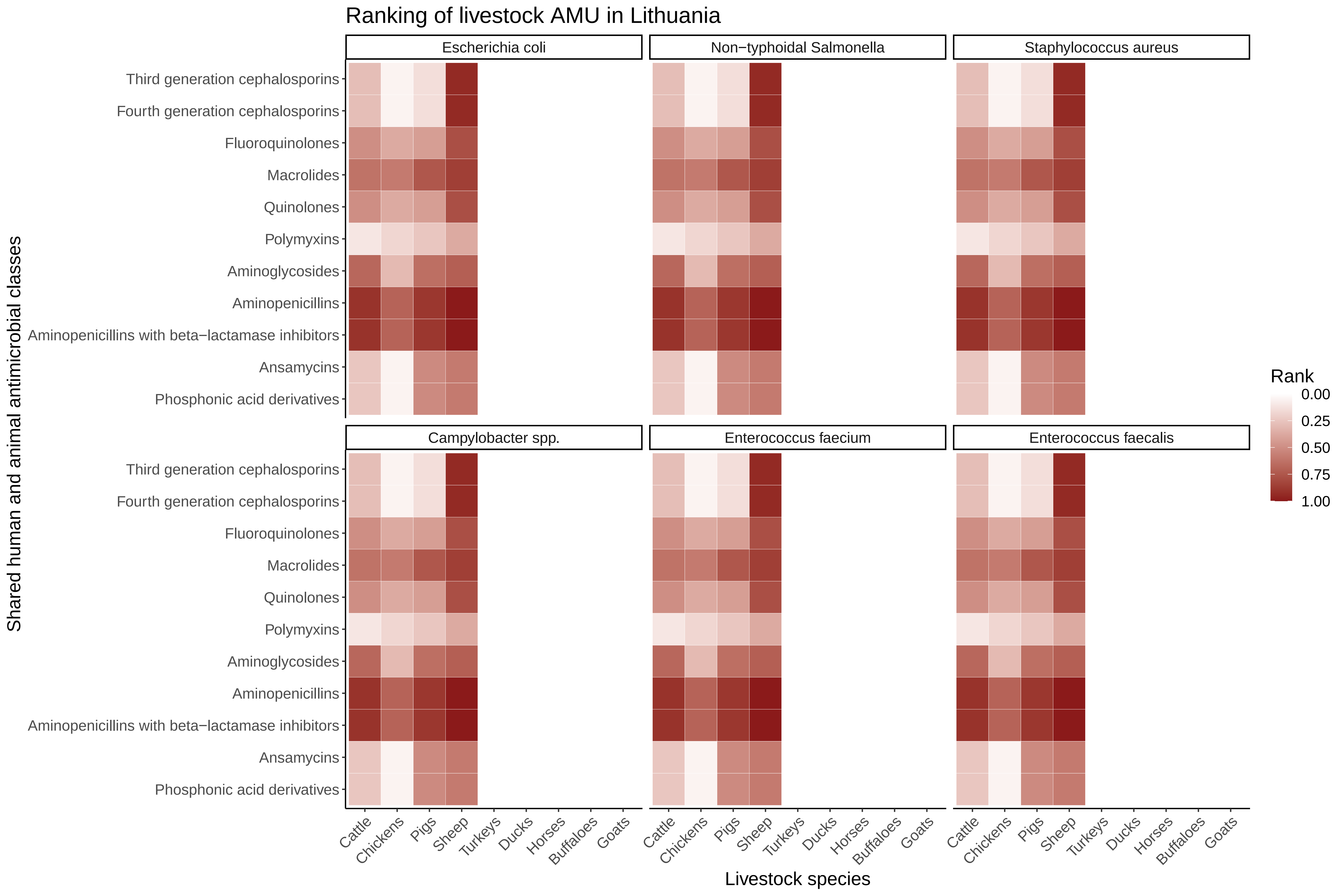

Ranking of livestock AMU in Lithuania
Escherichia coli
Non−typhoidal Salmonella
Staphylococcus aureus
Campylobacter spp.
Enterococcus faecium
Enterococcus faecalis
Shared human and animal antimicrobial classes
Third generation cephalosporins
Fourth generation cephalosporins
Fluoroquinolones
Macrolides
Quinolones
Polymyxins
Aminoglycosides
Aminopenicillins
Aminopenicillins with beta−lactamase inhibitors
Ansamycins
Phosphonic acid derivatives
Cattle
Chickens
Pigs
Sheep
Turkeys
Ducks
Horses
Buffaloes
Goats
Livestock species
Rank
0.00
0.25
0.50
0.75
1.00

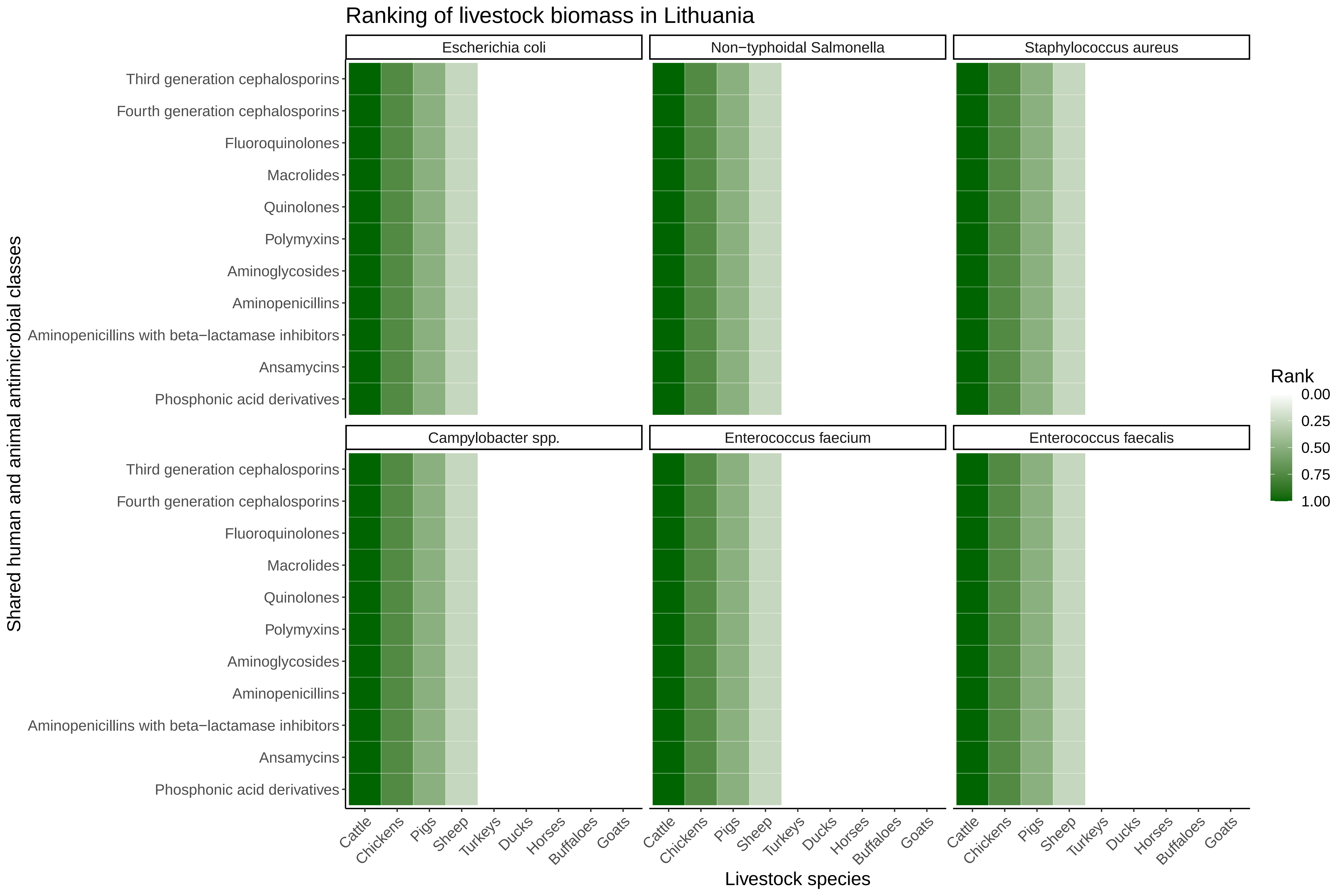
Ranking of livestock biomass in Lithuania
Escherichia coli
Non−typhoidal Salmonella
Staphylococcus aureus
Campylobacter spp.
Enterococcus faecium
Enterococcus faecalis
Third generation cephalosporins
Fourth generation cephalosporins
Fluoroquinolones
Macrolides
Quinolones
Polymyxins
Aminoglycosides
Aminopenicillins
Aminopenicillins with beta−lactamase inhibitors
Ansamycins
Phosphonic acid derivatives
Shared human and animal antimicrobial classes
Cattle
Chickens
Pigs
Sheep
Turkeys
Ducks
Horses
Buffaloes
Goats
Livestock species
Rank
0.00
0.25
0.50
0.75
1.00

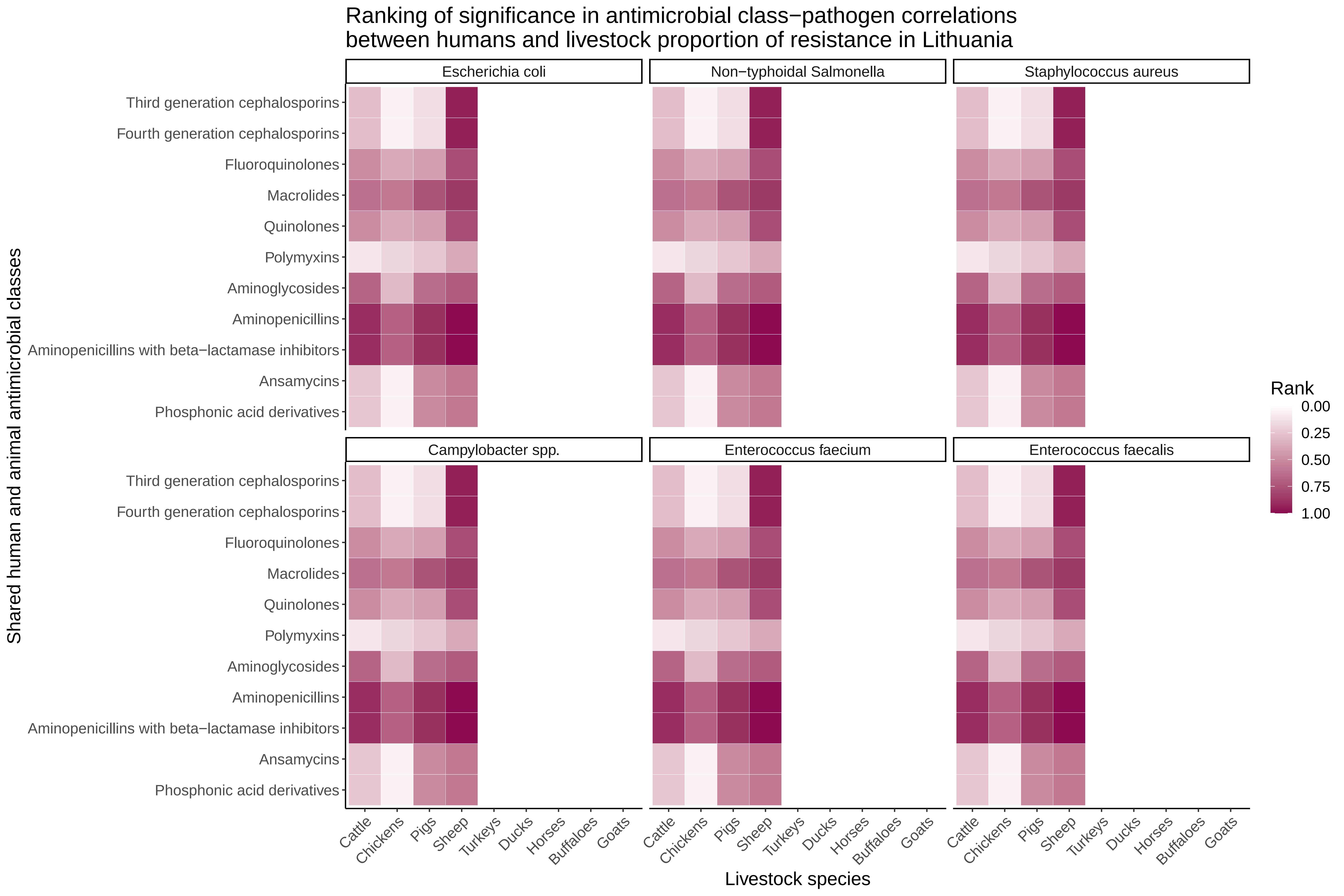

Ranking of significance in antimicrobial class−pathogen correlations
between humans and livestock proportion of resistance in Lithuania
Escherichia coli
Non−typhoidal Salmonella
Staphylococcus aureus
Campylobacter spp.
Enterococcus faecium
Enterococcus faecalis
Third generation cephalosporins
Fourth generation cephalosporins
Fluoroquinolones
Macrolides
Quinolones
Polymyxins
Aminoglycosides
Aminopenicillins
Aminopenicillins with beta−lactamase inhibitors
Ansamycins
Phosphonic acid derivatives
Shared human and animal antimicrobial classes
Cattle
Chickens
Pigs
Sheep
Turkeys
Ducks
Horses
Buffaloes
Goats
Livestock species
Rank
0.00
0.25
0.50
0.75
1.00

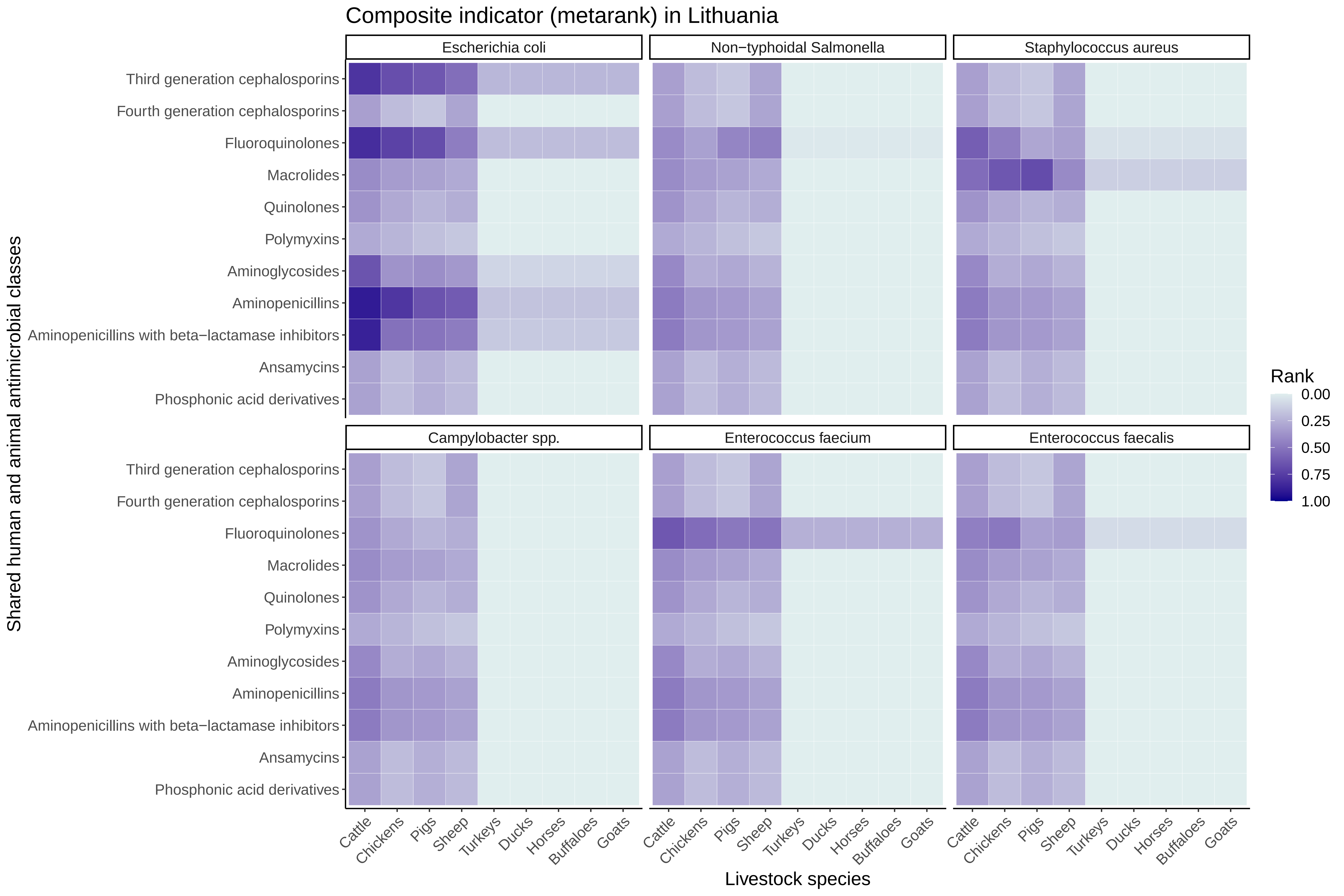

Composite indicator (metarank) in Lithuania
Escherichia coli
Non−typhoidal Salmonella
Staphylococcus aureus
Campylobacter spp.
Enterococcus faecium
Enterococcus faecalis
Shared human and animal antimicrobial classes
Third generation cephalosporins
Fourth generation cephalosporins
Fluoroquinolones
Macrolides
Quinolones
Polymyxins
Aminoglycosides
Aminopenicillins
Aminopenicillins with beta−lactamase inhibitors
Ansamycins
Phosphonic acid derivatives
Cattle
Chickens
Pigs
Sheep
Turkeys
Ducks
Horses
Buffaloes
Goats
Livestock species
Rank
0.00
0.25
0.50
0.75
1.00

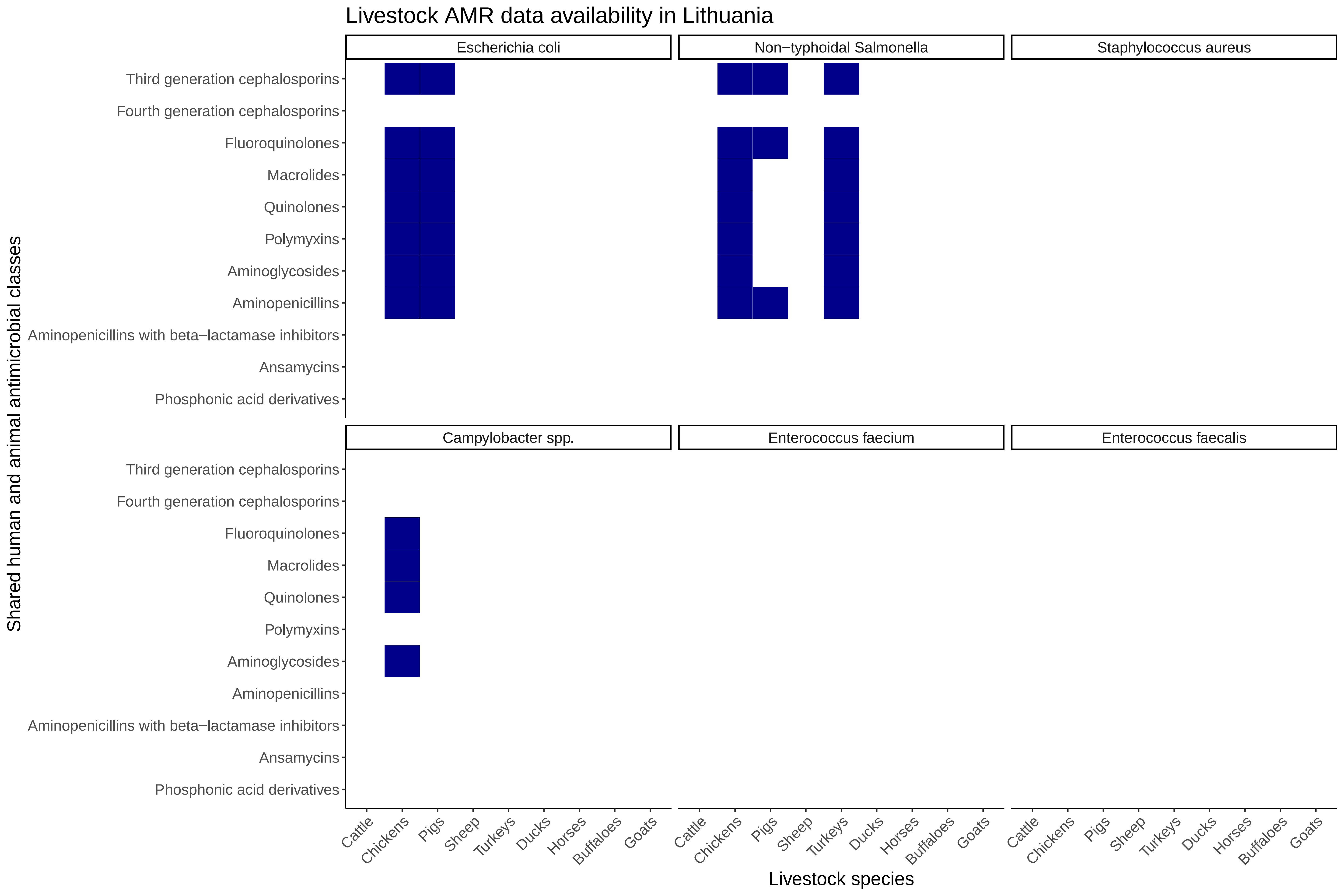

Livestock AMR data availability in Lithuania
Escherichia coli
Non−typhoidal Salmonella
Staphylococcus aureus
Campylobacter spp.
Enterococcus faecium
Enterococcus faecalis
Third generation cephalosporins
Fourth generation cephalosporins
Fluoroquinolones
Macrolides
Quinolones
Polymyxins
Aminoglycosides
Aminopenicillins
Aminopenicillins with beta−lactamase inhibitors
Ansamycins
Phosphonic acid derivatives
Shared human and animal antimicrobial classes
Cattle
Chickens
Pigs
Sheep
Turkeys
Ducks
Horses
Buffaloes
Goats
Livestock species

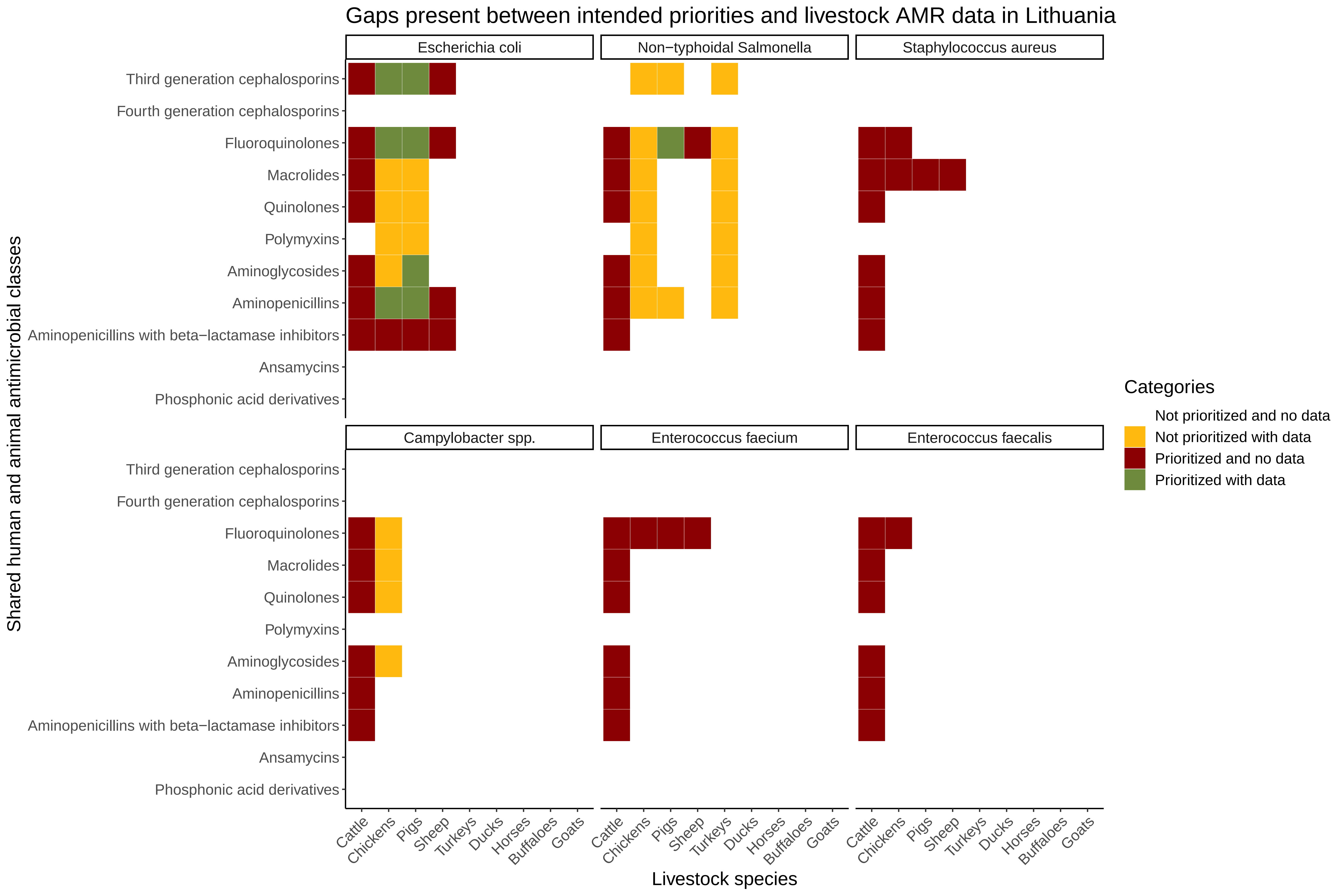

Gaps present between intended priorities and livestock AMR data in Lithuania
Escherichia coli
Non−typhoidal Salmonella
Staphylococcus aureus
Campylobacter spp.
Enterococcus faecium
Enterococcus faecalis
Shared human and animal antimicrobial classes
Third generation cephalosporins
Fourth generation cephalosporins
Fluoroquinolones
Macrolides
Quinolones
Polymyxins
Aminoglycosides
Aminopenicillins
Aminopenicillins with beta−lactamase inhibitors
Ansamycins
Phosphonic acid derivatives
Cattle
Chickens
Pigs
Sheep
Turkeys
Ducks
Horses
Buffaloes
Goats
Livestock species
Categories
Not prioritized and no data
Not prioritized with data
Prioritized and no data
Prioritized with data

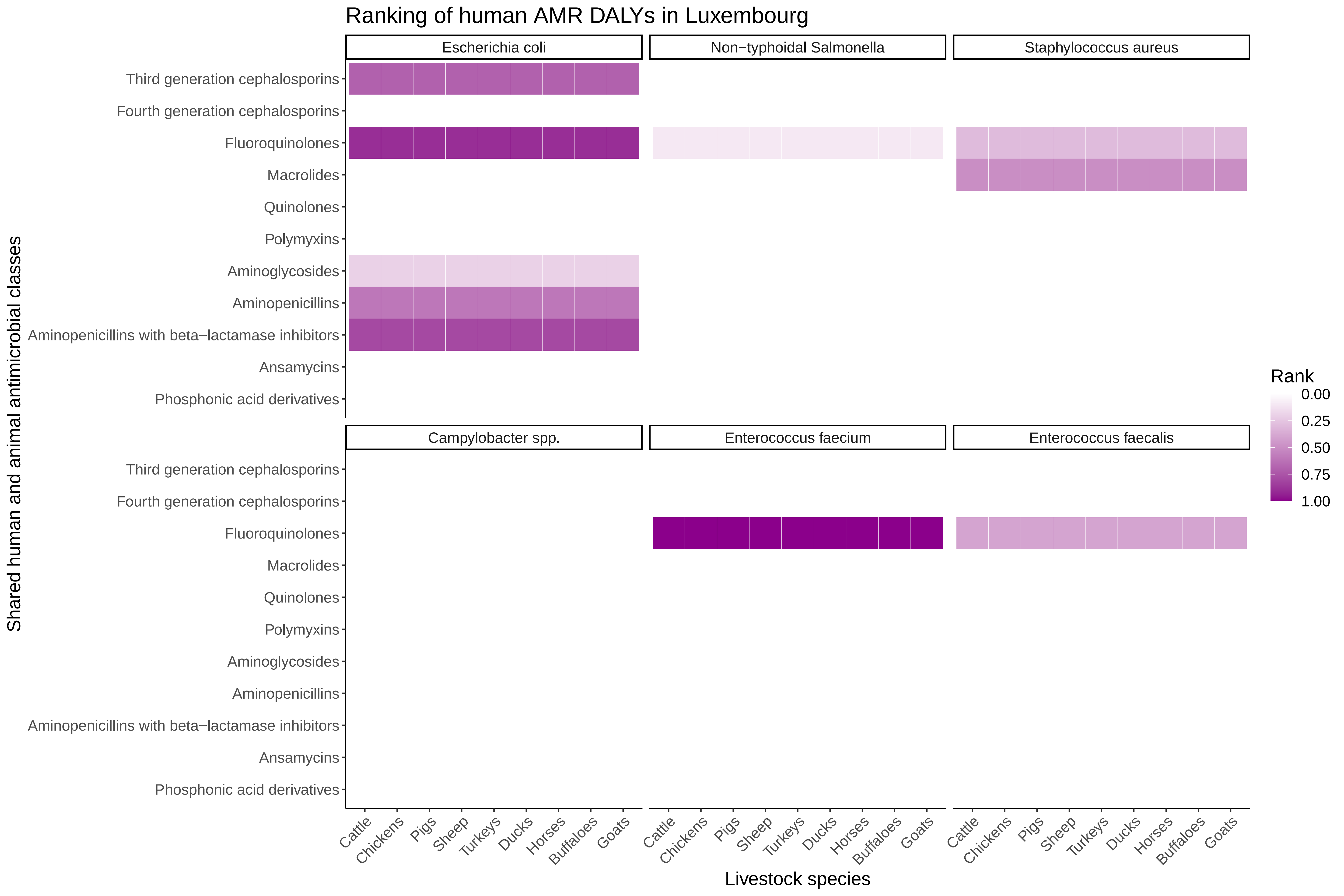

Ranking of human AMR DALYs in Luxembourg
Escherichia coli
Non−typhoidal Salmonella
Staphylococcus aureus
Campylobacter spp.
Enterococcus faecium
Enterococcus faecalis
Third generation cephalosporins
Fourth generation cephalosporins
Fluoroquinolones
Macrolides
Quinolones
Polymyxins
Aminoglycosides
Aminopenicillins
Aminopenicillins with beta−lactamase inhibitors
Ansamycins
Phosphonic acid derivatives
Shared human and animal antimicrobial classes
Cattle
Chickens
Pigs
Sheep
Turkeys
Ducks
Horses
Buffaloes
Goats
Livestock species
Rank
0.00
0.25
0.50
0.75
1.00

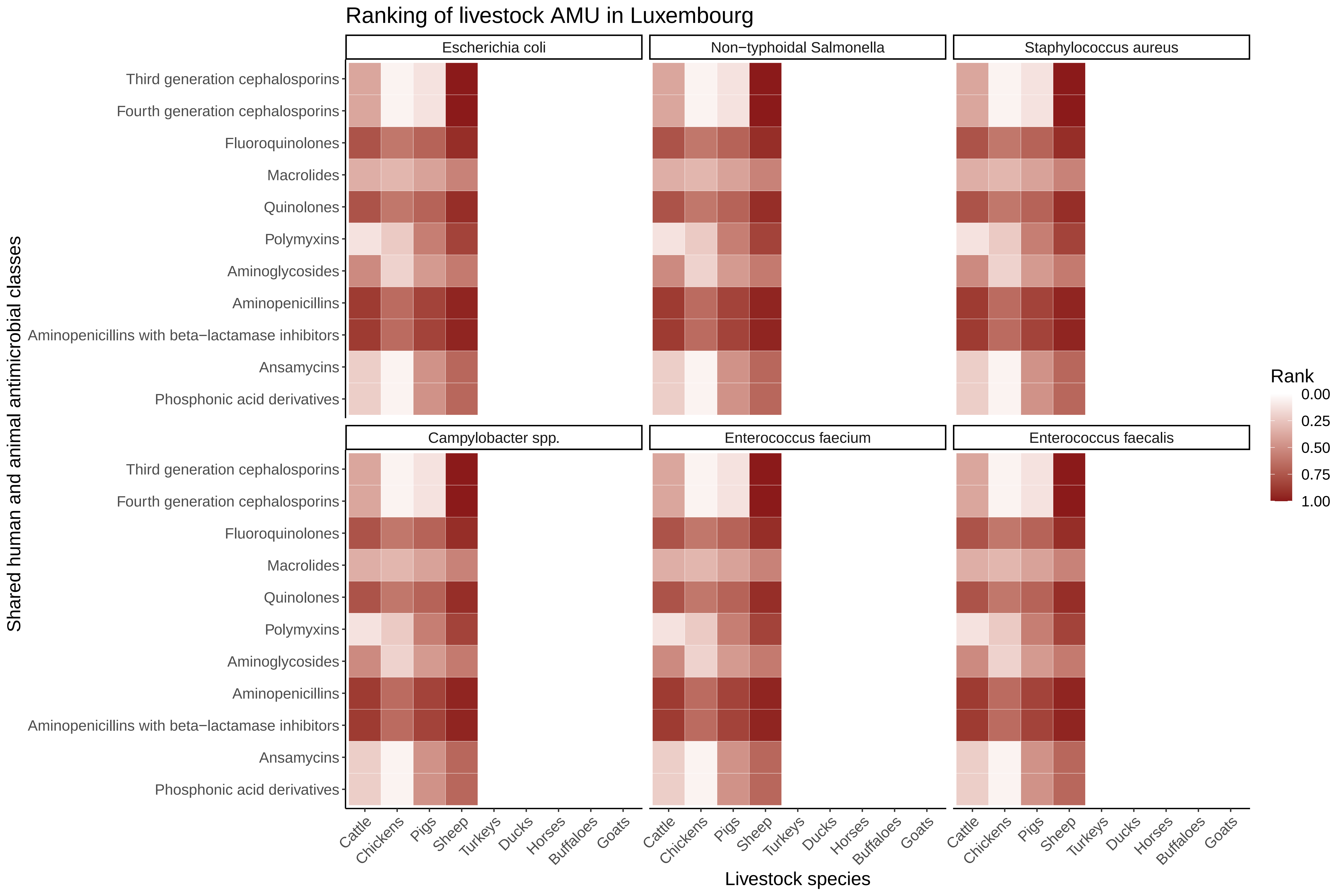
Ranking of livestock AMU in Luxembourg
Escherichia coli
Non−typhoidal Salmonella
Staphylococcus aureus
Campylobacter spp.
Enterococcus faecium
Enterococcus faecalis
Shared human and animal antimicrobial classes
Third generation cephalosporins
Fourth generation cephalosporins
Fluoroquinolones
Macrolides
Quinolones
Polymyxins
Aminoglycosides
Aminopenicillins
Aminopenicillins with beta−lactamase inhibitors
Ansamycins
Phosphonic acid derivatives
Cattle
Chickens
Pigs
Sheep
Turkeys
Ducks
Horses
Buffaloes
Goats
Livestock species
Rank
0.00
0.25
0.50
0.75
1.00

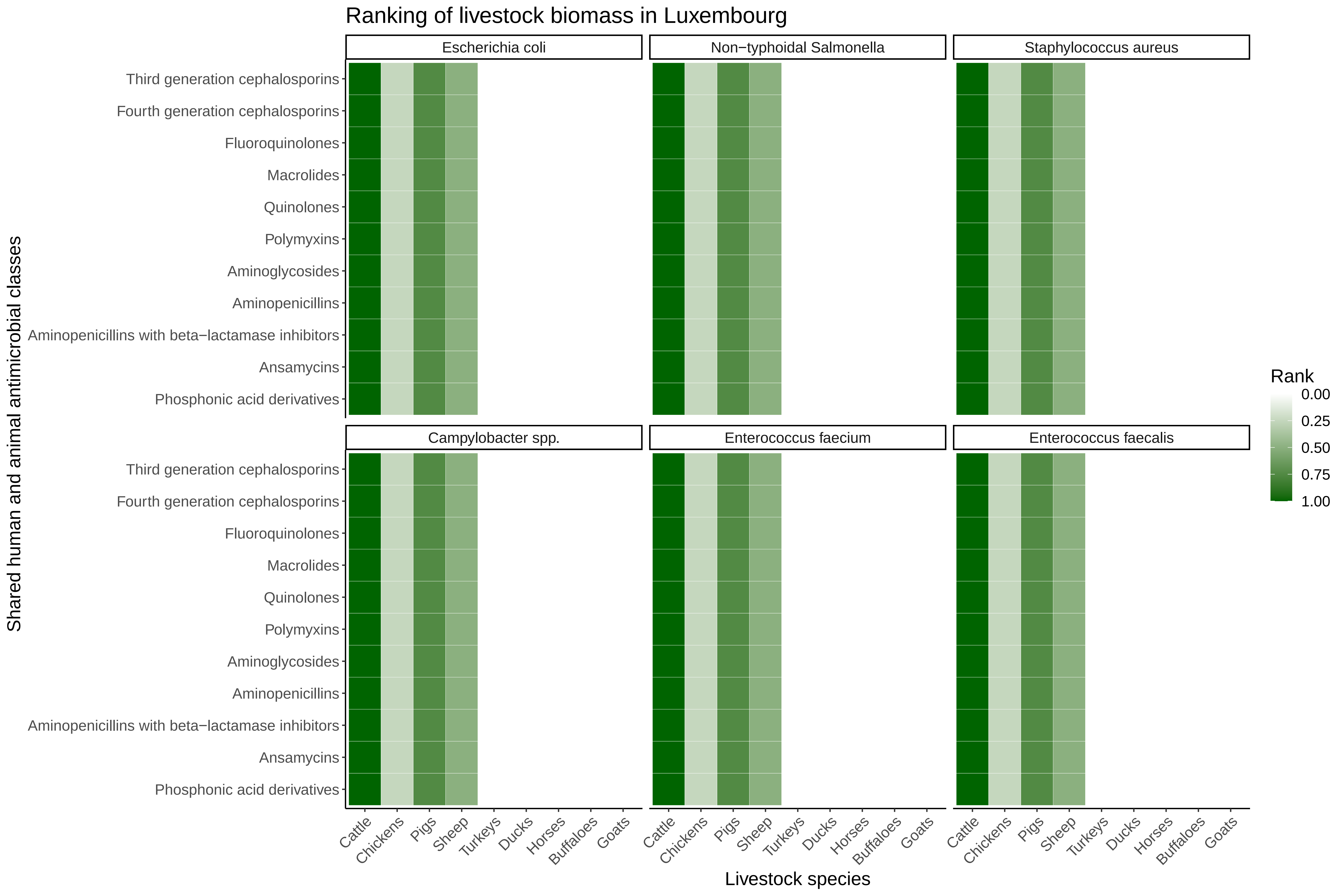

Ranking of livestock biomass in Luxembourg
Escherichia coli
Non−typhoidal Salmonella
Staphylococcus aureus
Campylobacter spp.
Enterococcus faecium
Enterococcus faecalis
Third generation cephalosporins
Fourth generation cephalosporins
Fluoroquinolones
Macrolides
Quinolones
Polymyxins
Aminoglycosides
Aminopenicillins
Aminopenicillins with beta−lactamase inhibitors
Ansamycins
Phosphonic acid derivatives
Shared human and animal antimicrobial classes
Cattle
Chickens
Pigs
Sheep
Turkeys
Ducks
Horses
Buffaloes
Goats
Livestock species
Rank
0.00
0.25
0.50
0.75
1.00

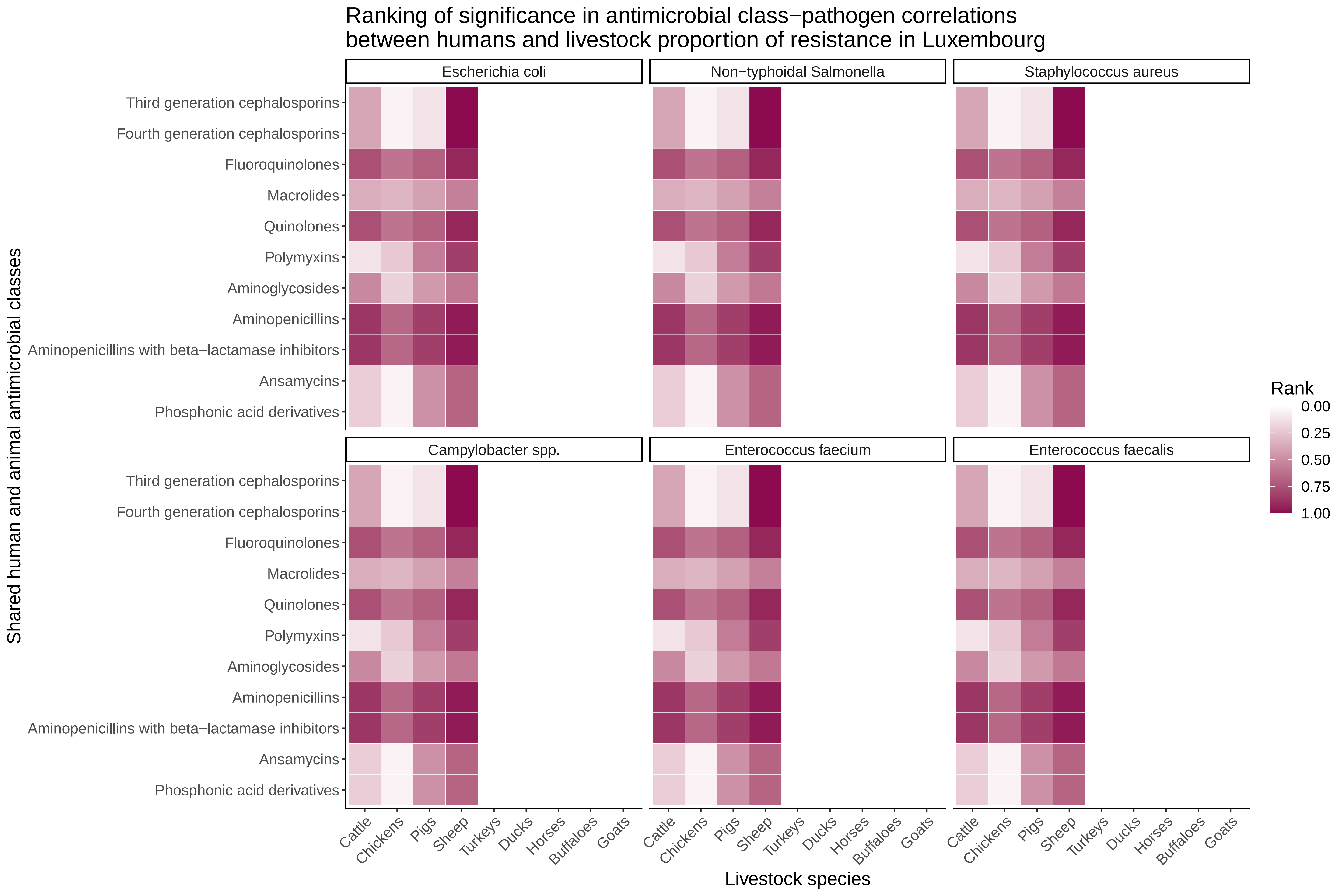

Ranking of significance in antimicrobial class−pathogen correlations
between humans and livestock proportion of resistance in Luxembourg
Escherichia coli
Non−typhoidal Salmonella
Staphylococcus aureus
Campylobacter spp.
Enterococcus faecium
Enterococcus faecalis
Third generation cephalosporins
Fourth generation cephalosporins
Fluoroquinolones
Macrolides
Quinolones
Polymyxins
Aminoglycosides
Aminopenicillins
Aminopenicillins with beta−lactamase inhibitors
Ansamycins
Phosphonic acid derivatives
Shared human and animal antimicrobial classes
Cattle
Chickens
Pigs
Sheep
Turkeys
Ducks
Horses
Buffaloes
Goats
Livestock species
Rank
0.00
0.25
0.50
0.75
1.00

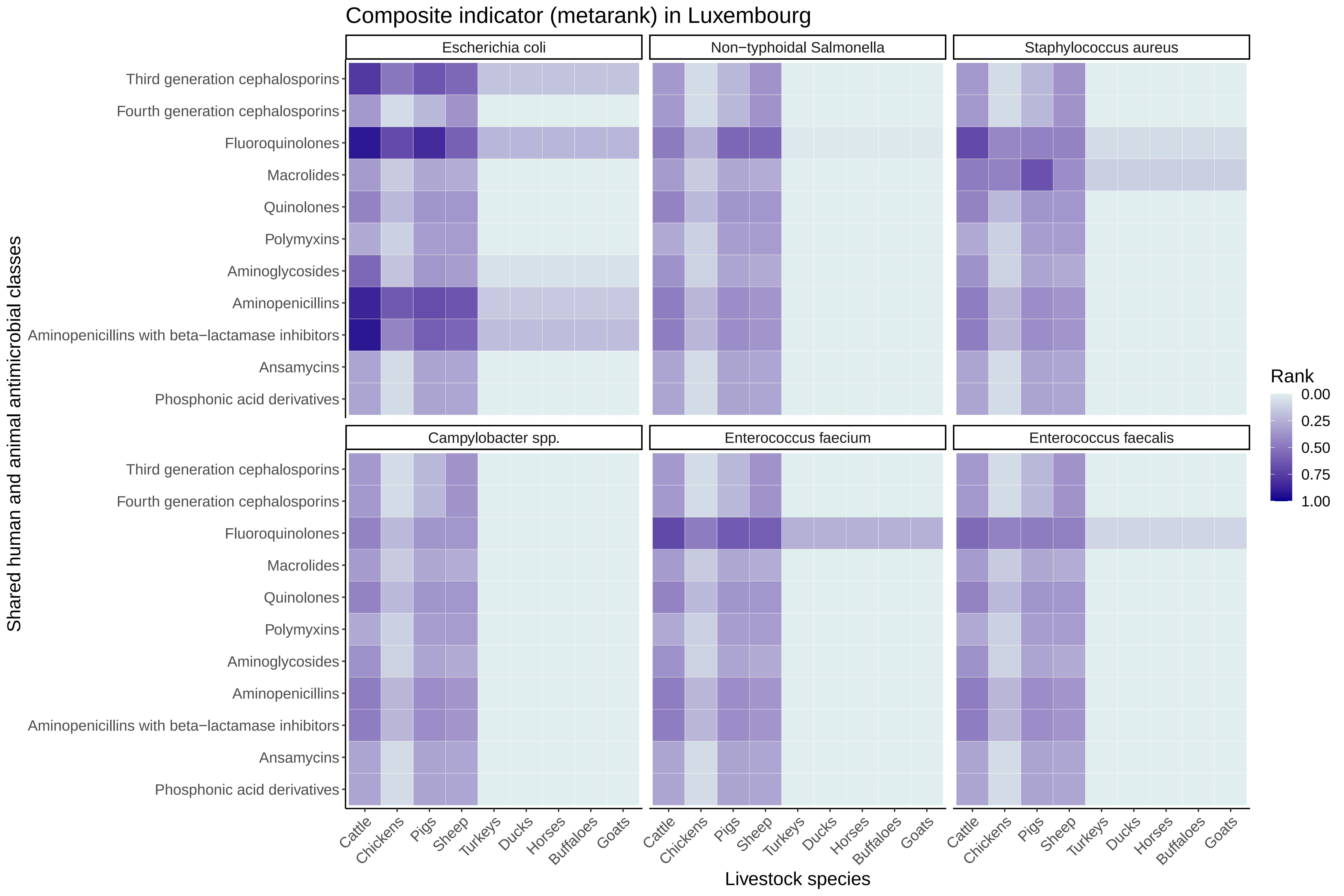

Composite indicator (metarank) in Luxembourg
Escherichia coli
Non−typhoidal Salmonella
Staphylococcus aureus
Campylobacter spp.
Enterococcus faecium
Enterococcus faecalis
Shared human and animal antimicrobial classes
Third generation cephalosporins
Fourth generation cephalosporins
Fluoroquinolones
Macrolides
Quinolones
Polymyxins
Aminoglycosides
Aminopenicillins
Aminopenicillins with beta−lactamase inhibitors
Ansamycins
Phosphonic acid derivatives
Livestock species
Cattle
Chickens
Pigs
Sheep
Turkeys
Ducks
Horses
Buffaloes
Goats
Rank
0.00
0.25
0.50
0.75
1.00

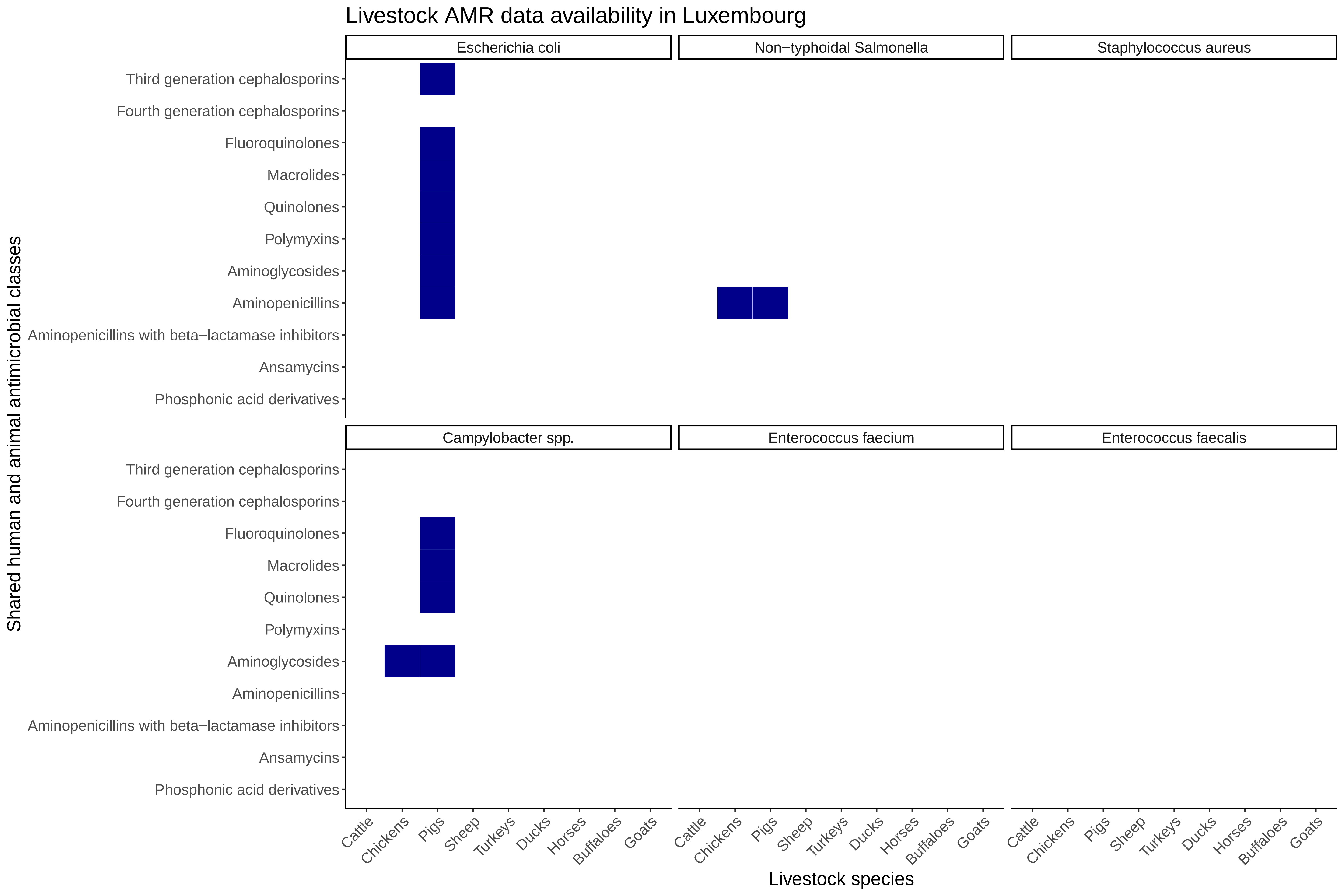

Livestock AMR data availability in Luxembourg
Escherichia coli
Non−typhoidal Salmonella
Staphylococcus aureus
Campylobacter spp.
Enterococcus faecium
Enterococcus faecalis
Shared human and animal antimicrobial classes
Third generation cephalosporins
Fourth generation cephalosporins
Fluoroquinolones
Macrolides
Quinolones
Polymyxins
Aminoglycosides
Aminopenicillins
Aminopenicillins with beta−lactamase inhibitors
Ansamycins
Phosphonic acid derivatives
Cattle
Chickens
Pigs
Sheep
Turkeys
Ducks
Horses
Buffaloes
Goats
Livestock species

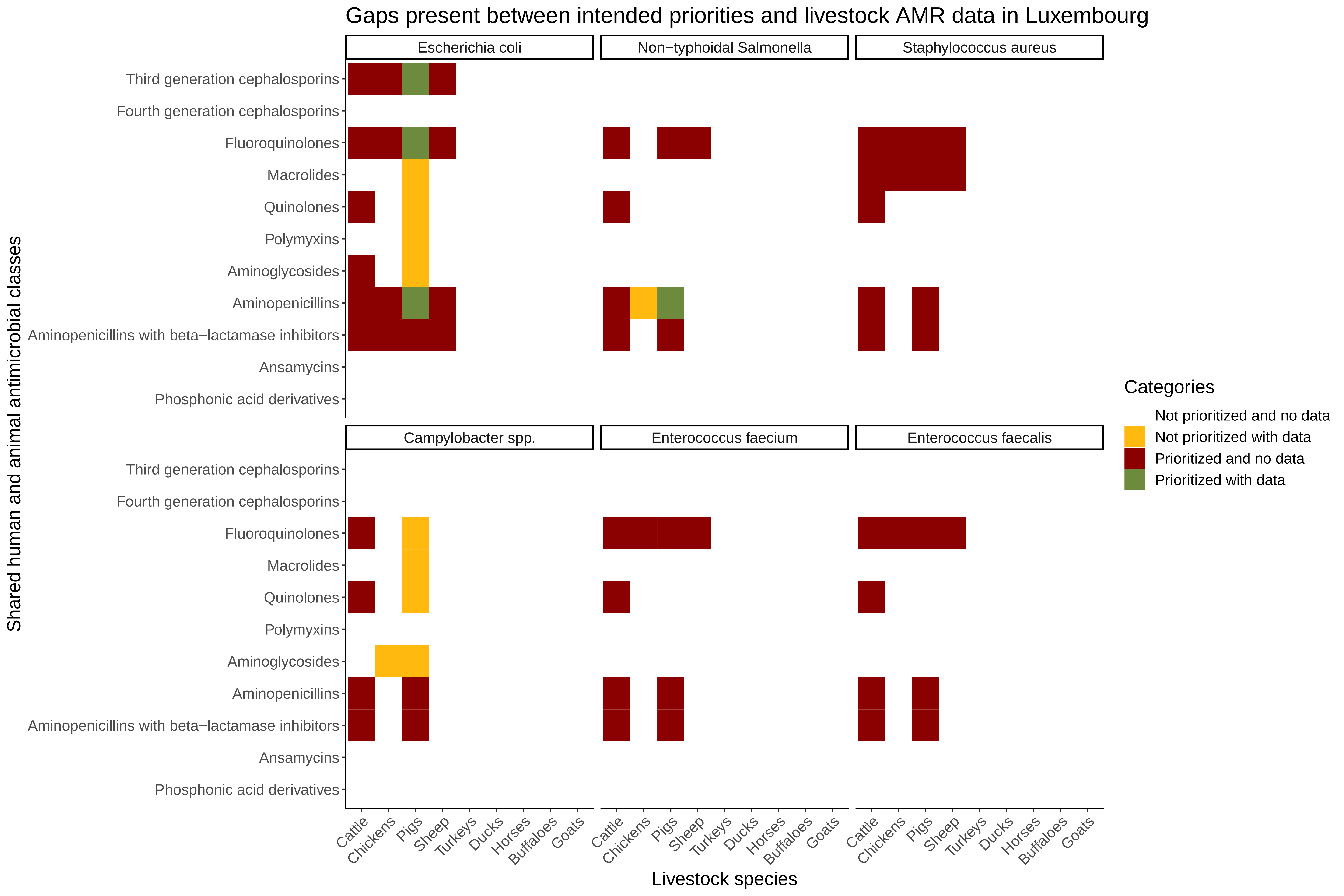

Gaps present between intended priorities and livestock AMR data in Luxembourg
Escherichia coli
Non−typhoidal Salmonella
Staphylococcus aureus
Campylobacter spp.
Enterococcus faecium
Enterococcus faecalis
Shared human and animal antimicrobial classes
Third generation cephalosporins
Fourth generation cephalosporins
Fluoroquinolones
Macrolides
Quinolones
Polymyxins
Aminoglycosides
Aminopenicillins
Aminopenicillins with beta−lactamase inhibitors
Ansamycins
Phosphonic acid derivatives
Cattle
Chickens
Pigs
Sheep
Turkeys
Ducks
Horses
Buffaloes
Goats
Livestock species
Categories
Not prioritized and no data
Not prioritized with data
Prioritized and no data
Prioritized with data

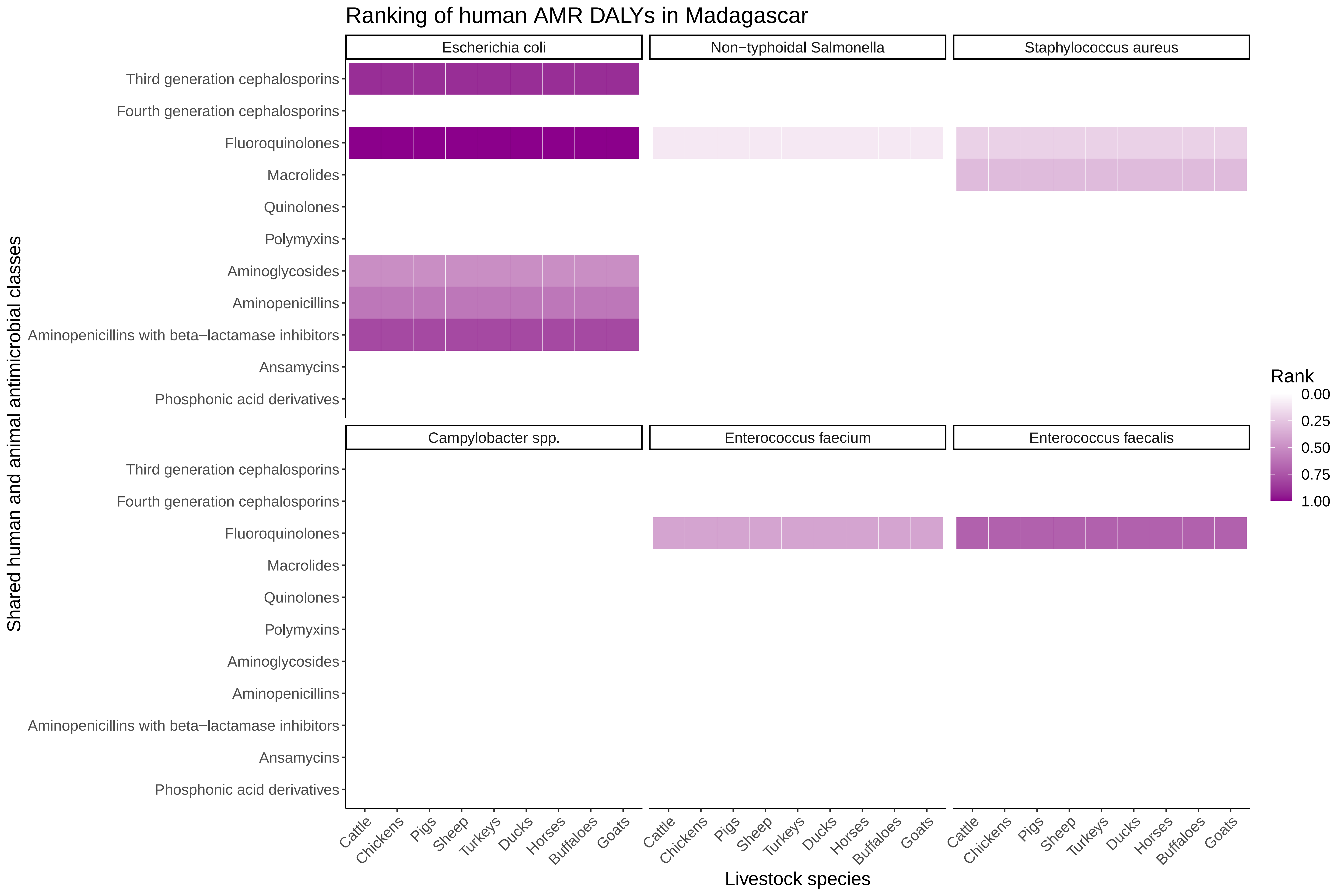

Ranking of human AMR DALYs in Madagascar
Escherichia coli
Non−typhoidal Salmonella
Staphylococcus aureus
Campylobacter spp.
Enterococcus faecium
Enterococcus faecalis
Third generation cephalosporins
Fourth generation cephalosporins
Fluoroquinolones
Macrolides
Quinolones
Polymyxins
Aminoglycosides
Aminopenicillins
Aminopenicillins with beta−lactamase inhibitors
Ansamycins
Phosphonic acid derivatives
Shared human and animal antimicrobial classes
Cattle
Chickens
Pigs
Sheep
Turkeys
Ducks
Horses
Buffaloes
Goats
Livestock species
Rank
0.00
0.25
0.50
0.75
1.00

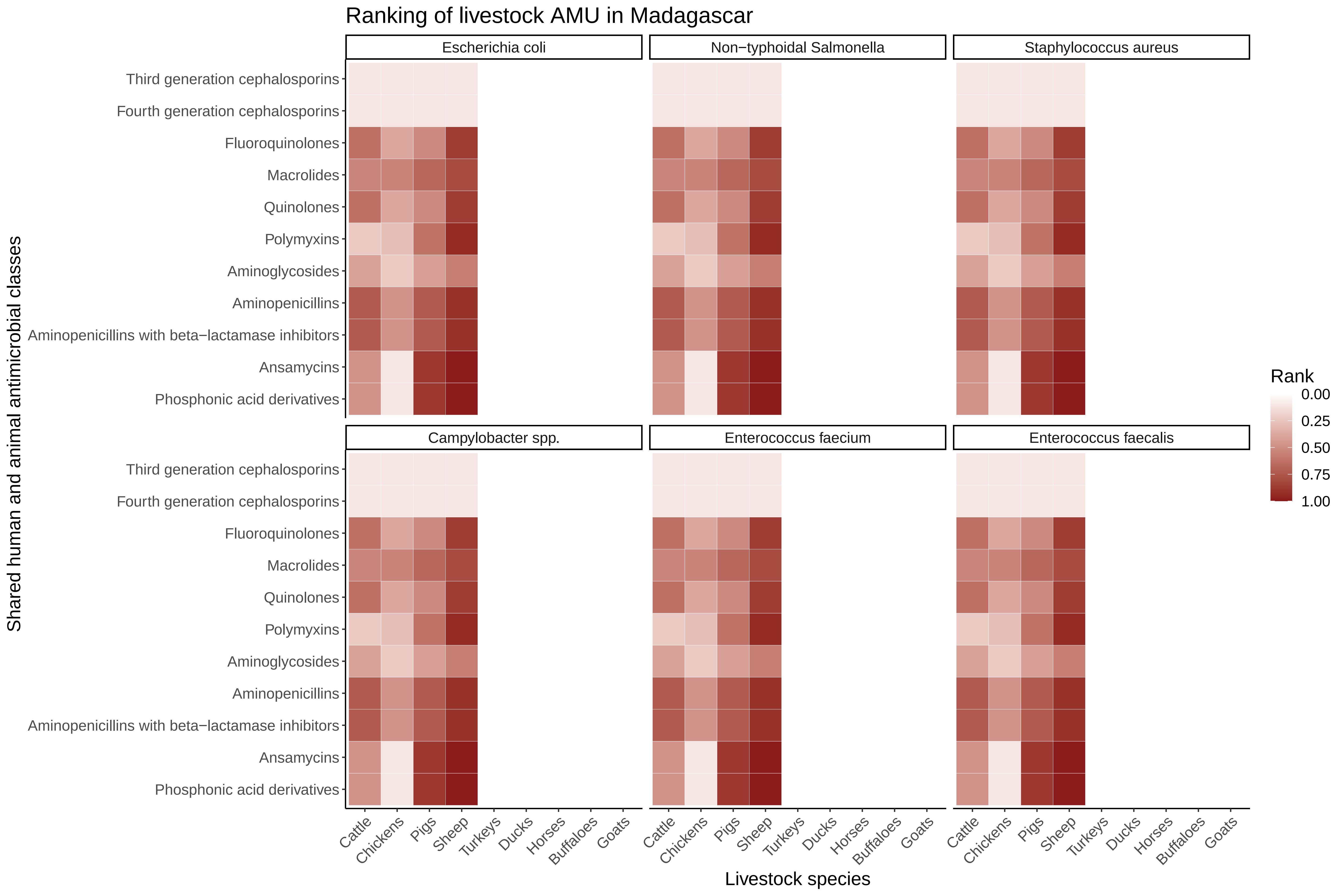

Ranking of livestock AMU in Madagascar
Escherichia coli
Non−typhoidal Salmonella
Staphylococcus aureus
Campylobacter spp.
Enterococcus faecium
Enterococcus faecalis
Shared human and animal antimicrobial classes
Third generation cephalosporins
Fourth generation cephalosporins
Fluoroquinolones
Macrolides
Quinolones
Polymyxins
Aminoglycosides
Aminopenicillins
Aminopenicillins with beta−lactamase inhibitors
Ansamycins
Phosphonic acid derivatives
Cattle
Chickens
Pigs
Sheep
Turkeys
Ducks
Horses
Buffaloes
Goats
Livestock species
Rank
0.00
0.25
0.50
0.75
1.00

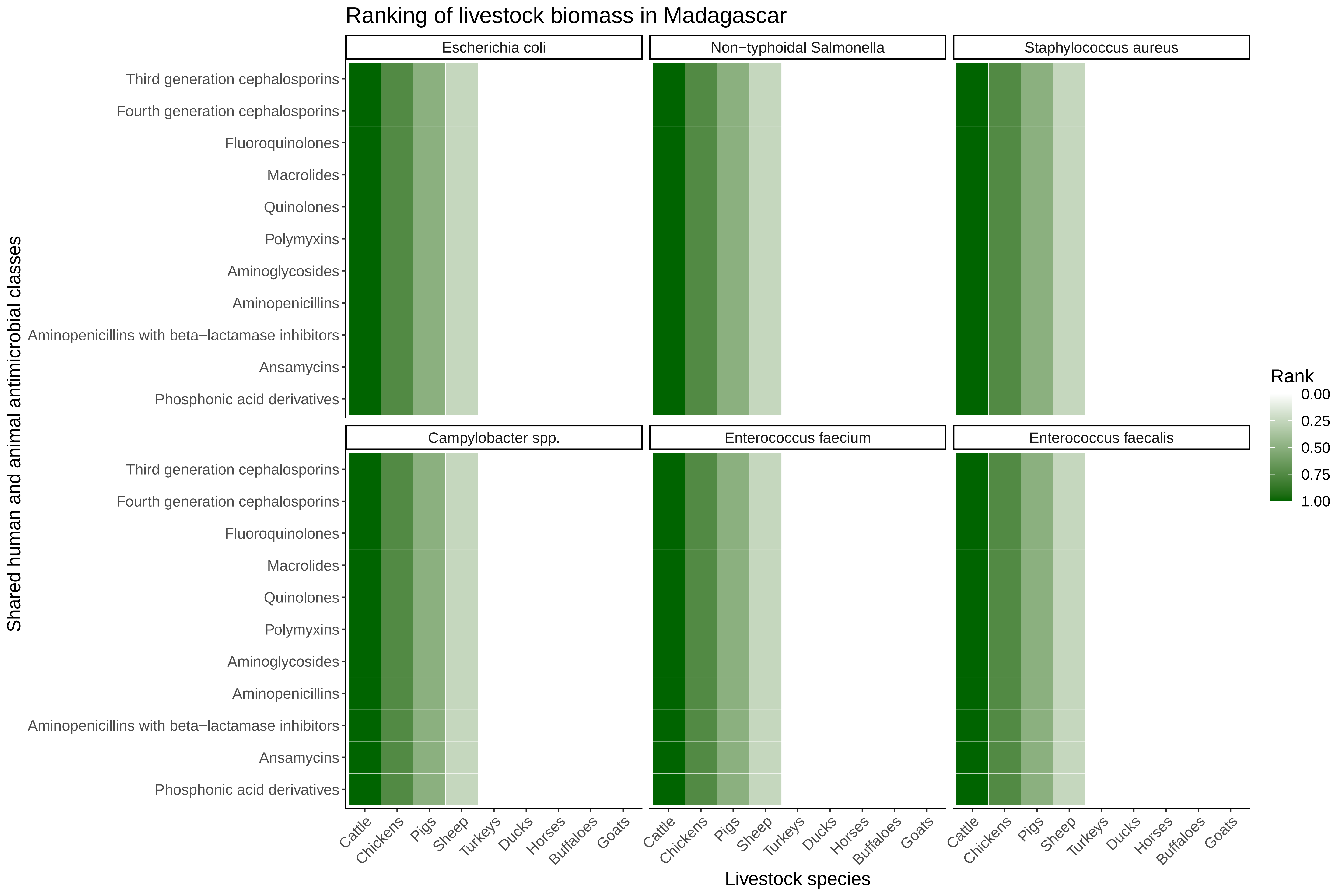

Ranking of livestock biomass in Madagascar
Escherichia coli
Non−typhoidal Salmonella
Staphylococcus aureus
Campylobacter spp.
Enterococcus faecium
Enterococcus faecalis
Shared human and animal antimicrobial classes
Third generation cephalosporins
Fourth generation cephalosporins
Fluoroquinolones
Macrolides
Quinolones
Polymyxins
Aminoglycosides
Aminopenicillins
Aminopenicillins with beta−lactamase inhibitors
Ansamycins
Phosphonic acid derivatives
Cattle
Chickens
Pigs
Sheep
Turkeys
Ducks
Horses
Buffaloes
Goats
Livestock species
Rank
0.00
0.25
0.50
0.75
1.00

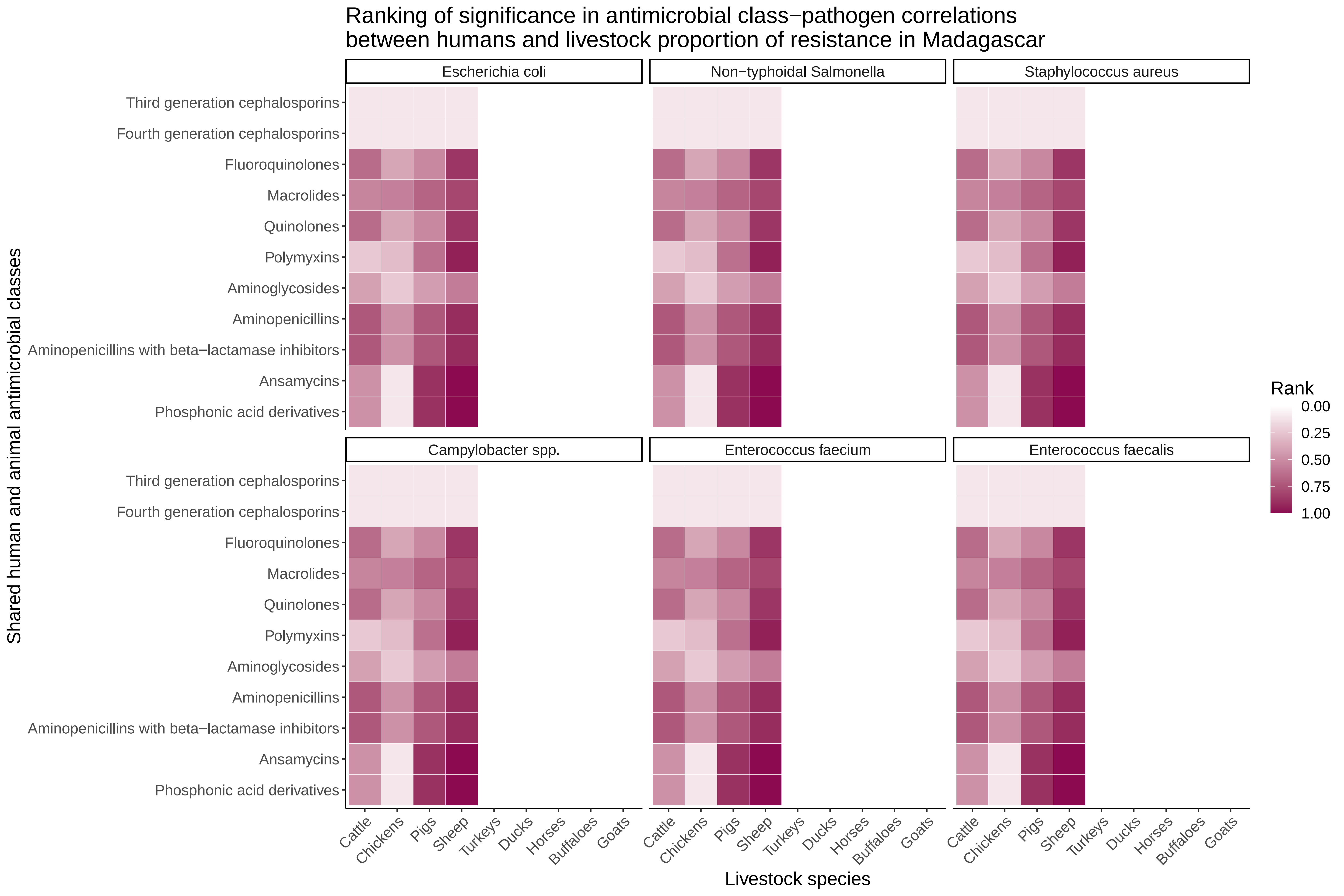
Ranking of significance in antimicrobial class−pathogen correlations
between humans and livestock proportion of resistance in Madagascar
Escherichia coli
Non−typhoidal Salmonella
Staphylococcus aureus
Campylobacter spp.
Enterococcus faecium
Enterococcus faecalis
Third generation cephalosporins
Fourth generation cephalosporins
Fluoroquinolones
Macrolides
Quinolones
Polymyxins
Aminoglycosides
Aminopenicillins
Aminopenicillins with beta−lactamase inhibitors
Ansamycins
Phosphonic acid derivatives
Shared human and animal antimicrobial classes
Cattle
Chickens
Pigs
Sheep
Turkeys
Ducks
Horses
Buffaloes
Goats
Livestock species
Rank
0.00
0.25
0.50
0.75
1.00

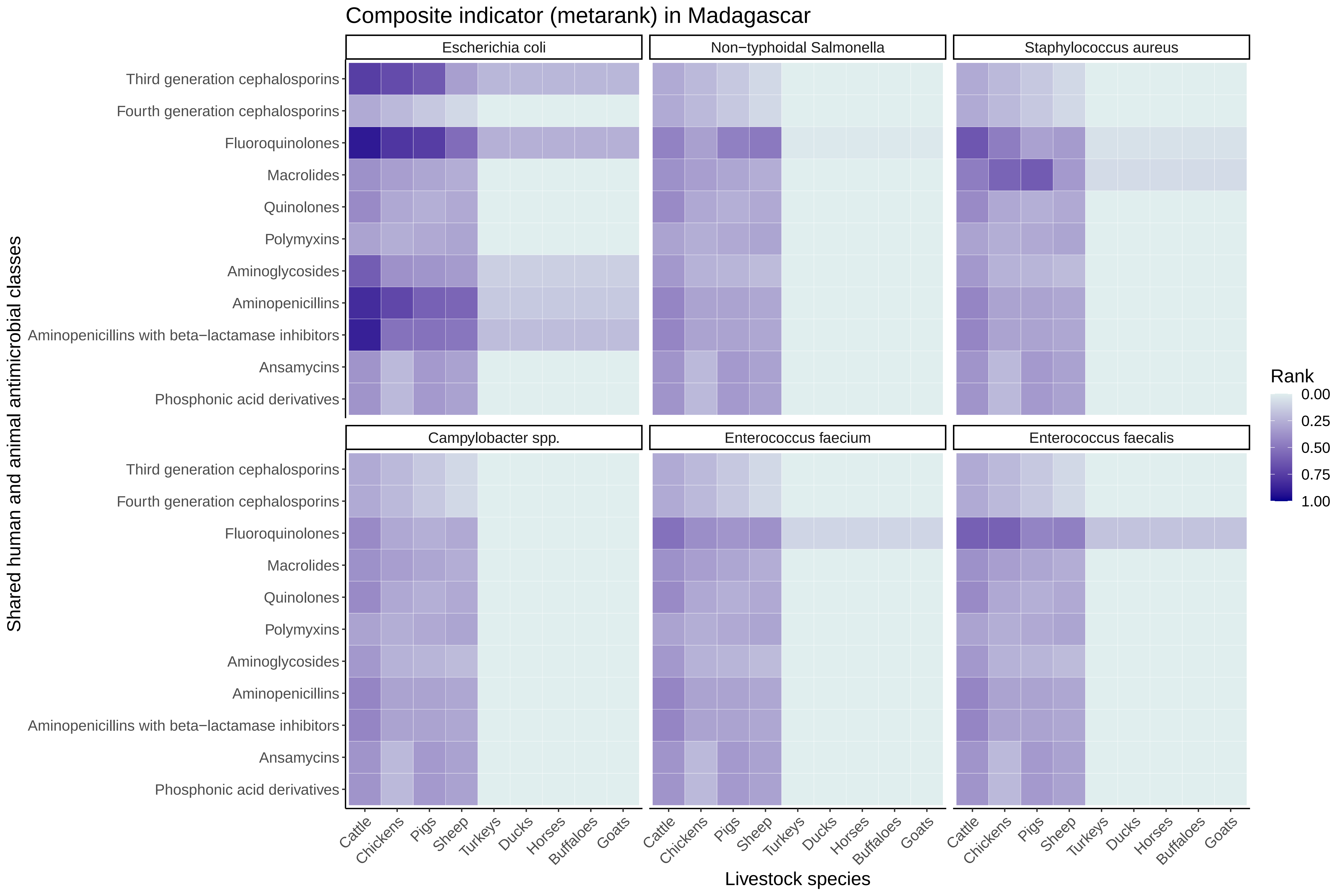

Composite indicator (metarank) in Madagascar
Escherichia coli
Non−typhoidal Salmonella
Staphylococcus aureus
Campylobacter spp.
Enterococcus faecium
Enterococcus faecalis
Shared human and animal antimicrobial classes
Third generation cephalosporins
Fourth generation cephalosporins
Fluoroquinolones
Macrolides
Quinolones
Polymyxins
Aminoglycosides
Aminopenicillins
Aminopenicillins with beta−lactamase inhibitors
Ansamycins
Phosphonic acid derivatives
Cattle
Chickens
Pigs
Sheep
Turkeys
Ducks
Horses
Buffaloes
Goats
Livestock species
Rank
0.00
0.25
0.50
0.75
1.00

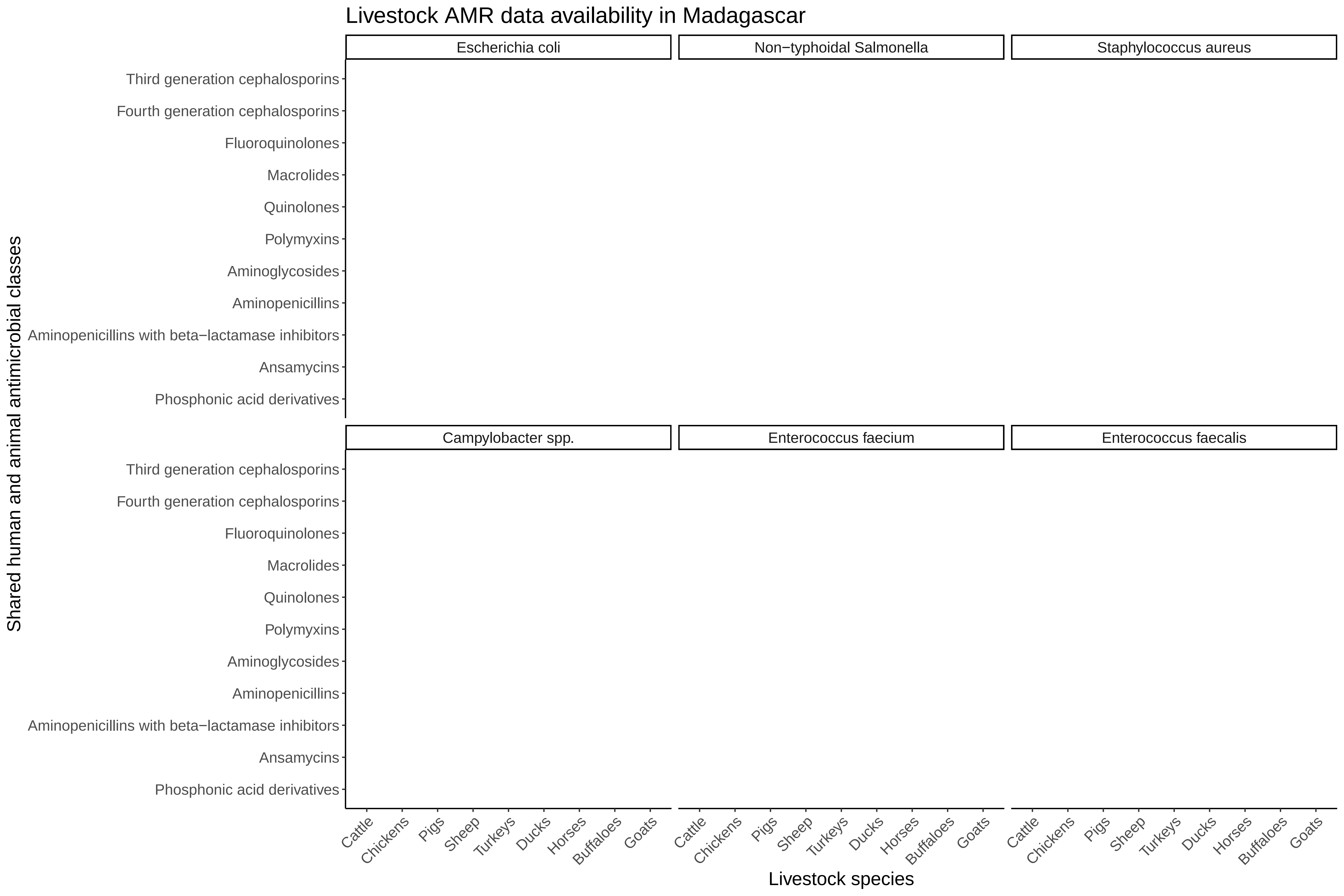
Livestock AMR data availability in Madagascar
Escherichia coli
Non−typhoidal Salmonella
Staphylococcus aureus
Campylobacter spp.
Enterococcus faecium
Enterococcus faecalis
Shared human and animal antimicrobial classes
Third generation cephalosporins
Fourth generation cephalosporins
Fluoroquinolones
Macrolides
Quinolones
Polymyxins
Aminoglycosides
Aminopenicillins
Aminopenicillins with beta−lactamase inhibitors
Ansamycins
Phosphonic acid derivatives
Cattle
Chickens
Pigs
Sheep
Turkeys
Ducks
Horses
Buffaloes
Goats
Livestock species

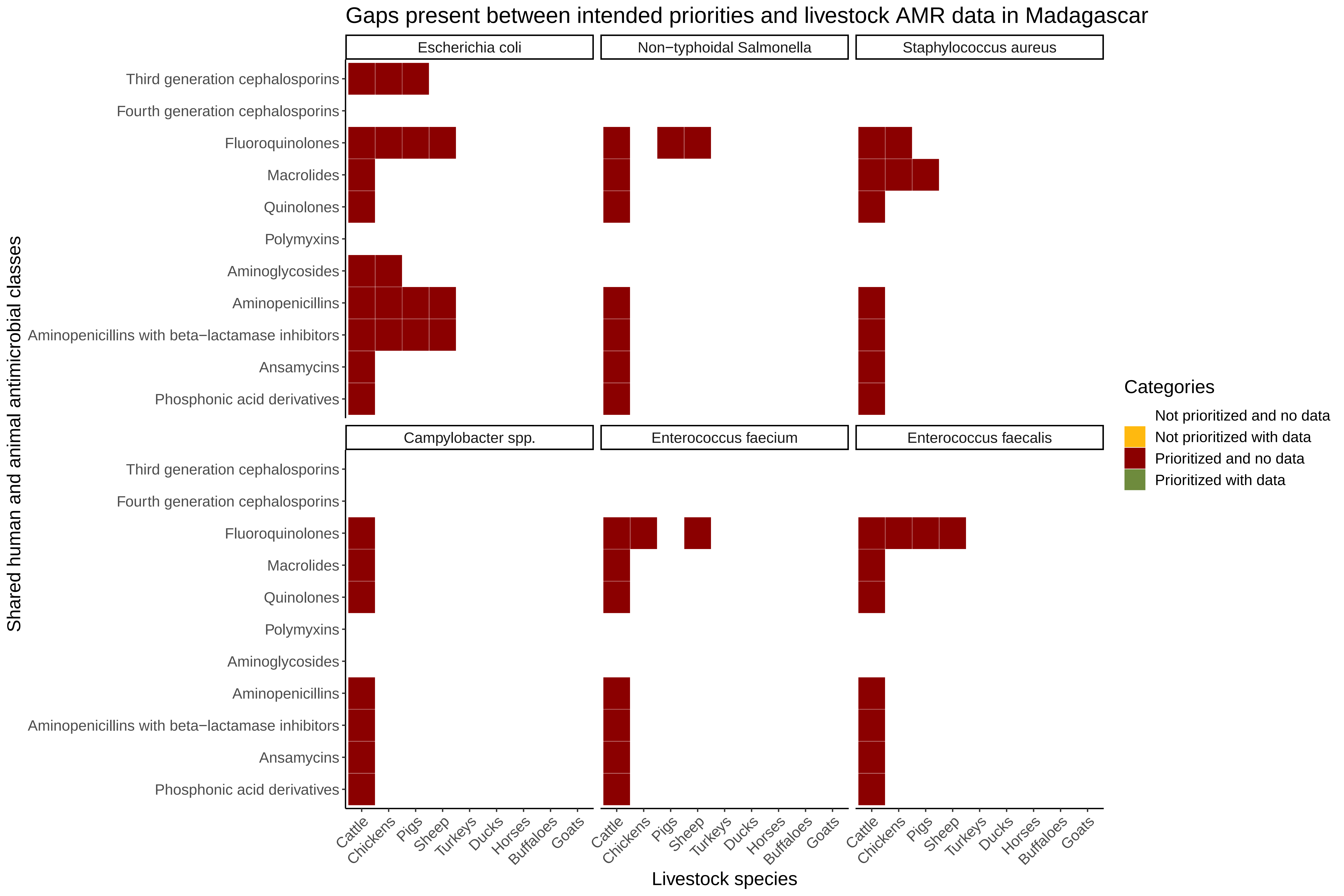

Gaps present between intended priorities and livestock AMR data in Madagascar
Escherichia coli
Non−typhoidal Salmonella
Staphylococcus aureus
Campylobacter spp.
Enterococcus faecium
Enterococcus faecalis
Shared human and animal antimicrobial classes
Third generation cephalosporins
Fourth generation cephalosporins
Fluoroquinolones
Macrolides
Quinolones
Polymyxins
Aminoglycosides
Aminopenicillins
Aminopenicillins with beta−lactamase inhibitors
Ansamycins
Phosphonic acid derivatives
Livestock species
Cattle
Chickens
Pigs
Sheep
Turkeys
Ducks
Horses
Buffaloes
Goats
Categories
Not prioritized and no data
Not prioritized with data
Prioritized and no data
Prioritized with data

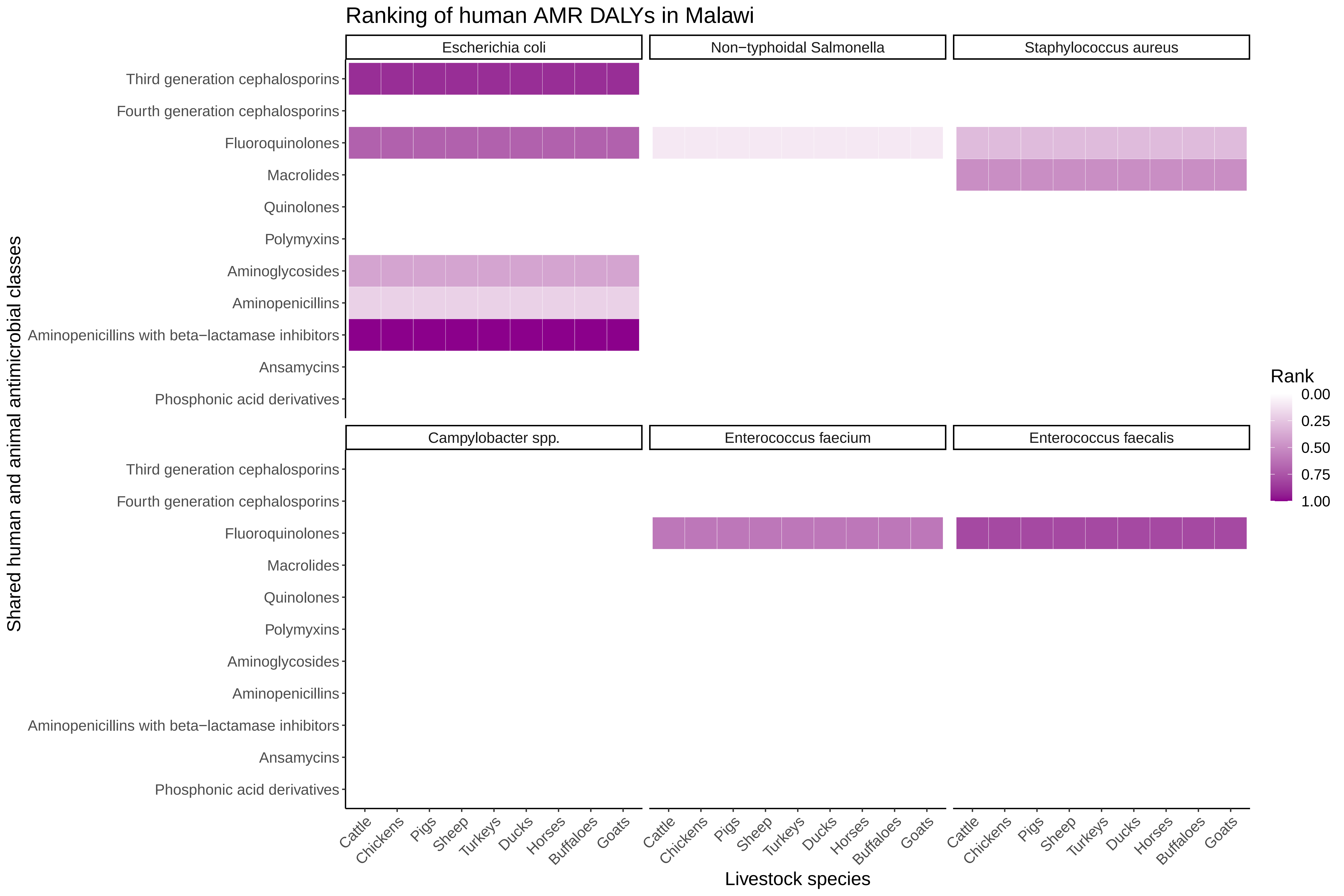

Ranking of human AMR DALYs in Malawi
Escherichia coli
Non−typhoidal Salmonella
Staphylococcus aureus
Campylobacter spp.
Enterococcus faecium
Enterococcus faecalis
Third generation cephalosporins
Fourth generation cephalosporins
Fluoroquinolones
Macrolides
Quinolones
Polymyxins
Aminoglycosides
Aminopenicillins
Aminopenicillins with beta−lactamase inhibitors
Ansamycins
Phosphonic acid derivatives
Shared human and animal antimicrobial classes
Cattle
Chickens
Pigs
Sheep
Turkeys
Ducks
Horses
Buffaloes
Goats
Livestock species
Rank
0.00
0.25
0.50
0.75
1.00

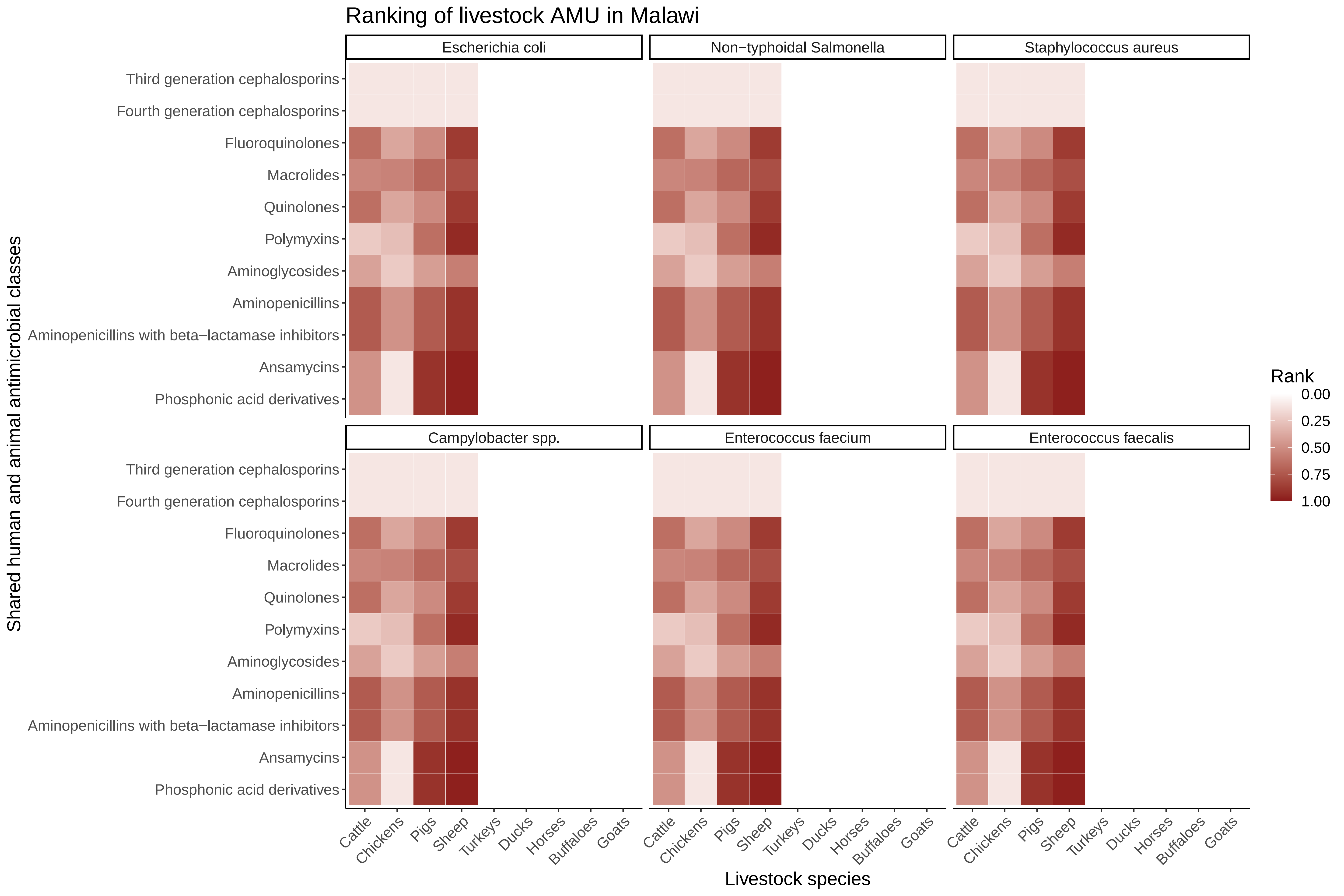
Ranking of livestock AMU in Malawi
Escherichia coli
Non−typhoidal Salmonella
Staphylococcus aureus
Campylobacter spp.
Enterococcus faecium
Enterococcus faecalis
Shared human and animal antimicrobial classes
Third generation cephalosporins
Fourth generation cephalosporins
Fluoroquinolones
Macrolides
Quinolones
Polymyxins
Aminoglycosides
Aminopenicillins
Aminopenicillins with beta−lactamase inhibitors
Ansamycins
Phosphonic acid derivatives
Livestock species
Cattle
Chickens
Pigs
Sheep
Turkeys
Ducks
Horses
Buffaloes
Goats
Rank
0.00
0.25
0.50
0.75
1.00

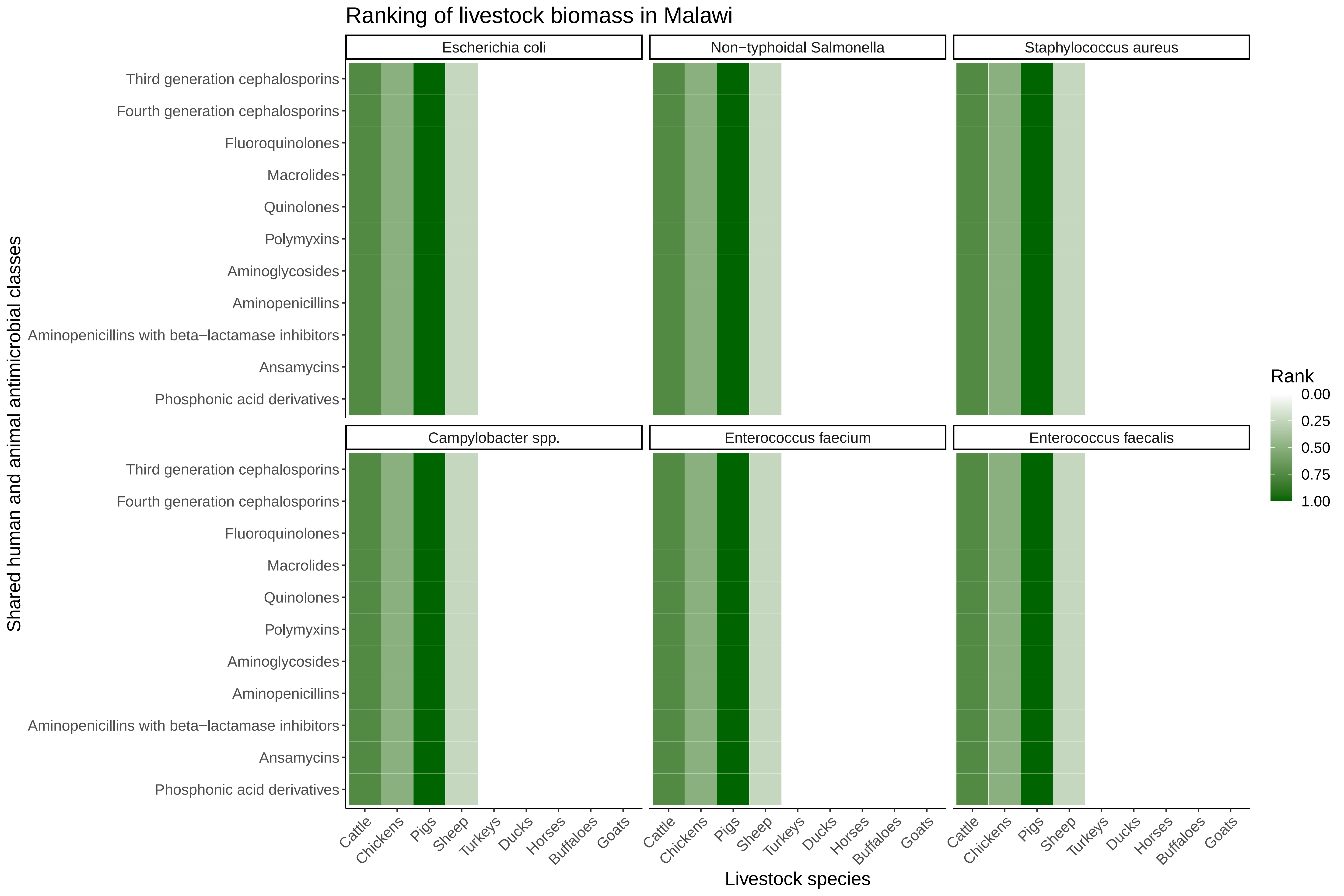
Ranking of livestock biomass in Malawi
Escherichia coli
Non−typhoidal Salmonella
Staphylococcus aureus
Campylobacter spp.
Enterococcus faecium
Enterococcus faecalis
Shared human and animal antimicrobial classes
Third generation cephalosporins
Fourth generation cephalosporins
Fluoroquinolones
Macrolides
Quinolones
Polymyxins
Aminoglycosides
Aminopenicillins
Aminopenicillins with beta−lactamase inhibitors
Ansamycins
Phosphonic acid derivatives
Livestock species
Cattle
Chickens
Pigs
Sheep
Turkeys
Ducks
Horses
Buffaloes
Goats
Rank
0.00
0.25
0.50
0.75
1.00

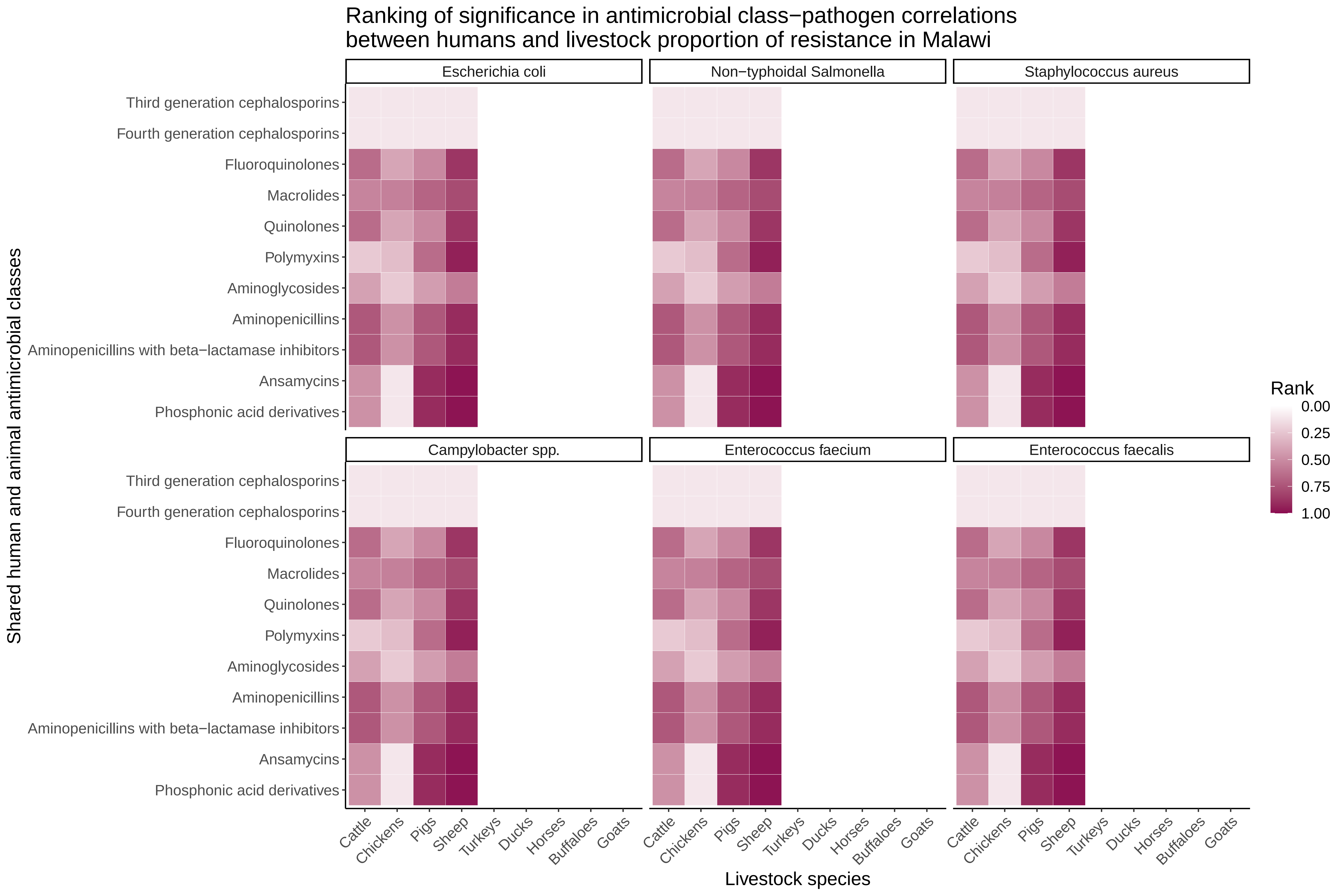
Ranking of significance in antimicrobial class−pathogen correlations
between humans and livestock proportion of resistance in Malawi
Escherichia coli
Non−typhoidal Salmonella
Staphylococcus aureus
Campylobacter spp.
Enterococcus faecium
Enterococcus faecalis
Third generation cephalosporins
Fourth generation cephalosporins
Fluoroquinolones
Macrolides
Quinolones
Polymyxins
Aminoglycosides
Aminopenicillins
Aminopenicillins with beta−lactamase inhibitors
Ansamycins
Phosphonic acid derivatives
Shared human and animal antimicrobial classes
Cattle
Chickens
Pigs
Sheep
Turkeys
Ducks
Horses
Buffaloes
Goats
Livestock species
Rank
0.00
0.25
0.50
0.75
1.00

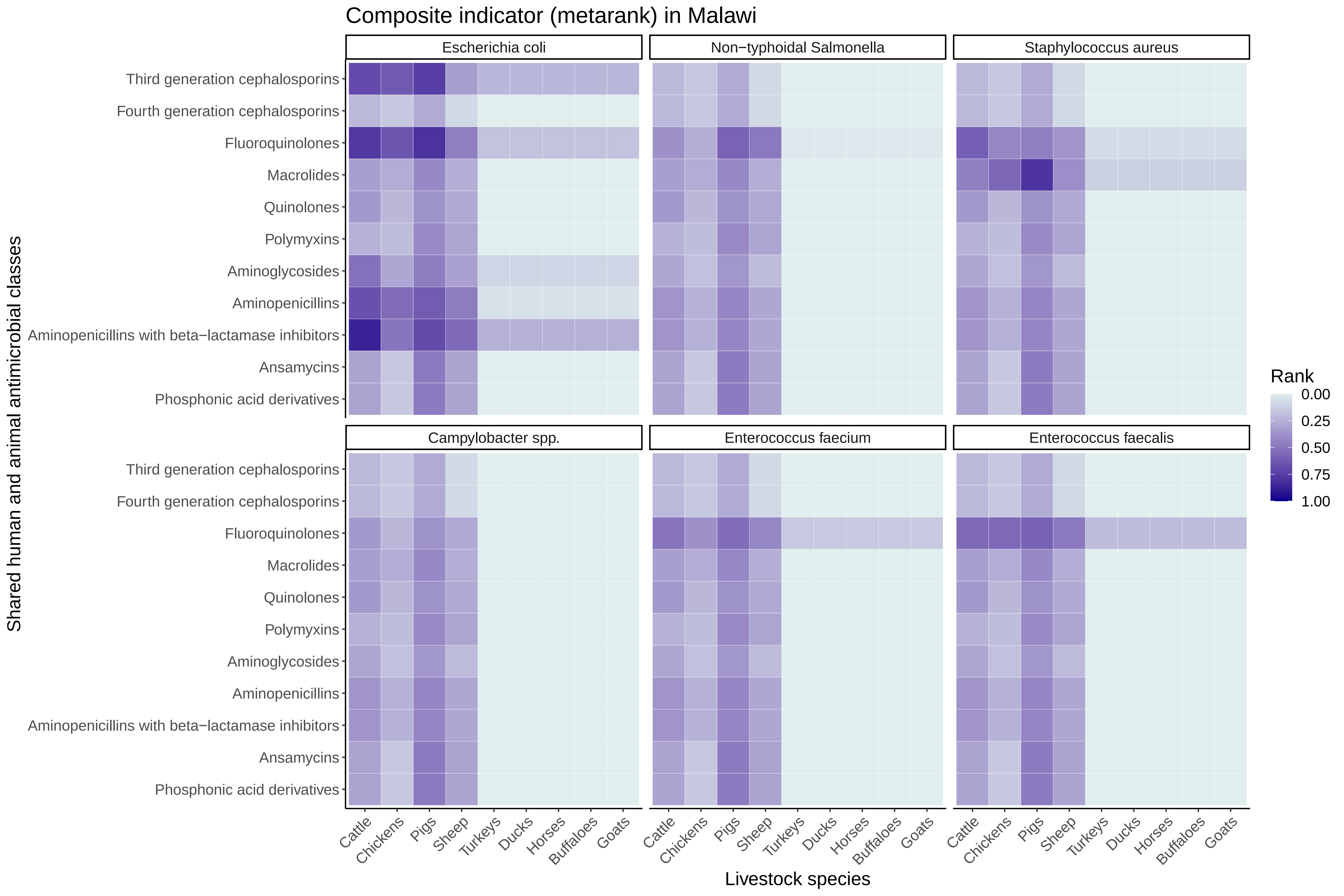
Composite indicator (metarank) in Malawi
Escherichia coli
Non−typhoidal Salmonella
Staphylococcus aureus
Campylobacter spp.
Enterococcus faecium
Enterococcus faecalis
Shared human and animal antimicrobial classes
Third generation cephalosporins
Fourth generation cephalosporins
Fluoroquinolones
Macrolides
Quinolones
Polymyxins
Aminoglycosides
Aminopenicillins
Aminopenicillins with beta−lactamase inhibitors
Ansamycins
Phosphonic acid derivatives
Cattle
Chickens
Pigs
Sheep
Turkeys
Ducks
Horses
Buffaloes
Goats
Livestock species
Rank
0.00
0.25
0.50
0.75
1.00

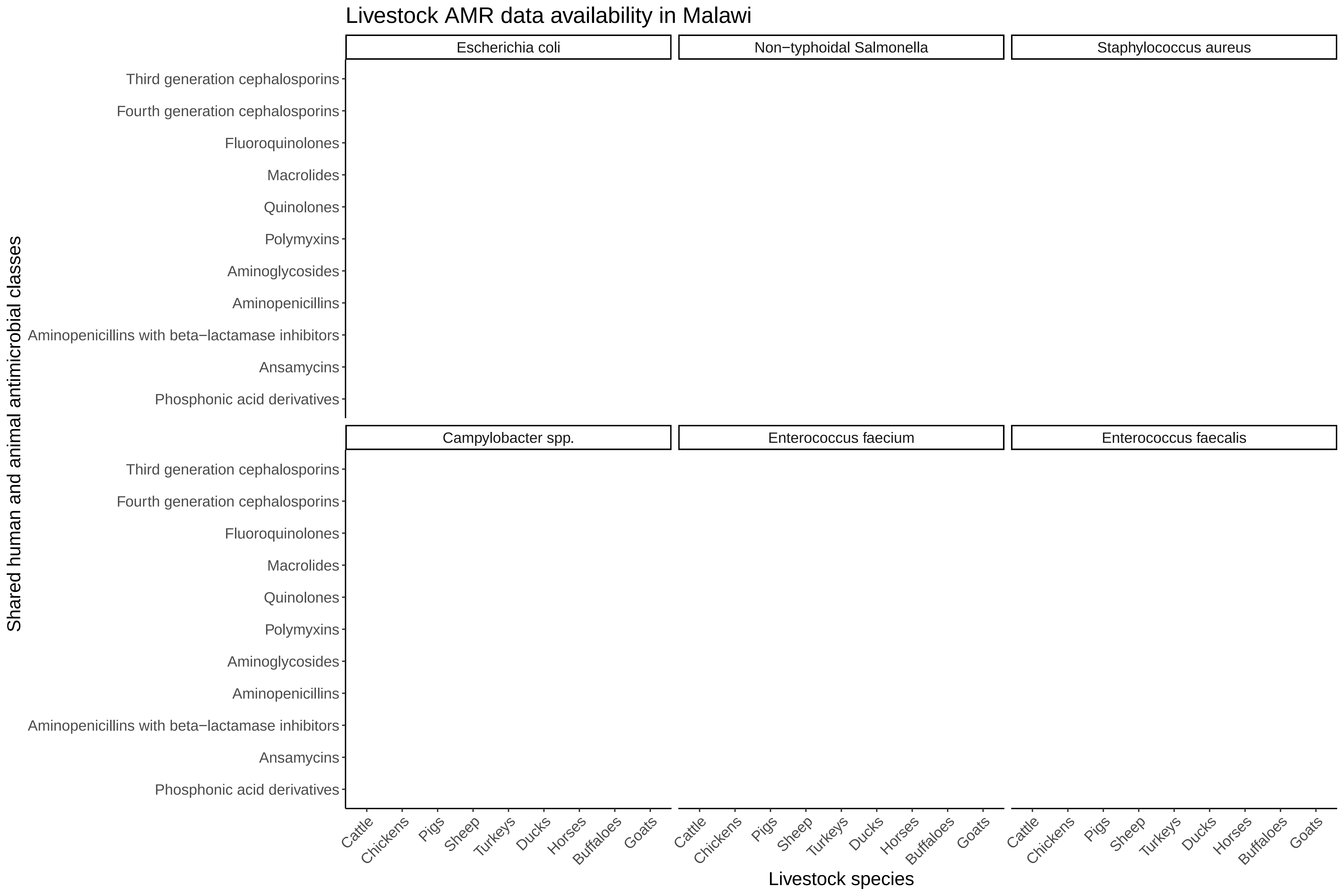
Livestock AMR data availability in Malawi
Escherichia coli
Non−typhoidal Salmonella
Staphylococcus aureus
Campylobacter spp.
Enterococcus faecium
Enterococcus faecalis
Third generation cephalosporins
Fourth generation cephalosporins
Fluoroquinolones
Macrolides
Quinolones
Polymyxins
Aminoglycosides
Aminopenicillins
Aminopenicillins with beta−lactamase inhibitors
Ansamycins
Phosphonic acid derivatives
Shared human and animal antimicrobial classes
Cattle
Chickens
Pigs
Sheep
Turkeys
Ducks
Horses
Buffaloes
Goats
Livestock species

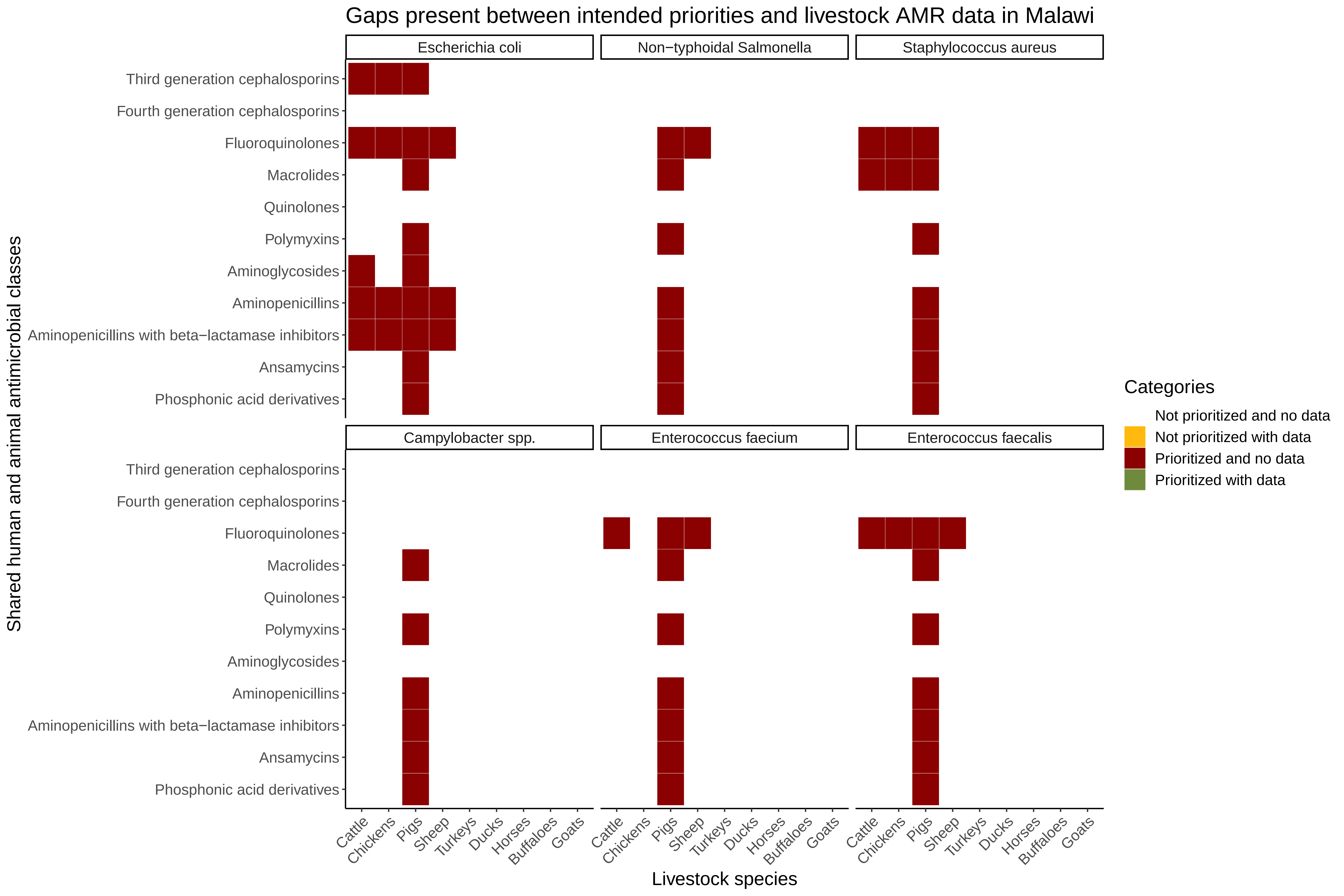
Gaps present between intended priorities and livestock AMR data in Malawi
Escherichia coli
Non−typhoidal Salmonella
Staphylococcus aureus
Campylobacter spp.
Enterococcus faecium
Enterococcus faecalis
Third generation cephalosporins
Fourth generation cephalosporins
Fluoroquinolones
Macrolides
Quinolones
Polymyxins
Aminoglycosides
Aminopenicillins
Aminopenicillins with beta−lactamase inhibitors
Ansamycins
Phosphonic acid derivatives
Shared human and animal antimicrobial classes
Cattle
Chickens
Pigs
Sheep
Turkeys
Ducks
Horses
Buffaloes
Goats
Livestock species
Categories
Not prioritized and no data
Not prioritized with data
Prioritized and no data
Prioritized with data

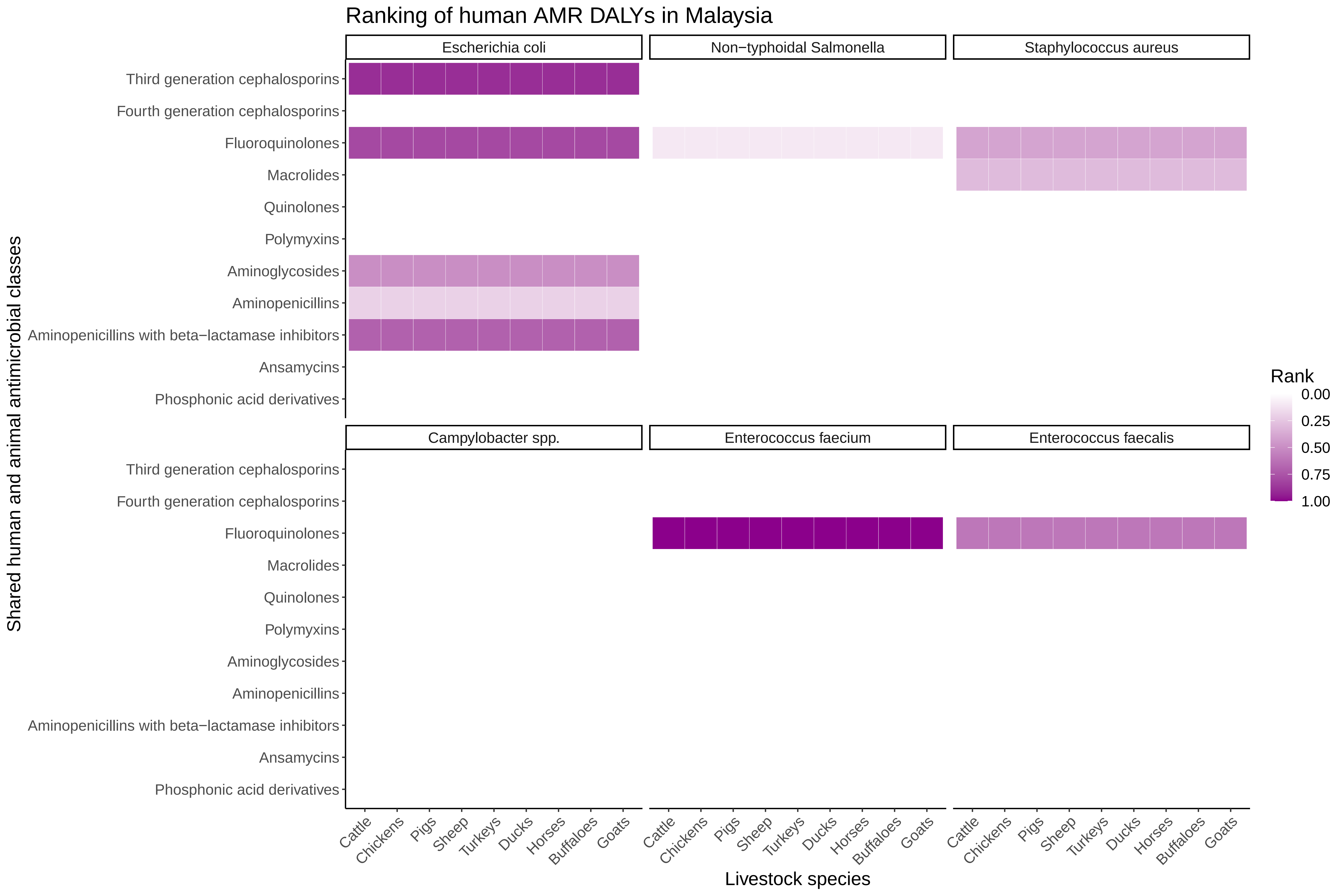

Ranking of human AMR DALYs in Malaysia
Escherichia coli
Non−typhoidal Salmonella
Staphylococcus aureus
Campylobacter spp.
Enterococcus faecium
Enterococcus faecalis
Third generation cephalosporins
Fourth generation cephalosporins
Fluoroquinolones
Macrolides
Quinolones
Polymyxins
Aminoglycosides
Aminopenicillins
Aminopenicillins with beta−lactamase inhibitors
Ansamycins
Phosphonic acid derivatives
Shared human and animal antimicrobial classes
Cattle
Chickens
Pigs
Sheep
Turkeys
Ducks
Horses
Buffaloes
Goats
Livestock species
Rank
0.00
0.25
0.50
0.75
1.00

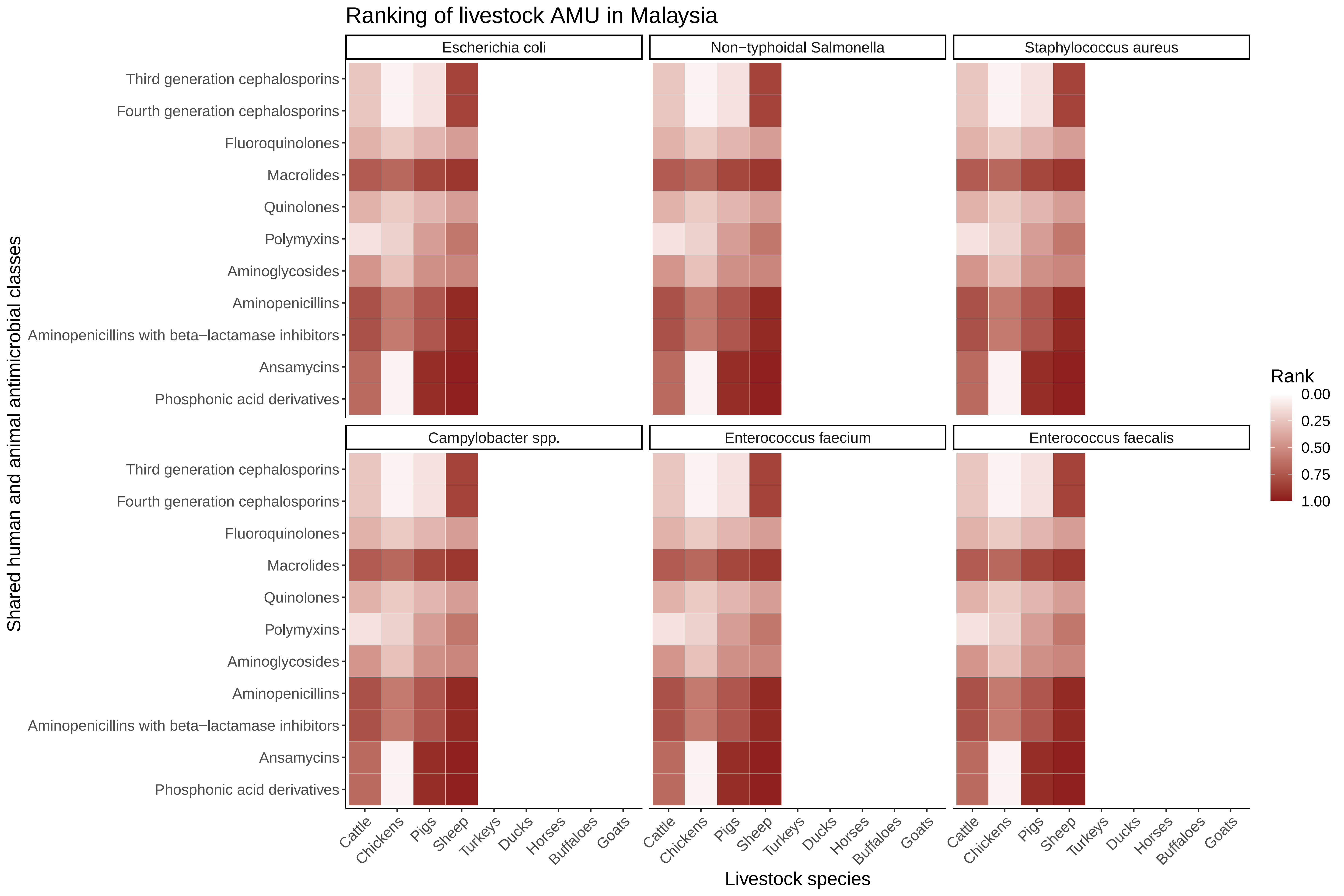

Ranking of livestock AMU in Malaysia
Escherichia coli
Non−typhoidal Salmonella
Staphylococcus aureus
Campylobacter spp.
Enterococcus faecium
Enterococcus faecalis
Third generation cephalosporins
Fourth generation cephalosporins
Fluoroquinolones
Macrolides
Quinolones
Polymyxins
Aminoglycosides
Aminopenicillins
Aminopenicillins with beta−lactamase inhibitors
Ansamycins
Phosphonic acid derivatives
Shared human and animal antimicrobial classes
Cattle
Chickens
Pigs
Sheep
Turkeys
Ducks
Horses
Buffaloes
Goats
Livestock species
Rank
0.00
0.25
0.50
0.75
1.00

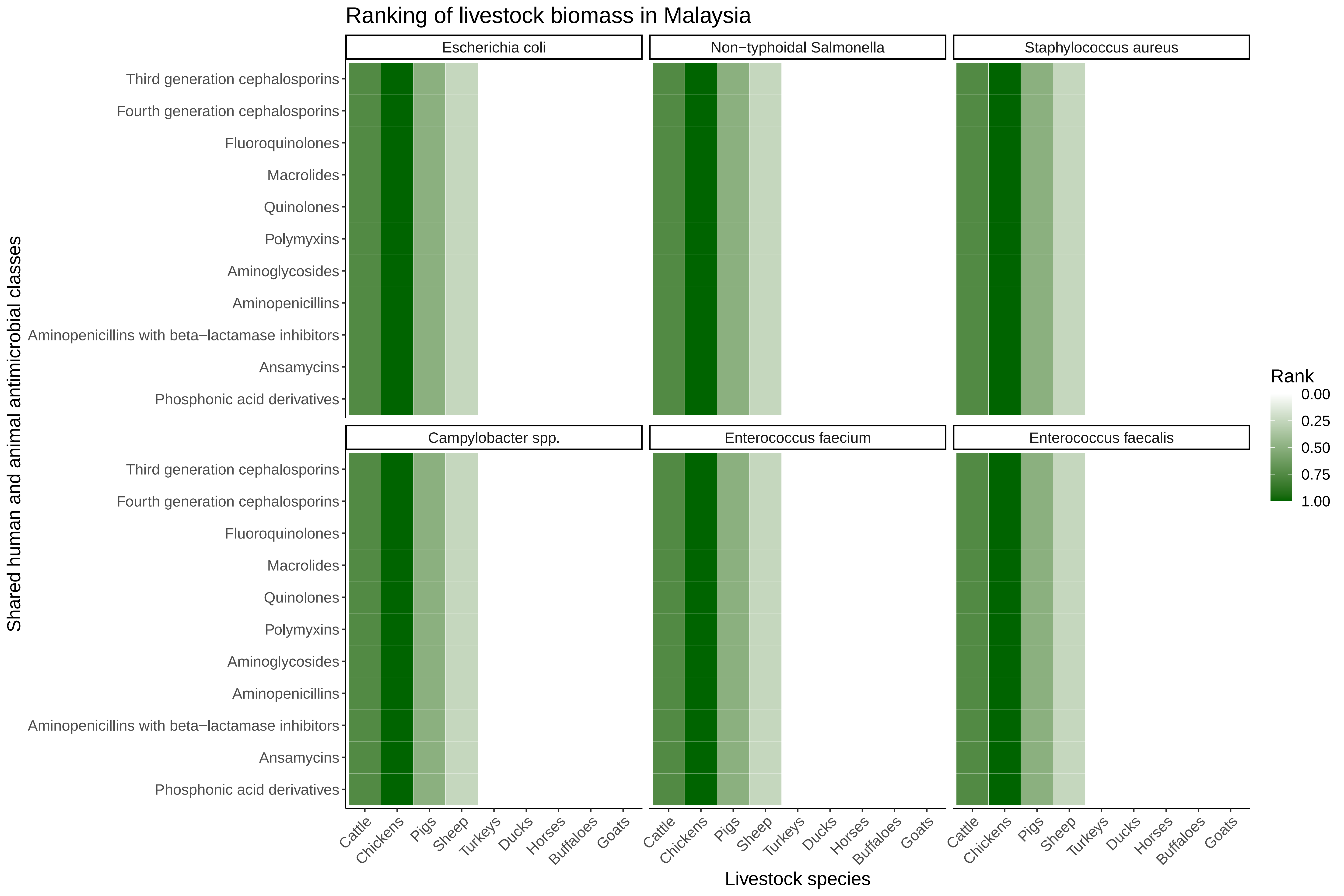

Ranking of livestock biomass in Malaysia
Escherichia coli
Non−typhoidal Salmonella
Staphylococcus aureus
Campylobacter spp.
Enterococcus faecium
Enterococcus faecalis
Third generation cephalosporins
Fourth generation cephalosporins
Fluoroquinolones
Macrolides
Quinolones
Polymyxins
Aminoglycosides
Aminopenicillins
Aminopenicillins with beta−lactamase inhibitors
Ansamycins
Phosphonic acid derivatives
Shared human and animal antimicrobial classes
Cattle
Chickens
Pigs
Sheep
Turkeys
Ducks
Horses
Buffaloes
Goats
Livestock species
Rank
0.00
0.25
0.50
0.75
1.00

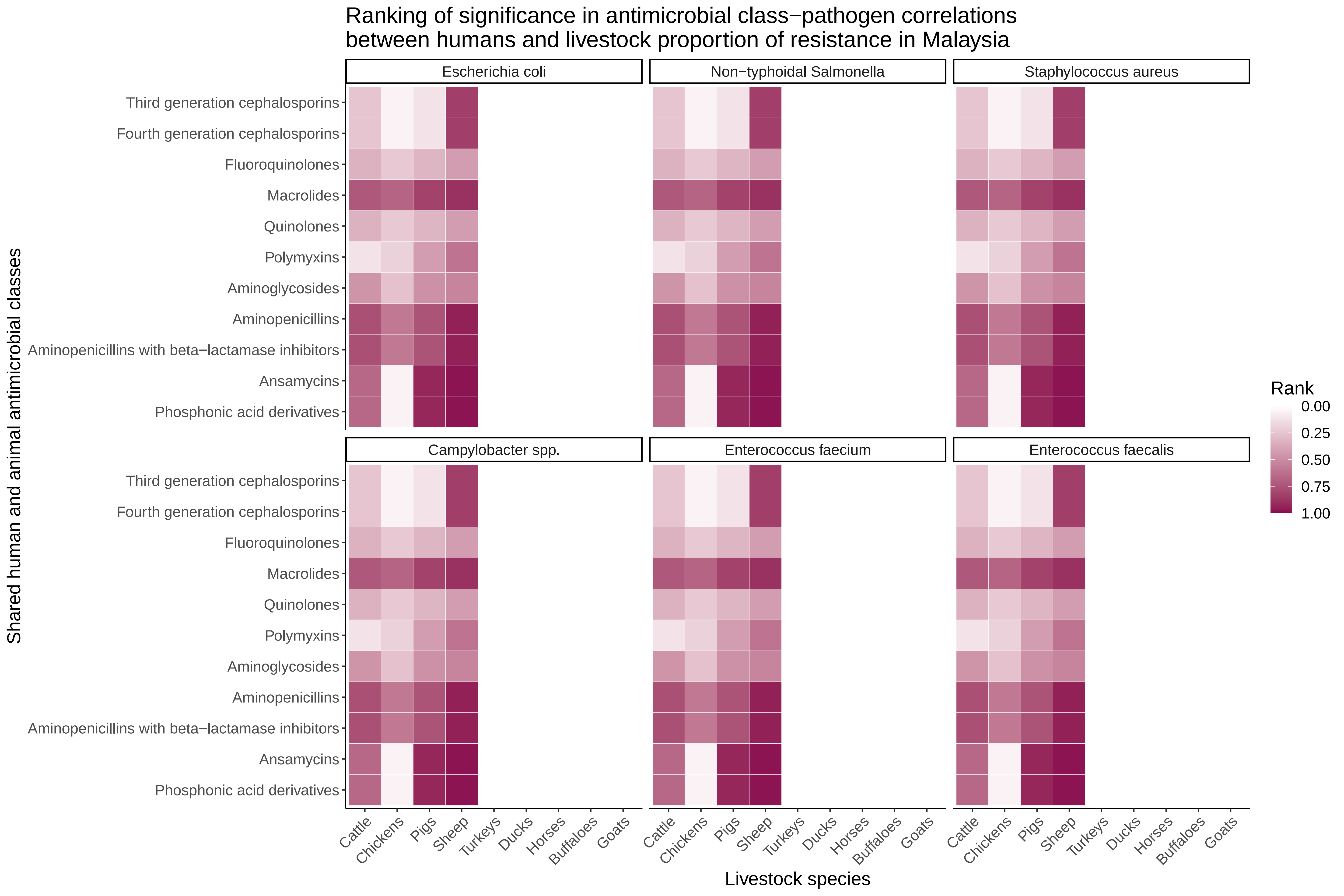
Ranking of significance in antimicrobial class−pathogen correlations
between humans and livestock proportion of resistance in Malaysia
Escherichia coli
Non−typhoidal Salmonella
Staphylococcus aureus
Campylobacter spp.
Enterococcus faecium
Enterococcus faecalis
Third generation cephalosporins
Fourth generation cephalosporins
Fluoroquinolones
Macrolides
Quinolones
Polymyxins
Aminoglycosides
Aminopenicillins
Aminopenicillins with beta−lactamase inhibitors
Ansamycins
Phosphonic acid derivatives
Shared human and animal antimicrobial classes
Cattle
Chickens
Pigs
Sheep
Turkeys
Ducks
Horses
Buffaloes
Goats
Livestock species
Rank
0.00
0.25
0.50
0.75
1.00

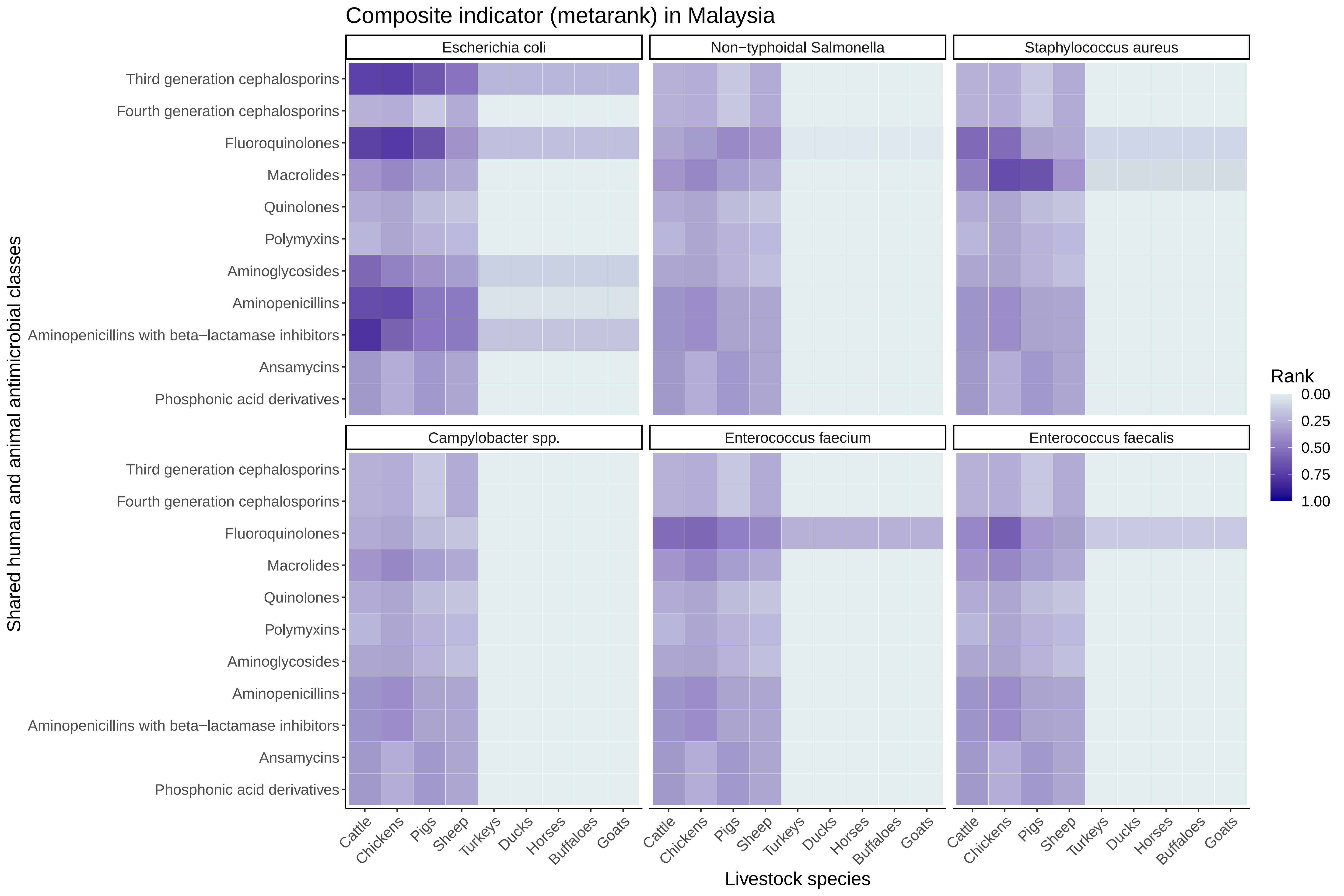

Composite indicator (metarank) in Malaysia
Escherichia coli
Non−typhoidal Salmonella
Staphylococcus aureus
Campylobacter spp.
Enterococcus faecium
Enterococcus faecalis
Shared human and animal antimicrobial classes
Third generation cephalosporins
Fourth generation cephalosporins
Fluoroquinolones
Macrolides
Quinolones
Polymyxins
Aminoglycosides
Aminopenicillins
Aminopenicillins with beta−lactamase inhibitors
Ansamycins
Phosphonic acid derivatives
Cattle
Chickens
Pigs
Sheep
Turkeys
Ducks
Horses
Buffaloes
Goats
Livestock species
Rank
0.00
0.25
0.50
0.75
1.00

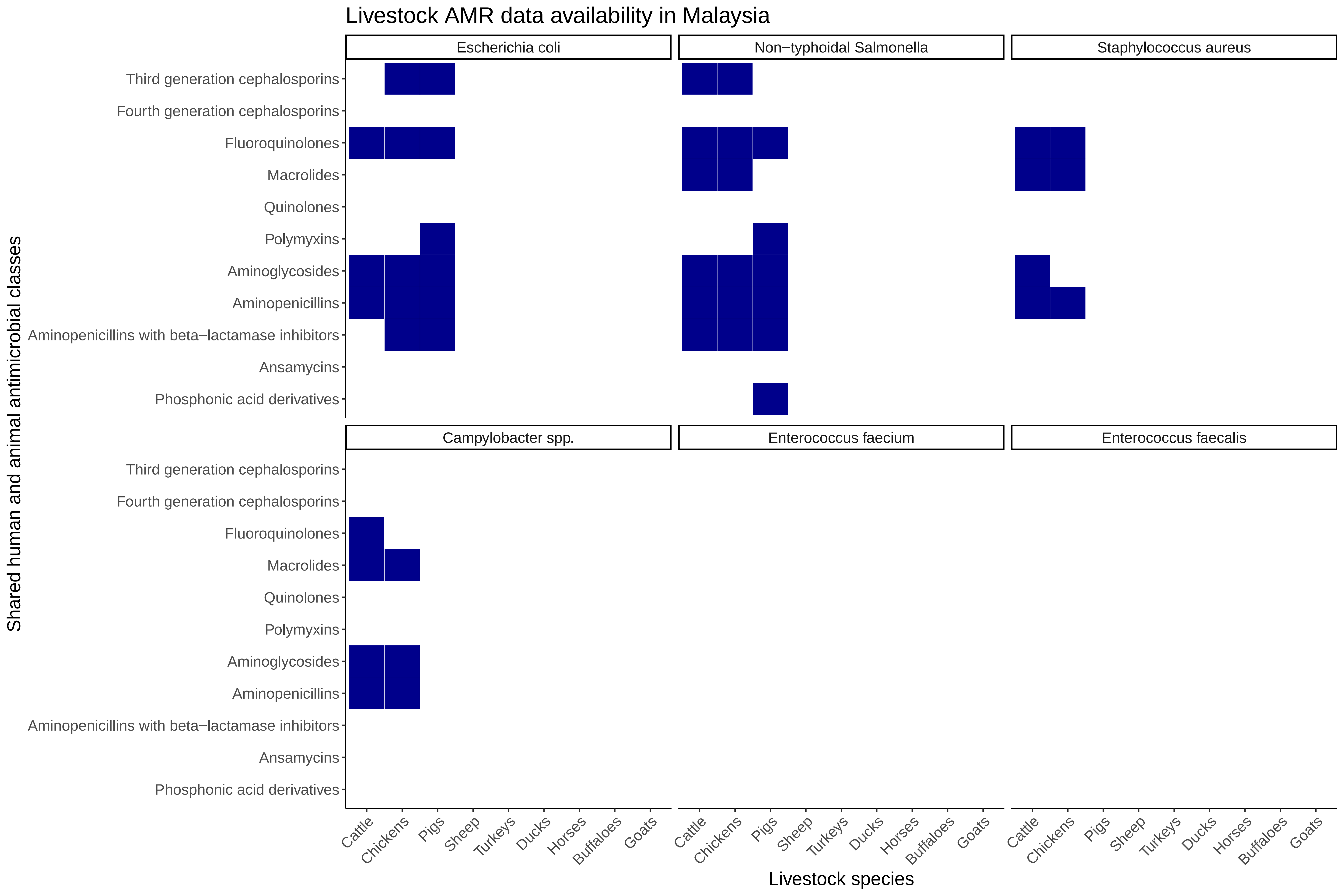

Livestock AMR data availability in Malaysia
Escherichia coli
Non−typhoidal Salmonella
Staphylococcus aureus
Campylobacter spp.
Enterococcus faecium
Enterococcus faecalis
Third generation cephalosporins
Fourth generation cephalosporins
Fluoroquinolones
Macrolides
Quinolones
Polymyxins
Aminoglycosides
Aminopenicillins
Aminopenicillins with beta−lactamase inhibitors
Ansamycins
Phosphonic acid derivatives
Shared human and animal antimicrobial classes
Cattle
Chickens
Pigs
Sheep
Turkeys
Ducks
Horses
Buffaloes
Goats
Livestock species

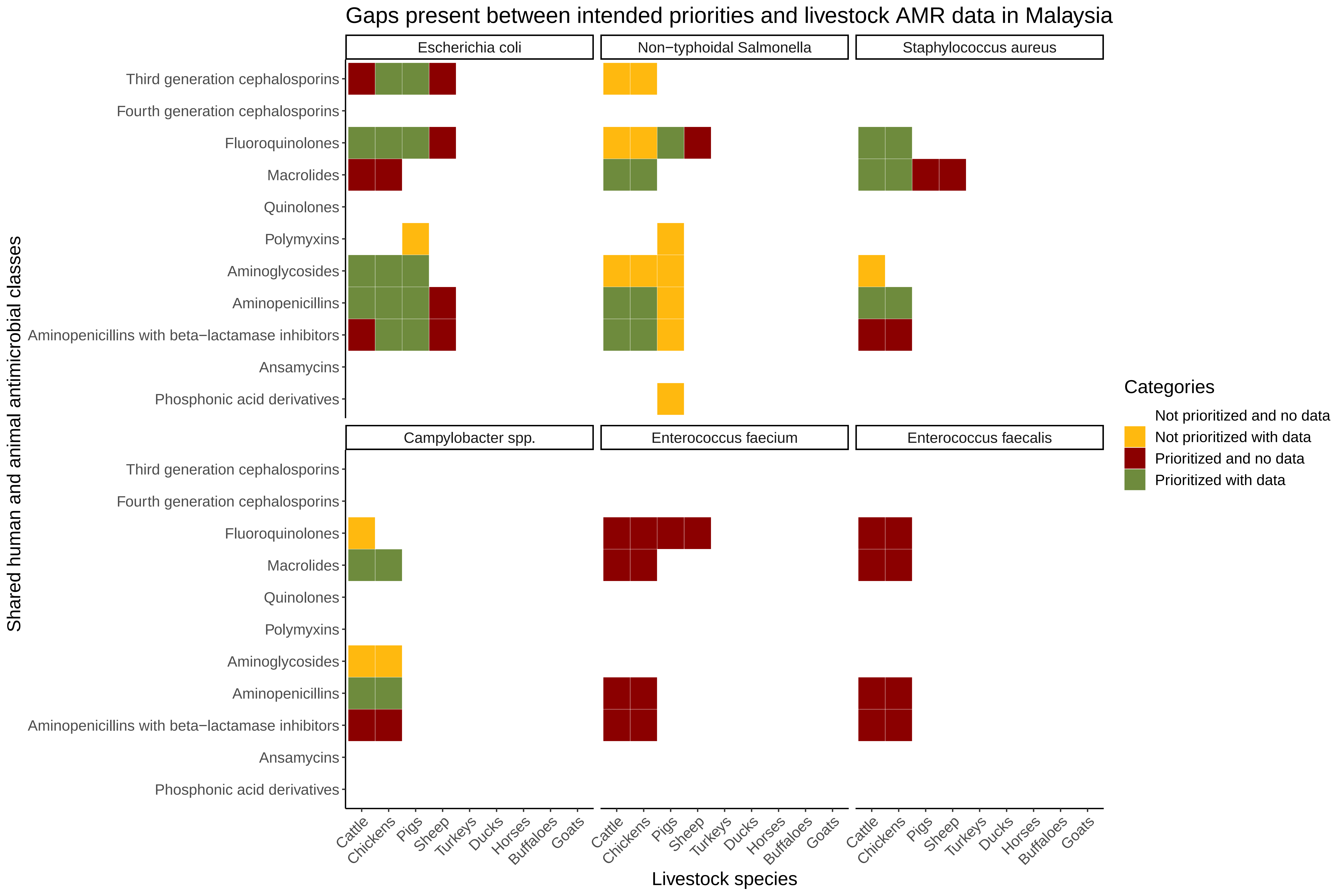

Gaps present between intended priorities and livestock AMR data in Malaysia
Escherichia coli
Non−typhoidal Salmonella
Staphylococcus aureus
Campylobacter spp.
Enterococcus faecium
Enterococcus faecalis
Third generation cephalosporins
Fourth generation cephalosporins
Fluoroquinolones
Macrolides
Quinolones
Polymyxins
Aminoglycosides
Aminopenicillins
Aminopenicillins with beta−lactamase inhibitors
Ansamycins
Phosphonic acid derivatives
Shared human and animal antimicrobial classes
Cattle
Chickens
Pigs
Sheep
Turkeys
Ducks
Horses
Buffaloes
Goats
Livestock species
Categories
Not prioritized and no data
Not prioritized with data
Prioritized and no data
Prioritized with data

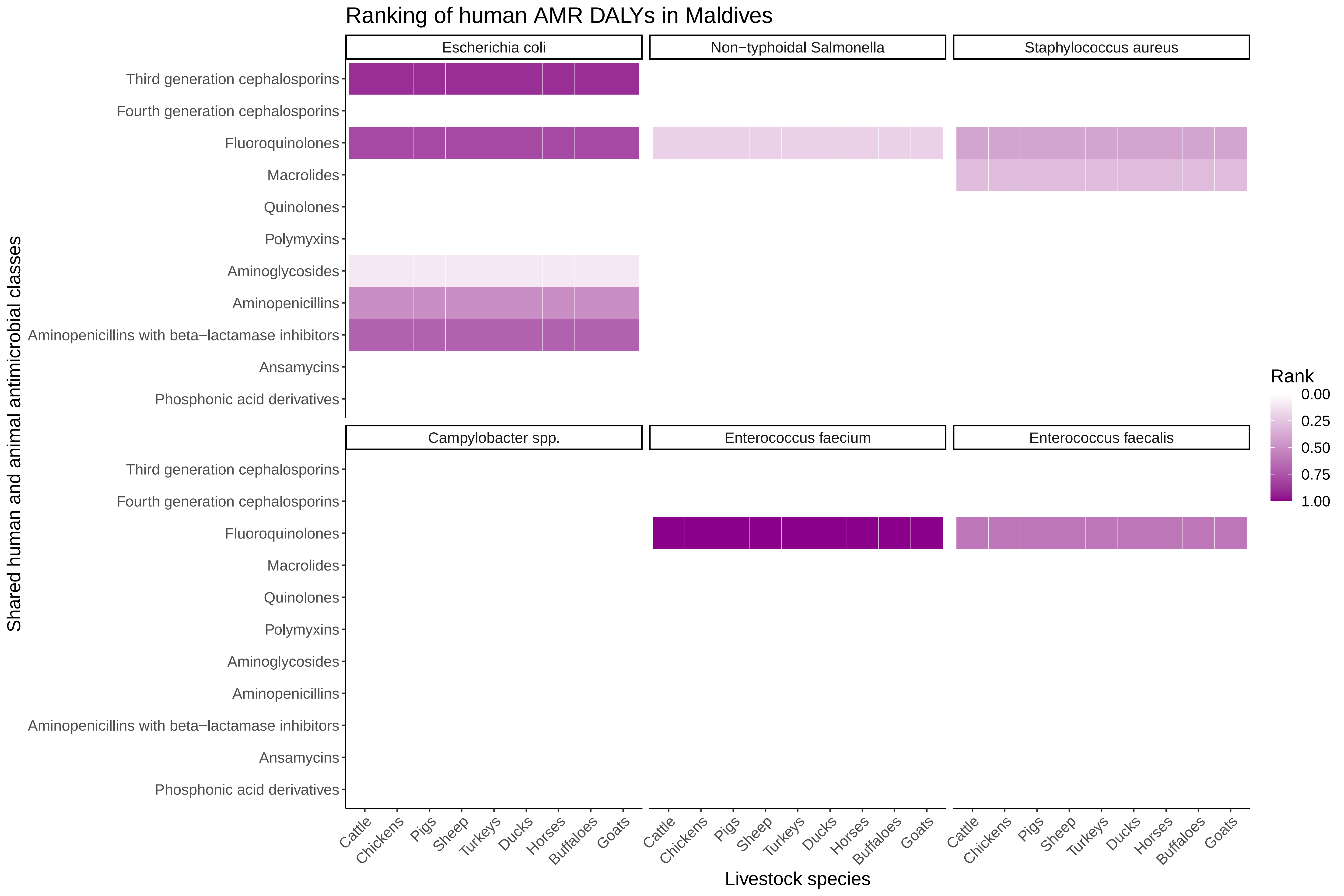

Ranking of human AMR DALYs in Maldives
Escherichia coli
Non−typhoidal Salmonella
Staphylococcus aureus
Campylobacter spp.
Enterococcus faecium
Enterococcus faecalis
Shared human and animal antimicrobial classes
Third generation cephalosporins
Fourth generation cephalosporins
Fluoroquinolones
Macrolides
Quinolones
Polymyxins
Aminoglycosides
Aminopenicillins
Aminopenicillins with beta−lactamase inhibitors
Ansamycins
Phosphonic acid derivatives
Livestock species
Cattle
Chickens
Pigs
Sheep
Turkeys
Ducks
Horses
Buffaloes
Goats
Rank
0.00
0.25
0.50
0.75
1.00

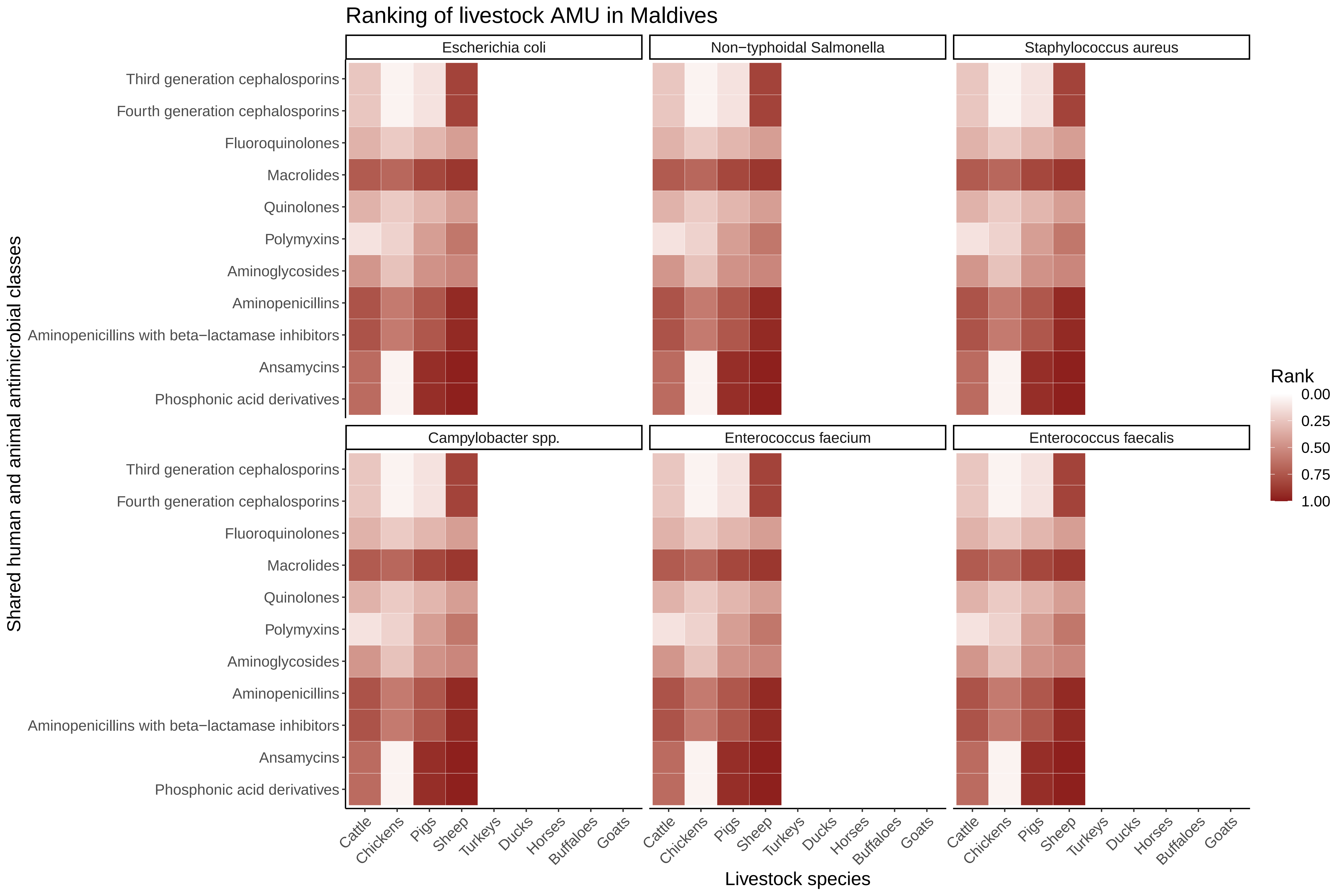
Ranking of livestock AMU in Maldives
Escherichia coli
Non−typhoidal Salmonella
Staphylococcus aureus
Campylobacter spp.
Enterococcus faecium
Enterococcus faecalis
Shared human and animal antimicrobial classes
Third generation cephalosporins
Fourth generation cephalosporins
Fluoroquinolones
Macrolides
Quinolones
Polymyxins
Aminoglycosides
Aminopenicillins
Aminopenicillins with beta−lactamase inhibitors
Ansamycins
Phosphonic acid derivatives
Cattle
Chickens
Pigs
Sheep
Turkeys
Ducks
Horses
Buffaloes
Goats
Livestock species
Rank
0.00
0.25
0.50
0.75
1.00

Ranking of livestock biomass in Maldives

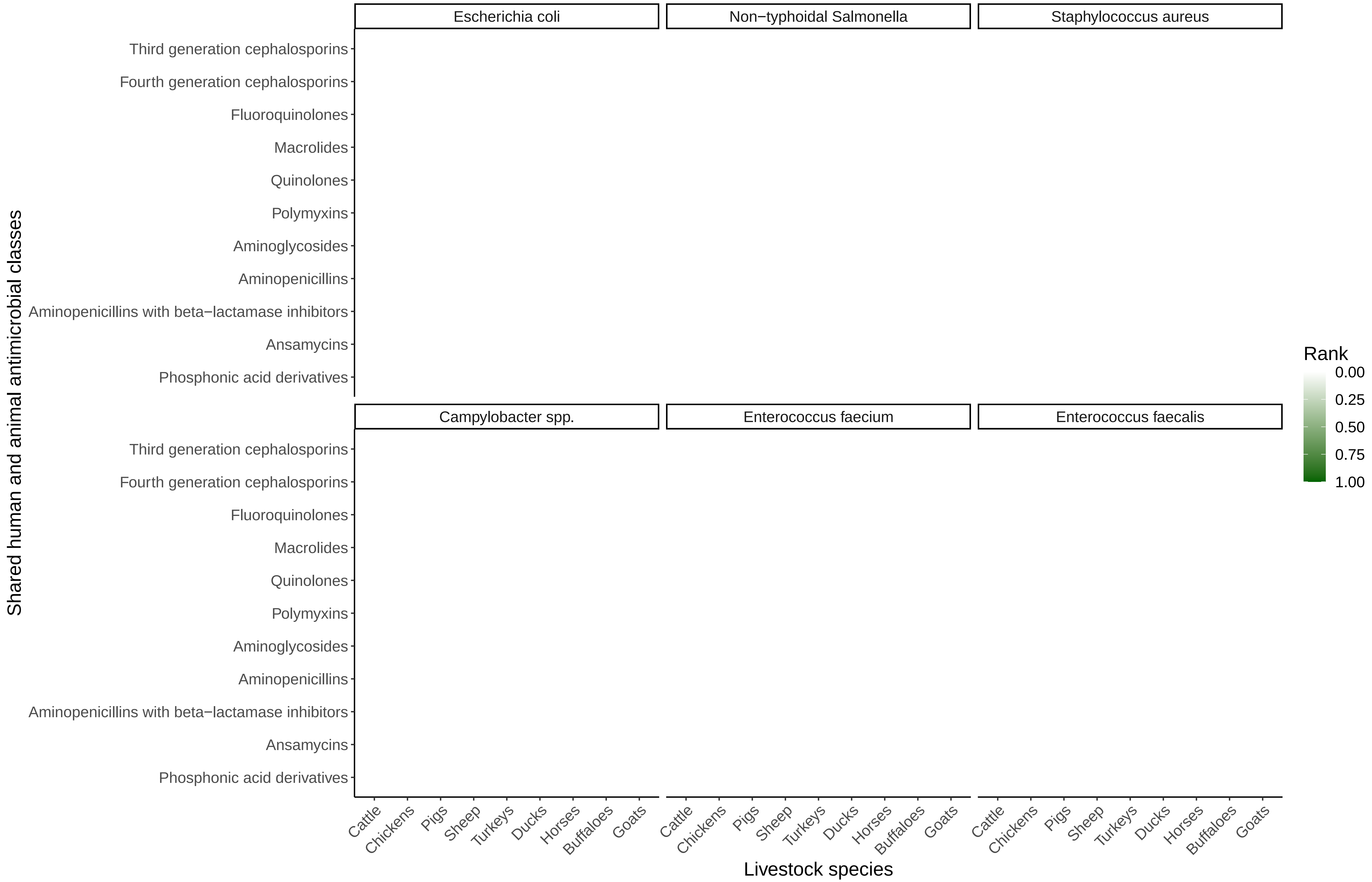

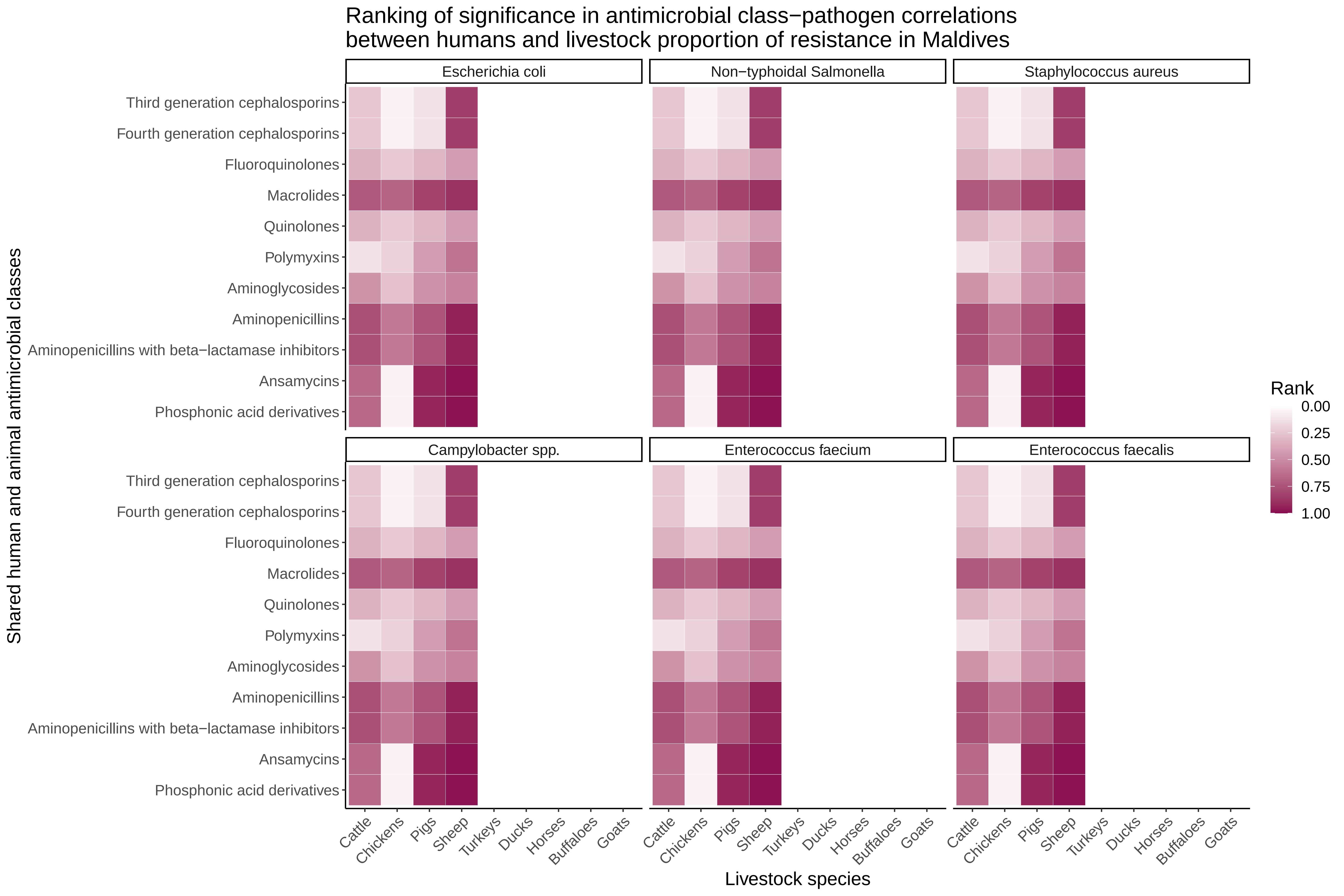

Ranking of significance in antimicrobial class−pathogen correlations
between humans and livestock proportion of resistance in Maldives
Escherichia coli
Non−typhoidal Salmonella
Staphylococcus aureus
Campylobacter spp.
Enterococcus faecium
Enterococcus faecalis
Third generation cephalosporins
Fourth generation cephalosporins
Fluoroquinolones
Macrolides
Quinolones
Polymyxins
Aminoglycosides
Aminopenicillins
Aminopenicillins with beta−lactamase inhibitors
Ansamycins
Phosphonic acid derivatives
Shared human and animal antimicrobial classes
Cattle
Chickens
Pigs
Sheep
Turkeys
Ducks
Horses
Buffaloes
Goats
Livestock species
Rank
0.00
0.25
0.50
0.75
1.00

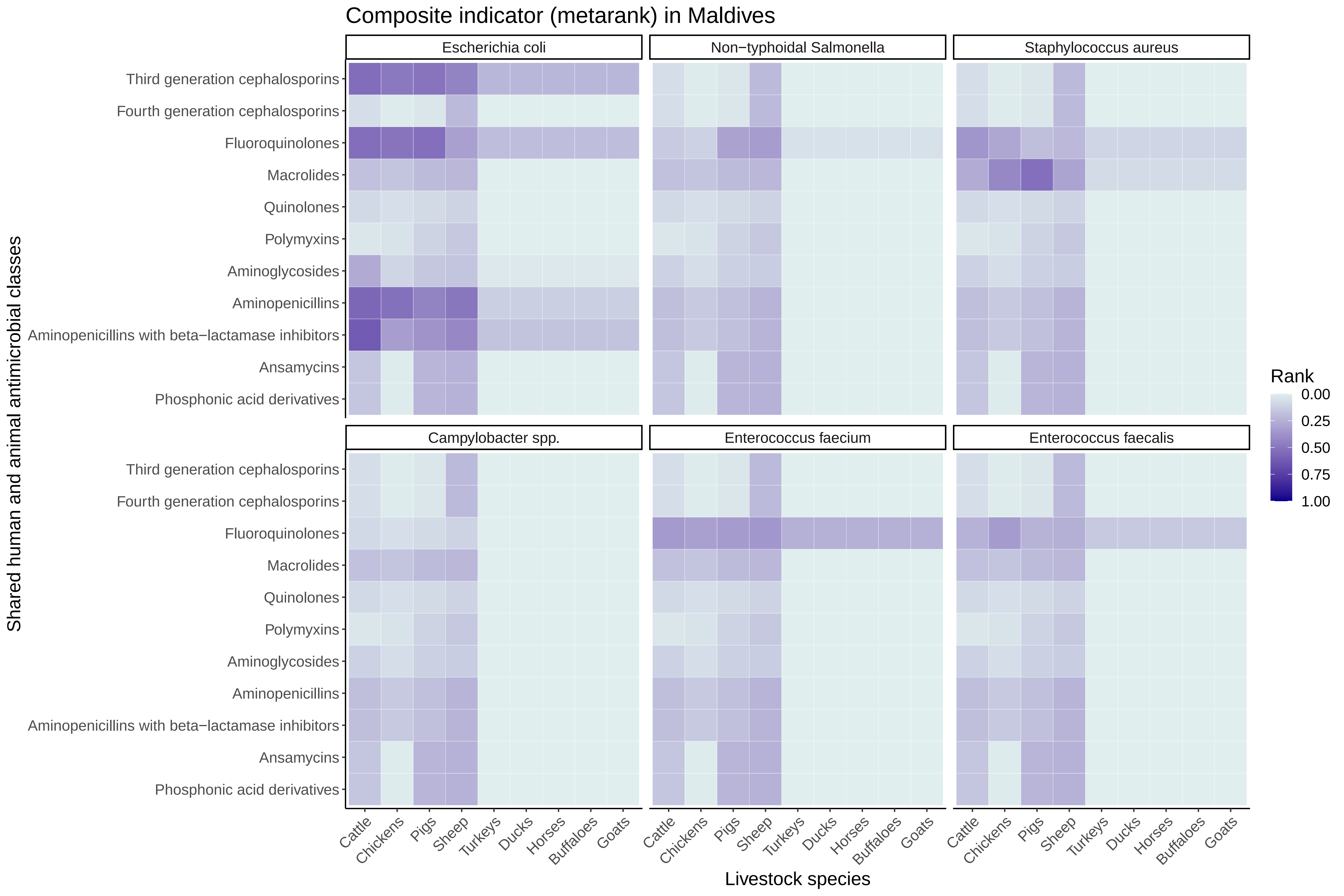

Composite indicator (metarank) in Maldives
Escherichia coli
Non−typhoidal Salmonella
Staphylococcus aureus
Campylobacter spp.
Enterococcus faecium
Enterococcus faecalis
Shared human and animal antimicrobial classes
Third generation cephalosporins
Fourth generation cephalosporins
Fluoroquinolones
Macrolides
Quinolones
Polymyxins
Aminoglycosides
Aminopenicillins
Aminopenicillins with beta−lactamase inhibitors
Ansamycins
Phosphonic acid derivatives
Livestock species
Cattle
Chickens
Pigs
Sheep
Turkeys
Ducks
Horses
Buffaloes
Goats
Rank
0.00
0.25
0.50
0.75
1.00

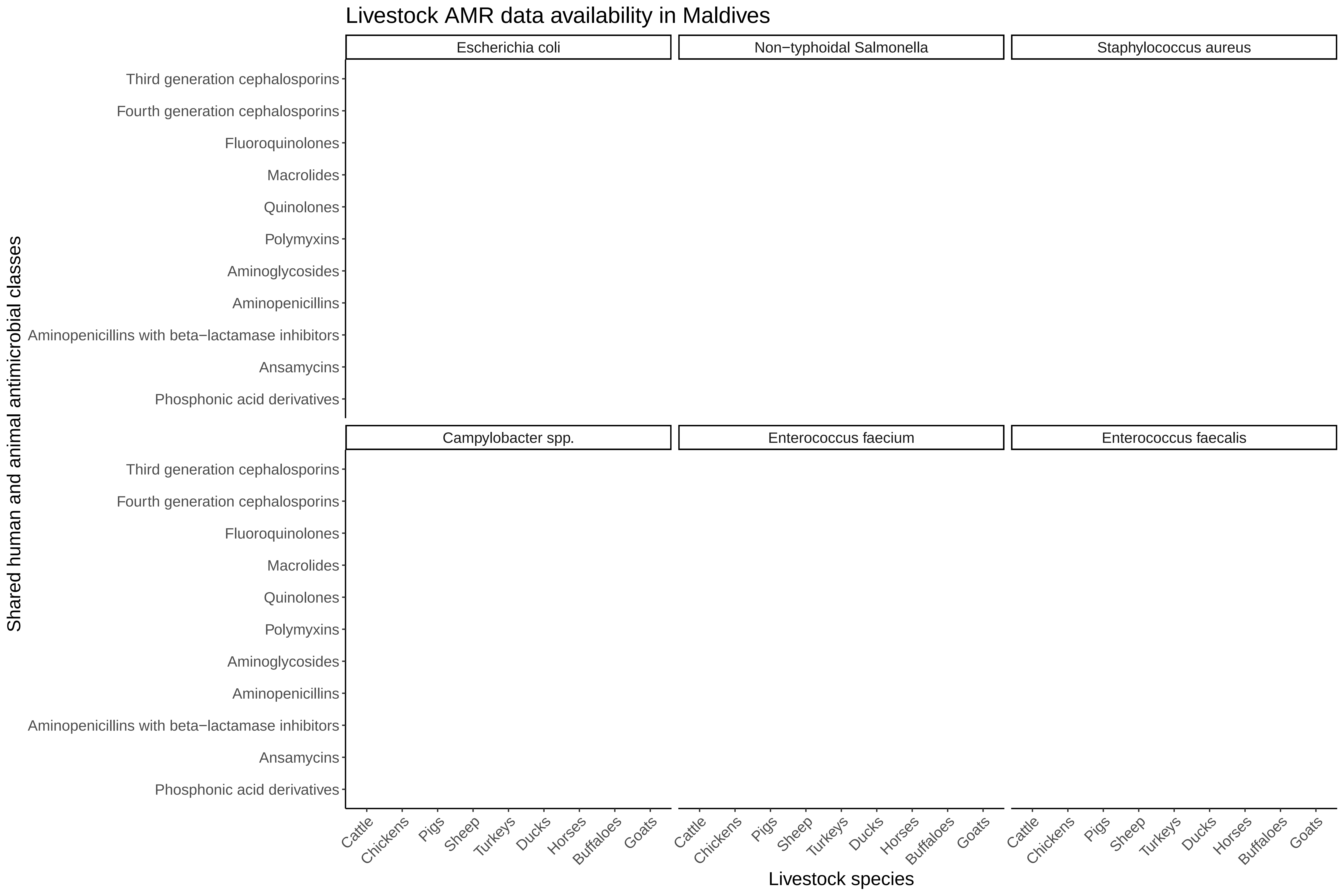
Livestock AMR data availability in Maldives
Escherichia coli
Non−typhoidal Salmonella
Staphylococcus aureus
Campylobacter spp.
Enterococcus faecium
Enterococcus faecalis
Shared human and animal antimicrobial classes
Third generation cephalosporins
Fourth generation cephalosporins
Fluoroquinolones
Macrolides
Quinolones
Polymyxins
Aminoglycosides
Aminopenicillins
Aminopenicillins with beta−lactamase inhibitors
Ansamycins
Phosphonic acid derivatives
Cattle
Chickens
Pigs
Sheep
Turkeys
Ducks
Horses
Buffaloes
Goats
Livestock species

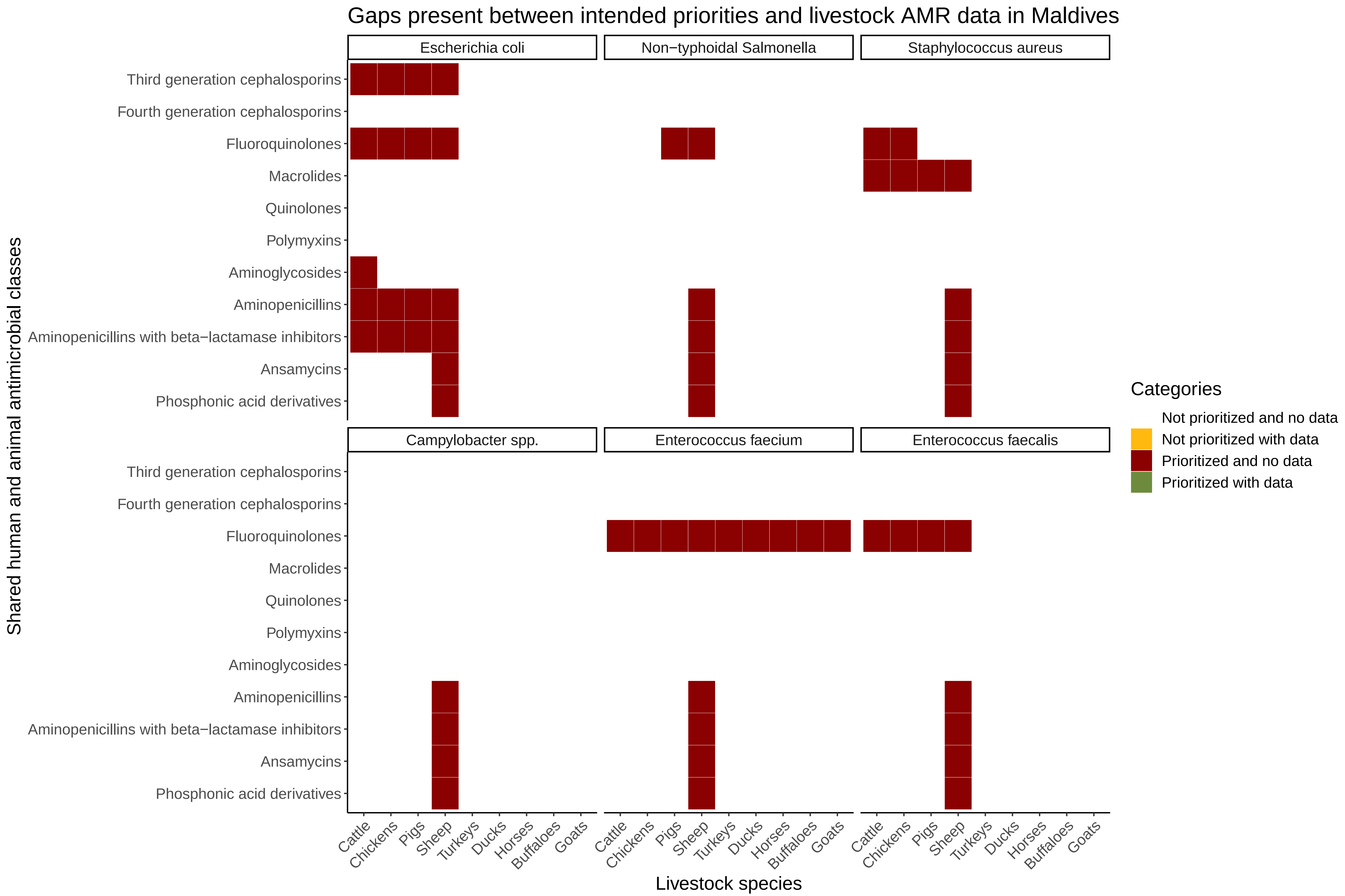
Gaps present between intended priorities and livestock AMR data in Maldives
Escherichia coli
Non−typhoidal Salmonella
Staphylococcus aureus
Campylobacter spp.
Enterococcus faecium
Enterococcus faecalis
Shared human and animal antimicrobial classes
Third generation cephalosporins
Fourth generation cephalosporins
Fluoroquinolones
Macrolides
Quinolones
Polymyxins
Aminoglycosides
Aminopenicillins
Aminopenicillins with beta−lactamase inhibitors
Ansamycins
Phosphonic acid derivatives
Livestock species
Cattle
Chickens
Pigs
Sheep
Turkeys
Ducks
Horses
Buffaloes
Goats
Categories
Not prioritized and no data
Not prioritized with data
Prioritized and no data
Prioritized with data

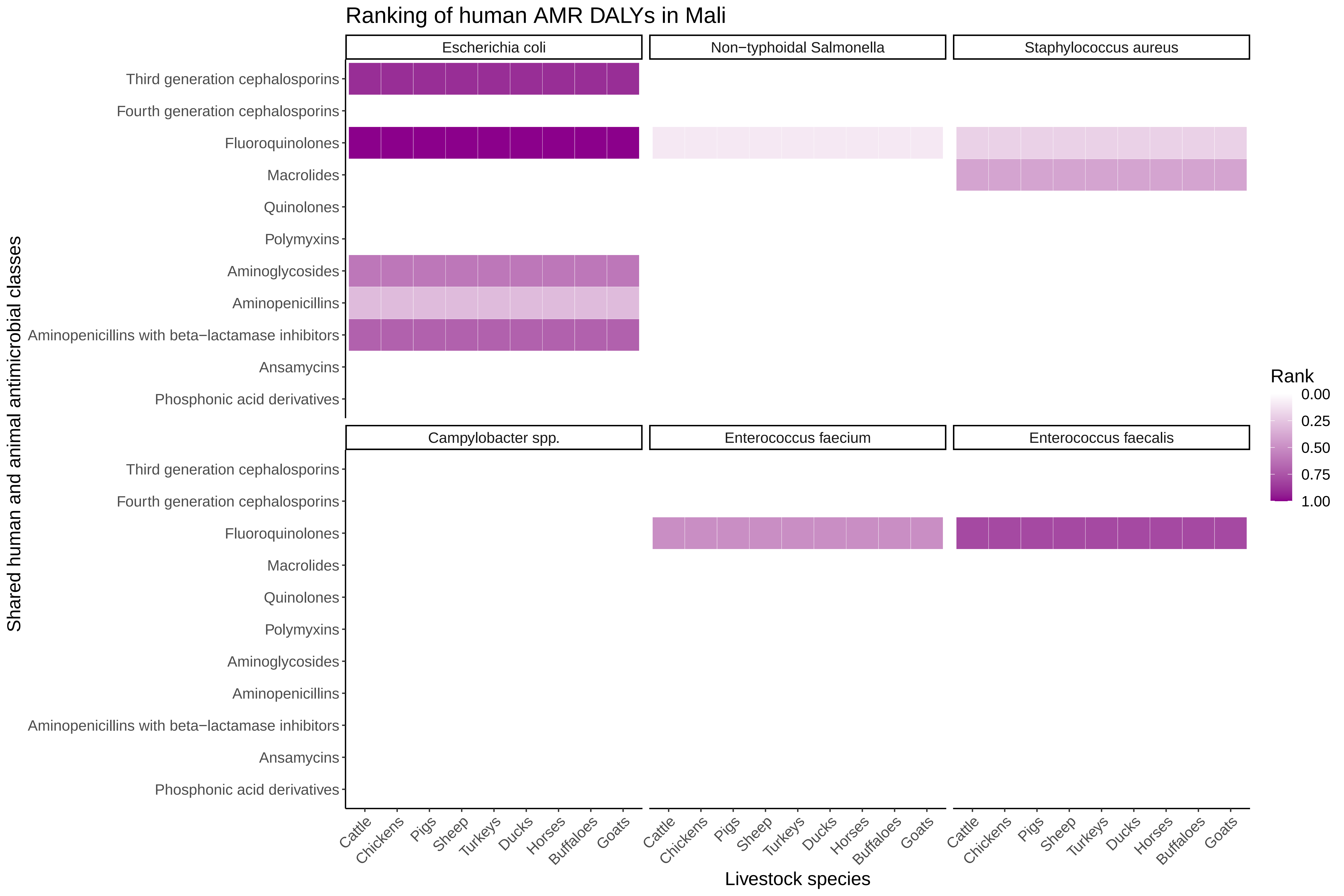

Ranking of human AMR DALYs in Mali
Escherichia coli
Non−typhoidal Salmonella
Staphylococcus aureus
Campylobacter spp.
Enterococcus faecium
Enterococcus faecalis
Third generation cephalosporins
Fourth generation cephalosporins
Fluoroquinolones
Macrolides
Quinolones
Polymyxins
Aminoglycosides
Aminopenicillins
Aminopenicillins with beta−lactamase inhibitors
Ansamycins
Phosphonic acid derivatives
Shared human and animal antimicrobial classes
Cattle
Chickens
Pigs
Sheep
Turkeys
Ducks
Horses
Buffaloes
Goats
Livestock species
Rank
0.00
0.25
0.50
0.75
1.00

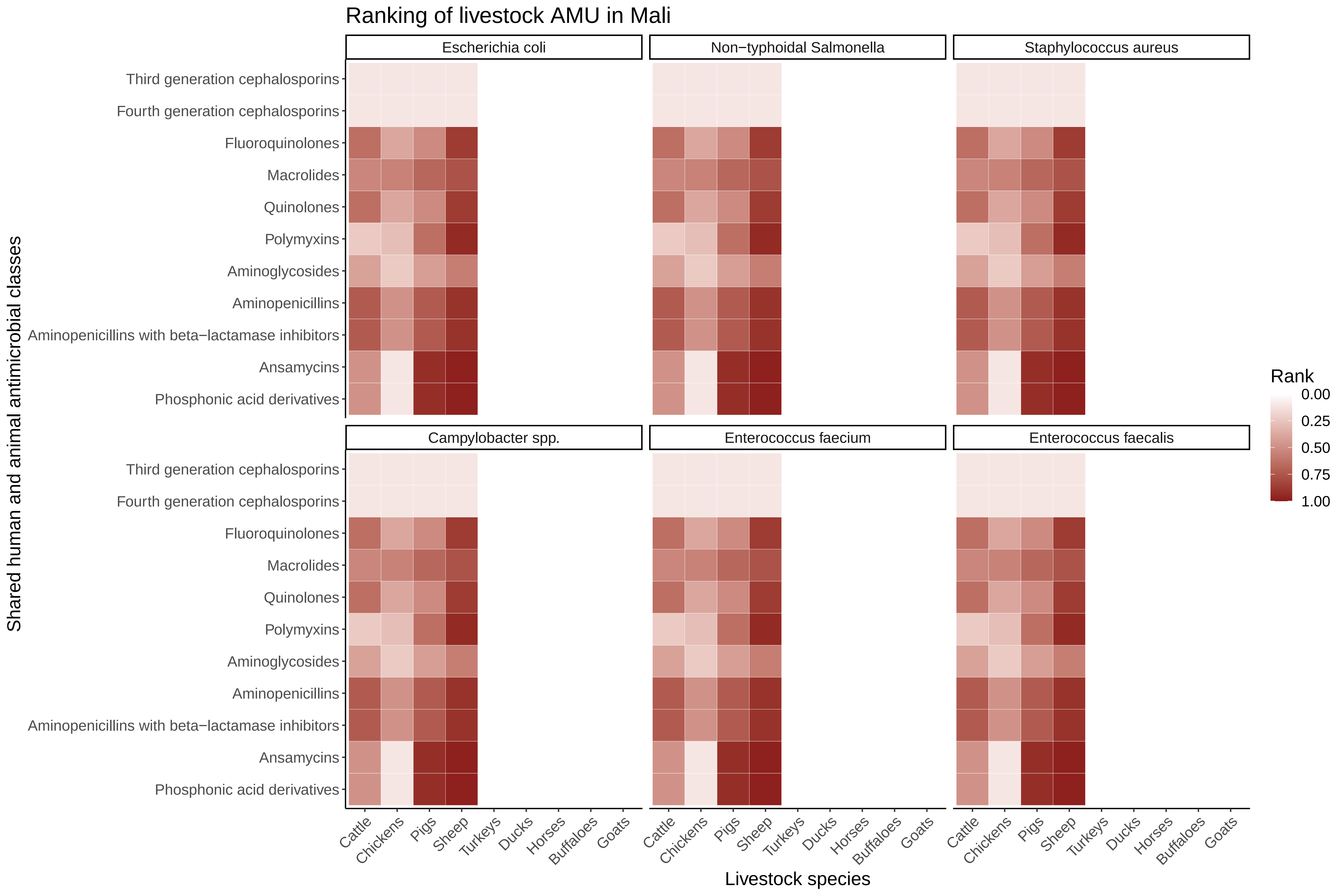

Ranking of livestock AMU in Mali
Escherichia coli
Non−typhoidal Salmonella
Staphylococcus aureus
Campylobacter spp.
Enterococcus faecium
Enterococcus faecalis
Third generation cephalosporins
Fourth generation cephalosporins
Fluoroquinolones
Macrolides
Quinolones
Polymyxins
Aminoglycosides
Aminopenicillins
Aminopenicillins with beta−lactamase inhibitors
Ansamycins
Phosphonic acid derivatives
Shared human and animal antimicrobial classes
Cattle
Chickens
Pigs
Sheep
Turkeys
Ducks
Horses
Buffaloes
Goats
Livestock species
Rank
0.00
0.25
0.50
0.75
1.00

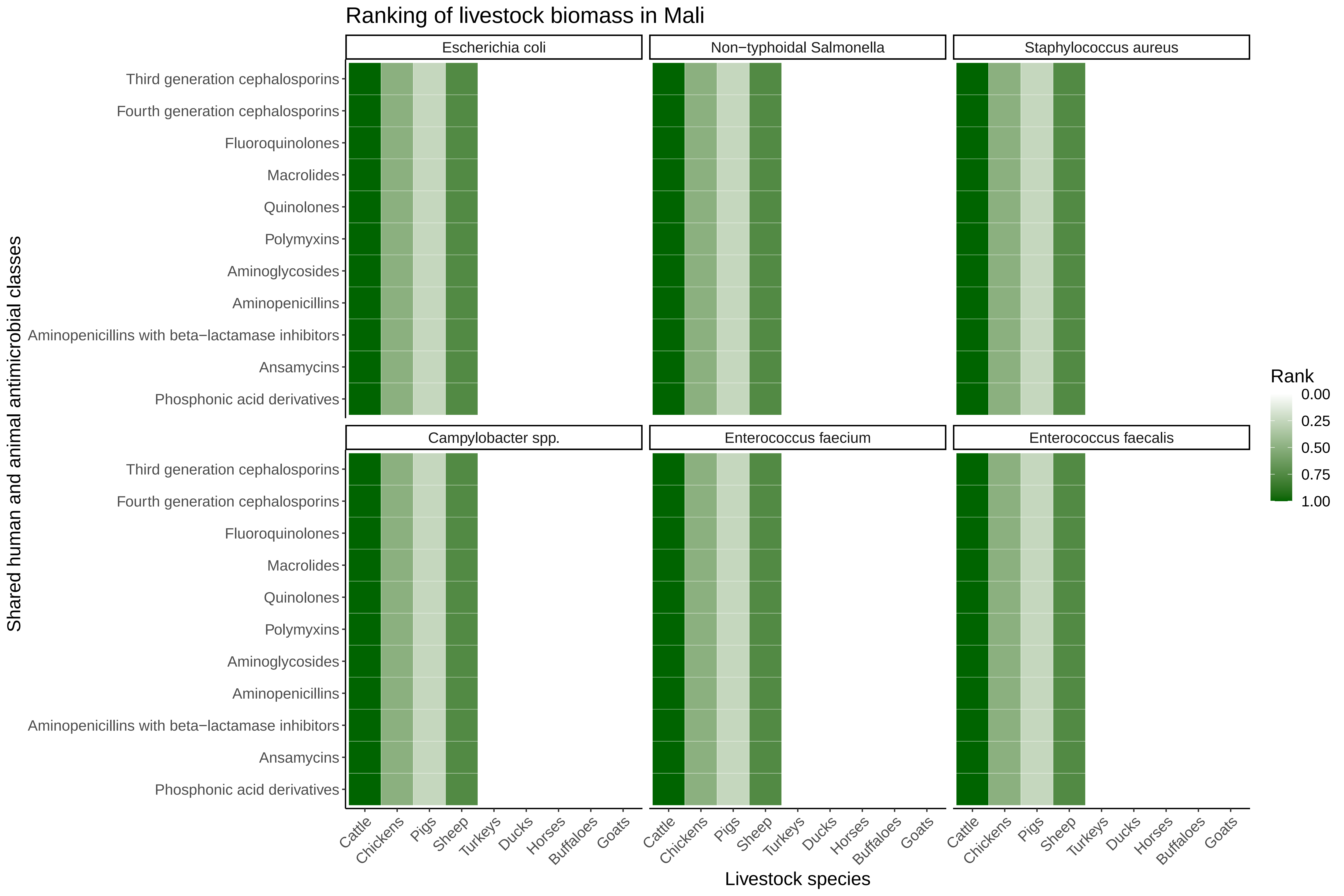

Ranking of livestock biomass in Mali
Escherichia coli
Non−typhoidal Salmonella
Staphylococcus aureus
Campylobacter spp.
Enterococcus faecium
Enterococcus faecalis
Third generation cephalosporins
Fourth generation cephalosporins
Fluoroquinolones
Macrolides
Quinolones
Polymyxins
Aminoglycosides
Aminopenicillins
Aminopenicillins with beta−lactamase inhibitors
Ansamycins
Phosphonic acid derivatives
Shared human and animal antimicrobial classes
Cattle
Chickens
Pigs
Sheep
Turkeys
Ducks
Horses
Buffaloes
Goats
Livestock species
Rank
0.00
0.25
0.50
0.75
1.00

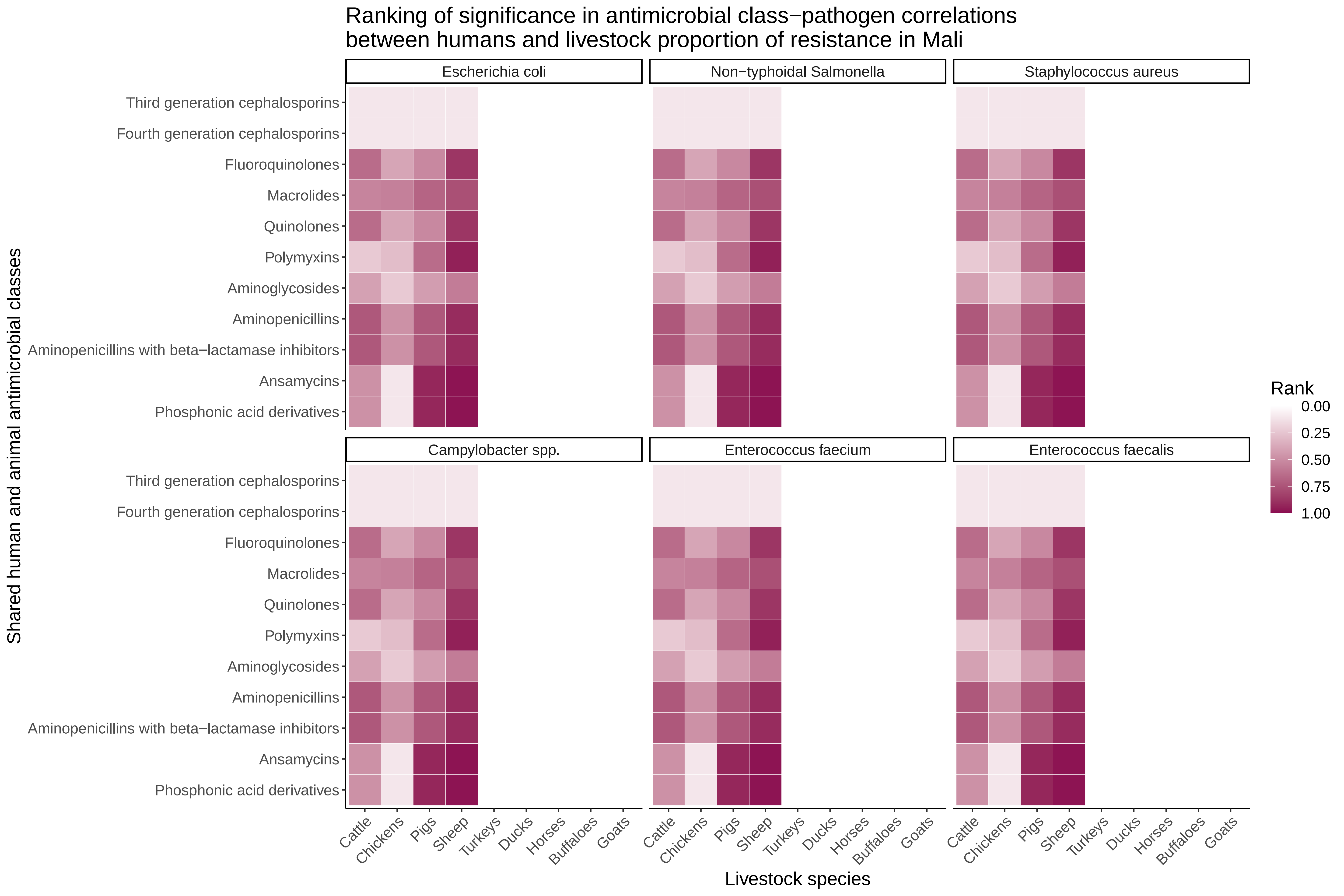
Ranking of significance in antimicrobial class−pathogen correlations between humans and livestock proportion of resistance in Mali
Escherichia coli
Non−typhoidal Salmonella
Staphylococcus aureus
Campylobacter spp.
Enterococcus faecium
Enterococcus faecalis
Shared human and animal antimicrobial classes
Third generation cephalosporins
Fourth generation cephalosporins
Fluoroquinolones
Macrolides
Quinolones
Polymyxins
Aminoglycosides
Aminopenicillins
Aminopenicillins with beta−lactamase inhibitors
Ansamycins
Phosphonic acid derivatives
Livestock species
Cattle
Chickens
Pigs
Sheep
Turkeys
Ducks
Horses
Buffaloes
Goats
Rank
0.00
0.25
0.50
0.75
1.00

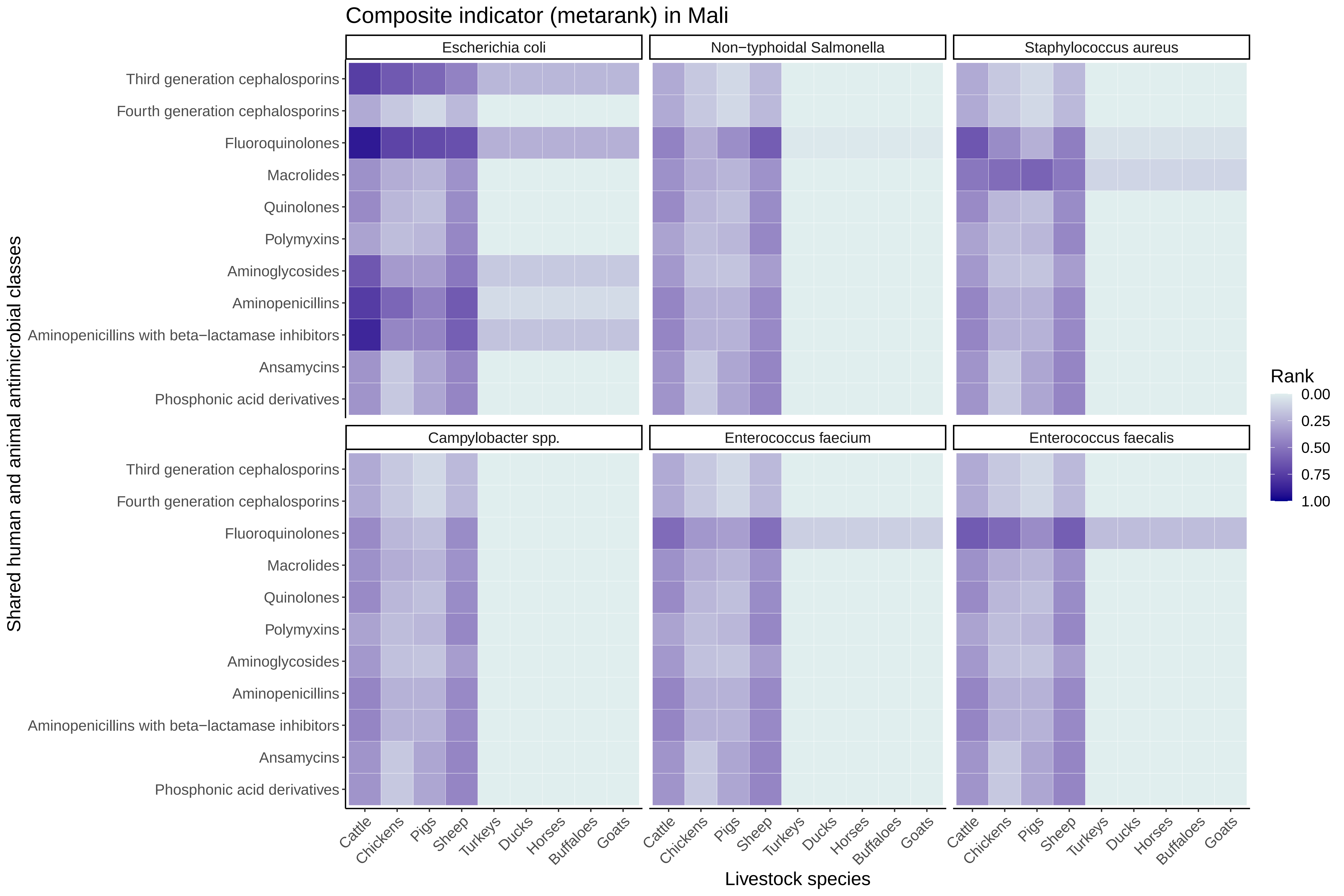

Composite indicator (metarank) in Mali
Escherichia coli
Non−typhoidal Salmonella
Staphylococcus aureus
Campylobacter spp.
Enterococcus faecium
Enterococcus faecalis
Third generation cephalosporins
Fourth generation cephalosporins
Fluoroquinolones
Macrolides
Quinolones
Polymyxins
Aminoglycosides
Aminopenicillins
Aminopenicillins with beta−lactamase inhibitors
Ansamycins
Phosphonic acid derivatives
Shared human and animal antimicrobial classes
Cattle
Chickens
Pigs
Sheep
Turkeys
Ducks
Horses
Buffaloes
Goats
Livestock species
Rank
0.00
0.25
0.50
0.75
1.00

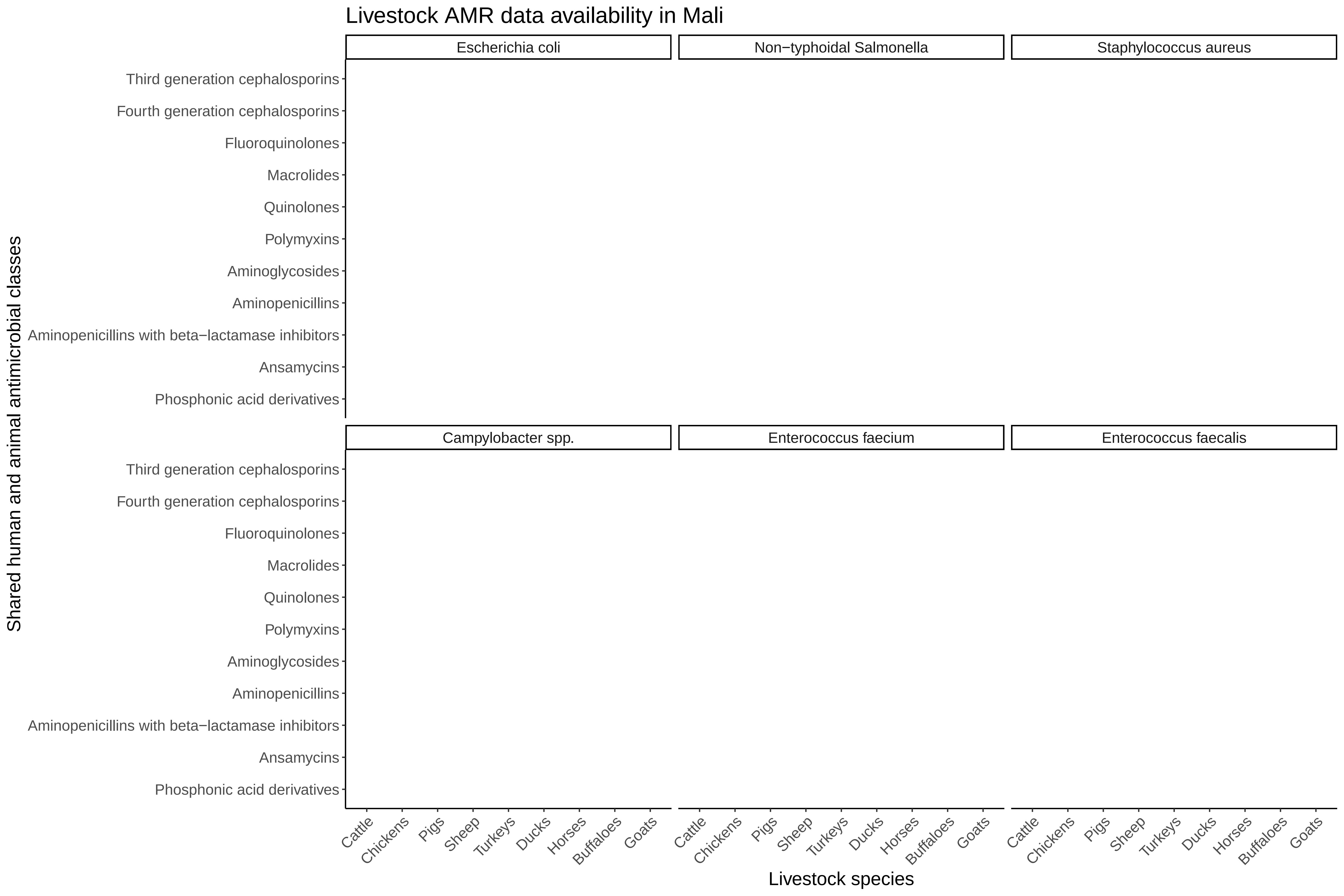

Livestock AMR data availability in Mali
Escherichia coli
Non−typhoidal Salmonella
Staphylococcus aureus
Campylobacter spp.
Enterococcus faecium
Enterococcus faecalis
Third generation cephalosporins
Fourth generation cephalosporins
Fluoroquinolones
Macrolides
Quinolones
Polymyxins
Aminoglycosides
Aminopenicillins
Aminopenicillins with beta−lactamase inhibitors
Ansamycins
Phosphonic acid derivatives
Shared human and animal antimicrobial classes
Cattle
Chickens
Pigs
Sheep
Turkeys
Ducks
Horses
Buffaloes
Goats
Livestock species

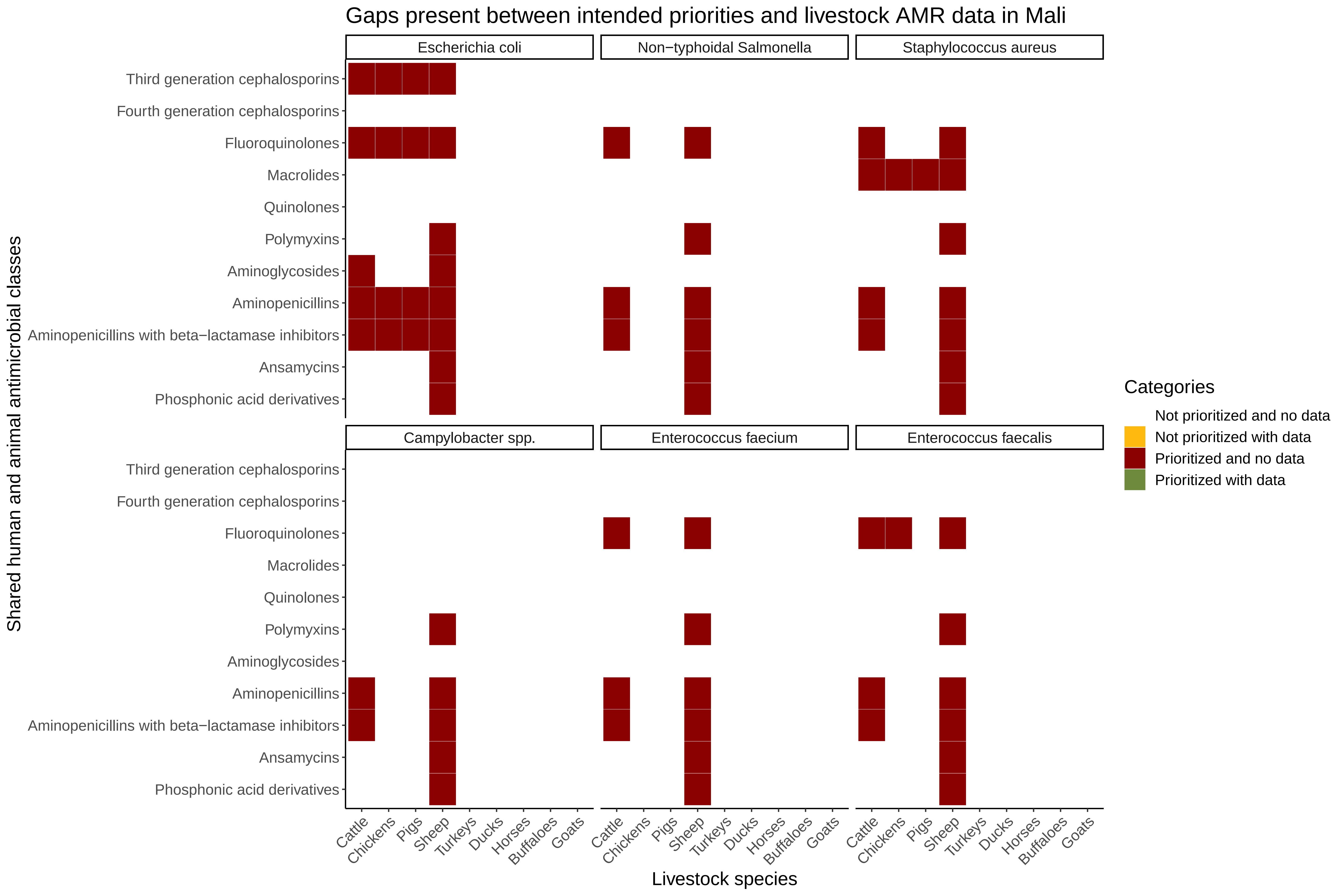

Gaps present between intended priorities and livestock AMR data in Mali
Escherichia coli
Non−typhoidal Salmonella
Staphylococcus aureus
Campylobacter spp.
Enterococcus faecium
Enterococcus faecalis
Third generation cephalosporins
Fourth generation cephalosporins
Fluoroquinolones
Macrolides
Quinolones
Polymyxins
Aminoglycosides
Aminopenicillins
Aminopenicillins with beta−lactamase inhibitors
Ansamycins
Phosphonic acid derivatives
Shared human and animal antimicrobial classes
Cattle
Chickens
Pigs
Sheep
Turkeys
Ducks
Horses
Buffaloes
Goats
Livestock species
Categories
Not prioritized and no data
Not prioritized with data
Prioritized and no data
Prioritized with data

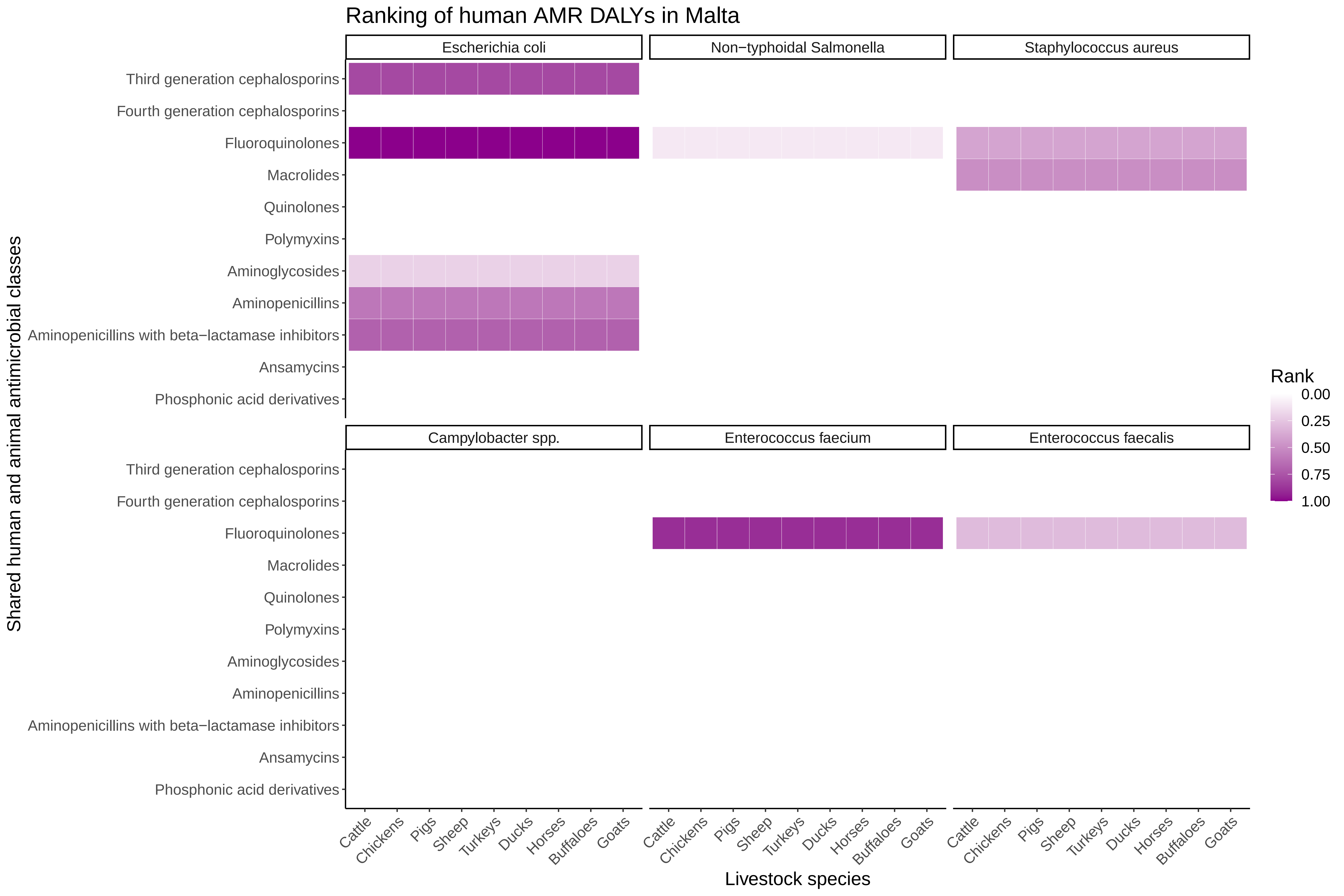

Ranking of human AMR DALYs in Malta
Escherichia coli
Non−typhoidal Salmonella
Staphylococcus aureus
Campylobacter spp.
Enterococcus faecium
Enterococcus faecalis
Shared human and animal antimicrobial classes
Third generation cephalosporins
Fourth generation cephalosporins
Fluoroquinolones
Macrolides
Quinolones
Polymyxins
Aminoglycosides
Aminopenicillins
Aminopenicillins with beta−lactamase inhibitors
Ansamycins
Phosphonic acid derivatives
Livestock species
Cattle
Chickens
Pigs
Sheep
Turkeys
Ducks
Horses
Buffaloes
Goats
Rank
0.00
0.25
0.50
0.75
1.00

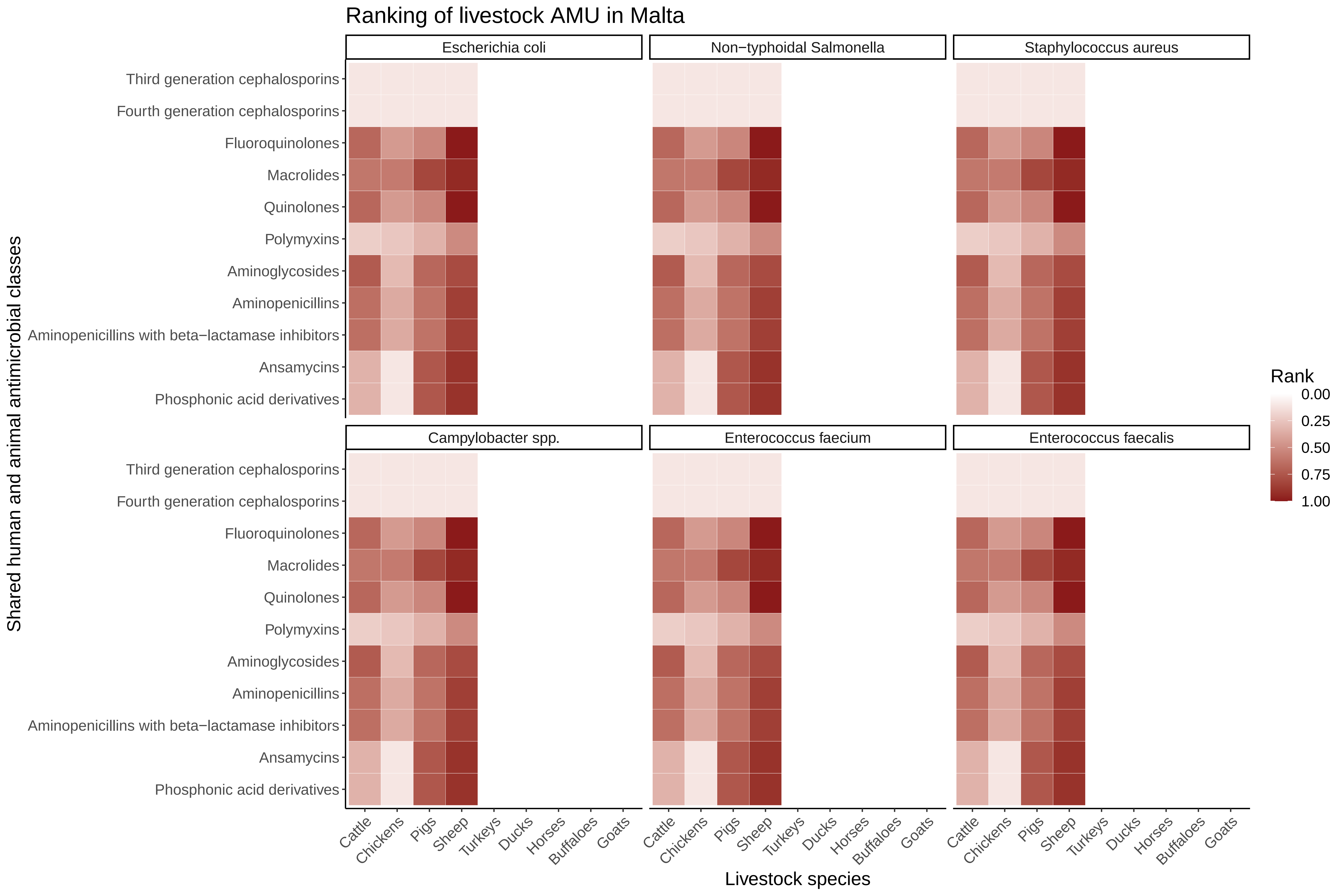

Ranking of livestock AMU in Malta
Escherichia coli
Non−typhoidal Salmonella
Staphylococcus aureus
Campylobacter spp.
Enterococcus faecium
Enterococcus faecalis
Third generation cephalosporins
Fourth generation cephalosporins
Fluoroquinolones
Macrolides
Quinolones
Polymyxins
Aminoglycosides
Aminopenicillins
Aminopenicillins with beta−lactamase inhibitors
Ansamycins
Phosphonic acid derivatives
Shared human and animal antimicrobial classes
Cattle
Chickens
Pigs
Sheep
Turkeys
Ducks
Horses
Buffaloes
Goats
Livestock species
Rank
0.00
0.25
0.50
0.75
1.00

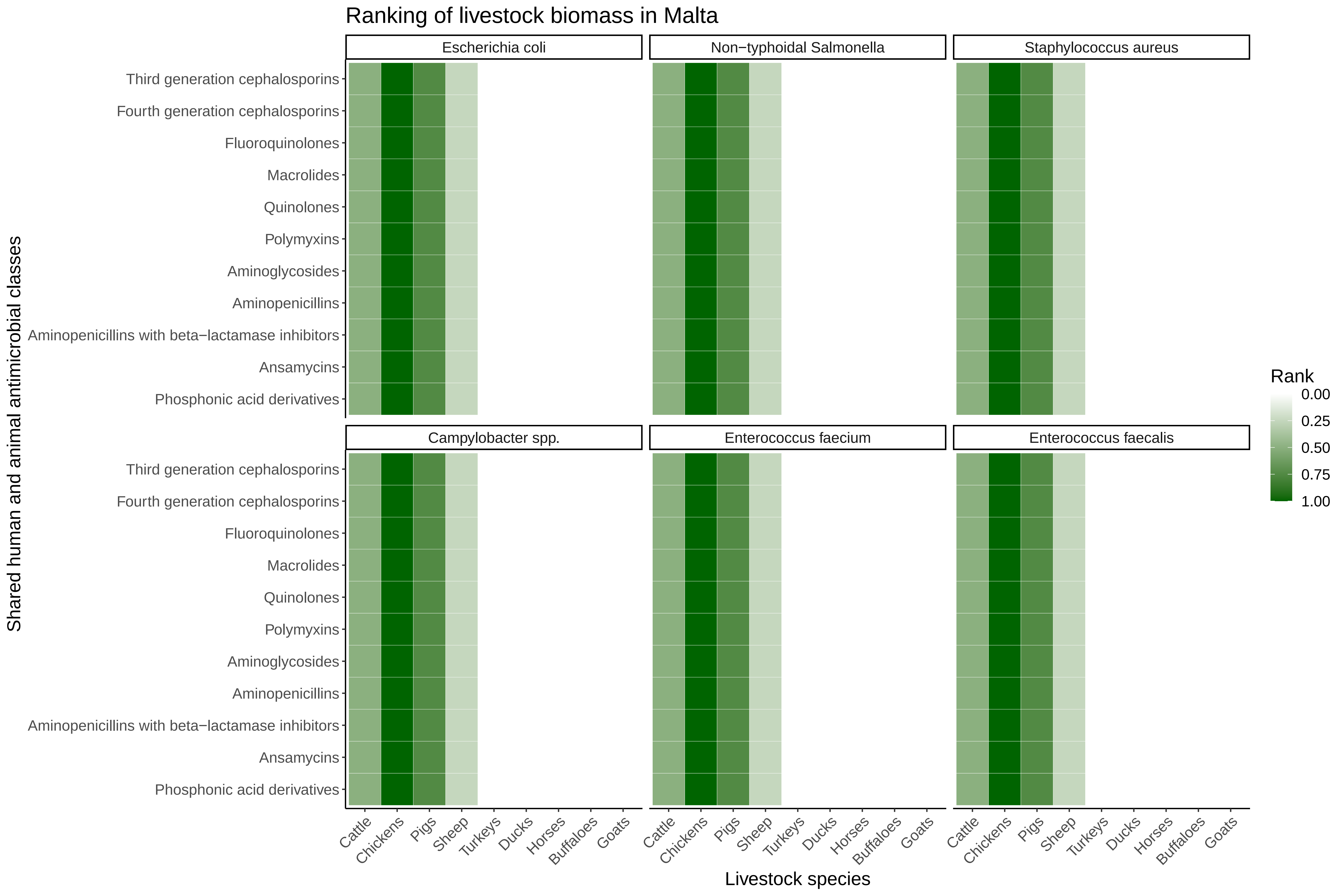

Ranking of livestock biomass in Malta
Escherichia coli
Non−typhoidal Salmonella
Staphylococcus aureus
Campylobacter spp.
Enterococcus faecium
Enterococcus faecalis
Third generation cephalosporins
Fourth generation cephalosporins
Fluoroquinolones
Macrolides
Quinolones
Polymyxins
Aminoglycosides
Aminopenicillins
Aminopenicillins with beta−lactamase inhibitors
Ansamycins
Phosphonic acid derivatives
Shared human and animal antimicrobial classes
Cattle
Chickens
Pigs
Sheep
Turkeys
Ducks
Horses
Buffaloes
Goats
Livestock species
Rank
0.00
0.25
0.50
0.75
1.00

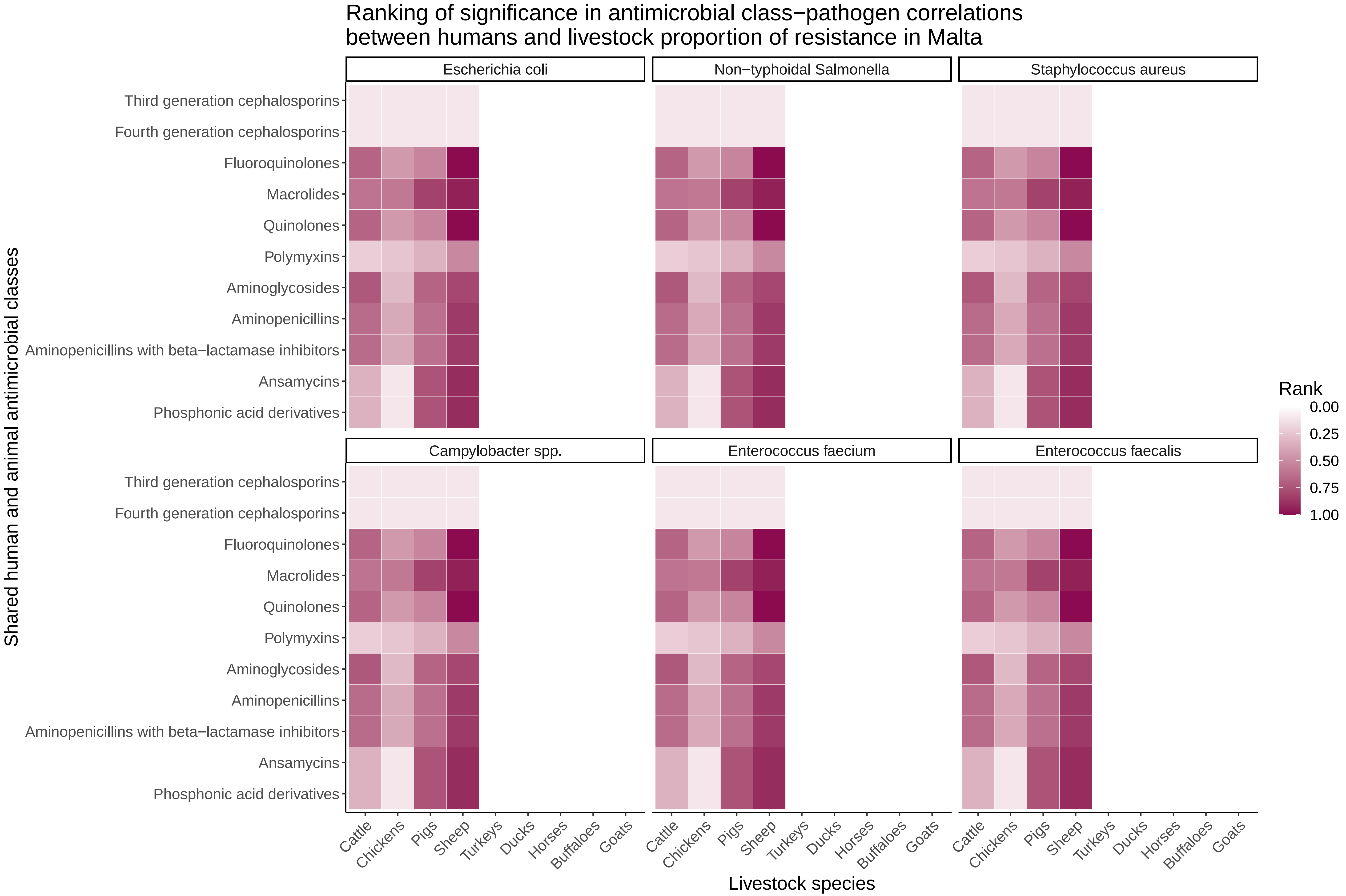

Ranking of significance in antimicrobial class−pathogen correlations
between humans and livestock proportion of resistance in Malta
Escherichia coli
Non−typhoidal Salmonella
Staphylococcus aureus
Campylobacter spp.
Enterococcus faecium
Enterococcus faecalis
Shared human and animal antimicrobial classes
Third generation cephalosporins
Fourth generation cephalosporins
Fluoroquinolones
Macrolides
Quinolones
Polymyxins
Aminoglycosides
Aminopenicillins
Aminopenicillins with beta−lactamase inhibitors
Ansamycins
Phosphonic acid derivatives
Livestock species
Cattle
Chickens
Pigs
Sheep
Turkeys
Ducks
Horses
Buffaloes
Goats
Rank
0.00
0.25
0.50
0.75
1.00

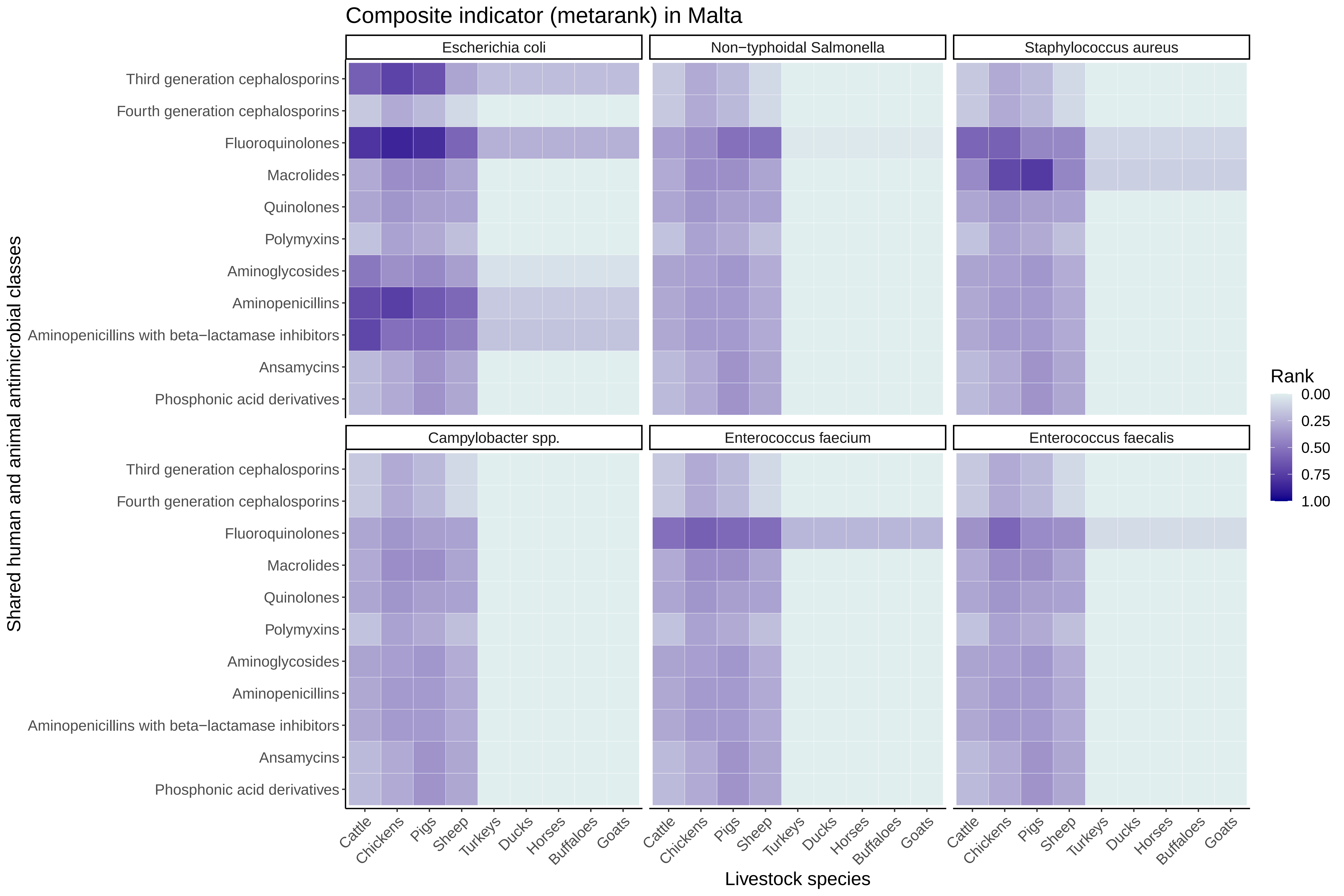

Composite indicator (metarank) in Malta
Escherichia coli
Non−typhoidal Salmonella
Staphylococcus aureus
Campylobacter spp.
Enterococcus faecium
Enterococcus faecalis
Third generation cephalosporins
Fourth generation cephalosporins
Fluoroquinolones
Macrolides
Quinolones
Polymyxins
Aminoglycosides
Aminopenicillins
Aminopenicillins with beta−lactamase inhibitors
Ansamycins
Phosphonic acid derivatives
Shared human and animal antimicrobial classes
Cattle
Chickens
Pigs
Sheep
Turkeys
Ducks
Horses
Buffaloes
Goats
Livestock species
Rank
0.00
0.25
0.50
0.75
1.00

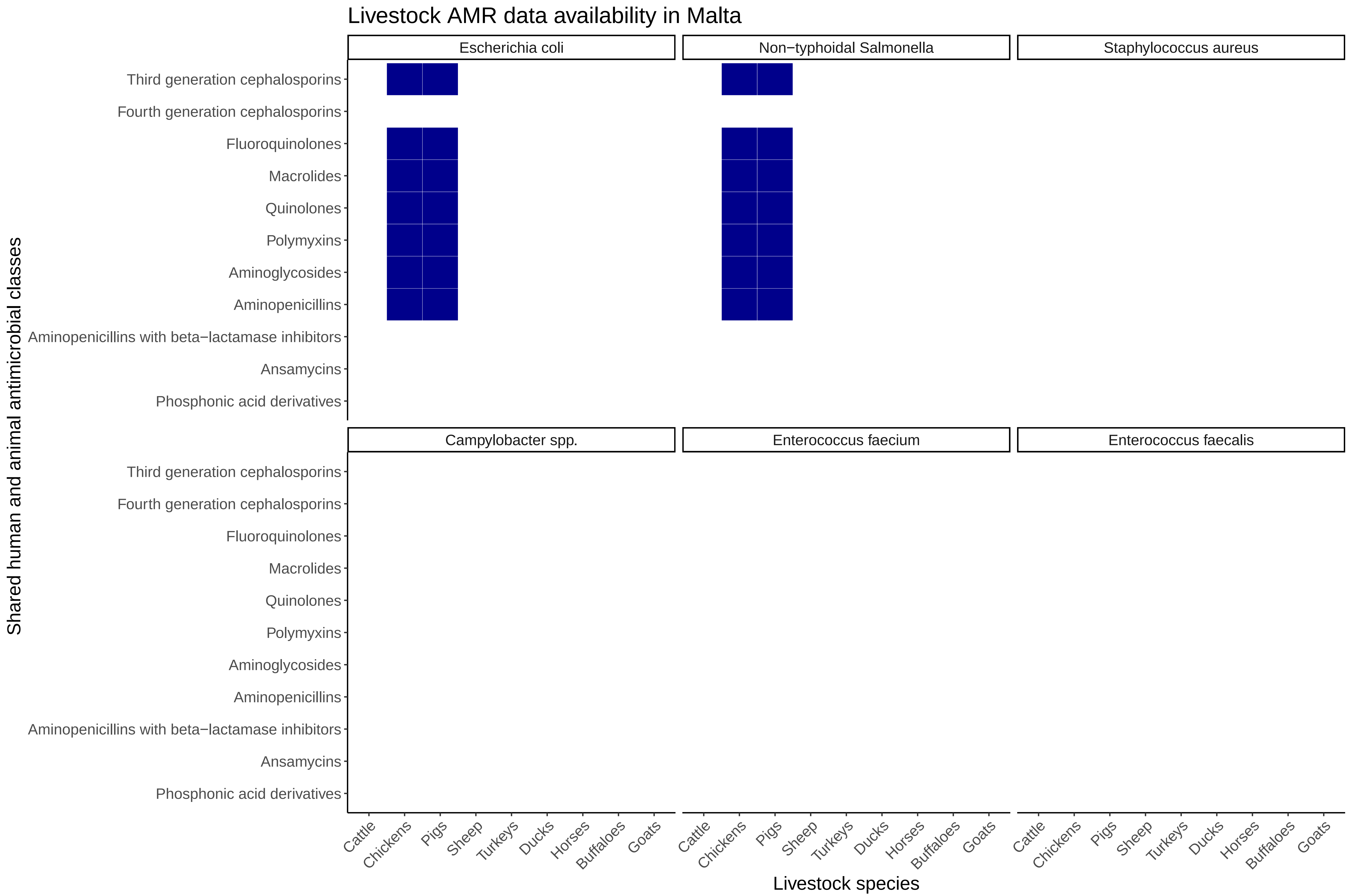

Livestock AMR data availability in Malta
Escherichia coli
Non−typhoidal Salmonella
Staphylococcus aureus
Campylobacter spp.
Enterococcus faecium
Enterococcus faecalis
Shared human and animal antimicrobial classes
Third generation cephalosporins
Fourth generation cephalosporins
Fluoroquinolones
Macrolides
Quinolones
Polymyxins
Aminoglycosides
Aminopenicillins
Aminopenicillins with beta−lactamase inhibitors
Ansamycins
Phosphonic acid derivatives
Cattle
Chickens
Pigs
Sheep
Turkeys
Ducks
Horses
Buffaloes
Goats
Livestock species

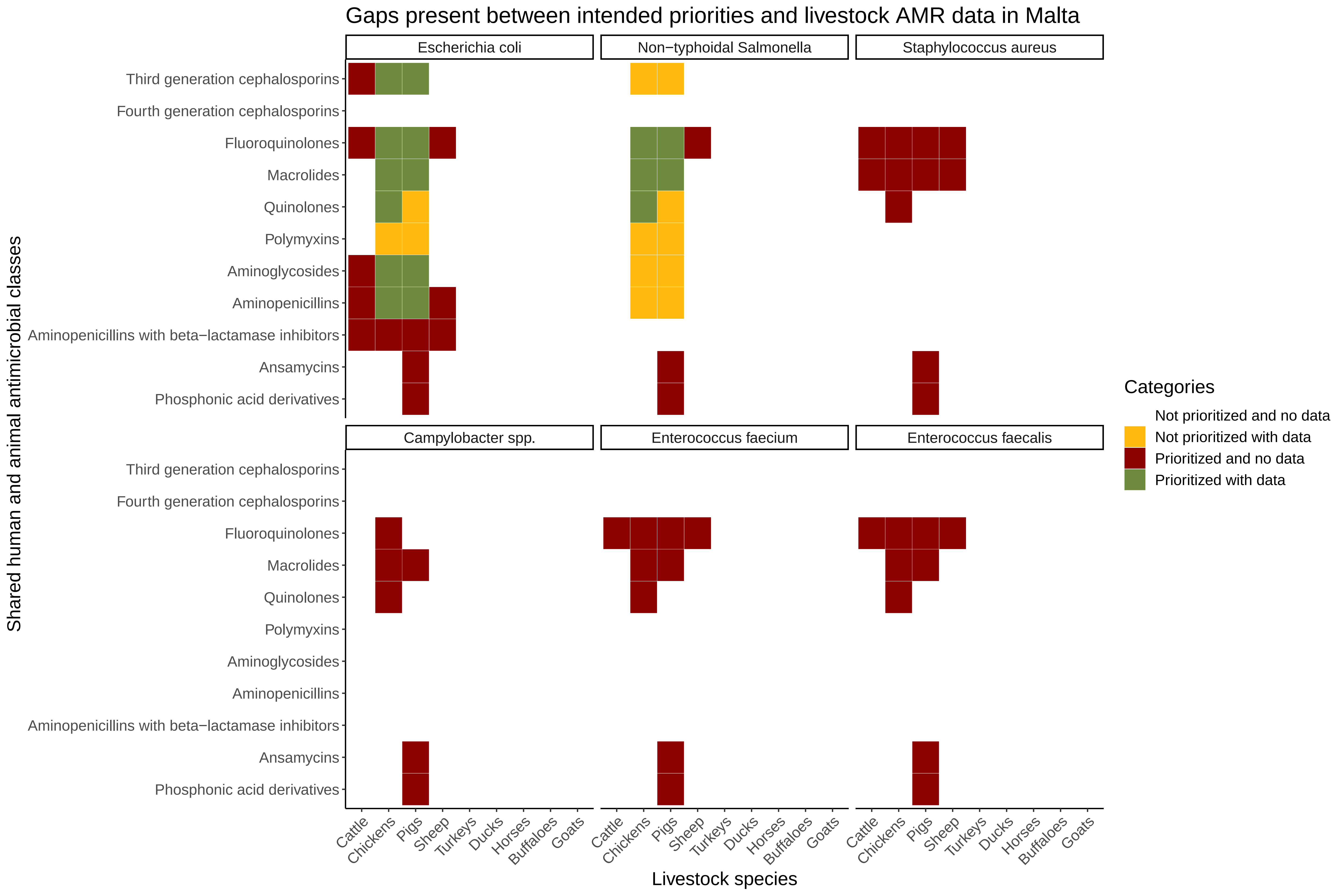
Gaps present between intended priorities and livestock AMR data in Malta
Escherichia coli
Non−typhoidal Salmonella
Staphylococcus aureus
Campylobacter spp.
Enterococcus faecium
Enterococcus faecalis
Third generation cephalosporins
Fourth generation cephalosporins
Fluoroquinolones
Macrolides
Quinolones
Polymyxins
Aminoglycosides
Aminopenicillins
Aminopenicillins with beta−lactamase inhibitors
Ansamycins
Phosphonic acid derivatives
Shared human and animal antimicrobial classes
Cattle
Chickens
Pigs
Sheep
Turkeys
Ducks
Horses
Buffaloes
Goats
Livestock species
Categories
Not prioritized and no data
Not prioritized with data
Prioritized and no data
Prioritized with data

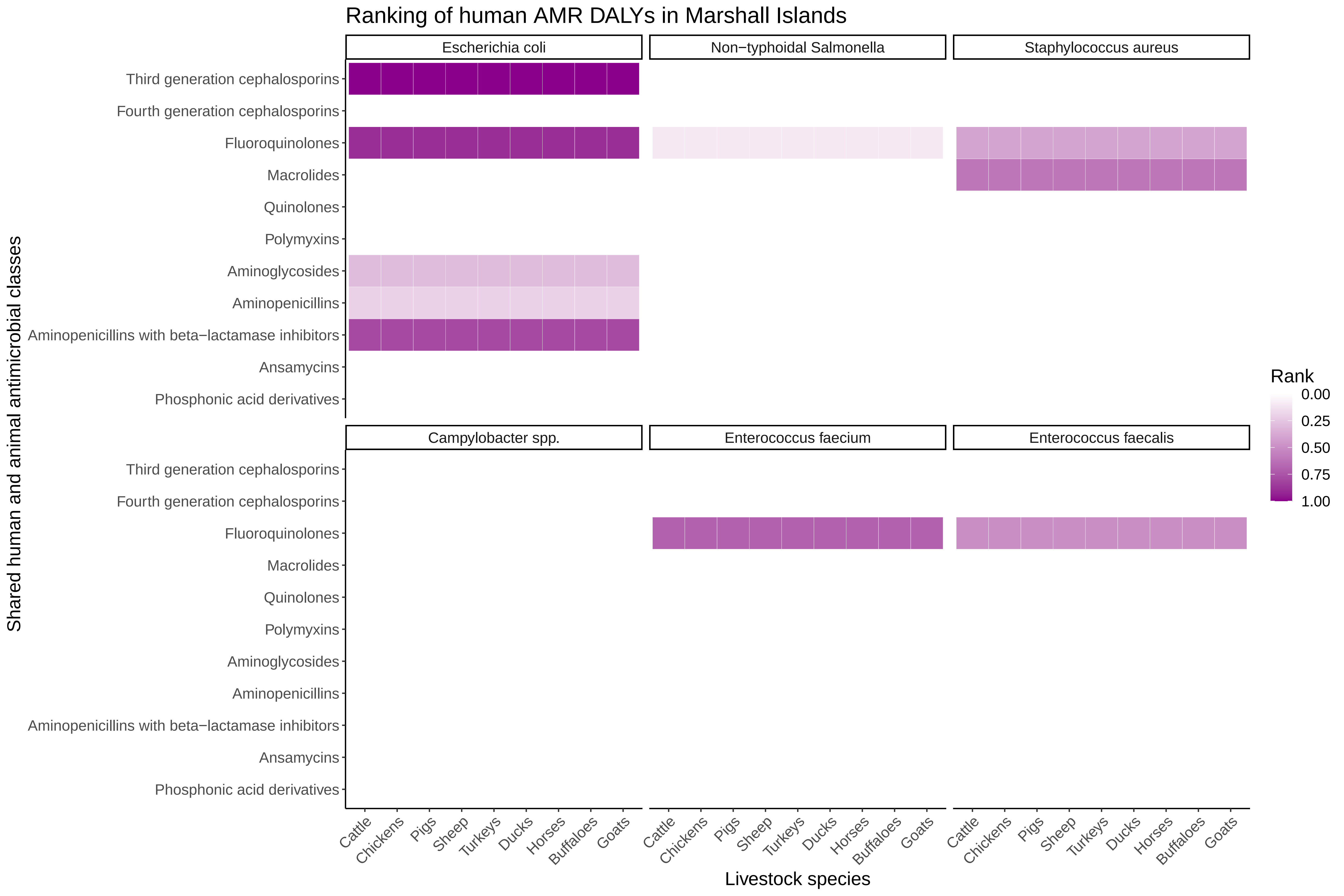

Ranking of human AMR DALYs in Marshall Islands
Escherichia coli
Non−typhoidal Salmonella
Staphylococcus aureus
Campylobacter spp.
Enterococcus faecium
Enterococcus faecalis
Third generation cephalosporins
Fourth generation cephalosporins
Fluoroquinolones
Macrolides
Quinolones
Polymyxins
Aminoglycosides
Aminopenicillins
Aminopenicillins with beta−lactamase inhibitors
Ansamycins
Phosphonic acid derivatives
Shared human and animal antimicrobial classes
Cattle
Chickens
Pigs
Sheep
Turkeys
Ducks
Horses
Buffaloes
Goats
Livestock species
Rank
0.00
0.25
0.50
0.75
1.00

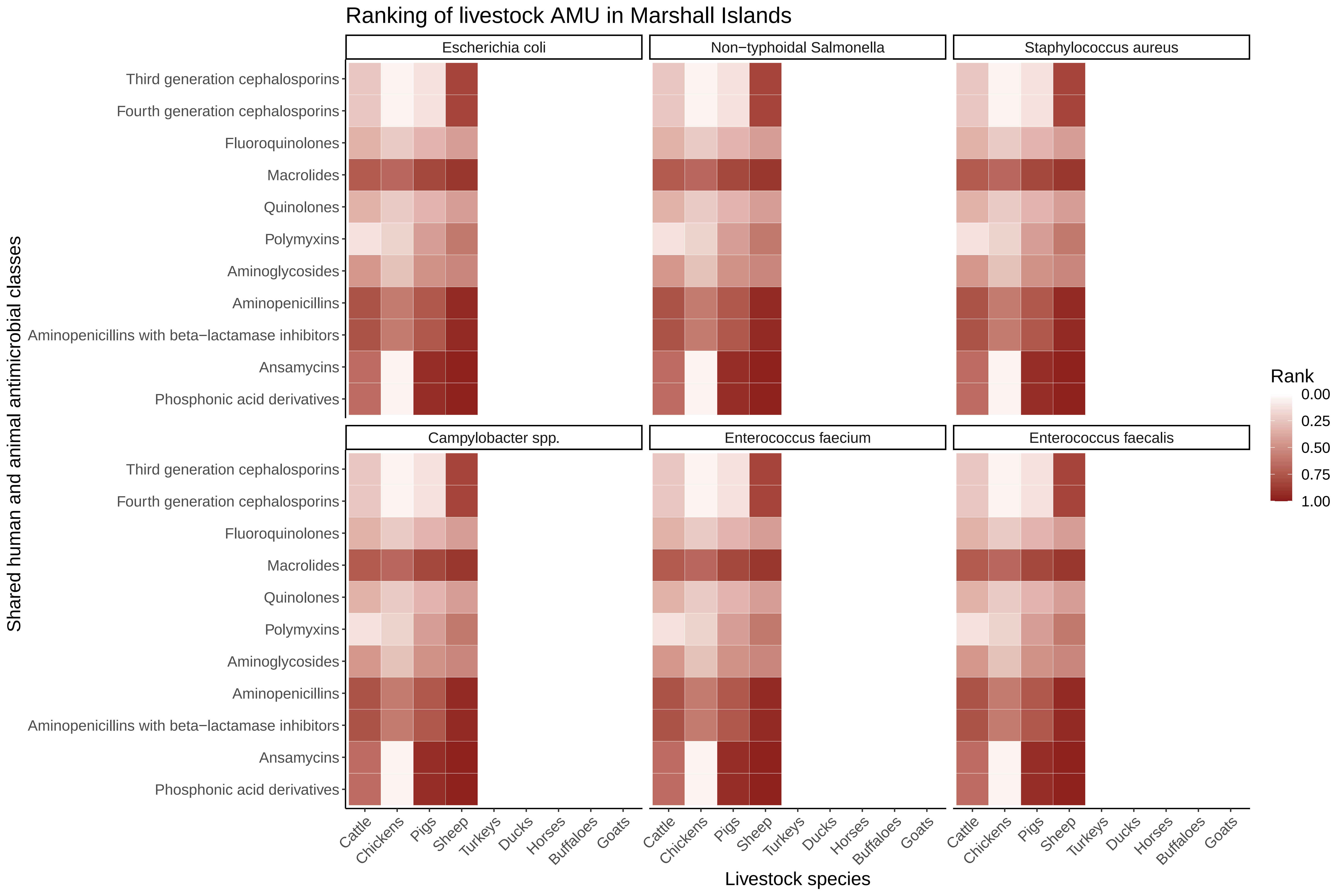

Ranking of livestock AMU in Marshall Islands
Escherichia coli
Non−typhoidal Salmonella
Staphylococcus aureus
Campylobacter spp.
Enterococcus faecium
Enterococcus faecalis
Third generation cephalosporins
Fourth generation cephalosporins
Fluoroquinolones
Macrolides
Quinolones
Polymyxins
Aminoglycosides
Aminopenicillins
Aminopenicillins with beta−lactamase inhibitors
Ansamycins
Phosphonic acid derivatives
Shared human and animal antimicrobial classes
Cattle
Chickens
Pigs
Sheep
Turkeys
Ducks
Horses
Buffaloes
Goats
Livestock species
Rank
0.00
0.25
0.50
0.75
1.00

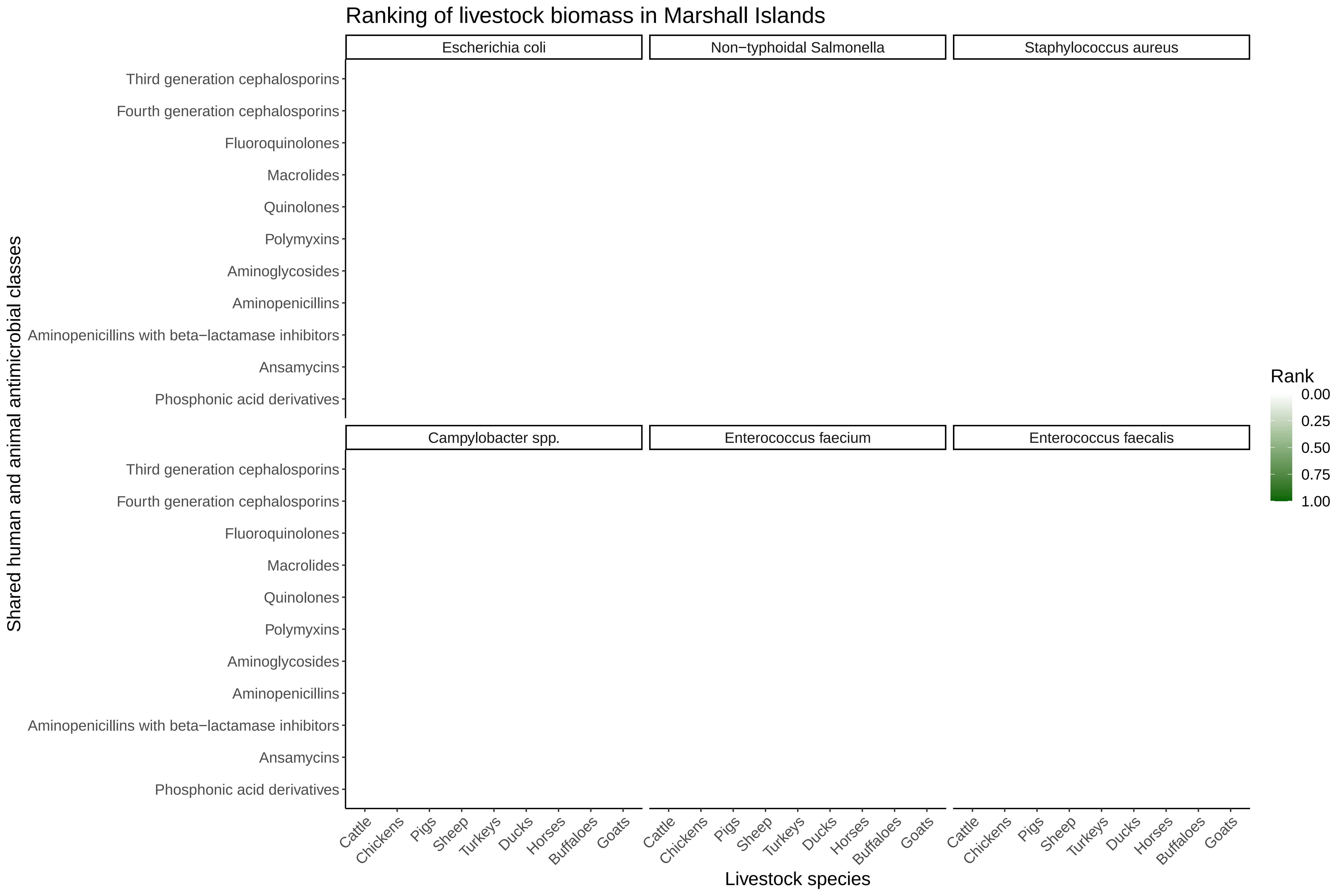
Ranking of livestock biomass in Marshall Islands
Escherichia coli
Non−typhoidal Salmonella
Staphylococcus aureus
Campylobacter spp.
Enterococcus faecium
Enterococcus faecalis
Third generation cephalosporins
Fourth generation cephalosporins
Fluoroquinolones
Macrolides
Quinolones
Polymyxins
Aminoglycosides
Aminopenicillins
Aminopenicillins with beta−lactamase inhibitors
Ansamycins
Phosphonic acid derivatives
Shared human and animal antimicrobial classes
Cattle
Chickens
Pigs
Sheep
Turkeys
Ducks
Horses
Buffaloes
Goats
Livestock species
Rank
0.00
0.25
0.50
0.75
1.00

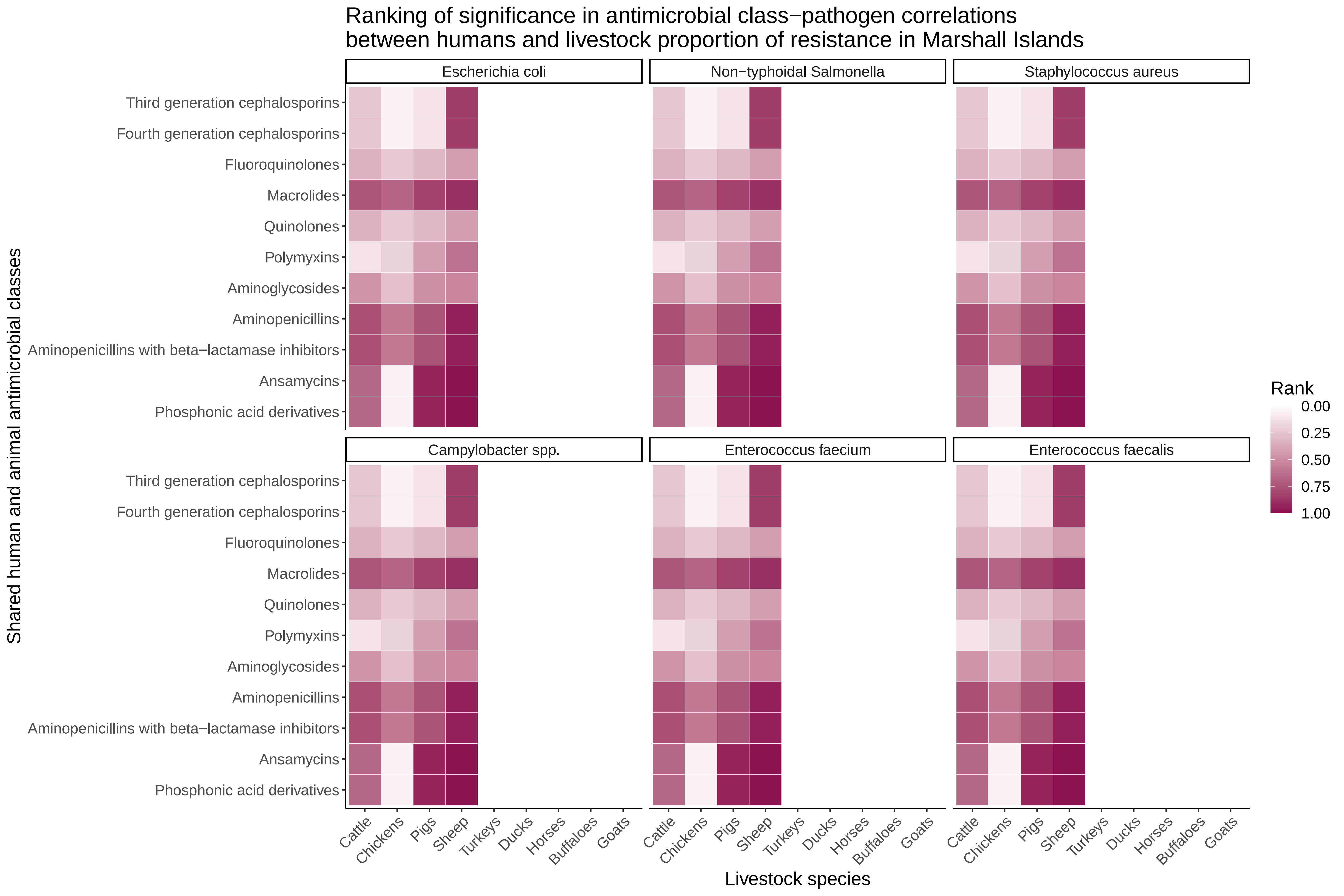

Ranking of significance in antimicrobial class−pathogen correlations
between humans and livestock proportion of resistance in Marshall Islands
Escherichia coli
Non−typhoidal Salmonella
Staphylococcus aureus
Campylobacter spp.
Enterococcus faecium
Enterococcus faecalis
Third generation cephalosporins
Fourth generation cephalosporins
Fluoroquinolones
Macrolides
Quinolones
Polymyxins
Aminoglycosides
Aminopenicillins
Aminopenicillins with beta−lactamase inhibitors
Ansamycins
Phosphonic acid derivatives
Shared human and animal antimicrobial classes
Cattle
Chickens
Pigs
Sheep
Turkeys
Ducks
Horses
Buffaloes
Goats
Livestock species
Rank
0.00
0.25
0.50
0.75
1.00

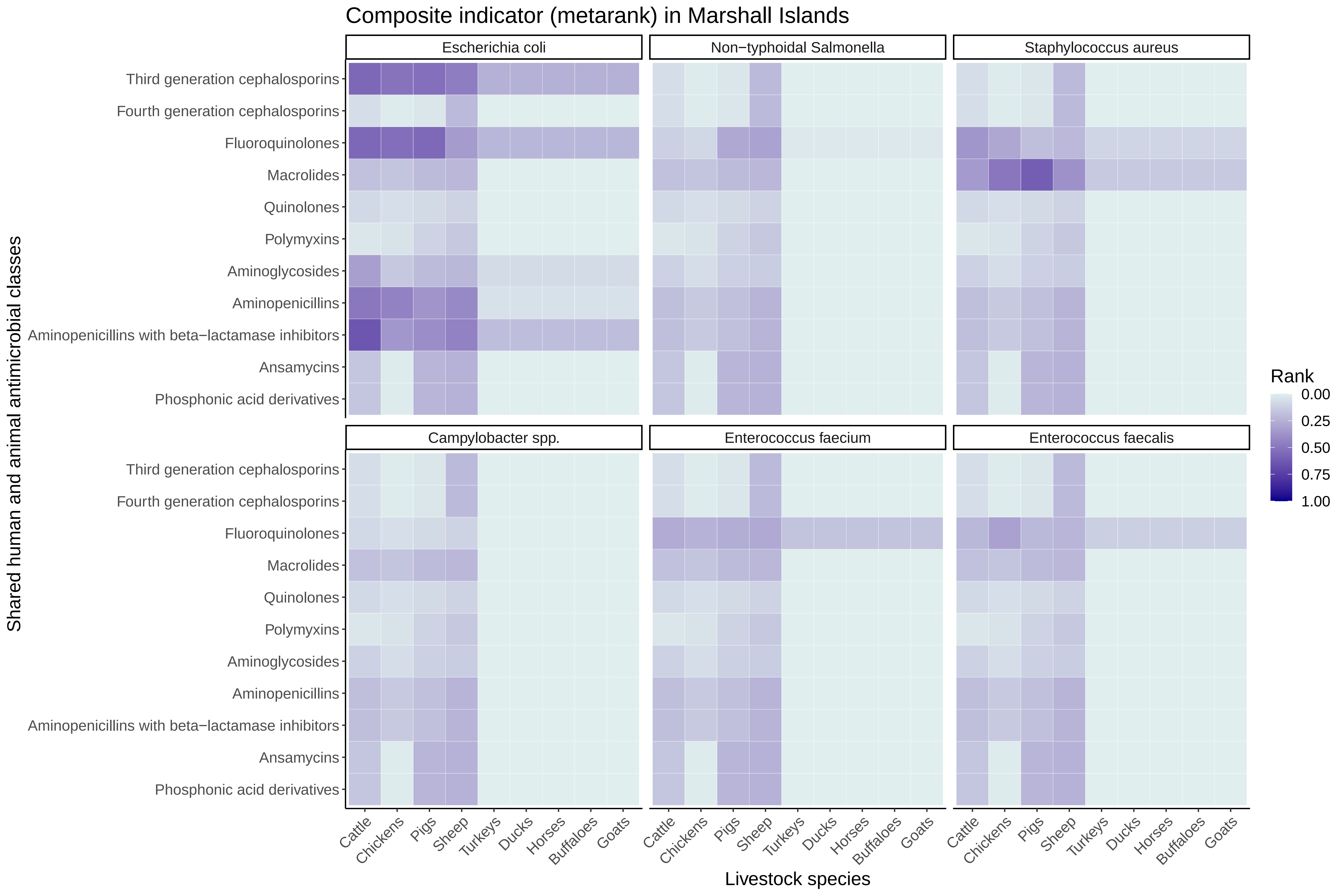

Composite indicator (metarank) in Marshall Islands
Escherichia coli
Non−typhoidal Salmonella
Staphylococcus aureus
Campylobacter spp.
Enterococcus faecium
Enterococcus faecalis
Third generation cephalosporins
Fourth generation cephalosporins
Fluoroquinolones
Macrolides
Quinolones
Polymyxins
Aminoglycosides
Aminopenicillins
Aminopenicillins with beta−lactamase inhibitors
Ansamycins
Phosphonic acid derivatives
Shared human and animal antimicrobial classes
Cattle
Chickens
Pigs
Sheep
Turkeys
Ducks
Horses
Buffaloes
Goats
Livestock species
Rank
0.00
0.25
0.50
0.75
1.00

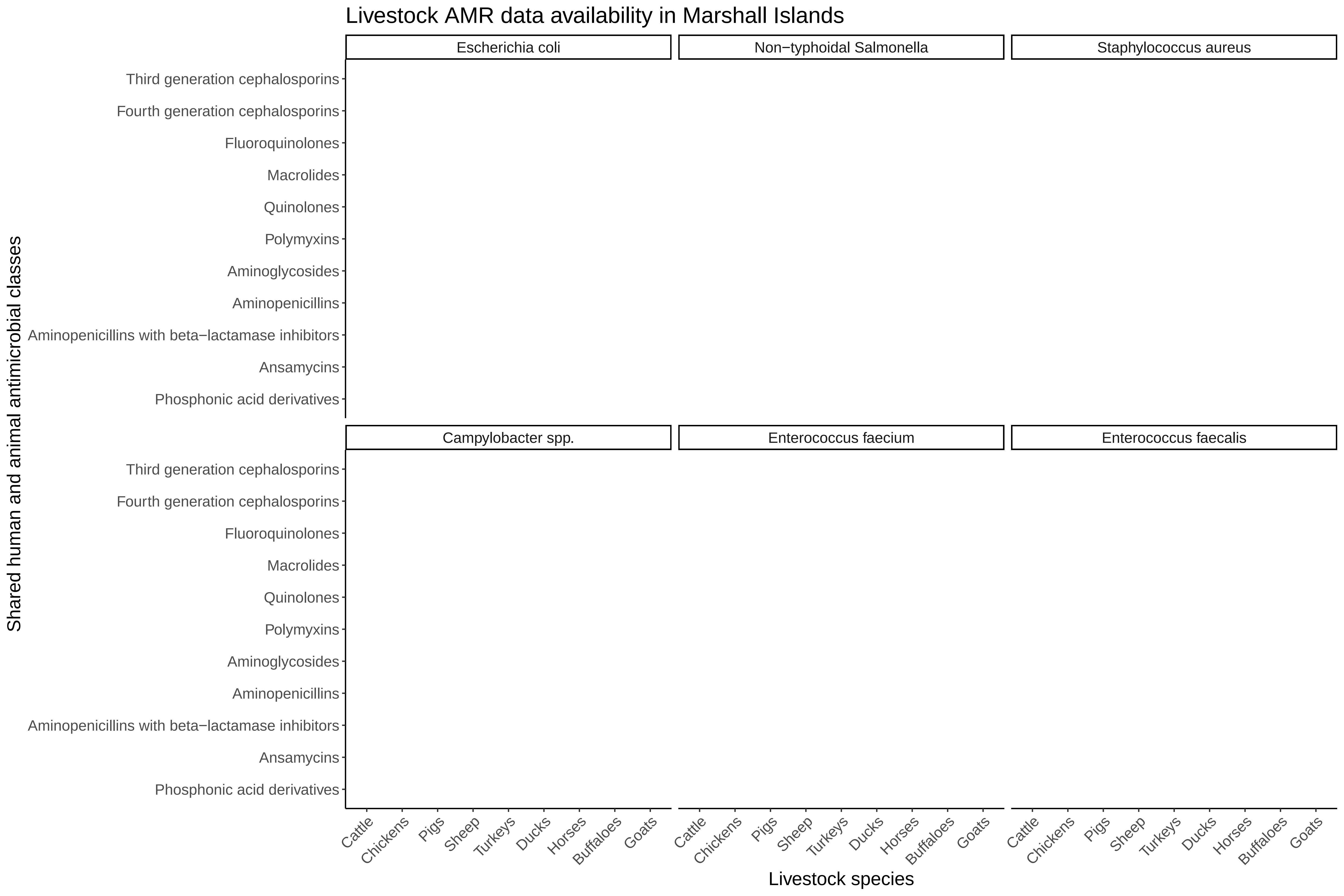
Livestock AMR data availability in Marshall Islands
Escherichia coli
Non−typhoidal Salmonella
Staphylococcus aureus
Campylobacter spp.
Enterococcus faecium
Enterococcus faecalis
Third generation cephalosporins
Fourth generation cephalosporins
Fluoroquinolones
Macrolides
Quinolones
Polymyxins
Aminoglycosides
Aminopenicillins
Aminopenicillins with beta−lactamase inhibitors
Ansamycins
Phosphonic acid derivatives
Shared human and animal antimicrobial classes
Cattle
Chickens
Pigs
Sheep
Turkeys
Ducks
Horses
Buffaloes
Goats
Livestock species

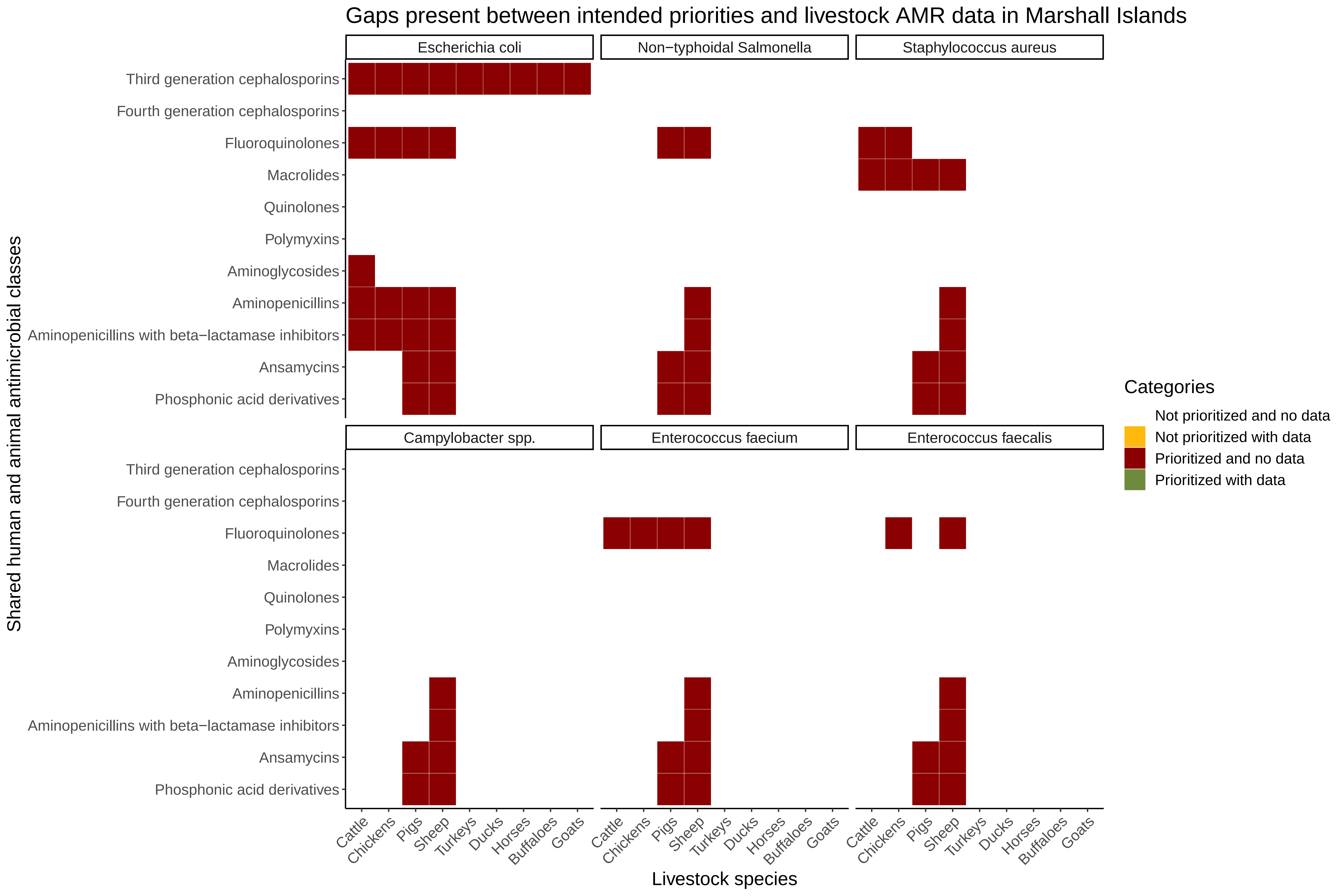

Gaps present between intended priorities and livestock AMR data in Marshall Islands
Escherichia coli
Non−typhoidal Salmonella
Staphylococcus aureus
Campylobacter spp.
Enterococcus faecium
Enterococcus faecalis
Third generation cephalosporins
Fourth generation cephalosporins
Fluoroquinolones
Macrolides
Quinolones
Polymyxins
Aminoglycosides
Aminopenicillins
Aminopenicillins with beta−lactamase inhibitors
Ansamycins
Phosphonic acid derivatives
Shared human and animal antimicrobial classes
Cattle
Chickens
Pigs
Sheep
Turkeys
Ducks
Horses
Buffaloes
Goats
Livestock species
Categories
Not prioritized and no data
Not prioritized with data
Prioritized and no data
Prioritized with data

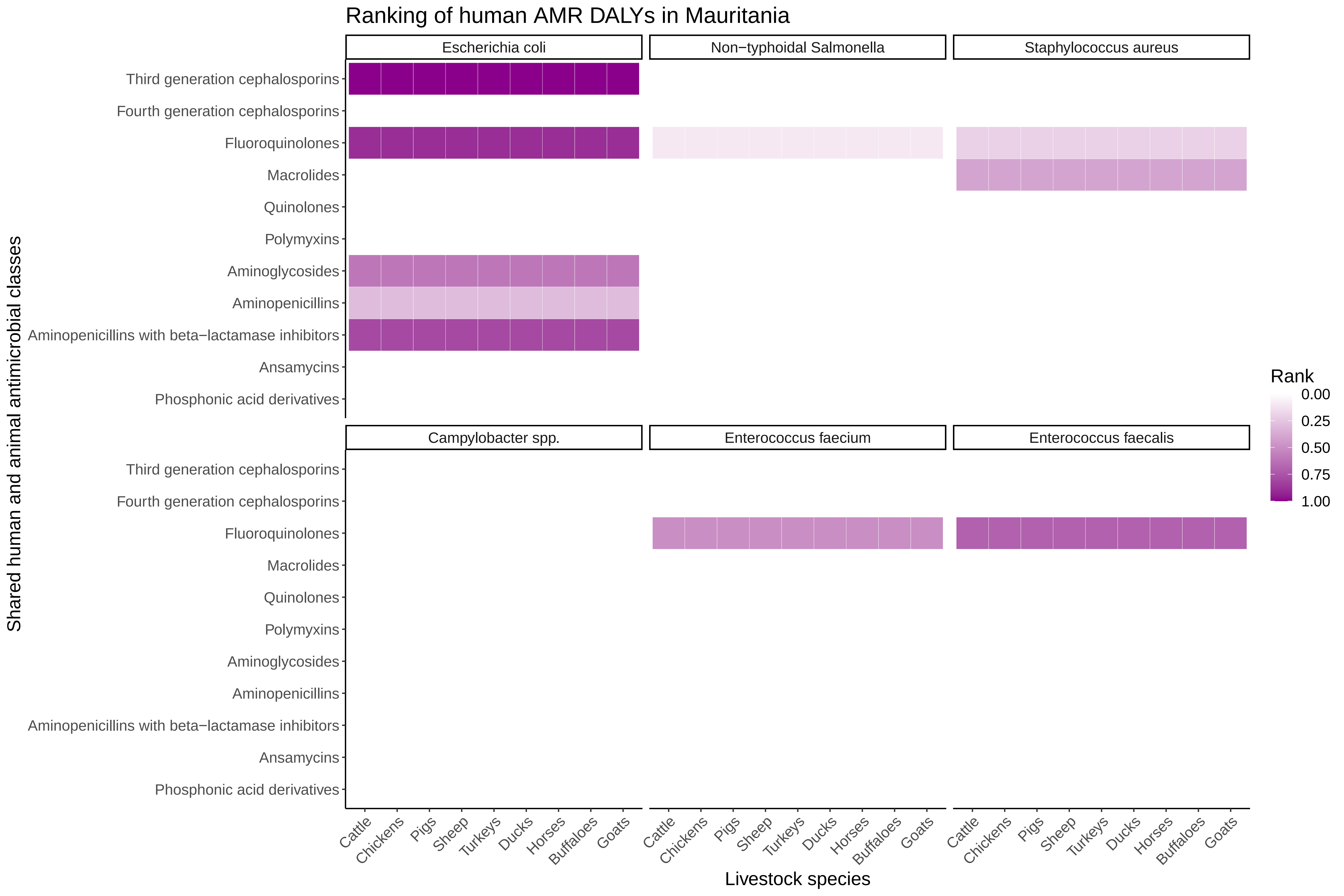

Ranking of human AMR DALYs in Mauritania
Escherichia coli
Non−typhoidal Salmonella
Staphylococcus aureus
Campylobacter spp.
Enterococcus faecium
Enterococcus faecalis
Shared human and animal antimicrobial classes
Third generation cephalosporins
Fourth generation cephalosporins
Fluoroquinolones
Macrolides
Quinolones
Polymyxins
Aminoglycosides
Aminopenicillins
Aminopenicillins with beta−lactamase inhibitors
Ansamycins
Phosphonic acid derivatives
Livestock species
Cattle
Chickens
Pigs
Sheep
Turkeys
Ducks
Horses
Buffaloes
Goats
Rank
0.00
0.25
0.50
0.75
1.00

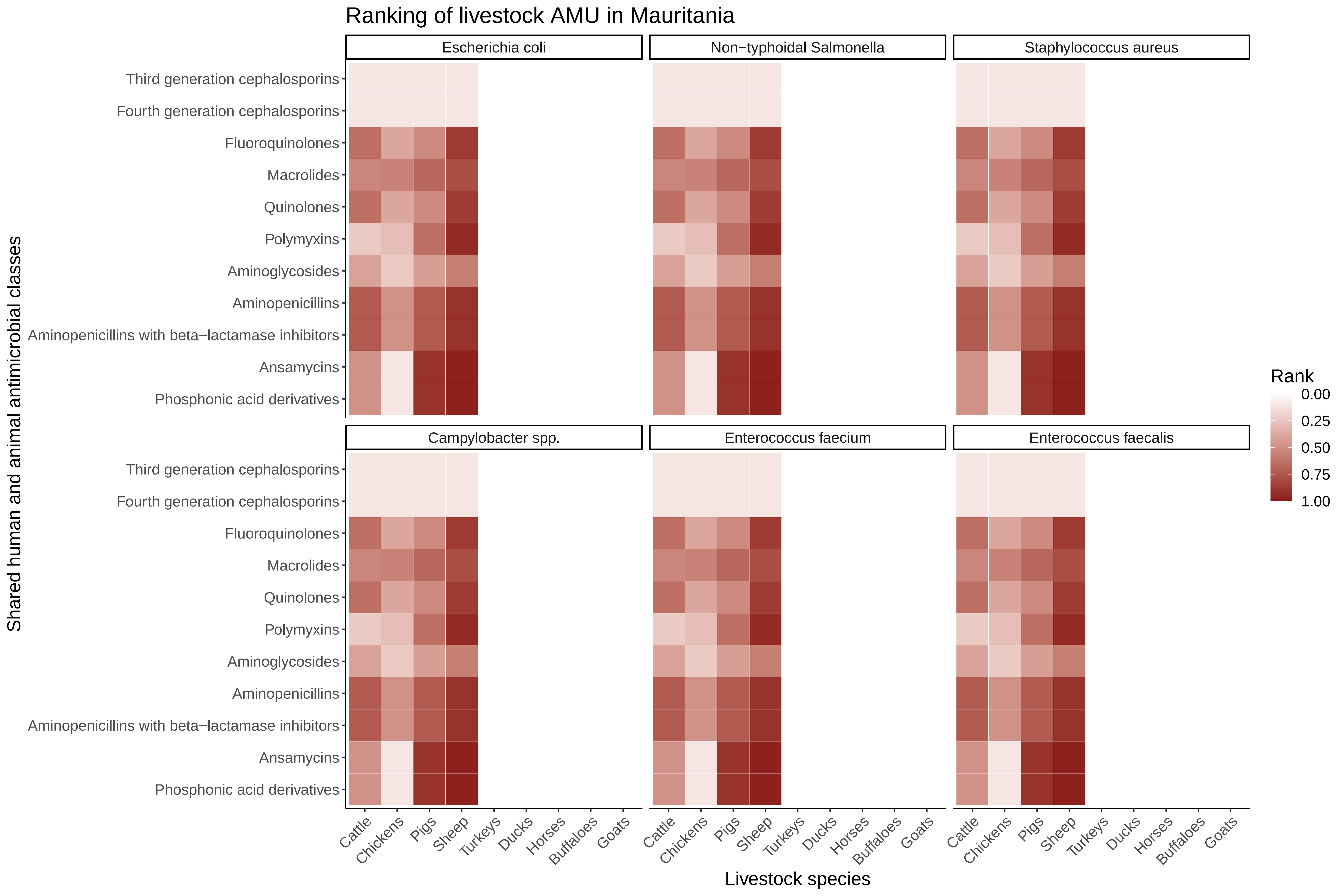

Ranking of livestock AMU in Mauritania
Escherichia coli
Non−typhoidal Salmonella
Staphylococcus aureus
Campylobacter spp.
Enterococcus faecium
Enterococcus faecalis
Third generation cephalosporins
Fourth generation cephalosporins
Fluoroquinolones
Macrolides
Quinolones
Polymyxins
Aminoglycosides
Aminopenicillins
Aminopenicillins with beta−lactamase inhibitors
Ansamycins
Phosphonic acid derivatives
Shared human and animal antimicrobial classes
Cattle
Chickens
Pigs
Sheep
Turkeys
Ducks
Horses
Buffaloes
Goats
Livestock species
Rank
0.00
0.25
0.50
0.75
1.00

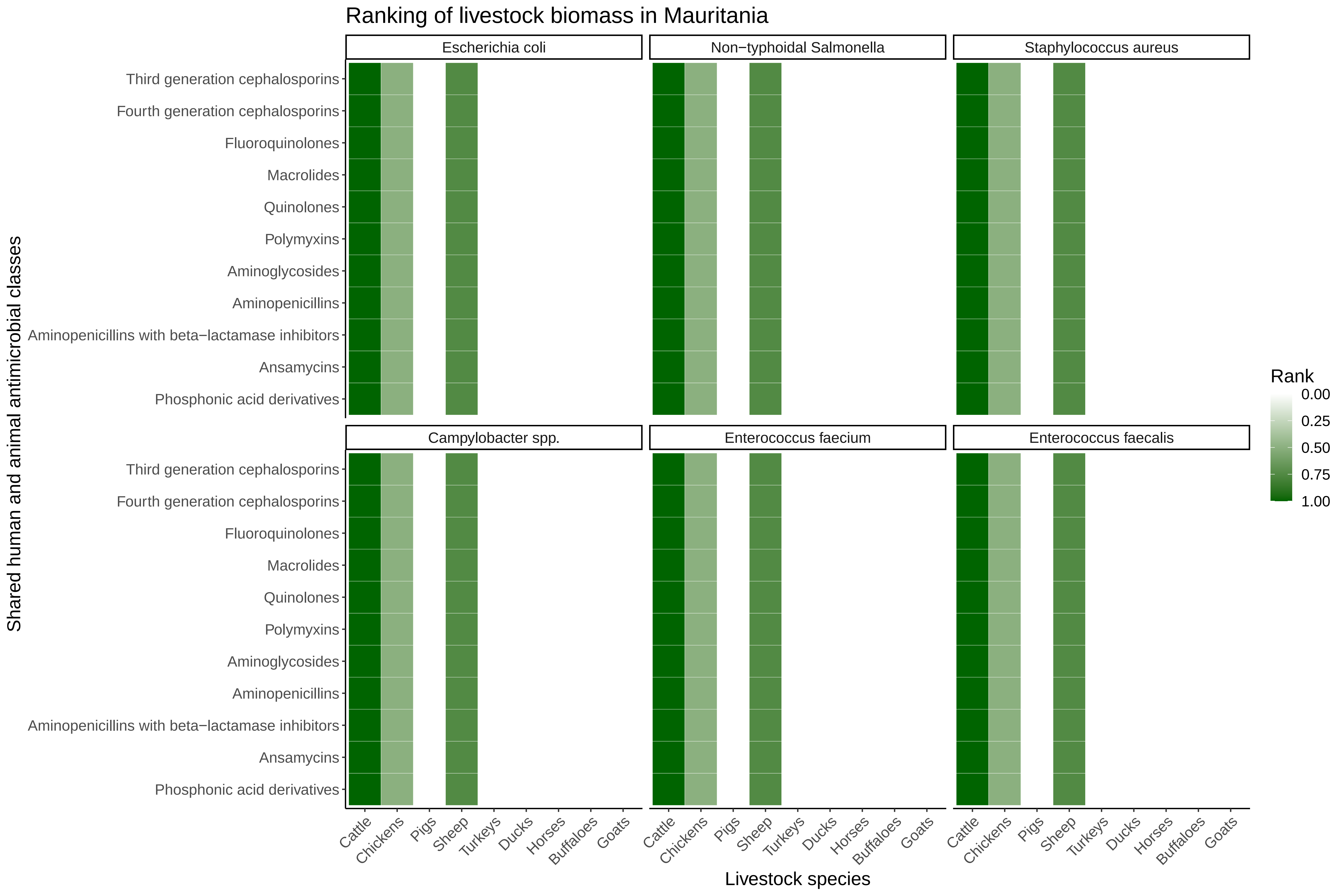

Ranking of livestock biomass in Mauritania
Escherichia coli
Non−typhoidal Salmonella
Staphylococcus aureus
Campylobacter spp.
Enterococcus faecium
Enterococcus faecalis
Third generation cephalosporins
Fourth generation cephalosporins
Fluoroquinolones
Macrolides
Quinolones
Polymyxins
Aminoglycosides
Aminopenicillins
Aminopenicillins with beta−lactamase inhibitors
Ansamycins
Phosphonic acid derivatives
Shared human and animal antimicrobial classes
Cattle
Chickens
Pigs
Sheep
Turkeys
Ducks
Horses
Buffaloes
Goats
Livestock species
Rank
0.00
0.25
0.50
0.75
1.00

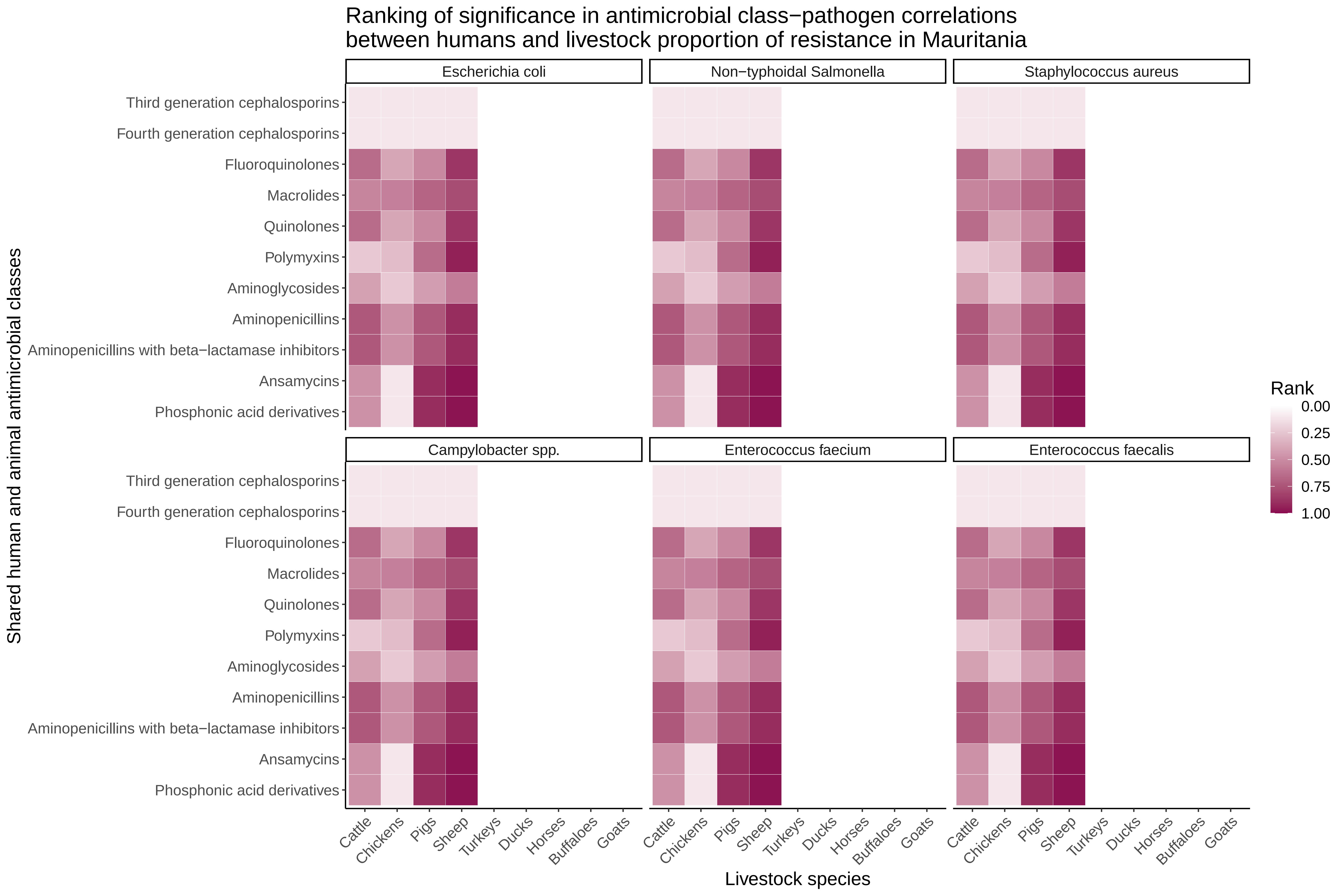
Ranking of significance in antimicrobial class−pathogen correlations
between humans and livestock proportion of resistance in Mauritania
Escherichia coli
Non−typhoidal Salmonella
Staphylococcus aureus
Campylobacter spp.
Enterococcus faecium
Enterococcus faecalis
Third generation cephalosporins
Fourth generation cephalosporins
Fluoroquinolones
Macrolides
Quinolones
Polymyxins
Aminoglycosides
Aminopenicillins
Aminopenicillins with beta−lactamase inhibitors
Ansamycins
Phosphonic acid derivatives
Shared human and animal antimicrobial classes
Cattle
Chickens
Pigs
Sheep
Turkeys
Ducks
Horses
Buffaloes
Goats
Livestock species
Rank
0.00
0.25
0.50
0.75
1.00

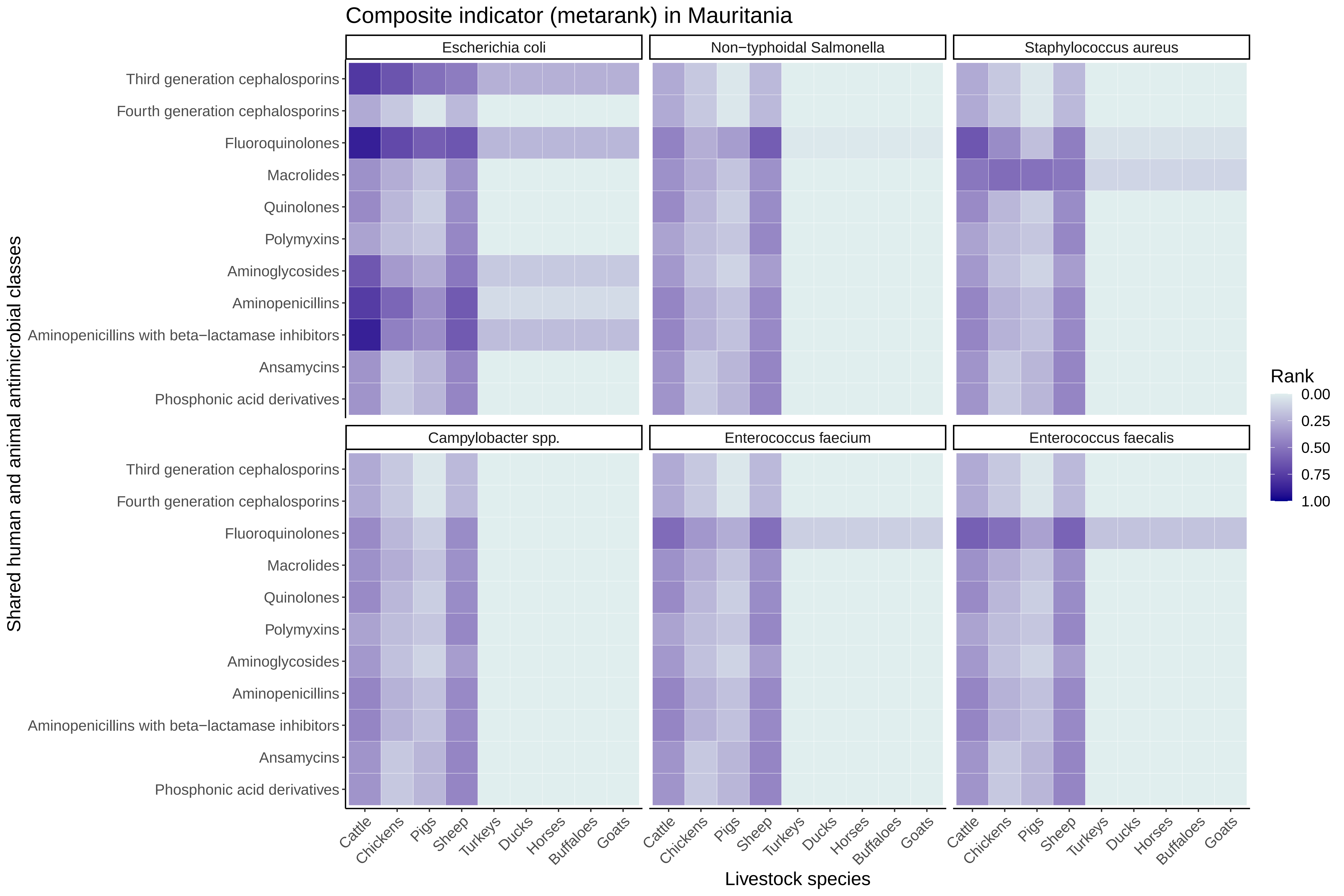
Composite indicator (metarank) in Mauritania
Escherichia coli
Non−typhoidal Salmonella
Staphylococcus aureus
Campylobacter spp.
Enterococcus faecium
Enterococcus faecalis
Shared human and animal antimicrobial classes
Third generation cephalosporins
Fourth generation cephalosporins
Fluoroquinolones
Macrolides
Quinolones
Polymyxins
Aminoglycosides
Aminopenicillins
Aminopenicillins with beta−lactamase inhibitors
Ansamycins
Phosphonic acid derivatives
Cattle
Chickens
Pigs
Sheep
Turkeys
Ducks
Horses
Buffaloes
Goats
Livestock species
Rank
0.00
0.25
0.50
0.75
1.00

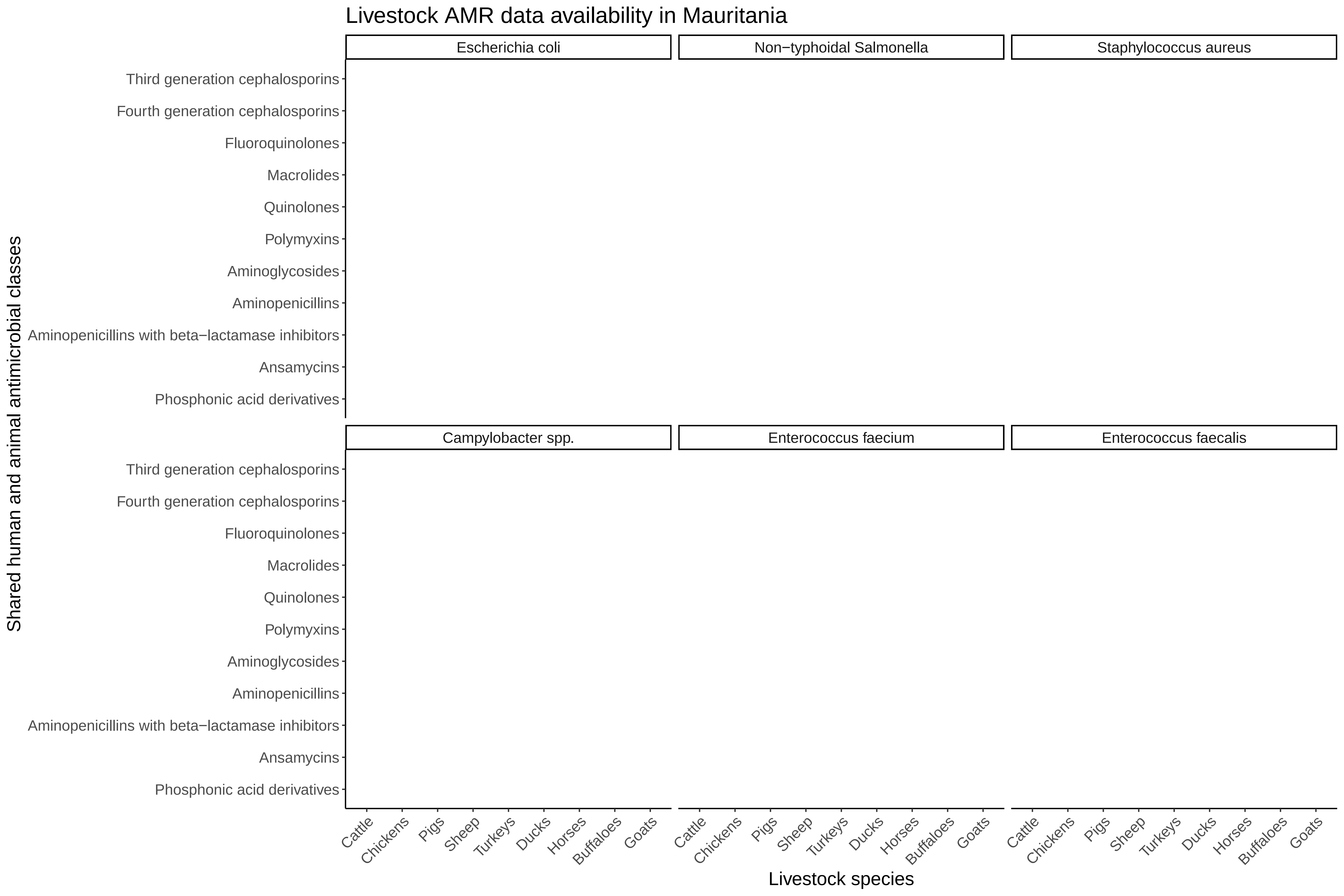
Livestock AMR data availability in Mauritania
Escherichia coli
Non−typhoidal Salmonella
Staphylococcus aureus
Campylobacter spp.
Enterococcus faecium
Enterococcus faecalis
Shared human and animal antimicrobial classes
Third generation cephalosporins
Fourth generation cephalosporins
Fluoroquinolones
Macrolides
Quinolones
Polymyxins
Aminoglycosides
Aminopenicillins
Aminopenicillins with beta−lactamase inhibitors
Ansamycins
Phosphonic acid derivatives
Cattle
Chickens
Pigs
Sheep
Turkeys
Ducks
Horses
Buffaloes
Goats
Livestock species

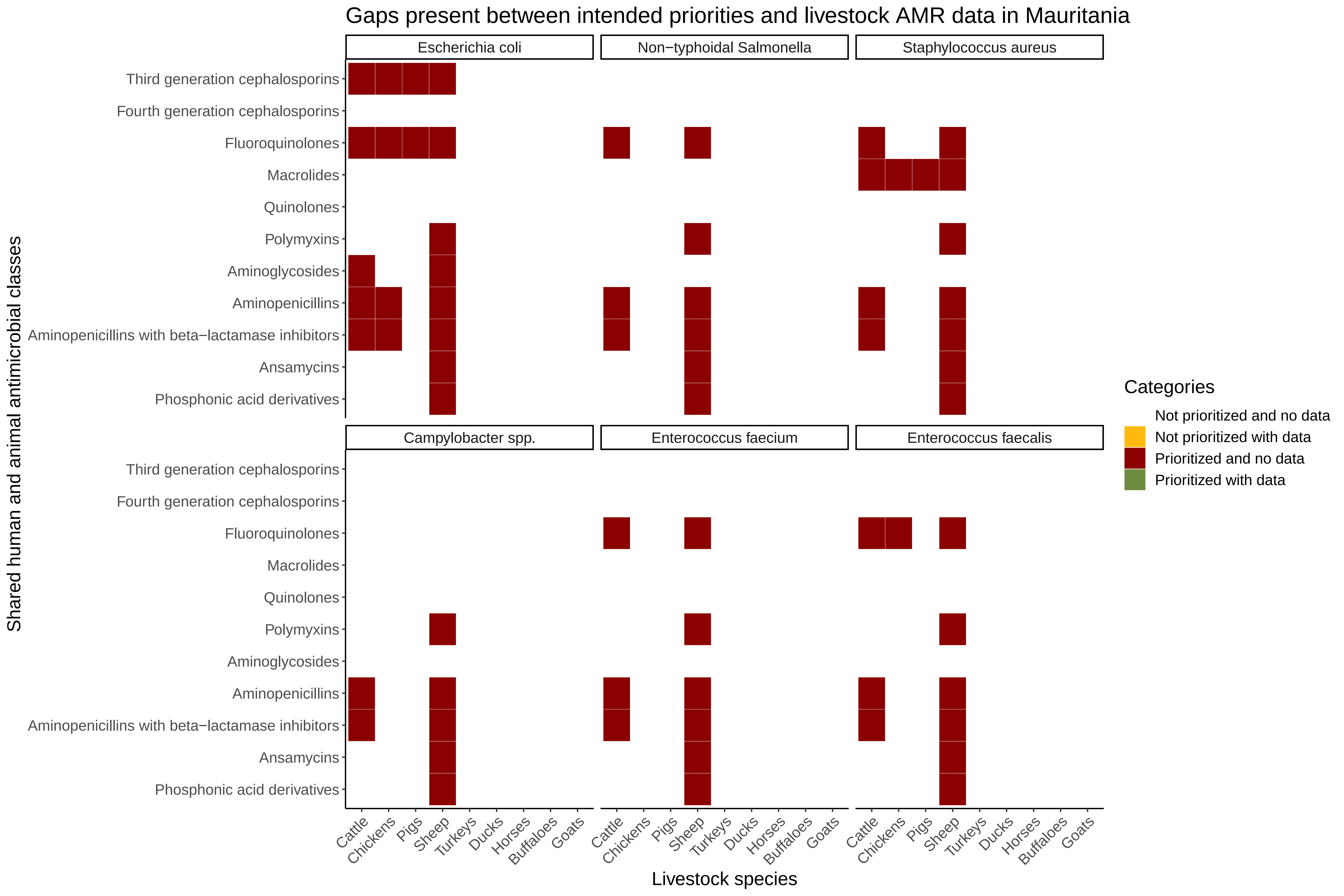

Gaps present between intended priorities and livestock AMR data in Mauritania
Escherichia coli
Non−typhoidal Salmonella
Staphylococcus aureus
Campylobacter spp.
Enterococcus faecium
Enterococcus faecalis
Third generation cephalosporins
Fourth generation cephalosporins
Fluoroquinolones
Macrolides
Quinolones
Polymyxins
Aminoglycosides
Aminopenicillins
Aminopenicillins with beta−lactamase inhibitors
Ansamycins
Phosphonic acid derivatives
Shared human and animal antimicrobial classes
Cattle
Chickens
Pigs
Sheep
Turkeys
Ducks
Horses
Buffaloes
Goats
Livestock species
Categories
Not prioritized and no data
Not prioritized with data
Prioritized and no data
Prioritized with data

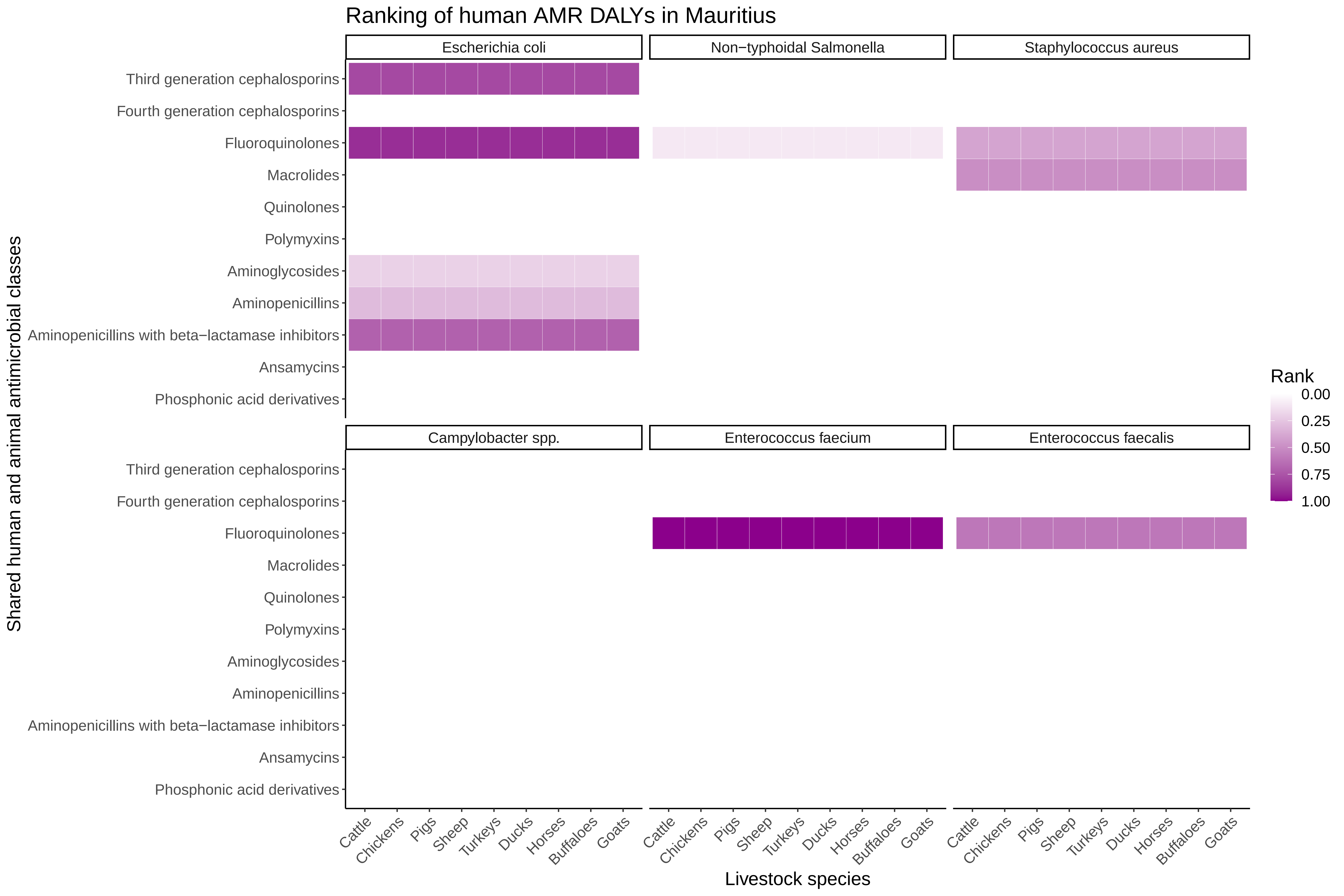

Ranking of human AMR DALYs in Mauritius
Escherichia coli
Non−typhoidal Salmonella
Staphylococcus aureus
Campylobacter spp.
Enterococcus faecium
Enterococcus faecalis
Third generation cephalosporins
Fourth generation cephalosporins
Fluoroquinolones
Macrolides
Quinolones
Polymyxins
Aminoglycosides
Aminopenicillins
Aminopenicillins with beta−lactamase inhibitors
Ansamycins
Phosphonic acid derivatives
Shared human and animal antimicrobial classes
Cattle
Chickens
Pigs
Sheep
Turkeys
Ducks
Horses
Buffaloes
Goats
Livestock species
Rank
0.00
0.25
0.50
0.75
1.00

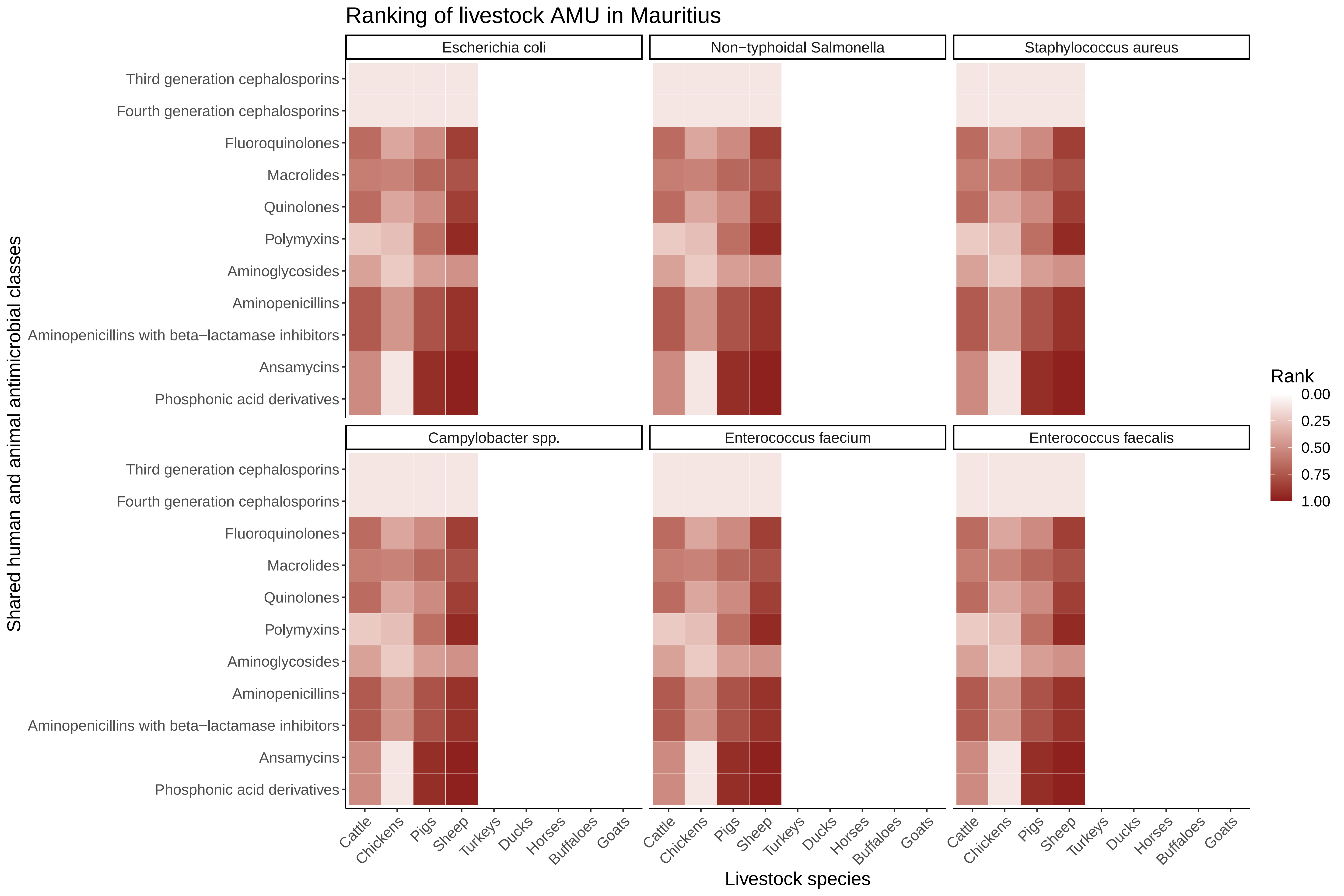

Ranking of livestock AMU in Mauritius
Escherichia coli
Non−typhoidal Salmonella
Staphylococcus aureus
Campylobacter spp.
Enterococcus faecium
Enterococcus faecalis
Shared human and animal antimicrobial classes
Third generation cephalosporins
Fourth generation cephalosporins
Fluoroquinolones
Macrolides
Quinolones
Polymyxins
Aminoglycosides
Aminopenicillins
Aminopenicillins with beta−lactamase inhibitors
Ansamycins
Phosphonic acid derivatives
Cattle
Chickens
Pigs
Sheep
Turkeys
Ducks
Horses
Buffaloes
Goats
Livestock species
Rank
0.00
0.25
0.50
0.75
1.00

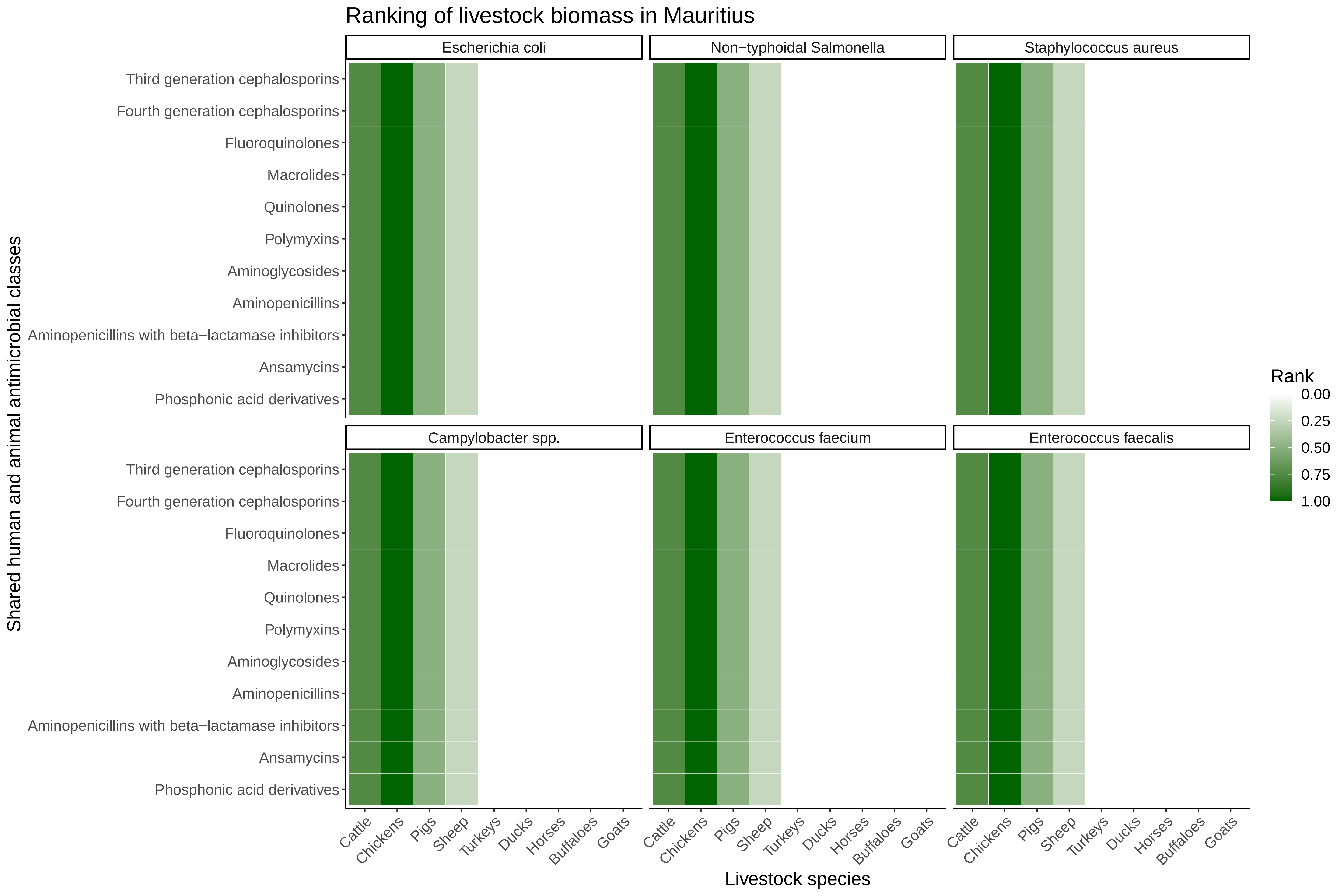

Ranking of livestock biomass in Mauritius
Escherichia coli
Non−typhoidal Salmonella
Staphylococcus aureus
Campylobacter spp.
Enterococcus faecium
Enterococcus faecalis
Shared human and animal antimicrobial classes
Third generation cephalosporins
Fourth generation cephalosporins
Fluoroquinolones
Macrolides
Quinolones
Polymyxins
Aminoglycosides
Aminopenicillins
Aminopenicillins with beta−lactamase inhibitors
Ansamycins
Phosphonic acid derivatives
Livestock species
Cattle
Chickens
Pigs
Sheep
Turkeys
Ducks
Horses
Buffaloes
Goats
Rank
0.00
0.25
0.50
0.75
1.00

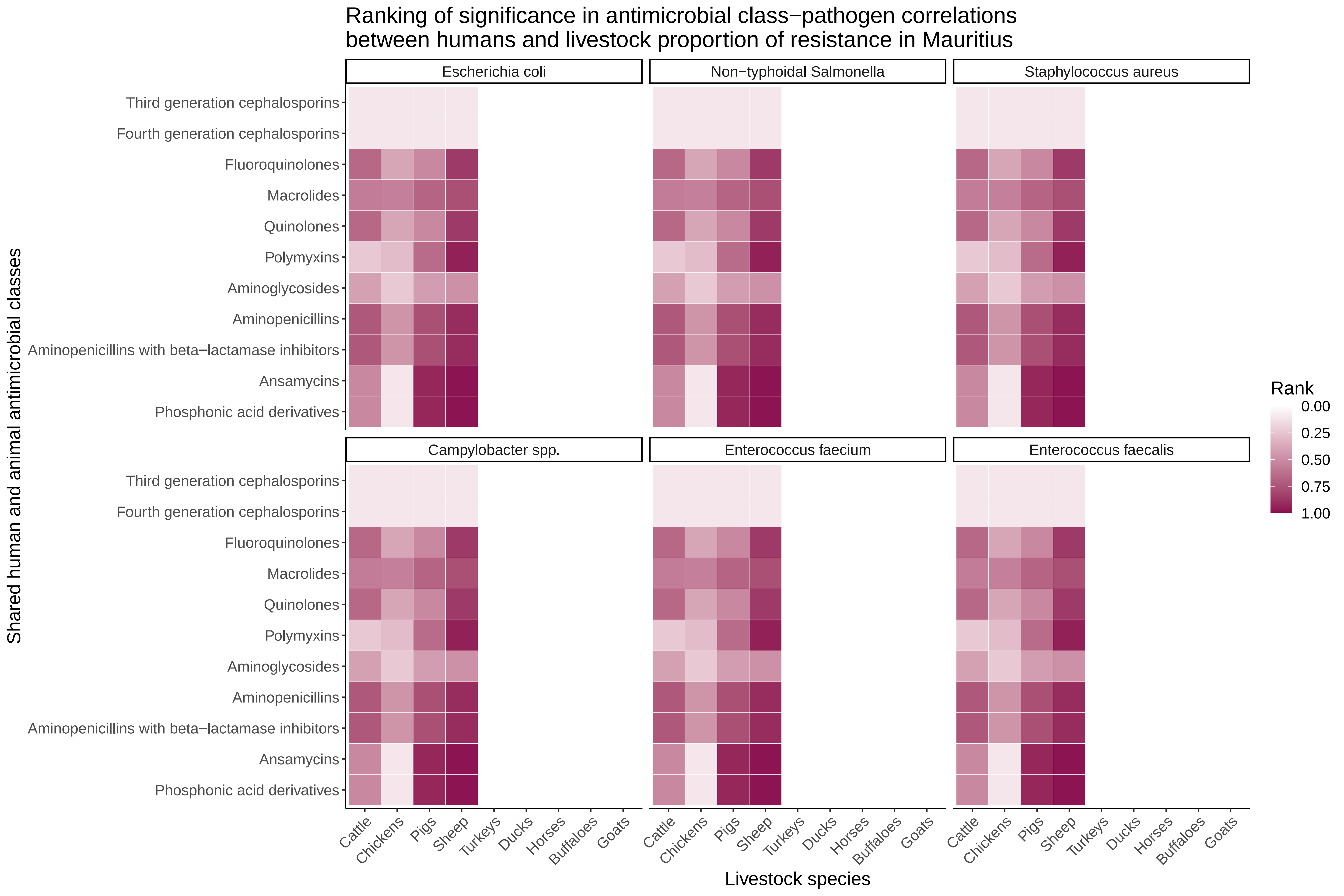

Ranking of significance in antimicrobial class−pathogen correlations between humans and livestock proportion of resistance in Mauritius
Escherichia coli
Non−typhoidal Salmonella
Staphylococcus aureus
Campylobacter spp.
Enterococcus faecium
Enterococcus faecalis
Third generation cephalosporins
Fourth generation cephalosporins
Fluoroquinolones
Macrolides
Quinolones
Polymyxins
Aminoglycosides
Aminopenicillins
Aminopenicillins with beta−lactamase inhibitors
Ansamycins
Phosphonic acid derivatives
Shared human and animal antimicrobial classes
Cattle
Chickens
Pigs
Sheep
Turkeys
Ducks
Horses
Buffaloes
Goats
Livestock species
Rank
0.00
0.25
0.50
0.75
1.00

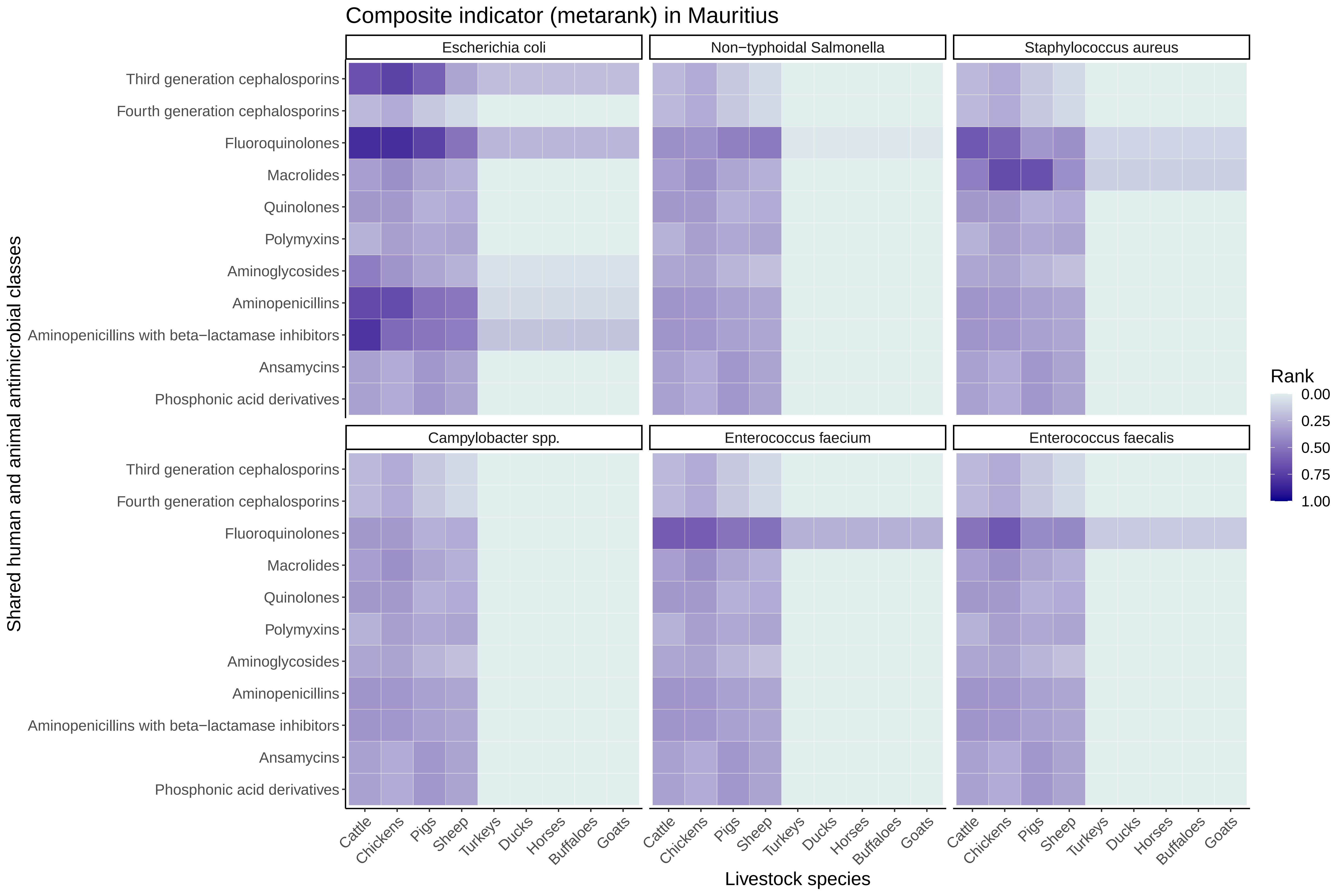
Composite indicator (metarank) in Mauritius
Escherichia coli
Non−typhoidal Salmonella
Staphylococcus aureus
Campylobacter spp.
Enterococcus faecium
Enterococcus faecalis
Shared human and animal antimicrobial classes
Third generation cephalosporins
Fourth generation cephalosporins
Fluoroquinolones
Macrolides
Quinolones
Polymyxins
Aminoglycosides
Aminopenicillins
Aminopenicillins with beta−lactamase inhibitors
Ansamycins
Phosphonic acid derivatives
Cattle
Chickens
Pigs
Sheep
Turkeys
Ducks
Horses
Buffaloes
Goats
Livestock species
Rank
0.00
0.25
0.50
0.75
1.00

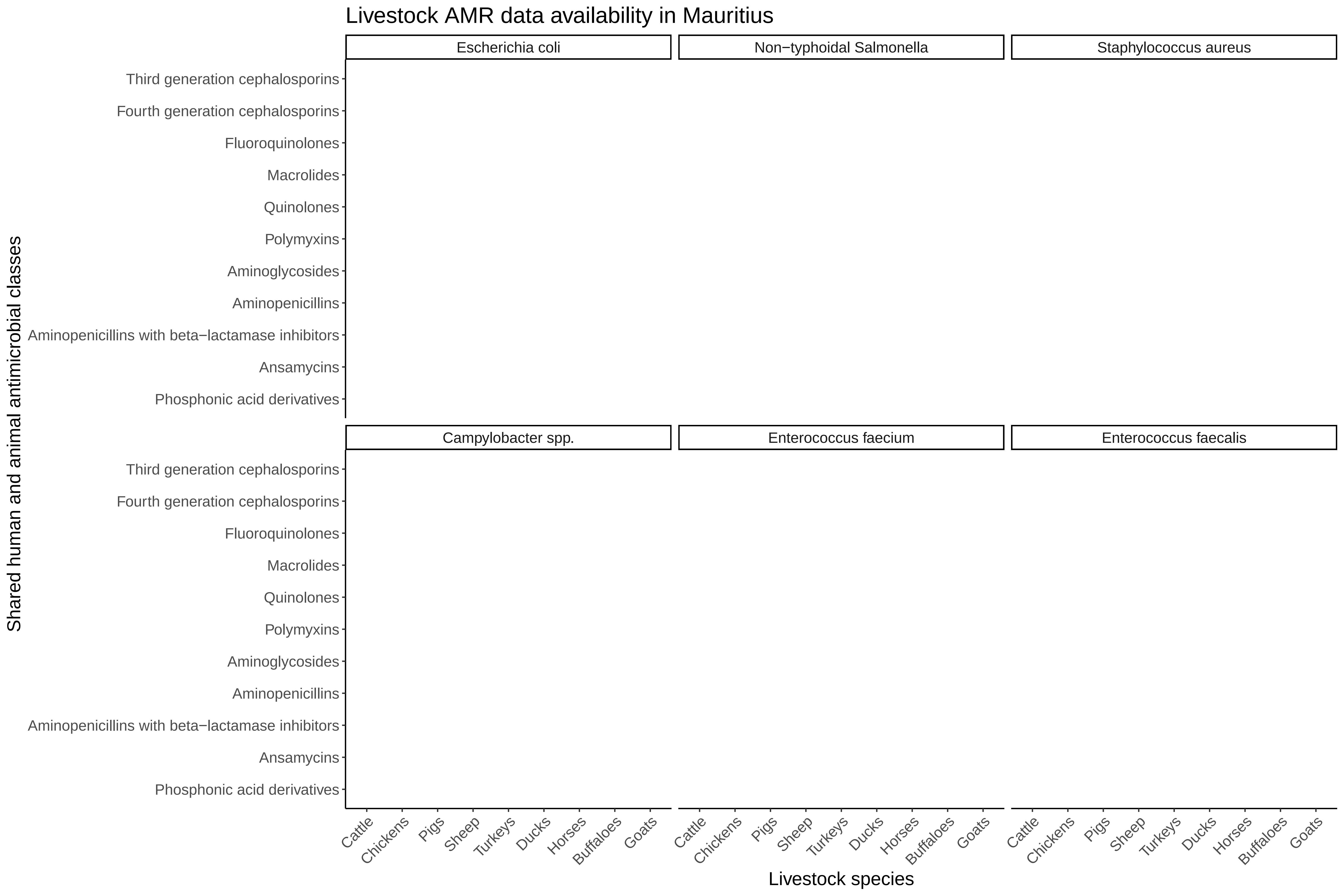
Livestock AMR data availability in Mauritius
Escherichia coli
Non−typhoidal Salmonella
Staphylococcus aureus
Campylobacter spp.
Enterococcus faecium
Enterococcus faecalis
Third generation cephalosporins
Fourth generation cephalosporins
Fluoroquinolones
Macrolides
Quinolones
Polymyxins
Aminoglycosides
Aminopenicillins
Aminopenicillins with beta−lactamase inhibitors
Ansamycins
Phosphonic acid derivatives
Shared human and animal antimicrobial classes
Cattle
Chickens
Pigs
Sheep
Turkeys
Ducks
Horses
Buffaloes
Goats
Livestock species

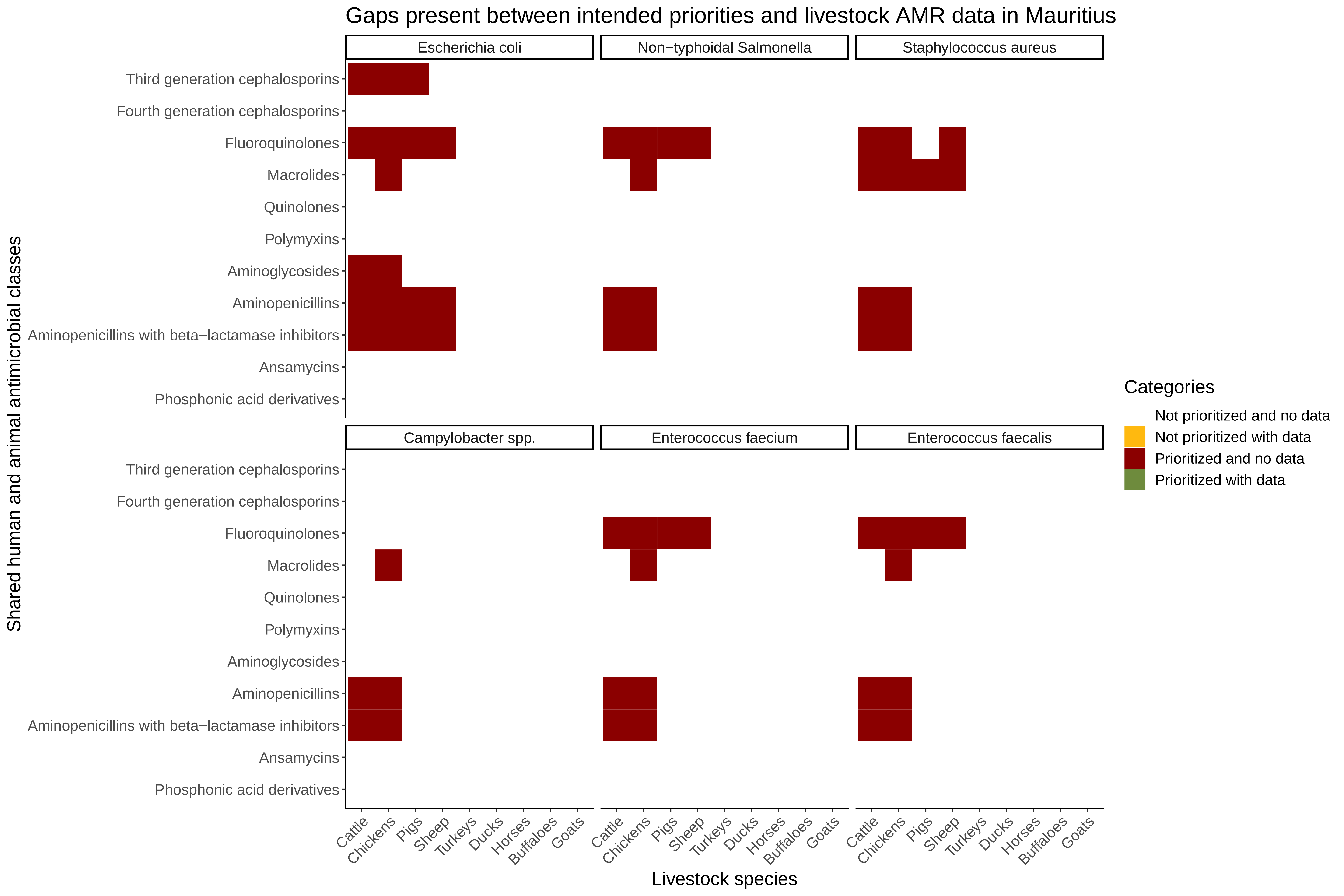

Gaps present between intended priorities and livestock AMR data in Mauritius
Escherichia coli
Non−typhoidal Salmonella
Staphylococcus aureus
Campylobacter spp.
Enterococcus faecium
Enterococcus faecalis
Third generation cephalosporins
Fourth generation cephalosporins
Fluoroquinolones
Macrolides
Quinolones
Polymyxins
Aminoglycosides
Aminopenicillins
Aminopenicillins with beta−lactamase inhibitors
Ansamycins
Phosphonic acid derivatives
Shared human and animal antimicrobial classes
Cattle
Chickens
Pigs
Sheep
Turkeys
Ducks
Horses
Buffaloes
Goats
Livestock species
Categories
Not prioritized and no data
Not prioritized with data
Prioritized and no data
Prioritized with data

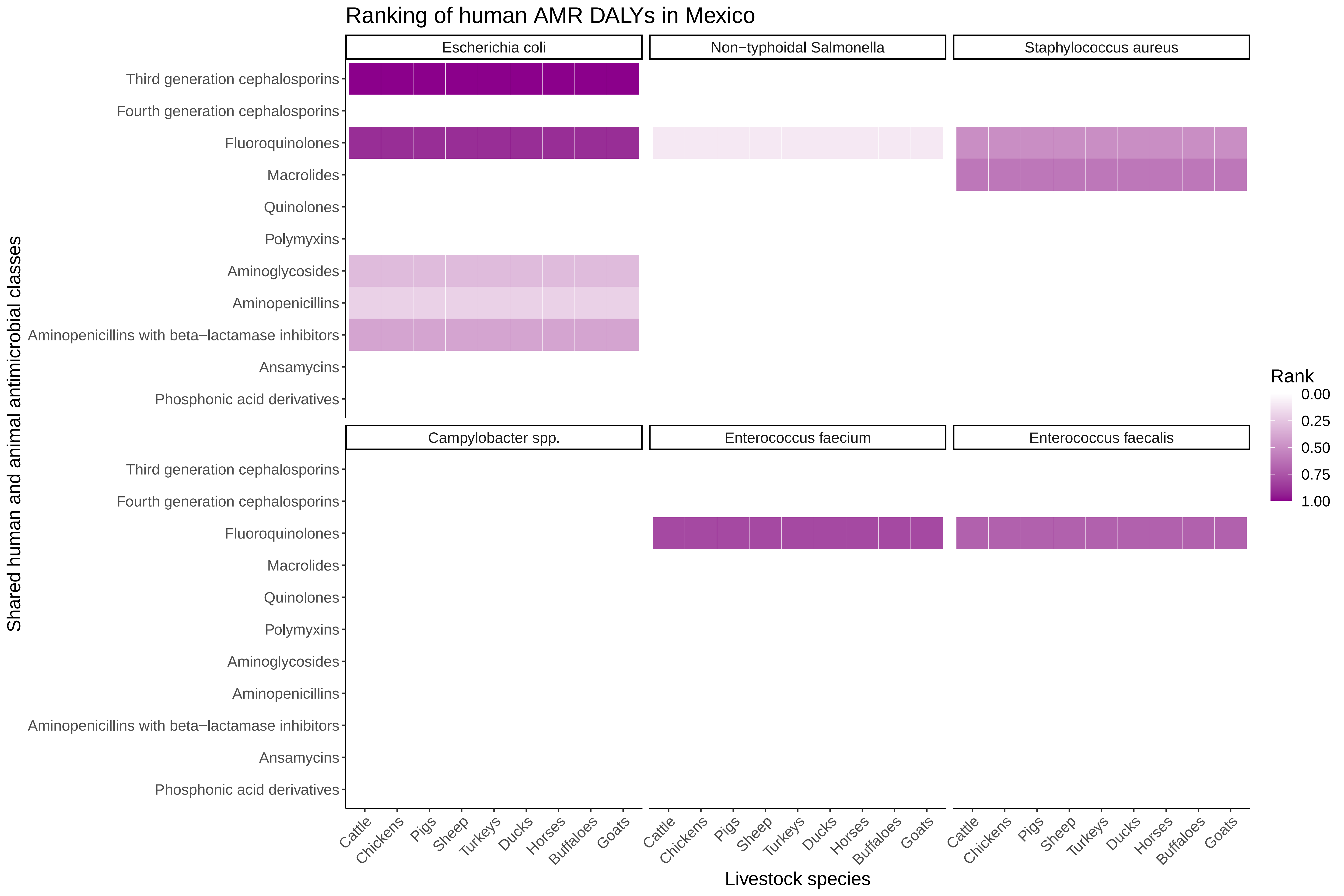

Ranking of human AMR DALYs in Mexico
Escherichia coli
Non−typhoidal Salmonella
Staphylococcus aureus
Campylobacter spp.
Enterococcus faecium
Enterococcus faecalis
Shared human and animal antimicrobial classes
Third generation cephalosporins
Fourth generation cephalosporins
Fluoroquinolones
Macrolides
Quinolones
Polymyxins
Aminoglycosides
Aminopenicillins
Aminopenicillins with beta−lactamase inhibitors
Ansamycins
Phosphonic acid derivatives
Cattle
Chickens
Pigs
Sheep
Turkeys
Ducks
Horses
Buffaloes
Goats
Livestock species
Rank
0.00
0.25
0.50
0.75
1.00

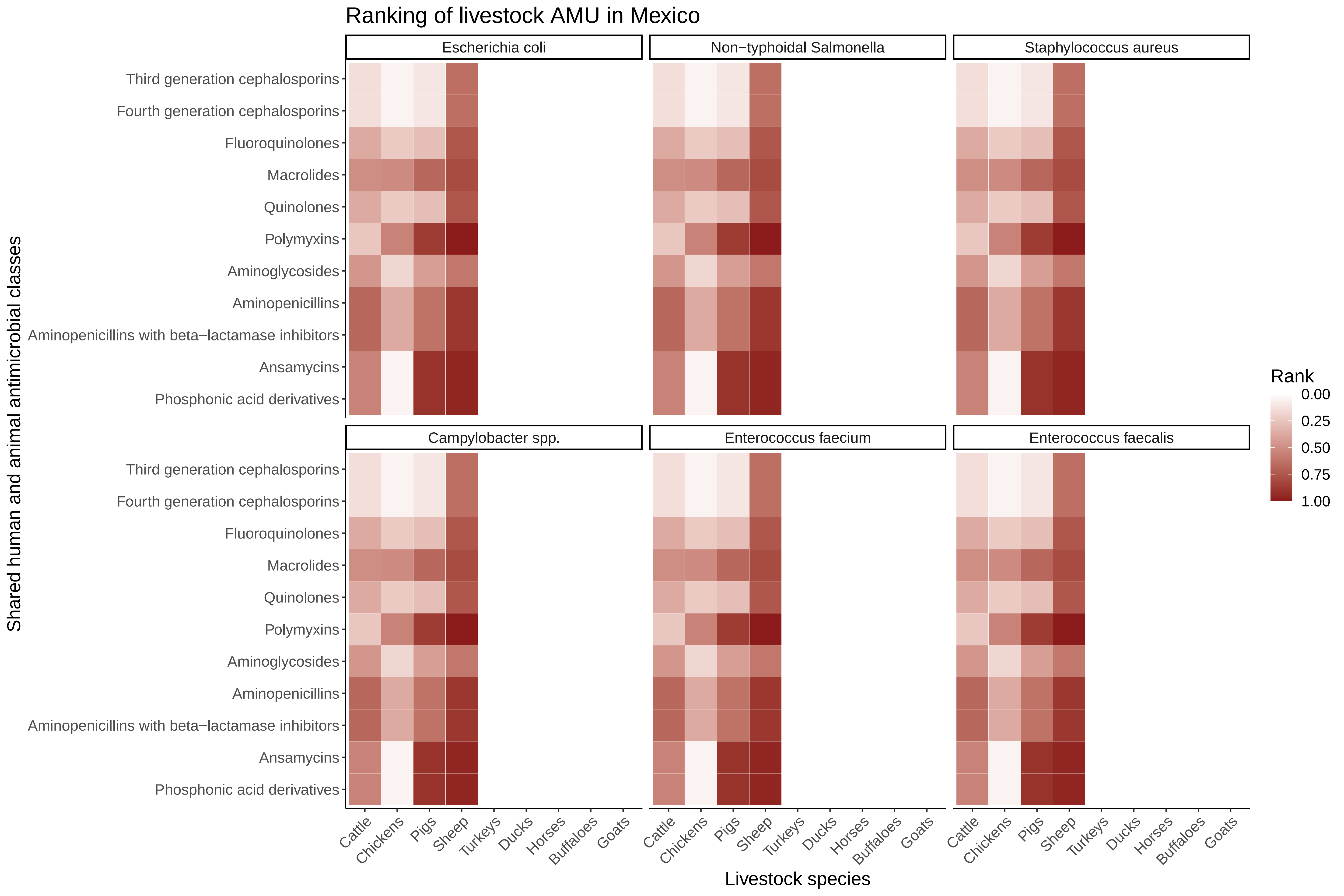

Ranking of livestock AMU in Mexico
Escherichia coli
Non−typhoidal Salmonella
Staphylococcus aureus
Campylobacter spp.
Enterococcus faecium
Enterococcus faecalis
Third generation cephalosporins
Fourth generation cephalosporins
Fluoroquinolones
Macrolides
Quinolones
Polymyxins
Aminoglycosides
Aminopenicillins
Aminopenicillins with beta−lactamase inhibitors
Ansamycins
Phosphonic acid derivatives
Shared human and animal antimicrobial classes
Cattle
Chickens
Pigs
Sheep
Turkeys
Ducks
Horses
Buffaloes
Goats
Livestock species
Rank
0.00
0.25
0.50
0.75
1.00

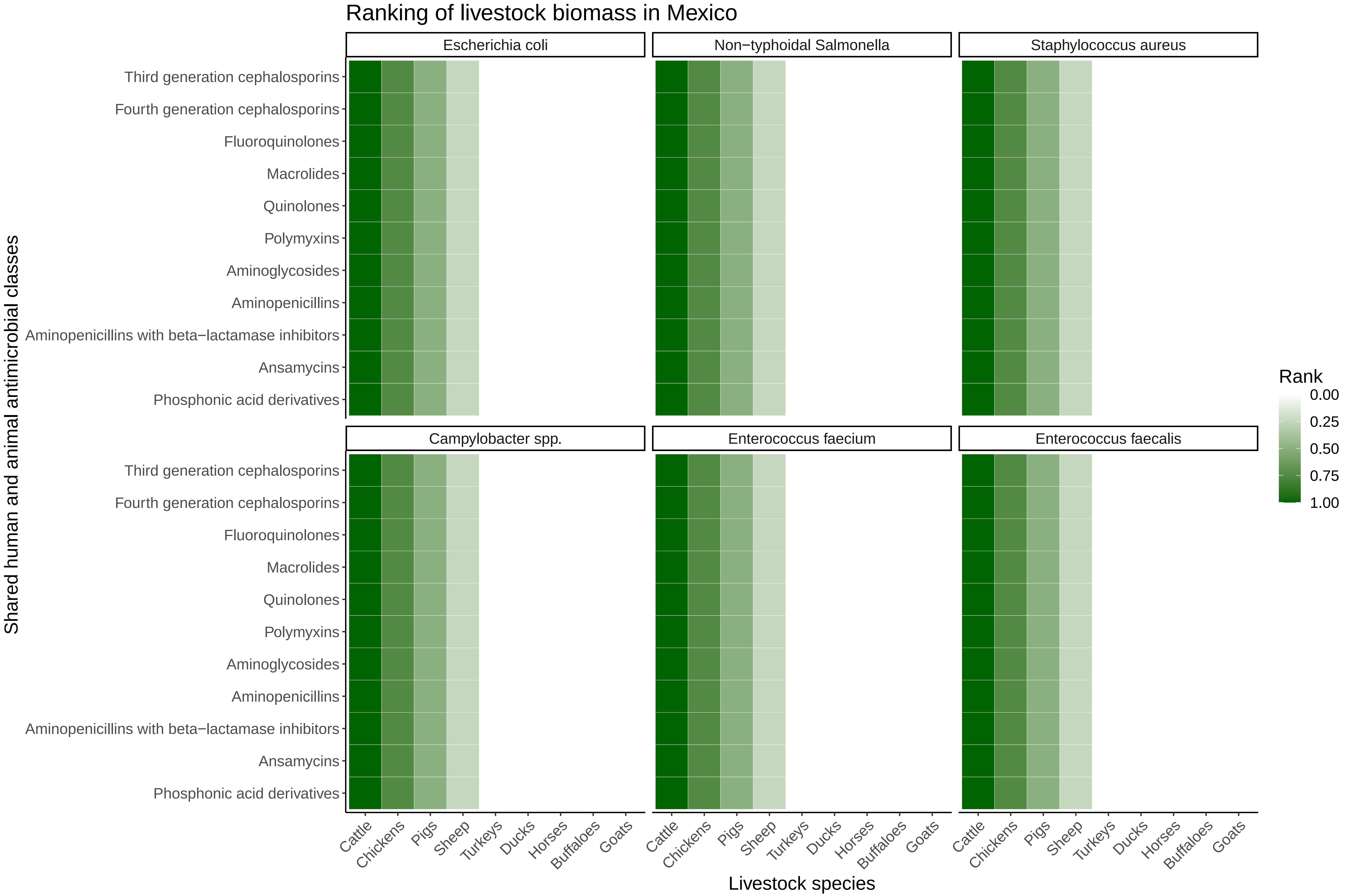
Ranking of livestock biomass in Mexico
Escherichia coli
Non−typhoidal Salmonella
Staphylococcus aureus
Campylobacter spp.
Enterococcus faecium
Enterococcus faecalis
Third generation cephalosporins
Fourth generation cephalosporins
Fluoroquinolones
Macrolides
Quinolones
Polymyxins
Aminoglycosides
Aminopenicillins
Aminopenicillins with beta−lactamase inhibitors
Ansamycins
Phosphonic acid derivatives
Shared human and animal antimicrobial classes
Cattle
Chickens
Pigs
Sheep
Turkeys
Ducks
Horses
Buffaloes
Goats
Livestock species
Rank
0.00
0.25
0.50
0.75
1.00

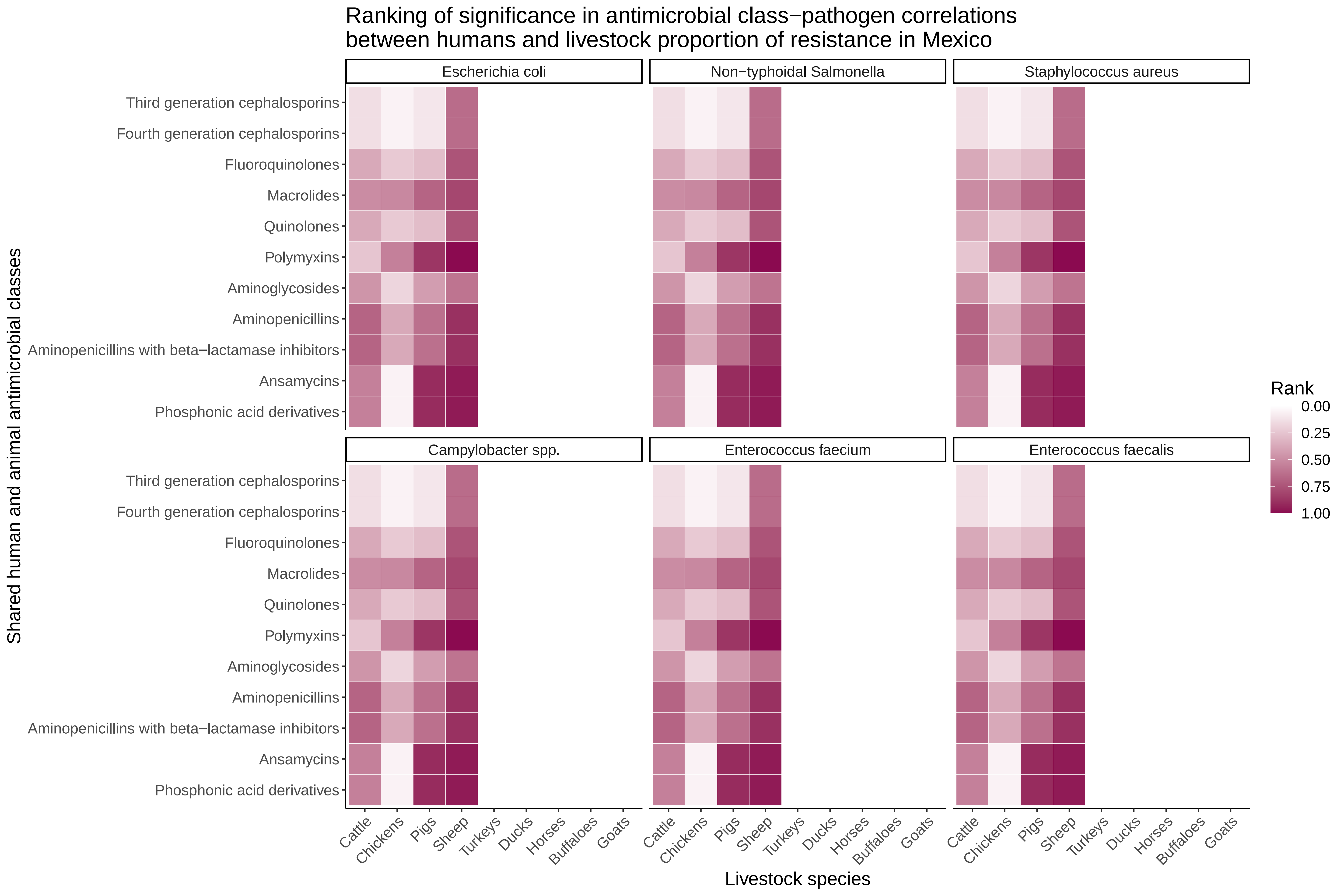
Ranking of significance in antimicrobial class−pathogen correlations
between humans and livestock proportion of resistance in Mexico
Escherichia coli
Non−typhoidal Salmonella
Staphylococcus aureus
Campylobacter spp.
Enterococcus faecium
Enterococcus faecalis
Third generation cephalosporins
Fourth generation cephalosporins
Fluoroquinolones
Macrolides
Quinolones
Polymyxins
Aminoglycosides
Aminopenicillins
Aminopenicillins with beta−lactamase inhibitors
Ansamycins
Phosphonic acid derivatives
Shared human and animal antimicrobial classes
Cattle
Chickens
Pigs
Sheep
Turkeys
Ducks
Horses
Buffaloes
Goats
Livestock species
Rank
0.00
0.25
0.50
0.75
1.00

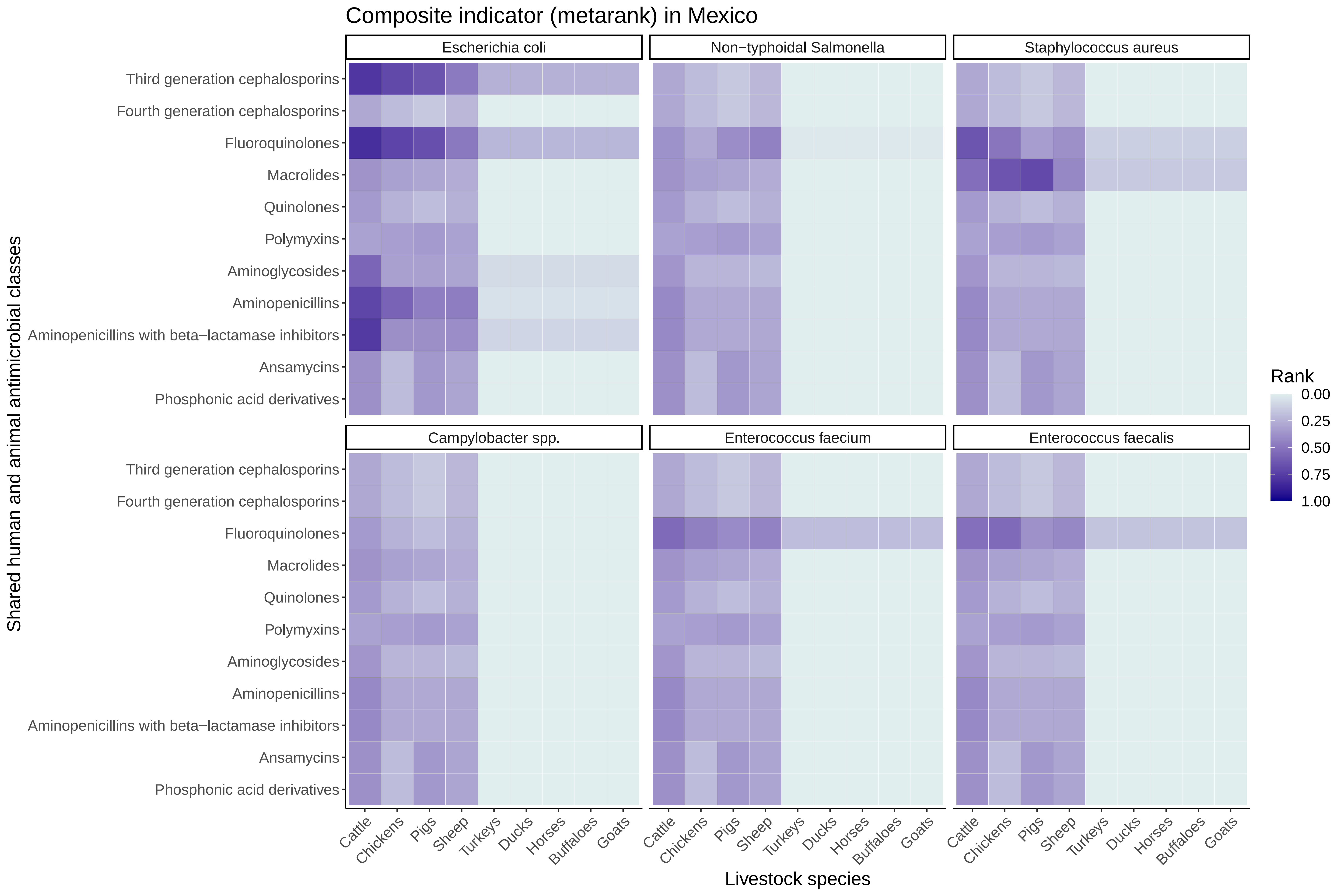
Composite indicator (metarank) in Mexico
Escherichia coli
Non−typhoidal Salmonella
Staphylococcus aureus
Campylobacter spp.
Enterococcus faecium
Enterococcus faecalis
Third generation cephalosporins
Fourth generation cephalosporins
Fluoroquinolones
Macrolides
Quinolones
Polymyxins
Aminoglycosides
Aminopenicillins
Aminopenicillins with beta−lactamase inhibitors
Ansamycins
Phosphonic acid derivatives
Shared human and animal antimicrobial classes
Cattle
Chickens
Pigs
Sheep
Turkeys
Ducks
Horses
Buffaloes
Goats
Livestock species
Rank
0.00
0.25
0.50
0.75
1.00

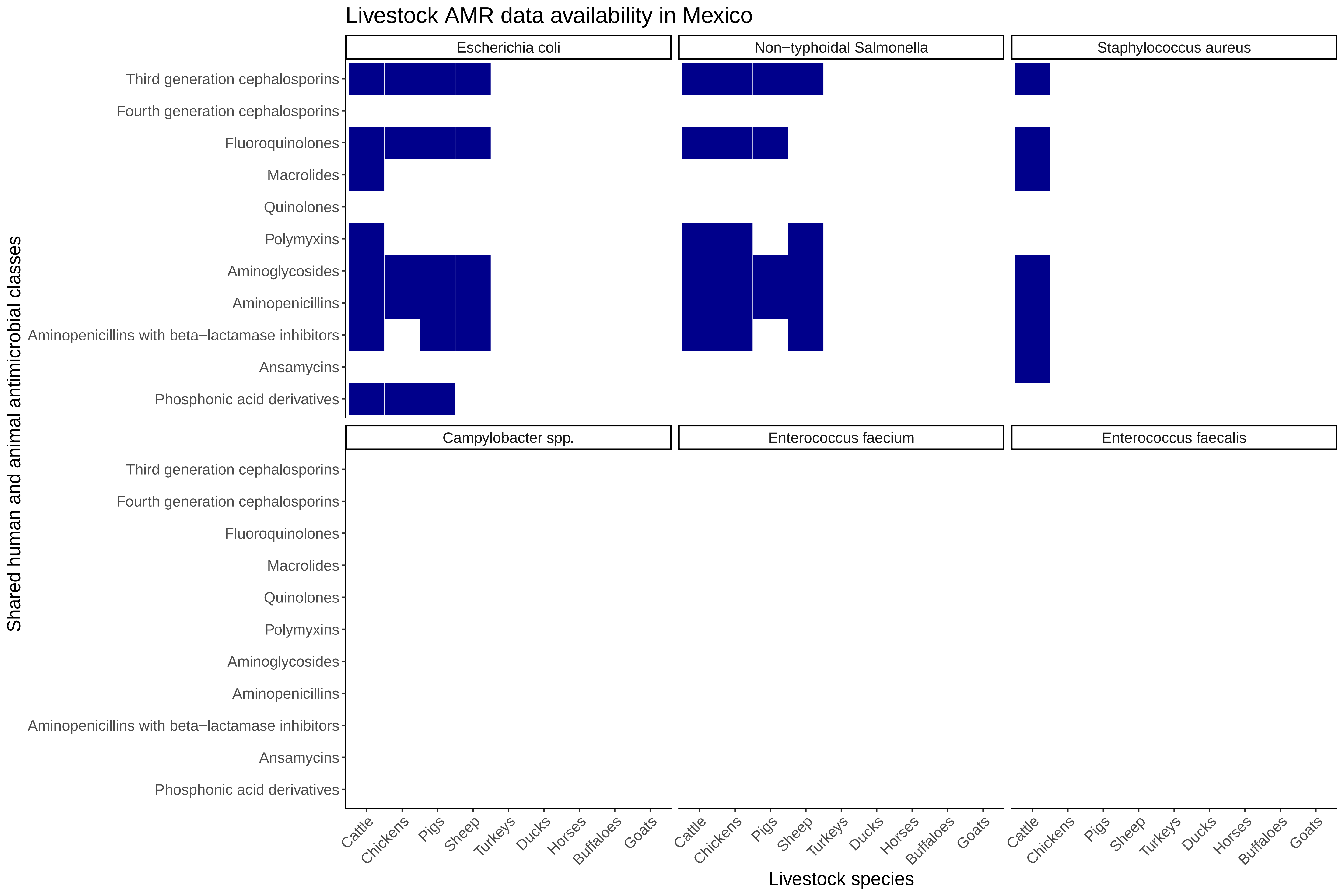

Livestock AMR data availability in Mexico
Escherichia coli
Non−typhoidal Salmonella
Staphylococcus aureus
Campylobacter spp.
Enterococcus faecium
Enterococcus faecalis
Shared human and animal antimicrobial classes
Third generation cephalosporins
Fourth generation cephalosporins
Fluoroquinolones
Macrolides
Quinolones
Polymyxins
Aminoglycosides
Aminopenicillins
Aminopenicillins with beta−lactamase inhibitors
Ansamycins
Phosphonic acid derivatives
Cattle
Chickens
Pigs
Sheep
Turkeys
Ducks
Horses
Buffaloes
Goats
Livestock species

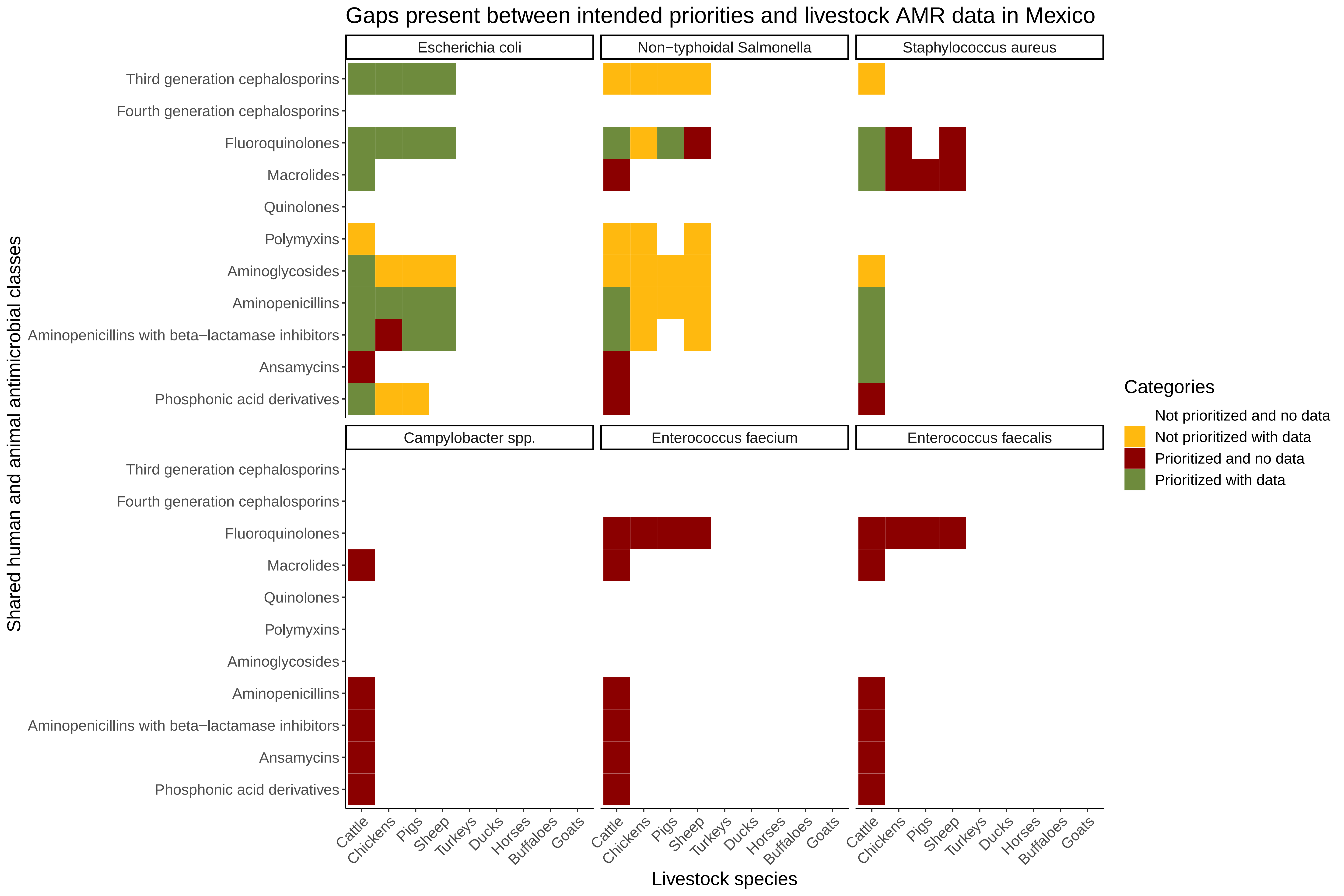

Gaps present between intended priorities and livestock AMR data in Mexico
Escherichia coli
Non−typhoidal Salmonella
Staphylococcus aureus
Campylobacter spp.
Enterococcus faecium
Enterococcus faecalis
Third generation cephalosporins
Fourth generation cephalosporins
Fluoroquinolones
Macrolides
Quinolones
Polymyxins
Aminoglycosides
Aminopenicillins
Aminopenicillins with beta−lactamase inhibitors
Ansamycins
Phosphonic acid derivatives
Shared human and animal antimicrobial classes
Cattle
Chickens
Pigs
Sheep
Turkeys
Ducks
Horses
Buffaloes
Goats
Livestock species
Categories
Not prioritized and no data
Not prioritized with data
Prioritized and no data
Prioritized with data

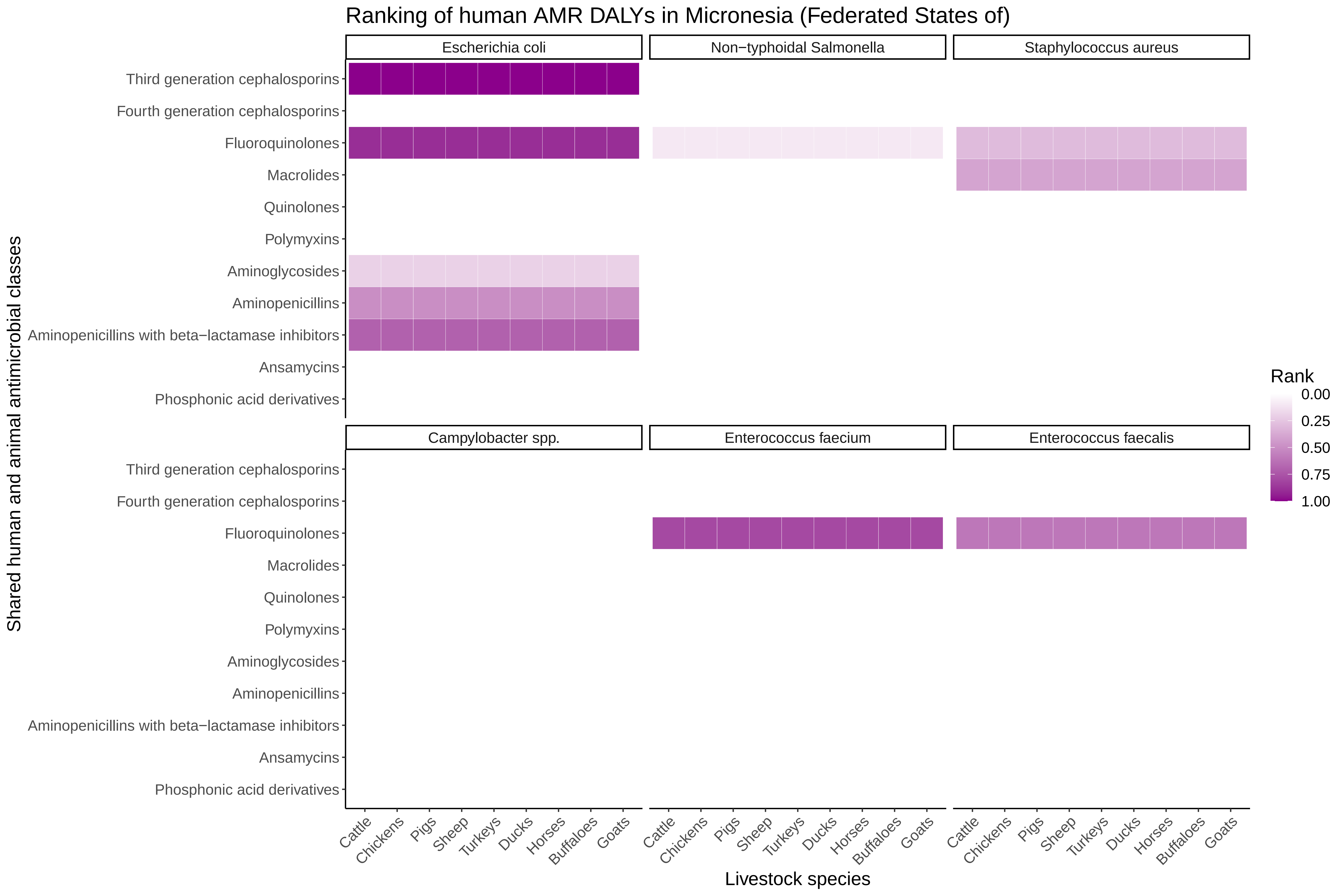

Ranking of human AMR DALYs in Micronesia (Federated States of)
Escherichia coli
Non−typhoidal Salmonella
Staphylococcus aureus
Campylobacter spp.
Enterococcus faecium
Enterococcus faecalis
Shared human and animal antimicrobial classes
Third generation cephalosporins
Fourth generation cephalosporins
Fluoroquinolones
Macrolides
Quinolones
Polymyxins
Aminoglycosides
Aminopenicillins
Aminopenicillins with beta−lactamase inhibitors
Ansamycins
Phosphonic acid derivatives
Livestock species
Cattle
Chickens
Pigs
Sheep
Turkeys
Ducks
Horses
Buffaloes
Goats
Rank
0.00
0.25
0.50
0.75
1.00

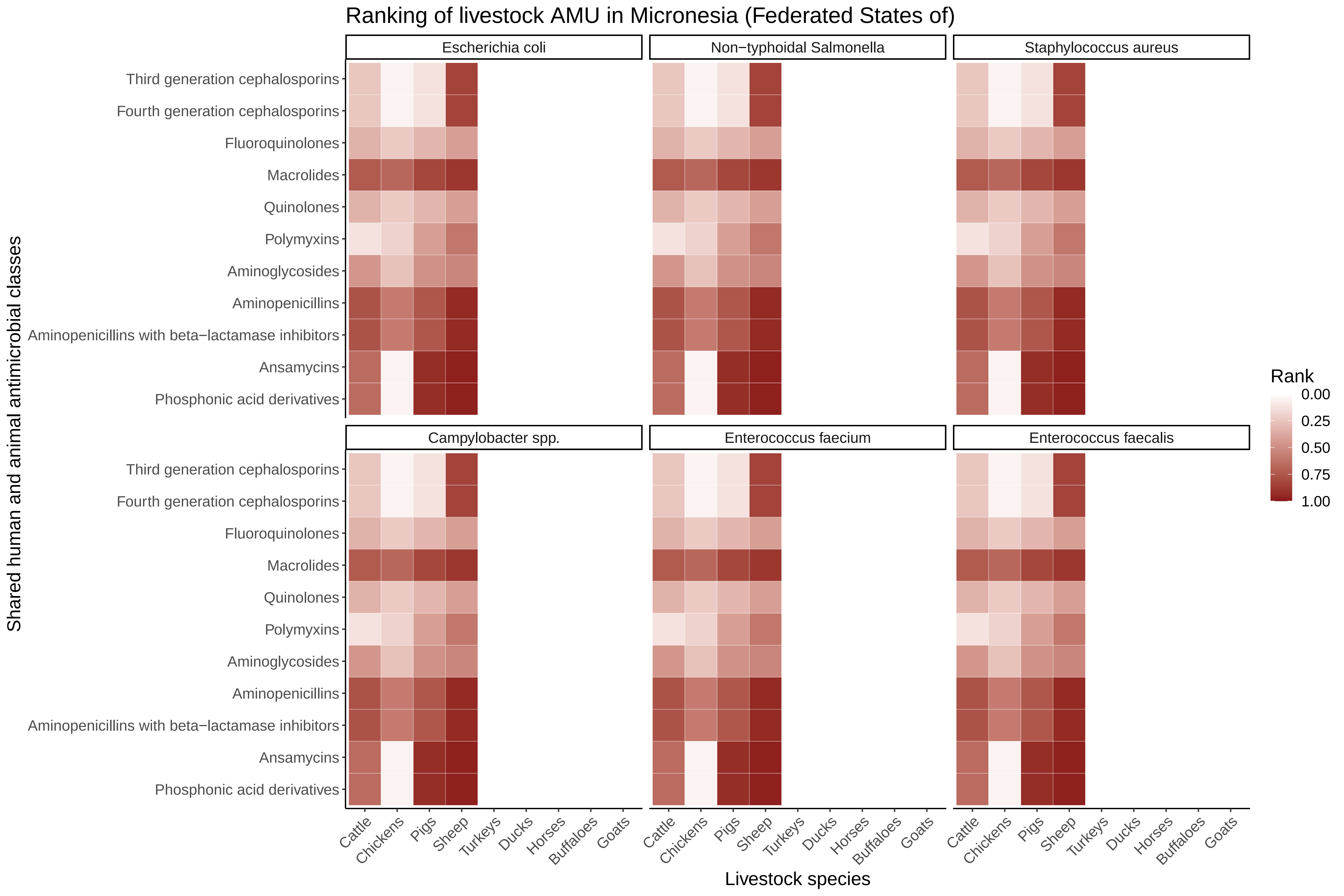

Ranking of livestock AMU in Micronesia (Federated States of)
Escherichia coli
Non−typhoidal Salmonella
Staphylococcus aureus
Campylobacter spp.
Enterococcus faecium
Enterococcus faecalis
Shared human and animal antimicrobial classes
Third generation cephalosporins
Fourth generation cephalosporins
Fluoroquinolones
Macrolides
Quinolones
Polymyxins
Aminoglycosides
Aminopenicillins
Aminopenicillins with beta−lactamase inhibitors
Ansamycins
Phosphonic acid derivatives
Livestock species
Cattle
Chickens
Pigs
Sheep
Turkeys
Ducks
Horses
Buffaloes
Goats
Rank
0.00
0.25
0.50
0.75
1.00

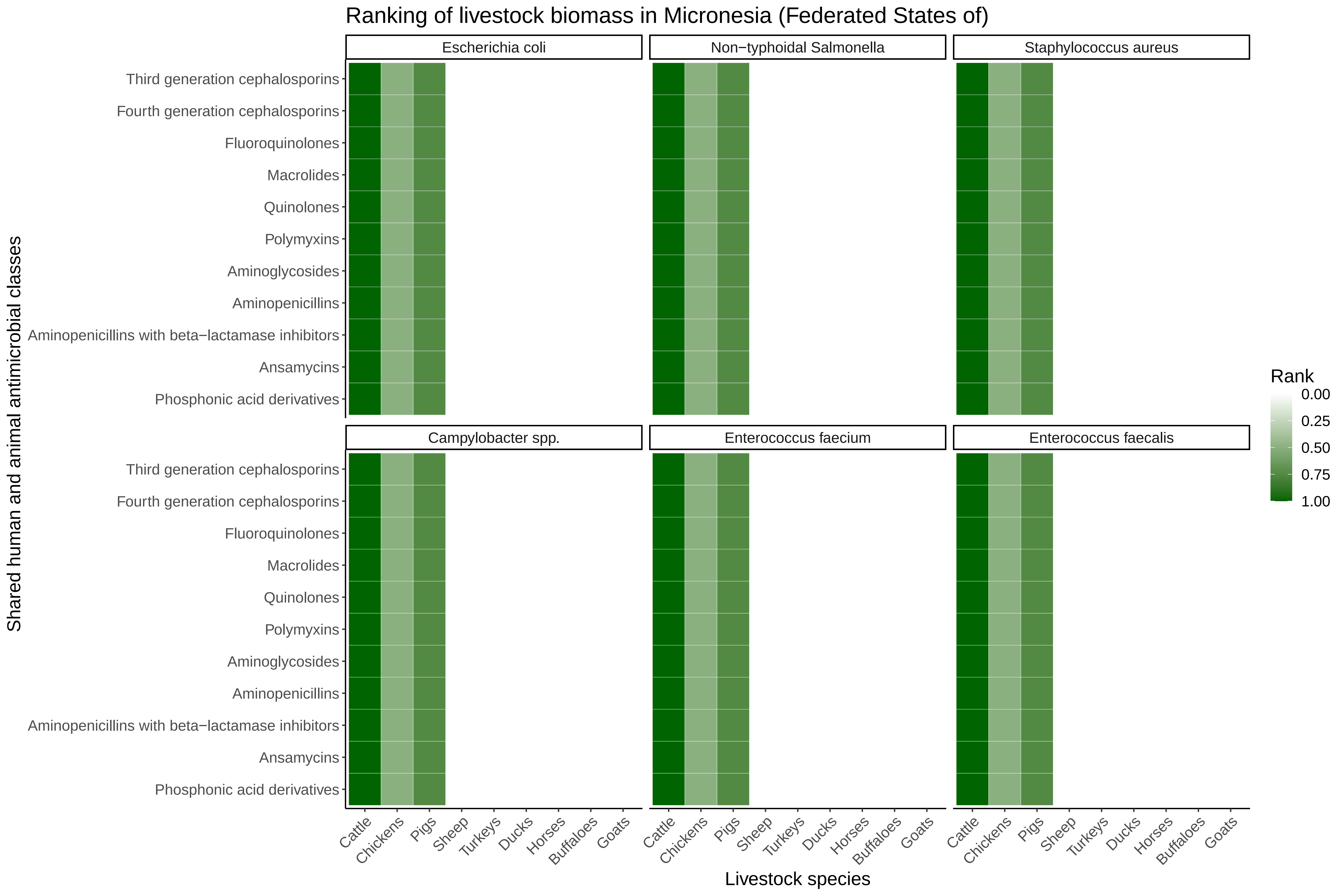

Ranking of livestock biomass in Micronesia (Federated States of)
Escherichia coli
Non−typhoidal Salmonella
Staphylococcus aureus
Campylobacter spp.
Enterococcus faecium
Enterococcus faecalis
Shared human and animal antimicrobial classes
Third generation cephalosporins
Fourth generation cephalosporins
Fluoroquinolones
Macrolides
Quinolones
Polymyxins
Aminoglycosides
Aminopenicillins
Aminopenicillins with beta−lactamase inhibitors
Ansamycins
Phosphonic acid derivatives
Livestock species
Cattle
Chickens
Pigs
Sheep
Turkeys
Ducks
Horses
Buffaloes
Goats
Rank
0.00
0.25
0.50
0.75
1.00

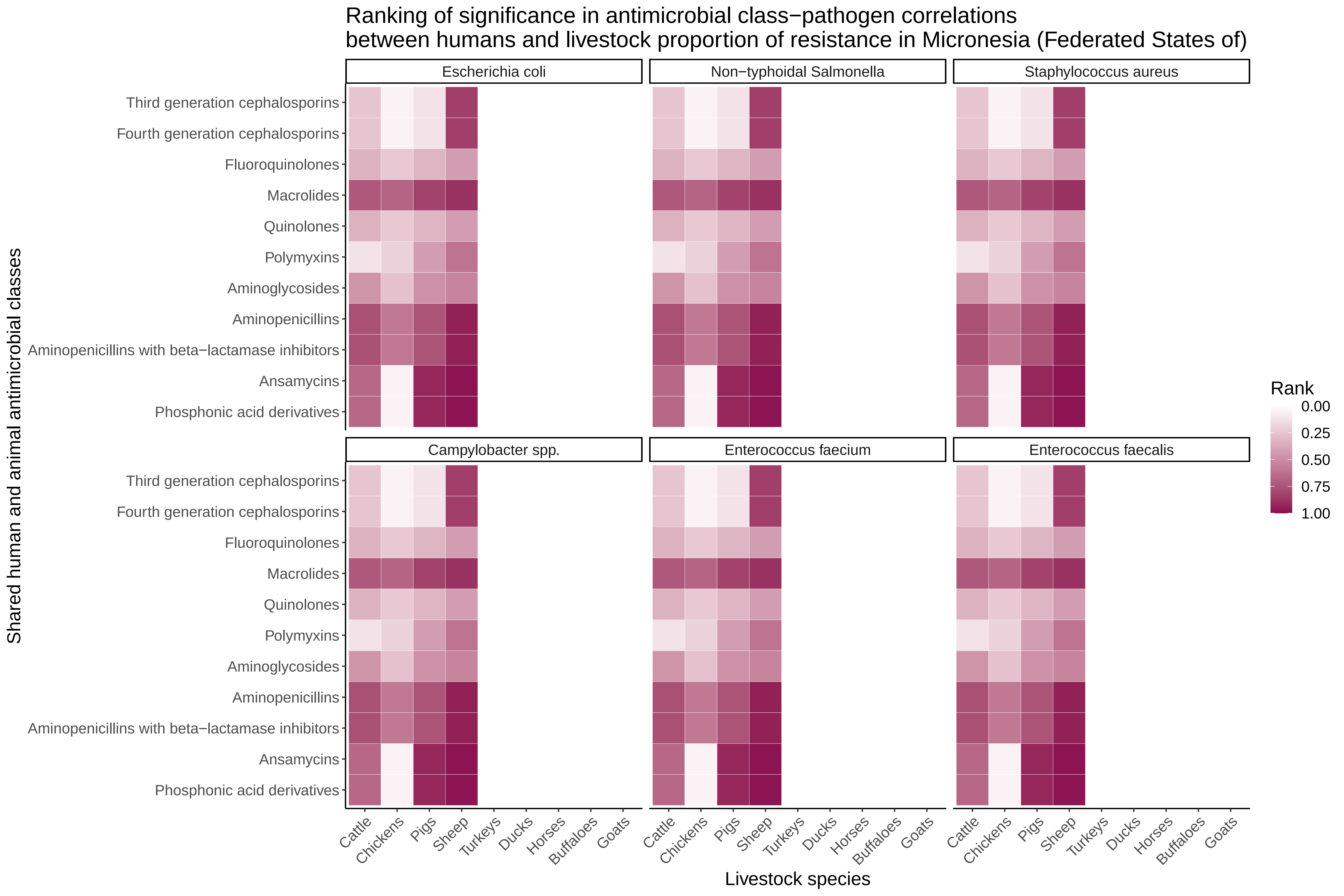
Ranking of significance in antimicrobial class−pathogen correlations
between humans and livestock proportion of resistance in Micronesia (Federated States of)
Escherichia coli
Non−typhoidal Salmonella
Staphylococcus aureus
Campylobacter spp.
Enterococcus faecium
Enterococcus faecalis
Third generation cephalosporins
Fourth generation cephalosporins
Fluoroquinolones
Macrolides
Quinolones
Polymyxins
Aminoglycosides
Aminopenicillins
Aminopenicillins with beta−lactamase inhibitors
Ansamycins
Phosphonic acid derivatives
Shared human and animal antimicrobial classes
Cattle
Chickens
Pigs
Sheep
Turkeys
Ducks
Horses
Buffaloes
Goats
Livestock species
Rank
0.00
0.25
0.50
0.75
1.00

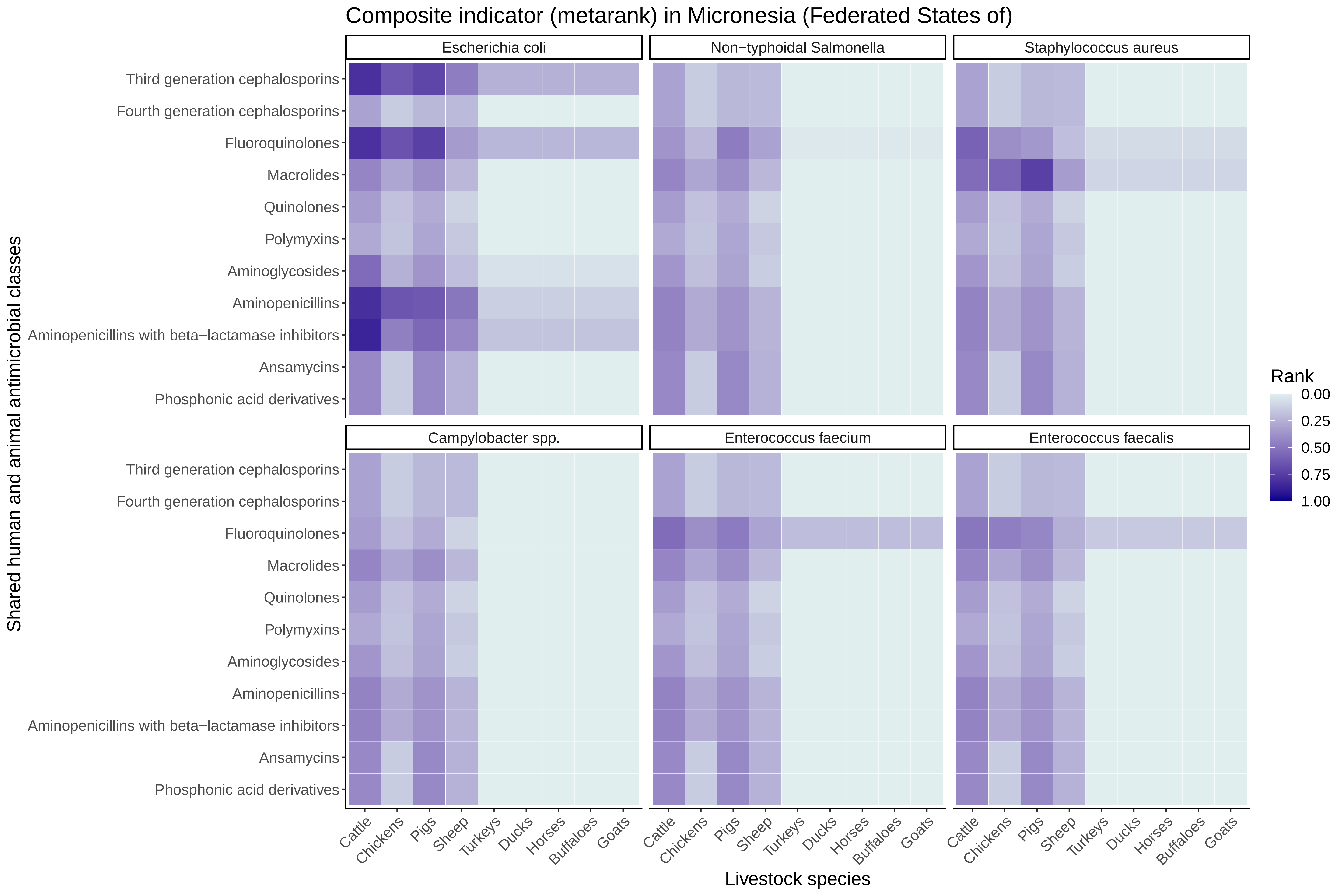

Composite indicator (metarank) in Micronesia (Federated States of)
Escherichia coli
Non−typhoidal Salmonella
Staphylococcus aureus
Campylobacter spp.
Enterococcus faecium
Enterococcus faecalis
Shared human and animal antimicrobial classes
Third generation cephalosporins
Fourth generation cephalosporins
Fluoroquinolones
Macrolides
Quinolones
Polymyxins
Aminoglycosides
Aminopenicillins
Aminopenicillins with beta−lactamase inhibitors
Ansamycins
Phosphonic acid derivatives
Livestock species
Cattle
Chickens
Pigs
Sheep
Turkeys
Ducks
Horses
Buffaloes
Goats
Rank
0.00
0.25
0.50
0.75
1.00

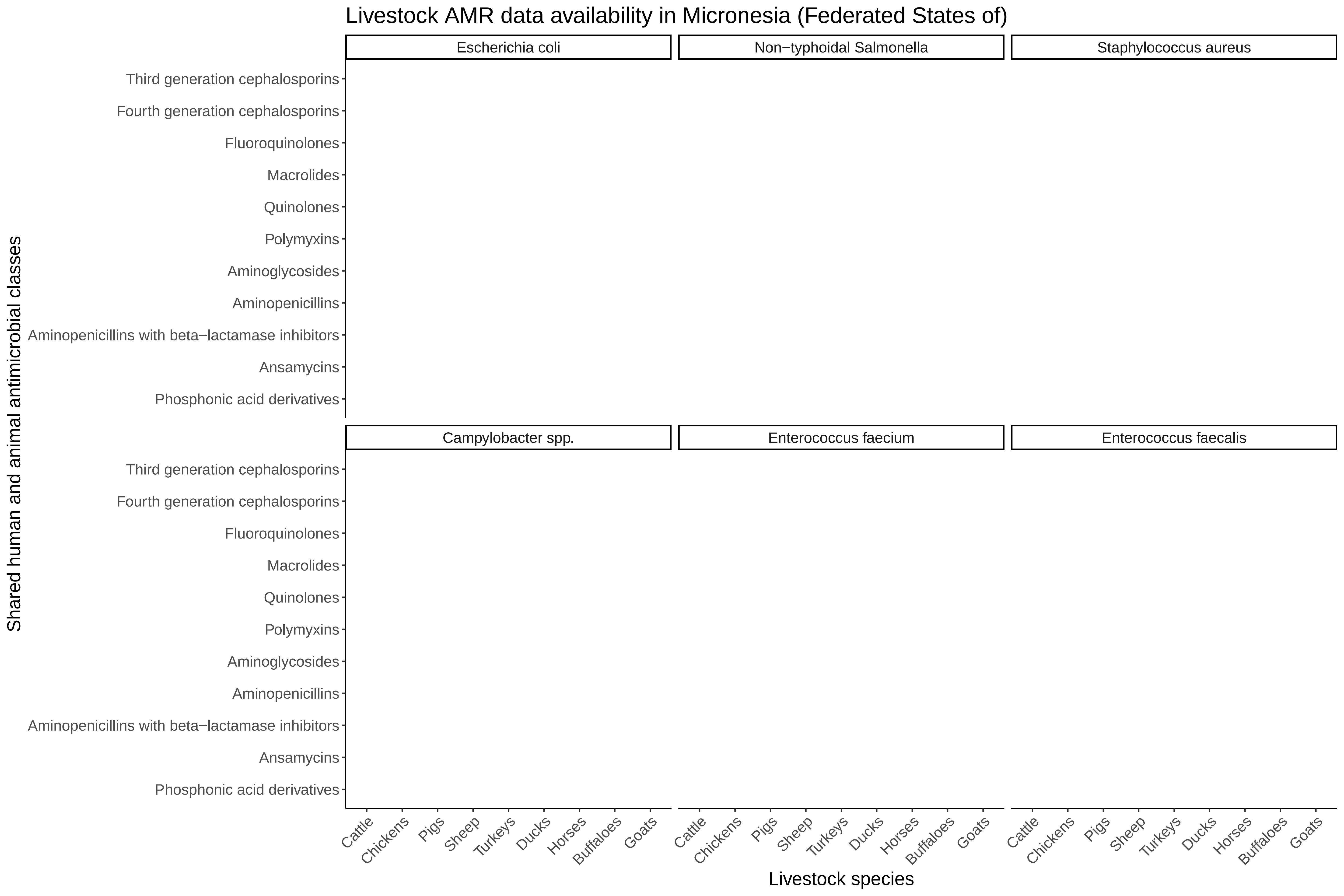

Livestock AMR data availability in Micronesia (Federated States of)
Escherichia coli
Non−typhoidal Salmonella
Staphylococcus aureus
Campylobacter spp.
Enterococcus faecium
Enterococcus faecalis
Third generation cephalosporins
Fourth generation cephalosporins
Fluoroquinolones
Macrolides
Quinolones
Polymyxins
Aminoglycosides
Aminopenicillins
Aminopenicillins with beta−lactamase inhibitors
Ansamycins
Phosphonic acid derivatives
Shared human and animal antimicrobial classes
Cattle
Chickens
Pigs
Sheep
Turkeys
Ducks
Horses
Buffaloes
Goats
Livestock species

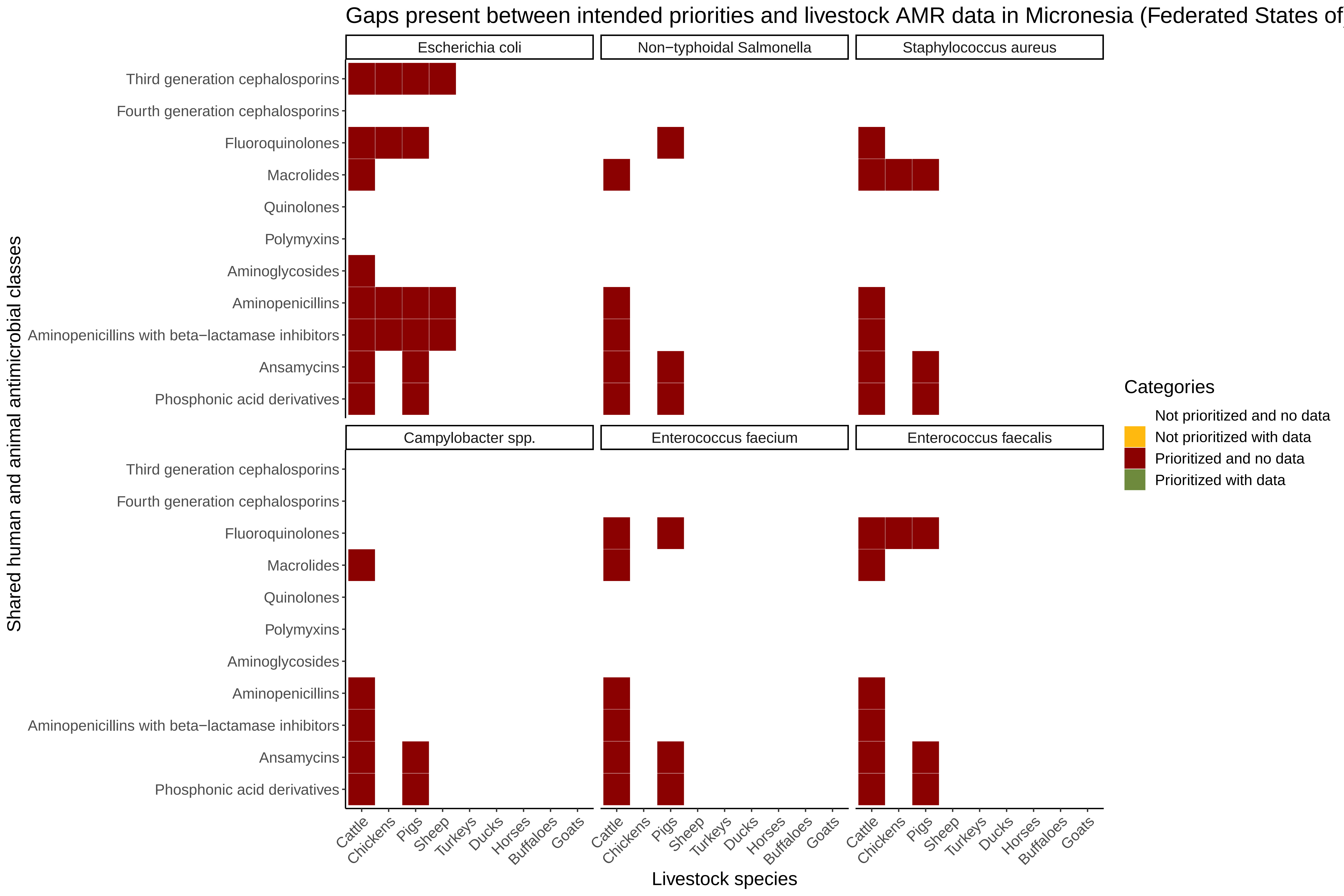
Gaps present between intended priorities and livestock AMR data in Micronesia (Federated States of
Escherichia coli
Non−typhoidal Salmonella
Staphylococcus aureus
Campylobacter spp.
Enterococcus faecium
Enterococcus faecalis
Shared human and animal antimicrobial classes
Third generation cephalosporins
Fourth generation cephalosporins
Fluoroquinolones
Macrolides
Quinolones
Polymyxins
Aminoglycosides
Aminopenicillins
Aminopenicillins with beta−lactamase inhibitors
Ansamycins
Phosphonic acid derivatives
Cattle
Chickens
Pigs
Sheep
Turkeys
Ducks
Horses
Buffaloes
Goats
Livestock species
Categories
Not prioritized and no data
Not prioritized with data
Prioritized and no data
Prioritized with data

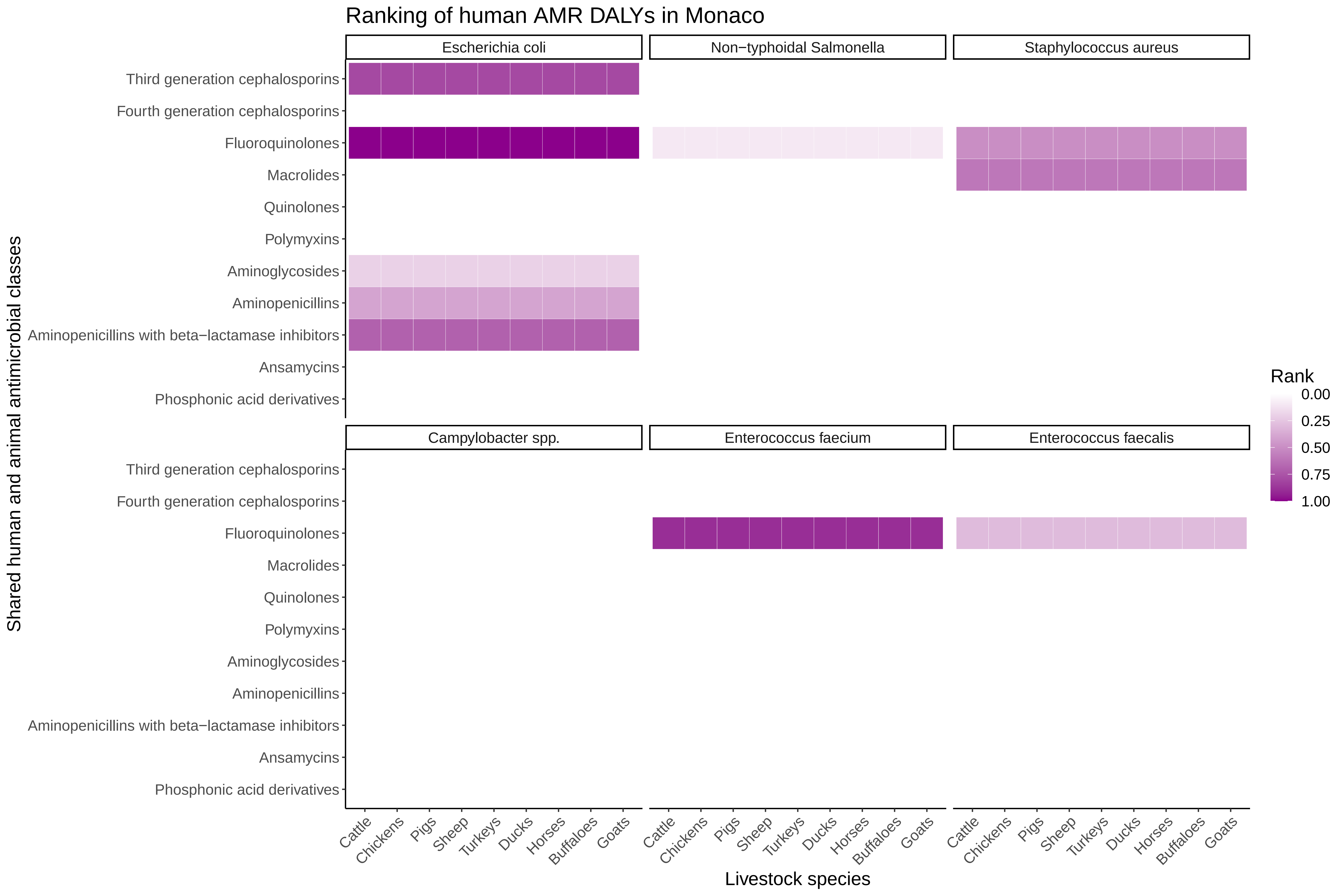
Ranking of human AMR DALYs in Monaco
Escherichia coli
Non−typhoidal Salmonella
Staphylococcus aureus
Campylobacter spp.
Enterococcus faecium
Enterococcus faecalis
Third generation cephalosporins
Fourth generation cephalosporins
Fluoroquinolones
Macrolides
Quinolones
Polymyxins
Aminoglycosides
Aminopenicillins
Aminopenicillins with beta−lactamase inhibitors
Ansamycins
Phosphonic acid derivatives
Shared human and animal antimicrobial classes
Cattle
Chickens
Pigs
Sheep
Turkeys
Ducks
Horses
Buffaloes
Goats
Livestock species
Rank
0.00
0.25
0.50
0.75
1.00

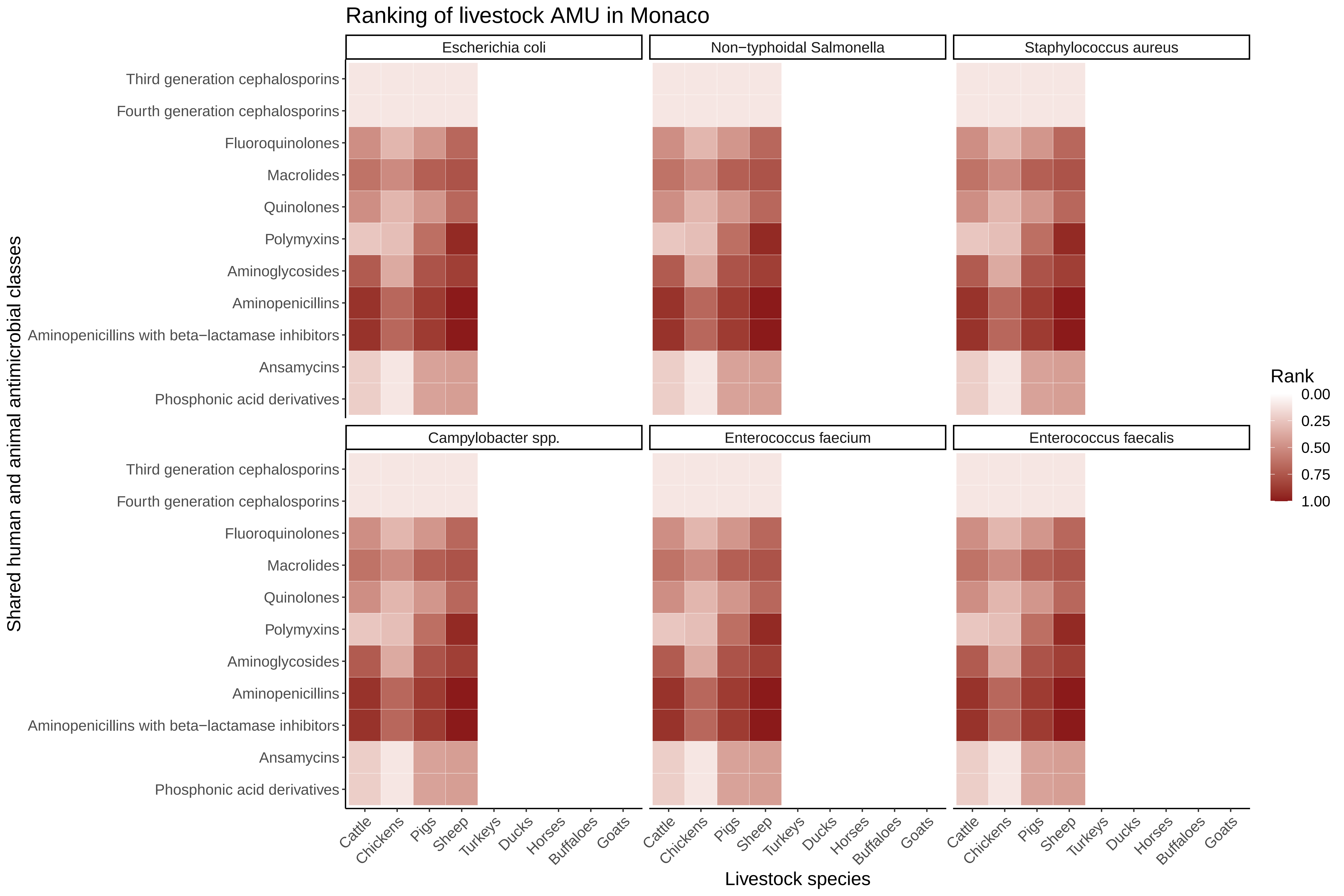
Ranking of livestock AMU in Monaco
Escherichia coli
Non−typhoidal Salmonella
Staphylococcus aureus
Campylobacter spp.
Enterococcus faecium
Enterococcus faecalis
Shared human and animal antimicrobial classes
Third generation cephalosporins
Fourth generation cephalosporins
Fluoroquinolones
Macrolides
Quinolones
Polymyxins
Aminoglycosides
Aminopenicillins
Aminopenicillins with beta−lactamase inhibitors
Ansamycins
Phosphonic acid derivatives
Cattle
Chickens
Pigs
Sheep
Turkeys
Ducks
Horses
Buffaloes
Goats
Livestock species
Rank
0.00
0.25
0.50
0.75
1.00

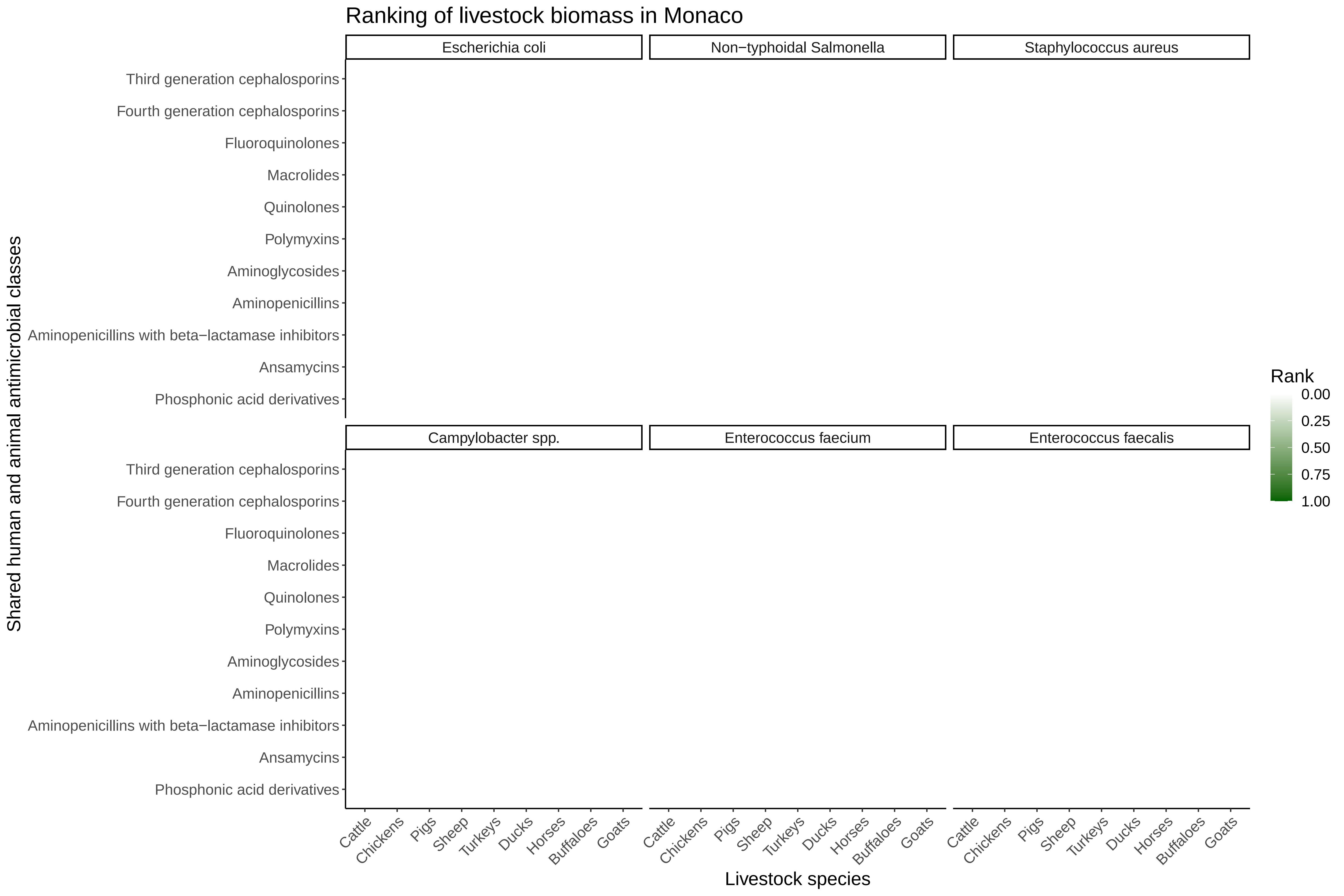

Ranking of livestock biomass in Monaco
Escherichia coli
Non−typhoidal Salmonella
Staphylococcus aureus
Campylobacter spp.
Enterococcus faecium
Enterococcus faecalis
Shared human and animal antimicrobial classes
Third generation cephalosporins
Fourth generation cephalosporins
Fluoroquinolones
Macrolides
Quinolones
Polymyxins
Aminoglycosides
Aminopenicillins
Aminopenicillins with beta−lactamase inhibitors
Ansamycins
Phosphonic acid derivatives
Cattle
Chickens
Pigs
Sheep
Turkeys
Ducks
Horses
Buffaloes
Goats
Livestock species
Rank
0.00
0.25
0.50
0.75
1.00

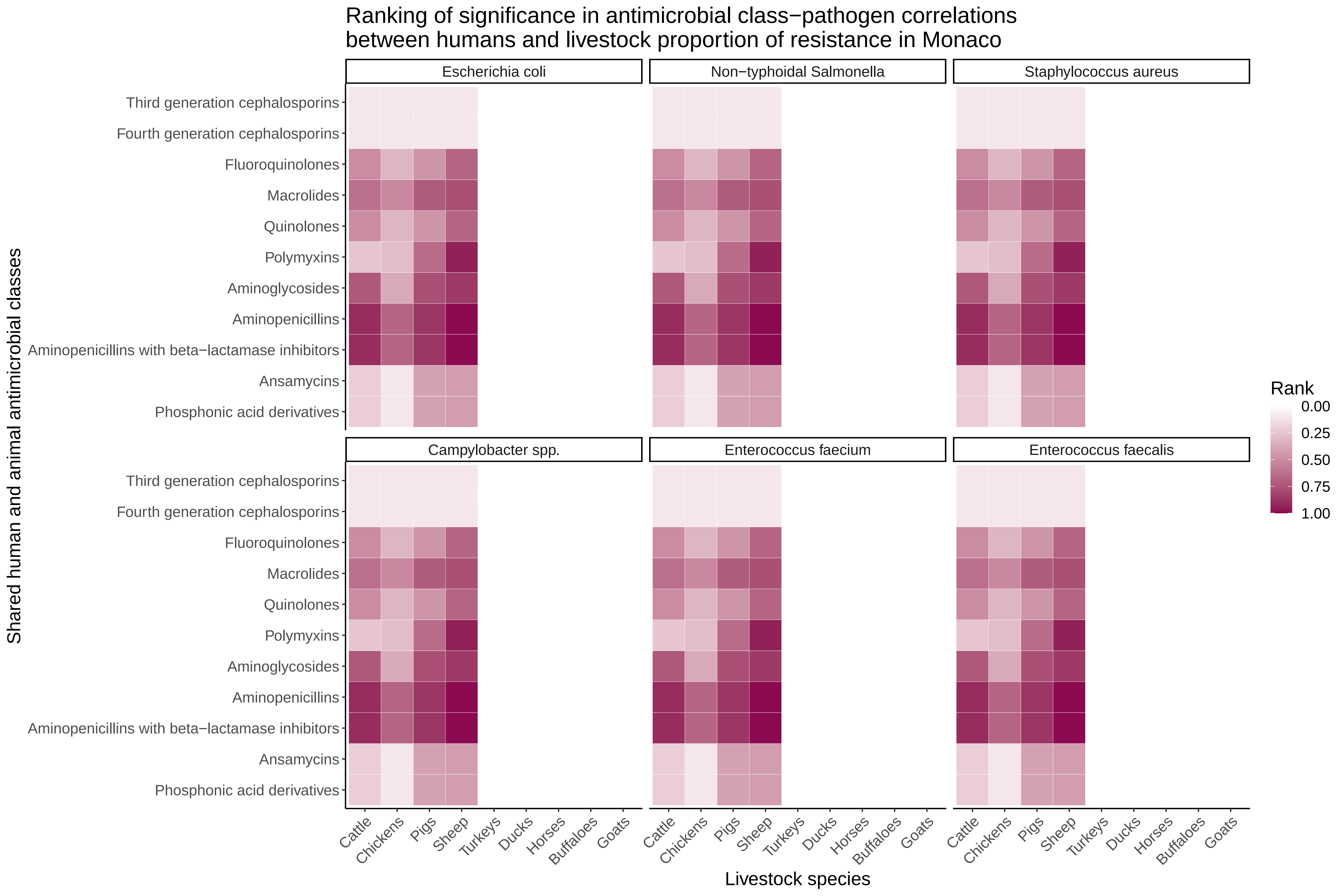
Ranking of significance in antimicrobial class−pathogen correlations
between humans and livestock proportion of resistance in Monaco
Escherichia coli
Non−typhoidal Salmonella
Staphylococcus aureus
Campylobacter spp.
Enterococcus faecium
Enterococcus faecalis
Shared human and animal antimicrobial classes
Third generation cephalosporins
Fourth generation cephalosporins
Fluoroquinolones
Macrolides
Quinolones
Polymyxins
Aminoglycosides
Aminopenicillins
Aminopenicillins with beta−lactamase inhibitors
Ansamycins
Phosphonic acid derivatives
Livestock species
Cattle
Chickens
Pigs
Sheep
Turkeys
Ducks
Horses
Buffaloes
Goats
Rank
0.00
0.25
0.50
0.75
1.00

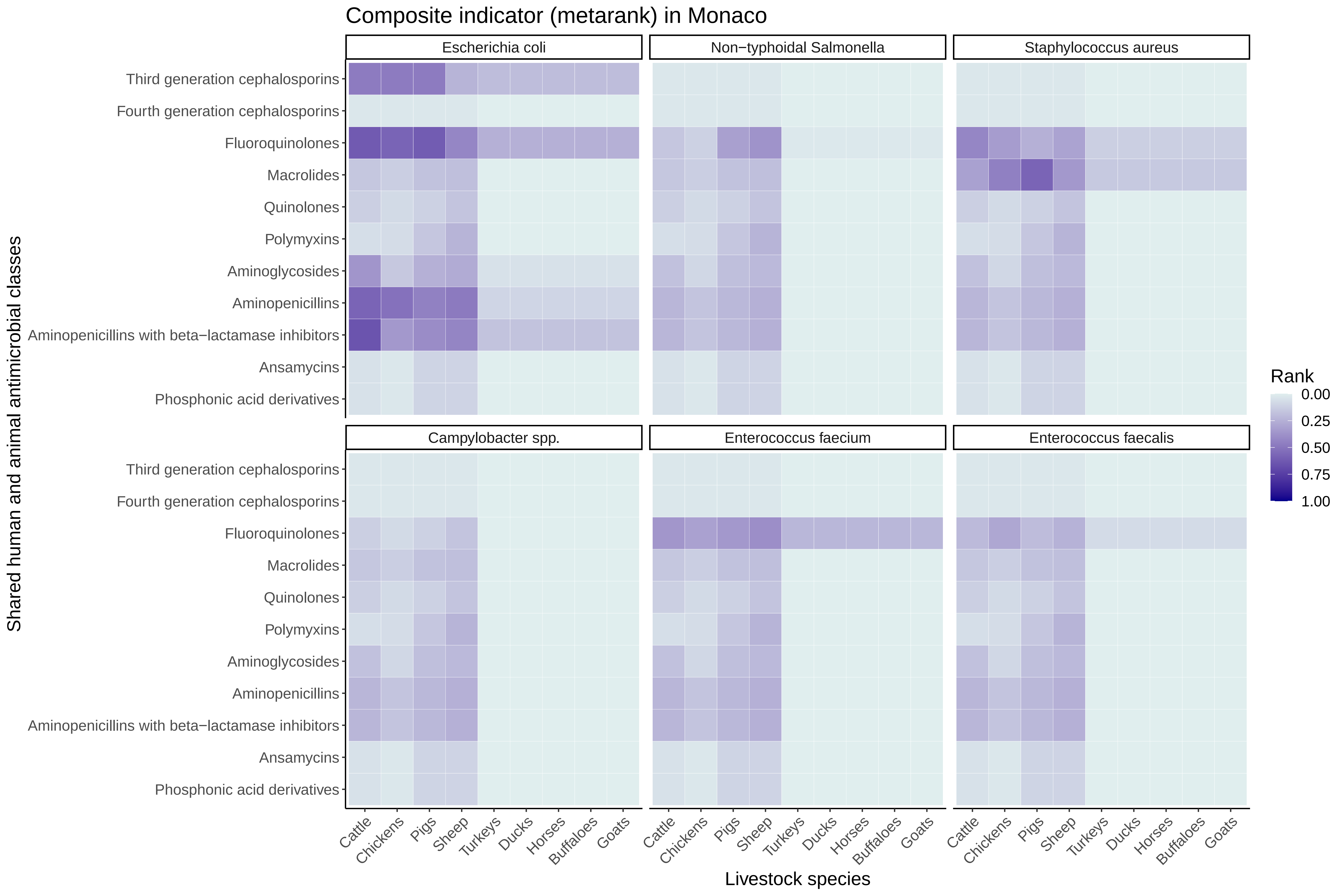

Composite indicator (metarank) in Monaco
Escherichia coli
Non−typhoidal Salmonella
Staphylococcus aureus
Campylobacter spp.
Enterococcus faecium
Enterococcus faecalis
Shared human and animal antimicrobial classes
Third generation cephalosporins
Fourth generation cephalosporins
Fluoroquinolones
Macrolides
Quinolones
Polymyxins
Aminoglycosides
Aminopenicillins
Aminopenicillins with beta−lactamase inhibitors
Ansamycins
Phosphonic acid derivatives
Rank
0.00
0.25
0.50
0.75
1.00
Cattle
Chickens
Pigs
Sheep
Turkeys
Ducks
Horses
Buffaloes
Goats
Livestock species

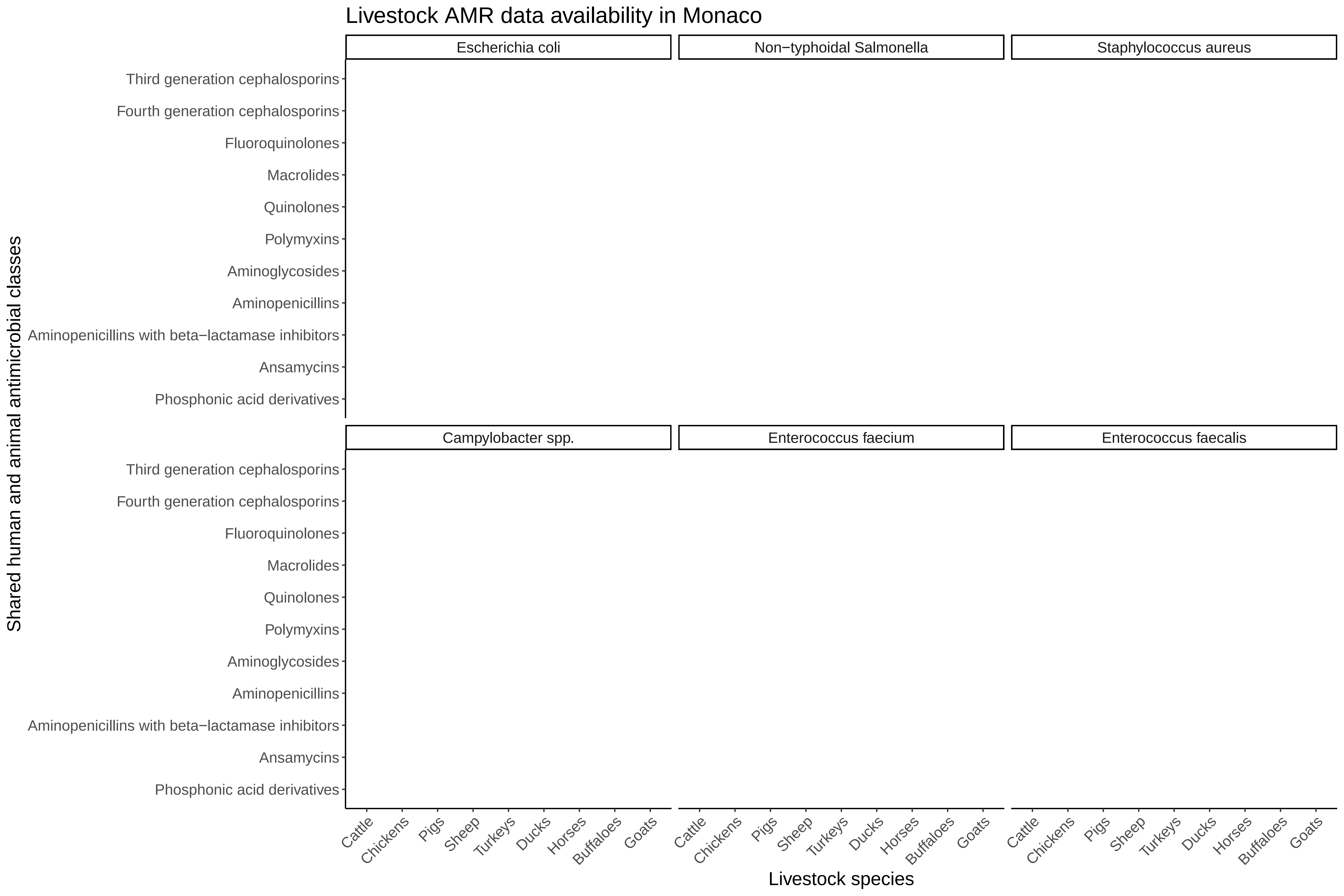

Livestock AMR data availability in Monaco
Escherichia coli
Non−typhoidal Salmonella
Staphylococcus aureus
Campylobacter spp.
Enterococcus faecium
Enterococcus faecalis
Shared human and animal antimicrobial classes
Third generation cephalosporins
Fourth generation cephalosporins
Fluoroquinolones
Macrolides
Quinolones
Polymyxins
Aminoglycosides
Aminopenicillins
Aminopenicillins with beta−lactamase inhibitors
Ansamycins
Phosphonic acid derivatives
Cattle
Chickens
Pigs
Sheep
Turkeys
Ducks
Horses
Buffaloes
Goats
Livestock species

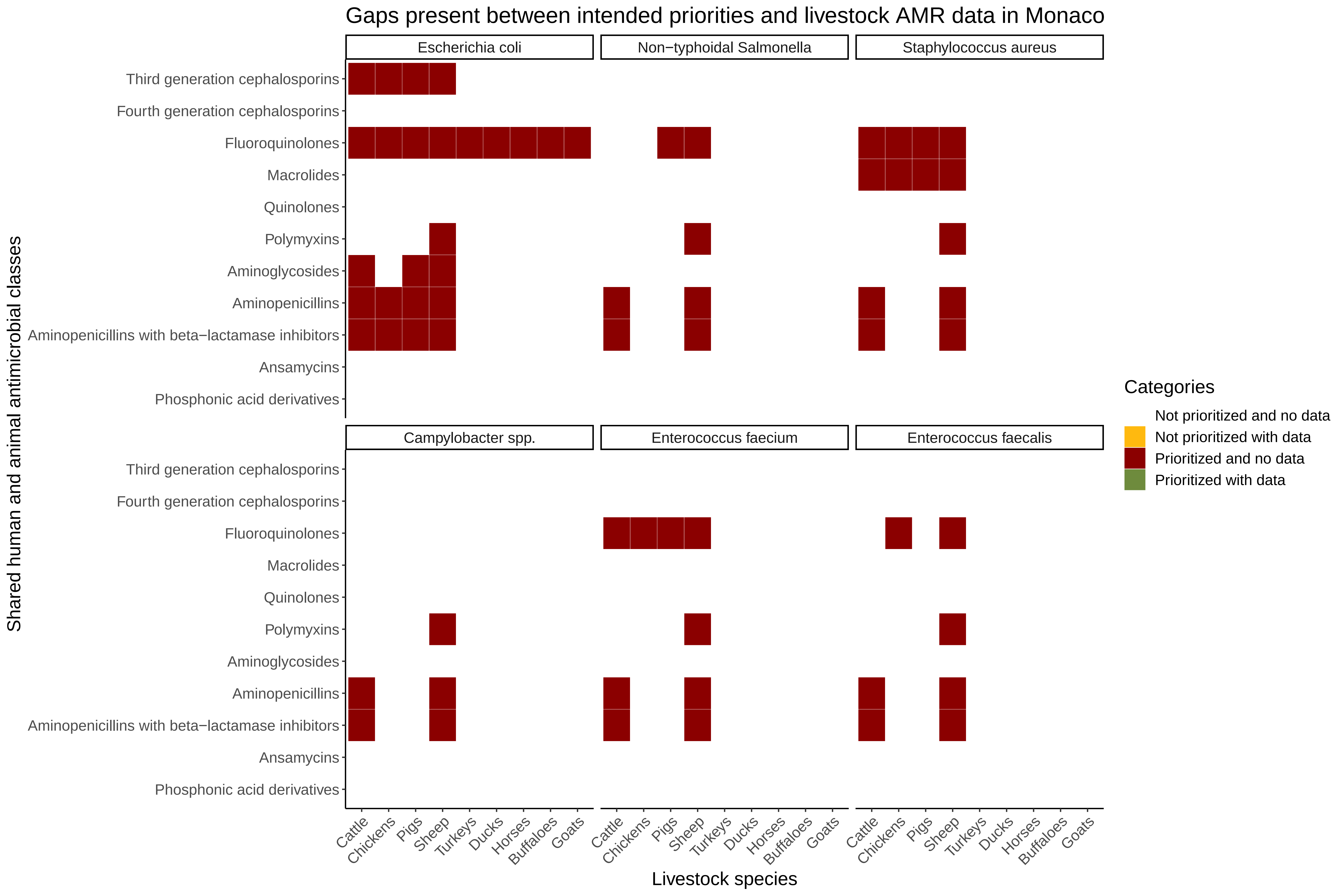

Gaps present between intended priorities and livestock AMR data in Monaco
Escherichia coli
Non−typhoidal Salmonella
Staphylococcus aureus
Campylobacter spp.
Enterococcus faecium
Enterococcus faecalis
Third generation cephalosporins
Fourth generation cephalosporins
Fluoroquinolones
Macrolides
Quinolones
Polymyxins
Aminoglycosides
Aminopenicillins
Aminopenicillins with beta−lactamase inhibitors
Ansamycins
Phosphonic acid derivatives
Shared human and animal antimicrobial classes
Cattle
Chickens
Pigs
Sheep
Turkeys
Ducks
Horses
Buffaloes
Goats
Livestock species
Categories
Not prioritized and no data
Not prioritized with data
Prioritized and no data
Prioritized with data

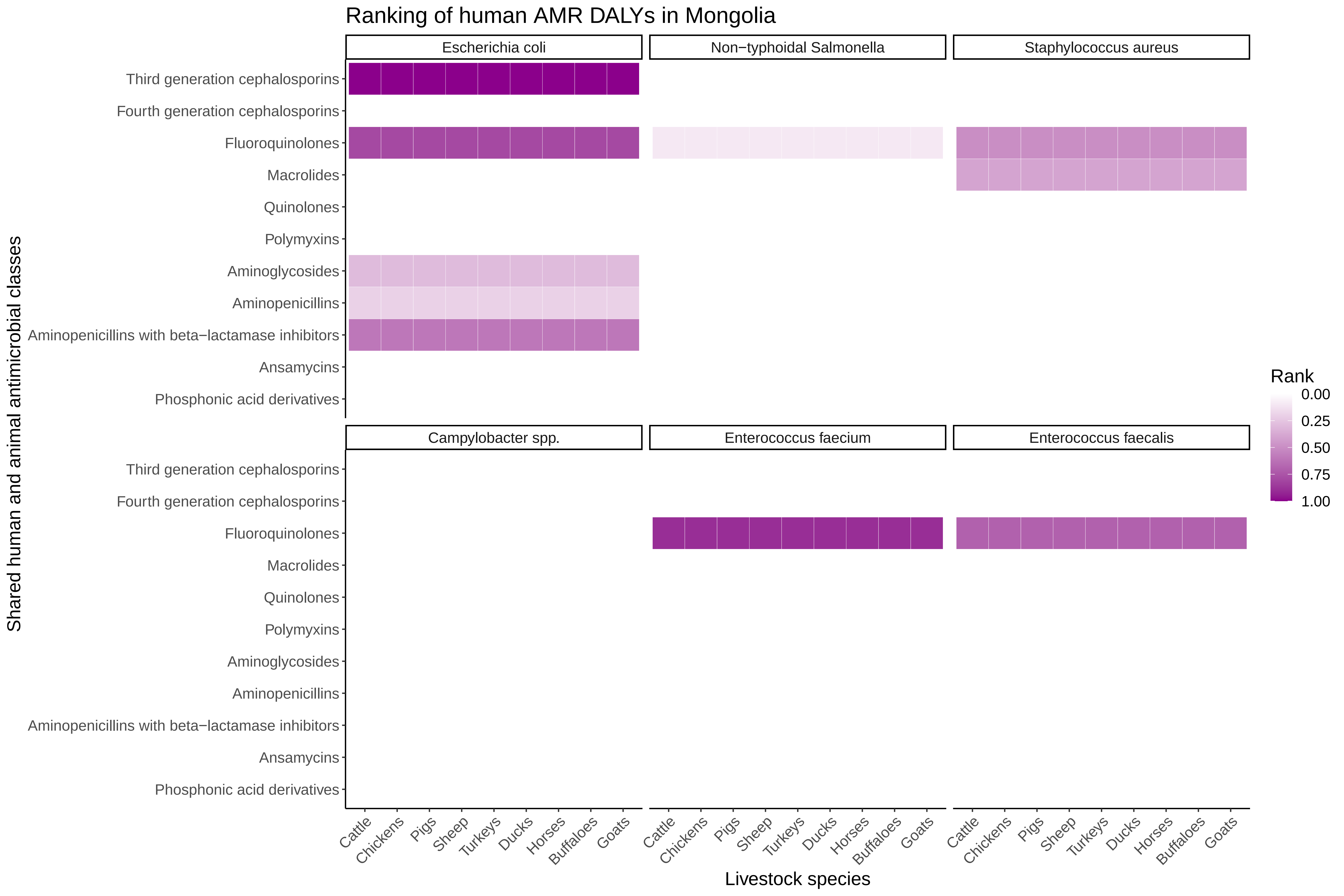

Ranking of human AMR DALYs in Mongolia
Escherichia coli
Non−typhoidal Salmonella
Staphylococcus aureus
Campylobacter spp.
Enterococcus faecium
Enterococcus faecalis
Third generation cephalosporins
Fourth generation cephalosporins
Fluoroquinolones
Macrolides
Quinolones
Polymyxins
Aminoglycosides
Aminopenicillins
Aminopenicillins with beta−lactamase inhibitors
Ansamycins
Phosphonic acid derivatives
Shared human and animal antimicrobial classes
Cattle
Chickens
Pigs
Sheep
Turkeys
Ducks
Horses
Buffaloes
Goats
Livestock species
Rank
0.00
0.25
0.50
0.75
1.00

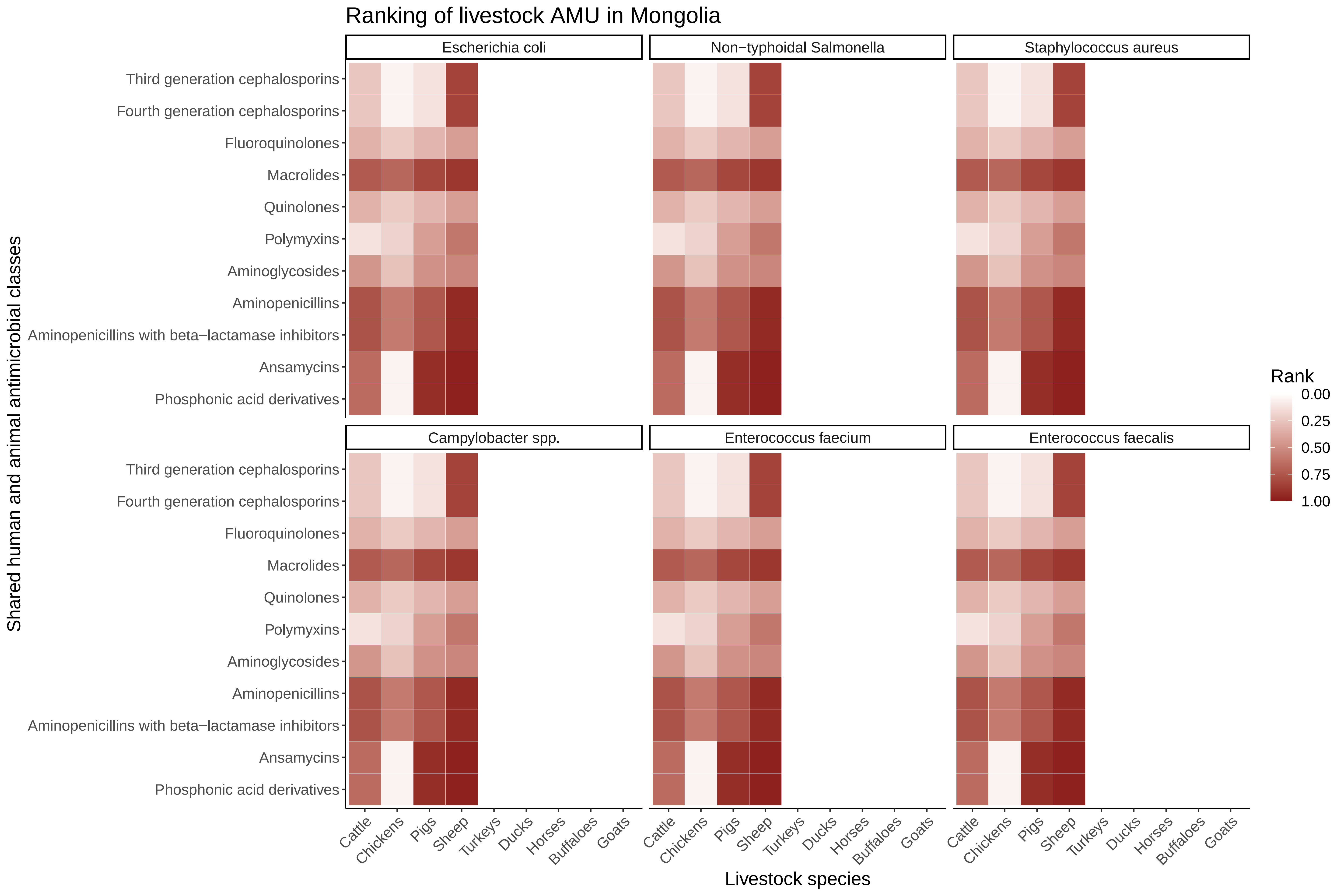
Ranking of livestock AMU in Mongolia
Escherichia coli
Non−typhoidal Salmonella
Staphylococcus aureus
Campylobacter spp.
Enterococcus faecium
Enterococcus faecalis
Third generation cephalosporins
Fourth generation cephalosporins
Fluoroquinolones
Macrolides
Quinolones
Polymyxins
Aminoglycosides
Aminopenicillins
Aminopenicillins with beta−lactamase inhibitors
Ansamycins
Phosphonic acid derivatives
Shared human and animal antimicrobial classes
Cattle
Chickens
Pigs
Sheep
Turkeys
Ducks
Horses
Buffaloes
Goats
Livestock species
Rank
0.00
0.25
0.50
0.75
1.00

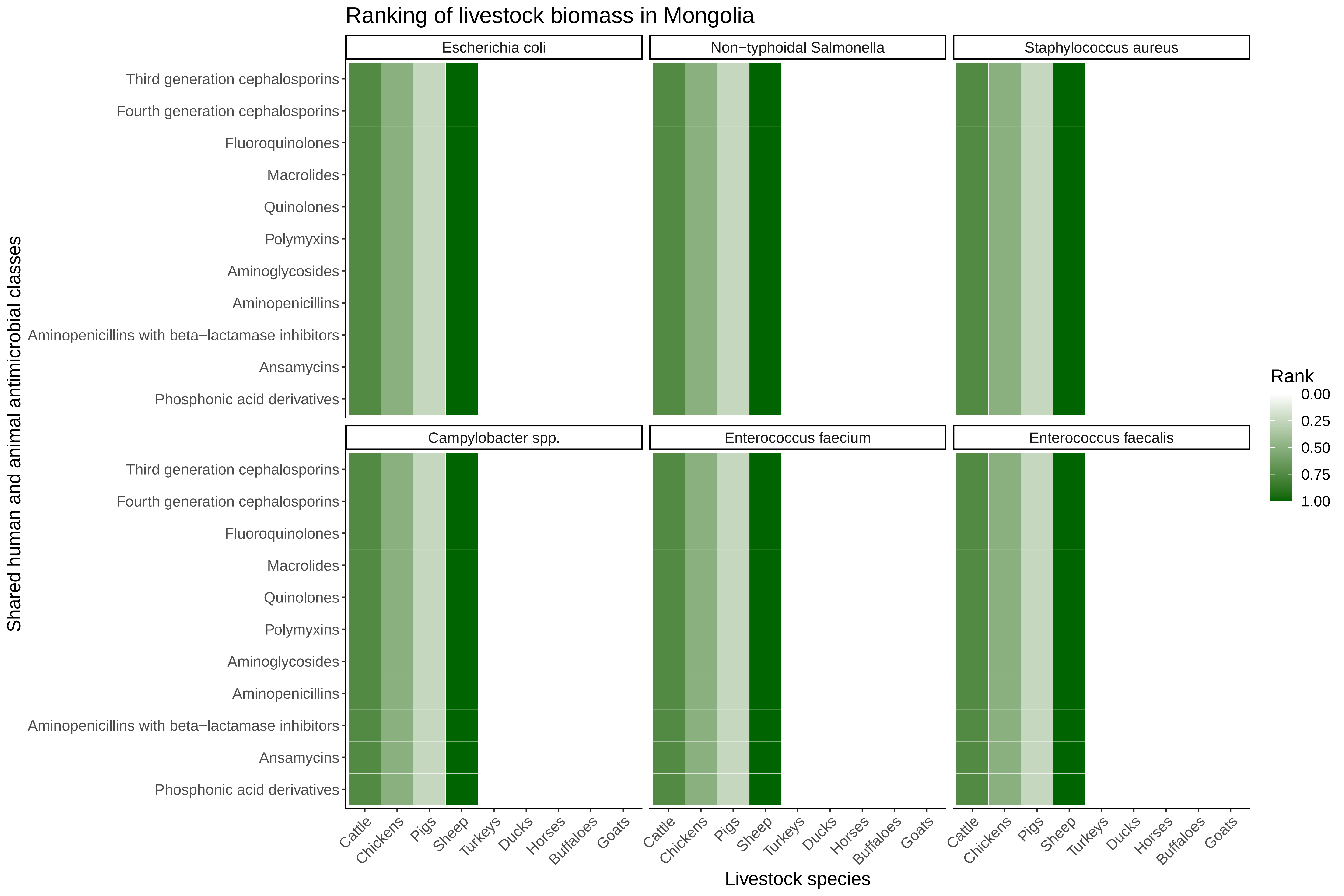

Ranking of livestock biomass in Mongolia
Escherichia coli
Non−typhoidal Salmonella
Staphylococcus aureus
Campylobacter spp.
Enterococcus faecium
Enterococcus faecalis
Third generation cephalosporins
Fourth generation cephalosporins
Fluoroquinolones
Macrolides
Quinolones
Polymyxins
Aminoglycosides
Aminopenicillins
Aminopenicillins with beta−lactamase inhibitors
Ansamycins
Phosphonic acid derivatives
Shared human and animal antimicrobial classes
Cattle
Chickens
Pigs
Sheep
Turkeys
Ducks
Horses
Buffaloes
Goats
Livestock species
Rank
0.00
0.25
0.50
0.75
1.00

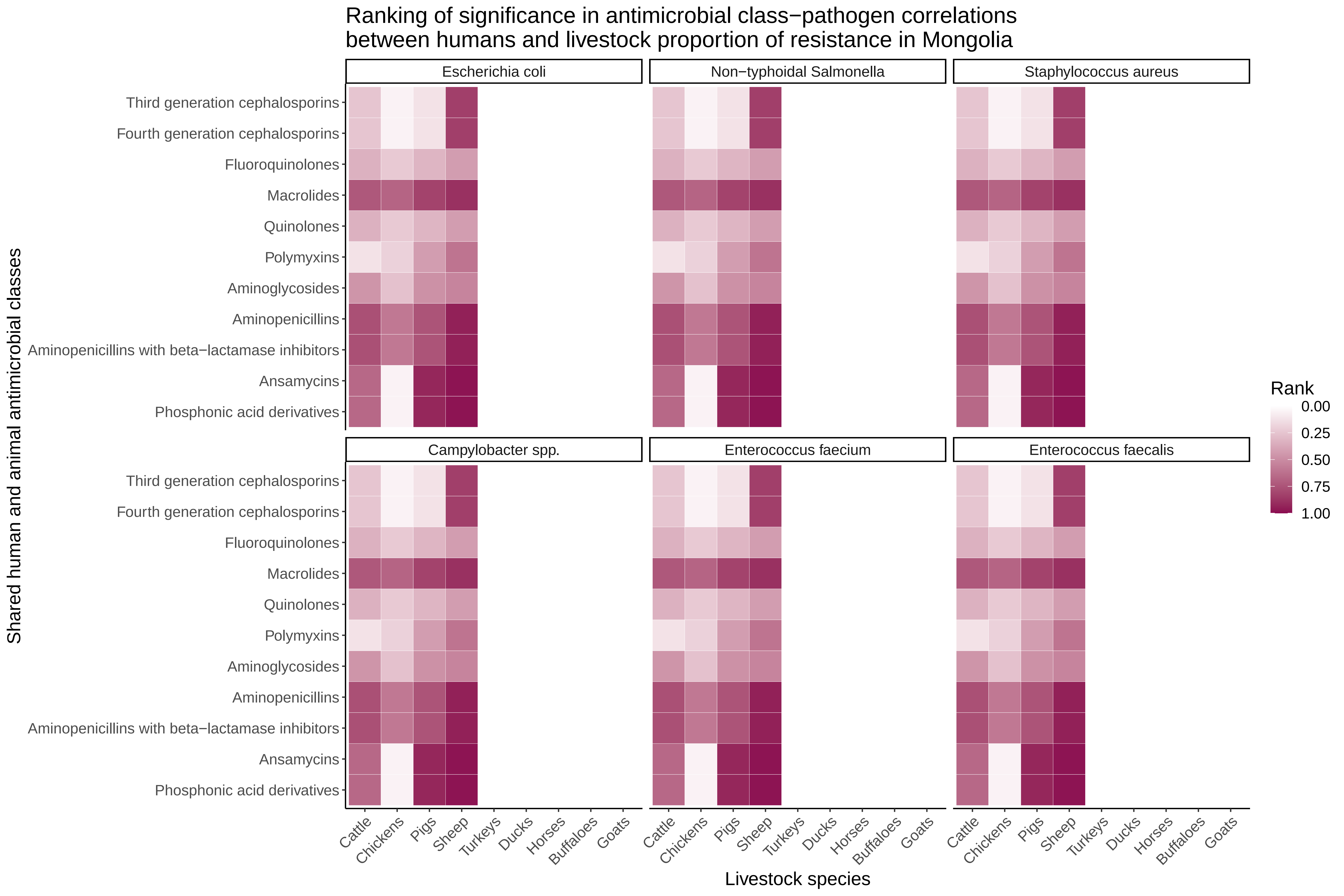
Ranking of significance in antimicrobial class−pathogen correlations
between humans and livestock proportion of resistance in Mongolia
Escherichia coli
Non−typhoidal Salmonella
Staphylococcus aureus
Campylobacter spp.
Enterococcus faecium
Enterococcus faecalis
Third generation cephalosporins
Fourth generation cephalosporins
Fluoroquinolones
Macrolides
Quinolones
Polymyxins
Aminoglycosides
Aminopenicillins
Aminopenicillins with beta−lactamase inhibitors
Ansamycins
Phosphonic acid derivatives
Shared human and animal antimicrobial classes
Cattle
Chickens
Pigs
Sheep
Turkeys
Ducks
Horses
Buffaloes
Goats
Livestock species
Rank
0.00
0.25
0.50
0.75
1.00

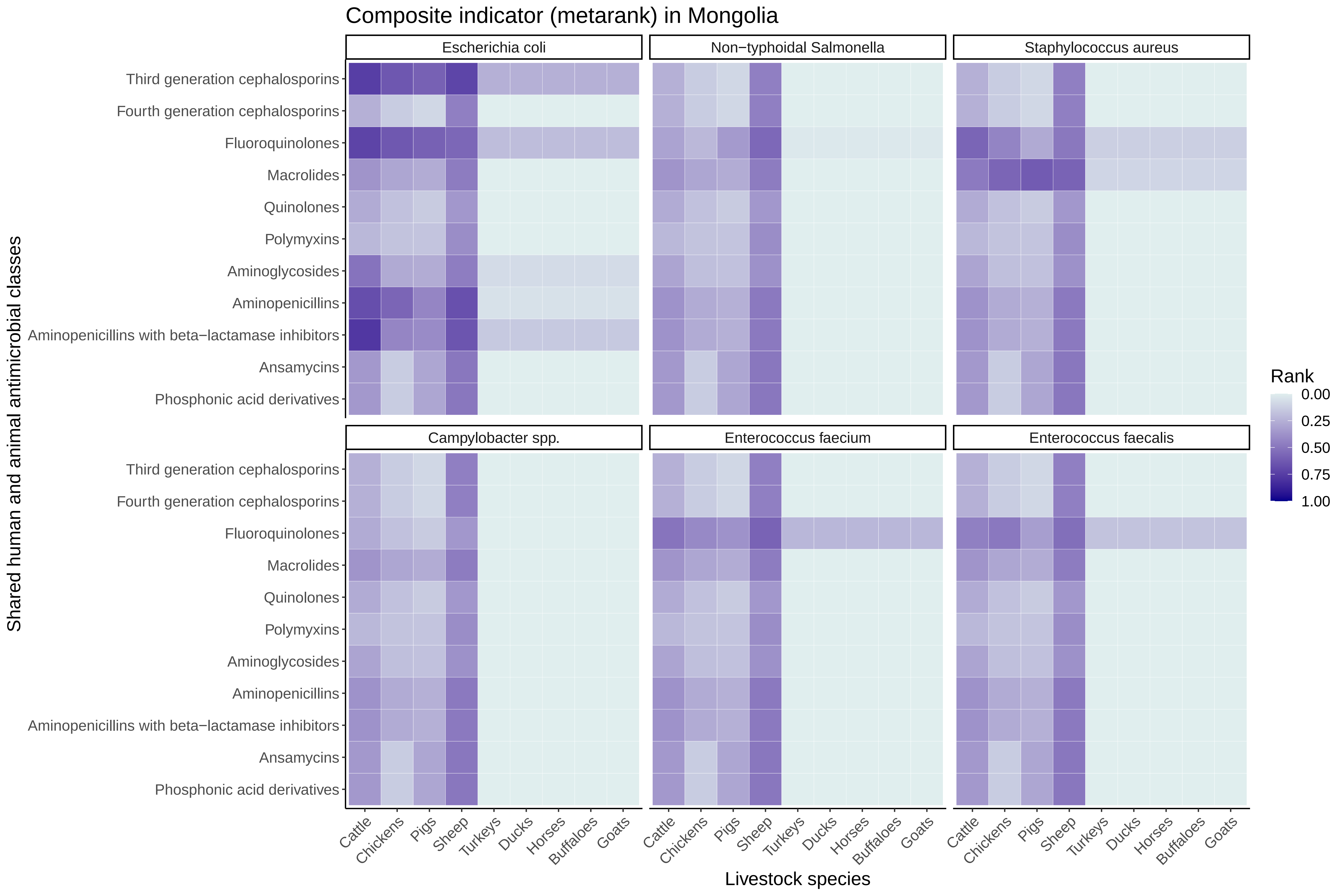

Composite indicator (metarank) in Mongolia
Escherichia coli
Non−typhoidal Salmonella
Staphylococcus aureus
Campylobacter spp.
Enterococcus faecium
Enterococcus faecalis
Shared human and animal antimicrobial classes
Third generation cephalosporins
Fourth generation cephalosporins
Fluoroquinolones
Macrolides
Quinolones
Polymyxins
Aminoglycosides
Aminopenicillins
Aminopenicillins with beta−lactamase inhibitors
Ansamycins
Phosphonic acid derivatives
Cattle
Chickens
Pigs
Sheep
Turkeys
Ducks
Horses
Buffaloes
Goats
Livestock species
Rank
0.00
0.25
0.50
0.75
1.00

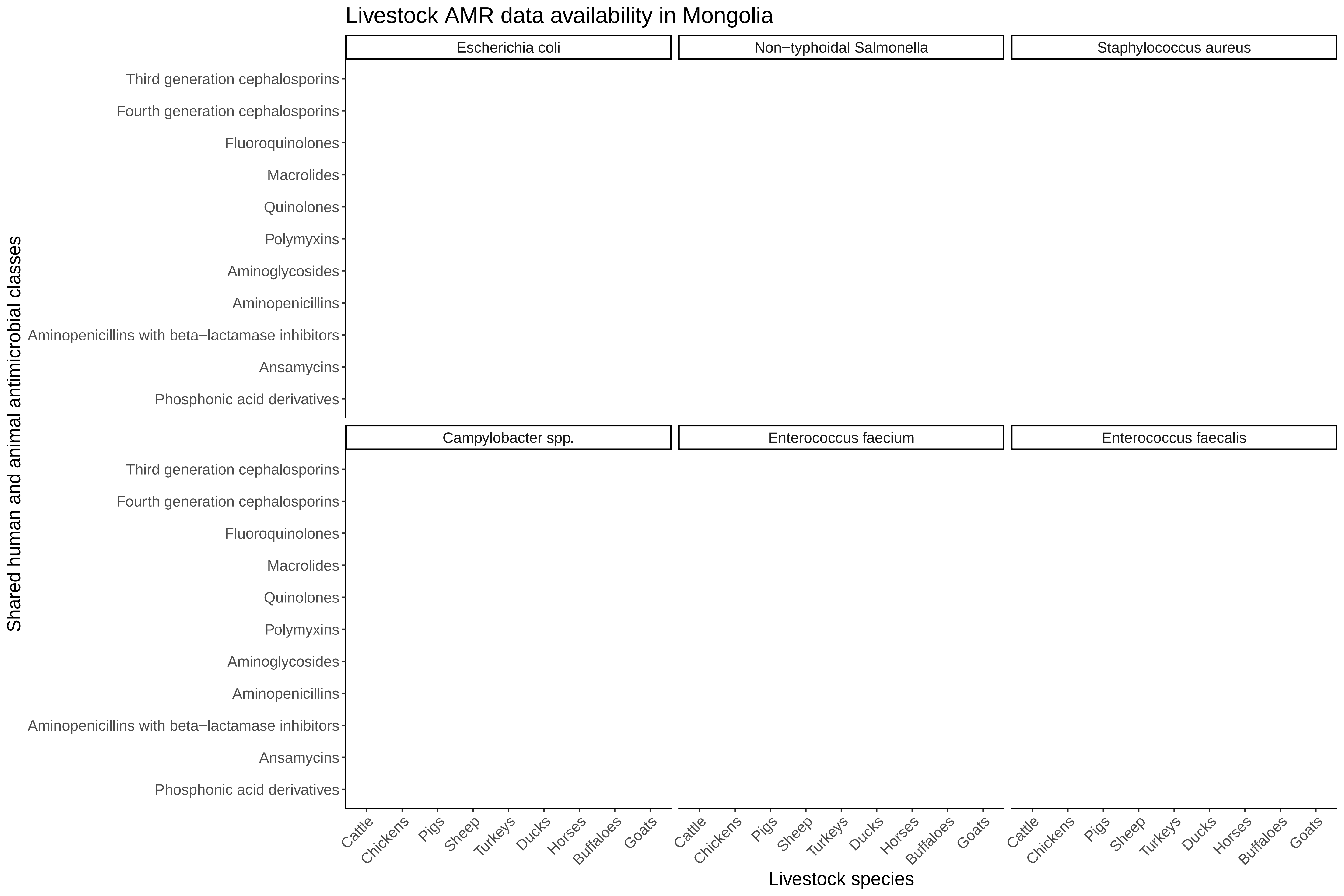
Livestock AMR data availability in Mongolia
Escherichia coli
Non−typhoidal Salmonella
Staphylococcus aureus
Campylobacter spp.
Enterococcus faecium
Enterococcus faecalis
Third generation cephalosporins
Fourth generation cephalosporins
Fluoroquinolones
Macrolides
Quinolones
Polymyxins
Aminoglycosides
Aminopenicillins
Aminopenicillins with beta−lactamase inhibitors
Ansamycins
Phosphonic acid derivatives
Shared human and animal antimicrobial classes
Cattle
Chickens
Pigs
Sheep
Turkeys
Ducks
Horses
Buffaloes
Goats
Livestock species

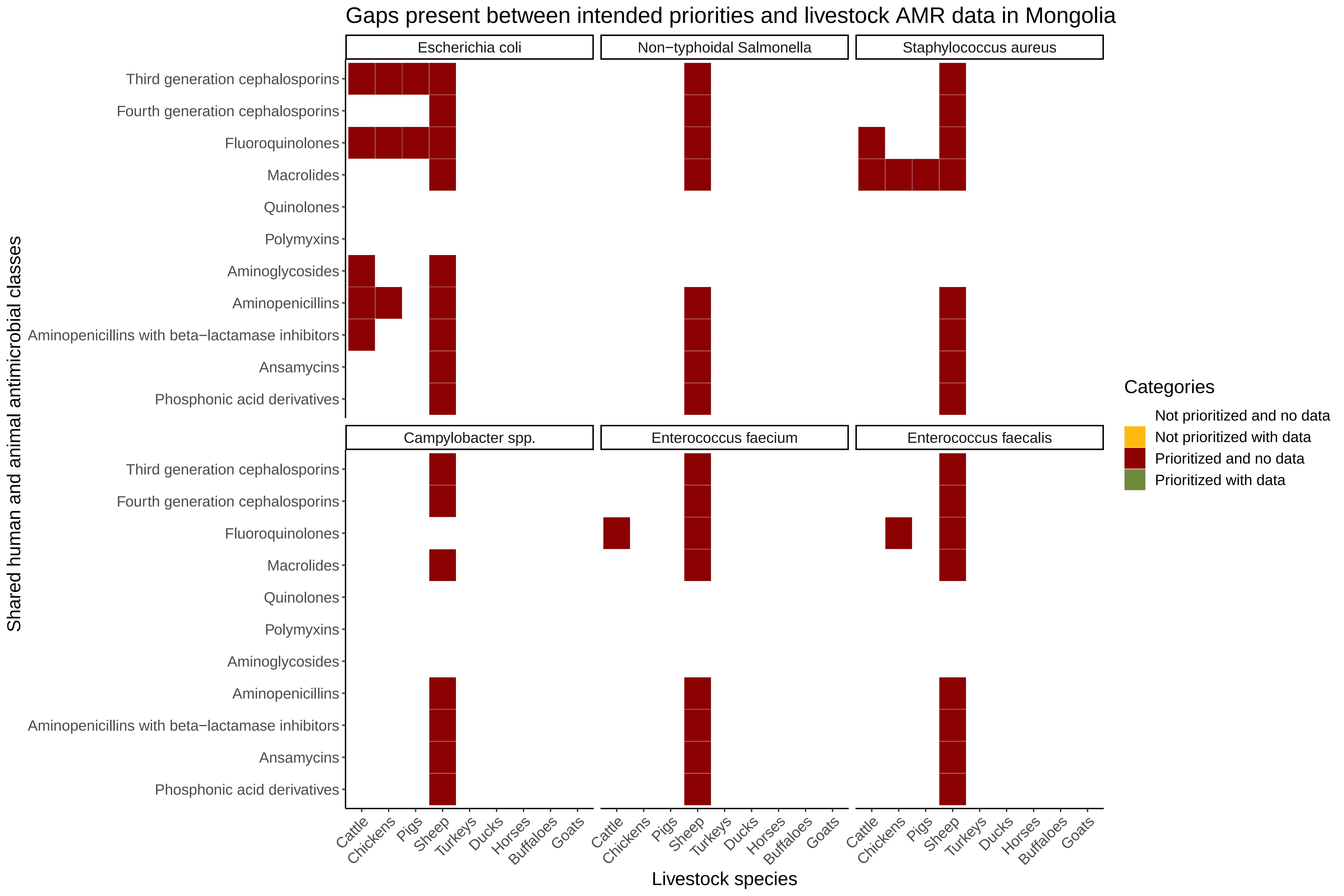

Gaps present between intended priorities and livestock AMR data in Mongolia
Escherichia coli
Non−typhoidal Salmonella
Staphylococcus aureus
Campylobacter spp.
Enterococcus faecium
Enterococcus faecalis
Shared human and animal antimicrobial classes
Third generation cephalosporins
Fourth generation cephalosporins
Fluoroquinolones
Macrolides
Quinolones
Polymyxins
Aminoglycosides
Aminopenicillins
Aminopenicillins with beta−lactamase inhibitors
Ansamycins
Phosphonic acid derivatives
Livestock species
Cattle
Chickens
Pigs
Sheep
Turkeys
Ducks
Horses
Buffaloes
Goats
Categories
Not prioritized and no data
Not prioritized with data
Prioritized and no data
Prioritized with data

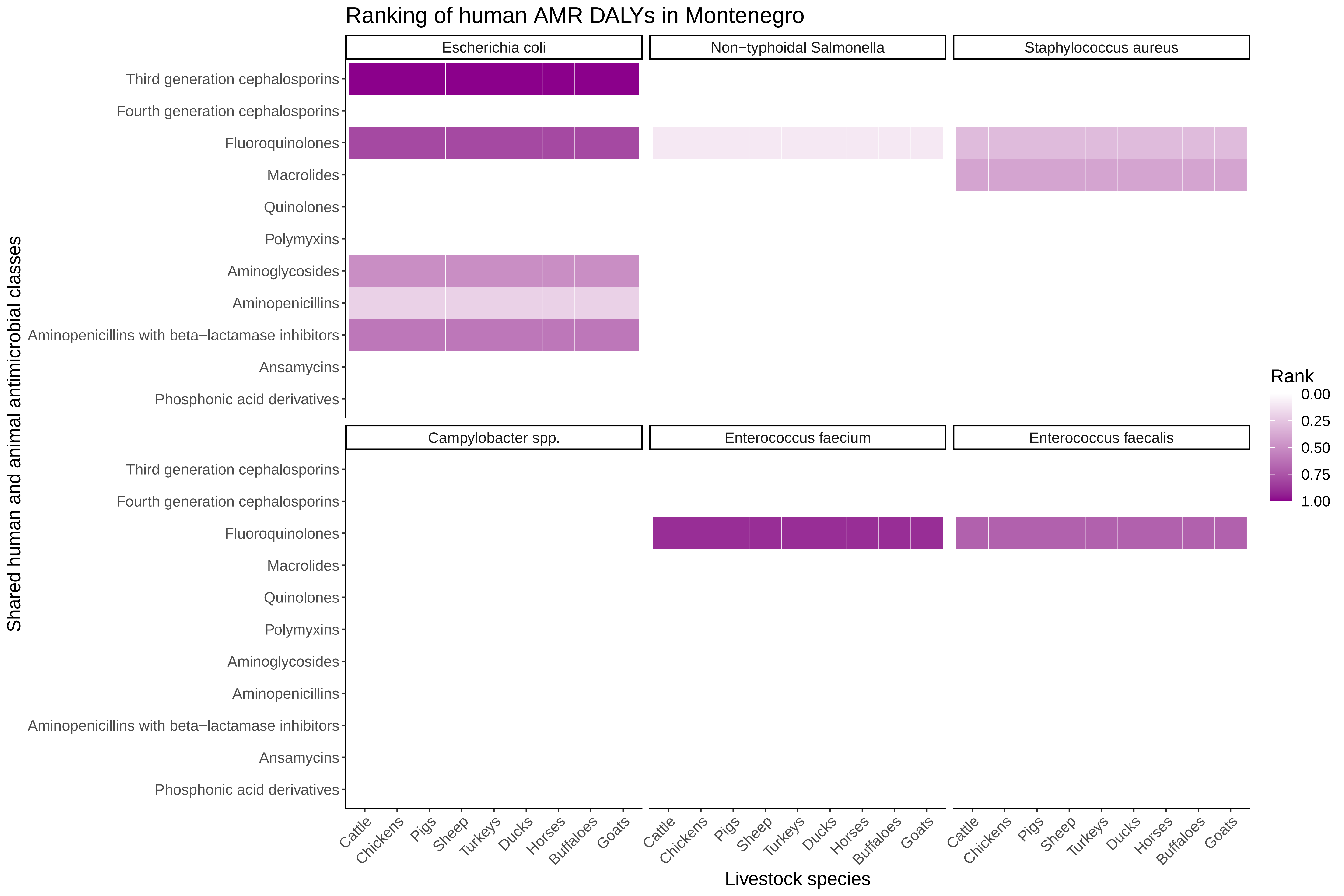

Ranking of human AMR DALYs in Montenegro
Escherichia coli
Non−typhoidal Salmonella
Staphylococcus aureus
Campylobacter spp.
Enterococcus faecium
Enterococcus faecalis
Shared human and animal antimicrobial classes
Third generation cephalosporins
Fourth generation cephalosporins
Fluoroquinolones
Macrolides
Quinolones
Polymyxins
Aminoglycosides
Aminopenicillins
Aminopenicillins with beta−lactamase inhibitors
Ansamycins
Phosphonic acid derivatives
Livestock species
Cattle
Chickens
Pigs
Sheep
Turkeys
Ducks
Horses
Buffaloes
Goats
Rank
0.00
0.25
0.50
0.75
1.00

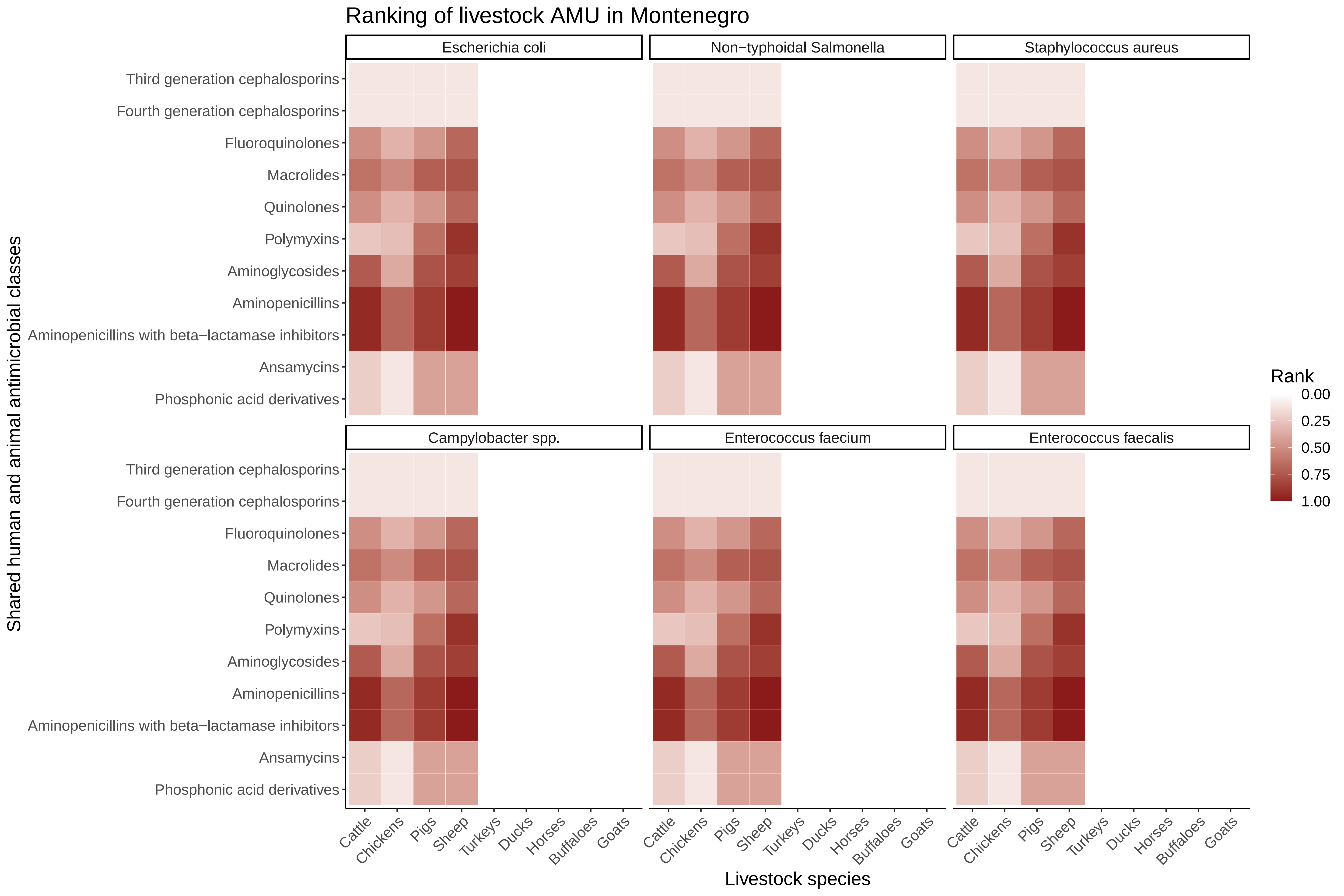

Ranking of livestock AMU in Montenegro
Escherichia coli
Non−typhoidal Salmonella
Staphylococcus aureus
Campylobacter spp.
Enterococcus faecium
Enterococcus faecalis
Shared human and animal antimicrobial classes
Third generation cephalosporins
Fourth generation cephalosporins
Fluoroquinolones
Macrolides
Quinolones
Polymyxins
Aminoglycosides
Aminopenicillins
Aminopenicillins with beta−lactamase inhibitors
Ansamycins
Phosphonic acid derivatives
Cattle
Chickens
Pigs
Sheep
Turkeys
Ducks
Horses
Buffaloes
Goats
Livestock species
Rank
0.00
0.25
0.50
0.75
1.00

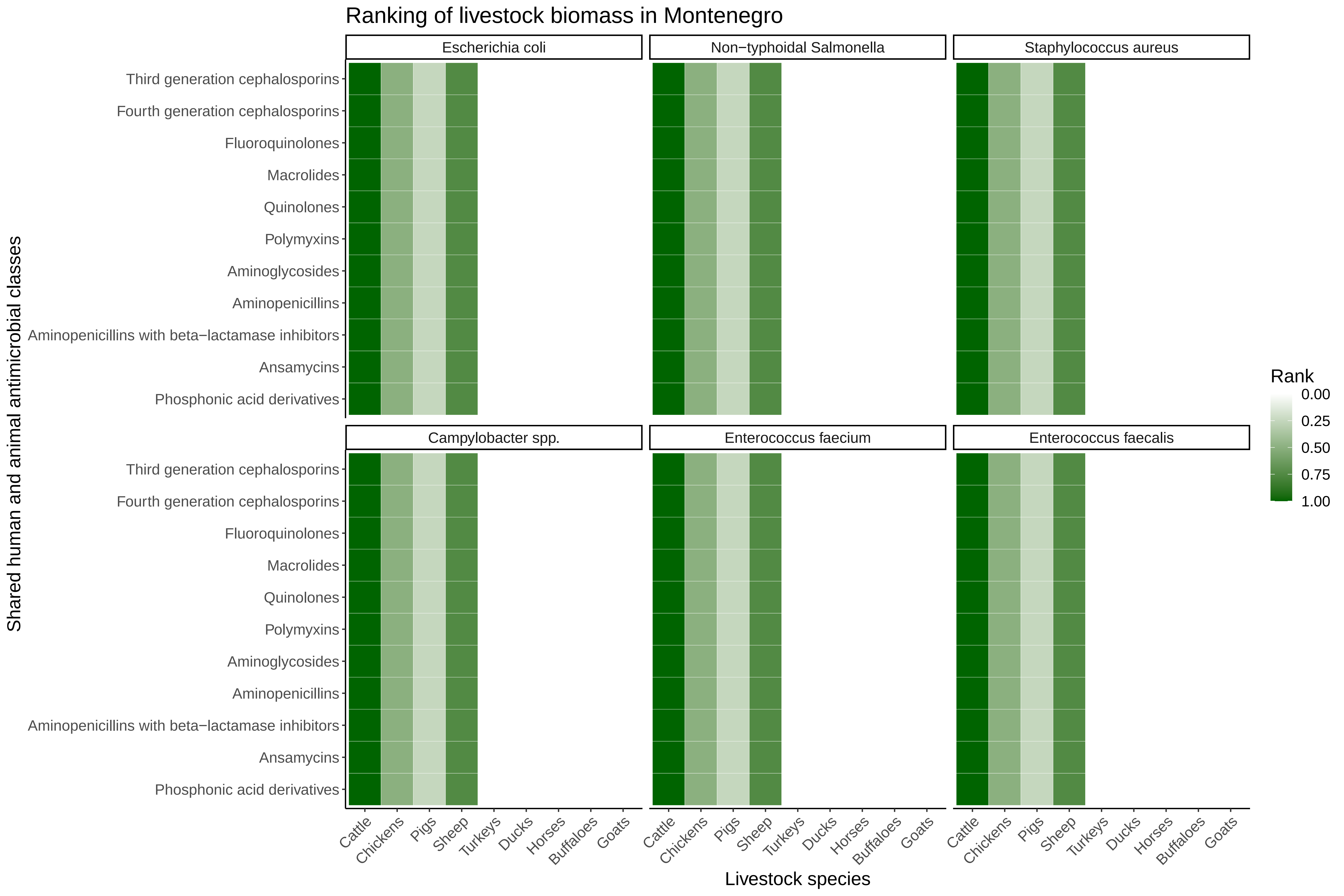

Ranking of livestock biomass in Montenegro
Escherichia coli
Non−typhoidal Salmonella
Staphylococcus aureus
Campylobacter spp.
Enterococcus faecium
Enterococcus faecalis
Shared human and animal antimicrobial classes
Third generation cephalosporins
Fourth generation cephalosporins
Fluoroquinolones
Macrolides
Quinolones
Polymyxins
Aminoglycosides
Aminopenicillins
Aminopenicillins with beta−lactamase inhibitors
Ansamycins
Phosphonic acid derivatives
Livestock species
Cattle
Chickens
Pigs
Sheep
Turkeys
Ducks
Horses
Buffaloes
Goats
Rank
0.00
0.25
0.50
0.75
1.00

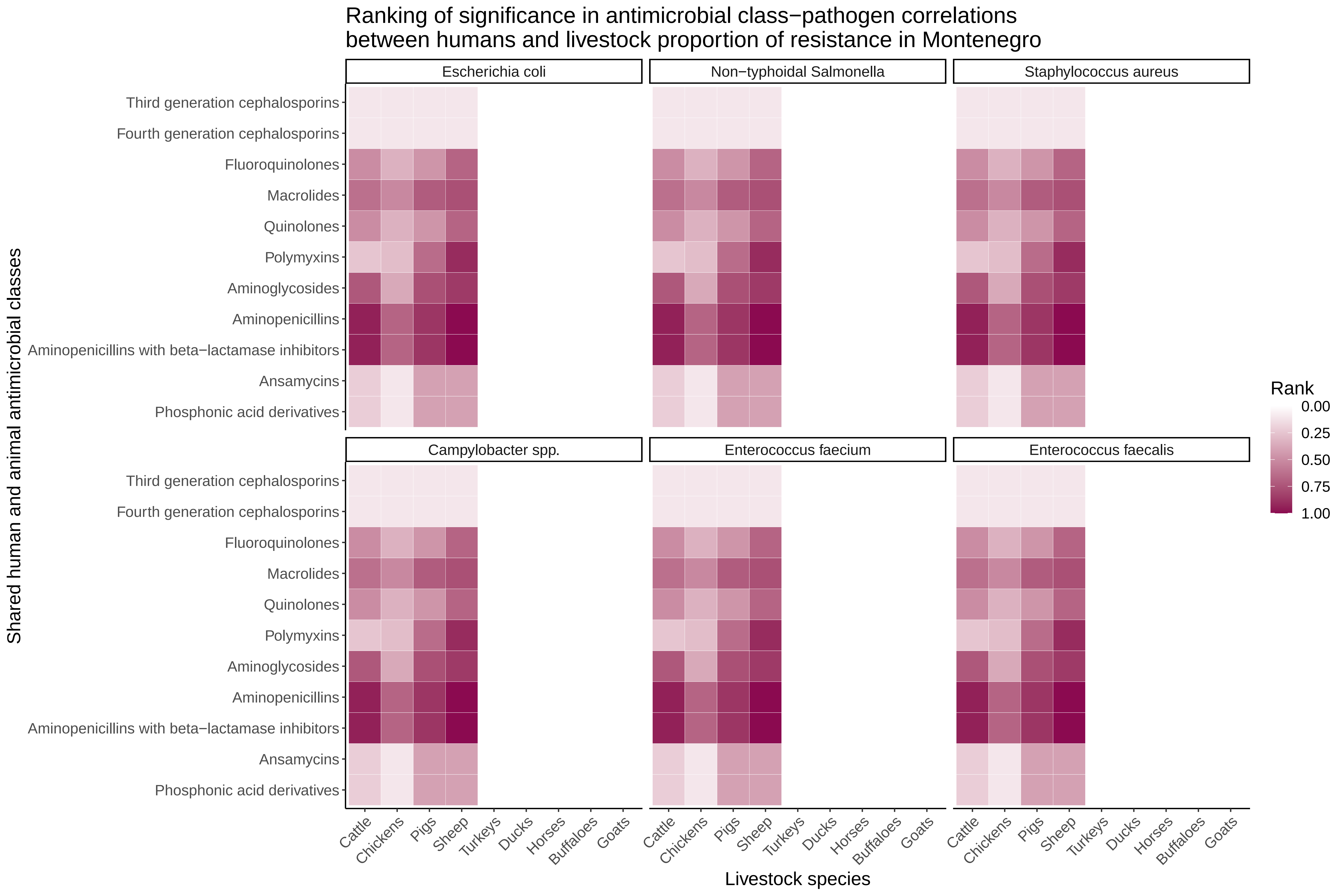

Ranking of significance in antimicrobial class−pathogen correlations
between humans and livestock proportion of resistance in Montenegro
Escherichia coli
Non−typhoidal Salmonella
Staphylococcus aureus
Campylobacter spp.
Enterococcus faecium
Enterococcus faecalis
Third generation cephalosporins
Fourth generation cephalosporins
Fluoroquinolones
Macrolides
Quinolones
Polymyxins
Aminoglycosides
Aminopenicillins
Aminopenicillins with beta−lactamase inhibitors
Ansamycins
Phosphonic acid derivatives
Shared human and animal antimicrobial classes
Cattle
Chickens
Pigs
Sheep
Turkeys
Ducks
Horses
Buffaloes
Goats
Livestock species
Rank
0.00
0.25
0.50
0.75
1.00

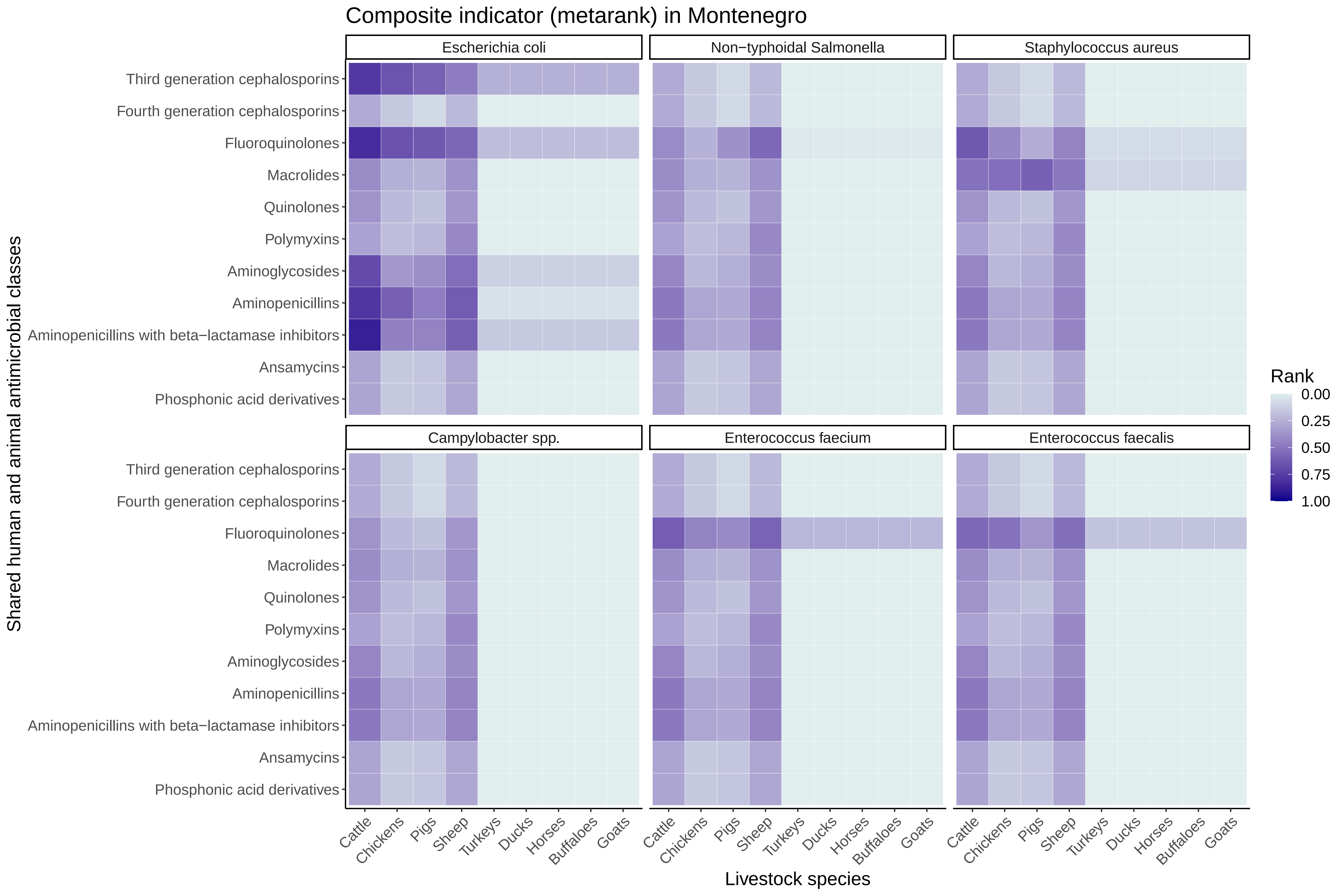

Composite indicator (metarank) in Montenegro
Escherichia coli
Non−typhoidal Salmonella
Staphylococcus aureus
Campylobacter spp.
Enterococcus faecium
Enterococcus faecalis
Shared human and animal antimicrobial classes
Third generation cephalosporins
Fourth generation cephalosporins
Fluoroquinolones
Macrolides
Quinolones
Polymyxins
Aminoglycosides
Aminopenicillins
Aminopenicillins with beta−lactamase inhibitors
Ansamycins
Phosphonic acid derivatives
Cattle
Chickens
Pigs
Sheep
Turkeys
Ducks
Horses
Buffaloes
Goats
Livestock species
Rank
0.00
0.25
0.50
0.75
1.00

Livestock AMR data availability in Montenegro

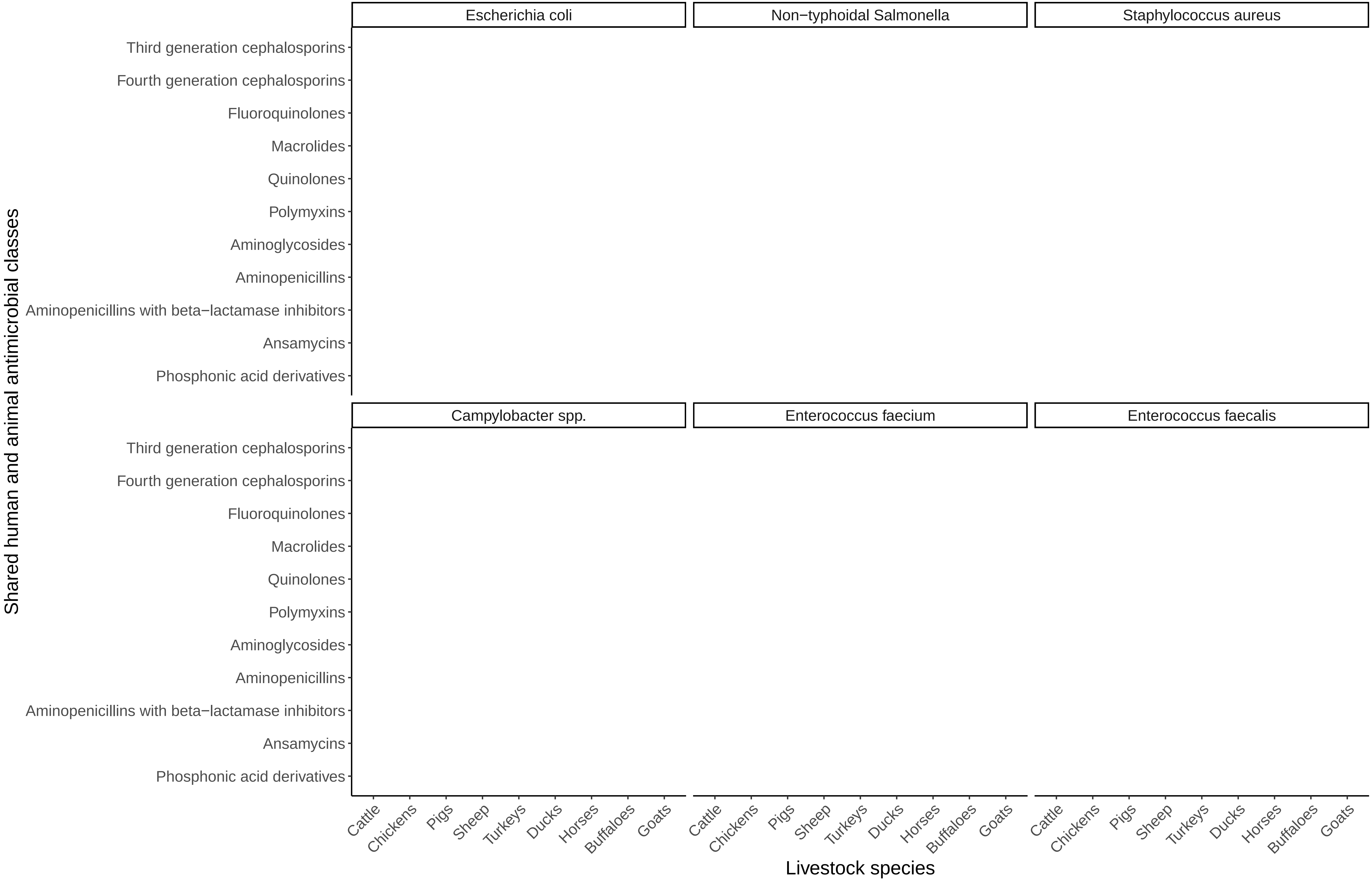

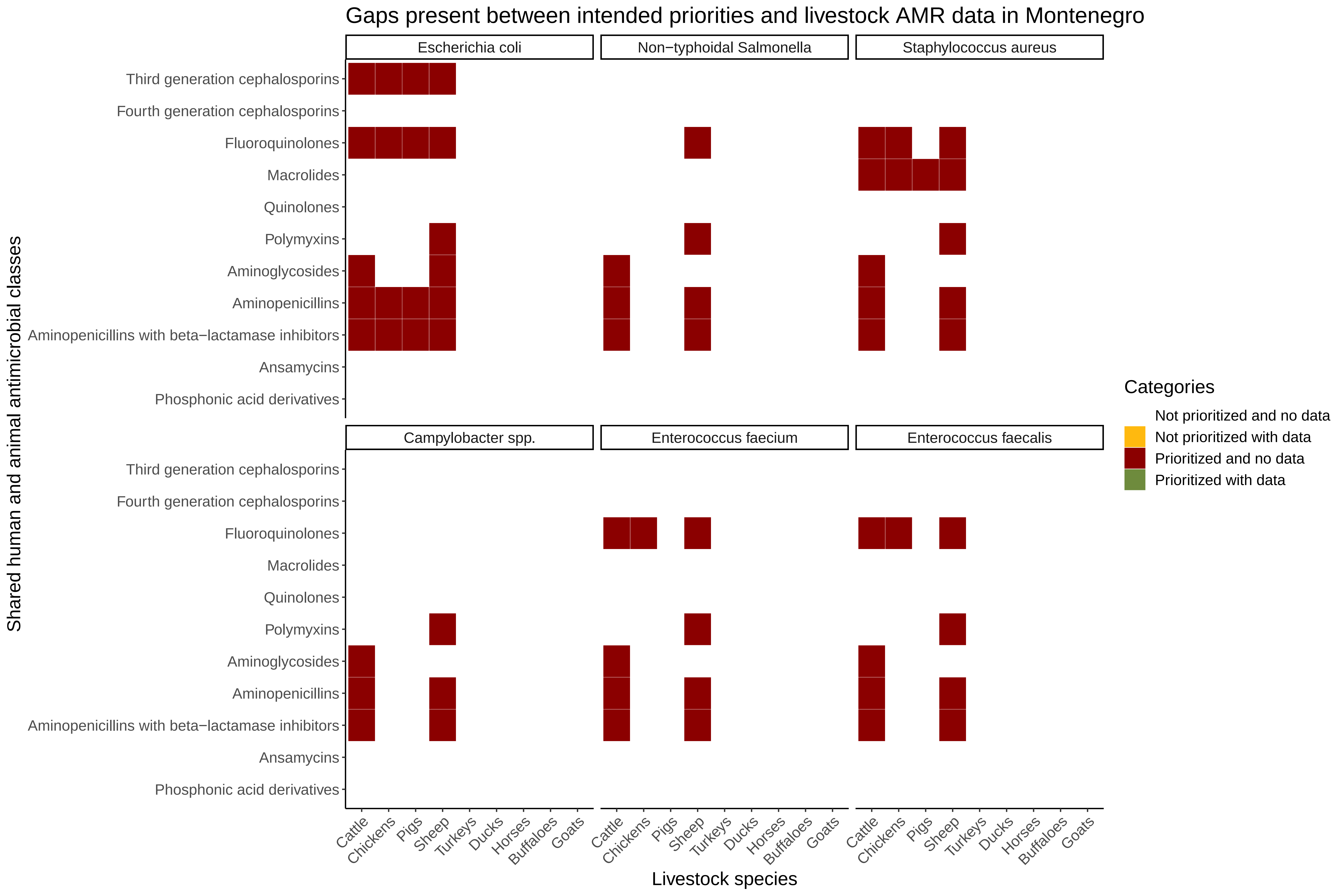

Gaps present between intended priorities and livestock AMR data in Montenegro
Escherichia coli
Non−typhoidal Salmonella
Staphylococcus aureus
Campylobacter spp.
Enterococcus faecium
Enterococcus faecalis
Third generation cephalosporins
Fourth generation cephalosporins
Fluoroquinolones
Macrolides
Quinolones
Polymyxins
Aminoglycosides
Aminopenicillins
Aminopenicillins with beta−lactamase inhibitors
Ansamycins
Phosphonic acid derivatives
Shared human and animal antimicrobial classes
Cattle
Chickens
Pigs
Sheep
Turkeys
Ducks
Horses
Buffaloes
Goats
Livestock species
Categories
Not prioritized and no data
Not prioritized with data
Prioritized and no data
Prioritized with data

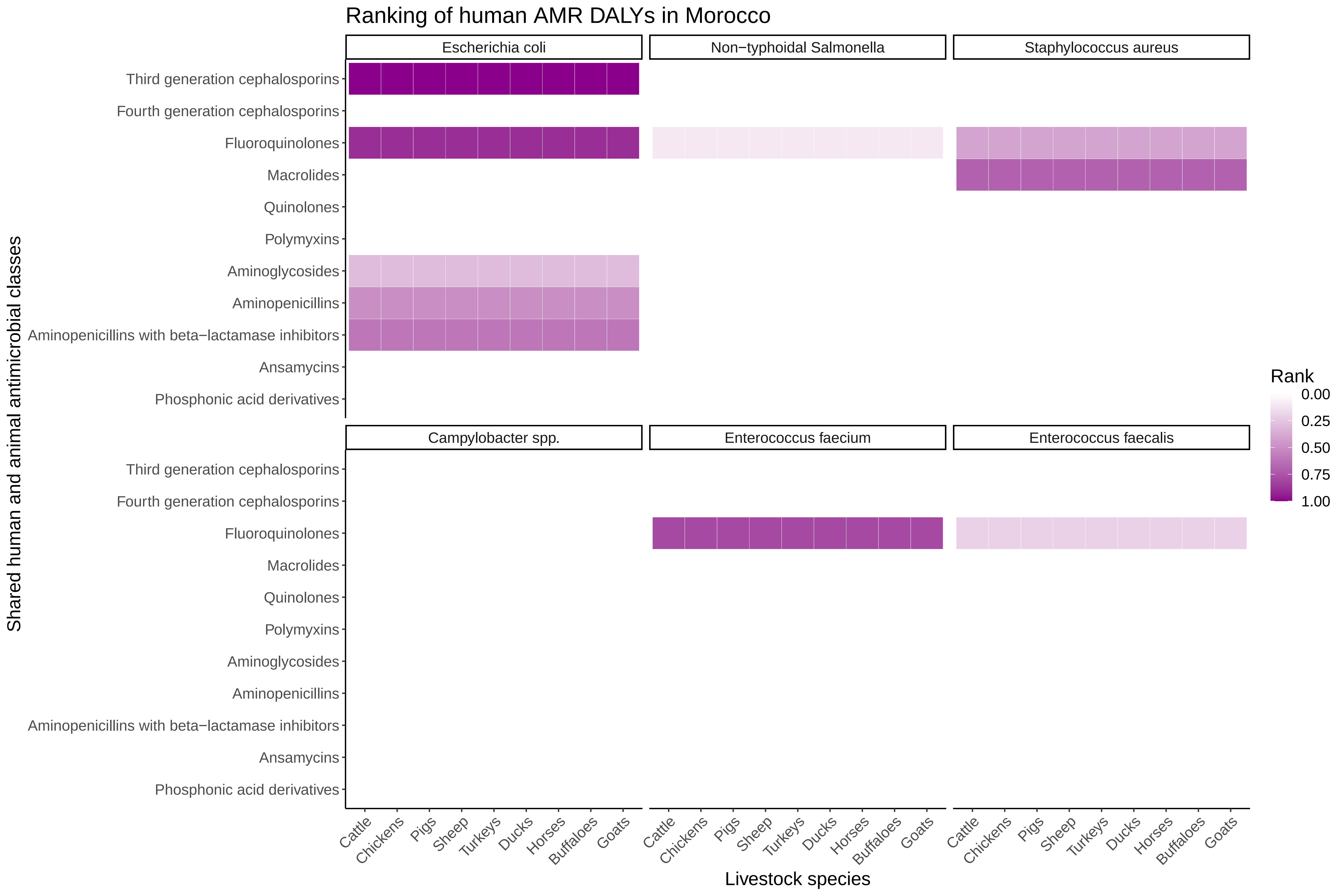

Ranking of human AMR DALYs in Morocco
Escherichia coli
Non−typhoidal Salmonella
Staphylococcus aureus
Campylobacter spp.
Enterococcus faecium
Enterococcus faecalis
Shared human and animal antimicrobial classes
Third generation cephalosporins
Fourth generation cephalosporins
Fluoroquinolones
Macrolides
Quinolones
Polymyxins
Aminoglycosides
Aminopenicillins
Aminopenicillins with beta−lactamase inhibitors
Ansamycins
Phosphonic acid derivatives
Cattle
Chickens
Pigs
Sheep
Turkeys
Ducks
Horses
Buffaloes
Goats
Livestock species
Rank
0.00
0.25
0.50
0.75
1.00

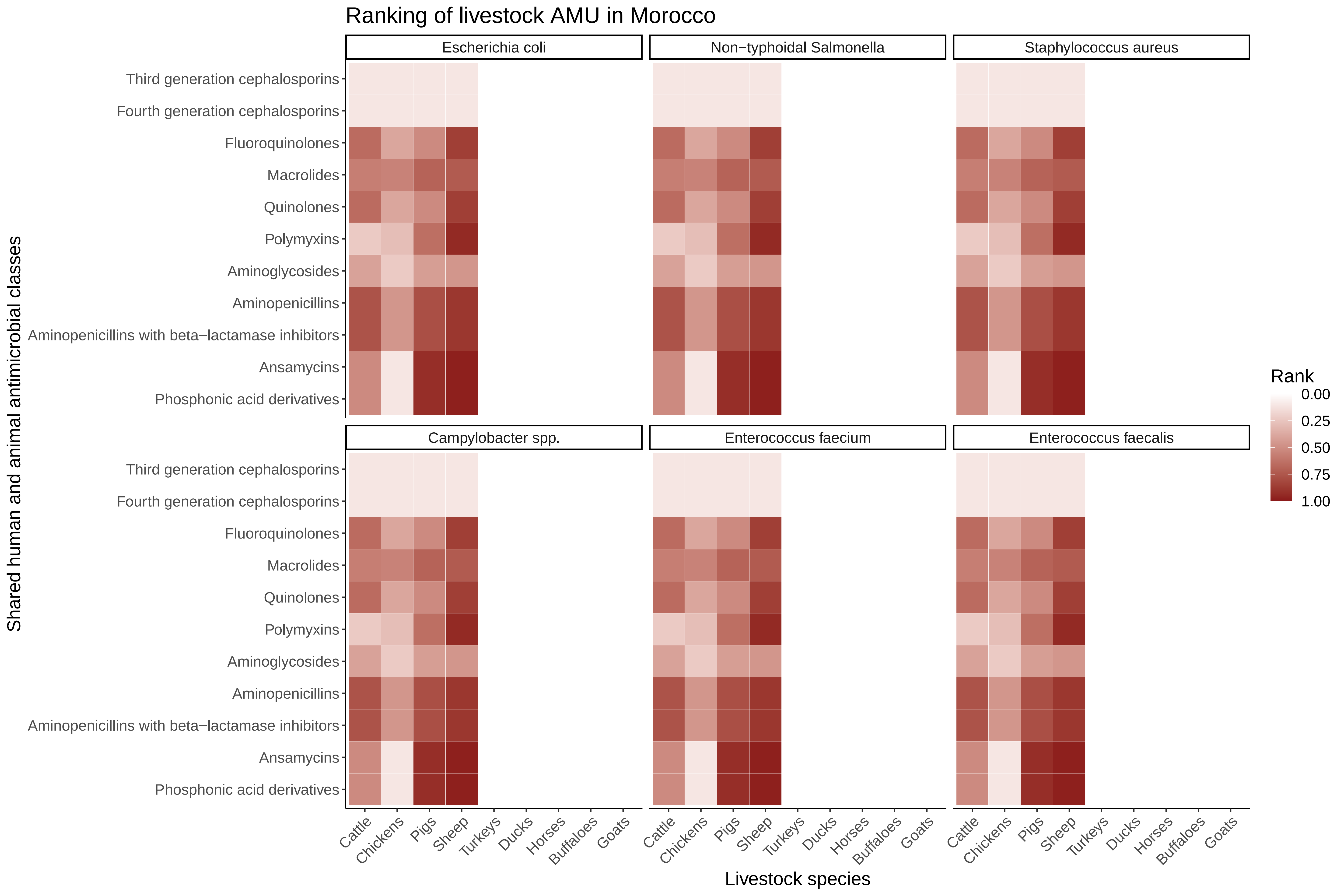
Ranking of livestock AMU in Morocco
Escherichia coli
Non−typhoidal Salmonella
Staphylococcus aureus
Campylobacter spp.
Enterococcus faecium
Enterococcus faecalis
Shared human and animal antimicrobial classes
Third generation cephalosporins
Fourth generation cephalosporins
Fluoroquinolones
Macrolides
Quinolones
Polymyxins
Aminoglycosides
Aminopenicillins
Aminopenicillins with beta−lactamase inhibitors
Ansamycins
Phosphonic acid derivatives
Livestock species
Cattle
Chickens
Pigs
Sheep
Turkeys
Ducks
Horses
Buffaloes
Goats
Rank
0.00
0.25
0.50
0.75
1.00

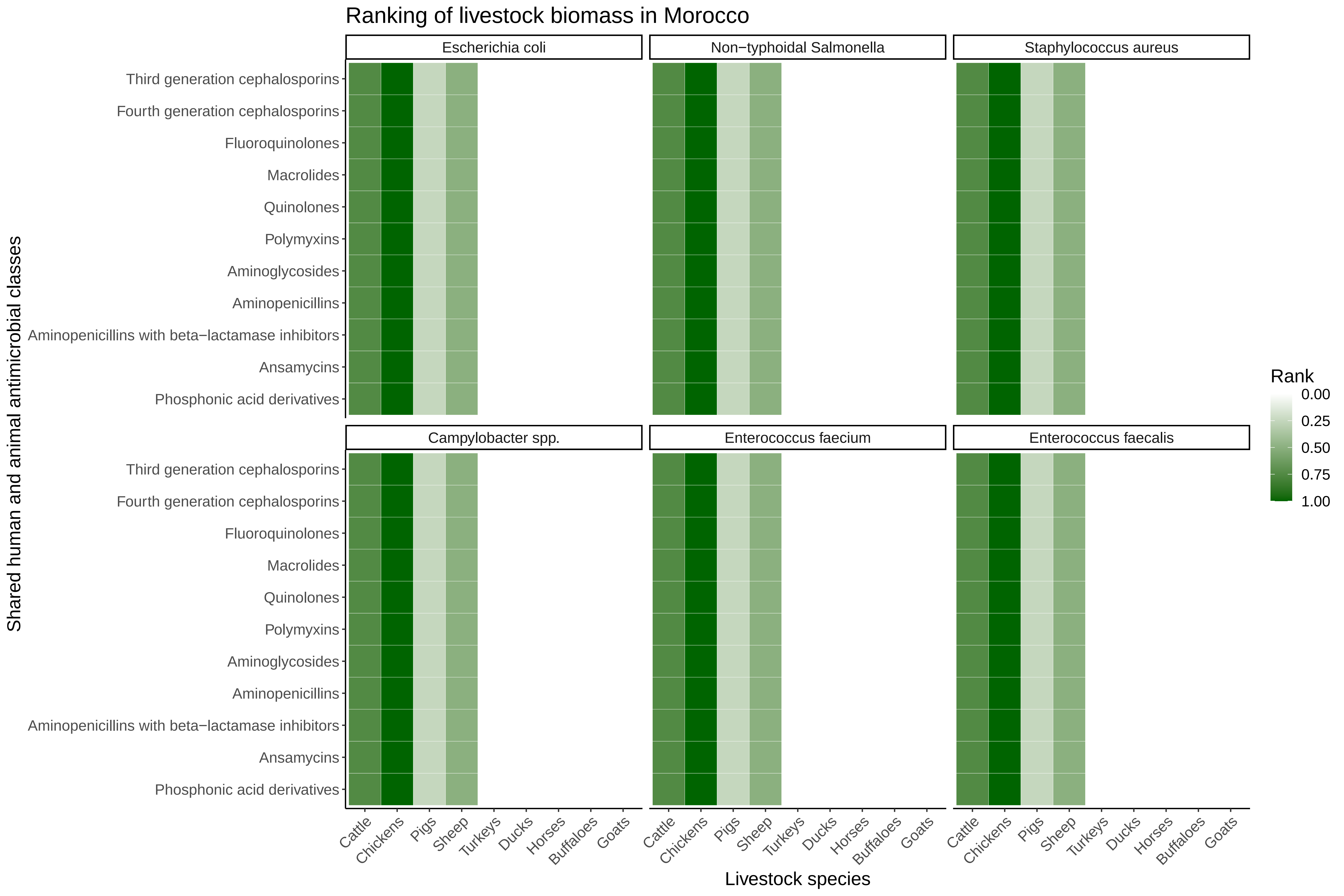
Ranking of livestock biomass in Morocco
Escherichia coli
Non−typhoidal Salmonella
Staphylococcus aureus
Campylobacter spp.
Enterococcus faecium
Enterococcus faecalis
Shared human and animal antimicrobial classes
Third generation cephalosporins
Fourth generation cephalosporins
Fluoroquinolones
Macrolides
Quinolones
Polymyxins
Aminoglycosides
Aminopenicillins
Aminopenicillins with beta−lactamase inhibitors
Ansamycins
Phosphonic acid derivatives
Livestock species
Cattle
Chickens
Pigs
Sheep
Turkeys
Ducks
Horses
Buffaloes
Goats
Rank
0.00
0.25
0.50
0.75
1.00

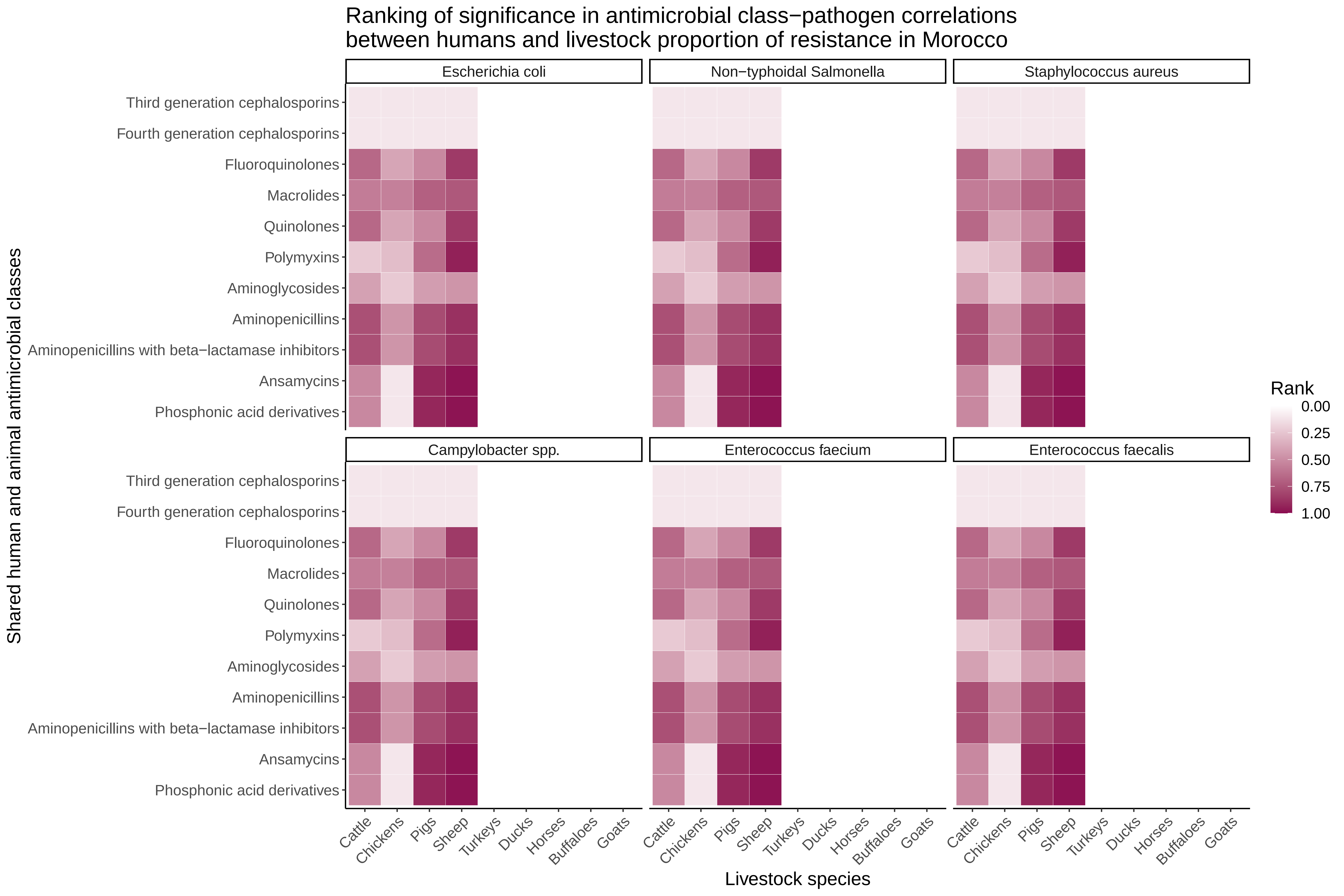
Ranking of significance in antimicrobial class−pathogen correlations
between humans and livestock proportion of resistance in Morocco
Escherichia coli
Non−typhoidal Salmonella
Staphylococcus aureus
Campylobacter spp.
Enterococcus faecium
Enterococcus faecalis
Third generation cephalosporins
Fourth generation cephalosporins
Fluoroquinolones
Macrolides
Quinolones
Polymyxins
Aminoglycosides
Aminopenicillins
Aminopenicillins with beta−lactamase inhibitors
Ansamycins
Phosphonic acid derivatives
Shared human and animal antimicrobial classes
Cattle
Chickens
Pigs
Sheep
Turkeys
Ducks
Horses
Buffaloes
Goats
Livestock species
Rank
0.00
0.25
0.50
0.75
1.00

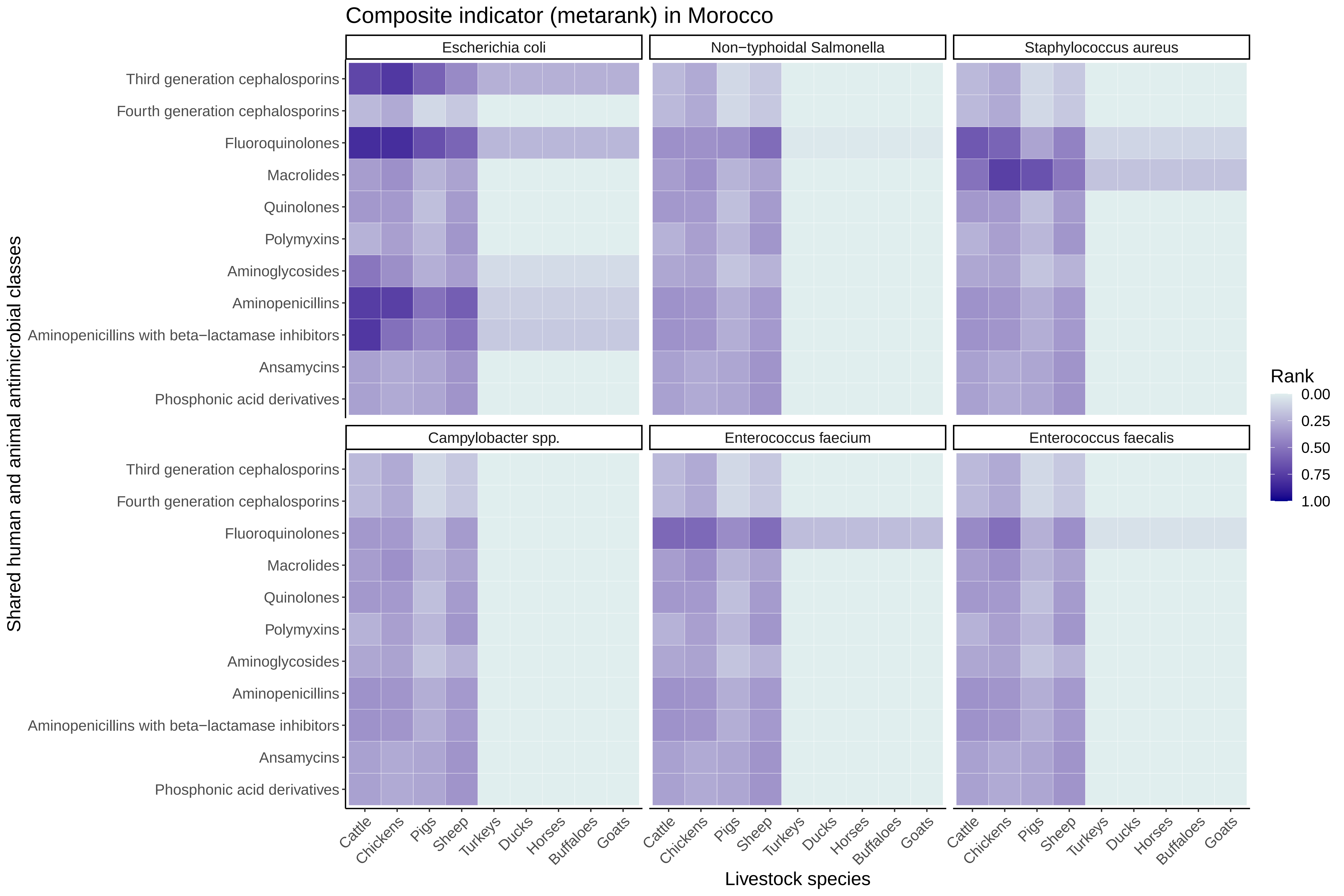
Composite indicator (metarank) in Morocco
Escherichia coli
Non−typhoidal Salmonella
Staphylococcus aureus
Campylobacter spp.
Enterococcus faecium
Enterococcus faecalis
Shared human and animal antimicrobial classes
Third generation cephalosporins
Fourth generation cephalosporins
Fluoroquinolones
Macrolides
Quinolones
Polymyxins
Aminoglycosides
Aminopenicillins
Aminopenicillins with beta−lactamase inhibitors
Ansamycins
Phosphonic acid derivatives
Cattle
Chickens
Pigs
Sheep
Turkeys
Ducks
Horses
Buffaloes
Goats
Livestock species
Rank
0.00
0.25
0.50
0.75
1.00

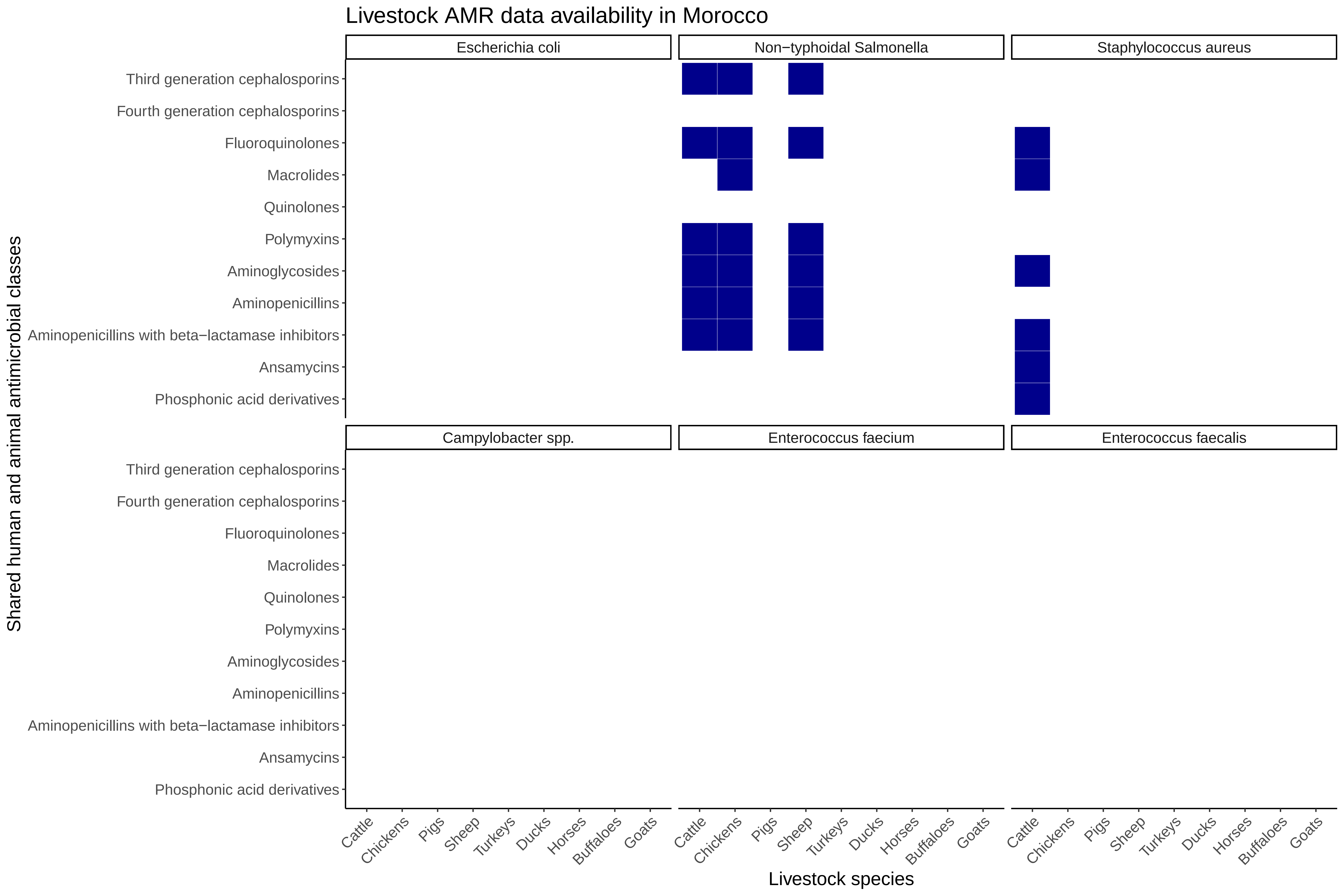

Livestock AMR data availability in Morocco
Escherichia coli
Non−typhoidal Salmonella
Staphylococcus aureus
Campylobacter spp.
Enterococcus faecium
Enterococcus faecalis
Shared human and animal antimicrobial classes
Third generation cephalosporins
Fourth generation cephalosporins
Fluoroquinolones
Macrolides
Quinolones
Polymyxins
Aminoglycosides
Aminopenicillins
Aminopenicillins with beta−lactamase inhibitors
Ansamycins
Phosphonic acid derivatives
Cattle
Chickens
Pigs
Sheep
Turkeys
Ducks
Horses
Buffaloes
Goats
Livestock species

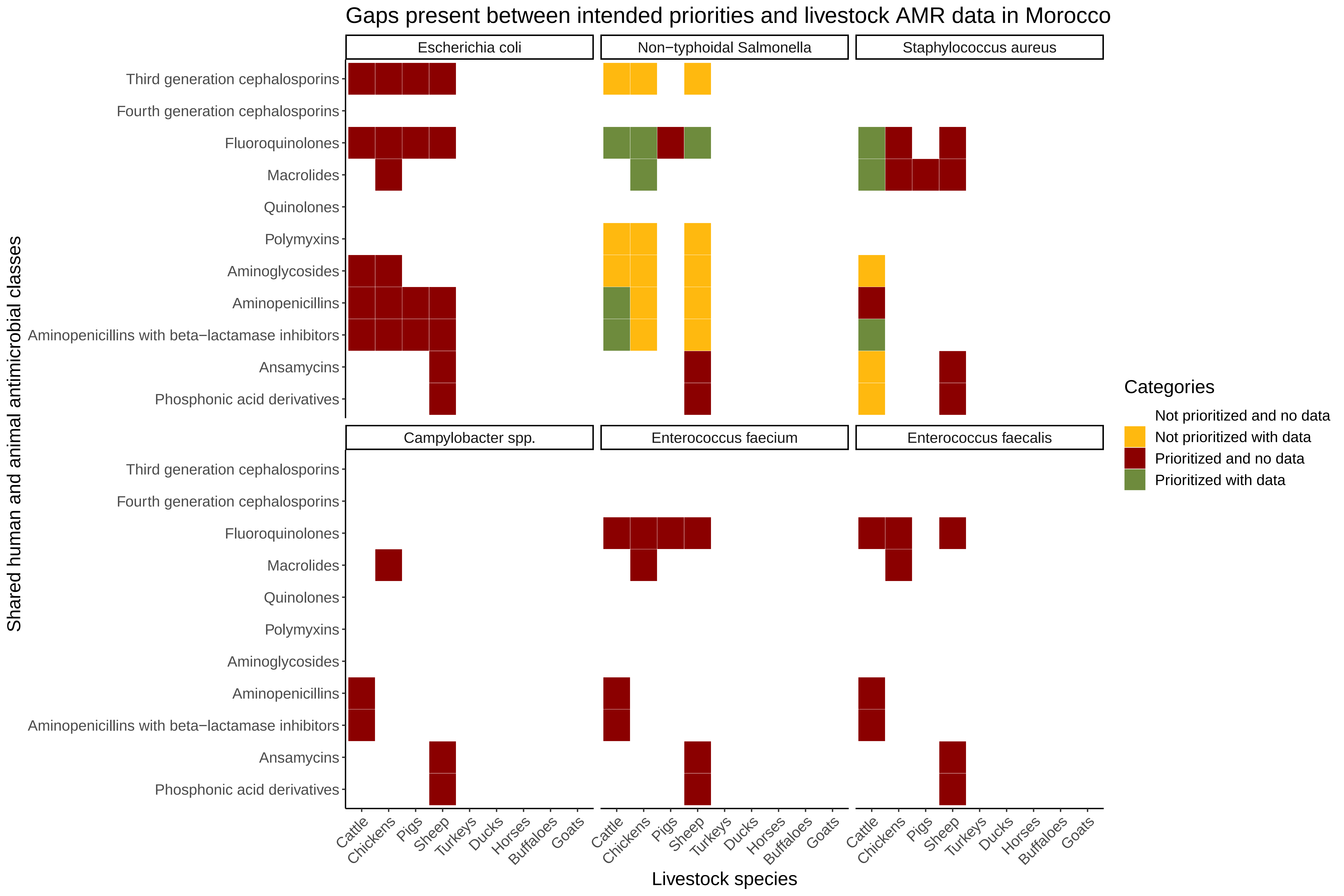

Gaps present between intended priorities and livestock AMR data in Morocco
Escherichia coli
Non−typhoidal Salmonella
Staphylococcus aureus
Campylobacter spp.
Enterococcus faecium
Enterococcus faecalis
Shared human and animal antimicrobial classes
Third generation cephalosporins
Fourth generation cephalosporins
Fluoroquinolones
Macrolides
Quinolones
Polymyxins
Aminoglycosides
Aminopenicillins
Aminopenicillins with beta−lactamase inhibitors
Ansamycins
Phosphonic acid derivatives
Livestock species
Cattle
Chickens
Pigs
Sheep
Turkeys
Ducks
Horses
Buffaloes
Goats
Categories
Not prioritized and no data
Not prioritized with data
Prioritized and no data
Prioritized with data

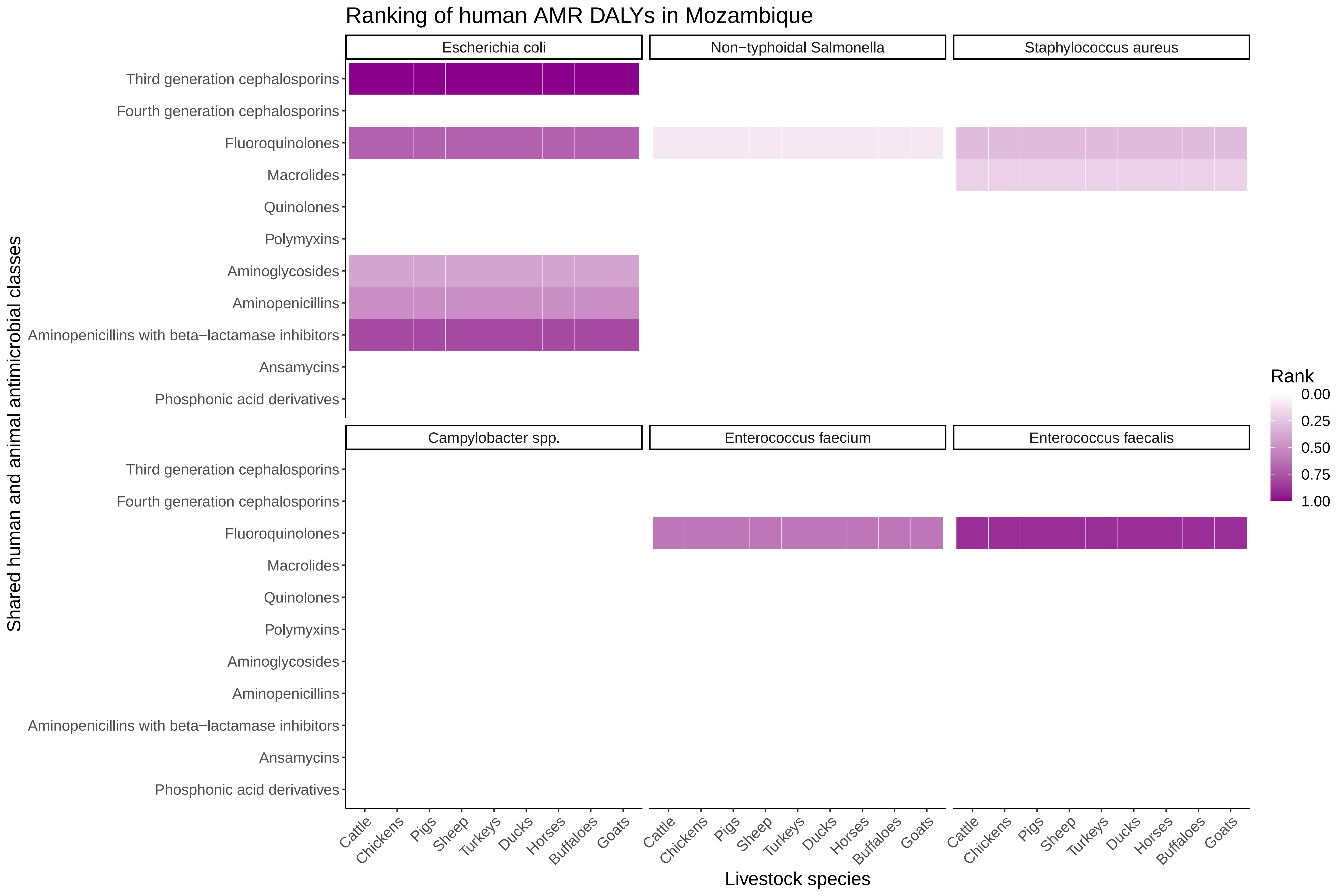

Ranking of human AMR DALYs in Mozambique
Escherichia coli
Non−typhoidal Salmonella
Staphylococcus aureus
Campylobacter spp.
Enterococcus faecium
Enterococcus faecalis
Third generation cephalosporins
Fourth generation cephalosporins
Fluoroquinolones
Macrolides
Quinolones
Polymyxins
Aminoglycosides
Aminopenicillins
Aminopenicillins with beta−lactamase inhibitors
Ansamycins
Phosphonic acid derivatives
Shared human and animal antimicrobial classes
Cattle
Chickens
Pigs
Sheep
Turkeys
Ducks
Horses
Buffaloes
Goats
Livestock species
Rank
0.00
0.25
0.50
0.75
1.00

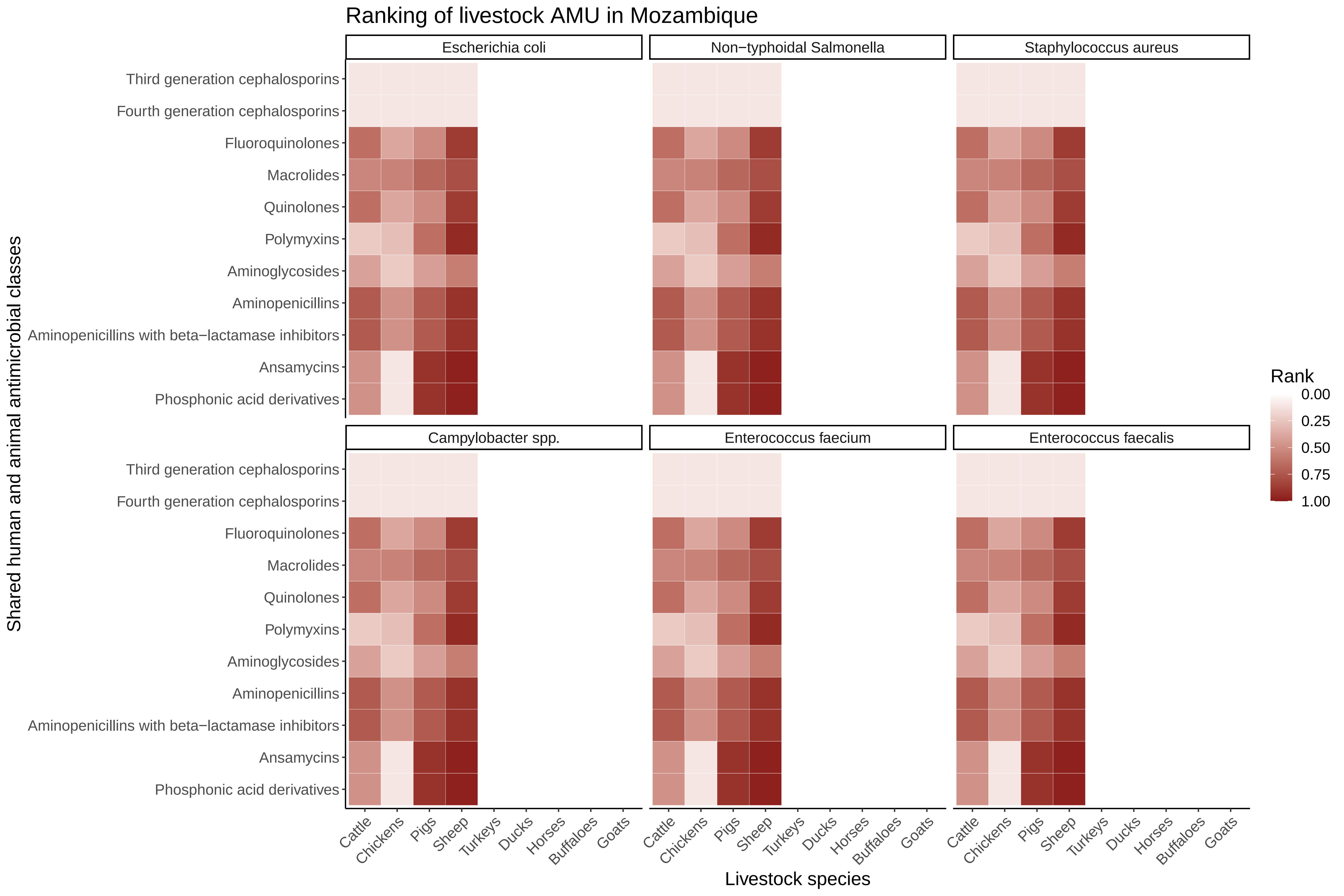

Ranking of livestock AMU in Mozambique
Escherichia coli
Non−typhoidal Salmonella
Staphylococcus aureus
Campylobacter spp.
Enterococcus faecium
Enterococcus faecalis
Third generation cephalosporins
Fourth generation cephalosporins
Fluoroquinolones
Macrolides
Quinolones
Polymyxins
Aminoglycosides
Aminopenicillins
Aminopenicillins with beta−lactamase inhibitors
Ansamycins
Phosphonic acid derivatives
Shared human and animal antimicrobial classes
Cattle
Chickens
Pigs
Sheep
Turkeys
Ducks
Horses
Buffaloes
Goats
Livestock species
Rank
0.00
0.25
0.50
0.75
1.00

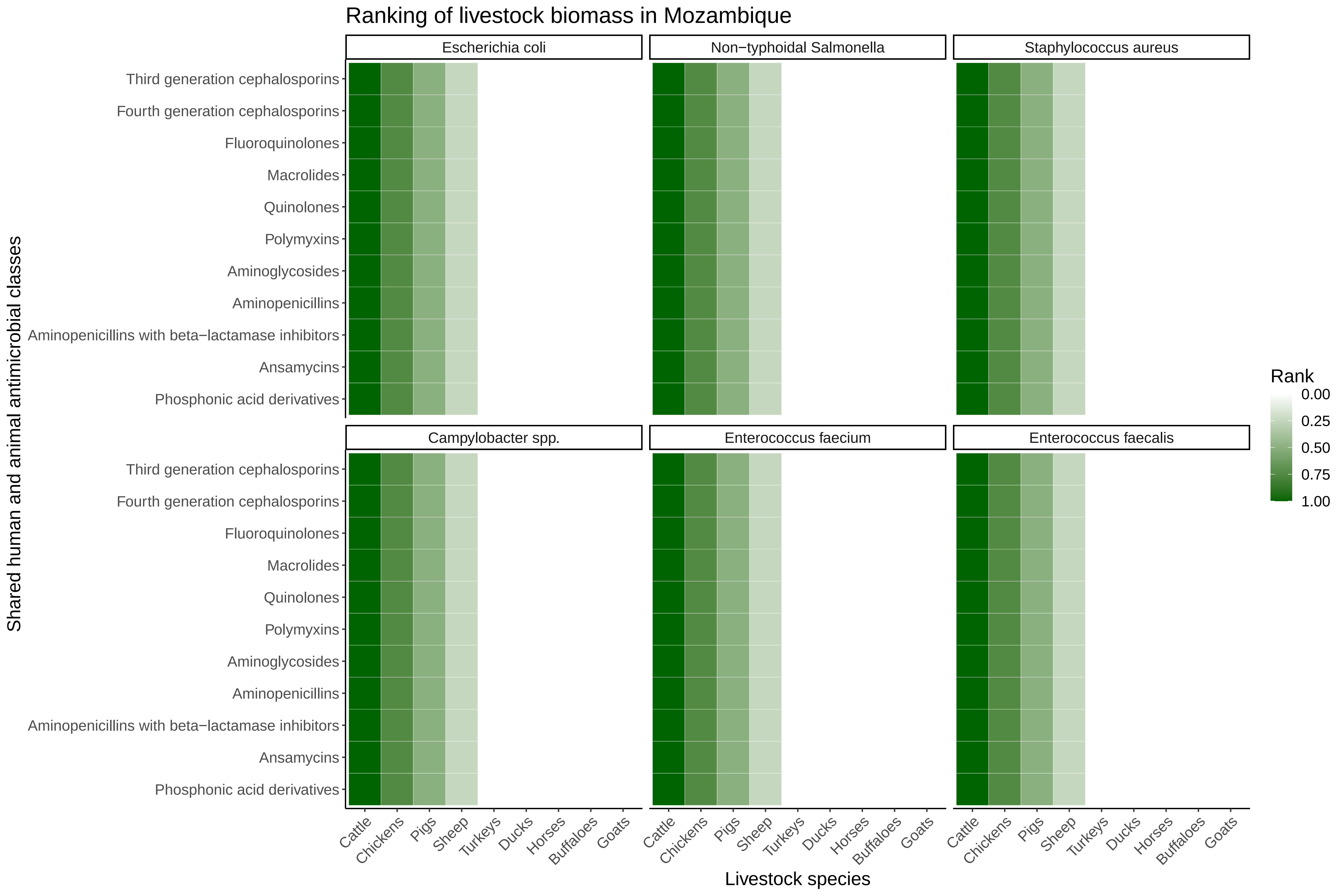
Ranking of livestock biomass in Mozambique
Escherichia coli
Non−typhoidal Salmonella
Staphylococcus aureus
Campylobacter spp.
Enterococcus faecium
Enterococcus faecalis
Third generation cephalosporins
Fourth generation cephalosporins
Fluoroquinolones
Macrolides
Quinolones
Polymyxins
Aminoglycosides
Aminopenicillins
Aminopenicillins with beta−lactamase inhibitors
Ansamycins
Phosphonic acid derivatives
Shared human and animal antimicrobial classes
Cattle
Chickens
Pigs
Sheep
Turkeys
Ducks
Horses
Buffaloes
Goats
Livestock species
Rank
0.00
0.25
0.50
0.75
1.00

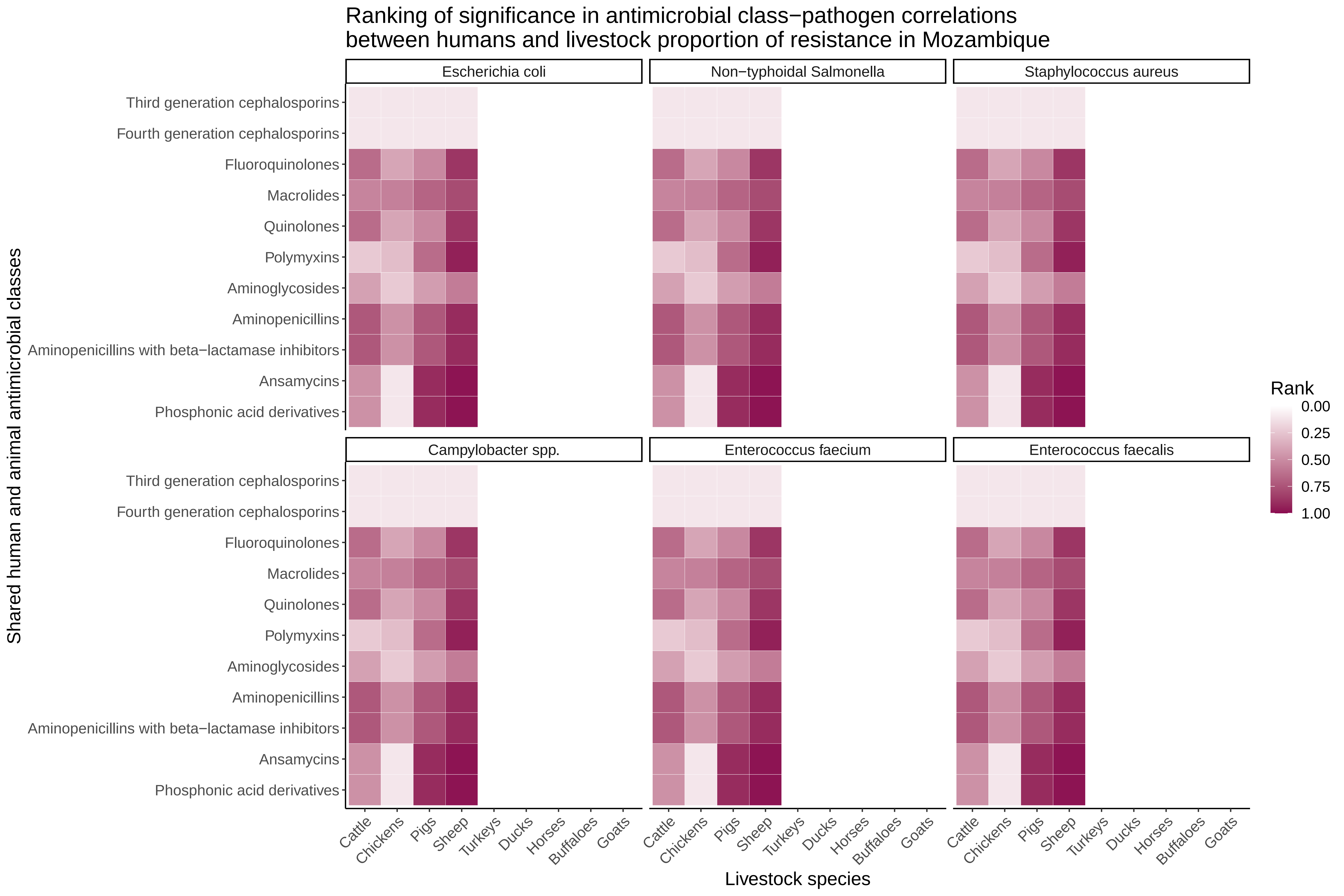

Ranking of significance in antimicrobial class−pathogen correlations
between humans and livestock proportion of resistance in Mozambique
Escherichia coli
Non−typhoidal Salmonella
Staphylococcus aureus
Campylobacter spp.
Enterococcus faecium
Enterococcus faecalis
Third generation cephalosporins
Fourth generation cephalosporins
Fluoroquinolones
Macrolides
Quinolones
Polymyxins
Aminoglycosides
Aminopenicillins
Aminopenicillins with beta−lactamase inhibitors
Ansamycins
Phosphonic acid derivatives
Shared human and animal antimicrobial classes
Cattle
Chickens
Pigs
Sheep
Turkeys
Ducks
Horses
Buffaloes
Goats
Livestock species
Rank
0.00
0.25
0.50
0.75
1.00

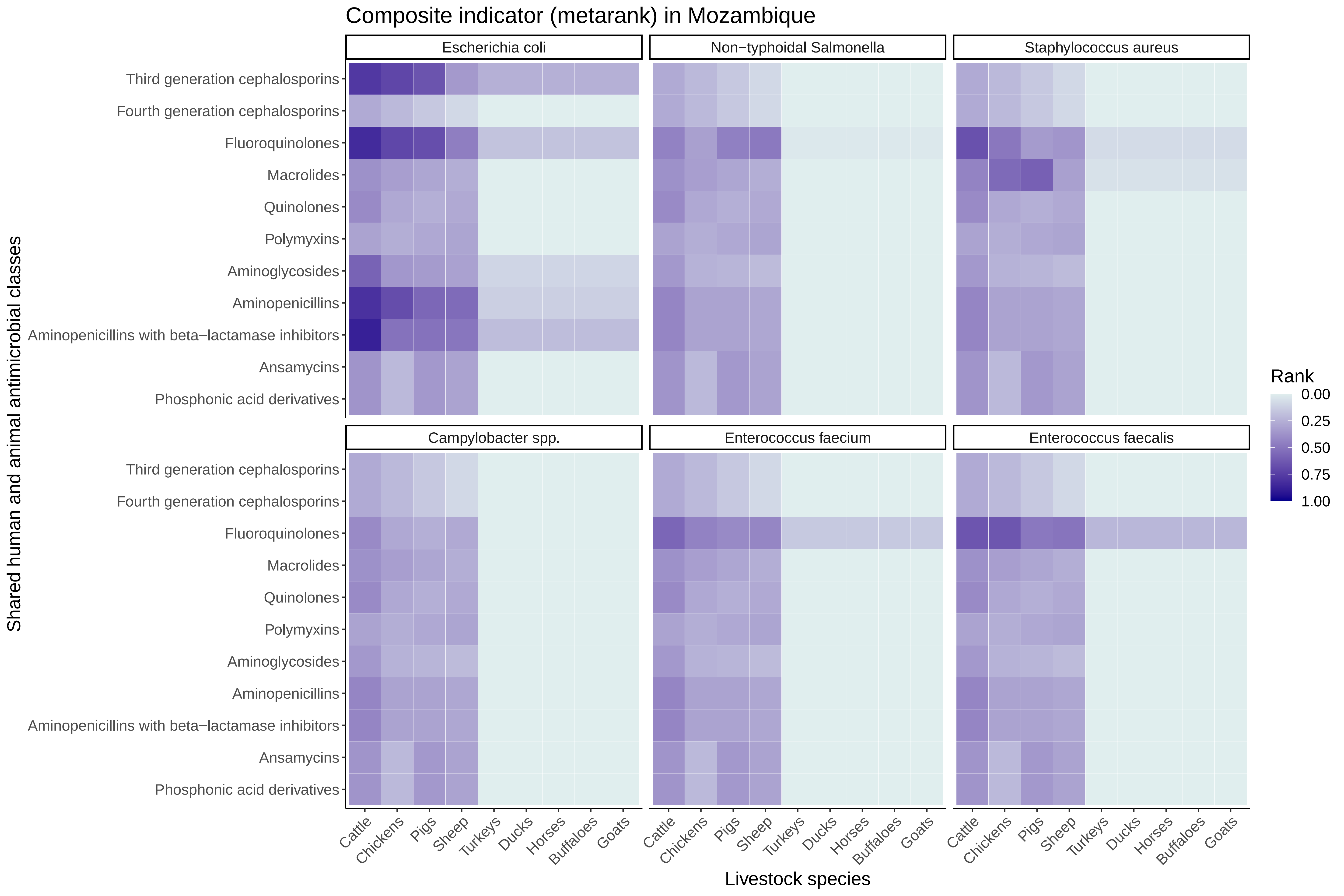

Composite indicator (metarank) in Mozambique
Escherichia coli
Non−typhoidal Salmonella
Staphylococcus aureus
Campylobacter spp.
Enterococcus faecium
Enterococcus faecalis
Third generation cephalosporins
Fourth generation cephalosporins
Fluoroquinolones
Macrolides
Quinolones
Polymyxins
Aminoglycosides
Aminopenicillins
Aminopenicillins with beta−lactamase inhibitors
Ansamycins
Phosphonic acid derivatives
Shared human and animal antimicrobial classes
Cattle
Chickens
Pigs
Sheep
Turkeys
Ducks
Horses
Buffaloes
Goats
Livestock species
Rank
0.00
0.25
0.50
0.75
1.00

Livestock AMR data availability in Mozambique

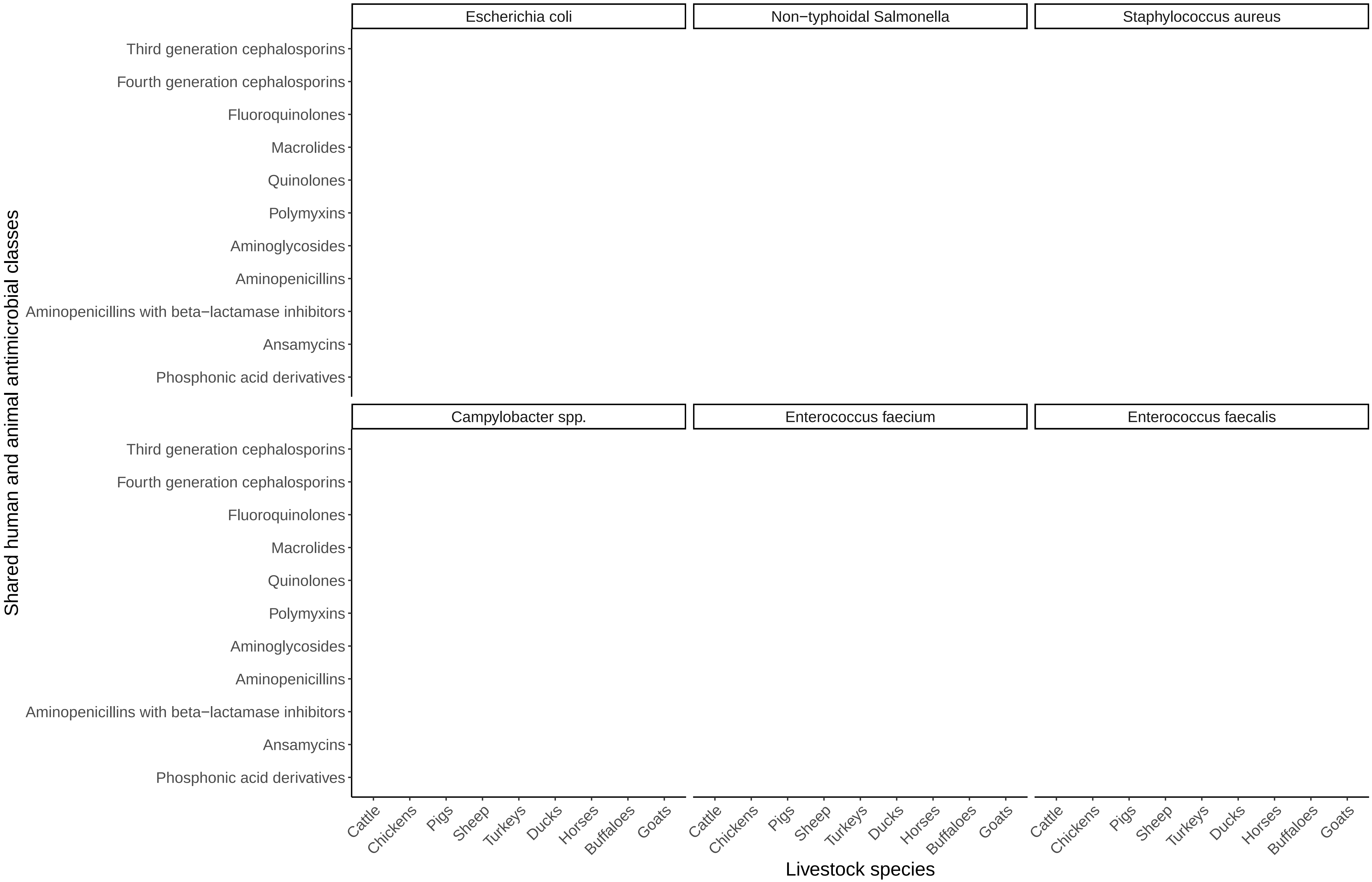

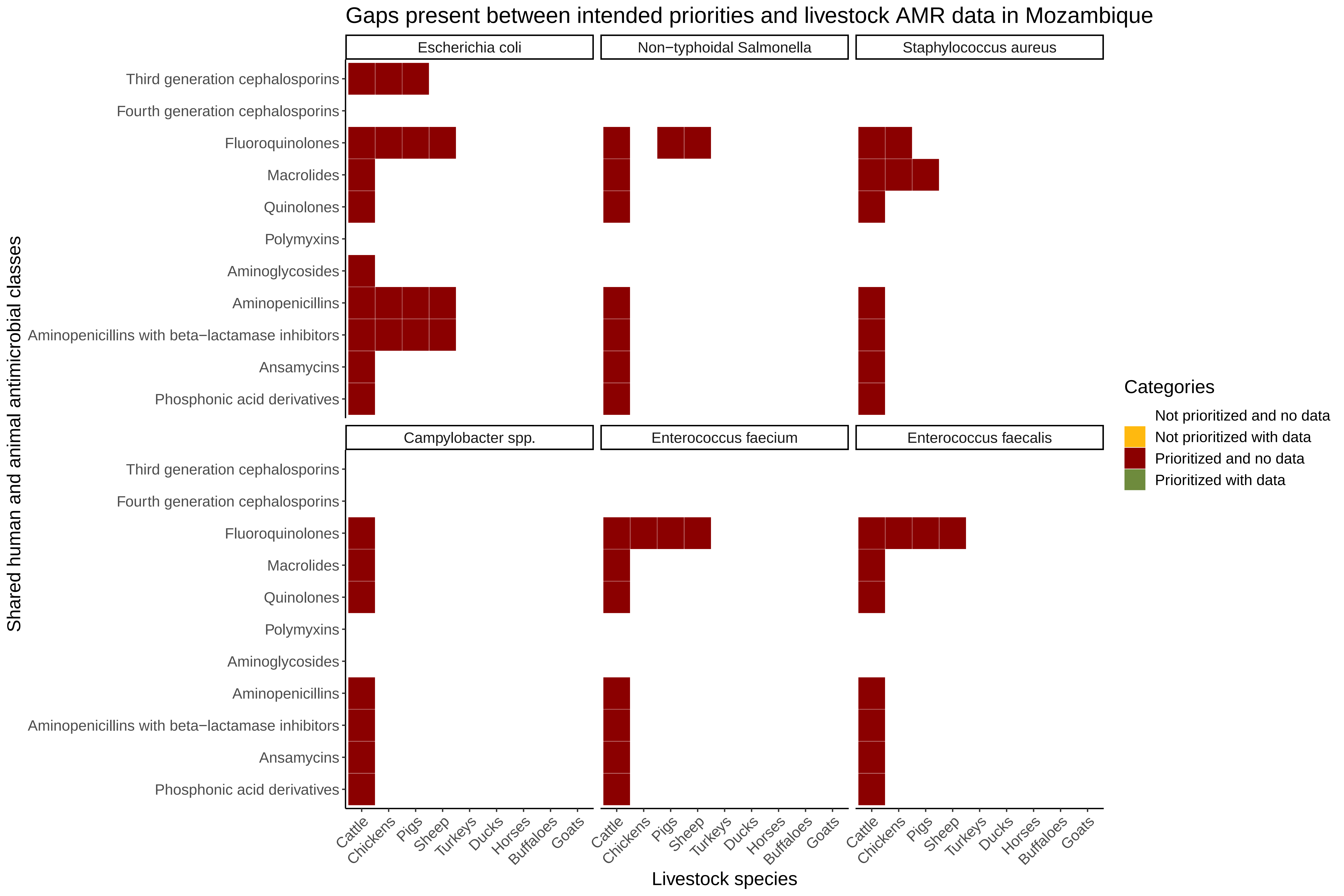

Gaps present between intended priorities and livestock AMR data in Mozambique
Escherichia coli
Non−typhoidal Salmonella
Staphylococcus aureus
Campylobacter spp.
Enterococcus faecium
Enterococcus faecalis
Third generation cephalosporins
Fourth generation cephalosporins
Fluoroquinolones
Macrolides
Quinolones
Polymyxins
Aminoglycosides
Aminopenicillins
Aminopenicillins with beta−lactamase inhibitors
Ansamycins
Phosphonic acid derivatives
Shared human and animal antimicrobial classes
Cattle
Chickens
Pigs
Sheep
Turkeys
Ducks
Horses
Buffaloes
Goats
Livestock species
Categories
Not prioritized and no data
Not prioritized with data
Prioritized and no data
Prioritized with data

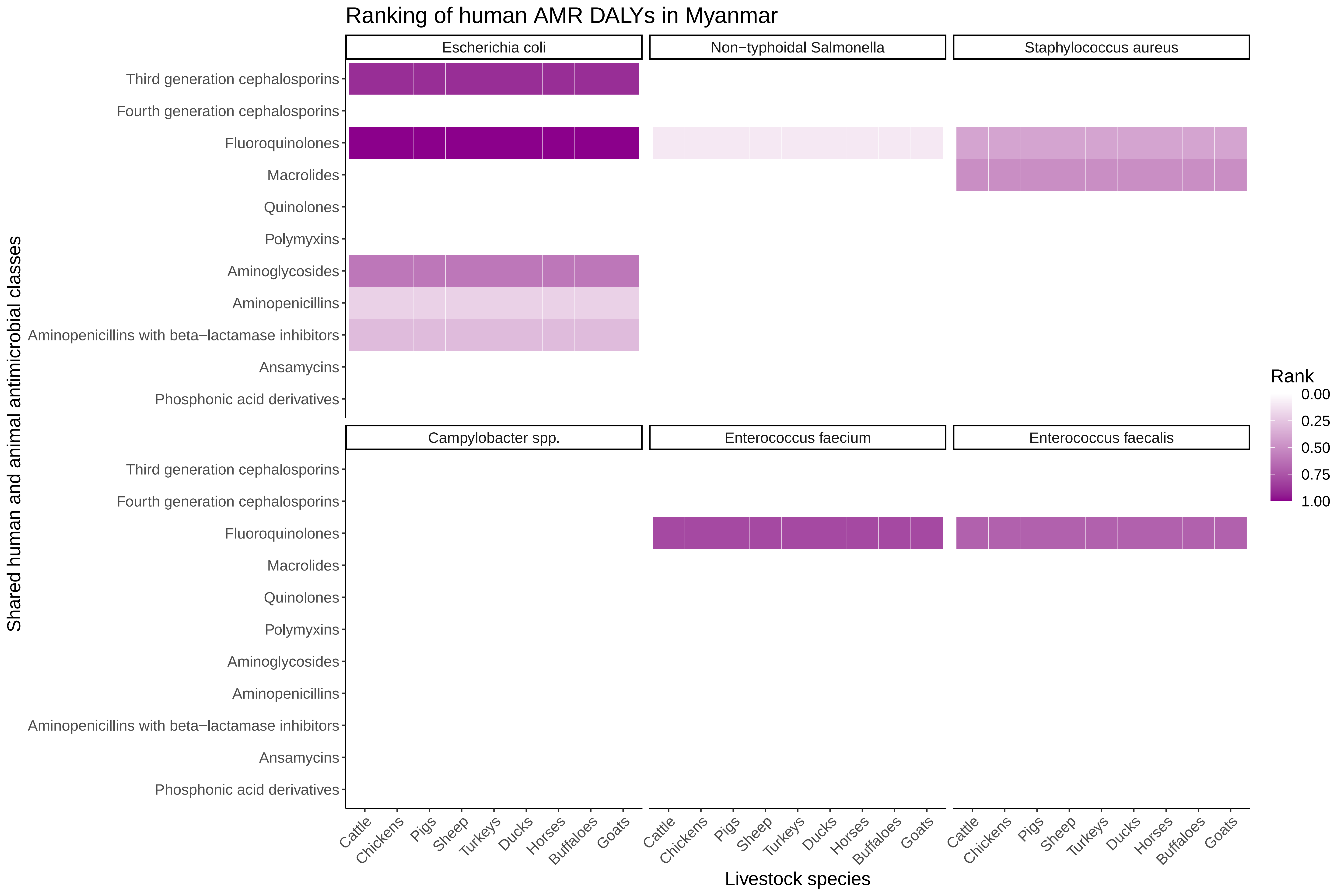
Ranking of human AMR DALYs in Myanmar
Escherichia coli
Non−typhoidal Salmonella
Staphylococcus aureus
Campylobacter spp.
Enterococcus faecium
Enterococcus faecalis
Shared human and animal antimicrobial classes
Third generation cephalosporins
Fourth generation cephalosporins
Fluoroquinolones
Macrolides
Quinolones
Polymyxins
Aminoglycosides
Aminopenicillins
Aminopenicillins with beta−lactamase inhibitors
Ansamycins
Phosphonic acid derivatives
Rank
0.00
0.25
0.50
0.75
1.00
Cattle
Chickens
Pigs
Sheep
Turkeys
Ducks
Horses
Buffaloes
Goats
Livestock species

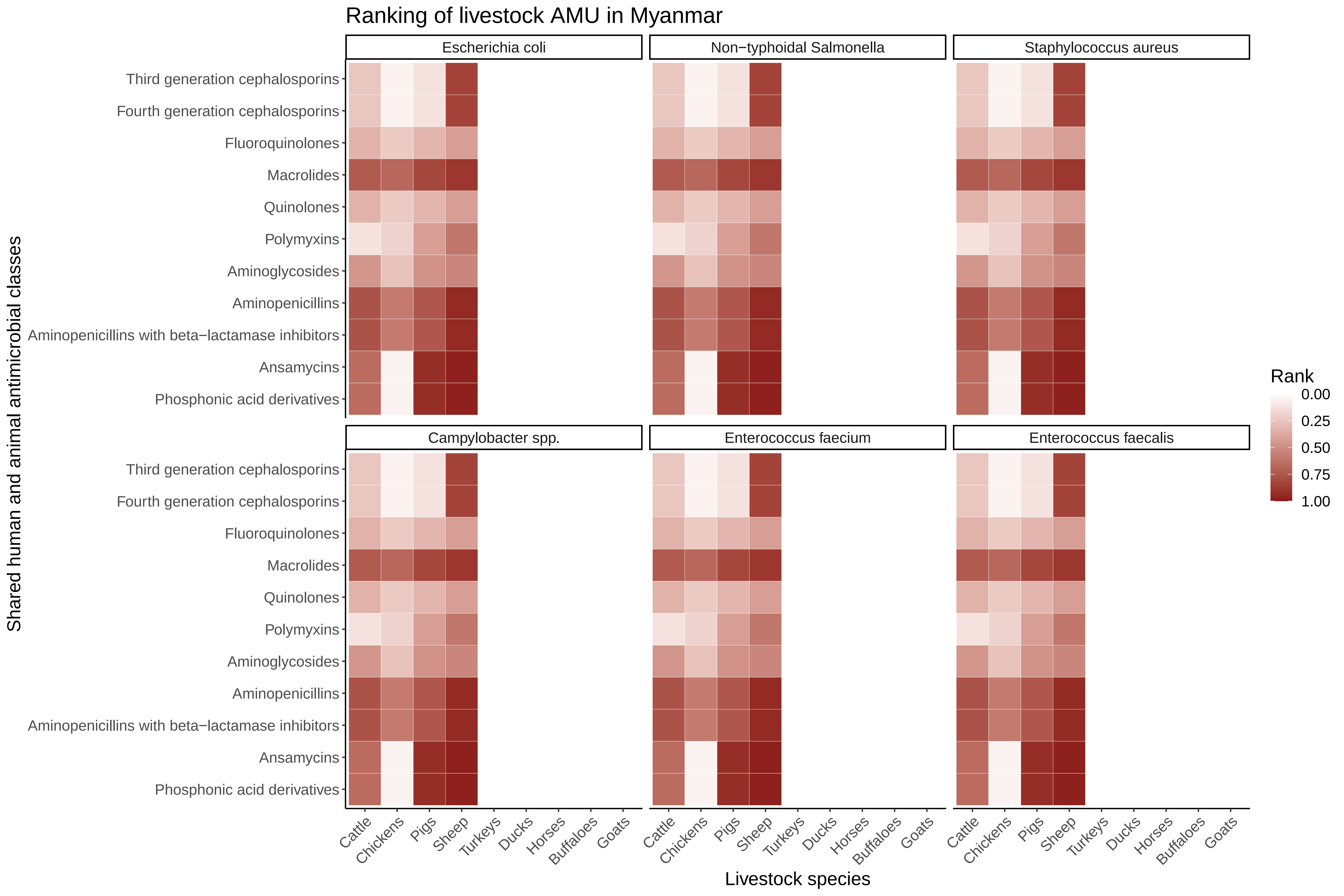
Ranking of livestock AMU in Myanmar
Escherichia coli
Non−typhoidal Salmonella
Staphylococcus aureus
Campylobacter spp.
Enterococcus faecium
Enterococcus faecalis
Shared human and animal antimicrobial classes
Third generation cephalosporins
Fourth generation cephalosporins
Fluoroquinolones
Macrolides
Quinolones
Polymyxins
Aminoglycosides
Aminopenicillins
Aminopenicillins with beta−lactamase inhibitors
Ansamycins
Phosphonic acid derivatives
Cattle
Chickens
Pigs
Sheep
Turkeys
Ducks
Horses
Buffaloes
Goats
Livestock species
Rank
0.00
0.25
0.50
0.75
1.00

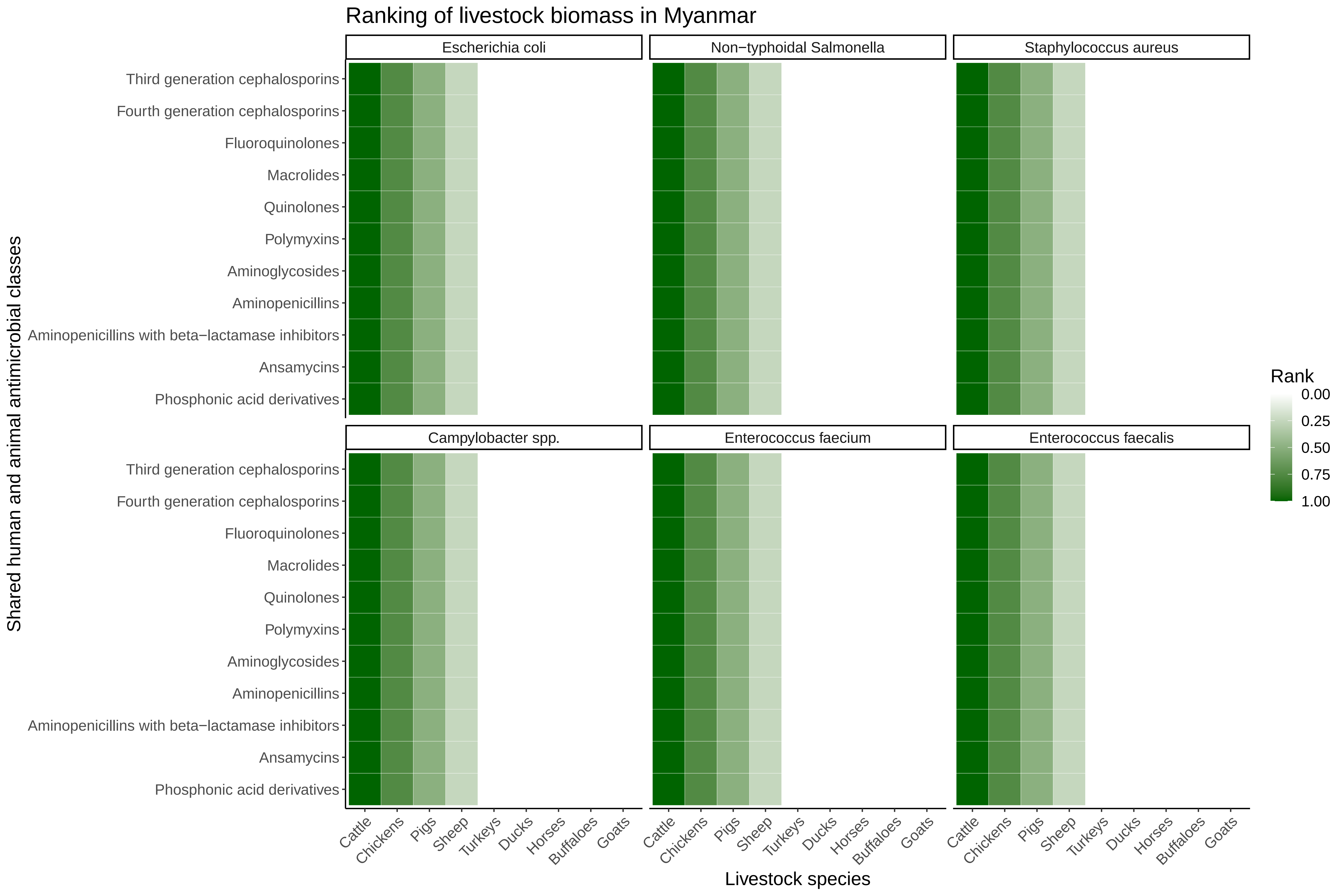

Ranking of livestock biomass in Myanmar
Escherichia coli
Non−typhoidal Salmonella
Staphylococcus aureus
Campylobacter spp.
Enterococcus faecium
Enterococcus faecalis
Shared human and animal antimicrobial classes
Third generation cephalosporins
Fourth generation cephalosporins
Fluoroquinolones
Macrolides
Quinolones
Polymyxins
Aminoglycosides
Aminopenicillins
Aminopenicillins with beta−lactamase inhibitors
Ansamycins
Phosphonic acid derivatives
Cattle
Chickens
Pigs
Sheep
Turkeys
Ducks
Horses
Buffaloes
Goats
Livestock species
Rank
0.00
0.25
0.50
0.75
1.00

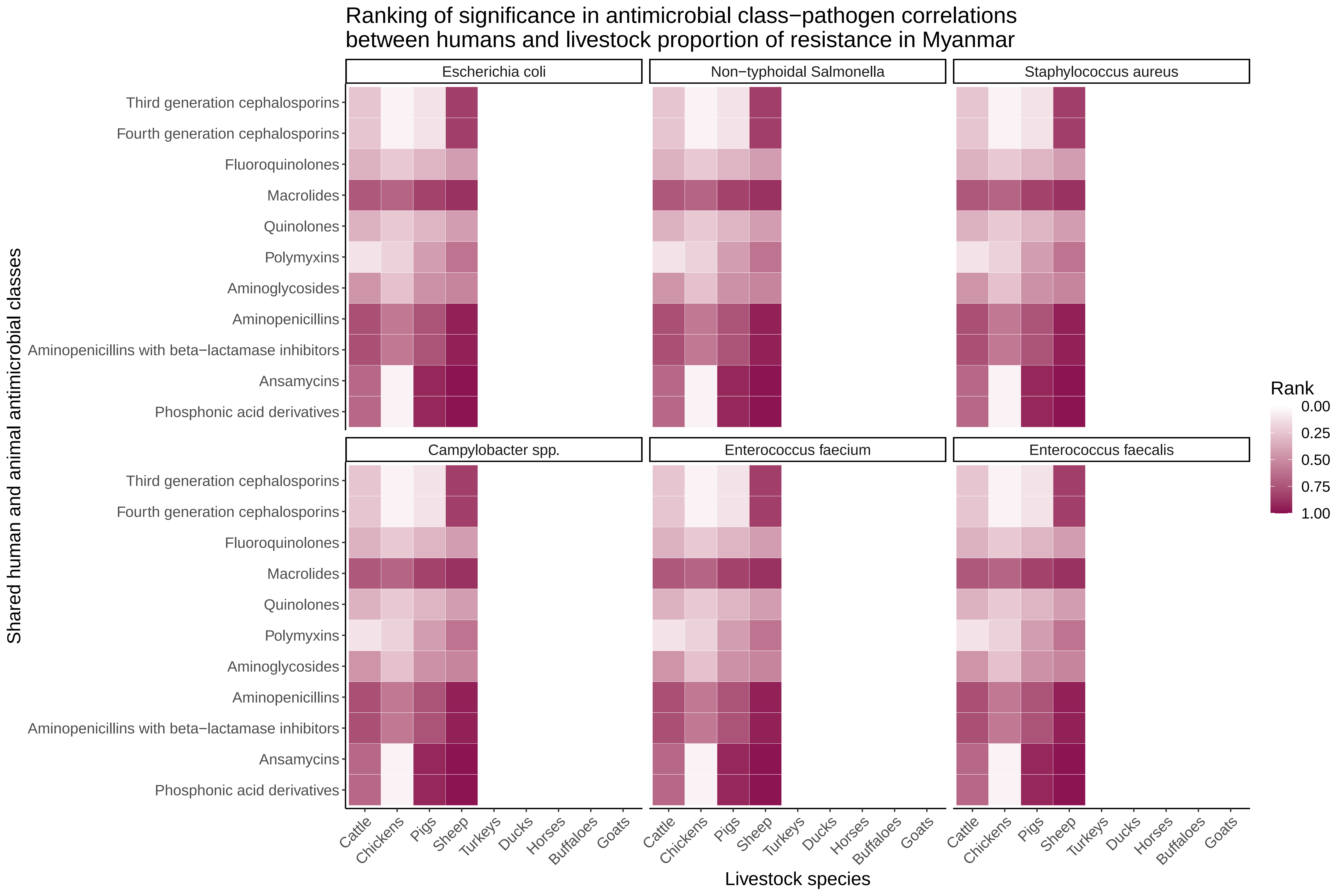

Ranking of significance in antimicrobial class−pathogen correlations
between humans and livestock proportion of resistance in Myanmar
Escherichia coli
Non−typhoidal Salmonella
Staphylococcus aureus
Campylobacter spp.
Enterococcus faecium
Enterococcus faecalis
Third generation cephalosporins
Fourth generation cephalosporins
Fluoroquinolones
Macrolides
Quinolones
Polymyxins
Aminoglycosides
Aminopenicillins
Aminopenicillins with beta−lactamase inhibitors
Ansamycins
Phosphonic acid derivatives
Shared human and animal antimicrobial classes
Cattle
Chickens
Pigs
Sheep
Turkeys
Ducks
Horses
Buffaloes
Goats
Livestock species
Rank
0.00
0.25
0.50
0.75
1.00

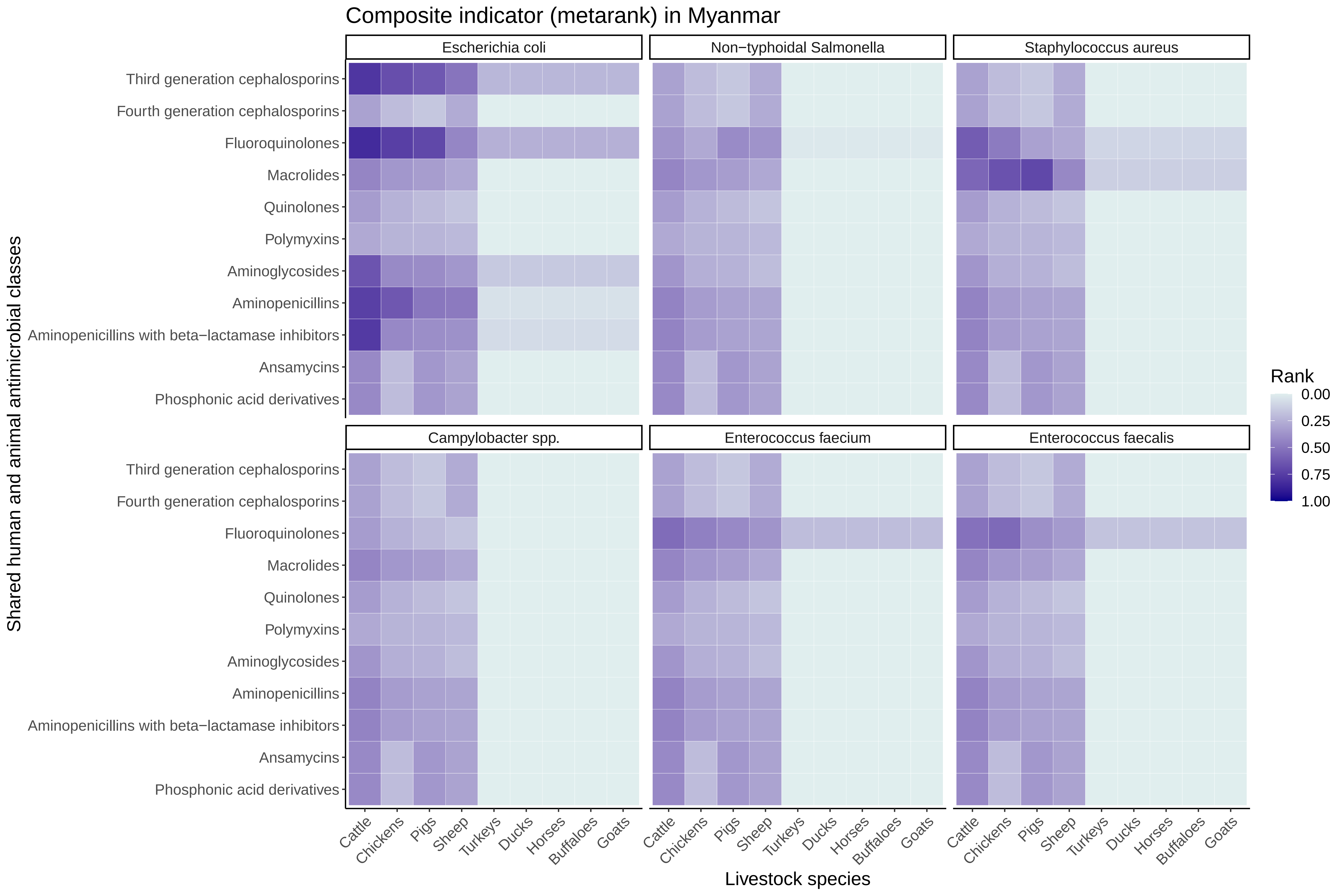

Composite indicator (metarank) in Myanmar
Escherichia coli
Non−typhoidal Salmonella
Staphylococcus aureus
Campylobacter spp.
Enterococcus faecium
Enterococcus faecalis
Shared human and animal antimicrobial classes
Third generation cephalosporins
Fourth generation cephalosporins
Fluoroquinolones
Macrolides
Quinolones
Polymyxins
Aminoglycosides
Aminopenicillins
Aminopenicillins with beta−lactamase inhibitors
Ansamycins
Phosphonic acid derivatives
Cattle
Chickens
Pigs
Sheep
Turkeys
Ducks
Horses
Buffaloes
Goats
Livestock species
Rank
0.00
0.25
0.50
0.75
1.00

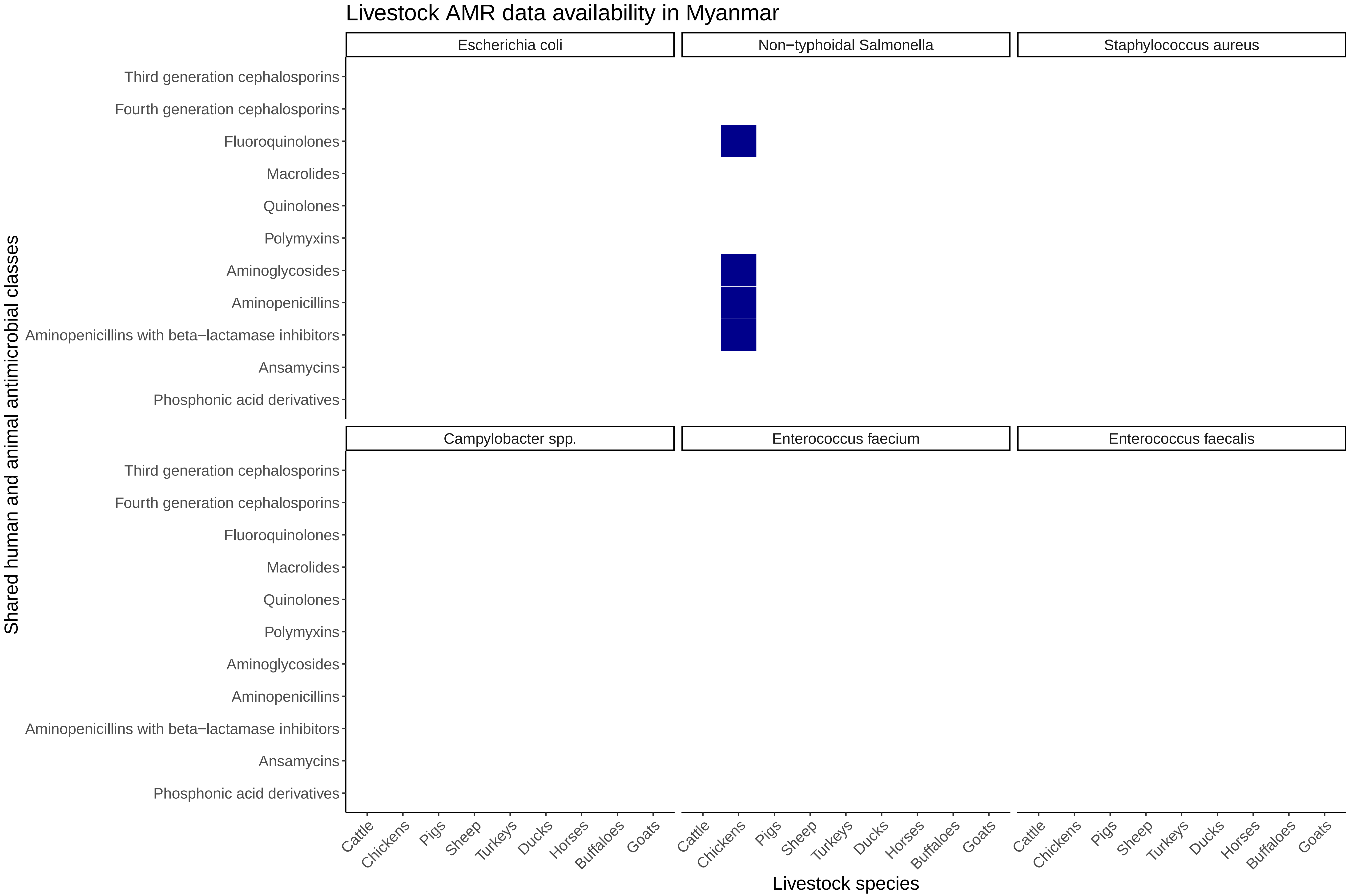
Livestock AMR data availability in Myanmar
Escherichia coli
Non−typhoidal Salmonella
Staphylococcus aureus
Campylobacter spp.
Enterococcus faecium
Enterococcus faecalis
Shared human and animal antimicrobial classes
Third generation cephalosporins
Fourth generation cephalosporins
Fluoroquinolones
Macrolides
Quinolones
Polymyxins
Aminoglycosides
Aminopenicillins
Aminopenicillins with beta−lactamase inhibitors
Ansamycins
Phosphonic acid derivatives
Cattle
Chickens
Pigs
Sheep
Turkeys
Ducks
Horses
Buffaloes
Goats
Livestock species

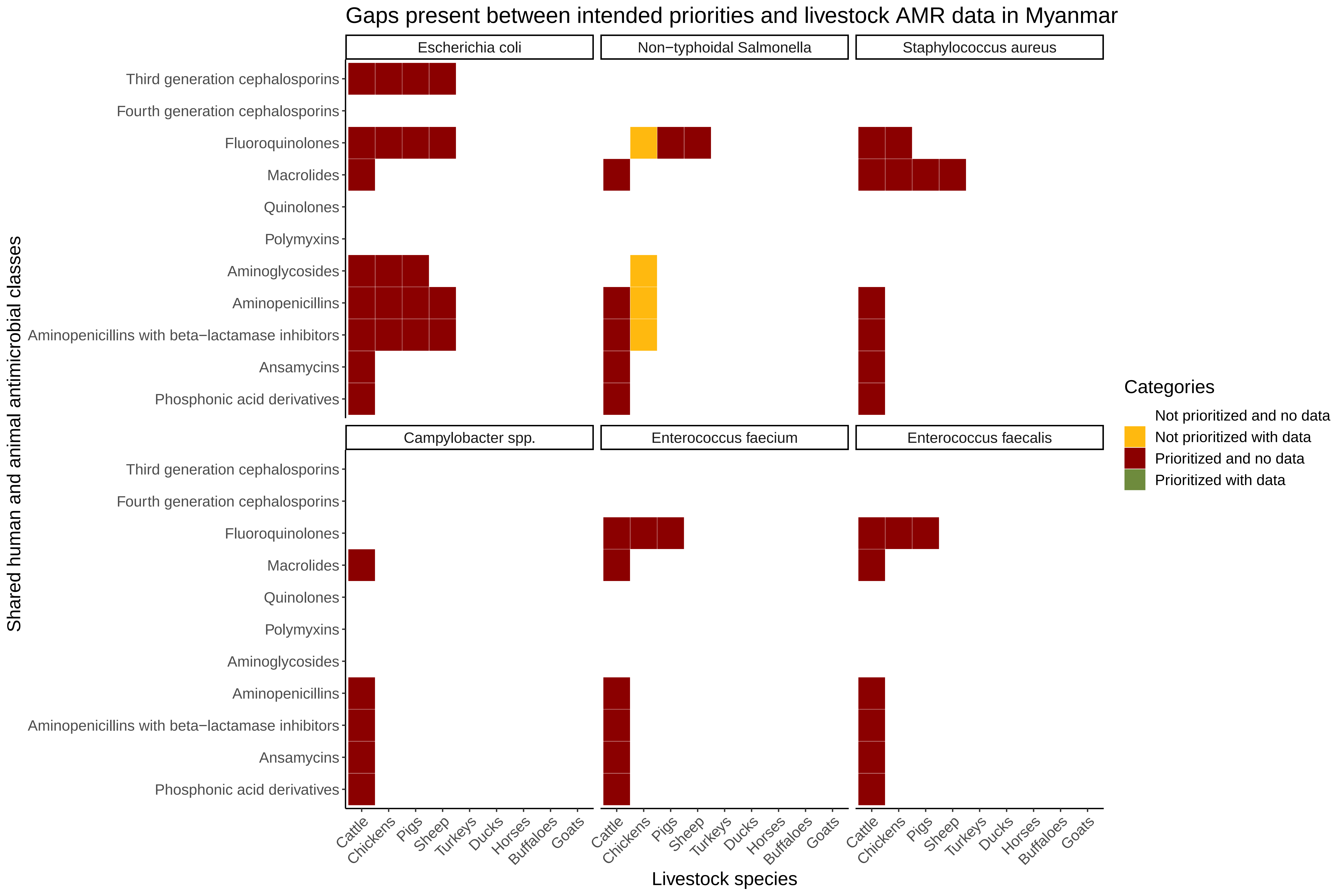

Gaps present between intended priorities and livestock AMR data in Myanmar
Escherichia coli
Non−typhoidal Salmonella
Staphylococcus aureus
Campylobacter spp.
Enterococcus faecium
Enterococcus faecalis
Third generation cephalosporins
Fourth generation cephalosporins
Fluoroquinolones
Macrolides
Quinolones
Polymyxins
Aminoglycosides
Aminopenicillins
Aminopenicillins with beta−lactamase inhibitors
Ansamycins
Phosphonic acid derivatives
Shared human and animal antimicrobial classes
Cattle
Chickens
Pigs
Sheep
Turkeys
Ducks
Horses
Buffaloes
Goats
Livestock species
Categories
Not prioritized and no data
Not prioritized with data
Prioritized and no data
Prioritized with data

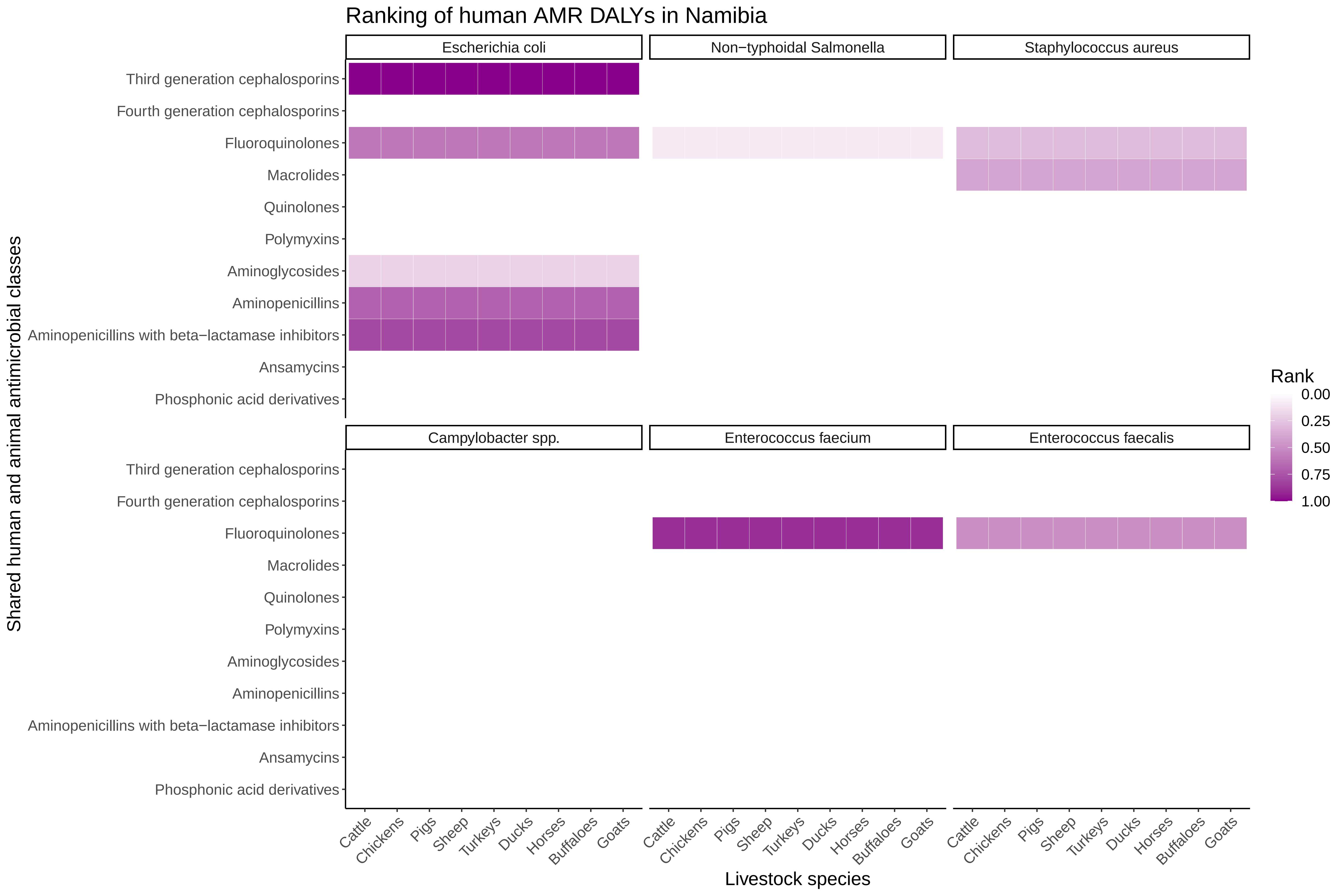

Ranking of human AMR DALYs in Namibia
Escherichia coli
Non−typhoidal Salmonella
Staphylococcus aureus
Campylobacter spp.
Enterococcus faecium
Enterococcus faecalis
Third generation cephalosporins
Fourth generation cephalosporins
Fluoroquinolones
Macrolides
Quinolones
Polymyxins
Aminoglycosides
Aminopenicillins
Aminopenicillins with beta−lactamase inhibitors
Ansamycins
Phosphonic acid derivatives
Shared human and animal antimicrobial classes
Cattle
Chickens
Pigs
Sheep
Turkeys
Ducks
Horses
Buffaloes
Goats
Livestock species
Rank
0.00
0.25
0.50
0.75
1.00

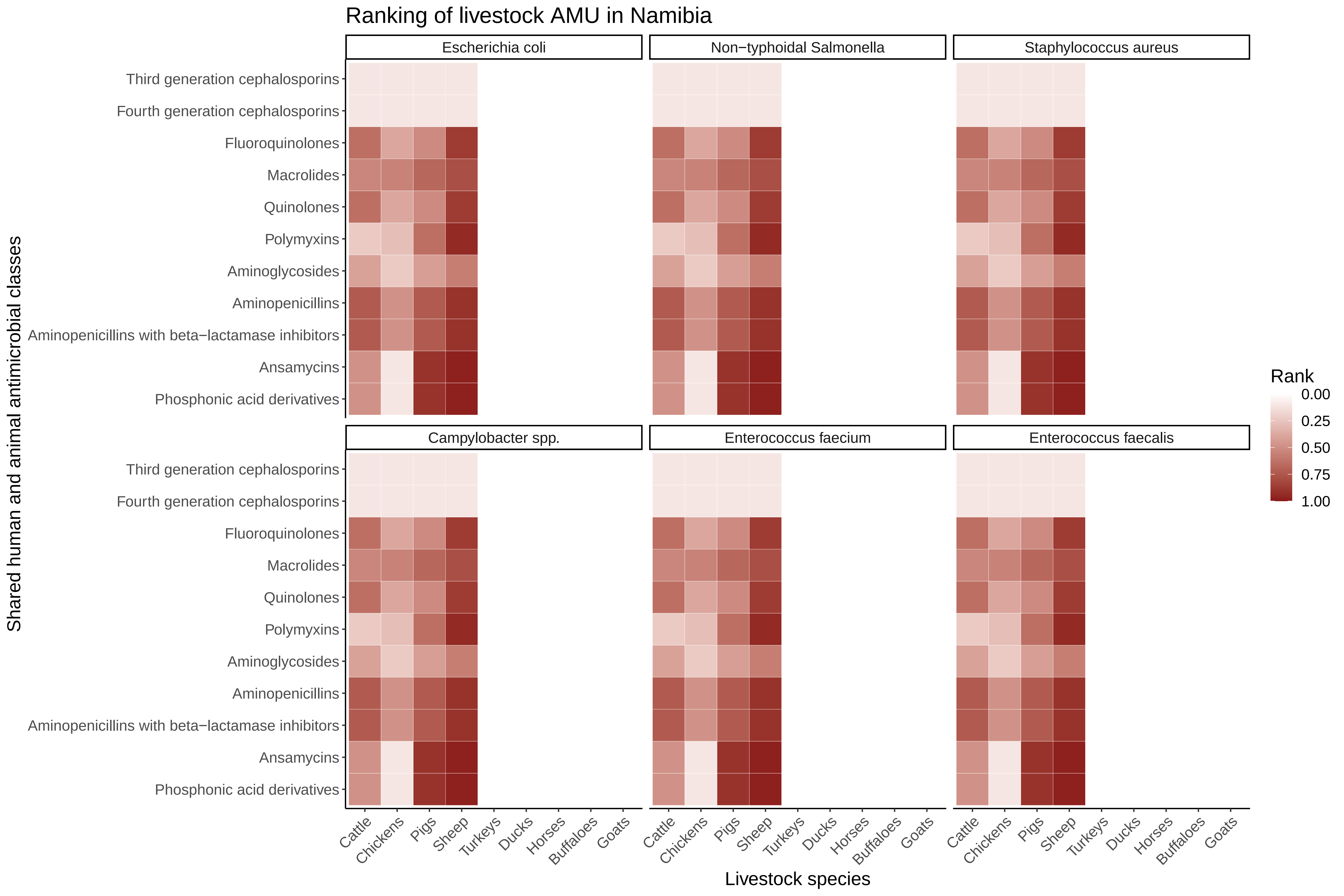
Ranking of livestock AMU in Namibia
Escherichia coli
Non−typhoidal Salmonella
Staphylococcus aureus
Campylobacter spp.
Enterococcus faecium
Enterococcus faecalis
Third generation cephalosporins
Fourth generation cephalosporins
Fluoroquinolones
Macrolides
Quinolones
Polymyxins
Aminoglycosides
Aminopenicillins
Aminopenicillins with beta−lactamase inhibitors
Ansamycins
Phosphonic acid derivatives
Shared human and animal antimicrobial classes
Cattle
Chickens
Pigs
Sheep
Turkeys
Ducks
Horses
Buffaloes
Goats
Livestock species
Rank
0.00
0.25
0.50
0.75
1.00

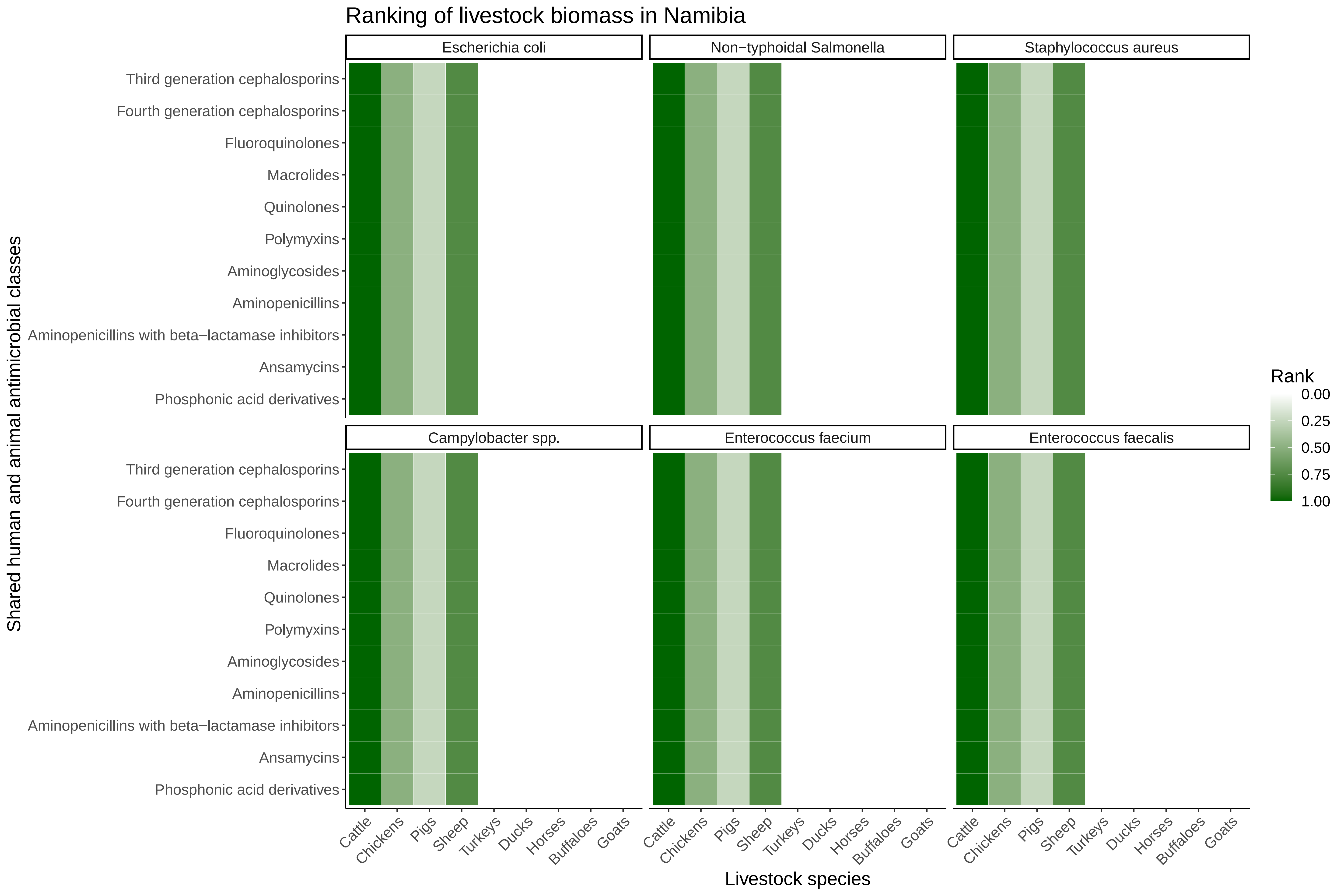

Ranking of livestock biomass in Namibia
Escherichia coli
Non−typhoidal Salmonella
Staphylococcus aureus
Campylobacter spp.
Enterococcus faecium
Enterococcus faecalis
Third generation cephalosporins
Fourth generation cephalosporins
Fluoroquinolones
Macrolides
Quinolones
Polymyxins
Aminoglycosides
Aminopenicillins
Aminopenicillins with beta−lactamase inhibitors
Ansamycins
Phosphonic acid derivatives
Shared human and animal antimicrobial classes
Cattle
Chickens
Pigs
Sheep
Turkeys
Ducks
Horses
Buffaloes
Goats
Livestock species
Rank
0.00
0.25
0.50
0.75
1.00

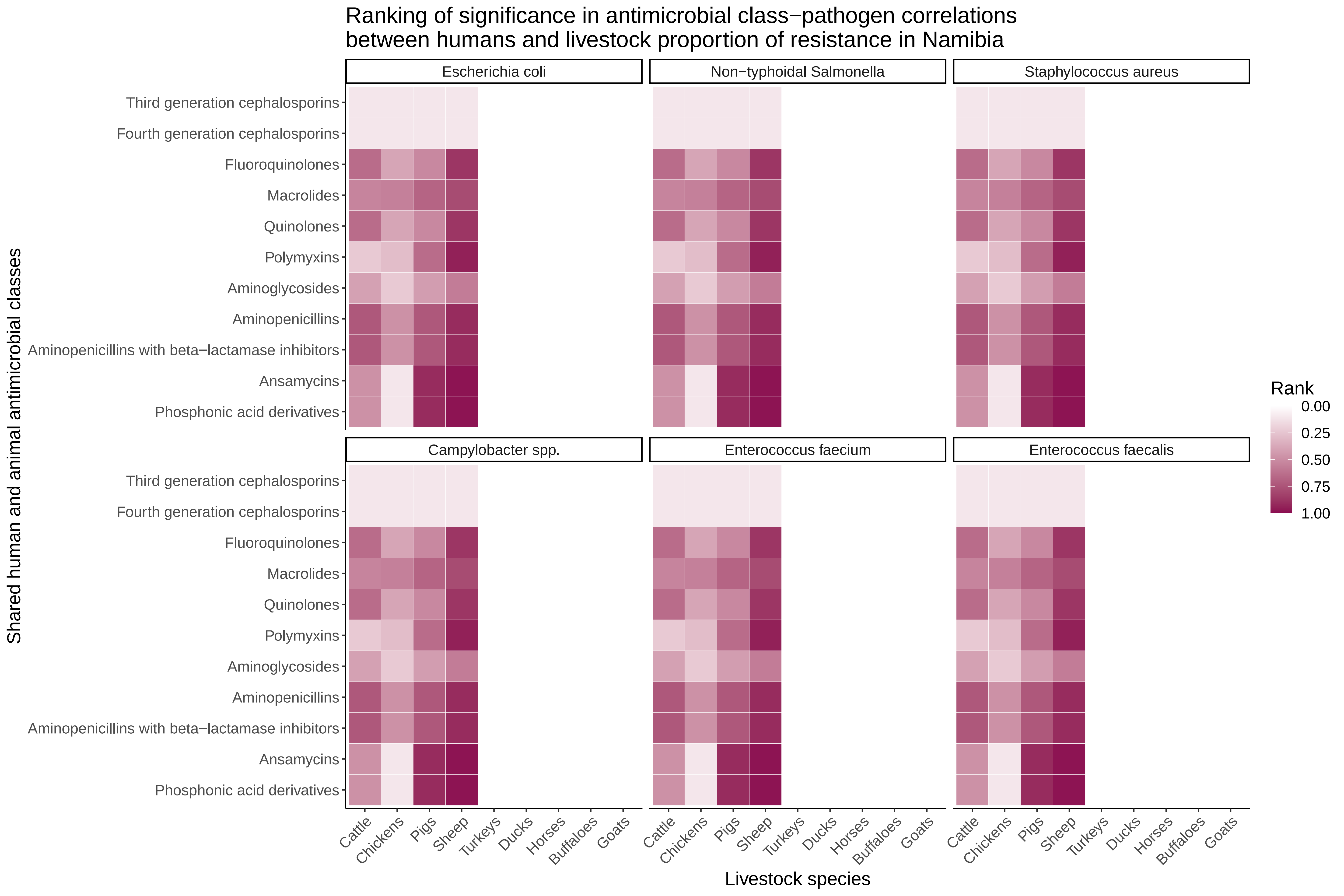

Ranking of significance in antimicrobial class−pathogen correlations between humans and livestock proportion of resistance in Namibia
Escherichia coli
Non−typhoidal Salmonella
Staphylococcus aureus
Campylobacter spp.
Enterococcus faecium
Enterococcus faecalis
Third generation cephalosporins
Fourth generation cephalosporins
Fluoroquinolones
Macrolides
Quinolones
Polymyxins
Aminoglycosides
Aminopenicillins
Aminopenicillins with beta−lactamase inhibitors
Ansamycins
Phosphonic acid derivatives
Shared human and animal antimicrobial classes
Cattle
Chickens
Pigs
Sheep
Turkeys
Ducks
Horses
Buffaloes
Goats
Livestock species
Rank
0.00
0.25
0.50
0.75
1.00

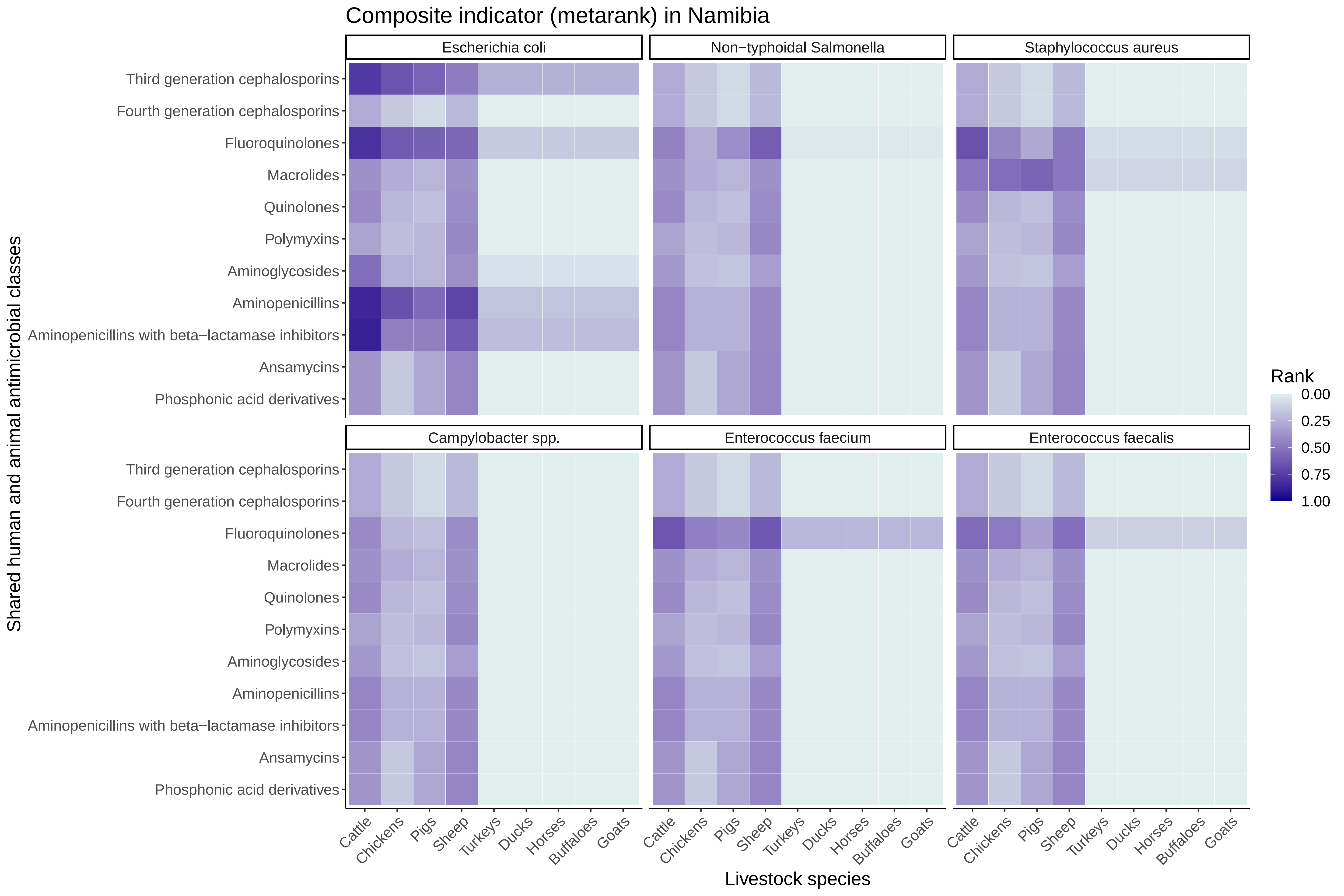

Composite indicator (metarank) in Namibia
Escherichia coli
Non−typhoidal Salmonella
Staphylococcus aureus
Campylobacter spp.
Enterococcus faecium
Enterococcus faecalis
Shared human and animal antimicrobial classes
Third generation cephalosporins
Fourth generation cephalosporins
Fluoroquinolones
Macrolides
Quinolones
Polymyxins
Aminoglycosides
Aminopenicillins
Aminopenicillins with beta−lactamase inhibitors
Ansamycins
Phosphonic acid derivatives
Cattle
Chickens
Pigs
Sheep
Turkeys
Ducks
Horses
Buffaloes
Goats
Livestock species
Rank
0.00
0.25
0.50
0.75
1.00

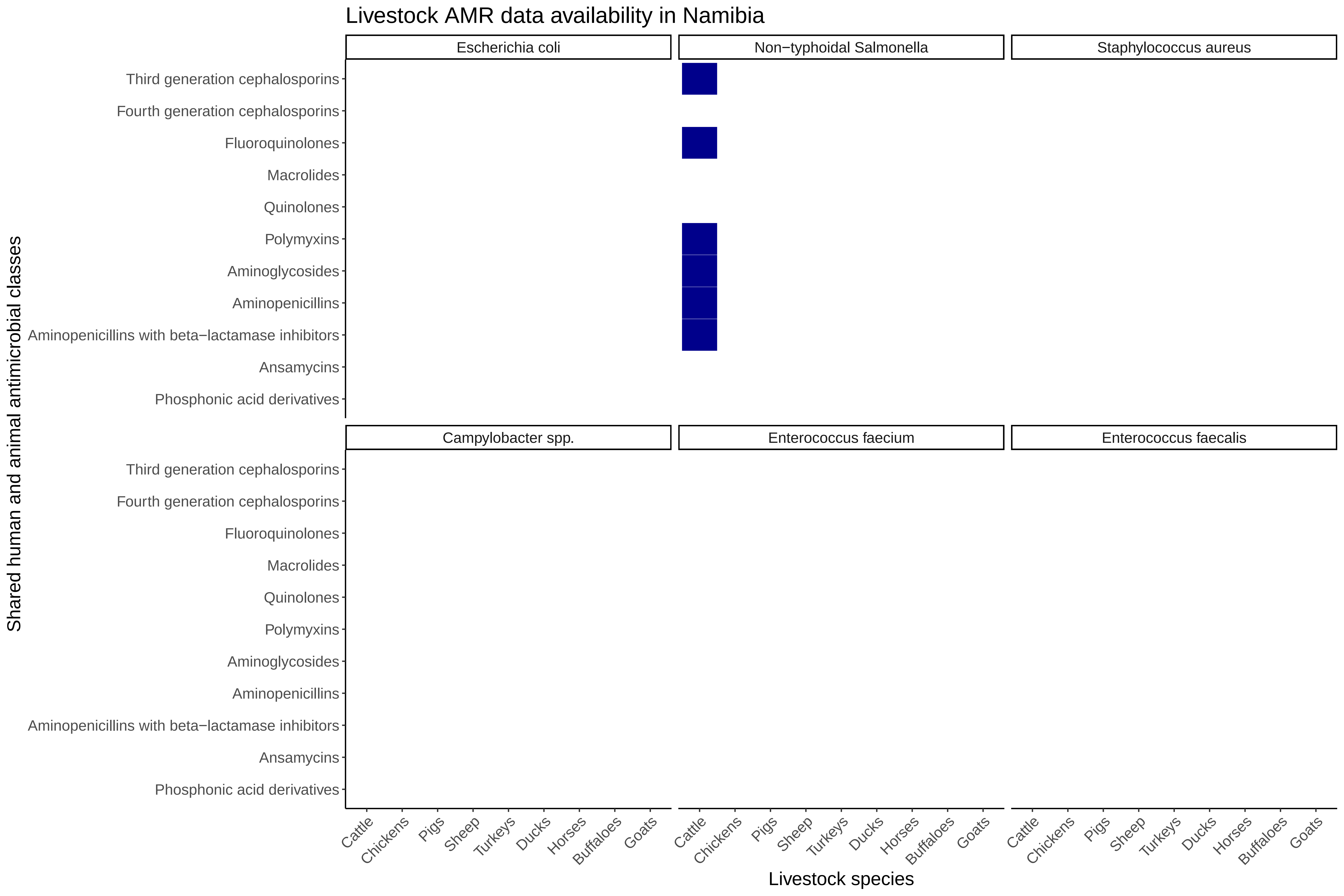

Livestock AMR data availability in Namibia
Escherichia coli
Non−typhoidal Salmonella
Staphylococcus aureus
Campylobacter spp.
Enterococcus faecium
Enterococcus faecalis
Shared human and animal antimicrobial classes
Third generation cephalosporins
Fourth generation cephalosporins
Fluoroquinolones
Macrolides
Quinolones
Polymyxins
Aminoglycosides
Aminopenicillins
Aminopenicillins with beta−lactamase inhibitors
Ansamycins
Phosphonic acid derivatives
Cattle
Chickens
Pigs
Sheep
Turkeys
Ducks
Horses
Buffaloes
Goats
Livestock species

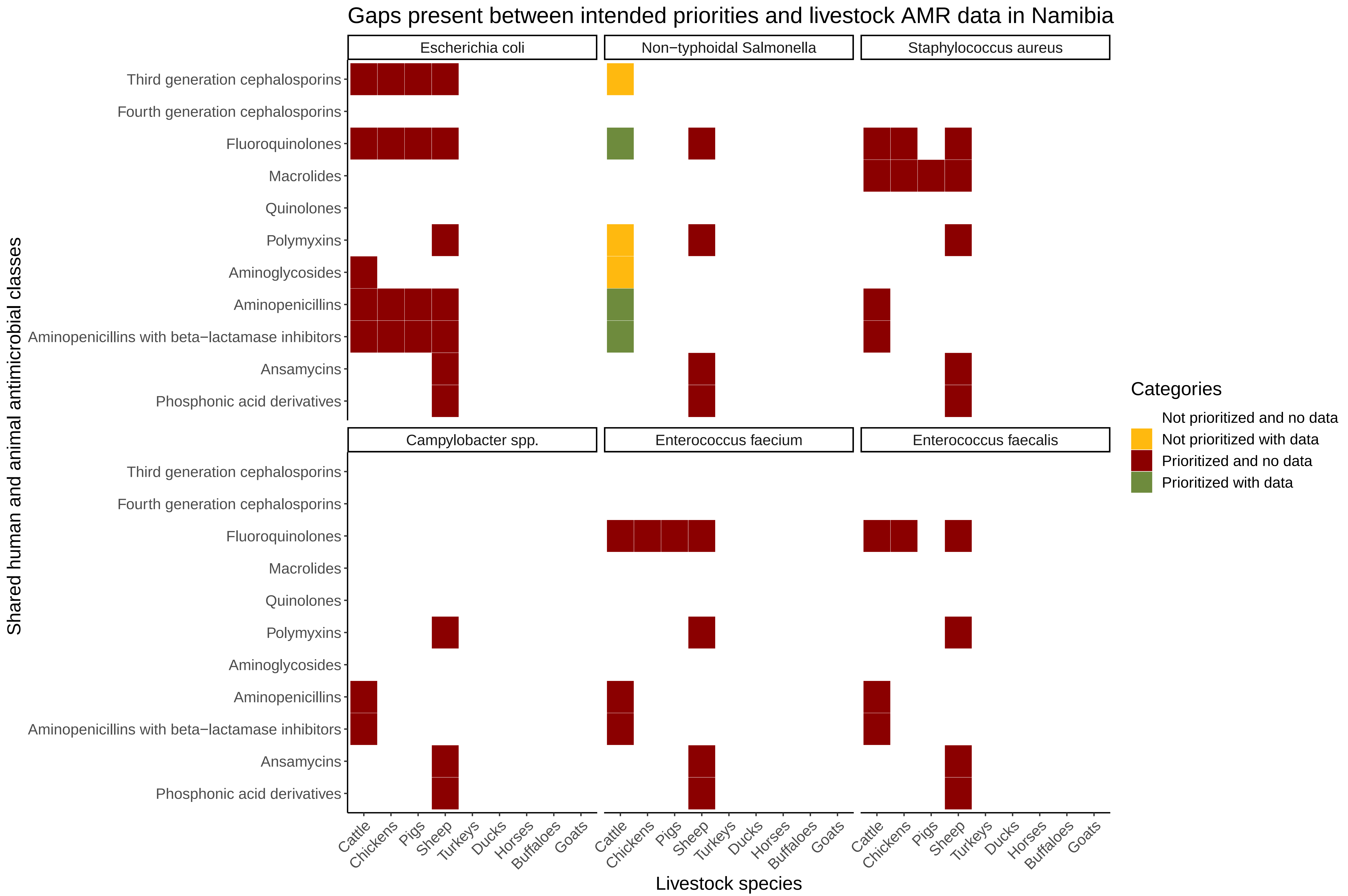

Gaps present between intended priorities and livestock AMR data in Namibia
Escherichia coli
Non−typhoidal Salmonella
Staphylococcus aureus
Campylobacter spp.
Enterococcus faecium
Enterococcus faecalis
Third generation cephalosporins
Fourth generation cephalosporins
Fluoroquinolones
Macrolides
Quinolones
Polymyxins
Aminoglycosides
Aminopenicillins
Aminopenicillins with beta−lactamase inhibitors
Ansamycins
Phosphonic acid derivatives
Shared human and animal antimicrobial classes
Cattle
Chickens
Pigs
Sheep
Turkeys
Ducks
Horses
Buffaloes
Goats
Livestock species
Categories
Not prioritized and no data
Not prioritized with data
Prioritized and no data
Prioritized with data

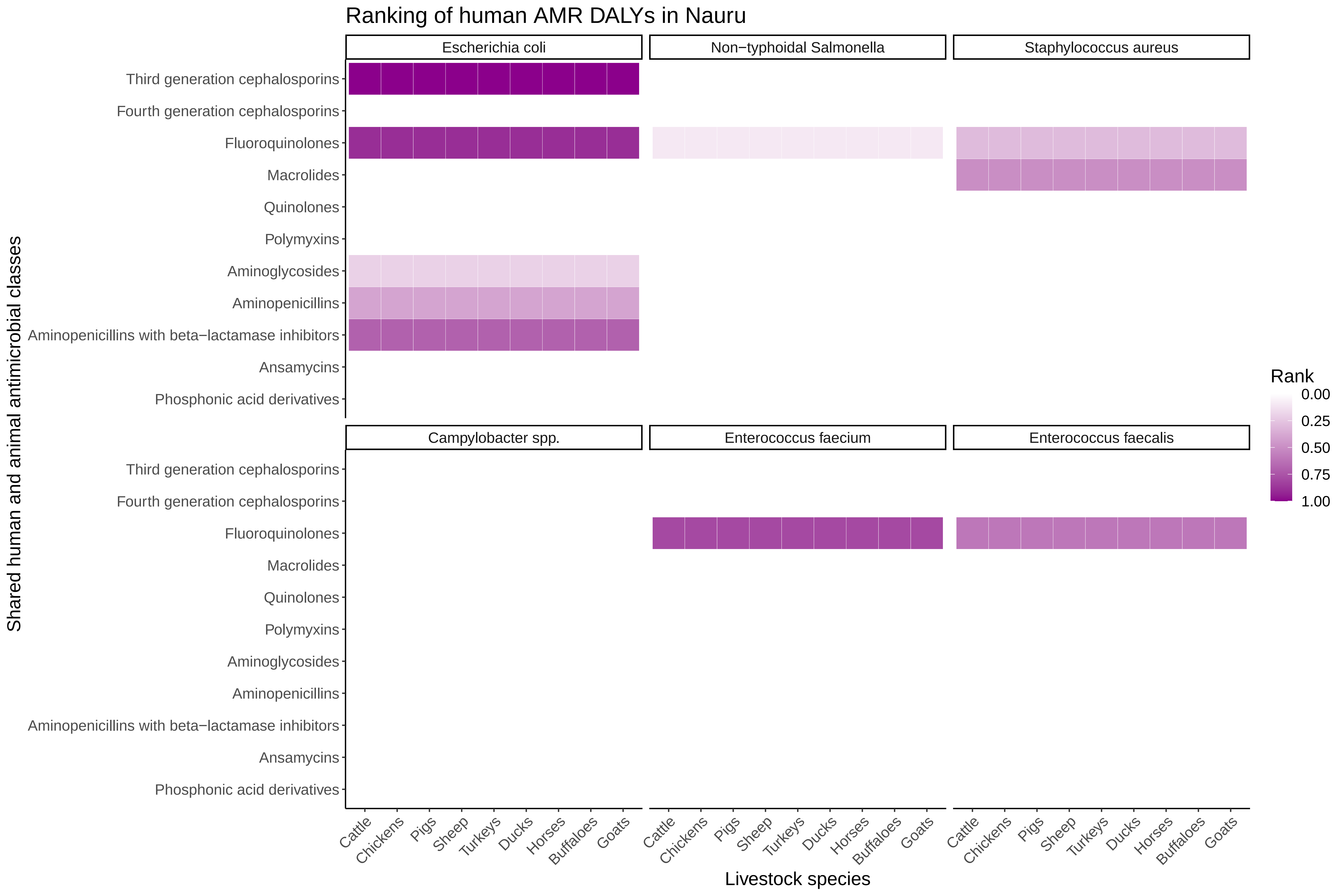
Ranking of human AMR DALYs in Nauru
Escherichia coli
Non−typhoidal Salmonella
Staphylococcus aureus
Campylobacter spp.
Enterococcus faecium
Enterococcus faecalis
Third generation cephalosporins
Fourth generation cephalosporins
Fluoroquinolones
Macrolides
Quinolones
Polymyxins
Aminoglycosides
Aminopenicillins
Aminopenicillins with beta−lactamase inhibitors
Ansamycins
Phosphonic acid derivatives
Shared human and animal antimicrobial classes
Cattle
Chickens
Pigs
Sheep
Turkeys
Ducks
Horses
Buffaloes
Goats
Livestock species
Rank
0.00
0.25
0.50
0.75
1.00

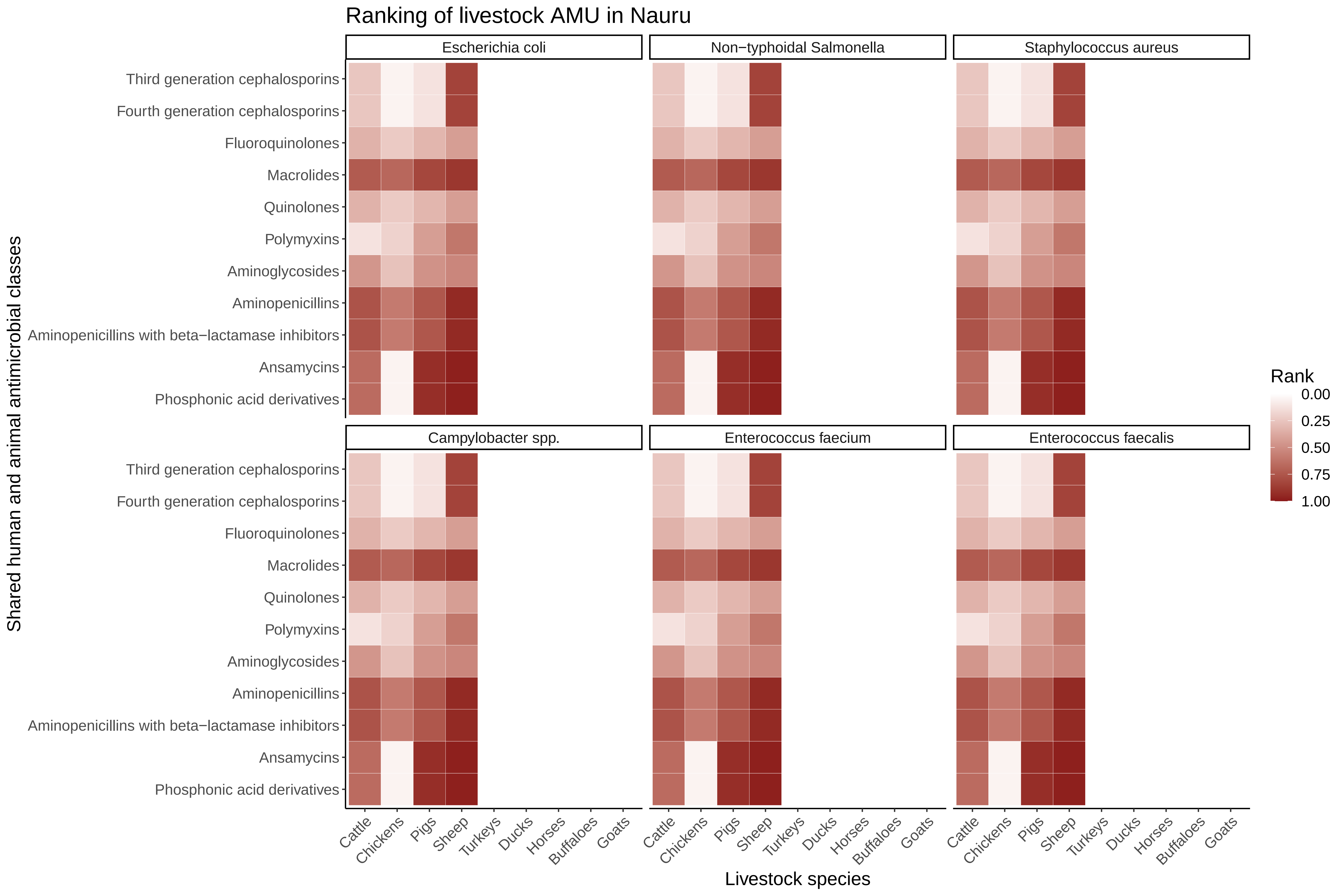
Ranking of livestock AMU in Nauru
Escherichia coli
Non−typhoidal Salmonella
Staphylococcus aureus
Campylobacter spp.
Enterococcus faecium
Enterococcus faecalis
Third generation cephalosporins
Fourth generation cephalosporins
Fluoroquinolones
Macrolides
Quinolones
Polymyxins
Aminoglycosides
Aminopenicillins
Aminopenicillins with beta−lactamase inhibitors
Ansamycins
Phosphonic acid derivatives
Shared human and animal antimicrobial classes
Cattle
Chickens
Pigs
Sheep
Turkeys
Ducks
Horses
Buffaloes
Goats
Livestock species
Rank
0.00
0.25
0.50
0.75
1.00

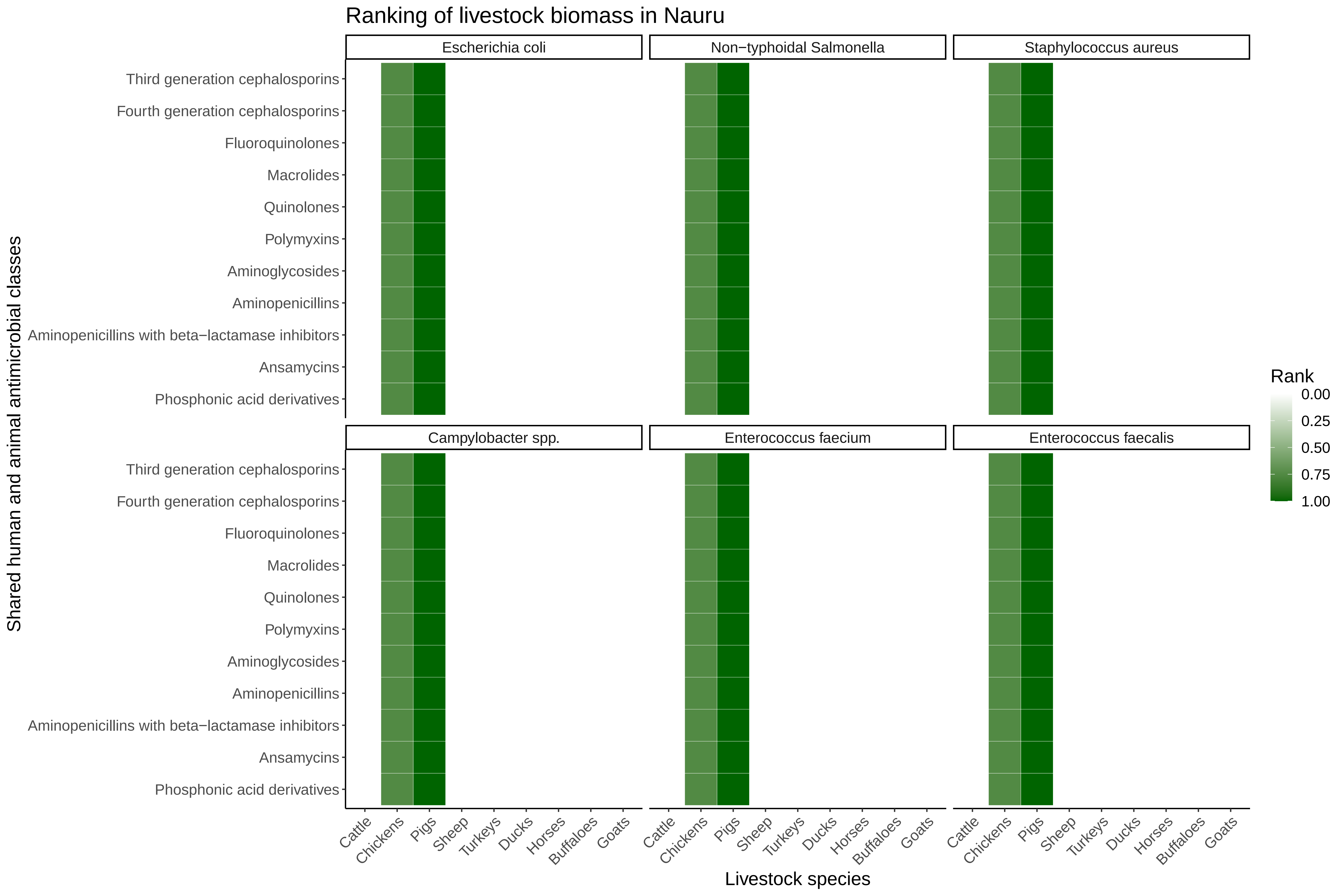

Ranking of livestock biomass in Nauru
Escherichia coli
Non−typhoidal Salmonella
Staphylococcus aureus
Campylobacter spp.
Enterococcus faecium
Enterococcus faecalis
Third generation cephalosporins
Fourth generation cephalosporins
Fluoroquinolones
Macrolides
Quinolones
Polymyxins
Aminoglycosides
Aminopenicillins
Aminopenicillins with beta−lactamase inhibitors
Ansamycins
Phosphonic acid derivatives
Shared human and animal antimicrobial classes
Cattle
Chickens
Pigs
Sheep
Turkeys
Ducks
Horses
Buffaloes
Goats
Livestock species
Rank
0.00
0.25
0.50
0.75
1.00

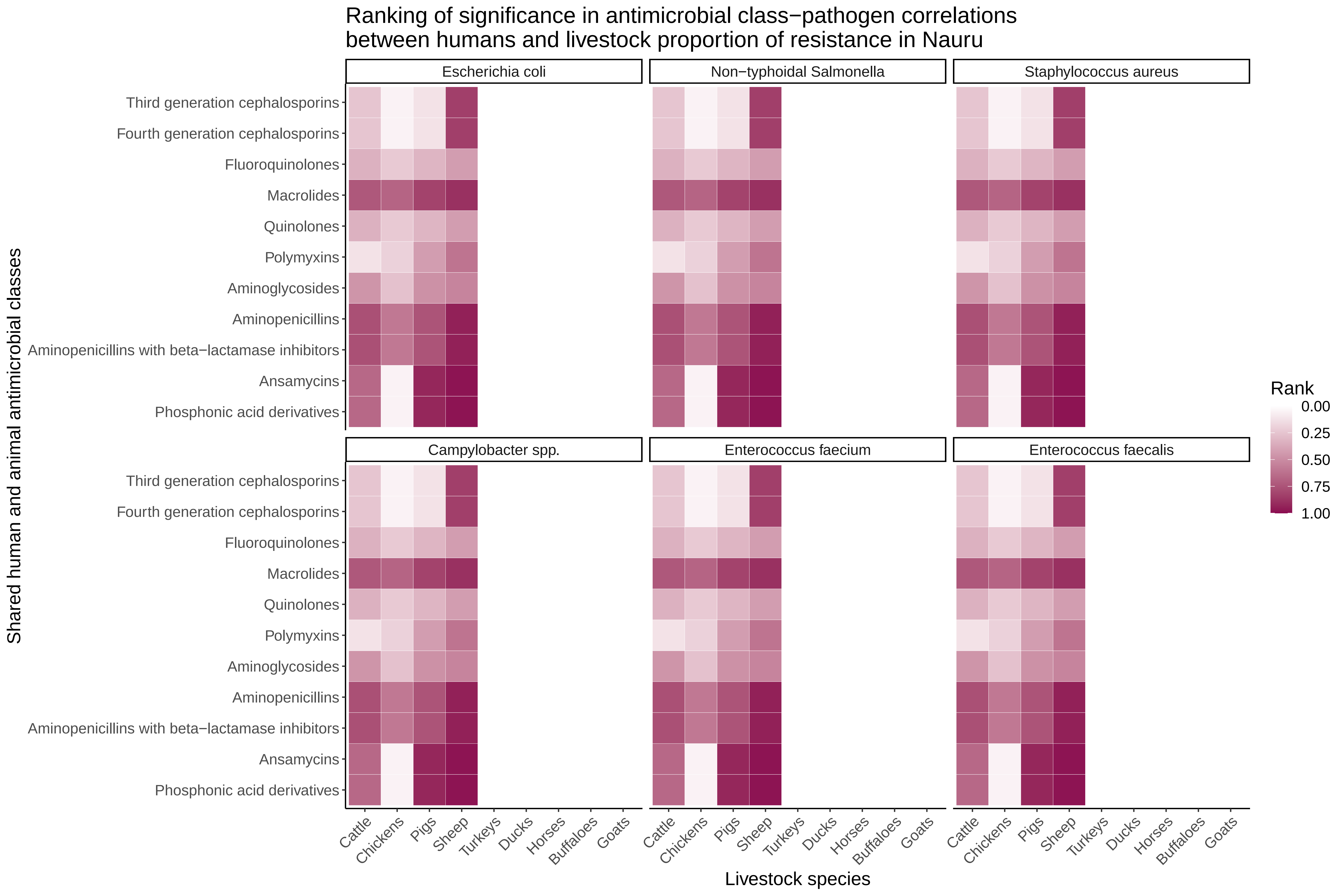

Ranking of significance in antimicrobial class−pathogen correlations
between humans and livestock proportion of resistance in Nauru
Escherichia coli
Non−typhoidal Salmonella
Staphylococcus aureus
Campylobacter spp.
Enterococcus faecium
Enterococcus faecalis
Shared human and animal antimicrobial classes
Third generation cephalosporins
Fourth generation cephalosporins
Fluoroquinolones
Macrolides
Quinolones
Polymyxins
Aminoglycosides
Aminopenicillins
Aminopenicillins with beta−lactamase inhibitors
Ansamycins
Phosphonic acid derivatives
Livestock species
Cattle
Chickens
Pigs
Sheep
Turkeys
Ducks
Horses
Buffaloes
Goats
Rank
0.00
0.25
0.50
0.75
1.00

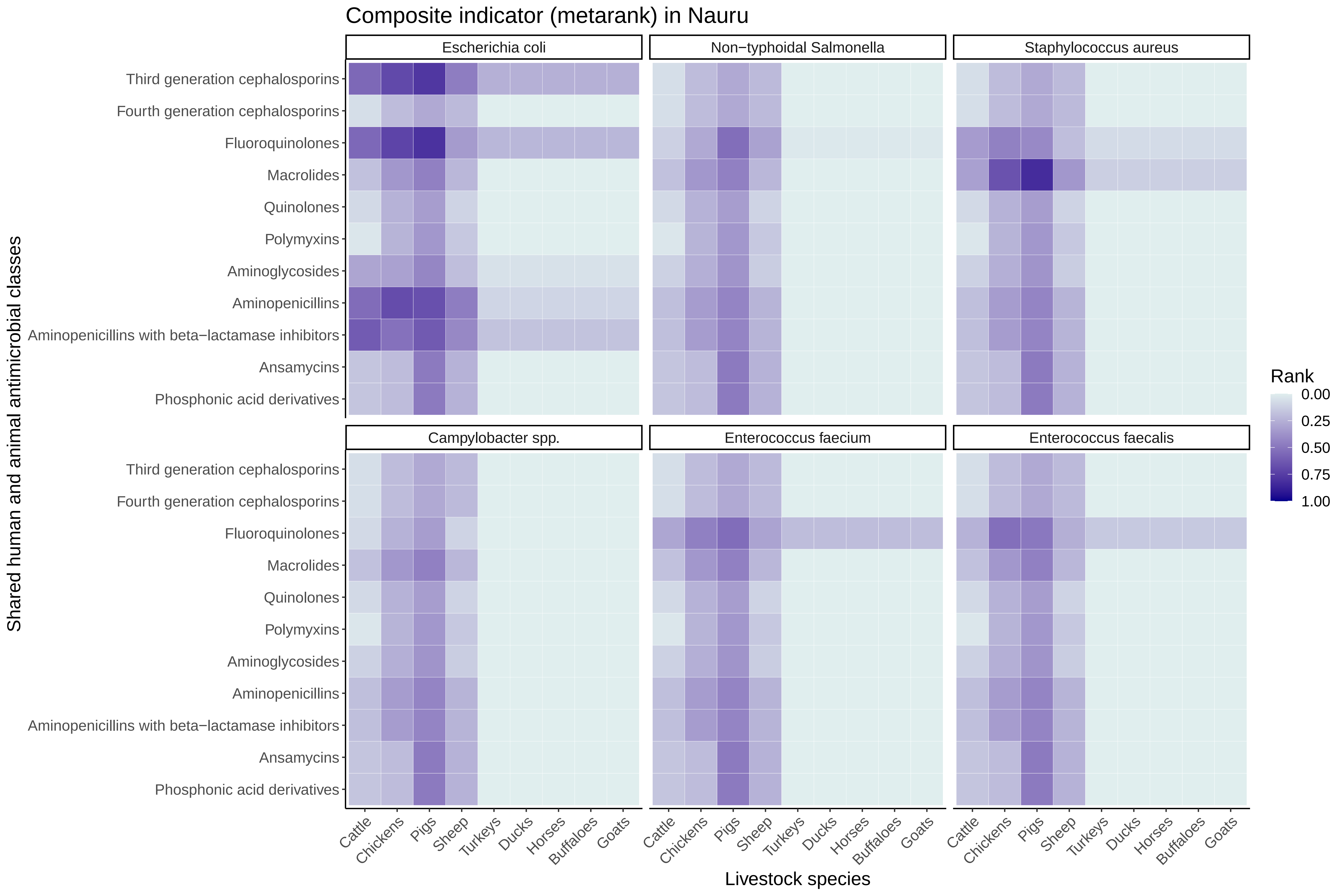

Composite indicator (metarank) in Nauru
Escherichia coli
Non−typhoidal Salmonella
Staphylococcus aureus
Campylobacter spp.
Enterococcus faecium
Enterococcus faecalis
Shared human and animal antimicrobial classes
Third generation cephalosporins
Fourth generation cephalosporins
Fluoroquinolones
Macrolides
Quinolones
Polymyxins
Aminoglycosides
Aminopenicillins
Aminopenicillins with beta−lactamase inhibitors
Ansamycins
Phosphonic acid derivatives
Cattle
Chickens
Pigs
Sheep
Turkeys
Ducks
Horses
Buffaloes
Goats
Livestock species
Rank
0.00
0.25
0.50
0.75
1.00

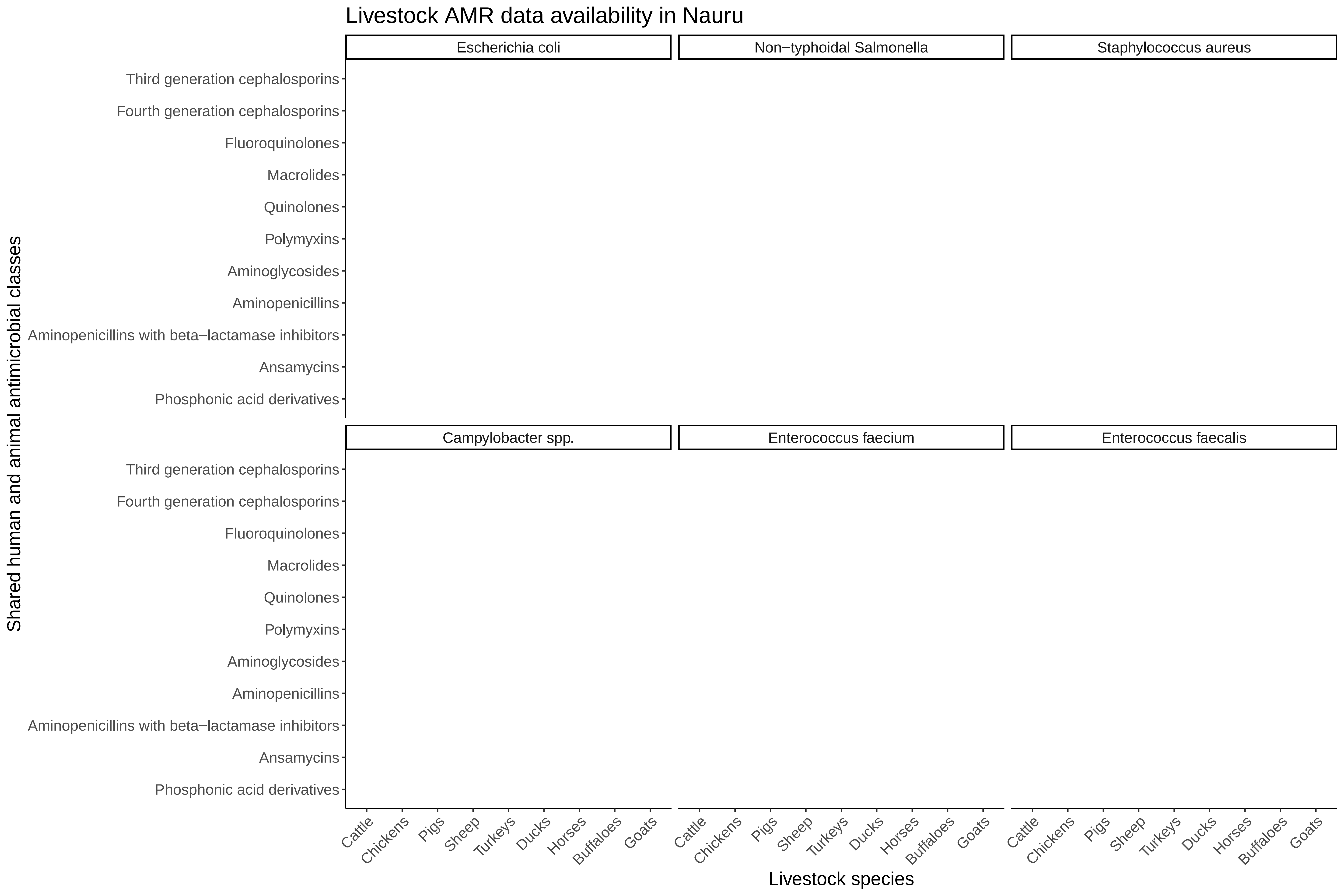
Livestock AMR data availability in Nauru
Escherichia coli
Non−typhoidal Salmonella
Staphylococcus aureus
Campylobacter spp.
Enterococcus faecium
Enterococcus faecalis
Shared human and animal antimicrobial classes
Third generation cephalosporins
Fourth generation cephalosporins
Fluoroquinolones
Macrolides
Quinolones
Polymyxins
Aminoglycosides
Aminopenicillins
Aminopenicillins with beta−lactamase inhibitors
Ansamycins
Phosphonic acid derivatives
Cattle
Chickens
Pigs
Sheep
Turkeys
Ducks
Horses
Buffaloes
Goats
Livestock species

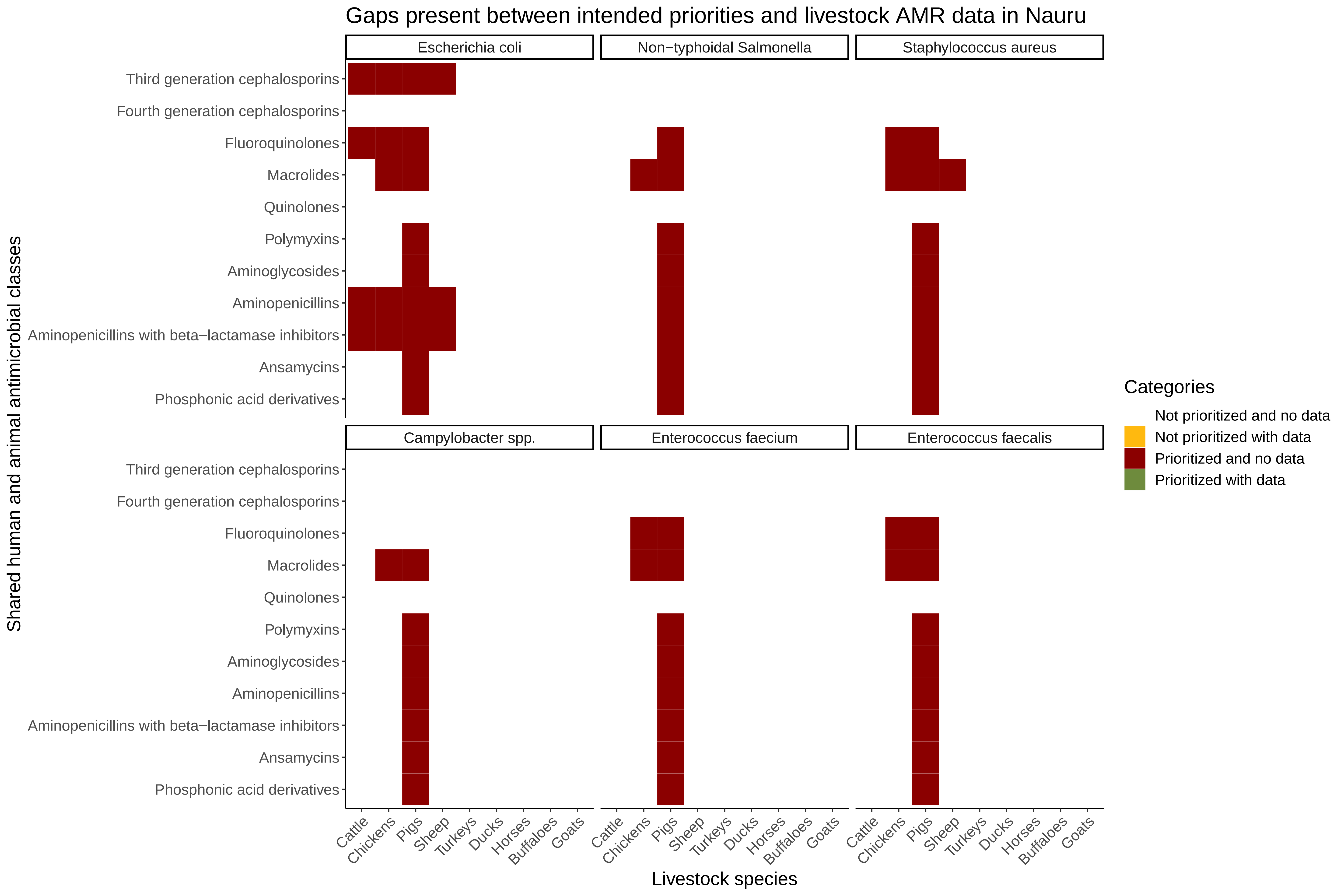

Gaps present between intended priorities and livestock AMR data in Nauru
Escherichia coli
Non−typhoidal Salmonella
Staphylococcus aureus
Campylobacter spp.
Enterococcus faecium
Enterococcus faecalis
Third generation cephalosporins
Fourth generation cephalosporins
Fluoroquinolones
Macrolides
Quinolones
Polymyxins
Aminoglycosides
Aminopenicillins
Aminopenicillins with beta−lactamase inhibitors
Ansamycins
Phosphonic acid derivatives
Shared human and animal antimicrobial classes
Cattle
Chickens
Pigs
Sheep
Turkeys
Ducks
Horses
Buffaloes
Goats
Livestock species
Categories
Not prioritized and no data
Not prioritized with data
Prioritized and no data
Prioritized with data

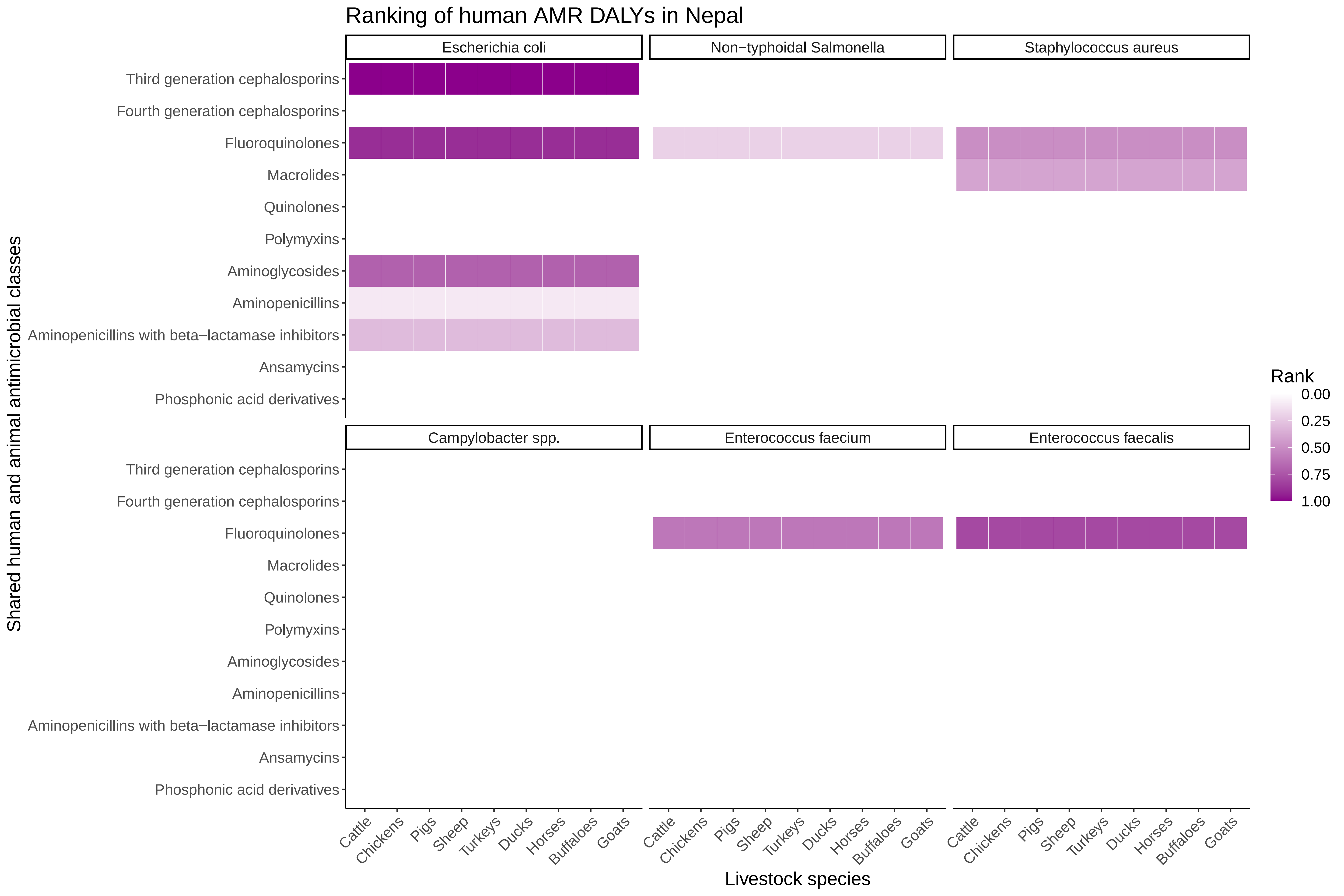
Ranking of human AMR DALYs in Nepal
Escherichia coli
Non−typhoidal Salmonella
Staphylococcus aureus
Campylobacter spp.
Enterococcus faecium
Enterococcus faecalis
Third generation cephalosporins
Fourth generation cephalosporins
Fluoroquinolones
Macrolides
Quinolones
Polymyxins
Aminoglycosides
Aminopenicillins
Aminopenicillins with beta−lactamase inhibitors
Ansamycins
Phosphonic acid derivatives
Shared human and animal antimicrobial classes
Cattle
Chickens
Pigs
Sheep
Turkeys
Ducks
Horses
Buffaloes
Goats
Livestock species
Rank
0.00
0.25
0.50
0.75
1.00

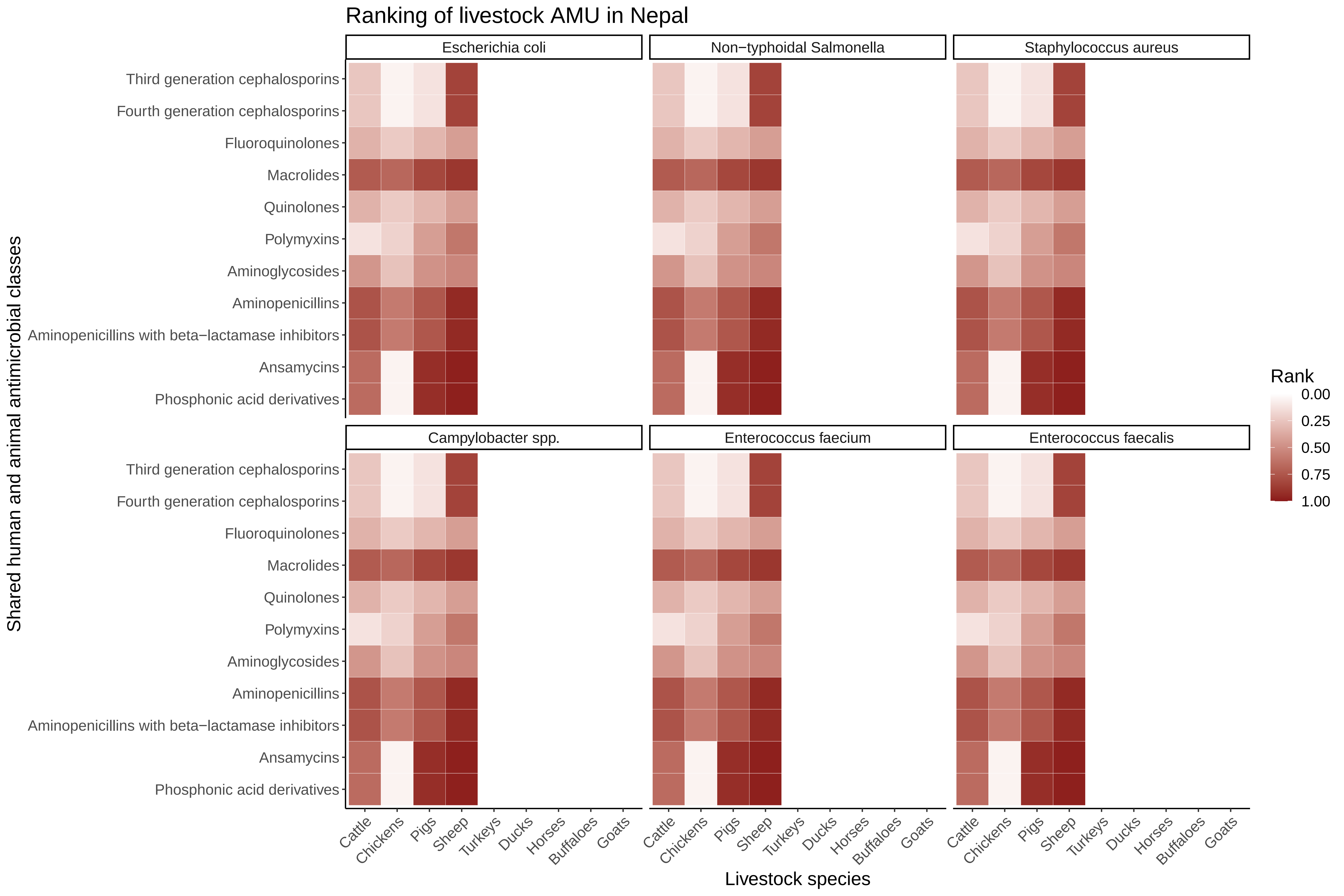
Ranking of livestock AMU in Nepal
Escherichia coli
Non−typhoidal Salmonella
Staphylococcus aureus
Campylobacter spp.
Enterococcus faecium
Enterococcus faecalis
Third generation cephalosporins
Fourth generation cephalosporins
Fluoroquinolones
Macrolides
Quinolones
Polymyxins
Aminoglycosides
Aminopenicillins
Aminopenicillins with beta−lactamase inhibitors
Ansamycins
Phosphonic acid derivatives
Shared human and animal antimicrobial classes
Cattle
Chickens
Pigs
Sheep
Turkeys
Ducks
Horses
Buffaloes
Goats
Livestock species
Rank
0.00
0.25
0.50
0.75
1.00

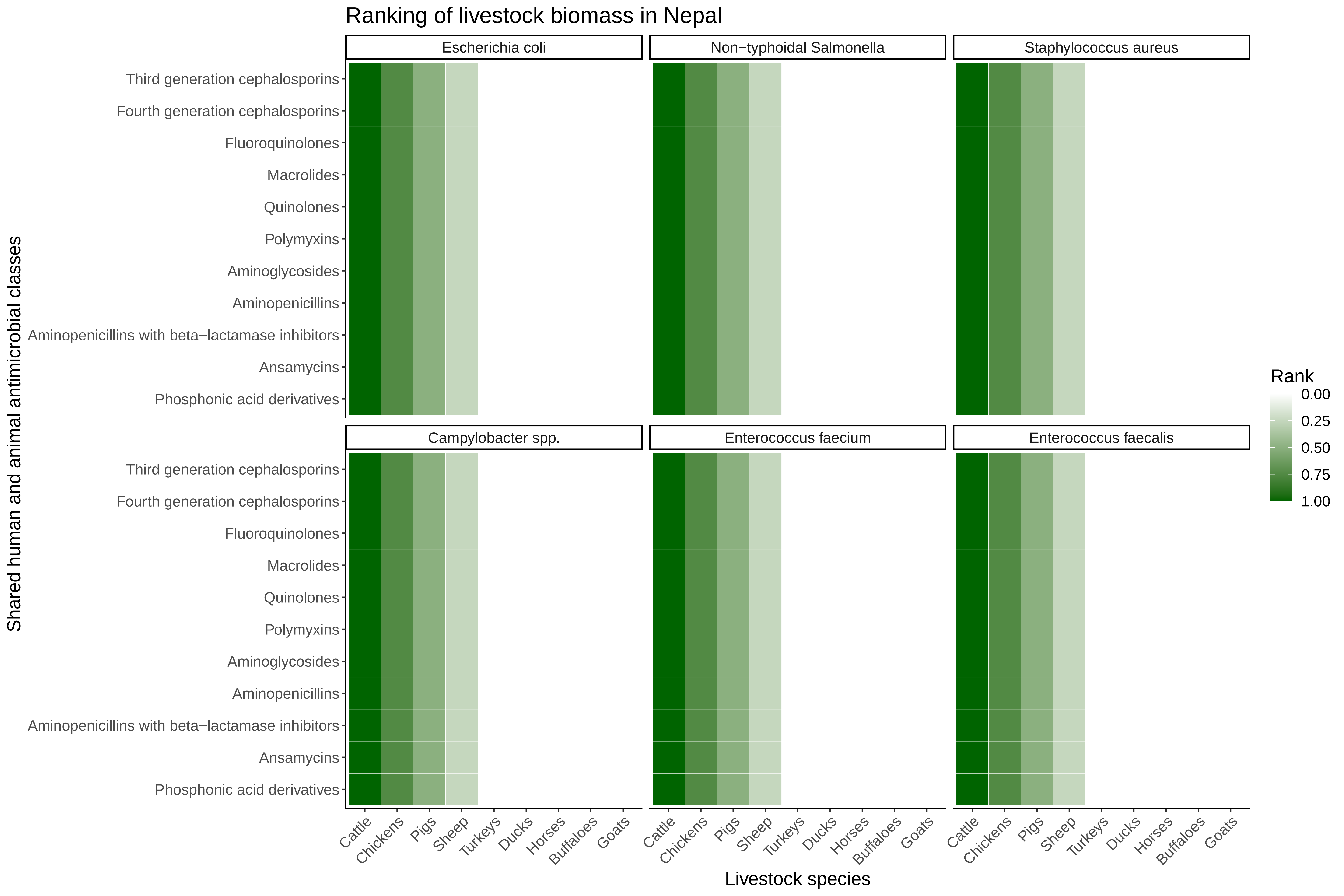
Ranking of livestock biomass in Nepal
Escherichia coli
Non−typhoidal Salmonella
Staphylococcus aureus
Campylobacter spp.
Enterococcus faecium
Enterococcus faecalis
Shared human and animal antimicrobial classes
Third generation cephalosporins
Fourth generation cephalosporins
Fluoroquinolones
Macrolides
Quinolones
Polymyxins
Aminoglycosides
Aminopenicillins
Aminopenicillins with beta−lactamase inhibitors
Ansamycins
Phosphonic acid derivatives
Livestock species
Cattle
Chickens
Pigs
Sheep
Turkeys
Ducks
Horses
Buffaloes
Goats
Rank
0.00
0.25
0.50
0.75
1.00

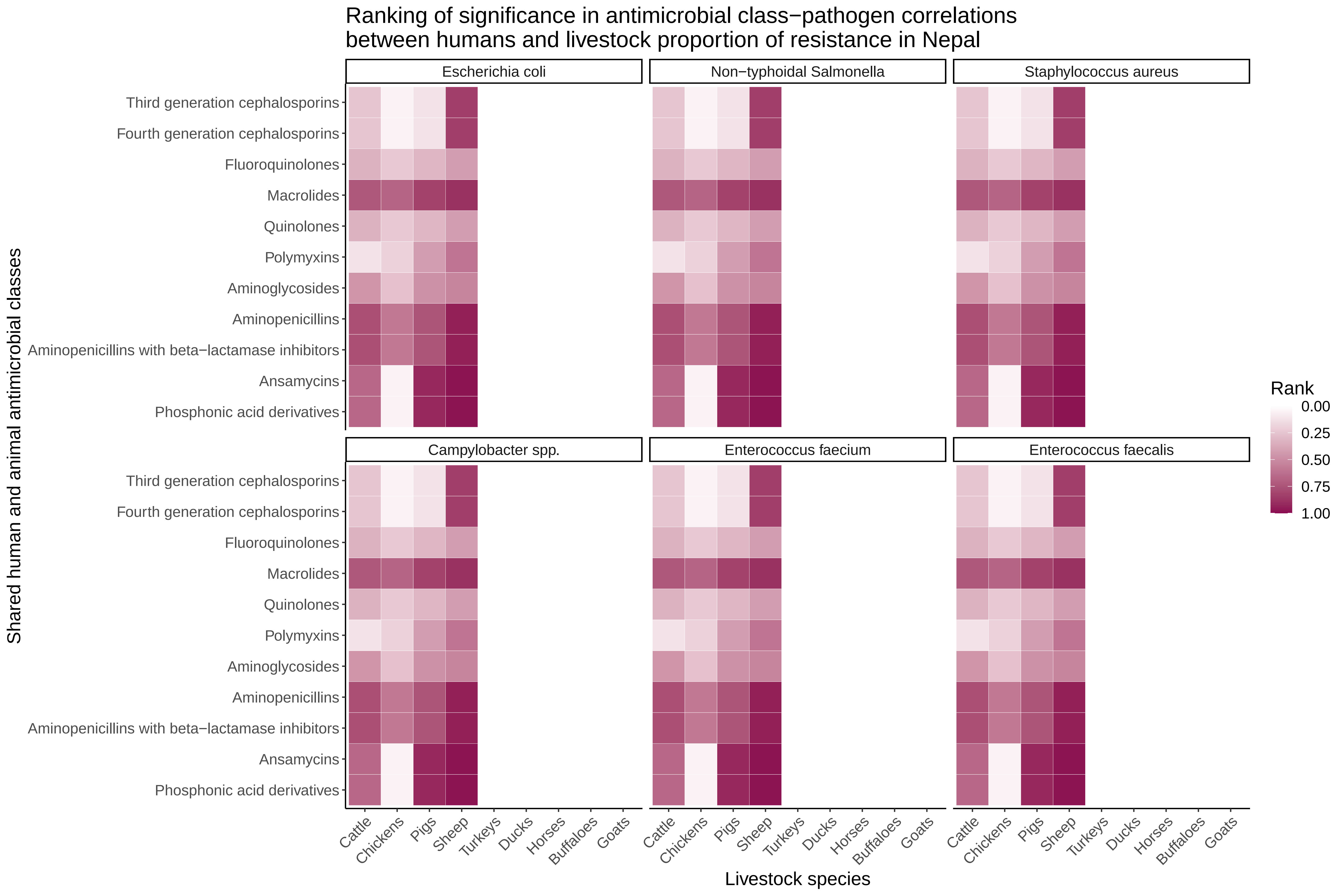

Ranking of significance in antimicrobial class−pathogen correlations
between humans and livestock proportion of resistance in Nepal
Escherichia coli
Non−typhoidal Salmonella
Staphylococcus aureus
Campylobacter spp.
Enterococcus faecium
Enterococcus faecalis
Third generation cephalosporins
Fourth generation cephalosporins
Fluoroquinolones
Macrolides
Quinolones
Polymyxins
Aminoglycosides
Aminopenicillins
Aminopenicillins with beta−lactamase inhibitors
Ansamycins
Phosphonic acid derivatives
Shared human and animal antimicrobial classes
Cattle
Chickens
Pigs
Sheep
Turkeys
Ducks
Horses
Buffaloes
Goats
Livestock species
Rank
0.00
0.25
0.50
0.75
1.00

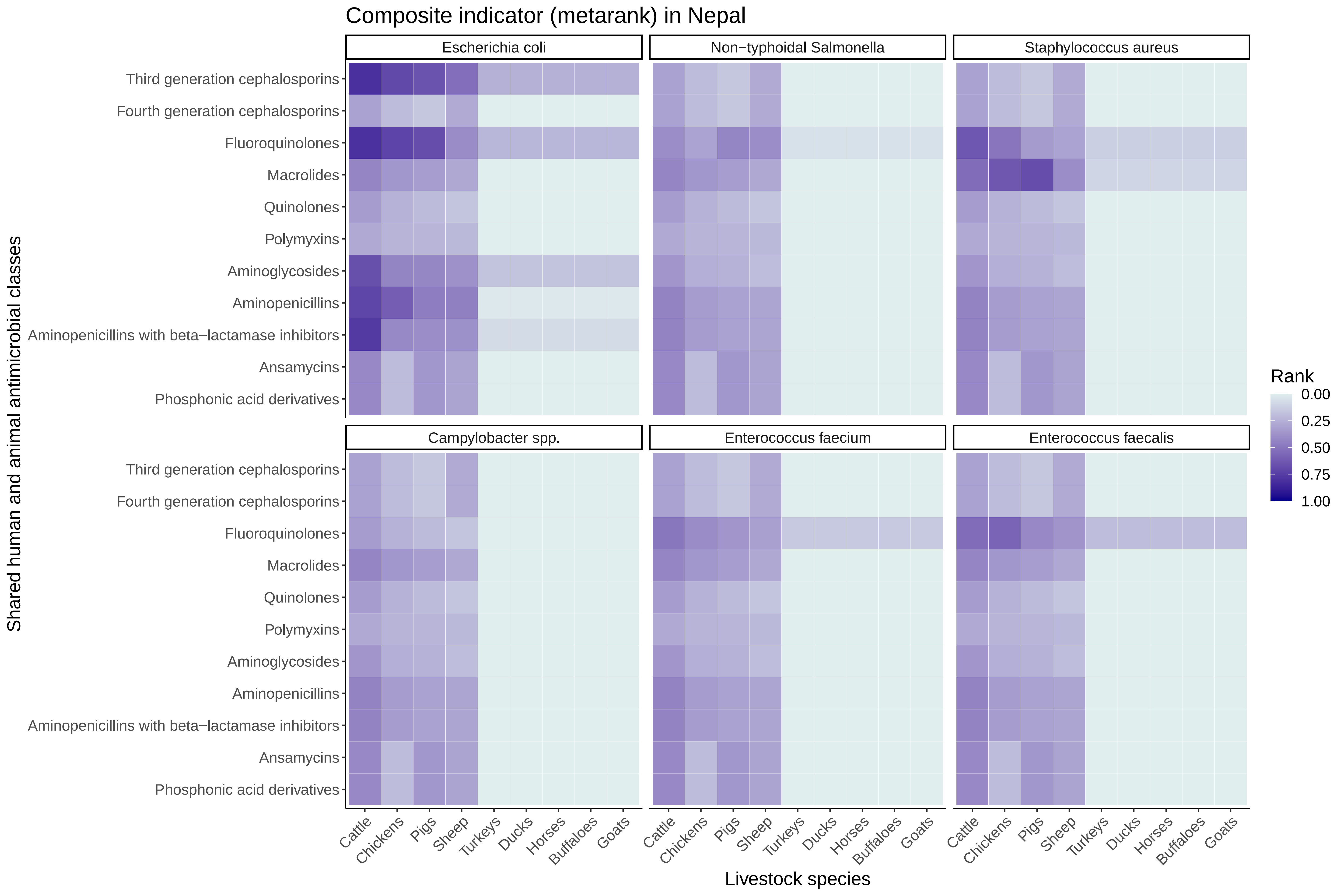

Composite indicator (metarank) in Nepal
Escherichia coli
Non−typhoidal Salmonella
Staphylococcus aureus
Campylobacter spp.
Enterococcus faecium
Enterococcus faecalis
Third generation cephalosporins
Fourth generation cephalosporins
Fluoroquinolones
Macrolides
Quinolones
Polymyxins
Aminoglycosides
Aminopenicillins
Aminopenicillins with beta−lactamase inhibitors
Ansamycins
Phosphonic acid derivatives
Shared human and animal antimicrobial classes
Cattle
Chickens
Pigs
Sheep
Turkeys
Ducks
Horses
Buffaloes
Goats
Livestock species
Rank
0.00
0.25
0.50
0.75
1.00

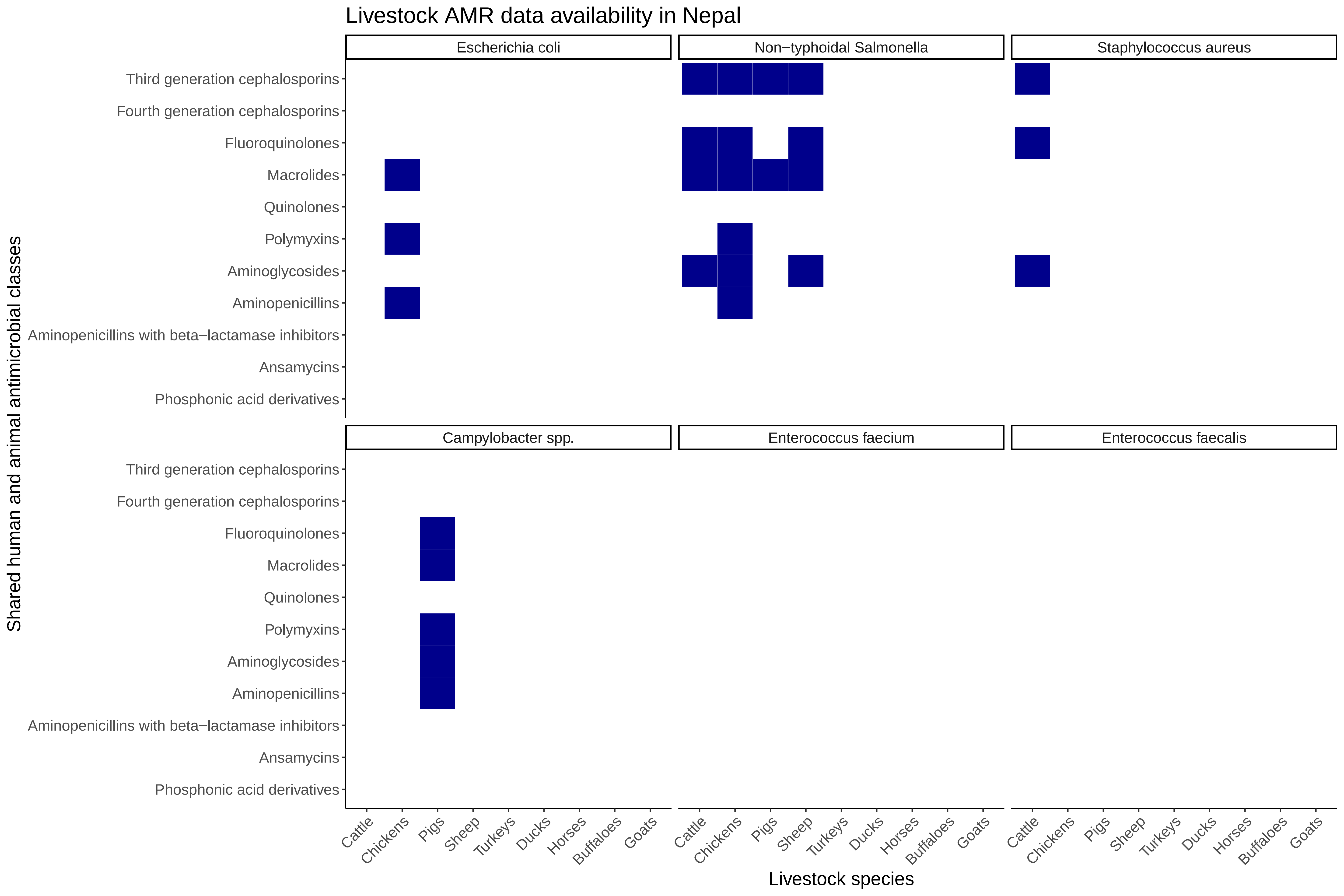

Livestock AMR data availability in Nepal
Escherichia coli
Non−typhoidal Salmonella
Staphylococcus aureus
Campylobacter spp.
Enterococcus faecium
Enterococcus faecalis
Shared human and animal antimicrobial classes
Third generation cephalosporins
Fourth generation cephalosporins
Fluoroquinolones
Macrolides
Quinolones
Polymyxins
Aminoglycosides
Aminopenicillins
Aminopenicillins with beta−lactamase inhibitors
Ansamycins
Phosphonic acid derivatives
Cattle
Chickens
Pigs
Sheep
Turkeys
Ducks
Horses
Buffaloes
Goats
Livestock species

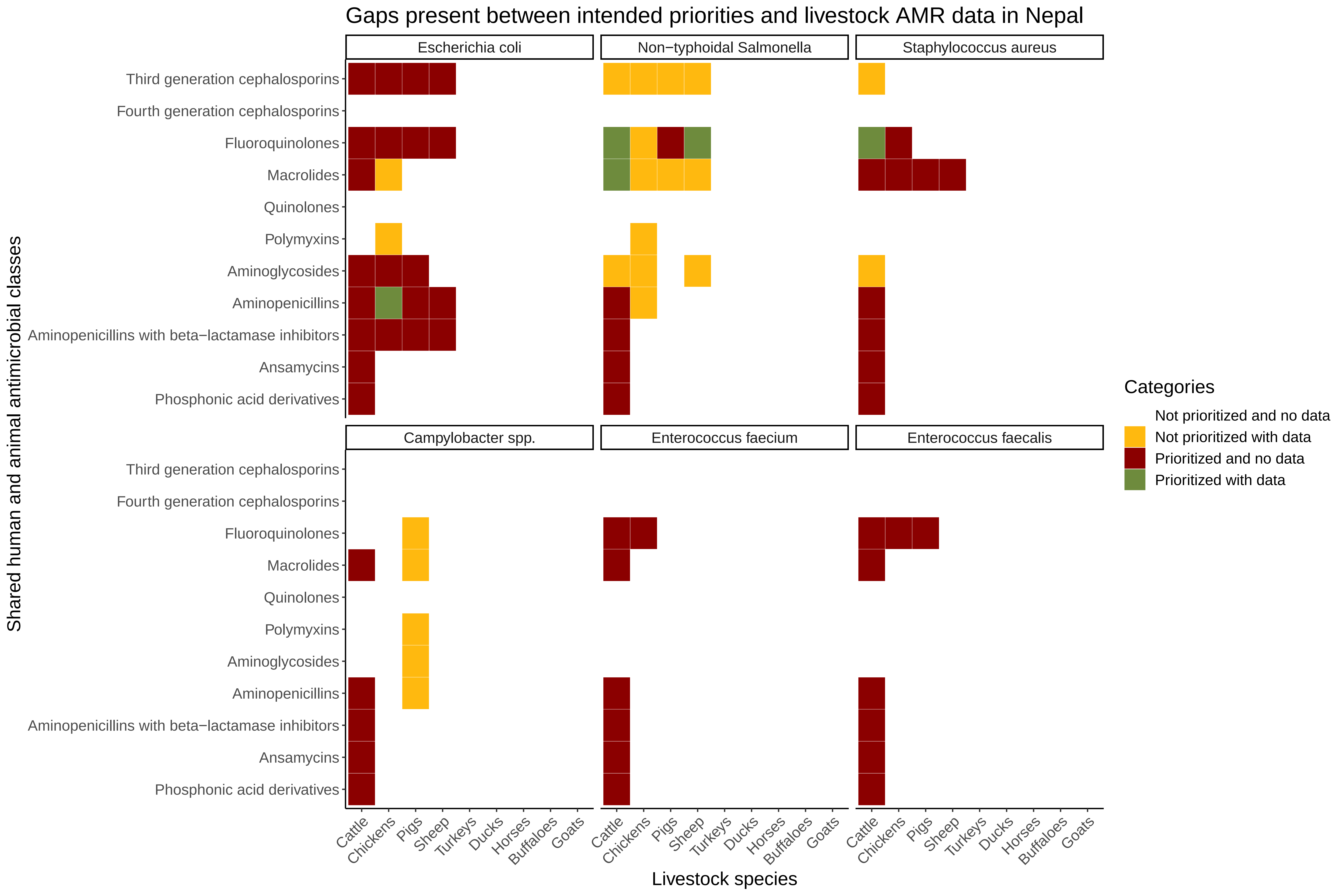

Gaps present between intended priorities and livestock AMR data in Nepal
Escherichia coli
Non−typhoidal Salmonella
Staphylococcus aureus
Campylobacter spp.
Enterococcus faecium
Enterococcus faecalis
Shared human and animal antimicrobial classes
Third generation cephalosporins
Fourth generation cephalosporins
Fluoroquinolones
Macrolides
Quinolones
Polymyxins
Aminoglycosides
Aminopenicillins
Aminopenicillins with beta−lactamase inhibitors
Ansamycins
Phosphonic acid derivatives
Livestock species
Cattle
Chickens
Pigs
Sheep
Turkeys
Ducks
Horses
Buffaloes
Goats
Categories
Not prioritized and no data
Not prioritized with data
Prioritized and no data
Prioritized with data

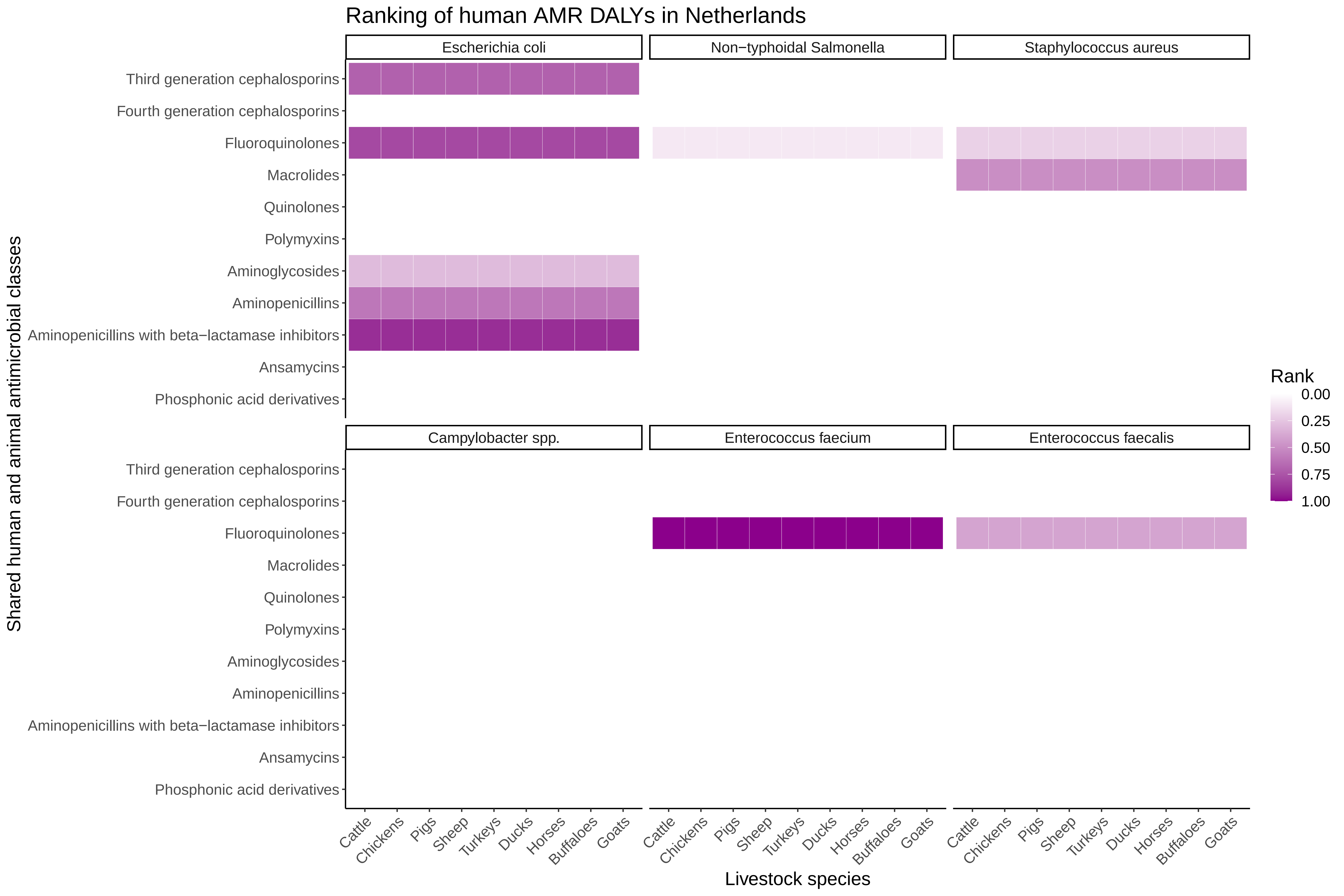

Ranking of human AMR DALYs in Netherlands
Escherichia coli
Non−typhoidal Salmonella
Staphylococcus aureus
Campylobacter spp.
Enterococcus faecium
Enterococcus faecalis
Shared human and animal antimicrobial classes
Third generation cephalosporins
Fourth generation cephalosporins
Fluoroquinolones
Macrolides
Quinolones
Polymyxins
Aminoglycosides
Aminopenicillins
Aminopenicillins with beta−lactamase inhibitors
Ansamycins
Phosphonic acid derivatives
Cattle
Chickens
Pigs
Sheep
Turkeys
Ducks
Horses
Buffaloes
Goats
Livestock species
Rank
0.00
0.25
0.50
0.75
1.00

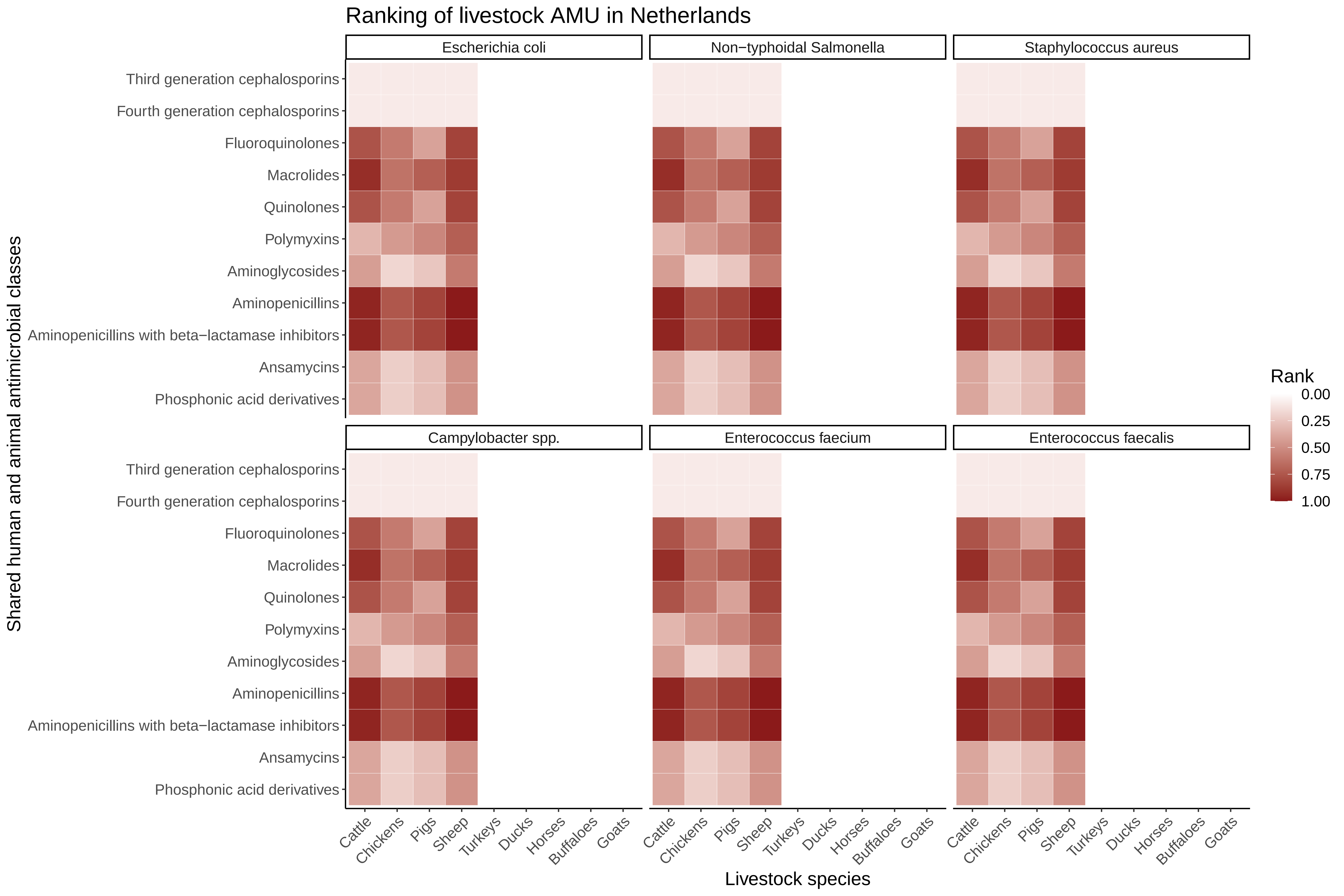

Ranking of livestock AMU in Netherlands
Escherichia coli
Non−typhoidal Salmonella
Staphylococcus aureus
Campylobacter spp.
Enterococcus faecium
Enterococcus faecalis
Third generation cephalosporins
Fourth generation cephalosporins
Fluoroquinolones
Macrolides
Quinolones
Polymyxins
Aminoglycosides
Aminopenicillins
Aminopenicillins with beta−lactamase inhibitors
Ansamycins
Phosphonic acid derivatives
Shared human and animal antimicrobial classes
Cattle
Chickens
Pigs
Sheep
Turkeys
Ducks
Horses
Buffaloes
Goats
Livestock species
Rank
0.00
0.25
0.50
0.75
1.00

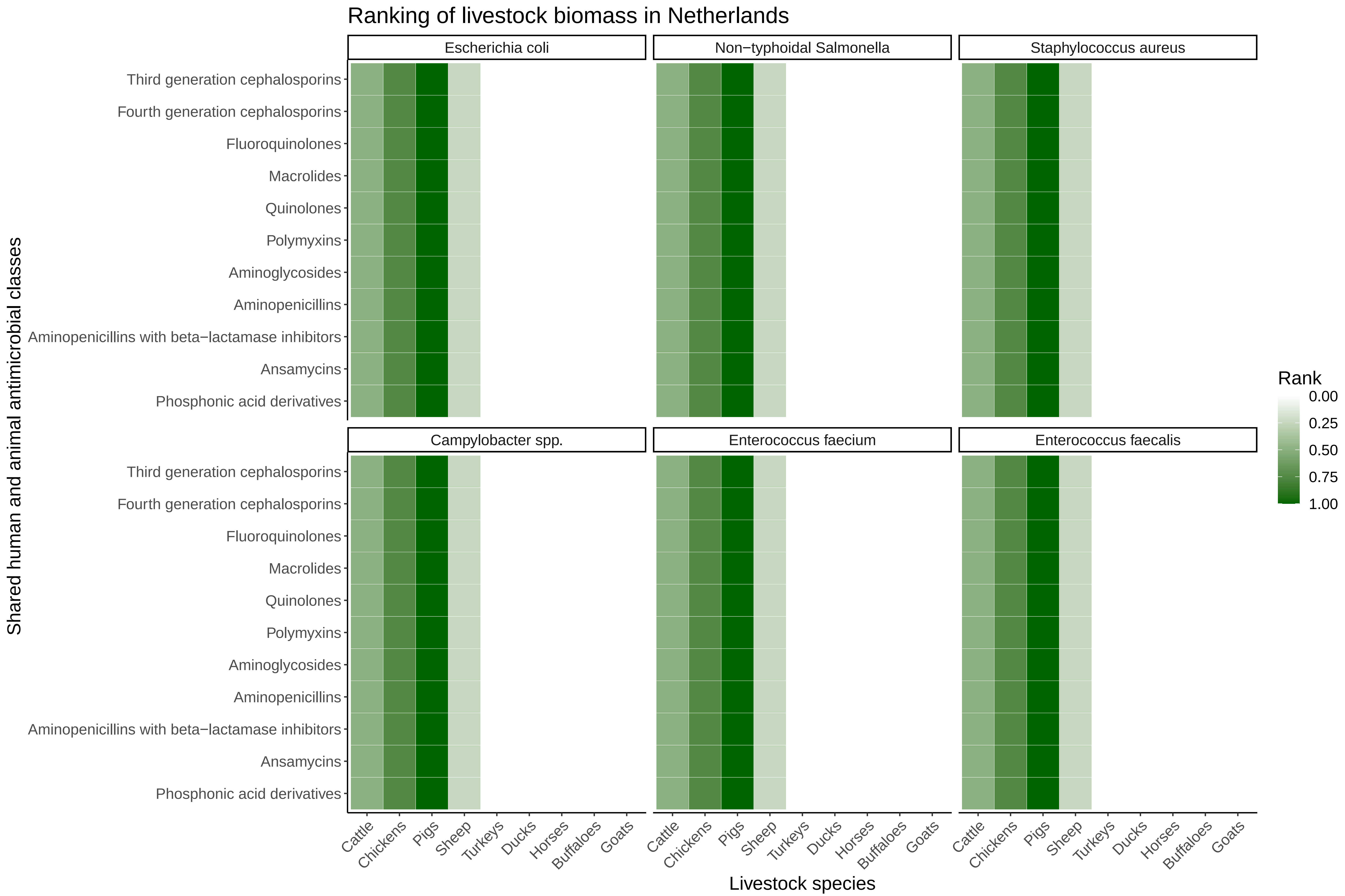
Ranking of livestock biomass in Netherlands
Escherichia coli
Non−typhoidal Salmonella
Staphylococcus aureus
Campylobacter spp.
Enterococcus faecium
Enterococcus faecalis
Third generation cephalosporins
Fourth generation cephalosporins
Fluoroquinolones
Macrolides
Quinolones
Polymyxins
Aminoglycosides
Aminopenicillins
Aminopenicillins with beta−lactamase inhibitors
Ansamycins
Phosphonic acid derivatives
Shared human and animal antimicrobial classes
Cattle
Chickens
Pigs
Sheep
Turkeys
Ducks
Horses
Buffaloes
Goats
Livestock species
Rank
0.00
0.25
0.50
0.75
1.00

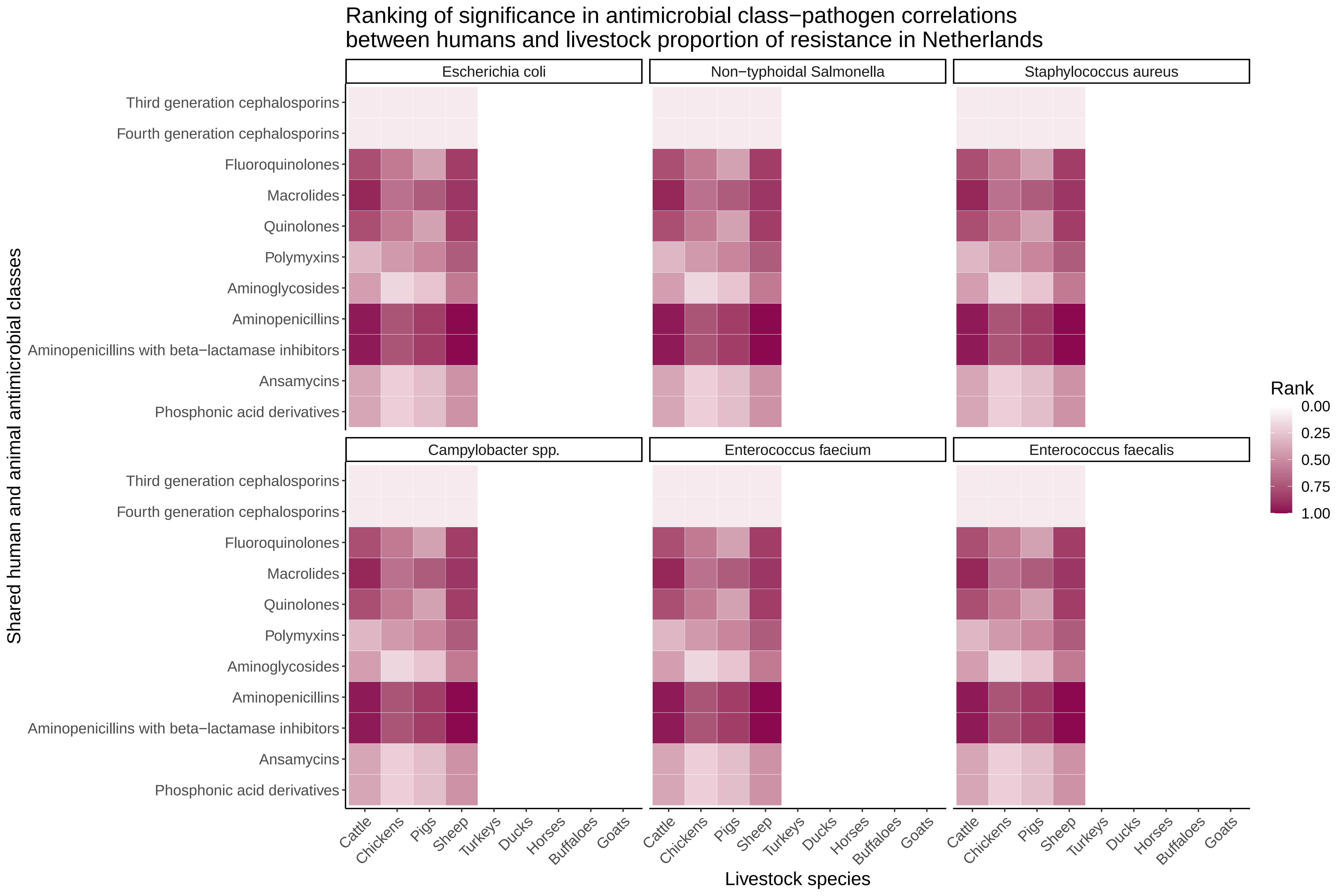

Ranking of significance in antimicrobial class−pathogen correlations
between humans and livestock proportion of resistance in Netherlands
Escherichia coli
Non−typhoidal Salmonella
Staphylococcus aureus
Campylobacter spp.
Enterococcus faecium
Enterococcus faecalis
Third generation cephalosporins
Fourth generation cephalosporins
Fluoroquinolones
Macrolides
Quinolones
Polymyxins
Aminoglycosides
Aminopenicillins
Aminopenicillins with beta−lactamase inhibitors
Ansamycins
Phosphonic acid derivatives
Shared human and animal antimicrobial classes
Cattle
Chickens
Pigs
Sheep
Turkeys
Ducks
Horses
Buffaloes
Goats
Livestock species
Rank
0.00
0.25
0.50
0.75
1.00

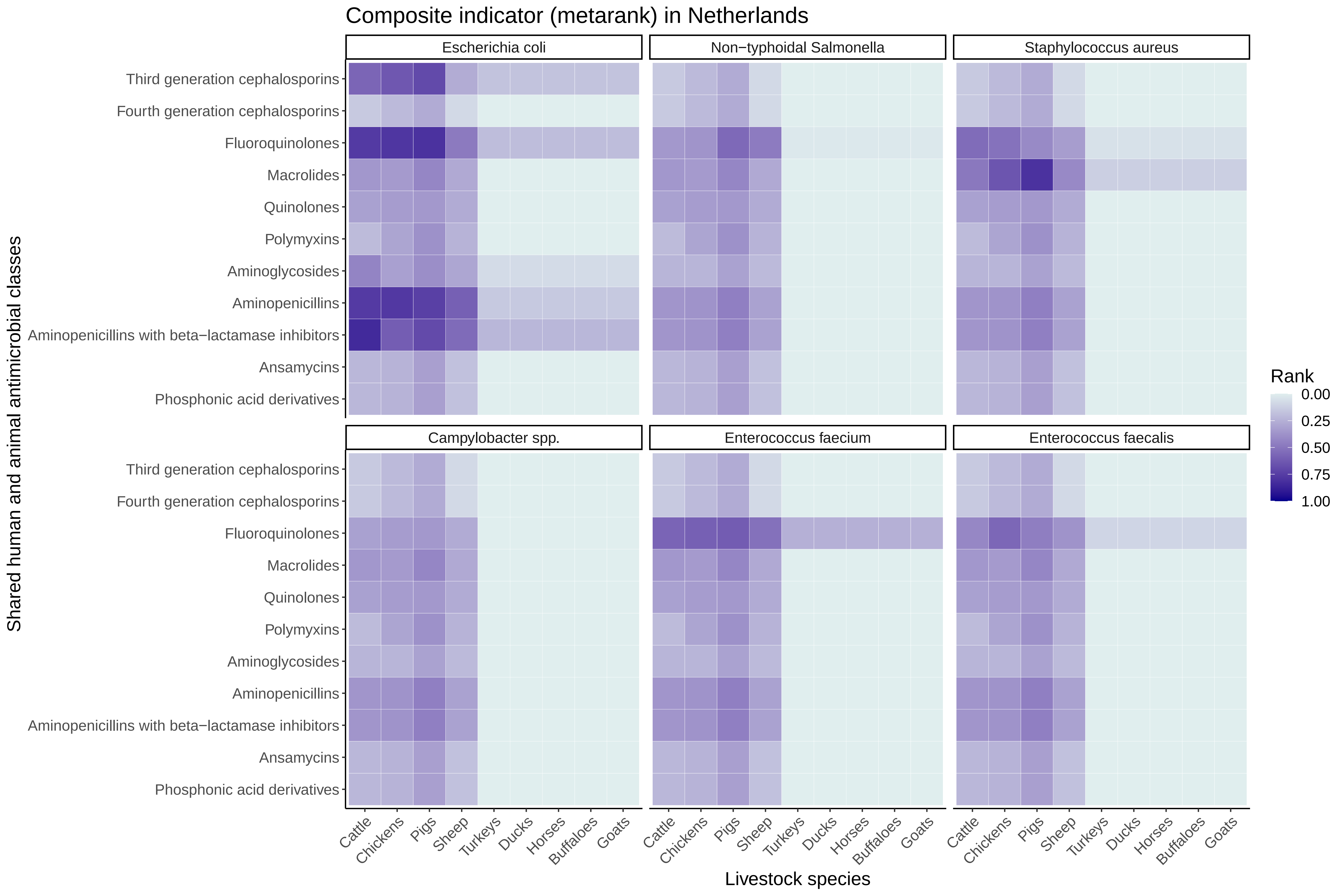

Composite indicator (metarank) in Netherlands
Escherichia coli
Non−typhoidal Salmonella
Staphylococcus aureus
Campylobacter spp.
Enterococcus faecium
Enterococcus faecalis
Shared human and animal antimicrobial classes
Third generation cephalosporins
Fourth generation cephalosporins
Fluoroquinolones
Macrolides
Quinolones
Polymyxins
Aminoglycosides
Aminopenicillins
Aminopenicillins with beta−lactamase inhibitors
Ansamycins
Phosphonic acid derivatives
Cattle
Chickens
Pigs
Sheep
Turkeys
Ducks
Horses
Buffaloes
Goats
Livestock species
Rank
0.00
0.25
0.50
0.75
1.00

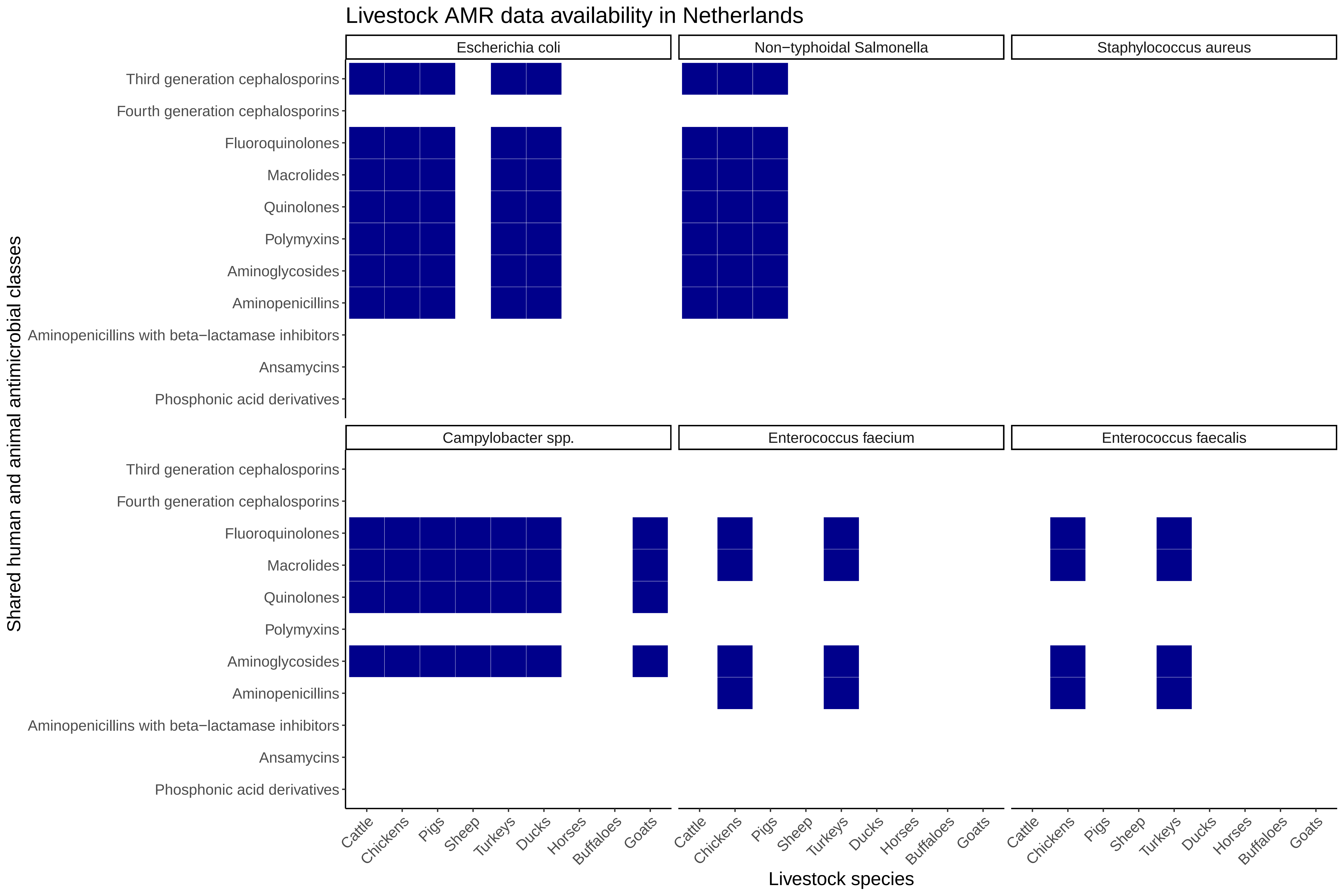
Livestock AMR data availability in Netherlands
Escherichia coli
Non−typhoidal Salmonella
Staphylococcus aureus
Campylobacter spp.
Enterococcus faecium
Enterococcus faecalis
Shared human and animal antimicrobial classes
Third generation cephalosporins
Fourth generation cephalosporins
Fluoroquinolones
Macrolides
Quinolones
Polymyxins
Aminoglycosides
Aminopenicillins
Aminopenicillins with beta−lactamase inhibitors
Ansamycins
Phosphonic acid derivatives
Livestock species
Cattle
Chickens
Pigs
Sheep
Turkeys
Ducks
Horses
Buffaloes
Goats

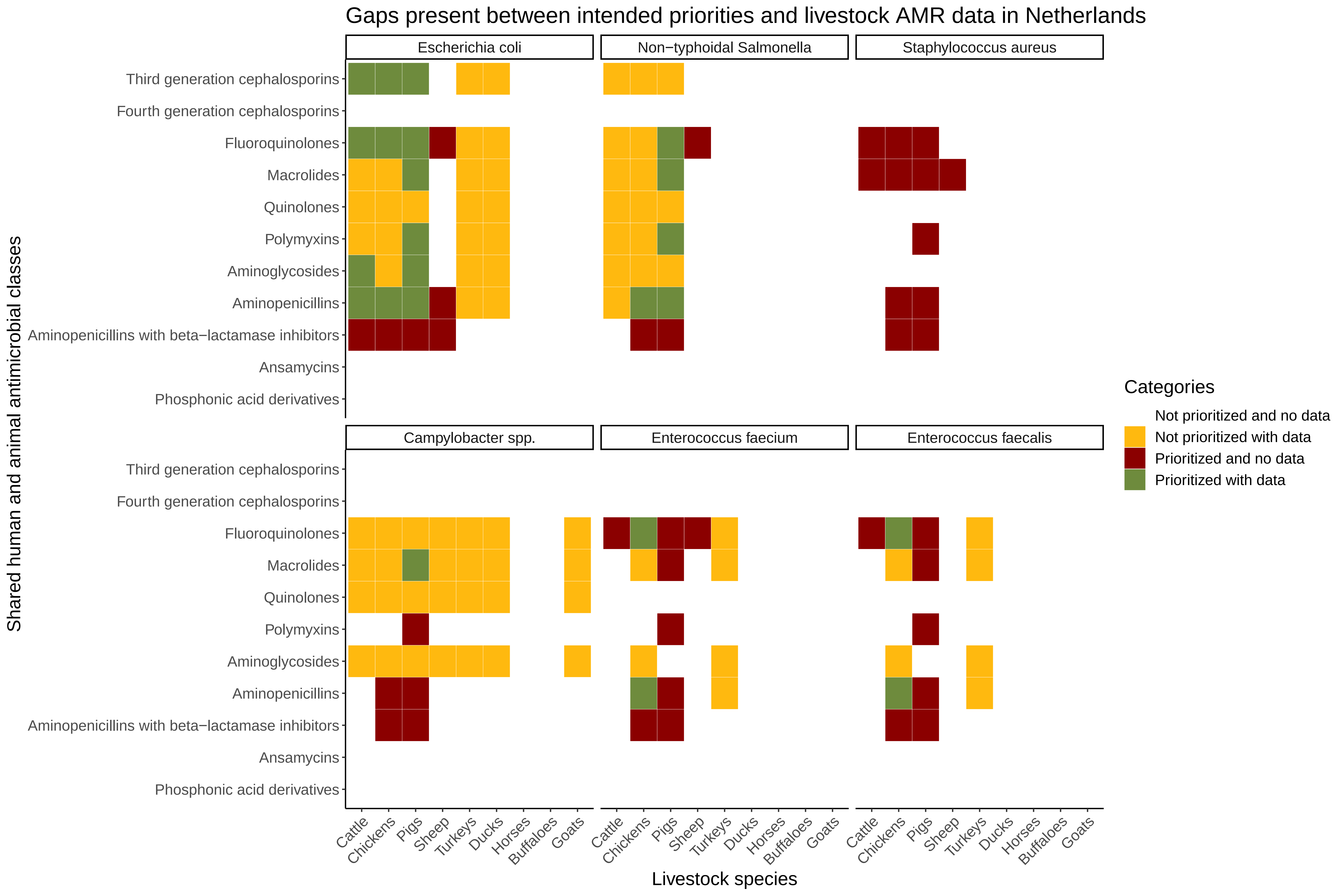
Gaps present between intended priorities and livestock AMR data in Netherlands
Escherichia coli
Non−typhoidal Salmonella
Staphylococcus aureus
Campylobacter spp.
Enterococcus faecium
Enterococcus faecalis
Third generation cephalosporins
Fourth generation cephalosporins
Fluoroquinolones
Macrolides
Quinolones
Polymyxins
Aminoglycosides
Aminopenicillins
Aminopenicillins with beta−lactamase inhibitors
Ansamycins
Phosphonic acid derivatives
Shared human and animal antimicrobial classes
Cattle
Chickens
Pigs
Sheep
Turkeys
Ducks
Horses
Buffaloes
Goats
Livestock species
Categories
Not prioritized and no data
Not prioritized with data
Prioritized and no data
Prioritized with data

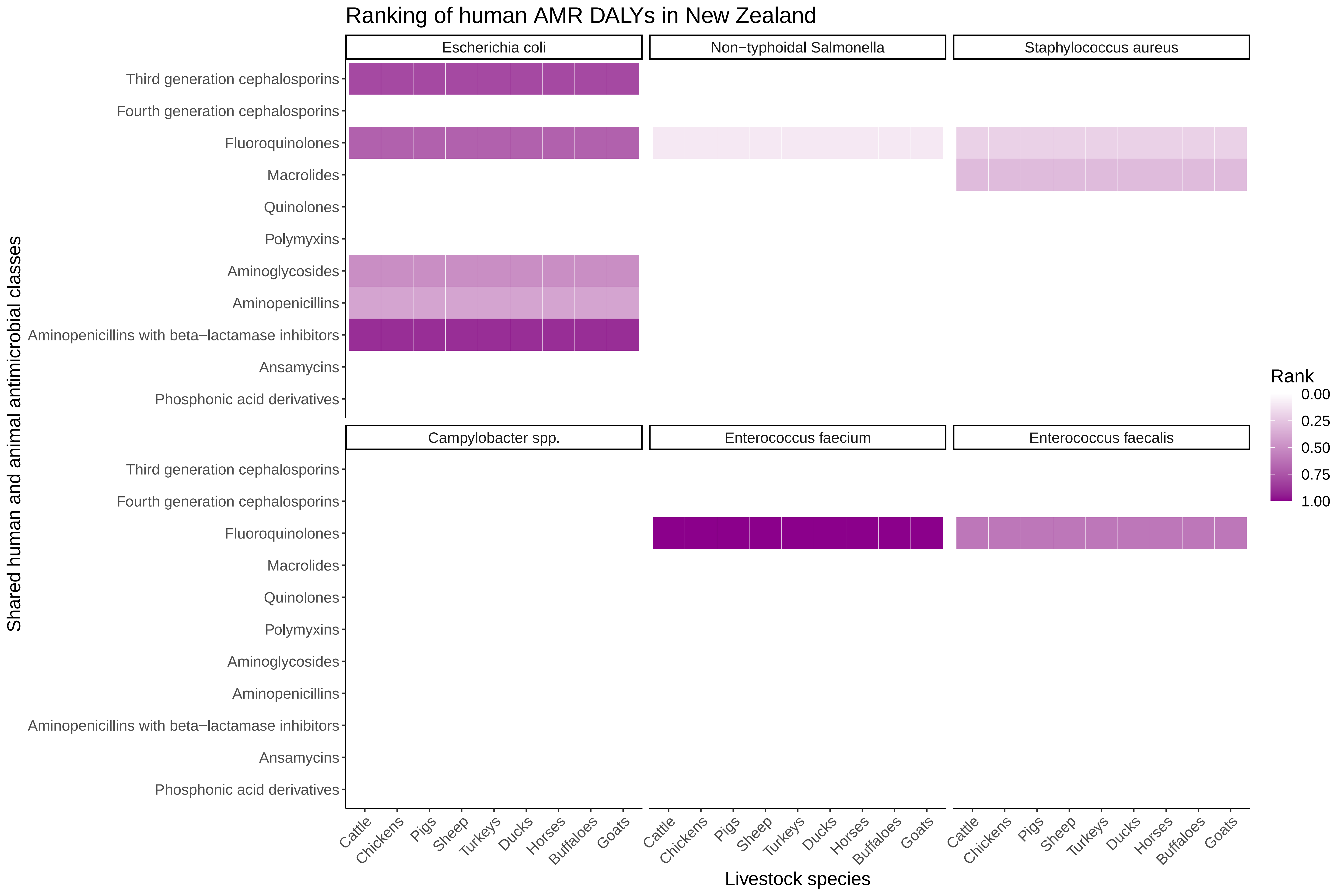

Ranking of human AMR DALYs in New Zealand
Escherichia coli
Non−typhoidal Salmonella
Staphylococcus aureus
Campylobacter spp.
Enterococcus faecium
Enterococcus faecalis
Shared human and animal antimicrobial classes
Third generation cephalosporins
Fourth generation cephalosporins
Fluoroquinolones
Macrolides
Quinolones
Polymyxins
Aminoglycosides
Aminopenicillins
Aminopenicillins with beta−lactamase inhibitors
Ansamycins
Phosphonic acid derivatives
Livestock species
Cattle
Chickens
Pigs
Sheep
Turkeys
Ducks
Horses
Buffaloes
Goats
Rank
0.00
0.25
0.50
0.75
1.00

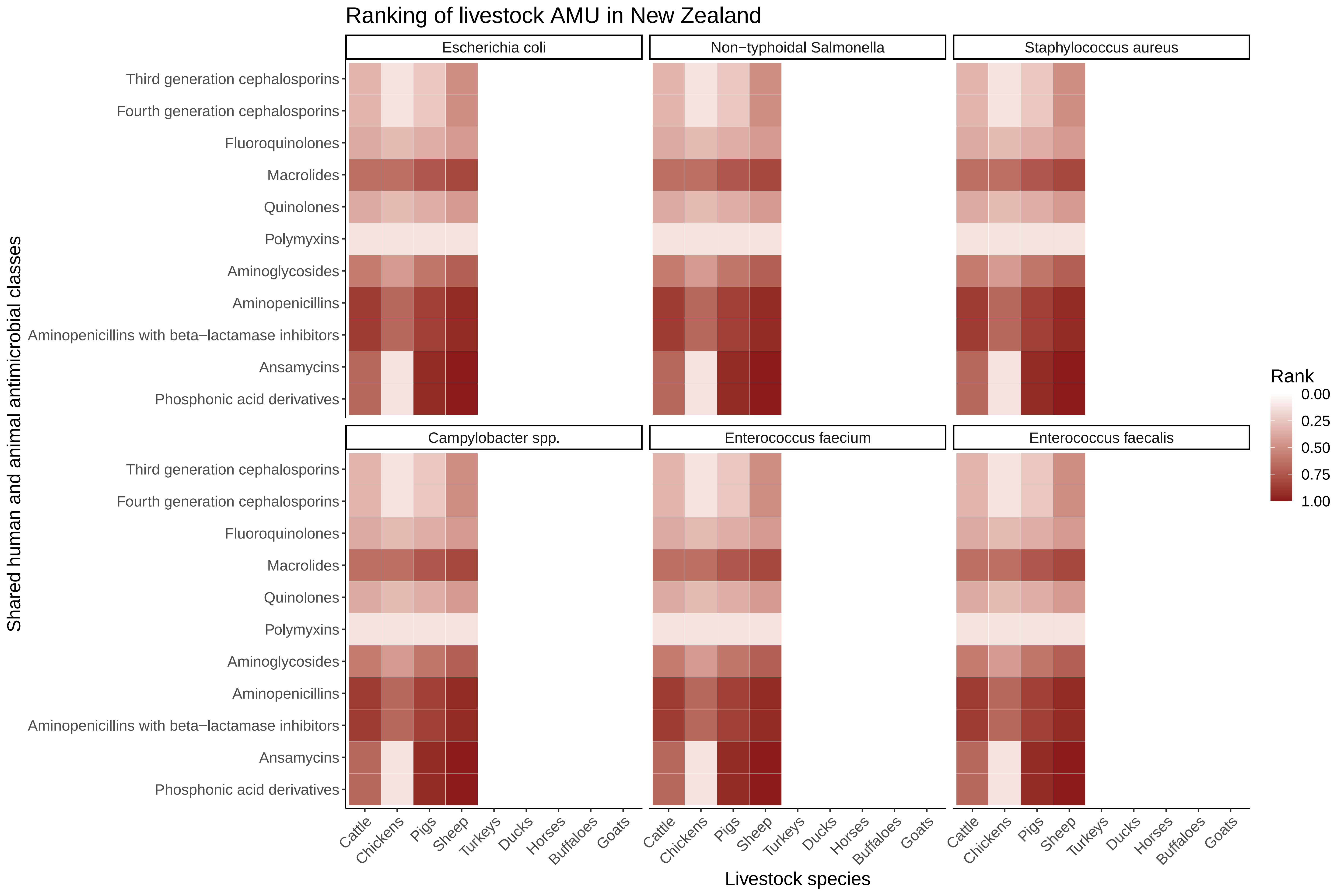

Ranking of livestock AMU in New Zealand
Escherichia coli
Non−typhoidal Salmonella
Staphylococcus aureus
Campylobacter spp.
Enterococcus faecium
Enterococcus faecalis
Shared human and animal antimicrobial classes
Third generation cephalosporins
Fourth generation cephalosporins
Fluoroquinolones
Macrolides
Quinolones
Polymyxins
Aminoglycosides
Aminopenicillins
Aminopenicillins with beta−lactamase inhibitors
Ansamycins
Phosphonic acid derivatives
Livestock species
Cattle
Chickens
Pigs
Sheep
Turkeys
Ducks
Horses
Buffaloes
Goats
Rank
0.00
0.25
0.50
0.75
1.00

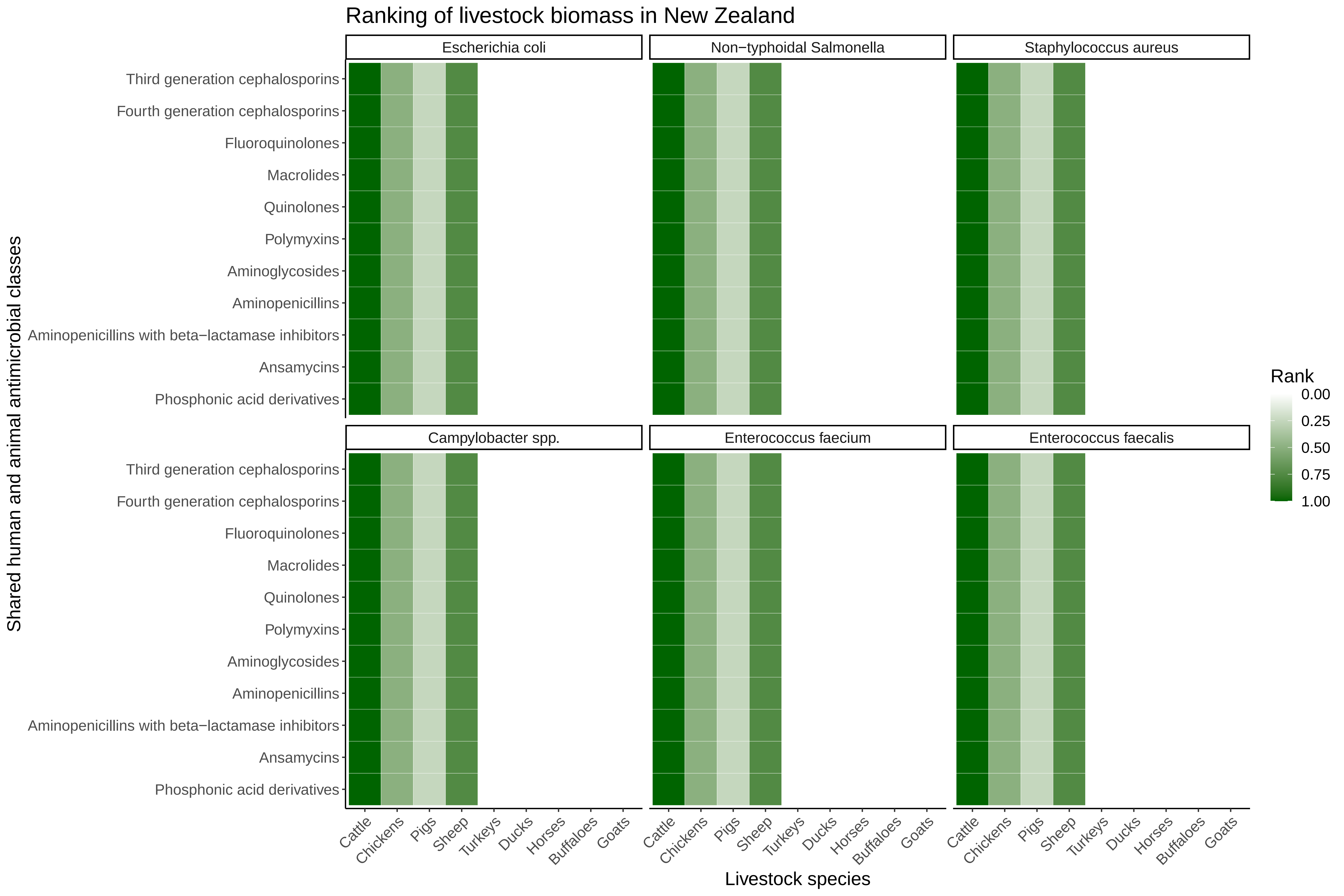

Ranking of livestock biomass in New Zealand
Escherichia coli
Non−typhoidal Salmonella
Staphylococcus aureus
Campylobacter spp.
Enterococcus faecium
Enterococcus faecalis
Shared human and animal antimicrobial classes
Third generation cephalosporins
Fourth generation cephalosporins
Fluoroquinolones
Macrolides
Quinolones
Polymyxins
Aminoglycosides
Aminopenicillins
Aminopenicillins with beta−lactamase inhibitors
Ansamycins
Phosphonic acid derivatives
Livestock species
Cattle
Chickens
Pigs
Sheep
Turkeys
Ducks
Horses
Buffaloes
Goats
Rank
0.00
0.25
0.50
0.75
1.00

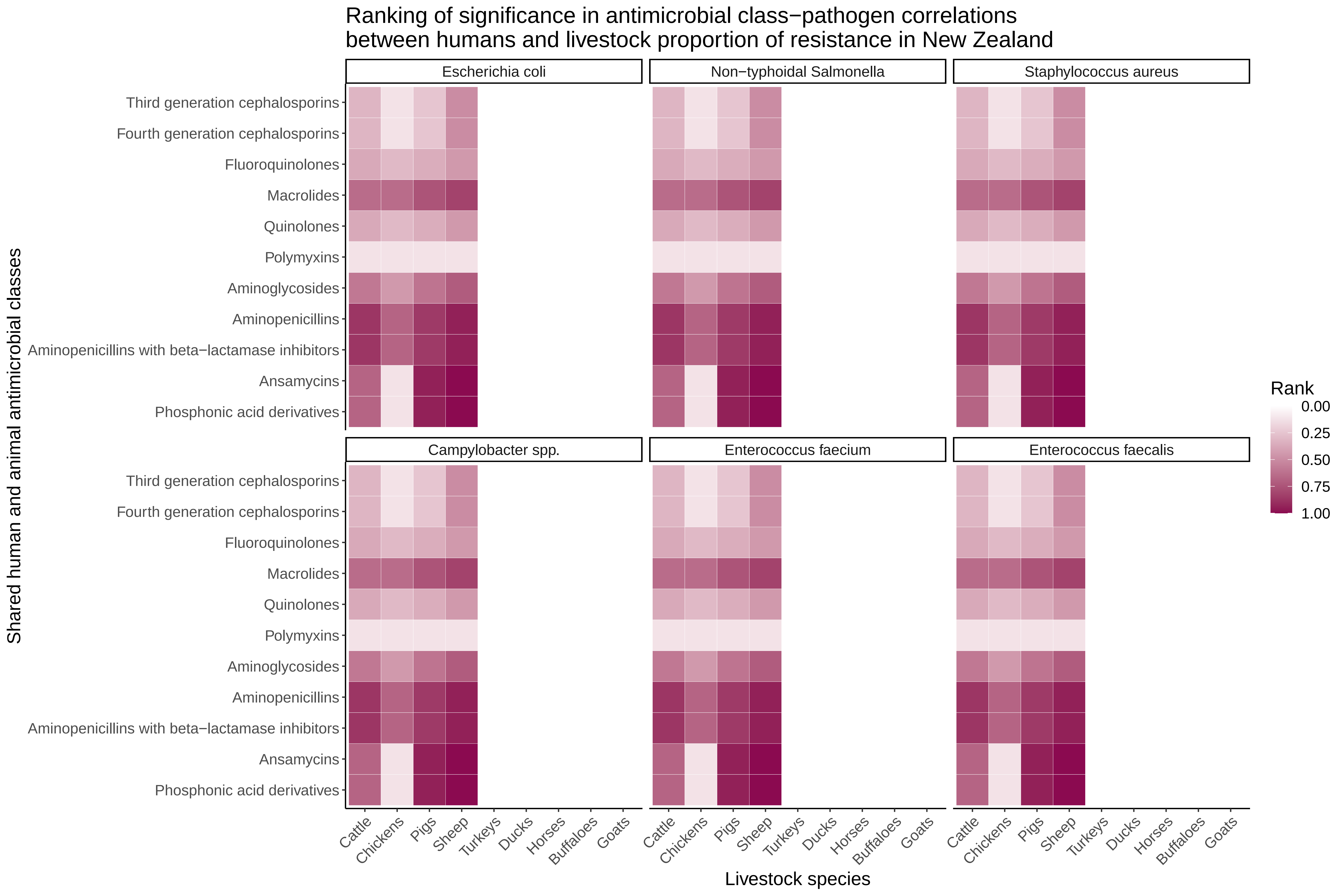

Ranking of significance in antimicrobial class−pathogen correlations
between humans and livestock proportion of resistance in New Zealand
Escherichia coli
Non−typhoidal Salmonella
Staphylococcus aureus
Campylobacter spp.
Enterococcus faecium
Enterococcus faecalis
Shared human and animal antimicrobial classes
Third generation cephalosporins
Fourth generation cephalosporins
Fluoroquinolones
Macrolides
Quinolones
Polymyxins
Aminoglycosides
Aminopenicillins
Aminopenicillins with beta−lactamase inhibitors
Ansamycins
Phosphonic acid derivatives
Livestock species
Cattle
Chickens
Pigs
Sheep
Turkeys
Ducks
Horses
Buffaloes
Goats
Rank
0.00
0.25
0.50
0.75
1.00

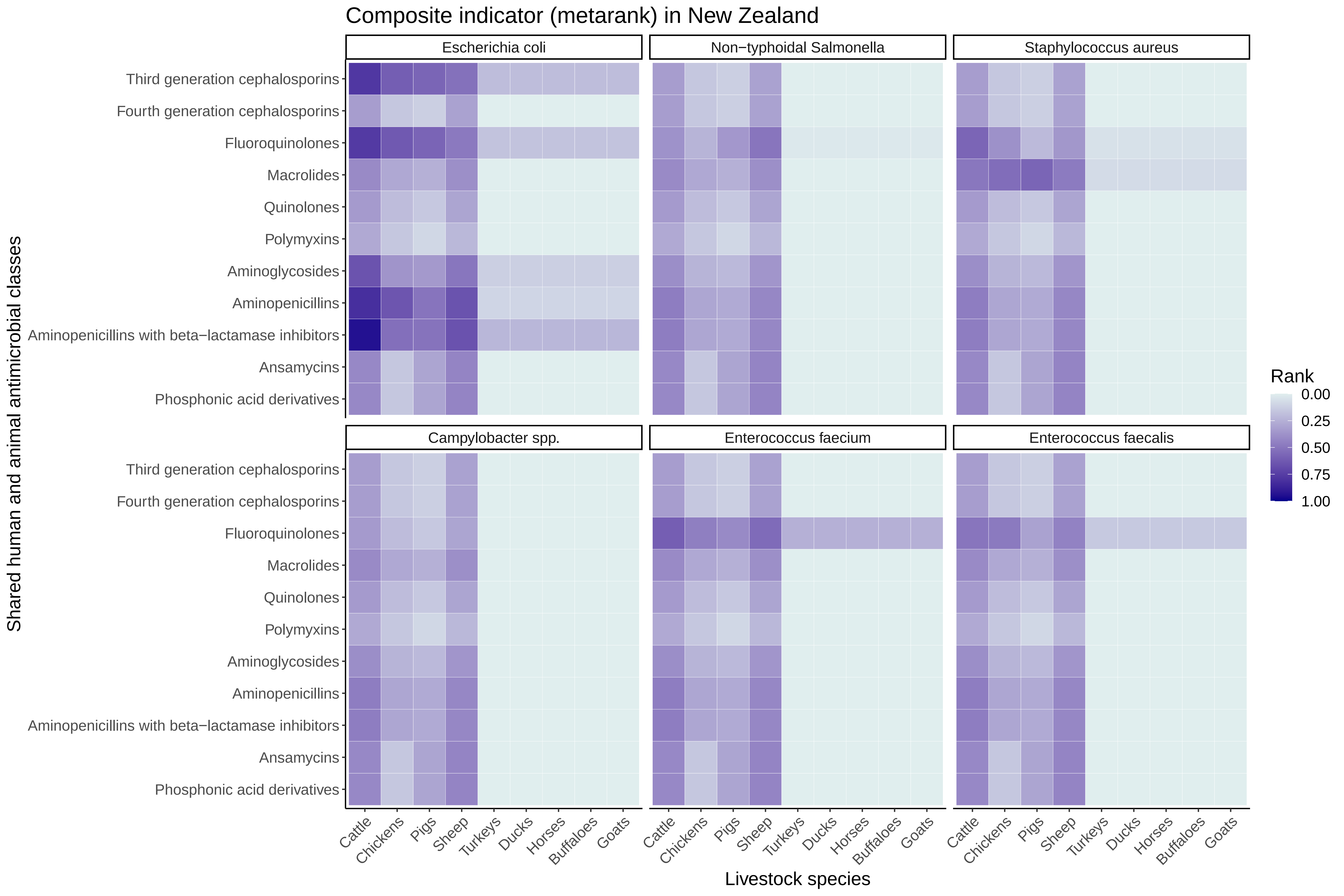

Composite indicator (metarank) in New Zealand
Escherichia coli
Non−typhoidal Salmonella
Staphylococcus aureus
Campylobacter spp.
Enterococcus faecium
Enterococcus faecalis
Shared human and animal antimicrobial classes
Third generation cephalosporins
Fourth generation cephalosporins
Fluoroquinolones
Macrolides
Quinolones
Polymyxins
Aminoglycosides
Aminopenicillins
Aminopenicillins with beta−lactamase inhibitors
Ansamycins
Phosphonic acid derivatives
Cattle
Chickens
Pigs
Sheep
Turkeys
Ducks
Horses
Buffaloes
Goats
Livestock species
Rank
0.00
0.25
0.50
0.75
1.00

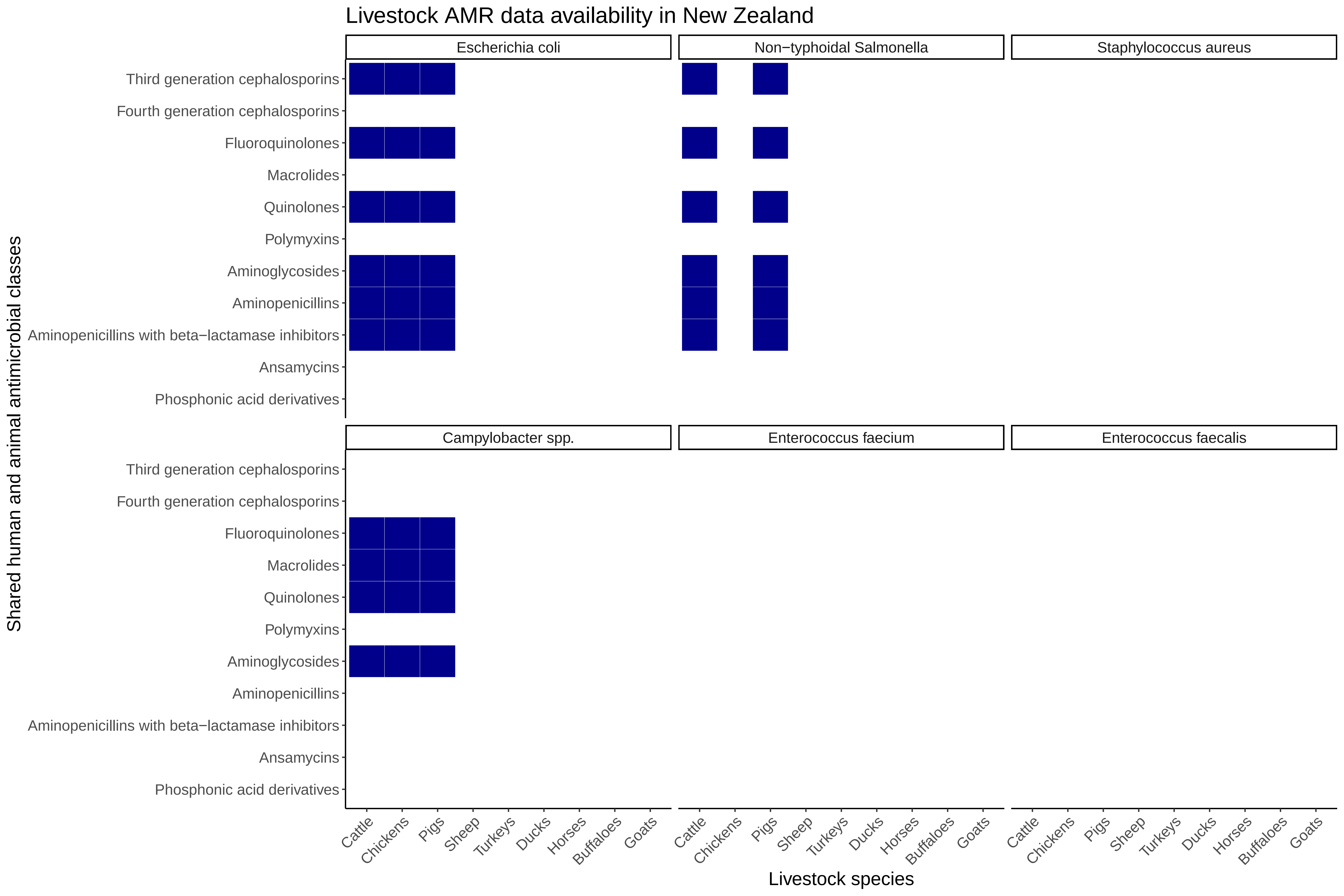
Livestock AMR data availability in New Zealand
Escherichia coli
Non−typhoidal Salmonella
Staphylococcus aureus
Campylobacter spp.
Enterococcus faecium
Enterococcus faecalis
Shared human and animal antimicrobial classes
Third generation cephalosporins
Fourth generation cephalosporins
Fluoroquinolones
Macrolides
Quinolones
Polymyxins
Aminoglycosides
Aminopenicillins
Aminopenicillins with beta−lactamase inhibitors
Ansamycins
Phosphonic acid derivatives
Cattle
Chickens
Pigs
Sheep
Turkeys
Ducks
Horses
Buffaloes
Goats
Livestock species

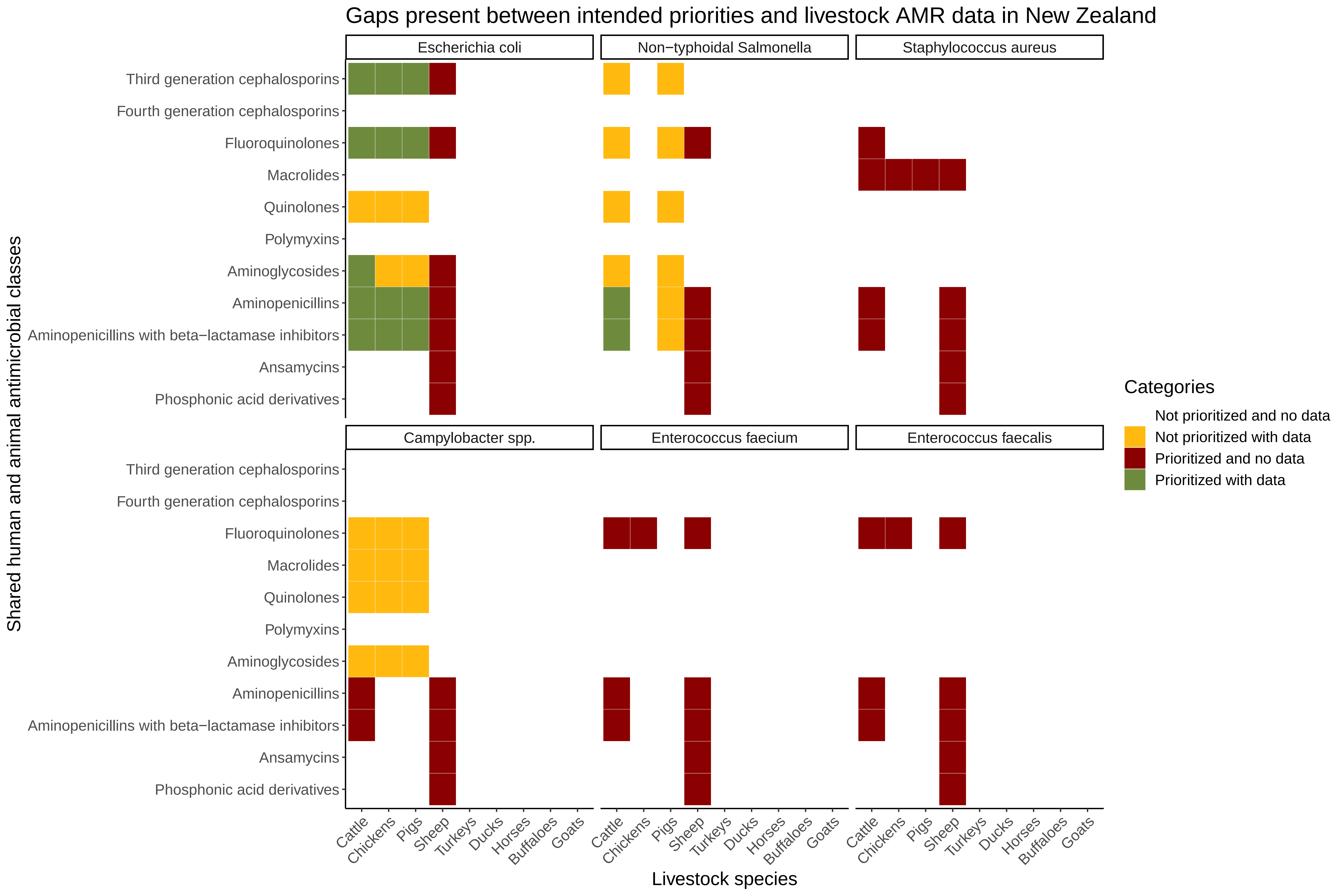

Gaps present between intended priorities and livestock AMR data in New Zealand
Escherichia coli
Non−typhoidal Salmonella
Staphylococcus aureus
Campylobacter spp.
Enterococcus faecium
Enterococcus faecalis
Third generation cephalosporins
Fourth generation cephalosporins
Fluoroquinolones
Macrolides
Quinolones
Polymyxins
Aminoglycosides
Aminopenicillins
Aminopenicillins with beta−lactamase inhibitors
Ansamycins
Phosphonic acid derivatives
Shared human and animal antimicrobial classes
Cattle
Chickens
Pigs
Sheep
Turkeys
Ducks
Horses
Buffaloes
Goats
Livestock species
Categories
Not prioritized and no data
Not prioritized with data
Prioritized and no data
Prioritized with data

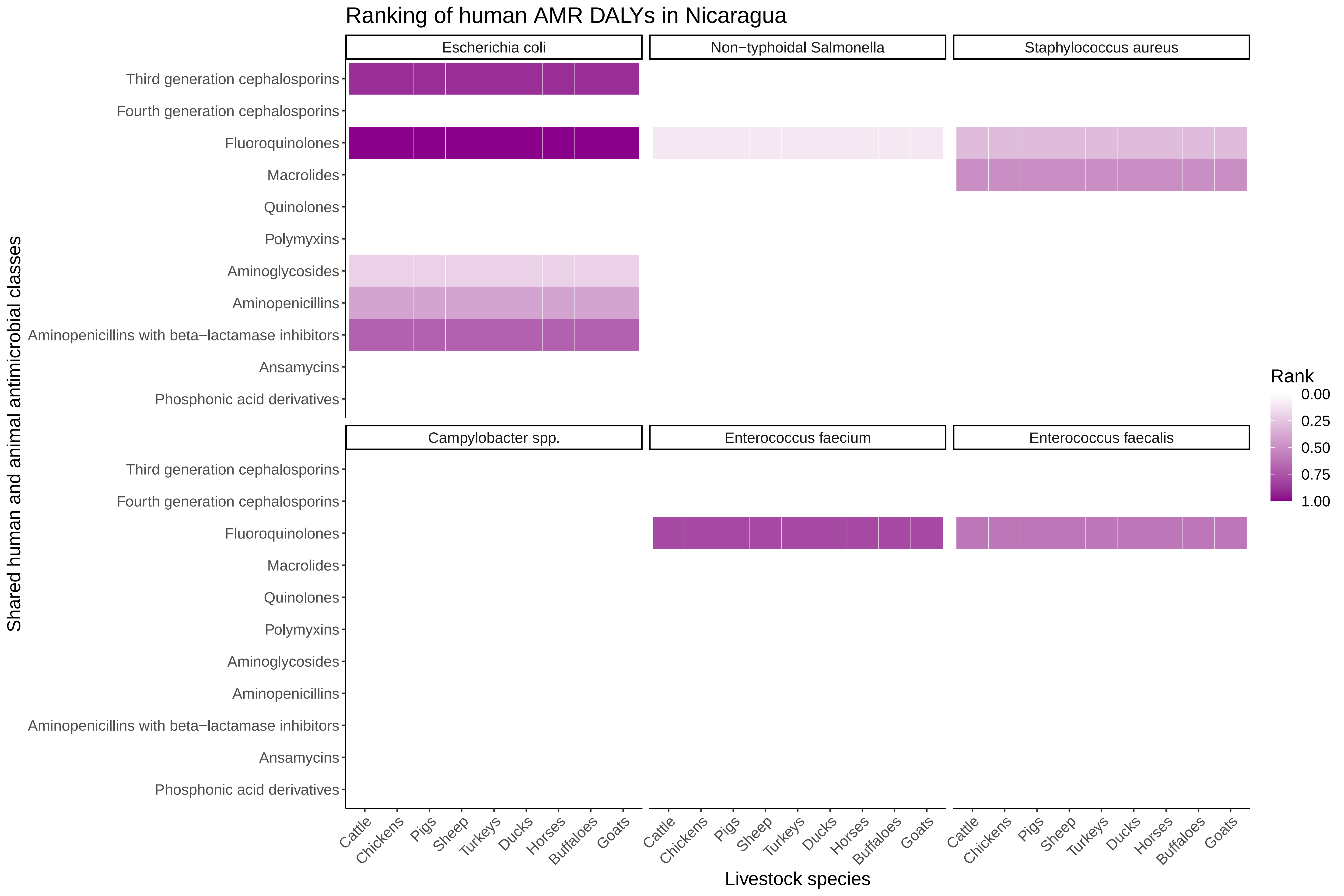

Ranking of human AMR DALYs in Nicaragua
Escherichia coli
Non−typhoidal Salmonella
Staphylococcus aureus
Campylobacter spp.
Enterococcus faecium
Enterococcus faecalis
Third generation cephalosporins
Fourth generation cephalosporins
Fluoroquinolones
Macrolides
Quinolones
Polymyxins
Aminoglycosides
Aminopenicillins
Aminopenicillins with beta−lactamase inhibitors
Ansamycins
Phosphonic acid derivatives
Shared human and animal antimicrobial classes
Cattle
Chickens
Pigs
Sheep
Turkeys
Ducks
Horses
Buffaloes
Goats
Livestock species
Rank
0.00
0.25
0.50
0.75
1.00

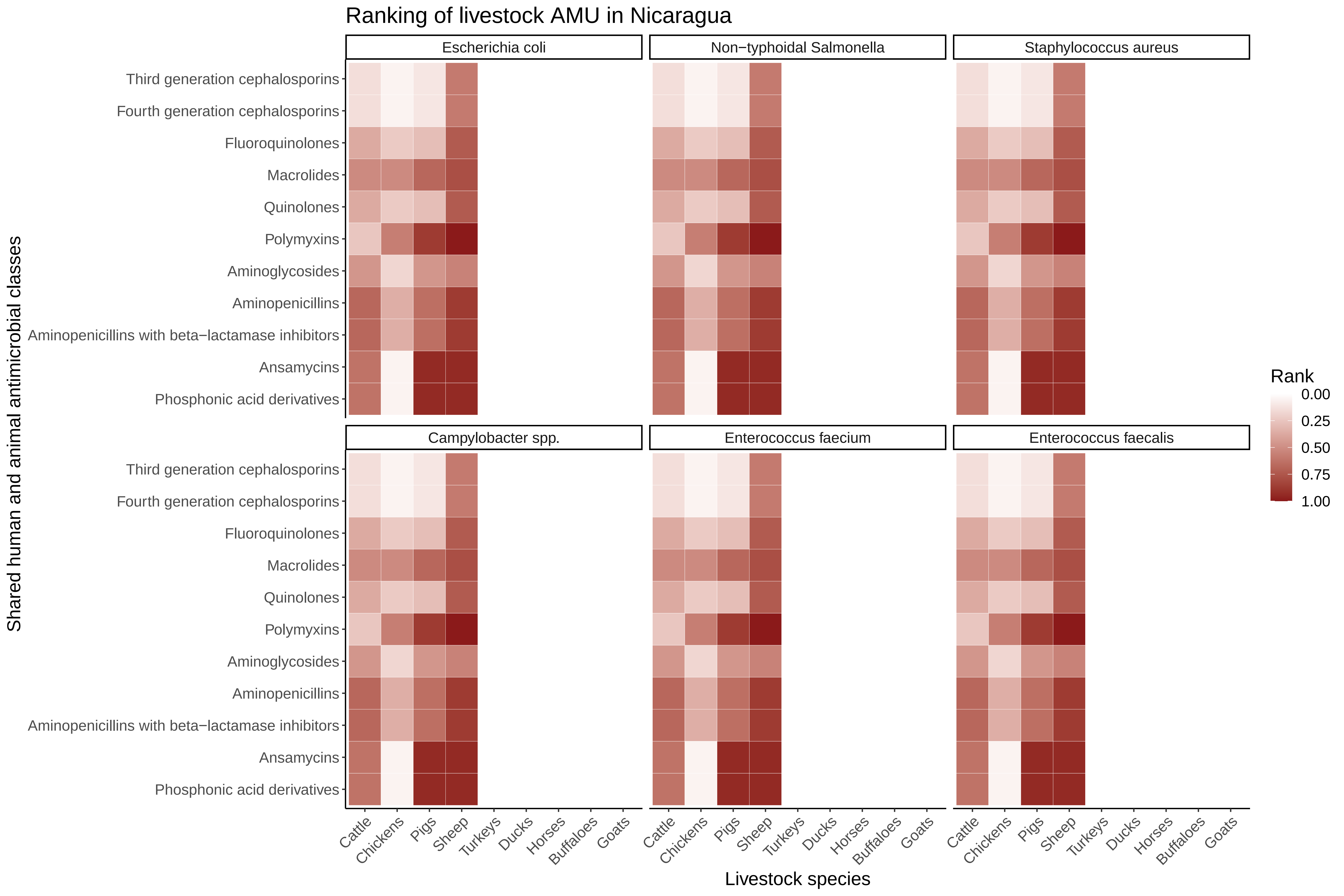

Ranking of livestock AMU in Nicaragua
Escherichia coli
Non−typhoidal Salmonella
Staphylococcus aureus
Campylobacter spp.
Enterococcus faecium
Enterococcus faecalis
Shared human and animal antimicrobial classes
Third generation cephalosporins
Fourth generation cephalosporins
Fluoroquinolones
Macrolides
Quinolones
Polymyxins
Aminoglycosides
Aminopenicillins
Aminopenicillins with beta−lactamase inhibitors
Ansamycins
Phosphonic acid derivatives
Rank
0.00
0.25
0.50
0.75
1.00
Cattle
Chickens
Pigs
Sheep
Turkeys
Ducks
Horses
Buffaloes
Goats
Livestock species

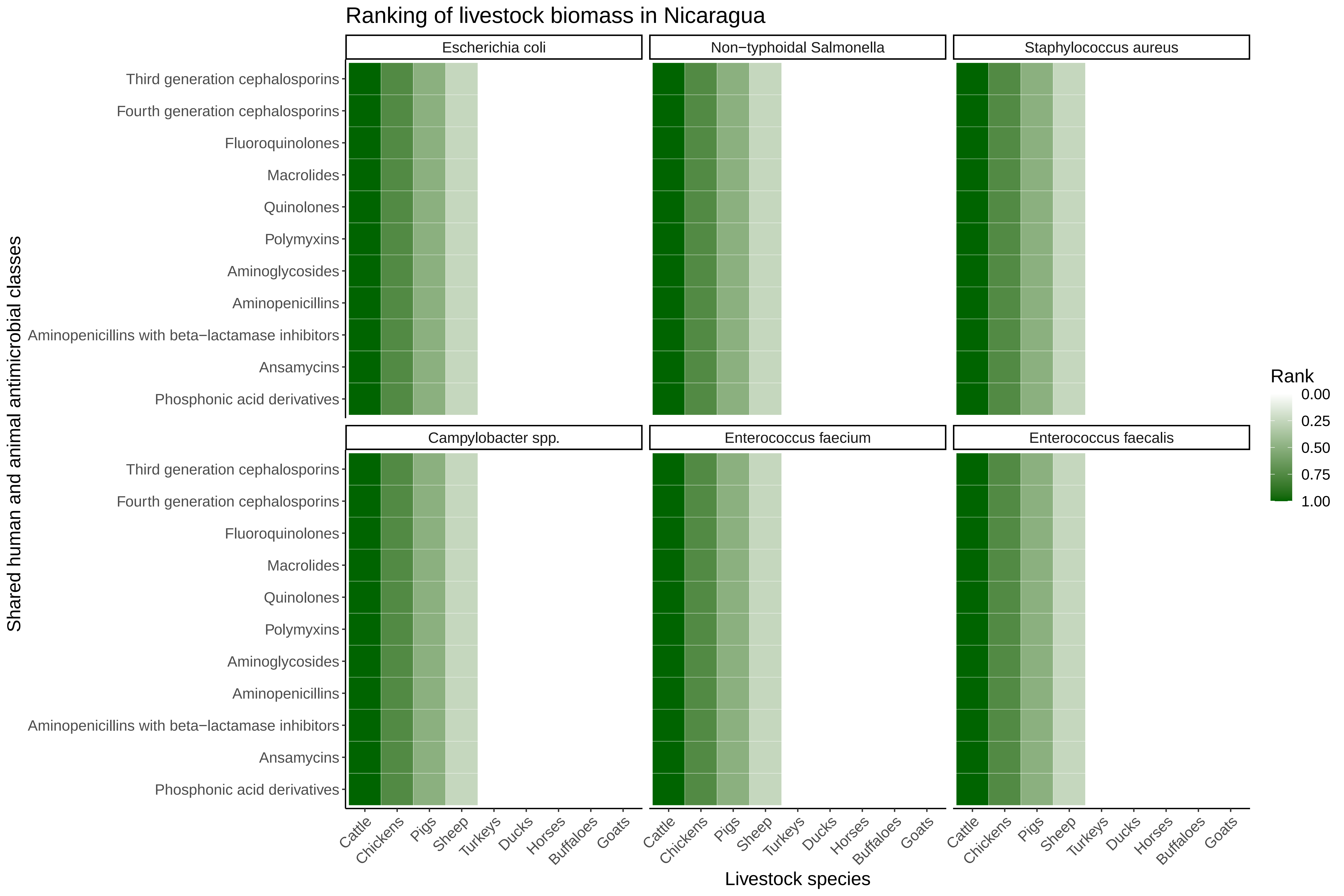

Ranking of livestock biomass in Nicaragua
Escherichia coli
Non−typhoidal Salmonella
Staphylococcus aureus
Campylobacter spp.
Enterococcus faecium
Enterococcus faecalis
Third generation cephalosporins
Fourth generation cephalosporins
Fluoroquinolones
Macrolides
Quinolones
Polymyxins
Aminoglycosides
Aminopenicillins
Aminopenicillins with beta−lactamase inhibitors
Ansamycins
Phosphonic acid derivatives
Shared human and animal antimicrobial classes
Cattle
Chickens
Pigs
Sheep
Turkeys
Ducks
Horses
Buffaloes
Goats
Livestock species
Rank
0.00
0.25
0.50
0.75
1.00

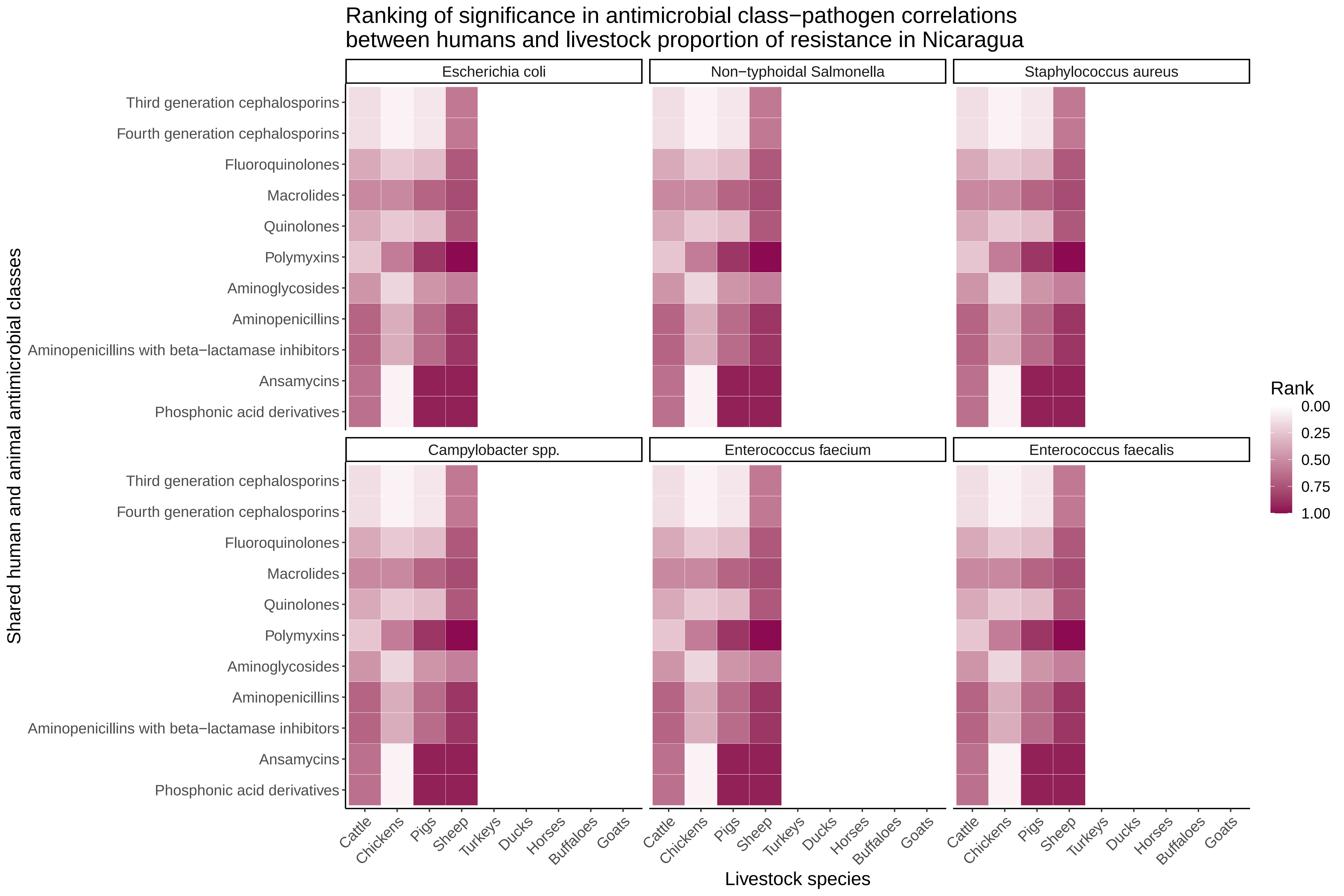

Ranking of significance in antimicrobial class−pathogen correlations
between humans and livestock proportion of resistance in Nicaragua
Escherichia coli
Non−typhoidal Salmonella
Staphylococcus aureus
Campylobacter spp.
Enterococcus faecium
Enterococcus faecalis
Third generation cephalosporins
Fourth generation cephalosporins
Fluoroquinolones
Macrolides
Quinolones
Polymyxins
Aminoglycosides
Aminopenicillins
Aminopenicillins with beta−lactamase inhibitors
Ansamycins
Phosphonic acid derivatives
Shared human and animal antimicrobial classes
Cattle
Chickens
Pigs
Sheep
Turkeys
Ducks
Horses
Buffaloes
Goats
Livestock species
Rank
0.00
0.25
0.50
0.75
1.00

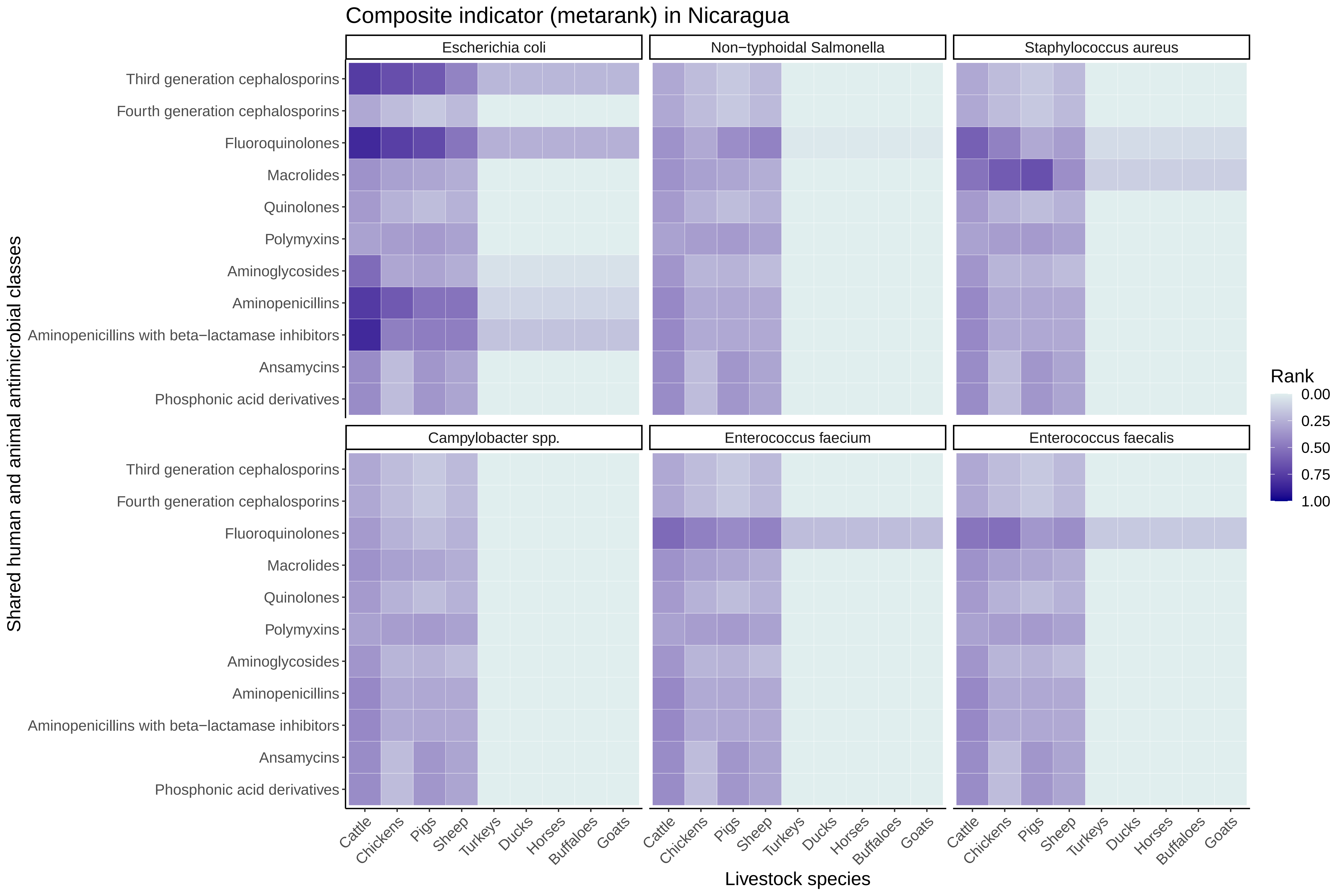

Composite indicator (metarank) in Nicaragua
Escherichia coli
Non−typhoidal Salmonella
Staphylococcus aureus
Campylobacter spp.
Enterococcus faecium
Enterococcus faecalis
Shared human and animal antimicrobial classes
Third generation cephalosporins
Fourth generation cephalosporins
Fluoroquinolones
Macrolides
Quinolones
Polymyxins
Aminoglycosides
Aminopenicillins
Aminopenicillins with beta−lactamase inhibitors
Ansamycins
Phosphonic acid derivatives
Livestock species
Cattle
Chickens
Pigs
Sheep
Turkeys
Ducks
Horses
Buffaloes
Goats
Rank
0.00
0.25
0.50
0.75
1.00

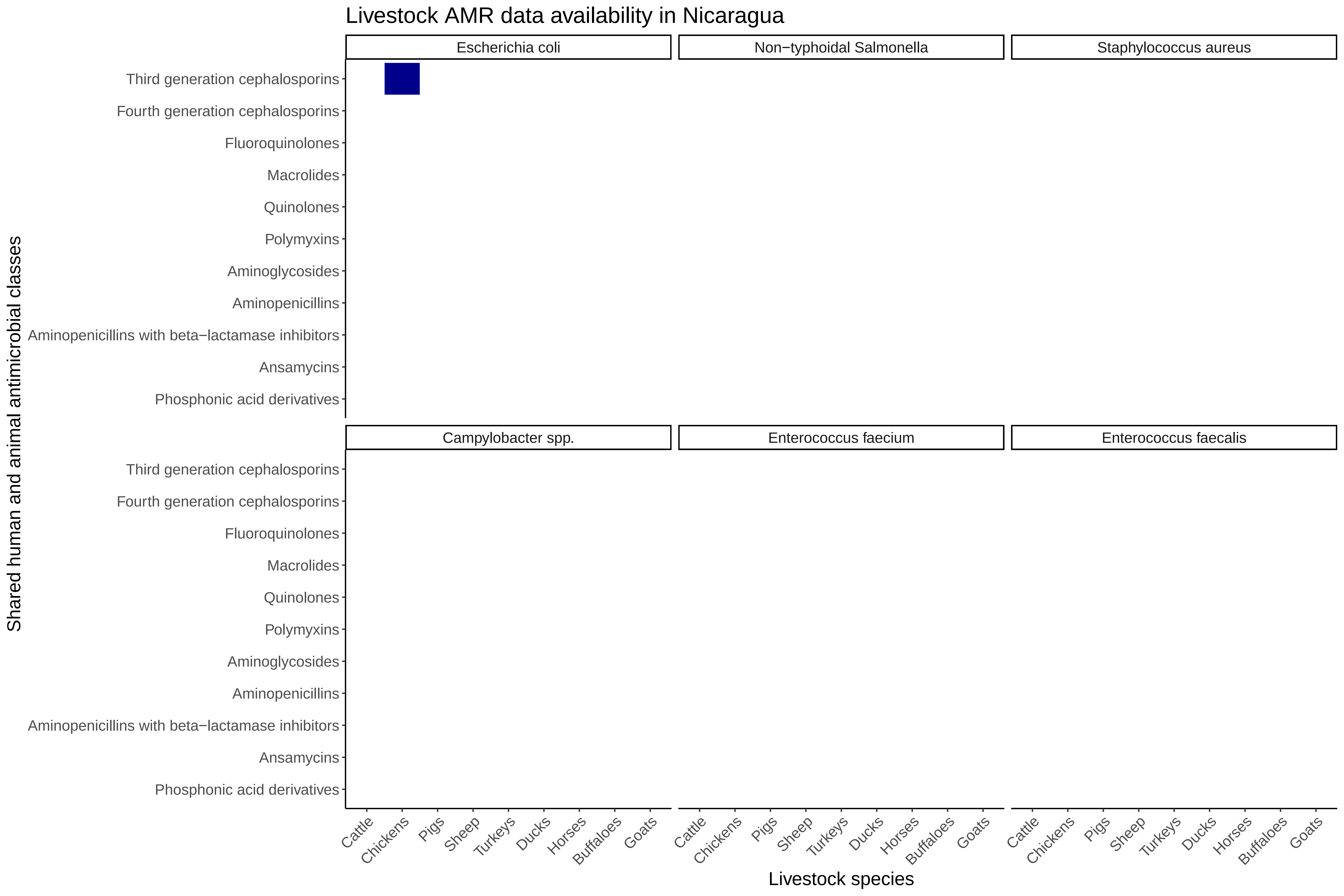

Livestock AMR data availability in Nicaragua
Escherichia coli
Non−typhoidal Salmonella
Staphylococcus aureus
Campylobacter spp.
Enterococcus faecium
Enterococcus faecalis
Shared human and animal antimicrobial classes
Third generation cephalosporins
Fourth generation cephalosporins
Fluoroquinolones
Macrolides
Quinolones
Polymyxins
Aminoglycosides
Aminopenicillins
Aminopenicillins with beta−lactamase inhibitors
Ansamycins
Phosphonic acid derivatives
Cattle
Chickens
Pigs
Sheep
Turkeys
Ducks
Horses
Buffaloes
Goats
Livestock species

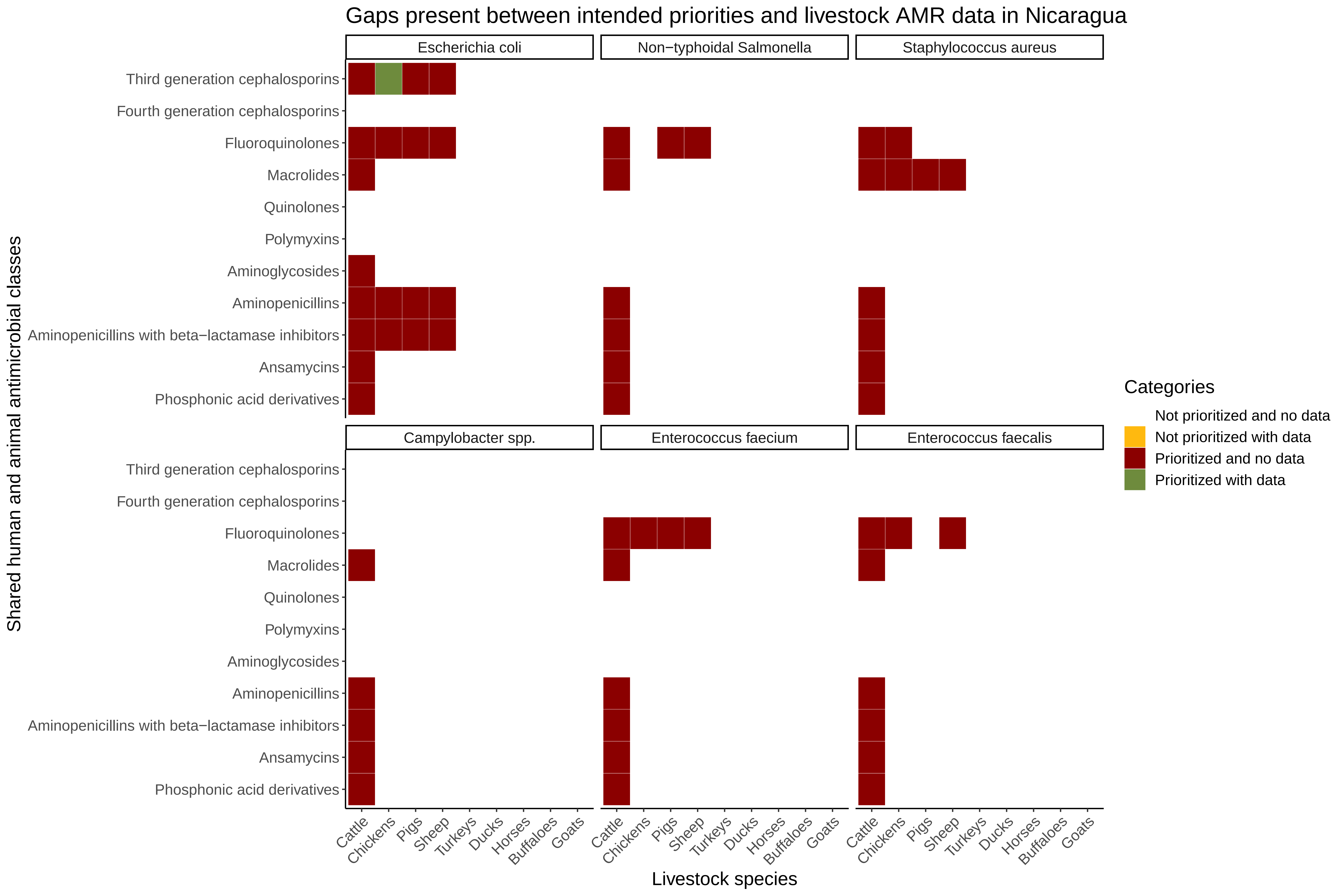
Gaps present between intended priorities and livestock AMR data in Nicaragua
Escherichia coli
Non−typhoidal Salmonella
Staphylococcus aureus
Campylobacter spp.
Enterococcus faecium
Enterococcus faecalis
Third generation cephalosporins
Fourth generation cephalosporins
Fluoroquinolones
Macrolides
Quinolones
Polymyxins
Aminoglycosides
Aminopenicillins
Aminopenicillins with beta−lactamase inhibitors
Ansamycins
Phosphonic acid derivatives
Shared human and animal antimicrobial classes
Cattle
Chickens
Pigs
Sheep
Turkeys
Ducks
Horses
Buffaloes
Goats
Livestock species
Categories
Not prioritized and no data
Not prioritized with data
Prioritized and no data
Prioritized with data

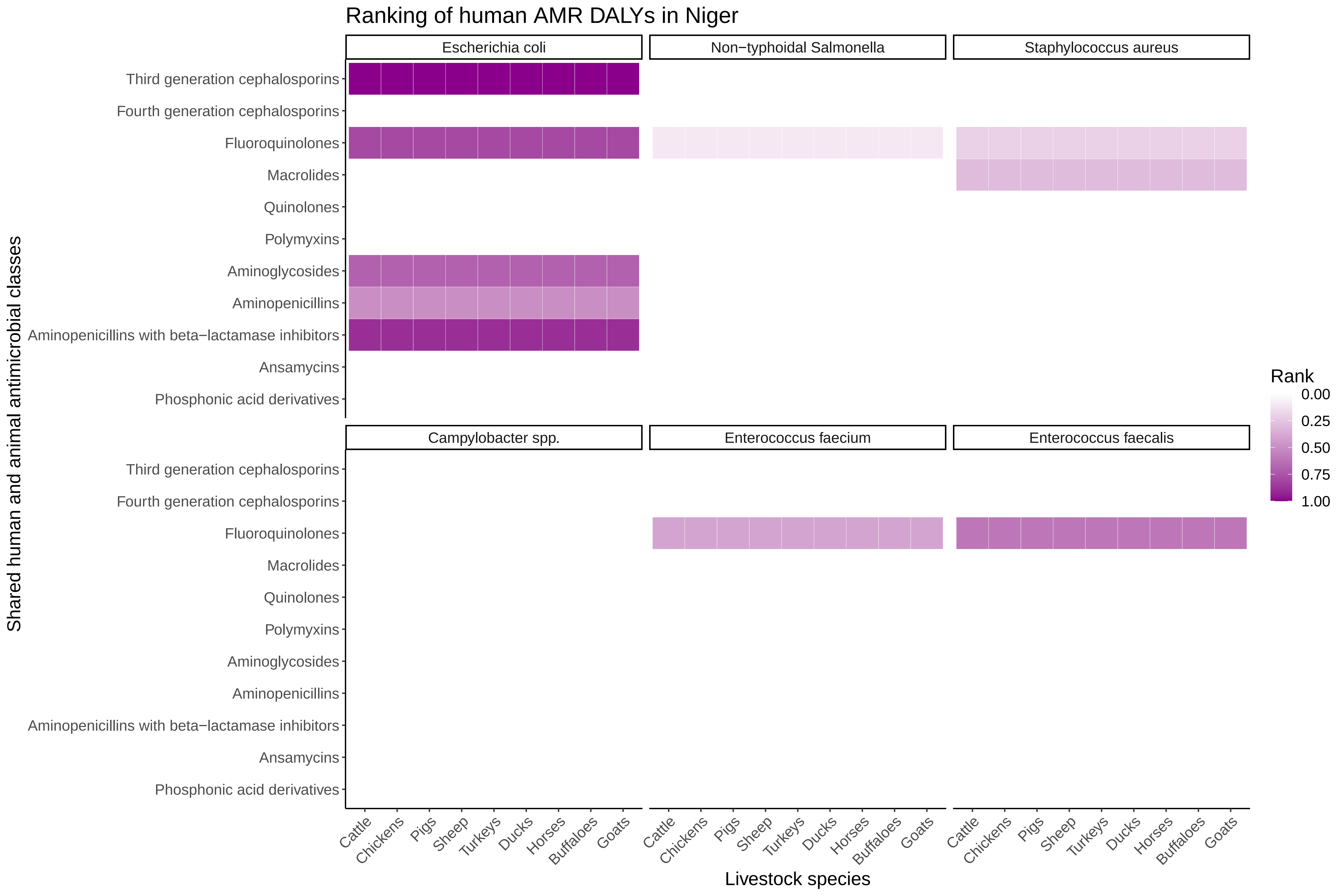

Ranking of human AMR DALYs in Niger
Escherichia coli
Non−typhoidal Salmonella
Staphylococcus aureus
Campylobacter spp.
Enterococcus faecium
Enterococcus faecalis
Third generation cephalosporins
Fourth generation cephalosporins
Fluoroquinolones
Macrolides
Quinolones
Polymyxins
Aminoglycosides
Aminopenicillins
Aminopenicillins with beta−lactamase inhibitors
Ansamycins
Phosphonic acid derivatives
Shared human and animal antimicrobial classes
Cattle
Chickens
Pigs
Sheep
Turkeys
Ducks
Horses
Buffaloes
Goats
Livestock species
Rank
0.00
0.25
0.50
0.75
1.00

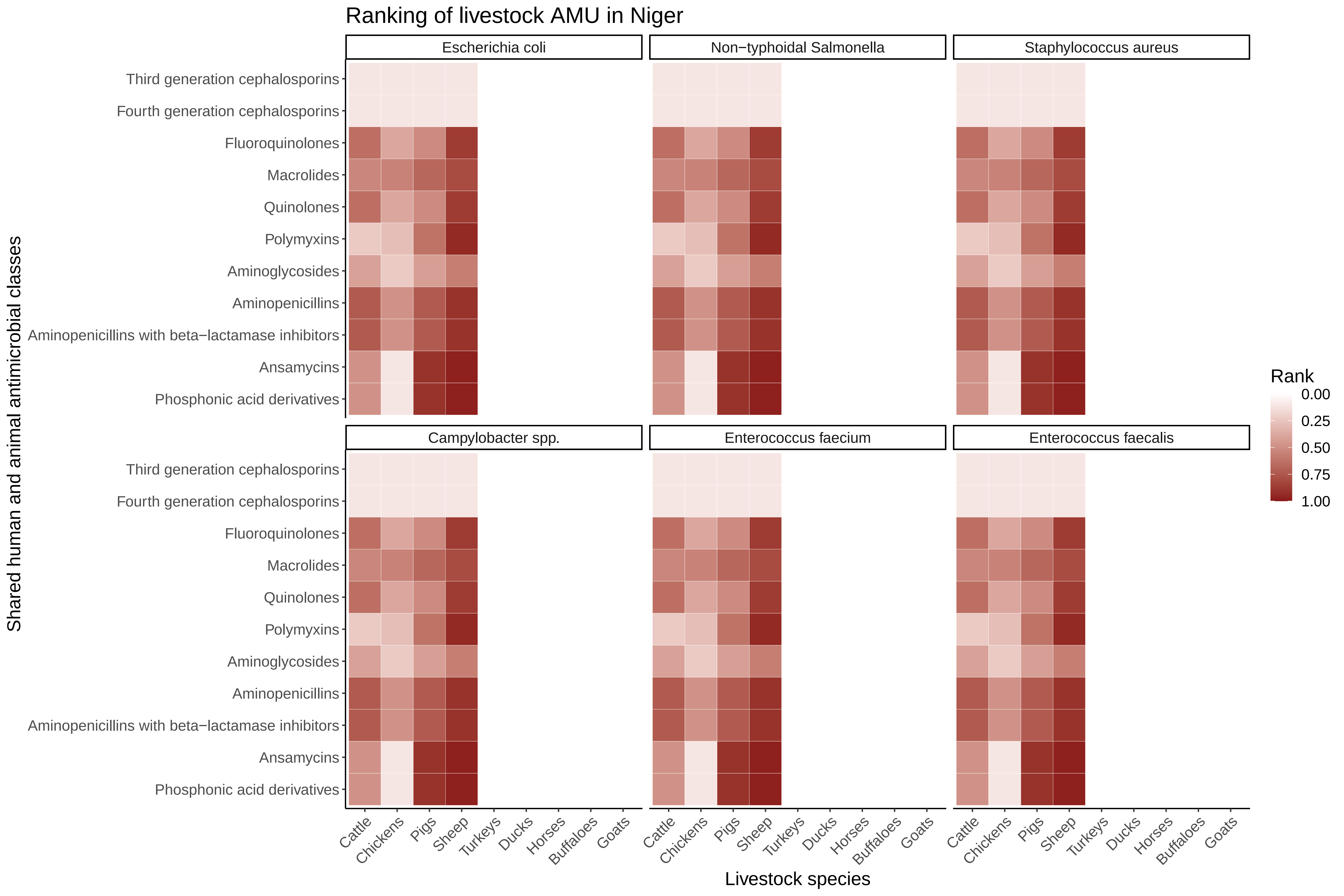

Ranking of livestock AMU in Niger
Escherichia coli
Non−typhoidal Salmonella
Staphylococcus aureus
Campylobacter spp.
Enterococcus faecium
Enterococcus faecalis
Shared human and animal antimicrobial classes
Third generation cephalosporins
Fourth generation cephalosporins
Fluoroquinolones
Macrolides
Quinolones
Polymyxins
Aminoglycosides
Aminopenicillins
Aminopenicillins with beta−lactamase inhibitors
Ansamycins
Phosphonic acid derivatives
Livestock species
Cattle
Chickens
Pigs
Sheep
Turkeys
Ducks
Horses
Buffaloes
Goats
Rank
0.00
0.25
0.50
0.75
1.00

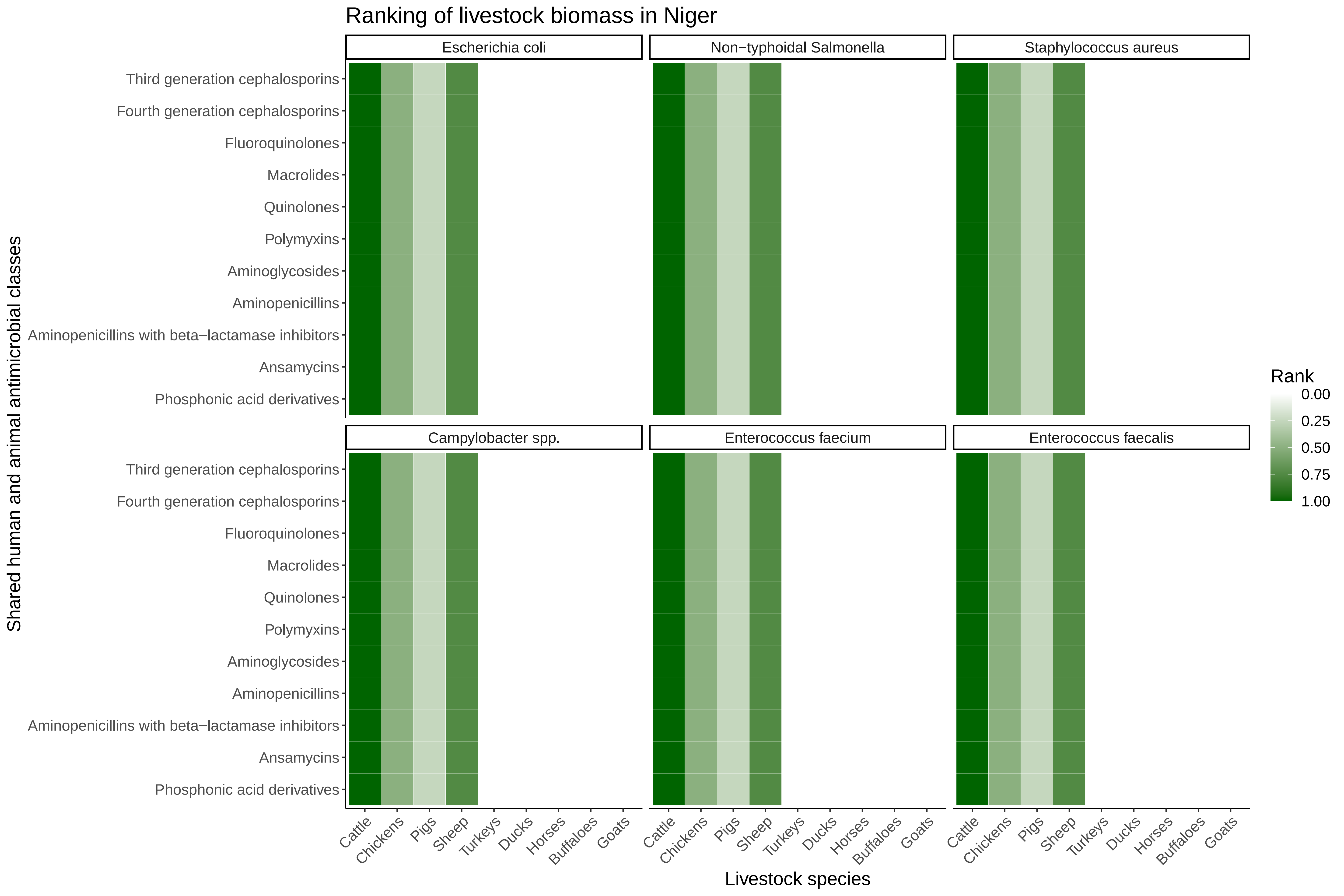
Ranking of livestock biomass in Niger
Escherichia coli
Non−typhoidal Salmonella
Staphylococcus aureus
Campylobacter spp.
Enterococcus faecium
Enterococcus faecalis
Third generation cephalosporins
Fourth generation cephalosporins
Fluoroquinolones
Macrolides
Quinolones
Polymyxins
Aminoglycosides
Aminopenicillins
Aminopenicillins with beta−lactamase inhibitors
Ansamycins
Phosphonic acid derivatives
Shared human and animal antimicrobial classes
Cattle
Chickens
Pigs
Sheep
Turkeys
Ducks
Horses
Buffaloes
Goats
Livestock species
Rank
0.00
0.25
0.50
0.75
1.00

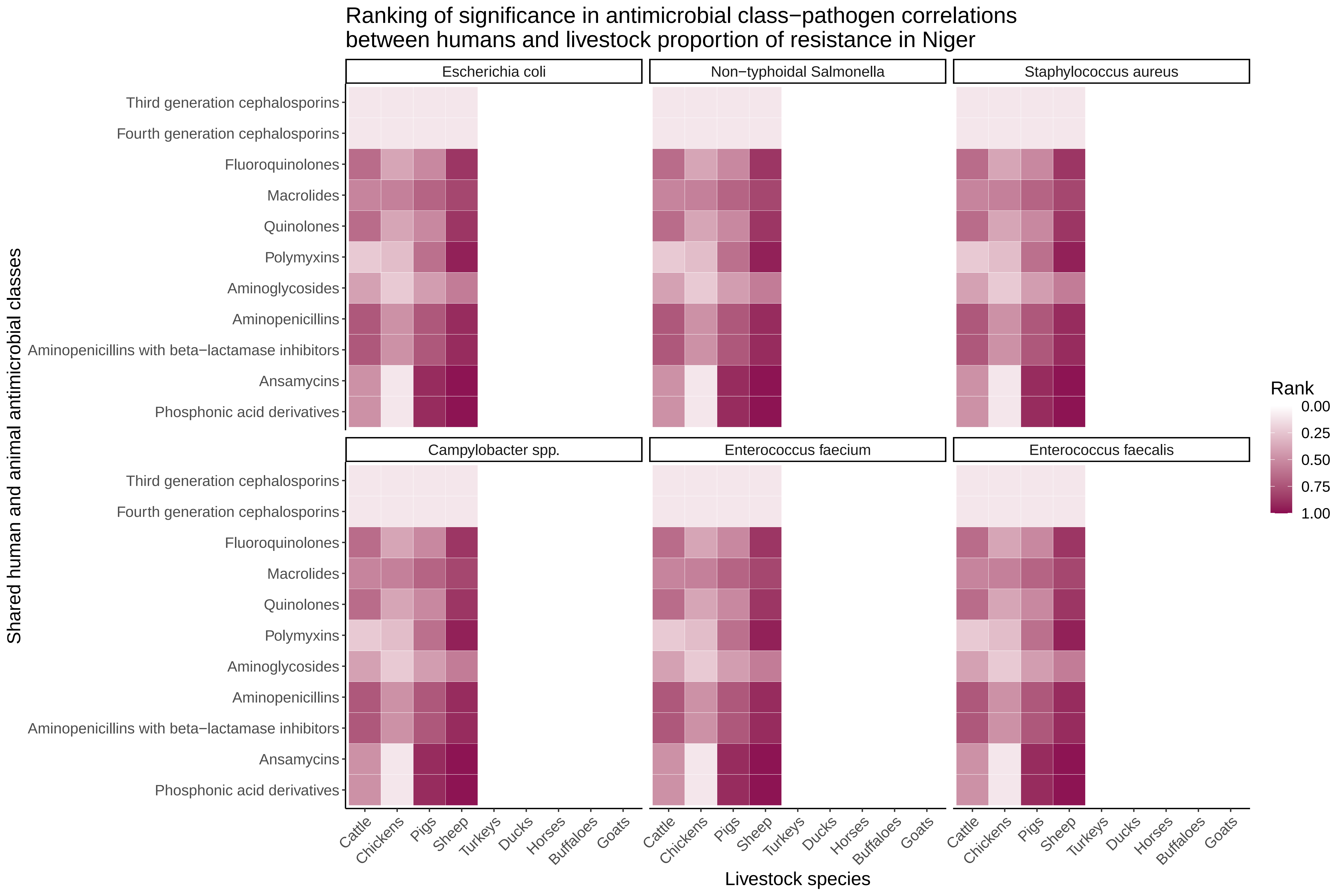
Ranking of significance in antimicrobial class−pathogen correlations
between humans and livestock proportion of resistance in Niger
Escherichia coli
Non−typhoidal Salmonella
Staphylococcus aureus
Campylobacter spp.
Enterococcus faecium
Enterococcus faecalis
Third generation cephalosporins
Fourth generation cephalosporins
Fluoroquinolones
Macrolides
Quinolones
Polymyxins
Aminoglycosides
Aminopenicillins
Aminopenicillins with beta−lactamase inhibitors
Ansamycins
Phosphonic acid derivatives
Shared human and animal antimicrobial classes
Cattle
Chickens
Pigs
Sheep
Turkeys
Ducks
Horses
Buffaloes
Goats
Livestock species
Rank
0.00
0.25
0.50
0.75
1.00

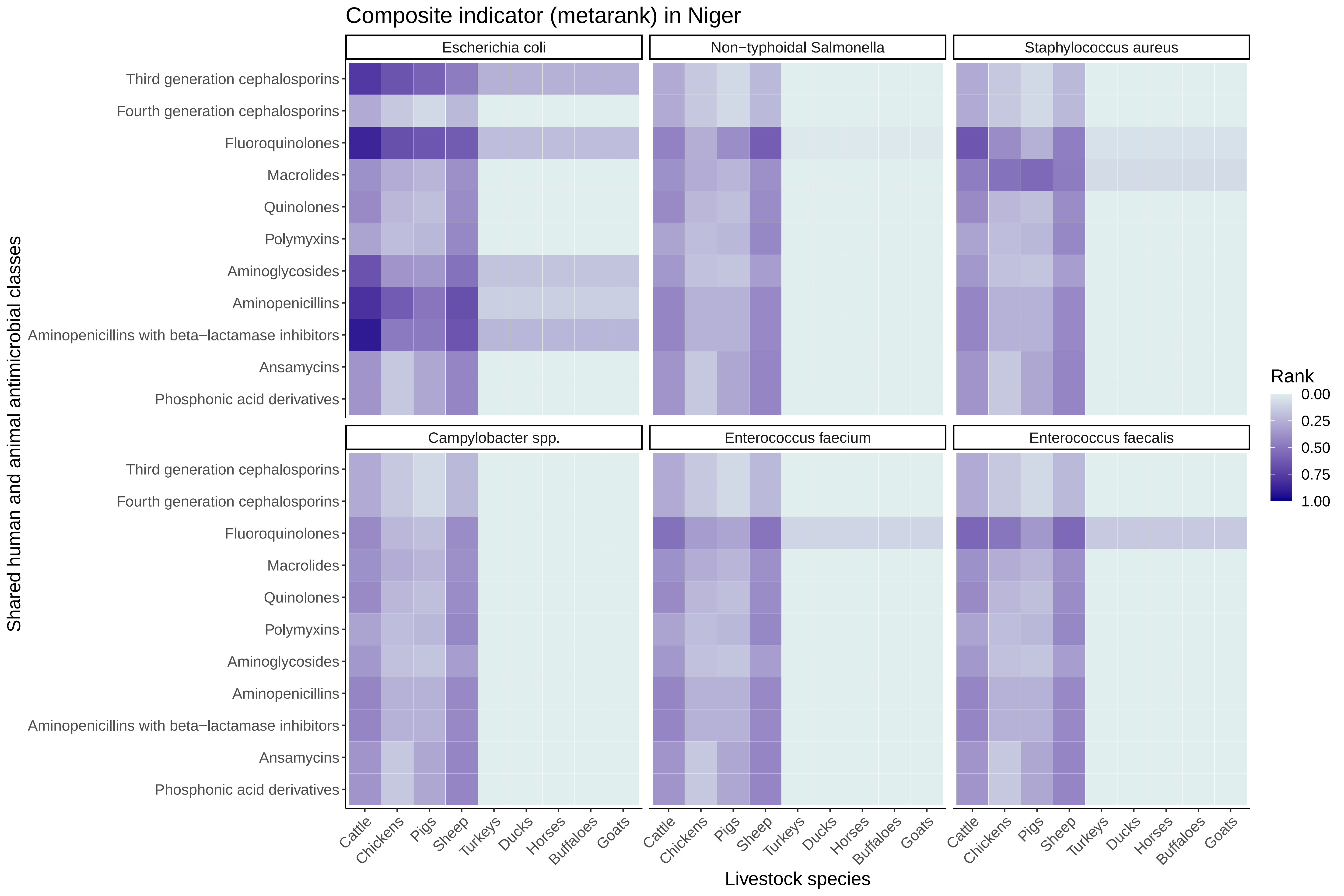

Composite indicator (metarank) in Niger
Escherichia coli
Non−typhoidal Salmonella
Staphylococcus aureus
Campylobacter spp.
Enterococcus faecium
Enterococcus faecalis
Shared human and animal antimicrobial classes
Third generation cephalosporins
Fourth generation cephalosporins
Fluoroquinolones
Macrolides
Quinolones
Polymyxins
Aminoglycosides
Aminopenicillins
Aminopenicillins with beta−lactamase inhibitors
Ansamycins
Phosphonic acid derivatives
Livestock species
Cattle
Chickens
Pigs
Sheep
Turkeys
Ducks
Horses
Buffaloes
Goats
Rank
0.00
0.25
0.50
0.75
1.00

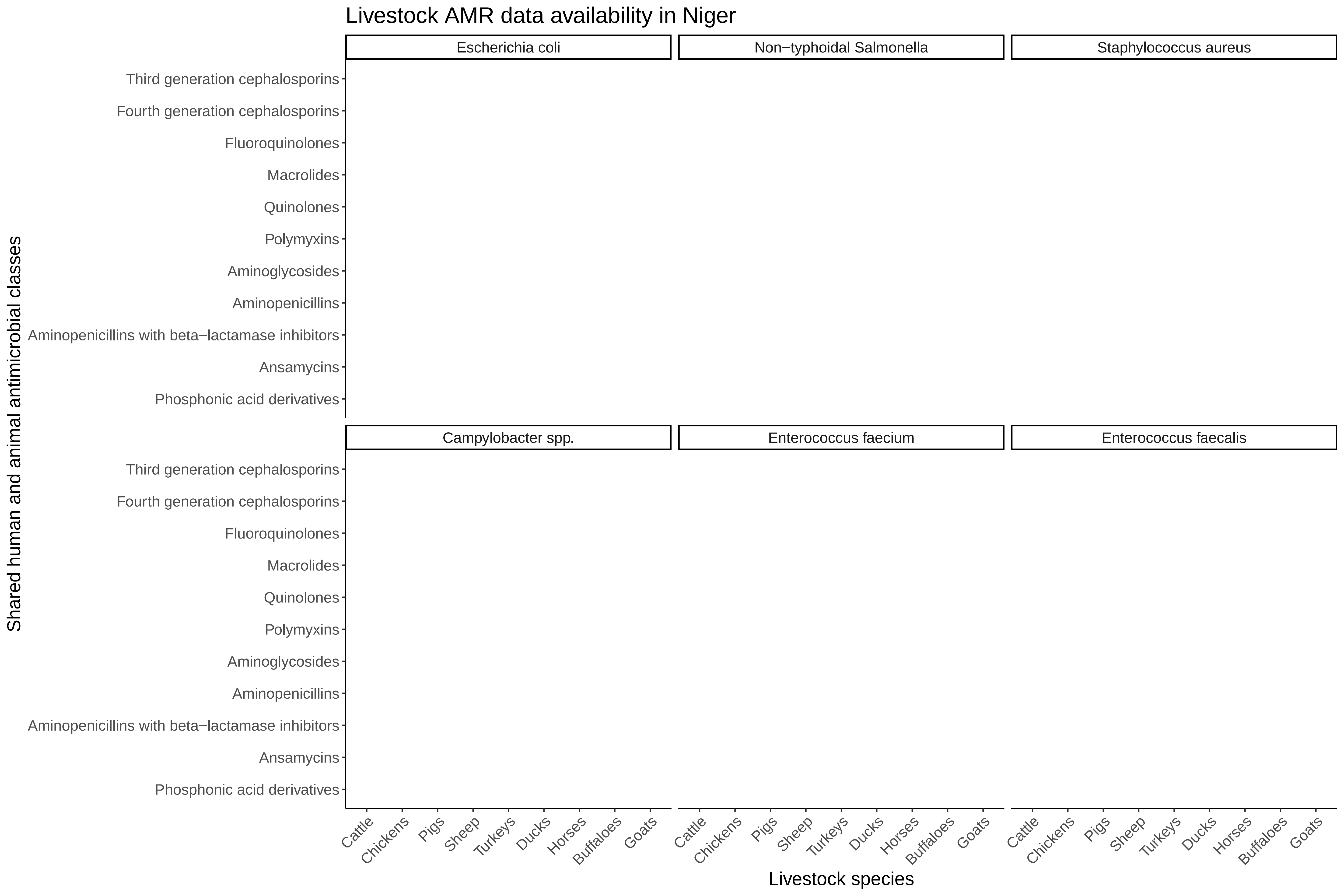
Livestock AMR data availability in Niger
Escherichia coli
Non−typhoidal Salmonella
Staphylococcus aureus
Campylobacter spp.
Enterococcus faecium
Enterococcus faecalis
Shared human and animal antimicrobial classes
Third generation cephalosporins
Fourth generation cephalosporins
Fluoroquinolones
Macrolides
Quinolones
Polymyxins
Aminoglycosides
Aminopenicillins
Aminopenicillins with beta−lactamase inhibitors
Ansamycins
Phosphonic acid derivatives
Cattle
Chickens
Pigs
Sheep
Turkeys
Ducks
Horses
Buffaloes
Goats
Livestock species

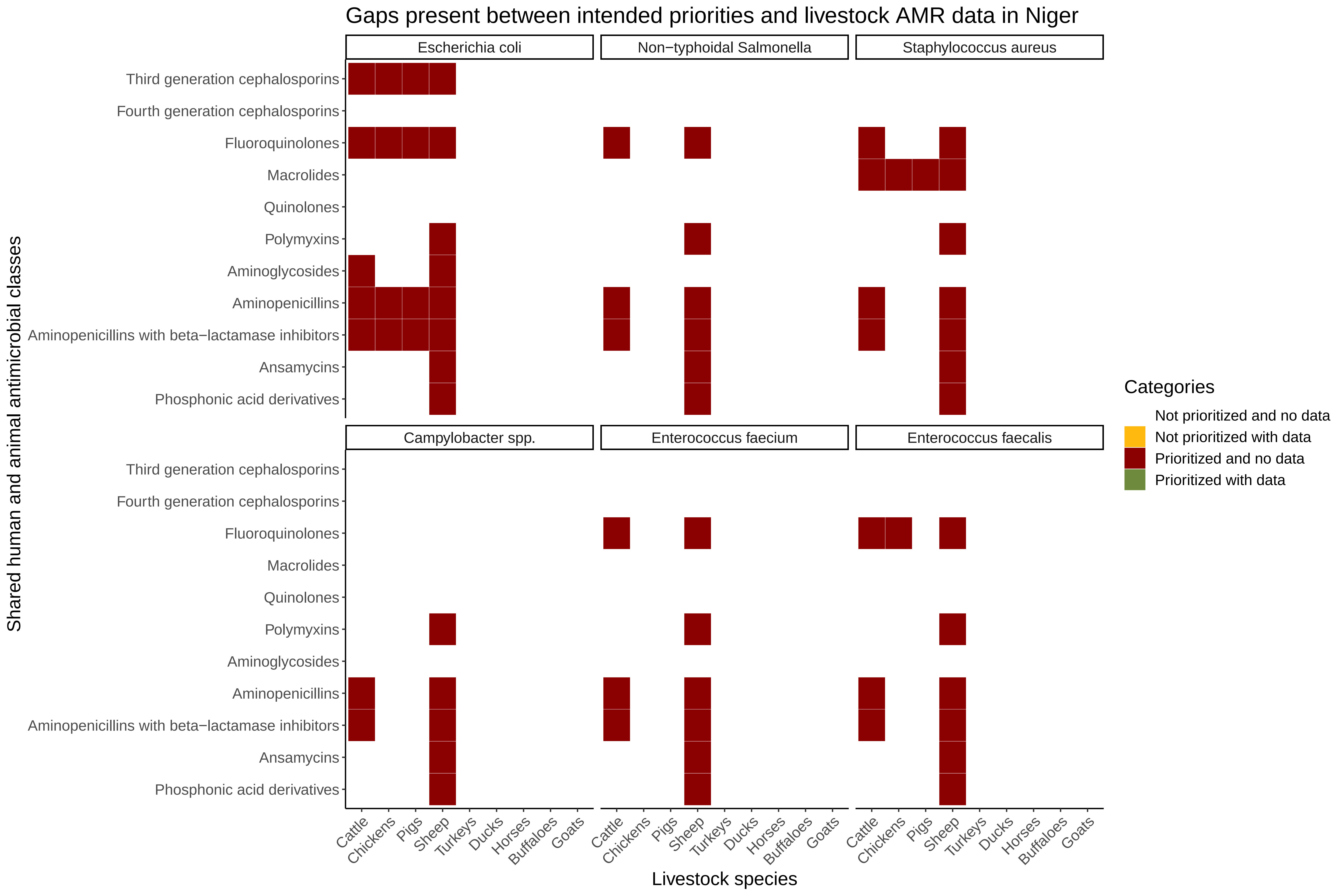

Gaps present between intended priorities and livestock AMR data in Niger
Escherichia coli
Non−typhoidal Salmonella
Staphylococcus aureus
Campylobacter spp.
Enterococcus faecium
Enterococcus faecalis
Third generation cephalosporins
Fourth generation cephalosporins
Fluoroquinolones
Macrolides
Quinolones
Polymyxins
Aminoglycosides
Aminopenicillins
Aminopenicillins with beta−lactamase inhibitors
Ansamycins
Phosphonic acid derivatives
Cattle
Chickens
Pigs
Sheep
Turkeys
Ducks
Horses
Buffaloes
Goats
Livestock species
Shared human and animal antimicrobial classes
Categories
Not prioritized and no data
Not prioritized with data
Prioritized and no data
Prioritized with data

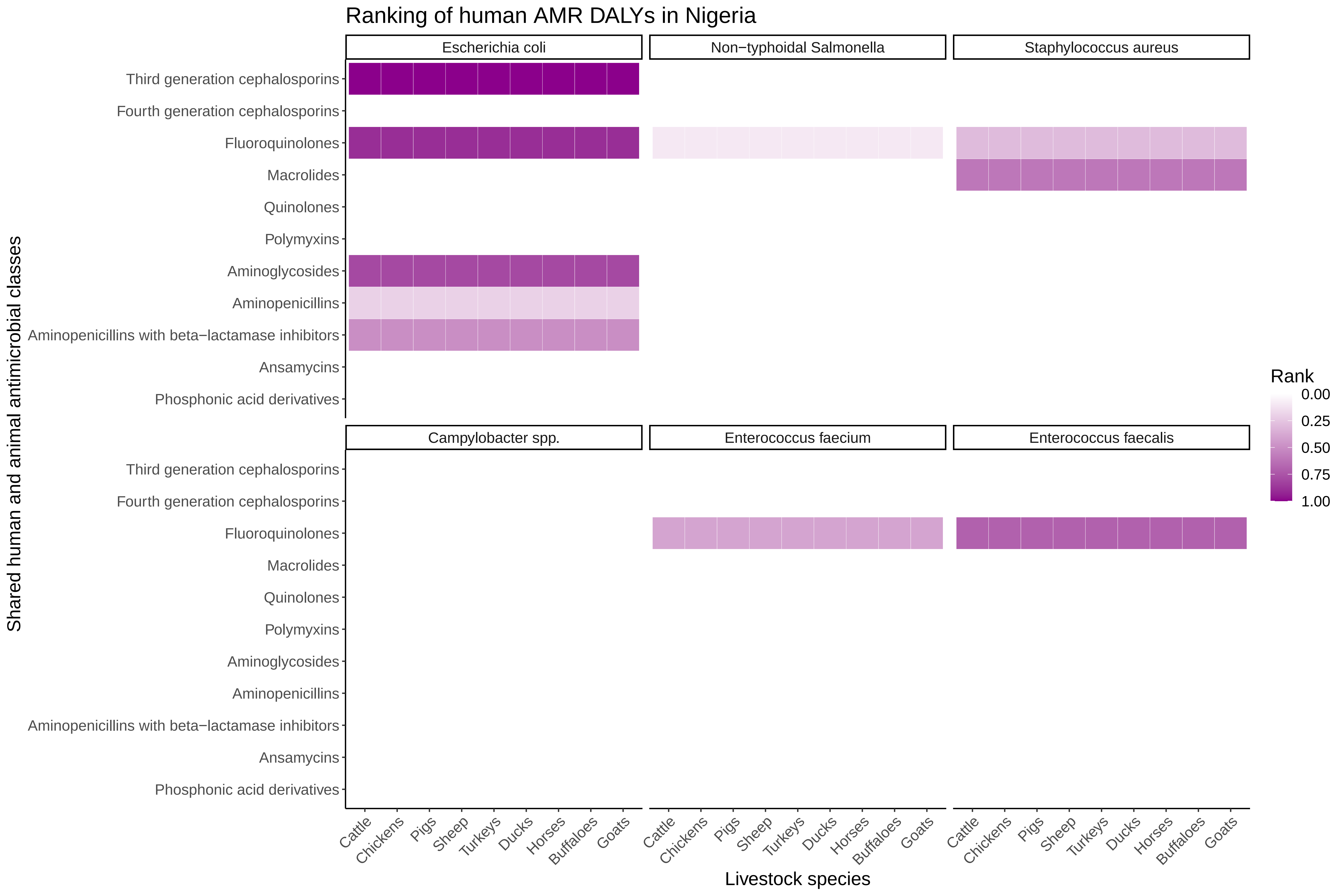
Ranking of human AMR DALYs in Nigeria
Escherichia coli
Non−typhoidal Salmonella
Staphylococcus aureus
Campylobacter spp.
Enterococcus faecium
Enterococcus faecalis
Third generation cephalosporins
Fourth generation cephalosporins
Fluoroquinolones
Macrolides
Quinolones
Polymyxins
Aminoglycosides
Aminopenicillins
Aminopenicillins with beta−lactamase inhibitors
Ansamycins
Phosphonic acid derivatives
Shared human and animal antimicrobial classes
Cattle
Chickens
Pigs
Sheep
Turkeys
Ducks
Horses
Buffaloes
Goats
Livestock species
Rank
0.00
0.25
0.50
0.75
1.00

Ranking of livestock AMU in Nigeria

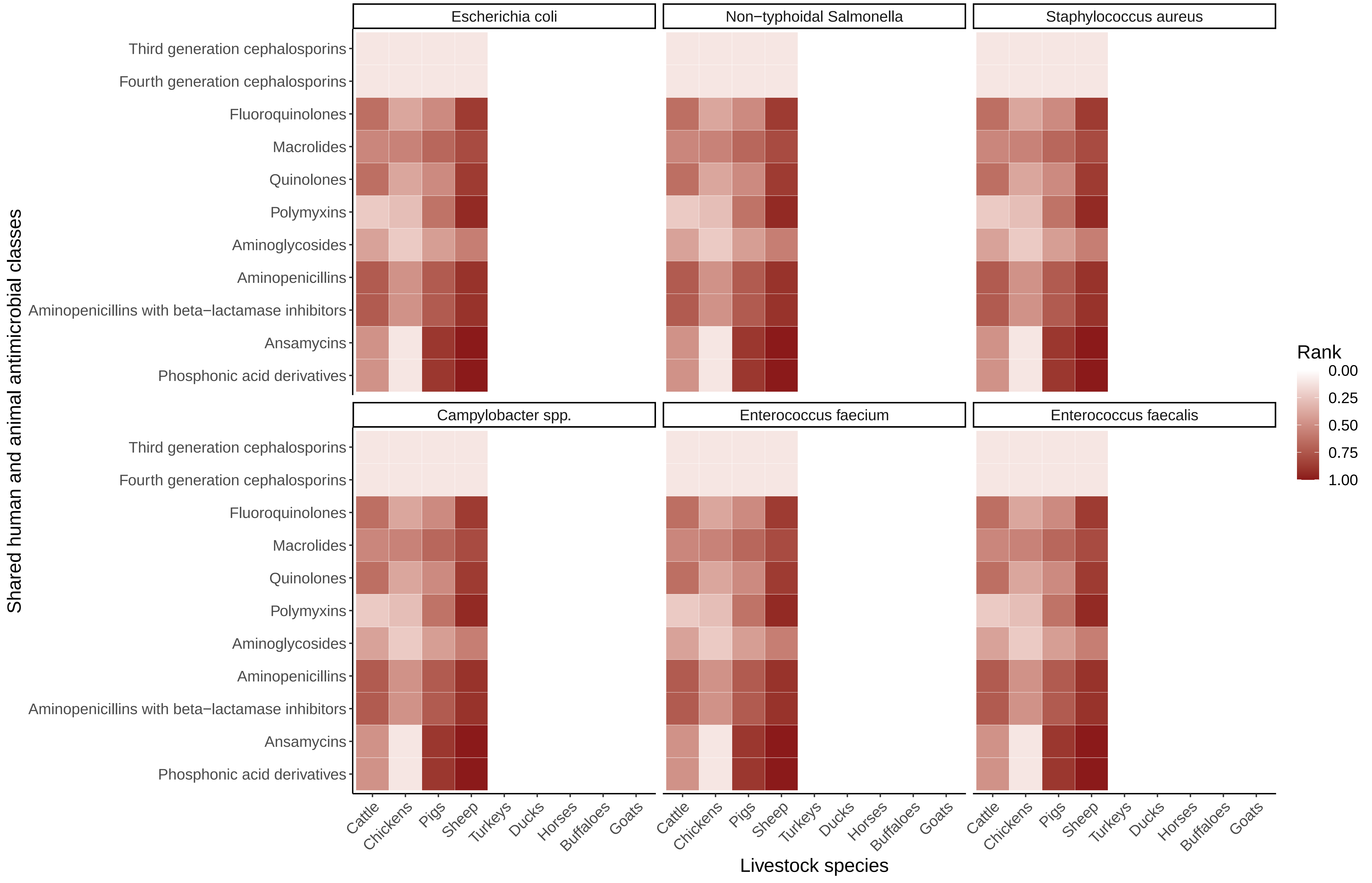

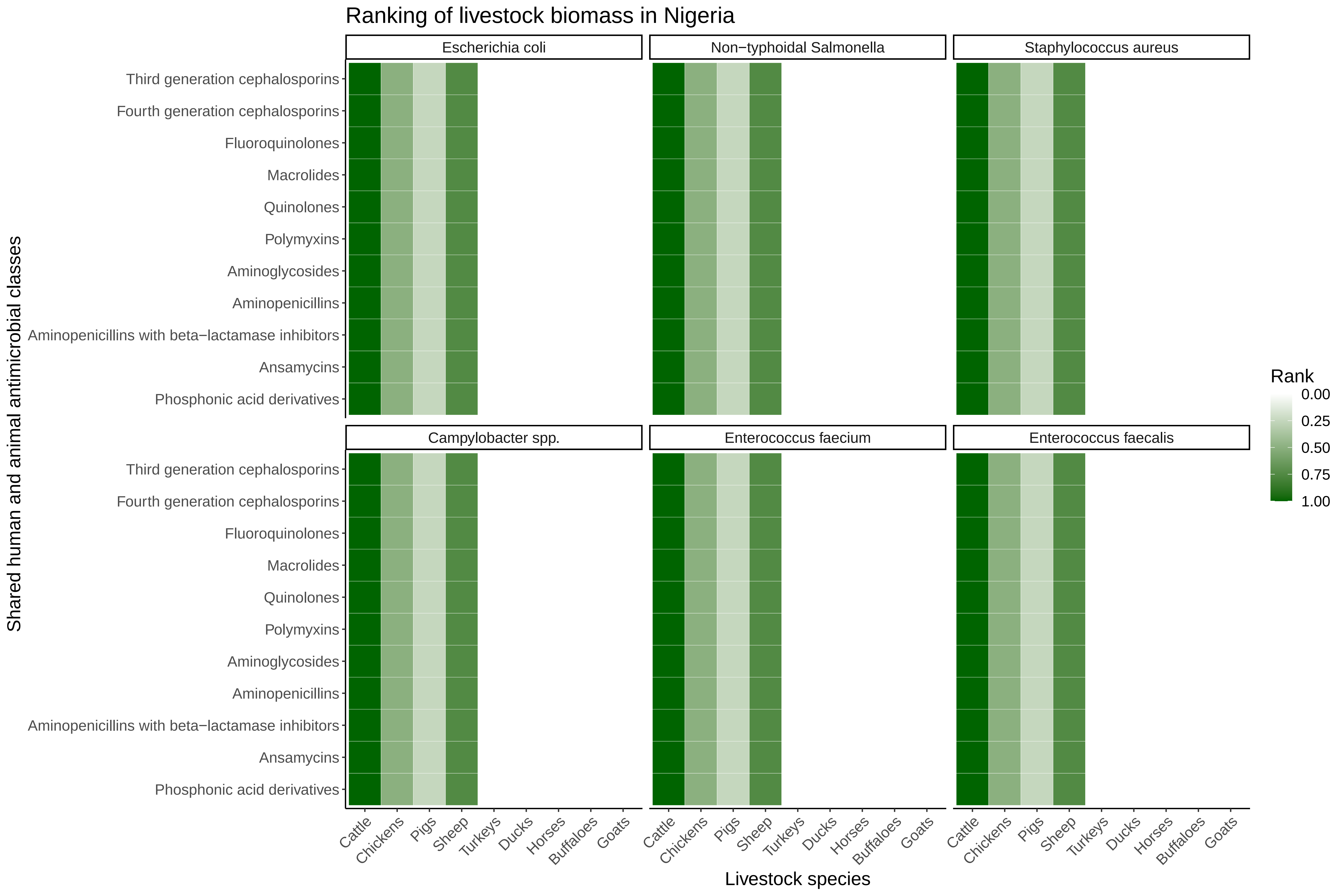

Ranking of livestock biomass in Nigeria
Escherichia coli
Non−typhoidal Salmonella
Staphylococcus aureus
Campylobacter spp.
Enterococcus faecium
Enterococcus faecalis
Third generation cephalosporins
Fourth generation cephalosporins
Fluoroquinolones
Macrolides
Quinolones
Polymyxins
Aminoglycosides
Aminopenicillins
Aminopenicillins with beta−lactamase inhibitors
Ansamycins
Phosphonic acid derivatives
Shared human and animal antimicrobial classes
Cattle
Chickens
Pigs
Sheep
Turkeys
Ducks
Horses
Buffaloes
Goats
Livestock species
Rank
0.00
0.25
0.50
0.75
1.00

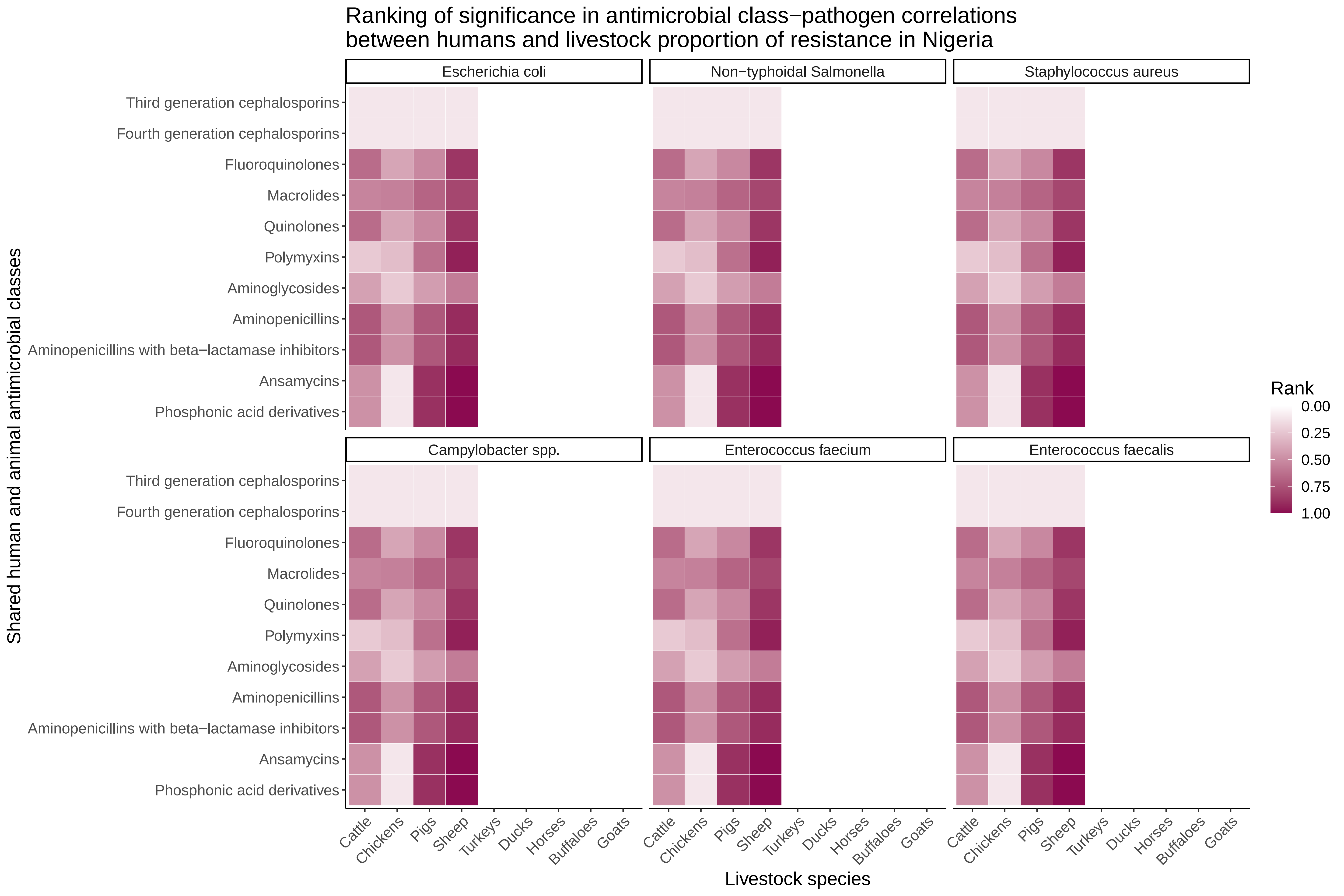

Ranking of significance in antimicrobial class−pathogen correlations
between humans and livestock proportion of resistance in Nigeria
Escherichia coli
Non−typhoidal Salmonella
Staphylococcus aureus
Campylobacter spp.
Enterococcus faecium
Enterococcus faecalis
Third generation cephalosporins
Fourth generation cephalosporins
Fluoroquinolones
Macrolides
Quinolones
Polymyxins
Aminoglycosides
Aminopenicillins
Aminopenicillins with beta−lactamase inhibitors
Ansamycins
Phosphonic acid derivatives
Shared human and animal antimicrobial classes
Cattle
Chickens
Pigs
Sheep
Turkeys
Ducks
Horses
Buffaloes
Goats
Livestock species
Rank
0.00
0.25
0.50
0.75
1.00

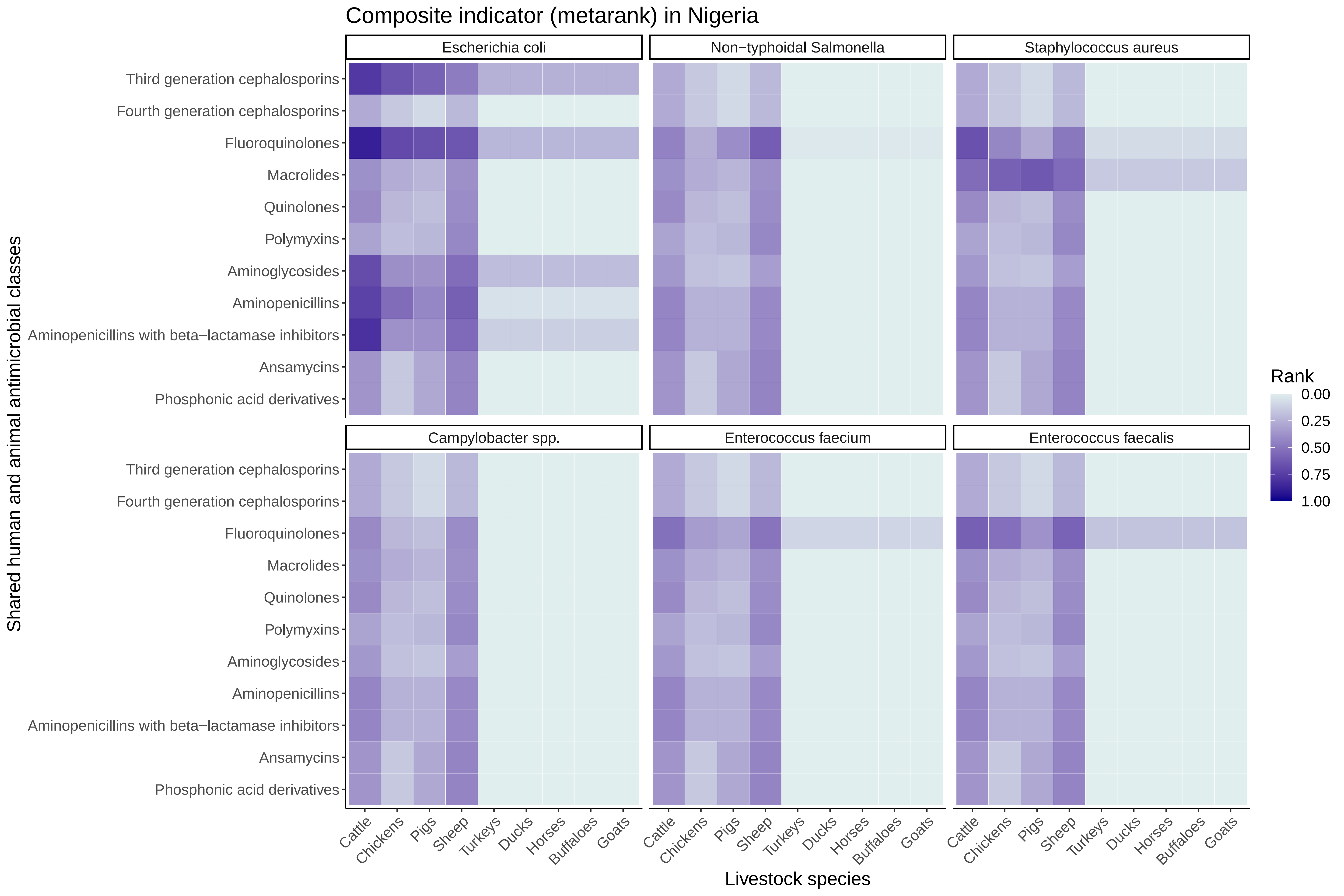

Composite indicator (metarank) in Nigeria
Escherichia coli
Non−typhoidal Salmonella
Staphylococcus aureus
Campylobacter spp.
Enterococcus faecium
Enterococcus faecalis
Shared human and animal antimicrobial classes
Third generation cephalosporins
Fourth generation cephalosporins
Fluoroquinolones
Macrolides
Quinolones
Polymyxins
Aminoglycosides
Aminopenicillins
Aminopenicillins with beta−lactamase inhibitors
Ansamycins
Phosphonic acid derivatives
Cattle
Chickens
Pigs
Sheep
Turkeys
Ducks
Horses
Buffaloes
Goats
Livestock species
Rank
0.00
0.25
0.50
0.75
1.00

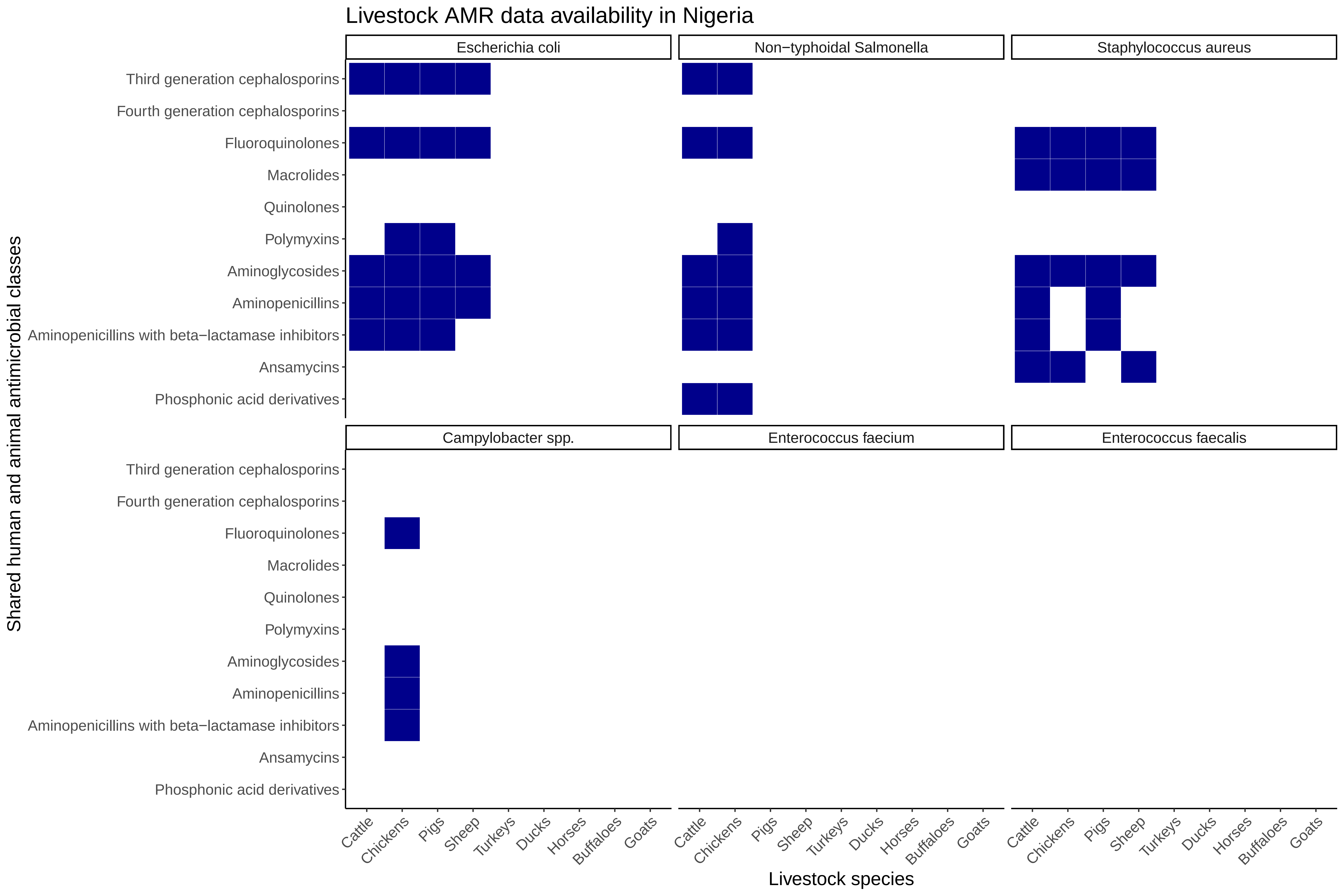

Livestock AMR data availability in Nigeria
Escherichia coli
Non−typhoidal Salmonella
Staphylococcus aureus
Campylobacter spp.
Enterococcus faecium
Enterococcus faecalis
Shared human and animal antimicrobial classes
Third generation cephalosporins
Fourth generation cephalosporins
Fluoroquinolones
Macrolides
Quinolones
Polymyxins
Aminoglycosides
Aminopenicillins
Aminopenicillins with beta−lactamase inhibitors
Ansamycins
Phosphonic acid derivatives
Cattle
Chickens
Pigs
Sheep
Turkeys
Ducks
Horses
Buffaloes
Goats
Livestock species

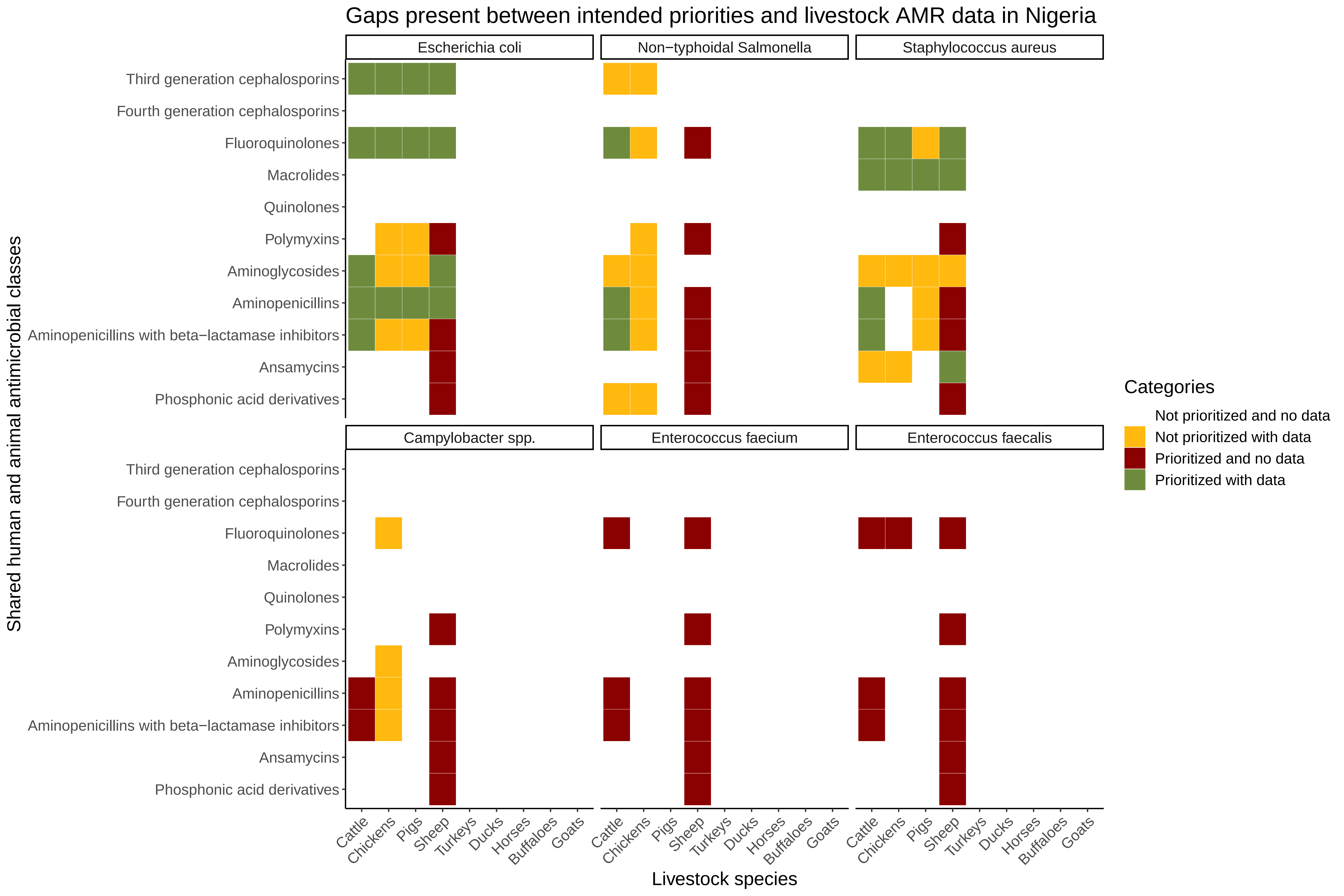
Gaps present between intended priorities and livestock AMR data in Nigeria
Escherichia coli
Non−typhoidal Salmonella
Staphylococcus aureus
Campylobacter spp.
Enterococcus faecium
Enterococcus faecalis
Third generation cephalosporins
Fourth generation cephalosporins
Fluoroquinolones
Macrolides
Quinolones
Polymyxins
Aminoglycosides
Aminopenicillins
Aminopenicillins with beta−lactamase inhibitors
Ansamycins
Phosphonic acid derivatives
Shared human and animal antimicrobial classes
Cattle
Chickens
Pigs
Sheep
Turkeys
Ducks
Horses
Buffaloes
Goats
Livestock species
Categories
Not prioritized and no data
Not prioritized with data
Prioritized and no data
Prioritized with data

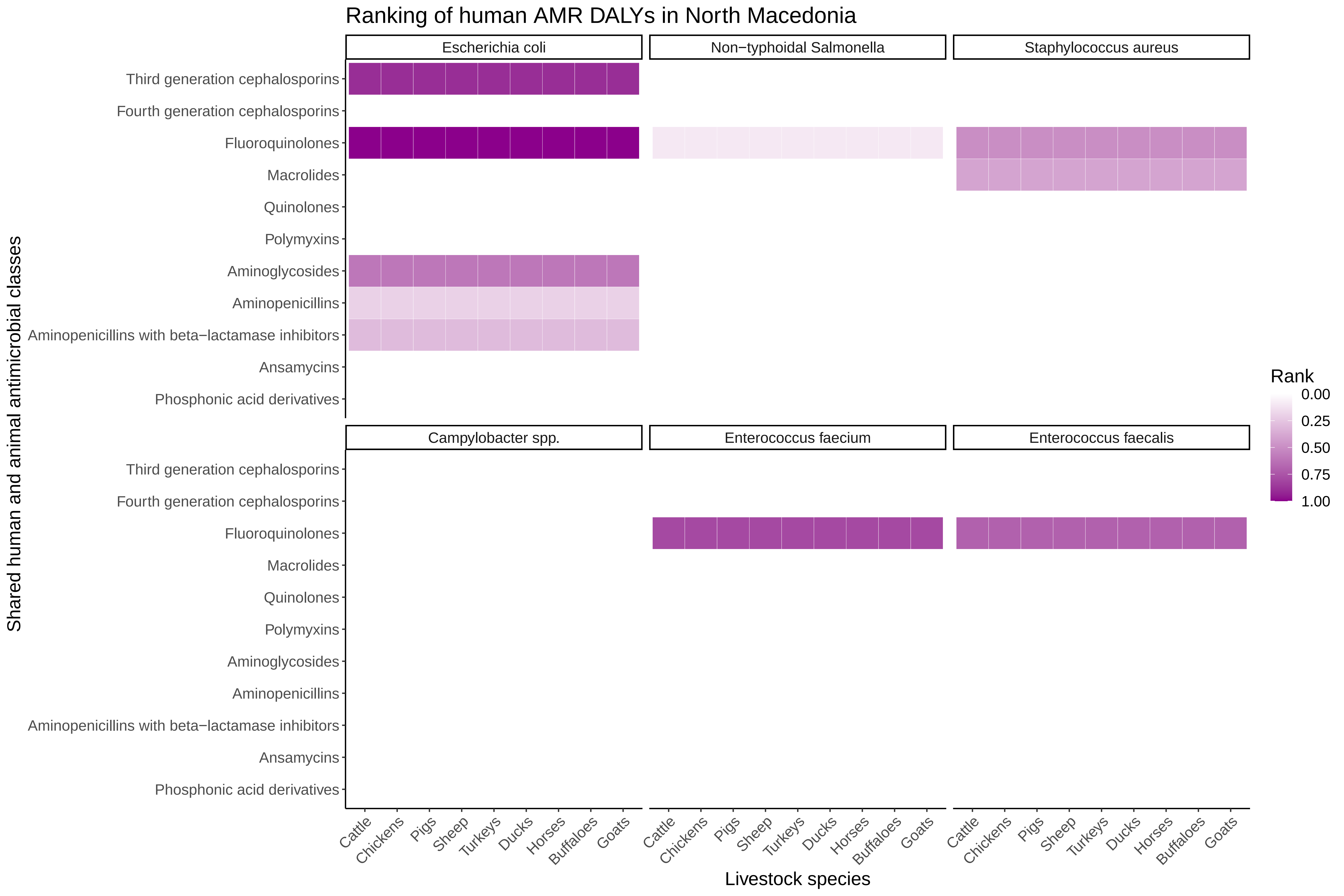

Ranking of human AMR DALYs in North Macedonia
Escherichia coli
Non−typhoidal Salmonella
Staphylococcus aureus
Campylobacter spp.
Enterococcus faecium
Enterococcus faecalis
Third generation cephalosporins
Fourth generation cephalosporins
Fluoroquinolones
Macrolides
Quinolones
Polymyxins
Aminoglycosides
Aminopenicillins
Aminopenicillins with beta−lactamase inhibitors
Ansamycins
Phosphonic acid derivatives
Shared human and animal antimicrobial classes
Cattle
Chickens
Pigs
Sheep
Turkeys
Ducks
Horses
Buffaloes
Goats
Livestock species
Rank
0.00
0.25
0.50
0.75
1.00

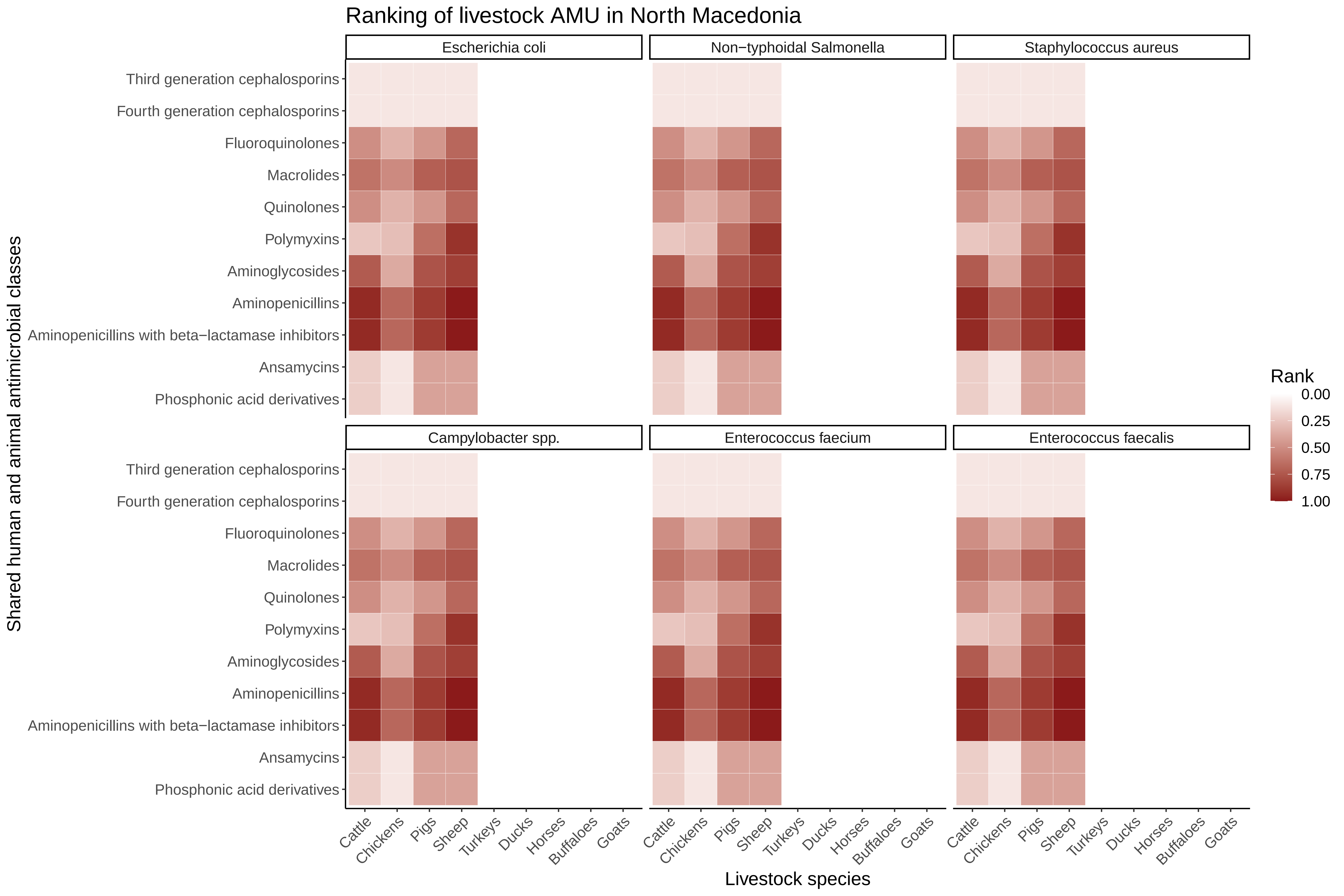
Ranking of livestock AMU in North Macedonia
Escherichia coli
Non−typhoidal Salmonella
Staphylococcus aureus
Campylobacter spp.
Enterococcus faecium
Enterococcus faecalis
Shared human and animal antimicrobial classes
Third generation cephalosporins
Fourth generation cephalosporins
Fluoroquinolones
Macrolides
Quinolones
Polymyxins
Aminoglycosides
Aminopenicillins
Aminopenicillins with beta−lactamase inhibitors
Ansamycins
Phosphonic acid derivatives
Cattle
Chickens
Pigs
Sheep
Turkeys
Ducks
Horses
Buffaloes
Goats
Livestock species
Rank
0.00
0.25
0.50
0.75
1.00

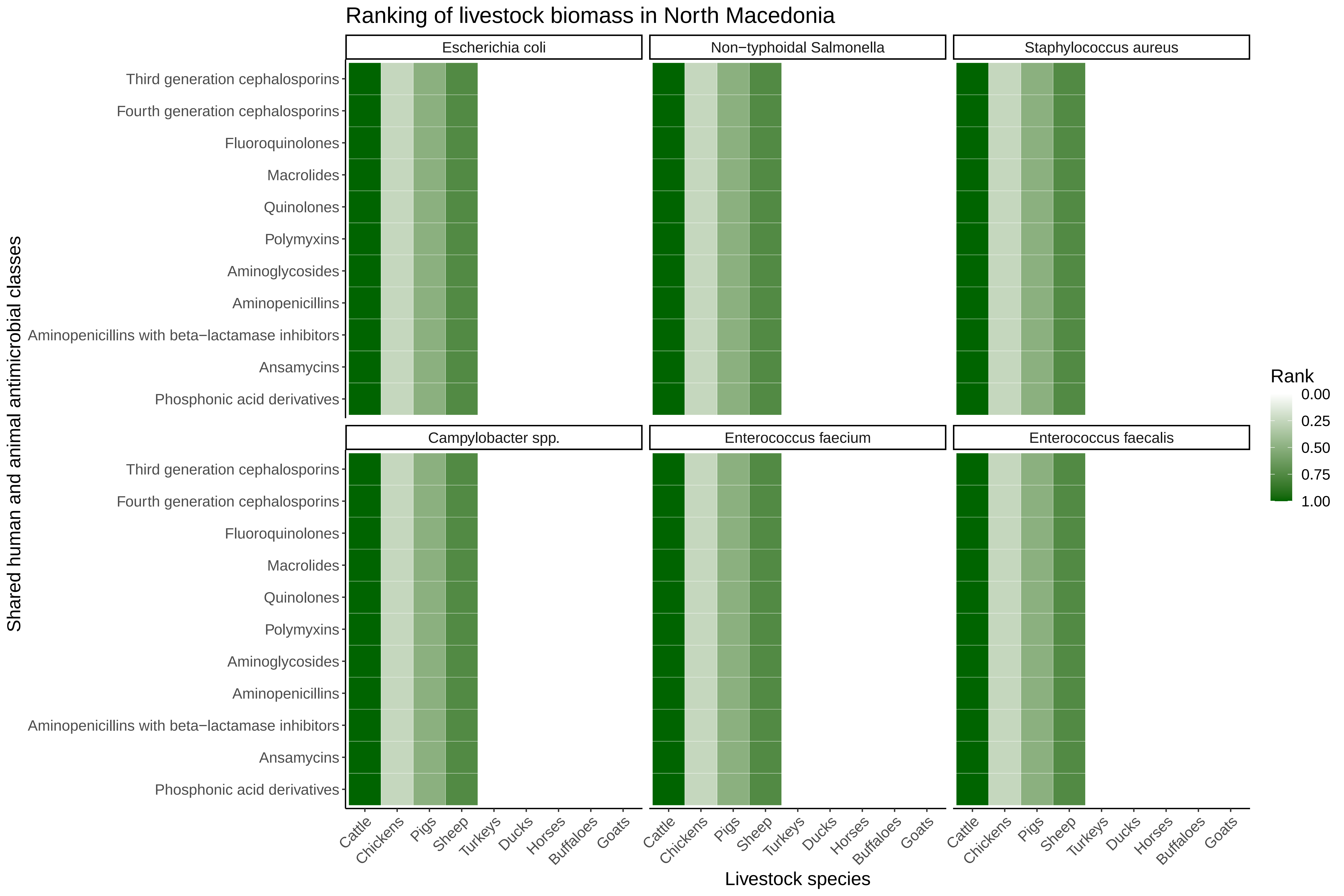

Ranking of livestock biomass in North Macedonia
Escherichia coli
Non−typhoidal Salmonella
Staphylococcus aureus
Campylobacter spp.
Enterococcus faecium
Enterococcus faecalis
Third generation cephalosporins
Fourth generation cephalosporins
Fluoroquinolones
Macrolides
Quinolones
Polymyxins
Aminoglycosides
Aminopenicillins
Aminopenicillins with beta−lactamase inhibitors
Ansamycins
Phosphonic acid derivatives
Shared human and animal antimicrobial classes
Cattle
Chickens
Pigs
Sheep
Turkeys
Ducks
Horses
Buffaloes
Goats
Livestock species
Rank
0.00
0.25
0.50
0.75
1.00

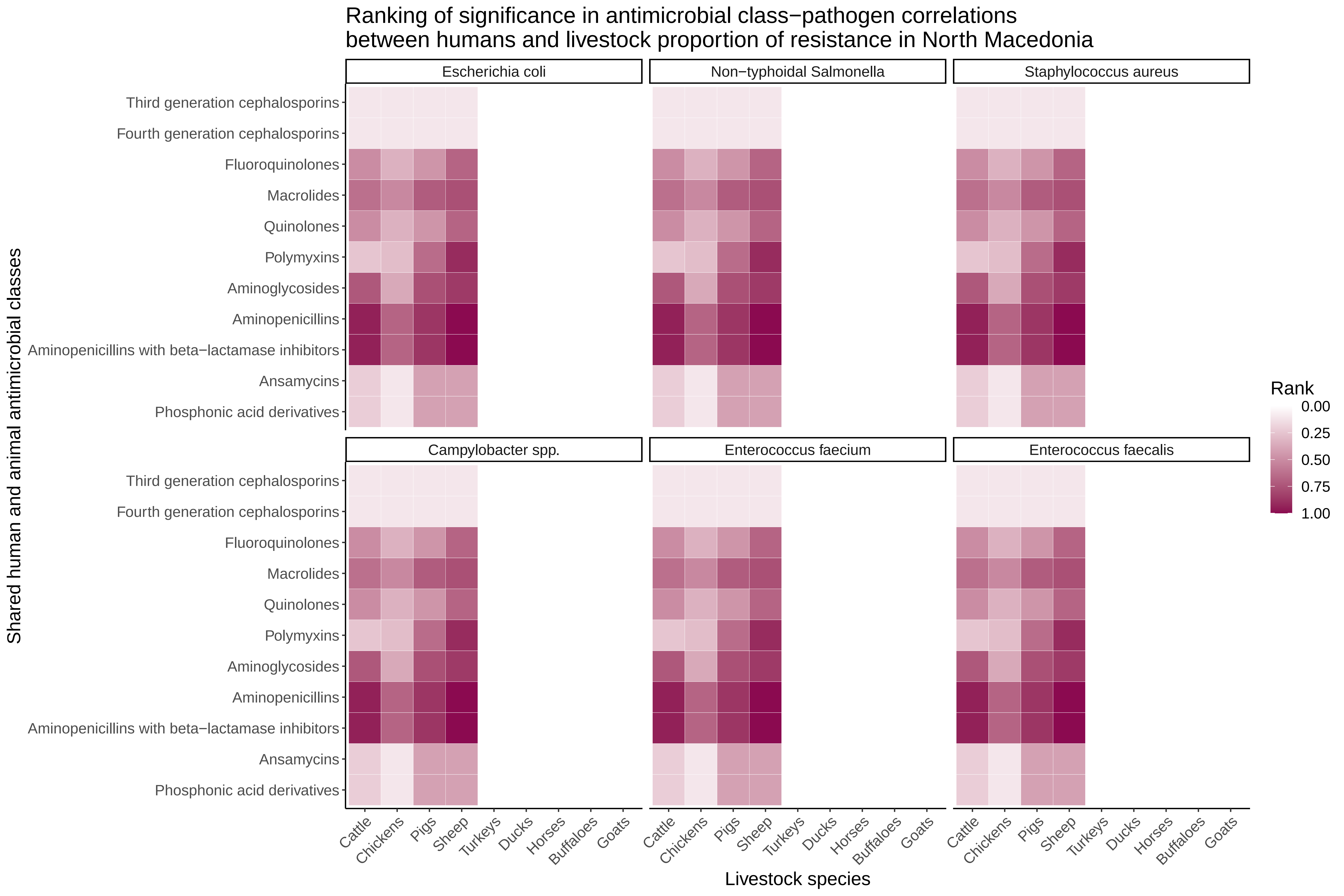

Ranking of significance in antimicrobial class−pathogen correlations
between humans and livestock proportion of resistance in North Macedonia
Escherichia coli
Non−typhoidal Salmonella
Staphylococcus aureus
Campylobacter spp.
Enterococcus faecium
Enterococcus faecalis
Third generation cephalosporins
Fourth generation cephalosporins
Fluoroquinolones
Macrolides
Quinolones
Polymyxins
Aminoglycosides
Aminopenicillins
Aminopenicillins with beta−lactamase inhibitors
Ansamycins
Phosphonic acid derivatives
Shared human and animal antimicrobial classes
Cattle
Chickens
Pigs
Sheep
Turkeys
Ducks
Horses
Buffaloes
Goats
Livestock species
Rank
0.00
0.25
0.50
0.75
1.00

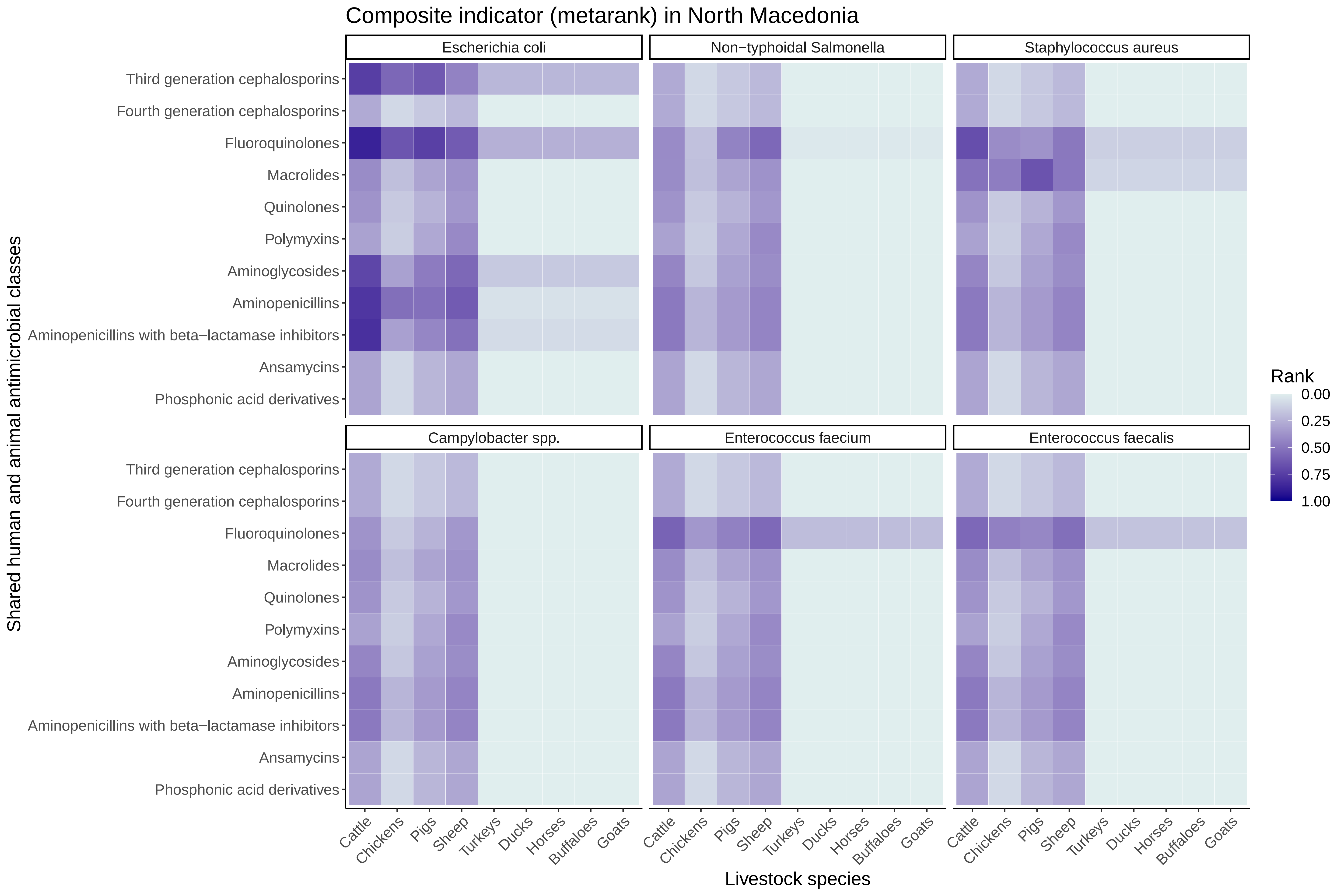

Composite indicator (metarank) in North Macedonia
Escherichia coli
Non−typhoidal Salmonella
Staphylococcus aureus
Campylobacter spp.
Enterococcus faecium
Enterococcus faecalis
Shared human and animal antimicrobial classes
Third generation cephalosporins
Fourth generation cephalosporins
Fluoroquinolones
Macrolides
Quinolones
Polymyxins
Aminoglycosides
Aminopenicillins
Aminopenicillins with beta−lactamase inhibitors
Ansamycins
Phosphonic acid derivatives
Cattle
Chickens
Pigs
Sheep
Turkeys
Ducks
Horses
Buffaloes
Goats
Livestock species
Rank
0.00
0.25
0.50
0.75
1.00

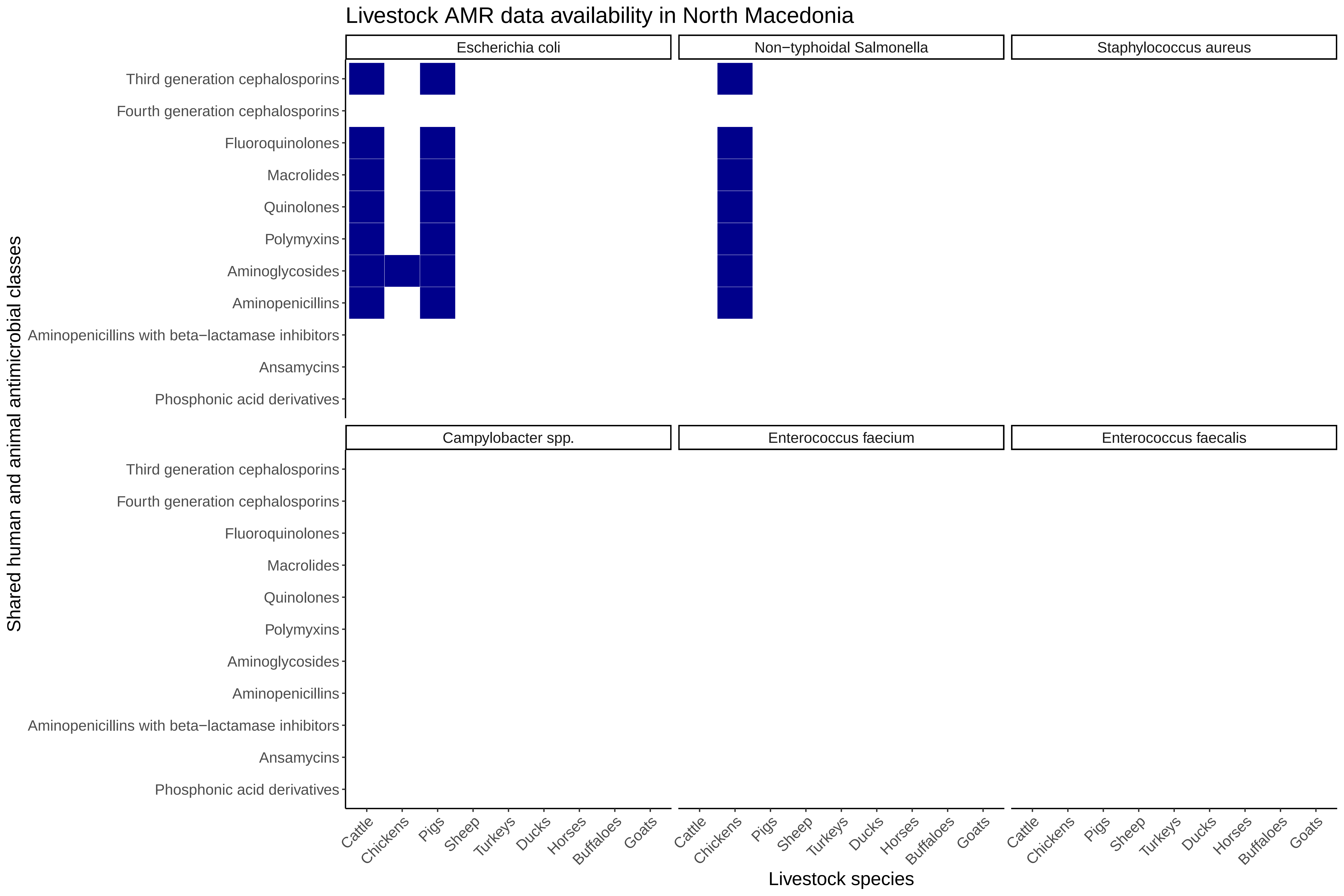

Livestock AMR data availability in North Macedonia
Escherichia coli
Non−typhoidal Salmonella
Staphylococcus aureus
Campylobacter spp.
Enterococcus faecium
Enterococcus faecalis
Shared human and animal antimicrobial classes
Third generation cephalosporins
Fourth generation cephalosporins
Fluoroquinolones
Macrolides
Quinolones
Polymyxins
Aminoglycosides
Aminopenicillins
Aminopenicillins with beta−lactamase inhibitors
Ansamycins
Phosphonic acid derivatives
Cattle
Chickens
Pigs
Sheep
Turkeys
Ducks
Horses
Buffaloes
Goats
Livestock species

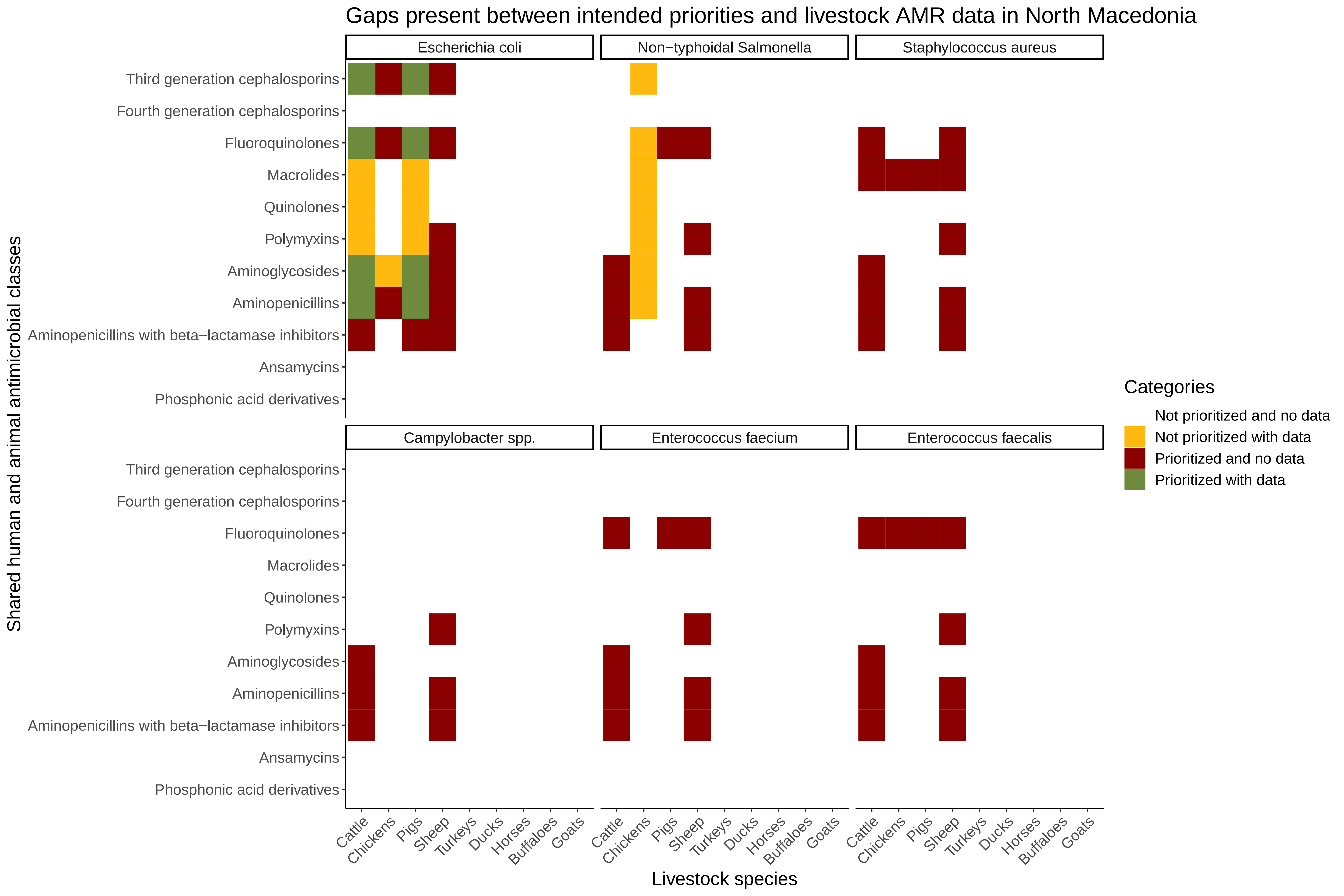

Gaps present between intended priorities and livestock AMR data in North Macedonia
Escherichia coli
Non−typhoidal Salmonella
Staphylococcus aureus
Campylobacter spp.
Enterococcus faecium
Enterococcus faecalis
Third generation cephalosporins
Fourth generation cephalosporins
Fluoroquinolones
Macrolides
Quinolones
Polymyxins
Aminoglycosides
Aminopenicillins
Aminopenicillins with beta−lactamase inhibitors
Ansamycins
Phosphonic acid derivatives
Shared human and animal antimicrobial classes
Cattle
Chickens
Pigs
Sheep
Turkeys
Ducks
Horses
Buffaloes
Goats
Livestock species
Categories
Not prioritized and no data
Not prioritized with data
Prioritized and no data
Prioritized with data

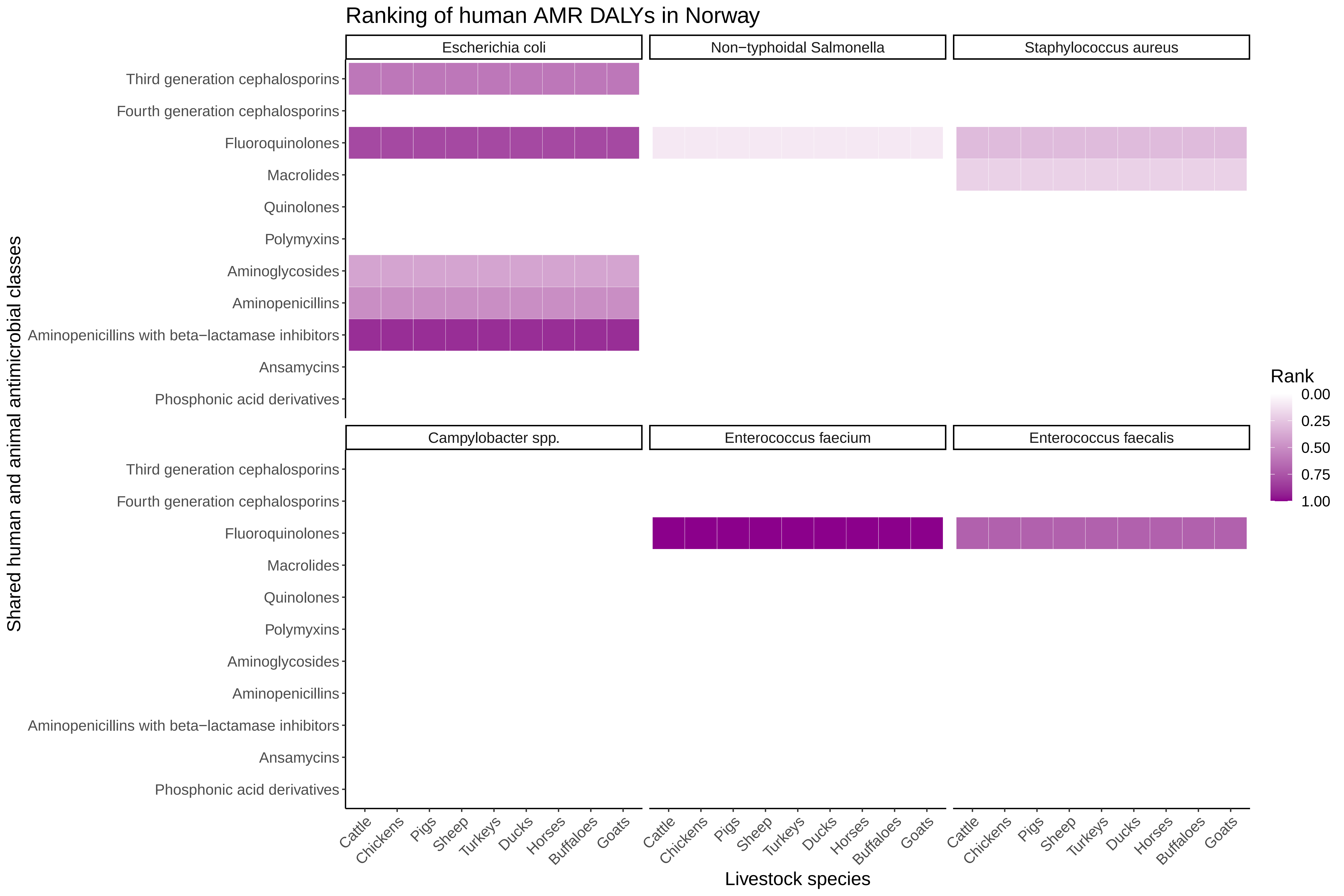
Ranking of human AMR DALYs in Norway
Escherichia coli
Non−typhoidal Salmonella
Staphylococcus aureus
Campylobacter spp.
Enterococcus faecium
Enterococcus faecalis
Third generation cephalosporins
Fourth generation cephalosporins
Fluoroquinolones
Macrolides
Quinolones
Polymyxins
Aminoglycosides
Aminopenicillins
Aminopenicillins with beta−lactamase inhibitors
Ansamycins
Phosphonic acid derivatives
Shared human and animal antimicrobial classes
Cattle
Chickens
Pigs
Sheep
Turkeys
Ducks
Horses
Buffaloes
Goats
Livestock species
Rank
0.00
0.25
0.50
0.75
1.00

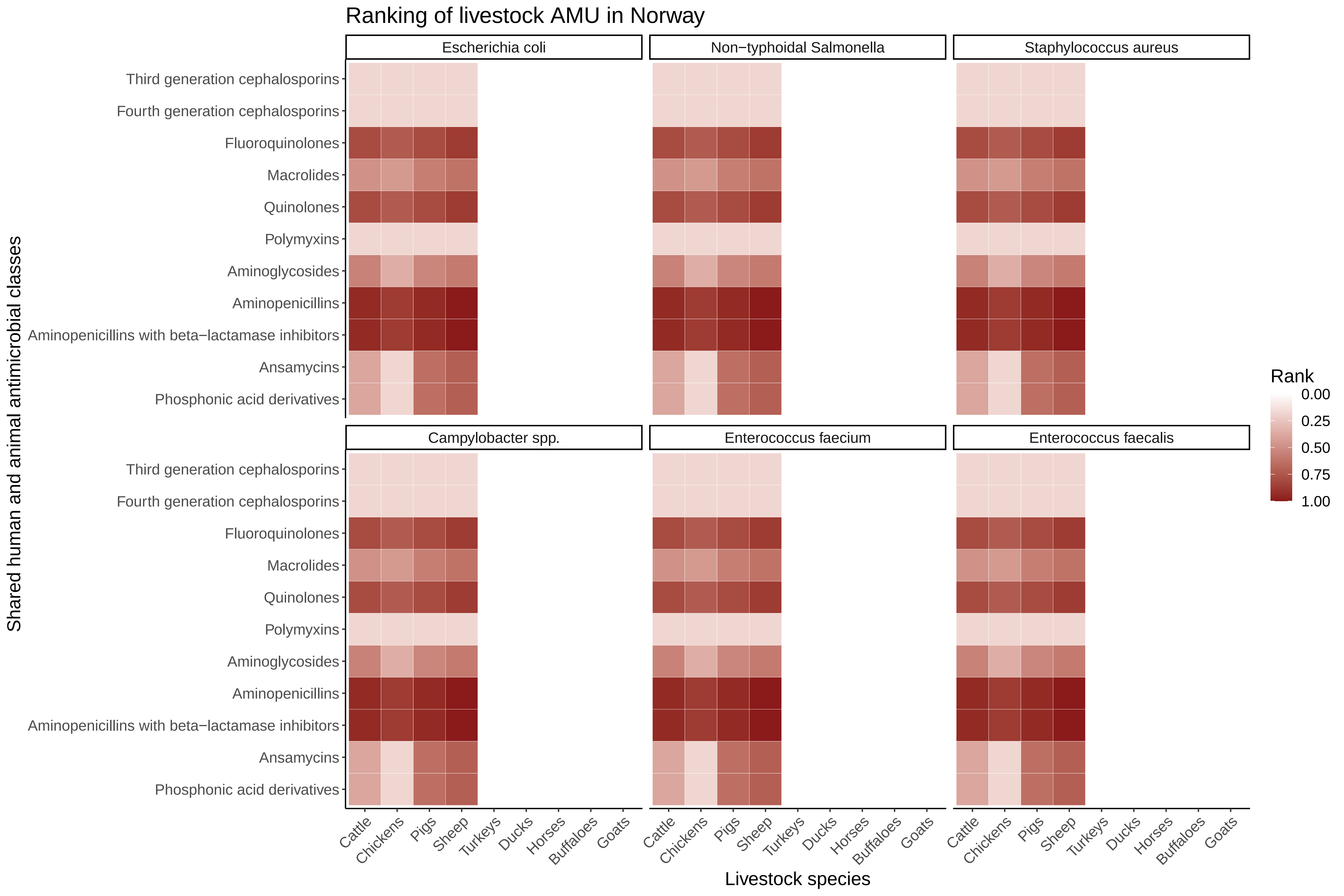

Ranking of livestock AMU in Norway
Escherichia coli
Non−typhoidal Salmonella
Staphylococcus aureus
Campylobacter spp.
Enterococcus faecium
Enterococcus faecalis
Shared human and animal antimicrobial classes
Third generation cephalosporins
Fourth generation cephalosporins
Fluoroquinolones
Macrolides
Quinolones
Polymyxins
Aminoglycosides
Aminopenicillins
Aminopenicillins with beta−lactamase inhibitors
Ansamycins
Phosphonic acid derivatives
Cattle
Chickens
Pigs
Sheep
Turkeys
Ducks
Horses
Buffaloes
Goats
Livestock species
Rank
0.00
0.25
0.50
0.75
1.00

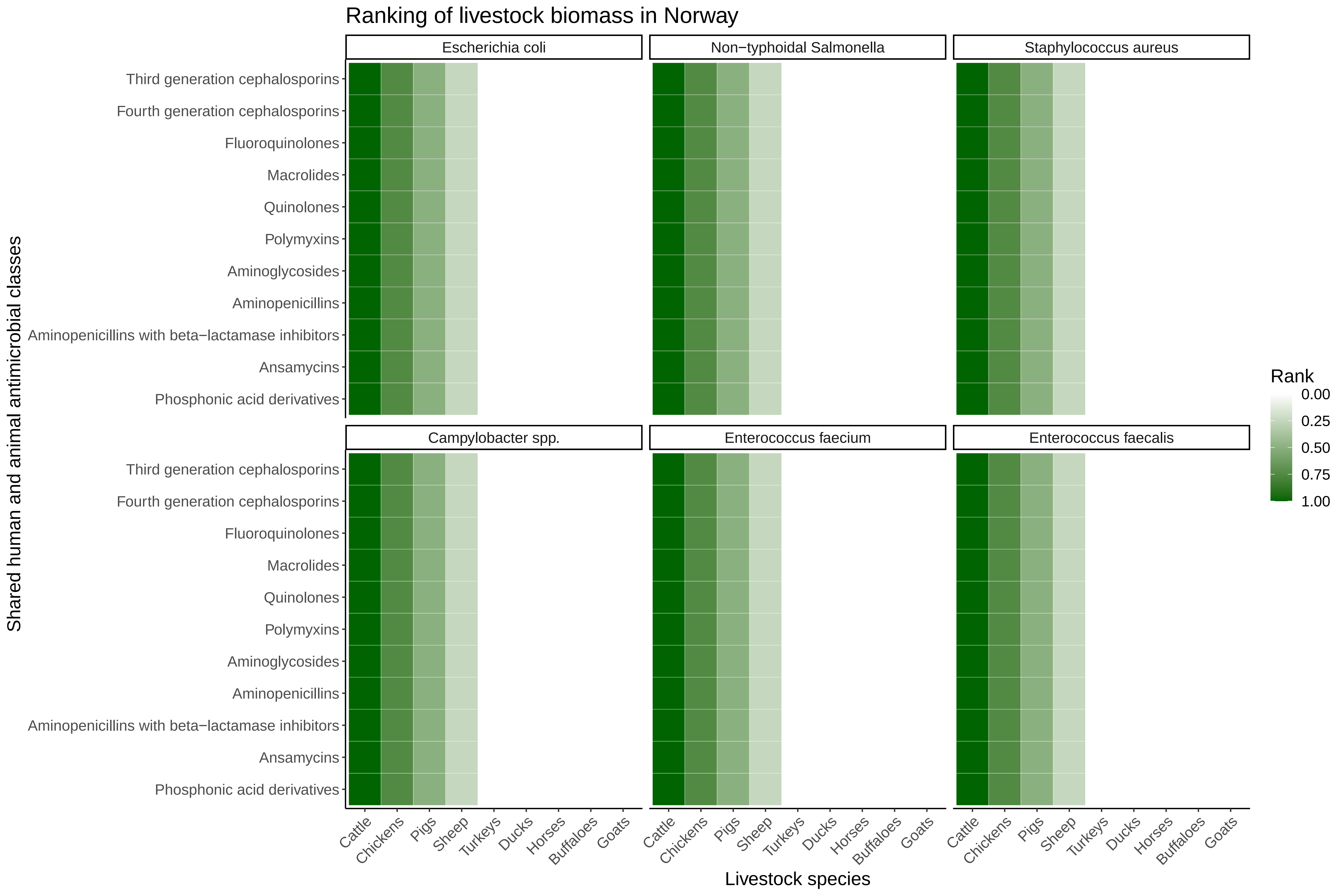
Ranking of livestock biomass in Norway
Escherichia coli
Non−typhoidal Salmonella
Staphylococcus aureus
Campylobacter spp.
Enterococcus faecium
Enterococcus faecalis
Third generation cephalosporins
Fourth generation cephalosporins
Fluoroquinolones
Macrolides
Quinolones
Polymyxins
Aminoglycosides
Aminopenicillins
Aminopenicillins with beta−lactamase inhibitors
Ansamycins
Phosphonic acid derivatives
Shared human and animal antimicrobial classes
Cattle
Chickens
Pigs
Sheep
Turkeys
Ducks
Horses
Buffaloes
Goats
Livestock species
Rank
0.00
0.25
0.50
0.75
1.00

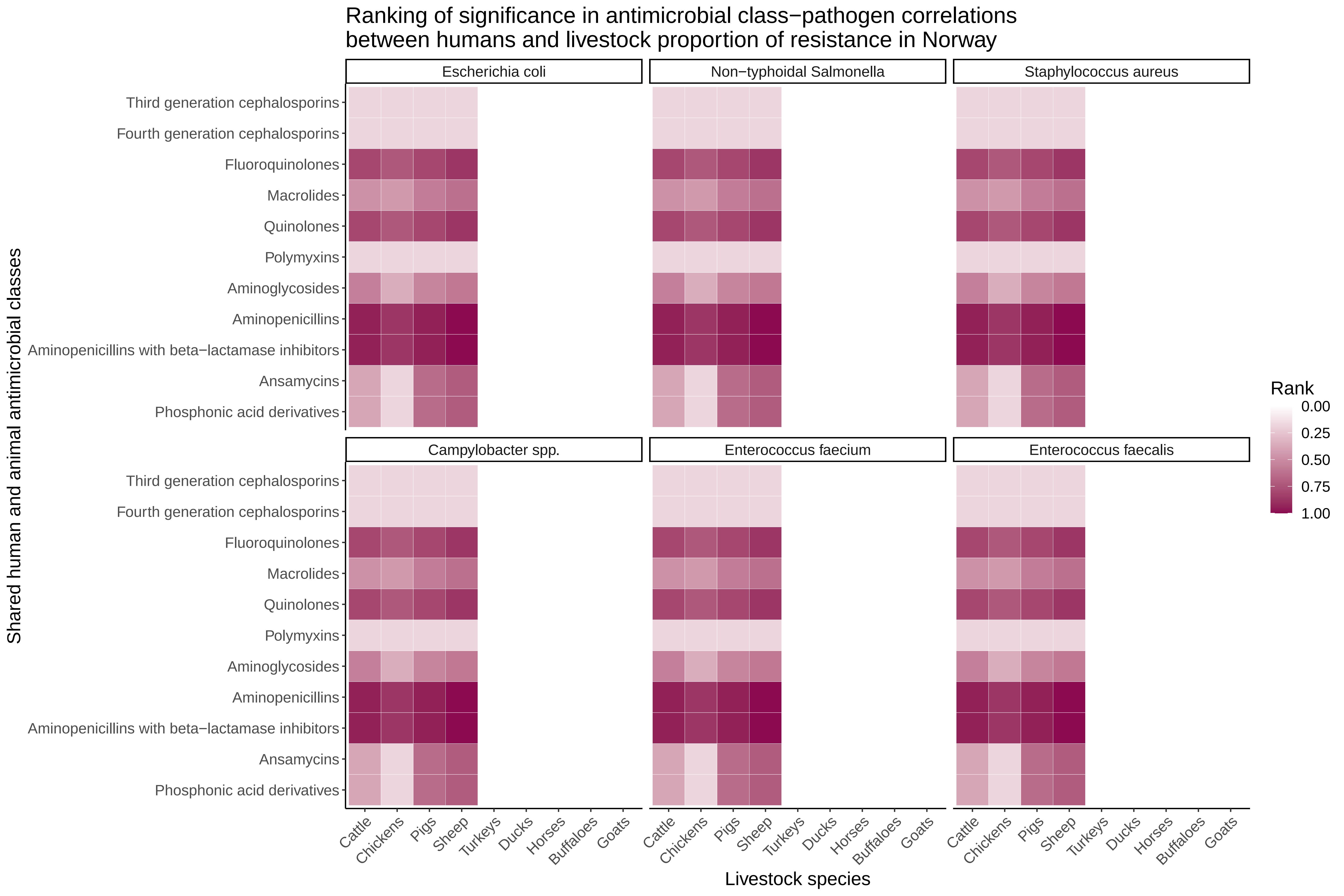
Ranking of significance in antimicrobial class−pathogen correlations
between humans and livestock proportion of resistance in Norway
Escherichia coli
Non−typhoidal Salmonella
Staphylococcus aureus
Campylobacter spp.
Enterococcus faecium
Enterococcus faecalis
Third generation cephalosporins
Fourth generation cephalosporins
Fluoroquinolones
Macrolides
Quinolones
Polymyxins
Aminoglycosides
Aminopenicillins
Aminopenicillins with beta−lactamase inhibitors
Ansamycins
Phosphonic acid derivatives
Shared human and animal antimicrobial classes
Cattle
Chickens
Pigs
Sheep
Turkeys
Ducks
Horses
Buffaloes
Goats
Livestock species
Rank
0.00
0.25
0.50
0.75
1.00

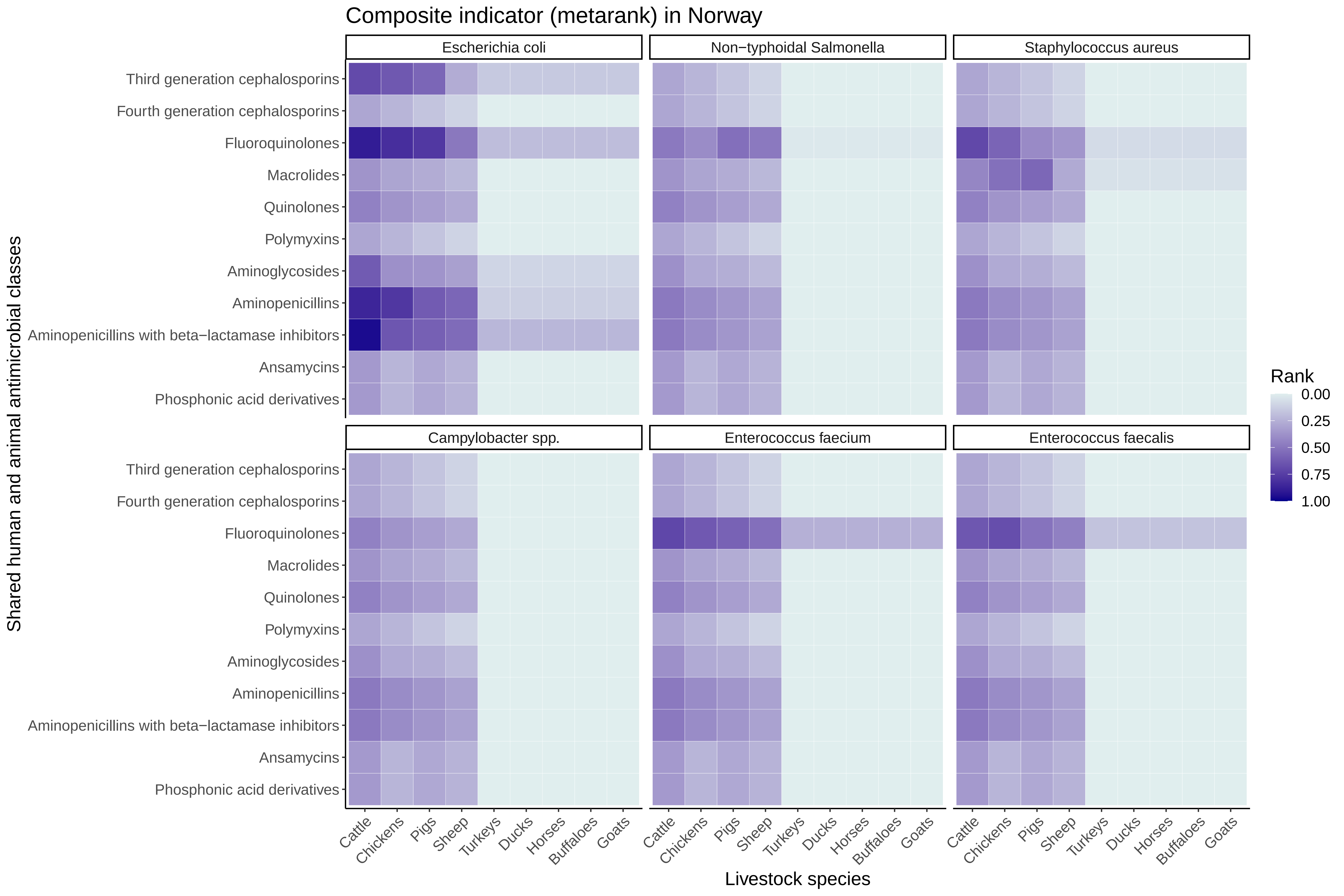

Composite indicator (metarank) in Norway
Escherichia coli
Non−typhoidal Salmonella
Staphylococcus aureus
Campylobacter spp.
Enterococcus faecium
Enterococcus faecalis
Shared human and animal antimicrobial classes
Third generation cephalosporins
Fourth generation cephalosporins
Fluoroquinolones
Macrolides
Quinolones
Polymyxins
Aminoglycosides
Aminopenicillins
Aminopenicillins with beta−lactamase inhibitors
Ansamycins
Phosphonic acid derivatives
Cattle
Chickens
Pigs
Sheep
Turkeys
Ducks
Horses
Buffaloes
Goats
Livestock species
Rank
0.00
0.25
0.50
0.75
1.00

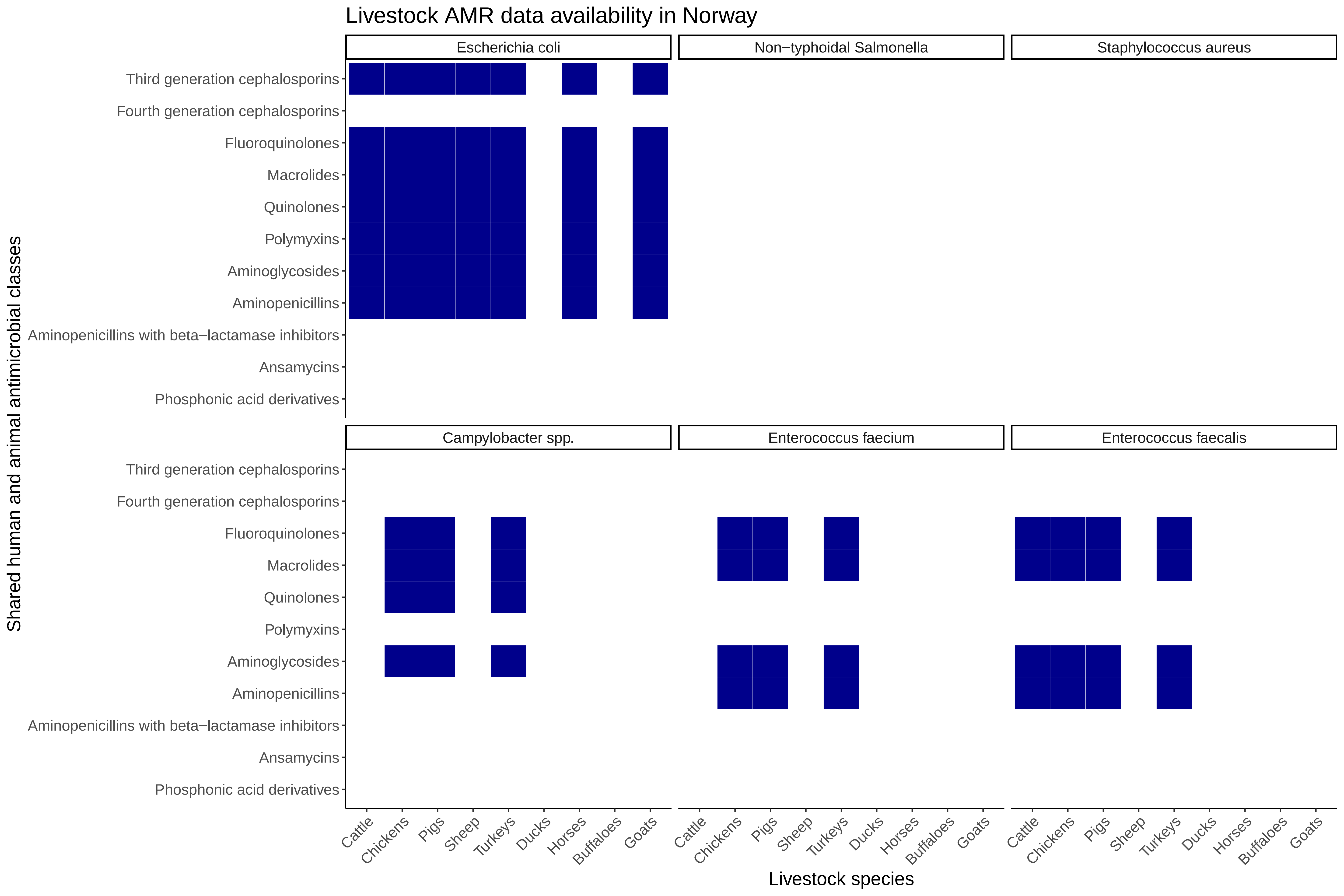

Livestock AMR data availability in Norway
Escherichia coli
Non−typhoidal Salmonella
Staphylococcus aureus
Campylobacter spp.
Enterococcus faecium
Enterococcus faecalis
Shared human and animal antimicrobial classes
Third generation cephalosporins
Fourth generation cephalosporins
Fluoroquinolones
Macrolides
Quinolones
Polymyxins
Aminoglycosides
Aminopenicillins
Aminopenicillins with beta−lactamase inhibitors
Ansamycins
Phosphonic acid derivatives
Cattle
Chickens
Pigs
Sheep
Turkeys
Ducks
Horses
Buffaloes
Goats
Livestock species

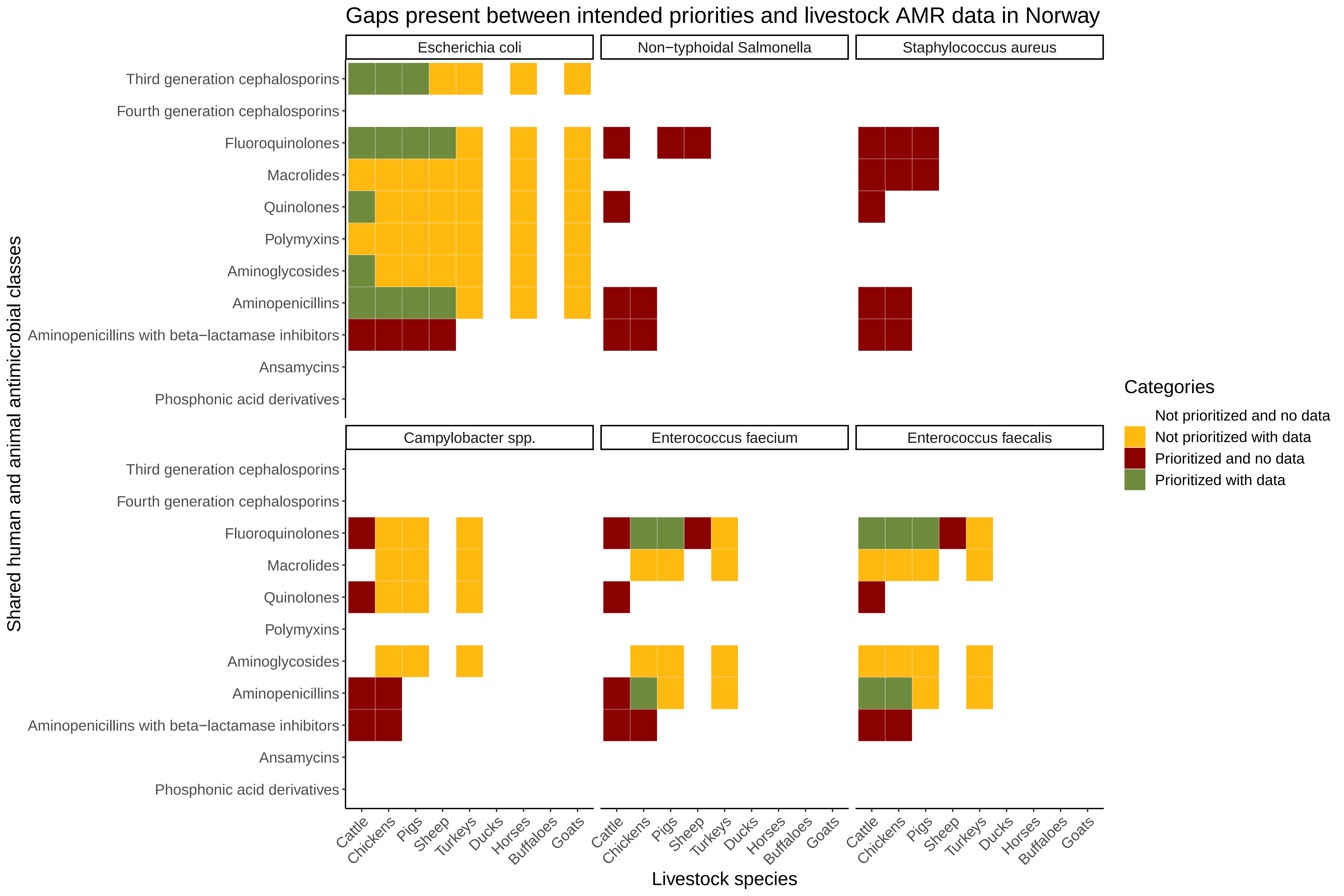

Gaps present between intended priorities and livestock AMR data in Norway
Escherichia coli
Non−typhoidal Salmonella
Staphylococcus aureus
Campylobacter spp.
Enterococcus faecium
Enterococcus faecalis
Third generation cephalosporins
Fourth generation cephalosporins
Fluoroquinolones
Macrolides
Quinolones
Polymyxins
Aminoglycosides
Aminopenicillins
Aminopenicillins with beta−lactamase inhibitors
Ansamycins
Phosphonic acid derivatives
Shared human and animal antimicrobial classes
Cattle
Chickens
Pigs
Sheep
Turkeys
Ducks
Horses
Buffaloes
Goats
Livestock species
Categories
Not prioritized and no data
Not prioritized with data
Prioritized and no data
Prioritized with data

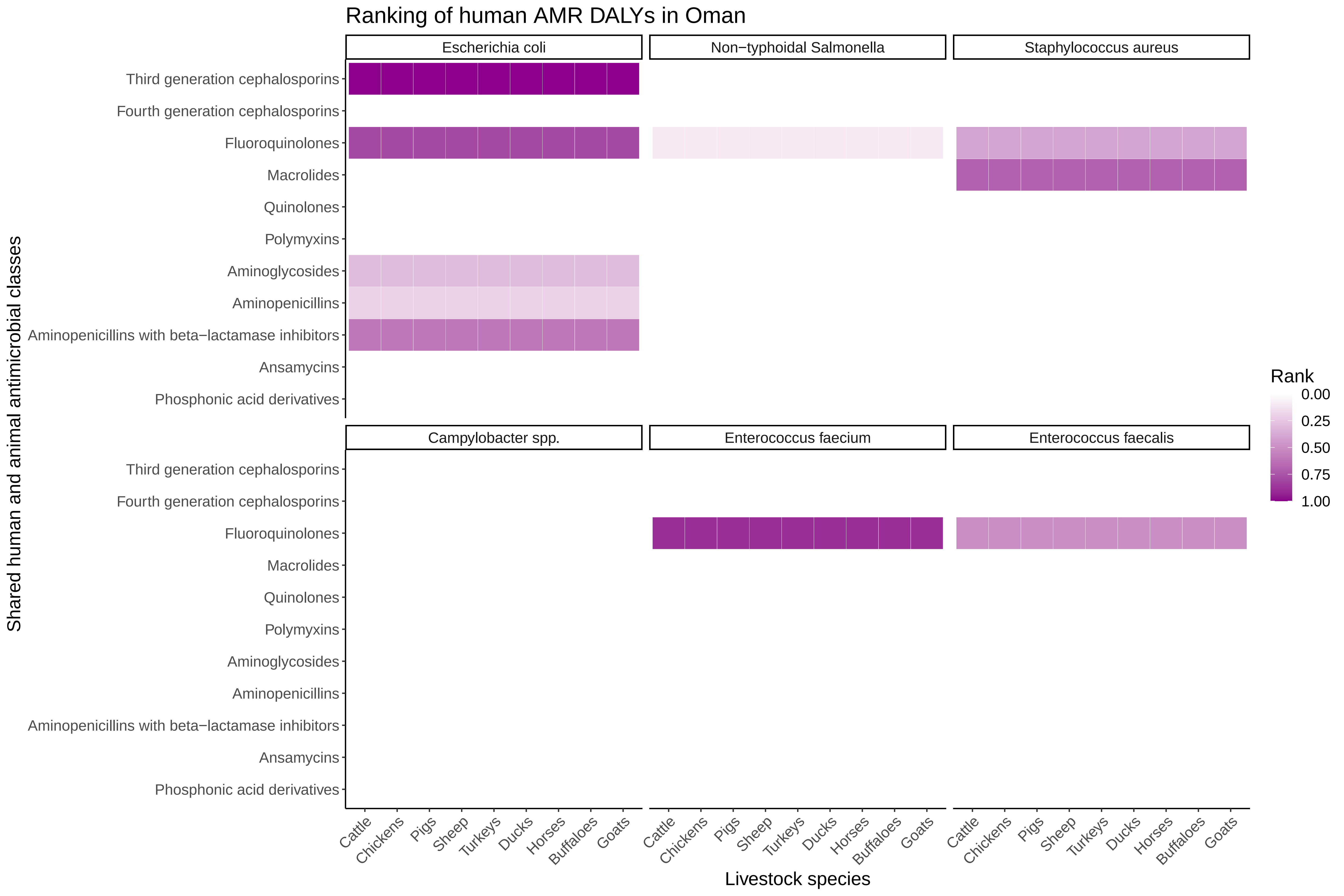

Ranking of human AMR DALYs in Oman
Escherichia coli
Non−typhoidal Salmonella
Staphylococcus aureus
Campylobacter spp.
Enterococcus faecium
Enterococcus faecalis
Third generation cephalosporins
Fourth generation cephalosporins
Fluoroquinolones
Macrolides
Quinolones
Polymyxins
Aminoglycosides
Aminopenicillins
Aminopenicillins with beta−lactamase inhibitors
Ansamycins
Phosphonic acid derivatives
Shared human and animal antimicrobial classes
Cattle
Chickens
Pigs
Sheep
Turkeys
Ducks
Horses
Buffaloes
Goats
Livestock species
Rank
0.00
0.25
0.50
0.75
1.00

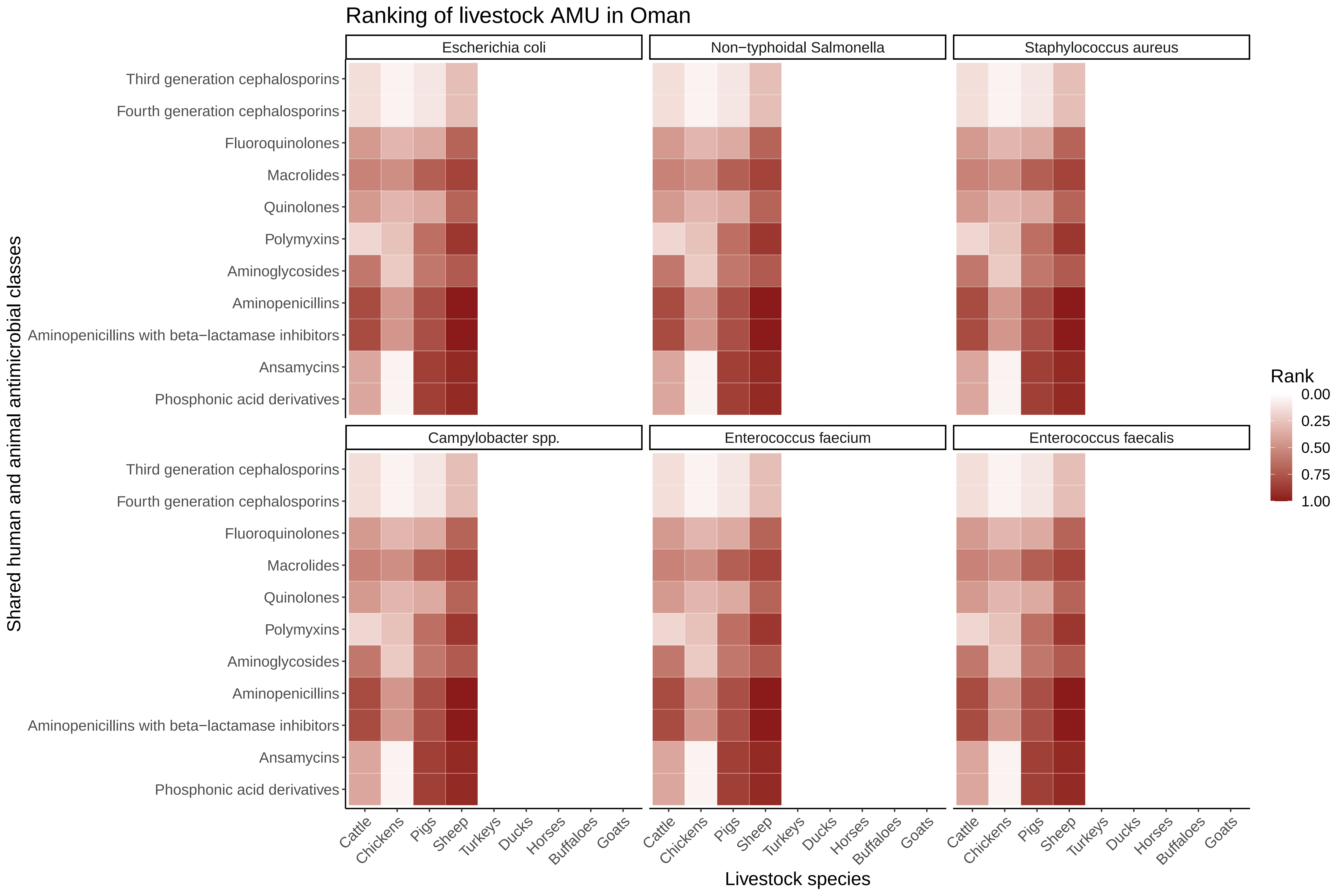

Ranking of livestock AMU in Oman
Escherichia coli
Non−typhoidal Salmonella
Staphylococcus aureus
Campylobacter spp.
Enterococcus faecium
Enterococcus faecalis
Third generation cephalosporins
Fourth generation cephalosporins
Fluoroquinolones
Macrolides
Quinolones
Polymyxins
Aminoglycosides
Aminopenicillins
Aminopenicillins with beta−lactamase inhibitors
Ansamycins
Phosphonic acid derivatives
Shared human and animal antimicrobial classes
Cattle
Chickens
Pigs
Sheep
Turkeys
Ducks
Horses
Buffaloes
Goats
Livestock species
Rank
0.00
0.25
0.50
0.75
1.00

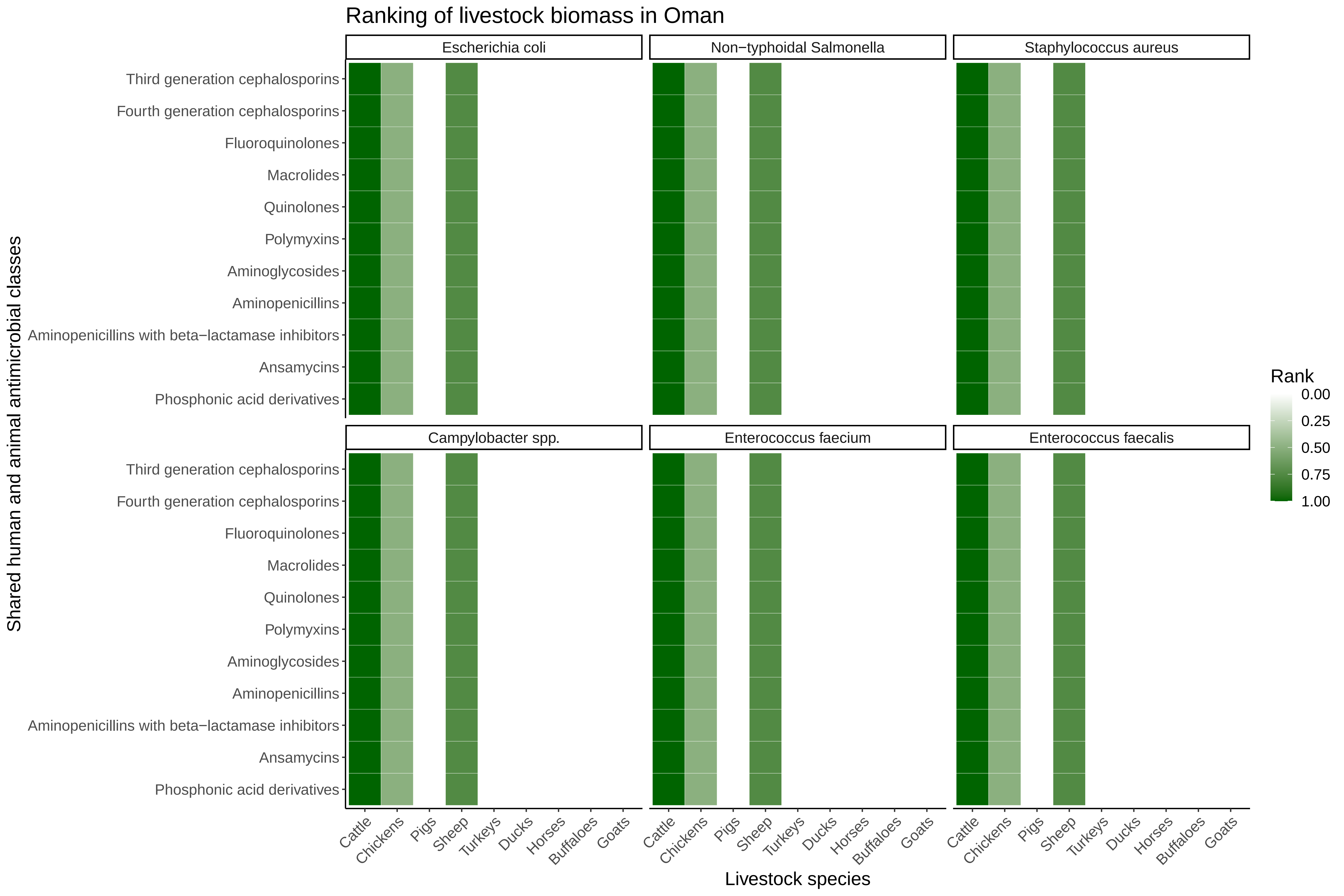
Ranking of livestock biomass in Oman
Escherichia coli
Non−typhoidal Salmonella
Staphylococcus aureus
Campylobacter spp.
Enterococcus faecium
Enterococcus faecalis
Shared human and animal antimicrobial classes
Third generation cephalosporins
Fourth generation cephalosporins
Fluoroquinolones
Macrolides
Quinolones
Polymyxins
Aminoglycosides
Aminopenicillins
Aminopenicillins with beta−lactamase inhibitors
Ansamycins
Phosphonic acid derivatives
Livestock species
Cattle
Chickens
Pigs
Sheep
Turkeys
Ducks
Horses
Buffaloes
Goats
Rank
0.00
0.25
0.50
0.75
1.00

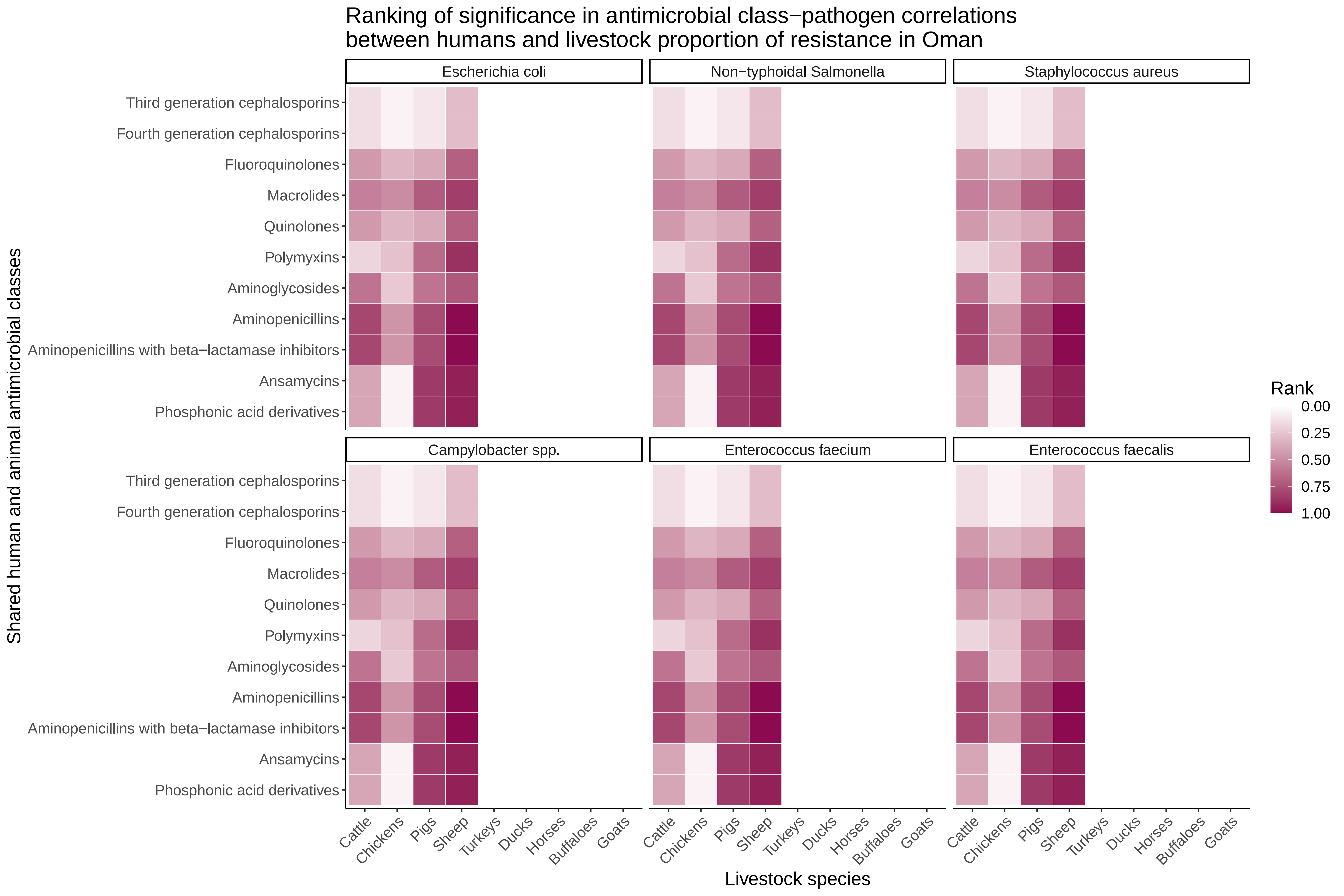

Ranking of significance in antimicrobial class−pathogen correlations
between humans and livestock proportion of resistance in Oman
Escherichia coli
Non−typhoidal Salmonella
Staphylococcus aureus
Campylobacter spp.
Enterococcus faecium
Enterococcus faecalis
Third generation cephalosporins
Fourth generation cephalosporins
Fluoroquinolones
Macrolides
Quinolones
Polymyxins
Aminoglycosides
Aminopenicillins
Aminopenicillins with beta−lactamase inhibitors
Ansamycins
Phosphonic acid derivatives
Shared human and animal antimicrobial classes
Cattle
Chickens
Pigs
Sheep
Turkeys
Ducks
Horses
Buffaloes
Goats
Livestock species
Rank
0.00
0.25
0.50
0.75
1.00

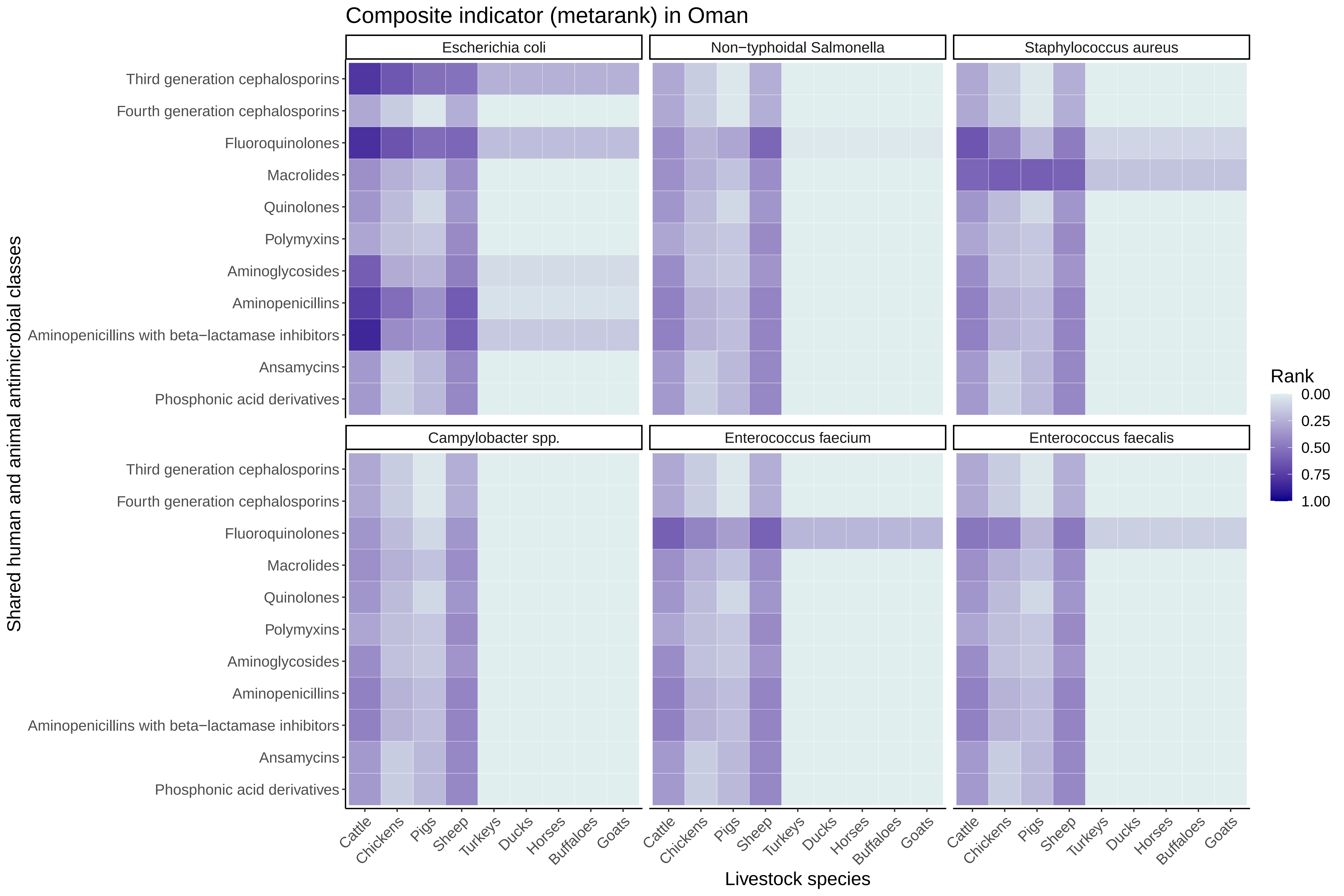

Composite indicator (metarank) in Oman
Escherichia coli
Non−typhoidal Salmonella
Staphylococcus aureus
Campylobacter spp.
Enterococcus faecium
Enterococcus faecalis
Third generation cephalosporins
Fourth generation cephalosporins
Fluoroquinolones
Macrolides
Quinolones
Polymyxins
Aminoglycosides
Aminopenicillins
Aminopenicillins with beta−lactamase inhibitors
Ansamycins
Phosphonic acid derivatives
Shared human and animal antimicrobial classes
Rank
0.00
0.25
0.50
0.75
1.00
Cattle
Chickens
Pigs
Sheep
Turkeys
Ducks
Horses
Buffaloes
Goats
Livestock species

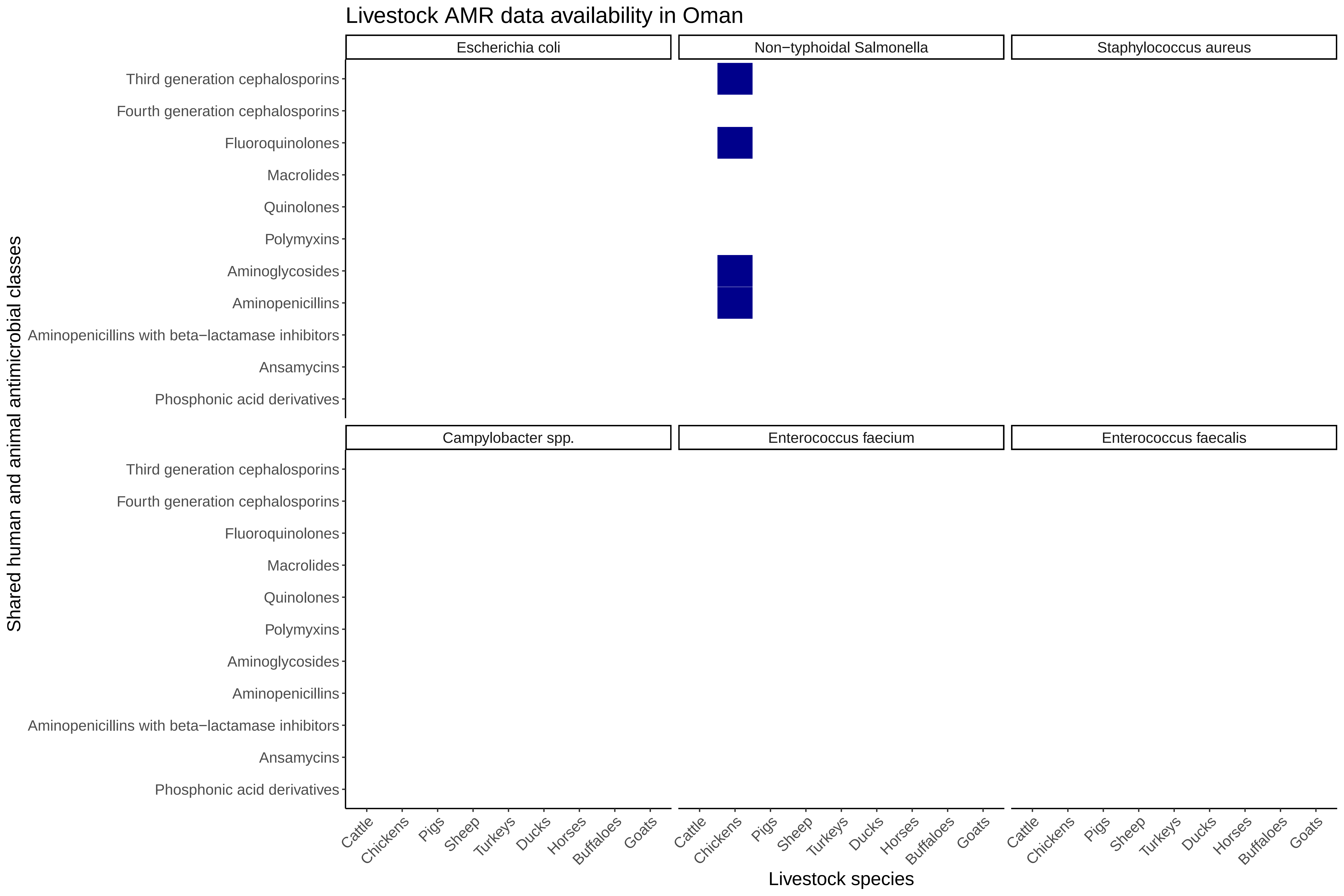

Livestock AMR data availability in Oman
Escherichia coli
Non−typhoidal Salmonella
Staphylococcus aureus
Campylobacter spp.
Enterococcus faecium
Enterococcus faecalis
Shared human and animal antimicrobial classes
Third generation cephalosporins
Fourth generation cephalosporins
Fluoroquinolones
Macrolides
Quinolones
Polymyxins
Aminoglycosides
Aminopenicillins
Aminopenicillins with beta−lactamase inhibitors
Ansamycins
Phosphonic acid derivatives
Cattle
Chickens
Pigs
Sheep
Turkeys
Ducks
Horses
Buffaloes
Goats
Livestock species

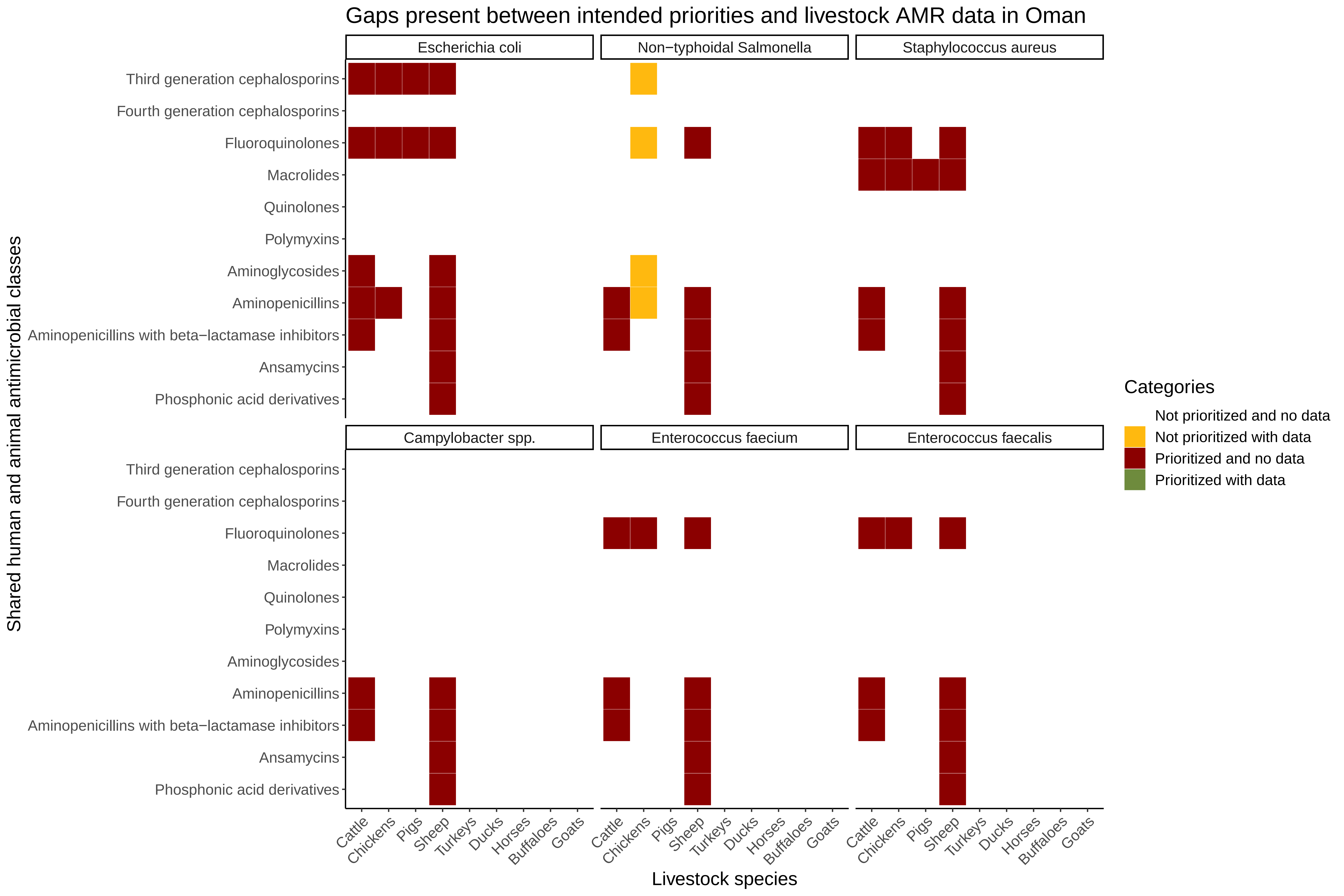

Gaps present between intended priorities and livestock AMR data in Oman
Escherichia coli
Non−typhoidal Salmonella
Staphylococcus aureus
Campylobacter spp.
Enterococcus faecium
Enterococcus faecalis
Third generation cephalosporins
Fourth generation cephalosporins
Fluoroquinolones
Macrolides
Quinolones
Polymyxins
Aminoglycosides
Aminopenicillins
Aminopenicillins with beta−lactamase inhibitors
Ansamycins
Phosphonic acid derivatives
Shared human and animal antimicrobial classes
Cattle
Chickens
Pigs
Sheep
Turkeys
Ducks
Horses
Buffaloes
Goats
Livestock species
Categories
Not prioritized and no data
Not prioritized with data
Prioritized and no data
Prioritized with data

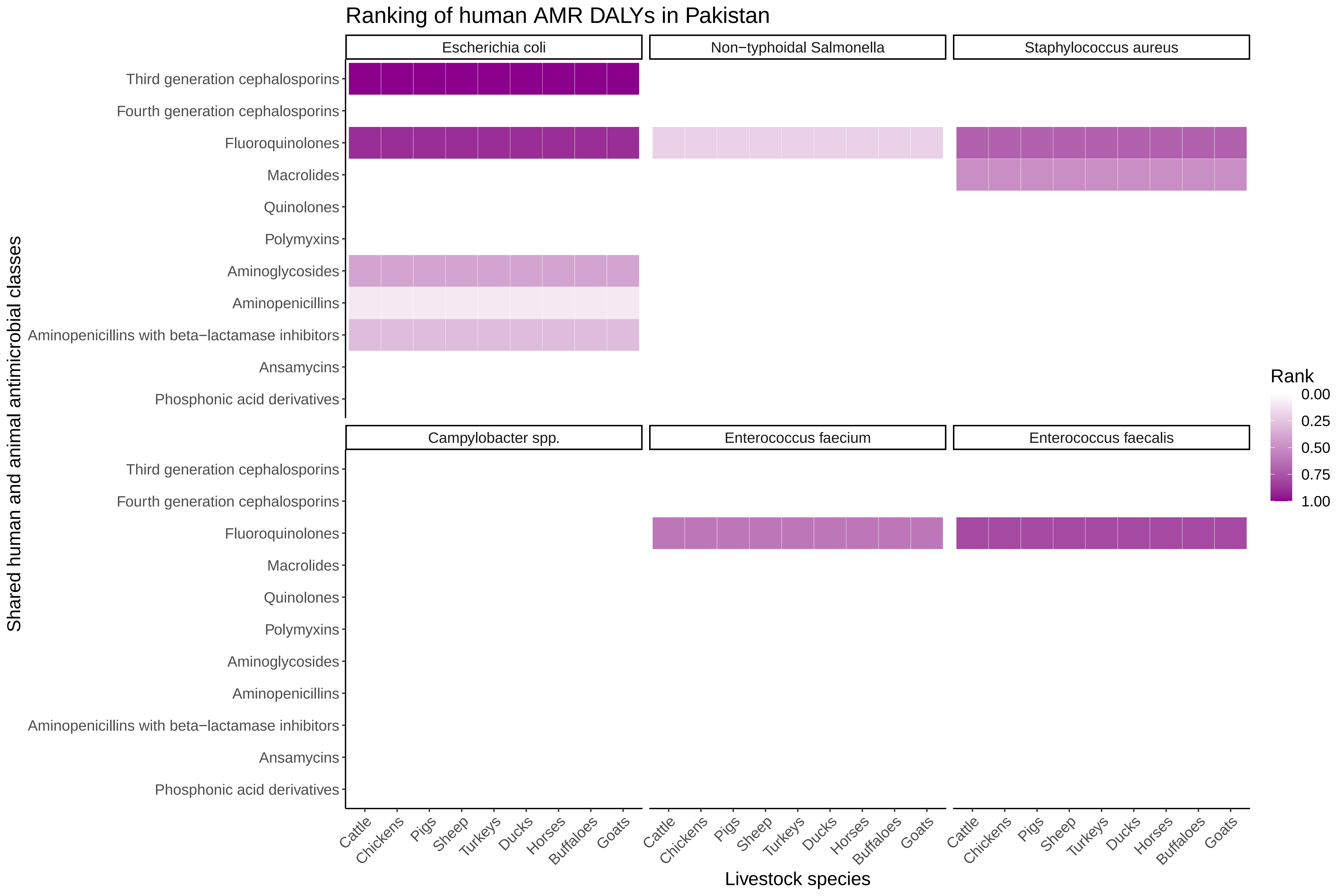

Ranking of human AMR DALYs in Pakistan
Escherichia coli
Non−typhoidal Salmonella
Staphylococcus aureus
Campylobacter spp.
Enterococcus faecium
Enterococcus faecalis
Shared human and animal antimicrobial classes
Third generation cephalosporins
Fourth generation cephalosporins
Fluoroquinolones
Macrolides
Quinolones
Polymyxins
Aminoglycosides
Aminopenicillins
Aminopenicillins with beta−lactamase inhibitors
Ansamycins
Phosphonic acid derivatives
Cattle
Chickens
Pigs
Sheep
Turkeys
Ducks
Horses
Buffaloes
Goats
Livestock species
Rank
0.00
0.25
0.50
0.75
1.00

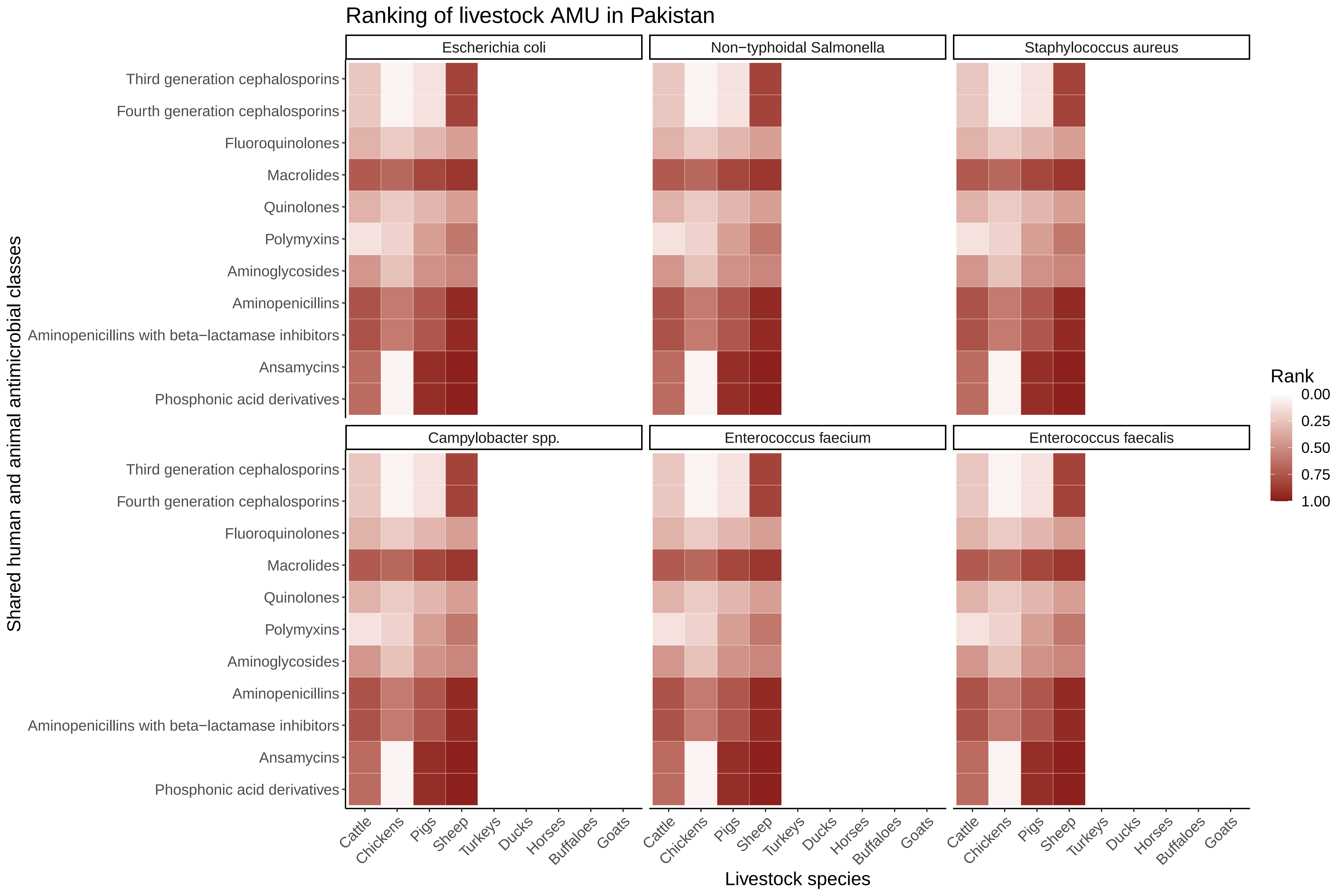
Ranking of livestock AMU in Pakistan
Escherichia coli
Non−typhoidal Salmonella
Staphylococcus aureus
Campylobacter spp.
Enterococcus faecium
Enterococcus faecalis
Shared human and animal antimicrobial classes
Third generation cephalosporins
Fourth generation cephalosporins
Fluoroquinolones
Macrolides
Quinolones
Polymyxins
Aminoglycosides
Aminopenicillins
Aminopenicillins with beta−lactamase inhibitors
Ansamycins
Phosphonic acid derivatives
Cattle
Chickens
Pigs
Sheep
Turkeys
Ducks
Horses
Buffaloes
Goats
Livestock species
Rank
0.00
0.25
0.50
0.75
1.00

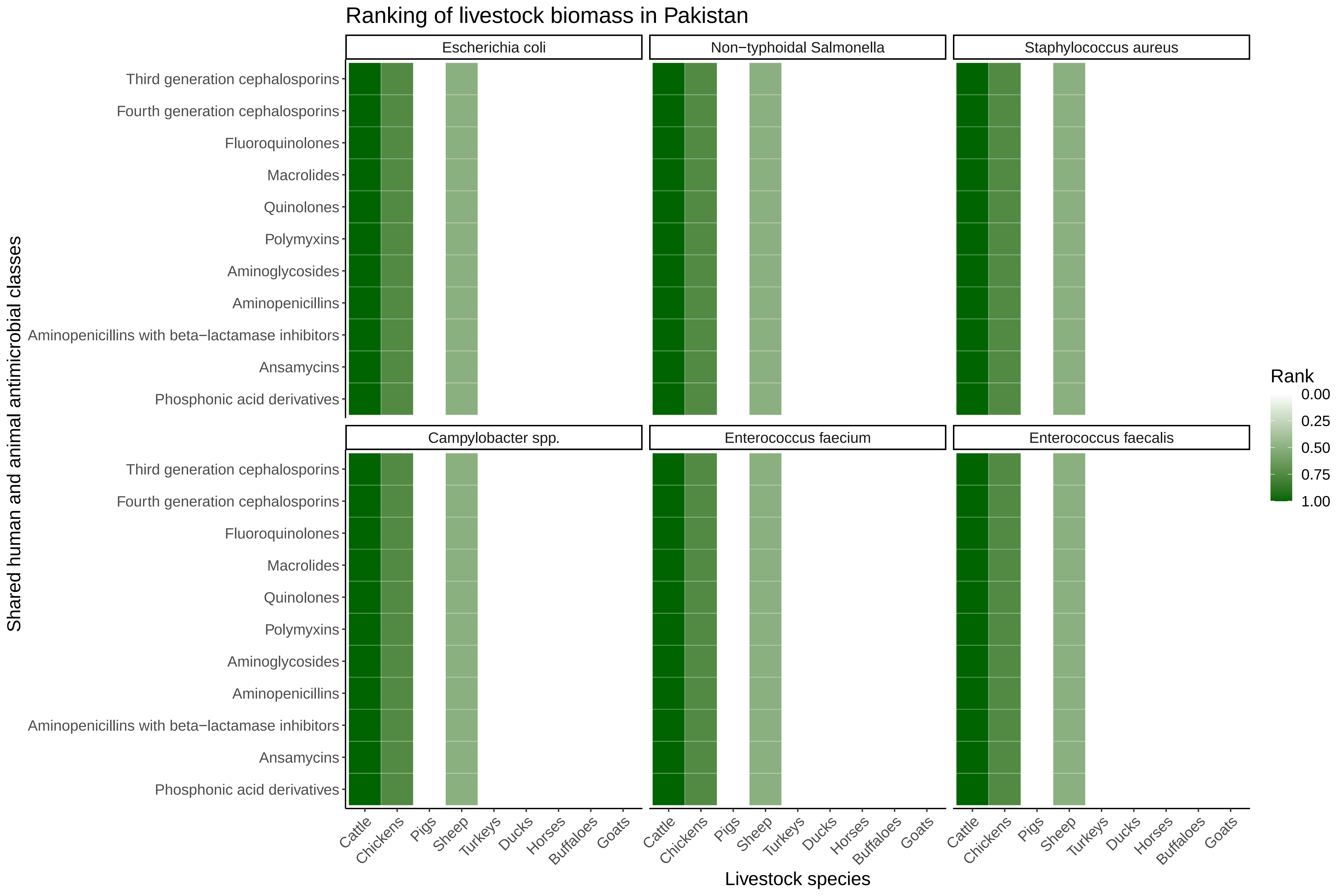

Ranking of livestock biomass in Pakistan
Escherichia coli
Non−typhoidal Salmonella
Staphylococcus aureus
Campylobacter spp.
Enterococcus faecium
Enterococcus faecalis
Shared human and animal antimicrobial classes
Third generation cephalosporins
Fourth generation cephalosporins
Fluoroquinolones
Macrolides
Quinolones
Polymyxins
Aminoglycosides
Aminopenicillins
Aminopenicillins with beta−lactamase inhibitors
Ansamycins
Phosphonic acid derivatives
Livestock species
Cattle
Chickens
Pigs
Sheep
Turkeys
Ducks
Horses
Buffaloes
Goats
Rank
0.00
0.25
0.50
0.75
1.00

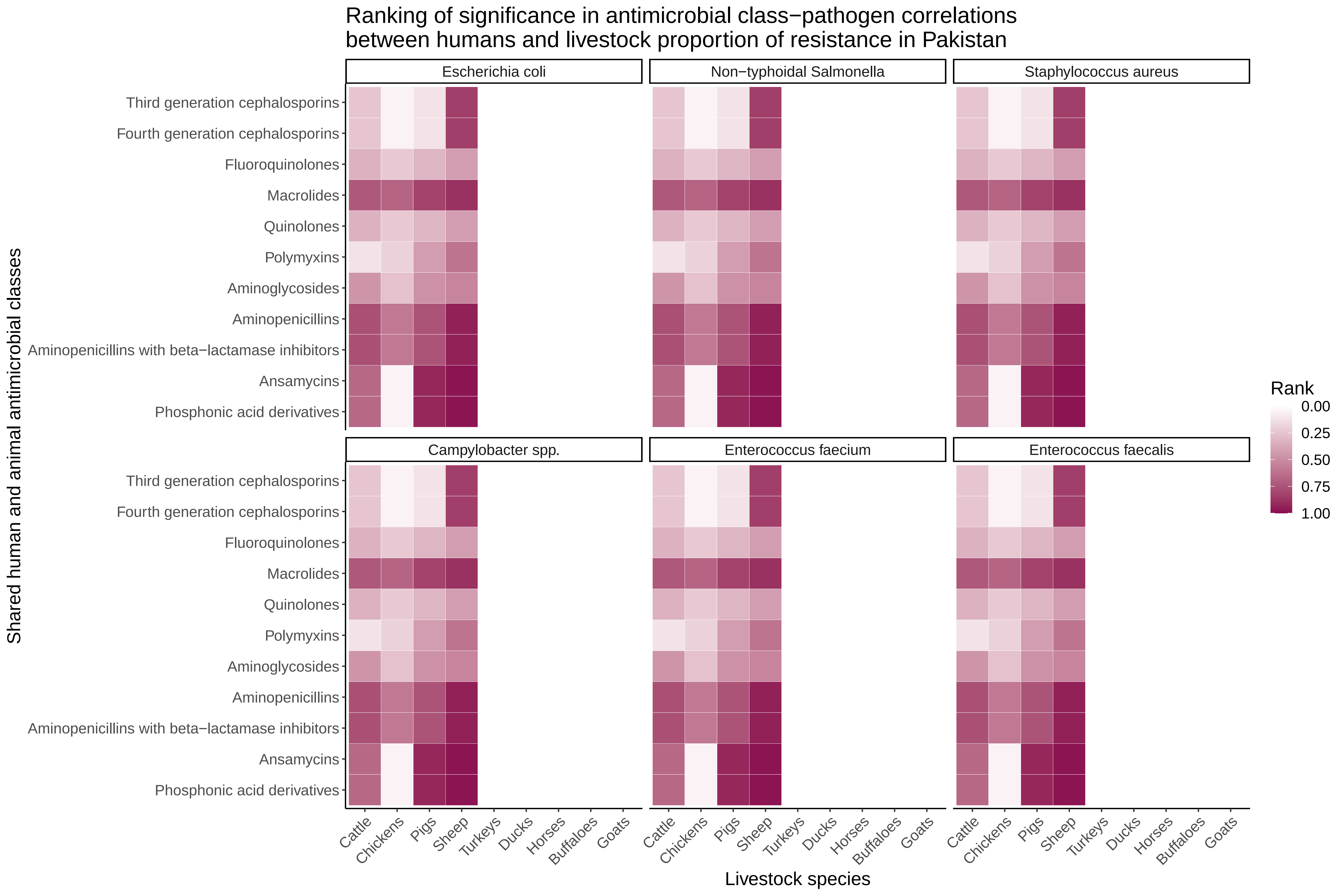

Ranking of significance in antimicrobial class−pathogen correlations
between humans and livestock proportion of resistance in Pakistan
Escherichia coli
Non−typhoidal Salmonella
Staphylococcus aureus
Campylobacter spp.
Enterococcus faecium
Enterococcus faecalis
Third generation cephalosporins
Fourth generation cephalosporins
Fluoroquinolones
Macrolides
Quinolones
Polymyxins
Aminoglycosides
Aminopenicillins
Aminopenicillins with beta−lactamase inhibitors
Ansamycins
Phosphonic acid derivatives
Shared human and animal antimicrobial classes
Cattle
Chickens
Pigs
Sheep
Turkeys
Ducks
Horses
Buffaloes
Goats
Livestock species
Rank
0.00
0.25
0.50
0.75
1.00

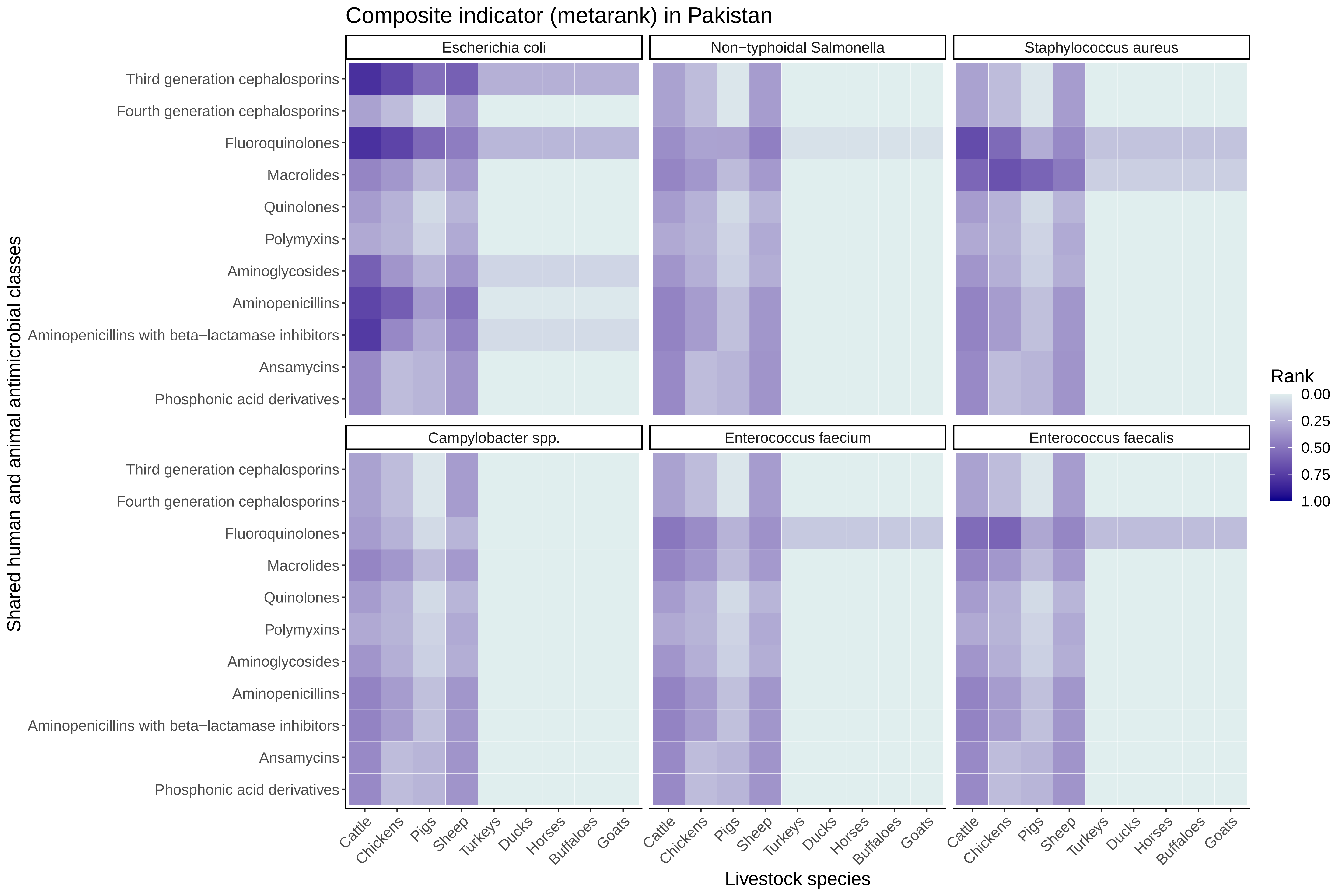

Composite indicator (metarank) in Pakistan
Escherichia coli
Non−typhoidal Salmonella
Staphylococcus aureus
Campylobacter spp.
Enterococcus faecium
Enterococcus faecalis
Third generation cephalosporins
Fourth generation cephalosporins
Fluoroquinolones
Macrolides
Quinolones
Polymyxins
Aminoglycosides
Aminopenicillins
Aminopenicillins with beta−lactamase inhibitors
Ansamycins
Phosphonic acid derivatives
Shared human and animal antimicrobial classes
Cattle
Chickens
Pigs
Sheep
Turkeys
Ducks
Horses
Buffaloes
Goats
Livestock species
Rank
0.00
0.25
0.50
0.75
1.00

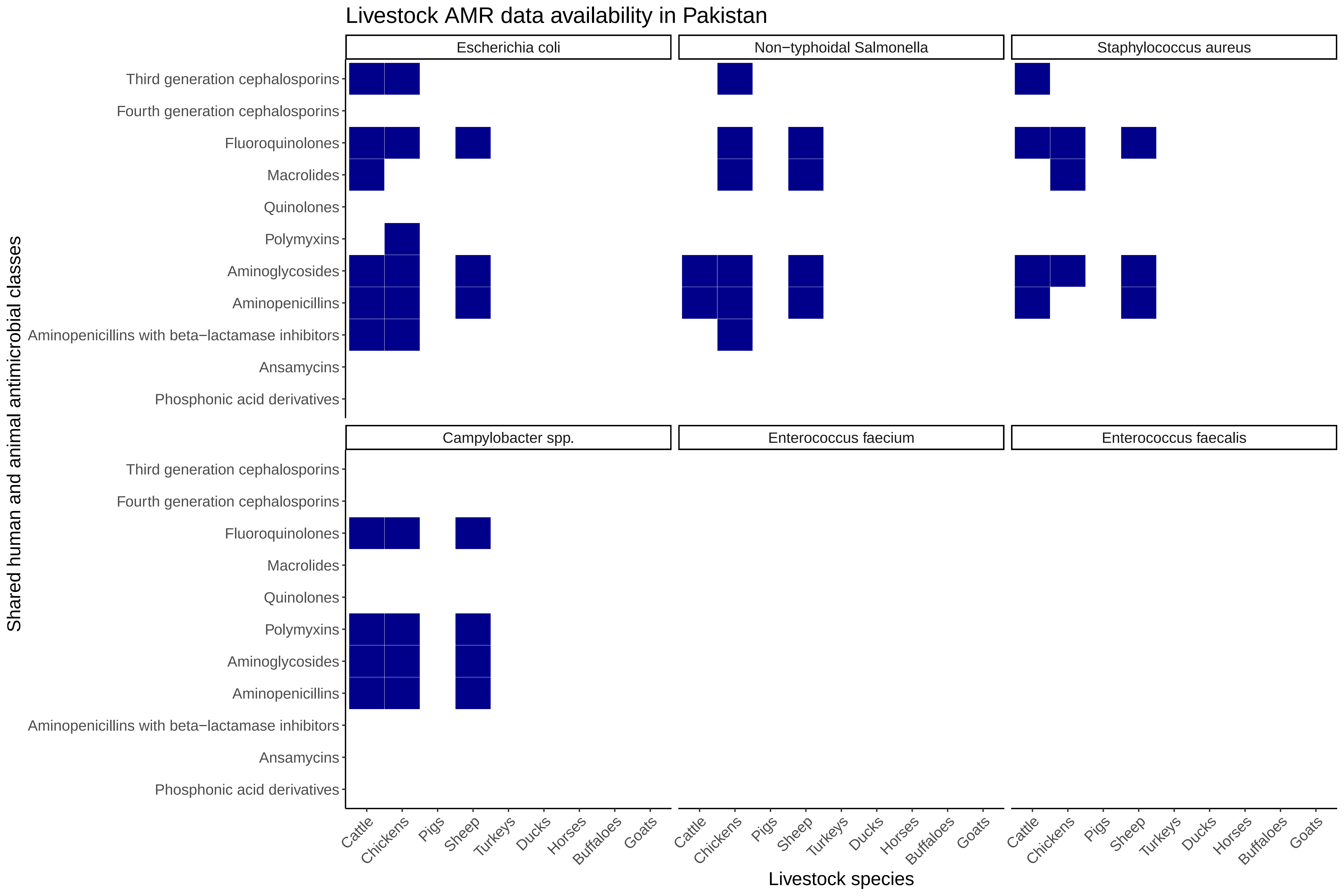

Livestock AMR data availability in Pakistan
Escherichia coli
Non−typhoidal Salmonella
Staphylococcus aureus
Campylobacter spp.
Enterococcus faecium
Enterococcus faecalis
Shared human and animal antimicrobial classes
Third generation cephalosporins
Fourth generation cephalosporins
Fluoroquinolones
Macrolides
Quinolones
Polymyxins
Aminoglycosides
Aminopenicillins
Aminopenicillins with beta−lactamase inhibitors
Ansamycins
Phosphonic acid derivatives
Cattle
Chickens
Pigs
Sheep
Turkeys
Ducks
Horses
Buffaloes
Goats
Livestock species

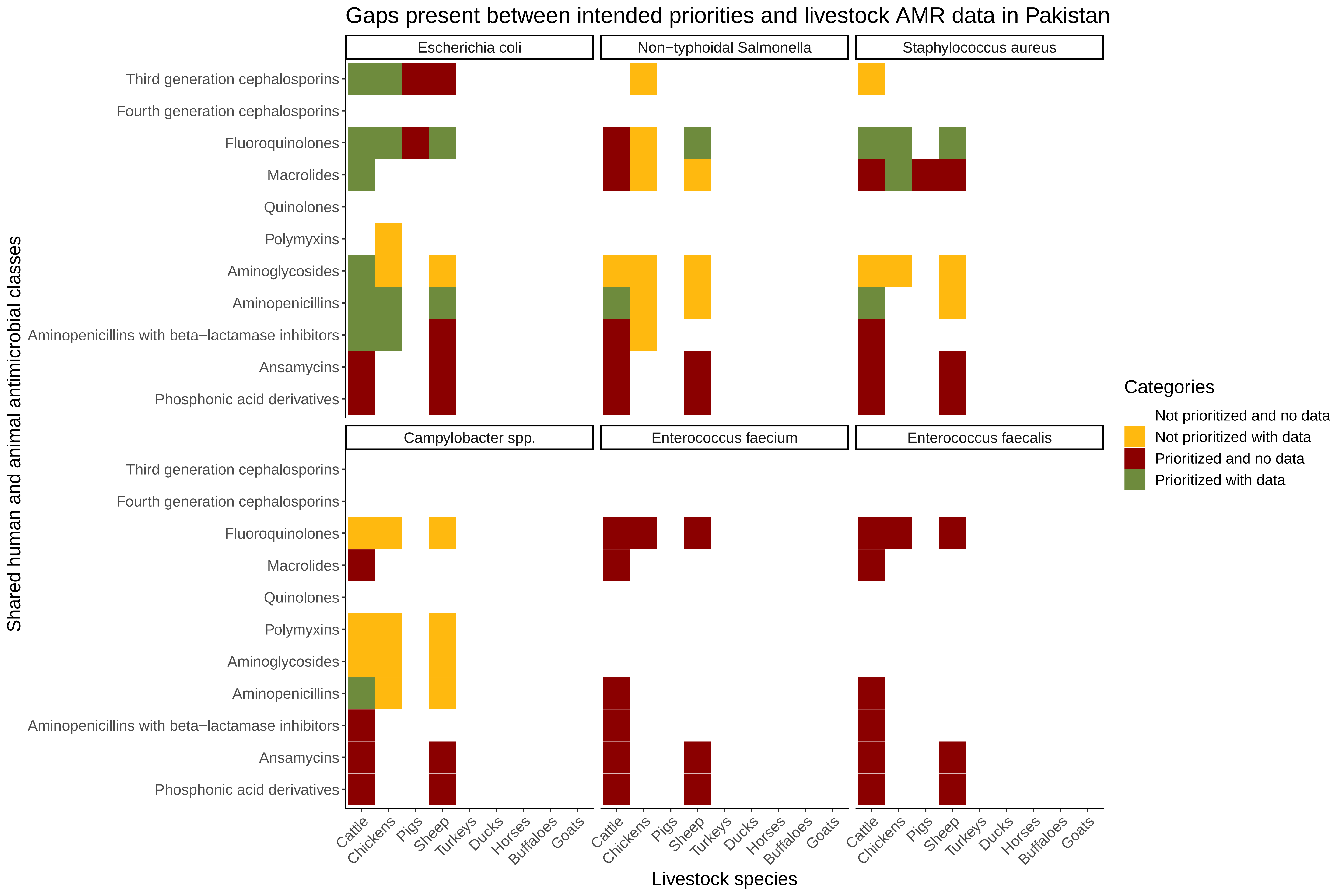
Gaps present between intended priorities and livestock AMR data in Pakistan
Escherichia coli
Non−typhoidal Salmonella
Staphylococcus aureus
Campylobacter spp.
Enterococcus faecium
Enterococcus faecalis
Third generation cephalosporins
Fourth generation cephalosporins
Fluoroquinolones
Macrolides
Quinolones
Polymyxins
Aminoglycosides
Aminopenicillins
Aminopenicillins with beta−lactamase inhibitors
Ansamycins
Phosphonic acid derivatives
Shared human and animal antimicrobial classes
Cattle
Chickens
Pigs
Sheep
Turkeys
Ducks
Horses
Buffaloes
Goats
Livestock species
Categories
Not prioritized and no data
Not prioritized with data
Prioritized and no data
Prioritized with data

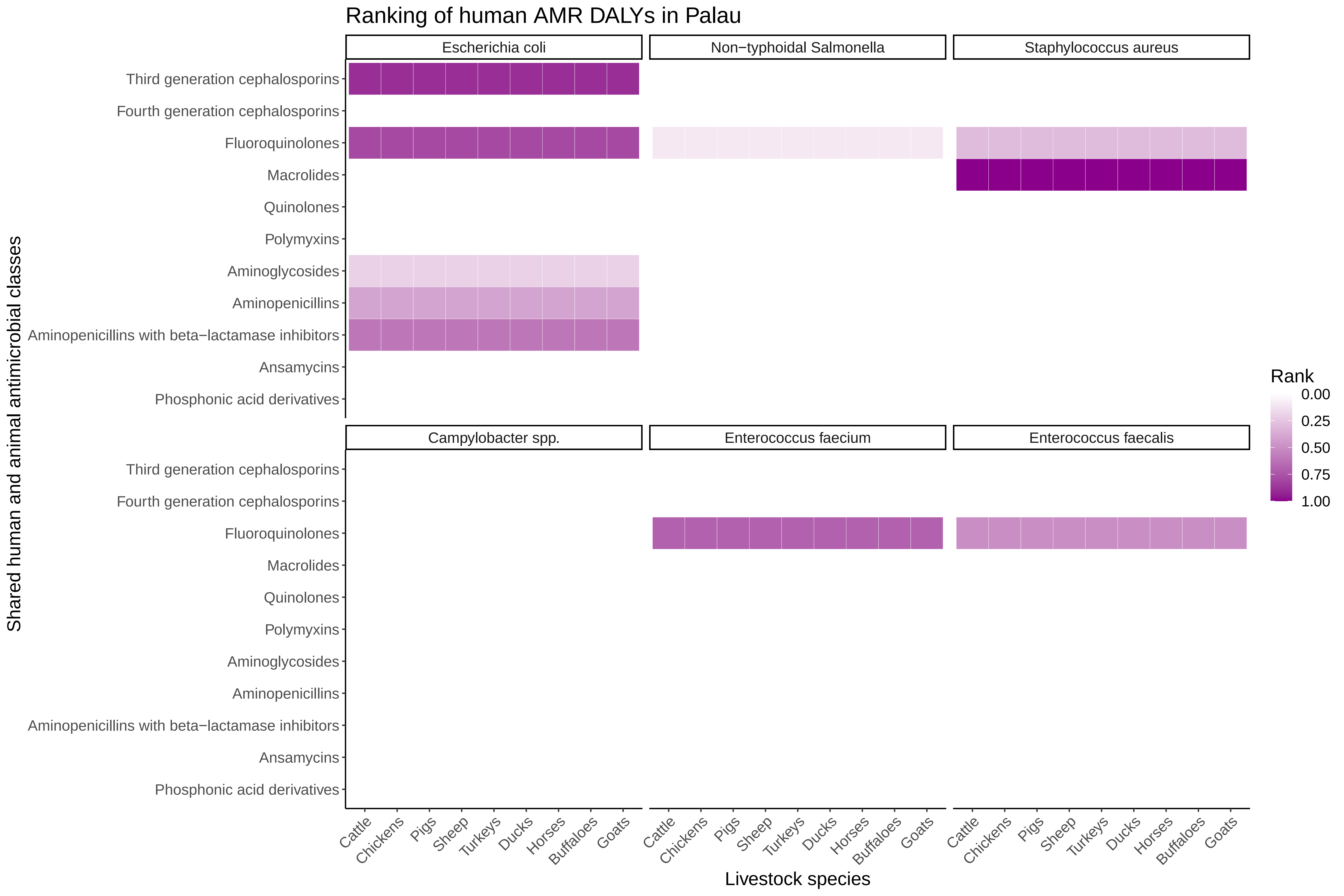

Ranking of human AMR DALYs in Palau
Escherichia coli
Non−typhoidal Salmonella
Staphylococcus aureus
Campylobacter spp.
Enterococcus faecium
Enterococcus faecalis
Third generation cephalosporins
Fourth generation cephalosporins
Fluoroquinolones
Macrolides
Quinolones
Polymyxins
Aminoglycosides
Aminopenicillins
Aminopenicillins with beta−lactamase inhibitors
Ansamycins
Phosphonic acid derivatives
Shared human and animal antimicrobial classes
Cattle
Chickens
Pigs
Sheep
Turkeys
Ducks
Horses
Buffaloes
Goats
Livestock species
Rank
0.00
0.25
0.50
0.75
1.00

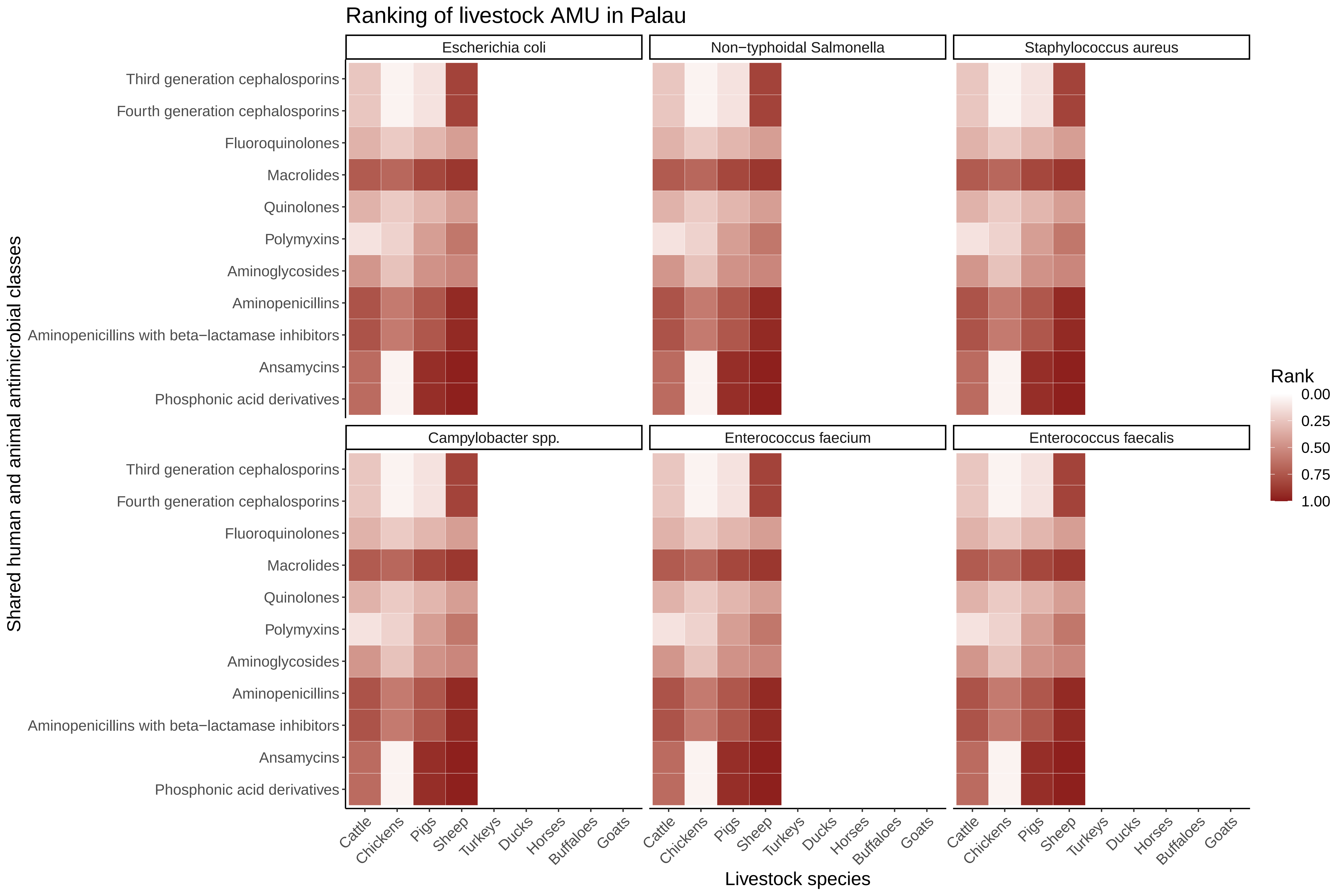
Ranking of livestock AMU in Palau
Escherichia coli
Non−typhoidal Salmonella
Staphylococcus aureus
Campylobacter spp.
Enterococcus faecium
Enterococcus faecalis
Third generation cephalosporins
Fourth generation cephalosporins
Fluoroquinolones
Macrolides
Quinolones
Polymyxins
Aminoglycosides
Aminopenicillins
Aminopenicillins with beta−lactamase inhibitors
Ansamycins
Phosphonic acid derivatives
Shared human and animal antimicrobial classes
Cattle
Chickens
Pigs
Sheep
Turkeys
Ducks
Horses
Buffaloes
Goats
Livestock species
Rank
0.00
0.25
0.50
0.75
1.00

Ranking of livestock biomass in Palau

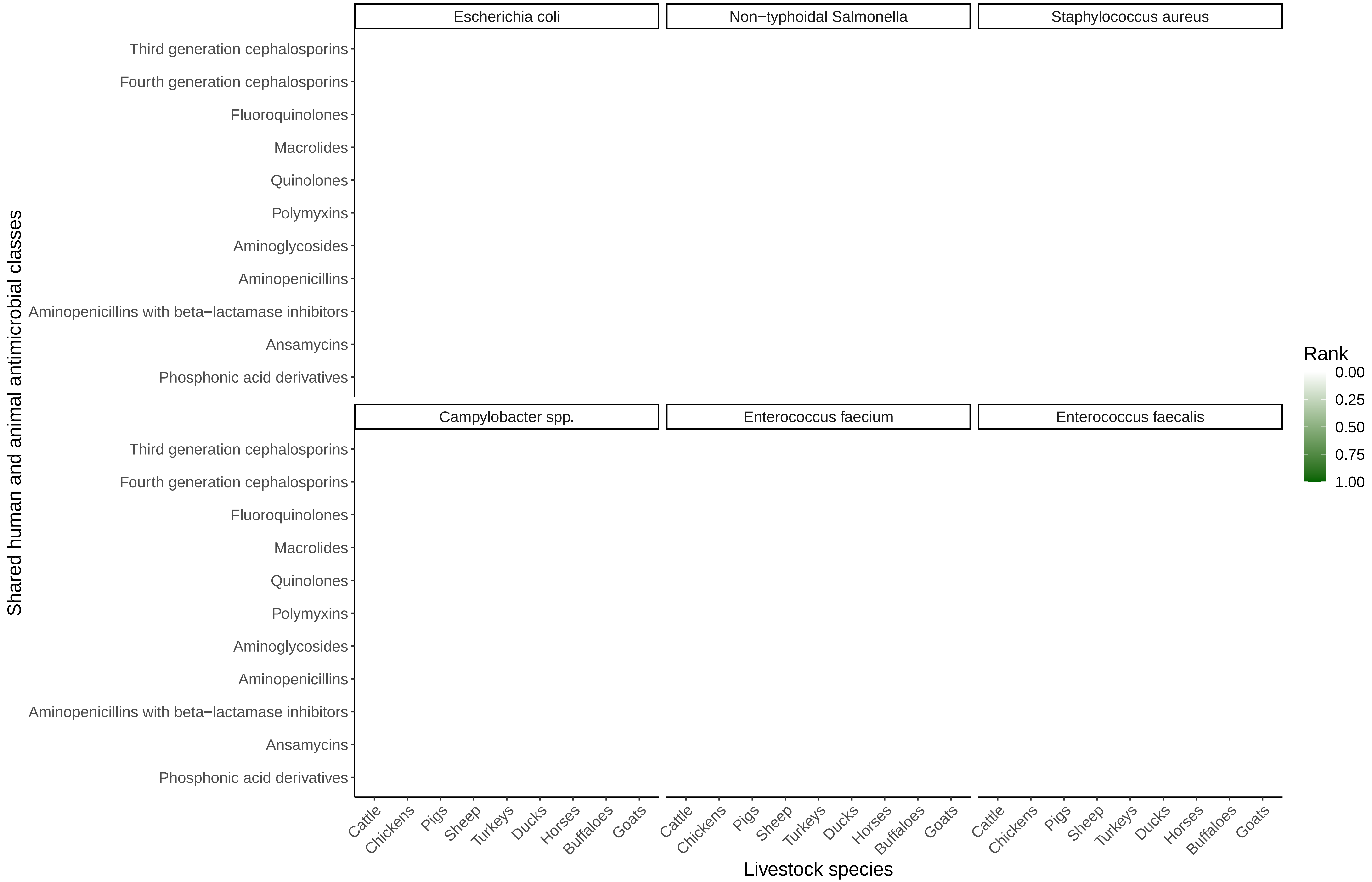

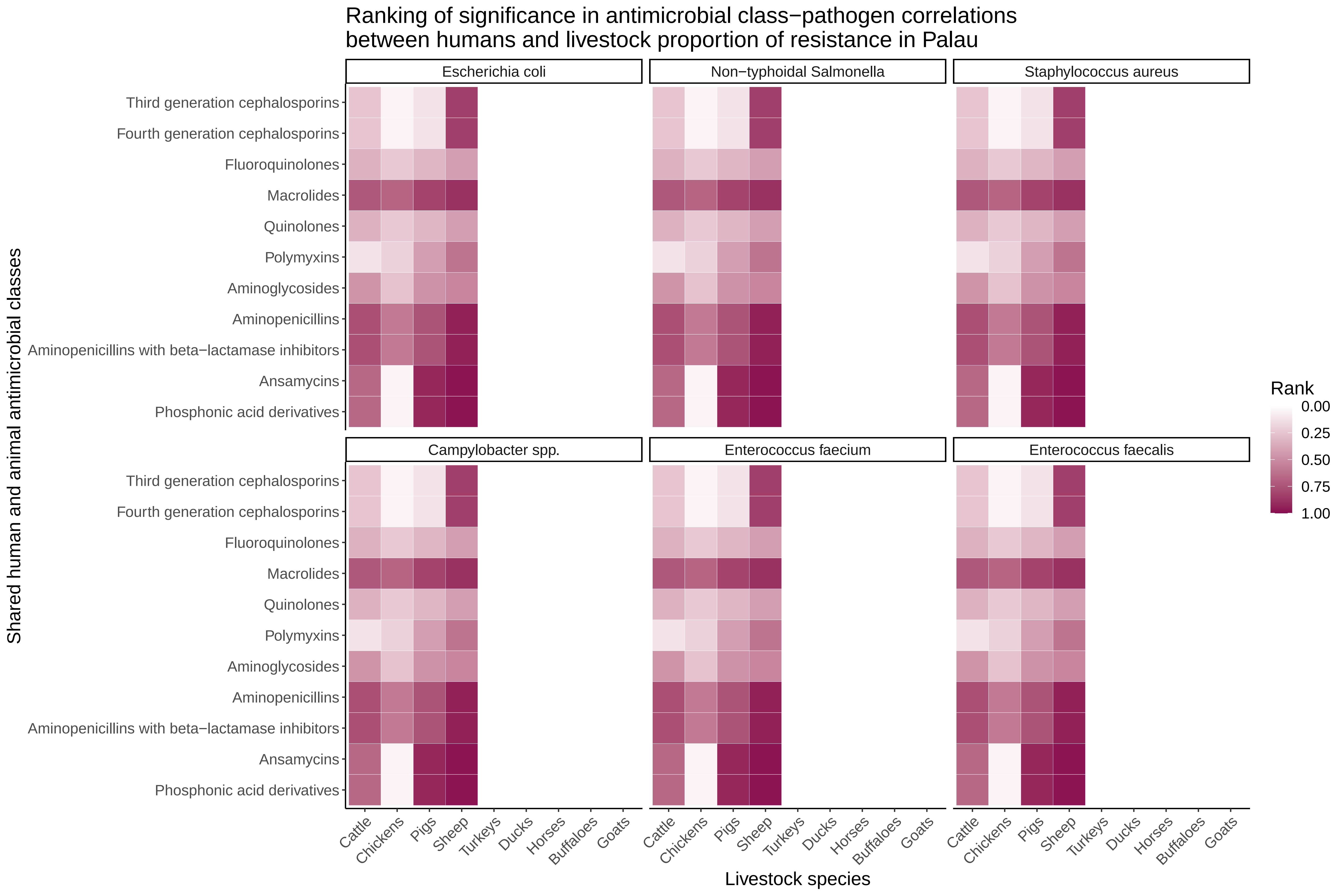

Ranking of significance in antimicrobial class−pathogen correlations
between humans and livestock proportion of resistance in Palau
Escherichia coli
Non−typhoidal Salmonella
Staphylococcus aureus
Campylobacter spp.
Enterococcus faecium
Enterococcus faecalis
Third generation cephalosporins
Fourth generation cephalosporins
Fluoroquinolones
Macrolides
Quinolones
Polymyxins
Aminoglycosides
Aminopenicillins
Aminopenicillins with beta−lactamase inhibitors
Ansamycins
Phosphonic acid derivatives
Shared human and animal antimicrobial classes
Cattle
Chickens
Pigs
Sheep
Turkeys
Ducks
Horses
Buffaloes
Goats
Livestock species
Rank
0.00
0.25
0.50
0.75
1.00

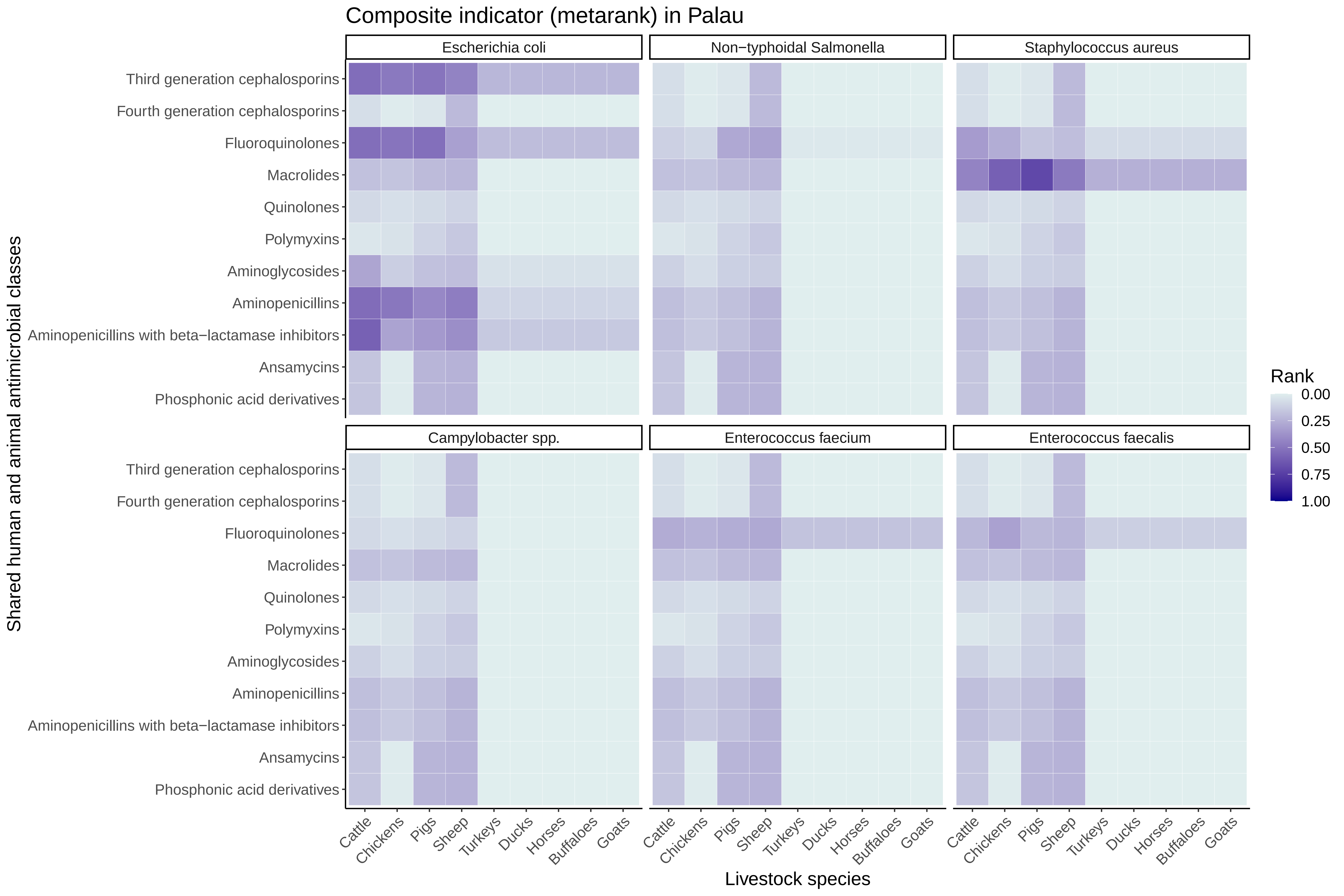

Composite indicator (metarank) in Palau
Escherichia coli
Non−typhoidal Salmonella
Staphylococcus aureus
Campylobacter spp.
Enterococcus faecium
Enterococcus faecalis
Shared human and animal antimicrobial classes
Third generation cephalosporins
Fourth generation cephalosporins
Fluoroquinolones
Macrolides
Quinolones
Polymyxins
Aminoglycosides
Aminopenicillins
Aminopenicillins with beta−lactamase inhibitors
Ansamycins
Phosphonic acid derivatives
Cattle
Chickens
Pigs
Sheep
Turkeys
Ducks
Horses
Buffaloes
Goats
Livestock species
Rank
0.00
0.25
0.50
0.75
1.00

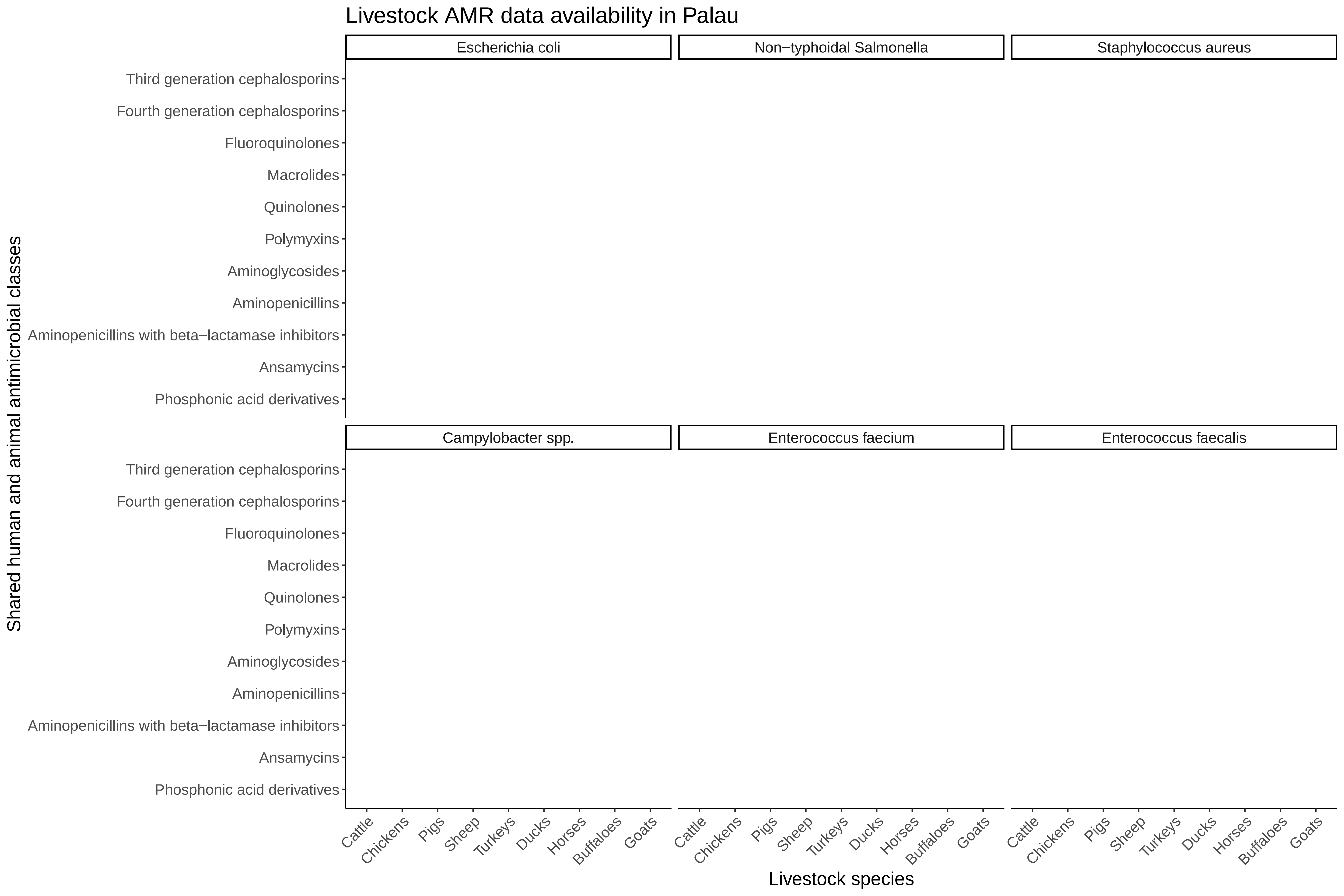
Livestock AMR data availability in Palau
Escherichia coli
Non−typhoidal Salmonella
Staphylococcus aureus
Campylobacter spp.
Enterococcus faecium
Enterococcus faecalis
Shared human and animal antimicrobial classes
Third generation cephalosporins
Fourth generation cephalosporins
Fluoroquinolones
Macrolides
Quinolones
Polymyxins
Aminoglycosides
Aminopenicillins
Aminopenicillins with beta−lactamase inhibitors
Ansamycins
Phosphonic acid derivatives
Cattle
Chickens
Pigs
Sheep
Turkeys
Ducks
Horses
Buffaloes
Goats
Livestock species

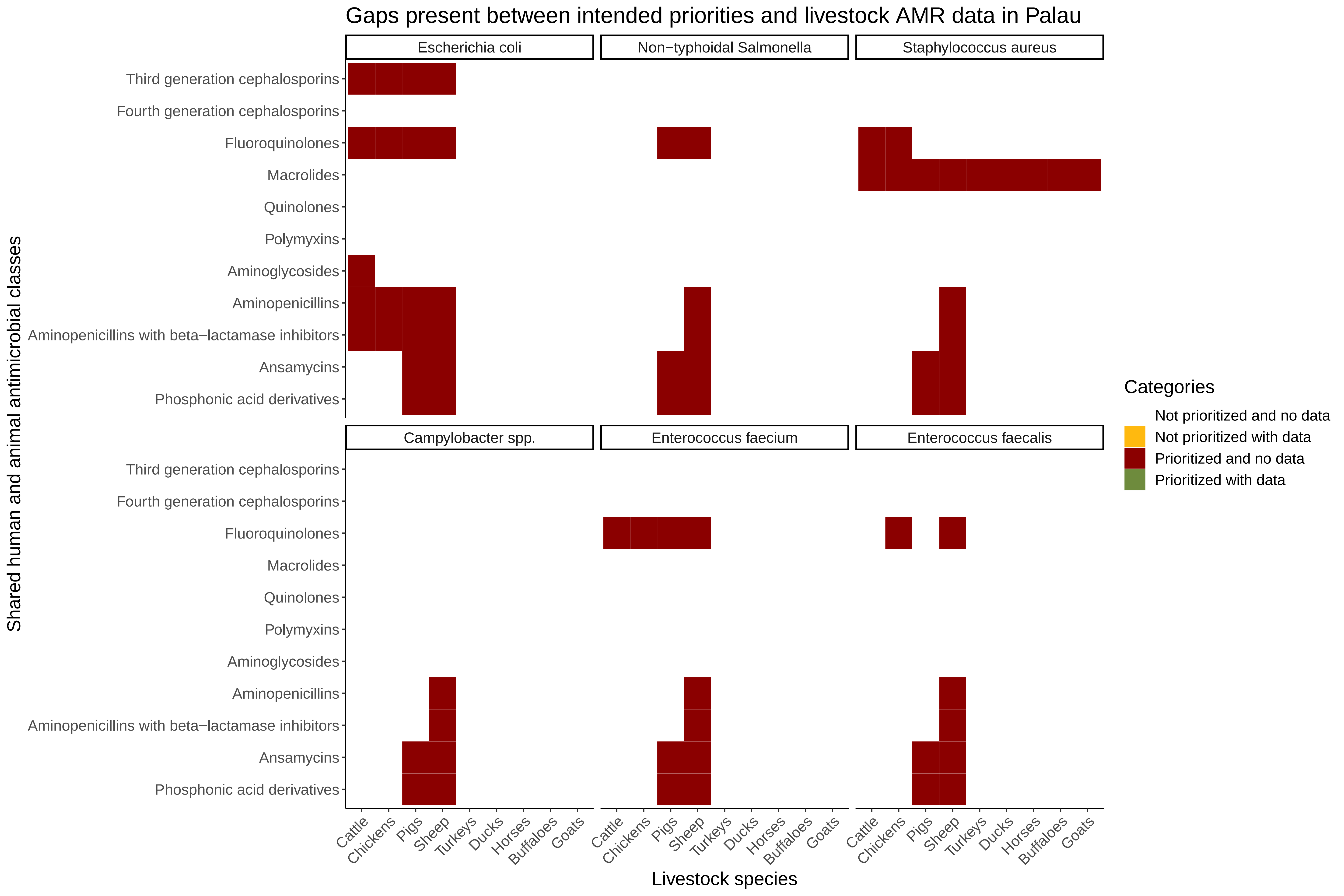
Gaps present between intended priorities and livestock AMR data in Palau
Escherichia coli
Non−typhoidal Salmonella
Staphylococcus aureus
Campylobacter spp.
Enterococcus faecium
Enterococcus faecalis
Shared human and animal antimicrobial classes
Third generation cephalosporins
Fourth generation cephalosporins
Fluoroquinolones
Macrolides
Quinolones
Polymyxins
Aminoglycosides
Aminopenicillins
Aminopenicillins with beta−lactamase inhibitors
Ansamycins
Phosphonic acid derivatives
Cattle
Chickens
Pigs
Sheep
Turkeys
Ducks
Horses
Buffaloes
Goats
Livestock species
Categories
Not prioritized and no data
Not prioritized with data
Prioritized and no data
Prioritized with data

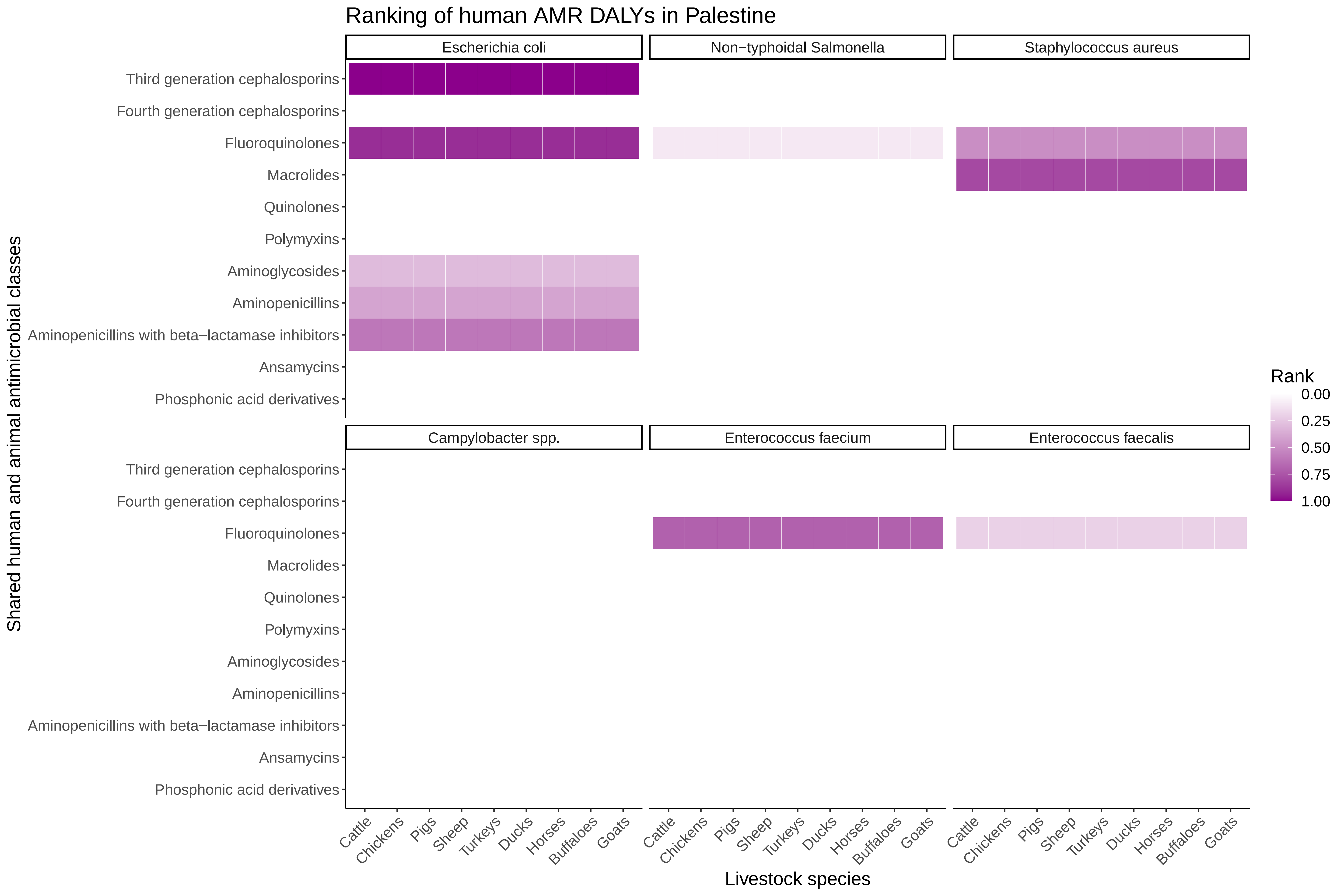
Ranking of human AMR DALYs in Palestine
Escherichia coli
Non−typhoidal Salmonella
Staphylococcus aureus
Campylobacter spp.
Enterococcus faecium
Enterococcus faecalis
Third generation cephalosporins
Fourth generation cephalosporins
Fluoroquinolones
Macrolides
Quinolones
Polymyxins
Aminoglycosides
Aminopenicillins
Aminopenicillins with beta−lactamase inhibitors
Ansamycins
Phosphonic acid derivatives
Shared human and animal antimicrobial classes
Cattle
Chickens
Pigs
Sheep
Turkeys
Ducks
Horses
Buffaloes
Goats
Livestock species
Rank
0.00
0.25
0.50
0.75
1.00

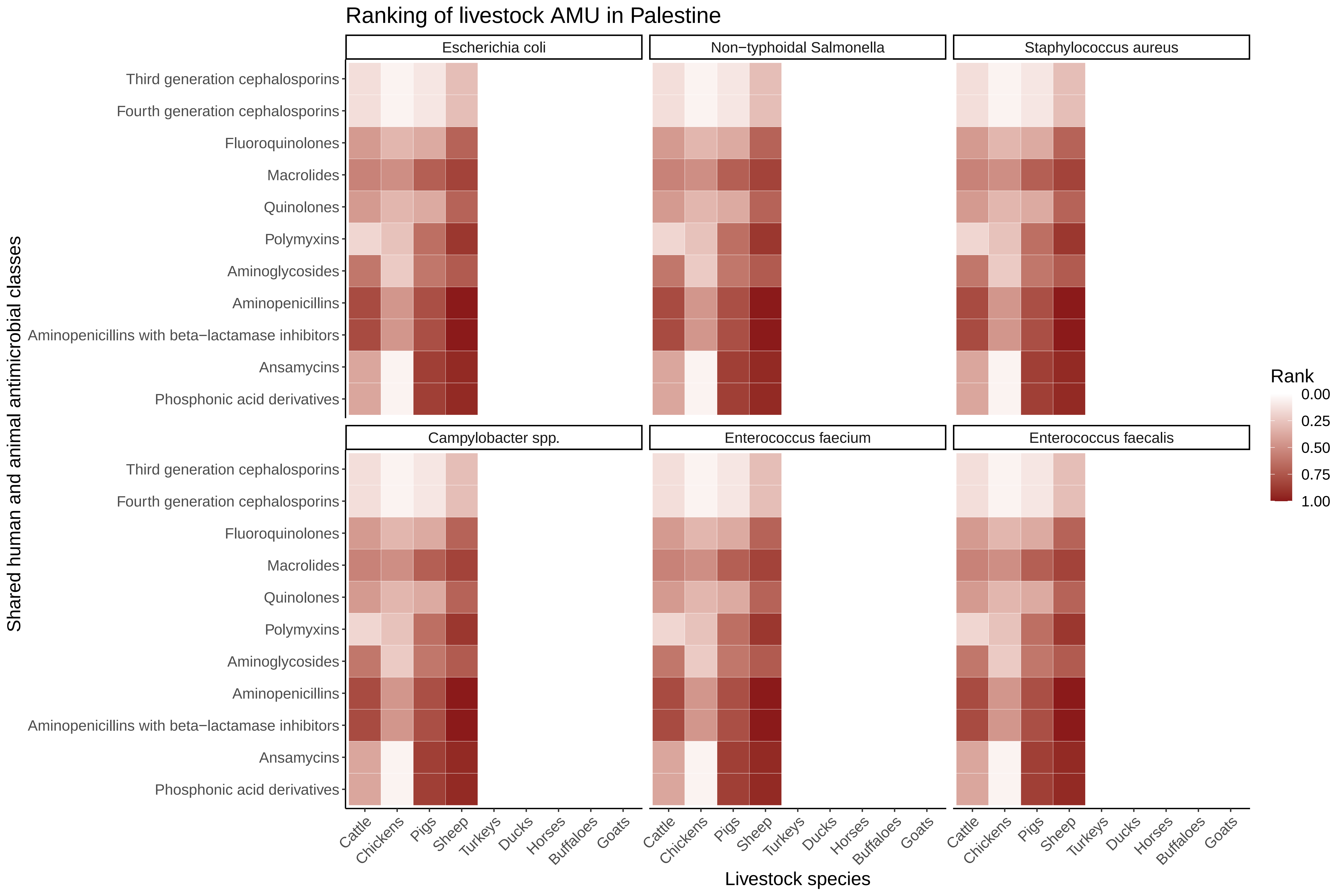

Ranking of livestock AMU in Palestine
Escherichia coli
Non−typhoidal Salmonella
Staphylococcus aureus
Campylobacter spp.
Enterococcus faecium
Enterococcus faecalis
Third generation cephalosporins
Fourth generation cephalosporins
Fluoroquinolones
Macrolides
Quinolones
Polymyxins
Aminoglycosides
Aminopenicillins
Aminopenicillins with beta−lactamase inhibitors
Ansamycins
Phosphonic acid derivatives
Shared human and animal antimicrobial classes
Cattle
Chickens
Pigs
Sheep
Turkeys
Ducks
Horses
Buffaloes
Goats
Livestock species
Rank
0.00
0.25
0.50
0.75
1.00

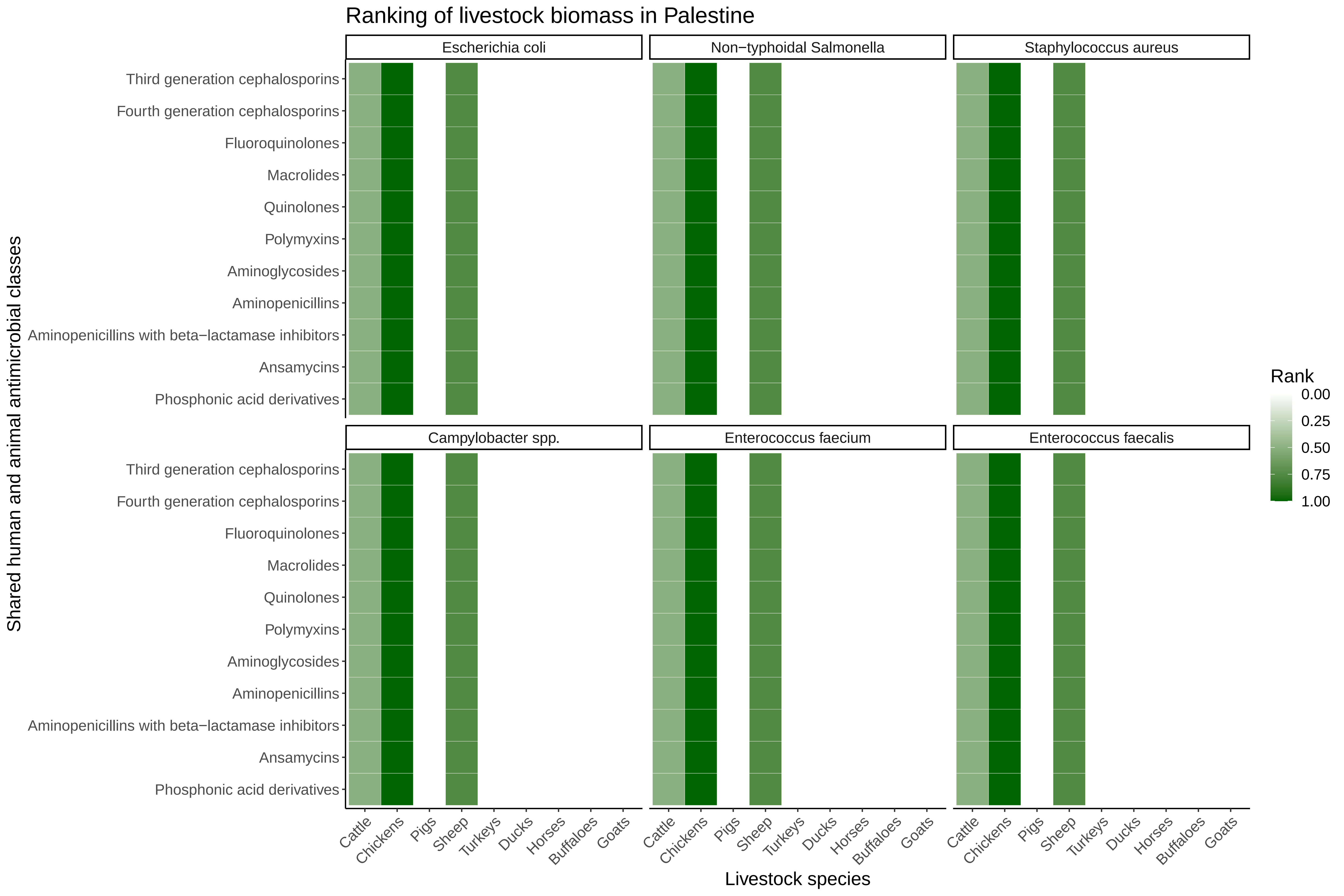
Ranking of livestock biomass in Palestine
Escherichia coli
Non−typhoidal Salmonella
Staphylococcus aureus
Campylobacter spp.
Enterococcus faecium
Enterococcus faecalis
Third generation cephalosporins
Fourth generation cephalosporins
Fluoroquinolones
Macrolides
Quinolones
Polymyxins
Aminoglycosides
Aminopenicillins
Aminopenicillins with beta−lactamase inhibitors
Ansamycins
Phosphonic acid derivatives
Shared human and animal antimicrobial classes
Cattle
Chickens
Pigs
Sheep
Turkeys
Ducks
Horses
Buffaloes
Goats
Livestock species
Rank
0.00
0.25
0.50
0.75
1.00

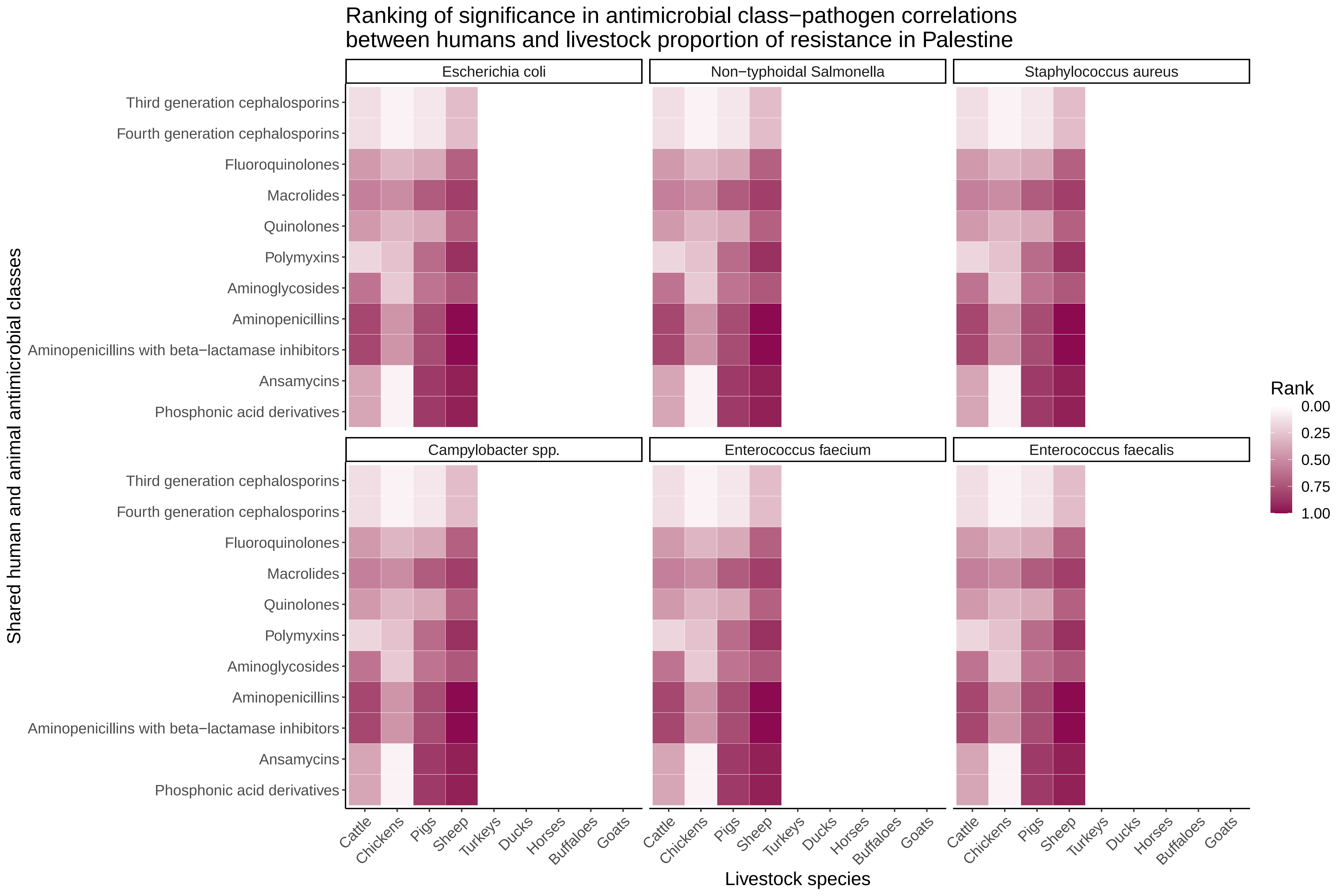

Ranking of significance in antimicrobial class−pathogen correlations
between humans and livestock proportion of resistance in Palestine
Escherichia coli
Non−typhoidal Salmonella
Staphylococcus aureus
Campylobacter spp.
Enterococcus faecium
Enterococcus faecalis
Third generation cephalosporins
Fourth generation cephalosporins
Fluoroquinolones
Macrolides
Quinolones
Polymyxins
Aminoglycosides
Aminopenicillins
Aminopenicillins with beta−lactamase inhibitors
Ansamycins
Phosphonic acid derivatives
Shared human and animal antimicrobial classes
Cattle
Chickens
Pigs
Sheep
Turkeys
Ducks
Horses
Buffaloes
Goats
Livestock species
Rank
0.00
0.25
0.50
0.75
1.00

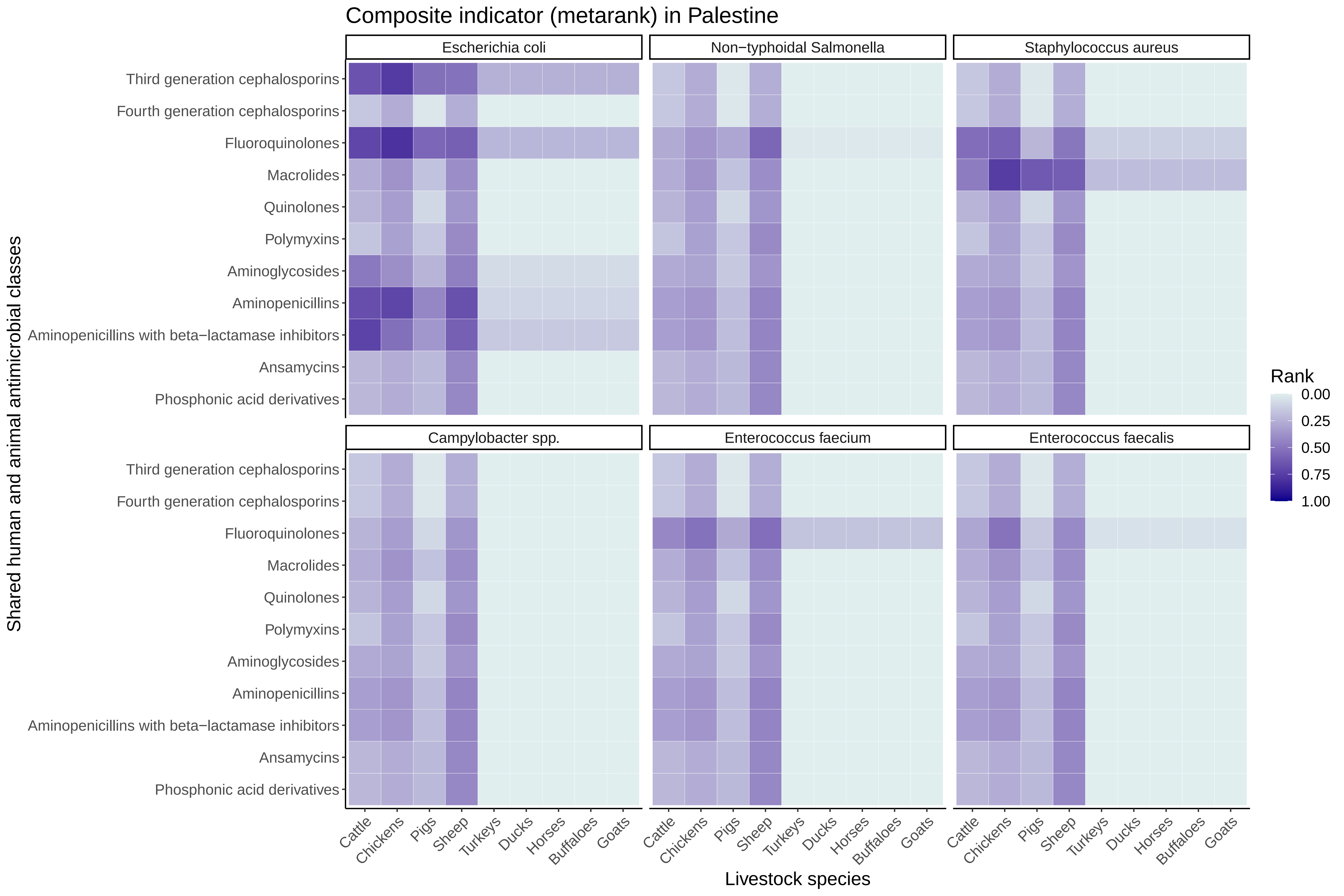

Composite indicator (metarank) in Palestine
Escherichia coli
Non−typhoidal Salmonella
Staphylococcus aureus
Campylobacter spp.
Enterococcus faecium
Enterococcus faecalis
Shared human and animal antimicrobial classes
Third generation cephalosporins
Fourth generation cephalosporins
Fluoroquinolones
Macrolides
Quinolones
Polymyxins
Aminoglycosides
Aminopenicillins
Aminopenicillins with beta−lactamase inhibitors
Ansamycins
Phosphonic acid derivatives
Cattle
Chickens
Pigs
Sheep
Turkeys
Ducks
Horses
Buffaloes
Goats
Livestock species
Rank
0.00
0.25
0.50
0.75
1.00

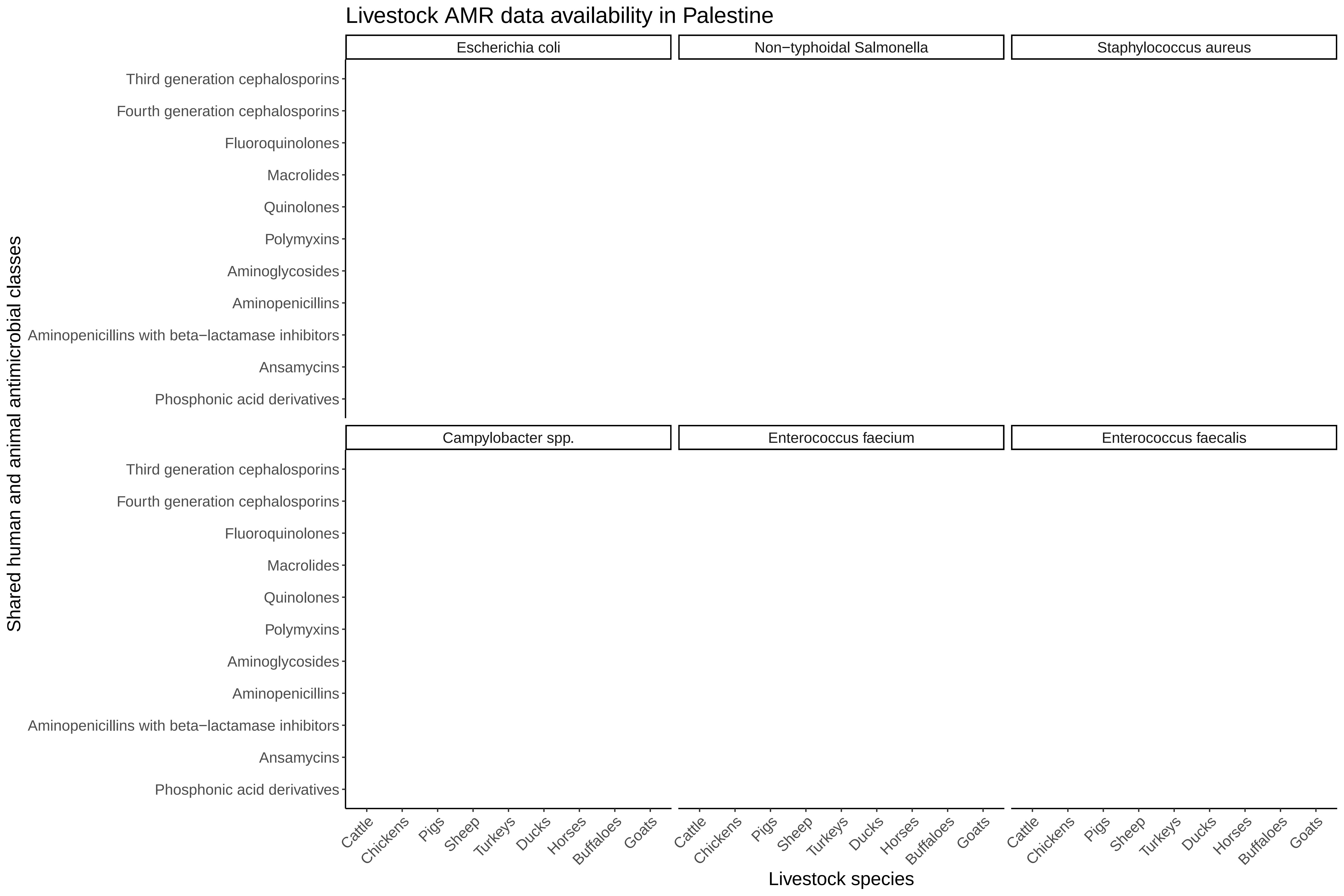
Livestock AMR data availability in Palestine
Escherichia coli
Non−typhoidal Salmonella
Staphylococcus aureus
Campylobacter spp.
Enterococcus faecium
Enterococcus faecalis
Shared human and animal antimicrobial classes
Third generation cephalosporins
Fourth generation cephalosporins
Fluoroquinolones
Macrolides
Quinolones
Polymyxins
Aminoglycosides
Aminopenicillins
Aminopenicillins with beta−lactamase inhibitors
Ansamycins
Phosphonic acid derivatives
Cattle
Chickens
Pigs
Sheep
Turkeys
Ducks
Horses
Buffaloes
Goats
Livestock species

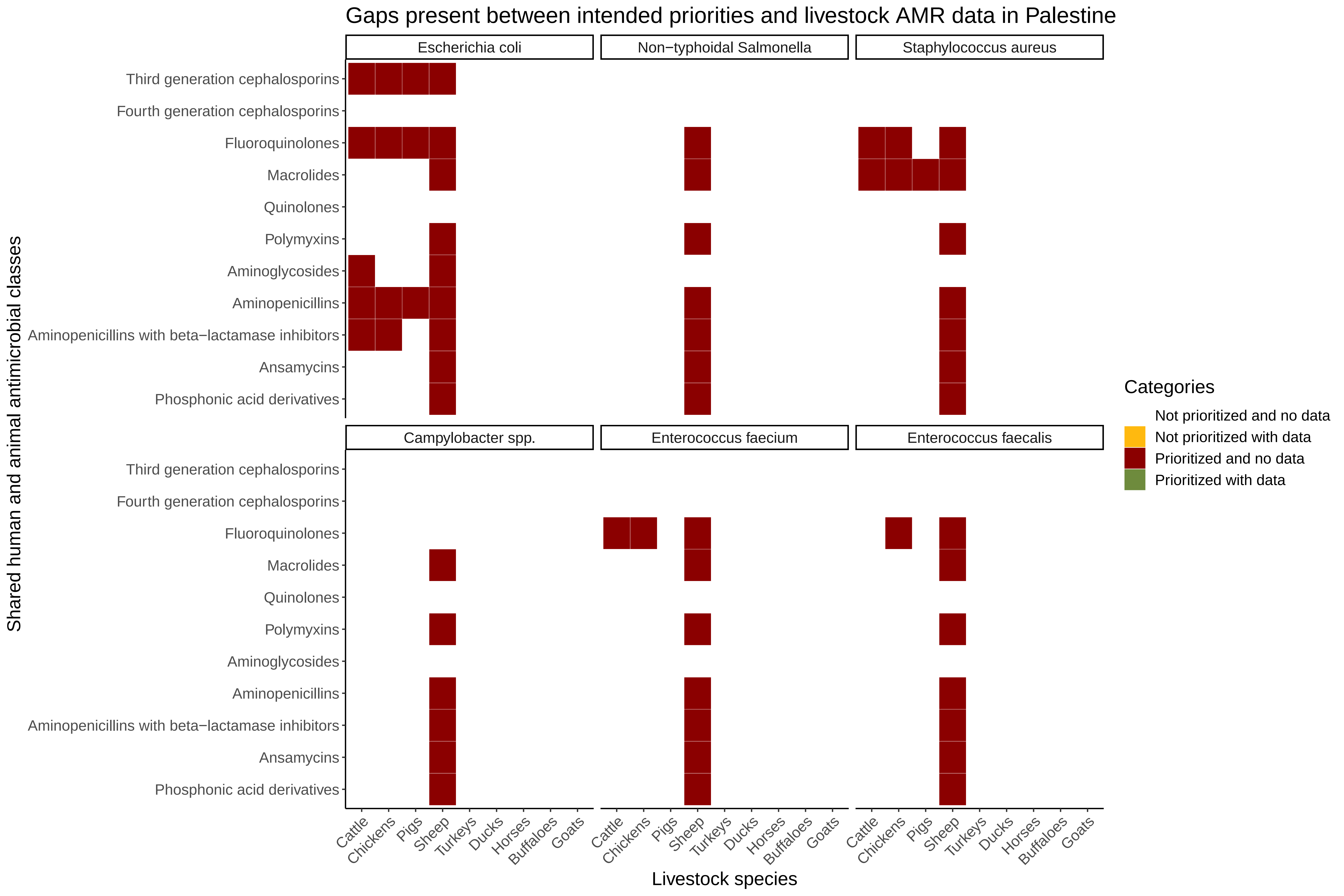
Gaps present between intended priorities and livestock AMR data in Palestine
Escherichia coli
Non−typhoidal Salmonella
Staphylococcus aureus
Campylobacter spp.
Enterococcus faecium
Enterococcus faecalis
Shared human and animal antimicrobial classes
Third generation cephalosporins
Fourth generation cephalosporins
Fluoroquinolones
Macrolides
Quinolones
Polymyxins
Aminoglycosides
Aminopenicillins
Aminopenicillins with beta−lactamase inhibitors
Ansamycins
Phosphonic acid derivatives
Cattle
Chickens
Pigs
Sheep
Turkeys
Ducks
Horses
Buffaloes
Goats
Livestock species
Categories
Not prioritized and no data
Not prioritized with data
Prioritized and no data
Prioritized with data

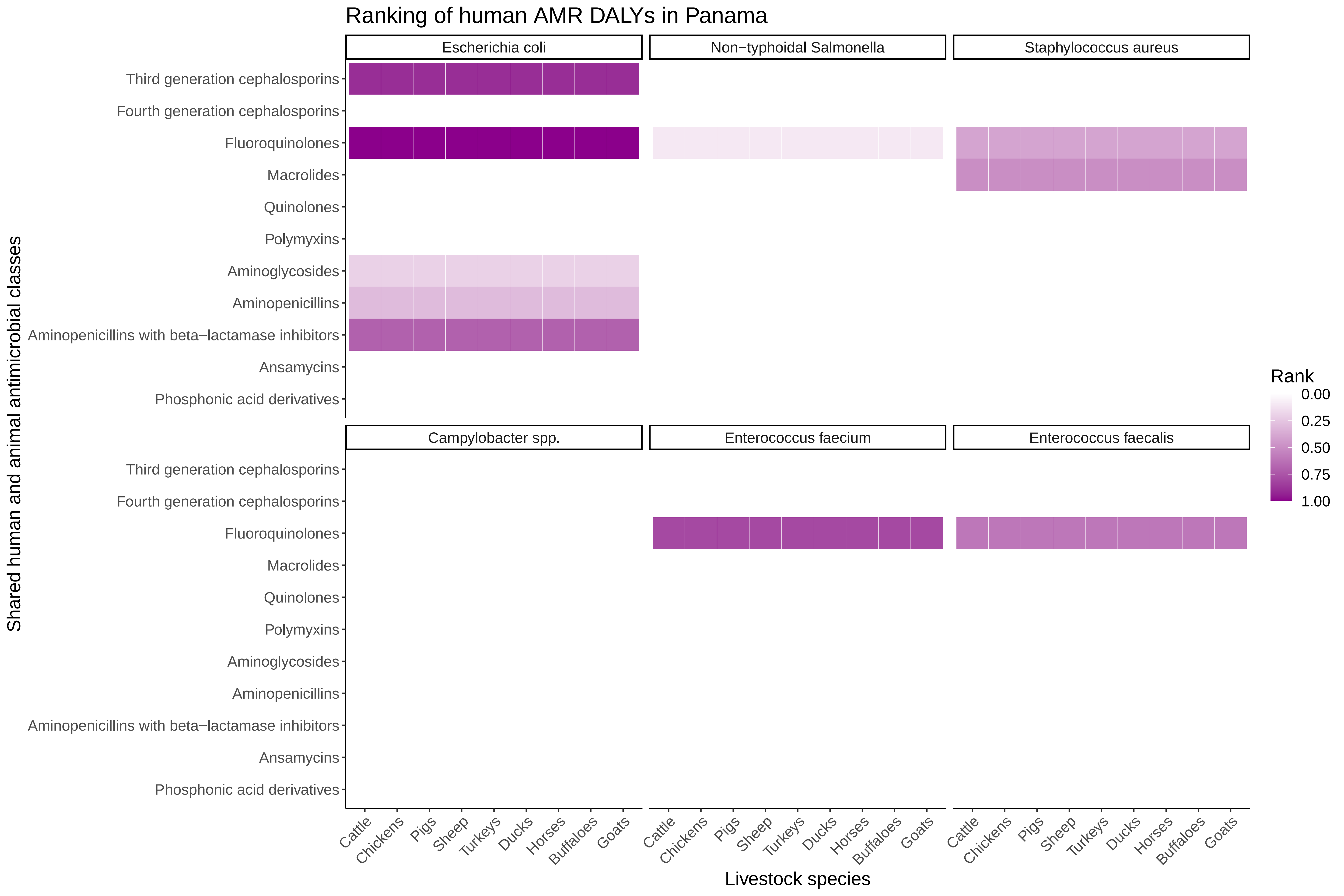

Ranking of human AMR DALYs in Panama
Escherichia coli
Non−typhoidal Salmonella
Staphylococcus aureus
Campylobacter spp.
Enterococcus faecium
Enterococcus faecalis
Third generation cephalosporins
Fourth generation cephalosporins
Fluoroquinolones
Macrolides
Quinolones
Polymyxins
Aminoglycosides
Aminopenicillins
Aminopenicillins with beta−lactamase inhibitors
Ansamycins
Phosphonic acid derivatives
Shared human and animal antimicrobial classes
Cattle
Chickens
Pigs
Sheep
Turkeys
Ducks
Horses
Buffaloes
Goats
Livestock species
Rank
0.00
0.25
0.50
0.75
1.00

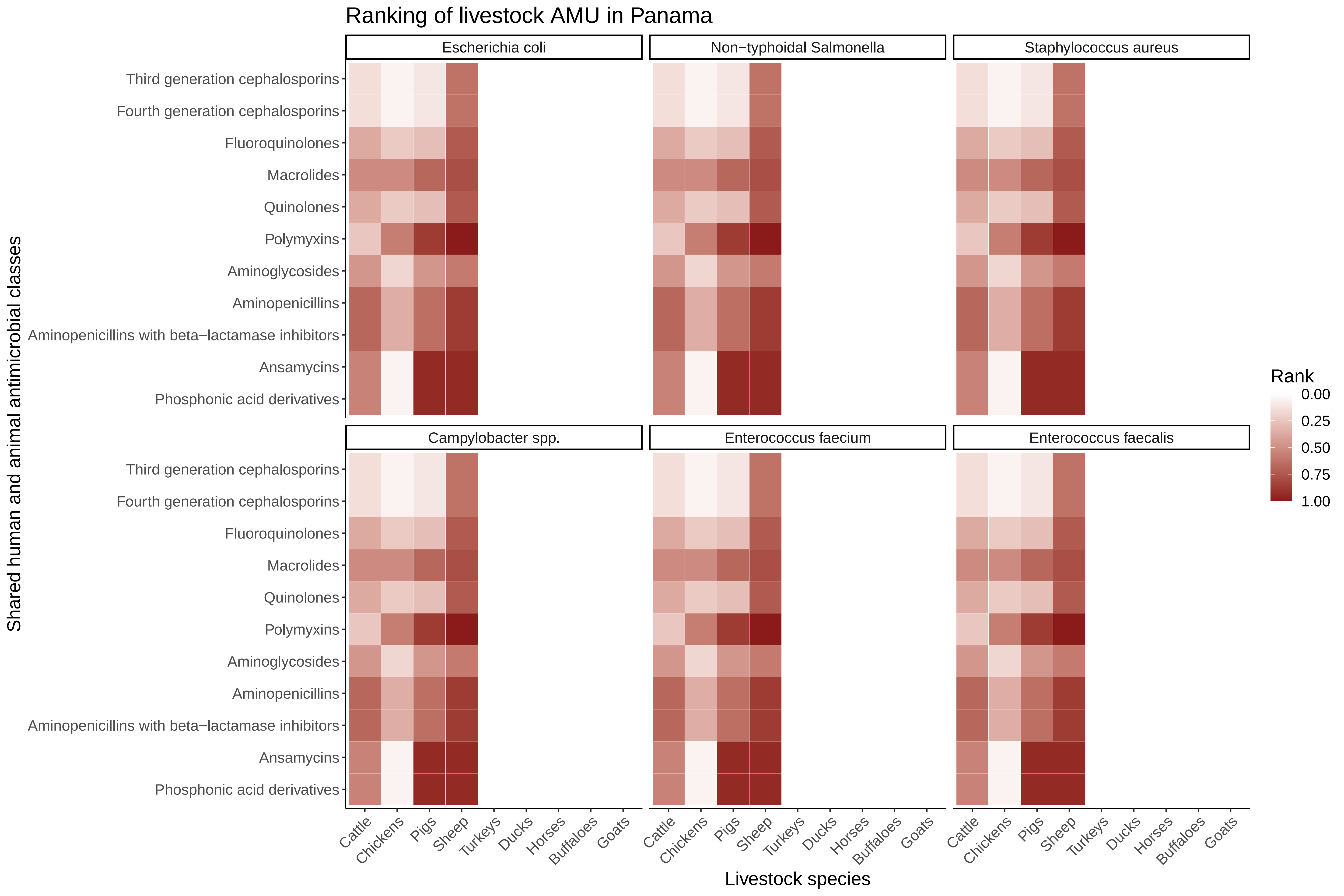

Ranking of livestock AMU in Panama
Escherichia coli
Non−typhoidal Salmonella
Staphylococcus aureus
Campylobacter spp.
Enterococcus faecium
Enterococcus faecalis
Third generation cephalosporins
Fourth generation cephalosporins
Fluoroquinolones
Macrolides
Quinolones
Polymyxins
Aminoglycosides
Aminopenicillins
Aminopenicillins with beta−lactamase inhibitors
Ansamycins
Phosphonic acid derivatives
Shared human and animal antimicrobial classes
Cattle
Chickens
Pigs
Sheep
Turkeys
Ducks
Horses
Buffaloes
Goats
Livestock species
Rank
0.00
0.25
0.50
0.75
1.00

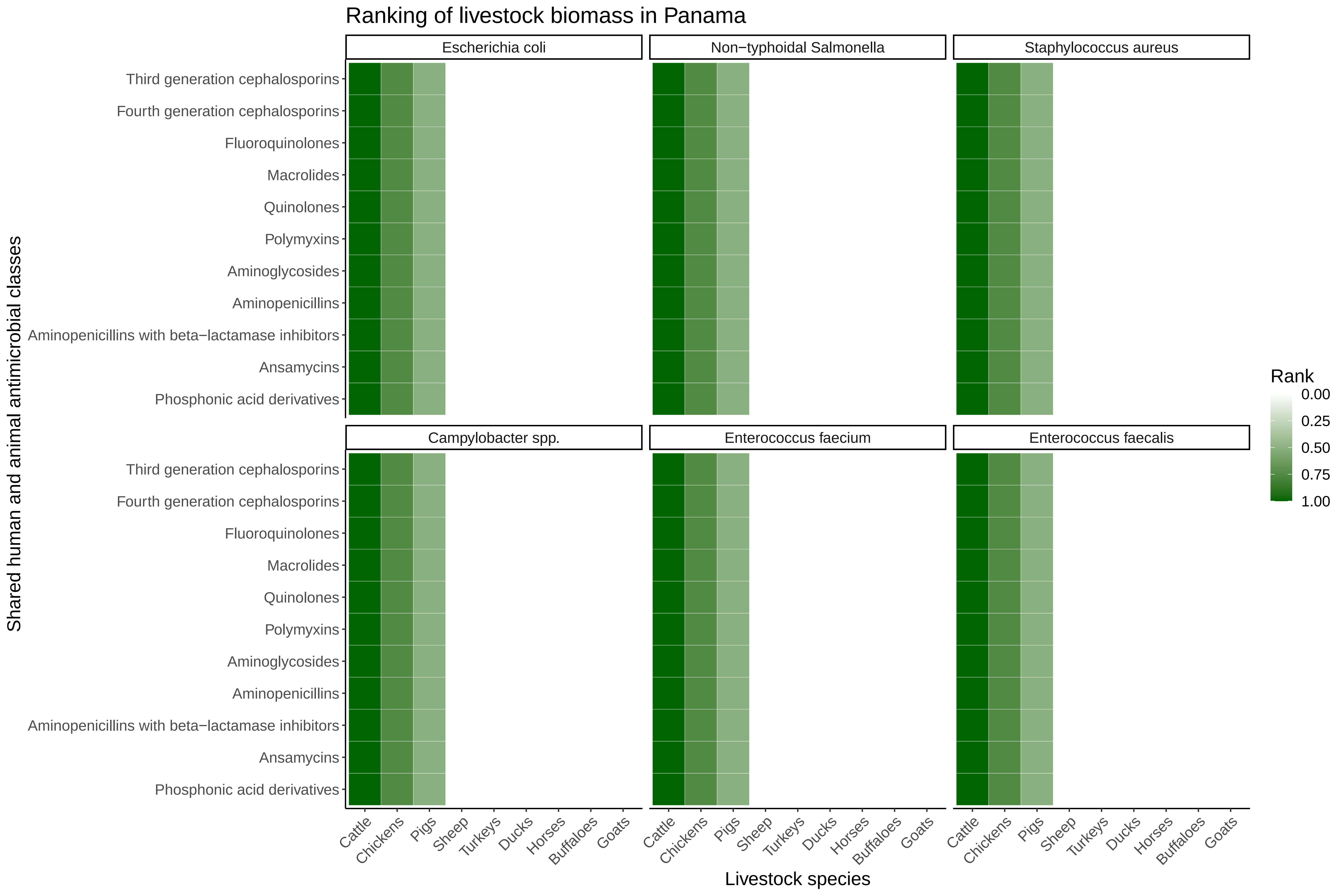

Ranking of livestock biomass in Panama
Escherichia coli
Non−typhoidal Salmonella
Staphylococcus aureus
Campylobacter spp.
Enterococcus faecium
Enterococcus faecalis
Shared human and animal antimicrobial classes
Third generation cephalosporins
Fourth generation cephalosporins
Fluoroquinolones
Macrolides
Quinolones
Polymyxins
Aminoglycosides
Aminopenicillins
Aminopenicillins with beta−lactamase inhibitors
Ansamycins
Phosphonic acid derivatives
Cattle
Chickens
Pigs
Sheep
Turkeys
Ducks
Horses
Buffaloes
Goats
Livestock species
Rank
0.00
0.25
0.50
0.75
1.00

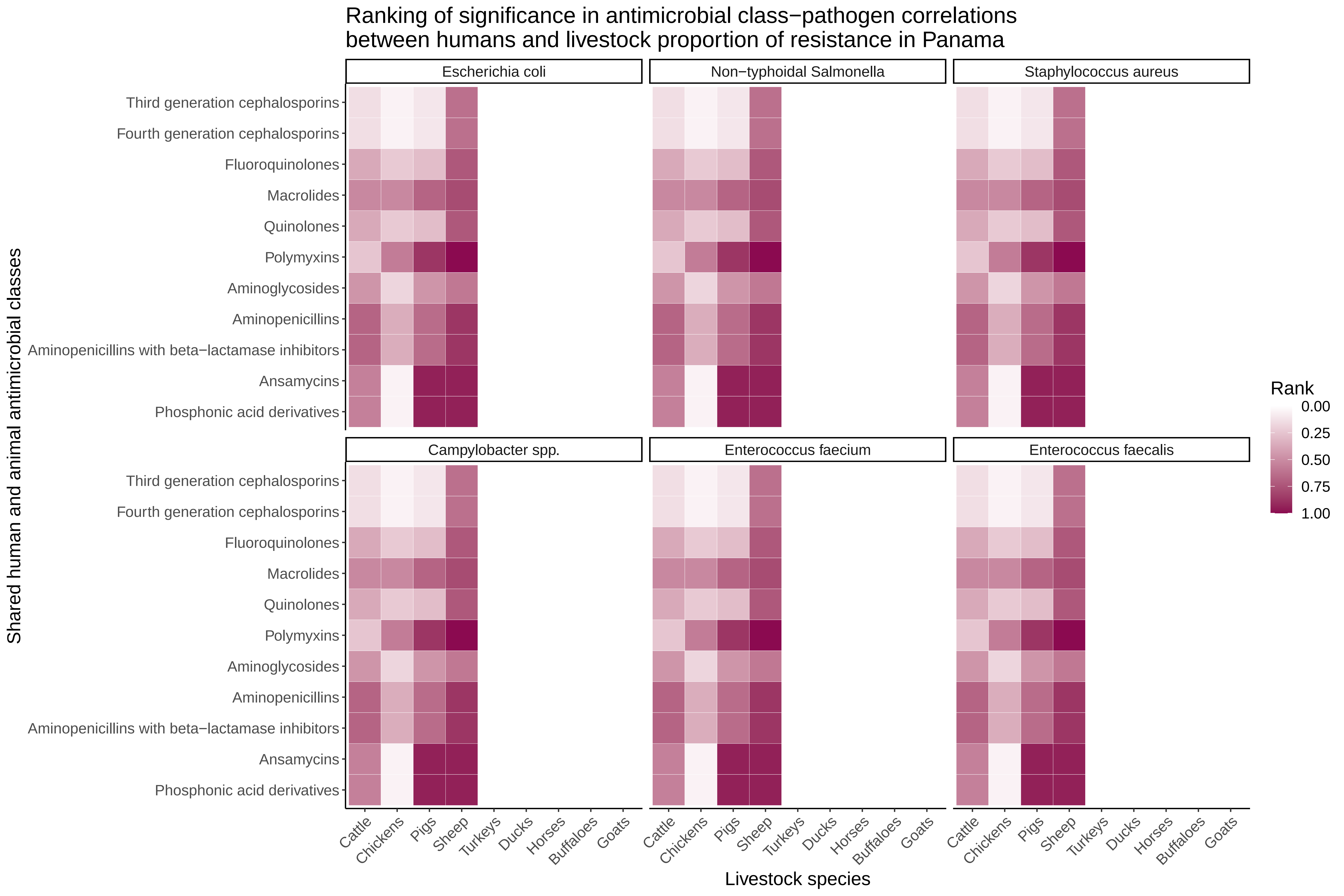

Ranking of significance in antimicrobial class−pathogen correlations
between humans and livestock proportion of resistance in Panama
Escherichia coli
Non−typhoidal Salmonella
Staphylococcus aureus
Campylobacter spp.
Enterococcus faecium
Enterococcus faecalis
Third generation cephalosporins
Fourth generation cephalosporins
Fluoroquinolones
Macrolides
Quinolones
Polymyxins
Aminoglycosides
Aminopenicillins
Aminopenicillins with beta−lactamase inhibitors
Ansamycins
Phosphonic acid derivatives
Shared human and animal antimicrobial classes
Cattle
Chickens
Pigs
Sheep
Turkeys
Ducks
Horses
Buffaloes
Goats
Livestock species
Rank
0.00
0.25
0.50
0.75
1.00

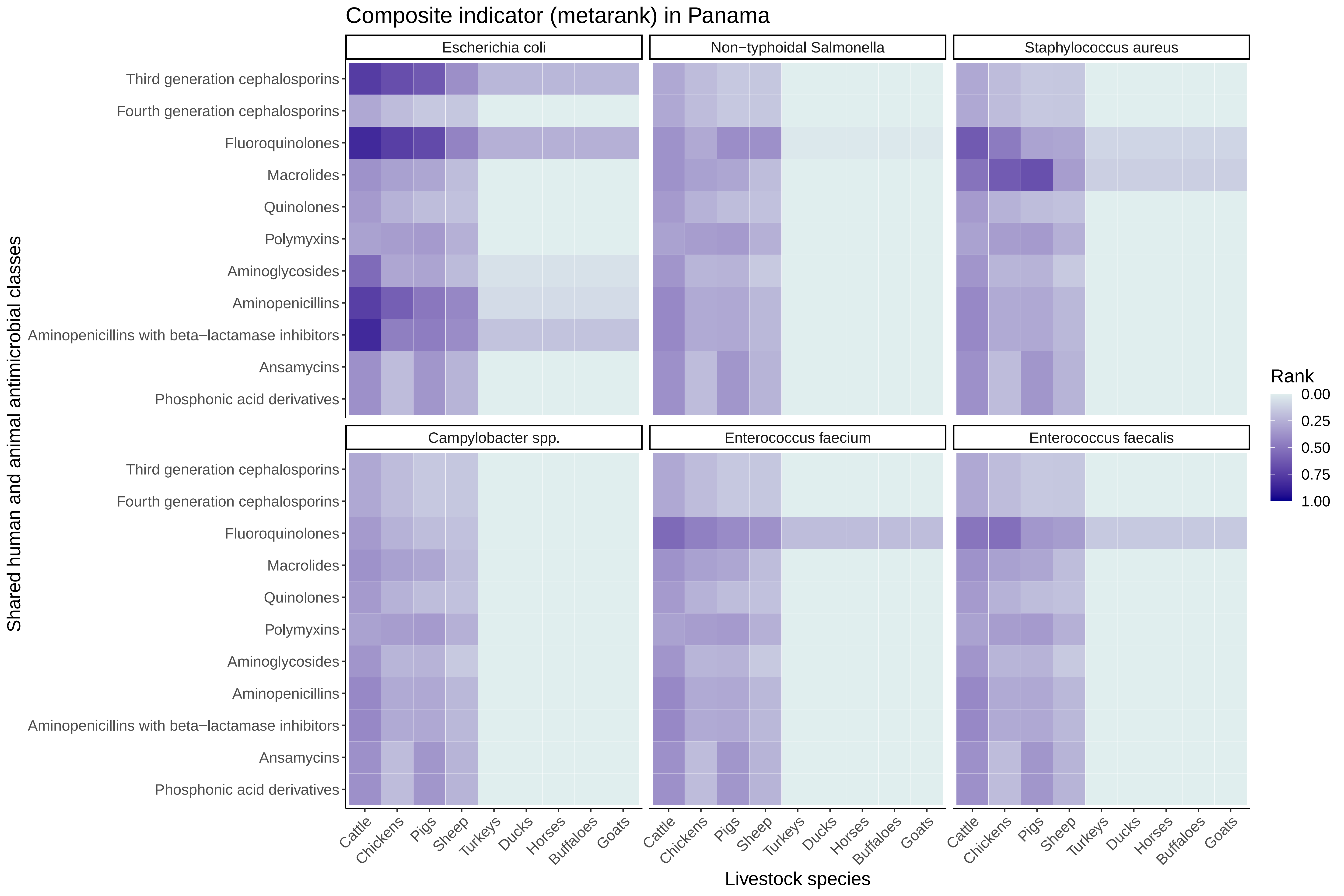
Composite indicator (metarank) in Panama
Escherichia coli
Non−typhoidal Salmonella
Staphylococcus aureus
Campylobacter spp.
Enterococcus faecium
Enterococcus faecalis
Shared human and animal antimicrobial classes
Third generation cephalosporins
Fourth generation cephalosporins
Fluoroquinolones
Macrolides
Quinolones
Polymyxins
Aminoglycosides
Aminopenicillins
Aminopenicillins with beta−lactamase inhibitors
Ansamycins
Phosphonic acid derivatives
Cattle
Chickens
Pigs
Sheep
Turkeys
Ducks
Horses
Buffaloes
Goats
Livestock species
Rank
0.00
0.25
0.50
0.75
1.00

Livestock AMR data availability in Panama

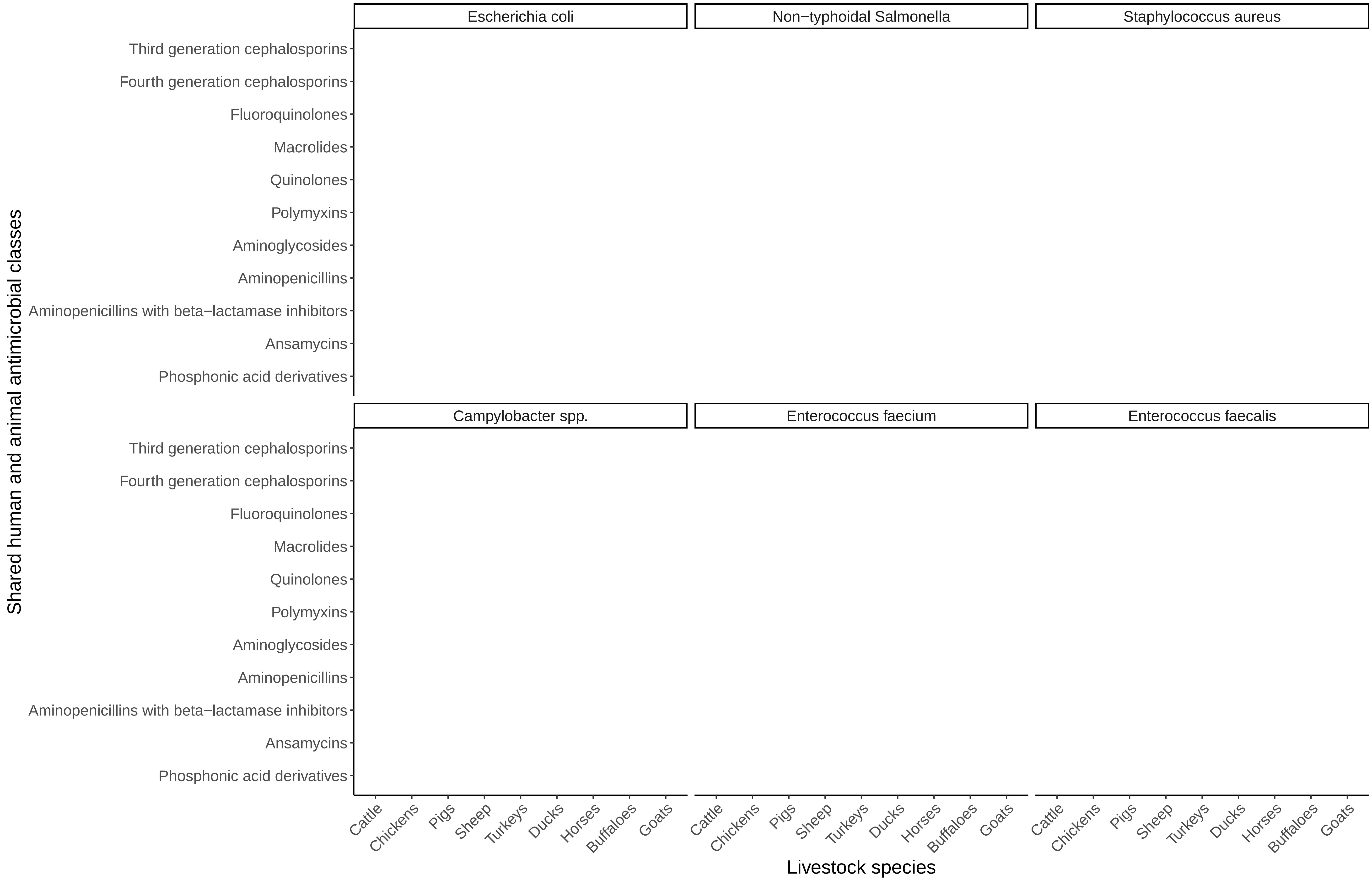

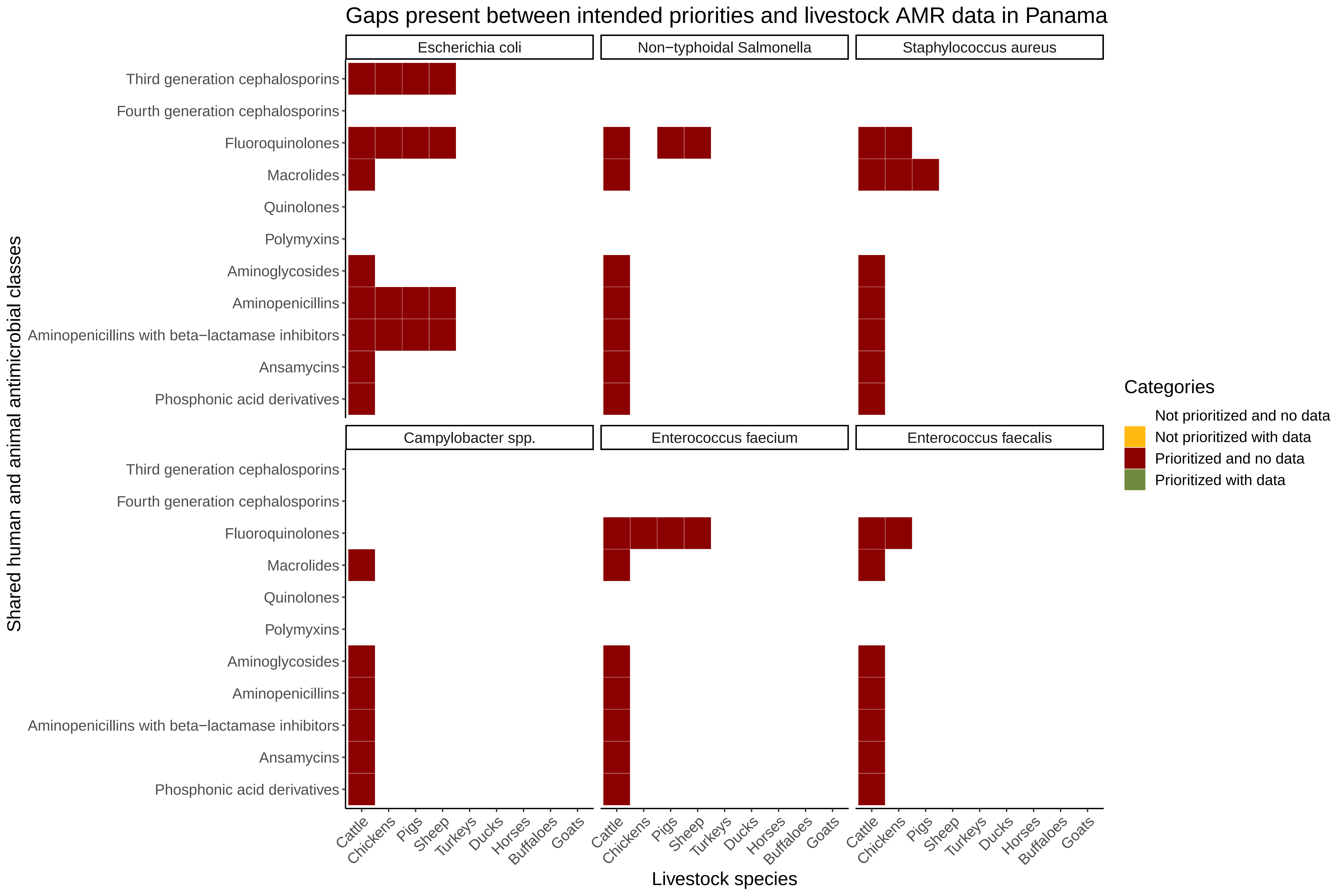

Gaps present between intended priorities and livestock AMR data in Panama
Escherichia coli
Non−typhoidal Salmonella
Staphylococcus aureus
Campylobacter spp.
Enterococcus faecium
Enterococcus faecalis
Third generation cephalosporins
Fourth generation cephalosporins
Fluoroquinolones
Macrolides
Quinolones
Polymyxins
Aminoglycosides
Aminopenicillins
Aminopenicillins with beta−lactamase inhibitors
Ansamycins
Phosphonic acid derivatives
Shared human and animal antimicrobial classes
Cattle
Chickens
Pigs
Sheep
Turkeys
Ducks
Horses
Buffaloes
Goats
Livestock species
Categories
Not prioritized and no data
Not prioritized with data
Prioritized and no data
Prioritized with data

Ranking of human AMR DALYs in Papua New Guinea

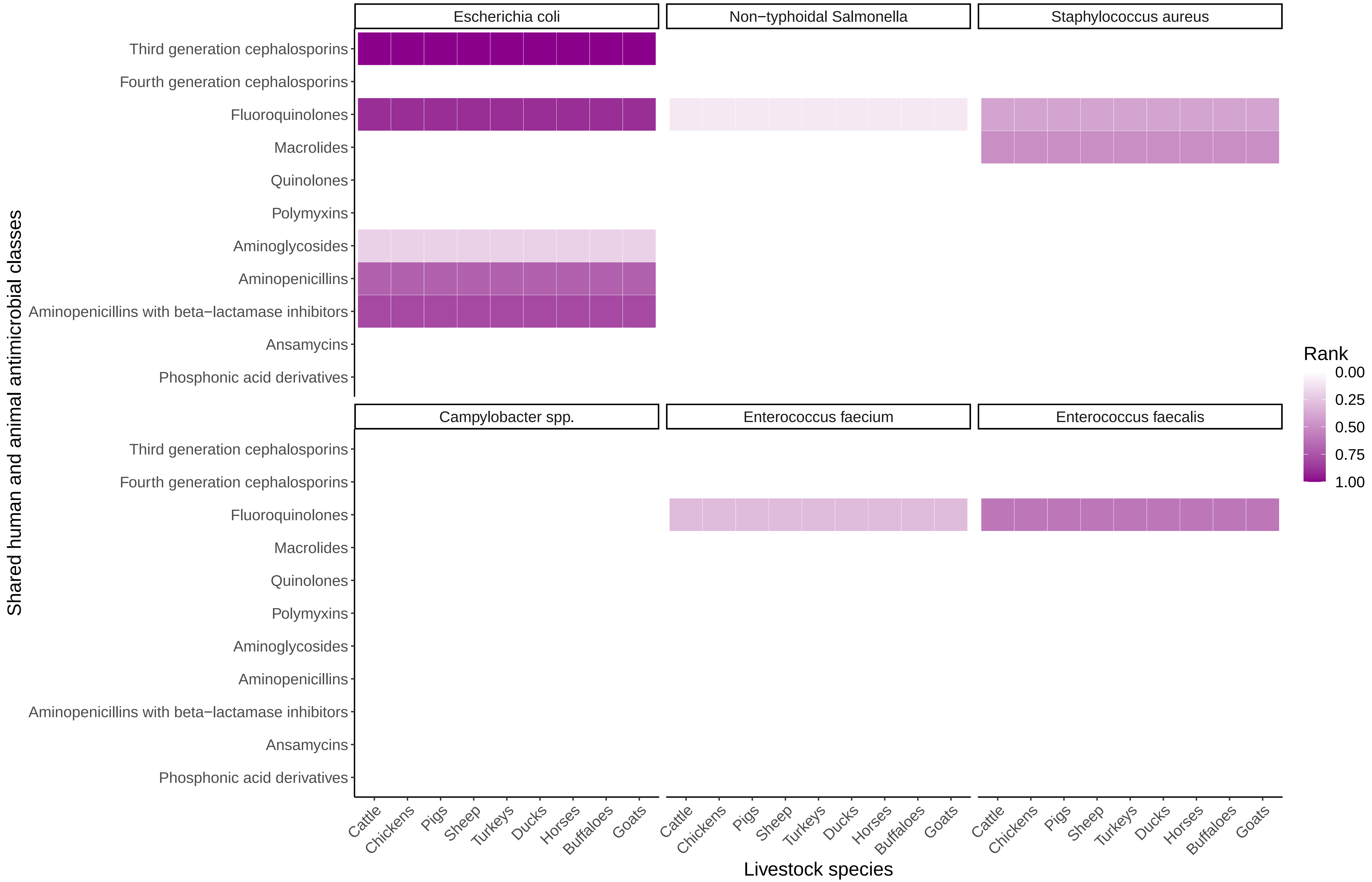

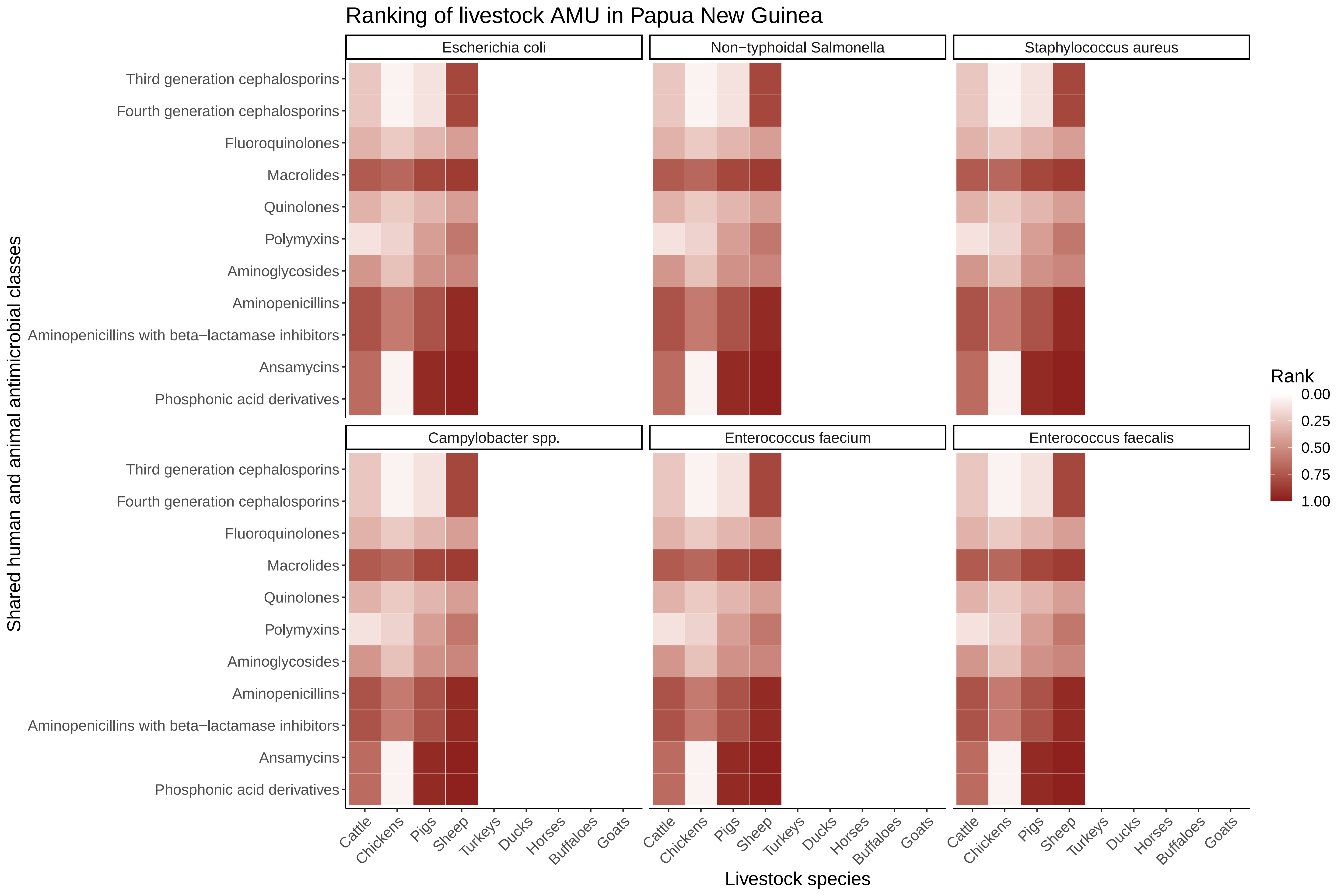
Ranking of livestock AMU in Papua New Guinea
Escherichia coli
Non−typhoidal Salmonella
Staphylococcus aureus
Campylobacter spp.
Enterococcus faecium
Enterococcus faecalis
Shared human and animal antimicrobial classes
Third generation cephalosporins
Fourth generation cephalosporins
Fluoroquinolones
Macrolides
Quinolones
Polymyxins
Aminoglycosides
Aminopenicillins
Aminopenicillins with beta−lactamase inhibitors
Ansamycins
Phosphonic acid derivatives
Cattle
Chickens
Pigs
Sheep
Turkeys
Ducks
Horses
Buffaloes
Goats
Livestock species
Rank
0.00
0.25
0.50
0.75
1.00

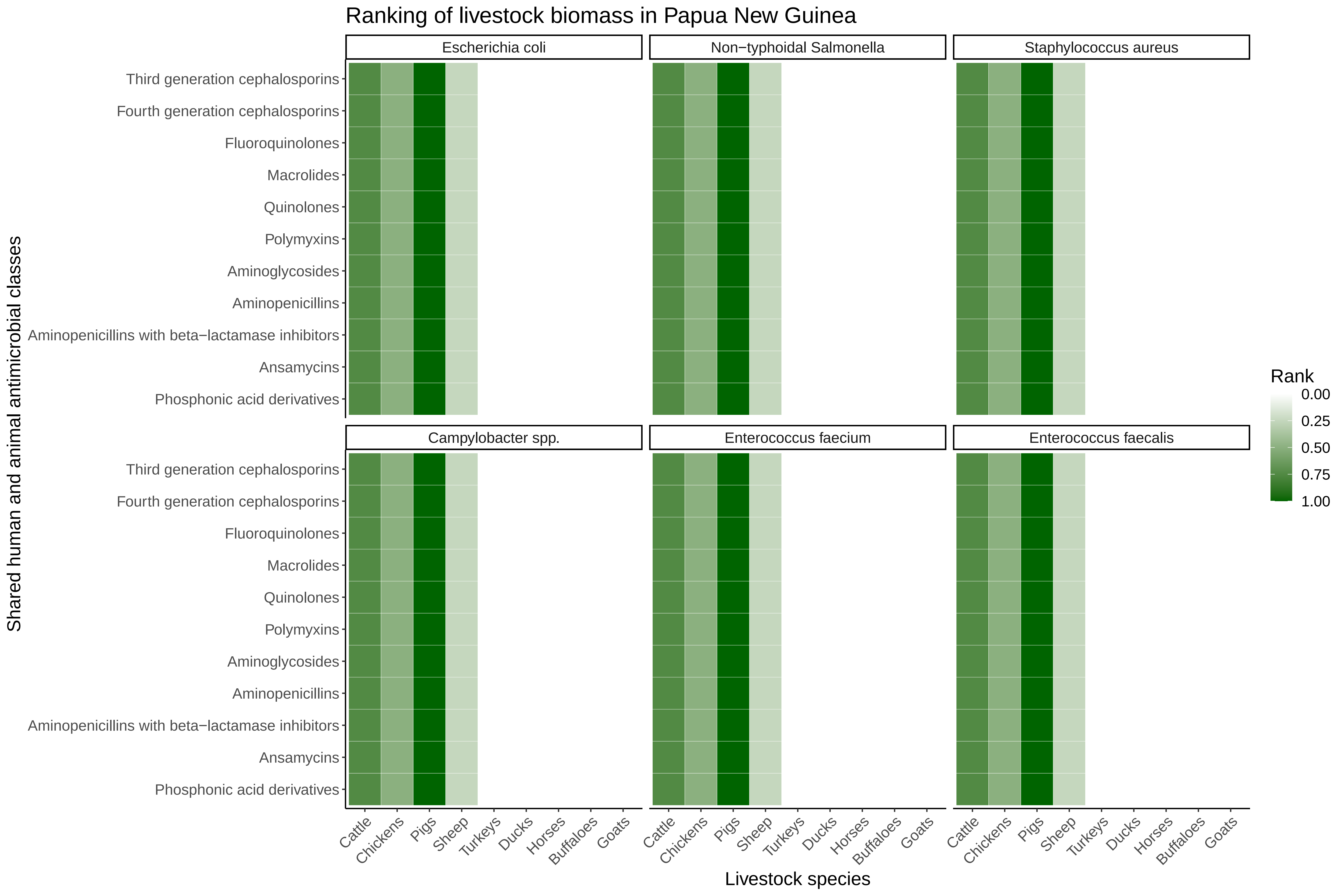

Ranking of livestock biomass in Papua New Guinea
Escherichia coli
Non−typhoidal Salmonella
Staphylococcus aureus
Campylobacter spp.
Enterococcus faecium
Enterococcus faecalis
Shared human and animal antimicrobial classes
Third generation cephalosporins
Fourth generation cephalosporins
Fluoroquinolones
Macrolides
Quinolones
Polymyxins
Aminoglycosides
Aminopenicillins
Aminopenicillins with beta−lactamase inhibitors
Ansamycins
Phosphonic acid derivatives
Cattle
Chickens
Pigs
Sheep
Turkeys
Ducks
Horses
Buffaloes
Goats
Livestock species
Rank
0.00
0.25
0.50
0.75
1.00

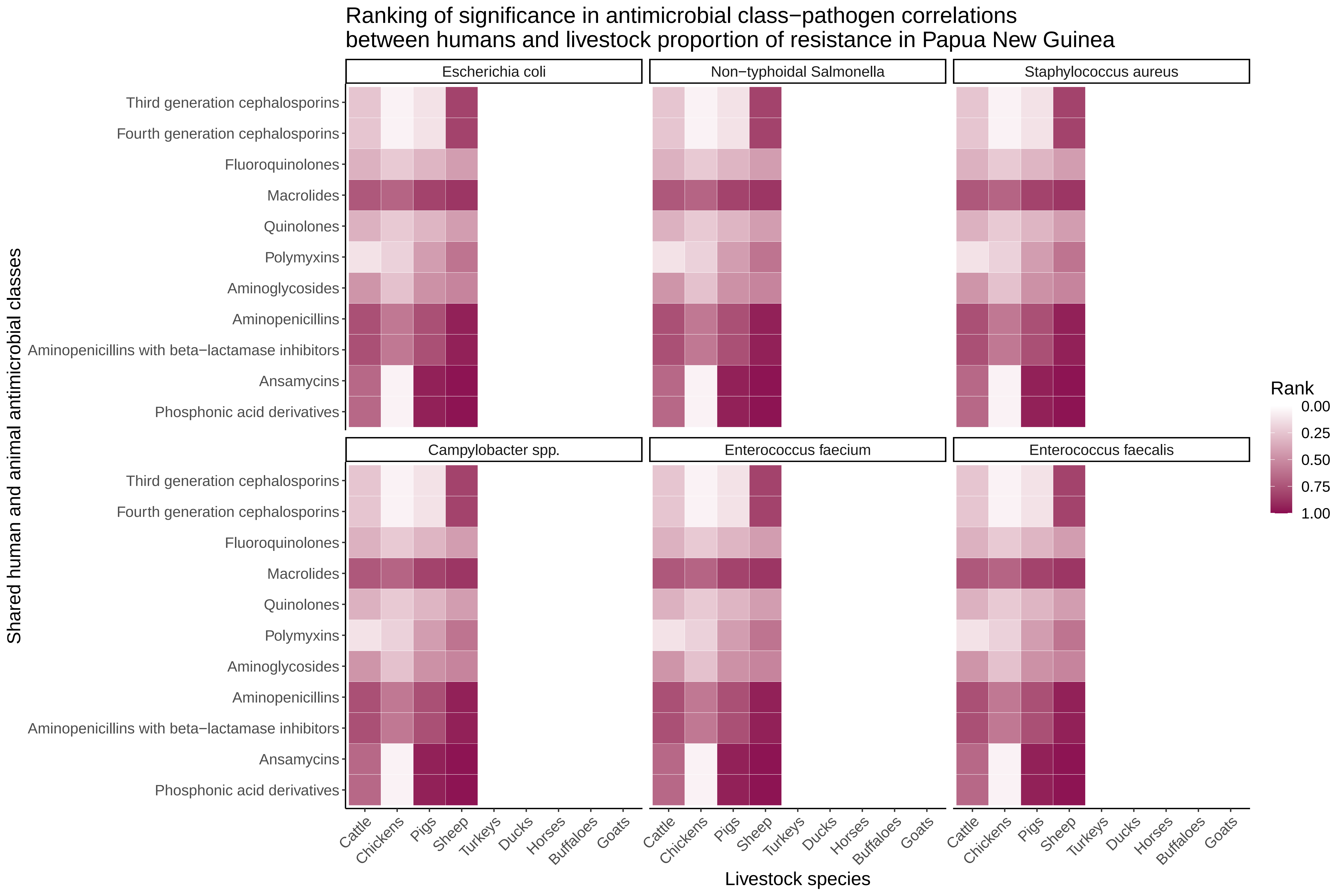

Ranking of significance in antimicrobial class−pathogen correlations
between humans and livestock proportion of resistance in Papua New Guinea
Escherichia coli
Non−typhoidal Salmonella
Staphylococcus aureus
Campylobacter spp.
Enterococcus faecium
Enterococcus faecalis
Third generation cephalosporins
Fourth generation cephalosporins
Fluoroquinolones
Macrolides
Quinolones
Polymyxins
Aminoglycosides
Aminopenicillins
Aminopenicillins with beta−lactamase inhibitors
Ansamycins
Phosphonic acid derivatives
Shared human and animal antimicrobial classes
Cattle
Chickens
Pigs
Sheep
Turkeys
Ducks
Horses
Buffaloes
Goats
Livestock species
Rank
0.00
0.25
0.50
0.75
1.00

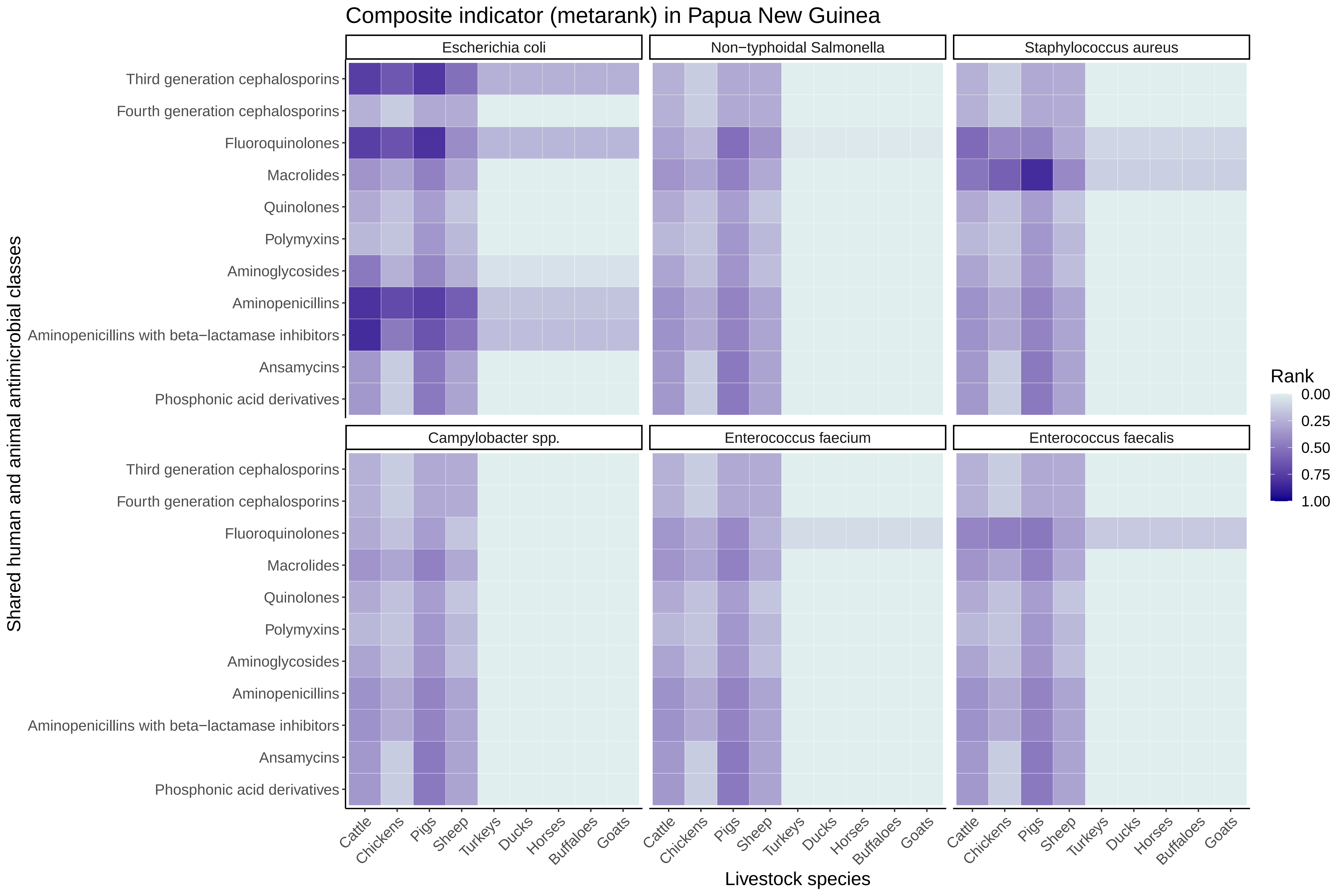
Composite indicator (metarank) in Papua New Guinea
Escherichia coli
Non−typhoidal Salmonella
Staphylococcus aureus
Campylobacter spp.
Enterococcus faecium
Enterococcus faecalis
Shared human and animal antimicrobial classes
Third generation cephalosporins
Fourth generation cephalosporins
Fluoroquinolones
Macrolides
Quinolones
Polymyxins
Aminoglycosides
Aminopenicillins
Aminopenicillins with beta−lactamase inhibitors
Ansamycins
Phosphonic acid derivatives
Livestock species
Cattle
Chickens
Pigs
Sheep
Turkeys
Ducks
Horses
Buffaloes
Goats
Rank
0.00
0.25
0.50
0.75
1.00

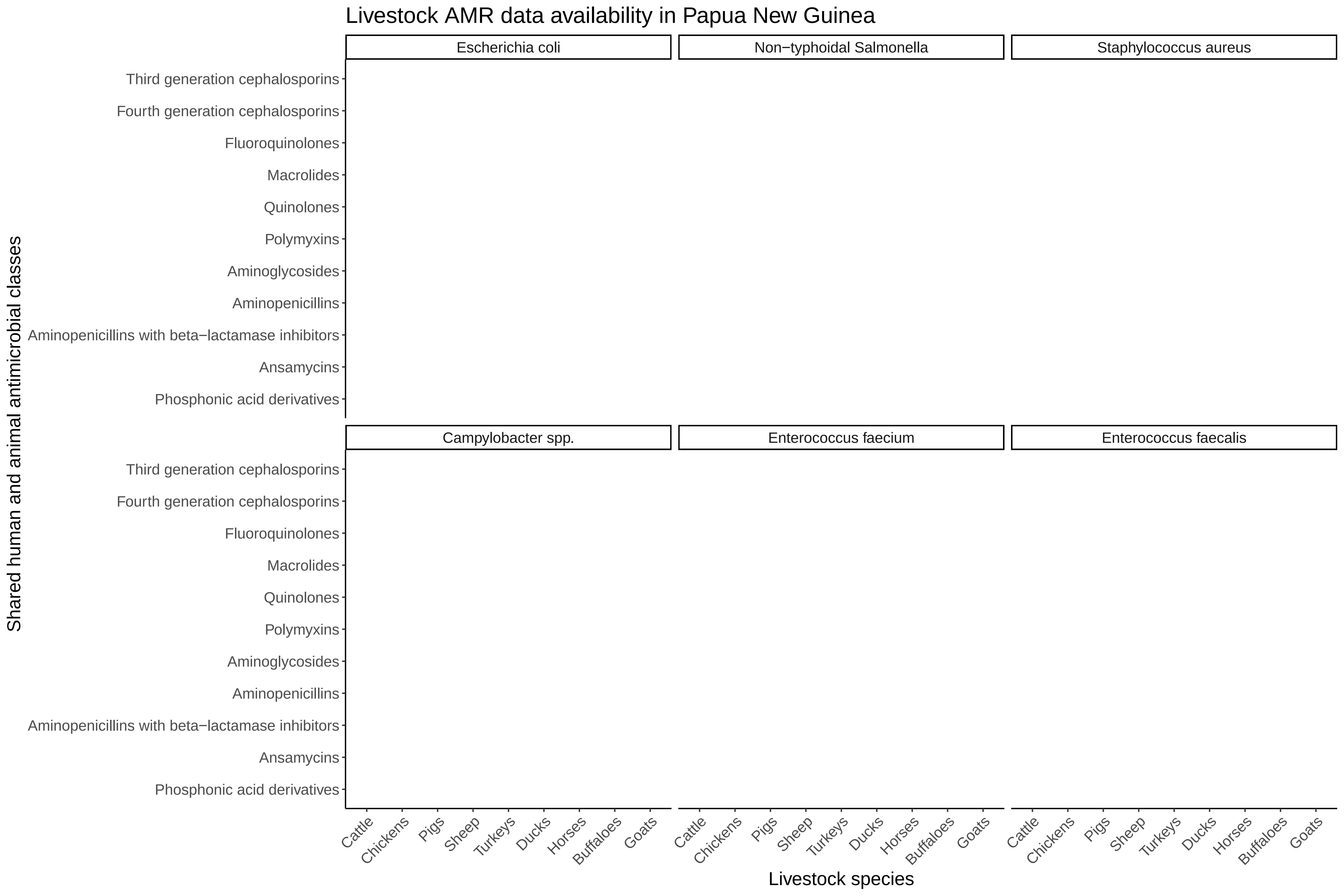

Livestock AMR data availability in Papua New Guinea
Escherichia coli
Non−typhoidal Salmonella
Staphylococcus aureus
Campylobacter spp.
Enterococcus faecium
Enterococcus faecalis
Shared human and animal antimicrobial classes
Third generation cephalosporins
Fourth generation cephalosporins
Fluoroquinolones
Macrolides
Quinolones
Polymyxins
Aminoglycosides
Aminopenicillins
Aminopenicillins with beta−lactamase inhibitors
Ansamycins
Phosphonic acid derivatives
Cattle
Chickens
Pigs
Sheep
Turkeys
Ducks
Horses
Buffaloes
Goats
Livestock species

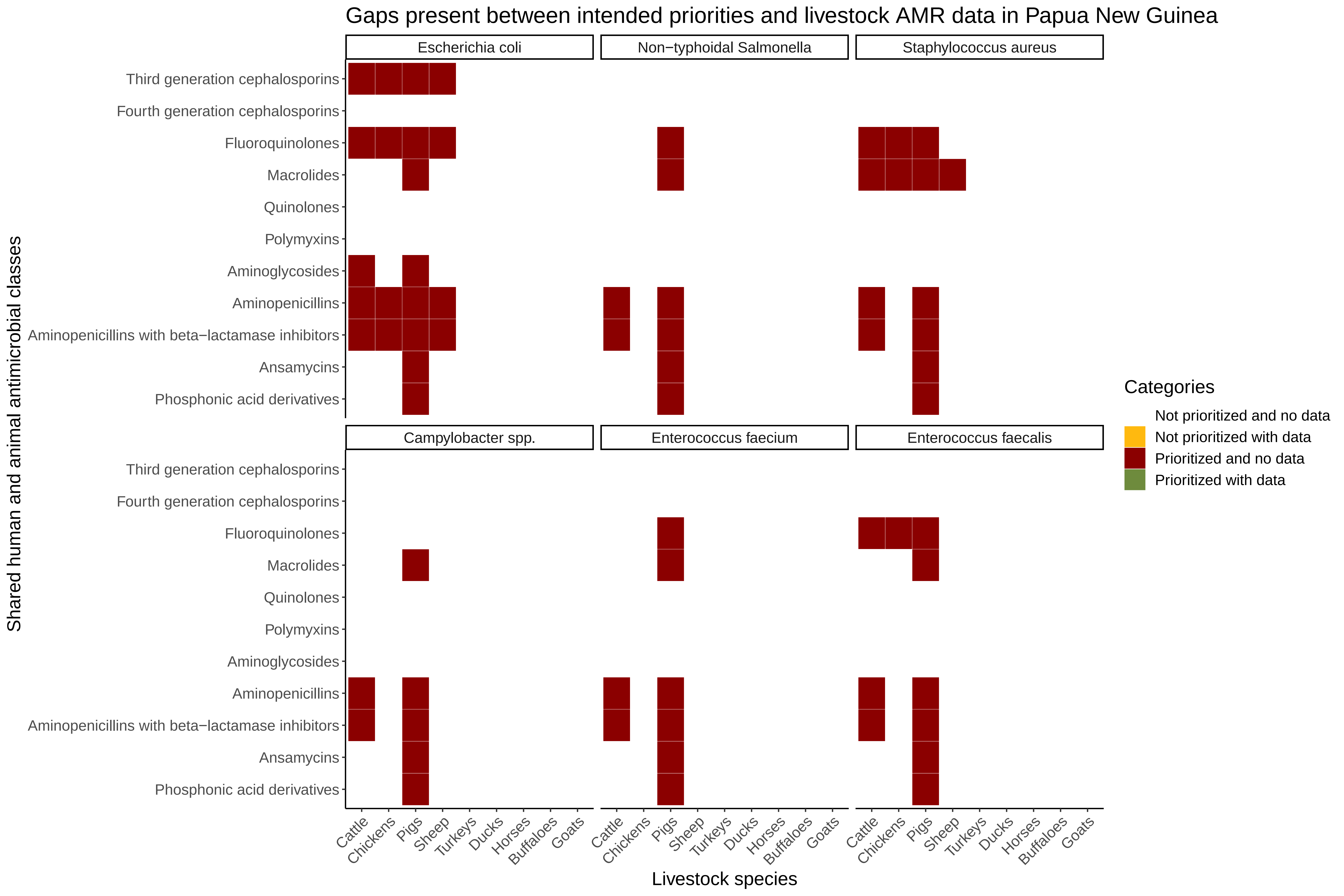

Gaps present between intended priorities and livestock AMR data in Papua New Guinea
Escherichia coli
Non−typhoidal Salmonella
Staphylococcus aureus
Campylobacter spp.
Enterococcus faecium
Enterococcus faecalis
Shared human and animal antimicrobial classes
Third generation cephalosporins
Fourth generation cephalosporins
Fluoroquinolones
Macrolides
Quinolones
Polymyxins
Aminoglycosides
Aminopenicillins
Aminopenicillins with beta−lactamase inhibitors
Ansamycins
Phosphonic acid derivatives
Cattle
Chickens
Pigs
Sheep
Turkeys
Ducks
Horses
Buffaloes
Goats
Livestock species
Categories
Not prioritized and no data
Not prioritized with data
Prioritized and no data
Prioritized with data

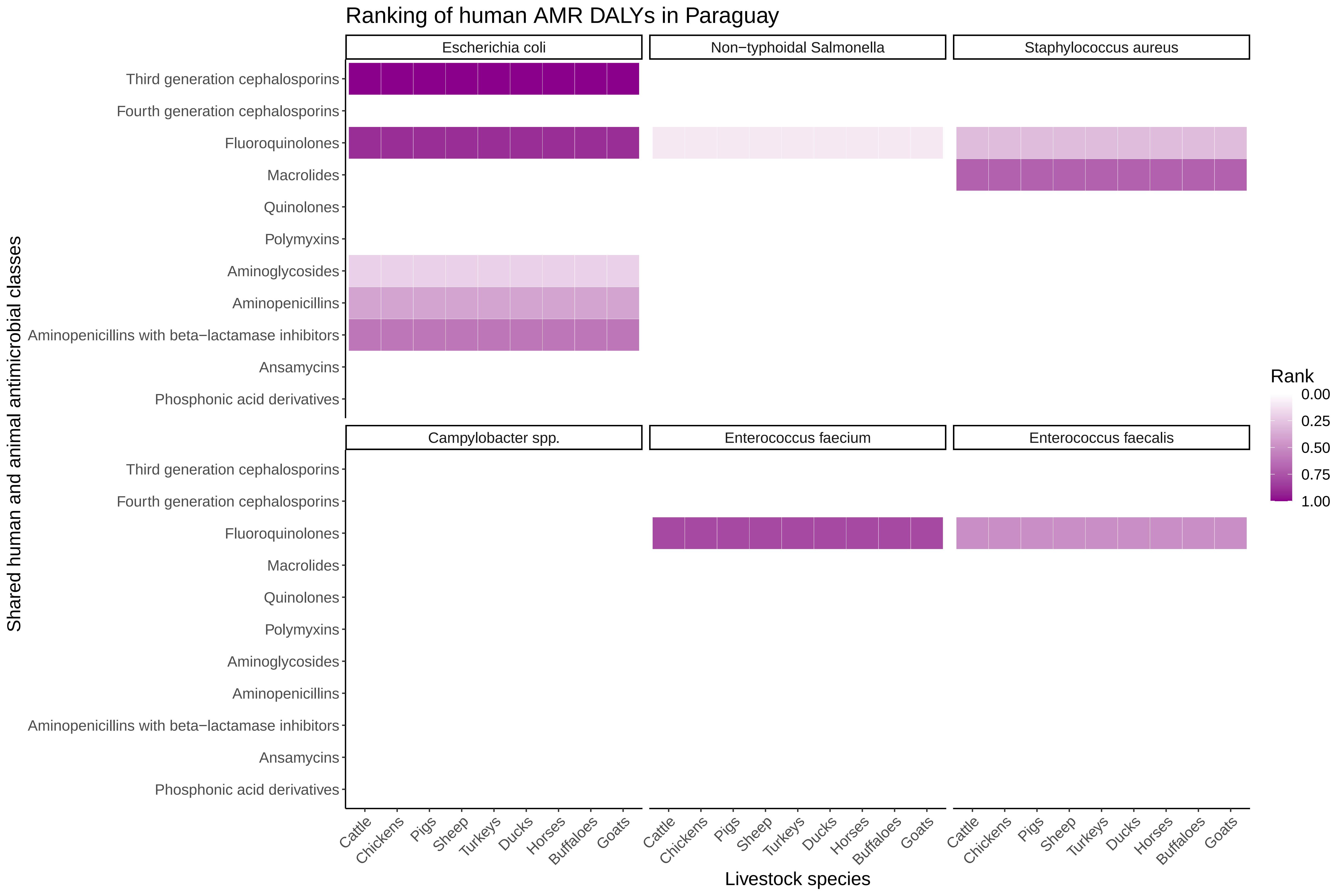
Ranking of human AMR DALYs in Paraguay
Escherichia coli
Non−typhoidal Salmonella
Staphylococcus aureus
Campylobacter spp.
Enterococcus faecium
Enterococcus faecalis
Shared human and animal antimicrobial classes
Third generation cephalosporins
Fourth generation cephalosporins
Fluoroquinolones
Macrolides
Quinolones
Polymyxins
Aminoglycosides
Aminopenicillins
Aminopenicillins with beta−lactamase inhibitors
Ansamycins
Phosphonic acid derivatives
Livestock species
Cattle
Chickens
Pigs
Sheep
Turkeys
Ducks
Horses
Buffaloes
Goats
Rank
0.00
0.25
0.50
0.75
1.00

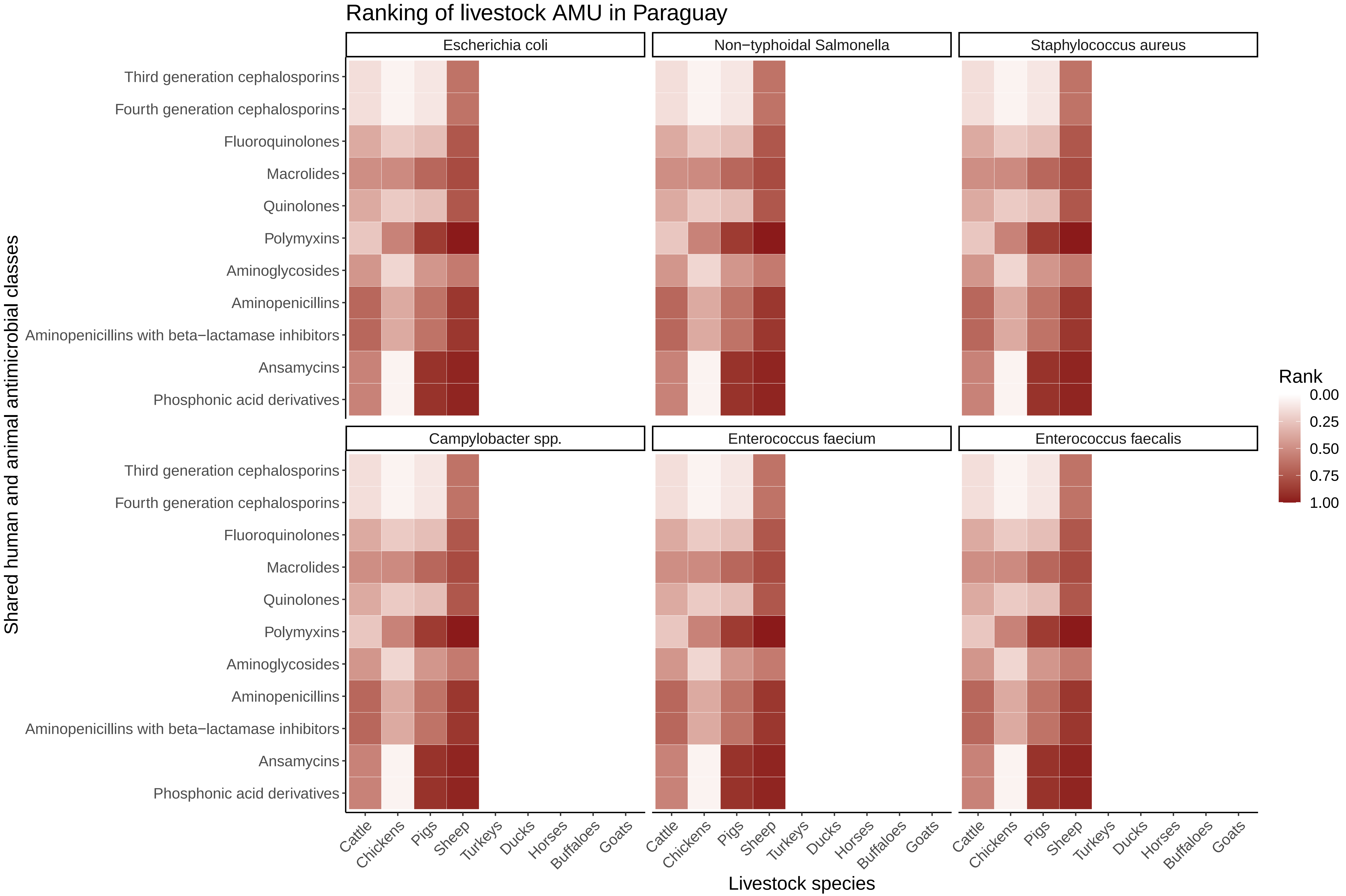

Ranking of livestock AMU in Paraguay
Escherichia coli
Non−typhoidal Salmonella
Staphylococcus aureus
Campylobacter spp.
Enterococcus faecium
Enterococcus faecalis
Shared human and animal antimicrobial classes
Third generation cephalosporins
Fourth generation cephalosporins
Fluoroquinolones
Macrolides
Quinolones
Polymyxins
Aminoglycosides
Aminopenicillins
Aminopenicillins with beta−lactamase inhibitors
Ansamycins
Phosphonic acid derivatives
Cattle
Chickens
Pigs
Sheep
Turkeys
Ducks
Horses
Buffaloes
Goats
Livestock species
Rank
0.00
0.25
0.50
0.75
1.00

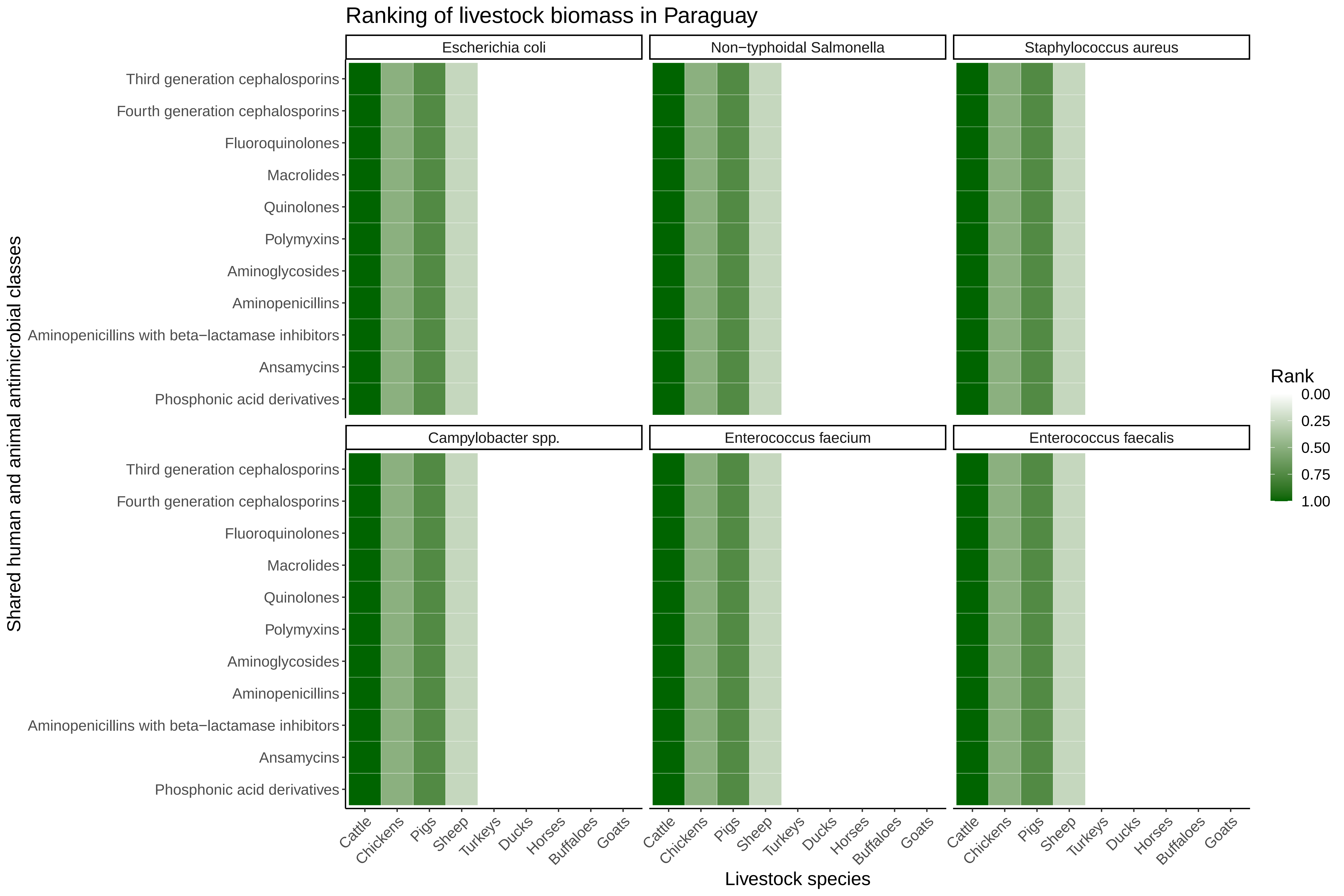

Ranking of livestock biomass in Paraguay
Escherichia coli
Non−typhoidal Salmonella
Staphylococcus aureus
Campylobacter spp.
Enterococcus faecium
Enterococcus faecalis
Third generation cephalosporins
Fourth generation cephalosporins
Fluoroquinolones
Macrolides
Quinolones
Polymyxins
Aminoglycosides
Aminopenicillins
Aminopenicillins with beta−lactamase inhibitors
Ansamycins
Phosphonic acid derivatives
Shared human and animal antimicrobial classes
Cattle
Chickens
Pigs
Sheep
Turkeys
Ducks
Horses
Buffaloes
Goats
Livestock species
Rank
0.00
0.25
0.50
0.75
1.00

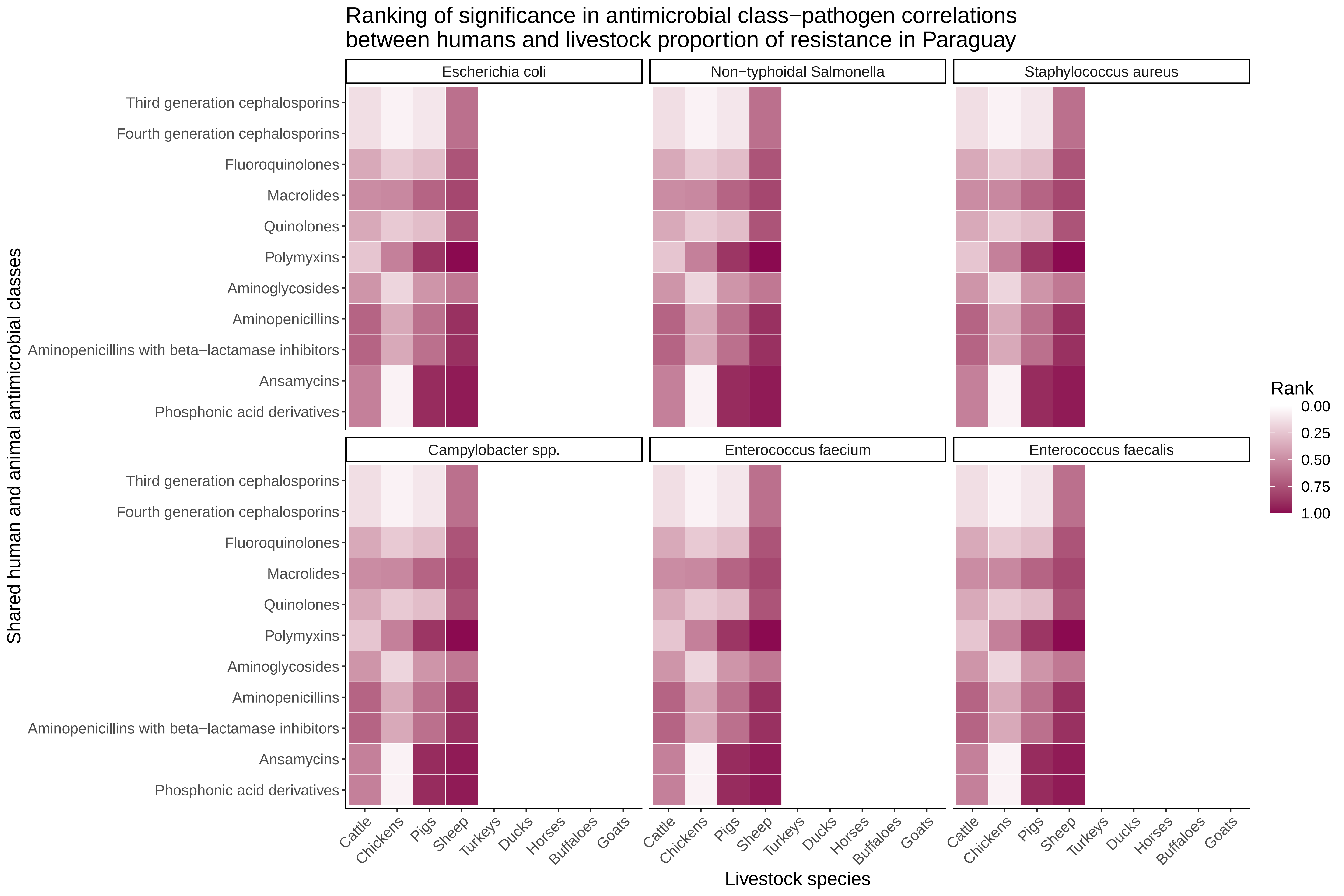

Ranking of significance in antimicrobial class−pathogen correlations
between humans and livestock proportion of resistance in Paraguay
Escherichia coli
Non−typhoidal Salmonella
Staphylococcus aureus
Campylobacter spp.
Enterococcus faecium
Enterococcus faecalis
Third generation cephalosporins
Fourth generation cephalosporins
Fluoroquinolones
Macrolides
Quinolones
Polymyxins
Aminoglycosides
Aminopenicillins
Aminopenicillins with beta−lactamase inhibitors
Ansamycins
Phosphonic acid derivatives
Shared human and animal antimicrobial classes
Cattle
Chickens
Pigs
Sheep
Turkeys
Ducks
Horses
Buffaloes
Goats
Livestock species
Rank
0.00
0.25
0.50
0.75
1.00

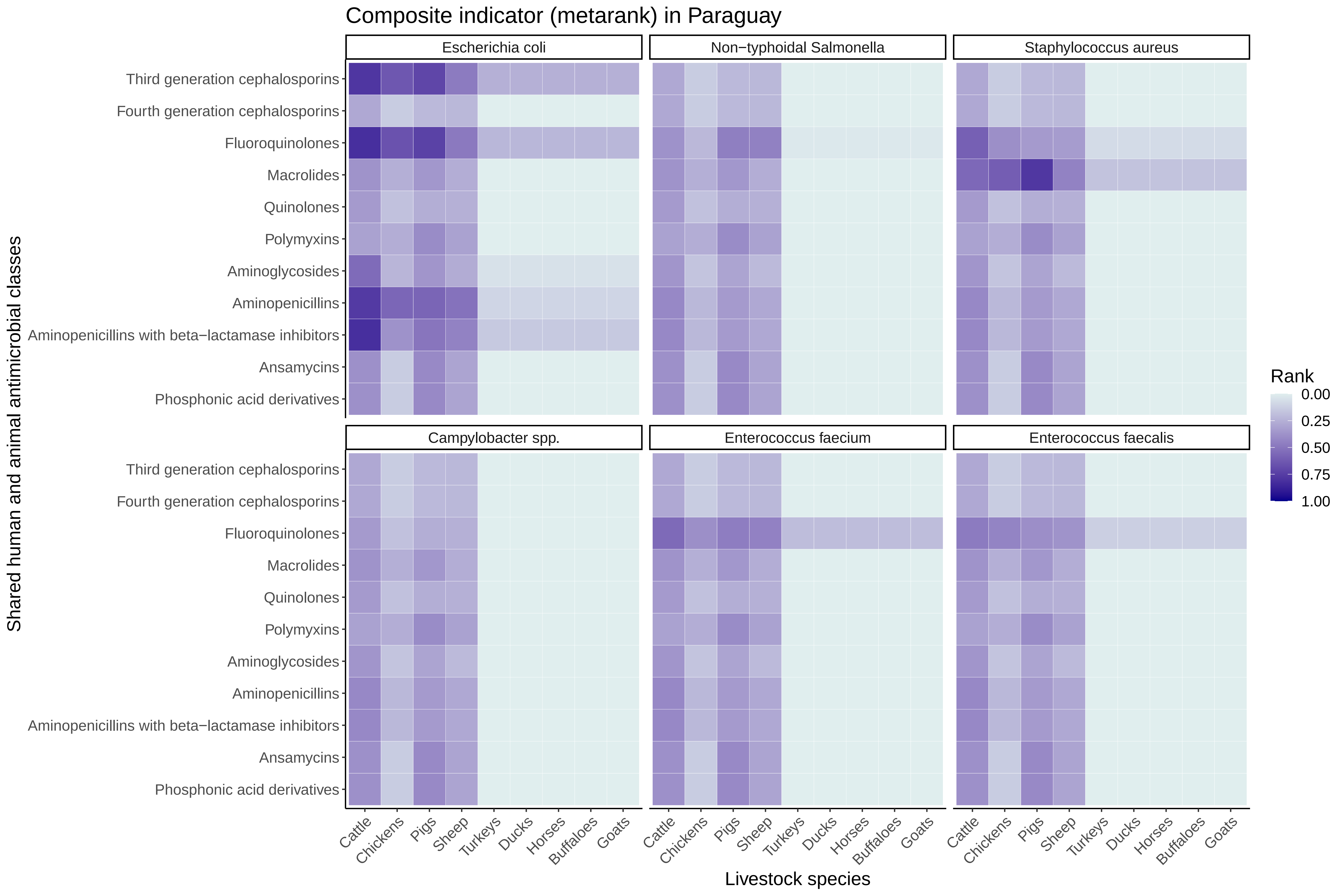

Composite indicator (metarank) in Paraguay
Escherichia coli
Non−typhoidal Salmonella
Staphylococcus aureus
Campylobacter spp.
Enterococcus faecium
Enterococcus faecalis
Shared human and animal antimicrobial classes
Third generation cephalosporins
Fourth generation cephalosporins
Fluoroquinolones
Macrolides
Quinolones
Polymyxins
Aminoglycosides
Aminopenicillins
Aminopenicillins with beta−lactamase inhibitors
Ansamycins
Phosphonic acid derivatives
Livestock species
Cattle
Chickens
Pigs
Sheep
Turkeys
Ducks
Horses
Buffaloes
Goats
Rank
0.00
0.25
0.50
0.75
1.00

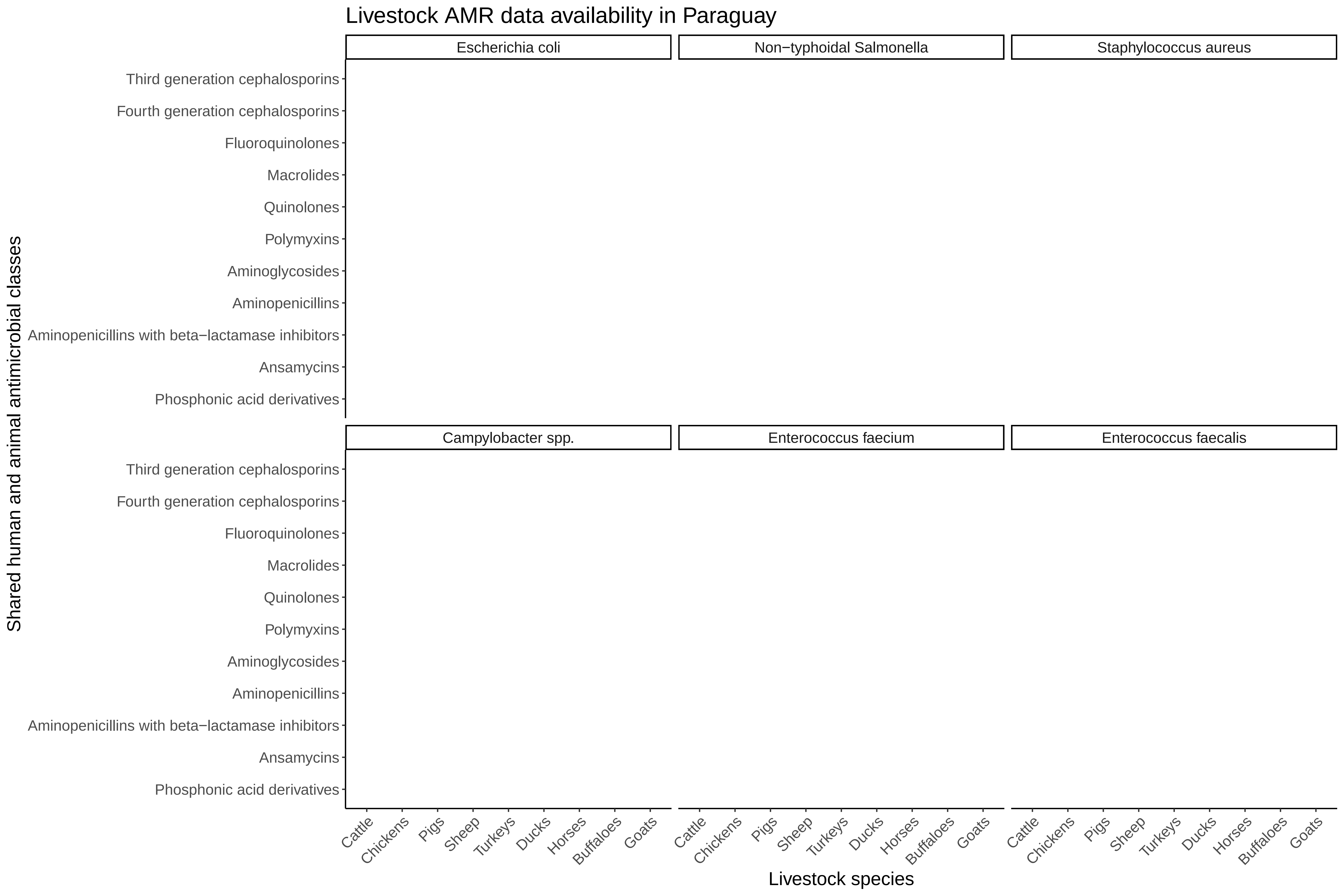
Livestock AMR data availability in Paraguay
Escherichia coli
Non−typhoidal Salmonella
Staphylococcus aureus
Campylobacter spp.
Enterococcus faecium
Enterococcus faecalis
Shared human and animal antimicrobial classes
Third generation cephalosporins
Fourth generation cephalosporins
Fluoroquinolones
Macrolides
Quinolones
Polymyxins
Aminoglycosides
Aminopenicillins
Aminopenicillins with beta−lactamase inhibitors
Ansamycins
Phosphonic acid derivatives
Cattle
Chickens
Pigs
Sheep
Turkeys
Ducks
Horses
Buffaloes
Goats
Livestock species

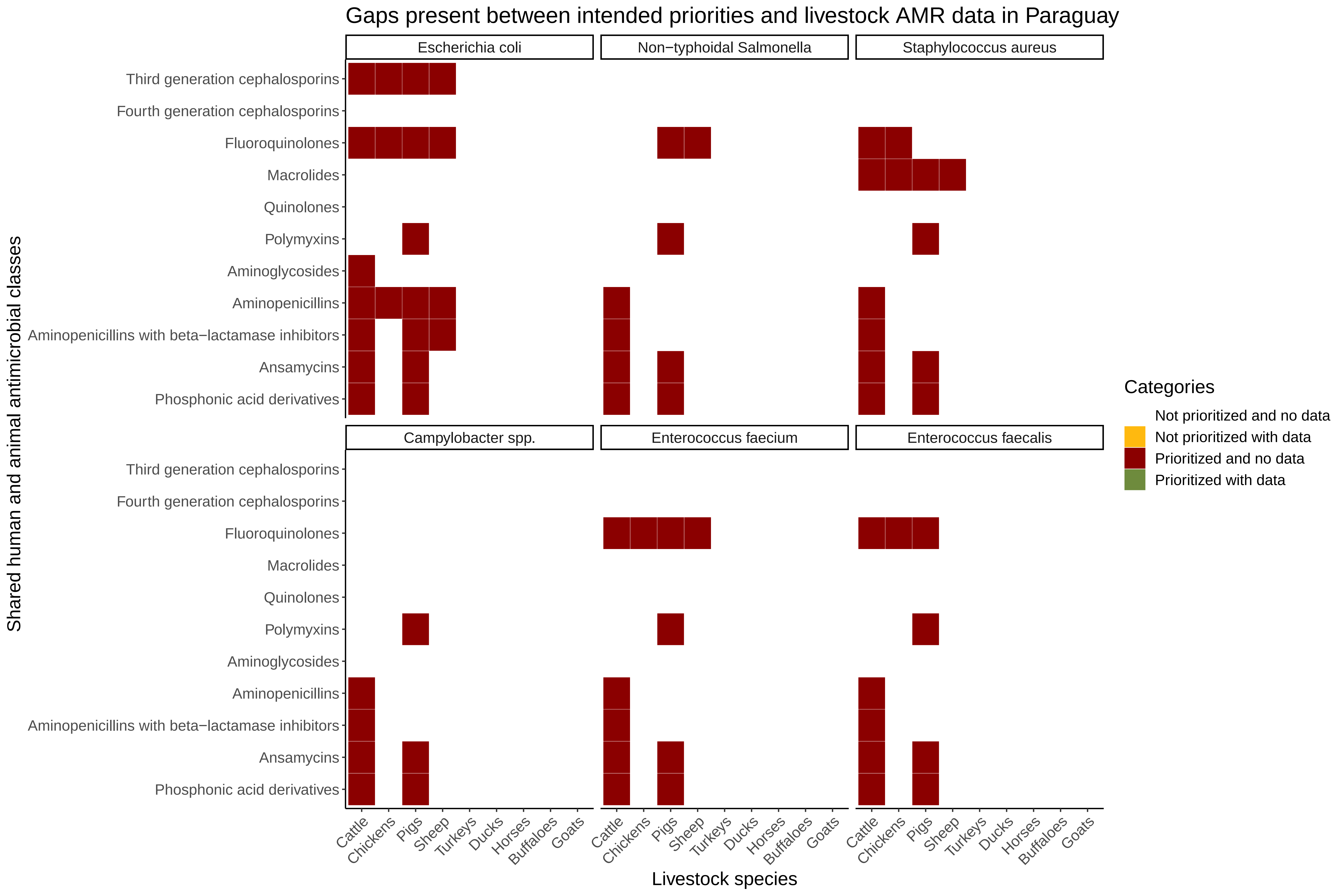

Gaps present between intended priorities and livestock AMR data in Paraguay
Escherichia coli
Non−typhoidal Salmonella
Staphylococcus aureus
Campylobacter spp.
Enterococcus faecium
Enterococcus faecalis
Third generation cephalosporins
Fourth generation cephalosporins
Fluoroquinolones
Macrolides
Quinolones
Polymyxins
Aminoglycosides
Aminopenicillins
Aminopenicillins with beta−lactamase inhibitors
Ansamycins
Phosphonic acid derivatives
Shared human and animal antimicrobial classes
Cattle
Chickens
Pigs
Sheep
Turkeys
Ducks
Horses
Buffaloes
Goats
Livestock species
Categories
Not prioritized and no data
Not prioritized with data
Prioritized and no data
Prioritized with data

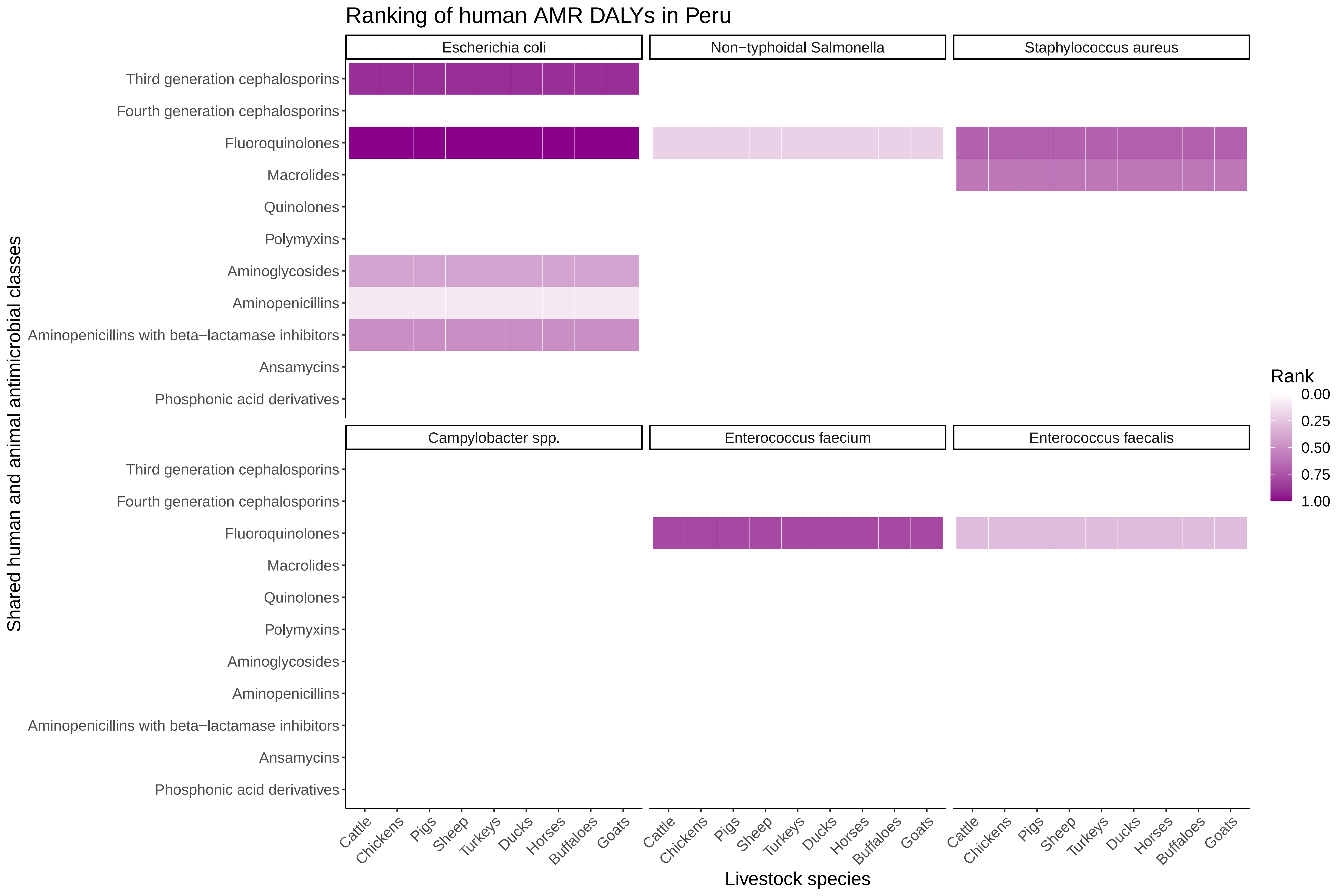

Ranking of human AMR DALYs in Peru
Escherichia coli
Non−typhoidal Salmonella
Staphylococcus aureus
Campylobacter spp.
Enterococcus faecium
Enterococcus faecalis
Third generation cephalosporins
Fourth generation cephalosporins
Fluoroquinolones
Macrolides
Quinolones
Polymyxins
Aminoglycosides
Aminopenicillins
Aminopenicillins with beta−lactamase inhibitors
Ansamycins
Phosphonic acid derivatives
Shared human and animal antimicrobial classes
Cattle
Chickens
Pigs
Sheep
Turkeys
Ducks
Horses
Buffaloes
Goats
Livestock species
Rank
0.00
0.25
0.50
0.75
1.00

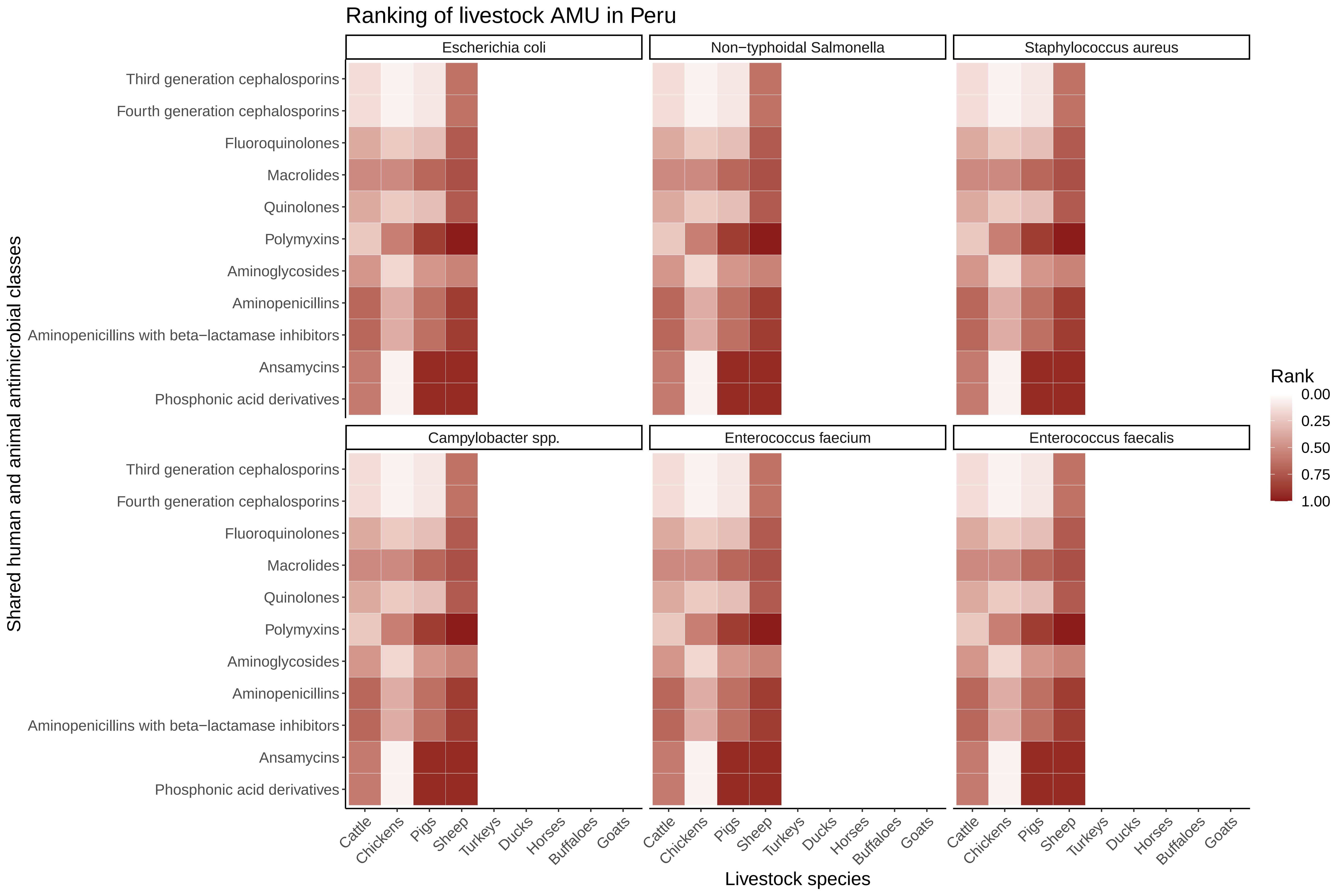

Ranking of livestock AMU in Peru
Escherichia coli
Non−typhoidal Salmonella
Staphylococcus aureus
Campylobacter spp.
Enterococcus faecium
Enterococcus faecalis
Shared human and animal antimicrobial classes
Third generation cephalosporins
Fourth generation cephalosporins
Fluoroquinolones
Macrolides
Quinolones
Polymyxins
Aminoglycosides
Aminopenicillins
Aminopenicillins with beta−lactamase inhibitors
Ansamycins
Phosphonic acid derivatives
Livestock species
Cattle
Chickens
Pigs
Sheep
Turkeys
Ducks
Horses
Buffaloes
Goats
Rank
0.00
0.25
0.50
0.75
1.00

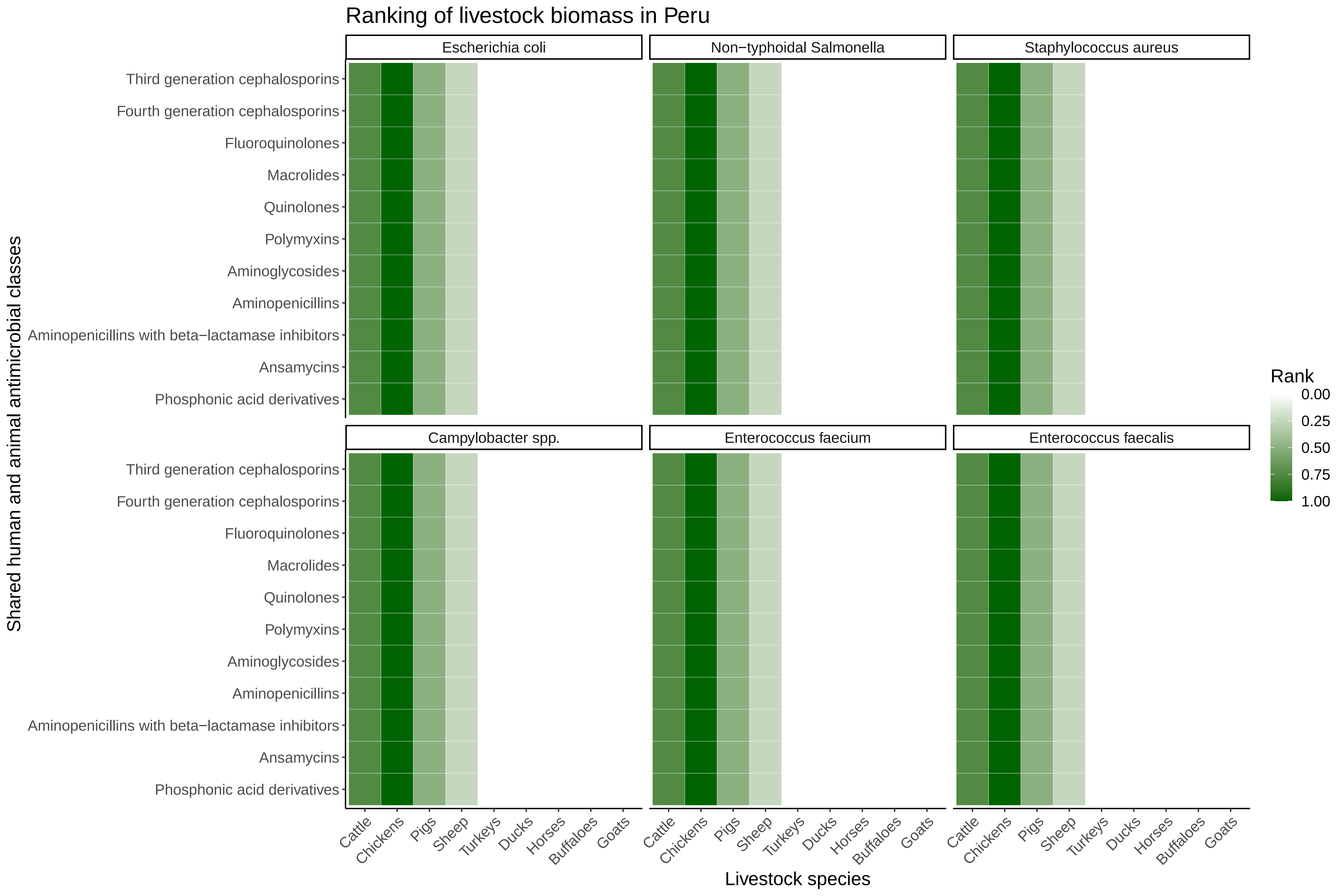
Ranking of livestock biomass in Peru
Escherichia coli
Non−typhoidal Salmonella
Staphylococcus aureus
Campylobacter spp.
Enterococcus faecium
Enterococcus faecalis
Shared human and animal antimicrobial classes
Third generation cephalosporins
Fourth generation cephalosporins
Fluoroquinolones
Macrolides
Quinolones
Polymyxins
Aminoglycosides
Aminopenicillins
Aminopenicillins with beta−lactamase inhibitors
Ansamycins
Phosphonic acid derivatives
Cattle
Chickens
Pigs
Sheep
Turkeys
Ducks
Horses
Buffaloes
Goats
Livestock species
Rank
0.00
0.25
0.50
0.75
1.00

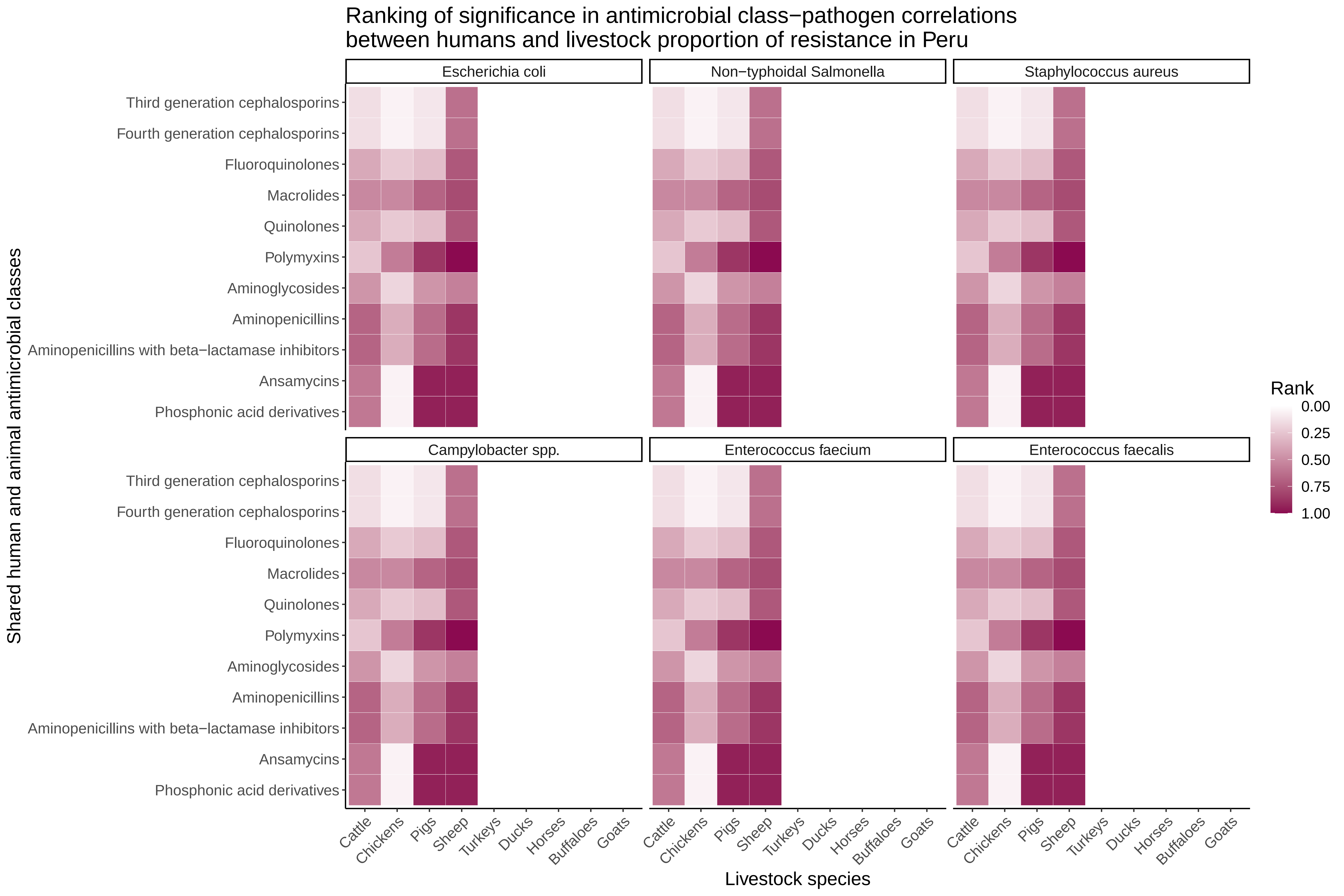
Ranking of significance in antimicrobial class−pathogen correlations
between humans and livestock proportion of resistance in Peru
Escherichia coli
Non−typhoidal Salmonella
Staphylococcus aureus
Campylobacter spp.
Enterococcus faecium
Enterococcus faecalis
Third generation cephalosporins
Fourth generation cephalosporins
Fluoroquinolones
Macrolides
Quinolones
Polymyxins
Aminoglycosides
Aminopenicillins
Aminopenicillins with beta−lactamase inhibitors
Ansamycins
Phosphonic acid derivatives
Shared human and animal antimicrobial classes
Cattle
Chickens
Pigs
Sheep
Turkeys
Ducks
Horses
Buffaloes
Goats
Livestock species
Rank
0.00
0.25
0.50
0.75
1.00

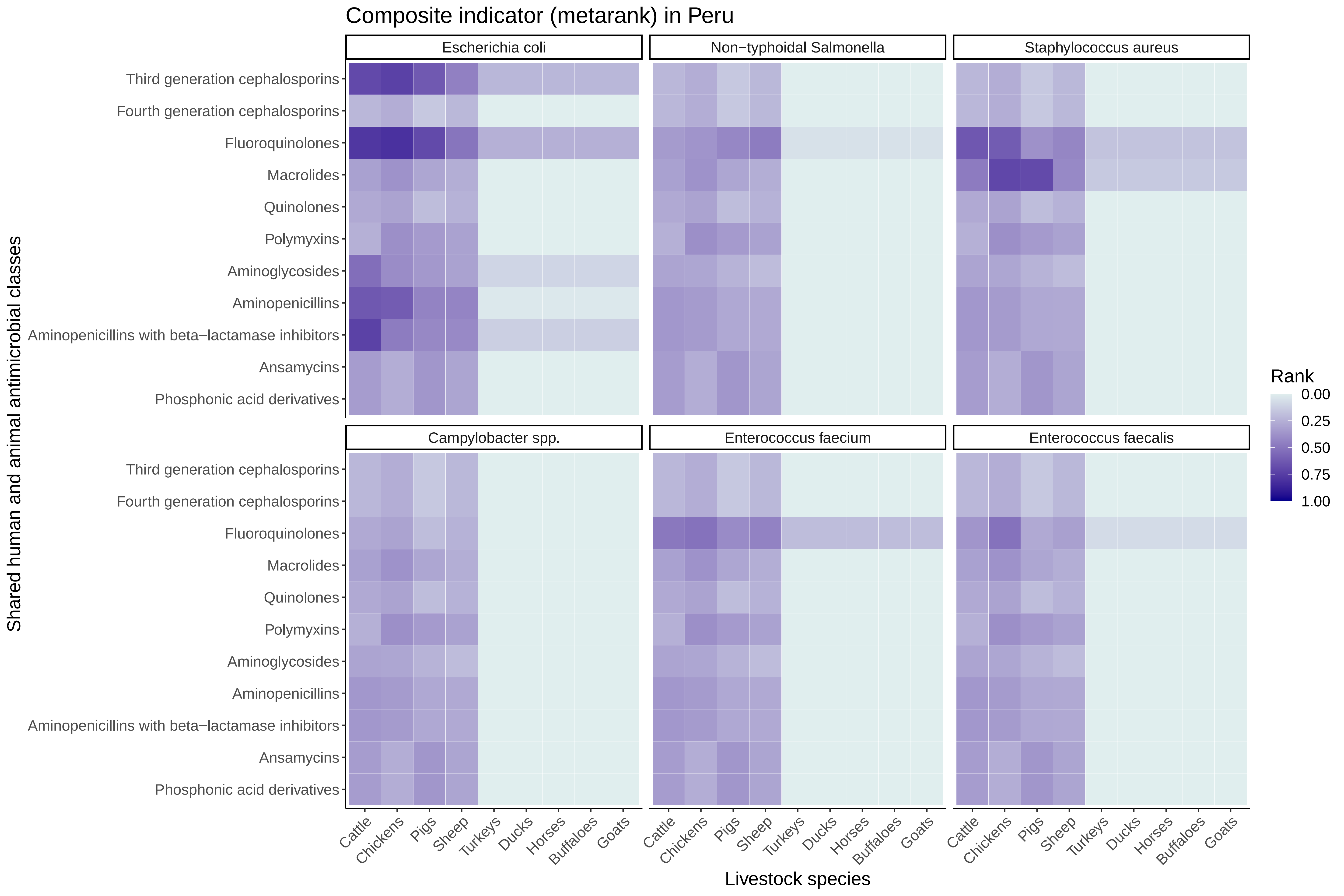

Composite indicator (metarank) in Peru
Escherichia coli
Non−typhoidal Salmonella
Staphylococcus aureus
Campylobacter spp.
Enterococcus faecium
Enterococcus faecalis
Shared human and animal antimicrobial classes
Third generation cephalosporins
Fourth generation cephalosporins
Fluoroquinolones
Macrolides
Quinolones
Polymyxins
Aminoglycosides
Aminopenicillins
Aminopenicillins with beta−lactamase inhibitors
Ansamycins
Phosphonic acid derivatives
Cattle
Chickens
Pigs
Sheep
Turkeys
Ducks
Horses
Buffaloes
Goats
Livestock species
Rank
0.00
0.25
0.50
0.75
1.00

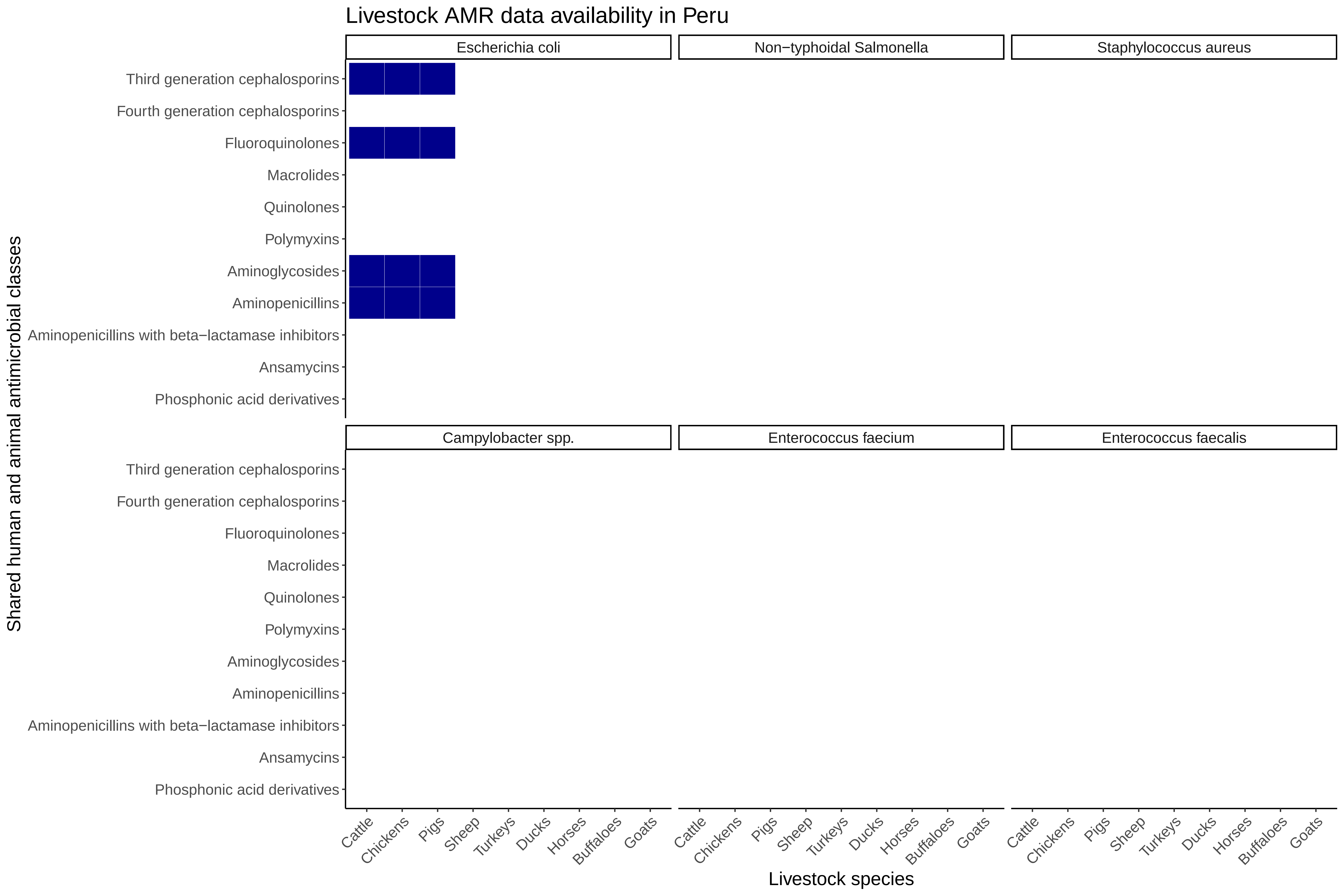

Livestock AMR data availability in Peru
Escherichia coli
Non−typhoidal Salmonella
Staphylococcus aureus
Campylobacter spp.
Enterococcus faecium
Enterococcus faecalis
Third generation cephalosporins
Fourth generation cephalosporins
Fluoroquinolones
Macrolides
Quinolones
Polymyxins
Aminoglycosides
Aminopenicillins
Aminopenicillins with beta−lactamase inhibitors
Ansamycins
Phosphonic acid derivatives
Shared human and animal antimicrobial classes
Cattle
Chickens
Pigs
Sheep
Turkeys
Ducks
Horses
Buffaloes
Goats
Livestock species

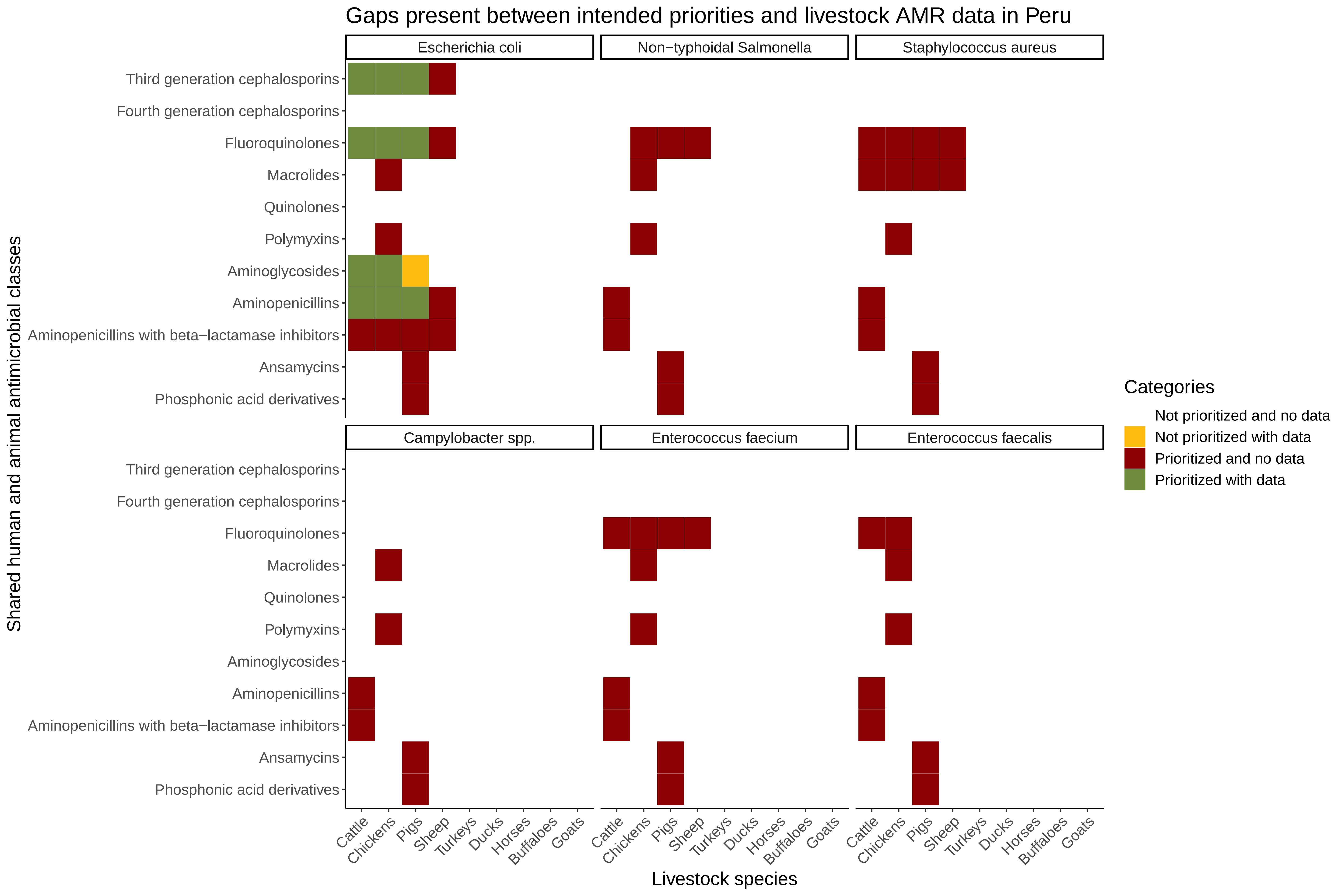

Gaps present between intended priorities and livestock AMR data in Peru
Escherichia coli
Non−typhoidal Salmonella
Staphylococcus aureus
Campylobacter spp.
Enterococcus faecium
Enterococcus faecalis
Shared human and animal antimicrobial classes
Third generation cephalosporins
Fourth generation cephalosporins
Fluoroquinolones
Macrolides
Quinolones
Polymyxins
Aminoglycosides
Aminopenicillins
Aminopenicillins with beta−lactamase inhibitors
Ansamycins
Phosphonic acid derivatives
Livestock species
Cattle
Chickens
Pigs
Sheep
Turkeys
Ducks
Horses
Buffaloes
Goats
Categories
Not prioritized and no data
Not prioritized with data
Prioritized and no data
Prioritized with data

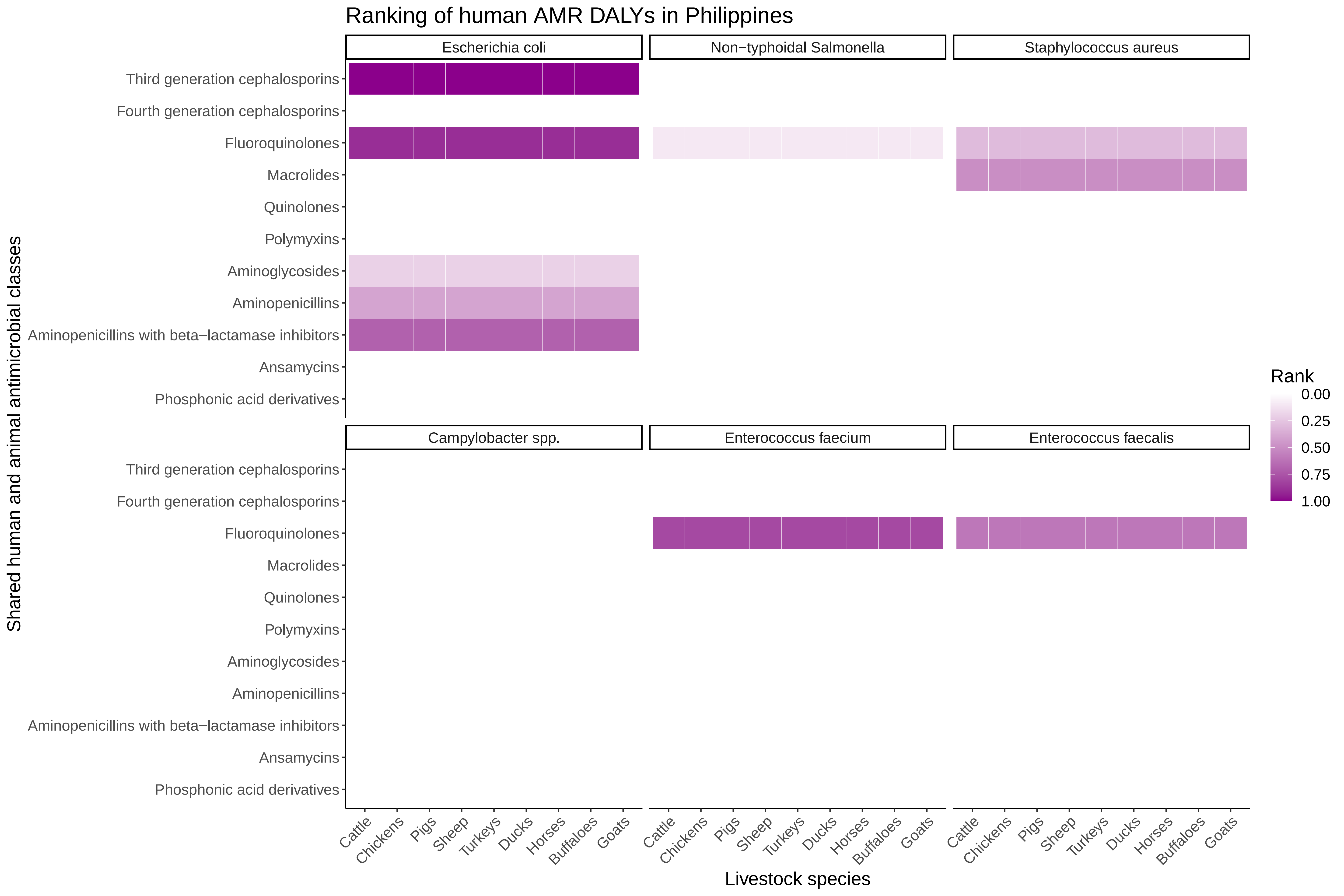
Ranking of human AMR DALYs in Philippines
Escherichia coli
Non−typhoidal Salmonella
Staphylococcus aureus
Campylobacter spp.
Enterococcus faecium
Enterococcus faecalis
Third generation cephalosporins
Fourth generation cephalosporins
Fluoroquinolones
Macrolides
Quinolones
Polymyxins
Aminoglycosides
Aminopenicillins
Aminopenicillins with beta−lactamase inhibitors
Ansamycins
Phosphonic acid derivatives
Shared human and animal antimicrobial classes
Cattle
Chickens
Pigs
Sheep
Turkeys
Ducks
Horses
Buffaloes
Goats
Livestock species
Rank
0.00
0.25
0.50
0.75
1.00

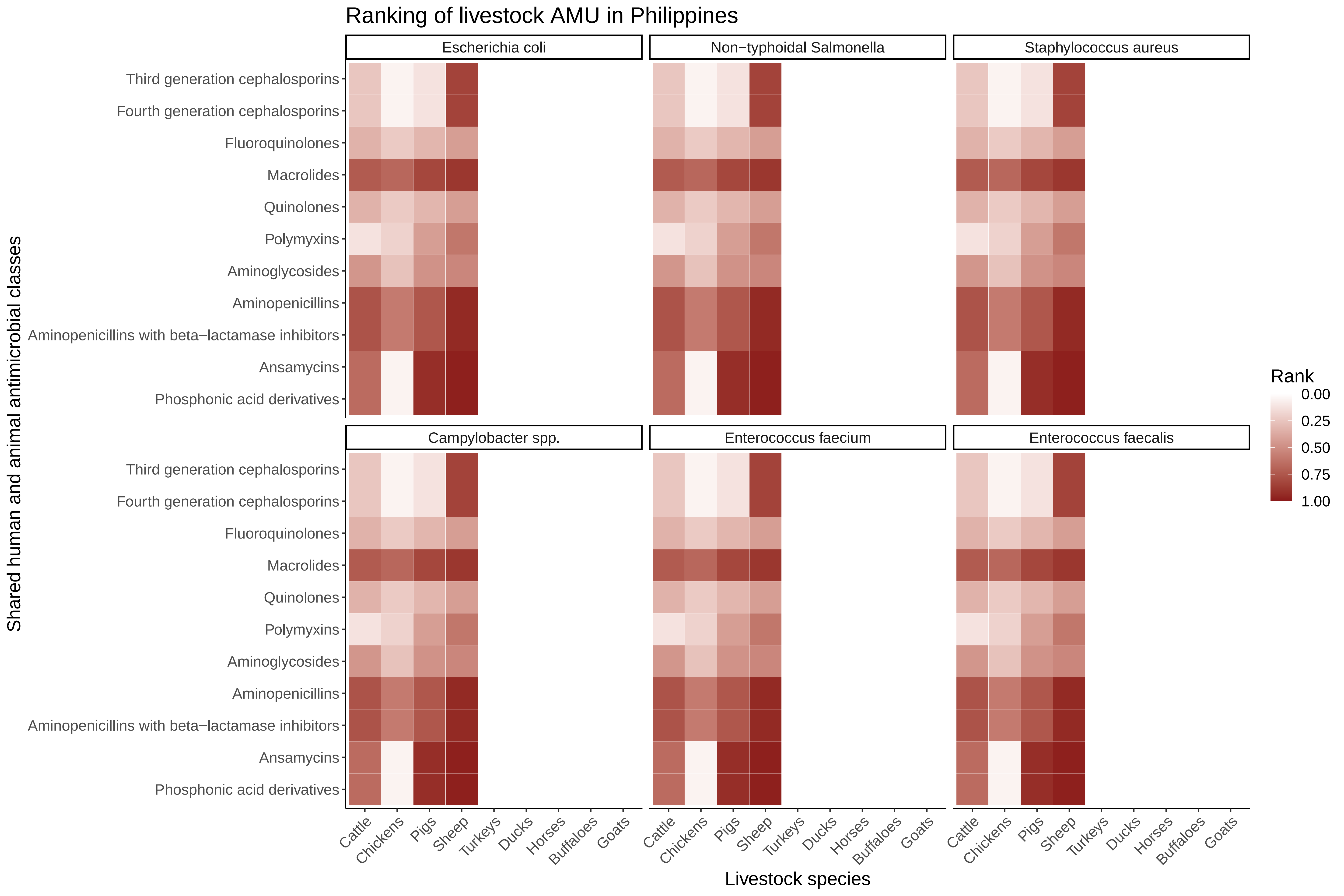

Ranking of livestock AMU in Philippines
Escherichia coli
Non−typhoidal Salmonella
Staphylococcus aureus
Campylobacter spp.
Enterococcus faecium
Enterococcus faecalis
Third generation cephalosporins
Fourth generation cephalosporins
Fluoroquinolones
Macrolides
Quinolones
Polymyxins
Aminoglycosides
Aminopenicillins
Aminopenicillins with beta−lactamase inhibitors
Ansamycins
Phosphonic acid derivatives
Shared human and animal antimicrobial classes
Cattle
Chickens
Pigs
Sheep
Turkeys
Ducks
Horses
Buffaloes
Goats
Livestock species
Rank
0.00
0.25
0.50
0.75
1.00

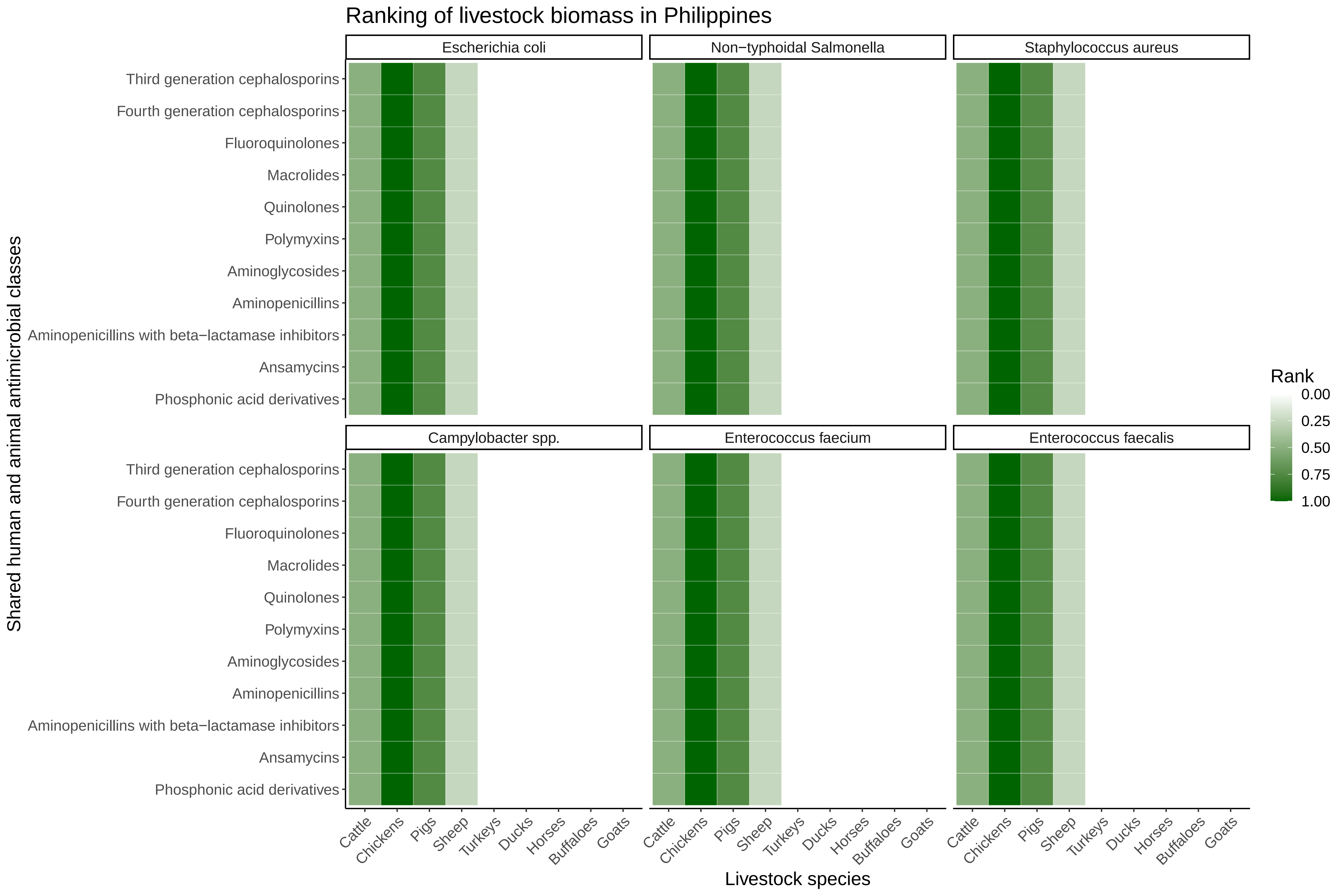

Ranking of livestock biomass in Philippines
Escherichia coli
Non−typhoidal Salmonella
Staphylococcus aureus
Campylobacter spp.
Enterococcus faecium
Enterococcus faecalis
Shared human and animal antimicrobial classes
Third generation cephalosporins
Fourth generation cephalosporins
Fluoroquinolones
Macrolides
Quinolones
Polymyxins
Aminoglycosides
Aminopenicillins
Aminopenicillins with beta−lactamase inhibitors
Ansamycins
Phosphonic acid derivatives
Cattle
Chickens
Pigs
Sheep
Turkeys
Ducks
Horses
Buffaloes
Goats
Livestock species
Rank
0.00
0.25
0.50
0.75
1.00

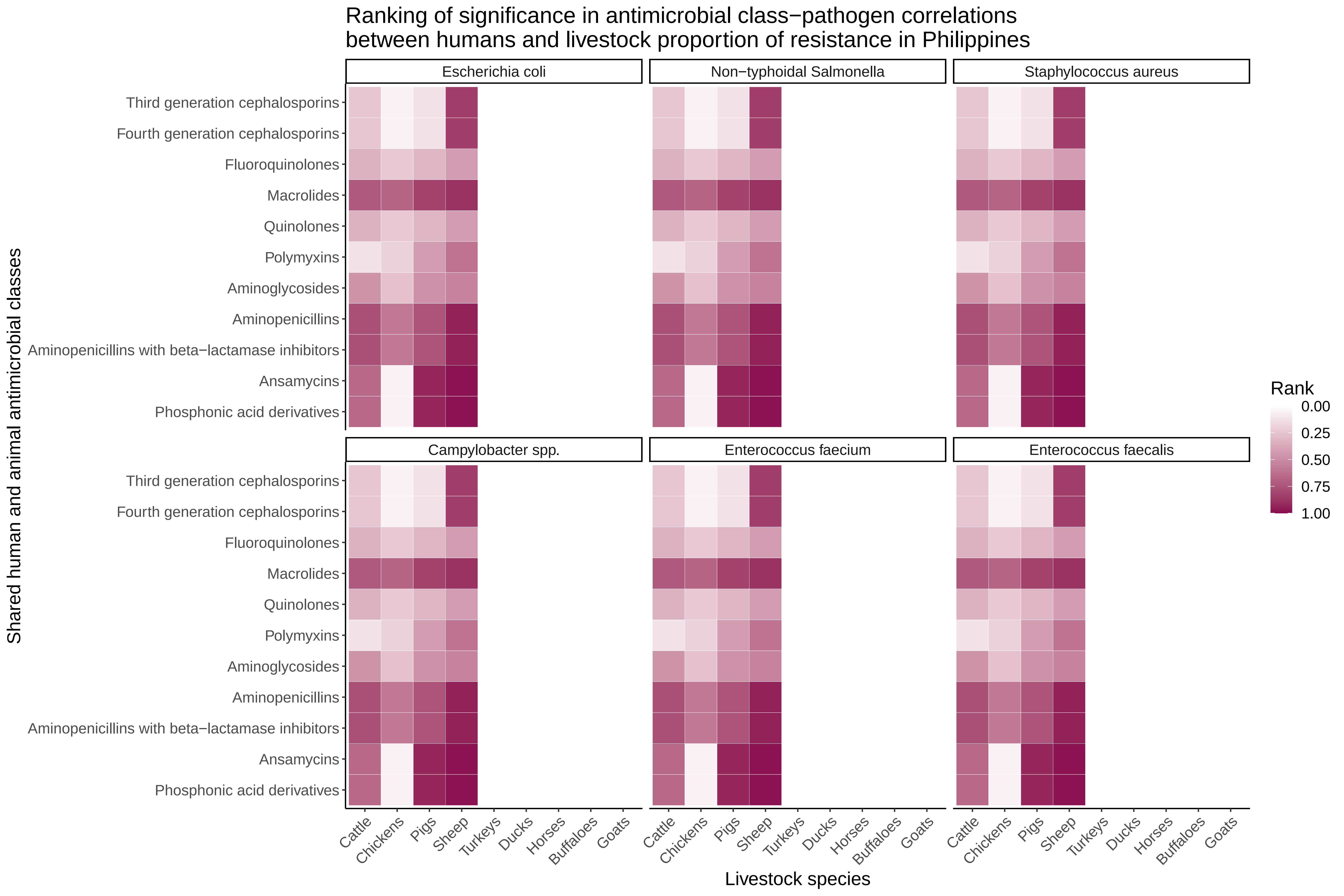

Ranking of significance in antimicrobial class−pathogen correlations
between humans and livestock proportion of resistance in Philippines
Escherichia coli
Non−typhoidal Salmonella
Staphylococcus aureus
Campylobacter spp.
Enterococcus faecium
Enterococcus faecalis
Third generation cephalosporins
Fourth generation cephalosporins
Fluoroquinolones
Macrolides
Quinolones
Polymyxins
Aminoglycosides
Aminopenicillins
Aminopenicillins with beta−lactamase inhibitors
Ansamycins
Phosphonic acid derivatives
Shared human and animal antimicrobial classes
Cattle
Chickens
Pigs
Sheep
Turkeys
Ducks
Horses
Buffaloes
Goats
Livestock species
Rank
0.00
0.25
0.50
0.75
1.00

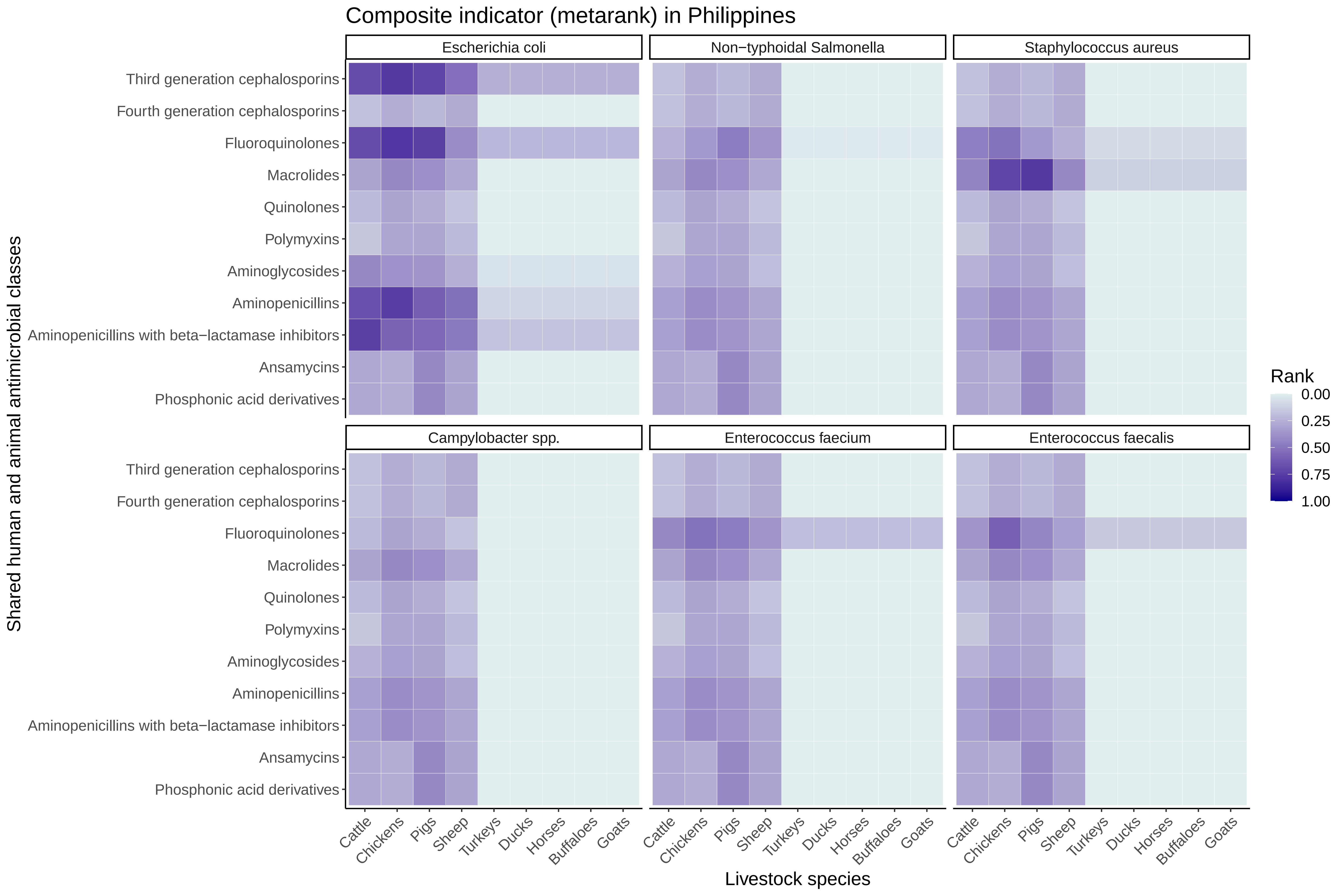

Composite indicator (metarank) in Philippines
Escherichia coli
Non−typhoidal Salmonella
Staphylococcus aureus
Campylobacter spp.
Enterococcus faecium
Enterococcus faecalis
Shared human and animal antimicrobial classes
Third generation cephalosporins
Fourth generation cephalosporins
Fluoroquinolones
Macrolides
Quinolones
Polymyxins
Aminoglycosides
Aminopenicillins
Aminopenicillins with beta−lactamase inhibitors
Ansamycins
Phosphonic acid derivatives
Livestock species
Cattle
Chickens
Pigs
Sheep
Turkeys
Ducks
Horses
Buffaloes
Goats
Rank
0.00
0.25
0.50
0.75
1.00

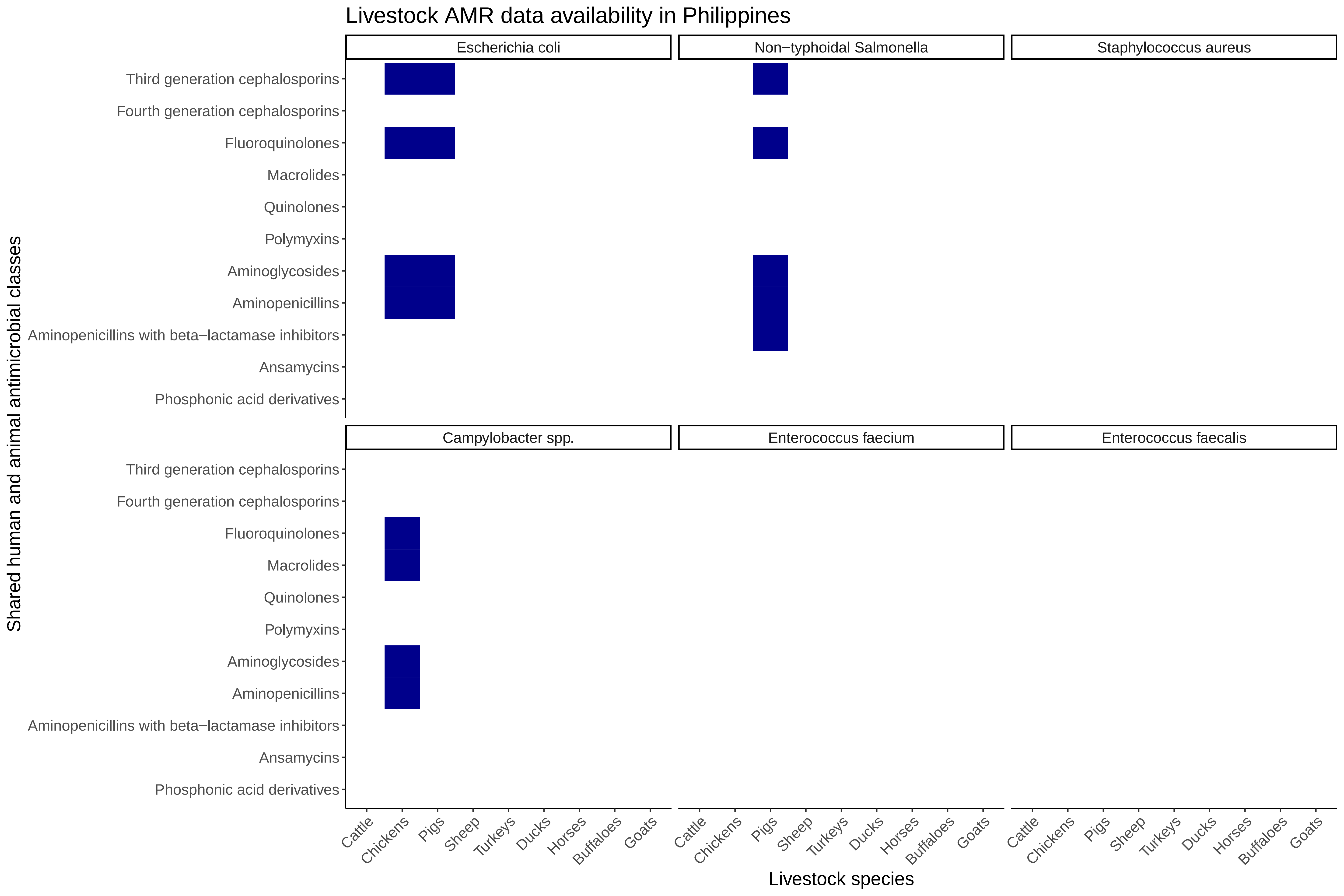

Livestock AMR data availability in Philippines
Escherichia coli
Non−typhoidal Salmonella
Staphylococcus aureus
Campylobacter spp.
Enterococcus faecium
Enterococcus faecalis
Shared human and animal antimicrobial classes
Third generation cephalosporins
Fourth generation cephalosporins
Fluoroquinolones
Macrolides
Quinolones
Polymyxins
Aminoglycosides
Aminopenicillins
Aminopenicillins with beta−lactamase inhibitors
Ansamycins
Phosphonic acid derivatives
Cattle
Chickens
Pigs
Sheep
Turkeys
Ducks
Horses
Buffaloes
Goats
Livestock species

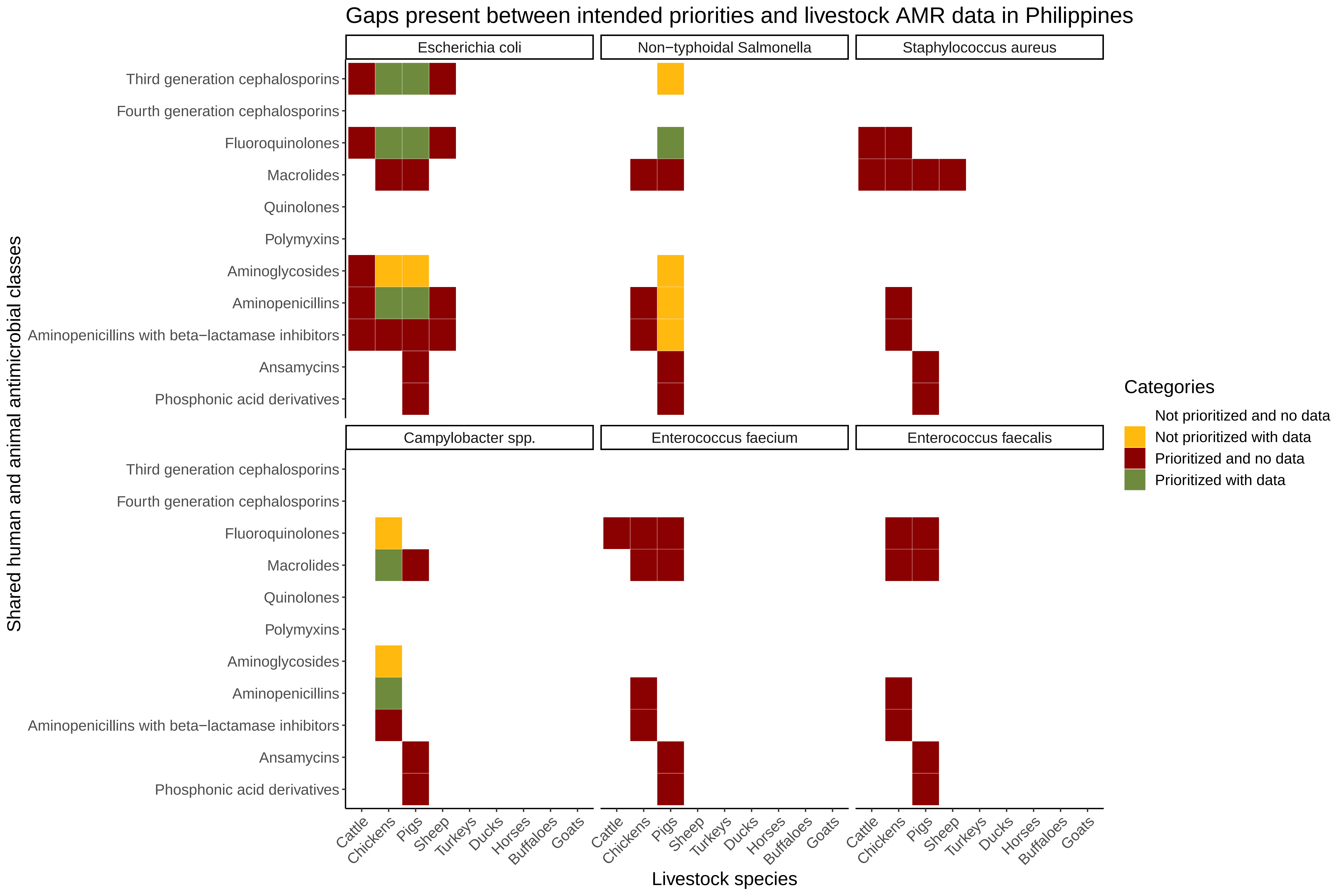
Gaps present between intended priorities and livestock AMR data in Philippines
Escherichia coli
Non−typhoidal Salmonella
Staphylococcus aureus
Campylobacter spp.
Enterococcus faecium
Enterococcus faecalis
Shared human and animal antimicrobial classes
Third generation cephalosporins
Fourth generation cephalosporins
Fluoroquinolones
Macrolides
Quinolones
Polymyxins
Aminoglycosides
Aminopenicillins
Aminopenicillins with beta−lactamase inhibitors
Ansamycins
Phosphonic acid derivatives
Cattle
Chickens
Pigs
Sheep
Turkeys
Ducks
Horses
Buffaloes
Goats
Livestock species
Categories
Not prioritized and no data
Not prioritized with data
Prioritized and no data
Prioritized with data

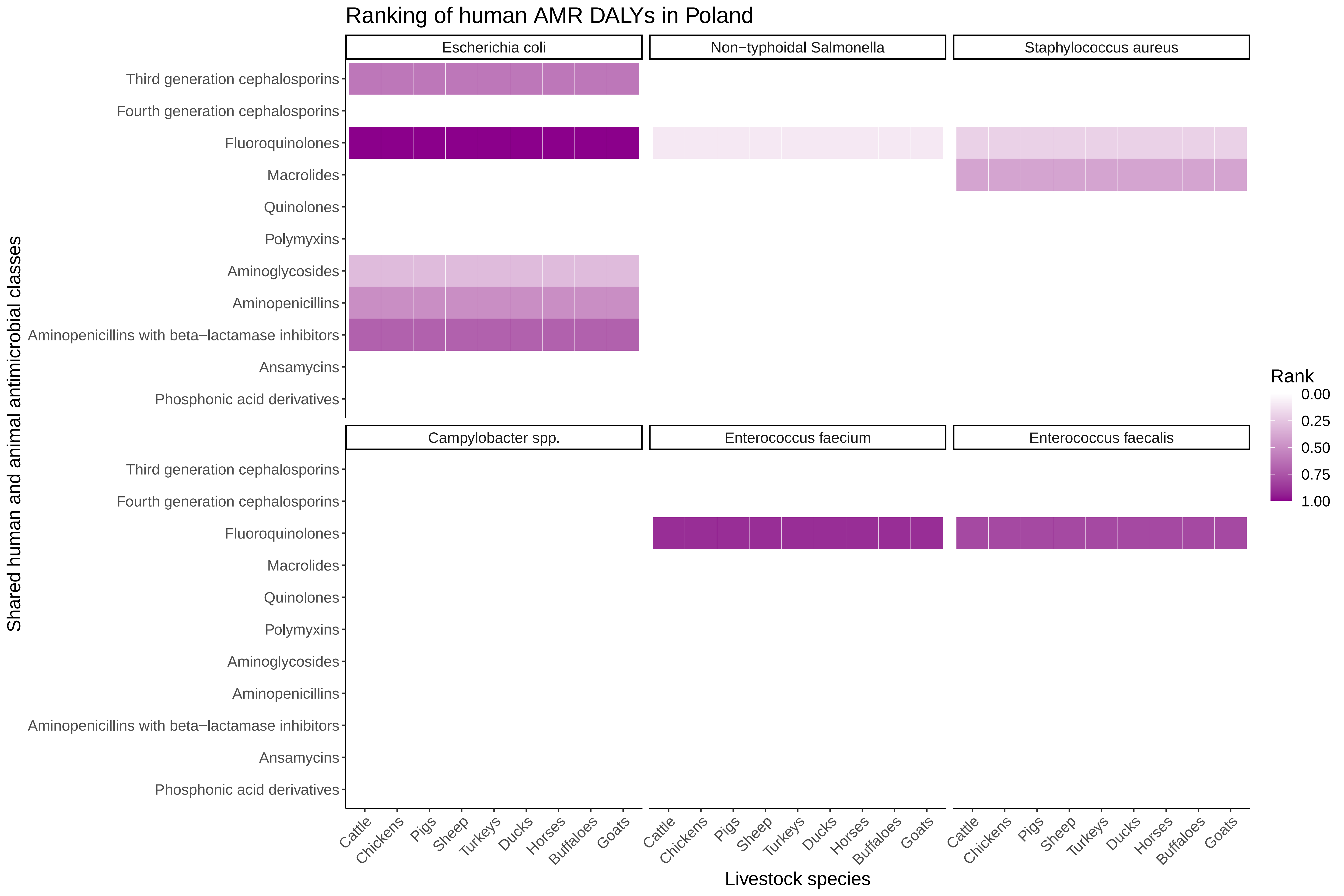

Ranking of human AMR DALYs in Poland
Escherichia coli
Non−typhoidal Salmonella
Staphylococcus aureus
Campylobacter spp.
Enterococcus faecium
Enterococcus faecalis
Shared human and animal antimicrobial classes
Third generation cephalosporins
Fourth generation cephalosporins
Fluoroquinolones
Macrolides
Quinolones
Polymyxins
Aminoglycosides
Aminopenicillins
Aminopenicillins with beta−lactamase inhibitors
Ansamycins
Phosphonic acid derivatives
Livestock species
Cattle
Chickens
Pigs
Sheep
Turkeys
Ducks
Horses
Buffaloes
Goats
Rank
0.00
0.25
0.50
0.75
1.00

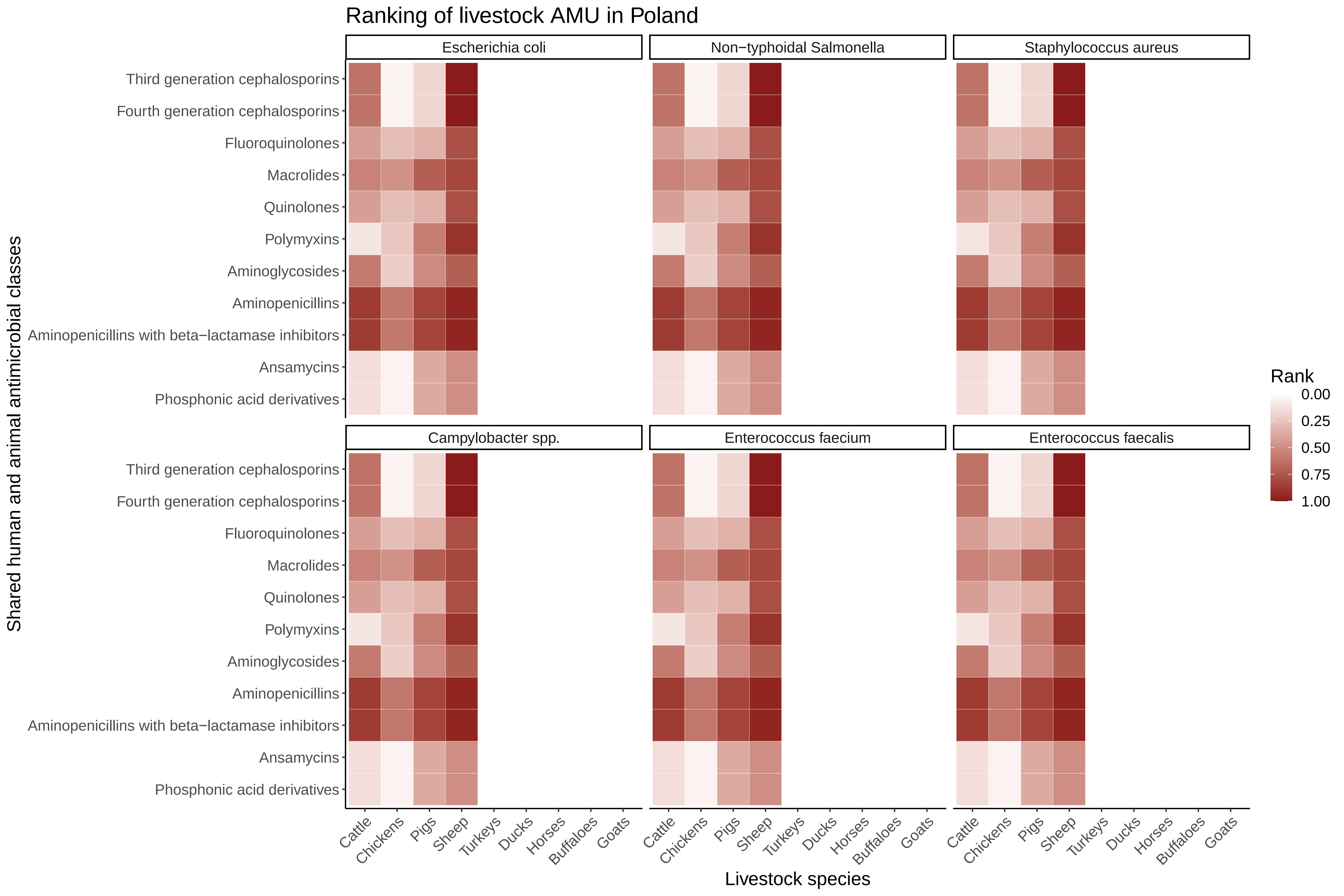

Ranking of livestock AMU in Poland
Escherichia coli
Non−typhoidal Salmonella
Staphylococcus aureus
Campylobacter spp.
Enterococcus faecium
Enterococcus faecalis
Third generation cephalosporins
Fourth generation cephalosporins
Fluoroquinolones
Macrolides
Quinolones
Polymyxins
Aminoglycosides
Aminopenicillins
Aminopenicillins with beta−lactamase inhibitors
Ansamycins
Phosphonic acid derivatives
Shared human and animal antimicrobial classes
Cattle
Chickens
Pigs
Sheep
Turkeys
Ducks
Horses
Buffaloes
Goats
Livestock species
Rank
0.00
0.25
0.50
0.75
1.00

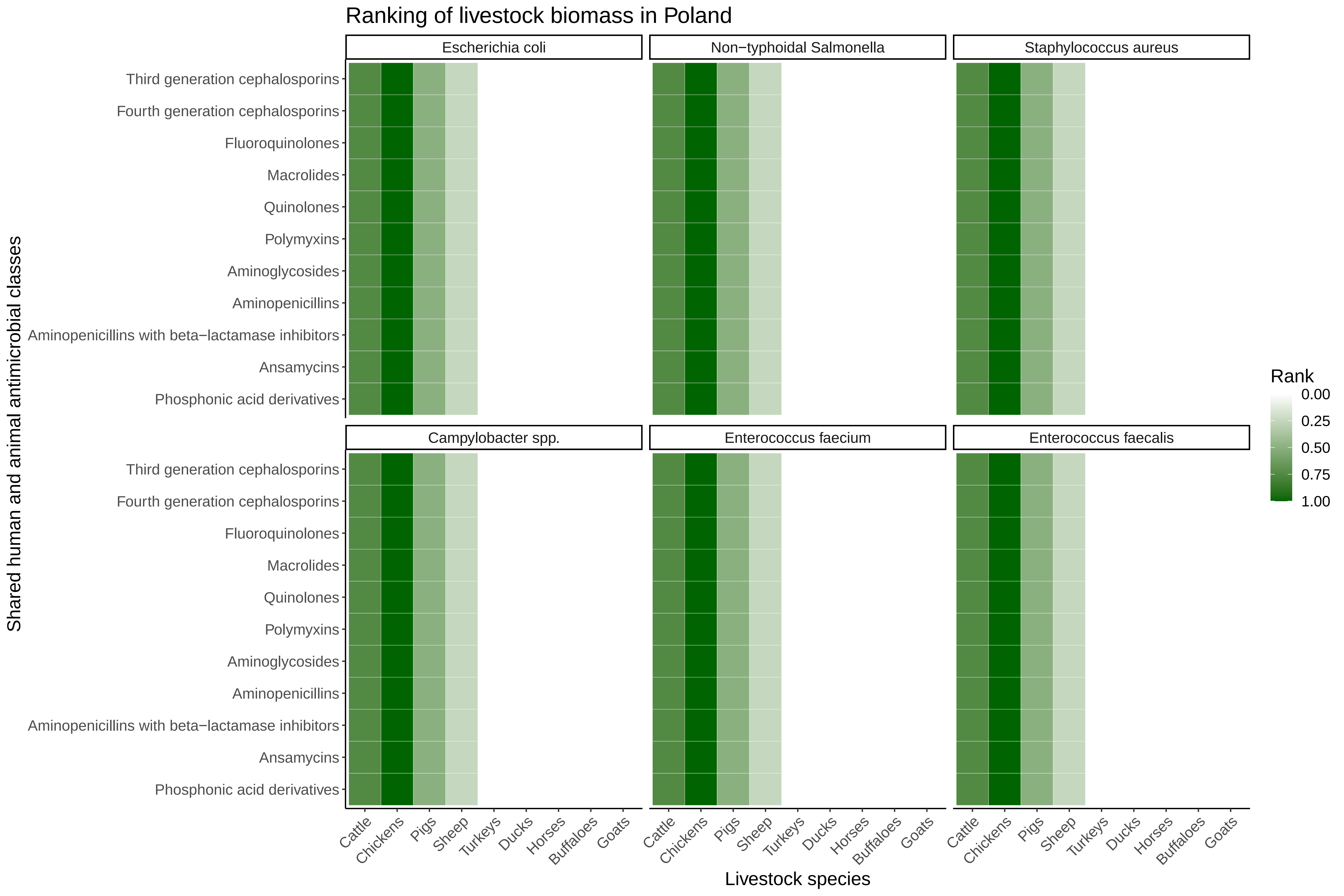
Ranking of livestock biomass in Poland
Escherichia coli
Non−typhoidal Salmonella
Staphylococcus aureus
Campylobacter spp.
Enterococcus faecium
Enterococcus faecalis
Third generation cephalosporins
Fourth generation cephalosporins
Fluoroquinolones
Macrolides
Quinolones
Polymyxins
Aminoglycosides
Aminopenicillins
Aminopenicillins with beta−lactamase inhibitors
Ansamycins
Phosphonic acid derivatives
Shared human and animal antimicrobial classes
Cattle
Chickens
Pigs
Sheep
Turkeys
Ducks
Horses
Buffaloes
Goats
Livestock species
Rank
0.00
0.25
0.50
0.75
1.00

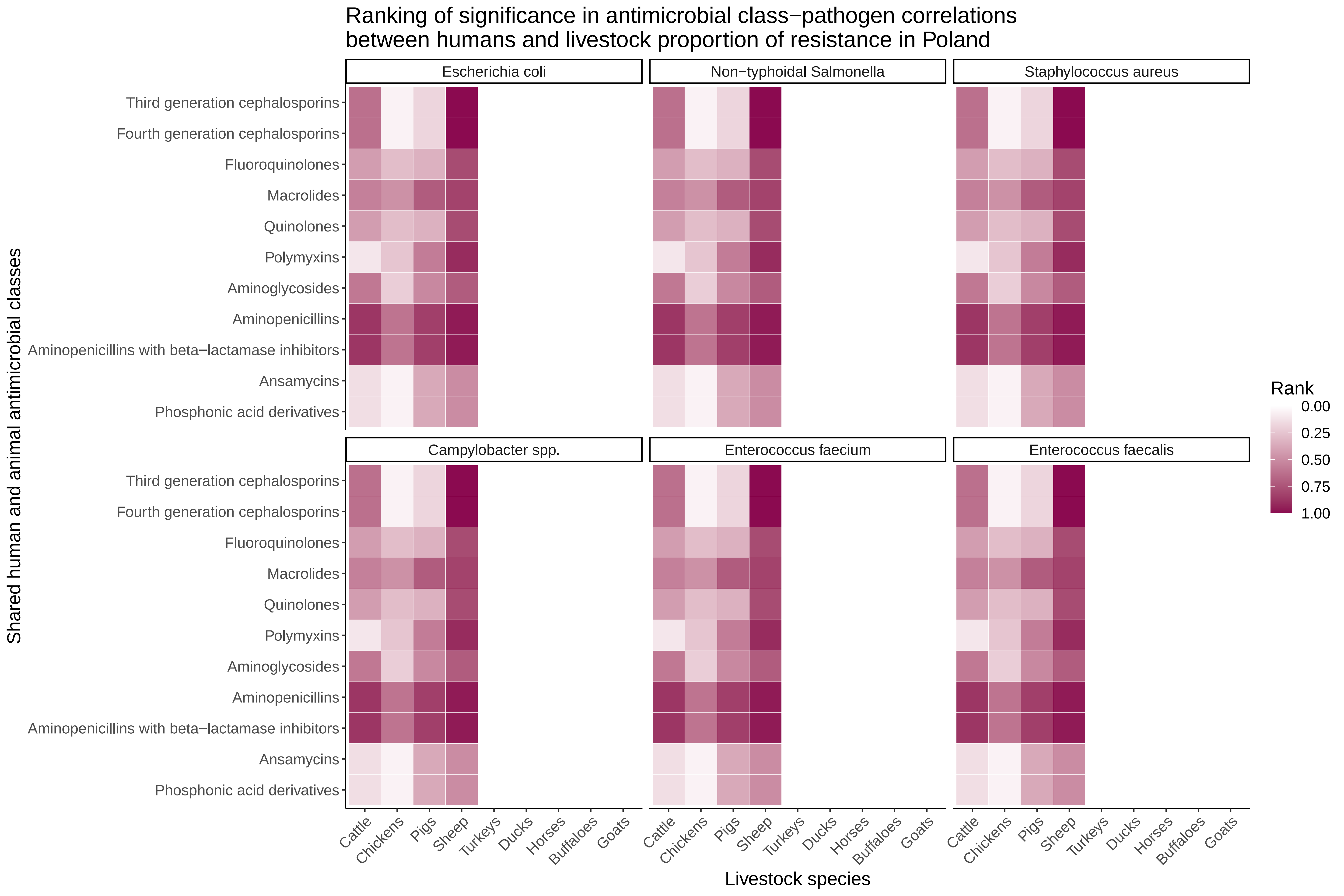

Ranking of significance in antimicrobial class−pathogen correlations
between humans and livestock proportion of resistance in Poland
Escherichia coli
Non−typhoidal Salmonella
Staphylococcus aureus
Campylobacter spp.
Enterococcus faecium
Enterococcus faecalis
Third generation cephalosporins
Fourth generation cephalosporins
Fluoroquinolones
Macrolides
Quinolones
Polymyxins
Aminoglycosides
Aminopenicillins
Aminopenicillins with beta−lactamase inhibitors
Ansamycins
Phosphonic acid derivatives
Shared human and animal antimicrobial classes
Cattle
Chickens
Pigs
Sheep
Turkeys
Ducks
Horses
Buffaloes
Goats
Livestock species
Rank
0.00
0.25
0.50
0.75
1.00

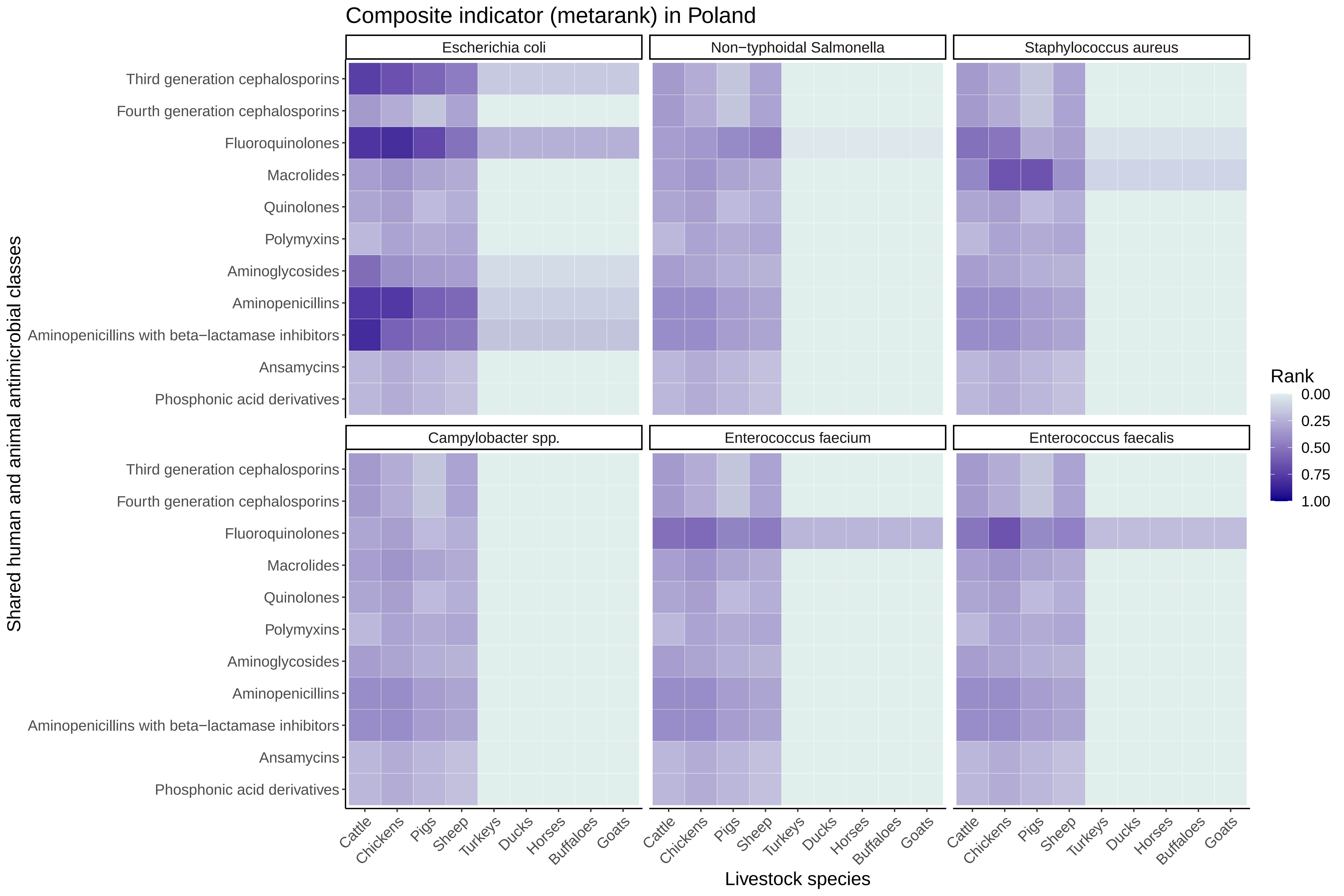

Composite indicator (metarank) in Poland
Escherichia coli
Non−typhoidal Salmonella
Staphylococcus aureus
Campylobacter spp.
Enterococcus faecium
Enterococcus faecalis
Shared human and animal antimicrobial classes
Third generation cephalosporins
Fourth generation cephalosporins
Fluoroquinolones
Macrolides
Quinolones
Polymyxins
Aminoglycosides
Aminopenicillins
Aminopenicillins with beta−lactamase inhibitors
Ansamycins
Phosphonic acid derivatives
Cattle
Chickens
Pigs
Sheep
Turkeys
Ducks
Horses
Buffaloes
Goats
Livestock species
Rank
0.00
0.25
0.50
0.75
1.00

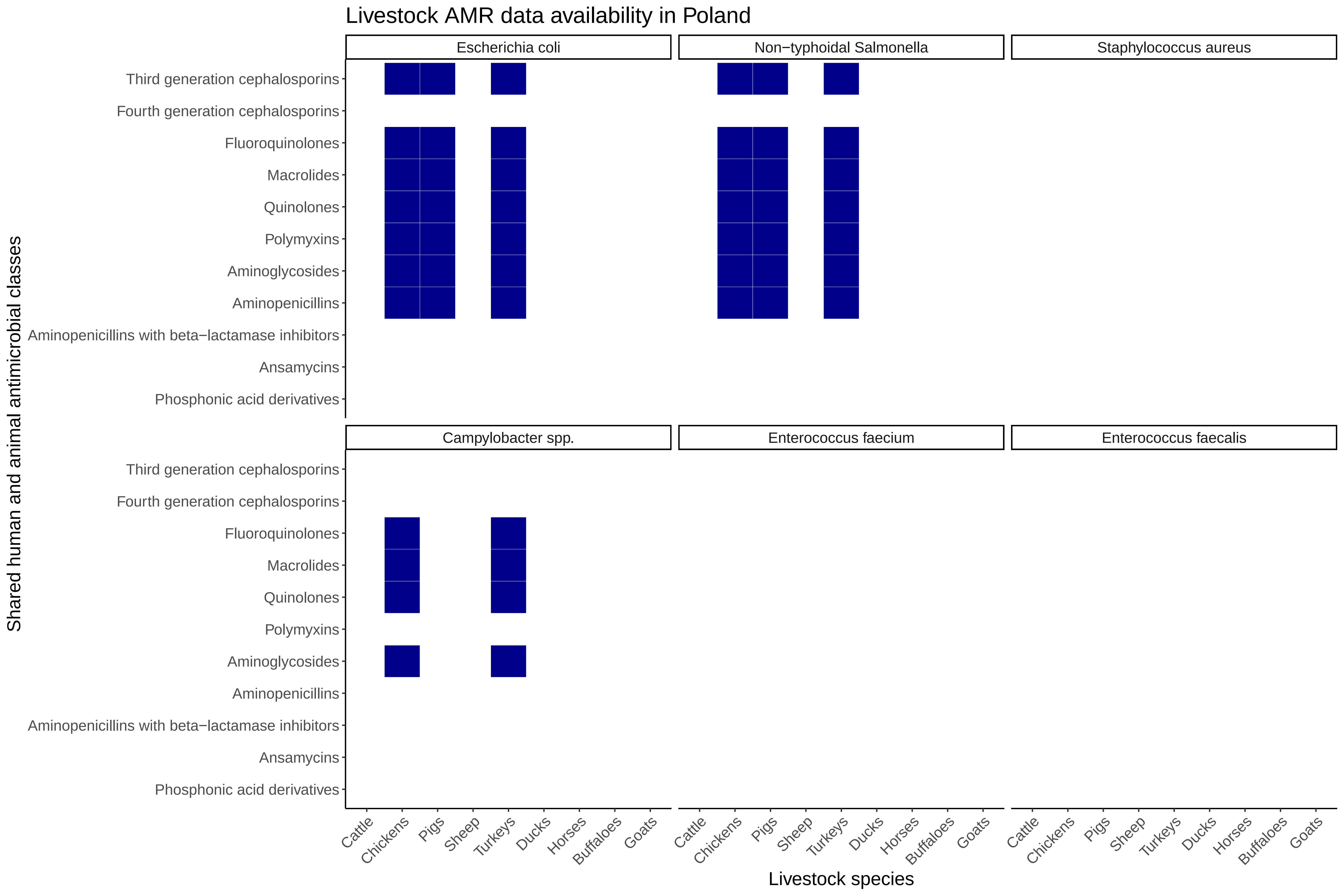

Livestock AMR data availability in Poland
Escherichia coli
Non−typhoidal Salmonella
Staphylococcus aureus
Campylobacter spp.
Enterococcus faecium
Enterococcus faecalis
Shared human and animal antimicrobial classes
Third generation cephalosporins
Fourth generation cephalosporins
Fluoroquinolones
Macrolides
Quinolones
Polymyxins
Aminoglycosides
Aminopenicillins
Aminopenicillins with beta−lactamase inhibitors
Ansamycins
Phosphonic acid derivatives
Cattle
Chickens
Pigs
Sheep
Turkeys
Ducks
Horses
Buffaloes
Goats
Livestock species

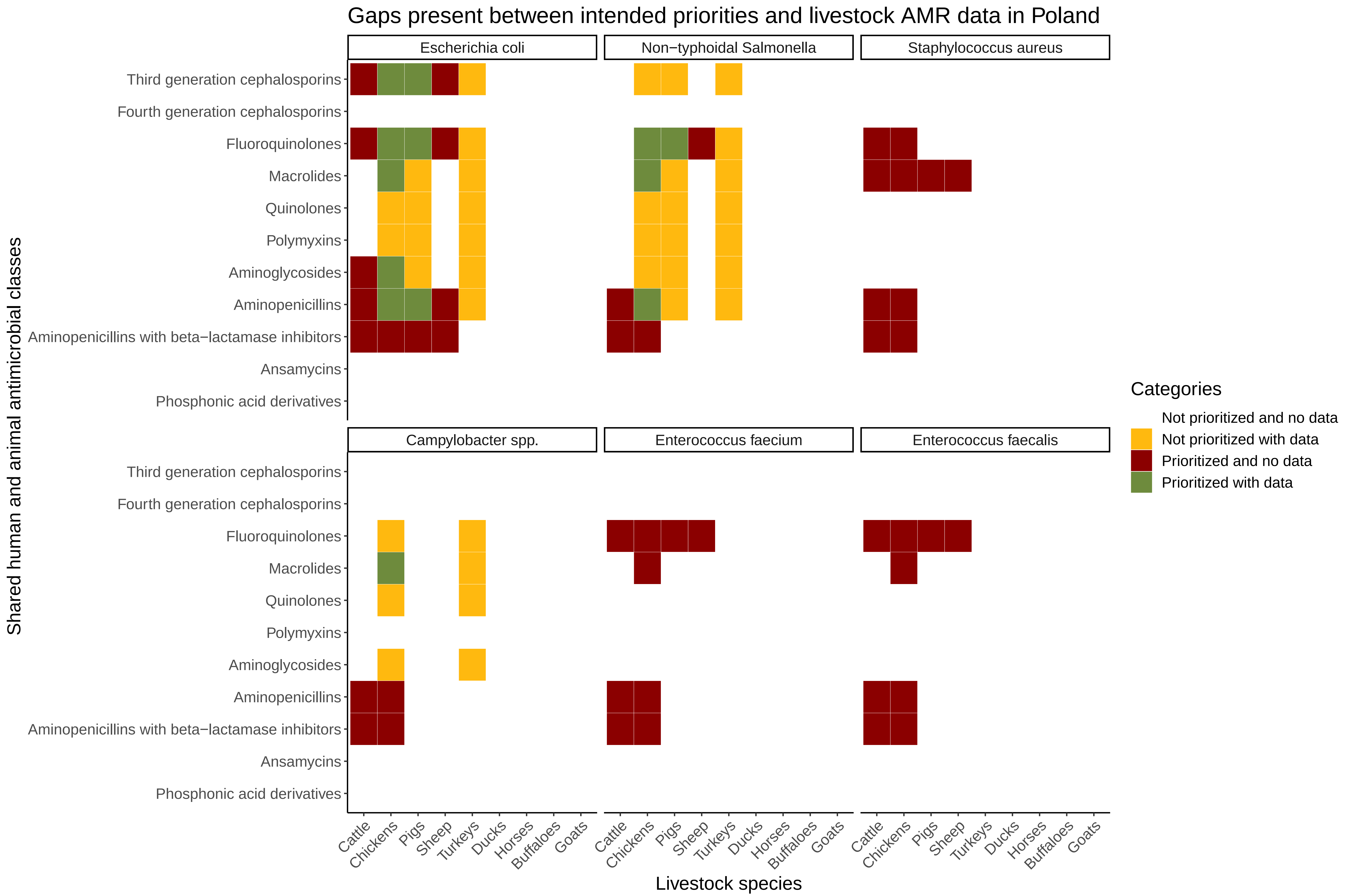

Gaps present between intended priorities and livestock AMR data in Poland
Escherichia coli
Non−typhoidal Salmonella
Staphylococcus aureus
Campylobacter spp.
Enterococcus faecium
Enterococcus faecalis
Third generation cephalosporins
Fourth generation cephalosporins
Fluoroquinolones
Macrolides
Quinolones
Polymyxins
Aminoglycosides
Aminopenicillins
Aminopenicillins with beta−lactamase inhibitors
Ansamycins
Phosphonic acid derivatives
Shared human and animal antimicrobial classes
Cattle
Chickens
Pigs
Sheep
Turkeys
Ducks
Horses
Buffaloes
Goats
Livestock species
Categories
Not prioritized and no data
Not prioritized with data
Prioritized and no data
Prioritized with data

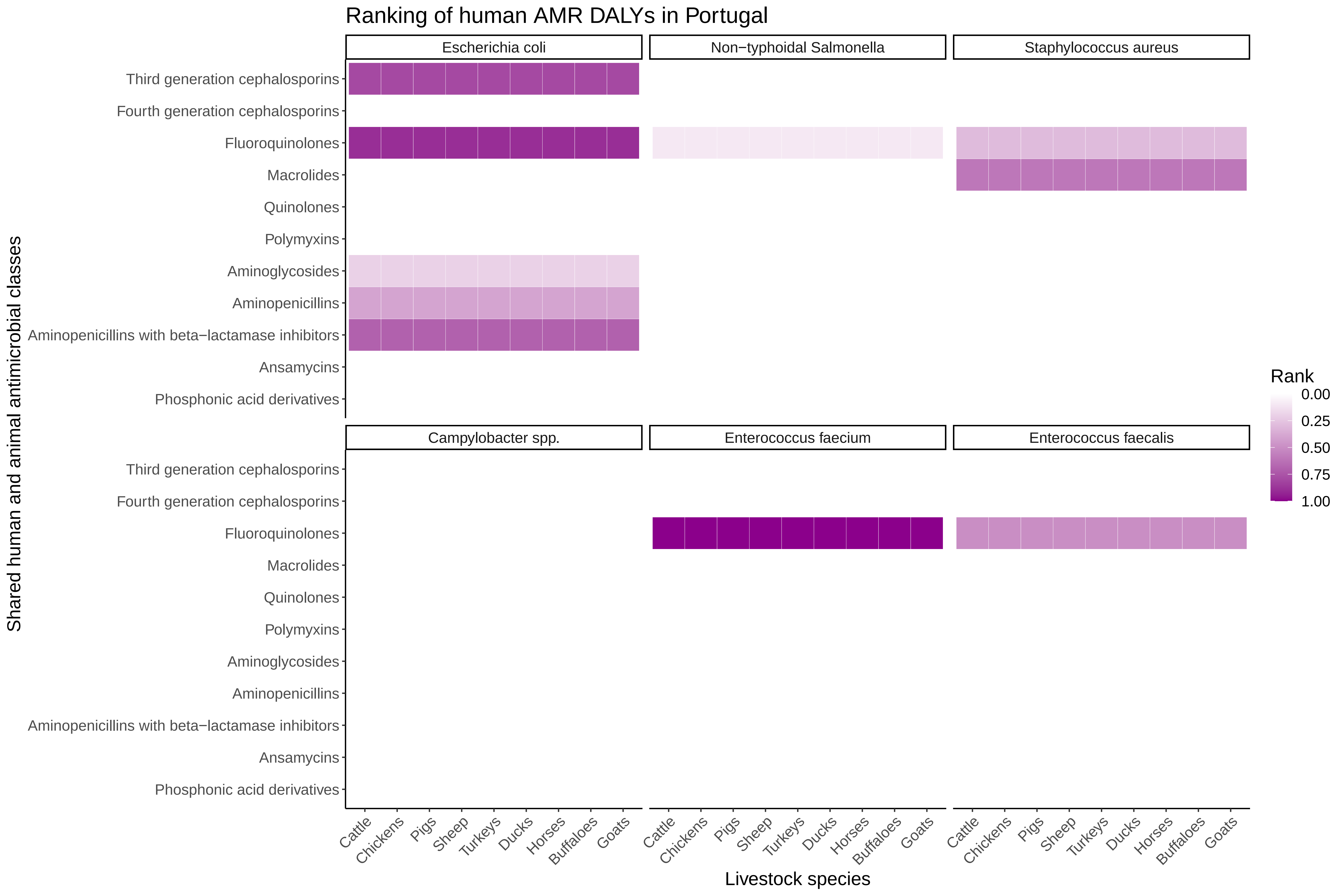

Ranking of human AMR DALYs in Portugal
Escherichia coli
Non−typhoidal Salmonella
Staphylococcus aureus
Campylobacter spp.
Enterococcus faecium
Enterococcus faecalis
Third generation cephalosporins
Fourth generation cephalosporins
Fluoroquinolones
Macrolides
Quinolones
Polymyxins
Aminoglycosides
Aminopenicillins
Aminopenicillins with beta−lactamase inhibitors
Ansamycins
Phosphonic acid derivatives
Shared human and animal antimicrobial classes
Cattle
Chickens
Pigs
Sheep
Turkeys
Ducks
Horses
Buffaloes
Goats
Livestock species
Rank
0.00
0.25
0.50
0.75
1.00

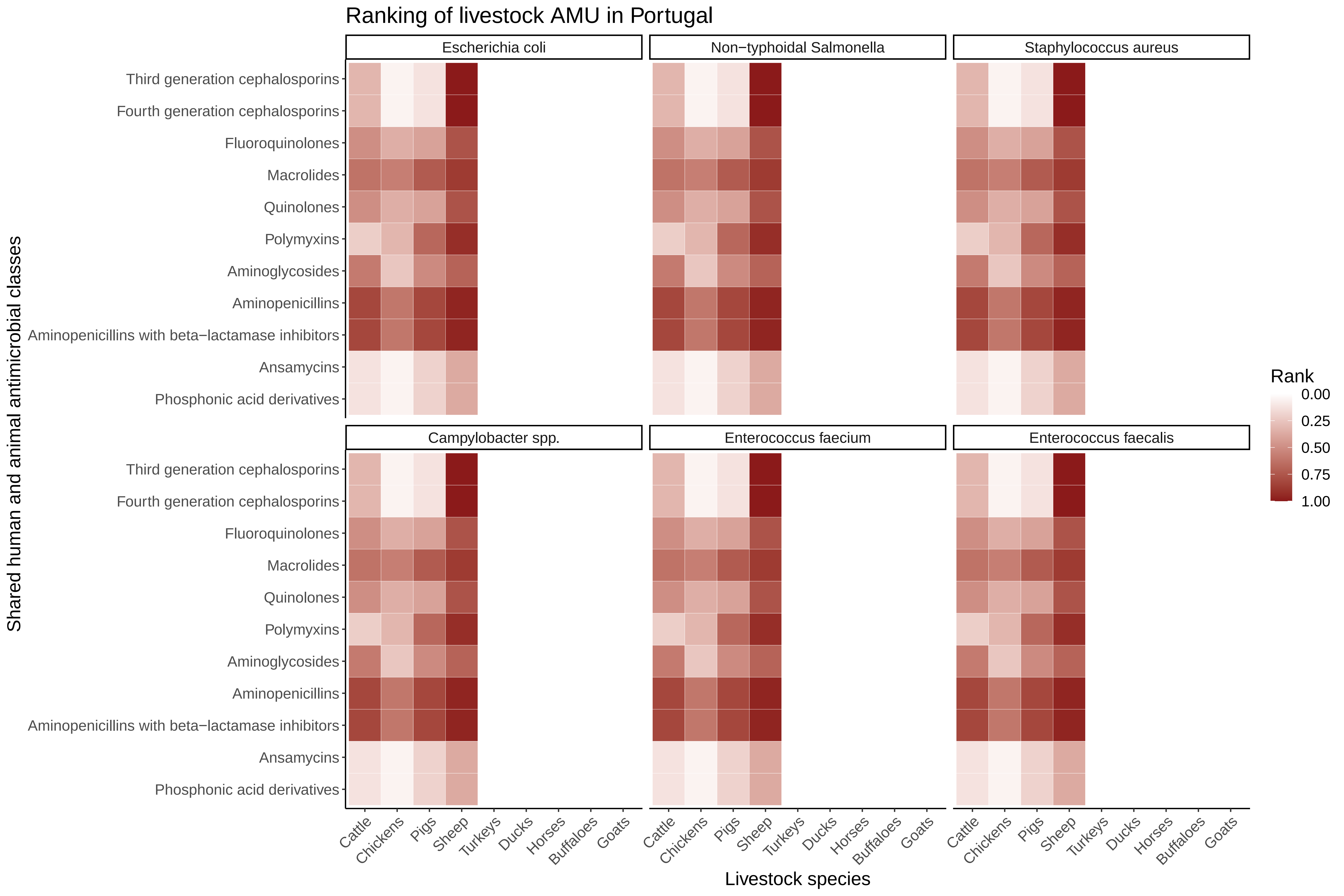

Ranking of livestock AMU in Portugal
Escherichia coli
Non−typhoidal Salmonella
Staphylococcus aureus
Campylobacter spp.
Enterococcus faecium
Enterococcus faecalis
Shared human and animal antimicrobial classes
Third generation cephalosporins
Fourth generation cephalosporins
Fluoroquinolones
Macrolides
Quinolones
Polymyxins
Aminoglycosides
Aminopenicillins
Aminopenicillins with beta−lactamase inhibitors
Ansamycins
Phosphonic acid derivatives
Cattle
Chickens
Pigs
Sheep
Turkeys
Ducks
Horses
Buffaloes
Goats
Livestock species
Rank
0.00
0.25
0.50
0.75
1.00

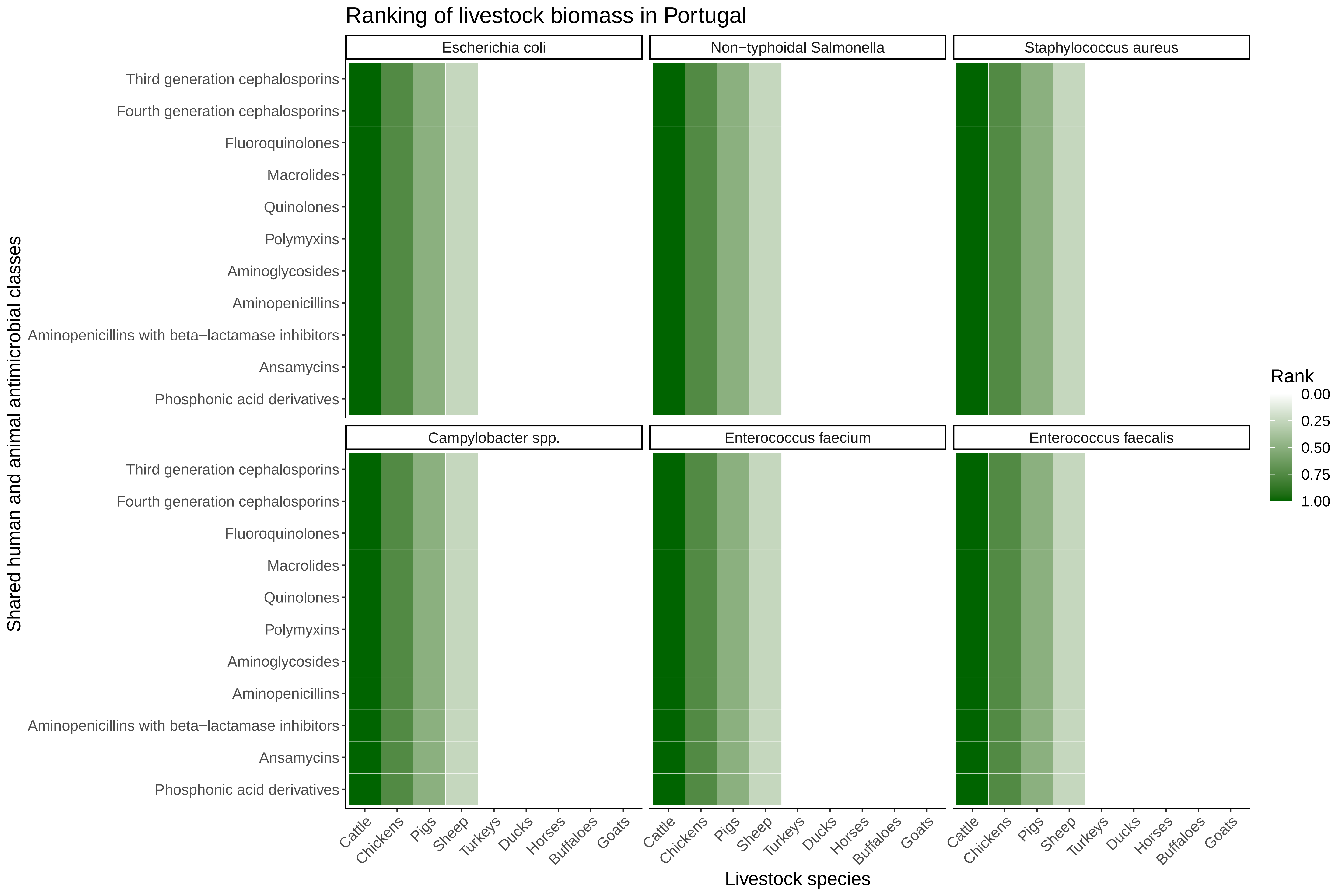
Ranking of livestock biomass in Portugal
Escherichia coli
Non−typhoidal Salmonella
Staphylococcus aureus
Campylobacter spp.
Enterococcus faecium
Enterococcus faecalis
Third generation cephalosporins
Fourth generation cephalosporins
Fluoroquinolones
Macrolides
Quinolones
Polymyxins
Aminoglycosides
Aminopenicillins
Aminopenicillins with beta−lactamase inhibitors
Ansamycins
Phosphonic acid derivatives
Shared human and animal antimicrobial classes
Cattle
Chickens
Pigs
Sheep
Turkeys
Ducks
Horses
Buffaloes
Goats
Livestock species
Rank
0.00
0.25
0.50
0.75
1.00

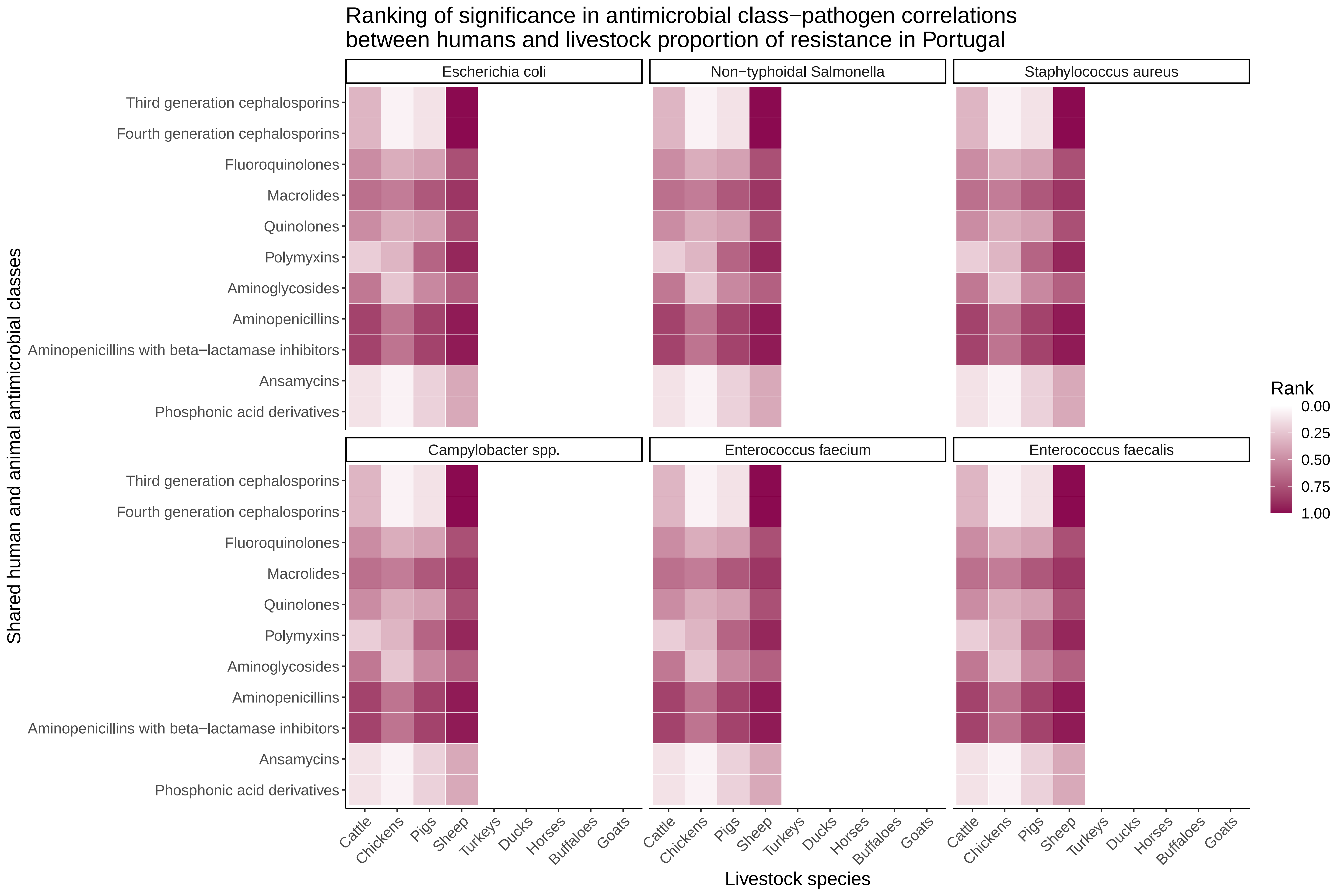

Ranking of significance in antimicrobial class−pathogen correlations
between humans and livestock proportion of resistance in Portugal
Escherichia coli
Non−typhoidal Salmonella
Staphylococcus aureus
Campylobacter spp.
Enterococcus faecium
Enterococcus faecalis
Third generation cephalosporins
Fourth generation cephalosporins
Fluoroquinolones
Macrolides
Quinolones
Polymyxins
Aminoglycosides
Aminopenicillins
Aminopenicillins with beta−lactamase inhibitors
Ansamycins
Phosphonic acid derivatives
Shared human and animal antimicrobial classes
Cattle
Chickens
Pigs
Sheep
Turkeys
Ducks
Horses
Buffaloes
Goats
Livestock species
Rank
0.00
0.25
0.50
0.75
1.00

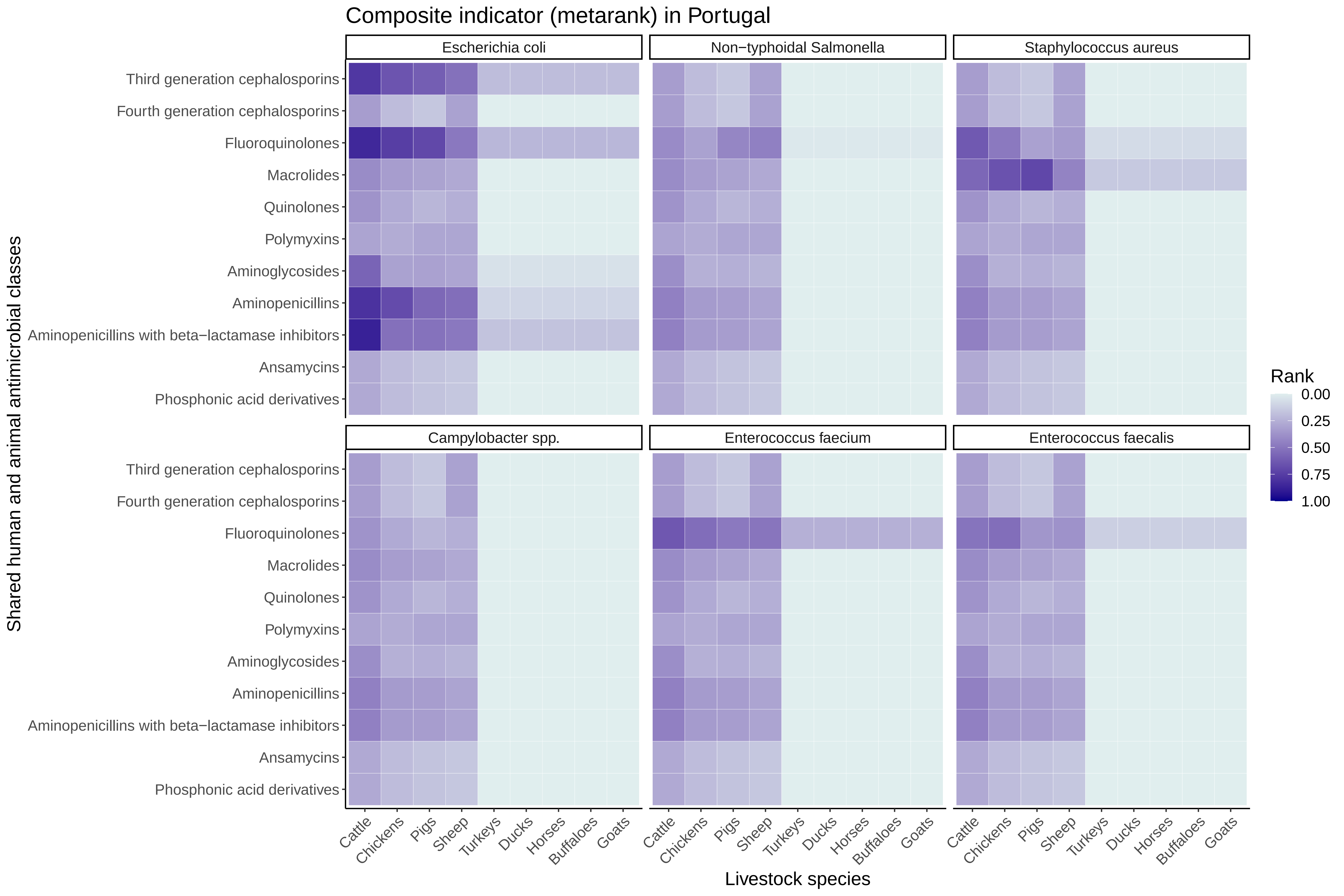

Composite indicator (metarank) in Portugal
Escherichia coli
Non−typhoidal Salmonella
Staphylococcus aureus
Campylobacter spp.
Enterococcus faecium
Enterococcus faecalis
Third generation cephalosporins
Fourth generation cephalosporins
Fluoroquinolones
Macrolides
Quinolones
Polymyxins
Aminoglycosides
Aminopenicillins
Aminopenicillins with beta−lactamase inhibitors
Ansamycins
Phosphonic acid derivatives
Shared human and animal antimicrobial classes
Cattle
Chickens
Pigs
Sheep
Turkeys
Ducks
Horses
Buffaloes
Goats
Livestock species
Rank
0.00
0.25
0.50
0.75
1.00

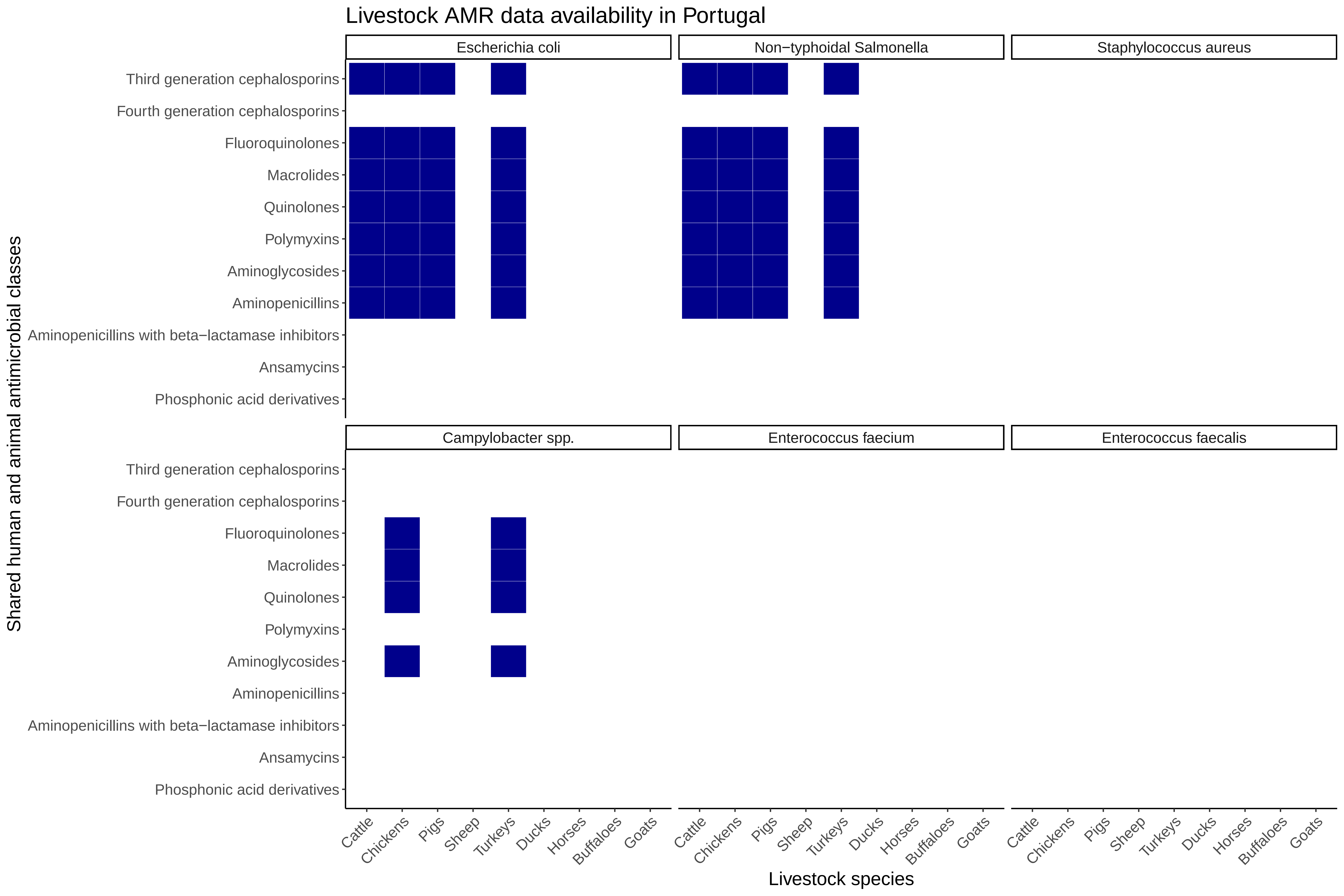

Livestock AMR data availability in Portugal
Escherichia coli
Non−typhoidal Salmonella
Staphylococcus aureus
Campylobacter spp.
Enterococcus faecium
Enterococcus faecalis
Shared human and animal antimicrobial classes
Third generation cephalosporins
Fourth generation cephalosporins
Fluoroquinolones
Macrolides
Quinolones
Polymyxins
Aminoglycosides
Aminopenicillins
Aminopenicillins with beta−lactamase inhibitors
Ansamycins
Phosphonic acid derivatives
Cattle
Chickens
Pigs
Sheep
Turkeys
Ducks
Horses
Buffaloes
Goats
Livestock species

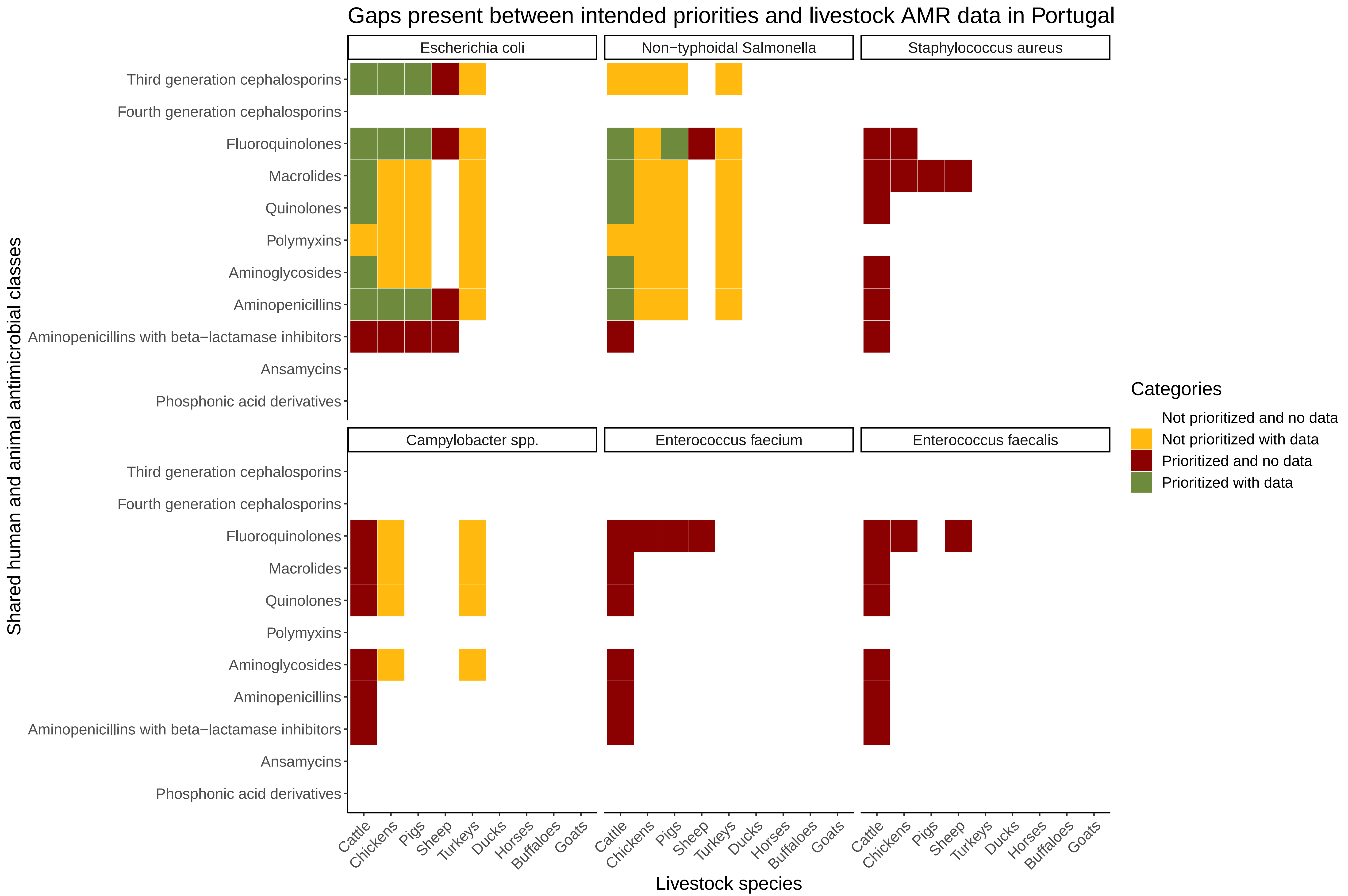

Gaps present between intended priorities and livestock AMR data in Portugal
Escherichia coli
Non−typhoidal Salmonella
Staphylococcus aureus
Campylobacter spp.
Enterococcus faecium
Enterococcus faecalis
Shared human and animal antimicrobial classes
Third generation cephalosporins
Fourth generation cephalosporins
Fluoroquinolones
Macrolides
Quinolones
Polymyxins
Aminoglycosides
Aminopenicillins
Aminopenicillins with beta−lactamase inhibitors
Ansamycins
Phosphonic acid derivatives
Cattle
Chickens
Pigs
Sheep
Turkeys
Ducks
Horses
Buffaloes
Goats
Livestock species
Categories
Not prioritized and no data
Not prioritized with data
Prioritized and no data
Prioritized with data

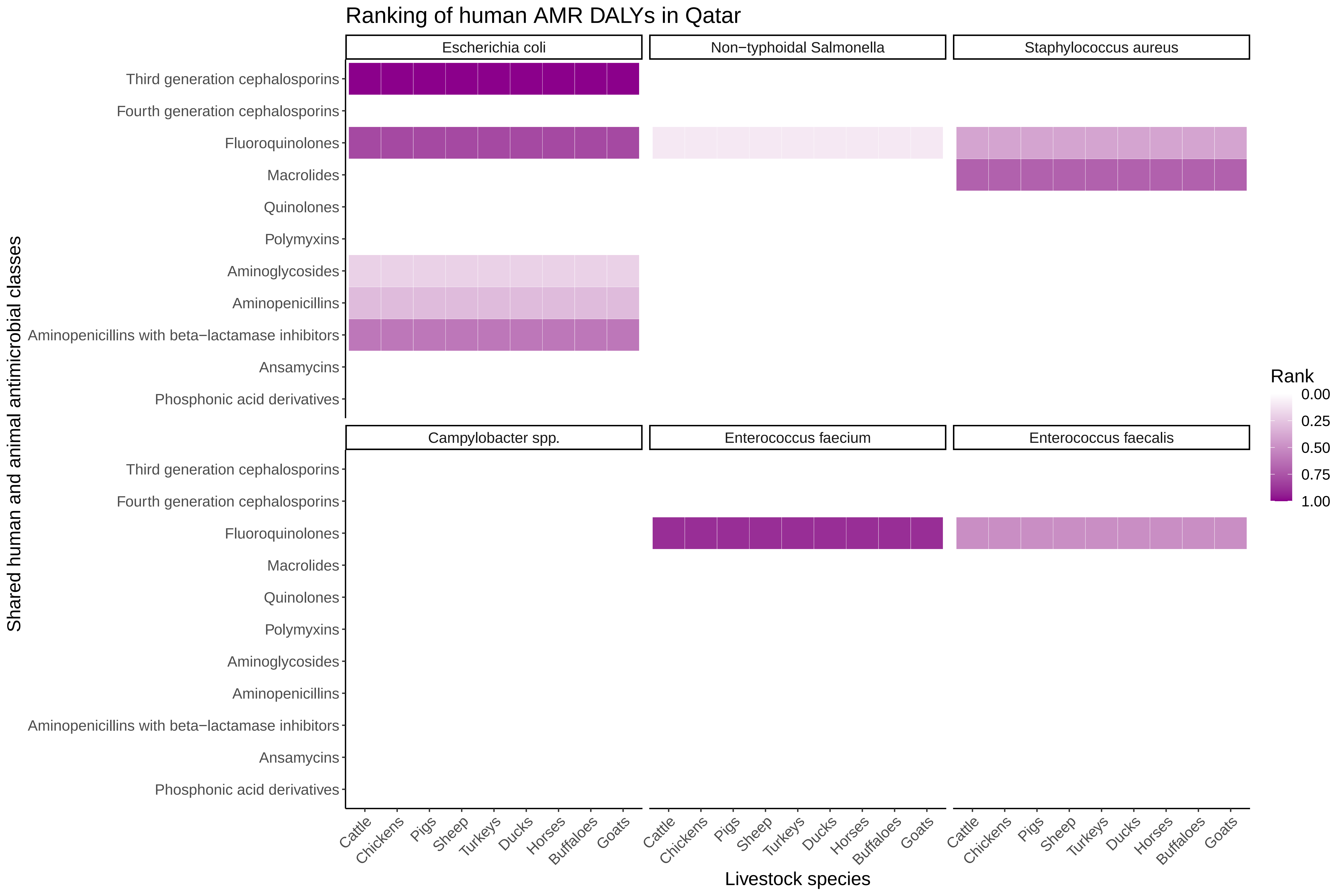

Ranking of human AMR DALYs in Qatar
Escherichia coli
Non−typhoidal Salmonella
Staphylococcus aureus
Campylobacter spp.
Enterococcus faecium
Enterococcus faecalis
Third generation cephalosporins
Fourth generation cephalosporins
Fluoroquinolones
Macrolides
Quinolones
Polymyxins
Aminoglycosides
Aminopenicillins
Aminopenicillins with beta−lactamase inhibitors
Ansamycins
Phosphonic acid derivatives
Shared human and animal antimicrobial classes
Cattle
Chickens
Pigs
Sheep
Turkeys
Ducks
Horses
Buffaloes
Goats
Livestock species
Rank
0.00
0.25
0.50
0.75
1.00

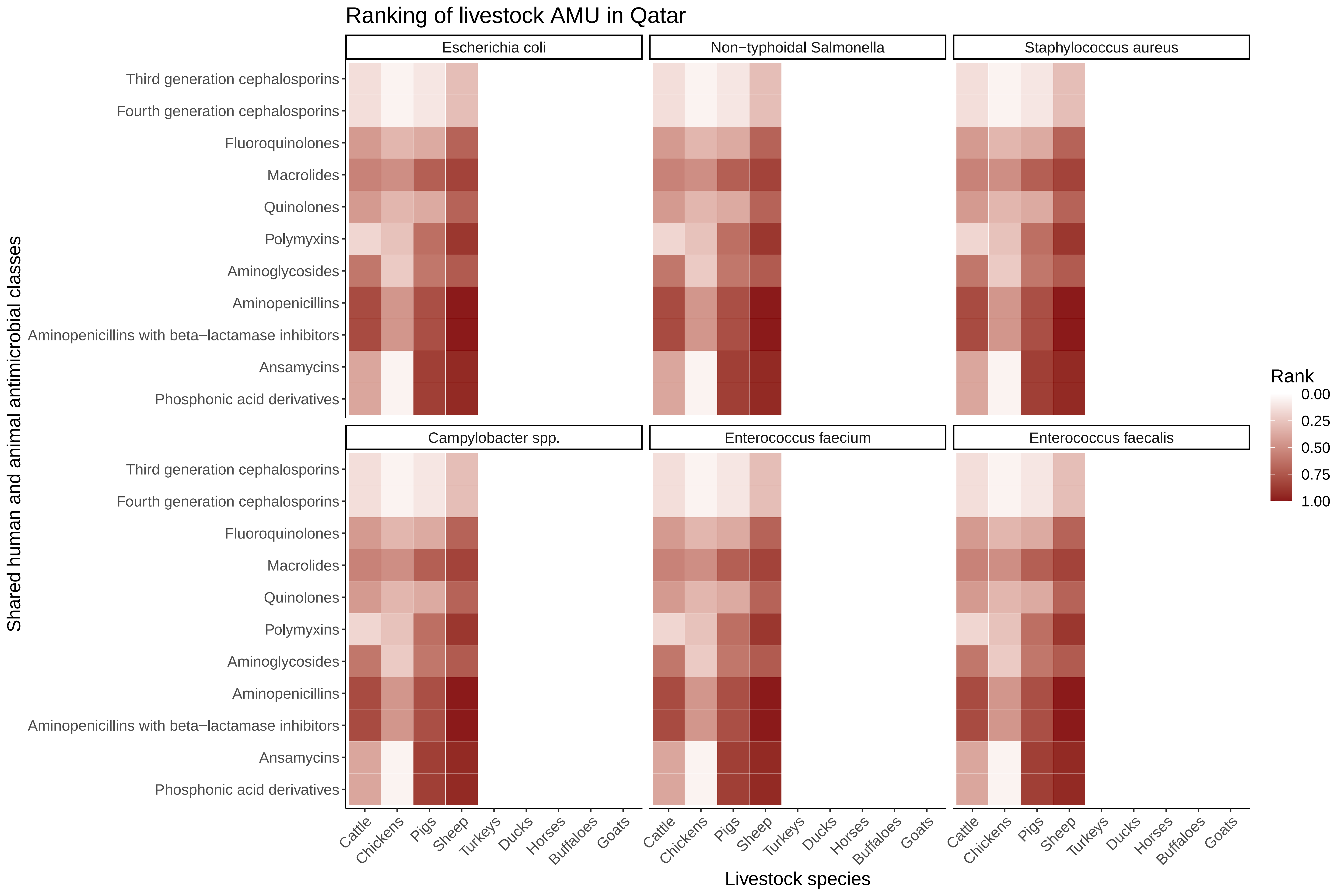

Ranking of livestock AMU in Qatar
Escherichia coli
Non−typhoidal Salmonella
Staphylococcus aureus
Campylobacter spp.
Enterococcus faecium
Enterococcus faecalis
Shared human and animal antimicrobial classes
Third generation cephalosporins
Fourth generation cephalosporins
Fluoroquinolones
Macrolides
Quinolones
Polymyxins
Aminoglycosides
Aminopenicillins
Aminopenicillins with beta−lactamase inhibitors
Ansamycins
Phosphonic acid derivatives
Cattle
Chickens
Pigs
Sheep
Turkeys
Ducks
Horses
Buffaloes
Goats
Livestock species
Rank
0.00
0.25
0.50
0.75
1.00

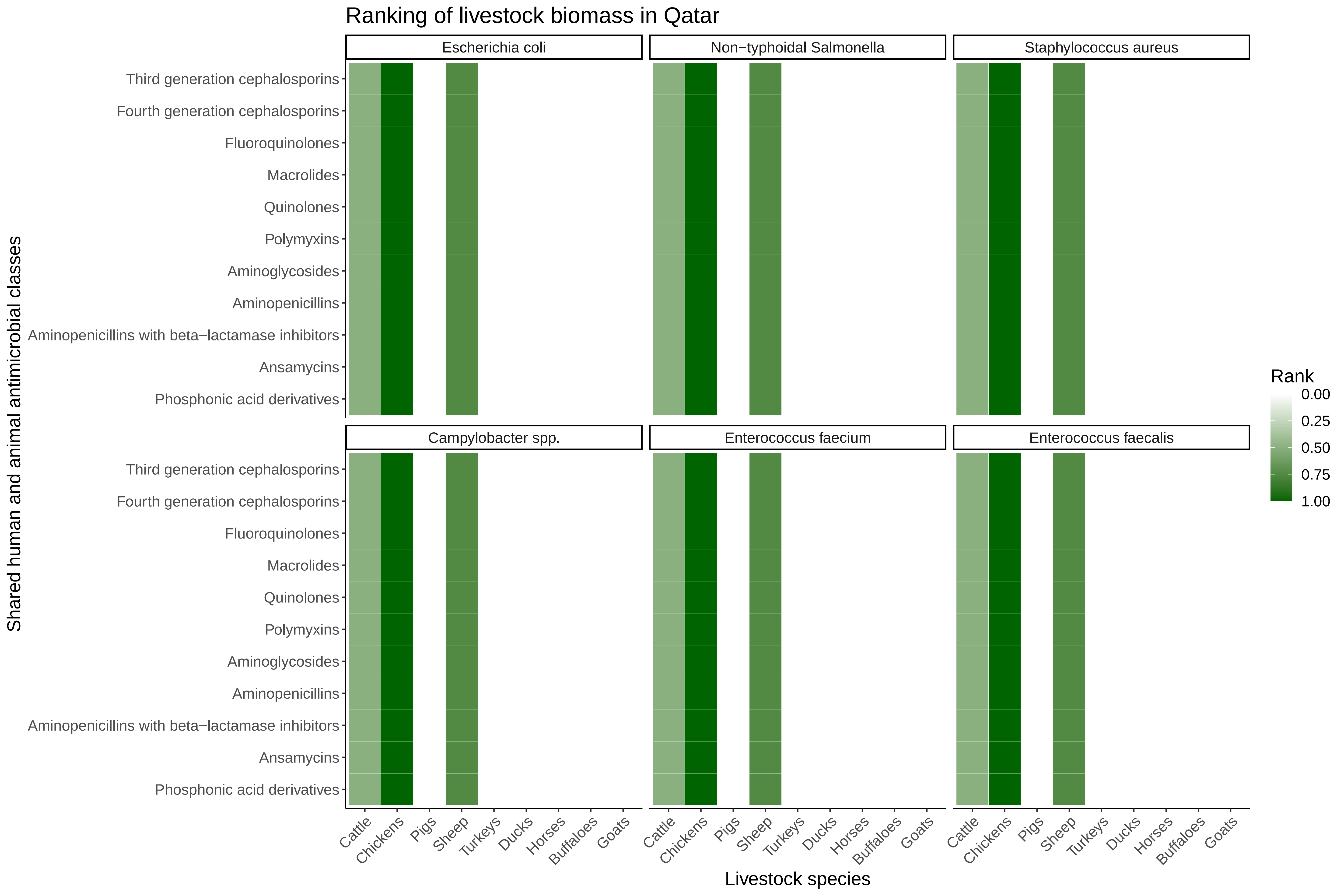

Ranking of livestock biomass in Qatar
Escherichia coli
Non−typhoidal Salmonella
Staphylococcus aureus
Campylobacter spp.
Enterococcus faecium
Enterococcus faecalis
Third generation cephalosporins
Fourth generation cephalosporins
Fluoroquinolones
Macrolides
Quinolones
Polymyxins
Aminoglycosides
Aminopenicillins
Aminopenicillins with beta−lactamase inhibitors
Ansamycins
Phosphonic acid derivatives
Shared human and animal antimicrobial classes
Cattle
Chickens
Pigs
Sheep
Turkeys
Ducks
Horses
Buffaloes
Goats
Livestock species
Rank
0.00
0.25
0.50
0.75
1.00

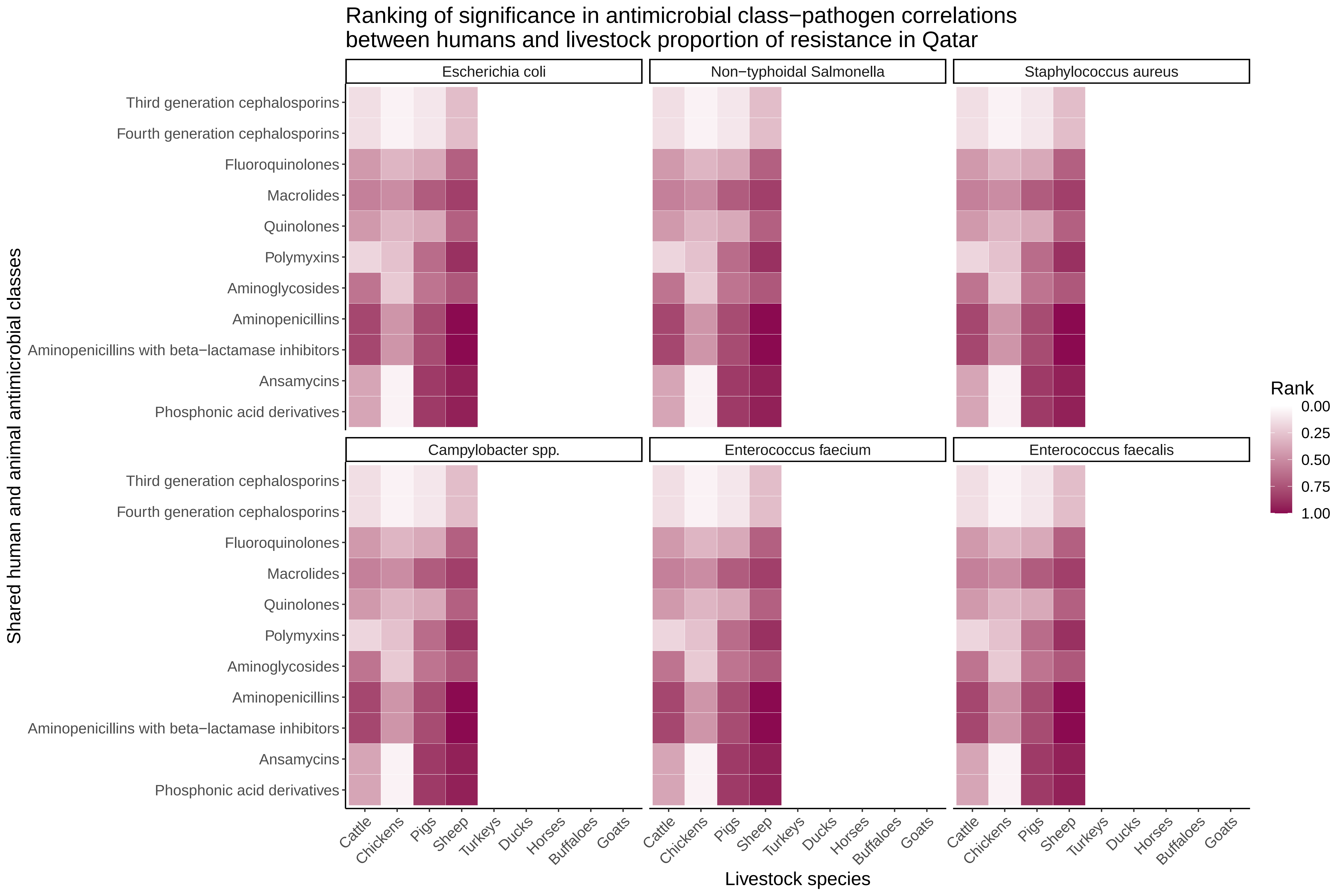

Ranking of significance in antimicrobial class−pathogen correlations
between humans and livestock proportion of resistance in Qatar
Escherichia coli
Non−typhoidal Salmonella
Staphylococcus aureus
Campylobacter spp.
Enterococcus faecium
Enterococcus faecalis
Third generation cephalosporins
Fourth generation cephalosporins
Fluoroquinolones
Macrolides
Quinolones
Polymyxins
Aminoglycosides
Aminopenicillins
Aminopenicillins with beta−lactamase inhibitors
Ansamycins
Phosphonic acid derivatives
Shared human and animal antimicrobial classes
Cattle
Chickens
Pigs
Sheep
Turkeys
Ducks
Horses
Buffaloes
Goats
Livestock species
Rank
0.00
0.25
0.50
0.75
1.00

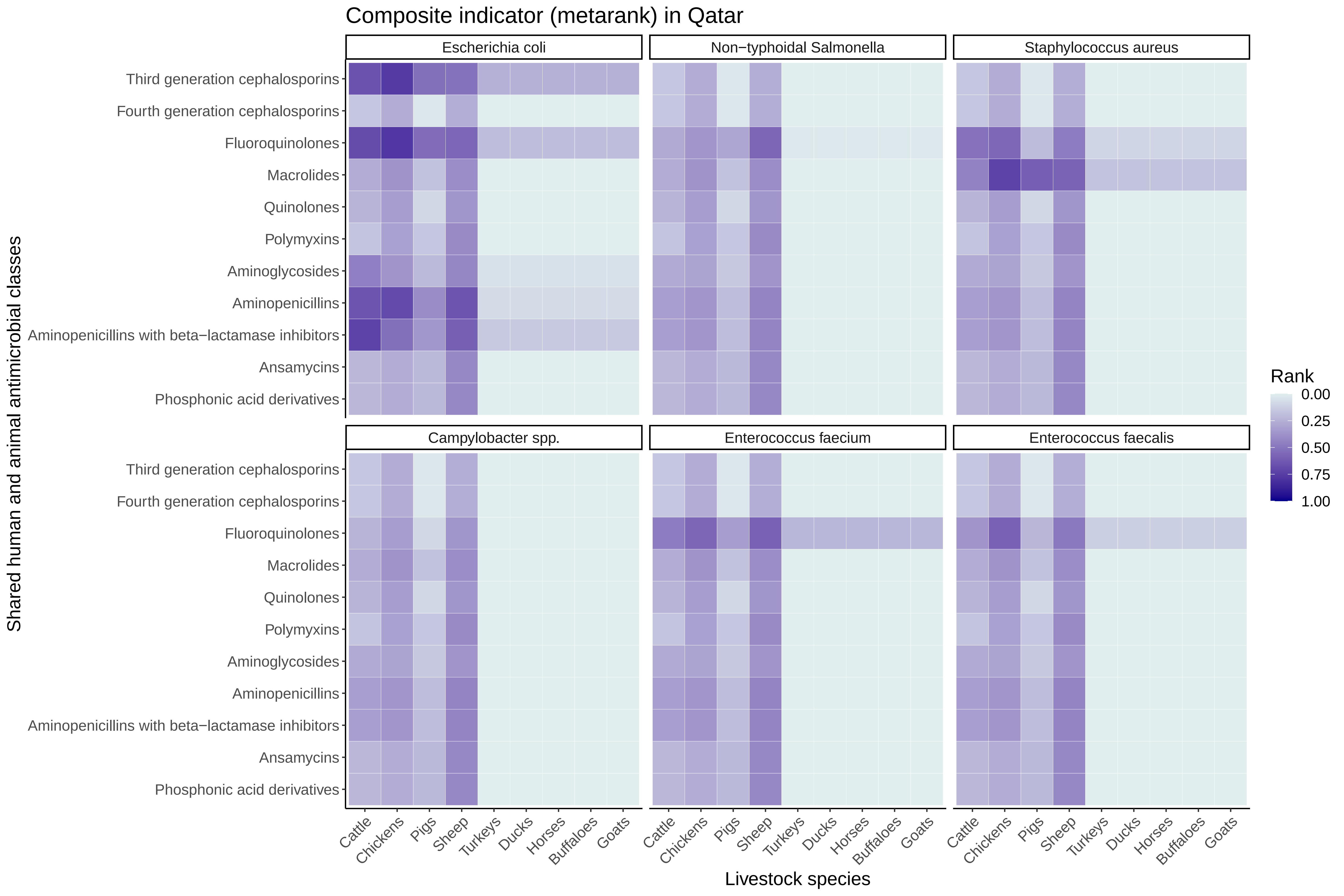

Composite indicator (metarank) in Qatar
Escherichia coli
Non−typhoidal Salmonella
Staphylococcus aureus
Campylobacter spp.
Enterococcus faecium
Enterococcus faecalis
Third generation cephalosporins
Fourth generation cephalosporins
Fluoroquinolones
Macrolides
Quinolones
Polymyxins
Aminoglycosides
Aminopenicillins
Aminopenicillins with beta−lactamase inhibitors
Ansamycins
Phosphonic acid derivatives
Cattle
Chickens
Pigs
Sheep
Turkeys
Ducks
Horses
Buffaloes
Goats
Livestock species
Shared human and animal antimicrobial classes
Rank
0.00
0.25
0.50
0.75
1.00

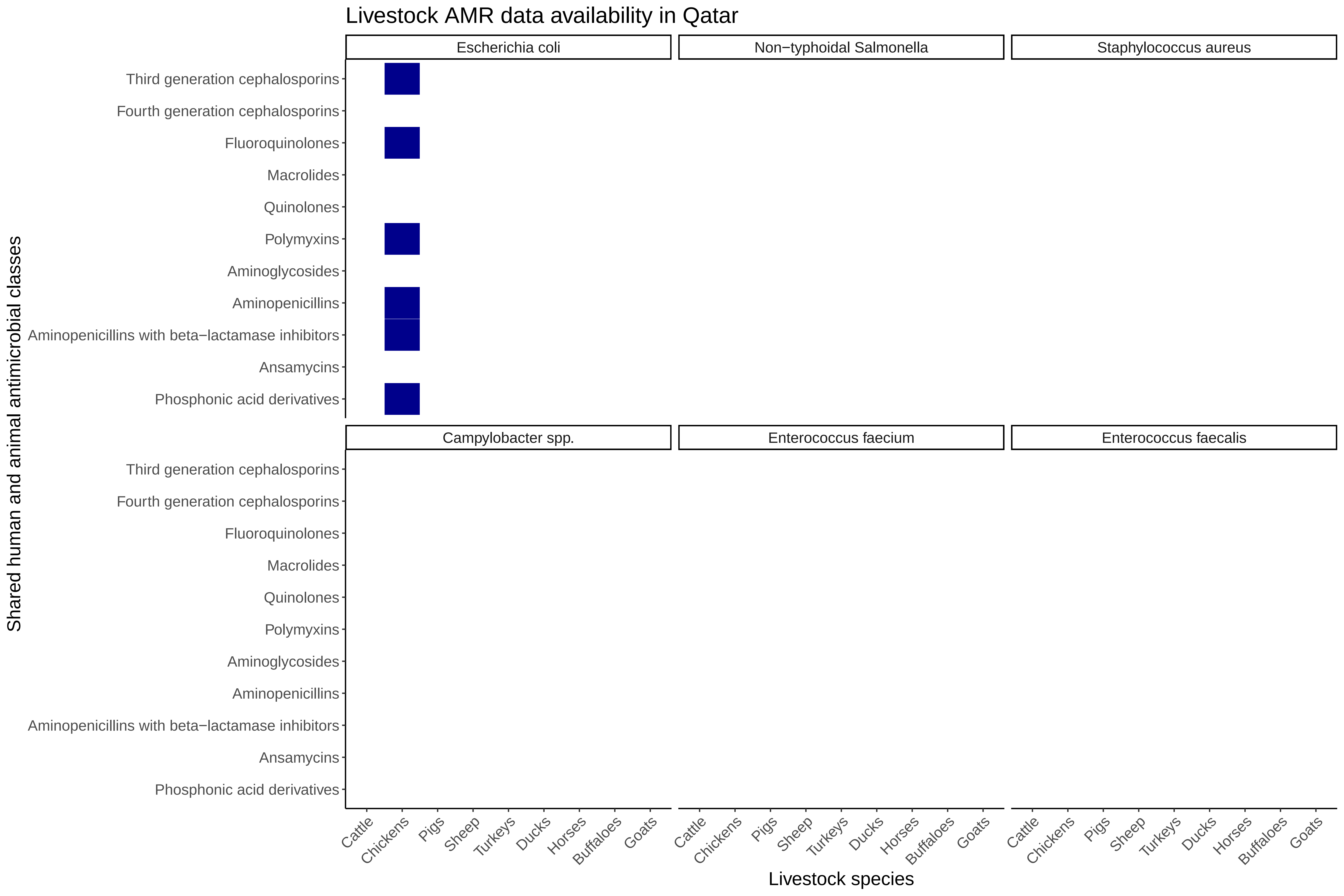

Livestock AMR data availability in Qatar
Escherichia coli
Non−typhoidal Salmonella
Staphylococcus aureus
Campylobacter spp.
Enterococcus faecium
Enterococcus faecalis
Third generation cephalosporins
Fourth generation cephalosporins
Fluoroquinolones
Macrolides
Quinolones
Polymyxins
Aminoglycosides
Aminopenicillins
Aminopenicillins with beta−lactamase inhibitors
Ansamycins
Phosphonic acid derivatives
Shared human and animal antimicrobial classes
Cattle
Chickens
Pigs
Sheep
Turkeys
Ducks
Horses
Buffaloes
Goats
Livestock species

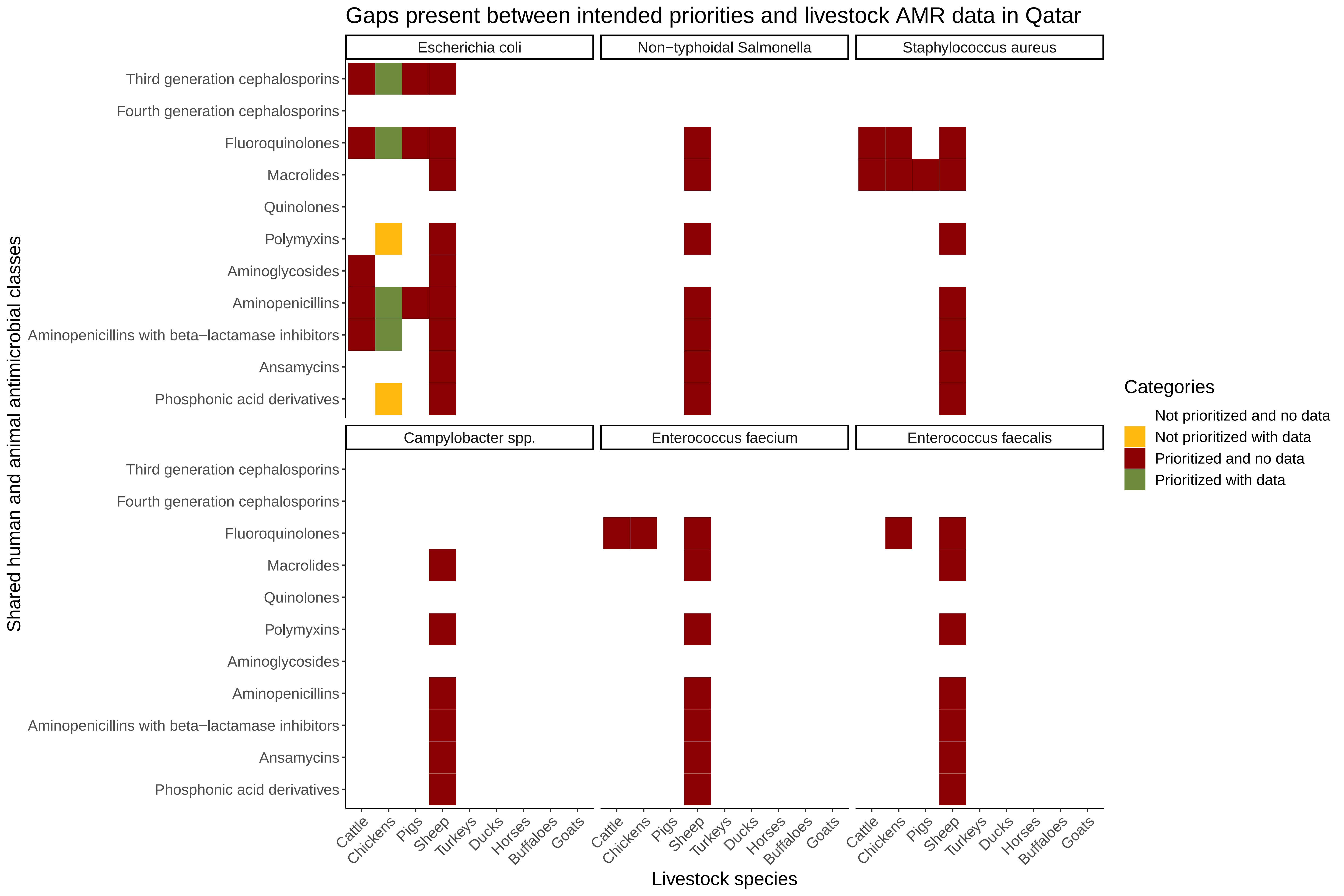

Gaps present between intended priorities and livestock AMR data in Qatar
Escherichia coli
Non−typhoidal Salmonella
Staphylococcus aureus
Campylobacter spp.
Enterococcus faecium
Enterococcus faecalis
Third generation cephalosporins
Fourth generation cephalosporins
Fluoroquinolones
Macrolides
Quinolones
Polymyxins
Aminoglycosides
Aminopenicillins
Aminopenicillins with beta−lactamase inhibitors
Ansamycins
Phosphonic acid derivatives
Shared human and animal antimicrobial classes
Cattle
Chickens
Pigs
Sheep
Turkeys
Ducks
Horses
Buffaloes
Goats
Livestock species
Categories
Not prioritized and no data
Not prioritized with data
Prioritized and no data
Prioritized with data

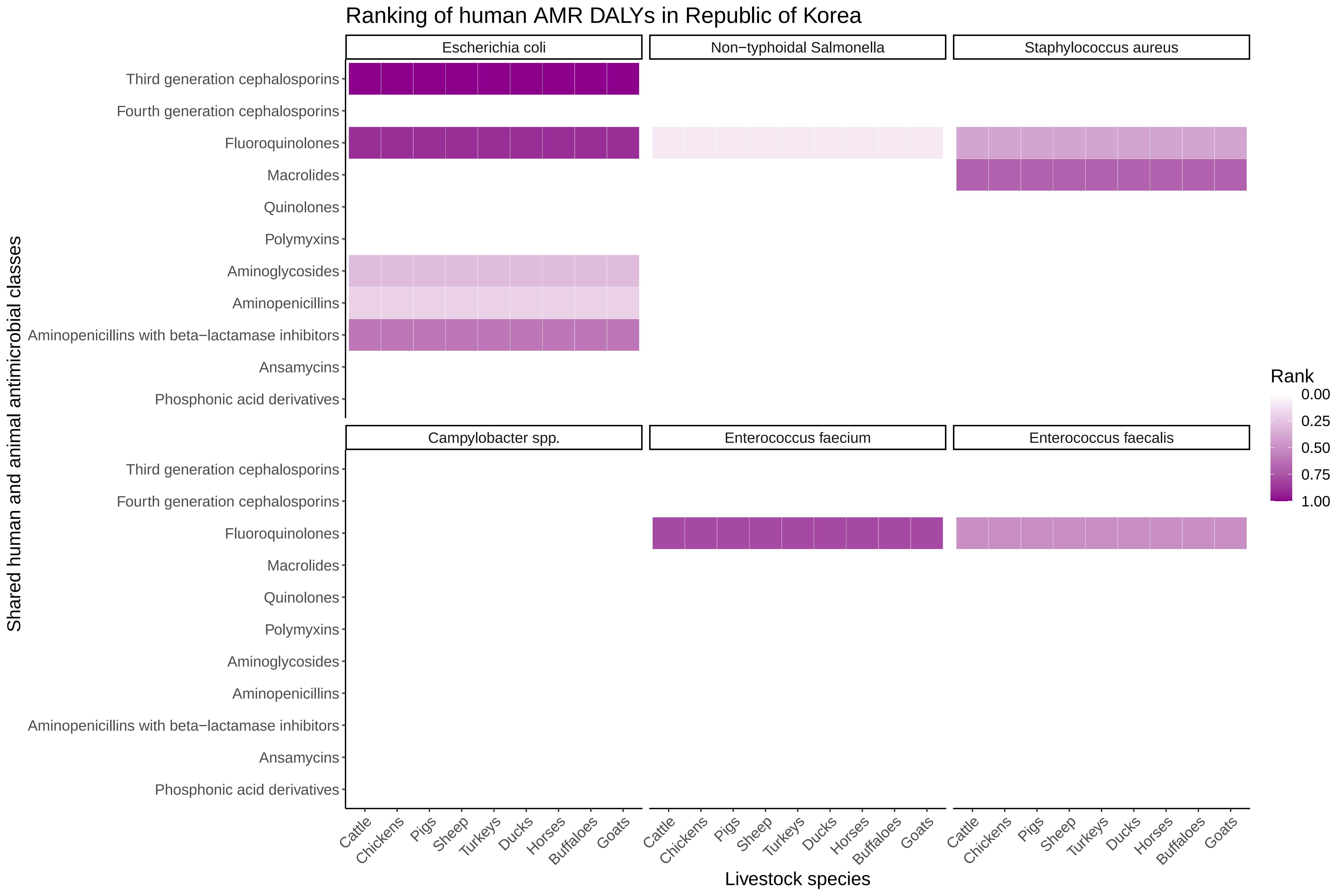
Ranking of human AMR DALYs in Republic of Korea
Escherichia coli
Non−typhoidal Salmonella
Staphylococcus aureus
Campylobacter spp.
Enterococcus faecium
Enterococcus faecalis
Shared human and animal antimicrobial classes
Third generation cephalosporins
Fourth generation cephalosporins
Fluoroquinolones
Macrolides
Quinolones
Polymyxins
Aminoglycosides
Aminopenicillins
Aminopenicillins with beta−lactamase inhibitors
Ansamycins
Phosphonic acid derivatives
Cattle
Chickens
Pigs
Sheep
Turkeys
Ducks
Horses
Buffaloes
Goats
Livestock species
Rank
0.00
0.25
0.50
0.75
1.00

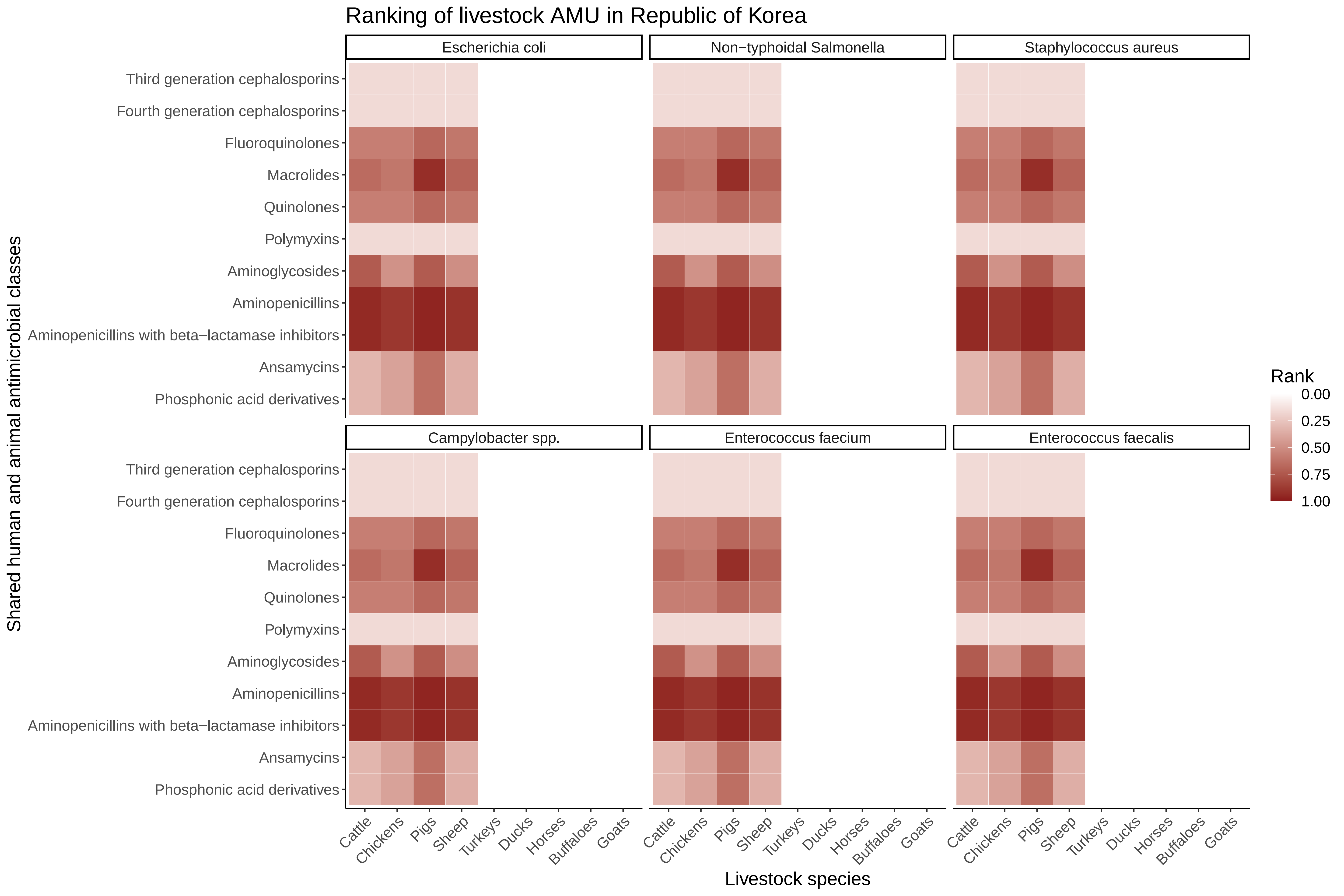
Ranking of livestock AMU in Republic of Korea
Escherichia coli
Non−typhoidal Salmonella
Staphylococcus aureus
Campylobacter spp.
Enterococcus faecium
Enterococcus faecalis
Shared human and animal antimicrobial classes
Third generation cephalosporins
Fourth generation cephalosporins
Fluoroquinolones
Macrolides
Quinolones
Polymyxins
Aminoglycosides
Aminopenicillins
Aminopenicillins with beta−lactamase inhibitors
Ansamycins
Phosphonic acid derivatives
Cattle
Chickens
Pigs
Sheep
Turkeys
Ducks
Horses
Buffaloes
Goats
Livestock species
Rank
0.00
0.25
0.50
0.75
1.00

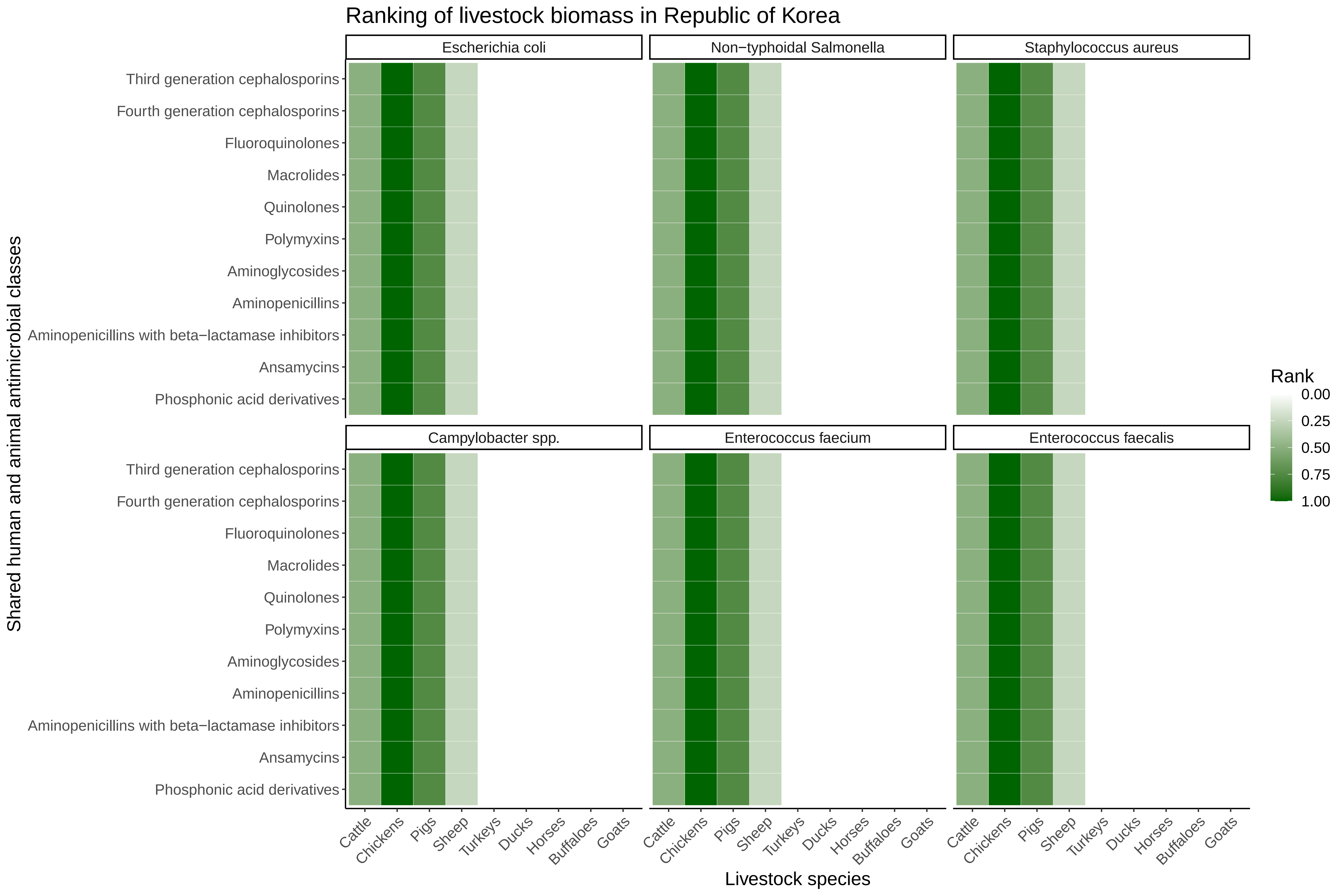

Ranking of livestock biomass in Republic of Korea
Escherichia coli
Non−typhoidal Salmonella
Staphylococcus aureus
Campylobacter spp.
Enterococcus faecium
Enterococcus faecalis
Shared human and animal antimicrobial classes
Third generation cephalosporins
Fourth generation cephalosporins
Fluoroquinolones
Macrolides
Quinolones
Polymyxins
Aminoglycosides
Aminopenicillins
Aminopenicillins with beta−lactamase inhibitors
Ansamycins
Phosphonic acid derivatives
Cattle
Chickens
Pigs
Sheep
Turkeys
Ducks
Horses
Buffaloes
Goats
Livestock species
Rank
0.00
0.25
0.50
0.75
1.00

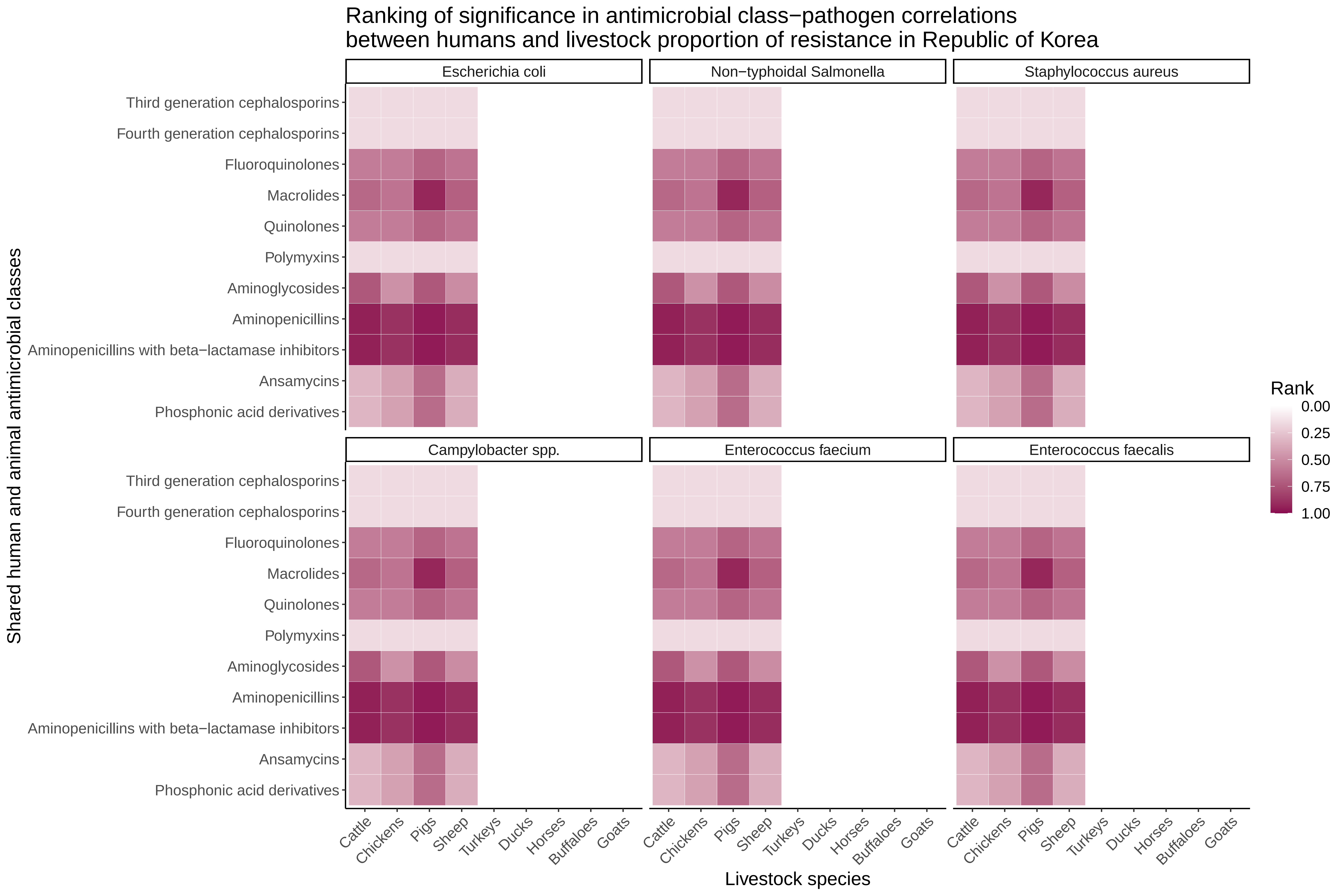

Ranking of significance in antimicrobial class−pathogen correlations
between humans and livestock proportion of resistance in Republic of Korea
Escherichia coli
Non−typhoidal Salmonella
Staphylococcus aureus
Campylobacter spp.
Enterococcus faecium
Enterococcus faecalis
Third generation cephalosporins
Fourth generation cephalosporins
Fluoroquinolones
Macrolides
Quinolones
Polymyxins
Aminoglycosides
Aminopenicillins
Aminopenicillins with beta−lactamase inhibitors
Ansamycins
Phosphonic acid derivatives
Shared human and animal antimicrobial classes
Cattle
Chickens
Pigs
Sheep
Turkeys
Ducks
Horses
Buffaloes
Goats
Livestock species
Rank
0.00
0.25
0.50
0.75
1.00

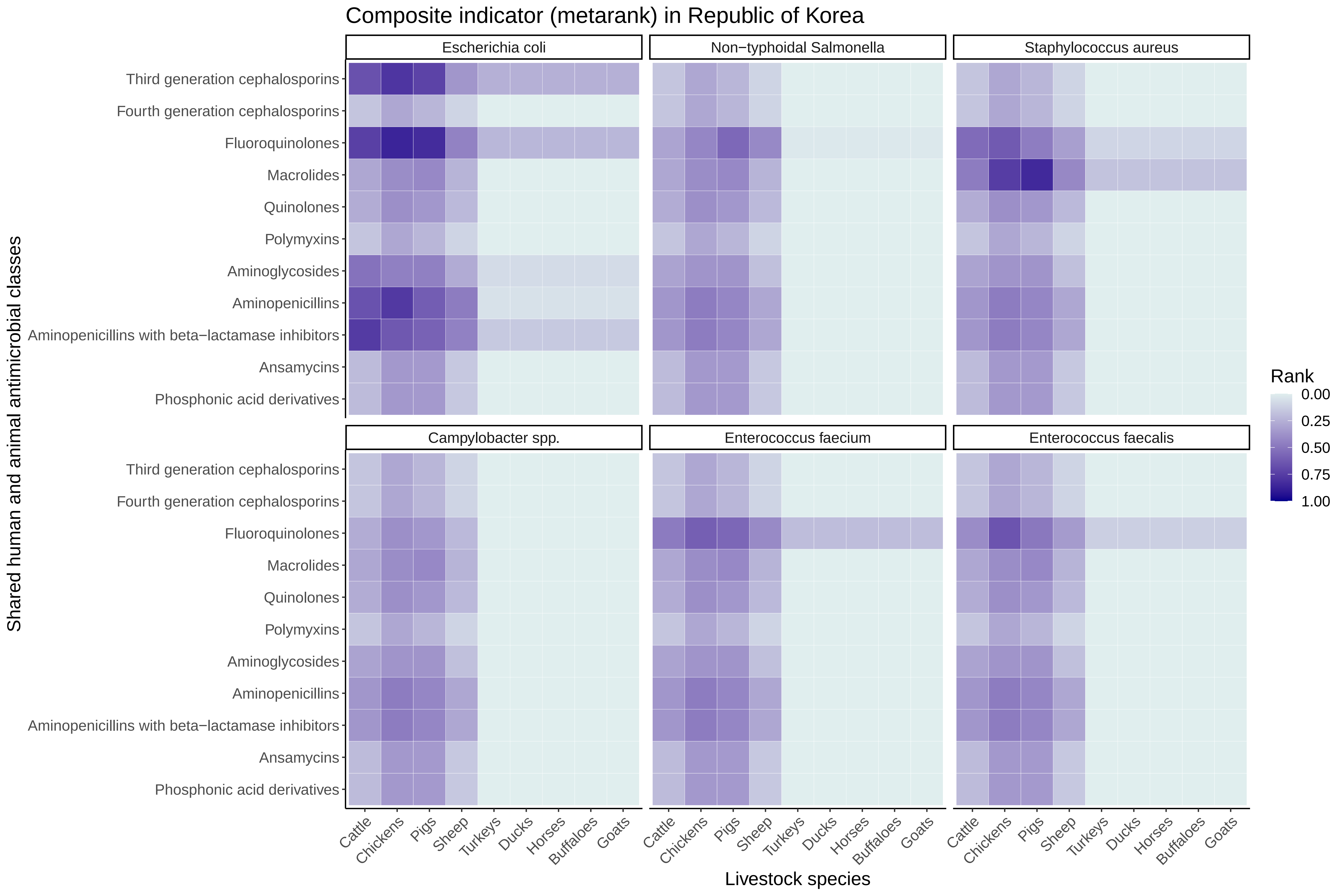

Composite indicator (metarank) in Republic of Korea
Escherichia coli
Non−typhoidal Salmonella
Staphylococcus aureus
Campylobacter spp.
Enterococcus faecium
Enterococcus faecalis
Third generation cephalosporins
Fourth generation cephalosporins
Fluoroquinolones
Macrolides
Quinolones
Polymyxins
Aminoglycosides
Aminopenicillins
Aminopenicillins with beta−lactamase inhibitors
Ansamycins
Phosphonic acid derivatives
Shared human and animal antimicrobial classes
Cattle
Chickens
Pigs
Sheep
Turkeys
Ducks
Horses
Buffaloes
Goats
Livestock species
Rank
0.00
0.25
0.50
0.75
1.00

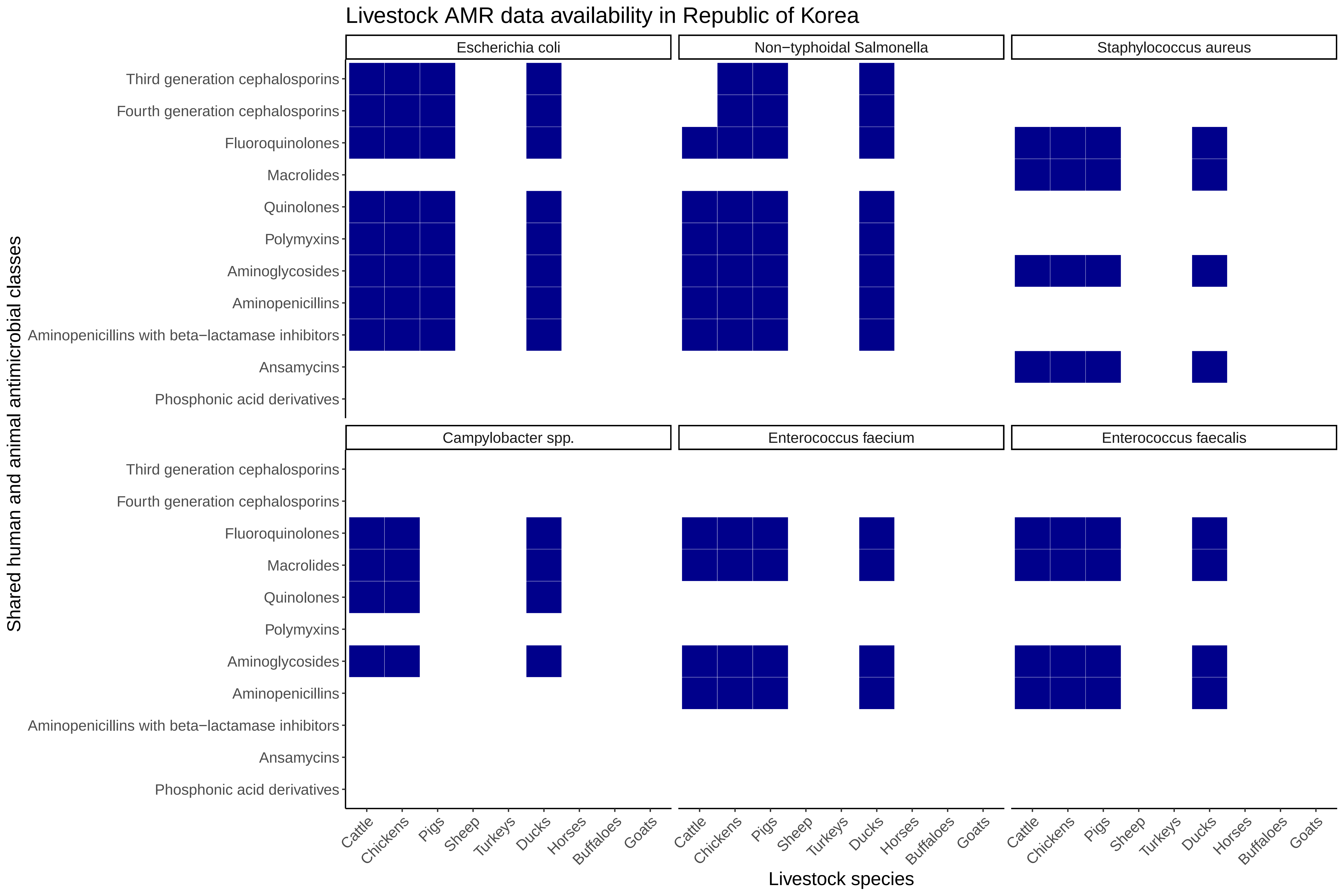

Livestock AMR data availability in Republic of Korea
Escherichia coli
Non−typhoidal Salmonella
Staphylococcus aureus
Campylobacter spp.
Enterococcus faecium
Enterococcus faecalis
Shared human and animal antimicrobial classes
Third generation cephalosporins
Fourth generation cephalosporins
Fluoroquinolones
Macrolides
Quinolones
Polymyxins
Aminoglycosides
Aminopenicillins
Aminopenicillins with beta−lactamase inhibitors
Ansamycins
Phosphonic acid derivatives
Cattle
Chickens
Pigs
Sheep
Turkeys
Ducks
Horses
Buffaloes
Goats
Livestock species

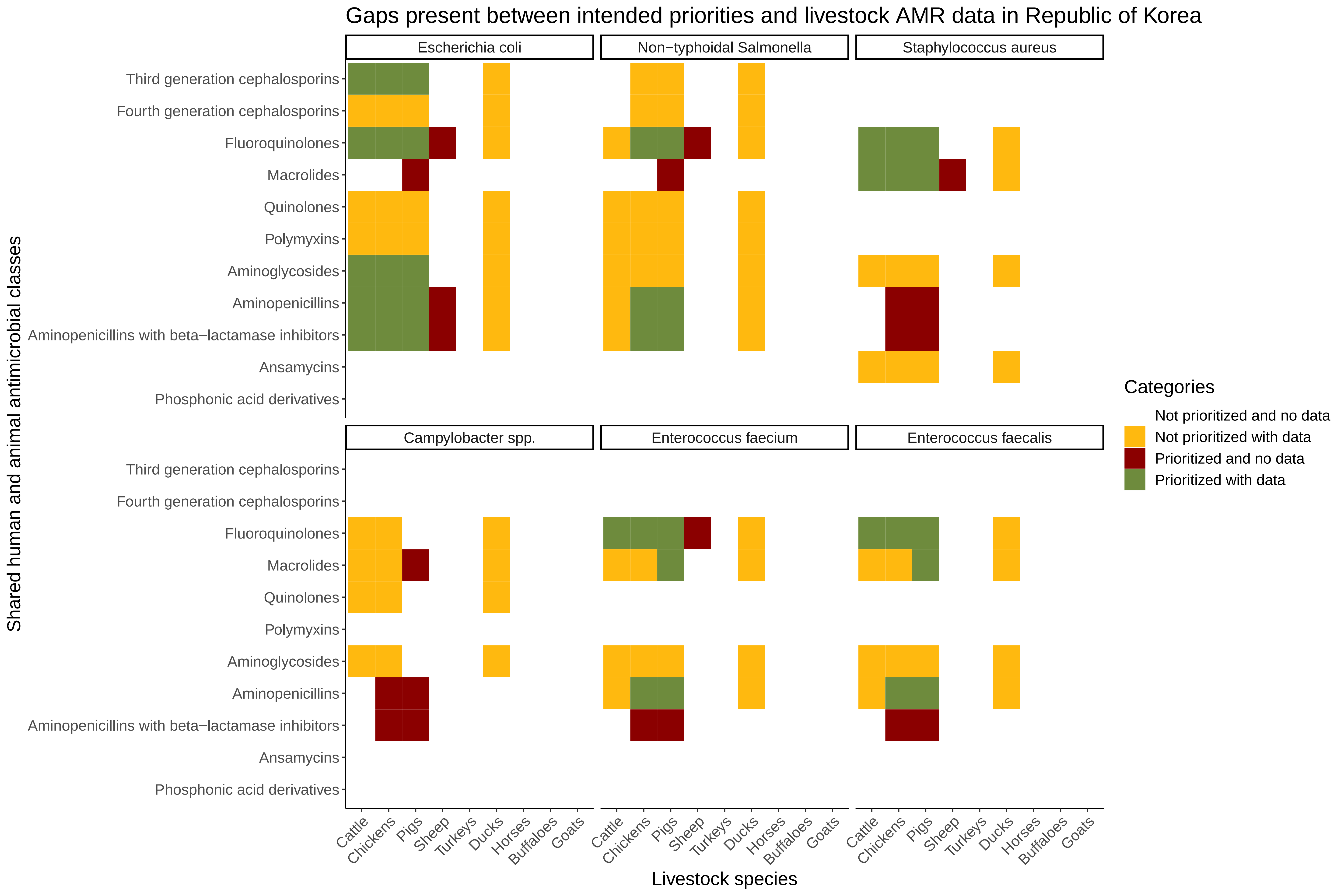
Gaps present between intended priorities and livestock AMR data in Republic of Korea
Escherichia coli
Non−typhoidal Salmonella
Staphylococcus aureus
Campylobacter spp.
Enterococcus faecium
Enterococcus faecalis
Third generation cephalosporins
Fourth generation cephalosporins
Fluoroquinolones
Macrolides
Quinolones
Polymyxins
Aminoglycosides
Aminopenicillins
Aminopenicillins with beta−lactamase inhibitors
Ansamycins
Phosphonic acid derivatives
Shared human and animal antimicrobial classes
Cattle
Chickens
Pigs
Sheep
Turkeys
Ducks
Horses
Buffaloes
Goats
Livestock species
Categories
Not prioritized and no data
Not prioritized with data
Prioritized and no data
Prioritized with data

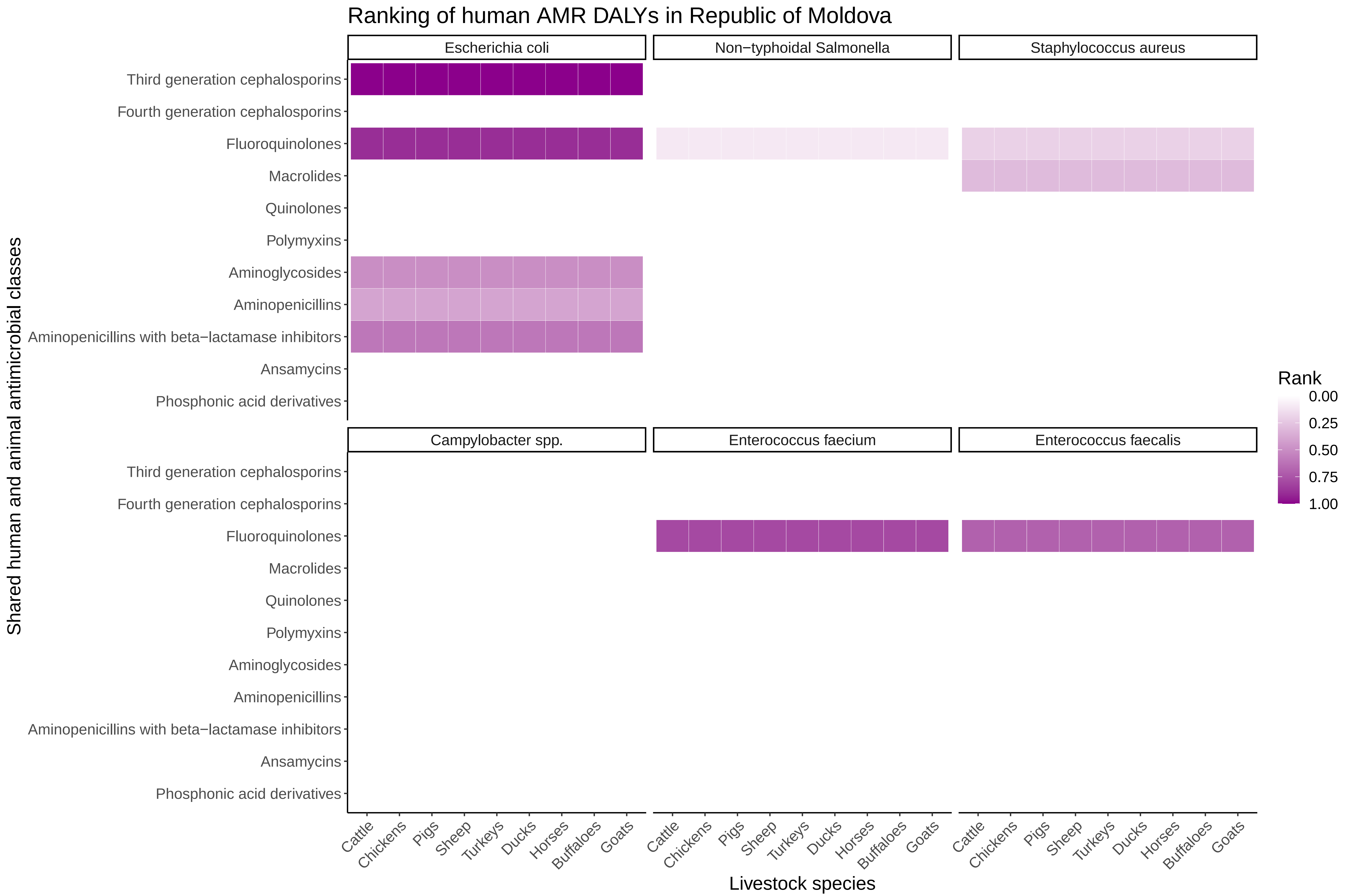

Ranking of human AMR DALYs in Republic of Moldova
Escherichia coli
Non−typhoidal Salmonella
Staphylococcus aureus
Campylobacter spp.
Enterococcus faecium
Enterococcus faecalis
Third generation cephalosporins
Fourth generation cephalosporins
Fluoroquinolones
Macrolides
Quinolones
Polymyxins
Aminoglycosides
Aminopenicillins
Aminopenicillins with beta−lactamase inhibitors
Ansamycins
Phosphonic acid derivatives
Shared human and animal antimicrobial classes
Cattle
Chickens
Pigs
Sheep
Turkeys
Ducks
Horses
Buffaloes
Goats
Livestock species
Rank
0.00
0.25
0.50
0.75
1.00

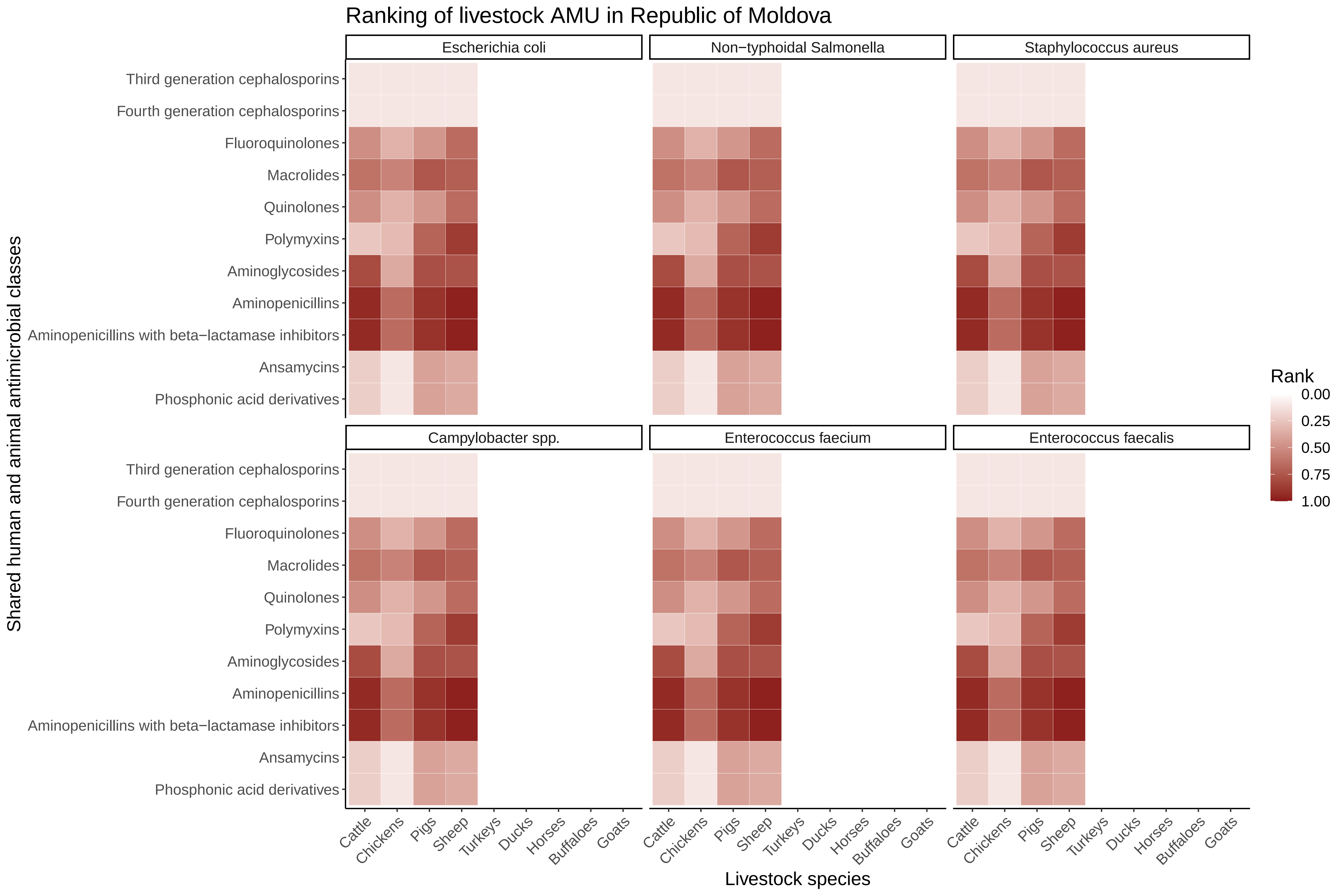

Ranking of livestock AMU in Republic of Moldova
Escherichia coli
Non−typhoidal Salmonella
Staphylococcus aureus
Campylobacter spp.
Enterococcus faecium
Enterococcus faecalis
Third generation cephalosporins
Fourth generation cephalosporins
Fluoroquinolones
Macrolides
Quinolones
Polymyxins
Aminoglycosides
Aminopenicillins
Aminopenicillins with beta−lactamase inhibitors
Ansamycins
Phosphonic acid derivatives
Shared human and animal antimicrobial classes
Cattle
Chickens
Pigs
Sheep
Turkeys
Ducks
Horses
Buffaloes
Goats
Livestock species
Rank
0.00
0.25
0.50
0.75
1.00

Ranking of livestock biomass in Republic of Moldova

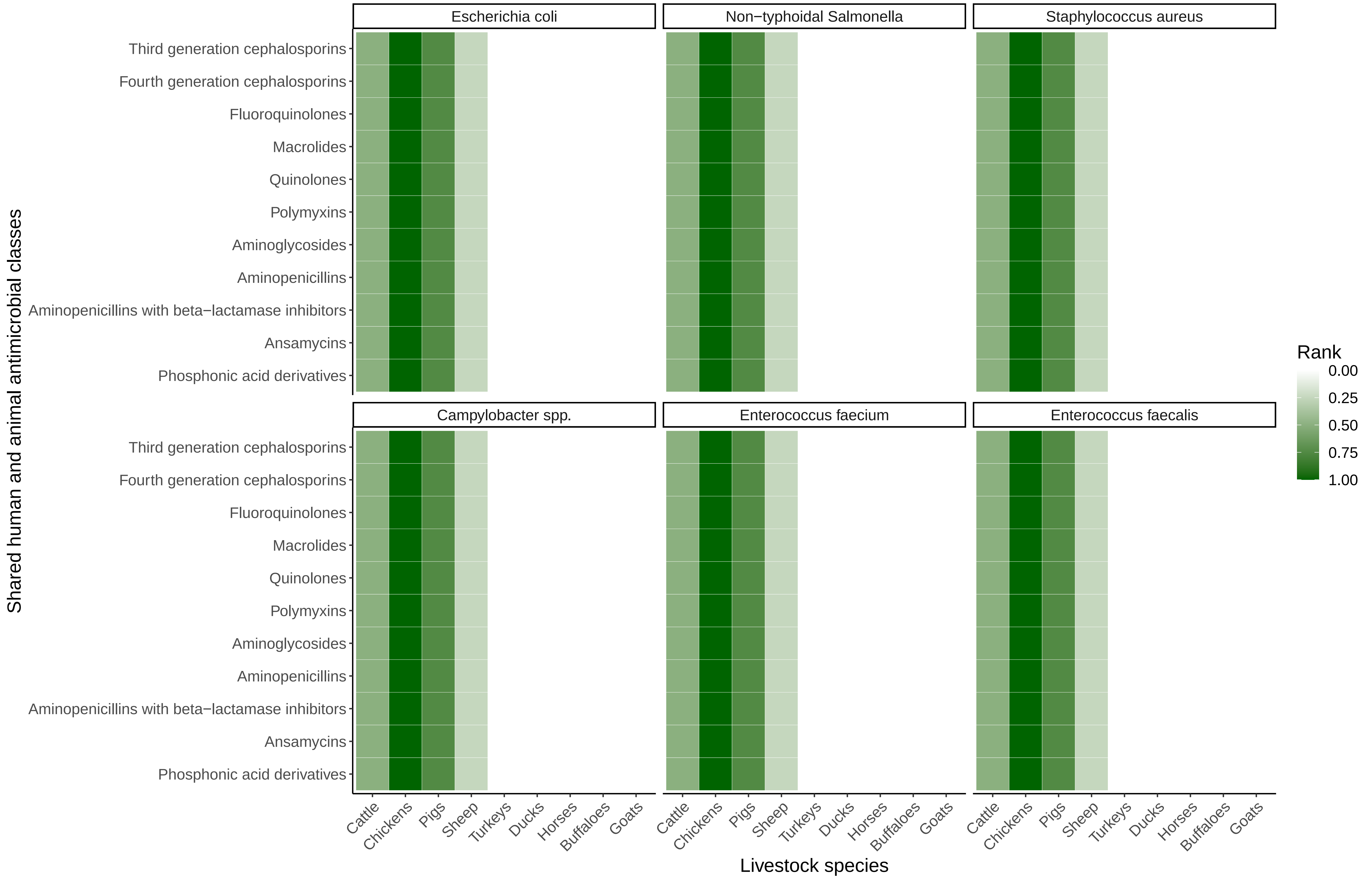

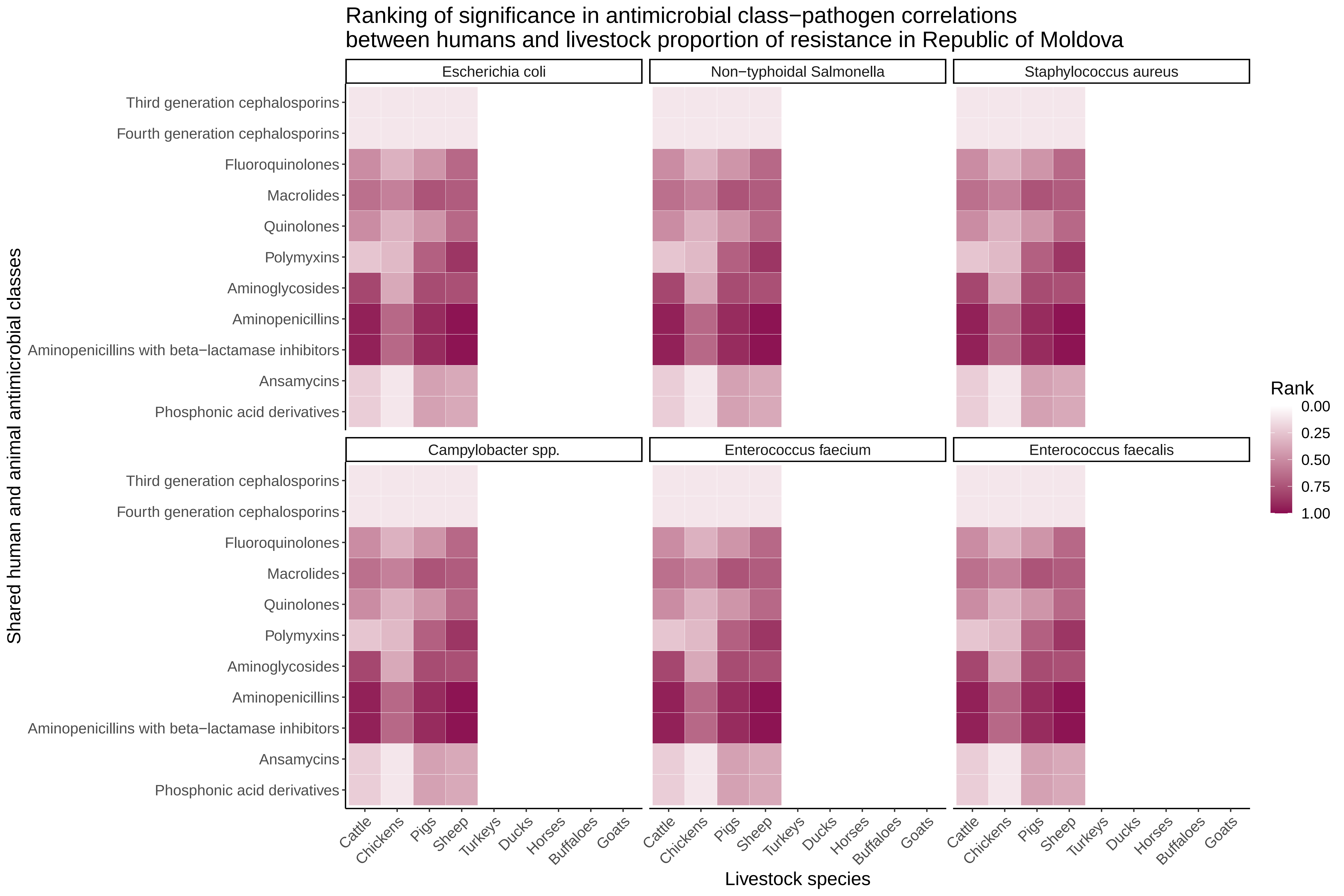

Ranking of significance in antimicrobial class−pathogen correlations
between humans and livestock proportion of resistance in Republic of Moldova
Escherichia coli
Non−typhoidal Salmonella
Staphylococcus aureus
Campylobacter spp.
Enterococcus faecium
Enterococcus faecalis
Third generation cephalosporins
Fourth generation cephalosporins
Fluoroquinolones
Macrolides
Quinolones
Polymyxins
Aminoglycosides
Aminopenicillins
Aminopenicillins with beta−lactamase inhibitors
Ansamycins
Phosphonic acid derivatives
Shared human and animal antimicrobial classes
Cattle
Chickens
Pigs
Sheep
Turkeys
Ducks
Horses
Buffaloes
Goats
Livestock species
Rank
0.00
0.25
0.50
0.75
1.00

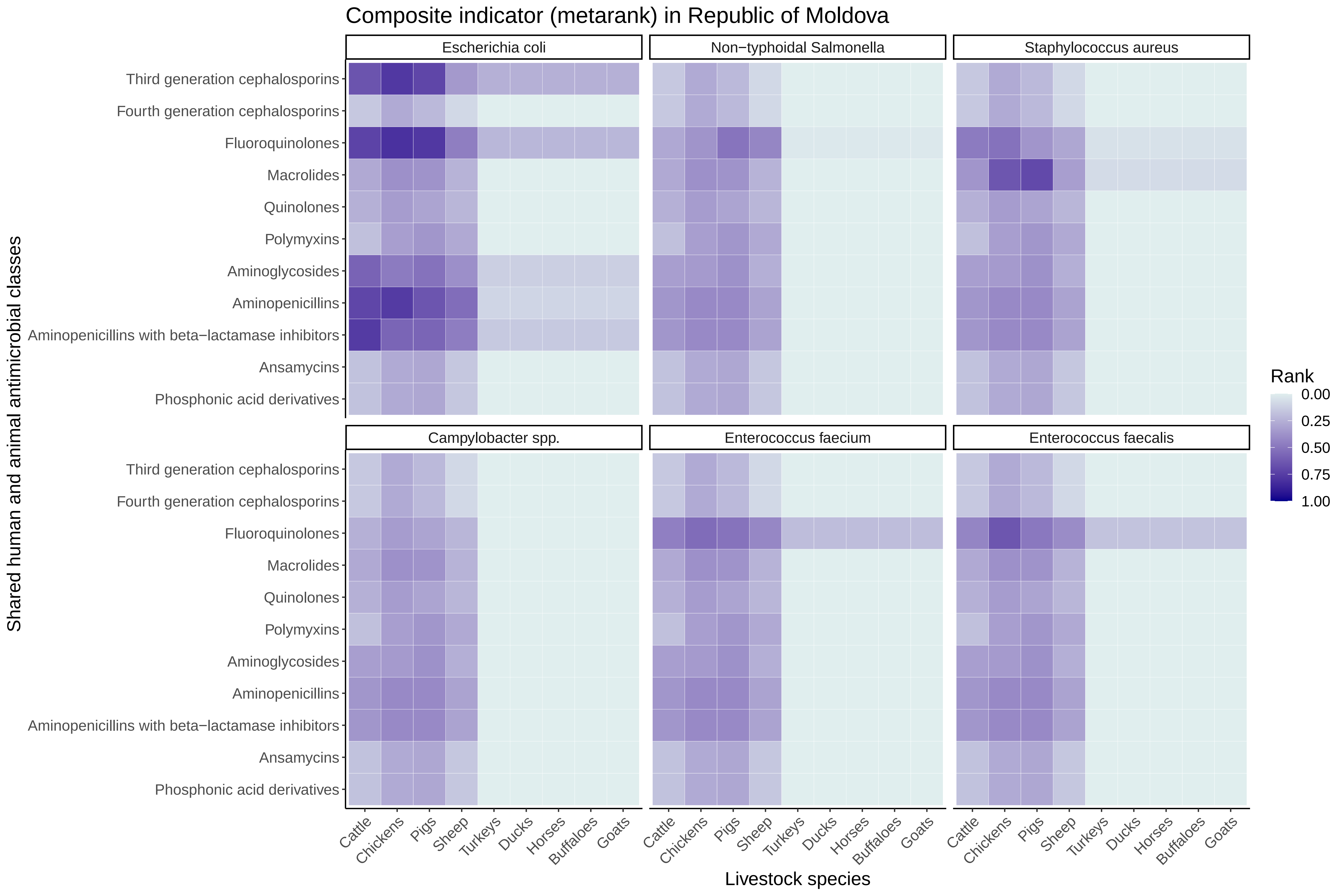

Composite indicator (metarank) in Republic of Moldova
Escherichia coli
Non−typhoidal Salmonella
Staphylococcus aureus
Campylobacter spp.
Enterococcus faecium
Enterococcus faecalis
Third generation cephalosporins
Fourth generation cephalosporins
Fluoroquinolones
Macrolides
Quinolones
Polymyxins
Aminoglycosides
Aminopenicillins
Aminopenicillins with beta−lactamase inhibitors
Ansamycins
Phosphonic acid derivatives
Shared human and animal antimicrobial classes
Cattle
Chickens
Pigs
Sheep
Turkeys
Ducks
Horses
Buffaloes
Goats
Livestock species
Rank
0.00
0.25
0.50
0.75
1.00

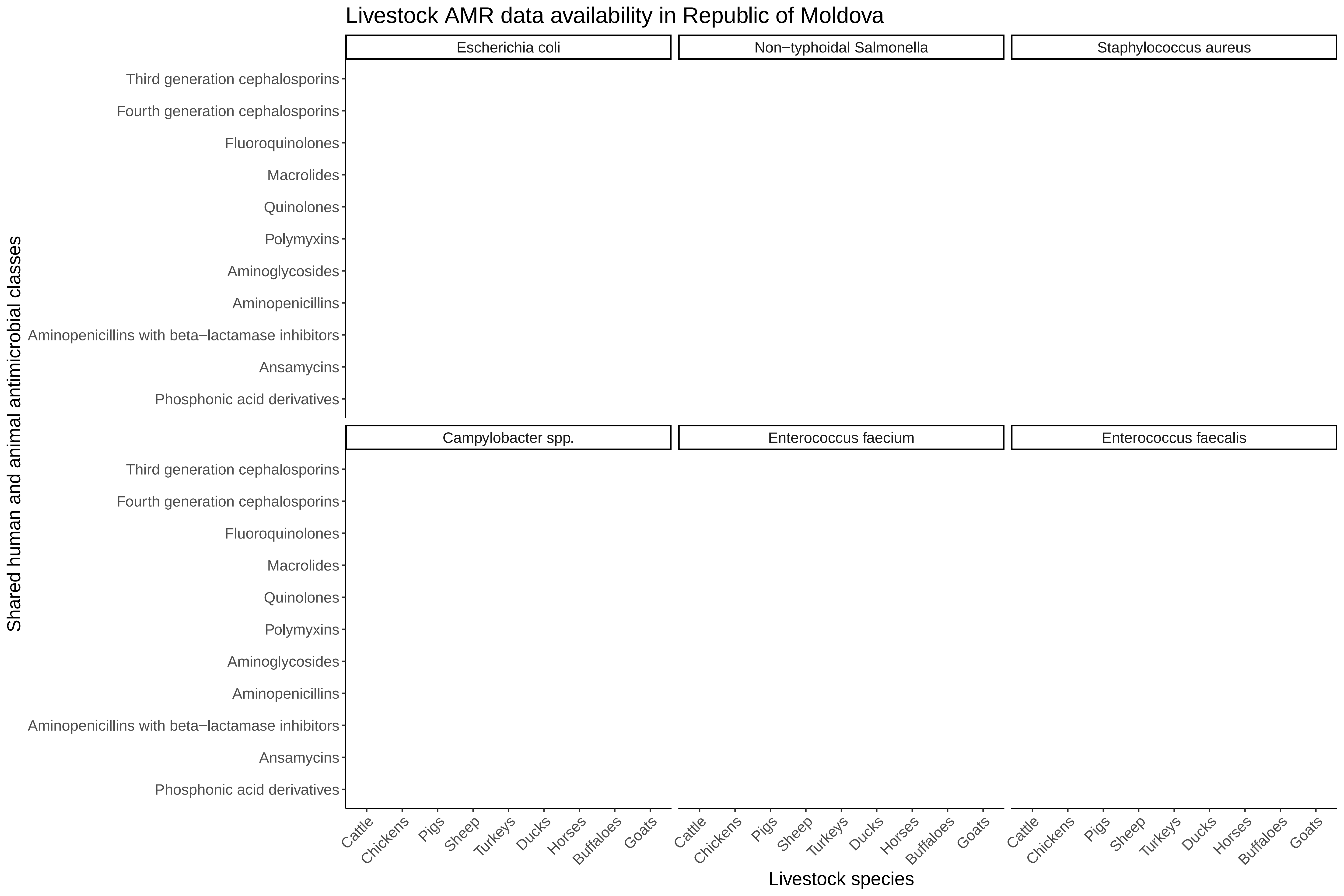
Livestock AMR data availability in Republic of Moldova
Escherichia coli
Non−typhoidal Salmonella
Staphylococcus aureus
Campylobacter spp.
Enterococcus faecium
Enterococcus faecalis
Shared human and animal antimicrobial classes
Third generation cephalosporins
Fourth generation cephalosporins
Fluoroquinolones
Macrolides
Quinolones
Polymyxins
Aminoglycosides
Aminopenicillins
Aminopenicillins with beta−lactamase inhibitors
Ansamycins
Phosphonic acid derivatives
Cattle
Chickens
Pigs
Sheep
Turkeys
Ducks
Horses
Buffaloes
Goats
Livestock species

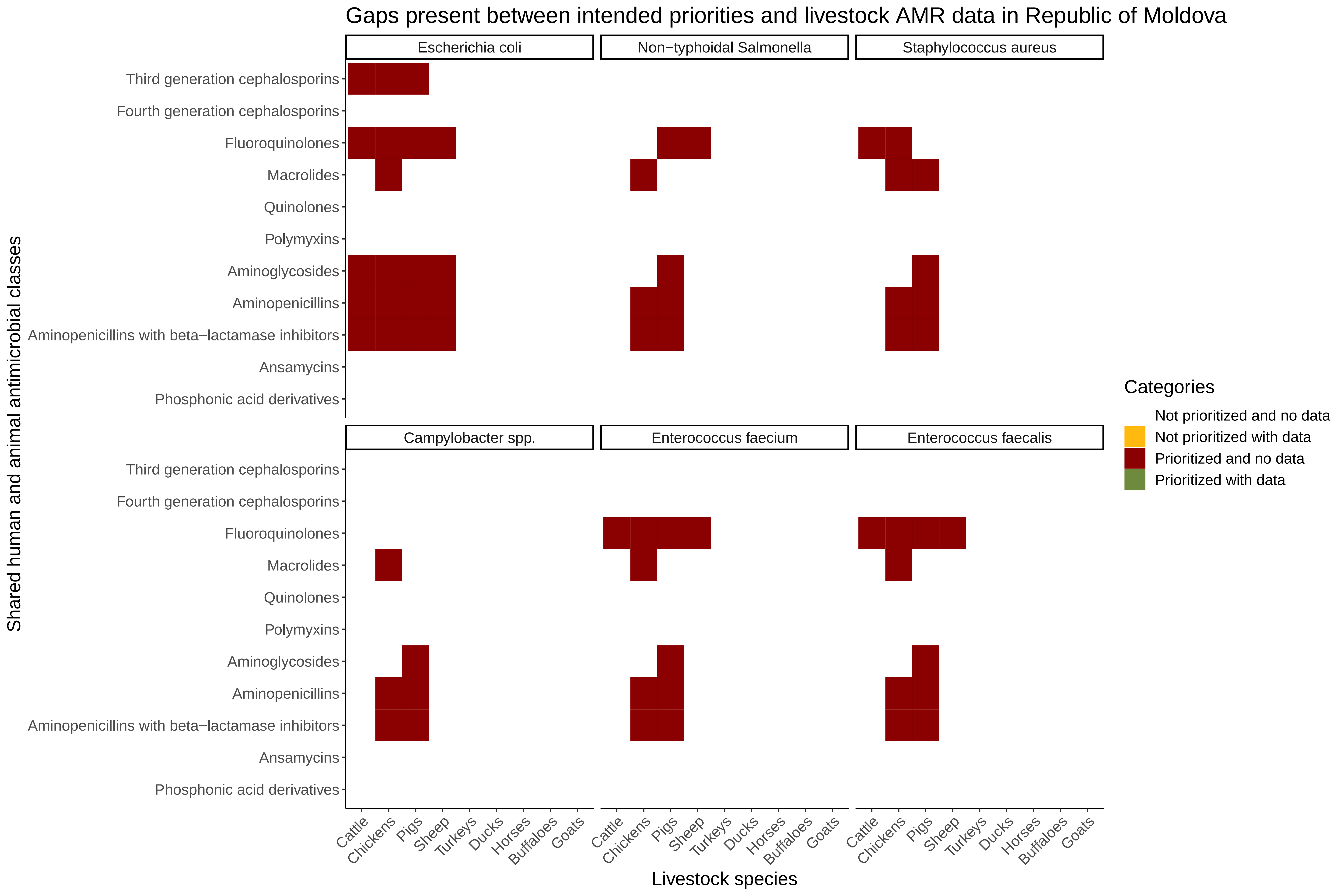
Gaps present between intended priorities and livestock AMR data in Republic of Moldova
Escherichia coli
Non−typhoidal Salmonella
Staphylococcus aureus
Campylobacter spp.
Enterococcus faecium
Enterococcus faecalis
Shared human and animal antimicrobial classes
Third generation cephalosporins
Fourth generation cephalosporins
Fluoroquinolones
Macrolides
Quinolones
Polymyxins
Aminoglycosides
Aminopenicillins
Aminopenicillins with beta−lactamase inhibitors
Ansamycins
Phosphonic acid derivatives
Livestock species
Cattle
Chickens
Pigs
Sheep
Turkeys
Ducks
Horses
Buffaloes
Goats
Categories
Not prioritized and no data
Not prioritized with data
Prioritized and no data
Prioritized with data

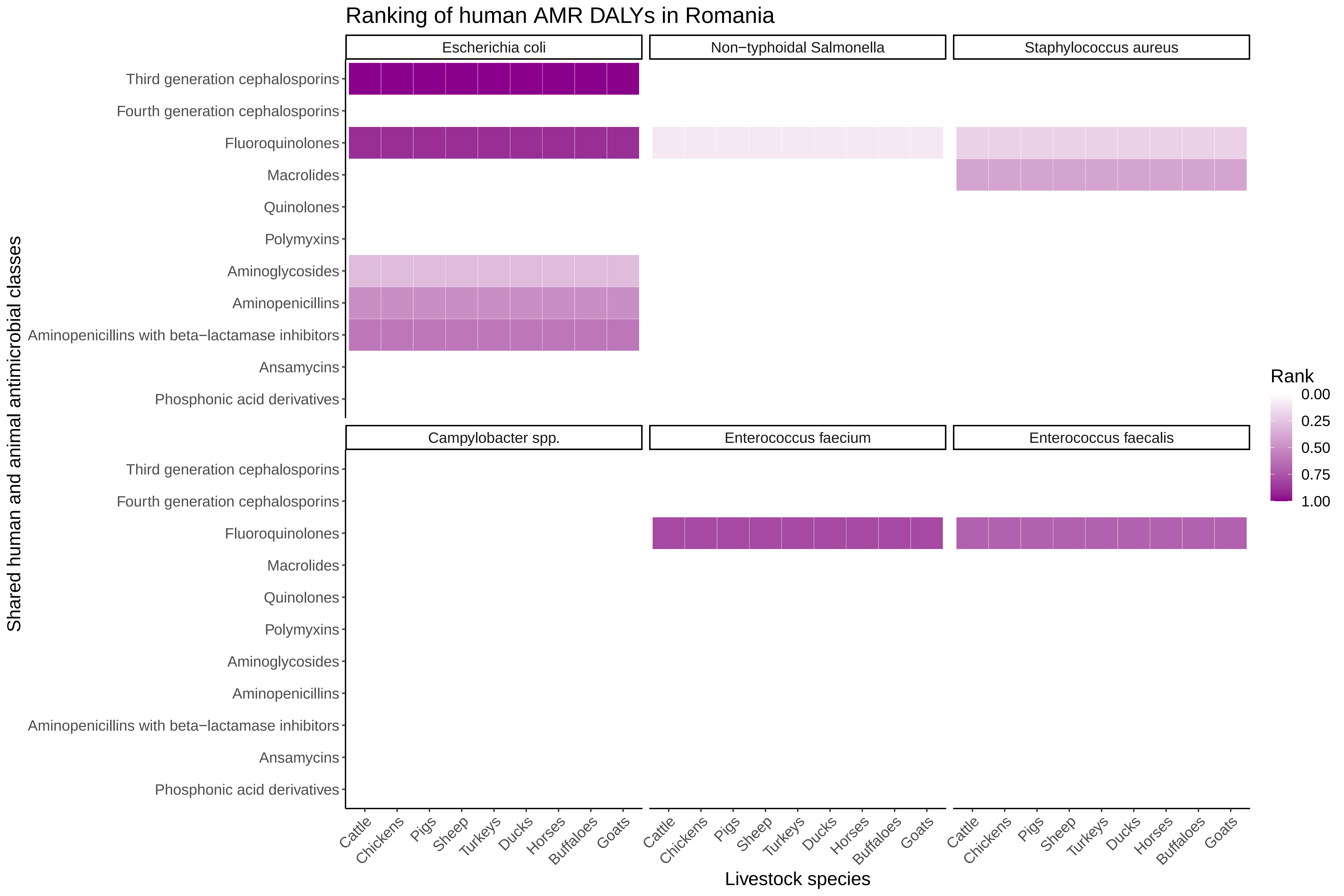
Ranking of human AMR DALYs in Romania
Escherichia coli
Non−typhoidal Salmonella
Staphylococcus aureus
Campylobacter spp.
Enterococcus faecium
Enterococcus faecalis
Third generation cephalosporins
Fourth generation cephalosporins
Fluoroquinolones
Macrolides
Quinolones
Polymyxins
Aminoglycosides
Aminopenicillins
Aminopenicillins with beta−lactamase inhibitors
Ansamycins
Phosphonic acid derivatives
Shared human and animal antimicrobial classes
Cattle
Chickens
Pigs
Sheep
Turkeys
Ducks
Horses
Buffaloes
Goats
Livestock species
Rank
0.00
0.25
0.50
0.75
1.00

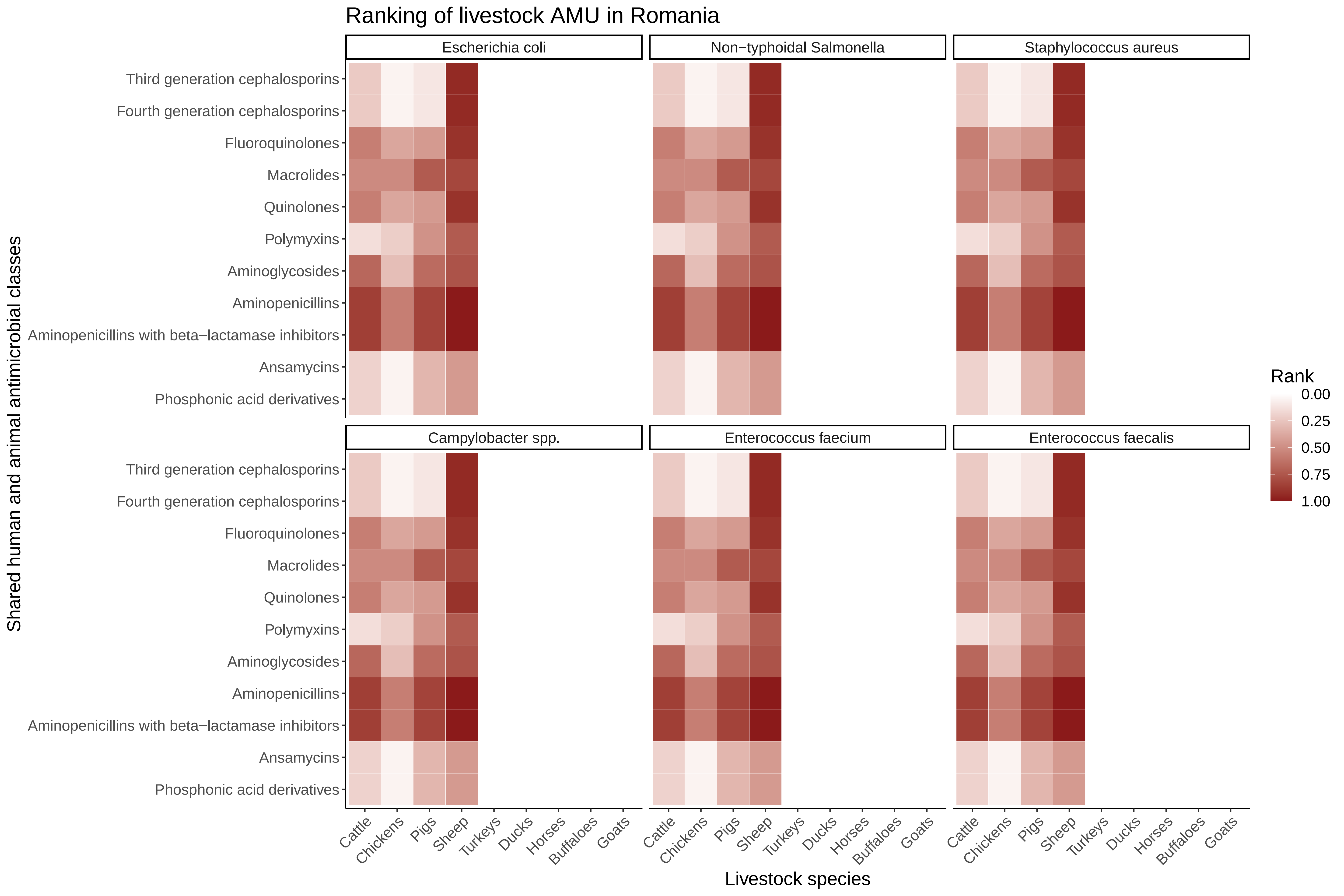

Ranking of livestock AMU in Romania
Escherichia coli
Non−typhoidal Salmonella
Staphylococcus aureus
Campylobacter spp.
Enterococcus faecium
Enterococcus faecalis
Third generation cephalosporins
Fourth generation cephalosporins
Fluoroquinolones
Macrolides
Quinolones
Polymyxins
Aminoglycosides
Aminopenicillins
Aminopenicillins with beta−lactamase inhibitors
Ansamycins
Phosphonic acid derivatives
Shared human and animal antimicrobial classes
Cattle
Chickens
Pigs
Sheep
Turkeys
Ducks
Horses
Buffaloes
Goats
Livestock species
Rank
0.00
0.25
0.50
0.75
1.00

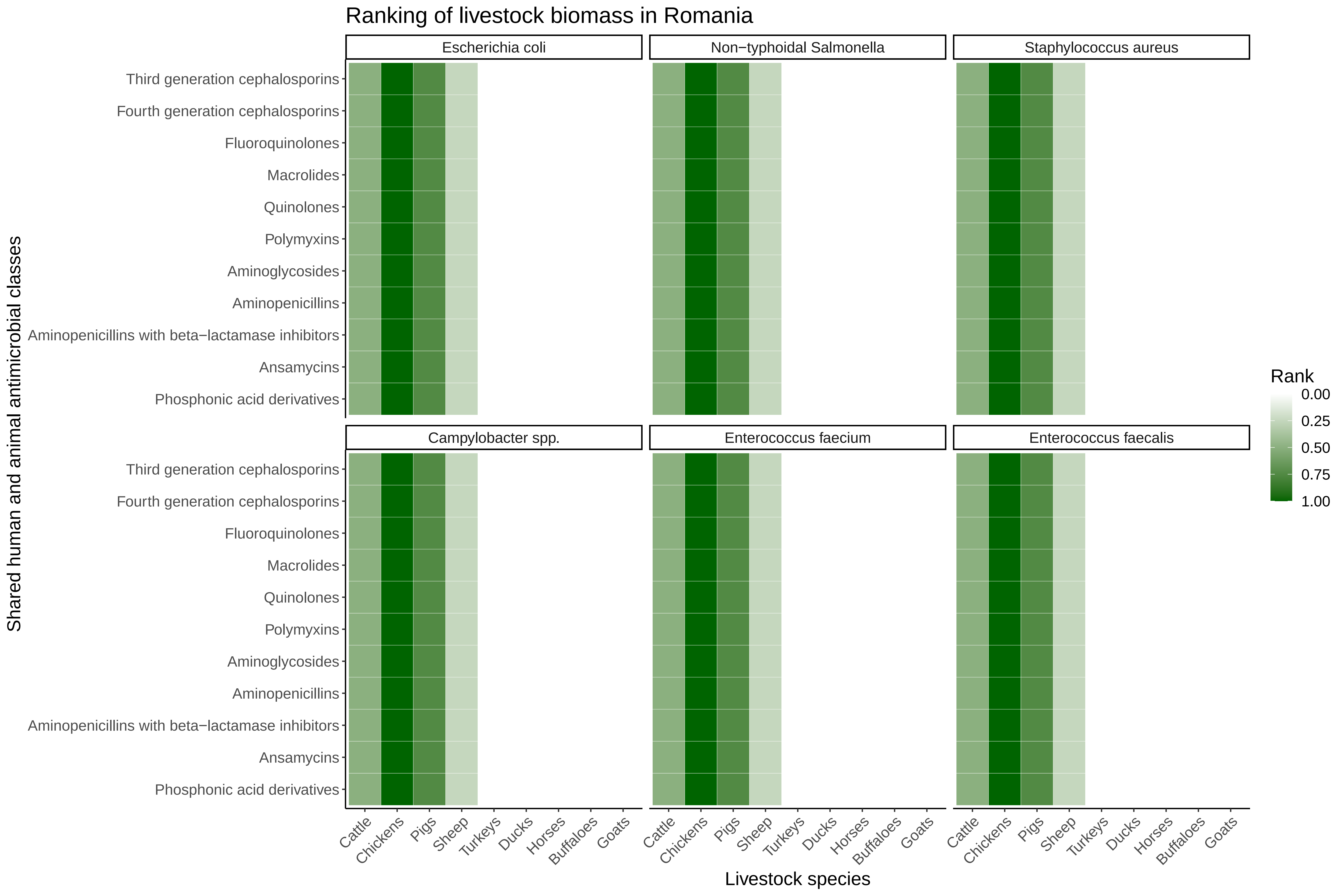
Ranking of livestock biomass in Romania
Escherichia coli
Non−typhoidal Salmonella
Staphylococcus aureus
Campylobacter spp.
Enterococcus faecium
Enterococcus faecalis
Shared human and animal antimicrobial classes
Third generation cephalosporins
Fourth generation cephalosporins
Fluoroquinolones
Macrolides
Quinolones
Polymyxins
Aminoglycosides
Aminopenicillins
Aminopenicillins with beta−lactamase inhibitors
Ansamycins
Phosphonic acid derivatives
Livestock species
Cattle
Chickens
Pigs
Sheep
Turkeys
Ducks
Horses
Buffaloes
Goats
Rank
0.00
0.25
0.50
0.75
1.00

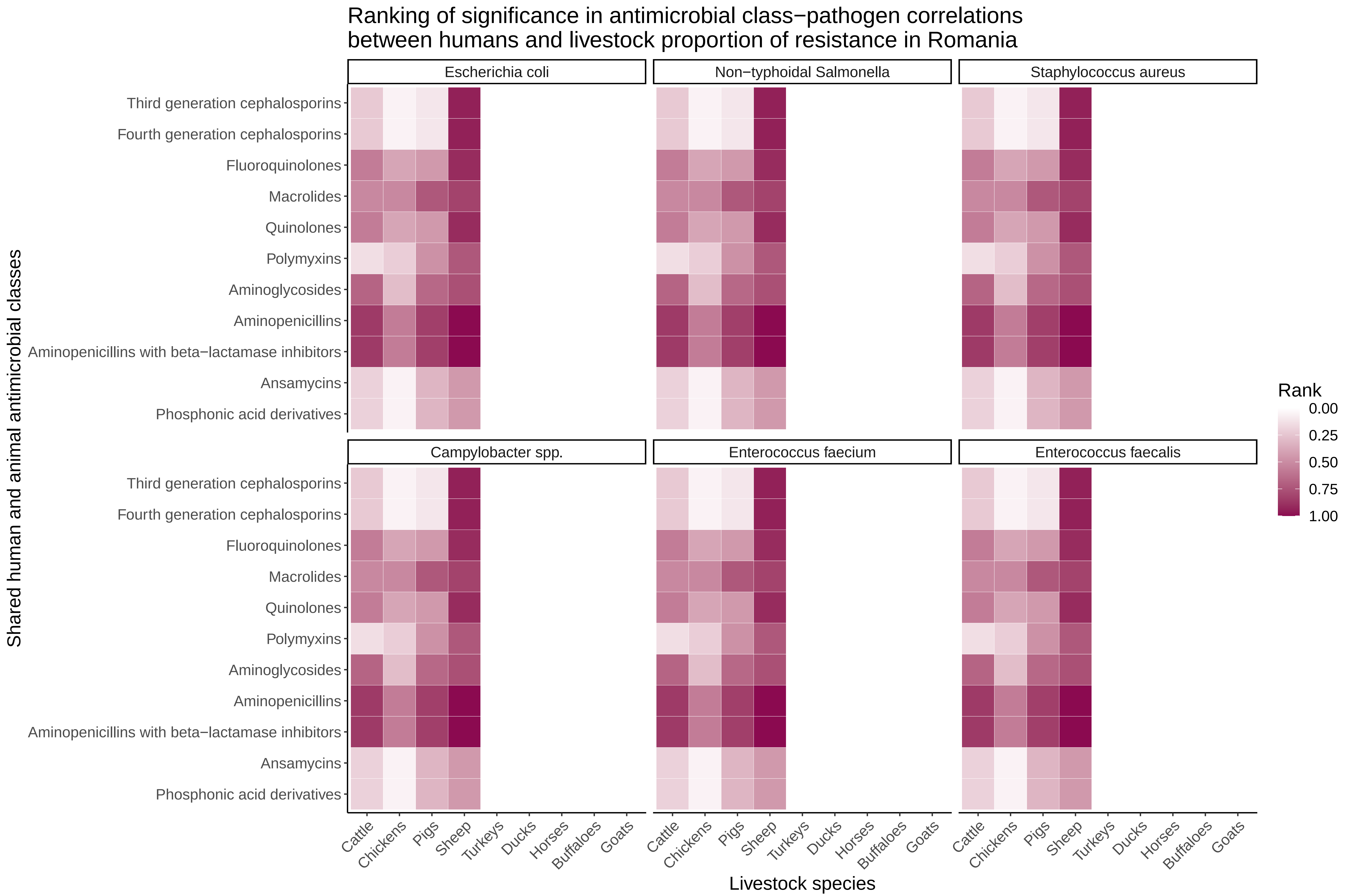

Ranking of significance in antimicrobial class−pathogen correlations
between humans and livestock proportion of resistance in Romania
Escherichia coli
Non−typhoidal Salmonella
Staphylococcus aureus
Campylobacter spp.
Enterococcus faecium
Enterococcus faecalis
Third generation cephalosporins
Fourth generation cephalosporins
Fluoroquinolones
Macrolides
Quinolones
Polymyxins
Aminoglycosides
Aminopenicillins
Aminopenicillins with beta−lactamase inhibitors
Ansamycins
Phosphonic acid derivatives
Shared human and animal antimicrobial classes
Cattle
Chickens
Pigs
Sheep
Turkeys
Ducks
Horses
Buffaloes
Goats
Livestock species
Rank
0.00
0.25
0.50
0.75
1.00

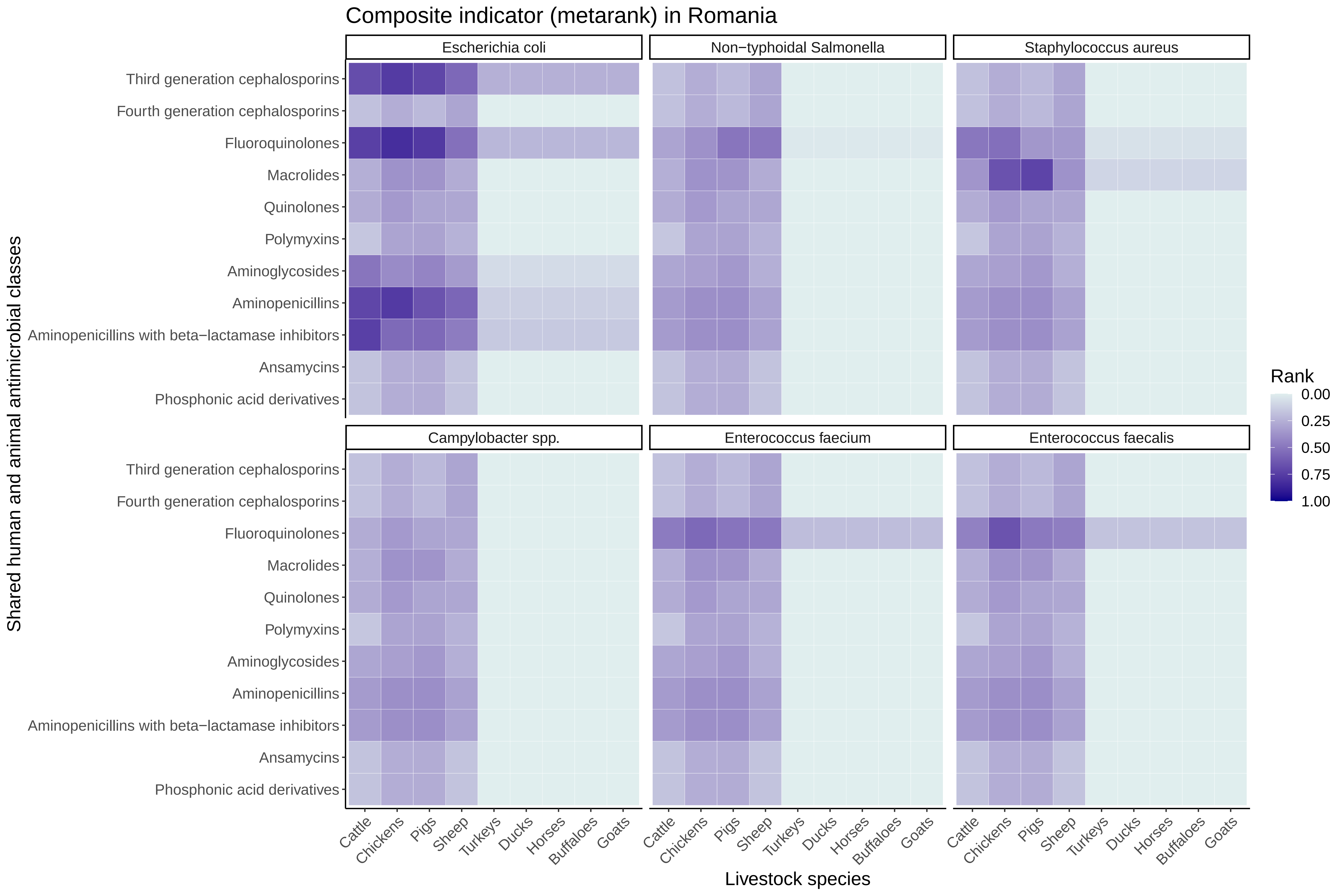

Composite indicator (metarank) in Romania
Escherichia coli
Non−typhoidal Salmonella
Staphylococcus aureus
Campylobacter spp.
Enterococcus faecium
Enterococcus faecalis
Shared human and animal antimicrobial classes
Third generation cephalosporins
Fourth generation cephalosporins
Fluoroquinolones
Macrolides
Quinolones
Polymyxins
Aminoglycosides
Aminopenicillins
Aminopenicillins with beta−lactamase inhibitors
Ansamycins
Phosphonic acid derivatives
Cattle
Chickens
Pigs
Sheep
Turkeys
Ducks
Horses
Buffaloes
Goats
Livestock species
Rank
0.00
0.25
0.50
0.75
1.00

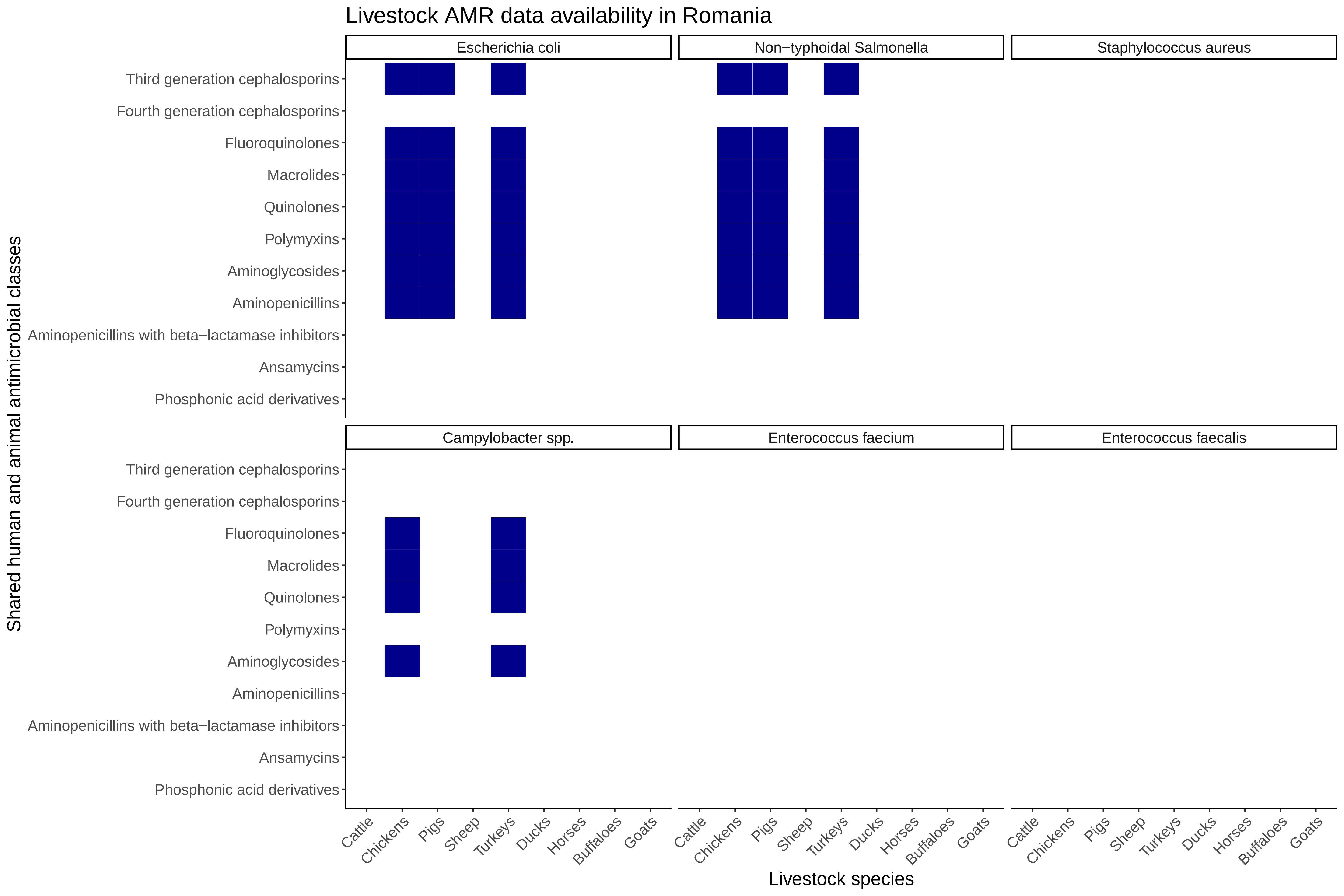
Livestock AMR data availability in Romania
Escherichia coli
Non−typhoidal Salmonella
Staphylococcus aureus
Campylobacter spp.
Enterococcus faecium
Enterococcus faecalis
Shared human and animal antimicrobial classes
Third generation cephalosporins
Fourth generation cephalosporins
Fluoroquinolones
Macrolides
Quinolones
Polymyxins
Aminoglycosides
Aminopenicillins
Aminopenicillins with beta−lactamase inhibitors
Ansamycins
Phosphonic acid derivatives
Cattle
Chickens
Pigs
Sheep
Turkeys
Ducks
Horses
Buffaloes
Goats
Livestock species

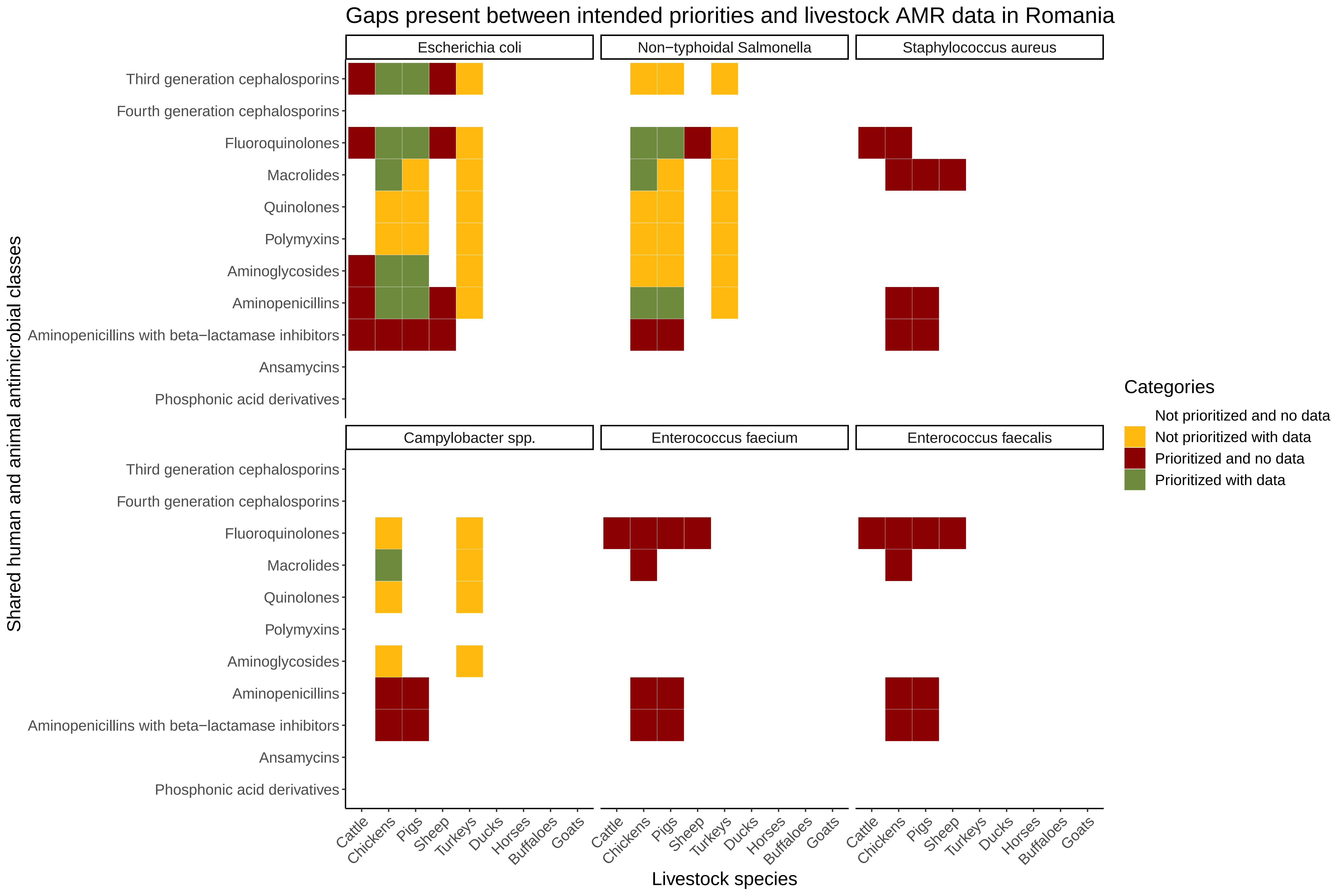

Gaps present between intended priorities and livestock AMR data in Romania
Escherichia coli
Non−typhoidal Salmonella
Staphylococcus aureus
Campylobacter spp.
Enterococcus faecium
Enterococcus faecalis
Third generation cephalosporins
Fourth generation cephalosporins
Fluoroquinolones
Macrolides
Quinolones
Polymyxins
Aminoglycosides
Aminopenicillins
Aminopenicillins with beta−lactamase inhibitors
Ansamycins
Phosphonic acid derivatives
Shared human and animal antimicrobial classes
Cattle
Chickens
Pigs
Sheep
Turkeys
Ducks
Horses
Buffaloes
Goats
Livestock species
Categories
Not prioritized and no data
Not prioritized with data
Prioritized and no data
Prioritized with data

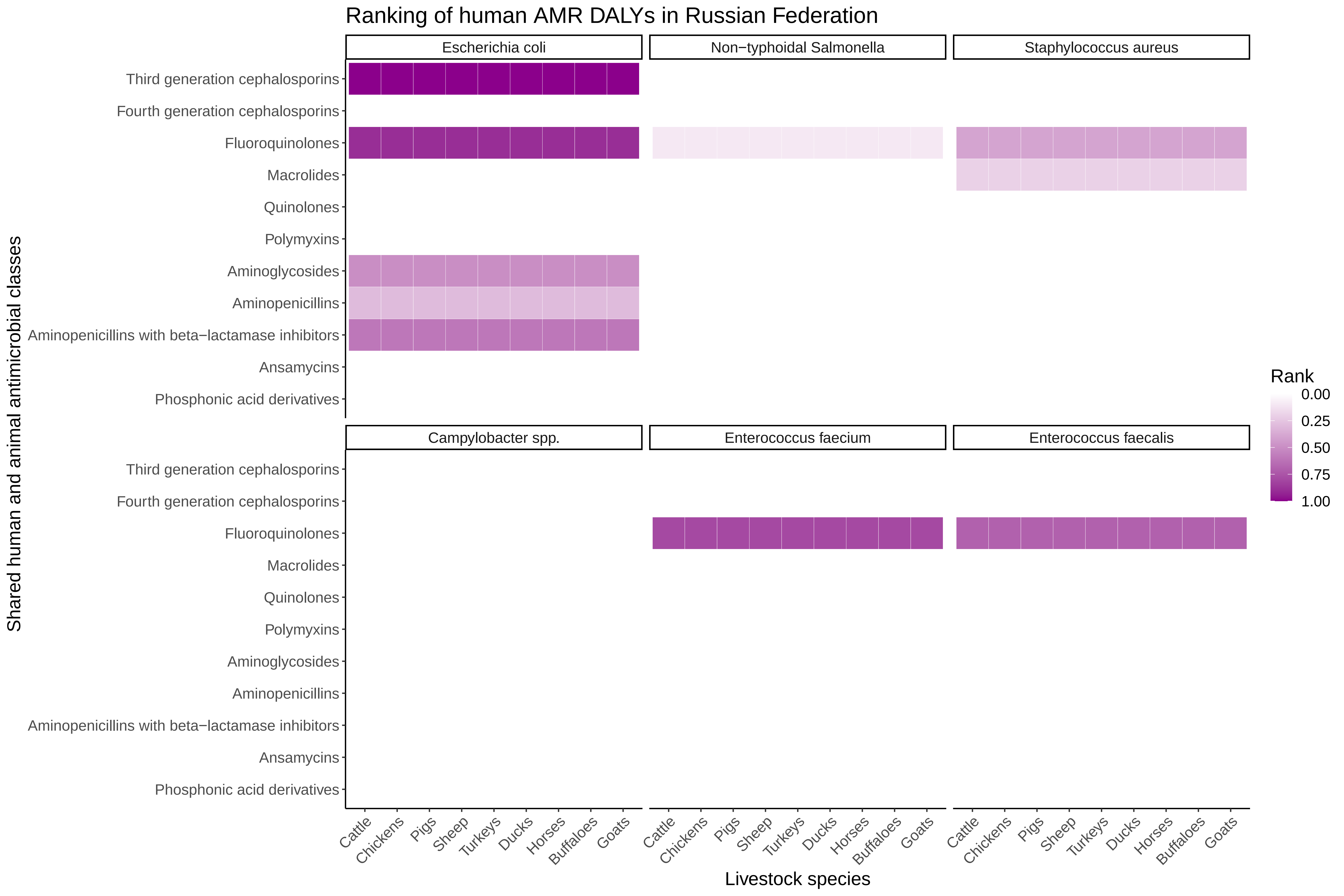

Ranking of human AMR DALYs in Russian Federation
Escherichia coli
Non−typhoidal Salmonella
Staphylococcus aureus
Campylobacter spp.
Enterococcus faecium
Enterococcus faecalis
Shared human and animal antimicrobial classes
Third generation cephalosporins
Fourth generation cephalosporins
Fluoroquinolones
Macrolides
Quinolones
Polymyxins
Aminoglycosides
Aminopenicillins
Aminopenicillins with beta−lactamase inhibitors
Ansamycins
Phosphonic acid derivatives
Cattle
Chickens
Pigs
Sheep
Turkeys
Ducks
Horses
Buffaloes
Goats
Livestock species
Rank
0.00
0.25
0.50
0.75
1.00

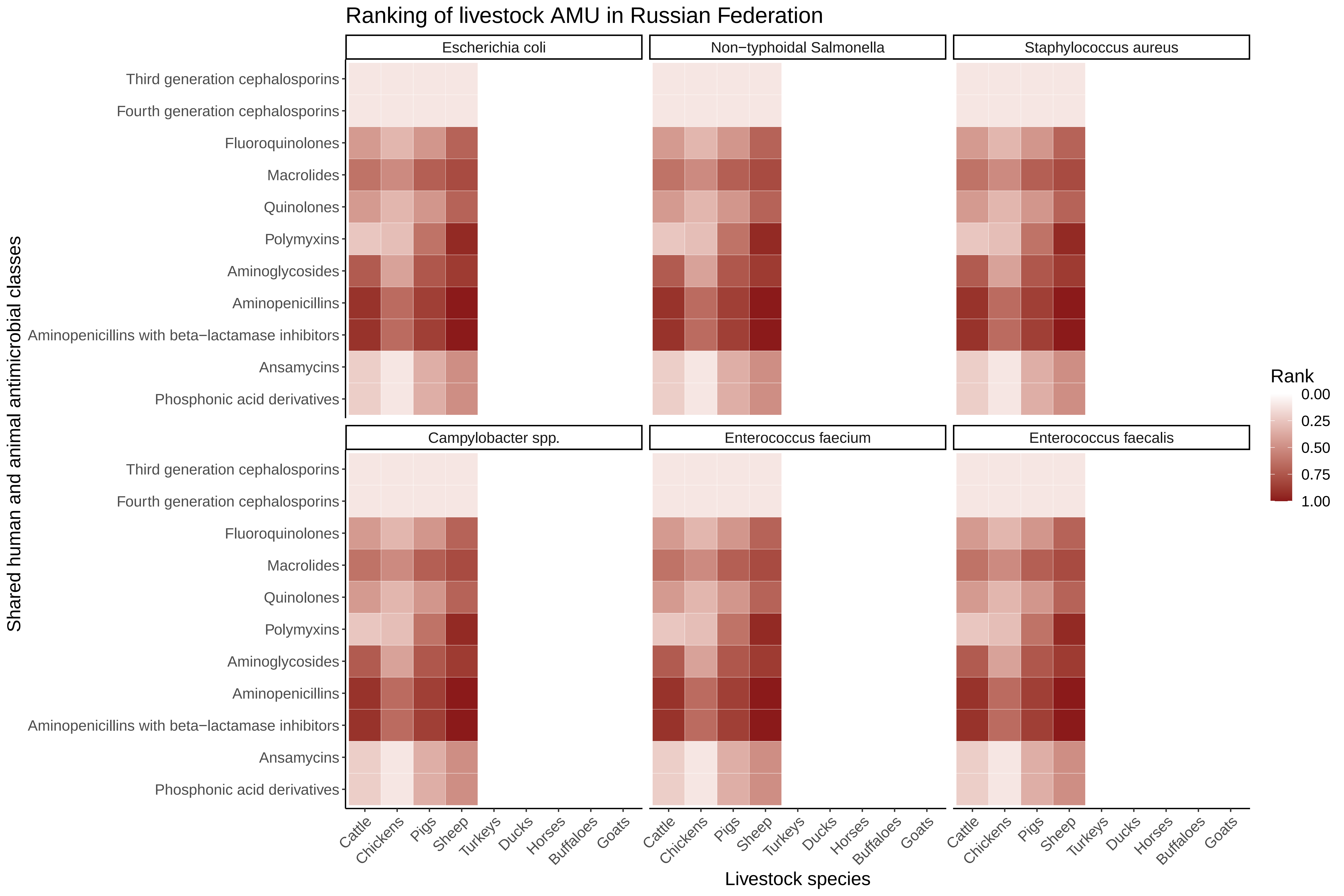

Ranking of livestock AMU in Russian Federation
Escherichia coli
Non−typhoidal Salmonella
Staphylococcus aureus
Campylobacter spp.
Enterococcus faecium
Enterococcus faecalis
Third generation cephalosporins
Fourth generation cephalosporins
Fluoroquinolones
Macrolides
Quinolones
Polymyxins
Aminoglycosides
Aminopenicillins
Aminopenicillins with beta−lactamase inhibitors
Ansamycins
Phosphonic acid derivatives
Shared human and animal antimicrobial classes
Cattle
Chickens
Pigs
Sheep
Turkeys
Ducks
Horses
Buffaloes
Goats
Livestock species
Rank
0.00
0.25
0.50
0.75
1.00

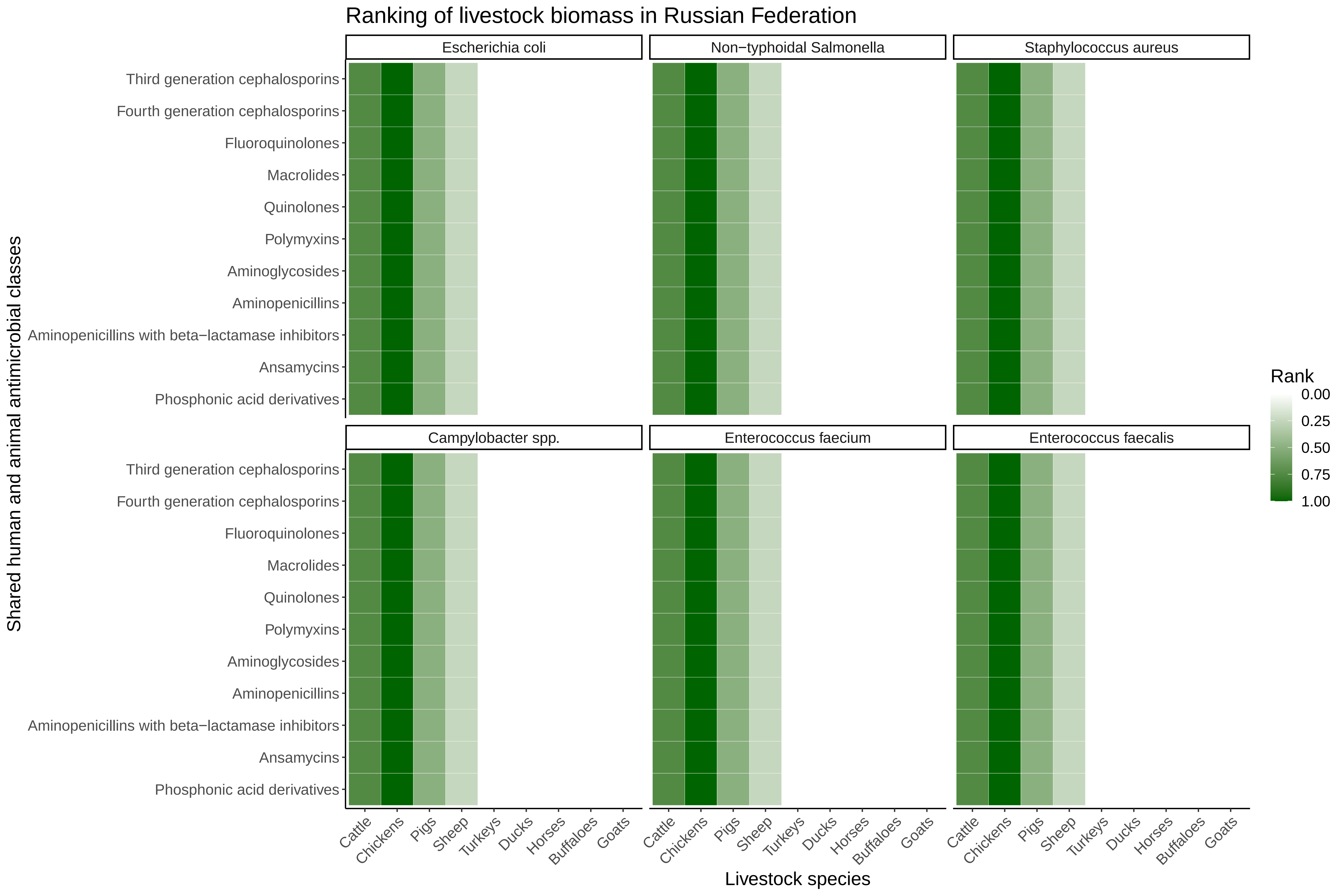
Ranking of livestock biomass in Russian Federation
Escherichia coli
Non−typhoidal Salmonella
Staphylococcus aureus
Campylobacter spp.
Enterococcus faecium
Enterococcus faecalis
Third generation cephalosporins
Fourth generation cephalosporins
Fluoroquinolones
Macrolides
Quinolones
Polymyxins
Aminoglycosides
Aminopenicillins
Aminopenicillins with beta−lactamase inhibitors
Ansamycins
Phosphonic acid derivatives
Shared human and animal antimicrobial classes
Cattle
Chickens
Pigs
Sheep
Turkeys
Ducks
Horses
Buffaloes
Goats
Livestock species
Rank
0.00
0.25
0.50
0.75
1.00

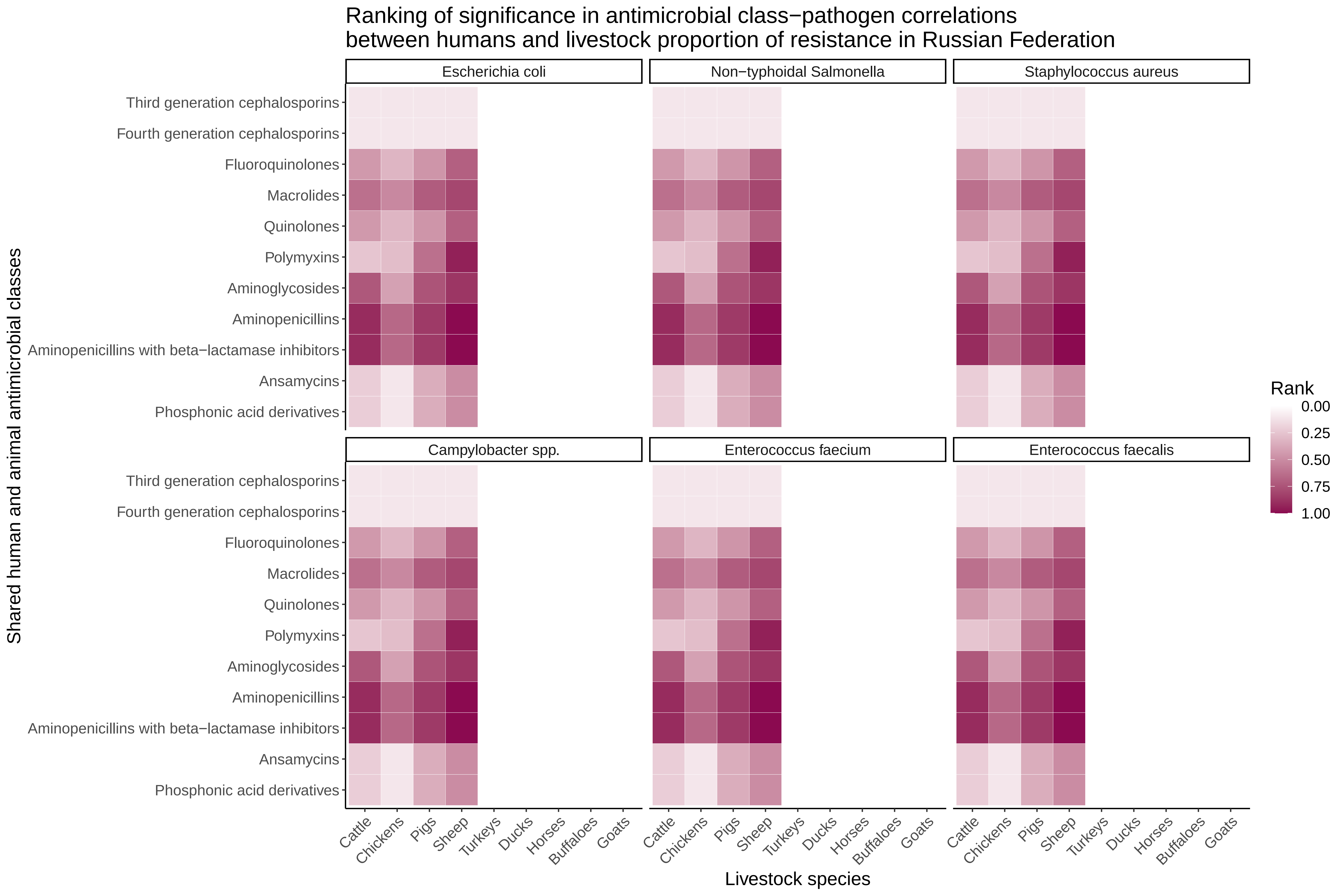
Ranking of significance in antimicrobial class−pathogen correlations
between humans and livestock proportion of resistance in Russian Federation
Escherichia coli
Non−typhoidal Salmonella
Staphylococcus aureus
Campylobacter spp.
Enterococcus faecium
Enterococcus faecalis
Shared human and animal antimicrobial classes
Third generation cephalosporins
Fourth generation cephalosporins
Fluoroquinolones
Macrolides
Quinolones
Polymyxins
Aminoglycosides
Aminopenicillins
Aminopenicillins with beta−lactamase inhibitors
Ansamycins
Phosphonic acid derivatives
Cattle
Chickens
Pigs
Sheep
Turkeys
Ducks
Horses
Buffaloes
Goats
Livestock species
Rank
0.00
0.25
0.50
0.75
1.00

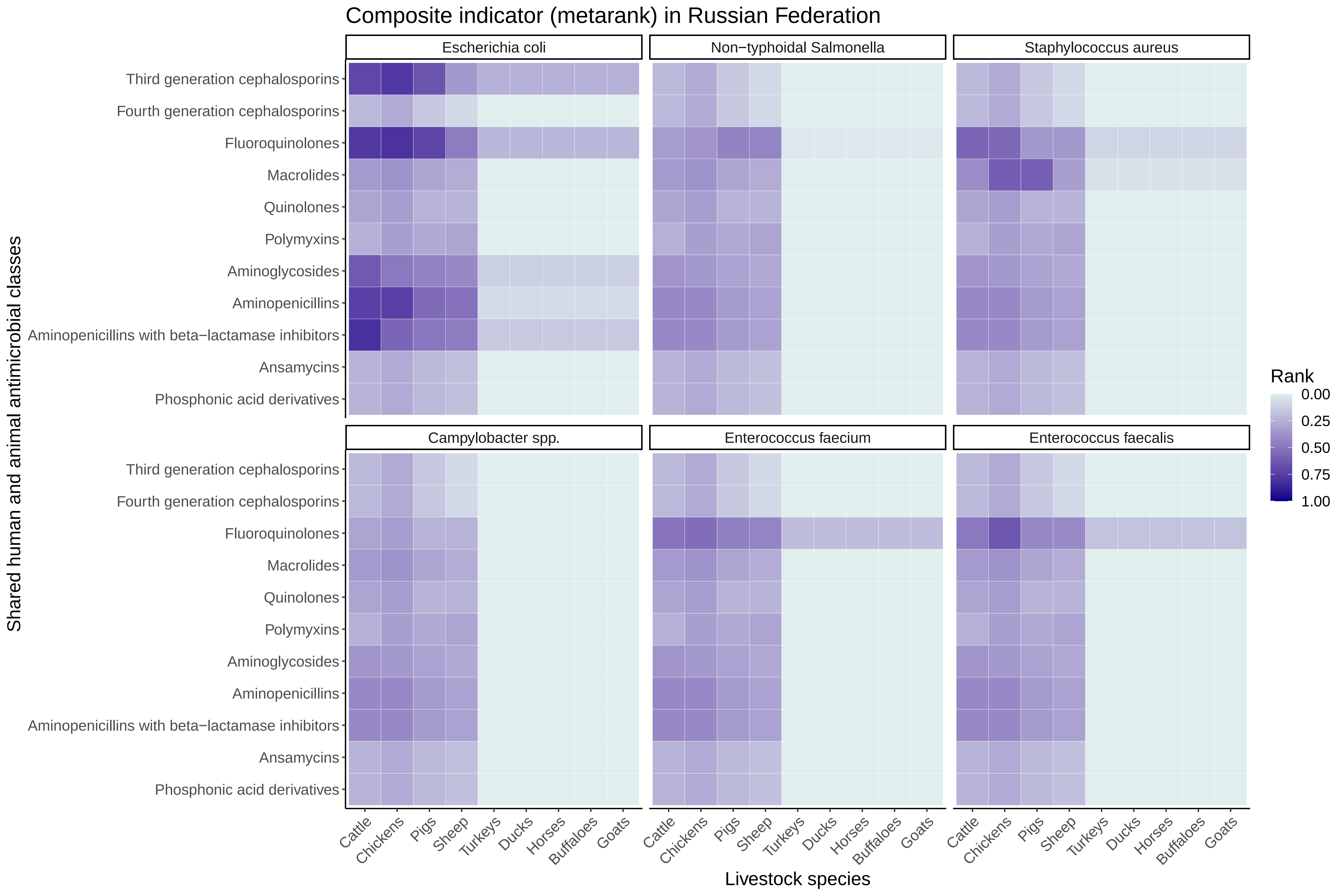

Composite indicator (metarank) in Russian Federation
Escherichia coli
Non−typhoidal Salmonella
Staphylococcus aureus
Campylobacter spp.
Enterococcus faecium
Enterococcus faecalis
Shared human and animal antimicrobial classes
Third generation cephalosporins
Fourth generation cephalosporins
Fluoroquinolones
Macrolides
Quinolones
Polymyxins
Aminoglycosides
Aminopenicillins
Aminopenicillins with beta−lactamase inhibitors
Ansamycins
Phosphonic acid derivatives
Livestock species
Cattle
Chickens
Pigs
Sheep
Turkeys
Ducks
Horses
Buffaloes
Goats
Rank
0.00
0.25
0.50
0.75
1.00

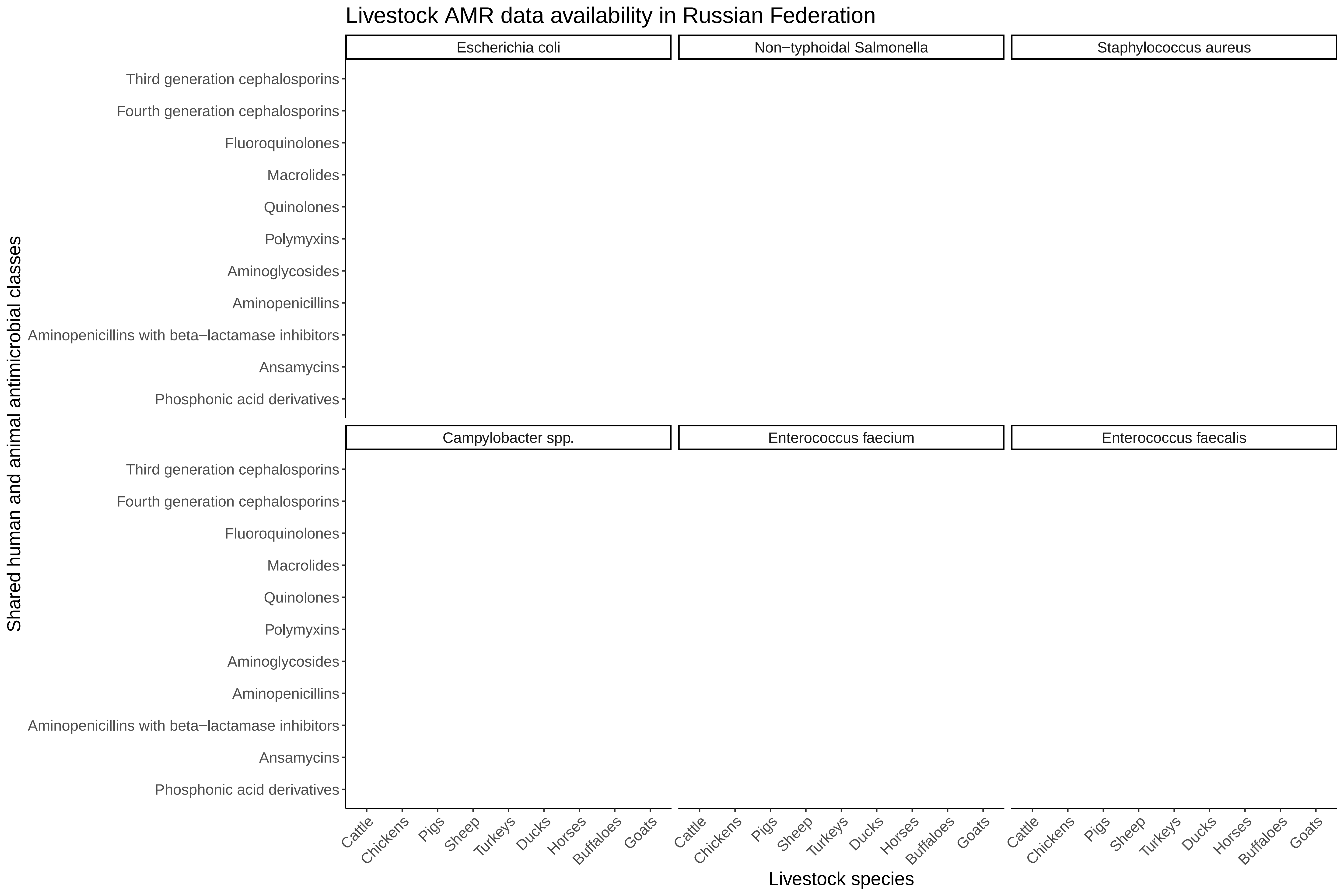
Livestock AMR data availability in Russian Federation
Escherichia coli
Non−typhoidal Salmonella
Staphylococcus aureus
Campylobacter spp.
Enterococcus faecium
Enterococcus faecalis
Third generation cephalosporins
Fourth generation cephalosporins
Fluoroquinolones
Macrolides
Quinolones
Polymyxins
Aminoglycosides
Aminopenicillins
Aminopenicillins with beta−lactamase inhibitors
Ansamycins
Phosphonic acid derivatives
Shared human and animal antimicrobial classes
Cattle
Chickens
Pigs
Sheep
Turkeys
Ducks
Horses
Buffaloes
Goats
Livestock species

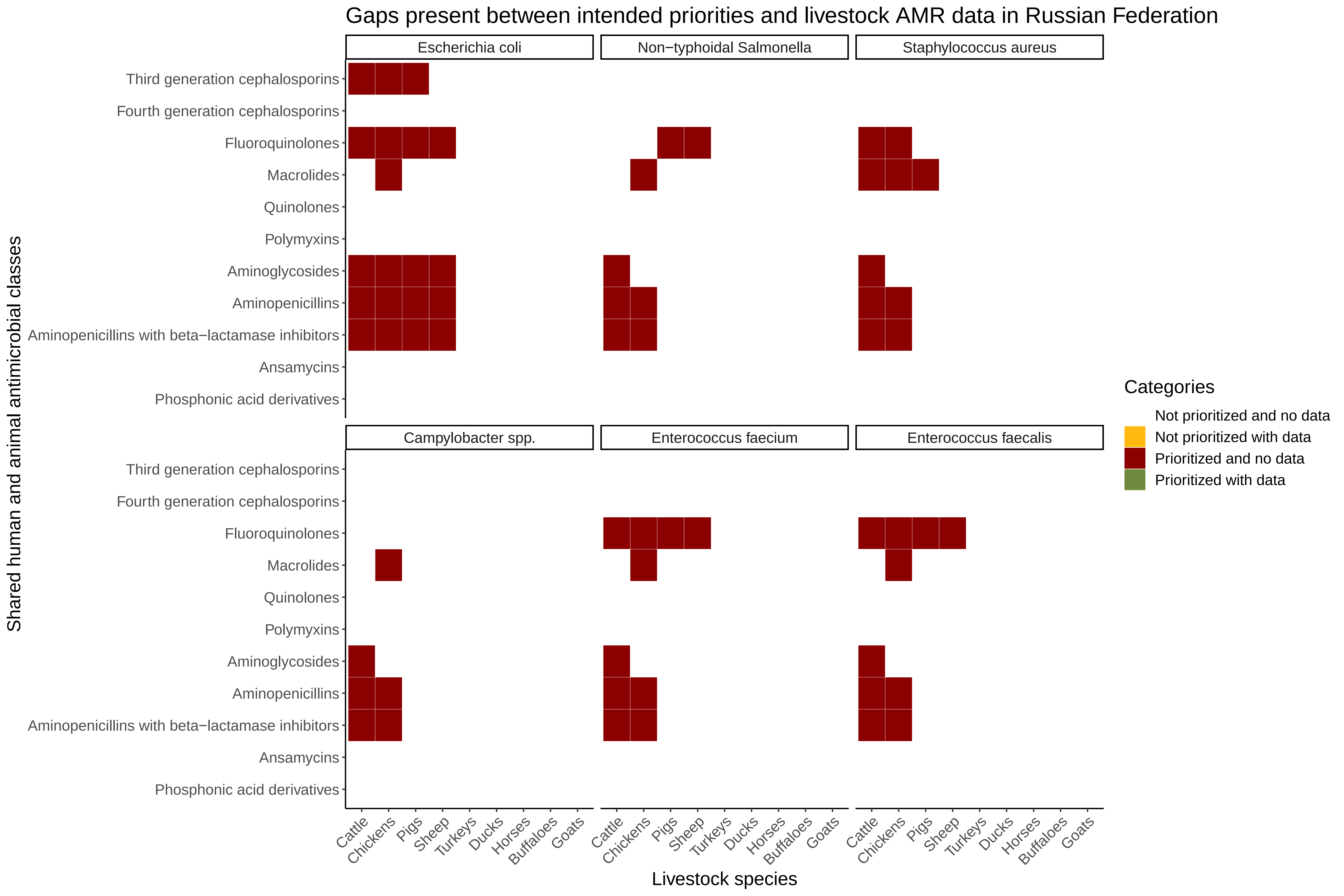

Gaps present between intended priorities and livestock AMR data in Russian Federation
Escherichia coli
Non−typhoidal Salmonella
Staphylococcus aureus
Campylobacter spp.
Enterococcus faecium
Enterococcus faecalis
Shared human and animal antimicrobial classes
Third generation cephalosporins
Fourth generation cephalosporins
Fluoroquinolones
Macrolides
Quinolones
Polymyxins
Aminoglycosides
Aminopenicillins
Aminopenicillins with beta−lactamase inhibitors
Ansamycins
Phosphonic acid derivatives
Cattle
Chickens
Pigs
Sheep
Turkeys
Ducks
Horses
Buffaloes
Goats
Livestock species
Categories
Not prioritized and no data
Not prioritized with data
Prioritized and no data
Prioritized with data

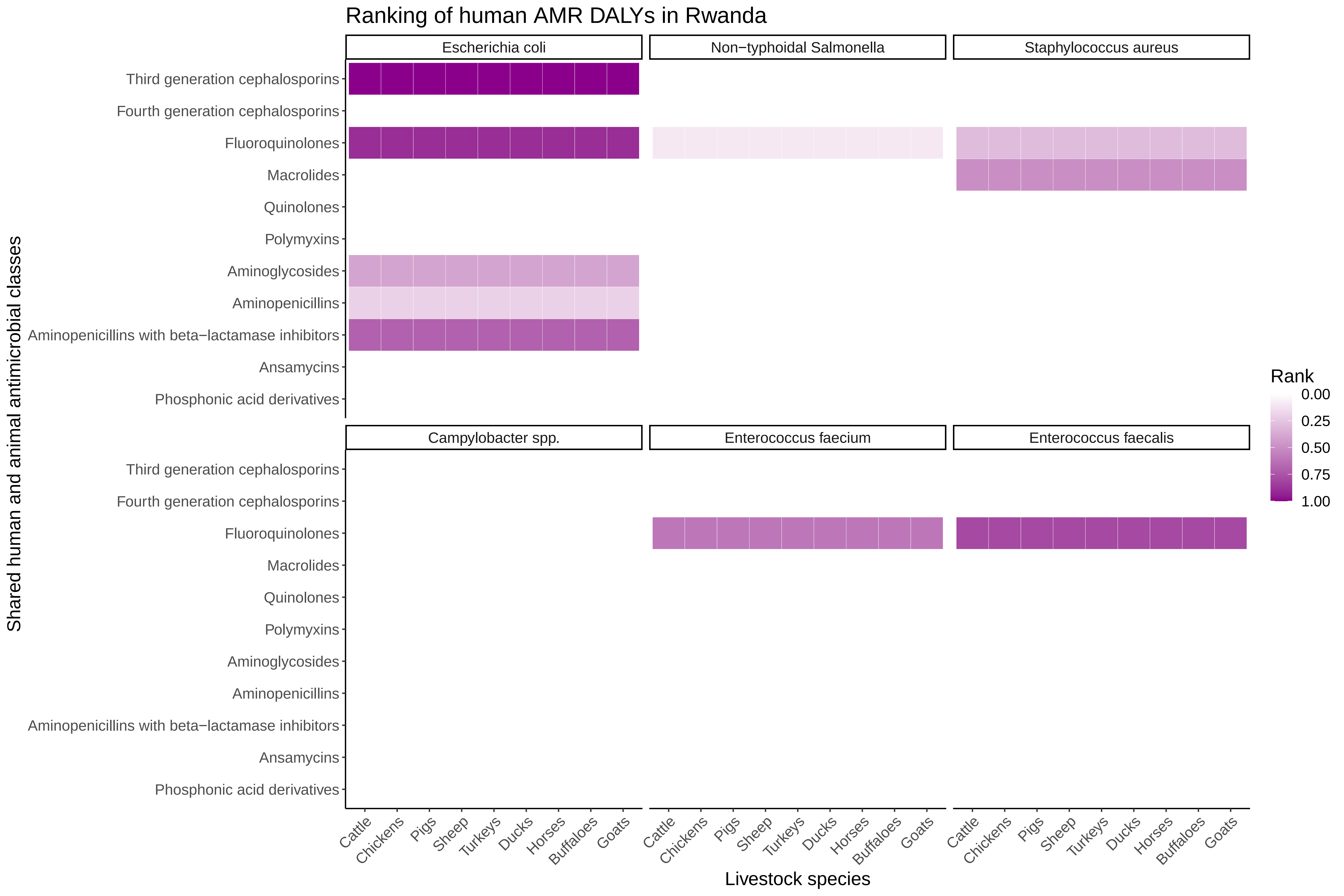

Ranking of human AMR DALYs in Rwanda
Escherichia coli
Non−typhoidal Salmonella
Staphylococcus aureus
Campylobacter spp.
Enterococcus faecium
Enterococcus faecalis
Third generation cephalosporins
Fourth generation cephalosporins
Fluoroquinolones
Macrolides
Quinolones
Polymyxins
Aminoglycosides
Aminopenicillins
Aminopenicillins with beta−lactamase inhibitors
Ansamycins
Phosphonic acid derivatives
Shared human and animal antimicrobial classes
Cattle
Chickens
Pigs
Sheep
Turkeys
Ducks
Horses
Buffaloes
Goats
Livestock species
Rank
0.00
0.25
0.50
0.75
1.00

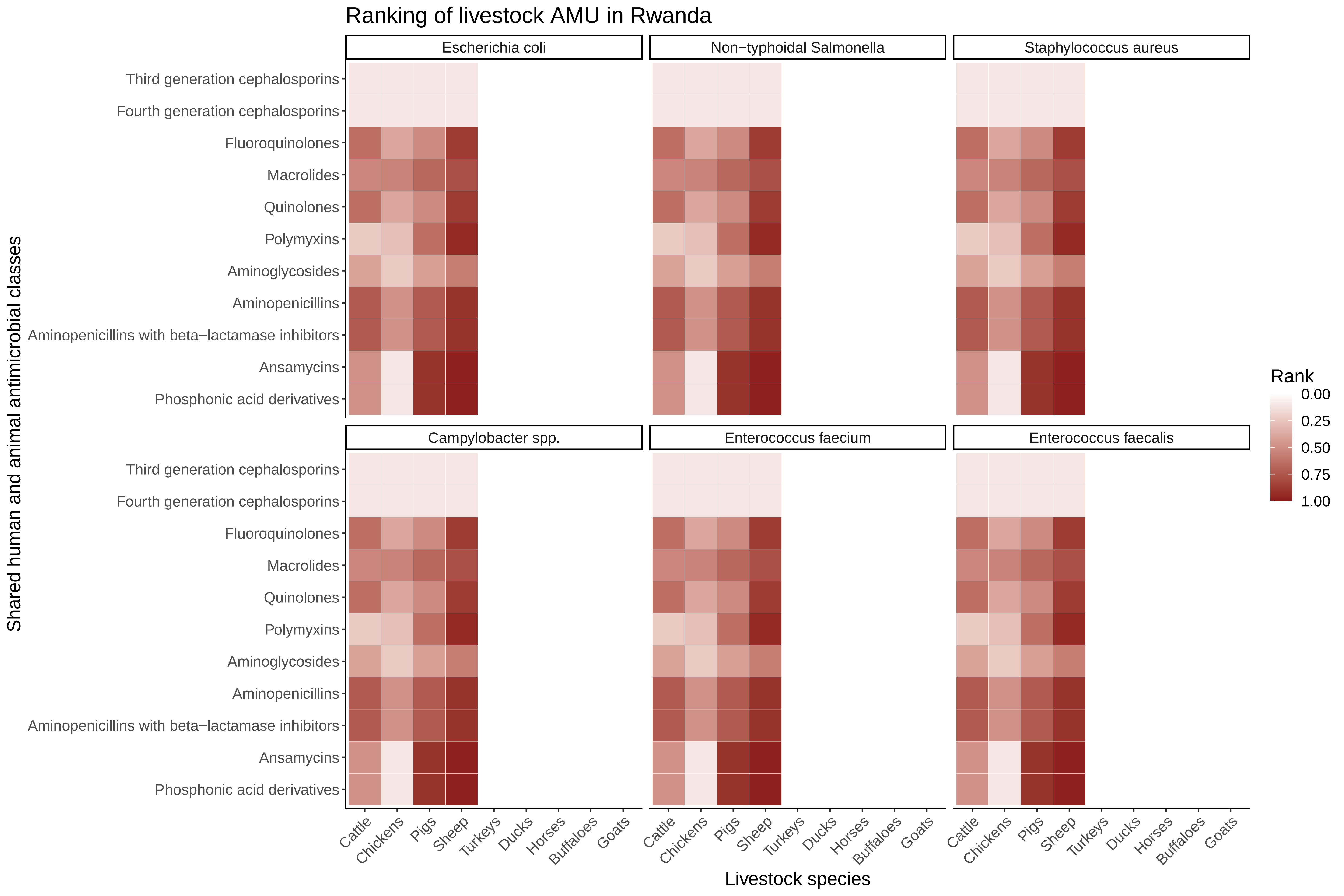

Ranking of livestock AMU in Rwanda
Escherichia coli
Non−typhoidal Salmonella
Staphylococcus aureus
Campylobacter spp.
Enterococcus faecium
Enterococcus faecalis
Shared human and animal antimicrobial classes
Third generation cephalosporins
Fourth generation cephalosporins
Fluoroquinolones
Macrolides
Quinolones
Polymyxins
Aminoglycosides
Aminopenicillins
Aminopenicillins with beta−lactamase inhibitors
Ansamycins
Phosphonic acid derivatives
Cattle
Chickens
Pigs
Sheep
Turkeys
Ducks
Horses
Buffaloes
Goats
Livestock species
Rank
0.00
0.25
0.50
0.75
1.00

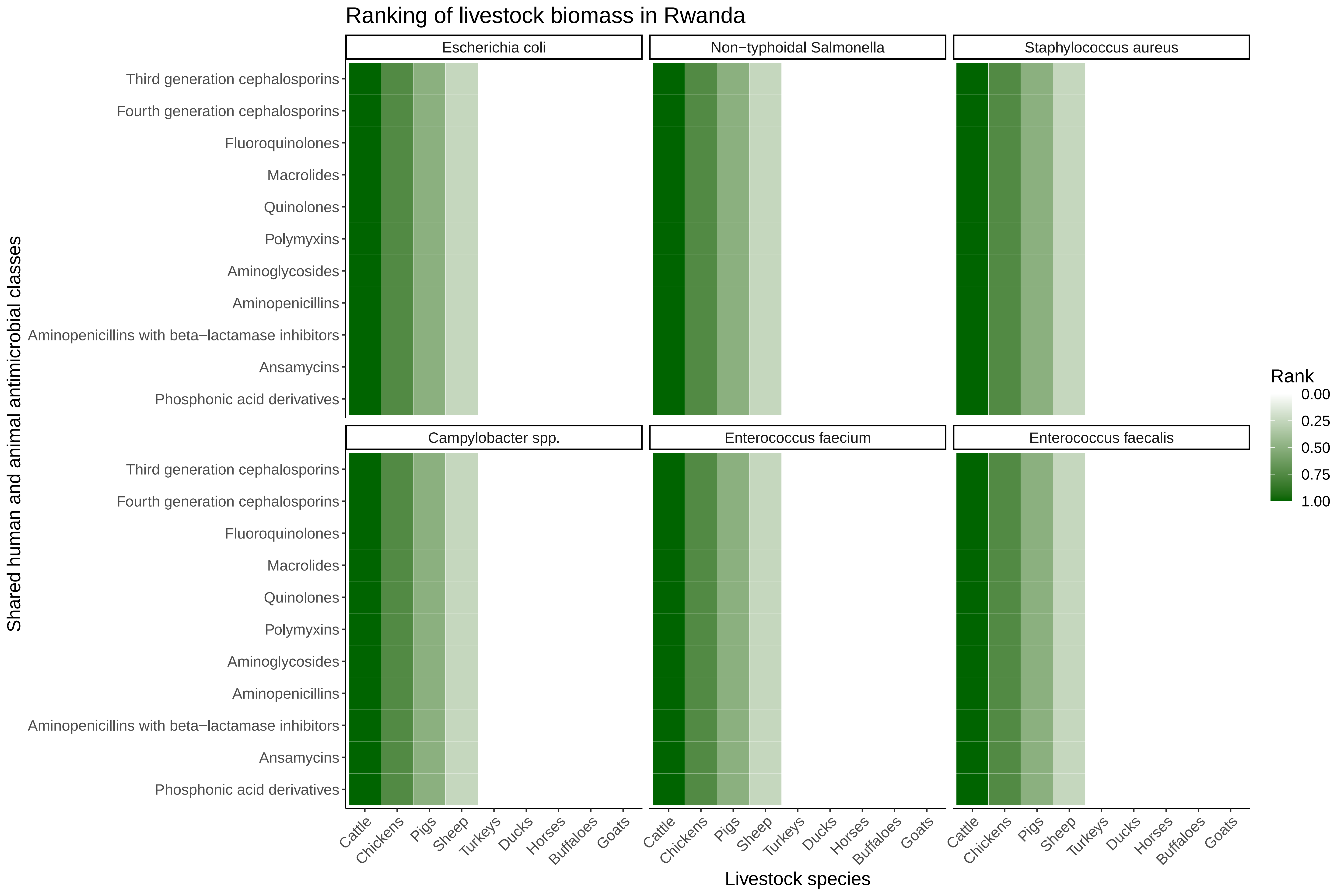

Ranking of livestock biomass in Rwanda
Escherichia coli
Non−typhoidal Salmonella
Staphylococcus aureus
Campylobacter spp.
Enterococcus faecium
Enterococcus faecalis
Shared human and animal antimicrobial classes
Third generation cephalosporins
Fourth generation cephalosporins
Fluoroquinolones
Macrolides
Quinolones
Polymyxins
Aminoglycosides
Aminopenicillins
Aminopenicillins with beta−lactamase inhibitors
Ansamycins
Phosphonic acid derivatives
Cattle
Chickens
Pigs
Sheep
Turkeys
Ducks
Horses
Buffaloes
Goats
Livestock species
Rank
0.00
0.25
0.50
0.75
1.00

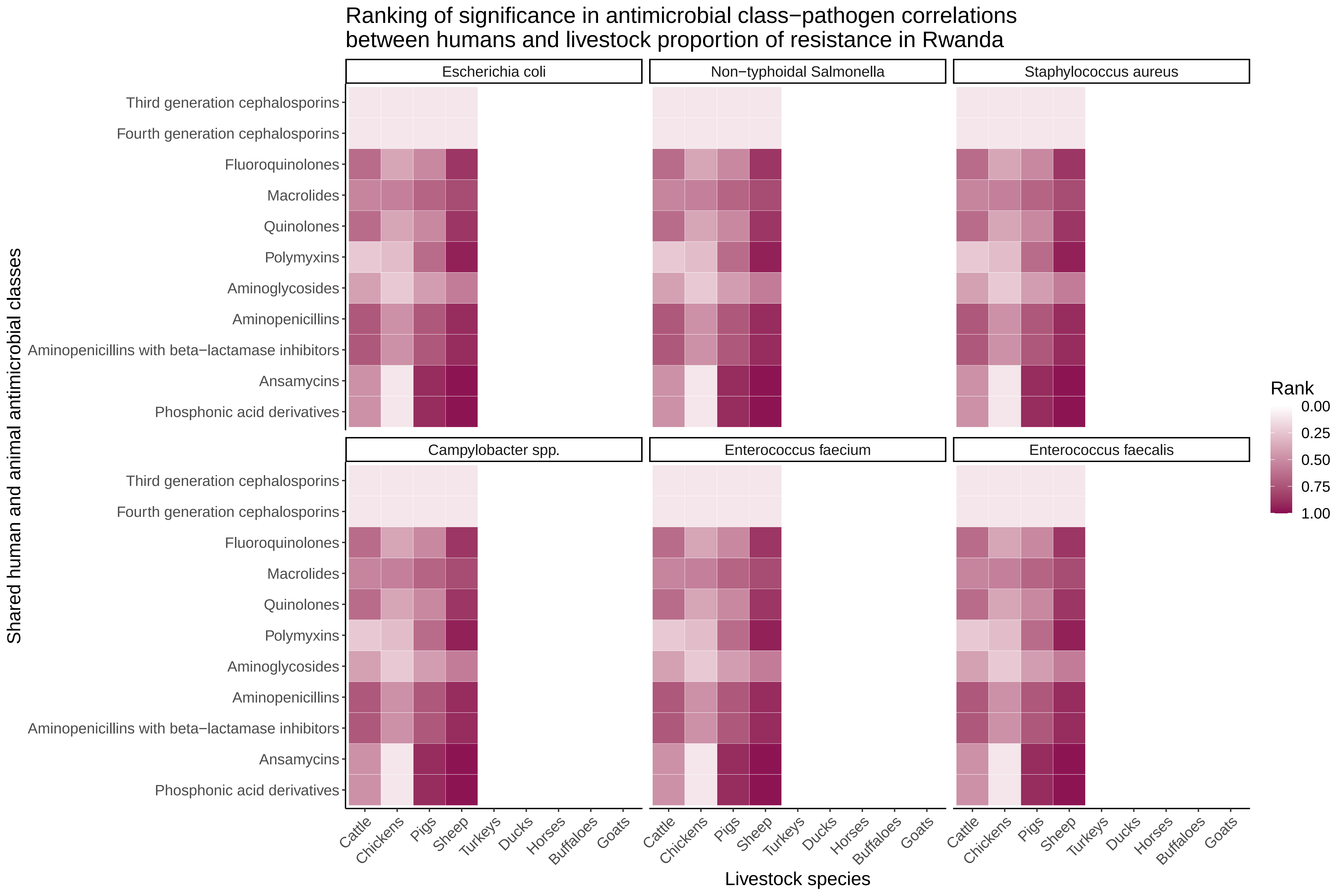
Ranking of significance in antimicrobial class−pathogen correlations
between humans and livestock proportion of resistance in Rwanda
Escherichia coli
Non−typhoidal Salmonella
Staphylococcus aureus
Campylobacter spp.
Enterococcus faecium
Enterococcus faecalis
Third generation cephalosporins
Fourth generation cephalosporins
Fluoroquinolones
Macrolides
Quinolones
Polymyxins
Aminoglycosides
Aminopenicillins
Aminopenicillins with beta−lactamase inhibitors
Ansamycins
Phosphonic acid derivatives
Shared human and animal antimicrobial classes
Cattle
Chickens
Pigs
Sheep
Turkeys
Ducks
Horses
Buffaloes
Goats
Livestock species
Rank
0.00
0.25
0.50
0.75
1.00

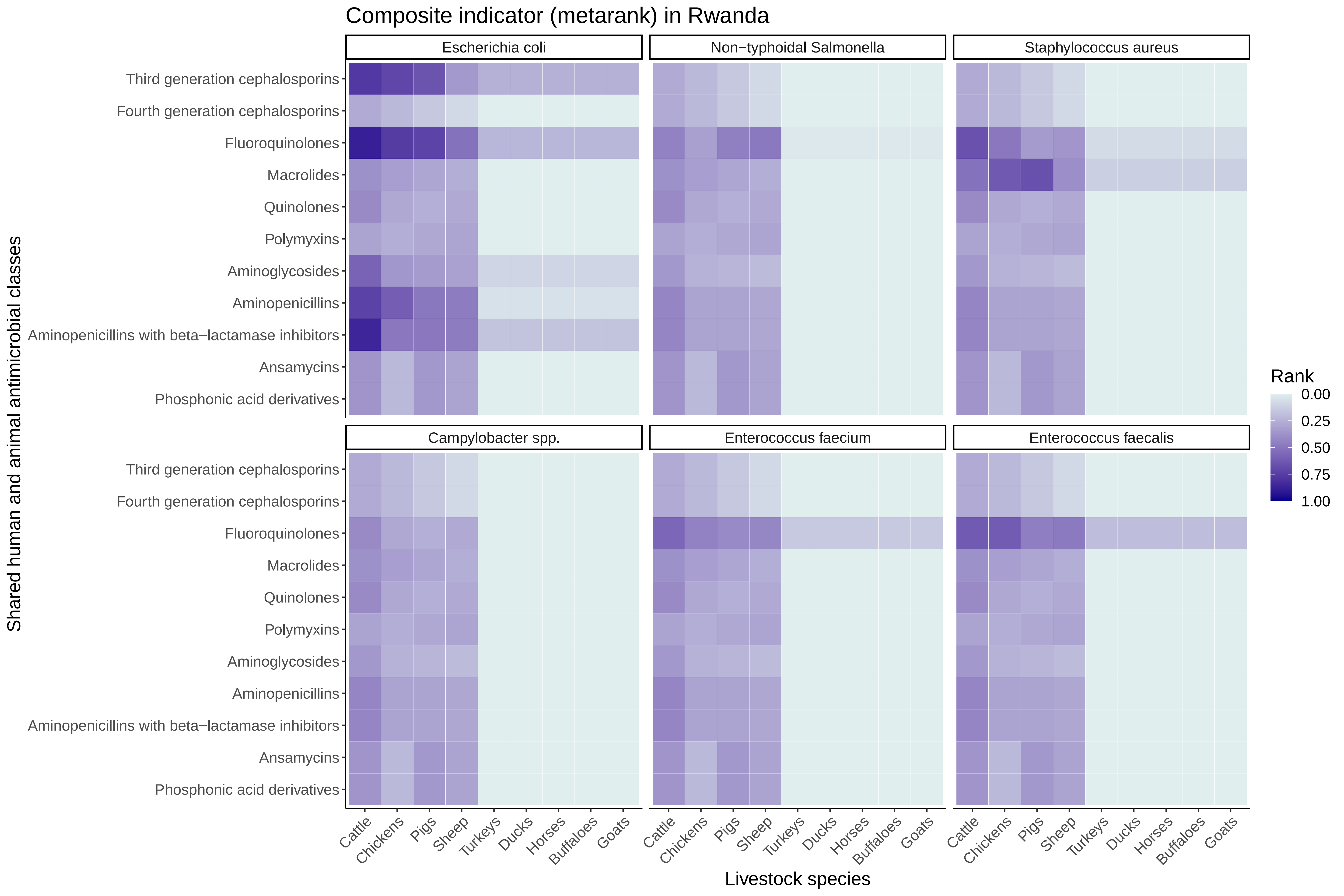

Composite indicator (metarank) in Rwanda
Escherichia coli
Non−typhoidal Salmonella
Staphylococcus aureus
Campylobacter spp.
Enterococcus faecium
Enterococcus faecalis
Shared human and animal antimicrobial classes
Third generation cephalosporins
Fourth generation cephalosporins
Fluoroquinolones
Macrolides
Quinolones
Polymyxins
Aminoglycosides
Aminopenicillins
Aminopenicillins with beta−lactamase inhibitors
Ansamycins
Phosphonic acid derivatives
Cattle
Chickens
Pigs
Sheep
Turkeys
Ducks
Horses
Buffaloes
Goats
Livestock species
Rank
0.00
0.25
0.50
0.75
1.00

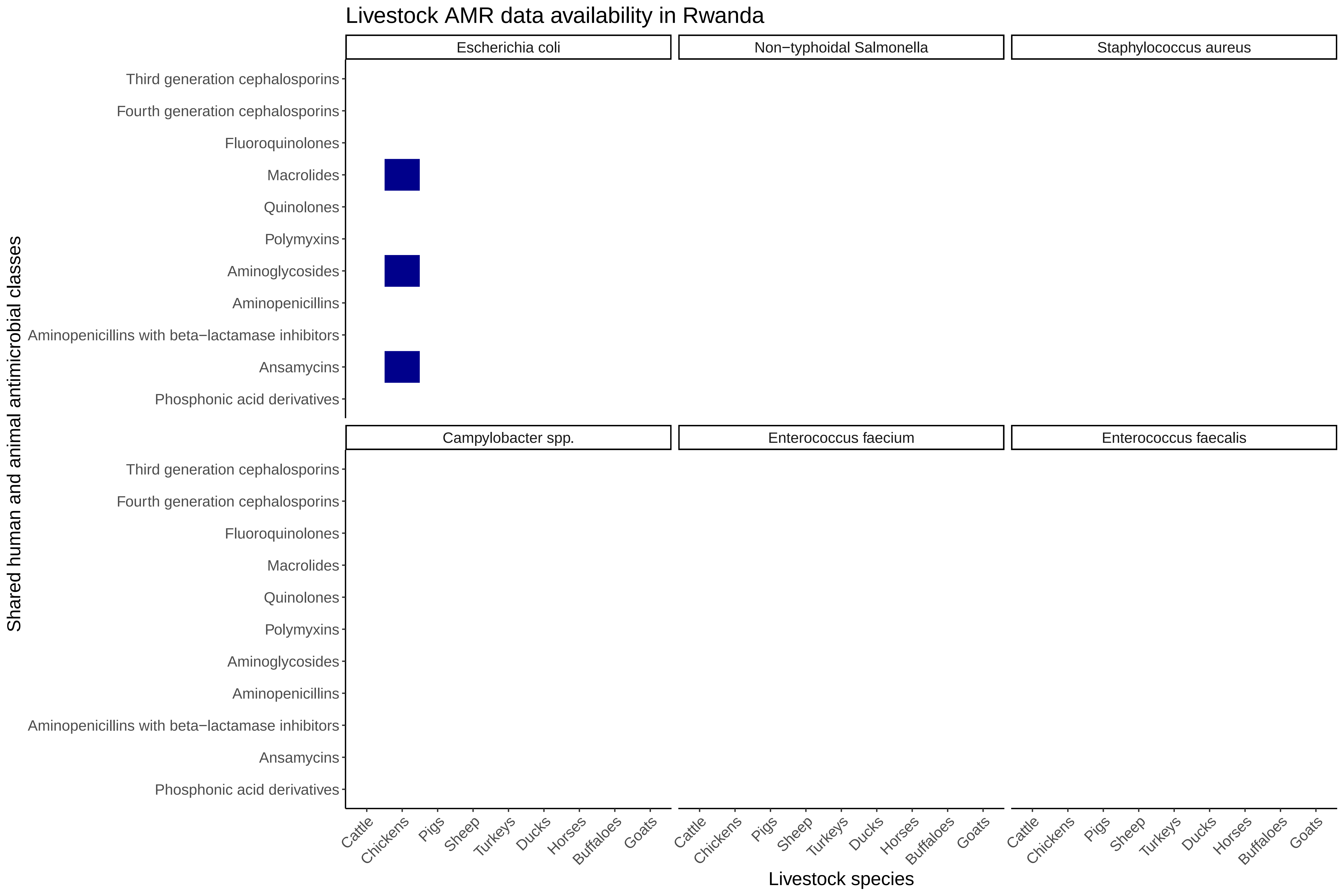

Livestock AMR data availability in Rwanda
Escherichia coli
Non−typhoidal Salmonella
Staphylococcus aureus
Campylobacter spp.
Enterococcus faecium
Enterococcus faecalis
Shared human and animal antimicrobial classes
Third generation cephalosporins
Fourth generation cephalosporins
Fluoroquinolones
Macrolides
Quinolones
Polymyxins
Aminoglycosides
Aminopenicillins
Aminopenicillins with beta−lactamase inhibitors
Ansamycins
Phosphonic acid derivatives
Cattle
Chickens
Pigs
Sheep
Turkeys
Ducks
Horses
Buffaloes
Goats
Livestock species

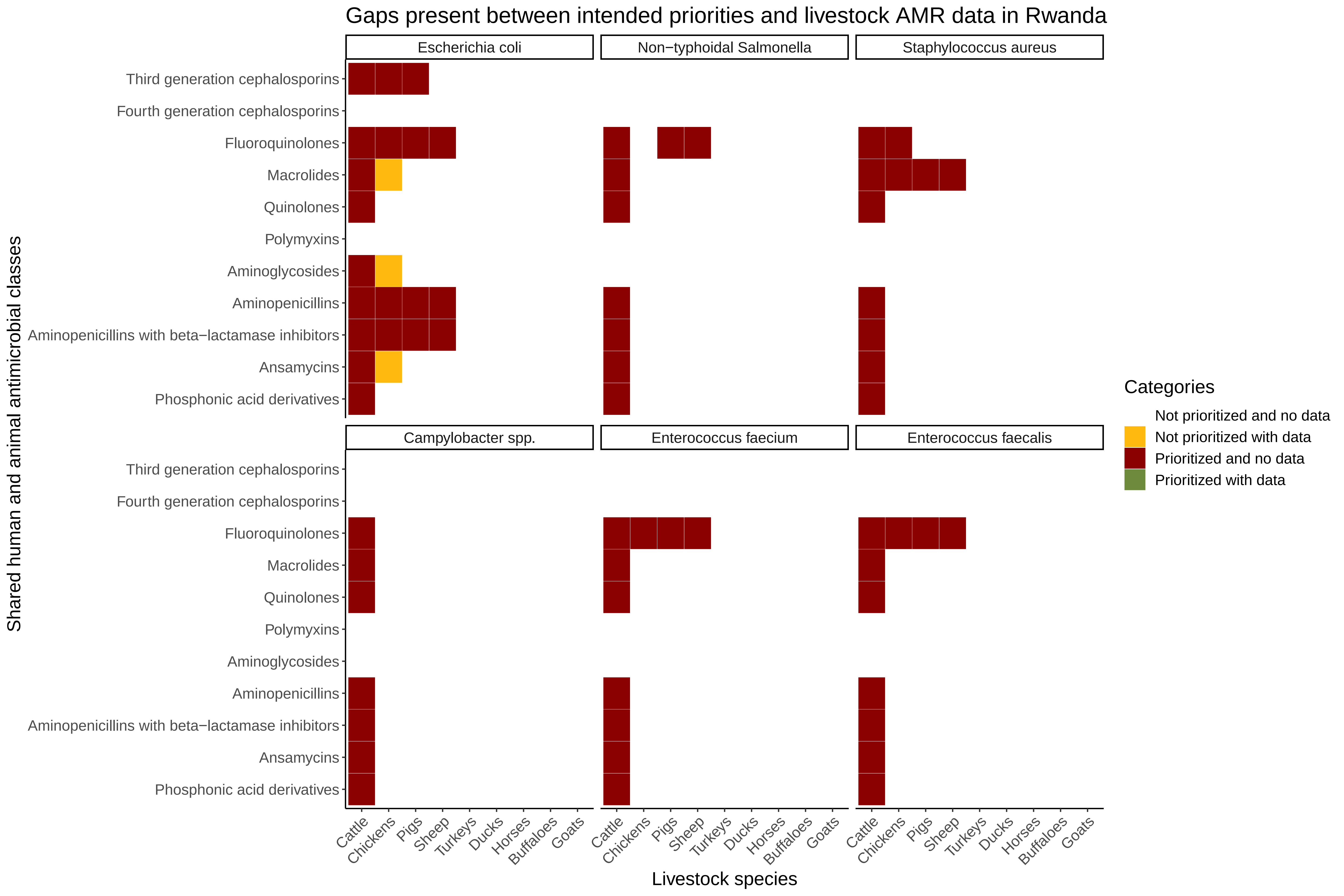
Gaps present between intended priorities and livestock AMR data in Rwanda
Escherichia coli
Non−typhoidal Salmonella
Staphylococcus aureus
Campylobacter spp.
Enterococcus faecium
Enterococcus faecalis
Shared human and animal antimicrobial classes
Third generation cephalosporins
Fourth generation cephalosporins
Fluoroquinolones
Macrolides
Quinolones
Polymyxins
Aminoglycosides
Aminopenicillins
Aminopenicillins with beta−lactamase inhibitors
Ansamycins
Phosphonic acid derivatives
Livestock species
Cattle
Chickens
Pigs
Sheep
Turkeys
Ducks
Horses
Buffaloes
Goats
Categories
Not prioritized and no data
Not prioritized with data
Prioritized and no data
Prioritized with data

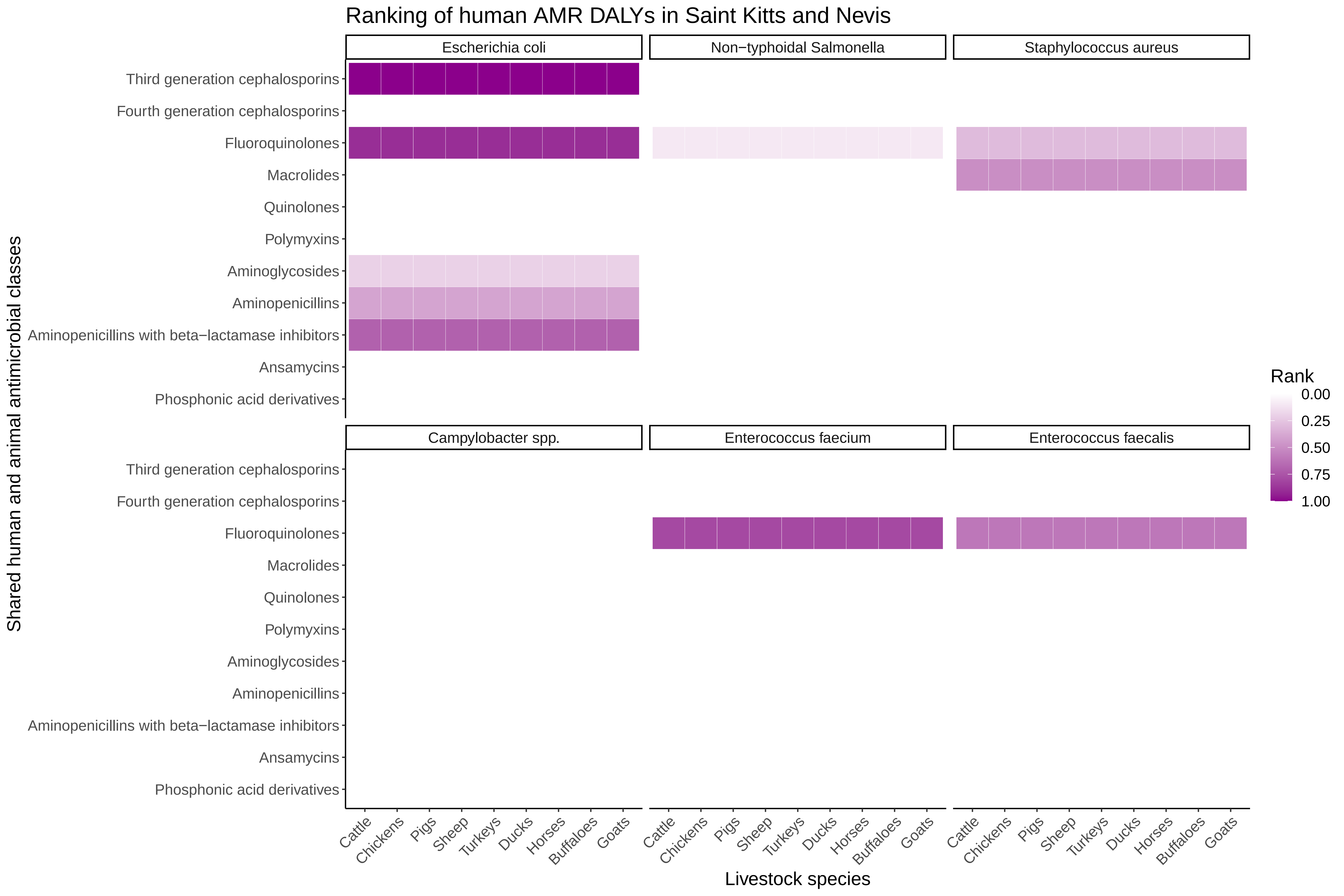

Ranking of human AMR DALYs in Saint Kitts and Nevis
Escherichia coli
Non−typhoidal Salmonella
Staphylococcus aureus
Campylobacter spp.
Enterococcus faecium
Enterococcus faecalis
Third generation cephalosporins
Fourth generation cephalosporins
Fluoroquinolones
Macrolides
Quinolones
Polymyxins
Aminoglycosides
Aminopenicillins
Aminopenicillins with beta−lactamase inhibitors
Ansamycins
Phosphonic acid derivatives
Shared human and animal antimicrobial classes
Cattle
Chickens
Pigs
Sheep
Turkeys
Ducks
Horses
Buffaloes
Goats
Livestock species
Rank
0.00
0.25
0.50
0.75
1.00

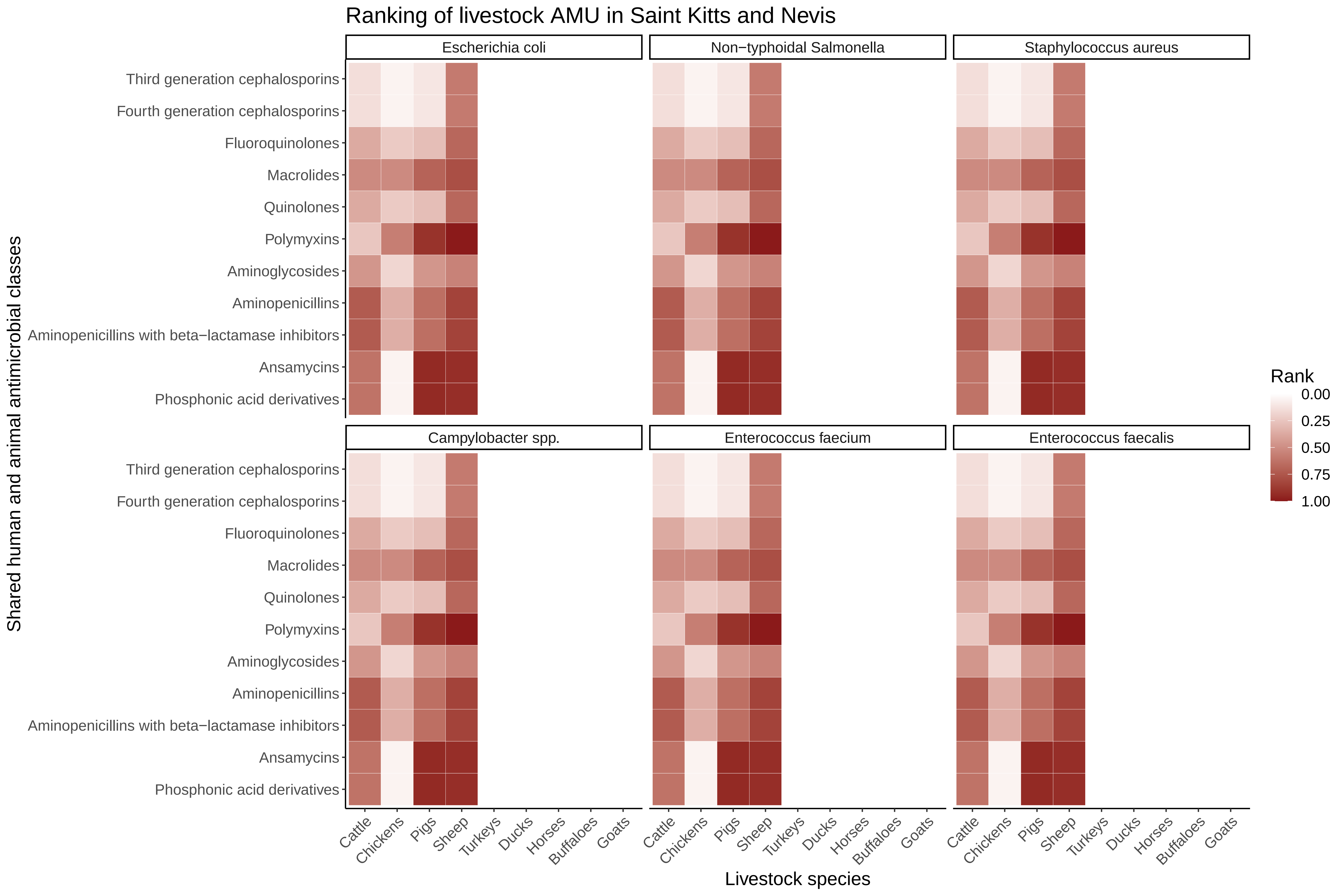

Ranking of livestock AMU in Saint Kitts and Nevis
Escherichia coli
Non−typhoidal Salmonella
Staphylococcus aureus
Campylobacter spp.
Enterococcus faecium
Enterococcus faecalis
Third generation cephalosporins
Fourth generation cephalosporins
Fluoroquinolones
Macrolides
Quinolones
Polymyxins
Aminoglycosides
Aminopenicillins
Aminopenicillins with beta−lactamase inhibitors
Ansamycins
Phosphonic acid derivatives
Shared human and animal antimicrobial classes
Cattle
Chickens
Pigs
Sheep
Turkeys
Ducks
Horses
Buffaloes
Goats
Livestock species
Rank
0.00
0.25
0.50
0.75
1.00

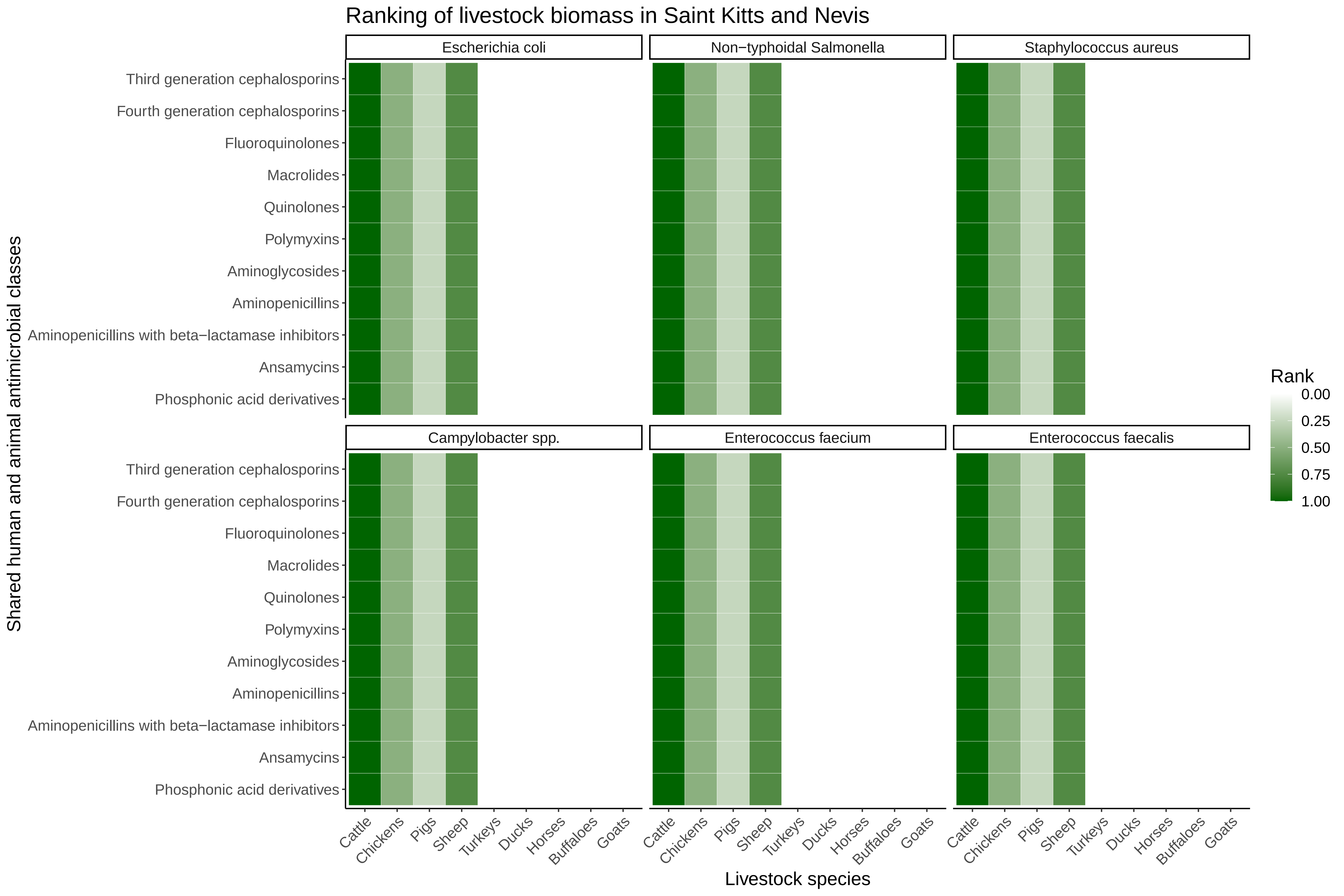

Ranking of livestock biomass in Saint Kitts and Nevis
Escherichia coli
Non−typhoidal Salmonella
Staphylococcus aureus
Campylobacter spp.
Enterococcus faecium
Enterococcus faecalis
Third generation cephalosporins
Fourth generation cephalosporins
Fluoroquinolones
Macrolides
Quinolones
Polymyxins
Aminoglycosides
Aminopenicillins
Aminopenicillins with beta−lactamase inhibitors
Ansamycins
Phosphonic acid derivatives
Shared human and animal antimicrobial classes
Cattle
Chickens
Pigs
Sheep
Turkeys
Ducks
Horses
Buffaloes
Goats
Livestock species
Rank
0.00
0.25
0.50
0.75
1.00

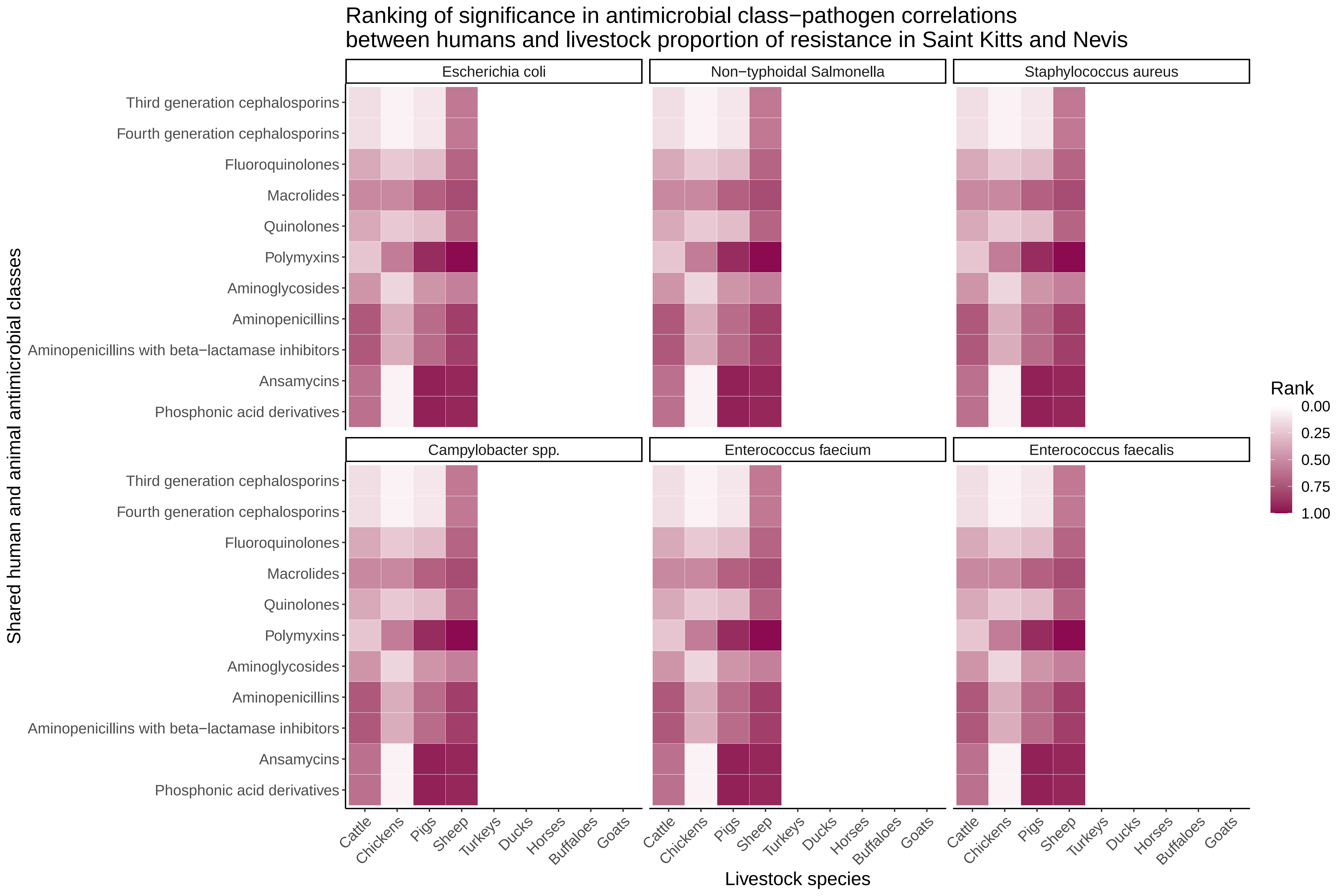

Ranking of significance in antimicrobial class−pathogen correlations
between humans and livestock proportion of resistance in Saint Kitts and Nevis
Escherichia coli
Non−typhoidal Salmonella
Staphylococcus aureus
Campylobacter spp.
Enterococcus faecium
Enterococcus faecalis
Third generation cephalosporins
Fourth generation cephalosporins
Fluoroquinolones
Macrolides
Quinolones
Polymyxins
Aminoglycosides
Aminopenicillins
Aminopenicillins with beta−lactamase inhibitors
Ansamycins
Phosphonic acid derivatives
Shared human and animal antimicrobial classes
Cattle
Chickens
Pigs
Sheep
Turkeys
Ducks
Horses
Buffaloes
Goats
Livestock species
Rank
0.00
0.25
0.50
0.75
1.00

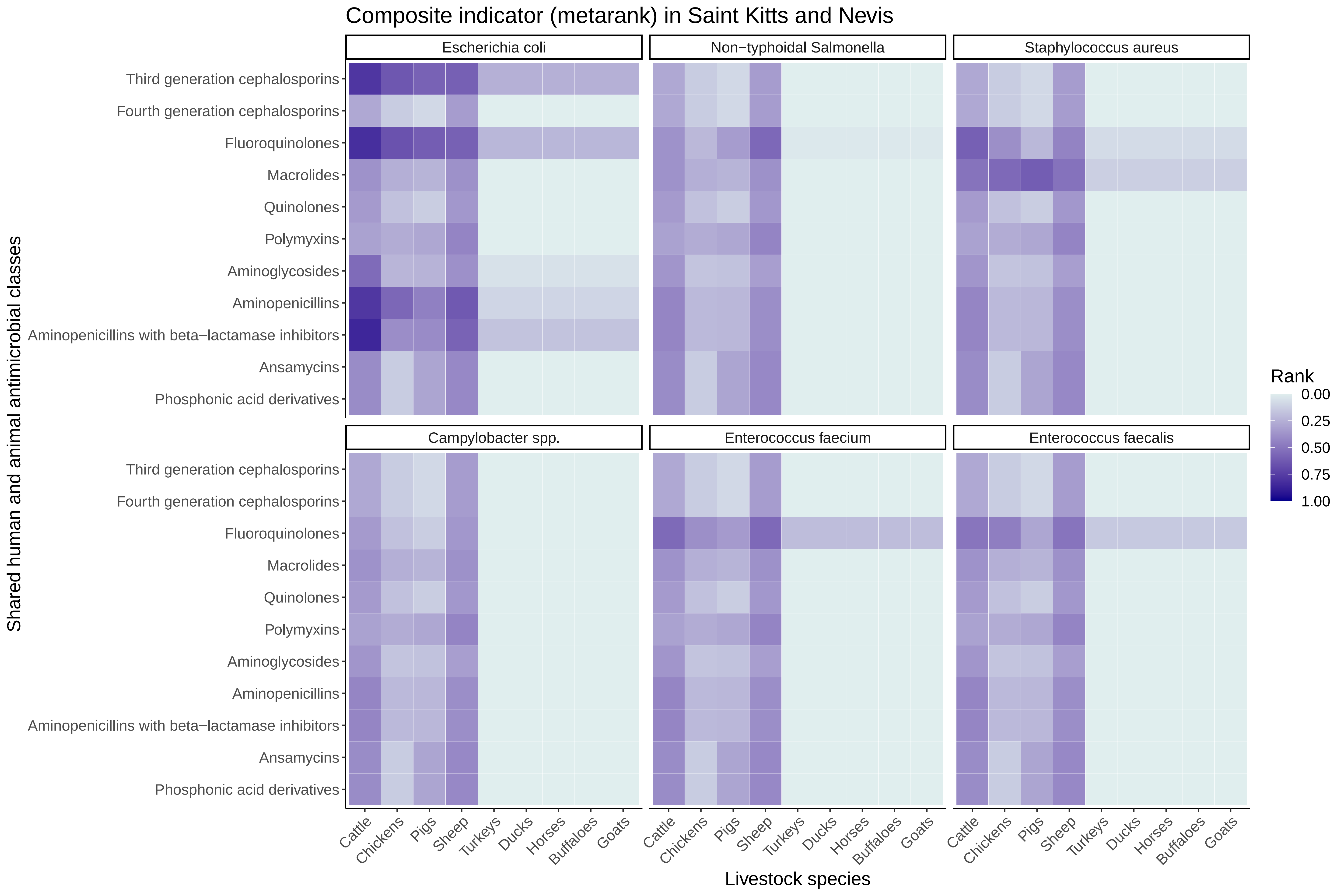

Composite indicator (metarank) in Saint Kitts and Nevis
Escherichia coli
Non−typhoidal Salmonella
Staphylococcus aureus
Campylobacter spp.
Enterococcus faecium
Enterococcus faecalis
Shared human and animal antimicrobial classes
Third generation cephalosporins
Fourth generation cephalosporins
Fluoroquinolones
Macrolides
Quinolones
Polymyxins
Aminoglycosides
Aminopenicillins
Aminopenicillins with beta−lactamase inhibitors
Ansamycins
Phosphonic acid derivatives
Cattle
Chickens
Pigs
Sheep
Turkeys
Ducks
Horses
Buffaloes
Goats
Livestock species
Rank
0.00
0.25
0.50
0.75
1.00

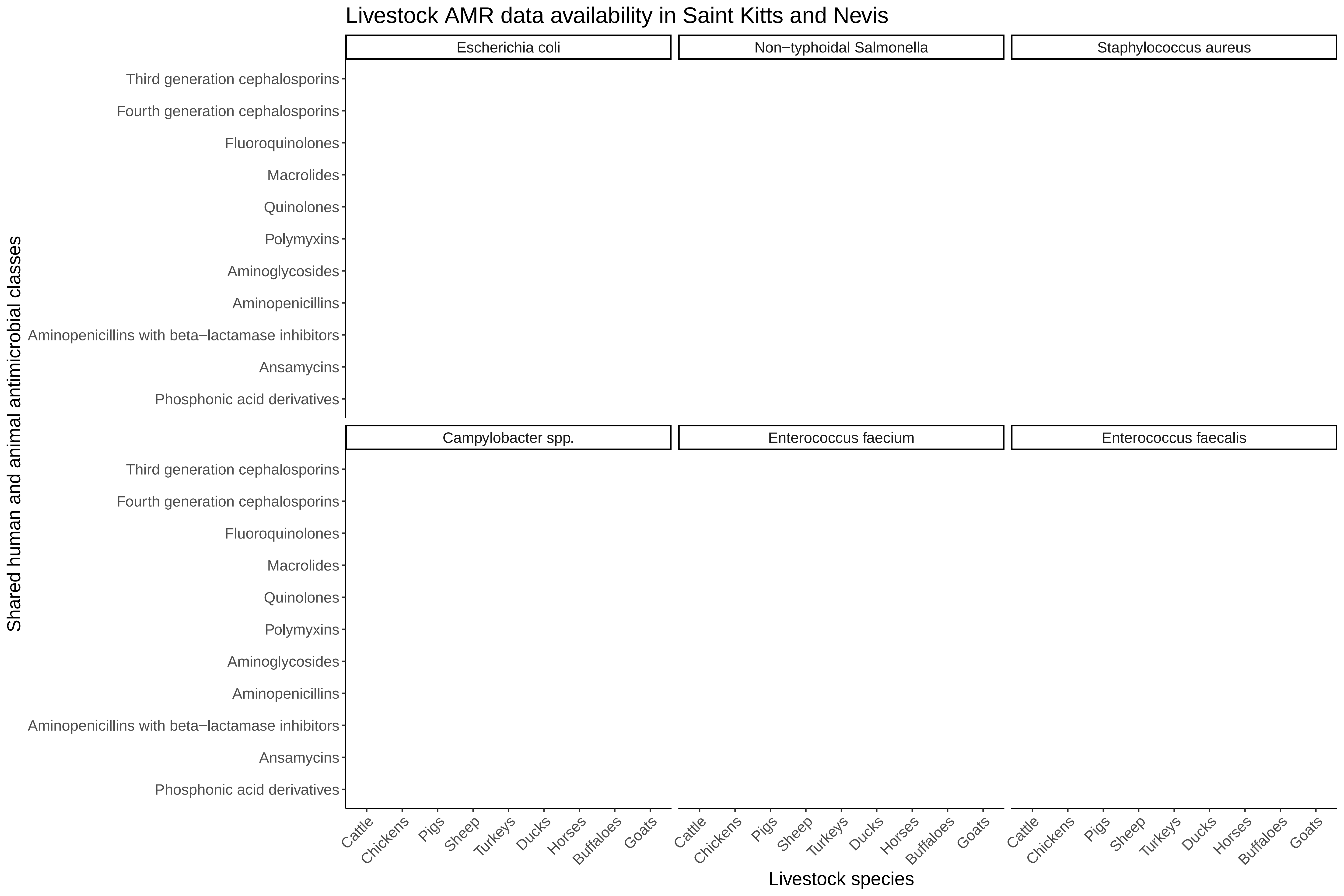
Livestock AMR data availability in Saint Kitts and Nevis
Escherichia coli
Non−typhoidal Salmonella
Staphylococcus aureus
Campylobacter spp.
Enterococcus faecium
Enterococcus faecalis
Shared human and animal antimicrobial classes
Third generation cephalosporins
Fourth generation cephalosporins
Fluoroquinolones
Macrolides
Quinolones
Polymyxins
Aminoglycosides
Aminopenicillins
Aminopenicillins with beta−lactamase inhibitors
Ansamycins
Phosphonic acid derivatives
Cattle
Chickens
Pigs
Sheep
Turkeys
Ducks
Horses
Buffaloes
Goats
Livestock species

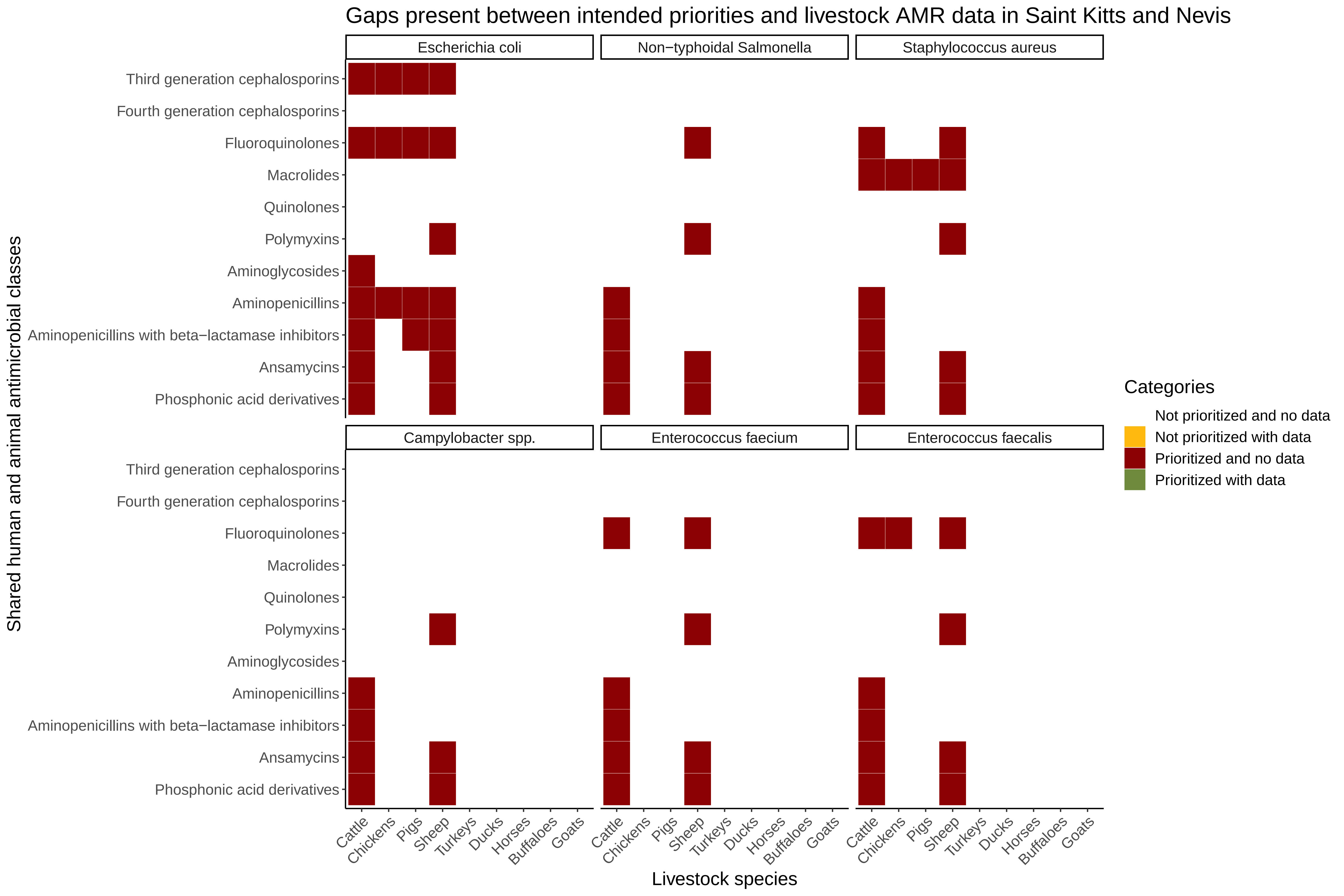
Gaps present between intended priorities and livestock AMR data in Saint Kitts and Nevis
Escherichia coli
Non−typhoidal Salmonella
Staphylococcus aureus
Campylobacter spp.
Enterococcus faecium
Enterococcus faecalis
Third generation cephalosporins
Fourth generation cephalosporins
Fluoroquinolones
Macrolides
Quinolones
Polymyxins
Aminoglycosides
Aminopenicillins
Aminopenicillins with beta−lactamase inhibitors
Ansamycins
Phosphonic acid derivatives
Shared human and animal antimicrobial classes
Cattle
Chickens
Pigs
Sheep
Turkeys
Ducks
Horses
Buffaloes
Goats
Livestock species
Categories
Not prioritized and no data
Not prioritized with data
Prioritized and no data
Prioritized with data

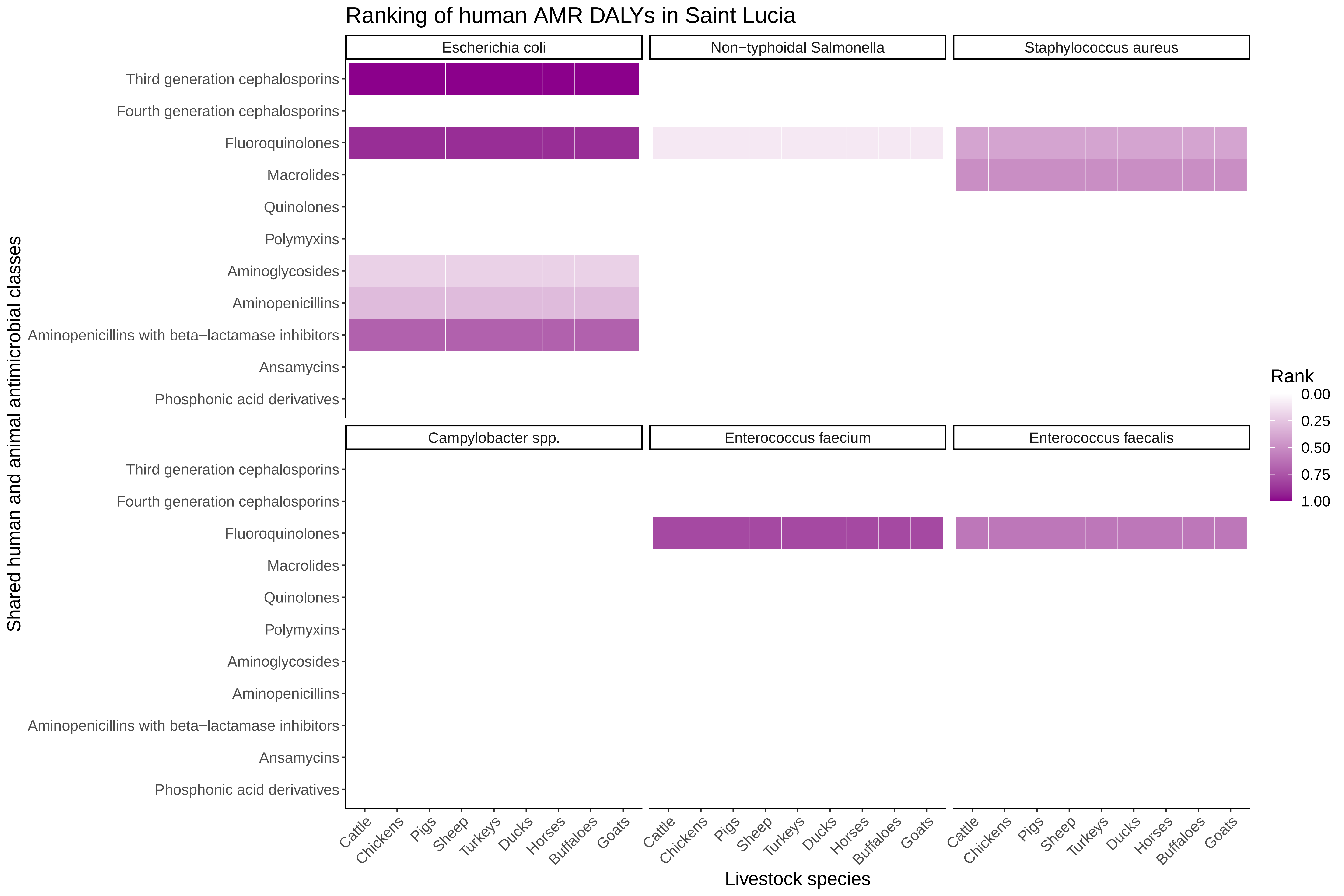

Ranking of human AMR DALYs in Saint Lucia
Escherichia coli
Non−typhoidal Salmonella
Staphylococcus aureus
Campylobacter spp.
Enterococcus faecium
Enterococcus faecalis
Third generation cephalosporins
Fourth generation cephalosporins
Fluoroquinolones
Macrolides
Quinolones
Polymyxins
Aminoglycosides
Aminopenicillins
Aminopenicillins with beta−lactamase inhibitors
Ansamycins
Phosphonic acid derivatives
Shared human and animal antimicrobial classes
Cattle
Chickens
Pigs
Sheep
Turkeys
Ducks
Horses
Buffaloes
Goats
Livestock species
Rank
0.00
0.25
0.50
0.75
1.00

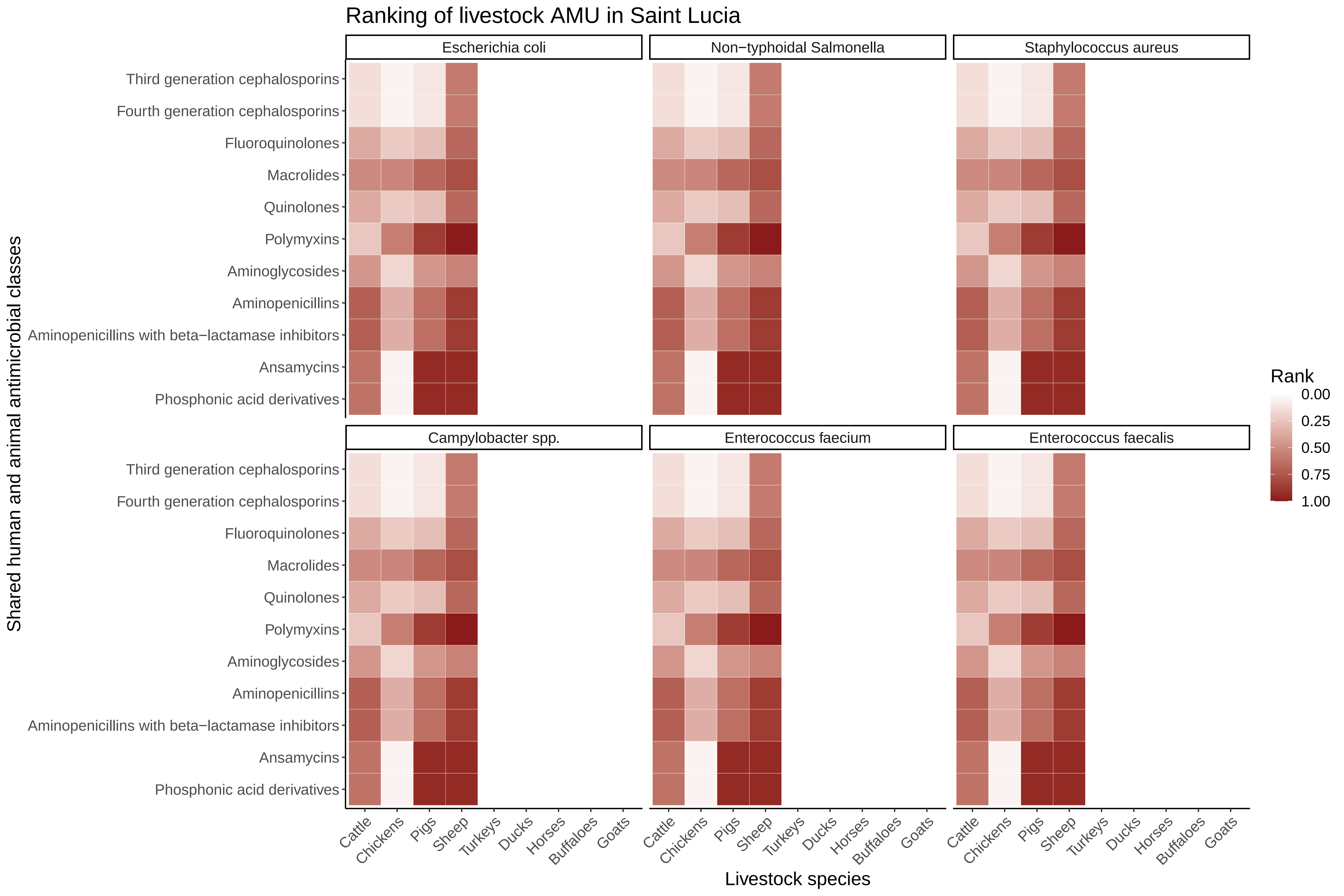

Ranking of livestock AMU in Saint Lucia
Escherichia coli
Non−typhoidal Salmonella
Staphylococcus aureus
Campylobacter spp.
Enterococcus faecium
Enterococcus faecalis
Third generation cephalosporins
Fourth generation cephalosporins
Fluoroquinolones
Macrolides
Quinolones
Polymyxins
Aminoglycosides
Aminopenicillins
Aminopenicillins with beta−lactamase inhibitors
Ansamycins
Phosphonic acid derivatives
Shared human and animal antimicrobial classes
Cattle
Chickens
Pigs
Sheep
Turkeys
Ducks
Horses
Buffaloes
Goats
Livestock species
Rank
0.00
0.25
0.50
0.75
1.00

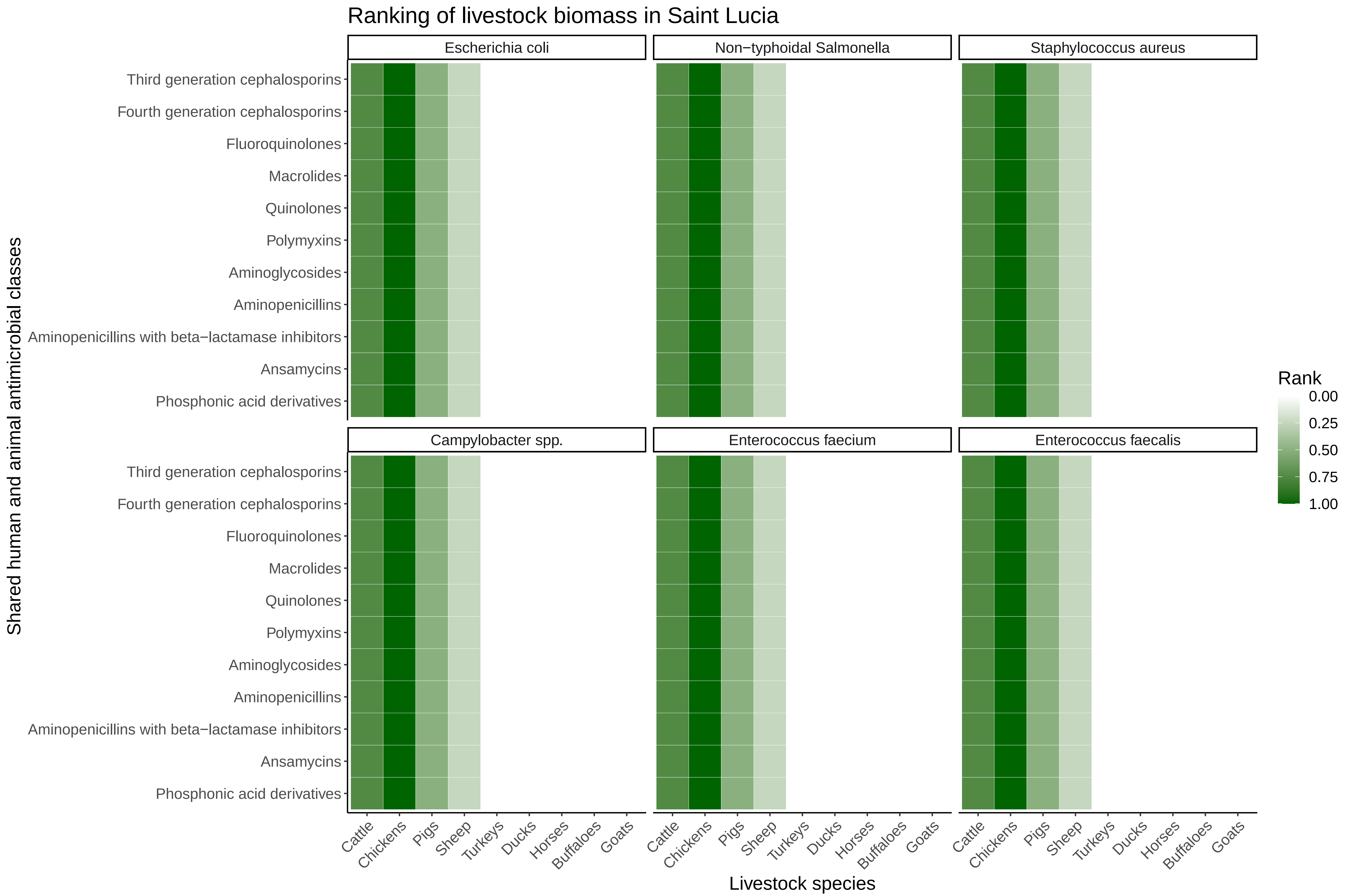

Ranking of livestock biomass in Saint Lucia
Escherichia coli
Non−typhoidal Salmonella
Staphylococcus aureus
Campylobacter spp.
Enterococcus faecium
Enterococcus faecalis
Shared human and animal antimicrobial classes
Third generation cephalosporins
Fourth generation cephalosporins
Fluoroquinolones
Macrolides
Quinolones
Polymyxins
Aminoglycosides
Aminopenicillins
Aminopenicillins with beta−lactamase inhibitors
Ansamycins
Phosphonic acid derivatives
Cattle
Chickens
Pigs
Sheep
Turkeys
Ducks
Horses
Buffaloes
Goats
Livestock species
Rank
0.00
0.25
0.50
0.75
1.00

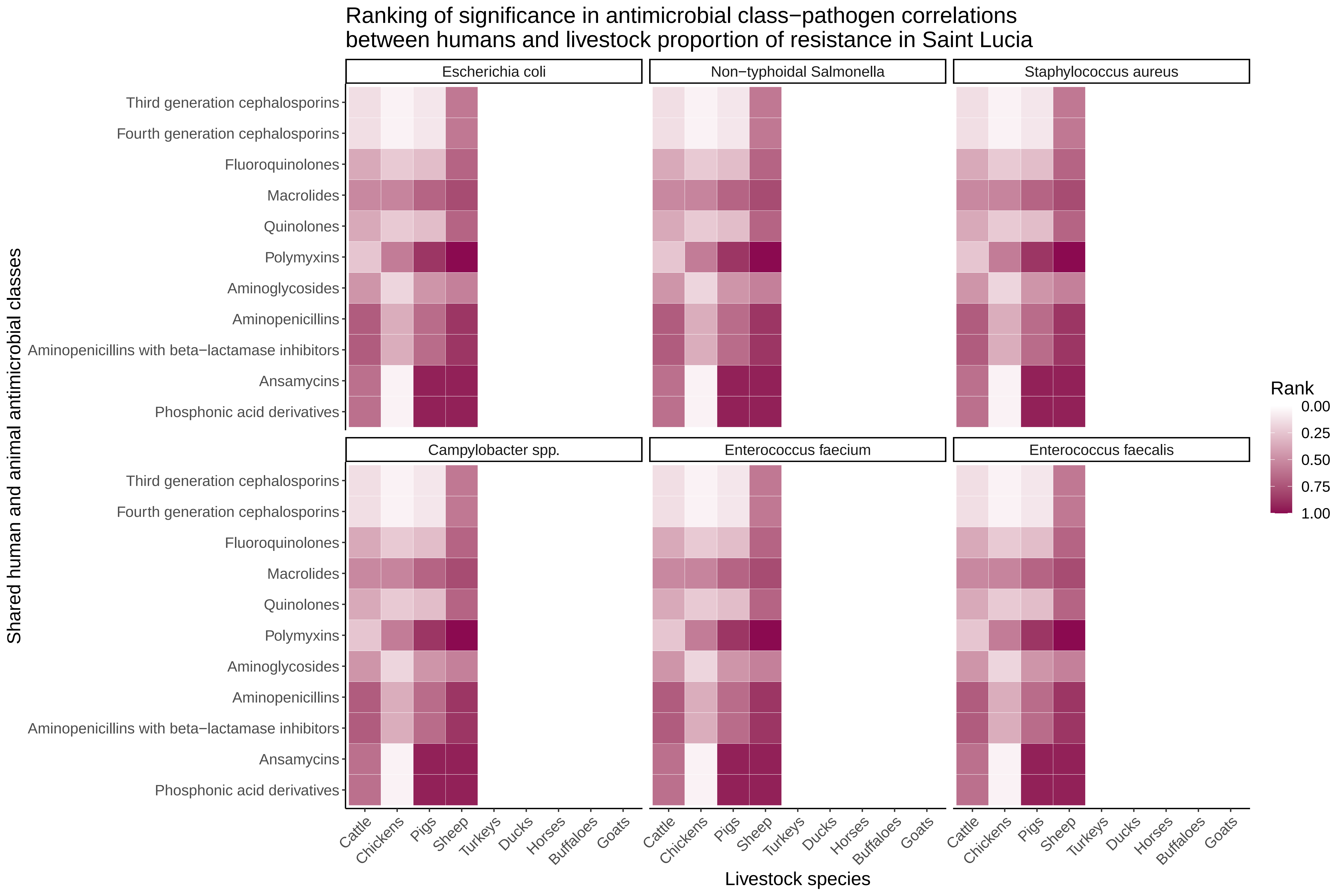

Ranking of significance in antimicrobial class−pathogen correlations
between humans and livestock proportion of resistance in Saint Lucia
Escherichia coli
Non−typhoidal Salmonella
Staphylococcus aureus
Campylobacter spp.
Enterococcus faecium
Enterococcus faecalis
Shared human and animal antimicrobial classes
Third generation cephalosporins
Fourth generation cephalosporins
Fluoroquinolones
Macrolides
Quinolones
Polymyxins
Aminoglycosides
Aminopenicillins
Aminopenicillins with beta−lactamase inhibitors
Ansamycins
Phosphonic acid derivatives
Livestock species
Cattle
Chickens
Pigs
Sheep
Turkeys
Ducks
Horses
Buffaloes
Goats
Rank
0.00
0.25
0.50
0.75
1.00

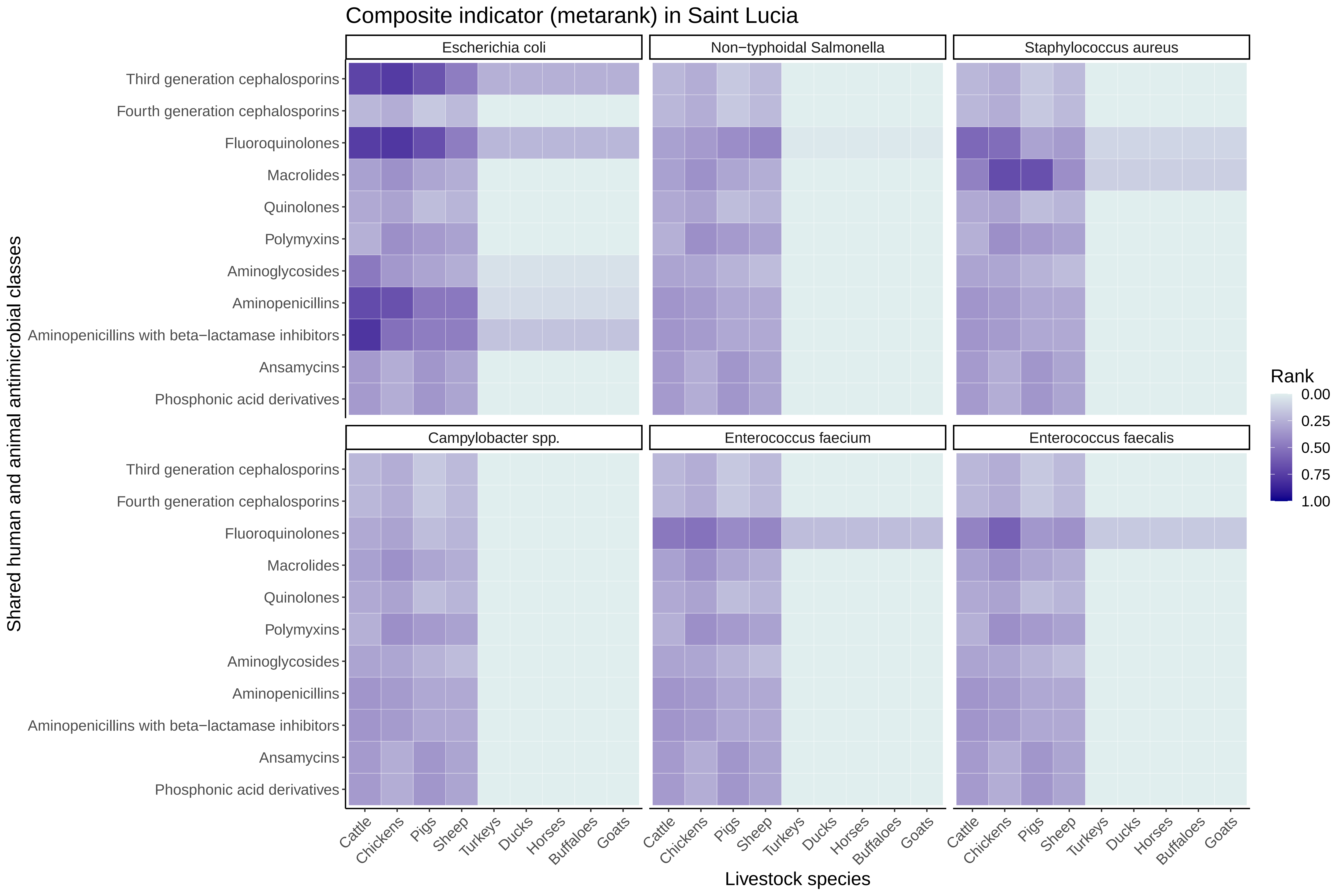

Composite indicator (metarank) in Saint Lucia
Escherichia coli
Non−typhoidal Salmonella
Staphylococcus aureus
Campylobacter spp.
Enterococcus faecium
Enterococcus faecalis
Third generation cephalosporins
Fourth generation cephalosporins
Fluoroquinolones
Macrolides
Quinolones
Polymyxins
Aminoglycosides
Aminopenicillins
Aminopenicillins with beta−lactamase inhibitors
Ansamycins
Phosphonic acid derivatives
Shared human and animal antimicrobial classes
Cattle
Chickens
Pigs
Sheep
Turkeys
Ducks
Horses
Buffaloes
Goats
Livestock species
Rank
0.00
0.25
0.50
0.75
1.00

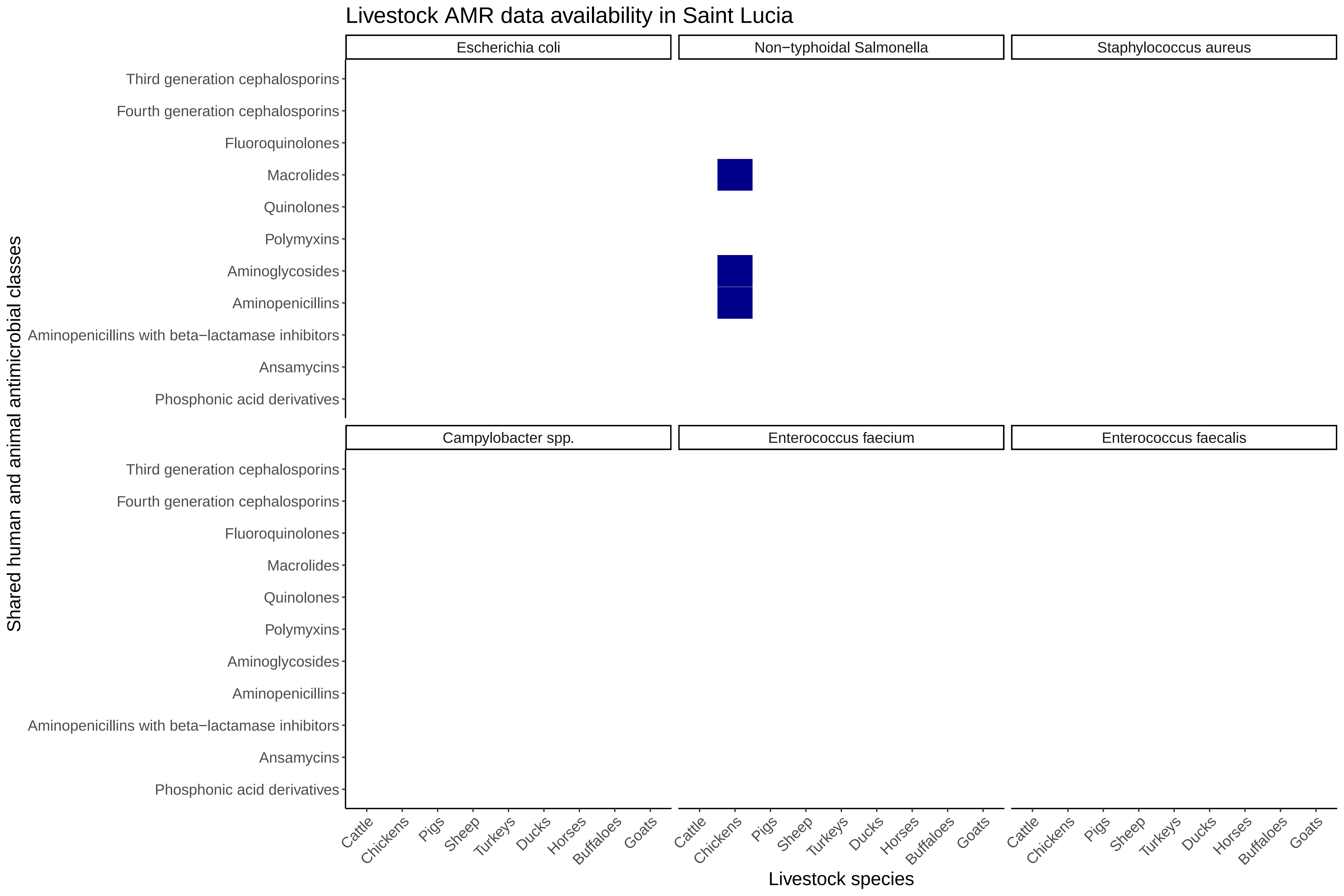

Livestock AMR data availability in Saint Lucia
Escherichia coli
Non−typhoidal Salmonella
Staphylococcus aureus
Campylobacter spp.
Enterococcus faecium
Enterococcus faecalis
Shared human and animal antimicrobial classes
Third generation cephalosporins
Fourth generation cephalosporins
Fluoroquinolones
Macrolides
Quinolones
Polymyxins
Aminoglycosides
Aminopenicillins
Aminopenicillins with beta−lactamase inhibitors
Ansamycins
Phosphonic acid derivatives
Cattle
Chickens
Pigs
Sheep
Turkeys
Ducks
Horses
Buffaloes
Goats
Livestock species

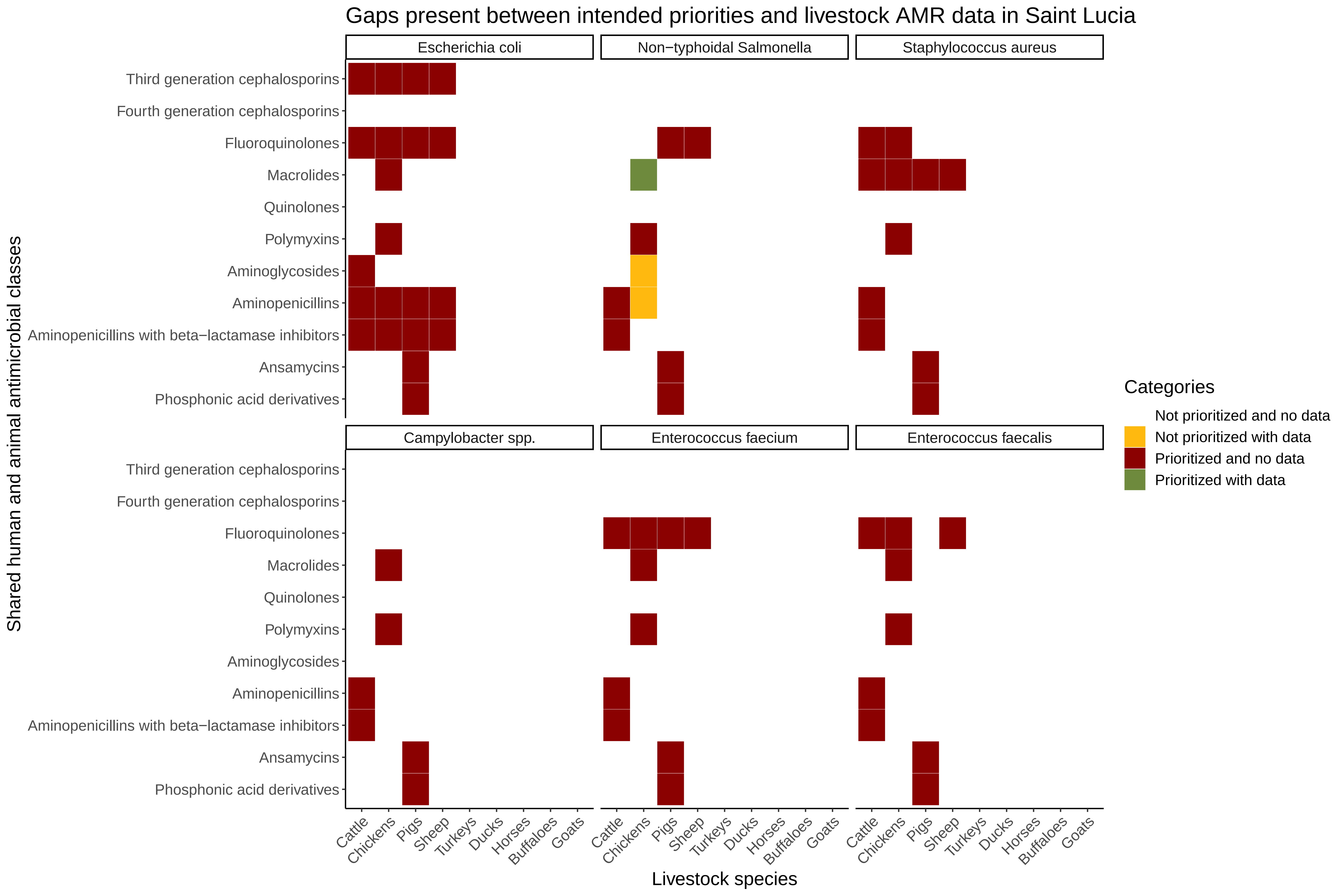

Gaps present between intended priorities and livestock AMR data in Saint Lucia
Escherichia coli
Non−typhoidal Salmonella
Staphylococcus aureus
Campylobacter spp.
Enterococcus faecium
Enterococcus faecalis
Shared human and animal antimicrobial classes
Third generation cephalosporins
Fourth generation cephalosporins
Fluoroquinolones
Macrolides
Quinolones
Polymyxins
Aminoglycosides
Aminopenicillins
Aminopenicillins with beta−lactamase inhibitors
Ansamycins
Phosphonic acid derivatives
Livestock species
Cattle
Chickens
Pigs
Sheep
Turkeys
Ducks
Horses
Buffaloes
Goats
Categories
Not prioritized and no data
Not prioritized with data
Prioritized and no data
Prioritized with data

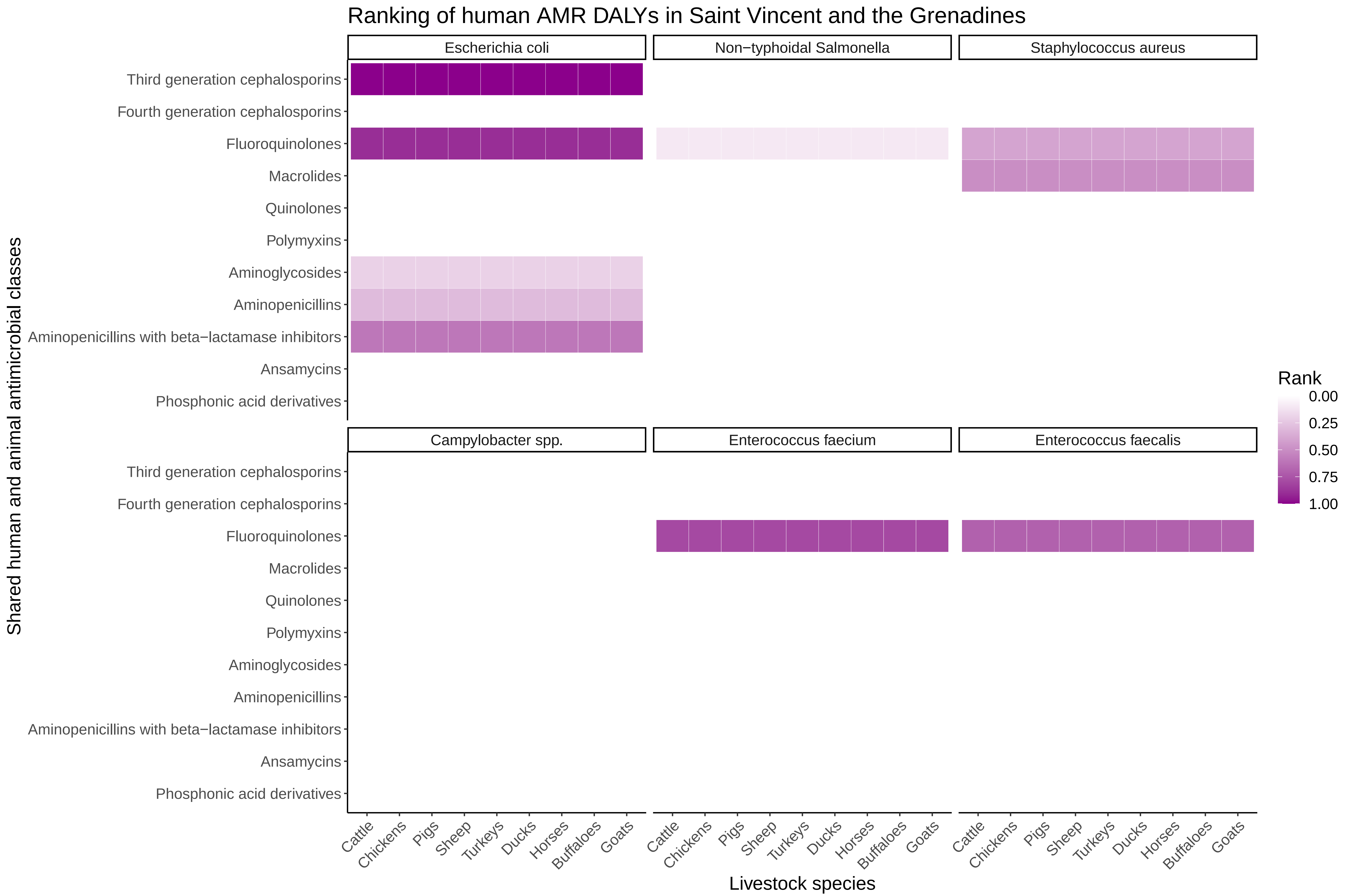
Ranking of human AMR DALYs in Saint Vincent and the Grenadines
Escherichia coli
Non−typhoidal Salmonella
Staphylococcus aureus
Campylobacter spp.
Enterococcus faecium
Enterococcus faecalis
Third generation cephalosporins
Fourth generation cephalosporins
Fluoroquinolones
Macrolides
Quinolones
Polymyxins
Aminoglycosides
Aminopenicillins
Aminopenicillins with beta−lactamase inhibitors
Ansamycins
Phosphonic acid derivatives
Shared human and animal antimicrobial classes
Cattle
Chickens
Pigs
Sheep
Turkeys
Ducks
Horses
Buffaloes
Goats
Livestock species
Rank
0.00
0.25
0.50
0.75
1.00

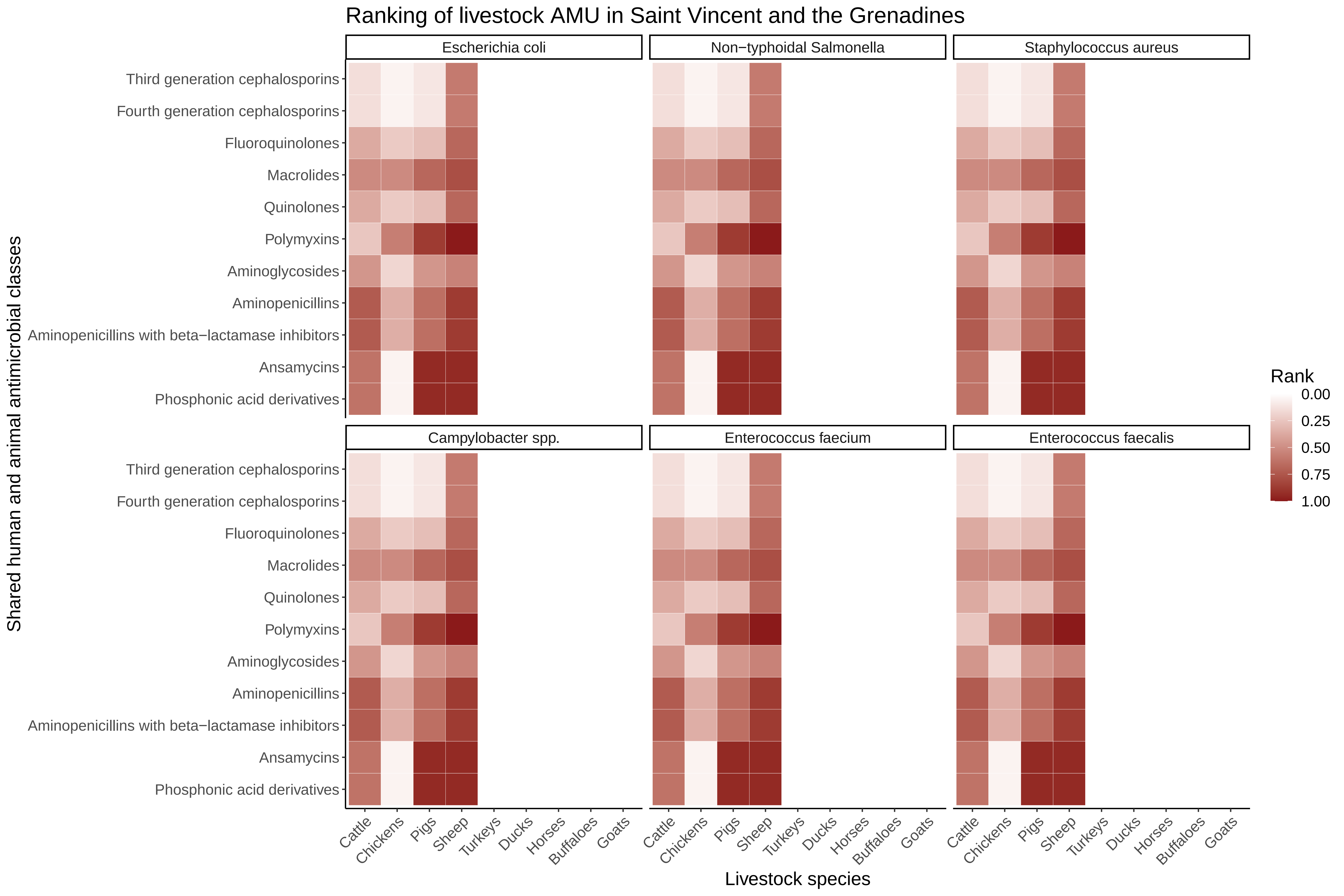

Ranking of livestock AMU in Saint Vincent and the Grenadines
Escherichia coli
Non−typhoidal Salmonella
Staphylococcus aureus
Campylobacter spp.
Enterococcus faecium
Enterococcus faecalis
Third generation cephalosporins
Fourth generation cephalosporins
Fluoroquinolones
Macrolides
Quinolones
Polymyxins
Aminoglycosides
Aminopenicillins
Aminopenicillins with beta−lactamase inhibitors
Ansamycins
Phosphonic acid derivatives
Cattle
Chickens
Pigs
Sheep
Turkeys
Ducks
Horses
Buffaloes
Goats
Livestock species
Shared human and animal antimicrobial classes
Rank
0.00
0.25
0.50
0.75
1.00

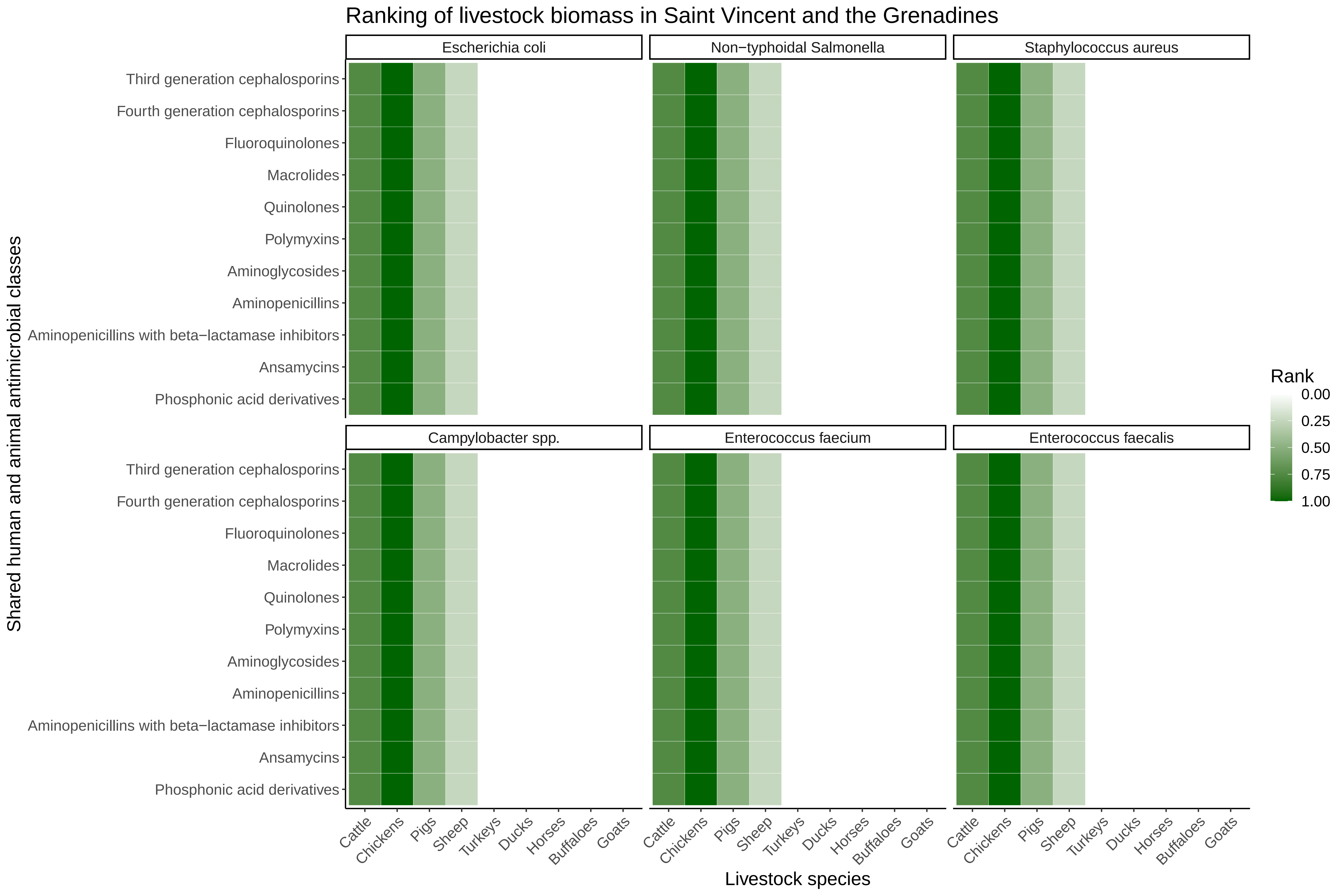
Ranking of livestock biomass in Saint Vincent and the Grenadines
Escherichia coli
Non−typhoidal Salmonella
Staphylococcus aureus
Campylobacter spp.
Enterococcus faecium
Enterococcus faecalis
Third generation cephalosporins
Fourth generation cephalosporins
Fluoroquinolones
Macrolides
Quinolones
Polymyxins
Aminoglycosides
Aminopenicillins
Aminopenicillins with beta−lactamase inhibitors
Ansamycins
Phosphonic acid derivatives
Shared human and animal antimicrobial classes
Cattle
Chickens
Pigs
Sheep
Turkeys
Ducks
Horses
Buffaloes
Goats
Livestock species
Rank
0.00
0.25
0.50
0.75
1.00

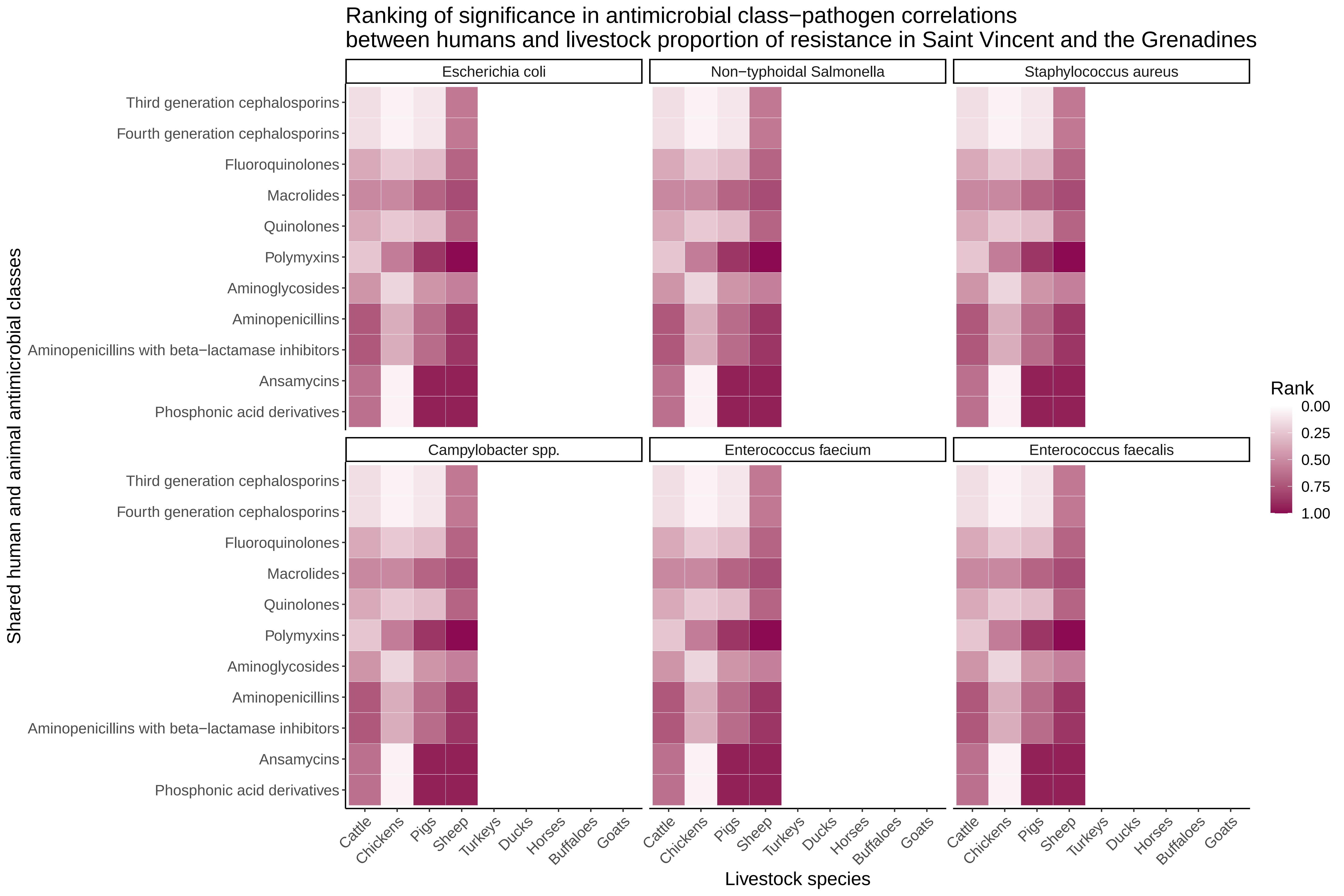

Ranking of significance in antimicrobial class−pathogen correlations
between humans and livestock proportion of resistance in Saint Vincent and the Grenadines
Escherichia coli
Non−typhoidal Salmonella
Staphylococcus aureus
Campylobacter spp.
Enterococcus faecium
Enterococcus faecalis
Third generation cephalosporins
Fourth generation cephalosporins
Fluoroquinolones
Macrolides
Quinolones
Polymyxins
Aminoglycosides
Aminopenicillins
Aminopenicillins with beta−lactamase inhibitors
Ansamycins
Phosphonic acid derivatives
Shared human and animal antimicrobial classes
Cattle
Chickens
Pigs
Sheep
Turkeys
Ducks
Horses
Buffaloes
Goats
Livestock species
Rank
0.00
0.25
0.50
0.75
1.00

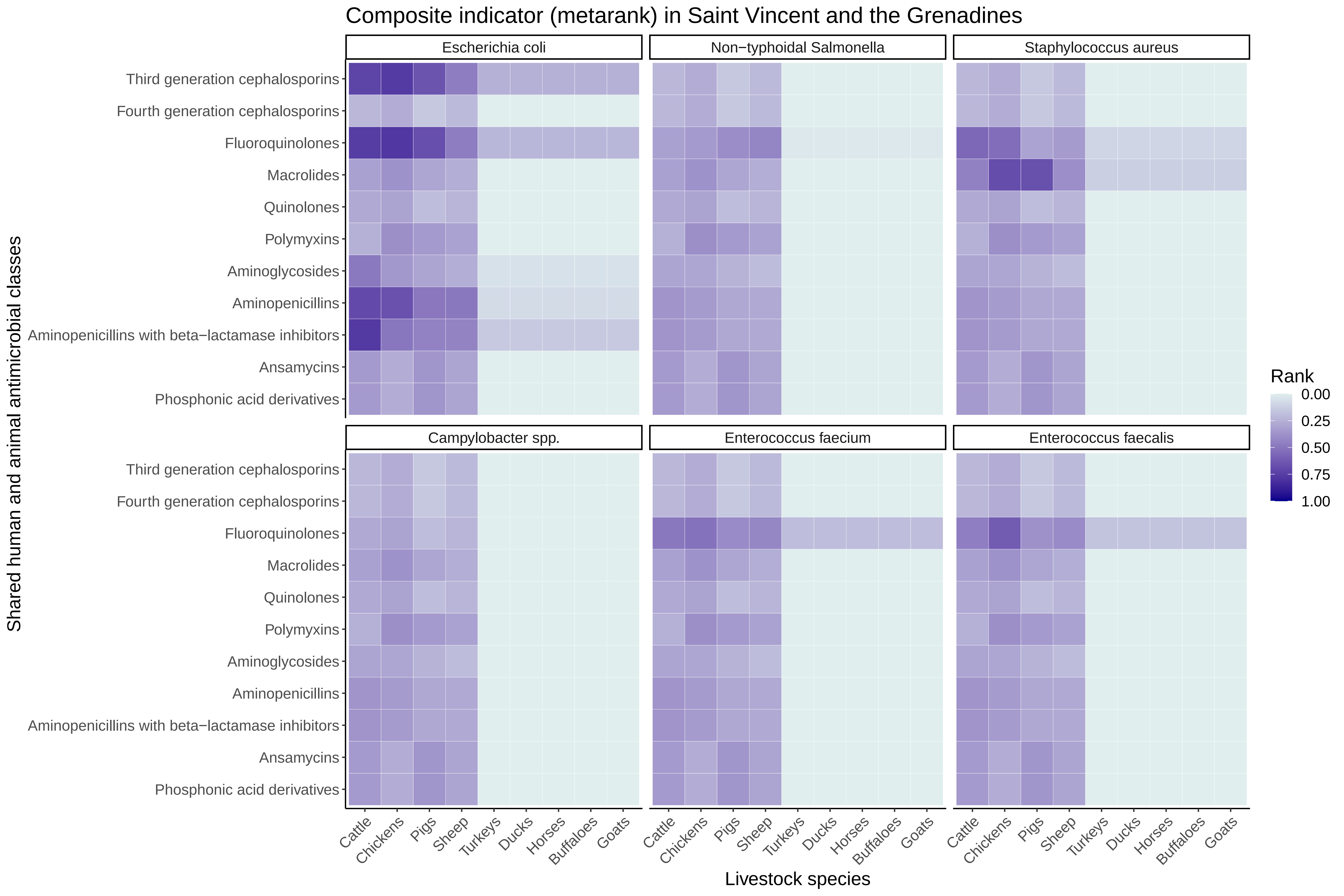
Composite indicator (metarank) in Saint Vincent and the Grenadines
Escherichia coli
Non−typhoidal Salmonella
Staphylococcus aureus
Campylobacter spp.
Enterococcus faecium
Enterococcus faecalis
Third generation cephalosporins
Fourth generation cephalosporins
Fluoroquinolones
Macrolides
Quinolones
Polymyxins
Aminoglycosides
Aminopenicillins
Aminopenicillins with beta−lactamase inhibitors
Ansamycins
Phosphonic acid derivatives
Shared human and animal antimicrobial classes
Cattle
Chickens
Pigs
Sheep
Turkeys
Ducks
Horses
Buffaloes
Goats
Livestock species
Rank
0.00
0.25
0.50
0.75
1.00

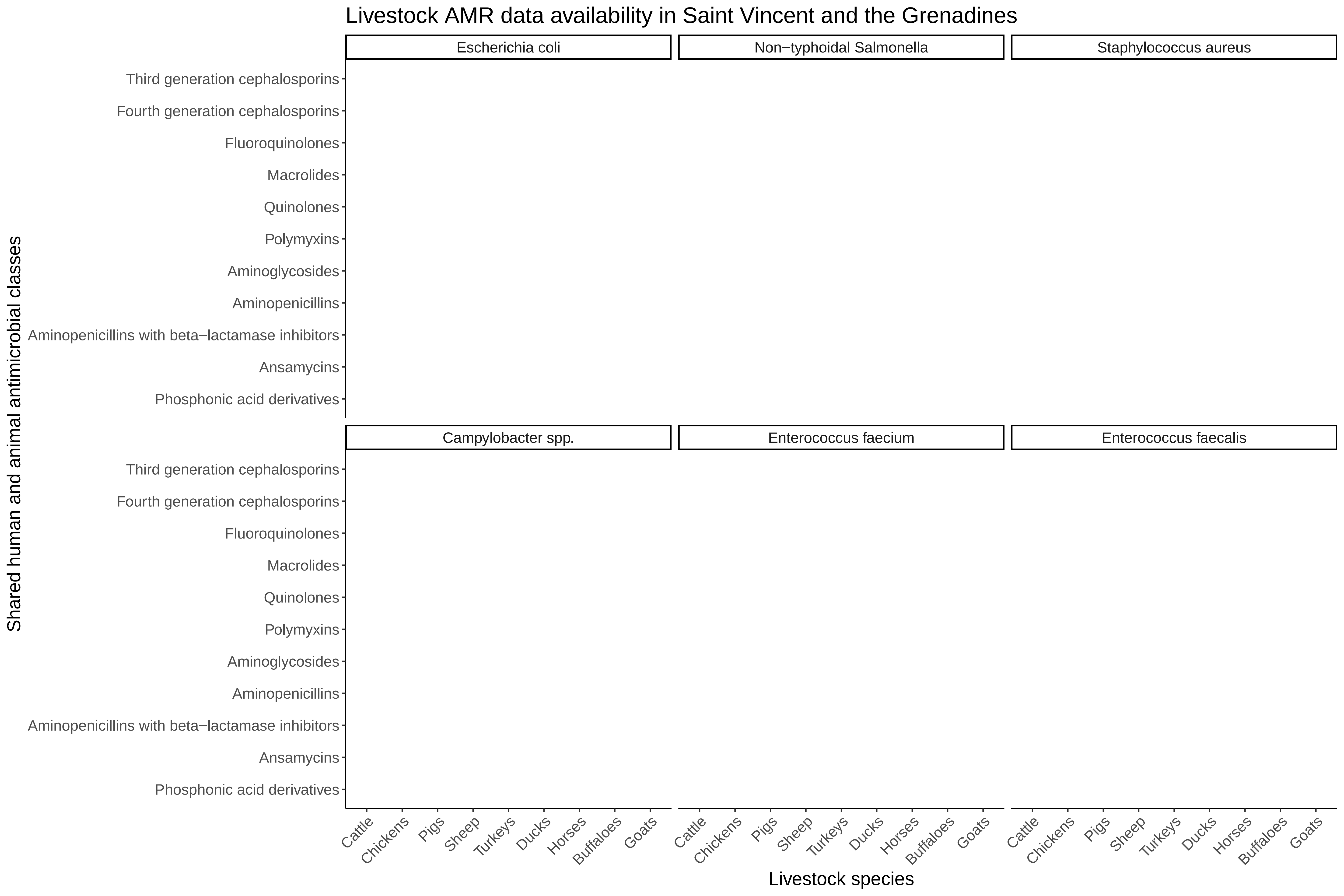

Livestock AMR data availability in Saint Vincent and the Grenadines
Escherichia coli
Non−typhoidal Salmonella
Staphylococcus aureus
Campylobacter spp.
Enterococcus faecium
Enterococcus faecalis
Shared human and animal antimicrobial classes
Third generation cephalosporins
Fourth generation cephalosporins
Fluoroquinolones
Macrolides
Quinolones
Polymyxins
Aminoglycosides
Aminopenicillins
Aminopenicillins with beta−lactamase inhibitors
Ansamycins
Phosphonic acid derivatives
Cattle
Chickens
Pigs
Sheep
Turkeys
Ducks
Horses
Buffaloes
Goats
Livestock species

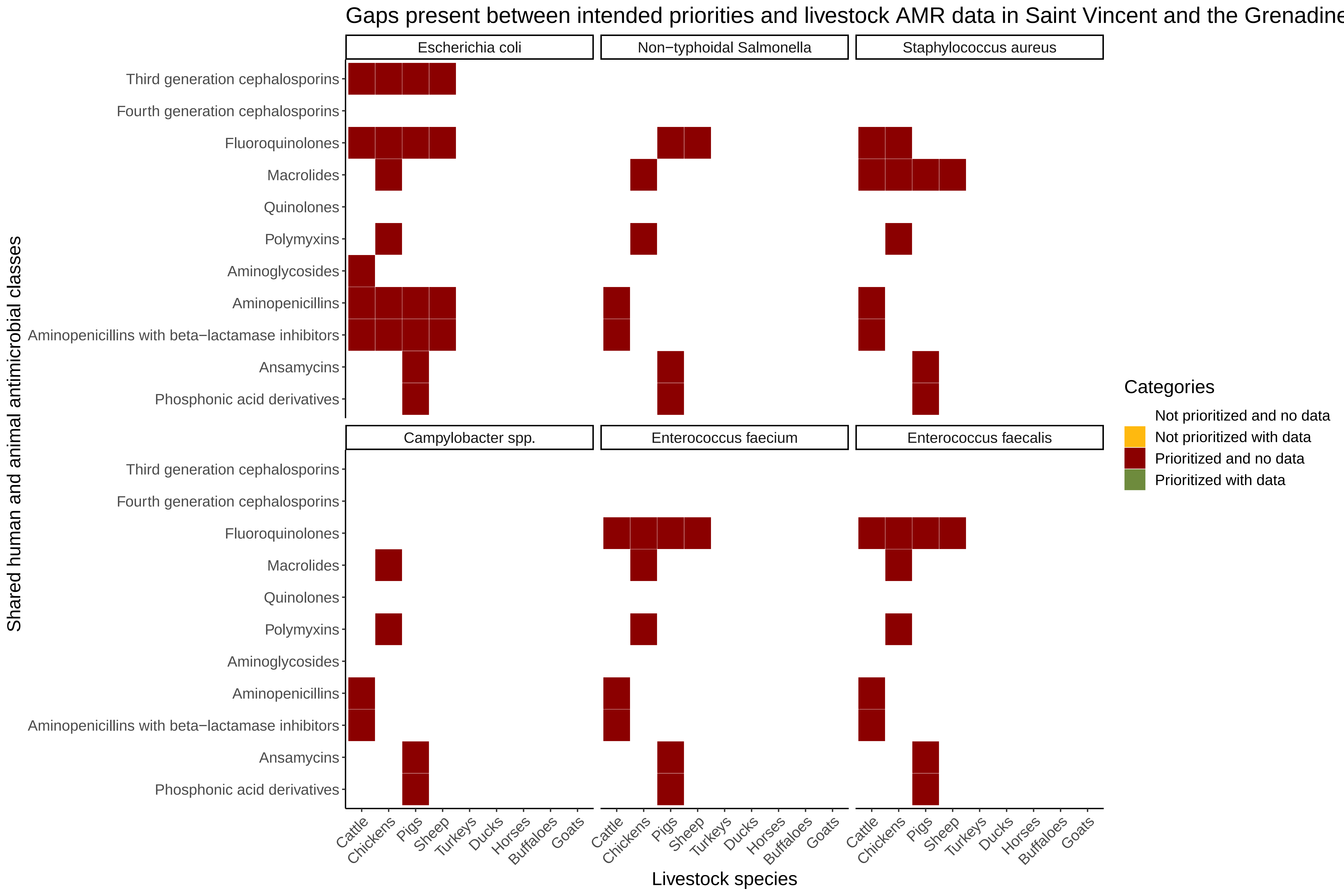

Gaps present between intended priorities and livestock AMR data in Saint Vincent and the Grenadine
Escherichia coli
Non−typhoidal Salmonella
Staphylococcus aureus
Campylobacter spp.
Enterococcus faecium
Enterococcus faecalis
Third generation cephalosporins
Fourth generation cephalosporins
Fluoroquinolones
Macrolides
Quinolones
Polymyxins
Aminoglycosides
Aminopenicillins
Aminopenicillins with beta−lactamase inhibitors
Ansamycins
Phosphonic acid derivatives
Shared human and animal antimicrobial classes
Cattle
Chickens
Pigs
Sheep
Turkeys
Ducks
Horses
Buffaloes
Goats
Livestock species
Categories
Not prioritized and no data
Not prioritized with data
Prioritized and no data
Prioritized with data

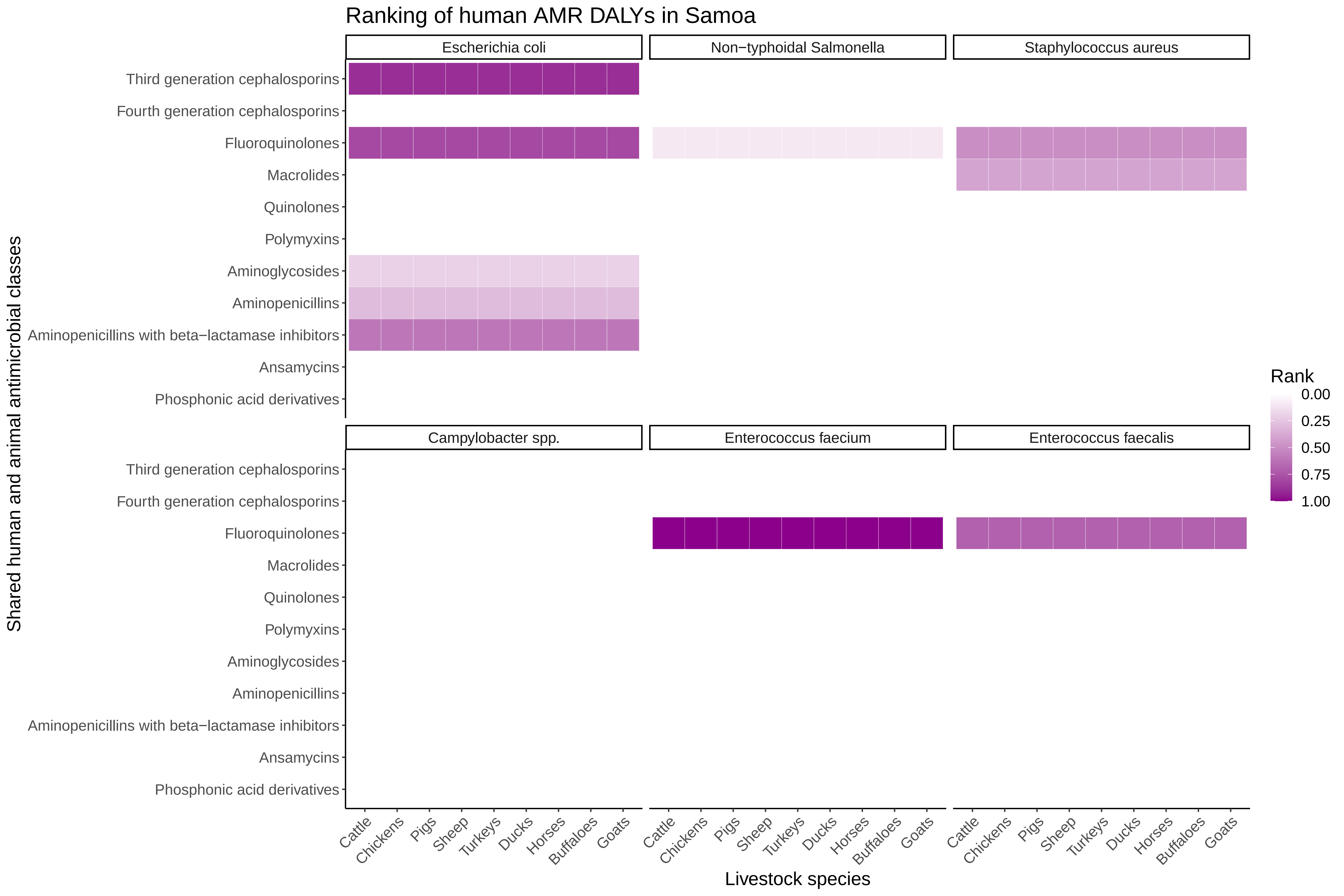
Ranking of human AMR DALYs in Samoa
Escherichia coli
Non−typhoidal Salmonella
Staphylococcus aureus
Campylobacter spp.
Enterococcus faecium
Enterococcus faecalis
Third generation cephalosporins
Fourth generation cephalosporins
Fluoroquinolones
Macrolides
Quinolones
Polymyxins
Aminoglycosides
Aminopenicillins
Aminopenicillins with beta−lactamase inhibitors
Ansamycins
Phosphonic acid derivatives
Shared human and animal antimicrobial classes
Cattle
Chickens
Pigs
Sheep
Turkeys
Ducks
Horses
Buffaloes
Goats
Livestock species
Rank
0.00
0.25
0.50
0.75
1.00

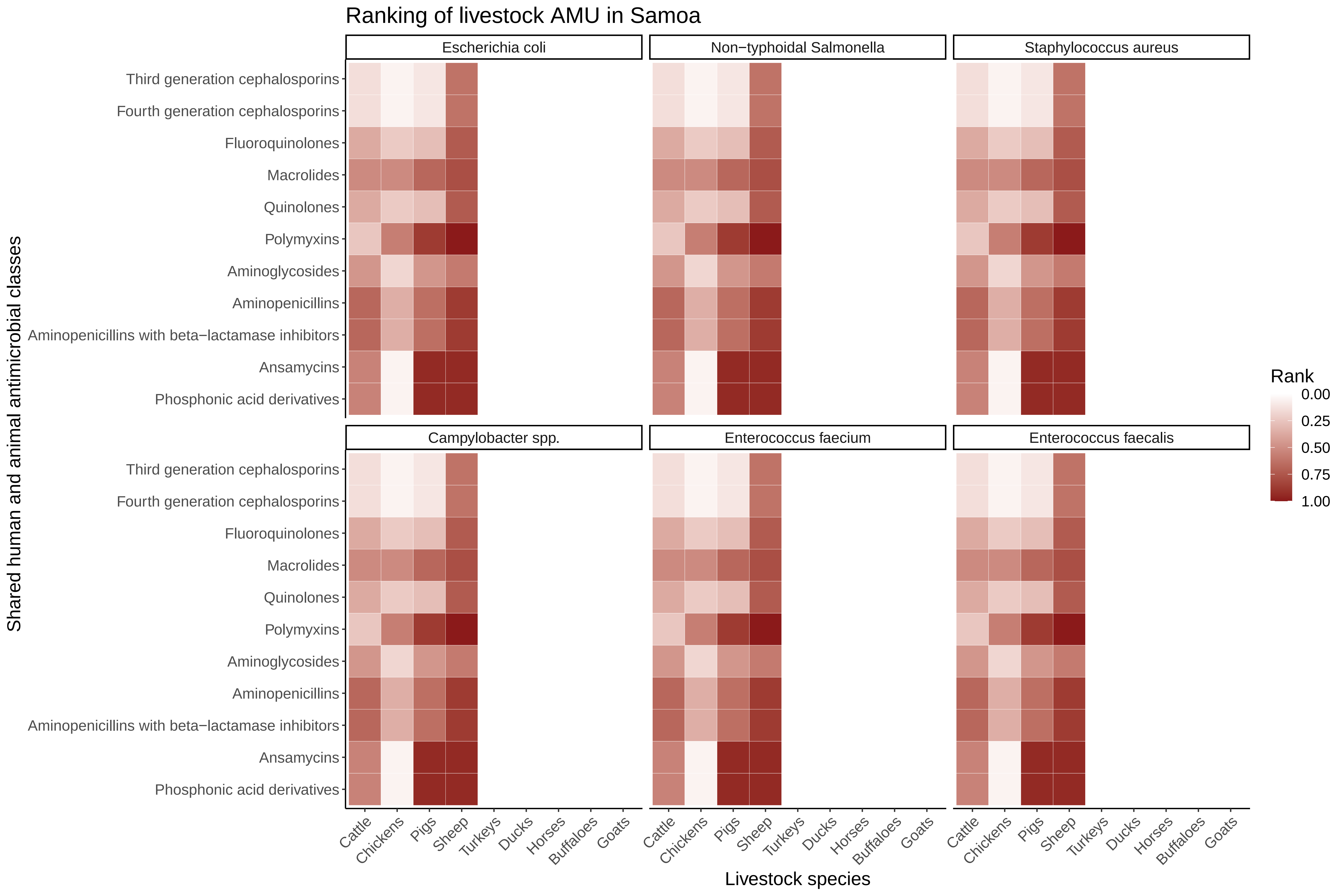
Ranking of livestock AMU in Samoa
Escherichia coli
Non−typhoidal Salmonella
Staphylococcus aureus
Campylobacter spp.
Enterococcus faecium
Enterococcus faecalis
Shared human and animal antimicrobial classes
Third generation cephalosporins
Fourth generation cephalosporins
Fluoroquinolones
Macrolides
Quinolones
Polymyxins
Aminoglycosides
Aminopenicillins
Aminopenicillins with beta−lactamase inhibitors
Ansamycins
Phosphonic acid derivatives
Rank
0.00
0.25
0.50
0.75
1.00
Cattle
Chickens
Pigs
Sheep
Turkeys
Ducks
Horses
Buffaloes
Goats
Livestock species

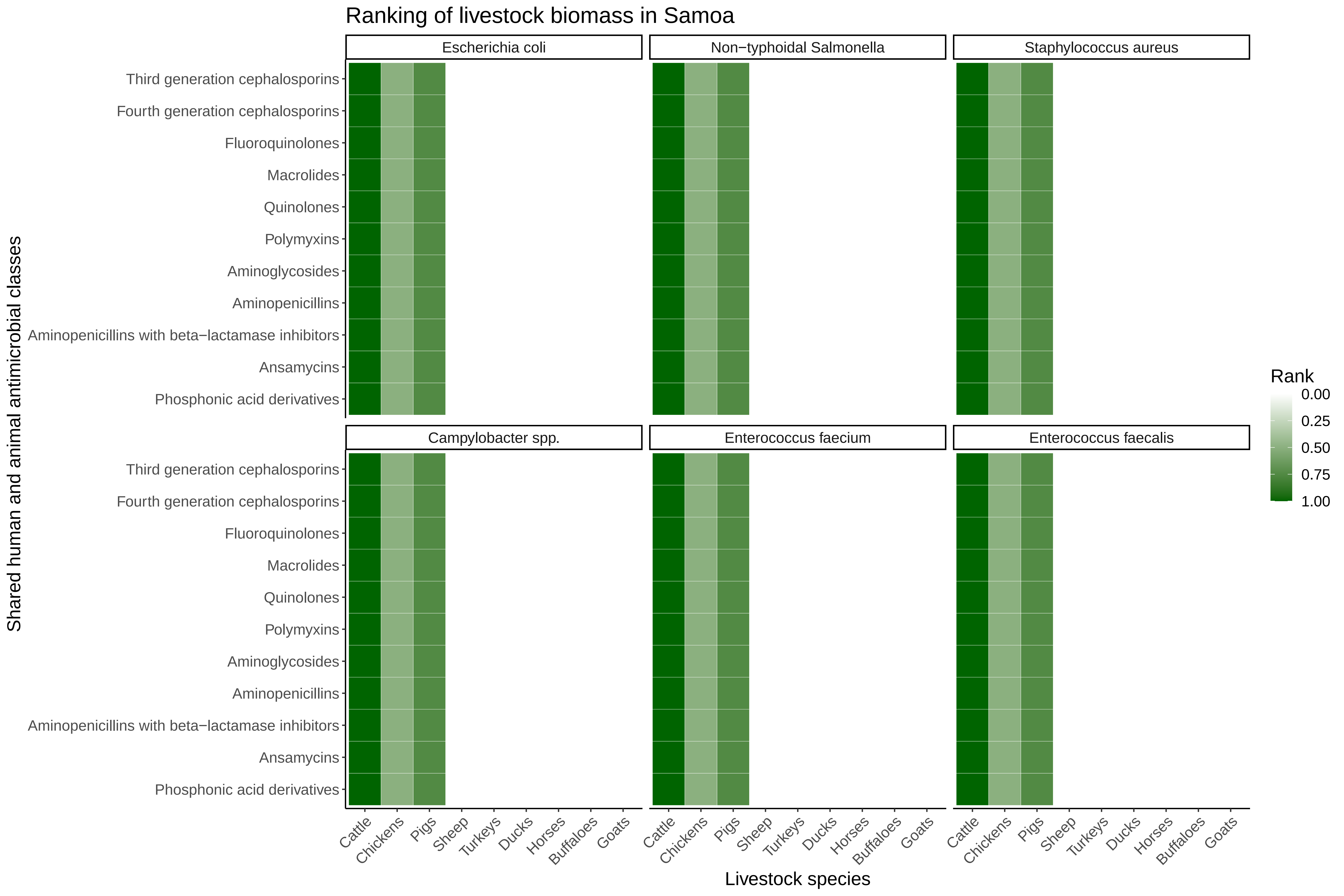

Ranking of livestock biomass in Samoa
Escherichia coli
Non−typhoidal Salmonella
Staphylococcus aureus
Campylobacter spp.
Enterococcus faecium
Enterococcus faecalis
Third generation cephalosporins
Fourth generation cephalosporins
Fluoroquinolones
Macrolides
Quinolones
Polymyxins
Aminoglycosides
Aminopenicillins
Aminopenicillins with beta−lactamase inhibitors
Ansamycins
Phosphonic acid derivatives
Shared human and animal antimicrobial classes
Cattle
Chickens
Pigs
Sheep
Turkeys
Ducks
Horses
Buffaloes
Goats
Livestock species
Rank
0.00
0.25
0.50
0.75
1.00

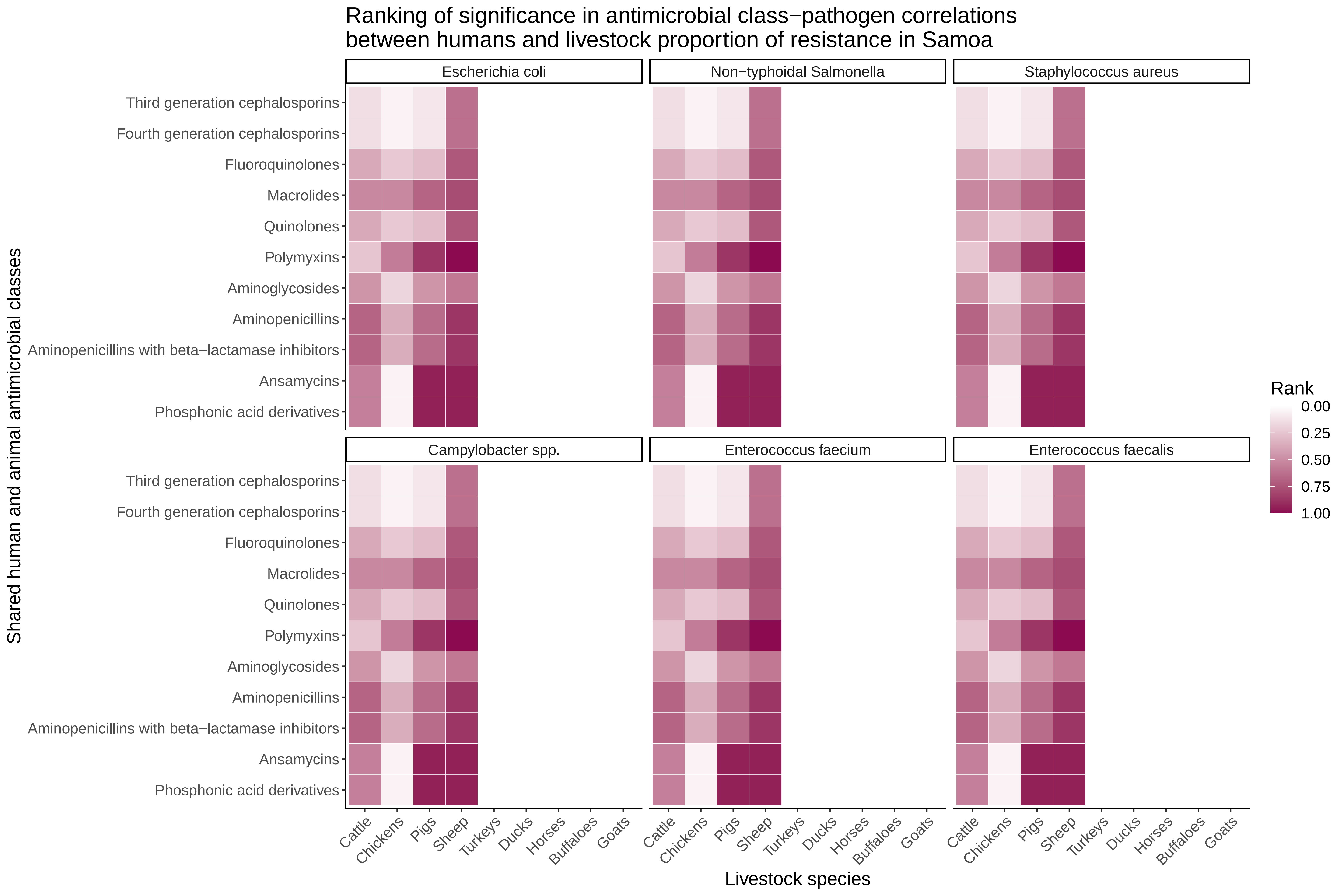

Ranking of significance in antimicrobial class−pathogen correlations
between humans and livestock proportion of resistance in Samoa
Escherichia coli
Non−typhoidal Salmonella
Staphylococcus aureus
Campylobacter spp.
Enterococcus faecium
Enterococcus faecalis
Third generation cephalosporins
Fourth generation cephalosporins
Fluoroquinolones
Macrolides
Quinolones
Polymyxins
Aminoglycosides
Aminopenicillins
Aminopenicillins with beta−lactamase inhibitors
Ansamycins
Phosphonic acid derivatives
Shared human and animal antimicrobial classes
Cattle
Chickens
Pigs
Sheep
Turkeys
Ducks
Horses
Buffaloes
Goats
Livestock species
Rank
0.00
0.25
0.50
0.75
1.00

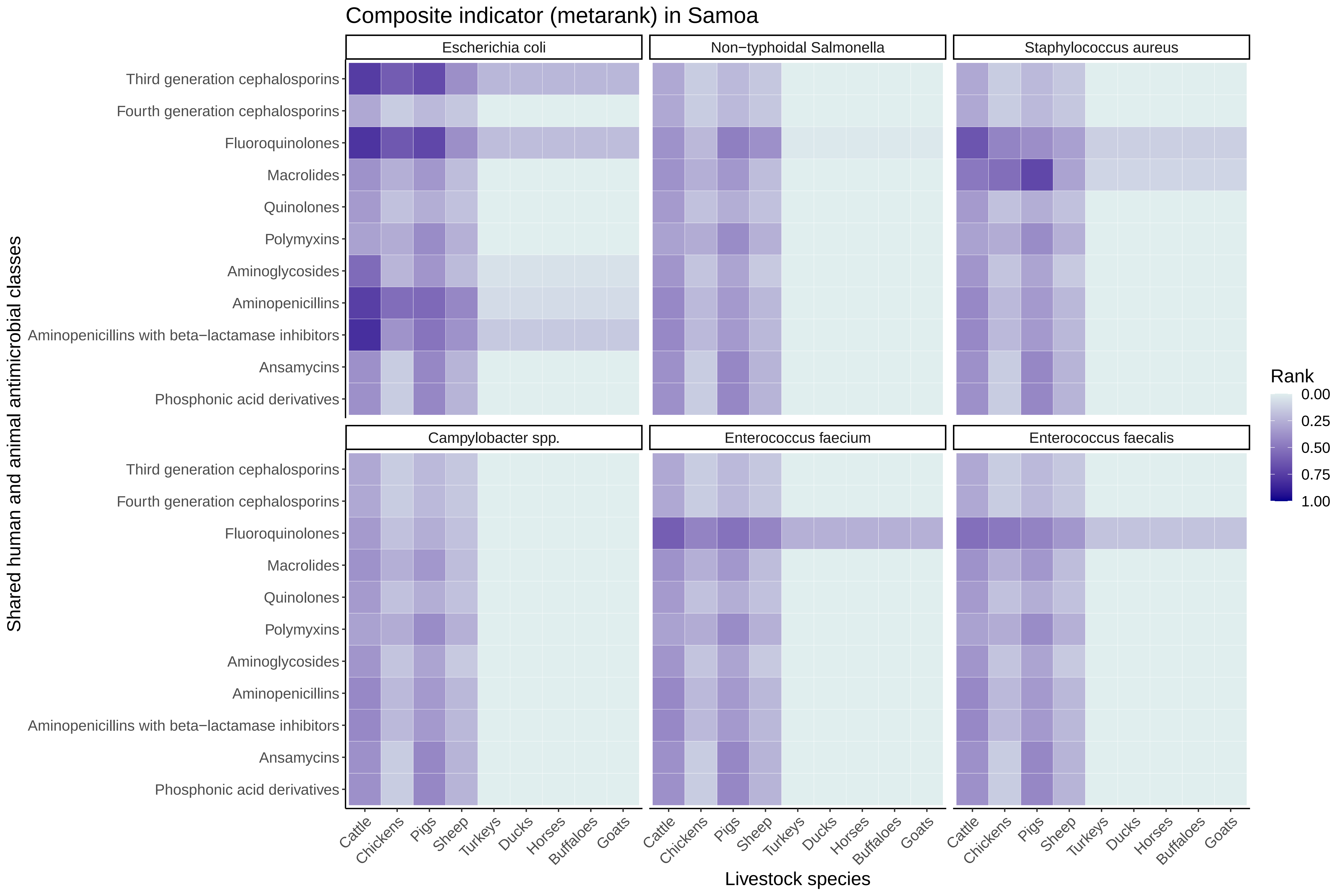
Composite indicator (metarank) in Samoa
Escherichia coli
Non−typhoidal Salmonella
Staphylococcus aureus
Campylobacter spp.
Enterococcus faecium
Enterococcus faecalis
Shared human and animal antimicrobial classes
Third generation cephalosporins
Fourth generation cephalosporins
Fluoroquinolones
Macrolides
Quinolones
Polymyxins
Aminoglycosides
Aminopenicillins
Aminopenicillins with beta−lactamase inhibitors
Ansamycins
Phosphonic acid derivatives
Cattle
Chickens
Pigs
Sheep
Turkeys
Ducks
Horses
Buffaloes
Goats
Livestock species
Rank
0.00
0.25
0.50
0.75
1.00

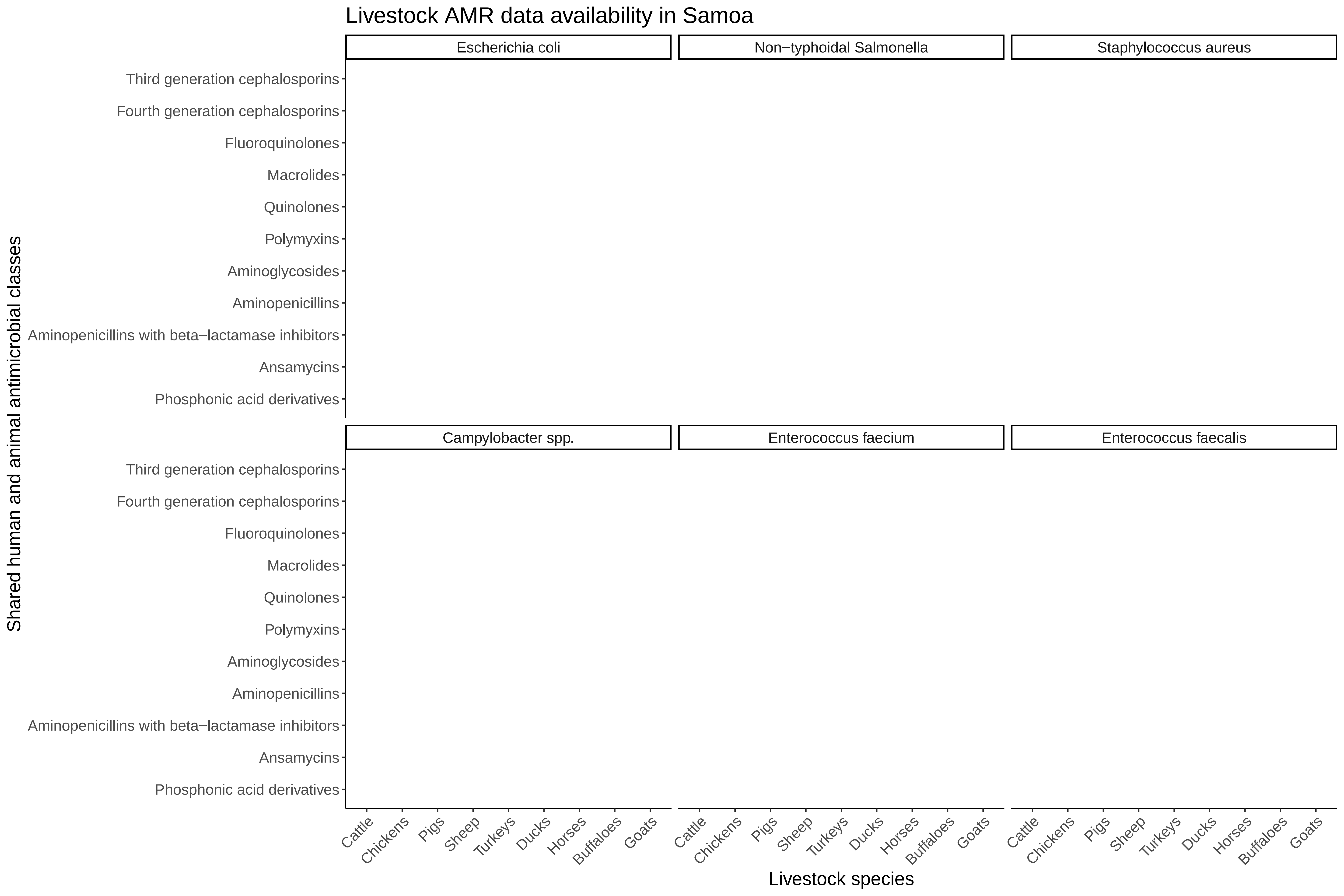

Livestock AMR data availability in Samoa
Escherichia coli
Non−typhoidal Salmonella
Staphylococcus aureus
Campylobacter spp.
Enterococcus faecium
Enterococcus faecalis
Shared human and animal antimicrobial classes
Third generation cephalosporins
Fourth generation cephalosporins
Fluoroquinolones
Macrolides
Quinolones
Polymyxins
Aminoglycosides
Aminopenicillins
Aminopenicillins with beta−lactamase inhibitors
Ansamycins
Phosphonic acid derivatives
Cattle
Chickens
Pigs
Sheep
Turkeys
Ducks
Horses
Buffaloes
Goats
Livestock species

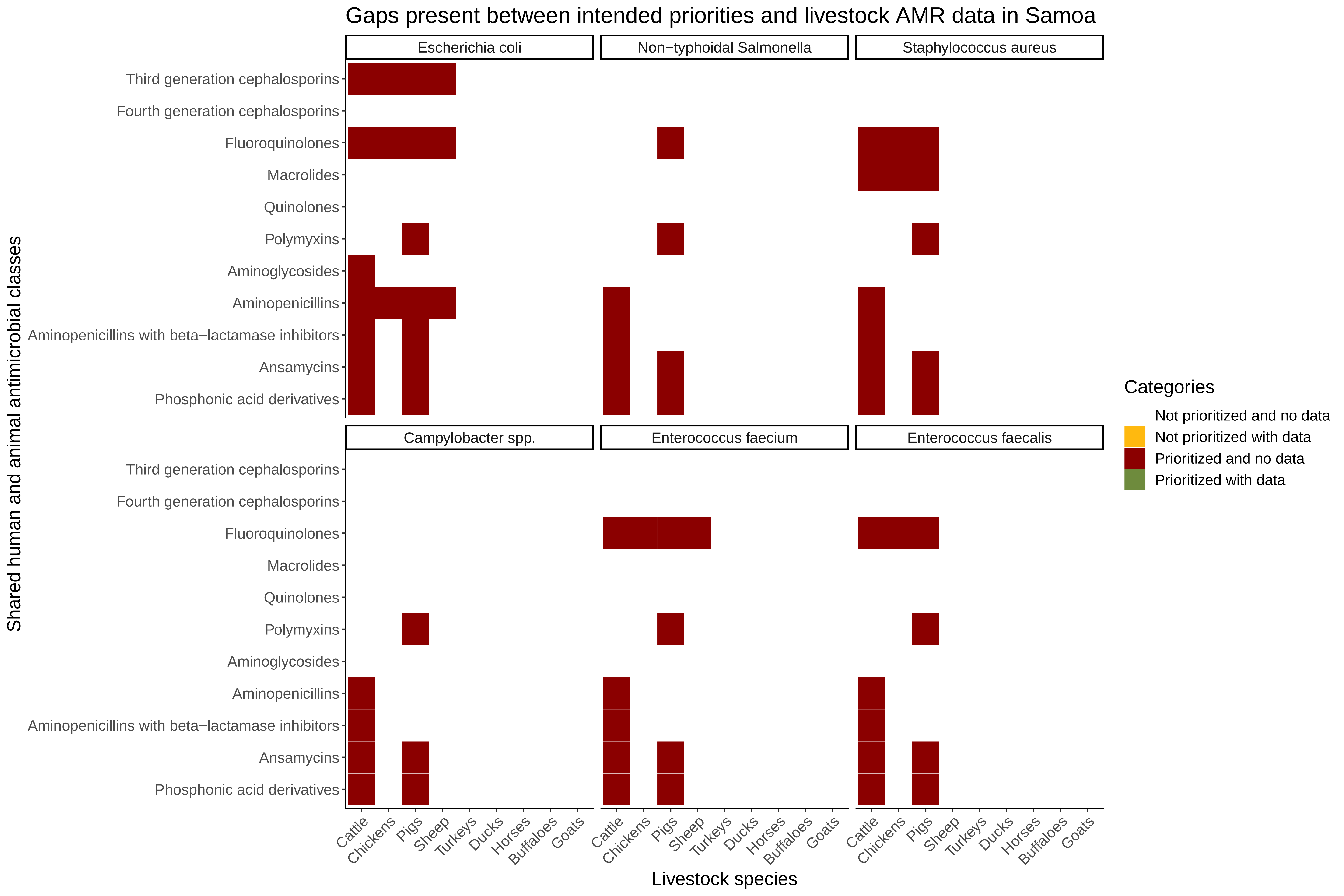
Gaps present between intended priorities and livestock AMR data in Samoa
Escherichia coli
Non−typhoidal Salmonella
Staphylococcus aureus
Campylobacter spp.
Enterococcus faecium
Enterococcus faecalis
Shared human and animal antimicrobial classes
Third generation cephalosporins
Fourth generation cephalosporins
Fluoroquinolones
Macrolides
Quinolones
Polymyxins
Aminoglycosides
Aminopenicillins
Aminopenicillins with beta−lactamase inhibitors
Ansamycins
Phosphonic acid derivatives
Cattle
Chickens
Pigs
Sheep
Turkeys
Ducks
Horses
Buffaloes
Goats
Livestock species
Categories
Not prioritized and no data
Not prioritized with data
Prioritized and no data
Prioritized with data

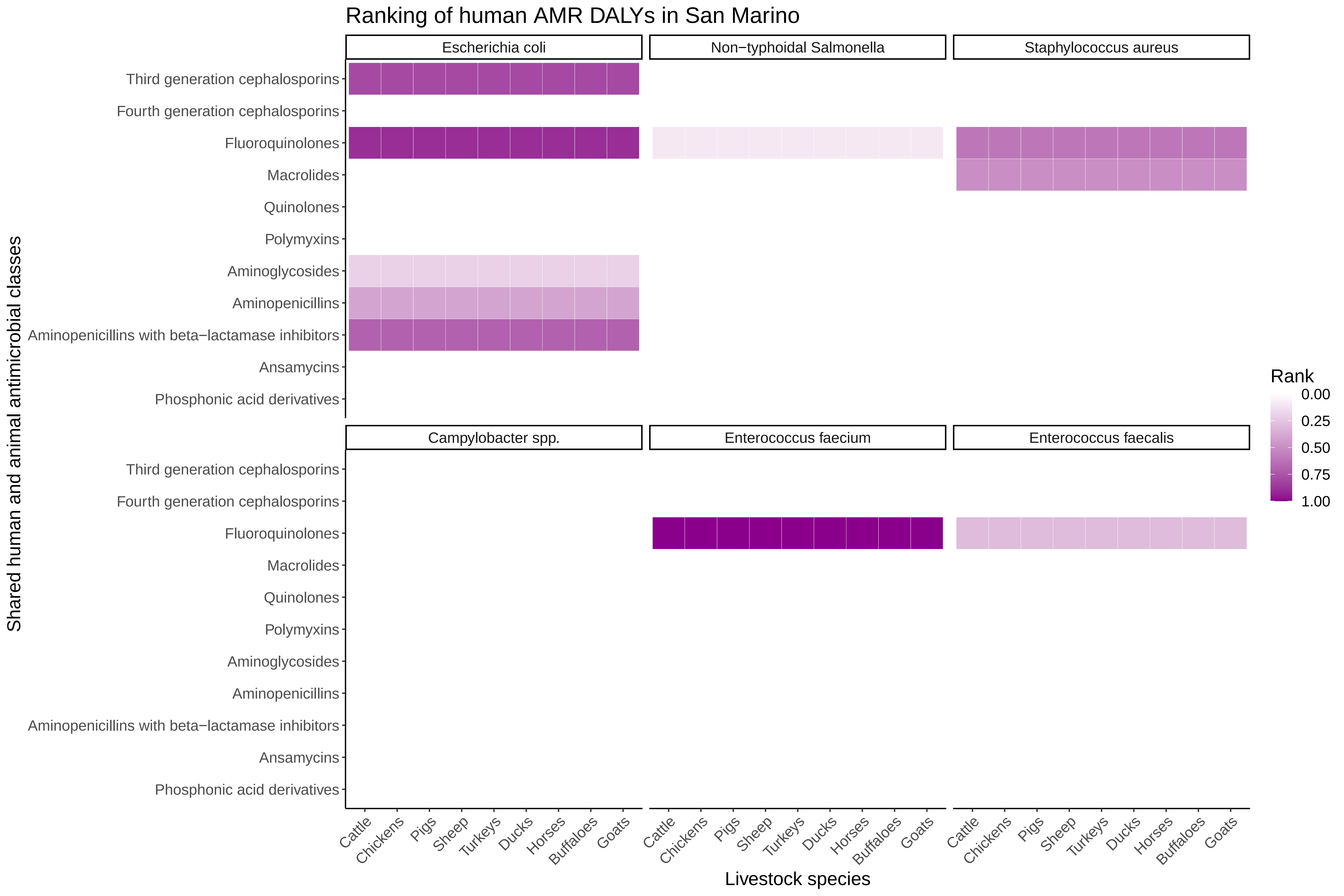

Ranking of human AMR DALYs in San Marino
Escherichia coli
Non−typhoidal Salmonella
Staphylococcus aureus
Campylobacter spp.
Enterococcus faecium
Enterococcus faecalis
Shared human and animal antimicrobial classes
Third generation cephalosporins
Fourth generation cephalosporins
Fluoroquinolones
Macrolides
Quinolones
Polymyxins
Aminoglycosides
Aminopenicillins
Aminopenicillins with beta−lactamase inhibitors
Ansamycins
Phosphonic acid derivatives
Cattle
Chickens
Pigs
Sheep
Turkeys
Ducks
Horses
Buffaloes
Goats
Livestock species
Rank
0.00
0.25
0.50
0.75
1.00

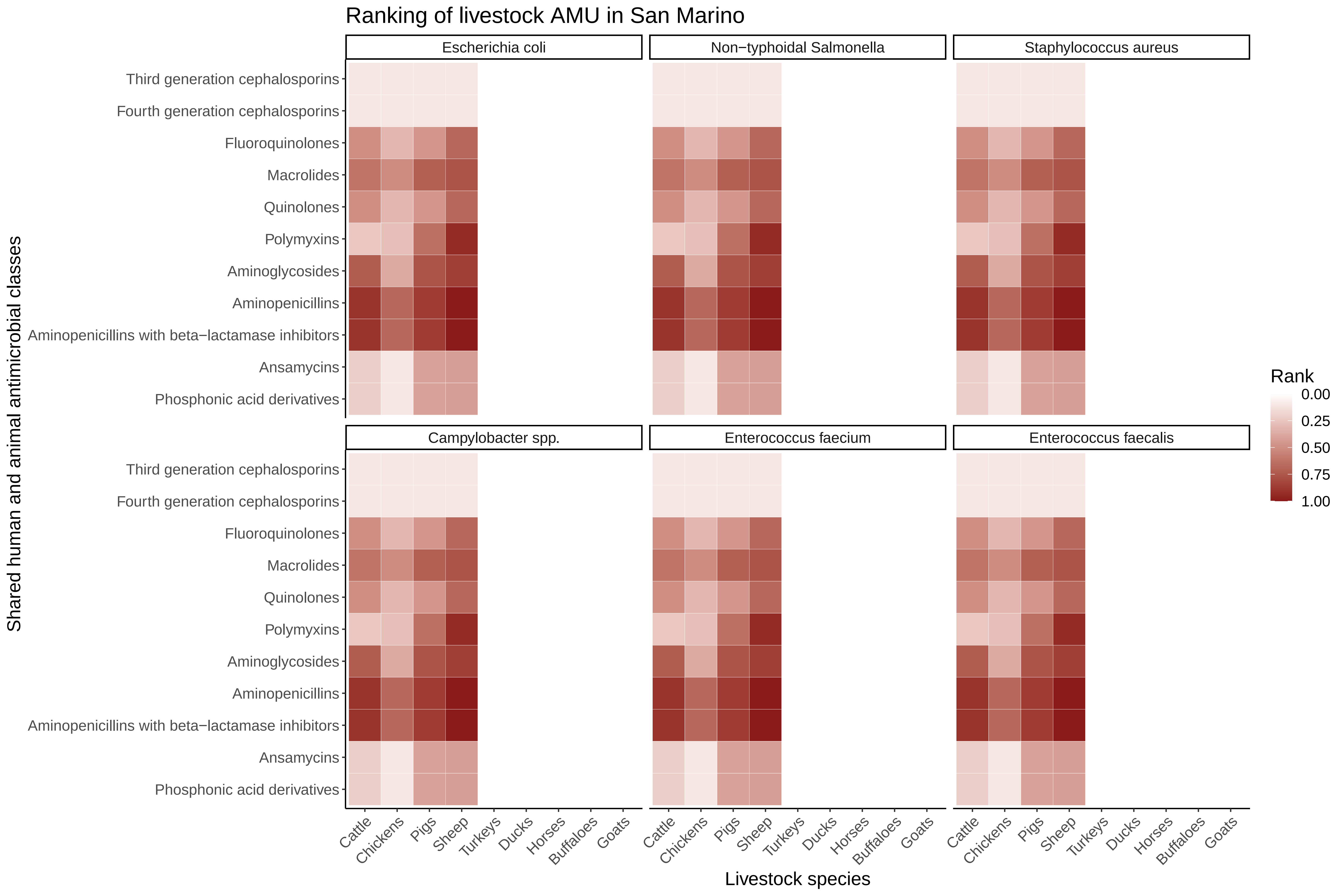
Ranking of livestock AMU in San Marino
Escherichia coli
Non−typhoidal Salmonella
Staphylococcus aureus
Campylobacter spp.
Enterococcus faecium
Enterococcus faecalis
Third generation cephalosporins
Fourth generation cephalosporins
Fluoroquinolones
Macrolides
Quinolones
Polymyxins
Aminoglycosides
Aminopenicillins
Aminopenicillins with beta−lactamase inhibitors
Ansamycins
Phosphonic acid derivatives
Shared human and animal antimicrobial classes
Cattle
Chickens
Pigs
Sheep
Turkeys
Ducks
Horses
Buffaloes
Goats
Livestock species
Rank
0.00
0.25
0.50
0.75
1.00

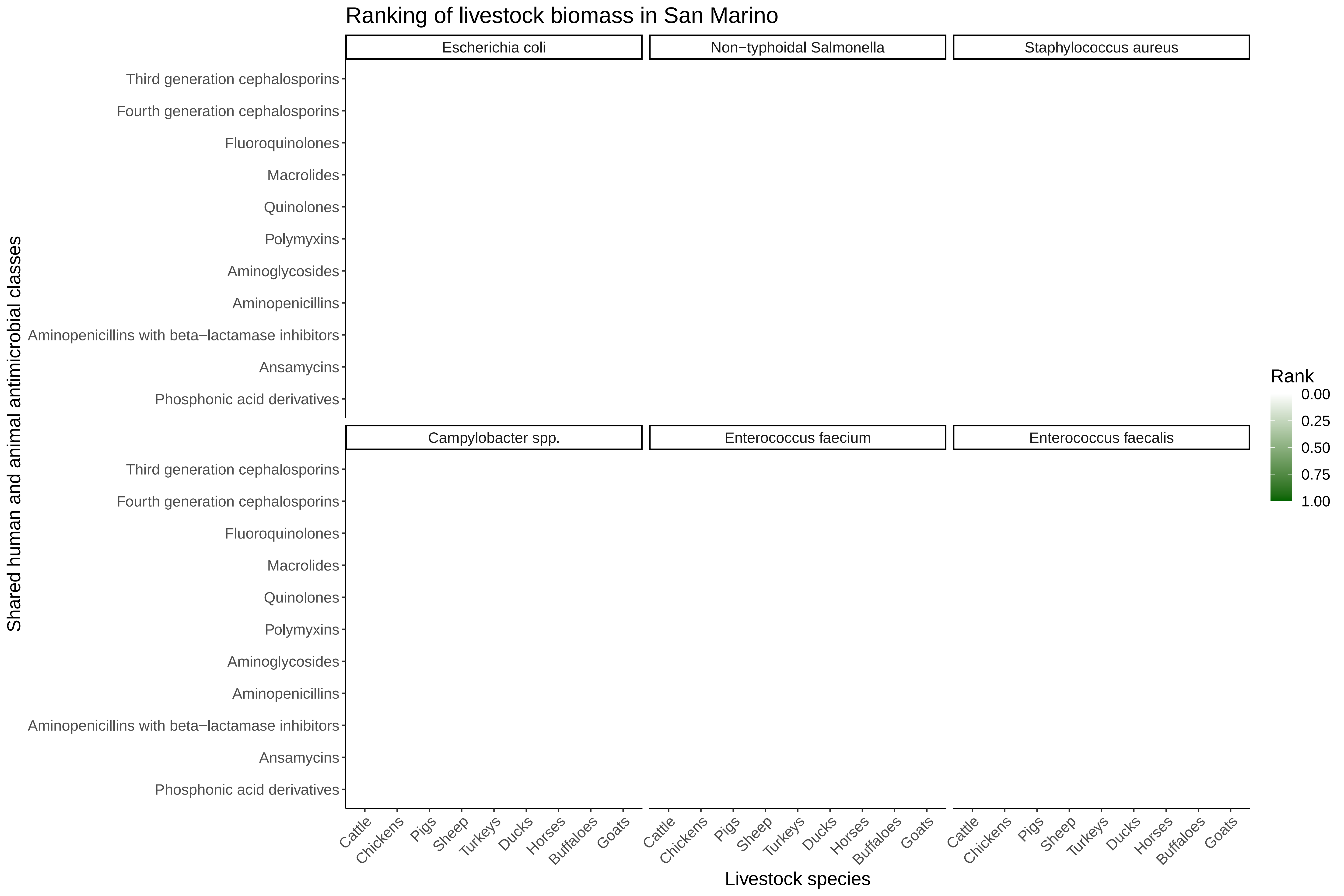

Ranking of livestock biomass in San Marino
Escherichia coli
Non−typhoidal Salmonella
Staphylococcus aureus
Campylobacter spp.
Enterococcus faecium
Enterococcus faecalis
Third generation cephalosporins
Fourth generation cephalosporins
Fluoroquinolones
Macrolides
Quinolones
Polymyxins
Aminoglycosides
Aminopenicillins
Aminopenicillins with beta−lactamase inhibitors
Ansamycins
Phosphonic acid derivatives
Shared human and animal antimicrobial classes
Cattle
Chickens
Pigs
Sheep
Turkeys
Ducks
Horses
Buffaloes
Goats
Livestock species
Rank
0.00
0.25
0.50
0.75
1.00

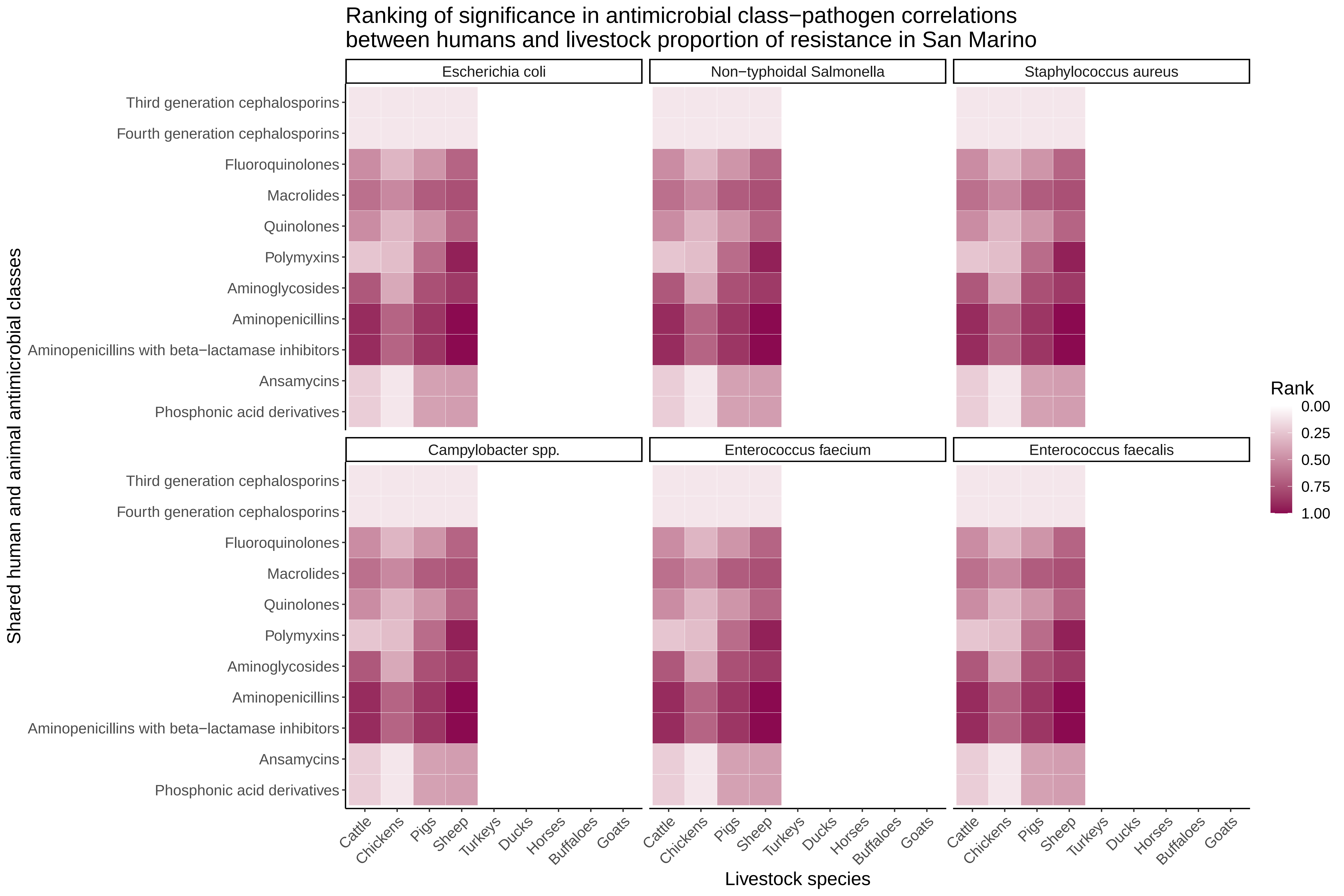

Ranking of significance in antimicrobial class−pathogen correlations
between humans and livestock proportion of resistance in San Marino
Escherichia coli
Non−typhoidal Salmonella
Staphylococcus aureus
Campylobacter spp.
Enterococcus faecium
Enterococcus faecalis
Third generation cephalosporins
Fourth generation cephalosporins
Fluoroquinolones
Macrolides
Quinolones
Polymyxins
Aminoglycosides
Aminopenicillins
Aminopenicillins with beta−lactamase inhibitors
Ansamycins
Phosphonic acid derivatives
Shared human and animal antimicrobial classes
Cattle
Chickens
Pigs
Sheep
Turkeys
Ducks
Horses
Buffaloes
Goats
Livestock species
Rank
0.00
0.25
0.50
0.75
1.00

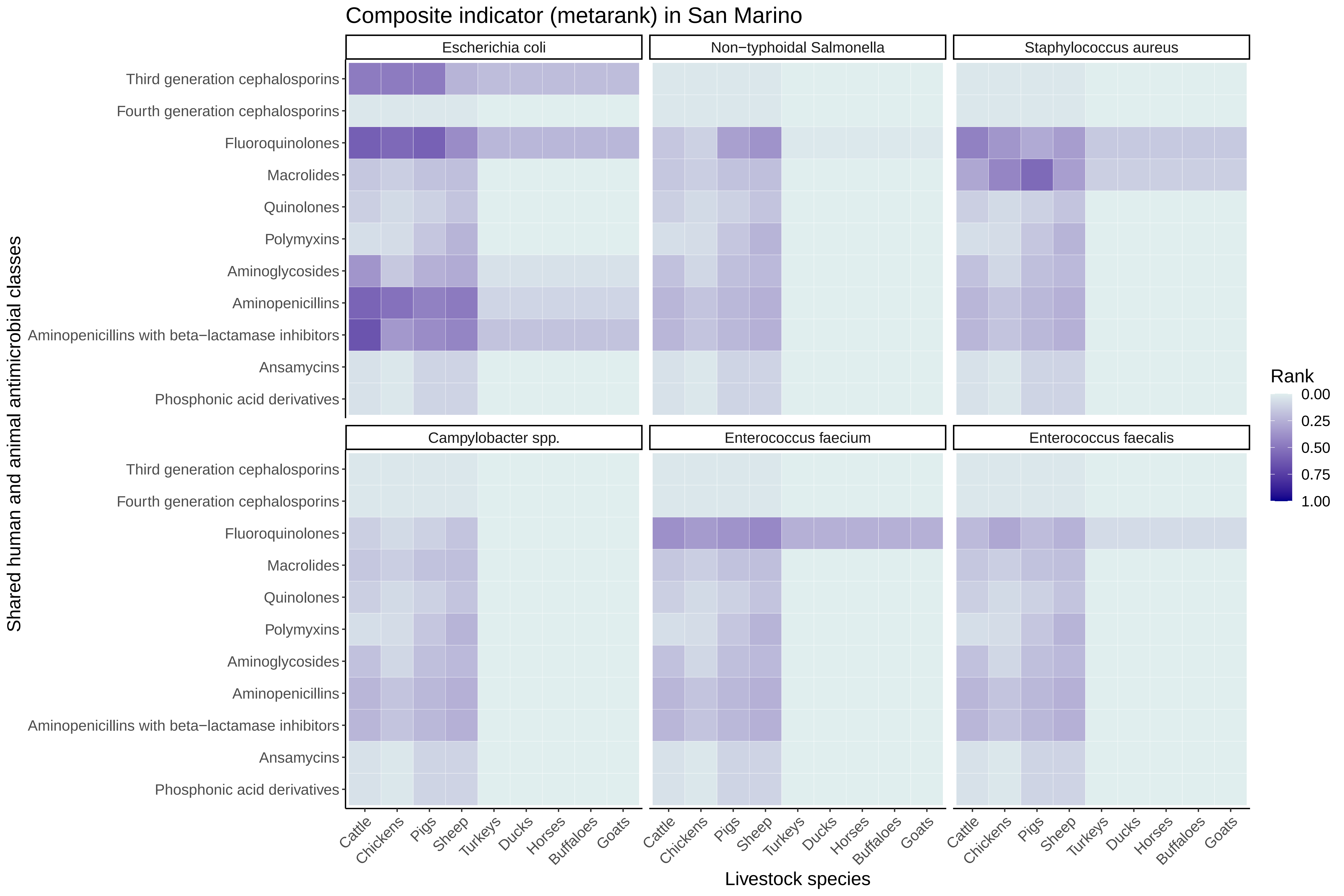
Composite indicator (metarank) in San Marino
Escherichia coli
Non−typhoidal Salmonella
Staphylococcus aureus
Campylobacter spp.
Enterococcus faecium
Enterococcus faecalis
Shared human and animal antimicrobial classes
Third generation cephalosporins
Fourth generation cephalosporins
Fluoroquinolones
Macrolides
Quinolones
Polymyxins
Aminoglycosides
Aminopenicillins
Aminopenicillins with beta−lactamase inhibitors
Ansamycins
Phosphonic acid derivatives
Cattle
Chickens
Pigs
Sheep
Turkeys
Ducks
Horses
Buffaloes
Goats
Livestock species
Rank
0.00
0.25
0.50
0.75
1.00

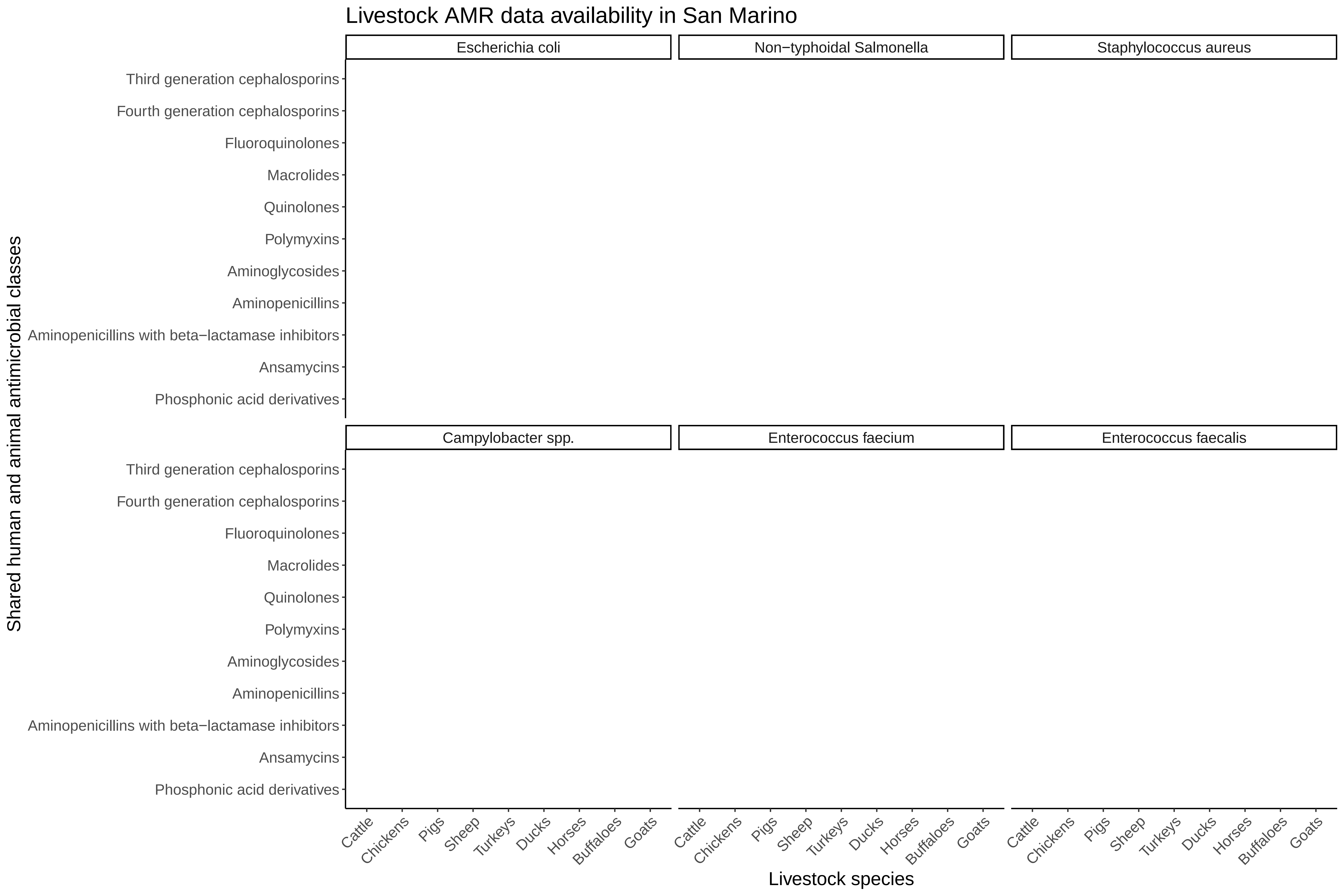

Livestock AMR data availability in San Marino
Escherichia coli
Non−typhoidal Salmonella
Staphylococcus aureus
Campylobacter spp.
Enterococcus faecium
Enterococcus faecalis
Shared human and animal antimicrobial classes
Third generation cephalosporins
Fourth generation cephalosporins
Fluoroquinolones
Macrolides
Quinolones
Polymyxins
Aminoglycosides
Aminopenicillins
Aminopenicillins with beta−lactamase inhibitors
Ansamycins
Phosphonic acid derivatives
Cattle
Chickens
Pigs
Sheep
Turkeys
Ducks
Horses
Buffaloes
Goats
Livestock species

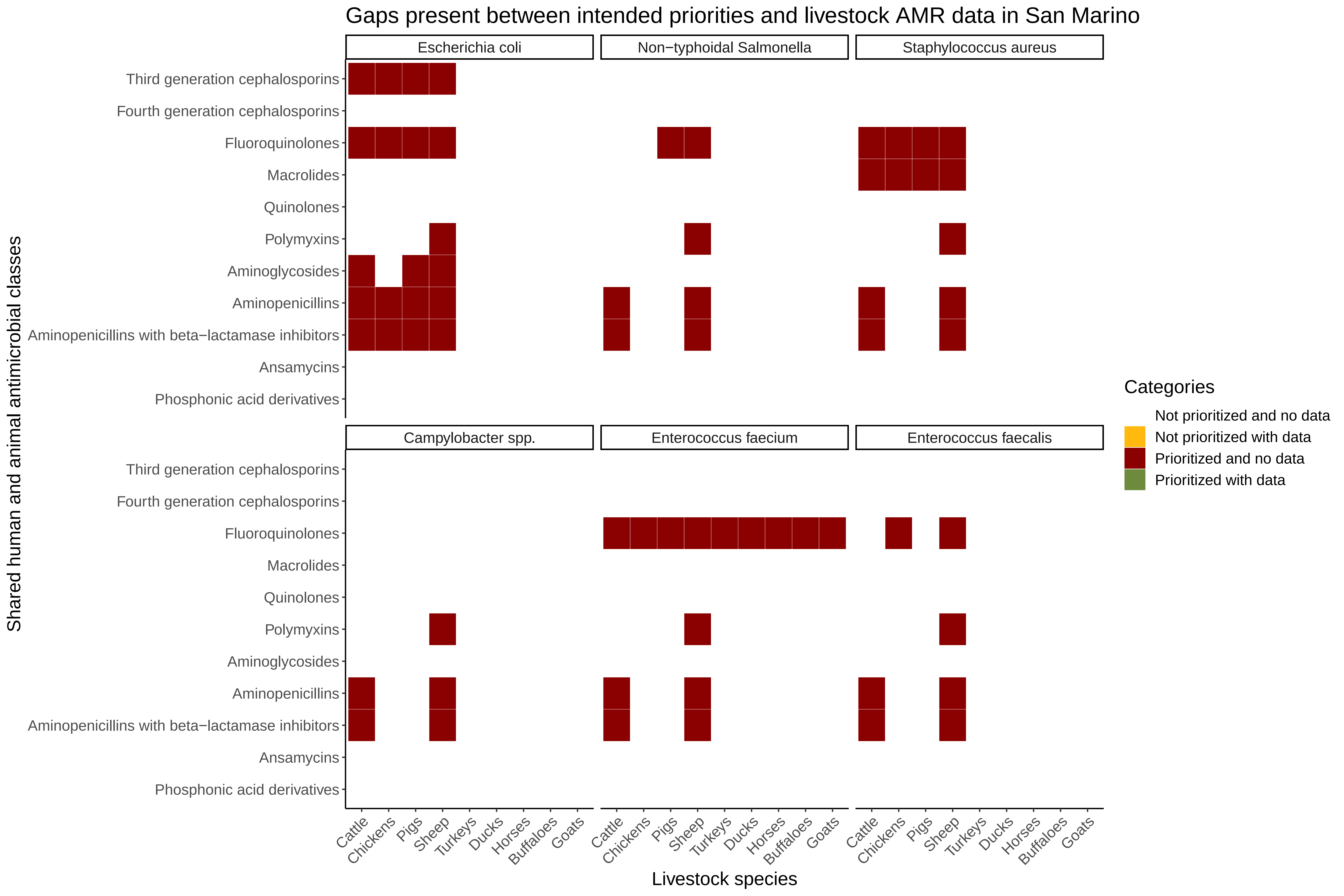
Gaps present between intended priorities and livestock AMR data in San Marino
Escherichia coli
Non−typhoidal Salmonella
Staphylococcus aureus
Campylobacter spp.
Enterococcus faecium
Enterococcus faecalis
Third generation cephalosporins
Fourth generation cephalosporins
Fluoroquinolones
Macrolides
Quinolones
Polymyxins
Aminoglycosides
Aminopenicillins
Aminopenicillins with beta−lactamase inhibitors
Ansamycins
Phosphonic acid derivatives
Shared human and animal antimicrobial classes
Cattle
Chickens
Pigs
Sheep
Turkeys
Ducks
Horses
Buffaloes
Goats
Livestock species
Categories
Not prioritized and no data
Not prioritized with data
Prioritized and no data
Prioritized with data

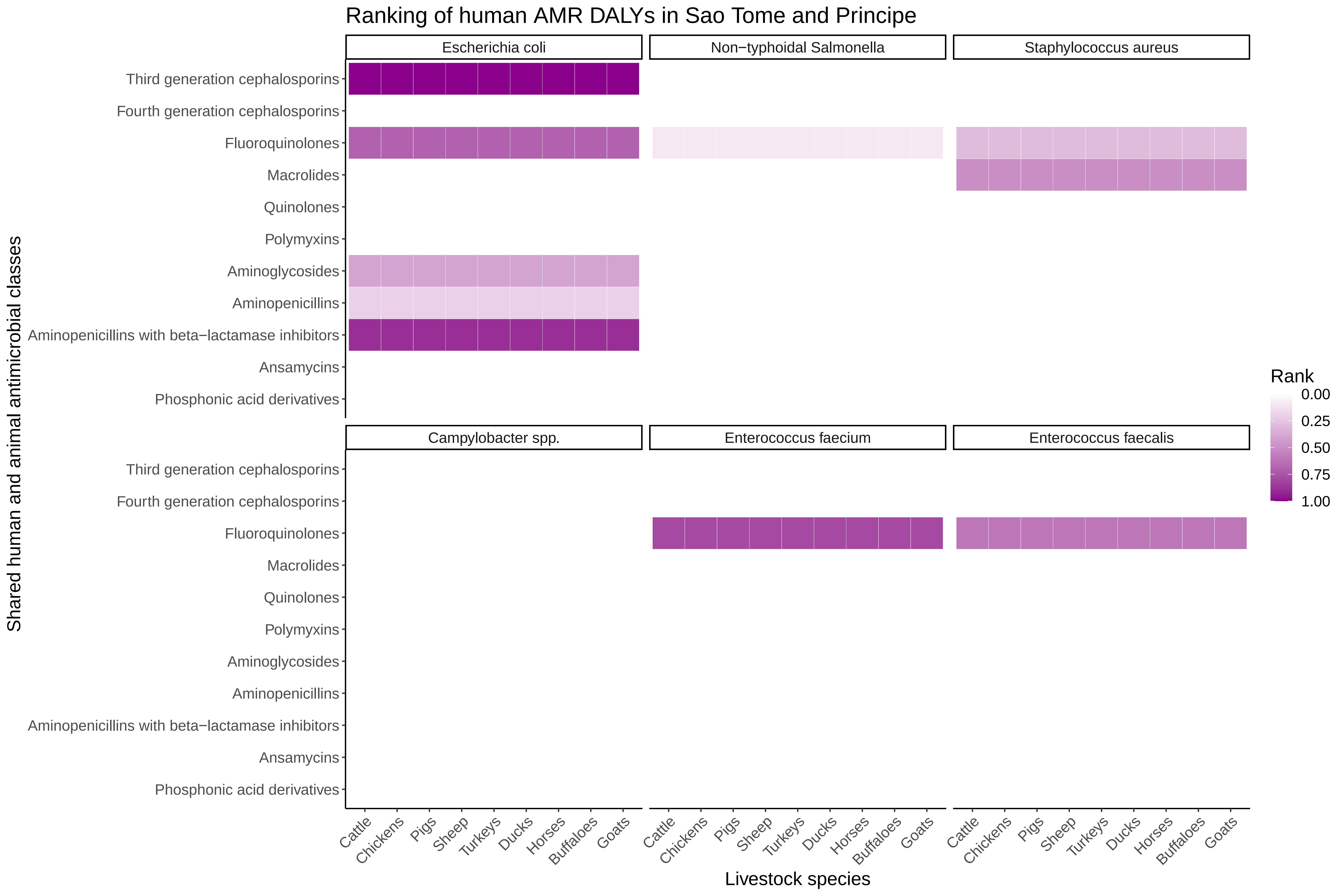

Ranking of human AMR DALYs in Sao Tome and Principe
Escherichia coli
Non−typhoidal Salmonella
Staphylococcus aureus
Campylobacter spp.
Enterococcus faecium
Enterococcus faecalis
Third generation cephalosporins
Fourth generation cephalosporins
Fluoroquinolones
Macrolides
Quinolones
Polymyxins
Aminoglycosides
Aminopenicillins
Aminopenicillins with beta−lactamase inhibitors
Ansamycins
Phosphonic acid derivatives
Shared human and animal antimicrobial classes
Cattle
Chickens
Pigs
Sheep
Turkeys
Ducks
Horses
Buffaloes
Goats
Livestock species
Rank
0.00
0.25
0.50
0.75
1.00

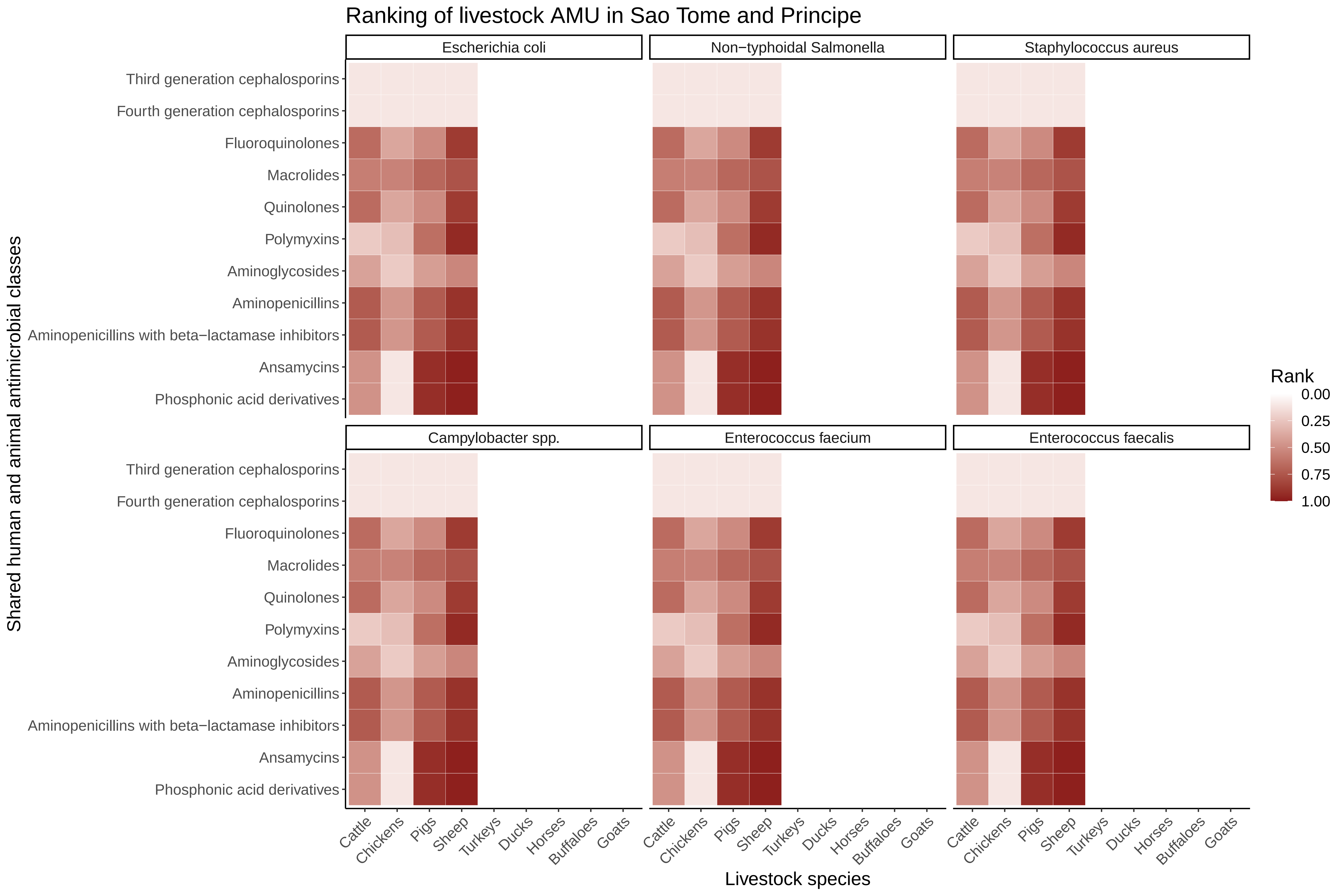

Ranking of livestock AMU in Sao Tome and Principe
Escherichia coli
Non−typhoidal Salmonella
Staphylococcus aureus
Campylobacter spp.
Enterococcus faecium
Enterococcus faecalis
Third generation cephalosporins
Fourth generation cephalosporins
Fluoroquinolones
Macrolides
Quinolones
Polymyxins
Aminoglycosides
Aminopenicillins
Aminopenicillins with beta−lactamase inhibitors
Ansamycins
Phosphonic acid derivatives
Shared human and animal antimicrobial classes
Cattle
Chickens
Pigs
Sheep
Turkeys
Ducks
Horses
Buffaloes
Goats
Livestock species
Rank
0.00
0.25
0.50
0.75
1.00

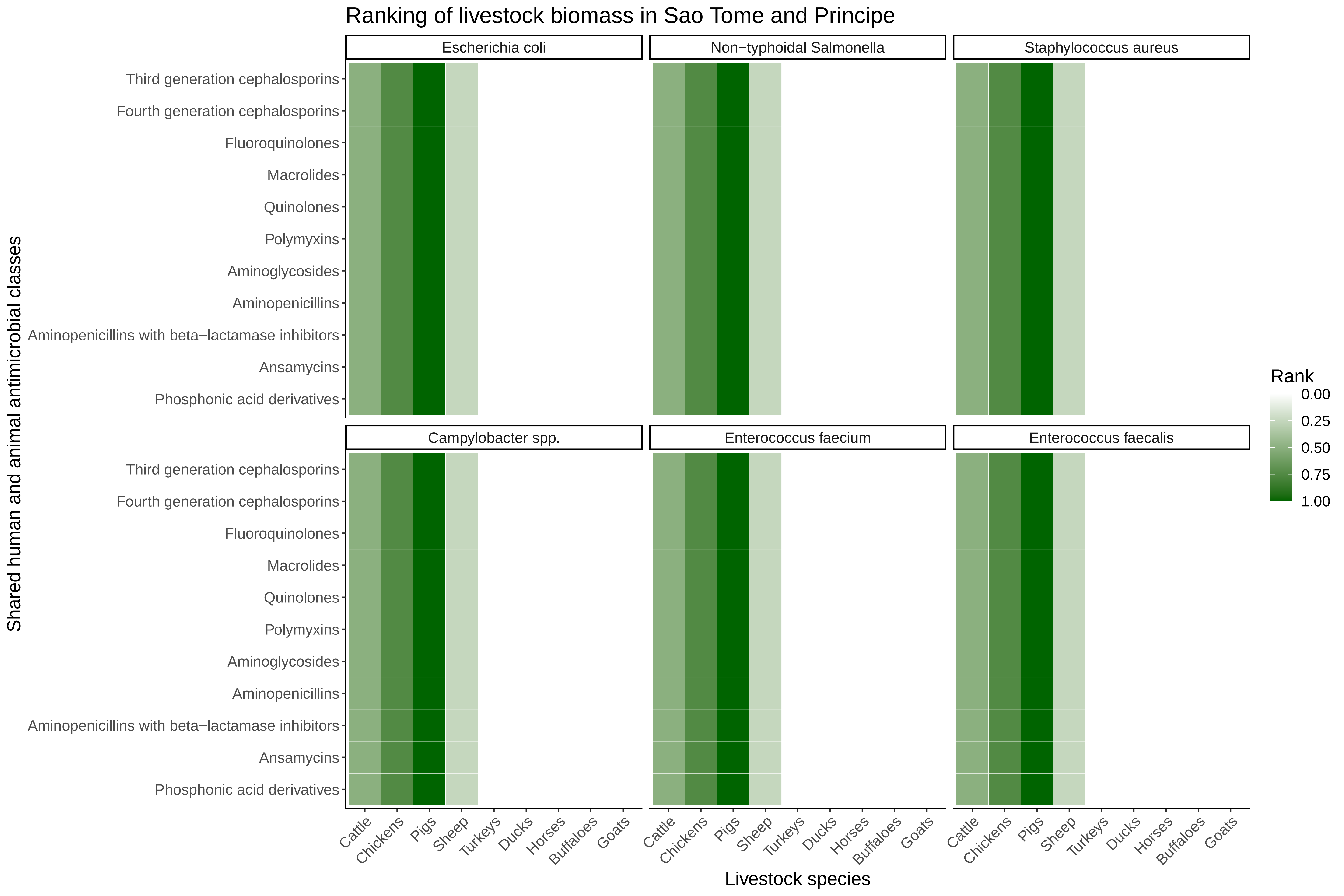

Ranking of livestock biomass in Sao Tome and Principe
Escherichia coli
Non−typhoidal Salmonella
Staphylococcus aureus
Campylobacter spp.
Enterococcus faecium
Enterococcus faecalis
Third generation cephalosporins
Fourth generation cephalosporins
Fluoroquinolones
Macrolides
Quinolones
Polymyxins
Aminoglycosides
Aminopenicillins
Aminopenicillins with beta−lactamase inhibitors
Ansamycins
Phosphonic acid derivatives
Shared human and animal antimicrobial classes
Cattle
Chickens
Pigs
Sheep
Turkeys
Ducks
Horses
Buffaloes
Goats
Livestock species
Rank
0.00
0.25
0.50
0.75
1.00

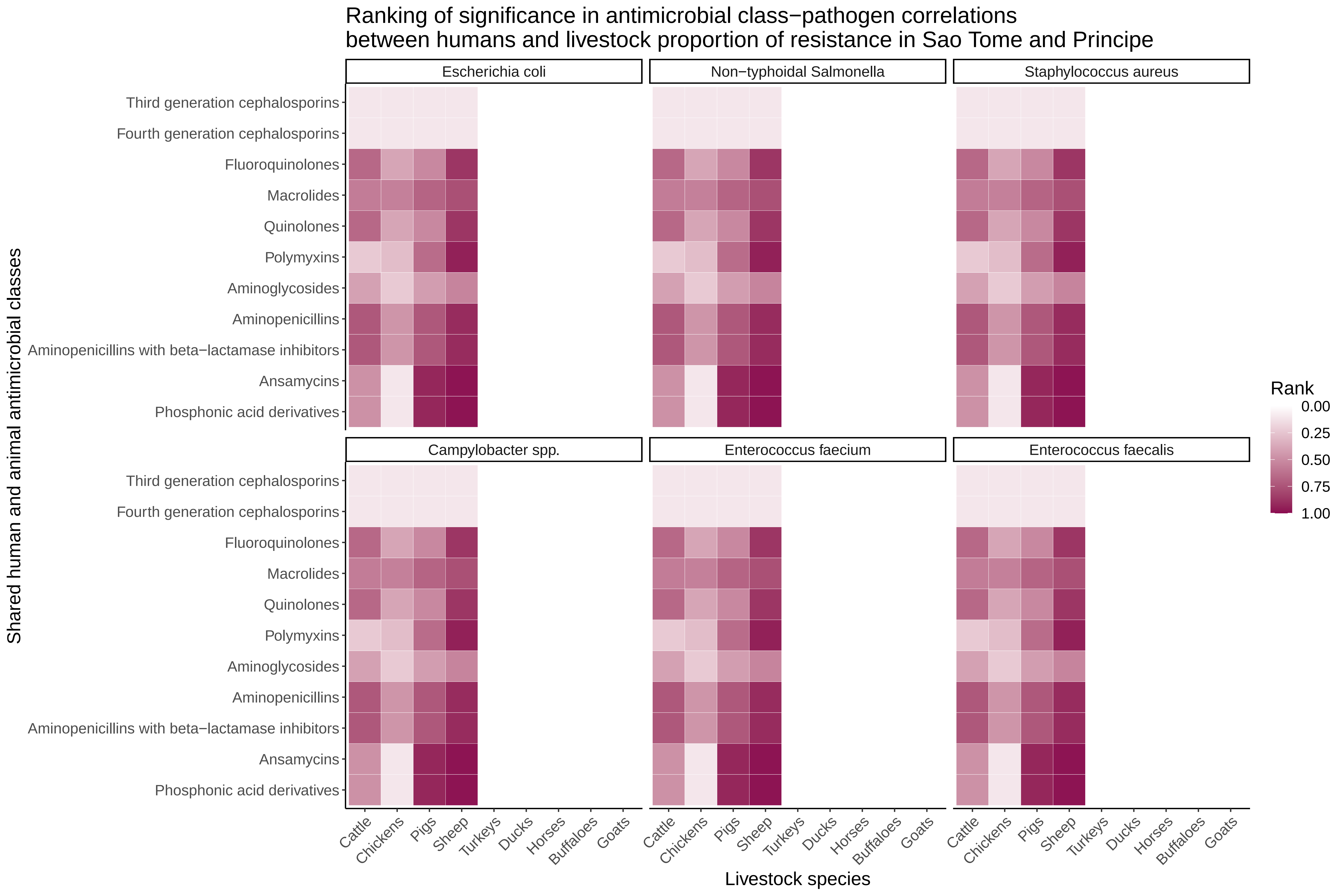

Ranking of significance in antimicrobial class−pathogen correlations
between humans and livestock proportion of resistance in Sao Tome and Principe
Escherichia coli
Non−typhoidal Salmonella
Staphylococcus aureus
Campylobacter spp.
Enterococcus faecium
Enterococcus faecalis
Third generation cephalosporins
Fourth generation cephalosporins
Fluoroquinolones
Macrolides
Quinolones
Polymyxins
Aminoglycosides
Aminopenicillins
Aminopenicillins with beta−lactamase inhibitors
Ansamycins
Phosphonic acid derivatives
Shared human and animal antimicrobial classes
Cattle
Chickens
Pigs
Sheep
Turkeys
Ducks
Horses
Buffaloes
Goats
Livestock species
Rank
0.00
0.25
0.50
0.75
1.00

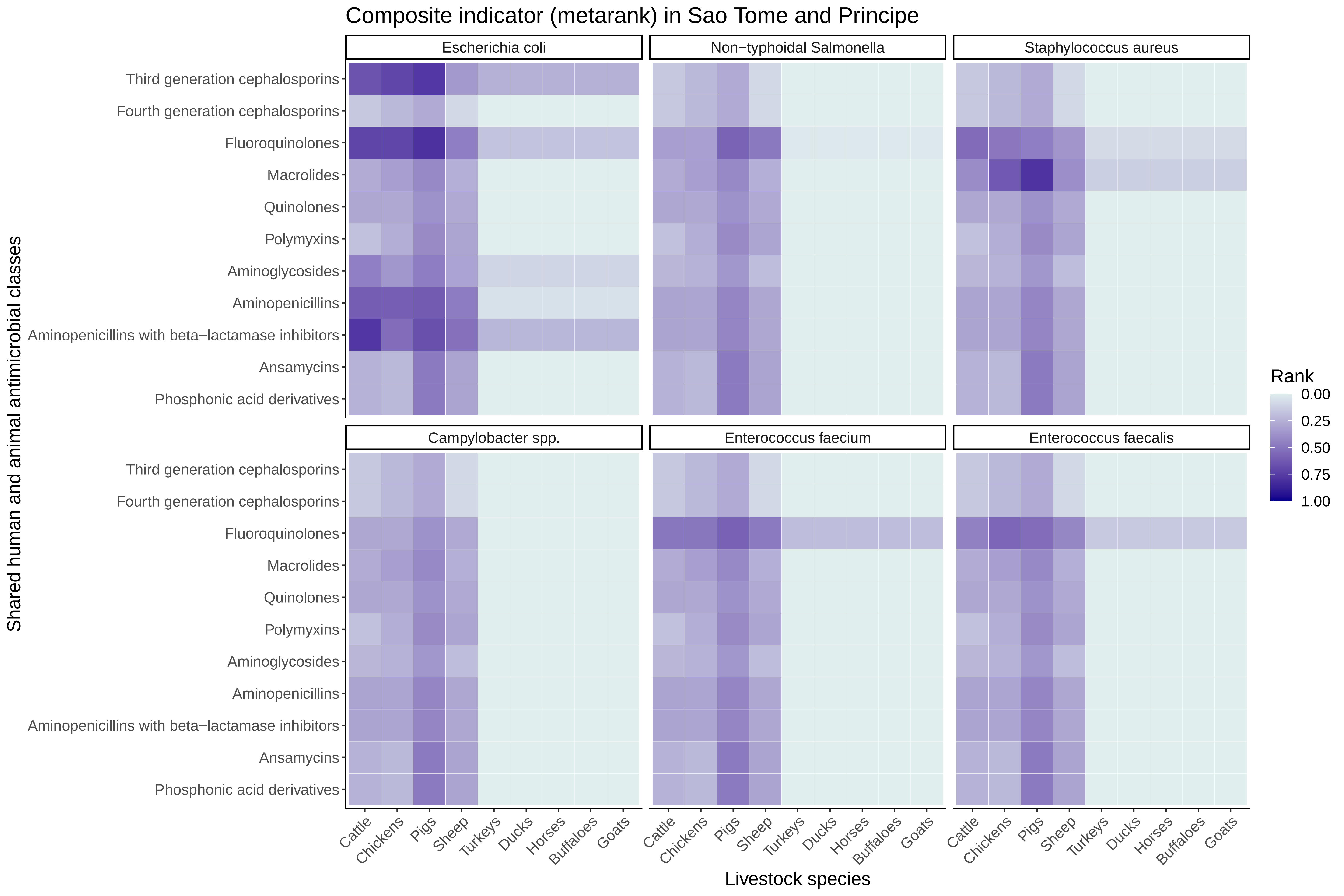

Composite indicator (metarank) in Sao Tome and Principe
Escherichia coli
Non−typhoidal Salmonella
Staphylococcus aureus
Campylobacter spp.
Enterococcus faecium
Enterococcus faecalis
Third generation cephalosporins
Fourth generation cephalosporins
Fluoroquinolones
Macrolides
Quinolones
Polymyxins
Aminoglycosides
Aminopenicillins
Aminopenicillins with beta−lactamase inhibitors
Ansamycins
Phosphonic acid derivatives
Shared human and animal antimicrobial classes
Cattle
Chickens
Pigs
Sheep
Turkeys
Ducks
Horses
Buffaloes
Goats
Livestock species
Rank
0.00
0.25
0.50
0.75
1.00

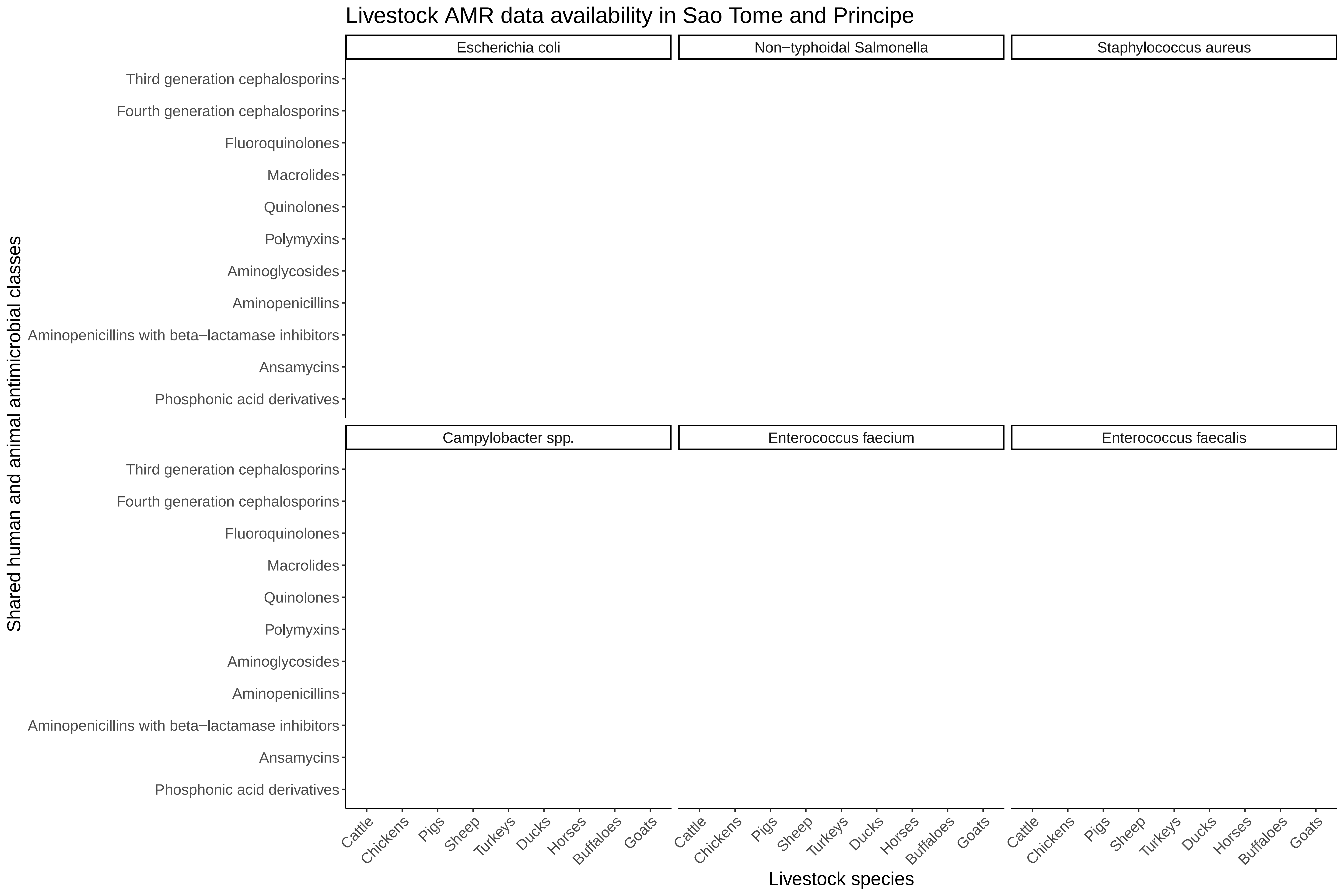
Livestock AMR data availability in Sao Tome and Principe
Escherichia coli
Non−typhoidal Salmonella
Staphylococcus aureus
Campylobacter spp.
Enterococcus faecium
Enterococcus faecalis
Shared human and animal antimicrobial classes
Third generation cephalosporins
Fourth generation cephalosporins
Fluoroquinolones
Macrolides
Quinolones
Polymyxins
Aminoglycosides
Aminopenicillins
Aminopenicillins with beta−lactamase inhibitors
Ansamycins
Phosphonic acid derivatives
Cattle
Chickens
Pigs
Sheep
Turkeys
Ducks
Horses
Buffaloes
Goats
Livestock species

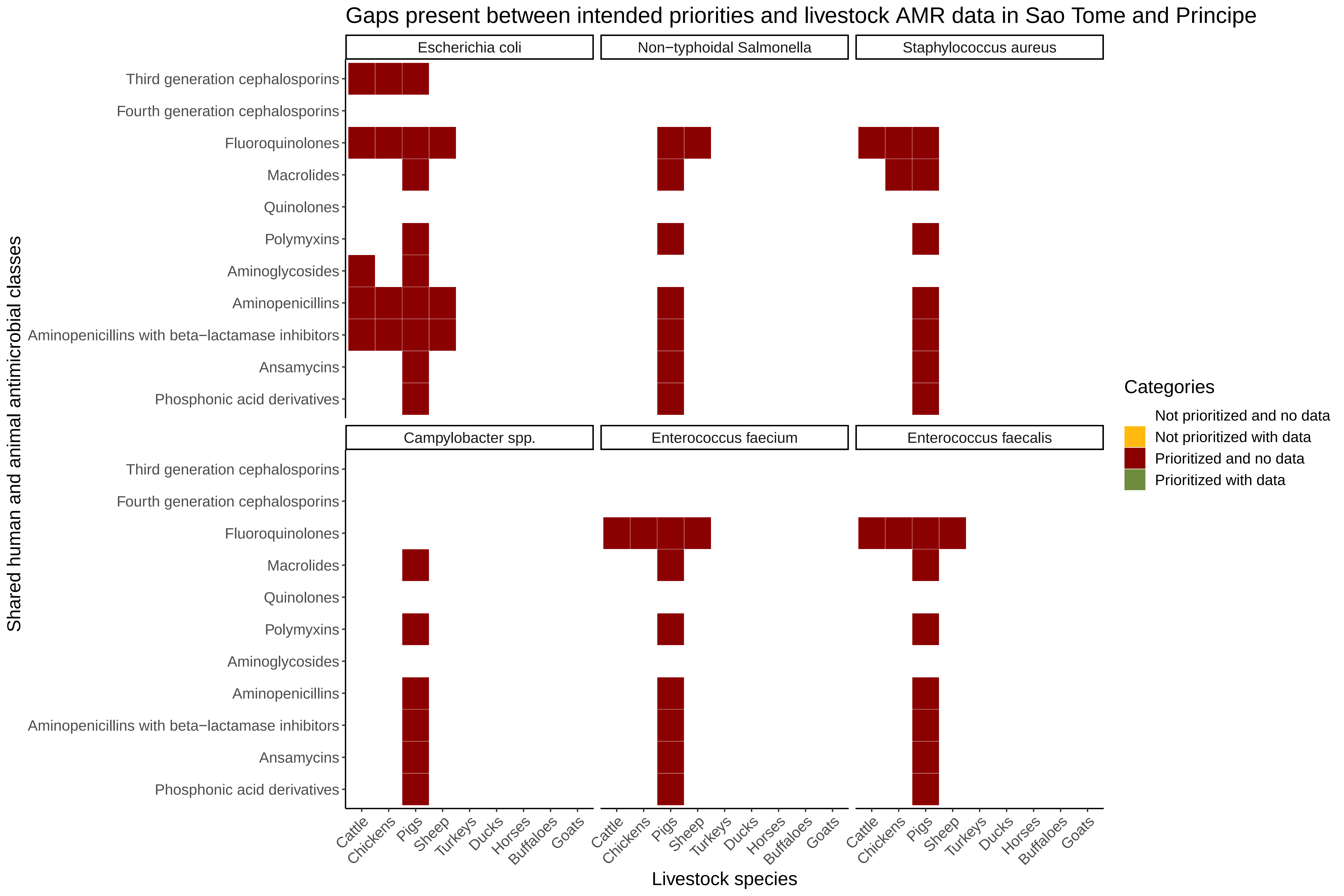

Gaps present between intended priorities and livestock AMR data in Sao Tome and Principe
Escherichia coli
Non−typhoidal Salmonella
Staphylococcus aureus
Campylobacter spp.
Enterococcus faecium
Enterococcus faecalis
Third generation cephalosporins
Fourth generation cephalosporins
Fluoroquinolones
Macrolides
Quinolones
Polymyxins
Aminoglycosides
Aminopenicillins
Aminopenicillins with beta−lactamase inhibitors
Ansamycins
Phosphonic acid derivatives
Shared human and animal antimicrobial classes
Cattle
Chickens
Pigs
Sheep
Turkeys
Ducks
Horses
Buffaloes
Goats
Livestock species
Categories
Not prioritized and no data
Not prioritized with data
Prioritized and no data
Prioritized with data

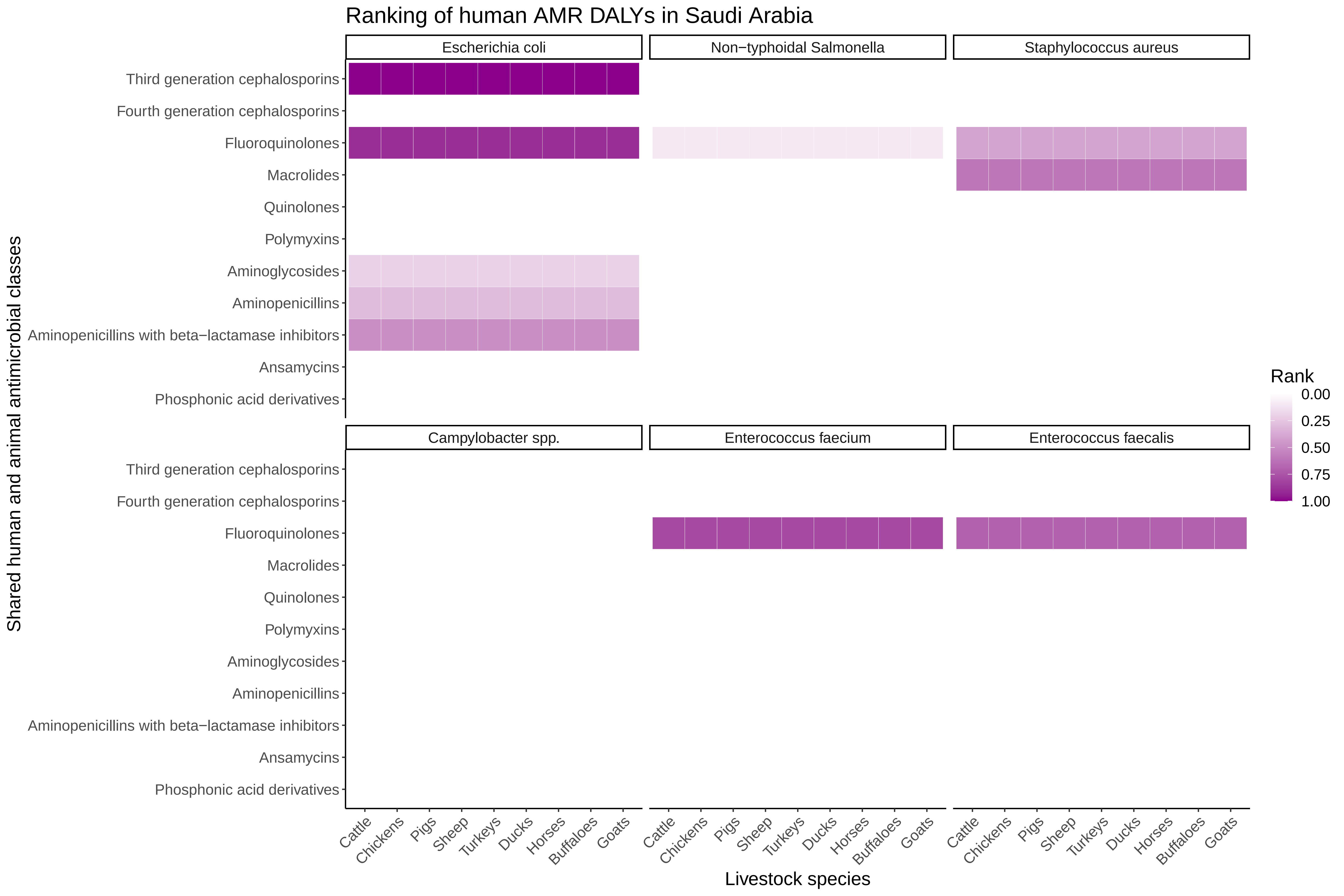
Ranking of human AMR DALYs in Saudi Arabia
Escherichia coli
Non−typhoidal Salmonella
Staphylococcus aureus
Campylobacter spp.
Enterococcus faecium
Enterococcus faecalis
Third generation cephalosporins
Fourth generation cephalosporins
Fluoroquinolones
Macrolides
Quinolones
Polymyxins
Aminoglycosides
Aminopenicillins
Aminopenicillins with beta−lactamase inhibitors
Ansamycins
Phosphonic acid derivatives
Shared human and animal antimicrobial classes
Cattle
Chickens
Pigs
Sheep
Turkeys
Ducks
Horses
Buffaloes
Goats
Livestock species
Rank
0.00
0.25
0.50
0.75
1.00

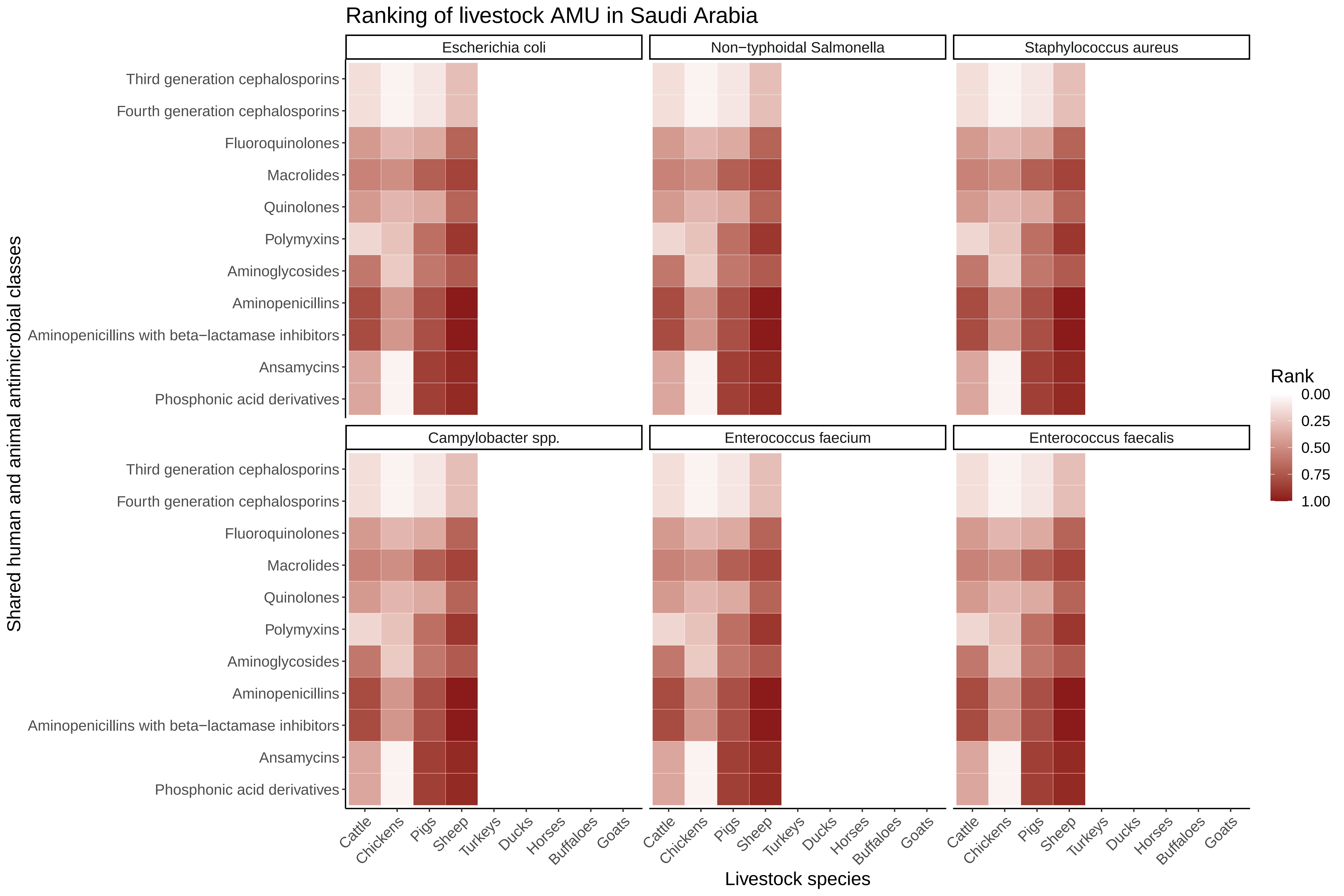

Ranking of livestock AMU in Saudi Arabia
Escherichia coli
Non−typhoidal Salmonella
Staphylococcus aureus
Campylobacter spp.
Enterococcus faecium
Enterococcus faecalis
Third generation cephalosporins
Fourth generation cephalosporins
Fluoroquinolones
Macrolides
Quinolones
Polymyxins
Aminoglycosides
Aminopenicillins
Aminopenicillins with beta−lactamase inhibitors
Ansamycins
Phosphonic acid derivatives
Shared human and animal antimicrobial classes
Cattle
Chickens
Pigs
Sheep
Turkeys
Ducks
Horses
Buffaloes
Goats
Livestock species
Rank
0.00
0.25
0.50
0.75
1.00

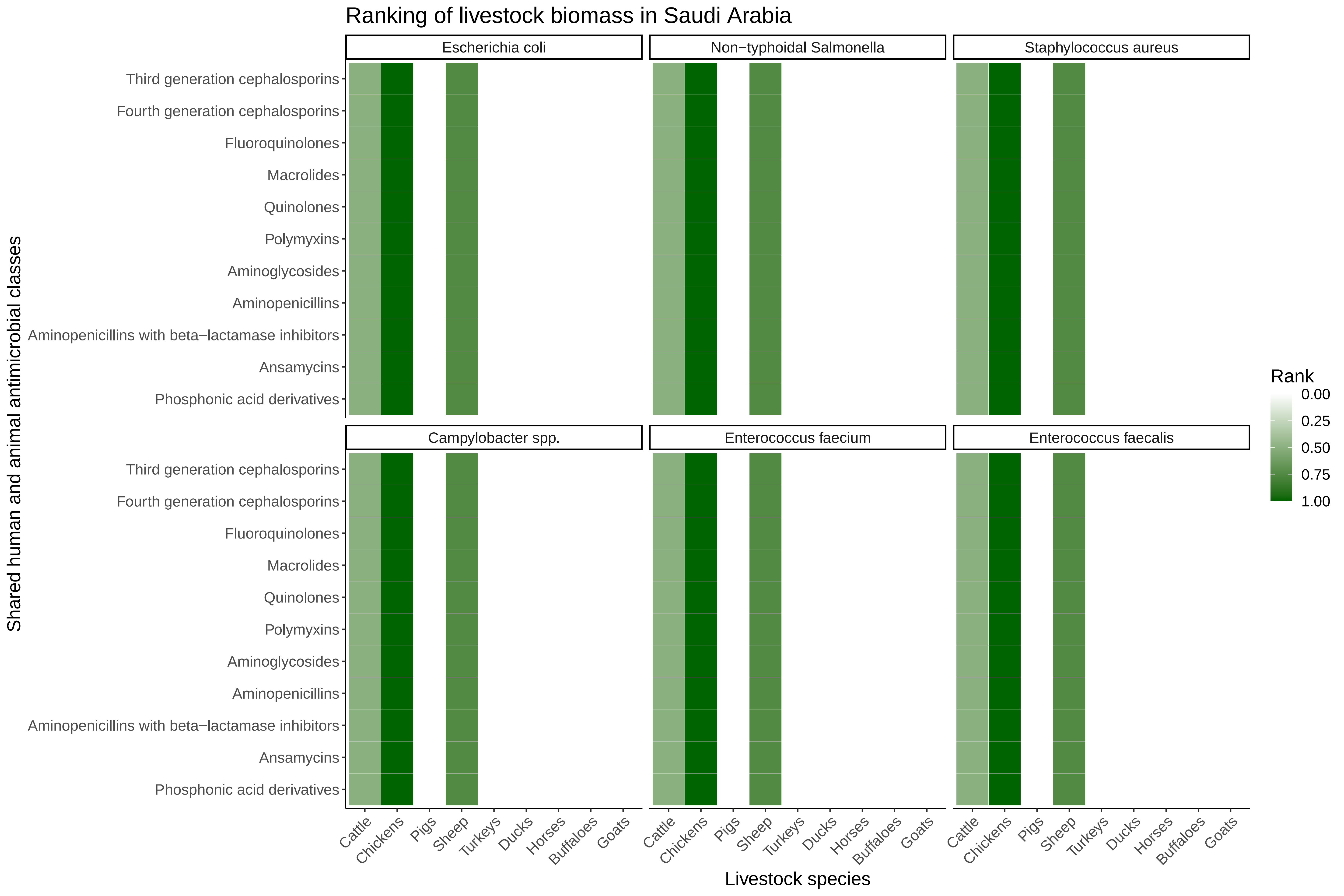

Ranking of livestock biomass in Saudi Arabia
Escherichia coli
Non−typhoidal Salmonella
Staphylococcus aureus
Campylobacter spp.
Enterococcus faecium
Enterococcus faecalis
Shared human and animal antimicrobial classes
Third generation cephalosporins
Fourth generation cephalosporins
Fluoroquinolones
Macrolides
Quinolones
Polymyxins
Aminoglycosides
Aminopenicillins
Aminopenicillins with beta−lactamase inhibitors
Ansamycins
Phosphonic acid derivatives
Cattle
Chickens
Pigs
Sheep
Turkeys
Ducks
Horses
Buffaloes
Goats
Livestock species
Rank
0.00
0.25
0.50
0.75
1.00

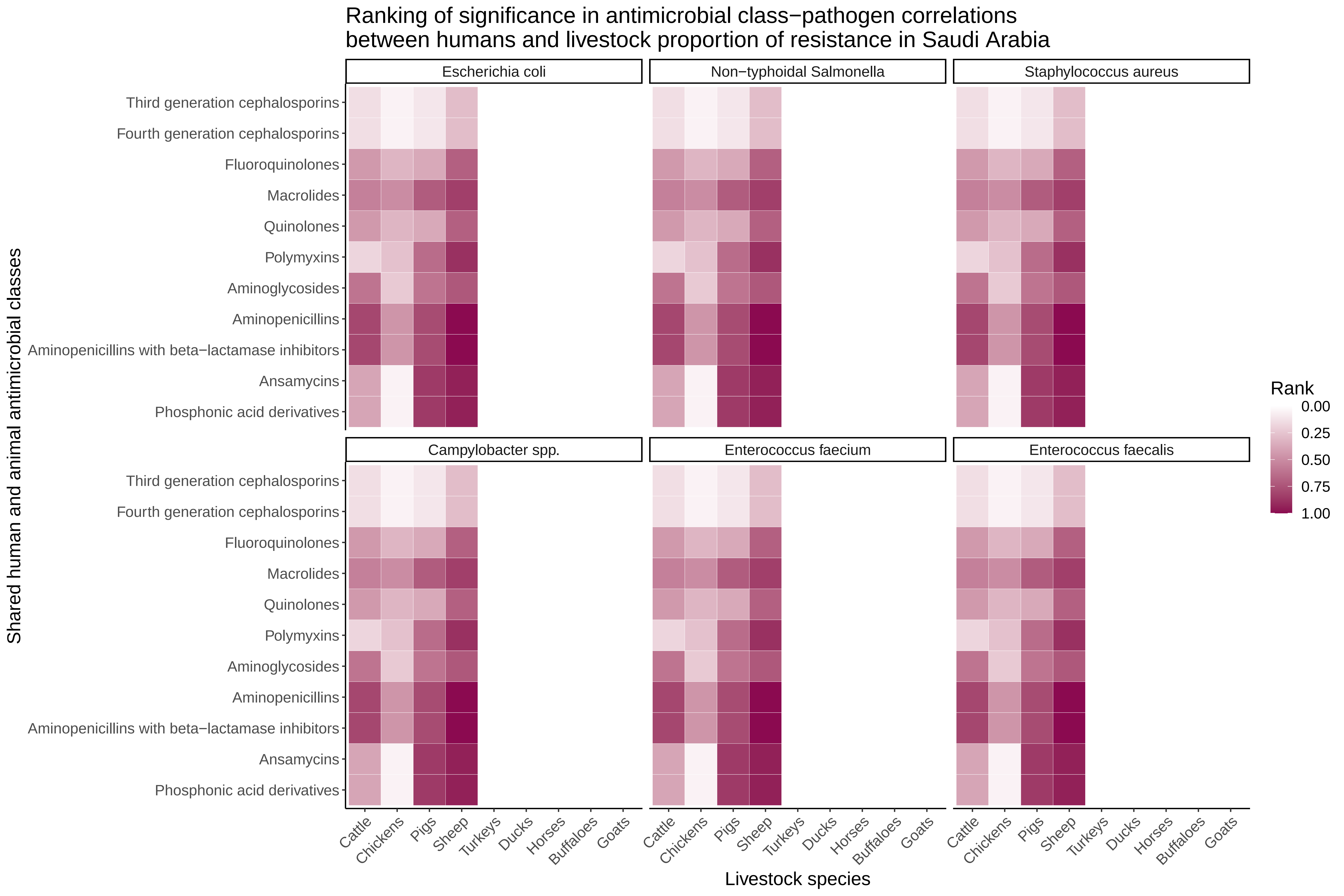

Ranking of significance in antimicrobial class−pathogen correlations
between humans and livestock proportion of resistance in Saudi Arabia
Escherichia coli
Non−typhoidal Salmonella
Staphylococcus aureus
Campylobacter spp.
Enterococcus faecium
Enterococcus faecalis
Shared human and animal antimicrobial classes
Third generation cephalosporins
Fourth generation cephalosporins
Fluoroquinolones
Macrolides
Quinolones
Polymyxins
Aminoglycosides
Aminopenicillins
Aminopenicillins with beta−lactamase inhibitors
Ansamycins
Phosphonic acid derivatives
Livestock species
Cattle
Chickens
Pigs
Sheep
Turkeys
Ducks
Horses
Buffaloes
Goats
Rank
0.00
0.25
0.50
0.75
1.00

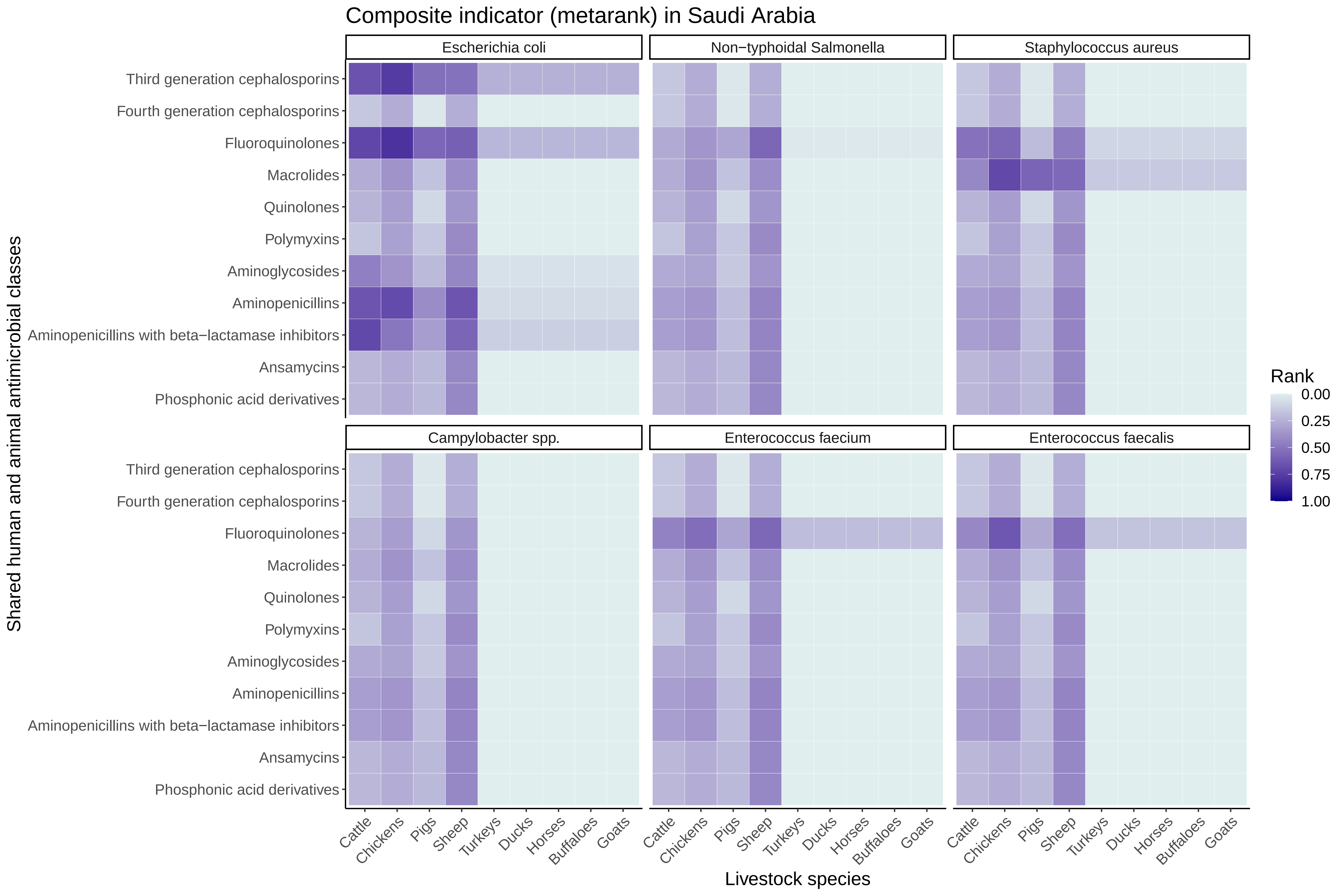

Composite indicator (metarank) in Saudi Arabia
Escherichia coli
Non−typhoidal Salmonella
Staphylococcus aureus
Campylobacter spp.
Enterococcus faecium
Enterococcus faecalis
Third generation cephalosporins
Fourth generation cephalosporins
Fluoroquinolones
Macrolides
Quinolones
Polymyxins
Aminoglycosides
Aminopenicillins
Aminopenicillins with beta−lactamase inhibitors
Ansamycins
Phosphonic acid derivatives
Shared human and animal antimicrobial classes
Cattle
Chickens
Pigs
Sheep
Turkeys
Ducks
Horses
Buffaloes
Goats
Livestock species
Rank
0.00
0.25
0.50
0.75
1.00

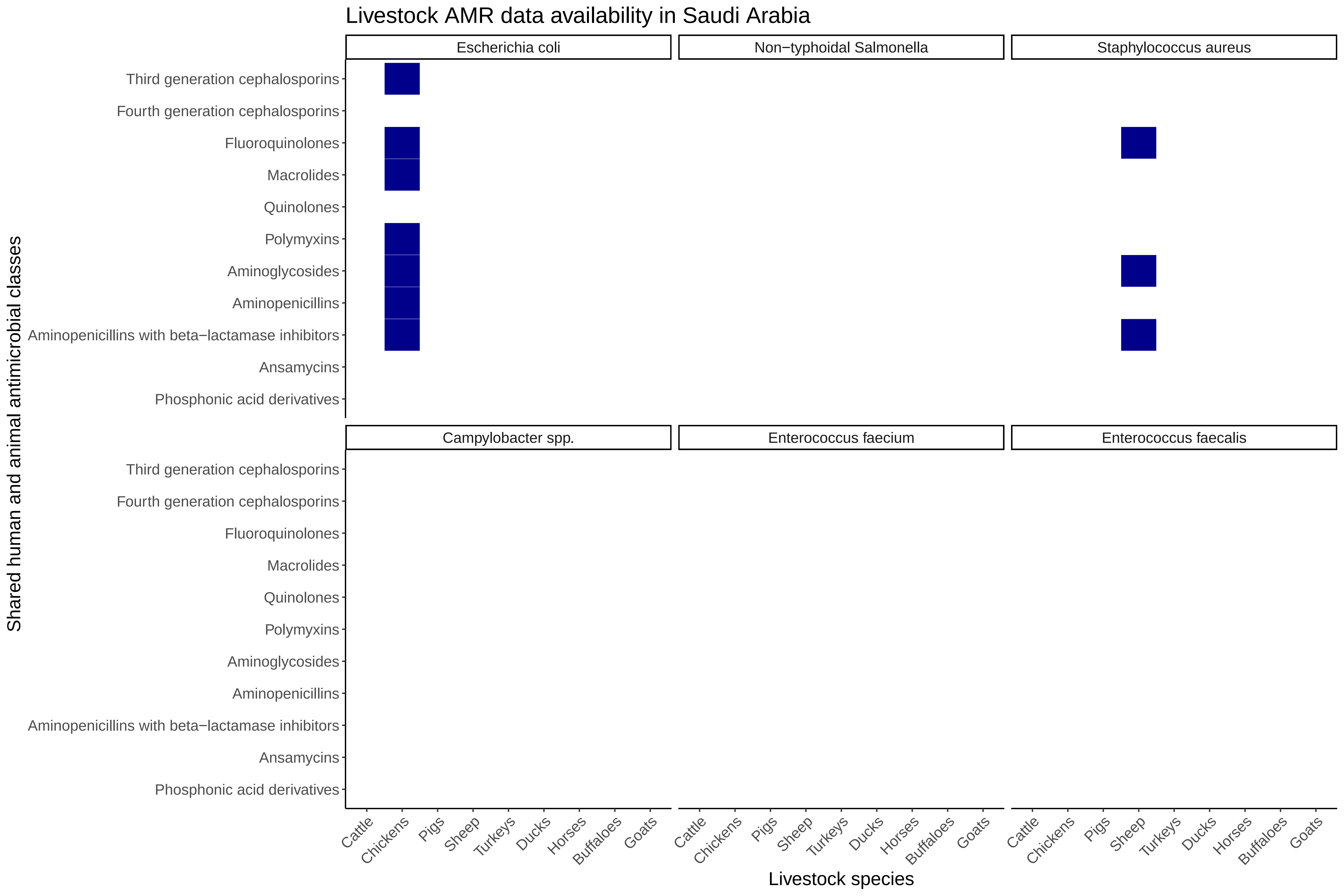

Livestock AMR data availability in Saudi Arabia
Escherichia coli
Non−typhoidal Salmonella
Staphylococcus aureus
Campylobacter spp.
Enterococcus faecium
Enterococcus faecalis
Third generation cephalosporins
Fourth generation cephalosporins
Fluoroquinolones
Macrolides
Quinolones
Polymyxins
Aminoglycosides
Aminopenicillins
Aminopenicillins with beta−lactamase inhibitors
Ansamycins
Phosphonic acid derivatives
Shared human and animal antimicrobial classes
Cattle
Chickens
Pigs
Sheep
Turkeys
Ducks
Horses
Buffaloes
Goats
Livestock species

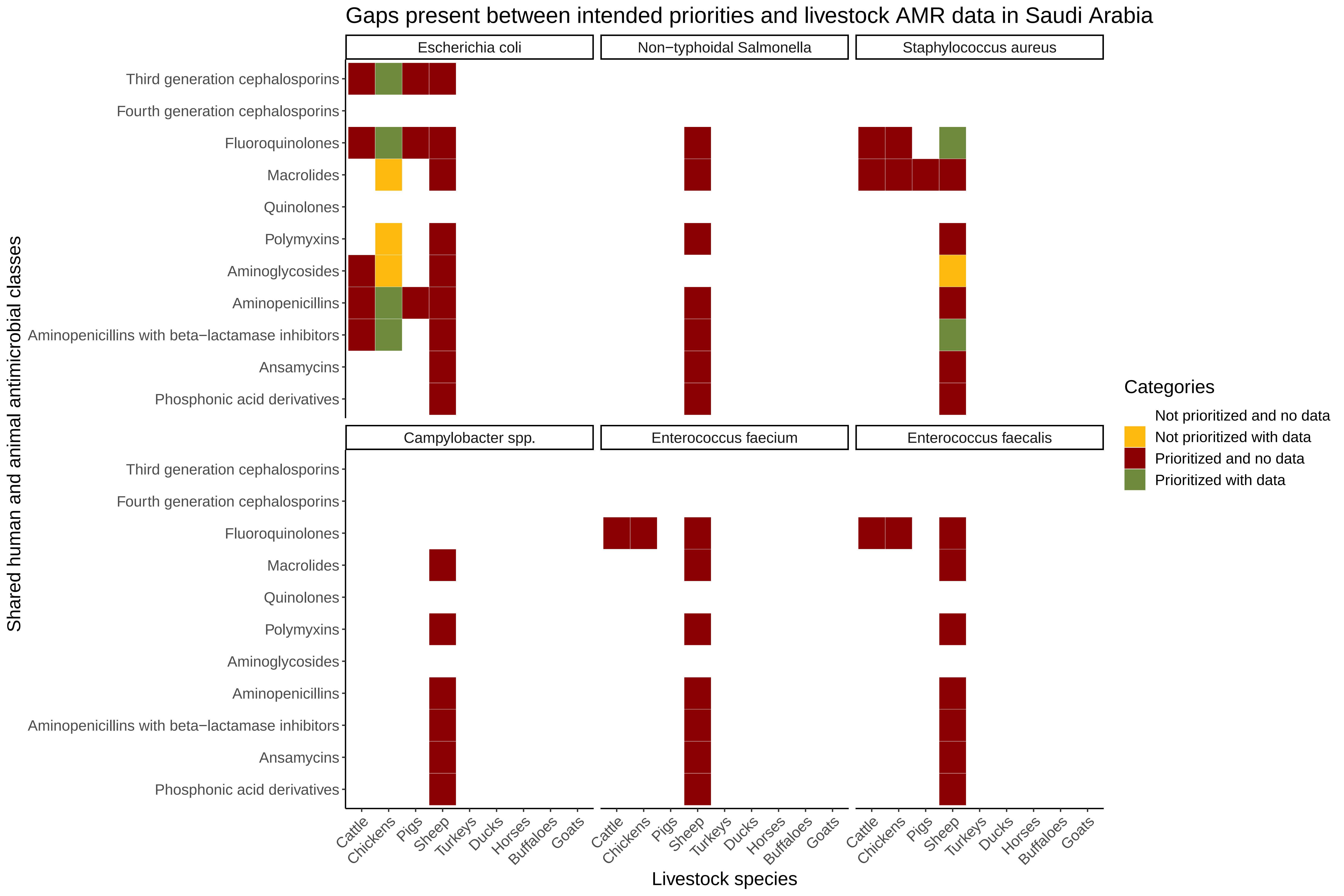

Gaps present between intended priorities and livestock AMR data in Saudi Arabia
Escherichia coli
Non−typhoidal Salmonella
Staphylococcus aureus
Campylobacter spp.
Enterococcus faecium
Enterococcus faecalis
Third generation cephalosporins
Fourth generation cephalosporins
Fluoroquinolones
Macrolides
Quinolones
Polymyxins
Aminoglycosides
Aminopenicillins
Aminopenicillins with beta−lactamase inhibitors
Ansamycins
Phosphonic acid derivatives
Shared human and animal antimicrobial classes
Cattle
Chickens
Pigs
Sheep
Turkeys
Ducks
Horses
Buffaloes
Goats
Livestock species
Categories
Not prioritized and no data
Not prioritized with data
Prioritized and no data
Prioritized with data

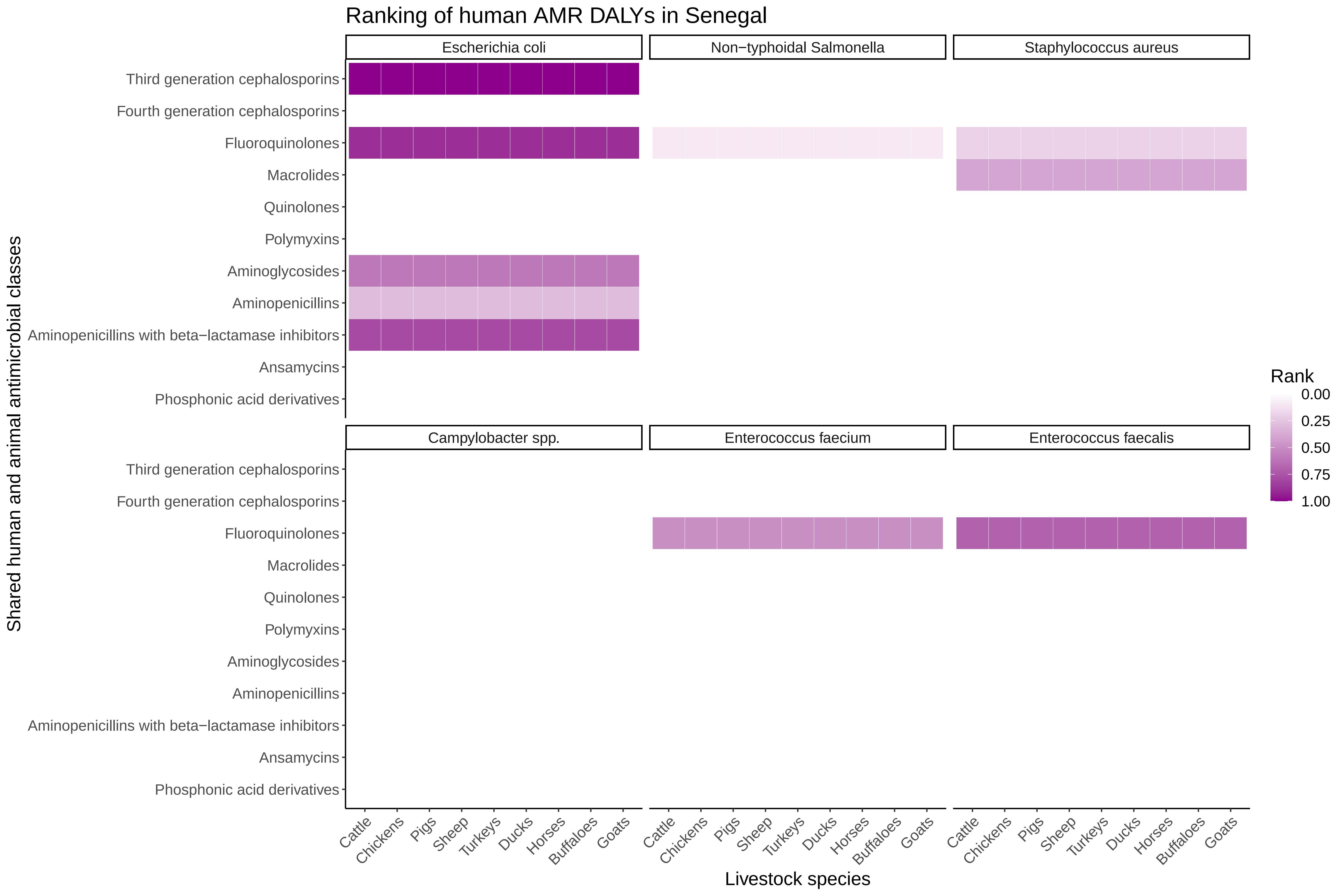
Ranking of human AMR DALYs in Senegal
Escherichia coli
Non−typhoidal Salmonella
Staphylococcus aureus
Campylobacter spp.
Enterococcus faecium
Enterococcus faecalis
Third generation cephalosporins
Fourth generation cephalosporins
Fluoroquinolones
Macrolides
Quinolones
Polymyxins
Aminoglycosides
Aminopenicillins
Aminopenicillins with beta−lactamase inhibitors
Ansamycins
Phosphonic acid derivatives
Shared human and animal antimicrobial classes
Cattle
Chickens
Pigs
Sheep
Turkeys
Ducks
Horses
Buffaloes
Goats
Livestock species
Rank
0.00
0.25
0.50
0.75
1.00

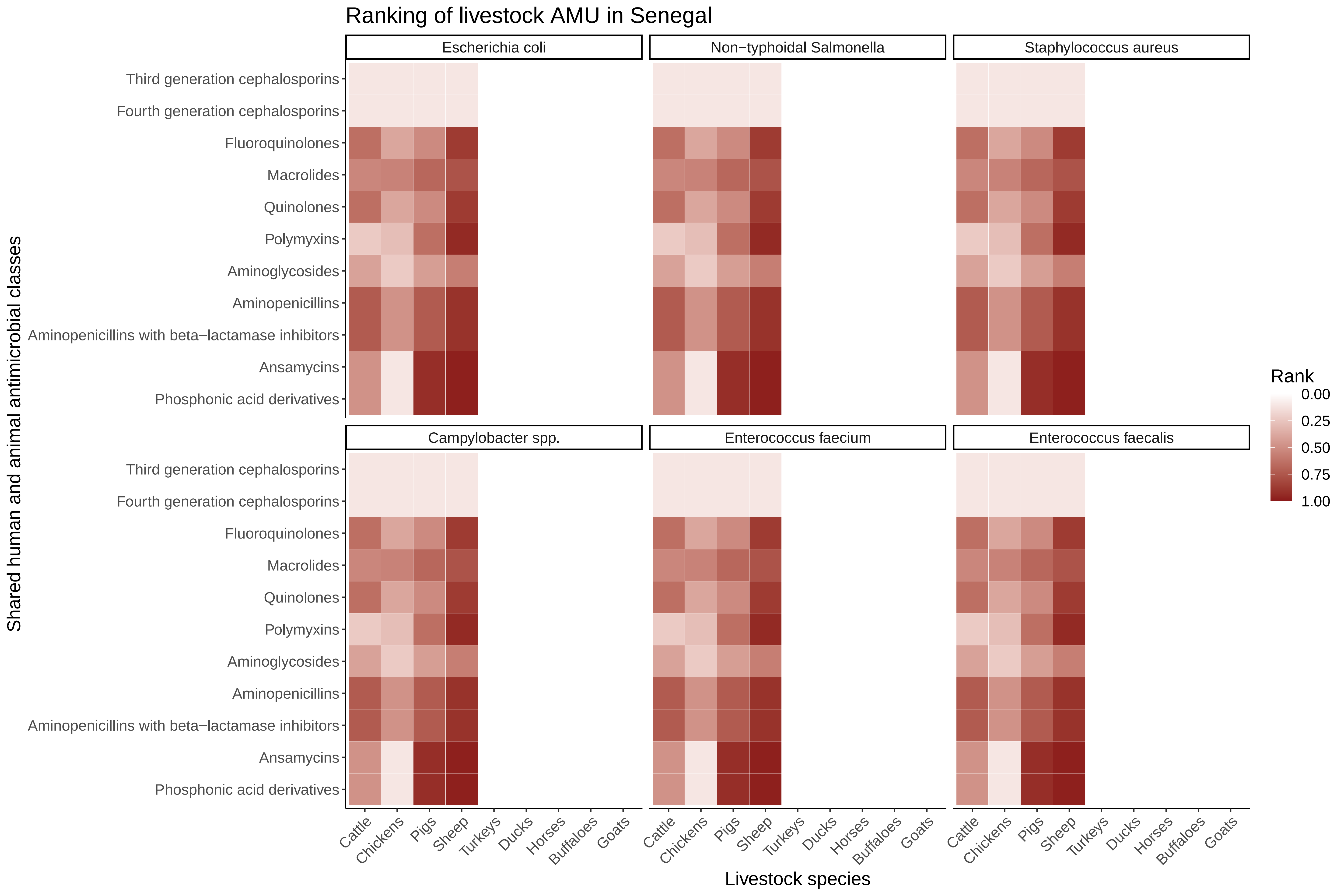
Ranking of livestock AMU in Senegal
Escherichia coli
Non−typhoidal Salmonella
Staphylococcus aureus
Campylobacter spp.
Enterococcus faecium
Enterococcus faecalis
Shared human and animal antimicrobial classes
Third generation cephalosporins
Fourth generation cephalosporins
Fluoroquinolones
Macrolides
Quinolones
Polymyxins
Aminoglycosides
Aminopenicillins
Aminopenicillins with beta−lactamase inhibitors
Ansamycins
Phosphonic acid derivatives
Rank
0.00
0.25
0.50
0.75
1.00
Cattle
Chickens
Pigs
Sheep
Turkeys
Ducks
Horses
Buffaloes
Goats
Livestock species

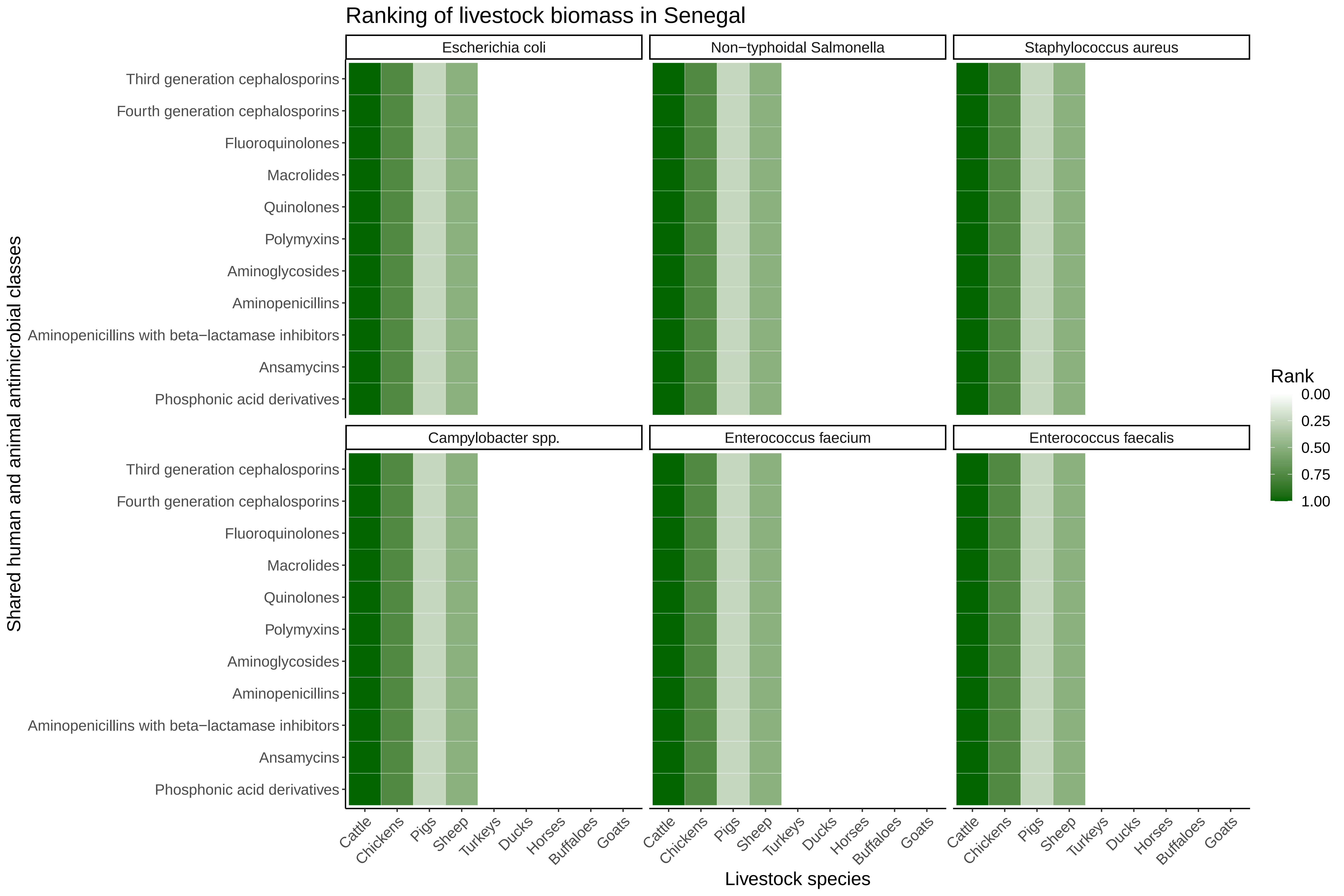

Ranking of livestock biomass in Senegal
Escherichia coli
Non−typhoidal Salmonella
Staphylococcus aureus
Campylobacter spp.
Enterococcus faecium
Enterococcus faecalis
Third generation cephalosporins
Fourth generation cephalosporins
Fluoroquinolones
Macrolides
Quinolones
Polymyxins
Aminoglycosides
Aminopenicillins
Aminopenicillins with beta−lactamase inhibitors
Ansamycins
Phosphonic acid derivatives
Shared human and animal antimicrobial classes
Cattle
Chickens
Pigs
Sheep
Turkeys
Ducks
Horses
Buffaloes
Goats
Livestock species
Rank
0.00
0.25
0.50
0.75
1.00

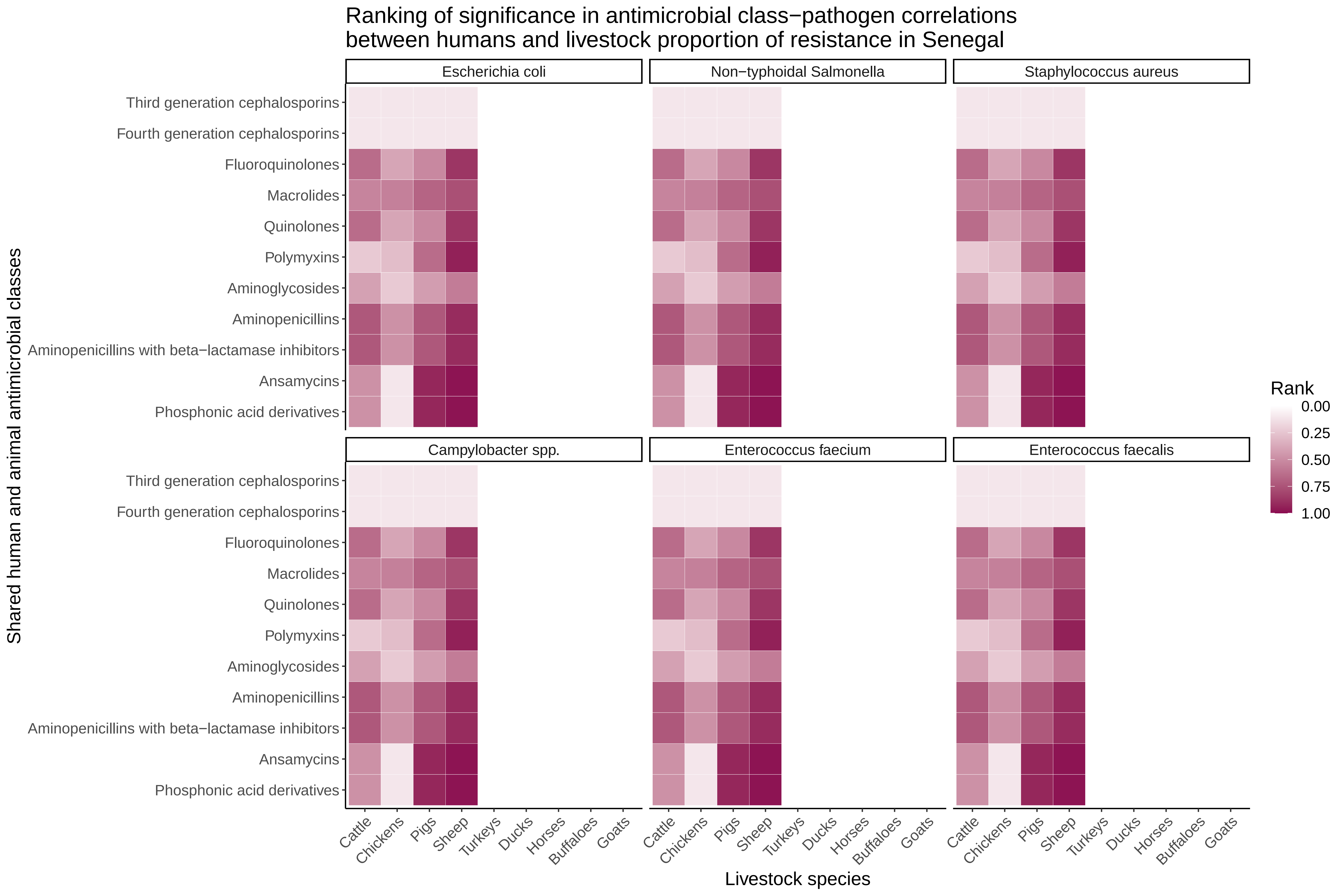
Ranking of significance in antimicrobial class−pathogen correlations
between humans and livestock proportion of resistance in Senegal
Escherichia coli
Non−typhoidal Salmonella
Staphylococcus aureus
Campylobacter spp.
Enterococcus faecium
Enterococcus faecalis
Third generation cephalosporins
Fourth generation cephalosporins
Fluoroquinolones
Macrolides
Quinolones
Polymyxins
Aminoglycosides
Aminopenicillins
Aminopenicillins with beta−lactamase inhibitors
Ansamycins
Phosphonic acid derivatives
Shared human and animal antimicrobial classes
Cattle
Chickens
Pigs
Sheep
Turkeys
Ducks
Horses
Buffaloes
Goats
Livestock species
Rank
0.00
0.25
0.50
0.75
1.00

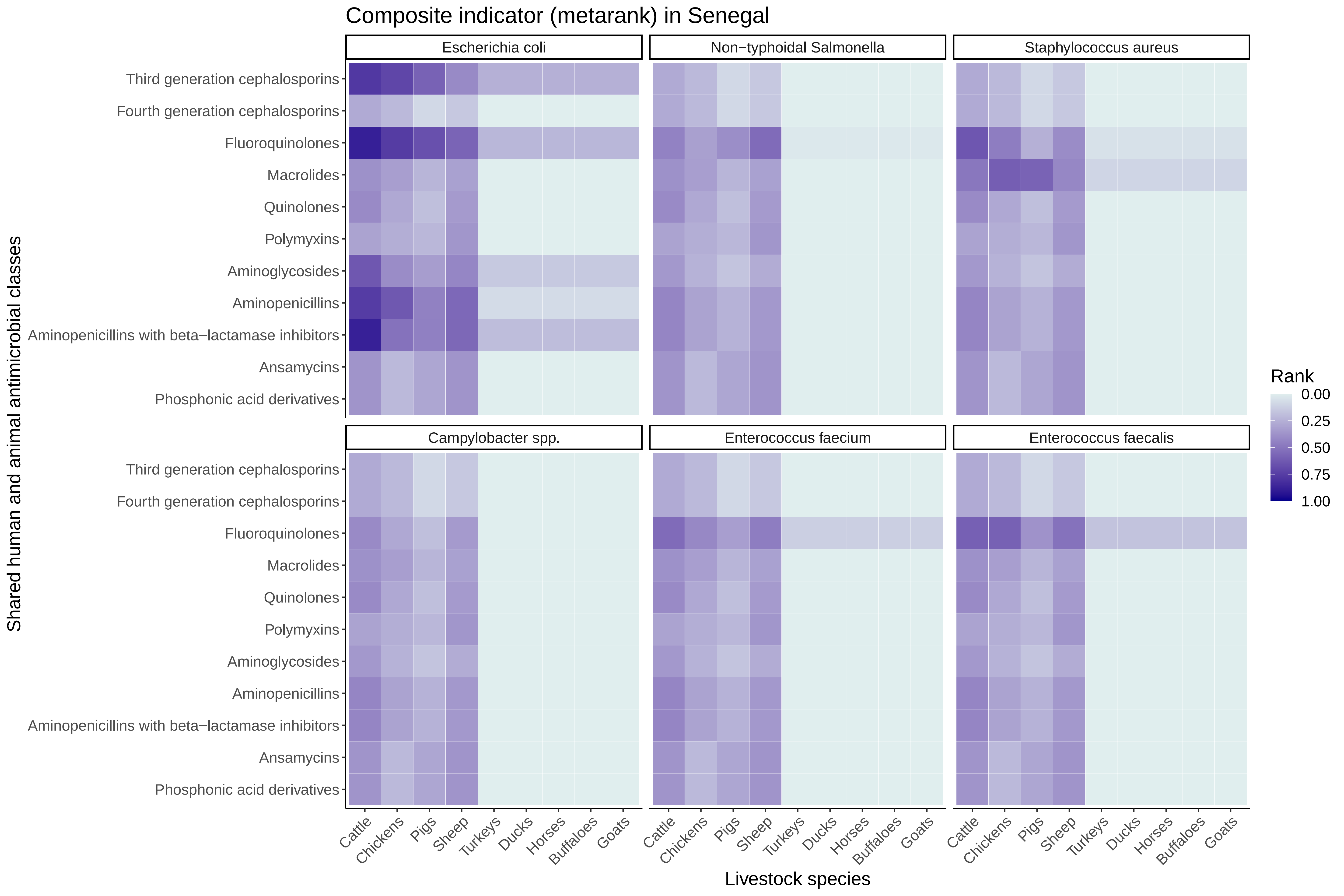

Composite indicator (metarank) in Senegal
Escherichia coli
Non−typhoidal Salmonella
Staphylococcus aureus
Campylobacter spp.
Enterococcus faecium
Enterococcus faecalis
Shared human and animal antimicrobial classes
Third generation cephalosporins
Fourth generation cephalosporins
Fluoroquinolones
Macrolides
Quinolones
Polymyxins
Aminoglycosides
Aminopenicillins
Aminopenicillins with beta−lactamase inhibitors
Ansamycins
Phosphonic acid derivatives
Cattle
Chickens
Pigs
Sheep
Turkeys
Ducks
Horses
Buffaloes
Goats
Livestock species
Rank
0.00
0.25
0.50
0.75
1.00

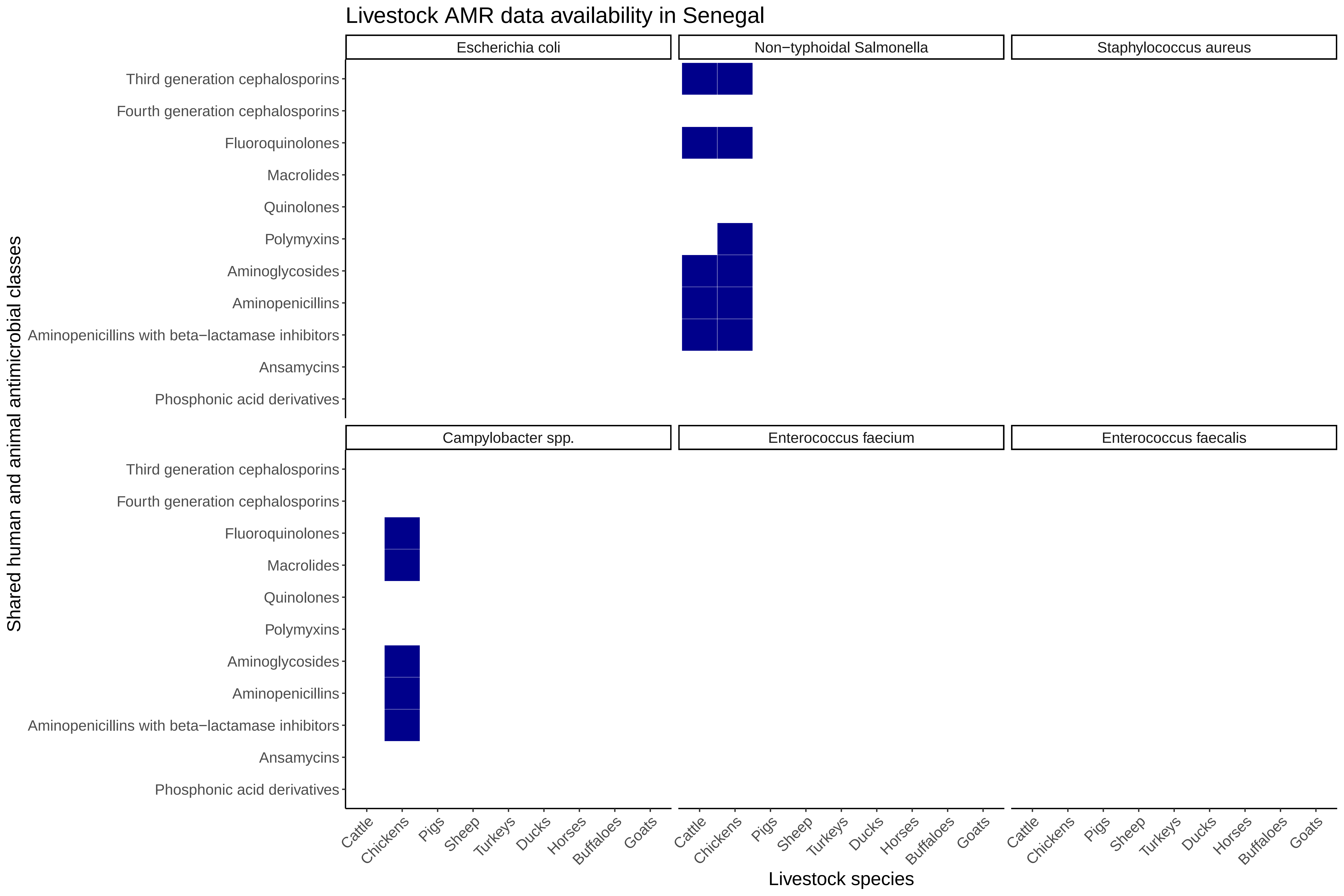

Livestock AMR data availability in Senegal
Escherichia coli
Non−typhoidal Salmonella
Staphylococcus aureus
Campylobacter spp.
Enterococcus faecium
Enterococcus faecalis
Shared human and animal antimicrobial classes
Third generation cephalosporins
Fourth generation cephalosporins
Fluoroquinolones
Macrolides
Quinolones
Polymyxins
Aminoglycosides
Aminopenicillins
Aminopenicillins with beta−lactamase inhibitors
Ansamycins
Phosphonic acid derivatives
Cattle
Chickens
Pigs
Sheep
Turkeys
Ducks
Horses
Buffaloes
Goats
Livestock species

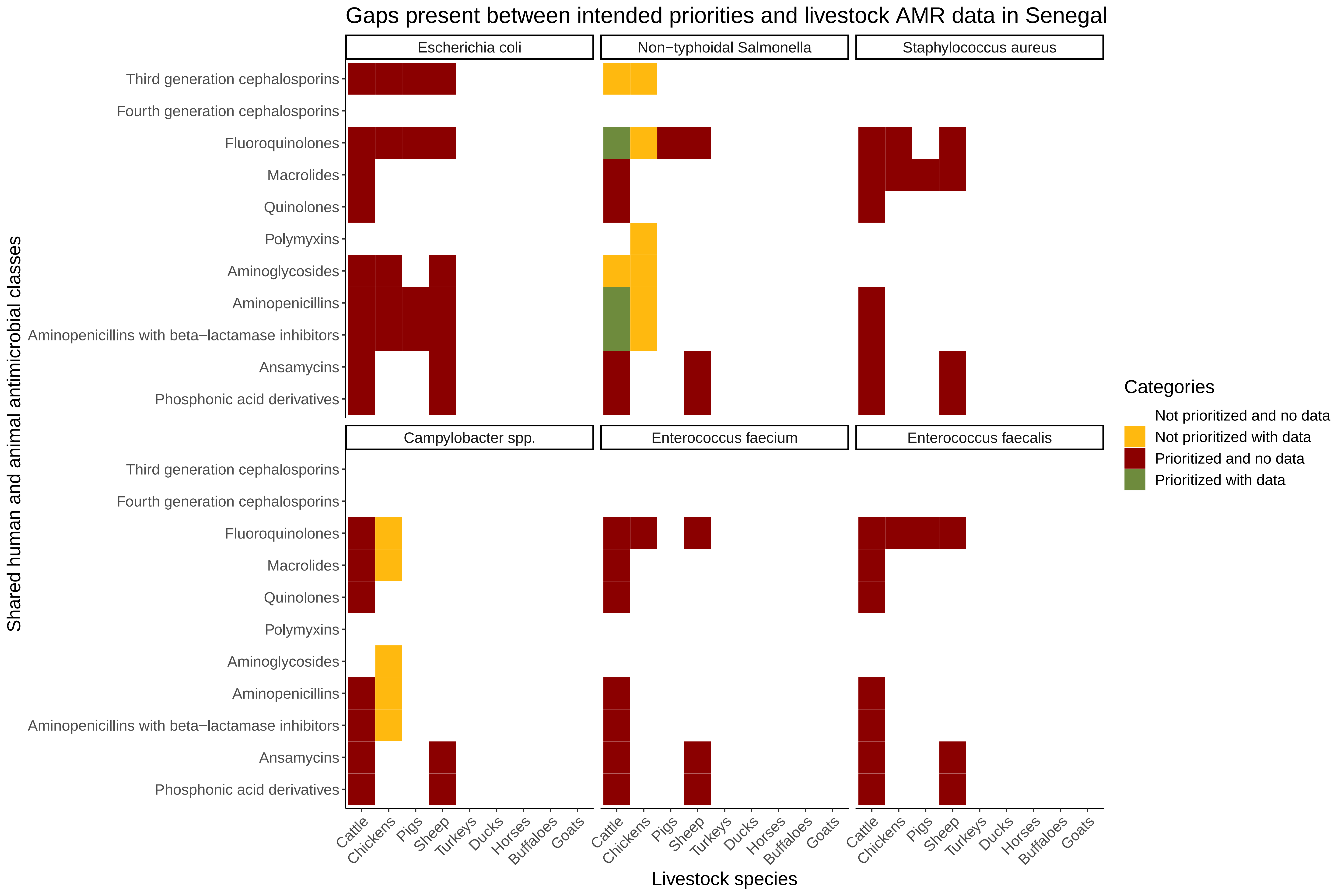

Gaps present between intended priorities and livestock AMR data in Senegal
Escherichia coli
Non−typhoidal Salmonella
Staphylococcus aureus
Campylobacter spp.
Enterococcus faecium
Enterococcus faecalis
Shared human and animal antimicrobial classes
Third generation cephalosporins
Fourth generation cephalosporins
Fluoroquinolones
Macrolides
Quinolones
Polymyxins
Aminoglycosides
Aminopenicillins
Aminopenicillins with beta−lactamase inhibitors
Ansamycins
Phosphonic acid derivatives
Livestock species
Cattle
Chickens
Pigs
Sheep
Turkeys
Ducks
Horses
Buffaloes
Goats
Categories
Not prioritized and no data
Not prioritized with data
Prioritized and no data
Prioritized with data

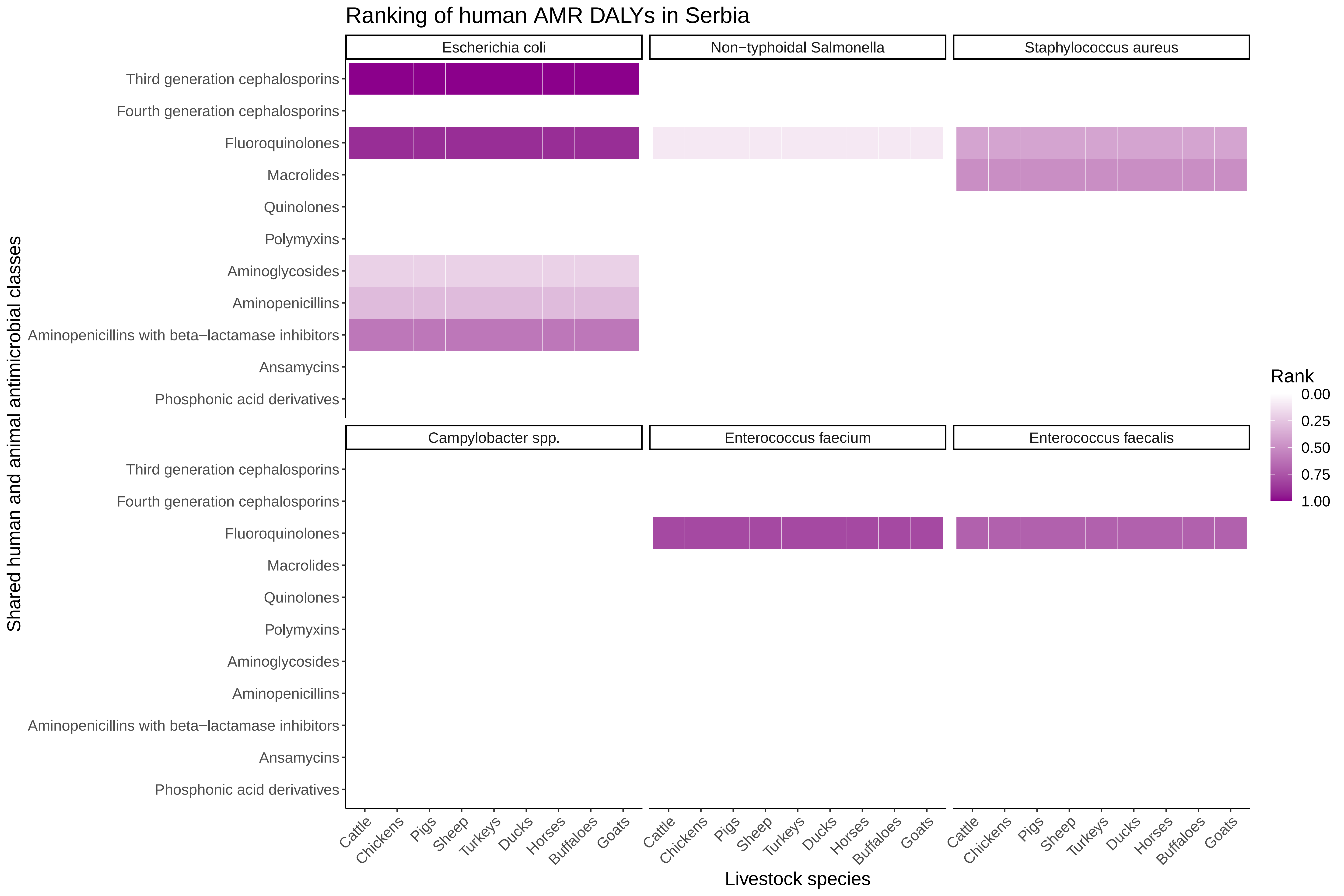

Ranking of human AMR DALYs in Serbia
Escherichia coli
Non−typhoidal Salmonella
Staphylococcus aureus
Campylobacter spp.
Enterococcus faecium
Enterococcus faecalis
Third generation cephalosporins
Fourth generation cephalosporins
Fluoroquinolones
Macrolides
Quinolones
Polymyxins
Aminoglycosides
Aminopenicillins
Aminopenicillins with beta−lactamase inhibitors
Ansamycins
Phosphonic acid derivatives
Shared human and animal antimicrobial classes
Cattle
Chickens
Pigs
Sheep
Turkeys
Ducks
Horses
Buffaloes
Goats
Livestock species
Rank
0.00
0.25
0.50
0.75
1.00

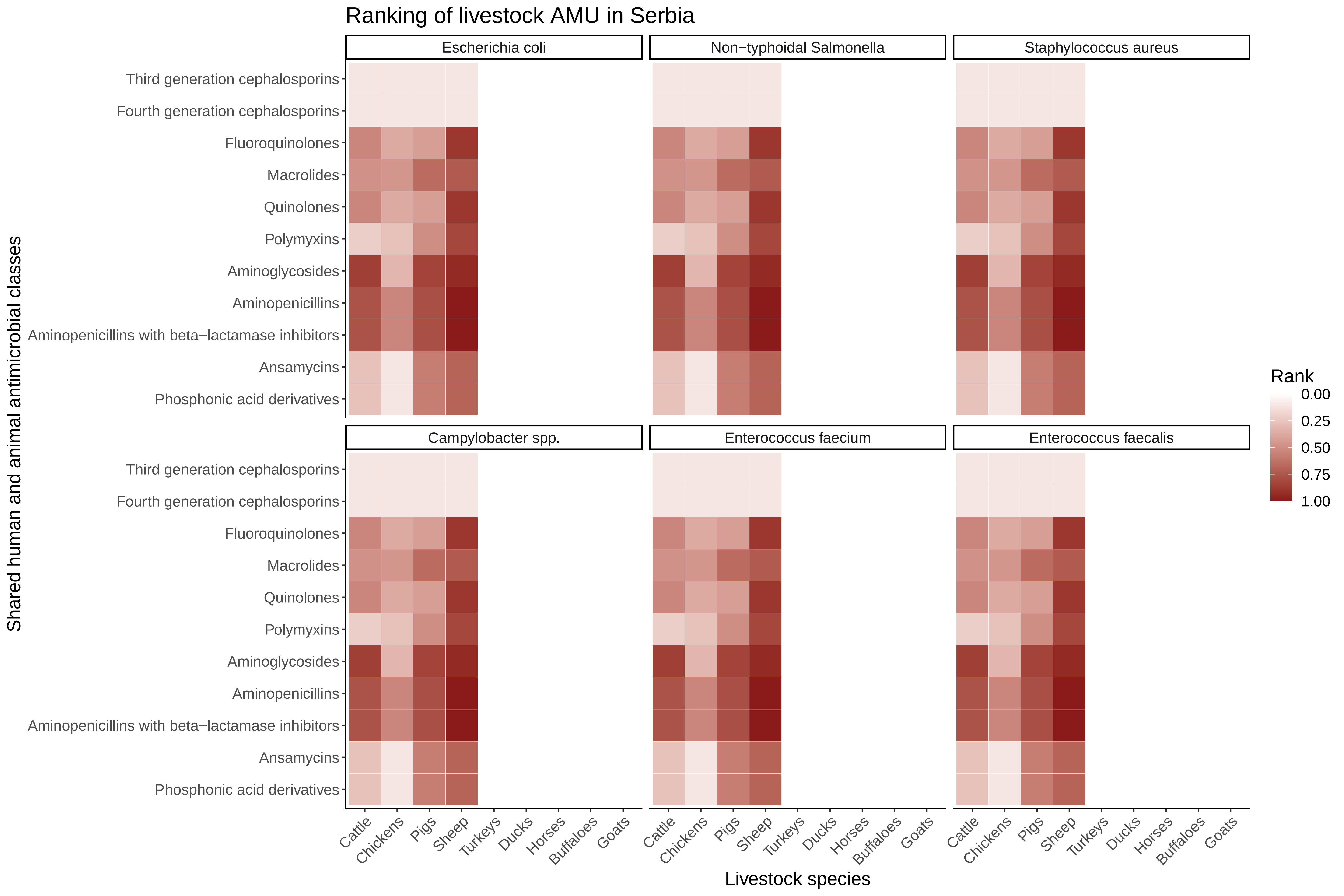

Ranking of livestock AMU in Serbia
Escherichia coli
Non−typhoidal Salmonella
Staphylococcus aureus
Campylobacter spp.
Enterococcus faecium
Enterococcus faecalis
Third generation cephalosporins
Fourth generation cephalosporins
Fluoroquinolones
Macrolides
Quinolones
Polymyxins
Aminoglycosides
Aminopenicillins
Aminopenicillins with beta−lactamase inhibitors
Ansamycins
Phosphonic acid derivatives
Shared human and animal antimicrobial classes
Cattle
Chickens
Pigs
Sheep
Turkeys
Ducks
Horses
Buffaloes
Goats
Livestock species
Rank
0.00
0.25
0.50
0.75
1.00

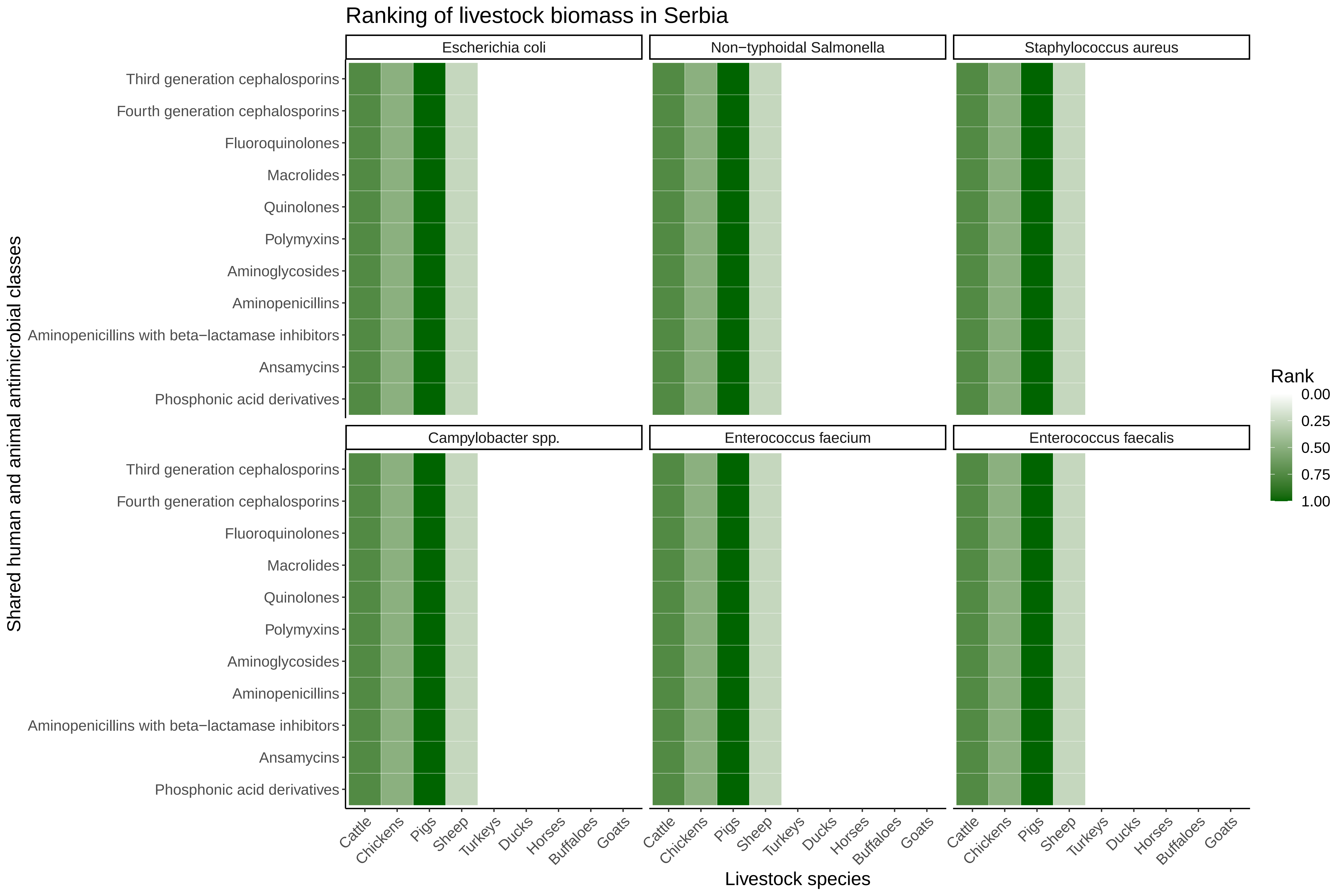

Ranking of livestock biomass in Serbia
Escherichia coli
Non−typhoidal Salmonella
Staphylococcus aureus
Campylobacter spp.
Enterococcus faecium
Enterococcus faecalis
Third generation cephalosporins
Fourth generation cephalosporins
Fluoroquinolones
Macrolides
Quinolones
Polymyxins
Aminoglycosides
Aminopenicillins
Aminopenicillins with beta−lactamase inhibitors
Ansamycins
Phosphonic acid derivatives
Shared human and animal antimicrobial classes
Cattle
Chickens
Pigs
Sheep
Turkeys
Ducks
Horses
Buffaloes
Goats
Livestock species
Rank
0.00
0.25
0.50
0.75
1.00

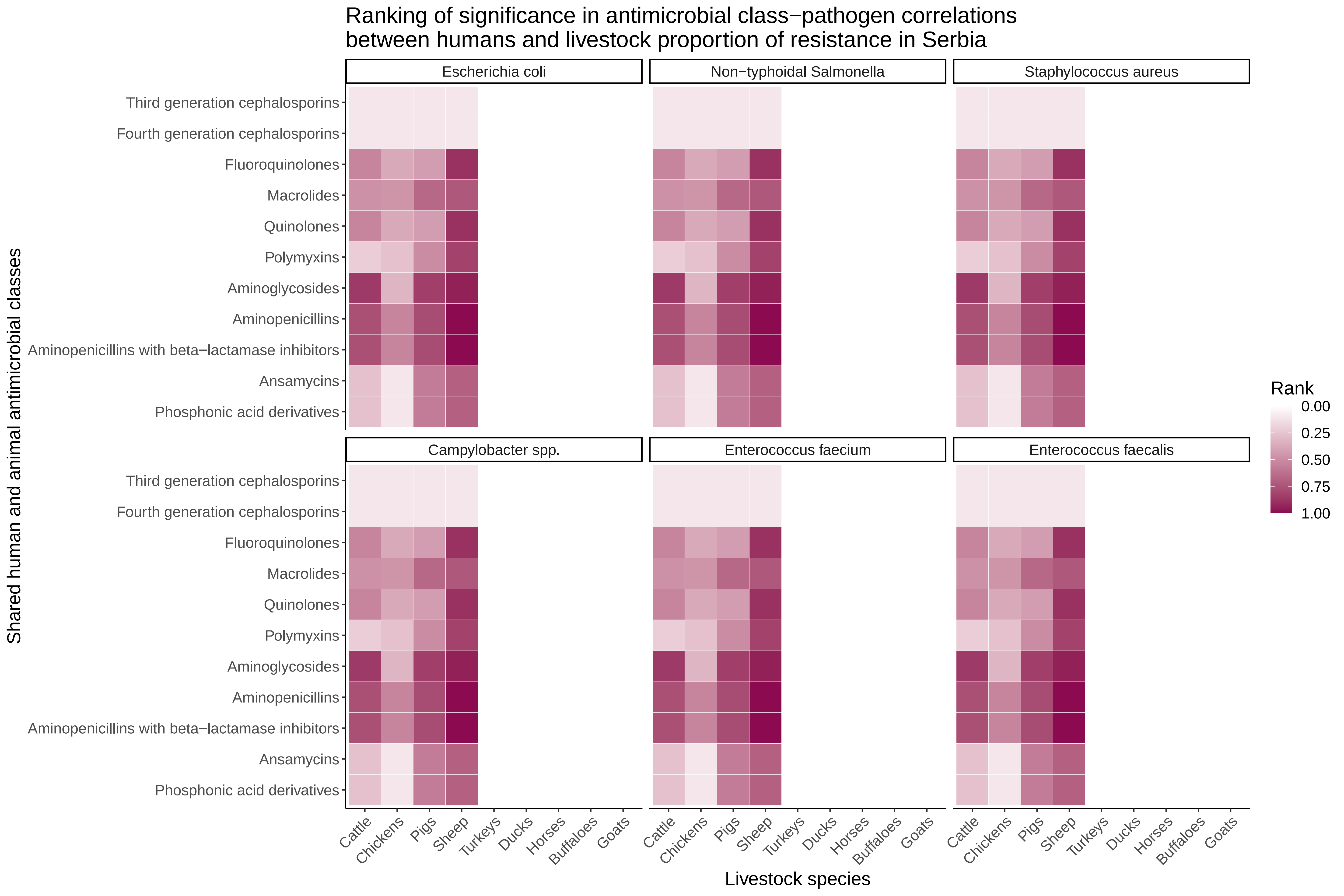

Ranking of significance in antimicrobial class−pathogen correlations
between humans and livestock proportion of resistance in Serbia
Escherichia coli
Non−typhoidal Salmonella
Staphylococcus aureus
Campylobacter spp.
Enterococcus faecium
Enterococcus faecalis
Third generation cephalosporins
Fourth generation cephalosporins
Fluoroquinolones
Macrolides
Quinolones
Polymyxins
Aminoglycosides
Aminopenicillins
Aminopenicillins with beta−lactamase inhibitors
Ansamycins
Phosphonic acid derivatives
Shared human and animal antimicrobial classes
Cattle
Chickens
Pigs
Sheep
Turkeys
Ducks
Horses
Buffaloes
Goats
Livestock species
Rank
0.00
0.25
0.50
0.75
1.00

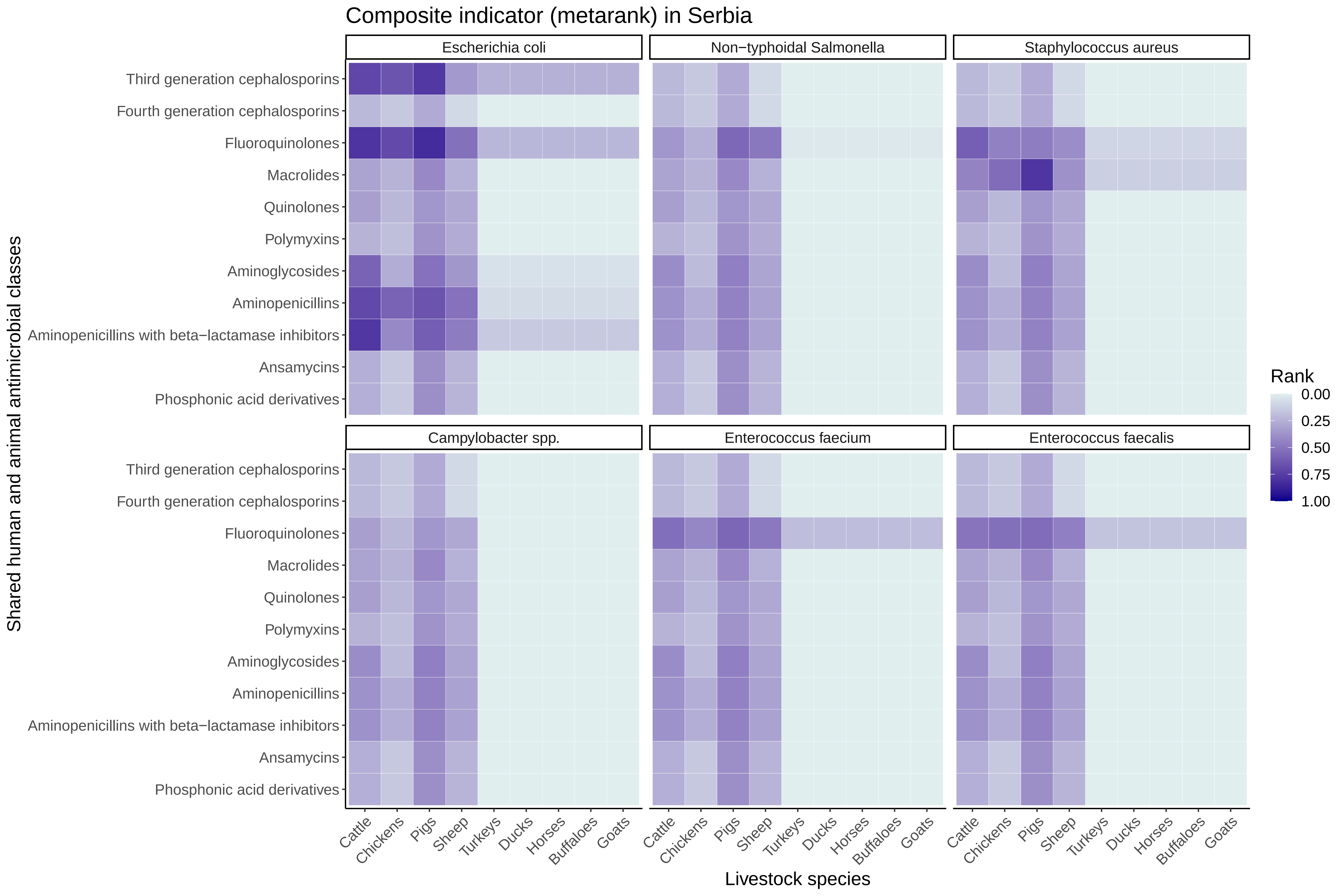
Composite indicator (metarank) in Serbia
Escherichia coli
Non−typhoidal Salmonella
Staphylococcus aureus
Campylobacter spp.
Enterococcus faecium
Enterococcus faecalis
Shared human and animal antimicrobial classes
Third generation cephalosporins
Fourth generation cephalosporins
Fluoroquinolones
Macrolides
Quinolones
Polymyxins
Aminoglycosides
Aminopenicillins
Aminopenicillins with beta−lactamase inhibitors
Ansamycins
Phosphonic acid derivatives
Livestock species
Cattle
Chickens
Pigs
Sheep
Turkeys
Ducks
Horses
Buffaloes
Goats
Rank
0.00
0.25
0.50
0.75
1.00

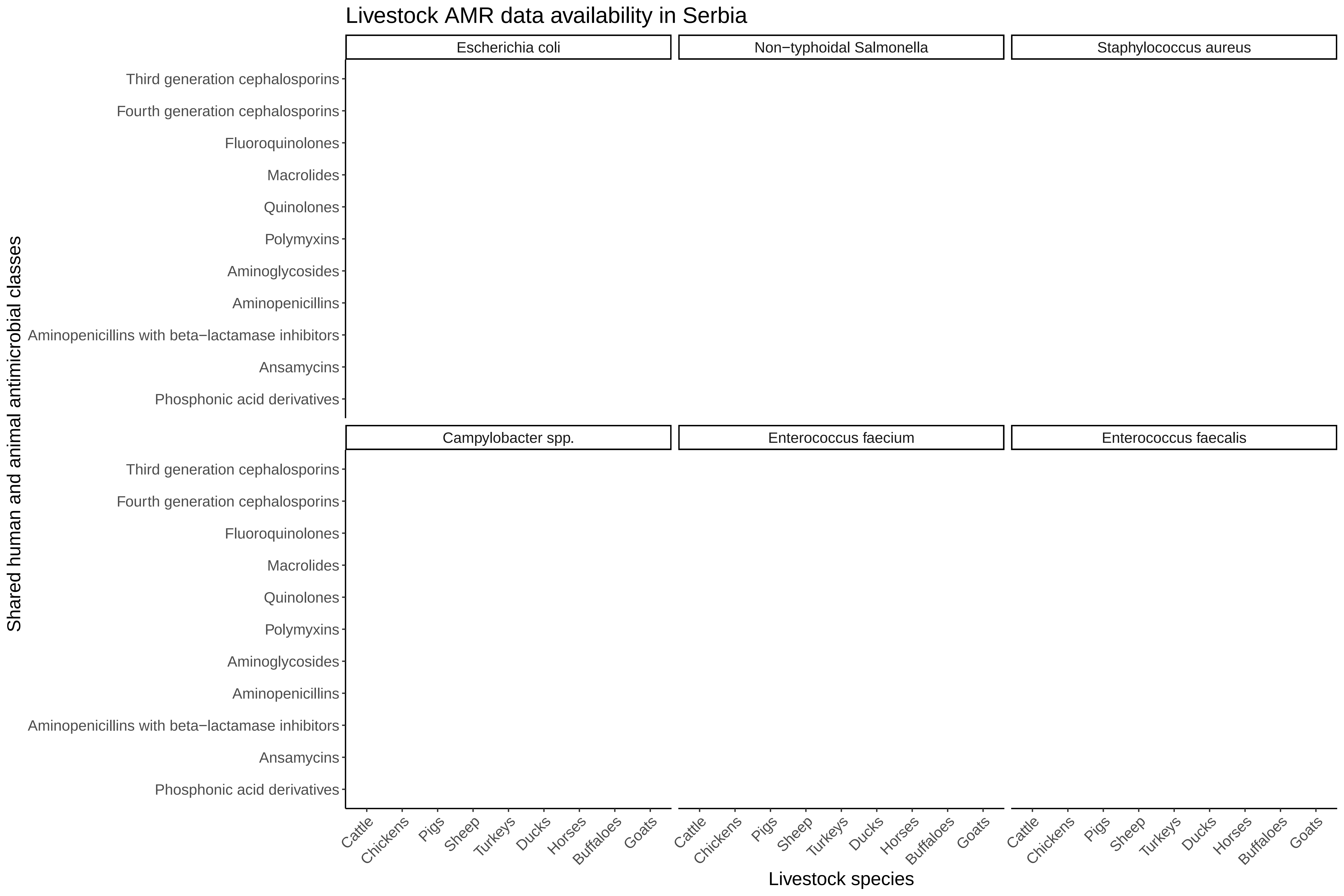
Livestock AMR data availability in Serbia
Escherichia coli
Non−typhoidal Salmonella
Staphylococcus aureus
Campylobacter spp.
Enterococcus faecium
Enterococcus faecalis
Shared human and animal antimicrobial classes
Third generation cephalosporins
Fourth generation cephalosporins
Fluoroquinolones
Macrolides
Quinolones
Polymyxins
Aminoglycosides
Aminopenicillins
Aminopenicillins with beta−lactamase inhibitors
Ansamycins
Phosphonic acid derivatives
Cattle
Chickens
Pigs
Sheep
Turkeys
Ducks
Horses
Buffaloes
Goats
Livestock species

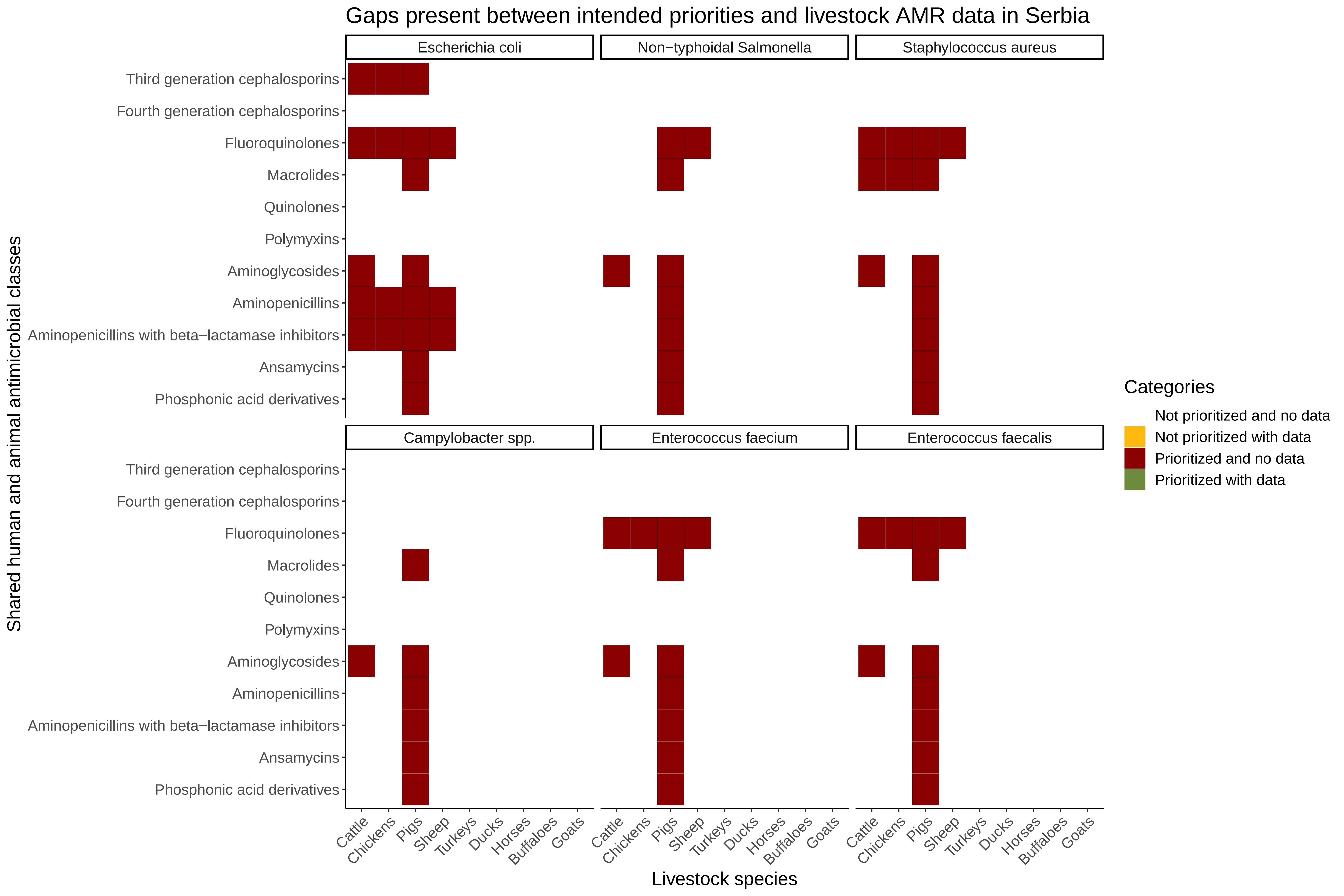
Gaps present between intended priorities and livestock AMR data in Serbia
Escherichia coli
Non−typhoidal Salmonella
Staphylococcus aureus
Campylobacter spp.
Enterococcus faecium
Enterococcus faecalis
Shared human and animal antimicrobial classes
Third generation cephalosporins
Fourth generation cephalosporins
Fluoroquinolones
Macrolides
Quinolones
Polymyxins
Aminoglycosides
Aminopenicillins
Aminopenicillins with beta−lactamase inhibitors
Ansamycins
Phosphonic acid derivatives
Livestock species
Cattle
Chickens
Pigs
Sheep
Turkeys
Ducks
Horses
Buffaloes
Goats
Categories
Not prioritized and no data
Not prioritized with data
Prioritized and no data
Prioritized with data

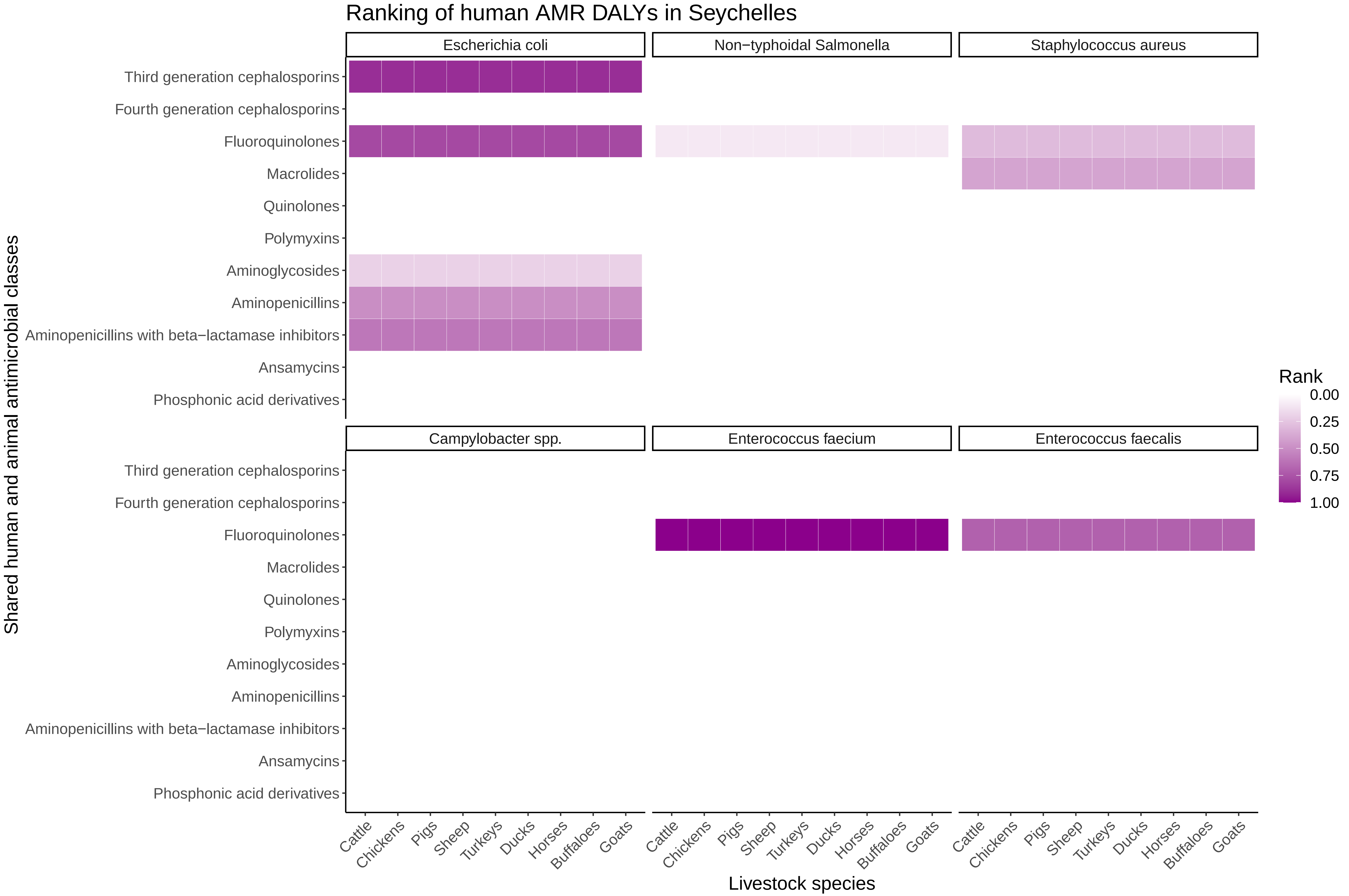
Ranking of human AMR DALYs in Seychelles
Escherichia coli
Non−typhoidal Salmonella
Staphylococcus aureus
Campylobacter spp.
Enterococcus faecium
Enterococcus faecalis
Third generation cephalosporins
Fourth generation cephalosporins
Fluoroquinolones
Macrolides
Quinolones
Polymyxins
Aminoglycosides
Aminopenicillins
Aminopenicillins with beta−lactamase inhibitors
Ansamycins
Phosphonic acid derivatives
Shared human and animal antimicrobial classes
Cattle
Chickens
Pigs
Sheep
Turkeys
Ducks
Horses
Buffaloes
Goats
Livestock species
Rank
0.00
0.25
0.50
0.75
1.00

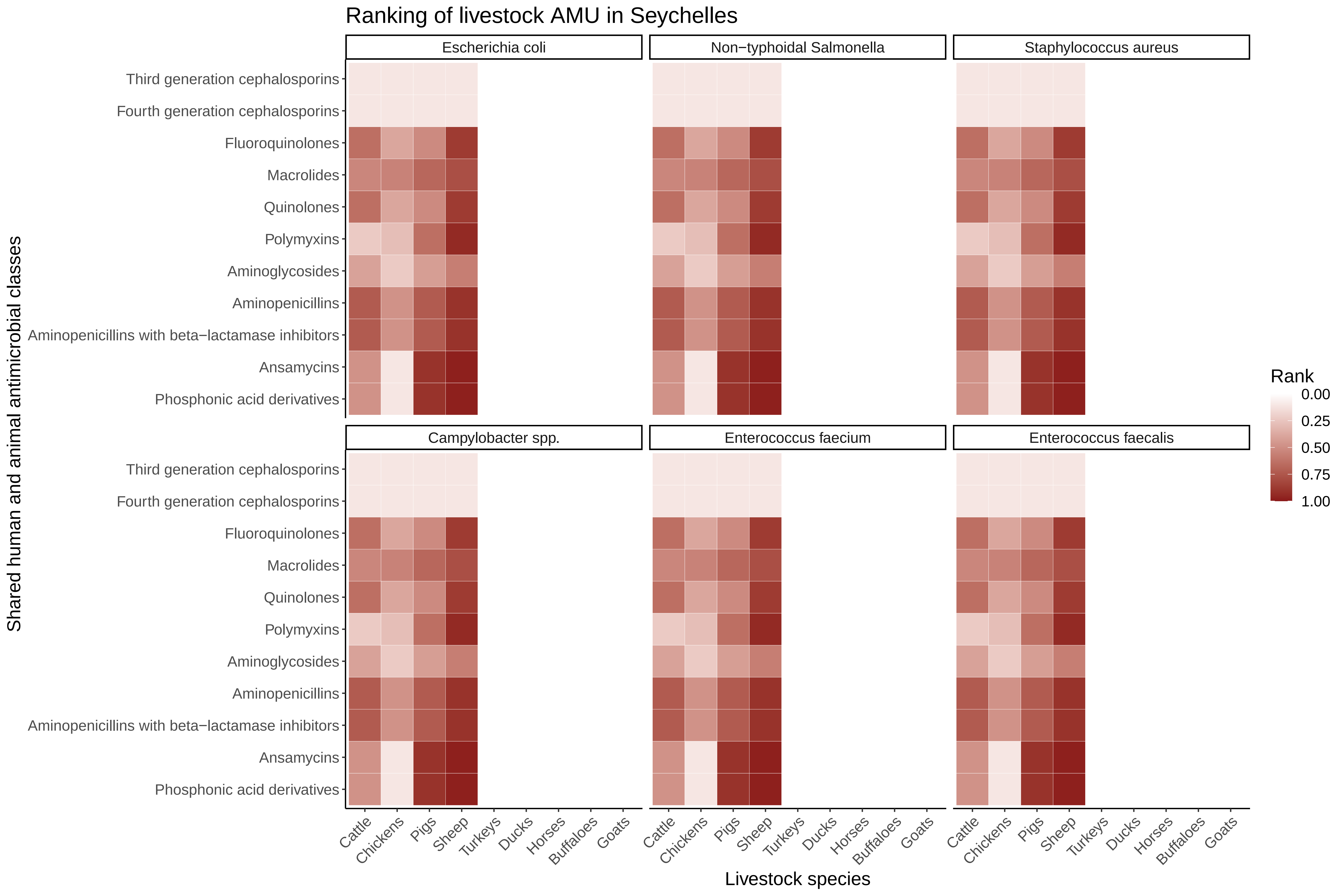
Ranking of livestock AMU in Seychelles
Escherichia coli
Non−typhoidal Salmonella
Staphylococcus aureus
Campylobacter spp.
Enterococcus faecium
Enterococcus faecalis
Third generation cephalosporins
Fourth generation cephalosporins
Fluoroquinolones
Macrolides
Quinolones
Polymyxins
Aminoglycosides
Aminopenicillins
Aminopenicillins with beta−lactamase inhibitors
Ansamycins
Phosphonic acid derivatives
Shared human and animal antimicrobial classes
Cattle
Chickens
Pigs
Sheep
Turkeys
Ducks
Horses
Buffaloes
Goats
Livestock species
Rank
0.00
0.25
0.50
0.75
1.00

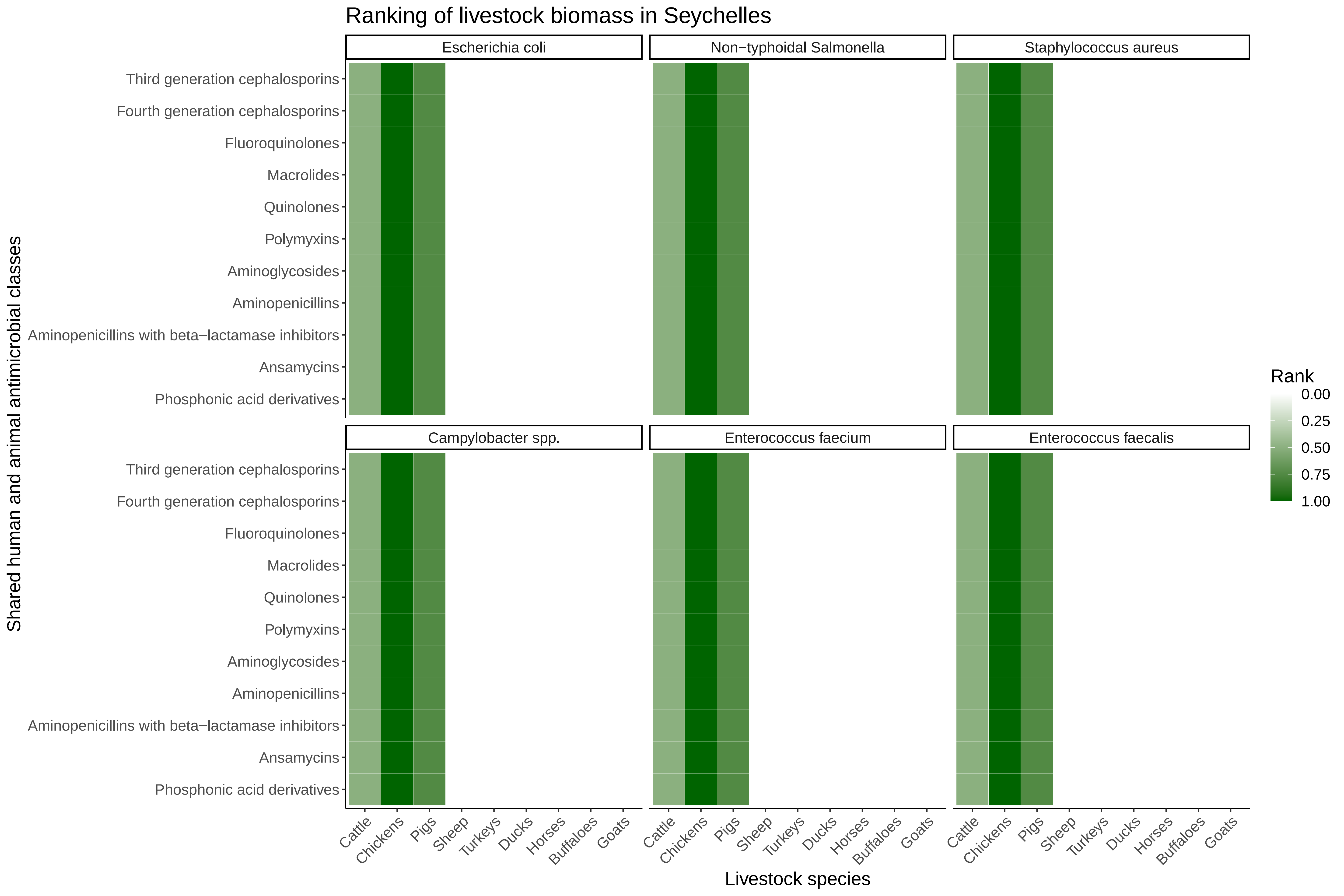
Ranking of livestock biomass in Seychelles
Escherichia coli
Non−typhoidal Salmonella
Staphylococcus aureus
Campylobacter spp.
Enterococcus faecium
Enterococcus faecalis
Third generation cephalosporins
Fourth generation cephalosporins
Fluoroquinolones
Macrolides
Quinolones
Polymyxins
Aminoglycosides
Aminopenicillins
Aminopenicillins with beta−lactamase inhibitors
Ansamycins
Phosphonic acid derivatives
Shared human and animal antimicrobial classes
Cattle
Chickens
Pigs
Sheep
Turkeys
Ducks
Horses
Buffaloes
Goats
Livestock species
Rank
0.00
0.25
0.50
0.75
1.00

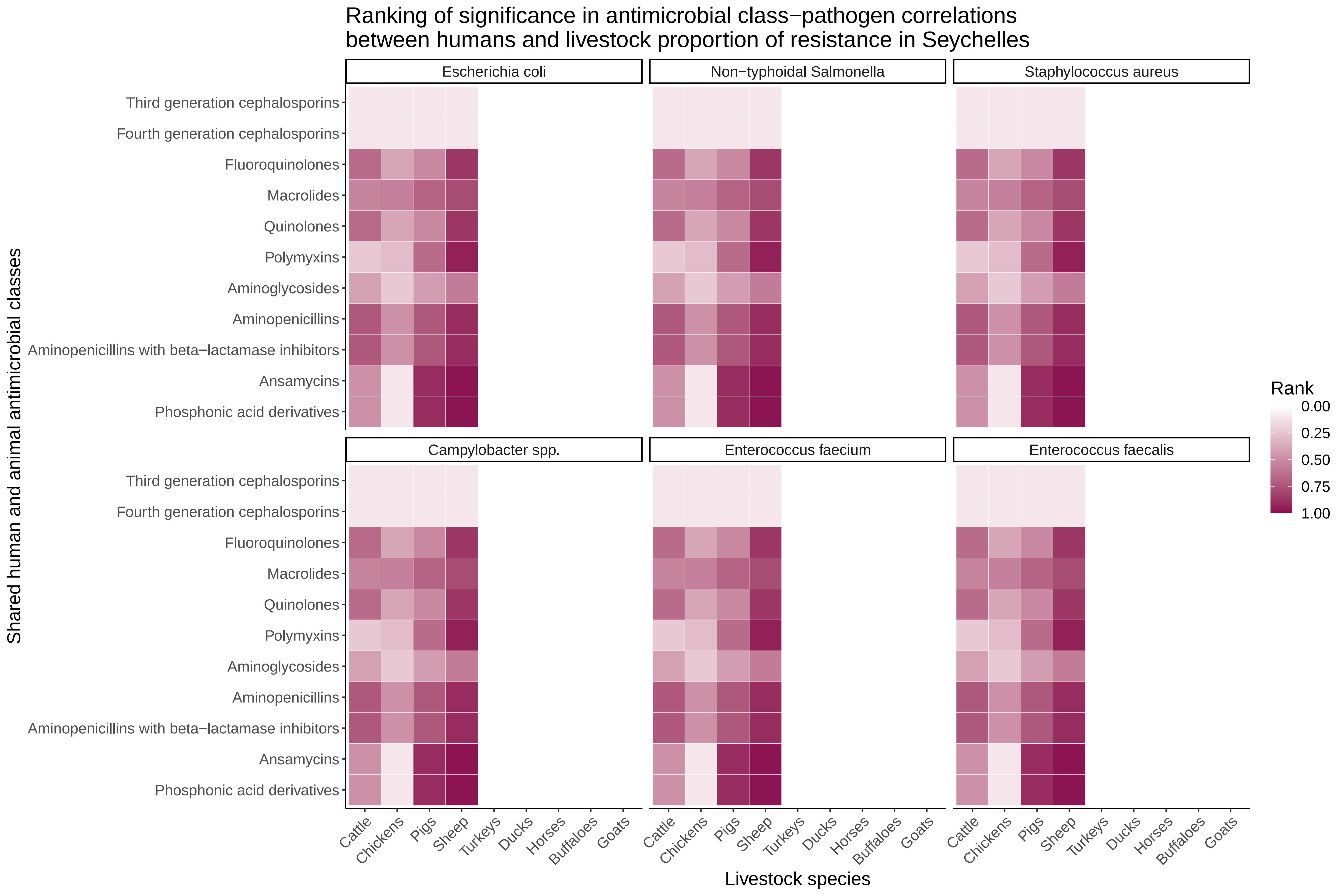
Ranking of significance in antimicrobial class−pathogen correlations
between humans and livestock proportion of resistance in Seychelles
Escherichia coli
Non−typhoidal Salmonella
Staphylococcus aureus
Campylobacter spp.
Enterococcus faecium
Enterococcus faecalis
Third generation cephalosporins
Fourth generation cephalosporins
Fluoroquinolones
Macrolides
Quinolones
Polymyxins
Aminoglycosides
Aminopenicillins
Aminopenicillins with beta−lactamase inhibitors
Ansamycins
Phosphonic acid derivatives
Shared human and animal antimicrobial classes
Cattle
Chickens
Pigs
Sheep
Turkeys
Ducks
Horses
Buffaloes
Goats
Livestock species
Rank
0.00
0.25
0.50
0.75
1.00

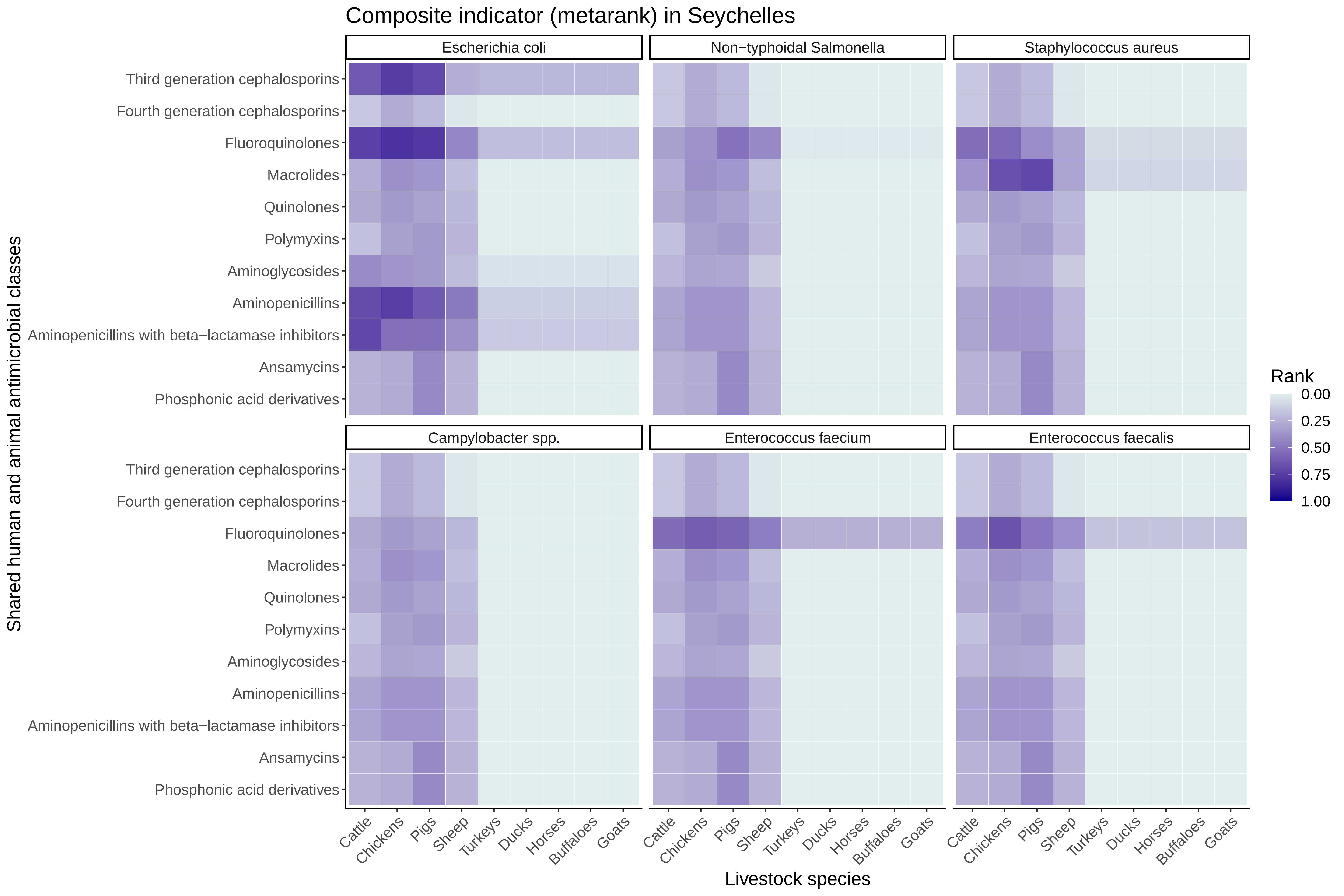

Composite indicator (metarank) in Seychelles
Escherichia coli
Non−typhoidal Salmonella
Staphylococcus aureus
Campylobacter spp.
Enterococcus faecium
Enterococcus faecalis
Third generation cephalosporins
Fourth generation cephalosporins
Fluoroquinolones
Macrolides
Quinolones
Polymyxins
Aminoglycosides
Aminopenicillins
Aminopenicillins with beta−lactamase inhibitors
Ansamycins
Phosphonic acid derivatives
Shared human and animal antimicrobial classes
Cattle
Chickens
Pigs
Sheep
Turkeys
Ducks
Horses
Buffaloes
Goats
Livestock species
Rank
0.00
0.25
0.50
0.75
1.00

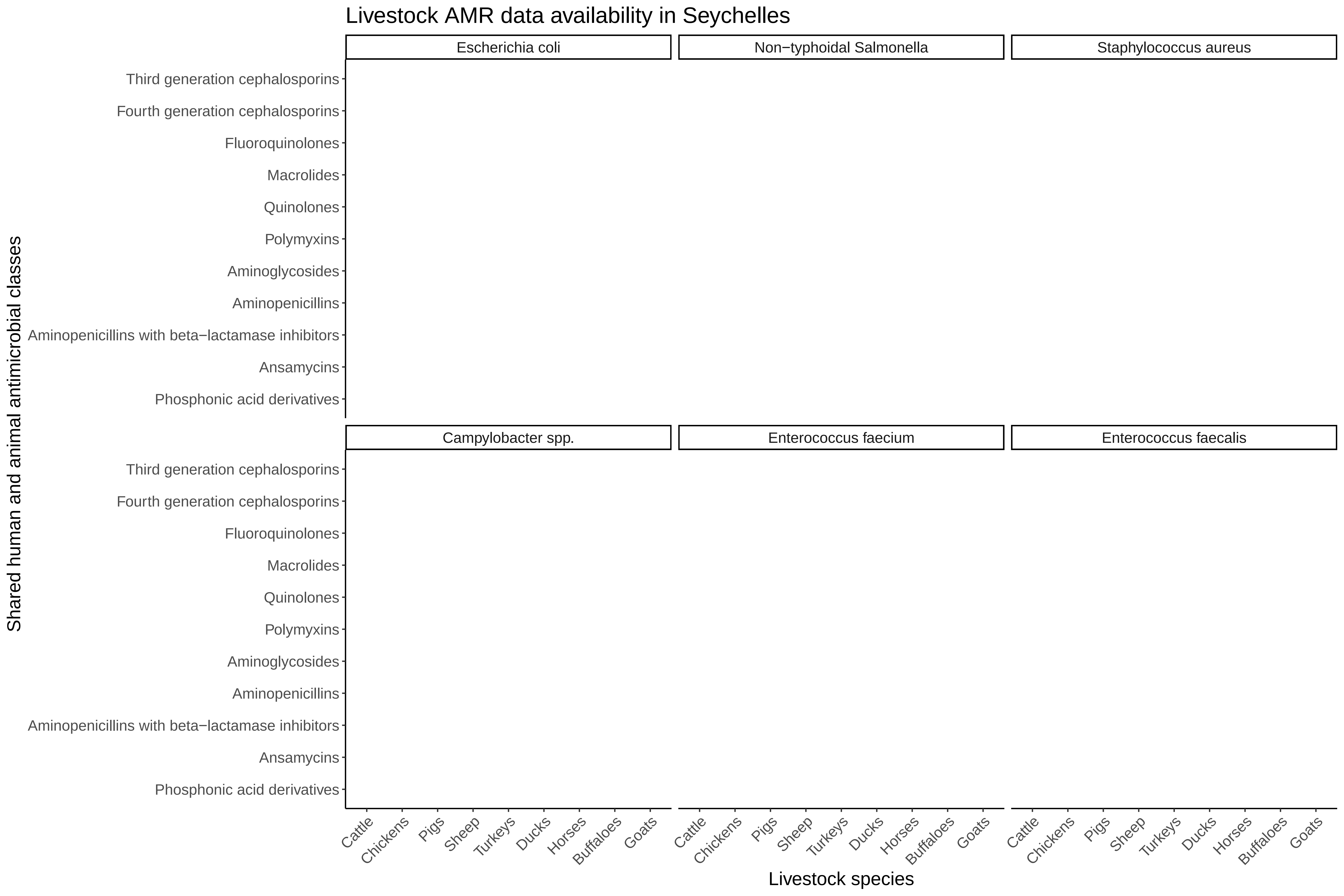
Livestock AMR data availability in Seychelles
Escherichia coli
Non−typhoidal Salmonella
Staphylococcus aureus
Campylobacter spp.
Enterococcus faecium
Enterococcus faecalis
Shared human and animal antimicrobial classes
Third generation cephalosporins
Fourth generation cephalosporins
Fluoroquinolones
Macrolides
Quinolones
Polymyxins
Aminoglycosides
Aminopenicillins
Aminopenicillins with beta−lactamase inhibitors
Ansamycins
Phosphonic acid derivatives
Cattle
Chickens
Pigs
Sheep
Turkeys
Ducks
Horses
Buffaloes
Goats
Livestock species

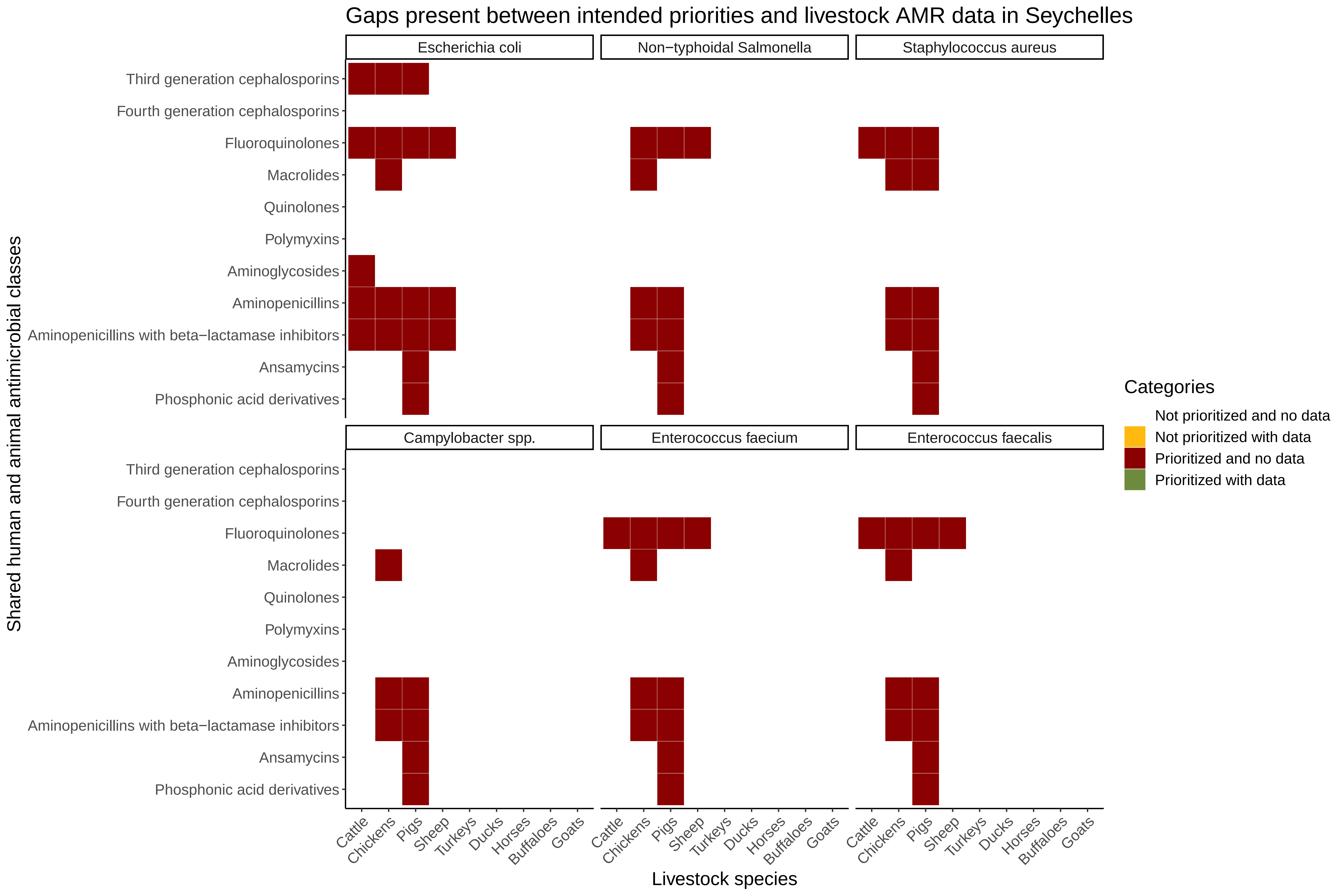
Gaps present between intended priorities and livestock AMR data in Seychelles
Escherichia coli
Non−typhoidal Salmonella
Staphylococcus aureus
Campylobacter spp.
Enterococcus faecium
Enterococcus faecalis
Third generation cephalosporins
Fourth generation cephalosporins
Fluoroquinolones
Macrolides
Quinolones
Polymyxins
Aminoglycosides
Aminopenicillins
Aminopenicillins with beta−lactamase inhibitors
Ansamycins
Phosphonic acid derivatives
Shared human and animal antimicrobial classes
Cattle
Chickens
Pigs
Sheep
Turkeys
Ducks
Horses
Buffaloes
Goats
Livestock species
Categories
Not prioritized and no data
Not prioritized with data
Prioritized and no data
Prioritized with data

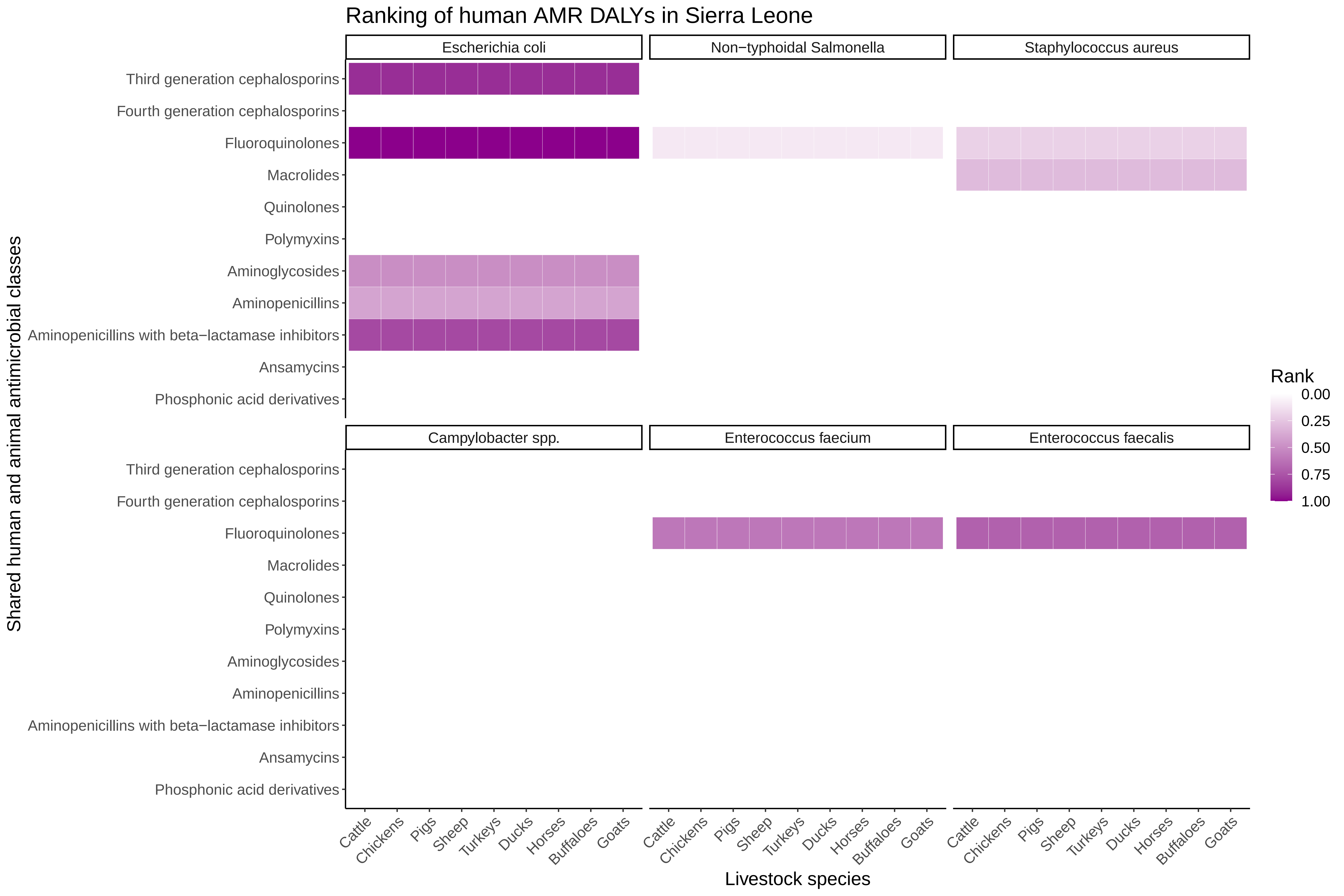
Ranking of human AMR DALYs in Sierra Leone
Escherichia coli
Non−typhoidal Salmonella
Staphylococcus aureus
Campylobacter spp.
Enterococcus faecium
Enterococcus faecalis
Third generation cephalosporins
Fourth generation cephalosporins
Fluoroquinolones
Macrolides
Quinolones
Polymyxins
Aminoglycosides
Aminopenicillins
Aminopenicillins with beta−lactamase inhibitors
Ansamycins
Phosphonic acid derivatives
Shared human and animal antimicrobial classes
Cattle
Chickens
Pigs
Sheep
Turkeys
Ducks
Horses
Buffaloes
Goats
Livestock species
Rank
0.00
0.25
0.50
0.75
1.00

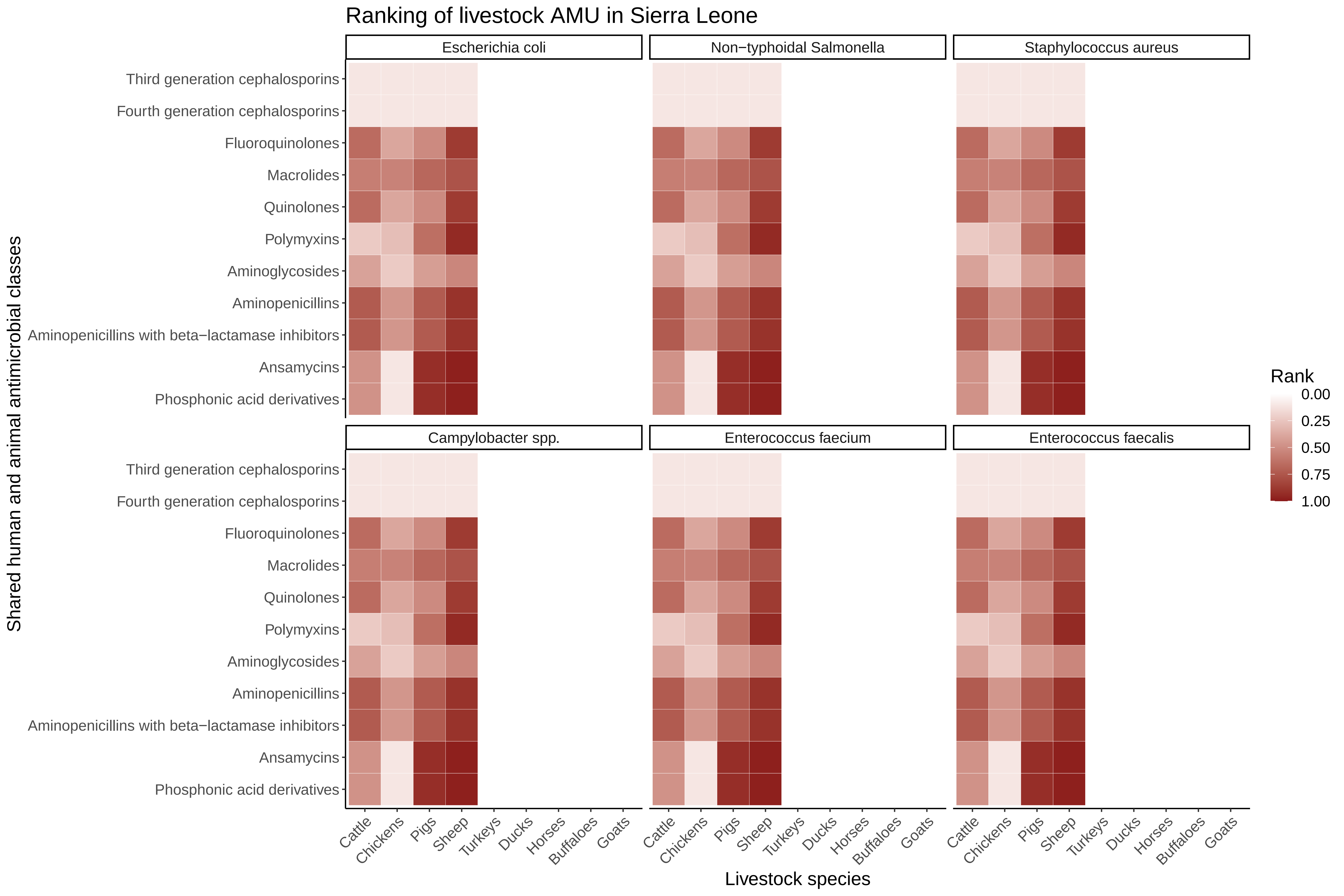
Ranking of livestock AMU in Sierra Leone
Escherichia coli
Non−typhoidal Salmonella
Staphylococcus aureus
Campylobacter spp.
Enterococcus faecium
Enterococcus faecalis
Shared human and animal antimicrobial classes
Third generation cephalosporins
Fourth generation cephalosporins
Fluoroquinolones
Macrolides
Quinolones
Polymyxins
Aminoglycosides
Aminopenicillins
Aminopenicillins with beta−lactamase inhibitors
Ansamycins
Phosphonic acid derivatives
Cattle
Chickens
Pigs
Sheep
Turkeys
Ducks
Horses
Buffaloes
Goats
Livestock species
Rank
0.00
0.25
0.50
0.75
1.00

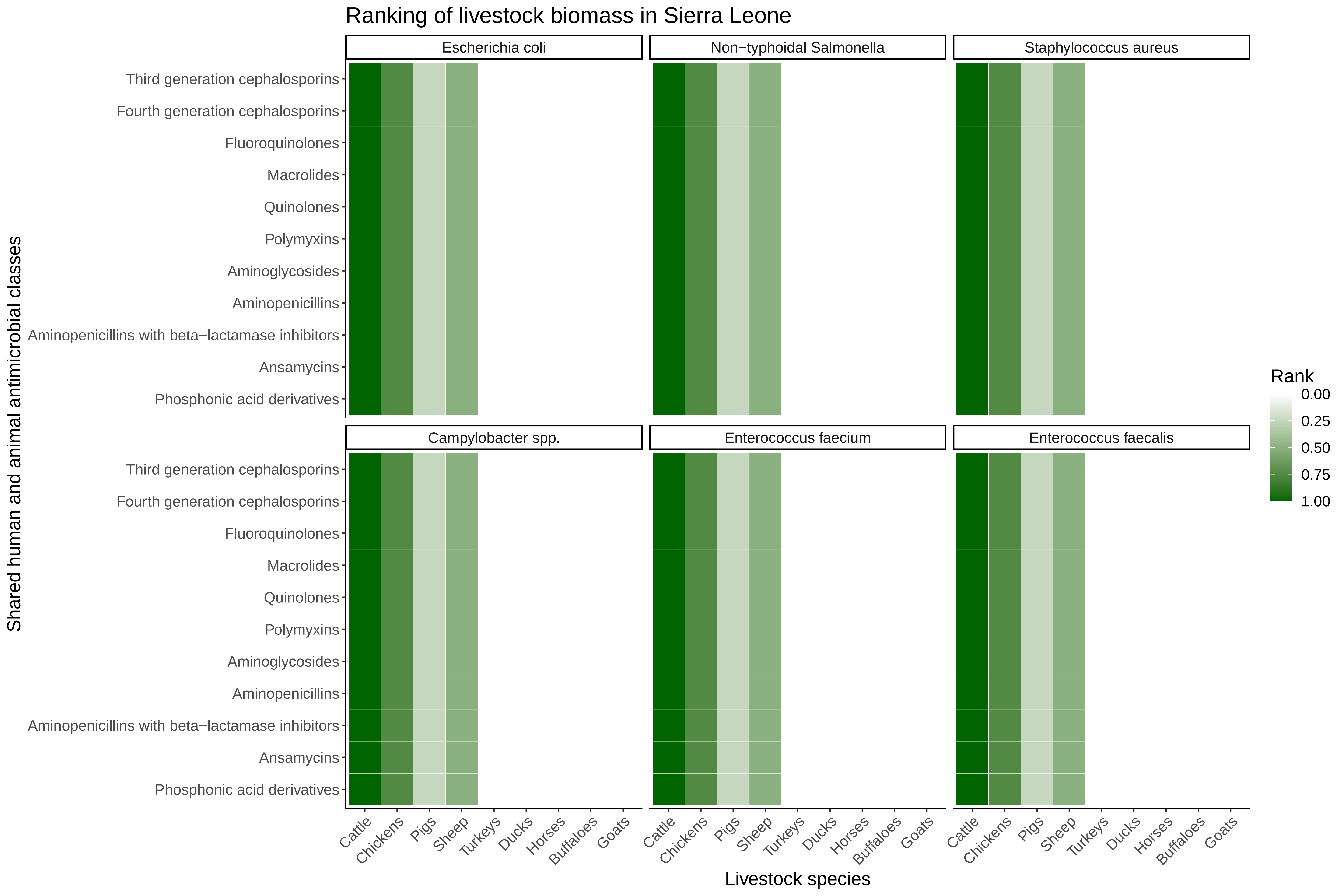

Ranking of livestock biomass in Sierra Leone
Escherichia coli
Non−typhoidal Salmonella
Staphylococcus aureus
Campylobacter spp.
Enterococcus faecium
Enterococcus faecalis
Third generation cephalosporins
Fourth generation cephalosporins
Fluoroquinolones
Macrolides
Quinolones
Polymyxins
Aminoglycosides
Aminopenicillins
Aminopenicillins with beta−lactamase inhibitors
Ansamycins
Phosphonic acid derivatives
Shared human and animal antimicrobial classes
Cattle
Chickens
Pigs
Sheep
Turkeys
Ducks
Horses
Buffaloes
Goats
Livestock species
Rank
0.00
0.25
0.50
0.75
1.00

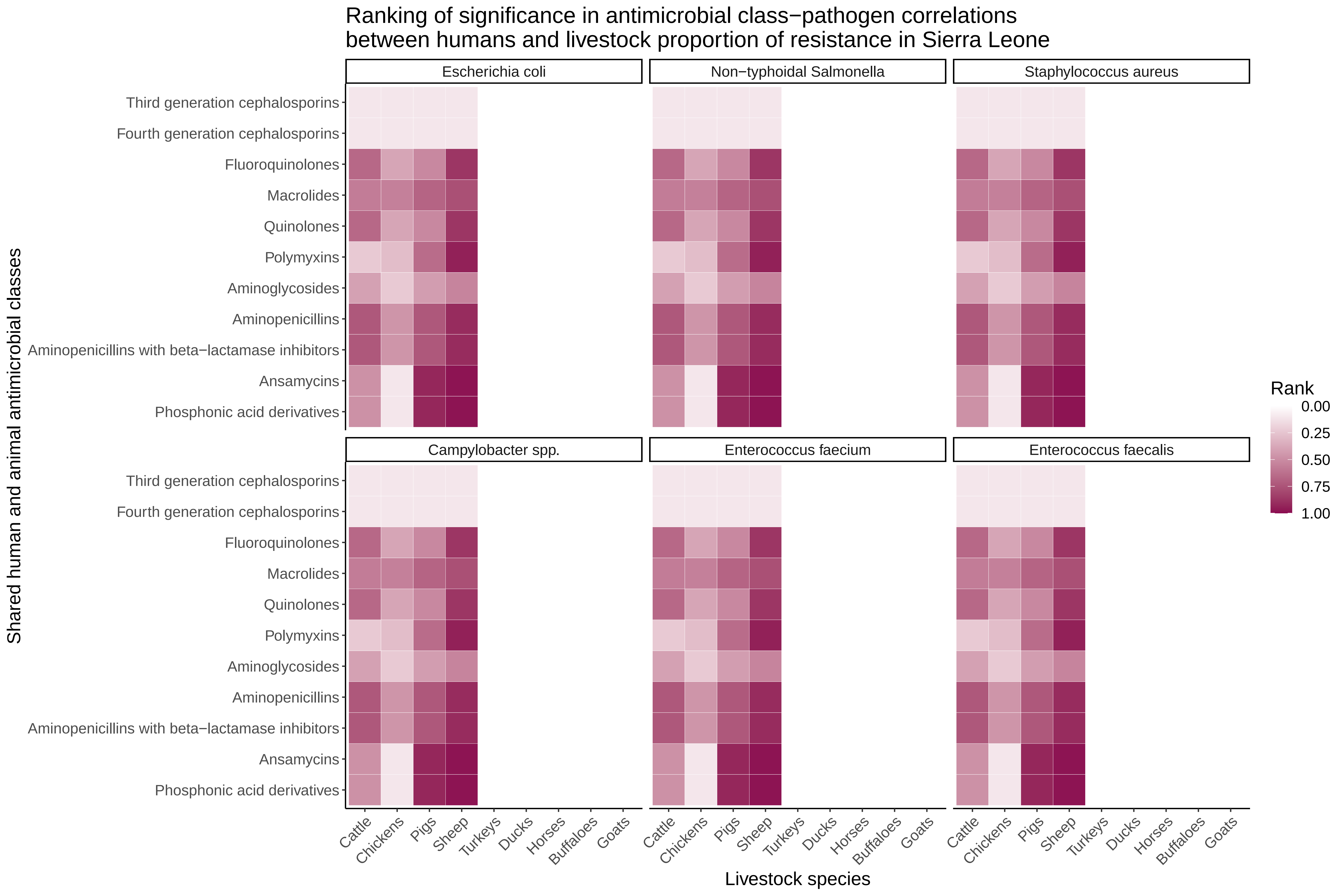
Ranking of significance in antimicrobial class−pathogen correlations
between humans and livestock proportion of resistance in Sierra Leone
Escherichia coli
Non−typhoidal Salmonella
Staphylococcus aureus
Campylobacter spp.
Enterococcus faecium
Enterococcus faecalis
Shared human and animal antimicrobial classes
Third generation cephalosporins
Fourth generation cephalosporins
Fluoroquinolones
Macrolides
Quinolones
Polymyxins
Aminoglycosides
Aminopenicillins
Aminopenicillins with beta−lactamase inhibitors
Ansamycins
Phosphonic acid derivatives
Livestock species
Cattle
Chickens
Pigs
Sheep
Turkeys
Ducks
Horses
Buffaloes
Goats
Rank
0.00
0.25
0.50
0.75
1.00

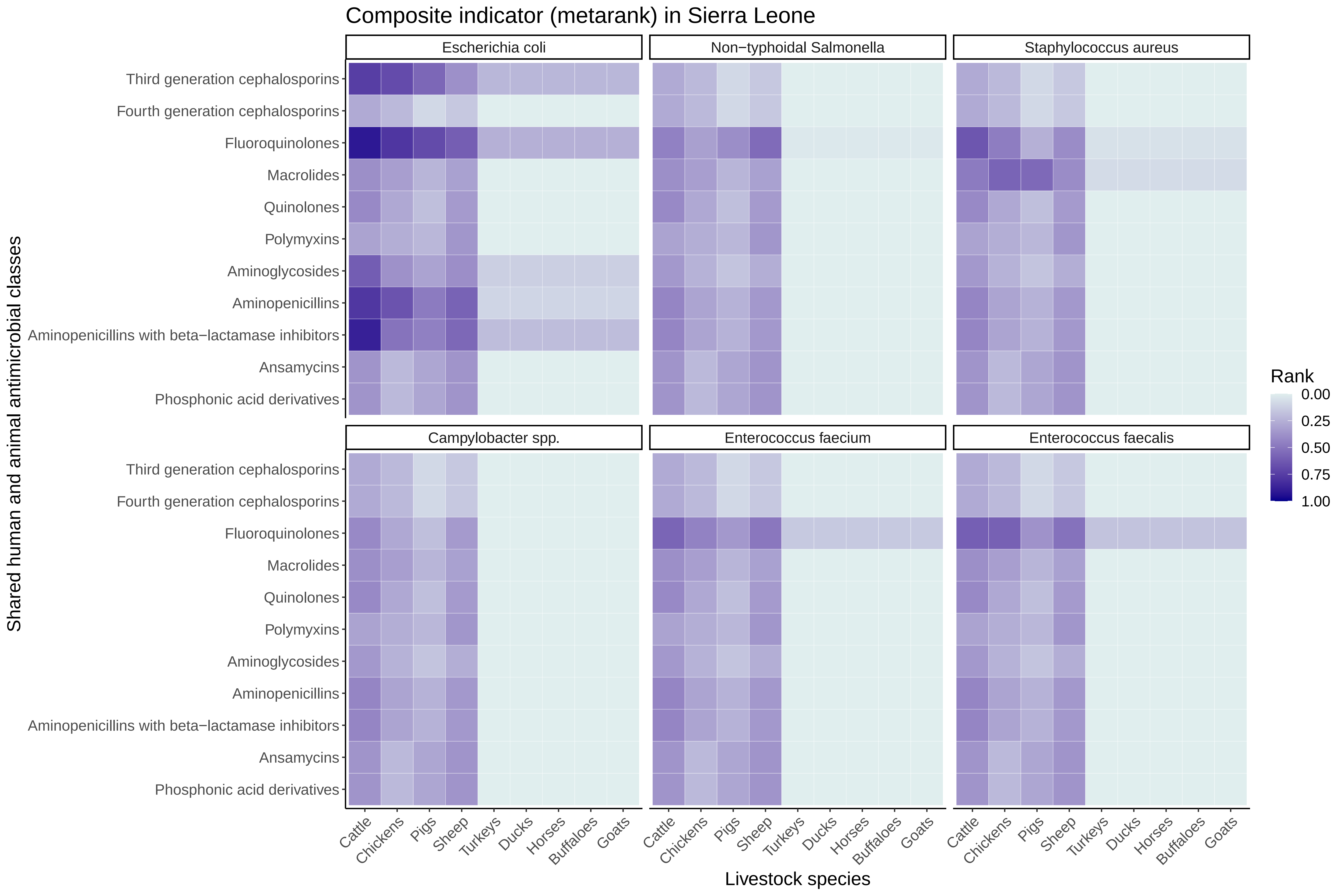

Composite indicator (metarank) in Sierra Leone
Escherichia coli
Non−typhoidal Salmonella
Staphylococcus aureus
Campylobacter spp.
Enterococcus faecium
Enterococcus faecalis
Third generation cephalosporins
Fourth generation cephalosporins
Fluoroquinolones
Macrolides
Quinolones
Polymyxins
Aminoglycosides
Aminopenicillins
Aminopenicillins with beta−lactamase inhibitors
Ansamycins
Phosphonic acid derivatives
Shared human and animal antimicrobial classes
Cattle
Chickens
Pigs
Sheep
Turkeys
Ducks
Horses
Buffaloes
Goats
Livestock species
Rank
0.00
0.25
0.50
0.75
1.00

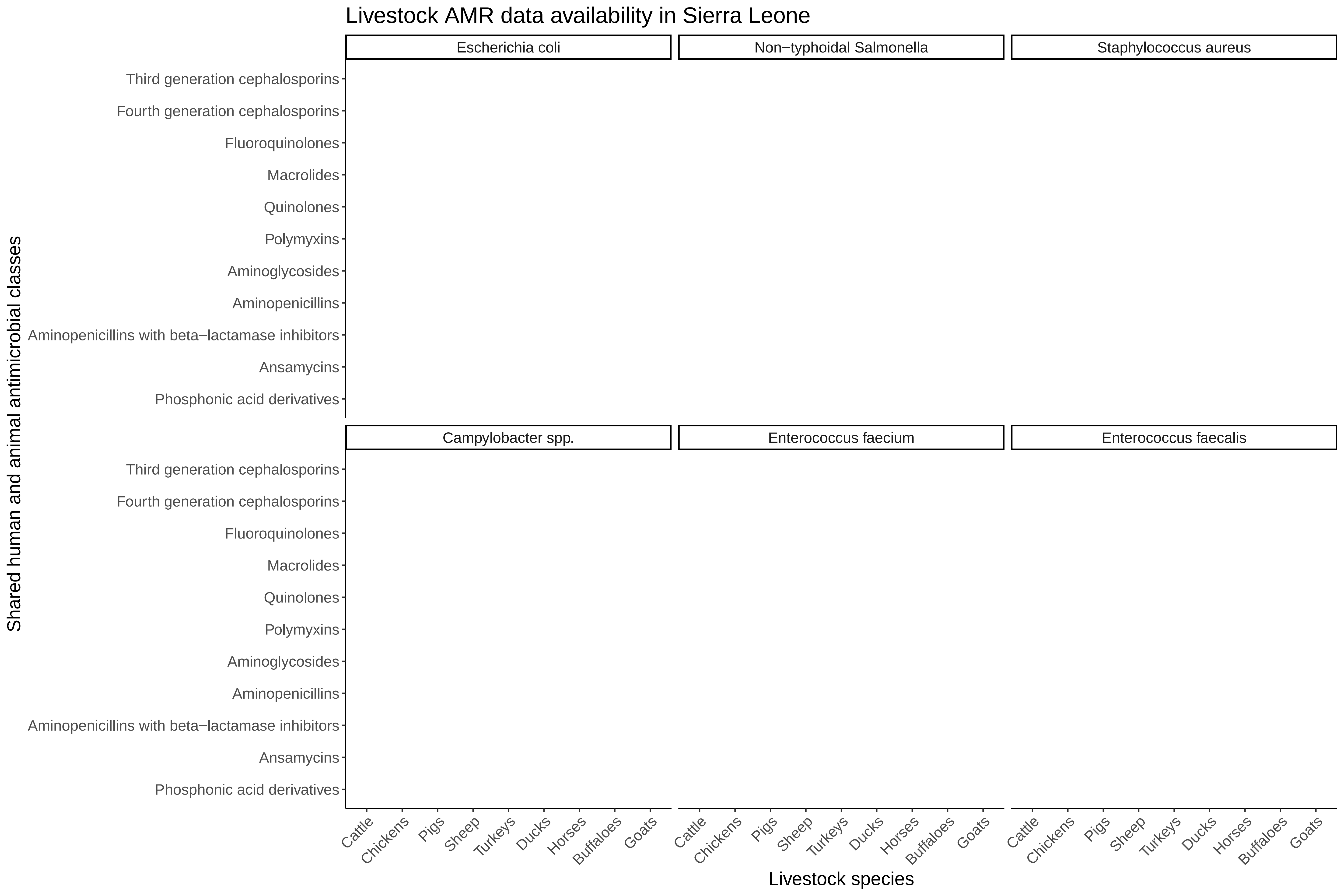

Livestock AMR data availability in Sierra Leone
Escherichia coli
Non−typhoidal Salmonella
Staphylococcus aureus
Campylobacter spp.
Enterococcus faecium
Enterococcus faecalis
Third generation cephalosporins
Fourth generation cephalosporins
Fluoroquinolones
Macrolides
Quinolones
Polymyxins
Aminoglycosides
Aminopenicillins
Aminopenicillins with beta−lactamase inhibitors
Ansamycins
Phosphonic acid derivatives
Shared human and animal antimicrobial classes
Cattle
Chickens
Pigs
Sheep
Turkeys
Ducks
Horses
Buffaloes
Goats
Livestock species

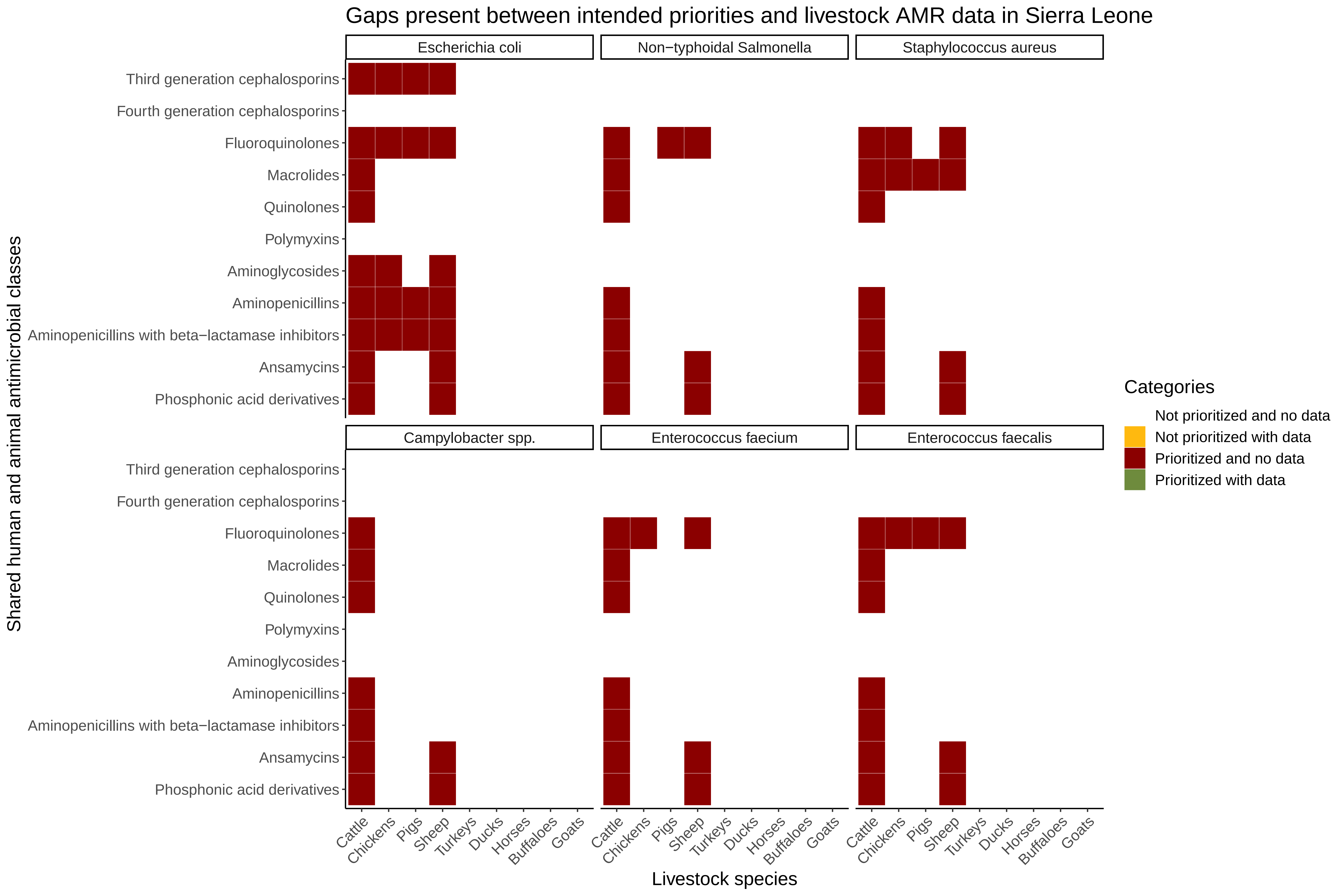
Gaps present between intended priorities and livestock AMR data in Sierra Leone
Escherichia coli
Non−typhoidal Salmonella
Staphylococcus aureus
Campylobacter spp.
Enterococcus faecium
Enterococcus faecalis
Shared human and animal antimicrobial classes
Third generation cephalosporins
Fourth generation cephalosporins
Fluoroquinolones
Macrolides
Quinolones
Polymyxins
Aminoglycosides
Aminopenicillins
Aminopenicillins with beta−lactamase inhibitors
Ansamycins
Phosphonic acid derivatives
Cattle
Chickens
Pigs
Sheep
Turkeys
Ducks
Horses
Buffaloes
Goats
Livestock species
Categories
Not prioritized and no data
Not prioritized with data
Prioritized and no data
Prioritized with data

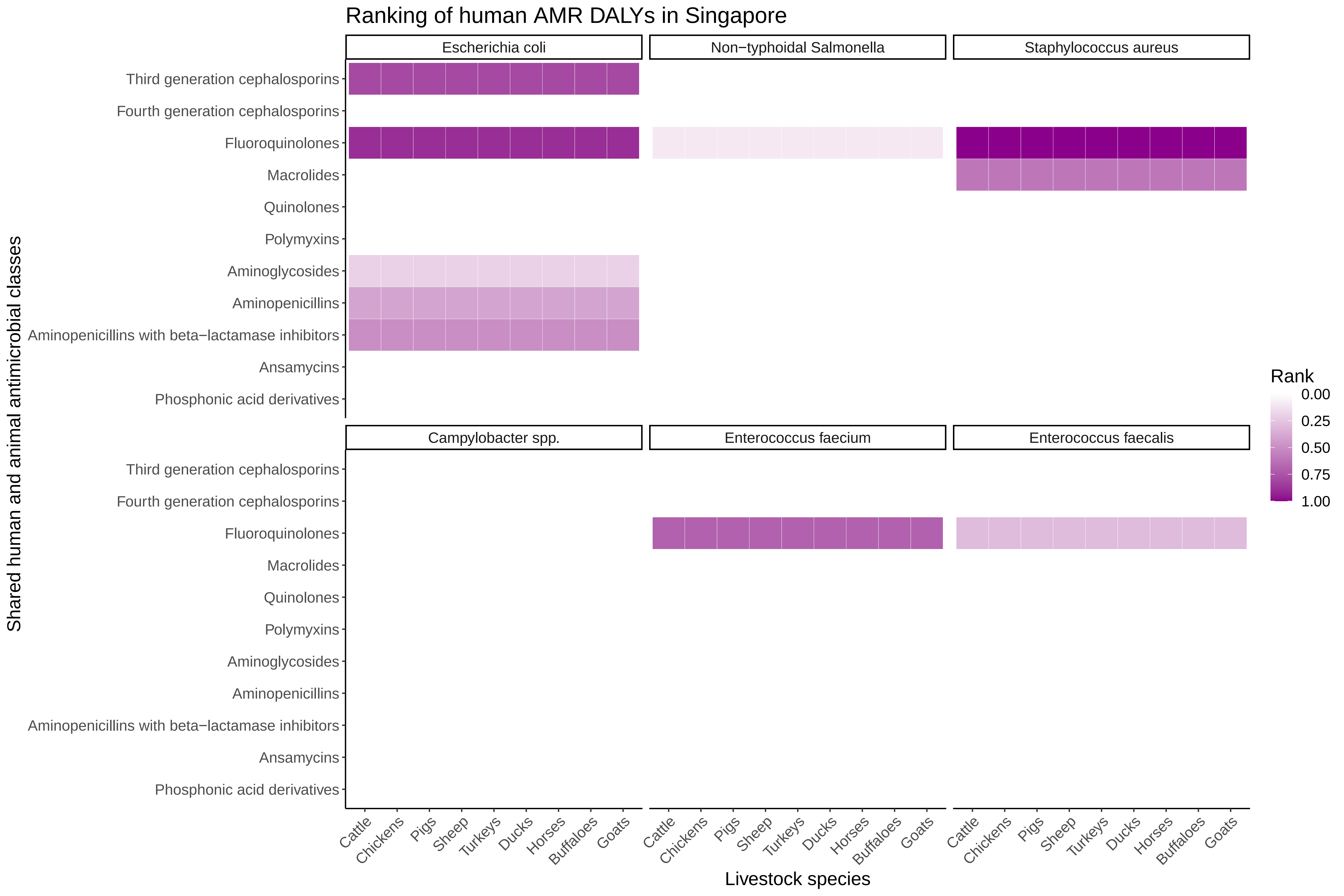

Ranking of human AMR DALYs in Singapore
Escherichia coli
Non−typhoidal Salmonella
Staphylococcus aureus
Campylobacter spp.
Enterococcus faecium
Enterococcus faecalis
Third generation cephalosporins
Fourth generation cephalosporins
Fluoroquinolones
Macrolides
Quinolones
Polymyxins
Aminoglycosides
Aminopenicillins
Aminopenicillins with beta−lactamase inhibitors
Ansamycins
Phosphonic acid derivatives
Shared human and animal antimicrobial classes
Cattle
Chickens
Pigs
Sheep
Turkeys
Ducks
Horses
Buffaloes
Goats
Livestock species
Rank
0.00
0.25
0.50
0.75
1.00

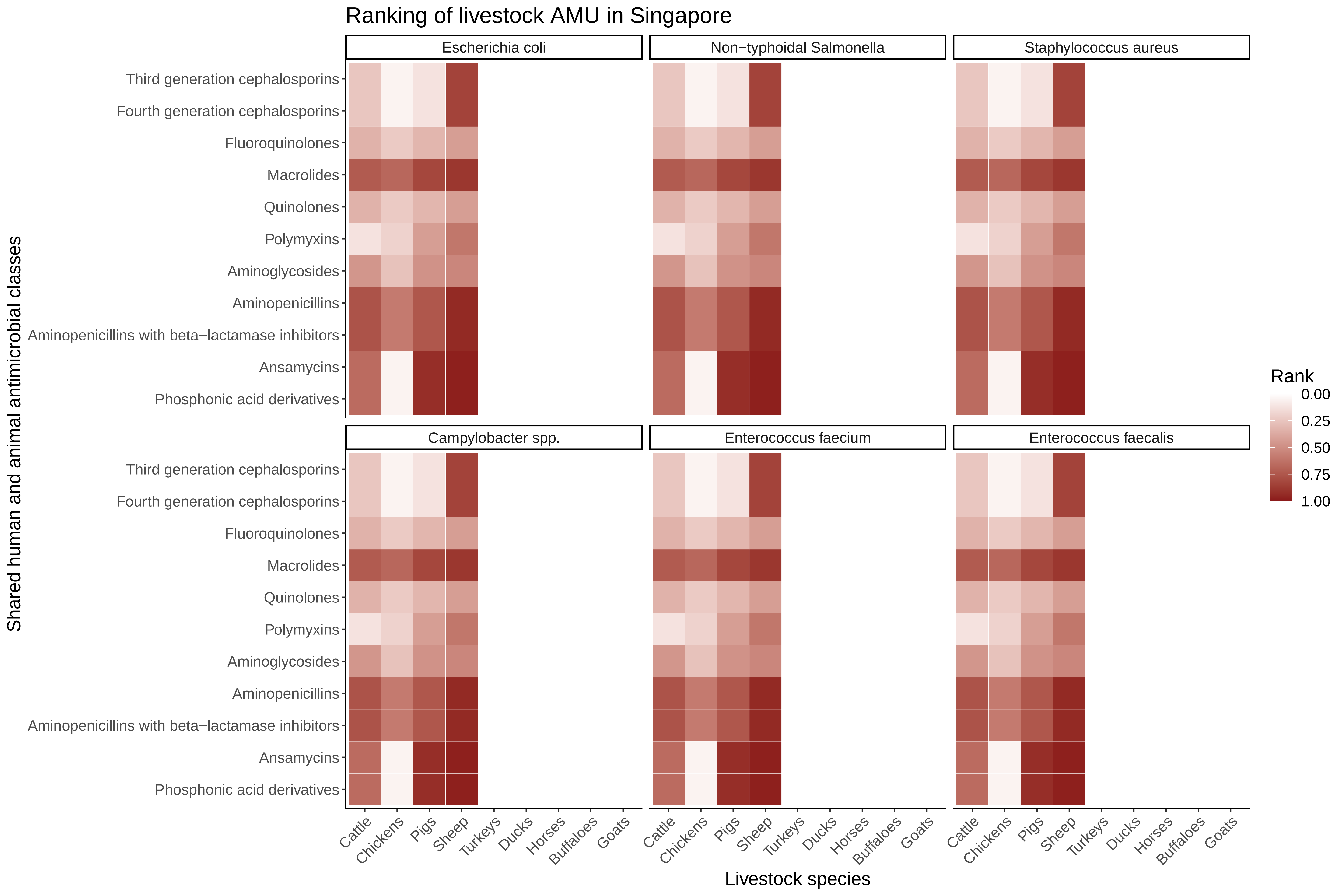
Ranking of livestock AMU in Singapore
Escherichia coli
Non−typhoidal Salmonella
Staphylococcus aureus
Campylobacter spp.
Enterococcus faecium
Enterococcus faecalis
Shared human and animal antimicrobial classes
Third generation cephalosporins
Fourth generation cephalosporins
Fluoroquinolones
Macrolides
Quinolones
Polymyxins
Aminoglycosides
Aminopenicillins
Aminopenicillins with beta−lactamase inhibitors
Ansamycins
Phosphonic acid derivatives
Cattle
Chickens
Pigs
Sheep
Turkeys
Ducks
Horses
Buffaloes
Goats
Livestock species
Rank
0.00
0.25
0.50
0.75
1.00

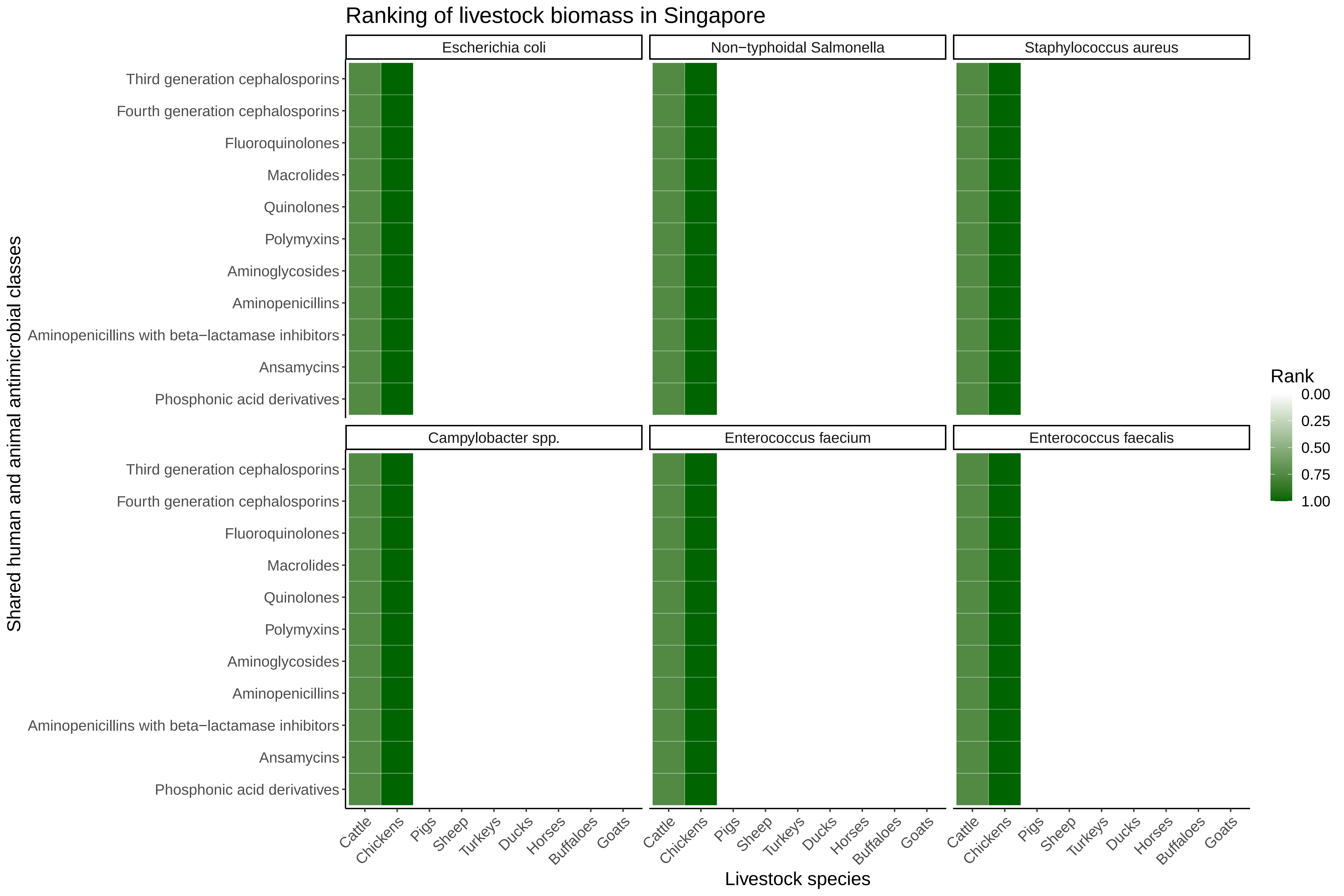

Ranking of livestock biomass in Singapore
Escherichia coli
Non−typhoidal Salmonella
Staphylococcus aureus
Campylobacter spp.
Enterococcus faecium
Enterococcus faecalis
Shared human and animal antimicrobial classes
Third generation cephalosporins
Fourth generation cephalosporins
Fluoroquinolones
Macrolides
Quinolones
Polymyxins
Aminoglycosides
Aminopenicillins
Aminopenicillins with beta−lactamase inhibitors
Ansamycins
Phosphonic acid derivatives
Cattle
Chickens
Pigs
Sheep
Turkeys
Ducks
Horses
Buffaloes
Goats
Livestock species
Rank
0.00
0.25
0.50
0.75
1.00

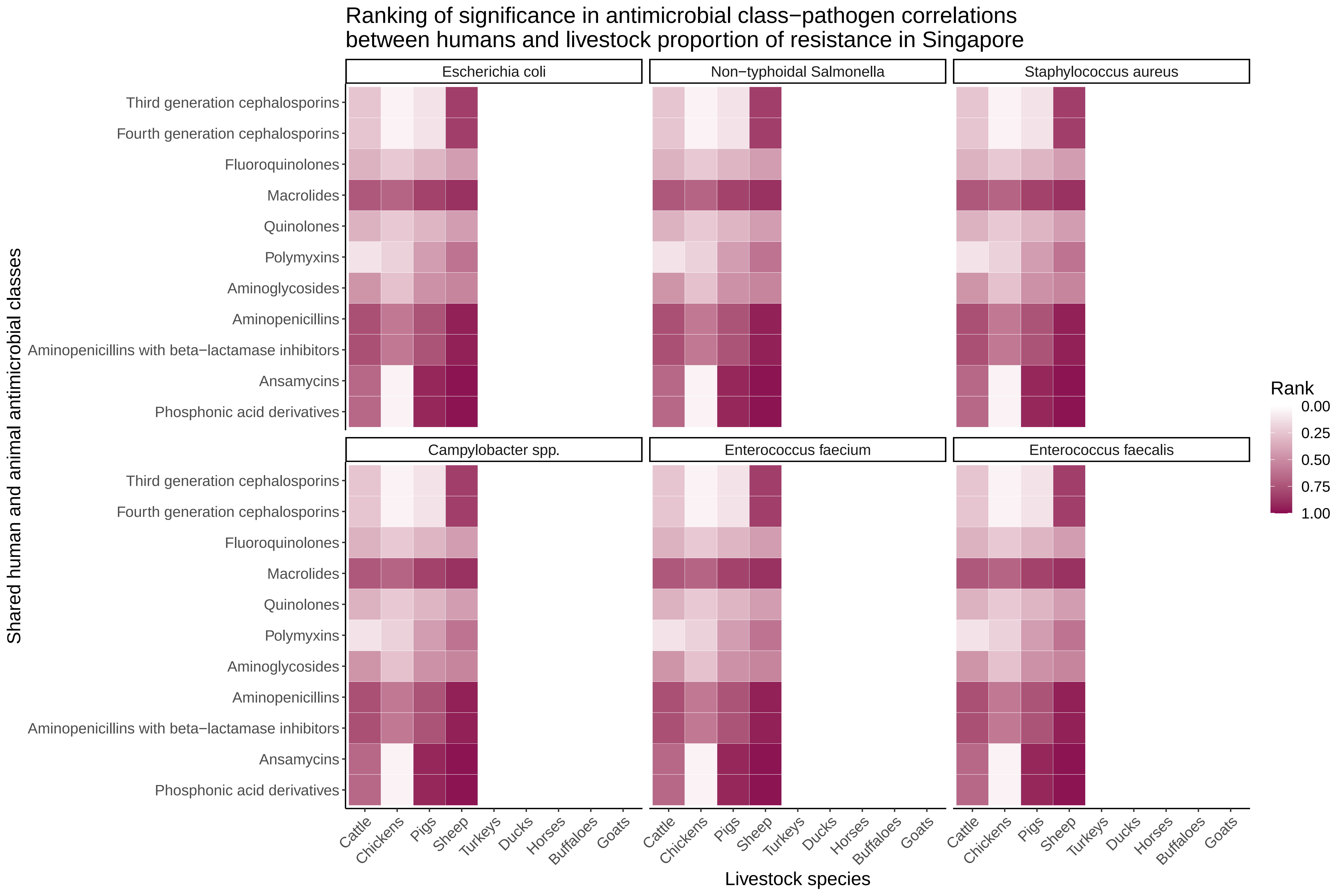
Ranking of significance in antimicrobial class−pathogen correlations
between humans and livestock proportion of resistance in Singapore
Escherichia coli
Non−typhoidal Salmonella
Staphylococcus aureus
Campylobacter spp.
Enterococcus faecium
Enterococcus faecalis
Third generation cephalosporins
Fourth generation cephalosporins
Fluoroquinolones
Macrolides
Quinolones
Polymyxins
Aminoglycosides
Aminopenicillins
Aminopenicillins with beta−lactamase inhibitors
Ansamycins
Phosphonic acid derivatives
Shared human and animal antimicrobial classes
Cattle
Chickens
Pigs
Sheep
Turkeys
Ducks
Horses
Buffaloes
Goats
Livestock species
Rank
0.00
0.25
0.50
0.75
1.00

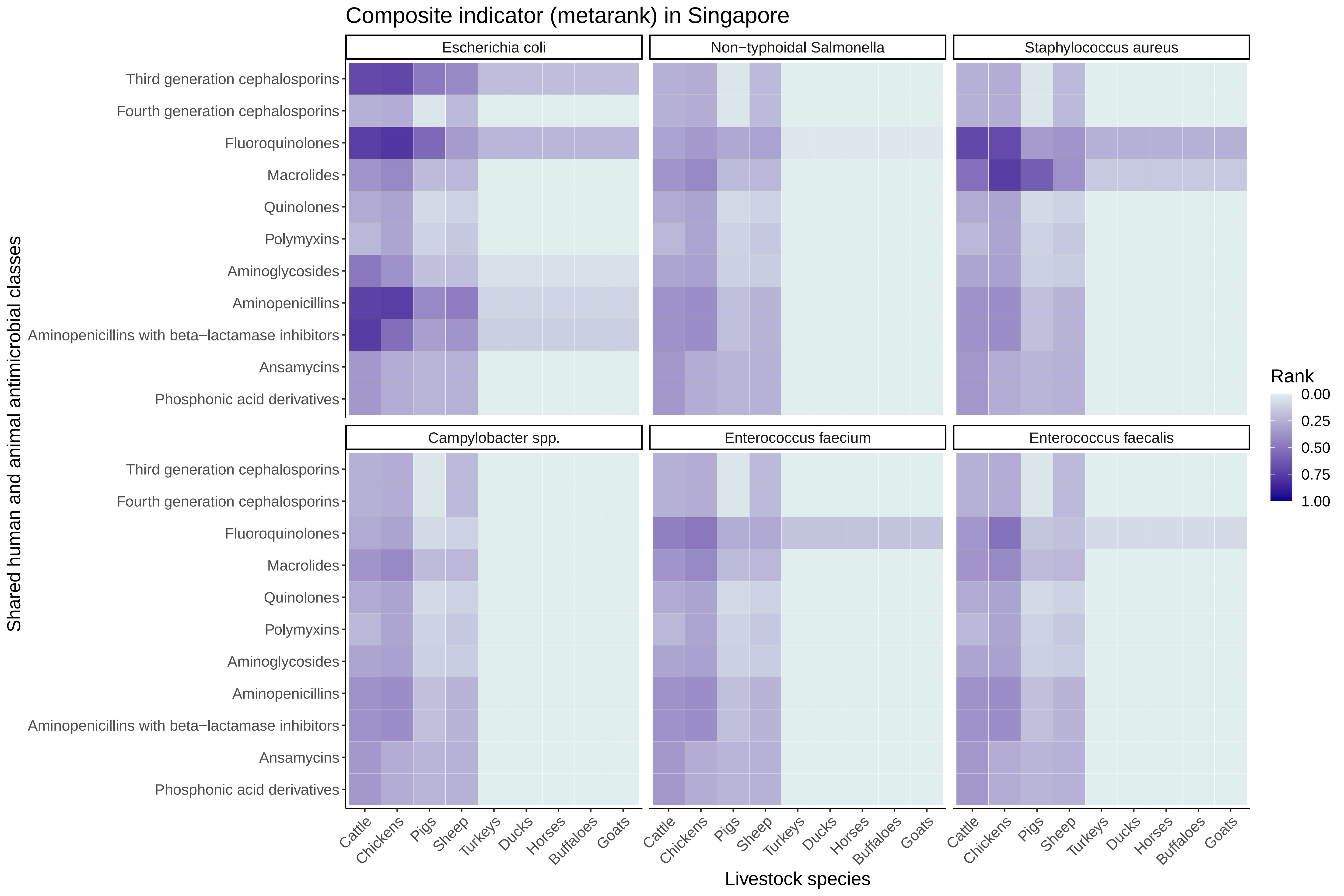

Composite indicator (metarank) in Singapore
Escherichia coli
Non−typhoidal Salmonella
Staphylococcus aureus
Campylobacter spp.
Enterococcus faecium
Enterococcus faecalis
Shared human and animal antimicrobial classes
Third generation cephalosporins
Fourth generation cephalosporins
Fluoroquinolones
Macrolides
Quinolones
Polymyxins
Aminoglycosides
Aminopenicillins
Aminopenicillins with beta−lactamase inhibitors
Ansamycins
Phosphonic acid derivatives
Livestock species
Cattle
Chickens
Pigs
Sheep
Turkeys
Ducks
Horses
Buffaloes
Goats
Rank
0.00
0.25
0.50
0.75
1.00

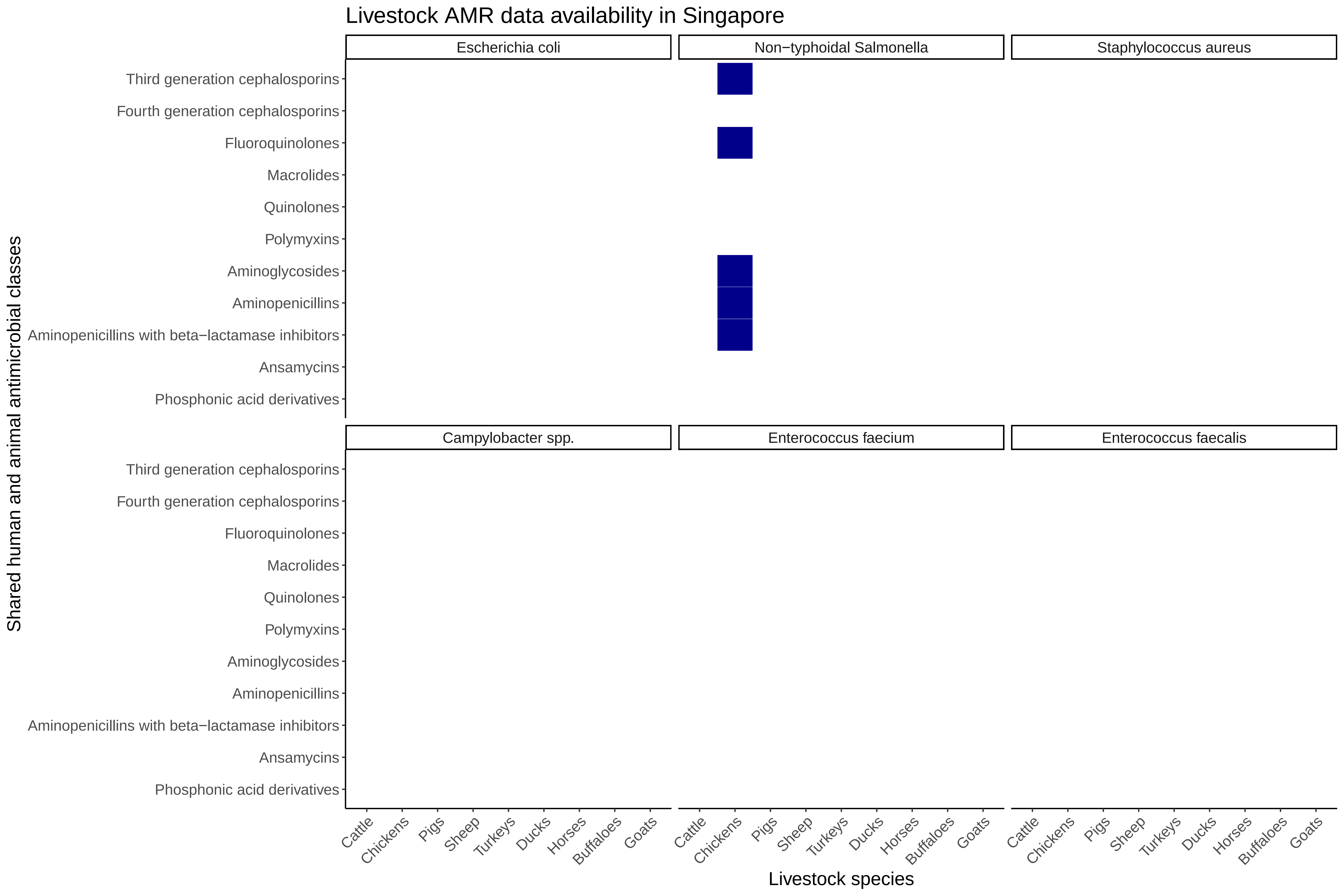

Livestock AMR data availability in Singapore
Escherichia coli
Non−typhoidal Salmonella
Staphylococcus aureus
Campylobacter spp.
Enterococcus faecium
Enterococcus faecalis
Third generation cephalosporins
Fourth generation cephalosporins
Fluoroquinolones
Macrolides
Quinolones
Polymyxins
Aminoglycosides
Aminopenicillins
Aminopenicillins with beta−lactamase inhibitors
Ansamycins
Phosphonic acid derivatives
Shared human and animal antimicrobial classes
Cattle
Chickens
Pigs
Sheep
Turkeys
Ducks
Horses
Buffaloes
Goats
Livestock species

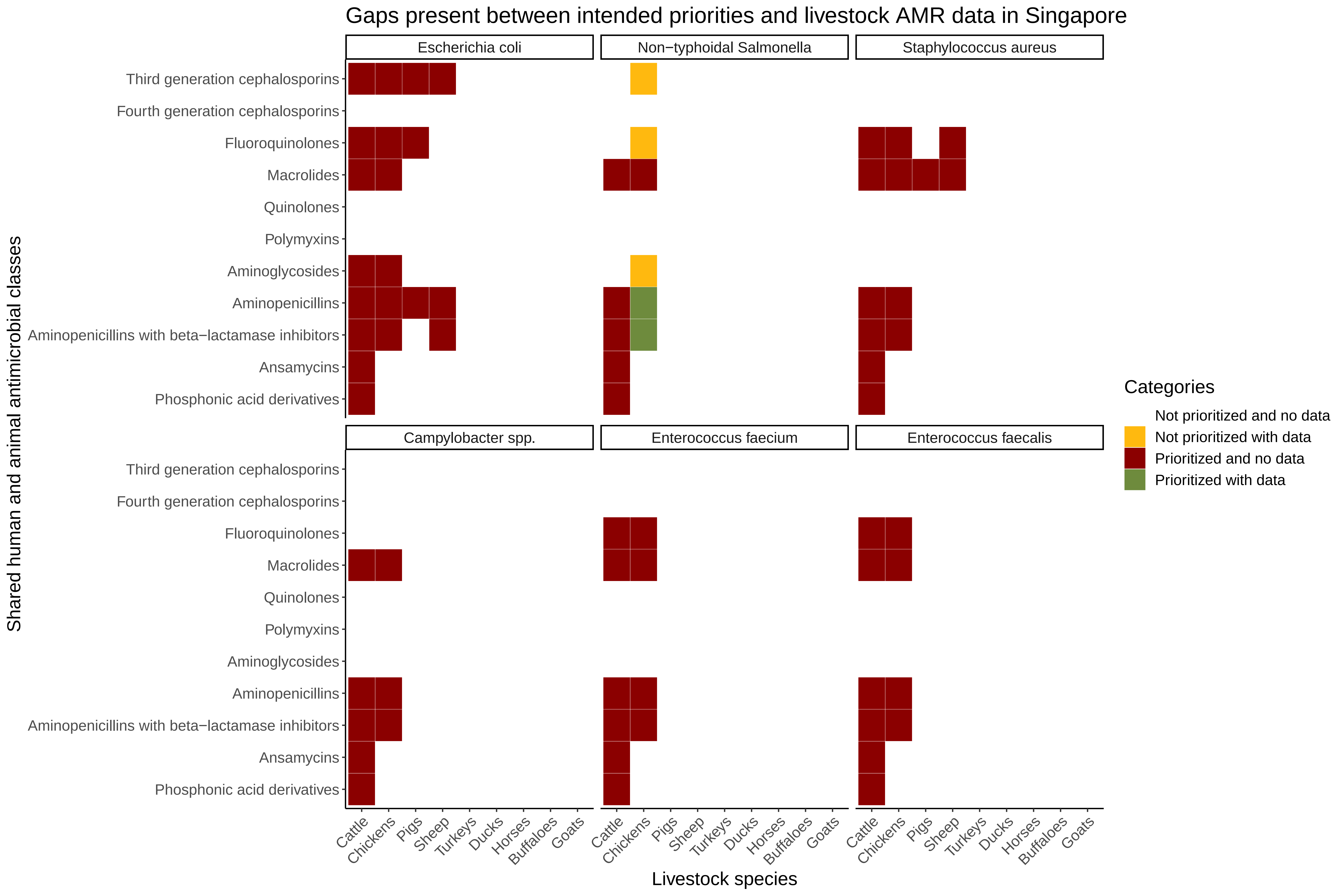
Gaps present between intended priorities and livestock AMR data in Singapore
Escherichia coli
Non−typhoidal Salmonella
Staphylococcus aureus
Campylobacter spp.
Enterococcus faecium
Enterococcus faecalis
Third generation cephalosporins
Fourth generation cephalosporins
Fluoroquinolones
Macrolides
Quinolones
Polymyxins
Aminoglycosides
Aminopenicillins
Aminopenicillins with beta−lactamase inhibitors
Ansamycins
Phosphonic acid derivatives
Shared human and animal antimicrobial classes
Cattle
Chickens
Pigs
Sheep
Turkeys
Ducks
Horses
Buffaloes
Goats
Livestock species
Categories
Not prioritized and no data
Not prioritized with data
Prioritized and no data
Prioritized with data

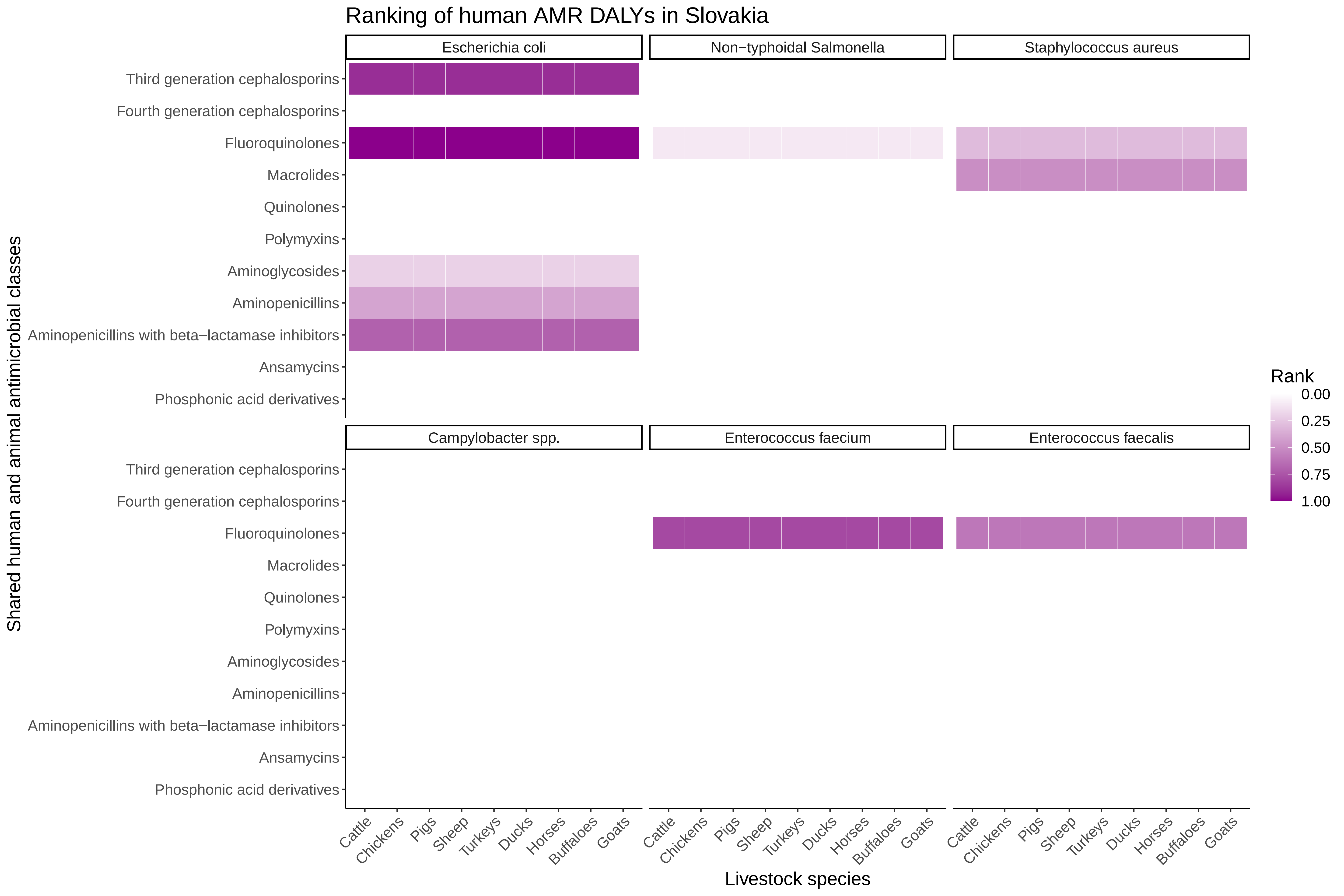
Ranking of human AMR DALYs in Slovakia
Escherichia coli
Non−typhoidal Salmonella
Staphylococcus aureus
Campylobacter spp.
Enterococcus faecium
Enterococcus faecalis
Third generation cephalosporins
Fourth generation cephalosporins
Fluoroquinolones
Macrolides
Quinolones
Polymyxins
Aminoglycosides
Aminopenicillins
Aminopenicillins with beta−lactamase inhibitors
Ansamycins
Phosphonic acid derivatives
Shared human and animal antimicrobial classes
Cattle
Chickens
Pigs
Sheep
Turkeys
Ducks
Horses
Buffaloes
Goats
Livestock species
Rank
0.00
0.25
0.50
0.75
1.00

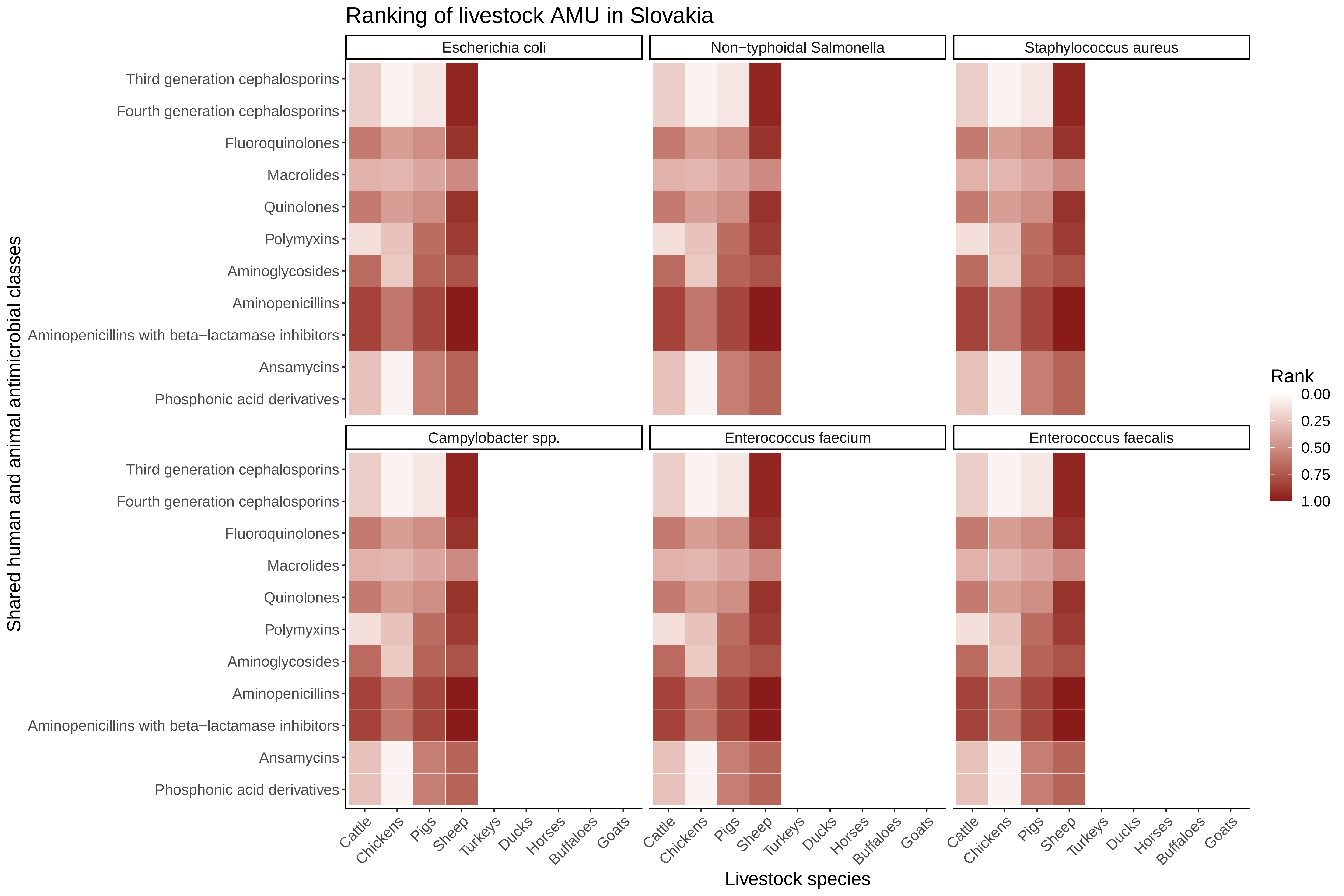

Ranking of livestock AMU in Slovakia
Escherichia coli
Non−typhoidal Salmonella
Staphylococcus aureus
Campylobacter spp.
Enterococcus faecium
Enterococcus faecalis
Third generation cephalosporins
Fourth generation cephalosporins
Fluoroquinolones
Macrolides
Quinolones
Polymyxins
Aminoglycosides
Aminopenicillins
Aminopenicillins with beta−lactamase inhibitors
Ansamycins
Phosphonic acid derivatives
Shared human and animal antimicrobial classes
Cattle
Chickens
Pigs
Sheep
Turkeys
Ducks
Horses
Buffaloes
Goats
Livestock species
Rank
0.00
0.25
0.50
0.75
1.00

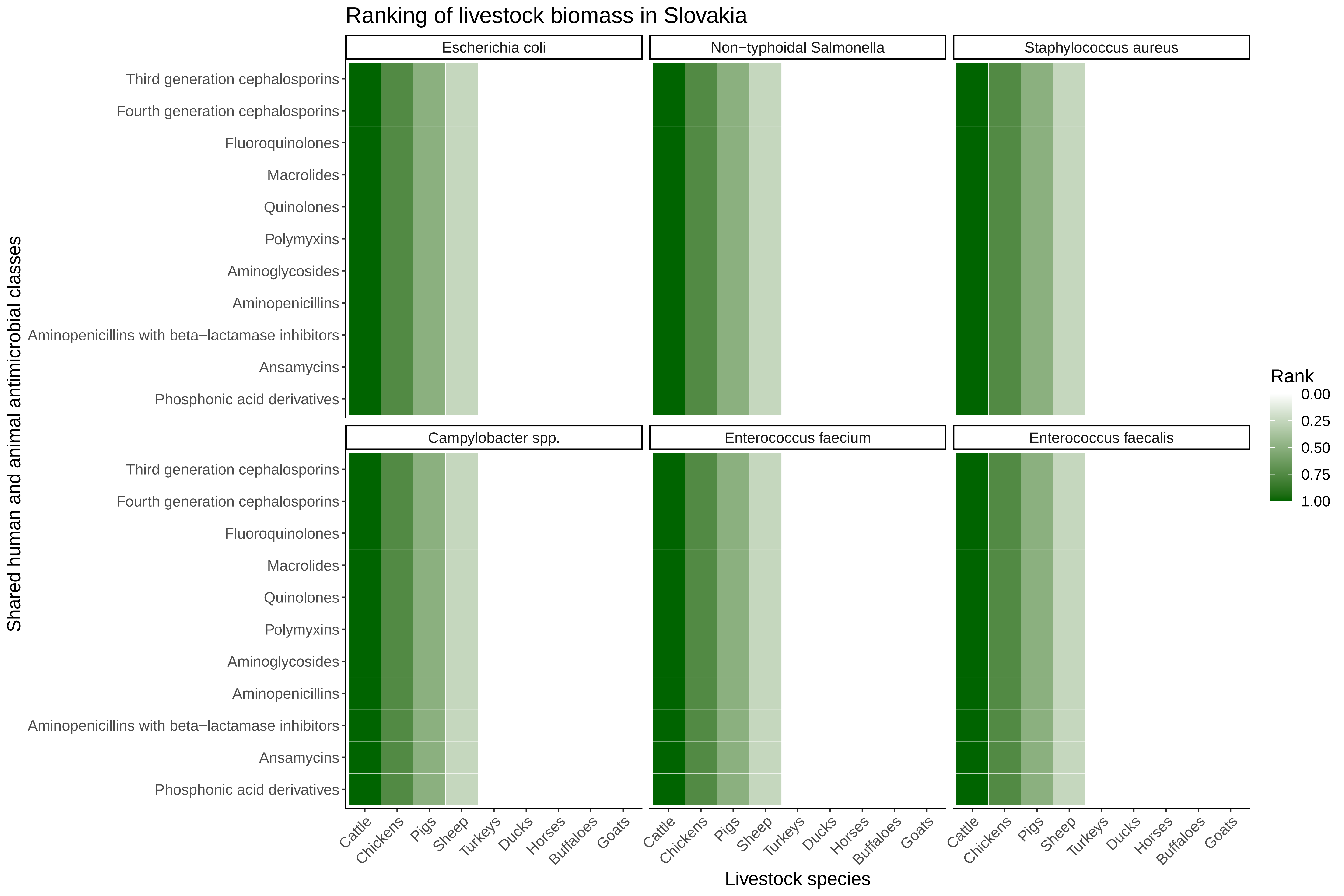

Ranking of livestock biomass in Slovakia
Escherichia coli
Non−typhoidal Salmonella
Staphylococcus aureus
Campylobacter spp.
Enterococcus faecium
Enterococcus faecalis
Third generation cephalosporins
Fourth generation cephalosporins
Fluoroquinolones
Macrolides
Quinolones
Polymyxins
Aminoglycosides
Aminopenicillins
Aminopenicillins with beta−lactamase inhibitors
Ansamycins
Phosphonic acid derivatives
Shared human and animal antimicrobial classes
Cattle
Chickens
Pigs
Sheep
Turkeys
Ducks
Horses
Buffaloes
Goats
Livestock species
Rank
0.00
0.25
0.50
0.75
1.00

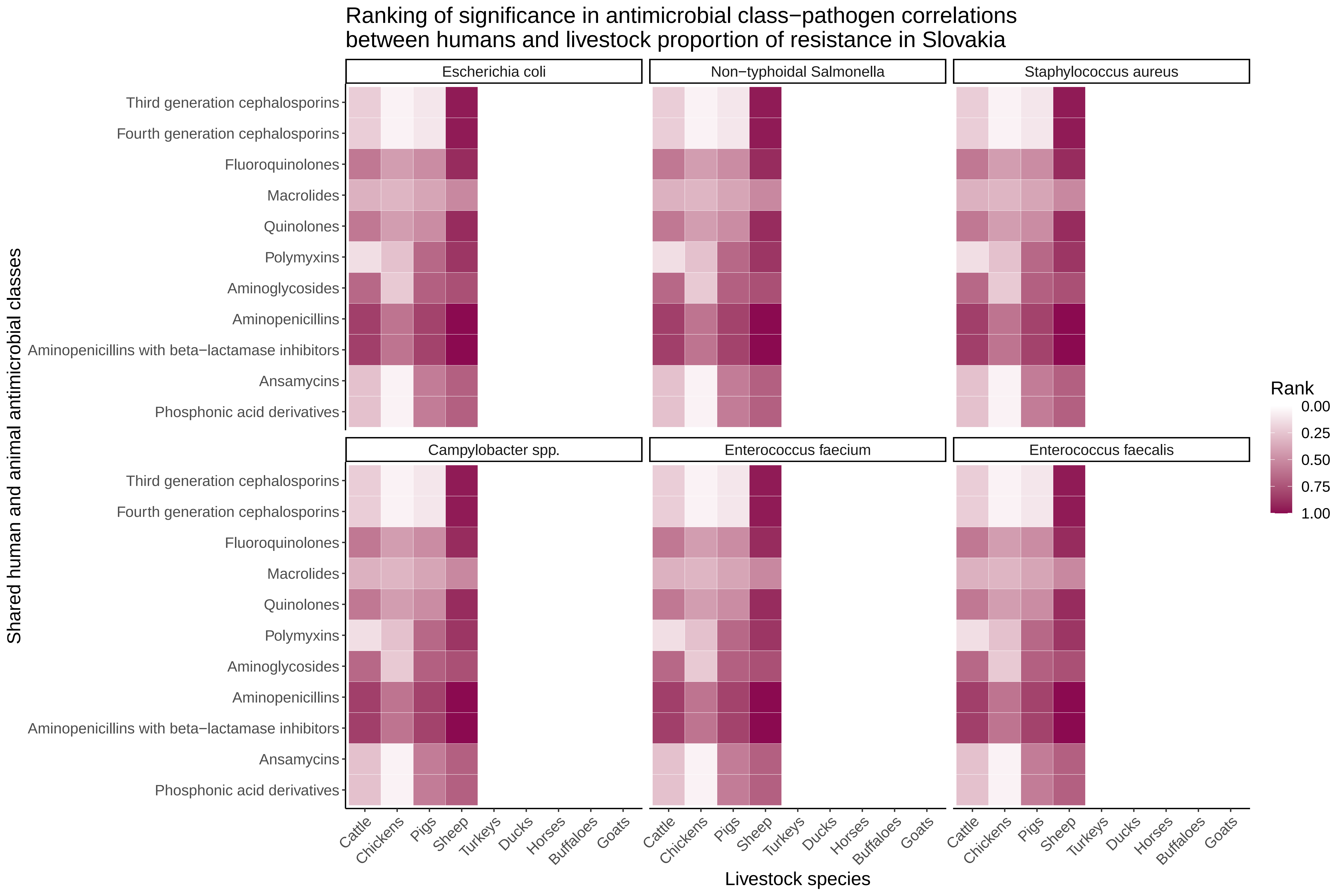

Ranking of significance in antimicrobial class−pathogen correlations
between humans and livestock proportion of resistance in Slovakia
Escherichia coli
Non−typhoidal Salmonella
Staphylococcus aureus
Campylobacter spp.
Enterococcus faecium
Enterococcus faecalis
Third generation cephalosporins
Fourth generation cephalosporins
Fluoroquinolones
Macrolides
Quinolones
Polymyxins
Aminoglycosides
Aminopenicillins
Aminopenicillins with beta−lactamase inhibitors
Ansamycins
Phosphonic acid derivatives
Shared human and animal antimicrobial classes
Cattle
Chickens
Pigs
Sheep
Turkeys
Ducks
Horses
Buffaloes
Goats
Livestock species
Rank
0.00
0.25
0.50
0.75
1.00

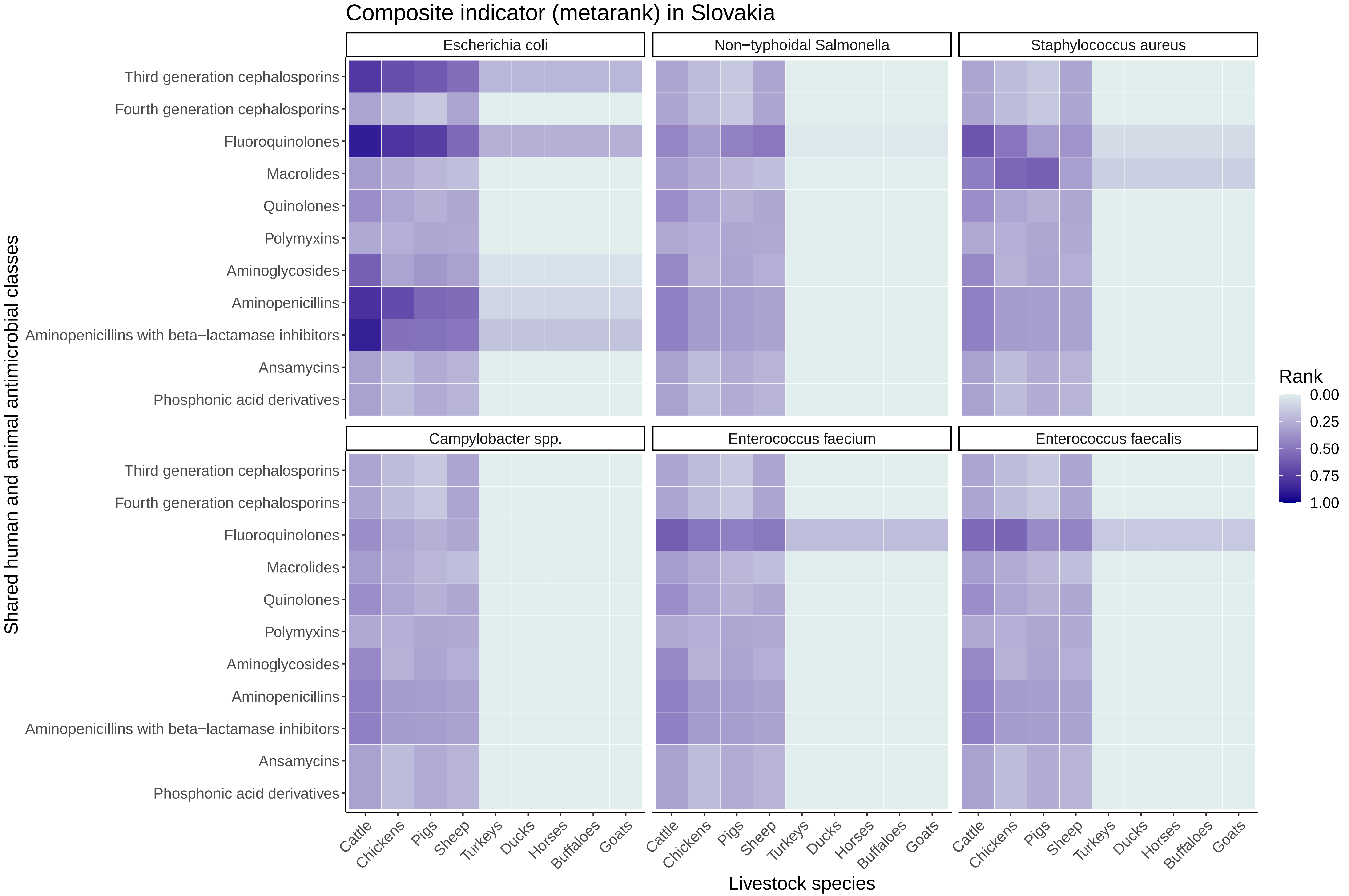

Composite indicator (metarank) in Slovakia
Escherichia coli
Non−typhoidal Salmonella
Staphylococcus aureus
Campylobacter spp.
Enterococcus faecium
Enterococcus faecalis
Shared human and animal antimicrobial classes
Third generation cephalosporins
Fourth generation cephalosporins
Fluoroquinolones
Macrolides
Quinolones
Polymyxins
Aminoglycosides
Aminopenicillins
Aminopenicillins with beta−lactamase inhibitors
Ansamycins
Phosphonic acid derivatives
Rank
0.00
0.25
0.50
0.75
1.00
Cattle
Chickens
Pigs
Sheep
Turkeys
Ducks
Horses
Buffaloes
Goats
Livestock species

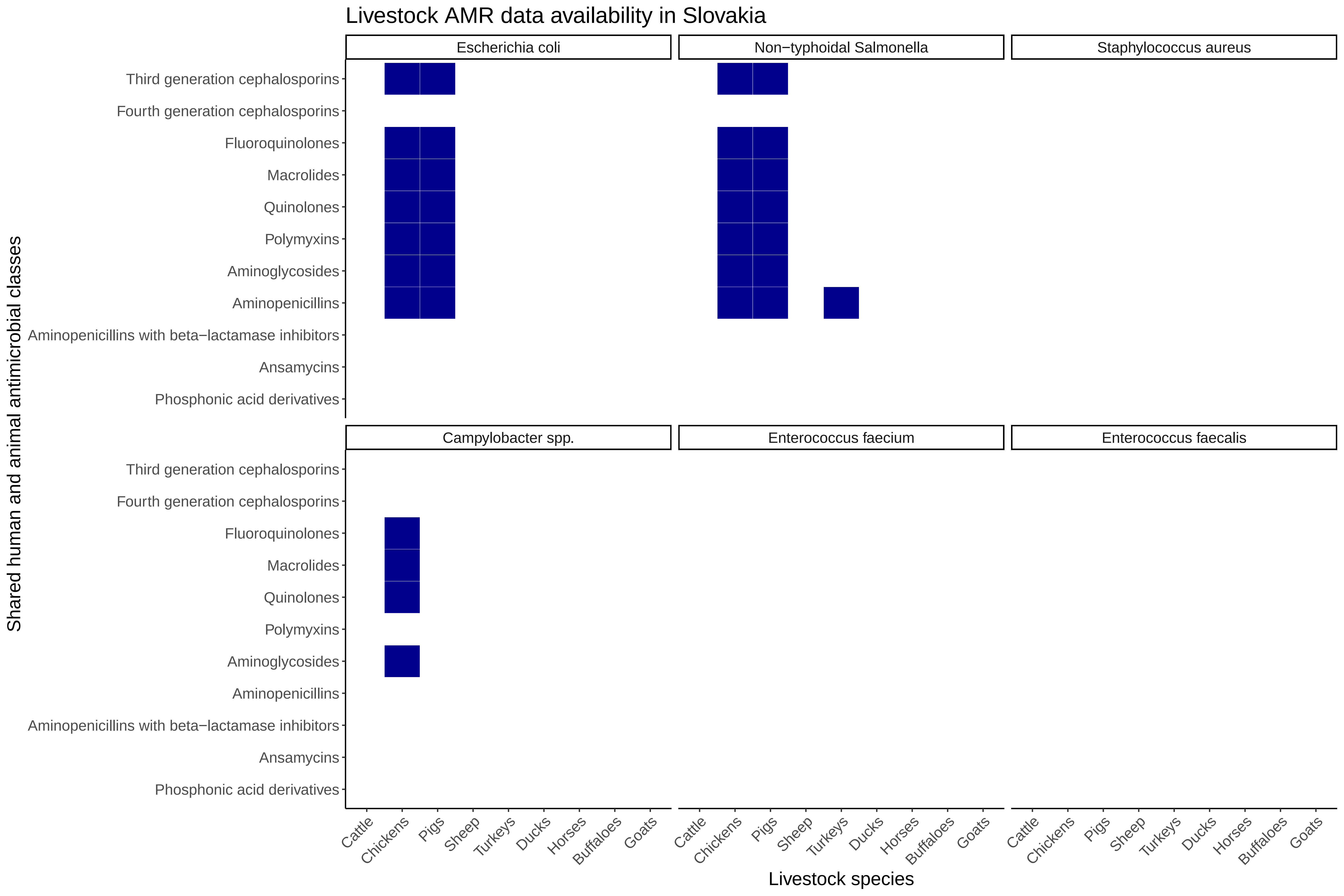

Livestock AMR data availability in Slovakia
Escherichia coli
Non−typhoidal Salmonella
Staphylococcus aureus
Campylobacter spp.
Enterococcus faecium
Enterococcus faecalis
Shared human and animal antimicrobial classes
Third generation cephalosporins
Fourth generation cephalosporins
Fluoroquinolones
Macrolides
Quinolones
Polymyxins
Aminoglycosides
Aminopenicillins
Aminopenicillins with beta−lactamase inhibitors
Ansamycins
Phosphonic acid derivatives
Cattle
Chickens
Pigs
Sheep
Turkeys
Ducks
Horses
Buffaloes
Goats
Livestock species

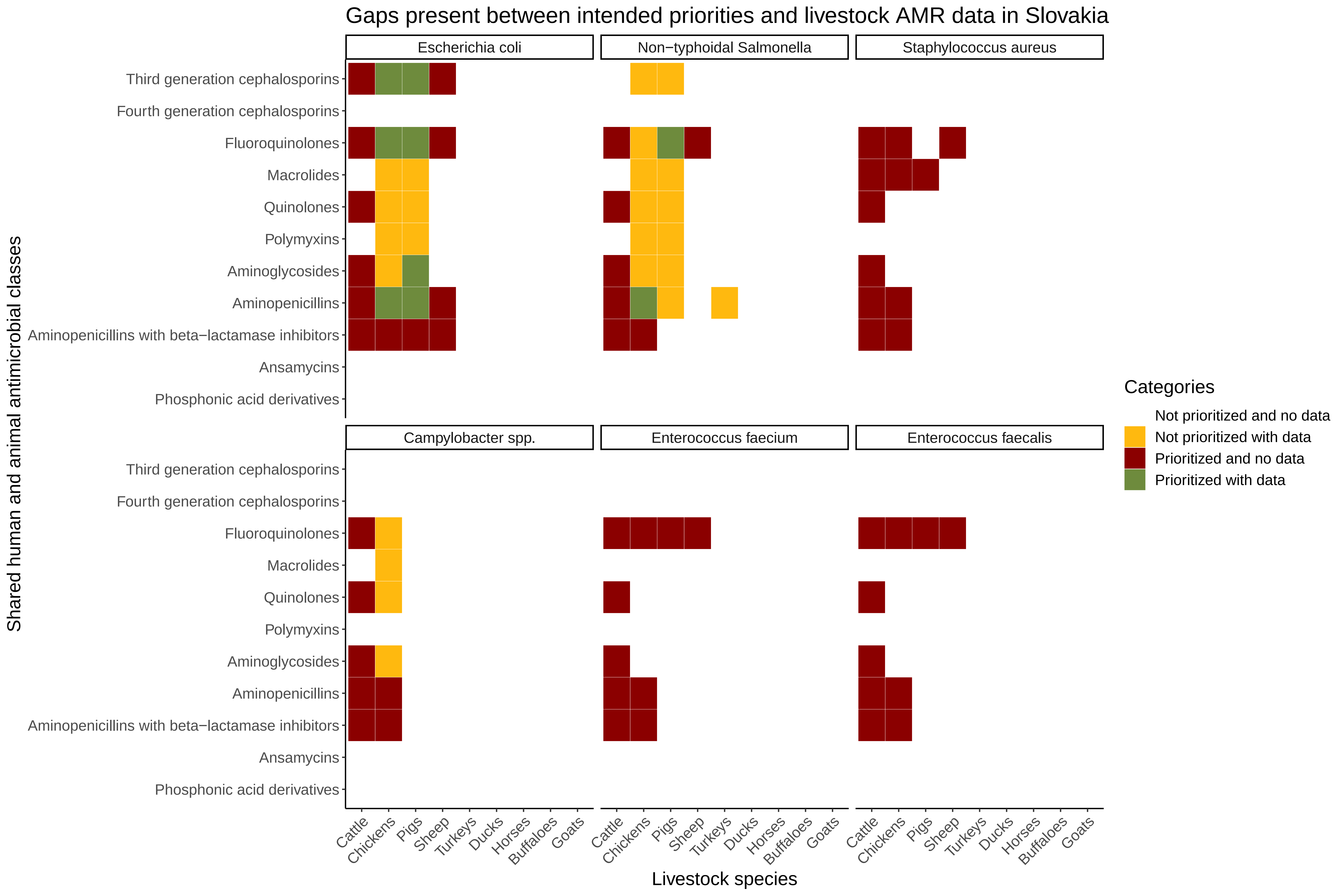

Gaps present between intended priorities and livestock AMR data in Slovakia
Escherichia coli
Non−typhoidal Salmonella
Staphylococcus aureus
Campylobacter spp.
Enterococcus faecium
Enterococcus faecalis
Third generation cephalosporins
Fourth generation cephalosporins
Fluoroquinolones
Macrolides
Quinolones
Polymyxins
Aminoglycosides
Aminopenicillins
Aminopenicillins with beta−lactamase inhibitors
Ansamycins
Phosphonic acid derivatives
Shared human and animal antimicrobial classes
Cattle
Chickens
Pigs
Sheep
Turkeys
Ducks
Horses
Buffaloes
Goats
Livestock species
Categories
Not prioritized and no data
Not prioritized with data
Prioritized and no data
Prioritized with data

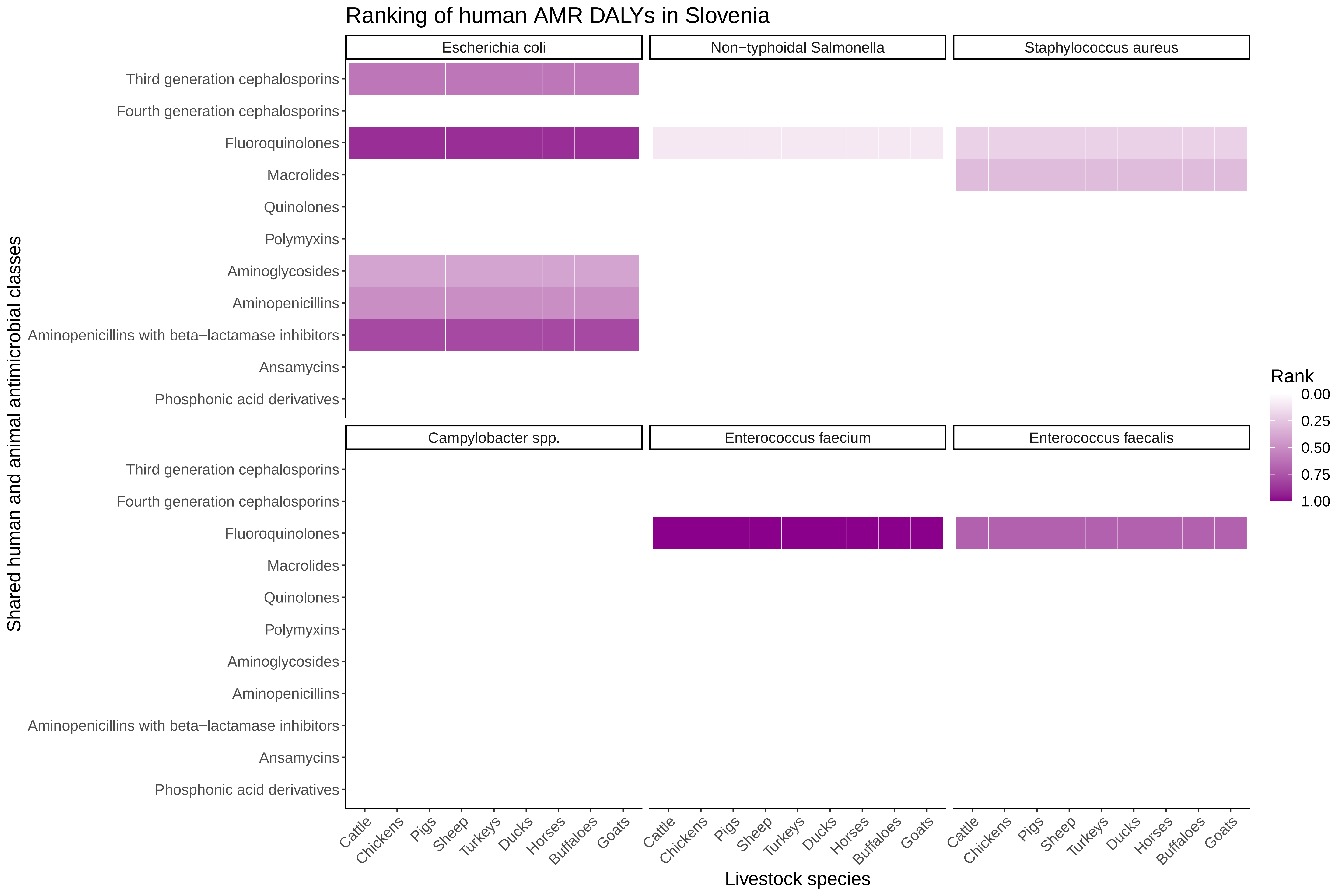
Ranking of human AMR DALYs in Slovenia
Escherichia coli
Non−typhoidal Salmonella
Staphylococcus aureus
Campylobacter spp.
Enterococcus faecium
Enterococcus faecalis
Third generation cephalosporins
Fourth generation cephalosporins
Fluoroquinolones
Macrolides
Quinolones
Polymyxins
Aminoglycosides
Aminopenicillins
Aminopenicillins with beta−lactamase inhibitors
Ansamycins
Phosphonic acid derivatives
Shared human and animal antimicrobial classes
Cattle
Chickens
Pigs
Sheep
Turkeys
Ducks
Horses
Buffaloes
Goats
Livestock species
Rank
0.00
0.25
0.50
0.75
1.00

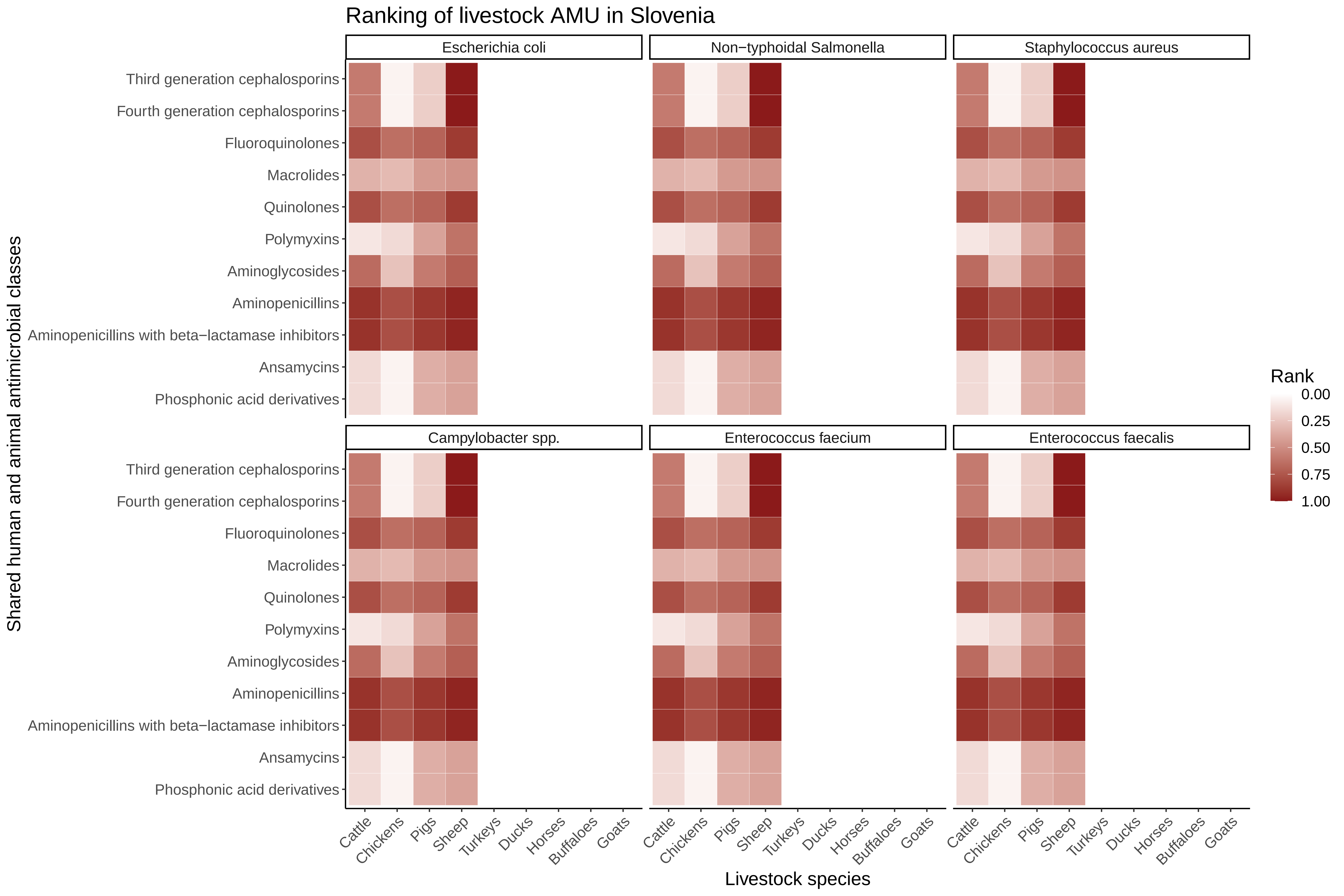

Ranking of livestock AMU in Slovenia
Escherichia coli
Non−typhoidal Salmonella
Staphylococcus aureus
Campylobacter spp.
Enterococcus faecium
Enterococcus faecalis
Third generation cephalosporins
Fourth generation cephalosporins
Fluoroquinolones
Macrolides
Quinolones
Polymyxins
Aminoglycosides
Aminopenicillins
Aminopenicillins with beta−lactamase inhibitors
Ansamycins
Phosphonic acid derivatives
Shared human and animal antimicrobial classes
Cattle
Chickens
Pigs
Sheep
Turkeys
Ducks
Horses
Buffaloes
Goats
Livestock species
Rank
0.00
0.25
0.50
0.75
1.00

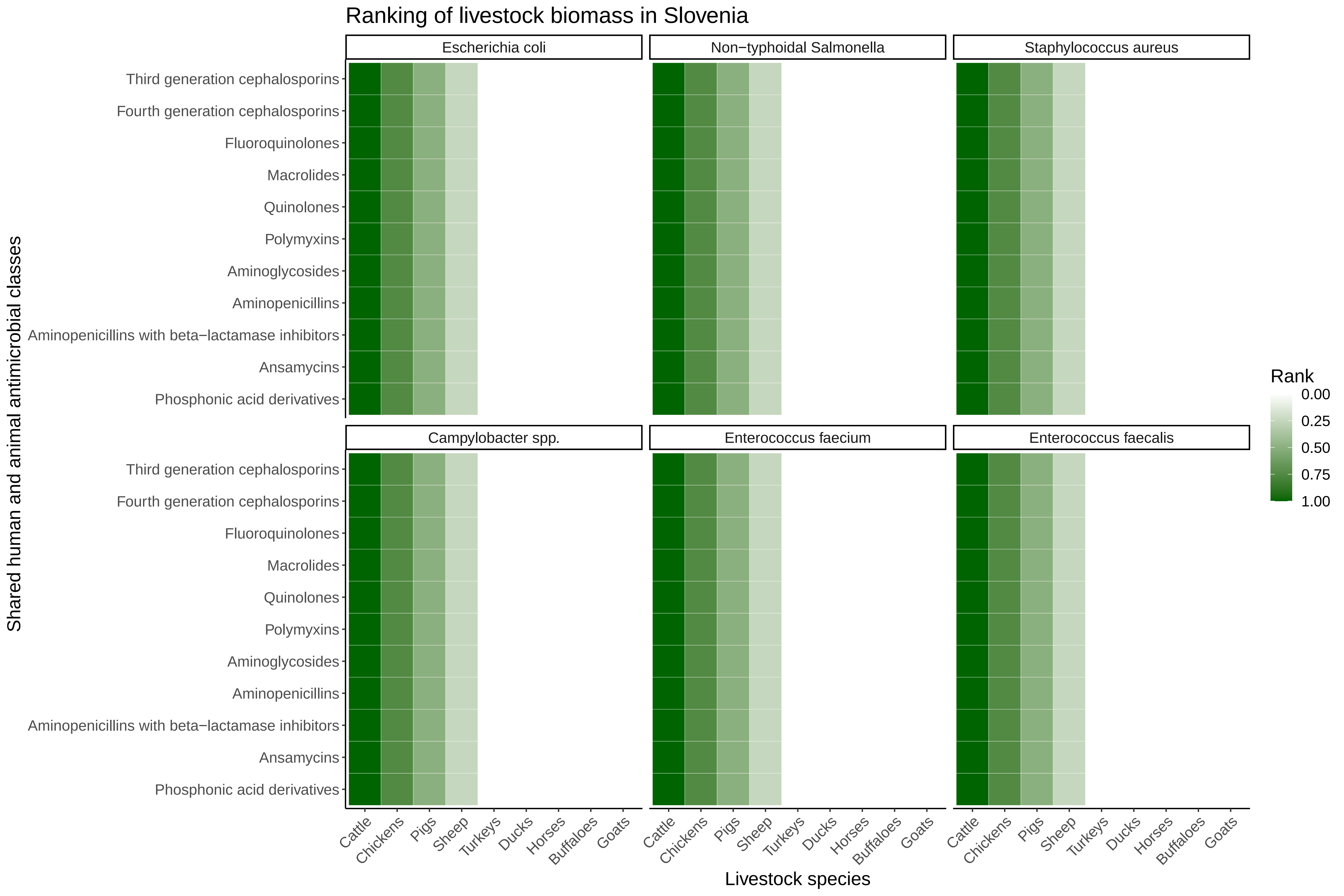
Ranking of livestock biomass in Slovenia
Escherichia coli
Non−typhoidal Salmonella
Staphylococcus aureus
Campylobacter spp.
Enterococcus faecium
Enterococcus faecalis
Third generation cephalosporins
Fourth generation cephalosporins
Fluoroquinolones
Macrolides
Quinolones
Polymyxins
Aminoglycosides
Aminopenicillins
Aminopenicillins with beta−lactamase inhibitors
Ansamycins
Phosphonic acid derivatives
Shared human and animal antimicrobial classes
Cattle
Chickens
Pigs
Sheep
Turkeys
Ducks
Horses
Buffaloes
Goats
Livestock species
Rank
0.00
0.25
0.50
0.75
1.00

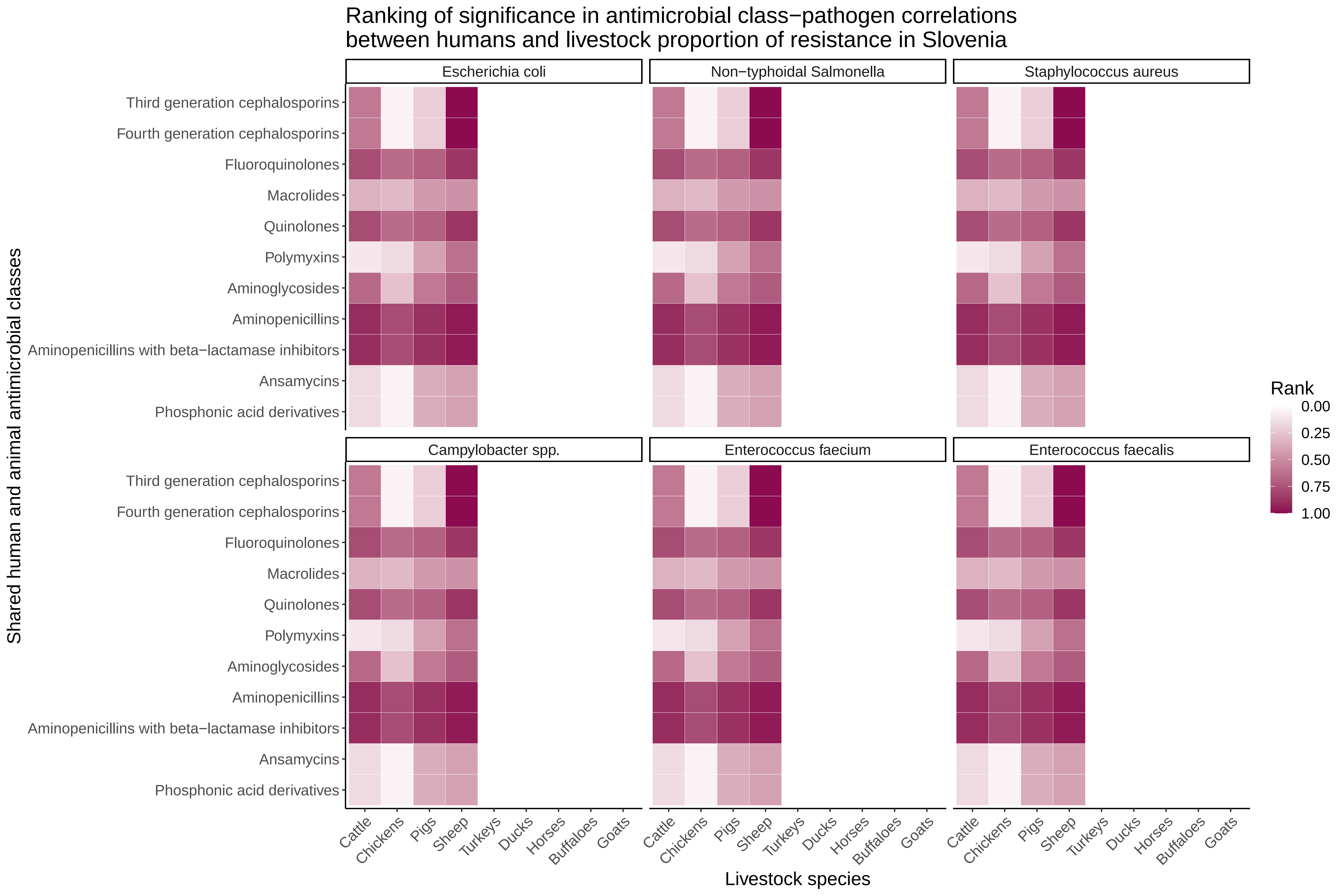
Ranking of significance in antimicrobial class−pathogen correlations
between humans and livestock proportion of resistance in Slovenia
Escherichia coli
Non−typhoidal Salmonella
Staphylococcus aureus
Campylobacter spp.
Enterococcus faecium
Enterococcus faecalis
Third generation cephalosporins
Fourth generation cephalosporins
Fluoroquinolones
Macrolides
Quinolones
Polymyxins
Aminoglycosides
Aminopenicillins
Aminopenicillins with beta−lactamase inhibitors
Ansamycins
Phosphonic acid derivatives
Shared human and animal antimicrobial classes
Cattle
Chickens
Pigs
Sheep
Turkeys
Ducks
Horses
Buffaloes
Goats
Livestock species
Rank
0.00
0.25
0.50
0.75
1.00

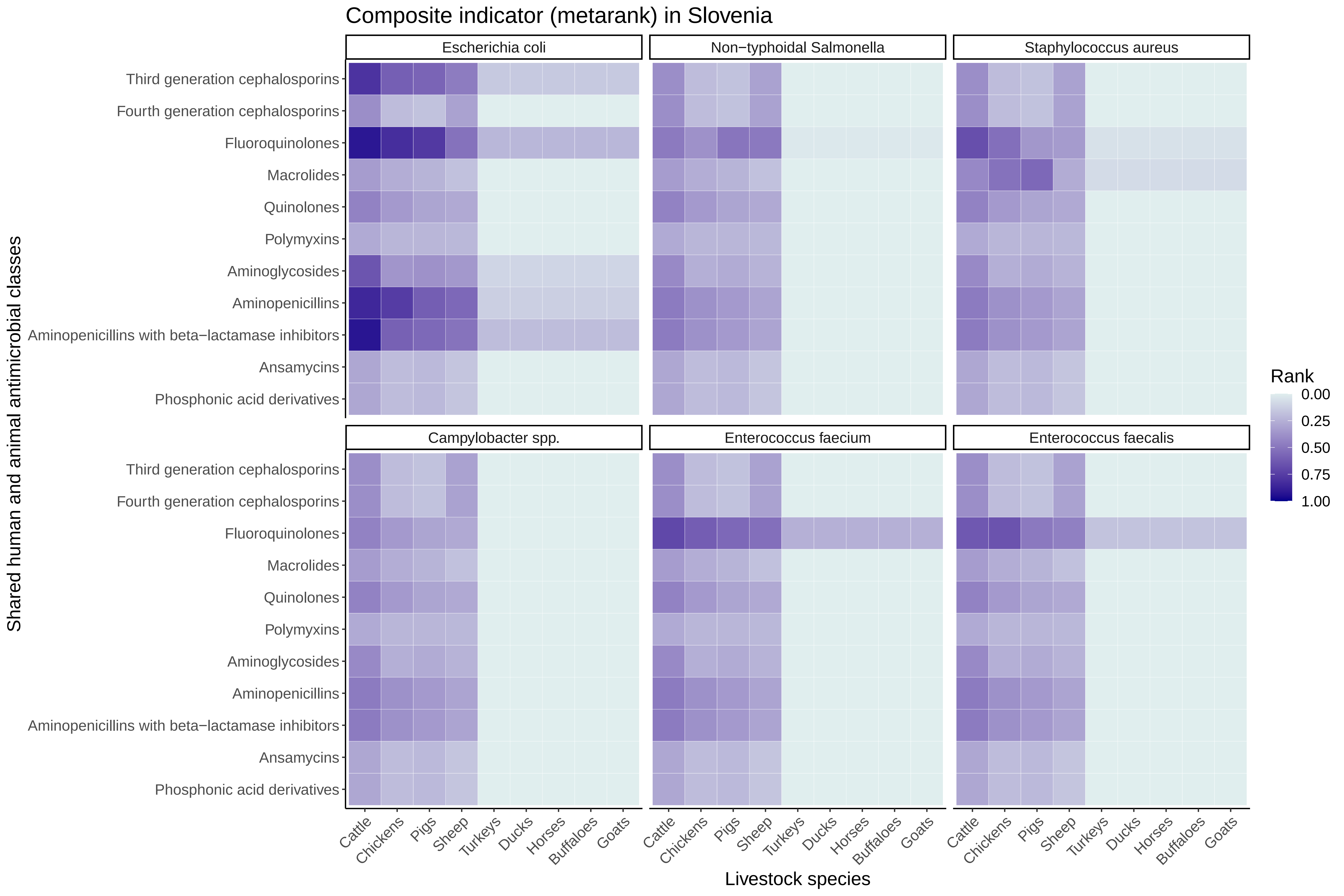
Composite indicator (metarank) in Slovenia
Escherichia coli
Non−typhoidal Salmonella
Staphylococcus aureus
Campylobacter spp.
Enterococcus faecium
Enterococcus faecalis
Shared human and animal antimicrobial classes
Third generation cephalosporins
Fourth generation cephalosporins
Fluoroquinolones
Macrolides
Quinolones
Polymyxins
Aminoglycosides
Aminopenicillins
Aminopenicillins with beta−lactamase inhibitors
Ansamycins
Phosphonic acid derivatives
Cattle
Chickens
Pigs
Sheep
Turkeys
Ducks
Horses
Buffaloes
Goats
Livestock species
Rank
0.00
0.25
0.50
0.75
1.00

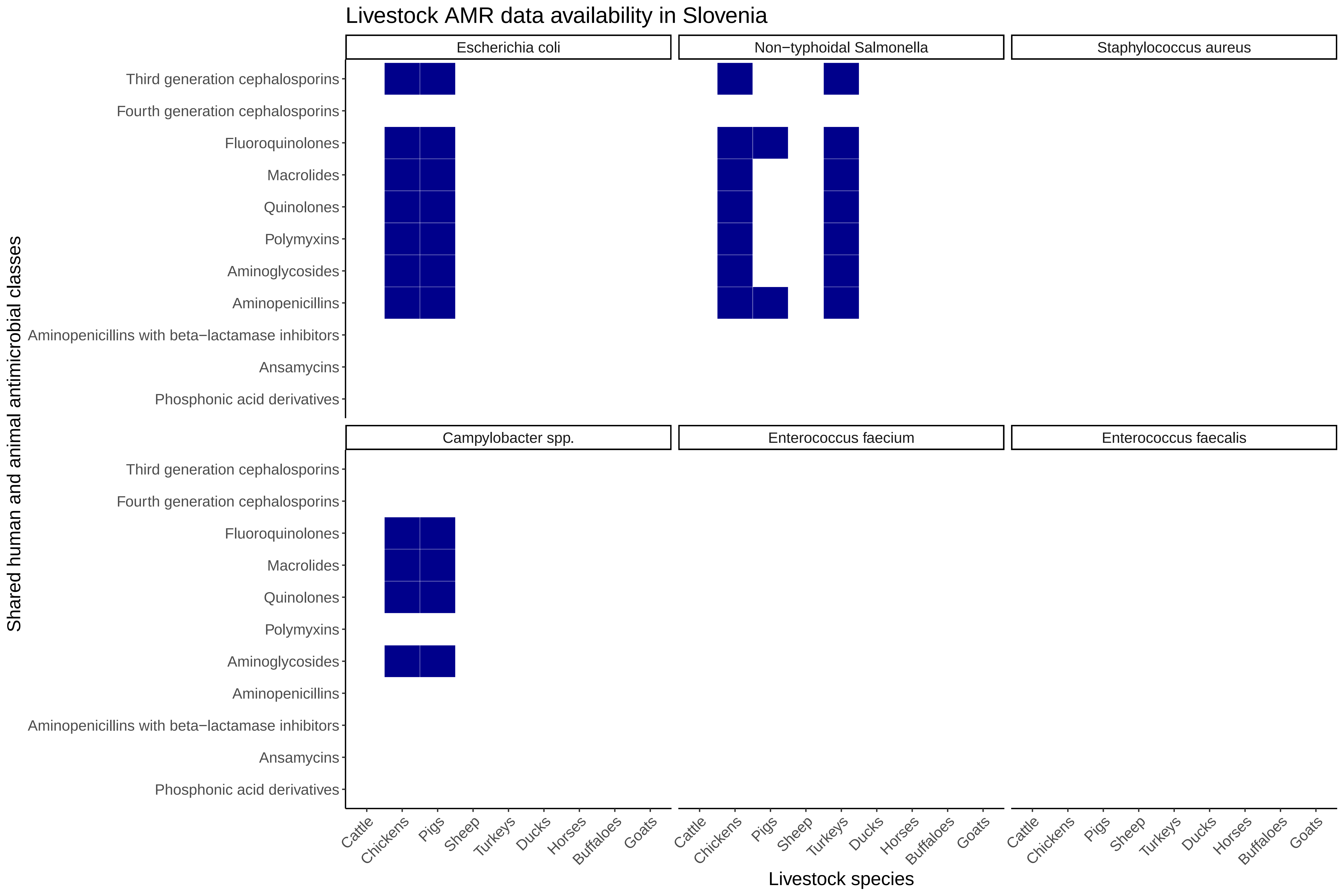

Livestock AMR data availability in Slovenia
Escherichia coli
Non−typhoidal Salmonella
Staphylococcus aureus
Campylobacter spp.
Enterococcus faecium
Enterococcus faecalis
Shared human and animal antimicrobial classes
Third generation cephalosporins
Fourth generation cephalosporins
Fluoroquinolones
Macrolides
Quinolones
Polymyxins
Aminoglycosides
Aminopenicillins
Aminopenicillins with beta−lactamase inhibitors
Ansamycins
Phosphonic acid derivatives
Cattle
Chickens
Pigs
Sheep
Turkeys
Ducks
Horses
Buffaloes
Goats
Livestock species

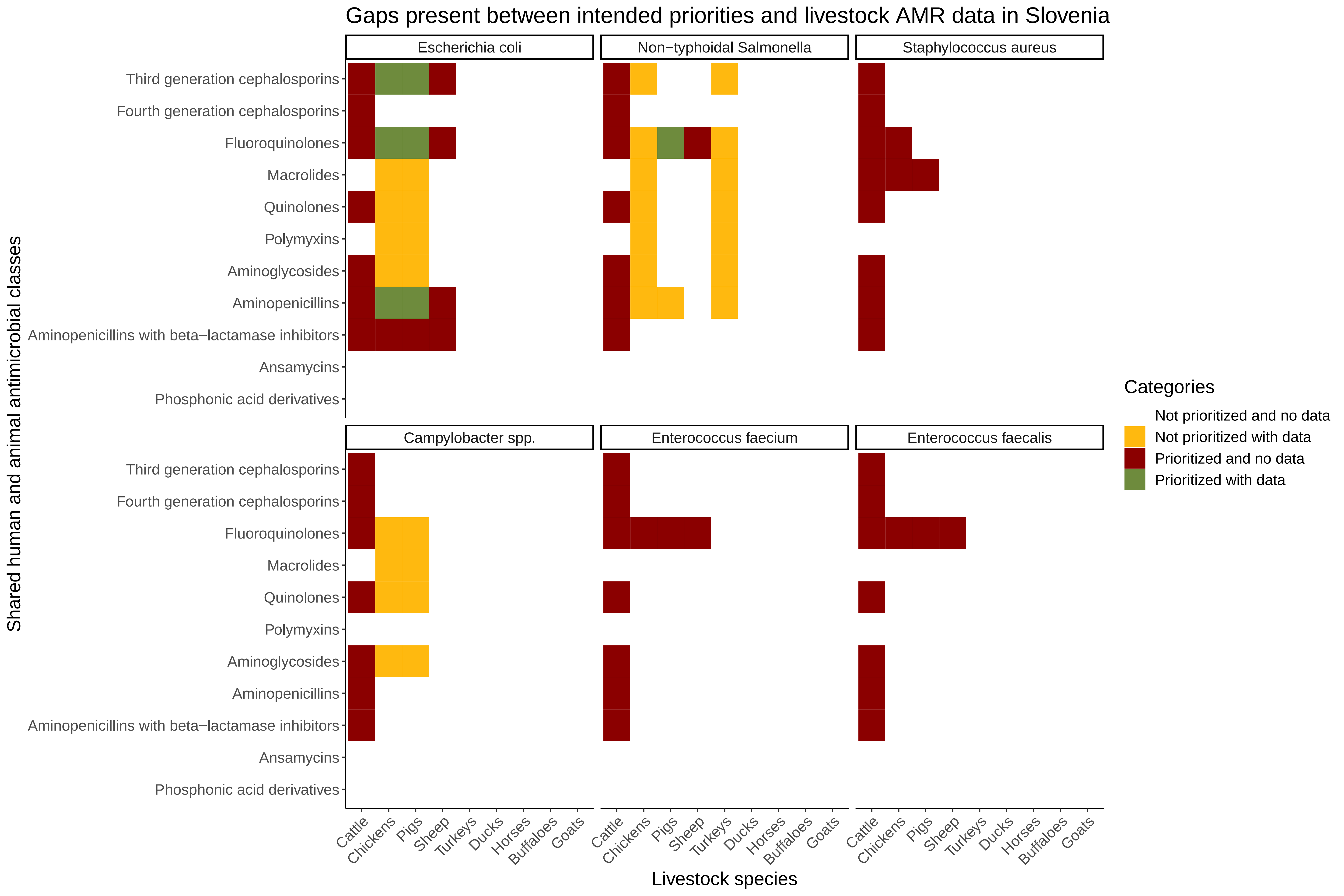

Gaps present between intended priorities and livestock AMR data in Slovenia
Escherichia coli
Non−typhoidal Salmonella
Staphylococcus aureus
Campylobacter spp.
Enterococcus faecium
Enterococcus faecalis
Shared human and animal antimicrobial classes
Third generation cephalosporins
Fourth generation cephalosporins
Fluoroquinolones
Macrolides
Quinolones
Polymyxins
Aminoglycosides
Aminopenicillins
Aminopenicillins with beta−lactamase inhibitors
Ansamycins
Phosphonic acid derivatives
Livestock species
Cattle
Chickens
Pigs
Sheep
Turkeys
Ducks
Horses
Buffaloes
Goats
Categories
Not prioritized and no data
Not prioritized with data
Prioritized and no data
Prioritized with data

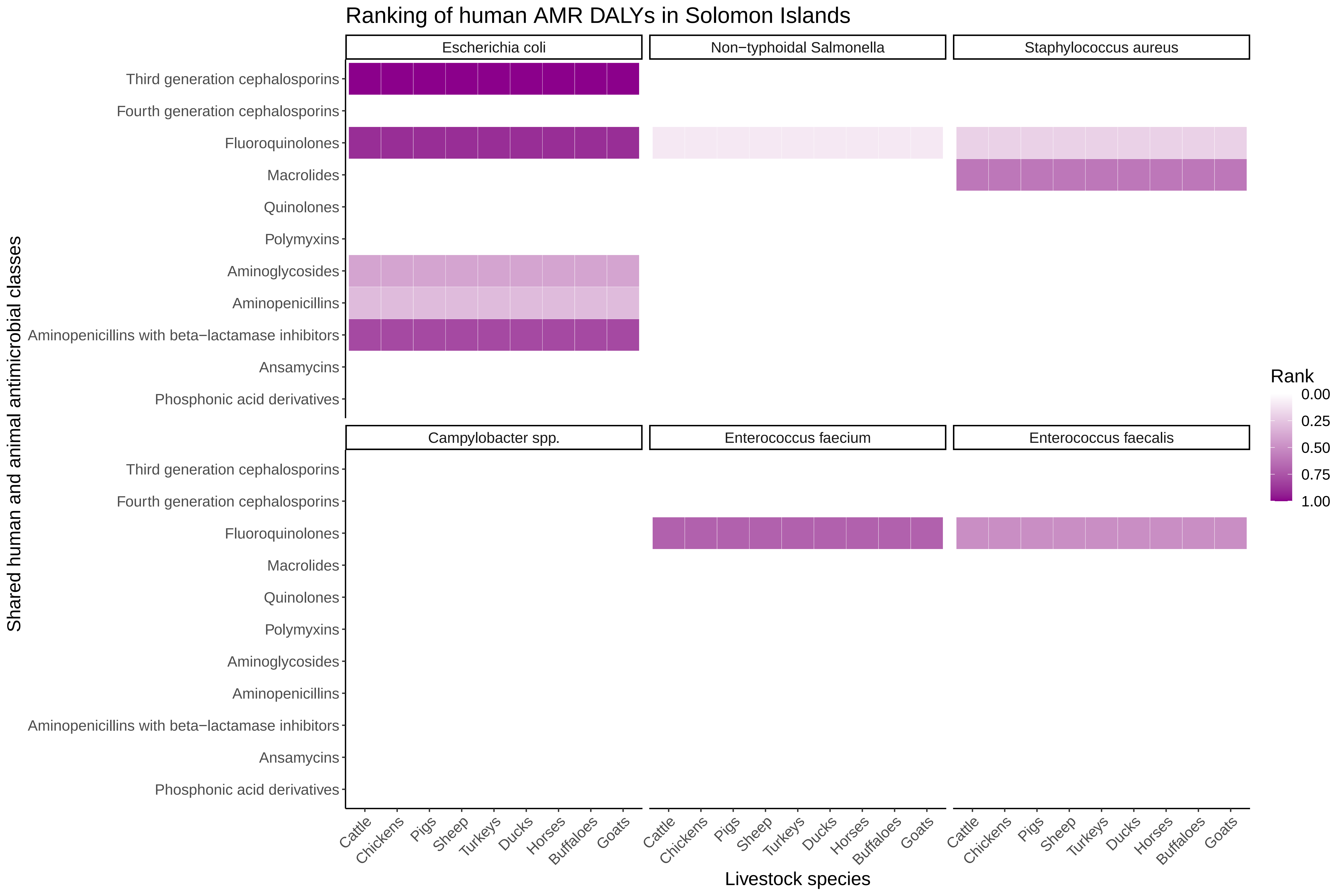
Ranking of human AMR DALYs in Solomon Islands
Escherichia coli
Non−typhoidal Salmonella
Staphylococcus aureus
Campylobacter spp.
Enterococcus faecium
Enterococcus faecalis
Third generation cephalosporins
Fourth generation cephalosporins
Fluoroquinolones
Macrolides
Quinolones
Polymyxins
Aminoglycosides
Aminopenicillins
Aminopenicillins with beta−lactamase inhibitors
Ansamycins
Phosphonic acid derivatives
Shared human and animal antimicrobial classes
Cattle
Chickens
Pigs
Sheep
Turkeys
Ducks
Horses
Buffaloes
Goats
Livestock species
Rank
0.00
0.25
0.50
0.75
1.00

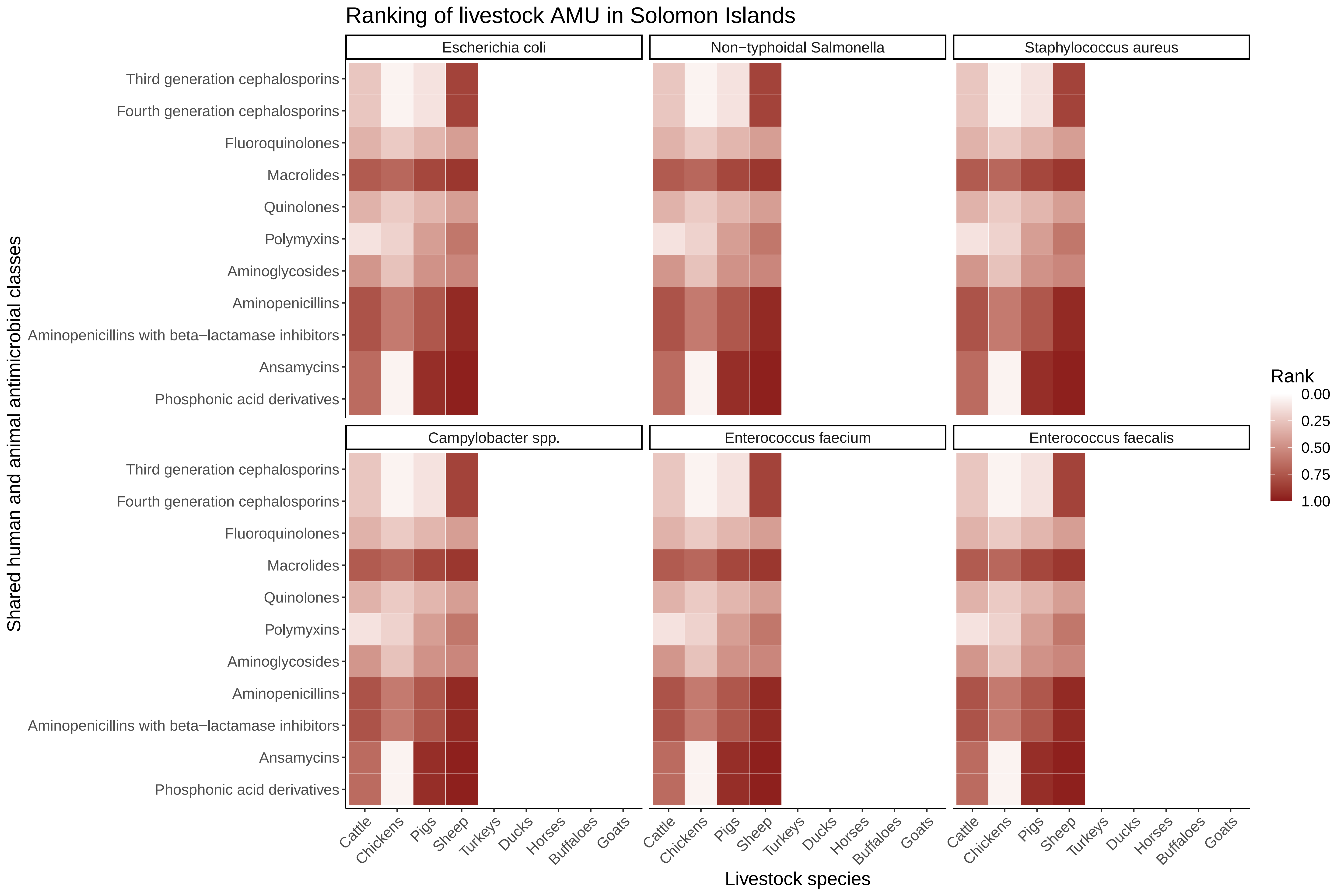
Ranking of livestock AMU in Solomon Islands
Escherichia coli
Non−typhoidal Salmonella
Staphylococcus aureus
Campylobacter spp.
Enterococcus faecium
Enterococcus faecalis
Shared human and animal antimicrobial classes
Third generation cephalosporins
Fourth generation cephalosporins
Fluoroquinolones
Macrolides
Quinolones
Polymyxins
Aminoglycosides
Aminopenicillins
Aminopenicillins with beta−lactamase inhibitors
Ansamycins
Phosphonic acid derivatives
Cattle
Chickens
Pigs
Sheep
Turkeys
Ducks
Horses
Buffaloes
Goats
Livestock species
Rank
0.00
0.25
0.50
0.75
1.00

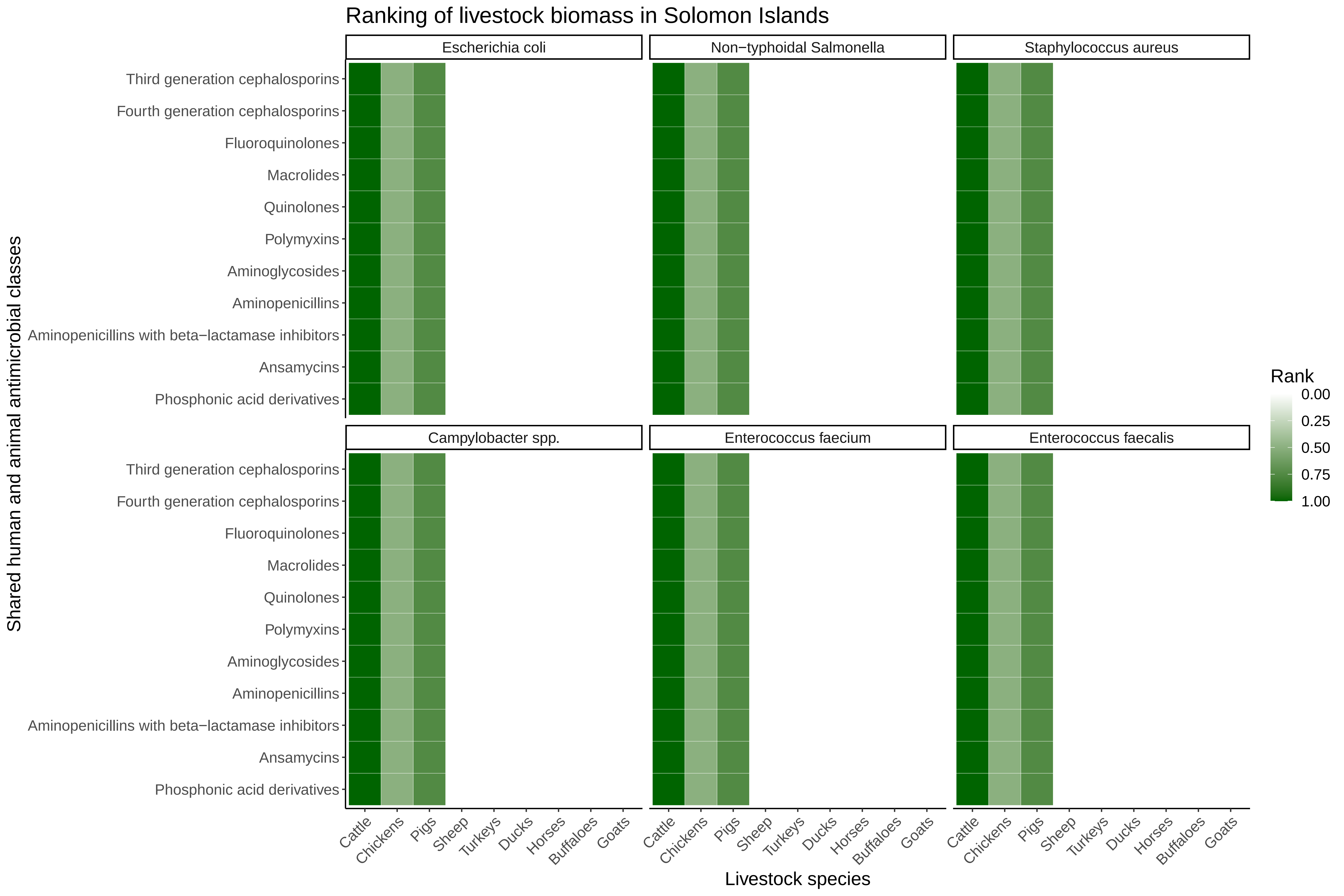

Ranking of livestock biomass in Solomon Islands
Escherichia coli
Non−typhoidal Salmonella
Staphylococcus aureus
Campylobacter spp.
Enterococcus faecium
Enterococcus faecalis
Third generation cephalosporins
Fourth generation cephalosporins
Fluoroquinolones
Macrolides
Quinolones
Polymyxins
Aminoglycosides
Aminopenicillins
Aminopenicillins with beta−lactamase inhibitors
Ansamycins
Phosphonic acid derivatives
Shared human and animal antimicrobial classes
Cattle
Chickens
Pigs
Sheep
Turkeys
Ducks
Horses
Buffaloes
Goats
Livestock species
Rank
0.00
0.25
0.50
0.75
1.00

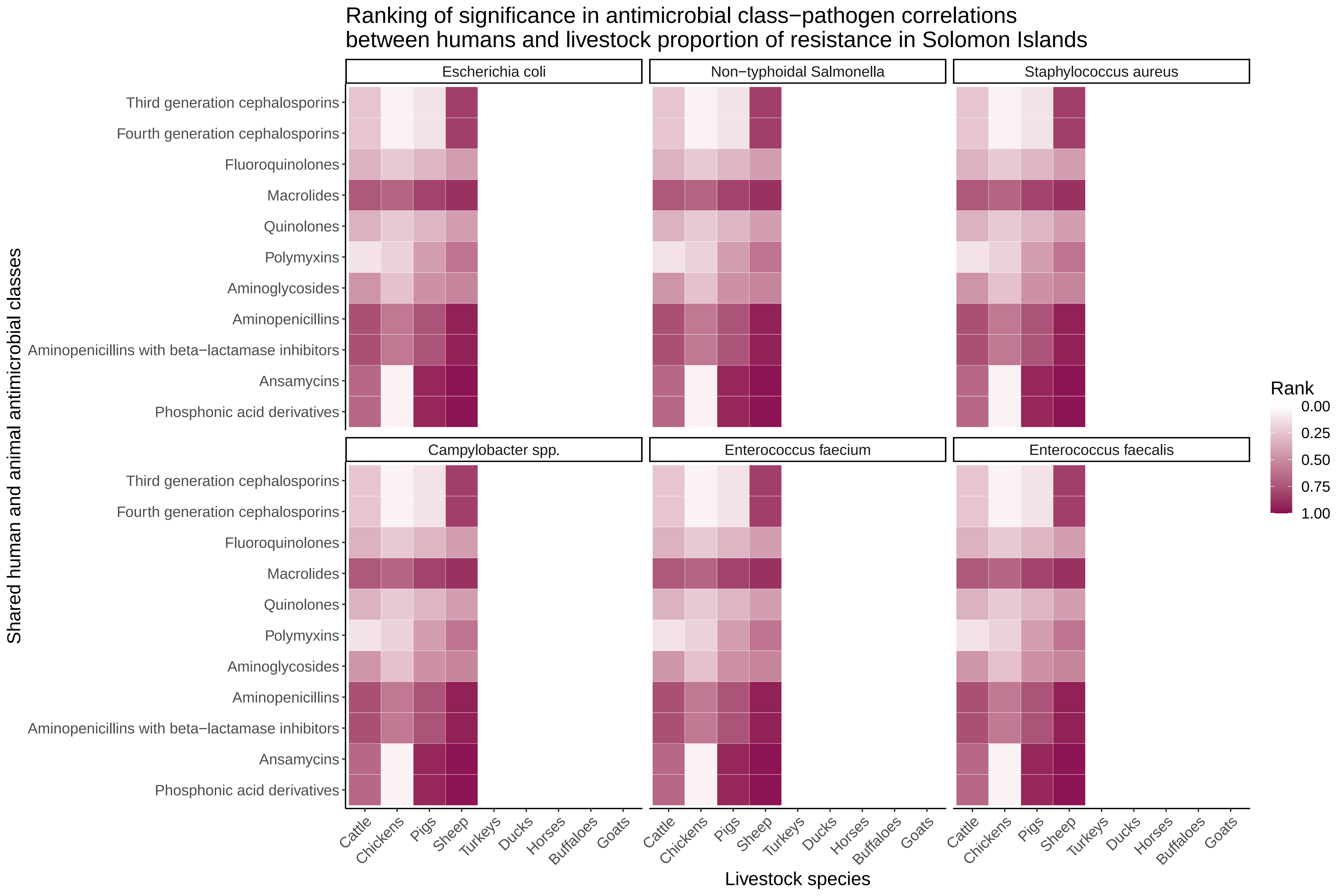

Ranking of significance in antimicrobial class−pathogen correlations
between humans and livestock proportion of resistance in Solomon Islands
Escherichia coli
Non−typhoidal Salmonella
Staphylococcus aureus
Campylobacter spp.
Enterococcus faecium
Enterococcus faecalis
Third generation cephalosporins
Fourth generation cephalosporins
Fluoroquinolones
Macrolides
Quinolones
Polymyxins
Aminoglycosides
Aminopenicillins
Aminopenicillins with beta−lactamase inhibitors
Ansamycins
Phosphonic acid derivatives
Shared human and animal antimicrobial classes
Cattle
Chickens
Pigs
Sheep
Turkeys
Ducks
Horses
Buffaloes
Goats
Livestock species
Rank
0.00
0.25
0.50
0.75
1.00

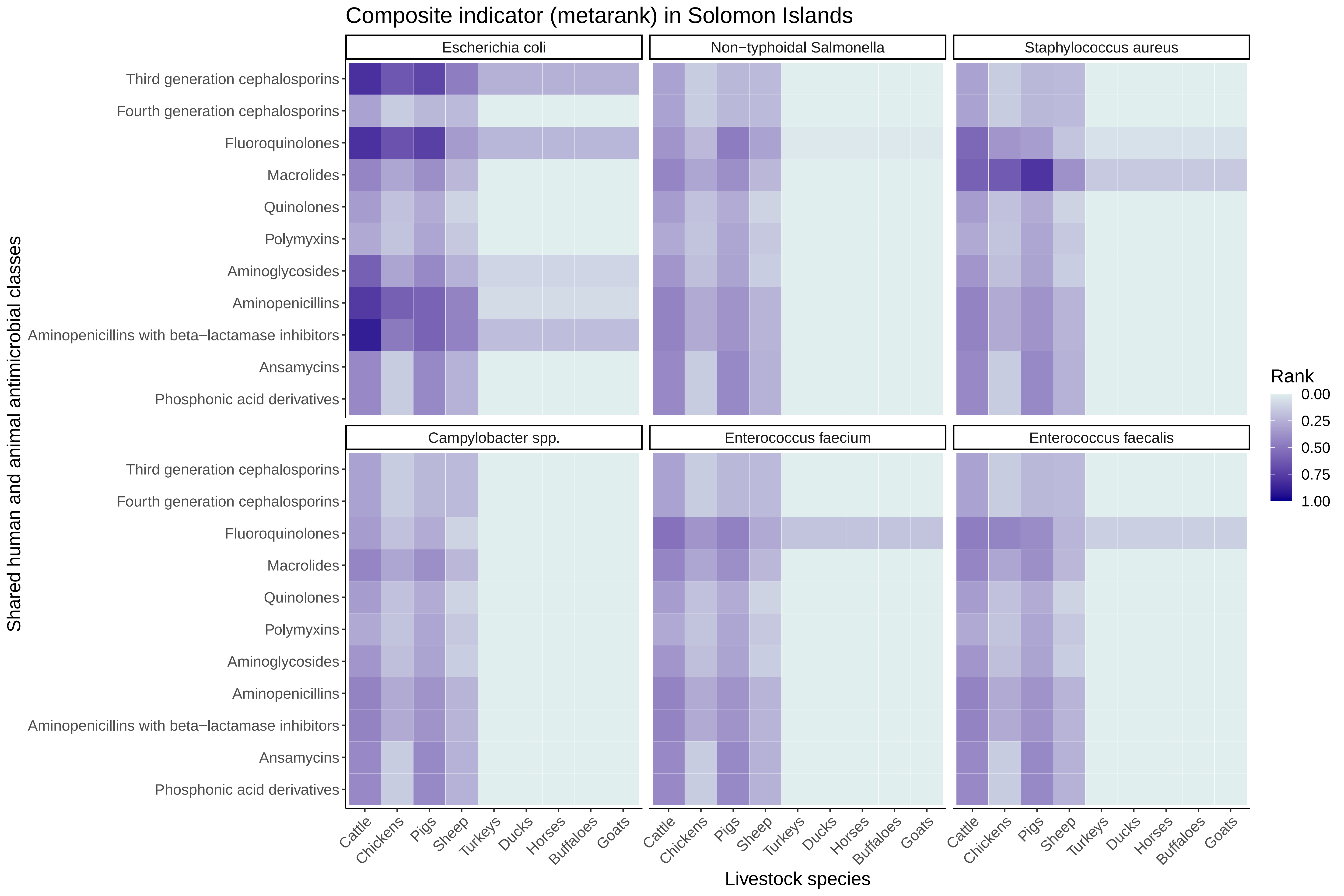
Composite indicator (metarank) in Solomon Islands
Escherichia coli
Non−typhoidal Salmonella
Staphylococcus aureus
Campylobacter spp.
Enterococcus faecium
Enterococcus faecalis
Shared human and animal antimicrobial classes
Third generation cephalosporins
Fourth generation cephalosporins
Fluoroquinolones
Macrolides
Quinolones
Polymyxins
Aminoglycosides
Aminopenicillins
Aminopenicillins with beta−lactamase inhibitors
Ansamycins
Phosphonic acid derivatives
Cattle
Chickens
Pigs
Sheep
Turkeys
Ducks
Horses
Buffaloes
Goats
Livestock species
Rank
0.00
0.25
0.50
0.75
1.00

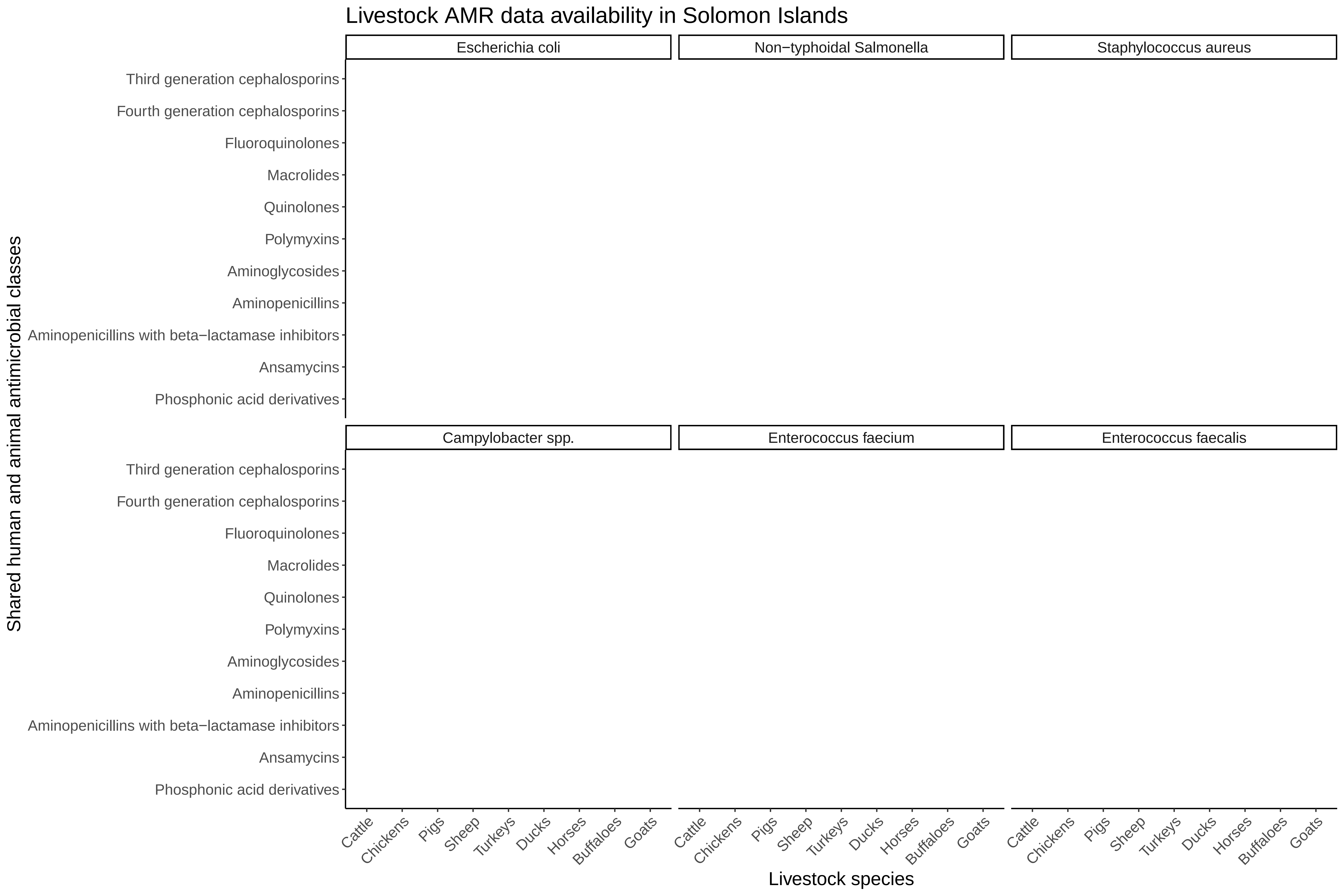
Livestock AMR data availability in Solomon Islands
Escherichia coli
Non−typhoidal Salmonella
Staphylococcus aureus
Campylobacter spp.
Enterococcus faecium
Enterococcus faecalis
Shared human and animal antimicrobial classes
Third generation cephalosporins
Fourth generation cephalosporins
Fluoroquinolones
Macrolides
Quinolones
Polymyxins
Aminoglycosides
Aminopenicillins
Aminopenicillins with beta−lactamase inhibitors
Ansamycins
Phosphonic acid derivatives
Cattle
Chickens
Pigs
Sheep
Turkeys
Ducks
Horses
Buffaloes
Goats
Livestock species

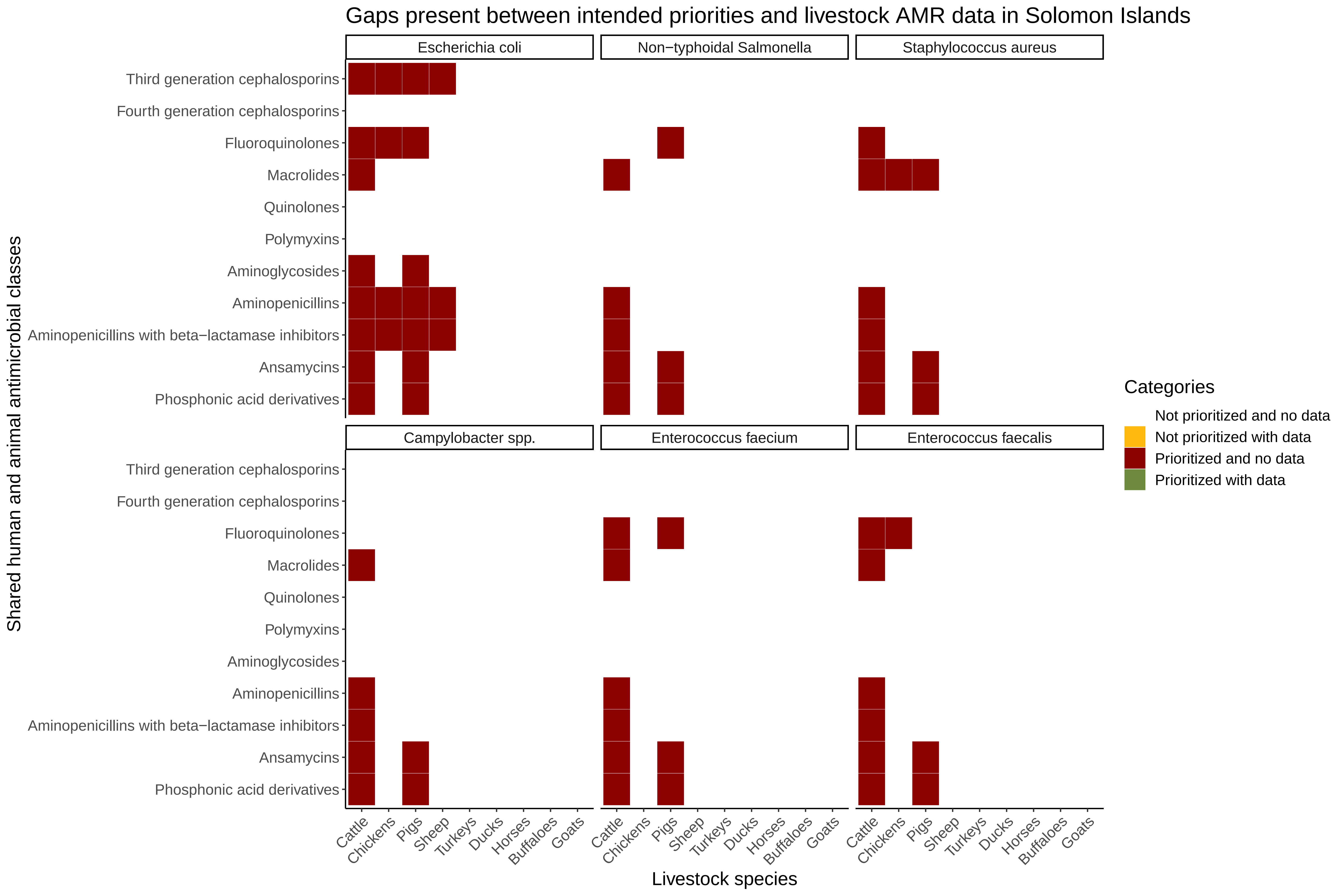

Gaps present between intended priorities and livestock AMR data in Solomon Islands
Escherichia coli
Non−typhoidal Salmonella
Staphylococcus aureus
Campylobacter spp.
Enterococcus faecium
Enterococcus faecalis
Third generation cephalosporins
Fourth generation cephalosporins
Fluoroquinolones
Macrolides
Quinolones
Polymyxins
Aminoglycosides
Aminopenicillins
Aminopenicillins with beta−lactamase inhibitors
Ansamycins
Phosphonic acid derivatives
Cattle
Chickens
Pigs
Sheep
Turkeys
Ducks
Horses
Buffaloes
Goats
Shared human and animal antimicrobial classes
Livestock species
Categories
Not prioritized and no data
Not prioritized with data
Prioritized and no data
Prioritized with data

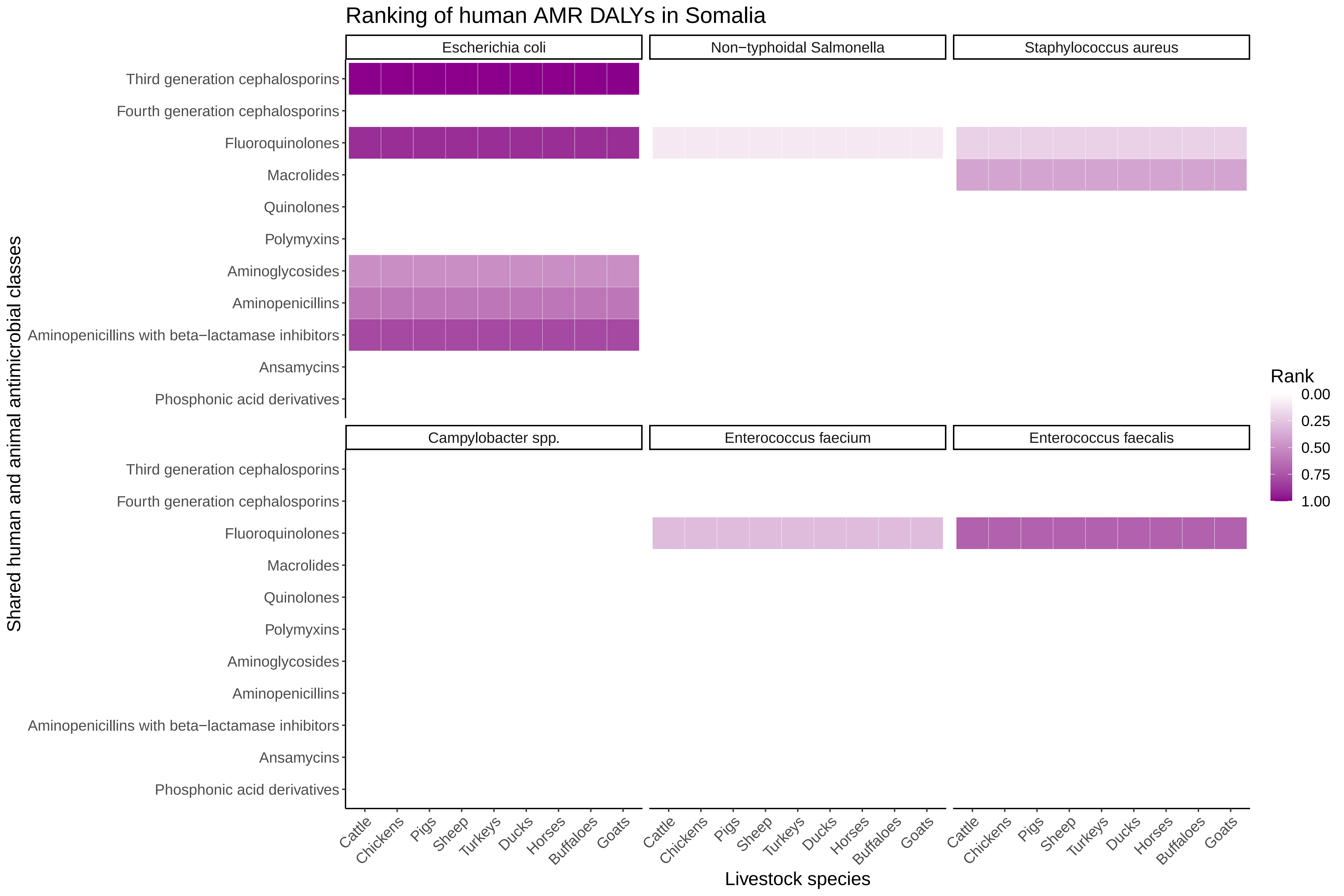
Ranking of human AMR DALYs in Somalia
Escherichia coli
Non−typhoidal Salmonella
Staphylococcus aureus
Campylobacter spp.
Enterococcus faecium
Enterococcus faecalis
Shared human and animal antimicrobial classes
Third generation cephalosporins
Fourth generation cephalosporins
Fluoroquinolones
Macrolides
Quinolones
Polymyxins
Aminoglycosides
Aminopenicillins
Aminopenicillins with beta−lactamase inhibitors
Ansamycins
Phosphonic acid derivatives
Livestock species
Cattle
Chickens
Pigs
Sheep
Turkeys
Ducks
Horses
Buffaloes
Goats
Rank
0.00
0.25
0.50
0.75
1.00

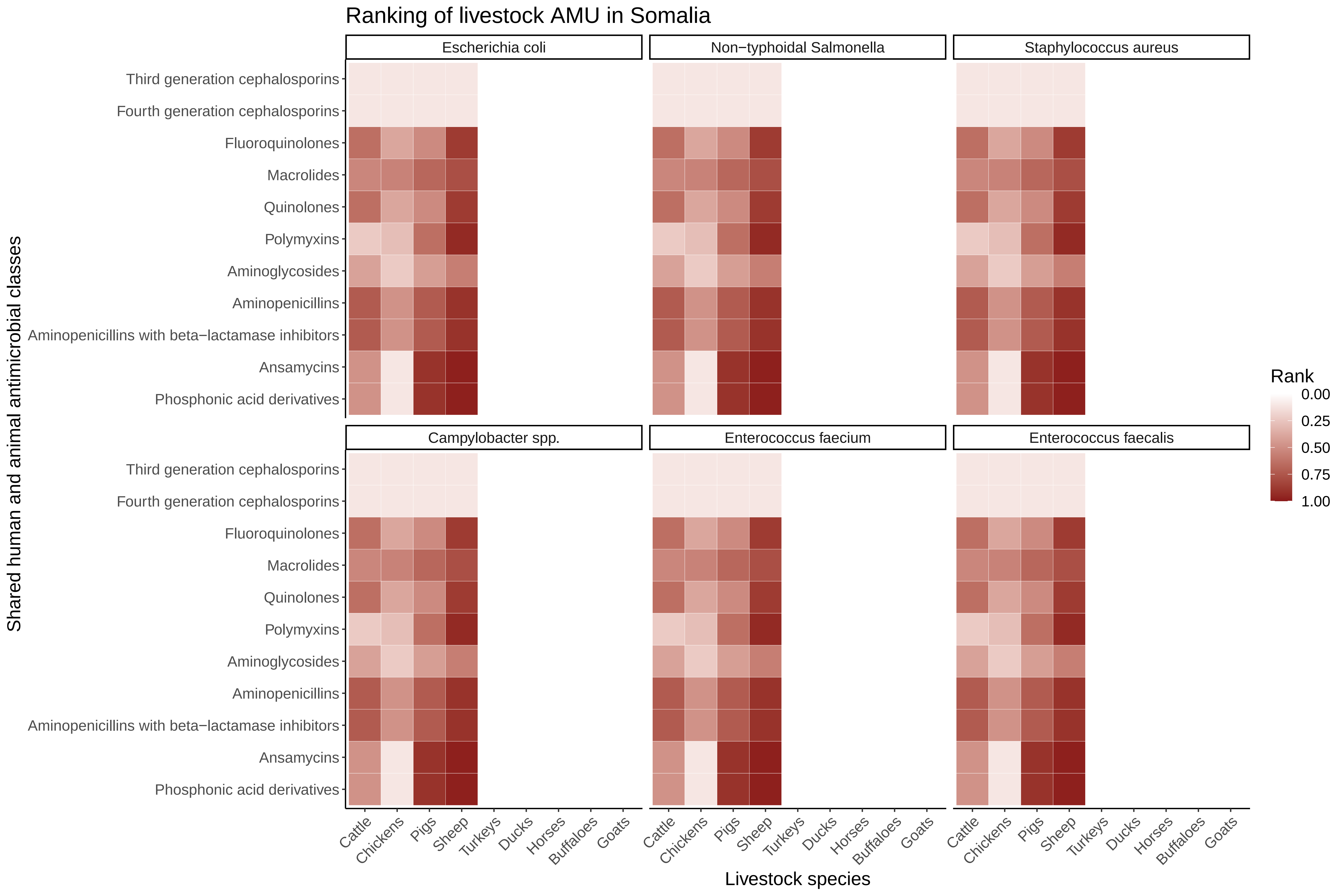

Ranking of livestock AMU in Somalia
Escherichia coli
Non−typhoidal Salmonella
Staphylococcus aureus
Campylobacter spp.
Enterococcus faecium
Enterococcus faecalis
Third generation cephalosporins
Fourth generation cephalosporins
Fluoroquinolones
Macrolides
Quinolones
Polymyxins
Aminoglycosides
Aminopenicillins
Aminopenicillins with beta−lactamase inhibitors
Ansamycins
Phosphonic acid derivatives
Shared human and animal antimicrobial classes
Cattle
Chickens
Pigs
Sheep
Turkeys
Ducks
Horses
Buffaloes
Goats
Livestock species
Rank
0.00
0.25
0.50
0.75
1.00

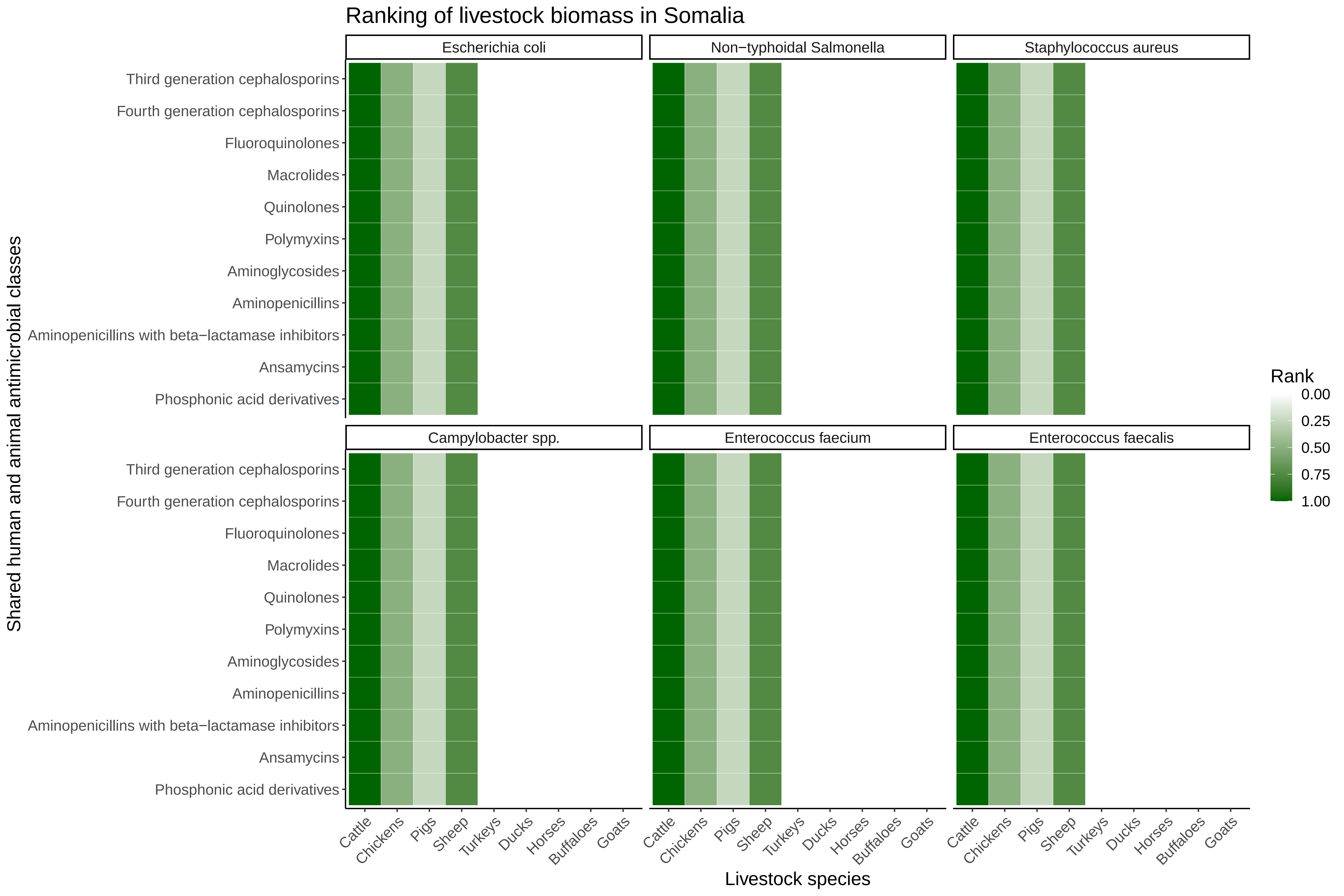
Ranking of livestock biomass in Somalia
Escherichia coli
Non−typhoidal Salmonella
Staphylococcus aureus
Campylobacter spp.
Enterococcus faecium
Enterococcus faecalis
Third generation cephalosporins
Fourth generation cephalosporins
Fluoroquinolones
Macrolides
Quinolones
Polymyxins
Aminoglycosides
Aminopenicillins
Aminopenicillins with beta−lactamase inhibitors
Ansamycins
Phosphonic acid derivatives
Shared human and animal antimicrobial classes
Cattle
Chickens
Pigs
Sheep
Turkeys
Ducks
Horses
Buffaloes
Goats
Livestock species
Rank
0.00
0.25
0.50
0.75
1.00

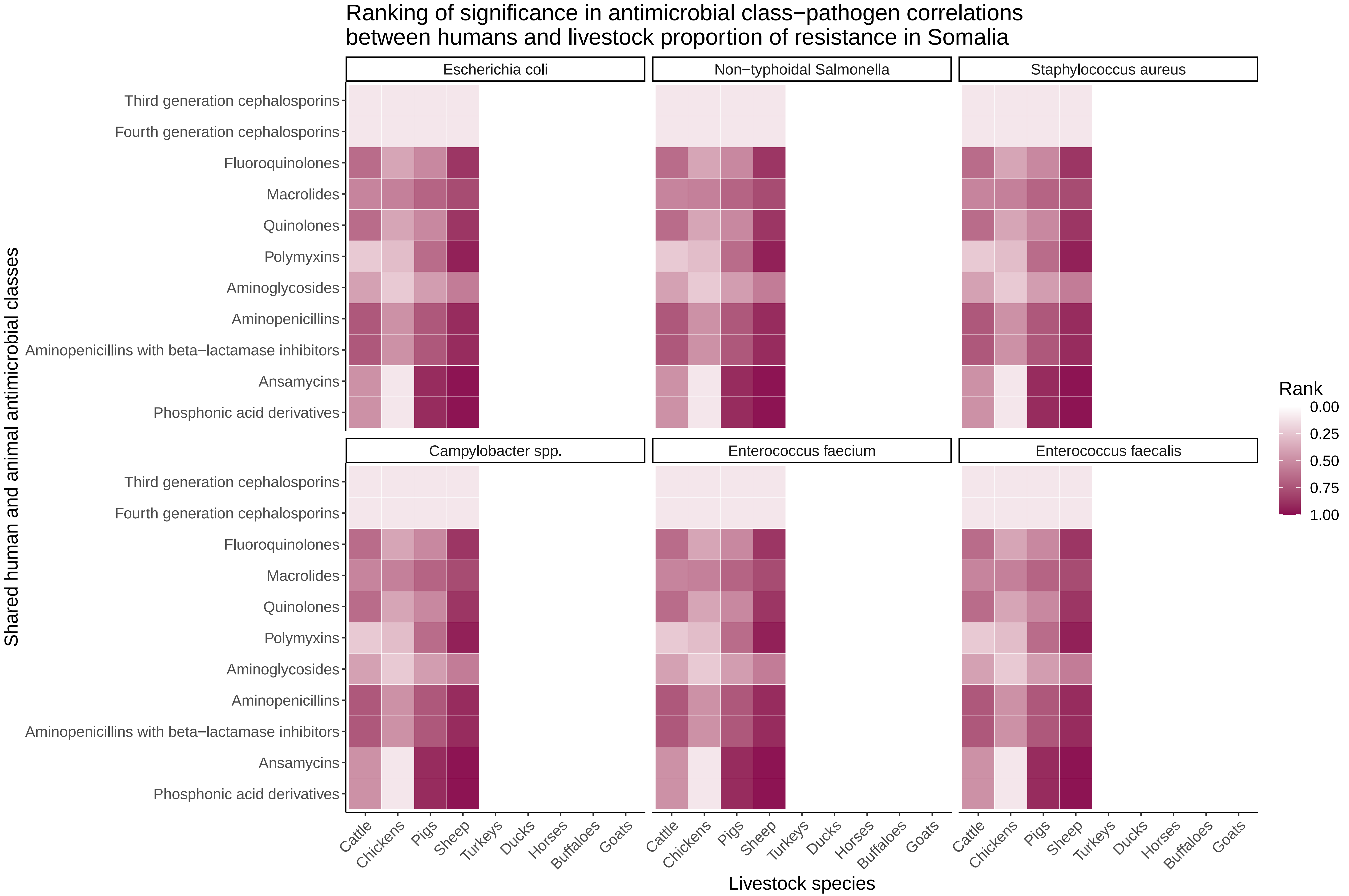
Ranking of significance in antimicrobial class−pathogen correlations between humans and livestock proportion of resistance in Somalia
Escherichia coli
Non−typhoidal Salmonella
Staphylococcus aureus
Campylobacter spp.
Enterococcus faecium
Enterococcus faecalis
Third generation cephalosporins
Fourth generation cephalosporins
Fluoroquinolones
Macrolides
Quinolones
Polymyxins
Aminoglycosides
Aminopenicillins
Aminopenicillins with beta−lactamase inhibitors
Ansamycins
Phosphonic acid derivatives
Shared human and animal antimicrobial classes
Cattle
Chickens
Pigs
Sheep
Turkeys
Ducks
Horses
Buffaloes
Goats
Livestock species
Rank
0.00
0.25
0.50
0.75
1.00

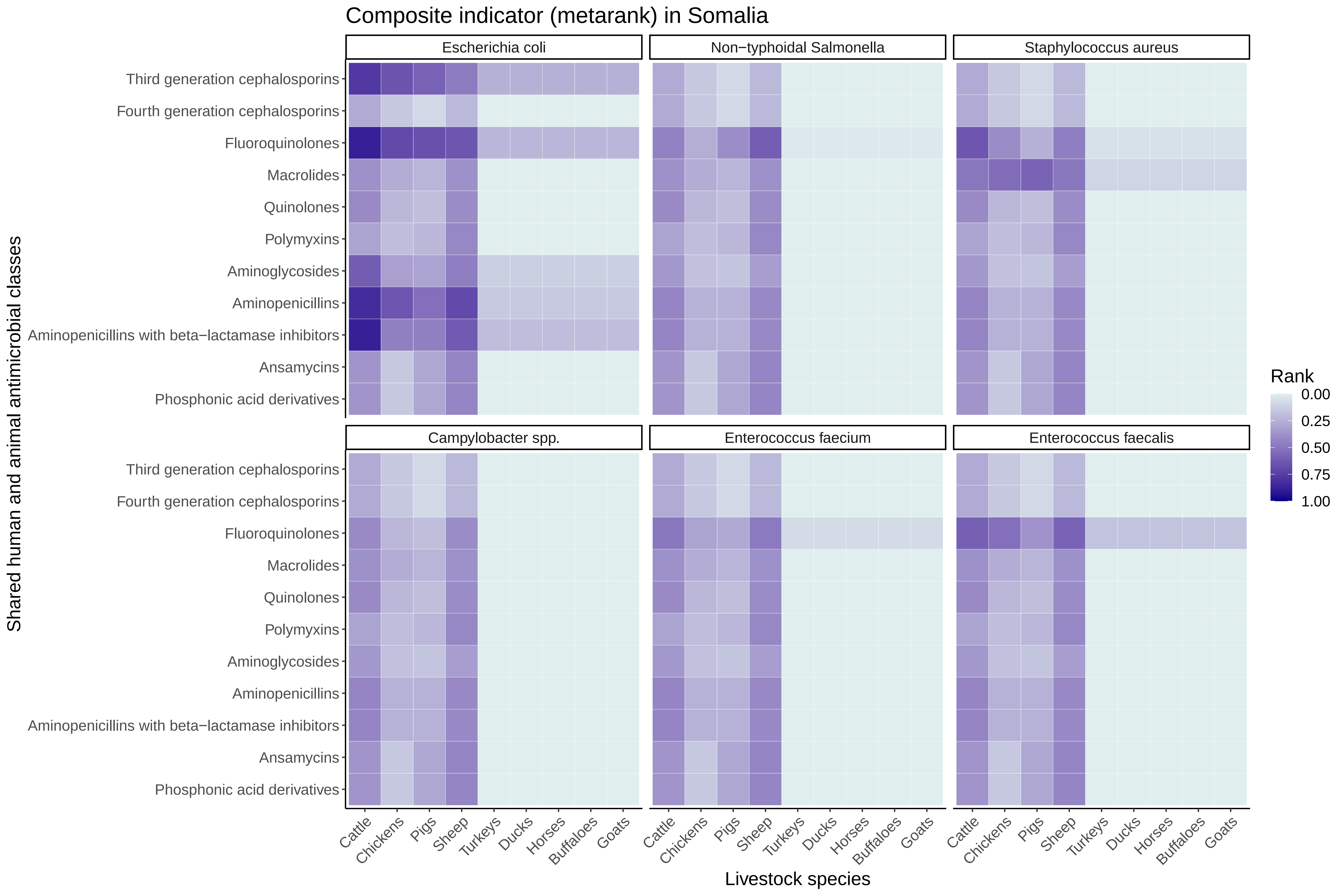

Composite indicator (metarank) in Somalia
Escherichia coli
Non−typhoidal Salmonella
Staphylococcus aureus
Campylobacter spp.
Enterococcus faecium
Enterococcus faecalis
Shared human and animal antimicrobial classes
Third generation cephalosporins
Fourth generation cephalosporins
Fluoroquinolones
Macrolides
Quinolones
Polymyxins
Aminoglycosides
Aminopenicillins
Aminopenicillins with beta−lactamase inhibitors
Ansamycins
Phosphonic acid derivatives
Cattle
Chickens
Pigs
Sheep
Turkeys
Ducks
Horses
Buffaloes
Goats
Livestock species
Rank
0.00
0.25
0.50
0.75
1.00

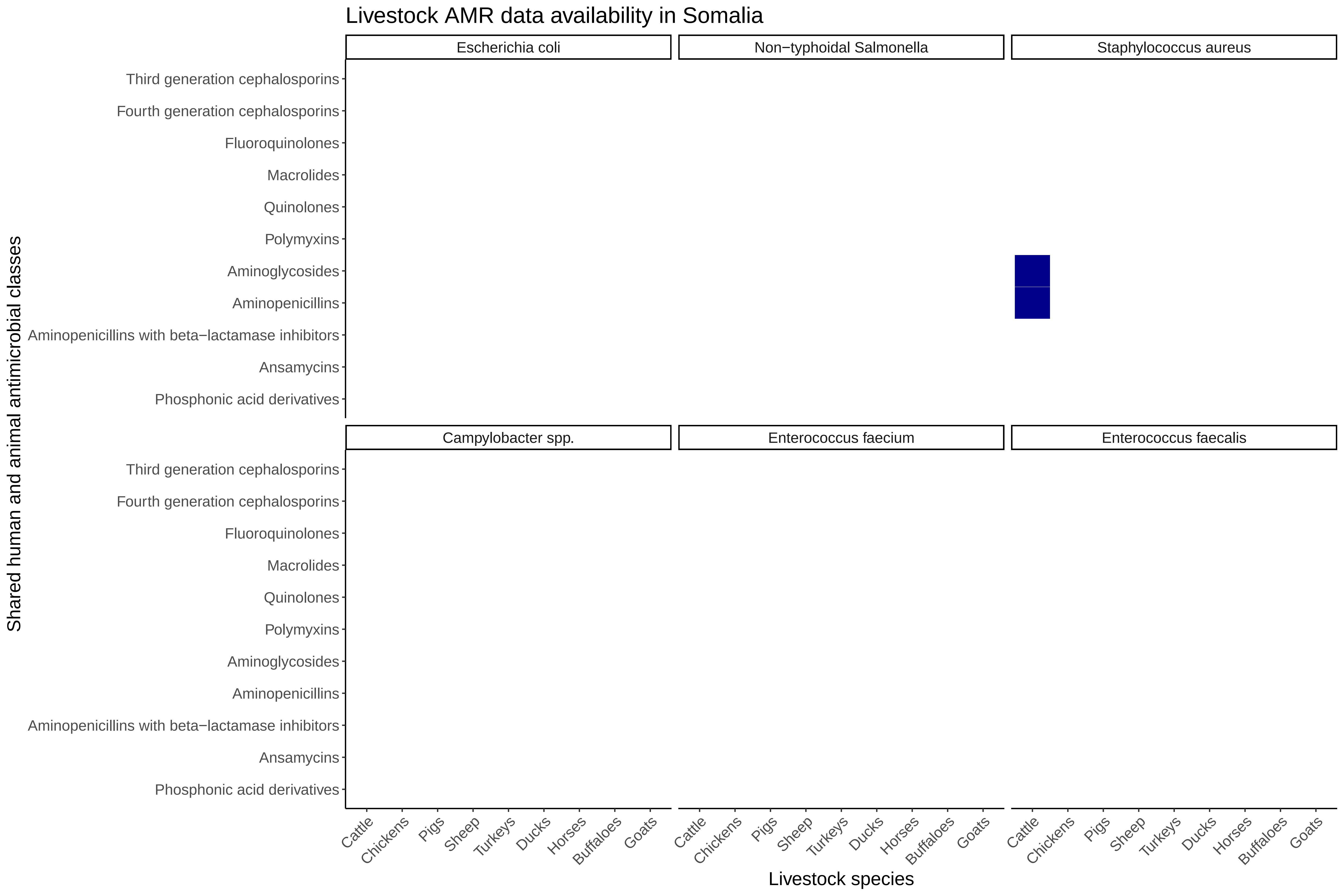
Livestock AMR data availability in Somalia
Escherichia coli
Non−typhoidal Salmonella
Staphylococcus aureus
Campylobacter spp.
Enterococcus faecium
Enterococcus faecalis
Shared human and animal antimicrobial classes
Third generation cephalosporins
Fourth generation cephalosporins
Fluoroquinolones
Macrolides
Quinolones
Polymyxins
Aminoglycosides
Aminopenicillins
Aminopenicillins with beta−lactamase inhibitors
Ansamycins
Phosphonic acid derivatives
Cattle
Chickens
Pigs
Sheep
Turkeys
Ducks
Horses
Buffaloes
Goats
Livestock species

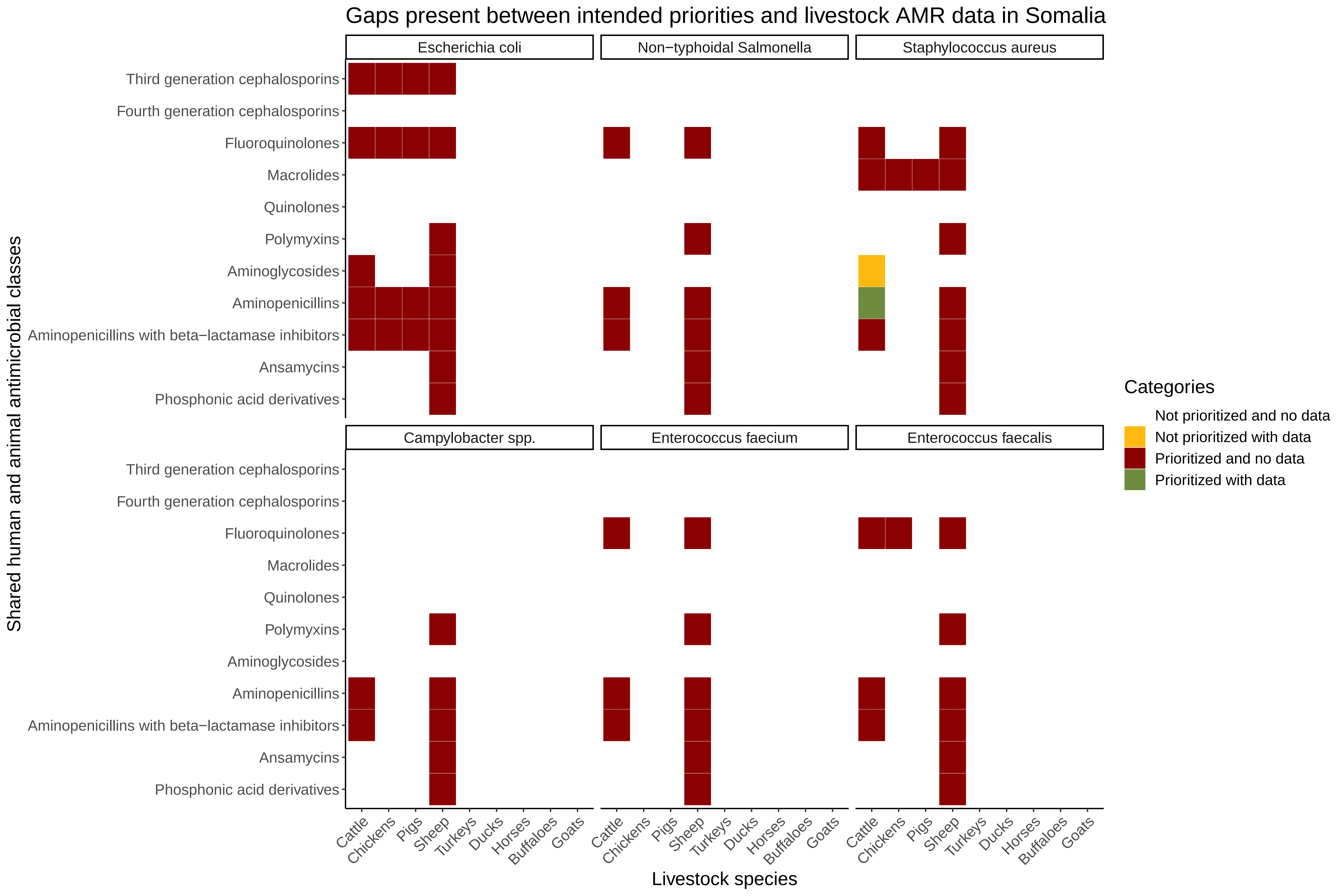

Gaps present between intended priorities and livestock AMR data in Somalia
Escherichia coli
Non−typhoidal Salmonella
Staphylococcus aureus
Campylobacter spp.
Enterococcus faecium
Enterococcus faecalis
Third generation cephalosporins
Fourth generation cephalosporins
Fluoroquinolones
Macrolides
Quinolones
Polymyxins
Aminoglycosides
Aminopenicillins
Aminopenicillins with beta−lactamase inhibitors
Ansamycins
Phosphonic acid derivatives
Shared human and animal antimicrobial classes
Cattle
Chickens
Pigs
Sheep
Turkeys
Ducks
Horses
Buffaloes
Goats
Livestock species
Categories
Not prioritized and no data
Not prioritized with data
Prioritized and no data
Prioritized with data

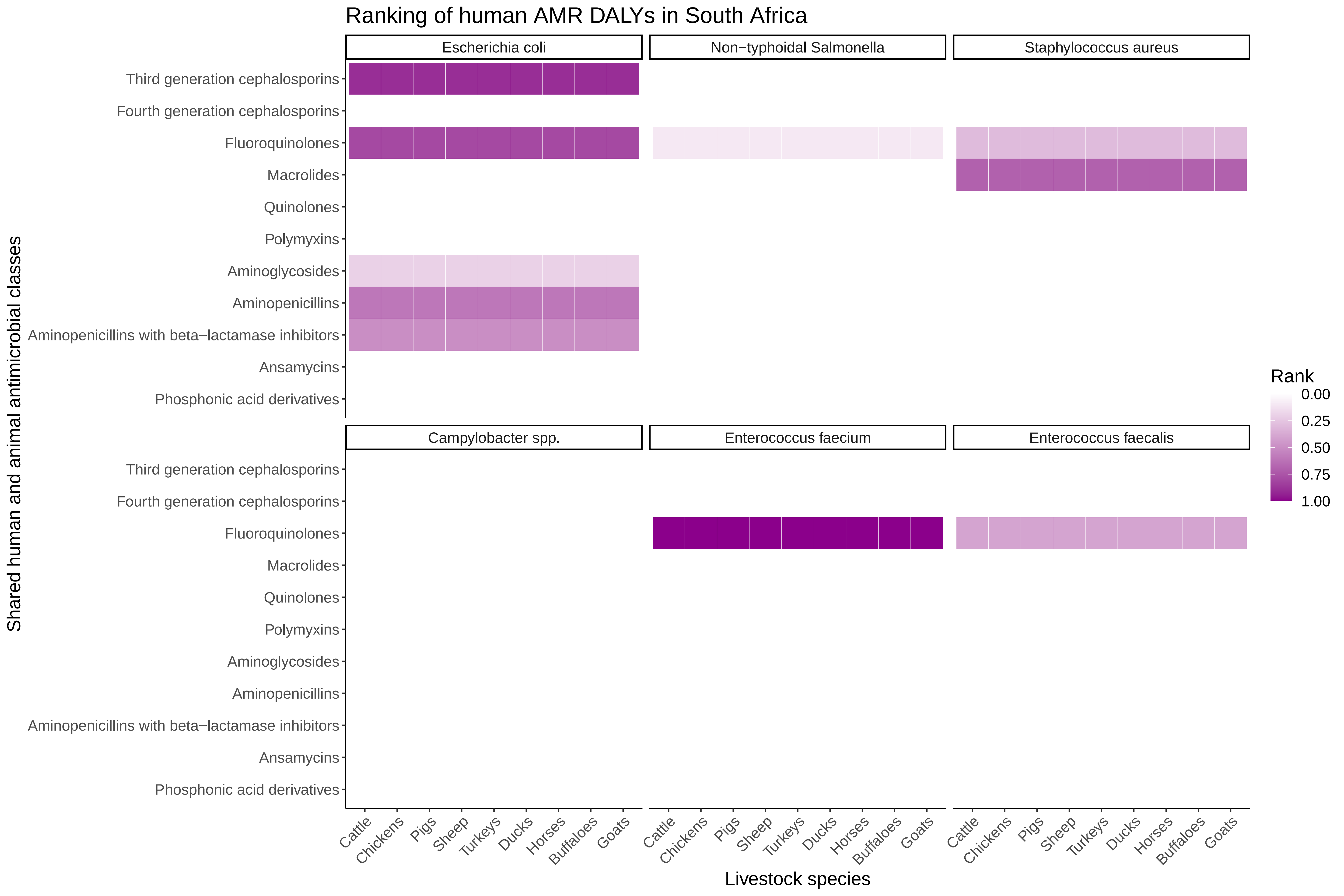

Ranking of human AMR DALYs in South Africa
Escherichia coli
Non−typhoidal Salmonella
Staphylococcus aureus
Campylobacter spp.
Enterococcus faecium
Enterococcus faecalis
Third generation cephalosporins
Fourth generation cephalosporins
Fluoroquinolones
Macrolides
Quinolones
Polymyxins
Aminoglycosides
Aminopenicillins
Aminopenicillins with beta−lactamase inhibitors
Ansamycins
Phosphonic acid derivatives
Shared human and animal antimicrobial classes
Cattle
Chickens
Pigs
Sheep
Turkeys
Ducks
Horses
Buffaloes
Goats
Livestock species
Rank
0.00
0.25
0.50
0.75
1.00

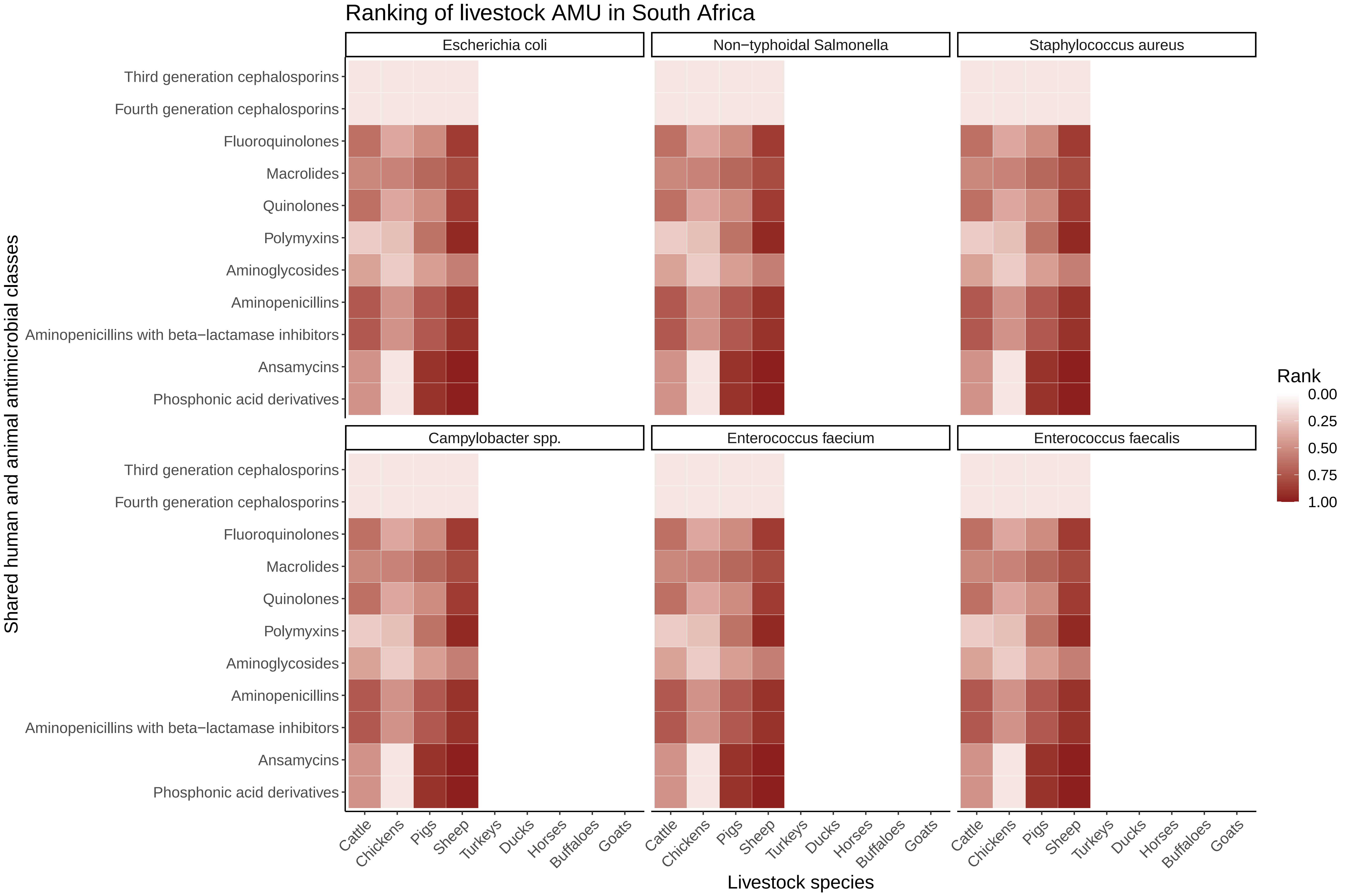
Ranking of livestock AMU in South Africa
Escherichia coli
Non−typhoidal Salmonella
Staphylococcus aureus
Campylobacter spp.
Enterococcus faecium
Enterococcus faecalis
Shared human and animal antimicrobial classes
Third generation cephalosporins
Fourth generation cephalosporins
Fluoroquinolones
Macrolides
Quinolones
Polymyxins
Aminoglycosides
Aminopenicillins
Aminopenicillins with beta−lactamase inhibitors
Ansamycins
Phosphonic acid derivatives
Cattle
Chickens
Pigs
Sheep
Turkeys
Ducks
Horses
Buffaloes
Goats
Livestock species
Rank
0.00
0.25
0.50
0.75
1.00

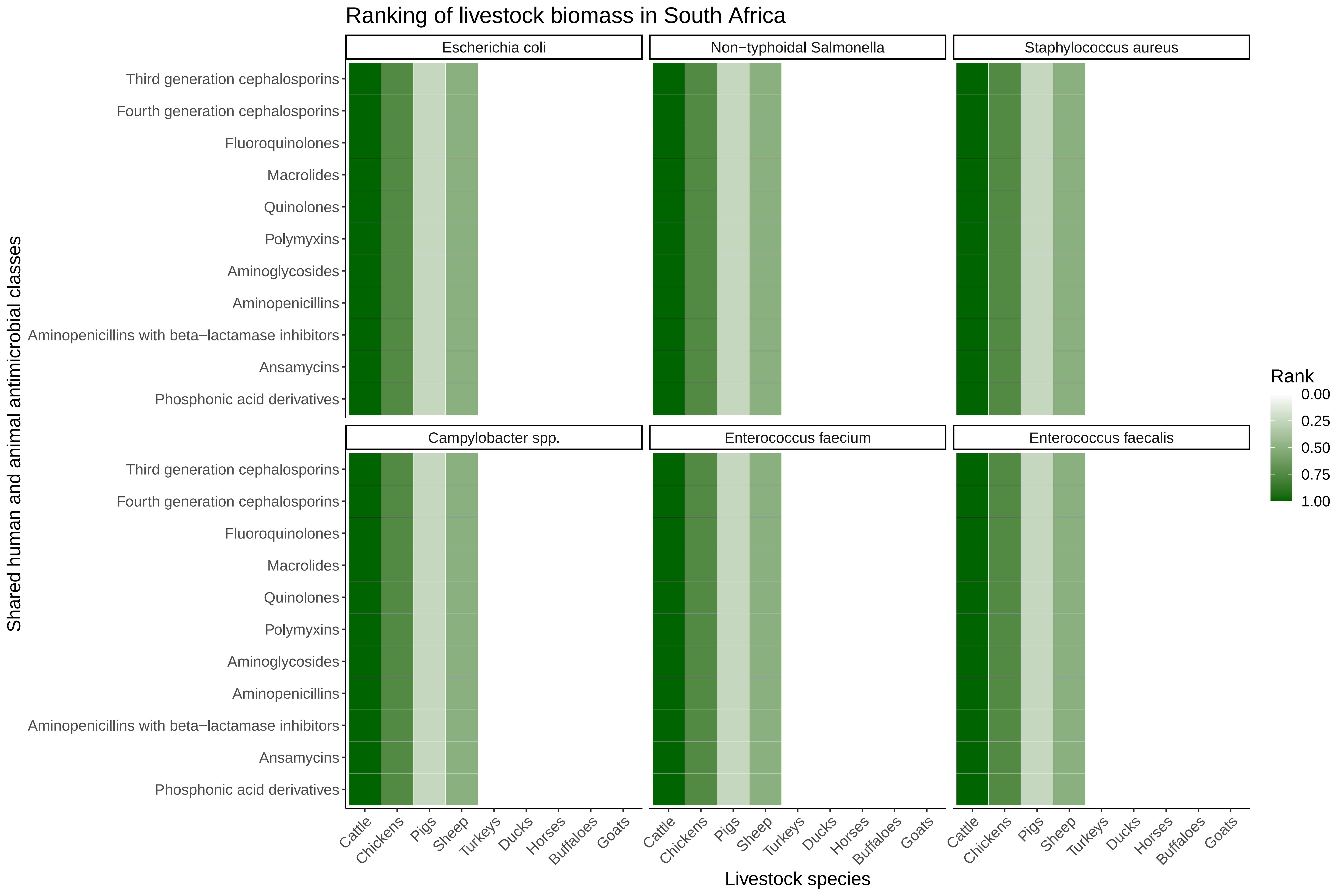

Ranking of livestock biomass in South Africa
Escherichia coli
Non−typhoidal Salmonella
Staphylococcus aureus
Campylobacter spp.
Enterococcus faecium
Enterococcus faecalis
Third generation cephalosporins
Fourth generation cephalosporins
Fluoroquinolones
Macrolides
Quinolones
Polymyxins
Aminoglycosides
Aminopenicillins
Aminopenicillins with beta−lactamase inhibitors
Ansamycins
Phosphonic acid derivatives
Shared human and animal antimicrobial classes
Cattle
Chickens
Pigs
Sheep
Turkeys
Ducks
Horses
Buffaloes
Goats
Livestock species
Rank
0.00
0.25
0.50
0.75
1.00

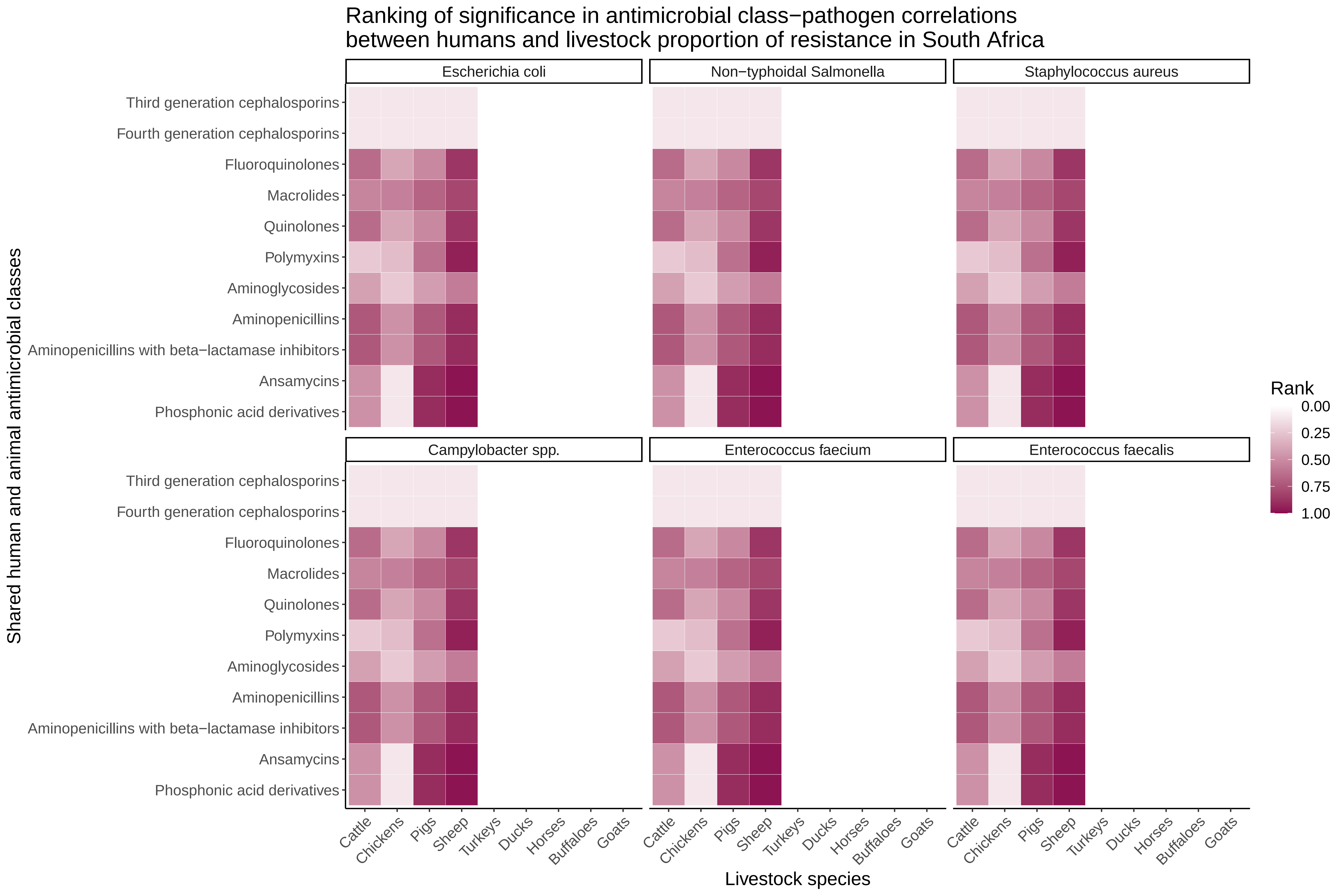

Ranking of significance in antimicrobial class−pathogen correlations
between humans and livestock proportion of resistance in South Africa
Escherichia coli
Non−typhoidal Salmonella
Staphylococcus aureus
Campylobacter spp.
Enterococcus faecium
Enterococcus faecalis
Third generation cephalosporins
Fourth generation cephalosporins
Fluoroquinolones
Macrolides
Quinolones
Polymyxins
Aminoglycosides
Aminopenicillins
Aminopenicillins with beta−lactamase inhibitors
Ansamycins
Phosphonic acid derivatives
Shared human and animal antimicrobial classes
Cattle
Chickens
Pigs
Sheep
Turkeys
Ducks
Horses
Buffaloes
Goats
Livestock species
Rank
0.00
0.25
0.50
0.75
1.00

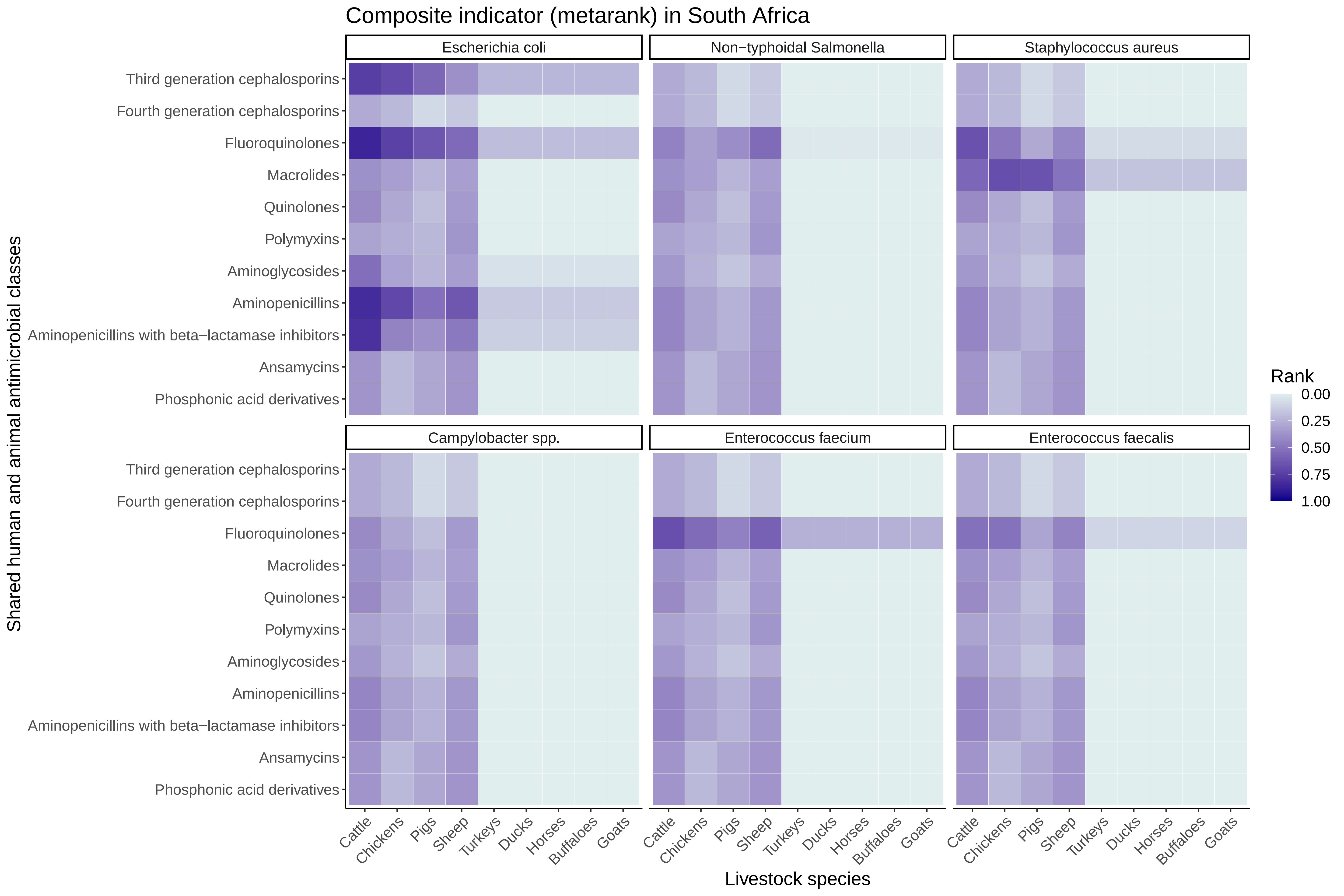

Composite indicator (metarank) in South Africa
Escherichia coli
Non−typhoidal Salmonella
Staphylococcus aureus
Campylobacter spp.
Enterococcus faecium
Enterococcus faecalis
Shared human and animal antimicrobial classes
Third generation cephalosporins
Fourth generation cephalosporins
Fluoroquinolones
Macrolides
Quinolones
Polymyxins
Aminoglycosides
Aminopenicillins
Aminopenicillins with beta−lactamase inhibitors
Ansamycins
Phosphonic acid derivatives
Cattle
Chickens
Pigs
Sheep
Turkeys
Ducks
Horses
Buffaloes
Goats
Livestock species
Rank
0.00
0.25
0.50
0.75
1.00

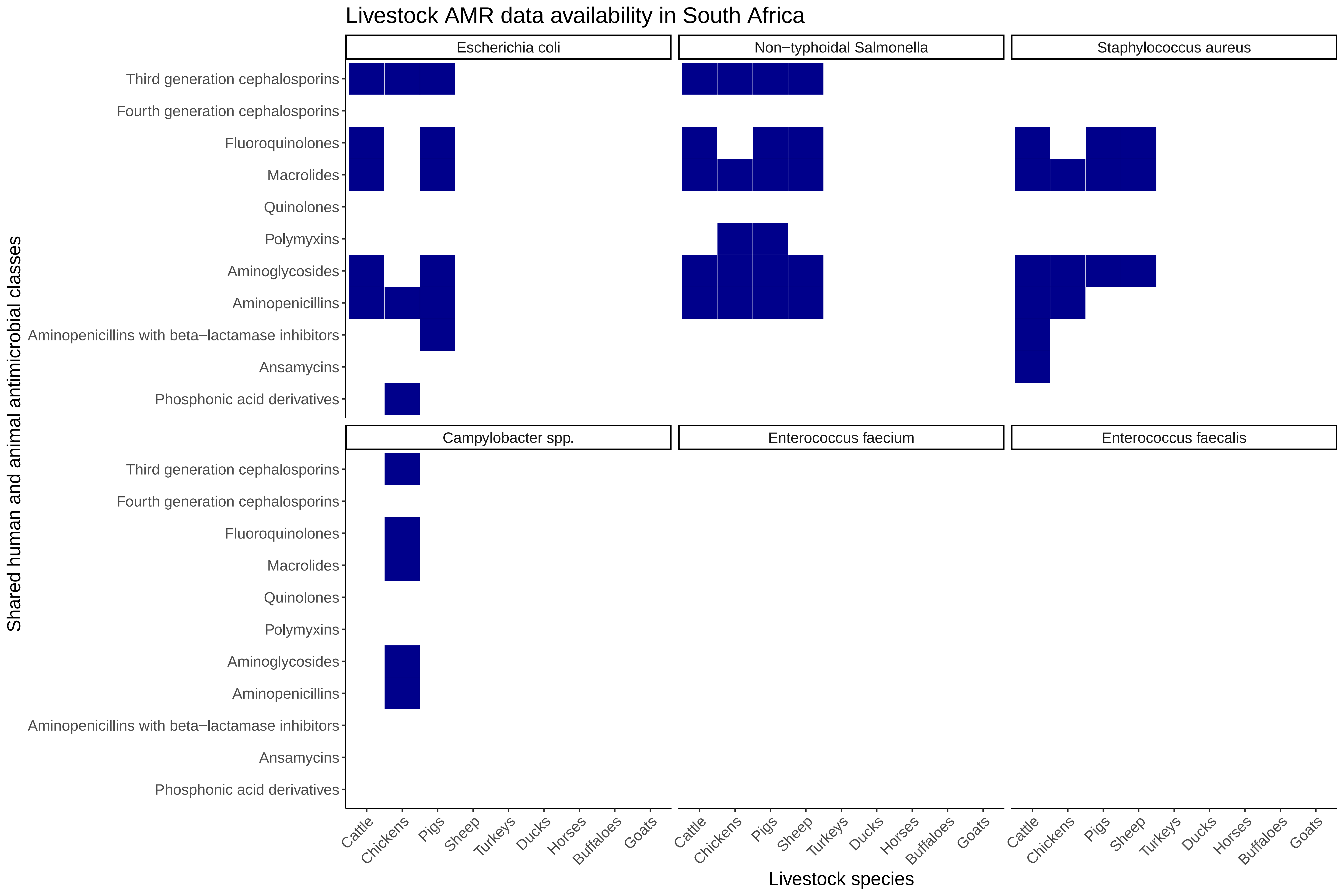

Livestock AMR data availability in South Africa
Escherichia coli
Non−typhoidal Salmonella
Staphylococcus aureus
Campylobacter spp.
Enterococcus faecium
Enterococcus faecalis
Shared human and animal antimicrobial classes
Third generation cephalosporins
Fourth generation cephalosporins
Fluoroquinolones
Macrolides
Quinolones
Polymyxins
Aminoglycosides
Aminopenicillins
Aminopenicillins with beta−lactamase inhibitors
Ansamycins
Phosphonic acid derivatives
Cattle
Chickens
Pigs
Sheep
Turkeys
Ducks
Horses
Buffaloes
Goats
Livestock species

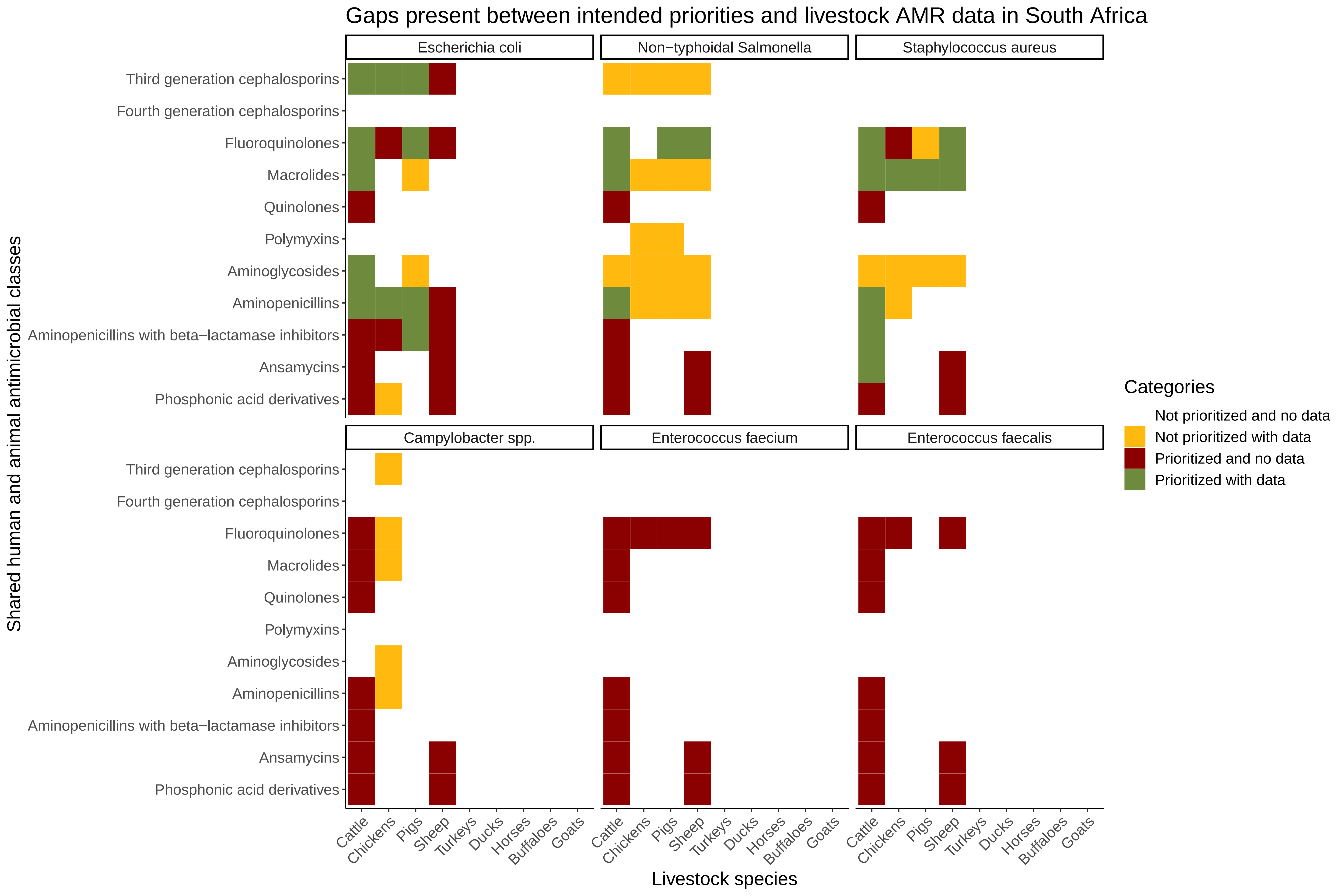

Gaps present between intended priorities and livestock AMR data in South Africa
Escherichia coli
Non−typhoidal Salmonella
Staphylococcus aureus
Campylobacter spp.
Enterococcus faecium
Enterococcus faecalis
Third generation cephalosporins
Fourth generation cephalosporins
Fluoroquinolones
Macrolides
Quinolones
Polymyxins
Aminoglycosides
Aminopenicillins
Aminopenicillins with beta−lactamase inhibitors
Ansamycins
Phosphonic acid derivatives
Shared human and animal antimicrobial classes
Cattle
Chickens
Pigs
Sheep
Turkeys
Ducks
Horses
Buffaloes
Goats
Livestock species
Categories
Not prioritized and no data
Not prioritized with data
Prioritized and no data
Prioritized with data

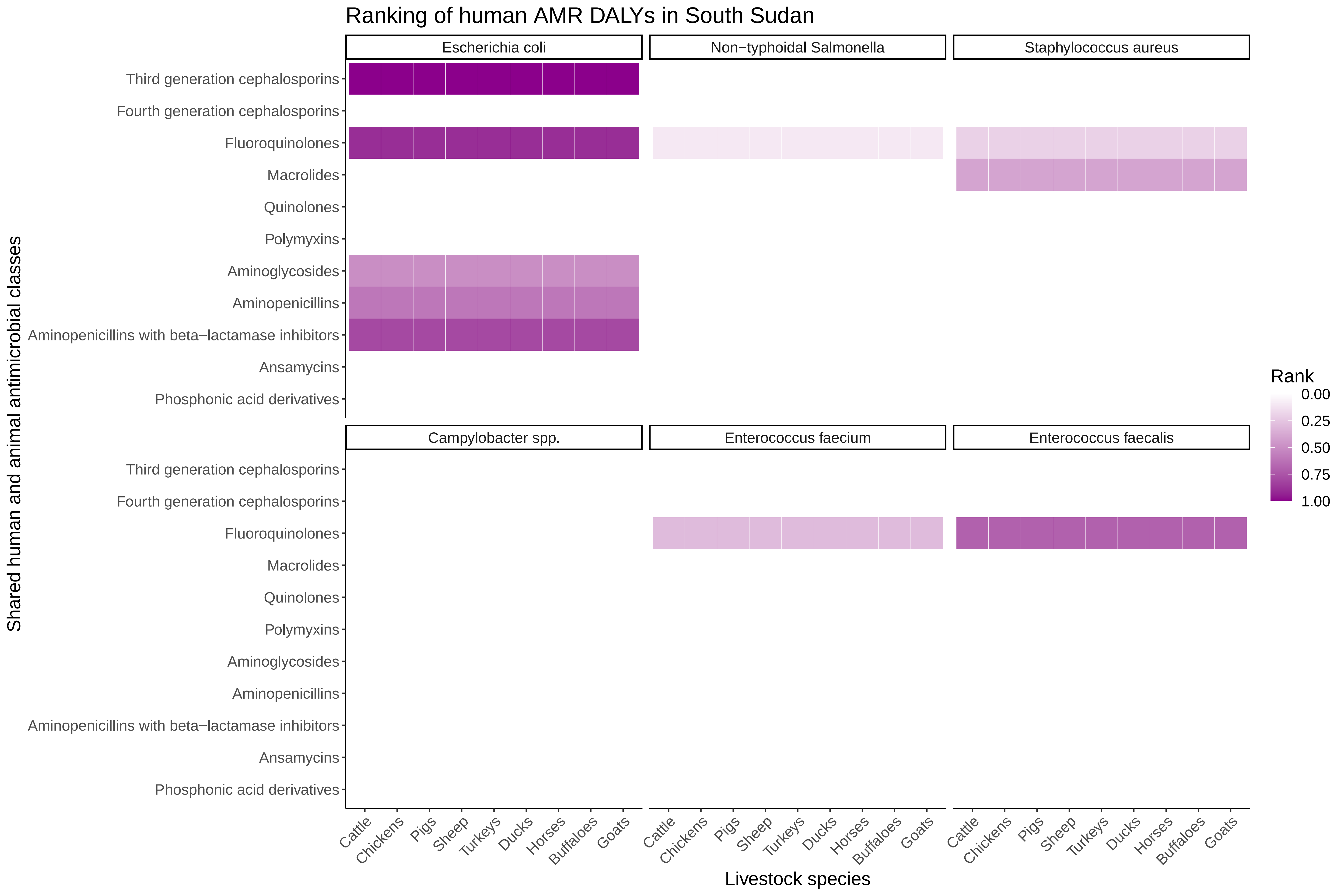
Ranking of human AMR DALYs in South Sudan
Escherichia coli
Non−typhoidal Salmonella
Staphylococcus aureus
Campylobacter spp.
Enterococcus faecium
Enterococcus faecalis
Third generation cephalosporins
Fourth generation cephalosporins
Fluoroquinolones
Macrolides
Quinolones
Polymyxins
Aminoglycosides
Aminopenicillins
Aminopenicillins with beta−lactamase inhibitors
Ansamycins
Phosphonic acid derivatives
Shared human and animal antimicrobial classes
Cattle
Chickens
Pigs
Sheep
Turkeys
Ducks
Horses
Buffaloes
Goats
Livestock species
Rank
0.00
0.25
0.50
0.75
1.00

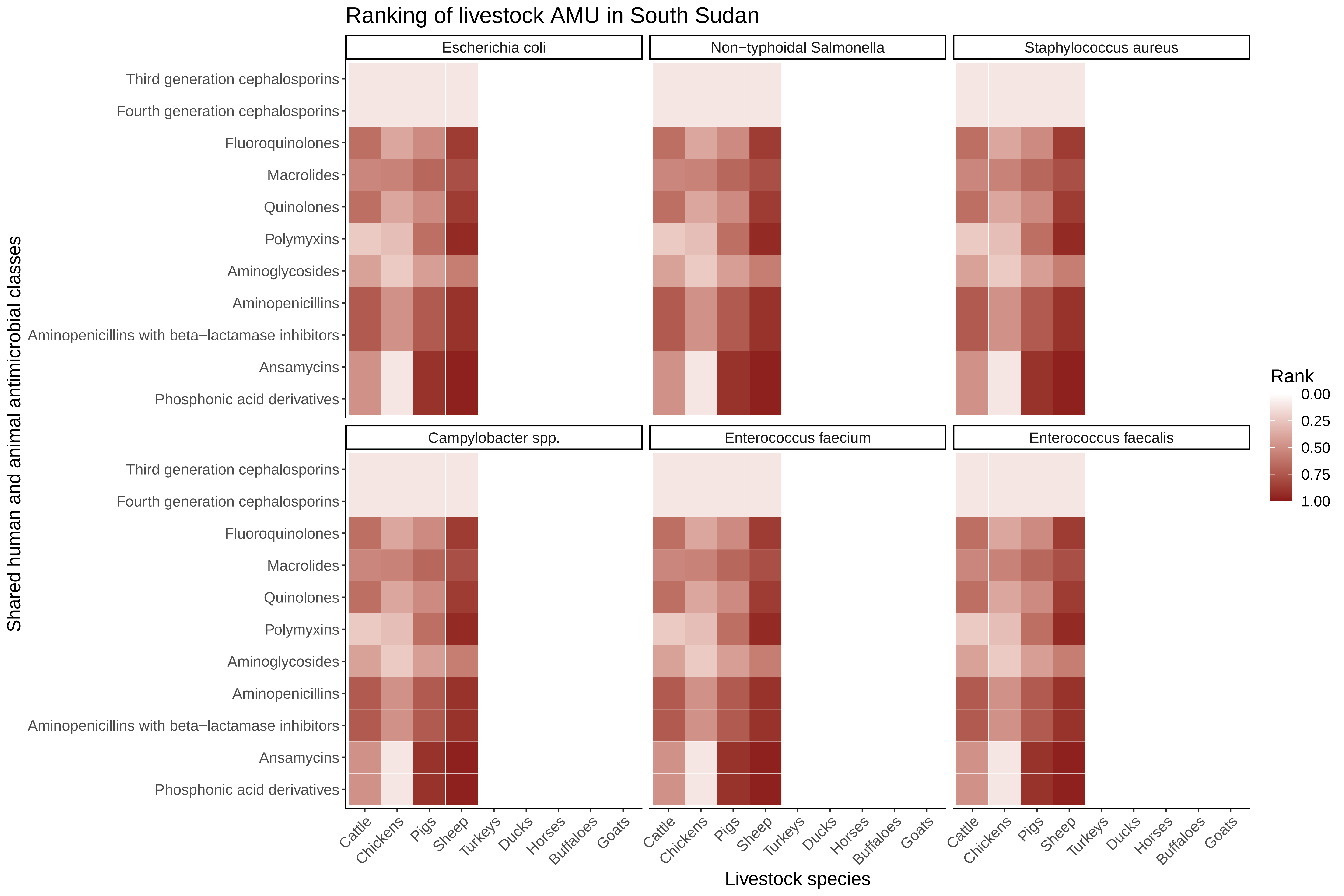

Ranking of livestock AMU in South Sudan
Escherichia coli
Non−typhoidal Salmonella
Staphylococcus aureus
Campylobacter spp.
Enterococcus faecium
Enterococcus faecalis
Third generation cephalosporins
Fourth generation cephalosporins
Fluoroquinolones
Macrolides
Quinolones
Polymyxins
Aminoglycosides
Aminopenicillins
Aminopenicillins with beta−lactamase inhibitors
Ansamycins
Phosphonic acid derivatives
Shared human and animal antimicrobial classes
Cattle
Chickens
Pigs
Sheep
Turkeys
Ducks
Horses
Buffaloes
Goats
Livestock species
Rank
0.00
0.25
0.50
0.75
1.00

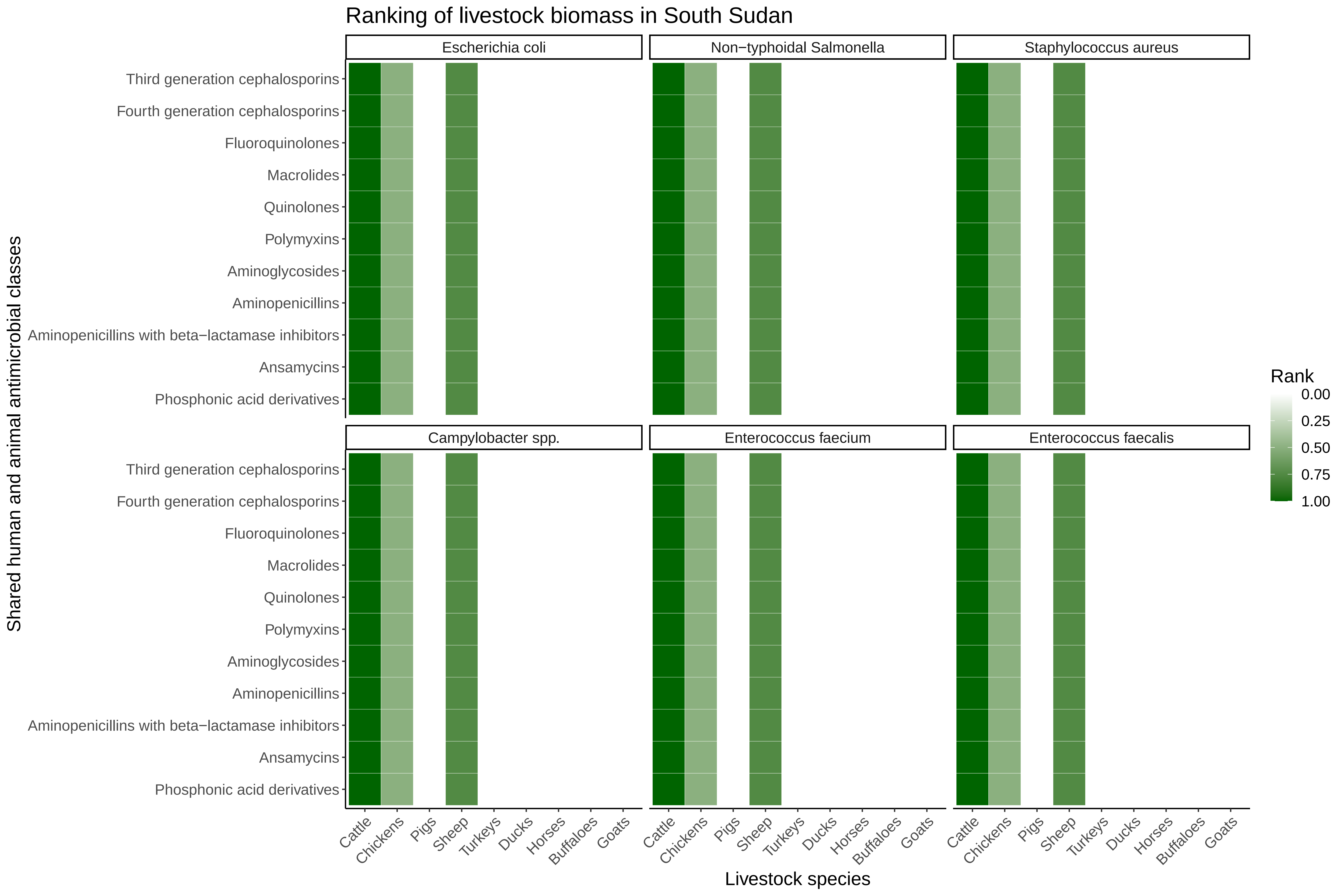
Ranking of livestock biomass in South Sudan
Escherichia coli
Non−typhoidal Salmonella
Staphylococcus aureus
Campylobacter spp.
Enterococcus faecium
Enterococcus faecalis
Third generation cephalosporins
Fourth generation cephalosporins
Fluoroquinolones
Macrolides
Quinolones
Polymyxins
Aminoglycosides
Aminopenicillins
Aminopenicillins with beta−lactamase inhibitors
Ansamycins
Phosphonic acid derivatives
Shared human and animal antimicrobial classes
Cattle
Chickens
Pigs
Sheep
Turkeys
Ducks
Horses
Buffaloes
Goats
Livestock species
Rank
0.00
0.25
0.50
0.75
1.00

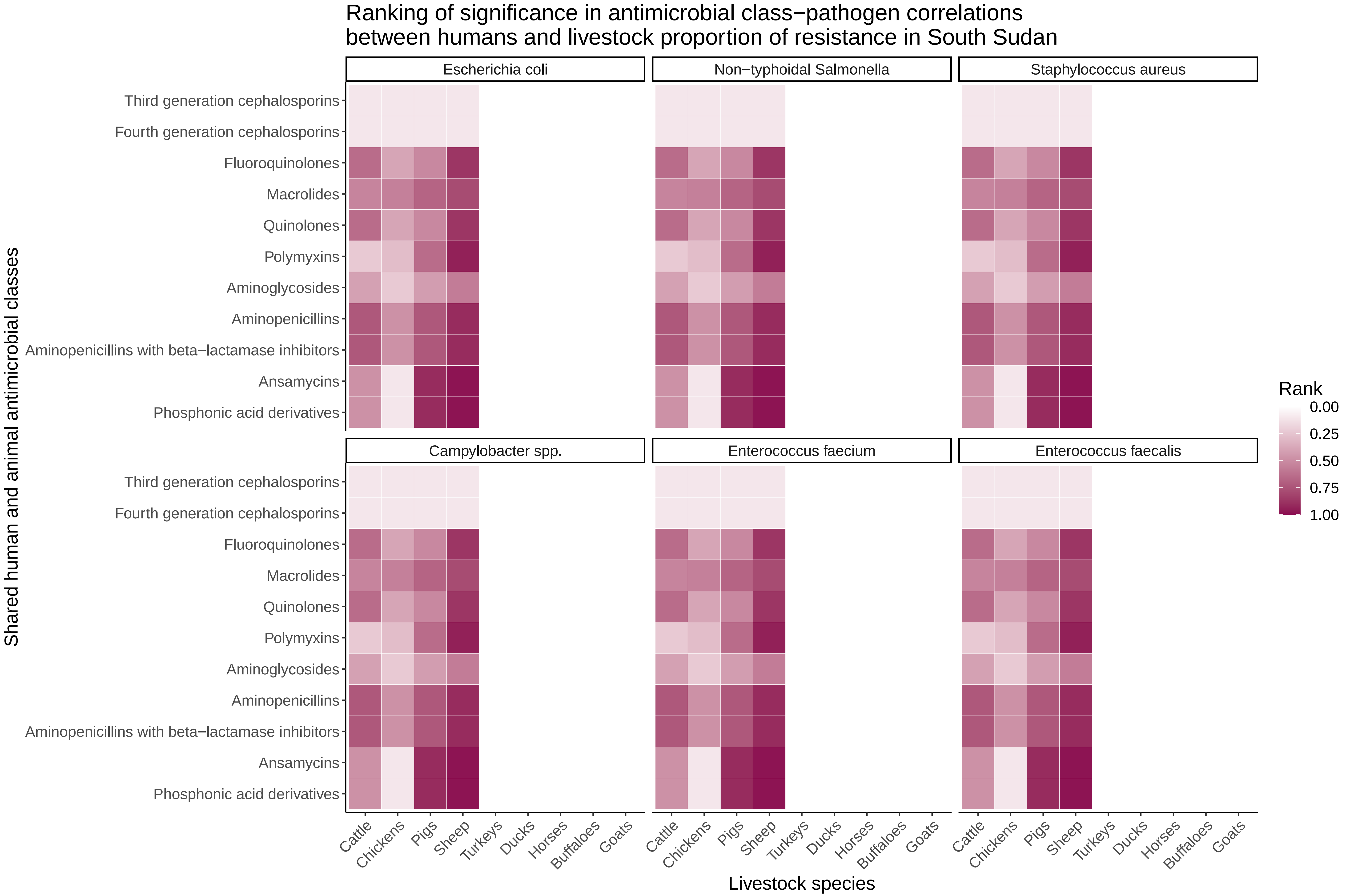

Ranking of significance in antimicrobial class−pathogen correlations
between humans and livestock proportion of resistance in South Sudan
Escherichia coli
Non−typhoidal Salmonella
Staphylococcus aureus
Campylobacter spp.
Enterococcus faecium
Enterococcus faecalis
Third generation cephalosporins
Fourth generation cephalosporins
Fluoroquinolones
Macrolides
Quinolones
Polymyxins
Aminoglycosides
Aminopenicillins
Aminopenicillins with beta−lactamase inhibitors
Ansamycins
Phosphonic acid derivatives
Shared human and animal antimicrobial classes
Cattle
Chickens
Pigs
Sheep
Turkeys
Ducks
Horses
Buffaloes
Goats
Livestock species
Rank
0.00
0.25
0.50
0.75
1.00

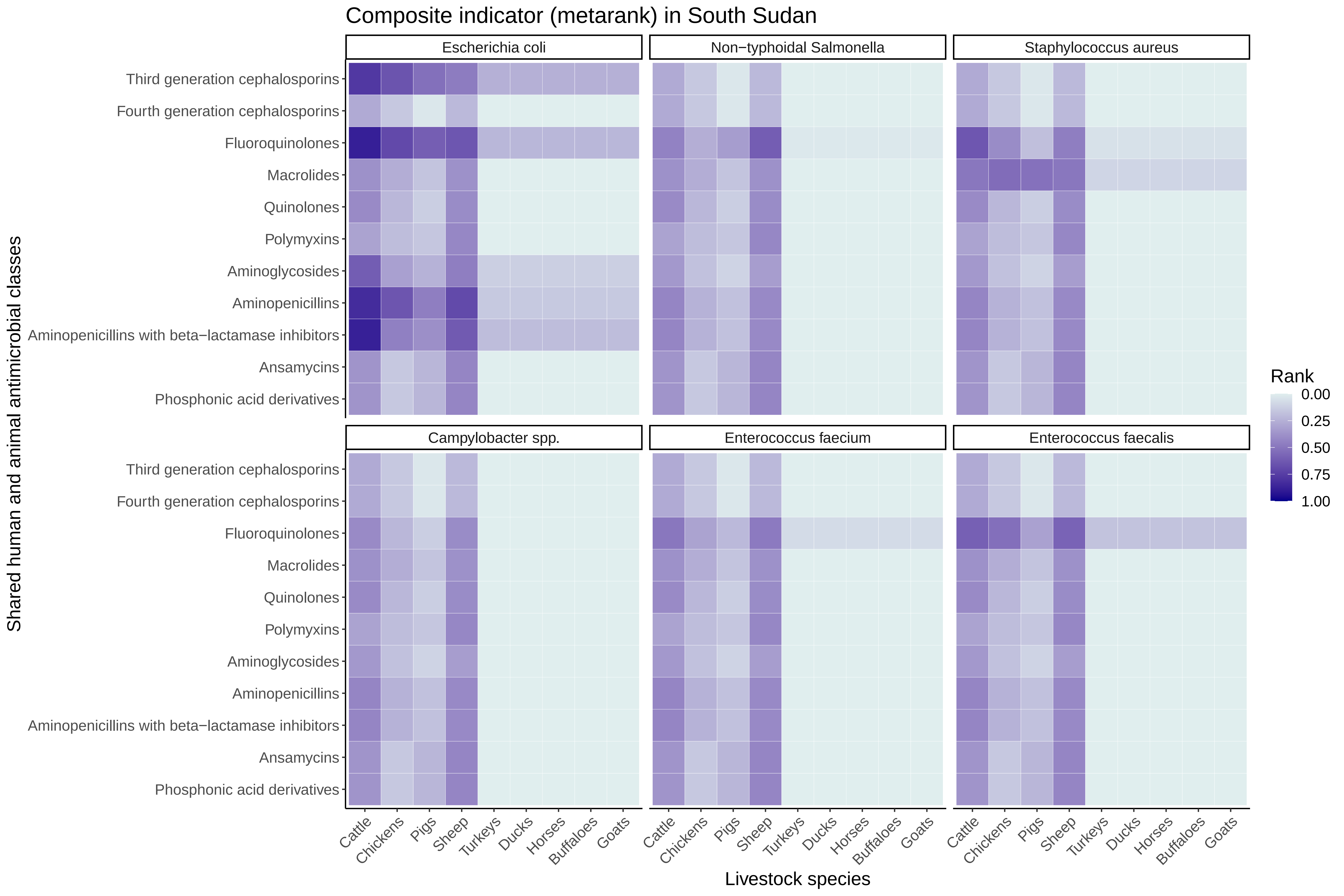

Composite indicator (metarank) in South Sudan
Escherichia coli
Non−typhoidal Salmonella
Staphylococcus aureus
Campylobacter spp.
Enterococcus faecium
Enterococcus faecalis
Third generation cephalosporins
Fourth generation cephalosporins
Fluoroquinolones
Macrolides
Quinolones
Polymyxins
Aminoglycosides
Aminopenicillins
Aminopenicillins with beta−lactamase inhibitors
Ansamycins
Phosphonic acid derivatives
Shared human and animal antimicrobial classes
Cattle
Chickens
Pigs
Sheep
Turkeys
Ducks
Horses
Buffaloes
Goats
Livestock species
Rank
0.00
0.25
0.50
0.75
1.00

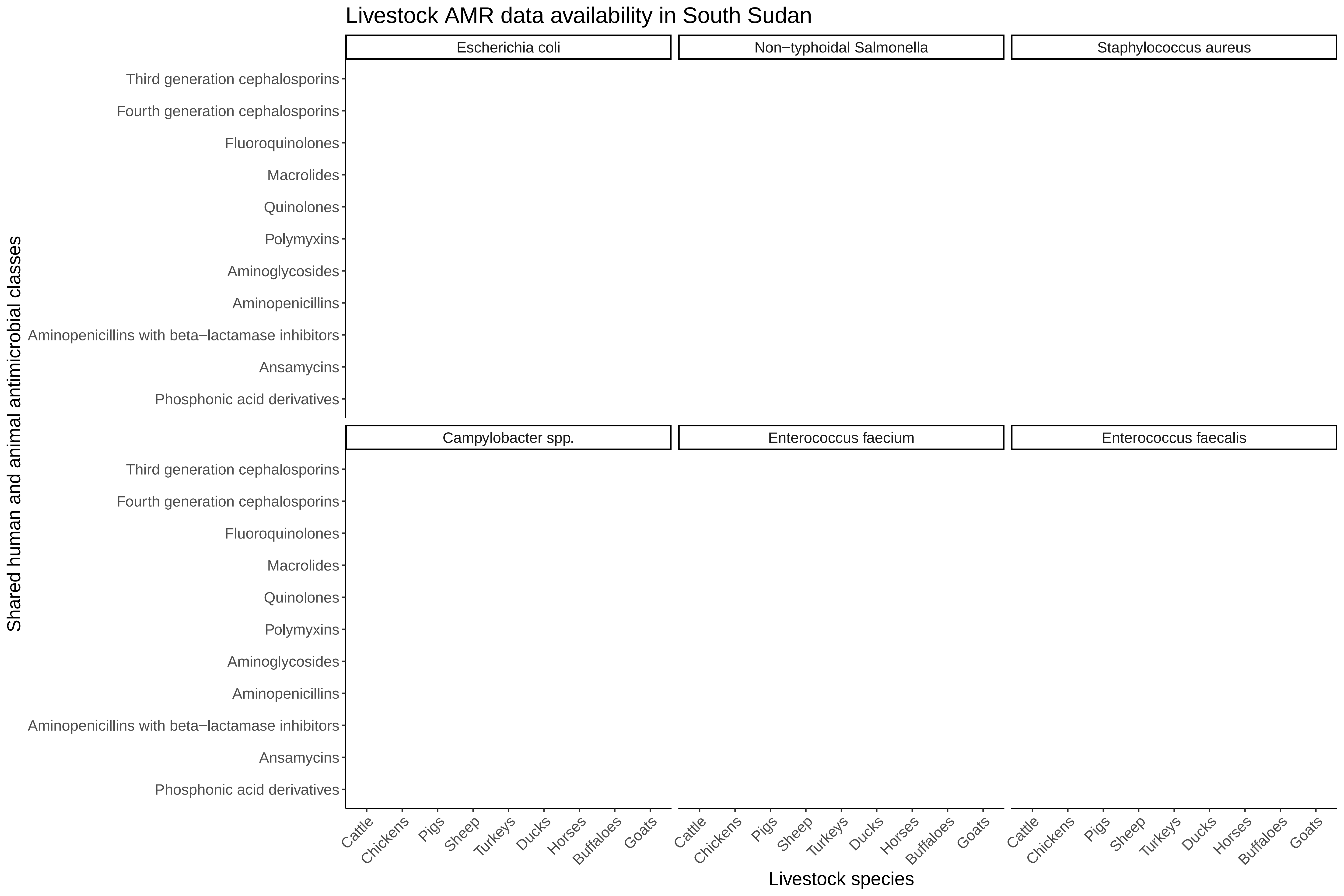
Livestock AMR data availability in South Sudan
Escherichia coli
Non−typhoidal Salmonella
Staphylococcus aureus
Campylobacter spp.
Enterococcus faecium
Enterococcus faecalis
Third generation cephalosporins
Fourth generation cephalosporins
Fluoroquinolones
Macrolides
Quinolones
Polymyxins
Aminoglycosides
Aminopenicillins
Aminopenicillins with beta−lactamase inhibitors
Ansamycins
Phosphonic acid derivatives
Shared human and animal antimicrobial classes
Cattle
Chickens
Pigs
Sheep
Turkeys
Ducks
Horses
Buffaloes
Goats
Livestock species

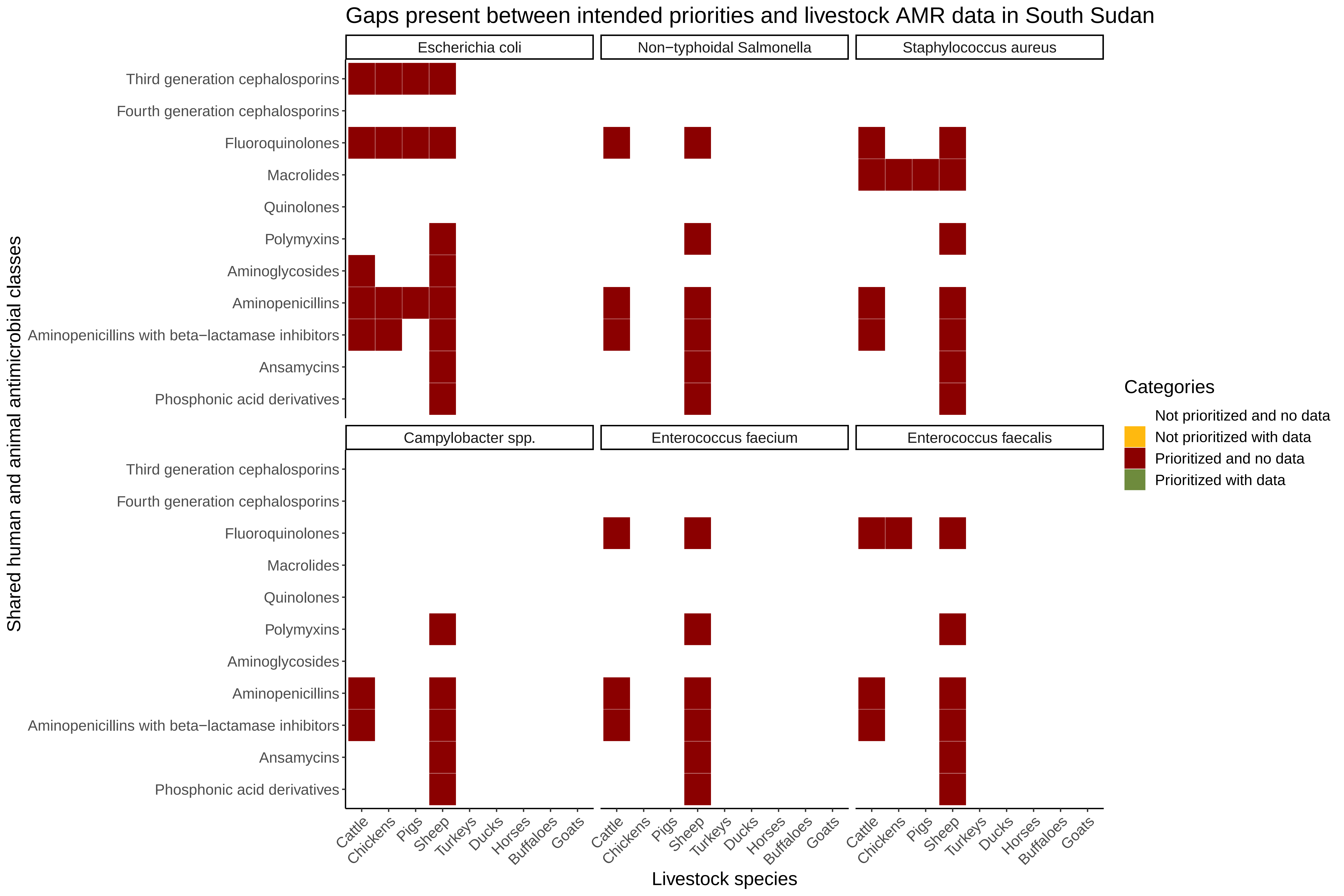

Gaps present between intended priorities and livestock AMR data in South Sudan
Escherichia coli
Non−typhoidal Salmonella
Staphylococcus aureus
Campylobacter spp.
Enterococcus faecium
Enterococcus faecalis
Third generation cephalosporins
Fourth generation cephalosporins
Fluoroquinolones
Macrolides
Quinolones
Polymyxins
Aminoglycosides
Aminopenicillins
Aminopenicillins with beta−lactamase inhibitors
Ansamycins
Phosphonic acid derivatives
Shared human and animal antimicrobial classes
Cattle
Chickens
Pigs
Sheep
Turkeys
Ducks
Horses
Buffaloes
Goats
Livestock species
Categories
Not prioritized and no data
Not prioritized with data
Prioritized and no data
Prioritized with data

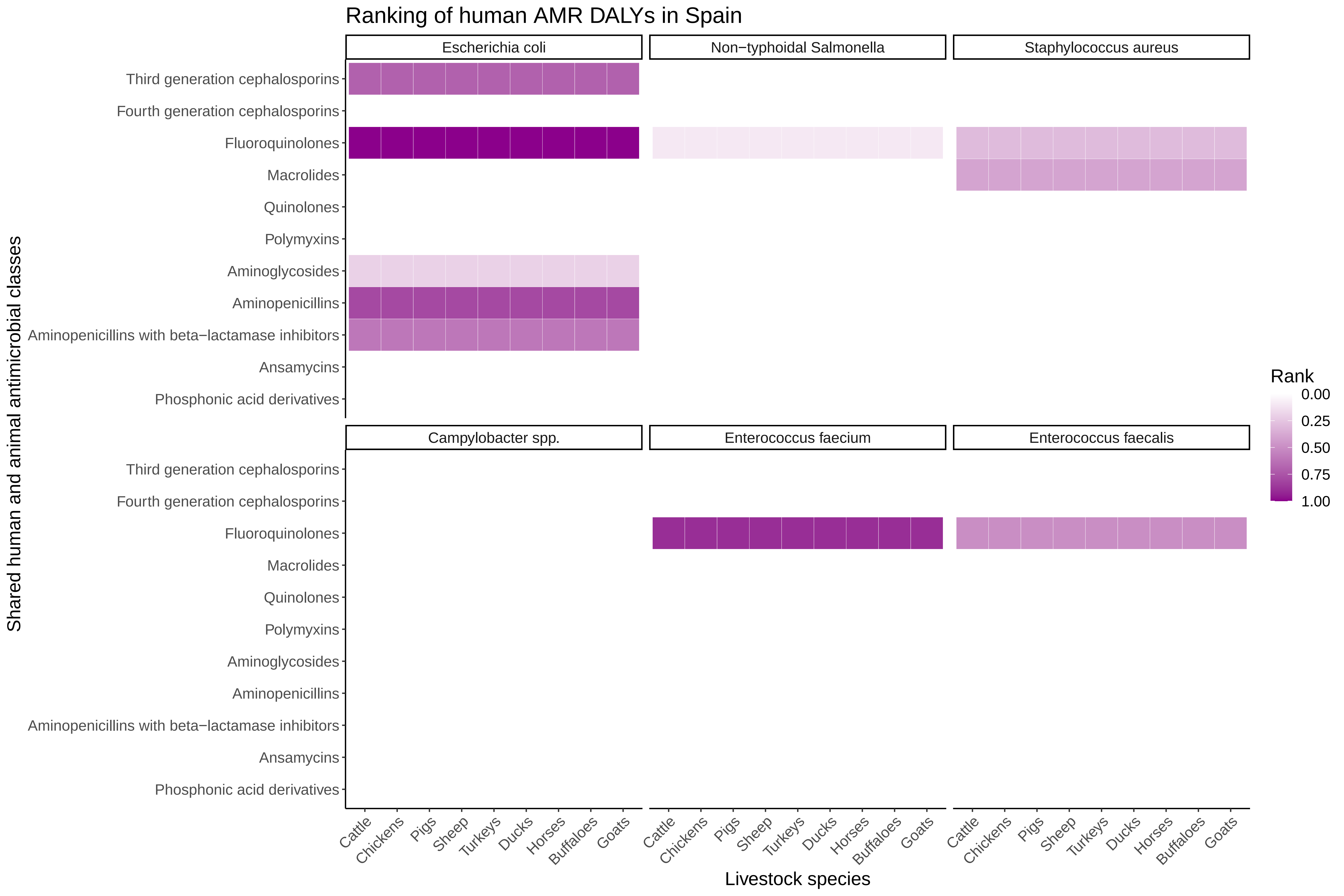

Ranking of human AMR DALYs in Spain
Escherichia coli
Non−typhoidal Salmonella
Staphylococcus aureus
Campylobacter spp.
Enterococcus faecium
Enterococcus faecalis
Third generation cephalosporins
Fourth generation cephalosporins
Fluoroquinolones
Macrolides
Quinolones
Polymyxins
Aminoglycosides
Aminopenicillins
Aminopenicillins with beta−lactamase inhibitors
Ansamycins
Phosphonic acid derivatives
Shared human and animal antimicrobial classes
Cattle
Chickens
Pigs
Sheep
Turkeys
Ducks
Horses
Buffaloes
Goats
Livestock species
Rank
0.00
0.25
0.50
0.75
1.00

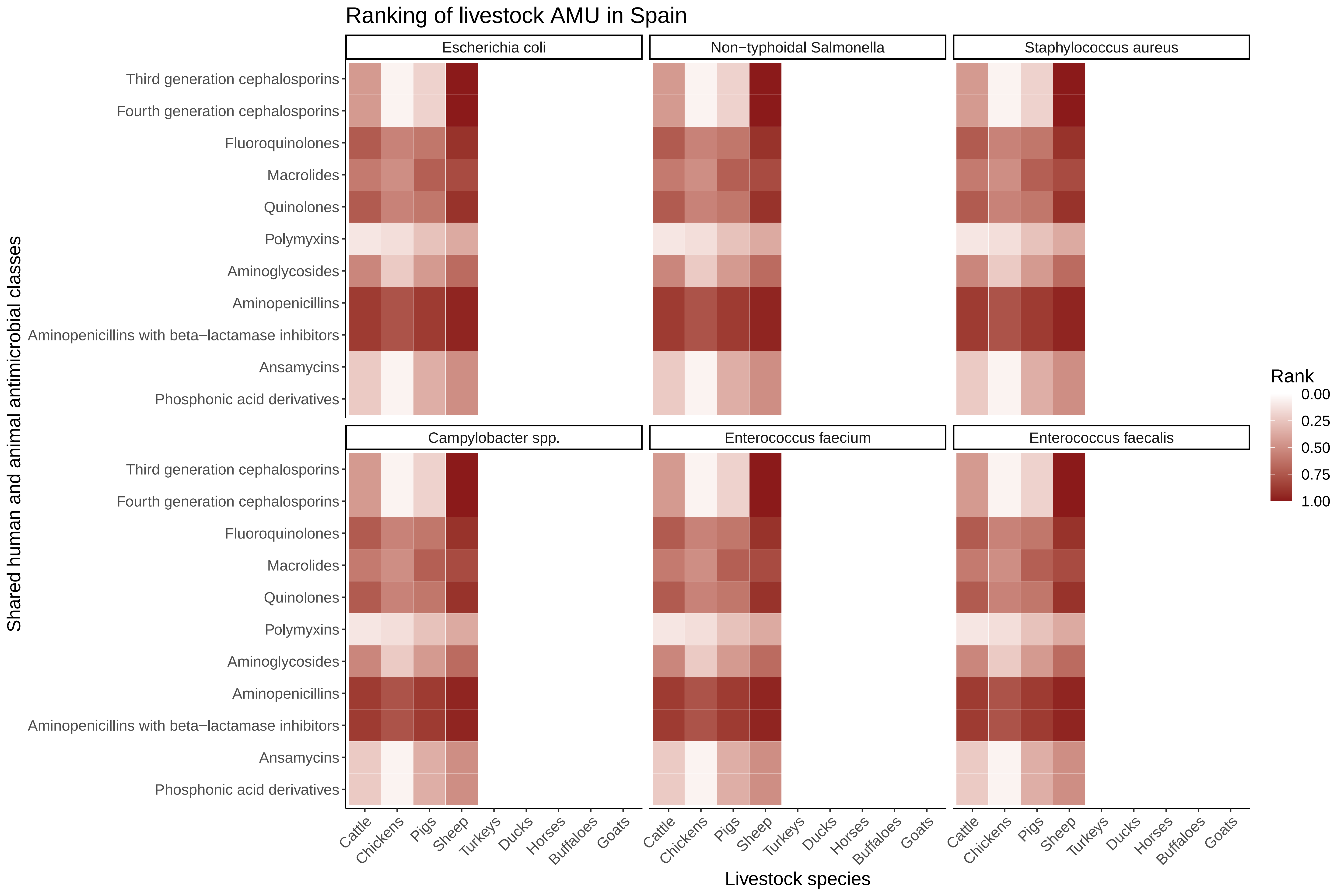
Ranking of livestock AMU in Spain
Escherichia coli
Non−typhoidal Salmonella
Staphylococcus aureus
Campylobacter spp.
Enterococcus faecium
Enterococcus faecalis
Shared human and animal antimicrobial classes
Third generation cephalosporins
Fourth generation cephalosporins
Fluoroquinolones
Macrolides
Quinolones
Polymyxins
Aminoglycosides
Aminopenicillins
Aminopenicillins with beta−lactamase inhibitors
Ansamycins
Phosphonic acid derivatives
Cattle
Chickens
Pigs
Sheep
Turkeys
Ducks
Horses
Buffaloes
Goats
Livestock species
Rank
0.00
0.25
0.50
0.75
1.00

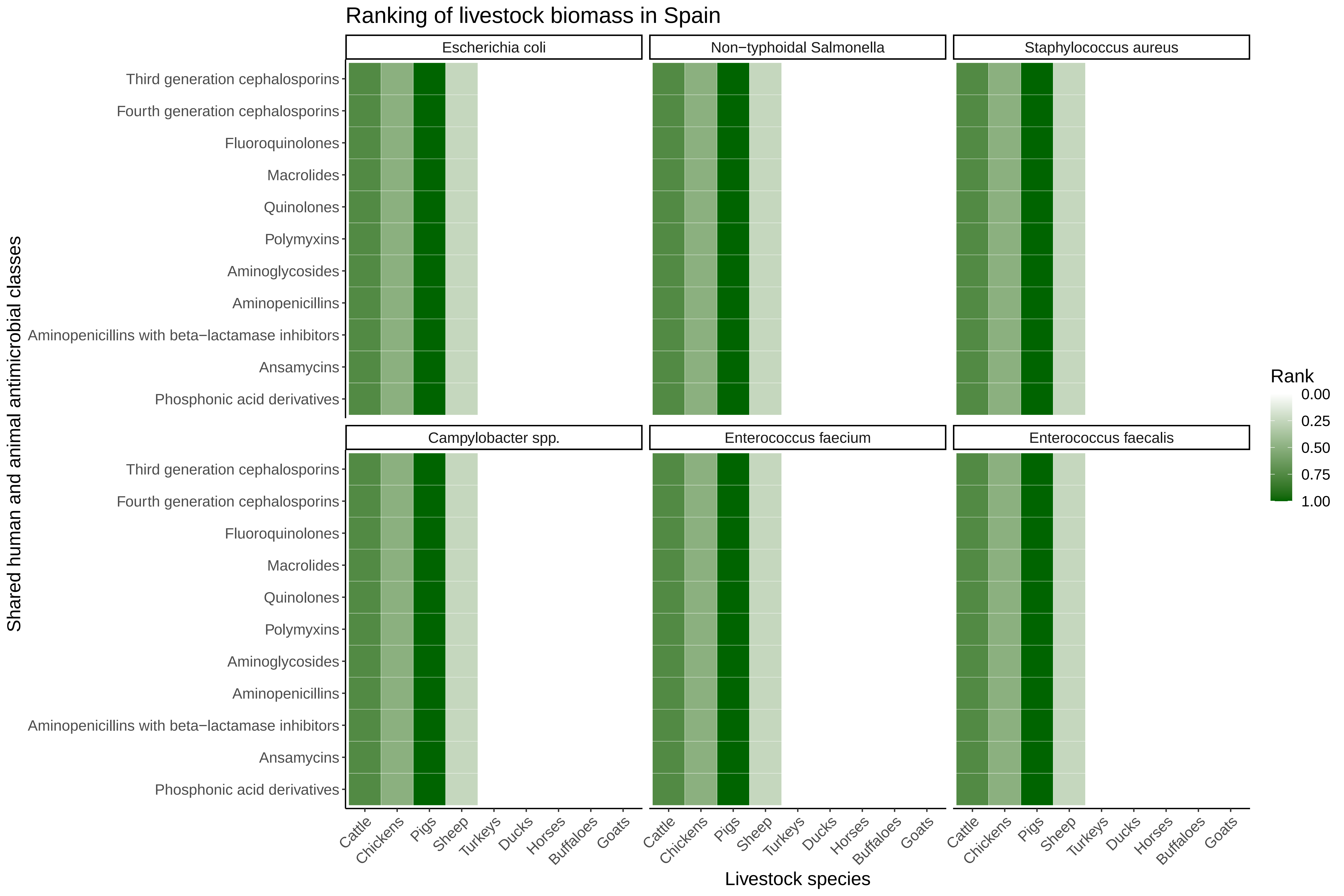
Ranking of livestock biomass in Spain
Escherichia coli
Non−typhoidal Salmonella
Staphylococcus aureus
Campylobacter spp.
Enterococcus faecium
Enterococcus faecalis
Shared human and animal antimicrobial classes
Third generation cephalosporins
Fourth generation cephalosporins
Fluoroquinolones
Macrolides
Quinolones
Polymyxins
Aminoglycosides
Aminopenicillins
Aminopenicillins with beta−lactamase inhibitors
Ansamycins
Phosphonic acid derivatives
Livestock species
Cattle
Chickens
Pigs
Sheep
Turkeys
Ducks
Horses
Buffaloes
Goats
Rank
0.00
0.25
0.50
0.75
1.00

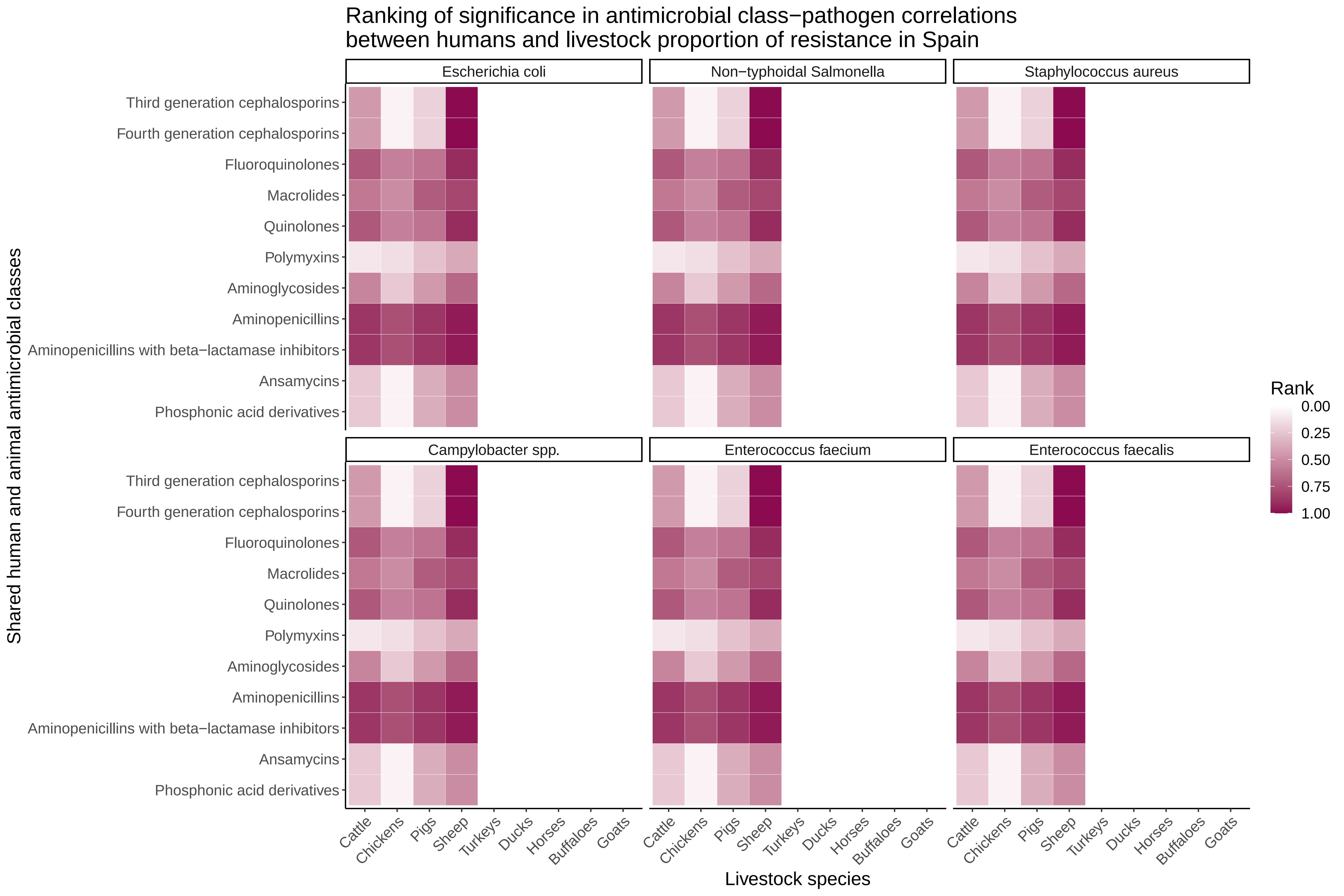

Ranking of significance in antimicrobial class−pathogen correlations
between humans and livestock proportion of resistance in Spain
Escherichia coli
Non−typhoidal Salmonella
Staphylococcus aureus
Campylobacter spp.
Enterococcus faecium
Enterococcus faecalis
Shared human and animal antimicrobial classes
Third generation cephalosporins
Fourth generation cephalosporins
Fluoroquinolones
Macrolides
Quinolones
Polymyxins
Aminoglycosides
Aminopenicillins
Aminopenicillins with beta−lactamase inhibitors
Ansamycins
Phosphonic acid derivatives
Livestock species
Cattle
Chickens
Pigs
Sheep
Turkeys
Ducks
Horses
Buffaloes
Goats
Rank
0.00
0.25
0.50
0.75
1.00

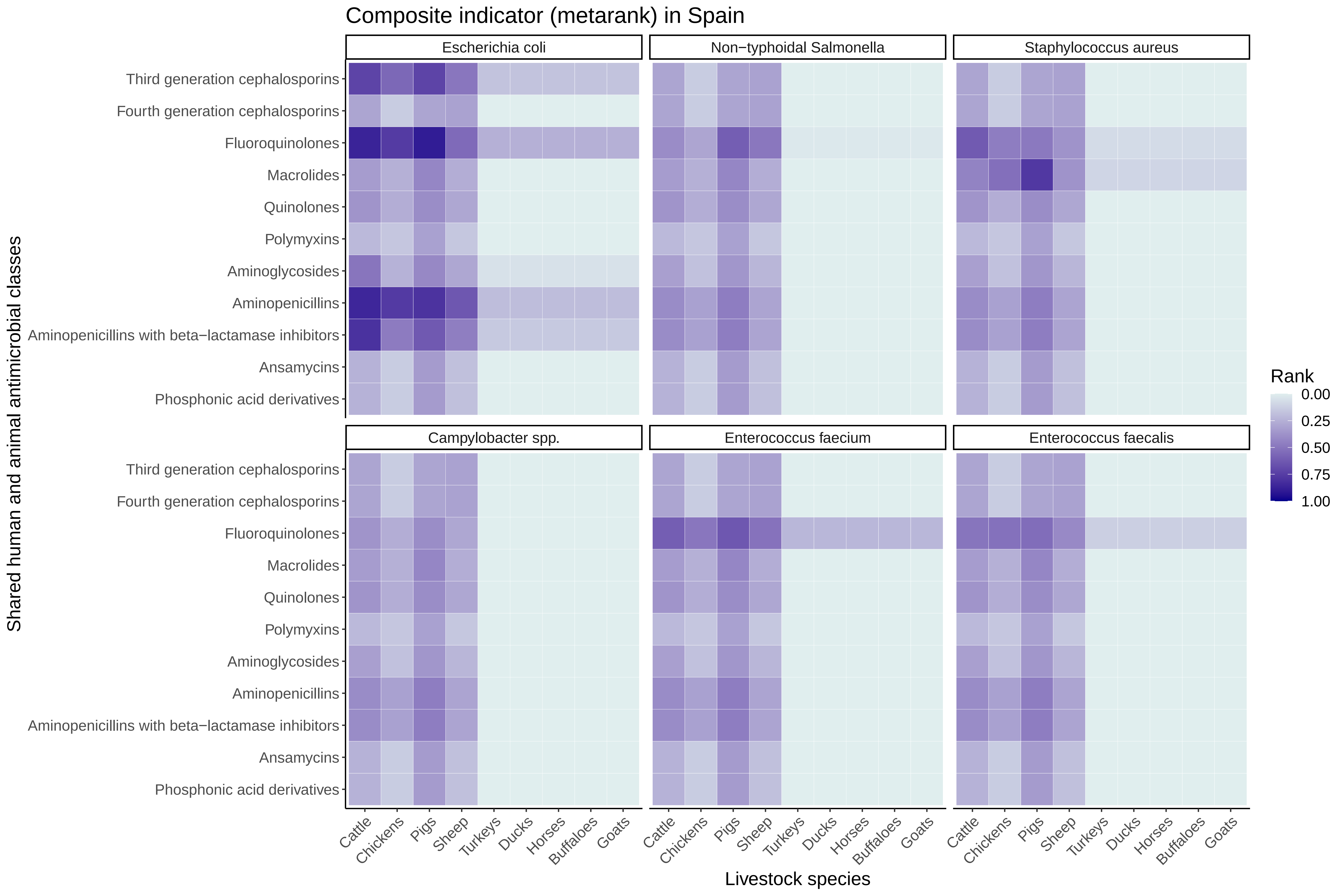

Composite indicator (metarank) in Spain
Escherichia coli
Non−typhoidal Salmonella
Staphylococcus aureus
Campylobacter spp.
Enterococcus faecium
Enterococcus faecalis
Shared human and animal antimicrobial classes
Third generation cephalosporins
Fourth generation cephalosporins
Fluoroquinolones
Macrolides
Quinolones
Polymyxins
Aminoglycosides
Aminopenicillins
Aminopenicillins with beta−lactamase inhibitors
Ansamycins
Phosphonic acid derivatives
Cattle
Chickens
Pigs
Sheep
Turkeys
Ducks
Horses
Buffaloes
Goats
Livestock species
Rank
0.00
0.25
0.50
0.75
1.00

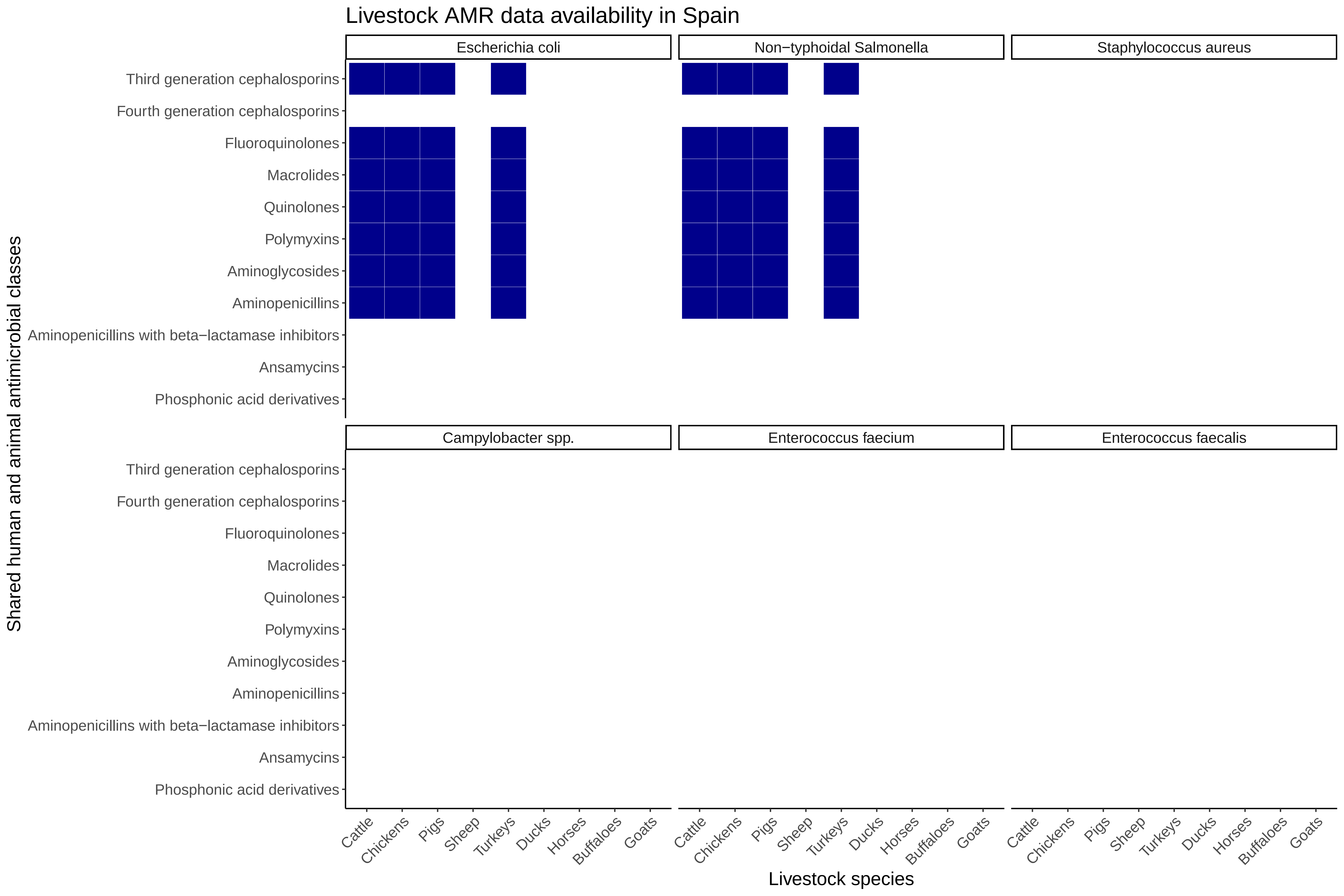
Livestock AMR data availability in Spain
Escherichia coli
Non−typhoidal Salmonella
Staphylococcus aureus
Campylobacter spp.
Enterococcus faecium
Enterococcus faecalis
Shared human and animal antimicrobial classes
Third generation cephalosporins
Fourth generation cephalosporins
Fluoroquinolones
Macrolides
Quinolones
Polymyxins
Aminoglycosides
Aminopenicillins
Aminopenicillins with beta−lactamase inhibitors
Ansamycins
Phosphonic acid derivatives
Cattle
Chickens
Pigs
Sheep
Turkeys
Ducks
Horses
Buffaloes
Goats
Livestock species

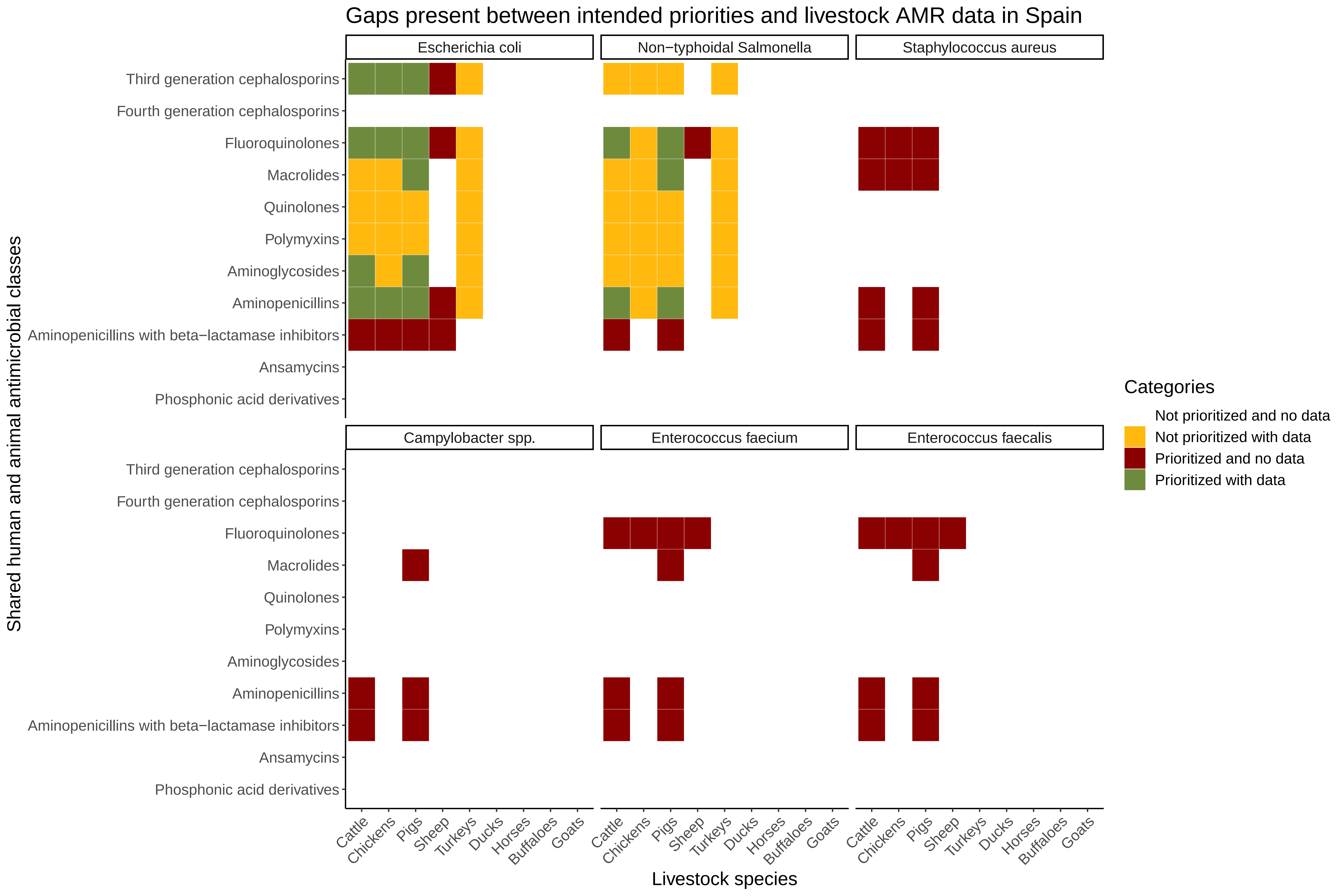

Gaps present between intended priorities and livestock AMR data in Spain
Escherichia coli
Non−typhoidal Salmonella
Staphylococcus aureus
Campylobacter spp.
Enterococcus faecium
Enterococcus faecalis
Third generation cephalosporins
Fourth generation cephalosporins
Fluoroquinolones
Macrolides
Quinolones
Polymyxins
Aminoglycosides
Aminopenicillins
Aminopenicillins with beta−lactamase inhibitors
Ansamycins
Phosphonic acid derivatives
Shared human and animal antimicrobial classes
Cattle
Chickens
Pigs
Sheep
Turkeys
Ducks
Horses
Buffaloes
Goats
Livestock species
Categories
Not prioritized and no data
Not prioritized with data
Prioritized and no data
Prioritized with data

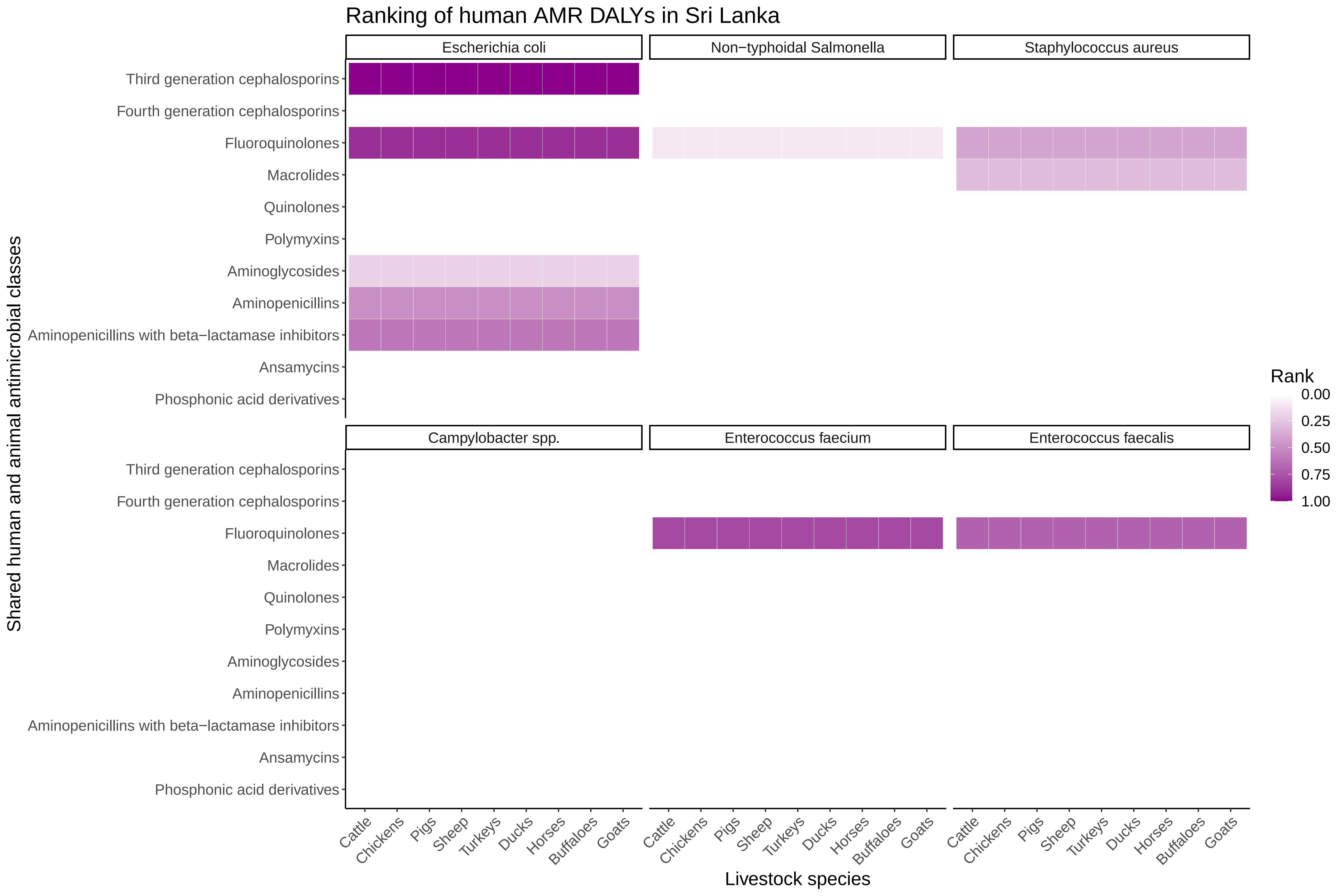
Ranking of human AMR DALYs in Sri Lanka
Escherichia coli
Non−typhoidal Salmonella
Staphylococcus aureus
Campylobacter spp.
Enterococcus faecium
Enterococcus faecalis
Third generation cephalosporins
Fourth generation cephalosporins
Fluoroquinolones
Macrolides
Quinolones
Polymyxins
Aminoglycosides
Aminopenicillins
Aminopenicillins with beta−lactamase inhibitors
Ansamycins
Phosphonic acid derivatives
Shared human and animal antimicrobial classes
Cattle
Chickens
Pigs
Sheep
Turkeys
Ducks
Horses
Buffaloes
Goats
Livestock species
Rank
0.00
0.25
0.50
0.75
1.00

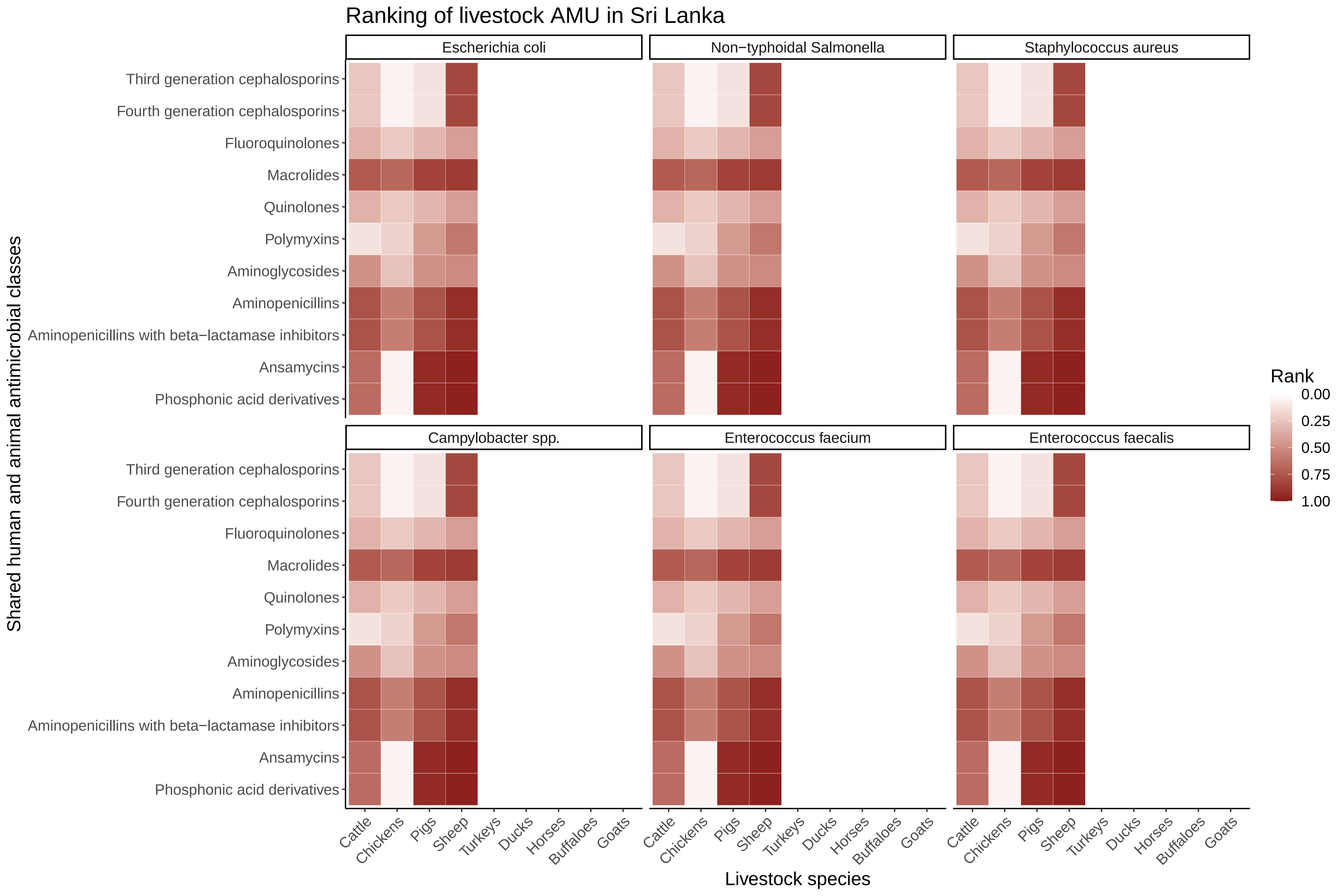

Ranking of livestock AMU in Sri Lanka
Escherichia coli
Non−typhoidal Salmonella
Staphylococcus aureus
Campylobacter spp.
Enterococcus faecium
Enterococcus faecalis
Third generation cephalosporins
Fourth generation cephalosporins
Fluoroquinolones
Macrolides
Quinolones
Polymyxins
Aminoglycosides
Aminopenicillins
Aminopenicillins with beta−lactamase inhibitors
Ansamycins
Phosphonic acid derivatives
Shared human and animal antimicrobial classes
Cattle
Chickens
Pigs
Sheep
Turkeys
Ducks
Horses
Buffaloes
Goats
Livestock species
Rank
0.00
0.25
0.50
0.75
1.00

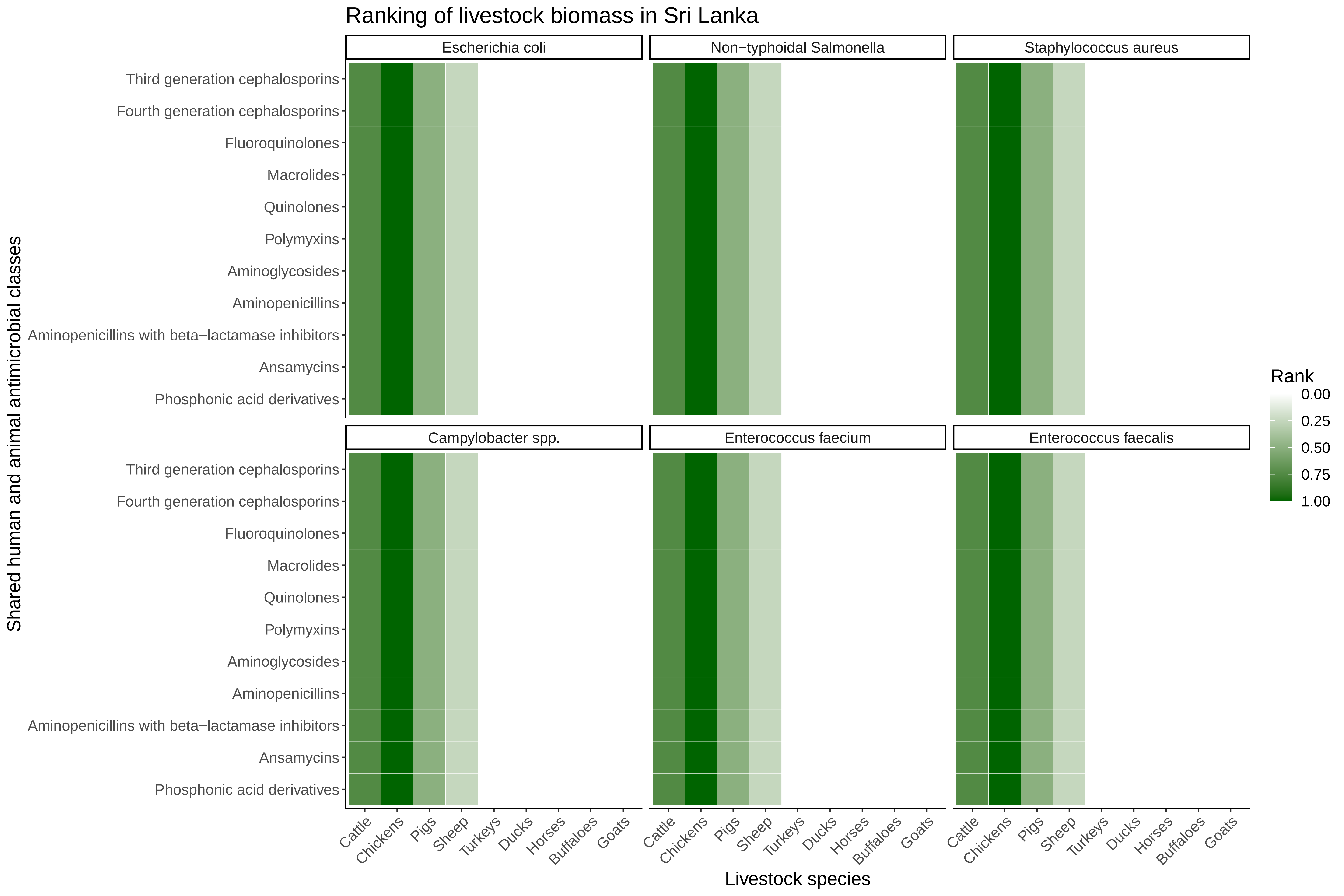

Ranking of livestock biomass in Sri Lanka
Escherichia coli
Non−typhoidal Salmonella
Staphylococcus aureus
Campylobacter spp.
Enterococcus faecium
Enterococcus faecalis
Third generation cephalosporins
Fourth generation cephalosporins
Fluoroquinolones
Macrolides
Quinolones
Polymyxins
Aminoglycosides
Aminopenicillins
Aminopenicillins with beta−lactamase inhibitors
Ansamycins
Phosphonic acid derivatives
Shared human and animal antimicrobial classes
Cattle
Chickens
Pigs
Sheep
Turkeys
Ducks
Horses
Buffaloes
Goats
Livestock species
Rank
0.00
0.25
0.50
0.75
1.00

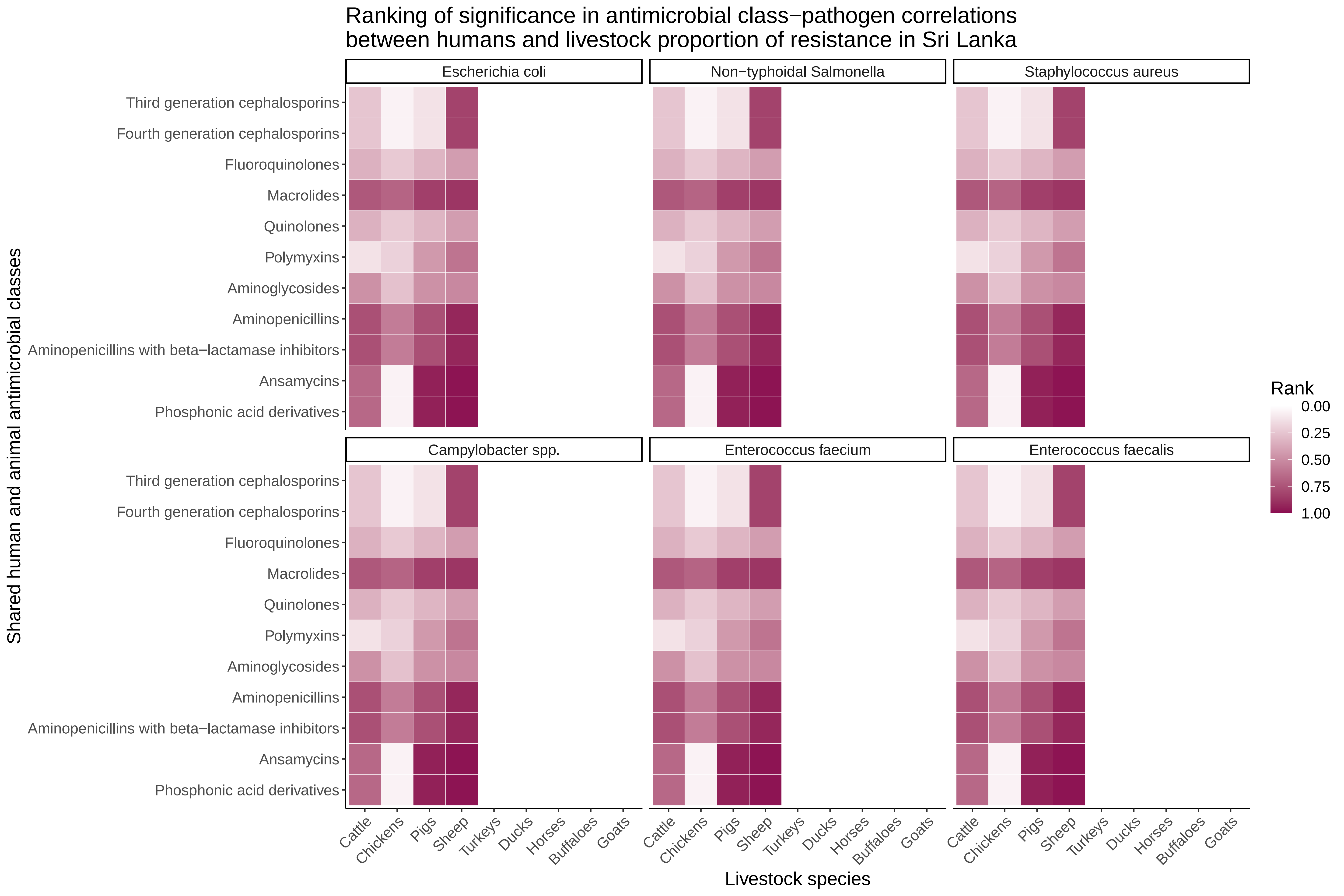

Ranking of significance in antimicrobial class−pathogen correlations
between humans and livestock proportion of resistance in Sri Lanka
Escherichia coli
Non−typhoidal Salmonella
Staphylococcus aureus
Campylobacter spp.
Enterococcus faecium
Enterococcus faecalis
Third generation cephalosporins
Fourth generation cephalosporins
Fluoroquinolones
Macrolides
Quinolones
Polymyxins
Aminoglycosides
Aminopenicillins
Aminopenicillins with beta−lactamase inhibitors
Ansamycins
Phosphonic acid derivatives
Shared human and animal antimicrobial classes
Cattle
Chickens
Pigs
Sheep
Turkeys
Ducks
Horses
Buffaloes
Goats
Livestock species
Rank
0.00
0.25
0.50
0.75
1.00

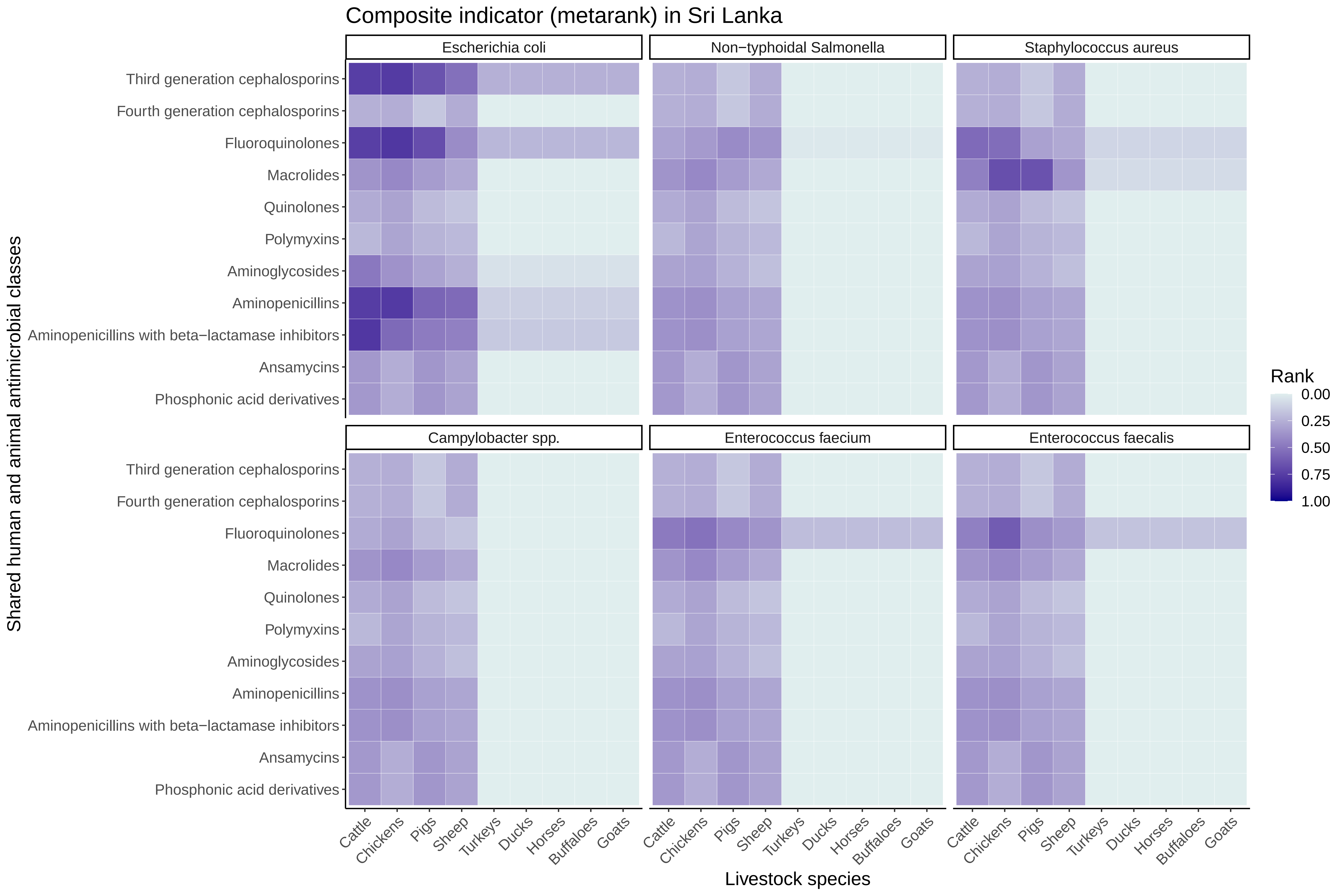

Composite indicator (metarank) in Sri Lanka
Escherichia coli
Non−typhoidal Salmonella
Staphylococcus aureus
Campylobacter spp.
Enterococcus faecium
Enterococcus faecalis
Shared human and animal antimicrobial classes
Third generation cephalosporins
Fourth generation cephalosporins
Fluoroquinolones
Macrolides
Quinolones
Polymyxins
Aminoglycosides
Aminopenicillins
Aminopenicillins with beta−lactamase inhibitors
Ansamycins
Phosphonic acid derivatives
Cattle
Chickens
Pigs
Sheep
Turkeys
Ducks
Horses
Buffaloes
Goats
Livestock species
Rank
0.00
0.25
0.50
0.75
1.00

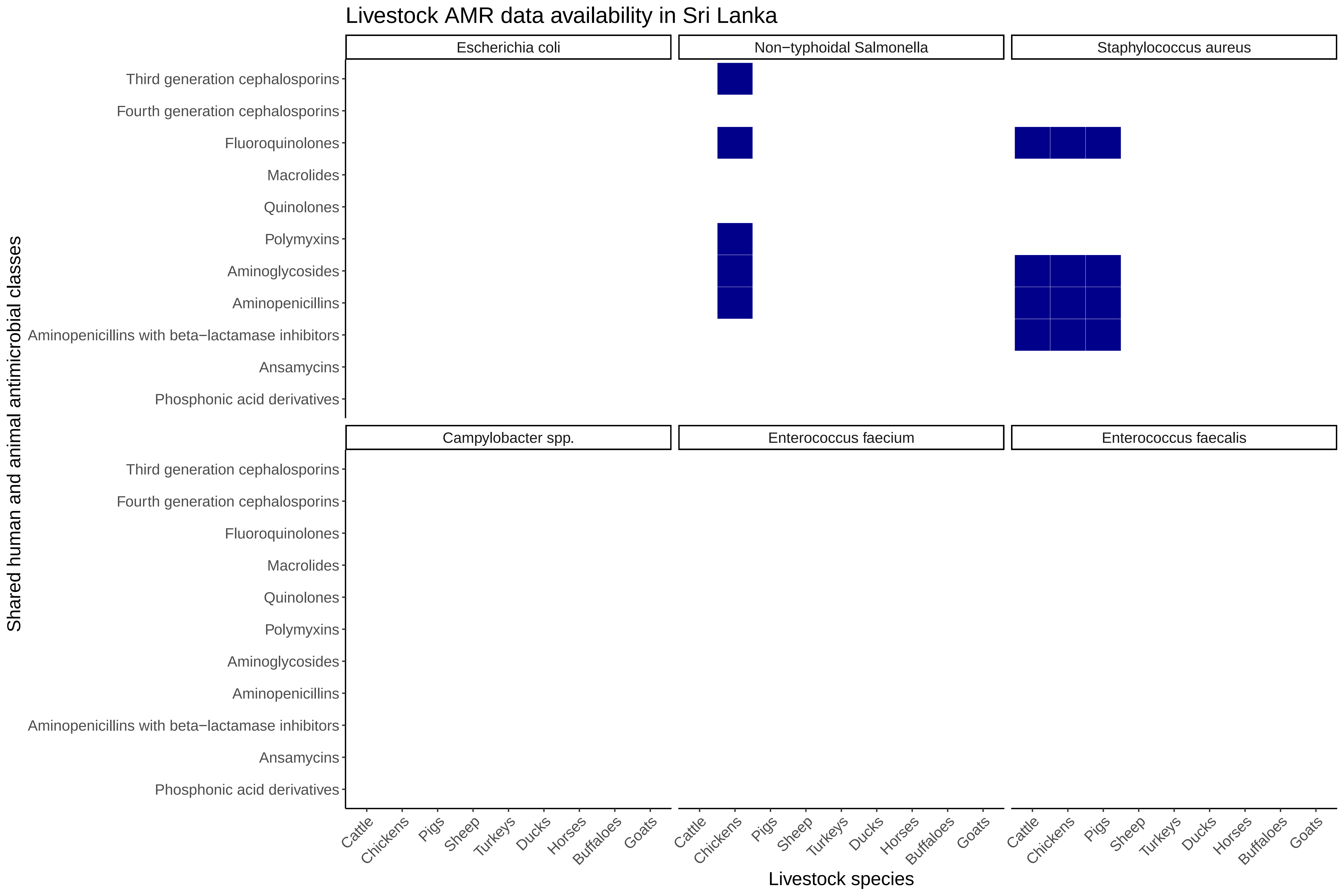
Livestock AMR data availability in Sri Lanka
Escherichia coli
Non−typhoidal Salmonella
Staphylococcus aureus
Campylobacter spp.
Enterococcus faecium
Enterococcus faecalis
Third generation cephalosporins
Fourth generation cephalosporins
Fluoroquinolones
Macrolides
Quinolones
Polymyxins
Aminoglycosides
Aminopenicillins
Aminopenicillins with beta−lactamase inhibitors
Ansamycins
Phosphonic acid derivatives
Shared human and animal antimicrobial classes
Cattle
Chickens
Pigs
Sheep
Turkeys
Ducks
Horses
Buffaloes
Goats
Livestock species

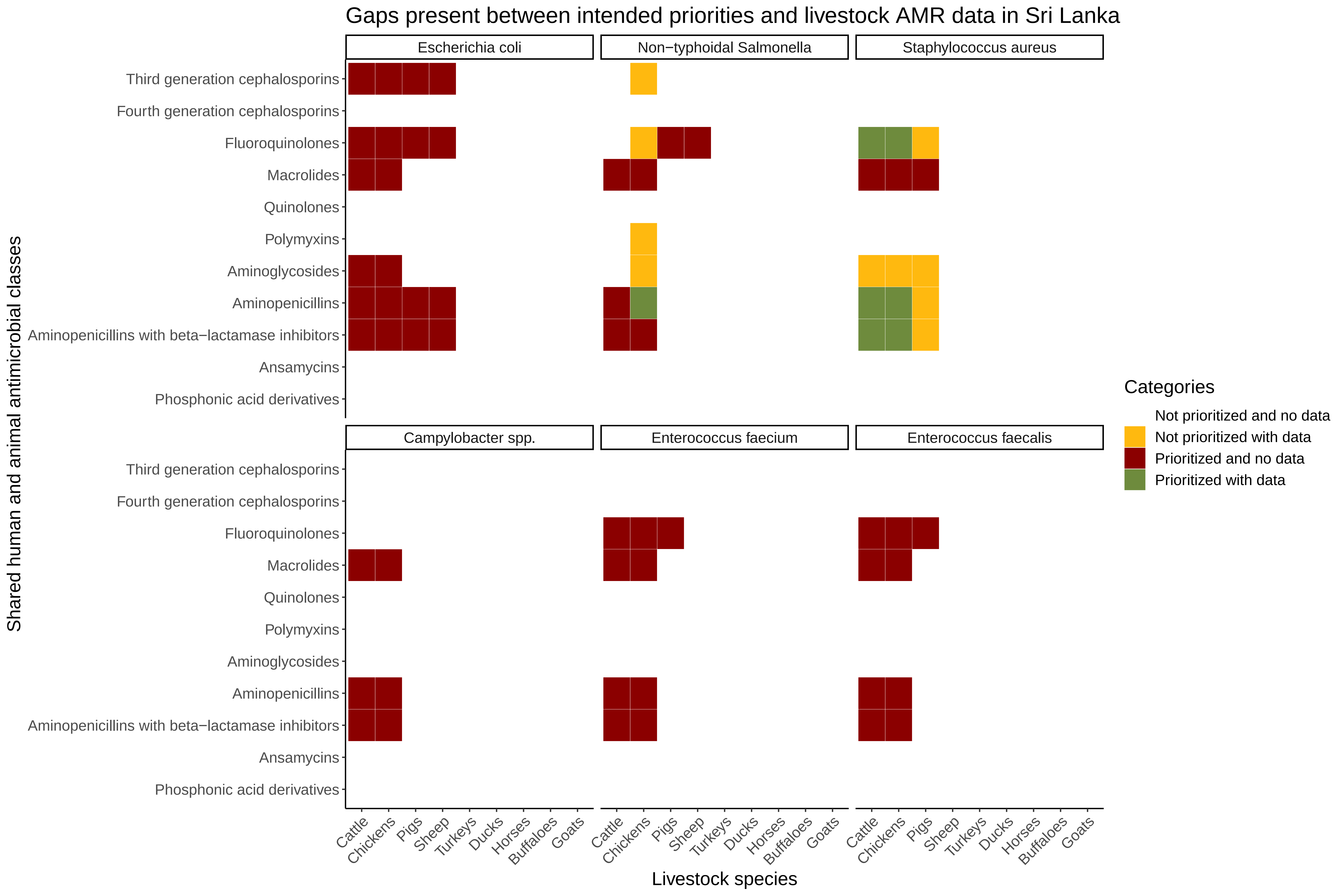
Gaps present between intended priorities and livestock AMR data in Sri Lanka
Escherichia coli
Non−typhoidal Salmonella
Staphylococcus aureus
Campylobacter spp.
Enterococcus faecium
Enterococcus faecalis
Shared human and animal antimicrobial classes
Third generation cephalosporins
Fourth generation cephalosporins
Fluoroquinolones
Macrolides
Quinolones
Polymyxins
Aminoglycosides
Aminopenicillins
Aminopenicillins with beta−lactamase inhibitors
Ansamycins
Phosphonic acid derivatives
Cattle
Chickens
Pigs
Sheep
Turkeys
Ducks
Horses
Buffaloes
Goats
Livestock species
Categories
Not prioritized and no data
Not prioritized with data
Prioritized and no data
Prioritized with data

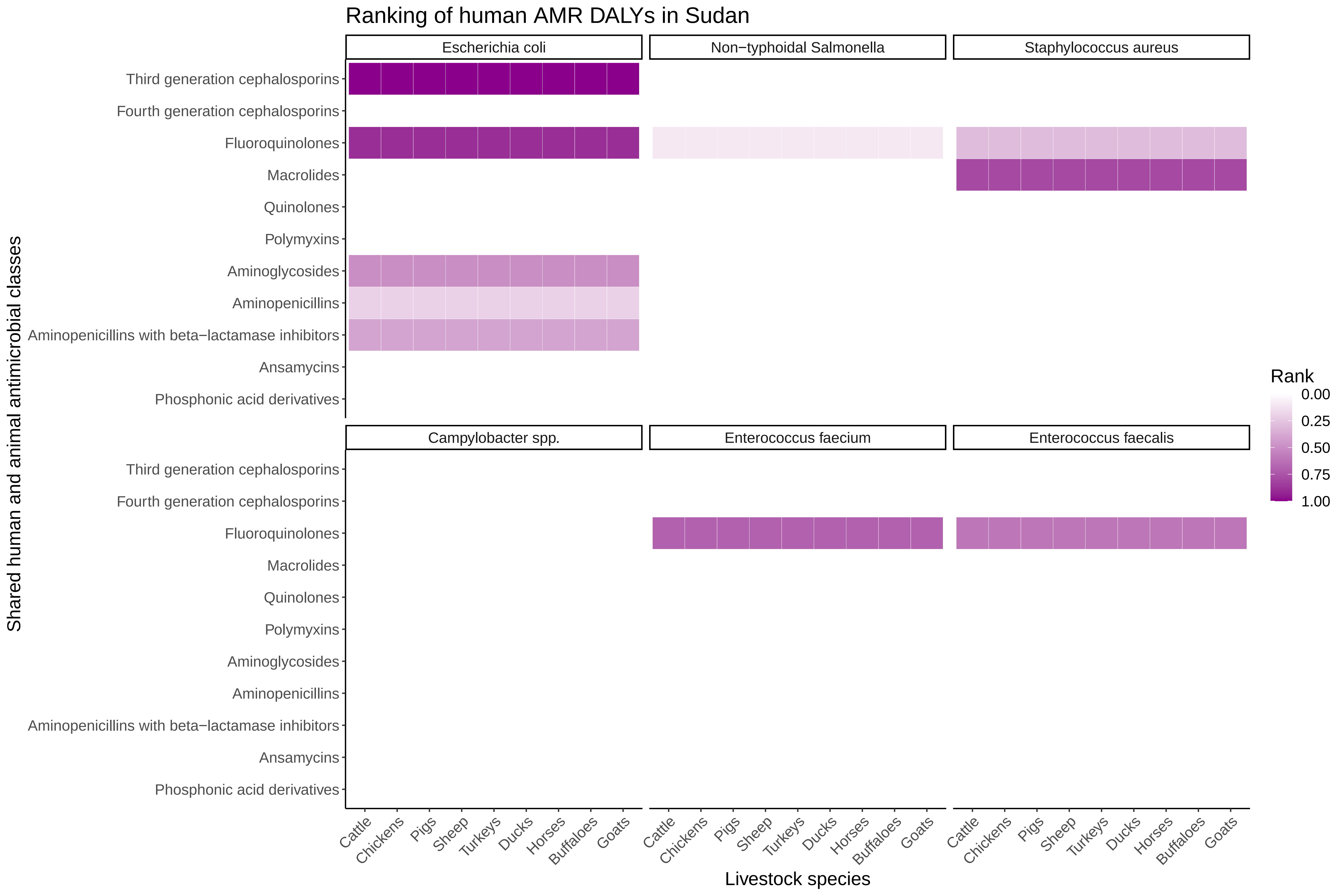
Ranking of human AMR DALYs in Sudan
Escherichia coli
Non−typhoidal Salmonella
Staphylococcus aureus
Campylobacter spp.
Enterococcus faecium
Enterococcus faecalis
Third generation cephalosporins
Fourth generation cephalosporins
Fluoroquinolones
Macrolides
Quinolones
Polymyxins
Aminoglycosides
Aminopenicillins
Aminopenicillins with beta−lactamase inhibitors
Ansamycins
Phosphonic acid derivatives
Shared human and animal antimicrobial classes
Cattle
Chickens
Pigs
Sheep
Turkeys
Ducks
Horses
Buffaloes
Goats
Livestock species
Rank
0.00
0.25
0.50
0.75
1.00

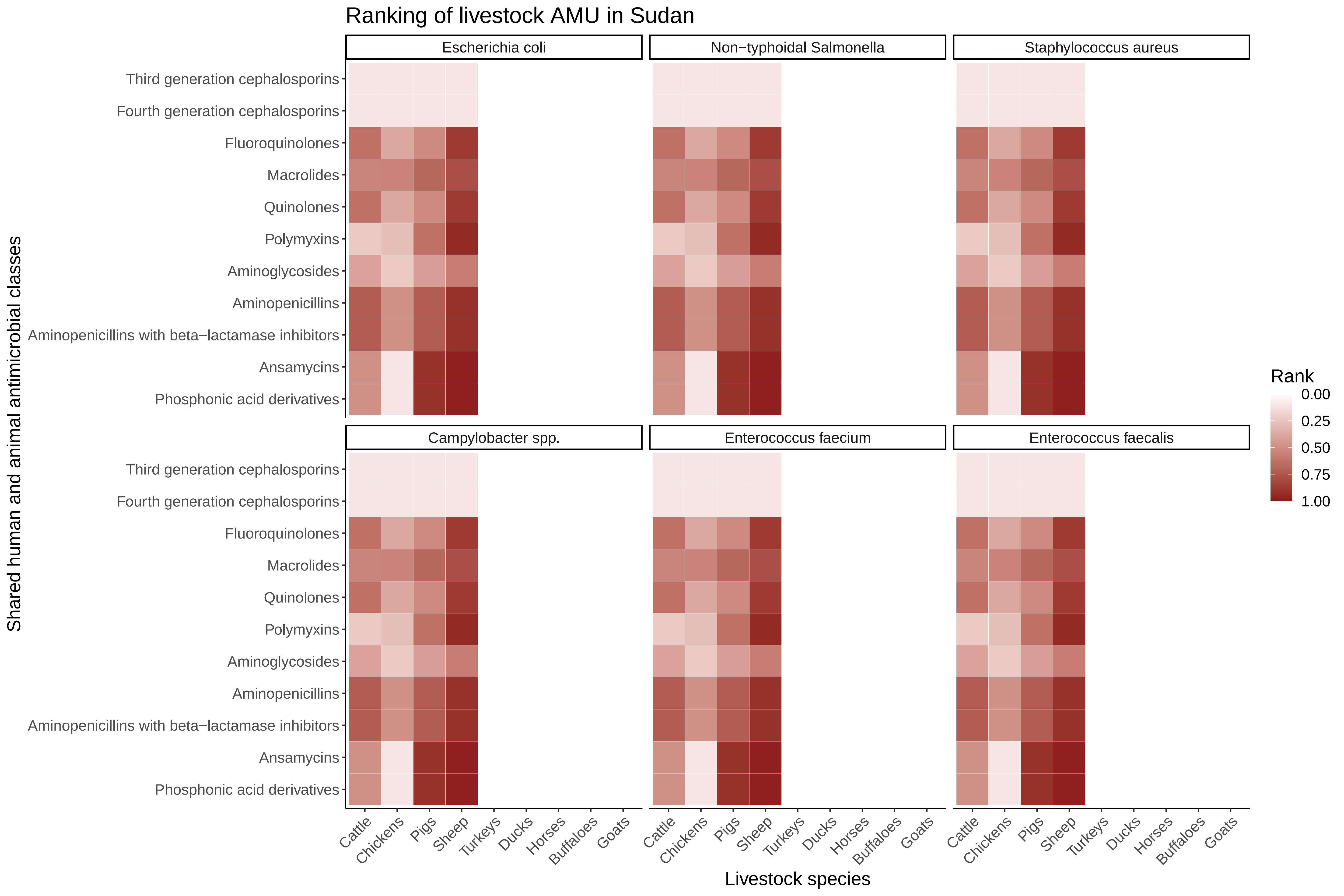

Ranking of livestock AMU in Sudan
Escherichia coli
Non−typhoidal Salmonella
Staphylococcus aureus
Campylobacter spp.
Enterococcus faecium
Enterococcus faecalis
Shared human and animal antimicrobial classes
Third generation cephalosporins
Fourth generation cephalosporins
Fluoroquinolones
Macrolides
Quinolones
Polymyxins
Aminoglycosides
Aminopenicillins
Aminopenicillins with beta−lactamase inhibitors
Ansamycins
Phosphonic acid derivatives
Cattle
Chickens
Pigs
Sheep
Turkeys
Ducks
Horses
Buffaloes
Goats
Livestock species
Rank
0.00
0.25
0.50
0.75
1.00

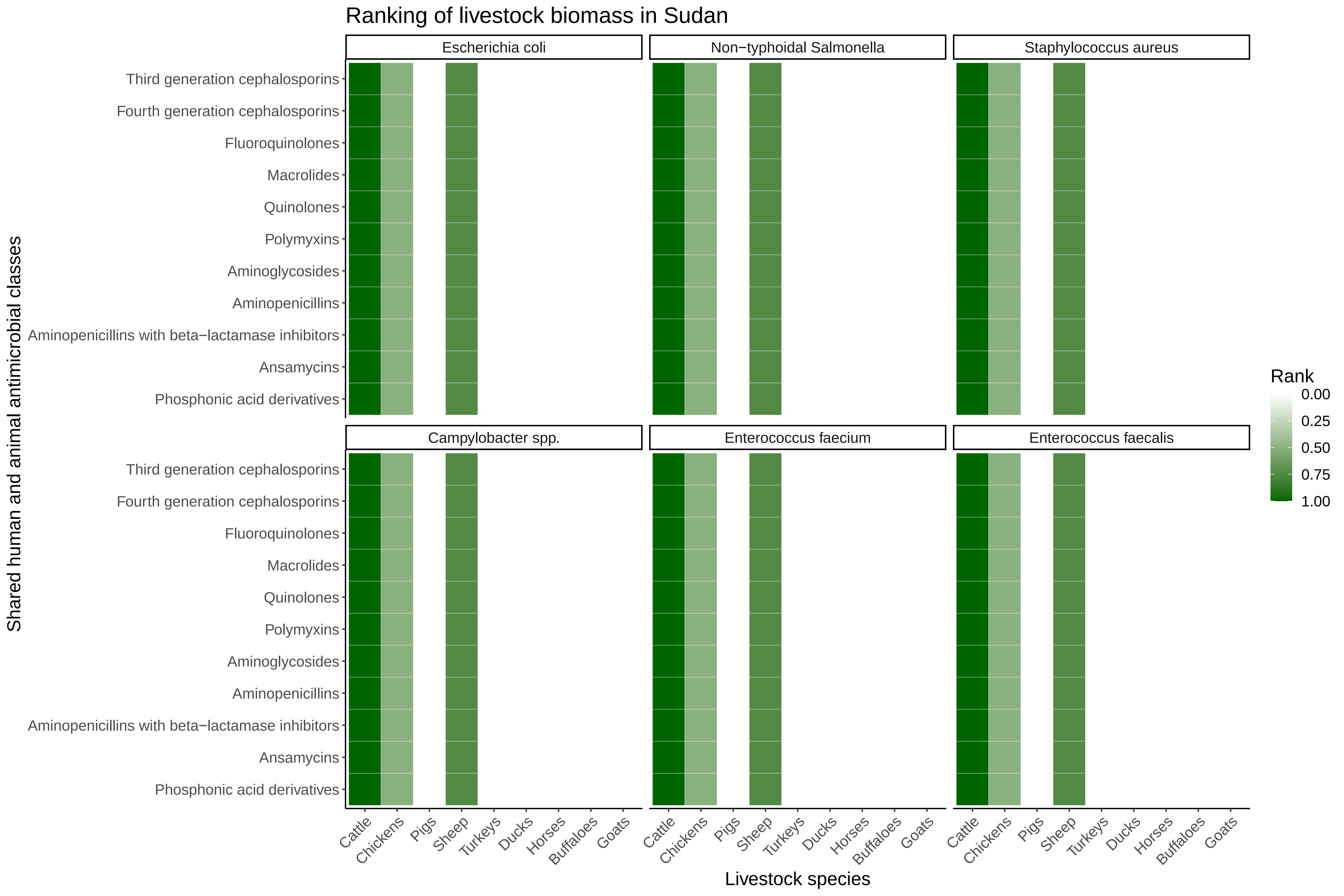

Ranking of livestock biomass in Sudan
Escherichia coli
Non−typhoidal Salmonella
Staphylococcus aureus
Campylobacter spp.
Enterococcus faecium
Enterococcus faecalis
Shared human and animal antimicrobial classes
Third generation cephalosporins
Fourth generation cephalosporins
Fluoroquinolones
Macrolides
Quinolones
Polymyxins
Aminoglycosides
Aminopenicillins
Aminopenicillins with beta−lactamase inhibitors
Ansamycins
Phosphonic acid derivatives
Livestock species
Cattle
Chickens
Pigs
Sheep
Turkeys
Ducks
Horses
Buffaloes
Goats
Rank
0.00
0.25
0.50
0.75
1.00

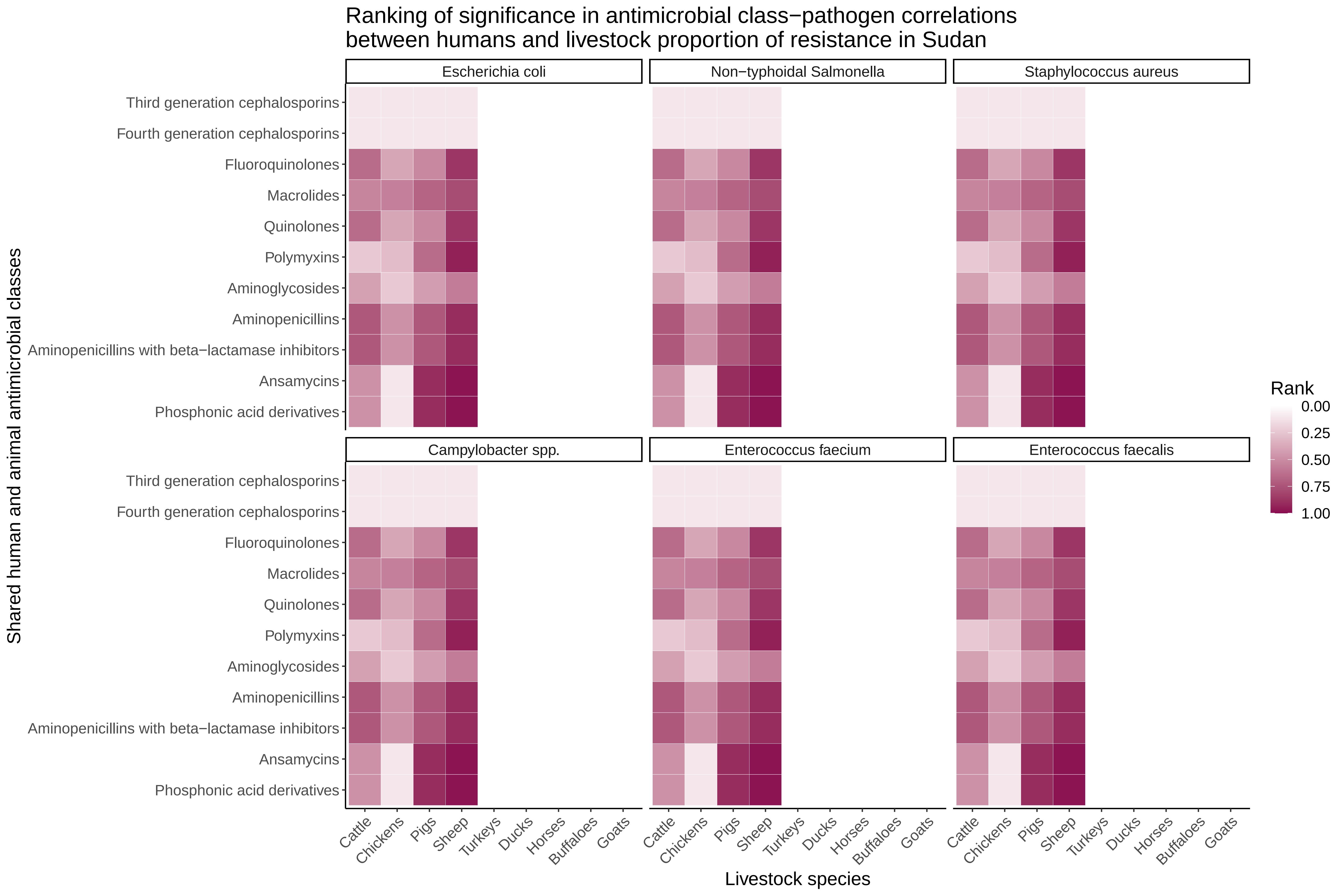

Ranking of significance in antimicrobial class−pathogen correlations
between humans and livestock proportion of resistance in Sudan
Escherichia coli
Non−typhoidal Salmonella
Staphylococcus aureus
Campylobacter spp.
Enterococcus faecium
Enterococcus faecalis
Third generation cephalosporins
Fourth generation cephalosporins
Fluoroquinolones
Macrolides
Quinolones
Polymyxins
Aminoglycosides
Aminopenicillins
Aminopenicillins with beta−lactamase inhibitors
Ansamycins
Phosphonic acid derivatives
Shared human and animal antimicrobial classes
Cattle
Chickens
Pigs
Sheep
Turkeys
Ducks
Horses
Buffaloes
Goats
Livestock species
Rank
0.00
0.25
0.50
0.75
1.00

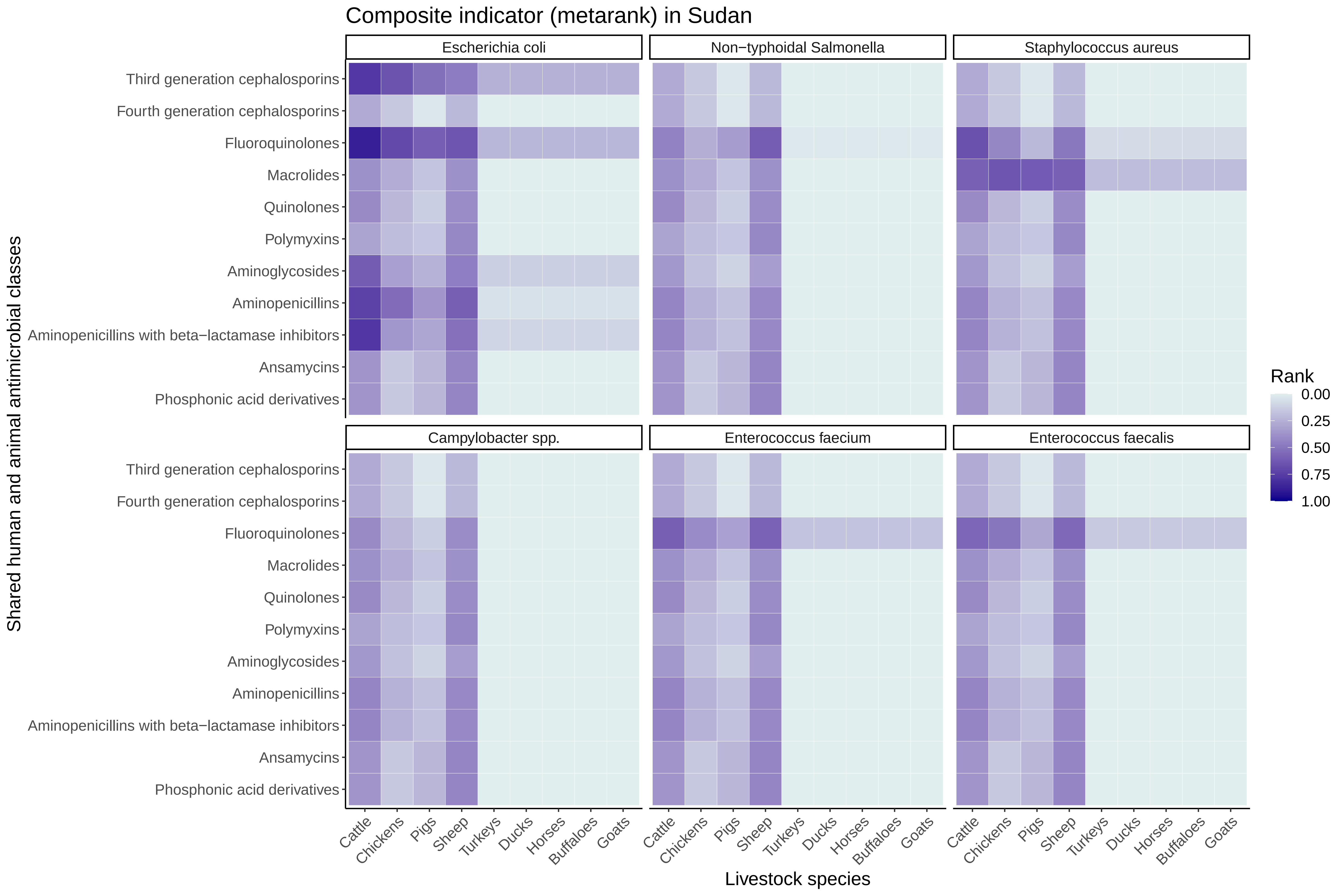

Composite indicator (metarank) in Sudan
Escherichia coli
Non−typhoidal Salmonella
Staphylococcus aureus
Campylobacter spp.
Enterococcus faecium
Enterococcus faecalis
Third generation cephalosporins
Fourth generation cephalosporins
Fluoroquinolones
Macrolides
Quinolones
Polymyxins
Aminoglycosides
Aminopenicillins
Aminopenicillins with beta−lactamase inhibitors
Ansamycins
Phosphonic acid derivatives
Shared human and animal antimicrobial classes
Cattle
Chickens
Pigs
Sheep
Turkeys
Ducks
Horses
Buffaloes
Goats
Livestock species
Rank
0.00
0.25
0.50
0.75
1.00

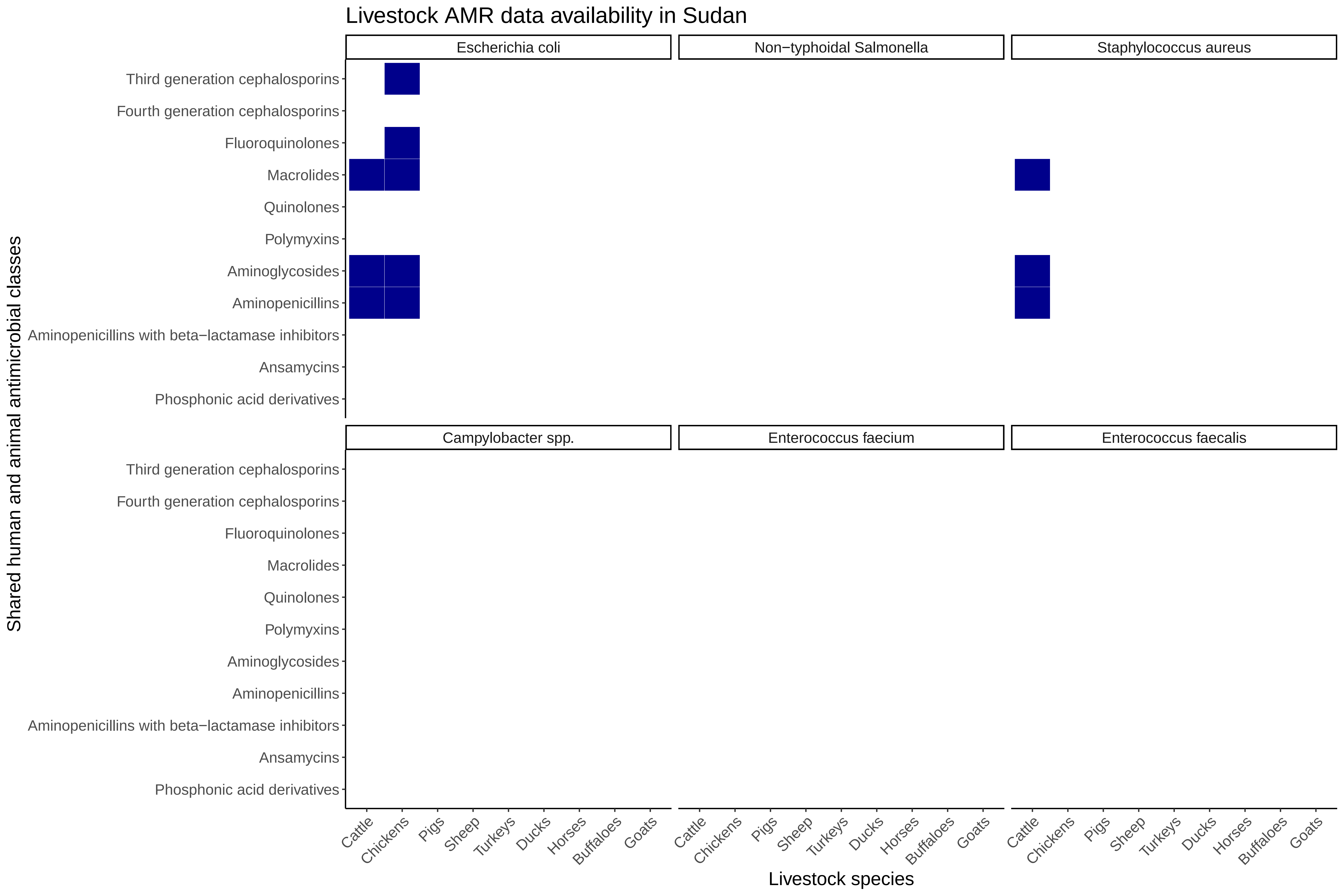

Livestock AMR data availability in Sudan
Escherichia coli
Non−typhoidal Salmonella
Staphylococcus aureus
Campylobacter spp.
Enterococcus faecium
Enterococcus faecalis
Shared human and animal antimicrobial classes
Third generation cephalosporins
Fourth generation cephalosporins
Fluoroquinolones
Macrolides
Quinolones
Polymyxins
Aminoglycosides
Aminopenicillins
Aminopenicillins with beta−lactamase inhibitors
Ansamycins
Phosphonic acid derivatives
Cattle
Chickens
Pigs
Sheep
Turkeys
Ducks
Horses
Buffaloes
Goats
Livestock species

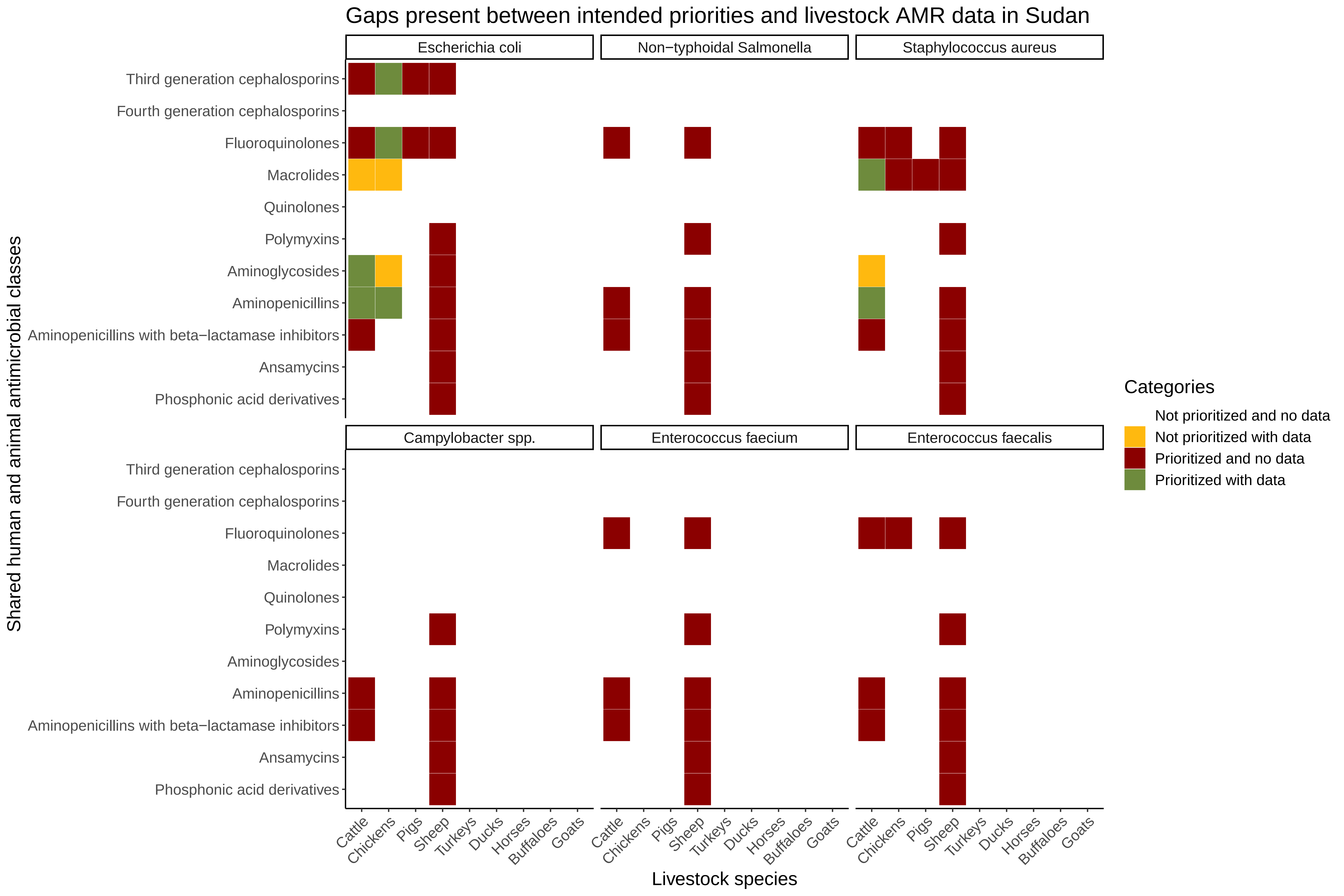

Gaps present between intended priorities and livestock AMR data in Sudan
Escherichia coli
Non−typhoidal Salmonella
Staphylococcus aureus
Campylobacter spp.
Enterococcus faecium
Enterococcus faecalis
Third generation cephalosporins
Fourth generation cephalosporins
Fluoroquinolones
Macrolides
Quinolones
Polymyxins
Aminoglycosides
Aminopenicillins
Aminopenicillins with beta−lactamase inhibitors
Ansamycins
Phosphonic acid derivatives
Shared human and animal antimicrobial classes
Cattle
Chickens
Pigs
Sheep
Turkeys
Ducks
Horses
Buffaloes
Goats
Livestock species
Categories
Not prioritized and no data
Not prioritized with data
Prioritized and no data
Prioritized with data

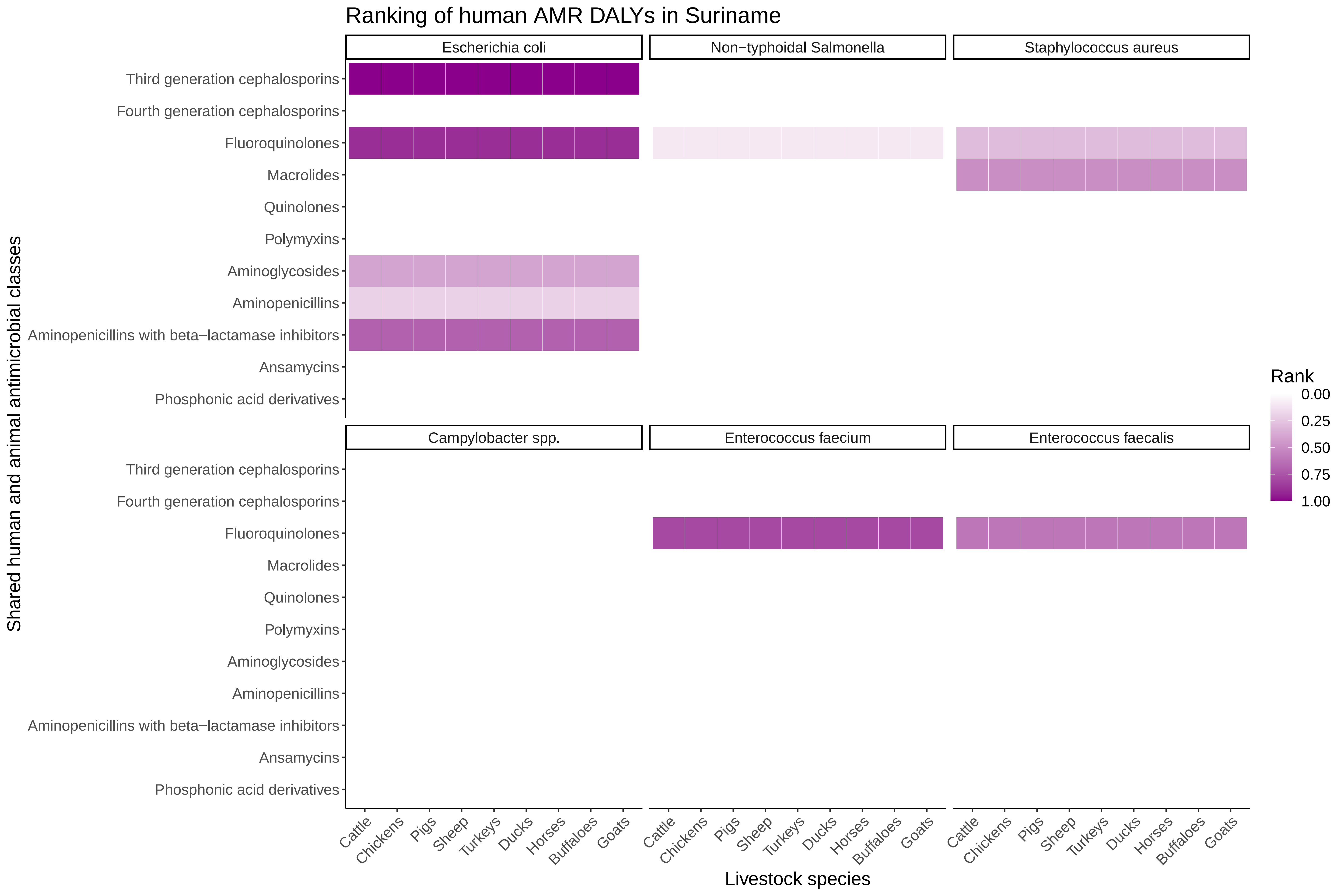
Ranking of human AMR DALYs in Suriname
Escherichia coli
Non−typhoidal Salmonella
Staphylococcus aureus
Campylobacter spp.
Enterococcus faecium
Enterococcus faecalis
Third generation cephalosporins
Fourth generation cephalosporins
Fluoroquinolones
Macrolides
Quinolones
Polymyxins
Aminoglycosides
Aminopenicillins
Aminopenicillins with beta−lactamase inhibitors
Ansamycins
Phosphonic acid derivatives
Shared human and animal antimicrobial classes
Cattle
Chickens
Pigs
Sheep
Turkeys
Ducks
Horses
Buffaloes
Goats
Livestock species
Rank
0.00
0.25
0.50
0.75
1.00

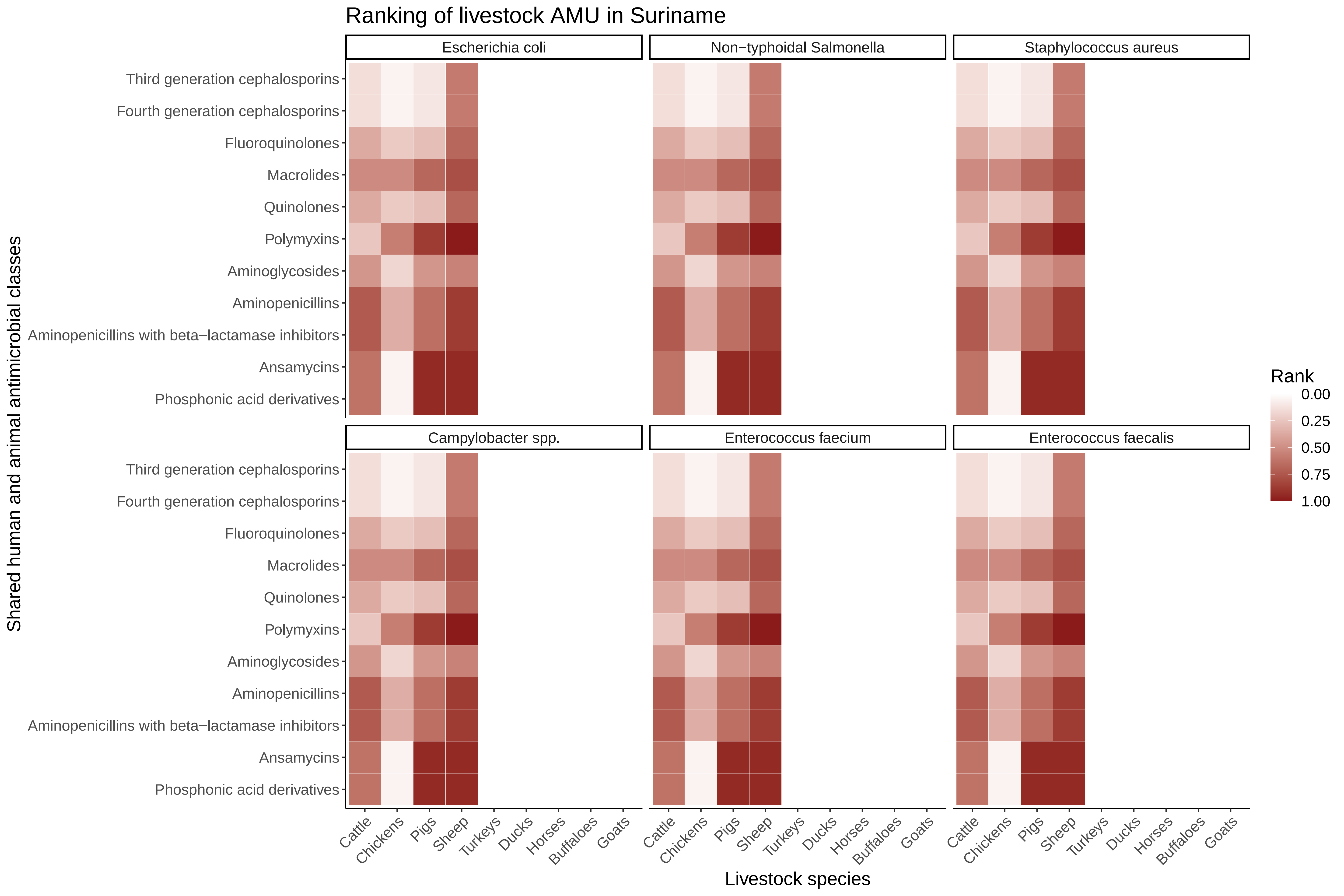

Ranking of livestock AMU in Suriname
Escherichia coli
Non−typhoidal Salmonella
Staphylococcus aureus
Campylobacter spp.
Enterococcus faecium
Enterococcus faecalis
Shared human and animal antimicrobial classes
Third generation cephalosporins
Fourth generation cephalosporins
Fluoroquinolones
Macrolides
Quinolones
Polymyxins
Aminoglycosides
Aminopenicillins
Aminopenicillins with beta−lactamase inhibitors
Ansamycins
Phosphonic acid derivatives
Livestock species
Cattle
Chickens
Pigs
Sheep
Turkeys
Ducks
Horses
Buffaloes
Goats
Rank
0.00
0.25
0.50
0.75
1.00

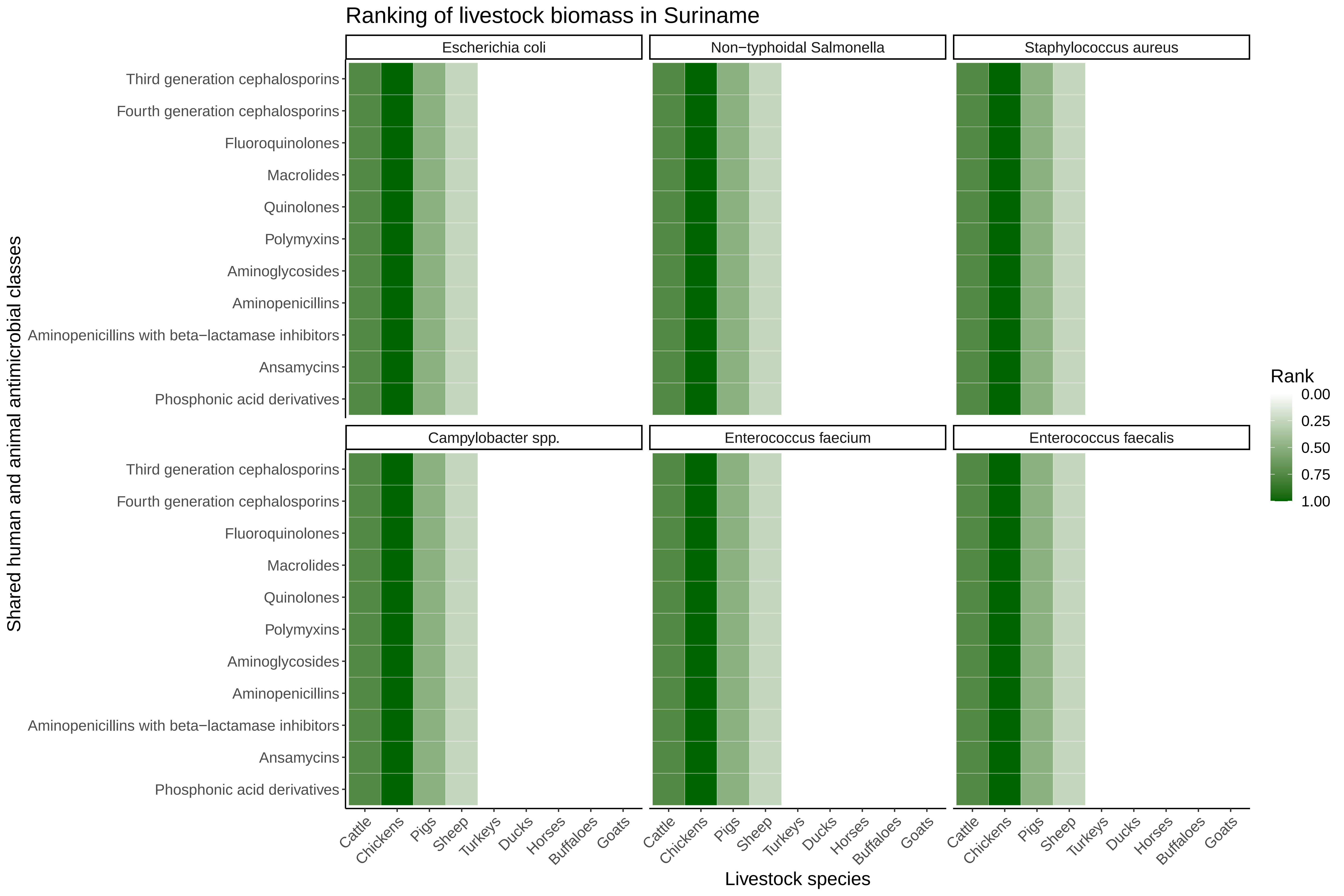

Ranking of livestock biomass in Suriname
Escherichia coli
Non−typhoidal Salmonella
Staphylococcus aureus
Campylobacter spp.
Enterococcus faecium
Enterococcus faecalis
Third generation cephalosporins
Fourth generation cephalosporins
Fluoroquinolones
Macrolides
Quinolones
Polymyxins
Aminoglycosides
Aminopenicillins
Aminopenicillins with beta−lactamase inhibitors
Ansamycins
Phosphonic acid derivatives
Shared human and animal antimicrobial classes
Cattle
Chickens
Pigs
Sheep
Turkeys
Ducks
Horses
Buffaloes
Goats
Livestock species
Rank
0.00
0.25
0.50
0.75
1.00

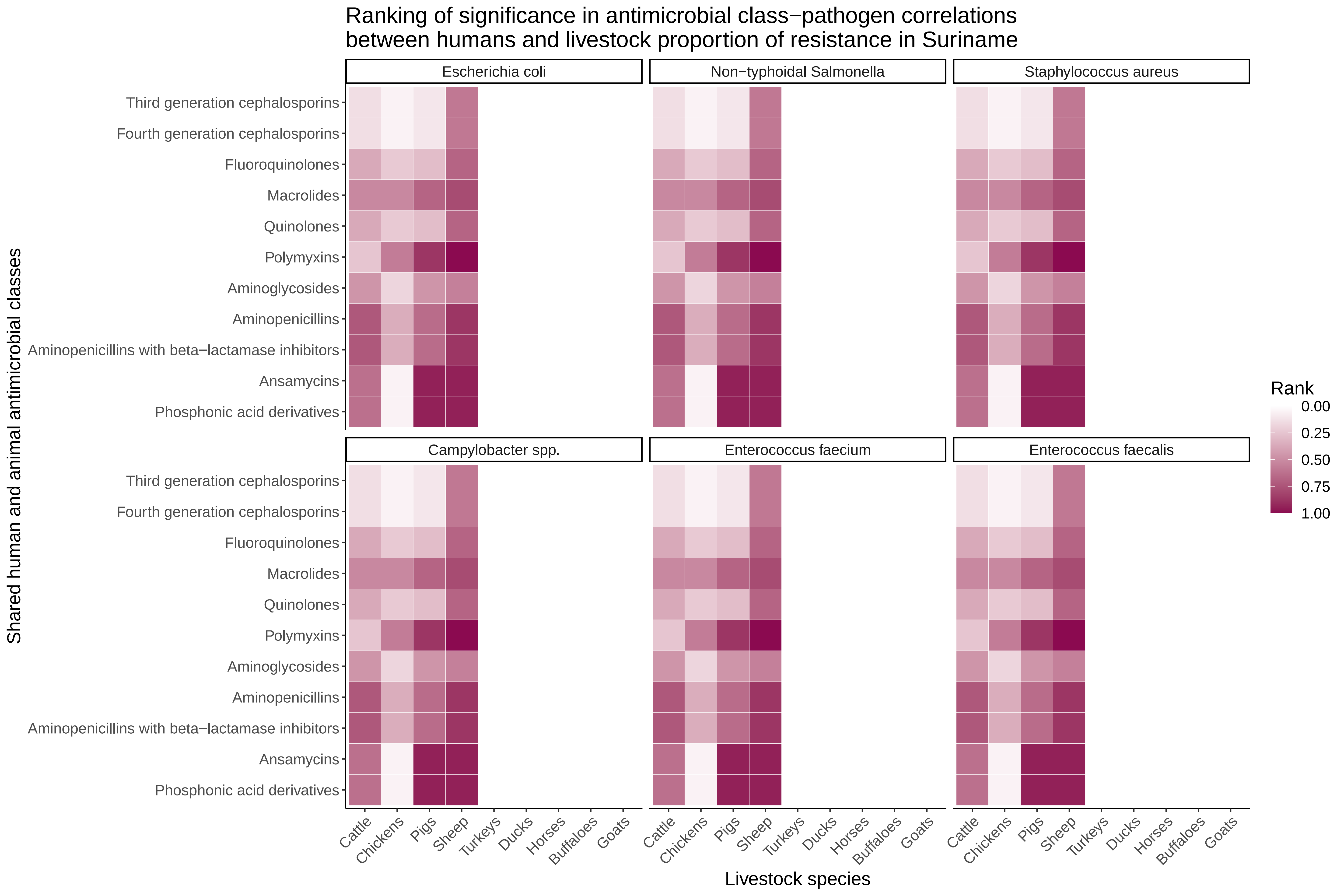

Ranking of significance in antimicrobial class−pathogen correlations
between humans and livestock proportion of resistance in Suriname
Escherichia coli
Non−typhoidal Salmonella
Staphylococcus aureus
Campylobacter spp.
Enterococcus faecium
Enterococcus faecalis
Third generation cephalosporins
Fourth generation cephalosporins
Fluoroquinolones
Macrolides
Quinolones
Polymyxins
Aminoglycosides
Aminopenicillins
Aminopenicillins with beta−lactamase inhibitors
Ansamycins
Phosphonic acid derivatives
Shared human and animal antimicrobial classes
Cattle
Chickens
Pigs
Sheep
Turkeys
Ducks
Horses
Buffaloes
Goats
Livestock species
Rank
0.00
0.25
0.50
0.75
1.00

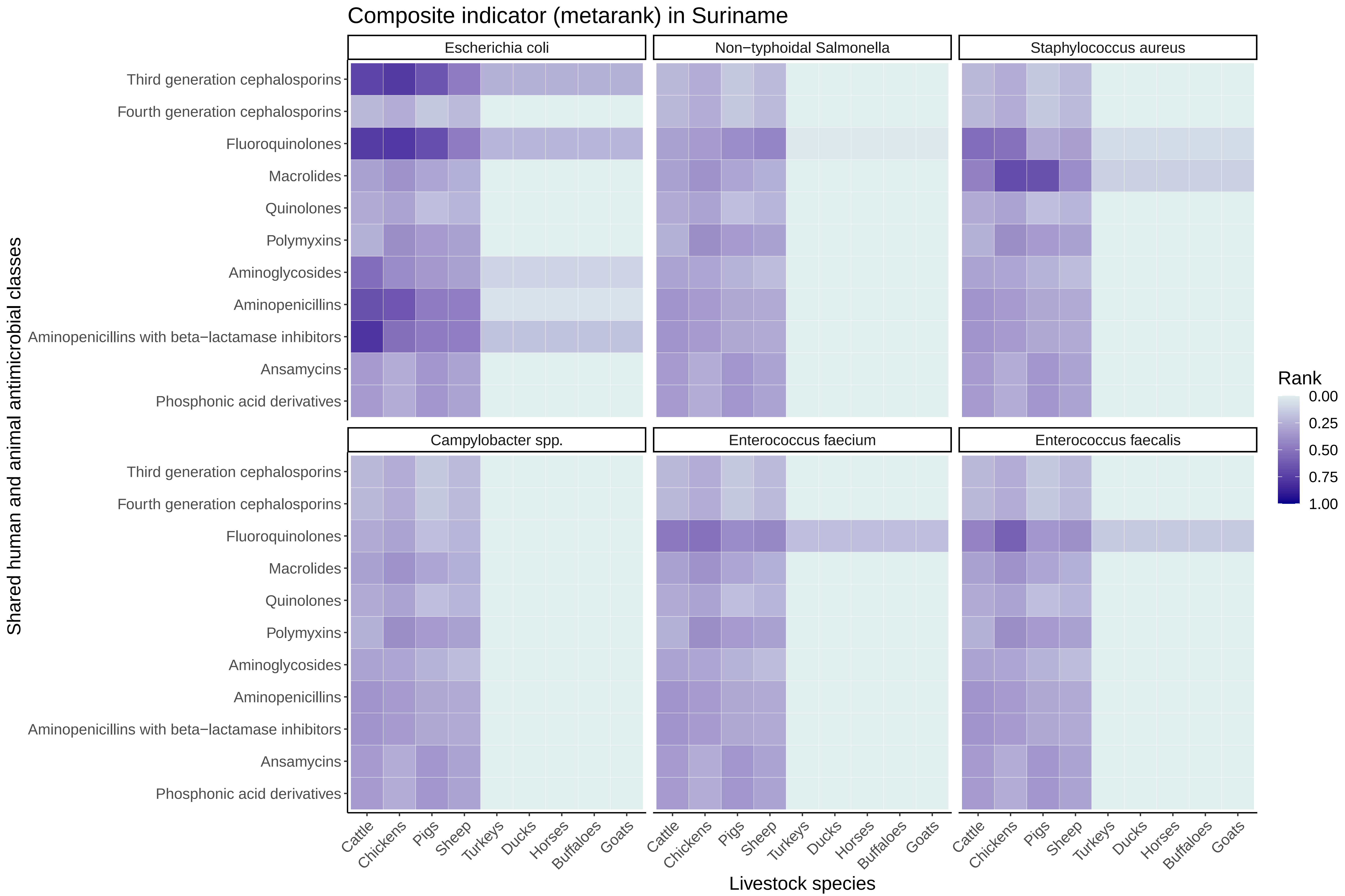

Composite indicator (metarank) in Suriname
Escherichia coli
Non−typhoidal Salmonella
Staphylococcus aureus
Campylobacter spp.
Enterococcus faecium
Enterococcus faecalis
Shared human and animal antimicrobial classes
Third generation cephalosporins
Fourth generation cephalosporins
Fluoroquinolones
Macrolides
Quinolones
Polymyxins
Aminoglycosides
Aminopenicillins
Aminopenicillins with beta−lactamase inhibitors
Ansamycins
Phosphonic acid derivatives
Cattle
Chickens
Pigs
Sheep
Turkeys
Ducks
Horses
Buffaloes
Goats
Livestock species
Rank
0.00
0.25
0.50
0.75
1.00

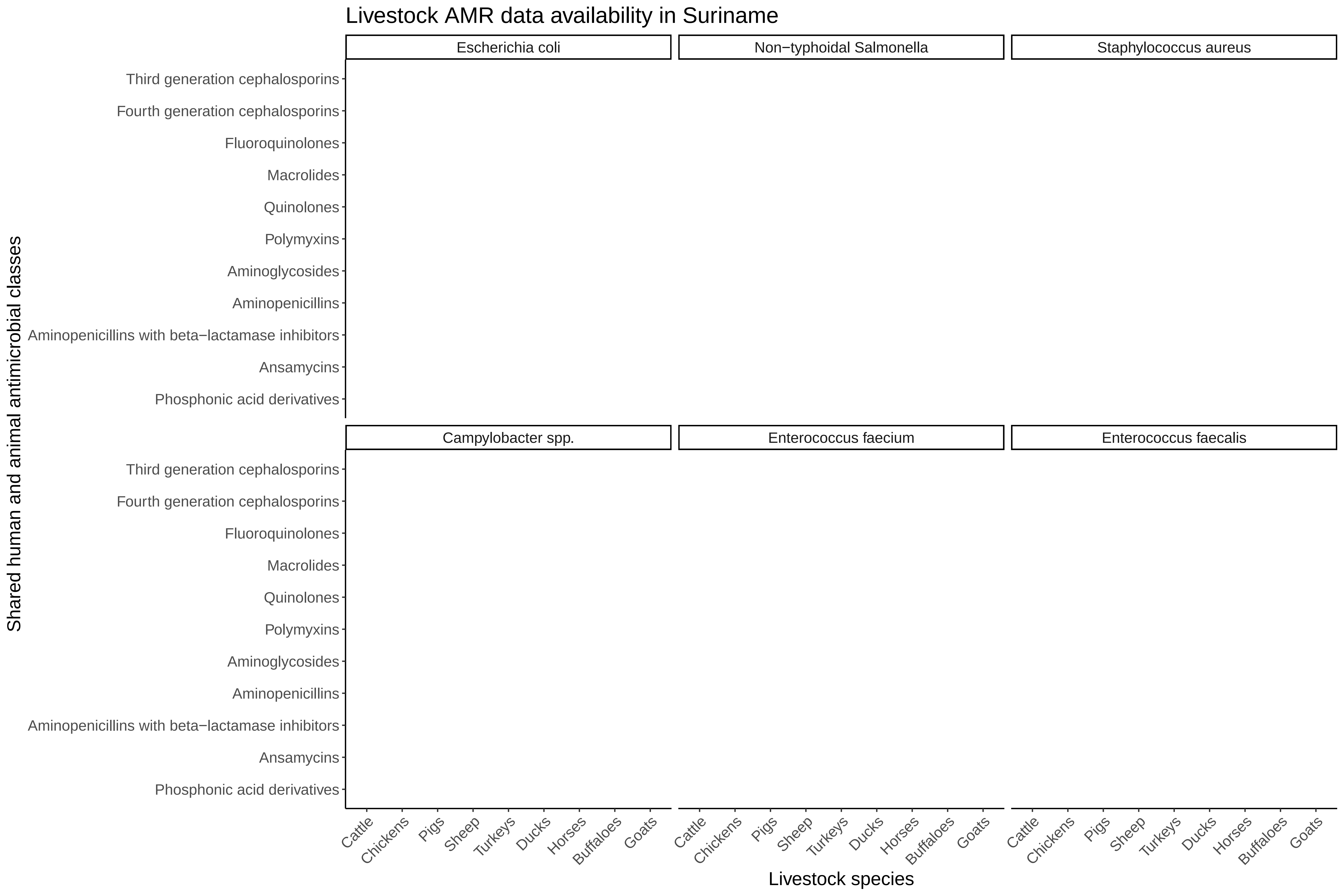
Livestock AMR data availability in Suriname
Escherichia coli
Non−typhoidal Salmonella
Staphylococcus aureus
Campylobacter spp.
Enterococcus faecium
Enterococcus faecalis
Shared human and animal antimicrobial classes
Third generation cephalosporins
Fourth generation cephalosporins
Fluoroquinolones
Macrolides
Quinolones
Polymyxins
Aminoglycosides
Aminopenicillins
Aminopenicillins with beta−lactamase inhibitors
Ansamycins
Phosphonic acid derivatives
Cattle
Chickens
Pigs
Sheep
Turkeys
Ducks
Horses
Buffaloes
Goats
Livestock species

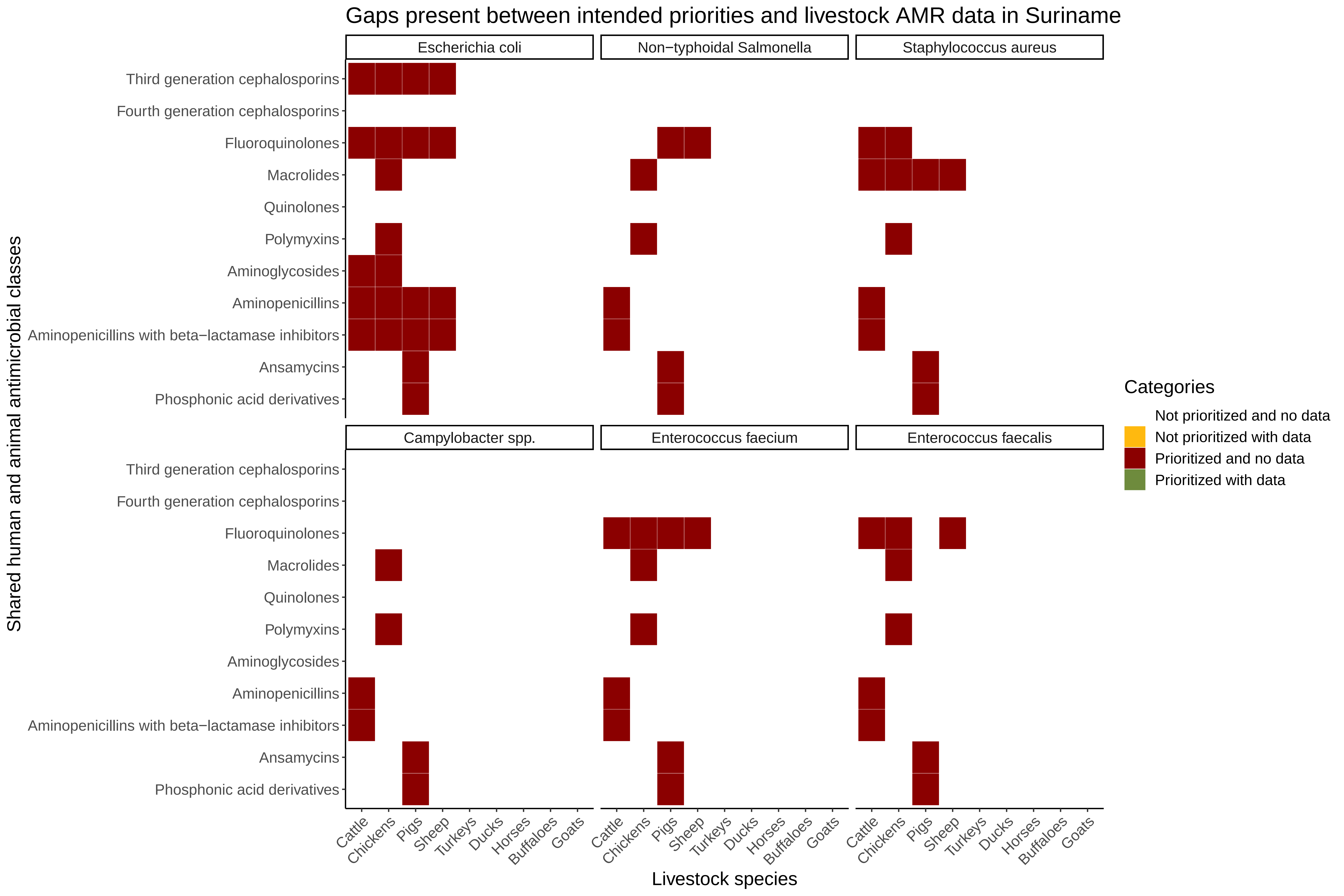

Gaps present between intended priorities and livestock AMR data in Suriname
Escherichia coli
Non−typhoidal Salmonella
Staphylococcus aureus
Campylobacter spp.
Enterococcus faecium
Enterococcus faecalis
Third generation cephalosporins
Fourth generation cephalosporins
Fluoroquinolones
Macrolides
Quinolones
Polymyxins
Aminoglycosides
Aminopenicillins
Aminopenicillins with beta−lactamase inhibitors
Ansamycins
Phosphonic acid derivatives
Shared human and animal antimicrobial classes
Cattle
Chickens
Pigs
Sheep
Turkeys
Ducks
Horses
Buffaloes
Goats
Livestock species
Categories
Not prioritized and no data
Not prioritized with data
Prioritized and no data
Prioritized with data

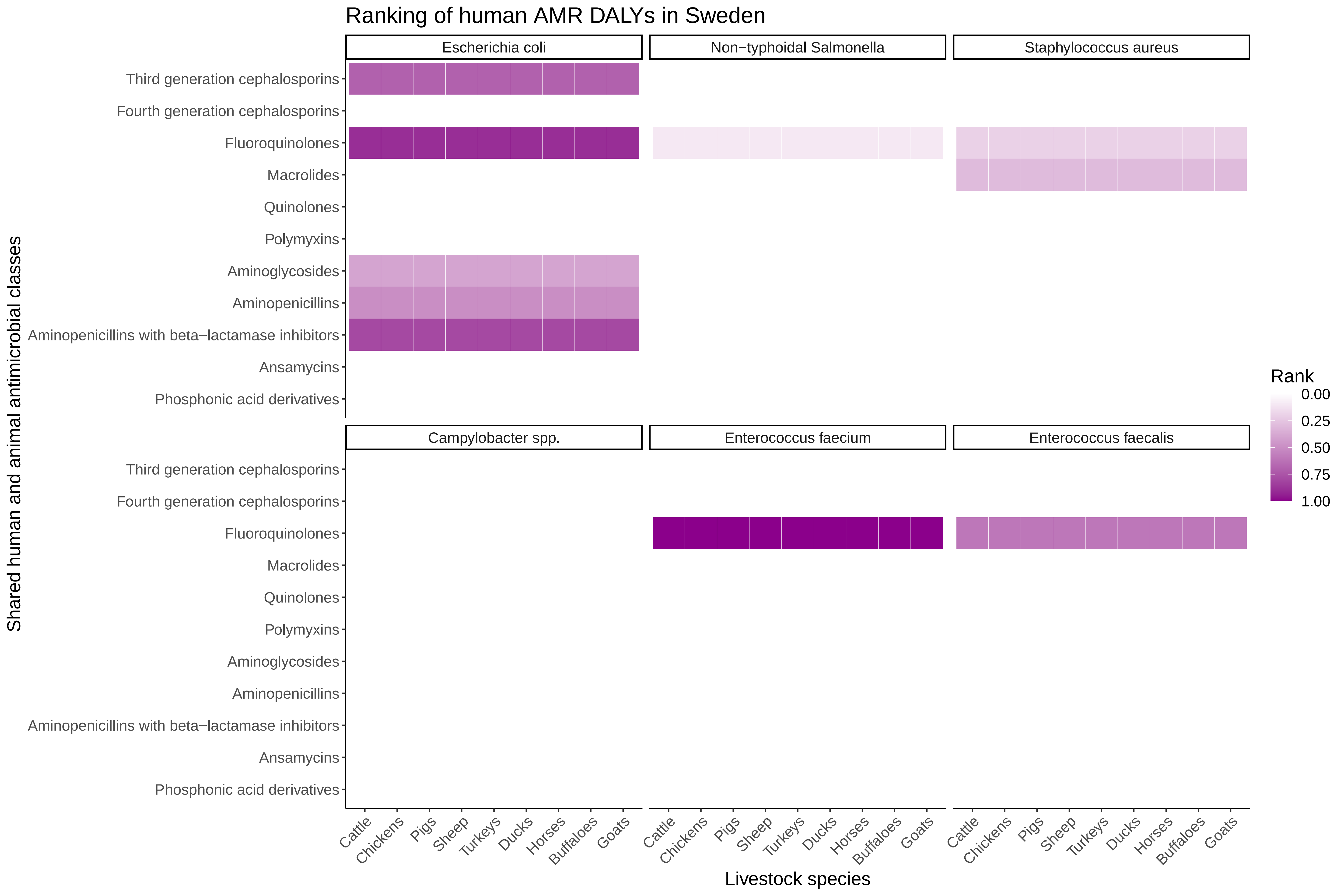

Ranking of human AMR DALYs in Sweden
Escherichia coli
Non−typhoidal Salmonella
Staphylococcus aureus
Campylobacter spp.
Enterococcus faecium
Enterococcus faecalis
Third generation cephalosporins
Fourth generation cephalosporins
Fluoroquinolones
Macrolides
Quinolones
Polymyxins
Aminoglycosides
Aminopenicillins
Aminopenicillins with beta−lactamase inhibitors
Ansamycins
Phosphonic acid derivatives
Shared human and animal antimicrobial classes
Cattle
Chickens
Pigs
Sheep
Turkeys
Ducks
Horses
Buffaloes
Goats
Livestock species
Rank
0.00
0.25
0.50
0.75
1.00

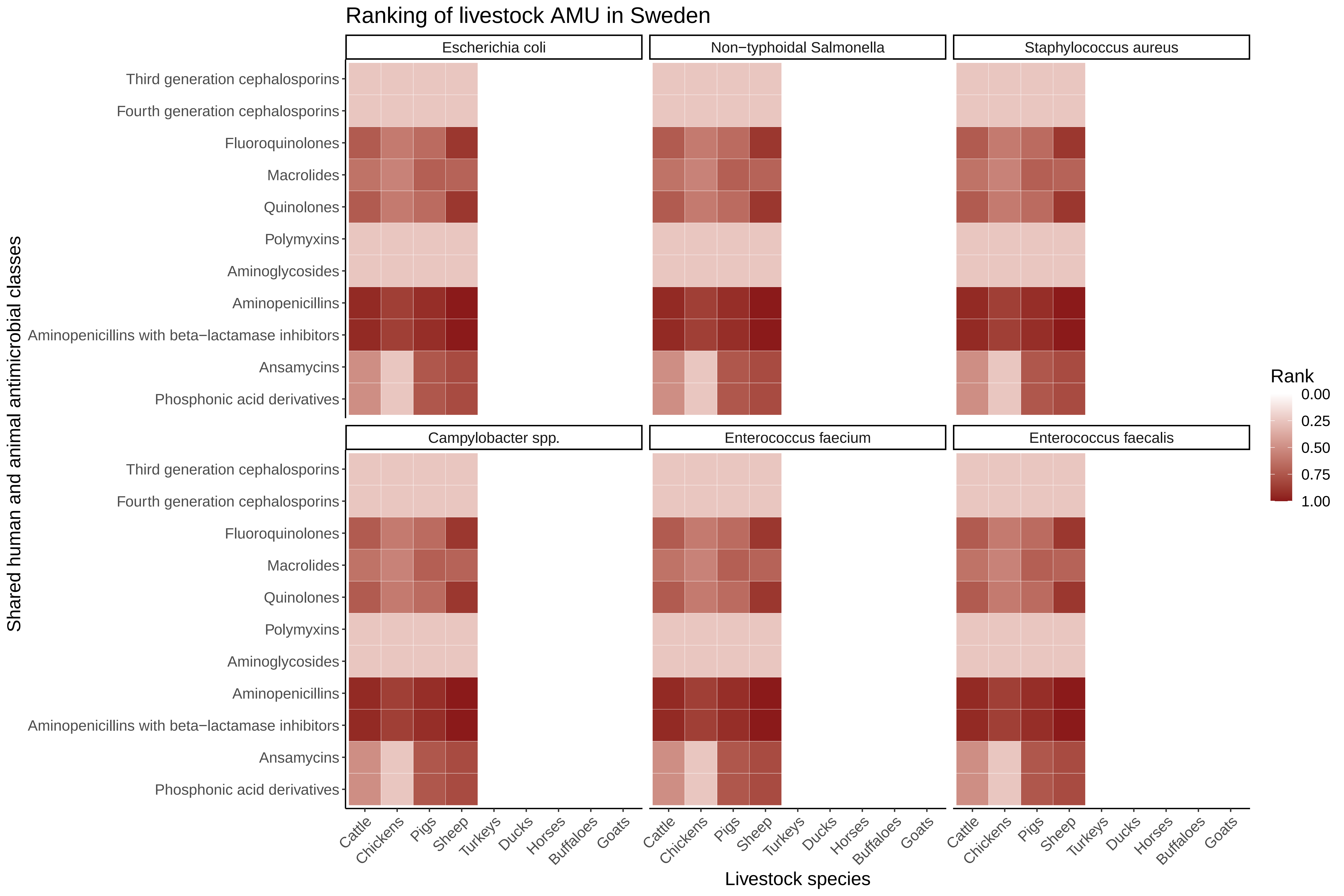

Ranking of livestock AMU in Sweden
Escherichia coli
Non−typhoidal Salmonella
Staphylococcus aureus
Campylobacter spp.
Enterococcus faecium
Enterococcus faecalis
Third generation cephalosporins
Fourth generation cephalosporins
Fluoroquinolones
Macrolides
Quinolones
Polymyxins
Aminoglycosides
Aminopenicillins
Aminopenicillins with beta−lactamase inhibitors
Ansamycins
Phosphonic acid derivatives
Shared human and animal antimicrobial classes
Cattle
Chickens
Pigs
Sheep
Turkeys
Ducks
Horses
Buffaloes
Goats
Livestock species
Rank
0.00
0.25
0.50
0.75
1.00

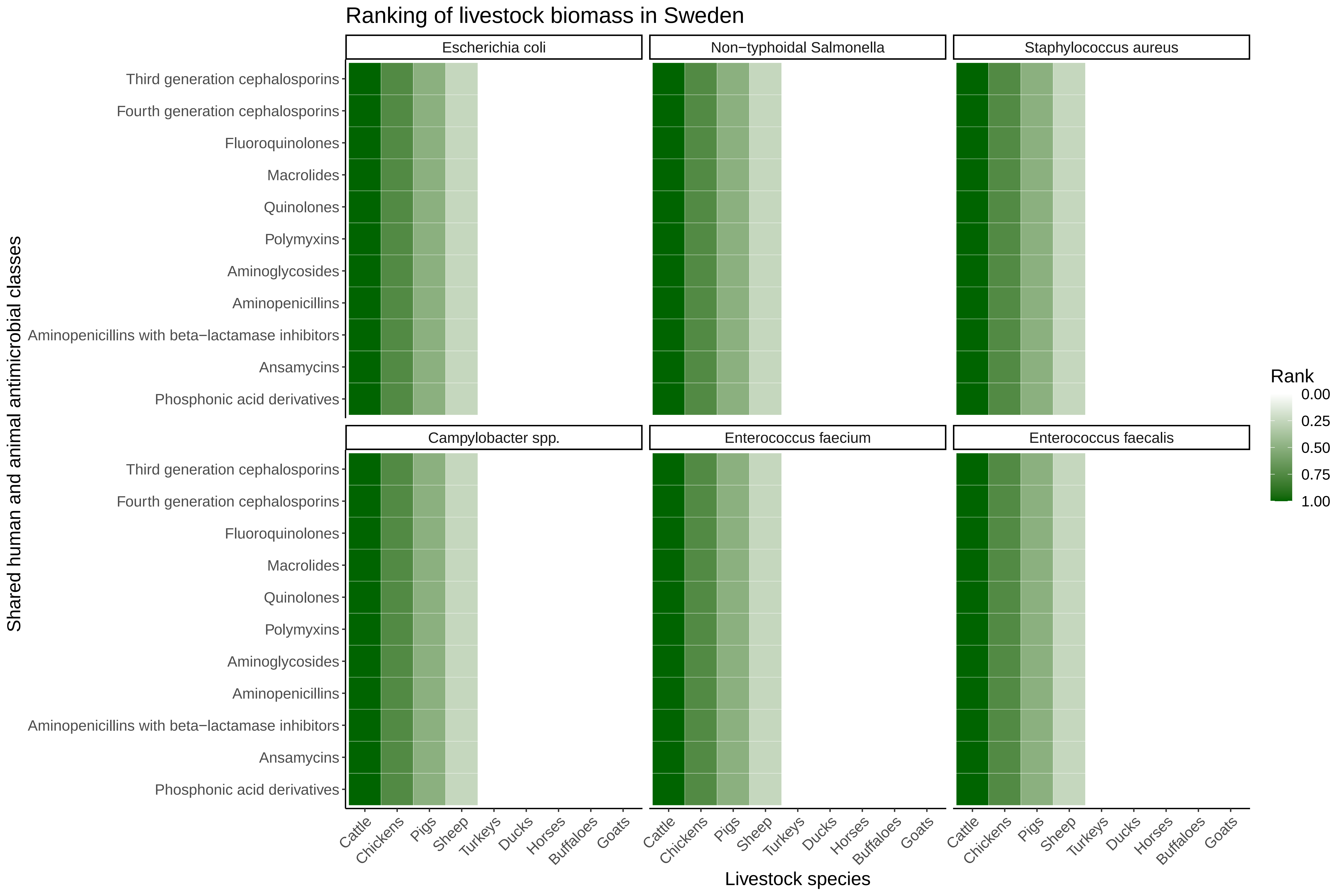
Ranking of livestock biomass in Sweden
Escherichia coli
Non−typhoidal Salmonella
Staphylococcus aureus
Campylobacter spp.
Enterococcus faecium
Enterococcus faecalis
Shared human and animal antimicrobial classes
Third generation cephalosporins
Fourth generation cephalosporins
Fluoroquinolones
Macrolides
Quinolones
Polymyxins
Aminoglycosides
Aminopenicillins
Aminopenicillins with beta−lactamase inhibitors
Ansamycins
Phosphonic acid derivatives
Cattle
Chickens
Pigs
Sheep
Turkeys
Ducks
Horses
Buffaloes
Goats
Livestock species
Rank
0.00
0.25
0.50
0.75
1.00

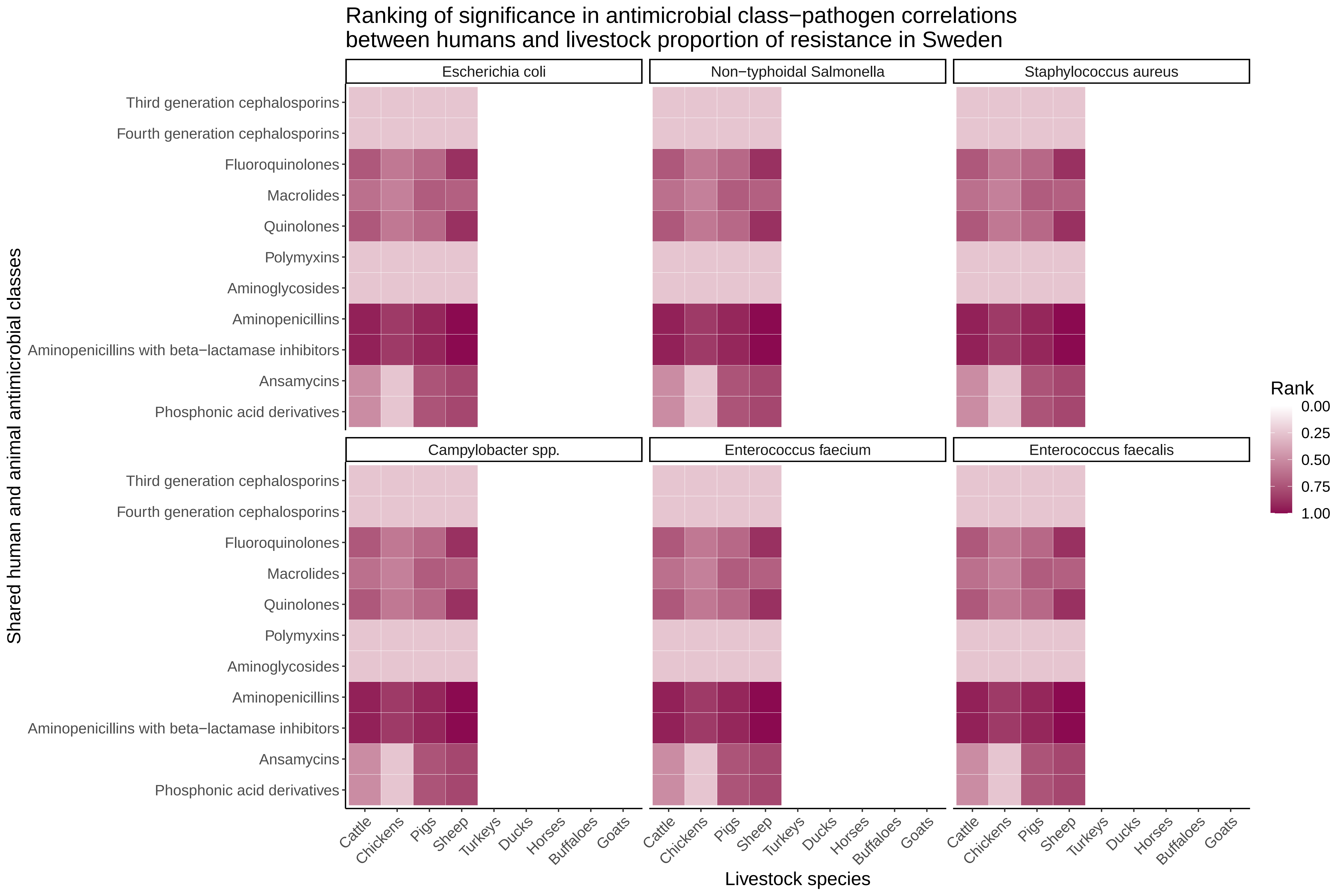

Ranking of significance in antimicrobial class−pathogen correlations
between humans and livestock proportion of resistance in Sweden
Escherichia coli
Non−typhoidal Salmonella
Staphylococcus aureus
Campylobacter spp.
Enterococcus faecium
Enterococcus faecalis
Third generation cephalosporins
Fourth generation cephalosporins
Fluoroquinolones
Macrolides
Quinolones
Polymyxins
Aminoglycosides
Aminopenicillins
Aminopenicillins with beta−lactamase inhibitors
Ansamycins
Phosphonic acid derivatives
Shared human and animal antimicrobial classes
Cattle
Chickens
Pigs
Sheep
Turkeys
Ducks
Horses
Buffaloes
Goats
Livestock species
Rank
0.00
0.25
0.50
0.75
1.00

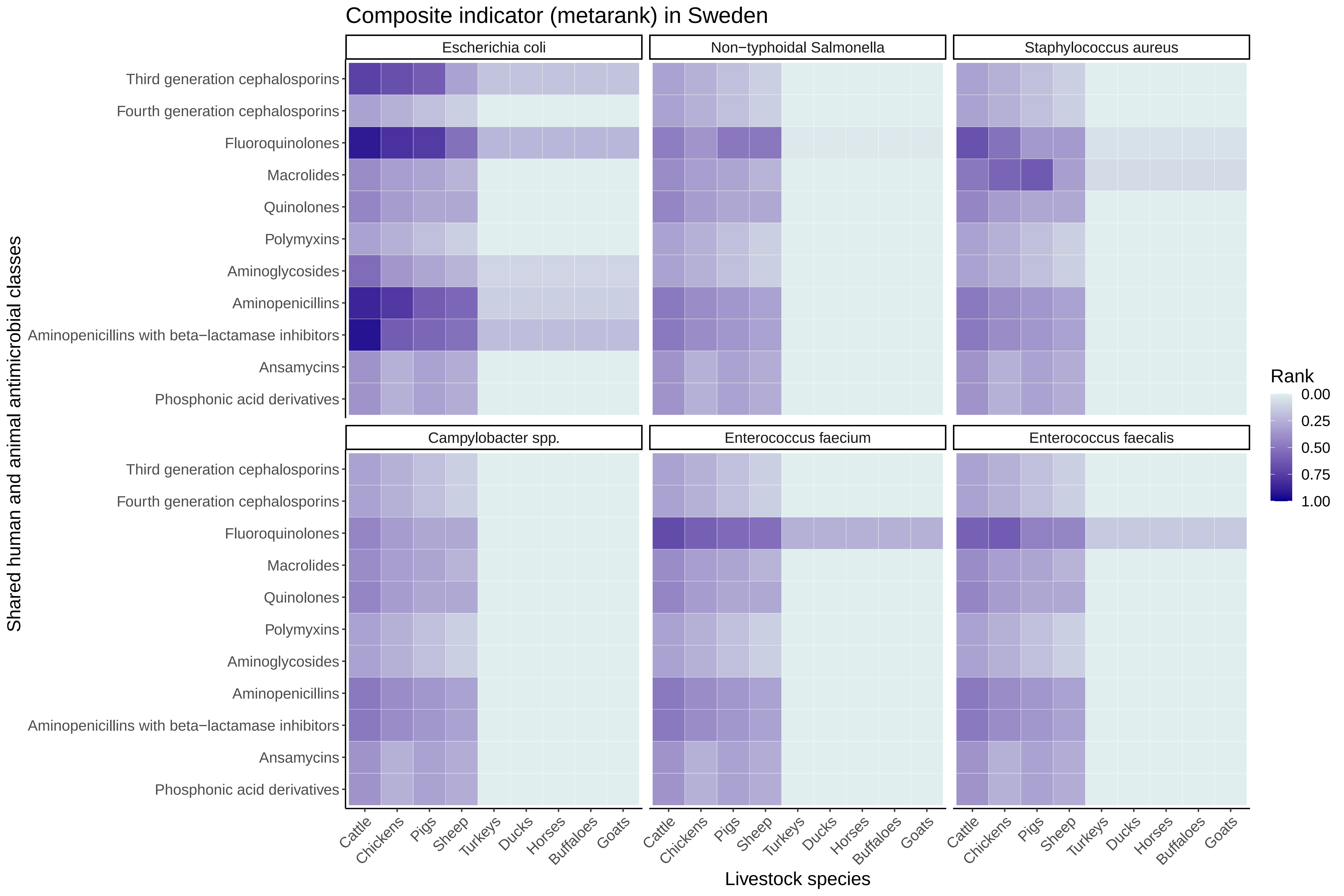

Composite indicator (metarank) in Sweden
Escherichia coli
Non−typhoidal Salmonella
Staphylococcus aureus
Campylobacter spp.
Enterococcus faecium
Enterococcus faecalis
Third generation cephalosporins
Fourth generation cephalosporins
Fluoroquinolones
Macrolides
Quinolones
Polymyxins
Aminoglycosides
Aminopenicillins
Aminopenicillins with beta−lactamase inhibitors
Ansamycins
Phosphonic acid derivatives
Shared human and animal antimicrobial classes
Cattle
Chickens
Pigs
Sheep
Turkeys
Ducks
Horses
Buffaloes
Goats
Livestock species
Rank
0.00
0.25
0.50
0.75
1.00

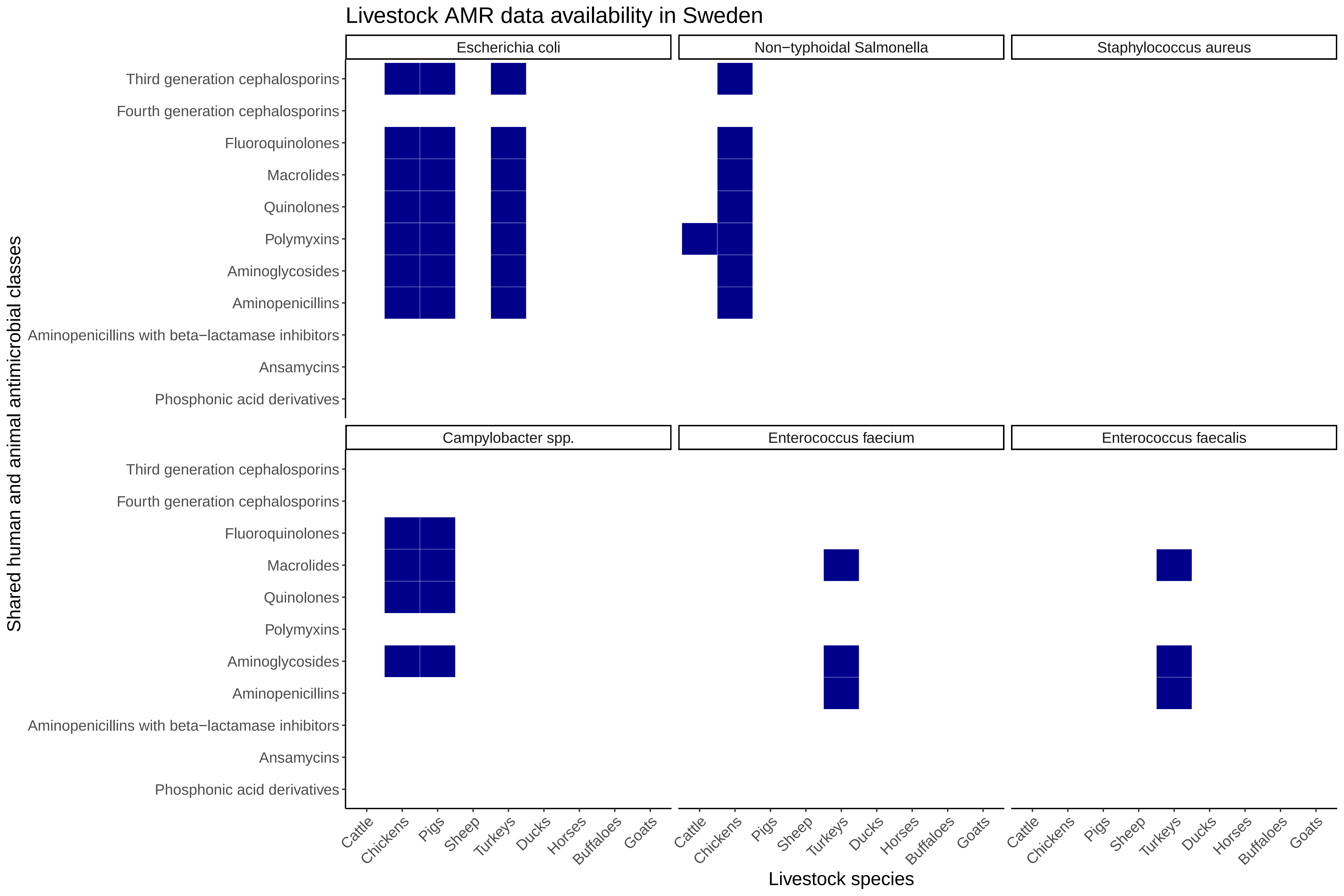
Livestock AMR data availability in Sweden
Escherichia coli
Non−typhoidal Salmonella
Staphylococcus aureus
Campylobacter spp.
Enterococcus faecium
Enterococcus faecalis
Shared human and animal antimicrobial classes
Third generation cephalosporins
Fourth generation cephalosporins
Fluoroquinolones
Macrolides
Quinolones
Polymyxins
Aminoglycosides
Aminopenicillins
Aminopenicillins with beta−lactamase inhibitors
Ansamycins
Phosphonic acid derivatives
Cattle
Chickens
Pigs
Sheep
Turkeys
Ducks
Horses
Buffaloes
Goats
Livestock species

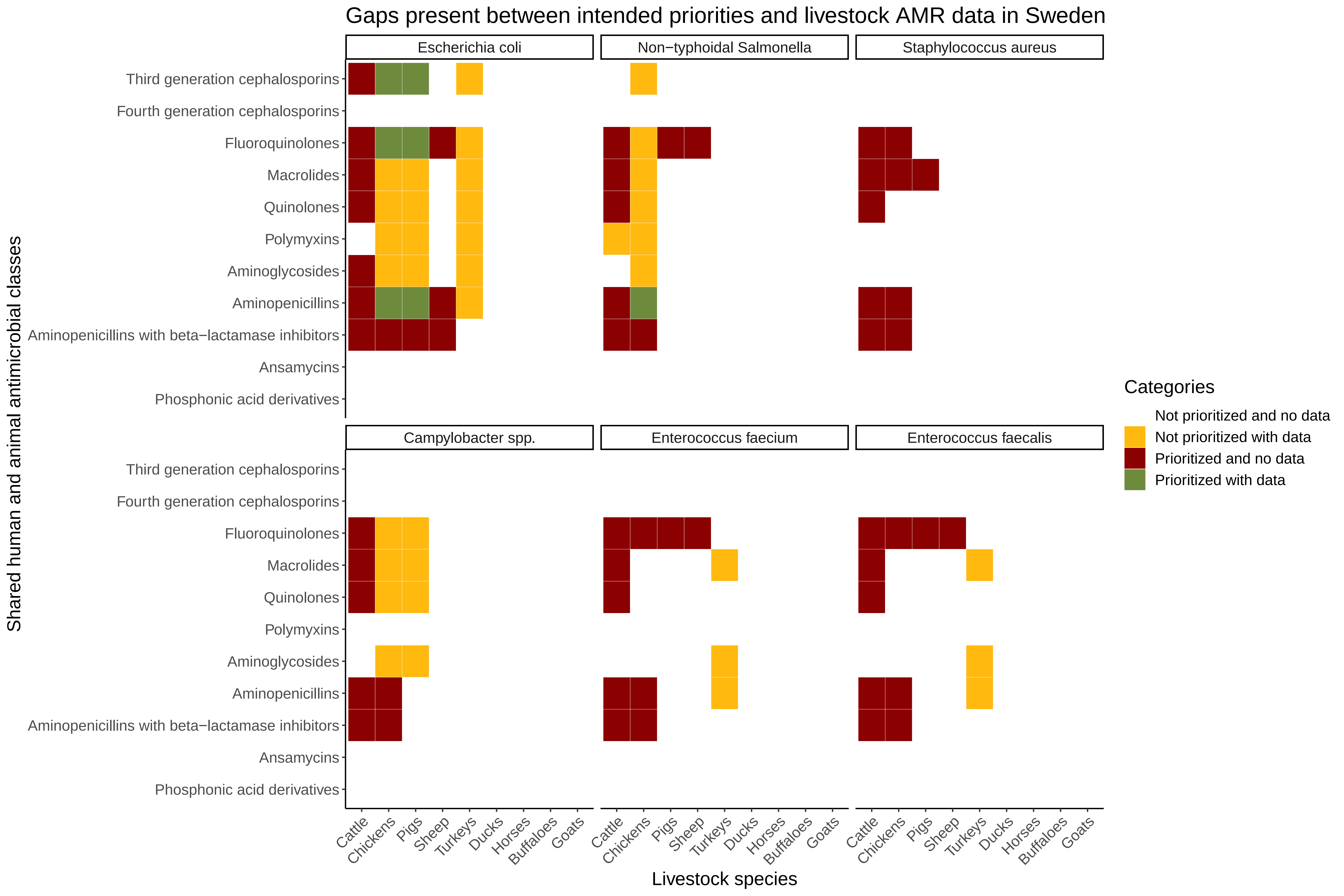

Gaps present between intended priorities and livestock AMR data in Sweden
Escherichia coli
Non−typhoidal Salmonella
Staphylococcus aureus
Campylobacter spp.
Enterococcus faecium
Enterococcus faecalis
Third generation cephalosporins
Fourth generation cephalosporins
Fluoroquinolones
Macrolides
Quinolones
Polymyxins
Aminoglycosides
Aminopenicillins
Aminopenicillins with beta−lactamase inhibitors
Ansamycins
Phosphonic acid derivatives
Shared human and animal antimicrobial classes
Cattle
Chickens
Pigs
Sheep
Turkeys
Ducks
Horses
Buffaloes
Goats
Livestock species
Categories
Not prioritized and no data
Not prioritized with data
Prioritized and no data
Prioritized with data

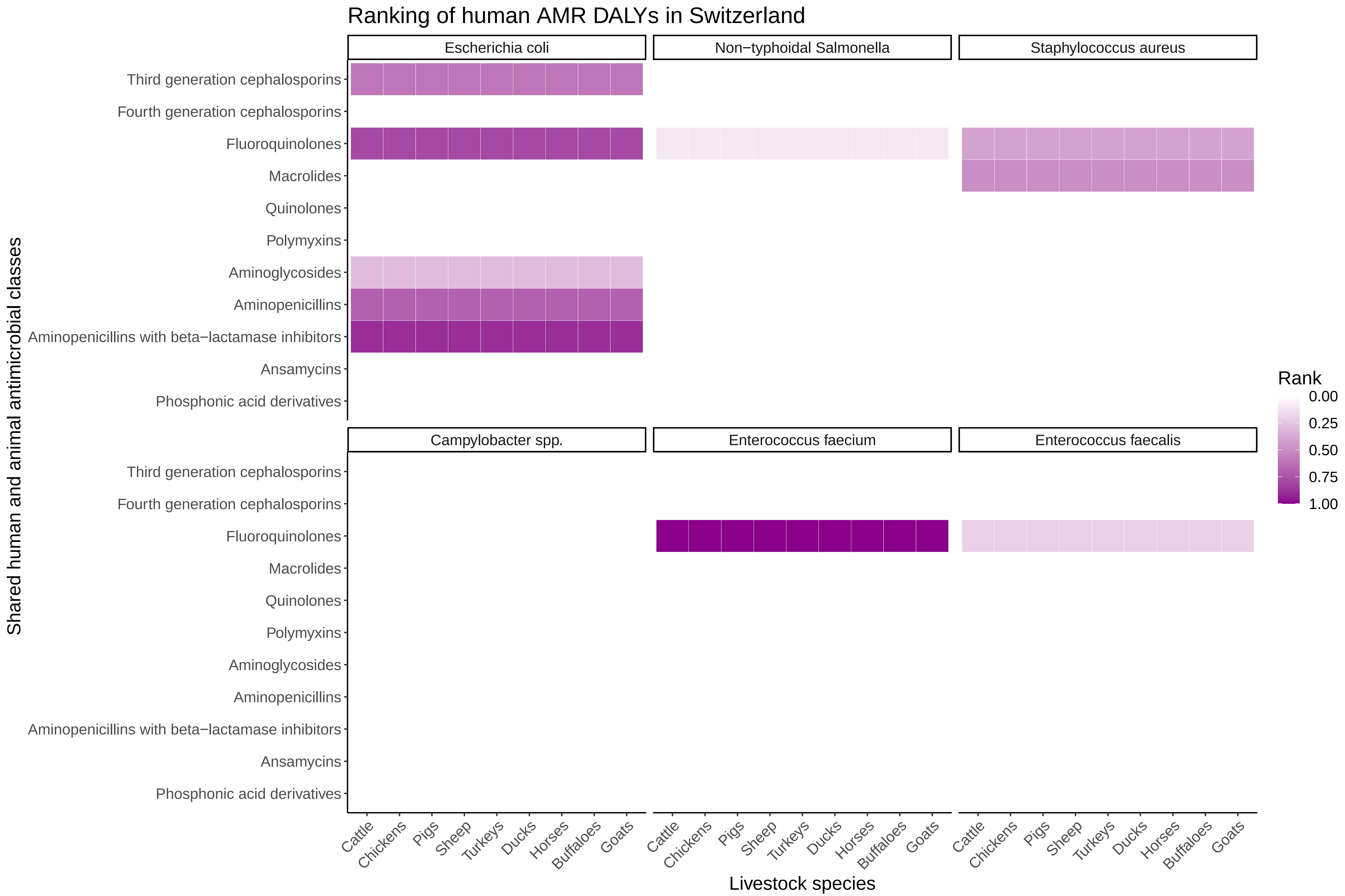

Ranking of human AMR DALYs in Switzerland
Escherichia coli
Non−typhoidal Salmonella
Staphylococcus aureus
Campylobacter spp.
Enterococcus faecium
Enterococcus faecalis
Third generation cephalosporins
Fourth generation cephalosporins
Fluoroquinolones
Macrolides
Quinolones
Polymyxins
Aminoglycosides
Aminopenicillins
Aminopenicillins with beta−lactamase inhibitors
Ansamycins
Phosphonic acid derivatives
Shared human and animal antimicrobial classes
Cattle
Chickens
Pigs
Sheep
Turkeys
Ducks
Horses
Buffaloes
Goats
Livestock species
Rank
0.00
0.25
0.50
0.75
1.00

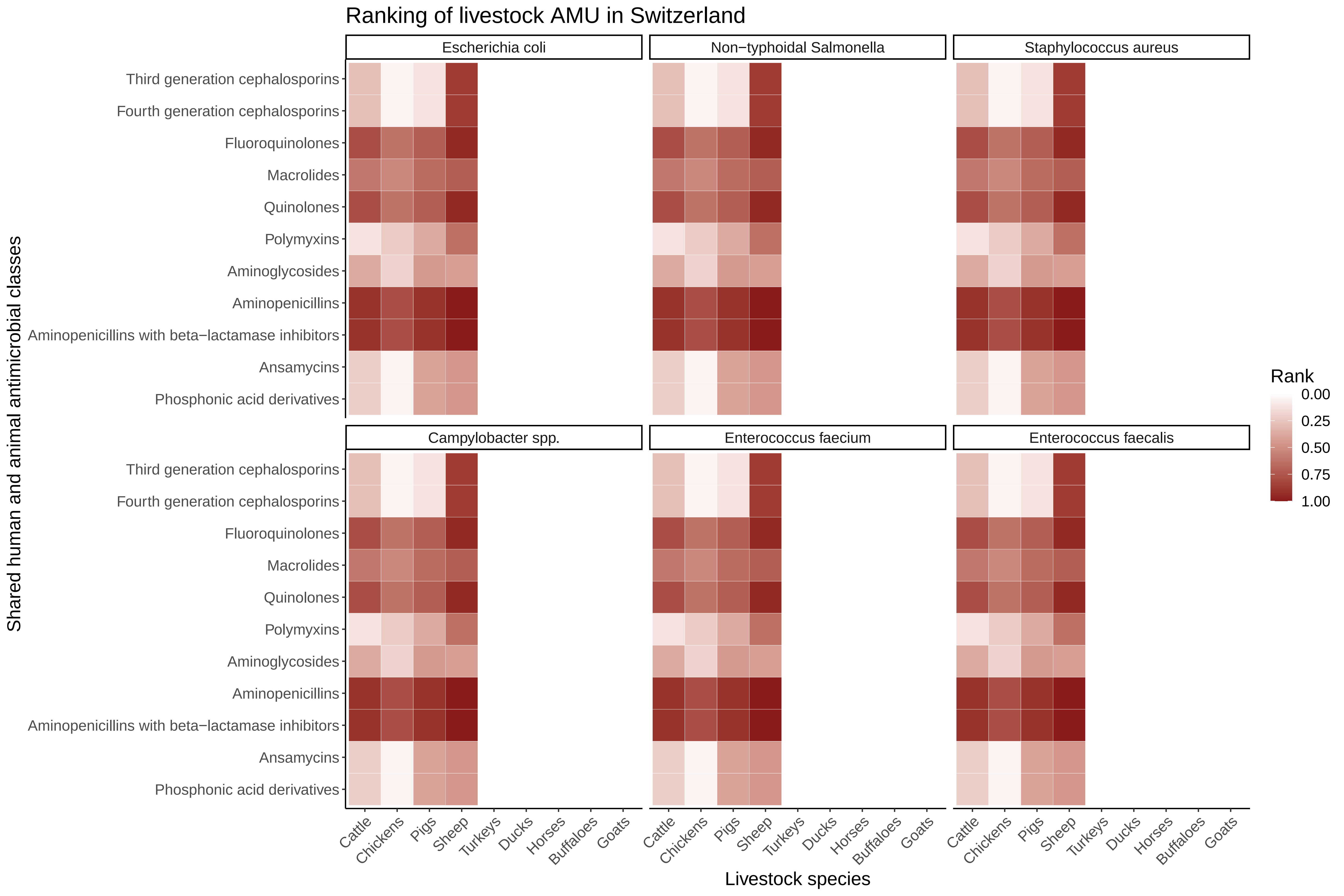

Ranking of livestock AMU in Switzerland
Escherichia coli
Non−typhoidal Salmonella
Staphylococcus aureus
Campylobacter spp.
Enterococcus faecium
Enterococcus faecalis
Third generation cephalosporins
Fourth generation cephalosporins
Fluoroquinolones
Macrolides
Quinolones
Polymyxins
Aminoglycosides
Aminopenicillins
Aminopenicillins with beta−lactamase inhibitors
Ansamycins
Phosphonic acid derivatives
Shared human and animal antimicrobial classes
Cattle
Chickens
Pigs
Sheep
Turkeys
Ducks
Horses
Buffaloes
Goats
Livestock species
Rank
0.00
0.25
0.50
0.75
1.00

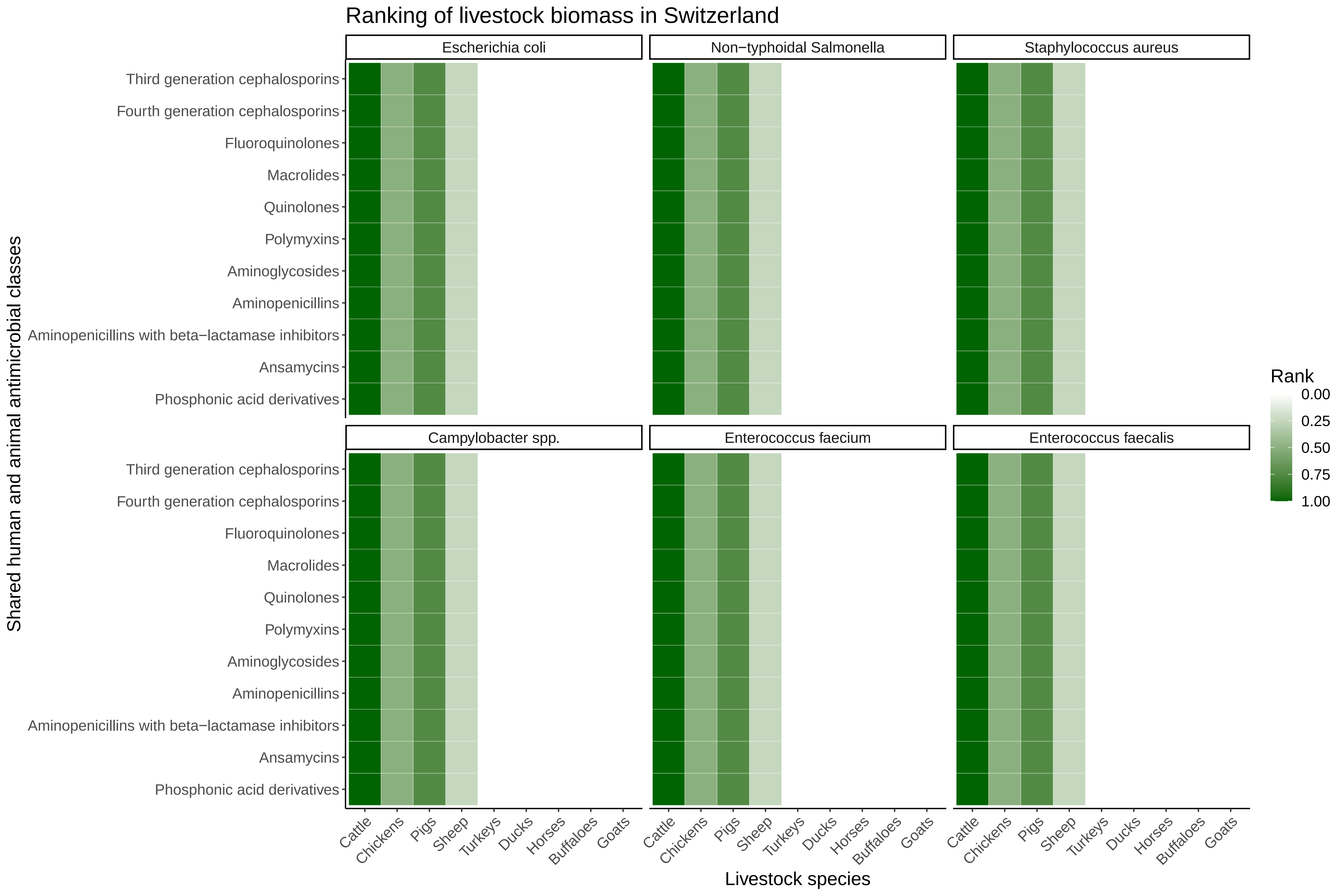
Ranking of livestock biomass in Switzerland
Escherichia coli
Non−typhoidal Salmonella
Staphylococcus aureus
Campylobacter spp.
Enterococcus faecium
Enterococcus faecalis
Third generation cephalosporins
Fourth generation cephalosporins
Fluoroquinolones
Macrolides
Quinolones
Polymyxins
Aminoglycosides
Aminopenicillins
Aminopenicillins with beta−lactamase inhibitors
Ansamycins
Phosphonic acid derivatives
Shared human and animal antimicrobial classes
Cattle
Chickens
Pigs
Sheep
Turkeys
Ducks
Horses
Buffaloes
Goats
Livestock species
Rank
0.00
0.25
0.50
0.75
1.00

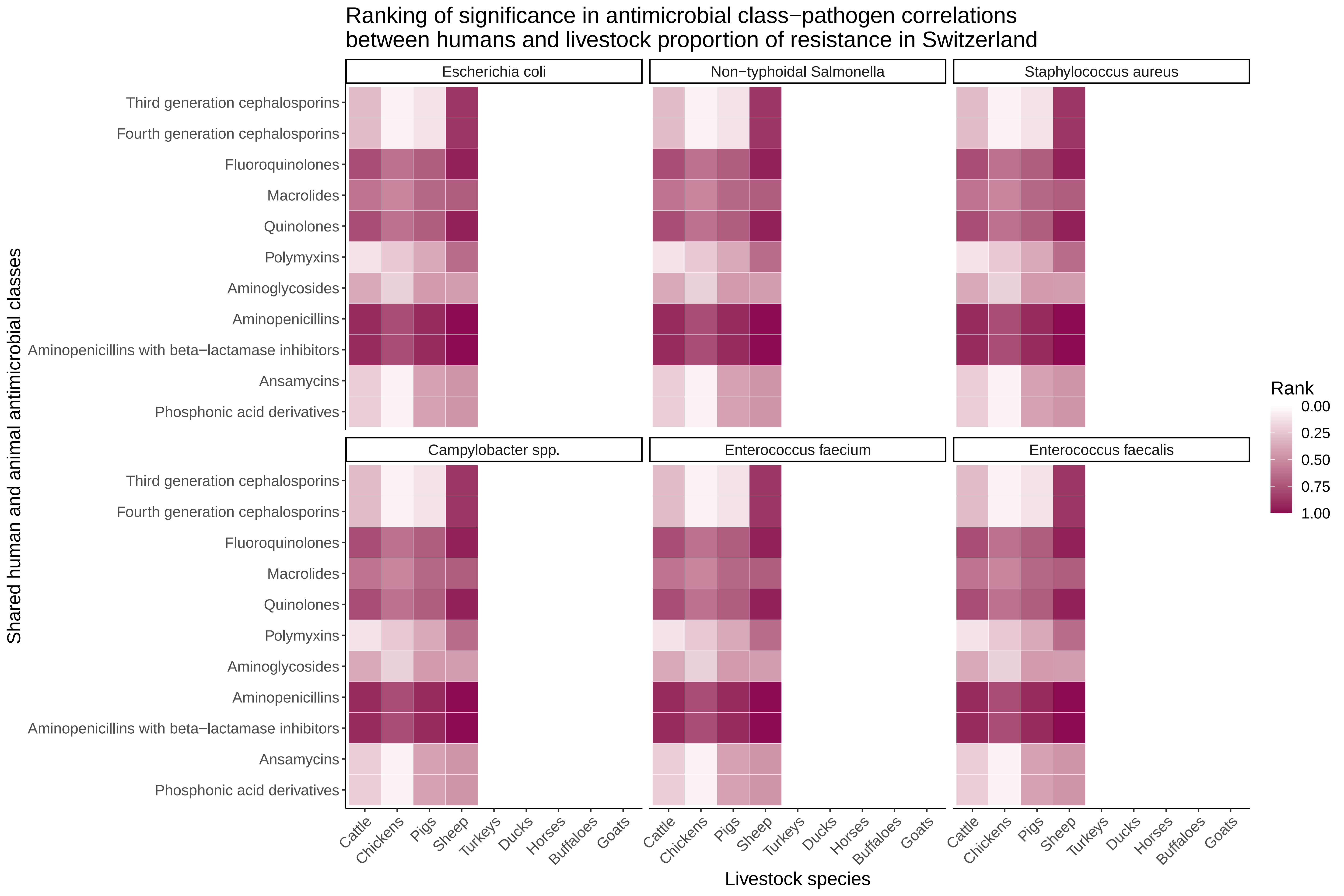
Ranking of significance in antimicrobial class−pathogen correlations
between humans and livestock proportion of resistance in Switzerland
Escherichia coli
Non−typhoidal Salmonella
Staphylococcus aureus
Campylobacter spp.
Enterococcus faecium
Enterococcus faecalis
Third generation cephalosporins
Fourth generation cephalosporins
Fluoroquinolones
Macrolides
Quinolones
Polymyxins
Aminoglycosides
Aminopenicillins
Aminopenicillins with beta−lactamase inhibitors
Ansamycins
Phosphonic acid derivatives
Shared human and animal antimicrobial classes
Cattle
Chickens
Pigs
Sheep
Turkeys
Ducks
Horses
Buffaloes
Goats
Livestock species
Rank
0.00
0.25
0.50
0.75
1.00

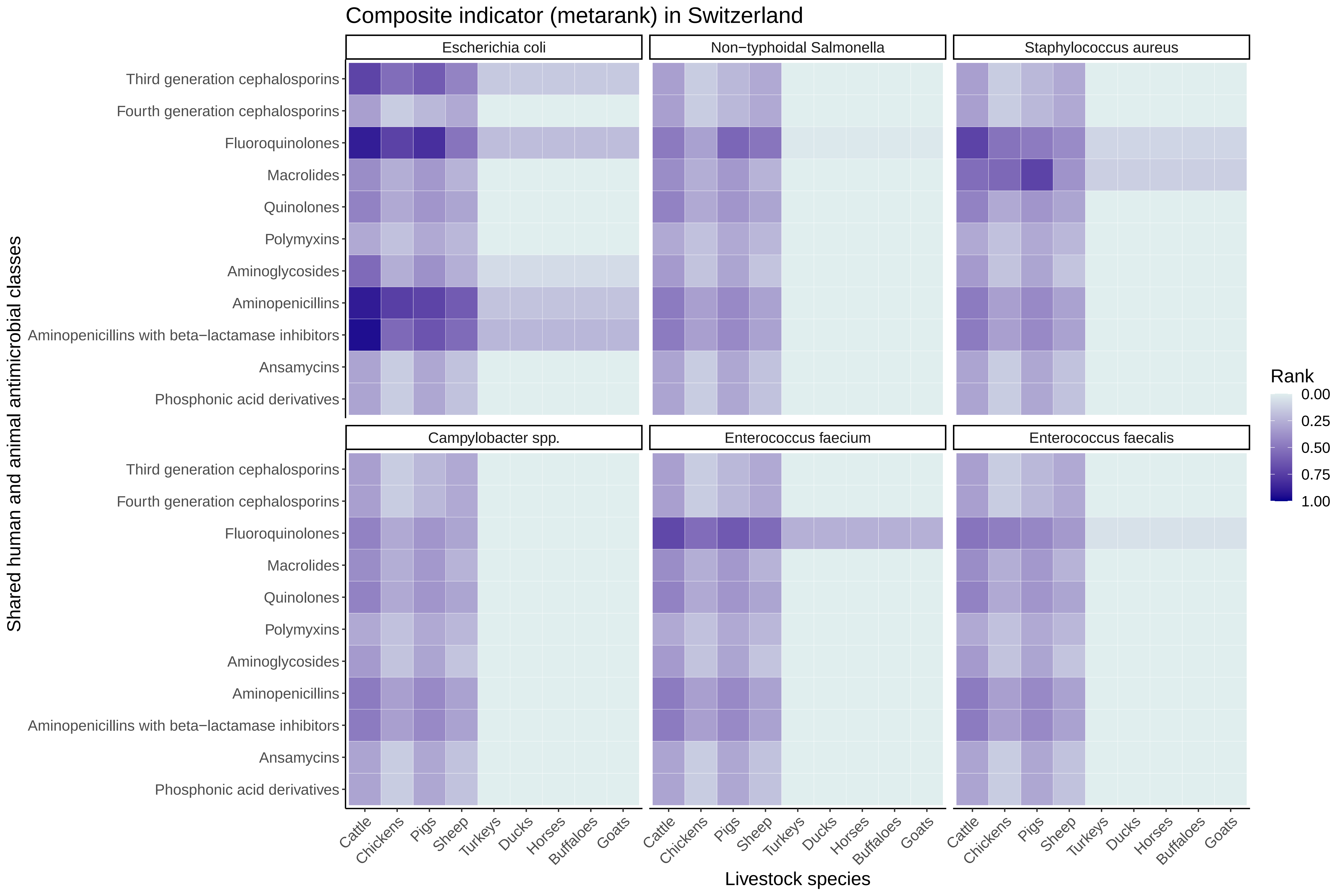
Composite indicator (metarank) in Switzerland
Escherichia coli
Non−typhoidal Salmonella
Staphylococcus aureus
Campylobacter spp.
Enterococcus faecium
Enterococcus faecalis
Third generation cephalosporins
Fourth generation cephalosporins
Fluoroquinolones
Macrolides
Quinolones
Polymyxins
Aminoglycosides
Aminopenicillins
Aminopenicillins with beta−lactamase inhibitors
Ansamycins
Phosphonic acid derivatives
Shared human and animal antimicrobial classes
Cattle
Chickens
Pigs
Sheep
Turkeys
Ducks
Horses
Buffaloes
Goats
Livestock species
Rank
0.00
0.25
0.50
0.75
1.00

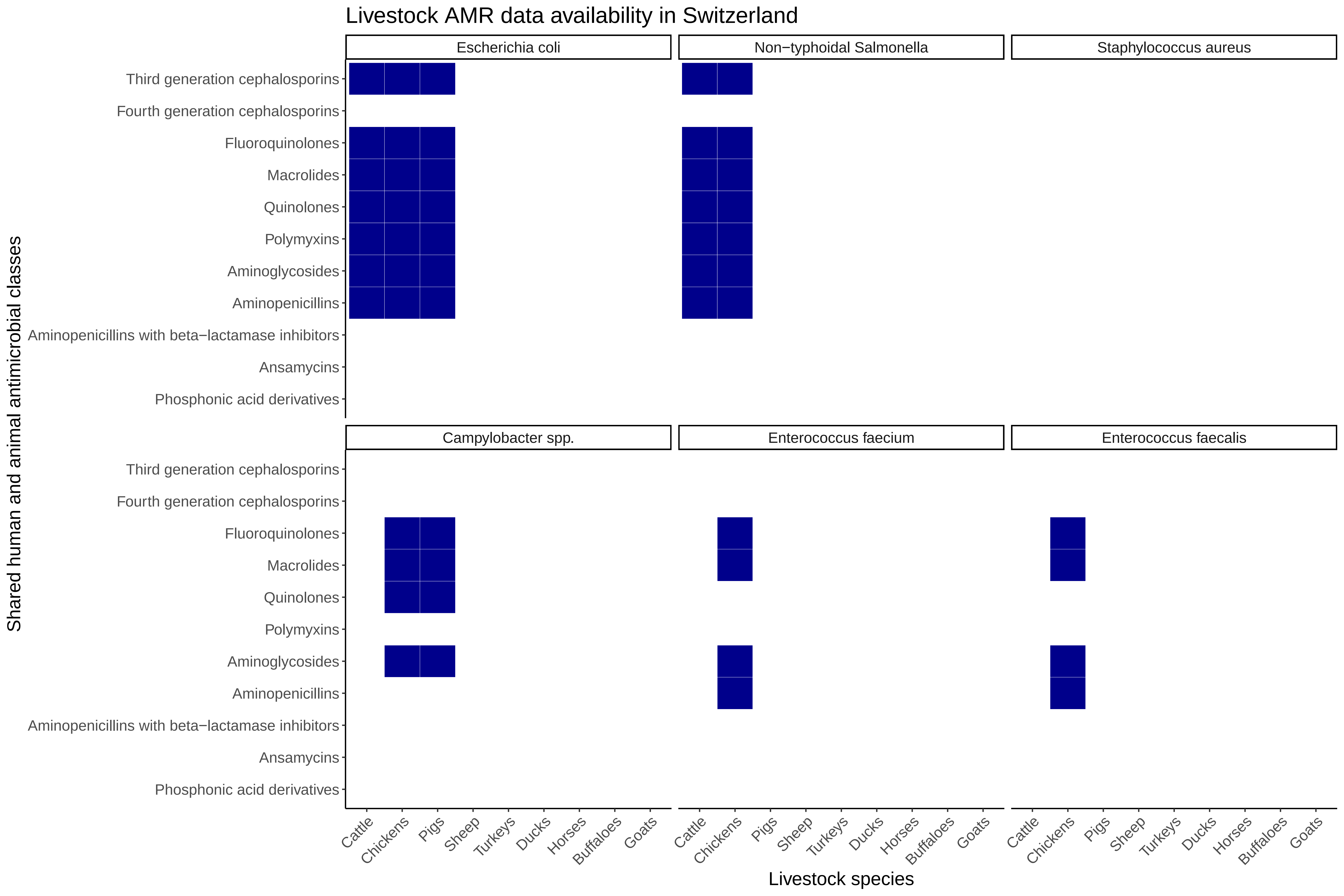
Livestock AMR data availability in Switzerland
Escherichia coli
Non−typhoidal Salmonella
Staphylococcus aureus
Campylobacter spp.
Enterococcus faecium
Enterococcus faecalis
Shared human and animal antimicrobial classes
Third generation cephalosporins
Fourth generation cephalosporins
Fluoroquinolones
Macrolides
Quinolones
Polymyxins
Aminoglycosides
Aminopenicillins
Aminopenicillins with beta−lactamase inhibitors
Ansamycins
Phosphonic acid derivatives
Livestock species
Cattle
Chickens
Pigs
Sheep
Turkeys
Ducks
Horses
Buffaloes
Goats

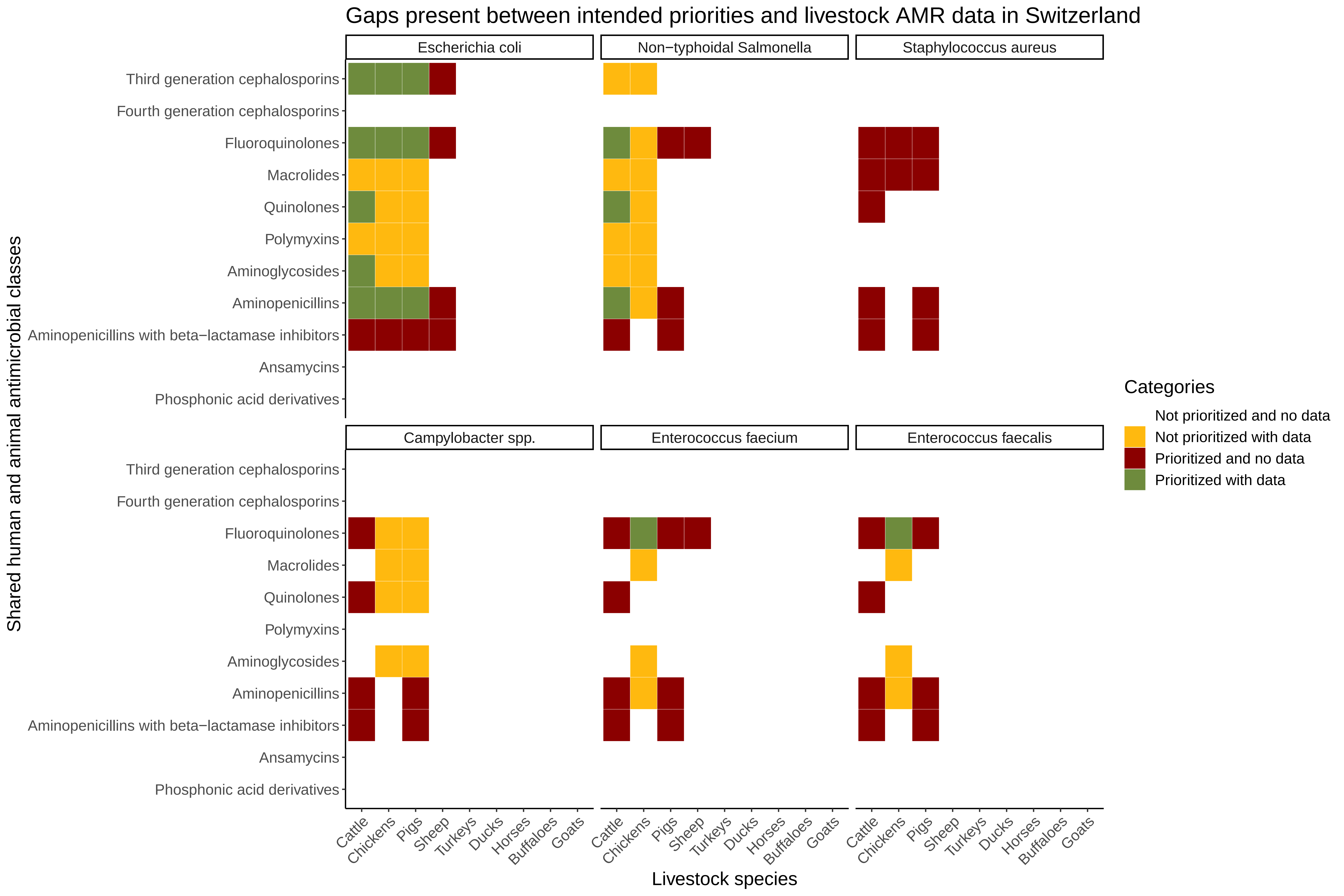
Gaps present between intended priorities and livestock AMR data in Switzerland
Escherichia coli
Non−typhoidal Salmonella
Staphylococcus aureus
Campylobacter spp.
Enterococcus faecium
Enterococcus faecalis
Shared human and animal antimicrobial classes
Third generation cephalosporins
Fourth generation cephalosporins
Fluoroquinolones
Macrolides
Quinolones
Polymyxins
Aminoglycosides
Aminopenicillins
Aminopenicillins with beta−lactamase inhibitors
Ansamycins
Phosphonic acid derivatives
Cattle
Chickens
Pigs
Sheep
Turkeys
Ducks
Horses
Buffaloes
Goats
Livestock species
Categories
Not prioritized and no data
Not prioritized with data
Prioritized and no data
Prioritized with data

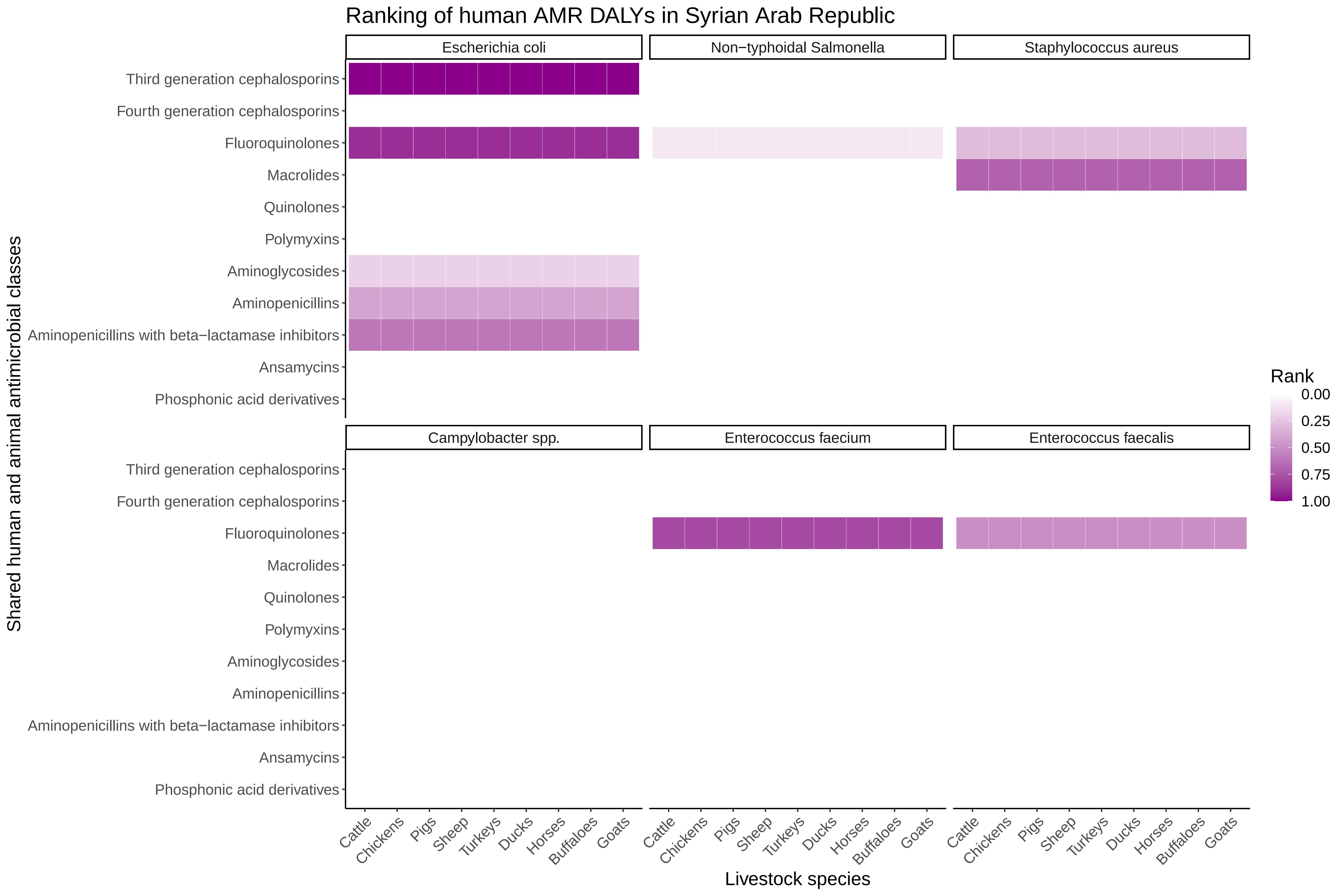
Ranking of human AMR DALYs in Syrian Arab Republic
Escherichia coli
Non−typhoidal Salmonella
Staphylococcus aureus
Campylobacter spp.
Enterococcus faecium
Enterococcus faecalis
Third generation cephalosporins
Fourth generation cephalosporins
Fluoroquinolones
Macrolides
Quinolones
Polymyxins
Aminoglycosides
Aminopenicillins
Aminopenicillins with beta−lactamase inhibitors
Ansamycins
Phosphonic acid derivatives
Shared human and animal antimicrobial classes
Cattle
Chickens
Pigs
Sheep
Turkeys
Ducks
Horses
Buffaloes
Goats
Livestock species
Rank
0.00
0.25
0.50
0.75
1.00

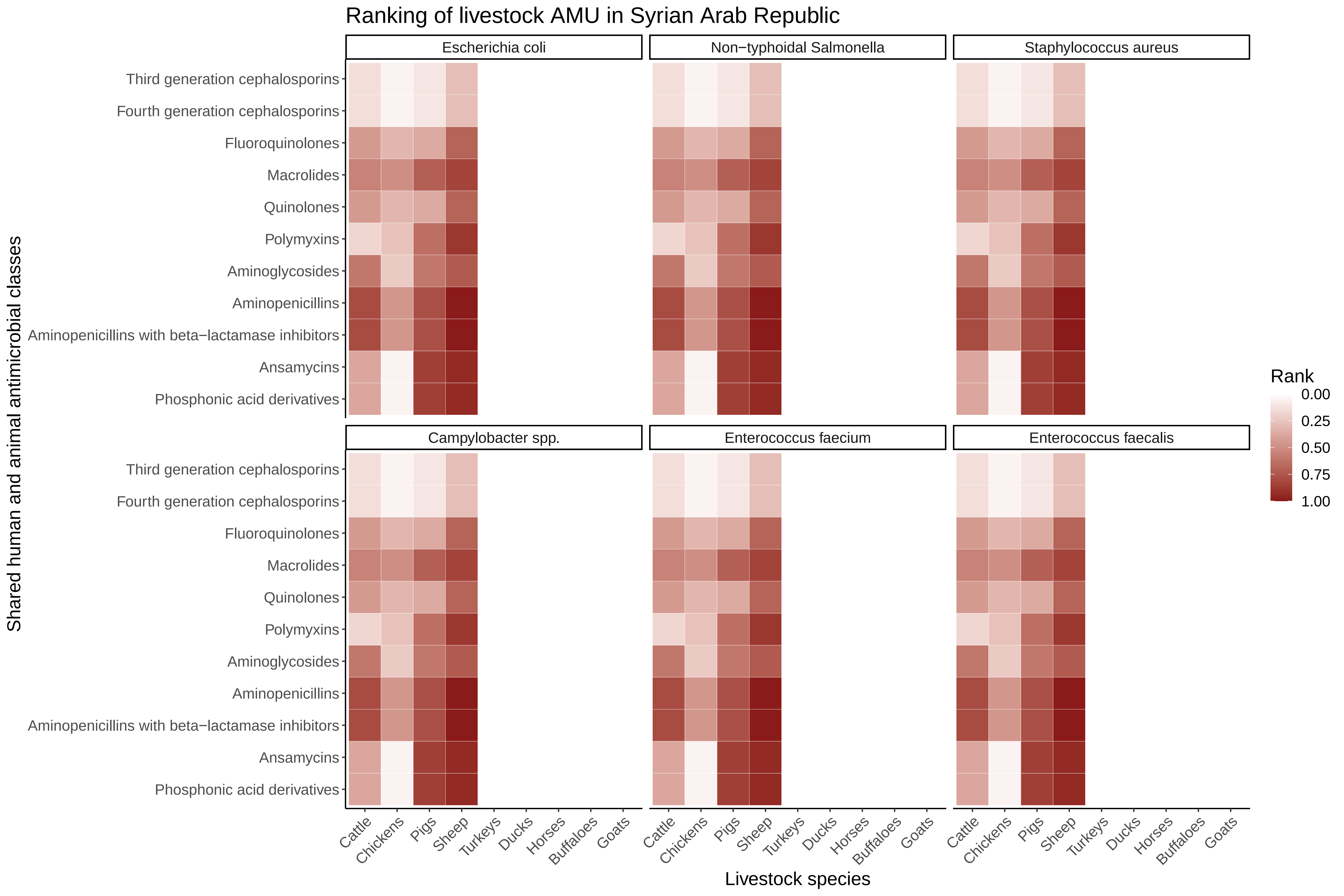

Ranking of livestock AMU in Syrian Arab Republic
Escherichia coli
Non−typhoidal Salmonella
Staphylococcus aureus
Campylobacter spp.
Enterococcus faecium
Enterococcus faecalis
Shared human and animal antimicrobial classes
Third generation cephalosporins
Fourth generation cephalosporins
Fluoroquinolones
Macrolides
Quinolones
Polymyxins
Aminoglycosides
Aminopenicillins
Aminopenicillins with beta−lactamase inhibitors
Ansamycins
Phosphonic acid derivatives
Livestock species
Cattle
Chickens
Pigs
Sheep
Turkeys
Ducks
Horses
Buffaloes
Goats
Rank
0.00
0.25
0.50
0.75
1.00

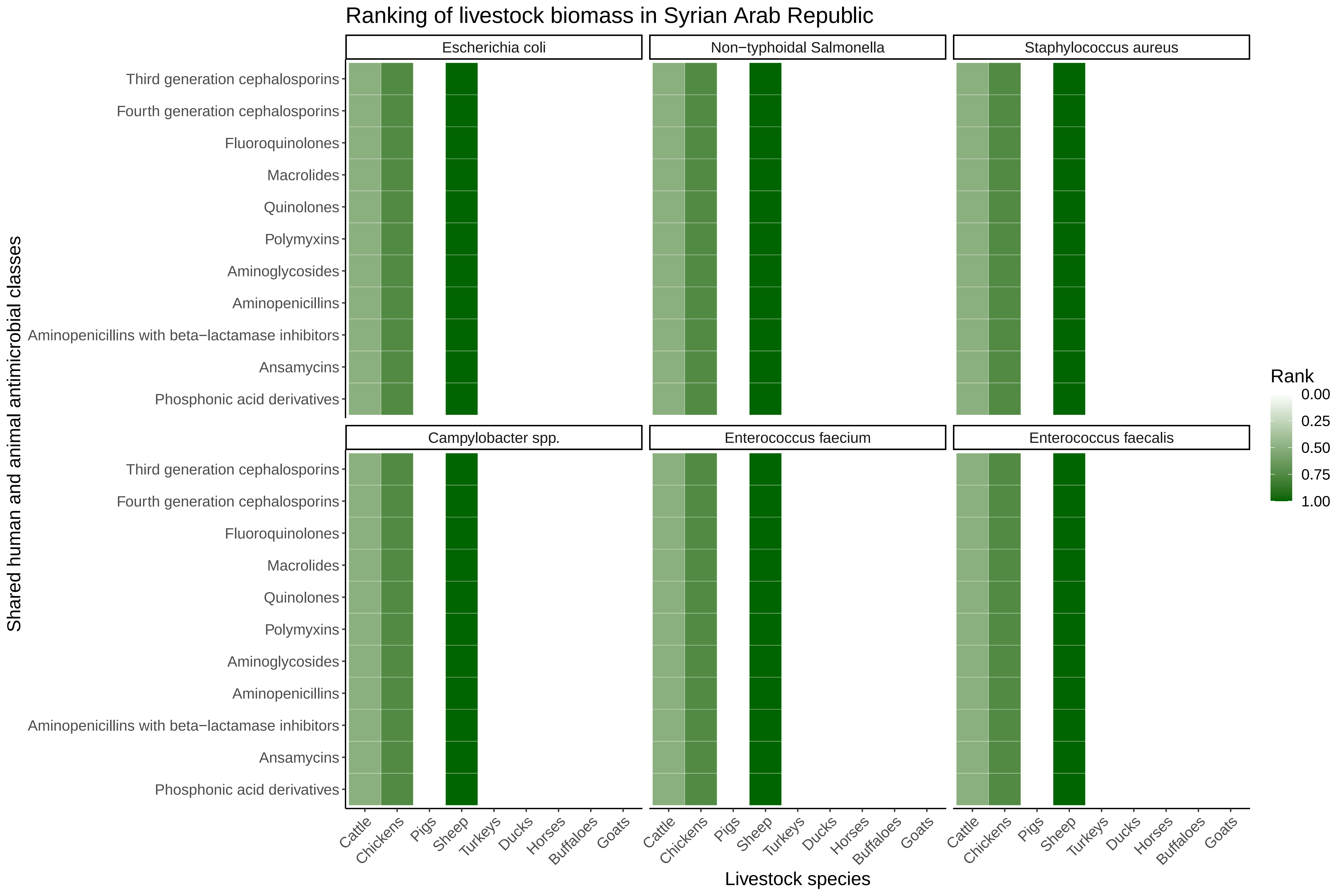

Ranking of livestock biomass in Syrian Arab Republic
Escherichia coli
Non−typhoidal Salmonella
Staphylococcus aureus
Campylobacter spp.
Enterococcus faecium
Enterococcus faecalis
Shared human and animal antimicrobial classes
Third generation cephalosporins
Fourth generation cephalosporins
Fluoroquinolones
Macrolides
Quinolones
Polymyxins
Aminoglycosides
Aminopenicillins
Aminopenicillins with beta−lactamase inhibitors
Ansamycins
Phosphonic acid derivatives
Livestock species
Cattle
Chickens
Pigs
Sheep
Turkeys
Ducks
Horses
Buffaloes
Goats
Rank
0.00
0.25
0.50
0.75
1.00

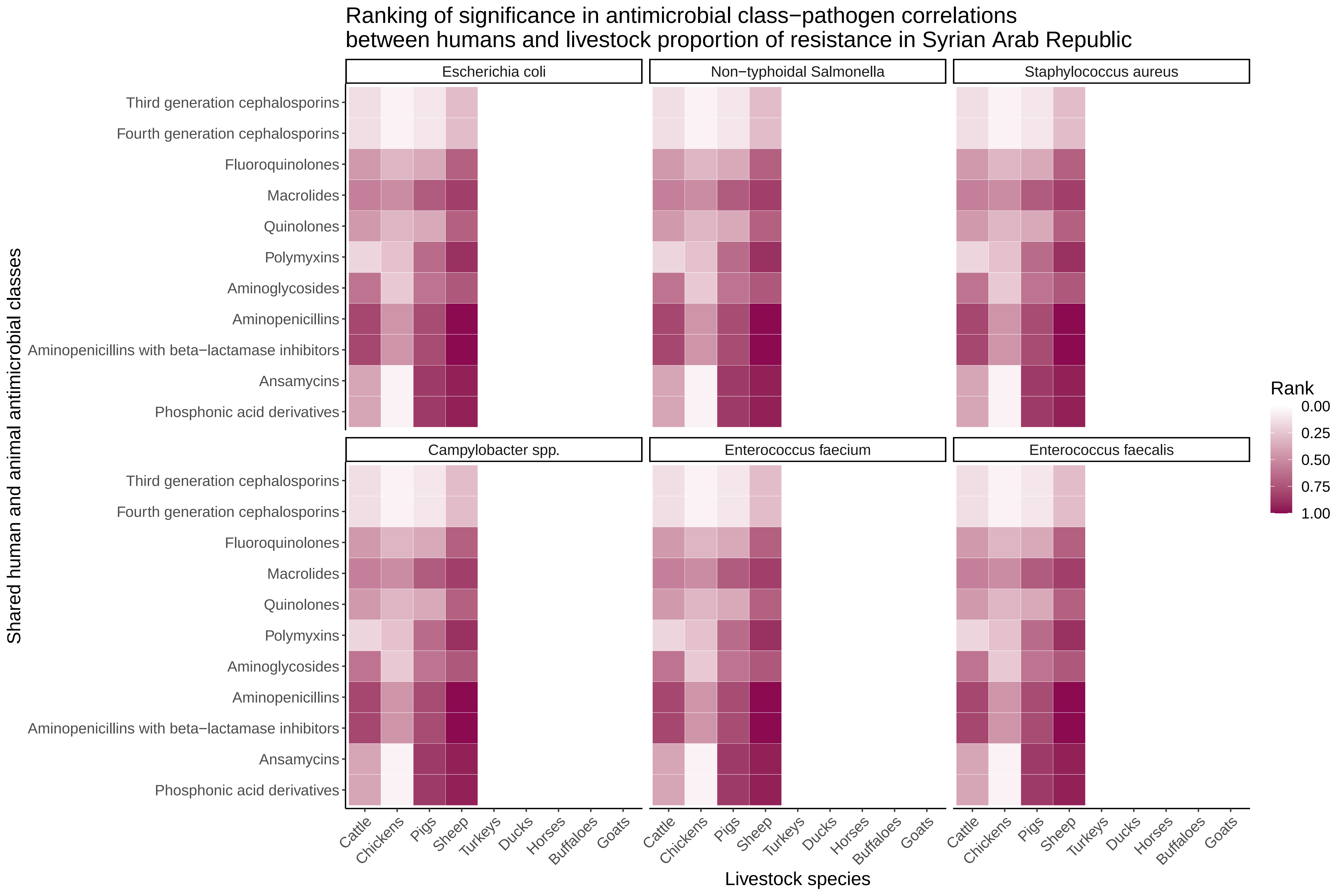

Ranking of significance in antimicrobial class−pathogen correlations
between humans and livestock proportion of resistance in Syrian Arab Republic
Escherichia coli
Non−typhoidal Salmonella
Staphylococcus aureus
Campylobacter spp.
Enterococcus faecium
Enterococcus faecalis
Third generation cephalosporins
Fourth generation cephalosporins
Fluoroquinolones
Macrolides
Quinolones
Polymyxins
Aminoglycosides
Aminopenicillins
Aminopenicillins with beta−lactamase inhibitors
Ansamycins
Phosphonic acid derivatives
Shared human and animal antimicrobial classes
Cattle
Chickens
Pigs
Sheep
Turkeys
Ducks
Horses
Buffaloes
Goats
Livestock species
Rank
0.00
0.25
0.50
0.75
1.00

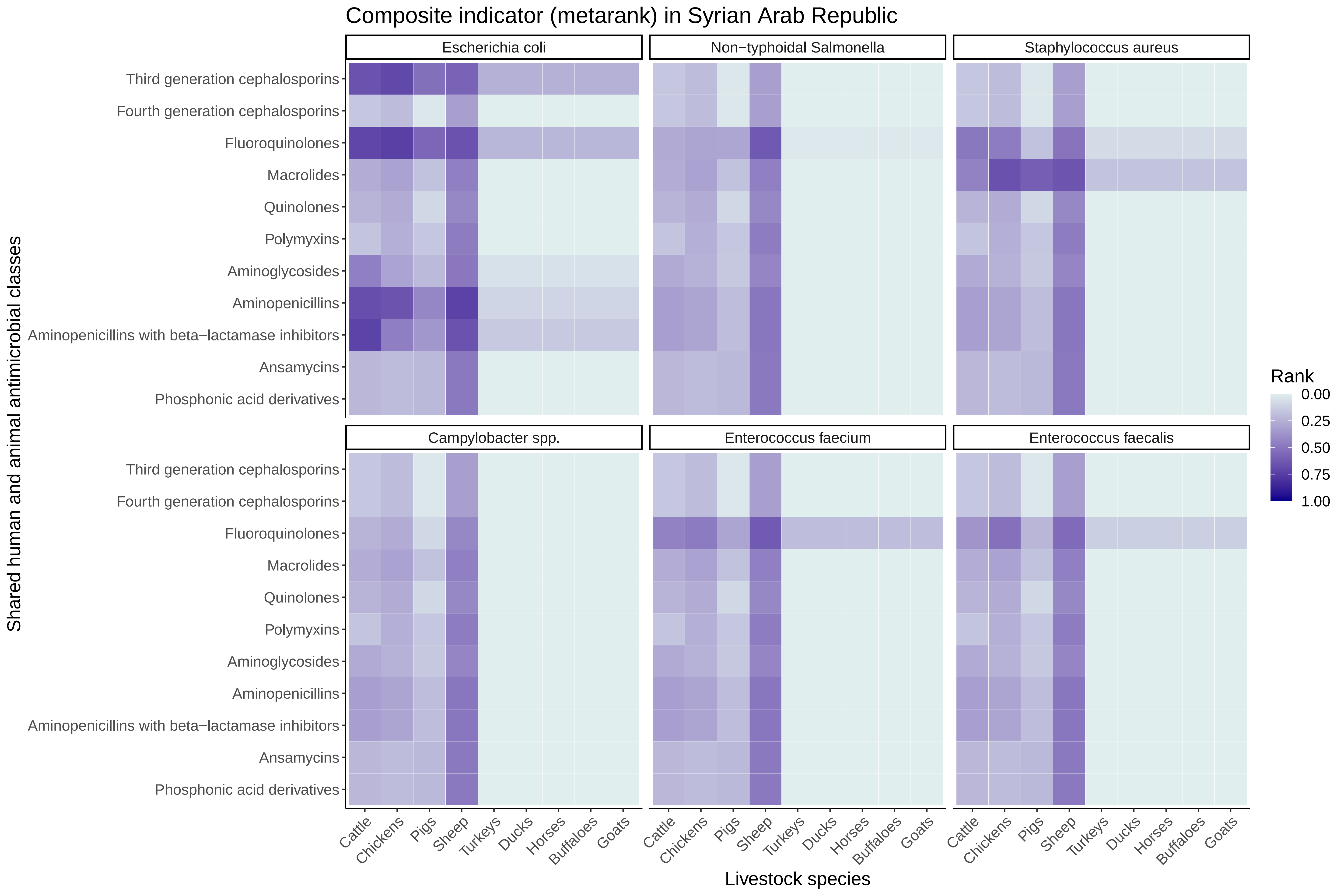
Composite indicator (metarank) in Syrian Arab Republic
Escherichia coli
Non−typhoidal Salmonella
Staphylococcus aureus
Campylobacter spp.
Enterococcus faecium
Enterococcus faecalis
Shared human and animal antimicrobial classes
Third generation cephalosporins
Fourth generation cephalosporins
Fluoroquinolones
Macrolides
Quinolones
Polymyxins
Aminoglycosides
Aminopenicillins
Aminopenicillins with beta−lactamase inhibitors
Ansamycins
Phosphonic acid derivatives
Cattle
Chickens
Pigs
Sheep
Turkeys
Ducks
Horses
Buffaloes
Goats
Livestock species
Rank
0.00
0.25
0.50
0.75
1.00

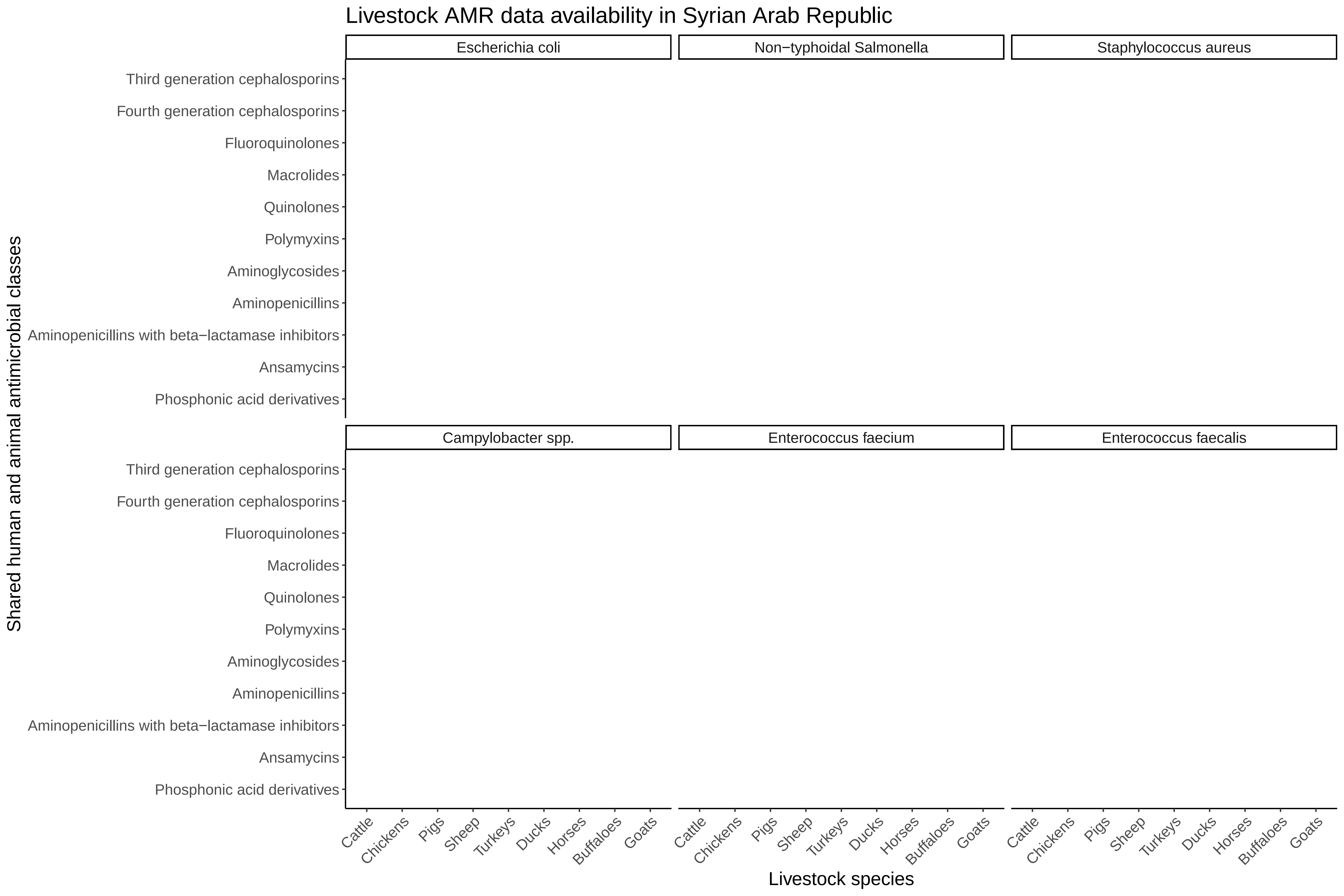
Livestock AMR data availability in Syrian Arab Republic
Escherichia coli
Non−typhoidal Salmonella
Staphylococcus aureus
Campylobacter spp.
Enterococcus faecium
Enterococcus faecalis
Shared human and animal antimicrobial classes
Third generation cephalosporins
Fourth generation cephalosporins
Fluoroquinolones
Macrolides
Quinolones
Polymyxins
Aminoglycosides
Aminopenicillins
Aminopenicillins with beta−lactamase inhibitors
Ansamycins
Phosphonic acid derivatives
Cattle
Chickens
Pigs
Sheep
Turkeys
Ducks
Horses
Buffaloes
Goats
Livestock species

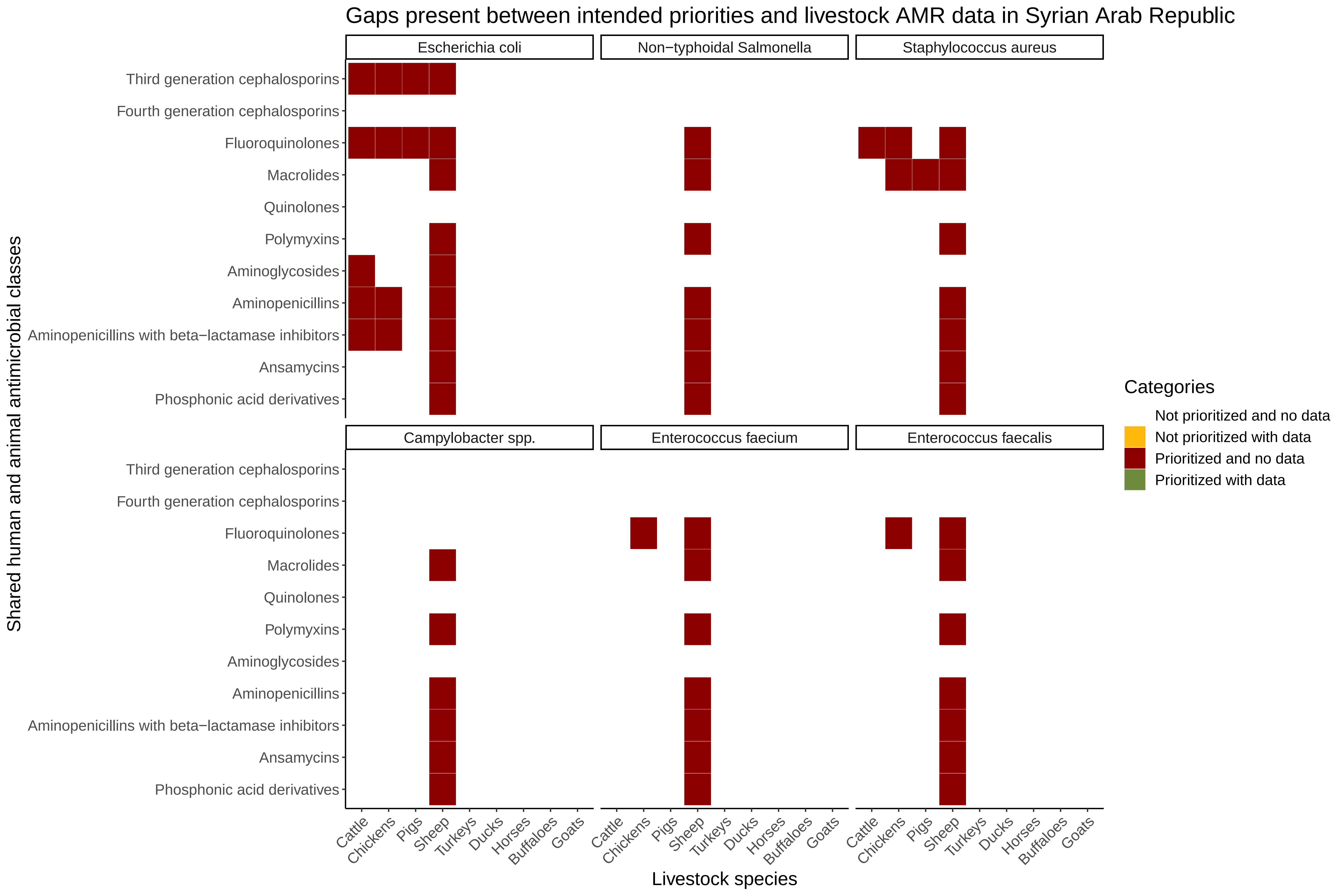
Gaps present between intended priorities and livestock AMR data in Syrian Arab Republic
Escherichia coli
Non−typhoidal Salmonella
Staphylococcus aureus
Campylobacter spp.
Enterococcus faecium
Enterococcus faecalis
Shared human and animal antimicrobial classes
Third generation cephalosporins
Fourth generation cephalosporins
Fluoroquinolones
Macrolides
Quinolones
Polymyxins
Aminoglycosides
Aminopenicillins
Aminopenicillins with beta−lactamase inhibitors
Ansamycins
Phosphonic acid derivatives
Livestock species
Cattle
Chickens
Pigs
Sheep
Turkeys
Ducks
Horses
Buffaloes
Goats
Categories
Not prioritized and no data
Not prioritized with data
Prioritized and no data
Prioritized with data

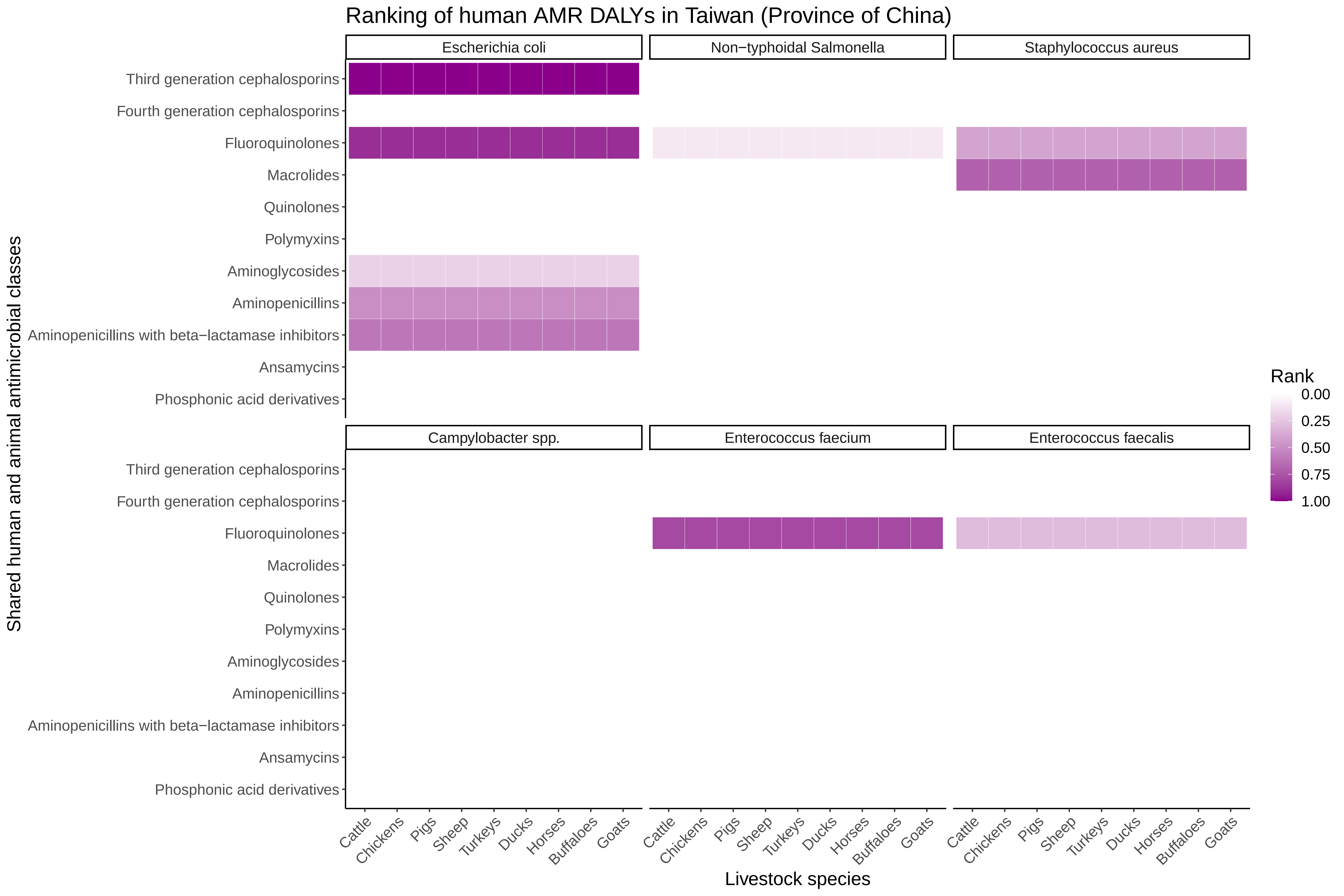
Ranking of human AMR DALYs in Taiwan (Province of China)
Escherichia coli
Non−typhoidal Salmonella
Staphylococcus aureus
Campylobacter spp.
Enterococcus faecium
Enterococcus faecalis
Third generation cephalosporins
Fourth generation cephalosporins
Fluoroquinolones
Macrolides
Quinolones
Polymyxins
Aminoglycosides
Aminopenicillins
Aminopenicillins with beta−lactamase inhibitors
Ansamycins
Phosphonic acid derivatives
Shared human and animal antimicrobial classes
Cattle
Chickens
Pigs
Sheep
Turkeys
Ducks
Horses
Buffaloes
Goats
Livestock species
Rank
0.00
0.25
0.50
0.75
1.00

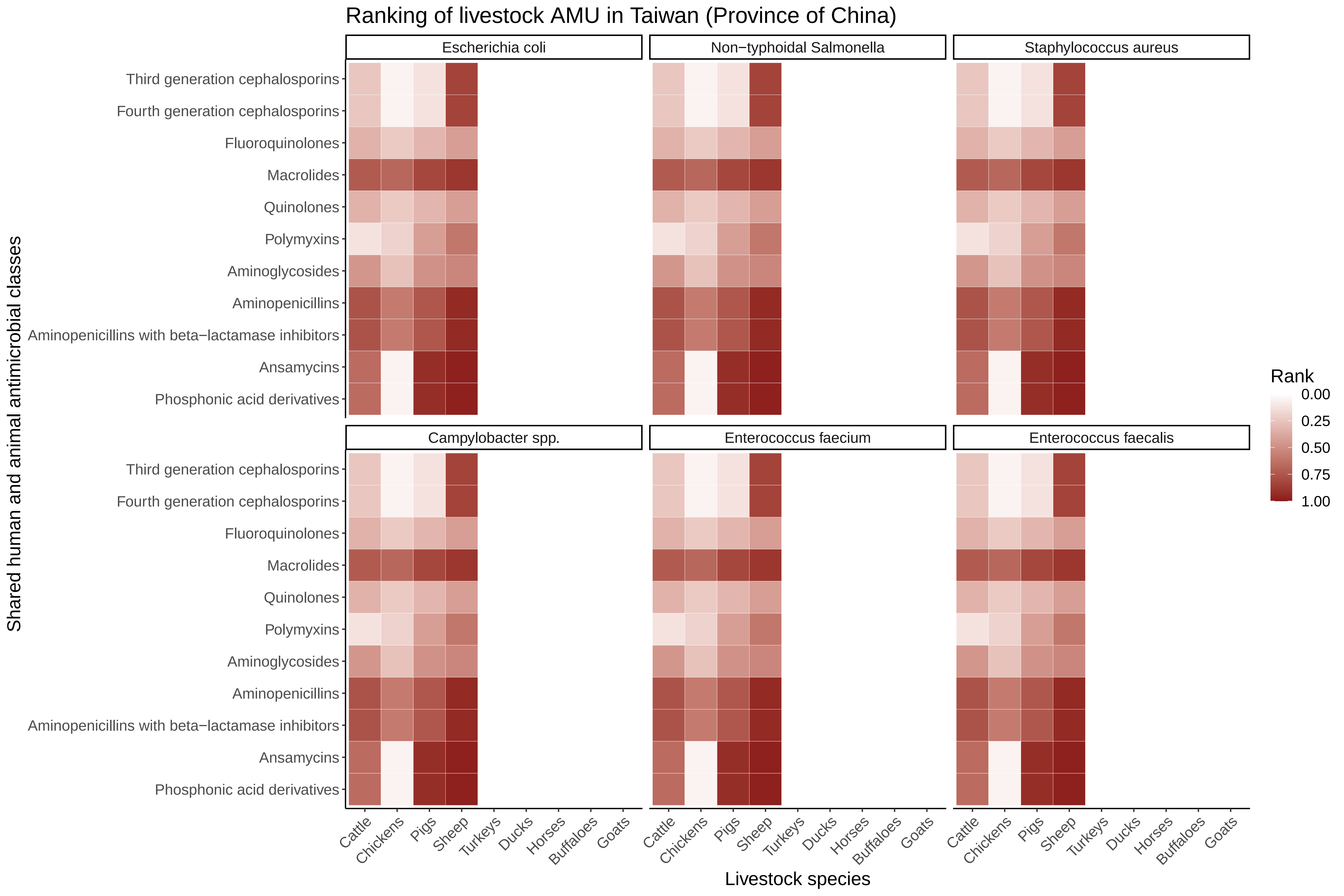

Ranking of livestock AMU in Taiwan (Province of China)
Escherichia coli
Non−typhoidal Salmonella
Staphylococcus aureus
Campylobacter spp.
Enterococcus faecium
Enterococcus faecalis
Shared human and animal antimicrobial classes
Third generation cephalosporins
Fourth generation cephalosporins
Fluoroquinolones
Macrolides
Quinolones
Polymyxins
Aminoglycosides
Aminopenicillins
Aminopenicillins with beta−lactamase inhibitors
Ansamycins
Phosphonic acid derivatives
Livestock species
Cattle
Chickens
Pigs
Sheep
Turkeys
Ducks
Horses
Buffaloes
Goats
Rank
0.00
0.25
0.50
0.75
1.00

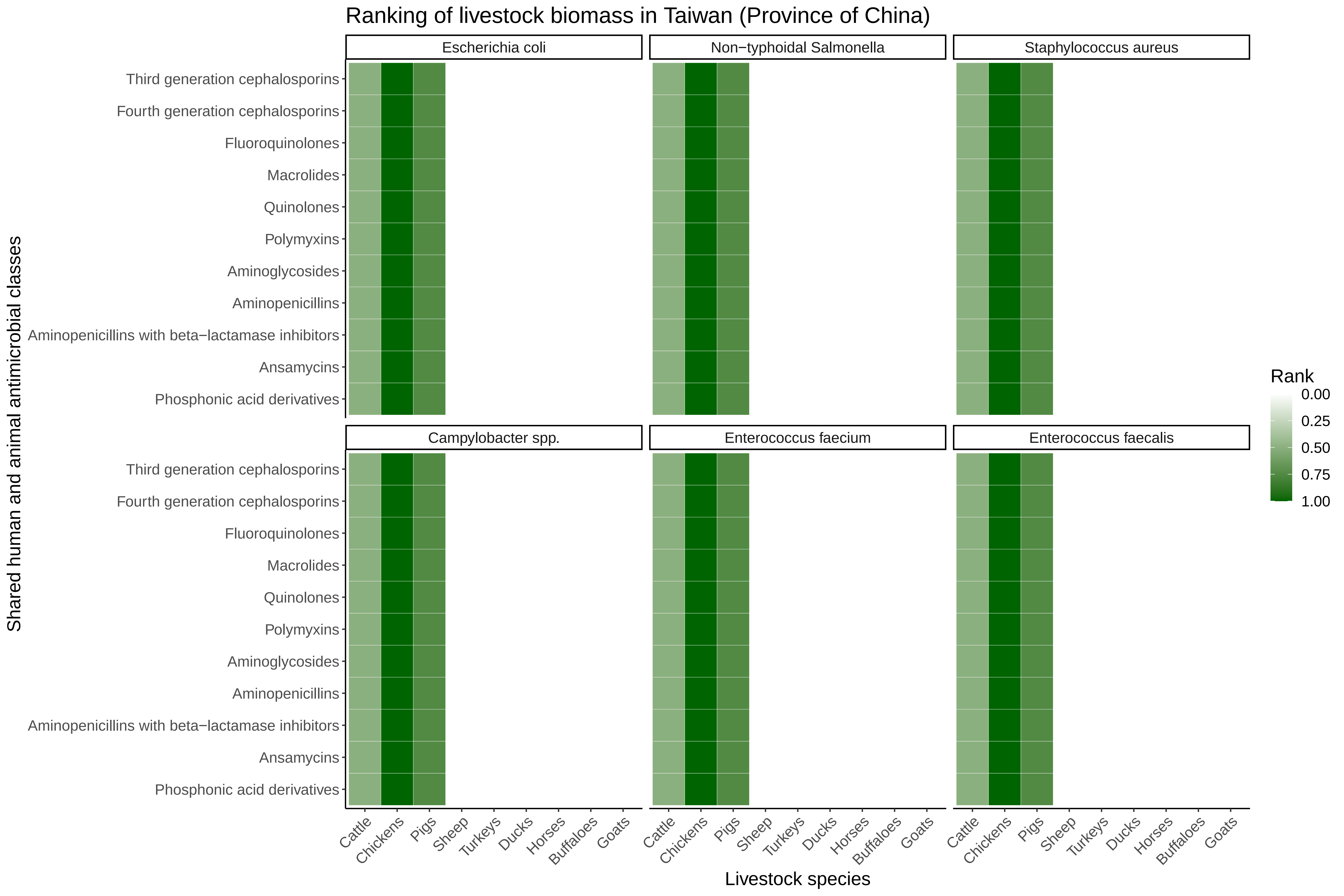

Ranking of livestock biomass in Taiwan (Province of China)
Escherichia coli
Non−typhoidal Salmonella
Staphylococcus aureus
Campylobacter spp.
Enterococcus faecium
Enterococcus faecalis
Third generation cephalosporins
Fourth generation cephalosporins
Fluoroquinolones
Macrolides
Quinolones
Polymyxins
Aminoglycosides
Aminopenicillins
Aminopenicillins with beta−lactamase inhibitors
Ansamycins
Phosphonic acid derivatives
Cattle
Chickens
Pigs
Sheep
Turkeys
Ducks
Horses
Buffaloes
Goats
Livestock species
Shared human and animal antimicrobial classes
Rank
0.00
0.25
0.50
0.75
1.00

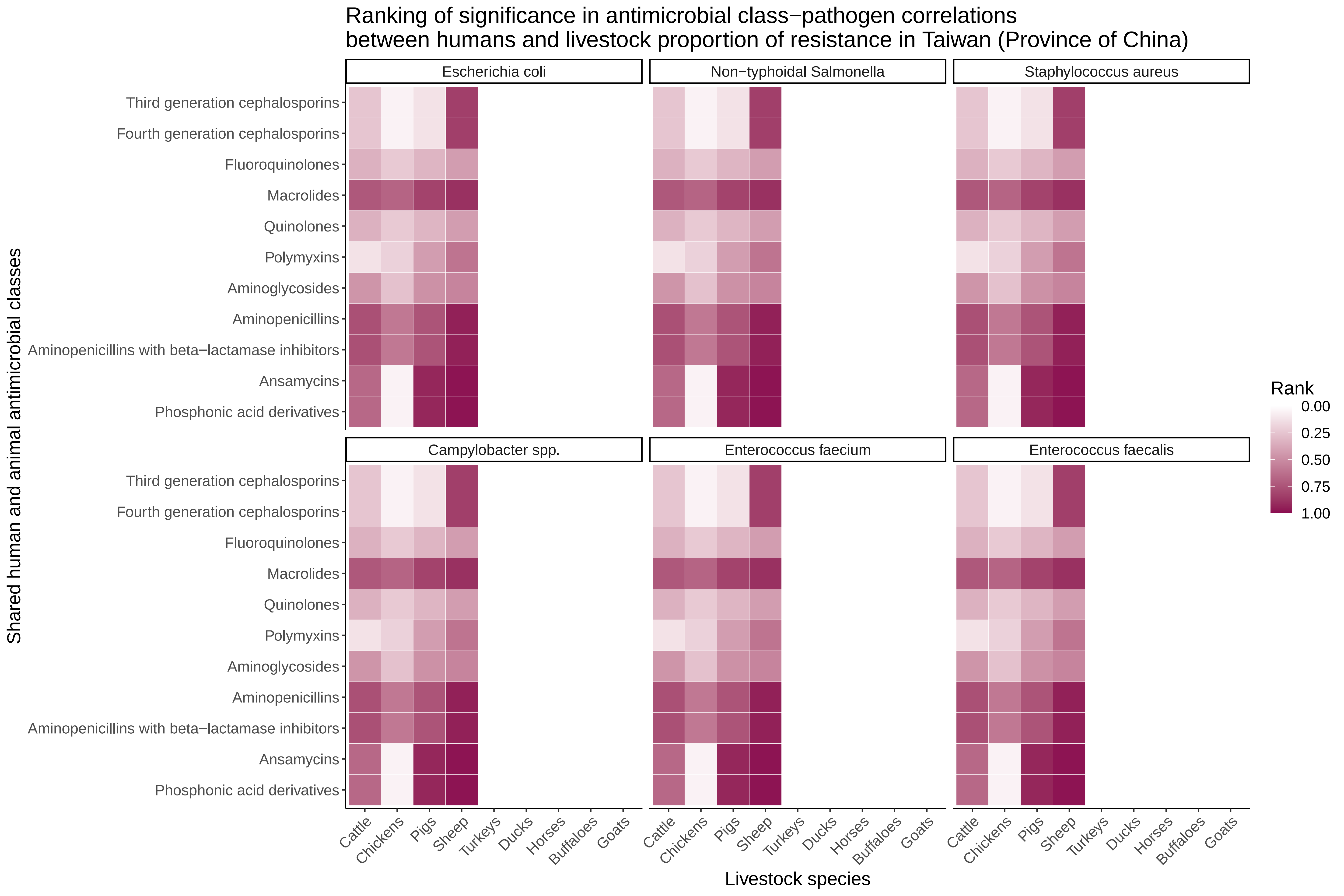
Ranking of significance in antimicrobial class−pathogen correlations
between humans and livestock proportion of resistance in Taiwan (Province of China)
Escherichia coli
Non−typhoidal Salmonella
Staphylococcus aureus
Campylobacter spp.
Enterococcus faecium
Enterococcus faecalis
Third generation cephalosporins
Fourth generation cephalosporins
Fluoroquinolones
Macrolides
Quinolones
Polymyxins
Aminoglycosides
Aminopenicillins
Aminopenicillins with beta−lactamase inhibitors
Ansamycins
Phosphonic acid derivatives
Shared human and animal antimicrobial classes
Cattle
Chickens
Pigs
Sheep
Turkeys
Ducks
Horses
Buffaloes
Goats
Livestock species
Rank
0.00
0.25
0.50
0.75
1.00

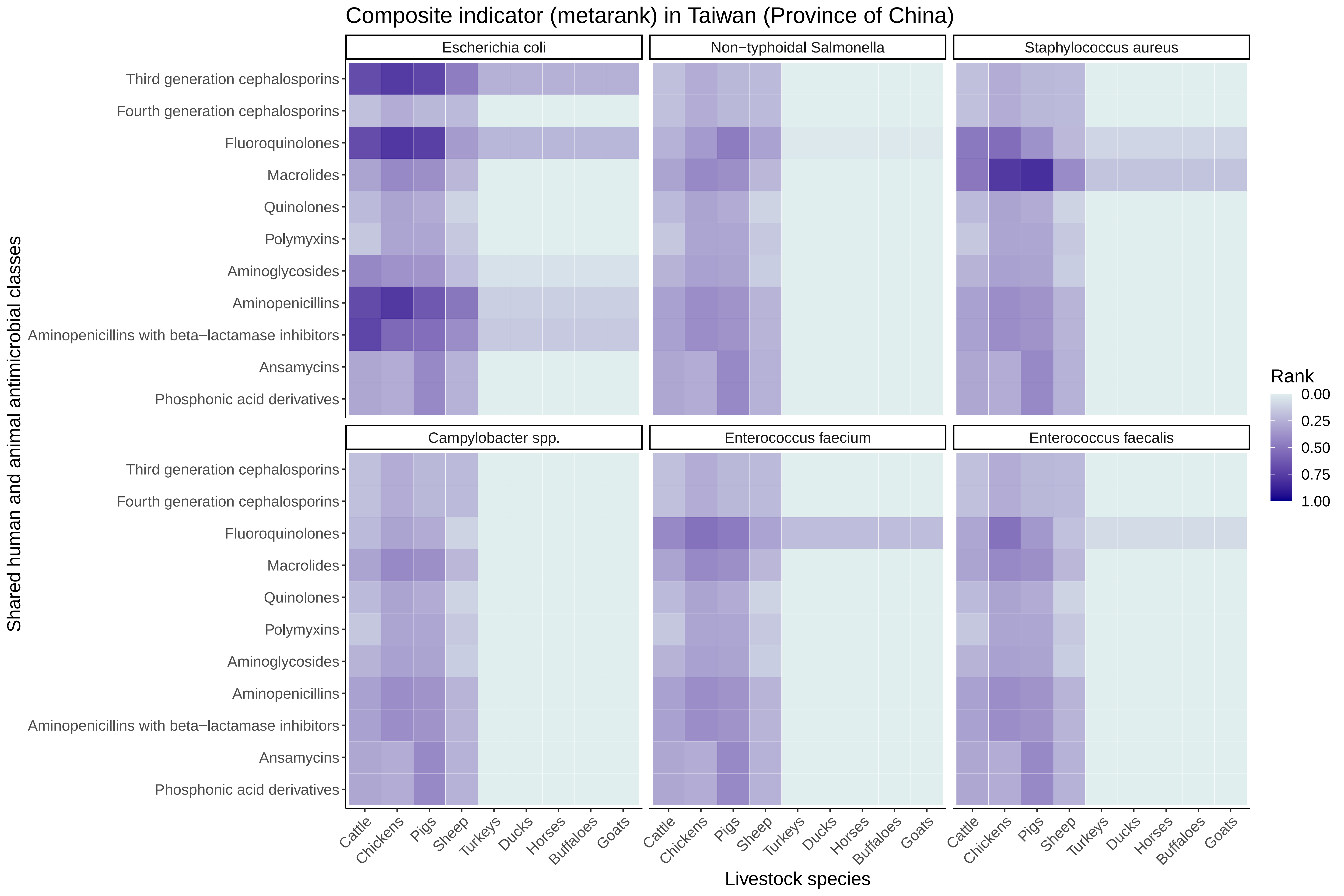

Composite indicator (metarank) in Taiwan (Province of China)
Escherichia coli
Non−typhoidal Salmonella
Staphylococcus aureus
Campylobacter spp.
Enterococcus faecium
Enterococcus faecalis
Third generation cephalosporins
Fourth generation cephalosporins
Fluoroquinolones
Macrolides
Quinolones
Polymyxins
Aminoglycosides
Aminopenicillins
Aminopenicillins with beta−lactamase inhibitors
Ansamycins
Phosphonic acid derivatives
Shared human and animal antimicrobial classes
Cattle
Chickens
Pigs
Sheep
Turkeys
Ducks
Horses
Buffaloes
Goats
Livestock species
Rank
0.00
0.25
0.50
0.75
1.00

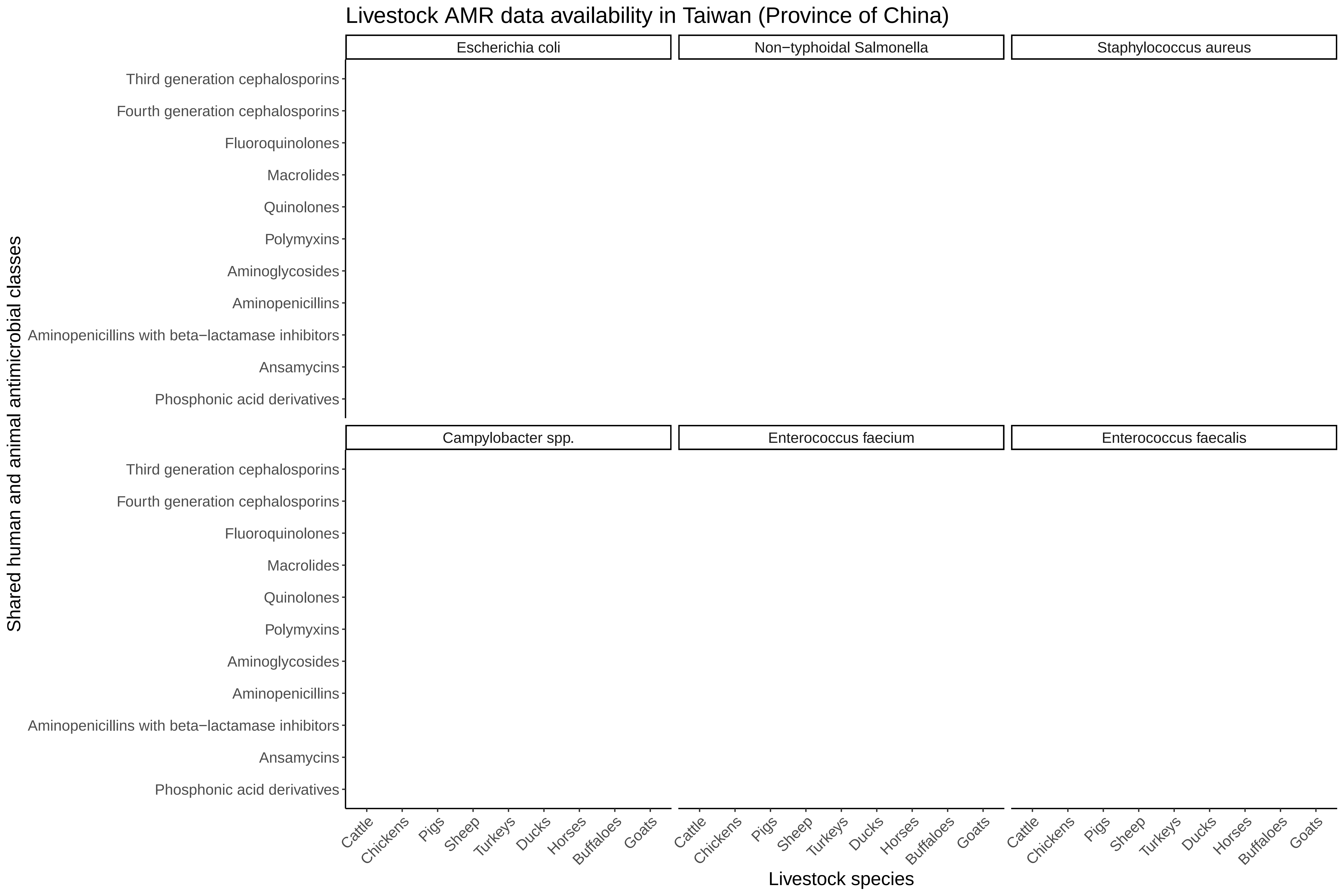

Livestock AMR data availability in Taiwan (Province of China)
Escherichia coli
Non−typhoidal Salmonella
Staphylococcus aureus
Campylobacter spp.
Enterococcus faecium
Enterococcus faecalis
Third generation cephalosporins
Fourth generation cephalosporins
Fluoroquinolones
Macrolides
Quinolones
Polymyxins
Aminoglycosides
Aminopenicillins
Aminopenicillins with beta−lactamase inhibitors
Ansamycins
Phosphonic acid derivatives
Shared human and animal antimicrobial classes
Cattle
Chickens
Pigs
Sheep
Turkeys
Ducks
Horses
Buffaloes
Goats
Livestock species

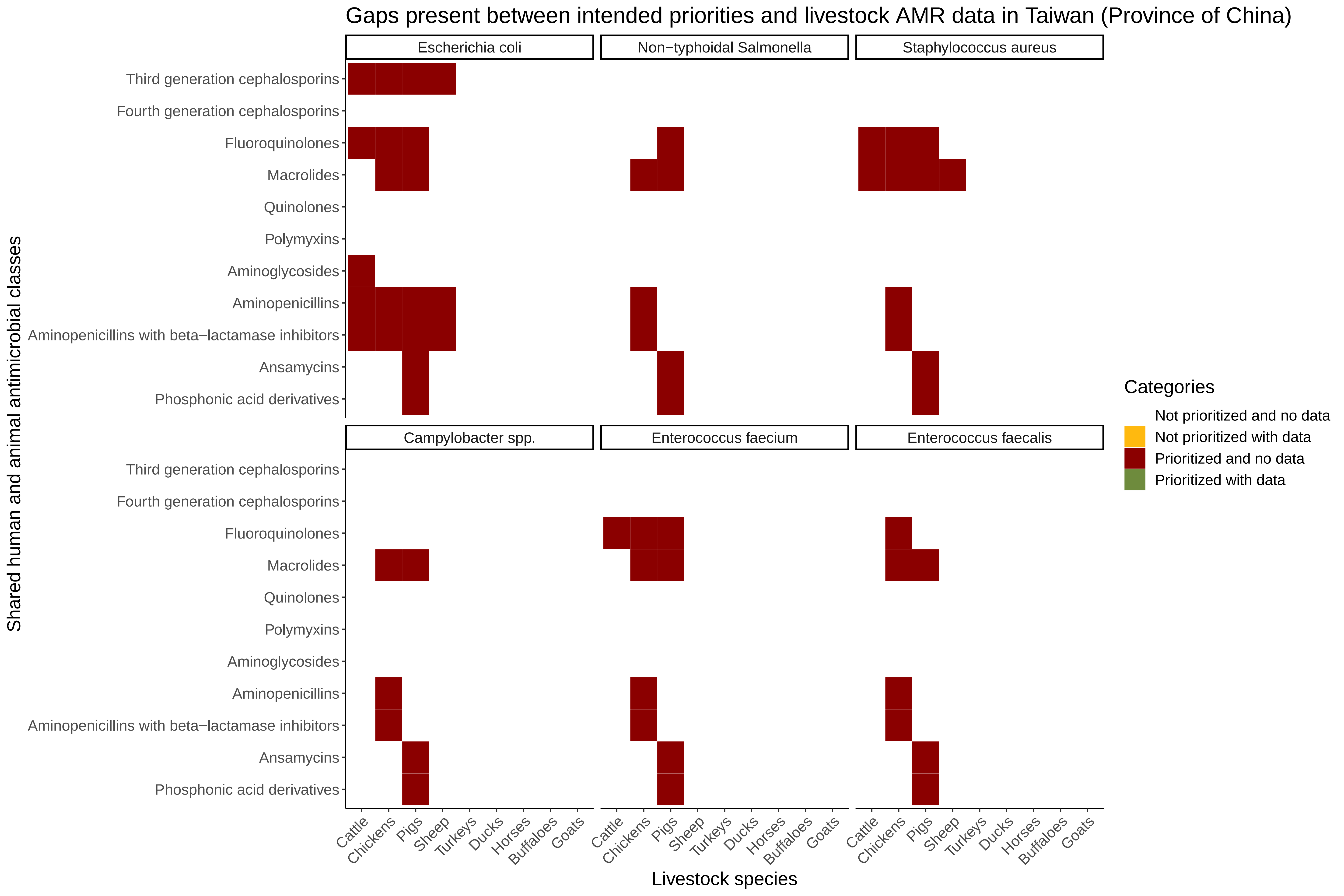

Gaps present between intended priorities and livestock AMR data in Taiwan (Province of China)
Escherichia coli
Non−typhoidal Salmonella
Staphylococcus aureus
Campylobacter spp.
Enterococcus faecium
Enterococcus faecalis
Third generation cephalosporins
Fourth generation cephalosporins
Fluoroquinolones
Macrolides
Quinolones
Polymyxins
Aminoglycosides
Aminopenicillins
Aminopenicillins with beta−lactamase inhibitors
Ansamycins
Phosphonic acid derivatives
Shared human and animal antimicrobial classes
Cattle
Chickens
Pigs
Sheep
Turkeys
Ducks
Horses
Buffaloes
Goats
Livestock species
Categories
Not prioritized and no data
Not prioritized with data
Prioritized and no data
Prioritized with data

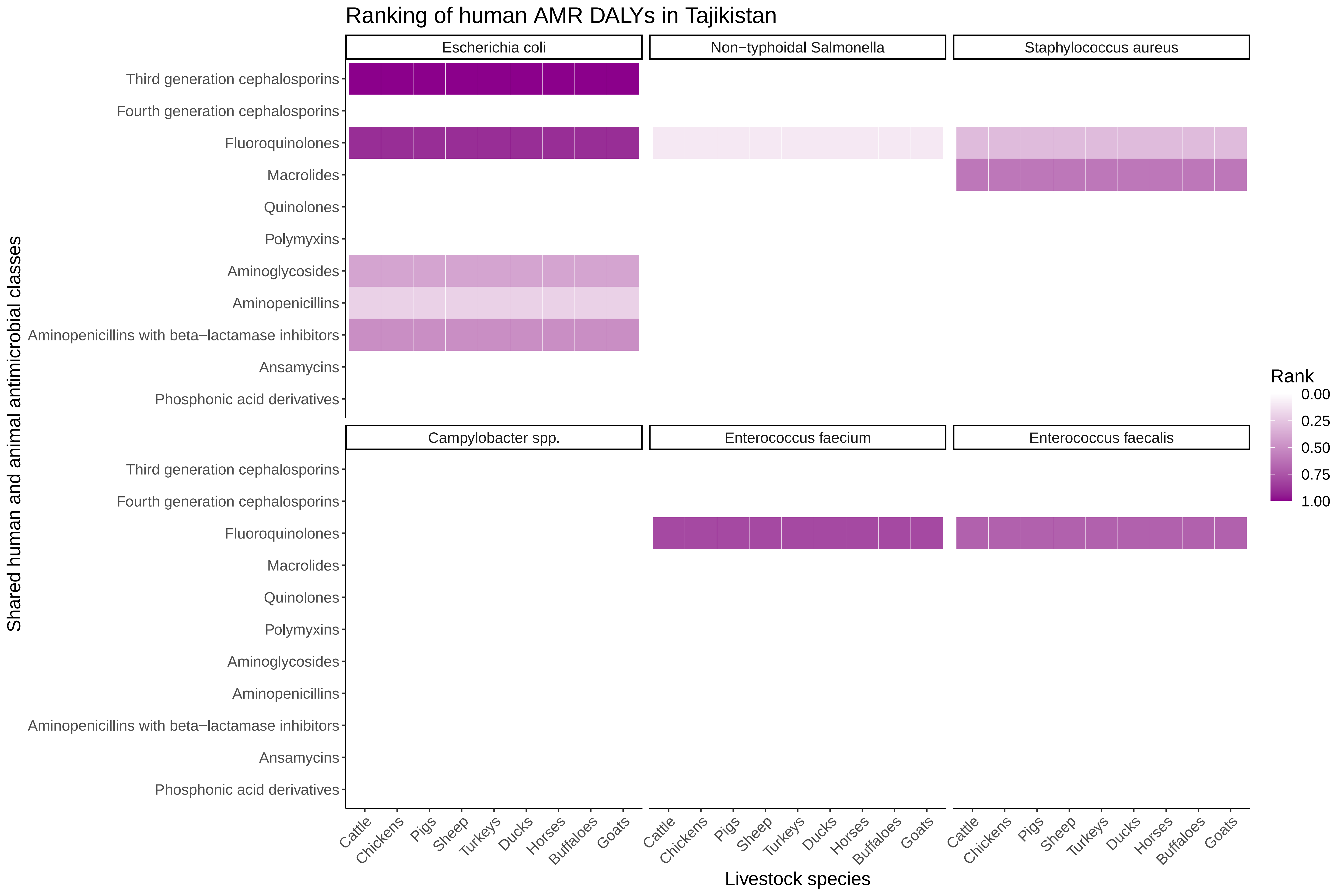
Ranking of human AMR DALYs in Tajikistan
Escherichia coli
Non−typhoidal Salmonella
Staphylococcus aureus
Campylobacter spp.
Enterococcus faecium
Enterococcus faecalis
Third generation cephalosporins
Fourth generation cephalosporins
Fluoroquinolones
Macrolides
Quinolones
Polymyxins
Aminoglycosides
Aminopenicillins
Aminopenicillins with beta−lactamase inhibitors
Ansamycins
Phosphonic acid derivatives
Shared human and animal antimicrobial classes
Cattle
Chickens
Pigs
Sheep
Turkeys
Ducks
Horses
Buffaloes
Goats
Livestock species
Rank
0.00
0.25
0.50
0.75
1.00

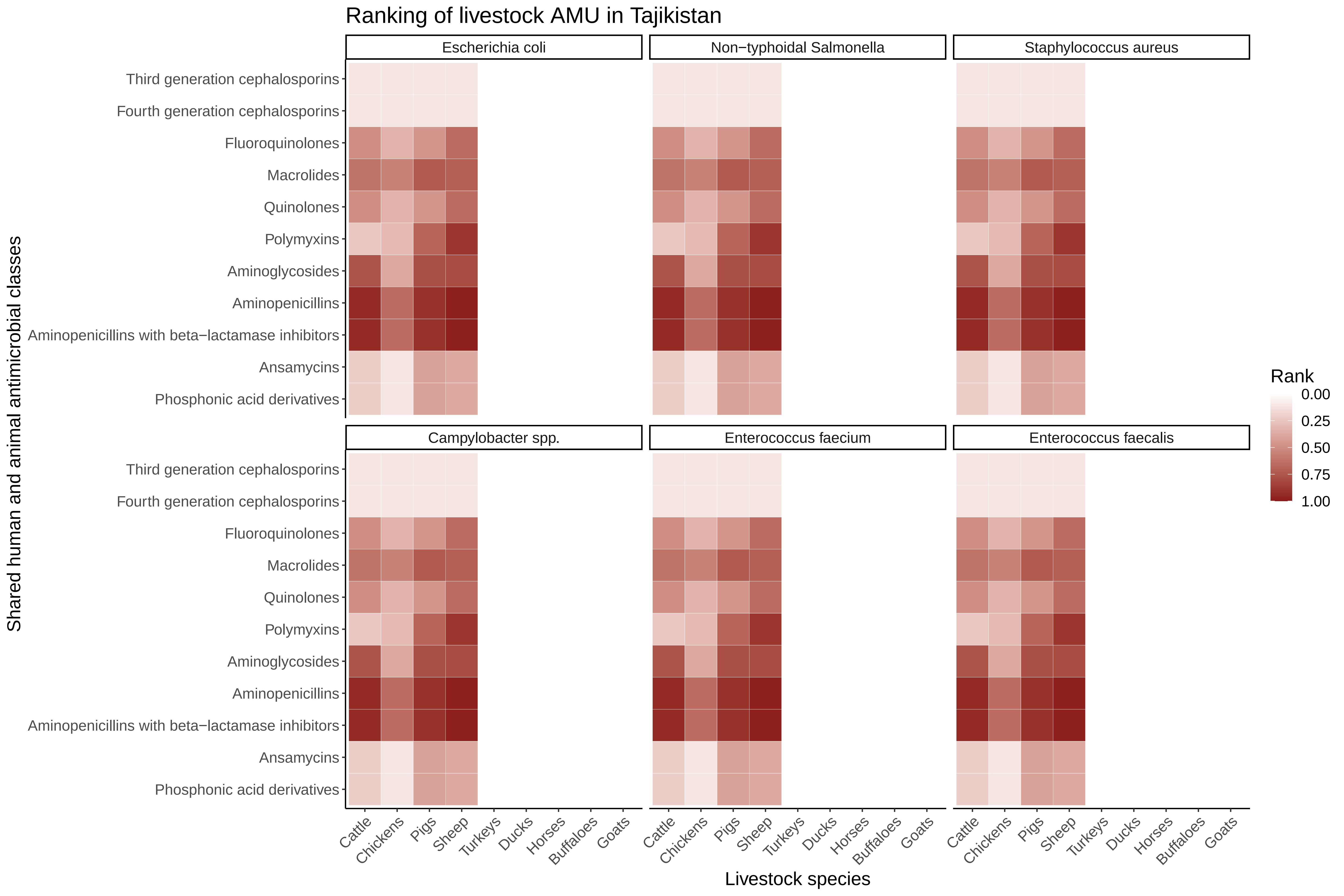

Ranking of livestock AMU in Tajikistan
Escherichia coli
Non−typhoidal Salmonella
Staphylococcus aureus
Campylobacter spp.
Enterococcus faecium
Enterococcus faecalis
Third generation cephalosporins
Fourth generation cephalosporins
Fluoroquinolones
Macrolides
Quinolones
Polymyxins
Aminoglycosides
Aminopenicillins
Aminopenicillins with beta−lactamase inhibitors
Ansamycins
Phosphonic acid derivatives
Shared human and animal antimicrobial classes
Cattle
Chickens
Pigs
Sheep
Turkeys
Ducks
Horses
Buffaloes
Goats
Livestock species
Rank
0.00
0.25
0.50
0.75
1.00

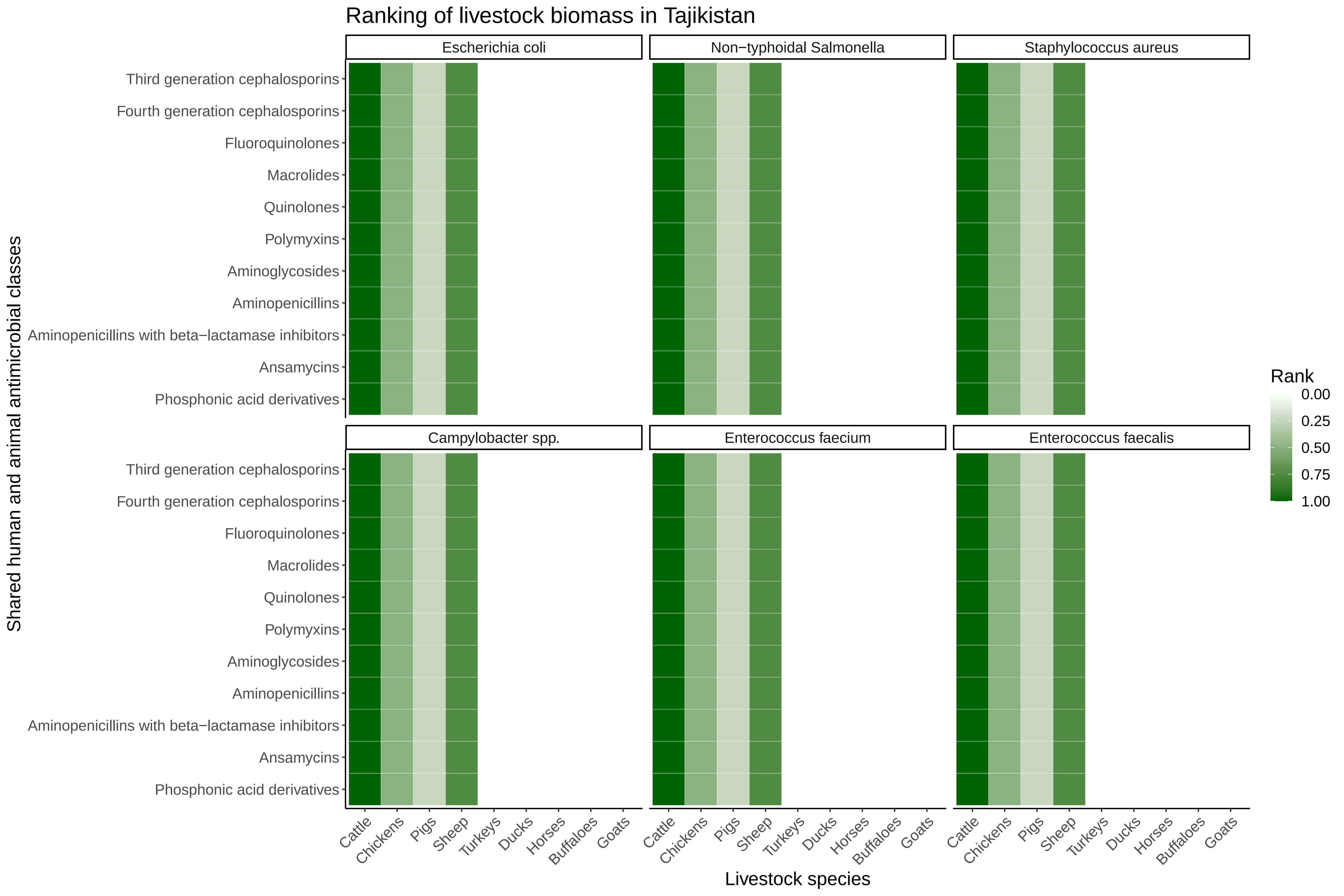

Ranking of livestock biomass in Tajikistan
Escherichia coli
Non−typhoidal Salmonella
Staphylococcus aureus
Campylobacter spp.
Enterococcus faecium
Enterococcus faecalis
Third generation cephalosporins
Fourth generation cephalosporins
Fluoroquinolones
Macrolides
Quinolones
Polymyxins
Aminoglycosides
Aminopenicillins
Aminopenicillins with beta−lactamase inhibitors
Ansamycins
Phosphonic acid derivatives
Shared human and animal antimicrobial classes
Cattle
Chickens
Pigs
Sheep
Turkeys
Ducks
Horses
Buffaloes
Goats
Livestock species
Rank
0.00
0.25
0.50
0.75
1.00

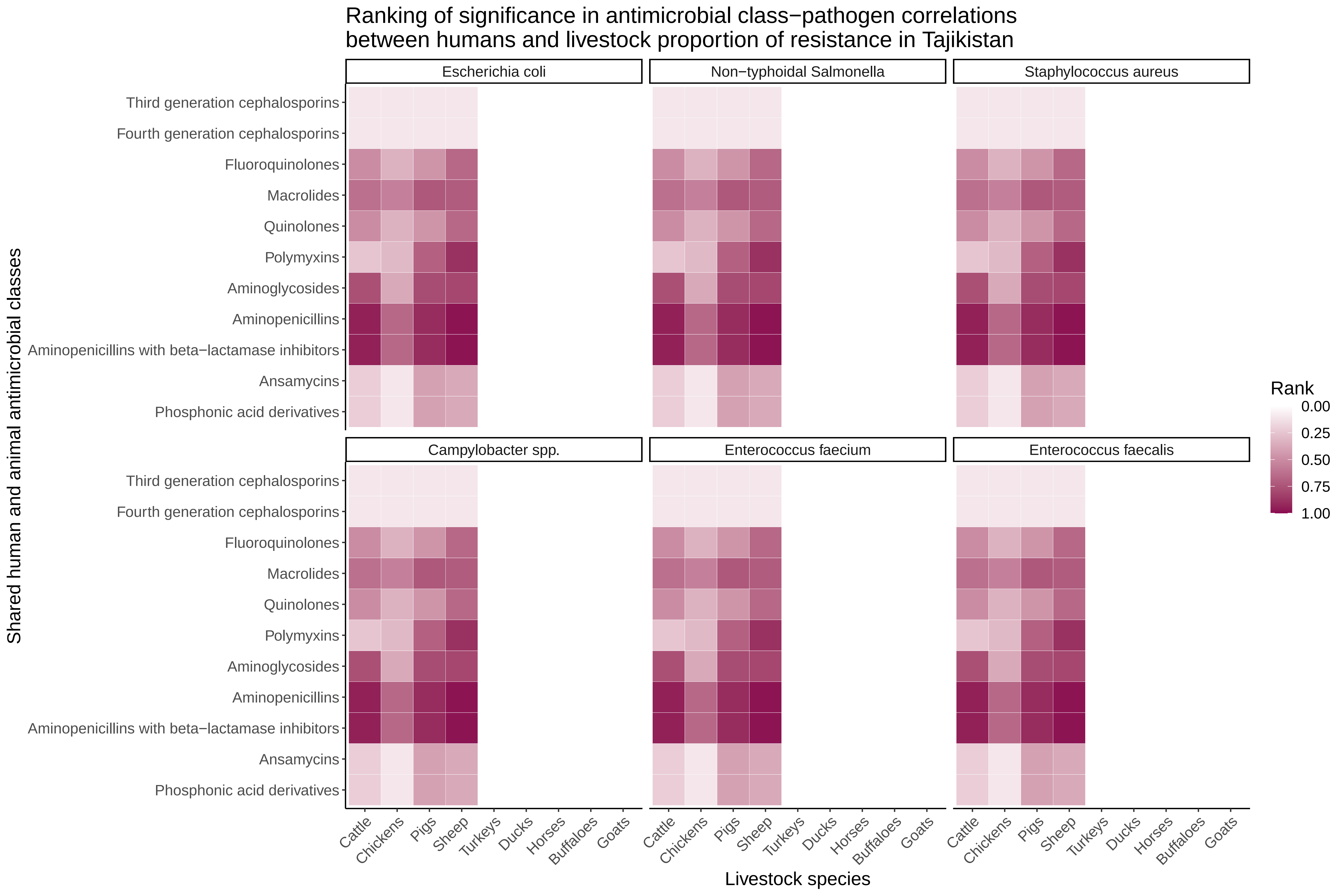
Ranking of significance in antimicrobial class−pathogen correlations
between humans and livestock proportion of resistance in Tajikistan
Escherichia coli
Non−typhoidal Salmonella
Staphylococcus aureus
Campylobacter spp.
Enterococcus faecium
Enterococcus faecalis
Third generation cephalosporins
Fourth generation cephalosporins
Fluoroquinolones
Macrolides
Quinolones
Polymyxins
Aminoglycosides
Aminopenicillins
Aminopenicillins with beta−lactamase inhibitors
Ansamycins
Phosphonic acid derivatives
Shared human and animal antimicrobial classes
Cattle
Chickens
Pigs
Sheep
Turkeys
Ducks
Horses
Buffaloes
Goats
Livestock species
Rank
0.00
0.25
0.50
0.75
1.00

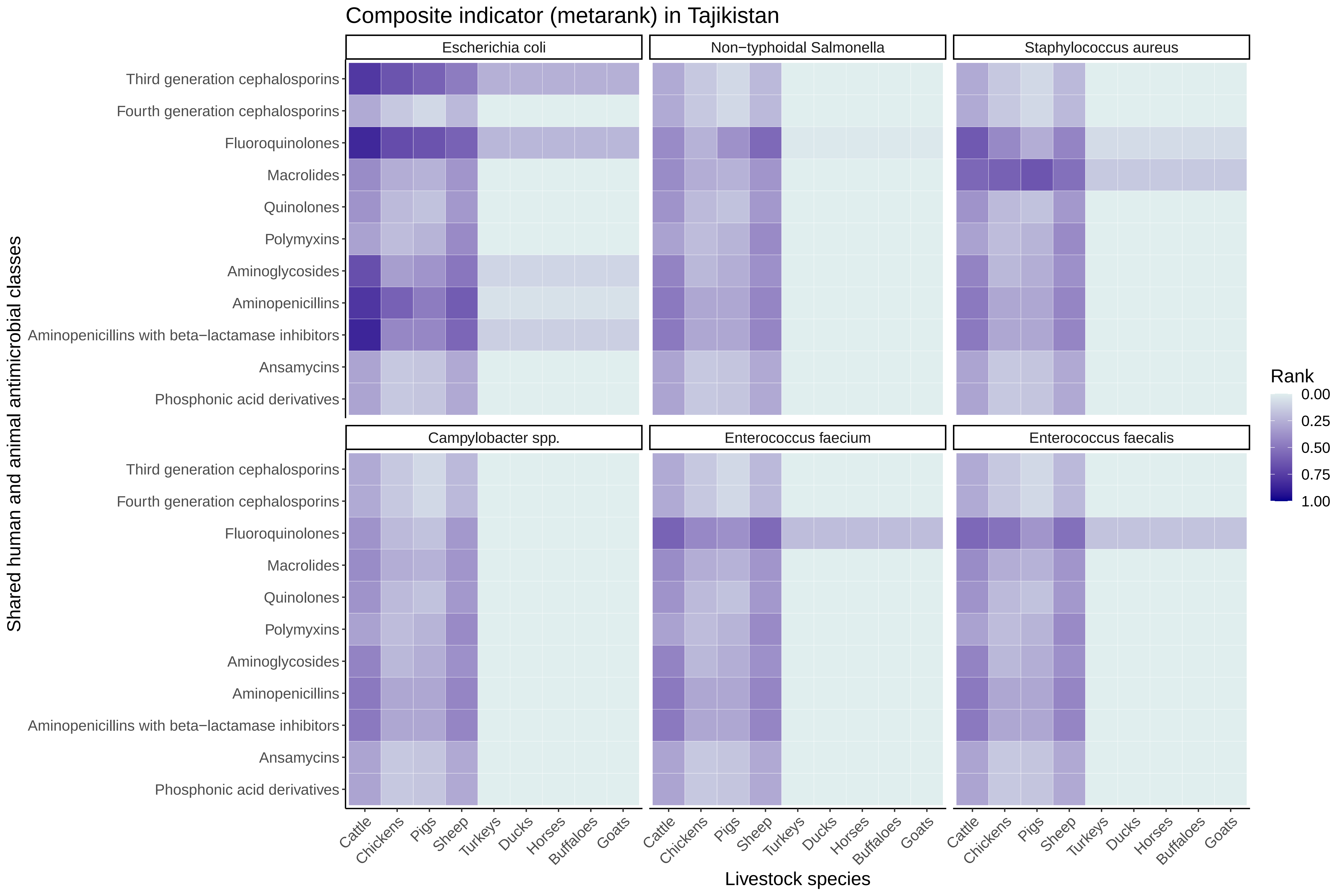
Composite indicator (metarank) in Tajikistan
Escherichia coli
Non−typhoidal Salmonella
Staphylococcus aureus
Campylobacter spp.
Enterococcus faecium
Enterococcus faecalis
Shared human and animal antimicrobial classes
Third generation cephalosporins
Fourth generation cephalosporins
Fluoroquinolones
Macrolides
Quinolones
Polymyxins
Aminoglycosides
Aminopenicillins
Aminopenicillins with beta−lactamase inhibitors
Ansamycins
Phosphonic acid derivatives
Cattle
Chickens
Pigs
Sheep
Turkeys
Ducks
Horses
Buffaloes
Goats
Livestock species
Rank
0.00
0.25
0.50
0.75
1.00

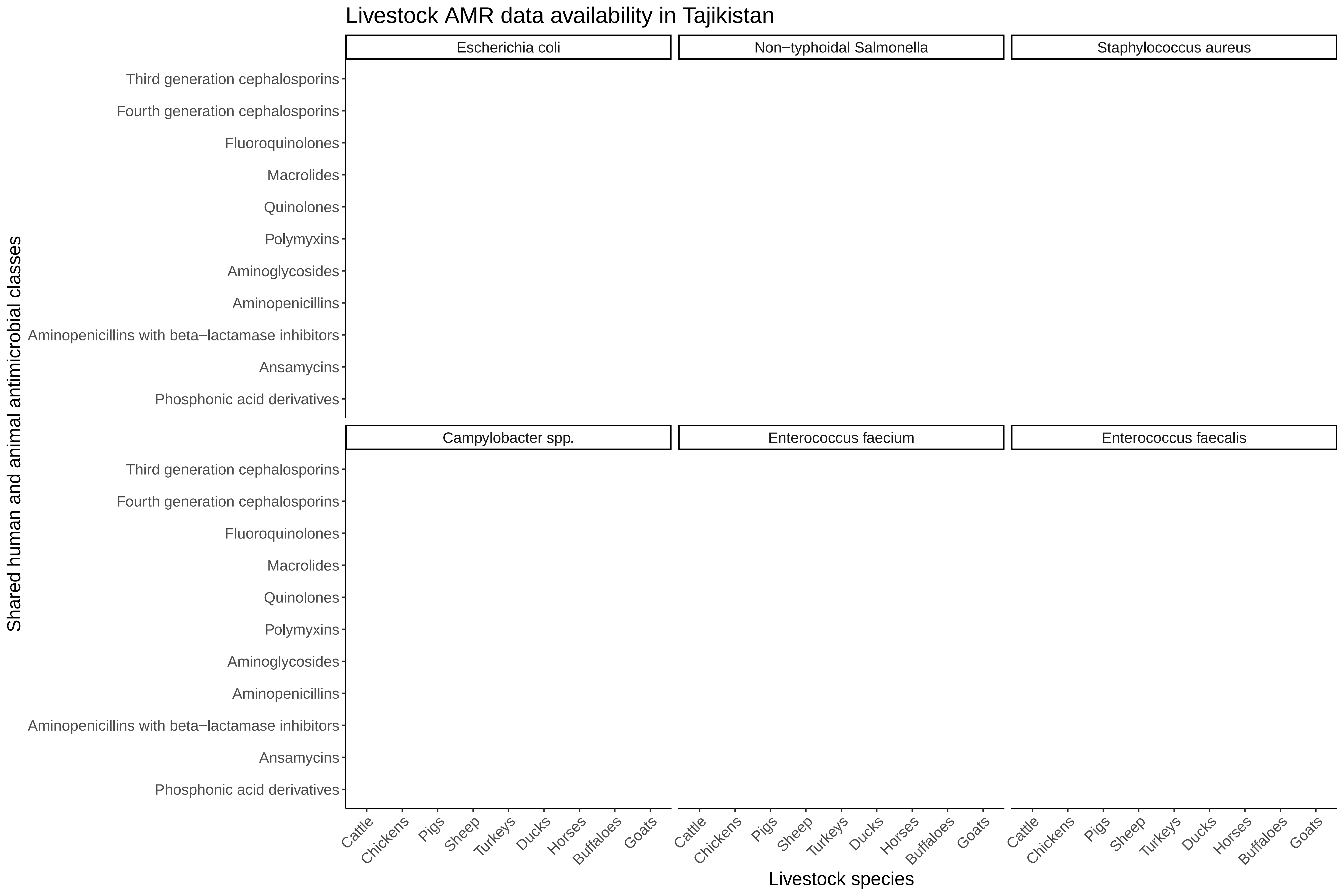

Livestock AMR data availability in Tajikistan
Escherichia coli
Non−typhoidal Salmonella
Staphylococcus aureus
Campylobacter spp.
Enterococcus faecium
Enterococcus faecalis
Shared human and animal antimicrobial classes
Third generation cephalosporins
Fourth generation cephalosporins
Fluoroquinolones
Macrolides
Quinolones
Polymyxins
Aminoglycosides
Aminopenicillins
Aminopenicillins with beta−lactamase inhibitors
Ansamycins
Phosphonic acid derivatives
Cattle
Chickens
Pigs
Sheep
Turkeys
Ducks
Horses
Buffaloes
Goats
Livestock species

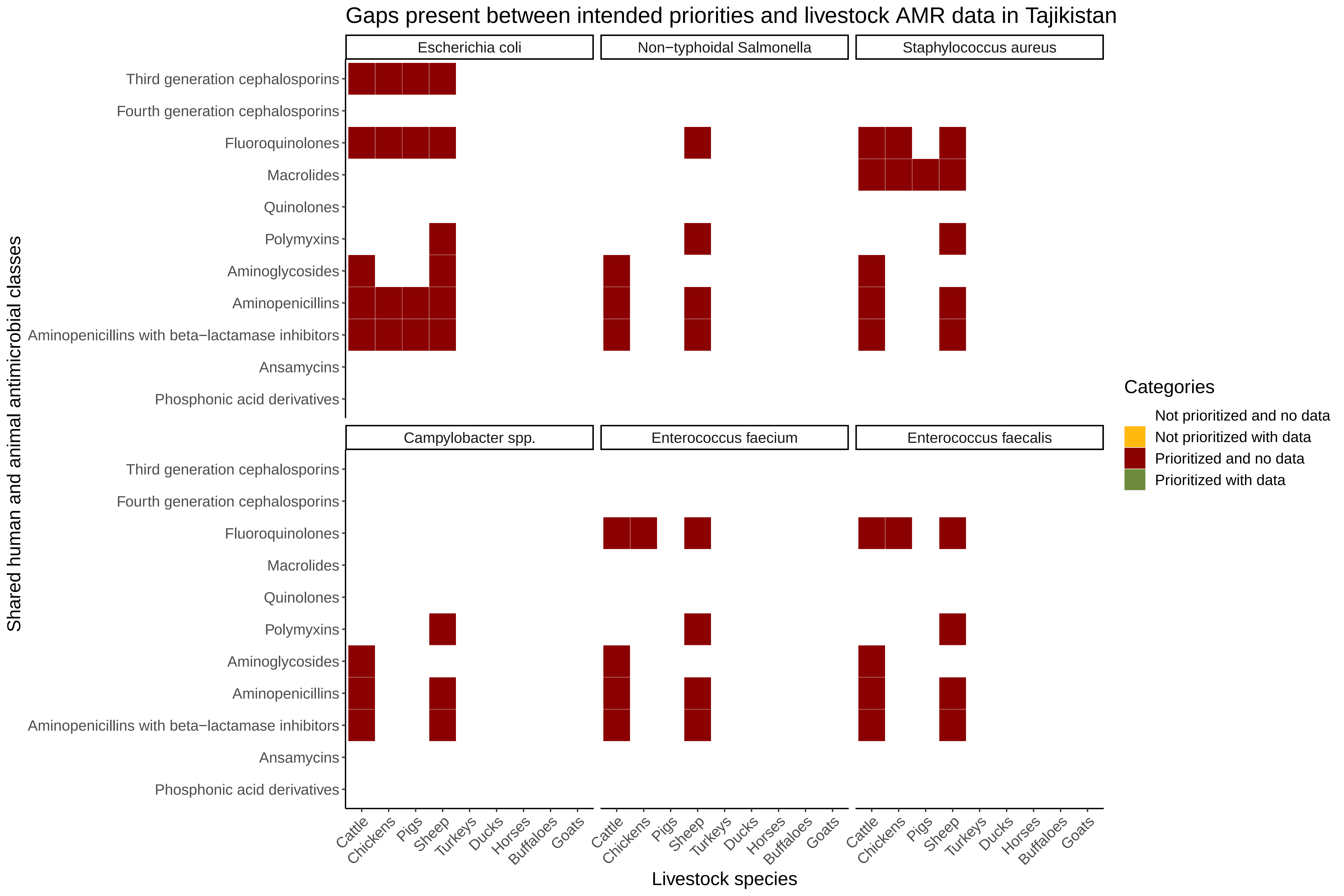

Gaps present between intended priorities and livestock AMR data in Tajikistan
Escherichia coli
Non−typhoidal Salmonella
Staphylococcus aureus
Campylobacter spp.
Enterococcus faecium
Enterococcus faecalis
Third generation cephalosporins
Fourth generation cephalosporins
Fluoroquinolones
Macrolides
Quinolones
Polymyxins
Aminoglycosides
Aminopenicillins
Aminopenicillins with beta−lactamase inhibitors
Ansamycins
Phosphonic acid derivatives
Shared human and animal antimicrobial classes
Cattle
Chickens
Pigs
Sheep
Turkeys
Ducks
Horses
Buffaloes
Goats
Livestock species
Categories
Not prioritized and no data
Not prioritized with data
Prioritized and no data
Prioritized with data

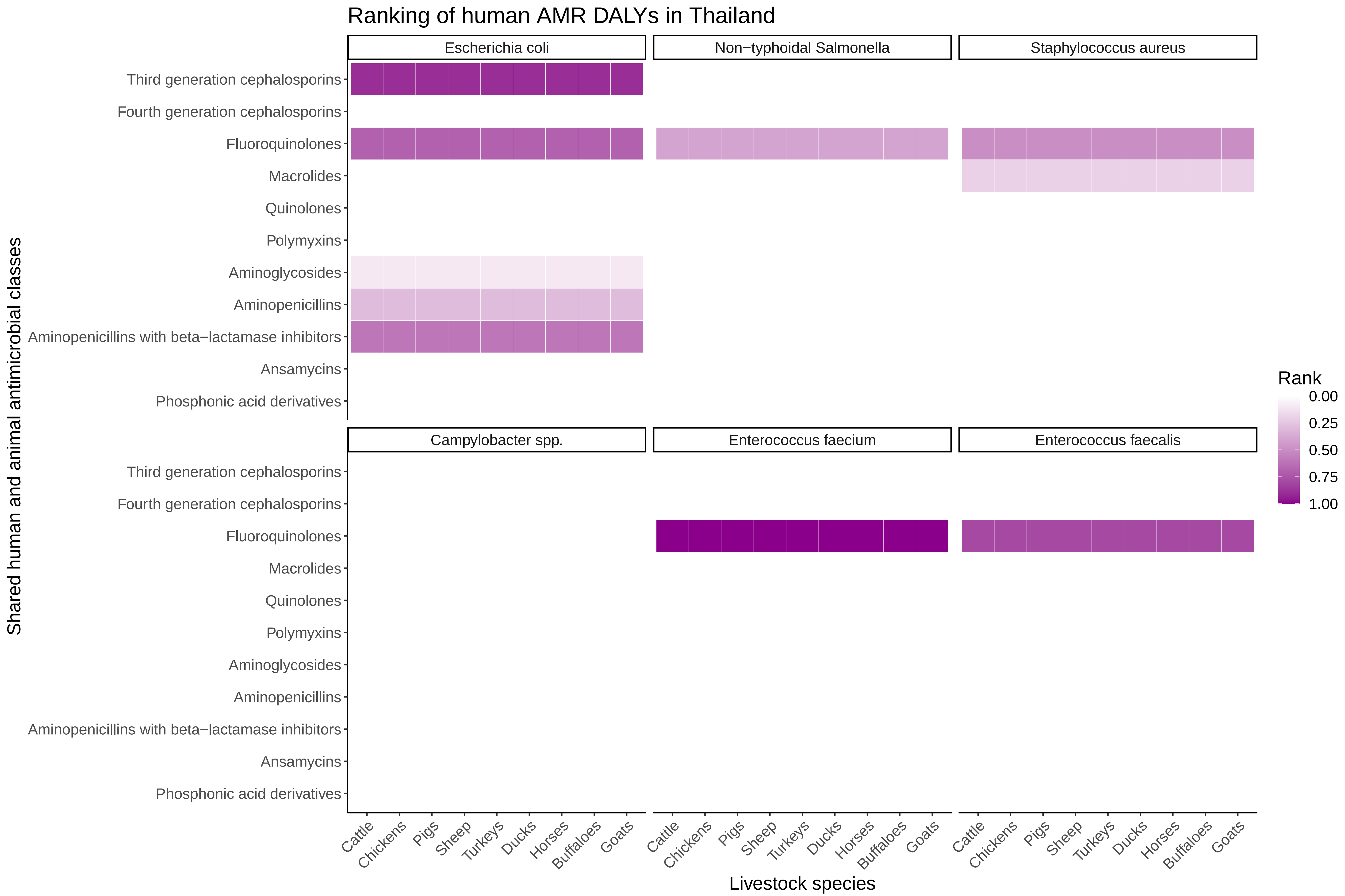
Ranking of human AMR DALYs in Thailand
Escherichia coli
Non−typhoidal Salmonella
Staphylococcus aureus
Campylobacter spp.
Enterococcus faecium
Enterococcus faecalis
Third generation cephalosporins
Fourth generation cephalosporins
Fluoroquinolones
Macrolides
Quinolones
Polymyxins
Aminoglycosides
Aminopenicillins
Aminopenicillins with beta−lactamase inhibitors
Ansamycins
Phosphonic acid derivatives
Shared human and animal antimicrobial classes
Cattle
Chickens
Pigs
Sheep
Turkeys
Ducks
Horses
Buffaloes
Goats
Livestock species
Rank
0.00
0.25
0.50
0.75
1.00

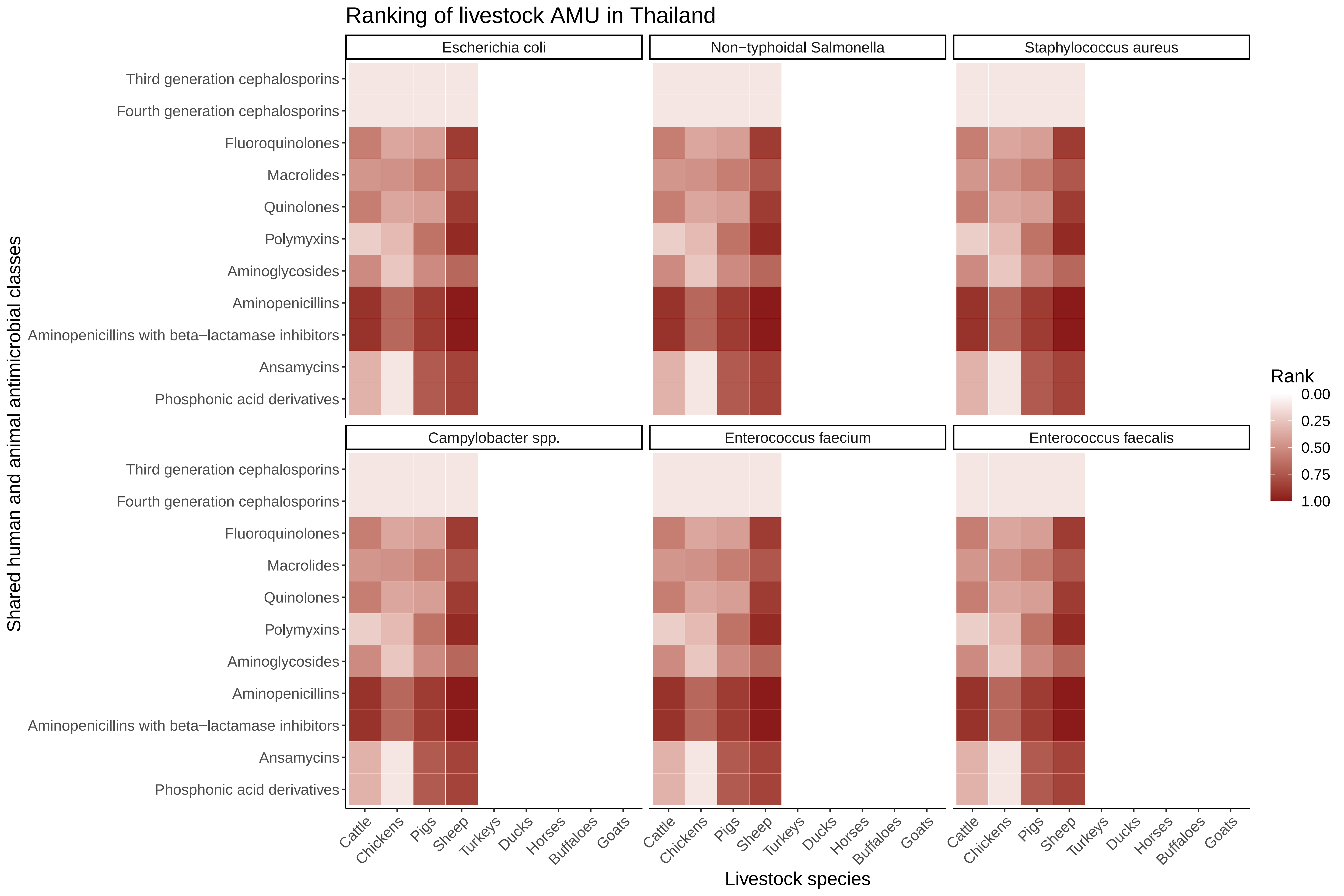

Ranking of livestock AMU in Thailand
Escherichia coli
Non−typhoidal Salmonella
Staphylococcus aureus
Campylobacter spp.
Enterococcus faecium
Enterococcus faecalis
Shared human and animal antimicrobial classes
Third generation cephalosporins
Fourth generation cephalosporins
Fluoroquinolones
Macrolides
Quinolones
Polymyxins
Aminoglycosides
Aminopenicillins
Aminopenicillins with beta−lactamase inhibitors
Ansamycins
Phosphonic acid derivatives
Livestock species
Cattle
Chickens
Pigs
Sheep
Turkeys
Ducks
Horses
Buffaloes
Goats
Rank
0.00
0.25
0.50
0.75
1.00

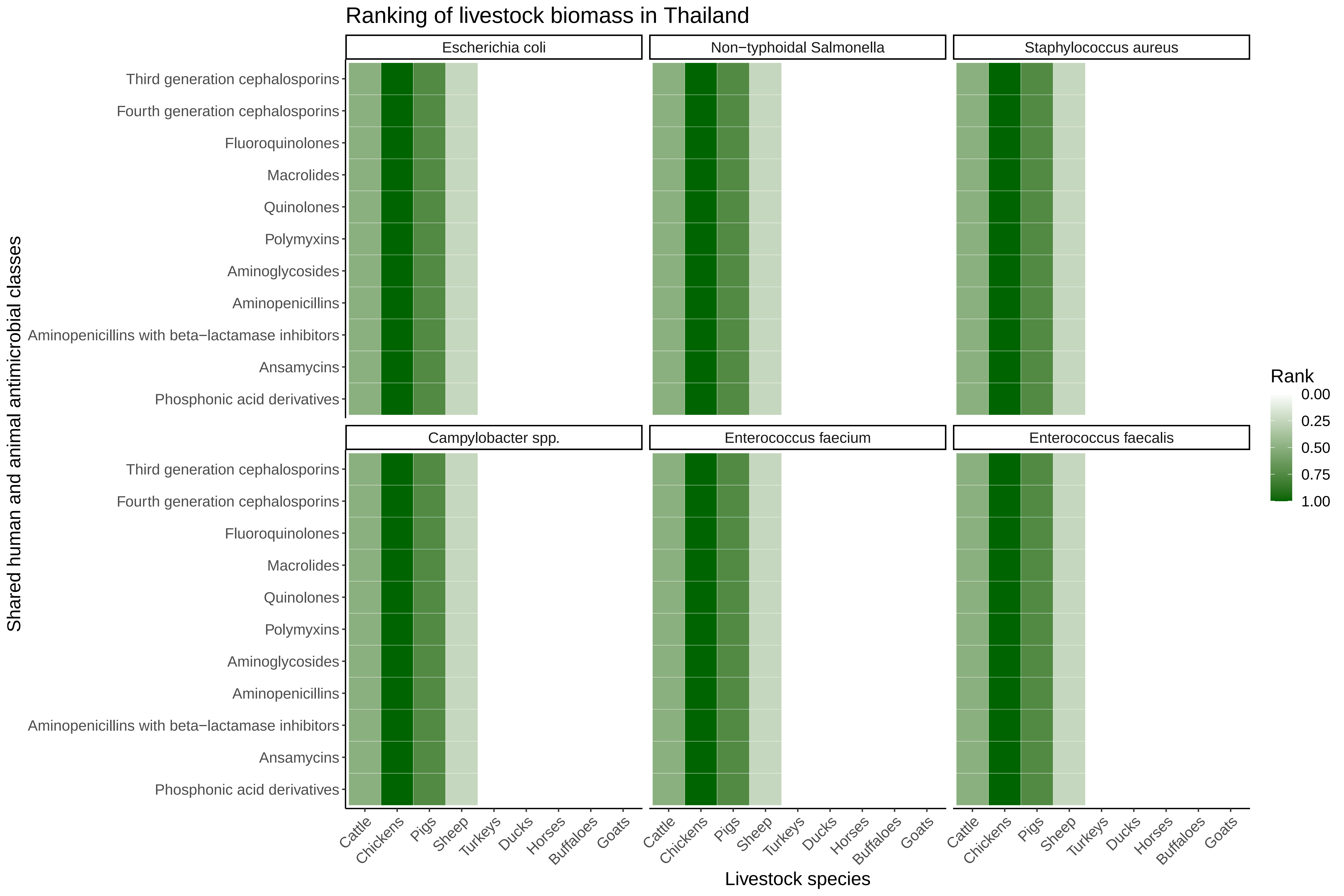

Ranking of livestock biomass in Thailand
Escherichia coli
Non−typhoidal Salmonella
Staphylococcus aureus
Campylobacter spp.
Enterococcus faecium
Enterococcus faecalis
Third generation cephalosporins
Fourth generation cephalosporins
Fluoroquinolones
Macrolides
Quinolones
Polymyxins
Aminoglycosides
Aminopenicillins
Aminopenicillins with beta−lactamase inhibitors
Ansamycins
Phosphonic acid derivatives
Shared human and animal antimicrobial classes
Cattle
Chickens
Pigs
Sheep
Turkeys
Ducks
Horses
Buffaloes
Goats
Livestock species
Rank
0.00
0.25
0.50
0.75
1.00

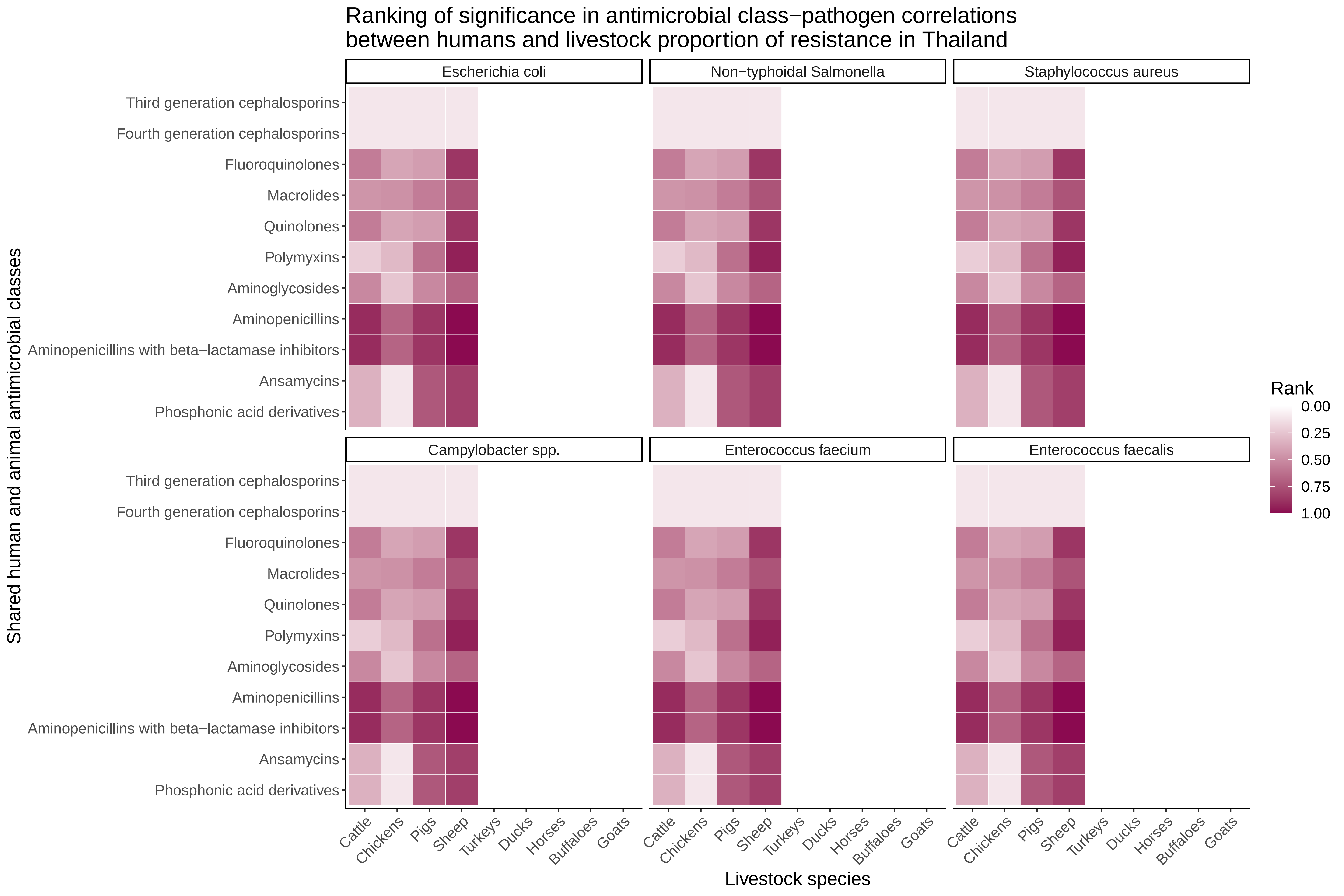
Ranking of significance in antimicrobial class−pathogen correlations between humans and livestock proportion of resistance in Thailand
Escherichia coli
Non−typhoidal Salmonella
Staphylococcus aureus
Campylobacter spp.
Enterococcus faecium
Enterococcus faecalis
Third generation cephalosporins
Fourth generation cephalosporins
Fluoroquinolones
Macrolides
Quinolones
Polymyxins
Aminoglycosides
Aminopenicillins
Aminopenicillins with beta−lactamase inhibitors
Ansamycins
Phosphonic acid derivatives
Shared human and animal antimicrobial classes
Cattle
Chickens
Pigs
Sheep
Turkeys
Ducks
Horses
Buffaloes
Goats
Livestock species
Rank
0.00
0.25
0.50
0.75
1.00

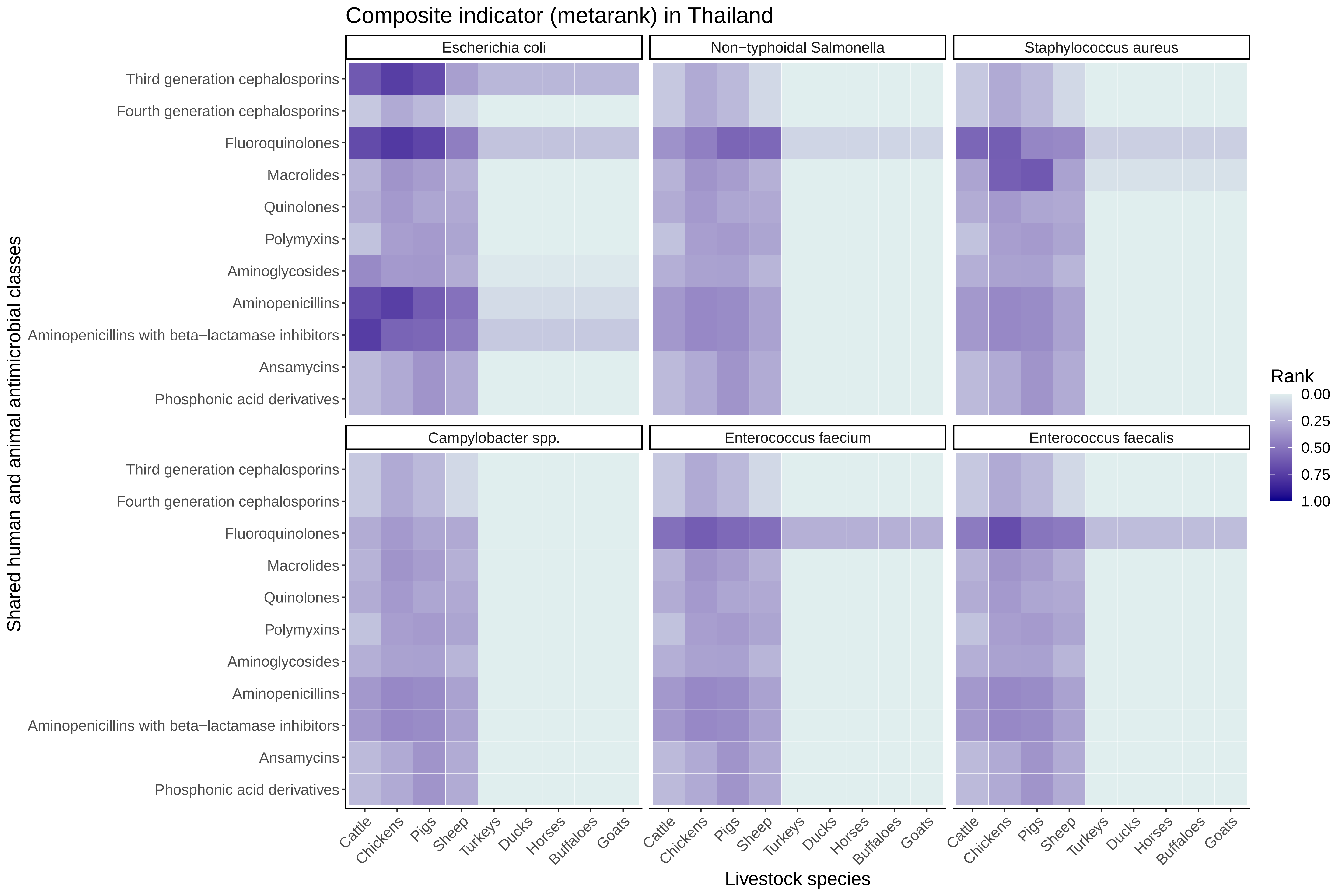
Composite indicator (metarank) in Thailand
Escherichia coli
Non−typhoidal Salmonella
Staphylococcus aureus
Campylobacter spp.
Enterococcus faecium
Enterococcus faecalis
Shared human and animal antimicrobial classes
Third generation cephalosporins
Fourth generation cephalosporins
Fluoroquinolones
Macrolides
Quinolones
Polymyxins
Aminoglycosides
Aminopenicillins
Aminopenicillins with beta−lactamase inhibitors
Ansamycins
Phosphonic acid derivatives
Cattle
Chickens
Pigs
Sheep
Turkeys
Ducks
Horses
Buffaloes
Goats
Livestock species
Rank
0.00
0.25
0.50
0.75
1.00

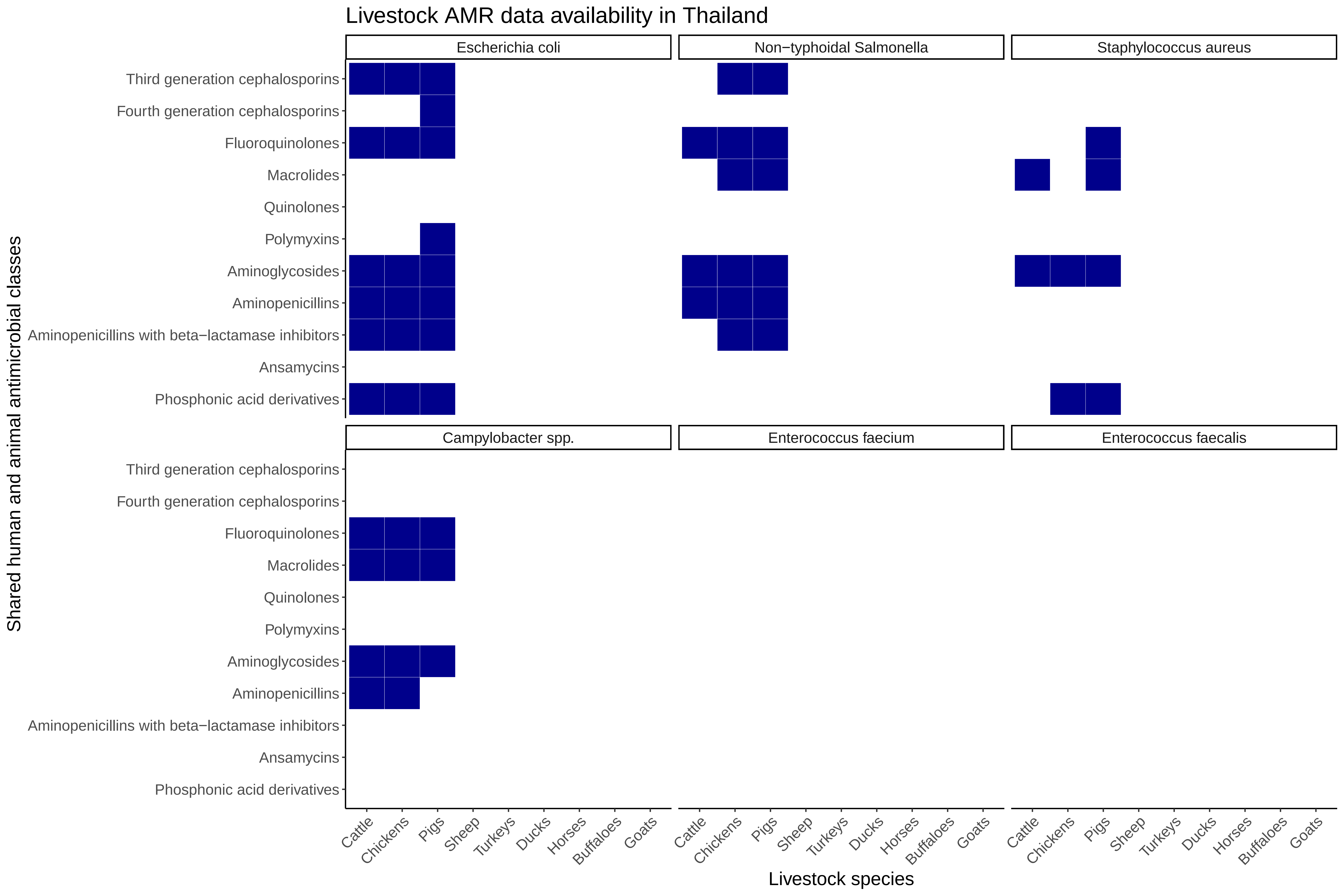

Livestock AMR data availability in Thailand
Escherichia coli
Non−typhoidal Salmonella
Staphylococcus aureus
Campylobacter spp.
Enterococcus faecium
Enterococcus faecalis
Shared human and animal antimicrobial classes
Third generation cephalosporins
Fourth generation cephalosporins
Fluoroquinolones
Macrolides
Quinolones
Polymyxins
Aminoglycosides
Aminopenicillins
Aminopenicillins with beta−lactamase inhibitors
Ansamycins
Phosphonic acid derivatives
Cattle
Chickens
Pigs
Sheep
Turkeys
Ducks
Horses
Buffaloes
Goats
Livestock species

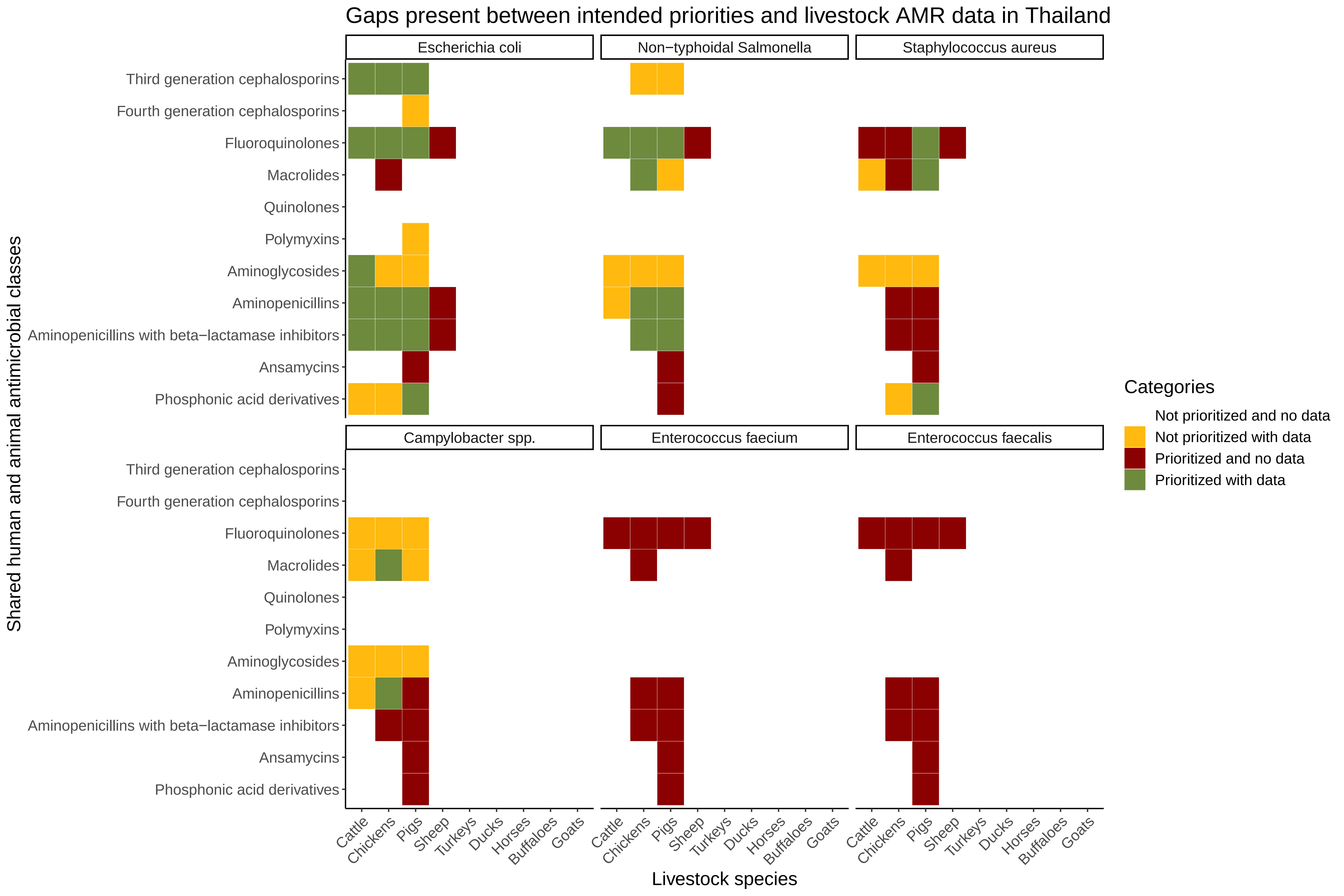
Gaps present between intended priorities and livestock AMR data in Thailand
Escherichia coli
Non−typhoidal Salmonella
Staphylococcus aureus
Campylobacter spp.
Enterococcus faecium
Enterococcus faecalis
Third generation cephalosporins
Fourth generation cephalosporins
Fluoroquinolones
Macrolides
Quinolones
Polymyxins
Aminoglycosides
Aminopenicillins
Aminopenicillins with beta−lactamase inhibitors
Ansamycins
Phosphonic acid derivatives
Shared human and animal antimicrobial classes
Cattle
Chickens
Pigs
Sheep
Turkeys
Ducks
Horses
Buffaloes
Goats
Livestock species
Categories
Not prioritized and no data
Not prioritized with data
Prioritized and no data
Prioritized with data

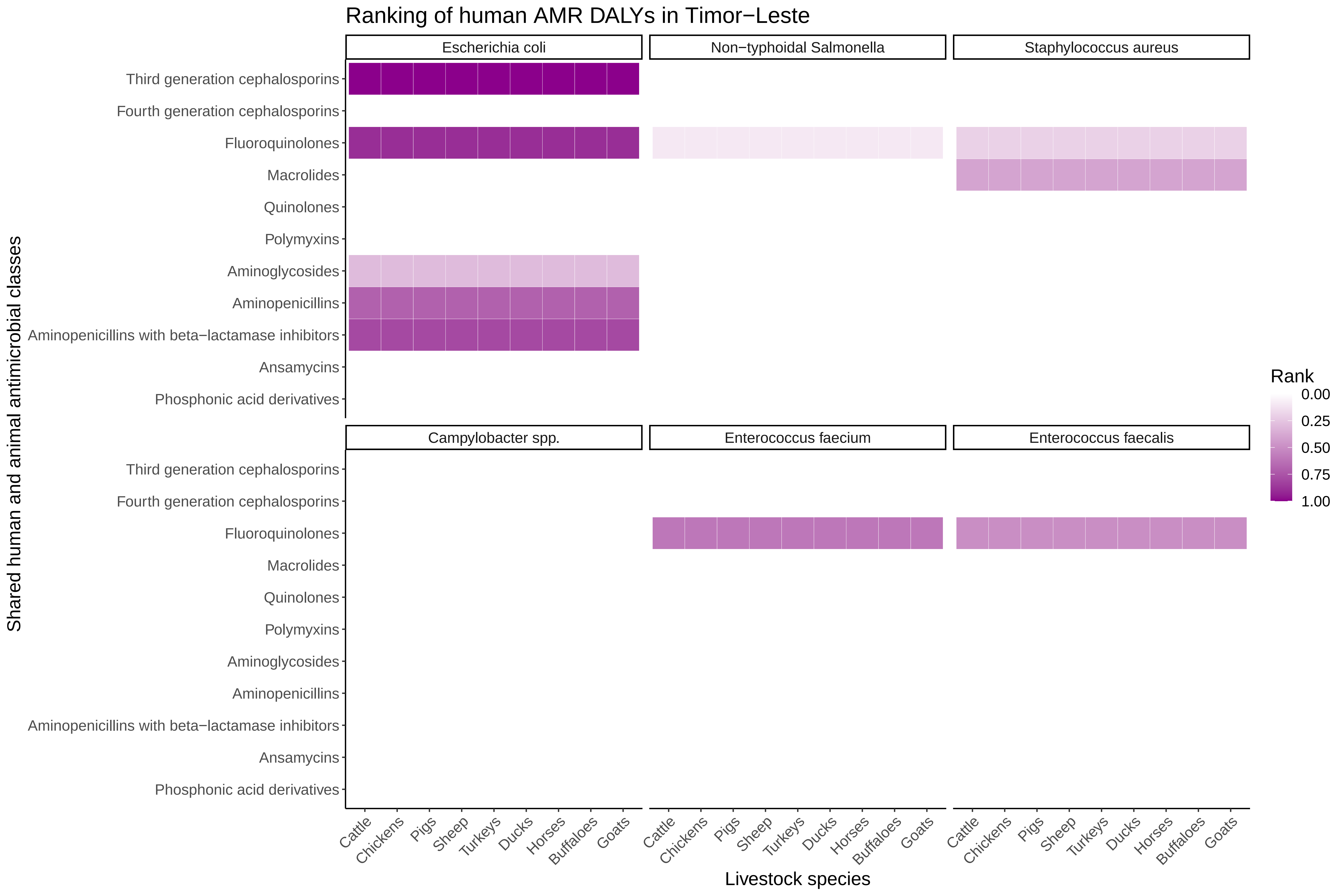

Ranking of human AMR DALYs in Timor−Leste
Escherichia coli
Non−typhoidal Salmonella
Staphylococcus aureus
Campylobacter spp.
Enterococcus faecium
Enterococcus faecalis
Third generation cephalosporins
Fourth generation cephalosporins
Fluoroquinolones
Macrolides
Quinolones
Polymyxins
Aminoglycosides
Aminopenicillins
Aminopenicillins with beta−lactamase inhibitors
Ansamycins
Phosphonic acid derivatives
Shared human and animal antimicrobial classes
Cattle
Chickens
Pigs
Sheep
Turkeys
Ducks
Horses
Buffaloes
Goats
Livestock species
Rank
0.00
0.25
0.50
0.75
1.00

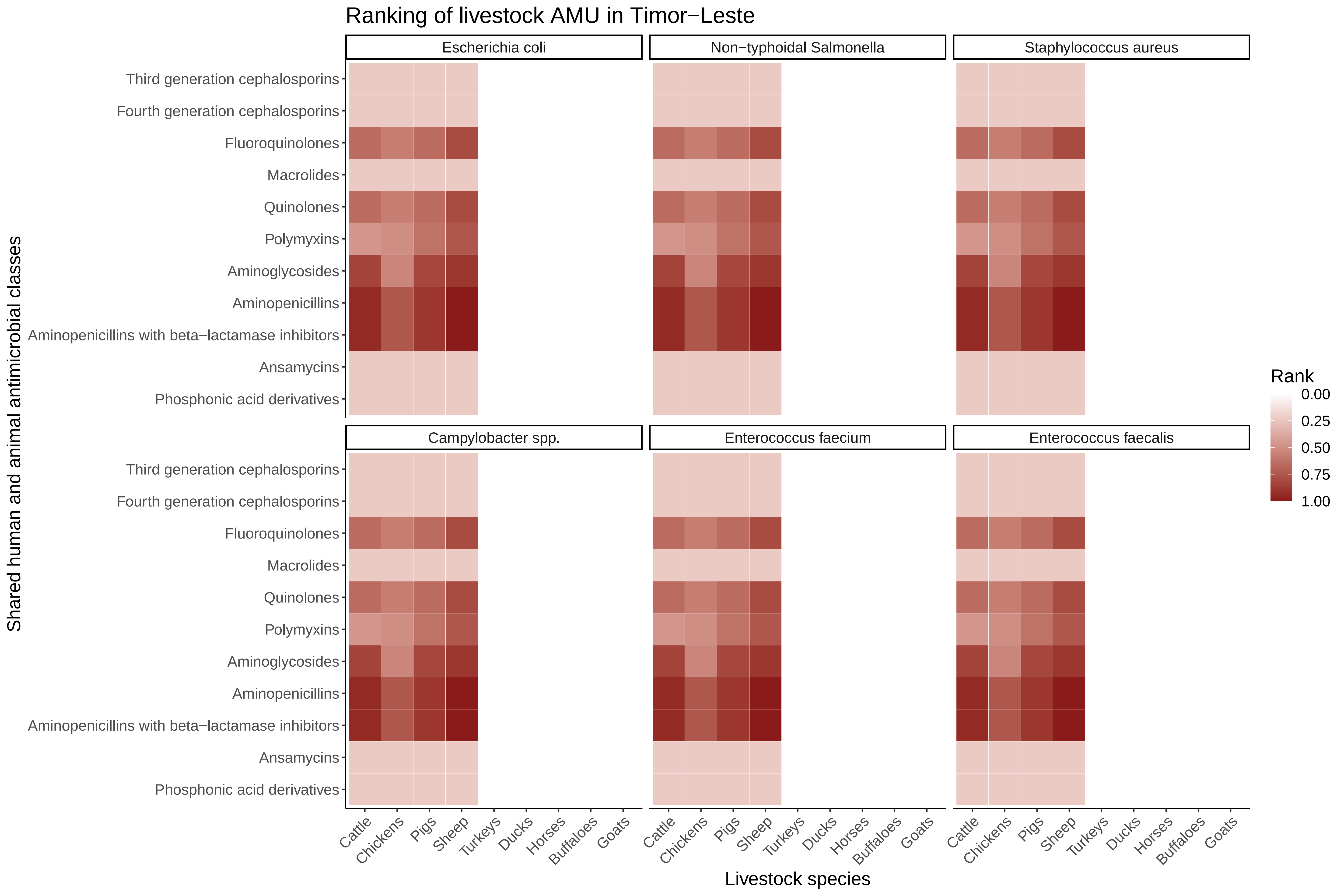
Ranking of livestock AMU in Timor−Leste
Escherichia coli
Non−typhoidal Salmonella
Staphylococcus aureus
Campylobacter spp.
Enterococcus faecium
Enterococcus faecalis
Third generation cephalosporins
Fourth generation cephalosporins
Fluoroquinolones
Macrolides
Quinolones
Polymyxins
Aminoglycosides
Aminopenicillins
Aminopenicillins with beta−lactamase inhibitors
Ansamycins
Phosphonic acid derivatives
Shared human and animal antimicrobial classes
Cattle
Chickens
Pigs
Sheep
Turkeys
Ducks
Horses
Buffaloes
Goats
Livestock species
Rank
0.00
0.25
0.50
0.75
1.00

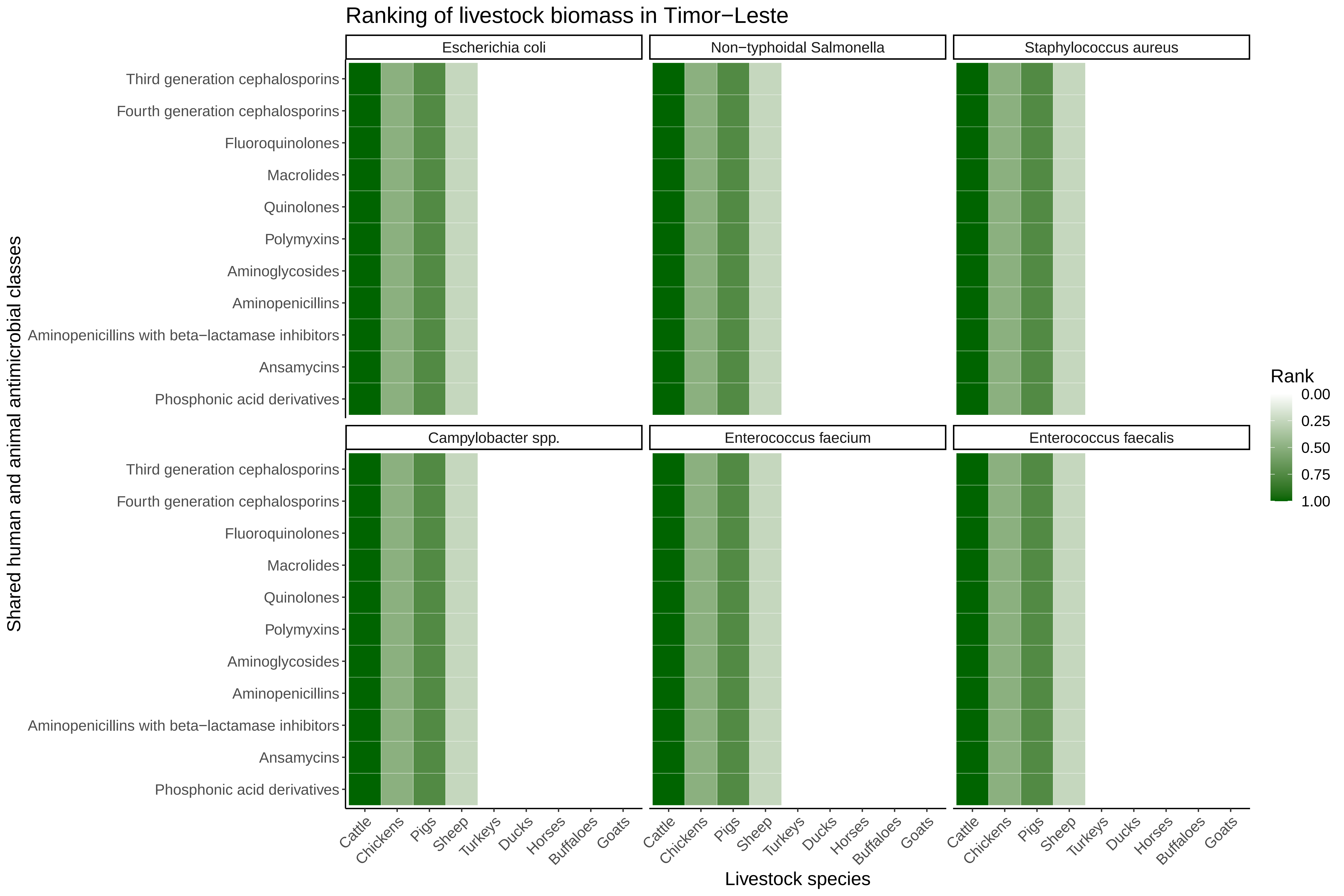
Ranking of livestock biomass in Timor−Leste
Escherichia coli
Non−typhoidal Salmonella
Staphylococcus aureus
Campylobacter spp.
Enterococcus faecium
Enterococcus faecalis
Third generation cephalosporins
Fourth generation cephalosporins
Fluoroquinolones
Macrolides
Quinolones
Polymyxins
Aminoglycosides
Aminopenicillins
Aminopenicillins with beta−lactamase inhibitors
Ansamycins
Phosphonic acid derivatives
Shared human and animal antimicrobial classes
Cattle
Chickens
Pigs
Sheep
Turkeys
Ducks
Horses
Buffaloes
Goats
Livestock species
Rank
0.00
0.25
0.50
0.75
1.00

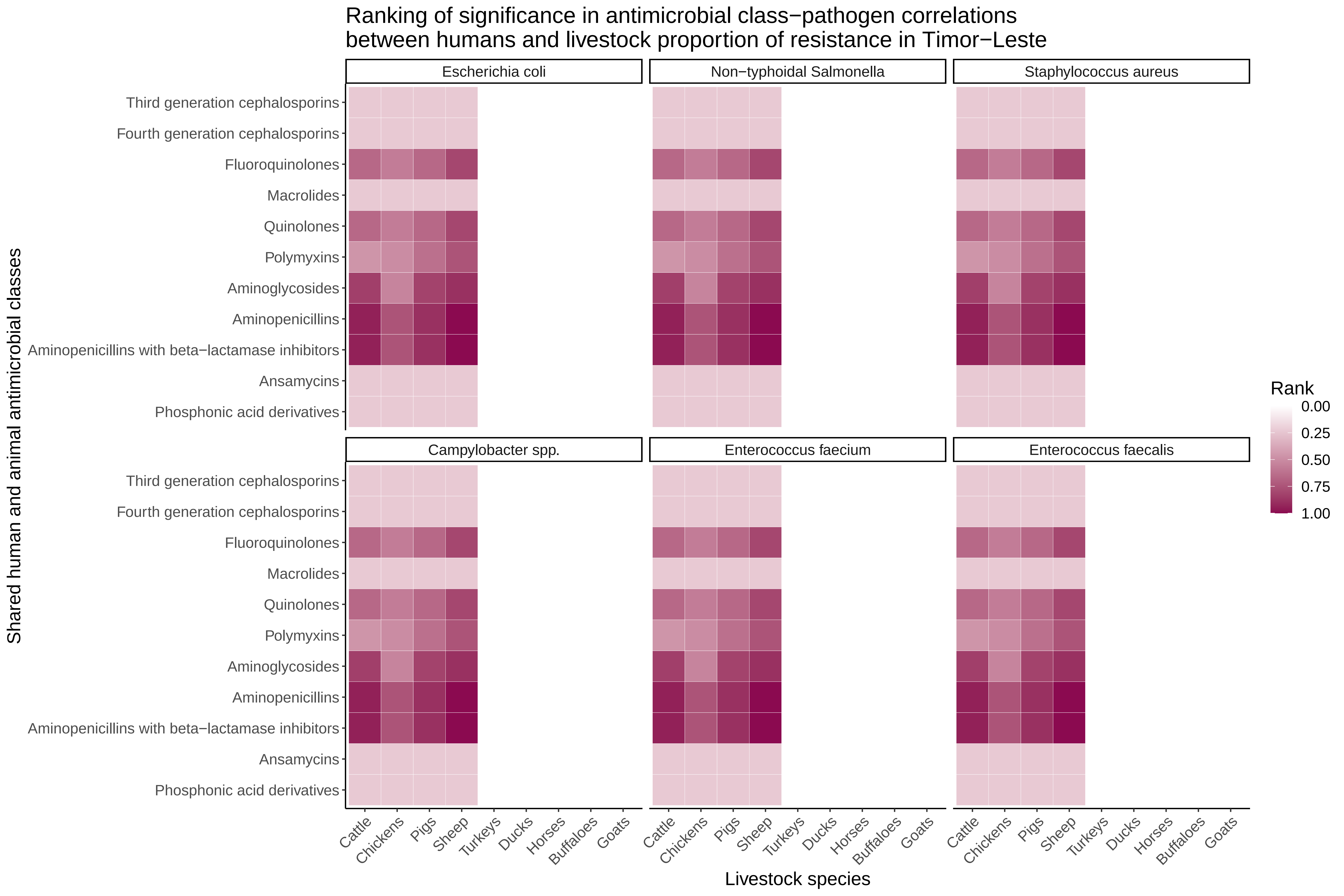
Ranking of significance in antimicrobial class−pathogen correlations
between humans and livestock proportion of resistance in Timor−Leste
Escherichia coli
Non−typhoidal Salmonella
Staphylococcus aureus
Campylobacter spp.
Enterococcus faecium
Enterococcus faecalis
Third generation cephalosporins
Fourth generation cephalosporins
Fluoroquinolones
Macrolides
Quinolones
Polymyxins
Aminoglycosides
Aminopenicillins
Aminopenicillins with beta−lactamase inhibitors
Ansamycins
Phosphonic acid derivatives
Shared human and animal antimicrobial classes
Cattle
Chickens
Pigs
Sheep
Turkeys
Ducks
Horses
Buffaloes
Goats
Livestock species
Rank
0.00
0.25
0.50
0.75
1.00

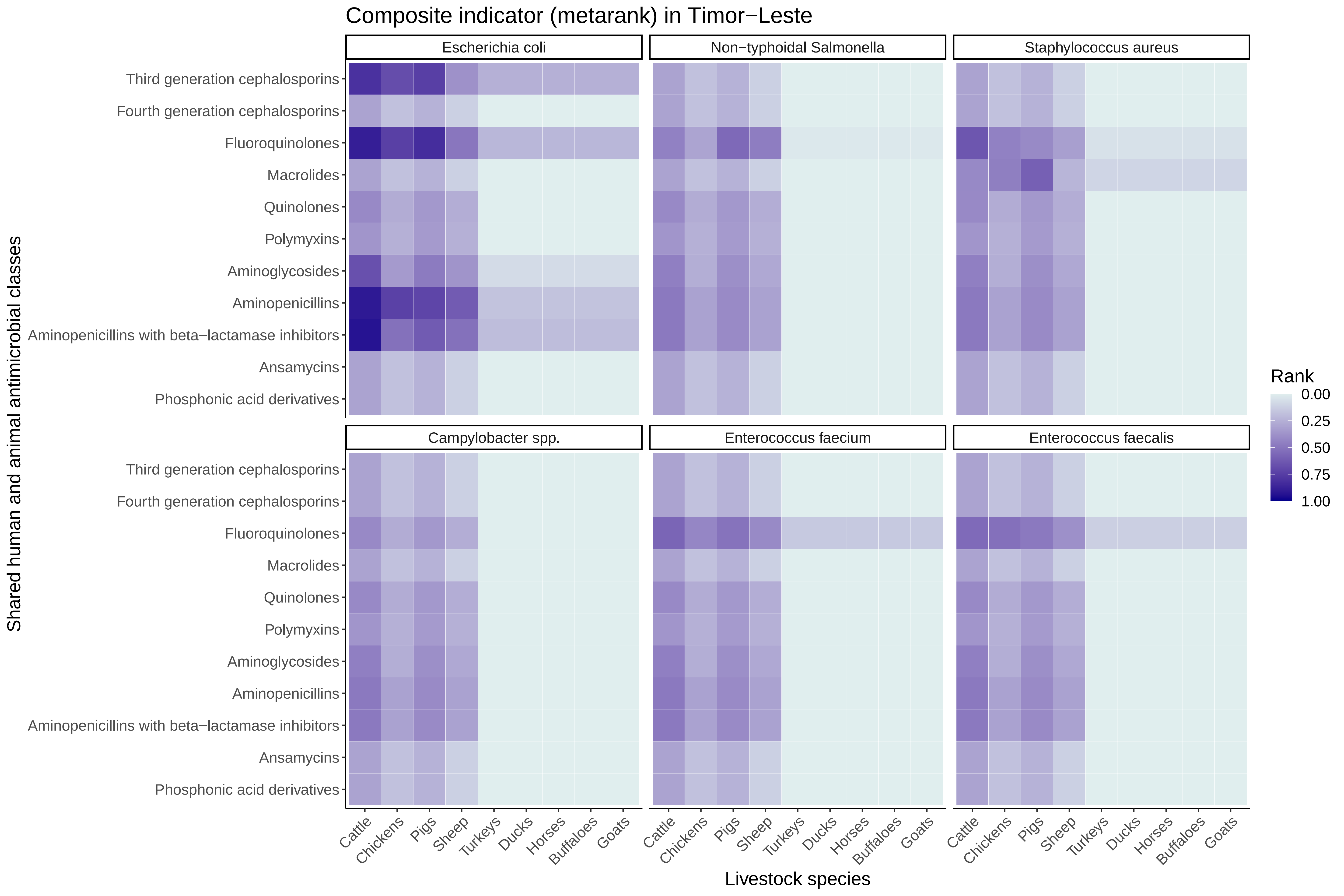

Composite indicator (metarank) in Timor−Leste
Escherichia coli
Non−typhoidal Salmonella
Staphylococcus aureus
Campylobacter spp.
Enterococcus faecium
Enterococcus faecalis
Shared human and animal antimicrobial classes
Third generation cephalosporins
Fourth generation cephalosporins
Fluoroquinolones
Macrolides
Quinolones
Polymyxins
Aminoglycosides
Aminopenicillins
Aminopenicillins with beta−lactamase inhibitors
Ansamycins
Phosphonic acid derivatives
Cattle
Chickens
Pigs
Sheep
Turkeys
Ducks
Horses
Buffaloes
Goats
Livestock species
Rank
0.00
0.25
0.50
0.75
1.00

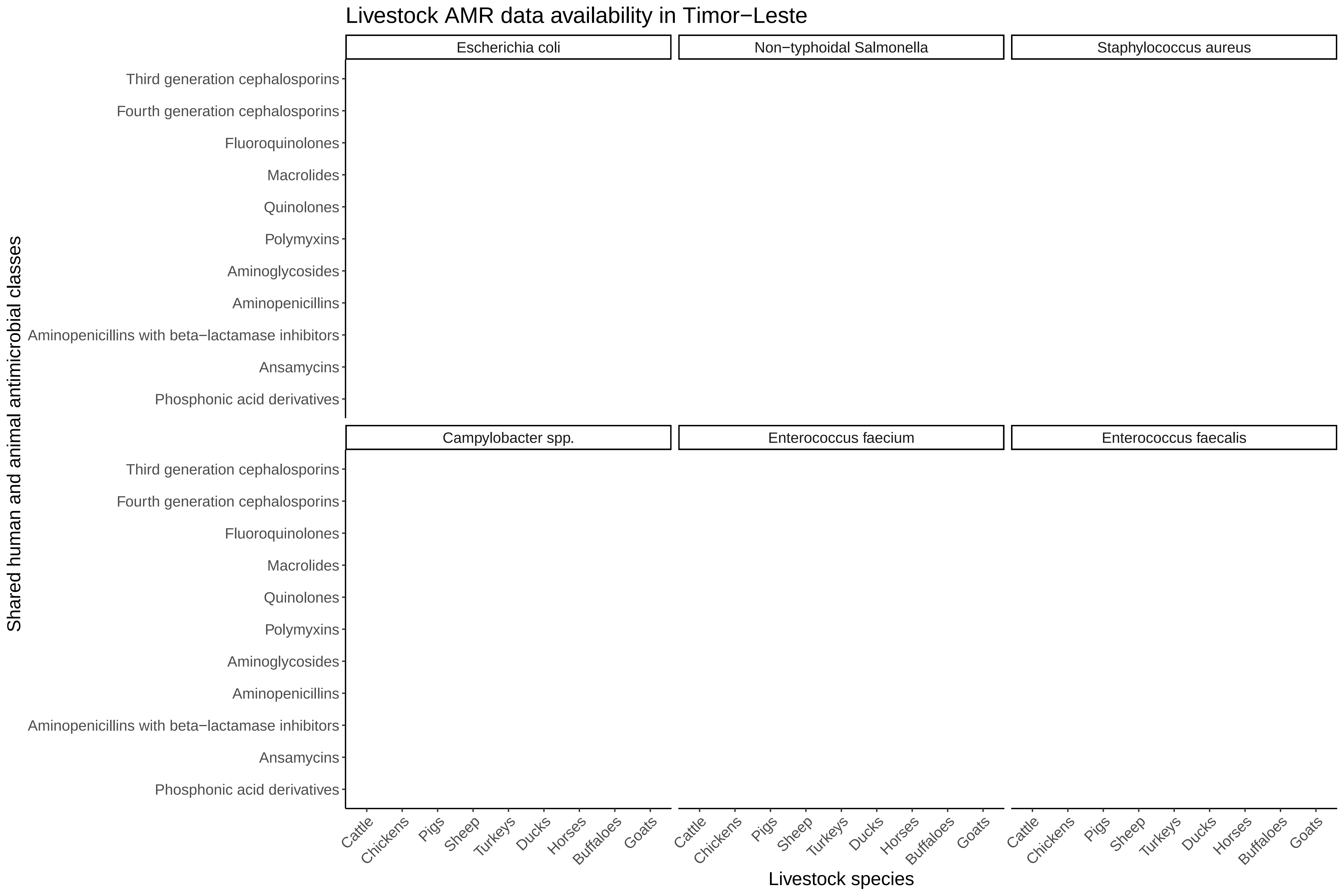
Livestock AMR data availability in Timor−Leste
Escherichia coli
Non−typhoidal Salmonella
Staphylococcus aureus
Campylobacter spp.
Enterococcus faecium
Enterococcus faecalis
Third generation cephalosporins
Fourth generation cephalosporins
Fluoroquinolones
Macrolides
Quinolones
Polymyxins
Aminoglycosides
Aminopenicillins
Aminopenicillins with beta−lactamase inhibitors
Ansamycins
Phosphonic acid derivatives
Shared human and animal antimicrobial classes
Cattle
Chickens
Pigs
Sheep
Turkeys
Ducks
Horses
Buffaloes
Goats
Livestock species

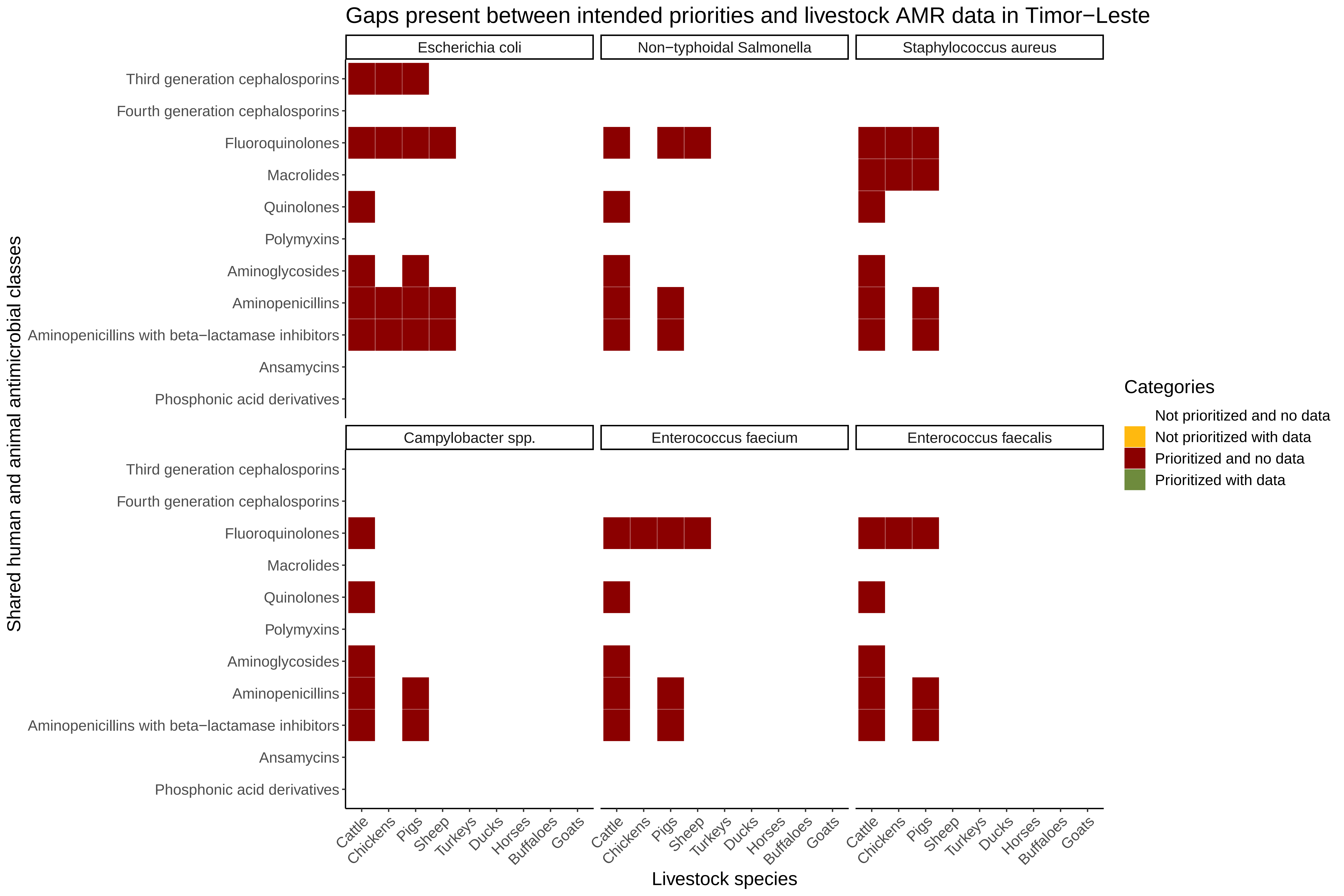
Gaps present between intended priorities and livestock AMR data in Timor−Leste
Escherichia coli
Non−typhoidal Salmonella
Staphylococcus aureus
Campylobacter spp.
Enterococcus faecium
Enterococcus faecalis
Third generation cephalosporins
Fourth generation cephalosporins
Fluoroquinolones
Macrolides
Quinolones
Polymyxins
Aminoglycosides
Aminopenicillins
Aminopenicillins with beta−lactamase inhibitors
Ansamycins
Phosphonic acid derivatives
Shared human and animal antimicrobial classes
Cattle
Chickens
Pigs
Sheep
Turkeys
Ducks
Horses
Buffaloes
Goats
Livestock species
Categories
Not prioritized and no data
Not prioritized with data
Prioritized and no data
Prioritized with data

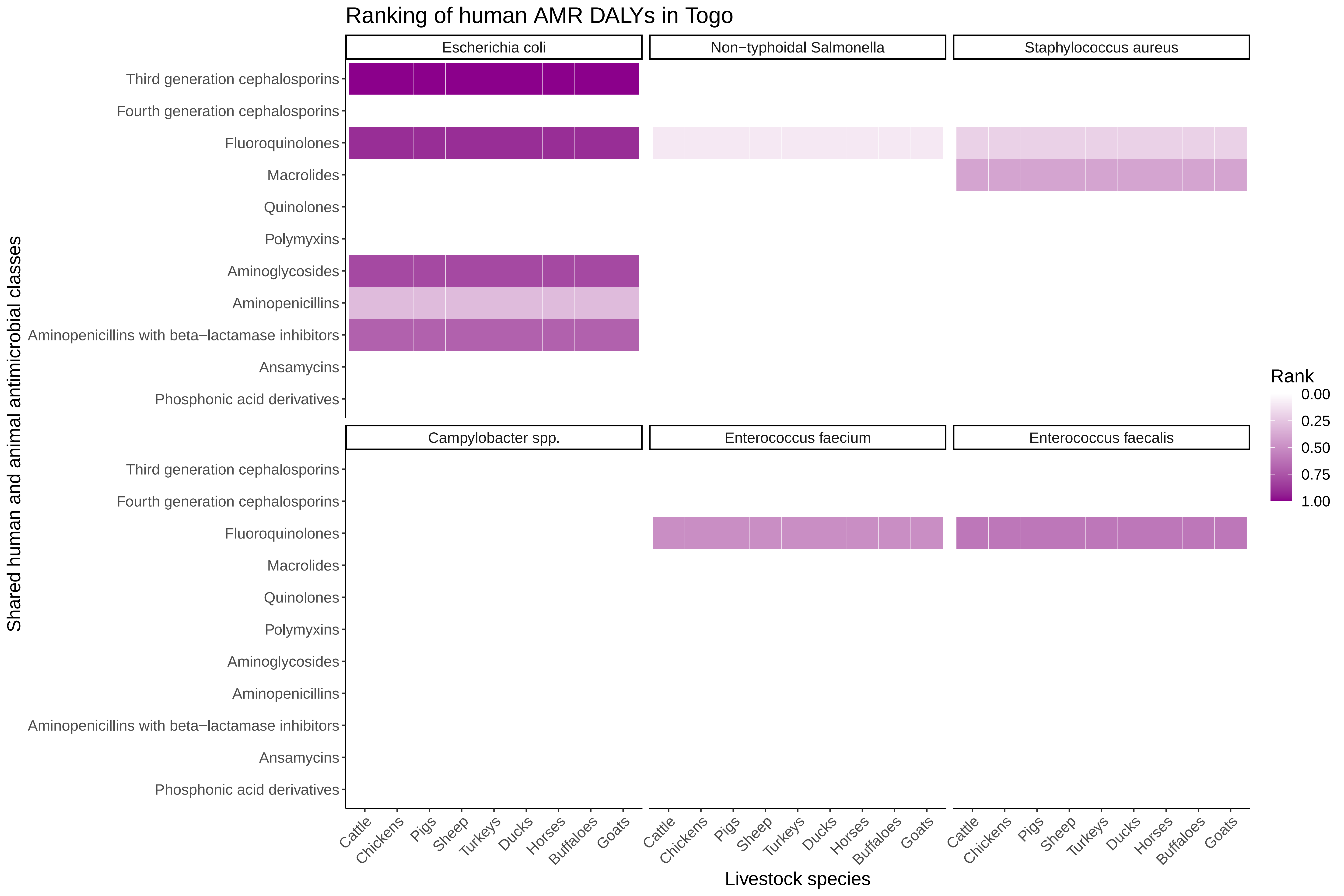

Ranking of human AMR DALYs in Togo
Escherichia coli
Non−typhoidal Salmonella
Staphylococcus aureus
Campylobacter spp.
Enterococcus faecium
Enterococcus faecalis
Third generation cephalosporins
Fourth generation cephalosporins
Fluoroquinolones
Macrolides
Quinolones
Polymyxins
Aminoglycosides
Aminopenicillins
Aminopenicillins with beta−lactamase inhibitors
Ansamycins
Phosphonic acid derivatives
Shared human and animal antimicrobial classes
Cattle
Chickens
Pigs
Sheep
Turkeys
Ducks
Horses
Buffaloes
Goats
Livestock species
Rank
0.00
0.25
0.50
0.75
1.00

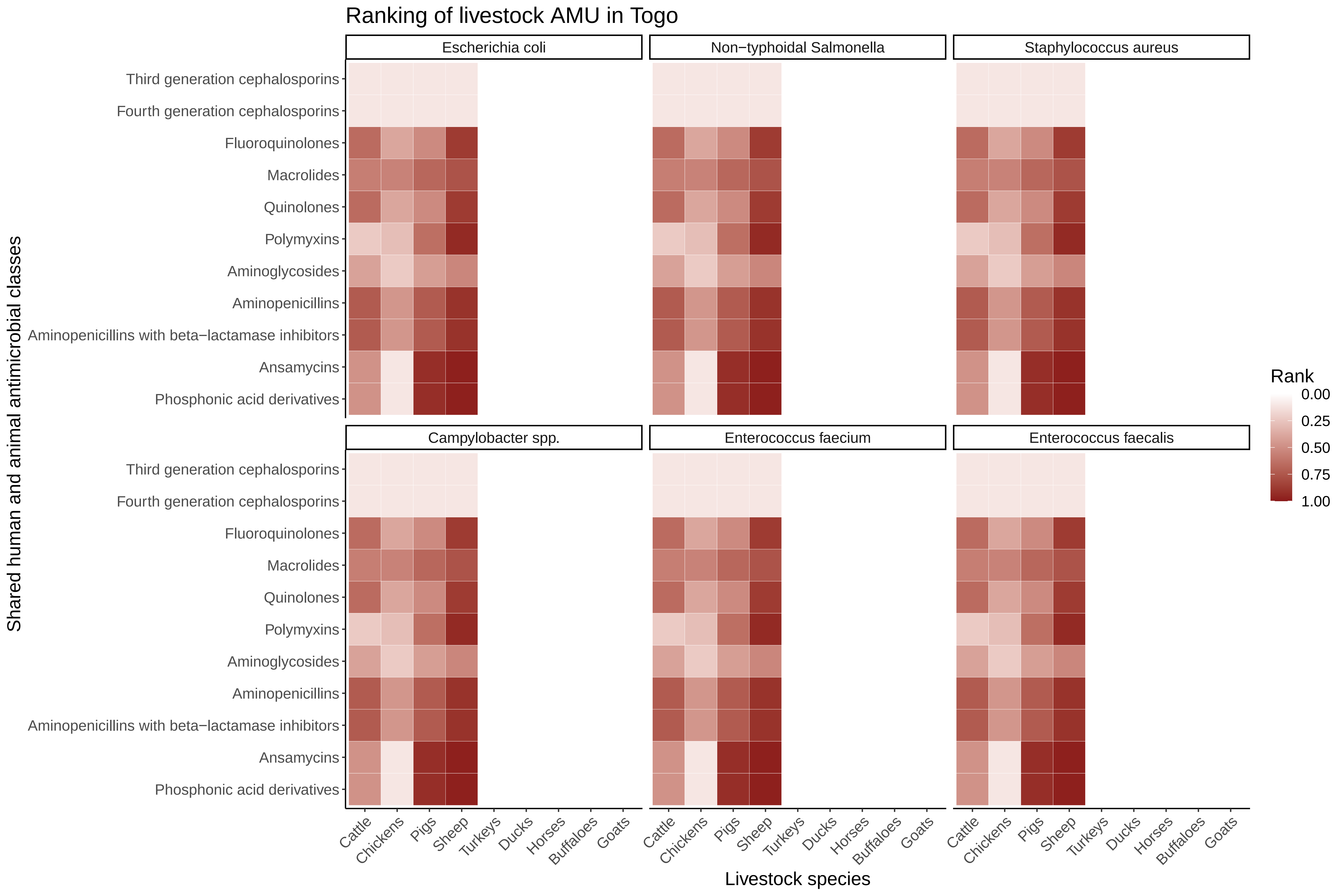

Ranking of livestock AMU in Togo
Escherichia coli
Non−typhoidal Salmonella
Staphylococcus aureus
Campylobacter spp.
Enterococcus faecium
Enterococcus faecalis
Shared human and animal antimicrobial classes
Third generation cephalosporins
Fourth generation cephalosporins
Fluoroquinolones
Macrolides
Quinolones
Polymyxins
Aminoglycosides
Aminopenicillins
Aminopenicillins with beta−lactamase inhibitors
Ansamycins
Phosphonic acid derivatives
Rank
0.00
0.25
0.50
0.75
1.00
Cattle
Chickens
Pigs
Sheep
Turkeys
Ducks
Horses
Buffaloes
Goats
Livestock species

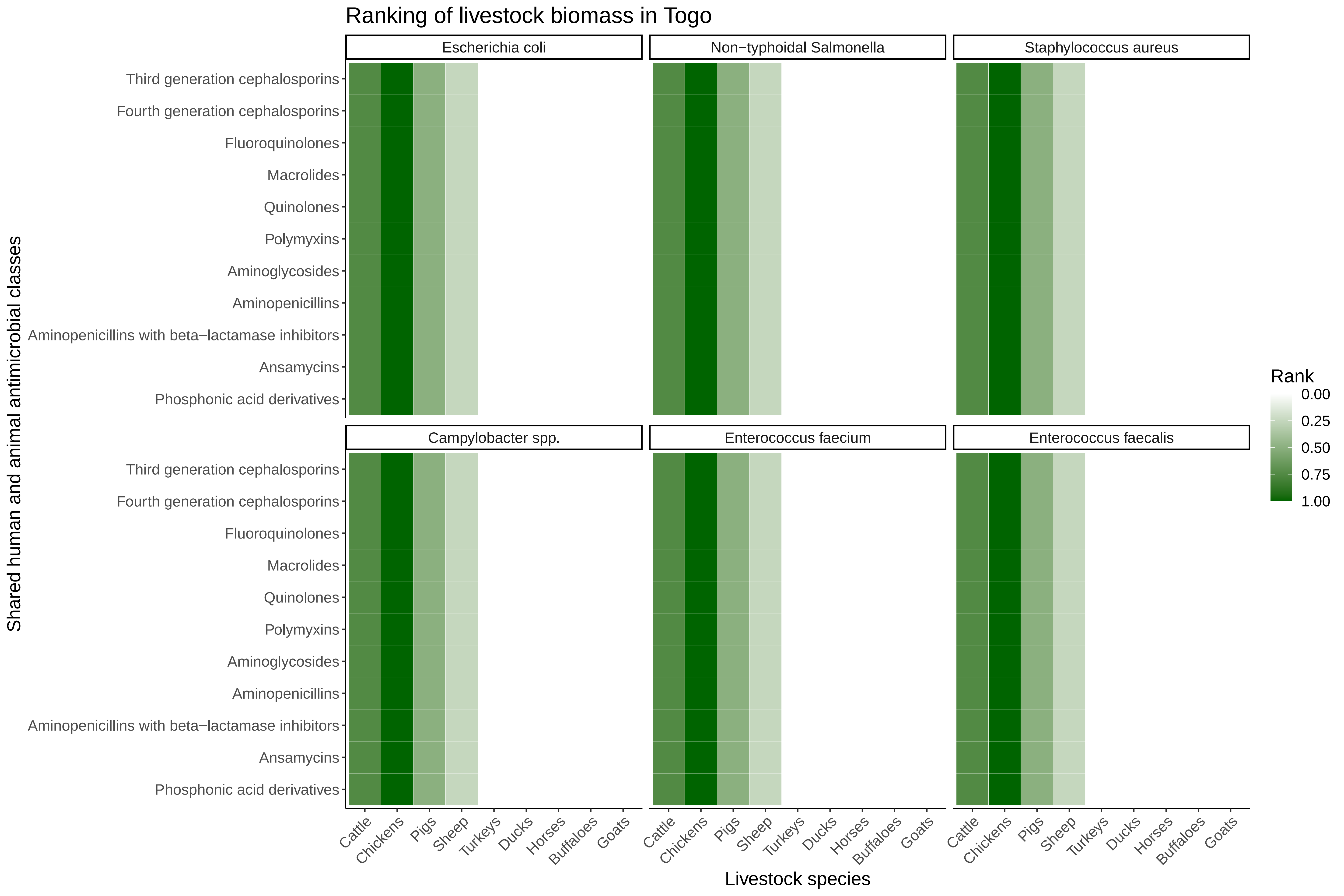
Ranking of livestock biomass in Togo
Escherichia coli
Non−typhoidal Salmonella
Staphylococcus aureus
Campylobacter spp.
Enterococcus faecium
Enterococcus faecalis
Third generation cephalosporins
Fourth generation cephalosporins
Fluoroquinolones
Macrolides
Quinolones
Polymyxins
Aminoglycosides
Aminopenicillins
Aminopenicillins with beta−lactamase inhibitors
Ansamycins
Phosphonic acid derivatives
Shared human and animal antimicrobial classes
Cattle
Chickens
Pigs
Sheep
Turkeys
Ducks
Horses
Buffaloes
Goats
Livestock species
Rank
0.00
0.25
0.50
0.75
1.00

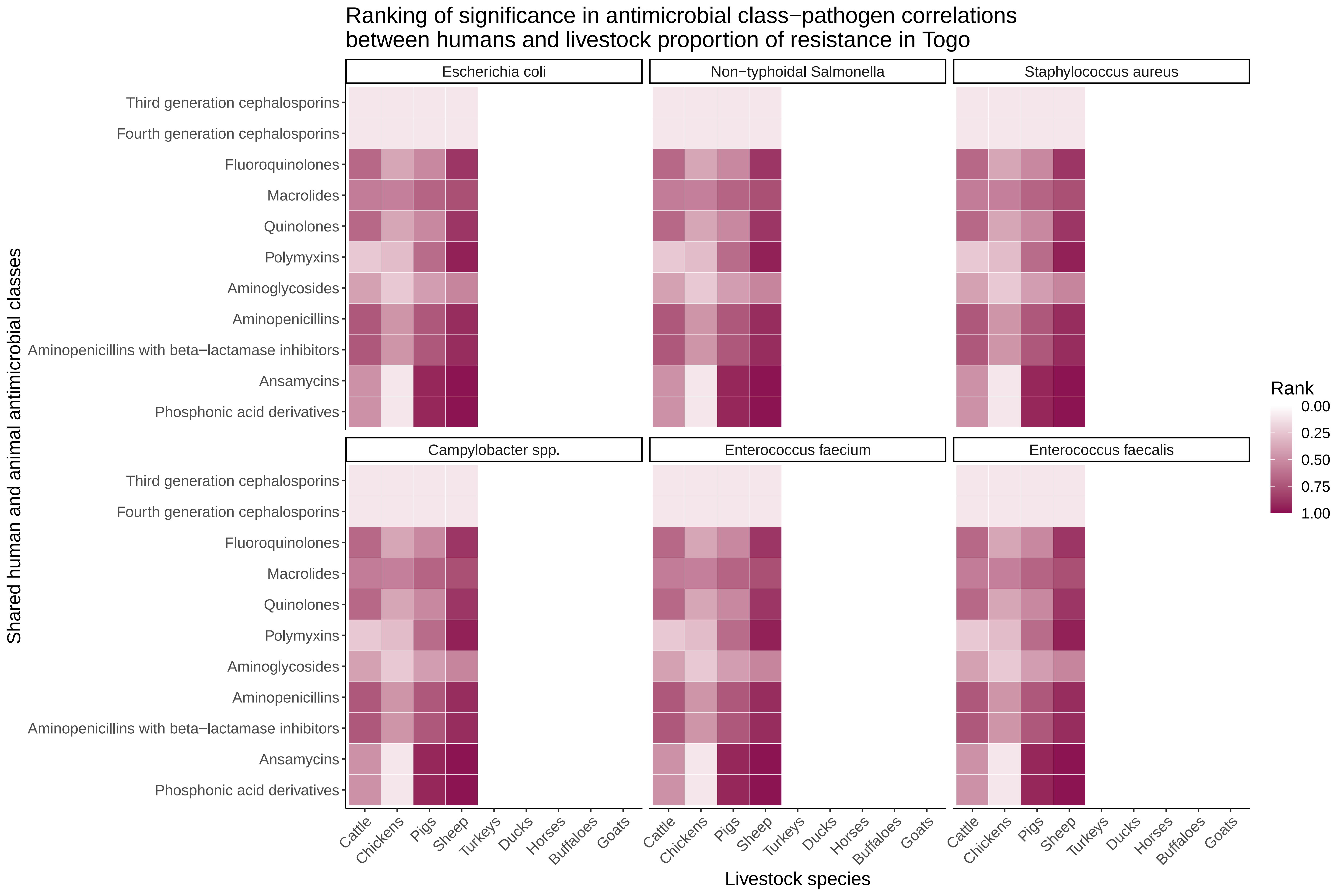
Ranking of significance in antimicrobial class−pathogen correlations
between humans and livestock proportion of resistance in Togo
Escherichia coli
Non−typhoidal Salmonella
Staphylococcus aureus
Campylobacter spp.
Enterococcus faecium
Enterococcus faecalis
Third generation cephalosporins
Fourth generation cephalosporins
Fluoroquinolones
Macrolides
Quinolones
Polymyxins
Aminoglycosides
Aminopenicillins
Aminopenicillins with beta−lactamase inhibitors
Ansamycins
Phosphonic acid derivatives
Shared human and animal antimicrobial classes
Cattle
Chickens
Pigs
Sheep
Turkeys
Ducks
Horses
Buffaloes
Goats
Livestock species
Rank
0.00
0.25
0.50
0.75
1.00

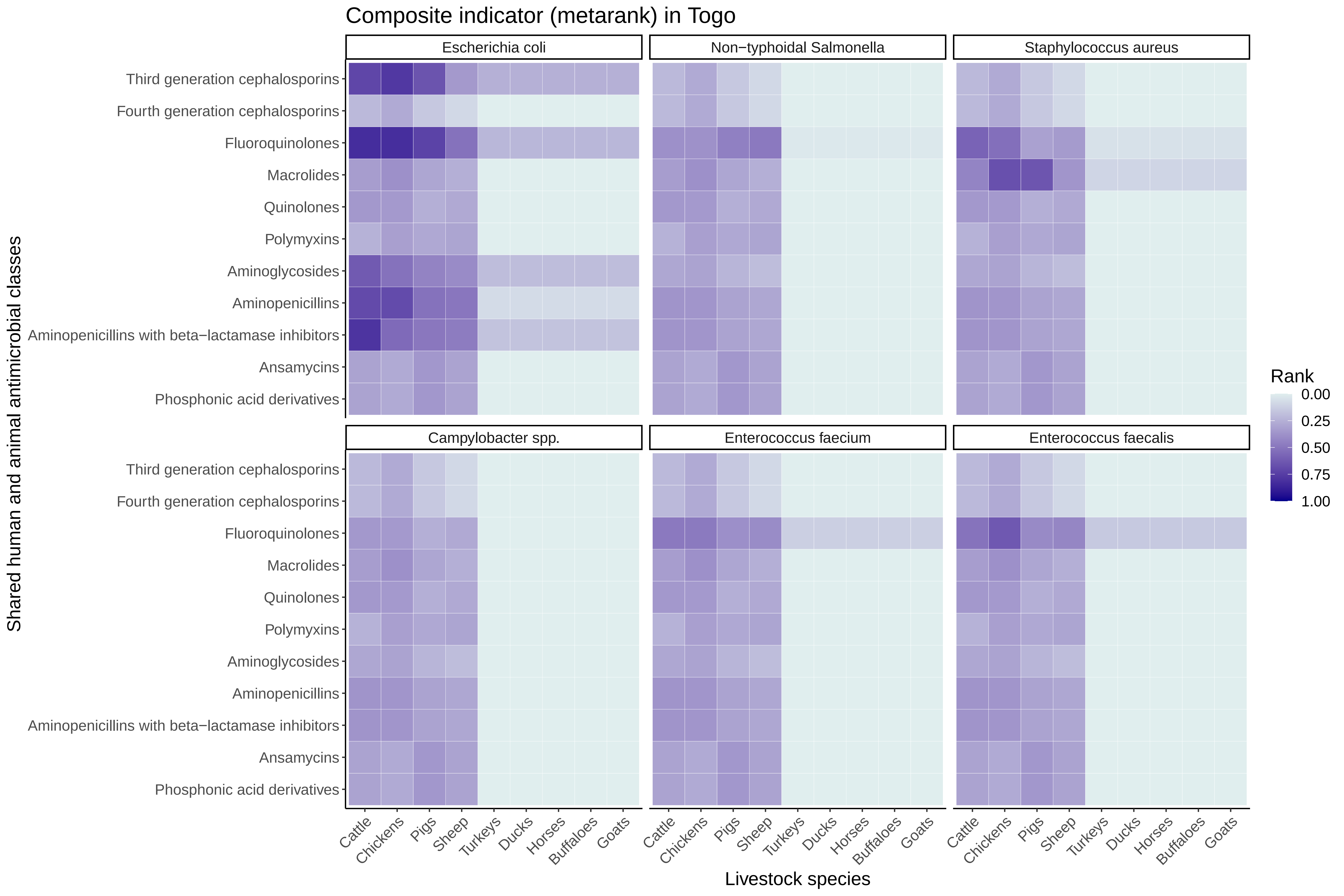

Composite indicator (metarank) in Togo
Escherichia coli
Non−typhoidal Salmonella
Staphylococcus aureus
Campylobacter spp.
Enterococcus faecium
Enterococcus faecalis
Third generation cephalosporins
Fourth generation cephalosporins
Fluoroquinolones
Macrolides
Quinolones
Polymyxins
Aminoglycosides
Aminopenicillins
Aminopenicillins with beta−lactamase inhibitors
Ansamycins
Phosphonic acid derivatives
Shared human and animal antimicrobial classes
Cattle
Chickens
Pigs
Sheep
Turkeys
Ducks
Horses
Buffaloes
Goats
Livestock species
Rank
0.00
0.25
0.50
0.75
1.00

Livestock AMR data availability in Togo

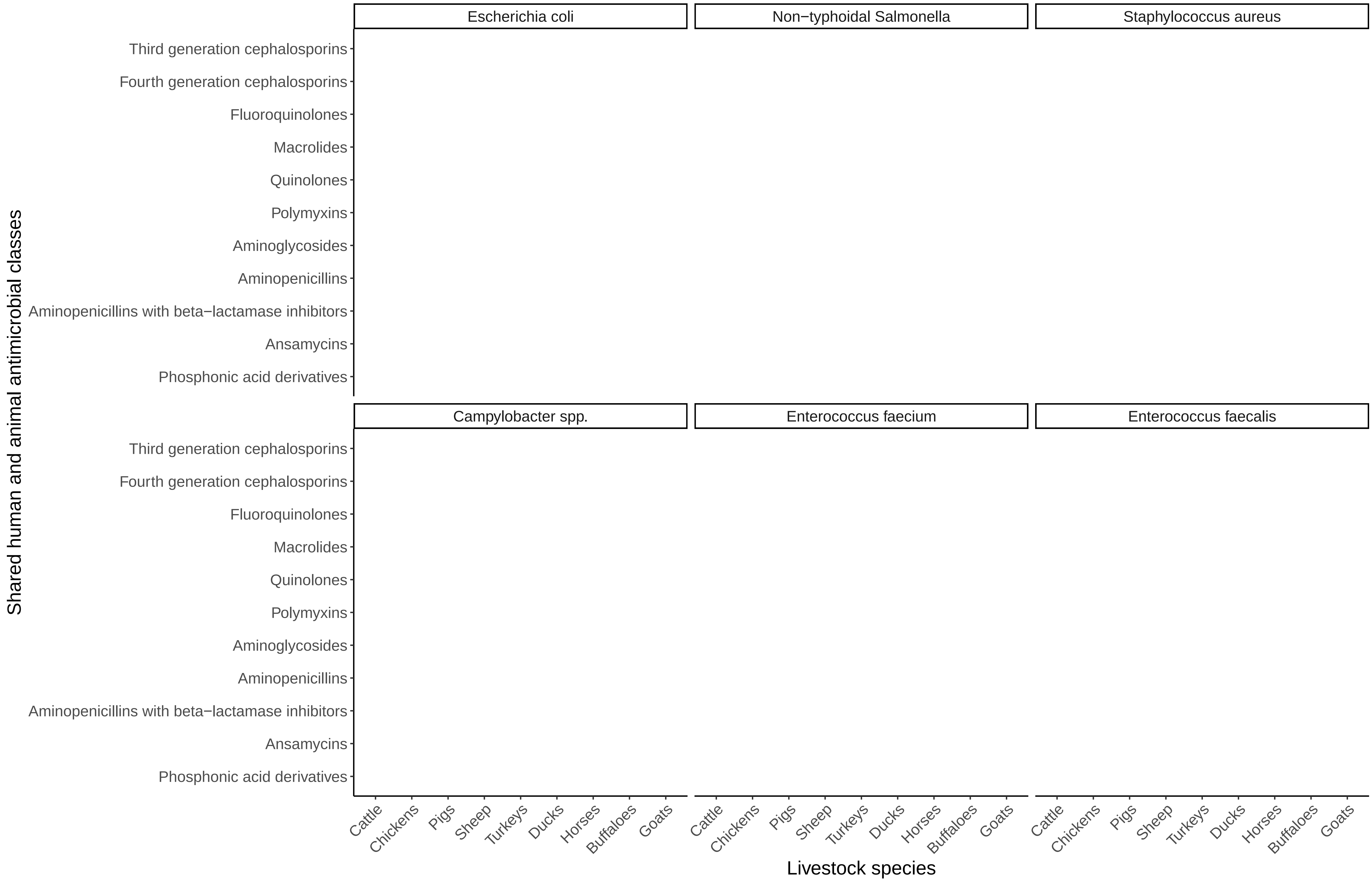

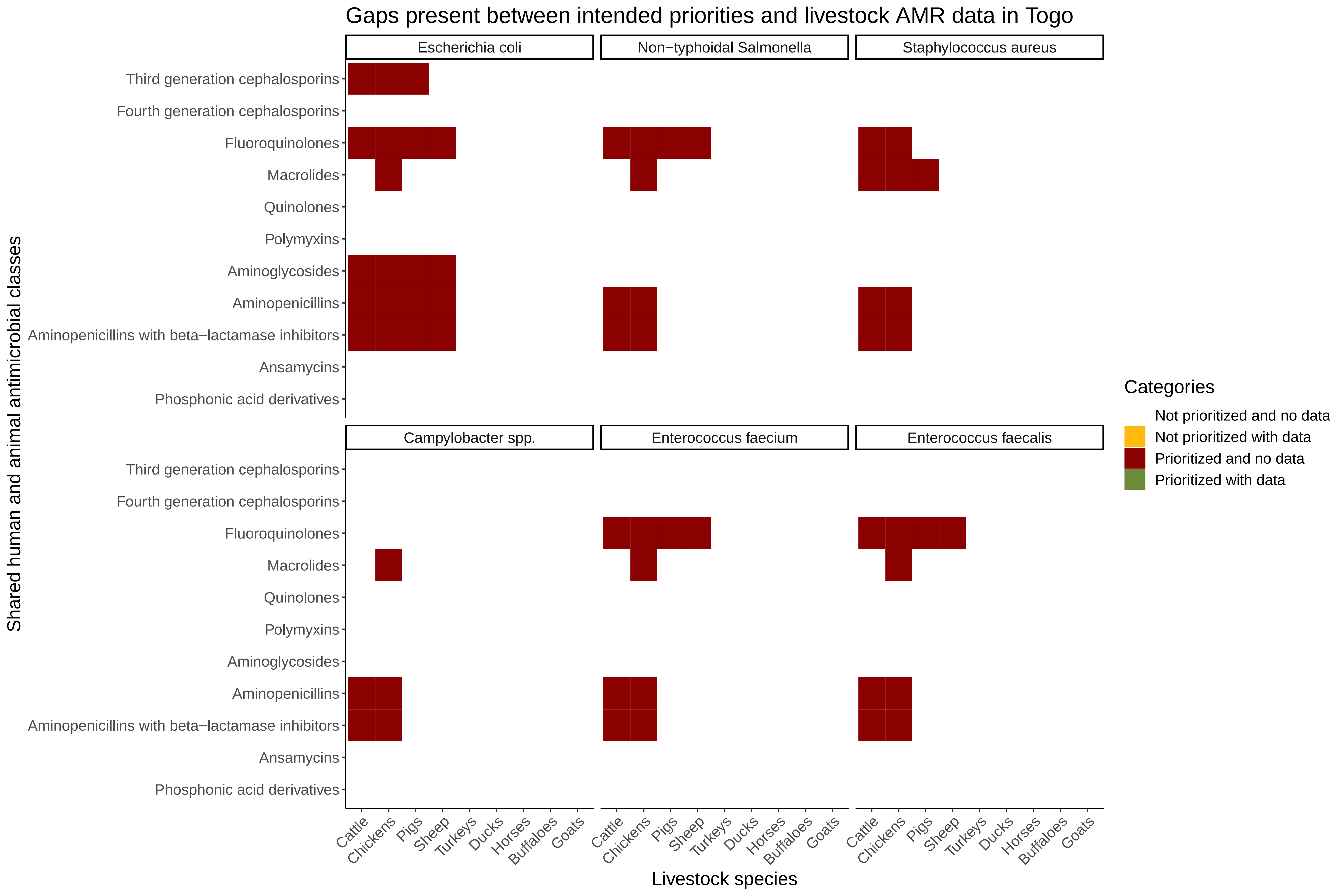

Gaps present between intended priorities and livestock AMR data in Togo
Escherichia coli
Non−typhoidal Salmonella
Staphylococcus aureus
Campylobacter spp.
Enterococcus faecium
Enterococcus faecalis
Third generation cephalosporins
Fourth generation cephalosporins
Fluoroquinolones
Macrolides
Quinolones
Polymyxins
Aminoglycosides
Aminopenicillins
Aminopenicillins with beta−lactamase inhibitors
Ansamycins
Phosphonic acid derivatives
Shared human and animal antimicrobial classes
Cattle
Chickens
Pigs
Sheep
Turkeys
Ducks
Horses
Buffaloes
Goats
Livestock species
Categories
Not prioritized and no data
Not prioritized with data
Prioritized and no data
Prioritized with data

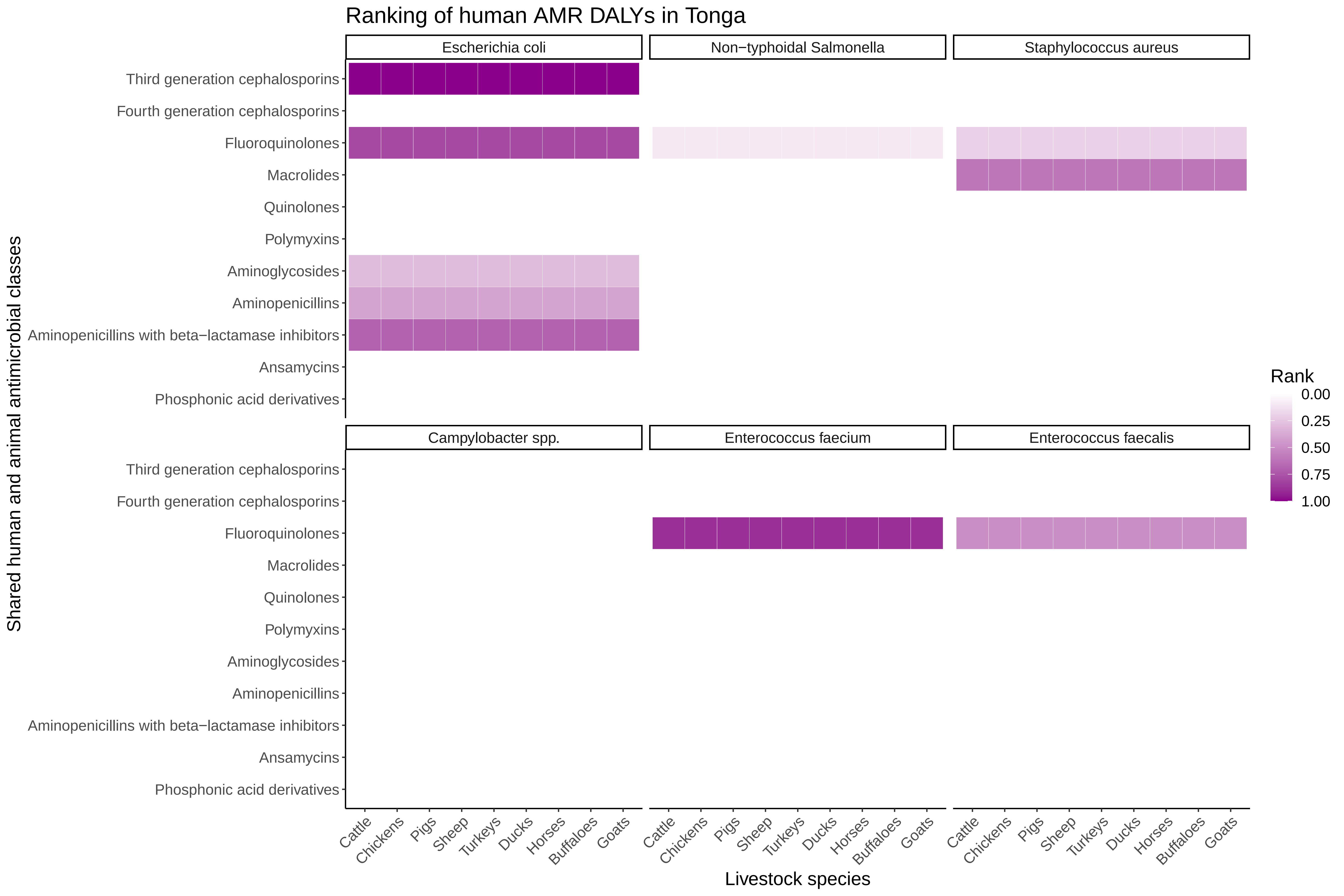

Ranking of human AMR DALYs in Tonga
Escherichia coli
Non−typhoidal Salmonella
Staphylococcus aureus
Campylobacter spp.
Enterococcus faecium
Enterococcus faecalis
Shared human and animal antimicrobial classes
Third generation cephalosporins
Fourth generation cephalosporins
Fluoroquinolones
Macrolides
Quinolones
Polymyxins
Aminoglycosides
Aminopenicillins
Aminopenicillins with beta−lactamase inhibitors
Ansamycins
Phosphonic acid derivatives
Cattle
Chickens
Pigs
Sheep
Turkeys
Ducks
Horses
Buffaloes
Goats
Livestock species
Rank
0.00
0.25
0.50
0.75
1.00

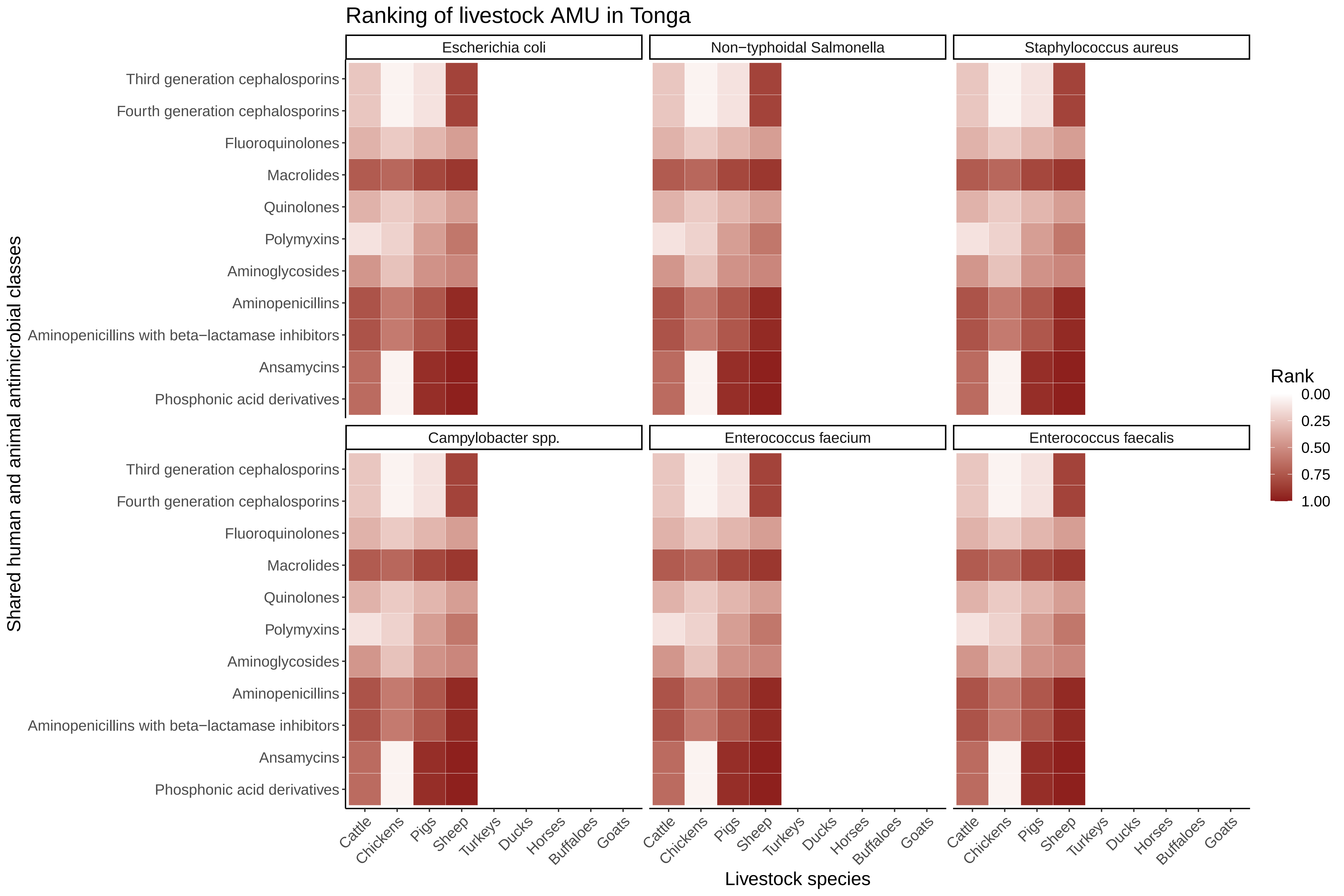

Ranking of livestock AMU in Tonga
Escherichia coli
Non−typhoidal Salmonella
Staphylococcus aureus
Campylobacter spp.
Enterococcus faecium
Enterococcus faecalis
Third generation cephalosporins
Fourth generation cephalosporins
Fluoroquinolones
Macrolides
Quinolones
Polymyxins
Aminoglycosides
Aminopenicillins
Aminopenicillins with beta−lactamase inhibitors
Ansamycins
Phosphonic acid derivatives
Shared human and animal antimicrobial classes
Cattle
Chickens
Pigs
Sheep
Turkeys
Ducks
Horses
Buffaloes
Goats
Livestock species
Rank
0.00
0.25
0.50
0.75
1.00

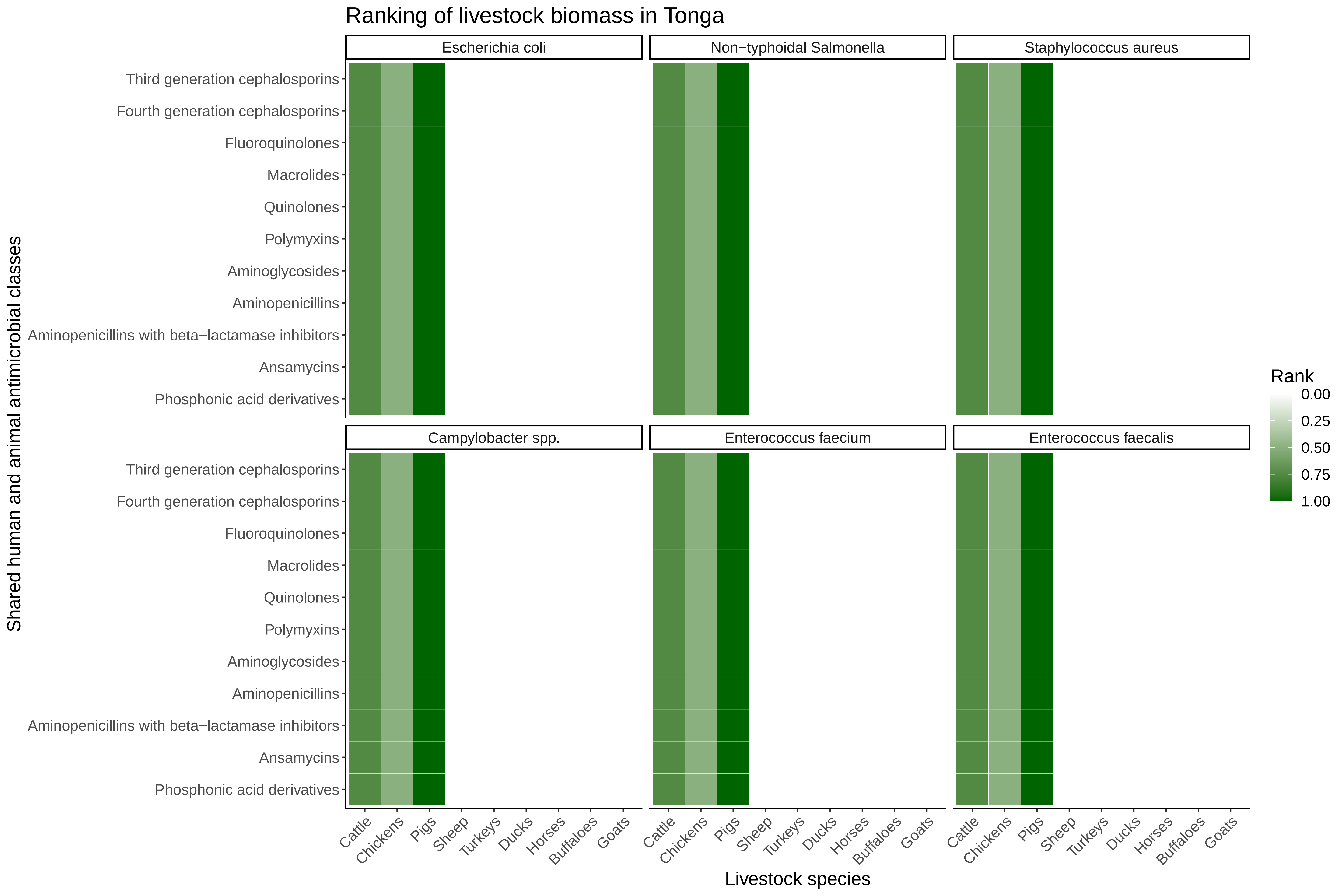

Ranking of livestock biomass in Tonga
Escherichia coli
Non−typhoidal Salmonella
Staphylococcus aureus
Campylobacter spp.
Enterococcus faecium
Enterococcus faecalis
Third generation cephalosporins
Fourth generation cephalosporins
Fluoroquinolones
Macrolides
Quinolones
Polymyxins
Aminoglycosides
Aminopenicillins
Aminopenicillins with beta−lactamase inhibitors
Ansamycins
Phosphonic acid derivatives
Shared human and animal antimicrobial classes
Cattle
Chickens
Pigs
Sheep
Turkeys
Ducks
Horses
Buffaloes
Goats
Livestock species
Rank
0.00
0.25
0.50
0.75
1.00

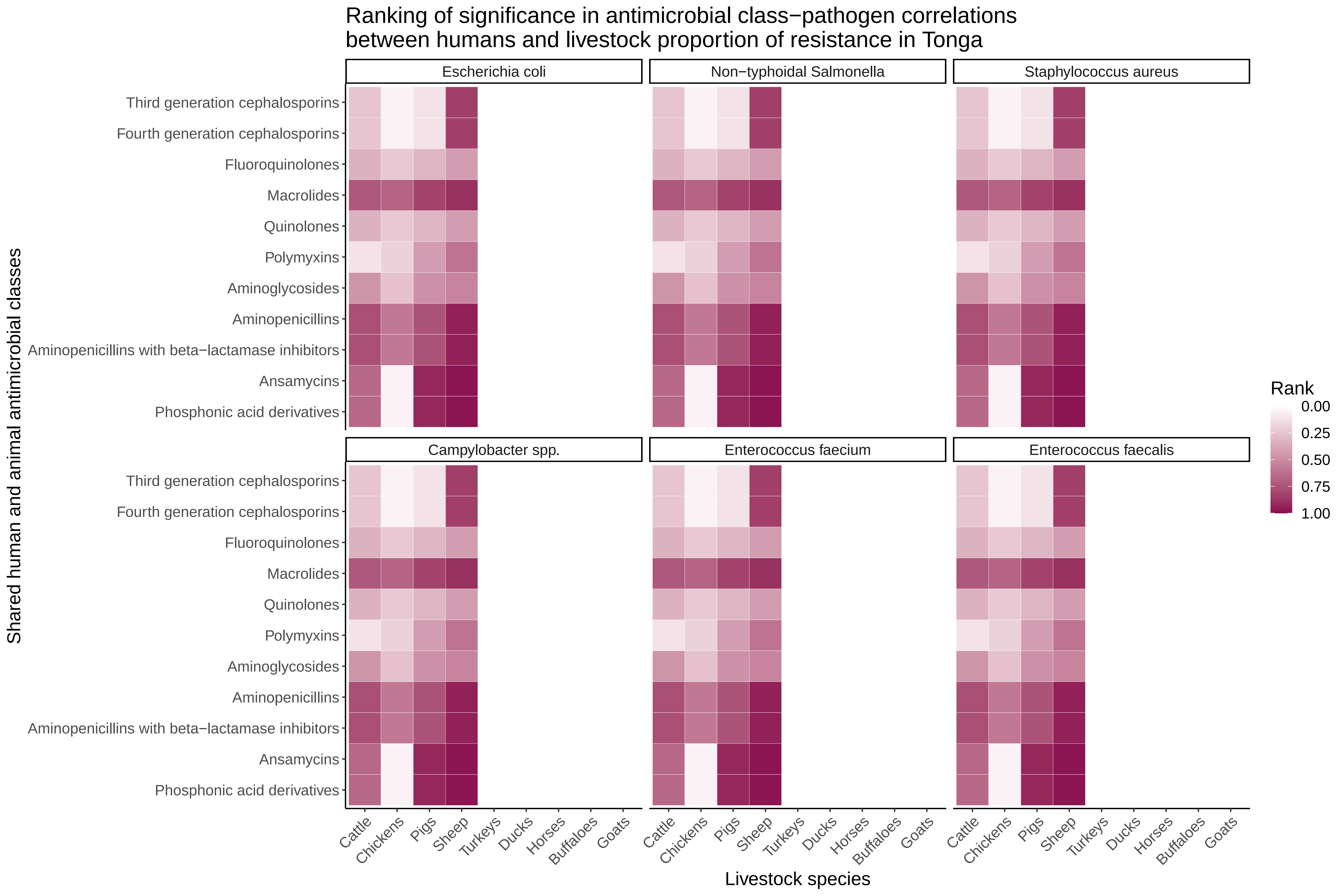

Ranking of significance in antimicrobial class−pathogen correlations between humans and livestock proportion of resistance in Tonga
Escherichia coli
Non−typhoidal Salmonella
Staphylococcus aureus
Campylobacter spp.
Enterococcus faecium
Enterococcus faecalis
Third generation cephalosporins
Fourth generation cephalosporins
Fluoroquinolones
Macrolides
Quinolones
Polymyxins
Aminoglycosides
Aminopenicillins
Aminopenicillins with beta−lactamase inhibitors
Ansamycins
Phosphonic acid derivatives
Shared human and animal antimicrobial classes
Cattle
Chickens
Pigs
Sheep
Turkeys
Ducks
Horses
Buffaloes
Goats
Livestock species
Rank
0.00
0.25
0.50
0.75
1.00

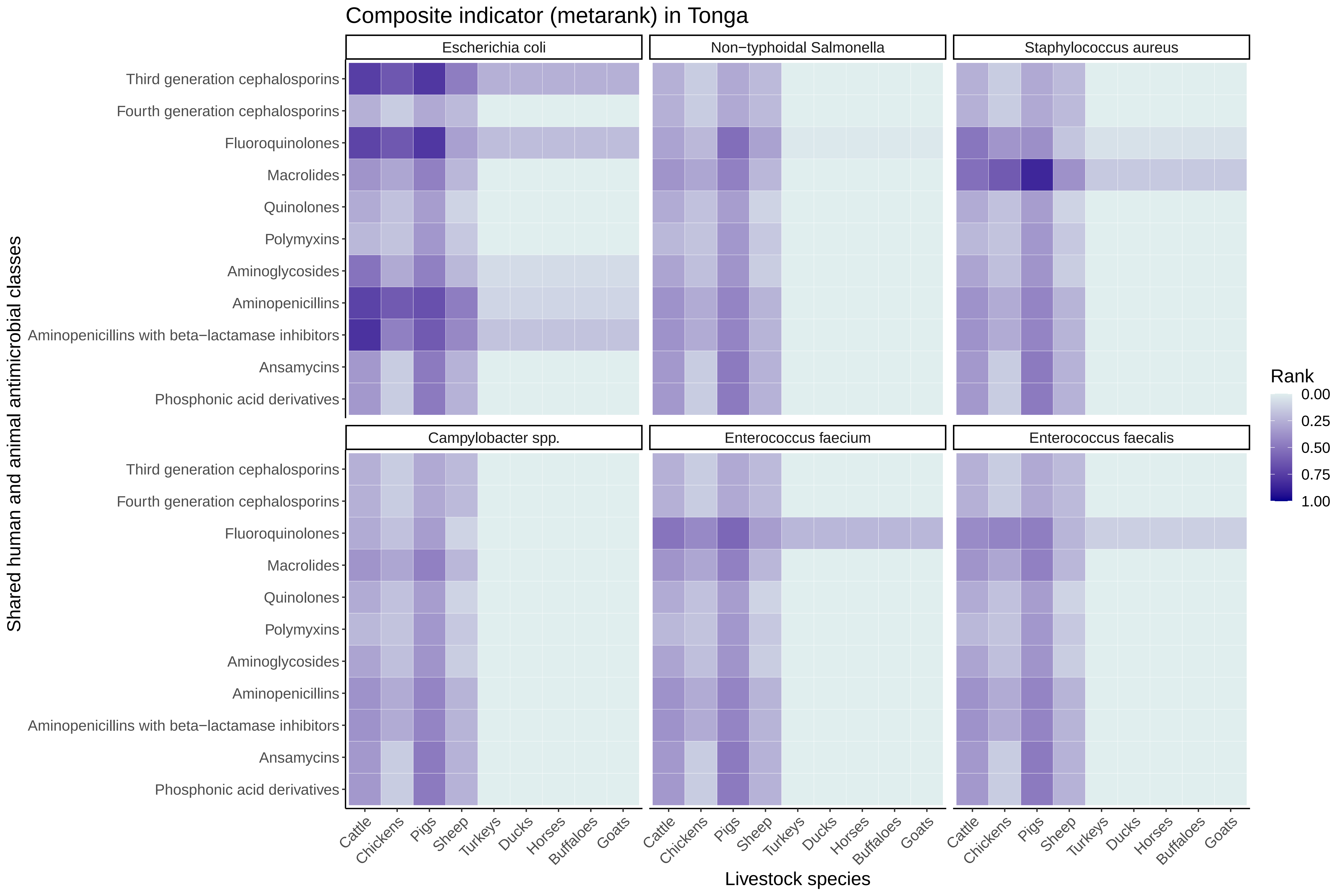
Composite indicator (metarank) in Tonga
Escherichia coli
Non−typhoidal Salmonella
Staphylococcus aureus
Campylobacter spp.
Enterococcus faecium
Enterococcus faecalis
Shared human and animal antimicrobial classes
Third generation cephalosporins
Fourth generation cephalosporins
Fluoroquinolones
Macrolides
Quinolones
Polymyxins
Aminoglycosides
Aminopenicillins
Aminopenicillins with beta−lactamase inhibitors
Ansamycins
Phosphonic acid derivatives
Cattle
Chickens
Pigs
Sheep
Turkeys
Ducks
Horses
Buffaloes
Goats
Livestock species
Rank
0.00
0.25
0.50
0.75
1.00

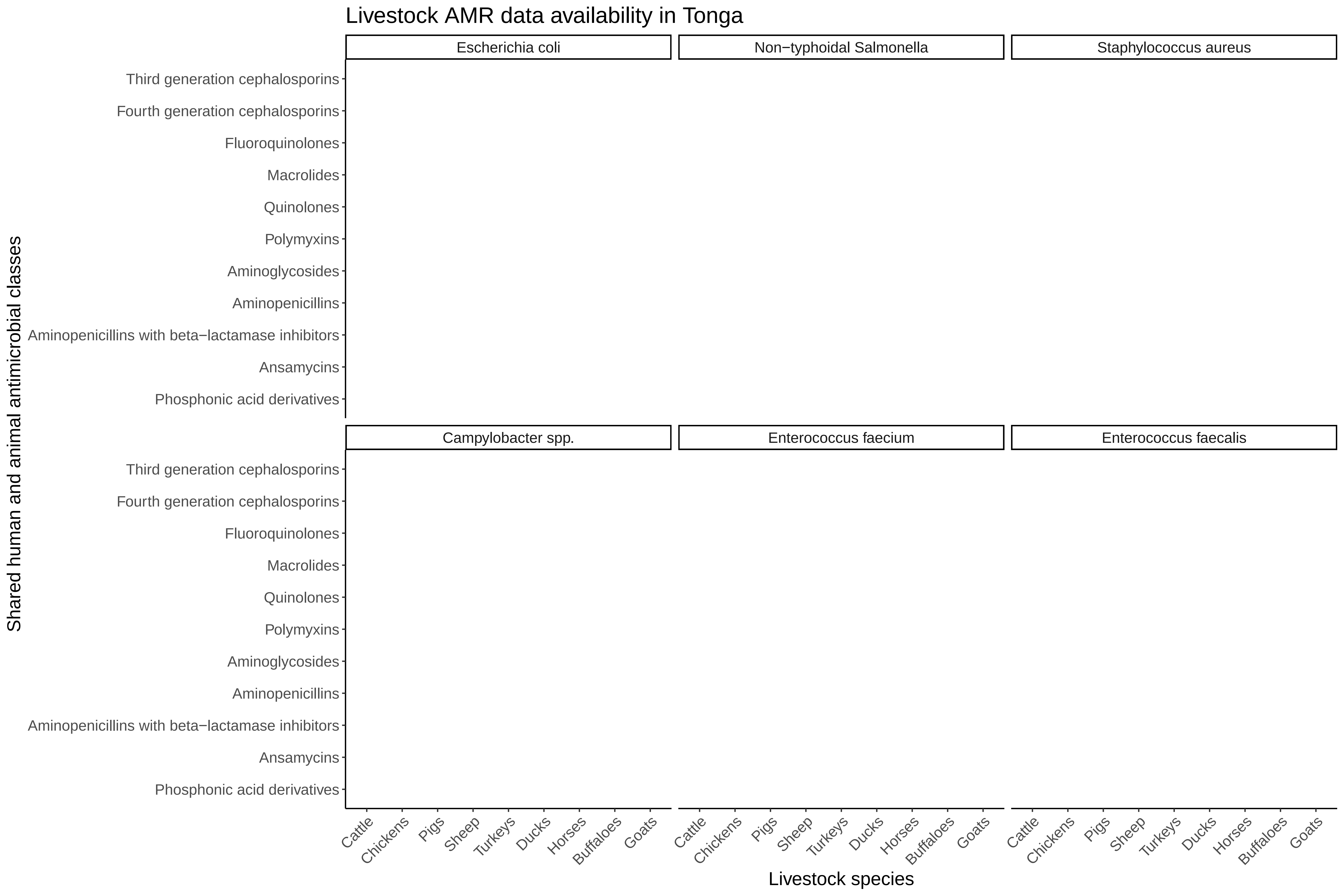

Livestock AMR data availability in Tonga
Escherichia coli
Non−typhoidal Salmonella
Staphylococcus aureus
Campylobacter spp.
Enterococcus faecium
Enterococcus faecalis
Third generation cephalosporins
Fourth generation cephalosporins
Fluoroquinolones
Macrolides
Quinolones
Polymyxins
Aminoglycosides
Aminopenicillins
Aminopenicillins with beta−lactamase inhibitors
Ansamycins
Phosphonic acid derivatives
Shared human and animal antimicrobial classes
Cattle
Chickens
Pigs
Sheep
Turkeys
Ducks
Horses
Buffaloes
Goats
Livestock species

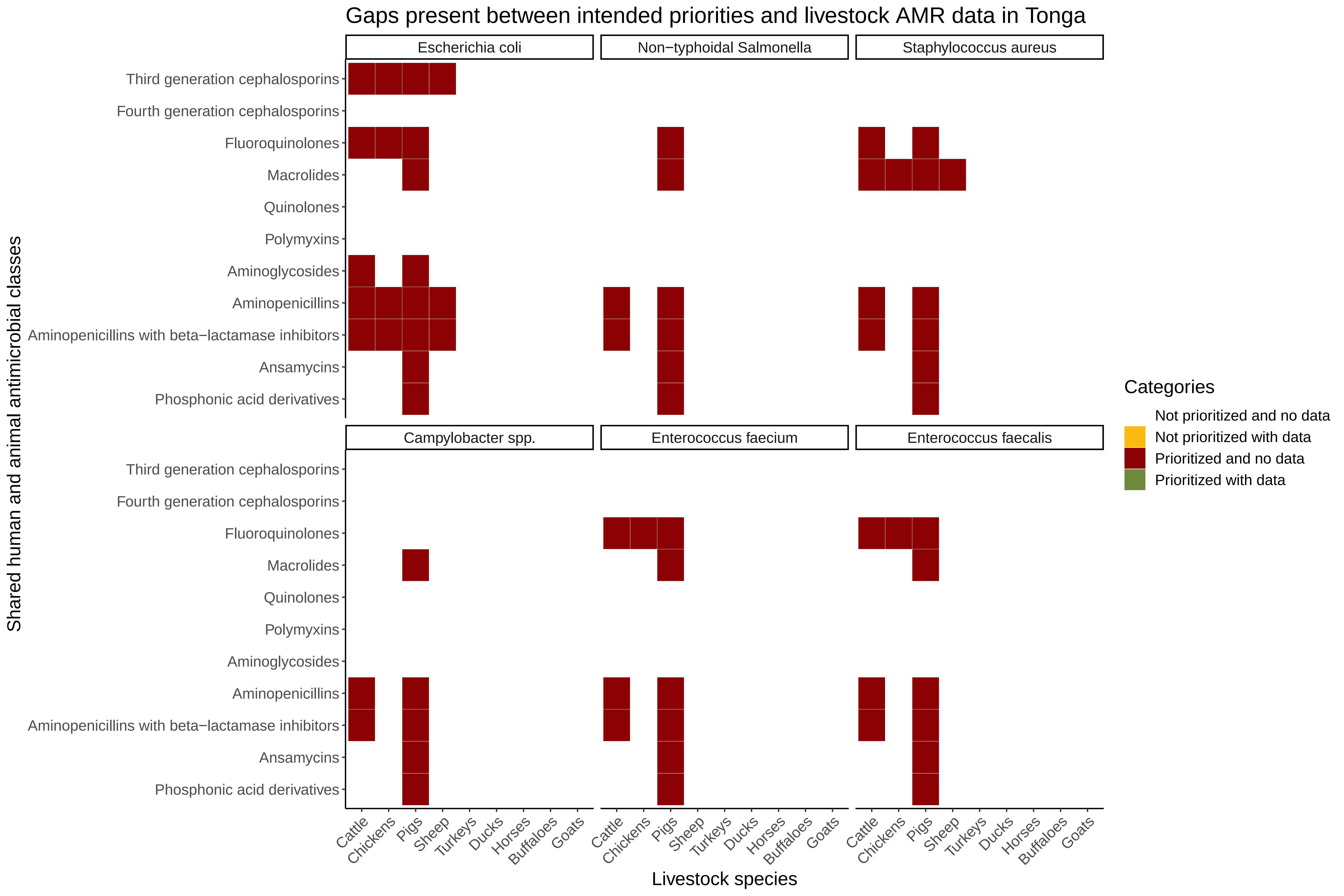
Gaps present between intended priorities and livestock AMR data in Tonga
Escherichia coli
Non−typhoidal Salmonella
Staphylococcus aureus
Campylobacter spp.
Enterococcus faecium
Enterococcus faecalis
Third generation cephalosporins
Fourth generation cephalosporins
Fluoroquinolones
Macrolides
Quinolones
Polymyxins
Aminoglycosides
Aminopenicillins
Aminopenicillins with beta−lactamase inhibitors
Ansamycins
Phosphonic acid derivatives
Shared human and animal antimicrobial classes
Cattle
Chickens
Pigs
Sheep
Turkeys
Ducks
Horses
Buffaloes
Goats
Livestock species
Categories
Not prioritized and no data
Not prioritized with data
Prioritized and no data
Prioritized with data

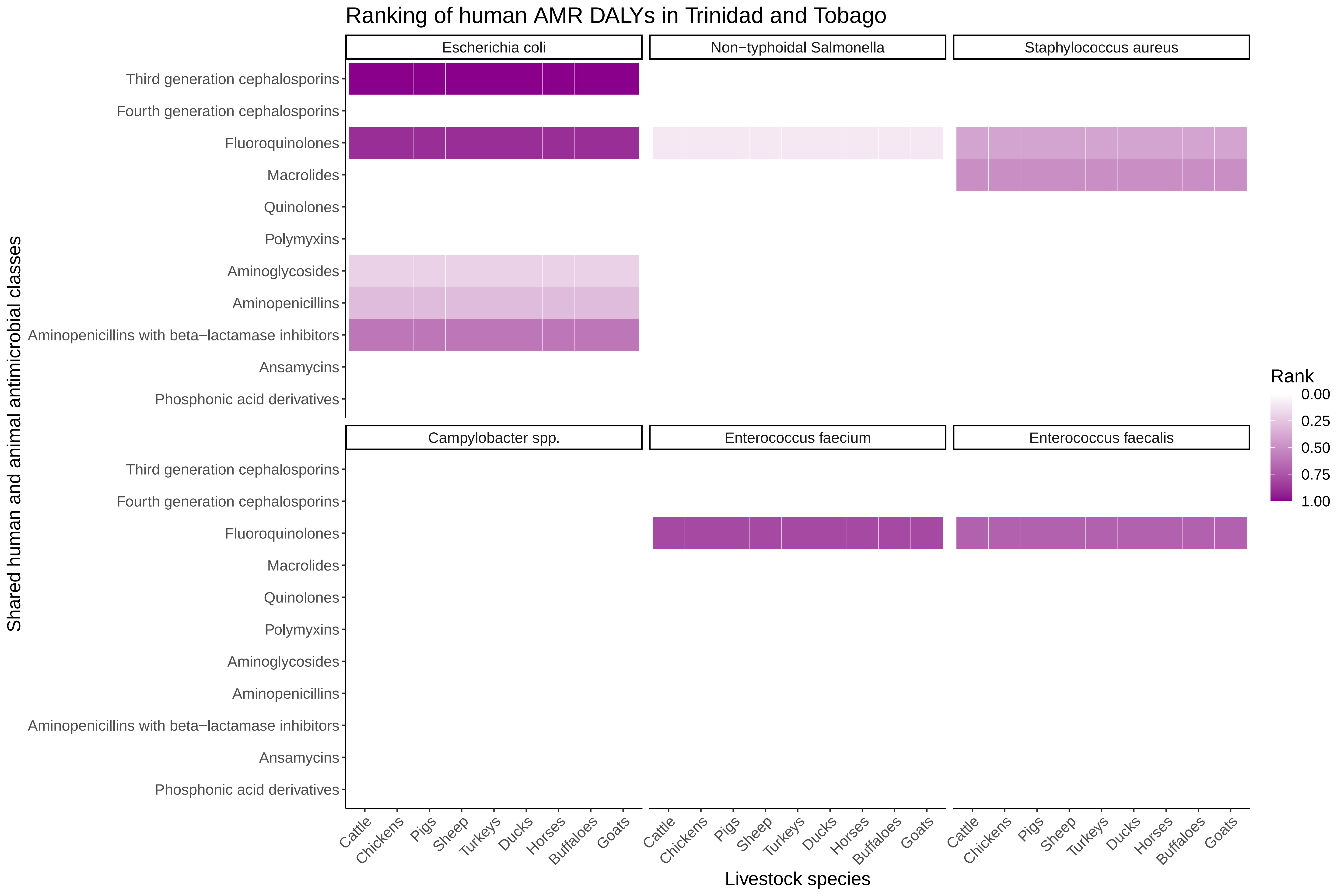

Ranking of human AMR DALYs in Trinidad and Tobago
Escherichia coli
Non−typhoidal Salmonella
Staphylococcus aureus
Campylobacter spp.
Enterococcus faecium
Enterococcus faecalis
Shared human and animal antimicrobial classes
Third generation cephalosporins
Fourth generation cephalosporins
Fluoroquinolones
Macrolides
Quinolones
Polymyxins
Aminoglycosides
Aminopenicillins
Aminopenicillins with beta−lactamase inhibitors
Ansamycins
Phosphonic acid derivatives
Cattle
Chickens
Pigs
Sheep
Turkeys
Ducks
Horses
Buffaloes
Goats
Livestock species
Rank
0.00
0.25
0.50
0.75
1.00

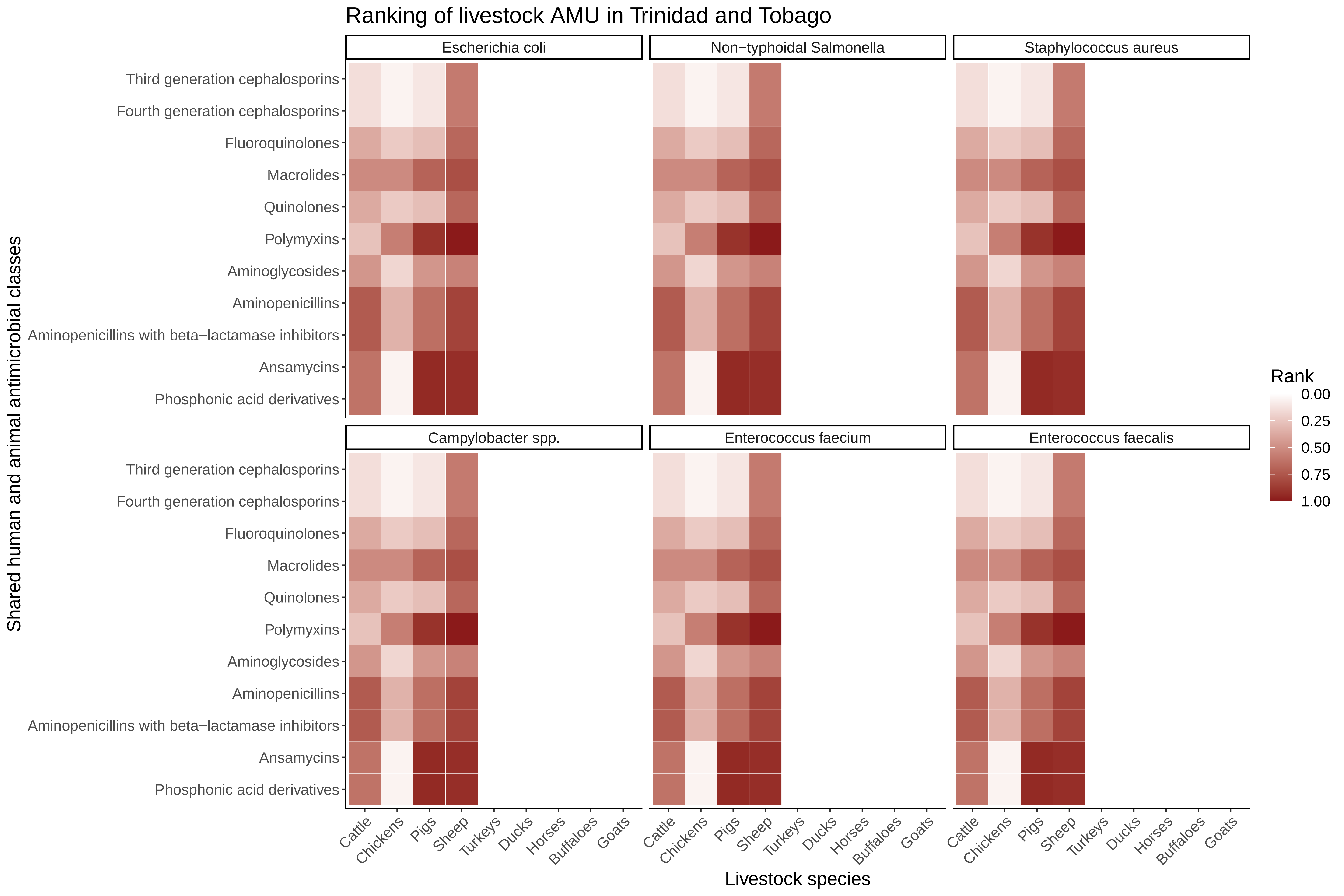

Ranking of livestock AMU in Trinidad and Tobago
Escherichia coli
Non−typhoidal Salmonella
Staphylococcus aureus
Campylobacter spp.
Enterococcus faecium
Enterococcus faecalis
Shared human and animal antimicrobial classes
Third generation cephalosporins
Fourth generation cephalosporins
Fluoroquinolones
Macrolides
Quinolones
Polymyxins
Aminoglycosides
Aminopenicillins
Aminopenicillins with beta−lactamase inhibitors
Ansamycins
Phosphonic acid derivatives
Cattle
Chickens
Pigs
Sheep
Turkeys
Ducks
Horses
Buffaloes
Goats
Livestock species
Rank
0.00
0.25
0.50
0.75
1.00

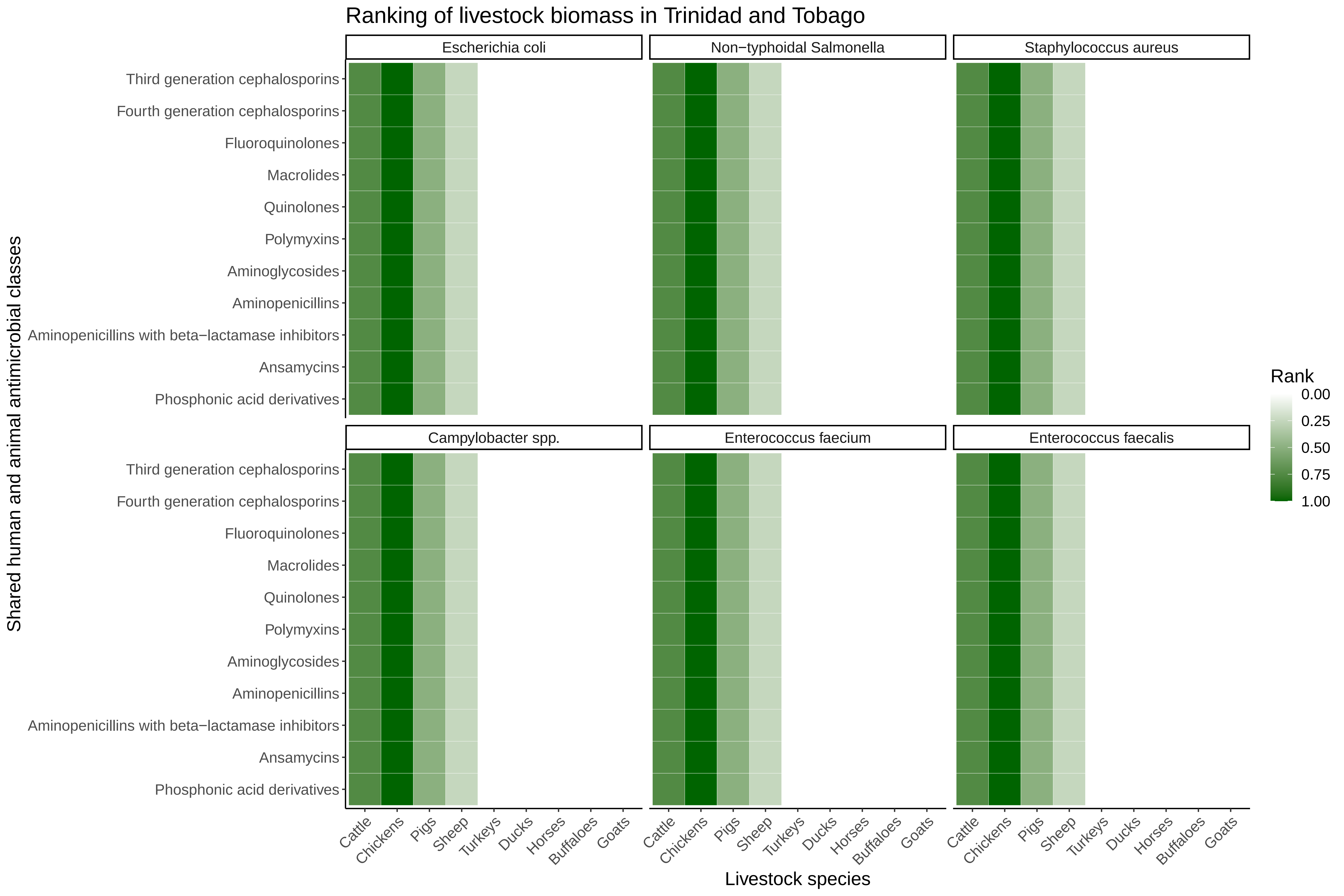

Ranking of livestock biomass in Trinidad and Tobago
Escherichia coli
Non−typhoidal Salmonella
Staphylococcus aureus
Campylobacter spp.
Enterococcus faecium
Enterococcus faecalis
Third generation cephalosporins
Fourth generation cephalosporins
Fluoroquinolones
Macrolides
Quinolones
Polymyxins
Aminoglycosides
Aminopenicillins
Aminopenicillins with beta−lactamase inhibitors
Ansamycins
Phosphonic acid derivatives
Shared human and animal antimicrobial classes
Cattle
Chickens
Pigs
Sheep
Turkeys
Ducks
Horses
Buffaloes
Goats
Livestock species
Rank
0.00
0.25
0.50
0.75
1.00

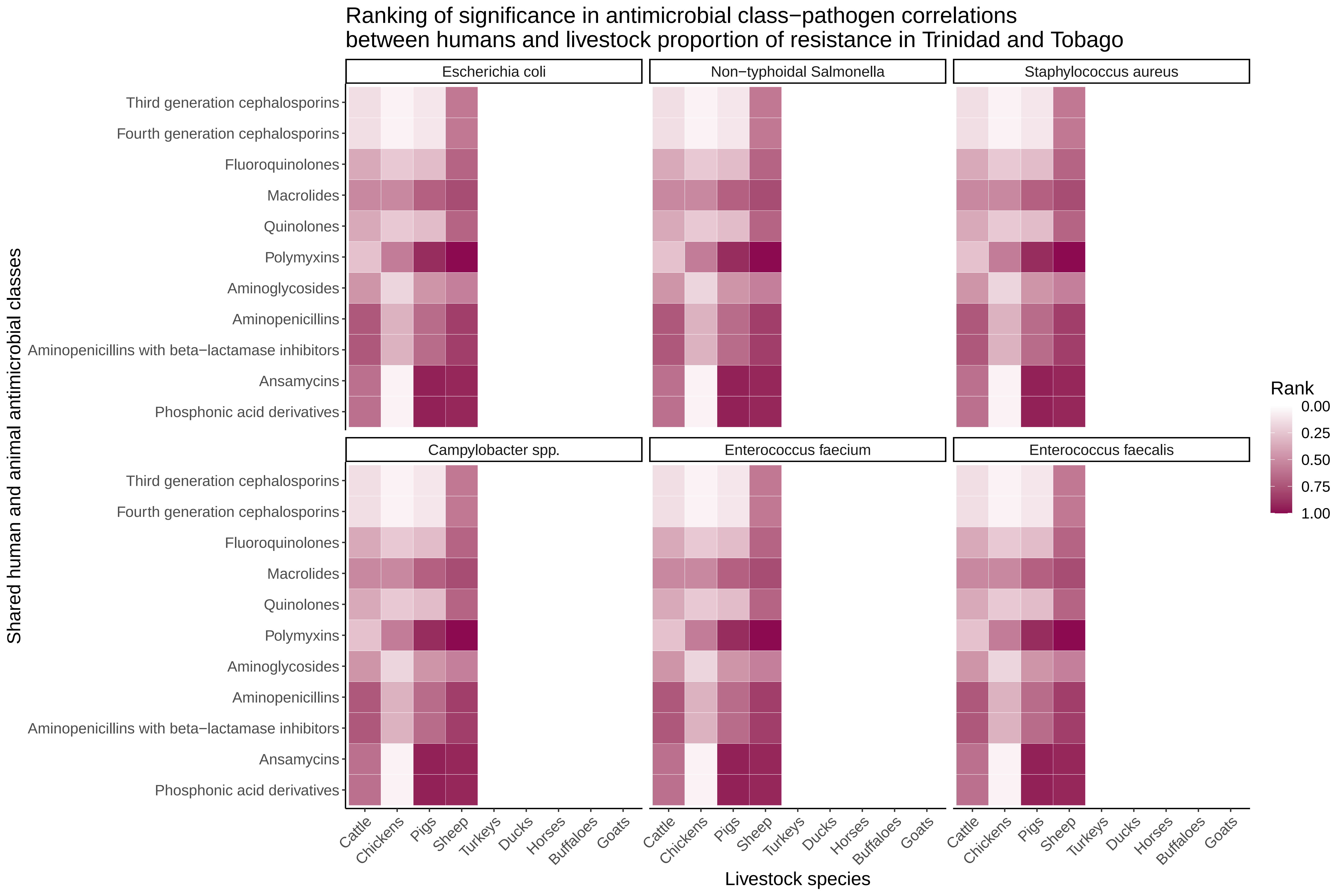
Ranking of significance in antimicrobial class−pathogen correlations
between humans and livestock proportion of resistance in Trinidad and Tobago
Escherichia coli
Non−typhoidal Salmonella
Staphylococcus aureus
Campylobacter spp.
Enterococcus faecium
Enterococcus faecalis
Third generation cephalosporins
Fourth generation cephalosporins
Fluoroquinolones
Macrolides
Quinolones
Polymyxins
Aminoglycosides
Aminopenicillins
Aminopenicillins with beta−lactamase inhibitors
Ansamycins
Phosphonic acid derivatives
Shared human and animal antimicrobial classes
Cattle
Chickens
Pigs
Sheep
Turkeys
Ducks
Horses
Buffaloes
Goats
Livestock species
Rank
0.00
0.25
0.50
0.75
1.00

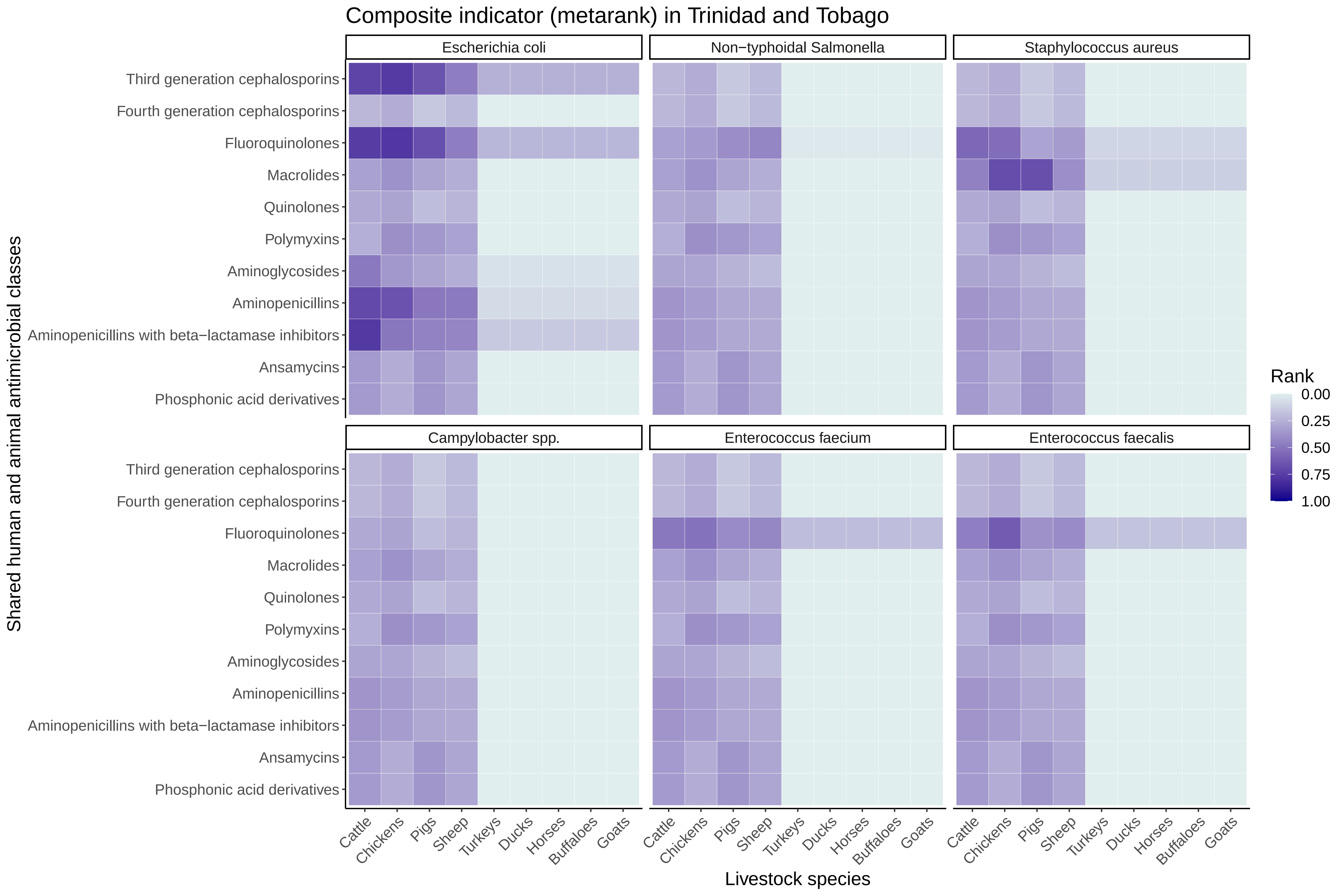
Composite indicator (metarank) in Trinidad and Tobago
Escherichia coli
Non−typhoidal Salmonella
Staphylococcus aureus
Campylobacter spp.
Enterococcus faecium
Enterococcus faecalis
Shared human and animal antimicrobial classes
Third generation cephalosporins
Fourth generation cephalosporins
Fluoroquinolones
Macrolides
Quinolones
Polymyxins
Aminoglycosides
Aminopenicillins
Aminopenicillins with beta−lactamase inhibitors
Ansamycins
Phosphonic acid derivatives
Cattle
Chickens
Pigs
Sheep
Turkeys
Ducks
Horses
Buffaloes
Goats
Livestock species
Rank
0.00
0.25
0.50
0.75
1.00

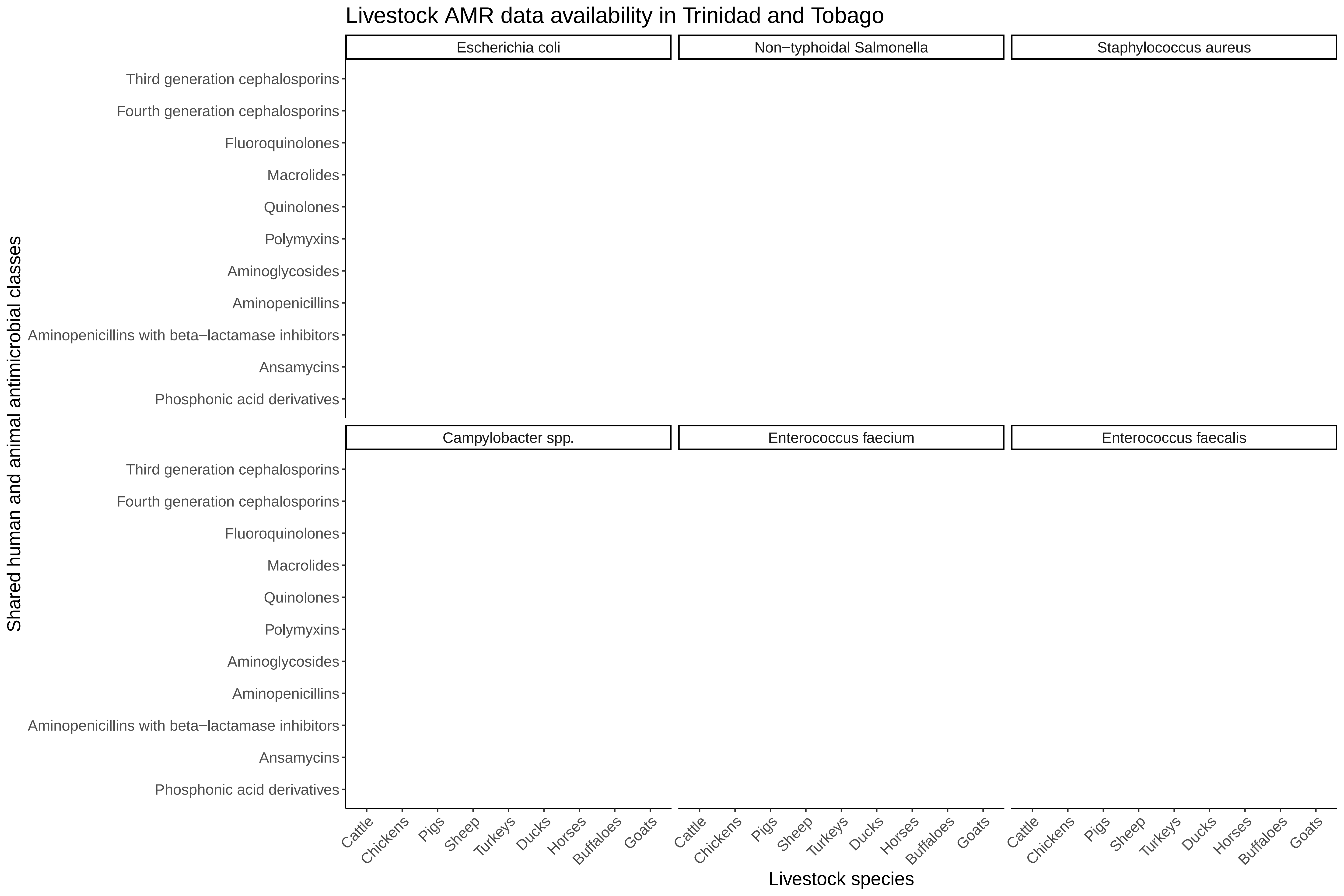

Livestock AMR data availability in Trinidad and Tobago
Escherichia coli
Non−typhoidal Salmonella
Staphylococcus aureus
Campylobacter spp.
Enterococcus faecium
Enterococcus faecalis
Third generation cephalosporins
Fourth generation cephalosporins
Fluoroquinolones
Macrolides
Quinolones
Polymyxins
Aminoglycosides
Aminopenicillins
Aminopenicillins with beta−lactamase inhibitors
Ansamycins
Phosphonic acid derivatives
Shared human and animal antimicrobial classes
Cattle
Chickens
Pigs
Sheep
Turkeys
Ducks
Horses
Buffaloes
Goats
Livestock species

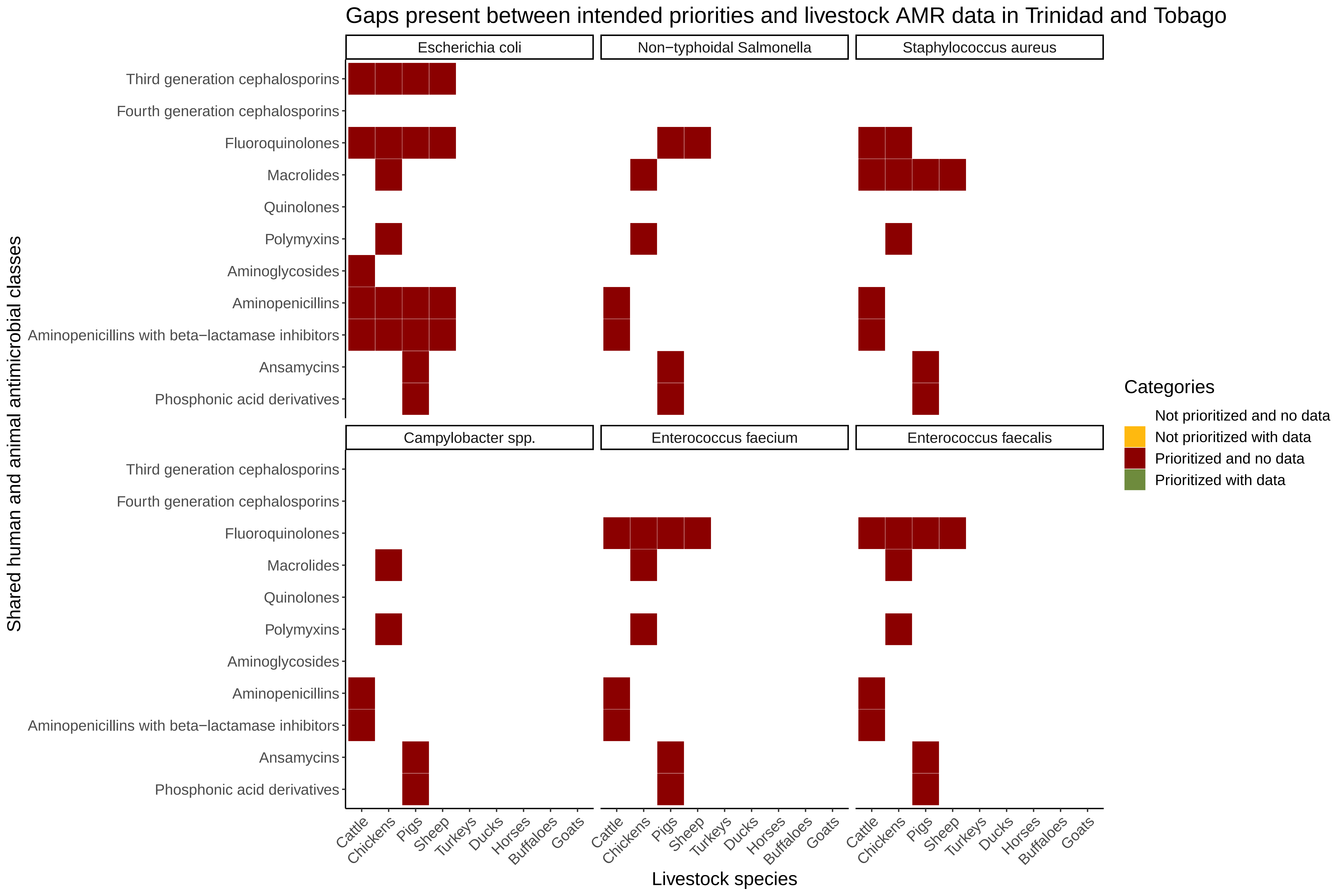

Gaps present between intended priorities and livestock AMR data in Trinidad and Tobago
Escherichia coli
Non−typhoidal Salmonella
Staphylococcus aureus
Campylobacter spp.
Enterococcus faecium
Enterococcus faecalis
Third generation cephalosporins
Fourth generation cephalosporins
Fluoroquinolones
Macrolides
Quinolones
Polymyxins
Aminoglycosides
Aminopenicillins
Aminopenicillins with beta−lactamase inhibitors
Ansamycins
Phosphonic acid derivatives
Shared human and animal antimicrobial classes
Cattle
Chickens
Pigs
Sheep
Turkeys
Ducks
Horses
Buffaloes
Goats
Livestock species
Categories
Not prioritized and no data
Not prioritized with data
Prioritized and no data
Prioritized with data

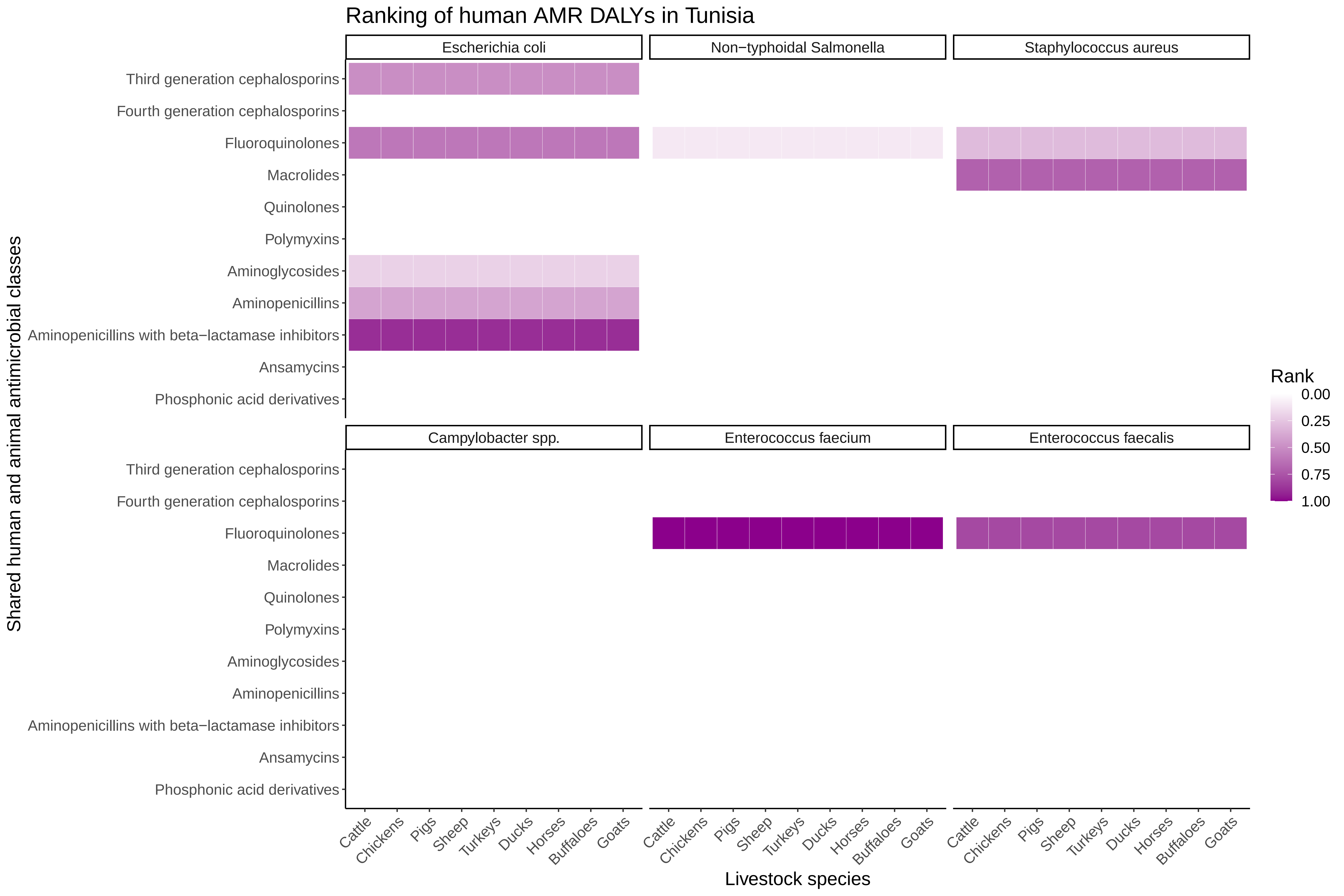

Ranking of human AMR DALYs in Tunisia
Escherichia coli
Non−typhoidal Salmonella
Staphylococcus aureus
Campylobacter spp.
Enterococcus faecium
Enterococcus faecalis
Third generation cephalosporins
Fourth generation cephalosporins
Fluoroquinolones
Macrolides
Quinolones
Polymyxins
Aminoglycosides
Aminopenicillins
Aminopenicillins with beta−lactamase inhibitors
Ansamycins
Phosphonic acid derivatives
Shared human and animal antimicrobial classes
Cattle
Chickens
Pigs
Sheep
Turkeys
Ducks
Horses
Buffaloes
Goats
Livestock species
Rank
0.00
0.25
0.50
0.75
1.00

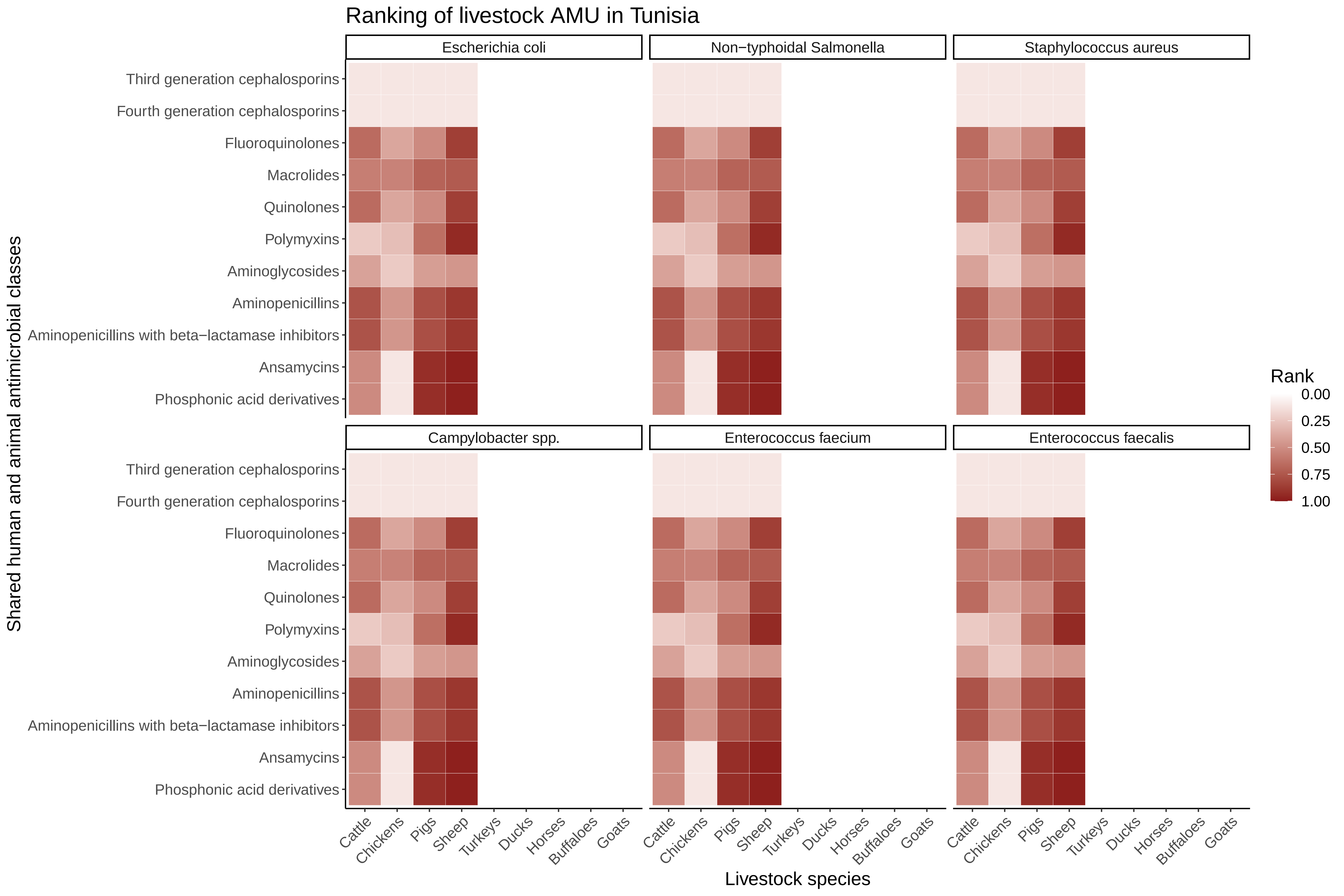

Ranking of livestock AMU in Tunisia
Escherichia coli
Non−typhoidal Salmonella
Staphylococcus aureus
Campylobacter spp.
Enterococcus faecium
Enterococcus faecalis
Third generation cephalosporins
Fourth generation cephalosporins
Fluoroquinolones
Macrolides
Quinolones
Polymyxins
Aminoglycosides
Aminopenicillins
Aminopenicillins with beta−lactamase inhibitors
Ansamycins
Phosphonic acid derivatives
Shared human and animal antimicrobial classes
Cattle
Chickens
Pigs
Sheep
Turkeys
Ducks
Horses
Buffaloes
Goats
Livestock species
Rank
0.00
0.25
0.50
0.75
1.00

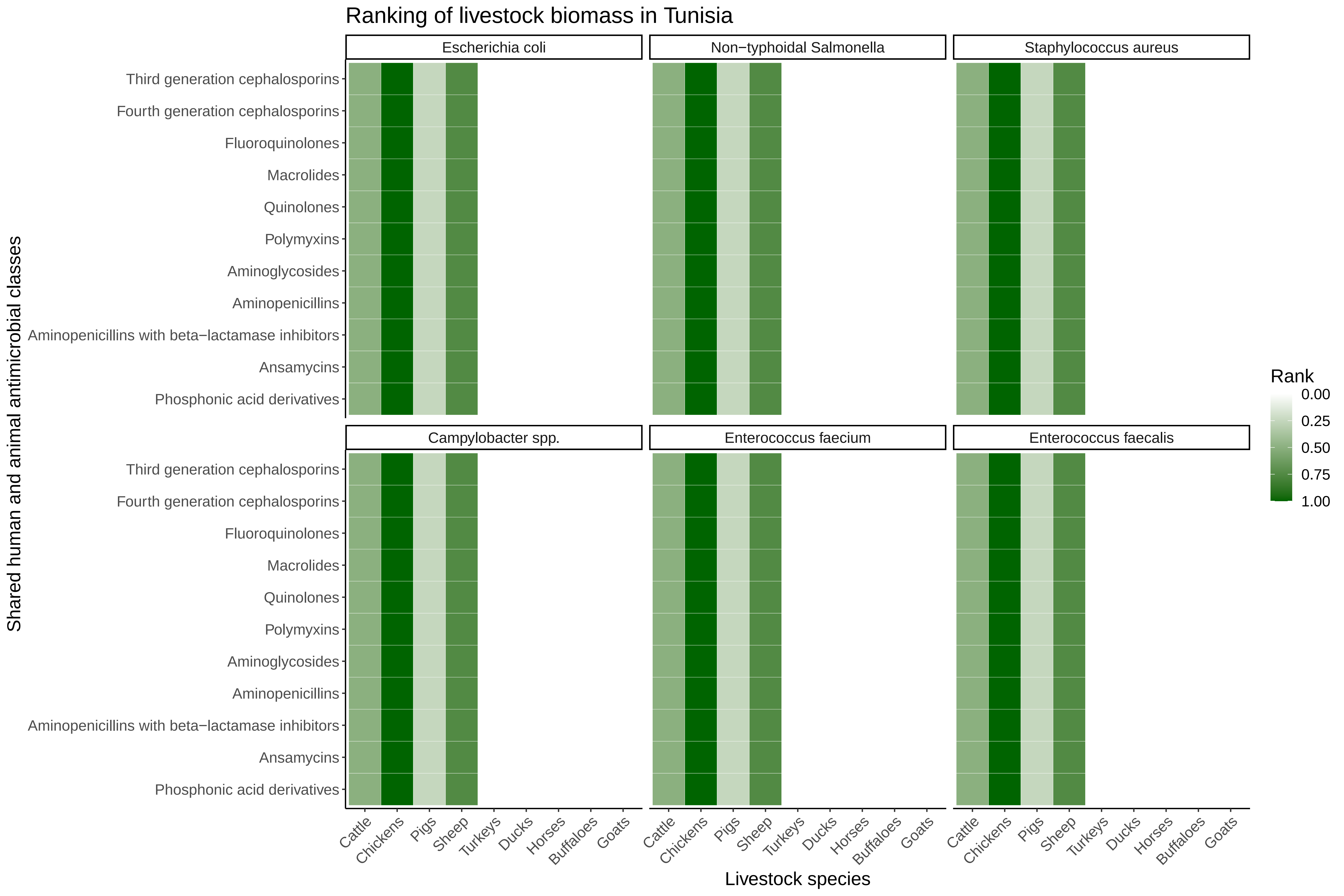

Ranking of livestock biomass in Tunisia
Escherichia coli
Non−typhoidal Salmonella
Staphylococcus aureus
Campylobacter spp.
Enterococcus faecium
Enterococcus faecalis
Third generation cephalosporins
Fourth generation cephalosporins
Fluoroquinolones
Macrolides
Quinolones
Polymyxins
Aminoglycosides
Aminopenicillins
Aminopenicillins with beta−lactamase inhibitors
Ansamycins
Phosphonic acid derivatives
Shared human and animal antimicrobial classes
Cattle
Chickens
Pigs
Sheep
Turkeys
Ducks
Horses
Buffaloes
Goats
Livestock species
Rank
0.00
0.25
0.50
0.75
1.00

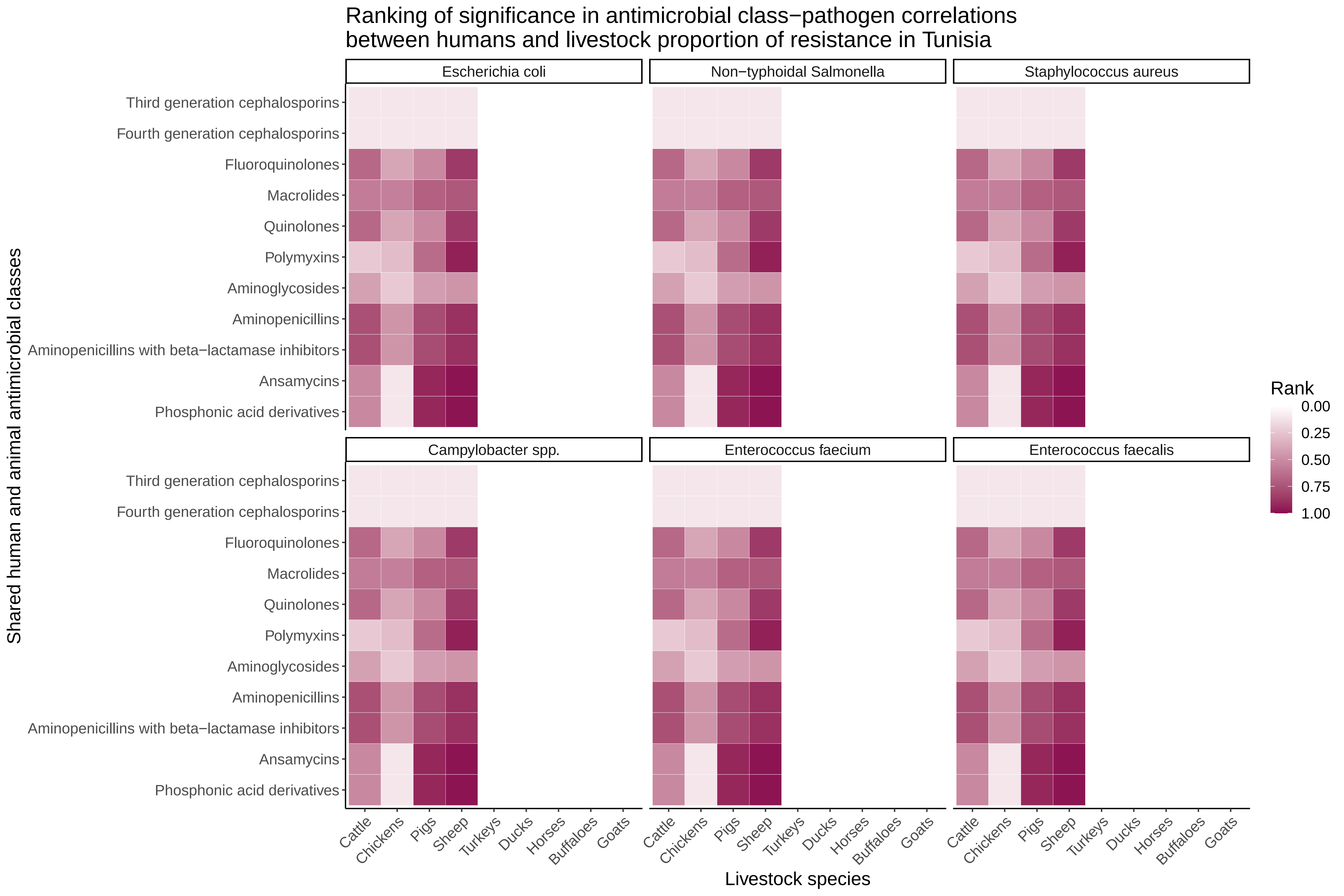
Ranking of significance in antimicrobial class−pathogen correlations
between humans and livestock proportion of resistance in Tunisia
Escherichia coli
Non−typhoidal Salmonella
Staphylococcus aureus
Campylobacter spp.
Enterococcus faecium
Enterococcus faecalis
Third generation cephalosporins
Fourth generation cephalosporins
Fluoroquinolones
Macrolides
Quinolones
Polymyxins
Aminoglycosides
Aminopenicillins
Aminopenicillins with beta−lactamase inhibitors
Ansamycins
Phosphonic acid derivatives
Shared human and animal antimicrobial classes
Cattle
Chickens
Pigs
Sheep
Turkeys
Ducks
Horses
Buffaloes
Goats
Livestock species
Rank
0.00
0.25
0.50
0.75
1.00

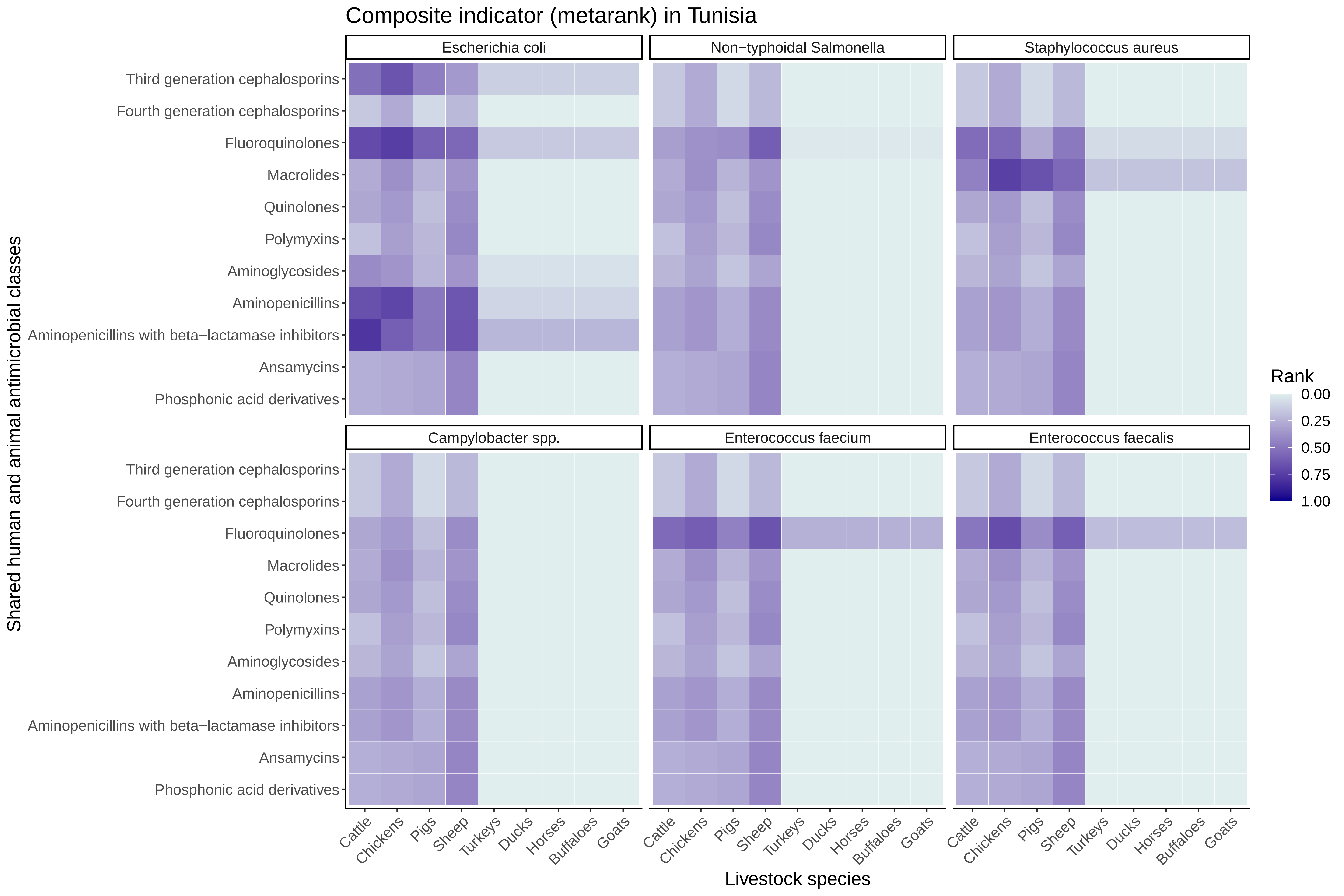
Composite indicator (metarank) in Tunisia
Escherichia coli
Non−typhoidal Salmonella
Staphylococcus aureus
Campylobacter spp.
Enterococcus faecium
Enterococcus faecalis
Shared human and animal antimicrobial classes
Third generation cephalosporins
Fourth generation cephalosporins
Fluoroquinolones
Macrolides
Quinolones
Polymyxins
Aminoglycosides
Aminopenicillins
Aminopenicillins with beta−lactamase inhibitors
Ansamycins
Phosphonic acid derivatives
Cattle
Chickens
Pigs
Sheep
Turkeys
Ducks
Horses
Buffaloes
Goats
Livestock species
Rank
0.00
0.25
0.50
0.75
1.00

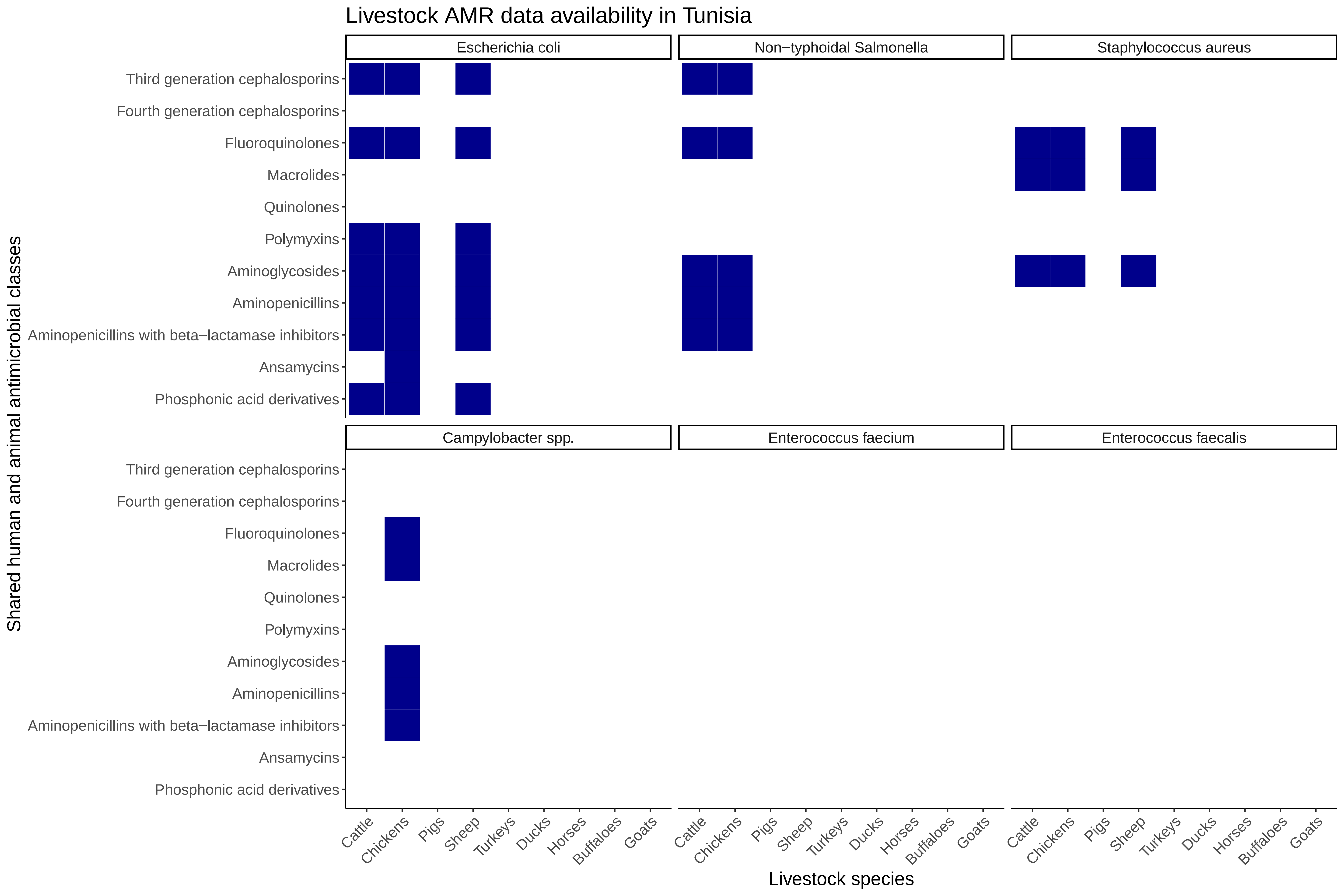

Livestock AMR data availability in Tunisia
Escherichia coli
Non−typhoidal Salmonella
Staphylococcus aureus
Campylobacter spp.
Enterococcus faecium
Enterococcus faecalis
Shared human and animal antimicrobial classes
Third generation cephalosporins
Fourth generation cephalosporins
Fluoroquinolones
Macrolides
Quinolones
Polymyxins
Aminoglycosides
Aminopenicillins
Aminopenicillins with beta−lactamase inhibitors
Ansamycins
Phosphonic acid derivatives
Cattle
Chickens
Pigs
Sheep
Turkeys
Ducks
Horses
Buffaloes
Goats
Livestock species

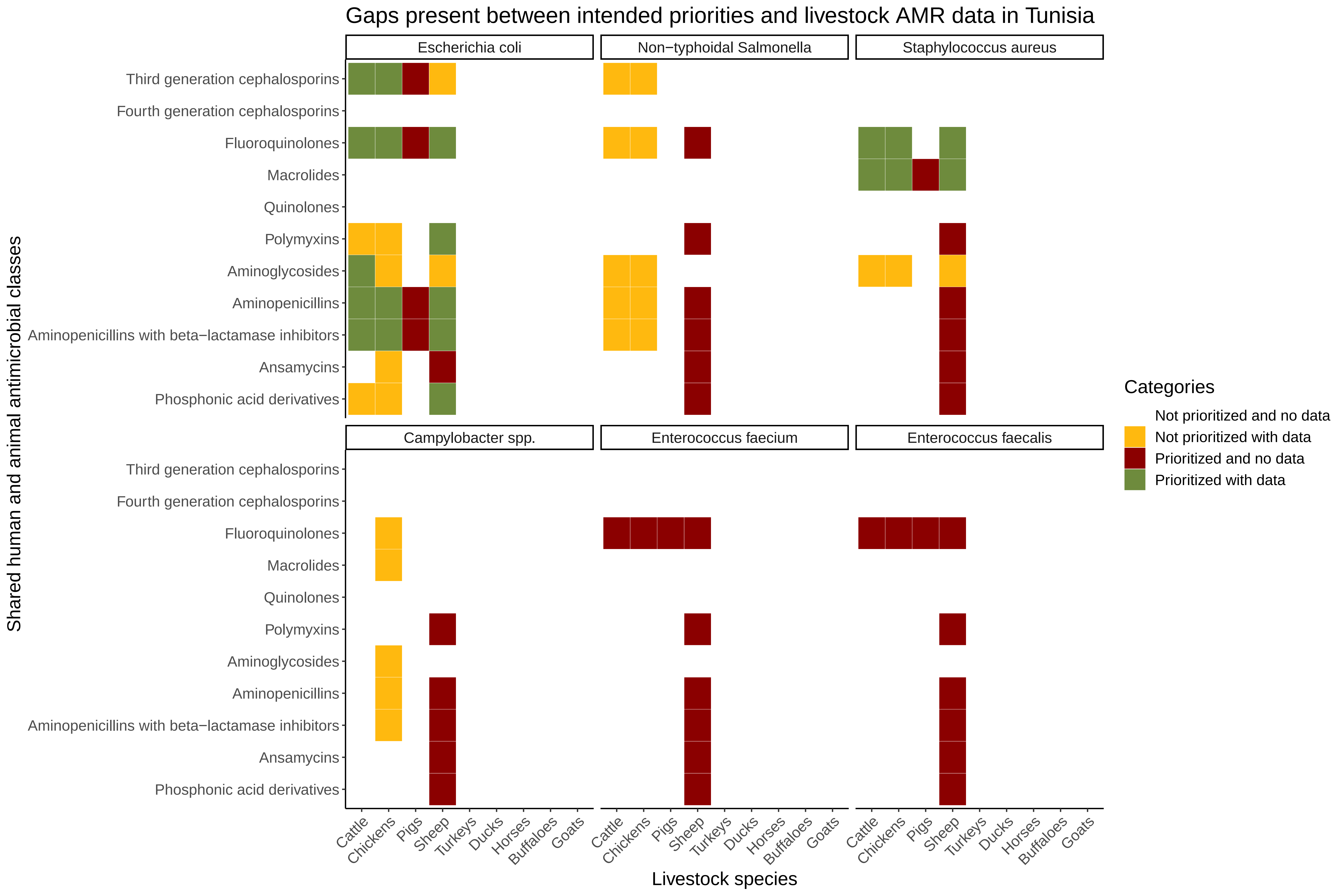

Gaps present between intended priorities and livestock AMR data in Tunisia
Escherichia coli
Non−typhoidal Salmonella
Staphylococcus aureus
Campylobacter spp.
Enterococcus faecium
Enterococcus faecalis
Third generation cephalosporins
Fourth generation cephalosporins
Fluoroquinolones
Macrolides
Quinolones
Polymyxins
Aminoglycosides
Aminopenicillins
Aminopenicillins with beta−lactamase inhibitors
Ansamycins
Phosphonic acid derivatives
Shared human and animal antimicrobial classes
Cattle
Chickens
Pigs
Sheep
Turkeys
Ducks
Horses
Buffaloes
Goats
Livestock species
Categories
Not prioritized and no data
Not prioritized with data
Prioritized and no data
Prioritized with data

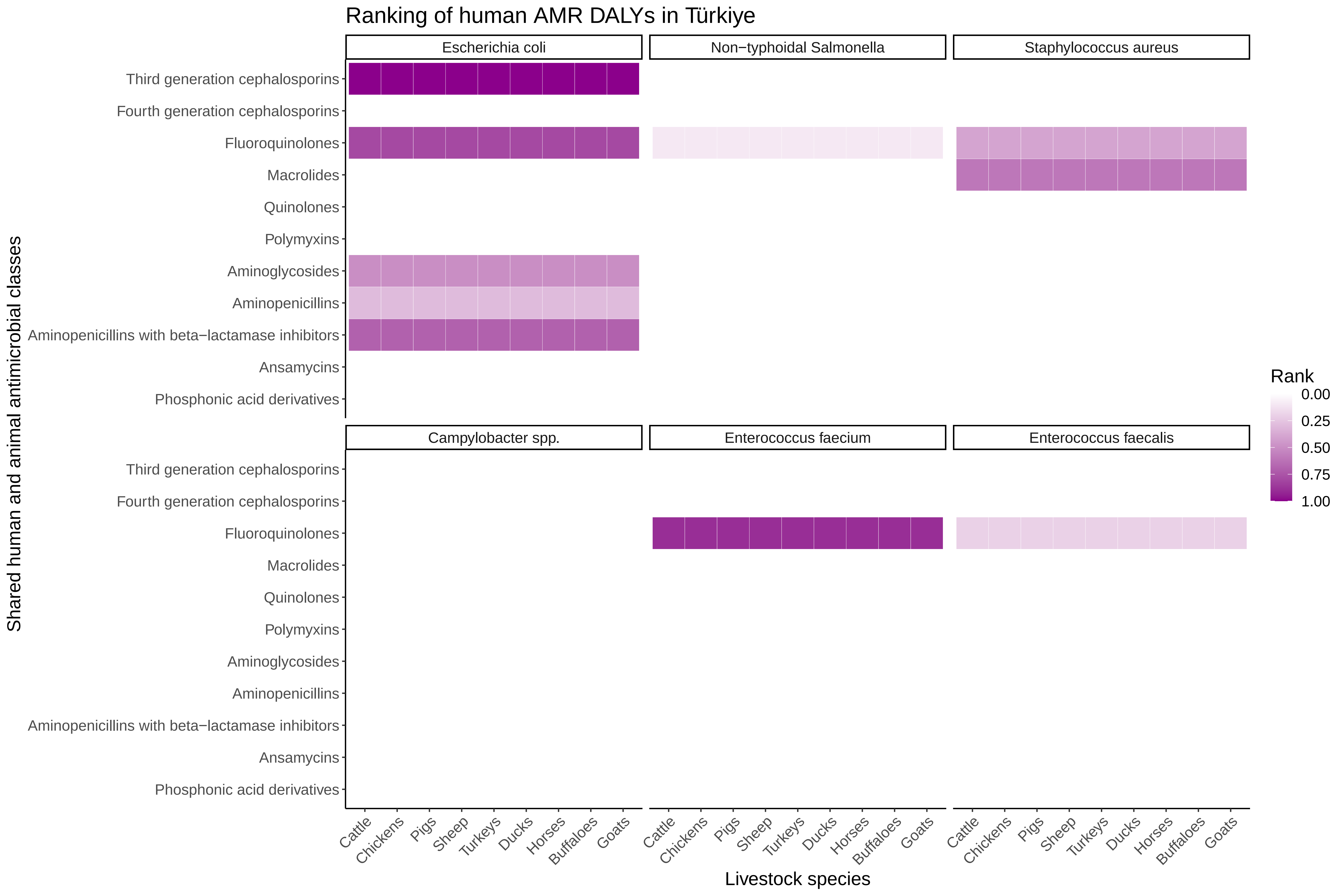

Ranking of human AMR DALYs in Türkiye
Escherichia coli
Non−typhoidal Salmonella
Staphylococcus aureus
Campylobacter spp.
Enterococcus faecium
Enterococcus faecalis
Third generation cephalosporins
Fourth generation cephalosporins
Fluoroquinolones
Macrolides
Quinolones
Polymyxins
Aminoglycosides
Aminopenicillins
Aminopenicillins with beta−lactamase inhibitors
Ansamycins
Phosphonic acid derivatives
Shared human and animal antimicrobial classes
Rank
0.00
0.25
0.50
0.75
1.00
Cattle
Chickens
Pigs
Sheep
Turkeys
Ducks
Horses
Buffaloes
Goats
Livestock species

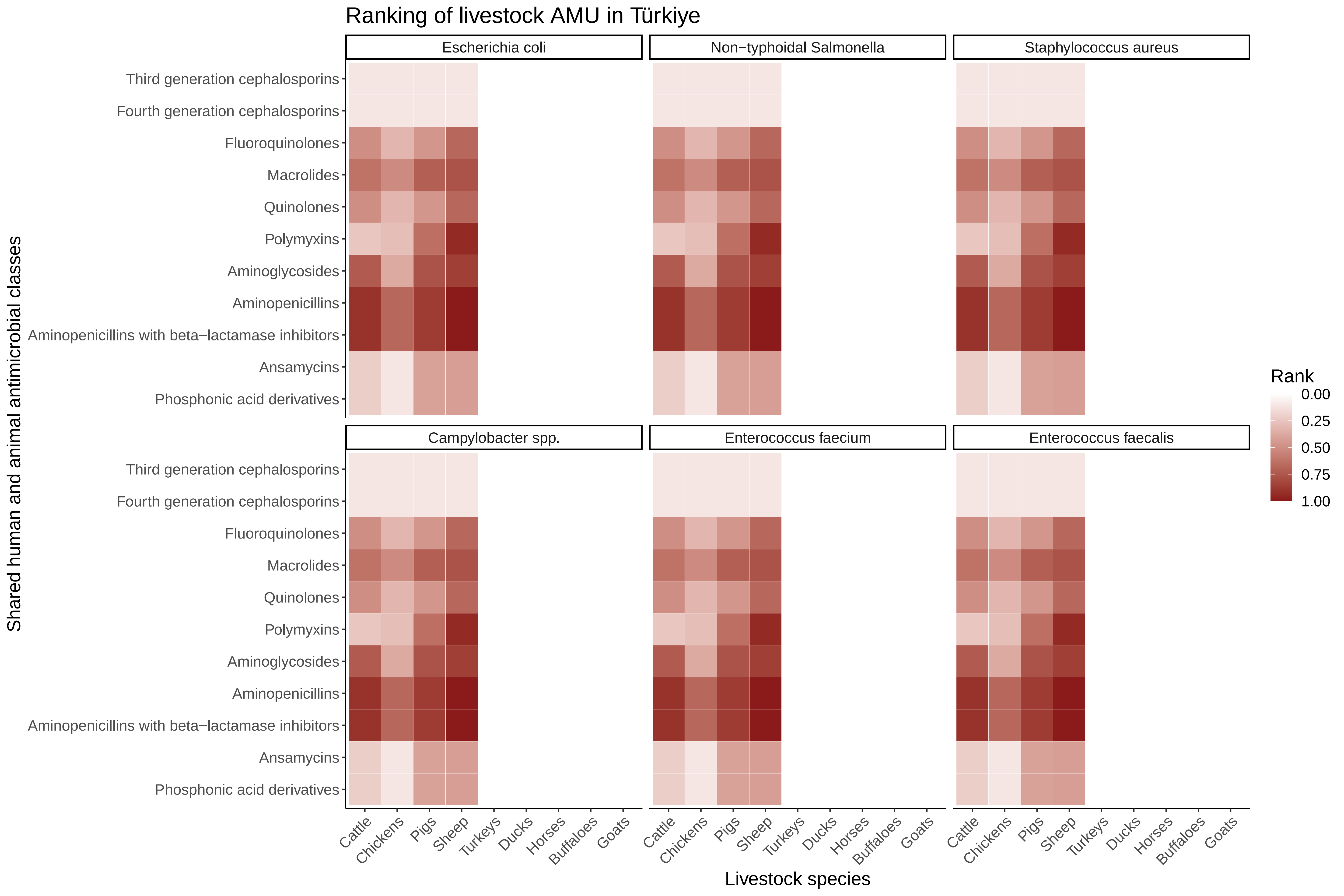
Ranking of livestock AMU in Türkiye
Escherichia coli
Non−typhoidal Salmonella
Staphylococcus aureus
Campylobacter spp.
Enterococcus faecium
Enterococcus faecalis
Third generation cephalosporins
Fourth generation cephalosporins
Fluoroquinolones
Macrolides
Quinolones
Polymyxins
Aminoglycosides
Aminopenicillins
Aminopenicillins with beta−lactamase inhibitors
Ansamycins
Phosphonic acid derivatives
Shared human and animal antimicrobial classes
Cattle
Chickens
Pigs
Sheep
Turkeys
Ducks
Horses
Buffaloes
Goats
Livestock species
Rank
0.00
0.25
0.50
0.75
1.00

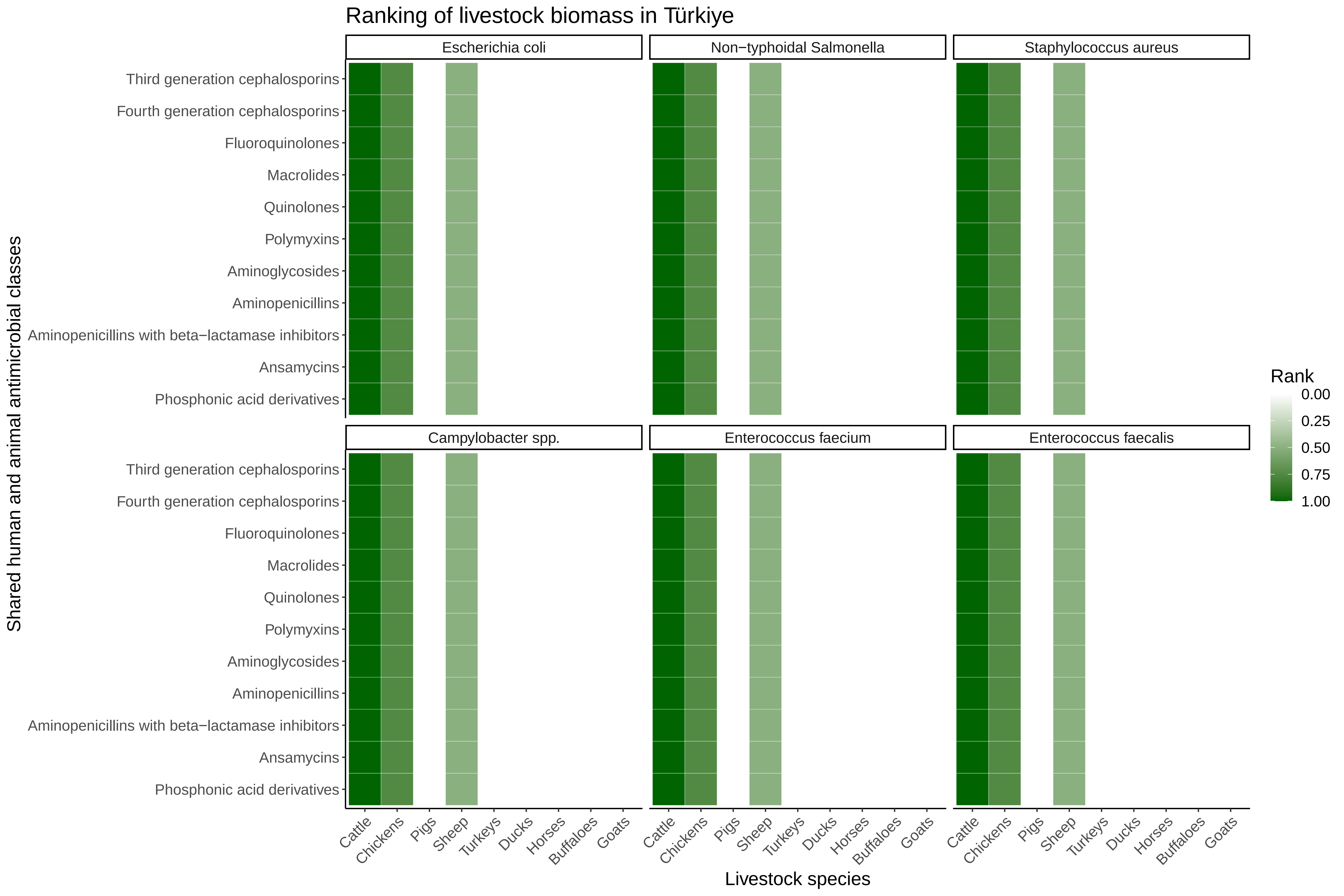

Ranking of livestock biomass in Türkiye
Escherichia coli
Non−typhoidal Salmonella
Staphylococcus aureus
Campylobacter spp.
Enterococcus faecium
Enterococcus faecalis
Shared human and animal antimicrobial classes
Third generation cephalosporins
Fourth generation cephalosporins
Fluoroquinolones
Macrolides
Quinolones
Polymyxins
Aminoglycosides
Aminopenicillins
Aminopenicillins with beta−lactamase inhibitors
Ansamycins
Phosphonic acid derivatives
Cattle
Chickens
Pigs
Sheep
Turkeys
Ducks
Horses
Buffaloes
Goats
Livestock species
Rank
0.00
0.25
0.50
0.75
1.00

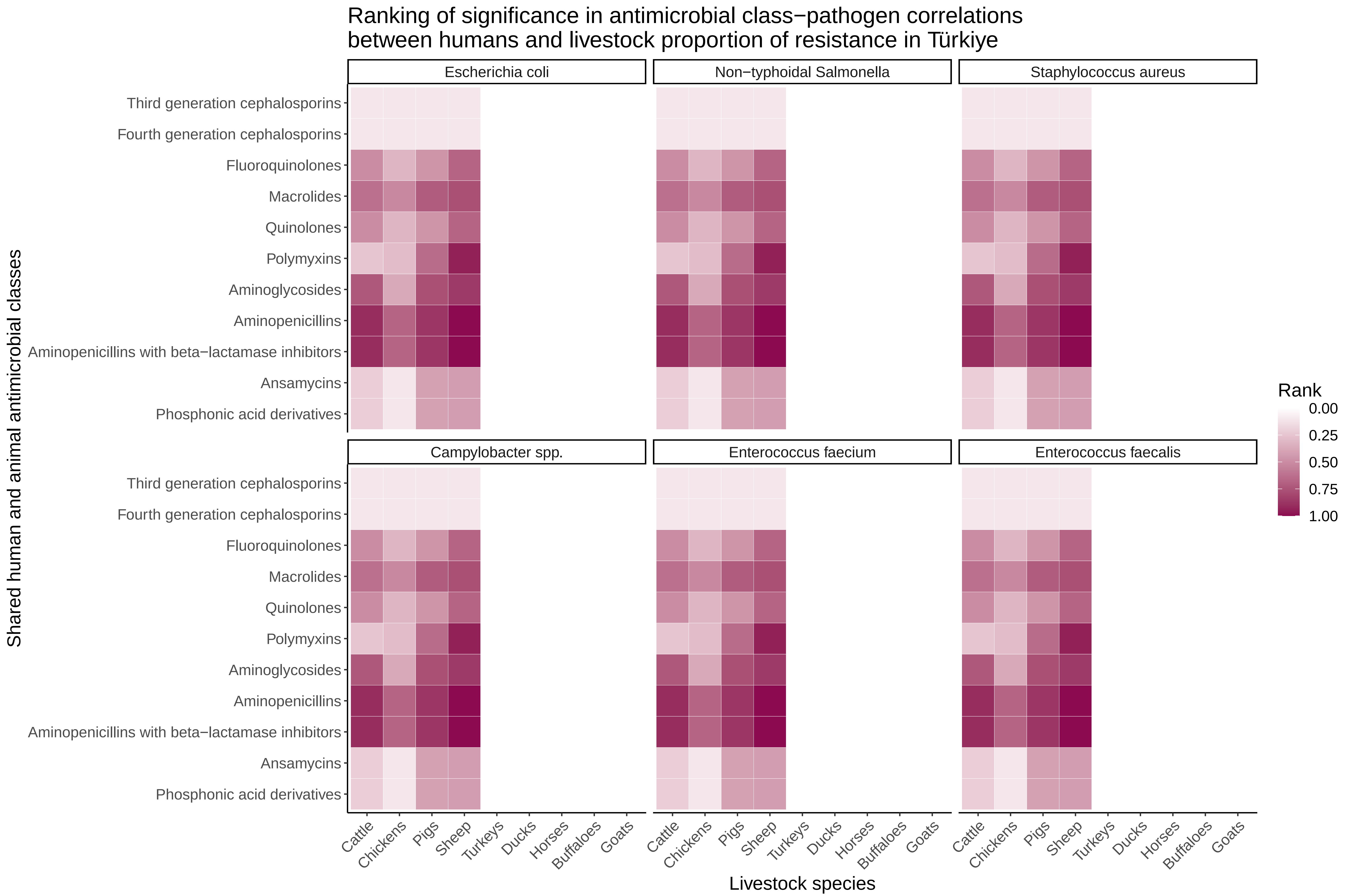

Ranking of significance in antimicrobial class−pathogen correlations
between humans and livestock proportion of resistance in Türkiye
Escherichia coli
Non−typhoidal Salmonella
Staphylococcus aureus
Campylobacter spp.
Enterococcus faecium
Enterococcus faecalis
Third generation cephalosporins
Fourth generation cephalosporins
Fluoroquinolones
Macrolides
Quinolones
Polymyxins
Aminoglycosides
Aminopenicillins
Aminopenicillins with beta−lactamase inhibitors
Ansamycins
Phosphonic acid derivatives
Shared human and animal antimicrobial classes
Cattle
Chickens
Pigs
Sheep
Turkeys
Ducks
Horses
Buffaloes
Goats
Livestock species
Rank
0.00
0.25
0.50
0.75
1.00

Composite indicator (metarank) in Türkiye

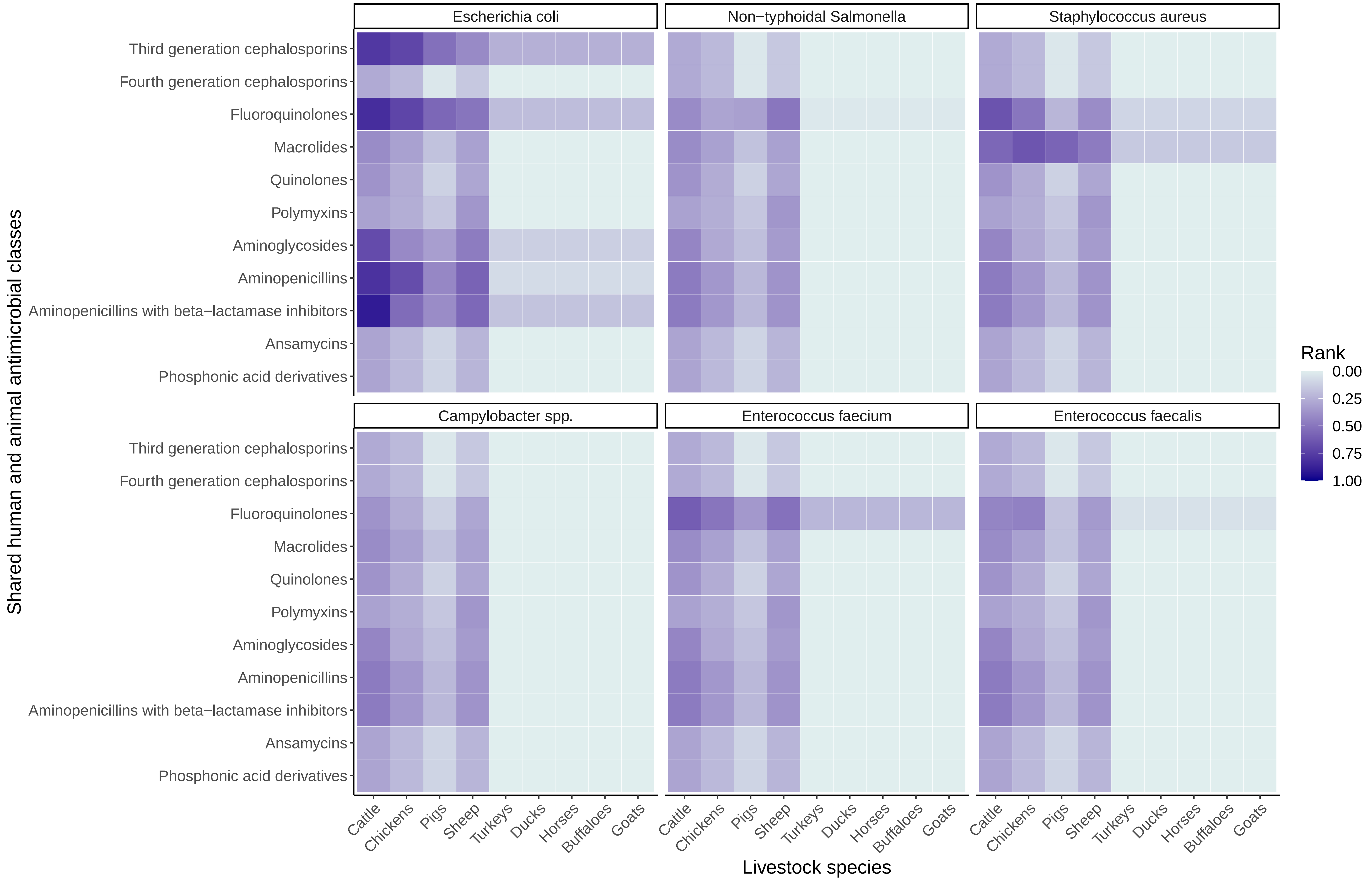

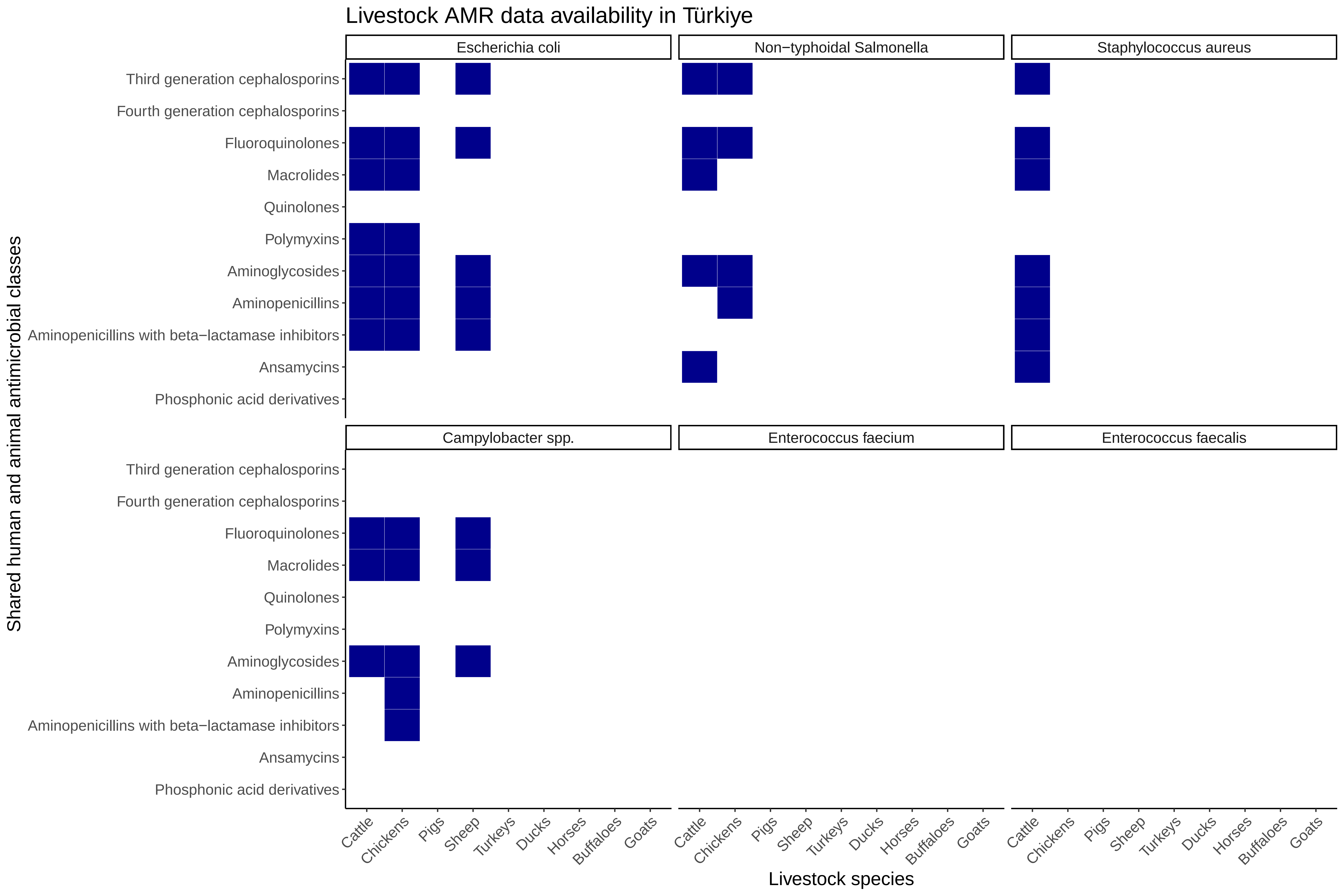

Livestock AMR data availability in Türkiye
Escherichia coli
Non−typhoidal Salmonella
Staphylococcus aureus
Campylobacter spp.
Enterococcus faecium
Enterococcus faecalis
Shared human and animal antimicrobial classes
Third generation cephalosporins
Fourth generation cephalosporins
Fluoroquinolones
Macrolides
Quinolones
Polymyxins
Aminoglycosides
Aminopenicillins
Aminopenicillins with beta−lactamase inhibitors
Ansamycins
Phosphonic acid derivatives
Cattle
Chickens
Pigs
Sheep
Turkeys
Ducks
Horses
Buffaloes
Goats
Livestock species

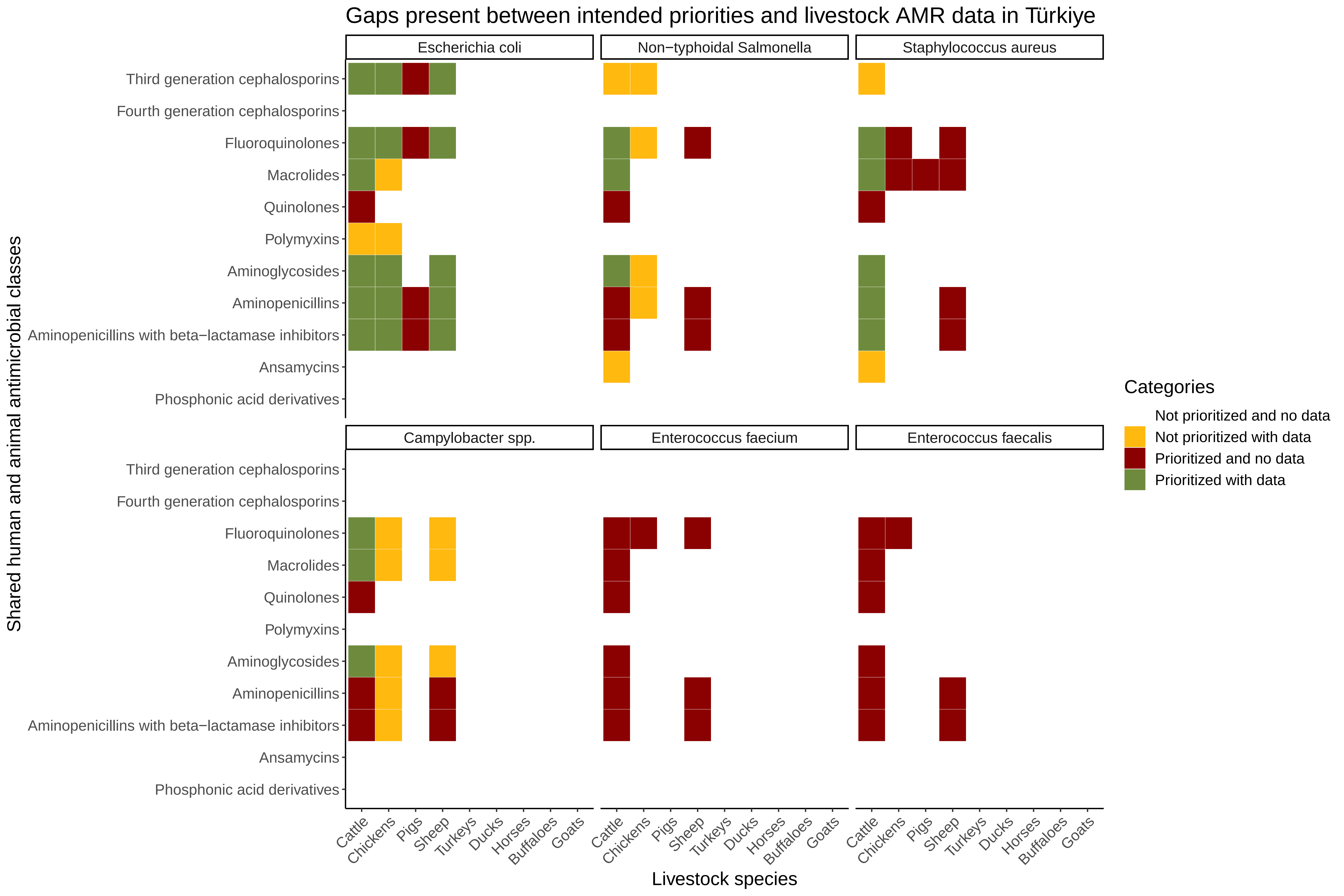
Gaps present between intended priorities and livestock AMR data in Türkiye
Escherichia coli
Non−typhoidal Salmonella
Staphylococcus aureus
Campylobacter spp.
Enterococcus faecium
Enterococcus faecalis
Shared human and animal antimicrobial classes
Third generation cephalosporins
Fourth generation cephalosporins
Fluoroquinolones
Macrolides
Quinolones
Polymyxins
Aminoglycosides
Aminopenicillins
Aminopenicillins with beta−lactamase inhibitors
Ansamycins
Phosphonic acid derivatives
Livestock species
Cattle
Chickens
Pigs
Sheep
Turkeys
Ducks
Horses
Buffaloes
Goats
Categories
Not prioritized and no data
Not prioritized with data
Prioritized and no data
Prioritized with data

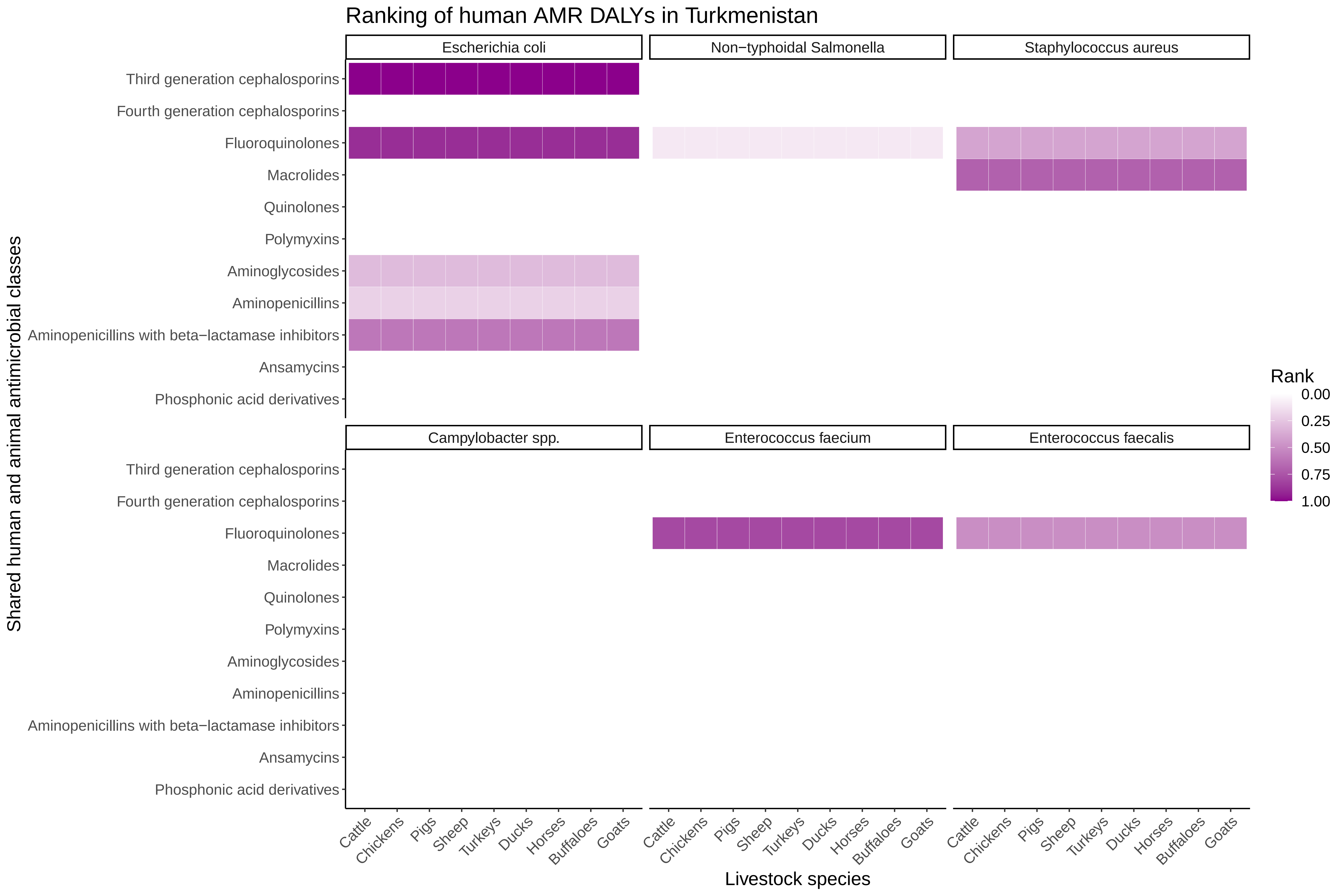

Ranking of human AMR DALYs in Turkmenistan
Escherichia coli
Non−typhoidal Salmonella
Staphylococcus aureus
Campylobacter spp.
Enterococcus faecium
Enterococcus faecalis
Third generation cephalosporins
Fourth generation cephalosporins
Fluoroquinolones
Macrolides
Quinolones
Polymyxins
Aminoglycosides
Aminopenicillins
Aminopenicillins with beta−lactamase inhibitors
Ansamycins
Phosphonic acid derivatives
Shared human and animal antimicrobial classes
Cattle
Chickens
Pigs
Sheep
Turkeys
Ducks
Horses
Buffaloes
Goats
Livestock species
Rank
0.00
0.25
0.50
0.75
1.00

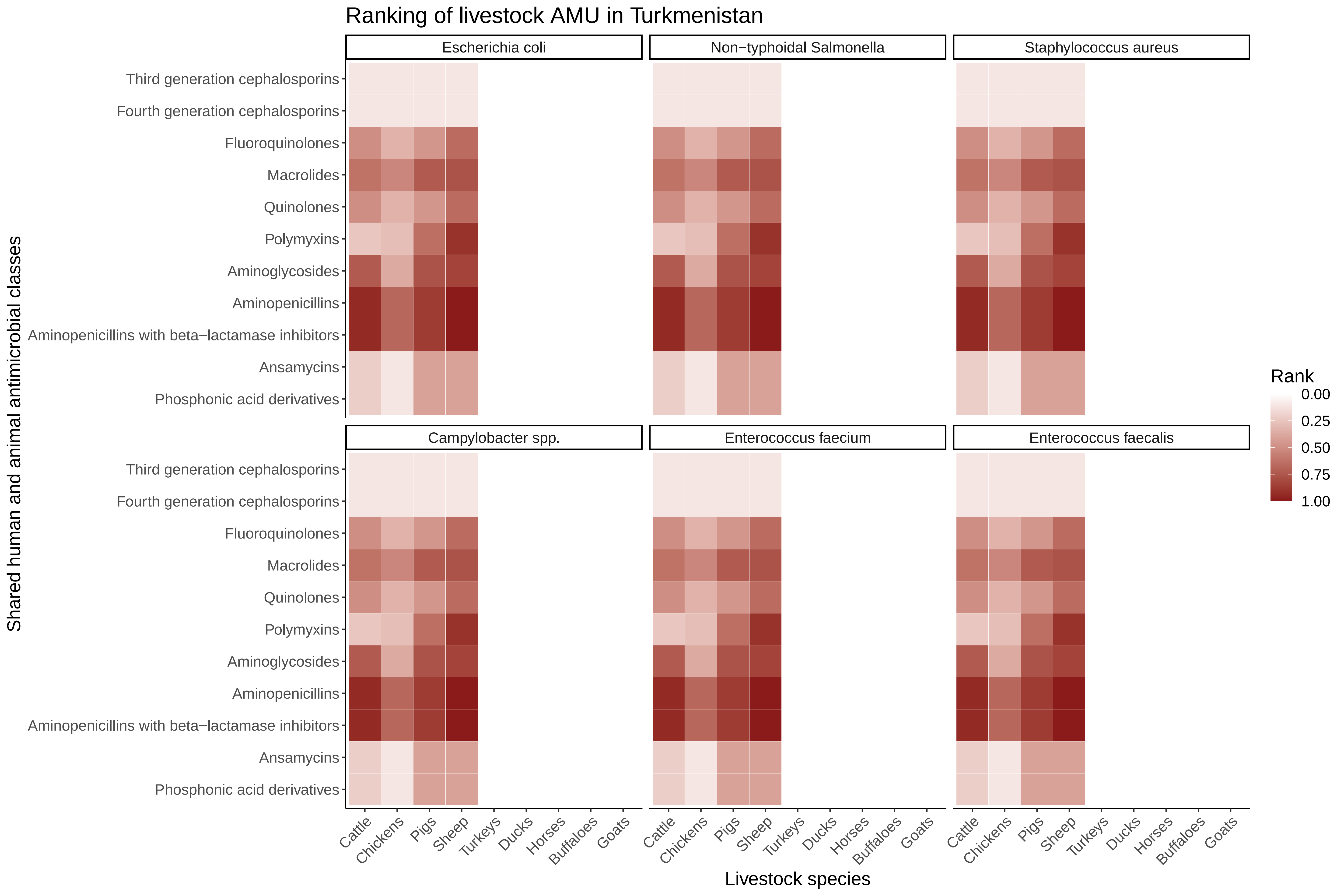

Ranking of livestock AMU in Turkmenistan
Escherichia coli
Non−typhoidal Salmonella
Staphylococcus aureus
Campylobacter spp.
Enterococcus faecium
Enterococcus faecalis
Shared human and animal antimicrobial classes
Third generation cephalosporins
Fourth generation cephalosporins
Fluoroquinolones
Macrolides
Quinolones
Polymyxins
Aminoglycosides
Aminopenicillins
Aminopenicillins with beta−lactamase inhibitors
Ansamycins
Phosphonic acid derivatives
Cattle
Chickens
Pigs
Sheep
Turkeys
Ducks
Horses
Buffaloes
Goats
Livestock species
Rank
0.00
0.25
0.50
0.75
1.00

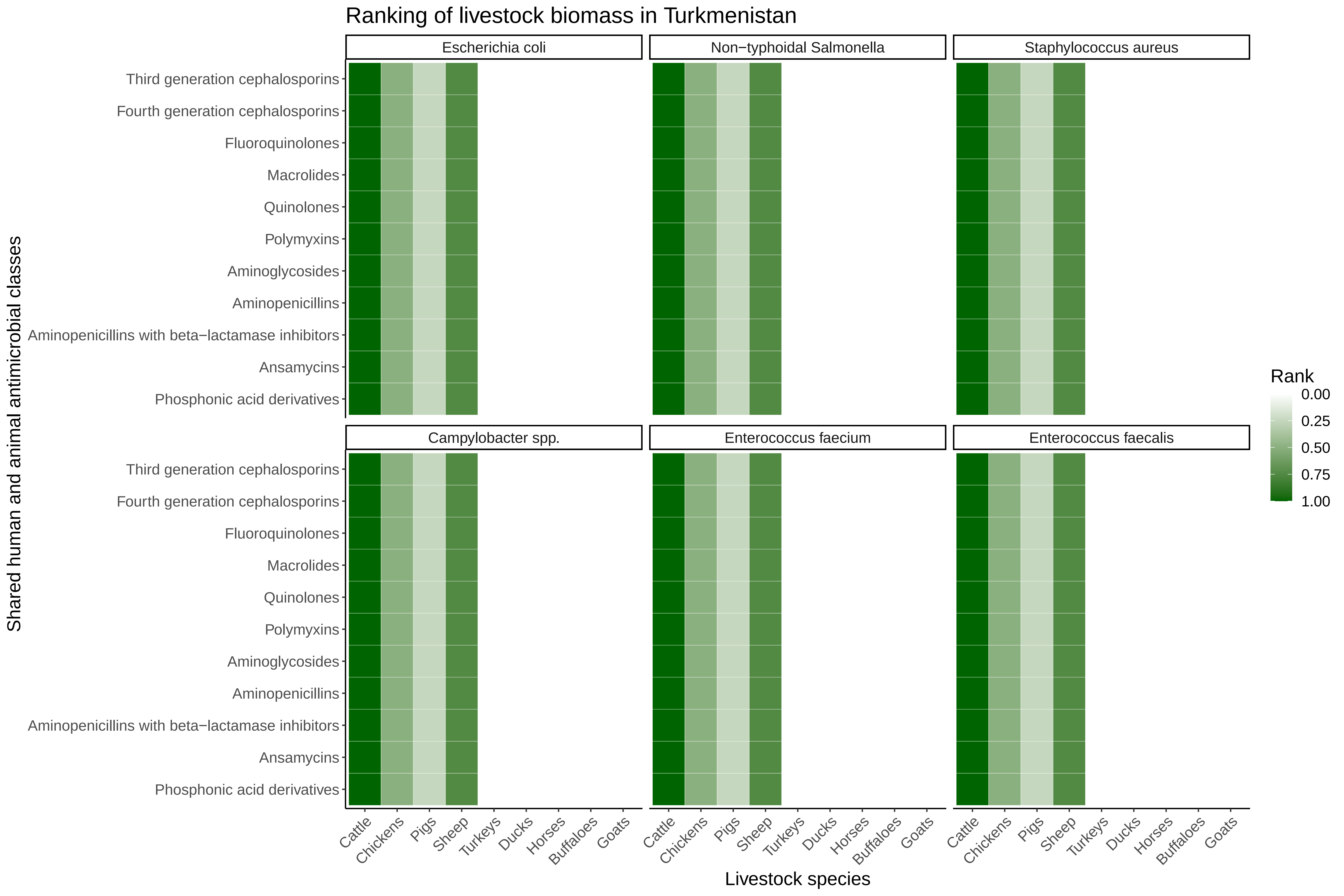
Ranking of livestock biomass in Turkmenistan
Escherichia coli
Non−typhoidal Salmonella
Staphylococcus aureus
Campylobacter spp.
Enterococcus faecium
Enterococcus faecalis
Third generation cephalosporins
Fourth generation cephalosporins
Fluoroquinolones
Macrolides
Quinolones
Polymyxins
Aminoglycosides
Aminopenicillins
Aminopenicillins with beta−lactamase inhibitors
Ansamycins
Phosphonic acid derivatives
Shared human and animal antimicrobial classes
Cattle
Chickens
Pigs
Sheep
Turkeys
Ducks
Horses
Buffaloes
Goats
Livestock species
Rank
0.00
0.25
0.50
0.75
1.00

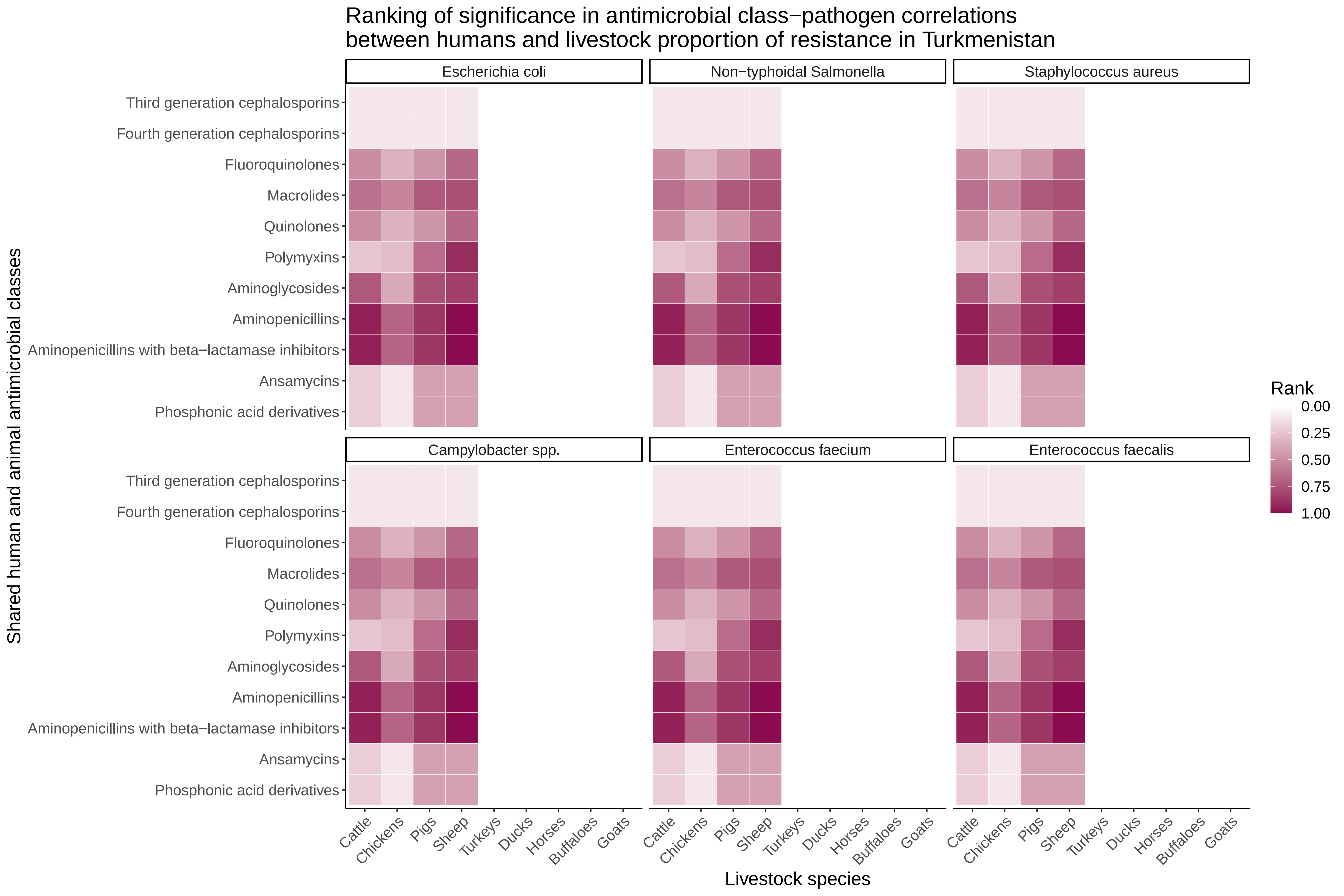
Ranking of significance in antimicrobial class−pathogen correlations
between humans and livestock proportion of resistance in Turkmenistan
Escherichia coli
Non−typhoidal Salmonella
Staphylococcus aureus
Campylobacter spp.
Enterococcus faecium
Enterococcus faecalis
Shared human and animal antimicrobial classes
Third generation cephalosporins
Fourth generation cephalosporins
Fluoroquinolones
Macrolides
Quinolones
Polymyxins
Aminoglycosides
Aminopenicillins
Aminopenicillins with beta−lactamase inhibitors
Ansamycins
Phosphonic acid derivatives
Livestock species
Cattle
Chickens
Pigs
Sheep
Turkeys
Ducks
Horses
Buffaloes
Goats
Rank
0.00
0.25
0.50
0.75
1.00

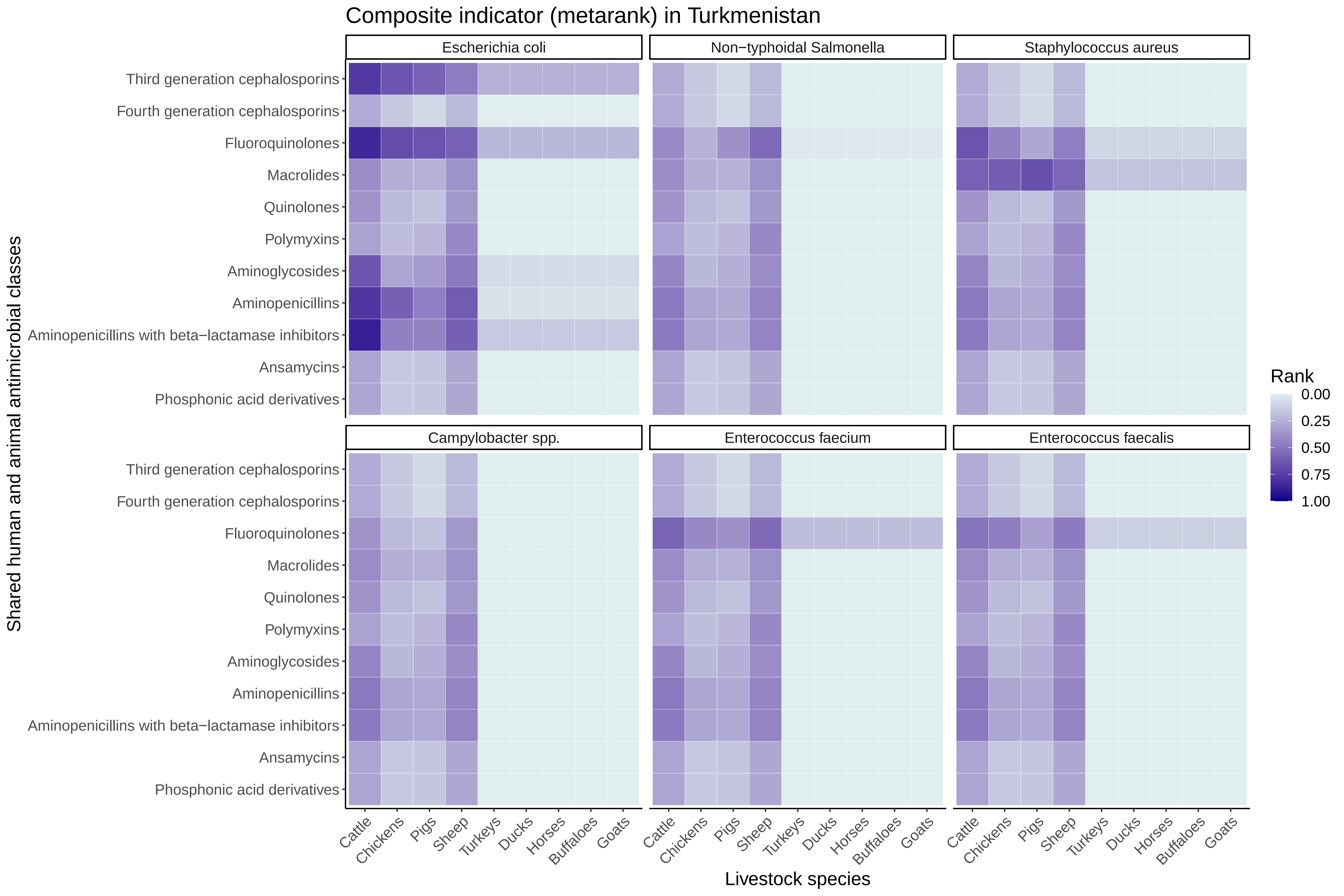
Composite indicator (metarank) in Turkmenistan
Escherichia coli
Non−typhoidal Salmonella
Staphylococcus aureus
Campylobacter spp.
Enterococcus faecium
Enterococcus faecalis
Third generation cephalosporins
Fourth generation cephalosporins
Fluoroquinolones
Macrolides
Quinolones
Polymyxins
Aminoglycosides
Aminopenicillins
Aminopenicillins with beta−lactamase inhibitors
Ansamycins
Phosphonic acid derivatives
Shared human and animal antimicrobial classes
Cattle
Chickens
Pigs
Sheep
Turkeys
Ducks
Horses
Buffaloes
Goats
Livestock species
Rank
0.00
0.25
0.50
0.75
1.00

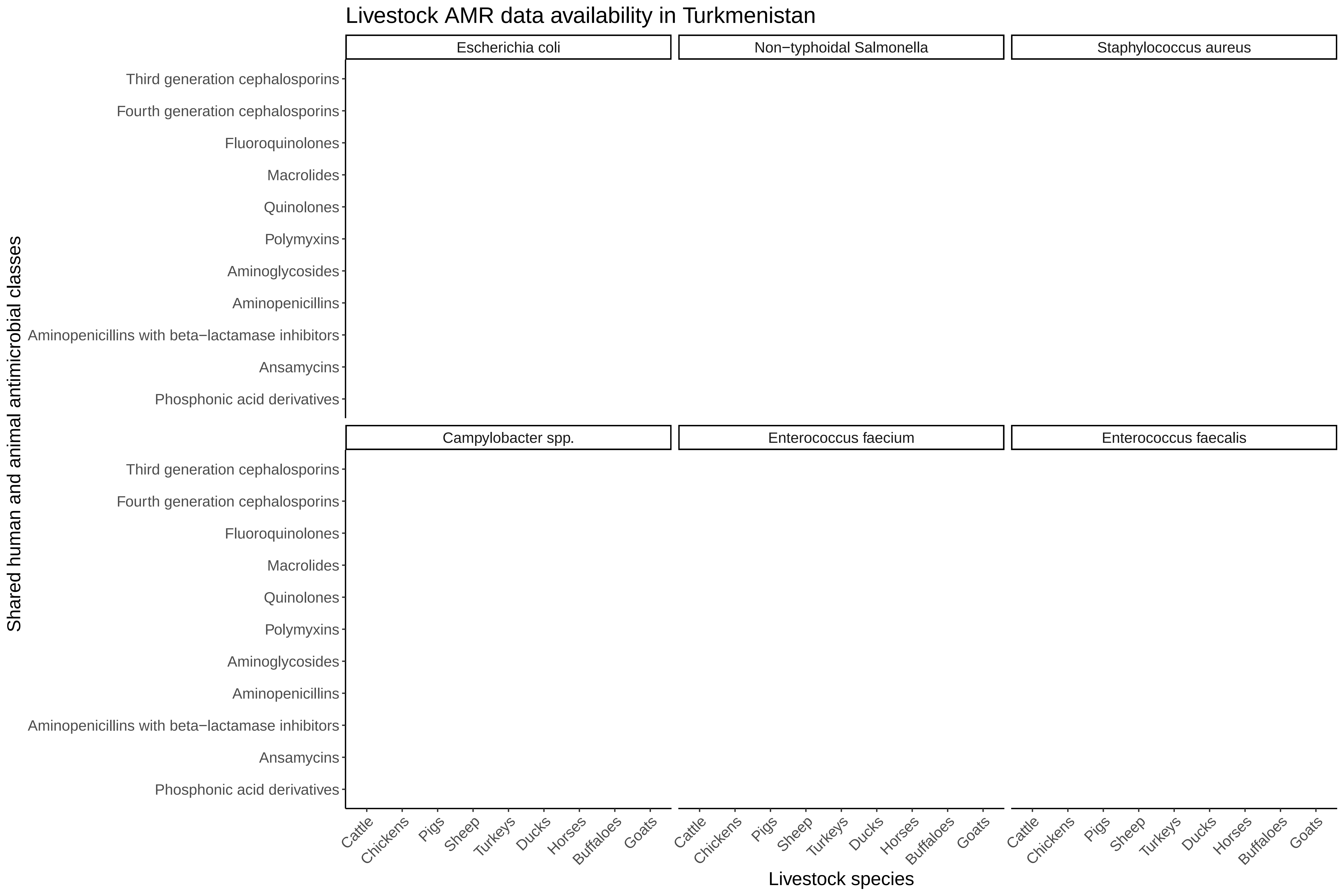
Livestock AMR data availability in Turkmenistan
Escherichia coli
Non−typhoidal Salmonella
Staphylococcus aureus
Campylobacter spp.
Enterococcus faecium
Enterococcus faecalis
Shared human and animal antimicrobial classes
Third generation cephalosporins
Fourth generation cephalosporins
Fluoroquinolones
Macrolides
Quinolones
Polymyxins
Aminoglycosides
Aminopenicillins
Aminopenicillins with beta−lactamase inhibitors
Ansamycins
Phosphonic acid derivatives
Cattle
Chickens
Pigs
Sheep
Turkeys
Ducks
Horses
Buffaloes
Goats
Livestock species

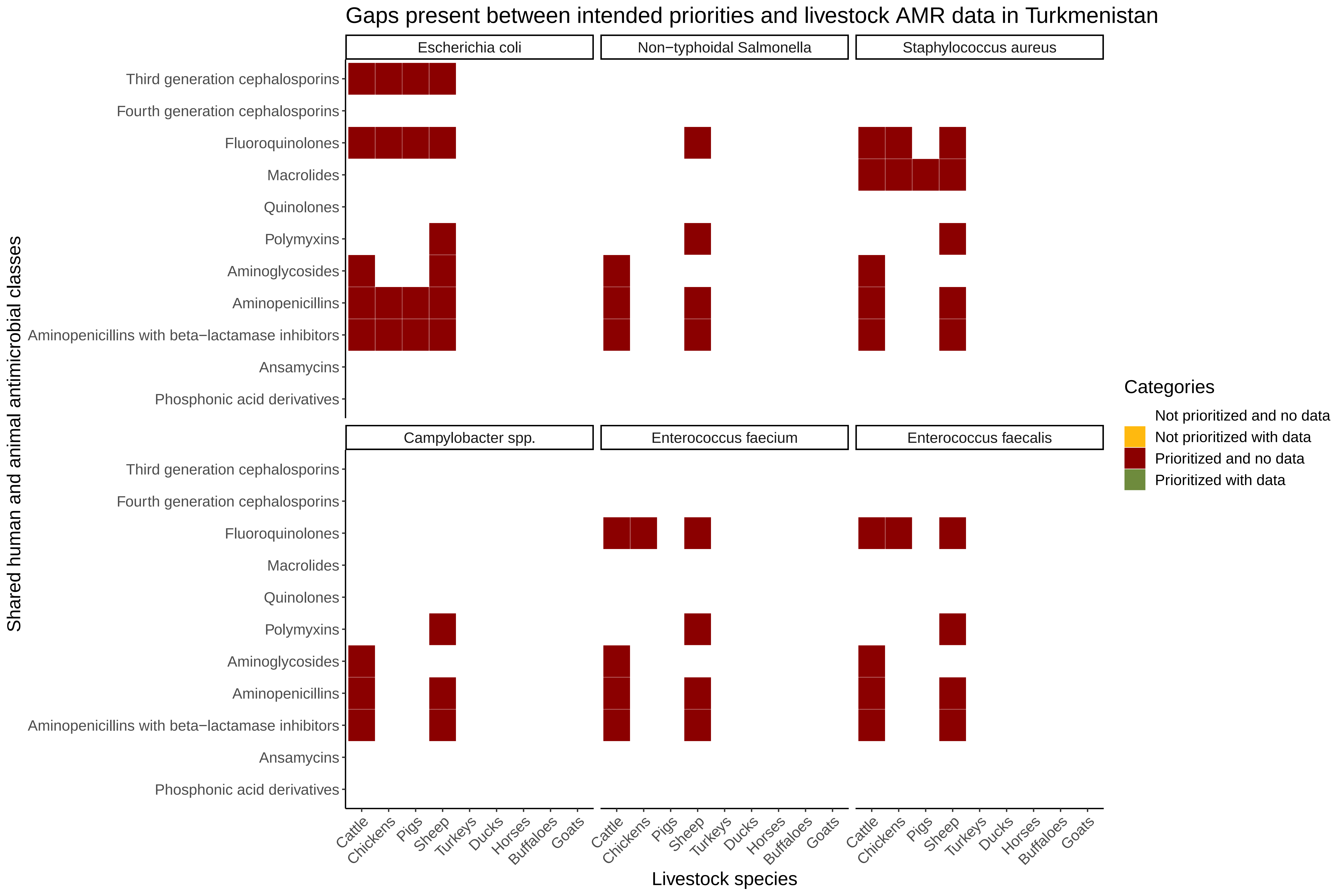

Gaps present between intended priorities and livestock AMR data in Turkmenistan
Escherichia coli
Non−typhoidal Salmonella
Staphylococcus aureus
Campylobacter spp.
Enterococcus faecium
Enterococcus faecalis
Shared human and animal antimicrobial classes
Third generation cephalosporins
Fourth generation cephalosporins
Fluoroquinolones
Macrolides
Quinolones
Polymyxins
Aminoglycosides
Aminopenicillins
Aminopenicillins with beta−lactamase inhibitors
Ansamycins
Phosphonic acid derivatives
Cattle
Chickens
Pigs
Sheep
Turkeys
Ducks
Horses
Buffaloes
Goats
Livestock species
Categories
Not prioritized and no data
Not prioritized with data
Prioritized and no data
Prioritized with data

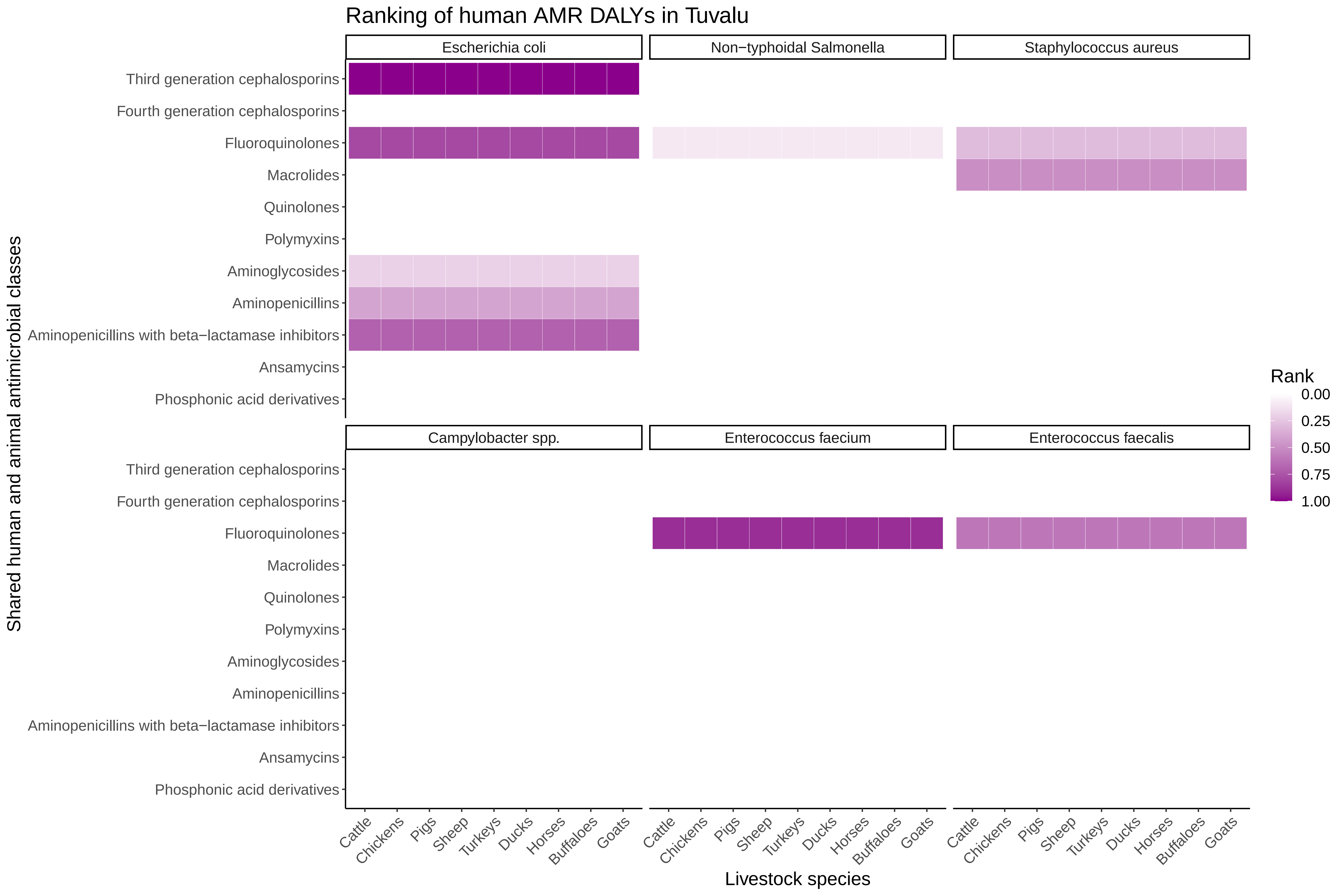

Ranking of human AMR DALYs in Tuvalu
Escherichia coli
Non−typhoidal Salmonella
Staphylococcus aureus
Campylobacter spp.
Enterococcus faecium
Enterococcus faecalis
Third generation cephalosporins
Fourth generation cephalosporins
Fluoroquinolones
Macrolides
Quinolones
Polymyxins
Aminoglycosides
Aminopenicillins
Aminopenicillins with beta−lactamase inhibitors
Ansamycins
Phosphonic acid derivatives
Shared human and animal antimicrobial classes
Cattle
Chickens
Pigs
Sheep
Turkeys
Ducks
Horses
Buffaloes
Goats
Livestock species
Rank
0.00
0.25
0.50
0.75
1.00

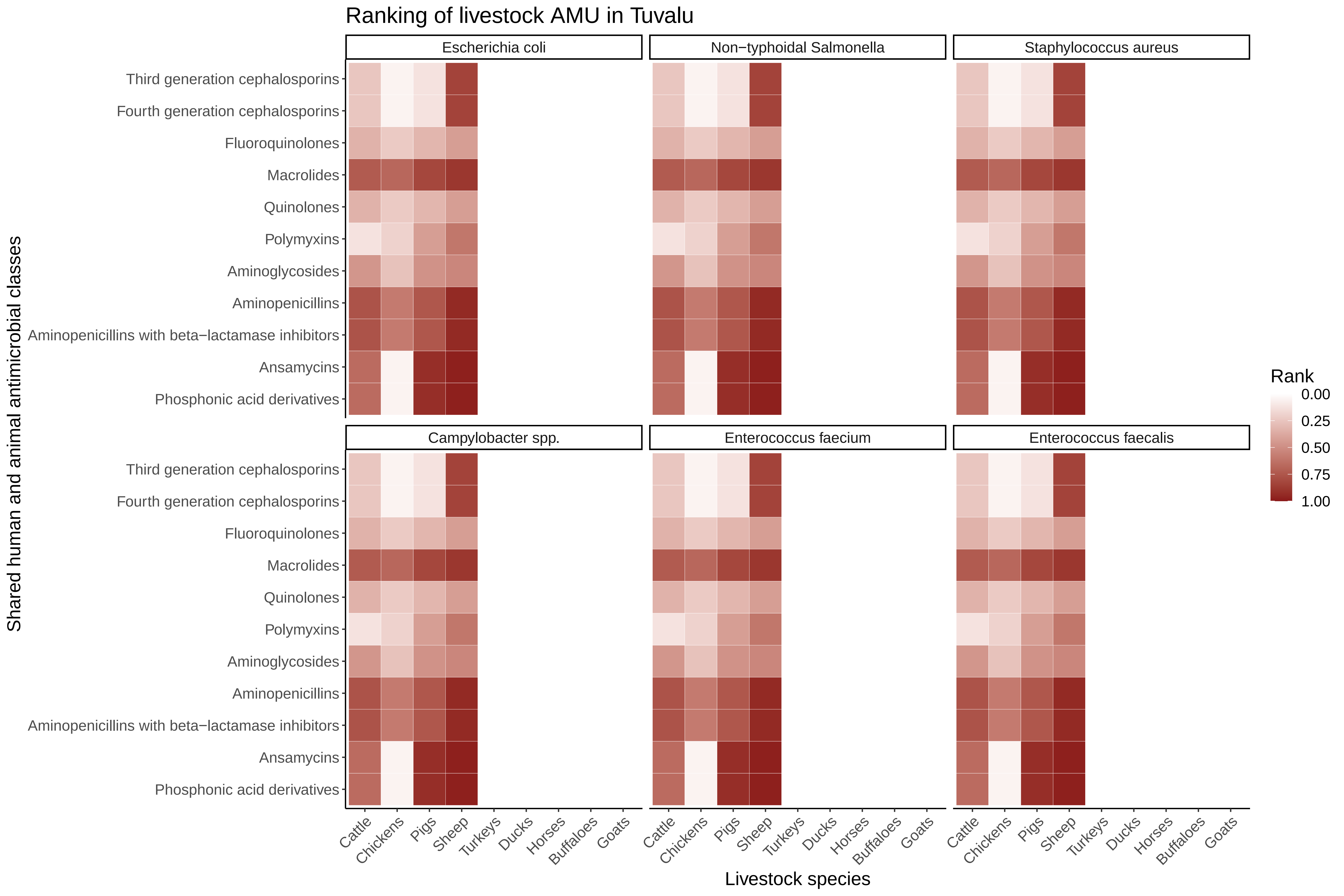

Ranking of livestock AMU in Tuvalu
Escherichia coli
Non−typhoidal Salmonella
Staphylococcus aureus
Campylobacter spp.
Enterococcus faecium
Enterococcus faecalis
Shared human and animal antimicrobial classes
Third generation cephalosporins
Fourth generation cephalosporins
Fluoroquinolones
Macrolides
Quinolones
Polymyxins
Aminoglycosides
Aminopenicillins
Aminopenicillins with beta−lactamase inhibitors
Ansamycins
Phosphonic acid derivatives
Cattle
Chickens
Pigs
Sheep
Turkeys
Ducks
Horses
Buffaloes
Goats
Livestock species
Rank
0.00
0.25
0.50
0.75
1.00

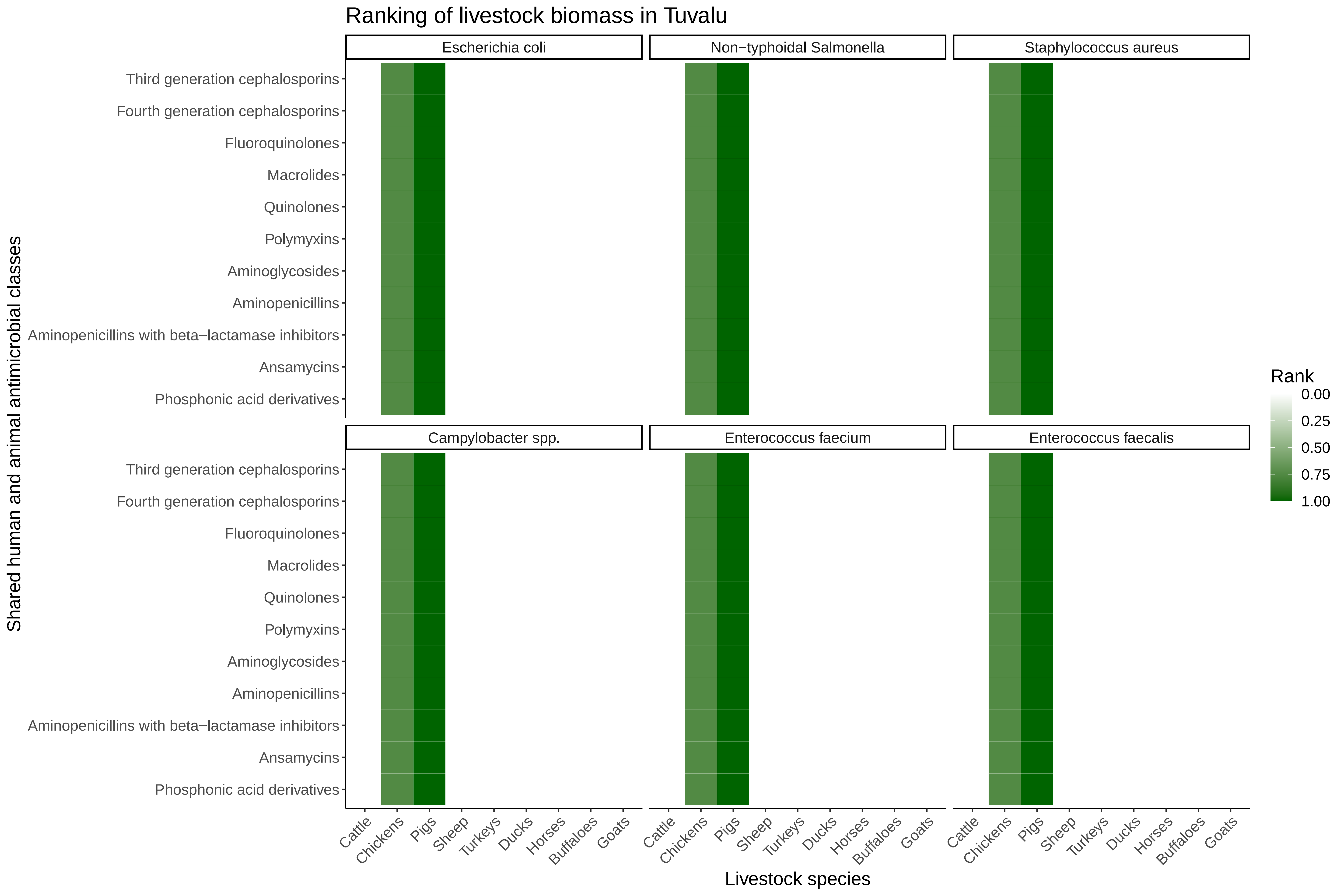
Ranking of livestock biomass in Tuvalu
Escherichia coli
Non−typhoidal Salmonella
Staphylococcus aureus
Campylobacter spp.
Enterococcus faecium
Enterococcus faecalis
Third generation cephalosporins
Fourth generation cephalosporins
Fluoroquinolones
Macrolides
Quinolones
Polymyxins
Aminoglycosides
Aminopenicillins
Aminopenicillins with beta−lactamase inhibitors
Ansamycins
Phosphonic acid derivatives
Shared human and animal antimicrobial classes
Cattle
Chickens
Pigs
Sheep
Turkeys
Ducks
Horses
Buffaloes
Goats
Livestock species
Rank
0.00
0.25
0.50
0.75
1.00

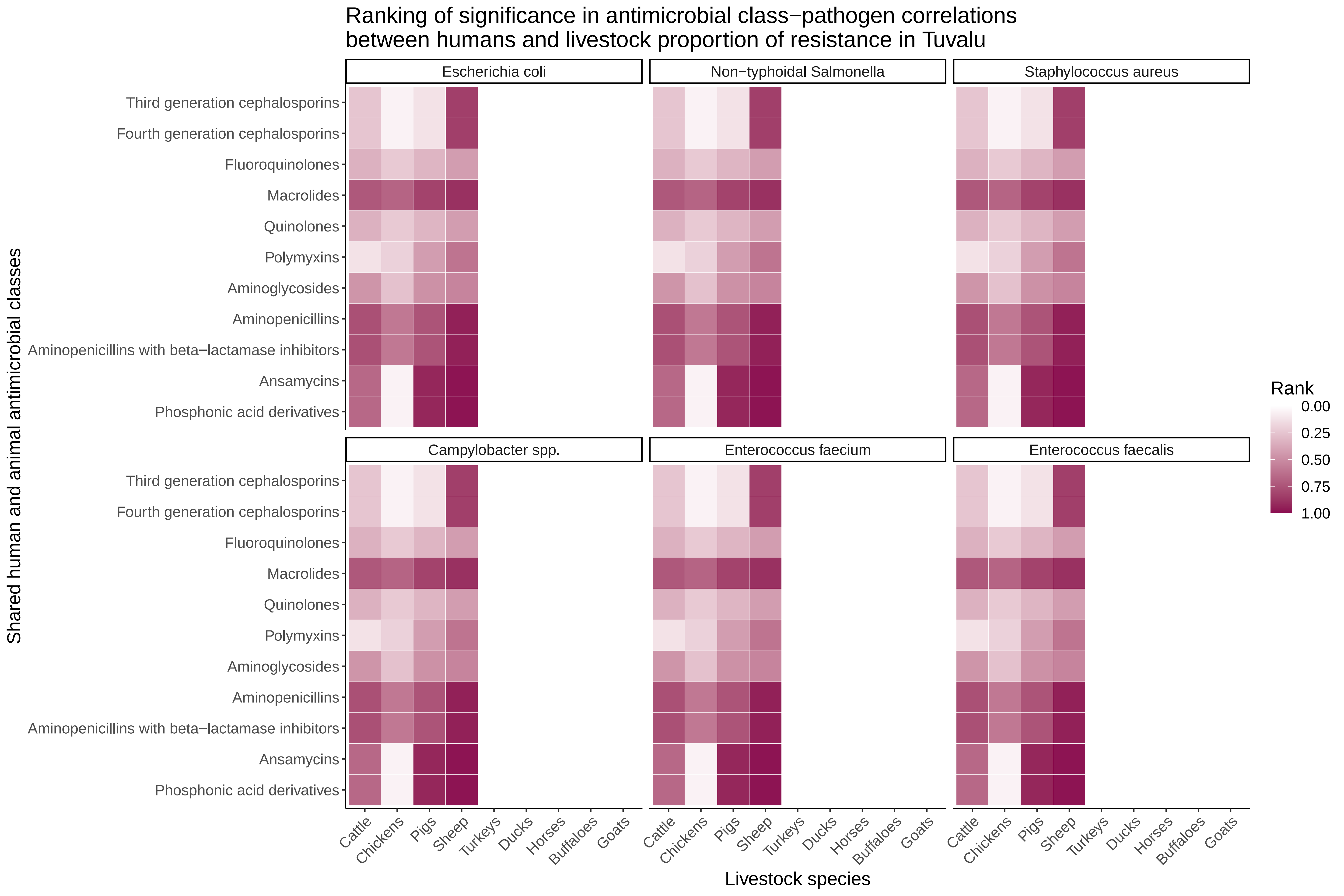
Ranking of significance in antimicrobial class−pathogen correlations
between humans and livestock proportion of resistance in Tuvalu
Escherichia coli
Non−typhoidal Salmonella
Staphylococcus aureus
Campylobacter spp.
Enterococcus faecium
Enterococcus faecalis
Shared human and animal antimicrobial classes
Third generation cephalosporins
Fourth generation cephalosporins
Fluoroquinolones
Macrolides
Quinolones
Polymyxins
Aminoglycosides
Aminopenicillins
Aminopenicillins with beta−lactamase inhibitors
Ansamycins
Phosphonic acid derivatives
Livestock species
Cattle
Chickens
Pigs
Sheep
Turkeys
Ducks
Horses
Buffaloes
Goats
Rank
0.00
0.25
0.50
0.75
1.00

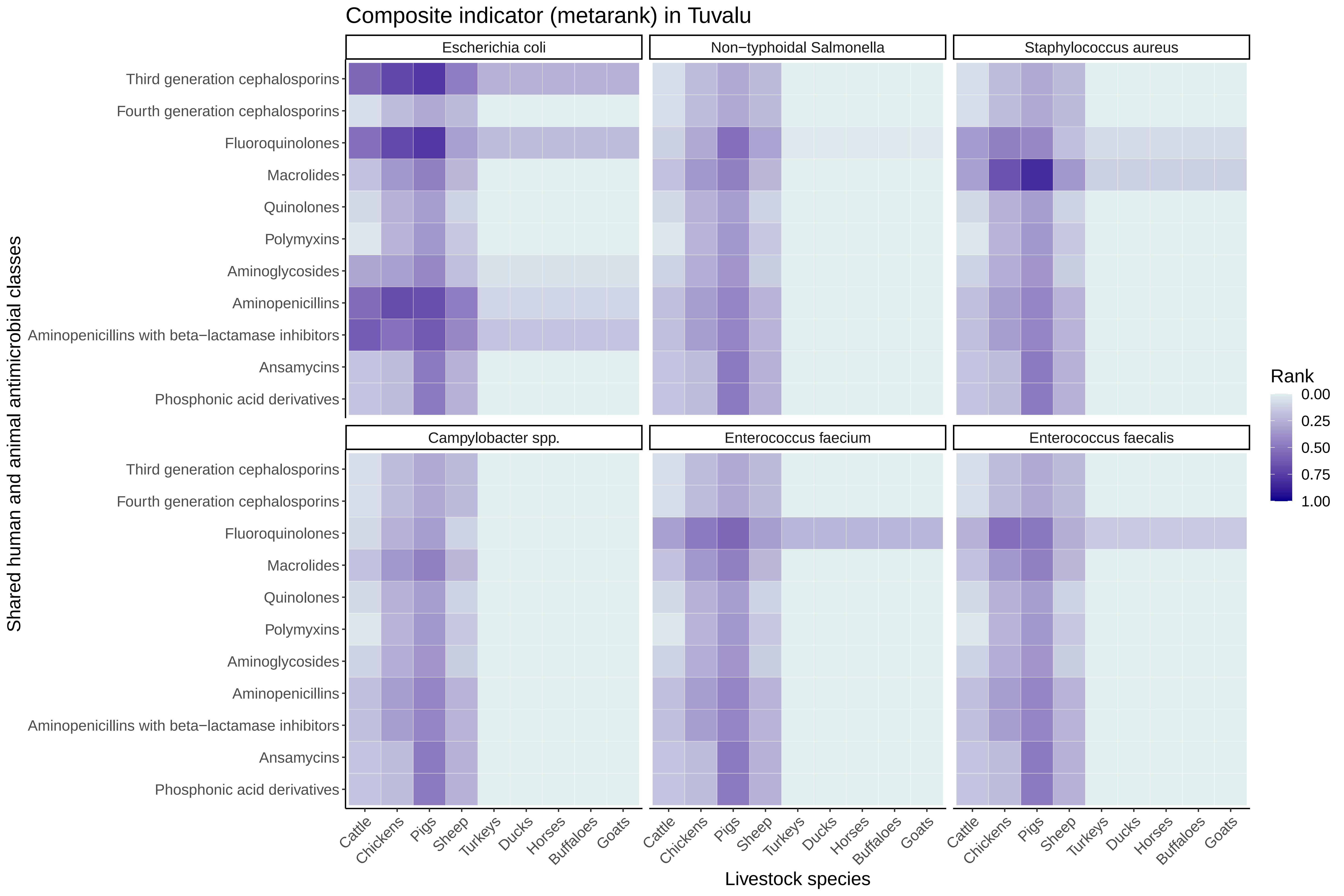
Composite indicator (metarank) in Tuvalu
Escherichia coli
Non−typhoidal Salmonella
Staphylococcus aureus
Campylobacter spp.
Enterococcus faecium
Enterococcus faecalis
Third generation cephalosporins
Fourth generation cephalosporins
Fluoroquinolones
Macrolides
Quinolones
Polymyxins
Aminoglycosides
Aminopenicillins
Aminopenicillins with beta−lactamase inhibitors
Ansamycins
Phosphonic acid derivatives
Shared human and animal antimicrobial classes
Cattle
Chickens
Pigs
Sheep
Turkeys
Ducks
Horses
Buffaloes
Goats
Livestock species
Rank
0.00
0.25
0.50
0.75
1.00

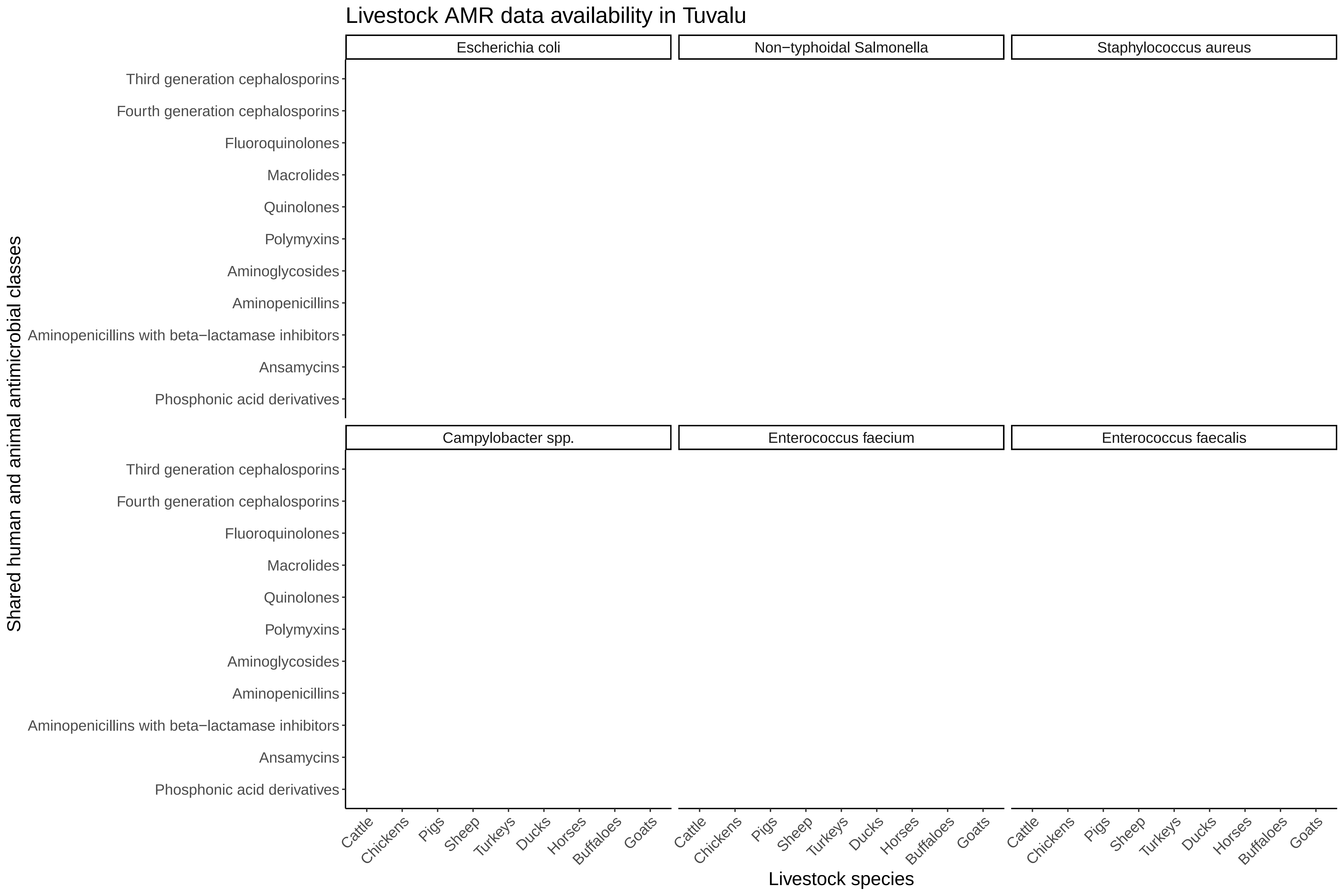
Livestock AMR data availability in Tuvalu
Escherichia coli
Non−typhoidal Salmonella
Staphylococcus aureus
Campylobacter spp.
Enterococcus faecium
Enterococcus faecalis
Third generation cephalosporins
Fourth generation cephalosporins
Fluoroquinolones
Macrolides
Quinolones
Polymyxins
Aminoglycosides
Aminopenicillins
Aminopenicillins with beta−lactamase inhibitors
Ansamycins
Phosphonic acid derivatives
Shared human and animal antimicrobial classes
Cattle
Chickens
Pigs
Sheep
Turkeys
Ducks
Horses
Buffaloes
Goats
Livestock species

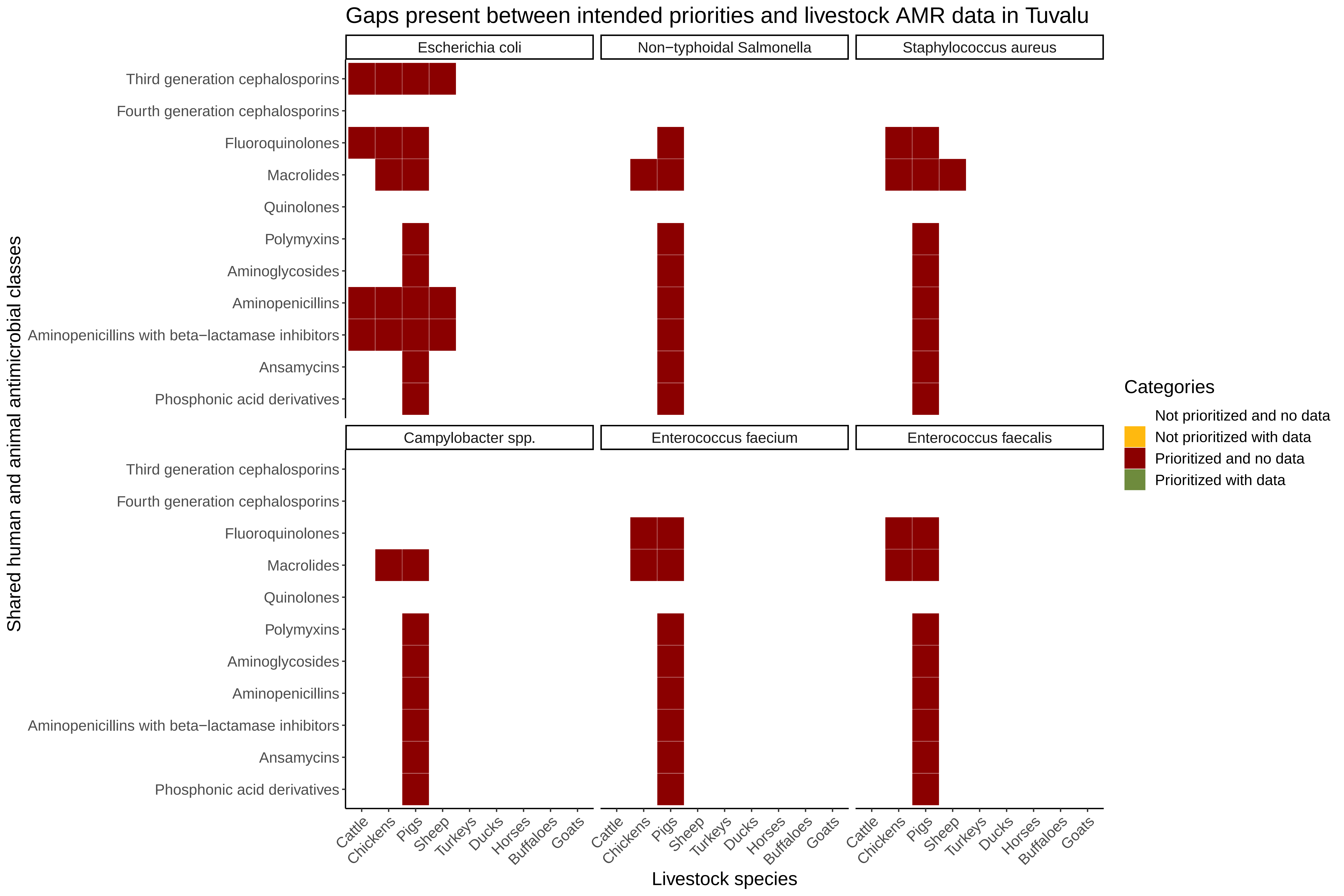

Gaps present between intended priorities and livestock AMR data in Tuvalu
Escherichia coli
Non−typhoidal Salmonella
Staphylococcus aureus
Campylobacter spp.
Enterococcus faecium
Enterococcus faecalis
Third generation cephalosporins
Fourth generation cephalosporins
Fluoroquinolones
Macrolides
Quinolones
Polymyxins
Aminoglycosides
Aminopenicillins
Aminopenicillins with beta−lactamase inhibitors
Ansamycins
Phosphonic acid derivatives
Shared human and animal antimicrobial classes
Cattle
Chickens
Pigs
Sheep
Turkeys
Ducks
Horses
Buffaloes
Goats
Livestock species
Categories
Not prioritized and no data
Not prioritized with data
Prioritized and no data
Prioritized with data

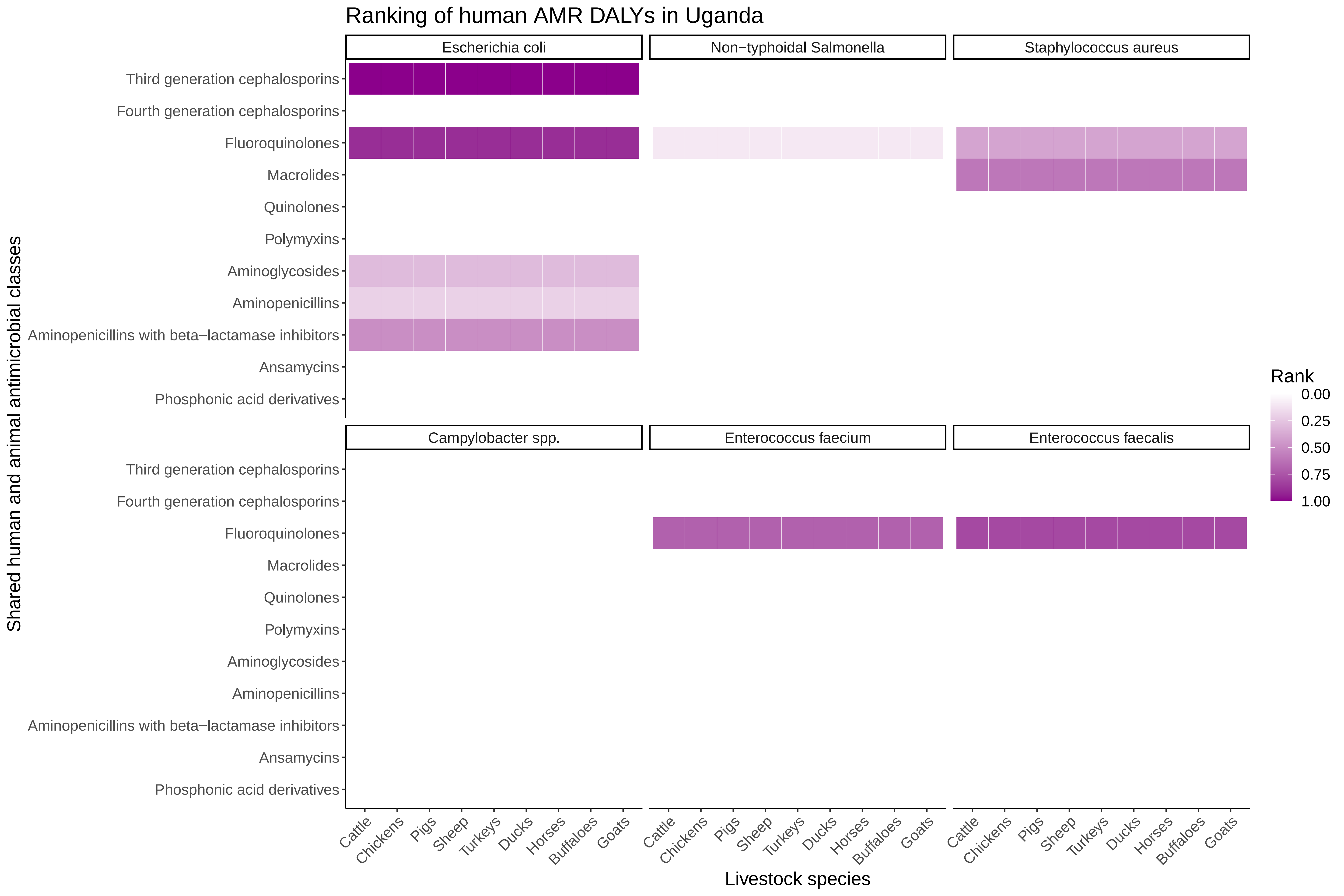
Ranking of human AMR DALYs in Uganda
Escherichia coli
Non−typhoidal Salmonella
Staphylococcus aureus
Campylobacter spp.
Enterococcus faecium
Enterococcus faecalis
Third generation cephalosporins
Fourth generation cephalosporins
Fluoroquinolones
Macrolides
Quinolones
Polymyxins
Aminoglycosides
Aminopenicillins
Aminopenicillins with beta−lactamase inhibitors
Ansamycins
Phosphonic acid derivatives
Shared human and animal antimicrobial classes
Cattle
Chickens
Pigs
Sheep
Turkeys
Ducks
Horses
Buffaloes
Goats
Livestock species
Rank
0.00
0.25
0.50
0.75
1.00

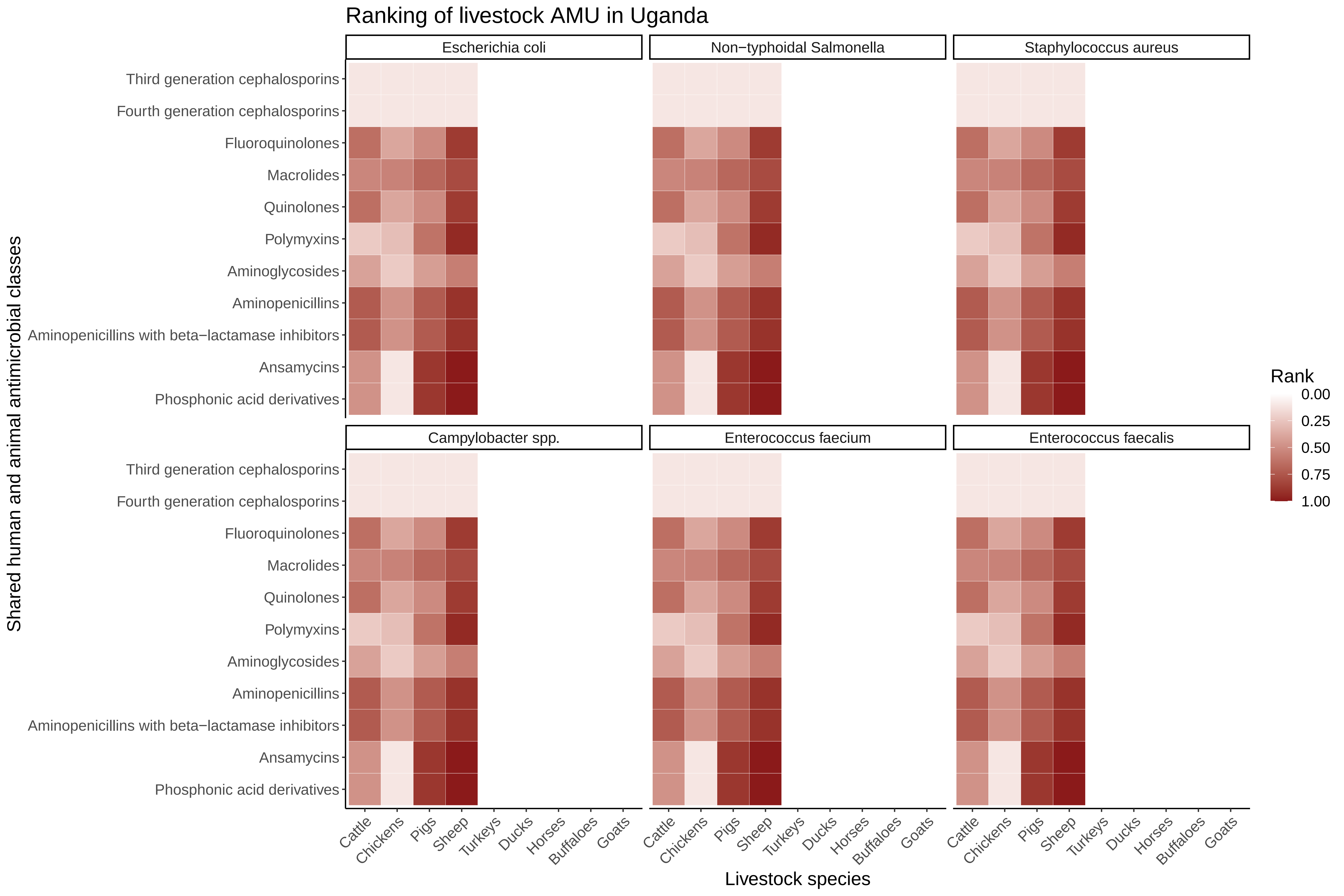
Ranking of livestock AMU in Uganda
Escherichia coli
Non−typhoidal Salmonella
Staphylococcus aureus
Campylobacter spp.
Enterococcus faecium
Enterococcus faecalis
Third generation cephalosporins
Fourth generation cephalosporins
Fluoroquinolones
Macrolides
Quinolones
Polymyxins
Aminoglycosides
Aminopenicillins
Aminopenicillins with beta−lactamase inhibitors
Ansamycins
Phosphonic acid derivatives
Shared human and animal antimicrobial classes
Cattle
Chickens
Pigs
Sheep
Turkeys
Ducks
Horses
Buffaloes
Goats
Livestock species
Rank
0.00
0.25
0.50
0.75
1.00

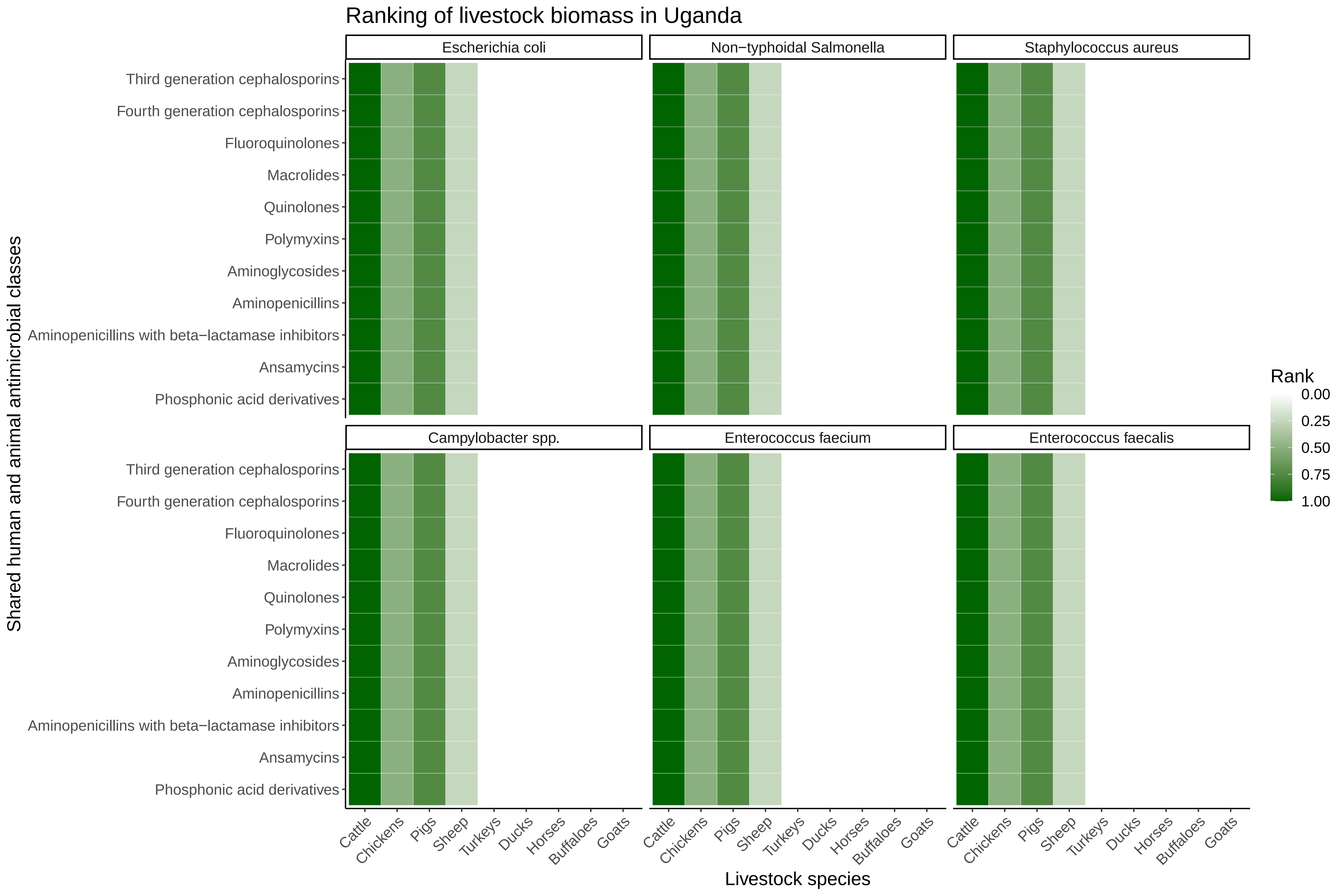
Ranking of livestock biomass in Uganda
Escherichia coli
Non−typhoidal Salmonella
Staphylococcus aureus
Campylobacter spp.
Enterococcus faecium
Enterococcus faecalis
Shared human and animal antimicrobial classes
Third generation cephalosporins
Fourth generation cephalosporins
Fluoroquinolones
Macrolides
Quinolones
Polymyxins
Aminoglycosides
Aminopenicillins
Aminopenicillins with beta−lactamase inhibitors
Ansamycins
Phosphonic acid derivatives
Livestock species
Cattle
Chickens
Pigs
Sheep
Turkeys
Ducks
Horses
Buffaloes
Goats
Rank
0.00
0.25
0.50
0.75
1.00

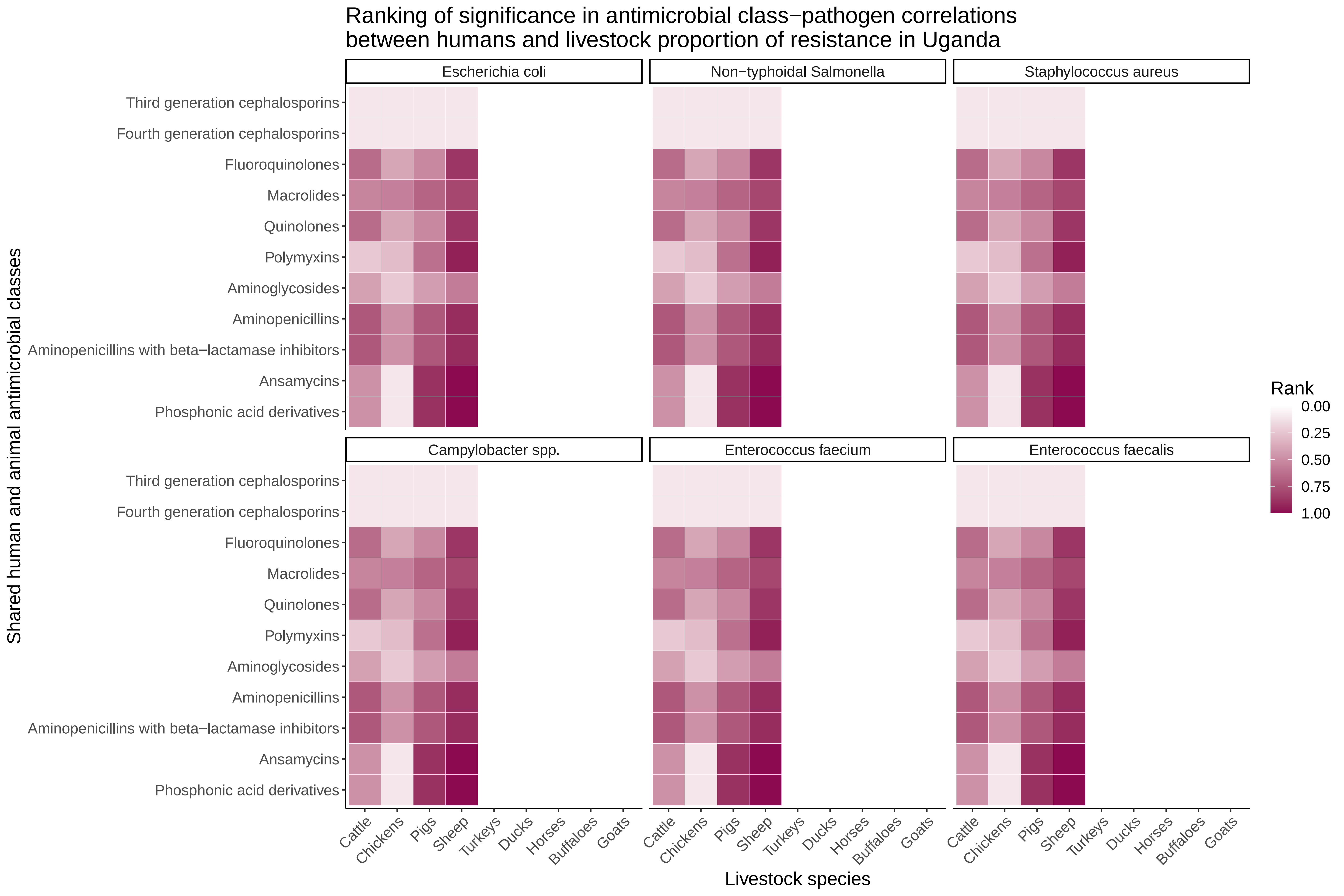

Ranking of significance in antimicrobial class−pathogen correlations
between humans and livestock proportion of resistance in Uganda
Escherichia coli
Non−typhoidal Salmonella
Staphylococcus aureus
Campylobacter spp.
Enterococcus faecium
Enterococcus faecalis
Third generation cephalosporins
Fourth generation cephalosporins
Fluoroquinolones
Macrolides
Quinolones
Polymyxins
Aminoglycosides
Aminopenicillins
Aminopenicillins with beta−lactamase inhibitors
Ansamycins
Phosphonic acid derivatives
Shared human and animal antimicrobial classes
Cattle
Chickens
Pigs
Sheep
Turkeys
Ducks
Horses
Buffaloes
Goats
Livestock species
Rank
0.00
0.25
0.50
0.75
1.00

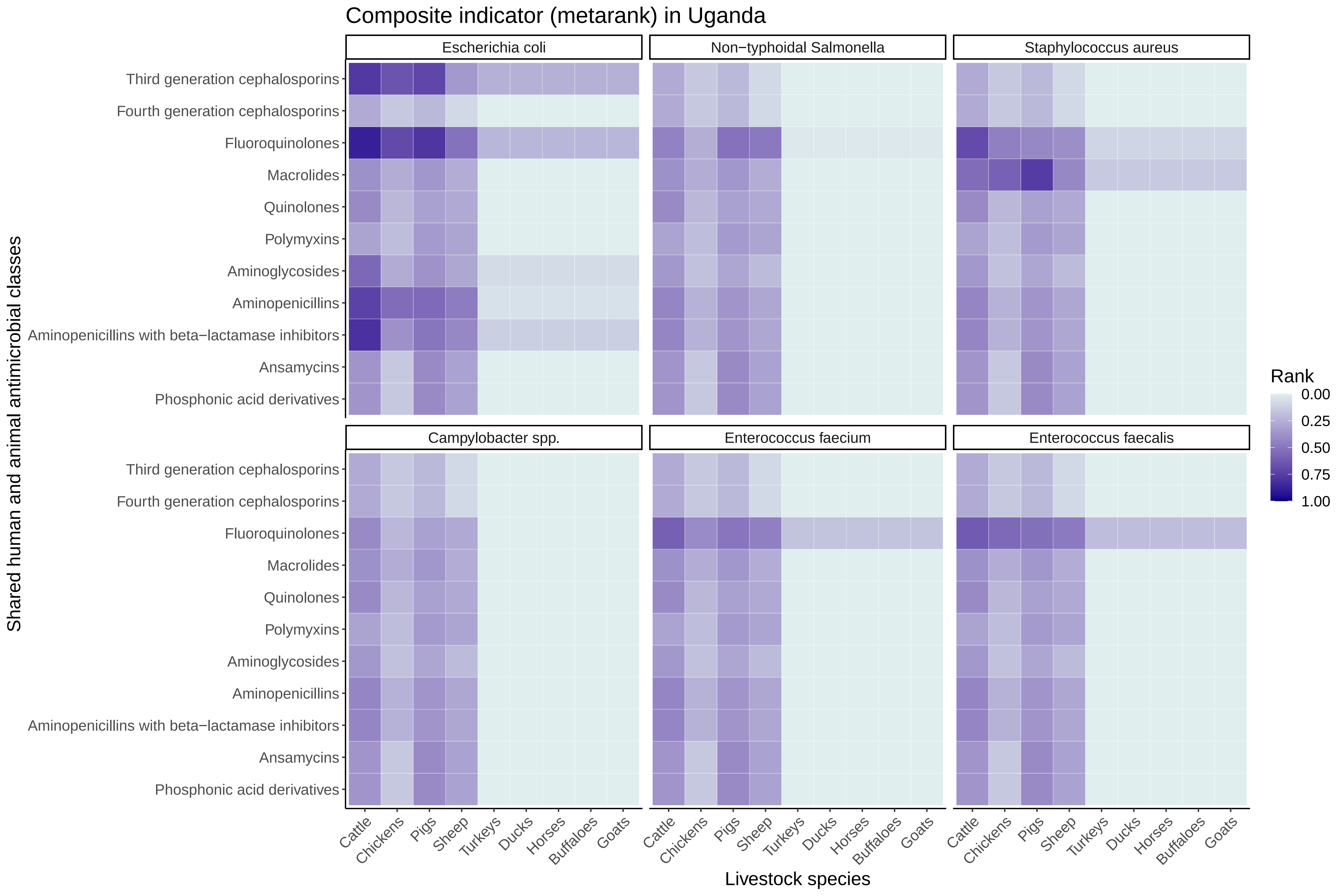

Composite indicator (metarank) in Uganda
Escherichia coli
Non−typhoidal Salmonella
Staphylococcus aureus
Campylobacter spp.
Enterococcus faecium
Enterococcus faecalis
Shared human and animal antimicrobial classes
Third generation cephalosporins
Fourth generation cephalosporins
Fluoroquinolones
Macrolides
Quinolones
Polymyxins
Aminoglycosides
Aminopenicillins
Aminopenicillins with beta−lactamase inhibitors
Ansamycins
Phosphonic acid derivatives
Livestock species
Cattle
Chickens
Pigs
Sheep
Turkeys
Ducks
Horses
Buffaloes
Goats
Rank
0.00
0.25
0.50
0.75
1.00

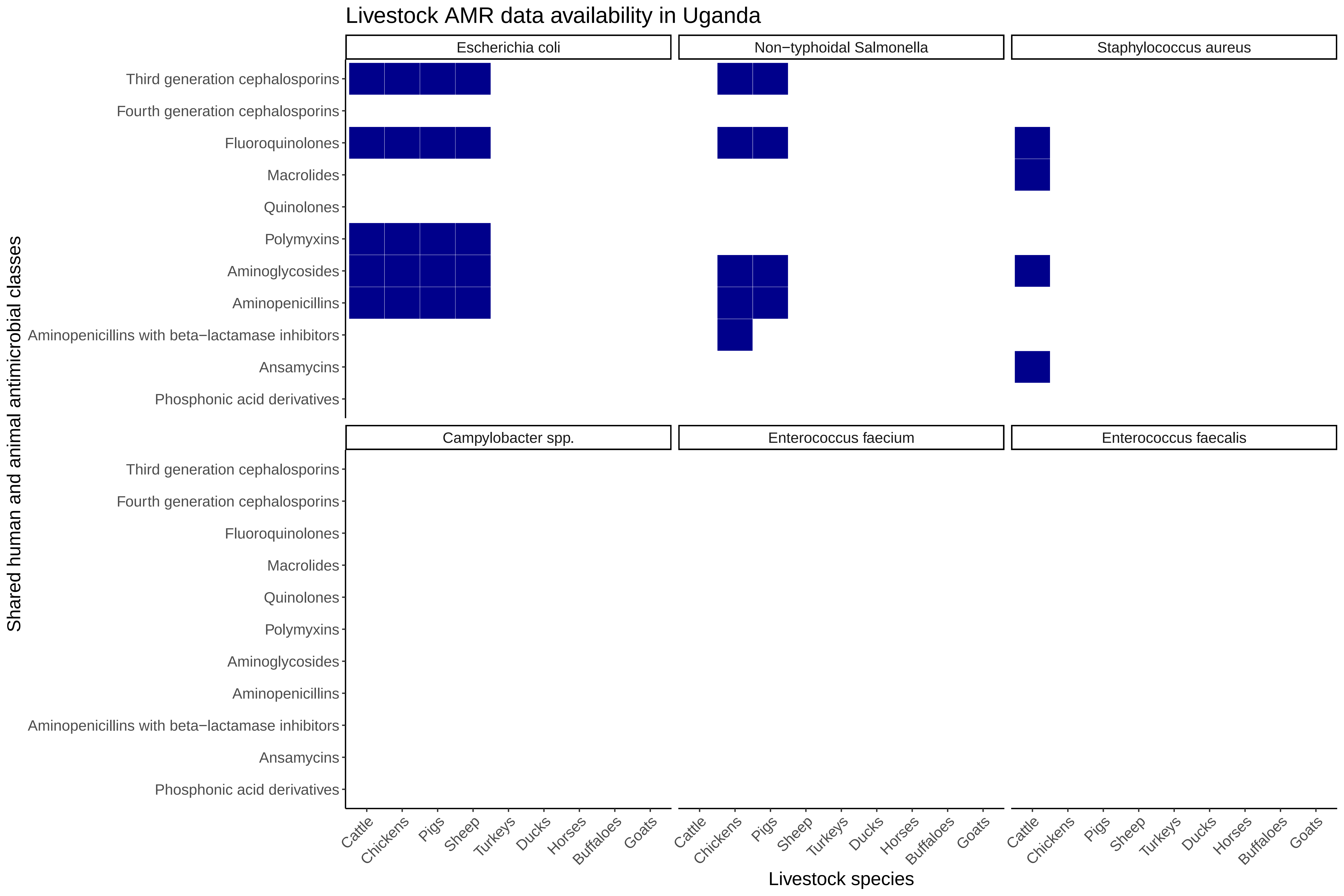

Livestock AMR data availability in Uganda
Escherichia coli
Non−typhoidal Salmonella
Staphylococcus aureus
Campylobacter spp.
Enterococcus faecium
Enterococcus faecalis
Third generation cephalosporins
Fourth generation cephalosporins
Fluoroquinolones
Macrolides
Quinolones
Polymyxins
Aminoglycosides
Aminopenicillins
Aminopenicillins with beta−lactamase inhibitors
Ansamycins
Phosphonic acid derivatives
Shared human and animal antimicrobial classes
Cattle
Chickens
Pigs
Sheep
Turkeys
Ducks
Horses
Buffaloes
Goats
Livestock species

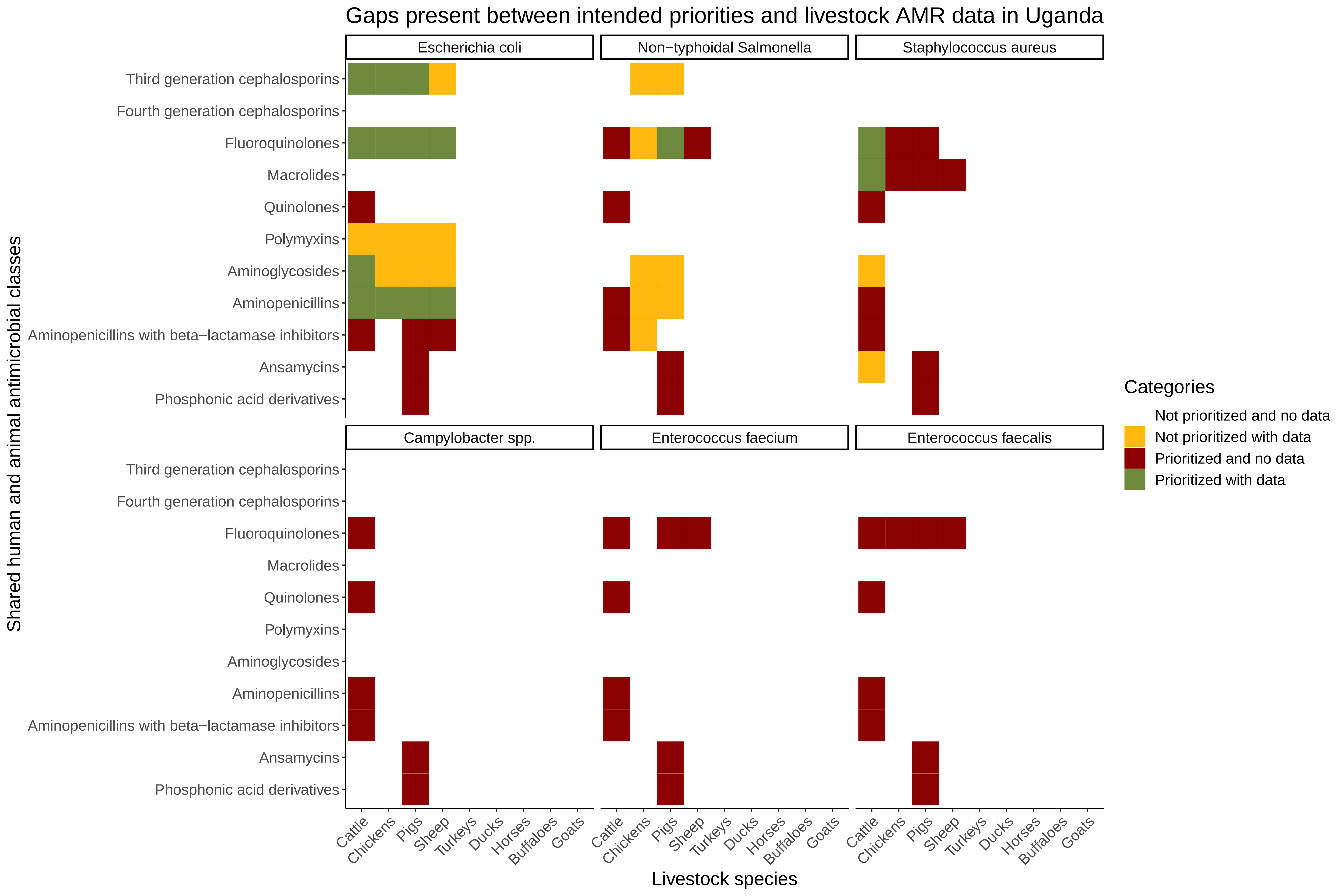
Gaps present between intended priorities and livestock AMR data in Uganda
Escherichia coli
Non−typhoidal Salmonella
Staphylococcus aureus
Campylobacter spp.
Enterococcus faecium
Enterococcus faecalis
Third generation cephalosporins
Fourth generation cephalosporins
Fluoroquinolones
Macrolides
Quinolones
Polymyxins
Aminoglycosides
Aminopenicillins
Aminopenicillins with beta−lactamase inhibitors
Ansamycins
Phosphonic acid derivatives
Cattle
Chickens
Pigs
Sheep
Turkeys
Ducks
Horses
Buffaloes
Goats
Shared human and animal antimicrobial classes
Livestock species
Categories
Not prioritized and no data
Not prioritized with data
Prioritized and no data
Prioritized with data

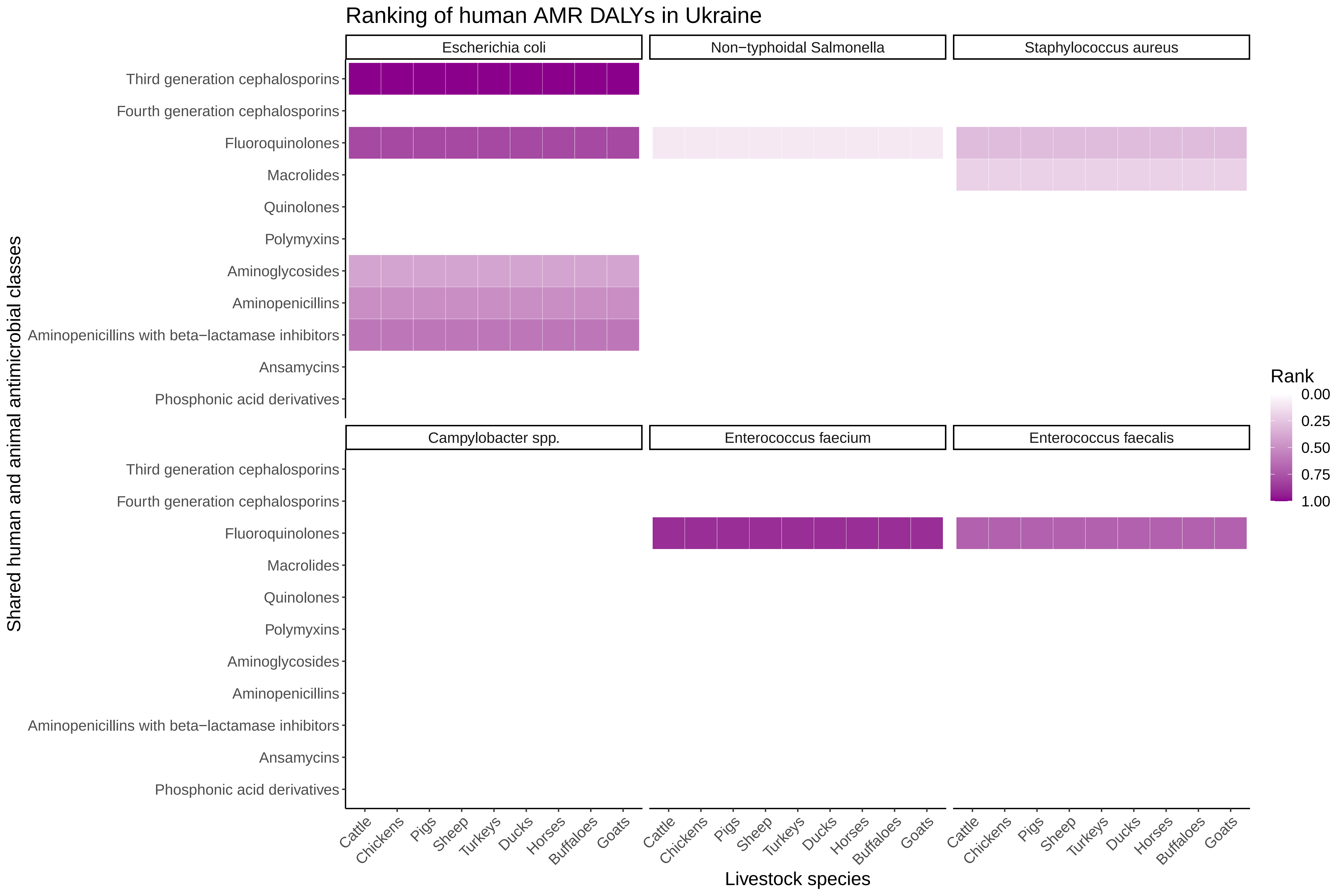
Ranking of human AMR DALYs in Ukraine
Escherichia coli
Non−typhoidal Salmonella
Staphylococcus aureus
Campylobacter spp.
Enterococcus faecium
Enterococcus faecalis
Shared human and animal antimicrobial classes
Third generation cephalosporins
Fourth generation cephalosporins
Fluoroquinolones
Macrolides
Quinolones
Polymyxins
Aminoglycosides
Aminopenicillins
Aminopenicillins with beta−lactamase inhibitors
Ansamycins
Phosphonic acid derivatives
Cattle
Chickens
Pigs
Sheep
Turkeys
Ducks
Horses
Buffaloes
Goats
Livestock species
Rank
0.00
0.25
0.50
0.75
1.00

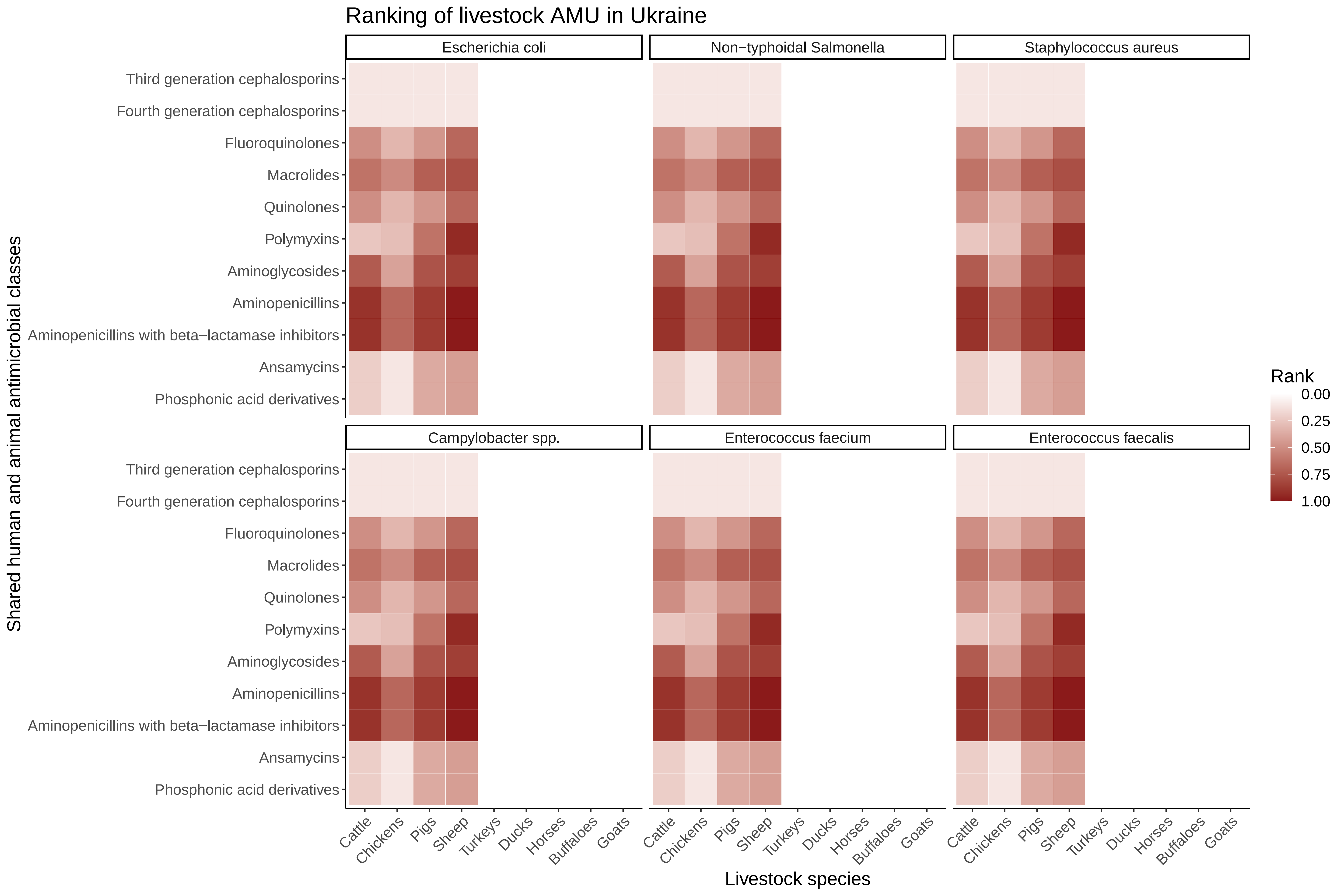

Ranking of livestock AMU in Ukraine
Escherichia coli
Non−typhoidal Salmonella
Staphylococcus aureus
Campylobacter spp.
Enterococcus faecium
Enterococcus faecalis
Third generation cephalosporins
Fourth generation cephalosporins
Fluoroquinolones
Macrolides
Quinolones
Polymyxins
Aminoglycosides
Aminopenicillins
Aminopenicillins with beta−lactamase inhibitors
Ansamycins
Phosphonic acid derivatives
Shared human and animal antimicrobial classes
Cattle
Chickens
Pigs
Sheep
Turkeys
Ducks
Horses
Buffaloes
Goats
Livestock species
Rank
0.00
0.25
0.50
0.75
1.00

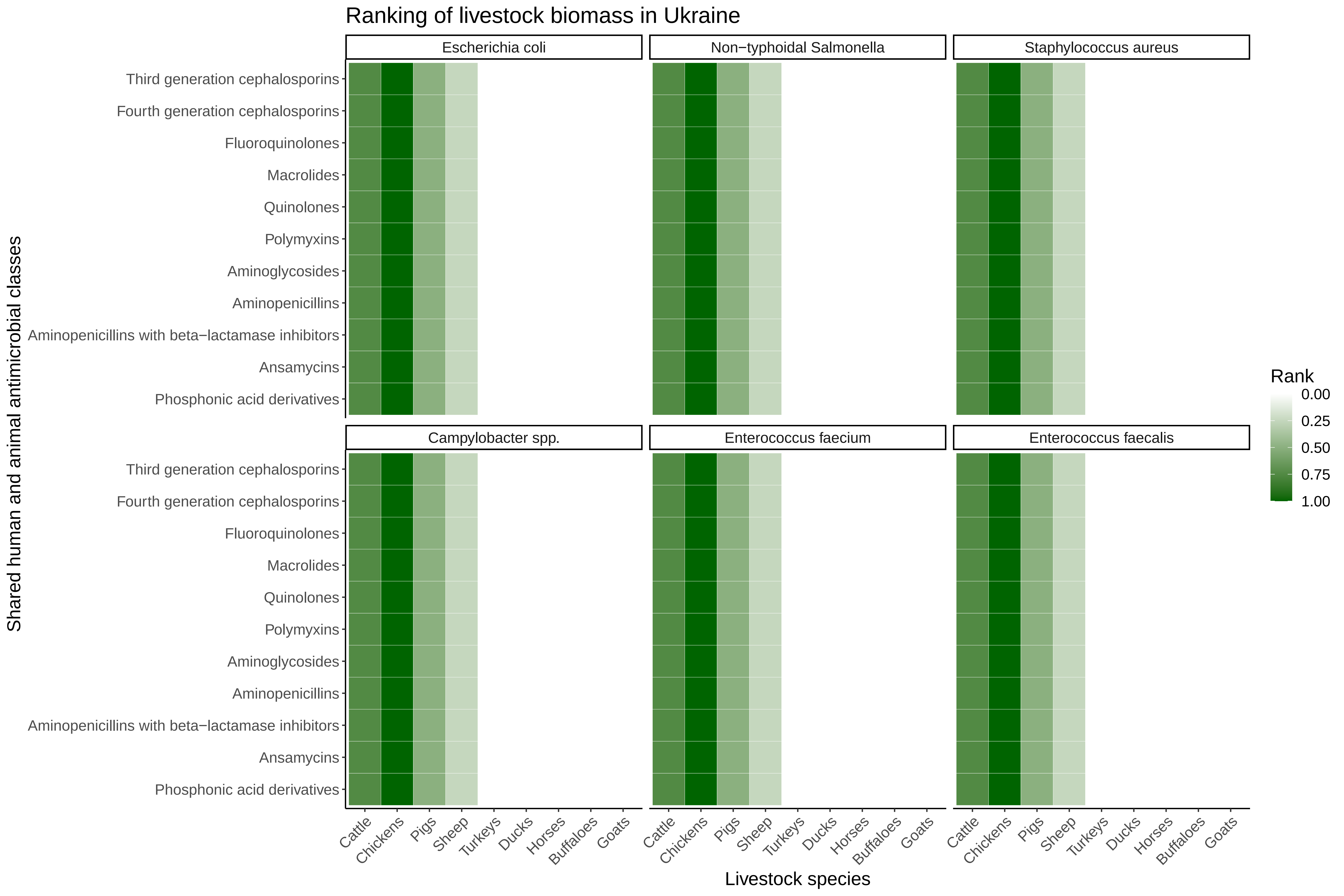

Ranking of livestock biomass in Ukraine
Escherichia coli
Non−typhoidal Salmonella
Staphylococcus aureus
Campylobacter spp.
Enterococcus faecium
Enterococcus faecalis
Third generation cephalosporins
Fourth generation cephalosporins
Fluoroquinolones
Macrolides
Quinolones
Polymyxins
Aminoglycosides
Aminopenicillins
Aminopenicillins with beta−lactamase inhibitors
Ansamycins
Phosphonic acid derivatives
Shared human and animal antimicrobial classes
Cattle
Chickens
Pigs
Sheep
Turkeys
Ducks
Horses
Buffaloes
Goats
Livestock species
Rank
0.00
0.25
0.50
0.75
1.00

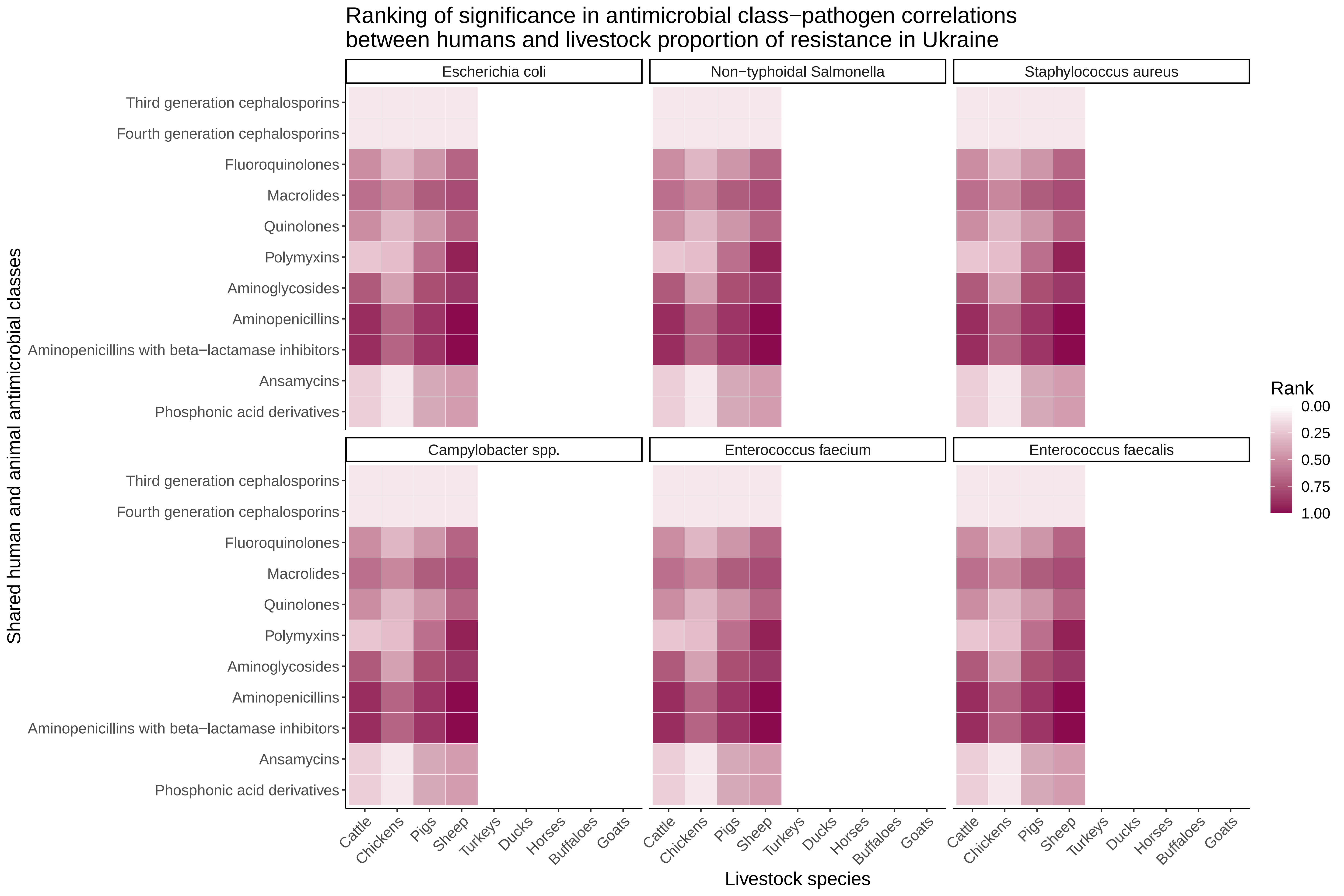

Ranking of significance in antimicrobial class−pathogen correlations
between humans and livestock proportion of resistance in Ukraine
Escherichia coli
Non−typhoidal Salmonella
Staphylococcus aureus
Campylobacter spp.
Enterococcus faecium
Enterococcus faecalis
Third generation cephalosporins
Fourth generation cephalosporins
Fluoroquinolones
Macrolides
Quinolones
Polymyxins
Aminoglycosides
Aminopenicillins
Aminopenicillins with beta−lactamase inhibitors
Ansamycins
Phosphonic acid derivatives
Shared human and animal antimicrobial classes
Cattle
Chickens
Pigs
Sheep
Turkeys
Ducks
Horses
Buffaloes
Goats
Livestock species
Rank
0.00
0.25
0.50
0.75
1.00

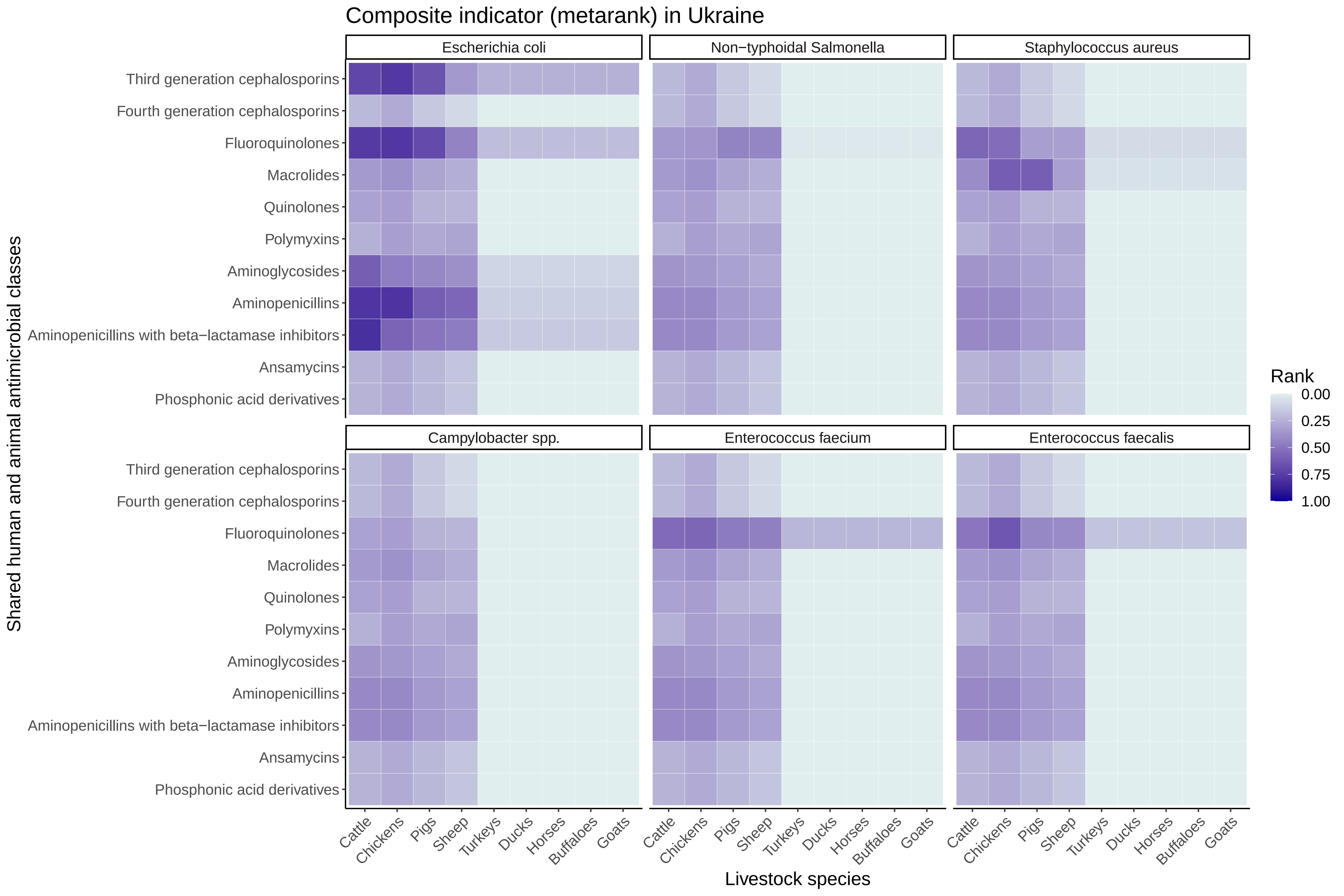

Composite indicator (metarank) in Ukraine
Escherichia coli
Non−typhoidal Salmonella
Staphylococcus aureus
Campylobacter spp.
Enterococcus faecium
Enterococcus faecalis
Third generation cephalosporins
Fourth generation cephalosporins
Fluoroquinolones
Macrolides
Quinolones
Polymyxins
Aminoglycosides
Aminopenicillins
Aminopenicillins with beta−lactamase inhibitors
Ansamycins
Phosphonic acid derivatives
Shared human and animal antimicrobial classes
Cattle
Chickens
Pigs
Sheep
Turkeys
Ducks
Horses
Buffaloes
Goats
Livestock species
Rank
0.00
0.25
0.50
0.75
1.00

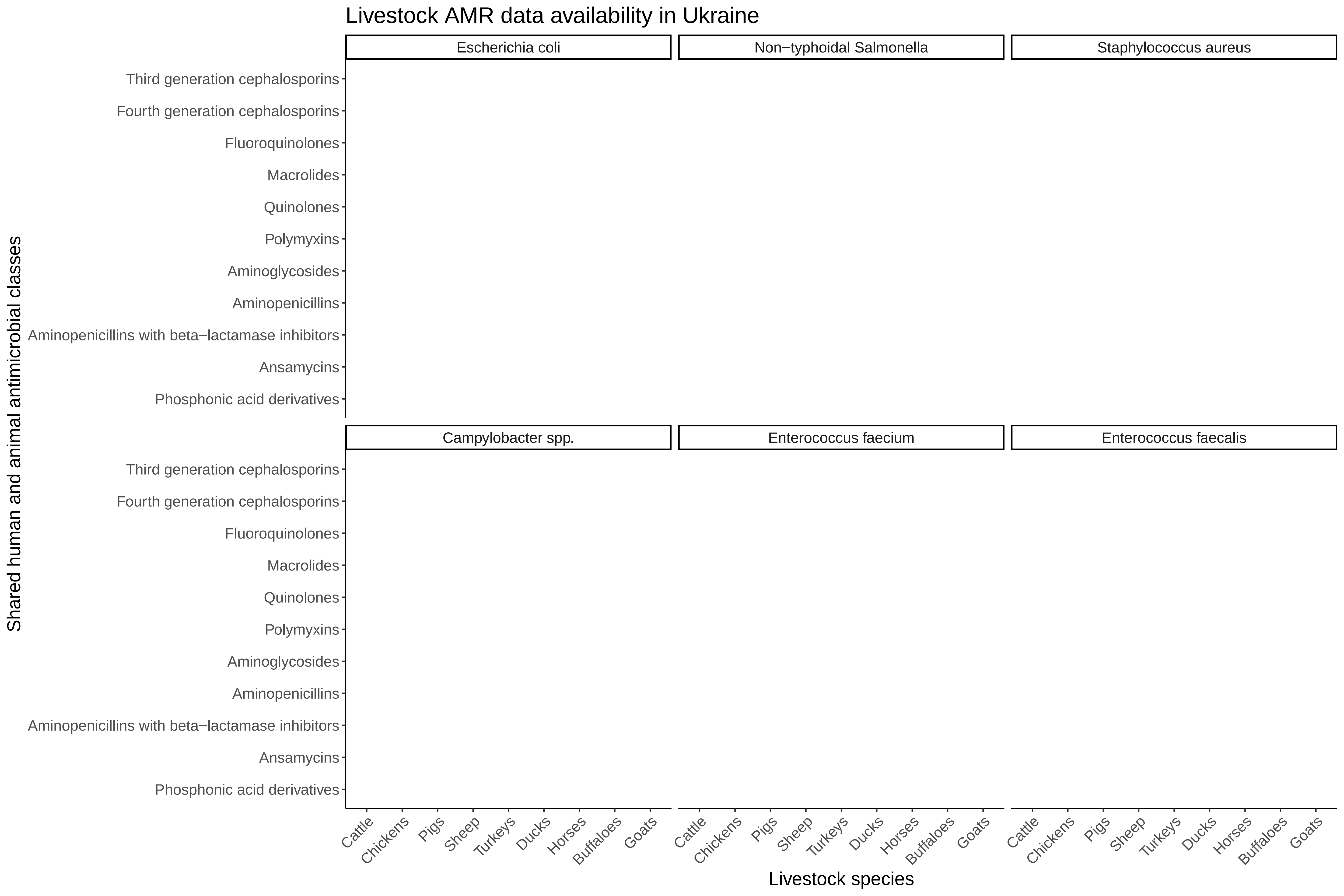
Livestock AMR data availability in Ukraine
Escherichia coli
Non−typhoidal Salmonella
Staphylococcus aureus
Campylobacter spp.
Enterococcus faecium
Enterococcus faecalis
Shared human and animal antimicrobial classes
Third generation cephalosporins
Fourth generation cephalosporins
Fluoroquinolones
Macrolides
Quinolones
Polymyxins
Aminoglycosides
Aminopenicillins
Aminopenicillins with beta−lactamase inhibitors
Ansamycins
Phosphonic acid derivatives
Cattle
Chickens
Pigs
Sheep
Turkeys
Ducks
Horses
Buffaloes
Goats
Livestock species

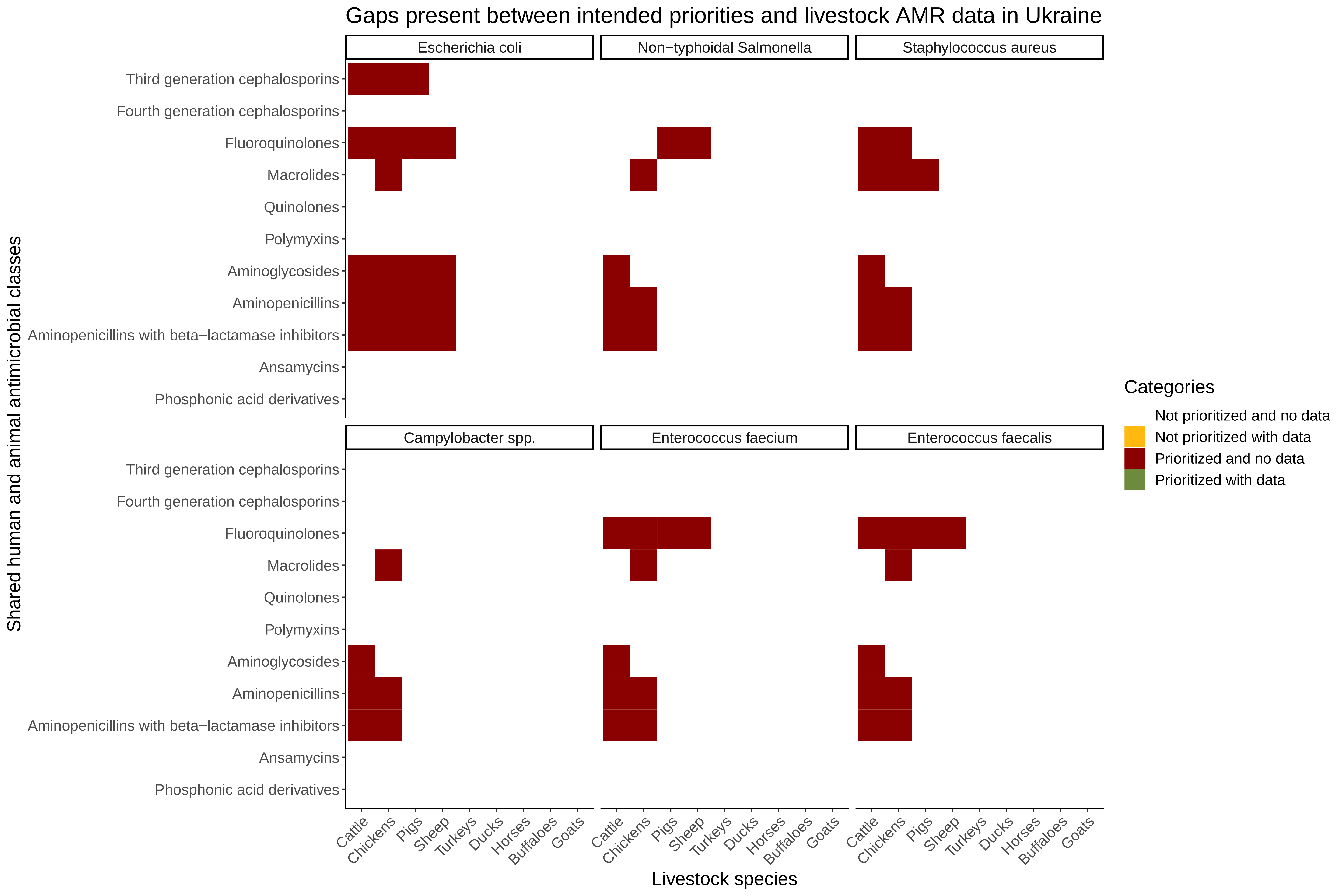
Gaps present between intended priorities and livestock AMR data in Ukraine
Escherichia coli
Non−typhoidal Salmonella
Staphylococcus aureus
Campylobacter spp.
Enterococcus faecium
Enterococcus faecalis
Third generation cephalosporins
Fourth generation cephalosporins
Fluoroquinolones
Macrolides
Quinolones
Polymyxins
Aminoglycosides
Aminopenicillins
Aminopenicillins with beta−lactamase inhibitors
Ansamycins
Phosphonic acid derivatives
Shared human and animal antimicrobial classes
Cattle
Chickens
Pigs
Sheep
Turkeys
Ducks
Horses
Buffaloes
Goats
Livestock species
Categories
Not prioritized and no data
Not prioritized with data
Prioritized and no data
Prioritized with data

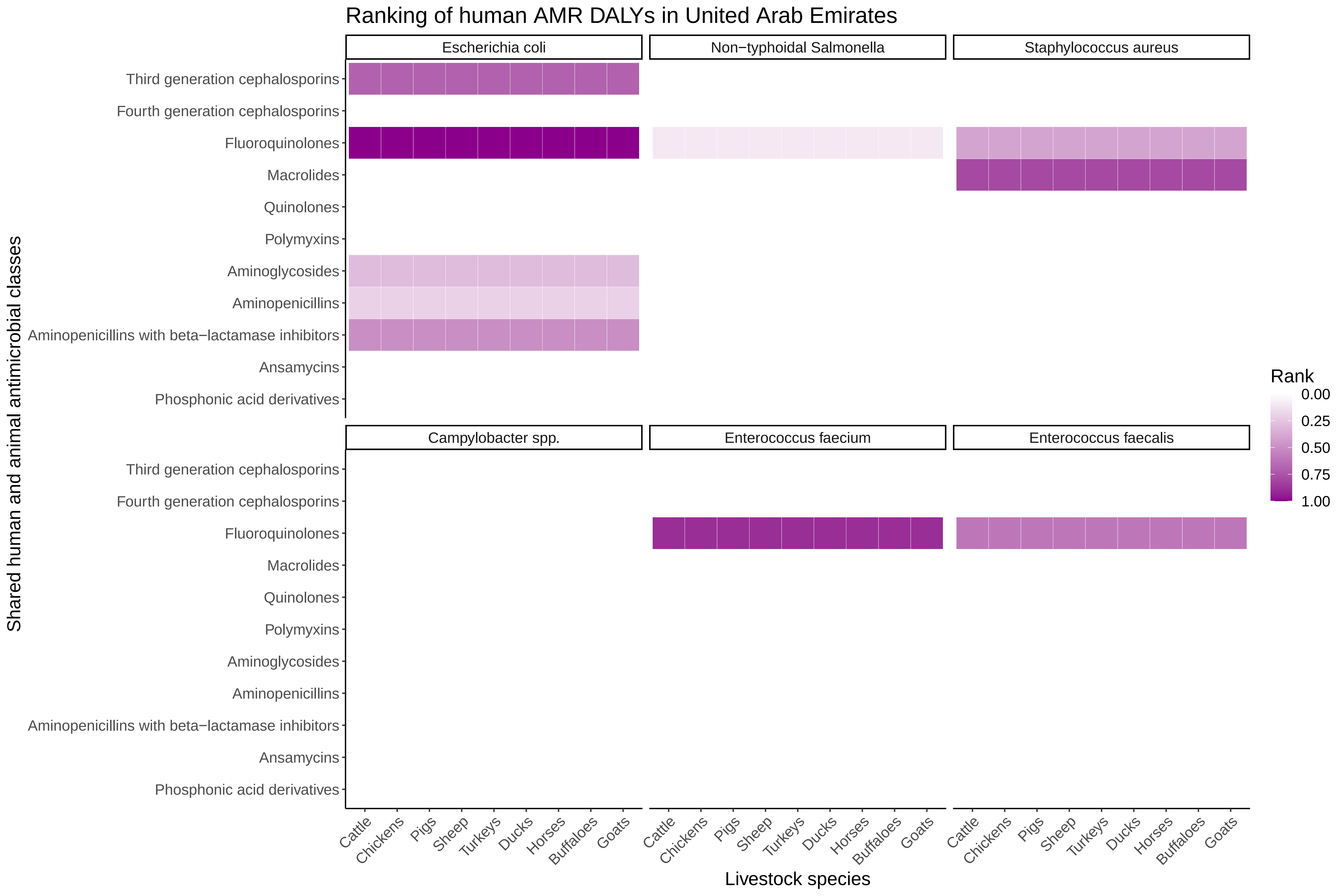

Ranking of human AMR DALYs in United Arab Emirates
Escherichia coli
Non−typhoidal Salmonella
Staphylococcus aureus
Campylobacter spp.
Enterococcus faecium
Enterococcus faecalis
Third generation cephalosporins
Fourth generation cephalosporins
Fluoroquinolones
Macrolides
Quinolones
Polymyxins
Aminoglycosides
Aminopenicillins
Aminopenicillins with beta−lactamase inhibitors
Ansamycins
Phosphonic acid derivatives
Shared human and animal antimicrobial classes
Cattle
Chickens
Pigs
Sheep
Turkeys
Ducks
Horses
Buffaloes
Goats
Livestock species
Rank
0.00
0.25
0.50
0.75
1.00

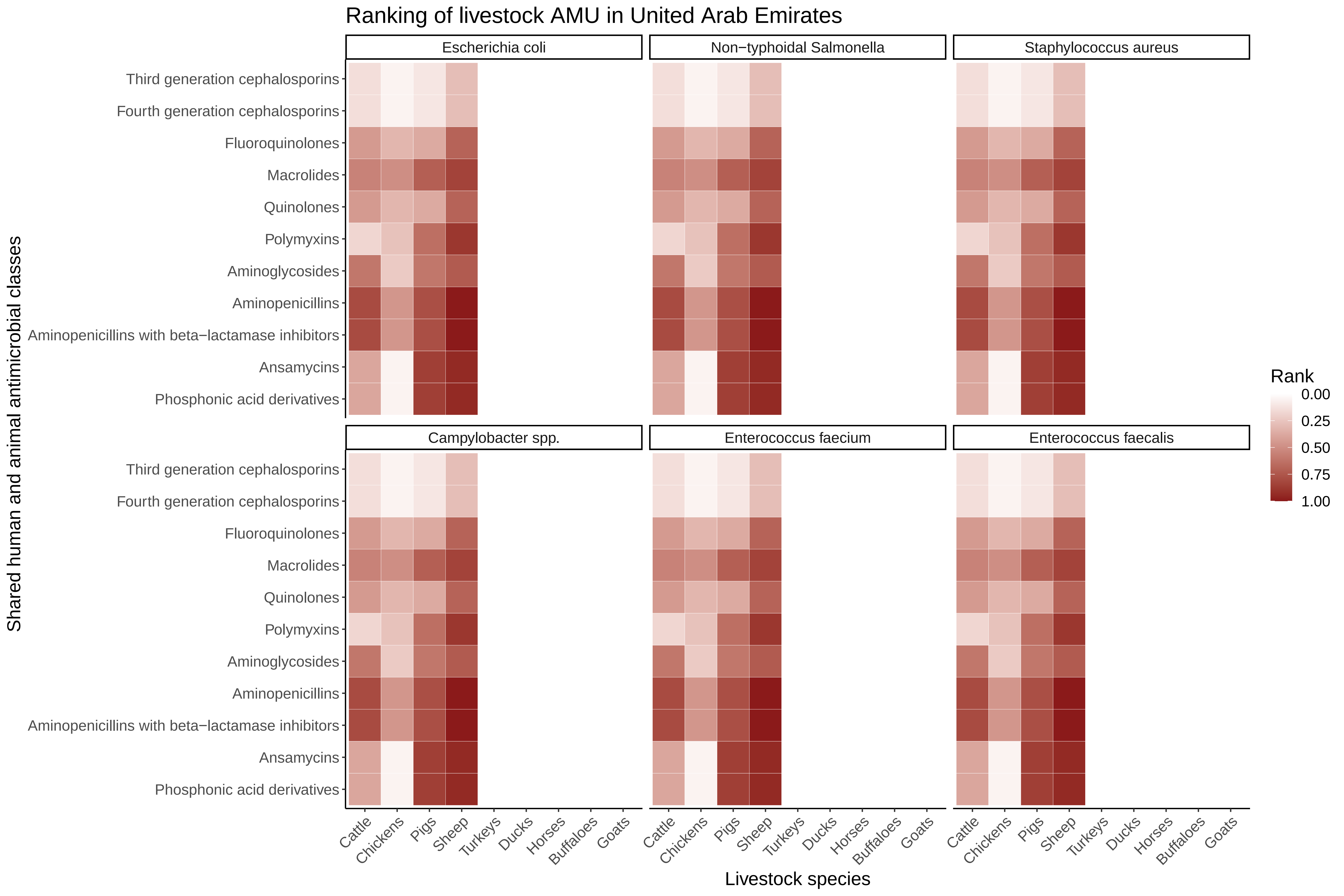

Ranking of livestock AMU in United Arab Emirates
Escherichia coli
Non−typhoidal Salmonella
Staphylococcus aureus
Campylobacter spp.
Enterococcus faecium
Enterococcus faecalis
Shared human and animal antimicrobial classes
Third generation cephalosporins
Fourth generation cephalosporins
Fluoroquinolones
Macrolides
Quinolones
Polymyxins
Aminoglycosides
Aminopenicillins
Aminopenicillins with beta−lactamase inhibitors
Ansamycins
Phosphonic acid derivatives
Cattle
Chickens
Pigs
Sheep
Turkeys
Ducks
Horses
Buffaloes
Goats
Livestock species
Rank
0.00
0.25
0.50
0.75
1.00

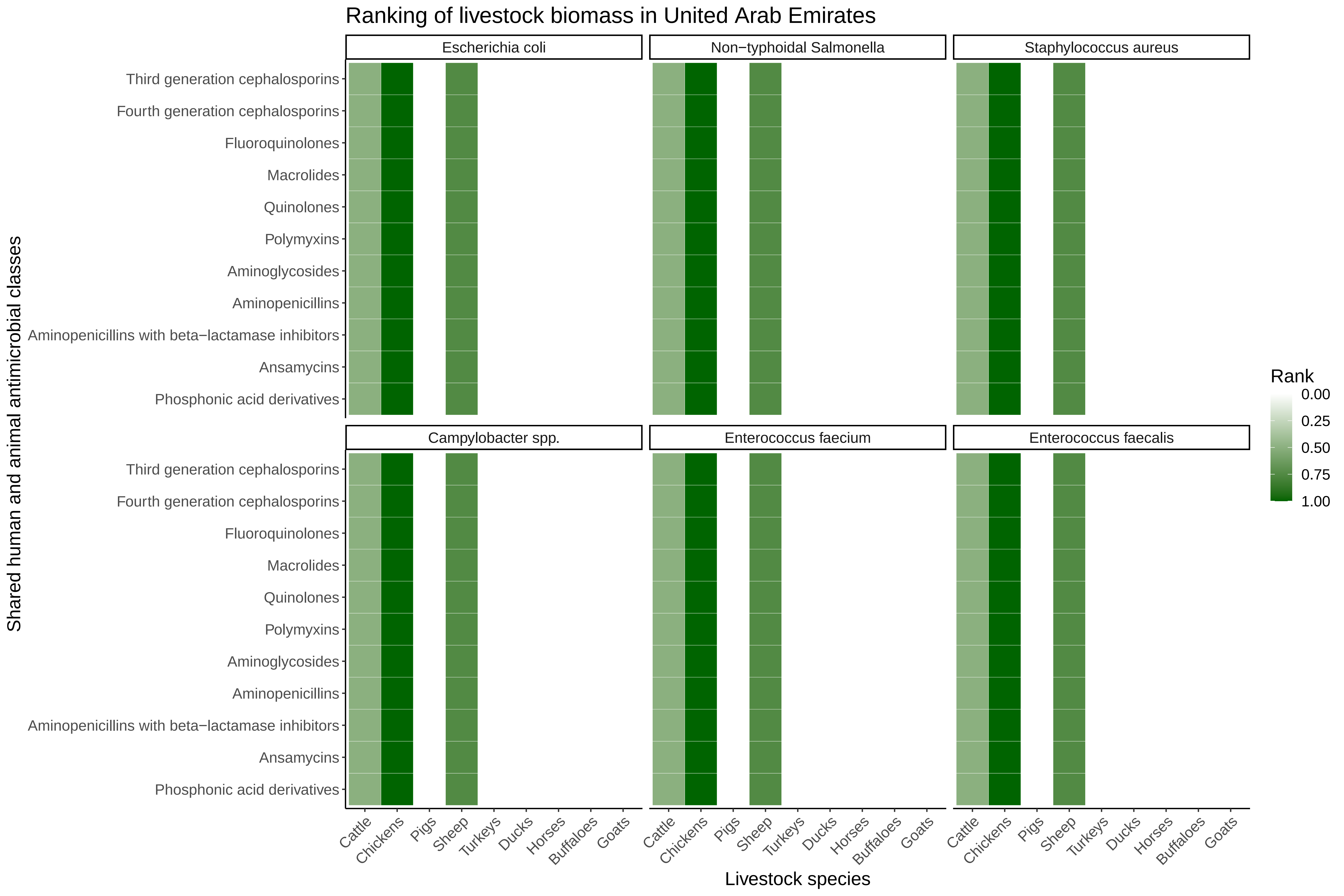

Ranking of livestock biomass in United Arab Emirates
Escherichia coli
Non−typhoidal Salmonella
Staphylococcus aureus
Campylobacter spp.
Enterococcus faecium
Enterococcus faecalis
Shared human and animal antimicrobial classes
Third generation cephalosporins
Fourth generation cephalosporins
Fluoroquinolones
Macrolides
Quinolones
Polymyxins
Aminoglycosides
Aminopenicillins
Aminopenicillins with beta−lactamase inhibitors
Ansamycins
Phosphonic acid derivatives
Cattle
Chickens
Pigs
Sheep
Turkeys
Ducks
Horses
Buffaloes
Goats
Livestock species
Rank
0.00
0.25
0.50
0.75
1.00

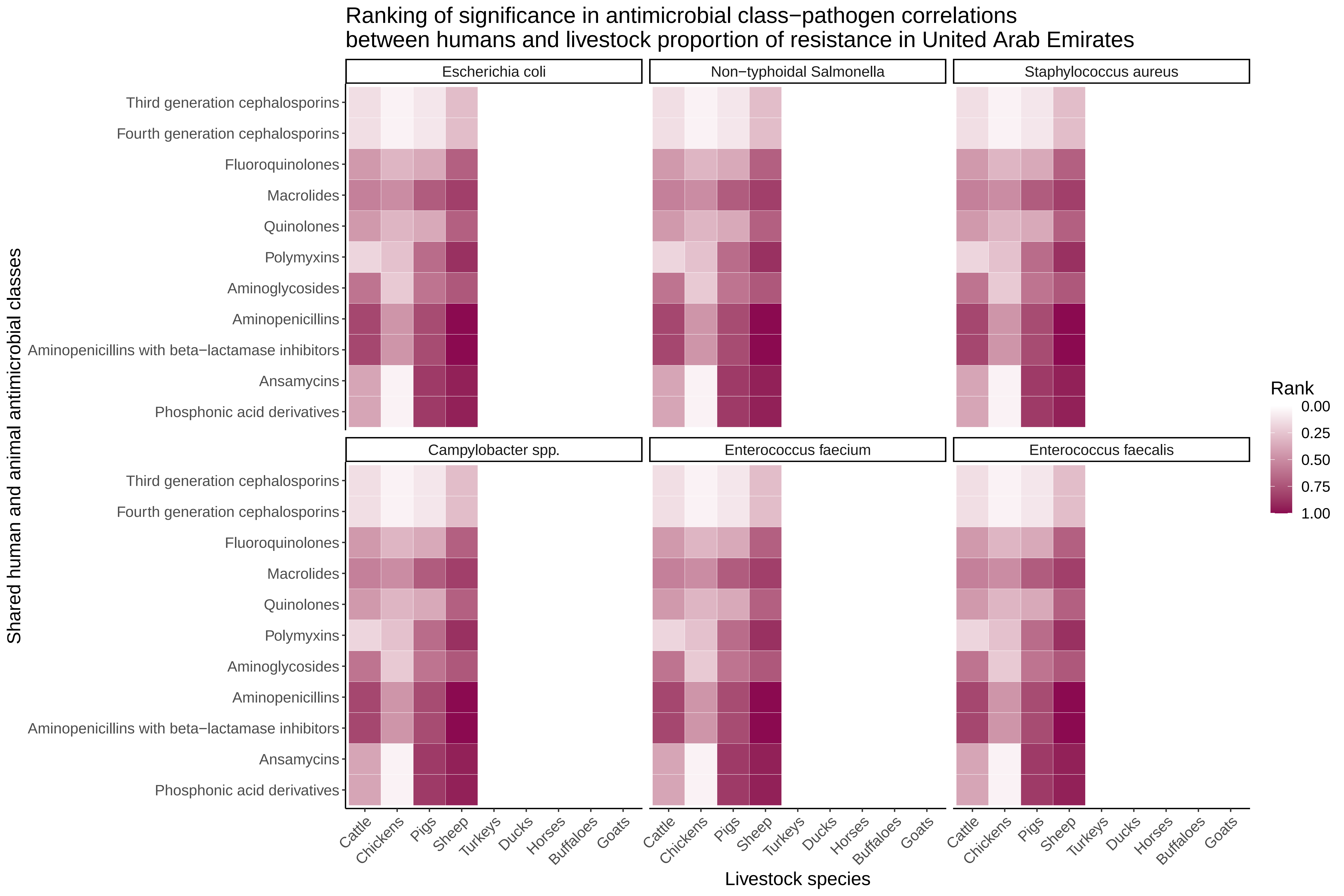

Ranking of significance in antimicrobial class−pathogen correlations
between humans and livestock proportion of resistance in United Arab Emirates
Escherichia coli
Non−typhoidal Salmonella
Staphylococcus aureus
Campylobacter spp.
Enterococcus faecium
Enterococcus faecalis
Third generation cephalosporins
Fourth generation cephalosporins
Fluoroquinolones
Macrolides
Quinolones
Polymyxins
Aminoglycosides
Aminopenicillins
Aminopenicillins with beta−lactamase inhibitors
Ansamycins
Phosphonic acid derivatives
Shared human and animal antimicrobial classes
Cattle
Chickens
Pigs
Sheep
Turkeys
Ducks
Horses
Buffaloes
Goats
Livestock species
Rank
0.00
0.25
0.50
0.75
1.00

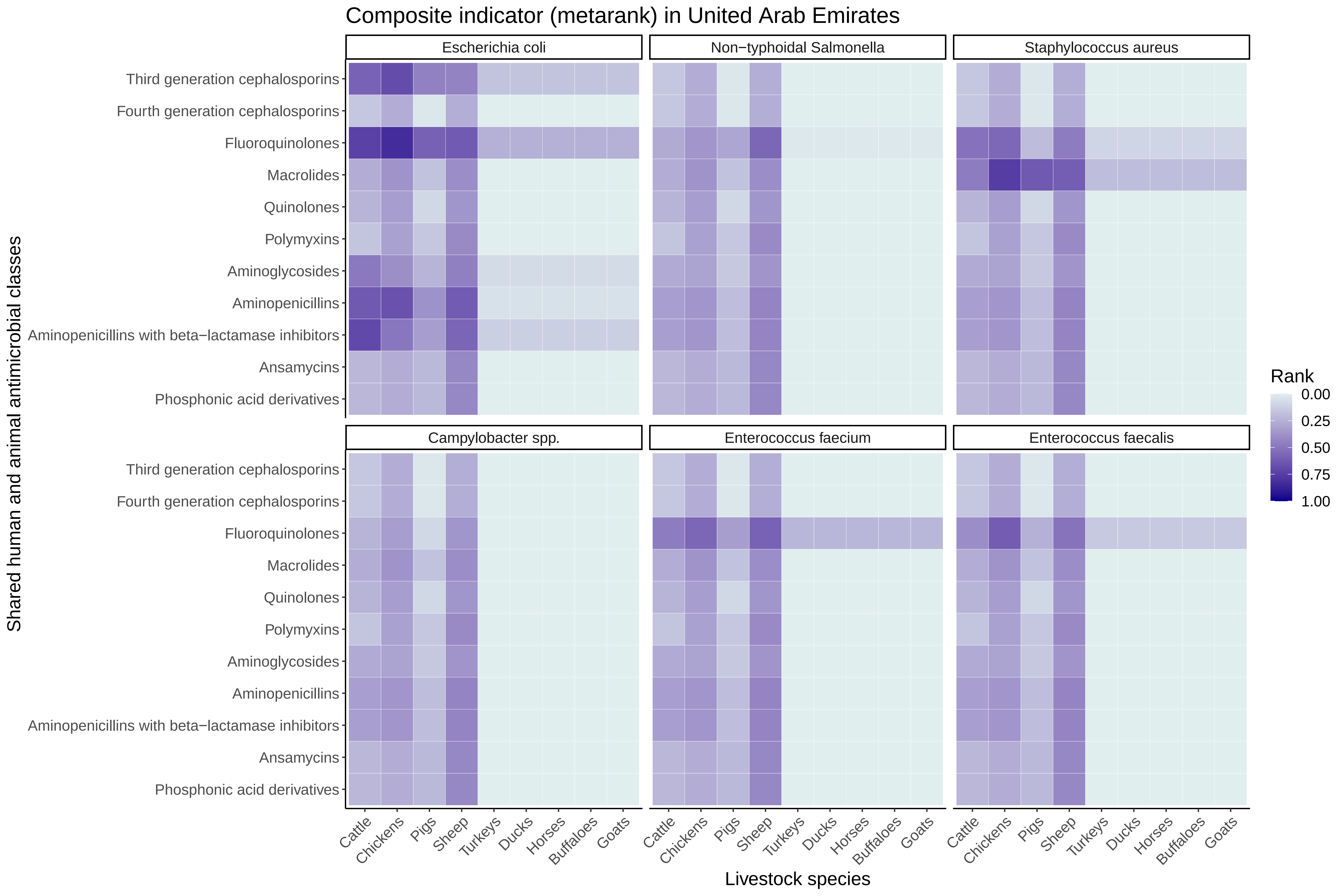

Composite indicator (metarank) in United Arab Emirates
Escherichia coli
Non−typhoidal Salmonella
Staphylococcus aureus
Campylobacter spp.
Enterococcus faecium
Enterococcus faecalis
Third generation cephalosporins
Fourth generation cephalosporins
Fluoroquinolones
Macrolides
Quinolones
Polymyxins
Aminoglycosides
Aminopenicillins
Aminopenicillins with beta−lactamase inhibitors
Ansamycins
Phosphonic acid derivatives
Shared human and animal antimicrobial classes
Cattle
Chickens
Pigs
Sheep
Turkeys
Ducks
Horses
Buffaloes
Goats
Livestock species
Rank
0.00
0.25
0.50
0.75
1.00

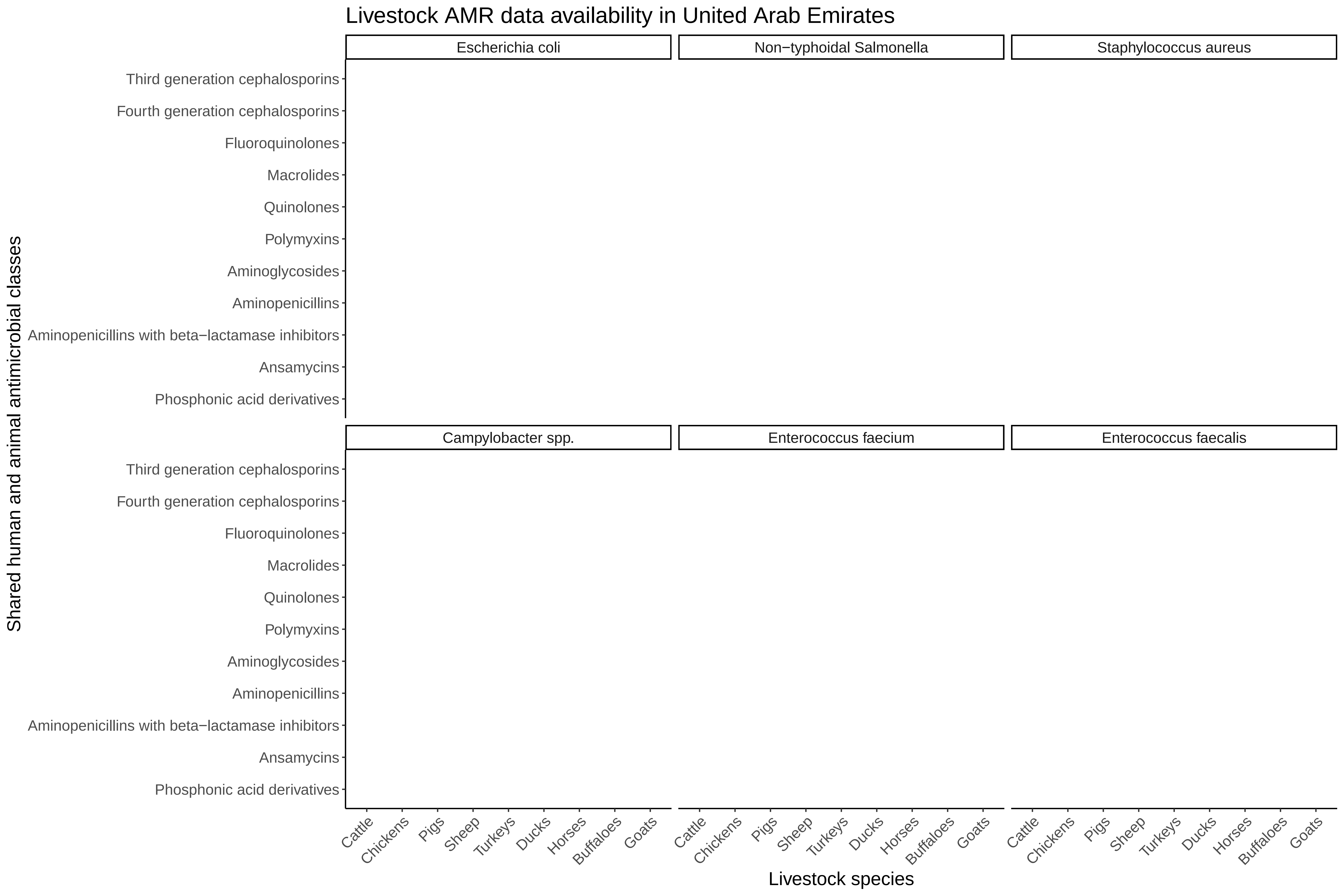
Livestock AMR data availability in United Arab Emirates
Escherichia coli
Non−typhoidal Salmonella
Staphylococcus aureus
Campylobacter spp.
Enterococcus faecium
Enterococcus faecalis
Third generation cephalosporins
Fourth generation cephalosporins
Fluoroquinolones
Macrolides
Quinolones
Polymyxins
Aminoglycosides
Aminopenicillins
Aminopenicillins with beta−lactamase inhibitors
Ansamycins
Phosphonic acid derivatives
Shared human and animal antimicrobial classes
Cattle
Chickens
Pigs
Sheep
Turkeys
Ducks
Horses
Buffaloes
Goats
Livestock species

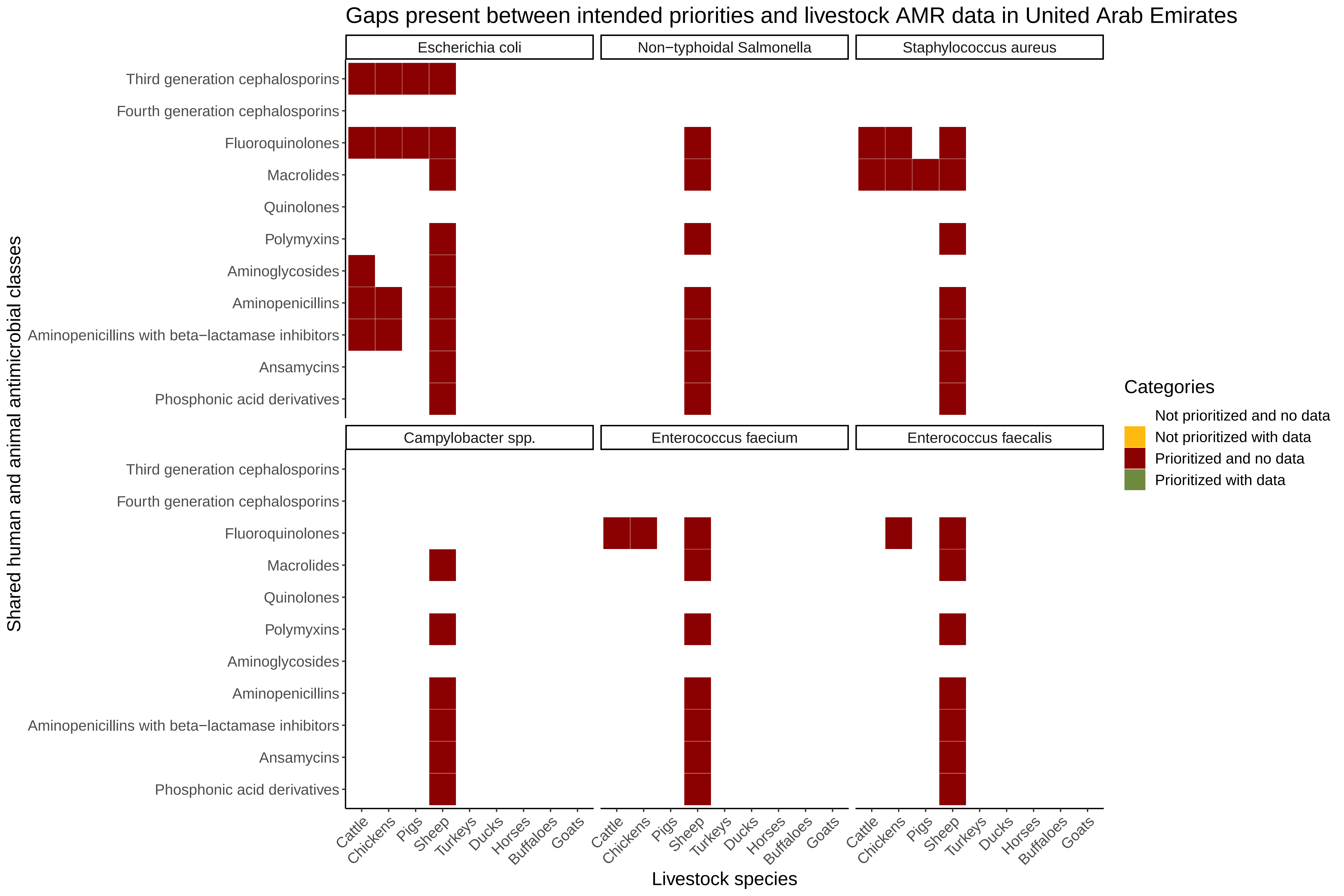

Gaps present between intended priorities and livestock AMR data in United Arab Emirates
Escherichia coli
Non−typhoidal Salmonella
Staphylococcus aureus
Campylobacter spp.
Enterococcus faecium
Enterococcus faecalis
Third generation cephalosporins
Fourth generation cephalosporins
Fluoroquinolones
Macrolides
Quinolones
Polymyxins
Aminoglycosides
Aminopenicillins
Aminopenicillins with beta−lactamase inhibitors
Ansamycins
Phosphonic acid derivatives
Shared human and animal antimicrobial classes
Cattle
Chickens
Pigs
Sheep
Turkeys
Ducks
Horses
Buffaloes
Goats
Livestock species
Categories
Not prioritized and no data
Not prioritized with data
Prioritized and no data
Prioritized with data

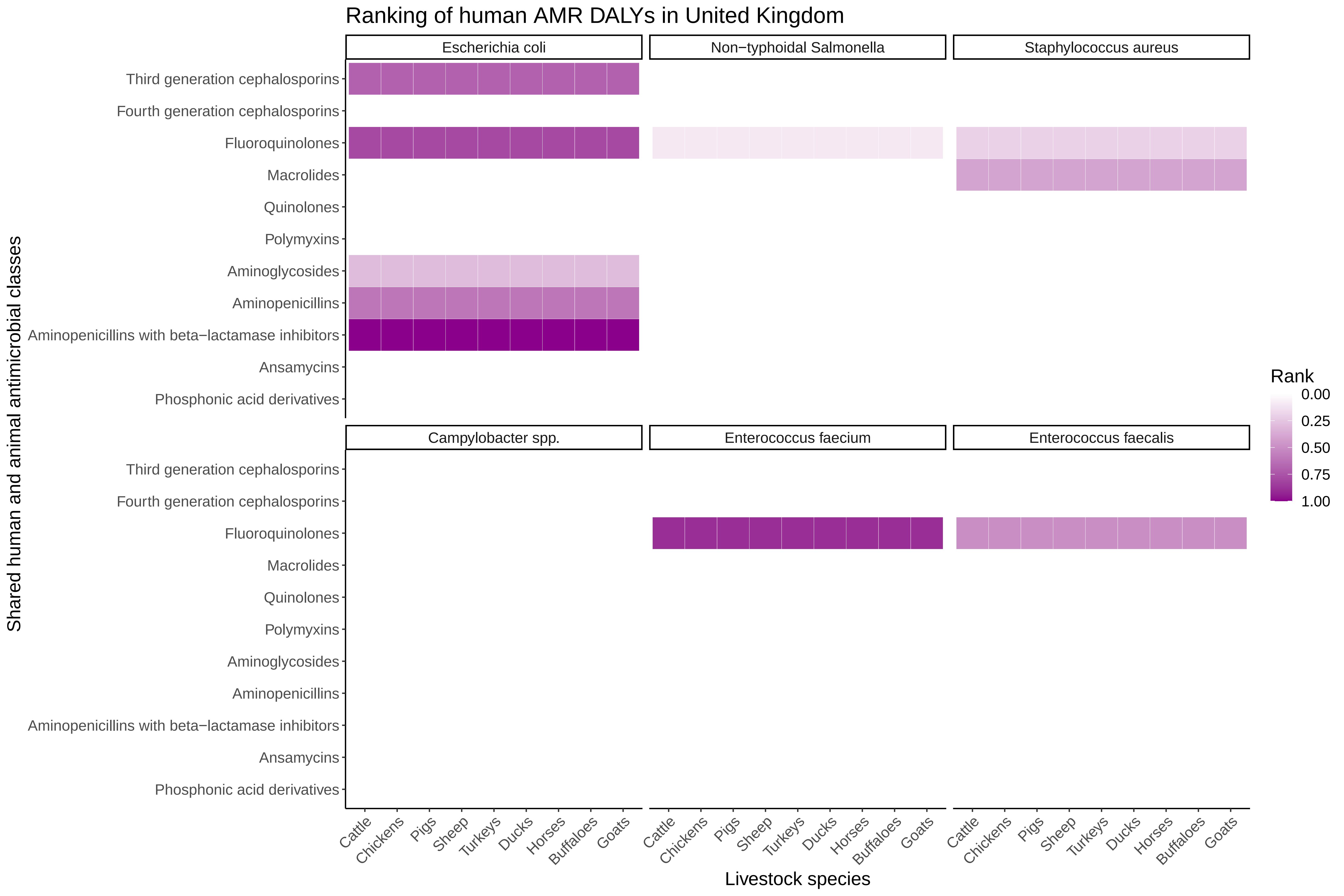

Ranking of human AMR DALYs in United Kingdom
Escherichia coli
Non−typhoidal Salmonella
Staphylococcus aureus
Campylobacter spp.
Enterococcus faecium
Enterococcus faecalis
Shared human and animal antimicrobial classes
Third generation cephalosporins
Fourth generation cephalosporins
Fluoroquinolones
Macrolides
Quinolones
Polymyxins
Aminoglycosides
Aminopenicillins
Aminopenicillins with beta−lactamase inhibitors
Ansamycins
Phosphonic acid derivatives
Livestock species
Cattle
Chickens
Pigs
Sheep
Turkeys
Ducks
Horses
Buffaloes
Goats
Rank
0.00
0.25
0.50
0.75
1.00

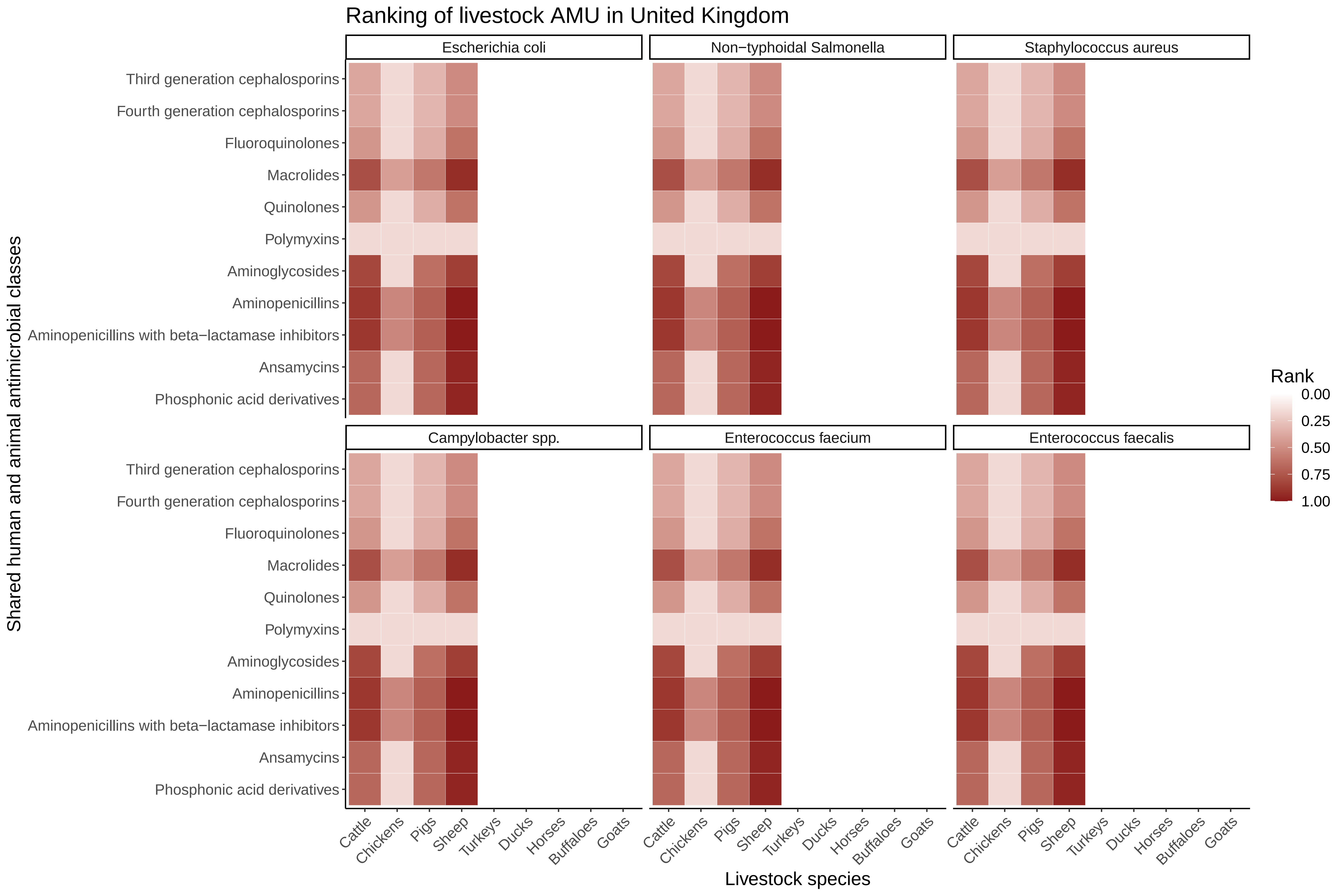

Ranking of livestock AMU in United Kingdom
Escherichia coli
Non−typhoidal Salmonella
Staphylococcus aureus
Campylobacter spp.
Enterococcus faecium
Enterococcus faecalis
Third generation cephalosporins
Fourth generation cephalosporins
Fluoroquinolones
Macrolides
Quinolones
Polymyxins
Aminoglycosides
Aminopenicillins
Aminopenicillins with beta−lactamase inhibitors
Ansamycins
Phosphonic acid derivatives
Shared human and animal antimicrobial classes
Cattle
Chickens
Pigs
Sheep
Turkeys
Ducks
Horses
Buffaloes
Goats
Livestock species
Rank
0.00
0.25
0.50
0.75
1.00

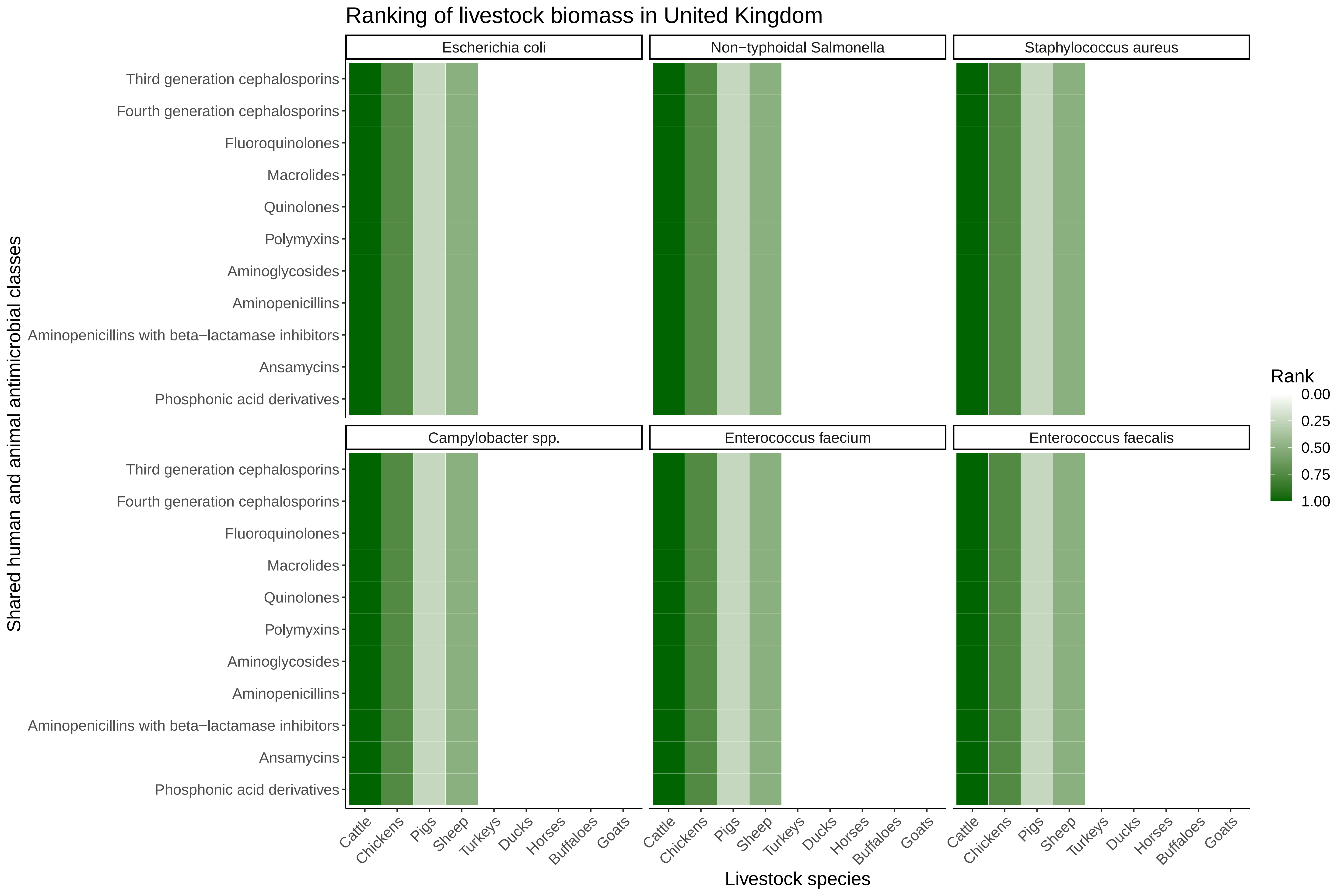

Ranking of livestock biomass in United Kingdom
Escherichia coli
Non−typhoidal Salmonella
Staphylococcus aureus
Campylobacter spp.
Enterococcus faecium
Enterococcus faecalis
Third generation cephalosporins
Fourth generation cephalosporins
Fluoroquinolones
Macrolides
Quinolones
Polymyxins
Aminoglycosides
Aminopenicillins
Aminopenicillins with beta−lactamase inhibitors
Ansamycins
Phosphonic acid derivatives
Shared human and animal antimicrobial classes
Cattle
Chickens
Pigs
Sheep
Turkeys
Ducks
Horses
Buffaloes
Goats
Livestock species
Rank
0.00
0.25
0.50
0.75
1.00

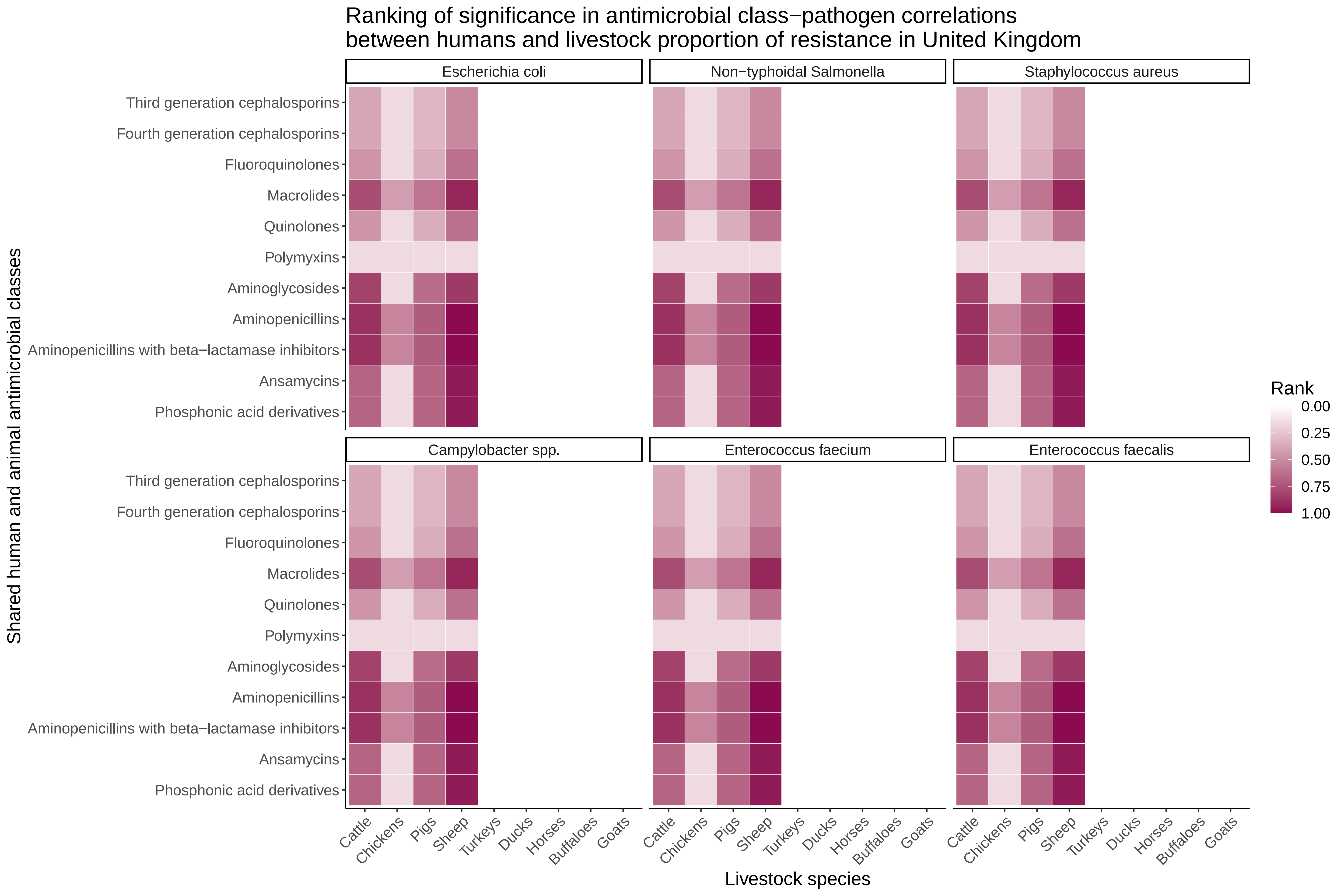

Ranking of significance in antimicrobial class−pathogen correlations
between humans and livestock proportion of resistance in United Kingdom
Escherichia coli
Non−typhoidal Salmonella
Staphylococcus aureus
Campylobacter spp.
Enterococcus faecium
Enterococcus faecalis
Third generation cephalosporins
Fourth generation cephalosporins
Fluoroquinolones
Macrolides
Quinolones
Polymyxins
Aminoglycosides
Aminopenicillins
Aminopenicillins with beta−lactamase inhibitors
Ansamycins
Phosphonic acid derivatives
Shared human and animal antimicrobial classes
Cattle
Chickens
Pigs
Sheep
Turkeys
Ducks
Horses
Buffaloes
Goats
Livestock species
Rank
0.00
0.25
0.50
0.75
1.00

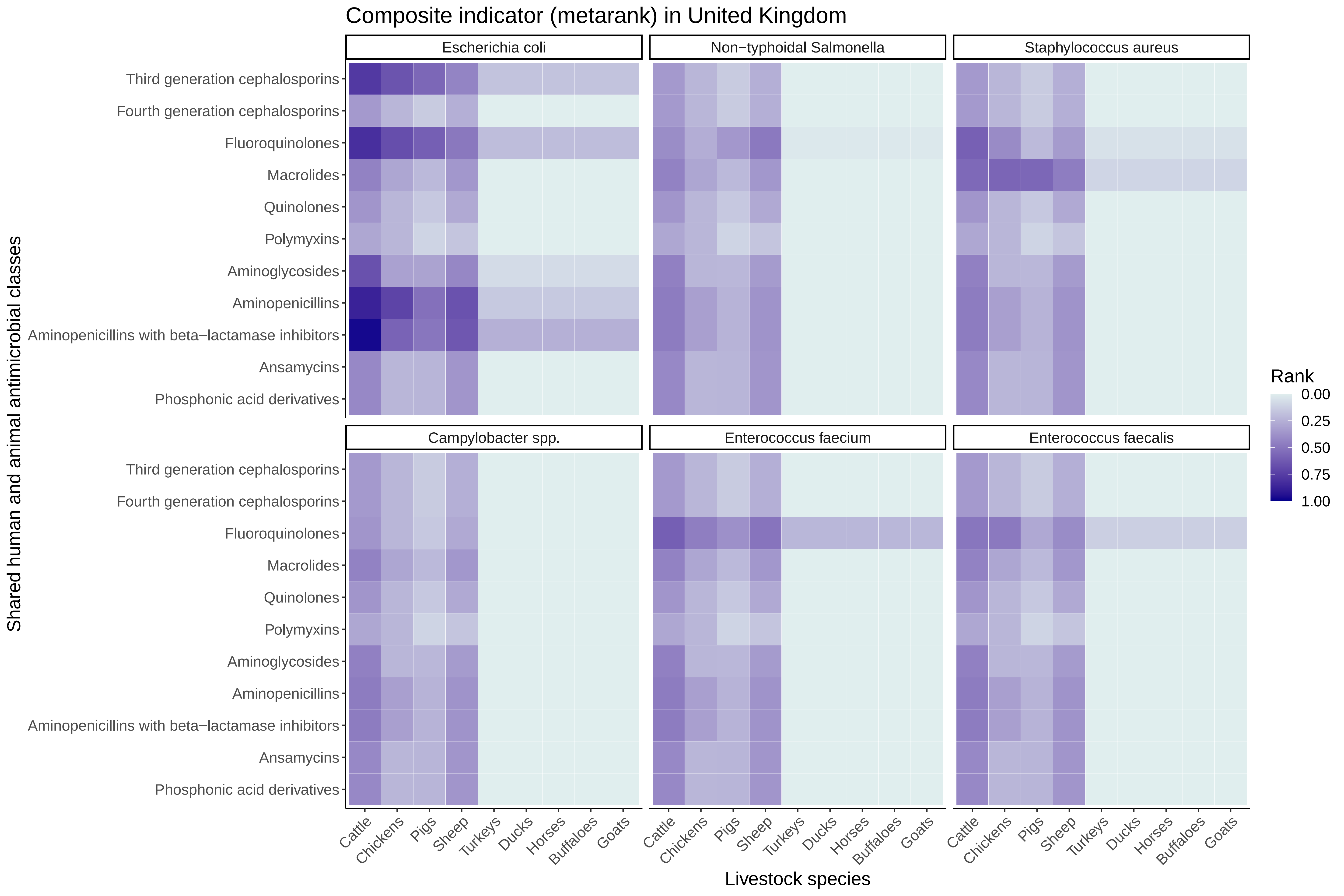
Composite indicator (metarank) in United Kingdom
Escherichia coli
Non−typhoidal Salmonella
Staphylococcus aureus
Campylobacter spp.
Enterococcus faecium
Enterococcus faecalis
Third generation cephalosporins
Fourth generation cephalosporins
Fluoroquinolones
Macrolides
Quinolones
Polymyxins
Aminoglycosides
Aminopenicillins
Aminopenicillins with beta−lactamase inhibitors
Ansamycins
Phosphonic acid derivatives
Shared human and animal antimicrobial classes
Cattle
Chickens
Pigs
Sheep
Turkeys
Ducks
Horses
Buffaloes
Goats
Livestock species
Rank
0.00
0.25
0.50
0.75
1.00

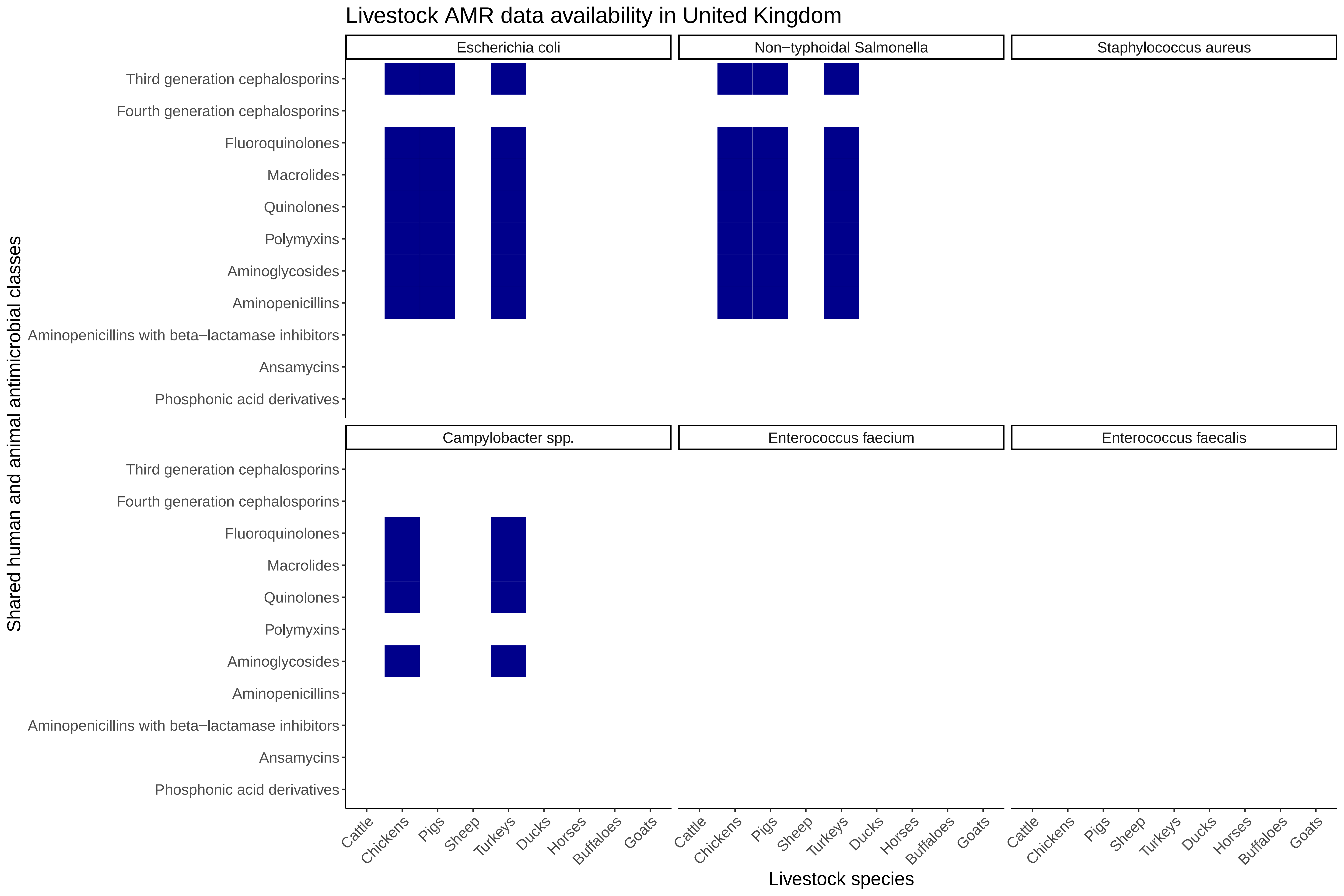

Livestock AMR data availability in United Kingdom
Escherichia coli
Non−typhoidal Salmonella
Staphylococcus aureus
Campylobacter spp.
Enterococcus faecium
Enterococcus faecalis
Shared human and animal antimicrobial classes
Third generation cephalosporins
Fourth generation cephalosporins
Fluoroquinolones
Macrolides
Quinolones
Polymyxins
Aminoglycosides
Aminopenicillins
Aminopenicillins with beta−lactamase inhibitors
Ansamycins
Phosphonic acid derivatives
Cattle
Chickens
Pigs
Sheep
Turkeys
Ducks
Horses
Buffaloes
Goats
Livestock species

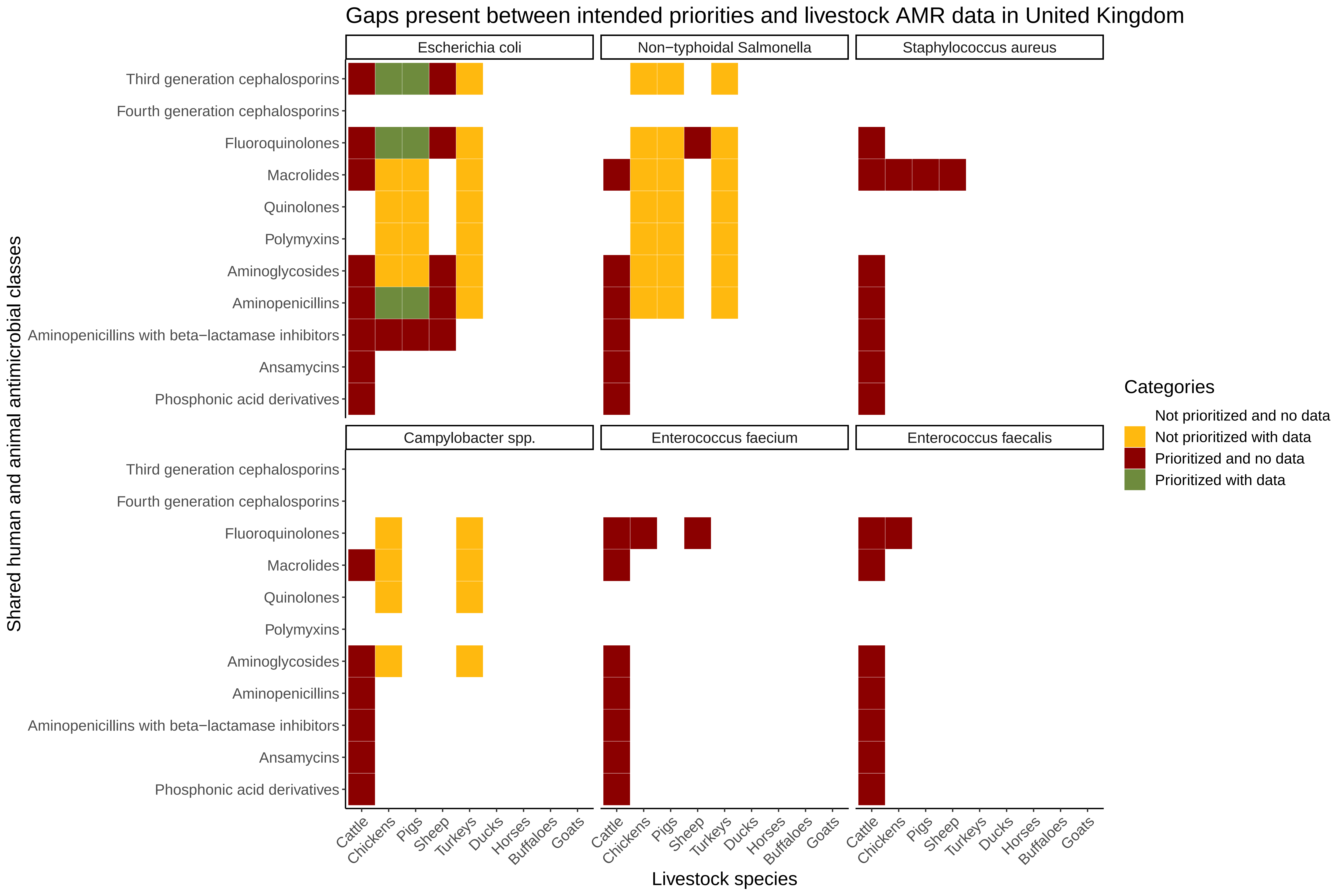

Gaps present between intended priorities and livestock AMR data in United Kingdom
Escherichia coli
Non−typhoidal Salmonella
Staphylococcus aureus
Campylobacter spp.
Enterococcus faecium
Enterococcus faecalis
Shared human and animal antimicrobial classes
Third generation cephalosporins
Fourth generation cephalosporins
Fluoroquinolones
Macrolides
Quinolones
Polymyxins
Aminoglycosides
Aminopenicillins
Aminopenicillins with beta−lactamase inhibitors
Ansamycins
Phosphonic acid derivatives
Cattle
Chickens
Pigs
Sheep
Turkeys
Ducks
Horses
Buffaloes
Goats
Livestock species
Categories
Not prioritized and no data
Not prioritized with data
Prioritized and no data
Prioritized with data

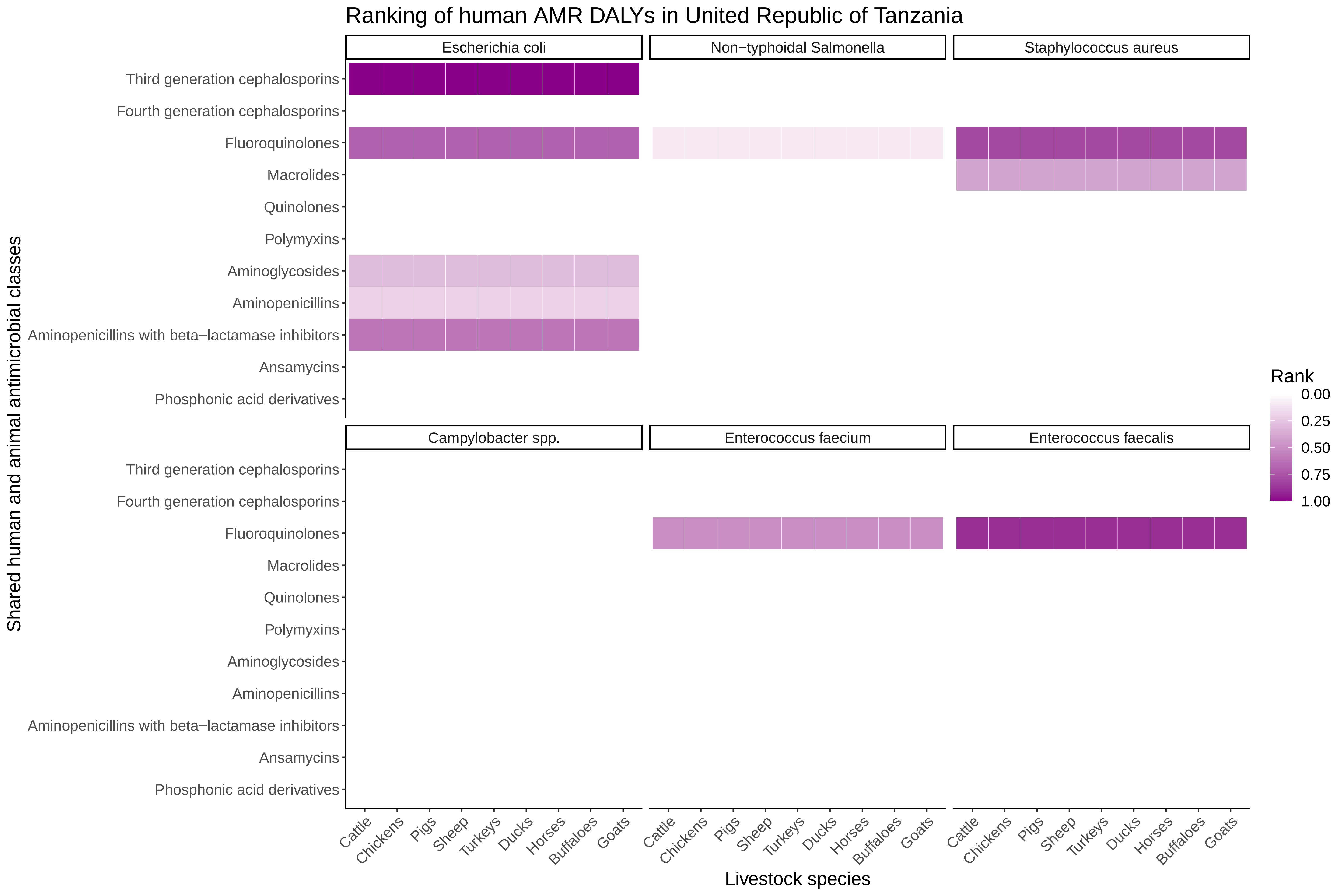

Ranking of human AMR DALYs in United Republic of Tanzania
Escherichia coli
Non−typhoidal Salmonella
Staphylococcus aureus
Campylobacter spp.
Enterococcus faecium
Enterococcus faecalis
Third generation cephalosporins
Fourth generation cephalosporins
Fluoroquinolones
Macrolides
Quinolones
Polymyxins
Aminoglycosides
Aminopenicillins
Aminopenicillins with beta−lactamase inhibitors
Ansamycins
Phosphonic acid derivatives
Shared human and animal antimicrobial classes
Cattle
Chickens
Pigs
Sheep
Turkeys
Ducks
Horses
Buffaloes
Goats
Livestock species
Rank
0.00
0.25
0.50
0.75
1.00

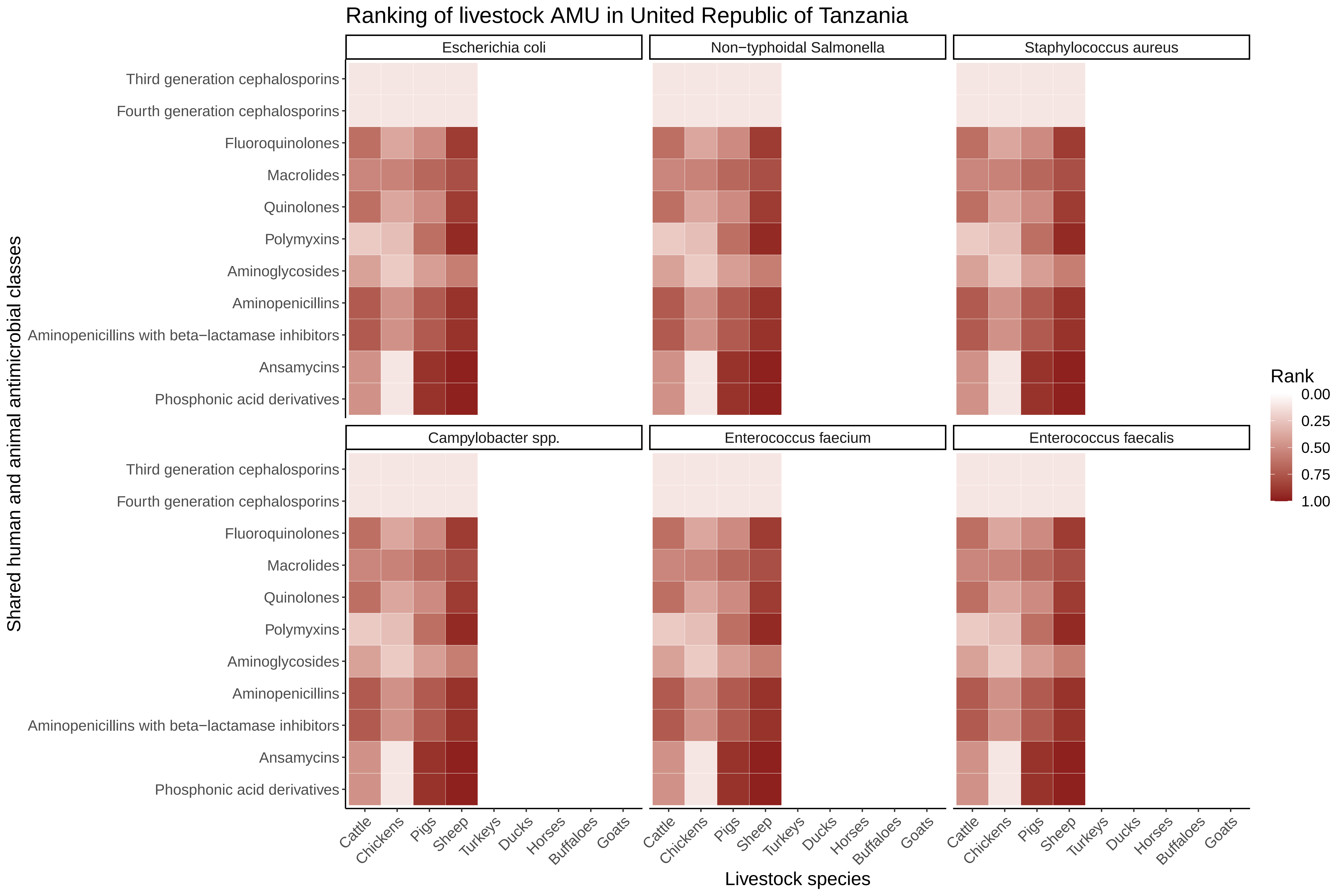

Ranking of livestock AMU in United Republic of Tanzania
Escherichia coli
Non−typhoidal Salmonella
Staphylococcus aureus
Campylobacter spp.
Enterococcus faecium
Enterococcus faecalis
Third generation cephalosporins
Fourth generation cephalosporins
Fluoroquinolones
Macrolides
Quinolones
Polymyxins
Aminoglycosides
Aminopenicillins
Aminopenicillins with beta−lactamase inhibitors
Ansamycins
Phosphonic acid derivatives
Shared human and animal antimicrobial classes
Cattle
Chickens
Pigs
Sheep
Turkeys
Ducks
Horses
Buffaloes
Goats
Livestock species
Rank
0.00
0.25
0.50
0.75
1.00

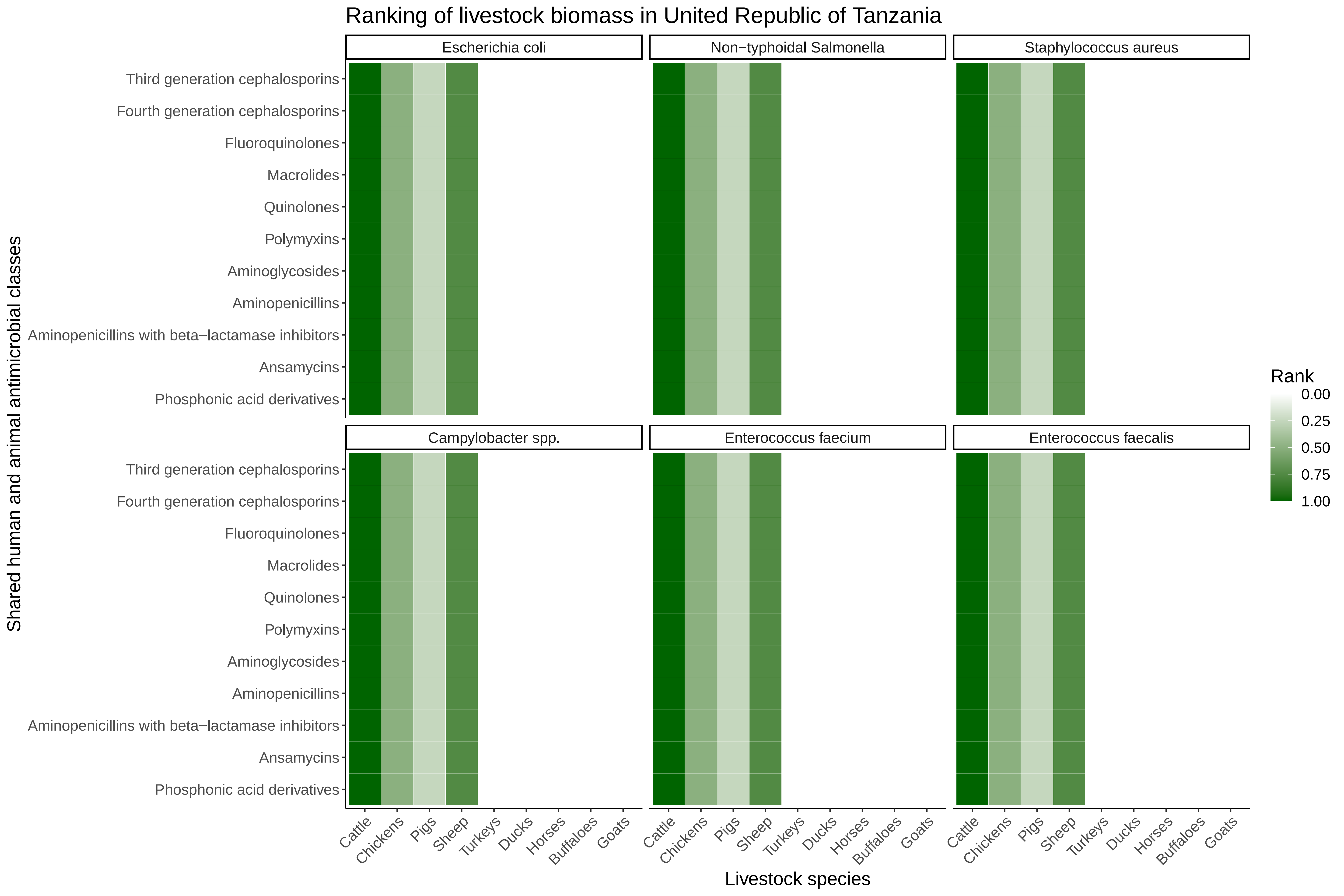

Ranking of livestock biomass in United Republic of Tanzania
Escherichia coli
Non−typhoidal Salmonella
Staphylococcus aureus
Campylobacter spp.
Enterococcus faecium
Enterococcus faecalis
Third generation cephalosporins
Fourth generation cephalosporins
Fluoroquinolones
Macrolides
Quinolones
Polymyxins
Aminoglycosides
Aminopenicillins
Aminopenicillins with beta−lactamase inhibitors
Ansamycins
Phosphonic acid derivatives
Shared human and animal antimicrobial classes
Cattle
Chickens
Pigs
Sheep
Turkeys
Ducks
Horses
Buffaloes
Goats
Livestock species
Rank
0.00
0.25
0.50
0.75
1.00

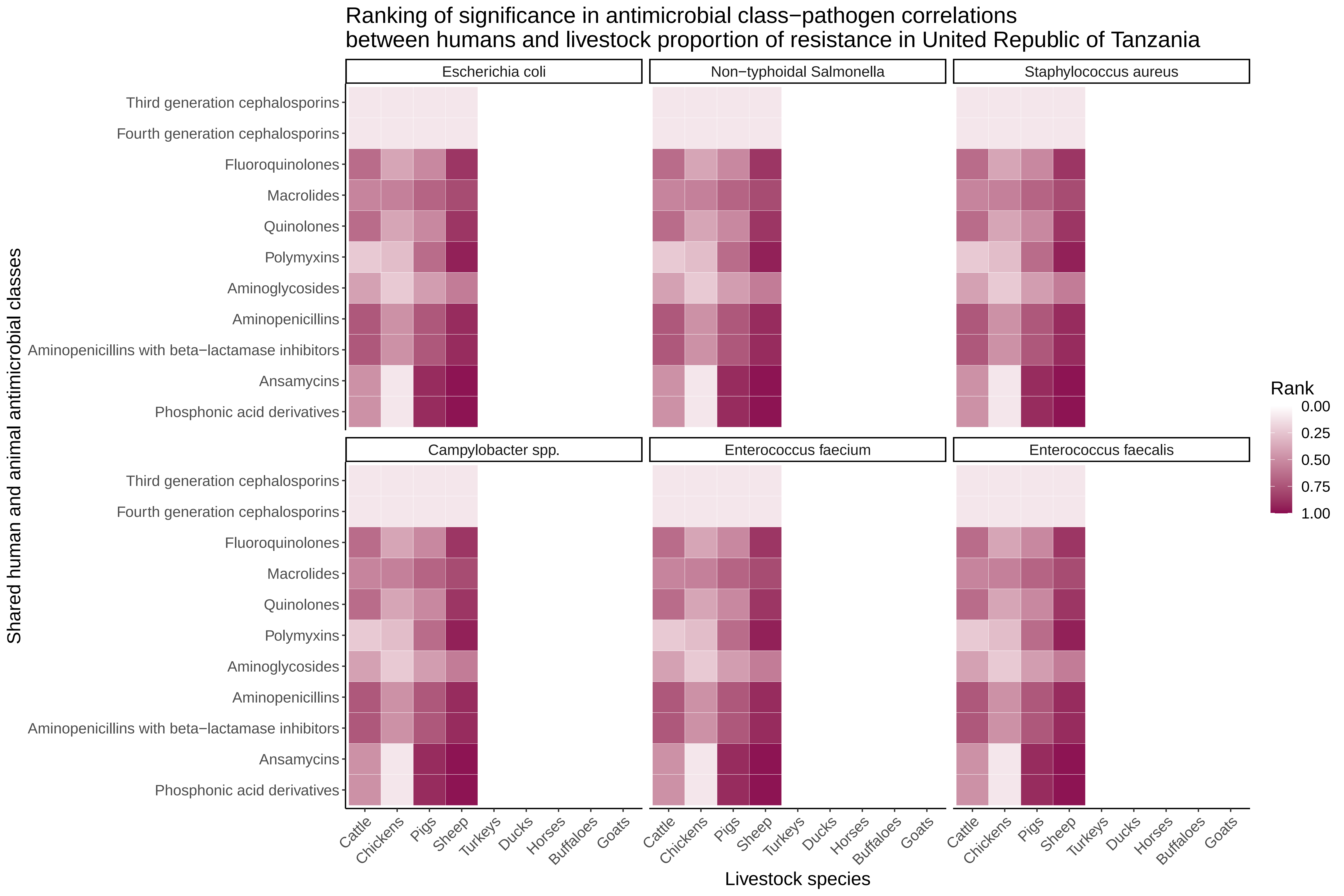

Ranking of significance in antimicrobial class−pathogen correlations
between humans and livestock proportion of resistance in United Republic of Tanzania
Escherichia coli
Non−typhoidal Salmonella
Staphylococcus aureus
Campylobacter spp.
Enterococcus faecium
Enterococcus faecalis
Shared human and animal antimicrobial classes
Third generation cephalosporins
Fourth generation cephalosporins
Fluoroquinolones
Macrolides
Quinolones
Polymyxins
Aminoglycosides
Aminopenicillins
Aminopenicillins with beta−lactamase inhibitors
Ansamycins
Phosphonic acid derivatives
Cattle
Chickens
Pigs
Sheep
Turkeys
Ducks
Horses
Buffaloes
Goats
Livestock species
Rank
0.00
0.25
0.50
0.75
1.00

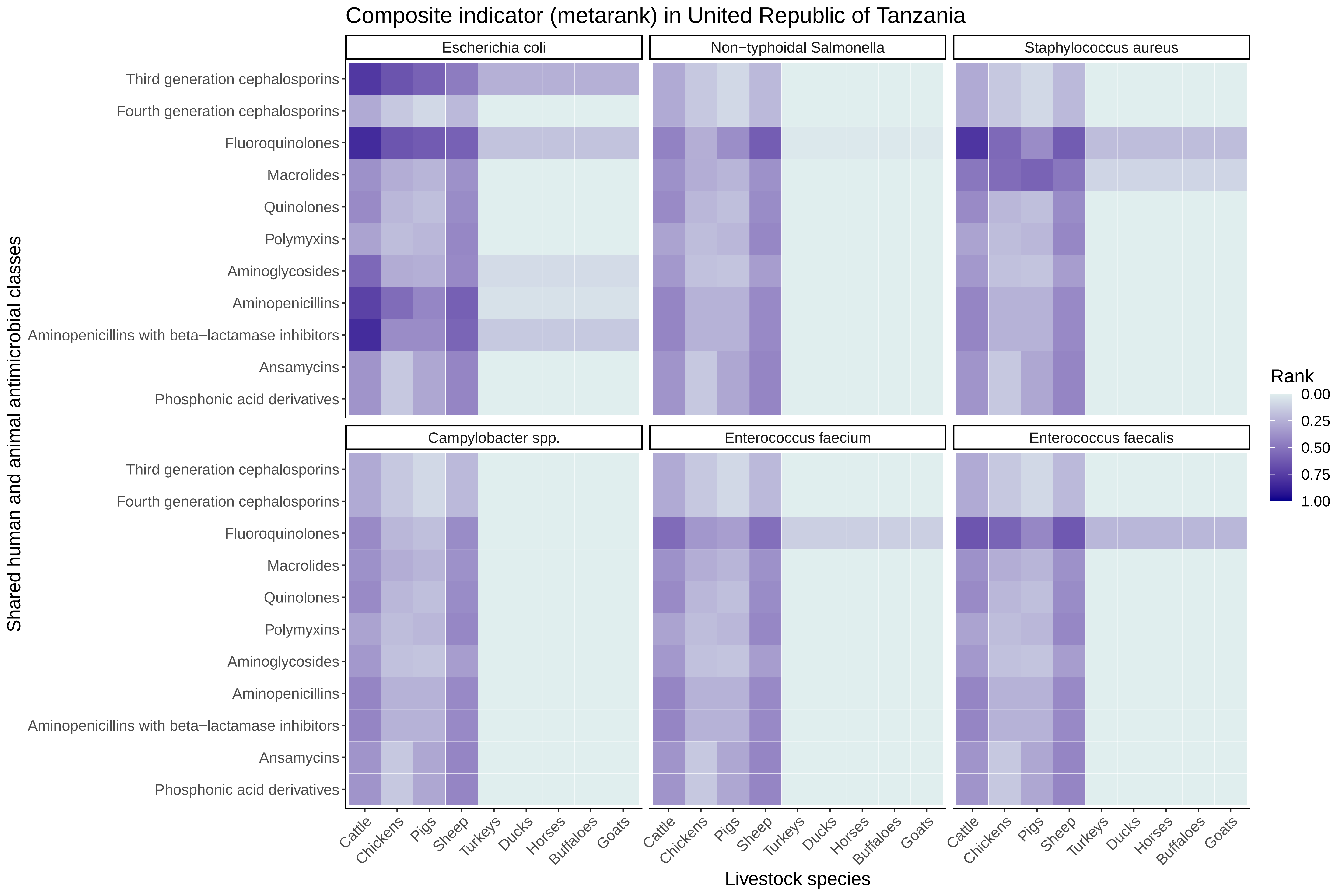

Composite indicator (metarank) in United Republic of Tanzania
Escherichia coli
Non−typhoidal Salmonella
Staphylococcus aureus
Campylobacter spp.
Enterococcus faecium
Enterococcus faecalis
Shared human and animal antimicrobial classes
Third generation cephalosporins
Fourth generation cephalosporins
Fluoroquinolones
Macrolides
Quinolones
Polymyxins
Aminoglycosides
Aminopenicillins
Aminopenicillins with beta−lactamase inhibitors
Ansamycins
Phosphonic acid derivatives
Livestock species
Cattle
Chickens
Pigs
Sheep
Turkeys
Ducks
Horses
Buffaloes
Goats
Rank
0.00
0.25
0.50
0.75
1.00

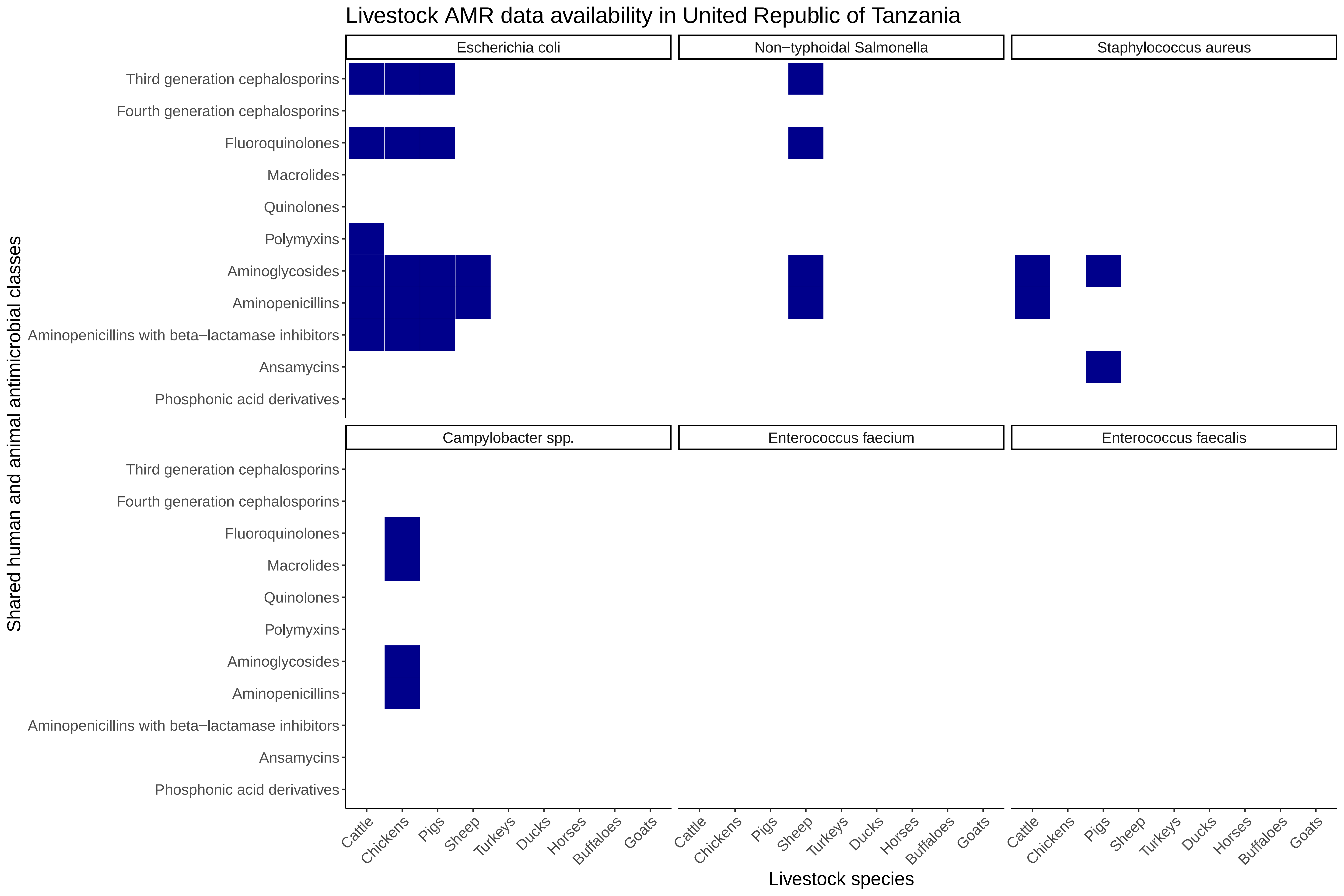
Livestock AMR data availability in United Republic of Tanzania
Escherichia coli
Non−typhoidal Salmonella
Staphylococcus aureus
Campylobacter spp.
Enterococcus faecium
Enterococcus faecalis
Third generation cephalosporins
Fourth generation cephalosporins
Fluoroquinolones
Macrolides
Quinolones
Polymyxins
Aminoglycosides
Aminopenicillins
Aminopenicillins with beta−lactamase inhibitors
Ansamycins
Phosphonic acid derivatives
Shared human and animal antimicrobial classes
Cattle
Chickens
Pigs
Sheep
Turkeys
Ducks
Horses
Buffaloes
Goats
Livestock species

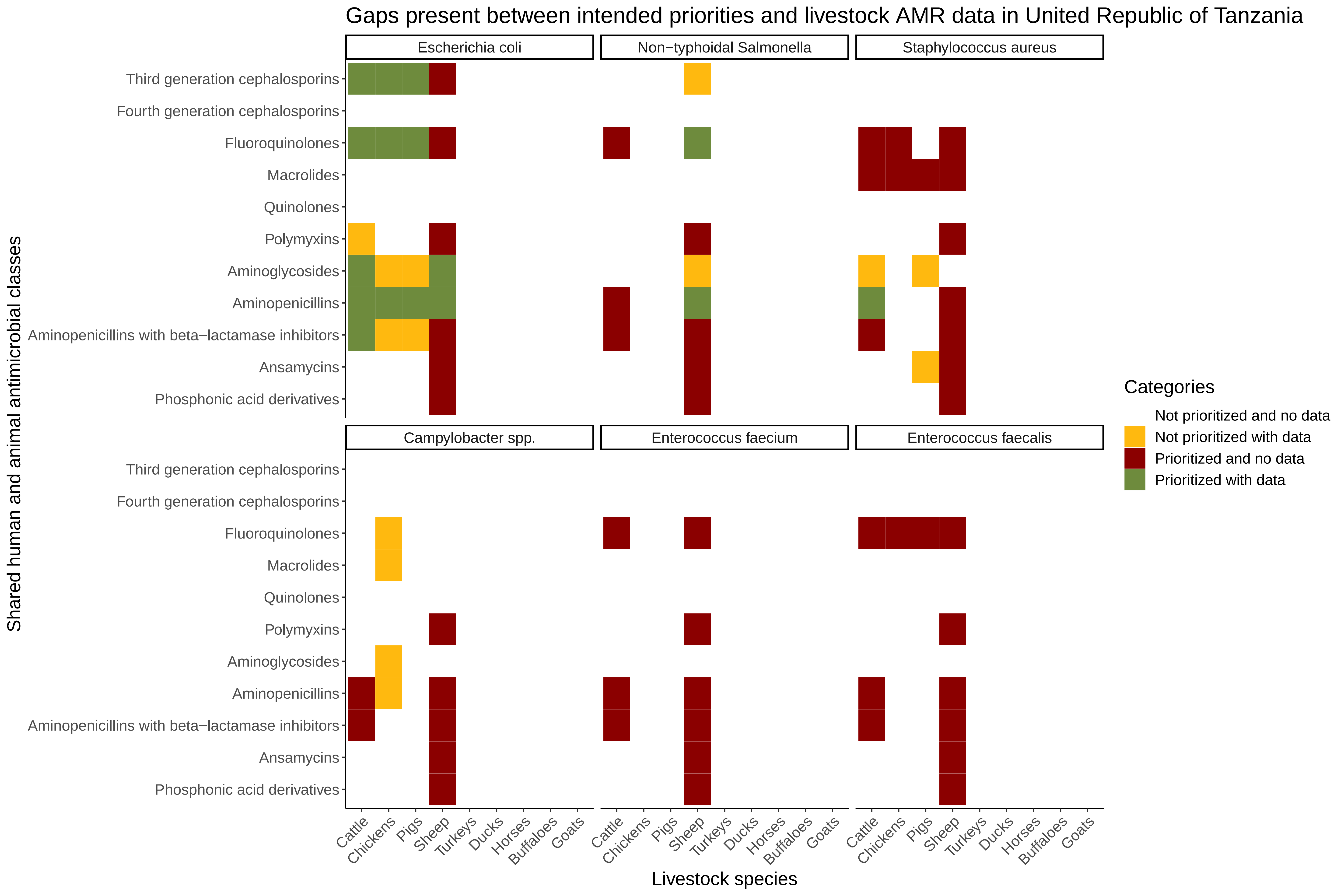

Gaps present between intended priorities and livestock AMR data in United Republic of Tanzania
Escherichia coli
Non−typhoidal Salmonella
Staphylococcus aureus
Campylobacter spp.
Enterococcus faecium
Enterococcus faecalis
Third generation cephalosporins
Fourth generation cephalosporins
Fluoroquinolones
Macrolides
Quinolones
Polymyxins
Aminoglycosides
Aminopenicillins
Aminopenicillins with beta−lactamase inhibitors
Ansamycins
Phosphonic acid derivatives
Shared human and animal antimicrobial classes
Cattle
Chickens
Pigs
Sheep
Turkeys
Ducks
Horses
Buffaloes
Goats
Livestock species
Categories
Not prioritized and no data
Not prioritized with data
Prioritized and no data
Prioritized with data

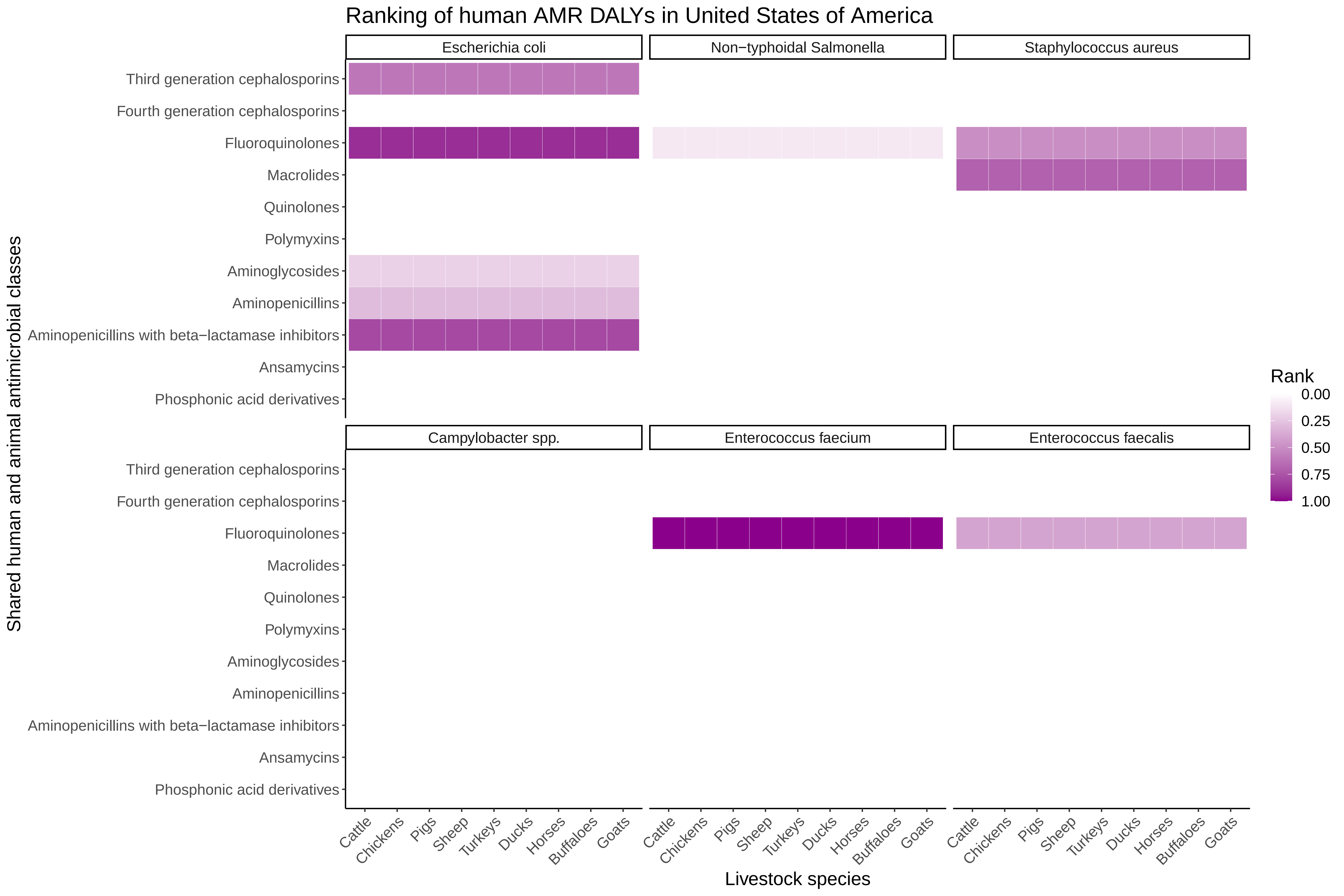

Ranking of human AMR DALYs in United States of America
Escherichia coli
Non−typhoidal Salmonella
Staphylococcus aureus
Campylobacter spp.
Enterococcus faecium
Enterococcus faecalis
Third generation cephalosporins
Fourth generation cephalosporins
Fluoroquinolones
Macrolides
Quinolones
Polymyxins
Aminoglycosides
Aminopenicillins
Aminopenicillins with beta−lactamase inhibitors
Ansamycins
Phosphonic acid derivatives
Shared human and animal antimicrobial classes
Cattle
Chickens
Pigs
Sheep
Turkeys
Ducks
Horses
Buffaloes
Goats
Livestock species
Rank
0.00
0.25
0.50
0.75
1.00

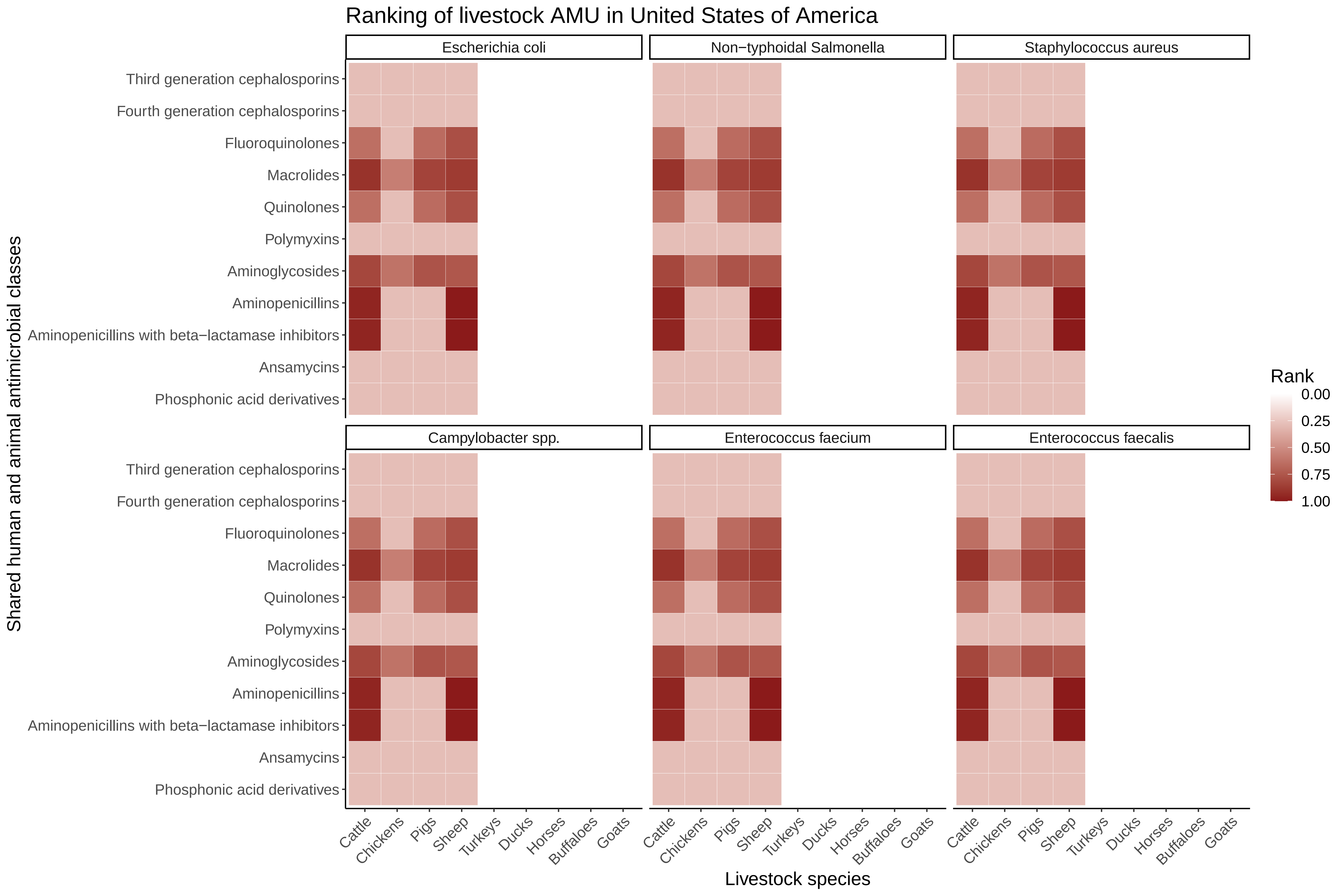

Ranking of livestock AMU in United States of America
Escherichia coli
Non−typhoidal Salmonella
Staphylococcus aureus
Campylobacter spp.
Enterococcus faecium
Enterococcus faecalis
Shared human and animal antimicrobial classes
Third generation cephalosporins
Fourth generation cephalosporins
Fluoroquinolones
Macrolides
Quinolones
Polymyxins
Aminoglycosides
Aminopenicillins
Aminopenicillins with beta−lactamase inhibitors
Ansamycins
Phosphonic acid derivatives
Cattle
Chickens
Pigs
Sheep
Turkeys
Ducks
Horses
Buffaloes
Goats
Livestock species
Rank
0.00
0.25
0.50
0.75
1.00

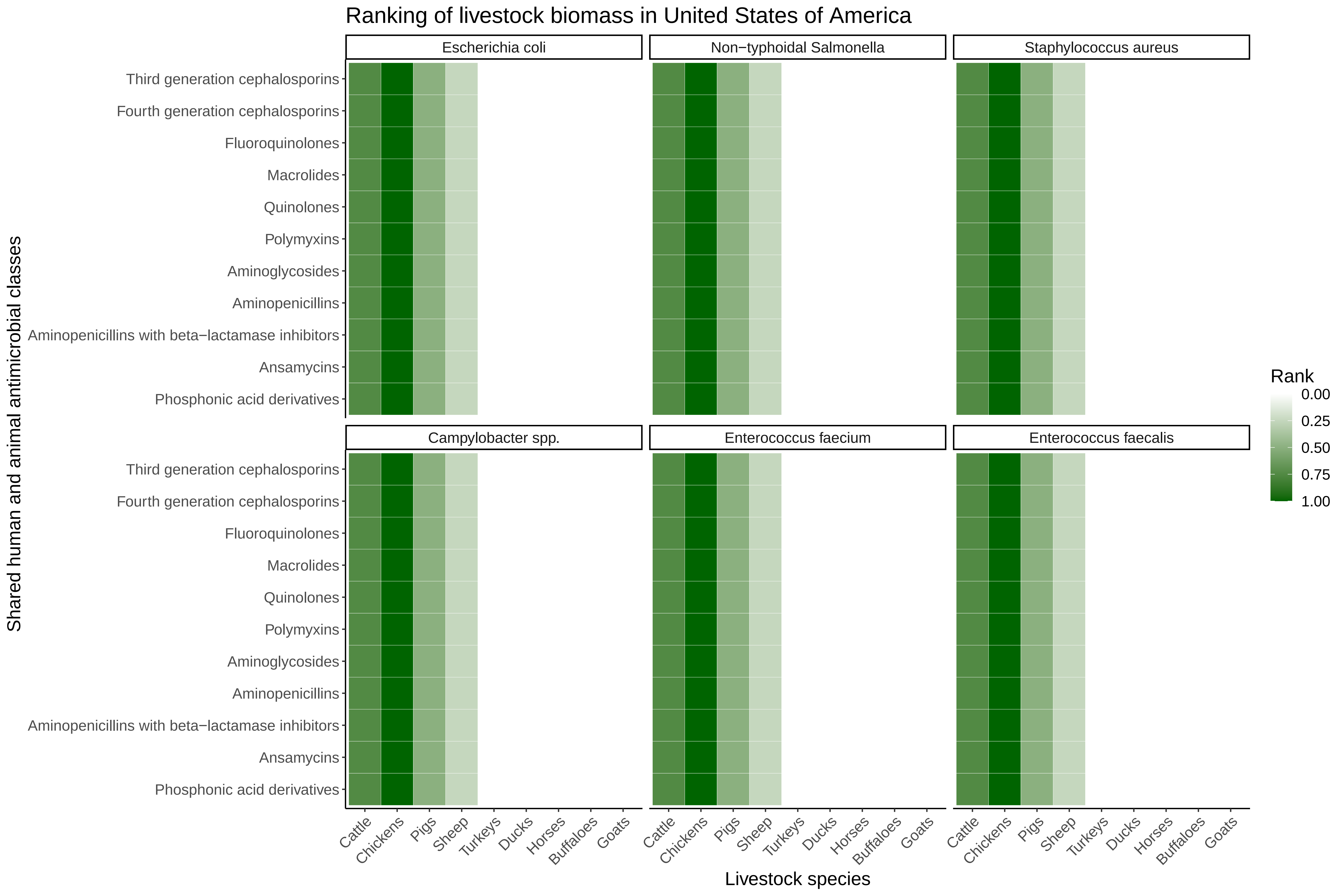

Ranking of livestock biomass in United States of America
Escherichia coli
Non−typhoidal Salmonella
Staphylococcus aureus
Campylobacter spp.
Enterococcus faecium
Enterococcus faecalis
Third generation cephalosporins
Fourth generation cephalosporins
Fluoroquinolones
Macrolides
Quinolones
Polymyxins
Aminoglycosides
Aminopenicillins
Aminopenicillins with beta−lactamase inhibitors
Ansamycins
Phosphonic acid derivatives
Shared human and animal antimicrobial classes
Cattle
Chickens
Pigs
Sheep
Turkeys
Ducks
Horses
Buffaloes
Goats
Livestock species
Rank
0.00
0.25
0.50
0.75
1.00

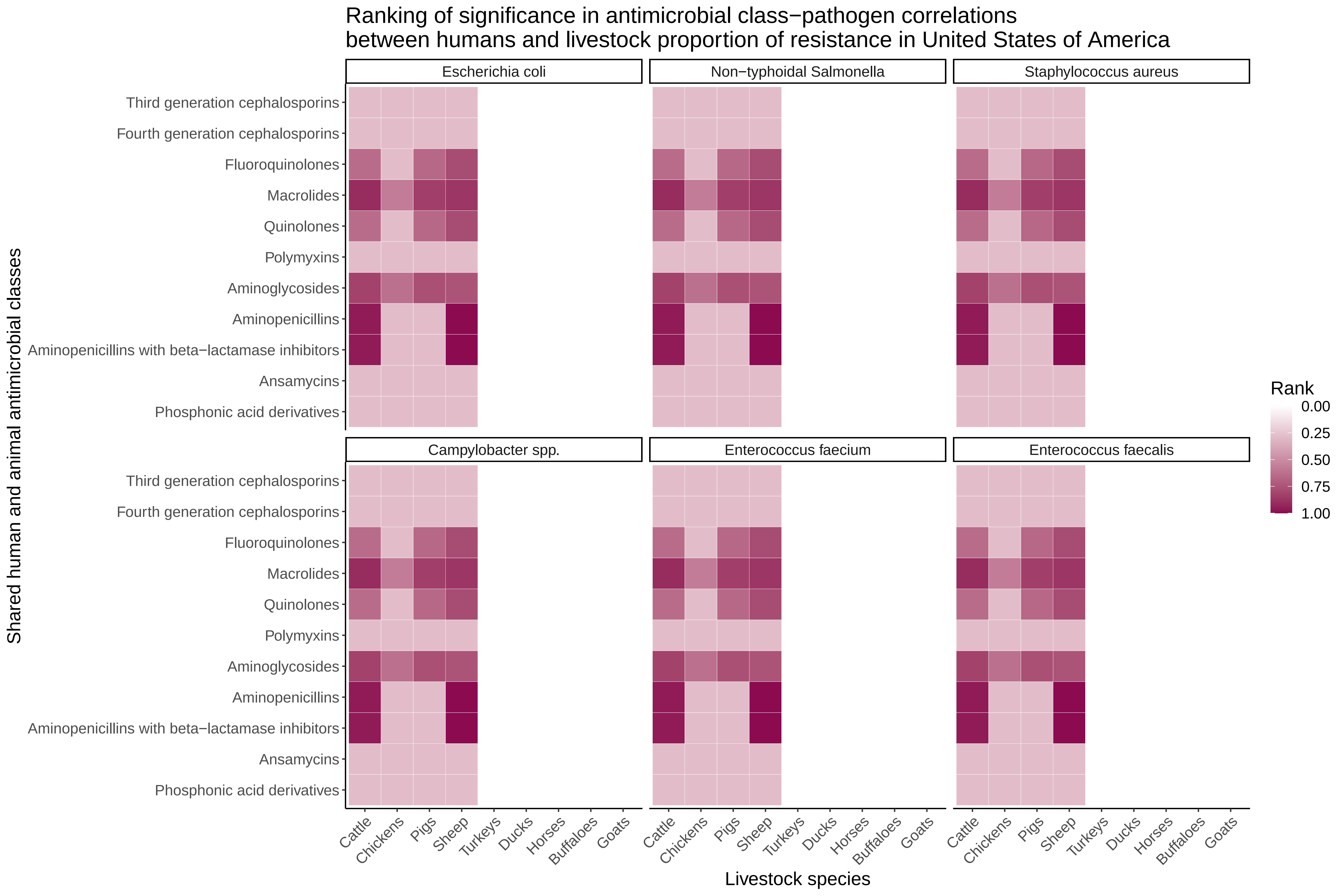
Ranking of significance in antimicrobial class−pathogen correlations
between humans and livestock proportion of resistance in United States of America
Escherichia coli
Non−typhoidal Salmonella
Staphylococcus aureus
Campylobacter spp.
Enterococcus faecium
Enterococcus faecalis
Third generation cephalosporins
Fourth generation cephalosporins
Fluoroquinolones
Macrolides
Quinolones
Polymyxins
Aminoglycosides
Aminopenicillins
Aminopenicillins with beta−lactamase inhibitors
Ansamycins
Phosphonic acid derivatives
Shared human and animal antimicrobial classes
Cattle
Chickens
Pigs
Sheep
Turkeys
Ducks
Horses
Buffaloes
Goats
Livestock species
Rank
0.00
0.25
0.50
0.75
1.00

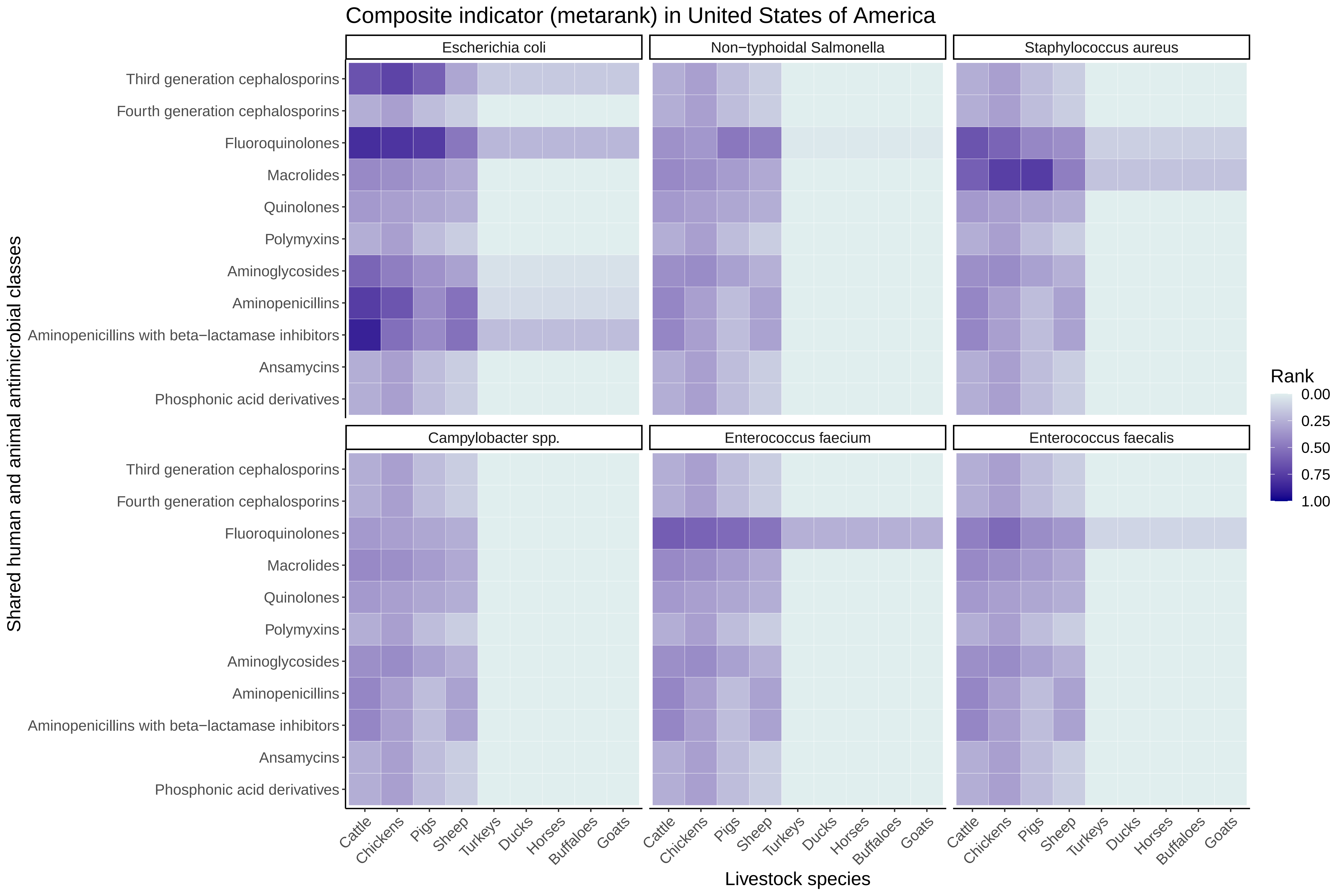
Composite indicator (metarank) in United States of America
Escherichia coli
Non−typhoidal Salmonella
Staphylococcus aureus
Campylobacter spp.
Enterococcus faecium
Enterococcus faecalis
Third generation cephalosporins
Fourth generation cephalosporins
Fluoroquinolones
Macrolides
Quinolones
Polymyxins
Aminoglycosides
Aminopenicillins
Aminopenicillins with beta−lactamase inhibitors
Ansamycins
Phosphonic acid derivatives
Shared human and animal antimicrobial classes
Cattle
Chickens
Pigs
Sheep
Turkeys
Ducks
Horses
Buffaloes
Goats
Livestock species
Rank
0.00
0.25
0.50
0.75
1.00

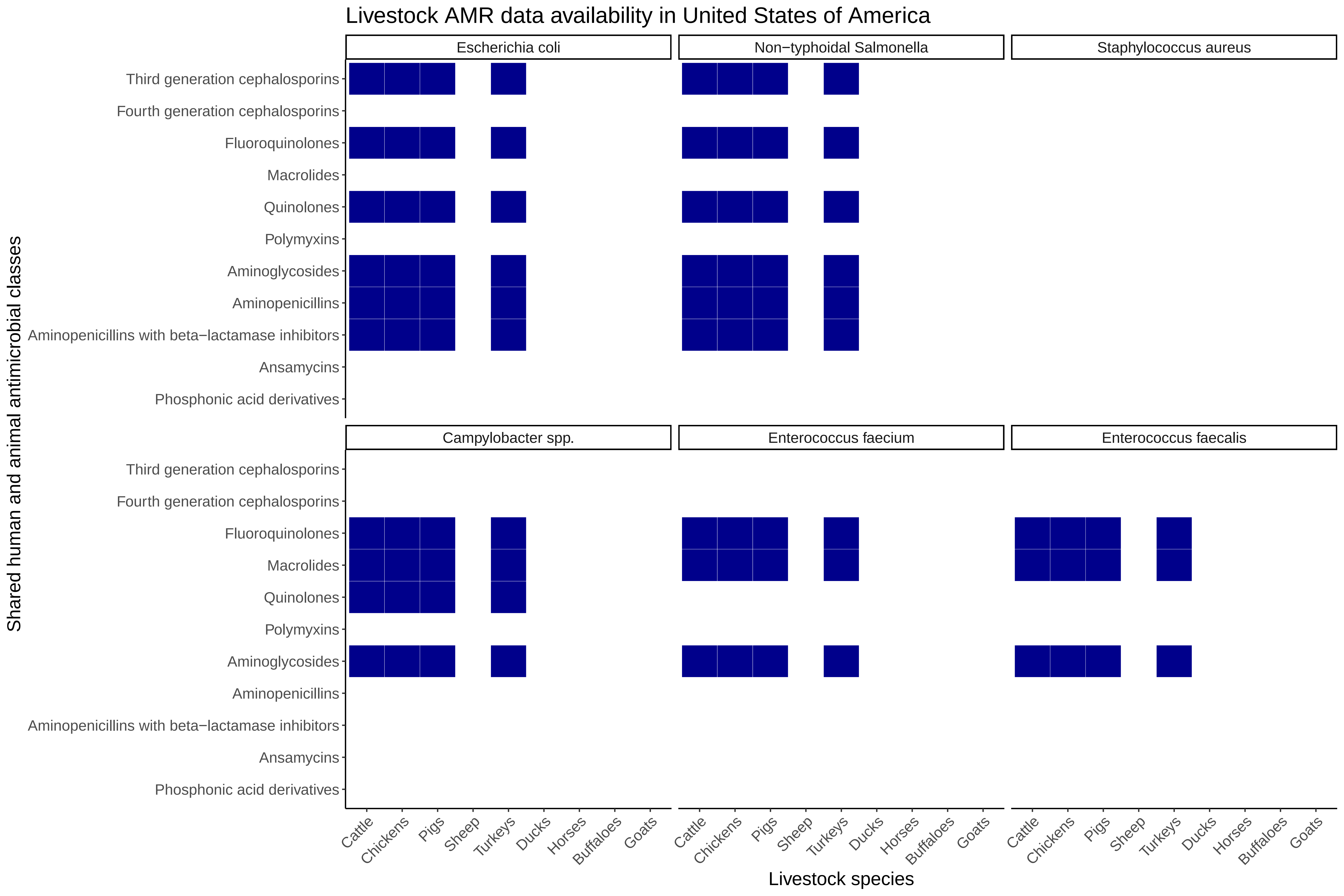

Livestock AMR data availability in United States of America
Escherichia coli
Non−typhoidal Salmonella
Staphylococcus aureus
Campylobacter spp.
Enterococcus faecium
Enterococcus faecalis
Shared human and animal antimicrobial classes
Third generation cephalosporins
Fourth generation cephalosporins
Fluoroquinolones
Macrolides
Quinolones
Polymyxins
Aminoglycosides
Aminopenicillins
Aminopenicillins with beta−lactamase inhibitors
Ansamycins
Phosphonic acid derivatives
Cattle
Chickens
Pigs
Sheep
Turkeys
Ducks
Horses
Buffaloes
Goats
Livestock species

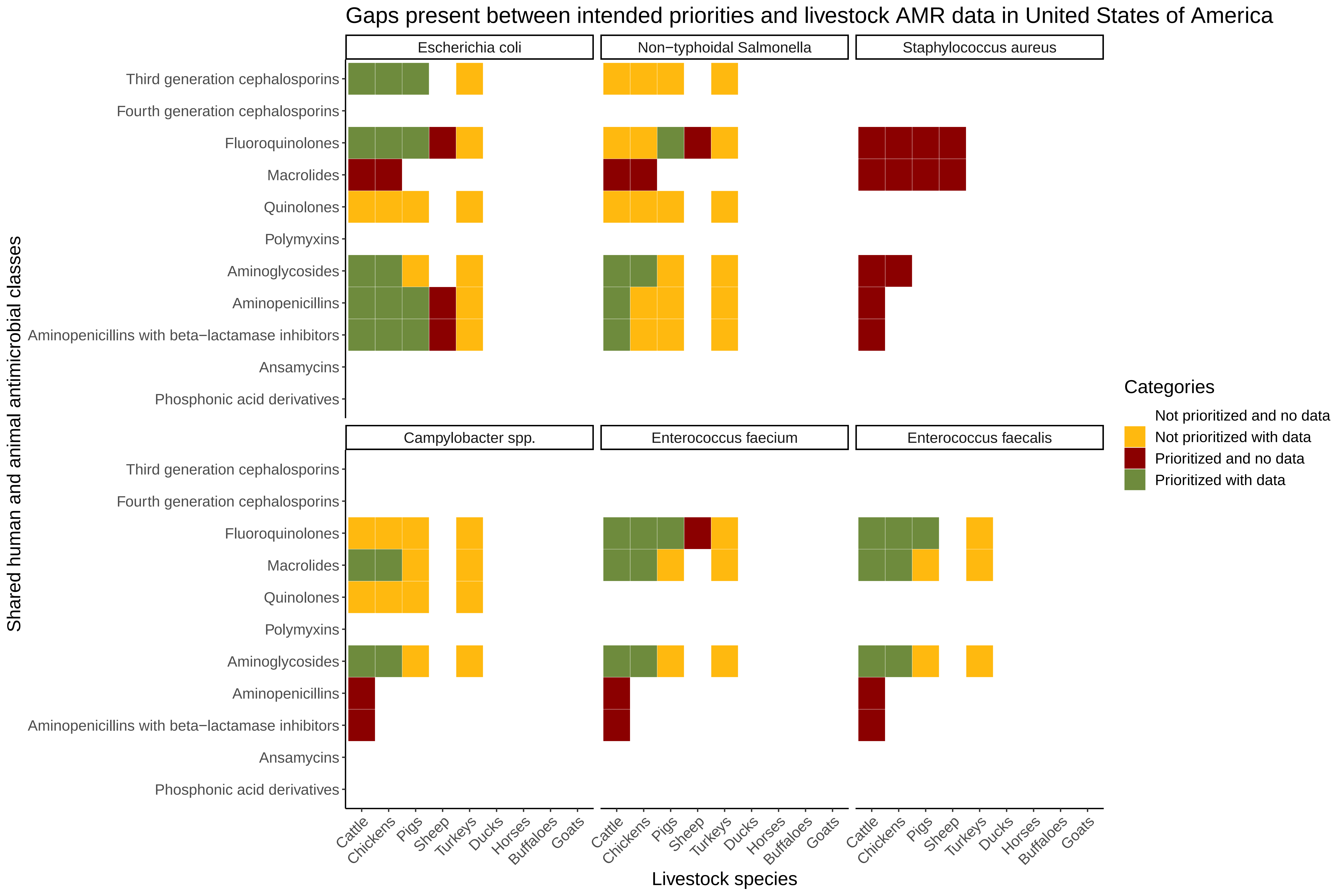

Gaps present between intended priorities and livestock AMR data in United States of America
Escherichia coli
Non−typhoidal Salmonella
Staphylococcus aureus
Campylobacter spp.
Enterococcus faecium
Enterococcus faecalis
Third generation cephalosporins
Fourth generation cephalosporins
Fluoroquinolones
Macrolides
Quinolones
Polymyxins
Aminoglycosides
Aminopenicillins
Aminopenicillins with beta−lactamase inhibitors
Ansamycins
Phosphonic acid derivatives
Shared human and animal antimicrobial classes
Cattle
Chickens
Pigs
Sheep
Turkeys
Ducks
Horses
Buffaloes
Goats
Livestock species
Categories
Not prioritized and no data
Not prioritized with data
Prioritized and no data
Prioritized with data

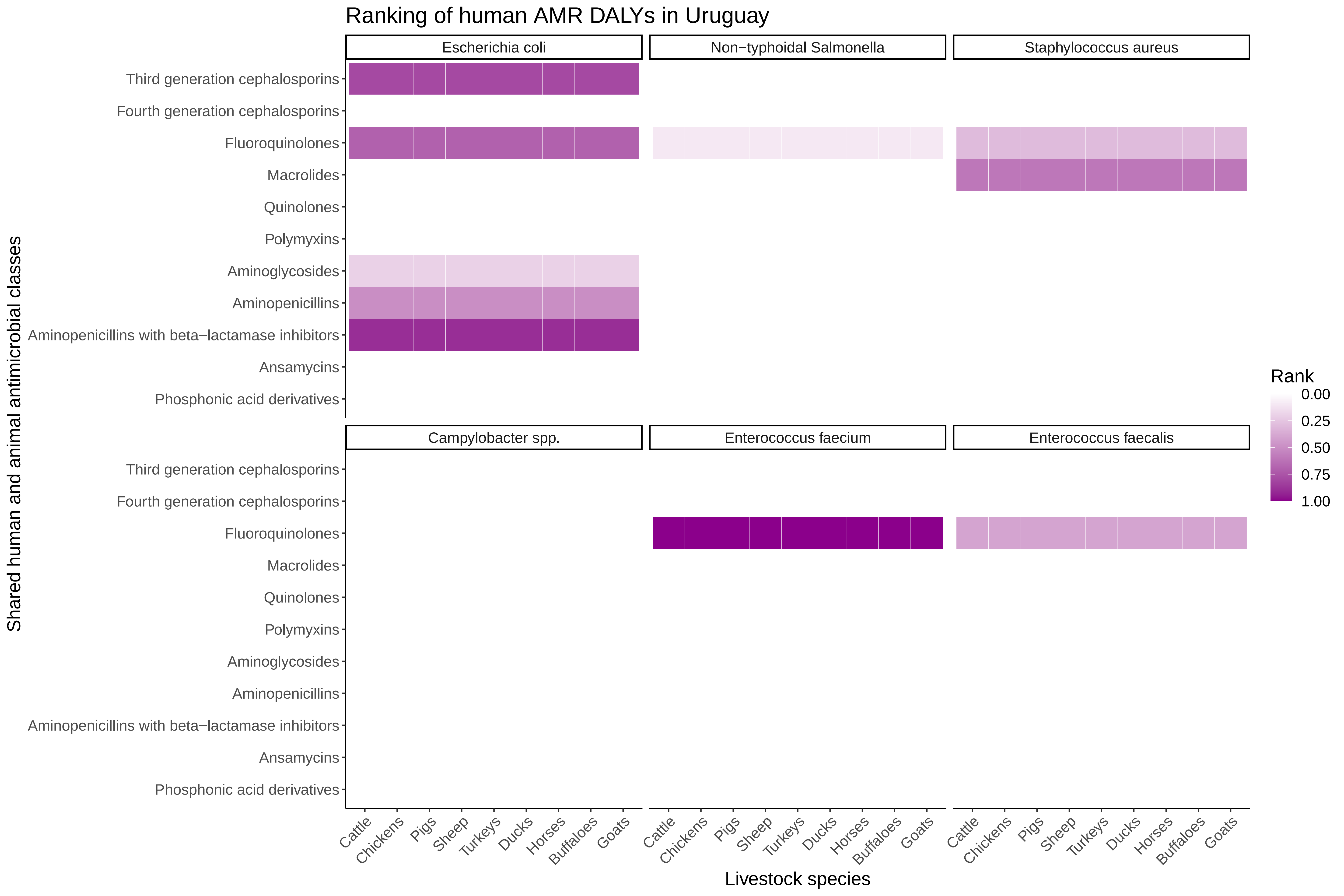
Ranking of human AMR DALYs in Uruguay
Escherichia coli
Non−typhoidal Salmonella
Staphylococcus aureus
Campylobacter spp.
Enterococcus faecium
Enterococcus faecalis
Third generation cephalosporins
Fourth generation cephalosporins
Fluoroquinolones
Macrolides
Quinolones
Polymyxins
Aminoglycosides
Aminopenicillins
Aminopenicillins with beta−lactamase inhibitors
Ansamycins
Phosphonic acid derivatives
Shared human and animal antimicrobial classes
Cattle
Chickens
Pigs
Sheep
Turkeys
Ducks
Horses
Buffaloes
Goats
Livestock species
Rank
0.00
0.25
0.50
0.75
1.00

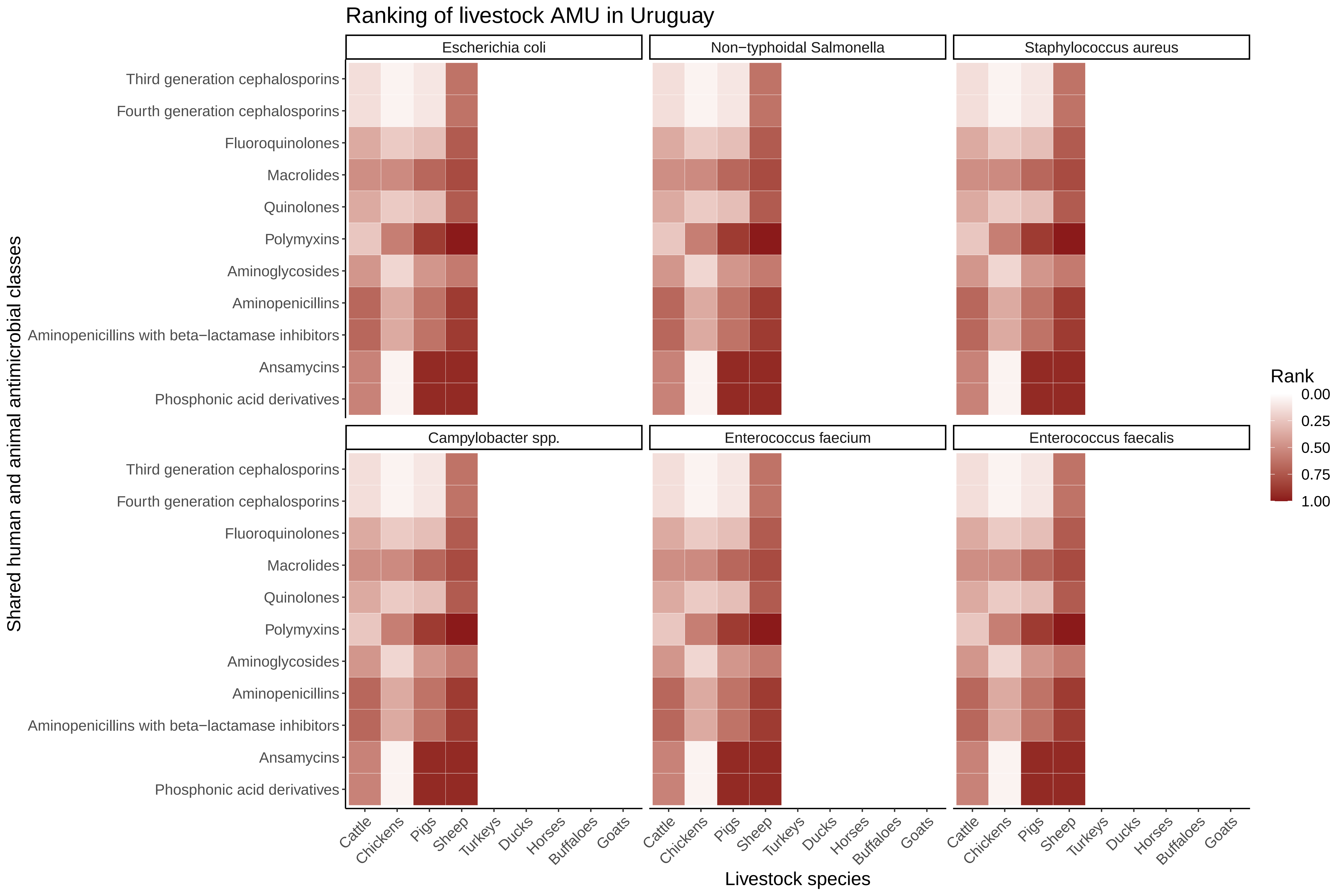
Ranking of livestock AMU in Uruguay
Escherichia coli
Non−typhoidal Salmonella
Staphylococcus aureus
Campylobacter spp.
Enterococcus faecium
Enterococcus faecalis
Shared human and animal antimicrobial classes
Third generation cephalosporins
Fourth generation cephalosporins
Fluoroquinolones
Macrolides
Quinolones
Polymyxins
Aminoglycosides
Aminopenicillins
Aminopenicillins with beta−lactamase inhibitors
Ansamycins
Phosphonic acid derivatives
Cattle
Chickens
Pigs
Sheep
Turkeys
Ducks
Horses
Buffaloes
Goats
Livestock species
Rank
0.00
0.25
0.50
0.75
1.00

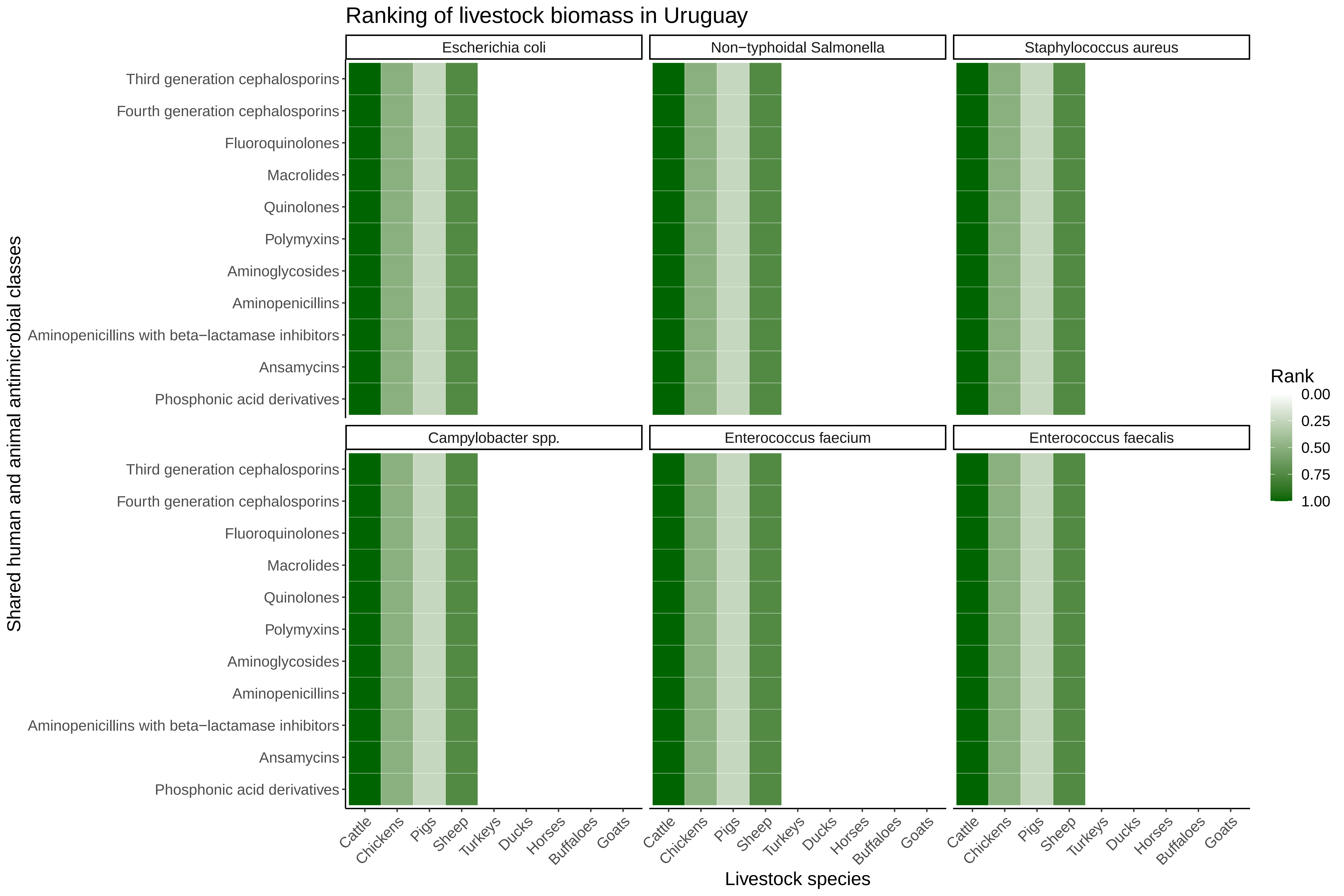

Ranking of livestock biomass in Uruguay
Escherichia coli
Non−typhoidal Salmonella
Staphylococcus aureus
Campylobacter spp.
Enterococcus faecium
Enterococcus faecalis
Shared human and animal antimicrobial classes
Third generation cephalosporins
Fourth generation cephalosporins
Fluoroquinolones
Macrolides
Quinolones
Polymyxins
Aminoglycosides
Aminopenicillins
Aminopenicillins with beta−lactamase inhibitors
Ansamycins
Phosphonic acid derivatives
Livestock species
Cattle
Chickens
Pigs
Sheep
Turkeys
Ducks
Horses
Buffaloes
Goats
Rank
0.00
0.25
0.50
0.75
1.00

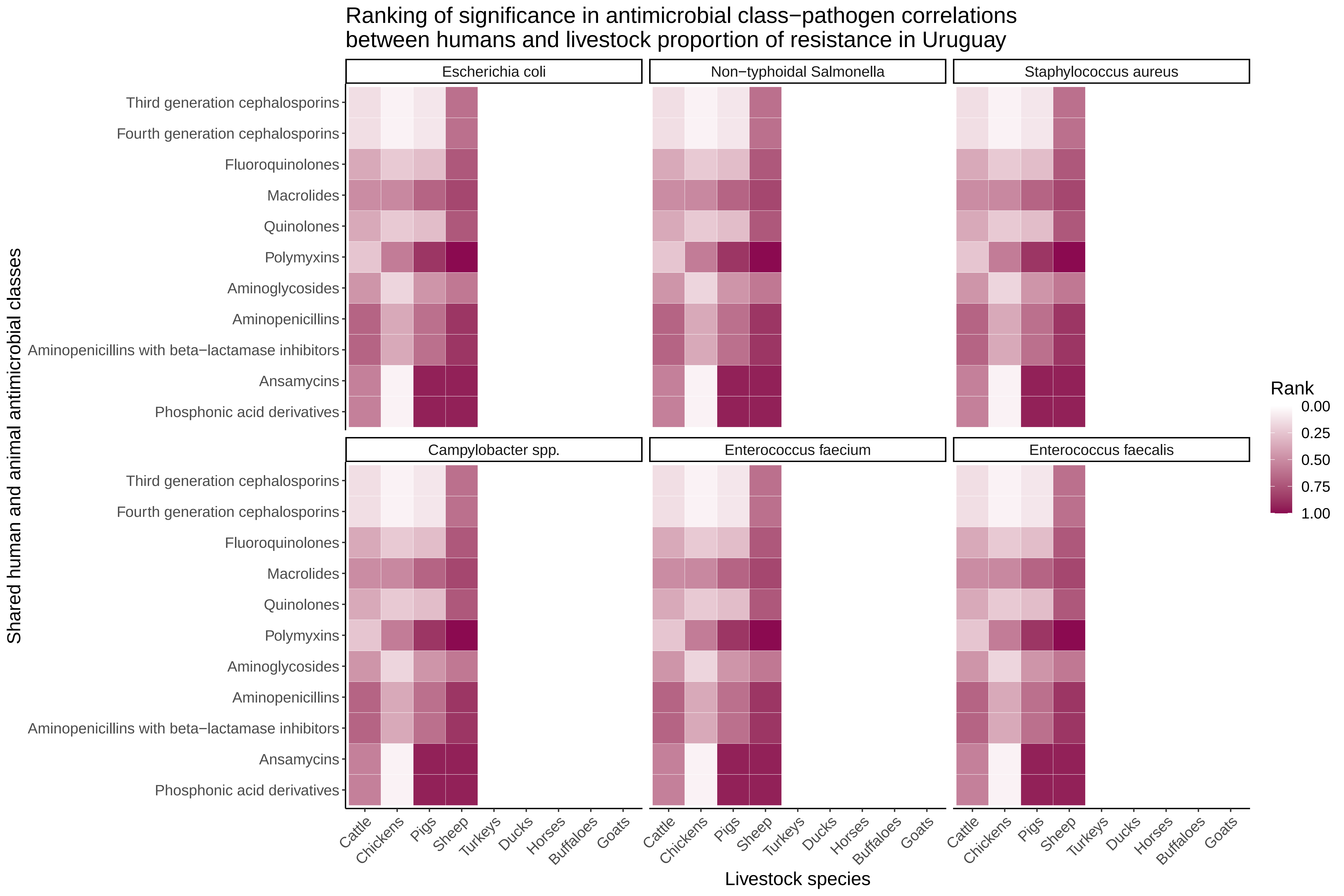

Ranking of significance in antimicrobial class−pathogen correlations
between humans and livestock proportion of resistance in Uruguay
Escherichia coli
Non−typhoidal Salmonella
Staphylococcus aureus
Campylobacter spp.
Enterococcus faecium
Enterococcus faecalis
Third generation cephalosporins
Fourth generation cephalosporins
Fluoroquinolones
Macrolides
Quinolones
Polymyxins
Aminoglycosides
Aminopenicillins
Aminopenicillins with beta−lactamase inhibitors
Ansamycins
Phosphonic acid derivatives
Shared human and animal antimicrobial classes
Cattle
Chickens
Pigs
Sheep
Turkeys
Ducks
Horses
Buffaloes
Goats
Livestock species
Rank
0.00
0.25
0.50
0.75
1.00

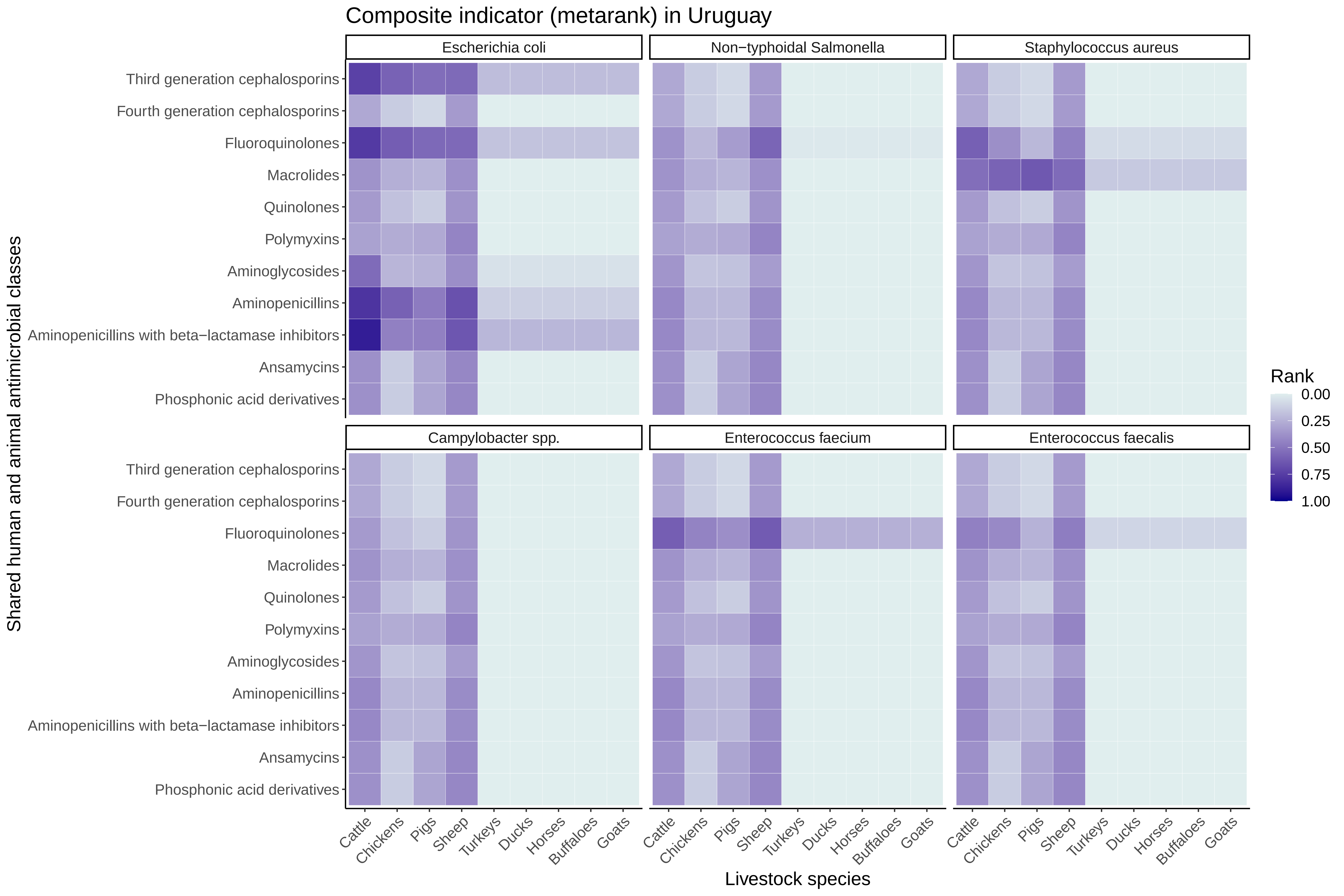

Composite indicator (metarank) in Uruguay
Escherichia coli
Non−typhoidal Salmonella
Staphylococcus aureus
Campylobacter spp.
Enterococcus faecium
Enterococcus faecalis
Third generation cephalosporins
Fourth generation cephalosporins
Fluoroquinolones
Macrolides
Quinolones
Polymyxins
Aminoglycosides
Aminopenicillins
Aminopenicillins with beta−lactamase inhibitors
Ansamycins
Phosphonic acid derivatives
Shared human and animal antimicrobial classes
Cattle
Chickens
Pigs
Sheep
Turkeys
Ducks
Horses
Buffaloes
Goats
Livestock species
Rank
0.00
0.25
0.50
0.75
1.00

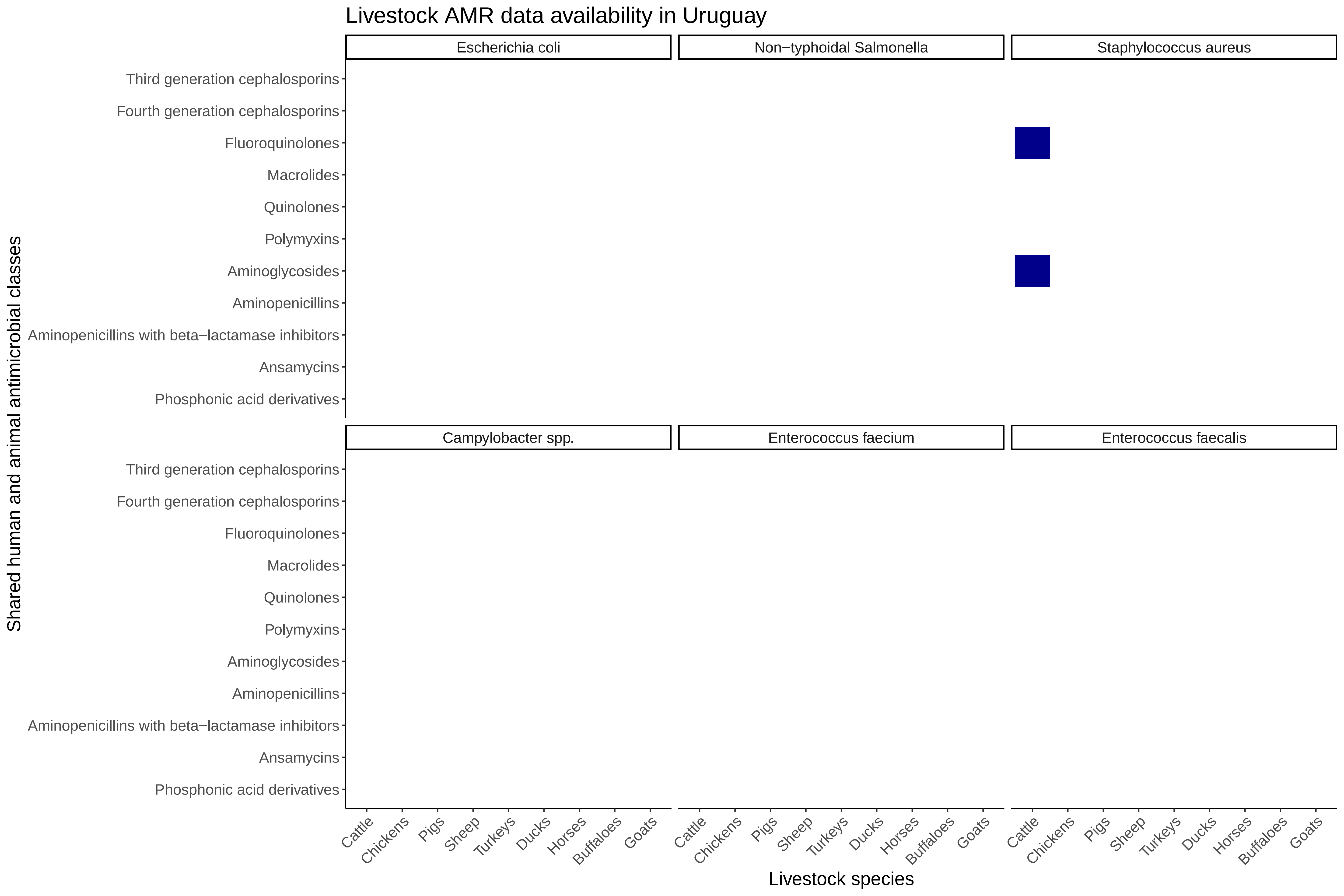
Livestock AMR data availability in Uruguay
Escherichia coli
Non−typhoidal Salmonella
Staphylococcus aureus
Campylobacter spp.
Enterococcus faecium
Enterococcus faecalis
Third generation cephalosporins
Fourth generation cephalosporins
Fluoroquinolones
Macrolides
Quinolones
Polymyxins
Aminoglycosides
Aminopenicillins
Aminopenicillins with beta−lactamase inhibitors
Ansamycins
Phosphonic acid derivatives
Shared human and animal antimicrobial classes
Cattle
Chickens
Pigs
Sheep
Turkeys
Ducks
Horses
Buffaloes
Goats
Livestock species

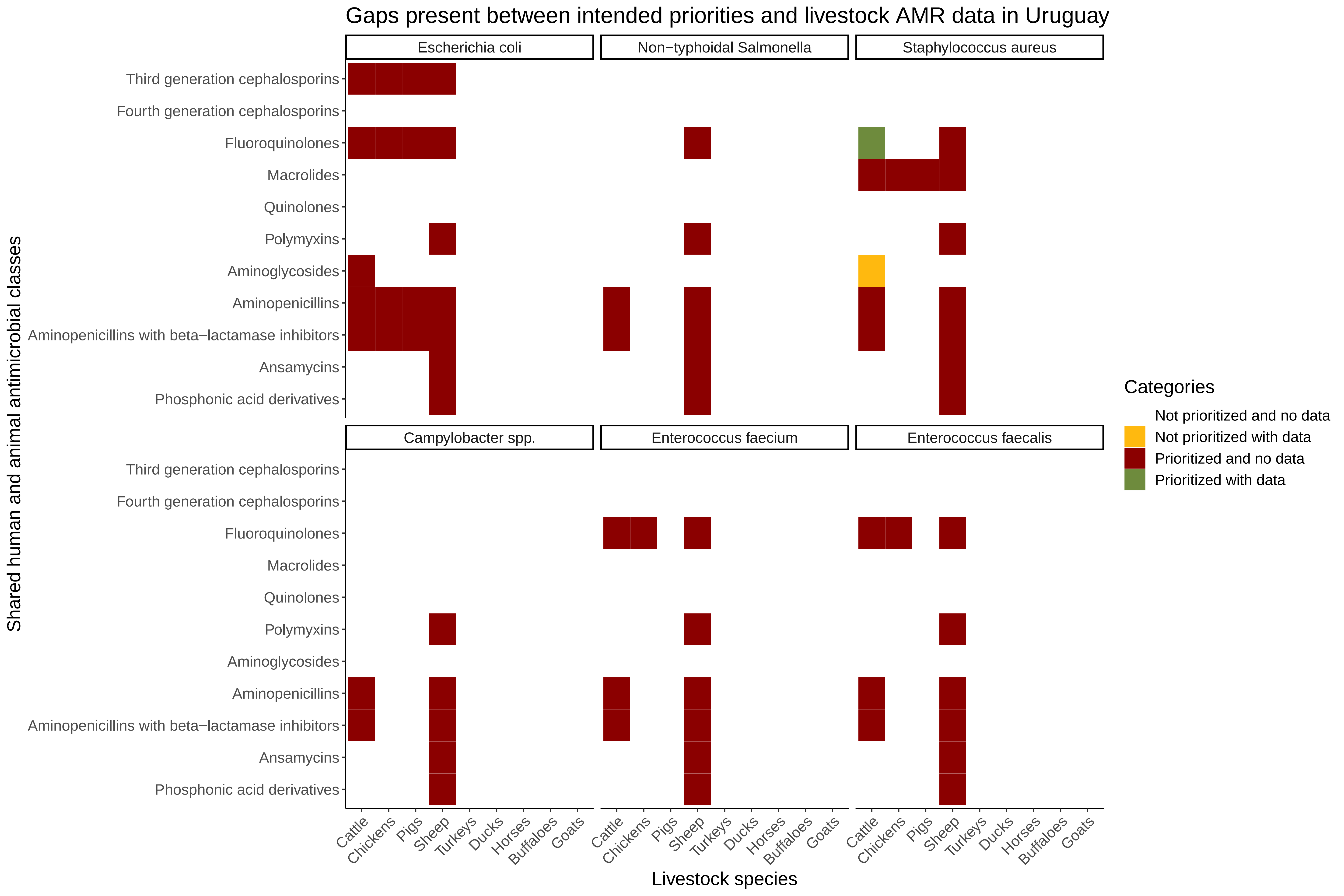
Gaps present between intended priorities and livestock AMR data in Uruguay
Escherichia coli
Non−typhoidal Salmonella
Staphylococcus aureus
Campylobacter spp.
Enterococcus faecium
Enterococcus faecalis
Third generation cephalosporins
Fourth generation cephalosporins
Fluoroquinolones
Macrolides
Quinolones
Polymyxins
Aminoglycosides
Aminopenicillins
Aminopenicillins with beta−lactamase inhibitors
Ansamycins
Phosphonic acid derivatives
Shared human and animal antimicrobial classes
Cattle
Chickens
Pigs
Sheep
Turkeys
Ducks
Horses
Buffaloes
Goats
Livestock species
Categories
Not prioritized and no data
Not prioritized with data
Prioritized and no data
Prioritized with data

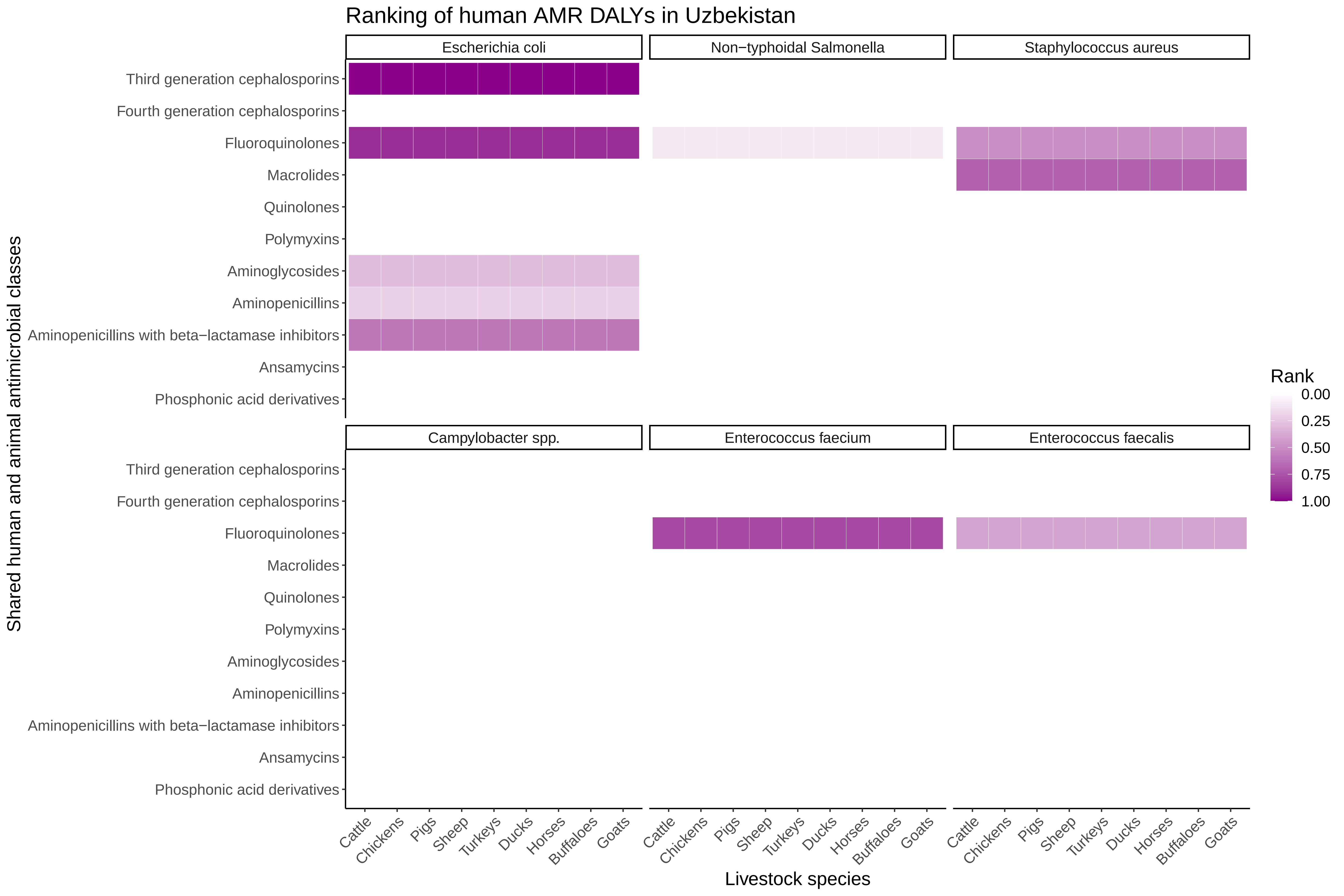

Ranking of human AMR DALYs in Uzbekistan
Escherichia coli
Non−typhoidal Salmonella
Staphylococcus aureus
Campylobacter spp.
Enterococcus faecium
Enterococcus faecalis
Third generation cephalosporins
Fourth generation cephalosporins
Fluoroquinolones
Macrolides
Quinolones
Polymyxins
Aminoglycosides
Aminopenicillins
Aminopenicillins with beta−lactamase inhibitors
Ansamycins
Phosphonic acid derivatives
Shared human and animal antimicrobial classes
Cattle
Chickens
Pigs
Sheep
Turkeys
Ducks
Horses
Buffaloes
Goats
Livestock species
Rank
0.00
0.25
0.50
0.75
1.00

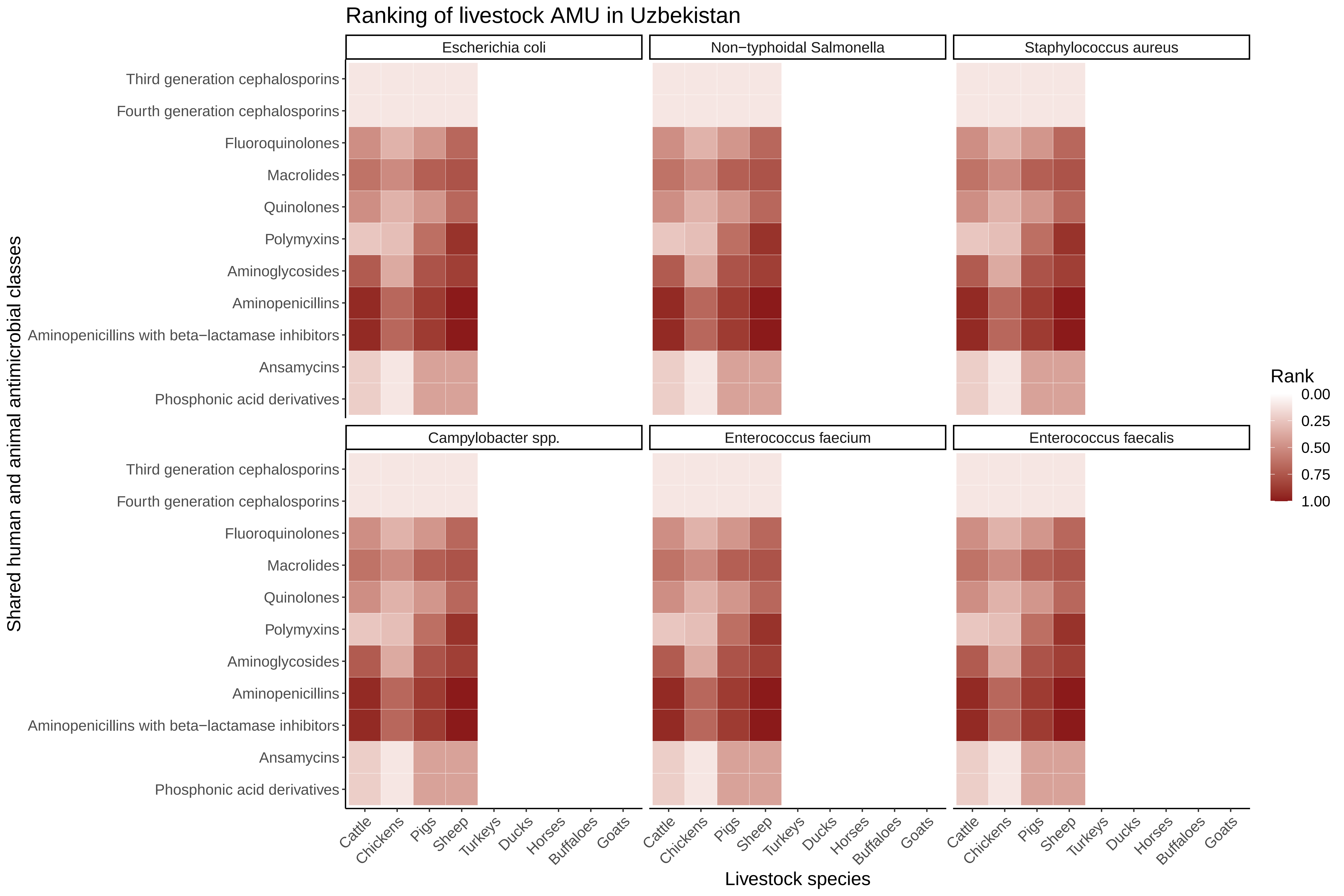

Ranking of livestock AMU in Uzbekistan
Escherichia coli
Non−typhoidal Salmonella
Staphylococcus aureus
Campylobacter spp.
Enterococcus faecium
Enterococcus faecalis
Third generation cephalosporins
Fourth generation cephalosporins
Fluoroquinolones
Macrolides
Quinolones
Polymyxins
Aminoglycosides
Aminopenicillins
Aminopenicillins with beta−lactamase inhibitors
Ansamycins
Phosphonic acid derivatives
Shared human and animal antimicrobial classes
Cattle
Chickens
Pigs
Sheep
Turkeys
Ducks
Horses
Buffaloes
Goats
Livestock species
Rank
0.00
0.25
0.50
0.75
1.00

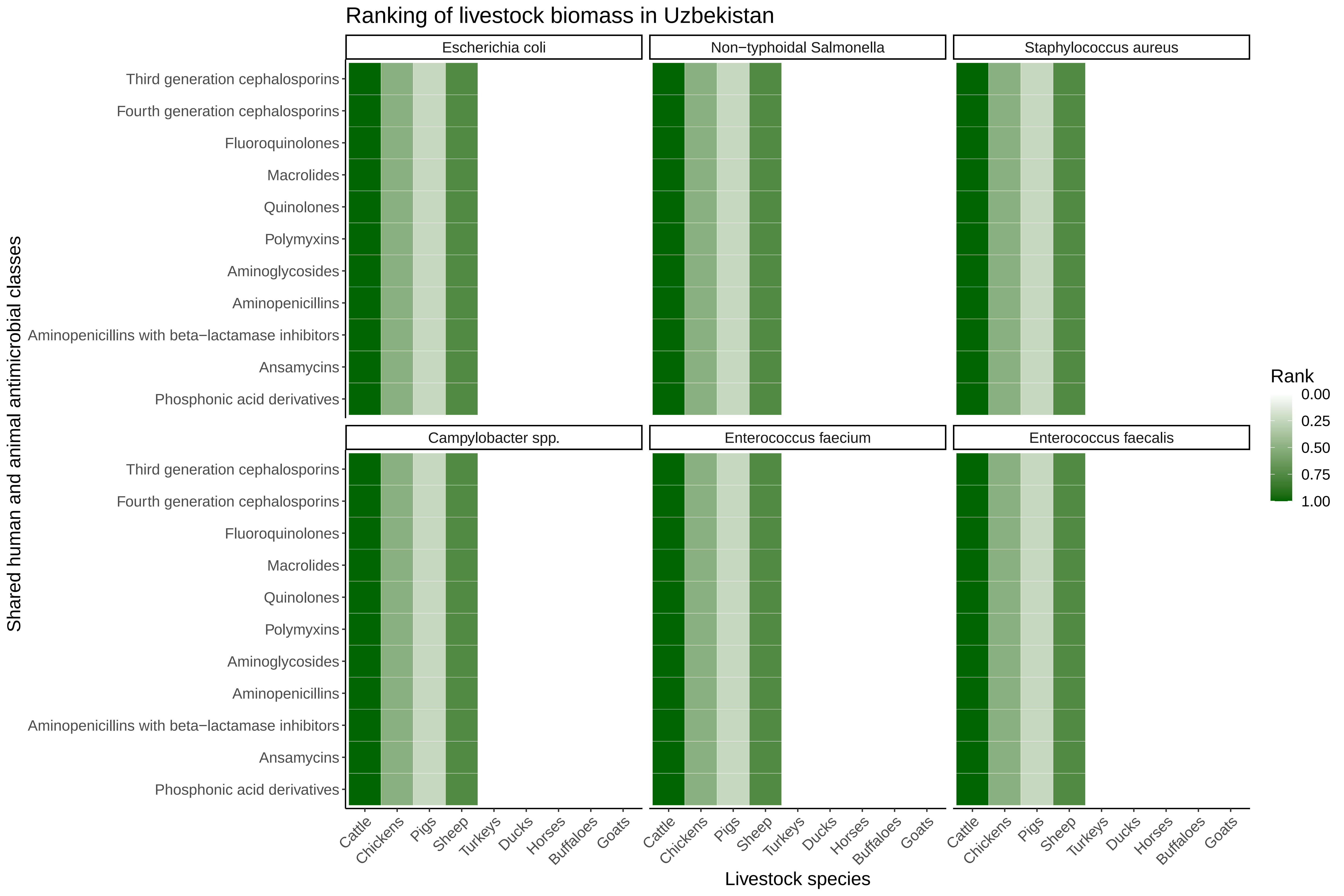

Ranking of livestock biomass in Uzbekistan
Escherichia coli
Non−typhoidal Salmonella
Staphylococcus aureus
Campylobacter spp.
Enterococcus faecium
Enterococcus faecalis
Third generation cephalosporins
Fourth generation cephalosporins
Fluoroquinolones
Macrolides
Quinolones
Polymyxins
Aminoglycosides
Aminopenicillins
Aminopenicillins with beta−lactamase inhibitors
Ansamycins
Phosphonic acid derivatives
Shared human and animal antimicrobial classes
Cattle
Chickens
Pigs
Sheep
Turkeys
Ducks
Horses
Buffaloes
Goats
Livestock species
Rank
0.00
0.25
0.50
0.75
1.00

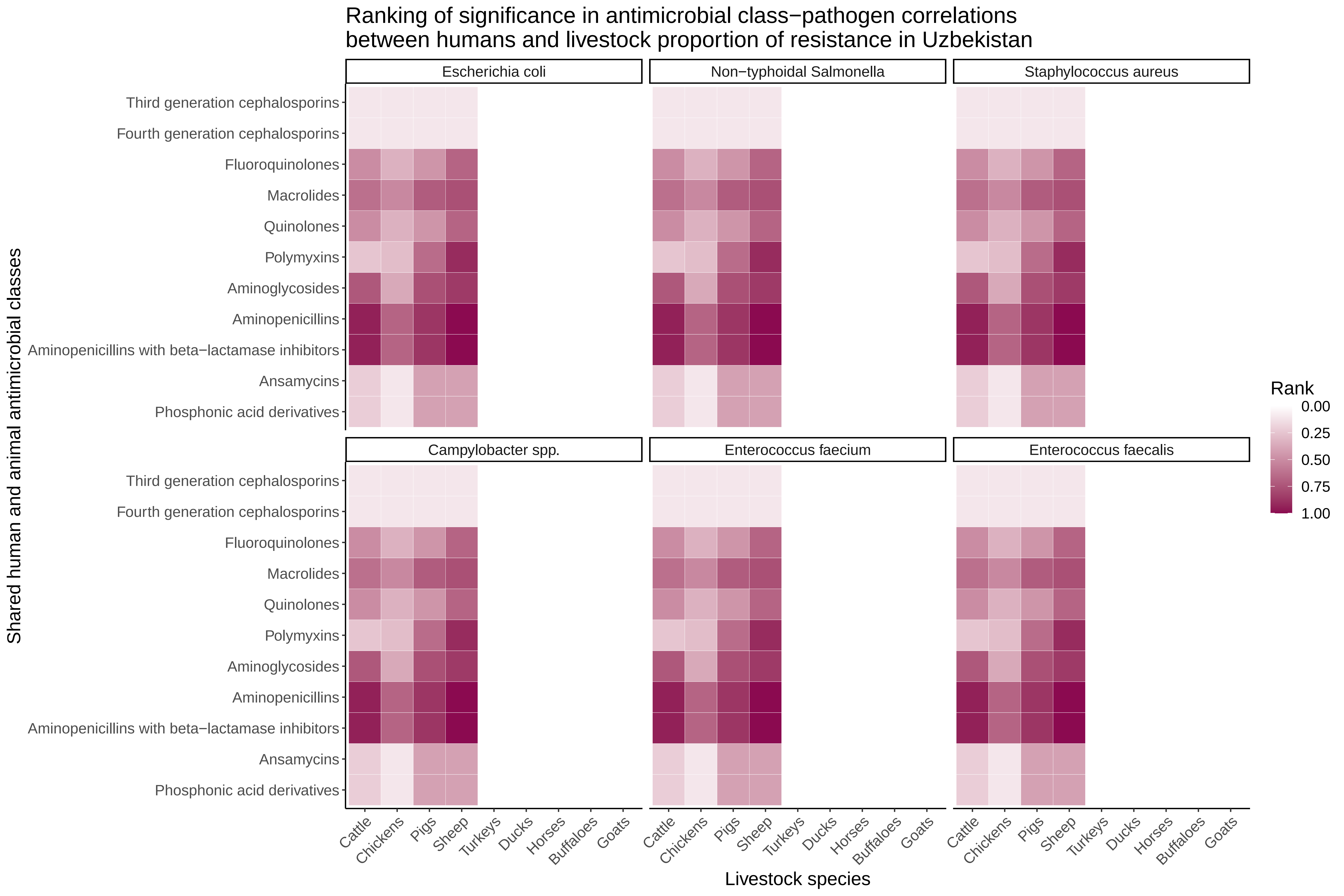

Ranking of significance in antimicrobial class−pathogen correlations
between humans and livestock proportion of resistance in Uzbekistan
Escherichia coli
Non−typhoidal Salmonella
Staphylococcus aureus
Campylobacter spp.
Enterococcus faecium
Enterococcus faecalis
Shared human and animal antimicrobial classes
Third generation cephalosporins
Fourth generation cephalosporins
Fluoroquinolones
Macrolides
Quinolones
Polymyxins
Aminoglycosides
Aminopenicillins
Aminopenicillins with beta−lactamase inhibitors
Ansamycins
Phosphonic acid derivatives
Cattle
Chickens
Pigs
Sheep
Turkeys
Ducks
Horses
Buffaloes
Goats
Livestock species
Rank
0.00
0.25
0.50
0.75
1.00

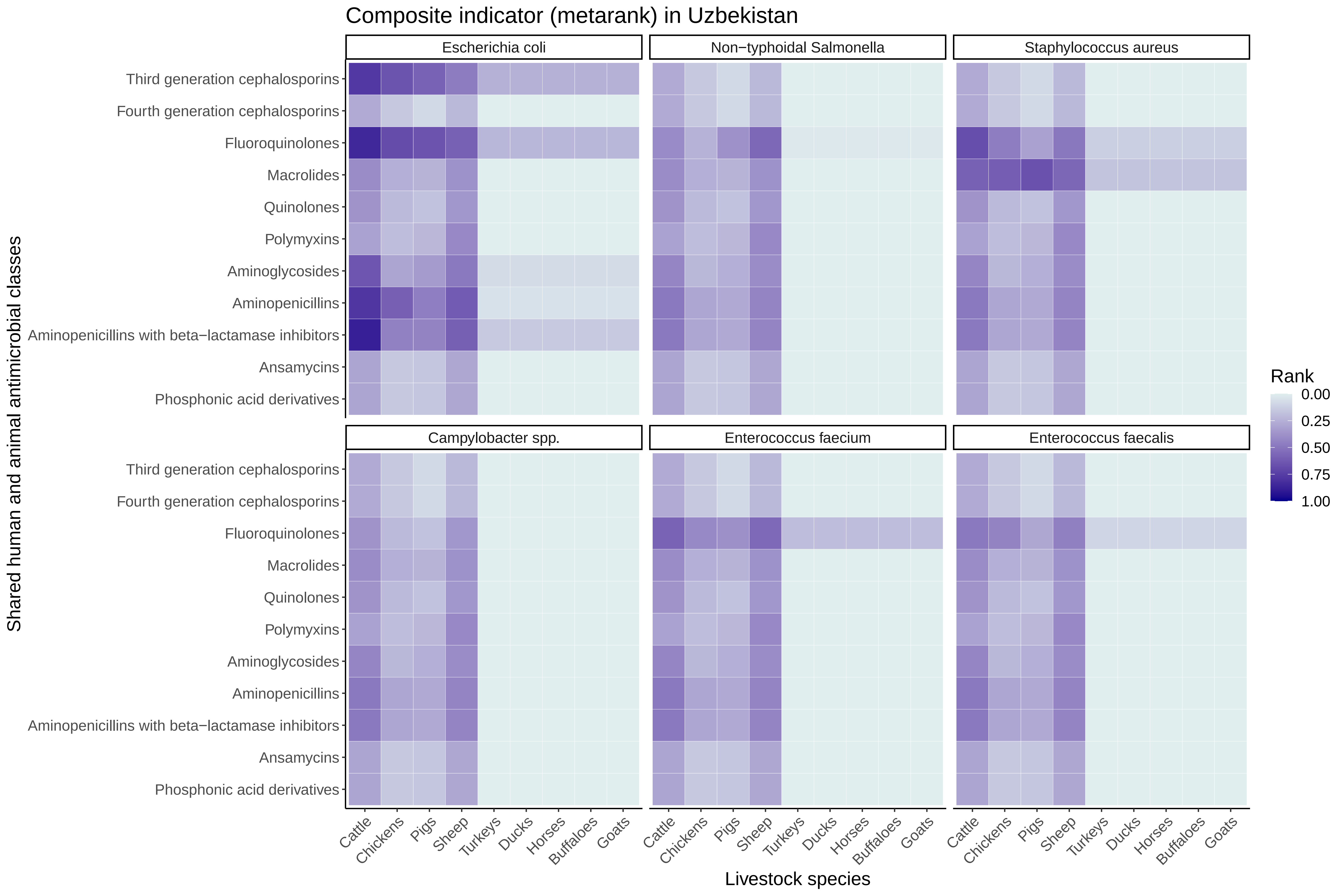

Composite indicator (metarank) in Uzbekistan
Escherichia coli
Non−typhoidal Salmonella
Staphylococcus aureus
Campylobacter spp.
Enterococcus faecium
Enterococcus faecalis
Third generation cephalosporins
Fourth generation cephalosporins
Fluoroquinolones
Macrolides
Quinolones
Polymyxins
Aminoglycosides
Aminopenicillins
Aminopenicillins with beta−lactamase inhibitors
Ansamycins
Phosphonic acid derivatives
Shared human and animal antimicrobial classes
Cattle
Chickens
Pigs
Sheep
Turkeys
Ducks
Horses
Buffaloes
Goats
Livestock species
Rank
0.00
0.25
0.50
0.75
1.00

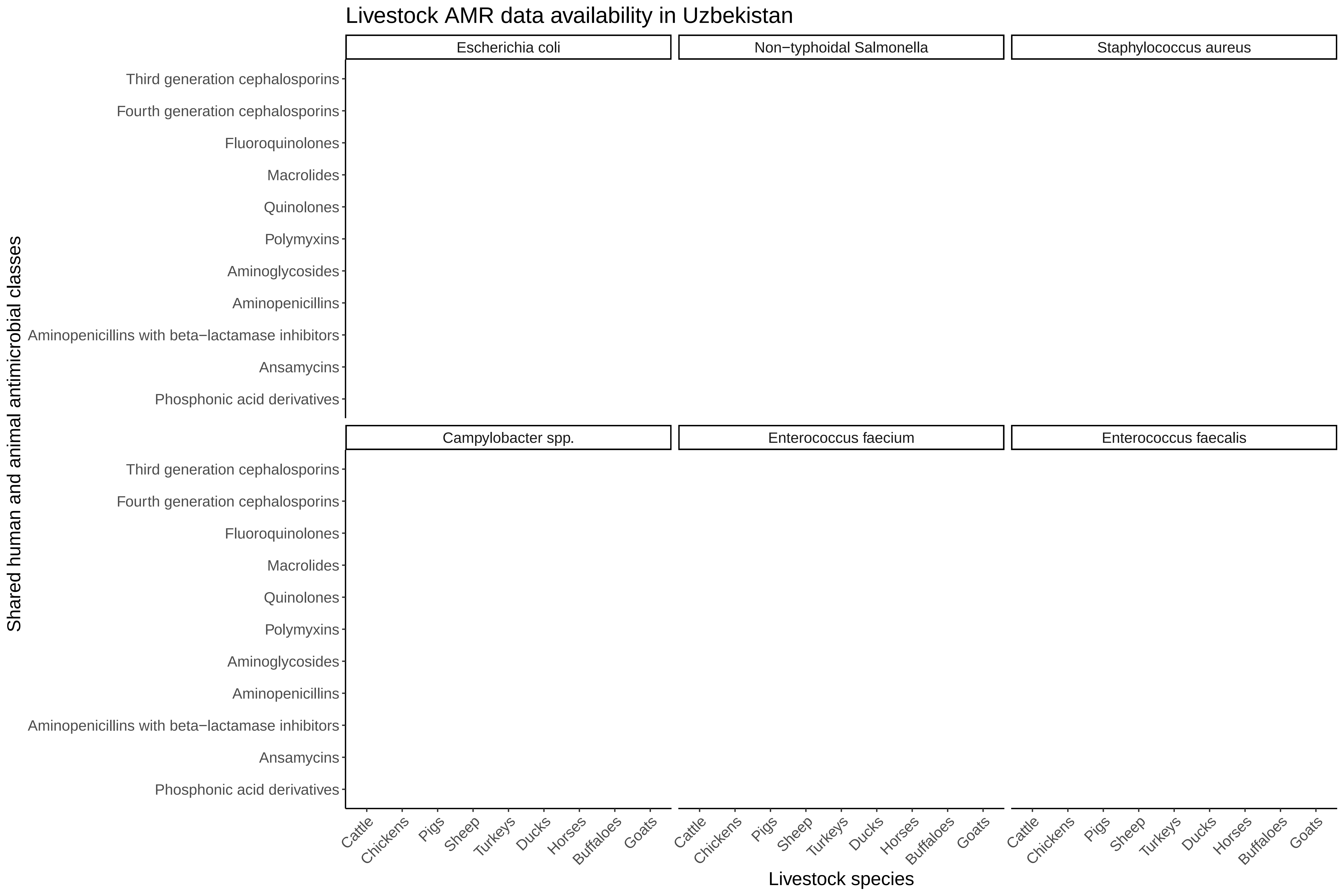
Livestock AMR data availability in Uzbekistan
Escherichia coli
Non−typhoidal Salmonella
Staphylococcus aureus
Campylobacter spp.
Enterococcus faecium
Enterococcus faecalis
Third generation cephalosporins
Fourth generation cephalosporins
Fluoroquinolones
Macrolides
Quinolones
Polymyxins
Aminoglycosides
Aminopenicillins
Aminopenicillins with beta−lactamase inhibitors
Ansamycins
Phosphonic acid derivatives
Cattle
Chickens
Pigs
Sheep
Turkeys
Ducks
Horses
Buffaloes
Goats
Shared human and animal antimicrobial classes
Livestock species

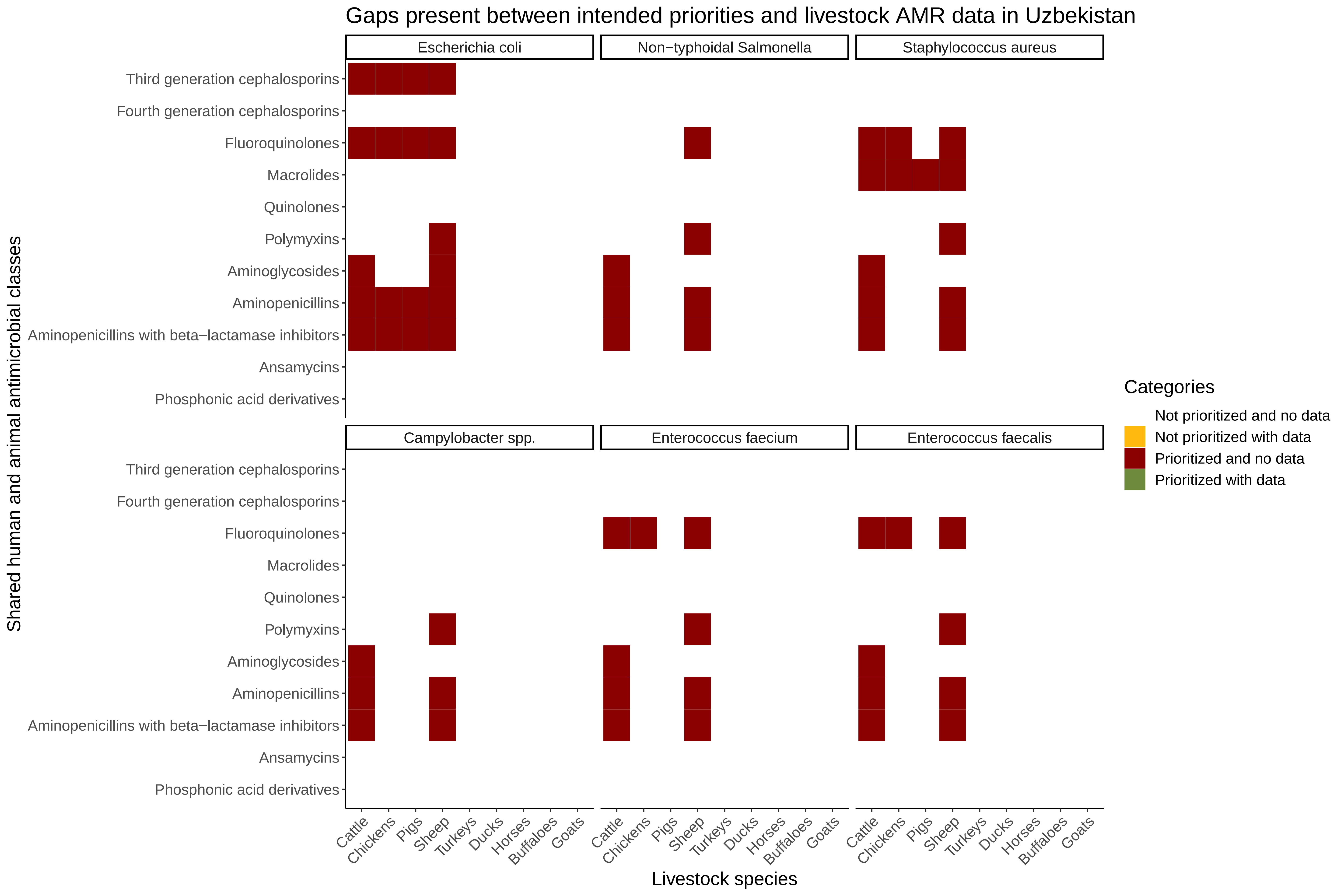

Gaps present between intended priorities and livestock AMR data in Uzbekistan
Escherichia coli
Non−typhoidal Salmonella
Staphylococcus aureus
Campylobacter spp.
Enterococcus faecium
Enterococcus faecalis
Third generation cephalosporins
Fourth generation cephalosporins
Fluoroquinolones
Macrolides
Quinolones
Polymyxins
Aminoglycosides
Aminopenicillins
Aminopenicillins with beta−lactamase inhibitors
Ansamycins
Phosphonic acid derivatives
Shared human and animal antimicrobial classes
Cattle
Chickens
Pigs
Sheep
Turkeys
Ducks
Horses
Buffaloes
Goats
Livestock species
Categories
Not prioritized and no data
Not prioritized with data
Prioritized and no data
Prioritized with data

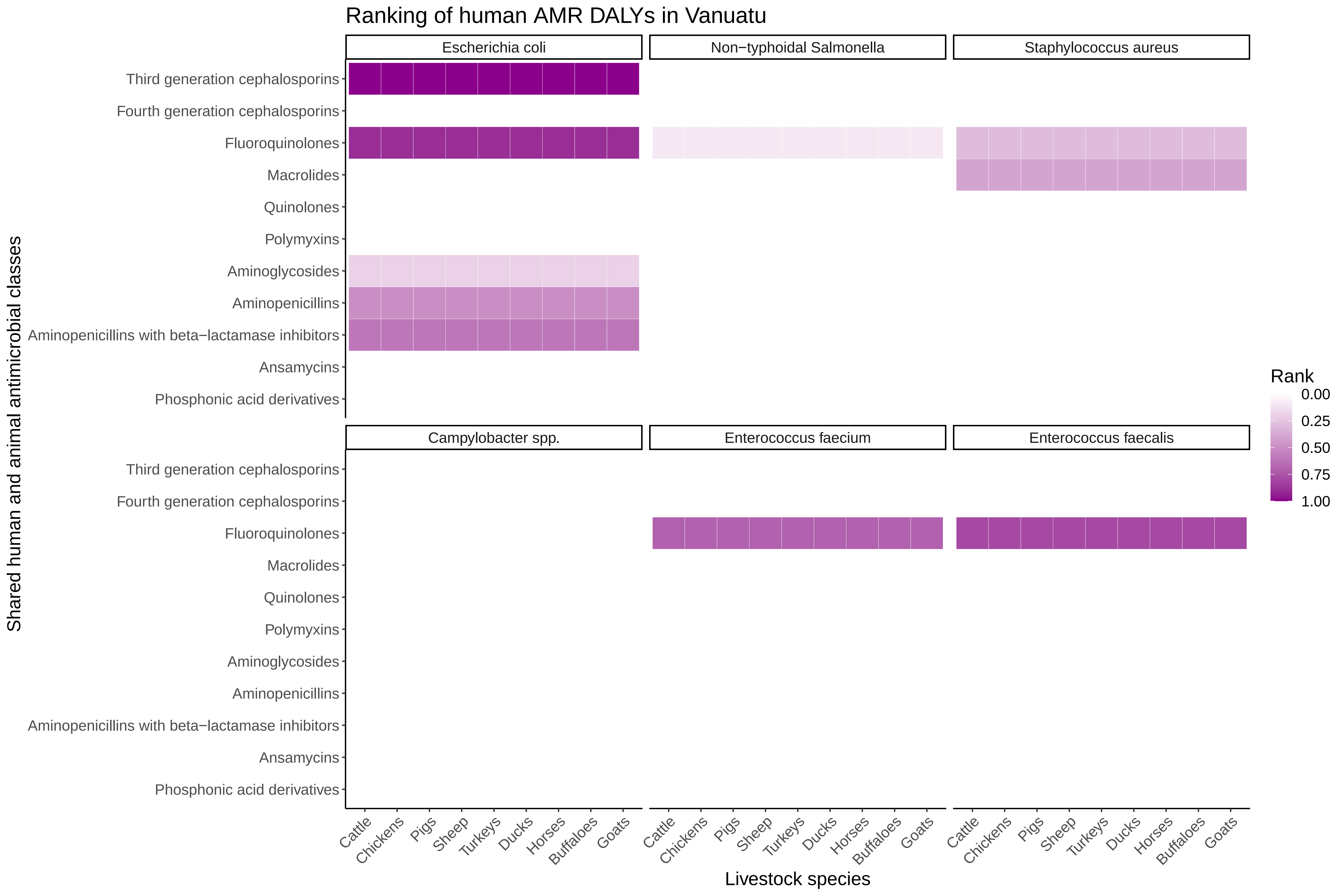

Ranking of human AMR DALYs in Vanuatu
Escherichia coli
Non−typhoidal Salmonella
Staphylococcus aureus
Campylobacter spp.
Enterococcus faecium
Enterococcus faecalis
Third generation cephalosporins
Fourth generation cephalosporins
Fluoroquinolones
Macrolides
Quinolones
Polymyxins
Aminoglycosides
Aminopenicillins
Aminopenicillins with beta−lactamase inhibitors
Ansamycins
Phosphonic acid derivatives
Shared human and animal antimicrobial classes
Cattle
Chickens
Pigs
Sheep
Turkeys
Ducks
Horses
Buffaloes
Goats
Livestock species
Rank
0.00
0.25
0.50
0.75
1.00

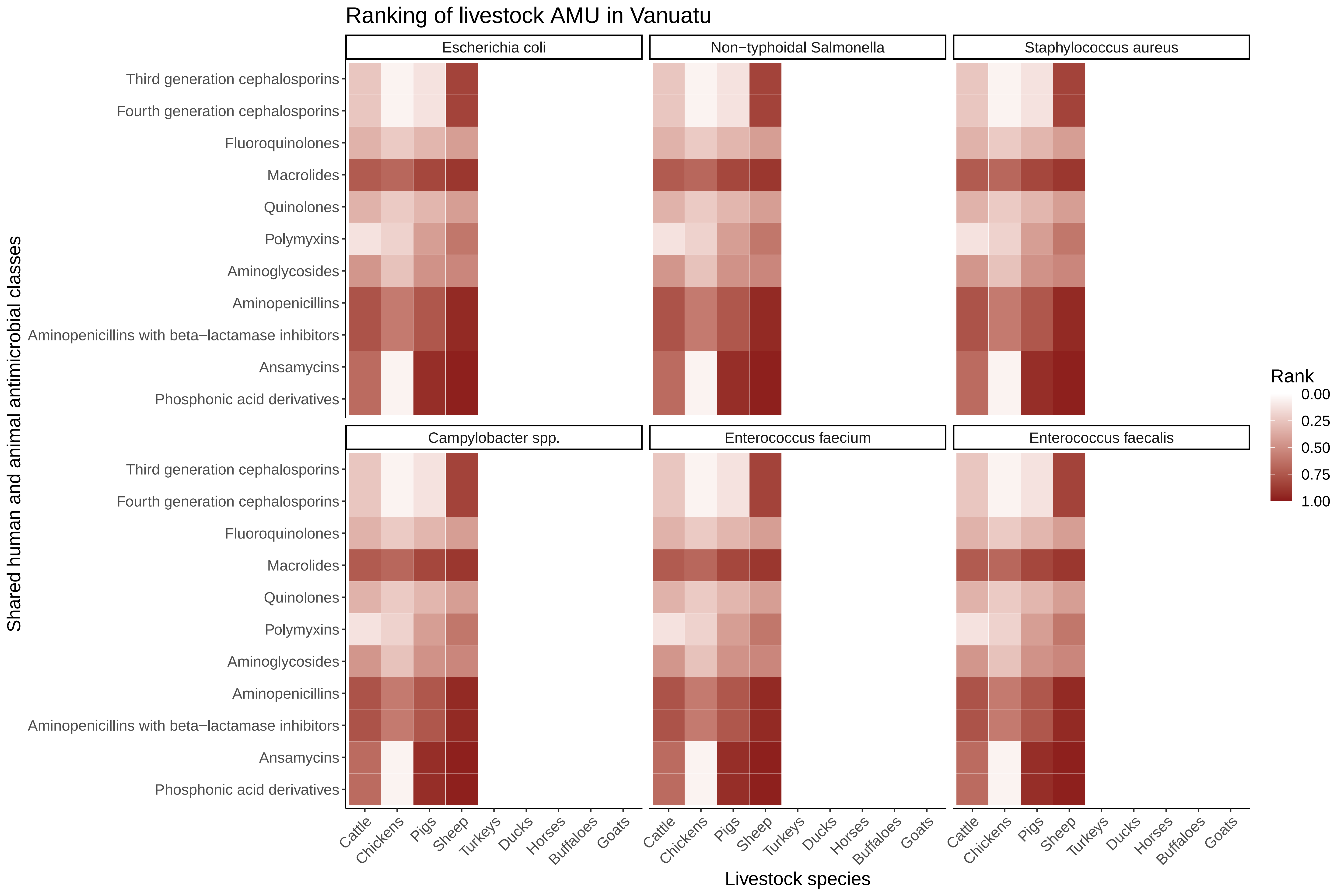
Ranking of livestock AMU in Vanuatu
Escherichia coli
Non−typhoidal Salmonella
Staphylococcus aureus
Campylobacter spp.
Enterococcus faecium
Enterococcus faecalis
Shared human and animal antimicrobial classes
Third generation cephalosporins
Fourth generation cephalosporins
Fluoroquinolones
Macrolides
Quinolones
Polymyxins
Aminoglycosides
Aminopenicillins
Aminopenicillins with beta−lactamase inhibitors
Ansamycins
Phosphonic acid derivatives
Cattle
Chickens
Pigs
Sheep
Turkeys
Ducks
Horses
Buffaloes
Goats
Livestock species
Rank
0.00
0.25
0.50
0.75
1.00

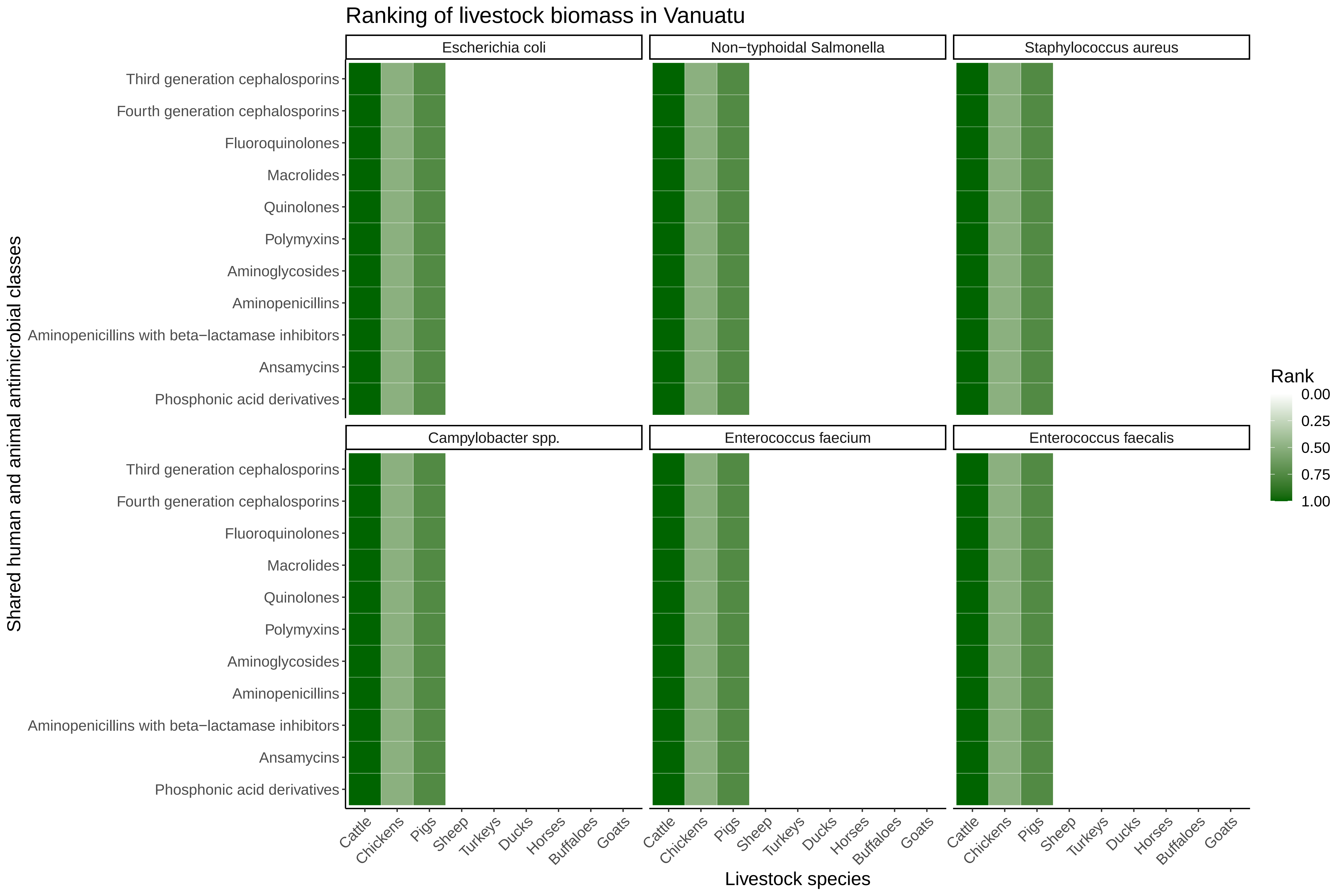

Ranking of livestock biomass in Vanuatu
Escherichia coli
Non−typhoidal Salmonella
Staphylococcus aureus
Campylobacter spp.
Enterococcus faecium
Enterococcus faecalis
Third generation cephalosporins
Fourth generation cephalosporins
Fluoroquinolones
Macrolides
Quinolones
Polymyxins
Aminoglycosides
Aminopenicillins
Aminopenicillins with beta−lactamase inhibitors
Ansamycins
Phosphonic acid derivatives
Shared human and animal antimicrobial classes
Cattle
Chickens
Pigs
Sheep
Turkeys
Ducks
Horses
Buffaloes
Goats
Livestock species
Rank
0.00
0.25
0.50
0.75
1.00

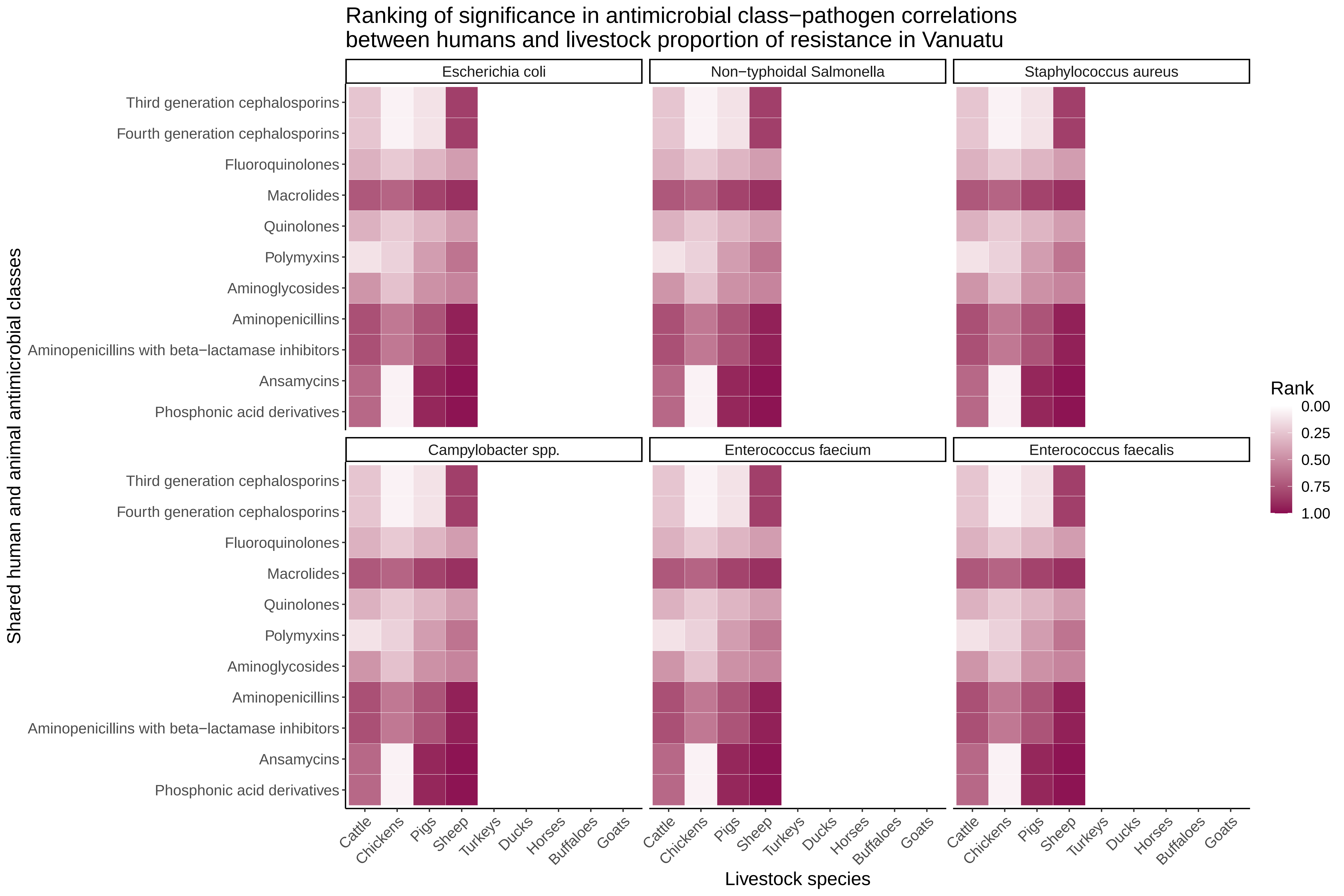

Ranking of significance in antimicrobial class−pathogen correlations
between humans and livestock proportion of resistance in Vanuatu
Escherichia coli
Non−typhoidal Salmonella
Staphylococcus aureus
Campylobacter spp.
Enterococcus faecium
Enterococcus faecalis
Third generation cephalosporins
Fourth generation cephalosporins
Fluoroquinolones
Macrolides
Quinolones
Polymyxins
Aminoglycosides
Aminopenicillins
Aminopenicillins with beta−lactamase inhibitors
Ansamycins
Phosphonic acid derivatives
Shared human and animal antimicrobial classes
Cattle
Chickens
Pigs
Sheep
Turkeys
Ducks
Horses
Buffaloes
Goats
Livestock species
Rank
0.00
0.25
0.50
0.75
1.00

Composite indicator (metarank) in Vanuatu

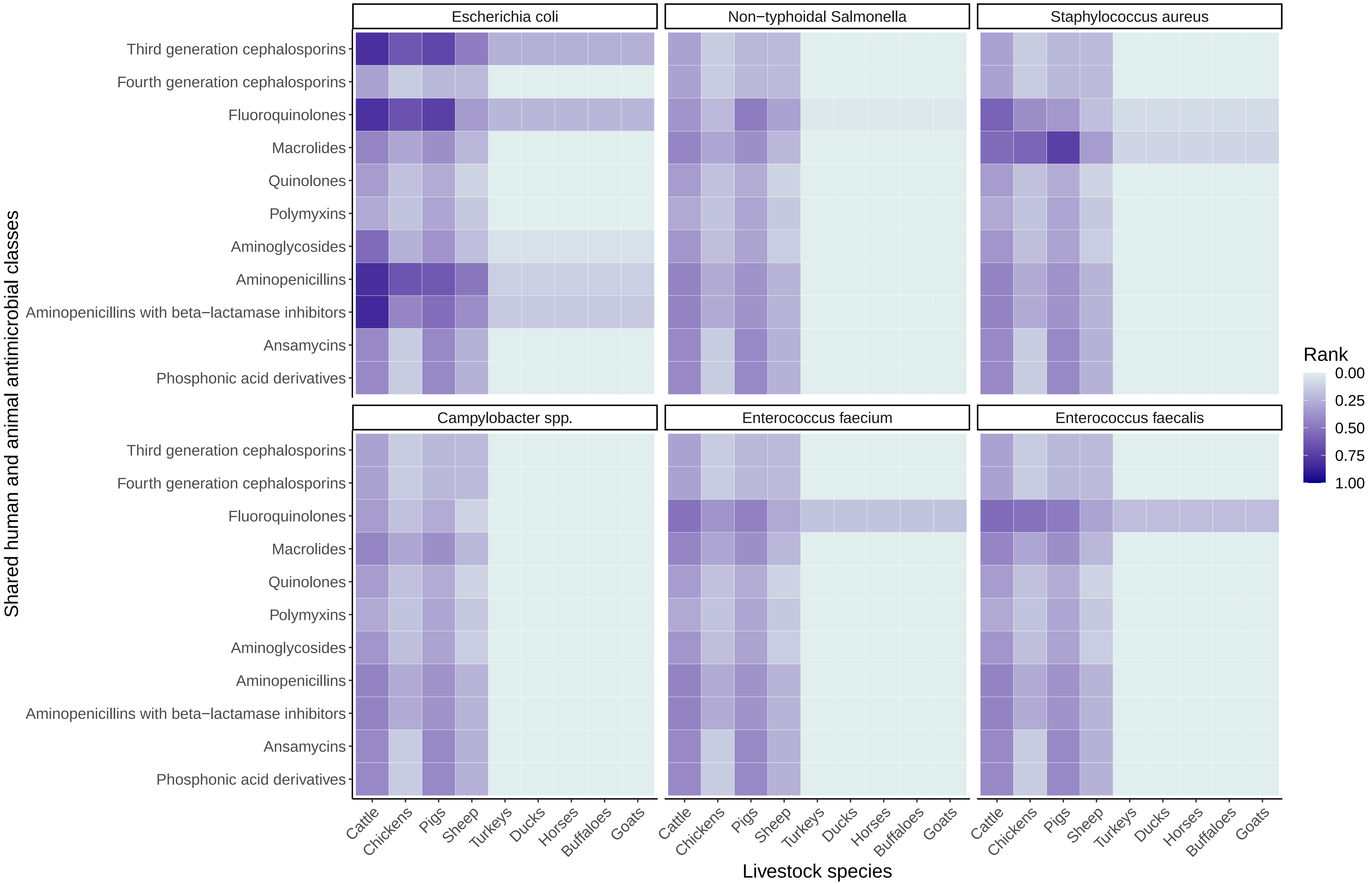

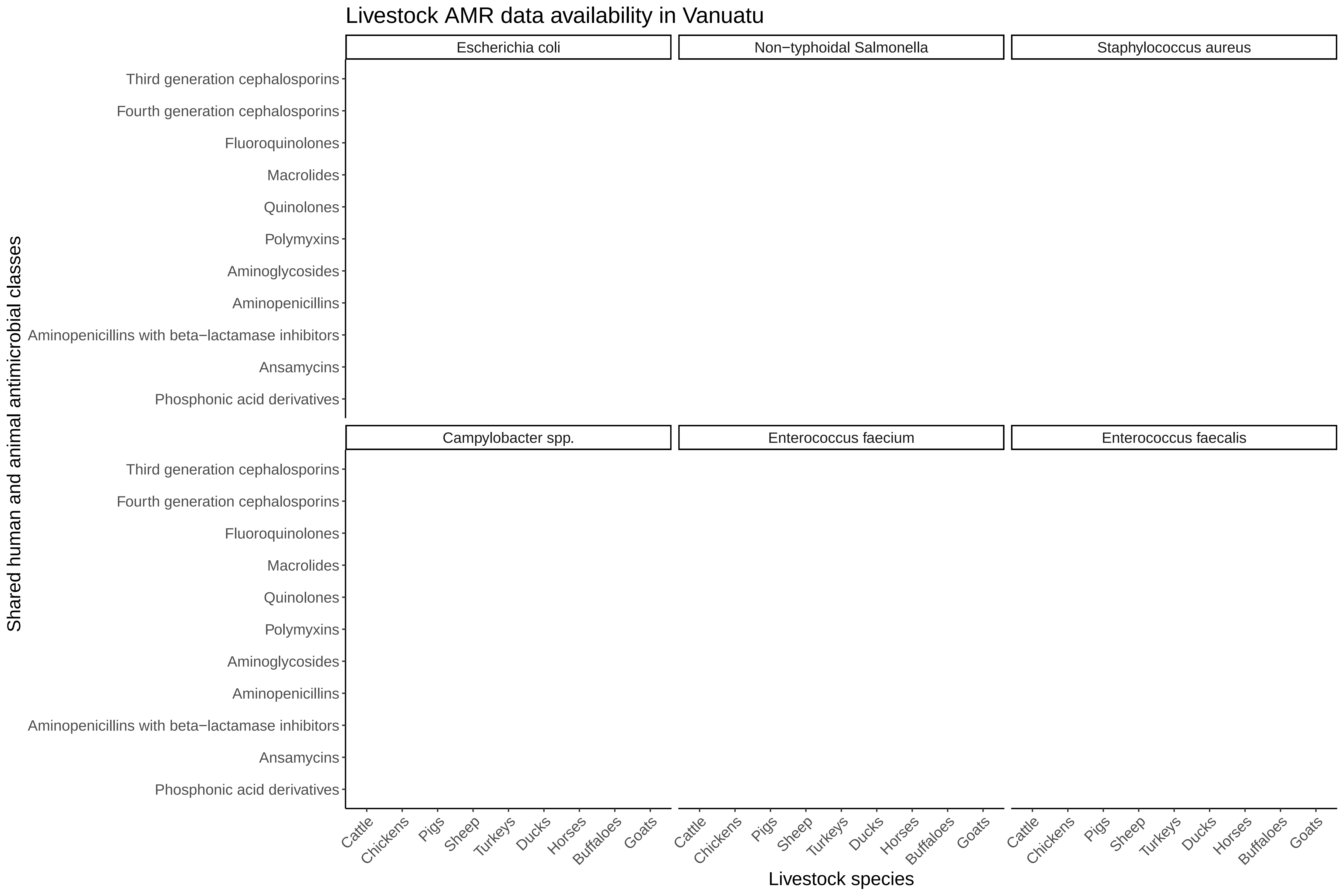

Livestock AMR data availability in Vanuatu
Escherichia coli
Non−typhoidal Salmonella
Staphylococcus aureus
Campylobacter spp.
Enterococcus faecium
Enterococcus faecalis
Third generation cephalosporins
Fourth generation cephalosporins
Fluoroquinolones
Macrolides
Quinolones
Polymyxins
Aminoglycosides
Aminopenicillins
Aminopenicillins with beta−lactamase inhibitors
Ansamycins
Phosphonic acid derivatives
Shared human and animal antimicrobial classes
Cattle
Chickens
Pigs
Sheep
Turkeys
Ducks
Horses
Buffaloes
Goats
Livestock species

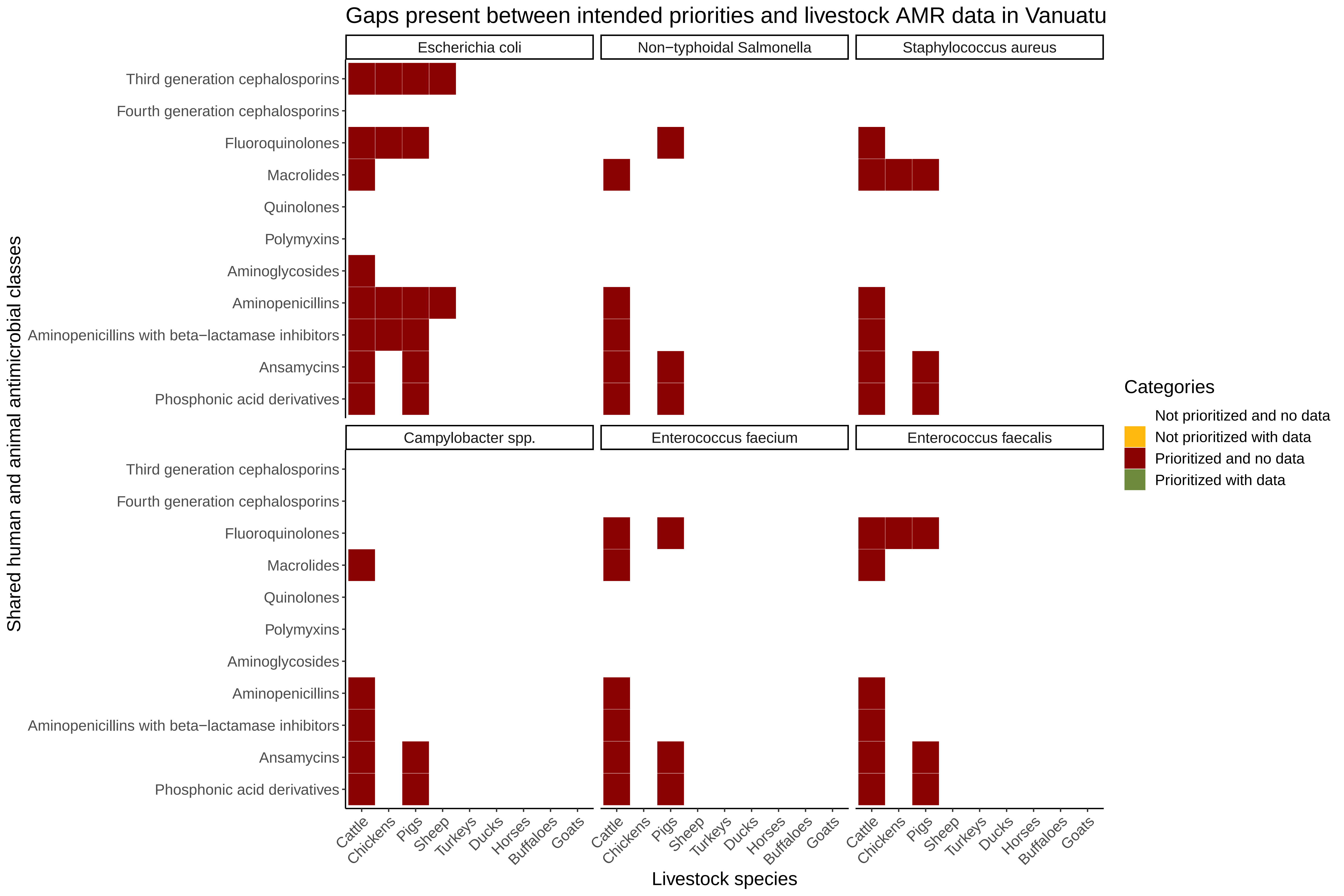

Gaps present between intended priorities and livestock AMR data in Vanuatu
Escherichia coli
Non−typhoidal Salmonella
Staphylococcus aureus
Campylobacter spp.
Enterococcus faecium
Enterococcus faecalis
Shared human and animal antimicrobial classes
Third generation cephalosporins
Fourth generation cephalosporins
Fluoroquinolones
Macrolides
Quinolones
Polymyxins
Aminoglycosides
Aminopenicillins
Aminopenicillins with beta−lactamase inhibitors
Ansamycins
Phosphonic acid derivatives
Cattle
Chickens
Pigs
Sheep
Turkeys
Ducks
Horses
Buffaloes
Goats
Livestock species
Categories
Not prioritized and no data
Not prioritized with data
Prioritized and no data
Prioritized with data

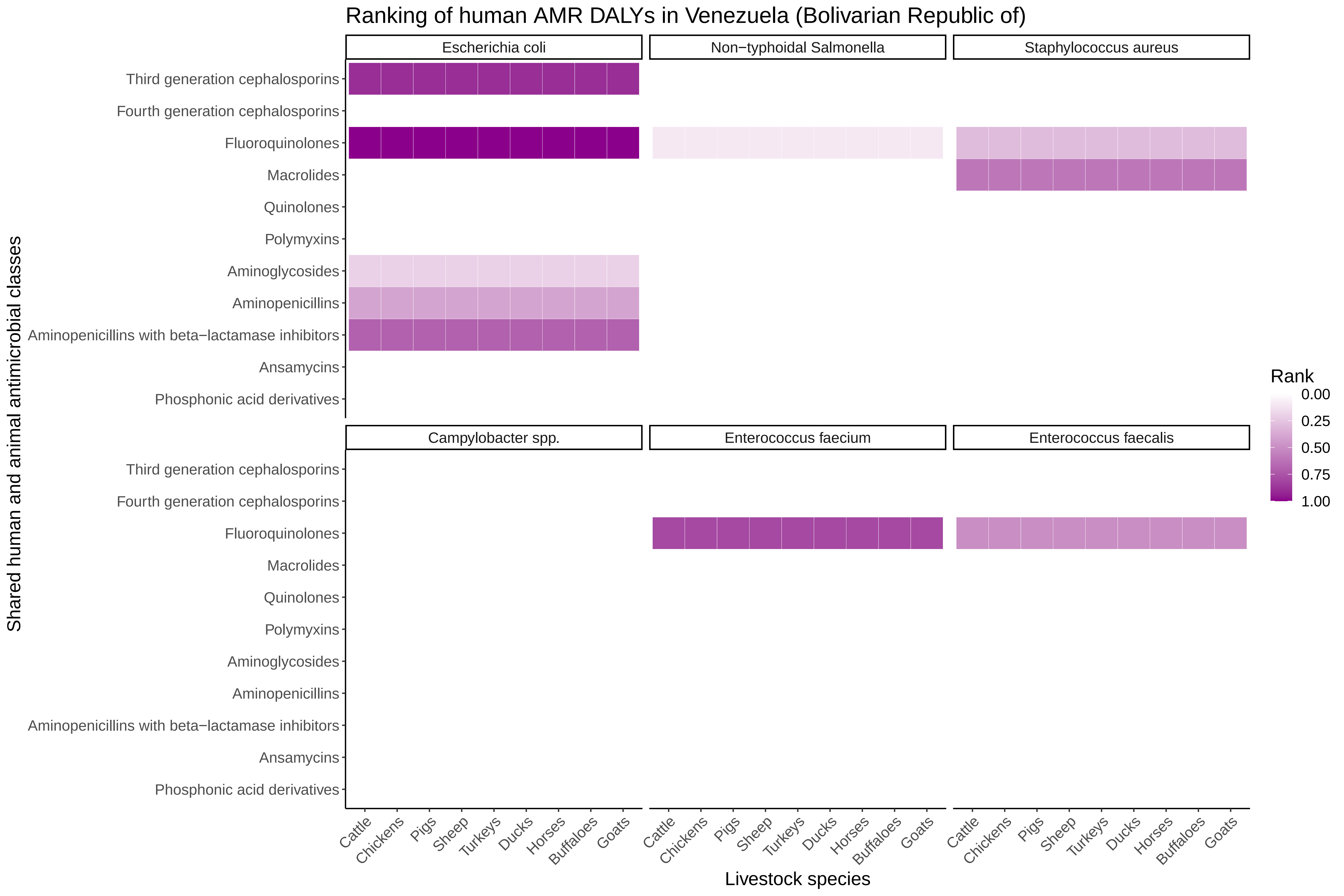
Ranking of human AMR DALYs in Venezuela (Bolivarian Republic of)
Escherichia coli
Non−typhoidal Salmonella
Staphylococcus aureus
Campylobacter spp.
Enterococcus faecium
Enterococcus faecalis
Third generation cephalosporins
Fourth generation cephalosporins
Fluoroquinolones
Macrolides
Quinolones
Polymyxins
Aminoglycosides
Aminopenicillins
Aminopenicillins with beta−lactamase inhibitors
Ansamycins
Phosphonic acid derivatives
Shared human and animal antimicrobial classes
Cattle
Chickens
Pigs
Sheep
Turkeys
Ducks
Horses
Buffaloes
Goats
Livestock species
Rank
0.00
0.25
0.50
0.75
1.00

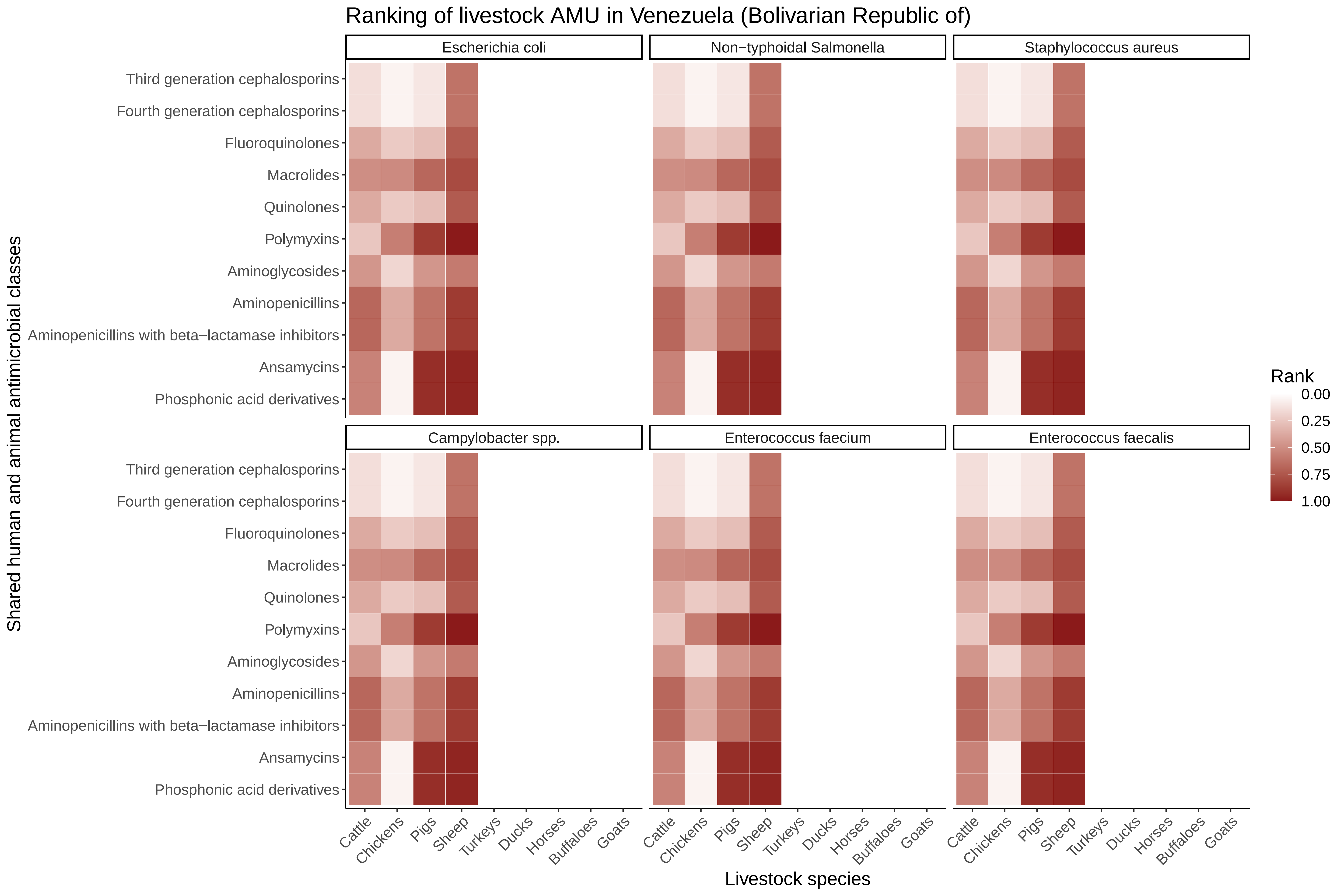

Ranking of livestock AMU in Venezuela (Bolivarian Republic of)
Escherichia coli
Non−typhoidal Salmonella
Staphylococcus aureus
Campylobacter spp.
Enterococcus faecium
Enterococcus faecalis
Shared human and animal antimicrobial classes
Third generation cephalosporins
Fourth generation cephalosporins
Fluoroquinolones
Macrolides
Quinolones
Polymyxins
Aminoglycosides
Aminopenicillins
Aminopenicillins with beta−lactamase inhibitors
Ansamycins
Phosphonic acid derivatives
Livestock species
Cattle
Chickens
Pigs
Sheep
Turkeys
Ducks
Horses
Buffaloes
Goats
Rank
0.00
0.25
0.50
0.75
1.00

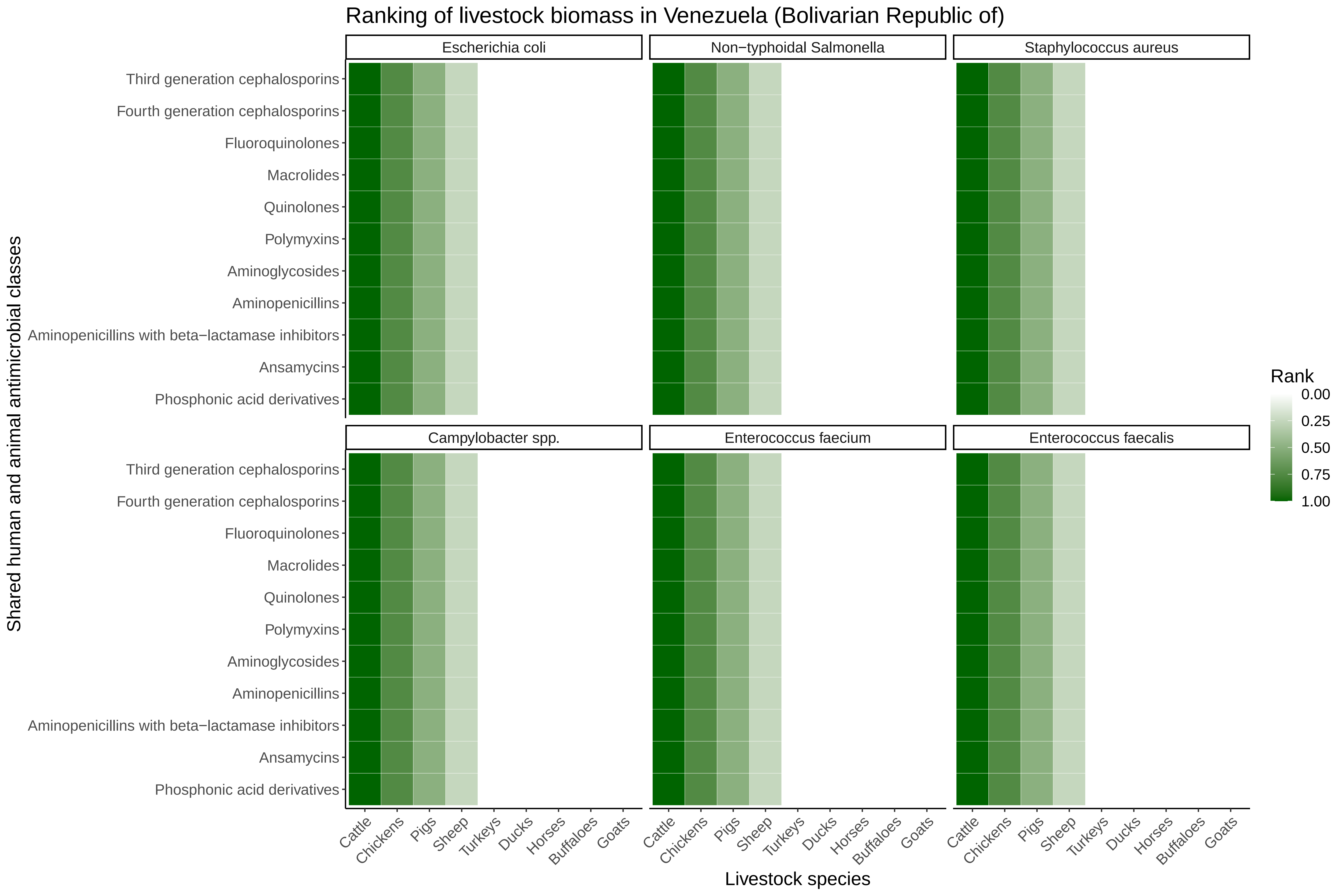
Ranking of livestock biomass in Venezuela (Bolivarian Republic of)
Escherichia coli
Non−typhoidal Salmonella
Staphylococcus aureus
Campylobacter spp.
Enterococcus faecium
Enterococcus faecalis
Third generation cephalosporins
Fourth generation cephalosporins
Fluoroquinolones
Macrolides
Quinolones
Polymyxins
Aminoglycosides
Aminopenicillins
Aminopenicillins with beta−lactamase inhibitors
Ansamycins
Phosphonic acid derivatives
Shared human and animal antimicrobial classes
Cattle
Chickens
Pigs
Sheep
Turkeys
Ducks
Horses
Buffaloes
Goats
Livestock species
Rank
0.00
0.25
0.50
0.75
1.00

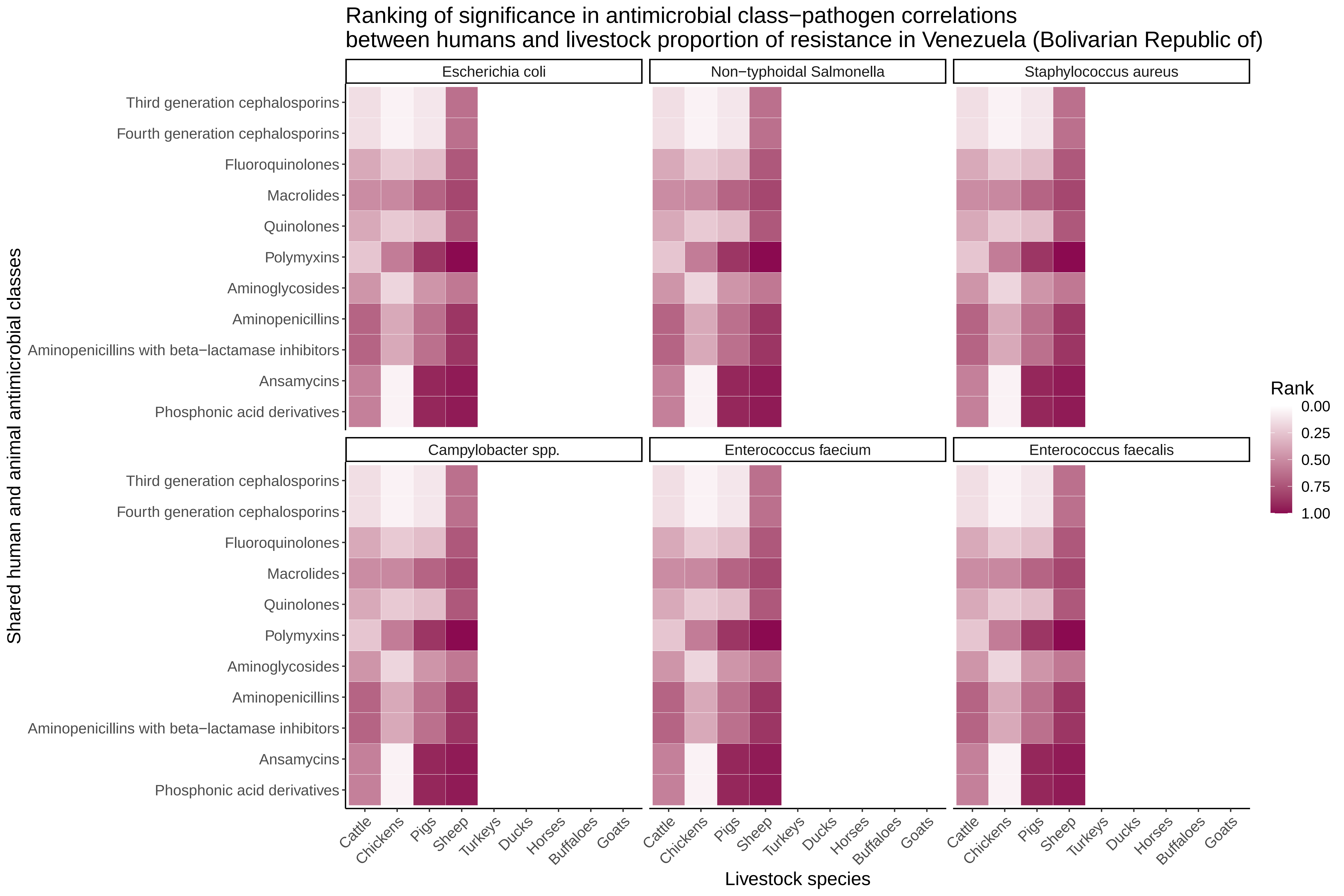

Ranking of significance in antimicrobial class−pathogen correlations
between humans and livestock proportion of resistance in Venezuela (Bolivarian Republic of)
Escherichia coli
Non−typhoidal Salmonella
Staphylococcus aureus
Campylobacter spp.
Enterococcus faecium
Enterococcus faecalis
Third generation cephalosporins
Fourth generation cephalosporins
Fluoroquinolones
Macrolides
Quinolones
Polymyxins
Aminoglycosides
Aminopenicillins
Aminopenicillins with beta−lactamase inhibitors
Ansamycins
Phosphonic acid derivatives
Shared human and animal antimicrobial classes
Cattle
Chickens
Pigs
Sheep
Turkeys
Ducks
Horses
Buffaloes
Goats
Livestock species
Rank
0.00
0.25
0.50
0.75
1.00

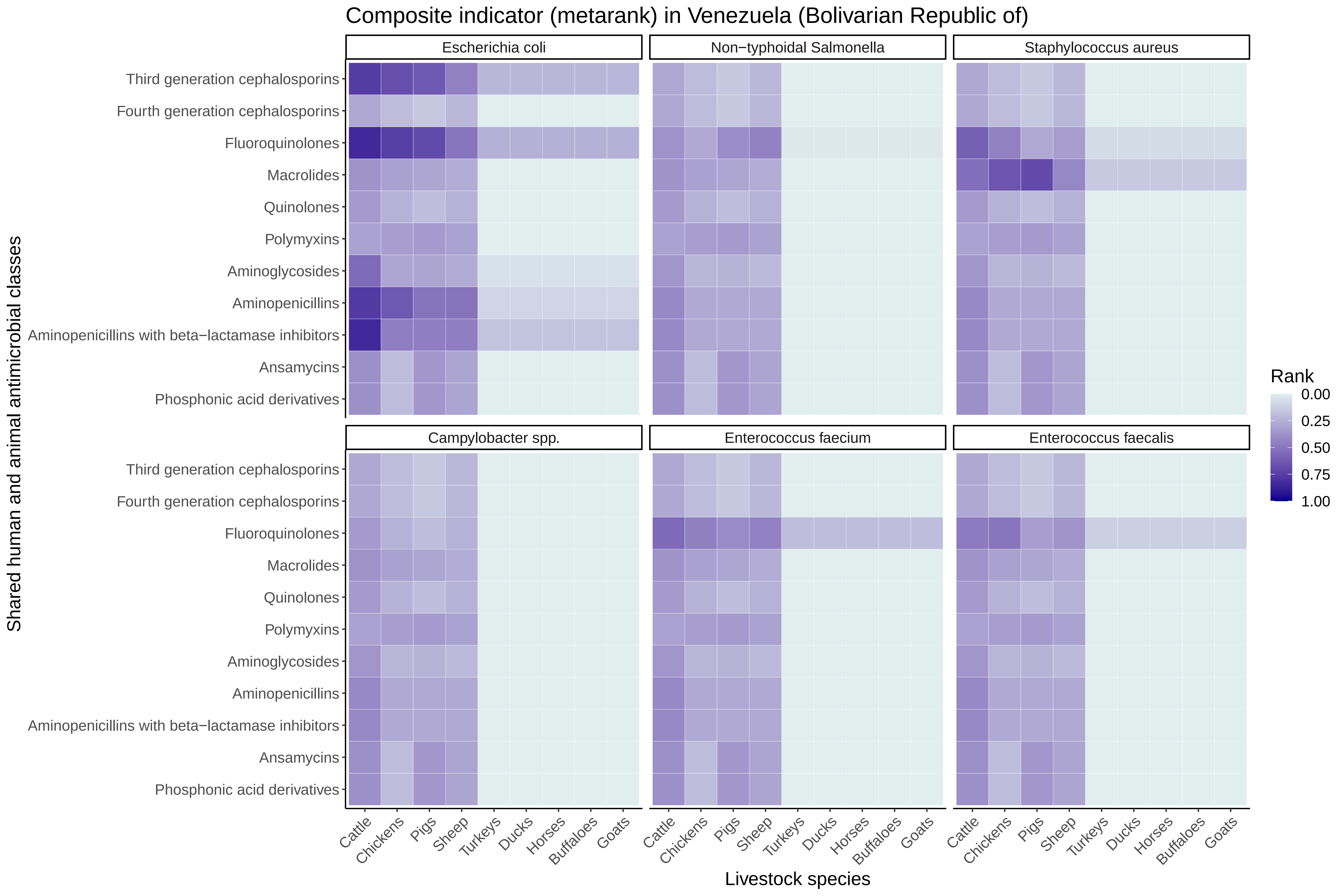

Composite indicator (metarank) in Venezuela (Bolivarian Republic of)
Escherichia coli
Non−typhoidal Salmonella
Staphylococcus aureus
Campylobacter spp.
Enterococcus faecium
Enterococcus faecalis
Shared human and animal antimicrobial classes
Third generation cephalosporins
Fourth generation cephalosporins
Fluoroquinolones
Macrolides
Quinolones
Polymyxins
Aminoglycosides
Aminopenicillins
Aminopenicillins with beta−lactamase inhibitors
Ansamycins
Phosphonic acid derivatives
Livestock species
Cattle
Chickens
Pigs
Sheep
Turkeys
Ducks
Horses
Buffaloes
Goats
Rank
0.00
0.25
0.50
0.75
1.00

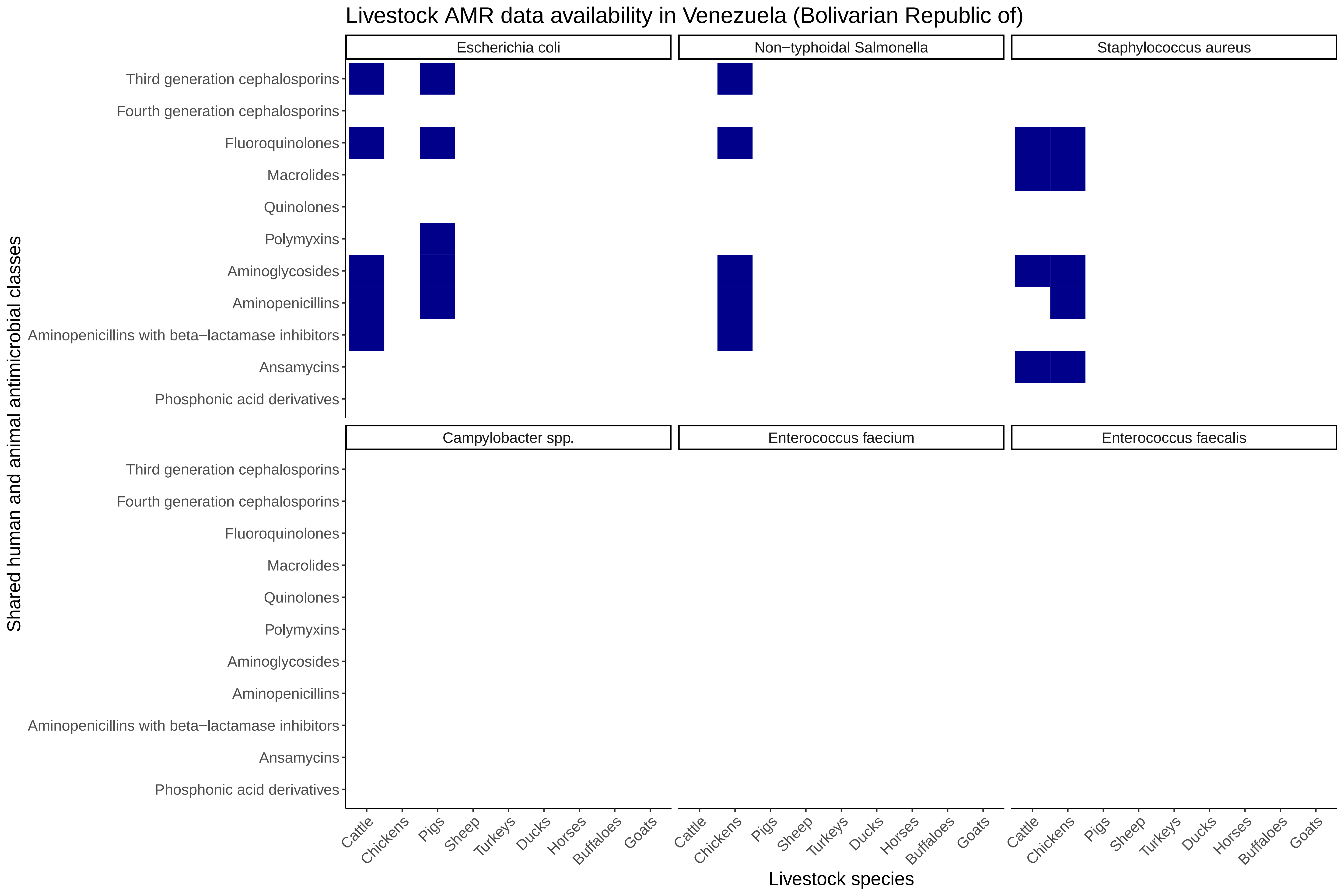
Livestock AMR data availability in Venezuela (Bolivarian Republic of)
Escherichia coli
Non−typhoidal Salmonella
Staphylococcus aureus
Campylobacter spp.
Enterococcus faecium
Enterococcus faecalis
Shared human and animal antimicrobial classes
Third generation cephalosporins
Fourth generation cephalosporins
Fluoroquinolones
Macrolides
Quinolones
Polymyxins
Aminoglycosides
Aminopenicillins
Aminopenicillins with beta−lactamase inhibitors
Ansamycins
Phosphonic acid derivatives
Cattle
Chickens
Pigs
Sheep
Turkeys
Ducks
Horses
Buffaloes
Goats
Livestock species

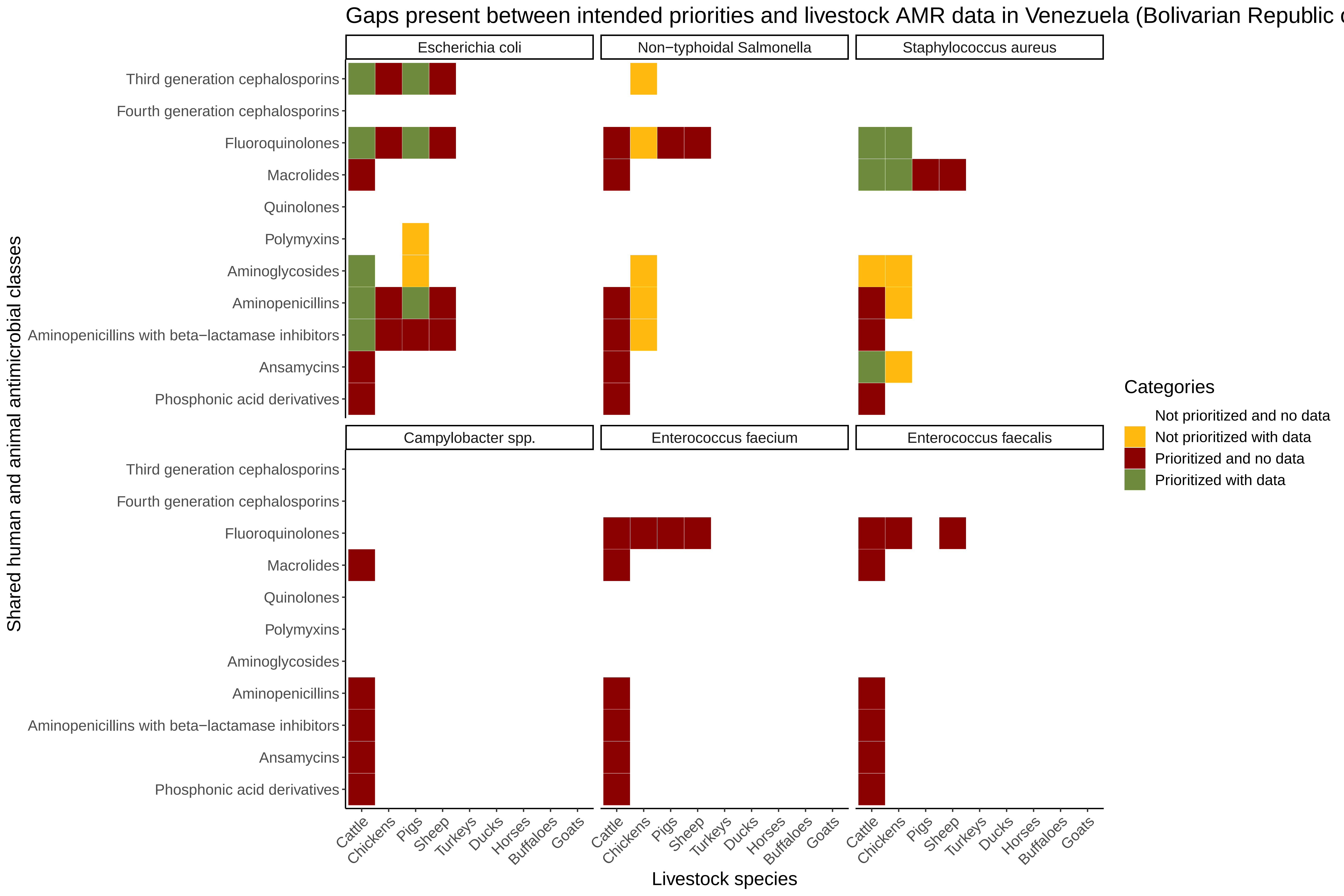

Gaps present between intended priorities and livestock AMR data in Venezuela (Bolivarian Republic o
Escherichia coli
Non−typhoidal Salmonella
Staphylococcus aureus
Campylobacter spp.
Enterococcus faecium
Enterococcus faecalis
Third generation cephalosporins
Fourth generation cephalosporins
Fluoroquinolones
Macrolides
Quinolones
Polymyxins
Aminoglycosides
Aminopenicillins
Aminopenicillins with beta−lactamase inhibitors
Ansamycins
Phosphonic acid derivatives
Shared human and animal antimicrobial classes
Cattle
Chickens
Pigs
Sheep
Turkeys
Ducks
Horses
Buffaloes
Goats
Livestock species
Categories
Not prioritized and no data
Not prioritized with data
Prioritized and no data
Prioritized with data

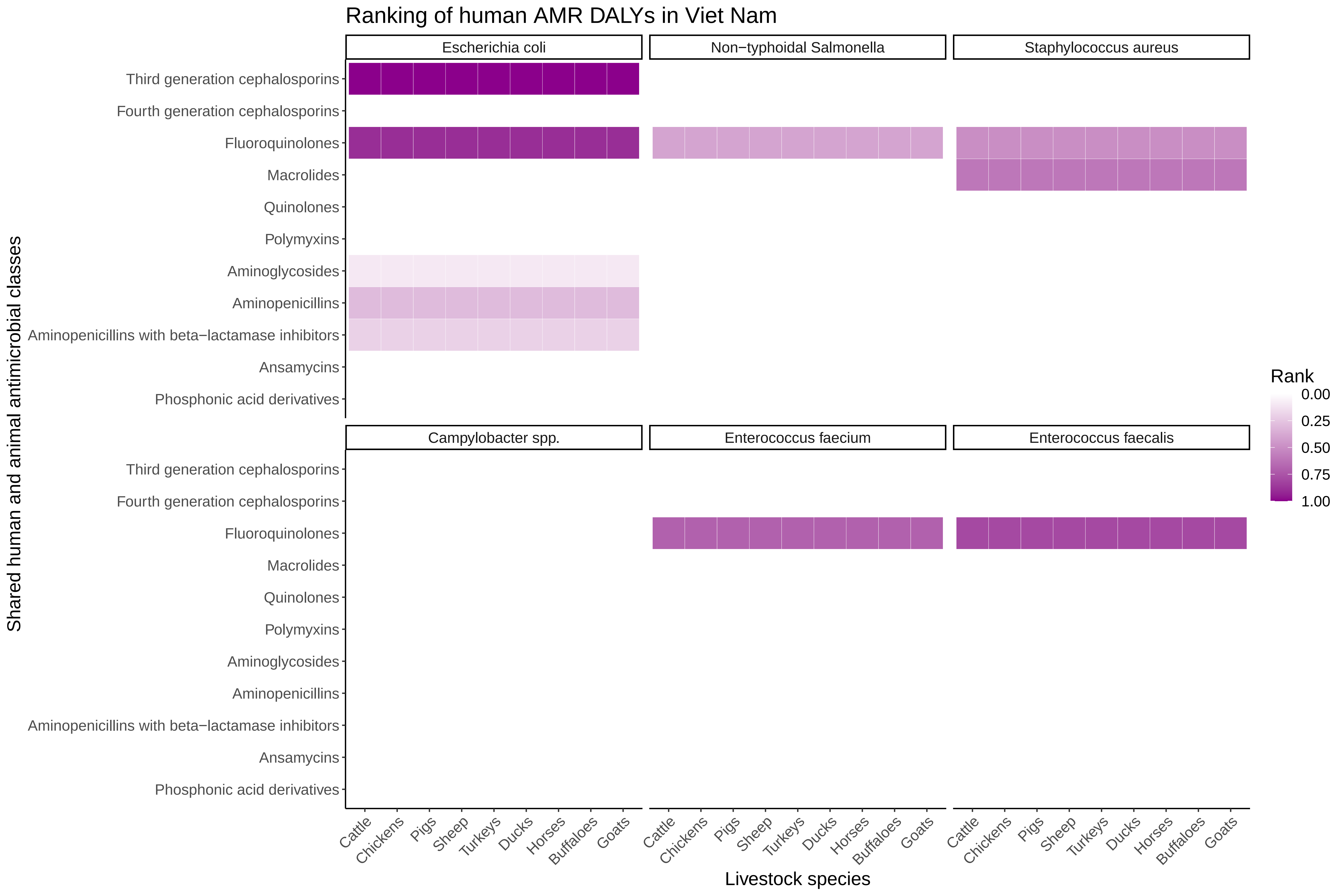
Ranking of human AMR DALYs in Viet Nam
Escherichia coli
Non−typhoidal Salmonella
Staphylococcus aureus
Campylobacter spp.
Enterococcus faecium
Enterococcus faecalis
Third generation cephalosporins
Fourth generation cephalosporins
Fluoroquinolones
Macrolides
Quinolones
Polymyxins
Aminoglycosides
Aminopenicillins
Aminopenicillins with beta−lactamase inhibitors
Ansamycins
Phosphonic acid derivatives
Shared human and animal antimicrobial classes
Cattle
Chickens
Pigs
Sheep
Turkeys
Ducks
Horses
Buffaloes
Goats
Livestock species
Rank
0.00
0.25
0.50
0.75
1.00

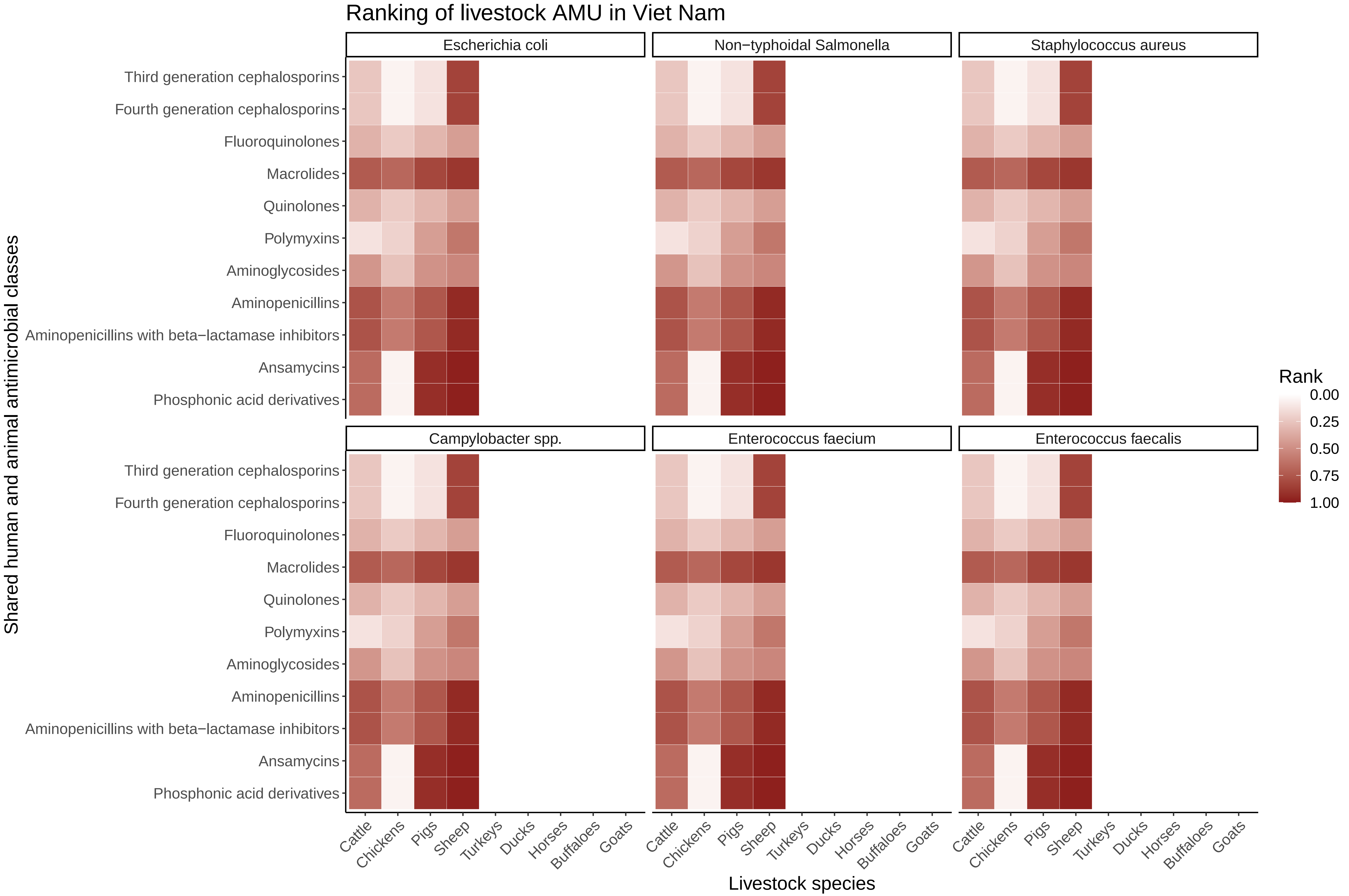
Ranking of livestock AMU in Viet Nam
Escherichia coli
Non−typhoidal Salmonella
Staphylococcus aureus
Campylobacter spp.
Enterococcus faecium
Enterococcus faecalis
Shared human and animal antimicrobial classes
Third generation cephalosporins
Fourth generation cephalosporins
Fluoroquinolones
Macrolides
Quinolones
Polymyxins
Aminoglycosides
Aminopenicillins
Aminopenicillins with beta−lactamase inhibitors
Ansamycins
Phosphonic acid derivatives
Cattle
Chickens
Pigs
Sheep
Turkeys
Ducks
Horses
Buffaloes
Goats
Livestock species
Rank
0.00
0.25
0.50
0.75
1.00

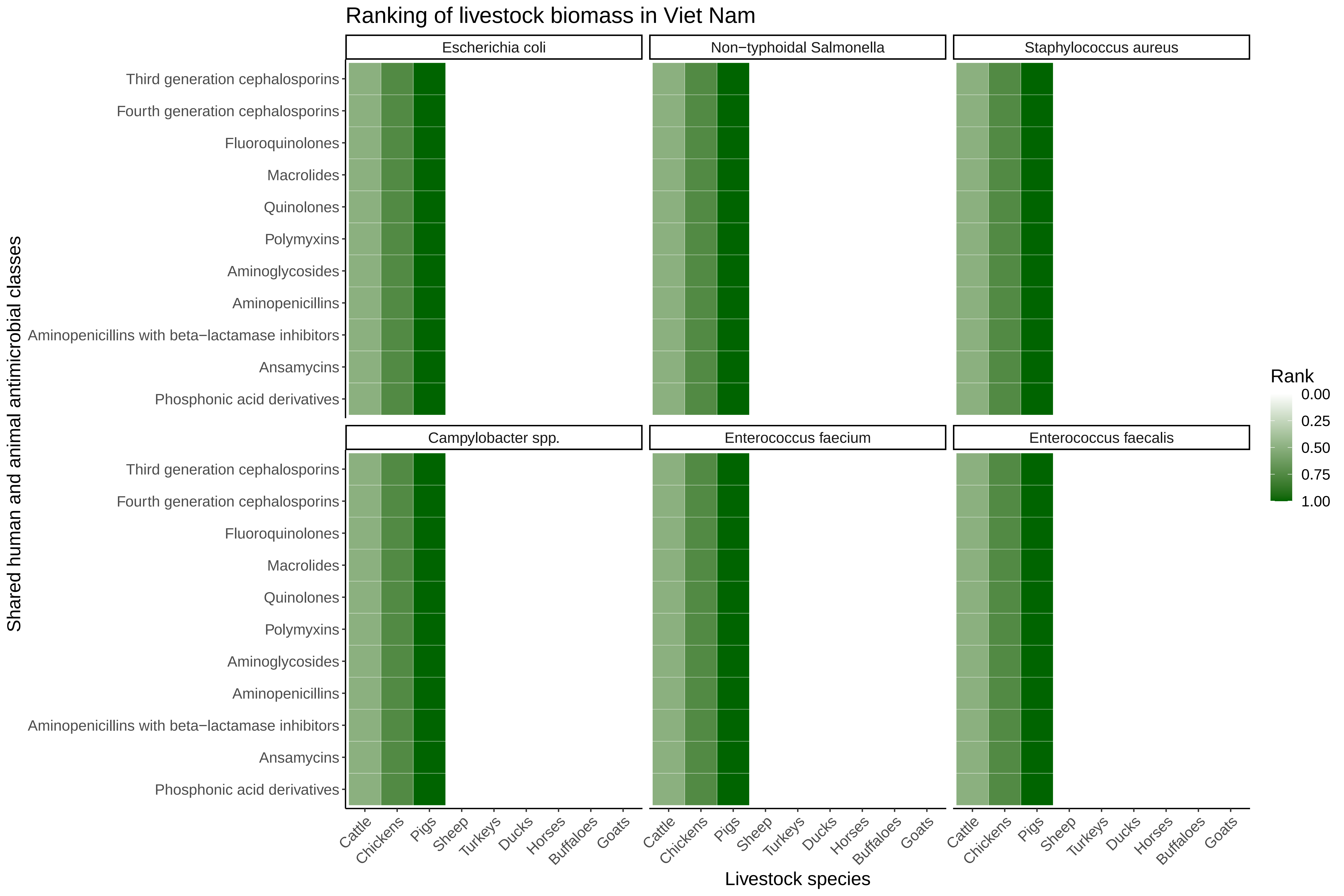
Ranking of livestock biomass in Viet Nam
Escherichia coli
Non−typhoidal Salmonella
Staphylococcus aureus
Campylobacter spp.
Enterococcus faecium
Enterococcus faecalis
Shared human and animal antimicrobial classes
Third generation cephalosporins
Fourth generation cephalosporins
Fluoroquinolones
Macrolides
Quinolones
Polymyxins
Aminoglycosides
Aminopenicillins
Aminopenicillins with beta−lactamase inhibitors
Ansamycins
Phosphonic acid derivatives
Cattle
Chickens
Pigs
Sheep
Turkeys
Ducks
Horses
Buffaloes
Goats
Livestock species
Rank
0.00
0.25
0.50
0.75
1.00

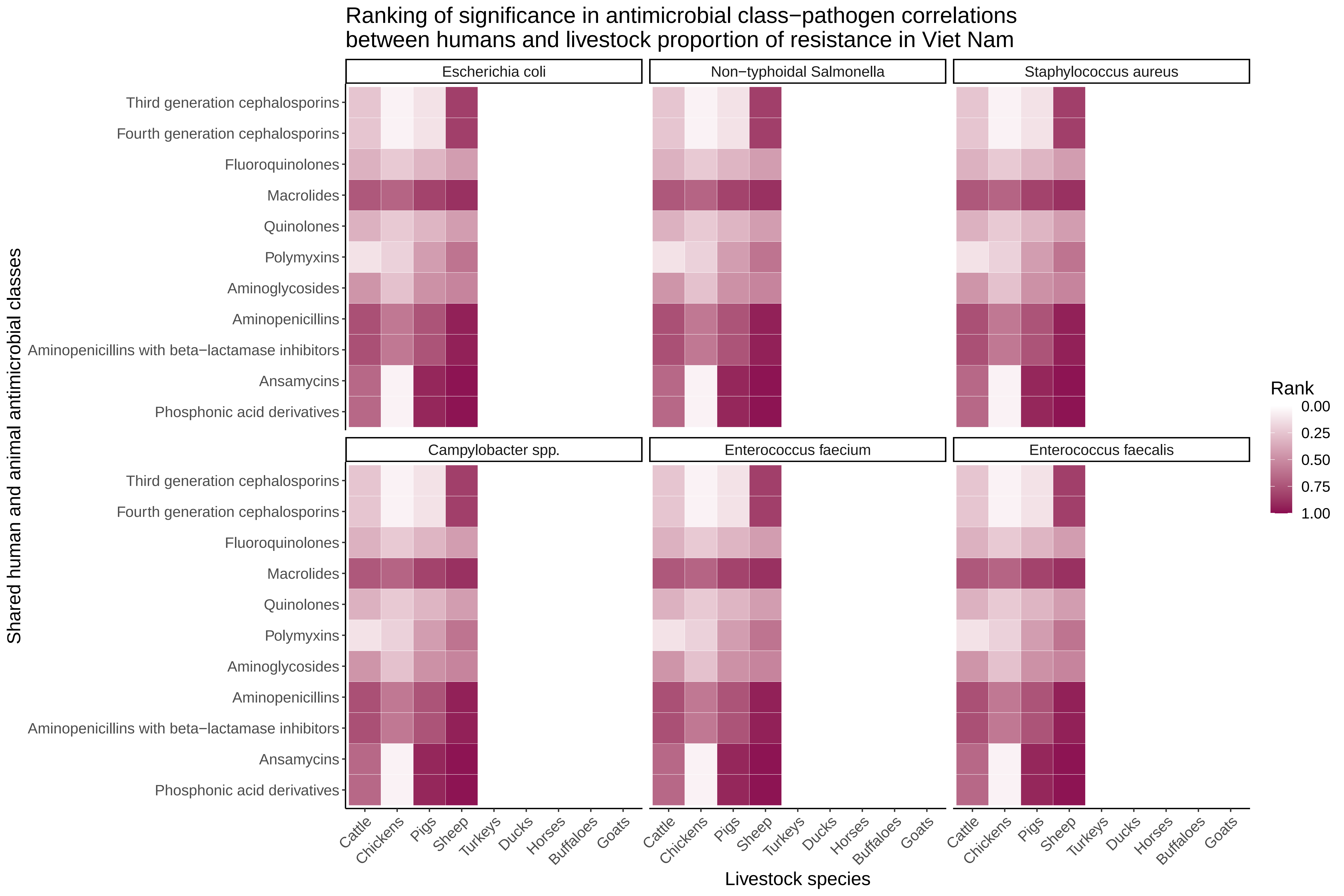
Ranking of significance in antimicrobial class−pathogen correlations
between humans and livestock proportion of resistance in Viet Nam
Escherichia coli
Non−typhoidal Salmonella
Staphylococcus aureus
Campylobacter spp.
Enterococcus faecium
Enterococcus faecalis
Third generation cephalosporins
Fourth generation cephalosporins
Fluoroquinolones
Macrolides
Quinolones
Polymyxins
Aminoglycosides
Aminopenicillins
Aminopenicillins with beta−lactamase inhibitors
Ansamycins
Phosphonic acid derivatives
Shared human and animal antimicrobial classes
Cattle
Chickens
Pigs
Sheep
Turkeys
Ducks
Horses
Buffaloes
Goats
Livestock species
Rank
0.00
0.25
0.50
0.75
1.00

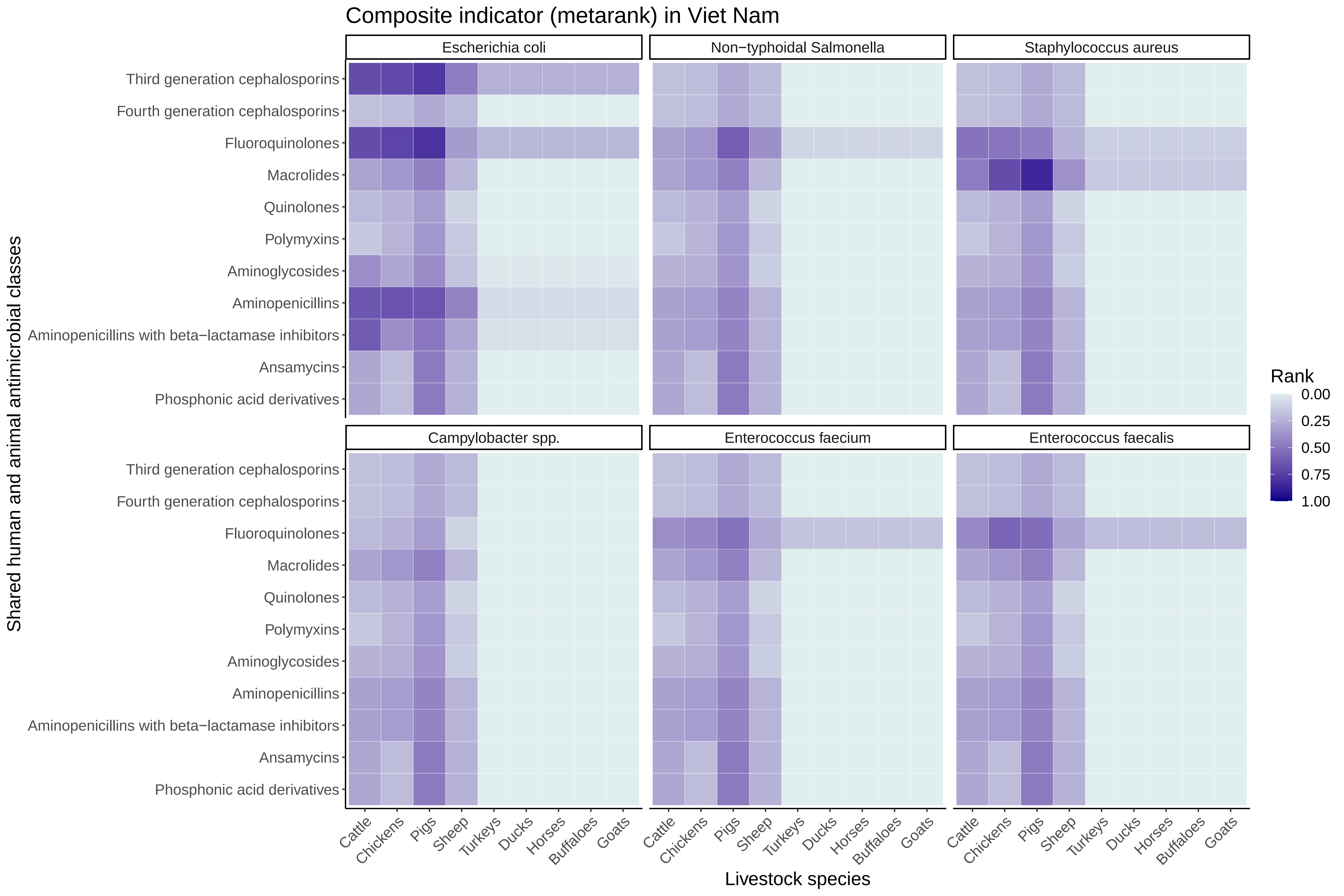
Composite indicator (metarank) in Viet Nam
Escherichia coli
Non−typhoidal Salmonella
Staphylococcus aureus
Campylobacter spp.
Enterococcus faecium
Enterococcus faecalis
Third generation cephalosporins
Fourth generation cephalosporins
Fluoroquinolones
Macrolides
Quinolones
Polymyxins
Aminoglycosides
Aminopenicillins
Aminopenicillins with beta−lactamase inhibitors
Ansamycins
Phosphonic acid derivatives
Shared human and animal antimicrobial classes
Cattle
Chickens
Pigs
Sheep
Turkeys
Ducks
Horses
Buffaloes
Goats
Livestock species
Rank
0.00
0.25
0.50
0.75
1.00

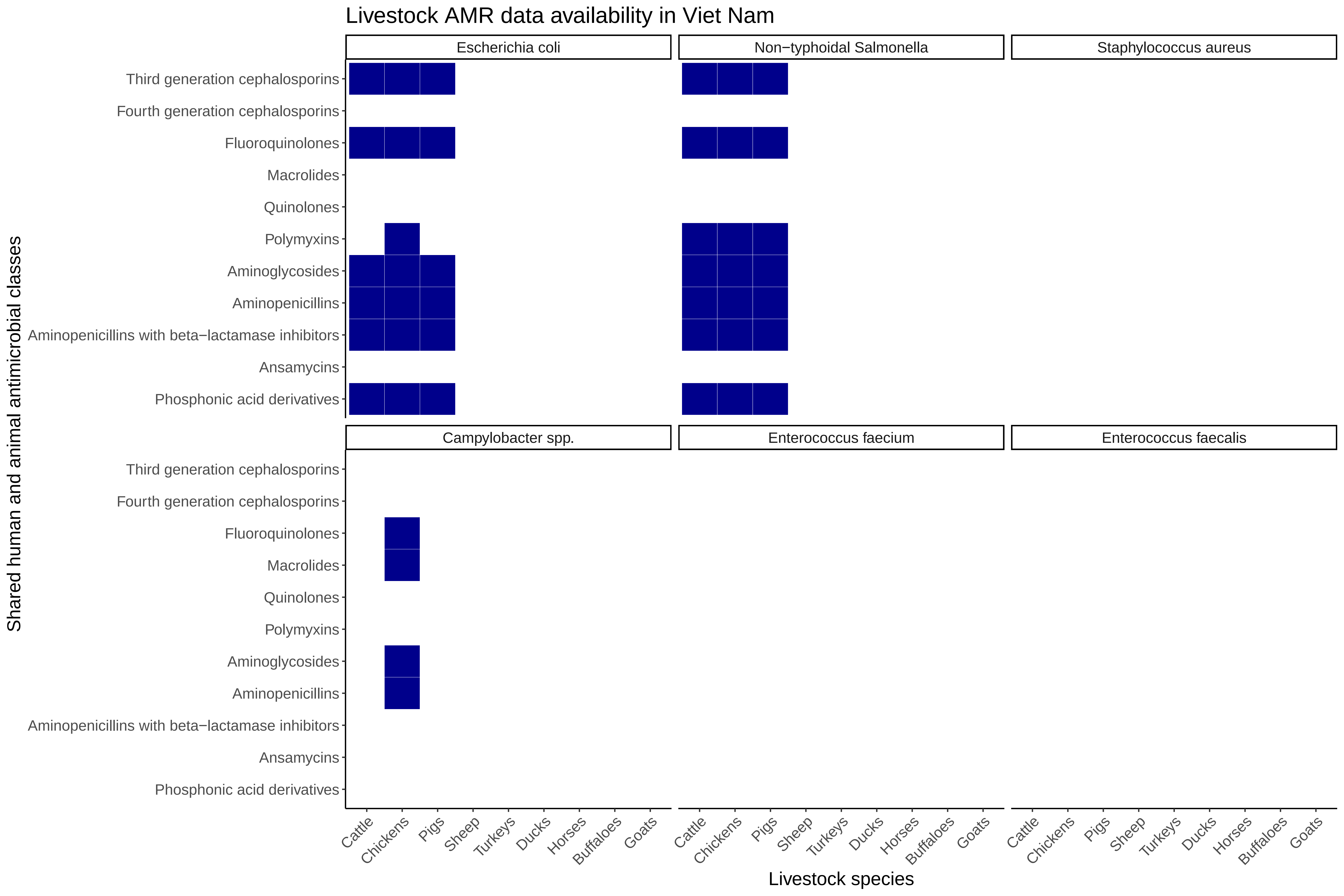

Livestock AMR data availability in Viet Nam
Escherichia coli
Non−typhoidal Salmonella
Staphylococcus aureus
Campylobacter spp.
Enterococcus faecium
Enterococcus faecalis
Third generation cephalosporins
Fourth generation cephalosporins
Fluoroquinolones
Macrolides
Quinolones
Polymyxins
Aminoglycosides
Aminopenicillins
Aminopenicillins with beta−lactamase inhibitors
Ansamycins
Phosphonic acid derivatives
Shared human and animal antimicrobial classes
Cattle
Chickens
Pigs
Sheep
Turkeys
Ducks
Horses
Buffaloes
Goats
Livestock species

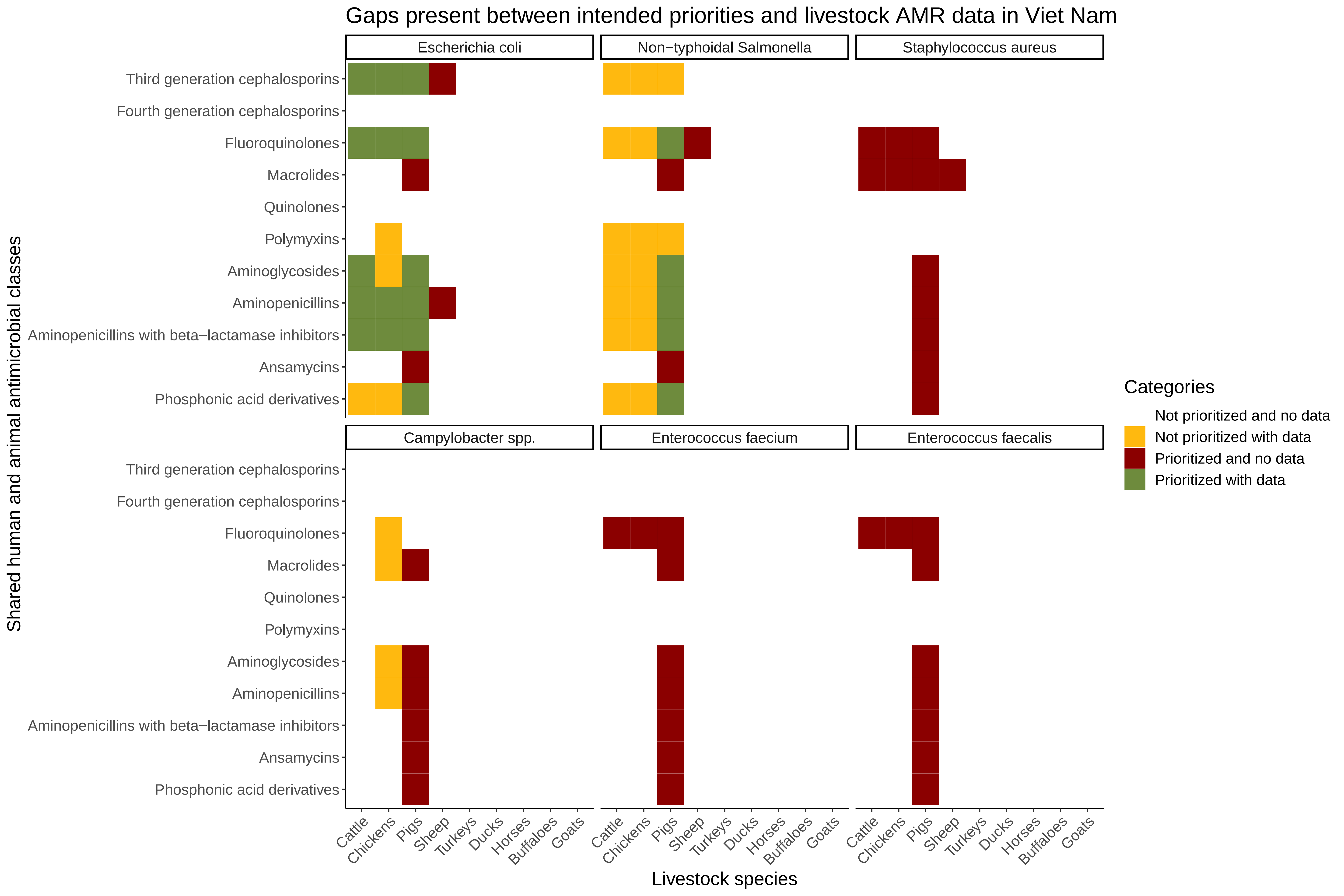

Gaps present between intended priorities and livestock AMR data in Viet Nam
Escherichia coli
Non−typhoidal Salmonella
Staphylococcus aureus
Campylobacter spp.
Enterococcus faecium
Enterococcus faecalis
Third generation cephalosporins
Fourth generation cephalosporins
Fluoroquinolones
Macrolides
Quinolones
Polymyxins
Aminoglycosides
Aminopenicillins
Aminopenicillins with beta−lactamase inhibitors
Ansamycins
Phosphonic acid derivatives
Shared human and animal antimicrobial classes
Cattle
Chickens
Pigs
Sheep
Turkeys
Ducks
Horses
Buffaloes
Goats
Livestock species
Categories
Not prioritized and no data
Not prioritized with data
Prioritized and no data
Prioritized with data

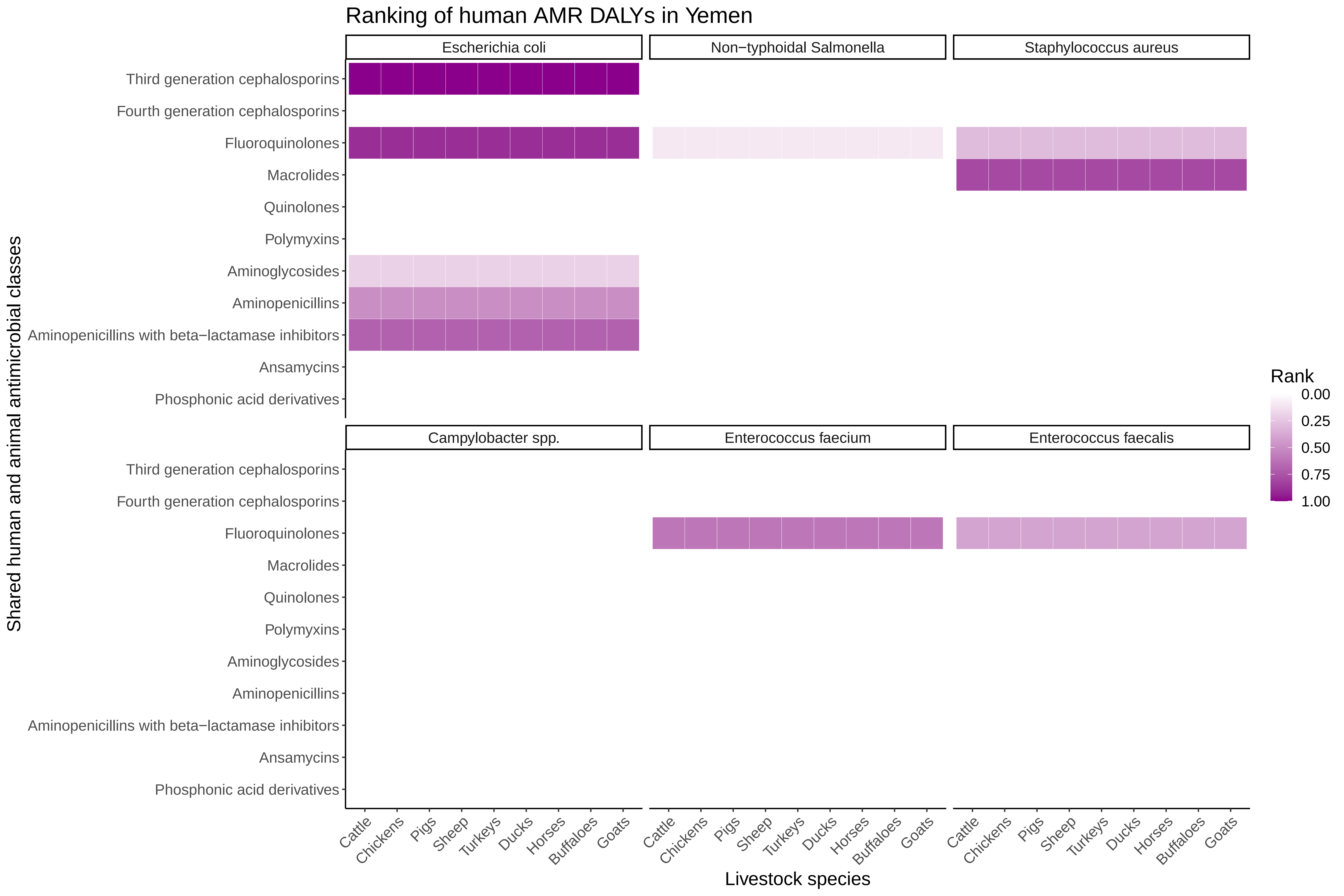

Ranking of human AMR DALYs in Yemen
Escherichia coli
Non−typhoidal Salmonella
Staphylococcus aureus
Campylobacter spp.
Enterococcus faecium
Enterococcus faecalis
Third generation cephalosporins
Fourth generation cephalosporins
Fluoroquinolones
Macrolides
Quinolones
Polymyxins
Aminoglycosides
Aminopenicillins
Aminopenicillins with beta−lactamase inhibitors
Ansamycins
Phosphonic acid derivatives
Shared human and animal antimicrobial classes
Cattle
Chickens
Pigs
Sheep
Turkeys
Ducks
Horses
Buffaloes
Goats
Livestock species
Rank
0.00
0.25
0.50
0.75
1.00

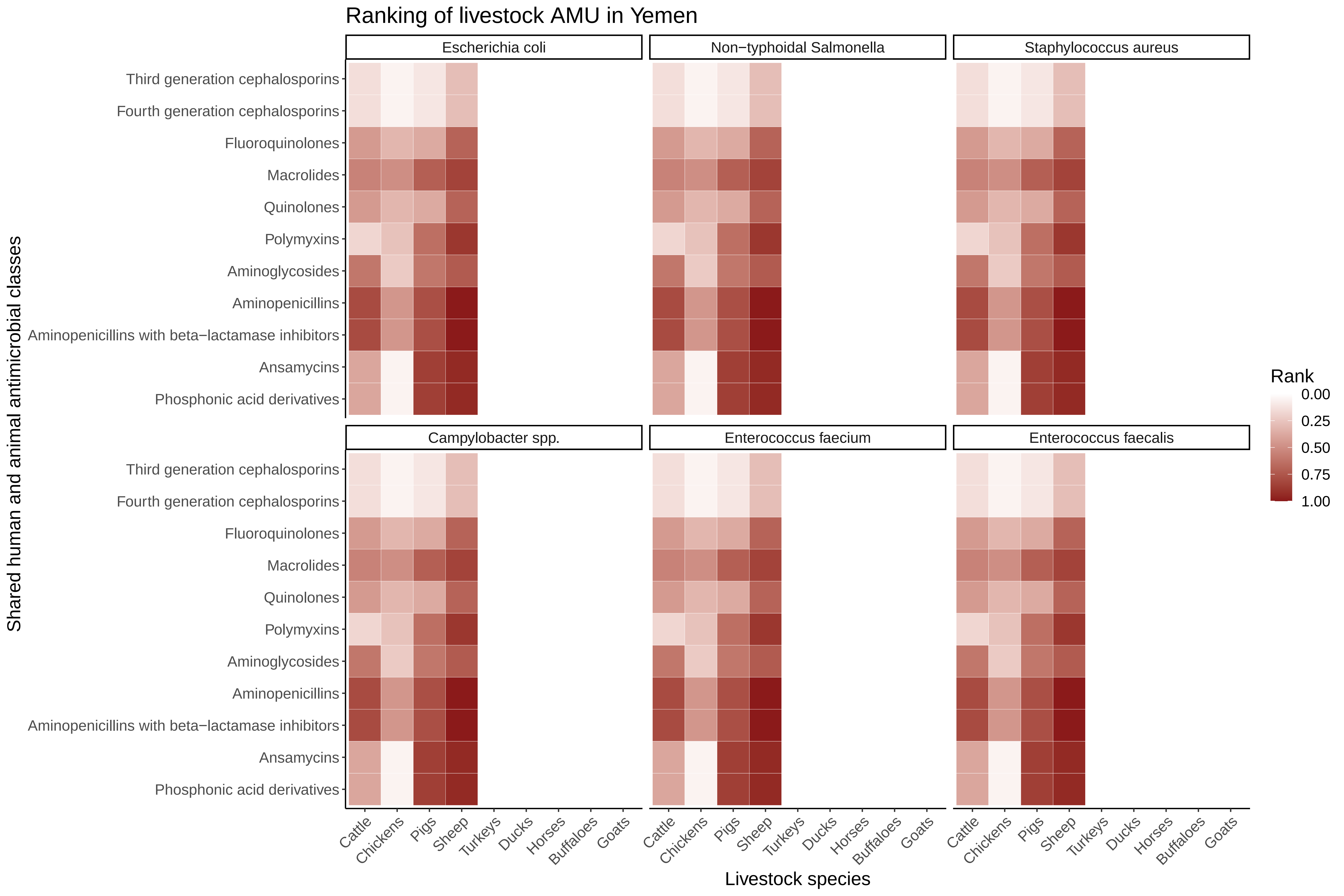
Ranking of livestock AMU in Yemen
Escherichia coli
Non−typhoidal Salmonella
Staphylococcus aureus
Campylobacter spp.
Enterococcus faecium
Enterococcus faecalis
Third generation cephalosporins
Fourth generation cephalosporins
Fluoroquinolones
Macrolides
Quinolones
Polymyxins
Aminoglycosides
Aminopenicillins
Aminopenicillins with beta−lactamase inhibitors
Ansamycins
Phosphonic acid derivatives
Shared human and animal antimicrobial classes
Cattle
Chickens
Pigs
Sheep
Turkeys
Ducks
Horses
Buffaloes
Goats
Livestock species
Rank
0.00
0.25
0.50
0.75
1.00

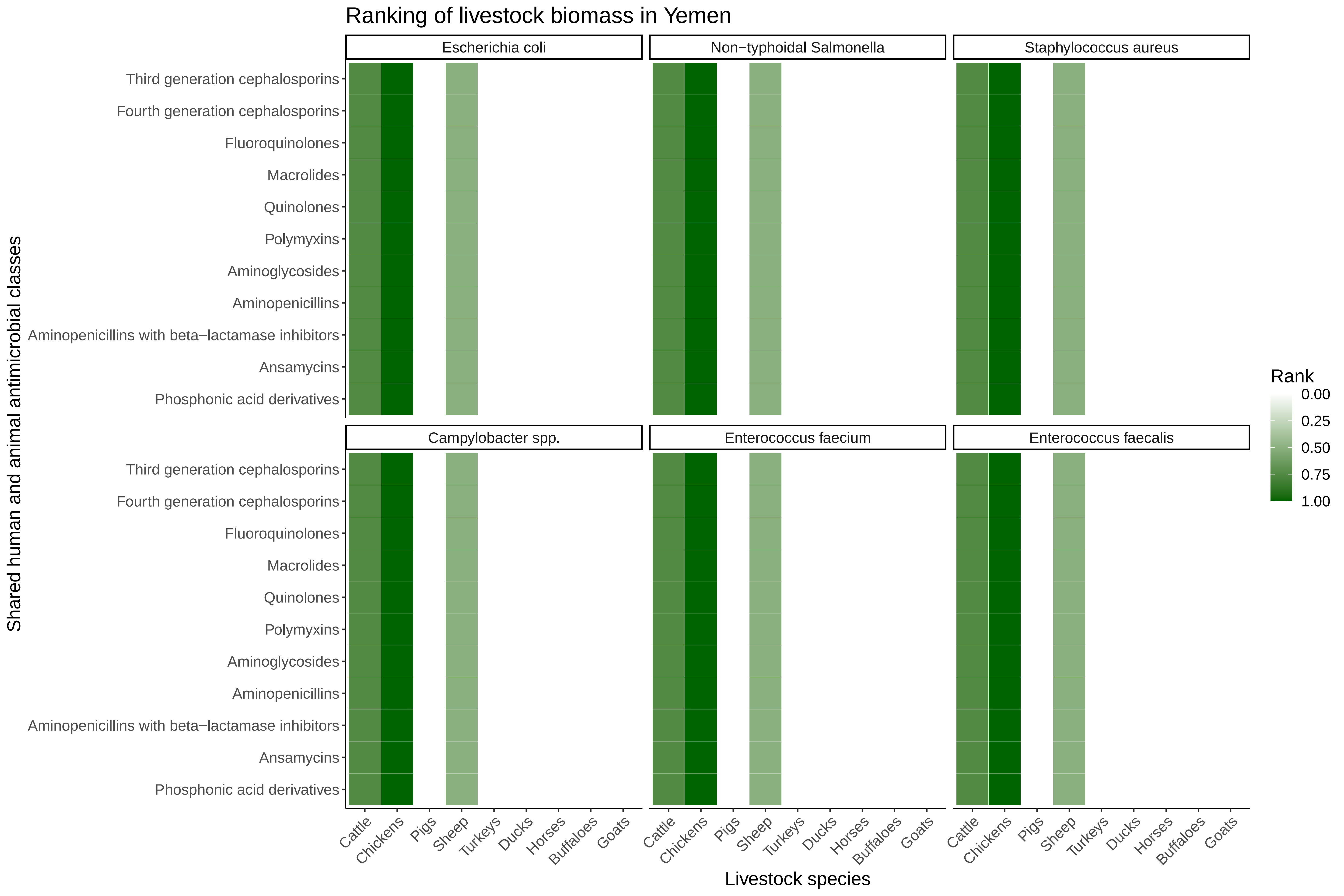

Ranking of livestock biomass in Yemen
Escherichia coli
Non−typhoidal Salmonella
Staphylococcus aureus
Campylobacter spp.
Enterococcus faecium
Enterococcus faecalis
Shared human and animal antimicrobial classes
Third generation cephalosporins
Fourth generation cephalosporins
Fluoroquinolones
Macrolides
Quinolones
Polymyxins
Aminoglycosides
Aminopenicillins
Aminopenicillins with beta−lactamase inhibitors
Ansamycins
Phosphonic acid derivatives
Cattle
Chickens
Pigs
Sheep
Turkeys
Ducks
Horses
Buffaloes
Goats
Livestock species
Rank
0.00
0.25
0.50
0.75
1.00

Ranking of significance in antimicrobial class−pathogen correlations between humans and livestock proportion of resistance in Yemen

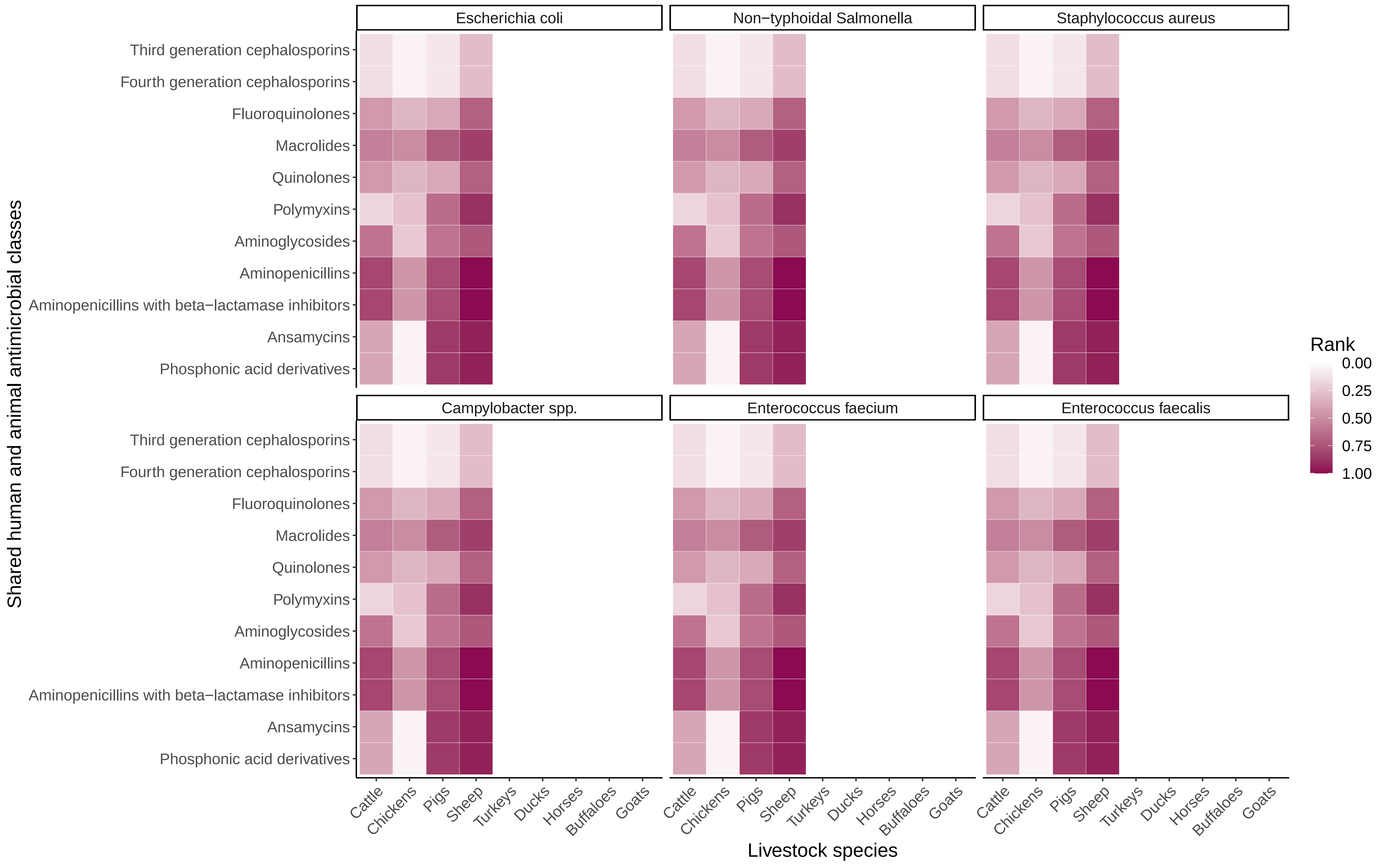

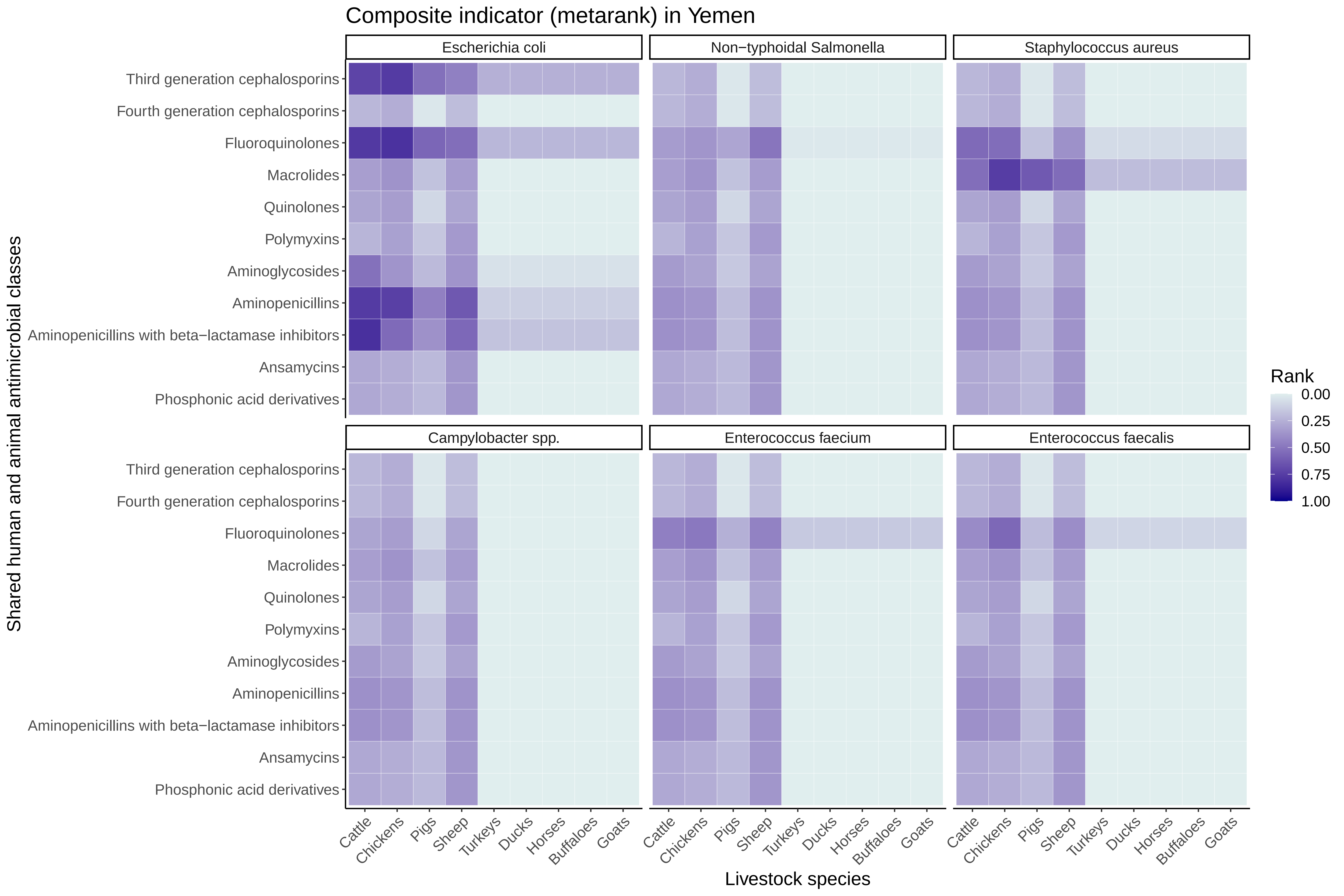

Composite indicator (metarank) in Yemen
Escherichia coli
Non−typhoidal Salmonella
Staphylococcus aureus
Campylobacter spp.
Enterococcus faecium
Enterococcus faecalis
Third generation cephalosporins
Fourth generation cephalosporins
Fluoroquinolones
Macrolides
Quinolones
Polymyxins
Aminoglycosides
Aminopenicillins
Aminopenicillins with beta−lactamase inhibitors
Ansamycins
Phosphonic acid derivatives
Shared human and animal antimicrobial classes
Cattle
Chickens
Pigs
Sheep
Turkeys
Ducks
Horses
Buffaloes
Goats
Livestock species
Rank
0.00
0.25
0.50
0.75
1.00

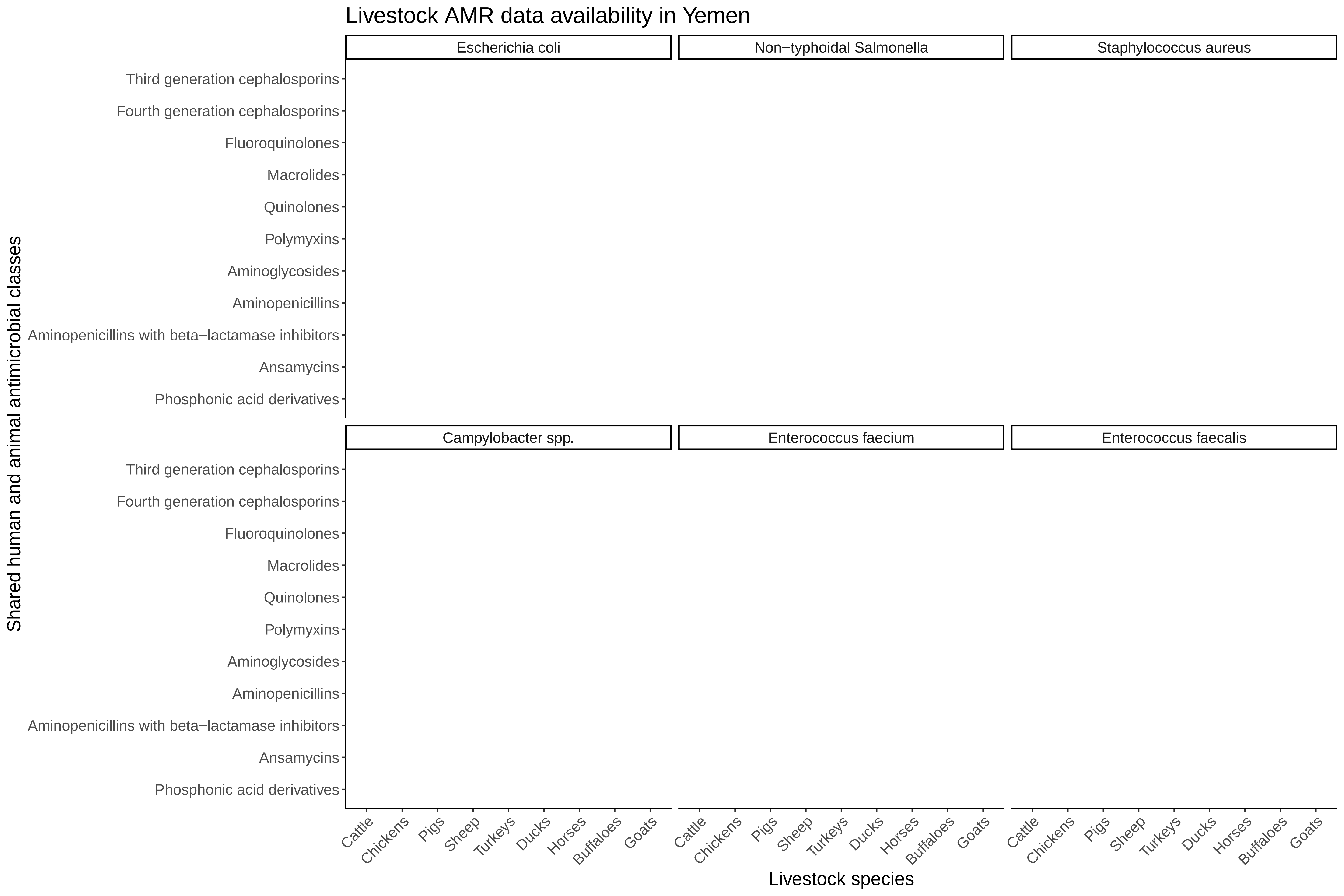
Livestock AMR data availability in Yemen
Escherichia coli
Non−typhoidal Salmonella
Staphylococcus aureus
Campylobacter spp.
Enterococcus faecium
Enterococcus faecalis
Shared human and animal antimicrobial classes
Third generation cephalosporins
Fourth generation cephalosporins
Fluoroquinolones
Macrolides
Quinolones
Polymyxins
Aminoglycosides
Aminopenicillins
Aminopenicillins with beta−lactamase inhibitors
Ansamycins
Phosphonic acid derivatives
Cattle
Chickens
Pigs
Sheep
Turkeys
Ducks
Horses
Buffaloes
Goats
Livestock species

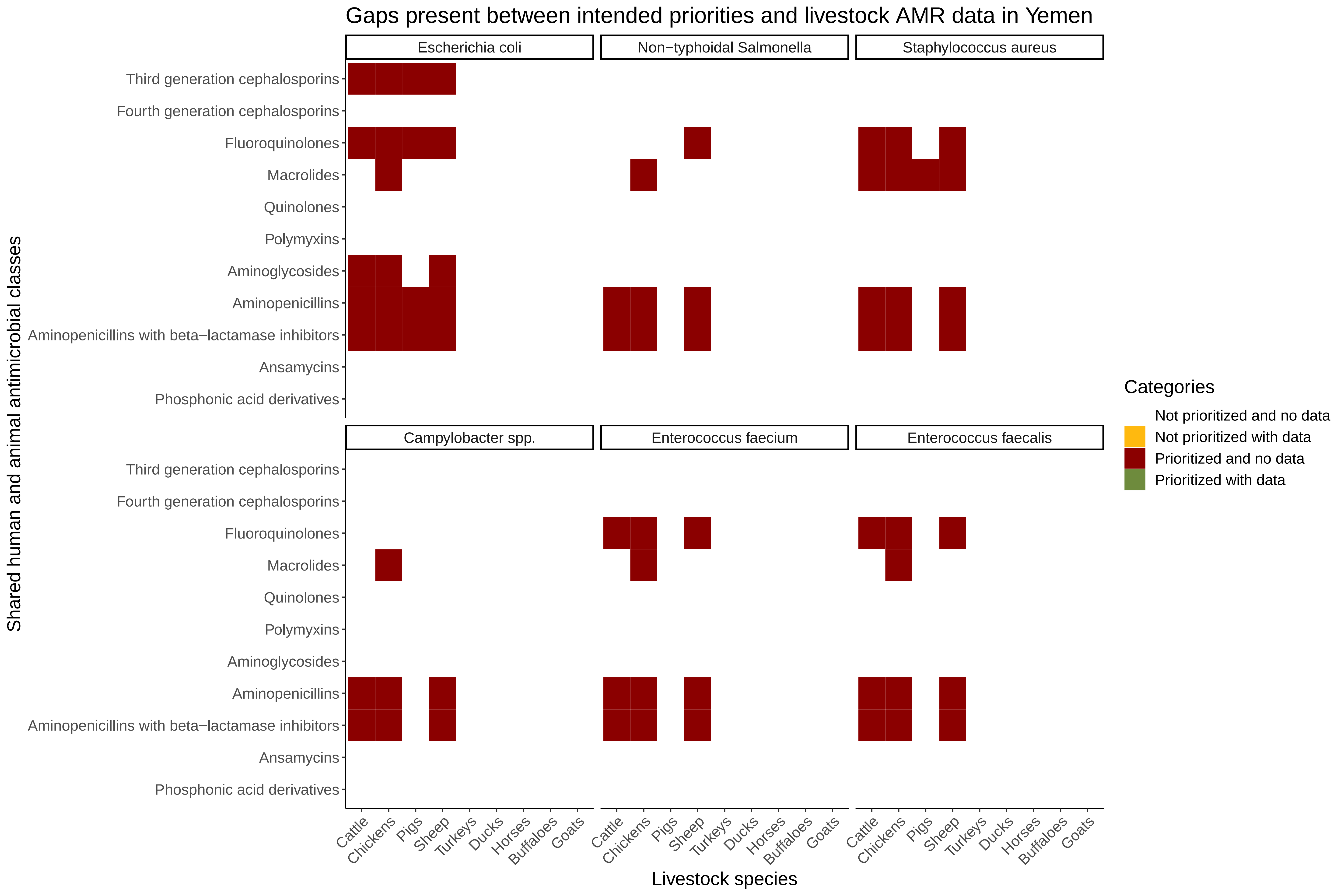

Gaps present between intended priorities and livestock AMR data in Yemen
Escherichia coli
Non−typhoidal Salmonella
Staphylococcus aureus
Campylobacter spp.
Enterococcus faecium
Enterococcus faecalis
Third generation cephalosporins
Fourth generation cephalosporins
Fluoroquinolones
Macrolides
Quinolones
Polymyxins
Aminoglycosides
Aminopenicillins
Aminopenicillins with beta−lactamase inhibitors
Ansamycins
Phosphonic acid derivatives
Shared human and animal antimicrobial classes
Cattle
Chickens
Pigs
Sheep
Turkeys
Ducks
Horses
Buffaloes
Goats
Livestock species
Categories
Not prioritized and no data
Not prioritized with data
Prioritized and no data
Prioritized with data

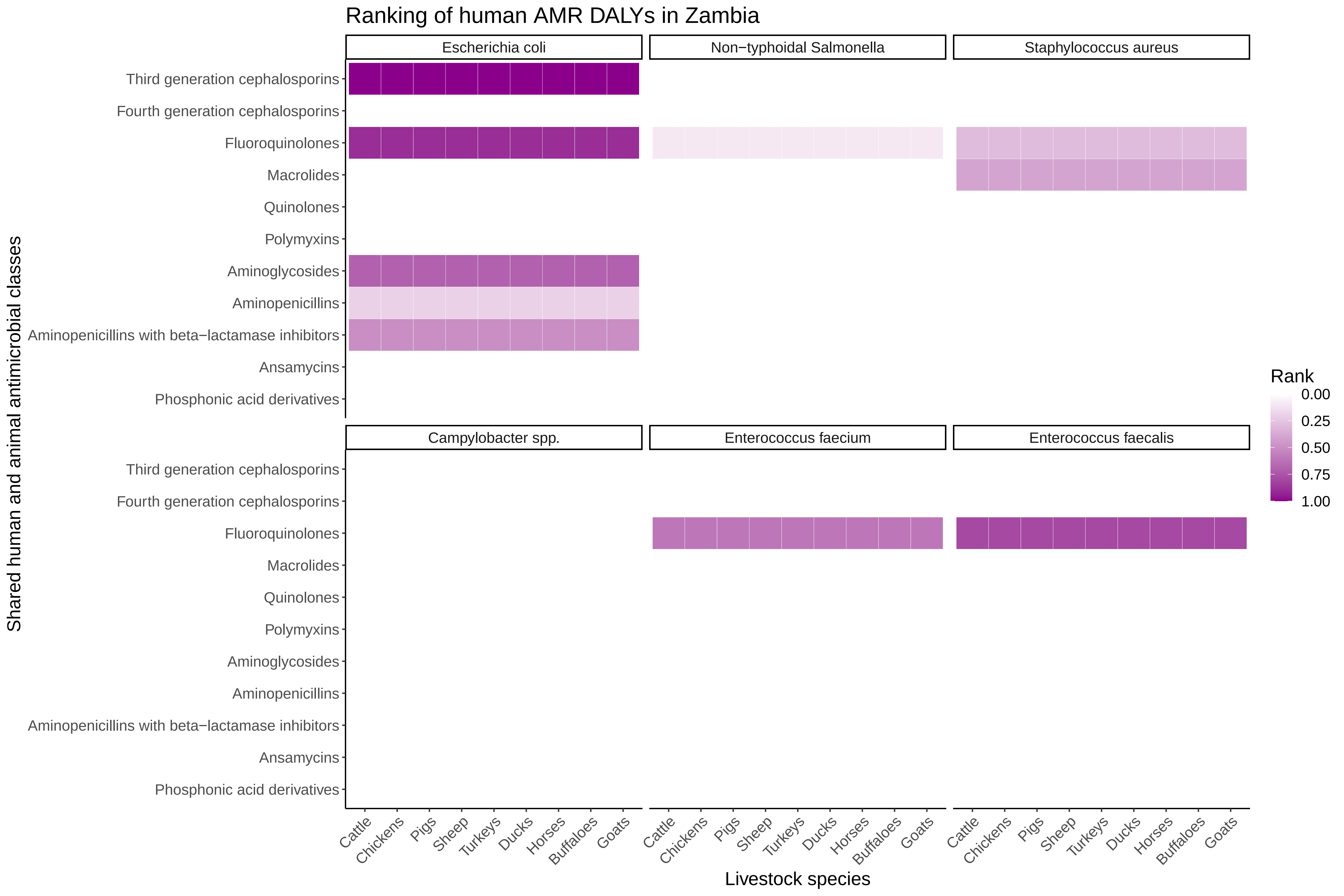
Ranking of human AMR DALYs in Zambia
Escherichia coli
Non−typhoidal Salmonella
Staphylococcus aureus
Campylobacter spp.
Enterococcus faecium
Enterococcus faecalis
Shared human and animal antimicrobial classes
Third generation cephalosporins
Fourth generation cephalosporins
Fluoroquinolones
Macrolides
Quinolones
Polymyxins
Aminoglycosides
Aminopenicillins
Aminopenicillins with beta−lactamase inhibitors
Ansamycins
Phosphonic acid derivatives
Cattle
Chickens
Pigs
Sheep
Turkeys
Ducks
Horses
Buffaloes
Goats
Livestock species
Rank
0.00
0.25
0.50
0.75
1.00

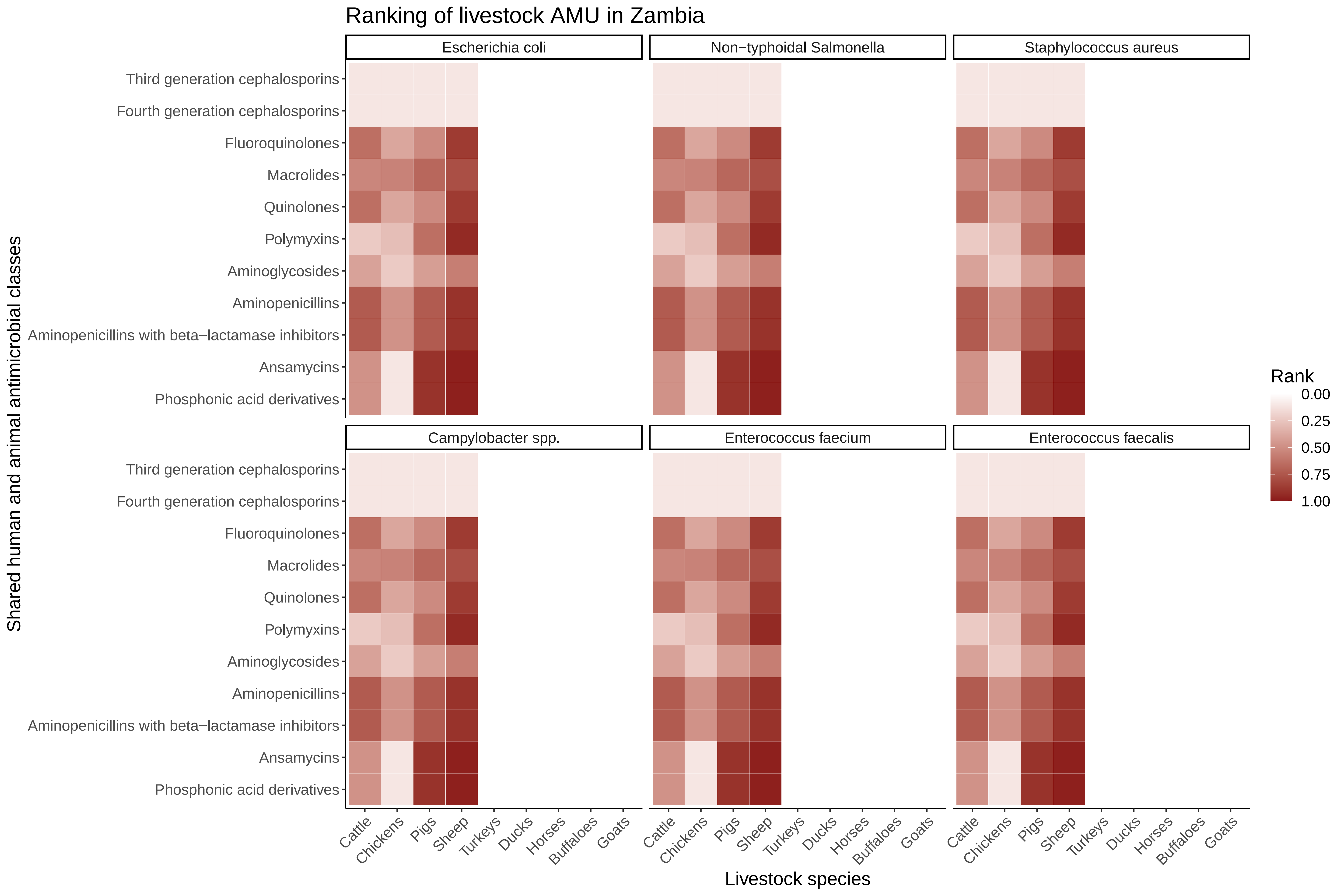

Ranking of livestock AMU in Zambia
Escherichia coli
Non−typhoidal Salmonella
Staphylococcus aureus
Campylobacter spp.
Enterococcus faecium
Enterococcus faecalis
Third generation cephalosporins
Fourth generation cephalosporins
Fluoroquinolones
Macrolides
Quinolones
Polymyxins
Aminoglycosides
Aminopenicillins
Aminopenicillins with beta−lactamase inhibitors
Ansamycins
Phosphonic acid derivatives
Shared human and animal antimicrobial classes
Cattle
Chickens
Pigs
Sheep
Turkeys
Ducks
Horses
Buffaloes
Goats
Livestock species
Rank
0.00
0.25
0.50
0.75
1.00

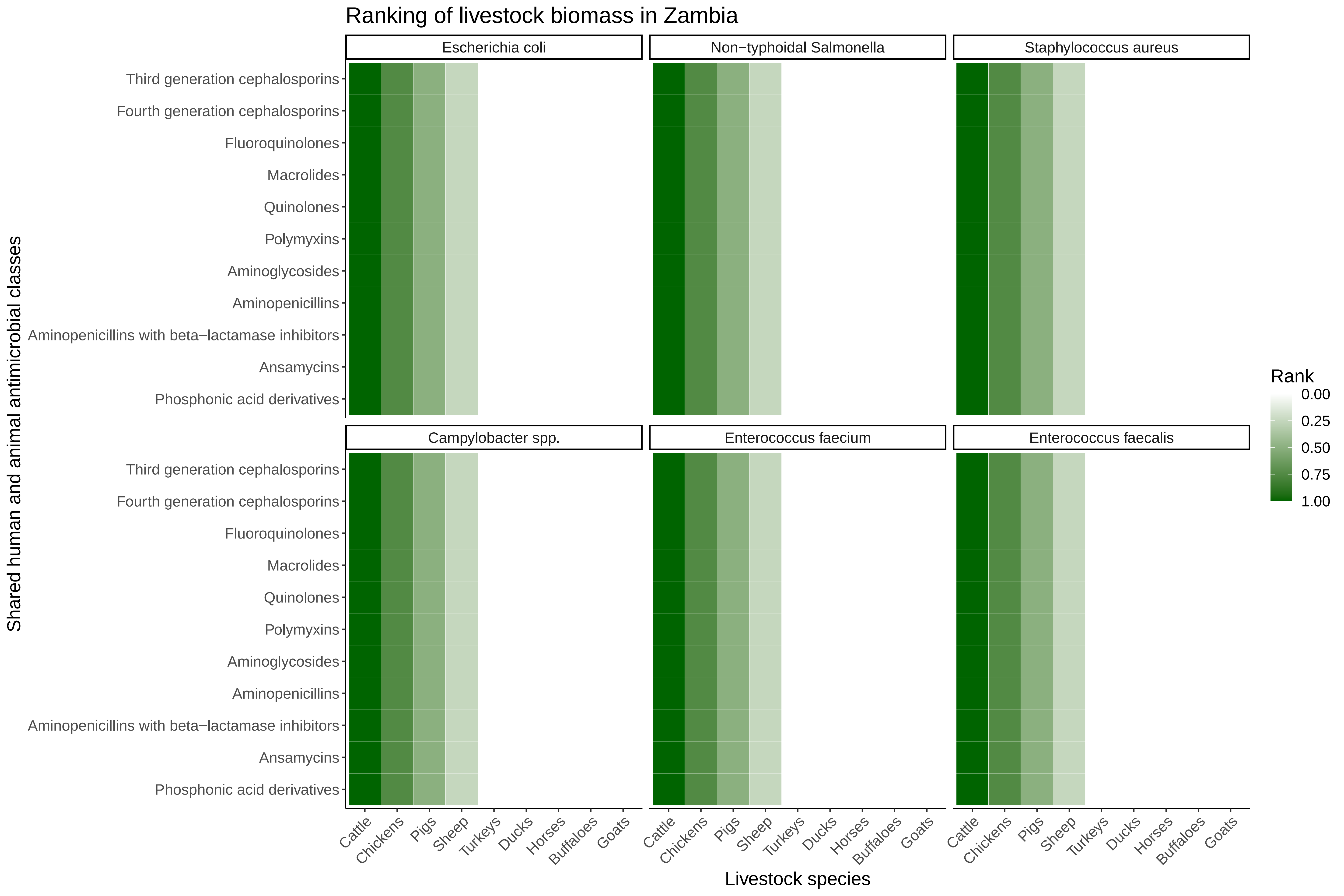

Ranking of livestock biomass in Zambia
Escherichia coli
Non−typhoidal Salmonella
Staphylococcus aureus
Campylobacter spp.
Enterococcus faecium
Enterococcus faecalis
Third generation cephalosporins
Fourth generation cephalosporins
Fluoroquinolones
Macrolides
Quinolones
Polymyxins
Aminoglycosides
Aminopenicillins
Aminopenicillins with beta−lactamase inhibitors
Ansamycins
Phosphonic acid derivatives
Shared human and animal antimicrobial classes
Cattle
Chickens
Pigs
Sheep
Turkeys
Ducks
Horses
Buffaloes
Goats
Livestock species
Rank
0.00
0.25
0.50
0.75
1.00

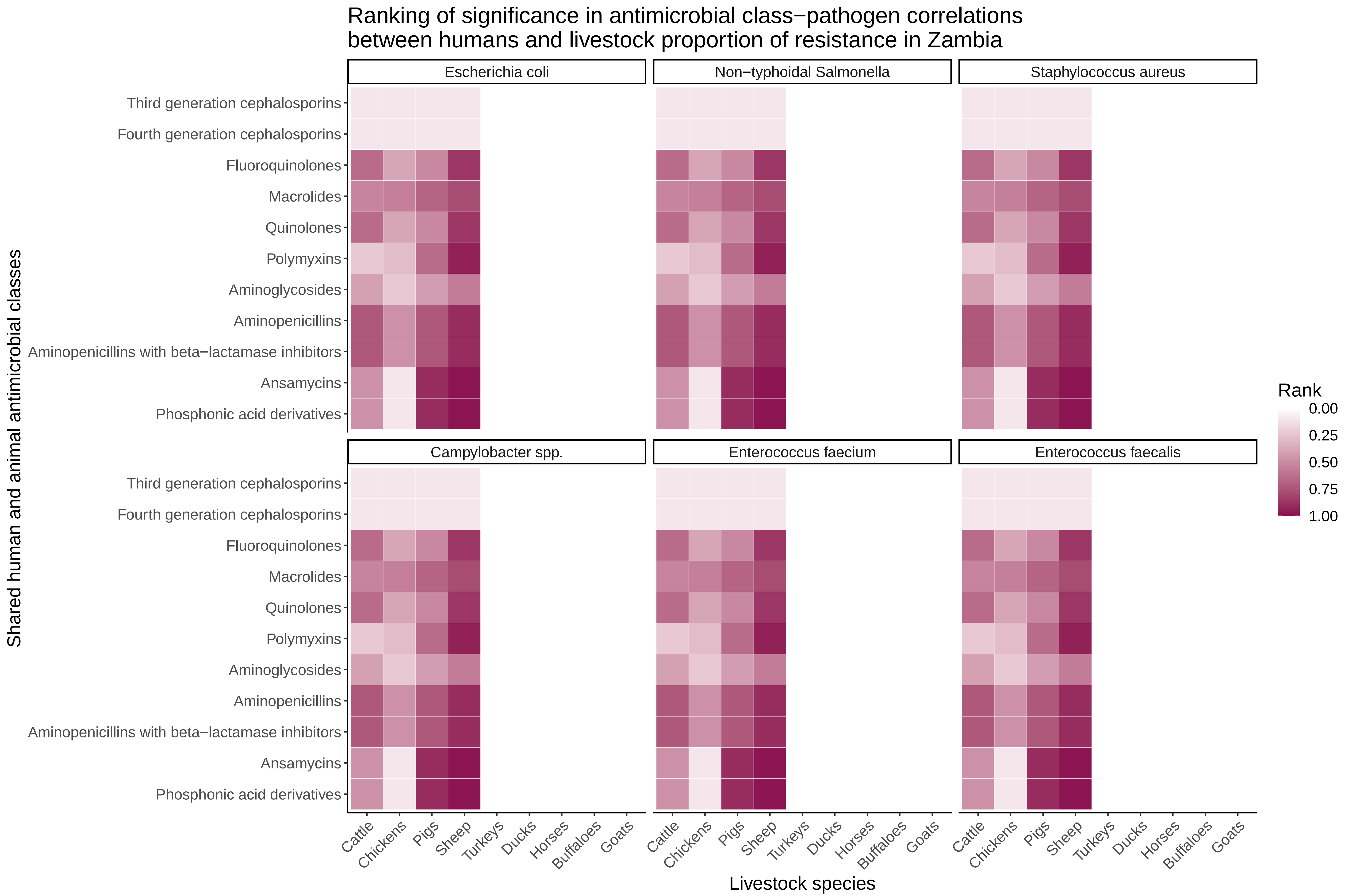

Ranking of significance in antimicrobial class−pathogen correlations between humans and livestock proportion of resistance in Zambia
Escherichia coli
Non−typhoidal Salmonella
Staphylococcus aureus
Campylobacter spp.
Enterococcus faecium
Enterococcus faecalis
Third generation cephalosporins
Fourth generation cephalosporins
Fluoroquinolones
Macrolides
Quinolones
Polymyxins
Aminoglycosides
Aminopenicillins
Aminopenicillins with beta−lactamase inhibitors
Ansamycins
Phosphonic acid derivatives
Shared human and animal antimicrobial classes
Cattle
Chickens
Pigs
Sheep
Turkeys
Ducks
Horses
Buffaloes
Goats
Livestock species
Rank
0.00
0.25
0.50
0.75
1.00

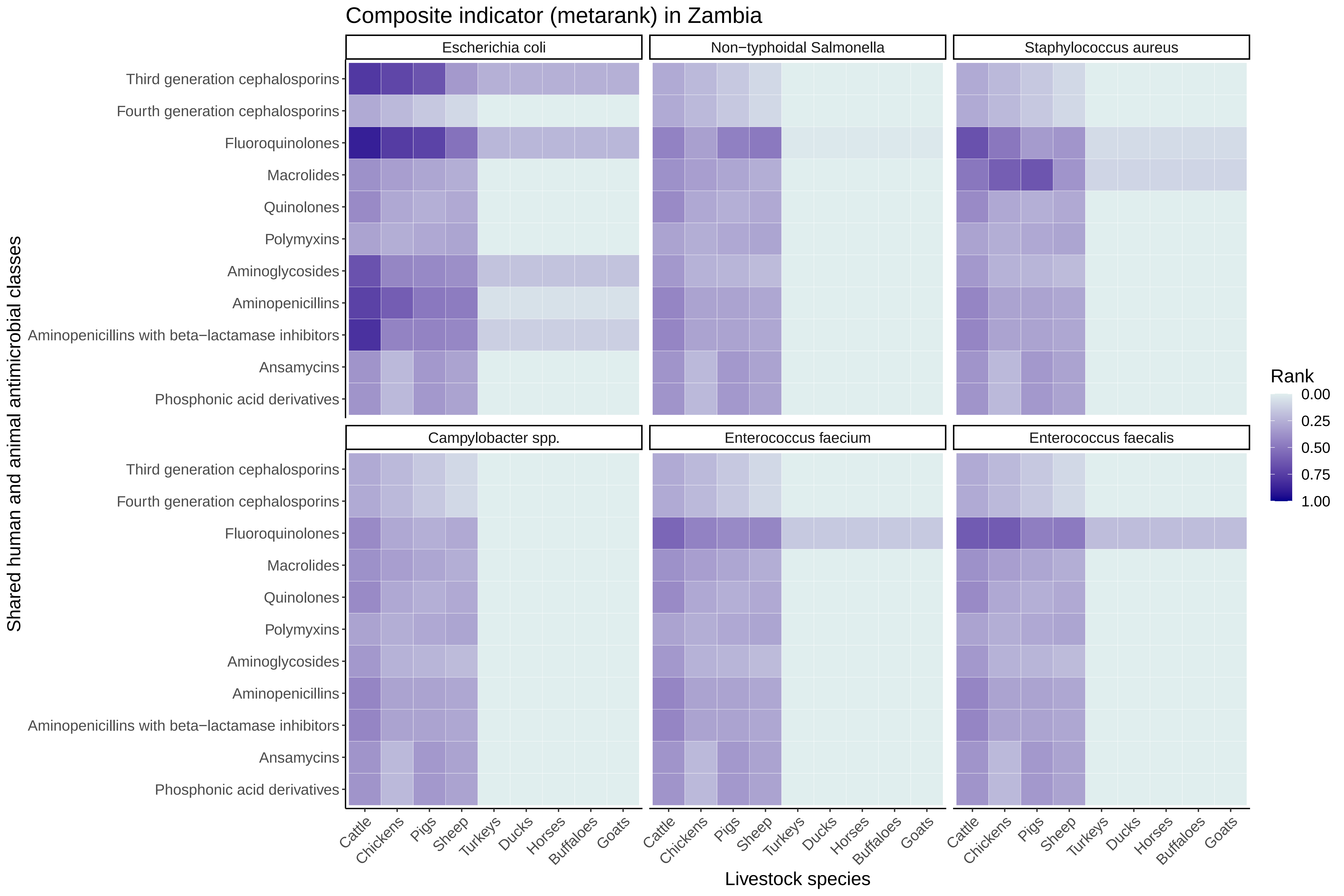
Composite indicator (metarank) in Zambia
Escherichia coli
Non−typhoidal Salmonella
Staphylococcus aureus
Campylobacter spp.
Enterococcus faecium
Enterococcus faecalis
Shared human and animal antimicrobial classes
Third generation cephalosporins
Fourth generation cephalosporins
Fluoroquinolones
Macrolides
Quinolones
Polymyxins
Aminoglycosides
Aminopenicillins
Aminopenicillins with beta−lactamase inhibitors
Ansamycins
Phosphonic acid derivatives
Cattle
Chickens
Pigs
Sheep
Turkeys
Ducks
Horses
Buffaloes
Goats
Livestock species
Rank
0.00
0.25
0.50
0.75
1.00

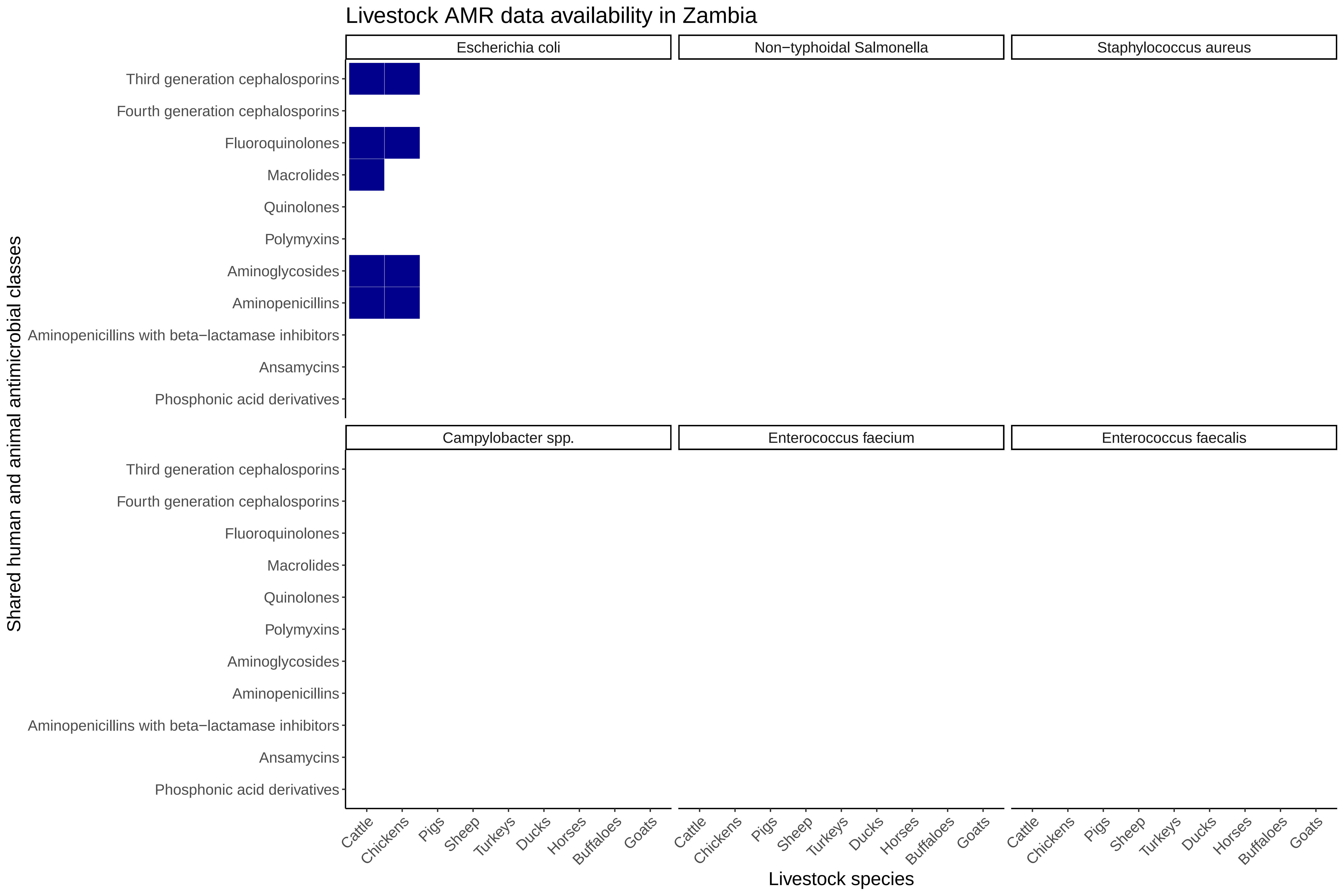

Livestock AMR data availability in Zambia
Escherichia coli
Non−typhoidal Salmonella
Staphylococcus aureus
Campylobacter spp.
Enterococcus faecium
Enterococcus faecalis
Shared human and animal antimicrobial classes
Third generation cephalosporins
Fourth generation cephalosporins
Fluoroquinolones
Macrolides
Quinolones
Polymyxins
Aminoglycosides
Aminopenicillins
Aminopenicillins with beta−lactamase inhibitors
Ansamycins
Phosphonic acid derivatives
Cattle
Chickens
Pigs
Sheep
Turkeys
Ducks
Horses
Buffaloes
Goats
Livestock species

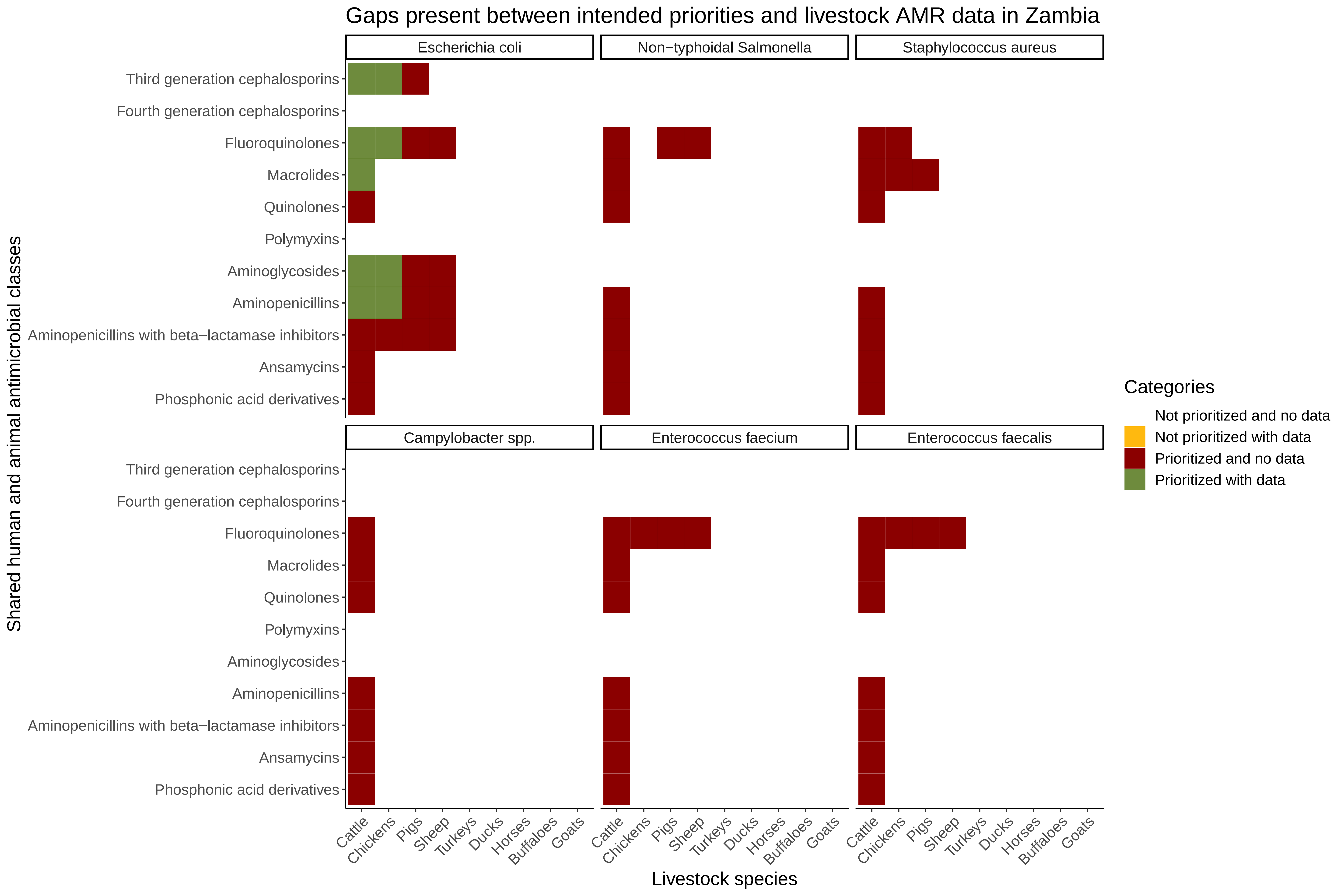

Gaps present between intended priorities and livestock AMR data in Zambia
Escherichia coli
Non−typhoidal Salmonella
Staphylococcus aureus
Campylobacter spp.
Enterococcus faecium
Enterococcus faecalis
Shared human and animal antimicrobial classes
Third generation cephalosporins
Fourth generation cephalosporins
Fluoroquinolones
Macrolides
Quinolones
Polymyxins
Aminoglycosides
Aminopenicillins
Aminopenicillins with beta−lactamase inhibitors
Ansamycins
Phosphonic acid derivatives
Cattle
Chickens
Pigs
Sheep
Turkeys
Ducks
Horses
Buffaloes
Goats
Livestock species
Categories
Not prioritized and no data
Not prioritized with data
Prioritized and no data
Prioritized with data

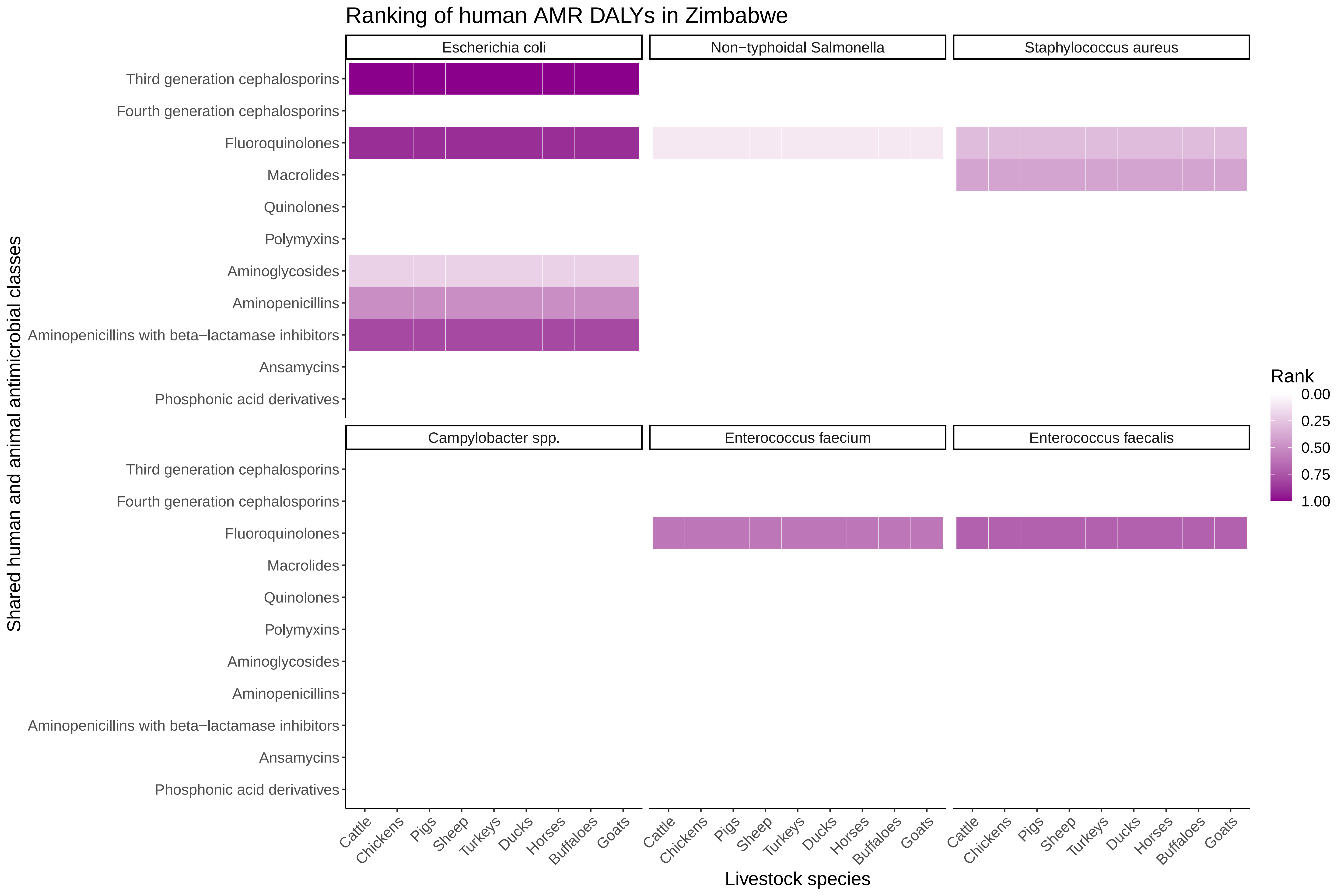

Ranking of human AMR DALYs in Zimbabwe
Escherichia coli
Non−typhoidal Salmonella
Staphylococcus aureus
Campylobacter spp.
Enterococcus faecium
Enterococcus faecalis
Third generation cephalosporins
Fourth generation cephalosporins
Fluoroquinolones
Macrolides
Quinolones
Polymyxins
Aminoglycosides
Aminopenicillins
Aminopenicillins with beta−lactamase inhibitors
Ansamycins
Phosphonic acid derivatives
Shared human and animal antimicrobial classes
Cattle
Chickens
Pigs
Sheep
Turkeys
Ducks
Horses
Buffaloes
Goats
Livestock species
Rank
0.00
0.25
0.50
0.75
1.00

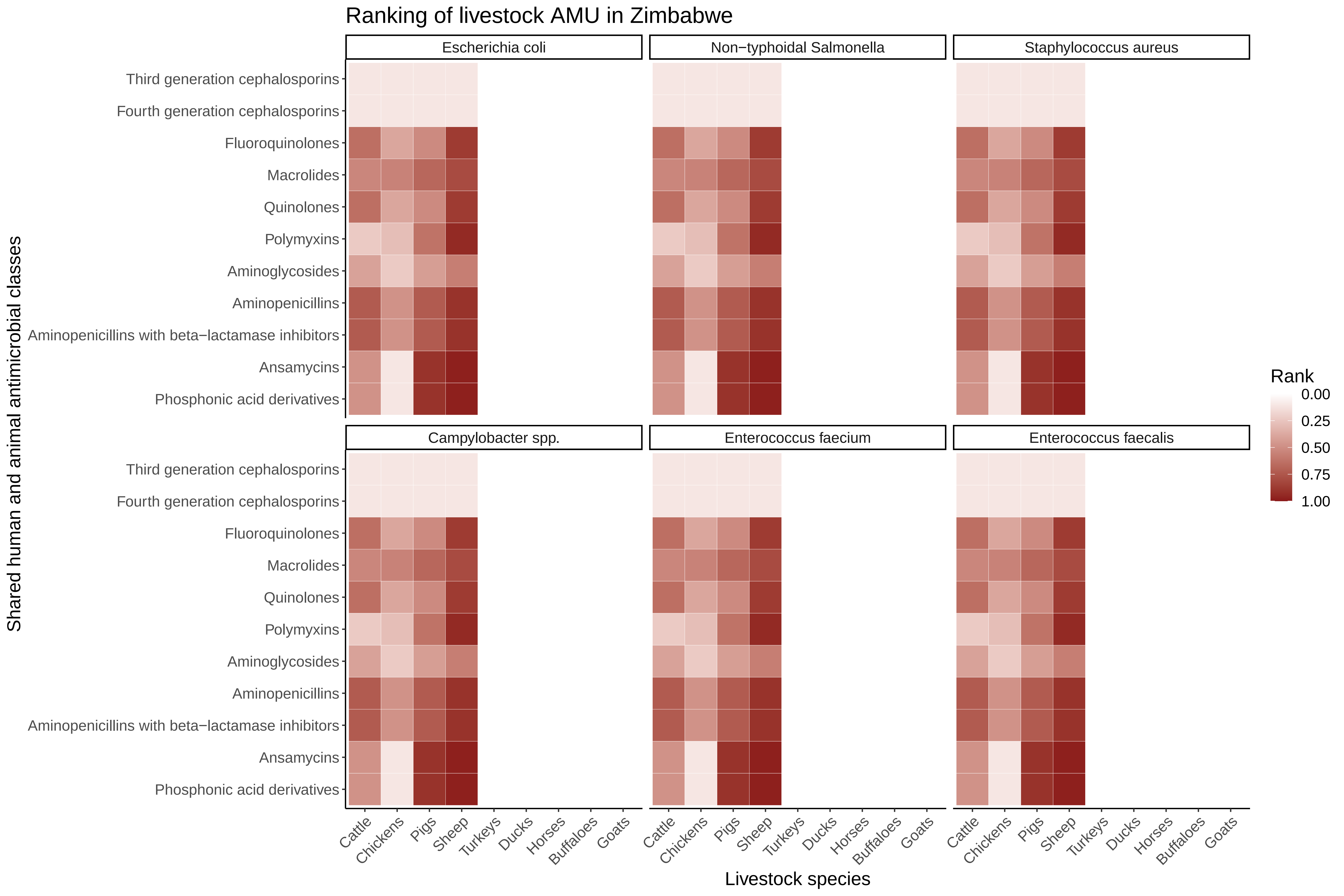
Ranking of livestock AMU in Zimbabwe
Escherichia coli
Non−typhoidal Salmonella
Staphylococcus aureus
Campylobacter spp.
Enterococcus faecium
Enterococcus faecalis
Third generation cephalosporins
Fourth generation cephalosporins
Fluoroquinolones
Macrolides
Quinolones
Polymyxins
Aminoglycosides
Aminopenicillins
Aminopenicillins with beta−lactamase inhibitors
Ansamycins
Phosphonic acid derivatives
Shared human and animal antimicrobial classes
Cattle
Chickens
Pigs
Sheep
Turkeys
Ducks
Horses
Buffaloes
Goats
Livestock species
Rank
0.00
0.25
0.50
0.75
1.00

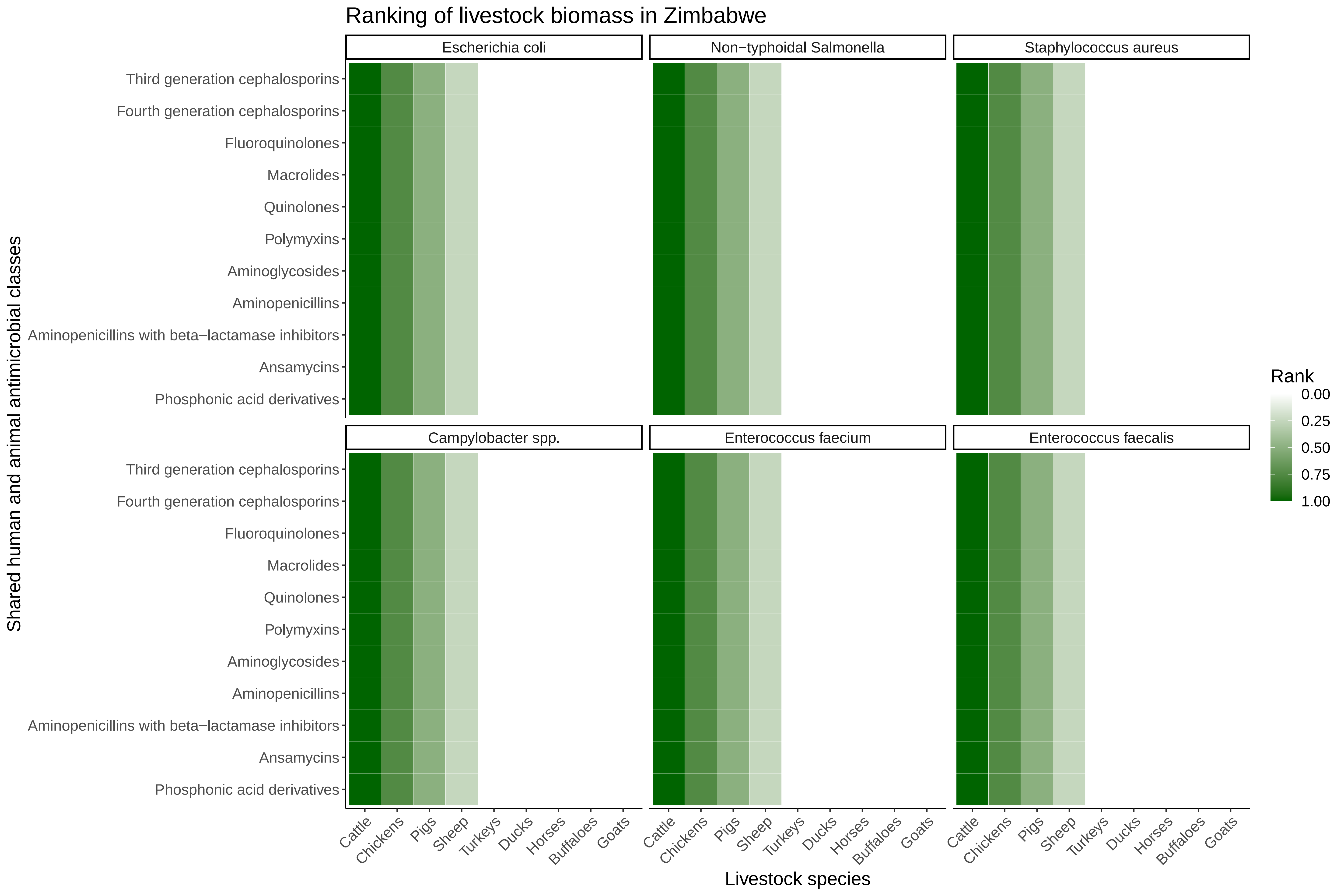
Ranking of livestock biomass in Zimbabwe
Escherichia coli
Non−typhoidal Salmonella
Staphylococcus aureus
Campylobacter spp.
Enterococcus faecium
Enterococcus faecalis
Third generation cephalosporins
Fourth generation cephalosporins
Fluoroquinolones
Macrolides
Quinolones
Polymyxins
Aminoglycosides
Aminopenicillins
Aminopenicillins with beta−lactamase inhibitors
Ansamycins
Phosphonic acid derivatives
Shared human and animal antimicrobial classes
Cattle
Chickens
Pigs
Sheep
Turkeys
Ducks
Horses
Buffaloes
Goats
Livestock species
Rank
0.00
0.25
0.50
0.75
1.00

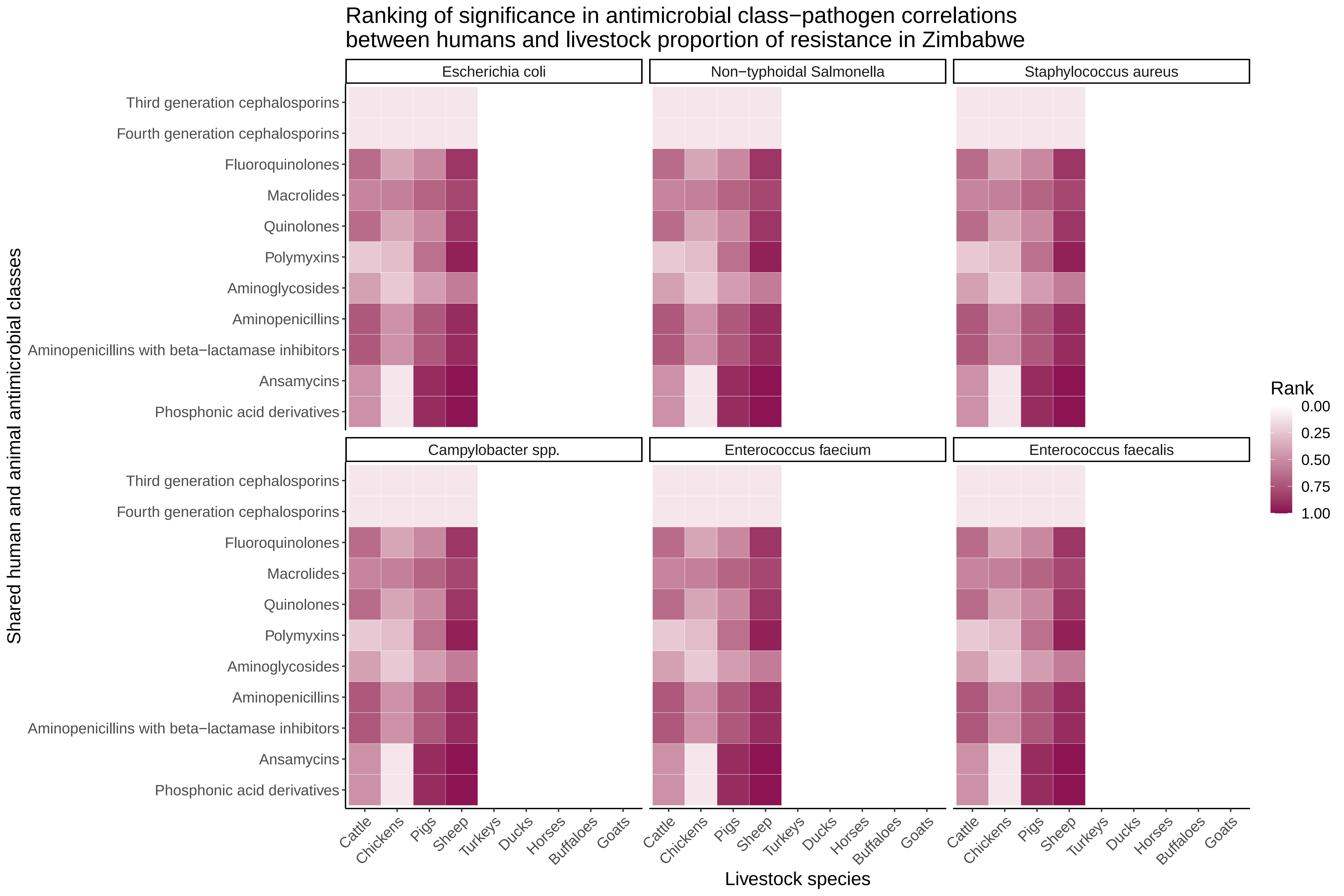
Ranking of significance in antimicrobial class−pathogen correlations
between humans and livestock proportion of resistance in Zimbabwe
Escherichia coli
Non−typhoidal Salmonella
Staphylococcus aureus
Campylobacter spp.
Enterococcus faecium
Enterococcus faecalis
Shared human and animal antimicrobial classes
Third generation cephalosporins
Fourth generation cephalosporins
Fluoroquinolones
Macrolides
Quinolones
Polymyxins
Aminoglycosides
Aminopenicillins
Aminopenicillins with beta−lactamase inhibitors
Ansamycins
Phosphonic acid derivatives
Livestock species
Cattle
Chickens
Pigs
Sheep
Turkeys
Ducks
Horses
Buffaloes
Goats
Rank
0.00
0.25
0.50
0.75
1.00

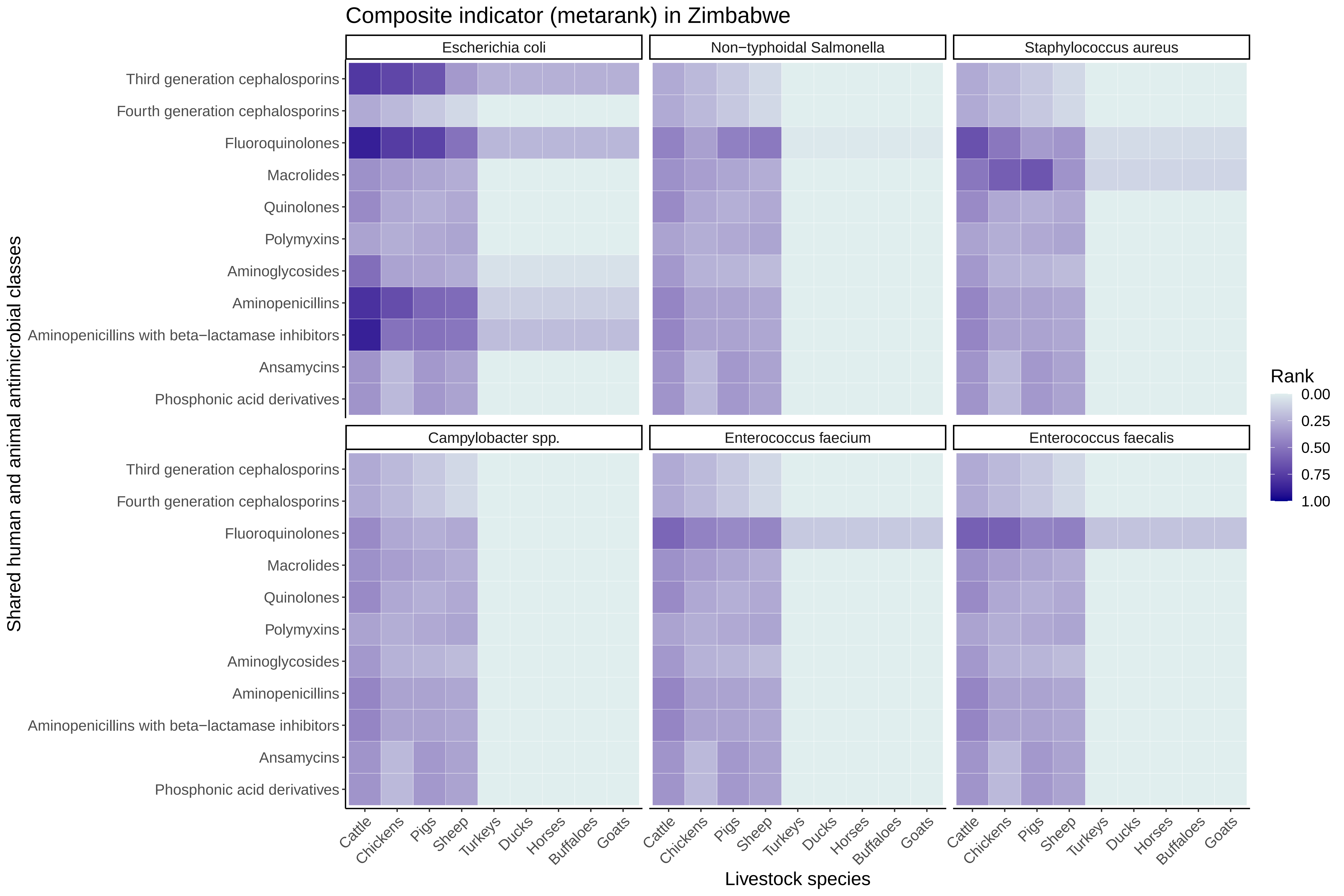

Composite indicator (metarank) in Zimbabwe
Escherichia coli
Non−typhoidal Salmonella
Staphylococcus aureus
Campylobacter spp.
Enterococcus faecium
Enterococcus faecalis
Shared human and animal antimicrobial classes
Third generation cephalosporins
Fourth generation cephalosporins
Fluoroquinolones
Macrolides
Quinolones
Polymyxins
Aminoglycosides
Aminopenicillins
Aminopenicillins with beta−lactamase inhibitors
Ansamycins
Phosphonic acid derivatives
Cattle
Chickens
Pigs
Sheep
Turkeys
Ducks
Horses
Buffaloes
Goats
Livestock species
Rank
0.00
0.25
0.50
0.75
1.00

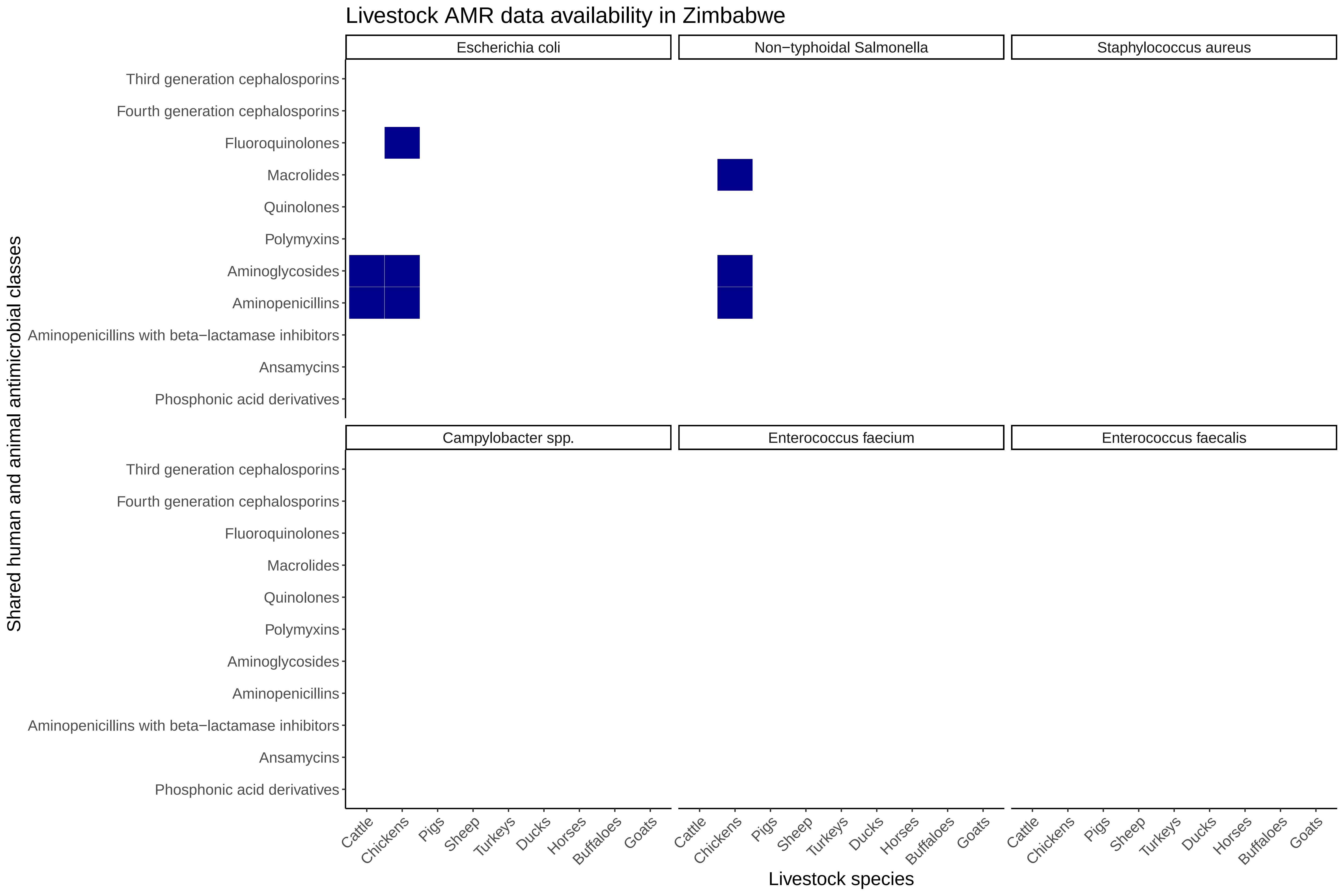

Livestock AMR data availability in Zimbabwe
Escherichia coli
Non−typhoidal Salmonella
Staphylococcus aureus
Campylobacter spp.
Enterococcus faecium
Enterococcus faecalis
Third generation cephalosporins
Fourth generation cephalosporins
Fluoroquinolones
Macrolides
Quinolones
Polymyxins
Aminoglycosides
Aminopenicillins
Aminopenicillins with beta−lactamase inhibitors
Ansamycins
Phosphonic acid derivatives
Shared human and animal antimicrobial classes
Cattle
Chickens
Pigs
Sheep
Turkeys
Ducks
Horses
Buffaloes
Goats
Livestock species

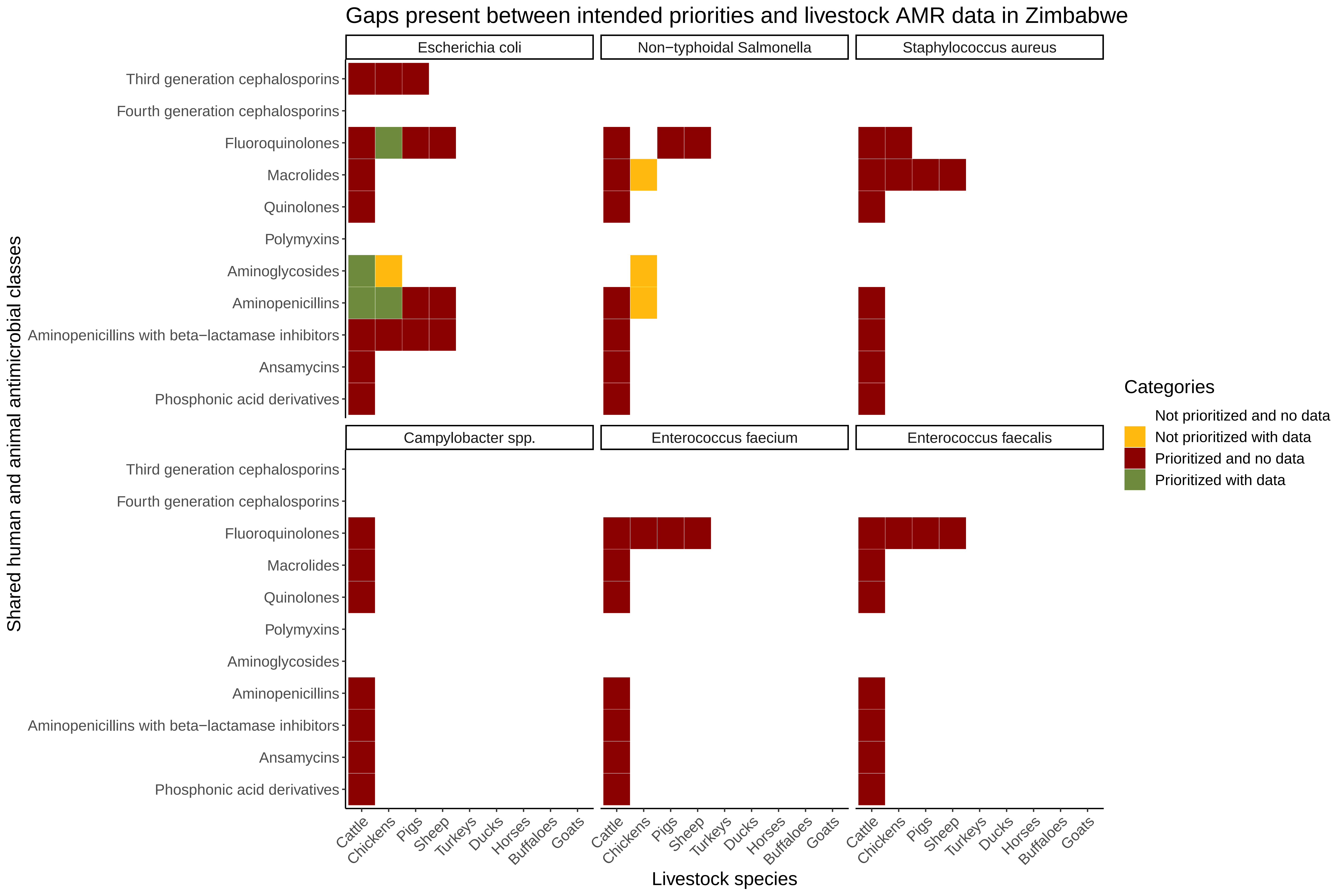

Gaps present between intended priorities and livestock AMR data in Zimbabwe
Escherichia coli
Non−typhoidal Salmonella
Staphylococcus aureus
Campylobacter spp.
Enterococcus faecium
Enterococcus faecalis
Third generation cephalosporins
Fourth generation cephalosporins
Fluoroquinolones
Macrolides
Quinolones
Polymyxins
Aminoglycosides
Aminopenicillins
Aminopenicillins with beta−lactamase inhibitors
Ansamycins
Phosphonic acid derivatives
Shared human and animal antimicrobial classes
Cattle
Chickens
Pigs
Sheep
Turkeys
Ducks
Horses
Buffaloes
Goats
Livestock species
Categories
Not prioritized and no data
Not prioritized with data
Prioritized and no data
Prioritized with data
